# Supplementary material for: Genomic effects of population collapse in a critically endangered ironwood tree Ostrya rehderiana
Source: Nat Commun. 2018 Dec 21;9:5449. doi: 10.1038/s41467-018-07913-4 (PMC6303402; doi:10.1038/s41467-018-07913-4)
Supplement: Supplementary file 1 — Supplementary Information [file 41467_2018_7913_MOESM1_ESM.pdf]

Supplementary Information

Genomic effects of population collapse in a critically endangered ironwood tree *Ostrya rehderiana*

Yang and Ma et al.

Supplementary Figures 2

Supplementary Tables..28

Supplementary Methods 82

Supplementary Notes..86

Supplementary References 93

Supplementary Figures

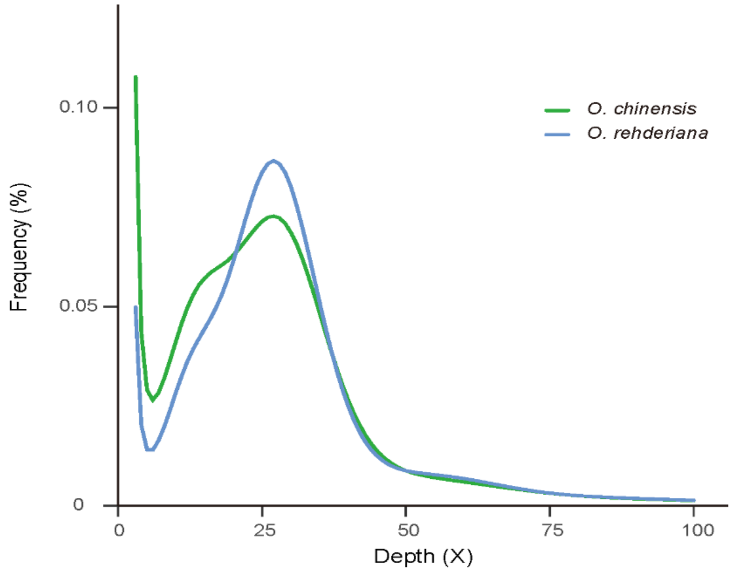

Supplementary Figure 1. 17-mer-based analysis to estimate the genome sizes of *O. rehderiana* and *O. chinensis* respectively.

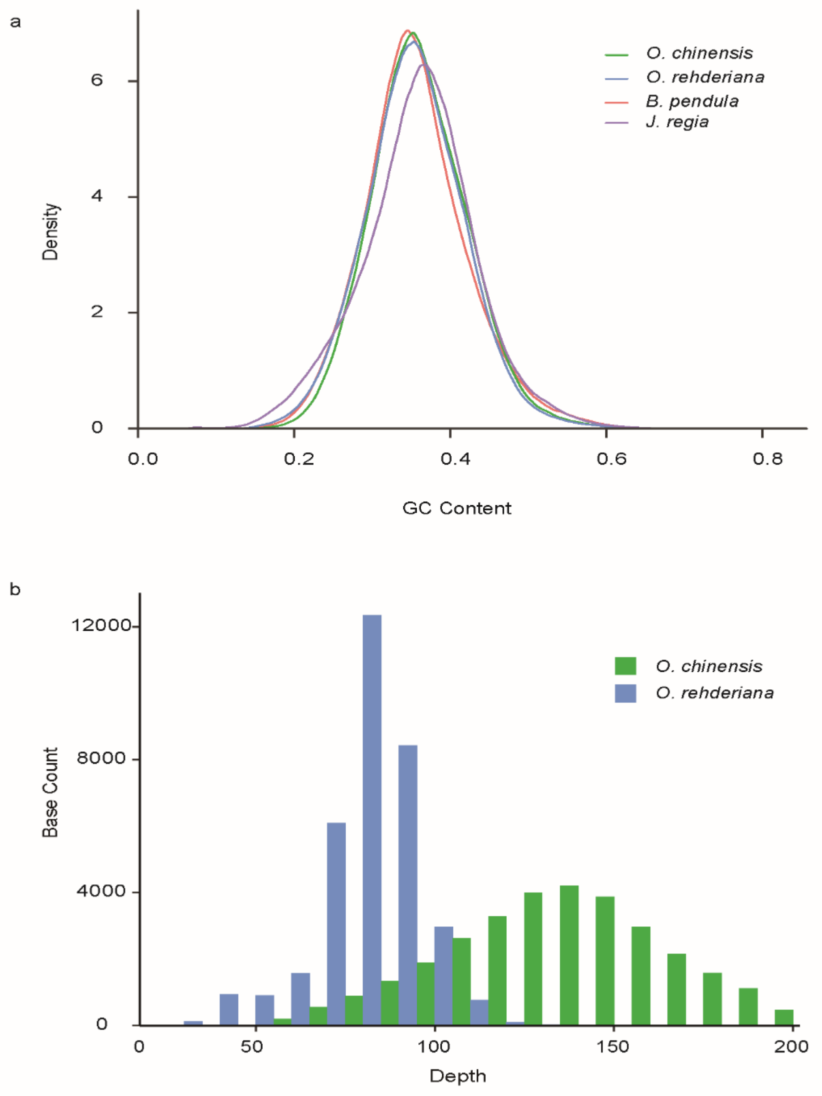

Supplementary Figure 2. GC-content and sequencing depth analyses in the *O. rehderiana* and *O. chinensis* genomes. (a) The GC content distribution for the genomes of *O. rehderiana*, *O. chinensis*, *B. pendula* and *J. regia*, established by 500 bp non-overlapping sliding windows. (b) Sequencing depth distribution of *O. rehderiana* and *O. chinensis* genomes. The sequencing reads from all small-insert libraries were realigned onto the assembly using BWA-MEM. The sequencing depth of each base was calculated and plotted.

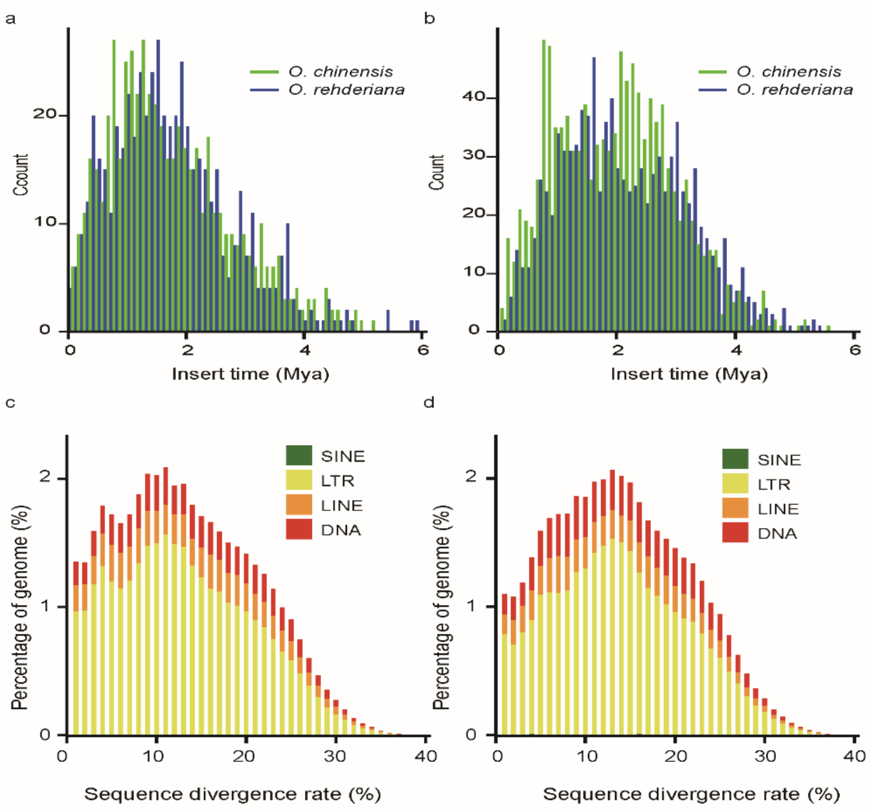

Supplementary Figure 3. Dating the LTR retrotransposon insertion time and the sequence divergence rate of different repetitive elements in the *O. rehderiana* and *O. chinensis* genomes. (a)-(b) The distribution insertion time of LTR retrotransposon of Copia (a) and Gypsy (b) in the *O. rehderiana* and *O. chinensis* genomes. (c)-(d) Divergence rate was calculated between the identified TES in the genome by the RepeatMasker and the consensus sequence in the TE library of *O. rehderiana (c)* and *O. chinensis* (d). DNA, DNA elements; LINE, long interspersed nuclear elements; LTR, long terminal repeat transposable element; SINE, short interspersed nuclear elements.

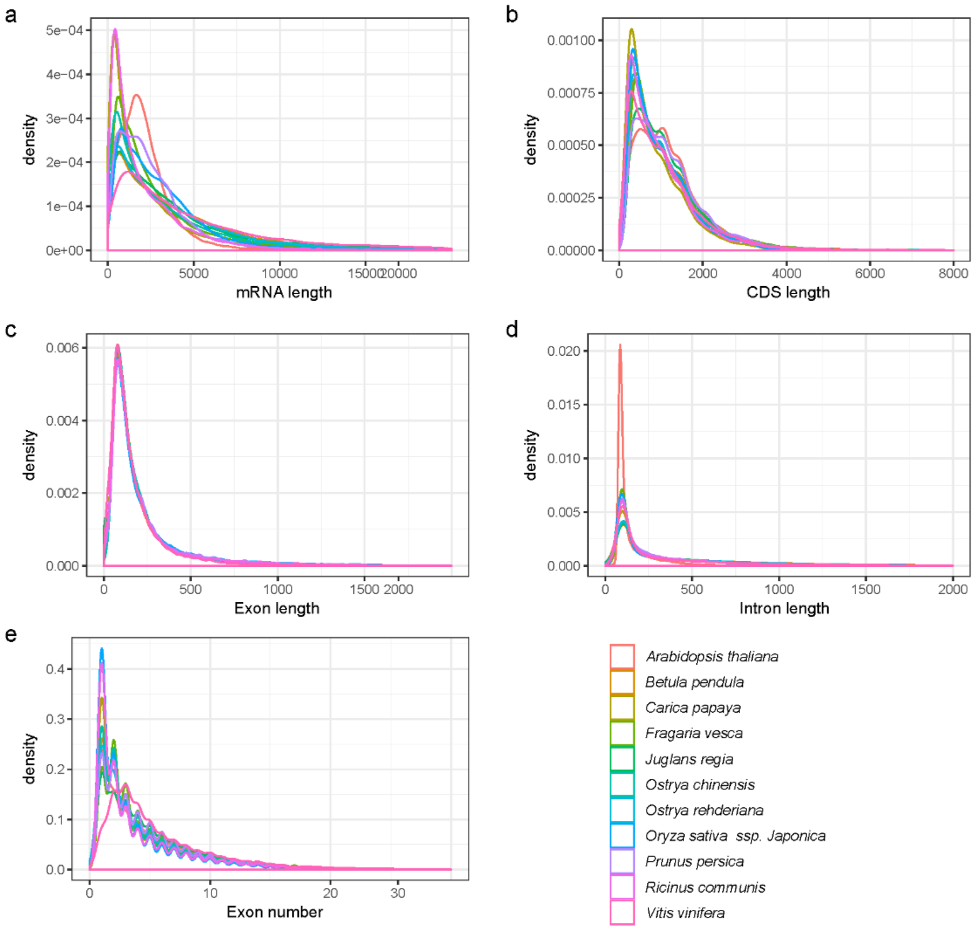

Supplementary Figure 4. Comparison of mRNA length (a), CDS length (b), Exon length (c), Intron length (d), and Exon number per gene (e) between *O. rehderiana*, *O. chinensis*, *Arabidopsis thaliana*, *Carica papaya*, *Fragaria vesca*, *Juglans regia*, *Oryza sativa ssp. Japonica*, *Prunus persica*, *Ricinus communis* and *Vitis vinifera*. The *x*-axis represents length or number and the *y*-axis represents the density of genes.

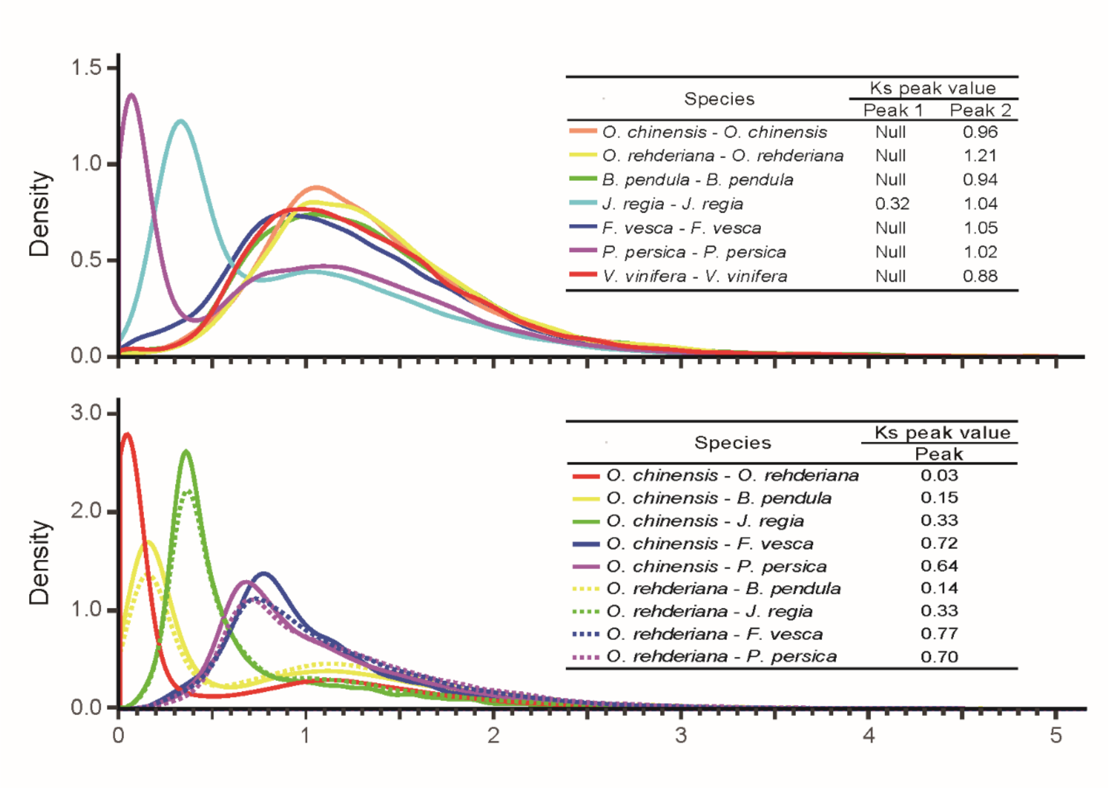

Supplementary Figure 5. Density distribution of Ks for synteny homologous genes within each species or between two species.

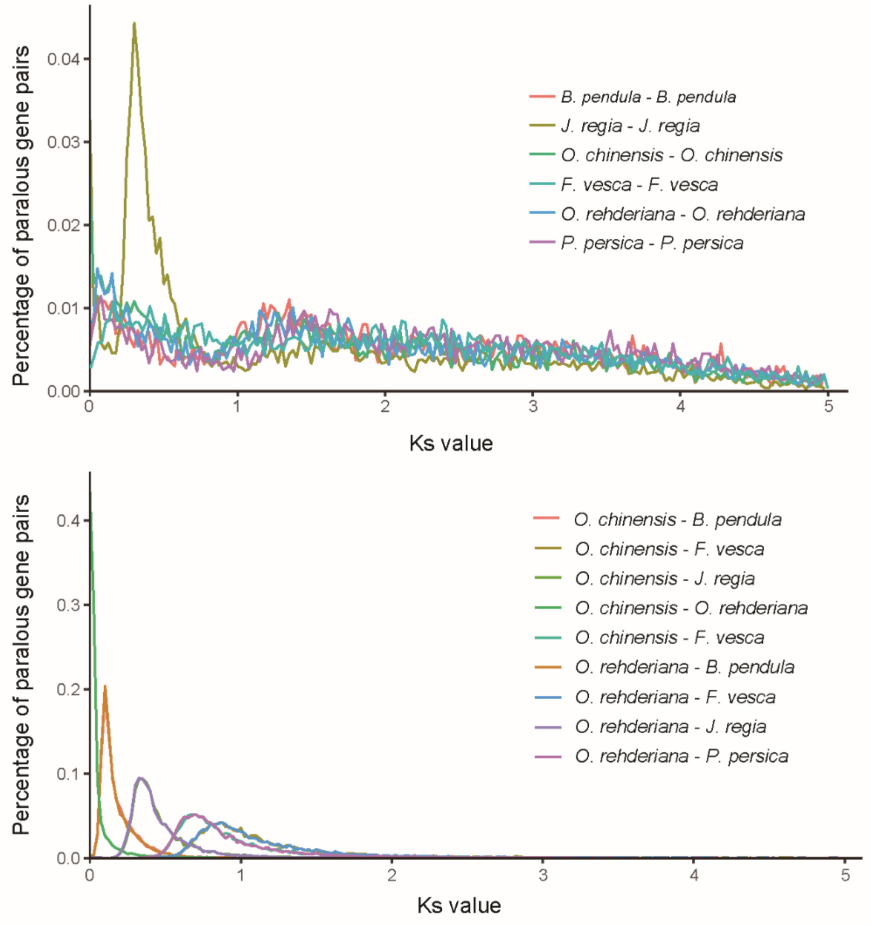

Supplementary Figure 6. Density distribution of Ks for genome homologous genes within each species or between two species.

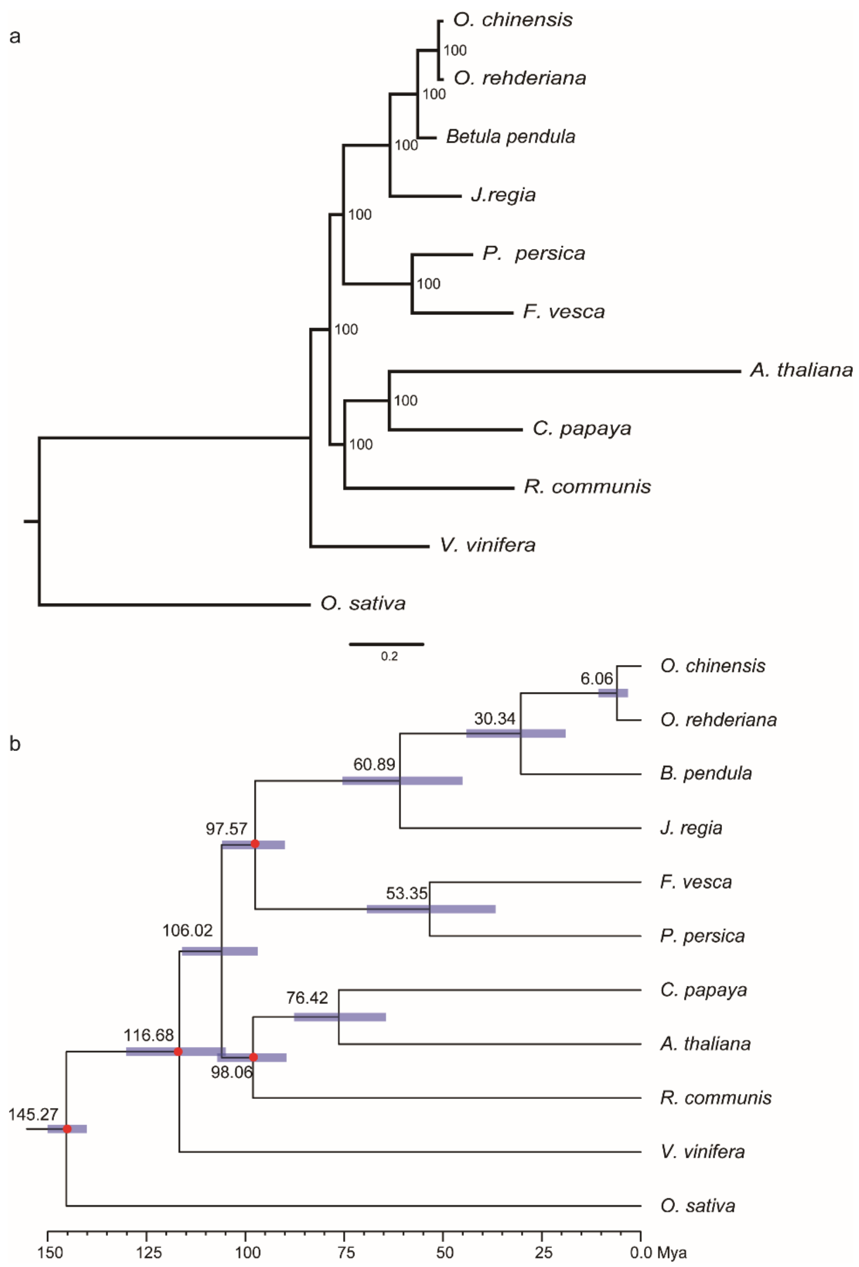

Supplementary Figure 7. Phylogenetic and divergence time analyses. (a) Phylogenetic tree was constructed by the single-copy genes from 11 species using RaxML and the bootstrap supports for all nodes were 100%. (b) Divergence estimates for all 11 species were generated by MCMCtree and the 4-fold degenerate sites. The red dots correspond to calibration points as mentioned in the methods. Divergence estimates (Mya, million years ago) are indicated above each node and the blue nodal bars show the 95% confidence intervals.

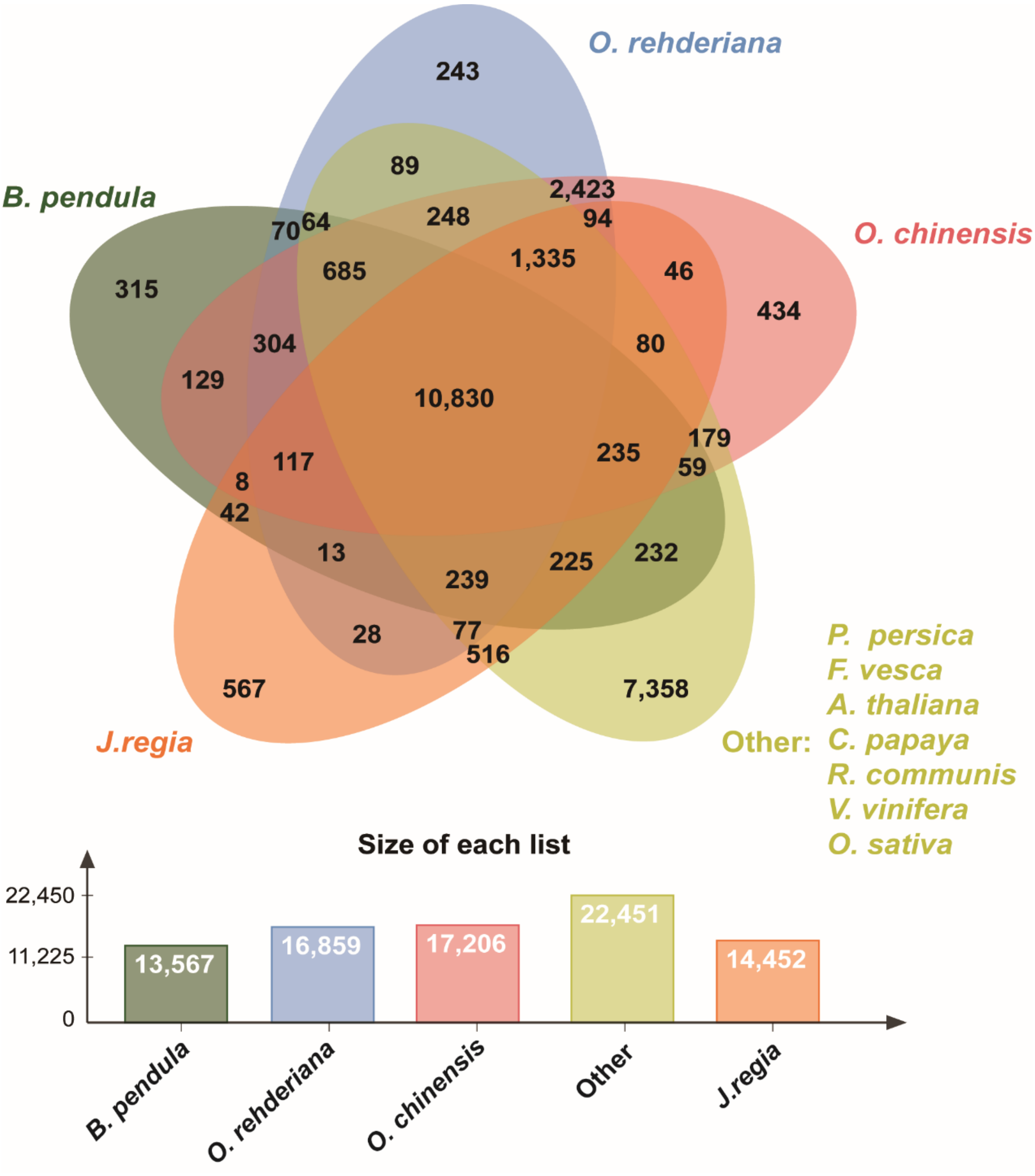

Supplementary Figure 8. Gene family comparison among 11 species. Each number represents a gene family number.

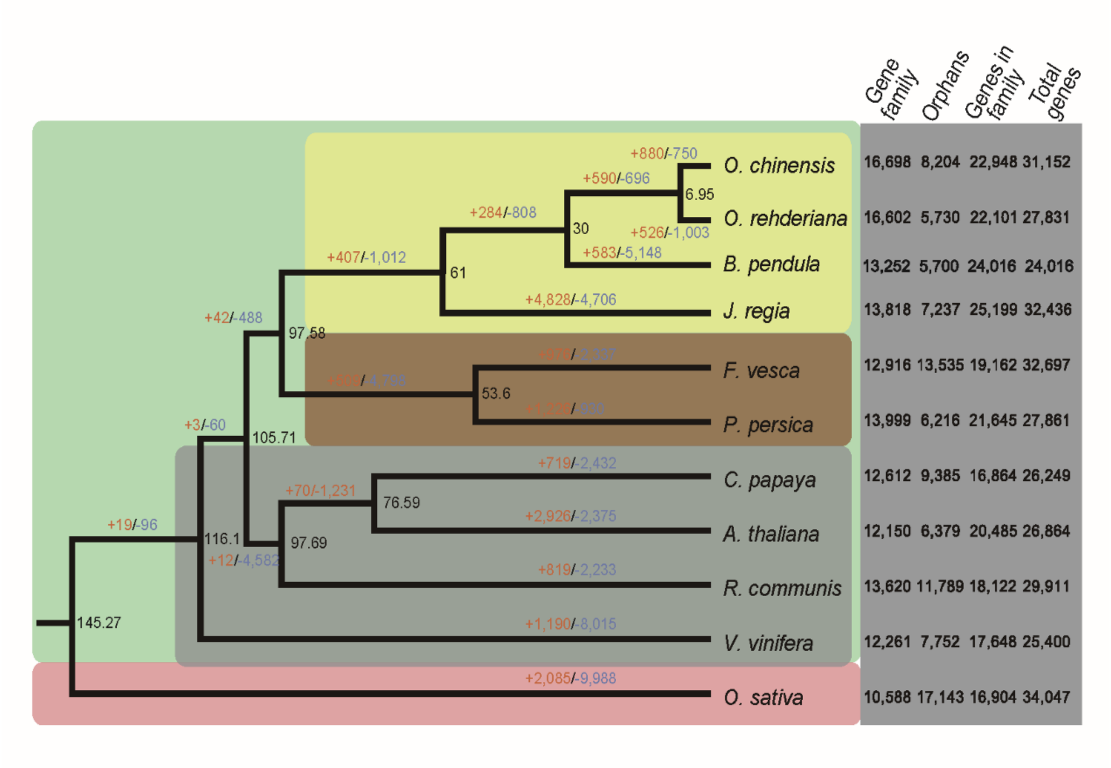

Supplementary Figure 9. Gene expansion and contraction along each lineage based on the phylogenetic tree of 11 species (constructed by the single-copy orthologous genes). The number in each node indicates the divergence time (Fig. S7). The values above each branch denote the gain/loss (red/blue) number of the gene family along each lineage.

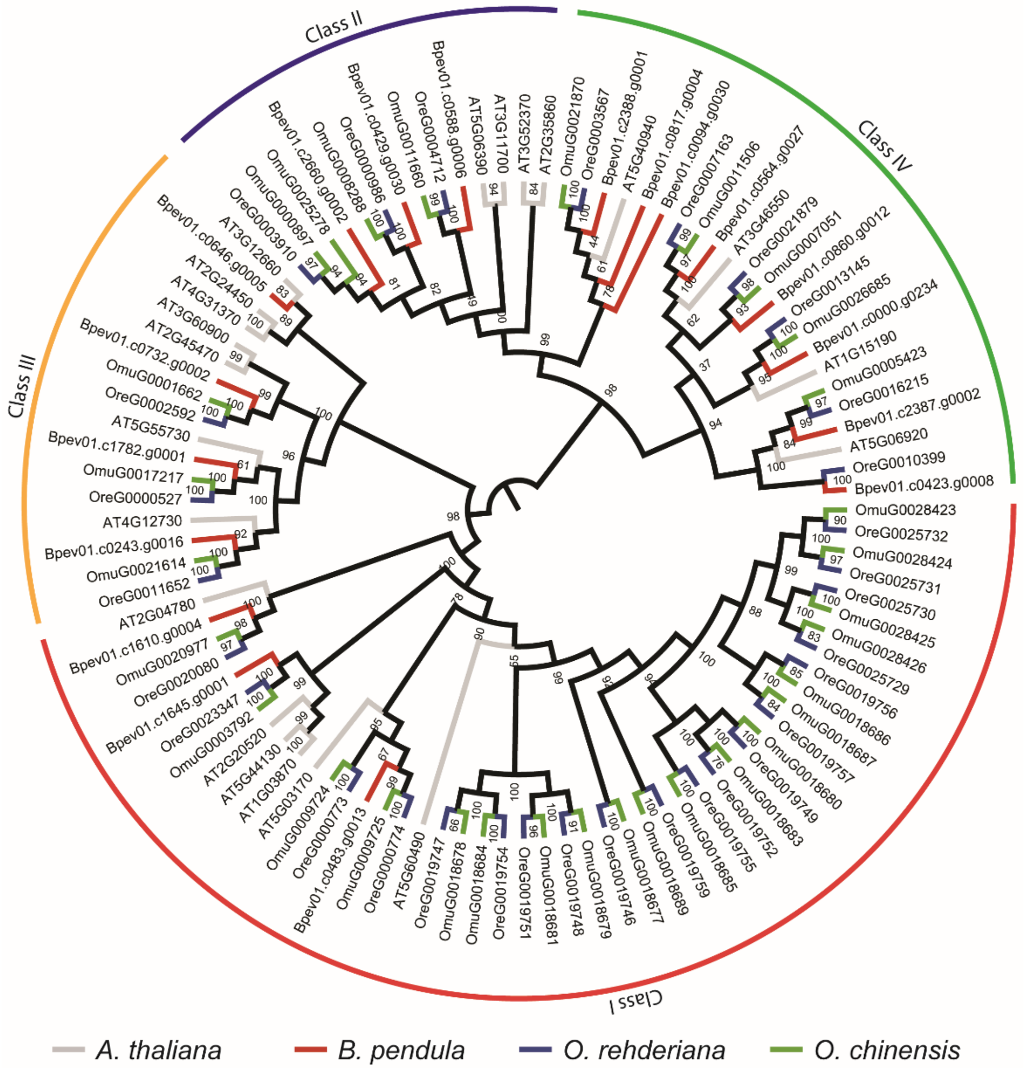

Supplementary Figure 10. The phylogenetic tree of FLAs proteins from *A. thaliana* (grey branches), *B. pendula* (red branches), *O. rehderiana* (blue branches) and *O. chinensis* (green branches). The circular segments represent the subfamily types classified in *A. thaliana*: Class I (red), Class II (blue), Class III (yellow) and Class IV (green).

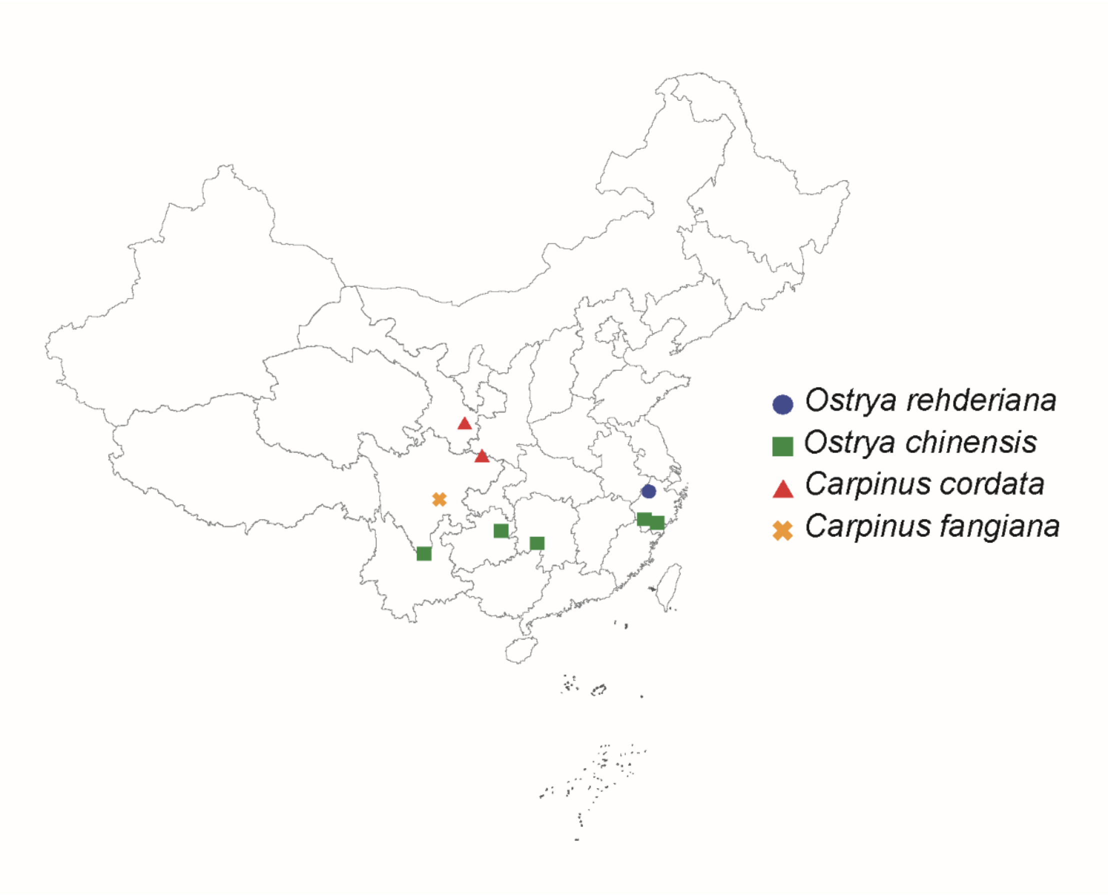

Supplementary Figure 11. The geographic distribution of the sampling locations for *O. rehderiana* (blue), *O. chinensis* (green), *C. cordata* (red), *C. cordata* (yellow) and *O. nobilis* (black). The basic China map was generated by the maps packages in R.

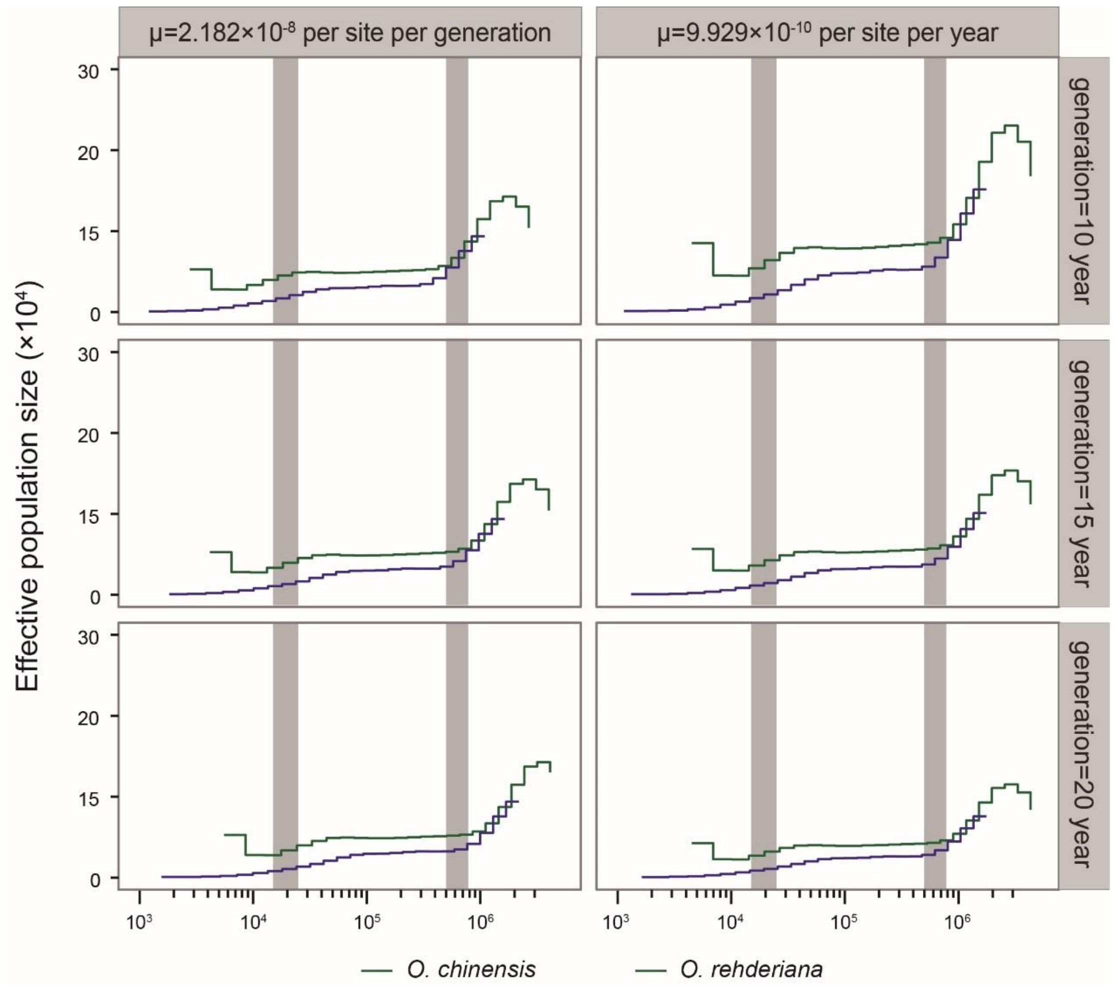

Supplementary Figure 12. Historical effective population size estimates in two iron-wood species with two different mutation rates (2.182×10^{-8} per site per generation and 9.929×10^{-10} per site per year) and three different generation time estimates (10, 15 and 20 years).

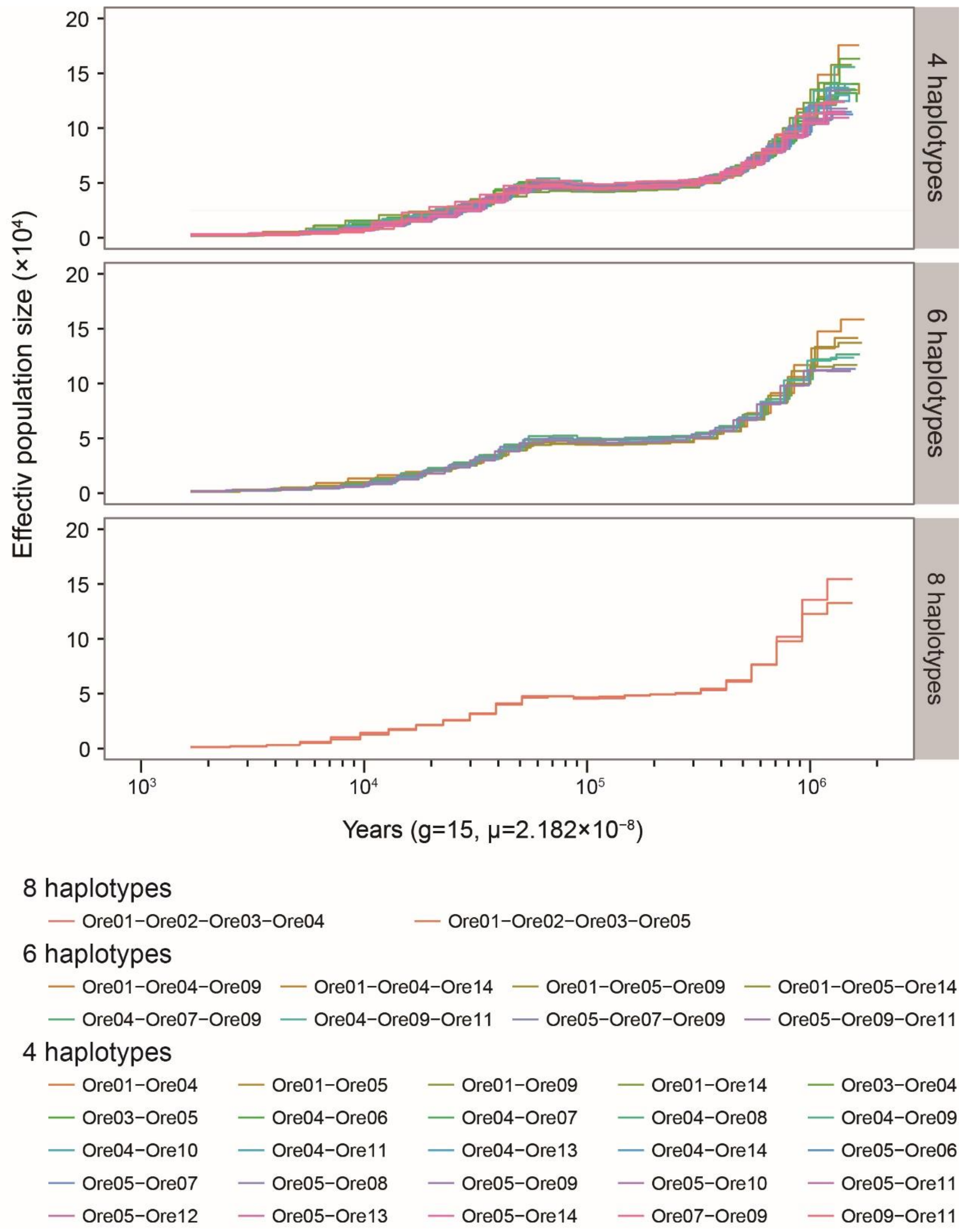

Supplementary Figure 13. Demographic history inferred by MSMC with different haplotypes in *O. rehderiana.*

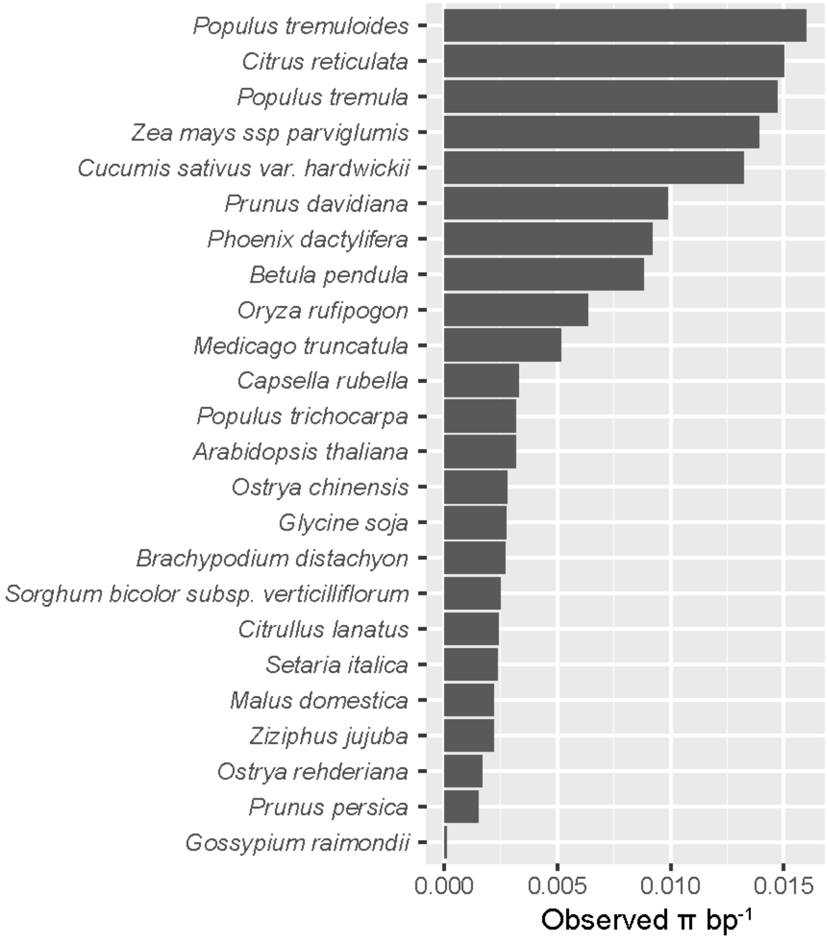

Supplementary Figure 14. Sequence diversity (π) for 24 plant species with genomes sequenced.

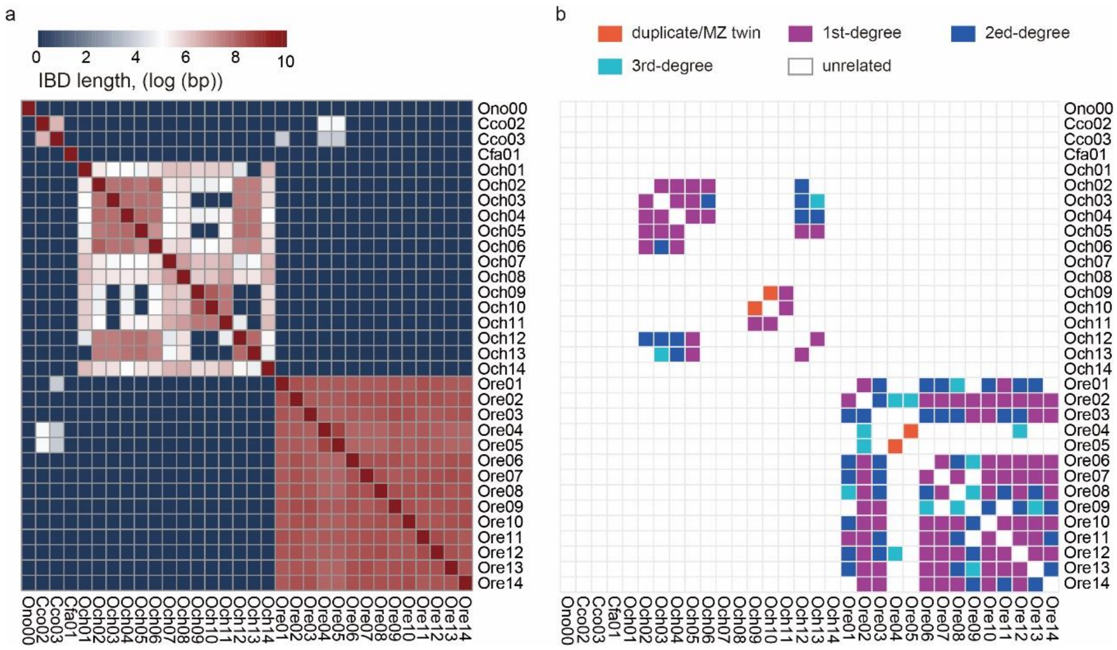

Supplementary Figure 15. Relationship between all sequenced individuals. (a) Total genome sequence shared between haplotypes in different individuals. Heatmap colors represent the total length of IBD blocks for each pairwise comparison. (b) Estimated relatedness between individuals.

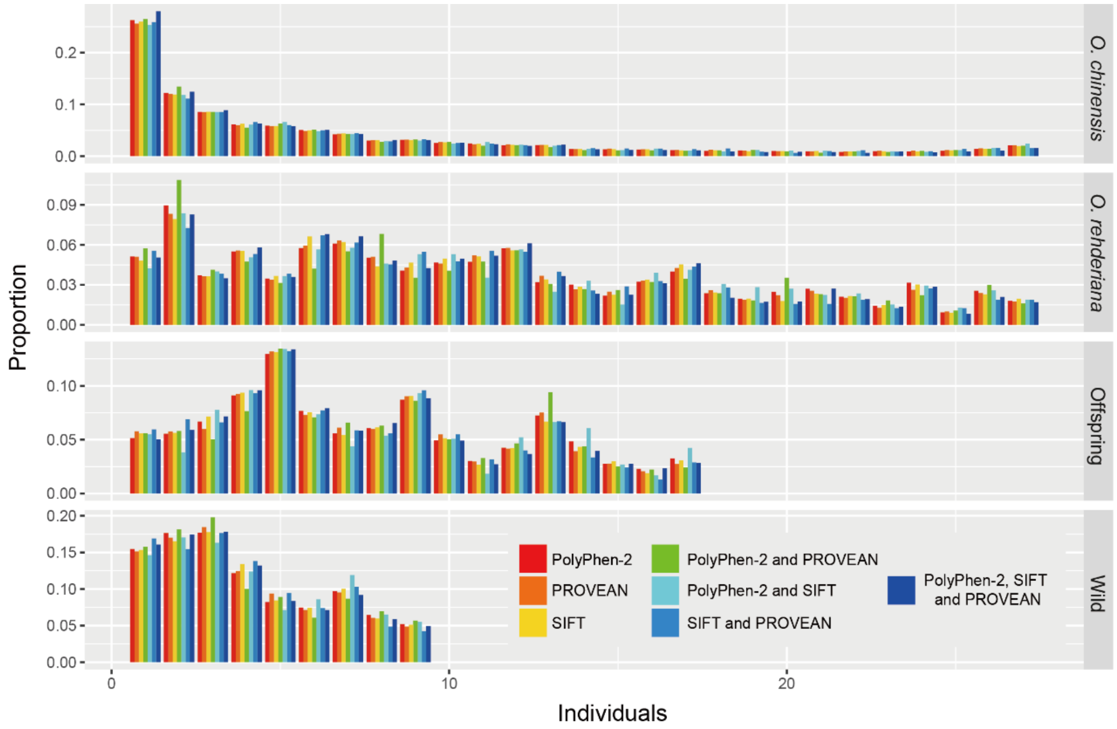

Supplementary Figure 16. Number of derived allele (unfolded) frequency spectra for SNPs predicted to be deleterious by different approaches. 'Wild' represents the only five large natural individuals of *O. reheriana* while 'Offspring' indicates the nine young individuals artificially planted.

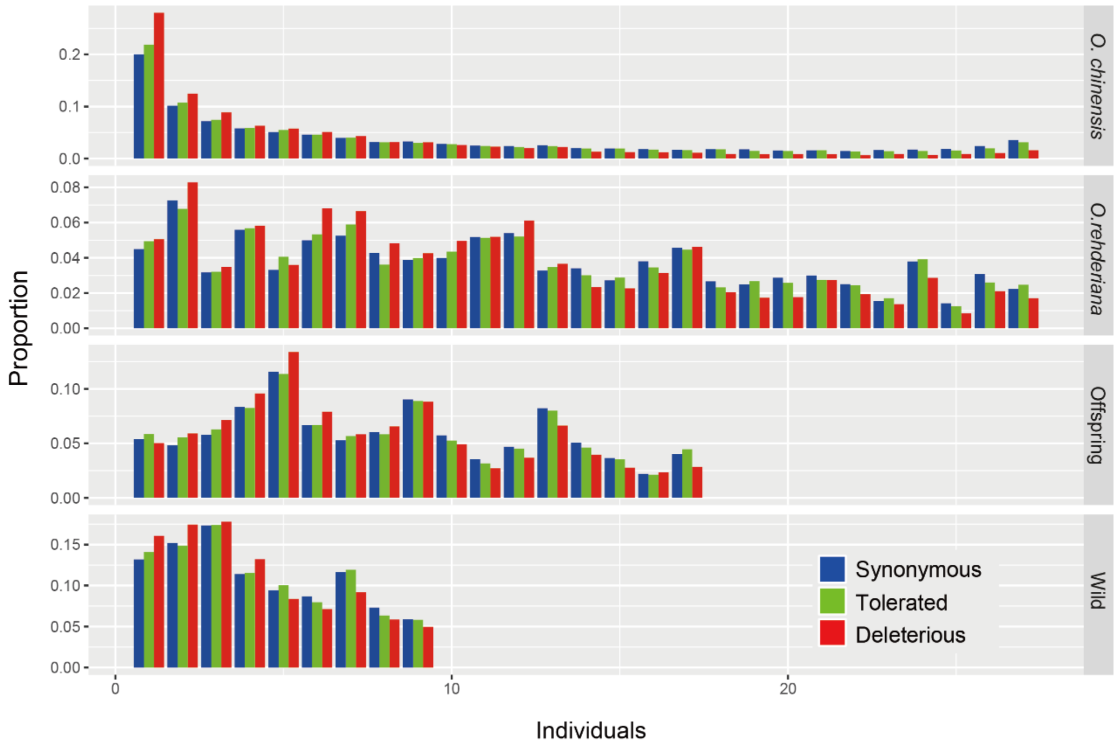

Supplementary Figure 17. The derived allele (unfolded) frequency spectra for coding regions, indicating deleterious, tolerated and synonymous SNPs. 'Wild' represents the only five large natural individuals of *O. reheriana* while 'Offspring' indicates nine young individuals artificially planted.

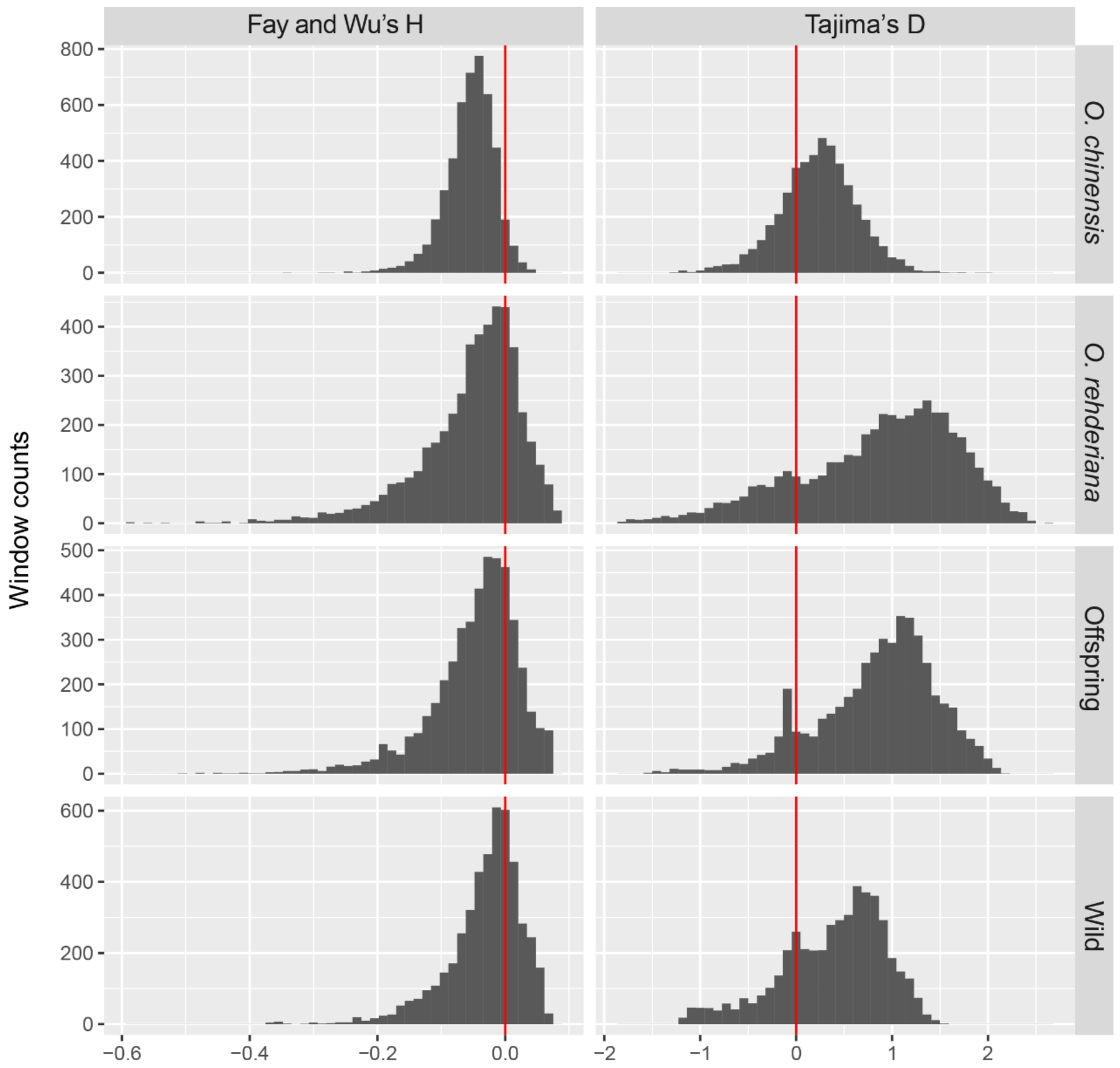

Supplementary Figure 18. The distribution of Tajima's D and Fay and Wu's values. 'Wild' represents the only five large natural individuals of *O. reheriana* while 'Offspring' indicates nine young individuals artificially planted.

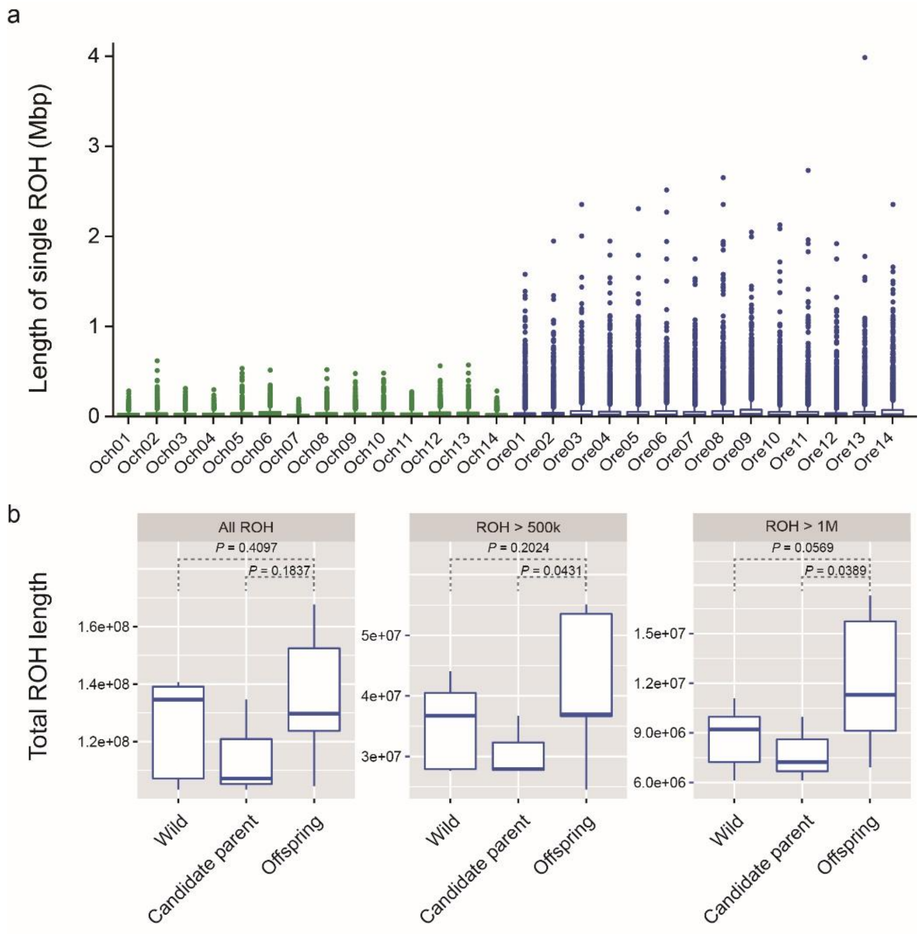

c

	Ore06	Ore09	Ore10	Ore13	Ore14
**Ore01**	1.2E-04	-	-	-	-
**Ore02**	2.8E-03	3.2E-07	3.2E-07	4.7E-02	1.4E-05
**Ore03**	-	3.7E-04	3.7E-04	8.5E-01	5.4E-02

Supplementary Figure 19. The ROH length distributions. (a) The ROH length of each exampled tree from two species (Och = *O. chinensis*; Ore = *O. rehderiana*). (b) The length distributions in different types of ROH among *O. rehderiana*. 'All ROH' represent all the ROH without any further filtering. 'ROH > 500k' and 'ROH > 1M' represent the ROHs with single length larger than 500kb and 1Mb, respectively. 'Wild' represent all the five wild individuals. 'Candidate parent' represent the candidate parent (Ore01, Ore02 and Ore03) of the offspring. 'Offspring' represent the nine young trees. The *P* values represent the t-test (one tailed) results between each group. (c) The comparison of ROH length between the offspring and their identified parents (by the KING software). The *P* values were calculated by the paired sample t-test (one tailed) of the total ROH lengths in each scaffold between offspring and its parent.

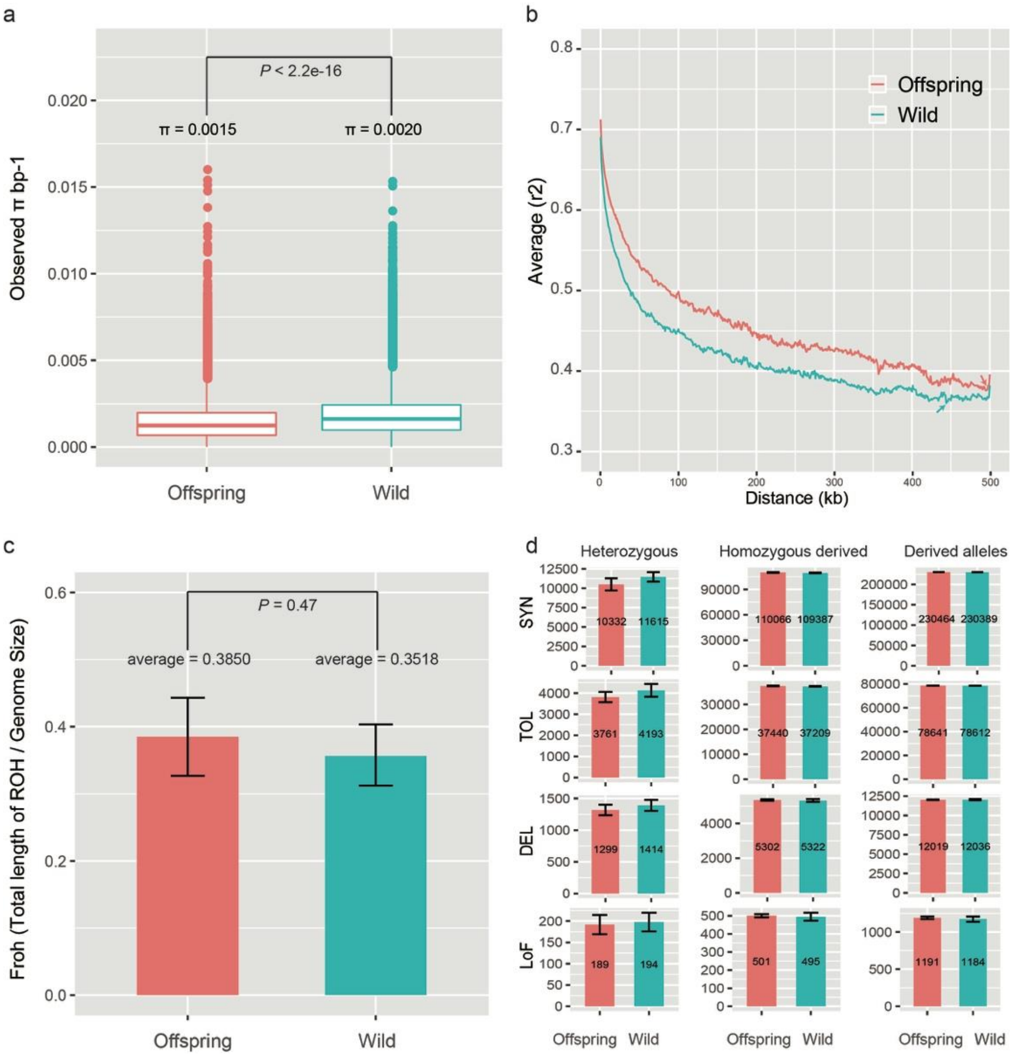

Supplementary Figure 20. Comparison of the π, LD, Froh and derived alleles between nine young trees (Offspring) and four old (Wild) ones (excluding one likely twins, Ore05) of *O. rehderiana*.

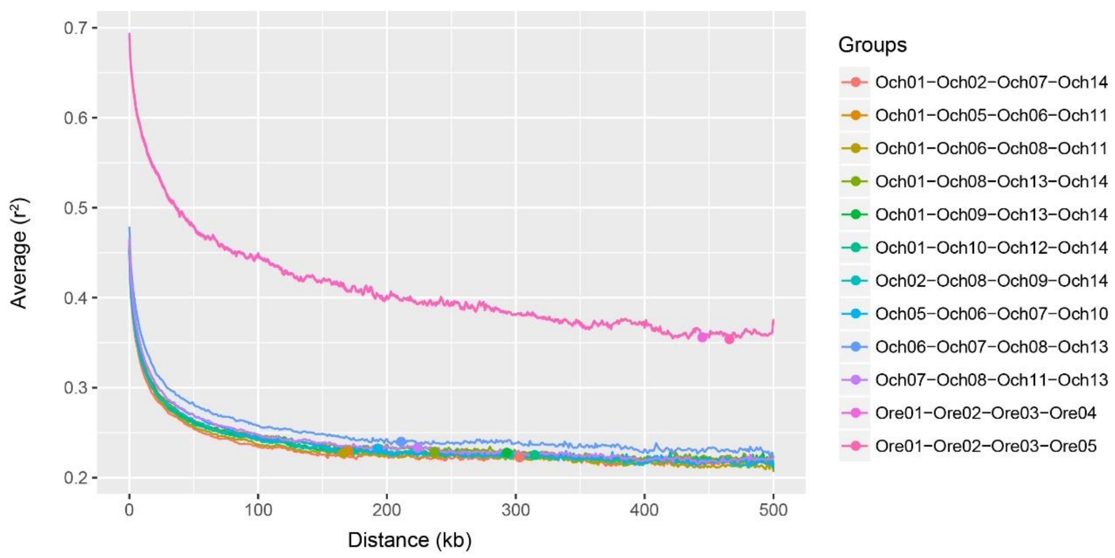

Supplementary Figure 21. The LD decay within four wild *O. rehderiana* individuals (including only one likely twins, Ore04 or 0re05, see Supplementary Figure 15) and four randomly selected *O. chinensis* individuals.

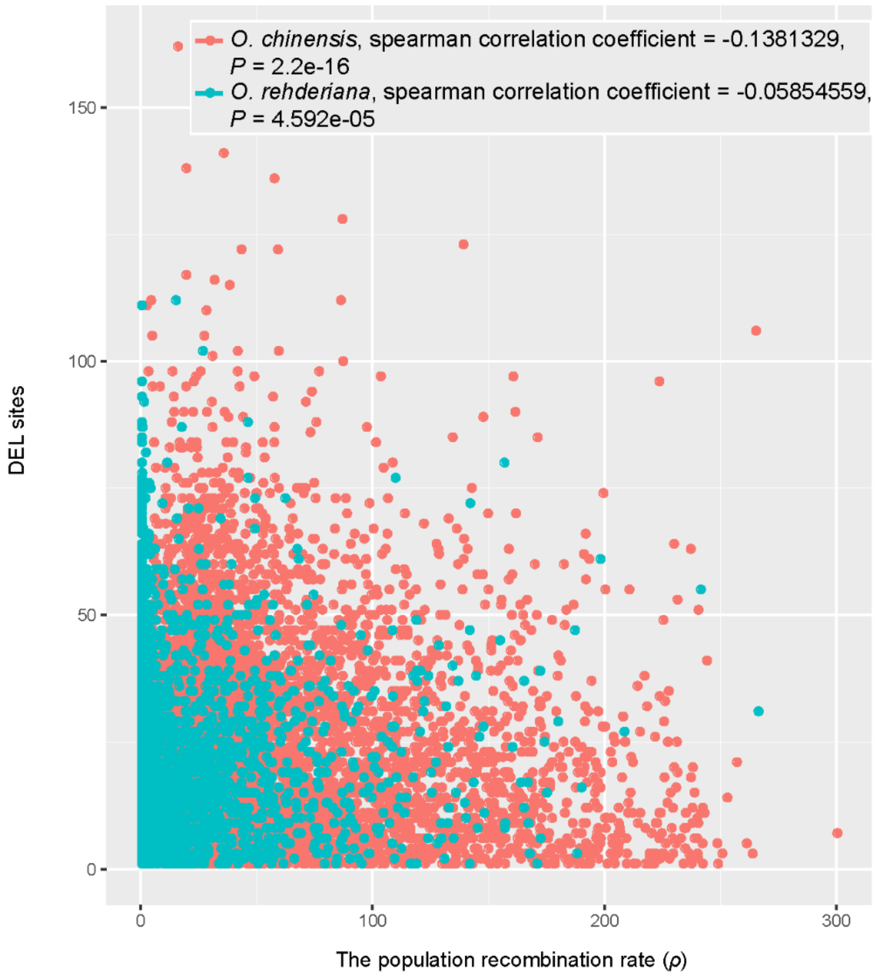

Supplementary Figure 22. The correlations between the population recombination rate (*ρ*) and the load of deleterious mutations of two iron-wood species.

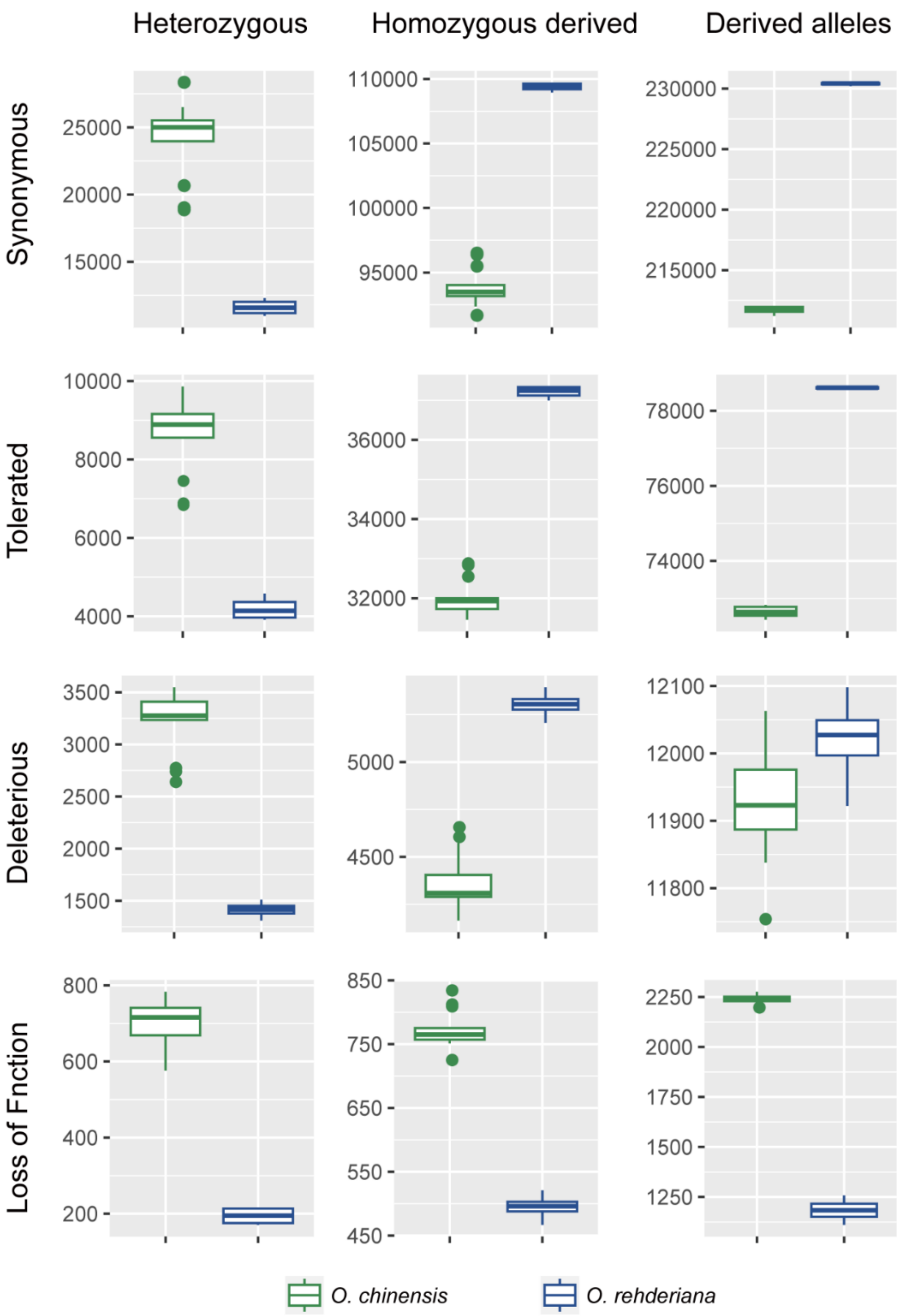

Supplementary Figure 23. The deleterious genetic variation of four wild *O. rehderiana* individuals (including one likely twins, Ore04, see Fig S14) and all *O. chinensis* trees.

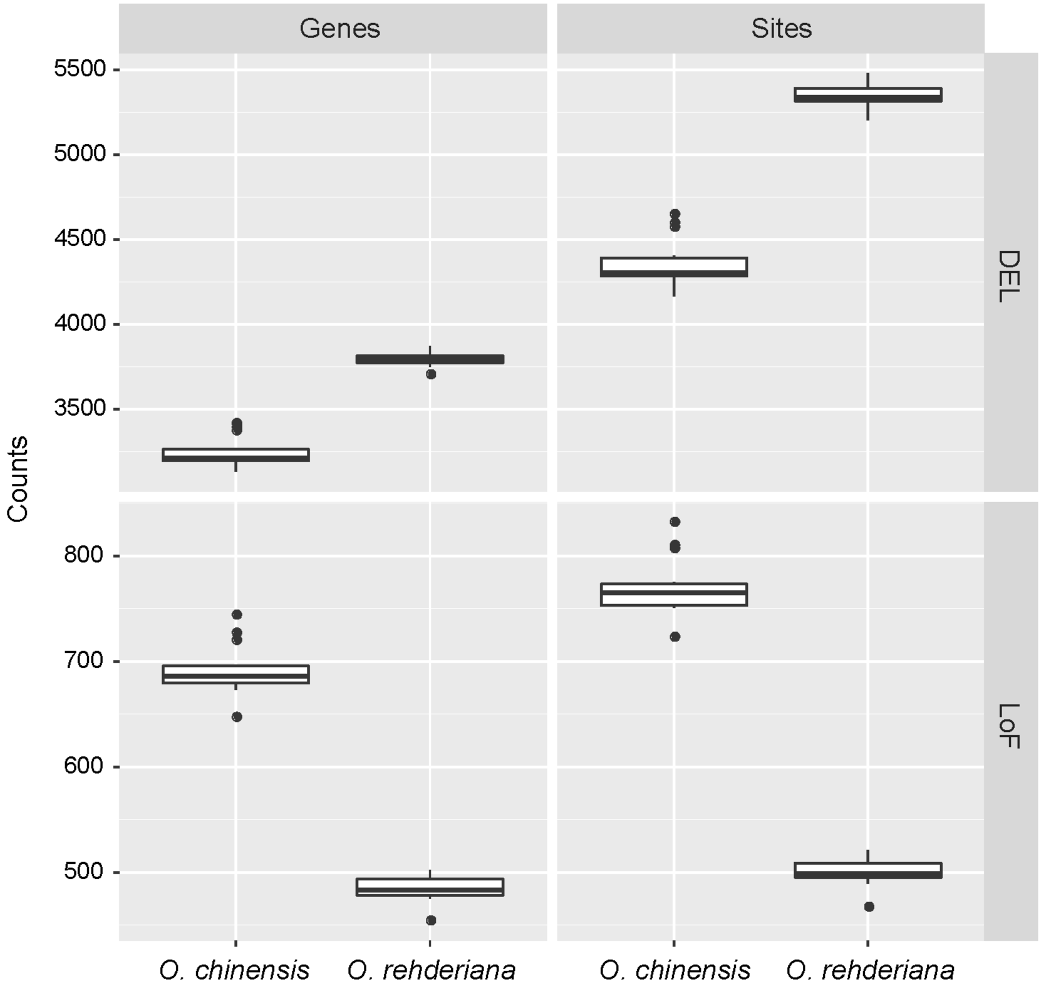

Supplementary Figure 24. The homozygous derived DEL/LoF variants and the affected gene numbers in each individual.

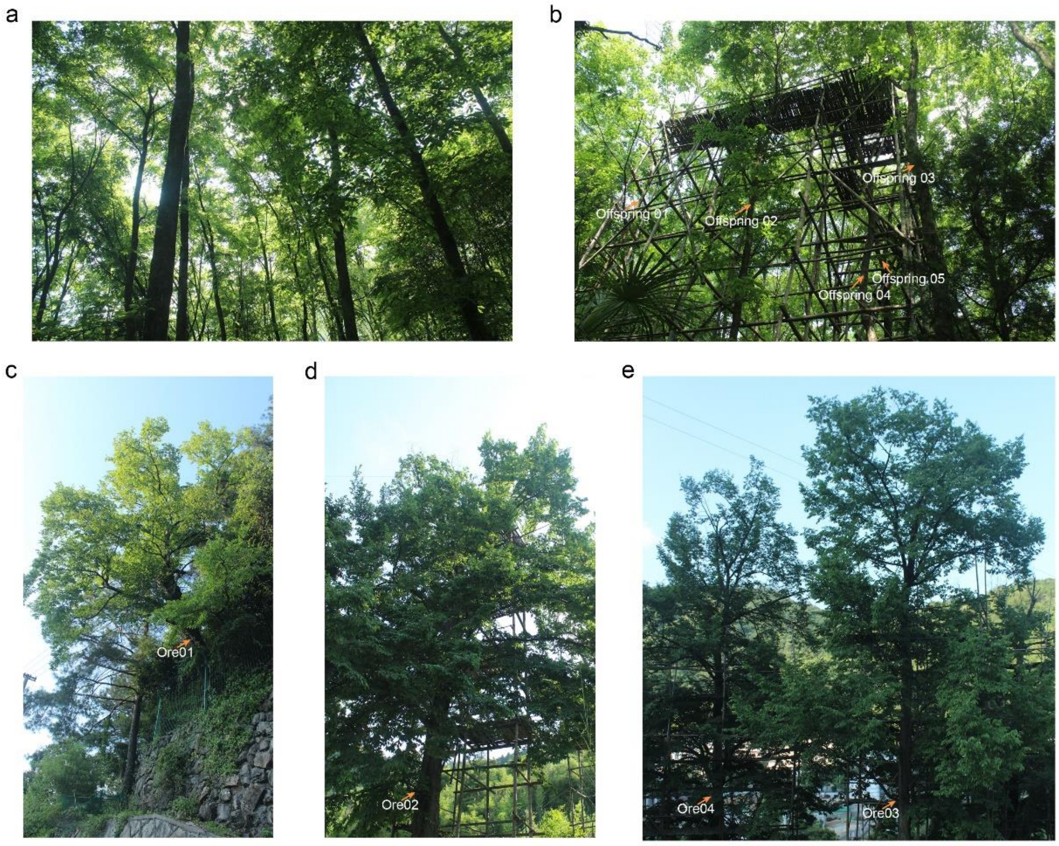

Supplementary Figure 25. **The artificial scaffolds for observing reproductive fitness of the five extant old wild trees and the young trees (seeds from five olds) of *O. rehderiana*.** (a) The young trees were transplanted in 1990 and the seedlings were artificially cultivated from seeds from five old trees in 1987. (b), (c), (d) and (e) showed the scaffolds built for four old (Ore01, Ore02, Ore03 and Ore04 with orange arrow) and a few young trees. All the photos were pictured by Yongzhi Yang and Zhiqiang Lu with the Canon camera.

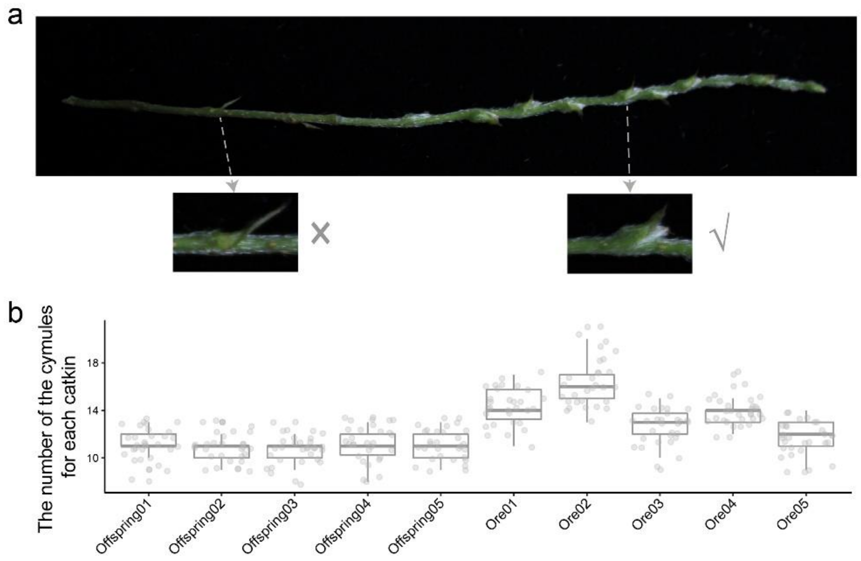

Supplementary Figure 26. The number of the developed cymules for each catkin in five old and five randomly selected young trees (Supplementary note 7). The cymules are very small and each contain 2-3 naked flowers subtended by a primary bracts. (a) The position where only the primary bract was retained after a cymule fell off (marked by ×) versus a developed cymule with the primary bract, secondary bract, and the wrapped ovaries after pollination (marked by √). (b) A box plot for the number of the developed cymules for each catkin in each of ten trees. All catkins were collected and examined on the main tree crowns (12~16m) where our artificial scaffolds could reach on May 17, 2018. All the photos were pictured by Yongzhi Yang and Zhiqiang Lu with the Canon camera.

Supplementary Tables

Supplementary Table 1. Whole-genome shotgun (WGS) reads used in assembling the *O. rehderiana* and *O. chinensis* genomes.

	Library Insert Size	Raw Reads				Qualified Reads[1]			
		Total Data (Gb)	Read Length (bp)	Sequence Coverage[2] (×)	Physical Coverage[2] (×)	Total Data (Gb)	Read Length (bp)	Sequence Coverage[2] (×)	Physical Coverage[2] (×)
O. rehderiana	170 bp	17.47	100	45.24	38.47	17.04	99.37	44.15	37.76
	500 bp	12.41	100	32.13	80.38	12.10	99.22	31.34	78.98
	800 bp	3.14	100	8.12	32.54	2.97	98.28	7.68	31.32
	2 kb	8.84	49	22.90	467.38	8.84	49	22.90	467.38
	5 kb	3.38	49	8.76	446.76	3.38	49	8.76	446.76
	10 kb	4.11	49	10.64	1086.50	4.11	49	10.64	1086.50
	20 kb	1.14	49	2.94	602.73	1.14	49	2.94	602.73
	**Total**	**50.47**	**-**	**130.75**	**2754.75**	**49.56**	**-**	**128.39**	**2751.42**
O. chinensis	180bp	31.41	126	81.39	58.13	29.52	125.47	76.46	54.85
	500 bp	31.31	126	81.12	160.95	28.69	125.50	74.33	148.07
	2 kb	30.22	126	78.30	621.41	27.57	126	71.42	566.86
	5 kb	42.75	126	110.75	2197.44	30.28	126	78.45	1556.46
	10 kb	17.85	126	46.24	1835.08	17.30	126	44.82	1778.52
	**Total**	**153.55**	**-**	**397.80**	**4873.02**	**133.36**	**-**	**345.48**	**4104.75**

[1]Qualified reads were generated by filtering the low quality reads, base-calling duplicate and adapter contamination from the raw reads.

[2]Coverage was calculated under the assumption of a genome size of 386 Mb for *O. rehderiana* and *O. chinensis*. Sequence coverage refers to the total length of generated reads, and physical coverage refers to the total cloned DNA used for the paired reads.

Supplementary Table 2. Genome sizes estimated based on 17-mer statistics.

Species	K-mer Value	K-mer Number	K-mer Depth	Genome Size (bp)	Used Bases	Used Reads	Depth (X)
O. rehderiana	17	10,419,236,548	27	385,897,650	12,405,673,800	124,056,738	32
O. chinensis	17	10,429,000,485	27	386,259,277	12,425,492,400	124,254,924	32

Supplementary Table 3. Statistics for the final assemblies of the *O. rehderiana* and *O. chinensis* genomes.

	O. rehderiana				***O. chinensis***			
	**Contig**		**Scaffold**		**Contig**		**Scaffold**	
	**Size (bp)**	**Number**	**Size (bp)**	**Number**	**Size (bp)**	**Number**	**Size (bp)**	**Number**
**N90**	4,996	17,279	304,887	203	2,740	29,101	82,340	617
**N50**[1]	21,957	4,738	2,307,550	49	13,648	7,607	808,709	136
**Longest**	194,973		10,886,402		160,091		4,342,237	
**Total Size**	359,702,000		366,195,549		362,921,557		371,640,940	
**Total Number (>100 bp)**	65,883		34,624		82,258		39,187	
**Total Number (>2 kb)**	23,132		1,534		33,229		2,888	

[1]N50 refers to the size above which 50% of the total length of the sequence assembly can be found.

Supplementary Table 4. Numbers of reads mapped to the assembled *O. rehderiana* and *O. chinensis* genomes.

	Library Insert Size	Total pairs (PE) reads	Pair end mapped reads	Pair end mapped ratio (%)	Single end mapped reads	Single end mapped ratio (%)	Total mapped reads	Total mapped ratio (%)
O. rehderiana	170 bp	171,474,978	170,347,902	99.34	299,797	0.17	170,647,699	99.52
	500 bp	121,912,322	121,062,970	99.30	372,782	0.31	121,435,752	99.61
	800 bp	30,174,024	29,987,428	99.38	83,018	0.28	30,070,446	99.66
	2 kb	180,371,244	172,928,184	95.87	2,461,121	1.36	175,389,305	97.24
	5 kb	69,001,616	66,040,020	95.71	1,022,812	1.48	67,062,832	97.19
	10 kb	83,798,202	78,807,482	94.04	2,074,463	2.48	80,881,945	96.52
	20 kb	23,177,286	21,321,852	91.99	729,115	3.15	22,050,967	95.14
	**Total**	**679,909,672**	**660,495,838**	**97.14**	**7,043,108**	**1.04**	**667,538,946**	**98.18**
O. chinensis	180bp	235,228,468	229,335,622	97.49	137,904	0.06	229,473,526	97.55
	500 bp	228,682,488	225,591,046	98.65	292,383	0.13	225,883,429	98.78
	2 kb	218,784,748	213,295,216	97.49	1,853,122	0.85	215,148,338	98.34
	5 kb	240,286,932	232,701,602	96.84	2,562,210	1.07	235,263,812	97.91
	10 kb	137,303,036	130,718,790	95.20	1,939,148	1.41	132,657,938	96.62
	**Total**	**1,060,285,672**	**1,031,642,276**	**97.30**	**6,784,767**	**0.64**	**1,038,427,043**	**97.94**

Supplementary Table 5. Completeness of the genomes based on 248 ultra-conserved core eukaryotic genes using Core Eukaryotic Genes Mapping Approach (CEGMA).

Description	Fully mapped CEGs		Fully + partially mapped CEGs	
	O. rehderiana	***O. chinensis***	***O. rehderiana***	***O. chinensis***
**Number of CEGs present in the assembly**	215	219	244	242
**Completeness of the genome (%)**	86.69	88.31	98.39	97.58
**Average number of orthologs per CEG**	1.71	1.67	1.97	1.98
**CEGs with more than one ortholog (%)**	46.98	43.84	57.79	57.85

Supplementary Table 6. Genome completeness measured by Benchmarking Universal Single-Copy Orthologs (BUSCO).

Description	Number of genes		% Percentage	
	O. rehderiana	***O. chinensis***	***O. rehderiana***	***O. chinensis***
**Complete BUSCOs**	1,397	1,385	97.00	96.20
**Complete and single-copy BUSCOs**	1,356	1,341	94.20	93.10
**Complete and duplicated BUSCOs**	41	44	2.80	3.10
**Fragmented BUSCOs**	14	17	1.00	1.20
**Missing BUSCOs**	29	38	2.00	2.60

Supplementary Table 7. Evaluation of the *O. rehderiana* genome completeness using data set of RNA transcripts.

Data set	Length type	Number	Total length	Covered by assembly (%)	With >90% sequence in one scaffold		With >50% sequence in on scaffold	
					Number	Percentage (%)	Number	Percentage (%)
**All**	>200 bp	59,296	91,850,284	97.91	55,017	92.78	57,870	97.60
	>500 bp	45,784	87,517,489	98.73	42,586	93.02	45,083	98.47
	>1,000 bp	34,422	79,122,708	99.33	32,024	93.03	34,127	99.14
**Leaf**	>200 bp	54,715	62,428,416	96.34	50,893	93.01	52,585	96.11
	>500 bp	33,182	56,039,735	98.95	31,407	94.65	32,773	98.77
	>1,000 bp	23,562	48,976,928	99.64	22,325	94.75	23,441	99.49
**Root**	>200 bp	54,924	62,799,312	85.55	44,972	81.88	46,794	85.20
	>500 bp	33,081	56,520,737	95.33	29,942	90.51	31,425	94.99
	>1,000 bp	23,426	49,436,464	97.76	21,623	92.30	22,850	97.54
**Xylem**	>200 bp	57,916	70,319,900	99.41	55,615	96.03	57,520	99.32
	>500 bp	35,566	63,598,418	99.86	33,929	95.40	35,480	99.76
	>1,000 bp	25,445	56,226,517	99.97	24,131	94.84	25,412	99.87
**Phloem**	>200 bp	54,715	62,428,416	96.34	50,893	93.01	52,585	96.11
	>500 bp	33,182	56,039,735	98.95	31,407	94.65	32,773	98.77
	>1,000 bp	23,562	48,976,928	99.64	22,325	94.75	23,441	99.49

Supplementary Table 8. Prediction of repetitive elements in the assembled *O. rehderiana* and *O. chinensis* genomes.

Type	Repeat Size (bp)		% of genome	
	O. rehderiana	***O. chinensis***	***O. rehderiana***	***O. chinensis***
**TRF**	13,633,633	13,020,183	3.72	3.50
**RepeatMasker**	31,416,145	32,022,152	8.58	8.62
**RepeatProteinMask**	40,907,626	42,371,740	11.17	11.40
De novo	181,224,551	183,851,974	49.49	49.47
**Total**	187,133,693	189,821,037	51.10	51.08

Supplementary Table 9. Classification of the interspersed repeats in the assembled *O. rehderiana* and *O. chinensis* genomes.

Type[a]	*O. rehderiana*			*O. chinensis*		
	**Length (bp)**	**% of repeat**	**% of genome**	**Length (bp)**	**% of repeat**	**% of genome**
**SINE**	552,359	0.30	0.15	612,101	0.32	0.16
**LINE**	25,380,276	13.65	6.93	27,755,860	14.71	7.47
L1	24,130,118	12.98	6.59	26,292,675	13.93	7.07
L2	360,853	0.19	0.10	395,349	0.21	0.11
**LTR**	116,518,141	62.66	31.82	122,805,924	65.08	33.04
Copia	35,604,453	19.15	9.72	35,527,156	18.83	9.56
Gypsy	61,789,325	33.23	16.87	65,191,871	34.55	17.54
**DNA**	34,947,755	18.79	9.54	30,275,098	16.04	8.15
CMC-EnSpm	5,887,770	3.17	1.61	5,127,038	2.72	1.38
hAT-Ac	6,151,680	3.31	1.68	6,002,022	3.18	1.61
hAT-Tip100	3,874,651	2.08	1.06	4,132,930	2.19	1.11
MuDR	4,425,381	2.38	1.21	2,651,837	1.41	0.71
PIF-Harbinger	2,654,643	1.43	0.72	1,595,941	0.85	0.43
**Unclassified**	9,292,144	5.00	2.54	8,803,498	4.67	2.37
**Satellites**	738,352	0.40	0.20	630,706	0.33	0.17
**Simple repeats**	18,156,130	9.76	4.96	17,846,958	9.46	4.80
**Small RNA**	479,158	0.26	0.13	162,570	0.09	0.04
**Total repeats**	**185,965,830**	**100**	**50.78**	**188,701,450**	**100**	**50.77**
**Low complexity sequence**	1,380,022	-	0.38	1,299,891	-	0.35

[a]All repeat types were assigned according to homology to the Repbase database (http://www.girinst.org/repbase).

Supplementary Table 10. Prediction of protein-coding genes in the *O. rehderiana* and *O. chinensis* genomes.

Gene set		*O. rehderiana*						*O. chinensis*					
		Total Genes Predicted	Average Gene Length (bp)	Average CDS Length (bp)	Average Exons per Gene	Average Exon Length (bp)	Average Intron Length (bp)	Total Genes Predicted	Average Gene Length (bp)	Average CDS Length (bp)	Average Exons per Gene	Average Exon Length (bp)	Average Intron Length (bp)
De novo	AUGUSTUS	24,134	4,759.09	1,298.63	5.50	236.24	766.62	24,503	4,730.70	1,270.91	5.32	238.90	792.61
	GLIMMERHMM	37,656	6,103.95	854.61	3.28	260.38	2,300.11	40,390	7,333.65	903.72	4.18	216.09	2,020.66
	GENEMARK	31,888	4,346.90	1,117.39	5.04	221.58	798.83	33,524	4,293.60	1,090.30	4.94	220.56	812.32
	SNAP	41,237	3,281.19	807.63	4.25	190.19	761.94	43,771	3,298.45	823.06	4.29	191.72	751.68
Homolog	*A. thaliana*	19,841	3,539.35	1,118.77	4.73	236.28	653.32	19,960	3,442.99	1,114.27	4.68	237.85	649.02
	C. papaya	22,774	2,837.61	979.84	3.99	245.56	626.99	22,932	2,773.36	974.47	3.97	245.63	623.86
	F. vesca	20,514	3,588.02	1,131.73	4.54	249.30	699.57	20,686	3,469.09	1,119.27	4.48	249.99	692.81
	P. persica	22,555	3,410.25	1,107.03	4.58	241.79	648.22	22,774	3,313.93	1,095.78	4.51	243.16	648.99
	V. vinifera	21,504	3,577.71	1,065.71	4.89	217.90	651.90	21,849	3,463.38	1,050.18	4.79	219.04	649.27
RNA-seq		22,871	6,023.56	989.61	4.80	404.01	1,076.06	–	–	–	–	–	–
EVM		28,608	4,715.59	1,094.38	4.61	237.60	822.34	32,318	3,606.85	1,041.09	4.55	228.67	722.17
Final set		27,831	4,755.26	1,108.09	4.74	233.59	769.82	31,152	3,690.14	1,061.70	4.64	228.65	721.45

Supplementary Table 11. Comparison of gene space of the *O. chinensis* and *O. rehderiana* genomes with other genomes.

Species	Total Genes	Average Gene Length[a] (bp)	Average CDS Length[a] (bp)	Average Exons per Gene[a]	Average Exon Length[a] (bp)	Average Intron Length[a] (bp)
Arabidopsis thaliana	27,416	2,192.47	1,218.40	5.13	237.57	157.92
Betula pendula	24,854	4,676.44	1,166.24	4.82	241.92	793.17
Carica papaya	27,736	2,358.73	893.34	4.06	220.23	479.42
Fragaria vesca	32,831	2,867.35	1,185.40	5.09	232.66	410.75
Juglans regia	32,496	4,359.82	1,209.31	5.27	229.53	729.82
Oryza sativa ssp. Japonica	37,869	2,986.76	982.71	3.74	262.43	426.67
Ostrya chinensis	31,152	3,691.19	1,061.86	4.64	228.64	721.50
Ostrya rehderiana	27,831	4,468.97	1,108.90	4.75	233.59	755.30
Prunus persica	27,864	2,604.79	1,214.53	4.91	247.52	315.14
Ricinus communis	31,221	2,261.54	1,004.06	4.14	242.46	377.56
Vitis vinifera	26,346	6,454.02	1,137.11	5.95	191.10	969.55

[a]The longest translation form was chosen to represent each gene.

Supplementary Table 12. List of non-coding RNAs genes in the O. rehderiana and O.chinensis genomes.

	Type	Cope number	Average length(bp)	Total length(bp)	% of genome
O. rehderian	**miRNA**	204	122.61	25,012	0.0068
	**snRNA**	113	141.70	16,012	0.0044
	**rRNA**	566	73.79	41,766	0.0114
	**tRNA**	125	320.15	40,018	0.0109
O.chinensis	**miRNA**	221	127.17	28,104	0.0076
	**snRNA**	109	138.44	15,090	0.0041
	**rRNA**	552	73.76	40,715	0.0110
	**tRNA**	129	319.37	41,199	0.0111

Supplementary Table 13. Functional annotation of the predicted genes for *O. rehderiana* and *O. chinensis*.

	Database	*O. rehderiana*		*O. chinensis*	
		**Number**	**Percent (%)**	**Number**	**Percent (%)**
**Total**		27,831	100.00	31,152	100.00
	**InterPro**	20,629	74.12	22,114	70.99
	**GO**	19,240	69.13	20,629	66.22
**Annotated**	**Swissprot**	19,725	70.87	21,035	67.52
	**TrEMBL**	19,815	71.20	26,085	83.73
	**KEGG**	13,126	47.16	13,930	44.72
**Unannotated**		3,879	13.94	4,890	15.70

Supplementary Table 14. Summary of gene family clustering.

Species	Total genes	Genes in families	Unclustered genes	Families	Unique families	Genes per family	Maximum gene family size
A. thaliana	26,864	23,171	3,693	12,861	711	1.80	137
B. pendula	24,854	19,124	5,730	13,567	315	1.41	81
C. papaya	26,249	19,112	7,137	13,122	510	1.46	193
F. vesca	32,697	24,780	7,917	14,262	1,346	1.74	161
J.regia	32,436	26,885	5,551	14,408	567	1.87	90
O. chinensis	31,152	24,284	6,868	17,152	434	1.42	108
O. rehderiana	27,831	22,902	4,929	16,857	243	1.36	81
O. sativa	34,047	22,463	11,584	12,271	1,683	1.83	67
P. persica	27,861	24,505	3,356	14,427	428	1.70	750
R. communis	29,911	20,128	9,783	14,307	687	1.41	56
V. vinifera	25,400	19,341	6,059	12,853	592	1.50	79
**All**	319,302	246,695	72,607	26,756	-	9.22	-

Supplementary Table 15. Gene ontology (GO) enrichment analysis of the unique gene families in *O. rehderiana* and *O. chinensis*, respectively.

Type[1]	GO ID	GO Terms	Number of enriched genes	Number of genes in background	Adjusted P-value
O. rehderiana					
MF	GO:0004568	chitinase activity	14	71	1.082E-10
MF	GO:0004553	hydrolase activity, hydrolyzing O-glycosyl compounds	22	468	5.141E-05
MF	GO:0016798	hydrolase activity, acting on glycosyl bonds	22	495	1.366E-04
MF	GO:0005179	hormone activity	4	6	2.941E-04
MF	GO:0004401	histidinol-phosphatase activity	4	7	6.797E-04
MF	GO:0008843	endochitinase activity	4	13	1.312E-02
MF	GO:0035885	exochitinase activity	4	13	1.312E-02
BP	GO:0006030	chitin metabolic process	14	71	1.082E-10
BP	GO:0006032	chitin catabolic process	14	71	1.082E-10
BP	GO:0046348	amino sugar catabolic process	14	71	1.082E-10
BP	GO:1901072	glucosamine-containing compound catabolic process	14	71	1.082E-10
BP	GO:1901071	glucosamine-containing compound metabolic process	14	72	1.330E-10
BP	GO:0006026	aminoglycan catabolic process	14	101	1.681E-08
BP	GO:0006040	amino sugar metabolic process	14	112	7.012E-08
BP	GO:1901136	carbohydrate derivative catabolic process	14	122	2.246E-07
BP	GO:0006022	aminoglycan metabolic process	14	138	1.169E-06
BP	GO:0016998	cell wall macromolecule catabolic process	8	70	1.850E-03
BP	GO:0005975	carbohydrate metabolic process	41	1686	9.095E-03
O. chinensis					
MF	GO:0016772	transferase activity, transferring phosphorus-containing groups	78	2254	1.063E-03
MF	GO:0004402	histone acetyltransferase activity	7	33	4.459E-03
MF	GO:0061733	peptide-lysine-N-acetyltransferase activity	7	33	4.459E-03
MF	GO:0034212	peptide N-acetyltransferase activity	7	36	8.275E-03
MF	GO:0004672	protein kinase activity	52	1409	1.748E-02
MF	GO:0004109	coproporphyrinogen oxidase activity	4	9	2.493E-02
MF	GO:0016301	kinase activity	64	1912	3.542E-02
CC	GO:0031931	TORC1 complex	4	8	1.407E-02
CC	GO:0038201	TOR complex	4	8	1.407E-02
BP	GO:0048527	lateral root development	9	48	5.270E-04
BP	GO:0048528	post-embryonic root development	9	52	1.078E-03
BP	GO:0031929	TOR signaling	4	8	1.407E-02
BP	GO:0006475	internal protein amino acid acetylation	7	42	2.413E-02
BP	GO:0016573	histone acetylation	7	42	2.413E-02
BP	GO:0018393	internal peptidyl-lysine acetylation	7	42	2.413E-02
BP	GO:0018394	peptidyl-lysine acetylation	7	42	2.413E-02
BP	GO:0006473	protein acetylation	7	43	2.833E-02

BP	GO:0010084	specification of organ axis polarity	4	10	4.089E-02
BP	GO:1900618	regulation of shoot system morphogenesis	4	10	4.089E-02
BP	GO:1901371	regulation of leaf morphogenesis	4	10	4.089E-02
BP	GO:2000025	regulation of leaf formation	4	10	4.089E-02

[1]MF: molecular function; BP: biological process; CC: cellular component.

Supplementary Table 16. Gene ontology (GO) enrichment analysis of the expanded and contracted gene families of *O. rehderiana* among 11 species.

Type[1]	GO ID	GO Terms	Number of enriched genes	Number of genes in background	Adjusted P-value
**Expansion**					
MF	GO:0016740	transferase activity	494	4,108	8.663E-28
MF	GO:0005216	ion channel activity	52	121	1.735E-23
MF	GO:0022838	substrate-specific channel activity	52	131	1.648E-21
MF	GO:0015267	channel activity	52	132	2.523E-21
MF	GO:0022803	passive transmembrane transporter activity	52	132	2.523E-21
MF	GO:0015276	ligand-gated ion channel activity	34	56	5.254E-21
MF	GO:0022834	ligand-gated channel activity	34	56	5.254E-21
MF	GO:0032559	adenyl ribonucleotide binding	293	2,192	1.167E-20
MF	GO:0030554	adenyl nucleotide binding	293	2,196	1.564E-20
MF	GO:0022836	gated channel activity	42	93	1.317E-19
MF	GO:0032555	purine ribonucleotide binding	305	2,421	1.487E-17
MF	GO:0017076	purine nucleotide binding	305	2,426	2.050E-17
MF	GO:0001882	nucleoside binding	304	2,429	4.872E-17
MF	GO:0001883	purine nucleoside binding	303	2,419	5.064E-17
MF	GO:0032550	purine ribonucleoside binding	303	2,419	5.064E-17
MF	GO:0032549	ribonucleoside binding	303	2,428	8.937E-17
MF	GO:0032553	ribonucleotide binding	305	2,459	1.639E-16
MF	GO:0097367	carbohydrate derivative binding	306	2,488	5.040E-16
MF	GO:0004970	ionotropic glutamate receptor activity	26	42	6.982E-16
MF	GO:0005230	extracellular ligand-gated ion channel activity	26	42	6.982E-16
MF	GO:0022824	transmitter-gated ion channel activity	26	42	6.982E-16
MF	GO:0022835	transmitter-gated channel activity	26	42	6.982E-16
MF	GO:0030594	neurotransmitter receptor activity	26	42	6.982E-16
MF	GO:0008066	glutamate receptor activity	26	43	1.640E-15
MF	GO:0004672	protein kinase activity	185	1,274	2.383E-15
MF	GO:0022857	transmembrane transporter activity	137	851	2.395E-14
MF	GO:0043531	ADP binding	35	93	5.899E-13
MF	GO:0005524	ATP binding	258	2,095	6.420E-13
MF	GO:0016758	transferase activity, transferring hexosyl groups	92	493	8.364E-13
MF	GO:0016301	kinase activity	223	1,742	1.538E-12
MF	GO:0036094	small molecule binding	362	3,260	1.903E-12
MF	GO:0016773	phosphotransferase activity, alcohol group as acceptor	189	1,419	8.013E-12
MF	GO:0016757	transferase activity, transferring glycosyl groups	104	638	1.158E-10
MF	GO:0016772	transferase activity, transferring phosphorus-containing groups	243	2,038	2.363E-10
MF	GO:0000166	nucleotide binding	339	3,107	2.782E-10
MF	GO:1901265	nucleoside phosphate binding	339	3,107	2.782E-10
MF	GO:0035639	purine ribonucleoside triphosphate binding	268	2,322	4.463E-10

MF	GO:0022891	substrate-specific transmembrane transporter activity	102	646	1.574E-09
MF	GO:0003674	molecular_function	1,339	16,622	6.332E-09
MF	GO:0005261	cation channel activity	21	46	6.576E-09
MF	GO:0003824	catalytic activity	878	9,973	1.711E-08
MF	GO:0099600	transmembrane receptor activity	34	124	5.315E-08
MF	GO:0008171	O-methyltransferase activity	27	82	5.929E-08
MF	GO:0005215	transporter activity	147	1,132	9.085E-08
MF	GO:0022892	substrate-specific transporter activity	105	719	9.280E-08
MF	GO:0015075	ion transmembrane transporter activity	79	482	1.073E-07
MF	GO:0035671	enone reductase activity	10	11	1.474E-07
MF	GO:0005249	voltage-gated potassium channel activity	15	30	1.786E-06
MF	GO:0004372	glycine hydroxymethyltransferase activity	8	8	2.543E-06
MF	GO:0022843	voltage-gated cation channel activity	15	31	3.218E-06
MF	GO:0004872	receptor activity	35	154	7.084E-06
MF	GO:0030551	cyclic nucleotide binding	8	9	2.136E-05
MF	GO:0005267	potassium channel activity	15	35	2.603E-05
MF	GO:0042626	ATPase activity, coupled to transmembrane movement of substances	36	170	3.123E-05
MF	GO:0022804	active transmembrane transporter activity	59	364	4.761E-05
MF	GO:0016820	hydrolase activity, acting on acid anhydrides, catalyzing transmembrane movement of substances	36	174	5.916E-05
MF	GO:0004683	calmodulin-dependent protein kinase activity	16	43	9.521E-05
MF	GO:0015399	primary active transmembrane transporter activity	36	182	1.980E-04
MF	GO:0015405	P-P-bond-hydrolysis-driven transmembrane transporter activity	36	182	1.980E-04
MF	GO:0047216	inositol 3-alpha-galactosyltransferase activity	7	8	2.524E-04
MF	GO:0043492	ATPase activity, coupled to movement of substances	36	185	3.045E-04
MF	GO:0004888	transmembrane signaling receptor activity	26	110	3.245E-04
MF	GO:0004525	ribonuclease III activity	10	18	3.578E-04
MF	GO:0032296	double-stranded RNA-specific ribonuclease activity	10	18	3.578E-04
MF	GO:0005221	intracellular cyclic nucleotide activated cation channel activity	8	12	9.547E-04
MF	GO:0043855	cyclic nucleotide-gated ion channel activity	8	12	9.547E-04
MF	GO:0008559	xenobiotic-transporting ATPase activity	10	20	1.312E-03
MF	GO:0042910	xenobiotic transporter activity	10	20	1.312E-03
MF	GO:0005217	intracellular ligand-gated ion channel activity	8	13	2.317E-03
MF	GO:0005244	voltage-gated ion channel activity	15	47	2.530E-03
MF	GO:0022832	voltage-gated channel activity	15	47	2.530E-03
MF	GO:0015114	phosphate ion transmembrane transporter activity	10	22	3.991E-03
MF	GO:0016742	hydroxymethyl-, formyl- and related transferase activity	8	14	5.046E-03
MF	GO:0047787	delta4-3-oxosteroid 5beta-reductase activity	5	5	5.948E-03
MF	GO:0010285	L,L-diaminopimelate aminotransferase activity	5	5	5.948E-03
MF	GO:0038023	signaling receptor activity	27	136	7.422E-03
MF	GO:0005242	inward rectifier potassium channel activity	7	11	8.491E-03
MF	GO:0052736	beta-glucanase activity	7	11	8.491E-03
MF	GO:0052861	glucan endo-1,3-beta-glucanase activity, C-3 substituted reducing group	7	11	8.491E-03
MF	GO:0052862	glucan endo-1,4-beta-glucanase activity, C-3 substituted reducing group	7	11	8.491E-03

MF	GO:0004540	ribonuclease activity	18	72	1.151E-02
MF	GO:0016759	cellulose synthase activity	14	47	1.444E-02
MF	GO:0016760	cellulose synthase (UDP-forming) activity	14	47	1.444E-02
MF	GO:0060089	molecular transducer activity	42	270	1.492E-02
MF	GO:0005315	inorganic phosphate transmembrane transporter activity	8	16	1.884E-02
MF	GO:0080019	fatty-acyl-CoA reductase (alcohol-forming) activity	8	17	3.323E-02
MF	GO:0030170	pyridoxal phosphate binding	22	107	3.324E-02
MF	GO:0070279	vitamin B6 binding	22	107	3.324E-02
MF	GO:0004096	catalase activity	5	6	3.344E-02
MF	GO:0015079	potassium ion transmembrane transporter activity	15	57	3.615E-02
MF	GO:0016887	ATPase activity	60	456	4.406E-02
CC	GO:0008076	voltage-gated potassium channel complex	15	27	2.488E-07
CC	GO:0034705	potassium channel complex	15	27	2.488E-07
CC	GO:0034703	cation channel complex	16	31	2.581E-07
CC	GO:0034702	ion channel complex	16	32	4.800E-07
CC	GO:1902495	transmembrane transporter complex	16	35	2.607E-06
CC	GO:1990351	transporter complex	16	35	2.607E-06
CC	GO:0017071	intracellular cyclic nucleotide activated cation channel complex	8	12	9.547E-04
CC	GO:0005887	integral component of plasma membrane	15	44	9.611E-04
CC	GO:0043235	receptor complex	8	14	5.046E-03
BP	GO:0007215	glutamate receptor signaling pathway	20	26	1.187E-14
BP	GO:0007268	chemical synaptic transmission	20	26	1.187E-14
BP	GO:0098916	anterograde trans-synaptic signaling	20	26	1.187E-14
BP	GO:0099536	synaptic signaling	20	26	1.187E-14
BP	GO:0099537	trans-synaptic signaling	20	26	1.187E-14
BP	GO:0035235	ionotropic glutamate receptor signaling pathway	20	26	1.187E-14
BP	GO:0007267	cell-cell signaling	30	64	5.406E-14
BP	GO:0006468	protein phosphorylation	169	1,292	1.387E-09
BP	GO:0008037	cell recognition	32	114	1.046E-07
BP	GO:0048544	recognition of pollen	32	114	1.046E-07
BP	GO:0009875	pollen-pistil interaction	32	115	1.346E-07
BP	GO:0055085	transmembrane transport	129	1,000	2.430E-06
BP	GO:0006817	phosphate ion transport	14	33	9.469E-05
BP	GO:0016310	phosphorylation	211	1,973	1.766E-04
BP	GO:0035999	tetrahydrofolate interconversion	8	11	3.410E-04
BP	GO:0044765	single-organism transport	202	1,894	4.317E-04
BP	GO:1902578	single-organism localization	202	1,920	1.161E-03
BP	GO:0042908	xenobiotic transport	10	20	1.312E-03
BP	GO:0006816	calcium ion transport	21	86	2.888E-03
BP	GO:0010216	maintenance of DNA methylation	5	5	5.948E-03
BP	GO:0006811	ion transport	107	915	8.723E-03
BP	GO:0009699	phenylpropanoid biosynthetic process	36	213	1.014E-02
BP	GO:1901699	cellular response to nitrogen compound	24	115	1.046E-02
BP	GO:0009856	pollination	35	205	1.087E-02

BP	GO:0044706	multi-multicellular organism process	35	205	1.087E-02
BP	GO:1901698	response to nitrogen compound	41	262	1.650E-02
BP	GO:0009718	anthocyanin-containing compound biosynthetic process	10	26	2.476E-02
BP	GO:0006813	potassium ion transport	18	76	2.603E-02
BP	GO:0010337	regulation of salicylic acid metabolic process	6	9	3.084E-02
BP	GO:0014075	response to amine	5	6	3.344E-02
BP	GO:0043200	response to amino acid	5	6	3.344E-02
BP	GO:0071230	cellular response to amino acid stimulus	5	6	3.344E-02
BP	GO:0071418	cellular response to amine stimulus	5	6	3.344E-02
BP	GO:0006544	glycine metabolic process	21	100	3.688E-02
BP	GO:0006464	cellular protein modification process	226	2,305	3.873E-02
BP	GO:0036211	protein modification process	226	2,305	3.873E-02
**Decrease**					
MF	GO:0052716	hydroquinone:oxygen oxidoreductase activity	36	45	2.608E-38
MF	GO:0016682	oxidoreductase activity, acting on diphenols and related substances as donors, oxygen as acceptor	36	69	5.922E-28
MF	GO:0016679	oxidoreductase activity, acting on diphenols and related substances as donors	36	75	2.869E-26
MF	GO:0004252	serine-type endopeptidase activity	46	136	5.921E-26
MF	GO:0016491	oxidoreductase activity	195	2,140	7.361E-23
MF	GO:0035251	UDP-glucosyltransferase activity	42	141	5.030E-21
MF	GO:0046527	glucosyltransferase activity	42	145	1.711E-20
MF	GO:0003824	catalytic activity	560	9,973	4.525E-20
MF	GO:0008236	serine-type peptidase activity	47	220	4.530E-17
MF	GO:0017171	serine hydrolase activity	47	220	4.530E-17
MF	GO:0016759	cellulose synthase activity	22	47	6.061E-15
MF	GO:0016760	cellulose synthase (UDP-forming) activity	22	47	6.061E-15
MF	GO:0015079	potassium ion transmembrane transporter activity	23	57	6.231E-14
MF	GO:0032440	2-alkenal reductase [NAD(P)] activity	42	213	1.103E-13
MF	GO:0005507	copper ion binding	37	169	2.317E-13
MF	GO:0005524	ATP binding	166	2,095	1.128E-12
MF	GO:0036094	small molecule binding	228	3,260	1.720E-12
MF	GO:0008194	UDP-glycosyltransferase activity	43	241	2.127E-12
MF	GO:0035639	purine ribonucleoside triphosphate binding	176	2,322	8.160E-12
MF	GO:0042623	ATPase activity, coupled	53	372	1.872E-11
MF	GO:0016628	oxidoreductase activity, acting on the CH-CH group of donors, NAD or NADP as acceptor	43	264	6.123E-11
MF	GO:0032559	adenyl ribonucleotide binding	166	2,192	6.845E-11
MF	GO:0030554	adenyl nucleotide binding	166	2,196	8.041E-11
MF	GO:0032553	ribonucleotide binding	180	2,459	9.756E-11
MF	GO:0000166	nucleotide binding	214	3,107	1.025E-10
MF	GO:1901265	nucleoside phosphate binding	214	3,107	1.025E-10
MF	GO:0097367	carbohydrate derivative binding	181	2,488	1.440E-10
MF	GO:0016627	oxidoreductase activity, acting on the CH-CH group of donors	50	360	3.012E-10

MF	GO:0001883	purine nucleoside binding	176	2,419	3.568E-10
MF	GO:0032550	purine ribonucleoside binding	176	2,419	3.568E-10
MF	GO:0032555	purine ribonucleotide binding	176	2,421	3.843E-10
MF	GO:0017076	purine nucleotide binding	176	2,426	4.626E-10
MF	GO:0032549	ribonucleoside binding	176	2,428	4.981E-10
MF	GO:0001882	nucleoside binding	176	2,429	5.168E-10
MF	GO:0016887	ATPase activity	57	456	5.479E-10
MF	GO:0004175	endopeptidase activity	47	329	5.837E-10
MF	GO:0005215	transporter activity	101	1,132	1.629E-09
MF	GO:0022804	active transmembrane transporter activity	49	364	1.755E-09
MF	GO:0042626	ATPase activity, coupled to transmembrane movement of substances	32	170	2.319E-09
MF	GO:0003843	1,3-beta-D-glucan synthase activity	10	12	2.516E-09
MF	GO:0017111	nucleoside-triphosphatase activity	80	809	2.976E-09
MF	GO:0016820	hydrolase activity, acting on acid anhydrides, catalyzing transmembrane movement of substances	32	174	4.483E-09
MF	GO:0046873	metal ion transmembrane transporter activity	29	148	9.493E-09
MF	GO:0015399	primary active transmembrane transporter activity	32	182	1.577E-08
MF	GO:0015405	P-P-bond-hydrolysis-driven transmembrane transporter activity	32	182	1.577E-08
MF	GO:0016462	pyrophosphatase activity	80	841	2.164E-08
MF	GO:0043492	ATPase activity, coupled to movement of substances	32	185	2.477E-08
MF	GO:0004672	protein kinase activity	106	1,274	3.118E-08
MF	GO:0016818	hydrolase activity, acting on acid anhydrides, in phosphorus-containing anhydrides	80	854	4.668E-08
MF	GO:0003674	molecular_function	763	16,622	6.801E-08
MF	GO:0015077	monovalent inorganic cation transmembrane transporter activity	30	171	7.596E-08
MF	GO:0016817	hydrolase activity, acting on acid anhydrides	80	864	8.312E-08
MF	GO:0022857	transmembrane transporter activity	79	851	9.816E-08
MF	GO:0008271	secondary active sulfate transmembrane transporter activity	8	9	2.025E-07
MF	GO:0004674	protein serine/threonine kinase activity	60	600	1.450E-06
MF	GO:0016773	phosphotransferase activity, alcohol group as acceptor	108	1,419	3.618E-06
MF	GO:0022890	inorganic cation transmembrane transporter activity	35	263	4.708E-06
MF	GO:0016787	hydrolase activity	206	3,346	1.164E-05
MF	GO:0008568	microtubule-severing ATPase activity	21	109	1.198E-05
MF	GO:0047268	galactinol-raffinose galactosyltransferase activity	6	6	1.319E-05
MF	GO:0015116	sulfate transmembrane transporter activity	8	13	2.487E-05
MF	GO:1901682	sulfur compound transmembrane transporter activity	8	15	1.152E-04
MF	GO:0016758	transferase activity, transferring hexosyl groups	48	493	1.791E-04
MF	GO:0005488	binding	532	10,836	1.873E-04
MF	GO:0008324	cation transmembrane transporter activity	35	306	2.347E-04
MF	GO:0005515	protein binding	225	3,930	8.243E-04
MF	GO:0046577	long-chain-alcohol oxidase activity	5	6	1.811E-03
MF	GO:0015198	oligopeptide transporter activity	5	6	1.811E-03
MF	GO:0070011	peptidase activity, acting on L-amino acid peptides	49	551	1.997E-03

MF	GO:0016705	oxidoreductase activity, acting on paired donors, with incorporation or reduction of molecular oxygen	49	561	3.351E-03
MF	GO:0015075	ion transmembrane transporter activity	44	482	3.637E-03
MF	GO:0043167	ion binding	180	3,082	5.586E-03
MF	GO:0008553	hydrogen-exporting ATPase activity, phosphorylative mechanism	5	7	6.117E-03
MF	GO:0016301	kinase activity	113	1,742	6.747E-03
MF	GO:0004033	aldo-keto reductase (NADP) activity	6	12	9.780E-03
MF	GO:0015662	ATPase activity, coupled to transmembrane movement of ions, phosphorylative mechanism	8	25	1.327E-02
MF	GO:0020037	heme binding	40	445	1.567E-02
MF	GO:0008601	protein phosphatase type 2A regulator activity	6	13	1.751E-02
MF	GO:0050660	flavin adenine dinucleotide binding	17	116	1.805E-02
MF	GO:0046914	transition metal ion binding	108	1,682	1.814E-02
MF	GO:0003779	actin binding	11	53	2.566E-02
MF	GO:0016837	carbon-oxygen lyase activity, acting on polysaccharides	6	14	2.954E-02
MF	GO:0030570	pectate lyase activity	6	14	2.954E-02
MF	GO:0008233	peptidase activity	50	626	3.184E-02
MF	GO:0046872	metal ion binding	166	2,879	3.268E-02
MF	GO:0043169	cation binding	166	2,890	4.051E-02
CC	GO:0016021	integral component of membrane	172	1,915	7.280E-19
CC	GO:0044425	membrane part	189	2,274	2.659E-17
CC	GO:0031224	intrinsic component of membrane	172	1,983	3.028E-17
CC	GO:0048046	apoplast	41	173	2.241E-16
CC	GO:0005576	extracellular region	59	415	5.887E-13
CC	GO:0016020	membrane	254	3,770	1.727E-12
CC	GO:0000148	1,3-beta-D-glucan synthase complex	10	12	2.516E-09
CC	GO:0016459	myosin complex	11	34	2.013E-04
CC	GO:0071944	cell periphery	81	1,081	9.330E-04
CC	GO:0098797	plasma membrane protein complex	13	63	4.417E-03
CC	GO:0005886	plasma membrane	61	787	9.417E-03
CC	GO:0000159	protein phosphatase type 2A complex	6	13	1.751E-02
CC	GO:0015629	actin cytoskeleton	11	54	3.101E-02
BP	GO:0046274	lignin catabolic process	36	45	2.608E-38
BP	GO:0046271	phenylpropanoid catabolic process	36	46	1.152E-37
BP	GO:0009808	lignin metabolic process	44	97	3.052E-31
BP	GO:0044699	single-organism process	554	9,883	2.572E-19
BP	GO:0051274	beta-glucan biosynthetic process	36	114	1.098E-18
BP	GO:0006011	UDP-glucose metabolic process	22	40	6.142E-17
BP	GO:0055114	oxidation-reduction process	188	2,303	2.560E-16
BP	GO:0071804	cellular potassium ion transport	24	53	4.613E-16
BP	GO:0071805	potassium ion transmembrane transport	24	53	4.613E-16
BP	GO:0009698	phenylpropanoid metabolic process	51	275	7.433E-16
BP	GO:0051273	beta-glucan metabolic process	37	161	4.226E-14
BP	GO:0044763	single-organism cellular process	406	6,863	6.300E-14

BP	GO:0008150	biological_process	727	15,029	1.262E-13
BP	GO:0044710	single-organism metabolic process	421	7,248	2.391E-13
BP	GO:0098660	inorganic ion transmembrane transport	41	214	7.581E-13
BP	GO:0019439	aromatic compound catabolic process	43	238	1.328E-12
BP	GO:1901361	organic cyclic compound catabolic process	44	249	1.418E-12
BP	GO:0055085	transmembrane transport	100	1,000	1.600E-12
BP	GO:0009225	nucleotide-sugar metabolic process	22	61	5.147E-12
BP	GO:0006813	potassium ion transport	24	76	8.819E-12
BP	GO:0019748	secondary metabolic process	62	494	3.866E-11
BP	GO:0005985	sucrose metabolic process	48	325	8.988E-11
BP	GO:0030244	cellulose biosynthetic process	26	102	1.902E-10
BP	GO:0009250	glucan biosynthetic process	39	229	2.265E-10
BP	GO:0016137	glycoside metabolic process	56	441	4.630E-10
BP	GO:0006074	(1->3)-beta-D-glucan metabolic process	10	12	2.516E-09
BP	GO:0006075	(1->3)-beta-D-glucan biosynthetic process	10	12	2.516E-09
BP	GO:0098662	inorganic cation transmembrane transport	33	188	7.747E-09
BP	GO:0008152	metabolic process	587	11,673	9.371E-09
BP	GO:1901657	glycosyl compound metabolic process	78	798	1.044E-08
BP	GO:0034220	ion transmembrane transport	42	299	1.950E-08
BP	GO:0006468	protein phosphorylation	106	1,292	7.211E-08
BP	GO:0044042	glucan metabolic process	60	557	7.405E-08
BP	GO:0000271	polysaccharide biosynthetic process	46	366	9.869E-08
BP	GO:0034637	cellular carbohydrate biosynthetic process	45	353	9.997E-08
BP	GO:0009311	oligosaccharide metabolic process	50	424	1.429E-07
BP	GO:0005984	disaccharide metabolic process	48	406	3.184E-07
BP	GO:0030243	cellulose metabolic process	27	149	3.419E-07
BP	GO:0005976	polysaccharide metabolic process	67	694	6.282E-07
BP	GO:0044712	single-organism catabolic process	85	985	6.970E-07
BP	GO:0033692	cellular polysaccharide biosynthetic process	41	323	8.439E-07
BP	GO:0044765	single-organism transport	136	1,894	9.854E-07
BP	GO:0005982	starch metabolic process	48	424	1.403E-06
BP	GO:1902578	single-organism localization	136	1,920	2.424E-06
BP	GO:0098655	cation transmembrane transport	33	232	2.452E-06
BP	GO:0006857	oligopeptide transport	15	52	5.421E-06
BP	GO:0016051	carbohydrate biosynthetic process	58	597	8.307E-06
BP	GO:0015833	peptide transport	15	56	1.678E-05
BP	GO:0006073	cellular glucan metabolic process	53	533	1.817E-05
BP	GO:1902358	sulfate transmembrane transport	8	13	2.487E-05
BP	GO:0009069	serine family amino acid metabolic process	69	807	5.310E-05
BP	GO:0008272	sulfate transport	8	14	5.586E-05
BP	GO:1901575	organic substance catabolic process	109	1,513	6.030E-05
BP	GO:0006796	phosphate-containing compound metabolic process	181	2,924	9.638E-05
BP	GO:0006793	phosphorus metabolic process	181	2,929	1.091E-04
BP	GO:0042886	amide transport	15	65	1.499E-04

BP	GO:0044264	cellular polysaccharide metabolic process	55	624	5.646E-04
BP	GO:0009056	catabolic process	113	1,656	5.660E-04
BP	GO:0071704	organic substance metabolic process	433	8,546	5.802E-04
BP	GO:1901605	alpha-amino acid metabolic process	91	1,254	7.096E-04
BP	GO:0002237	response to molecule of bacterial origin	10	31	8.101E-04
BP	GO:0015672	monovalent inorganic cation transport	32	282	9.861E-04
BP	GO:0072348	sulfur compound transport	8	19	1.163E-03
BP	GO:0016310	phosphorylation	128	1,973	1.306E-03
BP	GO:0006810	transport	162	2,685	3.079E-03
BP	GO:0048443	stamen development	16	93	3.904E-03
BP	GO:0048466	androecium development	16	93	3.904E-03
BP	GO:0006520	cellular amino acid metabolic process	95	1,383	4.352E-03
BP	GO:0044281	small molecule metabolic process	189	3,266	5.247E-03
BP	GO:0051234	establishment of localization	163	2,745	7.393E-03
BP	GO:0009987	cellular process	499	10,278	7.419E-03
BP	GO:0006754	ATP biosynthetic process	9	31	8.513E-03
BP	GO:0034047	regulation of protein phosphatase type 2A activity	6	12	9.780E-03
BP	GO:0007167	enzyme linked receptor protein signaling pathway	18	125	1.267E-02
BP	GO:0051179	localization	165	2,826	1.710E-02
BP	GO:0098661	inorganic anion transmembrane transport	8	26	1.845E-02
BP	GO:0044262	cellular carbohydrate metabolic process	56	719	2.043E-02
BP	GO:0030001	metal ion transport	38	423	2.686E-02
BP	GO:0042451	purine nucleoside biosynthetic process	12	64	3.101E-02
BP	GO:0046129	purine ribonucleoside biosynthetic process	12	64	3.101E-02
BP	GO:1901659	glycosyl compound biosynthetic process	24	214	3.274E-02
BP	GO:0007178	transmembrane receptor protein serine/threonine kinase signaling pathway	14	86	3.336E-02

[1]MF: molecular function; BP: biological process; CC: cellular component.

Supplementary Table 17. Gene ontology (GO) enrichment analysis of the expanded and contracted gene families in *O. chinensis* among 11 species.

Type[1]	GO ID	GO Terms	Number of enriched genes	Number of genes in background	Adjusted P-value
**Expansion**					
MF	GO:0016491	oxidoreductase activity	542	2,483	1.753E-35
MF	GO:0052716	hydroquinone:oxygen oxidoreductase activity	39	48	3.237E-23
MF	GO:0003824	catalytic activity	1,676	10,991	2.898E-21
MF	GO:0003674	molecular_function	2,479	17,905	1.785E-18
MF	GO:0016682	oxidoreductase activity, acting on diphenols and related substances as donors, oxygen as acceptor	45	74	1.854E-18
MF	GO:0016679	oxidoreductase activity, acting on diphenols and related substances as donors	45	82	5.859E-16
MF	GO:0005215	transporter activity	271	1,236	8.425E-16
MF	GO:0030246	carbohydrate binding	95	294	1.476E-14
MF	GO:0004252	serine-type endopeptidase activity	53	135	4.652E-11
MF	GO:0016705	oxidoreductase activity, acting on paired donors, with incorporation or reduction of molecular oxygen	154	658	2.826E-10
MF	GO:0004565	beta-galactosidase activity	18	22	1.325E-09
MF	GO:0001871	pattern binding	44	108	1.676E-09
MF	GO:0030247	polysaccharide binding	44	108	1.676E-09
MF	GO:0020037	heme binding	126	529	1.471E-08
MF	GO:0032440	2-alkenal reductase [NAD(P)] activity	68	230	7.573E-08
MF	GO:0015925	galactosidase activity	19	28	8.401E-08
MF	GO:0016628	oxidoreductase activity, acting on the CH-CH group of donors, NAD or NADP as acceptor	78	285	1.840E-07
MF	GO:0035251	UDP-glucosyltransferase activity	51	155	3.388E-07
MF	GO:0008236	serine-type peptidase activity	66	228	4.036E-07
MF	GO:0017171	serine hydrolase activity	66	228	4.036E-07
MF	GO:0046906	tetrapyrrole binding	126	559	7.081E-07
MF	GO:0022857	transmembrane transporter activity	186	921	8.551E-07
MF	GO:0046527	glucosyltransferase activity	51	159	9.491E-07
MF	GO:0005507	copper ion binding	54	175	1.454E-06
MF	GO:0050660	flavin adenine dinucleotide binding	47	146	4.036E-06
MF	GO:0016759	cellulose synthase activity	27	62	8.333E-06
MF	GO:0016760	cellulose synthase (UDP-forming) activity	27	62	8.333E-06
MF	GO:0003968	RNA-directed RNA polymerase activity	14	20	1.942E-05
MF	GO:0008762	UDP-N-acetylmuramate dehydrogenase activity	15	23	2.451E-05
MF	GO:0005506	iron ion binding	113	514	2.641E-05
MF	GO:0016758	transferase activity, transferring hexosyl groups	120	564	6.681E-05
MF	GO:0050662	coenzyme binding	91	400	1.376E-04
MF	GO:0015079	potassium ion transmembrane transporter activity	24	59	3.057E-04

MF	GO:0071949	FAD binding	13	21	6.115E-04
MF	GO:0004553	hydrolase activity, hydrolyzing O-glycosyl compounds	100	466	6.360E-04
MF	GO:0016614	oxidoreductase activity, acting on CH-OH group of donors	75	323	8.648E-04
MF	GO:0046577	long-chain-alcohol oxidase activity	7	7	1.749E-03
MF	GO:0015198	oligopeptide transporter activity	7	7	1.749E-03
MF	GO:0008756	o-succinylbenzoate-CoA ligase activity	8	9	1.808E-03
MF	GO:0016706	oxidoreductase activity, acting on paired donors, with incorporation or reduction of molecular oxygen, 2-oxoglutarate as one donor, and incorporation of one atom each of oxygen into both donors	43	155	2.218E-03
MF	GO:0033773	isoflavone 2'-hydroxylase activity	10	14	2.322E-03
MF	GO:0046873	metal ion transmembrane transporter activity	46	173	3.456E-03
MF	GO:0046914	transition metal ion binding	307	1,845	4.377E-03
MF	GO:0004672	protein kinase activity	242	1,409	6.017E-03
MF	GO:0045552	dihydrokaempferol 4-reductase activity	12	21	6.040E-03
MF	GO:0045300	acyl-[acyl-carrier-protein] desaturase activity	10	15	6.149E-03
MF	GO:0003987	acetate-CoA ligase activity	11	18	6.537E-03
MF	GO:0004175	endopeptidase activity	73	330	8.384E-03
MF	GO:0016627	oxidoreductase activity, acting on the CH-CH group of donors	82	385	1.027E-02
MF	GO:0008194	UDP-glycosyltransferase activity	61	263	1.098E-02
MF	GO:0016616	oxidoreductase activity, acting on the CH-OH group of donors, NAD or NADP as acceptor	65	287	1.261E-02
MF	GO:0045548	phenylalanine ammonia-lyase activity	6	6	1.346E-02
MF	GO:0008061	chitin binding	11	19	1.369E-02
MF	GO:0016798	hydrolase activity, acting on glycosyl bonds	101	504	1.449E-02
MF	GO:0016405	CoA-ligase activity	15	34	2.256E-02
MF	GO:0016878	acid-thiol ligase activity	15	34	2.256E-02
MF	GO:0016899	oxidoreductase activity, acting on the CH-OH group of donors, oxygen as acceptor	11	20	2.684E-02
MF	GO:0048037	cofactor binding	106	544	3.102E-02
MF	GO:0016773	phosphotransferase activity, alcohol group as acceptor	258	1,555	3.973E-02
MF	GO:0016772	transferase activity, transferring phosphorus-containing groups	358	2,254	4.680E-02
MF	GO:0004674	protein serine/threonine kinase activity	119	632	4.841E-02
MF	GO:0004351	glutamate decarboxylase activity	7	9	4.947E-02
CC	GO:0005576	extracellular region	120	464	1.175E-10
CC	GO:0009341	beta-galactosidase complex	18	22	1.325E-09
CC	GO:0016021	integral component of membrane	366	2,089	9.003E-07
CC	GO:0031224	intrinsic component of membrane	370	2,165	1.496E-05
CC	GO:0048046	apoplast	50	198	6.329E-03
BP	GO:0046274	lignin catabolic process	39	48	3.237E-23
BP	GO:0046271	phenylpropanoid catabolic process	39	49	1.387E-22
BP	GO:0055114	oxidation-reduction process	513	2,643	4.623E-20
BP	GO:0009698	phenylpropanoid metabolic process	100	300	1.622E-16
BP	GO:0009808	lignin metabolic process	52	105	4.208E-16
BP	GO:0055085	transmembrane transport	232	1,098	3.162E-11

BP	GO:0006857	oligopeptide transport	32	61	5.028E-10
BP	GO:0015833	peptide transport	32	64	2.834E-09
BP	GO:0019748	secondary metabolic process	126	532	2.217E-08
BP	GO:0042886	amide transport	32	73	2.374E-07
BP	GO:0006026	aminoglycan catabolic process	34	82	3.931E-07
BP	GO:0019439	aromatic compound catabolic process	67	240	1.614E-06
BP	GO:0006011	UDP-glucose metabolic process	26	55	1.825E-06
BP	GO:1901136	carbohydrate derivative catabolic process	38	106	5.029E-06
BP	GO:0006027	glycosaminoglycan catabolic process	18	30	5.516E-06
BP	GO:1901361	organic cyclic compound catabolic process	67	252	1.454E-05
BP	GO:0006022	aminoglycan metabolic process	38	122	3.860E-04
BP	GO:0006040	amino sugar metabolic process	33	99	4.287E-04
BP	GO:0071804	cellular potassium ion transport	24	62	9.245E-04
BP	GO:0071805	potassium ion transmembrane transport	24	62	9.245E-04
BP	GO:0006012	galactose metabolic process	27	77	1.807E-03
BP	GO:0006687	glycosphingolipid metabolic process	23	60	1.986E-03
BP	GO:0009225	nucleotide-sugar metabolic process	26	76	4.960E-03
BP	GO:0009800	cinnamic acid biosynthetic process	6	6	1.346E-02
BP	GO:0009803	cinnamic acid metabolic process	6	6	1.346E-02
BP	GO:0006813	potassium ion transport	26	81	1.895E-02
**Decrease**					
MF	GO:0022857	transmembrane transporter activity	86	921	2.403E-29
MF	GO:0032559	adenyl ribonucleotide binding	139	2,348	1.534E-28
MF	GO:0030554	adenyl nucleotide binding	139	2,353	1.921E-28
MF	GO:0005524	ATP binding	132	2,250	2.436E-26
MF	GO:0001883	purine nucleoside binding	140	2,578	7.078E-25
MF	GO:0032550	purine ribonucleoside binding	140	2,578	7.078E-25
MF	GO:0032555	purine ribonucleotide binding	140	2,578	7.078E-25
MF	GO:0017076	purine nucleotide binding	140	2,584	8.984E-25
MF	GO:0032549	ribonucleoside binding	140	2,586	9.725E-25
MF	GO:0001882	nucleoside binding	140	2,587	1.012E-24
MF	GO:0032553	ribonucleotide binding	140	2,621	3.839E-24
MF	GO:0097367	carbohydrate derivative binding	141	2,656	4.294E-24
MF	GO:0000166	nucleotide binding	162	3,367	6.048E-24
MF	GO:1901265	nucleoside phosphate binding	162	3,367	6.048E-24
MF	GO:0036094	small molecule binding	166	3,526	1.158E-23
MF	GO:0016301	kinase activity	115	1,912	4.363E-23
MF	GO:0035639	purine ribonucleoside triphosphate binding	133	2,480	9.338E-23
MF	GO:0005215	transporter activity	89	1,236	3.538E-22
MF	GO:0016772	transferase activity, transferring phosphorus-containing groups	119	2,254	3.721E-19
MF	GO:0004672	protein kinase activity	89	1,409	2.959E-18
MF	GO:0022804	active transmembrane transporter activity	46	428	9.802E-17
MF	GO:0042626	ATPase activity, coupled to transmembrane movement of substances	32	200	4.356E-16
MF	GO:0016773	phosphotransferase activity, alcohol group as acceptor	90	1,555	5.610E-16

MF	GO:0016820	hydrolase activity, acting on acid anhydrides, catalyzing transmembrane movement of substances	32	203	6.904E-16
MF	GO:0022891	substrate-specific transmembrane transporter activity	57	695	1.210E-15
MF	GO:0022892	substrate-specific transporter activity	60	769	1.486E-15
MF	GO:0015399	primary active transmembrane transporter activity	32	215	4.019E-15
MF	GO:0015405	P-P-bond-hydrolysis-driven transmembrane transporter activity	32	215	4.019E-15
MF	GO:0043492	ATPase activity, coupled to movement of substances	32	218	6.127E-15
MF	GO:0016887	ATPase activity	45	512	6.004E-13
MF	GO:0005216	ion channel activity	21	98	1.570E-12
MF	GO:0022838	substrate-specific channel activity	21	107	1.030E-11
MF	GO:0015267	channel activity	21	108	1.255E-11
MF	GO:0022803	passive transmembrane transporter activity	21	108	1.255E-11
MF	GO:0022836	gated channel activity	18	77	3.597E-11
MF	GO:0016740	transferase activity	162	4,505	6.292E-11
MF	GO:0008559	xenobiotic-transporting ATPase activity	11	21	1.458E-10
MF	GO:0042910	xenobiotic transporter activity	11	21	1.458E-10
MF	GO:1901363	heterocyclic compound binding	203	6,353	7.882E-10
MF	GO:0097159	organic cyclic compound binding	203	6,355	8.149E-10
MF	GO:0015075	ion transmembrane transporter activity	40	529	3.815E-09
MF	GO:0099600	transmembrane receptor activity	18	101	5.190E-09
MF	GO:0004888	transmembrane signaling receptor activity	17	90	7.862E-09
MF	GO:0004674	protein serine/threonine kinase activity	42	632	6.711E-08
MF	GO:0004872	receptor activity	18	132	5.284E-07
MF	GO:0038023	signaling receptor activity	17	118	6.892E-07
MF	GO:0042623	ATPase activity, coupled	32	424	7.008E-07
MF	GO:0060089	molecular transducer activity	25	267	7.508E-07
MF	GO:1901677	phosphate transmembrane transporter activity	13	65	1.261E-06
MF	GO:0004871	signal transducer activity	24	254	1.404E-06
MF	GO:0004970	ionotropic glutamate receptor activity	9	25	1.821E-06
MF	GO:0005230	extracellular ligand-gated ion channel activity	9	25	1.821E-06
MF	GO:0008066	glutamate receptor activity	9	25	1.821E-06
MF	GO:0022824	transmitter-gated ion channel activity	9	25	1.821E-06
MF	GO:0022835	transmitter-gated channel activity	9	25	1.821E-06
MF	GO:0030594	neurotransmitter receptor activity	9	25	1.821E-06
MF	GO:0015276	ligand-gated ion channel activity	10	36	3.830E-06
MF	GO:0022834	ligand-gated channel activity	10	36	3.830E-06
MF	GO:0015238	drug transmembrane transporter activity	14	86	4.808E-06
MF	GO:0090484	drug transporter activity	14	87	5.623E-06
MF	GO:0003855	3-dehydroquinate dehydratase activity	5	5	6.742E-06
MF	GO:0004764	shikimate 3-dehydrogenase (NADP+) activity	5	5	6.742E-06
MF	GO:0017111	nucleoside-triphosphatase activity	46	874	1.482E-05
MF	GO:0003824	catalytic activity	286	10,991	2.213E-05
MF	GO:0016462	pyrophosphatase activity	46	905	4.263E-05

MF	GO:0016818	hydrolase activity, acting on acid anhydrides, in phosphorus-containing anhydrides	46	917	6.318E-05
MF	GO:0005249	voltage-gated potassium channel activity	8	26	6.582E-05
MF	GO:0016817	hydrolase activity, acting on acid anhydrides	46	929	9.286E-05
MF	GO:0003674	molecular_function	407	17,905	9.385E-05
MF	GO:0022843	voltage-gated cation channel activity	8	28	1.262E-04
MF	GO:0005315	inorganic phosphate transmembrane transporter activity	6	13	2.105E-04
MF	GO:0005267	potassium channel activity	8	32	3.971E-04
MF	GO:0005261	cation channel activity	9	44	4.449E-04
MF	GO:0008509	anion transmembrane transporter activity	14	130	1.007E-03
MF	GO:0015416	organic phosphonate transmembrane-transporting ATPase activity	7	27	1.767E-03
MF	GO:0015604	organic phosphonate transmembrane transporter activity	7	27	1.767E-03
MF	GO:0015114	phosphate ion transmembrane transporter activity	6	18	2.085E-03
MF	GO:0005244	voltage-gated ion channel activity	8	45	6.419E-03
MF	GO:0022832	voltage-gated channel activity	8	45	6.419E-03
MF	GO:0004713	protein tyrosine kinase activity	12	117	1.077E-02
MF	GO:0003735	structural constituent of ribosome	21	334	1.393E-02
MF	GO:0043225	anion transmembrane-transporting ATPase activity	7	36	1.413E-02
MF	GO:0005275	amine transmembrane transporter activity	5	15	1.703E-02
MF	GO:0004345	glucose-6-phosphate dehydrogenase activity	4	8	2.138E-02
CC	GO:0016021	integral component of membrane	96	2,089	9.832E-11
CC	GO:0031224	intrinsic component of membrane	96	2,165	8.444E-10
CC	GO:0016020	membrane	141	4,098	2.159E-07
CC	GO:0044425	membrane part	97	2,478	7.585E-07
CC	GO:0034703	cation channel complex	9	30	1.163E-05
CC	GO:0034702	ion channel complex	9	31	1.609E-05
CC	GO:0008076	voltage-gated potassium channel complex	8	23	2.182E-05
CC	GO:0034705	potassium channel complex	8	23	2.182E-05
CC	GO:1902495	transmembrane transporter complex	9	35	5.232E-05
CC	GO:1990351	transporter complex	9	35	5.232E-05
CC	GO:0005887	integral component of plasma membrane	8	40	2.509E-03
CC	GO:0022626	cytosolic ribosome	8	57	3.955E-02
BP	GO:0055085	transmembrane transport	75	1,098	9.033E-17
BP	GO:0006468	protein phosphorylation	80	1,370	4.900E-14
BP	GO:0008037	cell recognition	23	118	6.039E-13
BP	GO:0048544	recognition of pollen	23	118	6.039E-13
BP	GO:0009875	pollen-pistil interaction	23	119	7.344E-13
BP	GO:0044765	single-organism transport	99	2,058	1.875E-12
BP	GO:1902578	single-organism localization	99	2,080	3.735E-12
BP	GO:0042908	xenobiotic transport	11	21	1.458E-10
BP	GO:0016310	phosphorylation	95	2,142	1.145E-09
BP	GO:0006855	drug transmembrane transport	16	87	5.165E-08
BP	GO:0009856	pollination	24	218	5.888E-08
BP	GO:0044706	multi-multicellular organism process	24	218	5.888E-08

BP	GO:0015893	drug transport	16	88	6.194E-08
BP	GO:0042493	response to drug	16	89	7.409E-08
BP	GO:0044267	cellular protein metabolic process	121	3,303	1.721E-07
BP	GO:0007166	cell surface receptor signaling pathway	21	174	1.809E-07
BP	GO:0006810	transport	110	2,892	1.890E-07
BP	GO:0051234	establishment of localization	110	2,957	7.136E-07
BP	GO:0051179	localization	111	3,039	1.696E-06
BP	GO:0044703	multi-organism reproductive process	27	343	6.928E-06
BP	GO:0043412	macromolecule modification	99	2,708	1.810E-05
BP	GO:0006464	cellular protein modification process	91	2,440	3.337E-05
BP	GO:0036211	protein modification process	91	2,440	3.337E-05
BP	GO:0009987	cellular process	285	10,981	3.501E-05
BP	GO:0019538	protein metabolic process	127	3,854	4.332E-05
BP	GO:0007215	glutamate receptor signaling pathway	6	11	5.873E-05
BP	GO:0007268	chemical synaptic transmission	6	11	5.873E-05
BP	GO:0098916	anterograde trans-synaptic signaling	6	11	5.873E-05
BP	GO:0099536	synaptic signaling	6	11	5.873E-05
BP	GO:0099537	trans-synaptic signaling	6	11	5.873E-05
BP	GO:0035235	ionotropic glutamate receptor signaling pathway	6	11	5.873E-05
BP	GO:0008643	carbohydrate transport	13	103	4.010E-04
BP	GO:0007154	cell communication	54	1,255	5.988E-04
BP	GO:0015716	organic phosphonate transport	7	27	1.767E-03
BP	GO:0006796	phosphate-containing compound metabolic process	104	3,181	1.962E-03
BP	GO:0006793	phosphorus metabolic process	104	3,187	2.144E-03
BP	GO:0018108	peptidyl-tyrosine phosphorylation	12	102	2.515E-03
BP	GO:0018212	peptidyl-tyrosine modification	12	102	2.515E-03
BP	GO:0044763	single-organism cellular process	200	7,364	3.641E-03
BP	GO:1901605	alpha-amino acid metabolic process	53	1,336	8.703E-03
BP	GO:0007167	enzyme linked receptor protein signaling pathway	13	136	9.738E-03
BP	GO:0006813	potassium ion transport	10	81	1.227E-02
BP	GO:0008150	biological process	373	16,227	1.599E-02
BP	GO:0006518	peptide metabolic process	30	611	2.514E-02
BP	GO:0006817	phosphate ion transport	6	28	3.545E-02

[1]MF: molecular function; BP: biological process; CC: cellular component.

Supplementary Table 18. Gene ontology (GO) enrichment analysis of the expanded and contracted gene families in the ancestral lineage of *O. rehderiana* and *O. chinensis* among 11 species.

Type[1]	GO ID	GO Terms	Number of enriched genes	Number of genes in background	Adjusted *P*-value
**Expansion**					
MF	GO:0003824	catalytic activity	1,393	9,973	2.881E-40
MF	GO:0003674	molecular_function	2,002	16,622	9.853E-36
MF	GO:0022857	transmembrane transporter activity	222	851	3.232E-33
MF	GO:0005215	transporter activity	265	1,132	1.534E-31
MF	GO:0004970	ionotropic glutamate receptor activity	36	42	1.699E-25
MF	GO:0005230	extracellular ligand-gated ion channel activity	36	42	1.699E-25
MF	GO:0022824	transmitter-gated ion channel activity	36	42	1.699E-25
MF	GO:0022835	transmitter-gated channel activity	36	42	1.699E-25
MF	GO:0030594	neurotransmitter receptor activity	36	42	1.699E-25
MF	GO:0008066	glutamate receptor activity	36	43	9.341E-25
MF	GO:0015276	ligand-gated ion channel activity	41	56	2.852E-24
MF	GO:0022834	ligand-gated channel activity	41	56	2.852E-24
MF	GO:0052716	hydroquinone:oxygen oxidoreductase activity	36	45	2.056E-23
MF	GO:0032559	adenyl ribonucleotide binding	397	2,192	5.464E-23
MF	GO:0030554	adenyl nucleotide binding	397	2,196	7.959E-23
MF	GO:0022891	substrate-specific transmembrane transporter activity	165	646	9.400E-23
MF	GO:0043531	ADP binding	51	93	9.701E-22
MF	GO:0042626	ATPase activity, coupled to transmembrane movement of substances	70	170	6.952E-21
MF	GO:0022804	active transmembrane transporter activity	110	364	1.537E-20
MF	GO:0022892	substrate-specific transporter activity	172	719	2.161E-20
MF	GO:0016820	hydrolase activity, acting on acid anhydrides, catalyzing transmembrane movement of substances	70	174	3.573E-20
MF	GO:0016887	ATPase activity	126	456	5.637E-20
MF	GO:0016491	oxidoreductase activity	379	2,140	7.788E-20
MF	GO:0016682	oxidoreductase activity, acting on diphenols and related substances as donors, oxygen as acceptor	41	69	6.925E-19
MF	GO:0015405	P-P-bond-hydrolysis-driven transmembrane transporter activity	70	182	7.875E-19
MF	GO:0015399	primary active transmembrane transporter activity	70	182	7.875E-19
MF	GO:0043492	ATPase activity, coupled to movement of substances	70	185	2.371E-18
MF	GO:0015075	ion transmembrane transporter activity	127	482	3.560E-18
MF	GO:0001883	purine nucleoside binding	406	2,419	4.886E-17
MF	GO:0032550	purine ribonucleoside binding	406	2,419	4.886E-17
MF	GO:0016679	oxidoreductase activity, acting on diphenols and related substances as donors	41	75	5.317E-17
MF	GO:0032555	purine ribonucleotide binding	406	2,421	5.702E-17

MF	GO:0017076	purine nucleotide binding	406	2,426	8.377E-17
MF	GO:0032549	ribonucleoside binding	406	2,428	9.764E-17
MF	GO:0001882	nucleoside binding	406	2,429	1.054E-16
MF	GO:0032553	ribonucleotide binding	409	2,459	1.827E-16
MF	GO:0097367	carbohydrate derivative binding	409	2,488	1.571E-15
MF	GO:0042623	ATPase activity, coupled	102	372	1.811E-15
MF	GO:0008236	serine-type peptidase activity	73	220	2.062E-15
MF	GO:0017171	serine hydrolase activity	73	220	2.062E-15
MF	GO:0022836	gated channel activity	41	93	1.225E-12
MF	GO:0099600	transmembrane receptor activity	48	124	2.139E-12
MF	GO:0005524	ATP binding	344	2,095	2.458E-12
MF	GO:0005216	ion channel activity	47	121	3.652E-12
MF	GO:0016787	hydrolase activity	500	3,346	1.377E-11
MF	GO:0004888	transmembrane signaling receptor activity	43	110	4.511E-11
MF	GO:0000166	nucleotide binding	467	3,107	5.888E-11
MF	GO:1901265	nucleoside phosphate binding	467	3,107	5.888E-11
MF	GO:0022838	substrate-specific channel activity	47	131	1.253E-10
MF	GO:0015267	channel activity	47	132	1.741E-10
MF	GO:0022803	passive transmembrane transporter activity	47	132	1.741E-10
MF	GO:0004872	receptor activity	51	154	4.110E-10
MF	GO:0016705	oxidoreductase activity, acting on paired donors, with incorporation or reduction of molecular oxygen	120	561	9.802E-10
MF	GO:0038023	signaling receptor activity	46	136	2.705E-09
MF	GO:0036094	small molecule binding	476	3,260	4.237E-09
MF	GO:0004553	hydrolase activity, hydrolyzing O-glycosyl compounds	103	468	7.906E-09
MF	GO:0016798	hydrolase activity, acting on glycosyl bonds	107	495	9.699E-09
MF	GO:0015077	monovalent inorganic cation transmembrane transporter activity	52	171	1.020E-08
MF	GO:0004672	protein kinase activity	219	1,274	1.096E-08
MF	GO:0004252	serine-type endopeptidase activity	45	136	1.137E-08
MF	GO:0008324	cation transmembrane transporter activity	75	306	3.686E-08
MF	GO:0005506	iron ion binding	96	435	3.729E-08
MF	GO:0035639	purine ribonucleoside triphosphate binding	353	2,322	5.224E-08
MF	GO:0070290	N-acylphosphatidylethanolamine-specific phospholipase D activity	11	11	7.651E-08
MF	GO:0005507	copper ion binding	50	169	8.065E-08
MF	GO:0015079	potassium ion transmembrane transporter activity	26	57	1.121E-07
MF	GO:0016712	oxidoreductase activity, acting on paired donors, with incorporation or reduction of molecular oxygen, reduced flavin or flavoprotein as one donor, and incorporation of one atom of oxygen	19	32	1.301E-07
MF	GO:0070330	aromatase activity	19	32	1.301E-07
MF	GO:0022890	inorganic cation transmembrane transporter activity	66	263	2.089E-07
MF	GO:0017111	nucleoside-triphosphatase activity	149	809	2.854E-07
MF	GO:0020037	heme binding	95	445	3.293E-07
MF	GO:0046906	tetrapyrrole binding	100	479	4.010E-07
MF	GO:0004565	beta-galactosidase activity	15	22	8.752E-07

MF	GO:0016462	pyrophosphatase activity	151	841	1.409E-06
MF	GO:0015298	solute:cation antiporter activity	22	47	1.912E-06
MF	GO:0015299	solute:proton antiporter activity	22	47	1.912E-06
MF	GO:0016818	hydrolase activity, acting on acid anhydrides, in phosphorus-containing anhydrides	151	854	4.252E-06
MF	GO:0016758	transferase activity, transferring hexosyl groups	99	493	4.503E-06
MF	GO:0016773	phosphotransferase activity, alcohol group as acceptor	227	1,419	4.553E-06
MF	GO:0004630	phospholipase D activity	11	13	4.832E-06
MF	GO:0060089	molecular transducer activity	64	270	4.956E-06
MF	GO:0016817	hydrolase activity, acting on acid anhydrides	151	864	9.659E-06
MF	GO:0015925	galactosidase activity	16	28	9.904E-06
MF	GO:0032440	2-alkenal reductase [NAD(P)] activity	53	213	2.070E-05
MF	GO:0004871	signal transducer activity	59	253	4.006E-05
MF	GO:0016628	oxidoreductase activity, acting on the CH-CH group of donors, NAD or NADP as acceptor	60	264	8.277E-05
MF	GO:0046873	metal ion transmembrane transporter activity	40	148	1.252E-04
MF	GO:0047213	anthocyanidin 3-O-glucosyltransferase activity	12	19	2.005E-04
MF	GO:0070011	peptidase activity, acting on L-amino acid peptides	102	551	2.096E-04
MF	GO:0004497	monooxygenase activity	69	329	2.363E-04
MF	GO:0004556	alpha-amylase activity	9	11	2.856E-04
MF	GO:0003968	RNA-directed RNA polymerase activity	11	17	5.030E-04
MF	GO:0004090	carbonyl reductase (NADPH) activity	7	7	5.325E-04
MF	GO:0016301	kinase activity	254	1,742	2.097E-03
MF	GO:0008756	o-succinylbenzoate-CoA ligase activity	8	10	2.139E-03
MF	GO:0008238	exopeptidase activity	30	110	4.741E-03
MF	GO:0008233	peptidase activity	107	626	5.698E-03
MF	GO:0004650	polygalacturonase activity	20	60	9.041E-03
MF	GO:0015297	antiporter activity	30	116	1.549E-02
MF	GO:0015291	secondary active transmembrane transporter activity	40	177	1.835E-02
MF	GO:0016772	transferase activity, transferring phosphorus-containing groups	284	2,038	2.043E-02
MF	GO:0016627	oxidoreductase activity, acting on the CH-CH group of donors	67	360	2.927E-02
MF	GO:0051213	dioxygenase activity	46	219	3.080E-02
MF	GO:0047787	delta4-3-oxosteroid 5beta-reductase activity	5	5	4.432E-02
MF	GO:0008559	xenobiotic-transporting ATPase activity	10	20	4.552E-02
MF	GO:0042910	xenobiotic transporter activity	10	20	4.552E-02
CC	GO:0009341	beta-galactosidase complex	15	22	8.752E-07
CC	GO:0016021	integral component of membrane	293	1,915	1.957E-06
CC	GO:0031224	intrinsic component of membrane	293	1,983	7.123E-05
CC	GO:0016020	membrane	509	3,770	1.186E-04
CC	GO:0048046	apoplast	44	173	1.918E-04
BP	GO:0046274	lignin catabolic process	36	45	2.056E-23
BP	GO:0046271	phenylpropanoid catabolic process	36	46	8.464E-23
BP	GO:0055085	transmembrane transport	218	1,000	7.101E-21
BP	GO:0009698	phenylpropanoid metabolic process	90	275	4.982E-19

BP	GO:0009808	lignin metabolic process	44	97	2.710E-14
BP	GO:0055114	oxidation-reduction process	374	2,303	5.687E-13
BP	GO:0008152	metabolic process	1,453	11,673	6.358E-13
BP	GO:0019748	secondary metabolic process	116	494	2.541E-12
BP	GO:0007215	glutamate receptor signaling pathway	20	26	2.013E-11
BP	GO:0007268	chemical synaptic transmission	20	26	2.013E-11
BP	GO:0098916	anterograde trans-synaptic signaling	20	26	2.013E-11
BP	GO:0099536	synaptic signaling	20	26	2.013E-11
BP	GO:0099537	trans-synaptic signaling	20	26	2.013E-11
BP	GO:0035235	ionotropic glutamate receptor signaling pathway	20	26	2.013E-11
BP	GO:0044765	single-organism transport	305	1,894	1.836E-09
BP	GO:1902578	single-organism localization	305	1,920	9.964E-09
BP	GO:0007267	cell-cell signaling	28	64	6.921E-08
BP	GO:0006468	protein phosphorylation	218	1,292	7.609E-08
BP	GO:0008037	cell recognition	39	114	9.598E-08
BP	GO:0048544	recognition of pollen	39	114	9.598E-08
BP	GO:0009875	pollen-pistil interaction	39	115	1.300E-07
BP	GO:0006810	transport	387	2,685	5.389E-06
BP	GO:0006813	potassium ion transport	28	76	8.214E-06
BP	GO:0009813	flavonoid biosynthetic process	38	126	1.026E-05
BP	GO:0009812	flavonoid metabolic process	38	129	2.117E-05
BP	GO:0015672	monovalent inorganic cation transport	63	282	7.494E-05
BP	GO:0051234	establishment of localization	387	2,745	7.774E-05
BP	GO:0071804	cellular potassium ion transport	21	53	1.765E-04
BP	GO:0071805	potassium ion transmembrane transport	21	53	1.765E-04
BP	GO:0051179	localization	393	2,826	2.875E-04
BP	GO:0006027	glycosaminoglycan catabolic process	15	30	3.369E-04
BP	GO:0009699	phenylpropanoid biosynthetic process	49	213	1.012E-03
BP	GO:0046470	phosphatidylcholine metabolic process	10	15	1.245E-03
BP	GO:0008150	biological_process	1,738	15,029	1.299E-03
BP	GO:0006811	ion transport	148	915	1.965E-03
BP	GO:0097164	ammonium ion metabolic process	10	16	2.991E-03
BP	GO:0006692	prostanoid metabolic process	9	13	3.019E-03
BP	GO:0006693	prostaglandin metabolic process	9	13	3.019E-03
BP	GO:0006687	glycosphingolipid metabolic process	20	58	4.948E-03
BP	GO:0007166	cell surface receptor signaling pathway	41	180	1.149E-02
BP	GO:0044550	secondary metabolite biosynthetic process	62	319	1.508E-02
BP	GO:0008643	carbohydrate transport	27	102	2.818E-02
BP	GO:0098662	inorganic cation transmembrane transport	41	188	3.548E-02
BP	GO:0006885	regulation of pH	15	41	4.249E-02
BP	GO:0042908	xenobiotic transport	10	20	4.552E-02
BP	GO:0055067	monovalent inorganic cation homeostasis	16	46	4.667E-02
BP	GO:1901361	organic cyclic compound catabolic process	50	249	4.783E-02
**Decrease**					

MF	GO:0003824	catalytic activity	310	9,973	3.022E-21
MF	GO:0016301	kinase activity	93	1,742	5.200E-14
MF	GO:0004672	protein kinase activity	76	1,274	2.110E-13
MF	GO:0016772	transferase activity, transferring phosphorus-containing groups	100	2,038	7.103E-13
MF	GO:0003843	1,3-beta-D-glucan synthase activity	10	12	1.891E-12
MF	GO:0016773	phosphotransferase activity, alcohol group as acceptor	76	1,419	6.806E-11
MF	GO:0035639	purine ribonucleoside triphosphate binding	103	2,322	1.772E-10
MF	GO:0036094	small molecule binding	129	3,260	2.330E-10
MF	GO:0005524	ATP binding	95	2,095	6.095E-10
MF	GO:0000166	nucleotide binding	123	3,107	1.128E-09
MF	GO:1901265	nucleoside phosphate binding	123	3,107	1.128E-09
MF	GO:0001883	purine nucleoside binding	103	2,419	2.330E-09
MF	GO:0032550	purine ribonucleoside binding	103	2,419	2.330E-09
MF	GO:0032555	purine ribonucleotide binding	103	2,421	2.452E-09
MF	GO:0017076	purine nucleotide binding	103	2,426	2.786E-09
MF	GO:0032549	ribonucleoside binding	103	2,428	2.931E-09
MF	GO:0001882	nucleoside binding	103	2,429	3.007E-09
MF	GO:0032553	ribonucleotide binding	103	2,459	6.388E-09
MF	GO:0032559	adenyl ribonucleotide binding	95	2,192	8.578E-09
MF	GO:0030554	adenyl nucleotide binding	95	2,196	9.524E-09
MF	GO:0097367	carbohydrate derivative binding	103	2,488	1.303E-08
MF	GO:0008447	L-ascorbate oxidase activity	8	13	8.129E-08
MF	GO:0005516	calmodulin binding	14	68	2.323E-07
MF	GO:0004012	phospholipid-translocating ATPase activity	8	15	3.916E-07
MF	GO:0005548	phospholipid transporter activity	8	15	3.916E-07
MF	GO:0016740	transferase activity	140	4,108	1.090E-06
MF	GO:0000287	magnesium ion binding	17	128	3.249E-06
MF	GO:0005319	lipid transporter activity	9	28	6.953E-06
MF	GO:0004497	monooxygenase activity	26	329	1.726E-05
MF	GO:0046527	glucosyltransferase activity	17	145	2.233E-05
MF	GO:0003674	MF	386	16,622	3.043E-05
MF	GO:1901363	heterocyclic compound binding	179	5,959	3.080E-05
MF	GO:0097159	organic cyclic compound binding	179	5,960	3.122E-05
MF	GO:0047215	indole-3-acetate beta-glucosyltransferase activity	6	11	6.750E-05
MF	GO:0035251	UDP-glucosyltransferase activity	16	141	9.588E-05
MF	GO:0043492	ATPase activity, coupled to movement of substances	18	185	1.552E-04
MF	GO:0030246	carbohydrate binding	21	251	1.795E-04
MF	GO:0015416	organic phosphonate transmembrane-transporting ATPase activity	7	21	2.998E-04
MF	GO:0015604	organic phosphonate transmembrane transporter activity	7	21	2.998E-04
MF	GO:0020037	heme binding	28	445	5.865E-04
MF	GO:0046906	tetrapyrrole binding	29	479	8.005E-04
MF	GO:0004058	aromatic-L-amino-acid decarboxylase activity	4	5	1.795E-03
MF	GO:0004674	protein serine/threonine kinase activity	32	600	3.238E-03
MF	GO:0043225	anion transmembrane-transporting ATPase activity	7	30	4.453E-03

MF	GO:0005506	iron ion binding	25	435	1.264E-02
MF	GO:0016758	transferase activity, transferring hexosyl groups	27	493	1.314E-02
MF	GO:0042625	ATPase coupled ion transmembrane transporter activity	10	81	1.465E-02
MF	GO:0010333	terpene synthase activity	7	36	1.637E-02
MF	GO:0022853	active ion transmembrane transporter activity	10	85	2.264E-02
MF	GO:0004345	glucose-6-phosphate dehydrogenase activity	4	8	2.389E-02
MF	GO:0016491	oxidoreductase activity	74	2,140	2.491E-02
MF	GO:0005507	copper ion binding	14	169	2.783E-02
MF	GO:0042623	ATPase activity, coupled	22	372	2.905E-02
MF	GO:0016634	oxidoreductase activity, acting on the CH-CH group of donors, oxygen as acceptor	6	27	3.245E-02
MF	GO:0008514	organic anion transmembrane transporter activity	7	40	3.398E-02
MF	GO:0043167	ion binding	97	3,082	4.429E-02
MF	GO:0016838	carbon-oxygen lyase activity, acting on phosphates	7	42	4.742E-02
CC	GO:0000148	1,3-beta-D-glucan synthase complex	10	12	1.891E-12
CC	GO:0071944	cell periphery	50	1,081	3.061E-04
CC	GO:0098797	plasma membrane protein complex	10	63	1.398E-03
CC	GO:0009505	plant-type cell wall	11	91	6.368E-03
CC	GO:0044459	plasma membrane part	12	119	1.561E-02
CC	GO:0005618	cell wall	20	309	1.926E-02
CC	GO:0030312	external encapsulating structure	20	313	2.324E-02
BP	GO:0006074	(1->3)-beta-D-glucan metabolic process	10	12	1.891E-12
BP	GO:0006075	(1->3)-beta-D-glucan biosynthetic process	10	12	1.891E-12
BP	GO:0009856	pollination	26	205	4.568E-10
BP	GO:0044706	multi-multicellular organism process	26	205	4.568E-10
BP	GO:0008037	cell recognition	20	114	5.829E-10
BP	GO:0048544	recognition of pollen	20	114	5.829E-10
BP	GO:0009875	pollen-pistil interaction	20	115	6.921E-10
BP	GO:0016310	phosphorylation	90	1,973	2.215E-09
BP	GO:0008152	metabolic process	309	11,673	6.606E-08
BP	GO:0006468	protein phosphorylation	65	1,292	1.002E-07
BP	GO:0015917	aminophospholipid transport	8	14	1.862E-07
BP	GO:0034204	lipid translocation	8	14	1.862E-07
BP	GO:0097035	regulation of membrane lipid distribution	8	14	1.862E-07
BP	GO:0045332	phospholipid translocation	8	14	1.862E-07
BP	GO:0015914	phospholipid transport	8	16	7.688E-07
BP	GO:0015748	organophosphate ester transport	15	92	1.617E-06
BP	GO:0006796	phosphate-containing compound metabolic process	108	2,924	3.240E-06
BP	GO:0006793	phosphorus metabolic process	108	2,929	3.571E-06
BP	GO:0044703	multi-organism reproductive process	26	329	1.726E-05
BP	GO:0008150	biological_process	360	15,029	6.579E-05
BP	GO:0015716	organic phosphonate transport	7	21	2.998E-04
BP	GO:0019852	L-ascorbic acid metabolic process	8	35	9.883E-04
BP	GO:0006869	lipid transport	12	96	1.591E-03

BP	GO:0071705	nitrogen compound transport	25	402	3.142E-03
BP	GO:0042431	indole metabolic process	7	29	3.477E-03
BP	GO:0042432	indole biosynthetic process	7	29	3.477E-03
BP	GO:0051704	multi-organism process	37	828	2.961E-02
BP	GO:0005985	sucrose metabolic process	20	325	4.006E-02

[1]MF: molecular function; BP: biological process; CC: cellular component.

Supplementary Table 19. Expanded gene families in two iron wood species compared to the silver birch.

Gene Family	*B. pendula*	*O. rehderiana*	*O. chinensis*
**Lignin biosynthesis**			
4CL	13	19	19
HCT	64	103	100
C3H	30	32	31
CSE	0	1	1
CCoAOMT	9	14	11
COMT	34	46	41
CAD	16	22	18
LAC	22	40	49
**Cellulose and Hemicellulose synthesis**			
CSL	39	50	63
GH1	20	32	34
GH10	2	4	5
GH28	40	56	53
GH3	12	18	23
GH35	14	20	22
GH79	4	5	5
GH9	21	25	26
GT8	33	36	37
XGT	34	35	44
**Cell wall structure protein**			
LRRP	9	21	18
FLAs	18	31	31
**Monosaccharide Inter-conversion**			
NSE	19	22	22
**Cell growth Related**			
Expansin	24	32	39

Supplementary Table 20. Overview of sample information and sequencing statistics.

Sample ID	Species	Description	Clean reads data (Gb)	*O. rehderiana*[3] Map Ratio	*O. rehderiana*[3] Genome Coverage	*O. rehderiana*[3] Efeective Depth	*O. chinensis*[4] Map Ratio	*O. chinensis*[4] Genome Coverage	*O. chinensis*[4] Efeective Depth
Ore01	*O. rehderiana*	Wild[1]; Tianmu Mountains, Zhejiang Province, PRC	11.25	0.96	1.00	29.51	0.96	0.91	28.55
Ore02	*O. rehderiana*	Wild[1]; Tianmu Mountains, Zhejiang Province, PRC	8.14	0.63	0.98	14.30	0.62	0.90	13.68
Ore03	*O. rehderiana*	Wild[1]; Tianmu Mountains, Zhejiang Province, PRC	8.12	0.97	0.98	21.83	0.96	0.90	20.98
Ore04	*O. rehderiana*	Wild[1]; Tianmu Mountains, Zhejiang Province, PRC	8.57	0.97	0.98	23.19	0.96	0.91	22.19
Ore05	*O. rehderiana*	Wild[1]; Tianmu Mountains, Zhejiang Province, PRC	4.86	0.94	0.97	12.68	0.95	0.88	12.49
Ore06	*O. rehderiana*	Offspring[2]; Tianmu Mountains, Zhejiang Province, PRC	4.11	0.98	0.97	11.32	0.98	0.88	10.97
Ore07	*O. rehderiana*	Offspring[2]; Tianmu Mountains, Zhejiang Province, PRC	4.13	0.98	0.97	11.39	0.97	0.88	11.04
Ore08	*O. rehderiana*	Offspring[2]; Tianmu Mountains, Zhejiang Province, PRC	3.69	0.98	0.97	10.25	0.97	0.88	9.87
Ore09	*O. rehderiana*	Offspring[2]; Tianmu Mountains, Zhejiang Province, PRC	8.15	0.98	0.97	22.47	0.97	0.90	21.73
Ore10	*O. rehderiana*	Offspring[2]; Tianmu Mountains, Zhejiang Province, PRC	3.68	0.98	0.97	10.15	0.97	0.88	9.77
Ore11	*O. rehderiana*	Offspring[2]; Tianmu Mountains, Zhejiang Province, PRC	3.70	0.98	0.97	10.15	0.97	0.88	9.74
Ore12	*O. rehderiana*	Offspring[2]; Tianmu Mountains, Zhejiang Province, PRC	3.63	0.97	0.97	9.89	0.97	0.88	9.60
Ore13	*O. rehderiana*	Offspring[2]; Tianmu Mountains, Zhejiang Province, PRC	3.61	0.98	0.97	9.93	0.97	0.88	9.52
Ore14	*O. rehderiana*	Offspring[2]; Tianmu Mountains, Zhejiang Province, PRC	4.09	0.98	0.97	11.22	0.97	0.88	10.82
Och01	*O. chinensis*	Wild[1]; Luquan County, Yunnan Province, PRC	25.62	0.96	0.93	66.73	0.98	1.00	70.75
Och02	*O. chinensis*	Wild[1]; Wencheng County, Zhejiang Province, PRC	6.85	0.93	0.90	17.40	0.94	0.95	18.21
Och03	*O. chinensis*	Wild[1]; Wencheng County, Zhejiang Province, PRC	5.63	0.94	0.90	14.48	0.96	0.95	15.16
Och04	*O. chinensis*	Wild[1]; Wencheng County, Zhejiang Province, PRC	5.05	0.90	0.92	36.38	0.91	0.96	38.14
Och05	*O. chinensis*	Wild[1]; Wencheng County, Zhejiang Province, PRC	4.26	0.88	0.92	29.99	0.89	0.95	31.54
Och06	*O. chinensis*	Wild[1]; Wencheng County, Zhejiang Province, PRC	4.45	0.90	0.91	32.06	0.92	0.95	33.74
Och07	*O. chinensis*	Wild[1]; Yinjiang County, Guizhou Province, PRC	5.71	0.91	0.90	14.15	0.93	0.95	14.86

Och08	*O. chinensis*	Wild[1]; Yinjiang County, Guizhou Province, PRC	4.87	0.87	0.89	11.61	0.89	0.94	12.24
Och09	*O. chinensis*	Wild[1]; Yinjiang County, Guizhou Province, PRC	6.59	0.91	0.91	16.24	0.92	0.95	17.05
Och10	*O. chinensis*	Wild[1]; Yinjiang County, Guizhou Province, PRC	8.68	0.92	0.92	40.58	0.94	0.96	42.84
Och11	*O. chinensis*	Wild[1]; Yinjiang County, Guizhou Province, PRC	7.22	0.94	0.92	35.88	0.95	0.95	37.72
Och12	*O. chinensis*	Wild[1]; Longquan Mountains, Zhejiang Province, PRC	3.69	0.97	0.87	9.74	0.98	0.93	10.19
Och13	*O. chinensis*	Wild[1]; Longquan Mountains, Zhejiang Province, PRC	4.09	0.97	0.88	10.90	0.99	0.93	11.34
Och14	*O. chinensis*	Wild[1]; Xinning County, Hunan Province, PRC	3.68	0.94	0.89	9.49	0.96	0.94	9.92
Cfa01	*C. fangiana*	Wild[1]; Ebian Yi Nationality Autonomous County, Sichuan Province, PRC	7.77	0.82	0.77	15.96	0.83	0.78	16.30
Cco02	*C. cordata*	Wild[1]; Tianshui City , Gansu Province, PRC	4.08	0.92	0.75	9.45	0.93	0.76	9.64
Cco03	*C. cordata*	Wild[1]; Ta-pieh Mountains, Anhui Province, PRC	4.05	0.92	0.75	9.34	0.92	0.76	9.53
Ono00	*O. nobilis*	Wild[1]; Yulong Naxi Autonomous County, Yunnan Province, PRC	7.76	0.78	0.58	14.06	0.79	0.59	14.49

[1]Represent the sample collected from the wild mature individuals.

[2]Represent the 9 *O. rehderiana* samples planted by human.

[3]Represent reads mapped to *O. rehderiana* genome.

[4]Represent reads mapped to *O. chineensis* genome.

Supplementary Table 21. Mutation rates estimated using the divergence time between *Ostryopsis* and *Ostrya*.

	O. rehderiana - *O.nobilis*	*O. chinensis* - *O.nobilis*
All sites	2,988,522	2,991,160
Diff sites	204,575	199,157
divergence time (million year)	34	34
mutation rate (per site per year)	1.00667E-09	9.79145E-10
**Average**	mutation rate: 9.9290E-10 per site per year	

Supplementary Table 22. Mutation rate estimated by pedigree sequencing.

Parents	Offspring	*de novo* mutation	mutation rate (per generation)
Ore01-Ore02	Ore06	2	7.2727E-09
Ore02-Ore03	Ore09	1	3.6364E-09
Ore02-Ore03	Ore10	7	2.5455E-08
Ore02-Ore03	Ore13	8	2.9091E-08
Ore02-Ore03	Ore14	12	4.3636E-08
**Average**		**6**	**2.18182E-08**

Supplementary Table 23. Genetic diversity of the plant species at the genomic level

Species name	Pi	Journal	Year	DOI	Title
Prunus persica	0.00150	Nature genetics	2012	10.1038/ng.2586	The high-quality draft genome of peach (*Prunus persica*) identifies unique patterns of genetic diversity, domestication and genome evolution
Phoenix dactylifera	0.00920	Nature communications	2015	10.1038/ncomms9824	Whole genome re-sequencing of date palms yields insights into diversification of a fruit tree crop
Gossypium raimondii	0.00012	Plos biology	2015	10.1371/journal.pbio.1002112	Natural Selection Constrains Neutral Diversity across A Wide Range of Species
Setaria italica	0.00233	Plos biology	2015	10.1371/journal.pbio.1002112	Natural Selection Constrains Neutral Diversity across A Wide Range of Species
Citrullus lanatus	0.00241	Plos biology	2015	10.1371/journal.pbio.1002112	Natural Selection Constrains Neutral Diversity across A Wide Range of Species
Sorghum bicolor subsp. verticilliflorum	0.00249	Plos biology	2015	10.1371/journal.pbio.1002112	Natural Selection Constrains Neutral Diversity across A Wide Range of Species
Brachypodium distachyon	0.00267	Plos biology	2015	10.1371/journal.pbio.1002112	Natural Selection Constrains Neutral Diversity across A Wide Range of Species
Glycine soja	0.00274	Plos biology	2015	10.1371/journal.pbio.1002112	Natural Selection Constrains Neutral Diversity across A Wide Range of Species
Arabidopsis thaliana	0.00315	Plos biology	2015	10.1371/journal.pbio.1002112	Natural Selection Constrains Neutral Diversity across A Wide Range of Species
Populus trichocarpa	0.00317	Plos biology	2015	10.1371/journal.pbio.1002112	Natural Selection Constrains Neutral Diversity across A Wide Range of Species
Capsella rubella	0.00327	Plos biology	2015	10.1371/journal.pbio.1002112	Natural Selection Constrains Neutral Diversity across A Wide Range of Species
Medicago truncatula	0.00514	Plos biology	2015	10.1371/journal.pbio.1002112	Natural Selection Constrains Neutral Diversity across A Wide Range of Species
Oryza rufipogon	0.00636	Plos biology	2015	10.1371/journal.pbio.1002112	Natural Selection Constrains Neutral Diversity across A Wide Range of Species
Prunus davidiana	0.00988	Plos biology	2015	10.1371/journal.pbio.1002112	Natural Selection Constrains Neutral Diversity across A Wide Range of Species
Cucumis sativus var. hardwickii	0.01321	Plos biology	2015	10.1371/journal.pbio.1002112	Natural Selection Constrains Neutral Diversity across A Wide Range of Species
Zea mays ssp parviglumis	0.01386	Plos biology	2015	10.1371/journal.pbio.1002112	Natural Selection Constrains Neutral Diversity across A Wide Range of Species
Citrus reticulata	0.01496	Plos biology	2015	10.1371/journal.pbio.1002112	Natural Selection Constrains Neutral Diversity across A Wide Range of Species
Populus tremula	0.01470	Molecular biology evolution	2016	10.1093/molbev/msw051	Variation in Linked Selection and Recombination Drive Genomic Divergence during Allopatric Speciation of European and American Aspens
Populus tremuloides	0.01600	Molecular biology evolution	2016	10.1093/molbev/msw051	Variation in Linked Selection and Recombination Drive Genomic Divergence during Allopatric Speciation of European and American Aspens
Ziziphus jujuba	0.00219	Plos genetics	2016	10.1371/journal.pgen.1006433	The Jujube Genome Provides Insights into Genome Evolution and the Domestication of Sweetness/Acidity Taste in Fruit Trees

Betula pendula	0.00880	Nature	2017	10.1038/ng.3862	Genome sequencing and population genomic analyses provide insights into the adaptive landscape of silver birch
Malus domestica	0.00220	Nature communications	2017	10.1038/s41467-017-00336-7	Genome re-sequencing reveals the history of apple and supports a two-stage model for fruit enlargement
Ostrya rehderiana	0.00166				This study
Ostrya chinensis	0.00279				

Supplementary Table 24. Genetic diversity (Pi) of *O. rehderiana* and *O. chinensis* based only on randomly selected four individuals.

Species	Combination	Mean Pi	95% CI
O. chinensis	(Och01, Och02, Och07, Och14)	0.00308	(0.00318, 0.00313)
O. chinensis	(Och01, Och05, Och06, Och11)	0.00313	(0.00323, 0.00318)
O. chinensis	(Och01, Och06, Och08, Och11)	0.00311	(0.00321, 0.00316)
O. chinensis	(Och01, Och08, Och13, Och14)	0.00293	(0.00302, 0.00298)
O. chinensis	(Och01, Och09, Och13, Och14)	0.00301	(0.00310, 0.00306)
O. chinensis	(Och01, Och10, Och12, Och14)	0.00297	(0.00306, 0.00302)
O. chinensis	(Och02, Och08, Och09, Och14)	0.00284	(0.00294, 0.00289)
O. chinensis	(Och05, Och06, Och07, Och10)	0.00296	(0.00306, 0.00301)
O. chinensis	(Och06, Och07, Och08, Och13)	0.00272	(0.00281, 0.00277)
O. chinensis	(Och07, Och08, Och11, Och13)	0.00281	(0.00290, 0.00285)
O. rehderiana	(Ore01, Ore02, Ore03, Ore04)	0.00201	(0.00197, 0.00204)
O. rehderiana	(Ore01, Ore02, Ore03, Ore05)	0.00197	(0.00194, 0.00201)

Supplementary Table 25. Statistics of whole genome heterozygosity rate of five species.

Sample ID	Species	SNVs	Available Sites	Heterozygosity Rate
Ore01[1]	*O. rehderiana*	1,202,276	348,380,310	0.00345
Ore02[1]	*O. rehderiana*	1,053,014	348,380,310	0.00302
Ore03[1]	*O. rehderiana*	1,008,837	348,380,310	0.00290
Ore04[1]	*O. rehderiana*	1,028,453	348,380,310	0.00295
Ore05[1]	*O. rehderiana*	849,675	348,380,310	0.00244
Ore06[1]	*O. rehderiana*	861,037	348,380,310	0.00247
Ore07[1]	*O. rehderiana*	917,111	348,380,310	0.00263
Ore08[1]	*O. rehderiana*	726,834	348,380,310	0.00209
Ore09[1]	*O. rehderiana*	798,670	348,380,310	0.00229
Ore10[1]	*O. rehderiana*	836,267	348,380,310	0.00240
Ore11[1]	*O. rehderiana*	803,818	348,380,310	0.00231
Ore12[1]	*O. rehderiana*	854,450	348,380,310	0.00245
Ore13[1]	*O. rehderiana*	725,514	348,380,310	0.00208
Ore14[1]	*O. rehderiana*	935,007	348,380,310	0.00268
Och01[1]	*O. chinensis*	1,586,252	347,152,468	0.00457
Och02[1]	*O. chinensis*	1,716,550	347,152,468	0.00494
Och03[1]	*O. chinensis*	1,663,273	347,152,468	0.00479
Och04[1]	*O. chinensis*	1,964,795	347,152,468	0.00566
Och05[1]	*O. chinensis*	1,895,360	347,152,468	0.00546
Och06[1]	*O. chinensis*	1,495,291	347,152,468	0.00431
Och07[1]	*O. chinensis*	1,936,542	347,152,468	0.00558
Och08[1]	*O. chinensis*	1,554,026	347,152,468	0.00448
Och09[1]	*O. chinensis*	1,907,822	347,152,468	0.00550
Och10[1]	*O. chinensis*	2,058,957	347,152,468	0.00593
Och11[1]	*O. chinensis*	1,855,620	347,152,468	0.00535
Och12[1]	*O. chinensis*	1,113,191	347,152,468	0.00321
Och13[1]	*O. chinensis*	1,120,341	347,152,468	0.00323
Och14[1]	*O. chinensis*	1,616,626	347,152,468	0.00466
Cfa01[2]	*C. fangiana*	1,376,177 / 1,385,189	260,070,399 / 262,657,893	0.00529 / 0.00527
Cco02[2]	*C. cordata*	1,292,338 / 1,295,496	269,048,165 / 271,978,226	0.00480 / 0.00476
Cco03[2]	*C. cordata*	1,244,986 / 1,218,357	262,354,431 / 265,266,689	0.00475 / 0.00459
Ono00[2]	*O. nobilis*	686,480 / 652,923	201,892,366 / 204,088,725	0.00340 / 0.00320

[1]Those samples were mapped to their genome reference, respectively. The heterozygosity were calculated as SNVs/Available sites, where the available sites is a genome size without unknown base pairs.
[2]Those four samples don't have genome reference, so we mapped their reads to *O. rehderiana* and *O. chinensis*, respectively. The heterozygosity were calculated as SNVs/Available_sites, where the available sites represent their covered sites.

Supplementary Table 26. Heterozygosity rate distribution across the *O. rehderiana* and *O. chinensis* genomes.

Sample ID	Species	Windows	Median Heterozgosity Rate	Mean Heterozgosity Rate
Ore01[1]	*O. rehderiana*	6,768	0.00286	0.00344
Ore02[1]	*O. rehderiana*	6,768	0.00236	0.00302
Ore03[1]	*O. rehderiana*	6,768	0.00212	0.00288
Ore04[1]	*O. rehderiana*	6,768	0.00212	0.00295
Ore05[1]	*O. rehderiana*	6,768	0.00173	0.00244
Ore06[1]	*O. rehderiana*	6,768	0.00182	0.00247
Ore07[1]	*O. rehderiana*	6,768	0.00204	0.00263
Ore08[1]	*O. rehderiana*	6,768	0.00135	0.00209
Ore09[1]	*O. rehderiana*	6,768	0.00142	0.00229
Ore10[1]	*O. rehderiana*	6,768	0.00173	0.00241
Ore11[1]	*O. rehderiana*	6,768	0.00178	0.00231
Ore12[1]	*O. rehderiana*	6,768	0.00196	0.00246
Ore13[1]	*O. rehderiana*	6,768	0.00208	0.00268
Ore14[1]	*O. rehderiana*	6,768	0.00134	0.00208
Och01[1]	*O. chinensis*	6,456	0.00391	0.00454
Och02[1]	*O. chinensis*	6,456	0.00395	0.00499
Och03[1]	*O. chinensis*	6,456	0.00400	0.00484
Och04[1]	*O. chinensis*	6,456	0.00448	0.00568
Och05[1]	*O. chinensis*	6,456	0.00444	0.00549
Och06[1]	*O. chinensis*	6,456	0.00305	0.00432
Och07[1]	*O. chinensis*	6,456	0.00469	0.00562
Och08[1]	*O. chinensis*	6,456	0.00381	0.00451
Och09[1]	*O. chinensis*	6,456	0.00460	0.00553
Och10[1]	*O. chinensis*	6,456	0.00489	0.00595
Och11[1]	*O. chinensis*	6,456	0.00437	0.00534
Och12[1]	*O. chinensis*	6,456	0.00238	0.00322
Och13[1]	*O. chinensis*	6,456	0.00233	0.00323
Och14[1]	*O. chinensis*	6,456	0.00384	0.00472

[1]Those samples were mapped to their genome reference, respectivly. The heterozygosity for each window was colculated as SNVs/Available sites, where the available sites is a genome size without unknown base pairs. We setted the window size to 50 kb and the avaliable sites at least to 30 kb.

Supplementary Table 27. A comparison of the number of SNVs between different *O. rehderiana* and *O. chinensis* individuals with different sequencing depths.

Thin sample id	Total SNVS	Overlaped with raw sample		Specific SNVs	
		Number	Percent	Number	Percent
Och01 (10 ×)	1,216,438	1,178,173	96.85%	38,265	3.15%
Och01 (20 ×)	1,458,011	1,407,718	96.55%	50,293	3.45%
Och04 (10 ×)	1,487,267	1,413,381	95.03%	73,886	4.97%
Och04 (20 ×)	1,820,301	1,741,467	95.67%	78,834	4.33%
Och05 (10 ×)	1,449,186	1,376,801	95.01%	72,385	4.99%
Och05 (20 ×)	1,786,790	1,712,446	95.84%	74,344	4.16%
Och06 (10 ×)	1,130,679	1,064,612	94.16%	66,067	5.84%
Och10 (10 ×)	1,554,425	1,477,979	95.08%	76,446	4.92%
Och10 (20 ×)	1,902,354	1,816,548	95.49%	85,806	4.51%
Och11 (10 ×)	1,433,686	1,368,464	95.45%	65,222	4.55%
Och11 (20 ×)	1,733,397	1,663,845	95.99%	69,552	4.01%
Ore01 (10 ×)	981,715	963,420	98.14%	18,295	1.86%
Ore03 (10 ×)	829,998	804,044	96.87%	25,954	3.13%
Ore04 (10 ×)	835,364	808,188	96.75%	27,176	3.25%
Ore09 (10 ×)	653,052	627,772	96.13%	25,280	3.87%

All the samples with a depth greater than 20 × and/or 30 × were down-sampled to 10 × and/or 20 × by the Picard software (https://broadinstitute.github.io/picard/), and SNVs were identified and filtered with the previous standard method we used in the raw samples.

Supplementary Table 28. The comparison of LD decay between *O. rehderiana* and *O. chinensis* with randomly selected four individuals.

Species	Combination	r^2 (half LD decay)	Distance (half LD decay)
O. chinensis	(Och01, Och02, Och07, Och14)	0.2224	302,000
O. chinensis	(Och01, Och05, Och06, Och11)	0.2295	169,000
O. chinensis	(Och01, Och06, Och08, Och11)	0.2270	165,000
O. chinensis	(Och01, Och08, Och13, Och14)	0.2286	236,000
O. chinensis	(Och01, Och09, Och13, Och14)	0.2272	292,000
O. chinensis	(Och01, Och10, Och12, Och14)	0.2251	314,000
O. chinensis	(Och02, Och08, Och09, Och14)	0.2258	294,000
O. chinensis	(Och05, Och06, Och07, Och10)	0.2319	192,000
O. chinensis	(Och06, Och07, Och08, Och13)	0.2394	210,000
O. chinensis	(Och07, Och08, Och11, Och13)	0.2331	223,000
O. rehderiana	(Ore01, Ore02, Ore03, Ore04)	0.3471	444,000
O. rehderiana	(Ore01, Ore02, Ore03, Ore05)	0.3452	465,000

Supplementary Table 29. Counting the different mutation sites in each individual.

Sample ID	Species	Synonymous			Tolerated			Deleterious			Loss of Function		
		Heterozygous	Homozygous Derived	All Derived[1]	Heterozygous	Homozygous Derived	All Derived[1]	Heterozygous	Homozygous Derived	All Derived[1]	Heterozygous	Homozygous Derived	All Derived[1]
Ore01	*O. rehderiana*	12,306	108,952	230,210	4,578	36,997	78,572	1,510	5,206	11,922	177	467	1,111
Ore02	*O. rehderiana*	11,247	109,603	230,453	3,986	37,329	78,644	1,402	5,310	12,022	213	495	1,203
Ore03	*O. rehderiana*	11,920	109,283	230,486	4,293	37,165	78,623	1,433	5,300	12,033	171	497	1,165
Ore04	*O. rehderiana*	10,985	109,710	230,405	3,915	37,346	78,607	1,312	5,393	12,098	216	521	1,258
Ore05	*O. rehderiana*	10,911	109,744	230,399	3,871	37,363	78,597	1,303	5,400	12,103	212	521	1,254
Ore06	*O. rehderiana*	10,570	109,972	230,514	3,827	37,418	78,663	1,372	5,347	12,066	209	501	1,211
Ore07	*O. rehderiana*	11,121	109,672	230,465	3,997	37,335	78,667	1,387	5,324	12,035	217	495	1,207
Ore08	*O. rehderiana*	9,844	110,306	230,456	3,563	37,549	78,661	1,258	5,392	12,042	177	513	1,203
Ore09	*O. rehderiana*	8,865	110,793	230,451	3,327	37,645	78,617	1,137	5,428	11,993	146	509	1,164
Ore10	*O. rehderiana*	10,747	109,827	230,401	4,052	37,292	78,636	1,353	5,336	12,025	183	505	1,193
Ore11	*O. rehderiana*	11,104	109,655	230,414	3,912	37,357	78,626	1,343	5,313	11,969	203	495	1,193
Ore12	*O. rehderiana*	11,015	109,747	230,509	3,900	37,369	78,638	1,350	5,328	12,006	201	489	1,179
Ore13	*O. rehderiana*	10,733	109,879	230,491	3,920	37,388	78,696	1,355	5,344	12,043	198	494	1,186
Ore14	*O. rehderiana*	8,989	110,744	230,477	3,354	37,605	78,564	1,134	5,483	12,100	164	508	1,180
Och01	*O. chinensis*	26,496	92,571	211,638	9,477	31,640	72,757	3,409	4,283	11,975	741	752	2,245
Och02	*O. chinensis*	23,962	94,021	212,004	8,636	32,001	72,638	3,235	4,405	12,045	697	775	2,247
Och03	*O. chinensis*	25,155	93,400	211,955	8,836	31,927	72,690	3,276	4,350	11,976	709	768	2,245
Och04	*O. chinensis*	25,309	93,289	211,887	9,162	31,729	72,620	3,417	4,309	12,035	721	765	2,251
Och05	*O. chinensis*	24,976	93,524	212,024	9,005	31,911	72,827	3,469	4,297	12,063	746	765	2,276
Och06	*O. chinensis*	18,988	96,469	211,926	6,822	32,861	72,544	2,727	4,598	11,923	610	807	2,224
Och07	*O. chinensis*	28,319	91,647	211,613	9,858	31,459	72,776	3,546	4,165	11,876	783	723	2,229
Och08	*O. chinensis*	24,188	93,529	211,246	8,557	31,940	72,437	3,235	4,335	11,905	669	761	2,191
Och09	*O. chinensis*	25,523	93,168	211,859	8,975	31,903	72,781	3,307	4,290	11,887	745	757	2,259
Och10	*O. chinensis*	25,757	93,062	211,881	9,070	31,860	72,790	3,357	4,270	11,897	765	751	2,267
Och11	*O. chinensis*	25,000	93,489	211,978	8,887	31,947	72,781	3,234	4,302	11,838	719	766	2,251

Och12	*O. chinensis*	20,632	95,458	211,548	7,438	32,530	72,498	2,760	4,573	11,906	623	810	2,243
Och13	*O. chinensis*	18,836	96,364	211,564	6,844	32,819	72,482	2,627	4,649	11,925	576	832	2,240
Och14	*O. chinensis*	26,498	92,368	211,234	9,316	31,628	72,572	3,344	4,204	11,752	716	751	2,218

[1]All Derived calculated by counting heterozygous genotypes once and homozygous-derived genotypes twice.

Supplementary Table 30. Gene ontology (GO) enrichment analysis of the DEL or LoF in at least one individual of *O. rehderiana*.

Type	GO ID	GO Terms	Number of enriched genes	Number of genes in background	*P*-value[1]
**DEL**					
MF	GO:0003674	molecular_function	2,966	16,622	3.151E-02
MF	GO:0003824	catalytic activity	1,849	9,973	2.388E-02
MF	GO:0005515	protein binding	852	3,930	2.388E-02
MF	GO:0008568	microtubule-severing ATPase activity	39	109	1.300E-02
**LoF**					
MF	GO:0003674	Molecular function	330	16,622	5.165E-03
MF	GO:0097159	organic cyclic compound binding	133	5,875	6.973E-03
MF	GO:1901363	heterocyclic compound binding	133	5,875	6.973E-03
MF	GO:0016740	transferase activity	90	3,954	2.727E-02
MF	GO:0036094	small molecule binding	80	3,175	3.394E-03
MF	GO:1901265	nucleoside phosphate binding	77	3,022	3.070E-03
MF	GO:0000166	nucleotide binding	77	3,022	3.070E-03
MF	GO:0017076	purine nucleotide binding	66	2,426	1.322E-03
MF	GO:0032553	ribonucleotide binding	66	2,421	1.254E-03
MF	GO:0032555	purine ribonucleotide binding	66	2,421	1.254E-03
MF	GO:0035639	purine ribonucleoside triphosphate binding	64	2,322	1.143E-03
MF	GO:0030554	adenyl nucleotide binding	62	2,196	7.590E-04
MF	GO:0032559	adenyl ribonucleotide binding	62	2,192	7.246E-04
MF	GO:0005524	ATP binding	60	2,095	6.555E-04
MF	GO:0016772	transferase activity, transferring phosphorus-containing groups	56	2,038	2.576E-03
MF	GO:0016301	kinase activity	48	1,741	4.958E-03
MF	GO:0016773	phosphotransferase activity, alcohol group as acceptor	40	1,419	7.117E-03
MF	GO:0004672	protein kinase activity	38	1,274	3.499E-03
MF	GO:0004674	protein serine/threonine kinase activity	18	600	3.652E-02
MF	GO:0016874	ligase activity	17	554	3.459E-02
MF	GO:0016879	ligase activity, forming carbon-nitrogen bonds	10	248	1.976E-02
MF	GO:0019787	ubiquitin-like protein transferase activity	7	160	3.239E-02
MF	GO:0004601	peroxidase activity	6	135	4.301E-02
MF	GO:0015297	antiporter activity	6	116	2.274E-02
MF	GO:0016684	oxidoreductase activity, acting on peroxide as acceptor	6	136	4.432E-02
MF	GO:0015238	drug transmembrane transporter activity	5	74	1.296E-02
MF	GO:0090484	drug transporter activity	5	77	1.519E-02
BP	GO:0043412	macromolecule modification	61	2,483	1.796E-02
BP	GO:0006464	cellular protein modification process	56	2,232	1.592E-02
BP	GO:0036211	protein modification process	56	2,232	1.592E-02
BP	GO:0016310	phosphorylation	42	1,712	4.734E-02
BP	GO:0006468	protein phosphorylation	36	1,211	4.690E-03
BP	GO:0048583	regulation of response to stimulus	13	392	3.590E-02
BP	GO:0080134	regulation of response to stress	10	229	1.194E-02
BP	GO:0006855	drug transmembrane transport	6	79	3.784E-03
BP	GO:0006804	obsolete peroxidase reaction	6	128	3.457E-02
BP	GO:0015893	drug transport	6	80	4.027E-03

[1]For DEL the P-value represent the adjusted *P*-vulae by the FDR correct, while for LoF is the raw *P*-value.

Supplementary Table 31. The number of the well-developed cymules of 30 randomly selected catkins for each of the five young (Offspring) and five old (Ore) trees.

Sample ID	The numer of the cymules for each catkin																													
	**1**	**2**	**3**	**4**	**5**	**6**	**7**	**8**	**9**	**10**	**11**	**12**	**13**	**14**	**15**	**16**	**17**	**18**	**19**	**20**	**21**	**22**	**23**	**24**	**25**	**26**	**27**	**28**	**29**	**30**
Offspring01	13	11	12	13	10	11	11	10	11	12	13	12	8	11	11	13	10	12	11	11	11	12	12	10	9	12	13	9	11	13
Offspring02	10	11	9	13	13	11	12	9	11	12	11	9	10	11	11	11	11	11	13	9	11	12	11	10	12	10	9	10	10	11
Offspring03	13	10	11	12	11	11	12	12	11	11	10	12	9	10	11	11	9	11	12	10	12	11	8	11	9	10	11	11	11	10
Offspring04	13	12	11	12	13	11	13	11	12	12	13	11	13	8	11	10	10	12	13	8	13	9	10	11	8	10	11	11	11	12
Offspring05	13	12	13	9	11	11	9	9	13	12	11	11	10	10	13	13	11	10	12	11	11	11	11	11	9	12	10	12	12	10
Ore01	13	16	12	17	15	15	15	14	14	17	12	16	15	14	14	13	14	15	16	13	15	14	14	11	16	13	16	16	12	14
Ore02	14	15	14	17	17	16	14	15	13	16	19	19	18	21	17	20	20	17	14	15	18	16	15	16	17	16	16	15	15	14
Ore03	13	15	13	12	13	9	12	14	10	11	15	13	12	14	11	14	14	14	12	13	11	14	13	13	13	11	12	12	13	13
Ore04	14	13	12	13	14	13	14	12	14	13	15	14	15	14	14	17	13	16	14	13	14	15	13	13	15	14	14	13	15	13
Ore05	11	10	12	14	12	12	13	13	12	13	13	12	13	14	13	11	11	12	13	12	11	11	11	9	11	10	9	9	9	11

Supplementary Table 32. Species and data used for comparative genomic analysis.

Species	Number of gene	Version	Number of original genes
Ostrya rehderiana	27,831	-	-
Ostrya chinensis	31,152	-	-
Arabidopsis thaliana	26,864	TAIR10[a]	27,416
Betula pendula	24,854	V1.4c Pseudochromosomes[b]	24,854
Carica papaya	26,249	ASGPBv0.4[a]	27,736
Juglans regia	32,436	-[c]	32,496
Fragaria vesca	32,697	V1.1[a]	32,831
Oryza sativa ssp. Japonica	34,047	V7.0[a]	37,869
Prunus persica	27,861	V2.1[a]	27,864
Ricinus communis	29,911	V0.1[a]	31,221
Vitis vinifera	25,400	Genoscope.12X[a]	26,346

[a]Genome annotations were download from JGI (PhytozomeV10)
[b]Genome annotation was download from https://genomevolution.org/CoGe/GenomeInfo.pl?gid=35080
[c]Genome annotations was download from http://http://dendrome.ucdavis.edu/ftp/Genome_Data/genome/Reju/

Supplementary Methods

Supplementary Method 1. RNA sequencing and assembly

Four tissues (roots, leaves, phloem and xylem) from the wild *O. rehderiana* (Ore01) were used to isolate RNA. Total RNA was extracted using a CTAB procedure[1]. The quality and integrity of the RNA samples were examined using the Agilent 2100 Bioanalyzer and their RIN (RNA Integrity Number) values ranged from 8.6 to 10.0, with no sign of degradation. About 20μg total RNA from each sample was used to construct cDNA libraries with a 200bp insert size, and the sequencing was done using Illumina Genome Analyzer platform. Raw reads were filtered for adaptors using SCYTHE (https://github.com/vsbuffalo/scythe) and low quality sequence by SICKLE (https://github.com/najoshi/sickle). After filtering, we assembled the RNA data by Trinity v2.0[2] for the combined reads and the each four tissues separately. The RSEM[3] package within Trinity was used to obtain abundance estimates for the transcriptome assemblies. The final assemblies retained transcripts with FPKM values greater than 1 in at least one tissue, to ensure that all of the included transcripts could be detected.

Supplementary Method 2. Estimation of genome size based on 17-mer analysis

We estimated the genome size using the k-mer method and the formula: G = k-mer_number/k-mer_depth, where the k-mer number is the total number of k-mers, and k-mer depth denotes the mode of the k-mer depth[4]. In this report, the k-mer size was 17, k-mer_number was 10,419,236,548 and 10,429,000,485 for *O. rehderiana* and *O. chinensis*, respectively. The k-mer_depth was 27 for both species, so the genome size was estimated to be 385,897,650 bp and 386,259,277 for *O. rehderiana* and *O. chinensis*, respectively (Supplementary Fig. 1, Supplementary Table 2).

Supplementary Method 3. Repeat annotation

Transposable elements were identified in the *O. rehderiana* and *O. chinensis* genomes by combining *de novo* and homology-based approaches. Transposable elements were identified at both DNA and protein levels, based on known sequences contained within the DNA repeat database (Repbase v21.01)[5], using both RepeatMasker v4.0.5[6] and RepeatProteinMask v4.0.5 (a package within RepeatMasker). We used three *de novo* prediction programs: LTR_FINDER v1.0.6[7], PILER v1.0[8] and RepeatModeler v1.0.8 (http://www.repeatmasker.org/RepeatModeler.html) to build *de novo* repeat database of each genome. After the raw *de novo* repeat library was constructed, cd-hit-est[9] with the parameter '-c 0.8' was used to remove redundancy. The RepeatClassifier module in the RepeatModeler package was used to classify the identified repetitive elements of the *de novo* repeat database based on Repbase. LTR retrotransposons identified by LTR_FINDER were classified as "unclassified LTR" if RepeatClassifier given an "Unknown" result. We then used RepeatMasker to identify repeats using both the *de novo* built repeat database and Repbase. Then we combined the

de novo prediction and the homolog prediction of transposable elements according to the coordination in the genome. Tandem repeats were annotated in each genome by RepeatMasker and Tandem Repeats Finder (TRF, v4.07)[10].

To infer the insertion time of LTRs, full-length LTRs were identified using LTR_FINDER and recovered in the *de novo* repeat library. Muscle[11] was used to align LTRs and the evolutionary distances between 5' and 3' solo LTRs were calculated with the Kimura two-parameter model by dnadist program implemented in the Phylip package (v3.695, http://evolution.genetics.washington.edu/phylip.html). The LTRs insertion time was estimated by the formula T=R/2×u (T, insertion time; R, substitution rate; u, mutation rate). The mutation rate of 1.5 ×1e-8 substitutions per site per year, 2-fold higher than the synonymous substitution rate of the coding region[12].

Supplementary Method 4. Gene prediction

We conducted the gene annotation in the *O. rehderiana* genome by combining homology information, *de novo* prediction, and RNA-seq. For homology-based prediction, protein sequences of five different species (*A. thaliana*, *C. papaya*, *F. vesca*, *P. persica*, *V. vinifera*) were mapped onto the repeat-masked genome using *TblastN* with an E-value cutoff 1e-5. The aligned sequences as well as their corresponding query proteins were then filtered and passed to GeneWise[13] to search for accurate spliced alignments. For RNA-seq, we used PASA[14] to align the transcripts from the combined tissue assemblies to the genome and build the PASA assemblies. Open reading frames (ORFs) were identified from these PASA assemblies, and we retained genes with complete structures while removing some redundant genes with 70% identity by cd-hit-est to generate the training set. Four *de novo* gene predictors were used on the repeat-masked genome. GlimerHMM v3.0.4[15], SNAP v2006-07-28[16], Augustus v3.2.1[17] and GeneMark-ET v3.49[18] were trained with the training set generated by PASA. EVidenceModeler (EVM)[19], which is an effective automated annotation combiner, was used to integrate the predicted genes with the *de novo*, homology-based search, and RNA-seq data to form a consensus gene set. The gene models were updated by PASA with two rounds to modify exons or gene models with added UTRs and identified alternatively splice isoforms. The genes which encode proteins with fewer than 50 amino acids were removed. Gene prediction for the *O. chinensis* genome employed the similar homology- and *de novo*- based approaches. For homology-based prediction, the same procedure was performed, and 3000 complete gene sets (with the start and end codons) were selected as the training set. The four *de novo* gene predictors were also used on the repeat-masked genome and trained with the homology-based training set. EVM integrated the predicted genes with the *de novo* and homology-based search.

Gene functions were assigned according to the best match of the alignments using *BLASTP* (E-value≤1e-5) searching against NCBI NR (National Center for Biotechnology Information nonredundant) databases, SwissProt and TrEMBL databases. Gene Ontology IDs for each gene were obtained from NR hits using the Blast2GO[20] program with default parameters. The motifs and

domains of genes were determined by InterProScan[21] against protein databases. The metabolic pathways were mapped using the KAAS[22] (KEGG Automatic Annotation Server) with a bi-directional best-hit strategy to assign KEGG orthology terms (KO) to each gene. The identified pathways were settled using their respective KO assignments.

The tRNA genes were identified by tRNAscan-SE[23]. For rRNA identification, we first downloaded the *Arabidopsis* rRNA sequences from NCBI (http://www.ncbi.nlm.nih.gov/guide/dna-rna/). Then rRNAs in the database were aligned against the genome using *BlastN* to identify possible rRNAs. Other noncoding RNAs, including miRNA, snRNA, were identified using INFERNAL[24] by searching against the Rfam database.

Supplementary Method 5. Genome synteny and whole genome duplication analysis

MCScanX[25] was employed to identify syntenic blocks within and between *O. rehderiana*, *O. chinensis*, *J. regia*, *P. persica* and *F. vesca* based on 'all-versus-all' *BLASTP* (E-value ≤ 1e-5) alignments. After filtering tandem duplications gene, syntenic blocks containing more than five genes were retained for the following analysis. For ortholog/paralog genes in syntenic blocks, pairwise synonymous substitutions per synonymous sites (KS) were estimated by the add-ka-and-ks-to-collinearity program in the MCscanX[25] software, with Nei-Gojobori statistics. A total of 20,683/19,512 genes were located in the syntenic blocks in the *O. rehderiana* and *O. chinensis* genomes, separately. Divergence time estimates were based on histogram peak Ks values of the youngest pairs and a molecular clock of 6.5 × 10e-9 synonymous substitutions per site per year[26]. We identified paralogs within each genome in a similar way as was conducted in the analysis of the Norway spruce genome[27]. We use *BlASTP* (identity ≥ 40%; E-value ≤ 1e-5; match length of more than 100 amino acids) to detect homologous genes within and between *O. rehderiana*, *O. chinensis*, *J. regia*, *P. persica* and *F. vesca*. OrthoMCL[28] was performed to detect gene families. For each paralogous gene pair, the distance over 100 kb was retained to mask the tandem duplications' effects. Ks estimates were obtained by the CODEML program from the PAML[29] package for each pair. For the paralogous gene pairs within one species, a further correction was performed using the method described by Maere et al[25]. Briefly, for each gene family, a tentative phylogenetic tree was constructed by average linkage hierarchical clustering, using Ks as a distance measure. For each split in the resulting tree corresponding to a duplication event, all Ks estimates between two child clades were added to the Ks distribution with a weight 1/m, so that the weights of all Ks estimates for a single duplication event sum up to one.

Supplementary Method 6. Inferring relationships

To identify the relationships between the sequenced samples, a neighbor-joining (NJ) tree were firstly constructed. PLINK v1.07[30] was used to obtain the matrix of pairwise genetic distances of all samples (dataset 3) and Phylip v3.695 was used to build the NJ tree. The identity-by-descent (IBD) blocks for all sampled individuals were identified by using the algorithm from BEAGLE v4.0[31] with

the following parameters: window = 100,000; overlap = 10,000; ibdtrim = 100. We expected no IBD block between *Ostryopsis* and *Ostrya*, *Carpinus*, which was satisfied using a minimum LOD score of 15. A further analysis of relatedness between the sampled individuals were performed using the KING[32] program, which infers degrees of relatedness based on a pairwise comparison of SNP data for individuals.

Supplementary Method 7. Estimated the inbreeding coefficient, LD decay and recombination rate

We identified the runs of homozygosity (ROH) for each individual for dataset 3 using the VCFtools v0.1.12b with "--LROH"[33,34]; ROH longer than 100 kb were retained. The individual genome-based inbreeding coefficient, denoted as FROH, was calculated as the fraction of total ROH length to genome effective length[35]. LD decay for *O. rehderiana* and *O. chinensis* (dataset 1 and dataset 2) were calculated within a range of 500 kb using Haploview[36]. Genome-wide LD decay was calculated by grouping SNP pairs into 1-kb bins and averaging the squared correlation coefficient (r^2) within bins. The ρ was calculated by the FastEPRR v1.0[37] for the two species with the sliding-window size of 50kb.

Supplementary Method 8. Demographic history

We inferred a demographic history for *O. rehderiana* and *O. chinensis* using the Multiple Sequentially Markovian Coalescence model (MSMC)[38] with multiple individuals. The MSMC method estimates the recent population size changes at a higher resolution when more haplotypes are used[38]. The mappability mask for *O. rehderiana* genome was generated by the SNPable (http://lh3lh3.users.sourceforge.net/snpable.shtml) program. All segregating sites (dataset 1 and dataset 2) in each population were generated with SAMtools mpileup[39] and phased and imputed with SHAPEIT[40]. Four individuals from each species with a relatively high coverage and low relatedness were selected to run MSMC. For *O. rehderiana*, Ore01, Ore02, Ore03 and Ore04 were selected, and for *O. chinensis*, Och01, Och05, Och06 and Och10 were selected. To detect the impact of relatedness on our demographic estimates, we compared different combinations of trees with different levels of relatedness within *O. rehderiana*. The combinations included: 1. eight haplotypes, which contained four wild trees (Ore1-Ore4, but excluding Ore5, a likely twin of Ore4); 2. six haplotypes, which contained three trees with less than a 3rd-degree relationship inferred by the King software[33]; 3. Four haplotypes, which contained two trees with less than a 3rd-degree relationship inferred by the King software.

Supplementary Notes

Supplementary Note 1

Higher heterozygosity was observed in *O. chinensis* than that in *O. rehderiana* by the k-mer analysis (Supplementary Fig. 1), which created greater complexity and difficulty for assembling the *O. chinensis* genome. Thus, we had to sequenced more NGS data to assemble the complicated *O. chinensis* (345.48×) than *O. rehderiana* (128.40×) genome, and selected Platanus v1.2.4[41] to assembly the genomes, which has a high performance when handling the high heterozygosity during the assembly. Short insert libraries (insert size < 2 kb) were used to construct the contigs using a de Brujin graph with a series of k-mers from 32 to 122 (step size was set 10). The long insert libraries (insert size >= 2 kb) were used in the scaffolding step to link the contigs made from the short insert libraries and close gaps using Platanus. We adopted a serial evaluation process on the two genome assemblies because the assemblies' quality were influenced by the sequencing depth and genome complexity. The assembled scaffolds represented 94.9% and 96.2% of *O. rehderiana* and *O. chinensis* genomes, respectively, and the GC content was 35.52% and 36.12%, which is similar to silver birch (35.67%)[42] and *J. regia* (36.32%)[43] (Fig. 1, Supplementary Figs. 1 and 2a). Mapping all of the paired-end reads to their genomes revealed that more than 95% of the sequence had a coverage depth greater than 20 × in both genomes (Supplementary Figs. 1 and 2b), ensuring a high single-base accuracy. Further quality analysis revealed that, 1) 97.94%-98.18% of all sequenced reads were mapped to their reference genomes (Supplementary Table 4), 2) more than 98% of the set of core eukaryotic genes (CEGMA)[44] were partially represented in the two genomes (Supplementary Table 5), 3) 96.20%-97.00% of all universal single-copy orthologs in the BUSCO's embryophyta benchmark set[45] were present and complete in both genomes (Supplementary Table 6), and 4) 85.55%-99.97% of the RNA-seq data set could be mapped onto the *O. rehderiana* genome (Supplementary Table 7). These results suggest that the *de novo* genomes of both species were well assembled with high contiguity, coverage and accuracy.

Using a combination of homology-based searches and *de novo* annotation, we found that ~51.10% and ~51.08% of the *O. rehderiana* and *O. chinensis* genomes, respectively, are composed of repetitive elements (Supplementary Fig. 1, Supplementary Table 8). Retrotransposable elements, known to be the dominant form of repeats in angiosperm genomes[46], constituted the most abundant subtypes in both genomes (LTR: 31.82% in *O. rehderiana* and 33.04% in *O. chinensis*; LINE/L1: 6.59% in *O. rehderiana* and 7.07% in *O. chinensis*; SINE: 0.15% in *O. rehderiana* and 0.16% in *O. chinensis*; Supplementary Table 9). In addition, the percentage of *de novo* predicted repeats was notably larger than that obtained for repeats based on Repbase (version 21.01)[5], indicating that both genomes of *O. rehderiana* and *O. chinensis* have many unique repeats compared to other sequenced plant genomes. Among these elements, long terminal repeats (LTRs) were the most dominant type and after calculating their times of insertion, we discovered that a burst of LTR activity occurred

during the last four million years. Most transposable elements had a divergence rate around 11% and 13% in the *O. rehderiana* or *O. chinensis* genome, respectively (Supplementary Fig. 3).

A total of 27,831 and 31,152 protein-coding genes were predicted to be present in the *O. rehderiana* and *O. chinensis* genomes, respectively (Supplementary Table 10). Both *O. rehderiana* and *O. chinensis* have, on average, longer genes than most other sequenced plant species, but similar to those of the *B. pendula* and *J. regia* genomes with longer average intron lengths (Supplementary Fig. 4, Supplementary Table 11). Therefore, this feature might be common in the genomes of Betulaceae and Juglandaceae. In addition, we identified 204/221 microRNAs, 125/129 transfer RNAs, 566/552 ribosomal RNAs, and 113/109 small nuclear RNAs in the *O. rehderiana* and *O. chinensis* genomes, respectively (Table 1, Supplementary Table 12). Functional annotation confirmed that 86.05% genes from *O. rehderiana* and 84.30% genes from *O. chinensis* had known homologues in protein databases, including InterPro, GO, Swissprot, TrEMBL and KEGG databases (Supplementary Table 13).

Supplementary Note 2

The distribution of synonymous substitutions per synonymous site (Ks) across all paralogous genes (regardless of gene order) and for duplicated genes lying in synteny blocks (Fig. 1) were calculated to explore the potential for whole-genome duplications (WGD). The results showed one strong peak in both genomes at Ks values 0.96 (*O. rehderiana*) and 1.21 (*O. chinensis*), suggesting no recent WGD has occurred in these two Betulaceae species (Supplementary Figs. 5 and 6). However, two peaks of *J. regia* at Ks values 0.33 and 1.04, suggest one more recent WGD, similar to the previous analysis[43]. The peak at the Ks value between 0.88-1.21 was shared by six species, *O. rehderiana*, *O. chinensis*, *J. regia*, *P. persica*, *F. vesca* and *V. vinifera*, suggesting a common WGD before their divergences. Dating of the WGDs suggests that the common WGD occurred 135-186 Mya, and the divergence between *O. rehderiana* and *O. chinensis* was ~4.6 Mya. We also used the phylogenetic analysis to estimate divergence times of 10 species based on genes extracted from a total of 1,896 single-copy families (Supplementary Fig. 7a). *O. rehderiana* and *O. chinensis* were closely related and diverged approximately 6.95 million years ago (Mya) (Supplementary Fig. 7b). The common ancestor of *O. rehderiana* and *O. chinensis* was closely related to *J. regia*, and our phylogeny suggests that the Juglandaceae and Betulaceae separated approximately 61 (44.8-76.2) Mya. The divergence time inferred from the phylogenetic analysis was approximate to that estimated from the WGD analysis.

Supplementary Note 3

A total of 10,830 gene families were shared among four Juglandaceae and Betulaceae species and at least one other species (Supplementary Fig. S8, Supplementary Table 14). The 243 unique gene

families in *O. rehderiana* were enriched in 19 GO categories with the main functions in hydrolase activities, cell wall macromolecule, and other sugar metabolic process (Supplementary Table 15). The 434 unique gene families in *O. chinensis* were significantly enriched in 21 GO categories with the main functions in TOR signaling, and leaf and root development (Supplementary Table 15). By analyzing orthologous gene families across 11 species, 526 ones expanded and 1,003 ones contracted for *O. rehderiana*, 880 ones expanded and 750 ones contracted for *O. chinensis*. In addition, 590 ones expanded and 696 ones contracted for the ancestor lineage of the two ironwood species (Supplementary Fig. 9). The expanded gene families in *O. rehderiana* and *O. chinensi* were enriched in 136 or 93 GO categories, which were mainly associated with the oxidoreductase activity, ion channel activity, signaling, cellulose/lignin metabolism and binding (Supplementary Tables 16 and 17). The expanded gene families in the ancestral lineage of two ironwood species were enriched in 158 GO categories (Supplementary Table 18) with a primary functions of lignin catabolism (GO:0046274, P=1.30E-03), hydrolase activity (GO:0016787, P=1.41E-06), β-galactosidase activity (GO:0009341, P=6.36E-13), and membrane (GO:0016020, P=3.23E-33). We further detected 43 families related to wood formation that were substantially expanded by tandem duplication within both iron-wood species compared with the silver birch (Supplementary Table 19). For example, eight gene families encoding key enzymes involved in the lignin biosynthesis (e.g. 4CL, HCT, COMT, LAC, C3H, CSE, CCoAOMT and CAD) were expanded in both ironwood species. These enzymes have key roles in the biosynthesis of three units of lignin, which provides the compression strength to woody cell walls[47,48]. Several gene families encoding glycoside hydrolases (e.g. GH1, GH10, GH28, GH3, GH35, GH79 and GH9) also had more copies in both ironwood species than in silver birch. These hydrolases play important roles in cellulose and hemicellulose biosynthesis, lignification, and modification of cell wall components[49-51]. Other expanded gene families include those encoding glycosyltransferase, such as GT8 and XGT, and genes involved in cell wall development, such as NSE, Expansin, LRRP and FLAs[52]. Two members of the FLA gene family, AtFLAs 11 and 12, were demonstrated to be critical in regulating biomechanical properties of plant stems, mutants of which resulted in reduced stem tensile strength and stiffness due to rapid changes in the molecular composition and architecture of the secondary cell wall[53]. The expansion of these gene families in both iron-wood species may account for the formation of the extremely hard wood of the genus *Ostrya*.

Supplementary Note 4

We used two methods to estimate the mutation rate of *Ostrya*. We first inferred the mutation rate based on the fossil-calibrated approach as: $\mu = D \times g / 2 \times T$, where D is the observed frequency of pairwise differences between two species, T is the estimated divergence time, and g is the estimated generation time for two species. To estimate the parameter D, the 1:1 orthologs between *O. nobilis* (no published gene data) and *O. rehderiana*, *O. chinensis* were identified by InParanoid[54] and the alignment for each single copy gene was generated by Muscle[11]. The four-fold degenerate sites were

extracted from the alignments and the frequency of pairwise differences between two species was calculated. The estimated divergence was assumed to be ~34 Myr based on a previous study on *Ostryopsis* and *Ostrya*[55,56]. These values yielded an estimated mutation rate of 10.07E-10/9.79E-10 per site per year for *O. rehderiana* and *O. chinensis*, respectively. We used the average mutation rate to represent the mutation rate of *O. rehderiana* and *O. chinensis* with a value of 9.93E-10 per site per year (Supplementary Table 21).

The second method that we used estimated the mutation rate between parents and offspring. We directly estimated the germline mutation rate in *O. rehderiana* based on the pedigree of the sequenced individuals. To gain insight into the relationships among the individuals, we first examined the kinship among the sampled individuals by using identical-by-descent (IBD) haplotypes using BEAGLE[57] and the relatedness analysis using the KING program[33]. Numerous IBD haplotypes were shared between *O. rehderiana* individuals while all sampled individuals from *O. chinensis* shared few IBD haplotypes (Supplementary Fig. 15a). Our KING relatedness analyses suggested that two large trees of *O. rehderiana*, Ore04 and Ore05, are twins from the same parents, and both are relatively distantly related to the others. The nine young trees (Ore06 – Ore14) appeared to share one parent, with a first-degree kinship with one old large tree, Ore02 (Fig. S15b). The other parent of five of these nine individuals was likely Ore01 (1 individual) or Ore03 (4 individuals) based on a first-degree kinship. The other parent of the remaining four offspring remained unclear.

We used the five confirmed F1 offspring and their two parents, Ore02 and Ore01 or Ore03, to estimate mutation rates. Variants were called with GATKs HaplotypCaller[58], and we applied an extensive set of hard filters to increase the likelihood of only calling true variants, which used in the bird germline mutation rate estimator[59]. Repetitive regions, which including transposable elements and tandem repeats were masked. Then each site had to pass GATKs CallableLoci threshold, which required a quality score of at least 20, mapping Quality of at least 30, and genotype quality (GQ) of at least 30. For the insertions and deletions (indels), after we set a cutoff quality score of 30, we identified 527,062 indels segregating in the pedigree. Since we only considered single nucleotide variants (SNV) in this study, all called insertion and deletions (indels) were masked and SNVs at or within 5bp from any indels were masked.

A hard coverage threshold of 6 (~50% of the average depth of all samples) to twice the average depth for each individual were used to minimize false variant calls due to insufficient read data. This represents a very stringent per-site coverage filter and was considered important to reduce the initial frequency of false positives before further quality control[59]. After the filtering we were left with 263-345 Mb per individual (average 275 Mb per individual).

Screening for new mutations represents a challenging task and has to be treated with the utmost care[60-62]. We applied extremely stringent filtering in attempts to minimize the false-discovery rate, which is similar to the previous studies[59,62]. For each offspring, heterozygous positions were extracted from the background and had to meet the following criteria to be considered as potential *de novo* mutations:

(1) At least 25% of the reads support the alternative allele,

(2) Does not overlap with known SNPs from all genomic resequencing of *O. rehderiana*,

(3) Both parents are homozygous and containing no alternative reads.

A total of 32 *de novo* mutations were identified, when considering the candidate five offspring and their parents, there was a mean of 6.2 mutation events per meiosis. The rate of mutation can thus be estimated to be 2.1818E-08 per site per meiosis (Supplementary Table 22). This mutation rate was slightly higher than *Arabidopsis*[62,63].

The *Ostrya* trees reach maturity at approximately 10-20 years and continue to produce seed and pollen throughout their lives (for example, one around 300-years-old wild *O. rehderiana* can still set seeds). Therefore, we chose three generation times of 10, 15, and 20 years and two-mutation rates of 2.18182E-08 per site per generation and 9.929×10^{-10} per site per year to investigate the demographic history of the two iron-wood species. Our results indicate that a mutation rate of 2.18182E-08 per site per generation and the generation time of 10 year resulted in the best fit relative to historical changes in climate (Supplementary Fig. 12). In our research, the parent–offspring mutation rate (2.18182E-08 per site per generation) was higher than the fossil-calibrated mutation rate (9.93E-10 per site per year) (Supplementary Tables 21 and 22). Differences in the estimated mutation rates between the fossil-calibrated approach and the germline sequencing approach are common, as the mutation rates are influenced by selection and drift[64-67] and each approach has different assumptions and shortcomings. The fossil-calibrated approach may overestimate the mutation rate because fossils always appear later than the origin of a lineage[68,69]. The direct parent–offspring sequencing mutation rate is also affected by random error, fast accumulation of deleterious variations, and varying mutations between heterozygous and homozygous individuals[62,70]. Trees are long-lived and produce seeds over a long time period, and therefore the generation time is particularly difficult to estimate precisely[71]. All these caveats may affect the precise estimations of the mutation rates.

Supplementary Note 5

We calculated the ROH regions along each individual by the VCFtools v0.1.12b[35]. Both the total ROH length (8.36~24.79 Mb) and the longest ROH length (0.19~0.63 Mb) in *O. chinensis* were shorter than that in *O. rehderiana* (total: 74.9~143.74 Mb, longest: 1.58~3.98 Mb, Supplementary Fig. 19a). We also compared the total ROH length between the wild trees and the offspring trees by considering three categories of the ROH length: all the identified ROH, ROH with lengths longer than 500 kb, and ROH with lengths longer than 1 Mb, and two comparisons: between all the wild individuals and offspring, or between the candidate parents (Ore01, Ore02 and Ore03) and their offspring. We found that the mean length of each type of ROH were longer in offspring than that in the wild individuals, but this difference is not significant (Supplementary Fig. 19b). When we

considered only the long ROH categories (larger than 500 kb or larger than 1 Mb) for candidate parents and offspring, the difference was statistically significant ($p < 0.05$, Fig. S19b). We also compared the total ROH length between the each predicted parent and its offspring, and most showed a significant difference ($p < 0.05$, Supplementary Fig. 19c). In summary, our results indicated with longer ROH in the offspring[72,73]. We also found that Ore04 and Ore05 exhibited a very long ROH relative to other comparisons, suggesting that they are the offspring of two closely related parents.

Supplementary Note 6

To address the impact of relatedness in the *O. rehderiana* individuals on our population parameters, we adopted a series of comparisons. For genetic load analyses, we only used the wild trees of two ironwood species, and the results were similar to those with all individuals of *O. rehderiana*: *O. chinensis* contained more derived synonymous, tolerated and deleterious variants, but fewer LoF variants than *O. chinensis*. For analyses of the genetic diversity (Pi) and LD decay, we deleted one of the likely twin individuals, and retained only four trees in *O. rehderiana* (“Ore01, Ore02, Ore03, Ore04” or “Ore01, Ore02, Ore03, Ore05”). For *O. chinensis*, we also randomly selected ten datasets with only four individuals for comparison. We found that all observed *Pi* mean values and 95% CI were lower in *O. rehderiana* than in *O. chinensis* (Supplementary Table 24), and all detected distances of the maximum r^2 were longer in *O. rehderiana* than in *O. chinensis*, indicating a lower LD decay in *O. rehderiana* (Supplementary Fig. 21, Supplementary Table 28). For analyses of the demography histories, we compared the following combinations to test the impact of relatedness in *O. rehderiana*: 1. eight haplotypes, which contained only four wild trees after deleting one likely twins; 2. six haplotypes, which included three diploid trees after deleting two trees with close relatedness to the sampled threes; 3. Four haplotypes, which included only two diploid trees with the highly distant relationship. All analyses showed a similar population demography trajectory (Supplementary Fig. 13).

Supplementary Note 7

We focused on DEL and LoF variants of *O. rehderiana*. A total of 6,305 DEL variants affecting 4,317 genes, and a total of 627 LoF variants affecting 598 genes were homozygous in at least one individual of *O. rehderiana* (Supplementary Table 29, Supplementary Data files 1 and 2). These genes were mainly enriched in GO categories related to molecular function, including catalytic activity, protein binding, ATP binding, and nucleotide binding (Supplementary Table 30). Among them, twos were associated with ion transport: *AtHIPP28* (*OreG0016322*) encoding a protein belonging to heavy metal transport/detoxification superfamily protein, which is involved in copper ion binding and transport; *AtMTP5* (*OreG0000577*) encodes a protein that interacts with *AtMTP12*

to form a complex to transport Zn into the Golgi. We also found that twos are associated with embryo defects: *AtDPG1* (*OreG0026000*), also known as *EMB1273*, plays an essential role in early chloroplast biogenesis, and its absence triggers chloroplast-to-nucleus retrograde signaling; *AtRRP44* (*OreG0003219*), known as *EMB2763*, is essential for viability and required for RNA processing and degradation. The loss function of these genes may partly contribute to the threatened status of *O. rehderiana*.

Supplementary Note 8

In order to compare components of reproductive fitness for the old and young trees, we built five large scaffolds to count the number of the developed cymules for each catkin in the Tianmu Mountain population of *O. rehderiana* in Zhejiang Province (Supplementary Fig. 25). All young trees transplanted in 1990 were normally producing both female and male catkins in 2018. Both female and male catkins flower from February and April. Thirty female catkins were used to calculate the differences in the number of the developed cymules (Supplementary Fig. 26) on each catkin after natural pollination between ten trees (all of the five old trees and five randomly selected young trees) on May 17, 2018. For each catkin, the cymule fell when without normal pollination and/or development while the cymule imprint and the primary bract were still retained (Supplementary Fig. 26). Therefore, we counted the well-developed cymules for each catkin. These cymules may develop into mature seeds in August. We statistically compared the number of the well-developed cymules after pollination based on the nested ANOVA and found the more well-developed cymules in old trees than in young trees (Supplementary Fig. 26, Supplementary Table 31), suggesting the serious consequence of inbreeding depression in the latter group when with the further reduced genetic diversity through mating between only two old trees (Supplementary note 4). Therefore, all results obtained in the present study for *O. rehderiana* likely underestimate the fitness consequences of inbreeding depressions in this endangered species.

Supplementary References

1. Tel-Zur, N., Abbo, S., Myslabodski, D. & Mizrahi, Y. Modified CTAB procedure for DNA isolation from epiphytic cacti of the genera Hylocereus and Selenicereus (Cactaceae). *Plant Molecular Biology Reporter* **17**, 249-254 (1999).
2. Kajitani, R. *et al.* Efficient de novo assembly of highly heterozygous genomes from whole-genome shotgun short reads. *Genome Res* **24**, 1384-95 (2014).
3. Li, B. & Dewey, C.N. RSEM: accurate transcript quantification from RNA-Seq data with or without a reference genome. *BMC Bioinformatics* **12**, 323 (2011).
4. Li, R. *et al.* The sequence and de novo assembly of the giant panda genome. *Nature* **463**, 311-7 (2010).
5. Bao, W., Kojima, K.K. & Kohany, O. Repbase Update, a database of repetitive elements in eukaryotic genomes. *Mob DNA* **6**, 11 (2015).
6. Tarailo-Graovac, M. & Chen, N. Using RepeatMasker to identify repetitive elements in genomic sequences. *Curr Protoc Bioinformatics* **Chapter 4**, Unit 4 10 (2009).
7. Xu, Z. & Wang, H. LTR_FINDER: an efficient tool for the prediction of full-length LTR retrotransposons. *Nucleic Acids Res* **35**, W265-8 (2007).
8. Edgar, R.C. & Myers, E.W. PILER: identification and classification of genomic repeats. *Bioinformatics* **21 Suppl 1**, i152-8 (2005).
9. Fu, L., Niu, B., Zhu, Z., Wu, S. & Li, W. CD-HIT: accelerated for clustering the next-generation sequencing data. *Bioinformatics* **28**, 3150-2 (2012).
10. Benson, G. Tandem repeats finder: a program to analyze DNA sequences. *Nucleic Acids Res* **27**, 573-80 (1999).
11. Edgar, R.C. MUSCLE: multiple sequence alignment with high accuracy and high throughput. *Nucleic Acids Res* **32**, 1792-7 (2004).
12. Ma, J. & Bennetzen, J.L. Rapid recent growth and divergence of rice nuclear genomes. *Proc Natl Acad Sci U S A* **101**, 12404-10 (2004).
13. Birney, E., Clamp, M. & Durbin, R. GeneWise and Genomewise. *Genome Res* **14**, 988-95 (2004).
14. Haas, B.J. *et al.* Improving the Arabidopsis genome annotation using maximal transcript alignment assemblies. *Nucleic Acids Research* **31**, 5654-5666 (2003).
15. Majoros, W.H., Pertea, M. & Salzberg, S.L. TigrScan and GlimmerHMM: two open source ab initio eukaryotic gene-finders. *Bioinformatics* **20**, 2878-2879 (2004).
16. Korf, I. Gene finding in novel genomes. *Bmc Bioinformatics* **5**(2004).
17. Stanke, M., Diekhans, M., Baertsch, R. & Haussler, D. Using native and syntenically mapped cDNA alignments to improve de novo gene finding. *Bioinformatics* **24**, 637-644 (2008).
18. Lomsadze, A., Burns, P.D. & Borodovsky, M. Integration of mapped RNA-Seq reads into automatic training of eukaryotic gene finding algorithm. *Nucleic Acids Research* **42**(2014).
19. Haas, B.J. *et al.* Automated eukaryotic gene structure annotation using EVidenceModeler and the Program to Assemble Spliced Alignments. *Genome biology* **9**, R7 (2008).
20. Conesa, A. *et al.* Blast2GO: a universal tool for annotation, visualization and analysis in functional genomics research. *Bioinformatics* **21**, 3674-6 (2005).
21. Quevillon, E. *et al.* InterProScan: protein domains identifier. *Nucleic Acids Res* **33**, W116-20 (2005).
22. Moriya, Y., Itoh, M., Okuda, S., Yoshizawa, A.C. & Kanehisa, M. KAAS: an automatic genome annotation and pathway reconstruction server. *Nucleic Acids Res* **35**, W182-5 (2007).
23. Lowe, T.M. & Eddy, S.R. tRNAscan-SE: a program for improved detection of transfer RNA genes in genomic sequence. *Nucleic Acids Res* **25**, 955-64 (1997).
24. Nawrocki, E.P., Kolbe, D.L. & Eddy, S.R. Infernal 1.0: inference of RNA alignments. *Bioinformatics* **25**, 1335-7 (2009).
25. Maere, S. *et al.* Modeling gene and genome duplications in eukaryotes. *Proc Natl Acad Sci U S A* **102**, 5454-9 (2005).
26. Gaut, B.S., Morton, B.R., McCaig, B.C. & Clegg, M.T. Substitution rate comparisons between grasses and palms: synonymous rate differences at the nuclear gene Adh parallel rate differences at the plastid gene rbcL. *Proc Natl Acad Sci U S A* **93**, 10274-9 (1996).
27. Nystedt, B. *et al.* The Norway spruce genome sequence and conifer genome evolution. *Nature*

497, 579-84 (2013).
28. Li, L., Stoeckert, C.J., Jr. & Roos, D.S. OrthoMCL: identification of ortholog groups for eukaryotic genomes. *Genome Res* **13**, 2178-89 (2003).
29. Yang, Z. PAML 4: phylogenetic analysis by maximum likelihood. *Mol Biol Evol* **24**, 1586-91 (2007).
30. Purcell, S. *et al.* PLINK: a tool set for whole-genome association and population-based linkage analyses. *Am J Hum Genet* **81**, 559-75 (2007).
31. Browning, B.L. & Browning, S.R. Improving the accuracy and efficiency of identity-by-descent detection in population data. *Genetics* **194**, 459-71 (2013).
32. Manichaikul, A. *et al.* Robust relationship inference in genome-wide association studies. *Bioinformatics* **26**, 2867-73 (2010).
33. Willi, Y., Van Buskirk, J. & Hoffmann, A.A. Limits to the adaptive potential of small populations. *Annual Review of Ecology Evolution and Systematics* **37**, 433-458 (2006).
34. Danecek, P. *et al.* The variant call format and VCFtools. *Bioinformatics* **27**, 2156-8 (2011).
35. Gazal, S. *et al.* Inbreeding coefficient estimation with dense SNP data: comparison of strategies and application to HapMap III. *Hum Hered* **77**, 49-62 (2014).
36. Barrett, J.C., Fry, B., Maller, J. & Daly, M.J. Haploview: analysis and visualization of LD and haplotype maps. *Bioinformatics* **21**, 263-5 (2005).
37. Gao, F., Ming, C., Hu, W. & Li, H. New Software for the Fast Estimation of Population Recombination Rates (FastEPRR) in the Genomic Era. *G3 (Bethesda)* **6**, 1563-71 (2016).
38. Schiffels, S. & Durbin, R. Inferring human population size and separation history from multiple genome sequences. *Nat Genet* **46**, 919-25 (2014).
39. Li, H. *et al.* The Sequence Alignment/Map format and SAMtools. *Bioinformatics* **25**, 2078-9 (2009).
40. Delaneau, O., Marchini, J. & Zagury, J.F. A linear complexity phasing method for thousands of genomes. *Nat Methods* **9**, 179-81 (2011).
41. Kajitani, R. *et al.* Efficient de novo assembly of highly heterozygous genomes from whole-genome shotgun short reads. *Genome research* **24**, 1384-1395 (2014).
42. Salojarvi, J. *et al.* Genome sequencing and population genomic analyses provide insights into the adaptive landscape of silver birch. *Nat Genet* **49**, 904-912 (2017).
43. Martinez-Garcia, P.J. *et al.* The walnut (*Juglans regia*) genome sequence reveals diversity in genes coding for the biosynthesis of non-structural polyphenols. *Plant J* **87**, 507-32 (2016).
44. Parra, G., Bradnam, K. & Korf, I. CEGMA: a pipeline to accurately annotate core genes in eukaryotic genomes. *Bioinformatics* **23**, 1061-7 (2007).
45. Simao, F.A., Waterhouse, R.M., Ioannidis, P., Kriventseva, E.V. & Zdobnov, E.M. BUSCO: assessing genome assembly and annotation completeness with single-copy orthologs. *Bioinformatics* **31**, 3210-2 (2015).
46. Oliver, K.R., McComb, J.A. & Greene, W.K. Transposable Elements: Powerful Contributors to Angiosperm Evolution and Diversity. *Genome Biology and Evolution* **5**, 1886-1901 (2013).
47. Kumar, M., Campbell, L. & Turner, S. Secondary cell walls: biosynthesis and manipulation. *J Exp Bot* **67**, 515-31 (2016).
48. Vanholme, R., Demedts, B., Morreel, K., Ralph, J. & Boerjan, W. Lignin biosynthesis and structure. *Plant Physiol* **153**, 895-905 (2010).
49. Eudes, A. *et al.* Purification, cloning and functional characterization of an endogenous beta-glucuronidase in Arabidopsis thaliana. *Plant Cell Physiol* **49**, 1331-41 (2008).
50. Xu, Z. *et al.* Functional genomic analysis of Arabidopsis thaliana glycoside hydrolase family 1. *Plant Mol Biol* **55**, 343-67 (2004).
51. Ahn, Y.O. *et al.* Functional genomic analysis of Arabidopsis thaliana glycoside hydrolase family 35. *Phytochemistry* **68**, 1510-20 (2007).
52. Xu, T., Ma, T., Hu, Q. & Liu, J. An integrated database of wood-formation related genes in plants. *Sci Rep* **5**, 11422 (2015).
53. MacMillan, C.P., Mansfield, S.D., Stachurski, Z.H., Evans, R. & Southerton, S.G. Fasciclin-like arabinogalactan proteins: specialization for stem biomechanics and cell wall architecture in Arabidopsis and Eucalyptus. *Plant J* **62**, 689-703 (2010).
54. Remm, M., Storm, C.E. & Sonnhammer, E.L. Automatic clustering of orthologs and in-paralogs from pairwise species comparisons. *J Mol Biol* **314**, 1041-52 (2001).
55. Grimm, G.W. & Renner, S.S. Harvesting Betulaceae sequences from GenBank to generate a new chronogram for the family. *Botanical Journal of the Linnean Society* **172**, 465-477 (2013).

56. Hedges, S.B., Marin, J., Suleski, M., Paymer, M. & Kumar, S. Tree of life reveals clock-like speciation and diversification. *Mol Biol Evol* **32**, 835-45 (2015).
57. Browning, S.R. & Browning, B.L. Rapid and accurate haplotype phasing and missing-data inference for whole-genome association studies by use of localized haplotype clustering. *Am J Hum Genet* **81**, 1084-97 (2007).
58. DePristo, M.A. *et al.* A framework for variation discovery and genotyping using next-generation DNA sequencing data. *Nature Genetics* **43**, 491-+ (2011).
59. Smeds, L., Qvarnstrom, A. & Ellegren, H. Direct estimate of the rate of germline mutation in a bird. *Genome Res* **26**, 1211-8 (2016).
60. Keightley, P.D., Ness, R.W., Halligan, D.L. & Haddrill, P.R. Estimation of the spontaneous mutation rate per nucleotide site in a Drosophila melanogaster full-sib family. *Genetics* **196**, 313-20 (2014).
61. Roach, J.C. *et al.* Analysis of genetic inheritance in a family quartet by whole-genome sequencing. *Science* **328**, 636-9 (2010).
62. Yang, S. *et al.* Parent-progeny sequencing indicates higher mutation rates in heterozygotes. *Nature* **523**, 463-7 (2015).
63. Koch, M.A., Haubold, B. & Mitchell-Olds, T. Comparative evolutionary analysis of chalcone synthase and alcohol dehydrogenase loci in Arabidopsis, Arabis, and related genera (Brassicaceae). *Mol Biol Evol* **17**, 1483-98 (2000).
64. Kaplan, R.W. Evolutionary adjustment of spontaneous mutation rates. *Humangenetik* **16**, 39-42 (1972).
65. Sniegowski, P.D., Gerrish, P.J., Johnson, T. & Shaver, A. The evolution of mutation rates: separating causes from consequences. *Bioessays* **22**, 1057-66 (2000).
66. Lynch, M. Evolution of the mutation rate. *Trends Genet* **26**, 345-52 (2010).
67. dos Reis, M., Donoghue, P.C. & Yang, Z. Bayesian molecular clock dating of species divergences in the genomics era. *Nat Rev Genet* **17**, 71-80 (2016).
68. Ho, S.Y. & Larson, G. Molecular clocks: when times are a-changin'. *Trends Genet* **22**, 79-83 (2006).
69. Mao, K. *et al.* Distribution of living Cupressaceae reflects the breakup of Pangea. *Proc Natl Acad Sci U S A* **109**, 7793-8 (2012).
70. Barrick, J.E. & Lenski, R.E. Genome dynamics during experimental evolution. *Nat Rev Genet* **14**, 827-39 (2013).
71. Petit, R.J. & Hampe, A. Some Evolutionary Consequences of Being a Tree. *Annual Review of Ecology, Evolution, and Systematics* **37**, 187-214 (2006).
72. Kardos, M., Taylor, H.R., Ellegren, H., Luikart, G. & Allendorf, F.W. Genomics advances the study of inbreeding depression in the wild. *Evol Appl* **9**, 1205-1218 (2016).
73. McQuillan, R. *et al.* Runs of homozygosity in European populations. *Am J Hum Genet* **83**, 359-72 (2008).
