# Supplementary material for: Genomic effects of population collapse in a critically endangered ironwood tree Ostrya rehderiana
Source: Nat Commun. 2018 Dec 21;9:5449. doi: 10.1038/s41467-018-07913-4 (PMC6303402; doi:10.1038/s41467-018-07913-4)
Supplement: Supplementary file 3 — Description of Additional Supplementary Files [file 41467_2018_7913_MOESM3_ESM.pdf]

Description of Additional Supplementary Files

File Name: Supplementary Data 1
Description: The position, distribution and the corresponding effected genes of the deleterious variant sites (DEL).

File Name: Supplementary Data 2
Description: The position, distribution and the corresponding effected genes of the loss of function variant sites (LOF).
