# Supplementary material for: Genomic effects of population collapse in a critically endangered ironwood tree Ostrya rehderiana
Source: Nat Commun. 2018 Dec 21;9:5449. doi: 10.1038/s41467-018-07913-4 (PMC6303402; doi:10.1038/s41467-018-07913-4)
Supplement: Supplementary file 4 — Supplementary Data 1 [file 41467_2018_7913_MOESM4_ESM.pdf]

Supplementary Data file 1. The deleterious variant sites (DEL)

Chr	Pos	Ancestral	Derived	Individuals carried homozygous LoF	Gene affectd
scaffold188	3592	C	T	Och09,Och10,Och11,Och14	*OreG0004622*
scaffold188	3642	G	A	Och09,Och10,Och11	*OreG0004622*
scaffold188	3661	C	T	Och01,Och02,Och03,Och04,Och05,Och06,Och07,Och08,Och09,Och10,Och11,Och12,Och13,Och14	*OreG0004622*
scaffold188	3667	C	A	Och04,Och05,Och12,Och13	*OreG0004622*
scaffold191	3359	G	C	Ore01,Ore02	*OreG0004902*
scaffold191	3389	C	A	Ore01,Ore02,Ore03,Ore04,Ore05,Ore06,Ore07,Ore08,Ore09,Ore10,Ore11,Ore12,Ore13,Ore14	*OreG0004902*
scaffold220	4748	C	T	NA	*OreG0006227*
scaffold220	16244	C	G	NA	*OreG0006229*
scaffold220	16681	A	G	NA	*OreG0006229*
scaffold220	26123	G	C	NA	*OreG0006231*
scaffold220	26131	C	A	NA	*OreG0006231*
scaffold220	59170	T	C	NA	*OreG0006238*
scaffold220	63489	A	T	NA	*OreG0006239*
scaffold220	63756	T	G	NA	*OreG0006239*
scaffold220	74653	C	T	NA	*OreG0006242*
scaffold333	489496	T	G	Och01,Och02,Och03,Och04,Och05,Och06,Och07,Och08,Och09,Och10,Och11,Och12,Och13,Och14,Ore01,Ore02,Ore03,Ore04,Ore05,Ore06,Ore07,Ore08,Ore09,Ore10,Ore11,Ore12,Ore13,Ore14	*OreG0018513*
scaffold333	489669	T	C	Och01,Och02,Och03,Och04,Och05,Och06,Och07,Och08,Och09,Och10,Och11,Och12,Och13,Och14	*OreG0018513*
scaffold333	550774	A	T	Ore01,Ore02,Ore03,Ore04,Ore05,Ore06,Ore07,Ore08,Ore09,Ore10,Ore11,Ore12,Ore13,Ore14	*OreG0018514*
scaffold333	551405	G	A	NA	*OreG0018514*
scaffold333	553216	G	A	NA	*OreG0018514*
scaffold333	736155	T	G	NA	*OreG0018522*
scaffold333	744351	A	G	NA	*OreG0018523*
scaffold333	744417	A	T	NA	*OreG0018523*
scaffold333	813055	G	A	NA	*OreG0018528*
scaffold333	844716	G	A	NA	*OreG0018531*
scaffold333	891960	T	A	Ore03,Ore06,Ore09,Ore11,Ore13,Ore14	*OreG0018533*
scaffold333	970006	C	T	NA	*OreG0018550*
scaffold333	976801	C	G	Ore03,Ore06,Ore09,Ore11,Ore13,Ore14	*OreG0018550*
scaffold399	92890	G	A	Och02,Och03,Och04,Och05,Och06,Och07,Och08,Och09,Och10,Och12,Och13	*OreG0019580*
scaffold399	102862	T	A	NA	*OreG0019582*
scaffold399	102884	C	T	NA	*OreG0019582*
scaffold399	104248	T	A	Och01,Och02,Och03,Och04,Och05,Och06,Och07,Och08,Och09,Och10,Och11,Och12,Och13,Och14,Ore01,Ore02,Ore03,Ore04,Ore05,Ore06,Ore07,Ore08,Ore09,Ore10,Ore11,Ore12,Ore13,Ore14	*OreG0019582*
scaffold399	110846	G	C	NA	*OreG0019582*
scaffold399	112087	G	T	NA	*OreG0019582*
scaffold399	112600	T	C	NA	*OreG0019582*
scaffold399	126539	C	T	Och01,Och02,Och03,Och04,Och05,Och06,Och09,Och10,Och11,Och12,Och13,Och14,Ore01,Ore02,Ore03,Ore04,Ore05,Ore06,Ore07,Ore08,Ore09,Ore10,Ore11,Ore12,Ore13,Ore14	*OreG0019583*
scaffold399	139890	G	A	NA	*OreG0019584*
scaffold399	140068	G	A	Och01,Och02,Och03,Och04,Och05,Och06,Och07,Och08,Och09,Och10,Och11,Och12,Och13,Och14,Ore01,Ore02,Ore03,Ore04,Ore05,Ore06,Ore07,Ore08,Ore09,Ore10,Ore11,Ore12,Ore13,Ore14	*OreG0019584*
scaffold399	140199	T	C	NA	*OreG0019584*
scaffold399	140392	C	T	NA	*OreG0019584*
scaffold399	140542	C	T	NA	*OreG0019584*
scaffold399	140604	G	A	NA	*OreG0019584*
scaffold399	140816	G	T	NA	*OreG0019584*
scaffold399	140865	C	G	NA	*OreG0019584*
scaffold399	141079	C	T	NA	*OreG0019584*
scaffold399	141109	G	A	NA	*OreG0019584*
scaffold399	141169	G	A	NA	*OreG0019584*
scaffold399	141180	A	G	NA	*OreG0019584*
scaffold399	141199	A	G	NA	*OreG0019584*
scaffold399	141202	C	G	NA	*OreG0019584*
scaffold399	141211	C	T	NA	*OreG0019584*
scaffold399	141241	C	T	NA	*OreG0019584*
scaffold399	141433	G	A	NA	*OreG0019584*
scaffold35	19175	G	A	NA	*OreG0018777*
scaffold35	21249	G	A	NA	*OreG0018777*
scaffold35	23965	G	A	NA	*OreG0018777*
scaffold35	23981	A	T	NA	*OreG0018777*
scaffold35	28953	C	T	Ore01,Ore02,Ore03,Ore04,Ore05,Ore06,Ore07,Ore08,Ore09,Ore10,Ore11,Ore12,Ore13,Ore14	*OreG0018777*
scaffold35	34799	G	A	NA	*OreG0018777*

scaffold35	45630	T	G	Ore04,Ore05	*OreG0018777*
scaffold35	47569	A	C	Och01,Och02,Och03,Och04,Och05,Och06,Och07,Och08,Och09,Och10,Och11,Och12,Och13,Och14	*OreG0018778*
scaffold35	52212	C	T	Och01,Och02,Och03,Och04,Och05,Och06,Och07,Och12,Och13	*OreG0018779*
scaffold35	73201	A	T	NA	*OreG0018781*
scaffold35	73213	C	A	NA	*OreG0018781*
scaffold35	73299	C	T	NA	*OreG0018781*
scaffold35	76580	C	A	NA	*OreG0018782*
scaffold35	88319	C	T	NA	*OreG0018783*
scaffold35	88960	C	T	NA	*OreG0018783*
scaffold35	96163	T	G	Och01,Och07,Och08,Och09,Och10,Och11,Och12,Och14	*OreG0018784*
scaffold35	118154	C	T	NA	*OreG0018785*
scaffold35	122124	G	C	Och08	*OreG0018785*
scaffold35	126326	G	T	Och03	*OreG0018786*
scaffold35	134631	C	G	Ore01,Ore02,Ore03,Ore04,Ore05,Ore06,Ore07,Ore08,Ore09,Ore10,Ore11,Ore12,Ore13,Ore14	*OreG0018786*
scaffold35	160378	G	C	NA	*OreG0018787*
scaffold35	186397	T	C	NA	*OreG0018792*
scaffold35	186431	G	A	Och01,Och02,Och03,Och04,Och05,Och06,Och07,Och08,Och09,Och10,Och11,Och12,Och13,Och14	*OreG0018792*
scaffold35	187978	C	A	NA	*OreG0018792*
scaffold35	188143	G	C	Ore01,Ore02,Ore03,Ore04,Ore05,Ore06,Ore07,Ore08,Ore09,Ore10,Ore11,Ore12,Ore13,Ore14	*OreG0018792*
scaffold35	203837	A	G	NA	*OreG0018794*
scaffold35	205204	G	A	Ore01,Ore02,Ore03,Ore04,Ore05,Ore06,Ore07,Ore08,Ore09,Ore10,Ore11,Ore12,Ore13,Ore14	*OreG0018794*
scaffold35	207225	C	A	NA	*OreG0018795*
scaffold35	207336	T	C	NA	*OreG0018795*
scaffold35	207455	G	A	NA	*OreG0018795*
scaffold35	208065	G	A	NA	*OreG0018795*
scaffold35	208101	G	A	NA	*OreG0018795*
scaffold35	209955	T	A	NA	*OreG0018796*
scaffold35	211127	G	T	Ore01,Ore02,Ore03,Ore04,Ore05,Ore06,Ore07,Ore08,Ore09,Ore10,Ore11,Ore12,Ore13,Ore14	*OreG0018796*
scaffold35	211460	G	A	NA	*OreG0018796*
scaffold35	212454	A	T	Och12,Och14	*OreG0018797*
scaffold35	212803	C	T	Och01,Och02,Och03,Och04,Och05,Och06,Och07,Och08,Och09,Och10,Och11,Och12,Och13,Och14,Ore01,Ore02,Ore03,Ore04,Ore05,Ore06,Ore07,Ore08,Ore09,Ore10,Ore11,Ore12,Ore13,Ore14	*OreG0018797*
scaffold35	213093	G	A	Och01,Och02,Och03,Och04,Och05,Och06,Och07,Och08,Och09,Och10,Och11,Och12,Och13,Och14,Ore01,Ore02,Ore03,Ore04,Ore05,Ore06,Ore07,Ore08,Ore09,Ore10,Ore11,Ore12,Ore13,Ore14	*OreG0018797*
scaffold35	213097	A	G	Och01,Och02,Och03,Och04,Och05,Och06,Och07,Och08,Och09,Och10,Och11,Och12,Och13,Och14,Ore01,Ore02,Ore03,Ore04,Ore05,Ore06,Ore07,Ore08,Ore09,Ore10,Ore11,Ore12,Ore13,Ore14	*OreG0018797*
scaffold35	219634	T	A	NA	*OreG0018797*
scaffold35	220294	A	T	Ore01,Ore02,Ore03,Ore04,Ore05,Ore06,Ore07,Ore08,Ore09,Ore10,Ore11,Ore12,Ore13,Ore14	*OreG0018797*
scaffold35	222411	T	G	Och01,Och02,Och03,Och04,Och05,Och06,Och07,Och08,Och09,Och10,Och11,Och12,Och13,Och14,Ore01,Ore02,Ore03,Ore04,Ore05,Ore06,Ore07,Ore08,Ore09,Ore10,Ore11,Ore12,Ore13,Ore14	*OreG0018798*
scaffold35	222956	G	A	Och01,Och02,Och03,Och04,Och05,Och06,Och07,Och08,Och09,Och10,Och11,Och12,Och13,Och14,Ore01,Ore02,Ore03,Ore04,Ore05,Ore06,Ore07,Ore08,Ore09,Ore10,Ore11,Ore12,Ore13,Ore14	*OreG0018798*
scaffold35	256587	G	A	Och02,Och03,Och04,Och06,Och07,Och13,Och14	*OreG0018800*
scaffold35	261263	G	T	Ore02,Ore04,Ore05,Ore08,Ore09,Ore10,Ore11,Ore13	*OreG0018801*
scaffold35	277269	G	T	Ore04,Ore05,Ore09,Ore10,Ore13	*OreG0018803*
scaffold35	277325	T	A	Och02,Och03,Och04,Och06,Och07,Och13,Och14	*OreG0018803*
scaffold35	277630	C	A	NA	*OreG0018803*
scaffold35	326606	A	G	Ore09,Ore10,Ore13	*OreG0018810*
scaffold35	329565	A	T	Och02,Och03,Och04,Och05,Och06,Och12,Och13	*OreG0018811*
scaffold35	330503	C	G	NA	*OreG0018811*
scaffold35	376361	G	T	NA	*OreG0018814*
scaffold35	379354	A	C	NA	*OreG0018814*
scaffold35	383015	T	G	Och01,Och02,Och03,Och04,Och05,Och06,Och07,Och08,Och09,Och10,Och11,Och12,Och13,Och14,Ore01,Ore02,Ore03,Ore04,Ore05,Ore06,Ore07,Ore08,Ore09,Ore10,Ore11,Ore12,Ore13,Ore14	*OreG0018814*
scaffold35	384576	C	T	Och01,Och02,Och03,Och04,Och05,Och06,Och07,Och08,Och09,Och10,Och11,Och12,Och13,Och14,Ore01,Ore02,Ore03,Ore04,Ore05,Ore06,Ore07,Ore08,Ore09,Ore10,Ore11,Ore12,Ore13,Ore14	*OreG0018814*
scaffold35	403698	C	A	NA	*OreG0018816*
scaffold35	415077	G	A	Och01,Och02,Och03,Och04,Och05,Och06,Och07,Och08,Och09,Och10,Och11,Och12,Och13,Och14,Ore01,Ore02,Ore03,Ore04,Ore05,Ore06,Ore07,Ore08,Ore09,Ore10,Ore11,Ore12,Ore13,Ore14	*OreG0018817*

scaffold35	415087	G	T	Och01,Och02,Och03,Och04,Och05,Och06,Och07,Och08,Och09,Och10,Och11,Och12,Och13,Och14,Ore01,Ore02,Ore03,Ore04,Ore05,Ore06,Ore07,Ore08,Ore09,Ore10,Ore11,Ore12,Ore13,Ore14	*OreG0018817*
scaffold35	417904	C	T	Och01,Och02,Och03,Och04,Och05,Och06,Och07,Och08,Och09,Och10,Och11,Och12,Och13,Och14,Ore01,Ore02,Ore03,Ore04,Ore05,Ore06,Ore07,Ore08,Ore09,Ore10,Ore11,Ore12,Ore13,Ore14	*OreG0018818*
scaffold35	438632	C	G	Ore01,Ore02,Ore03,Ore04,Ore05,Ore06,Ore07,Ore08,Ore09,Ore10,Ore11,Ore12,Ore13,Ore14	*OreG0018819*
scaffold35	441608	G	A	NA	*OreG0018820*
scaffold35	442562	G	A	Och01,Och02,Och03,Och04,Och05,Och06,Och07,Och08,Och09,Och10,Och11,Och12,Och13,Och14	*OreG0018820*
scaffold35	446157	C	G	Och01,Och02,Och03,Och04,Och05,Och06,Och07,Och08,Och09,Och10,Och11,Och12,Och13,Och14,Ore01,Ore02,Ore03,Ore04,Ore05,Ore06,Ore07,Ore08,Ore09,Ore10,Ore11,Ore12,Ore13,Ore14	*OreG0018821*
scaffold35	482213	C	T	Och01,Och02,Och03,Och04,Och05,Och06,Och07,Och08,Och09,Och10,Och11,Och12,Och13,Och14	*OreG0018825*
scaffold35	483167	T	A	NA	*OreG0018825*
scaffold35	490636	C	T	Och01,Och02,Och03,Och04,Och05,Och06,Och07,Och08,Och09,Och10,Och11,Och12,Och13,Och14,Ore01,Ore02,Ore03,Ore04,Ore05,Ore06,Ore07,Ore08,Ore09,Ore10,Ore11,Ore12,Ore13,Ore14	*OreG0018826*
scaffold35	493137	G	C	Ore01,Ore02,Ore03,Ore04,Ore05,Ore06,Ore07,Ore08,Ore09,Ore10,Ore11,Ore12,Ore13,Ore14	*OreG0018826*
scaffold35	517192	T	C	Ore01,Ore02,Ore03,Ore04,Ore05,Ore06,Ore07,Ore08,Ore09,Ore10,Ore11,Ore12,Ore13,Ore14	*OreG0018830*
scaffold35	517445	T	C	Ore02,Ore08,Ore09,Ore10,Ore11,Ore13	*OreG0018830*
scaffold35	531113	C	T	NA	*OreG0018831*
scaffold35	532171	A	C	NA	*OreG0018831*
scaffold35	533746	T	A	Och01,Och02,Och03,Och04,Och05,Och06,Och07,Och08,Och09,Och10,Och11,Och12,Och13,Och14	*OreG0018831*
scaffold35	533967	A	T	NA	*OreG0018831*
scaffold35	550979	G	T	Ore09,Ore10,Ore13	*OreG0018832*
scaffold35	551101	G	T	Och02,Och03,Och04,Och06,Och08,Och11,Och13	*OreG0018832*
scaffold35	551213	C	T	NA	*OreG0018832*
scaffold35	552158	A	C	Ore01,Ore02,Ore03,Ore04,Ore05,Ore06,Ore07,Ore08,Ore09,Ore10,Ore11,Ore12,Ore13,Ore14	*OreG0018832*
scaffold35	571736	C	T	NA	*OreG0018833*
scaffold35	605809	A	T	Och01,Och02,Och03,Och04,Och05,Och06,Och07,Och08,Och12,Och13,Och14	*OreG0018837*
scaffold35	612218	A	T	Och01,Och02,Och03,Och04,Och05,Och06,Och07,Och08,Och09,Och10,Och11,Och12,Och13,Och14,Ore01,Ore02,Ore03,Ore04,Ore05,Ore06,Ore07,Ore08,Ore09,Ore10,Ore11,Ore12,Ore13,Ore14	*OreG0018837*
scaffold35	612285	C	T	Och01,Och02,Och03,Och04,Och05,Och06,Och07,Och08,Och12,Och13,Och14	*OreG0018837*
scaffold35	779259	A	T	Och01,Och02,Och03,Och04,Och05,Och06,Och07,Och12,Och13,Och14	*OreG0018848*
scaffold35	788271	A	T	NA	*OreG0018849*
scaffold35	814072	C	A	NA	*OreG0018852*
scaffold35	843186	A	G	Ore01,Ore02,Ore03,Ore04,Ore05,Ore06,Ore07,Ore08,Ore09,Ore10,Ore11,Ore12,Ore13,Ore14	*OreG0018855*
scaffold35	846077	C	T	NA	*OreG0018855*
scaffold35	847034	C	T	Och07,Och08,Och09,Och10,Och11,Och12,Och14	*OreG0018855*
scaffold35	848882	T	A	Ore01,Ore02,Ore03,Ore04,Ore05,Ore06,Ore07,Ore08,Ore09,Ore10,Ore11,Ore12,Ore13,Ore14	*OreG0018855*
scaffold35	851935	T	A	Ore01,Ore02,Ore03,Ore04,Ore05,Ore06,Ore07,Ore08,Ore09,Ore10,Ore11,Ore12,Ore13,Ore14	*OreG0018855*
scaffold35	856542	C	T	NA	*OreG0018856*
scaffold35	859524	G	C	NA	*OreG0018857*
scaffold35	861203	G	T	NA	*OreG0018857*
scaffold35	879966	A	G	NA	*OreG0018860*
scaffold35	888506	C	T	Och01,Och02,Och03,Och04,Och05,Och06,Och08,Och09,Och10,Och11,Och12,Och13,Och14,Ore01,Ore02,Ore03,Ore04,Ore05,Ore06,Ore07,Ore08,Ore09,Ore10,Ore11,Ore12,Ore13,Ore14	*OreG0018861*
scaffold35	918451	A	G	NA	*OreG0018865*
scaffold35	941748	A	G	Och01	*OreG0018869*
scaffold35	943201	G	C	Och03,Och05,Och07,Och08,Och09,Och10,Och11,Och14	*OreG0018869*
scaffold35	943216	C	A	NA	*OreG0018869*
scaffold35	944173	A	G	NA	*OreG0018869*
scaffold35	948058	T	C	NA	*OreG0018870*
scaffold35	948388	T	G	NA	*OreG0018870*
scaffold35	948511	A	G	Och03	*OreG0018870*
scaffold35	948777	C	A	Och01,Och02,Och03,Och04,Och05,Och06,Och07,Och08,Och09,Och10,Och11,Och12,Och13,Och14,Ore01,Ore02,Ore03,Ore04,Ore05,Ore06,Ore07,Ore08,Ore09,Ore10,Ore11,Ore12,Ore13,Ore14	*OreG0018870*
scaffold35	954272	C	A	NA	*OreG0018871*
scaffold35	992341	T	G	Ore01,Ore02,Ore03,Ore04,Ore05,Ore06,Ore07,Ore08,Ore09,Ore10,Ore11,Ore12,Ore13,Ore14	*OreG0018874*
scaffold35	992982	T	C	Ore01,Ore02,Ore03,Ore04,Ore05,Ore06,Ore07,Ore08,Ore09,Ore10,Ore11,Ore12,Ore13,Ore14	*OreG0018874*

scaffold35	993711	G	T	Och03,Och05	*OreG0018874*
scaffold35	997729	A	C	Och01,Och02,Och03,Och04,Och05,Och06,Och07,Och08,Och09,Och10,Och11,Och12,Och13,Och14	*OreG0018875*
scaffold35	1004100	A	T	Och03,Och05,Och07,Och08	*OreG0018875*
scaffold35	1005255	T	C	Ore09,Ore10,Ore13	*OreG0018875*
scaffold35	1005585	T	C	NA	*OreG0018875*
scaffold35	1025249	T	C	NA	*OreG0018877*
scaffold35	1071638	T	G	Och01,Och02,Och03,Och04,Och05,Och06,Och07,Och08,Och09,Och10,Och11,Och12,Och13,Och14,Ore01,Ore02,Ore03,Ore04,Ore05,Ore06,Ore07,Ore08,Ore09,Ore10,Ore11,Ore12,Ore13,Ore14	*OreG0018880*
scaffold35	1084758	G	T	Och01,Och02,Och03,Och04,Och05,Och06,Och07,Och08,Och09,Och10,Och11,Och12,Och13,Och14,Ore01,Ore02,Ore03,Ore04,Ore05,Ore06,Ore07,Ore08,Ore09,Ore10,Ore11,Ore12,Ore13,Ore14	*OreG0018880*
scaffold35	1084770	C	G	Och01,Och02,Och03,Och04,Och05,Och06,Och07,Och08,Och09,Och10,Och11,Och12,Och13,Och14,Ore01,Ore02,Ore03,Ore04,Ore05,Ore06,Ore07,Ore08,Ore09,Ore10,Ore11,Ore12,Ore13,Ore14	*OreG0018880*
scaffold35	1087461	G	T	NA	*OreG0018880*
scaffold35	1105945	T	G	NA	*OreG0018882*
scaffold35	1127646	T	A	Och08,Och12	*OreG0018883*
scaffold35	1157631	G	T	NA	*OreG0018885*
scaffold35	1159431	G	A	Ore06,Ore07	*OreG0018885*
scaffold35	1221205	G	A	NA	*OreG0018890*
scaffold35	1221237	C	T	Och12	*OreG0018890*
scaffold35	1221250	G	T	NA	*OreG0018890*
scaffold35	1320302	T	A	NA	*OreG0018897*
scaffold35	1320310	G	T	Och01,Och02,Och03,Och04,Och05,Och06,Och07,Och08,Och09,Och10,Och11,Och12,Och13,Och14,Ore01,Ore02,Ore03,Ore04,Ore05,Ore06,Ore07,Ore08,Ore09,Ore10,Ore11,Ore12,Ore13,Ore14	*OreG0018897*
scaffold35	1320365	C	G	Ore01,Ore02,Ore03,Ore04,Ore05,Ore06,Ore07,Ore08,Ore09,Ore10,Ore11,Ore12,Ore13,Ore14	*OreG0018897*
scaffold35	1328189	C	T	Ore01,Ore02,Ore03,Ore04,Ore05,Ore06,Ore07,Ore08,Ore09,Ore10,Ore11,Ore12,Ore13,Ore14	*OreG0018899*
scaffold35	1328450	C	A	NA	*OreG0018899*
scaffold35	1328474	C	T	NA	*OreG0018899*
scaffold35	1329138	G	T	NA	*OreG0018899*
scaffold35	1337743	C	T	NA	*OreG0018900*
scaffold35	1368866	A	G	NA	*OreG0018903*
scaffold35	1391116	T	A	NA	*OreG0018907*
scaffold35	1391907	G	C	NA	*OreG0018907*
scaffold35	1392216	C	T	Och01,Och03,Och14	*OreG0018907*
scaffold53	26100	A	G	NA	*OreG0023584*
scaffold53	26111	A	G	NA	*OreG0023584*
scaffold53	253066	A	G	Och01,Och02,Och03,Och04,Och05,Och06,Och07,Och08,Och09,Och10,Och11,Och12,Och13,Och14,Ore01,Ore02,Ore03,Ore04,Ore05,Ore06,Ore07,Ore08,Ore09,Ore10,Ore11,Ore12,Ore13,Ore14	*OreG0023587*
scaffold53	253334	A	G	Och01,Och02,Och03,Och04,Och05,Och06,Och07,Och08,Och09,Och10,Och11,Och12,Och13,Och14	*OreG0023587*
scaffold53	253492	A	T	Och01,Och02,Och03,Och04,Och05,Och06,Och07,Och08,Och09,Och10,Och11,Och12,Och13,Och14,Ore01,Ore02,Ore03,Ore04,Ore05,Ore06,Ore07,Ore08,Ore09,Ore10,Ore11,Ore12,Ore13,Ore14	*OreG0023587*
scaffold53	384949	A	T	NA	*OreG0023589*
scaffold53	385753	A	G	Och02,Och06	*OreG0023589*
scaffold53	388341	G	A	Ore01,Ore02,Ore08	*OreG0023589*
scaffold53	478003	C	A	Och01,Och02,Och03,Och04,Och05,Och06,Och07,Och08,Och09,Och10,Och11,Och12,Och13,Och14,Ore01,Ore02,Ore03,Ore04,Ore05,Ore06,Ore07,Ore08,Ore09,Ore10,Ore11,Ore12,Ore13,Ore14	*OreG0023591*
scaffold53	537324	G	A	NA	*OreG0023593*
scaffold53	542302	A	G	Och01,Och02,Och03,Och04,Och05,Och06,Och07,Och08,Och09,Och10,Och11,Och12,Och13,Och14,Ore01,Ore02,Ore03,Ore04,Ore05,Ore06,Ore07,Ore08,Ore09,Ore10,Ore11,Ore12,Ore13,Ore14	*OreG0023593*
scaffold53	650065	C	A	Och01,Och02,Och03,Och04,Och05,Och06,Och07,Och08,Och09,Och10,Och11,Och12,Och13,Och14,Ore01,Ore02,Ore03,Ore04,Ore05,Ore06,Ore07,Ore08,Ore09,Ore10,Ore11,Ore12,Ore13,Ore14	*OreG0023595*
scaffold53	897975	G	A	Och01,Och02,Och03,Och04,Och05,Och06,Och07,Och08,Och09,Och10,Och11,Och12,Och13,Och14	*OreG0023598*
scaffold53	1044821	G	T	Och01,Och02,Och03,Och04,Och05,Och06,Och07,Och08,Och09,Och10,Och11,Och12,Och13,Och14,Ore01,Ore02,Ore03,Ore04,Ore05,Ore06,Ore07,Ore08,Ore09,Ore10,Ore11,Ore12,Ore13,Ore14	*OreG0023604*
scaffold53	1078061	C	T	Ore01,Ore02,Ore03,Ore04,Ore05,Ore06,Ore07,Ore08,Ore09,Ore10,Ore11,Ore12,Ore13,Ore14	*OreG0023605*
scaffold385	4156	T	C	Och06	*OreG0019436*
scaffold385	16720	T	C	NA	*OreG0019437*
scaffold385	35928	G	T	Och01,Och02,Och03,Och04,Och05,Och06,Och07,Och08,Och11,Och12,Och13,Och14	*OreG0019441*
scaffold385	36005	C	T	NA	*OreG0019441*

scaffold385	76969	G	A	Ore01,Ore02,Ore03,Ore04,Ore05,Ore06,Ore07,Ore08,Ore09,Ore10,Ore11,Ore12,Ore13,Ore14	*OreG0019443*
scaffold385	88842	T	G	NA	*OreG0019445*
scaffold385	89687	G	A	NA	*OreG0019445*
scaffold385	101899	C	T	NA	*OreG0019448*
scaffold385	104047	G	A	NA	*OreG0019448*
scaffold385	120457	G	T	NA	*OreG0019449*
scaffold385	120497	G	T	NA	*OreG0019449*
scaffold385	145460	A	T	Och01,Och02,Och03,Och04,Och05,Och06,Och07,Och08,Och09,Och10,Och11,Och12,Och13,Och14,Ore01,Ore02,Ore03,Ore04,Ore05,Ore06,Ore07,Ore08,Ore09,Ore10,Ore11,Ore12,Ore13,Ore14	*OreG0019453*
scaffold385	150065	C	A	Ore01,Ore02,Ore03,Ore04,Ore05,Ore06,Ore07,Ore08,Ore09,Ore10,Ore11,Ore12,Ore13,Ore14	*OreG0019453*
scaffold385	154109	C	A	Och01,Och02,Och03,Och04,Och05,Och06,Och07,Och08,Och09,Och10,Och11,Och12,Och13,Och14,Ore01,Ore02,Ore03,Ore04,Ore05,Ore06,Ore07,Ore08,Ore09,Ore10,Ore11,Ore12,Ore13,Ore14	*OreG0019454*
scaffold385	155506	C	T	NA	*OreG0019454*
scaffold385	163976	C	T	Och01,Och02,Och03,Och04,Och05,Och06,Och07,Och08,Och09,Och10,Och11,Och12,Och13,Och14,Ore01,Ore02,Ore03,Ore04,Ore05,Ore06,Ore07,Ore08,Ore09,Ore10,Ore11,Ore12,Ore13,Ore14	*OreG0019455*
scaffold385	165416	T	C	Ore01,Ore02,Ore03,Ore04,Ore05,Ore06,Ore07,Ore08,Ore09,Ore10,Ore11,Ore12,Ore13,Ore14	*OreG0019455*
scaffold385	172314	C	A	Och08	*OreG0019456*
scaffold385	173570	C	T	NA	*OreG0019456*
scaffold385	173613	A	T	NA	*OreG0019456*
scaffold385	209403	G	T	Och02,Och03,Och04,Och05,Och06,Och07,Och08,Och09,Och10,Och11,Och12,Och14	*OreG0019459*
scaffold385	209823	T	A	NA	*OreG0019459*
scaffold385	213135	G	T	NA	*OreG0019459*
scaffold385	220788	G	A	Och02,Och03,Och04,Och05,Och06,Och07,Och08,Och09,Och10,Och11,Och12,Och13,Och14	*OreG0019460*
scaffold385	257489	A	G	Och02,Och03,Och04,Och05,Och06,Och07,Och08,Och09,Och10,Och11,Och12,Och13,Och14	*OreG0019464*
scaffold385	258391	C	T	Ore01,Ore02,Ore03,Ore04,Ore05,Ore06,Ore07,Ore08,Ore09,Ore10,Ore11,Ore12,Ore13,Ore14	*OreG0019464*
scaffold385	258850	T	A	NA	*OreG0019464*
scaffold385	259154	G	A	NA	*OreG0019465*
scaffold385	263186	G	A	Och07,Och08	*OreG0019466*
scaffold385	263363	T	A	Ore01,Ore02,Ore03,Ore04,Ore05,Ore06,Ore07,Ore08,Ore09,Ore10,Ore11,Ore12,Ore13,Ore14	*OreG0019466*
scaffold385	269987	C	T	NA	*OreG0019467*
scaffold385	303625	T	A	NA	*OreG0019470*
scaffold385	305228	C	A	NA	*OreG0019470*
scaffold385	305452	C	T	NA	*OreG0019470*
scaffold385	348385	C	G	Och01,Och02,Och03,Och04,Och05,Och06,Och07,Och08,Och09,Och10,Och11,Och12,Och13,Och14,Ore01,Ore02,Ore03,Ore04,Ore05,Ore06,Ore07,Ore08,Ore09,Ore10,Ore11,Ore12,Ore13,Ore14	*OreG0019473*
scaffold385	348516	A	T	Ore01,Ore02,Ore03,Ore04,Ore05,Ore06,Ore07,Ore08,Ore09,Ore10,Ore11,Ore12,Ore13,Ore14	*OreG0019473*
scaffold385	349013	A	T	Och01,Och02,Och03,Och04,Och05,Och06,Och07,Och08,Och09,Och10,Och11,Och12,Och13,Och14,Ore01,Ore02,Ore03,Ore04,Ore05,Ore06,Ore07,Ore08,Ore09,Ore10,Ore11,Ore12,Ore13,Ore14	*OreG0019473*
scaffold385	349531	A	G	NA	*OreG0019473*
scaffold385	349756	T	C	Ore01,Ore02,Ore03,Ore04,Ore05,Ore06,Ore07,Ore08,Ore09,Ore10,Ore11,Ore12,Ore13,Ore14	*OreG0019473*
scaffold385	353268	A	G	NA	*OreG0019473*
scaffold385	390686	C	A	Och02,Och03,Och04,Och05,Och06,Och07,Och11,Och12,Och13	*OreG0019476*
scaffold385	420389	T	C	Ore01,Ore02,Ore03,Ore04,Ore05,Ore06,Ore07,Ore08,Ore09,Ore10,Ore11,Ore12,Ore13,Ore14	*OreG0019480*
scaffold385	422502	G	A	NA	*OreG0019480*
scaffold385	425291	G	T	NA	*OreG0019481*
scaffold385	426776	A	G	Ore02,Ore03,Ore09,Ore10,Ore14	*OreG0019481*
scaffold385	427798	G	A	Ore01,Ore02,Ore03,Ore04,Ore05,Ore06,Ore07,Ore08,Ore09,Ore10,Ore11,Ore12,Ore13,Ore14	*OreG0019481*
scaffold385	430691	G	A	Ore01,Ore02,Ore03,Ore04,Ore05,Ore06,Ore07,Ore08,Ore09,Ore10,Ore11,Ore12,Ore13,Ore14	*OreG0019481*
scaffold385	477426	C	A	NA	*OreG0019487*
scaffold385	477438	C	A	Och01,Och02,Och03,Och04,Och05,Och06,Och07,Och08,Och09,Och10,Och11,Och12,Och13,Och14,Ore01,Ore02,Ore03,Ore04,Ore05,Ore06,Ore07,Ore08,Ore09,Ore10,Ore11,Ore12,Ore13,Ore14	*OreG0019487*
scaffold385	656495	T	A	NA	*OreG0019496*
scaffold385	657538	T	C	Och01,Och02,Och03,Och04,Och05,Och06,Och07,Och08,Och09,Och10,Och11,Och12,Och13,Och14	*OreG0019496*
scaffold385	665403	C	G	NA	*OreG0019497*
scaffold385	690779	A	C	NA	*OreG0019498*
scaffold385	690802	G	A	NA	*OreG0019498*

scaffold385	691462	C	T	NA	*OreG0019498*
scaffold385	705094	G	C	Ore09	*OreG0019500*
scaffold385	708190	C	A	Och01	*OreG0019501*
scaffold385	709391	G	A	NA	*OreG0019501*
scaffold385	709936	G	A	Och01,Och02,Och03,Och04,Och05,Och06,Och07,Och08,Och09,Och10,Och11,Och12,Och13,Och14,Ore01,Ore02,Ore03,Ore04,Ore05,Ore06,Ore07,Ore08,Ore09,Ore10,Ore11,Ore12,Ore13,Ore14	*OreG0019501*
scaffold385	738519	C	G	Och01,Och06,Och07,Och08,Och11,Och14	*OreG0019504*
scaffold385	776825	C	A	NA	*OreG0019509*
scaffold385	777510	C	G	NA	*OreG0019509*
scaffold385	777520	C	T	NA	*OreG0019509*
scaffold385	777633	C	T	NA	*OreG0019509*
scaffold385	782507	G	C	NA	*OreG0019510*
scaffold385	787732	G	T	NA	*OreG0019510*
scaffold385	787735	G	A	NA	*OreG0019510*
scaffold385	787748	C	A	NA	*OreG0019510*
scaffold385	787774	G	A	NA	*OreG0019510*
scaffold385	836844	G	A	Ore01,Ore02,Ore03,Ore04,Ore05,Ore06,Ore07,Ore08,Ore09,Ore10,Ore11,Ore12,Ore13,Ore14	*OreG0019514*
scaffold385	854677	T	C	Ore09	*OreG0019515*
scaffold385	855363	C	G	NA	*OreG0019515*
scaffold385	856008	G	T	NA	*OreG0019515*
scaffold385	856415	A	T	NA	*OreG0019515*
scaffold385	861516	C	G	NA	*OreG0019516*
scaffold385	861695	A	T	NA	*OreG0019516*
scaffold385	868426	T	C	Ore01,Ore02,Ore03,Ore04,Ore05,Ore06,Ore07,Ore08,Ore09,Ore10,Ore11,Ore12,Ore13,Ore14	*OreG0019517*
scaffold385	868485	C	A	Och01,Och02,Och03,Och04,Och05,Och06,Och07,Och08,Och09,Och10,Och11,Och12,Och13,Och14	*OreG0019517*
scaffold385	868773	G	A	Och01,Och02,Och03,Och04,Och05,Och06,Och07,Och08,Och09,Och10,Och11,Och12,Och13,Och14,Ore01,Ore02,Ore03,Ore04,Ore05,Ore06,Ore07,Ore08,Ore09,Ore10,Ore11,Ore12,Ore13,Ore14	*OreG0019517*
scaffold385	868774	C	A	Och01,Och02,Och03,Och04,Och05,Och06,Och07,Och08,Och09,Och10,Och11,Och12,Och13,Och14,Ore01,Ore02,Ore03,Ore04,Ore05,Ore06,Ore07,Ore08,Ore09,Ore10,Ore11,Ore12,Ore13,Ore14	*OreG0019517*
scaffold385	881207	G	A	NA	*OreG0019518*
scaffold385	888347	G	A	NA	*OreG0019519*
scaffold385	897532	C	T	NA	*OreG0019520*
scaffold385	897616	A	G	NA	*OreG0019520*
scaffold385	897769	A	G	Ore01,Ore02,Ore03,Ore04,Ore05,Ore06,Ore07,Ore08,Ore09,Ore10,Ore11,Ore12,Ore13,Ore14	*OreG0019520*
scaffold385	897885	G	A	Och01,Och02,Och03,Och04,Och05,Och06,Och07,Och08,Och09,Och10,Och11,Och12,Och13,Och14,Ore01,Ore02,Ore03,Ore04,Ore05,Ore06,Ore07,Ore08,Ore09,Ore10,Ore11,Ore12,Ore13,Ore14	*OreG0019520*
scaffold385	935576	A	C	Och01,Och02,Och03,Och04,Och05,Och06,Och07,Och08,Och09,Och10,Och11,Och12,Och13,Och14,Ore01,Ore02,Ore03,Ore04,Ore05,Ore06,Ore07,Ore08,Ore09,Ore10,Ore11,Ore12,Ore13,Ore14	*OreG0019524*
scaffold385	957850	C	A	Och01,Och02,Och03,Och04,Och05,Och06,Och07,Och08,Och09,Och10,Och11,Och12,Och13,Och14,Ore01,Ore02,Ore03,Ore04,Ore05,Ore06,Ore07,Ore08,Ore09,Ore10,Ore11,Ore12,Ore13,Ore14	*OreG0019527*
scaffold385	958264	G	A	NA	*OreG0019527*
scaffold385	961661	T	A	Ore01,Ore02,Ore03,Ore04,Ore05,Ore06,Ore07,Ore08,Ore09,Ore10,Ore11,Ore12,Ore13,Ore14	*OreG0019527*
scaffold385	962548	T	C	Och03,Och05,Och11,Och12,Och13	*OreG0019527*
scaffold385	978927	C	A	Och08	*OreG0019528*
scaffold385	979318	G	C	Ore01,Ore02,Ore03,Ore04,Ore05,Ore06,Ore07,Ore08,Ore09,Ore10,Ore11,Ore12,Ore13,Ore14	*OreG0019528*
scaffold385	983936	T	A	NA	*OreG0019529*
scaffold385	1004690	T	A	NA	*OreG0019531*
scaffold385	1004906	G	T	NA	*OreG0019531*
scaffold385	1008749	G	A	Ore09	*OreG0019532*
scaffold385	1014041	G	A	Och09	*OreG0019534*
scaffold385	1014235	C	A	NA	*OreG0019534*
scaffold316	71301	G	A	Och01,Och02,Och03,Och04,Och05,Och06,Och07,Och08,Och09,Och10,Och11,Och12,Och13,Och14,Ore01,Ore02,Ore03,Ore04,Ore05,Ore06,Ore07,Ore08,Ore09,Ore10,Ore11,Ore12,Ore13,Ore14	*OreG0009509*
scaffold316	71826	C	G	Och14	*OreG0009509*
scaffold316	71912	G	A	NA	*OreG0009509*
scaffold316	76875	G	T	Ore04,Ore05,Ore06,Ore11	*OreG0009510*
scaffold316	76998	T	C	NA	*OreG0009510*
scaffold316	86509	G	A	Och01,Och04,Och07,Och08,Och09,Och10,Och11,Och13	*OreG0009511*
scaffold316	86756	C	T	NA	*OreG0009511*
scaffold316	87340	T	A	NA	*OreG0009511*
scaffold316	87398	C	T	Och01,Och04,Och07,Och08,Och09,Och10,Och11,Och13	*OreG0009511*

scaffold316	92772	G	T	Och01,Och02,Och03,Och04,Och05,Och06,Och07,Och08,Och09,Och10,Och11,Och12,Och13,Och14,Ore01,Ore02,Ore03,Ore04,Ore05,Ore06,Ore07,Ore08,Ore09,Ore10,Ore11,Ore12,Ore13,Ore14	*OreG0009512*
scaffold316	105273	C	T	Ore09,Ore13	*OreG0009514*
scaffold316	105330	A	T	Och05,Ore01,Ore02,Ore03,Ore04,Ore05,Ore06,Ore07,Ore08,Ore09,Ore10,Ore11,Ore12,Ore13,Ore14	*OreG0009514*
scaffold316	105409	T	A	Och05,Ore01,Ore02,Ore03,Ore04,Ore05,Ore06,Ore07,Ore08,Ore09,Ore10,Ore11,Ore12,Ore13,Ore14	*OreG0009514*
scaffold316	105673	C	T	Och02,Och09,Och10,Och11,Och13	*OreG0009514*
scaffold316	114182	T	C	Och01	*OreG0009515*
scaffold316	114198	C	A	Ore01,Ore02,Ore03,Ore04,Ore05,Ore06,Ore07,Ore08,Ore09,Ore10,Ore11,Ore12,Ore13,Ore14	*OreG0009515*
scaffold316	116723	T	C	Ore09,Ore13	*OreG0009516*
scaffold316	116894	A	G	Och06,Och08	*OreG0009516*
scaffold316	117570	C	A	Och01,Och02,Och03,Och04,Och05,Och06,Och07,Och08,Och09,Och10,Och11,Och12,Och13,Och14,Ore01,Ore02,Ore03,Ore04,Ore05,Ore06,Ore07,Ore08,Ore09,Ore10,Ore11,Ore12,Ore13,Ore14	*OreG0009516*
scaffold316	133988	T	G	Ore01,Ore02,Ore03,Ore04,Ore05,Ore06,Ore07,Ore08,Ore09,Ore10,Ore11,Ore12,Ore13,Ore14	*OreG0009518*
scaffold316	153734	T	C	NA	*OreG0009521*
scaffold316	157082	G	T	Ore09,Ore13	*OreG0009521*
scaffold316	295328	G	T	Och01,Och02,Och03,Och04,Och05,Och06,Och07,Och08,Och09,Och10,Och11,Och12,Och13,Och14,Ore01,Ore02,Ore03,Ore04,Ore05,Ore06,Ore07,Ore08,Ore09,Ore10,Ore11,Ore12,Ore13,Ore14	*OreG0009534*
scaffold316	314970	A	T	NA	*OreG0009536*
scaffold316	314972	C	T	NA	*OreG0009536*
scaffold316	334163	C	T	NA	*OreG0009538*
scaffold316	344833	G	A	NA	*OreG0009539*
scaffold316	350935	T	G	Och07,Och08,Och14	*OreG0009542*
scaffold316	351075	G	A	Ore01,Ore02,Ore03,Ore04,Ore05,Ore06,Ore07,Ore08,Ore09,Ore10,Ore11,Ore12,Ore13,Ore14	*OreG0009542*
scaffold316	380808	C	G	Och01,Och02,Och03,Och04,Och05,Och06,Och07,Och08,Och09,Och10,Och11,Och12,Och13,Och14	*OreG0009547*
scaffold316	382365	G	A	Och01,Och02,Och03,Och04,Och05,Och06,Och07,Och08,Och09,Och10,Och11,Och12,Och13,Och14	*OreG0009547*
scaffold316	387139	C	T	Och01,Och02,Och03,Och04,Och05,Och06,Och07,Och08,Och09,Och10,Och11,Och12,Och13,Och14	*OreG0009548*
scaffold316	402857	A	G	Och14	*OreG0009549*
scaffold316	402884	A	T	Och08	*OreG0009549*
scaffold316	424703	G	T	Och01	*OreG0009551*
scaffold316	425512	T	C	NA	*OreG0009551*
scaffold316	432760	T	A	Och01	*OreG0009552*
scaffold316	432839	G	A	Och01	*OreG0009552*
scaffold316	433028	A	C	NA	*OreG0009552*
scaffold316	457406	G	A	NA	*OreG0009557*
scaffold316	459050	C	G	Och09,Och10,Och11	*OreG0009557*
scaffold316	469842	T	G	Ore09,Ore13	*OreG0009559*
scaffold316	475826	G	A	NA	*OreG0009561*
scaffold316	475922	G	T	Och08	*OreG0009561*
scaffold316	475934	G	A	Och04,Och05,Och07,Och13,Ore04,Ore05,Ore06,Ore11	*OreG0009561*
scaffold316	478964	A	T	Och07,Och08,Och14	*OreG0009562*
scaffold316	479489	A	T	Och07,Och08,Och14	*OreG0009562*
scaffold316	517017	C	T	Och06	*OreG0009566*
scaffold316	517187	C	A	Och01,Och06,Och09,Och10,Och11	*OreG0009566*
scaffold316	521755	T	C	NA	*OreG0009566*
scaffold316	530666	G	A	Och01,Och02,Och03,Och04,Och05,Och06,Och07,Och08,Och09,Och10,Och11,Och12,Och13,Och14,Ore01,Ore02,Ore03,Ore04,Ore05,Ore06,Ore07,Ore08,Ore09,Ore10,Ore11,Ore12,Ore13,Ore14	*OreG0009567*
scaffold316	534336	T	G	Och05,Ore03,Ore04,Ore05,Ore09,Ore10,Ore13,Ore14	*OreG0009568*
scaffold316	534566	G	T	Och05,Ore04,Ore05	*OreG0009568*
scaffold316	829861	C	A	NA	*OreG0009589*
scaffold316	830566	A	G	NA	*OreG0009590*
scaffold316	997231	C	A	NA	*OreG0009601*
scaffold316	997635	G	A	Och02,Och03,Och04,Och05,Och06,Och07,Och08,Och12,Och13,Och14	*OreG0009601*
scaffold316	1001620	A	G	NA	*OreG0009602*
scaffold316	1073612	T	A	NA	*OreG0009609*
scaffold316	1093621	G	A	Och09,Och10	*OreG0009611*
scaffold316	1114232	C	T	Och01,Och02,Och03,Och04,Och05,Och06,Och07,Och08,Och09,Och10,Och11,Och12,Och13,Och14,Ore01,Ore02,Ore03,Ore04,Ore05,Ore06,Ore07,Ore08,Ore09,Ore10,Ore11,Ore12,Ore13,Ore14	*OreG0009613*
scaffold316	1164081	G	A	Och12,Och13	*OreG0009617*
scaffold316	1164298	A	G	Ore01,Ore02,Ore03,Ore04,Ore05,Ore06,Ore07,Ore08,Ore09,Ore10,Ore11,Ore12,Ore13,Ore14	*OreG0009617*
scaffold316	1165777	G	A	NA	*OreG0009617*

scaffold316	1176398	T	C	Och01,Och02,Och03,Och04,Och05,Och06,Och07,Och08,Och09,Och10,Och11,Och12,Och13,Och14,Ore01,Ore02,Ore03,Ore04,Ore05,Ore06,Ore07,Ore08,Ore09,Ore10,Ore11,Ore12,Ore13,Ore14	*OreG0009620*
scaffold316	1177408	G	C	NA	*OreG0009620*
scaffold316	1178500	G	T	NA	*OreG0009620*
scaffold316	1188422	A	T	Ore01,Ore02,Ore03,Ore04,Ore05,Ore06,Ore07,Ore08,Ore09,Ore10,Ore11,Ore12,Ore13,Ore14	*OreG0009622*
scaffold316	1191365	C	T	NA	*OreG0009622*
scaffold316	1194395	A	C	Ore01,Ore02,Ore03,Ore04,Ore05,Ore06,Ore07,Ore08,Ore09,Ore10,Ore11,Ore12,Ore13,Ore14	*OreG0009623*
scaffold316	1194607	T	C	Och01,Och02,Och03,Och04,Och05,Och06,Och07,Och08,Och09,Och10,Och11,Och12,Och13,Och14	*OreG0009623*
scaffold316	1207204	G	C	NA	*OreG0009624*
scaffold316	1208635	A	G	Och11	*OreG0009624*
scaffold316	1211488	A	G	NA	*OreG0009624*
scaffold316	1212277	G	A	Och01,Och02,Och03,Och04,Och05,Och06,Och07,Och08,Och09,Och10,Och11,Och12,Och13,Och14,Ore01,Ore02,Ore03,Ore04,Ore05,Ore06,Ore07,Ore08,Ore09,Ore10,Ore11,Ore12,Ore13,Ore14	*OreG0009624*
scaffold316	1212390	G	A	NA	*OreG0009624*
scaffold316	1218739	C	T	Och01,Och02,Och03,Och04,Och05,Och06,Och07,Och08,Och09,Och10,Och11,Och12,Och13,Och14,Ore01,Ore02,Ore03,Ore04,Ore05,Ore06,Ore07,Ore08,Ore09,Ore10,Ore11,Ore12,Ore13,Ore14	*OreG0009624*
scaffold316	1237802	G	T	Och01,Och02,Och03,Och04,Och05,Och06,Och07,Och08,Och09,Och10,Och11,Och12,Och13,Och14,Ore01,Ore02,Ore03,Ore04,Ore05,Ore06,Ore07,Ore08,Ore09,Ore10,Ore11,Ore12,Ore13,Ore14	*OreG0009625*
scaffold316	1238515	T	C	Ore01	*OreG0009625*
scaffold316	1238948	T	C	Och01,Och02,Och03,Och04,Och05,Och06,Och07,Och09,Och10,Och11,Och12,Och13,Och14,Ore01,Ore02,Ore03,Ore04,Ore05,Ore06,Ore07,Ore08,Ore09,Ore10,Ore11,Ore12,Ore13,Ore14	*OreG0009625*
scaffold316	1243851	G	C	Och11,Och12,Och13	*OreG0009626*
scaffold316	1243918	A	G	Och03,Och06,Och07,Och08,Och11,Och12,Och13,Ore01,Ore02,Ore03,Ore04,Ore05,Ore06,Ore07,Ore08,Ore09,Ore10,Ore11,Ore12,Ore13,Ore14	*OreG0009626*
scaffold316	1243931	A	G	NA	*OreG0009626*
scaffold316	1247971	C	T	NA	*OreG0009627*
scaffold316	1249240	G	T	NA	*OreG0009627*
scaffold316	1251881	T	A	NA	*OreG0009627*
scaffold316	1272593	G	T	Och03,Och06,Och12,Och13,Och14,Ore03,Ore04,Ore05,Ore09,Ore14	*OreG0009629*
scaffold316	1289887	T	A	Och06	*OreG0009632*
scaffold316	1290324	A	G	NA	*OreG0009632*
scaffold316	1290400	A	T	Och01,Och02,Och03,Och04,Och05,Och06,Och07,Och08,Och09,Och10,Och11,Och12,Och13	*OreG0009632*
scaffold316	1293872	A	G	Och01,Och02,Och03,Och04,Och05,Och06,Och07,Och08,Och09,Och10,Och11,Och12,Och13	*OreG0009633*
scaffold17	88033	G	T	NA	*OreG0003466*
scaffold17	188929	G	C	NA	*OreG0003474*
scaffold17	207810	G	C	NA	*OreG0003474*
scaffold17	210848	C	T	NA	*OreG0003476*
scaffold17	211019	C	T	NA	*OreG0003476*
scaffold17	211198	A	T	Ore04,Ore05,Ore06,Ore09	*OreG0003476*
scaffold17	213469	T	A	NA	*OreG0003477*
scaffold17	213647	G	C	NA	*OreG0003477*
scaffold17	214251	C	A	Ore07,Ore08,Ore14	*OreG0003477*
scaffold17	214421	G	T	NA	*OreG0003477*
scaffold17	226548	T	A	NA	*OreG0003479*
scaffold17	226573	C	A	Ore04,Ore05,Ore06,Ore09	*OreG0003479*
scaffold17	227280	C	A	NA	*OreG0003479*
scaffold17	240839	T	A	NA	*OreG0003480*
scaffold17	260365	C	T	Och02,Och03,Och04,Och05,Och06,Och09,Och10,Och11,Och12,Och13	*OreG0003481*
scaffold17	305150	A	G	Och01,Och02,Och03,Och04,Och05,Och06,Och07,Och08,Och09,Och10,Och12,Och13,Och14	*OreG0003485*
scaffold17	313787	G	A	NA	*OreG0003486*
scaffold17	345958	C	T	Ore01,Ore02,Ore03,Ore04,Ore05,Ore06,Ore07,Ore08,Ore09,Ore10,Ore11,Ore12,Ore13,Ore14	*OreG0003488*
scaffold17	346387	C	T	Och01,Och02,Och06,Och07,Och08,Och09,Och10,Och11,Och14	*OreG0003488*
scaffold17	346662	A	T	NA	*OreG0003488*
scaffold17	352690	G	C	NA	*OreG0003489*
scaffold17	356270	G	A	NA	*OreG0003490*
scaffold17	356582	C	T	Och01,Och14	*OreG0003490*
scaffold17	357058	A	G	Ore07,Ore08,Ore14	*OreG0003490*
scaffold17	400822	G	A	Och01,Och02,Och03,Och04,Och05,Och06,Och07,Och08,Och09,Och10,Och11,Och12,Och13,Och14	*OreG0003495*
scaffold17	447345	C	T	NA	*OreG0003501*
scaffold17	447348	G	T	NA	*OreG0003501*
scaffold17	447497	C	G	NA	*OreG0003501*
scaffold17	467785	T	A	Och09,Och10	*OreG0003504*

scaffold17	469548	C	T	Och09,Och10	*OreG0003504*
scaffold17	531837	A	G	Och11	*OreG0003511*
scaffold17	532121	A	C	Och04,Och05,Och06,Och12,Och13	*OreG0003511*
scaffold17	532183	A	G	Och01,Och02,Och03,Och04,Och05,Och06,Och07,Och08,Och09,Och10,Och11,Och12,Och13,Och14	*OreG0003511*
scaffold17	532736	T	C	Ore01,Ore02,Ore03,Ore04,Ore05,Ore06,Ore07,Ore08,Ore09,Ore10,Ore11,Ore12,Ore13,Ore14	*OreG0003511*
scaffold17	532742	G	A	Och01,Och02,Och03,Och04,Och05,Och06,Och07,Och08,Och09,Och10,Och11,Och12,Och13,Och14	*OreG0003511*
scaffold17	532824	C	T	Ore01,Ore02,Ore03,Ore04,Ore05,Ore06,Ore07,Ore08,Ore09,Ore10,Ore11,Ore12,Ore13,Ore14	*OreG0003511*
scaffold17	532979	A	T	Ore01,Ore02,Ore03,Ore04,Ore05,Ore06,Ore07,Ore08,Ore09,Ore10,Ore11,Ore12,Ore13,Ore14	*OreG0003511*
scaffold17	549606	T	G	NA	*OreG0003514*
scaffold17	549658	G	C	NA	*OreG0003514*
scaffold17	550219	G	A	NA	*OreG0003514*
scaffold17	568218	G	T	Och01,Och02,Och03,Och04,Och05,Och06,Och07,Och08,Och09,Och10,Och11,Och12,Och13,Och14	*OreG0003520*
scaffold17	568253	C	G	NA	*OreG0003520*
scaffold17	578626	G	T	NA	*OreG0003521*
scaffold17	594432	C	T	NA	*OreG0003522*
scaffold17	595351	C	A	NA	*OreG0003522*
scaffold17	595405	G	T	Ore01,Ore02,Ore03,Ore04,Ore05,Ore06,Ore07,Ore08,Ore09,Ore10,Ore11,Ore12,Ore13,Ore14	*OreG0003522*
scaffold17	597818	T	A	NA	*OreG0003522*
scaffold17	598022	C	A	Ore01,Ore02,Ore03,Ore04,Ore05,Ore06,Ore07,Ore08,Ore09,Ore10,Ore11,Ore12,Ore13,Ore14	*OreG0003522*
scaffold17	602098	T	C	NA	*OreG0003524*
scaffold17	604531	C	T	NA	*OreG0003525*
scaffold17	604552	G	A	Ore06,Ore11	*OreG0003525*
scaffold17	605349	C	T	NA	*OreG0003525*
scaffold17	605580	A	C	NA	*OreG0003525*
scaffold17	606430	G	A	Och07,Ore01,Ore02,Ore03,Ore04,Ore05,Ore06,Ore07,Ore08,Ore09,Ore10,Ore11,Ore12,Ore13,Ore14	*OreG0003525*
scaffold17	606487	T	G	NA	*OreG0003525*
scaffold17	608282	A	G	NA	*OreG0003525*
scaffold17	616194	G	A	Och01,Och02,Och03,Och04,Och05,Och06,Och07,Och08,Och09,Och10,Och11,Och12,Och13,Och14,Ore01,Ore02,Ore03,Ore04,Ore05,Ore06,Ore07,Ore08,Ore09,Ore10,Ore11,Ore12,Ore13,Ore14	*OreG0003526*
scaffold109	17680	T	A	Och07,Och12	*OreG0000618*
scaffold109	18419	A	T	Och07,Och12,Och13	*OreG0000618*
scaffold109	18699	G	A	Och02,Och03,Och05,Och08,Ore06,Ore07,Ore08,Ore12,Ore13,Ore14	*OreG0000618*
scaffold109	20544	C	T	Och01,Och02,Och03,Och04,Och05,Och06,Och07,Och08,Och09,Och10,Och11,Och12,Och13,Och14,Ore01,Ore02,Ore03,Ore04,Ore05,Ore06,Ore07,Ore08,Ore09,Ore10,Ore11,Ore12,Ore13,Ore14	*OreG0000618*
scaffold109	196324	G	T	NA	*OreG0000628*
scaffold109	209106	C	A	Ore01	*OreG0000629*
scaffold109	218952	G	T	Och02,Och05	*OreG0000630*
scaffold109	219036	C	T	NA	*OreG0000630*
scaffold109	219072	C	T	NA	*OreG0000630*
scaffold109	219602	G	T	NA	*OreG0000630*
scaffold109	219605	A	G	NA	*OreG0000630*
scaffold109	246875	C	A	Och11	*OreG0000632*
scaffold109	248813	G	T	NA	*OreG0000633*
scaffold109	255081	G	A	Och02,Och04	*OreG0000633*
scaffold109	272471	G	A	NA	*OreG0000636*
scaffold109	321013	T	C	NA	*OreG0000642*
scaffold109	329521	G	A	NA	*OreG0000643*
scaffold109	329768	C	A	NA	*OreG0000643*
scaffold109	329786	A	G	NA	*OreG0000643*
scaffold109	329813	T	A	NA	*OreG0000643*
scaffold109	329902	G	T	NA	*OreG0000643*
scaffold109	330200	G	A	NA	*OreG0000643*
scaffold109	335546	C	T	Och08	*OreG0000644*
scaffold109	337068	T	A	NA	*OreG0000644*
scaffold109	337953	A	T	Och02,Och04,Och05,Och12,Och13	*OreG0000644*
scaffold109	363020	A	C	Och01,Och02,Och03,Och04,Och05,Och06,Och07,Och08,Och09,Och10,Och11,Och12,Och13,Och14,Ore01,Ore02,Ore03,Ore04,Ore05,Ore06,Ore07,Ore08,Ore09,Ore10,Ore11,Ore12,Ore13,Ore14	*OreG0000647*
scaffold109	430487	C	T	Och01,Och03,Och05,Och07,Och08,Och09,Och10,Och11,Och12,Och13,Och14	*OreG0000652*
scaffold109	436806	C	G	NA	*OreG0000654*
scaffold109	437718	T	A	NA	*OreG0000654*
scaffold109	472124	C	G	NA	*OreG0000656*
scaffold109	495112	C	G	Ore01,Ore02,Ore06,Ore07,Ore08,Ore09,Ore11,Ore12,Ore13,Ore14	*OreG0000658*
scaffold109	502182	C	A	NA	*OreG0000660*

scaffold109	502588	T	A	Och01,Och03,Och05,Och07,Och08,Och09,Och10,Och11,Och12,Och13,Och14,Ore01,Ore02,Ore06,Ore07,Ore08,Ore09,Ore11,Ore12,Ore13,Ore14	*OreG0000660*
scaffold109	505307	A	G	NA	*OreG0000661*
scaffold109	531720	G	T	NA	*OreG0000663*
scaffold109	538725	C	T	Ore01,Ore02,Ore06,Ore07,Ore08,Ore09,Ore11,Ore12,Ore13,Ore14	*OreG0000664*
scaffold109	596199	T	A	Ore01,Ore02,Ore03,Ore04,Ore05,Ore06,Ore07,Ore08,Ore09,Ore10,Ore11,Ore12,Ore13,Ore14	*OreG0000668*
scaffold109	596370	T	A	NA	*OreG0000668*
scaffold109	606936	C	A	Och06	*OreG0000669*
scaffold109	628428	T	A	Och08	*OreG0000670*
scaffold109	628712	C	T	NA	*OreG0000670*
scaffold109	635505	G	T	NA	*OreG0000671*
scaffold109	635616	T	G	NA	*OreG0000671*
scaffold109	635953	T	G	Och08	*OreG0000671*
scaffold109	636002	C	T	Och08,Ore01,Ore02,Ore03,Ore04,Ore05,Ore06,Ore07,Ore08,Ore09,Ore10,Ore11,Ore12,Ore13,Ore14	*OreG0000671*
scaffold109	636299	G	T	NA	*OreG0000671*
scaffold109	636320	C	T	Och01,Och12,Och14	*OreG0000671*
scaffold109	655743	A	T	Och01,Och02,Och03,Och04,Och05,Och06,Och07,Och08,Och09,Och10,Och11,Och12,Och13,Och14	*OreG0000673*
scaffold109	656071	T	C	Ore01,Ore02,Ore03,Ore04,Ore05,Ore06,Ore07,Ore08,Ore09,Ore10,Ore11,Ore12,Ore13,Ore14	*OreG0000674*
scaffold109	656212	G	T	Och01,Och02,Och03,Och04,Och05,Och06,Och07,Och08,Och09,Och10,Och11,Och12,Och13,Och14	*OreG0000674*
scaffold109	656264	C	G	Ore01,Ore02,Ore03,Ore04,Ore05,Ore06,Ore07,Ore08,Ore09,Ore10,Ore11,Ore12,Ore13,Ore14	*OreG0000674*
scaffold109	656697	A	C	Ore01,Ore02,Ore03,Ore04,Ore05,Ore06,Ore07,Ore08,Ore09,Ore10,Ore11,Ore12,Ore13,Ore14	*OreG0000674*
scaffold109	657637	G	A	Och01,Och02,Och03,Och04,Och05,Och06,Och07,Och08,Och09,Och10,Och11,Och12,Och13,Och14	*OreG0000675*
scaffold109	659579	A	T	Och01,Och02,Och03,Och04,Och05,Och06,Och07,Och08,Och09,Och10,Och11,Och12,Och13,Och14	*OreG0000675*
scaffold109	659582	C	T	NA	*OreG0000675*
scaffold109	666212	T	C	Och01,Och02,Och03,Och04,Och05,Och06,Och07,Och08,Och09,Och10,Och11,Och12,Och13,Och14	*OreG0000676*
scaffold109	666305	G	T	Och01,Och02,Och03,Och04,Och05,Och06,Och07,Och08,Och09,Och10,Och11,Och12,Och13,Och14,Ore01,Ore02,Ore03,Ore04,Ore05,Ore06,Ore07,Ore08,Ore09,Ore10,Ore11,Ore12,Ore13,Ore14	*OreG0000676*
scaffold109	667256	A	C	Ore01,Ore02,Ore03,Ore04,Ore05,Ore06,Ore07,Ore08,Ore09,Ore10,Ore11,Ore12,Ore13,Ore14	*OreG0000676*
scaffold109	682046	C	T	Och01,Och02,Och03,Och04,Och05,Och06,Och07,Och08,Och09,Och10,Och11,Och12,Och13,Och14	*OreG0000677*
scaffold109	805048	C	G	NA	*OreG0000685*
scaffold109	805124	T	C	NA	*OreG0000685*
scaffold109	826894	A	T	Och09,Och10,Och11	*OreG0000687*
scaffold109	827357	C	A	NA	*OreG0000687*
scaffold109	827849	C	T	NA	*OreG0000687*
scaffold109	833670	G	A	NA	*OreG0000689*
scaffold109	834656	A	G	NA	*OreG0000689*
scaffold109	835190	C	G	NA	*OreG0000689*
scaffold109	835405	G	T	NA	*OreG0000689*
scaffold109	835728	T	G	NA	*OreG0000689*
scaffold109	850382	T	G	Och02,Och04,Och06,Och07,Och08,Och13	*OreG0000691*
scaffold109	858347	A	T	Och01,Och02,Och03,Och04,Och05,Och06,Och07,Och08,Och09,Och10,Och11,Och12,Och13,Och14,Ore01,Ore02,Ore03,Ore04,Ore05,Ore06,Ore07,Ore08,Ore09,Ore10,Ore11,Ore12,Ore13,Ore14	*OreG0000694*
scaffold109	861369	A	G	Och01,Och02,Och04,Och06,Och13	*OreG0000694*
scaffold109	863469	A	T	Och01	*OreG0000694*
scaffold109	863538	A	T	Och13	*OreG0000694*
scaffold109	928995	A	C	Och01,Och02,Och03,Och04,Och05,Och06,Och07,Och08,Och09,Och10,Och11,Och12,Och13,Och14,Ore01,Ore02,Ore03,Ore04,Ore05,Ore06,Ore07,Ore08,Ore09,Ore10,Ore11,Ore12,Ore13,Ore14	*OreG0000700*
scaffold109	929549	C	T	NA	*OreG0000700*
scaffold109	972764	A	G	Och09,Och10,Och11	*OreG0000714*
scaffold109	973710	C	A	Ore01,Ore02,Ore03,Ore04,Ore05,Ore06,Ore07,Ore08,Ore09,Ore10,Ore11,Ore12,Ore13,Ore14	*OreG0000715*
scaffold109	974561	C	A	Och02,Och03,Och04,Och05,Och06,Och09,Och10,Och11,Och12,Och13	*OreG0000715*
scaffold109	975070	G	T	NA	*OreG0000715*
scaffold109	976816	G	A	Ore09	*OreG0000716*
scaffold109	982580	T	A	NA	*OreG0000716*
scaffold109	996969	G	A	NA	*OreG0000718*
scaffold109	997047	C	G	Och01,Och02,Och03,Och04,Och05,Och06,Och07,Och08,Och09,Och10,Och11,Och12,Och13,Och14	*OreG0000718*
scaffold109	998013	A	G	NA	*OreG0000718*
scaffold109	1003041	G	A	NA	*OreG0000719*

scaffold109	1004125	T	C	NA	*OreG0000719*
scaffold109	1007558	G	T	Ore01,Ore02,Ore03,Ore04,Ore05,Ore06,Ore07,Ore08,Ore09,Ore10,Ore11,Ore12,Ore13,Ore14	*OreG0000719*
scaffold109	1008979	G	T	Ore01,Ore02,Ore03,Ore04,Ore05,Ore06,Ore07,Ore08,Ore09,Ore10,Ore11,Ore12,Ore13,Ore14	*OreG0000719*
scaffold109	1019489	G	T	NA	*OreG0000720*
scaffold109	1044694	A	G	NA	*OreG0000721*
scaffold109	1045783	T	G	Och01,Och02,Och03,Och04,Och05,Och06,Och07,Och08,Och09,Och10,Och11,Och12,Och13,Och14	*OreG0000721*
scaffold109	1101214	C	T	Och01,Och02,Och03,Och04,Och05,Och06,Och07,Och08,Och09,Och10,Och11,Och12,Och13,Och14,Ore01,Ore02,Ore03,Ore04,Ore05,Ore06,Ore07,Ore08,Ore09,Ore10,Ore11,Ore12,Ore13,Ore14	*OreG0000727*
scaffold109	1150126	C	T	Ore01,Ore02,Ore03,Ore04,Ore05,Ore06,Ore07,Ore08,Ore09,Ore10,Ore11,Ore12,Ore13,Ore14	*OreG0000734*
scaffold109	1158174	C	T	Ore06,Ore11,Ore14	*OreG0000736*
scaffold109	1194670	A	G	Och01,Och02,Och03,Och04,Och05,Och06,Och07,Och08,Och09,Och10,Och11,Och12,Och13,Och14	*OreG0000740*
scaffold109	1212152	G	T	Och13	*OreG0000741*
scaffold109	1212334	C	A	Ore09	*OreG0000741*
scaffold109	1213575	T	C	NA	*OreG0000741*
scaffold109	1214327	G	C	Ore01,Ore02,Ore03,Ore04,Ore05,Ore06,Ore07,Ore08,Ore09,Ore10,Ore11,Ore12,Ore13,Ore14	*OreG0000741*
scaffold109	1216126	T	C	Ore02,Ore03,Ore06,Ore07,Ore08,Ore09,Ore10,Ore11,Ore12,Ore13,Ore14	*OreG0000743*
scaffold109	1216423	C	T	Ore01,Ore02,Ore03,Ore04,Ore05,Ore06,Ore07,Ore08,Ore09,Ore10,Ore11,Ore12,Ore13,Ore14	*OreG0000743*
scaffold109	1216771	T	C	NA	*OreG0000743*
scaffold109	1217234	T	A	NA	*OreG0000743*
scaffold109	1220342	C	A	Och01,Och02,Och03,Och04,Och05,Och06,Och07,Och08,Och09,Och10,Och11,Och12,Och13,Och14	*OreG0000742*
scaffold109	1240866	C	A	NA	*OreG0000747*
scaffold109	1244217	G	T	NA	*OreG0000747*
scaffold109	1250574	G	T	Ore01,Ore02,Ore03,Ore04,Ore05,Ore06,Ore07,Ore08,Ore09,Ore10,Ore11,Ore12,Ore13,Ore14	*OreG0000748*
scaffold109	1274965	G	T	Och05,Och08,Och12,Och13	*OreG0000751*
scaffold109	1274995	T	C	Ore04,Ore05,Ore06,Ore11,Ore14	*OreG0000751*
scaffold109	1305111	C	G	Och08	*OreG0000754*
scaffold109	1306786	A	G	NA	*OreG0000754*
scaffold109	1329874	A	G	NA	*OreG0000757*
scaffold109	1330243	C	A	Ore09	*OreG0000757*
scaffold109	1340273	A	C	Ore01,Ore02,Ore03,Ore04,Ore05,Ore06,Ore07,Ore08,Ore09,Ore10,Ore11,Ore12,Ore13,Ore14	*OreG0000758*
scaffold109	1488418	C	G	Ore01,Ore02,Ore03,Ore04,Ore05,Ore06,Ore07,Ore08,Ore09,Ore10,Ore11,Ore12,Ore13,Ore14	*OreG0000770*
scaffold109	1488422	T	C	NA	*OreG0000770*
scaffold109	1488439	C	A	Och03,Och05,Och12,Och13,Och14	*OreG0000770*
scaffold109	1488593	G	C	Och08	*OreG0000770*
scaffold109	1488670	T	C	Ore02,Ore03,Ore04,Ore05,Ore06,Ore08,Ore09,Ore10,Ore14	*OreG0000770*
scaffold109	1501109	T	C	NA	*OreG0000771*
scaffold109	1501981	A	T	Och08	*OreG0000771*
scaffold109	1533667	G	T	Och01,Och02,Och03,Och04,Och05,Och06,Och07,Och08,Och09,Och10,Och11,Och12,Och13,Och14	*OreG0000774*
scaffold109	1540750	T	C	Ore01,Ore02,Ore03,Ore04,Ore05,Ore06,Ore07,Ore08,Ore09,Ore10,Ore11,Ore12,Ore13,Ore14	*OreG0000775*
scaffold109	1542120	C	T	Ore06	*OreG0000775*
scaffold109	1549783	G	A	NA	*OreG0000776*
scaffold109	1665166	C	G	Och01,Och02,Och03,Och04,Och05,Och06,Och07,Och08,Och09,Och10,Och11,Och12,Och13,Och14,Ore01,Ore02,Ore03,Ore04,Ore05,Ore06,Ore07,Ore08,Ore09,Ore10,Ore11,Ore12,Ore13,Ore14	*OreG0000780*
scaffold109	1734764	G	C	NA	*OreG0000784*
scaffold109	1787833	C	T	Och01,Och02,Och03,Och04,Och05,Och06,Och07,Och08,Och09,Och10,Och11,Och12,Och13,Och14	*OreG0000785*
scaffold508	1923	C	T	NA	*OreG0023553*
scaffold508	2332	G	C	Och01,Och02,Och03,Och04,Och05,Och06,Och07,Och08,Och09,Och10,Och11,Och12,Och13,Och14,Ore01,Ore02,Ore03,Ore04,Ore05,Ore06,Ore07,Ore08,Ore09,Ore10,Ore11,Ore12,Ore13,Ore14	*OreG0023553*
scaffold45	30723	C	A	Ore01,Ore02,Ore03,Ore04,Ore05,Ore06,Ore07,Ore08,Ore09,Ore10,Ore11,Ore12,Ore13,Ore14	*OreG0020496*
scaffold45	30945	C	T	Ore01,Ore02,Ore03,Ore04,Ore05,Ore06,Ore07,Ore08,Ore09,Ore10,Ore11,Ore12,Ore13,Ore14	*OreG0020496*
scaffold45	30978	G	A	Och01,Och02,Och03,Och04,Och05,Och06,Och07,Och08,Och09,Och10,Och11,Och12,Och13,Och14	*OreG0020496*
scaffold45	31188	T	A	Ore01,Ore04,Ore05	*OreG0020496*
scaffold45	31203	A	T	Ore01,Ore02,Ore03,Ore04,Ore05,Ore06,Ore07,Ore08,Ore09,Ore10,Ore11,Ore12,Ore13,Ore14	*OreG0020496*
scaffold45	31580	T	C	Ore01,Ore02,Ore03,Ore04,Ore05,Ore06,Ore07,Ore08,Ore09,Ore10,Ore11,Ore12,Ore13,Ore14	*OreG0020496*

scaffold45	31815	G	A	Och02,Och04,Och06,Och07,Och09,Och10,Och14,Ore01,Ore02,Ore03,Ore04,Ore05,Ore06,Ore07,Ore08,Ore09,Ore10,Ore11,Ore12,Ore13,Ore14	*OreG0020496*
scaffold45	45887	C	T	Och09,Och10,Och13	*OreG0020499*
scaffold45	46298	C	T	NA	*OreG0020499*
scaffold45	46316	C	T	Ore01,Ore02,Ore03,Ore04,Ore05,Ore06,Ore07,Ore08,Ore09,Ore10,Ore11,Ore12,Ore13,Ore14	*OreG0020499*
scaffold45	46513	C	T	NA	*OreG0020499*
scaffold45	57272	C	A	Och01,Och02,Och03,Och04,Och05,Och06,Och07,Och08,Och09,Och10,Och11,Och12,Och13,Och14,Ore01,Ore02,Ore03,Ore04,Ore05,Ore06,Ore07,Ore08,Ore09,Ore10,Ore11,Ore12,Ore13,Ore14	*OreG0020501*
scaffold45	57282	A	T	NA	*OreG0020501*
scaffold45	62267	A	G	NA	*OreG0020502*
scaffold45	69683	A	C	Och01,Och02,Och03,Och04,Och05,Och06,Och07,Och08,Och09,Och10,Och11,Och12,Och13,Och14,Ore01,Ore02,Ore03,Ore04,Ore05,Ore06,Ore07,Ore08,Ore09,Ore10,Ore11,Ore12,Ore13,Ore14	*OreG0020502*
scaffold45	74601	G	A	Och01,Och02,Och03,Och04,Och05,Och06,Och07,Och08,Och09,Och10,Och11,Och12,Och13,Och14,Ore01,Ore02,Ore03,Ore04,Ore05,Ore06,Ore07,Ore08,Ore09,Ore10,Ore11,Ore12,Ore13,Ore14	*OreG0020502*
scaffold45	82289	G	A	Ore01,Ore02,Ore03,Ore04,Ore05,Ore06,Ore07,Ore08,Ore09,Ore10,Ore11,Ore12,Ore13,Ore14	*OreG0020503*
scaffold45	82398	G	T	Ore01,Ore02,Ore03,Ore04,Ore05,Ore06,Ore07,Ore08,Ore09,Ore10,Ore11,Ore12,Ore13,Ore14	*OreG0020503*
scaffold45	82828	G	A	Ore01,Ore02,Ore03,Ore04,Ore05,Ore06,Ore07,Ore08,Ore09,Ore10,Ore11,Ore12,Ore13,Ore14	*OreG0020504*
scaffold45	83059	G	T	NA	*OreG0020504*
scaffold45	99145	C	T	NA	*OreG0020506*
scaffold45	99277	A	T	Och01,Och02,Och03,Och04,Och05,Och06,Och07,Och08,Och09,Och10,Och11,Och12,Och13,Och14,Ore01,Ore02,Ore03,Ore04,Ore05,Ore06,Ore07,Ore08,Ore09,Ore10,Ore11,Ore12,Ore13,Ore14	*OreG0020506*
scaffold45	116318	A	T	Och04	*OreG0020507*
scaffold45	123264	G	T	Och01,Och13,Och14	*OreG0020510*
scaffold45	124333	C	A	NA	*OreG0020510*
scaffold45	124582	G	A	NA	*OreG0020510*
scaffold45	131718	T	A	Och04	*OreG0020511*
scaffold45	131742	C	A	Och01,Och02,Och03,Och04,Och05,Och06,Och07,Och08,Och09,Och10,Och11,Och12,Och13,Och14,Ore01,Ore02,Ore03,Ore04,Ore05,Ore06,Ore07,Ore08,Ore09,Ore10,Ore11,Ore12,Ore13,Ore14	*OreG0020511*
scaffold45	132194	C	A	Och07,Och08,Och09,Och10,Och11,Och13,Och14	*OreG0020511*
scaffold45	132651	T	C	NA	*OreG0020511*
scaffold45	132849	T	C	Och09,Och10	*OreG0020511*
scaffold45	179137	C	T	Och01,Och02,Och03,Och04,Och05,Och06,Och12,Och13	*OreG0020516*
scaffold45	181163	A	C	NA	*OreG0020516*
scaffold45	184222	G	A	NA	*OreG0020516*
scaffold45	184225	C	T	NA	*OreG0020516*
scaffold45	184228	C	T	NA	*OreG0020516*
scaffold45	272940	C	T	Ore06	*OreG0020525*
scaffold45	299841	C	T	NA	*OreG0020529*
scaffold45	300012	G	T	NA	*OreG0020529*
scaffold45	300036	T	G	NA	*OreG0020529*
scaffold45	300144	C	T	NA	*OreG0020529*
scaffold45	300175	G	A	NA	*OreG0020529*
scaffold45	300542	T	A	NA	*OreG0020529*
scaffold45	300875	A	G	NA	*OreG0020529*
scaffold45	301299	G	T	Och01	*OreG0020529*
scaffold45	301356	A	G	NA	*OreG0020529*
scaffold45	301431	T	C	NA	*OreG0020529*
scaffold45	301460	G	T	NA	*OreG0020529*
scaffold45	301521	C	T	NA	*OreG0020529*
scaffold45	301572	T	G	NA	*OreG0020529*
scaffold45	301579	C	A	NA	*OreG0020529*
scaffold45	308325	T	C	NA	*OreG0020530*
scaffold45	308519	T	C	NA	*OreG0020530*
scaffold45	308525	T	A	NA	*OreG0020530*
scaffold45	308684	A	G	NA	*OreG0020530*
scaffold45	308761	C	T	NA	*OreG0020530*
scaffold45	308935	C	T	Och14	*OreG0020530*
scaffold45	309022	G	T	NA	*OreG0020530*
scaffold45	313199	T	C	NA	*OreG0020530*
scaffold45	313229	G	A	NA	*OreG0020530*
scaffold45	316027	T	C	Och07,Och08,Och11,Och14,Ore01,Ore02,Ore03,Ore04,Ore05,Ore06,Ore07,Ore08,Ore09,Ore10,Ore11,Ore12,Ore13,Ore14	*OreG0020532*
scaffold45	316054	G	A	NA	*OreG0020532*
scaffold45	316716	G	A	NA	*OreG0020532*
scaffold45	316717	T	A	NA	*OreG0020532*
scaffold45	316996	C	T	NA	*OreG0020533*

scaffold45	317065	A	C	NA	*OreG0020533*
scaffold45	317104	T	C	NA	*OreG0020533*
scaffold45	333595	C	T	NA	*OreG0020535*
scaffold45	333636	A	T	Ore01,Ore02,Ore03,Ore04,Ore05,Ore06,Ore07,Ore08,Ore09,Ore10,Ore11,Ore12,Ore13,Ore14	*OreG0020535*
scaffold45	335234	A	G	Och02,Och03,Och04,Och05,Och06,Och12,Och13	*OreG0020536*
scaffold45	336133	C	T	NA	*OreG0020536*
scaffold45	337335	G	C	NA	*OreG0020536*
scaffold45	338635	T	A	Ore01,Ore02,Ore03,Ore04,Ore05,Ore06,Ore07,Ore08,Ore09,Ore10,Ore11,Ore12,Ore13,Ore14	*OreG0020536*
scaffold45	342641	C	A	NA	*OreG0020536*
scaffold45	352282	C	T	Och01	*OreG0020539*
scaffold45	352306	C	A	Ore01,Ore02,Ore03,Ore04,Ore05,Ore06,Ore07,Ore08,Ore09,Ore10,Ore11,Ore12,Ore13,Ore14	*OreG0020539*
scaffold45	353455	A	C	NA	*OreG0020539*
scaffold45	360551	G	A	NA	*OreG0020539*
scaffold45	360553	C	T	NA	*OreG0020539*
scaffold45	364800	G	T	Och01	*OreG0020541*
scaffold45	365317	A	G	NA	*OreG0020541*
scaffold45	365907	G	A	NA	*OreG0020541*
scaffold45	366246	G	A	Och01,Och02,Och03,Och04,Och05,Och06,Och07,Och08,Och09,Och10,Och11,Och12,Och13,Och14,Ore01,Ore02,Ore03,Ore04,Ore05,Ore06,Ore07,Ore08,Ore09,Ore10,Ore11,Ore12,Ore13,Ore14	*OreG0020541*
scaffold45	371229	T	C	Ore07,Ore08,Ore11,Ore12,Ore13,Ore14	*OreG0020542*
scaffold45	374559	C	G	NA	*OreG0020543*
scaffold45	374658	G	A	NA	*OreG0020543*
scaffold45	374666	G	A	NA	*OreG0020543*
scaffold45	382475	G	A	Ore07,Ore08,Ore14	*OreG0020543*
scaffold45	386598	A	G	NA	*OreG0020544*
scaffold45	386875	A	G	Ore01,Ore02,Ore03,Ore04,Ore05,Ore06,Ore07,Ore08,Ore09,Ore10,Ore11,Ore12,Ore13,Ore14	*OreG0020544*
scaffold45	387309	T	C	NA	*OreG0020544*
scaffold45	394484	T	C	NA	*OreG0020545*
scaffold45	394745	A	G	NA	*OreG0020545*
scaffold45	394904	T	A	NA	*OreG0020545*
scaffold45	419800	C	G	NA	*OreG0020547*
scaffold45	482836	C	T	Ore07,Ore08,Ore14	*OreG0020550*
scaffold45	542655	G	A	Ore01,Ore02,Ore03,Ore04,Ore05,Ore06,Ore07,Ore08,Ore09,Ore10,Ore11,Ore12,Ore13,Ore14	*OreG0020554*
scaffold45	542829	C	T	Och01,Och02,Och03,Och04,Och05,Och06,Och07,Och08,Och09,Och10,Och11,Och12,Och13,Och14	*OreG0020554*
scaffold45	543020	G	A	NA	*OreG0020554*
scaffold45	630671	A	G	Och02,Och06	*OreG0020558*
scaffold45	656350	C	T	NA	*OreG0020558*
scaffold323	47555	T	G	NA	*OreG0010424*
scaffold323	132037	C	G	Ore01,Ore02,Ore03,Ore04,Ore05,Ore06,Ore07,Ore08,Ore09,Ore10,Ore11,Ore12,Ore13,Ore14	*OreG0010429*
scaffold323	183580	C	G	NA	*OreG0010431*
scaffold323	264322	G	T	Och14	*OreG0010436*
scaffold323	474098	C	A	NA	*OreG0010450*
scaffold323	474305	C	A	Och01,Och02,Och03,Och04,Och05,Och06,Och07,Och08,Och09,Och10,Och11,Och12,Och13,Och14	*OreG0010450*
scaffold323	474308	T	C	Och01,Och02,Och03,Och04,Och05,Och06,Och07,Och08,Och09,Och10,Och11,Och12,Och13,Och14,Ore01,Ore02,Ore03,Ore04,Ore05,Ore06,Ore07,Ore08,Ore09,Ore10,Ore11,Ore12,Ore13,Ore14	*OreG0010450*
scaffold323	477487	T	A	Och01,Och02,Och03,Och04,Och05,Och06,Och07,Och08,Och09,Och10,Och11,Och12,Och13,Och14	*OreG0010451*
scaffold323	479040	A	G	Och12,Och13	*OreG0010451*
scaffold323	510151	T	C	NA	*OreG0010453*
scaffold323	511322	G	A	NA	*OreG0010453*
scaffold323	511346	G	A	Och01,Och05,Och13	*OreG0010453*
scaffold323	511392	A	C	Och06,Och08	*OreG0010453*
scaffold323	511401	G	A	Ore01,Ore02,Ore03,Ore04,Ore05,Ore06,Ore07,Ore08,Ore09,Ore10,Ore11,Ore12,Ore13,Ore14	*OreG0010453*
scaffold323	511446	A	T	Ore01,Ore02,Ore03,Ore04,Ore05,Ore06,Ore07,Ore08,Ore09,Ore10,Ore11,Ore12,Ore13,Ore14	*OreG0010453*
scaffold323	511518	C	T	Och06,Och08	*OreG0010453*
scaffold323	511559	G	A	Ore04,Ore05	*OreG0010453*
scaffold323	511647	C	T	NA	*OreG0010453*
scaffold323	511649	G	A	Och06,Och08	*OreG0010453*
scaffold323	518752	G	A	NA	*OreG0010454*
scaffold323	520402	G	A	NA	*OreG0010454*
scaffold323	522876	A	G	Och01,Och02,Och03,Och04,Och05,Och06,Och07,Och08,Och09,Och10,Och11,Och12,Och13,Och14	*OreG0010455*

scaffold323	522942	G	T	Ore01,Ore02,Ore03,Ore04,Ore05,Ore06,Ore07,Ore08,Ore09,Ore10,Ore11,Ore12,Ore13,Ore14	*OreG0010455*
scaffold323	524672	G	A	Ore04,Ore05	*OreG0010455*
scaffold323	524771	A	T	Ore01,Ore02,Ore03,Ore04,Ore05,Ore06,Ore07,Ore08,Ore09,Ore10,Ore11,Ore12,Ore13,Ore14	*OreG0010455*
scaffold323	534991	A	C	NA	*OreG0010456*
scaffold323	546417	G	T	Ore01,Ore02,Ore03,Ore04,Ore05,Ore06,Ore07,Ore08,Ore09,Ore10,Ore11,Ore12,Ore13,Ore14	*OreG0010457*
scaffold323	546475	G	T	NA	*OreG0010457*
scaffold323	618118	C	T	NA	*OreG0010464*
scaffold323	670815	C	G	Och01,Och06,Och07,Och08,Och09,Och10,Och11,Och12,Och13,Och14,Ore01,Ore02,Ore03,Ore04,Ore05,Ore06,Ore07,Ore08,Ore09,Ore10,Ore11,Ore12,Ore13,Ore14	*OreG0010469*
scaffold323	705138	G	T	NA	*OreG0010471*
scaffold323	705200	T	A	NA	*OreG0010471*
scaffold323	705408	C	T	NA	*OreG0010471*
scaffold323	705423	C	T	Och01,Och06,Och07,Och08,Och09,Och10,Och11,Och12,Och13	*OreG0010471*
scaffold323	711479	G	A	NA	*OreG0010472*
scaffold323	867623	A	G	NA	*OreG0010477*
scaffold323	889967	G	T	Och01,Och02,Och03,Och04,Och05,Och06,Och07,Och08,Och09,Och10,Och11,Och12,Och13,Och14	*OreG0010479*
scaffold323	890564	C	A	Ore01,Ore02,Ore03,Ore04,Ore05,Ore06,Ore07,Ore08,Ore09,Ore10,Ore11,Ore12,Ore13,Ore14	*OreG0010479*
scaffold323	906662	G	A	Och01,Och02,Och03,Och04,Och05,Och06,Och07,Och08,Och09,Och10,Och11,Och12,Och13,Och14,Ore01,Ore02,Ore03,Ore04,Ore05,Ore06,Ore07,Ore08,Ore09,Ore10,Ore11,Ore12,Ore13,Ore14	*OreG0010480*
scaffold323	963926	C	A	Ore01,Ore02,Ore03,Ore04,Ore05,Ore06,Ore07,Ore08,Ore09,Ore10,Ore11,Ore12,Ore13,Ore14	*OreG0010486*
scaffold323	993461	C	G	Och02,Och06	*OreG0010487*
scaffold323	1000296	G	A	Och09,Och10	*OreG0010489*
scaffold323	1050777	G	A	NA	*OreG0010492*
scaffold323	1066496	T	C	Och02,Och03,Och04,Och05,Och06,Och09,Och10,Och12,Och13,Ore01,Ore02,Ore03,Ore04,Ore05,Ore06,Ore07,Ore08,Ore09,Ore10,Ore11,Ore12,Ore13,Ore14	*OreG0010495*
scaffold323	1095555	A	C	NA	*OreG0010497*
scaffold323	1095642	A	T	NA	*OreG0010497*
scaffold323	1095691	C	G	Ore08	*OreG0010497*
scaffold323	1097130	C	T	Och09,Och10	*OreG0010497*
scaffold323	1097350	T	C	NA	*OreG0010497*
scaffold323	1097958	G	A	Och05	*OreG0010497*
scaffold323	1098138	G	A	Ore01,Ore02,Ore03,Ore04,Ore05,Ore06,Ore07,Ore08,Ore09,Ore10,Ore11,Ore12,Ore13,Ore14	*OreG0010497*
scaffold323	1106204	G	A	NA	*OreG0010499*
scaffold323	1106379	C	T	NA	*OreG0010499*
scaffold323	1107516	G	C	Och01,Och02,Och03,Och04,Och05,Och06,Och07,Och08,Och09,Och10,Och11,Och12,Och13,Och14	*OreG0010499*
scaffold323	1107651	C	T	Ore01,Ore02,Ore03,Ore04,Ore05,Ore06,Ore07,Ore08,Ore09,Ore10,Ore11,Ore12,Ore13,Ore14	*OreG0010499*
scaffold323	1107687	G	A	Och01,Och02,Och03,Och04,Och05,Och06,Och07,Och08,Och09,Och10,Och11,Och12,Och13,Och14,Ore01,Ore02,Ore03,Ore04,Ore05,Ore06,Ore07,Ore08,Ore09,Ore10,Ore11,Ore12,Ore13,Ore14	*OreG0010499*
scaffold323	1108965	G	T	NA	*OreG0010499*
scaffold323	1136277	C	T	Och02,Och06	*OreG0010501*
scaffold323	1173582	C	T	NA	*OreG0010503*
scaffold323	1191345	T	A	NA	*OreG0010505*
scaffold323	1289561	C	G	NA	*OreG0010518*
scaffold323	1290226	G	T	NA	*OreG0010518*
scaffold323	1292153	A	C	NA	*OreG0010519*
scaffold323	1292331	G	A	NA	*OreG0010519*
scaffold323	1293639	A	G	NA	*OreG0010520*
scaffold323	1293667	C	A	NA	*OreG0010520*
scaffold323	1293955	C	A	NA	*OreG0010520*
scaffold323	1293962	A	C	NA	*OreG0010520*
scaffold323	1294233	G	A	NA	*OreG0010520*
scaffold323	1294245	A	G	Och11,Och12	*OreG0010520*
scaffold323	1303019	T	A	Och11,Och12	*OreG0010521*
scaffold323	1303078	A	T	NA	*OreG0010521*
scaffold323	1303394	G	A	NA	*OreG0010521*
scaffold323	1309980	G	T	Och01,Och02,Och03,Och04,Och05,Och06,Och07,Och08,Och09,Och10,Och11,Och12,Och13,Och14,Ore01,Ore02,Ore03,Ore04,Ore05,Ore06,Ore07,Ore08,Ore09,Ore10,Ore11,Ore12,Ore13,Ore14	*OreG0010522*
scaffold323	1310000	G	C	NA	*OreG0010522*
scaffold323	1313364	A	T	NA	*OreG0010522*
scaffold323	1327942	T	C	NA	*OreG0010524*
scaffold323	1340760	G	A	NA	*OreG0010526*
scaffold323	1340934	G	A	Ore02,Ore06,Ore08,Ore09,Ore10,Ore12,Ore14	*OreG0010526*
scaffold323	1341274	A	G	NA	*OreG0010526*

scaffold323	1341307	G	A	NA	*OreG0010526*
scaffold323	1343611	A	C	Och01,Och02,Och03,Och04,Och05,Och06,Och08,Och11,Och12,Och13,Och14,Ore02,Ore06,Ore08,Ore09,Ore10,Ore12,Ore14	*OreG0010526*
scaffold323	1343710	A	G	NA	*OreG0010526*
scaffold323	1343868	C	A	Ore02,Ore06,Ore08,Ore09,Ore10,Ore12,Ore14	*OreG0010526*
scaffold323	1357604	G	A	Och05,Och13	*OreG0010528*
scaffold323	1359640	G	T	NA	*OreG0010529*
scaffold218	186335	C	A	Och01,Och02,Och03,Och04,Och05,Och06,Och07,Och08,Och09,Och10,Och11,Och12,Och13,Och14	*OreG0006134*
scaffold218	274698	G	T	Och01,Och02,Och03,Och04,Och05,Och06,Och07,Och08,Och09,Och10,Och11,Och12,Och13,Och14,Ore01,Ore02,Ore03,Ore04,Ore05,Ore06,Ore07,Ore08,Ore09,Ore10,Ore11,Ore12,Ore13,Ore14	*OreG0006135*
scaffold218	275828	A	C	Ore01,Ore02,Ore03,Ore04,Ore05,Ore06,Ore07,Ore08,Ore09,Ore10,Ore11,Ore12,Ore13,Ore14	*OreG0006135*
scaffold218	306029	A	G	Och01,Och02,Och03,Och04,Och05,Och06,Och07,Och08,Och09,Och10,Och11,Och12,Och13,Och14,Ore01,Ore02,Ore03,Ore04,Ore05,Ore06,Ore07,Ore08,Ore09,Ore10,Ore11,Ore12,Ore13,Ore14	*OreG0006136*
scaffold218	370897	T	G	NA	*OreG0006139*
scaffold218	371275	T	C	Ore01,Ore02,Ore03,Ore04,Ore05,Ore06,Ore07,Ore08,Ore09,Ore10,Ore11,Ore12,Ore13,Ore14	*OreG0006139*
scaffold218	371303	T	C	Och01,Och11,Och14	*OreG0006139*
scaffold218	372041	A	T	Ore03,Ore08,Ore09,Ore10,Ore14	*OreG0006139*
scaffold218	506853	C	T	NA	*OreG0006143*
scaffold218	507360	G	C	NA	*OreG0006143*
scaffold218	509158	C	T	NA	*OreG0006143*
scaffold218	559037	G	T	NA	*OreG0006145*
scaffold218	581991	C	A	NA	*OreG0006147*
scaffold218	584898	A	T	Ore01,Ore03,Ore06,Ore10,Ore11,Ore13	*OreG0006148*
scaffold218	584952	C	T	NA	*OreG0006148*
scaffold218	630887	C	T	Ore01,Ore03,Ore06,Ore10,Ore11,Ore13	*OreG0006151*
scaffold218	630926	T	C	NA	*OreG0006151*
scaffold218	630956	G	A	Och02,Och03,Och04,Och05,Och06,Och07,Och08,Och09,Och10,Och11,Och12,Och13,Och14	*OreG0006151*
scaffold218	686204	G	T	Ore01,Ore02,Ore03,Ore04,Ore05,Ore06,Ore07,Ore08,Ore09,Ore10,Ore11,Ore12,Ore13,Ore14	*OreG0006154*
scaffold218	686325	C	G	Och01,Och02,Och03,Och04,Och05,Och06,Och07,Och08,Och09,Och10,Och11,Och12,Och13,Och14,Ore01,Ore02,Ore03,Ore04,Ore05,Ore06,Ore07,Ore08,Ore09,Ore10,Ore11,Ore12,Ore13,Ore14	*OreG0006154*
scaffold218	710250	A	G	NA	*OreG0006156*
scaffold218	710520	G	T	NA	*OreG0006156*
scaffold218	897495	C	T	Ore01,Ore03,Ore06,Ore10,Ore11,Ore13	*OreG0006165*
scaffold218	898059	G	A	NA	*OreG0006165*
scaffold218	898837	C	T	NA	*OreG0006165*
scaffold218	903569	G	A	NA	*OreG0006165*
scaffold218	910502	T	A	NA	*OreG0006166*
scaffold218	910577	T	C	NA	*OreG0006166*
scaffold218	910586	A	T	Ore01,Ore02,Ore03,Ore04,Ore05,Ore06,Ore07,Ore08,Ore09,Ore10,Ore11,Ore12,Ore13,Ore14	*OreG0006166*
scaffold218	1019877	T	A	Och06	*OreG0006175*
scaffold218	1019967	T	G	NA	*OreG0006175*
scaffold454	72293	G	A	Ore05,Ore06,Ore11,Ore14	*OreG0021783*
scaffold664	4991	T	C	Och01,Och02,Och03,Och04,Och05,Och06,Och07,Och08,Och09,Och10,Och11,Och12,Och13,Och14,Ore01,Ore02,Ore03,Ore04,Ore05,Ore06,Ore07,Ore08,Ore09,Ore10,Ore11,Ore12,Ore13,Ore14	*OreG0024350*
scaffold664	8861	T	G	NA	*OreG0024351*
scaffold25	481745	G	A	Och01,Och02,Och03,Och04,Och05,Och06,Och07,Och08,Och09,Och10,Och11,Och12,Och13,Och14,Ore01,Ore02,Ore03,Ore04,Ore05,Ore06,Ore07,Ore08,Ore09,Ore10,Ore11,Ore12,Ore13,Ore14	*OreG0007567*
scaffold585	140008	C	T	NA	*OreG0023798*
scaffold585	158789	T	C	Och02,Och06	*OreG0023800*
scaffold585	159019	A	T	Ore01,Ore02,Ore03,Ore04,Ore05,Ore06,Ore07,Ore08,Ore09,Ore10,Ore11,Ore12,Ore13,Ore14	*OreG0023800*
scaffold585	160321	A	T	Ore01,Ore02,Ore03,Ore04,Ore05,Ore06,Ore07,Ore08,Ore09,Ore10,Ore11,Ore12,Ore13,Ore14	*OreG0023800*
scaffold585	160372	G	A	Ore01,Ore02,Ore03,Ore04,Ore05,Ore06,Ore07,Ore08,Ore09,Ore10,Ore11,Ore12,Ore13,Ore14	*OreG0023800*
scaffold585	160399	A	G	Ore01,Ore02,Ore03,Ore04,Ore05,Ore06,Ore07,Ore08,Ore09,Ore10,Ore11,Ore12,Ore13,Ore14	*OreG0023800*
scaffold585	160448	C	T	Och01,Och02,Och03,Och04,Och05,Och06,Och07,Och08,Och09,Och10,Och11,Och12,Och13,Och14,Ore01,Ore02,Ore03,Ore04,Ore05,Ore06,Ore07,Ore08,Ore09,Ore10,Ore11,Ore12,Ore13,Ore14	*OreG0023800*
scaffold585	162176	C	T	NA	*OreG0023800*
scaffold585	162183	C	T	NA	*OreG0023800*
scaffold585	162327	G	A	NA	*OreG0023800*

scaffold585	167020	G	A	Ore01,Ore02,Ore03,Ore04,Ore05,Ore06,Ore07,Ore08,Ore09,Ore10,Ore11,Ore12,Ore13,Ore14	*OreG0023801*
scaffold585	168189	G	C	Och01,Och03,Och04,Och07,Och13,Och14	*OreG0023801*
scaffold585	168215	C	G	NA	*OreG0023801*
scaffold585	168601	A	G	NA	*OreG0023801*
scaffold585	169639	G	A	Ore06	*OreG0023801*
scaffold232	896137	C	A	Ore01,Ore02,Ore03,Ore04,Ore05,Ore06,Ore07,Ore08,Ore09,Ore10,Ore11,Ore12,Ore13,Ore14	*OreG0007053*
scaffold232	896195	A	T	Ore01,Ore02,Ore03,Ore04,Ore05,Ore06,Ore07,Ore08,Ore09,Ore10,Ore11,Ore12,Ore13,Ore14	*OreG0007053*
scaffold232	896199	T	A	Och01,Och02,Och03,Och04,Och05,Och06,Och07,Och08,Och09,Och10,Och11,Och12,Och13,Och14,Ore01,Ore02,Ore03,Ore04,Ore05,Ore06,Ore07,Ore08,Ore09,Ore10,Ore11,Ore12,Ore13,Ore14	*OreG0007053*
scaffold232	896357	G	A	Och03,Och05,Och12,Och13,Ore01,Ore02,Ore03,Ore04,Ore05,Ore06,Ore07,Ore08,Ore09,Ore10,Ore11,Ore12,Ore13,Ore14	*OreG0007053*
scaffold232	896400	C	T	Ore03,Ore06,Ore07,Ore08,Ore12,Ore13,Ore14	*OreG0007053*
scaffold490	52107	G	A	Ore01,Ore02,Ore03,Ore04,Ore05,Ore06,Ore07,Ore08,Ore09,Ore10,Ore11,Ore12,Ore13,Ore14	*OreG0022990*
scaffold490	56641	G	T	Och01,Och02,Och03,Och04,Och05,Och06,Och07,Och12,Och13	*OreG0022990*
scaffold490	74929	C	A	Och11	*OreG0022991*
scaffold490	93874	C	A	Ore02,Ore04,Ore05	*OreG0022994*
scaffold490	103173	A	T	Ore01,Ore02,Ore03,Ore04,Ore05,Ore06,Ore07,Ore08,Ore09,Ore10,Ore11,Ore12,Ore13,Ore14	*OreG0022995*
scaffold490	103586	G	T	Ore03	*OreG0022995*
scaffold490	114607	T	C	Ore01,Ore02,Ore03,Ore04,Ore05,Ore06,Ore07,Ore08,Ore09,Ore10,Ore11,Ore12,Ore13,Ore14	*OreG0022995*
scaffold490	120800	C	T	Och11	*OreG0022996*
scaffold490	120848	C	A	NA	*OreG0022996*
scaffold490	136776	T	A	Och01,Och02,Och03,Och04,Och05,Och06,Och07,Och08,Och09,Och10,Och11,Och12,Och13,Och14,Ore01,Ore02,Ore03,Ore04,Ore05,Ore06,Ore07,Ore08,Ore09,Ore10,Ore11,Ore12,Ore13,Ore14	*OreG0022997*
scaffold490	164089	C	A	Och01,Och02,Och03,Och04,Och05,Och06,Och07,Och08,Och09,Och10,Och11,Och12,Och13,Och14,Ore01,Ore02,Ore03,Ore04,Ore05,Ore06,Ore07,Ore08,Ore09,Ore10,Ore11,Ore12,Ore13,Ore14	*OreG0023002*
scaffold490	168205	A	G	NA	*OreG0023003*
scaffold490	201362	C	T	Och02,Och03,Och04,Och05,Och06,Och07,Och08,Och09,Och10,Och11,Och12,Och13,Och14	*OreG0023004*
scaffold490	201512	G	T	Ore01,Ore02,Ore03,Ore04,Ore05,Ore06,Ore07,Ore08,Ore09,Ore10,Ore11,Ore12,Ore13,Ore14	*OreG0023004*
scaffold490	207490	G	C	Och01,Och02,Och03,Och04,Och05,Och06,Och07,Och08,Och09,Och10,Och11,Och12,Och13,Och14	*OreG0023005*
scaffold490	294535	G	C	NA	*OreG0023013*
scaffold490	308012	C	T	NA	*OreG0023013*
scaffold490	309686	T	A	NA	*OreG0023013*
scaffold490	312884	G	A	NA	*OreG0023014*
scaffold490	313426	G	A	NA	*OreG0023014*
scaffold490	316926	C	A	NA	*OreG0023014*
scaffold490	323246	A	T	Ore04,Ore05	*OreG0023015*
scaffold490	351993	C	T	Och01,Och02,Och03,Och04,Och05,Och06,Och07,Och08,Och09,Och10,Och11,Och12,Och13,Och14,Ore01,Ore02,Ore03,Ore04,Ore05,Ore06,Ore07,Ore08,Ore09,Ore10,Ore11,Ore12,Ore13,Ore14	*OreG0023016*
scaffold490	352005	G	A	Och05	*OreG0023016*
scaffold490	387018	G	A	Ore01,Ore02,Ore07,Ore08,Ore09,Ore12,Ore13	*OreG0023020*
scaffold490	389182	G	T	Och05	*OreG0023020*
scaffold490	392906	C	T	Och05	*OreG0023021*
scaffold386	61095	T	A	Och01,Och02,Och03,Och04,Och05,Och06,Och07,Och08,Och09,Och10,Och11,Och12,Och13,Och14,Ore01,Ore02,Ore03,Ore04,Ore05,Ore06,Ore07,Ore08,Ore09,Ore10,Ore11,Ore12,Ore13,Ore14	*OreG0019535*
scaffold386	61219	A	G	Och01,Och02,Och03,Och04,Och05,Och06,Och07,Och08,Och09,Och10,Och11,Och12,Och13,Och14	*OreG0019535*
scaffold386	272380	C	A	Ore01,Ore02,Ore03,Ore04,Ore05,Ore06,Ore07,Ore08,Ore09,Ore10,Ore11,Ore12,Ore13,Ore14	*OreG0019538*
scaffold386	272395	G	A	Ore01,Ore02,Ore03,Ore04,Ore05,Ore06,Ore07,Ore08,Ore09,Ore10,Ore11,Ore12,Ore13,Ore14	*OreG0019538*
scaffold386	272461	G	A	Och01,Och02,Och03,Och04,Och05,Och06,Och07,Och08,Och09,Och10,Och11,Och12,Och13,Och14	*OreG0019538*
scaffold386	273110	G	T	Och01,Och02,Och03,Och04,Och05,Och06,Och07,Och08,Och09,Och10,Och11,Och12,Och13,Och14,Ore01,Ore02,Ore03,Ore04,Ore05,Ore06,Ore07,Ore08,Ore09,Ore10,Ore11,Ore12,Ore13,Ore14	*OreG0019538*
scaffold386	728143	G	T	Och07	*OreG0019539*
scaffold386	737055	G	A	Och01,Och11	*OreG0019539*
scaffold386	738984	A	T	NA	*OreG0019539*
scaffold386	1007427	T	A	Och01,Och02,Och03,Och04,Och05,Och06,Och07,Och08,Och09,Och10,Och11,Och12,Och13,Och14,Ore01,Ore02,Ore03,Ore04,Ore05,Ore06,Ore07,Ore08,Ore09,Ore10,Ore11,Ore12,Ore13,Ore14	*OreG0019545*

scaffold386	1007500	C	A	Och01,Och02,Och03,Och04,Och05,Och06,Och07,Och08,Och09,Och10,Och11,Och12,Och13,Och14	*OreG0019545*
scaffold386	1012828	G	T	NA	*OreG0019546*
scaffold386	1012927	C	A	NA	*OreG0019546*
scaffold386	1504061	G	C	Ore01,Ore02,Ore03,Ore04,Ore05,Ore06,Ore07,Ore08,Ore09,Ore10,Ore11,Ore12,Ore13,Ore14	*OreG0019555*
scaffold386	1580084	C	T	NA	*OreG0019558*
scaffold386	1593468	T	A	NA	*OreG0019558*
scaffold187	118221	T	C	NA	*OreG0004280*
scaffold187	118443	C	T	NA	*OreG0004280*
scaffold187	118469	A	G	NA	*OreG0004280*
scaffold187	119799	G	C	NA	*OreG0004280*
scaffold187	121722	A	T	Och02,Och04,Och06	*OreG0004280*
scaffold187	121731	C	T	NA	*OreG0004280*
scaffold187	121808	C	T	NA	*OreG0004280*
scaffold187	121839	G	A	NA	*OreG0004280*
scaffold187	121857	T	C	NA	*OreG0004280*
scaffold187	123089	C	G	NA	*OreG0004280*
scaffold187	123099	C	G	NA	*OreG0004280*
scaffold187	123514	T	C	NA	*OreG0004280*
scaffold187	123626	T	C	NA	*OreG0004280*
scaffold187	123641	G	A	NA	*OreG0004280*
scaffold187	124182	A	T	NA	*OreG0004280*
scaffold187	147592	C	A	NA	*OreG0004281*
scaffold187	188121	G	T	Ore01,Ore02,Ore03,Ore06,Ore07,Ore08,Ore09,Ore10,Ore11,Ore12,Ore13,Ore14	*OreG0004285*
scaffold187	188418	G	T	Och02,Och03,Och04,Och05,Och06,Och12,Och13	*OreG0004285*
scaffold187	194108	C	T	Ore01,Ore02,Ore03,Ore04,Ore05,Ore06,Ore07,Ore08,Ore09,Ore10,Ore11,Ore12,Ore13,Ore14	*OreG0004285*
scaffold187	194168	A	C	NA	*OreG0004285*
scaffold187	209147	A	C	Och02,Och03,Och04,Och05,Och06,Och12,Och13	*OreG0004287*
scaffold187	248408	T	A	Och04,Och05,Och08,Och12,Och13	*OreG0004291*
scaffold187	263178	A	G	Ore01,Ore02,Ore03,Ore04,Ore05,Ore06,Ore07,Ore08,Ore09,Ore10,Ore11,Ore12,Ore13,Ore14	*OreG0004292*
scaffold187	264057	G	T	NA	*OreG0004292*
scaffold187	264353	A	T	Och11	*OreG0004292*
scaffold187	317121	C	T	NA	*OreG0004299*
scaffold187	319024	A	T	Ore01,Ore02,Ore03,Ore06,Ore07,Ore08,Ore09,Ore10,Ore11,Ore12,Ore13,Ore14	*OreG0004299*
scaffold187	330428	T	C	NA	*OreG0004301*
scaffold187	338073	T	C	Och12	*OreG0004302*
scaffold187	345145	T	C	NA	*OreG0004302*
scaffold187	345878	T	A	Och12	*OreG0004302*
scaffold187	363046	A	G	NA	*OreG0004303*
scaffold187	402046	A	T	Ore01,Ore02,Ore03,Ore04,Ore05,Ore06,Ore07,Ore08,Ore09,Ore10,Ore11,Ore12,Ore13,Ore14	*OreG0004309*
scaffold187	431826	A	G	Ore01,Ore02,Ore03,Ore04,Ore05,Ore06,Ore07,Ore08,Ore09,Ore10,Ore11,Ore12,Ore13,Ore14	*OreG0004312*
scaffold187	530960	G	A	Och02,Och06,Och09,Och10,Och14	*OreG0004319*
scaffold187	536082	T	G	NA	*OreG0004320*
scaffold187	541740	G	A	NA	*OreG0004321*
scaffold187	542802	A	G	NA	*OreG0004321*
scaffold187	542820	G	A	NA	*OreG0004321*
scaffold187	542830	C	T	Och01,Och03,Och05,Och08,Och09,Och10,Och11,Och12,Och13	*OreG0004321*
scaffold187	545067	G	A	NA	*OreG0004321*
scaffold187	569009	C	A	Ore01,Ore02,Ore03,Ore04,Ore05,Ore06,Ore07,Ore08,Ore09,Ore10,Ore11,Ore12,Ore13,Ore14	*OreG0004322*
scaffold187	609582	A	G	NA	*OreG0004325*
scaffold187	610126	A	T	Och04,Och05,Och12,Och13	*OreG0004325*
scaffold187	610664	T	C	Och01,Och02,Och03,Och04,Och05,Och06,Och07,Och08,Och09,Och10,Och11,Och12,Och13,Och14	*OreG0004325*
scaffold187	633767	T	C	Ore01,Ore02,Ore03,Ore04,Ore05,Ore06,Ore07,Ore08,Ore09,Ore10,Ore11,Ore12,Ore13,Ore14	*OreG0004328*
scaffold187	646147	A	G	Ore01,Ore02,Ore03,Ore04,Ore05,Ore06,Ore07,Ore08,Ore09,Ore10,Ore11,Ore12,Ore13,Ore14	*OreG0004329*
scaffold187	646327	C	T	NA	*OreG0004329*
scaffold187	646657	T	C	NA	*OreG0004329*
scaffold187	647537	T	A	Ore01,Ore02,Ore03,Ore04,Ore05,Ore06,Ore07,Ore08,Ore09,Ore10,Ore11,Ore12,Ore13,Ore14	*OreG0004329*
scaffold187	666658	G	A	Och01,Och02,Och03,Och04,Och05,Och06,Och07,Och08,Och09,Och10,Och11,Och12,Och13,Och14,Ore01,Ore02,Ore03,Ore04,Ore05,Ore06,Ore07,Ore08,Ore09,Ore10,Ore11,Ore12,Ore13,Ore14	*OreG0004331*
scaffold187	667030	C	T	Ore01,Ore02,Ore03,Ore04,Ore05,Ore06,Ore07,Ore08,Ore09,Ore10,Ore11,Ore12,Ore13,Ore14	*OreG0004331*
scaffold187	679757	G	T	NA	*OreG0004332*
scaffold187	682644	A	C	Ore01,Ore02,Ore03,Ore04,Ore05,Ore06,Ore07,Ore08,Ore09,Ore10,Ore11,Ore12,Ore13,Ore14	*OreG0004332*

scaffold187	682715	C	T	NA	*OreG0004332*
scaffold187	685850	C	G	Och01,Och02,Och03,Och04,Och05,Och06,Och07,Och08,Och09,Och10,Och11,Och12,Och13,Och14,Ore01,Ore02,Ore03,Ore04,Ore05,Ore06,Ore07,Ore08,Ore09,Ore10,Ore11,Ore12,Ore13,Ore14	*OreG0004332*
scaffold187	735777	G	T	Ore01,Ore02,Ore03,Ore04,Ore05,Ore06,Ore07,Ore08,Ore09,Ore10,Ore11,Ore12,Ore13,Ore14	*OreG0004334*
scaffold187	747620	G	A	NA	*OreG0004336*
scaffold187	747708	A	C	Och02,Och03,Och04,Och05,Och06,Och11,Och12,Och13,Och14,Ore01,Ore02,Ore03,Ore04,Ore05,Ore06,Ore07,Ore08,Ore09,Ore10,Ore11,Ore12,Ore13,Ore14	*OreG0004336*
scaffold187	801627	G	A	Och01	*OreG0004340*
scaffold187	840093	T	A	Och01,Och02,Och03,Och04,Och05,Och06,Och07,Och08,Och09,Och10,Och11,Och12,Och13,Och14,Ore01,Ore02,Ore03,Ore04,Ore05,Ore06,Ore07,Ore08,Ore09,Ore10,Ore11,Ore12,Ore13,Ore14	*OreG0004345*
scaffold187	884764	G	T	NA	*OreG0004348*
scaffold187	888159	C	A	NA	*OreG0004349*
scaffold187	967184	G	T	Och01,Och02,Och03,Och04,Och05,Och06,Och07,Och08,Och09,Och10,Och11,Och12,Och13,Och14,Ore01,Ore02,Ore03,Ore04,Ore05,Ore06,Ore07,Ore08,Ore09,Ore10,Ore11,Ore12,Ore13,Ore14	*OreG0004352*
scaffold187	996367	G	C	NA	*OreG0004354*
scaffold187	996713	T	G	NA	*OreG0004354*
scaffold187	1034965	A	G	Och01,Och02,Och03,Och04,Och05,Och06,Och07,Och08,Och09,Och10,Och11,Och12,Och13,Och14	*OreG0004357*
scaffold187	1036986	G	A	Och07	*OreG0004358*
scaffold187	1165581	A	T	NA	*OreG0004365*
scaffold187	1172970	A	G	Ore01,Ore02,Ore03,Ore04,Ore05,Ore06,Ore07,Ore08,Ore09,Ore10,Ore11,Ore12,Ore13,Ore14	*OreG0004366*
scaffold187	1190930	G	C	Ore01,Ore02,Ore03,Ore04,Ore05,Ore06,Ore07,Ore08,Ore09,Ore10,Ore11,Ore12,Ore13,Ore14	*OreG0004367*
scaffold187	1191513	T	G	NA	*OreG0004367*
scaffold187	1191698	A	T	Och01,Och02,Och03,Och04,Och05,Och06,Och07,Och08,Och09,Och10,Och11,Och12,Och13,Och14,Ore01,Ore02,Ore03,Ore04,Ore05,Ore06,Ore07,Ore08,Ore09,Ore10,Ore11,Ore12,Ore13,Ore14	*OreG0004367*
scaffold187	1210837	G	C	NA	*OreG0004368*
scaffold187	1210982	C	G	Och01,Och02,Och03,Och04,Och05,Och06,Och07,Och08,Och09,Och10,Och11,Och12,Och13,Och14,Ore01,Ore02,Ore03,Ore04,Ore05,Ore06,Ore07,Ore08,Ore09,Ore10,Ore11,Ore12,Ore13,Ore14	*OreG0004368*
scaffold187	1232834	A	G	Ore01,Ore02,Ore03,Ore04,Ore05,Ore06,Ore07,Ore08,Ore09,Ore10,Ore11,Ore12,Ore13,Ore14	*OreG0004371*
scaffold187	1233133	A	G	Och01	*OreG0004371*
scaffold187	1234879	G	A	Ore01,Ore02,Ore03,Ore04,Ore05,Ore06,Ore07,Ore08,Ore09,Ore10,Ore11,Ore12,Ore13,Ore14	*OreG0004371*
scaffold187	1240918	C	T	Och12	*OreG0004372*
scaffold187	1242011	T	A	Och03,Och07,Och11,Och12	*OreG0004373*
scaffold187	1244159	C	T	Ore01,Ore02,Ore03,Ore04,Ore05,Ore06,Ore07,Ore08,Ore09,Ore10,Ore11,Ore12,Ore13,Ore14	*OreG0004373*
scaffold187	1266060	T	C	NA	*OreG0004374*
scaffold187	1295496	C	A	Och01,Och02,Och03,Och04,Och05,Och06,Och07,Och08,Och09,Och10,Och11,Och12,Och13,Och14	*OreG0004376*
scaffold187	1311703	C	A	NA	*OreG0004376*
scaffold187	1313270	C	T	NA	*OreG0004376*
scaffold187	1323803	G	C	Ore01,Ore02,Ore03,Ore04,Ore05,Ore06,Ore07,Ore08,Ore09,Ore10,Ore11,Ore12,Ore13,Ore14	*OreG0004377*
scaffold187	1325575	A	G	Och01,Och02,Och03,Och04,Och05,Och06,Och07,Och08,Och09,Och10,Och11,Och12,Och13,Och14,Ore01,Ore02,Ore03,Ore04,Ore05,Ore06,Ore07,Ore08,Ore09,Ore10,Ore11,Ore12,Ore13,Ore14	*OreG0004377*
scaffold187	1384926	T	A	NA	*OreG0004381*
scaffold187	1414708	T	C	Ore01,Ore02,Ore03,Ore04,Ore05,Ore06,Ore07,Ore08,Ore09,Ore10,Ore11,Ore12,Ore13,Ore14	*OreG0004384*
scaffold187	1423435	G	A	Ore01,Ore02,Ore03,Ore04,Ore05,Ore06,Ore07,Ore08,Ore09,Ore10,Ore11,Ore12,Ore13,Ore14	*OreG0004385*
scaffold187	1451003	T	C	NA	*OreG0004389*
scaffold187	1451328	G	A	Och01,Och02,Och03,Och04,Och05,Och06,Och07,Och08,Och09,Och10,Och11,Och12,Och13,Och14,Ore01,Ore02,Ore03,Ore04,Ore05,Ore06,Ore07,Ore08,Ore09,Ore10,Ore11,Ore12,Ore13,Ore14	*OreG0004389*
scaffold187	1451347	G	T	NA	*OreG0004389*
scaffold187	1451374	C	T	Och01,Och02,Och03,Och04,Och05,Och06,Och07,Och08,Och09,Och10,Och11,Och12,Och13,Och14	*OreG0004389*
scaffold187	1451380	C	T	Ore01,Ore02,Ore03,Ore04,Ore05,Ore06,Ore07,Ore08,Ore09,Ore10,Ore11,Ore12,Ore13,Ore14	*OreG0004389*
scaffold187	1451384	C	A	Ore01,Ore02,Ore03,Ore04,Ore05,Ore06,Ore07,Ore08,Ore09,Ore10,Ore11,Ore12,Ore13,Ore14	*OreG0004389*
scaffold187	1461594	T	A	NA	*OreG0004390*
scaffold187	1474250	T	C	Och01,Och08,Och09,Och10,Och11	*OreG0004391*
scaffold187	1476289	G	A	Och01,Och08,Och09,Och10,Och11	*OreG0004391*
scaffold187	1476953	T	G	Och01,Och02,Och03,Och04,Och05,Och06,Och07,Och08,Och09,Och10,Och11,Och12,Och13,Och14	*OreG0004391*

scaffold187	1502641	C	A	Och01,Och02,Och03,Och04,Och05,Och06,Och07,Och08,Och09,Och10,Och11,Och12,Och13,Och14,Ore01,Ore02,Ore03,Ore04,Ore05,Ore06,Ore07,Ore08,Ore09,Ore10,Ore11,Ore12,Ore13,Ore14	*OreG0004392*
scaffold187	1502888	A	C	Ore01,Ore02,Ore03,Ore04,Ore05,Ore06,Ore07,Ore08,Ore09,Ore10,Ore11,Ore12,Ore13,Ore14	*OreG0004392*
scaffold187	1513535	G	A	Och01,Och02,Och03,Och04,Och05,Och06,Och07,Och08,Och09,Och10,Och11,Och12,Och13,Och14	*OreG0004393*
scaffold187	1528525	G	C	NA	*OreG0004396*
scaffold187	1529507	G	A	NA	*OreG0004396*
scaffold187	1559517	C	A	Och01,Och02,Och03,Och04,Och05,Och06,Och07,Och08,Och09,Och10,Och11,Och12,Och13,Och14	*OreG0004400*
scaffold187	1559526	C	G	NA	*OreG0004400*
scaffold187	1559835	G	T	Ore01,Ore02,Ore03,Ore04,Ore05,Ore06,Ore07,Ore08,Ore09,Ore10,Ore11,Ore12,Ore13,Ore14	*OreG0004400*
scaffold187	1581126	G	C	Och01,Och02,Och03,Och04,Och05,Och06,Och07,Och08,Och09,Och10,Och11,Och12,Och13,Och14	*OreG0004402*
scaffold187	1586369	G	C	NA	*OreG0004403*
scaffold187	1586804	T	C	NA	*OreG0004403*
scaffold187	1586932	T	A	Ore02,Ore04,Ore05,Ore06,Ore07,Ore08,Ore09,Ore10,Ore11,Ore12,Ore13,Ore14	*OreG0004403*
scaffold187	1599646	A	T	NA	*OreG0004404*
scaffold187	1599855	C	A	Ore01,Ore02,Ore03,Ore04,Ore05,Ore06,Ore07,Ore08,Ore09,Ore10,Ore11,Ore12,Ore13,Ore14	*OreG0004404*
scaffold187	1607951	C	T	NA	*OreG0004405*
scaffold187	1616637	G	A	NA	*OreG0004406*
scaffold187	1620431	G	A	Och01,Och02,Och03,Och04,Och05,Och06,Och07,Och08,Och09,Och10,Och11,Och12,Och13,Och14	*OreG0004406*
scaffold187	1625159	A	T	NA	*OreG0004407*
scaffold187	1625291	C	A	Och01,Och02,Och03,Och04,Och05,Och06,Och07,Och08,Och09,Och10,Och11,Och12,Och13,Och14	*OreG0004407*
scaffold187	1647452	G	A	NA	*OreG0004411*
scaffold187	1655325	T	A	Ore01,Ore02,Ore03,Ore04,Ore05,Ore06,Ore07,Ore08,Ore09,Ore10,Ore11,Ore12,Ore13,Ore14	*OreG0004413*
scaffold187	1679905	C	A	NA	*OreG0004416*
scaffold187	1706143	G	A	NA	*OreG0004418*
scaffold187	1716029	G	T	Och01,Och02,Och03,Och04,Och05,Och06,Och07,Och08,Och09,Och10,Och11,Och12,Och13	*OreG0004419*
scaffold187	1759550	C	G	NA	*OreG0004425*
scaffold187	1762516	C	T	NA	*OreG0004425*
scaffold187	1766101	G	T	Ore01,Ore02,Ore03,Ore04,Ore05,Ore06,Ore07,Ore08,Ore09,Ore10,Ore11,Ore12,Ore13,Ore14	*OreG0004425*
scaffold187	1781993	A	G	NA	*OreG0004426*
scaffold187	1848134	C	A	NA	*OreG0004432*
scaffold187	1848474	G	A	NA	*OreG0004432*
scaffold187	1849054	T	A	Och01,Och02,Och03,Och04,Och05,Och06,Och07,Och09,Och10,Och11,Och12,Och13,Och14	*OreG0004432*
scaffold187	1849250	T	C	NA	*OreG0004432*
scaffold187	1851518	T	C	NA	*OreG0004433*
scaffold187	1893621	G	C	NA	*OreG0004433*
scaffold187	1910457	T	G	Ore01,Ore02,Ore03,Ore04,Ore05,Ore06,Ore07,Ore08,Ore09,Ore10,Ore11,Ore12,Ore13,Ore14	*OreG0004436*
scaffold187	1914615	G	A	Ore01,Ore02,Ore03,Ore04,Ore05,Ore06,Ore07,Ore08,Ore09,Ore10,Ore11,Ore12,Ore13,Ore14	*OreG0004437*
scaffold187	1914723	A	T	Ore01,Ore02,Ore03,Ore04,Ore05,Ore06,Ore07,Ore08,Ore09,Ore10,Ore11,Ore12,Ore13,Ore14	*OreG0004437*
scaffold187	2003896	C	A	Ore01,Ore02,Ore03,Ore04,Ore05,Ore06,Ore07,Ore08,Ore09,Ore10,Ore11,Ore12,Ore13,Ore14	*OreG0004442*
scaffold187	2004148	T	A	Och01,Och02,Och03,Och04,Och05,Och06,Och07,Och08,Och09,Och10,Och11,Och12,Och13,Och14,Ore01,Ore02,Ore03,Ore04,Ore05,Ore06,Ore07,Ore08,Ore09,Ore10,Ore11,Ore12,Ore13,Ore14	*OreG0004442*
scaffold187	2055215	C	T	Ore01,Ore02,Ore03,Ore04,Ore05,Ore06,Ore07,Ore08,Ore09,Ore10,Ore11,Ore12,Ore13,Ore14	*OreG0004447*
scaffold187	2097291	C	T	Och01	*OreG0004450*
scaffold187	2097708	C	A	Och09,Och10,Och11	*OreG0004450*
scaffold187	2097724	A	T	Ore01,Ore02	*OreG0004450*
scaffold187	2097830	G	C	Ore01,Ore02,Ore03,Ore04,Ore05,Ore06,Ore07,Ore08,Ore09,Ore10,Ore11,Ore12,Ore13,Ore14	*OreG0004450*
scaffold187	2098187	G	A	Och04,Och05,Och12,Och13	*OreG0004451*
scaffold187	2098302	A	C	Ore01,Ore02,Ore03,Ore04,Ore05,Ore06,Ore07,Ore08,Ore09,Ore10,Ore11,Ore12,Ore13,Ore14	*OreG0004451*
scaffold187	2098465	A	C	Och04,Och05,Och12,Och13	*OreG0004451*
scaffold187	2099005	T	A	Och02,Och03,Och04,Och05,Och06,Och09,Och10,Och11,Och12,Och13	*OreG0004451*
scaffold187	2099107	G	A	Ore01,Ore02,Ore03,Ore04,Ore05,Ore06,Ore07,Ore08,Ore09,Ore10,Ore11,Ore12,Ore13,Ore14	*OreG0004451*
scaffold187	2099112	T	C	Ore01,Ore02,Ore03,Ore04,Ore05,Ore06,Ore07,Ore08,Ore09,Ore10,Ore11,Ore12,Ore13,Ore14	*OreG0004451*
scaffold187	2125389	G	T	NA	*OreG0004454*

scaffold187	2139071	G	A	NA	*OreG0004456*
scaffold187	2177945	C	T	Och07	*OreG0004458*
scaffold187	2178742	T	C	Och01	*OreG0004458*
scaffold187	2190816	C	T	Och01,Och02,Och03,Och04,Och05,Och06,Och07,Och08,Och09,Och10,Och11,Och12,Och13,Och14,Ore01,Ore02,Ore03,Ore04,Ore05,Ore06,Ore07,Ore08,Ore09,Ore10,Ore11,Ore12,Ore13,Ore14	*OreG0004460*
scaffold187	2192526	C	T	Ore01,Ore02,Ore03,Ore04,Ore05,Ore06,Ore07,Ore08,Ore09,Ore10,Ore11,Ore12,Ore13,Ore14	*OreG0004460*
scaffold187	2202364	T	C	NA	*OreG0004462*
scaffold187	2253415	A	T	Och03	*OreG0004468*
scaffold187	2306506	G	A	Och01	*OreG0004476*
scaffold187	2309589	T	A	NA	*OreG0004476*
scaffold187	2311641	T	G	Ore01,Ore02,Ore03,Ore04,Ore05,Ore06,Ore07,Ore08,Ore09,Ore10,Ore11,Ore12,Ore13,Ore14	*OreG0004476*
scaffold187	2314563	C	A	NA	*OreG0004476*
scaffold187	2314815	A	G	NA	*OreG0004476*
scaffold187	2314896	T	G	Ore01,Ore02,Ore03,Ore04,Ore05,Ore06,Ore07,Ore08,Ore09,Ore10,Ore11,Ore12,Ore13,Ore14	*OreG0004476*
scaffold187	2333505	C	T	NA	*OreG0004478*
scaffold187	2393496	C	T	NA	*OreG0004483*
scaffold187	2395770	G	A	Ore01,Ore02,Ore03,Ore04,Ore05,Ore06,Ore07,Ore08,Ore09,Ore10,Ore11,Ore12,Ore13,Ore14	*OreG0004483*
scaffold187	2396024	G	T	Och02,Och03,Och04,Och05,Och06,Och09,Och10,Och11,Och12,Och13,Och14	*OreG0004483*
scaffold187	2396035	C	T	Och07	*OreG0004483*
scaffold187	2436370	G	A	Ore01,Ore02,Ore03,Ore04,Ore05,Ore06,Ore07,Ore08,Ore09,Ore10,Ore11,Ore12,Ore13,Ore14	*OreG0004486*
scaffold187	2459505	C	A	Och01,Och02,Och03,Och04,Och05,Och06,Och08,Och09,Och11,Och12,Och13,Och14	*OreG0004488*
scaffold187	2489352	G	T	NA	*OreG0004492*
scaffold187	2623856	C	G	NA	*OreG0004504*
scaffold187	2845861	A	G	Och01,Och07,Och11	*OreG0004518*
scaffold187	2848112	C	A	NA	*OreG0004518*
scaffold187	2856802	T	C	Ore01,Ore02,Ore03,Ore04,Ore05,Ore06,Ore07,Ore08,Ore09,Ore10,Ore11,Ore12,Ore13,Ore14	*OreG0004518*
scaffold187	2878653	T	C	NA	*OreG0004524*
scaffold187	2884336	A	G	Och07,Och11	*OreG0004524*
scaffold187	2885852	G	T	NA	*OreG0004524*
scaffold187	2904989	A	T	NA	*OreG0004527*
scaffold187	2906965	G	A	Och01,Och02,Och03,Och04,Och05,Och06,Och07,Och08,Och09,Och10,Och11,Och12,Och13,Och14	*OreG0004527*
scaffold187	2929942	G	A	NA	*OreG0004529*
scaffold187	2963787	A	C	Ore01,Ore02,Ore03,Ore04,Ore05,Ore06,Ore07,Ore08,Ore09,Ore10,Ore11,Ore12,Ore13,Ore14	*OreG0004533*
scaffold187	2963802	G	A	NA	*OreG0004533*
scaffold187	2964875	T	C	Och02,Och03,Och04,Och05,Och06,Och11,Och12,Och13,Och14,Ore01,Ore02,Ore03,Ore04,Ore05,Ore06,Ore07,Ore08,Ore09,Ore10,Ore11,Ore12,Ore13,Ore14	*OreG0004533*
scaffold187	2966922	C	G	Och14	*OreG0004533*
scaffold187	2988230	A	G	Ore01,Ore02,Ore03,Ore04,Ore05,Ore06,Ore07,Ore08,Ore09,Ore10,Ore11,Ore12,Ore13,Ore14	*OreG0004534*
scaffold187	2989581	T	C	Och01,Och02,Och03,Och04,Och05,Och06,Och07,Och08,Och09,Och10,Och11,Och12,Och13,Och14	*OreG0004534*
scaffold187	3015287	A	C	Ore01,Ore02,Ore03,Ore04,Ore05,Ore06,Ore07,Ore08,Ore09,Ore10,Ore11,Ore12,Ore13,Ore14	*OreG0004538*
scaffold187	3018607	T	A	Ore01,Ore02,Ore03,Ore04,Ore05,Ore06,Ore07,Ore08,Ore09,Ore10,Ore11,Ore12,Ore13,Ore14	*OreG0004539*
scaffold187	3028339	C	A	NA	*OreG0004540*
scaffold187	3032680	G	T	Och01,Och02,Och03,Och04,Och05,Och06,Och07,Och08,Och09,Och10,Och11,Och12,Och13,Och14	*OreG0004541*
scaffold187	3046683	A	C	Och01,Och02,Och03,Och04,Och05,Och06,Och07,Och08,Och09,Och10,Och11,Och12,Och13,Och14,Ore01,Ore02,Ore03,Ore04,Ore05,Ore06,Ore07,Ore08,Ore09,Ore10,Ore11,Ore12,Ore13,Ore14	*OreG0004542*
scaffold187	3056817	G	A	Ore01,Ore02,Ore03,Ore04,Ore05,Ore06,Ore07,Ore08,Ore09,Ore10,Ore11,Ore12,Ore13,Ore14	*OreG0004543*
scaffold187	3060997	C	G	NA	*OreG0004544*
scaffold187	3074866	A	T	Och01,Och02,Och03,Och04,Och05,Och06,Och07,Och08,Och09,Och10,Och11,Och12,Och13,Och14,Ore01,Ore02,Ore03,Ore04,Ore05,Ore06,Ore07,Ore08,Ore09,Ore10,Ore11,Ore12,Ore13,Ore14	*OreG0004545*
scaffold187	3087994	G	A	NA	*OreG0004547*
scaffold187	3092209	A	T	NA	*OreG0004548*
scaffold187	3092263	C	T	Och01,Och02,Och03,Och04,Och05,Och06,Och07,Och08,Och09,Och10,Och11,Och12,Och13,Och14,Ore01,Ore02,Ore03,Ore04,Ore05,Ore06,Ore07,Ore08,Ore09,Ore10,Ore11,Ore12,Ore13,Ore14	*OreG0004548*
scaffold187	3094668	T	C	Och07,Och14	*OreG0004549*
scaffold187	3104961	G	A	Och03	*OreG0004550*
scaffold187	3174241	A	T	NA	*OreG0004556*

scaffold187	3184040	C	G	NA	*OreG0004557*
scaffold187	3191647	C	T	NA	*OreG0004557*
scaffold187	3196799	A	C	Och01,Och02,Och03,Och04,Och05,Och06,Och07,Och08,Och09,Och10,Och11,Och12,Och13,Och14	*OreG0004558*
scaffold187	3217519	C	A	NA	*OreG0004561*
scaffold187	3219285	A	T	Och01,Och02,Och03,Och04,Och05,Och06,Och07,Och08,Och09,Och10,Och11,Och12,Och13,Och14,Ore01,Ore02,Ore03,Ore04,Ore05,Ore06,Ore07,Ore08,Ore09,Ore10,Ore11,Ore12,Ore13,Ore14	*OreG0004562*
scaffold187	3220429	A	G	Och01,Och02,Och03,Och04,Och05,Och06,Och07,Och08,Och09,Och10,Och11,Och12,Och13,Och14,Ore01,Ore02,Ore03,Ore04,Ore05,Ore06,Ore07,Ore08,Ore09,Ore10,Ore11,Ore12,Ore13,Ore14	*OreG0004562*
scaffold187	3223035	T	C	Och01,Och02,Och03,Och04,Och05,Och06,Och07,Och08,Och09,Och10,Och11,Och12,Och13,Och14,Ore01,Ore02,Ore03,Ore04,Ore05,Ore06,Ore07,Ore08,Ore09,Ore10,Ore11,Ore12,Ore13,Ore14	*OreG0004563*
scaffold187	3227560	C	T	NA	*OreG0004563*
scaffold187	3229520	G	A	NA	*OreG0004563*
scaffold187	3229745	T	C	NA	*OreG0004563*
scaffold187	3248412	C	T	Ore01,Ore02,Ore03,Ore04,Ore05,Ore06,Ore07,Ore08,Ore09,Ore10,Ore11,Ore12,Ore13,Ore14	*OreG0004565*
scaffold187	3248469	C	T	NA	*OreG0004565*
scaffold187	3248667	G	A	Och01,Och09,Och10	*OreG0004565*
scaffold187	3251446	G	A	Och01,Och02,Och03,Och04,Och05,Och06,Och07,Och08,Och09,Och10,Och11,Och12,Och13,Och14,Ore01,Ore02,Ore03,Ore04,Ore05,Ore06,Ore07,Ore08,Ore09,Ore10,Ore11,Ore12,Ore13,Ore14	*OreG0004566*
scaffold187	3319567	T	G	Och03	*OreG0004571*
scaffold187	3368664	A	G	NA	*OreG0004575*
scaffold187	3391618	A	T	NA	*OreG0004580*
scaffold187	3391644	G	A	Och01,Och02,Och03,Och04,Och05,Och06,Och07,Och08,Och09,Och10,Och11,Och12,Och13,Och14,Ore01,Ore02,Ore03,Ore04,Ore05,Ore06,Ore07,Ore08,Ore09,Ore10,Ore11,Ore12,Ore13,Ore14	*OreG0004580*
scaffold187	3399900	T	A	NA	*OreG0004581*
scaffold187	3407067	T	C	NA	*OreG0004582*
scaffold187	3474748	A	G	NA	*OreG0004589*
scaffold187	3485121	C	G	Och03	*OreG0004590*
scaffold187	3486547	C	T	NA	*OreG0004590*
scaffold187	3558415	T	A	Och01,Och02,Och03,Och04,Och05,Och06,Och07,Och08,Och09,Och10,Och11,Och12,Och13,Och14,Ore01,Ore02,Ore03,Ore04,Ore05,Ore06,Ore07,Ore08,Ore09,Ore10,Ore11,Ore12,Ore13,Ore14	*OreG0004595*
scaffold187	3574559	C	T	Och03,Och14	*OreG0004596*
scaffold187	3574784	C	T	Ore01,Ore02,Ore03,Ore04,Ore05,Ore06,Ore07,Ore08,Ore09,Ore10,Ore11,Ore12,Ore13,Ore14	*OreG0004596*
scaffold187	3592212	C	T	Och01,Och02,Och03,Och04,Och05,Och06,Och07,Och08,Och09,Och10,Och11,Och12,Och13,Och14	*OreG0004600*
scaffold187	3592219	A	G	Och01,Och02,Och03,Och04,Och05,Och06,Och07,Och08,Och09,Och10,Och11,Och12,Och13,Och14,Ore01,Ore02,Ore03,Ore04,Ore05,Ore06,Ore07,Ore08,Ore09,Ore10,Ore11,Ore12,Ore13,Ore14	*OreG0004600*
scaffold187	3593511	C	T	Och01,Och02,Och03,Och04,Och05,Och06,Och07,Och08,Och09,Och10,Och11,Och12,Och13,Och14	*OreG0004600*
scaffold187	3593730	T	G	NA	*OreG0004600*
scaffold187	3594569	T	A	Och01,Och02,Och03,Och04,Och05,Och06,Och07,Och08,Och09,Och10,Och11,Och12,Och13,Och14	*OreG0004600*
scaffold187	3601717	C	T	NA	*OreG0004602*
scaffold187	3601759	T	A	NA	*OreG0004602*
scaffold187	3601857	A	G	NA	*OreG0004602*
scaffold187	3629224	G	A	NA	*OreG0004604*
scaffold187	3638749	T	A	NA	*OreG0004605*
scaffold187	3650104	C	T	Och01,Och02,Och03,Och04,Och05,Och06,Och07,Och08,Och09,Och10,Och11,Och12,Och13,Och14,Ore01,Ore02,Ore03,Ore04,Ore05,Ore06,Ore07,Ore08,Ore09,Ore10,Ore11,Ore12,Ore13,Ore14	*OreG0004606*
scaffold187	3650282	C	T	Ore01,Ore02,Ore03,Ore04,Ore05,Ore06,Ore07,Ore08,Ore09,Ore10,Ore11,Ore12,Ore13,Ore14	*OreG0004606*
scaffold187	3650316	G	A	Och01,Och02,Och03,Och04,Och05,Och06,Och07,Och08,Och09,Och10,Och11,Och12,Och13,Och14,Ore01,Ore02,Ore03,Ore04,Ore05,Ore06,Ore07,Ore08,Ore09,Ore10,Ore11,Ore12,Ore13,Ore14	*OreG0004606*
scaffold187	3650403	C	T	Och01,Och02,Och03,Och04,Och05,Och06,Och07,Och12,Och13	*OreG0004606*
scaffold187	3650405	G	A	Och09,Och10,Och11	*OreG0004606*
scaffold187	3650522	G	T	Och01,Och02,Och03,Och04,Och05,Och06,Och07,Och08,Och09,Och10,Och11,Och12,Och13,Och14	*OreG0004606*
scaffold187	3668521	T	A	NA	*OreG0004609*
scaffold187	3678641	A	G	Ore08	*OreG0004611*
scaffold187	3683175	C	T	Och01,Och02,Och03,Och04,Och05,Och06,Och07,Och08,Och09,Och10,Och11,Och12,Och13,Och14,Ore01,Ore02,Ore03,Ore04,Ore05,Ore06,Ore07,Ore08,Ore09,Ore10,Ore11,Ore12,Ore13,Ore14	*OreG0004612*
scaffold187	3683198	A	G	Ore01,Ore02,Ore03,Ore04,Ore05,Ore06,Ore07,Ore08,Ore09,Ore10,Ore11,Ore12,Ore13,Ore14	*OreG0004612*
scaffold187	3711235	C	G	NA	*OreG0004615*

scaffold187	3711475	G	T	Ore01,Ore02,Ore03,Ore04,Ore05,Ore06,Ore07,Ore08,Ore09,Ore10,Ore11,Ore12,Ore13,Ore14	*OreG0004615*
scaffold187	3711696	T	A	NA	*OreG0004615*
scaffold187	3717512	A	G	NA	*OreG0004615*
scaffold187	3733813	C	A	NA	*OreG0004618*
scaffold187	3741241	A	G	NA	*OreG0004619*
scaffold187	3741289	T	G	NA	*OreG0004619*
scaffold187	3741465	T	A	Ore01,Ore02,Ore03,Ore04,Ore05,Ore06,Ore07,Ore08,Ore09,Ore10,Ore11,Ore12,Ore13,Ore14	*OreG0004619*
scaffold187	3750826	A	G	Och01,Och02,Och03,Och04,Och05,Och06,Och07,Och08,Och09,Och10,Och11,Och12,Och13,Och14,Ore01,Ore02,Ore03,Ore04,Ore05,Ore06,Ore07,Ore08,Ore09,Ore10,Ore11,Ore12,Ore13,Ore14	*OreG0004620*
scaffold187	3751422	A	G	Och01,Och02,Och03,Och04,Och05,Och06,Och07,Och12,Och13,Och14	*OreG0004620*
scaffold187	3751425	G	T	NA	*OreG0004620*
scaffold187	3751515	A	G	NA	*OreG0004620*
scaffold187	3751744	C	T	Och01,Och02,Och03,Och04,Och05,Och06,Och07,Och08,Och12,Och13,Och14	*OreG0004620*
scaffold187	3751756	C	A	Och01,Och02,Och03,Och04,Och05,Och06,Och07,Och08,Och12,Och13,Och14	*OreG0004620*
scaffold187	3751791	G	C	Och01,Och02,Och03,Och04,Och05,Och06,Och07,Och08,Och12,Och13,Och14	*OreG0004620*
scaffold187	3751815	A	G	Och01,Och02,Och03,Och04,Och05,Och06,Och07,Och08,Och12,Och13,Och14	*OreG0004620*
scaffold187	3752143	T	A	NA	*OreG0004620*
scaffold187	3752305	G	T	NA	*OreG0004620*
scaffold187	3752333	C	T	Och01,Och02,Och03,Och04,Och05,Och06,Och12,Och13	*OreG0004620*
scaffold187	3752570	T	G	Och01,Och02,Och03,Och04,Och05,Och06,Och12,Och13	*OreG0004620*
scaffold141	263	C	T	Och01,Och02,Och03,Och04,Och05,Och06,Och07,Och08,Och09,Och10,Och11,Och12,Och13,Och14	*OreG0002154*
scaffold141	265	G	A	Ore01,Ore02,Ore03,Ore04,Ore05,Ore06,Ore07,Ore08,Ore09,Ore10,Ore11,Ore12,Ore13,Ore14	*OreG0002154*
scaffold141	768	C	A	NA	*OreG0002155*
scaffold141	777	T	C	Och01,Och02,Och03,Och04,Och06,Och07,Och08,Och09,Och10,Och11,Och13,Och14	*OreG0002155*
scaffold141	33018	G	A	Och08	*OreG0002159*
scaffold141	35573	G	T	Ore01,Ore02,Ore03,Ore04,Ore05,Ore06,Ore07,Ore08,Ore09,Ore10,Ore11,Ore12,Ore13,Ore14	*OreG0002160*
scaffold141	36969	C	T	Ore01,Ore02,Ore03,Ore04,Ore05,Ore06,Ore07,Ore08,Ore09,Ore10,Ore11,Ore12,Ore13,Ore14	*OreG0002160*
scaffold141	42069	G	C	Och01	*OreG0002161*
scaffold141	60369	C	G	Ore01,Ore02,Ore03,Ore04,Ore05,Ore06,Ore07,Ore08,Ore09,Ore10,Ore11,Ore12,Ore13,Ore14	*OreG0002162*
scaffold141	76496	A	T	NA	*OreG0002164*
scaffold141	89340	A	G	Ore01,Ore02,Ore03,Ore04,Ore05,Ore06,Ore07,Ore08,Ore09,Ore10,Ore11,Ore12,Ore13,Ore14	*OreG0002167*
scaffold141	99344	C	T	Och01,Och02,Och03,Och04,Och05,Och06,Och07,Och08,Och09,Och10,Och11,Och12,Och13,Och14,Ore01,Ore02,Ore03,Ore04,Ore05,Ore06,Ore07,Ore08,Ore09,Ore10,Ore11,Ore12,Ore13,Ore14	*OreG0002168*
scaffold141	101357	T	G	Och01,Och02,Och03,Och04,Och05,Och06,Och07,Och08,Och09,Och10,Och11,Och12,Och13,Och14	*OreG0002168*
scaffold141	103764	A	G	NA	*OreG0002169*
scaffold141	133537	G	A	Ore01,Ore02,Ore03,Ore04,Ore05,Ore06,Ore07,Ore08,Ore09,Ore10,Ore11,Ore12,Ore13,Ore14	*OreG0002172*
scaffold141	141497	G	A	Ore09,Ore10,Ore13	*OreG0002174*
scaffold141	141521	A	G	Ore01,Ore02,Ore03,Ore04,Ore05,Ore06,Ore07,Ore08,Ore09,Ore10,Ore11,Ore12,Ore13,Ore14	*OreG0002174*
scaffold141	171855	G	A	Ore01,Ore02,Ore03,Ore04,Ore05,Ore06,Ore07,Ore08,Ore09,Ore10,Ore11,Ore12,Ore13,Ore14	*OreG0002177*
scaffold141	173868	G	A	Och02,Och03,Och04,Och06,Och13,Och14	*OreG0002177*
scaffold141	174591	T	A	Och01,Och02,Och03,Och04,Och05,Och06,Och07,Och08,Och09,Och10,Och11,Och12,Och13,Och14,Ore01,Ore02,Ore03,Ore04,Ore05,Ore06,Ore07,Ore08,Ore09,Ore10,Ore11,Ore12,Ore13,Ore14	*OreG0002177*
scaffold141	174908	A	T	Och01,Och02,Och03,Och04,Och05,Och06,Och07,Och08,Och09,Och10,Och11,Och12,Och13,Och14,Ore01,Ore02,Ore03,Ore04,Ore05,Ore06,Ore07,Ore08,Ore09,Ore10,Ore11,Ore12,Ore13,Ore14	*OreG0002177*
scaffold141	176507	C	A	Ore01,Ore04,Ore05	*OreG0002177*
scaffold141	176547	C	A	NA	*OreG0002177*
scaffold141	177020	C	A	Och01,Och02,Och03,Och04,Och05,Och06,Och07,Och08,Och09,Och10,Och11,Och12,Och13,Och14,Ore01,Ore02,Ore03,Ore04,Ore05,Ore06,Ore07,Ore08,Ore09,Ore10,Ore11,Ore12,Ore13,Ore14	*OreG0002177*
scaffold141	179134	G	A	NA	*OreG0002177*
scaffold141	198163	T	C	Och12,Och14	*OreG0002179*
scaffold141	209154	C	G	Ore01,Ore02,Ore03,Ore04,Ore05,Ore06,Ore07,Ore08,Ore09,Ore10,Ore11,Ore12,Ore13,Ore14	*OreG0002180*
scaffold141	227572	T	C	Och01,Och02,Och03,Och04,Och05,Och06,Och07,Och08,Och09,Och10,Och11,Och12,Och13,Och14,Ore01,Ore02,Ore03,Ore04,Ore05,Ore06,Ore07,Ore08,Ore09,Ore10,Ore11,Ore12,Ore13,Ore14	*OreG0002183*
scaffold141	237013	T	G	NA	*OreG0002185*
scaffold141	272725	A	G	NA	*OreG0002190*

scaffold141	272842	C	T	Ore01,Ore02,Ore03,Ore04,Ore05,Ore06,Ore07,Ore08,Ore09,Ore10,Ore11,Ore12,Ore13,Ore14	*OreG0002190*
scaffold141	273027	T	C	Ore01,Ore02,Ore03,Ore04,Ore05,Ore06,Ore07,Ore08,Ore09,Ore10,Ore11,Ore12,Ore13,Ore14	*OreG0002190*
scaffold141	274056	C	T	Och01,Och02,Och03,Och04,Och05,Och06,Och07,Och08,Och09,Och10,Och11,Och12,Och13,Och14	*OreG0002190*
scaffold141	276420	G	T	NA	*OreG0002190*
scaffold141	334990	C	G	Och14	*OreG0002193*
scaffold141	335933	G	T	Och01,Och02,Och03,Och04,Och05,Och06,Och07,Och08,Och09,Och10,Och11,Och12,Och13,Och14,Ore01,Ore02,Ore03,Ore04,Ore05,Ore06,Ore07,Ore08,Ore09,Ore10,Ore11,Ore12,Ore13,Ore14	*OreG0002194*
scaffold141	336003	G	A	Och01,Och02,Och03,Och04,Och05,Och06,Och07,Och08,Och09,Och10,Och11,Och12,Och13,Och14	*OreG0002194*
scaffold141	336021	C	T	Och01,Och02,Och03,Och04,Och05,Och06,Och07,Och08,Och09,Och10,Och11,Och12,Och13,Och14	*OreG0002194*
scaffold141	343918	C	T	NA	*OreG0002195*
scaffold141	343990	G	A	NA	*OreG0002195*
scaffold141	346435	T	A	Ore01	*OreG0002195*
scaffold141	352995	C	T	Och08	*OreG0002196*
scaffold141	373340	C	G	Och01,Och02,Och03,Och04,Och05,Och06,Och07,Och08,Och09,Och10,Och11,Och12,Och13,Och14,Ore01,Ore02,Ore03,Ore04,Ore05,Ore06,Ore07,Ore08,Ore09,Ore10,Ore11,Ore12,Ore13,Ore14	*OreG0002198*
scaffold141	373606	G	A	Och09,Och10	*OreG0002198*
scaffold141	379588	C	A	NA	*OreG0002199*
scaffold141	397238	C	T	Ore01,Ore02,Ore03,Ore04,Ore05,Ore06,Ore07,Ore08,Ore09,Ore10,Ore11,Ore12,Ore13,Ore14	*OreG0002200*
scaffold141	397388	T	C	Ore01,Ore04,Ore05	*OreG0002200*
scaffold141	397430	C	A	NA	*OreG0002200*
scaffold141	409658	A	G	Ore08,Ore09,Ore10	*OreG0002202*
scaffold141	409670	C	T	NA	*OreG0002202*
scaffold141	434465	G	T	NA	*OreG0002205*
scaffold141	446877	T	G	Ore01,Ore02,Ore03,Ore04,Ore05,Ore06,Ore07,Ore08,Ore09,Ore10,Ore11,Ore12,Ore13,Ore14	*OreG0002207*
scaffold141	447291	A	G	Ore01,Ore02,Ore03,Ore04,Ore05,Ore06,Ore07,Ore08,Ore09,Ore10,Ore11,Ore12,Ore13,Ore14	*OreG0002207*
scaffold141	448236	G	A	Och02,Och04,Och06,Och07,Och08	*OreG0002207*
scaffold141	448576	T	A	NA	*OreG0002207*
scaffold141	448623	T	A	NA	*OreG0002207*
scaffold141	448873	A	G	Och04	*OreG0002207*
scaffold141	450397	G	A	Ore01,Ore02,Ore03,Ore04,Ore05,Ore06,Ore07,Ore08,Ore09,Ore10,Ore11,Ore12,Ore13,Ore14	*OreG0002209*
scaffold141	450413	T	A	NA	*OreG0002209*
scaffold141	450461	T	G	NA	*OreG0002209*
scaffold141	450572	A	G	Och02,Och04,Och06,Och07,Och08	*OreG0002209*
scaffold141	456526	C	T	Och02,Och04,Och06,Och07,Och08	*OreG0002210*
scaffold141	456596	G	T	Och13	*OreG0002210*
scaffold141	532458	C	T	Ore01,Ore02,Ore03,Ore06,Ore07,Ore08,Ore09,Ore10,Ore11,Ore12,Ore13,Ore14	*OreG0002217*
scaffold141	532484	G	A	Och02,Och04,Och06	*OreG0002217*
scaffold141	532502	T	C	Och02,Och04,Och06	*OreG0002217*
scaffold141	532532	G	A	Och02,Och04,Och06	*OreG0002217*
scaffold141	532554	G	A	Ore01,Ore02,Ore03,Ore04,Ore05,Ore06,Ore07,Ore08,Ore09,Ore10,Ore11,Ore12,Ore13,Ore14	*OreG0002217*
scaffold141	594388	C	T	Och01,Och02,Och03,Och04,Och05,Och06,Och07,Och08,Och09,Och10,Och11,Och12,Och13,Och14,Ore01,Ore02,Ore03,Ore04,Ore05,Ore06,Ore07,Ore08,Ore09,Ore10,Ore11,Ore12,Ore13,Ore14	*OreG0002226*
scaffold141	614842	C	G	NA	*OreG0002229*
scaffold141	615481	C	T	NA	*OreG0002229*
scaffold141	618362	A	G	NA	*OreG0002231*
scaffold141	665325	A	G	Ore01,Ore02,Ore03,Ore04,Ore05,Ore06,Ore07,Ore08,Ore09,Ore10,Ore11,Ore12,Ore13,Ore14	*OreG0002235*
scaffold141	666356	A	T	Ore01	*OreG0002235*
scaffold141	669823	C	G	Och01,Ore01,Ore02,Ore03,Ore04,Ore05,Ore06,Ore07,Ore08,Ore09,Ore10,Ore11,Ore12,Ore13,Ore14	*OreG0002236*
scaffold141	674696	C	T	Ore01,Ore02,Ore03,Ore04,Ore05,Ore06,Ore07,Ore08,Ore09,Ore10,Ore11,Ore12,Ore13,Ore14	*OreG0002238*
scaffold141	747772	C	A	Och08	*OreG0002247*
scaffold141	776904	G	C	NA	*OreG0002248*
scaffold141	814930	T	C	Och02,Och04,Och06	*OreG0002253*
scaffold141	816093	A	G	Och02,Och04,Och06	*OreG0002253*
scaffold141	836350	T	G	NA	*OreG0002257*
scaffold141	841141	T	A	Och01,Och08	*OreG0002257*
scaffold141	848420	A	G	Och08	*OreG0002258*
scaffold141	937945	A	G	NA	*OreG0002268*
scaffold141	938126	A	C	Ore01	*OreG0002268*

scaffold141	956358	T	G	Ore01,Ore02,Ore03,Ore04,Ore05,Ore06,Ore07,Ore08,Ore09,Ore10,Ore11,Ore12,Ore13,Ore14	*OreG0002269*
scaffold141	956502	G	A	Ore01,Ore02,Ore03,Ore04,Ore05,Ore06,Ore07,Ore08,Ore09,Ore10,Ore11,Ore12,Ore13,Ore14	*OreG0002269*
scaffold141	956609	T	A	Ore01	*OreG0002269*
scaffold141	1026771	A	T	Ore06,Ore08,Ore09,Ore10	*OreG0002277*
scaffold141	1192378	C	A	Och12,Och13	*OreG0002286*
scaffold141	1223856	C	T	Och12,Och13	*OreG0002290*
scaffold141	1223943	C	T	NA	*OreG0002290*
scaffold141	1237793	G	A	NA	*OreG0002292*
scaffold141	1239225	C	T	NA	*OreG0002292*
scaffold141	1275334	C	G	NA	*OreG0002298*
scaffold141	1275726	G	A	NA	*OreG0002298*
scaffold141	1278547	C	T	NA	*OreG0002298*
scaffold141	1307975	C	T	Och03	*OreG0002301*
scaffold141	1309028	T	C	NA	*OreG0002301*
scaffold141	1310659	A	C	Och01,Och03,Och05,Och07,Och08,Och09,Och10,Och11,Och12,Och13,Och14	*OreG0002302*
scaffold141	1311081	C	A	Ore01,Ore02,Ore03,Ore04,Ore05,Ore06,Ore07,Ore08,Ore09,Ore10,Ore11,Ore12,Ore13,Ore14	*OreG0002302*
scaffold141	1320466	C	T	NA	*OreG0002304*
scaffold141	1320470	A	T	NA	*OreG0002304*
scaffold141	1320784	C	T	NA	*OreG0002304*
scaffold141	1351376	G	C	Ore01,Ore02,Ore03,Ore04,Ore05,Ore06,Ore07,Ore08,Ore09,Ore10,Ore11,Ore12,Ore13,Ore14	*OreG0002308*
scaffold141	1351681	G	A	NA	*OreG0002308*
scaffold141	1351987	C	T	Ore01,Ore02,Ore03,Ore04,Ore05,Ore06,Ore07,Ore08,Ore09,Ore10,Ore11,Ore12,Ore13,Ore14	*OreG0002308*
scaffold141	1352200	G	T	NA	*OreG0002308*
scaffold141	1380327	G	T	Ore01,Ore02,Ore03,Ore04,Ore05,Ore06,Ore07,Ore08,Ore09,Ore10,Ore11,Ore12,Ore13,Ore14	*OreG0002312*
scaffold141	1380366	T	C	NA	*OreG0002312*
scaffold141	1397390	A	G	NA	*OreG0002314*
scaffold141	1406188	T	C	Ore03,Ore06,Ore07,Ore08,Ore10,Ore12,Ore13,Ore14	*OreG0002317*
scaffold141	1406192	C	G	NA	*OreG0002317*
scaffold141	1406279	C	T	Och07,Och08,Och09,Och10,Och14	*OreG0002317*
scaffold141	1407036	T	C	Och07,Och10,Och14	*OreG0002318*
scaffold141	1547423	G	T	Och01,Och03,Och05,Och07,Och08,Och09,Och10,Och11,Och12,Och13,Och14	*OreG0002321*
scaffold141	1547790	C	A	NA	*OreG0002321*
scaffold141	1547804	G	A	NA	*OreG0002321*
scaffold141	1547810	C	T	NA	*OreG0002321*
scaffold141	1547814	T	A	NA	*OreG0002321*
scaffold141	1547825	G	A	NA	*OreG0002321*
scaffold141	1551725	A	G	NA	*OreG0002322*
scaffold141	1551752	A	G	NA	*OreG0002322*
scaffold141	1551797	G	A	NA	*OreG0002322*
scaffold141	1551855	A	T	NA	*OreG0002322*
scaffold141	1575487	G	A	Och02,Och03,Och04,Och05,Och06,Och12,Och13	*OreG0002325*
scaffold141	1586829	C	T	Och03,Och05,Och12,Och13	*OreG0002327*
scaffold141	1588448	A	G	Ore06,Ore07,Ore10,Ore12,Ore13	*OreG0002327*
scaffold141	1588988	G	A	Ore06,Ore07,Ore10,Ore12,Ore13	*OreG0002327*
scaffold141	1589708	G	A	NA	*OreG0002327*
scaffold141	1590335	G	A	Ore01,Ore02,Ore04,Ore05,Ore06,Ore07,Ore08,Ore09,Ore10,Ore11,Ore12,Ore13	*OreG0002327*
scaffold141	1590437	C	A	Och01,Och02,Och03,Och04,Och05,Och06,Och07,Och08,Och09,Och10,Och11,Och12,Och13,Och14,Ore01,Ore02,Ore03,Ore04,Ore05,Ore06,Ore07,Ore08,Ore09,Ore10,Ore11,Ore12,Ore13,Ore14	*OreG0002327*
scaffold141	1590463	G	T	Ore01,Ore04,Ore05	*OreG0002327*
scaffold141	1593858	A	G	NA	*OreG0002327*
scaffold141	1607784	T	G	Ore01,Ore02,Ore03,Ore04,Ore05,Ore06,Ore07,Ore08,Ore09,Ore10,Ore11,Ore12,Ore13,Ore14	*OreG0002328*
scaffold141	1608350	A	G	Ore06,Ore07,Ore10,Ore12,Ore13	*OreG0002328*
scaffold141	1608390	T	C	Ore06,Ore07,Ore10,Ore12,Ore13	*OreG0002328*
scaffold141	1661958	G	T	Ore01,Ore02,Ore03,Ore04,Ore05,Ore06,Ore07,Ore08,Ore09,Ore10,Ore11,Ore12,Ore13,Ore14	*OreG0002331*
scaffold141	1701499	C	T	NA	*OreG0002333*
scaffold141	1704680	G	A	Ore01,Ore02,Ore04,Ore05,Ore06,Ore07,Ore08,Ore09,Ore10,Ore11,Ore12,Ore13	*OreG0002334*
scaffold141	1705215	T	A	NA	*OreG0002334*
scaffold141	1708636	G	A	NA	*OreG0002334*
scaffold141	1736188	T	A	Ore01,Ore02,Ore03,Ore04,Ore05,Ore06,Ore07,Ore08,Ore09,Ore10,Ore11,Ore12,Ore13,Ore14	*OreG0002337*
scaffold141	1736398	C	T	NA	*OreG0002337*
scaffold141	1739914	G	T	Ore01,Ore02,Ore03,Ore04,Ore05,Ore06,Ore07,Ore08,Ore09,Ore10,Ore11,Ore12,Ore13,Ore14	*OreG0002338*
scaffold141	1740595	A	G	Och01,Och02,Och03,Och04,Och05,Och06,Och07,Och08,Och09,Och10,Och11,Och12,Och13,Och14	*OreG0002338*
scaffold141	1743254	A	T	NA	*OreG0002339*

scaffold141	1822710	G	A	NA	*OreG0002345*
scaffold141	1824762	T	A	Och01,Och02,Och03,Och04,Och05,Och06,Och07,Och08,Och09,Och10,Och11,Och12,Och13,Och14,Ore01,Ore02,Ore03,Ore04,Ore05,Ore06,Ore07,Ore08,Ore09,Ore10,Ore11,Ore12,Ore13,Ore14	*OreG0002345*
scaffold141	1876907	G	T	Och01,Och02,Och03,Och04,Och05,Och06,Och07,Och08,Och09,Och10,Och11,Och12,Och13,Och14,Ore01,Ore02,Ore03,Ore04,Ore05,Ore06,Ore07,Ore08,Ore09,Ore10,Ore11,Ore12,Ore13,Ore14	*OreG0002352*
scaffold141	1882986	T	C	Och01,Och02,Och03,Och04,Och05,Och06,Och07,Och08,Och09,Och10,Och11,Och12,Och13,Och14,Ore01,Ore02,Ore03,Ore04,Ore05,Ore06,Ore07,Ore08,Ore09,Ore10,Ore11,Ore12,Ore13,Ore14	*OreG0002354*
scaffold141	1883537	A	G	Och01,Och02,Och03,Och04,Och05,Och06,Och07,Och08,Och09,Och10,Och11,Och12,Och13,Och14,Ore01,Ore02,Ore03,Ore04,Ore05,Ore06,Ore07,Ore08,Ore09,Ore10,Ore11,Ore12,Ore13,Ore14	*OreG0002354*
scaffold118	70393	A	C	Ore01,Ore02,Ore03,Ore04,Ore05,Ore06,Ore07,Ore08,Ore09,Ore10,Ore11,Ore12,Ore13,Ore14	*OreG0001082*
scaffold118	76119	T	A	Och14,Ore01,Ore02,Ore03,Ore04,Ore05,Ore06,Ore07,Ore08,Ore09,Ore10,Ore11,Ore12,Ore13,Ore14	*OreG0001083*
scaffold118	82393	T	C	NA	*OreG0001085*
scaffold118	83087	T	C	Och09,Och10	*OreG0001085*
scaffold118	114466	T	C	Och01,Och03,Och06,Och08,Och09,Och10,Och14	*OreG0001092*
scaffold118	114487	T	G	Ore03,Ore06,Ore07,Ore10,Ore12,Ore14	*OreG0001092*
scaffold118	115577	C	T	Och01,Och02,Och03,Och04,Och05,Och06,Och07,Och08,Och09,Och10,Och11,Och12,Och13,Och14	*OreG0001092*
scaffold118	118566	T	A	Och01,Och02,Och03,Och04,Och05,Och06,Och07,Och08,Och09,Och10,Och11,Och12,Och13,Och14	*OreG0001093*
scaffold118	120658	C	A	Ore01,Ore02,Ore03,Ore04,Ore05,Ore06,Ore07,Ore08,Ore09,Ore10,Ore11,Ore12,Ore13,Ore14	*OreG0001093*
scaffold118	141549	C	A	NA	*OreG0001096*
scaffold118	141555	C	T	NA	*OreG0001096*
scaffold118	141570	C	A	NA	*OreG0001096*
scaffold118	141636	C	T	NA	*OreG0001096*
scaffold118	141644	C	T	NA	*OreG0001096*
scaffold118	171107	T	C	NA	*OreG0001097*
scaffold118	171484	A	G	Och01,Och07,Och08,Och09,Och10,Och12,Och13	*OreG0001097*
scaffold118	190194	C	A	Och01	*OreG0001096*
scaffold118	225800	A	G	Ore04,Ore05,Ore08	*OreG0001101*
scaffold118	228788	C	A	Och01,Och02,Och03,Och04,Och05,Och06,Och07,Och08,Och09,Och10,Och11,Och12,Och13,Och14,Ore01,Ore02,Ore03,Ore04,Ore05,Ore06,Ore07,Ore08,Ore09,Ore10,Ore11,Ore12,Ore13,Ore14	*OreG0001101*
scaffold118	233159	A	T	Ore03,Ore06,Ore07,Ore10,Ore12,Ore14	*OreG0001101*
scaffold118	238881	T	G	NA	*OreG0001101*
scaffold118	244337	C	G	NA	*OreG0001103*
scaffold118	249783	C	T	NA	*OreG0001104*
scaffold118	268443	A	T	Och02,Och04,Och05,Och09,Och10,Och11,Och12,Och13	*OreG0001107*
scaffold118	268465	C	T	NA	*OreG0001107*
scaffold118	270176	C	A	NA	*OreG0001107*
scaffold118	270952	T	A	Ore01,Ore02,Ore03,Ore04,Ore05,Ore06,Ore07,Ore08,Ore09,Ore10,Ore11,Ore12,Ore13,Ore14	*OreG0001107*
scaffold118	272827	C	A	NA	*OreG0001107*
scaffold118	277700	T	A	Och01	*OreG0001107*
scaffold118	279171	G	C	Och09,Och10,Och12,Och13	*OreG0001107*
scaffold118	286967	C	T	Och01,Och02,Och03,Och04,Och05,Och06,Och07,Och08,Och09,Och10,Och11,Och12,Och13,Och14,Ore01,Ore02,Ore03,Ore04,Ore05,Ore06,Ore07,Ore08,Ore09,Ore10,Ore11,Ore12,Ore13,Ore14	*OreG0001109*
scaffold118	287467	G	C	Och01,Och02,Och03,Och04,Och05,Och06,Och07,Och08,Och09,Och10,Och11,Och12,Och13,Och14,Ore01,Ore02,Ore03,Ore04,Ore05,Ore06,Ore07,Ore08,Ore09,Ore10,Ore11,Ore12,Ore13,Ore14	*OreG0001109*
scaffold118	290815	T	A	NA	*OreG0001110*
scaffold118	298937	C	T	Och12,Och13	*OreG0001110*
scaffold118	307817	A	T	Ore01,Ore02,Ore03,Ore04,Ore05,Ore06,Ore07,Ore08,Ore09,Ore10,Ore11,Ore12,Ore13,Ore14	*OreG0001112*
scaffold118	314552	G	A	Och12,Och13	*OreG0001113*
scaffold118	314562	T	A	NA	*OreG0001113*
scaffold118	344219	C	G	Och14	*OreG0001115*
scaffold118	370001	G	A	Ore01,Ore02,Ore03,Ore04,Ore05,Ore06,Ore07,Ore08,Ore09,Ore10,Ore11,Ore12,Ore13,Ore14	*OreG0001119*
scaffold118	370282	T	A	Och01,Och02,Och03,Och04,Och05,Och06,Och07,Och09,Och10,Och11,Och12,Och13,Och14	*OreG0001119*
scaffold118	370738	G	A	Och01,Och02,Och03,Och04,Och05,Och06,Och07,Och09,Och10,Och11,Och12,Och13,Och14	*OreG0001119*
scaffold118	370742	G	C	NA	*OreG0001119*
scaffold118	383570	G	A	Och01	*OreG0001120*
scaffold118	395149	A	G	Och09,Och10	*OreG0001122*
scaffold118	395514	C	T	NA	*OreG0001122*
scaffold118	405383	A	T	NA	*OreG0001123*

scaffold118	416173	C	A	Ore03,Ore06,Ore07,Ore10,Ore12,Ore14	*OreG0001124*
scaffold118	463738	C	T	Ore01,Ore02,Ore03,Ore04,Ore05,Ore06,Ore07,Ore08,Ore09,Ore10,Ore11,Ore12,Ore13,Ore14	*OreG0001129*
scaffold118	477434	G	A	Ore04,Ore05,Ore08	*OreG0001131*
scaffold118	486068	T	G	Och01,Och02,Och03,Och04,Och05,Och06,Och07,Och08,Och09,Och10,Och11,Och12,Och13,Och14,Ore01,Ore02,Ore03,Ore04,Ore05,Ore06,Ore07,Ore08,Ore09,Ore10,Ore11,Ore12,Ore13,Ore14	*OreG0001132*
scaffold118	488036	T	A	Och01,Och02,Och03,Och04,Och05,Och06,Och07,Och08,Och09,Och10,Och11,Och12,Och13,Och14,Ore01,Ore02,Ore03,Ore04,Ore05,Ore06,Ore07,Ore08,Ore09,Ore10,Ore11,Ore12,Ore13,Ore14	*OreG0001133*
scaffold118	489307	G	A	NA	*OreG0001133*
scaffold118	489517	C	T	Ore04,Ore05,Ore08	*OreG0001133*
scaffold118	490531	G	T	Ore01,Ore02,Ore03,Ore04,Ore05,Ore06,Ore07,Ore08,Ore09,Ore10,Ore11,Ore12,Ore13,Ore14	*OreG0001133*
scaffold118	568183	C	G	Och14	*OreG0001140*
scaffold118	600304	A	T	NA	*OreG0001140*
scaffold118	600334	G	C	Och01,Och02,Och03,Och04,Och05,Och06,Och07,Och08,Och09,Och10,Och11,Och12,Och13,Och14,Ore01,Ore02,Ore03,Ore04,Ore05,Ore06,Ore07,Ore08,Ore09,Ore10,Ore11,Ore12,Ore13,Ore14	*OreG0001140*
scaffold118	600554	G	A	NA	*OreG0001140*
scaffold118	609424	C	T	Och01,Och02,Och03,Och04,Och05,Och06,Och07,Och08,Och09,Och10,Och11,Och12,Och13,Och14,Ore01,Ore02,Ore03,Ore04,Ore05,Ore06,Ore07,Ore08,Ore09,Ore10,Ore11,Ore12,Ore13,Ore14	*OreG0001144*
scaffold118	641763	A	G	Ore04,Ore05,Ore08	*OreG0001148*
scaffold118	642565	G	A	NA	*OreG0001148*
scaffold118	643238	G	A	Och01,Och02,Och03,Och04,Och05,Och06,Och07,Och08,Och09,Och10,Och11,Och12,Och13,Och14,Ore01,Ore02,Ore03,Ore04,Ore05,Ore06,Ore07,Ore08,Ore09,Ore10,Ore11,Ore12,Ore13,Ore14	*OreG0001148*
scaffold118	646433	A	G	Och02,Och03,Och05,Och07,Och09,Och10,Och11,Och12,Och13	*OreG0001149*
scaffold118	647985	C	G	NA	*OreG0001149*
scaffold118	648059	C	T	NA	*OreG0001149*
scaffold118	670590	T	C	NA	*OreG0001151*
scaffold118	671071	A	T	NA	*OreG0001151*
scaffold118	671108	G	A	NA	*OreG0001151*
scaffold118	686240	G	T	Ore04,Ore05	*OreG0001153*
scaffold118	687383	C	T	Ore06,Ore07,Ore10,Ore12,Ore13	*OreG0001153*
scaffold118	690519	G	C	Och01,Och02,Och03,Och04,Och05,Och06,Och07,Och08,Och09,Och10,Och11,Och12,Och13,Och14	*OreG0001153*
scaffold118	699505	G	T	NA	*OreG0001154*
scaffold118	700093	T	A	Och01,Och02,Och03,Och04,Och05,Och06,Och07,Och08,Och09,Och10,Och11,Och12,Och13,Och14	*OreG0001154*
scaffold118	706706	A	T	Och01,Och02,Och03,Och04,Och05,Och06,Och07,Och08,Och09,Och10,Och11,Och12,Och13,Och14	*OreG0001155*
scaffold118	716042	C	T	Och01,Och02,Och03,Och04,Och05,Och06,Och07,Och08,Och09,Och10,Och11,Och12,Och13,Och14	*OreG0001156*
scaffold118	726078	A	G	Och03	*OreG0001156*
scaffold118	726611	C	T	Och01,Och04,Och08,Och09,Och10,Och11,Och12,Och13	*OreG0001156*
scaffold118	730488	T	C	Och09,Och10	*OreG0001157*
scaffold118	730840	C	A	Och01,Och02,Och03,Och04,Och05,Och06,Och07,Och08,Och09,Och10,Och11,Och12,Och13,Och14,Ore01,Ore02,Ore03,Ore04,Ore05,Ore06,Ore07,Ore08,Ore09,Ore10,Ore11,Ore12,Ore13,Ore14	*OreG0001157*
scaffold118	745449	T	C	NA	*OreG0001160*
scaffold118	784067	T	A	NA	*OreG0001163*
scaffold118	784094	A	T	NA	*OreG0001163*
scaffold118	804492	T	A	NA	*OreG0001166*
scaffold118	825505	C	T	NA	*OreG0001168*
scaffold118	825590	C	T	NA	*OreG0001168*
scaffold118	837839	T	C	Och01,Och02,Och03,Och04,Och05,Och06,Och07,Och08,Och09,Och10,Och11,Och12,Och13,Och14	*OreG0001170*
scaffold118	930387	A	G	NA	*OreG0001179*
scaffold118	939961	C	G	NA	*OreG0001180*
scaffold118	939990	G	T	Ore01,Ore02,Ore03,Ore04,Ore05,Ore06,Ore07,Ore08,Ore09,Ore10,Ore11,Ore12,Ore13,Ore14	*OreG0001180*
scaffold118	945492	G	A	Och01,Och02,Och03,Och04,Och05,Och06,Och07,Och08,Och09,Och10,Och11,Och12,Och13,Och14,Ore01,Ore02,Ore03,Ore04,Ore05,Ore06,Ore07,Ore08,Ore09,Ore10,Ore11,Ore12,Ore13,Ore14	*OreG0001181*
scaffold118	945540	G	C	NA	*OreG0001181*
scaffold118	948557	C	T	Ore06,Ore07,Ore10,Ore12,Ore13	*OreG0001182*
scaffold118	951084	C	G	Och01,Och02,Och03,Och04,Och05,Och06,Och07,Och09,Och10,Och11,Och12,Och13,Och14	*OreG0001182*
scaffold118	957136	A	T	Ore01,Ore02,Ore03,Ore04,Ore05,Ore06,Ore07,Ore08,Ore09,Ore10,Ore11,Ore12,Ore13,Ore14	*OreG0001183*
scaffold118	960308	G	C	NA	*OreG0001184*
scaffold118	971858	C	G	Ore01,Ore02,Ore03,Ore04,Ore05,Ore06,Ore07,Ore08,Ore09,Ore10,Ore11,Ore12,Ore13,Ore14	*OreG0001186*

scaffold118	987883	C	G	Ore01,Ore02,Ore03,Ore04,Ore05,Ore06,Ore07,Ore08,Ore09,Ore10,Ore11,Ore12,Ore13,Ore14	*OreG0001188*
scaffold118	988086	T	G	Ore04,Ore05	*OreG0001188*
scaffold118	988328	T	C	Och02,Och03,Och04,Och05,Och06,Och07,Och08,Och12,Och13,Och14	*OreG0001188*
scaffold118	988472	A	G	NA	*OreG0001188*
scaffold118	988691	A	C	NA	*OreG0001189*
scaffold118	989137	A	C	Och01,Och09,Och10	*OreG0001189*
scaffold118	997042	A	G	NA	*OreG0001190*
scaffold118	1023986	A	C	NA	*OreG0001192*
scaffold118	1025211	A	C	NA	*OreG0001192*
scaffold118	1025317	C	A	Och01,Och02,Och03,Och04,Och05,Och06,Och07,Och08,Och09,Och10,Och11,Och12,Och13,Och14	*OreG0001192*
scaffold118	1029116	G	T	Och01,Och02,Och03,Och04,Och05,Och06,Och07,Och08,Och09,Och10,Och11,Och12,Och13,Och14,Ore01,Ore02,Ore03,Ore04,Ore05,Ore06,Ore07,Ore08,Ore09,Ore10,Ore11,Ore12,Ore13,Ore14	*OreG0001193*
scaffold118	1032634	G	A	Och01,Och02,Och03,Och04,Och05,Och06,Och07,Och08,Och09,Och10,Och11,Och12,Och13,Och14	*OreG0001194*
scaffold118	1037935	C	T	Och01,Och02,Och03,Och04,Och05,Och06,Och07,Och08,Och09,Och10,Och11,Och12,Och13,Och14,Ore01,Ore02,Ore03,Ore04,Ore05,Ore06,Ore07,Ore08,Ore09,Ore10,Ore11,Ore12,Ore13,Ore14	*OreG0001195*
scaffold118	1052924	G	T	Och02,Och03,Och04,Och05,Och06,Och07,Och08,Och09,Och10,Och11,Och12,Och13	*OreG0001196*
scaffold118	1053905	A	G	Och01,Och02,Och03,Och04,Och05,Och06,Och07,Och08,Och09,Och10,Och11,Och12,Och13,Och14	*OreG0001196*
scaffold118	1061641	C	G	Och04,Och06,Och09,Och10,Och11,Och12,Och13	*OreG0001197*
scaffold118	1062272	G	A	Och01,Och02,Och03,Och04,Och05,Och06,Och07,Och08,Och09,Och10,Och11,Och12,Och13,Och14	*OreG0001197*
scaffold118	1099738	G	T	Och01,Och02,Och03,Och04,Och05,Och06,Och07,Och08,Och09,Och10,Och11,Och12,Och13,Och14	*OreG0001199*
scaffold118	1102088	C	T	Ore01,Ore02,Ore03,Ore04,Ore05,Ore06,Ore07,Ore08,Ore09,Ore10,Ore11,Ore12,Ore13,Ore14	*OreG0001200*
scaffold118	1113653	C	A	Och01,Och02,Och03,Och04,Och05,Och06,Och08,Och09,Och10,Och11,Och12,Och13,Och14	*OreG0001202*
scaffold118	1114077	G	A	Ore01,Ore02,Ore03,Ore04,Ore05,Ore06,Ore07,Ore08,Ore09,Ore10,Ore11,Ore12,Ore13,Ore14	*OreG0001202*
scaffold118	1157351	C	T	NA	*OreG0001208*
scaffold118	1178600	G	A	Ore01,Ore04,Ore05	*OreG0001211*
scaffold118	1217295	G	A	NA	*OreG0001217*
scaffold118	1219162	T	C	Ore06,Ore07,Ore10,Ore12,Ore13	*OreG0001217*
scaffold118	1221042	A	G	NA	*OreG0001218*
scaffold118	1252778	C	A	Och01,Och02,Och03,Och04,Och05,Och06,Och07,Och08,Och09,Och10,Och11,Och12,Och13,Och14,Ore01,Ore02,Ore03,Ore04,Ore05,Ore06,Ore07,Ore08,Ore09,Ore10,Ore11,Ore12,Ore13,Ore14	*OreG0001220*
scaffold118	1294250	T	C	Ore06,Ore07,Ore10,Ore12,Ore13	*OreG0001224*
scaffold118	1315703	C	A	Och06	*OreG0001227*
scaffold118	1315876	T	A	Och01,Och02,Och03,Och04,Och05,Och06,Och07,Och08,Och09,Och10,Och11,Och12,Och13,Och14	*OreG0001227*
scaffold118	1326488	G	A	Och09,Och10	*OreG0001228*
scaffold118	1339186	A	C	Ore01,Ore02,Ore03,Ore04,Ore05,Ore06,Ore07,Ore08,Ore09,Ore10,Ore11,Ore12,Ore13,Ore14	*OreG0001229*
scaffold118	1339418	A	T	Ore01,Ore02,Ore03,Ore04,Ore05,Ore06,Ore07,Ore08,Ore09,Ore10,Ore11,Ore12,Ore13,Ore14	*OreG0001229*
scaffold118	1361802	A	C	Och01,Och02,Och03,Och04,Och05,Och06,Och07,Och08,Och09,Och10,Och11,Och12,Och13,Och14,Ore01,Ore02,Ore03,Ore04,Ore05,Ore06,Ore07,Ore08,Ore09,Ore10,Ore11,Ore12,Ore13,Ore14	*OreG0001232*
scaffold118	1367502	C	T	Och01,Och02,Och03,Och04,Och05,Och06,Och07,Och08,Och09,Och10,Och11,Och12,Och13,Och14,Ore01,Ore02,Ore03,Ore04,Ore05,Ore06,Ore07,Ore08,Ore09,Ore10,Ore11,Ore12,Ore13,Ore14	*OreG0001232*
scaffold118	1388374	T	A	Och01,Och02,Och03,Och04,Och05,Och06,Och07,Och08,Och09,Och10,Och11,Och12,Och13,Och14	*OreG0001235*
scaffold118	1418377	C	T	NA	*OreG0001240*
scaffold118	1420675	A	G	NA	*OreG0001241*
scaffold118	1420761	C	T	Ore01,Ore02,Ore03,Ore04,Ore05,Ore06,Ore07,Ore08,Ore09,Ore10,Ore11,Ore12,Ore13,Ore14	*OreG0001241*
scaffold118	1420849	C	A	Och01,Och02,Och03,Och04,Och05,Och06,Och07,Och08,Och09,Och10,Och11,Och12,Och13,Och14,Ore01,Ore02,Ore03,Ore04,Ore05,Ore06,Ore07,Ore08,Ore09,Ore10,Ore11,Ore12,Ore13,Ore14	*OreG0001241*
scaffold118	1420912	C	G	Ore06,Ore07,Ore10,Ore12,Ore13	*OreG0001241*
scaffold118	1421877	G	T	Ore01,Ore04,Ore05	*OreG0001242*
scaffold118	1422255	C	T	NA	*OreG0001242*
scaffold118	1425662	T	C	Och01,Och02,Och03,Och04,Och05,Och06,Och07,Och08,Och09,Och10,Och11,Och12,Och13,Och14	*OreG0001243*
scaffold118	1438166	G	C	Och01,Och02,Och03,Och04,Och05,Och06,Och07,Och08,Och09,Och10,Och11,Och12,Och13,Och14,Ore01,Ore02,Ore03,Ore04,Ore05,Ore06,Ore07,Ore08,Ore09,Ore10,Ore11,Ore12,Ore13,Ore14	*OreG0001244*
scaffold118	1447138	C	A	Och08,Och09,Och10	*OreG0001246*

scaffold118	1447152	C	T	Ore01,Ore02,Ore03,Ore04,Ore05,Ore06,Ore07,Ore08,Ore09,Ore10,Ore11,Ore12,Ore13,Ore14	*OreG0001246*
scaffold118	1460699	T	A	NA	*OreG0001247*
scaffold118	1546428	C	T	Och01,Och02,Och03,Och04,Och05,Och06,Och07,Och08,Och09,Och10,Och11,Och12,Och13,Och14,Ore01,Ore02,Ore03,Ore04,Ore05,Ore06,Ore07,Ore08,Ore09,Ore10,Ore11,Ore12,Ore13,Ore14	*OreG0001255*
scaffold118	1546447	C	T	Och01,Och02,Och03,Och04,Och05,Och06,Och07,Och08,Och09,Och10,Och11,Och12,Och13,Och14,Ore01,Ore02,Ore03,Ore04,Ore05,Ore06,Ore07,Ore08,Ore09,Ore10,Ore11,Ore12,Ore13,Ore14	*OreG0001255*
scaffold118	1551397	A	T	Ore01,Ore02,Ore03,Ore04,Ore05,Ore06,Ore07,Ore08,Ore09,Ore10,Ore11,Ore12,Ore13,Ore14	*OreG0001256*
scaffold118	1596896	T	A	NA	*OreG0001259*
scaffold118	1725031	G	A	Och01,Och02,Och03,Och04,Och05,Och06,Och07,Och08,Och09,Och10,Och11,Och12,Och13,Och14,Ore01,Ore02,Ore03,Ore04,Ore05,Ore06,Ore07,Ore08,Ore09,Ore10,Ore11,Ore12,Ore13,Ore14	*OreG0001263*
scaffold118	1750068	C	T	Och01,Och02,Och03,Och04,Och05,Och06,Och07,Och08,Och09,Och10,Och11,Och12,Och13,Och14,Ore01,Ore02,Ore03,Ore04,Ore05,Ore06,Ore07,Ore08,Ore09,Ore10,Ore11,Ore12,Ore13,Ore14	*OreG0001265*
scaffold118	1750564	A	T	Och01,Och02,Och03,Och04,Och05,Och06,Och07,Och08,Och09,Och10,Och11,Och12,Och13,Och14,Ore01,Ore02,Ore03,Ore04,Ore05,Ore06,Ore07,Ore08,Ore09,Ore10,Ore11,Ore12,Ore13,Ore14	*OreG0001265*
scaffold118	1750847	T	A	Och04,Och09,Och10,Och11,Och12,Och13	*OreG0001265*
scaffold118	1751725	G	T	Och03	*OreG0001265*
scaffold118	1751838	G	A	Ore01,Ore02,Ore03,Ore04,Ore05,Ore06,Ore07,Ore08,Ore09,Ore10,Ore11,Ore12,Ore13,Ore14	*OreG0001265*
scaffold118	1751840	T	C	Ore01,Ore04,Ore05	*OreG0001265*
scaffold118	1751855	A	G	Och04,Och07,Och08,Och09,Och10,Och11,Och12,Och13,Och14	*OreG0001265*
scaffold118	1815223	C	T	Och01,Och02,Och03,Och04,Och05,Och06,Och07,Och08,Och09,Och10,Och11,Och12,Och13,Och14	*OreG0001270*
scaffold118	1860536	C	T	NA	*OreG0001273*
scaffold118	1860598	T	A	Och09,Och10	*OreG0001273*
scaffold118	1872645	T	C	NA	*OreG0001276*
scaffold118	1872647	C	A	NA	*OreG0001276*
scaffold118	1872656	A	G	NA	*OreG0001276*
scaffold118	1872671	A	G	NA	*OreG0001276*
scaffold118	1876986	A	T	NA	*OreG0001276*
scaffold118	1877115	T	G	NA	*OreG0001276*
scaffold118	1879373	C	A	Och01,Och02,Och03,Och04,Och05,Och06,Och07,Och08,Och09,Och10,Och11,Och12,Och13,Och14,Ore01,Ore02,Ore03,Ore04,Ore05,Ore06,Ore07,Ore08,Ore09,Ore10,Ore11,Ore12,Ore13,Ore14	*OreG0001277*
scaffold118	1879482	G	C	NA	*OreG0001277*
scaffold118	1880340	G	A	Och01,Och02,Och03,Och04,Och05,Och06,Och07,Och08,Och09,Och10,Och11,Och12,Och13,Och14,Ore01,Ore02,Ore03,Ore04,Ore05,Ore06,Ore07,Ore08,Ore09,Ore10,Ore11,Ore12,Ore13,Ore14	*OreG0001277*
scaffold118	1881489	A	C	Ore01,Ore02,Ore03,Ore04,Ore05,Ore06,Ore07,Ore08,Ore09,Ore10,Ore11,Ore12,Ore13,Ore14	*OreG0001277*
scaffold118	1883173	T	C	Och01,Och02,Och03,Och04,Och05,Och06,Och07,Och08,Och09,Och10,Och11,Och12,Och13,Och14	*OreG0001277*
scaffold118	1931210	C	A	Och01,Och02,Och03,Och04,Och05,Och06,Och07,Och08,Och09,Och10,Och11,Och12,Och13,Och14,Ore01,Ore02,Ore03,Ore04,Ore05,Ore06,Ore07,Ore08,Ore09,Ore10,Ore11,Ore12,Ore13,Ore14	*OreG0001284*
scaffold118	1945326	G	A	NA	*OreG0001286*
scaffold118	1992488	C	T	NA	*OreG0001293*
scaffold118	1992560	C	T	NA	*OreG0001293*
scaffold118	1992632	T	C	NA	*OreG0001293*
scaffold118	1992698	C	T	NA	*OreG0001293*
scaffold118	1992716	C	T	NA	*OreG0001293*
scaffold118	1992731	C	T	NA	*OreG0001293*
scaffold118	1992769	A	G	NA	*OreG0001293*
scaffold118	1992794	C	T	NA	*OreG0001293*
scaffold118	1992863	C	T	NA	*OreG0001293*
scaffold118	1992950	G	A	NA	*OreG0001293*
scaffold118	2032371	T	A	NA	*OreG0001297*
scaffold118	2054129	C	A	Och05	*OreG0001300*
scaffold118	2054154	C	T	NA	*OreG0001300*
scaffold118	2054442	G	T	NA	*OreG0001300*
scaffold118	2054822	G	A	NA	*OreG0001300*
scaffold118	2054850	G	C	NA	*OreG0001300*
scaffold118	2055099	A	T	NA	*OreG0001300*
scaffold118	2059357	C	T	Och05	*OreG0001301*
scaffold118	2097616	T	A	Och05	*OreG0001306*
scaffold118	2099623	G	A	NA	*OreG0001306*
scaffold118	2100175	G	A	NA	*OreG0001306*
scaffold118	2107820	C	A	NA	*OreG0001306*
scaffold76	5921	G	A	Och06	*OreG0025293*

scaffold76	6089	C	T	Ore01,Ore02,Ore03,Ore04,Ore05,Ore06,Ore07,Ore08,Ore09,Ore10,Ore11,Ore12,Ore13,Ore14	*OreG0025293*
scaffold76	6241	A	T	Och03,Och04,Och05,Och06,Och07,Och08,Och09,Och10,Och12,Och13,Och14,Ore01,Ore02,Ore03,Ore04,Ore05,Ore06,Ore07,Ore08,Ore09,Ore10,Ore11,Ore12,Ore13,Ore14	*OreG0025293*
scaffold76	81537	A	T	Och09,Och10	*OreG0025298*
scaffold76	84227	C	T	NA	*OreG0025298*
scaffold76	84240	G	A	Och02,Och06	*OreG0025298*
scaffold76	124686	C	A	Ore01,Ore02,Ore03,Ore04,Ore05,Ore06,Ore07,Ore08,Ore09,Ore10,Ore11,Ore12,Ore13,Ore14	*OreG0025301*
scaffold76	125316	G	C	Och02,Och06	*OreG0025301*
scaffold76	126888	A	G	NA	*OreG0025301*
scaffold76	131500	A	T	NA	*OreG0025302*
scaffold76	132230	A	T	Ore01,Ore02,Ore03,Ore04,Ore05,Ore06,Ore07,Ore08,Ore09,Ore10,Ore11,Ore12,Ore13,Ore14	*OreG0025302*
scaffold76	141750	T	A	Och02,Och06,Och08	*OreG0025303*
scaffold76	142341	T	C	Och02,Och03,Och06,Och08	*OreG0025303*
scaffold76	142502	C	A	NA	*OreG0025303*
scaffold76	142679	T	A	Ore01,Ore02,Ore06,Ore07,Ore08,Ore11,Ore12,Ore13,Ore14	*OreG0025303*
scaffold76	167945	T	C	NA	*OreG0025304*
scaffold76	169467	A	G	Och02,Och03,Och04,Och05,Och06,Och08,Och12,Och13	*OreG0025304*
scaffold76	171066	C	T	NA	*OreG0025305*
scaffold76	171422	G	A	NA	*OreG0025305*
scaffold76	186729	T	C	Och01,Och02,Och03,Och04,Och05,Och06,Och07,Och08,Och09,Och10,Och11,Och12,Och13,Och14	*OreG0025307*
scaffold76	187065	C	T	NA	*OreG0025307*
scaffold76	187129	C	T	Och14	*OreG0025307*
scaffold76	192696	T	A	Ore01,Ore02,Ore03,Ore04,Ore05,Ore06,Ore07,Ore08,Ore09,Ore10,Ore11,Ore12,Ore13,Ore14	*OreG0025308*
scaffold76	193058	C	T	Ore03,Ore07,Ore08,Ore10,Ore11,Ore12,Ore13,Ore14	*OreG0025308*
scaffold76	193929	C	A	NA	*OreG0025308*
scaffold76	206044	G	T	NA	*OreG0025310*
scaffold76	241578	C	T	NA	*OreG0025312*
scaffold76	278197	T	C	Ore07,Ore08,Ore14	*OreG0025318*
scaffold76	278320	C	T	Ore01,Ore02,Ore03,Ore04,Ore05,Ore06,Ore07,Ore08,Ore09,Ore10,Ore11,Ore12,Ore13,Ore14	*OreG0025318*
scaffold76	278427	G	T	NA	*OreG0025318*
scaffold76	381344	A	C	Och01,Och02,Och03,Och04,Och05,Och06,Och07,Och08,Och09,Och10,Och11,Och12,Och13,Och14	*OreG0025322*
scaffold76	410523	C	A	Och11	*OreG0025325*
scaffold76	410650	G	A	Ore01,Ore02,Ore03,Ore04,Ore05,Ore06,Ore07,Ore08,Ore09,Ore10,Ore11,Ore12,Ore13,Ore14	*OreG0025325*
scaffold76	411459	G	A	NA	*OreG0025325*
scaffold76	419646	T	C	Och01,Och02,Och03,Och04,Och05,Och06,Och07,Och08,Och09,Och10,Och11,Och12,Och13,Och14,Ore01,Ore02,Ore03,Ore04,Ore05,Ore06,Ore07,Ore08,Ore09,Ore10,Ore11,Ore12,Ore13,Ore14	*OreG0025326*
scaffold76	420294	T	C	NA	*OreG0025326*
scaffold76	420930	G	A	Och01,Och02,Och03,Och04,Och05,Och06,Och07,Och08,Och09,Och10,Och11,Och12,Och13,Och14	*OreG0025326*
scaffold76	422890	T	A	NA	*OreG0025327*
scaffold76	471875	C	T	NA	*OreG0025335*
scaffold76	471896	C	T	NA	*OreG0025335*
scaffold76	471973	C	A	NA	*OreG0025335*
scaffold76	471977	G	A	Ore07,Ore08,Ore11,Ore12,Ore13,Ore14	*OreG0025335*
scaffold76	471983	C	T	NA	*OreG0025335*
scaffold76	471989	C	T	NA	*OreG0025335*
scaffold76	474957	T	A	NA	*OreG0025336*
scaffold76	474985	C	A	Och02,Och06	*OreG0025336*
scaffold76	480011	A	T	NA	*OreG0025336*
scaffold76	483713	G	A	NA	*OreG0025337*
scaffold76	489152	C	G	Och01,Och02,Och03,Och04,Och05,Och06,Och07,Och08,Och09,Och10,Och11,Och12,Och13,Och14	*OreG0025337*
scaffold76	490926	C	G	Ore04,Ore05,Ore06,Ore09	*OreG0025337*
scaffold76	540360	G	C	NA	*OreG0025340*
scaffold76	553788	C	G	Och01,Och02,Och03,Och04,Och05,Och06,Och07,Och08,Och09,Och10,Och11,Och12,Och13,Och14,Ore01,Ore02,Ore03,Ore04,Ore05,Ore06,Ore07,Ore08,Ore09,Ore10,Ore11,Ore12,Ore13,Ore14	*OreG0025342*
scaffold76	649459	G	A	NA	*OreG0025349*
scaffold76	678938	G	A	NA	*OreG0025352*
scaffold76	679001	T	C	NA	*OreG0025352*
scaffold76	679222	C	T	Och02,Och06	*OreG0025352*
scaffold76	679501	T	C	NA	*OreG0025352*
scaffold76	679533	G	A	NA	*OreG0025352*
scaffold76	679549	T	A	NA	*OreG0025352*
scaffold76	684331	G	A	NA	*OreG0025353*

scaffold76	685073	G	T	Ore01,Ore02,Ore03,Ore04,Ore05,Ore06,Ore07,Ore08,Ore09,Ore10,Ore11,Ore12,Ore13,Ore14	*OreG0025354*
scaffold76	685593	C	A	NA	*OreG0025354*
scaffold76	701526	T	A	NA	*OreG0025357*
scaffold76	702759	G	A	Och02,Och06	*OreG0025357*
scaffold76	707170	T	A	Och02,Och06	*OreG0025357*
scaffold76	745186	A	G	NA	*OreG0025360*
scaffold76	746325	C	A	NA	*OreG0025360*
scaffold76	788107	C	G	NA	*OreG0025362*
scaffold76	801509	C	A	NA	*OreG0025363*
scaffold76	804254	A	C	Och01,Och02,Och03,Och04,Och05,Och06,Och07,Och08,Och09,Och10,Och11,Och12,Och13,Och14,Ore01,Ore02,Ore03,Ore04,Ore05,Ore06,Ore07,Ore08,Ore09,Ore10,Ore11,Ore12,Ore13,Ore14	*OreG0025363*
scaffold76	815800	A	G	NA	*OreG0025364*
scaffold76	816028	T	A	Och02,Och06	*OreG0025364*
scaffold76	824892	T	A	Och01,Och02,Och03,Och04,Och05,Och06,Och07,Och08,Och09,Och10,Och11,Och12,Och13,Och14	*OreG0025366*
scaffold76	835531	C	T	Ore06	*OreG0025368*
scaffold76	835539	G	C	Och14	*OreG0025368*
scaffold76	948696	G	C	Ore01,Ore02,Ore03,Ore04,Ore05,Ore06,Ore07,Ore08,Ore09,Ore10,Ore11,Ore12,Ore13,Ore14	*OreG0025371*
scaffold76	965878	A	T	Ore07,Ore08,Ore14	*OreG0025372*
scaffold76	974213	G	A	Ore07,Ore08,Ore14	*OreG0025374*
scaffold76	979143	G	A	Och01,Och02,Och03,Och04,Och05,Och06,Och07,Och08,Och09,Och10,Och11,Och12,Och13,Och14,Ore01,Ore02,Ore03,Ore04,Ore05,Ore06,Ore07,Ore08,Ore09,Ore10,Ore11,Ore12,Ore13,Ore14	*OreG0025375*
scaffold76	1072141	C	T	NA	*OreG0025381*
scaffold76	1076213	A	C	Och01,Och02,Och03,Och04,Och05,Och06,Och07,Och08,Och09,Och10,Och11,Och14	*OreG0025381*
scaffold76	1080666	T	C	Och01,Och02,Och03,Och04,Och05,Och06,Och07,Och08,Och09,Och10,Och11,Och12,Och13,Och14,Ore01,Ore02,Ore03,Ore04,Ore05,Ore06,Ore07,Ore08,Ore09,Ore10,Ore11,Ore12,Ore13,Ore14	*OreG0025382*
scaffold76	1082200	A	G	NA	*OreG0025382*
scaffold76	1083695	A	G	Och01,Och02,Och03,Och04,Och05,Och06,Och07,Och08,Och09,Och10,Och11,Och12,Och13,Och14,Ore01,Ore02,Ore03,Ore04,Ore05,Ore06,Ore07,Ore08,Ore09,Ore10,Ore11,Ore12,Ore13,Ore14	*OreG0025382*
scaffold76	1098011	C	A	NA	*OreG0025383*
scaffold164	206112	G	A	Och02,Och03,Och04,Och05,Och06,Och12,Och13,Och14,Ore03	*OreG0003358*
scaffold164	206115	G	A	Och11	*OreG0003358*
scaffold164	206226	C	A	Och11	*OreG0003358*
scaffold164	207028	G	T	NA	*OreG0003358*
scaffold164	207271	G	A	NA	*OreG0003358*
scaffold164	207510	T	G	NA	*OreG0003358*
scaffold164	224482	G	T	Och02,Och03,Och04,Och05,Och06,Och08,Och11,Och12,Och13,Och14	*OreG0003360*
scaffold164	224717	G	A	NA	*OreG0003360*
scaffold164	228144	G	A	NA	*OreG0003361*
scaffold164	230933	T	A	Och01,Och02,Och03,Och04,Och05,Och06,Och07,Och08,Och09,Och10,Och11,Och12,Och13,Och14,Ore01,Ore02,Ore03,Ore04,Ore05,Ore06,Ore07,Ore08,Ore09,Ore10,Ore11,Ore12,Ore13,Ore14	*OreG0003361*
scaffold164	242448	C	G	Och01,Och02,Och03,Och04,Och05,Och06,Och07,Och08,Och09,Och10,Och11,Och12,Och13,Och14,Ore01,Ore02,Ore03,Ore04,Ore05,Ore06,Ore07,Ore08,Ore09,Ore10,Ore11,Ore12,Ore13,Ore14	*OreG0003362*
scaffold164	244428	C	T	NA	*OreG0003363*
scaffold164	248137	G	A	Och01,Och02,Och03,Och04,Och05,Och06,Och07,Och08,Och09,Och10,Och11,Och12,Och13,Och14,Ore03,Ore04,Ore05,Ore08,Ore10,Ore13,Ore14	*OreG0003363*
scaffold164	251137	C	G	NA	*OreG0003363*
scaffold164	254661	G	T	NA	*OreG0003364*
scaffold164	272020	T	C	Och01,Och02,Och03,Och04,Och05,Och06,Och07,Och08,Och09,Och10,Och11,Och12,Och13,Och14	*OreG0003365*
scaffold164	274832	C	T	Och04,Och06,Och08,Och11,Och13	*OreG0003366*
scaffold164	291115	C	T	NA	*OreG0003367*
scaffold164	321214	G	T	NA	*OreG0003369*
scaffold164	322761	A	T	Ore01,Ore02,Ore03,Ore04,Ore05,Ore06,Ore07,Ore08,Ore09,Ore10,Ore11,Ore12,Ore13,Ore14	*OreG0003369*
scaffold164	337187	C	A	Och08,Och11	*OreG0003370*
scaffold164	368542	G	A	Ore01,Ore02,Ore03,Ore04,Ore05,Ore06,Ore07,Ore08,Ore09,Ore10,Ore11,Ore12,Ore13,Ore14	*OreG0003374*
scaffold164	429408	G	T	NA	*OreG0003376*
scaffold164	439370	G	T	NA	*OreG0003377*
scaffold164	439432	T	C	NA	*OreG0003377*
scaffold164	523808	G	A	Ore01,Ore03,Ore06,Ore09,Ore13	*OreG0003384*
scaffold164	537590	A	G	Och14	*OreG0003386*
scaffold164	538325	G	A	Ore01,Ore02,Ore03,Ore04,Ore05,Ore06,Ore07,Ore08,Ore09,Ore10,Ore11,Ore12,Ore13,Ore14	*OreG0003386*

scaffold164	686618	C	T	Och01,Och02,Och03,Och04,Och05,Och06,Och07,Och08,Och09,Och11,Och12,Och13,Och14	*OreG0003394*
scaffold164	704372	A	G	Och13	*OreG0003396*
scaffold164	728685	G	A	NA	*OreG0003397*
scaffold164	746005	C	A	Och03,Och07,Och08,Och09,Och10,Och11,Ore01,Ore02,Ore03,Ore04,Ore05,Ore06,Ore07,Ore08,Ore09,Ore10,Ore11,Ore12,Ore13,Ore14	*OreG0003399*
scaffold164	746270	C	G	Och07,Och08,Och09,Och10,Och11,Ore01,Ore02,Ore03,Ore04,Ore05,Ore06,Ore07,Ore08,Ore09,Ore10,Ore11,Ore12,Ore13,Ore14	*OreG0003399*
scaffold164	754345	C	T	NA	*OreG0003400*
scaffold164	754362	A	G	NA	*OreG0003400*
scaffold164	754428	T	G	Och11	*OreG0003400*
scaffold164	754825	C	T	NA	*OreG0003400*
scaffold164	755011	T	A	Och07,Och08,Och09,Och10,Och11,Ore01,Ore02,Ore03,Ore04,Ore05,Ore06,Ore07,Ore08,Ore09,Ore10,Ore11,Ore12,Ore13,Ore14	*OreG0003400*
scaffold164	755835	A	G	NA	*OreG0003400*
scaffold164	755841	G	A	NA	*OreG0003400*
scaffold164	763334	C	T	NA	*OreG0003401*
scaffold164	763385	A	T	Ore03	*OreG0003401*
scaffold164	763418	T	G	Och01,Och02,Och03,Och04,Och05,Och06,Och07,Och08,Och09,Och10,Och11,Och12,Och13,Och14	*OreG0003401*
scaffold164	770401	T	A	Och01,Och02,Och03,Och04,Och05,Och06,Och07,Och08,Och09,Och10,Och11,Och12,Och13,Och14	*OreG0003402*
scaffold164	770423	A	G	Och05,Och13	*OreG0003402*
scaffold164	778615	G	T	NA	*OreG0003403*
scaffold164	778633	C	T	Ore03,Ore04,Ore05,Ore08,Ore14	*OreG0003403*
scaffold164	778786	A	G	Och01,Och11	*OreG0003403*
scaffold164	848832	G	A	NA	*OreG0003409*
scaffold164	879864	A	G	NA	*OreG0003409*
scaffold164	896382	G	T	Ore04,Ore05	*OreG0003412*
scaffold164	902864	G	A	Och01,Och02,Och03,Och04,Och05,Och06,Och07,Och08,Och09,Och10,Och11,Och12,Och13,Och14,Ore01,Ore02,Ore03,Ore04,Ore05,Ore06,Ore07,Ore08,Ore09,Ore10,Ore11,Ore12,Ore13,Ore14	*OreG0003413*
scaffold164	906011	T	G	Ore03	*OreG0003413*
scaffold164	908280	T	A	Ore04,Ore05	*OreG0003413*
scaffold164	908976	G	A	Och11,Och13	*OreG0003413*
scaffold164	925535	T	A	Och13	*OreG0003414*
scaffold164	934253	G	A	Och11	*OreG0003415*
scaffold164	937289	G	A	NA	*OreG0003416*
scaffold164	937576	T	A	Och04,Och06,Och07,Och08,Och09,Och10,Och13	*OreG0003416*
scaffold164	938117	T	A	Och06	*OreG0003416*
scaffold164	938131	G	T	Ore01,Ore02,Ore03,Ore04,Ore05,Ore06,Ore07,Ore08,Ore09,Ore10,Ore11,Ore12,Ore13,Ore14	*OreG0003416*
scaffold164	943664	C	T	Och11,Ore01,Ore02,Ore03,Ore04,Ore05,Ore06,Ore07,Ore08,Ore09,Ore10,Ore11,Ore12,Ore13,Ore14	*OreG0003417*
scaffold164	960665	G	A	NA	*OreG0003418*
scaffold164	960750	G	A	Och01,Och02,Och03,Och04,Och05,Och06,Och07,Och08,Och09,Och10,Och11,Och12,Och13,Och14	*OreG0003418*
scaffold164	960753	A	G	Ore01,Ore02,Ore03,Ore04,Ore05,Ore06,Ore07,Ore08,Ore09,Ore10,Ore11,Ore12,Ore13,Ore14	*OreG0003418*
scaffold164	960984	G	A	NA	*OreG0003418*
scaffold164	961172	C	T	NA	*OreG0003418*
scaffold164	961416	C	G	Och13	*OreG0003418*
scaffold164	961737	A	T	Och01,Och02,Och03,Och04,Och05,Och06,Och07,Och08,Och09,Och10,Och11,Och12,Och13,Och14	*OreG0003418*
scaffold164	962303	A	G	NA	*OreG0003418*
scaffold164	962478	T	A	Ore01,Ore02,Ore03,Ore04,Ore05,Ore06,Ore07,Ore08,Ore09,Ore10,Ore11,Ore12,Ore13,Ore14	*OreG0003418*
scaffold164	992917	A	G	NA	*OreG0003423*
scaffold164	996570	T	G	Och01,Och02,Och03,Och04,Och05,Och06,Och07,Och08,Och09,Och10,Och11,Och12,Och13,Och14,Ore01,Ore02,Ore03,Ore04,Ore05,Ore06,Ore07,Ore08,Ore09,Ore10,Ore11,Ore12,Ore13,Ore14	*OreG0003424*
scaffold565	42982	T	G	Och01,Och02,Och03,Och04,Och05,Och06,Och07,Och09,Och10,Och11,Och12,Och13,Ore02,Ore03,Ore06,Ore07,Ore08,Ore09,Ore10,Ore11,Ore12,Ore13,Ore14	*OreG0023623*
scaffold565	43062	C	A	NA	*OreG0023623*
scaffold565	43095	C	A	NA	*OreG0023623*
scaffold565	43887	T	G	NA	*OreG0023623*
scaffold565	44974	T	A	NA	*OreG0023623*
scaffold565	49693	C	G	NA	*OreG0023624*
scaffold565	49762	A	G	NA	*OreG0023624*
scaffold565	64051	G	A	NA	*OreG0023625*
scaffold565	113090	C	A	Och02,Och03,Och06,Och08,Och09,Och10,Och14	*OreG0023626*
scaffold565	113396	T	G	Och01,Och02,Och03,Och04,Och05,Och06,Och07,Och08,Och09,Och10,Och11,Och12,Och13,Och14,Ore01,Ore02,Ore03,Ore04,Ore05,Ore06,Ore07,Ore08,Ore09,Ore10,Ore11,Ore12,Ore13,Ore14	*OreG0023626*
scaffold565	119083	G	A	NA	*OreG0023626*

scaffold565	119452	T	A	NA	*OreG0023626*
scaffold565	119915	G	A	NA	*OreG0023626*
scaffold565	119950	G	A	Och02,Och03,Och06,Och08	*OreG0023626*
scaffold565	119971	G	A	Och01,Och02,Och03,Och04,Och05,Och06,Och07,Och08,Och09,Och10,Och11,Och12,Och13,Och14	*OreG0023626*
scaffold565	138173	G	A	Och11	*OreG0023629*
scaffold565	358486	G	A	NA	*OreG0023635*
scaffold565	360417	C	T	Och12,Och13	*OreG0023635*
scaffold565	394249	G	A	NA	*OreG0023640*
scaffold565	404646	C	A	NA	*OreG0023641*
scaffold565	410902	C	T	Och12,Och13	*OreG0023642*
scaffold565	418609	T	C	NA	*OreG0023642*
scaffold565	427583	G	T	NA	*OreG0023643*
scaffold565	427585	G	A	NA	*OreG0023643*
scaffold565	427845	T	A	Ore01,Ore02,Ore03,Ore04,Ore05,Ore06,Ore07,Ore08,Ore09,Ore10,Ore11,Ore12,Ore13,Ore14	*OreG0023643*
scaffold565	429325	T	C	NA	*OreG0023643*
scaffold565	433641	C	T	NA	*OreG0023644*
scaffold565	485662	T	G	Ore01,Ore02,Ore03,Ore04,Ore05,Ore06,Ore07,Ore08,Ore09,Ore10,Ore11,Ore12,Ore13,Ore14	*OreG0023647*
scaffold565	491484	G	C	NA	*OreG0023648*
scaffold565	493157	C	T	Ore01,Ore02,Ore03,Ore04,Ore05,Ore06,Ore07,Ore08,Ore09,Ore10,Ore11,Ore12,Ore13,Ore14	*OreG0023649*
scaffold565	497697	C	T	Och13,Ore01,Ore02,Ore03,Ore04,Ore05,Ore06,Ore07,Ore08,Ore09,Ore10,Ore11,Ore12,Ore13,Ore14	*OreG0023651*
scaffold565	514948	A	G	NA	*OreG0023654*
scaffold565	547518	G	A	NA	*OreG0023657*
scaffold565	548667	A	G	Och01,Och02,Och03,Och04,Och05,Och06,Och07,Och08,Och09,Och10,Och11,Och12,Och13,Och14	*OreG0023657*
scaffold565	549222	T	C	Ore01,Ore02,Ore03,Ore04,Ore05,Ore06,Ore07,Ore08,Ore09,Ore10,Ore11,Ore12,Ore13,Ore14	*OreG0023657*
scaffold565	569081	C	G	Och09,Och10	*OreG0023659*
scaffold565	569119	G	C	Och01,Och02,Och03,Och04,Och05,Och06,Och07,Och08,Och09,Och10,Och11,Och12,Och13,Och14,Ore01,Ore02,Ore03,Ore04,Ore05,Ore06,Ore07,Ore08,Ore09,Ore10,Ore11,Ore12,Ore13,Ore14	*OreG0023659*
scaffold565	569815	A	C	Ore01,Ore02,Ore03,Ore04,Ore05,Ore06,Ore07,Ore08,Ore09,Ore10,Ore11,Ore12,Ore13,Ore14	*OreG0023659*
scaffold565	570417	A	T	Ore01,Ore02,Ore03,Ore04,Ore05,Ore06,Ore07,Ore08,Ore09,Ore10,Ore11,Ore12,Ore13,Ore14	*OreG0023659*
scaffold565	571190	T	C	NA	*OreG0023659*
scaffold565	610941	C	T	NA	*OreG0023663*
scaffold565	611049	C	G	NA	*OreG0023663*
scaffold565	611217	C	A	NA	*OreG0023663*
scaffold565	611288	A	C	NA	*OreG0023663*
scaffold146	41267	C	T	NA	*OreG0002560*
scaffold146	61527	G	T	Och01,Och02,Och03,Och04,Och05,Och06,Och07,Och08,Och09,Och10,Och11,Och12,Och13,Och14,Ore01,Ore02,Ore03,Ore04,Ore05,Ore06,Ore07,Ore08,Ore09,Ore10,Ore11,Ore12,Ore13,Ore14	*OreG0002562*
scaffold146	61541	G	A	Och01,Och02,Och03,Och04,Och05,Och06,Och07,Och08,Och09,Och10,Och11,Och12,Och13,Och14,Ore01,Ore02,Ore03,Ore04,Ore05,Ore06,Ore07,Ore08,Ore09,Ore10,Ore11,Ore12,Ore13,Ore14	*OreG0002562*
scaffold146	63355	A	T	Och12,Och13	*OreG0002562*
scaffold146	64799	G	T	NA	*OreG0002562*
scaffold146	65001	C	G	Och12,Och13	*OreG0002562*
scaffold146	101976	C	A	NA	*OreG0002566*
scaffold146	102035	A	G	Och12,Och13,Ore01,Ore02,Ore03,Ore04,Ore05,Ore06,Ore07,Ore08,Ore09,Ore10,Ore11,Ore12,Ore13,Ore14	*OreG0002566*
scaffold146	102098	G	A	NA	*OreG0002566*
scaffold146	102335	T	C	Och01,Och02,Och03,Och06,Och07,Och08,Och09,Och10,Och11	*OreG0002566*
scaffold146	102345	G	A	NA	*OreG0002566*
scaffold146	102446	C	A	NA	*OreG0002566*
scaffold146	102584	T	C	Och01,Och02,Och03,Och04,Och05,Och06,Och07,Och08,Och09,Och10,Och11,Och12,Och13,Och14,Ore01,Ore02,Ore03,Ore04,Ore05,Ore06,Ore07,Ore08,Ore09,Ore10,Ore11,Ore12,Ore13,Ore14	*OreG0002566*
scaffold146	105551	C	G	Och12,Och13	*OreG0002566*
scaffold146	158344	C	T	NA	*OreG0002569*
scaffold146	159448	C	T	Och01,Och02,Och03,Och04,Och05,Och06,Och07,Och08,Och09,Och10,Och11,Och12,Och13,Och14,Ore01,Ore02,Ore03,Ore04,Ore05,Ore06,Ore07,Ore08,Ore09,Ore10,Ore11,Ore12,Ore13,Ore14	*OreG0002569*
scaffold146	170306	G	A	Och05,Och12,Och13	*OreG0002570*
scaffold146	212653	C	A	Ore01,Ore02,Ore03,Ore06,Ore07,Ore08,Ore09,Ore10,Ore11,Ore12,Ore13,Ore14	*OreG0002574*
scaffold146	245767	C	T	NA	*OreG0002575*
scaffold146	245776	T	C	NA	*OreG0002575*
scaffold146	389280	A	T	NA	*OreG0002581*
scaffold146	389739	A	G	NA	*OreG0002581*

scaffold146	390615	A	T	NA	*OreG0002581*
scaffold146	520556	G	A	Och01,Och02,Och03,Och04,Och05,Och06,Och07,Och08,Och09,Och10,Och11,Och12,Och13,Och14,Ore01,Ore02,Ore03,Ore04,Ore05,Ore06,Ore07,Ore08,Ore09,Ore10,Ore11,Ore12,Ore13,Ore14	*OreG0002587*
scaffold146	520558	C	T	NA	*OreG0002587*
scaffold146	561776	T	A	Ore01,Ore02,Ore03,Ore04,Ore05,Ore06,Ore07,Ore08,Ore09,Ore10,Ore11,Ore12,Ore13,Ore14	*OreG0002588*
scaffold146	561809	T	G	NA	*OreG0002588*
scaffold146	561951	C	G	NA	*OreG0002588*
scaffold146	568099	C	T	NA	*OreG0002589*
scaffold146	571607	A	G	NA	*OreG0002589*
scaffold146	583907	G	A	NA	*OreG0002591*
scaffold146	600552	T	C	NA	*OreG0002593*
scaffold146	605591	G	A	Och03,Och06,Och12,Ore02,Ore03,Ore04,Ore05,Ore06,Ore07,Ore08,Ore09,Ore10,Ore11,Ore12,Ore13,Ore14	*OreG0002594*
scaffold146	606554	T	C	NA	*OreG0002594*
scaffold146	608875	C	T	NA	*OreG0002594*
scaffold146	613422	G	A	NA	*OreG0002595*
scaffold146	613491	C	T	NA	*OreG0002595*
scaffold146	613509	G	T	NA	*OreG0002595*
scaffold146	627944	G	C	Och01,Och02,Och03,Och04,Och05,Och06,Och07,Och08,Och09,Och10,Och11,Och12,Och13,Och14,Ore01,Ore02,Ore03,Ore04,Ore05,Ore06,Ore07,Ore08,Ore09,Ore10,Ore11,Ore12,Ore13,Ore14	*OreG0002597*
scaffold146	628739	A	T	NA	*OreG0002597*
scaffold146	632137	A	C	NA	*OreG0002598*
scaffold146	718795	C	T	Ore01,Ore02,Ore03,Ore04,Ore05,Ore06,Ore07,Ore08,Ore09,Ore10,Ore11,Ore12,Ore13,Ore14	*OreG0002606*
scaffold146	739956	A	G	NA	*OreG0002608*
scaffold146	757715	A	T	Och01,Och02,Och03,Och04,Och05,Och06,Och07,Och08,Och09,Och10,Och11,Och12,Och13,Och14	*OreG0002610*
scaffold146	758995	C	T	NA	*OreG0002610*
scaffold146	795053	C	T	NA	*OreG0002613*
scaffold146	808155	T	A	Och04,Och09,Och10,Och14	*OreG0002614*
scaffold146	808275	C	T	NA	*OreG0002614*
scaffold146	808489	T	C	NA	*OreG0002614*
scaffold146	809469	C	T	NA	*OreG0002614*
scaffold146	809486	C	T	Och01,Och02,Och03,Och04,Och05,Och06,Och07,Och08,Och09,Och10,Och11,Och12,Och13,Och14,Ore01,Ore02,Ore03,Ore04,Ore05,Ore06,Ore07,Ore08,Ore09,Ore10,Ore11,Ore12,Ore13,Ore14	*OreG0002614*
scaffold146	812954	G	T	NA	*OreG0002614*
scaffold146	813580	G	T	NA	*OreG0002614*
scaffold146	814371	A	T	Ore01,Ore02,Ore03,Ore04,Ore05,Ore06,Ore07,Ore08,Ore09,Ore10,Ore11,Ore12,Ore13,Ore14	*OreG0002614*
scaffold146	814809	T	A	NA	*OreG0002614*
scaffold250	1042	T	C	NA	*OreG0007626*
scaffold250	1880	T	A	Ore01,Ore02,Ore03,Ore04,Ore05,Ore06,Ore07,Ore08,Ore09,Ore10,Ore11,Ore12,Ore13,Ore14	*OreG0007626*
scaffold250	123721	G	T	NA	*OreG0007633*
scaffold250	123742	A	T	NA	*OreG0007633*
scaffold250	123948	G	A	Och01,Och02,Och03,Och04,Och05,Och06,Och11,Och12,Och13,Och14,Ore01,Ore02,Ore03,Ore04,Ore05,Ore06,Ore07,Ore08,Ore09,Ore10,Ore11,Ore12,Ore13,Ore14	*OreG0007633*
scaffold250	124095	G	A	NA	*OreG0007633*
scaffold250	124189	C	T	Och01,Och02,Och03,Och04,Och05,Och06,Och11,Och12,Och13,Och14	*OreG0007633*
scaffold250	124309	C	A	Ore09	*OreG0007633*
scaffold250	124381	T	C	NA	*OreG0007633*
scaffold250	124390	T	A	NA	*OreG0007633*
scaffold250	124428	G	A	Och03,Och05,Och12,Och13	*OreG0007633*
scaffold250	124725	G	A	Ore04,Ore05	*OreG0007633*
scaffold250	124735	C	T	Och08	*OreG0007633*
scaffold250	125019	C	T	NA	*OreG0007633*
scaffold250	128102	C	T	Ore01,Ore02,Ore03,Ore04,Ore05,Ore06,Ore07,Ore08,Ore09,Ore10,Ore11,Ore12,Ore13,Ore14	*OreG0007633*
scaffold250	128189	C	T	Och08	*OreG0007633*
scaffold250	128346	G	A	Och08,Ore04,Ore05	*OreG0007633*
scaffold250	128353	C	T	Ore01,Ore02,Ore03,Ore04,Ore05,Ore06,Ore07,Ore08,Ore09,Ore10,Ore11,Ore12,Ore13,Ore14	*OreG0007633*
scaffold250	229673	C	G	NA	*OreG0007637*
scaffold250	229715	T	C	Och01,Och02,Och03,Och04,Och05,Och06,Och07,Och08,Och09,Och10,Och11,Och12,Och13,Och14,Ore01,Ore02,Ore03,Ore04,Ore05,Ore06,Ore07,Ore08,Ore09,Ore10,Ore11,Ore12,Ore13,Ore14	*OreG0007637*
scaffold250	412603	G	T	NA	*OreG0007651*
scaffold250	420665	A	T	Och06	*OreG0007651*
scaffold250	504670	G	A	NA	*OreG0007660*

scaffold250	506039	T	C	Och01,Och02,Och03,Och04,Och05,Och06,Och07,Och08,Och09,Och10,Och11,Och12,Och13,Och14,Ore01,Ore02,Ore03,Ore04,Ore05,Ore06,Ore07,Ore08,Ore09,Ore10,Ore11,Ore12,Ore13,Ore14	*OreG0007660*
scaffold250	509116	G	A	NA	*OreG0007660*
scaffold250	510389	T	A	Och01,Och02,Och03,Och04,Och05,Och06,Och07,Och08,Och12,Och13,Ore01,Ore02,Ore03,Ore06,Ore07,Ore08,Ore09,Ore10,Ore11,Ore12,Ore13,Ore14	*OreG0007660*
scaffold250	562650	T	A	Och01,Och02,Och03,Och04,Och05,Och06,Och07,Och08,Och12,Och13,Ore01,Ore02,Ore03,Ore04,Ore05,Ore06,Ore07,Ore08,Ore09,Ore10,Ore11,Ore12,Ore13,Ore14	*OreG0007667*
scaffold250	563541	G	A	NA	*OreG0007667*
scaffold250	564387	G	A	NA	*OreG0007667*
scaffold250	564787	T	A	NA	*OreG0007667*
scaffold250	569656	T	A	Och12,Och13,Ore01,Ore02,Ore03,Ore06,Ore07,Ore08,Ore09,Ore10,Ore11,Ore12,Ore13,Ore14	*OreG0007667*
scaffold250	959942	T	G	Och01,Och02,Och03,Och04,Och05,Och06,Och07,Och08,Och09,Och10,Och11,Och12,Och13,Och14,Ore01,Ore02,Ore03,Ore04,Ore05,Ore06,Ore07,Ore08,Ore09,Ore10,Ore11,Ore12,Ore13,Ore14	*OreG0007689*
scaffold250	981091	C	T	NA	*OreG0007692*
scaffold250	982511	G	C	NA	*OreG0007692*
scaffold250	982772	G	T	Och01,Och09,Och10	*OreG0007692*
scaffold250	1285204	G	T	Och08	*OreG0007728*
scaffold250	1300916	G	A	Ore01,Ore02,Ore03,Ore04,Ore05,Ore06,Ore07,Ore08,Ore09,Ore10,Ore11,Ore12,Ore13,Ore14	*OreG0007730*
scaffold250	1345126	C	G	NA	*OreG0007737*
scaffold250	1406981	C	T	Och01,Och07,Och14	*OreG0007744*
scaffold250	1408825	C	T	Ore01,Ore02,Ore03,Ore04,Ore05,Ore06,Ore07,Ore08,Ore09,Ore10,Ore11,Ore12,Ore13,Ore14	*OreG0007744*
scaffold250	1416905	C	T	Och03,Och05,Och08,Och12,Och13	*OreG0007744*
scaffold250	1440024	G	T	NA	*OreG0007747*
scaffold250	1440241	A	G	Och01,Och02,Och03,Och04,Och05,Och06,Och07,Och08,Och09,Och10,Och11,Och12,Och13,Och14,Ore01,Ore02,Ore03,Ore04,Ore05,Ore06,Ore07,Ore08,Ore09,Ore10,Ore11,Ore12,Ore13,Ore14	*OreG0007747*
scaffold250	1440291	C	A	Ore01,Ore02,Ore03,Ore04,Ore05,Ore06,Ore07,Ore08,Ore09,Ore10,Ore11,Ore12,Ore13,Ore14	*OreG0007747*
scaffold250	1449910	T	A	NA	*OreG0007748*
scaffold250	1450214	A	T	NA	*OreG0007748*
scaffold250	1467305	G	T	NA	*OreG0007750*
scaffold250	1541555	C	T	NA	*OreG0007757*
scaffold250	1544709	T	G	NA	*OreG0007757*
scaffold250	1545168	A	T	NA	*OreG0007757*
scaffold250	1549247	T	A	Och01,Och02,Och03,Och04,Och05,Och06,Och07,Och08,Och09,Och10,Och11,Och12,Och13,Och14,Ore01,Ore02,Ore03,Ore04,Ore05,Ore06,Ore07,Ore08,Ore09,Ore10,Ore11,Ore12,Ore13,Ore14	*OreG0007758*
scaffold250	1551429	C	T	Och01,Och02,Och03,Och04,Och05,Och06,Och07,Och08,Och09,Och10,Och11,Och12,Och13,Och14,Ore01,Ore02,Ore03,Ore04,Ore05,Ore06,Ore07,Ore08,Ore09,Ore10,Ore11,Ore12,Ore13,Ore14	*OreG0007758*
scaffold250	1715538	T	A	Ore01,Ore02,Ore03,Ore04,Ore05,Ore06,Ore07,Ore08,Ore09,Ore10,Ore11,Ore12,Ore13,Ore14	*OreG0007768*
scaffold250	1715824	G	T	Och02,Och03,Och04,Och05,Och06,Och08,Och09,Och10,Och11,Och12,Och13	*OreG0007768*
scaffold250	1716652	T	A	Och08	*OreG0007768*
scaffold250	1719625	T	C	Och01,Och02,Och03,Och04,Och05,Och06,Och07,Och08,Och09,Och10,Och11,Och12,Och13,Och14,Ore01,Ore02,Ore03,Ore04,Ore05,Ore06,Ore07,Ore08,Ore09,Ore10,Ore11,Ore12,Ore13,Ore14	*OreG0007770*
scaffold250	1721014	C	T	NA	*OreG0007770*
scaffold250	1721522	G	C	Ore01,Ore02,Ore06,Ore07,Ore08,Ore09,Ore10,Ore11,Ore12,Ore13,Ore14	*OreG0007770*
scaffold250	1721531	C	T	NA	*OreG0007770*
scaffold250	1726642	A	T	Och03,Och05,Och12,Och13	*OreG0007771*
scaffold250	1727426	G	T	Ore02,Ore09,Ore10,Ore14	*OreG0007771*
scaffold250	1727995	G	A	Ore01,Ore02,Ore03,Ore04,Ore05,Ore06,Ore07,Ore08,Ore09,Ore10,Ore11,Ore12,Ore13,Ore14	*OreG0007771*
scaffold579	80223	C	A	NA	*OreG0023686*
scaffold579	80268	C	A	Och01,Och02,Och03,Och04,Och05,Och06,Och07,Och08,Och09,Och10,Och11,Och12,Och13,Och14,Ore01,Ore02,Ore03,Ore04,Ore05,Ore06,Ore07,Ore08,Ore09,Ore10,Ore11,Ore12,Ore13,Ore14	*OreG0023686*
scaffold579	80515	C	T	NA	*OreG0023686*
scaffold579	120761	C	T	NA	*OreG0023691*
scaffold579	121610	T	C	NA	*OreG0023691*
scaffold579	132243	C	T	NA	*OreG0023692*
scaffold579	132254	G	T	NA	*OreG0023692*
scaffold579	132267	C	T	NA	*OreG0023692*
scaffold579	132282	C	T	NA	*OreG0023692*
scaffold579	132291	T	A	NA	*OreG0023692*
scaffold579	132497	G	C	NA	*OreG0023692*
scaffold579	134856	G	A	Ore06,Ore11,Ore14	*OreG0023693*
scaffold579	135019	C	A	Och02,Och03,Och04,Och05,Och06,Och08,Och12,Och13	*OreG0023693*
scaffold579	135307	G	C	Ore06,Ore11,Ore14	*OreG0023693*

scaffold579	143395	T	C	Och01,Och02,Och03,Och04,Och05,Och06,Och07,Och08,Och09,Och10,Och11,Och12,Och13,Och14	*OreG0023694*
scaffold579	143774	G	C	NA	*OreG0023694*
scaffold579	181826	G	A	Och08,Och09,Och10,Och11,Och14	*OreG0023698*
scaffold579	190014	A	C	NA	*OreG0023699*
scaffold579	243092	C	G	Ore04,Ore05,Ore06,Ore11,Ore14	*OreG0023705*
scaffold579	243227	A	G	NA	*OreG0023705*
scaffold579	243649	A	G	Och08,Ore09	*OreG0023705*
scaffold579	244525	A	C	Ore04,Ore05,Ore06,Ore11,Ore14	*OreG0023705*
scaffold579	245003	A	G	Ore04,Ore05,Ore06,Ore11,Ore14	*OreG0023705*
scaffold579	245032	T	A	Och01,Och02,Och03,Och04,Och05,Och06,Och07,Och08,Och09,Och10,Och11,Och12,Och13,Och14,Ore01,Ore02,Ore03,Ore04,Ore05,Ore06,Ore07,Ore08,Ore09,Ore10,Ore11,Ore12,Ore13,Ore14	*OreG0023705*
scaffold579	245616	G	A	NA	*OreG0023705*
scaffold579	246395	G	A	NA	*OreG0023705*
scaffold579	254566	A	G	Ore04,Ore05,Ore06,Ore11,Ore14	*OreG0023707*
scaffold579	254776	A	G	Ore04,Ore05,Ore06,Ore11,Ore14	*OreG0023707*
scaffold579	254954	C	T	Och08,Ore09	*OreG0023707*
scaffold579	308445	A	T	Och04,Och13,Ore09	*OreG0023711*
scaffold579	311523	G	A	Och01,Och07,Och08,Och09,Och10,Och11,Och14	*OreG0023711*
scaffold579	311568	C	T	Ore06,Ore11,Ore14	*OreG0023711*
scaffold579	326018	T	C	NA	*OreG0023713*
scaffold579	328275	G	C	Och01,Och02,Och03,Och04,Och05,Och06,Och07,Och08,Och09,Och10,Och11,Och12,Och13,Och14,Ore01,Ore02,Ore03,Ore04,Ore05,Ore06,Ore07,Ore08,Ore09,Ore10,Ore11,Ore12,Ore13,Ore14	*OreG0023713*
scaffold579	331226	C	A	Och08	*OreG0023713*
scaffold579	331730	C	A	NA	*OreG0023713*
scaffold579	336256	T	A	Och08	*OreG0023713*
scaffold579	336431	G	A	Och08	*OreG0023713*
scaffold579	336519	C	A	NA	*OreG0023713*
scaffold579	337693	A	T	Och01,Och02,Och03,Och04,Och05,Och06,Och07,Och08,Och09,Och10,Och11,Och12,Och13,Och14,Ore09	*OreG0023714*
scaffold579	337721	C	T	NA	*OreG0023714*
scaffold579	340223	C	A	Och01,Och07,Och08,Och14	*OreG0023715*
scaffold579	362173	G	T	NA	*OreG0023716*
scaffold579	362418	A	T	NA	*OreG0023716*
scaffold579	362548	G	A	NA	*OreG0023716*
scaffold579	362599	A	G	NA	*OreG0023716*
scaffold579	362788	A	G	NA	*OreG0023716*
scaffold579	362789	A	T	NA	*OreG0023716*
scaffold579	362797	C	T	NA	*OreG0023716*
scaffold579	362944	C	T	NA	*OreG0023716*
scaffold579	363060	G	A	Och04,Och08,Och13	*OreG0023716*
scaffold579	363091	G	A	NA	*OreG0023716*
scaffold579	363124	G	A	NA	*OreG0023716*
scaffold579	364630	T	C	NA	*OreG0023717*
scaffold579	366800	C	A	NA	*OreG0023717*
scaffold579	370472	C	G	NA	*OreG0023718*
scaffold579	386629	C	A	Och01,Och02,Och03,Och04,Och05,Och06,Och07,Och08,Och09,Och10,Och11,Och12,Och13,Och14	*OreG0023721*
scaffold579	396951	G	A	NA	*OreG0023722*
scaffold579	397209	A	T	Ore02,Ore03,Ore04,Ore05,Ore06,Ore07,Ore08,Ore09,Ore10,Ore11,Ore12,Ore13,Ore14	*OreG0023722*
scaffold579	398116	C	T	NA	*OreG0023722*
scaffold579	478342	G	T	Ore14	*OreG0023731*
scaffold579	481210	T	C	Ore01,Ore02,Ore03,Ore04,Ore05,Ore06,Ore07,Ore08,Ore09,Ore10,Ore11,Ore12,Ore13,Ore14	*OreG0023732*
scaffold579	481260	T	G	Och07,Och08	*OreG0023732*
scaffold579	482120	T	A	NA	*OreG0023732*
scaffold579	482166	A	C	NA	*OreG0023732*
scaffold579	500157	G	A	Och01,Och02,Och03,Och04,Och05,Och06,Och07,Och08,Och09,Och10,Och11,Och12,Och13,Och14	*OreG0023735*
scaffold579	500436	C	G	Och01,Och02,Och03,Och04,Och05,Och06,Och07,Och08,Och09,Och10,Och11,Och12,Och13,Och14	*OreG0023735*
scaffold579	500877	A	T	NA	*OreG0023735*
scaffold579	502836	G	T	Och01,Och02,Och03,Och04,Och05,Och06,Och07,Och08,Och09,Och10,Och11,Och12,Och13,Och14,Ore01,Ore02,Ore03,Ore04,Ore05,Ore06,Ore07,Ore08,Ore09,Ore10,Ore11,Ore12,Ore13,Ore14	*OreG0023736*
scaffold579	517194	G	T	NA	*OreG0023741*
scaffold579	517328	A	T	NA	*OreG0023741*
scaffold579	523506	C	A	NA	*OreG0023742*
scaffold579	523526	G	A	NA	*OreG0023742*
scaffold579	523572	T	C	NA	*OreG0023742*
scaffold579	550843	C	T	Och01,Och02,Och03,Och04,Och05,Och06,Och07,Och08,Och09,Och10,Och11,Och12,Och13,Och14	*OreG0023744*

scaffold579	559284	C	T	NA	*OreG0023745*
scaffold579	677436	C	A	NA	*OreG0023755*
scaffold579	677780	G	T	NA	*OreG0023755*
scaffold579	700160	C	T	NA	*OreG0023758*
scaffold579	719291	A	G	Och01,Och02,Och03,Och04,Och05,Och06,Och07,Och08,Och09,Och10,Och11,Och12,Och13,Och14,Ore01,Ore02,Ore03,Ore04,Ore05,Ore06,Ore07,Ore08,Ore09,Ore10,Ore11,Ore12,Ore13,Ore14	*OreG0023759*
scaffold579	722394	G	A	Ore09	*OreG0023760*
scaffold579	734517	C	A	Och01,Och02,Och03,Och04,Och05,Och06,Och07,Och08,Och09,Och10,Och11,Och12,Och13,Och14,Ore01,Ore02,Ore03,Ore04,Ore05,Ore06,Ore07,Ore08,Ore09,Ore10,Ore11,Ore12,Ore13,Ore14	*OreG0023761*
scaffold579	737936	C	A	NA	*OreG0023761*
scaffold579	744273	A	C	NA	*OreG0023761*
scaffold579	766699	A	G	NA	*OreG0023762*
scaffold579	772491	C	A	NA	*OreG0023763*
scaffold579	810220	C	T	NA	*OreG0023769*
scaffold579	825175	C	G	NA	*OreG0023771*
scaffold579	825587	A	G	NA	*OreG0023771*
scaffold579	828456	G	A	Ore02,Ore07,Ore08,Ore09,Ore12	*OreG0023772*
scaffold579	833677	C	T	NA	*OreG0023773*
scaffold579	833959	A	G	NA	*OreG0023773*
scaffold579	833965	C	T	NA	*OreG0023773*
scaffold579	841738	T	C	NA	*OreG0023774*
scaffold579	841882	A	G	Och01	*OreG0023774*
scaffold579	859192	G	A	Och13	*OreG0023777*
scaffold579	859196	C	T	NA	*OreG0023777*
scaffold579	859220	C	T	NA	*OreG0023777*
scaffold579	859355	C	T	NA	*OreG0023777*
scaffold579	859446	A	T	NA	*OreG0023777*
scaffold579	900930	T	A	NA	*OreG0023781*
scaffold579	901462	G	A	NA	*OreG0023781*
scaffold579	959794	T	A	NA	*OreG0023786*
scaffold579	959831	G	A	NA	*OreG0023786*
scaffold579	995044	C	T	NA	*OreG0023789*
scaffold1014	5145	G	T	Ore01,Ore02,Ore03,Ore04,Ore05,Ore06,Ore07,Ore08,Ore09,Ore10,Ore11,Ore12,Ore13,Ore14	*OreG0000061*
scaffold1014	5703	C	T	NA	*OreG0000061*
scaffold741	10771	G	A	NA	*OreG0024997*
scaffold741	37957	A	G	Och06	*OreG0024998*
scaffold741	82968	C	T	Ore01,Ore06	*OreG0025006*
scaffold741	85709	G	T	Och01,Och02,Och03,Och04,Och05,Och06,Och07,Och08,Och09,Och10,Och11,Och12,Och13,Och14,Ore01,Ore02,Ore03,Ore04,Ore05,Ore06,Ore07,Ore08,Ore09,Ore10,Ore11,Ore12,Ore13,Ore14	*OreG0025006*
scaffold741	118456	C	T	Och01,Och02,Och03,Och04,Och05,Och06,Och07,Och08,Och09,Och10,Och11,Och12,Och13,Och14,Ore01,Ore02,Ore03,Ore04,Ore05,Ore06,Ore07,Ore08,Ore09,Ore10,Ore11,Ore12,Ore13,Ore14	*OreG0025009*
scaffold741	136663	A	G	Och01,Och02,Och03,Och04,Och05,Och06,Och07,Och08,Och09,Och10,Och11,Och12,Och13,Och14,Ore01,Ore02,Ore03,Ore04,Ore05,Ore06,Ore07,Ore08,Ore09,Ore10,Ore11,Ore12,Ore13,Ore14	*OreG0025011*
scaffold741	188186	G	T	Och02,Och03,Och04,Och05,Och06,Och11,Och12,Och13,Ore02,Ore03,Ore04,Ore05,Ore08,Ore09,Ore10,Ore13,Ore14	*OreG0025017*
scaffold741	216449	T	C	NA	*OreG0025019*
scaffold741	217027	A	T	NA	*OreG0025019*
scaffold741	217083	G	A	Ore02,Ore03,Ore04,Ore05,Ore08,Ore09,Ore10,Ore12,Ore13,Ore14	*OreG0025019*
scaffold61	24115	T	C	Och02,Och04,Och06	*OreG0024158*
scaffold61	51385	C	T	NA	*OreG0024160*
scaffold61	129212	C	A	NA	*OreG0024162*
scaffold61	129260	G	T	NA	*OreG0024162*
scaffold61	129438	T	C	NA	*OreG0024162*
scaffold61	129699	T	A	NA	*OreG0024162*
scaffold61	138451	G	C	Och12	*OreG0024164*
scaffold61	208703	G	C	Och08	*OreG0024167*
scaffold61	246566	T	G	NA	*OreG0024170*
scaffold61	246627	C	T	NA	*OreG0024170*
scaffold61	246654	G	A	Och01,Och03,Och06,Och07,Och09,Och10	*OreG0024170*
scaffold61	328041	G	T	Ore01,Ore02,Ore03,Ore04,Ore05,Ore06,Ore07,Ore08,Ore09,Ore10,Ore11,Ore12,Ore13,Ore14	*OreG0024172*
scaffold61	328052	T	C	Ore01,Ore02,Ore03,Ore04,Ore05,Ore06,Ore07,Ore08,Ore09,Ore10,Ore11,Ore12,Ore13,Ore14	*OreG0024172*
scaffold61	392652	A	G	Och01,Och03,Och05,Och07,Och08,Och09,Och10,Och11,Och12,Och13,Och14,Ore01,Ore02,Ore03,Ore04,Ore05,Ore06,Ore07,Ore08,Ore09,Ore10,Ore11,Ore12,Ore13,Ore14	*OreG0024181*
scaffold61	392922	C	T	Och06	*OreG0024181*
scaffold61	395920	T	A	NA	*OreG0024182*

scaffold61	396286	G	T	Och01,Och02,Och03,Och04,Och05,Och06,Och07,Och08,Och09,Och10,Och11,Och12,Och13,Och14,Ore01,Ore02,Ore03,Ore04,Ore05,Ore06,Ore07,Ore08,Ore09,Ore10,Ore11,Ore12,Ore13,Ore14	*OreG0024182*
scaffold61	410764	T	G	Och01,Och02,Och03,Och04,Och05,Och06,Och07,Och08,Och09,Och10,Och11,Och12,Och13,Och14,Ore01,Ore02,Ore03,Ore04,Ore05,Ore06,Ore07,Ore08,Ore09,Ore10,Ore11,Ore12,Ore13,Ore14	*OreG0024183*
scaffold61	410851	C	T	NA	*OreG0024183*
scaffold61	410874	A	T	NA	*OreG0024183*
scaffold61	412681	T	C	NA	*OreG0024183*
scaffold61	416887	A	T	Och01,Och02,Och03,Och04,Och05,Och06,Och07,Och08,Och09,Och10,Och11,Och12,Och13,Och14,Ore01,Ore02,Ore03,Ore04,Ore05,Ore06,Ore07,Ore08,Ore09,Ore10,Ore11,Ore12,Ore13,Ore14	*OreG0024184*
scaffold61	417016	T	G	NA	*OreG0024184*
scaffold61	417136	G	A	NA	*OreG0024184*
scaffold61	420204	C	T	Och01,Och02,Och03,Och04,Och05,Och06,Och07,Och08,Och09,Och10,Och11,Och12,Och13,Och14,Ore01,Ore02,Ore03,Ore04,Ore05,Ore06,Ore07,Ore08,Ore09,Ore10,Ore11,Ore12,Ore13,Ore14	*OreG0024185*
scaffold61	433855	C	T	NA	*OreG0024185*
scaffold61	438144	T	C	NA	*OreG0024186*
scaffold61	439127	G	A	NA	*OreG0024186*
scaffold61	606877	T	G	NA	*OreG0024190*
scaffold61	638442	T	G	Och05	*OreG0024191*
scaffold61	638448	C	T	NA	*OreG0024191*
scaffold61	722749	C	T	Och01,Och02,Och03,Och04,Och05,Och06,Och07,Och08,Och09,Och10,Och11,Och12,Och13,Och14	*OreG0024196*
scaffold460	177540	G	A	NA	*OreG0021802*
scaffold460	177556	C	G	NA	*OreG0021802*
scaffold460	177600	C	T	NA	*OreG0021802*
scaffold460	177700	C	T	NA	*OreG0021802*
scaffold460	307612	G	A	Och01,Och02,Och03,Och04,Och05,Och06,Och07,Och08,Och10,Och11,Och12,Och13,Och14,Ore02	*OreG0021804*
scaffold460	334824	C	A	Och01,Och02,Och03,Och04,Och05,Och06,Och07,Och08,Och09,Och10,Och11,Och12,Och13,Och14,Ore01,Ore02,Ore03,Ore04,Ore05,Ore06,Ore07,Ore08,Ore09,Ore10,Ore11,Ore12,Ore13,Ore14	*OreG0021808*
scaffold460	364731	G	A	NA	*OreG0021810*
scaffold460	389889	A	T	Ore04,Ore05	*OreG0021812*
scaffold460	433960	C	T	NA	*OreG0021814*
scaffold460	453272	G	A	Ore04,Ore05	*OreG0021815*
scaffold460	478800	C	G	Och01,Och14	*OreG0021820*
scaffold460	479013	T	C	Och01,Och14	*OreG0021820*
scaffold460	479046	C	A	NA	*OreG0021820*
scaffold460	479224	T	C	Och09,Och10	*OreG0021820*
scaffold460	488581	T	A	Och05,Och07,Och08,Och13	*OreG0021822*
scaffold460	488608	C	A	Och01,Och14,Ore01,Ore02,Ore03,Ore04,Ore05,Ore06,Ore07,Ore08,Ore09,Ore10,Ore11,Ore12,Ore13,Ore14	*OreG0021822*
scaffold460	489644	T	C	Och09,Och10	*OreG0021822*
scaffold460	489899	A	T	Och08	*OreG0021822*
scaffold460	490902	T	A	Och01,Och06,Och09,Och10,Och14,Ore01,Ore02,Ore03,Ore04,Ore05,Ore06,Ore07,Ore08,Ore09,Ore10,Ore11,Ore12,Ore13,Ore14	*OreG0021822*
scaffold460	510609	A	T	Och01,Och09,Och10	*OreG0021824*
scaffold460	520214	C	T	Och14	*OreG0021824*
scaffold460	523887	T	A	Och13	*OreG0021824*
scaffold460	552537	C	G	Och01	*OreG0021826*
scaffold460	558396	T	G	Och01	*OreG0021827*
scaffold460	566799	C	T	Och01,Och02,Och03,Och04,Och05,Och06,Och07,Och08,Och09,Och10,Och11,Och12,Och13,Och14,Ore01,Ore02,Ore03,Ore04,Ore05,Ore06,Ore07,Ore08,Ore09,Ore10,Ore11,Ore12,Ore13,Ore14	*OreG0021828*
scaffold460	584790	G	A	Och01,Och02,Och03,Och04,Och05,Och06,Och07,Och08,Och09,Och10,Och11,Och12,Och13,Och14,Ore01,Ore02,Ore03,Ore04,Ore05,Ore06,Ore07,Ore08,Ore09,Ore10,Ore11,Ore12,Ore13,Ore14	*OreG0021829*
scaffold460	585220	C	T	NA	*OreG0021829*
scaffold460	585489	C	G	Och12,Och14	*OreG0021829*
scaffold460	585504	T	C	NA	*OreG0021829*
scaffold460	585566	C	G	Och01,Och02,Och03,Och04,Och05,Och06,Och07,Och08,Och09,Och10,Och11,Och12,Och13,Och14,Ore01,Ore02,Ore03,Ore04,Ore05,Ore06,Ore07,Ore08,Ore09,Ore10,Ore11,Ore12,Ore13,Ore14	*OreG0021829*
scaffold460	625432	C	T	Ore04,Ore05	*OreG0021832*
scaffold460	761840	T	A	Och01,Och02,Och03,Och04,Och05,Och06,Och07,Och08,Och09,Och10,Och11,Och12,Och13,Och14,Ore01,Ore02,Ore03,Ore04,Ore05,Ore06,Ore07,Ore08,Ore09,Ore10,Ore11,Ore12,Ore13,Ore14	*OreG0021843*
scaffold460	762138	C	T	NA	*OreG0021843*
scaffold460	838999	G	A	Ore01,Ore02,Ore03,Ore04,Ore05,Ore06,Ore07,Ore08,Ore09,Ore10,Ore11,Ore12,Ore13,Ore14	*OreG0021845*
scaffold460	839818	T	A	NA	*OreG0021845*
scaffold460	839916	C	A	NA	*OreG0021845*

scaffold460	839948	G	A	NA	*OreG0021845*
scaffold460	840335	C	T	NA	*OreG0021845*
scaffold460	840977	A	G	Ore01,Ore02,Ore03,Ore04,Ore05,Ore06,Ore07,Ore08,Ore09,Ore10,Ore11,Ore12,Ore13,Ore14	*OreG0021845*
scaffold460	841447	T	C	NA	*OreG0021845*
scaffold460	899056	T	A	NA	*OreG0021852*
scaffold460	904940	C	T	Och08	*OreG0021852*
scaffold460	1096089	T	A	Och08,Och09,Och10	*OreG0021860*
scaffold460	1140823	A	T	Ore01,Ore02,Ore03,Ore04,Ore05,Ore06,Ore07,Ore08,Ore09,Ore10,Ore11,Ore12,Ore13,Ore14	*OreG0021861*
scaffold460	1146305	T	G	NA	*OreG0021861*
scaffold460	1319750	C	T	NA	*OreG0021871*
scaffold460	1332786	C	T	NA	*OreG0021872*
scaffold460	1366938	C	T	Och01,Och02,Och03,Och04,Och05,Och06,Och07,Och08,Och09,Och10,Och11,Och12,Och13,Och14,Ore01,Ore02,Ore03,Ore04,Ore05,Ore06,Ore07,Ore08,Ore09,Ore10,Ore11,Ore12,Ore13,Ore14	*OreG0021875*
scaffold460	1370304	C	T	NA	*OreG0021875*
scaffold460	1371438	A	G	NA	*OreG0021875*
scaffold460	1371451	A	G	NA	*OreG0021875*
scaffold460	1382001	A	G	NA	*OreG0021876*
scaffold460	1382184	C	G	Ore02,Ore04,Ore05,Ore06,Ore07,Ore10,Ore11,Ore13	*OreG0021876*
scaffold460	1383951	T	C	Ore02,Ore04,Ore05,Ore06,Ore07,Ore10,Ore11,Ore13	*OreG0021876*
scaffold460	1384226	C	G	Ore02,Ore04,Ore05,Ore06,Ore07,Ore10,Ore11,Ore13	*OreG0021876*
scaffold460	1384248	T	G	Och01,Och02,Och09,Och10,Och11,Och12,Och13,Och14,Ore01,Ore02,Ore03,Ore04,Ore05,Ore06,Ore07,Ore08,Ore09,Ore10,Ore11,Ore12,Ore13,Ore14	*OreG0021876*
scaffold460	1417198	G	A	NA	*OreG0021880*
scaffold460	1419732	C	G	Och09,Och10	*OreG0021880*
scaffold4	229951	T	C	Ore01,Ore02,Ore03,Ore04,Ore05,Ore06,Ore07,Ore08,Ore09,Ore10,Ore11,Ore12,Ore13,Ore14	*OreG0019590*
scaffold4	248086	C	T	NA	*OreG0019592*
scaffold4	248759	T	G	NA	*OreG0019592*
scaffold4	248782	G	A	NA	*OreG0019592*
scaffold4	254590	C	T	Ore01,Ore02,Ore03,Ore04,Ore05,Ore06,Ore07,Ore08,Ore09,Ore10,Ore11,Ore12,Ore13,Ore14	*OreG0019593*
scaffold4	254592	T	C	Ore01,Ore02,Ore03,Ore04,Ore05,Ore06,Ore07,Ore08,Ore09,Ore10,Ore11,Ore12,Ore13,Ore14	*OreG0019593*
scaffold4	289211	G	T	NA	*OreG0019596*
scaffold4	295553	T	G	NA	*OreG0019596*
scaffold4	316348	C	G	Ore01,Ore02,Ore03,Ore04,Ore05,Ore06,Ore07,Ore08,Ore09,Ore10,Ore11,Ore12,Ore13,Ore14	*OreG0019599*
scaffold4	316387	C	A	Ore01,Ore02,Ore03,Ore04,Ore05,Ore06,Ore07,Ore08,Ore09,Ore10,Ore11,Ore12,Ore13,Ore14	*OreG0019599*
scaffold4	316469	T	A	Och01,Och02,Och03,Och04,Och05,Och06,Och09,Och10,Och12,Och13	*OreG0019599*
scaffold4	317116	G	A	Ore01,Ore02,Ore03,Ore04,Ore05,Ore06,Ore07,Ore08,Ore09,Ore10,Ore11,Ore12,Ore13,Ore14	*OreG0019599*
scaffold4	317138	C	T	Och01,Och02,Och03,Och04,Och05,Och06,Och07,Och08,Och09,Och10,Och11,Och12,Och13,Och14,Ore01,Ore02,Ore03,Ore04,Ore05,Ore06,Ore07,Ore08,Ore09,Ore10,Ore11,Ore12,Ore13,Ore14	*OreG0019599*
scaffold4	439680	A	G	NA	*OreG0019601*
scaffold4	583486	G	A	NA	*OreG0019610*
scaffold4	583549	A	T	NA	*OreG0019610*
scaffold4	583630	A	G	NA	*OreG0019610*
scaffold4	586169	G	A	NA	*OreG0019610*
scaffold4	586203	G	A	NA	*OreG0019610*
scaffold4	848683	G	A	Ore03,Ore08,Ore09,Ore10,Ore14	*OreG0019618*
scaffold4	850853	A	T	Och01,Och02,Och03,Och04,Och05,Och06,Och07,Och08,Och09,Och10,Och11,Och12,Och13,Och14,Ore01,Ore02,Ore03,Ore04,Ore05,Ore06,Ore07,Ore08,Ore09,Ore10,Ore11,Ore12,Ore13,Ore14	*OreG0019618*
scaffold4	853849	A	G	Och01,Och02,Och03,Och04,Och05,Och06,Och07,Och08,Och09,Och10,Och11,Och12,Och13,Och14,Ore01,Ore02,Ore03,Ore04,Ore05,Ore06,Ore07,Ore08,Ore09,Ore10,Ore11,Ore12,Ore13,Ore14	*OreG0019618*
scaffold4	1085876	T	C	Och01,Och02,Och03,Och04,Och05,Och06,Och07,Och08,Och09,Och10,Och11,Och12,Och13,Och14	*OreG0019625*
scaffold4	1107509	A	T	Och01,Och02,Och03,Och04,Och05,Och06,Och07,Och08,Och09,Och10,Och11,Och12,Och13,Och14,Ore01,Ore02,Ore03,Ore04,Ore05,Ore06,Ore07,Ore08,Ore09,Ore10,Ore11,Ore12,Ore13,Ore14	*OreG0019626*
scaffold4	1465344	T	G	NA	*OreG0019639*
scaffold4	1573150	G	A	Ore01,Ore02,Ore03,Ore04,Ore05,Ore06,Ore07,Ore08,Ore09,Ore10,Ore11,Ore12,Ore13,Ore14	*OreG0019642*
scaffold4	1628917	G	A	NA	*OreG0019646*
scaffold4	1628941	A	T	Ore01,Ore02,Ore03,Ore04,Ore05,Ore06,Ore07,Ore08,Ore09,Ore10,Ore11,Ore12,Ore13,Ore14	*OreG0019646*
scaffold4	1638694	C	T	NA	*OreG0019646*
scaffold4	1638721	C	T	NA	*OreG0019646*
scaffold424	34303	C	T	NA	*OreG0020105*

scaffold424	34387	A	C	Ore01,Ore03,Ore04,Ore05,Ore08,Ore09,Ore12,Ore13	*OreG0020105*
scaffold424	34714	C	A	NA	*OreG0020105*
scaffold424	96662	C	A	Och01,Och02,Och03,Och04,Och05,Och06,Och07,Och08,Och09,Och10,Och11,Och12,Och13,Och14	*OreG0020110*
scaffold424	96717	T	G	NA	*OreG0020110*
scaffold424	114962	C	T	NA	*OreG0020113*
scaffold424	115096	T	A	NA	*OreG0020113*
scaffold424	117989	T	C	NA	*OreG0020114*
scaffold424	132670	G	A	NA	*OreG0020116*
scaffold424	147918	G	T	Ore01,Ore02,Ore03,Ore04,Ore05,Ore06,Ore07,Ore08,Ore09,Ore10,Ore11,Ore12,Ore13,Ore14	*OreG0020119*
scaffold424	147940	G	T	Ore01,Ore02,Ore03,Ore04,Ore05,Ore06,Ore07,Ore08,Ore09,Ore10,Ore11,Ore12,Ore13,Ore14	*OreG0020119*
scaffold424	147943	A	G	Och07	*OreG0020119*
scaffold424	147949	C	T	Ore01,Ore02,Ore03,Ore04,Ore05,Ore06,Ore07,Ore08,Ore09,Ore10,Ore11,Ore12,Ore13,Ore14	*OreG0020119*
scaffold424	148201	C	T	NA	*OreG0020119*
scaffold424	153753	A	G	Och06,Och08	*OreG0020120*
scaffold424	184752	C	T	NA	*OreG0020124*
scaffold424	218461	A	G	NA	*OreG0020125*
scaffold424	223120	C	A	NA	*OreG0020125*
scaffold424	237953	T	C	NA	*OreG0020126*
scaffold424	237965	G	C	NA	*OreG0020126*
scaffold424	240335	G	T	Och03,Och05,Och07,Och08,Och09,Och10,Och11,Och14,Ore01,Ore02,Ore03,Ore04,Ore05,Ore06,Ore07,Ore08,Ore09,Ore10,Ore11,Ore12,Ore13,Ore14	*OreG0020127*
scaffold424	242922	A	T	Och07,Och09,Och10,Och11	*OreG0020127*
scaffold424	246984	G	T	Och01,Och02,Och03,Och04,Och05,Och06,Och07,Och09,Och10,Och12,Och13	*OreG0020129*
scaffold424	277828	T	C	NA	*OreG0020132*
scaffold424	277845	A	C	Och01,Och02,Och03,Och04,Och05,Och06,Och07,Och08,Och09,Och10,Och11,Och12,Och13,Och14	*OreG0020132*
scaffold424	278199	G	A	NA	*OreG0020132*
scaffold424	287217	T	G	NA	*OreG0020132*
scaffold424	312337	G	A	NA	*OreG0020135*
scaffold424	313160	G	C	NA	*OreG0020135*
scaffold424	313615	C	T	NA	*OreG0020135*
scaffold424	313911	A	T	NA	*OreG0020135*
scaffold424	314134	C	A	Och01,Och02,Och04,Och07,Och09,Och10,Och11,Och12,Och13,Ore01,Ore02,Ore03,Ore04,Ore05,Ore06,Ore07,Ore08,Ore09,Ore10,Ore11,Ore12,Ore13,Ore14	*OreG0020135*
scaffold424	318536	C	T	Ore03,Ore04,Ore05,Ore08,Ore09,Ore12,Ore13	*OreG0020136*
scaffold424	318556	T	C	NA	*OreG0020136*
scaffold424	318590	C	A	NA	*OreG0020136*
scaffold424	319003	G	A	NA	*OreG0020136*
scaffold424	319434	T	A	Och01,Och02,Och03,Och04,Och05,Och06,Och07,Och08,Och09,Och10,Och11,Och12,Och13,Och14	*OreG0020136*
scaffold424	319487	C	A	Ore01,Ore02,Ore03,Ore04,Ore05,Ore06,Ore07,Ore08,Ore09,Ore10,Ore11,Ore12,Ore13,Ore14	*OreG0020136*
scaffold424	319868	C	T	NA	*OreG0020136*
scaffold424	320616	G	C	Och01,Och02,Och04,Och07,Och09,Och10,Och11,Och12,Och13	*OreG0020136*
scaffold424	329994	A	G	Och01,Och02,Och03,Och04,Och05,Och06,Och07,Och08,Och09,Och10,Och11,Och12,Och13,Och14,Ore01,Ore02,Ore03,Ore04,Ore05,Ore06,Ore07,Ore08,Ore09,Ore10,Ore11,Ore12,Ore13,Ore14	*OreG0020138*
scaffold424	375928	T	A	NA	*OreG0020144*
scaffold424	375949	A	G	NA	*OreG0020144*
scaffold424	376196	G	C	Ore01,Ore02,Ore03,Ore04,Ore05,Ore06,Ore07,Ore08,Ore09,Ore10,Ore11,Ore12,Ore13,Ore14	*OreG0020144*
scaffold424	388661	T	C	Ore01,Ore02,Ore03,Ore04,Ore05,Ore06,Ore07,Ore08,Ore09,Ore10,Ore11,Ore12,Ore13,Ore14	*OreG0020147*
scaffold424	392222	A	T	NA	*OreG0020148*
scaffold424	428607	A	C	Ore01,Ore02,Ore03,Ore04,Ore05,Ore06,Ore07,Ore08,Ore09,Ore10,Ore11,Ore12,Ore13,Ore14	*OreG0020153*
scaffold424	428612	A	C	Ore01,Ore02,Ore03,Ore04,Ore05,Ore06,Ore07,Ore08,Ore09,Ore10,Ore11,Ore12,Ore13,Ore14	*OreG0020153*
scaffold424	445623	C	G	NA	*OreG0020156*
scaffold424	446355	C	T	NA	*OreG0020156*
scaffold424	454634	G	T	NA	*OreG0020157*
scaffold424	454653	G	T	Ore03,Ore04,Ore05,Ore08,Ore09,Ore12,Ore13	*OreG0020157*
scaffold424	454806	T	C	Ore01,Ore02,Ore03,Ore04,Ore05,Ore06,Ore07,Ore08,Ore09,Ore10,Ore11,Ore12,Ore13,Ore14	*OreG0020157*
scaffold424	461426	T	C	NA	*OreG0020158*
scaffold424	470045	G	A	Och07,Och09,Och10	*OreG0020160*
scaffold424	473499	A	T	Och09,Och10,Och14,Ore01,Ore02,Ore03,Ore04,Ore05,Ore06,Ore07,Ore08,Ore09,Ore10,Ore11,Ore12,Ore13,Ore14	*OreG0020161*
scaffold424	495726	C	G	NA	*OreG0020163*
scaffold424	817689	C	T	NA	*OreG0020172*
scaffold424	818238	A	T	Och06	*OreG0020172*

scaffold424	840254	T	C	Ore01,Ore02,Ore03,Ore04,Ore05,Ore06,Ore07,Ore08,Ore09,Ore10,Ore11,Ore12,Ore13,Ore14	*OreG0020174*
scaffold424	886266	G	C	NA	*OreG0020178*
scaffold424	886733	G	T	Och05,Och14	*OreG0020178*
scaffold424	886978	A	G	Ore01,Ore02,Ore03,Ore04,Ore05,Ore06,Ore07,Ore08,Ore09,Ore10,Ore11,Ore12,Ore13,Ore14	*OreG0020178*
scaffold424	887009	G	T	Och01,Och02,Och03,Och04,Och05,Och06,Och07,Och08,Och09,Och10,Och11,Och12,Och13,Och14,Ore01,Ore02,Ore03,Ore04,Ore05,Ore06,Ore07,Ore08,Ore09,Ore10,Ore11,Ore12,Ore13,Ore14	*OreG0020178*
scaffold424	888408	C	A	Och05,Och14	*OreG0020178*
scaffold424	888774	G	A	NA	*OreG0020178*
scaffold424	890995	A	T	Och01,Och02,Och03,Och04,Och05,Och06,Och07,Och08,Och09,Och10,Och11,Och12,Och13,Och14	*OreG0020179*
scaffold424	891070	G	C	NA	*OreG0020179*
scaffold424	893806	T	A	Och01,Och02,Och03,Och04,Och05,Och06,Och07,Och08,Och09,Och10,Och11,Och12,Och13,Och14,Ore01,Ore02,Ore03,Ore04,Ore05,Ore06,Ore07,Ore08,Ore09,Ore10,Ore11,Ore12,Ore13,Ore14	*OreG0020180*
scaffold424	893809	G	T	Och01,Och02,Och03,Och04,Och05,Och06,Och07,Och08,Och09,Och10,Och11,Och12,Och13,Och14,Ore01,Ore02,Ore03,Ore04,Ore05,Ore06,Ore07,Ore08,Ore09,Ore10,Ore11,Ore12,Ore13,Ore14	*OreG0020180*
scaffold424	894385	A	C	Och05	*OreG0020180*
scaffold424	900780	T	C	NA	*OreG0020182*
scaffold424	963334	C	T	Ore01,Ore02,Ore03,Ore04,Ore05,Ore06,Ore07,Ore08,Ore09,Ore10,Ore11,Ore12,Ore13,Ore14	*OreG0020185*
scaffold424	963450	G	A	NA	*OreG0020185*
scaffold424	963507	A	T	NA	*OreG0020185*
scaffold424	963631	C	G	NA	*OreG0020185*
scaffold424	963751	C	T	Och01,Och02,Och03,Och04,Och05,Och06,Och07,Och08,Och09,Och10,Och11,Och12,Och13,Och14,Ore01,Ore02,Ore03,Ore04,Ore05,Ore06,Ore07,Ore08,Ore09,Ore10,Ore11,Ore12,Ore13,Ore14	*OreG0020185*
scaffold424	964005	C	A	Och01,Och08,Och09,Och10	*OreG0020185*
scaffold424	964369	C	G	Och01,Och02,Och03,Och04,Och05,Och06,Och07,Och08,Och09,Och10,Och11,Och12,Och13,Och14,Ore01,Ore02,Ore03,Ore04,Ore05,Ore06,Ore07,Ore08,Ore09,Ore10,Ore11,Ore12,Ore13,Ore14	*OreG0020185*
scaffold424	967362	G	A	Och02,Och03,Och04,Och06,Och12,Och13,Och14	*OreG0020186*
scaffold424	968034	A	G	NA	*OreG0020186*
scaffold424	974935	G	A	Och01,Och02,Och03,Och04,Och05,Och06,Och07,Och08,Och09,Och10,Och11,Och12,Och13,Och14	*OreG0020187*
scaffold424	1004139	G	A	Ore01,Ore02,Ore03,Ore04,Ore05,Ore06,Ore07,Ore08,Ore09,Ore10,Ore11,Ore12,Ore13,Ore14	*OreG0020189*
scaffold424	1008745	G	A	NA	*OreG0020190*
scaffold424	1008914	C	A	NA	*OreG0020190*
scaffold424	1042417	C	A	NA	*OreG0020192*
scaffold424	1068346	C	T	NA	*OreG0020194*
scaffold424	1068349	C	T	NA	*OreG0020194*
scaffold424	1079021	A	G	NA	*OreG0020195*
scaffold424	1119882	T	A	NA	*OreG0020200*
scaffold424	1120123	G	A	NA	*OreG0020200*
scaffold424	1125980	A	C	NA	*OreG0020200*
scaffold424	1127328	G	A	NA	*OreG0020200*
scaffold424	1143148	G	A	Och01,Och02,Och03,Och04,Och05,Och06,Och07,Och08,Och09,Och10,Och11,Och12,Och13,Och14,Ore01,Ore02,Ore03,Ore04,Ore05,Ore06,Ore07,Ore08,Ore09,Ore10,Ore11,Ore12,Ore13,Ore14	*OreG0020202*
scaffold424	1157174	C	A	NA	*OreG0020204*
scaffold424	1167655	G	C	Och06,Och11,Och14	*OreG0020205*
scaffold424	1219505	A	T	NA	*OreG0020211*
scaffold424	1287436	A	G	Ore01,Ore02,Ore03,Ore04,Ore05,Ore06,Ore07,Ore08,Ore09,Ore10,Ore11,Ore12,Ore13,Ore14	*OreG0020218*
scaffold424	1292292	T	A	Ore01,Ore02,Ore03,Ore04,Ore05,Ore06,Ore07,Ore08,Ore09,Ore10,Ore11,Ore12,Ore13,Ore14	*OreG0020219*
scaffold424	1292626	C	A	Ore01,Ore02,Ore03,Ore04,Ore05,Ore06,Ore07,Ore08,Ore09,Ore10,Ore11,Ore12,Ore13,Ore14	*OreG0020219*
scaffold424	1298035	T	C	Och01,Och02,Och03,Och04,Och05,Och06,Och07,Och08,Och09,Och10,Och11,Och12,Och13,Och14,Ore01,Ore02,Ore03,Ore04,Ore05,Ore06,Ore07,Ore08,Ore09,Ore10,Ore11,Ore12,Ore13,Ore14	*OreG0020219*
scaffold424	1298104	G	A	Och01	*OreG0020219*
scaffold424	1323310	G	A	Ore01,Ore02,Ore03,Ore04,Ore05,Ore06,Ore07,Ore08,Ore09,Ore10,Ore11,Ore12,Ore13,Ore14	*OreG0020224*
scaffold424	1323549	G	T	Och01,Och02,Och03,Och04,Och06,Och07,Och12,Och13	*OreG0020224*
scaffold424	1325529	T	A	Ore01,Ore02,Ore03,Ore04,Ore05,Ore06,Ore07,Ore08,Ore09,Ore10,Ore11,Ore12,Ore13,Ore14	*OreG0020225*
scaffold424	1390164	G	A	NA	*OreG0020231*
scaffold424	1390203	G	A	NA	*OreG0020231*
scaffold424	1445638	C	G	Och01,Och02,Och03,Och04,Och05,Och06,Och07,Och08,Och09,Och10,Och11,Och12,Och13,Och14,Ore01,Ore02,Ore03,Ore04,Ore05,Ore06,Ore07,Ore08,Ore09,Ore10,Ore11,Ore12,Ore13,Ore14	*OreG0020233*

scaffold424	1445776	A	G	Ore01,Ore02,Ore03,Ore04,Ore05,Ore06,Ore07,Ore08,Ore09,Ore10,Ore11,Ore12,Ore13,Ore14	*OreG0020233*
scaffold424	1450860	A	G	NA	*OreG0020235*
scaffold424	1451102	T	G	Och01	*OreG0020235*
scaffold424	1505738	T	C	Och01,Och02,Och03,Och04,Och05,Och06,Och07,Och08,Och09,Och10,Och11,Och12,Och13,Och14	*OreG0020241*
scaffold424	1506532	C	A	NA	*OreG0020243*
scaffold424	1507909	G	T	Ore01,Ore02,Ore03,Ore04,Ore05,Ore06,Ore07,Ore08,Ore09,Ore10,Ore11,Ore12,Ore13,Ore14	*OreG0020244*
scaffold424	1508300	A	T	NA	*OreG0020244*
scaffold424	1513173	T	C	Ore01,Ore02,Ore03,Ore04,Ore05,Ore06,Ore07,Ore08,Ore09,Ore10,Ore11,Ore12,Ore13,Ore14	*OreG0020245*
scaffold424	1514597	A	T	Och08	*OreG0020245*
scaffold424	1514802	T	C	NA	*OreG0020245*
scaffold424	1549007	G	T	NA	*OreG0020247*
scaffold424	1559547	C	G	Ore01,Ore02,Ore03,Ore04,Ore05,Ore06,Ore07,Ore08,Ore09,Ore10,Ore11,Ore12,Ore13,Ore14	*OreG0020248*
scaffold424	1559900	G	A	Och01	*OreG0020248*
scaffold424	1576298	G	A	Ore02,Ore03,Ore04,Ore05,Ore06,Ore07,Ore08,Ore09,Ore10,Ore11,Ore12,Ore13,Ore14	*OreG0020250*
scaffold424	1577424	C	T	Och01,Och02,Och03,Och04,Och05,Och06,Och07,Och08,Och09,Och10,Och11,Och12,Och13,Och14,Ore01,Ore02,Ore03,Ore04,Ore05,Ore06,Ore07,Ore08,Ore09,Ore10,Ore11,Ore12,Ore13,Ore14	*OreG0020250*
scaffold424	1578064	C	T	Och01,Och02,Och03,Och04,Och05,Och06,Och07,Och08,Och09,Och10,Och11,Och12,Och13,Och14	*OreG0020250*
scaffold424	1587174	C	T	NA	*OreG0020251*
scaffold424	1596776	G	A	Ore02,Ore03,Ore04,Ore05,Ore06,Ore07,Ore08,Ore09,Ore10,Ore11,Ore12,Ore13,Ore14	*OreG0020253*
scaffold424	1713307	C	A	Ore01,Ore02,Ore03,Ore04,Ore05,Ore06,Ore07,Ore08,Ore09,Ore10,Ore11,Ore12,Ore13,Ore14	*OreG0020260*
scaffold424	1716254	A	G	NA	*OreG0020260*
scaffold424	1716693	G	T	Ore02,Ore03,Ore04,Ore05,Ore06,Ore07,Ore08,Ore09,Ore10,Ore11,Ore12,Ore13,Ore14	*OreG0020260*
scaffold424	1716708	A	G	Och14	*OreG0020260*
scaffold424	1726664	G	T	Och02,Och03,Och04,Och06,Och12,Och13	*OreG0020262*
scaffold424	1726801	A	T	NA	*OreG0020262*
scaffold424	1732877	G	A	Och01,Och02,Och03,Och04,Och05,Och06,Och07,Och08,Och09,Och10,Och11,Och12,Och13,Och14,Ore01,Ore02,Ore03,Ore04,Ore05,Ore06,Ore07,Ore08,Ore09,Ore10,Ore11,Ore12,Ore13,Ore14	*OreG0020263*
scaffold424	1735606	A	G	Och01,Och02,Och03,Och04,Och05,Och06,Och07,Och08,Och09,Och10,Och11,Och12,Och13,Och14	*OreG0020264*
scaffold424	1741065	T	G	Ore01,Ore02,Ore03,Ore04,Ore05,Ore06,Ore07,Ore08,Ore09,Ore10,Ore11,Ore12,Ore13,Ore14	*OreG0020265*
scaffold424	1741131	A	T	Och01,Och02,Och03,Och04,Och05,Och06,Och07,Och08,Och09,Och10,Och11,Och12,Och13,Och14,Ore01,Ore02,Ore03,Ore04,Ore05,Ore06,Ore07,Ore08,Ore09,Ore10,Ore11,Ore12,Ore13,Ore14	*OreG0020265*
scaffold424	1741178	C	T	NA	*OreG0020265*
scaffold424	1741191	A	G	Och01,Och02,Och03,Och04,Och05,Och06,Och07,Och08,Och09,Och10,Och11,Och12,Och13,Och14	*OreG0020265*
scaffold424	1751222	T	C	Och01,Och02,Och03,Och04,Och05,Och06,Och07,Och08,Och09,Och10,Och11,Och12,Och13,Och14,Ore01,Ore02,Ore03,Ore04,Ore05,Ore06,Ore07,Ore08,Ore09,Ore10,Ore11,Ore12,Ore13,Ore14	*OreG0020266*
scaffold424	1751468	T	G	Och01,Och02,Och03,Och04,Och05,Och06,Och07,Och08,Och09,Och10,Och11,Och12,Och13,Och14,Ore01,Ore02,Ore03,Ore04,Ore05,Ore06,Ore07,Ore08,Ore09,Ore10,Ore11,Ore12,Ore13,Ore14	*OreG0020266*
scaffold424	1752080	C	G	Och01,Och02,Och03,Och04,Och05,Och06,Och07,Och08,Och09,Och10,Och11,Och12,Och13,Och14,Ore01,Ore02,Ore03,Ore04,Ore05,Ore06,Ore07,Ore08,Ore09,Ore10,Ore11,Ore12,Ore13,Ore14	*OreG0020266*
scaffold424	1752232	A	G	Och01,Och02,Och03,Och04,Och05,Och06,Och07,Och08,Och09,Och10,Och11,Och12,Och13,Och14	*OreG0020266*
scaffold424	1774397	T	C	NA	*OreG0020268*
scaffold424	1774651	C	G	Ore01,Ore02,Ore03,Ore04,Ore05,Ore06,Ore07,Ore08,Ore09,Ore10,Ore11,Ore12,Ore13,Ore14	*OreG0020268*
scaffold424	1775663	C	G	Och01,Och09,Och10,Och11,Ore01,Ore02,Ore03,Ore04,Ore05,Ore06,Ore07,Ore08,Ore09,Ore10,Ore11,Ore12,Ore13,Ore14	*OreG0020268*
scaffold424	1786132	G	T	Och01,Och02,Och03,Och04,Och05,Och06,Och07,Och08,Och09,Och10,Och11,Och12,Och13,Och14,Ore01,Ore02,Ore03,Ore04,Ore05,Ore06,Ore07,Ore08,Ore09,Ore10,Ore11,Ore12,Ore13,Ore14	*OreG0020271*
scaffold424	1792962	G	A	Och01,Och02,Och03,Och04,Och05,Och06,Och07,Och08,Och09,Och10,Och11,Och12,Och13,Och14,Ore01,Ore02,Ore03,Ore04,Ore05,Ore06,Ore07,Ore08,Ore09,Ore10,Ore11,Ore12,Ore13,Ore14	*OreG0020271*
scaffold424	1812505	A	T	Ore01,Ore02,Ore03,Ore04,Ore05,Ore06,Ore07,Ore08,Ore09,Ore10,Ore11,Ore12,Ore13,Ore14	*OreG0020272*
scaffold424	1814261	G	T	Ore01,Ore02,Ore03,Ore04,Ore05,Ore06,Ore07,Ore08,Ore09,Ore10,Ore11,Ore12,Ore13,Ore14	*OreG0020272*

scaffold424	1869709	T	C	Ore01,Ore02,Ore03,Ore04,Ore05,Ore06,Ore07,Ore08,Ore09,Ore10,Ore11,Ore12,Ore13,Ore14	*OreG0020274*
scaffold424	1869711	C	T	NA	*OreG0020274*
scaffold424	1881760	A	C	NA	*OreG0020275*
scaffold424	1906055	A	G	NA	*OreG0020277*
scaffold424	2012219	C	T	NA	*OreG0020286*
scaffold424	2013894	A	T	Ore01,Ore02,Ore03,Ore04,Ore05,Ore06,Ore07,Ore08,Ore09,Ore10,Ore11,Ore12,Ore13,Ore14	*OreG0020286*
scaffold424	2014084	A	T	Och01,Och02,Och03,Och04,Och05,Och06,Och07,Och08,Och09,Och10,Och11,Och12,Och13,Och14	*OreG0020286*
scaffold424	2024877	G	T	NA	*OreG0020289*
scaffold424	2024932	G	T	Och01,Och02,Och03,Och04,Och05,Och06,Och07,Och08,Och09,Och10,Och11,Och12,Och13,Och14	*OreG0020289*
scaffold424	2061596	G	C	NA	*OreG0020294*
scaffold424	2090637	G	A	NA	*OreG0020297*
scaffold424	2090722	T	C	NA	*OreG0020297*
scaffold424	2095658	G	A	NA	*OreG0020297*
scaffold424	2101926	C	G	NA	*OreG0020299*
scaffold424	2106198	T	G	NA	*OreG0020299*
scaffold424	2113375	C	A	Och01,Och02,Och03,Och04,Och05,Och06,Och07,Och08,Och09,Och10,Och11,Och12,Och13,Och14,Ore01,Ore02,Ore03,Ore04,Ore05,Ore06,Ore07,Ore08,Ore09,Ore10,Ore11,Ore12,Ore13,Ore14	*OreG0020300*
scaffold424	2113387	A	T	Och01,Och02,Och03,Och04,Och05,Och06,Och07,Och08,Och09,Och10,Och11,Och12,Och13,Och14	*OreG0020300*
scaffold424	2114191	C	T	Ore01,Ore02,Ore03,Ore04,Ore05,Ore06,Ore07,Ore08,Ore09,Ore10,Ore11,Ore12,Ore13,Ore14	*OreG0020300*
scaffold424	2115657	T	C	Ore01,Ore02,Ore03,Ore04,Ore05,Ore06,Ore07,Ore08,Ore09,Ore10,Ore11,Ore12,Ore13,Ore14	*OreG0020300*
scaffold424	2115895	T	G	Och01,Och02,Och03,Och04,Och05,Och06,Och07,Och08,Och09,Och10,Och11,Och12,Och13,Och14	*OreG0020300*
scaffold424	2130343	T	A	NA	*OreG0020301*
scaffold424	2175470	T	C	Och01,Och06,Och08	*OreG0020304*
scaffold424	2195725	T	G	NA	*OreG0020310*
scaffold424	2196052	G	T	NA	*OreG0020310*
scaffold424	2197520	C	T	NA	*OreG0020310*
scaffold424	2210308	A	G	NA	*OreG0020311*
scaffold424	2210320	A	G	NA	*OreG0020311*
scaffold424	2210485	T	G	Ore02,Ore03,Ore04,Ore05,Ore06,Ore07,Ore08,Ore09,Ore10,Ore11,Ore12,Ore13,Ore14	*OreG0020311*
scaffold424	2240514	T	A	NA	*OreG0020313*
scaffold424	2241669	C	T	NA	*OreG0020313*
scaffold424	2243329	G	A	Ore01,Ore02,Ore03,Ore04,Ore05,Ore06,Ore07,Ore08,Ore09,Ore10,Ore11,Ore12,Ore13,Ore14	*OreG0020313*
scaffold424	2243979	A	T	Ore01,Ore02,Ore03,Ore04,Ore05,Ore06,Ore07,Ore08,Ore09,Ore10,Ore11,Ore12,Ore13,Ore14	*OreG0020313*
scaffold424	2244673	G	T	NA	*OreG0020313*
scaffold424	2244689	C	A	NA	*OreG0020313*
scaffold424	2258549	T	C	NA	*OreG0020314*
scaffold424	2265216	C	T	Ore01,Ore02,Ore03,Ore04,Ore05,Ore06,Ore07,Ore08,Ore09,Ore10,Ore11,Ore12,Ore13,Ore14	*OreG0020315*
scaffold424	2274217	G	T	Och01,Och02,Och03,Och04,Och05,Och06,Och07,Och08,Och09,Och10,Och11,Och12,Och13,Och14	*OreG0020317*
scaffold424	2277166	T	A	Och01,Och02,Och03,Och04,Och05,Och06,Och07,Och08,Och09,Och10,Och11,Och12,Och13,Och14,Ore01,Ore02,Ore03,Ore04,Ore05,Ore06,Ore07,Ore08,Ore09,Ore10,Ore11,Ore12,Ore13,Ore14	*OreG0020317*
scaffold424	2288665	G	A	NA	*OreG0020318*
scaffold424	2297072	C	T	Och01,Och02,Och03,Och04,Och05,Och06,Och07,Och08,Och09,Och10,Och11,Och12,Och13,Och14,Ore01,Ore02,Ore03,Ore04,Ore05,Ore06,Ore07,Ore08,Ore09,Ore10,Ore11,Ore12,Ore13,Ore14	*OreG0020319*
scaffold424	2312708	C	T	NA	*OreG0020321*
scaffold424	2312843	C	T	NA	*OreG0020321*
scaffold424	2312859	G	A	NA	*OreG0020321*
scaffold424	2313339	A	T	NA	*OreG0020321*
scaffold424	2313494	G	A	NA	*OreG0020321*
scaffold424	2313702	C	T	NA	*OreG0020321*
scaffold424	2322549	T	C	NA	*OreG0020323*
scaffold424	2322768	A	C	NA	*OreG0020323*
scaffold424	2352713	C	T	Och14	*OreG0020328*
scaffold424	2352914	C	T	NA	*OreG0020328*
scaffold424	2353078	G	A	NA	*OreG0020328*
scaffold424	2353147	C	T	NA	*OreG0020328*
scaffold424	2353250	C	T	NA	*OreG0020328*
scaffold424	2353258	C	T	NA	*OreG0020328*
scaffold424	2353318	C	G	Och14	*OreG0020328*
scaffold424	2353390	C	T	NA	*OreG0020328*

scaffold424	2353393	C	G	NA	*OreG0020328*
scaffold424	2354134	C	T	NA	*OreG0020328*
scaffold424	2354453	G	A	Och14	*OreG0020328*
scaffold424	2355979	C	A	NA	*OreG0020328*
scaffold424	2390355	G	T	NA	*OreG0020332*
scaffold424	2390569	C	T	NA	*OreG0020332*
scaffold424	2435421	G	A	Ore01,Ore02,Ore03,Ore04,Ore05,Ore06,Ore07,Ore08,Ore09,Ore10,Ore11,Ore12,Ore13,Ore14	*OreG0020336*
scaffold424	2459812	T	C	NA	*OreG0020337*
scaffold424	2467056	G	T	Och01,Och02,Och03,Och04,Och05,Och06,Och07,Och08,Och09,Och10,Och11,Och12,Och13,Och14	*OreG0020337*
scaffold424	2499960	G	A	NA	*OreG0020339*
scaffold618	69425	A	T	Och01,Och02,Och03,Och04,Och05,Och06,Och07,Och08,Och09,Och10,Och11,Och12,Och13,Och14,Ore01,Ore02,Ore03,Ore04,Ore05,Ore06,Ore07,Ore08,Ore09,Ore10,Ore11,Ore12,Ore13,Ore14	*OreG0024204*
scaffold618	131151	C	A	Och01,Och02,Och03,Och04,Och05,Och06,Och07,Och08,Och09,Och10,Och11,Och12,Och13,Och14	*OreG0024209*
scaffold618	182933	G	A	Och09,Och10	*OreG0024212*
scaffold618	199717	G	A	Ore01,Ore02,Ore08	*OreG0024214*
scaffold618	199924	G	C	NA	*OreG0024214*
scaffold618	199957	C	A	NA	*OreG0024214*
scaffold618	227689	G	A	NA	*OreG0024216*
scaffold618	228013	G	A	NA	*OreG0024216*
scaffold618	232799	A	C	NA	*OreG0024217*
scaffold618	240743	G	T	Och09,Och10	*OreG0024219*
scaffold618	242035	G	A	Och07,Och08,Och09,Och10	*OreG0024219*
scaffold618	251675	G	T	Och01,Och02,Och03,Och04,Och05,Och06,Och07,Och08,Och09,Och10,Och11,Och12,Och13,Och14,Ore01,Ore02,Ore03,Ore04,Ore05,Ore06,Ore07,Ore08,Ore09,Ore10,Ore11,Ore12,Ore13,Ore14	*OreG0024220*
scaffold618	265032	A	G	Och01,Och02,Och03,Och04,Och05,Och06,Och07,Och08,Och09,Och10,Och11,Och12,Och13,Och14	*OreG0024221*
scaffold618	267538	C	G	NA	*OreG0024221*
scaffold618	292800	T	C	NA	*OreG0024224*
scaffold618	317626	G	T	Och02,Och03,Och04,Och05,Och06,Och07,Och08,Och09,Och10,Och11,Och12,Och13,Och14,Ore01,Ore02,Ore03,Ore04,Ore05,Ore06,Ore07,Ore08,Ore09,Ore10,Ore11,Ore12,Ore13,Ore14	*OreG0024228*
scaffold618	360299	C	T	NA	*OreG0024234*
scaffold951	102733	T	C	NA	*OreG0026943*
scaffold951	103257	C	A	NA	*OreG0026943*
scaffold951	103645	C	G	NA	*OreG0026943*
scaffold951	103761	G	T	Och03	*OreG0026943*
scaffold951	104366	C	T	Och11	*OreG0026943*
scaffold951	104368	T	C	NA	*OreG0026943*
scaffold524	45726	C	T	NA	*OreG0023565*
scaffold524	45816	G	A	NA	*OreG0023565*
scaffold524	45822	C	T	NA	*OreG0023565*
scaffold606	28370	C	T	NA	*OreG0024053*
scaffold606	29563	A	G	Ore13	*OreG0024054*
scaffold606	29572	T	C	NA	*OreG0024054*
scaffold606	29608	C	A	NA	*OreG0024054*
scaffold606	29641	G	A	NA	*OreG0024054*
scaffold606	29992	T	A	NA	*OreG0024054*
scaffold606	30092	G	A	NA	*OreG0024054*
scaffold606	30233	C	T	NA	*OreG0024054*
scaffold606	40252	C	T	NA	*OreG0024055*
scaffold606	44073	G	T	NA	*OreG0024055*
scaffold606	47588	G	A	NA	*OreG0024056*
scaffold606	47597	A	G	Och01,Och02,Och03,Och04,Och05,Och06,Och07,Och08,Och09,Och10,Och11,Och12,Och13,Och14,Ore01,Ore02,Ore03,Ore04,Ore05,Ore06,Ore07,Ore08,Ore09,Ore10,Ore11,Ore12,Ore13,Ore14	*OreG0024056*
scaffold606	47600	T	A	NA	*OreG0024056*
scaffold606	47837	G	C	Och01,Och02,Och03,Och04,Och05,Och06,Och07,Och08,Och09,Och10,Och11,Och12,Och13,Och14,Ore01,Ore02,Ore03,Ore04,Ore05,Ore06,Ore07,Ore08,Ore09,Ore10,Ore11,Ore12,Ore13,Ore14	*OreG0024056*
scaffold606	60343	T	A	Och01,Och02,Och03,Och04,Och05,Och06,Och07,Och08,Och09,Och10,Och11,Och12,Och13,Och14,Ore01,Ore02,Ore03,Ore04,Ore05,Ore06,Ore07,Ore08,Ore09,Ore10,Ore11,Ore12,Ore13,Ore14	*OreG0024057*
scaffold606	60345	G	T	Och01,Och02,Och03,Och04,Och05,Och06,Och07,Och08,Och09,Och10,Och11,Och12,Och13,Och14,Ore01,Ore02,Ore03,Ore04,Ore05,Ore06,Ore07,Ore08,Ore09,Ore10,Ore11,Ore12,Ore13,Ore14	*OreG0024057*
scaffold606	60647	A	G	NA	*OreG0024057*
scaffold606	60713	T	C	Och01,Och02,Och03,Och04,Och05,Och06,Och07,Och08,Och09,Och10,Och11,Och12,Och13,Och14,Ore01,Ore02,Ore03,Ore04,Ore05,Ore06,Ore07,Ore08,Ore09,Ore10,Ore11,Ore12,Ore13,Ore14	*OreG0024057*
scaffold606	60741	G	T	NA	*OreG0024057*

scaffold606	60880	A	G	NA	*OreG0024057*
scaffold606	61408	T	G	NA	*OreG0024057*
scaffold606	67278	G	C	NA	*OreG0024060*
scaffold606	112450	G	A	NA	*OreG0024063*
scaffold606	113638	G	A	NA	*OreG0024063*
scaffold606	116011	T	A	Och01,Och02,Och03,Och04,Och05,Och06,Och07,Och08,Och09,Och10,Och11,Och12,Och13,Och14,Ore01,Ore02,Ore03,Ore04,Ore05,Ore06,Ore07,Ore08,Ore09,Ore10,Ore11,Ore12,Ore13,Ore14	*OreG0024063*
scaffold606	116099	G	T	Och01,Och02,Och03,Och04,Och05,Och06,Och07,Och09,Och10,Och11,Och12,Och13,Och14,Ore01,Ore02,Ore03,Ore04,Ore05,Ore06,Ore07,Ore08,Ore09,Ore10,Ore11,Ore12,Ore13,Ore14	*OreG0024063*
scaffold606	120747	C	A	NA	*OreG0024065*
scaffold606	121165	A	T	NA	*OreG0024065*
scaffold606	121346	C	T	Och12,Ore01,Ore02,Ore03,Ore04,Ore05,Ore06,Ore07,Ore08,Ore09,Ore10,Ore11,Ore12,Ore13,Ore14	*OreG0024065*
scaffold606	121349	C	A	NA	*OreG0024065*
scaffold606	121531	T	C	Och08,Ore01,Ore02,Ore03,Ore04,Ore05,Ore06,Ore07,Ore08,Ore09,Ore10,Ore11,Ore12,Ore13,Ore14	*OreG0024065*
scaffold606	121702	G	C	Och08	*OreG0024065*
scaffold606	121732	G	A	NA	*OreG0024065*
scaffold606	122260	C	T	NA	*OreG0024065*
scaffold606	122377	G	C	Och02,Och03,Och04,Och05,Och06,Och09,Och10,Och11,Och12,Och13,Och14,Ore01,Ore02,Ore03,Ore04,Ore05,Ore06,Ore07,Ore08,Ore09,Ore10,Ore11,Ore12,Ore13,Ore14	*OreG0024065*
scaffold606	127164	A	G	NA	*OreG0024066*
scaffold606	127430	T	G	NA	*OreG0024066*
scaffold606	127652	C	T	NA	*OreG0024066*
scaffold606	127661	C	T	NA	*OreG0024066*
scaffold606	127905	G	A	NA	*OreG0024066*
scaffold606	139038	T	G	NA	*OreG0024068*
scaffold606	186828	G	A	NA	*OreG0024077*
scaffold606	199715	C	T	NA	*OreG0024080*
scaffold606	202582	T	A	NA	*OreG0024080*
scaffold606	251581	G	T	NA	*OreG0024082*
scaffold606	262884	T	C	NA	*OreG0024085*
scaffold606	262953	G	C	NA	*OreG0024085*
scaffold606	263772	A	G	NA	*OreG0024085*
scaffold606	264311	T	G	NA	*OreG0024085*
scaffold606	264475	A	G	Och08	*OreG0024085*
scaffold606	264501	G	A	NA	*OreG0024085*
scaffold606	266951	A	G	NA	*OreG0024086*
scaffold606	266977	C	A	NA	*OreG0024086*
scaffold606	267224	C	T	NA	*OreG0024086*
scaffold606	268247	C	T	NA	*OreG0024086*
scaffold606	276974	A	C	NA	*OreG0024087*
scaffold606	356330	T	G	Och01,Och02,Och03,Och04,Och05,Och06,Och07,Och08,Och09,Och10,Och11,Och12,Och13,Och14,Ore01,Ore02,Ore03,Ore04,Ore05,Ore06,Ore07,Ore08,Ore09,Ore10,Ore11,Ore12,Ore13,Ore14	*OreG0024089*
scaffold606	378128	A	T	NA	*OreG0024093*
scaffold606	379222	C	T	NA	*OreG0024093*
scaffold606	379326	T	G	NA	*OreG0024093*
scaffold606	397323	G	A	Och07	*OreG0024095*
scaffold606	400411	C	A	NA	*OreG0024096*
scaffold606	400504	C	T	NA	*OreG0024096*
scaffold606	406764	T	A	NA	*OreG0024098*
scaffold606	418028	G	A	Ore01,Ore02,Ore03,Ore04,Ore05,Ore06,Ore07,Ore08,Ore09,Ore10,Ore11,Ore12,Ore13,Ore14	*OreG0024100*
scaffold606	418102	C	T	NA	*OreG0024100*
scaffold606	419912	C	A	NA	*OreG0024100*
scaffold606	432494	G	C	NA	*OreG0024102*
scaffold606	487320	G	T	Ore01,Ore03,Ore04,Ore05	*OreG0024113*
scaffold606	508891	C	T	NA	*OreG0024117*
scaffold606	532119	C	G	NA	*OreG0024118*
scaffold606	543693	G	C	NA	*OreG0024119*
scaffold606	543702	G	T	NA	*OreG0024119*
scaffold606	543730	C	G	Ore02,Ore03,Ore04,Ore05,Ore06,Ore07,Ore08,Ore09,Ore10,Ore11,Ore12,Ore13,Ore14	*OreG0024119*
scaffold606	545118	C	T	Och02,Och03,Och04,Och06,Och13,Och14	*OreG0024120*
scaffold606	545246	G	A	Ore04,Ore05	*OreG0024120*
scaffold606	545250	G	A	Ore02,Ore03,Ore04,Ore05,Ore06,Ore07,Ore08,Ore09,Ore10,Ore11,Ore12,Ore13,Ore14	*OreG0024120*
scaffold606	546170	G	A	Ore01,Ore02,Ore03,Ore04,Ore05,Ore06,Ore07,Ore08,Ore09,Ore10,Ore11,Ore12,Ore13,Ore14	*OreG0024120*
scaffold606	546387	A	G	Ore02,Ore04,Ore05,Ore06,Ore07,Ore08,Ore11,Ore12,Ore13,Ore14	*OreG0024120*

scaffold606	546741	T	A	Och01,Och02,Och03,Och04,Och05,Och06,Och07,Och08,Och09,Och10,Och11,Och12,Och13,Och14,Ore01,Ore02,Ore03,Ore04,Ore05,Ore06,Ore07,Ore08,Ore09,Ore10,Ore11,Ore12,Ore13,Ore14	*OreG0024120*
scaffold606	546855	T	A	NA	*OreG0024120*
scaffold606	548358	T	G	Och01,Och02,Och03,Och04,Och05,Och06,Och07,Och08,Och09,Och10,Och11,Och12,Och13,Och14	*OreG0024120*
scaffold606	548504	G	T	Ore02,Ore11,Ore13	*OreG0024120*
scaffold606	548970	G	A	NA	*OreG0024120*
scaffold606	549056	A	T	NA	*OreG0024120*
scaffold606	549412	T	C	Och13	*OreG0024121*
scaffold606	549418	T	C	Ore02,Ore11,Ore13	*OreG0024121*
scaffold606	552592	A	G	Och01,Och02,Och03,Och04,Och05,Och06,Och07,Och08,Och09,Och10,Och11,Och12,Och13,Och14,Ore01,Ore02,Ore03,Ore04,Ore05,Ore06,Ore07,Ore08,Ore09,Ore10,Ore11,Ore12,Ore13,Ore14	*OreG0024122*
scaffold606	573297	A	C	Ore01,Ore02,Ore03,Ore04,Ore05,Ore06,Ore07,Ore08,Ore09,Ore10,Ore11,Ore12,Ore13,Ore14	*OreG0024124*
scaffold606	576147	C	G	Ore02,Ore09,Ore10,Ore11,Ore13	*OreG0024125*
scaffold606	579644	C	T	NA	*OreG0024126*
scaffold606	592973	C	T	Och01,Och02,Och03,Och04,Och05,Och06,Och07,Och08,Och09,Och10,Och11,Och12,Och13,Och14,Ore01,Ore02,Ore03,Ore04,Ore05,Ore06,Ore07,Ore08,Ore09,Ore10,Ore11,Ore12,Ore13,Ore14	*OreG0024127*
scaffold606	598749	A	G	NA	*OreG0024128*
scaffold606	604363	G	A	NA	*OreG0024129*
scaffold606	623860	A	T	NA	*OreG0024132*
scaffold606	628596	C	A	NA	*OreG0024133*
scaffold606	641241	G	T	NA	*OreG0024135*
scaffold606	670858	G	T	Och01,Och02,Och03,Och04,Och05,Och06,Och07,Och08,Och09,Och10,Och11,Och12,Och13,Och14,Ore01,Ore02,Ore03,Ore04,Ore05,Ore06,Ore07,Ore08,Ore09,Ore10,Ore11,Ore12,Ore13,Ore14	*OreG0024139*
scaffold606	679071	T	C	NA	*OreG0024141*
scaffold606	679091	A	G	Och01,Och14	*OreG0024141*
scaffold606	679245	C	T	NA	*OreG0024141*
scaffold606	679275	A	G	Och01,Och14	*OreG0024141*
scaffold606	680502	C	G	Och02,Och03,Och04,Och05,Och06,Och07,Och08,Och12,Och13	*OreG0024142*
scaffold606	693710	G	A	Ore01,Ore02,Ore03,Ore04,Ore05,Ore06,Ore07,Ore08,Ore09,Ore10,Ore11,Ore12,Ore13,Ore14	*OreG0024143*
scaffold606	702132	G	A	Och01,Och02,Och03,Och04,Och05,Och06,Och07,Och08,Och09,Och10,Och11,Och12,Och13,Och14,Ore01,Ore02,Ore03,Ore04,Ore05,Ore06,Ore07,Ore08,Ore09,Ore10,Ore11,Ore12,Ore13,Ore14	*OreG0024143*
scaffold606	703268	G	T	Och05	*OreG0024143*
scaffold606	728146	T	C	NA	*OreG0024148*
scaffold606	757624	T	A	NA	*OreG0024153*
scaffold185	4913	A	G	Ore01,Ore02,Ore03,Ore04,Ore05,Ore06,Ore07,Ore08,Ore09,Ore10,Ore11,Ore12,Ore13,Ore14	*OreG0003980*
scaffold185	5123	A	T	Och12,Och13	*OreG0003980*
scaffold185	5230	G	A	Och12,Och13	*OreG0003980*
scaffold185	59265	A	G	Och06	*OreG0003983*
scaffold185	59371	T	G	Och12,Och13	*OreG0003983*
scaffold185	59701	T	A	NA	*OreG0003983*
scaffold185	59827	C	T	NA	*OreG0003983*
scaffold185	89578	A	C	NA	*OreG0003989*
scaffold185	102430	A	C	NA	*OreG0003991*
scaffold185	106062	A	T	NA	*OreG0003992*
scaffold185	106083	T	A	NA	*OreG0003992*
scaffold185	112115	C	T	NA	*OreG0003993*
scaffold185	112244	A	G	Ore02,Ore03,Ore06,Ore07,Ore09,Ore10,Ore11,Ore12,Ore13,Ore14	*OreG0003993*
scaffold185	112431	C	A	NA	*OreG0003993*
scaffold185	112466	G	C	NA	*OreG0003993*
scaffold185	117710	G	A	NA	*OreG0003994*
scaffold185	118549	T	C	NA	*OreG0003994*
scaffold185	120441	C	A	NA	*OreG0003994*
scaffold185	120482	T	A	Och09,Och10	*OreG0003994*
scaffold185	136931	C	T	Och02,Och04,Och06,Och12,Och13	*OreG0003996*
scaffold185	137826	T	C	NA	*OreG0003996*
scaffold185	137843	A	G	NA	*OreG0003996*
scaffold185	141046	C	T	Och02,Och03,Och04,Och05,Och06,Och08,Och09,Och10,Och11,Och12,Och13,Och14,Ore01,Ore02,Ore03,Ore04,Ore05,Ore06,Ore07,Ore08,Ore09,Ore10,Ore11,Ore12,Ore13,Ore14	*OreG0003997*
scaffold185	167293	G	A	Ore01,Ore02,Ore03,Ore04,Ore05,Ore06,Ore07,Ore08,Ore09,Ore10,Ore11,Ore12,Ore13,Ore14	*OreG0004000*
scaffold185	168012	T	A	Och01,Och02,Och03,Och04,Och05,Och06,Och07,Och08,Och09,Och10,Och11,Och12,Och13,Och14,Ore01,Ore02,Ore03,Ore04,Ore05,Ore06,Ore07,Ore08,Ore09,Ore10,Ore11,Ore12,Ore13,Ore14	*OreG0004000*

scaffold185	209226	A	T	Och01,Och02,Och03,Och04,Och05,Och06,Och07,Och08,Och09,Och10,Och11,Och12,Och13,Och14,Ore01,Ore02,Ore03,Ore04,Ore05,Ore06,Ore07,Ore08,Ore09,Ore10,Ore11,Ore12,Ore13,Ore14	*OreG0004007*
scaffold185	231825	G	T	Och01,Och02,Och03,Och04,Och05,Och06,Och07,Och08,Och09,Och10,Och11,Och12,Och13,Och14,Ore01,Ore02,Ore03,Ore04,Ore05,Ore06,Ore07,Ore08,Ore09,Ore10,Ore11,Ore12,Ore13,Ore14	*OreG0004010*
scaffold185	238328	A	C	NA	*OreG0004011*
scaffold185	240245	G	C	Ore01,Ore02,Ore03,Ore04,Ore05,Ore06,Ore07,Ore08,Ore09,Ore10,Ore11,Ore12,Ore13,Ore14	*OreG0004011*
scaffold185	240261	T	A	NA	*OreG0004011*
scaffold185	242139	G	A	NA	*OreG0004011*
scaffold185	256954	C	A	Och01,Och02,Och03,Och04,Och05,Och06,Och07,Och08,Och09,Och10,Och11,Och12,Och13,Och14,Ore01,Ore02,Ore03,Ore04,Ore05,Ore06,Ore07,Ore08,Ore09,Ore10,Ore11,Ore12,Ore13,Ore14	*OreG0004015*
scaffold185	263263	G	C	NA	*OreG0004016*
scaffold185	271269	G	T	NA	*OreG0004018*
scaffold185	272476	C	A	Och09,Och10	*OreG0004018*
scaffold185	272487	G	C	NA	*OreG0004018*
scaffold185	272548	C	A	Och12,Och13	*OreG0004018*
scaffold185	272836	T	C	Ore01,Ore02,Ore03,Ore04,Ore05,Ore06,Ore07,Ore08,Ore09,Ore10,Ore11,Ore12,Ore13,Ore14	*OreG0004018*
scaffold185	272847	G	T	Ore01,Ore02,Ore03,Ore04,Ore05,Ore06,Ore07,Ore08,Ore09,Ore10,Ore11,Ore12,Ore13,Ore14	*OreG0004018*
scaffold185	273321	G	T	Och09,Och10	*OreG0004018*
scaffold185	273410	G	A	Och12,Och13	*OreG0004018*
scaffold185	273426	T	C	Ore01,Ore02,Ore03,Ore04,Ore05,Ore06,Ore07,Ore08,Ore09,Ore10,Ore11,Ore12,Ore13,Ore14	*OreG0004018*
scaffold185	273456	C	T	Och05,Och12,Och13	*OreG0004018*
scaffold185	273483	C	T	Och09,Och10,Och12,Och13	*OreG0004018*
scaffold185	273564	C	T	Och01,Och09,Och10,Och12,Och13	*OreG0004018*
scaffold185	273569	G	A	Och09,Och10	*OreG0004018*
scaffold185	273588	C	T	Och09,Och10	*OreG0004018*
scaffold185	280560	T	C	NA	*OreG0004020*
scaffold185	283315	T	G	NA	*OreG0004020*
scaffold185	283488	T	C	NA	*OreG0004020*
scaffold185	283491	A	T	NA	*OreG0004020*
scaffold185	283607	A	G	Ore01,Ore02,Ore03,Ore04,Ore05,Ore06,Ore07,Ore08,Ore09,Ore10,Ore11,Ore12,Ore13,Ore14	*OreG0004020*
scaffold185	286551	G	A	Och01,Och02,Och03,Och04,Och05,Och06,Och07,Och08,Och09,Och10,Och11,Och12,Och13,Och14,Ore01,Ore02,Ore03,Ore04,Ore05,Ore06,Ore07,Ore08,Ore09,Ore10,Ore11,Ore12,Ore13,Ore14	*OreG0004021*
scaffold185	286744	T	A	Ore01,Ore02,Ore03,Ore04,Ore05,Ore06,Ore07,Ore08,Ore09,Ore10,Ore11,Ore12,Ore13,Ore14	*OreG0004021*
scaffold185	286872	C	T	Och01,Och02,Och03,Och04,Och05,Och06,Och07,Och08,Och09,Och10,Och11,Och12,Och13,Och14	*OreG0004021*
scaffold185	287508	C	G	Och01,Och02,Och03,Och04,Och05,Och06,Och07,Och08,Och09,Och10,Och11,Och12,Och13,Och14	*OreG0004021*
scaffold185	287549	A	C	NA	*OreG0004021*
scaffold185	287684	T	A	Ore01,Ore02,Ore03,Ore04,Ore05,Ore06,Ore07,Ore08,Ore09,Ore10,Ore11,Ore12,Ore13,Ore14	*OreG0004021*
scaffold185	287745	T	C	Ore01,Ore02,Ore03,Ore04,Ore05,Ore06,Ore07,Ore08,Ore09,Ore10,Ore11,Ore12,Ore13,Ore14	*OreG0004021*
scaffold185	290051	C	T	Och09,Och10,Och12,Och13	*OreG0004022*
scaffold185	290243	C	G	Ore01,Ore02,Ore03,Ore04,Ore05,Ore06,Ore07,Ore08,Ore09,Ore10,Ore11,Ore12,Ore13,Ore14	*OreG0004022*
scaffold185	290843	T	G	Och01,Och02,Och03,Och04,Och05,Och06,Och07,Och08,Och09,Och10,Och11,Och12,Och13,Och14	*OreG0004022*
scaffold185	290911	C	T	Och01,Och02,Och03,Och04,Och05,Och06,Och07,Och08,Och09,Och10,Och11,Och12,Och13,Och14	*OreG0004022*
scaffold185	291965	C	A	Och01,Och02,Och03,Och04,Och05,Och06,Och07,Och08,Och09,Och10,Och11,Och12,Och13,Och14,Ore01,Ore02,Ore03,Ore04,Ore05,Ore06,Ore07,Ore08,Ore09,Ore10,Ore11,Ore12,Ore13,Ore14	*OreG0004022*
scaffold185	292183	A	T	Och01,Och02,Och04,Och06,Och09,Och10,Och11,Och12,Och13	*OreG0004022*
scaffold185	301990	T	C	Ore01,Ore02,Ore03,Ore04,Ore05,Ore06,Ore07,Ore08,Ore09,Ore10,Ore11,Ore12,Ore13,Ore14	*OreG0004024*
scaffold185	302354	T	C	Och14	*OreG0004024*
scaffold185	313875	T	G	Och06,Och09,Och10,Och14	*OreG0004025*
scaffold185	349041	C	G	NA	*OreG0004029*
scaffold185	351693	G	T	NA	*OreG0004030*
scaffold185	351775	T	C	Och01,Och12,Och13	*OreG0004030*
scaffold185	351868	T	C	Ore01,Ore02,Ore03,Ore04,Ore05,Ore06,Ore07,Ore08,Ore09,Ore10,Ore11,Ore12,Ore13,Ore14	*OreG0004030*
scaffold185	359404	G	A	Och09,Och10	*OreG0004032*
scaffold185	359664	G	T	Och05,Och07,Och09,Och10,Och12,Och13	*OreG0004032*
scaffold185	431359	A	T	Och02,Och03,Och04,Och05,Och06,Och08,Och11,Och12,Och13,Och14	*OreG0004040*

scaffold185	435999	G	C	Och01,Och02,Och03,Och04,Och05,Och06,Och07,Och08,Och09,Och10,Och11,Och12,Och13,Och14,Ore01,Ore02,Ore03,Ore04,Ore05,Ore06,Ore07,Ore08,Ore09,Ore10,Ore11,Ore12,Ore13,Ore14	*OreG0004041*
scaffold185	436002	A	G	Och01,Och02,Och03,Och04,Och05,Och06,Och07,Och08,Och09,Och10,Och11,Och12,Och13,Och14,Ore01,Ore02,Ore03,Ore04,Ore05,Ore06,Ore07,Ore08,Ore09,Ore10,Ore11,Ore12,Ore13,Ore14	*OreG0004041*
scaffold185	438565	G	T	Och01,Och02,Och03,Och04,Och05,Och06,Och07,Och08,Och09,Och10,Och11,Och12,Och13,Och14,Ore01,Ore02,Ore03,Ore04,Ore05,Ore06,Ore07,Ore08,Ore09,Ore10,Ore11,Ore12,Ore13,Ore14	*OreG0004041*
scaffold185	446190	G	A	NA	*OreG0004042*
scaffold185	446291	A	C	Och07,Och09,Och10	*OreG0004042*
scaffold185	446769	G	C	Och09,Och10	*OreG0004042*
scaffold185	467559	G	T	NA	*OreG0004044*
scaffold185	467694	T	G	Och01,Och02,Och03,Och04,Och05,Och06,Och07,Och08,Och09,Och10,Och11,Och12,Och13,Och14,Ore01,Ore02,Ore03,Ore04,Ore05,Ore06,Ore07,Ore08,Ore09,Ore10,Ore11,Ore12,Ore13,Ore14	*OreG0004044*
scaffold185	469069	G	A	Ore02,Ore03,Ore06,Ore07,Ore09,Ore10,Ore11,Ore12,Ore13,Ore14	*OreG0004044*
scaffold185	473376	T	A	NA	*OreG0004044*
scaffold185	473482	C	T	Och02,Och03,Och04,Och05,Och06,Och08,Och11,Och12,Och13,Och14	*OreG0004044*
scaffold185	473573	C	T	Och03,Och06	*OreG0004044*
scaffold185	515671	C	T	Och12,Och13	*OreG0004046*
scaffold185	553478	G	A	Och01,Och02,Och03,Och04,Och05,Och06,Och07,Och08,Och09,Och10,Och11,Och12,Och13,Och14	*OreG0004049*
scaffold185	686864	G	A	Och08,Och12,Och13,Ore01,Ore02,Ore03,Ore04,Ore05,Ore06,Ore07,Ore08,Ore09,Ore10,Ore11,Ore12,Ore13,Ore14	*OreG0004056*
scaffold185	688179	C	T	NA	*OreG0004056*
scaffold185	688818	A	T	Ore01,Ore02,Ore03,Ore04,Ore05,Ore06,Ore07,Ore08,Ore09,Ore10,Ore11,Ore12,Ore13,Ore14	*OreG0004056*
scaffold185	721344	C	T	NA	*OreG0004058*
scaffold185	721353	G	A	NA	*OreG0004058*
scaffold185	721452	C	T	NA	*OreG0004058*
scaffold185	721454	G	A	NA	*OreG0004058*
scaffold185	721977	C	T	Och12,Och13	*OreG0004058*
scaffold185	722200	A	T	Och01,Och02,Och03,Och04,Och05,Och06,Och07,Och08,Och09,Och10,Och11,Och12,Och13,Och14,Ore01,Ore02,Ore03,Ore04,Ore05,Ore06,Ore07,Ore08,Ore09,Ore10,Ore11,Ore12,Ore13,Ore14	*OreG0004058*
scaffold185	790249	A	G	Och01,Och07,Och08,Och09,Och10,Och11,Och12,Och13	*OreG0004059*
scaffold185	823770	C	A	Ore01,Ore02,Ore03,Ore04,Ore05,Ore06,Ore07,Ore08,Ore09,Ore10,Ore11,Ore12,Ore13,Ore14	*OreG0004059*
scaffold185	844377	G	T	Ore01,Ore02,Ore03,Ore04,Ore05,Ore06,Ore07,Ore08,Ore09,Ore10,Ore11,Ore12,Ore13,Ore14	*OreG0004059*
scaffold185	857158	G	A	NA	*OreG0004061*
scaffold185	911567	C	T	Och06	*OreG0004065*
scaffold185	911570	A	T	Och06	*OreG0004065*
scaffold185	991873	A	G	Och01	*OreG0004069*
scaffold185	998423	G	C	Och03	*OreG0004069*
scaffold185	1162838	T	A	Och02,Och03,Och04,Och05,Och06,Och12,Och13,Och14	*OreG0004073*
scaffold185	1177285	T	A	Och01,Och02,Och03,Och04,Och05,Och06,Och07,Och08,Och09,Och10,Och11,Och12,Och13,Och14,Ore01,Ore02,Ore03,Ore04,Ore05,Ore06,Ore07,Ore08,Ore09,Ore10,Ore11,Ore12,Ore13,Ore14	*OreG0004073*
scaffold185	1243273	C	T	Och01,Och02,Och03,Och04,Och05,Och06,Och07,Och08,Och09,Och10,Och11,Och12,Och13,Och14,Ore01,Ore02,Ore03,Ore04,Ore05,Ore06,Ore07,Ore08,Ore09,Ore10,Ore11,Ore12,Ore13,Ore14	*OreG0004074*
scaffold185	1409566	C	A	NA	*OreG0004080*
scaffold185	1414547	G	T	NA	*OreG0004081*
scaffold185	1414567	G	T	NA	*OreG0004081*
scaffold185	1414697	C	T	NA	*OreG0004081*
scaffold185	1483734	G	T	Och12,Och13	*OreG0004088*
scaffold185	1507875	C	T	Och01,Och02,Och03,Och04,Och05,Och06,Och07,Och08,Och09,Och10,Och11,Och12,Och13,Och14	*OreG0004089*
scaffold185	1508498	A	T	Ore01,Ore02,Ore03,Ore04,Ore05,Ore06,Ore07,Ore08,Ore09,Ore10,Ore11,Ore12,Ore13,Ore14	*OreG0004089*
scaffold185	1560117	A	T	Ore01,Ore02,Ore03,Ore04,Ore05,Ore06,Ore07,Ore08,Ore09,Ore10,Ore11,Ore12,Ore13,Ore14	*OreG0004090*
scaffold185	1711730	T	A	NA	*OreG0004092*
scaffold185	1856661	G	T	NA	*OreG0004099*
scaffold185	1856695	G	A	NA	*OreG0004099*
scaffold185	1856776	G	A	NA	*OreG0004099*
scaffold185	1856802	G	A	NA	*OreG0004099*
scaffold185	1856967	G	A	NA	*OreG0004099*
scaffold185	1857018	C	T	NA	*OreG0004099*
scaffold185	1857030	C	T	NA	*OreG0004099*
scaffold185	1880360	A	C	Och01,Och02,Och03,Och04,Och05,Och06,Och07,Och08,Och09,Och10,Och11,Och12,Och13,Och14,Ore01,Ore02,Ore03,Ore04,Ore05,Ore06,Ore07,Ore08,Ore09,Ore10,Ore11,Ore12,Ore13,Ore14	*OreG0004100*

scaffold185	1906131	A	C	Ore01,Ore02,Ore03,Ore04,Ore05,Ore06,Ore07,Ore08,Ore09,Ore10,Ore11,Ore12,Ore13,Ore14	*OreG0004101*
scaffold185	1911692	C	G	NA	*OreG0004101*
scaffold185	1912991	A	G	NA	*OreG0004101*
scaffold185	1913223	C	T	Ore01,Ore02,Ore03,Ore04,Ore05,Ore06,Ore07,Ore08,Ore09,Ore10,Ore11,Ore12,Ore13,Ore14	*OreG0004101*
scaffold185	1943107	A	T	NA	*OreG0004102*
scaffold185	1943959	G	T	NA	*OreG0004102*
scaffold185	1944100	C	G	NA	*OreG0004102*
scaffold185	1944114	T	C	NA	*OreG0004102*
scaffold185	1944129	G	A	NA	*OreG0004102*
scaffold185	1944134	G	A	NA	*OreG0004102*
scaffold185	1944424	C	A	Ore01,Ore02,Ore03,Ore04,Ore05,Ore06,Ore08,Ore09,Ore10,Ore11,Ore12,Ore13,Ore14	*OreG0004102*
scaffold185	1949007	G	A	Och01,Och02,Och03,Och04,Och05,Och06,Och07,Och08,Och09,Och10,Och11,Och12,Och13,Och14,Ore01,Ore02,Ore03,Ore04,Ore05,Ore06,Ore07,Ore08,Ore09,Ore10,Ore11,Ore12,Ore13,Ore14	*OreG0004103*
scaffold185	1949033	G	T	Ore01,Ore02,Ore03,Ore04,Ore05,Ore06,Ore07,Ore08,Ore09,Ore10,Ore11,Ore12,Ore13,Ore14	*OreG0004103*
scaffold185	1949274	C	T	Och01,Och02,Och03,Och04,Och05,Och06,Och07,Och08,Och09,Och10,Och11,Och12,Och13,Och14,Ore01,Ore02,Ore03,Ore04,Ore05,Ore06,Ore07,Ore08,Ore09,Ore10,Ore11,Ore12,Ore13,Ore14	*OreG0004103*
scaffold185	1949277	G	T	Ore01,Ore02,Ore03,Ore04,Ore05,Ore06,Ore07,Ore08,Ore09,Ore10,Ore11,Ore12,Ore13,Ore14	*OreG0004103*
scaffold185	1969030	C	T	NA	*OreG0004105*
scaffold185	1969131	C	A	NA	*OreG0004105*
scaffold185	1969151	C	G	NA	*OreG0004105*
scaffold185	1969166	C	T	NA	*OreG0004105*
scaffold185	1976000	G	C	Ore01,Ore02,Ore03,Ore04,Ore05,Ore06,Ore07,Ore08,Ore09,Ore10,Ore11,Ore12,Ore13,Ore14	*OreG0004105*
scaffold185	2079486	G	C	NA	*OreG0004108*
scaffold185	2079765	G	T	Ore01,Ore02,Ore03,Ore04,Ore05,Ore06,Ore07,Ore08,Ore09,Ore10,Ore11,Ore12,Ore13,Ore14	*OreG0004108*
scaffold185	2079950	A	T	NA	*OreG0004108*
scaffold185	2080025	G	T	NA	*OreG0004108*
scaffold185	2282786	C	T	Och01,Och03,Och07,Och08,Och09,Och10,Och11	*OreG0004114*
scaffold185	2283413	G	A	Och01,Och02,Och03,Och04,Och05,Och06,Och07,Och08,Och09,Och10,Och11,Och12,Och13,Och14	*OreG0004114*
scaffold185	2283621	G	A	Och12,Och13	*OreG0004114*
scaffold185	2283839	G	A	Och01,Och03,Och07,Och08,Och09,Och10,Och11	*OreG0004114*
scaffold185	2287065	G	A	Ore01,Ore02,Ore03,Ore04,Ore05,Ore06,Ore07,Ore08,Ore09,Ore10,Ore11,Ore12,Ore13,Ore14	*OreG0004114*
scaffold185	2303488	G	T	NA	*OreG0004114*
scaffold185	2445967	C	T	Och12,Och13	*OreG0004116*
scaffold185	2564083	A	T	NA	*OreG0004125*
scaffold185	2572321	C	G	NA	*OreG0004125*
scaffold185	2577707	C	A	NA	*OreG0004126*
scaffold185	2586437	C	A	Och04,Och12,Och13	*OreG0004127*
scaffold185	2587216	T	C	NA	*OreG0004127*
scaffold185	2587682	T	G	Och14	*OreG0004127*
scaffold185	2587909	G	T	Ore01,Ore02,Ore03,Ore04,Ore05,Ore06,Ore07,Ore08,Ore09,Ore10,Ore11,Ore12,Ore13,Ore14	*OreG0004127*
scaffold185	2632501	T	A	Och05,Och12,Och13,Ore01,Ore02,Ore03,Ore04,Ore05,Ore06,Ore07,Ore08,Ore09,Ore10,Ore11,Ore12,Ore13,Ore14	*OreG0004129*
scaffold185	2693054	C	T	NA	*OreG0004140*
scaffold185	2696713	A	G	NA	*OreG0004139*
scaffold185	2701995	C	T	NA	*OreG0004139*
scaffold185	2705116	C	G	NA	*OreG0004139*
scaffold185	2705572	G	A	NA	*OreG0004139*
scaffold185	2705938	T	C	NA	*OreG0004139*
scaffold185	2706444	G	C	NA	*OreG0004139*
scaffold185	2706617	G	T	NA	*OreG0004139*
scaffold185	2720866	C	T	NA	*OreG0004139*
scaffold185	2720918	A	T	NA	*OreG0004139*
scaffold185	2758528	G	C	Och12,Och13	*OreG0004142*
scaffold185	2804288	C	A	Och06	*OreG0004146*
scaffold185	2812763	C	G	Och02,Och03,Och04,Och05,Och06,Och07,Och08,Och09,Och10,Och11,Och12,Och13,Och14,Ore01,Ore02,Ore03,Ore04,Ore05,Ore06,Ore07,Ore08,Ore09,Ore10,Ore11,Ore12,Ore13,Ore14	*OreG0004147*
scaffold185	2812855	G	A	Ore01,Ore03,Ore09,Ore10,Ore11	*OreG0004147*
scaffold185	2812895	C	T	Och07	*OreG0004147*
scaffold185	2812906	C	G	NA	*OreG0004147*
scaffold185	2813191	A	G	NA	*OreG0004147*
scaffold185	2813494	C	T	Och06,Och09,Och10,Och11	*OreG0004147*
scaffold185	2815117	T	C	NA	*OreG0004148*

scaffold185	2815388	C	G	NA	*OreG0004148*
scaffold185	2815501	G	T	NA	*OreG0004148*
scaffold185	2815601	T	A	NA	*OreG0004148*
scaffold185	2815619	G	T	Och12,Och13,Ore01,Ore03,Ore09,Ore10,Ore11	*OreG0004148*
scaffold185	2815734	T	A	NA	*OreG0004148*
scaffold185	2820179	C	T	NA	*OreG0004149*
scaffold185	2820197	A	G	NA	*OreG0004149*
scaffold185	2820710	T	C	Och02,Och03,Och06,Och07,Och08,Och09,Och10,Och11,Och14,Ore01,Ore02,Ore03,Ore04,Ore05,Ore06,Ore07,Ore08,Ore09,Ore10,Ore11,Ore12,Ore13,Ore14	*OreG0004149*
scaffold185	2820785	C	T	Ore01,Ore03,Ore09,Ore10,Ore11	*OreG0004149*
scaffold185	2820800	C	T	NA	*OreG0004149*
scaffold185	2820823	C	T	Och02,Och03,Och04,Och05,Och06,Och07,Och08,Och09,Och10,Och11,Och12,Och13,Och14,Ore01,Ore02,Ore03,Ore04,Ore05,Ore06,Ore07,Ore08,Ore09,Ore10,Ore11,Ore12,Ore13,Ore14	*OreG0004149*
scaffold185	2820845	C	A	NA	*OreG0004149*
scaffold185	2820878	T	A	NA	*OreG0004149*
scaffold185	2823369	T	G	Och12,Och13	*OreG0004150*
scaffold185	2823408	A	G	Och08,Och12,Och13,Ore01,Ore02,Ore03,Ore06,Ore07,Ore08,Ore09,Ore10,Ore11,Ore12,Ore13,Ore14	*OreG0004150*
scaffold185	2823684	T	C	NA	*OreG0004150*
scaffold185	2823690	C	G	NA	*OreG0004150*
scaffold185	2823913	G	T	Och12,Och13	*OreG0004150*
scaffold185	2823915	C	T	NA	*OreG0004150*
scaffold185	2824086	G	A	NA	*OreG0004150*
scaffold185	2828635	C	A	NA	*OreG0004151*
scaffold185	2832747	C	T	NA	*OreG0004152*
scaffold185	2832904	C	T	NA	*OreG0004152*
scaffold185	2832973	C	T	NA	*OreG0004152*
scaffold185	2833051	C	T	NA	*OreG0004152*
scaffold185	2833207	T	G	NA	*OreG0004152*
scaffold185	2833219	G	C	NA	*OreG0004152*
scaffold185	2833234	C	A	NA	*OreG0004152*
scaffold185	2833248	T	C	NA	*OreG0004152*
scaffold185	2833793	G	T	NA	*OreG0004152*
scaffold185	2864537	T	A	Och02,Och14	*OreG0004158*
scaffold185	2866343	G	A	Och02	*OreG0004159*
scaffold185	2866547	T	C	NA	*OreG0004159*
scaffold185	2866556	C	A	NA	*OreG0004159*
scaffold185	2867043	T	C	Och02,Och07,Och14	*OreG0004159*
scaffold185	2868444	G	T	NA	*OreG0004160*
scaffold185	2868904	G	A	NA	*OreG0004160*
scaffold185	2876894	T	A	NA	*OreG0004161*
scaffold185	2877079	C	T	Och01,Och02,Och03,Och04,Och05,Och06,Och07,Och08,Och09,Och10,Och11,Och12,Och13,Och14,Ore01,Ore02,Ore03,Ore04,Ore05,Ore06,Ore07,Ore08,Ore09,Ore10,Ore11,Ore12,Ore13,Ore14	*OreG0004161*
scaffold185	2877296	G	A	NA	*OreG0004161*
scaffold185	2877466	G	A	Ore01,Ore02,Ore03,Ore06,Ore07,Ore08,Ore09,Ore10,Ore11,Ore12,Ore13,Ore14	*OreG0004161*
scaffold185	2897595	G	A	NA	*OreG0004164*
scaffold185	2903158	C	G	NA	*OreG0004165*
scaffold185	2903332	C	T	NA	*OreG0004165*
scaffold185	2914429	G	T	NA	*OreG0004168*
scaffold185	2915153	A	G	NA	*OreG0004168*
scaffold185	2915208	G	T	Och01,Och02,Och03,Och04,Och05,Och06,Och07,Och08,Och09,Och10,Och11,Och12,Och13,Och14,Ore01,Ore02,Ore03,Ore04,Ore05,Ore06,Ore07,Ore08,Ore09,Ore10,Ore11,Ore12,Ore13,Ore14	*OreG0004168*
scaffold185	2915228	T	C	NA	*OreG0004168*
scaffold185	2924687	G	A	NA	*OreG0004169*
scaffold185	2953768	T	C	NA	*OreG0004172*
scaffold185	2962975	T	G	Och03,Och05,Och07,Och09,Och10,Och11,Och12,Och13	*OreG0004175*
scaffold185	2963507	C	G	Ore01,Ore02,Ore03,Ore04,Ore05,Ore06,Ore07,Ore08,Ore09,Ore10,Ore11,Ore12,Ore13,Ore14	*OreG0004175*
scaffold185	2964107	C	A	NA	*OreG0004175*
scaffold185	3045136	C	A	Och01,Och02,Och03,Och04,Och05,Och06,Och07,Och08,Och09,Och10,Och11,Och12,Och13,Och14,Ore01,Ore02,Ore03,Ore04,Ore05,Ore06,Ore07,Ore08,Ore09,Ore10,Ore11,Ore12,Ore13,Ore14	*OreG0004183*
scaffold185	3045194	C	T	Ore01,Ore02,Ore03,Ore04,Ore05,Ore06,Ore07,Ore08,Ore09,Ore10,Ore11,Ore12,Ore13,Ore14	*OreG0004183*
scaffold185	3050604	G	T	NA	*OreG0004186*
scaffold185	3050675	G	T	NA	*OreG0004186*
scaffold185	3068884	A	T	NA	*OreG0004188*
scaffold185	3069001	G	A	Och06,Och14	*OreG0004188*
scaffold185	3069268	C	A	Och06,Och14	*OreG0004188*
scaffold185	3069768	C	A	NA	*OreG0004189*
scaffold185	3123902	A	G	NA	*OreG0004193*

scaffold185	3251222	C	T	NA	*OreG0004202*
scaffold185	3254827	C	T	Och01,Och02,Och03,Och04,Och05,Och06,Och07,Och08,Och09,Och10,Och11,Och12,Och13,Och14,Ore01,Ore02,Ore03,Ore04,Ore05,Ore06,Ore07,Ore08,Ore09,Ore10,Ore11,Ore12,Ore13,Ore14	*OreG0004203*
scaffold185	3257202	A	C	Och01,Och02,Och03,Och04,Och05,Och06,Och07,Och08,Och09,Och10,Och11,Och12,Och13,Och14,Ore01,Ore02,Ore03,Ore04,Ore05,Ore06,Ore07,Ore08,Ore09,Ore10,Ore11,Ore12,Ore13,Ore14	*OreG0004204*
scaffold185	3257229	T	C	Och01,Och02,Och03,Och04,Och05,Och06,Och07,Och08,Och09,Och10,Och11,Och12,Och13,Och14,Ore01,Ore02,Ore03,Ore04,Ore05,Ore06,Ore07,Ore08,Ore09,Ore10,Ore11,Ore12,Ore13,Ore14	*OreG0004204*
scaffold185	3299155	A	T	NA	*OreG0004209*
scaffold185	3299595	A	T	NA	*OreG0004209*
scaffold185	3299906	A	G	NA	*OreG0004209*
scaffold185	3311392	A	T	Och01,Och02,Och03,Och04,Och05,Och06,Och07,Och08,Och09,Och10,Och11,Och12,Och13,Och14	*OreG0004211*
scaffold185	3320664	T	C	Ore01,Ore02,Ore03,Ore04,Ore05,Ore06,Ore07,Ore08,Ore09,Ore10,Ore11,Ore12,Ore13,Ore14	*OreG0004213*
scaffold185	3322751	C	T	NA	*OreG0004214*
scaffold185	3322838	T	A	NA	*OreG0004214*
scaffold185	3322862	A	T	Och01,Och02,Och03,Och04,Och05,Och06,Och07,Och08,Och11,Och12,Och13,Och14	*OreG0004214*
scaffold185	3326039	G	A	Och03,Och04,Och05,Och12,Och13	*OreG0004214*
scaffold185	3329451	A	T	Och03,Och04,Och05,Och12,Och13	*OreG0004215*
scaffold185	3329519	T	C	NA	*OreG0004215*
scaffold185	3329523	C	A	NA	*OreG0004215*
scaffold185	3338537	T	C	NA	*OreG0004217*
scaffold185	3343779	A	G	Och01,Och02,Och03,Och04,Och05,Och06,Och07,Och08,Och09,Och10,Och11,Och12,Och13,Och14,Ore01,Ore02,Ore03,Ore04,Ore05,Ore06,Ore07,Ore08,Ore09,Ore10,Ore11,Ore12,Ore13,Ore14	*OreG0004218*
scaffold185	3344089	G	T	NA	*OreG0004218*
scaffold185	3356411	C	T	NA	*OreG0004219*
scaffold185	3356962	A	G	NA	*OreG0004219*
scaffold185	3383895	C	T	NA	*OreG0004222*
scaffold185	3394466	C	T	Och03,Och04,Och05,Och12,Och13	*OreG0004224*
scaffold185	3475869	T	C	NA	*OreG0004232*
scaffold185	3517635	C	G	NA	*OreG0004236*
scaffold185	3531643	A	T	Och01,Och02,Och03,Och04,Och05,Och06,Och07,Och08,Och09,Och10,Och11,Och12,Och13,Och14	*OreG0004237*
scaffold185	3588171	T	C	Och01,Och02,Och03,Och04,Och05,Och06,Och07,Och08,Och09,Och10,Och11,Och12,Och13,Och14	*OreG0004239*
scaffold185	3621484	T	A	NA	*OreG0004243*
scaffold185	3643546	G	C	Och11	*OreG0004247*
scaffold185	3643979	T	C	NA	*OreG0004247*
scaffold185	3644506	G	C	Och01,Och02,Och03,Och04,Och05,Och06,Och07,Och08,Och09,Och10,Och11,Och12,Och13,Och14,Ore01,Ore02,Ore03,Ore04,Ore05,Ore06,Ore07,Ore08,Ore09,Ore10,Ore11,Ore12,Ore13,Ore14	*OreG0004247*
scaffold185	3658833	C	T	Och06,Och08,Och09,Och10	*OreG0004248*
scaffold185	3659799	C	T	NA	*OreG0004248*
scaffold185	3812548	A	G	NA	*OreG0004259*
scaffold185	3824907	C	G	Och01,Och02,Och03,Och04,Och05,Och06,Och07,Och08,Och09,Och10,Och11,Och12,Och13,Och14	*OreG0004260*
scaffold185	3893534	G	C	Ore01,Ore02,Ore03,Ore04,Ore05,Ore06,Ore07,Ore08,Ore09,Ore10,Ore11,Ore12,Ore13,Ore14	*OreG0004265*
scaffold185	3894263	A	T	Ore01,Ore02,Ore03,Ore04,Ore05,Ore06,Ore07,Ore08,Ore09,Ore10,Ore11,Ore12,Ore13,Ore14	*OreG0004265*
scaffold185	3894452	G	C	NA	*OreG0004265*
scaffold185	3899841	T	A	Och06	*OreG0004266*
scaffold185	3900087	G	T	NA	*OreG0004266*
scaffold185	3900692	T	C	Ore02,Ore03,Ore04,Ore05,Ore06,Ore07,Ore08,Ore09,Ore10,Ore11,Ore12,Ore13,Ore14	*OreG0004266*
scaffold185	3906045	C	T	NA	*OreG0004267*
scaffold185	3906694	A	G	Och07,Och09,Och10	*OreG0004267*
scaffold185	3918698	C	G	Och01,Och06,Och14,Ore01,Ore02,Ore03,Ore04,Ore05,Ore06,Ore07,Ore08,Ore09,Ore10,Ore11,Ore12,Ore13,Ore14	*OreG0004268*
scaffold185	3919262	T	C	NA	*OreG0004268*
scaffold629	185403	G	T	NA	*OreG0024243*
scaffold629	186050	C	A	Och01,Och02,Och03,Och04,Och05,Och06,Och07,Och08,Och09,Och10,Och11,Och12,Och13,Och14	*OreG0024243*
scaffold629	217577	C	A	NA	*OreG0024246*
scaffold629	217880	A	G	Och01,Och03,Och07,Och08,Och09,Och10,Och11	*OreG0024246*
scaffold629	282175	A	C	Och12,Och13	*OreG0024249*
scaffold629	282385	A	T	Och01,Och02,Och03,Och04,Och05,Och06,Och07,Och08,Och09,Och10,Och11,Och12,Och13,Och14	*OreG0024249*
scaffold1218	2171	C	A	NA	*OreG0001355*
scaffold1218	2177	C	A	NA	*OreG0001355*

scaffold733	7544	C	A	Och02,Och06	*OreG0024821*
scaffold494	5939	A	T	NA	*OreG0023027*
scaffold494	5988	A	C	NA	*OreG0023027*
scaffold494	6092	G	A	NA	*OreG0023027*
scaffold494	6218	G	A	NA	*OreG0023027*
scaffold494	6561	G	A	Ore01,Ore02,Ore03,Ore04,Ore05,Ore06,Ore07,Ore08,Ore09,Ore10,Ore11,Ore12,Ore13,Ore14	*OreG0023027*
scaffold494	9321	C	A	NA	*OreG0023028*
scaffold494	14752	C	G	Och01,Och02,Och03,Och04,Och05,Och06,Och08,Och09,Och10,Och11,Och12,Och13	*OreG0023028*
scaffold494	14768	C	T	NA	*OreG0023028*
scaffold494	49051	A	G	NA	*OreG0023032*
scaffold494	59933	T	C	NA	*OreG0023034*
scaffold494	61426	C	G	NA	*OreG0023034*
scaffold494	68314	T	A	Ore01,Ore02,Ore03,Ore04,Ore05,Ore06,Ore07,Ore08,Ore09,Ore10,Ore11,Ore12,Ore13,Ore14	*OreG0023035*
scaffold494	68392	A	C	Och01,Och02,Och03,Och04,Och05,Och06,Och07,Och08,Och09,Och10,Och11,Och12,Och13,Och14,Ore01,Ore02,Ore03,Ore04,Ore05,Ore06,Ore07,Ore08,Ore09,Ore10,Ore11,Ore12,Ore13,Ore14	*OreG0023035*
scaffold494	82155	G	A	Ore01,Ore02,Ore03,Ore04,Ore05,Ore06,Ore07,Ore08,Ore09,Ore10,Ore11,Ore12,Ore13,Ore14	*OreG0023036*
scaffold494	88488	G	T	Ore04,Ore05,Ore10,Ore12,Ore13,Ore14	*OreG0023037*
scaffold494	96271	C	G	Ore01,Ore02,Ore03,Ore04,Ore05,Ore06,Ore07,Ore08,Ore09,Ore10,Ore11,Ore12,Ore13,Ore14	*OreG0023038*
scaffold494	103169	G	T	NA	*OreG0023039*
scaffold494	110941	A	G	Och01,Och07,Och08,Och09,Och10,Och11,Och14	*OreG0023040*
scaffold494	112107	C	A	NA	*OreG0023040*
scaffold494	161880	C	A	Och04,Och06,Och12	*OreG0023045*
scaffold494	182496	G	A	Och11	*OreG0023049*
scaffold494	182824	C	T	Och04,Och06,Och12	*OreG0023049*
scaffold494	183768	C	T	Och07,Och08,Och09,Och10,Och11	*OreG0023049*
scaffold494	183781	C	A	Och01,Och02,Och03,Och04,Och05,Och06,Och07,Och08,Och09,Och10,Och11,Och12,Och13,Och14,Ore01,Ore02,Ore03,Ore04,Ore05,Ore06,Ore07,Ore08,Ore09,Ore10,Ore11,Ore12,Ore13,Ore14	*OreG0023049*
scaffold494	200550	C	G	NA	*OreG0023050*
scaffold494	219470	A	G	NA	*OreG0023052*
scaffold494	219593	G	A	NA	*OreG0023052*
scaffold494	219597	C	T	NA	*OreG0023052*
scaffold494	244972	G	A	NA	*OreG0023056*
scaffold494	258697	A	T	Och01,Och02,Och03,Och04,Och05,Och06,Och07,Och08,Och09,Och10,Och11,Och12,Och13,Och14,Ore01,Ore02,Ore03,Ore04,Ore05,Ore06,Ore07,Ore08,Ore09,Ore10,Ore11,Ore12,Ore13,Ore14	*OreG0023057*
scaffold494	258892	A	G	Och08	*OreG0023057*
scaffold494	259973	C	G	NA	*OreG0023057*
scaffold494	260579	C	A	Och02,Och03,Och04,Och05,Och06,Och12,Och13	*OreG0023057*
scaffold494	263444	C	T	Och02,Och03,Och04,Och05,Och06,Och12,Och13	*OreG0023058*
scaffold494	373731	T	C	NA	*OreG0023068*
scaffold494	373800	C	T	NA	*OreG0023068*
scaffold494	374806	G	A	NA	*OreG0023068*
scaffold494	405523	C	T	NA	*OreG0023072*
scaffold494	405698	G	T	NA	*OreG0023072*
scaffold494	407669	T	C	Och06	*OreG0023072*
scaffold494	409138	A	G	Och06	*OreG0023073*
scaffold494	409151	A	C	Ore01,Ore02,Ore03,Ore04,Ore05,Ore06,Ore07,Ore08,Ore09,Ore10,Ore11,Ore12,Ore13,Ore14	*OreG0023073*
scaffold494	410704	T	A	Ore01,Ore02,Ore03,Ore04,Ore05,Ore06,Ore07,Ore08,Ore09,Ore10,Ore11,Ore12,Ore13,Ore14	*OreG0023073*
scaffold494	417109	T	C	NA	*OreG0023074*
scaffold494	444098	C	T	NA	*OreG0023077*
scaffold494	450395	T	C	Ore01,Ore02,Ore03,Ore04,Ore05,Ore06,Ore07,Ore08,Ore09,Ore10,Ore11,Ore12,Ore13,Ore14	*OreG0023078*
scaffold494	450462	G	A	Och01,Och02,Och03,Och04,Och05,Och06,Och07,Och08,Och09,Och10,Och11,Och12,Och13,Och14,Ore01,Ore02,Ore03,Ore04,Ore05,Ore06,Ore07,Ore08,Ore09,Ore10,Ore11,Ore12,Ore13,Ore14	*OreG0023078*
scaffold494	474177	C	T	Och01,Och02,Och03,Och04,Och05,Och06,Och07,Och08,Och09,Och10,Och11,Och12,Och13,Och14,Ore01,Ore02,Ore03,Ore04,Ore05,Ore06,Ore07,Ore08,Ore09,Ore10,Ore11,Ore12,Ore13,Ore14	*OreG0023080*
scaffold494	481376	A	G	Ore01,Ore02,Ore03,Ore04,Ore05,Ore06,Ore07,Ore08,Ore09,Ore10,Ore11,Ore12,Ore13,Ore14	*OreG0023081*
scaffold494	490785	G	T	NA	*OreG0023082*
scaffold494	490878	C	A	NA	*OreG0023082*
scaffold494	492083	G	T	NA	*OreG0023082*
scaffold494	492111	G	T	Och02,Och03,Och04,Och05,Och06,Och07,Och08,Och09,Och10,Och11,Och12,Och13,Och14	*OreG0023082*
scaffold494	492117	G	C	NA	*OreG0023082*

scaffold494	492531	T	A	NA	*OreG0023082*
scaffold494	494588	T	C	NA	*OreG0023082*
scaffold494	494938	G	C	NA	*OreG0023082*
scaffold494	494981	G	C	NA	*OreG0023082*
scaffold494	496569	A	T	NA	*OreG0023082*
scaffold494	496961	T	A	Ore01,Ore02,Ore03,Ore04,Ore05,Ore06,Ore07,Ore08,Ore09,Ore10,Ore11,Ore12,Ore13,Ore14	*OreG0023082*
scaffold494	496966	G	T	NA	*OreG0023082*
scaffold494	496990	G	A	Och11	*OreG0023082*
scaffold494	498655	G	T	NA	*OreG0023082*
scaffold494	536248	A	G	NA	*OreG0023087*
scaffold494	536308	T	G	NA	*OreG0023087*
scaffold494	569480	T	C	Och01,Och02,Och03,Och04,Och05,Och06,Och07,Och08,Och09,Och10,Och11,Och12,Och13,Och14	*OreG0023093*
scaffold494	571102	C	G	Ore01,Ore02,Ore03,Ore04,Ore05,Ore06,Ore07,Ore08,Ore09,Ore10,Ore11,Ore12,Ore13,Ore14	*OreG0023093*
scaffold494	571196	A	C	Och05,Och07,Och08,Och09,Och10,Och11,Och14	*OreG0023093*
scaffold494	571728	G	T	Och01,Och02,Och03,Och04,Och05,Och06,Och07,Och08,Och09,Och10,Och11,Och12,Och13,Och14,Ore01,Ore02,Ore03,Ore04,Ore05,Ore06,Ore07,Ore08,Ore09,Ore10,Ore11,Ore12,Ore13,Ore14	*OreG0023093*
scaffold494	575677	G	T	Ore01,Ore02,Ore03,Ore04,Ore05,Ore06,Ore07,Ore08,Ore09,Ore10,Ore11,Ore12,Ore13,Ore14	*OreG0023094*
scaffold494	580125	A	G	NA	*OreG0023095*
scaffold494	594317	C	A	NA	*OreG0023097*
scaffold494	594456	T	C	NA	*OreG0023097*
scaffold494	597500	C	A	NA	*OreG0023098*
scaffold494	597562	T	C	NA	*OreG0023098*
scaffold494	604656	C	G	NA	*OreG0023100*
scaffold494	675015	T	C	NA	*OreG0023111*
scaffold494	683141	A	G	Och06	*OreG0023112*
scaffold494	683510	A	T	Ore02,Ore04,Ore05,Ore09,Ore10,Ore12,Ore13,Ore14	*OreG0023112*
scaffold494	774861	G	C	NA	*OreG0023122*
scaffold494	888501	A	T	Och06	*OreG0023135*
scaffold494	896990	A	G	Och02,Och03,Och04,Och05,Och06,Och07,Och09,Och10,Och11,Och12,Och13,Ore01,Ore02,Ore03,Ore04,Ore05,Ore06,Ore07,Ore08,Ore09,Ore10,Ore11,Ore12,Ore13,Ore14	*OreG0023137*
scaffold494	901279	C	T	Och05	*OreG0023138*
scaffold494	907140	C	G	Ore01,Ore02,Ore03,Ore04,Ore05,Ore06,Ore07,Ore08,Ore09,Ore10,Ore11,Ore12,Ore13,Ore14	*OreG0023138*
scaffold494	907158	A	C	NA	*OreG0023138*
scaffold494	979541	C	A	Ore01,Ore03	*OreG0023145*
scaffold494	987646	C	A	Och01,Och02,Och03,Och04,Och05,Och06,Och07,Och08,Och09,Och10,Och11,Och12,Och13,Och14,Ore01,Ore02,Ore03,Ore04,Ore05,Ore06,Ore07,Ore08,Ore09,Ore10,Ore11,Ore12,Ore13,Ore14	*OreG0023146*
scaffold494	1011231	T	C	Ore01,Ore03	*OreG0023148*
scaffold494	1011312	G	A	Och02,Och04,Och05,Och06,Och07,Och08,Och09,Och10,Och11,Och12,Och13,Och14	*OreG0023148*
scaffold494	1011588	A	C	NA	*OreG0023148*
scaffold494	1011590	T	A	NA	*OreG0023148*
scaffold494	1011593	T	A	Och01,Och02,Och03,Och04,Och05,Och06,Och07,Och08,Och09,Och10,Och11,Och12,Och13,Och14,Ore01,Ore02,Ore03,Ore04,Ore05,Ore06,Ore07,Ore08,Ore09,Ore10,Ore11,Ore12,Ore13,Ore14	*OreG0023148*
scaffold494	1011607	T	C	Ore02	*OreG0023148*
scaffold494	1081826	C	A	NA	*OreG0023158*
scaffold494	1088678	G	T	NA	*OreG0023161*
scaffold494	1143925	T	C	Ore01,Ore02,Ore03,Ore04,Ore05,Ore06,Ore07,Ore08,Ore09,Ore10,Ore11,Ore12,Ore13,Ore14	*OreG0023171*
scaffold494	1144352	G	T	Och01,Och02,Och03,Och04,Och05,Och06,Och07,Och08,Och09,Och10,Och11,Och12,Och13,Och14	*OreG0023171*
scaffold494	1144359	G	C	NA	*OreG0023171*
scaffold494	1144466	C	T	Ore01,Ore02,Ore03,Ore04,Ore05,Ore06,Ore07,Ore08,Ore09,Ore10,Ore11,Ore12,Ore13,Ore14	*OreG0023171*
scaffold494	1163688	G	T	NA	*OreG0023173*
scaffold494	1173036	T	C	Och01,Och02,Och03,Och04,Och05,Och06,Och07,Och08,Och09,Och10,Och11,Och12,Och13,Och14,Ore01,Ore02,Ore03,Ore04,Ore05,Ore06,Ore07,Ore08,Ore09,Ore10,Ore11,Ore12,Ore13,Ore14	*OreG0023175*
scaffold494	1183559	G	A	NA	*OreG0023176*
scaffold494	1183567	A	T	Och01	*OreG0023176*
scaffold494	1219738	A	T	Och07	*OreG0023179*
scaffold494	1238946	A	G	Och01,Och02,Och03,Och04,Och05,Och06,Och07,Och08,Och09,Och10,Och11,Och12,Och13,Och14,Ore01,Ore02,Ore03,Ore04,Ore05,Ore06,Ore07,Ore08,Ore09,Ore10,Ore11,Ore12,Ore13,Ore14	*OreG0023182*
scaffold494	1239272	G	C	NA	*OreG0023182*
scaffold494	1240790	C	T	Ore02	*OreG0023183*
scaffold494	1240849	G	T	Och03,Och04,Och06,Och12	*OreG0023183*

scaffold494	1241035	A	G	NA	*OreG0023183*
scaffold494	1241627	G	A	NA	*OreG0023183*
scaffold494	1265611	G	A	NA	*OreG0023186*
scaffold494	1271320	G	A	NA	*OreG0023186*
scaffold494	1341304	G	A	NA	*OreG0023192*
scaffold494	1343251	A	T	Och02,Och03,Och04,Och05,Och06,Och07,Och08,Och09,Och10,Och11,Och12,Och13,Och14	*OreG0023192*
scaffold494	1363882	G	A	NA	*OreG0023195*
scaffold494	1363896	G	A	NA	*OreG0023195*
scaffold494	1365994	C	G	NA	*OreG0023196*
scaffold494	1390038	C	T	NA	*OreG0023199*
scaffold494	1391813	G	A	Och05	*OreG0023199*
scaffold494	1398699	C	T	Och07,Och08,Och11	*OreG0023200*
scaffold494	1398706	C	A	NA	*OreG0023200*
scaffold494	1411490	A	G	NA	*OreG0023201*
scaffold494	1411875	G	A	Och01,Och02,Och03,Och04,Och05,Och06,Och07,Och08,Och09,Och10,Och11,Och12,Och13,Och14,Ore01,Ore02,Ore03,Ore04,Ore05,Ore06,Ore07,Ore08,Ore09,Ore10,Ore11,Ore12,Ore13,Ore14	*OreG0023202*
scaffold494	1411884	G	T	Ore01,Ore03,Ore04,Ore05,Ore07,Ore10,Ore11,Ore12,Ore13,Ore14	*OreG0023202*
scaffold494	1412023	T	A	Och06,Och08,Och14	*OreG0023202*
scaffold494	1425053	A	T	Och02,Och03,Och04,Och05,Och06,Och07,Och08,Och09,Och10,Och11,Och12,Och13,Ore01,Ore02,Ore03,Ore04,Ore05,Ore06,Ore07,Ore08,Ore09,Ore10,Ore11,Ore12,Ore13,Ore14	*OreG0023204*
scaffold494	1440449	C	G	NA	*OreG0023206*
scaffold494	1441822	G	A	NA	*OreG0023206*
scaffold494	1441868	T	A	NA	*OreG0023206*
scaffold494	1446344	G	T	Ore01,Ore02,Ore03,Ore04,Ore05,Ore06,Ore07,Ore08,Ore09,Ore10,Ore11,Ore12,Ore13,Ore14	*OreG0023207*
scaffold494	1450990	C	A	Och01,Och02,Och03,Och04,Och05,Och06,Och07,Och08,Och09,Och10,Och11,Och12,Och13,Och14,Ore01,Ore02,Ore03,Ore04,Ore05,Ore06,Ore07,Ore08,Ore09,Ore10,Ore11,Ore12,Ore13,Ore14	*OreG0023208*
scaffold494	1451237	C	T	Och01,Och02,Och03,Och04,Och05,Och06,Och07,Och08,Och09,Och10,Och11,Och12,Och13,Och14,Ore01,Ore02,Ore03,Ore04,Ore05,Ore06,Ore07,Ore08,Ore09,Ore10,Ore11,Ore12,Ore13,Ore14	*OreG0023208*
scaffold494	1451641	T	A	Och01,Och02,Och03,Och04,Och05,Och06,Och07,Och08,Och09,Och10,Och11,Och12,Och13,Och14,Ore01,Ore02,Ore03,Ore04,Ore05,Ore06,Ore07,Ore08,Ore09,Ore10,Ore11,Ore12,Ore13,Ore14	*OreG0023208*
scaffold494	1454651	G	C	Och01,Och02,Och03,Och04,Och05,Och06,Och07,Och08,Och09,Och10,Och11,Och12,Och13,Och14,Ore01,Ore02,Ore03,Ore04,Ore05,Ore06,Ore07,Ore08,Ore09,Ore10,Ore11,Ore12,Ore13,Ore14	*OreG0023209*
scaffold494	1527461	G	C	NA	*OreG0023218*
scaffold494	1545293	G	T	Och01,Och02,Och05,Och06,Och07,Och08,Och13,Och14	*OreG0023221*
scaffold494	1545406	T	G	NA	*OreG0023221*
scaffold494	1597233	C	T	NA	*OreG0023229*
scaffold494	1605673	A	G	Och01,Och02,Och03,Och04,Och05,Och06,Och07,Och08,Och09,Och10,Och11,Och12,Och13,Och14	*OreG0023230*
scaffold494	1634394	G	T	Och01,Och02,Och03,Och04,Och05,Och06,Och07,Och08,Och09,Och10,Och11,Och12,Och13	*OreG0023234*
scaffold494	1639788	T	C	NA	*OreG0023236*
scaffold494	1667004	A	C	Och01	*OreG0023242*
scaffold494	1688541	C	T	NA	*OreG0023244*
scaffold494	1693026	G	C	Och02,Och05,Och06,Och13	*OreG0023245*
scaffold494	1699874	A	T	NA	*OreG0023247*
scaffold494	1718312	T	C	NA	*OreG0023250*
scaffold494	1719790	A	G	Ore01,Ore02,Ore03,Ore04,Ore05,Ore06,Ore07,Ore08,Ore09,Ore10,Ore11,Ore12,Ore13,Ore14	*OreG0023251*
scaffold494	1719889	T	A	Och01,Och02,Och03,Och04,Och05,Och06,Och07,Och08,Och09,Och10,Och11,Och12,Och13,Och14	*OreG0023251*
scaffold494	1720208	C	T	Och01,Och02,Och03,Och04,Och05,Och06,Och07,Och08,Och09,Och10,Och11,Och13,Och14	*OreG0023251*
scaffold494	1721023	T	C	Ore01,Ore02,Ore03,Ore04,Ore05,Ore06,Ore07,Ore08,Ore09,Ore10,Ore11,Ore12,Ore13,Ore14	*OreG0023251*
scaffold494	1721396	G	A	NA	*OreG0023251*
scaffold494	1744586	G	T	Och01,Och02,Och03,Och04,Och05,Och06,Och07,Och08,Och09,Och10,Och11,Och12,Och13,Och14,Ore01,Ore02,Ore03,Ore04,Ore05,Ore06,Ore07,Ore08,Ore09,Ore10,Ore11,Ore12,Ore13,Ore14	*OreG0023254*
scaffold494	1746541	C	T	Och01,Och02,Och03,Och04,Och05,Och06,Och07,Och08,Och09,Och10,Och11,Och12,Och13,Och14,Ore01,Ore02,Ore03,Ore04,Ore05,Ore06,Ore07,Ore08,Ore09,Ore10,Ore11,Ore12,Ore13,Ore14	*OreG0023255*
scaffold494	1747510	T	C	Ore01,Ore02,Ore03,Ore04,Ore05,Ore06,Ore07,Ore08,Ore09,Ore10,Ore11,Ore12,Ore13,Ore14	*OreG0023255*
scaffold494	1756609	C	A	NA	*OreG0023257*
scaffold494	1759079	G	A	NA	*OreG0023258*
scaffold494	1759205	C	T	Och02,Och05,Och06,Och07,Och08,Och09,Och10,Och11,Och13	*OreG0023258*
scaffold494	1785228	C	T	Och01	*OreG0023262*

scaffold494	1822247	T	A	Och01,Och02,Och03,Och04,Och05,Och06,Och07,Och08,Och09,Och10,Och11,Och12,Och13,Och14	*OreG0023266*
scaffold494	1852663	G	T	NA	*OreG0023268*
scaffold494	1903878	G	A	Och04,Och05,Och12,Och13	*OreG0023273*
scaffold494	1903927	C	T	Ore01,Ore02,Ore03,Ore04,Ore05,Ore06,Ore07,Ore08,Ore09,Ore10,Ore11,Ore12,Ore13,Ore14	*OreG0023273*
scaffold494	1903933	C	T	Och06,Och08,Och14	*OreG0023273*
scaffold494	1903948	A	G	Och06,Och08	*OreG0023273*
scaffold494	1904073	T	C	Och06,Och08,Och14	*OreG0023273*
scaffold494	2056595	G	A	NA	*OreG0023286*
scaffold494	2064128	C	T	NA	*OreG0023287*
scaffold494	2098325	A	T	NA	*OreG0023288*
scaffold494	2098372	A	T	NA	*OreG0023288*
scaffold494	2099892	T	A	Och13	*OreG0023288*
scaffold494	2111621	C	T	NA	*OreG0023290*
scaffold494	2111877	G	A	NA	*OreG0023290*
scaffold494	2113532	C	A	NA	*OreG0023290*
scaffold494	2153955	C	A	Och01	*OreG0023294*
scaffold494	2153964	G	A	NA	*OreG0023294*
scaffold494	2153984	C	A	NA	*OreG0023294*
scaffold494	2158745	G	T	Ore09,Ore13,Ore14	*OreG0023294*
scaffold465	85889	G	A	Och09,Och10	*OreG0021898*
scaffold465	89813	A	G	Och09,Och10	*OreG0021899*
scaffold465	90027	G	A	NA	*OreG0021899*
scaffold465	92802	G	A	Ore02	*OreG0021899*
scaffold465	93251	C	A	NA	*OreG0021899*
scaffold465	135488	T	C	Och01,Och02,Och03,Och04,Och05,Och06,Och07,Och08,Och09,Och10,Och11,Och12,Och13,Och14	*OreG0021904*
scaffold465	149576	G	A	Ore03,Ore04,Ore05	*OreG0021905*
scaffold465	150595	G	A	Och01,Och09,Och10	*OreG0021905*
scaffold465	164869	C	T	Ore01,Ore02,Ore03,Ore04,Ore05,Ore06,Ore07,Ore08,Ore09,Ore10,Ore11,Ore12,Ore13,Ore14	*OreG0021907*
scaffold465	166401	G	A	NA	*OreG0021907*
scaffold465	166848	A	C	NA	*OreG0021907*
scaffold465	199770	C	T	Och02,Och03,Och04,Och05,Och06,Och07,Och08,Och09,Och10,Och11,Och12,Och13,Och14	*OreG0021912*
scaffold465	202040	A	C	NA	*OreG0021912*
scaffold465	202369	G	A	Och03	*OreG0021912*
scaffold465	232642	C	T	NA	*OreG0021916*
scaffold465	267561	G	T	NA	*OreG0021919*
scaffold465	306943	A	G	Och01,Och02,Och03,Och04,Och05,Och06,Och07,Och08,Och09,Och10,Och11,Och12,Och13,Och14,Ore01,Ore02,Ore03,Ore04,Ore05,Ore06,Ore07,Ore08,Ore09,Ore10,Ore11,Ore12,Ore13,Ore14	*OreG0021924*
scaffold465	308111	G	T	Och01,Och02,Och03,Och04,Och05,Och06,Och07,Och08,Och09,Och10,Och11,Och12,Och13,Och14,Ore01,Ore02,Ore03,Ore04,Ore05,Ore06,Ore07,Ore08,Ore09,Ore10,Ore11,Ore12,Ore13,Ore14	*OreG0021924*
scaffold465	308944	G	A	Och01,Och02,Och03,Och04,Och05,Och06,Och08,Och09,Och10,Och11,Och12,Och13,Ore01,Ore02,Ore03,Ore04,Ore05,Ore06,Ore07,Ore08,Ore09,Ore10,Ore11,Ore12,Ore13,Ore14	*OreG0021924*
scaffold465	368757	T	A	Ore01,Ore03,Ore04,Ore05,Ore08,Ore09,Ore12,Ore13,Ore14	*OreG0021927*
scaffold465	368910	C	T	NA	*OreG0021927*
scaffold465	373474	A	G	NA	*OreG0021928*
scaffold465	373502	C	T	NA	*OreG0021928*
scaffold465	373514	C	T	NA	*OreG0021928*
scaffold465	373667	G	A	NA	*OreG0021928*
scaffold465	373674	A	C	NA	*OreG0021928*
scaffold465	373798	A	T	NA	*OreG0021928*
scaffold465	398333	C	T	NA	*OreG0021929*
scaffold465	445271	T	A	Och01,Och02,Och03,Och04,Och05,Och06,Och07,Och08,Och09,Och10,Och11,Och12,Och13,Och14	*OreG0021929*
scaffold465	445541	C	G	Ore01,Ore02,Ore03,Ore04,Ore05,Ore06,Ore07,Ore08,Ore09,Ore10,Ore11,Ore12,Ore13,Ore14	*OreG0021929*
scaffold465	445547	G	A	Ore01,Ore02,Ore03,Ore04,Ore05,Ore06,Ore07,Ore08,Ore09,Ore10,Ore11,Ore12,Ore13,Ore14	*OreG0021929*
scaffold465	586235	G	C	NA	*OreG0021937*
scaffold465	586269	T	A	NA	*OreG0021937*
scaffold465	629361	G	T	Och01,Och02,Och03,Och04,Och05,Och06,Och07,Och08,Och09,Och10,Och11,Och12,Och13,Och14,Ore01,Ore02,Ore03,Ore04,Ore05,Ore06,Ore07,Ore08,Ore09,Ore10,Ore11,Ore12,Ore13,Ore14	*OreG0021940*
scaffold465	629364	G	A	Och01,Och02,Och03,Och04,Och05,Och06,Och07,Och08,Och09,Och10,Och11,Och12,Och13,Och14	*OreG0021940*
scaffold465	629716	G	T	Och01,Och02,Och03,Och04,Och05,Och06,Och07,Och08,Och09,Och10,Och11,Och12,Och13,Och14	*OreG0021940*
scaffold465	727226	C	T	Och02,Och03,Och04,Och05,Och06,Och07,Och08,Och09,Och10,Och11,Och12,Och14	*OreG0021946*

scaffold465	727869	G	A	NA	*OreG0021946*
scaffold465	728061	A	G	NA	*OreG0021946*
scaffold465	728180	G	A	Ore02	*OreG0021946*
scaffold465	728214	G	A	Ore01,Ore02,Ore03,Ore04,Ore05,Ore06,Ore07,Ore08,Ore09,Ore10,Ore11,Ore12,Ore13,Ore14	*OreG0021946*
scaffold465	728238	C	T	Ore03,Ore04,Ore05	*OreG0021946*
scaffold465	728262	G	T	Och01,Och02,Och03,Och04,Och05,Och06,Och07,Och08,Och09,Och10,Och11,Och12,Och13,Och14,Ore01,Ore02,Ore03,Ore04,Ore05,Ore06,Ore07,Ore08,Ore09,Ore10,Ore11,Ore12,Ore13,Ore14	*OreG0021946*
scaffold465	728280	G	A	Ore01,Ore02,Ore03,Ore04,Ore05,Ore06,Ore07,Ore08,Ore09,Ore10,Ore11,Ore12,Ore13,Ore14	*OreG0021946*
scaffold465	749126	G	A	Och01,Och02,Och03,Och04,Och05,Och06,Och07,Och08,Och09,Och10,Och11,Och12,Och13,Och14	*OreG0021950*
scaffold465	749153	G	A	Och01,Och02,Och03,Och04,Och05,Och06,Och07,Och08,Och09,Och10,Och11,Och12,Och13,Och14,Ore01,Ore02,Ore03,Ore04,Ore05,Ore06,Ore07,Ore08,Ore09,Ore10,Ore11,Ore12,Ore13,Ore14	*OreG0021950*
scaffold465	790904	A	G	NA	*OreG0021954*
scaffold465	790967	C	T	Och02,Och06,Och08,Och09,Och10,Och11,Och12	*OreG0021954*
scaffold465	791052	A	C	Och02,Och03,Och04,Och06,Och08,Och09,Och10,Och11,Och12,Och13	*OreG0021954*
scaffold465	795179	G	A	Och01,Och02,Och03,Och04,Och05,Och06,Och07,Och08,Och09,Och10,Och11,Och12,Och13,Och14,Ore01,Ore02,Ore03,Ore04,Ore05,Ore06,Ore07,Ore08,Ore09,Ore10,Ore11,Ore12,Ore13,Ore14	*OreG0021956*
scaffold465	826693	G	A	Och01,Och02,Och03,Och04,Och05,Och06,Och07,Och08,Och09,Och10,Och11,Och12,Och13,Och14	*OreG0021959*
scaffold465	828832	C	T	NA	*OreG0021959*
scaffold465	878003	G	C	Och01,Och02,Och03,Och04,Och05,Och06,Och07,Och08,Och09,Och10,Och11,Och12,Och13,Och14,Ore01,Ore02,Ore03,Ore04,Ore05,Ore06,Ore07,Ore08,Ore09,Ore10,Ore11,Ore12,Ore13,Ore14	*OreG0021963*
scaffold465	891737	G	A	NA	*OreG0021965*
scaffold465	900684	G	A	Ore03,Ore04,Ore05,Ore06,Ore07,Ore10,Ore11	*OreG0021967*
scaffold465	918095	T	A	NA	*OreG0021969*
scaffold465	940926	G	T	Och01,Och02,Och03,Och04,Och05,Och06,Och07,Och08,Och09,Och10,Och11,Och12,Och13,Och14,Ore01,Ore02,Ore03,Ore04,Ore05,Ore06,Ore07,Ore08,Ore09,Ore10,Ore11,Ore12,Ore13,Ore14	*OreG0021971*
scaffold465	941123	G	A	Och01,Och02,Och03,Och04,Och05,Och06,Och07,Och08,Och09,Och10,Och11,Och12,Och13,Och14,Ore01,Ore02,Ore03,Ore04,Ore05,Ore06,Ore07,Ore08,Ore09,Ore10,Ore11,Ore12,Ore13,Ore14	*OreG0021971*
scaffold465	957626	T	A	NA	*OreG0021974*
scaffold465	957656	C	A	Ore01,Ore02,Ore03,Ore04,Ore05,Ore06,Ore07,Ore08,Ore09,Ore10,Ore11,Ore12,Ore13,Ore14	*OreG0021974*
scaffold465	981917	T	C	Och03,Och05,Och14	*OreG0021975*
scaffold465	982004	C	T	Och01,Och02,Och03,Och04,Och05,Och06,Och07,Och08,Och09,Och10,Och11,Och12,Och13,Och14,Ore01,Ore02,Ore03,Ore04,Ore05,Ore06,Ore07,Ore08,Ore09,Ore10,Ore11,Ore12,Ore13,Ore14	*OreG0021975*
scaffold465	982085	G	A	NA	*OreG0021975*
scaffold465	982182	G	A	Och01,Och09,Och10,Och11	*OreG0021975*
scaffold465	982293	C	T	Och01,Och09,Och10,Och11	*OreG0021975*
scaffold465	982427	G	T	NA	*OreG0021975*
scaffold465	982473	A	T	NA	*OreG0021975*
scaffold465	982490	C	A	Ore03,Ore04,Ore05	*OreG0021975*
scaffold465	1020355	G	A	Och01,Och02,Och03,Och04,Och05,Och06,Och07,Och08,Och09,Och10,Och11,Och12,Och13,Och14,Ore01,Ore02,Ore03,Ore04,Ore05,Ore06,Ore07,Ore08,Ore09,Ore10,Ore11,Ore12,Ore13,Ore14	*OreG0021982*
scaffold465	1020431	C	G	Och01,Och02,Och03,Och04,Och05,Och06,Och07,Och08,Och09,Och10,Och11,Och12,Och13,Och14,Ore01,Ore02,Ore03,Ore04,Ore05,Ore06,Ore07,Ore08,Ore09,Ore10,Ore11,Ore12,Ore13,Ore14	*OreG0021982*
scaffold465	1020456	C	T	Och01,Och02,Och03,Och04,Och05,Och06,Och07,Och08,Och09,Och10,Och11,Och12,Och13,Och14,Ore01,Ore02,Ore03,Ore04,Ore05,Ore06,Ore07,Ore08,Ore09,Ore10,Ore11,Ore12,Ore13,Ore14	*OreG0021982*
scaffold465	1020484	C	T	Och01,Och07,Och09,Och10	*OreG0021982*
scaffold465	1057453	G	C	NA	*OreG0021988*
scaffold465	1057713	A	G	NA	*OreG0021988*
scaffold465	1082915	C	T	Och02,Och03,Och04,Och05,Och06,Och09,Och10,Och12,Och13	*OreG0021990*
scaffold465	1148949	G	A	Ore01,Ore02,Ore03,Ore04,Ore05,Ore06,Ore07,Ore08,Ore09,Ore10,Ore11,Ore12,Ore13,Ore14	*OreG0021997*
scaffold465	1172616	C	T	Och01,Och02,Och03,Och04,Och05,Och06,Och07,Och08,Och09,Och10,Och11,Och12,Och13,Och14,Ore01,Ore02,Ore03,Ore04,Ore05,Ore06,Ore07,Ore08,Ore09,Ore10,Ore11,Ore12,Ore13,Ore14	*OreG0022000*
scaffold465	1177477	C	A	Ore01,Ore02,Ore03,Ore04,Ore05,Ore06,Ore07,Ore08,Ore09,Ore10,Ore11,Ore12,Ore13,Ore14	*OreG0022002*
scaffold465	1177515	C	A	Ore01,Ore02,Ore03,Ore04,Ore05,Ore06,Ore07,Ore08,Ore09,Ore10,Ore11,Ore12,Ore13,Ore14	*OreG0022002*
scaffold465	1189892	C	T	Och06,Och07,Och08,Och09,Och10,Och11,Och14	*OreG0022003*
scaffold465	1190081	G	T	Och08	*OreG0022003*
scaffold465	1195550	G	A	NA	*OreG0022004*
scaffold465	1224091	C	T	Och09,Och10,Och14	*OreG0022008*

scaffold465	1233399	G	A	NA	*OreG0022010*
scaffold465	1271430	T	A	Ore01,Ore02,Ore03,Ore04,Ore05,Ore06,Ore07,Ore08,Ore09,Ore10,Ore11,Ore12,Ore13,Ore14	*OreG0022014*
scaffold465	1271462	G	T	NA	*OreG0022014*
scaffold465	1271531	T	A	NA	*OreG0022014*
scaffold465	1271534	G	A	NA	*OreG0022014*
scaffold465	1271582	A	G	Ore03,Ore04,Ore05	*OreG0022014*
scaffold465	1271585	A	T	Ore01,Ore02,Ore03,Ore04,Ore05,Ore06,Ore07,Ore08,Ore09,Ore10,Ore11,Ore12,Ore13,Ore14	*OreG0022014*
scaffold465	1316536	C	G	NA	*OreG0022020*
scaffold465	1448751	A	C	Och01,Och02,Och03,Och04,Och05,Och06,Och07,Och08,Och09,Och10,Och11,Och12,Och13,Och14,Ore01,Ore02,Ore03,Ore04,Ore05,Ore06,Ore07,Ore08,Ore09,Ore10,Ore11,Ore12,Ore13,Ore14	*OreG0022026*
scaffold465	1448812	A	C	NA	*OreG0022026*
scaffold465	1448832	A	T	Och01,Och02,Och03,Och04,Och05,Och06,Och07,Och08,Och09,Och10,Och11,Och12,Och13,Och14,Ore01,Ore02,Ore03,Ore04,Ore05,Ore06,Ore07,Ore08,Ore09,Ore10,Ore11,Ore12,Ore13,Ore14	*OreG0022026*
scaffold465	1467571	A	G	Och04,Och05,Och07,Och09,Och10,Och12,Och13,Och14	*OreG0022028*
scaffold465	1467800	C	T	Och04,Och05,Och07,Och09,Och10,Och12,Och13	*OreG0022028*
scaffold465	1475147	A	G	Och02,Och03,Och04,Och05,Och06,Och07,Och08,Och09,Och10,Och11,Och12,Och13,Och14	*OreG0022029*
scaffold465	1475222	G	A	Och01,Och02,Och03,Och04,Och05,Och06,Och07,Och08,Och09,Och10,Och11,Och12,Och13,Och14,Ore01,Ore02,Ore03,Ore04,Ore05,Ore06,Ore07,Ore08,Ore09,Ore10,Ore11,Ore12,Ore13,Ore14	*OreG0022029*
scaffold465	1475700	T	C	Och01,Och14,Ore01,Ore02,Ore03,Ore04,Ore05,Ore06,Ore07,Ore08,Ore09,Ore10,Ore11,Ore12,Ore13,Ore14	*OreG0022030*
scaffold465	1476078	G	A	NA	*OreG0022030*
scaffold465	1476183	A	C	NA	*OreG0022031*
scaffold465	1476500	C	T	Och11	*OreG0022031*
scaffold465	1476514	G	T	Och01,Och02,Och03,Och04,Och05,Och06,Och07,Och08,Och09,Och10,Och11,Och12,Och13,Ore01,Ore02,Ore03,Ore04,Ore05,Ore06,Ore07,Ore08,Ore09,Ore10,Ore11,Ore12,Ore13,Ore14	*OreG0022031*
scaffold465	1480474	A	G	Och01,Och14	*OreG0022033*
scaffold465	1481012	C	T	Och01,Och06,Och14	*OreG0022033*
scaffold465	1481048	T	C	Och01,Och06,Och14	*OreG0022033*
scaffold465	1481073	G	A	Och01,Och06,Och14	*OreG0022033*
scaffold465	1482983	A	G	Och06	*OreG0022034*
scaffold465	1505355	T	A	Och01,Och14,Ore01,Ore02,Ore03,Ore04,Ore05,Ore06,Ore07,Ore08,Ore09,Ore10,Ore11,Ore12,Ore13,Ore14	*OreG0022036*
scaffold465	1583125	C	A	Och11	*OreG0022040*
scaffold465	1583848	A	T	NA	*OreG0022040*
scaffold465	1584343	G	T	Och09,Och10,Och14	*OreG0022040*
scaffold465	1594229	G	A	Och01,Och02,Och03,Och04,Och05,Och06,Och07,Och08,Och09,Och10,Och11,Och12,Och13,Och14,Ore01,Ore02,Ore03,Ore04,Ore05,Ore06,Ore07,Ore08,Ore09,Ore10,Ore11,Ore12,Ore13,Ore14	*OreG0022041*
scaffold465	1594292	C	T	Och14	*OreG0022041*
scaffold465	1612616	C	T	NA	*OreG0022044*
scaffold465	1766732	G	A	NA	*OreG0022057*
scaffold465	1767741	C	T	NA	*OreG0022057*
scaffold465	1768044	A	C	NA	*OreG0022057*
scaffold465	1768142	C	T	NA	*OreG0022057*
scaffold465	1768184	C	G	NA	*OreG0022057*
scaffold465	1768410	T	C	NA	*OreG0022057*
scaffold465	1795273	A	T	NA	*OreG0022059*
scaffold465	1814989	G	T	Och01,Och02,Och03,Och04,Och05,Och06,Och07,Och08,Och09,Och10,Och11,Och12,Och13,Och14,Ore01,Ore02,Ore03,Ore04,Ore05,Ore06,Ore07,Ore08,Ore09,Ore10,Ore11,Ore12,Ore13,Ore14	*OreG0022061*
scaffold465	1815498	A	G	Och01,Och02,Och03,Och04,Och05,Och06,Och07,Och08,Och09,Och10,Och11,Och12,Och13,Och14,Ore01,Ore02,Ore03,Ore04,Ore05,Ore06,Ore07,Ore08,Ore09,Ore10,Ore11,Ore12,Ore13,Ore14	*OreG0022061*
scaffold465	1821006	T	A	Och01,Och02,Och03,Och04,Och05,Och06,Och07,Och08,Och09,Och10,Och11,Och12,Och13,Och14,Ore01,Ore02,Ore03,Ore04,Ore05,Ore06,Ore07,Ore08,Ore09,Ore10,Ore11,Ore12,Ore13,Ore14	*OreG0022062*
scaffold465	1853543	C	G	NA	*OreG0022064*
scaffold465	1853785	A	T	Ore02,Ore03,Ore06,Ore08,Ore09,Ore10,Ore11,Ore12,Ore14	*OreG0022064*
scaffold465	1853855	T	G	Ore02,Ore03,Ore06,Ore08,Ore09,Ore10,Ore11,Ore12,Ore14	*OreG0022064*
scaffold465	1853868	A	C	NA	*OreG0022064*
scaffold465	1863469	A	G	NA	*OreG0022065*
scaffold465	1863716	T	A	Och01,Och02,Och03,Och04,Och05,Och06,Och07,Och08,Och09,Och10,Och11,Och12,Och13,Och14,Ore01,Ore02,Ore03,Ore04,Ore05,Ore06,Ore07,Ore08,Ore09,Ore10,Ore11,Ore12,Ore13,Ore14	*OreG0022065*
scaffold465	1932723	G	T	Och02,Och03,Och04,Och05,Och06,Och07,Och08,Och09,Och10,Och11,Och12,Och13,Och14,Ore01,Ore02,Ore03,Ore04,Ore05,Ore06,Ore07,Ore08,Ore09,Ore10,Ore11,Ore12,Ore13,Ore14	*OreG0022071*

scaffold465	1933608	G	C	Ore01,Ore02,Ore03,Ore04,Ore05,Ore06,Ore07,Ore08,Ore09,Ore10,Ore11,Ore12,Ore13,Ore14	*OreG0022071*
scaffold465	1945967	C	T	NA	*OreG0022072*
scaffold465	2004169	T	C	Ore01,Ore02,Ore03,Ore04,Ore05,Ore06,Ore07,Ore08,Ore09,Ore10,Ore11,Ore12,Ore13,Ore14	*OreG0022075*
scaffold465	2016358	C	T	Och06,Och09,Och10,Och11,Och13	*OreG0022076*
scaffold465	2113002	C	T	Och13	*OreG0022081*
scaffold465	2158141	G	T	NA	*OreG0022084*
scaffold465	2159120	T	A	NA	*OreG0022084*
scaffold465	2170158	C	T	Ore01,Ore02,Ore03,Ore06,Ore08,Ore09,Ore10,Ore11,Ore12,Ore14	*OreG0022086*
scaffold465	2172675	T	A	NA	*OreG0022087*
scaffold465	2172699	T	C	NA	*OreG0022087*
scaffold465	2172869	G	A	NA	*OreG0022087*
scaffold465	2198935	A	T	NA	*OreG0022088*
scaffold465	2203321	C	A	NA	*OreG0022089*
scaffold465	2203591	G	A	Och01,Och04,Och05,Och09,Och10,Och12,Ore01,Ore02,Ore03,Ore04,Ore05,Ore06,Ore07,Ore08,Ore09,Ore10,Ore11,Ore12,Ore13,Ore14	*OreG0022089*
scaffold465	2213333	C	T	Och11	*OreG0022090*
scaffold465	2214893	G	A	Ore01,Ore02,Ore03,Ore04,Ore05,Ore06,Ore07,Ore08,Ore09,Ore10,Ore11,Ore12,Ore13,Ore14	*OreG0022090*
scaffold465	2215258	C	A	NA	*OreG0022090*
scaffold465	2215652	G	A	Och04,Och05,Och12	*OreG0022090*
scaffold465	2297449	G	T	Ore02,Ore03,Ore06,Ore08,Ore09,Ore10,Ore11,Ore12,Ore14	*OreG0022096*
scaffold465	2299942	C	T	Ore02,Ore03,Ore06,Ore08,Ore09,Ore10,Ore11,Ore12,Ore14	*OreG0022096*
scaffold465	2325964	G	T	Och07	*OreG0022098*
scaffold465	2326072	A	G	Och11	*OreG0022098*
scaffold465	2330181	G	A	Och11	*OreG0022098*
scaffold465	2330227	T	C	Och01,Och02,Och03,Och04,Och05,Och06,Och11,Och12,Och13	*OreG0022098*
scaffold465	2333952	G	A	Och01,Och02,Och03,Och04,Och05,Och06,Och11,Och12,Och13	*OreG0022098*
scaffold466	95700	A	T	NA	*OreG0022103*
scaffold466	96816	A	G	NA	*OreG0022103*
scaffold466	114060	A	T	Och09,Och10,Ore02,Ore04,Ore05,Ore06,Ore07,Ore08,Ore11,Ore12,Ore13	*OreG0022105*
scaffold466	117090	C	T	Och01,Och14	*OreG0022106*
scaffold466	126388	T	A	NA	*OreG0022108*
scaffold466	126626	A	G	Och09,Och10,Ore02,Ore04,Ore05,Ore06,Ore07,Ore08,Ore11,Ore12,Ore13	*OreG0022108*
scaffold466	127026	T	C	Och06	*OreG0022108*
scaffold466	135039	G	A	NA	*OreG0022109*
scaffold466	135250	C	T	NA	*OreG0022109*
scaffold466	135694	G	A	Ore02,Ore04,Ore05,Ore06,Ore07,Ore08,Ore11,Ore12,Ore13	*OreG0022109*
scaffold466	136415	C	A	Och06	*OreG0022110*
scaffold466	136468	A	C	NA	*OreG0022110*
scaffold466	139093	T	C	NA	*OreG0022111*
scaffold466	139233	A	G	Ore02,Ore04,Ore05,Ore06,Ore07,Ore08,Ore11,Ore12,Ore13	*OreG0022111*
scaffold466	139684	A	C	Och01,Och02,Och03,Och04,Och05,Och06,Och07,Och08,Och09,Och10,Och11,Och12,Och13,Och14,Ore01,Ore02,Ore03,Ore04,Ore05,Ore06,Ore07,Ore08,Ore09,Ore10,Ore11,Ore12,Ore13,Ore14	*OreG0022111*
scaffold466	149689	C	T	NA	*OreG0022113*
scaffold466	156514	A	T	NA	*OreG0022114*
scaffold466	168113	C	T	Ore02,Ore04,Ore05,Ore06,Ore07,Ore08,Ore11,Ore12,Ore13	*OreG0022116*
scaffold466	168347	T	A	NA	*OreG0022116*
scaffold466	173526	C	G	NA	*OreG0022117*
scaffold466	178901	C	A	NA	*OreG0022118*
scaffold466	180561	T	A	Och06	*OreG0022119*
scaffold466	180738	A	G	NA	*OreG0022119*
scaffold466	220437	C	A	Och02,Och03,Och05,Och07,Och08,Och09,Och10,Och12,Och13,Och14	*OreG0022122*
scaffold466	243696	C	A	NA	*OreG0022123*
scaffold466	243706	G	T	Och01,Och03,Och06,Och09,Och10	*OreG0022123*
scaffold466	243718	A	G	Och01,Och03,Och06,Och09,Och10,Och14	*OreG0022123*
scaffold466	290748	C	T	NA	*OreG0022128*
scaffold466	290932	C	G	Och01,Och02,Och03,Och04,Och05,Och06,Och07,Och08,Och09,Och10,Och11,Och12,Och13,Och14,Ore01,Ore02,Ore03,Ore04,Ore05,Ore06,Ore07,Ore08,Ore09,Ore10,Ore11,Ore12,Ore13,Ore14	*OreG0022128*
scaffold466	296579	C	A	NA	*OreG0022129*
scaffold466	300175	G	A	Ore03	*OreG0022130*
scaffold466	300190	T	G	Ore01,Ore02,Ore03,Ore04,Ore05,Ore06,Ore07,Ore08,Ore09,Ore10,Ore11,Ore12,Ore13,Ore14	*OreG0022130*
scaffold466	307456	C	T	Och06,Och08,Och09,Och10,Och11,Och14	*OreG0022131*
scaffold466	307497	G	A	Ore01,Ore02,Ore03,Ore04,Ore05,Ore06,Ore07,Ore08,Ore09,Ore10,Ore11,Ore12,Ore13,Ore14	*OreG0022131*
scaffold466	322017	A	G	Och06	*OreG0022132*
scaffold466	322107	G	C	Och01,Och02,Och03,Och04,Och05,Och06,Och07,Och08,Och09,Och10,Och11,Och12,Och13,Och14,Ore01,Ore02,Ore03,Ore04,Ore05,Ore06,Ore07,Ore08,Ore09,Ore10,Ore11,Ore12,Ore13,Ore14	*OreG0022132*

scaffold466	322150	C	T	Och01,Och02,Och03,Och04,Och05,Och06,Och07,Och08,Och09,Och10,Och11,Och12,Och13,Och14,Ore01,Ore02,Ore03,Ore04,Ore05,Ore06,Ore07,Ore08,Ore09,Ore10,Ore11,Ore12,Ore13,Ore14	*OreG0022132*
scaffold466	392985	T	C	NA	*OreG0022142*
scaffold466	399413	C	A	NA	*OreG0022143*
scaffold466	400558	G	T	NA	*OreG0022143*
scaffold466	401911	A	T	Och01,Och02,Och03,Och04,Och05,Och06,Och07,Och08,Och09,Och10,Och11,Och12,Och13,Och14,Ore01,Ore02,Ore03,Ore04,Ore05,Ore06,Ore07,Ore08,Ore09,Ore10,Ore11,Ore12,Ore13,Ore14	*OreG0022143*
scaffold466	417558	C	T	NA	*OreG0022146*
scaffold466	425136	T	C	Och01,Och02,Och03,Och04,Och05,Och06,Och07,Och08,Och09,Och10,Och11,Och12,Och13,Och14	*OreG0022148*
scaffold466	425741	G	A	Och06	*OreG0022148*
scaffold466	428515	T	A	NA	*OreG0022148*
scaffold466	458480	T	C	NA	*OreG0022151*
scaffold466	462770	G	T	NA	*OreG0022151*
scaffold466	484409	A	T	Och02,Och03,Och04,Och05,Och06,Och08,Och12,Och13	*OreG0022154*
scaffold466	497988	T	C	NA	*OreG0022156*
scaffold466	500995	T	G	NA	*OreG0022157*
scaffold466	508717	C	T	NA	*OreG0022158*
scaffold466	508803	T	G	NA	*OreG0022158*
scaffold466	509123	A	C	Och01,Och02,Och03,Och04,Och05,Och06,Och07,Och08,Och09,Och10,Och11,Och12,Och13,Och14,Ore01,Ore02,Ore03,Ore04,Ore05,Ore06,Ore07,Ore08,Ore09,Ore10,Ore11,Ore12,Ore13,Ore14	*OreG0022158*
scaffold466	511344	T	C	NA	*OreG0022158*
scaffold466	516384	T	C	NA	*OreG0022159*
scaffold466	545428	G	T	Ore01,Ore02,Ore03,Ore04,Ore05,Ore06,Ore07,Ore08,Ore09,Ore10,Ore11,Ore12,Ore13,Ore14	*OreG0022163*
scaffold466	547330	A	G	Ore01,Ore03,Ore10	*OreG0022164*
scaffold466	547656	A	G	NA	*OreG0022164*
scaffold466	547768	A	G	Och09,Och10	*OreG0022164*
scaffold466	549128	C	A	Ore01,Ore02,Ore03,Ore04,Ore05,Ore06,Ore07,Ore08,Ore09,Ore10,Ore11,Ore12,Ore13,Ore14	*OreG0022164*
scaffold466	549262	A	T	NA	*OreG0022164*
scaffold466	553333	G	A	Och13	*OreG0022165*
scaffold466	557066	A	G	NA	*OreG0022165*
scaffold466	557777	C	T	Och01,Och02,Och03,Och04,Och05,Och06,Och07,Och08,Och09,Och10,Och11,Och12,Och13,Och14,Ore01,Ore02,Ore03,Ore04,Ore05,Ore06,Ore07,Ore08,Ore09,Ore10,Ore11,Ore12,Ore13,Ore14	*OreG0022165*
scaffold466	588783	C	T	NA	*OreG0022169*
scaffold466	588810	A	G	Och13	*OreG0022169*
scaffold466	597755	C	A	Och01,Och02,Och03,Och04,Och05,Och06,Och07,Och08,Och09,Och10,Och11,Och12,Och13,Och14	*OreG0022170*
scaffold466	625362	C	T	Och09,Och10	*OreG0022175*
scaffold466	700291	C	T	Och01,Och08,Och14	*OreG0022183*
scaffold466	706089	A	G	Ore01,Ore02,Ore03,Ore04,Ore05,Ore06,Ore07,Ore08,Ore09,Ore10,Ore11,Ore12,Ore13,Ore14	*OreG0022184*
scaffold466	711017	A	C	Och02,Och03,Och04,Och05,Och06,Och07,Och08,Och09,Och10,Och12,Och13	*OreG0022185*
scaffold466	723389	A	T	Ore01,Ore02,Ore03,Ore04,Ore05,Ore06,Ore07,Ore08,Ore09,Ore10,Ore11,Ore12,Ore13,Ore14	*OreG0022189*
scaffold466	724115	A	T	NA	*OreG0022189*
scaffold466	724892	G	A	NA	*OreG0022189*
scaffold466	745972	T	G	Och09,Och10	*OreG0022194*
scaffold466	749501	C	G	NA	*OreG0022195*
scaffold466	784791	C	T	Och01,Och02,Och03,Och04,Och05,Och06,Och07,Och08,Och09,Och10,Och11,Och12,Och13,Och14	*OreG0022198*
scaffold466	785730	C	T	NA	*OreG0022198*
scaffold466	787184	G	T	Och01,Och02,Och03,Och04,Och05,Och06,Och07,Och08,Och09,Och10,Och11,Och12,Och13,Och14,Ore01,Ore02,Ore03,Ore04,Ore05,Ore06,Ore07,Ore08,Ore09,Ore10,Ore11,Ore12,Ore13,Ore14	*OreG0022198*
scaffold466	806492	G	C	NA	*OreG0022201*
scaffold466	819174	A	T	Ore03	*OreG0022204*
scaffold466	821079	A	G	Och01,Och02,Och03,Och04,Och05,Och06,Och07,Och08,Och09,Och10,Och11,Och12,Och13,Och14,Ore01,Ore02,Ore03,Ore04,Ore05,Ore06,Ore07,Ore08,Ore09,Ore10,Ore11,Ore12,Ore13,Ore14	*OreG0022205*
scaffold466	821748	G	A	NA	*OreG0022205*
scaffold466	838984	G	T	Ore01,Ore02,Ore03,Ore04,Ore05,Ore06,Ore07,Ore08,Ore09,Ore10,Ore11,Ore12,Ore13,Ore14	*OreG0022208*
scaffold466	839336	A	C	NA	*OreG0022208*
scaffold466	839351	T	A	NA	*OreG0022208*
scaffold466	839354	T	C	NA	*OreG0022208*
scaffold466	839399	G	A	NA	*OreG0022208*
scaffold466	839420	G	A	NA	*OreG0022208*
scaffold466	849326	T	C	NA	*OreG0022210*

scaffold466	849437	T	C	Och01,Och02,Och03,Och04,Och05,Och06,Och07,Och08,Och09,Och10,Och11,Och12,Och13,Och14,Ore01,Ore02,Ore03,Ore04,Ore05,Ore06,Ore07,Ore08,Ore09,Ore10,Ore11,Ore12,Ore13,Ore14	*OreG0022210*
scaffold466	849452	G	A	NA	*OreG0022210*
scaffold466	910768	C	A	NA	*OreG0022214*
scaffold466	911021	A	T	Och01,Och02,Och03,Och04,Och05,Och06,Och07,Och08,Och09,Och10,Och11,Och12,Och13,Och14,Ore01,Ore02,Ore03,Ore04,Ore05,Ore06,Ore07,Ore08,Ore09,Ore10,Ore11,Ore12,Ore13,Ore14	*OreG0022214*
scaffold466	912907	G	A	Och06	*OreG0022215*
scaffold466	1006979	C	A	Ore01,Ore02,Ore03,Ore04,Ore05,Ore06,Ore07,Ore08,Ore09,Ore10,Ore11,Ore12,Ore13,Ore14	*OreG0022222*
scaffold466	1009171	C	A	Och01,Och02,Och03,Och04,Och05,Och06,Och07,Och08,Och09,Och10,Och11,Och12,Och13,Och14,Ore01,Ore02,Ore03,Ore04,Ore05,Ore06,Ore07,Ore08,Ore09,Ore10,Ore11,Ore12,Ore13,Ore14	*OreG0022222*
scaffold466	1026614	T	C	NA	*OreG0022223*
scaffold466	1402270	C	G	Ore01,Ore02,Ore03,Ore04,Ore05,Ore06,Ore07,Ore08,Ore09,Ore10,Ore11,Ore12,Ore13,Ore14	*OreG0022238*
scaffold466	1425666	T	C	Och01,Och02,Och03,Och04,Och05,Och06,Och07,Och08,Och09,Och10,Och11,Och12,Och13,Och14,Ore01,Ore02,Ore03,Ore04,Ore05,Ore06,Ore07,Ore08,Ore09,Ore10,Ore11,Ore12,Ore13,Ore14	*OreG0022240*
scaffold466	1425678	A	G	Ore04,Ore05,Ore08	*OreG0022240*
scaffold466	1425687	T	C	Och01,Och02,Och03,Och04,Och05,Och06,Och07,Och08,Och09,Och10,Och11,Och12,Och13,Och14,Ore01,Ore02,Ore03,Ore04,Ore05,Ore06,Ore07,Ore08,Ore09,Ore10,Ore11,Ore12,Ore13,Ore14	*OreG0022240*
scaffold466	1425845	G	C	Och01,Och02,Och03,Och04,Och05,Och06,Och07,Och08,Och09,Och10,Och11,Och12,Och13,Och14,Ore01,Ore02,Ore03,Ore04,Ore05,Ore06,Ore07,Ore08,Ore09,Ore10,Ore11,Ore12,Ore13,Ore14	*OreG0022240*
scaffold466	1426091	T	C	Ore04,Ore05,Ore08	*OreG0022240*
scaffold466	1426197	A	G	NA	*OreG0022240*
scaffold466	1426358	T	A	Och14	*OreG0022240*
scaffold466	1426409	T	C	NA	*OreG0022240*
scaffold466	1426660	C	T	Och01,Och02,Och04,Och06,Och07,Och08,Och09,Och10,Och11,Och14	*OreG0022241*
scaffold466	1427065	C	G	NA	*OreG0022241*
scaffold466	1427203	A	T	NA	*OreG0022241*
scaffold466	1427304	G	A	NA	*OreG0022241*
scaffold466	1474926	G	T	NA	*OreG0022246*
scaffold466	1476952	G	A	NA	*OreG0022247*
scaffold466	1477197	A	G	Och06	*OreG0022247*
scaffold466	1477228	A	T	Ore01,Ore02,Ore03,Ore04,Ore05,Ore06,Ore07,Ore08,Ore09,Ore10,Ore11,Ore12,Ore13,Ore14	*OreG0022247*
scaffold466	1477909	C	G	NA	*OreG0022247*
scaffold466	1495719	T	A	Och01	*OreG0022249*
scaffold466	1496020	G	T	NA	*OreG0022249*
scaffold466	1504694	C	A	NA	*OreG0022250*
scaffold466	1507596	T	G	Och01,Och02,Och03,Och04,Och05,Och06,Och07,Och08,Och09,Och10,Och11,Och12,Och13,Och14	*OreG0022251*
scaffold466	1509936	T	A	NA	*OreG0022251*
scaffold466	1531530	C	A	NA	*OreG0022254*
scaffold466	1552296	G	T	Och01,Och02,Och03,Och04,Och05,Och06,Och07,Och09,Och10,Och11,Och12,Och13,Ore01,Ore02,Ore03,Ore04,Ore05,Ore06,Ore07,Ore08,Ore09,Ore10,Ore11,Ore12,Ore13,Ore14	*OreG0022257*
scaffold466	1552605	A	T	NA	*OreG0022257*
scaffold466	1667407	A	T	Och01,Och02,Och03,Och04,Och05,Och06,Och07,Och08,Och09,Och10,Och11,Och12,Och13,Och14	*OreG0022266*
scaffold466	1681316	A	T	Ore01,Ore02,Ore03,Ore04,Ore05,Ore06,Ore07,Ore08,Ore09,Ore10,Ore11,Ore12,Ore13,Ore14	*OreG0022268*
scaffold466	1681466	G	A	NA	*OreG0022268*
scaffold466	1681511	T	A	Och01,Och02,Och03,Och04,Och05,Och06,Och07,Och08,Och09,Och10,Och11,Och12,Och13,Och14	*OreG0022268*
scaffold466	1681516	A	G	Ore01,Ore02,Ore04,Ore05,Ore06,Ore07,Ore08,Ore11,Ore12,Ore13,Ore14	*OreG0022268*
scaffold466	1704282	G	C	Ore01,Ore02,Ore03,Ore04,Ore05,Ore06,Ore07,Ore08,Ore09,Ore10,Ore11,Ore12,Ore13,Ore14	*OreG0022273*
scaffold466	1704768	C	T	Och01,Och02,Och03,Och04,Och05,Och06,Och07,Och08,Och09,Och10,Och11,Och12,Och13,Och14,Ore01,Ore02,Ore03,Ore04,Ore05,Ore06,Ore07,Ore08,Ore09,Ore10,Ore11,Ore12,Ore13,Ore14	*OreG0022273*
scaffold466	1743034	C	T	Och01,Och02,Och03,Och04,Och05,Och06,Och07,Och08,Och09,Och10,Och11,Och12,Och13,Och14,Ore01,Ore02,Ore03,Ore04,Ore05,Ore06,Ore07,Ore08,Ore09,Ore10,Ore11,Ore12,Ore13,Ore14	*OreG0022278*
scaffold466	1744690	G	T	Och01,Och02,Och03,Och04,Och05,Och06,Och07,Och08,Och09,Och10,Och11,Och12,Och13,Och14,Ore01,Ore02,Ore03,Ore04,Ore05,Ore06,Ore07,Ore08,Ore09,Ore10,Ore11,Ore12,Ore13,Ore14	*OreG0022278*
scaffold466	1744722	A	T	NA	*OreG0022278*
scaffold466	1745000	A	T	Ore02,Ore03,Ore04,Ore05,Ore06,Ore07,Ore08,Ore09,Ore10,Ore11,Ore12,Ore13,Ore14	*OreG0022278*
scaffold466	1773902	C	A	NA	*OreG0022284*
scaffold466	1781550	C	A	Och01	*OreG0022285*

scaffold466	1800000	G	A	Ore02,Ore04,Ore05,Ore06,Ore07,Ore08,Ore11,Ore12,Ore13,Ore14	*OreG0022290*
scaffold466	1809701	T	G	Och01,Och02,Och03,Och04,Och05,Och06,Och07,Och08,Och09,Och10,Och11,Och12,Och13,Och14	*OreG0022291*
scaffold466	1810517	G	C	Och01,Och02,Och03,Och04,Och05,Och06,Och07,Och08,Och09,Och10,Och11,Och12,Och13,Och14	*OreG0022291*
scaffold466	1837681	T	A	Ore01,Ore02,Ore03,Ore04,Ore05,Ore06,Ore07,Ore08,Ore09,Ore10,Ore11,Ore12,Ore13,Ore14	*OreG0022294*
scaffold466	1875003	A	C	NA	*OreG0022301*
scaffold466	1908771	T	C	Ore01,Ore02,Ore03,Ore04,Ore05,Ore06,Ore07,Ore08,Ore09,Ore10,Ore11,Ore12,Ore13,Ore14	*OreG0022305*
scaffold466	1929518	T	A	NA	*OreG0022308*
scaffold466	1929835	A	G	NA	*OreG0022308*
scaffold466	1930578	G	A	NA	*OreG0022308*
scaffold466	1933275	C	T	NA	*OreG0022308*
scaffold466	1933368	C	T	NA	*OreG0022308*
scaffold466	1933531	G	A	NA	*OreG0022308*
scaffold466	1933548	T	C	Ore01,Ore02,Ore03,Ore04,Ore05,Ore06,Ore07,Ore08,Ore09,Ore10,Ore11,Ore12,Ore13,Ore14	*OreG0022308*
scaffold466	1941449	C	A	NA	*OreG0022308*
scaffold466	1944650	C	T	Och01,Och02,Och03,Och04,Och05,Och06,Och07,Och08,Och09,Och10,Och11,Och12,Och13,Och14,Ore01,Ore02,Ore03,Ore04,Ore05,Ore06,Ore07,Ore08,Ore09,Ore10,Ore11,Ore12,Ore13,Ore14	*OreG0022309*
scaffold466	1946722	C	T	NA	*OreG0022309*
scaffold466	1950074	T	C	Och01,Och02,Och03,Och04,Och05,Och06,Och07,Och08,Och09,Och10,Och11,Och12,Och13,Och14,Ore01,Ore02,Ore03,Ore04,Ore05,Ore06,Ore07,Ore08,Ore09,Ore10,Ore11,Ore12,Ore13,Ore14	*OreG0022310*
scaffold466	1957030	T	C	NA	*OreG0022312*
scaffold466	1958943	T	C	NA	*OreG0022312*
scaffold466	1976038	G	A	NA	*OreG0022315*
scaffold466	1996589	C	G	Ore01,Ore02,Ore03,Ore04,Ore05,Ore06,Ore07,Ore08,Ore09,Ore10,Ore11,Ore12,Ore13,Ore14	*OreG0022319*
scaffold466	1998168	C	T	Och01,Och02,Och03,Och04,Och05,Och06,Och07,Och08,Och09,Och10,Och11,Och12,Och13,Och14	*OreG0022320*
scaffold466	1998851	G	T	Och01,Och02,Och03,Och04,Och05,Och06,Och07,Och08,Och09,Och10,Och11,Och12,Och13,Och14	*OreG0022320*
scaffold466	1999064	G	A	Och01	*OreG0022320*
scaffold466	1999524	A	G	NA	*OreG0022321*
scaffold1318	5731	C	T	Ore01,Ore02,Ore03,Ore04,Ore05,Ore06,Ore07,Ore08,Ore09,Ore10,Ore11,Ore12,Ore13,Ore14	*OreG0001994*
scaffold1318	16474	G	T	NA	*OreG0001995*
scaffold928	139010	T	C	Och01,Och02,Och03,Och04,Och05,Och06,Och07,Och08,Och09,Och10,Och11,Och12,Och13,Och14,Ore01,Ore02,Ore03,Ore04,Ore05,Ore06,Ore07,Ore08,Ore09,Ore10,Ore11,Ore12,Ore13,Ore14	*OreG0026784*
scaffold928	244087	A	C	Ore03,Ore07,Ore08,Ore10,Ore11,Ore12,Ore13,Ore14	*OreG0026789*
scaffold928	245191	C	T	Och02	*OreG0026789*
scaffold928	247379	T	C	Och02	*OreG0026789*
scaffold928	301363	G	T	NA	*OreG0026793*
scaffold928	314767	T	C	NA	*OreG0026794*
scaffold928	314881	C	T	NA	*OreG0026794*
scaffold928	314902	C	T	NA	*OreG0026794*
scaffold928	454280	C	G	NA	*OreG0026799*
scaffold928	454288	A	G	NA	*OreG0026799*
scaffold928	454858	G	C	NA	*OreG0026799*
scaffold928	460707	G	T	Och02,Och03,Och04,Och05,Och06,Och07,Och08,Och09,Och10,Och11,Och12,Och13,Och14,Ore01,Ore02,Ore03,Ore04,Ore05,Ore06,Ore07,Ore08,Ore09,Ore10,Ore11,Ore12,Ore13,Ore14	*OreG0026799*
scaffold928	460725	G	A	NA	*OreG0026799*
scaffold928	460770	A	G	NA	*OreG0026799*
scaffold928	488094	G	A	Ore01,Ore02,Ore03,Ore04,Ore05,Ore06,Ore07,Ore08,Ore09,Ore10,Ore11,Ore12,Ore13,Ore14	*OreG0026802*
scaffold928	488354	G	A	Och02,Och05,Och07,Och12,Och13	*OreG0026802*
scaffold928	520184	C	T	NA	*OreG0026804*
scaffold928	520310	G	T	NA	*OreG0026804*
scaffold928	533566	C	A	NA	*OreG0026805*
scaffold928	536657	G	A	Ore01,Ore02,Ore03,Ore04,Ore05,Ore06,Ore07,Ore08,Ore09,Ore10,Ore11,Ore12,Ore13,Ore14	*OreG0026805*
scaffold928	582344	A	G	Och03,Och04,Och08,Och14,Ore01,Ore02,Ore03,Ore04,Ore05,Ore06,Ore07,Ore08,Ore09,Ore10,Ore11,Ore12,Ore13,Ore14	*OreG0026808*
scaffold928	582495	C	T	NA	*OreG0026808*
scaffold928	582587	A	C	Och01,Och02,Och07	*OreG0026808*
scaffold928	588783	A	G	Och02	*OreG0026809*
scaffold928	588800	A	G	NA	*OreG0026809*
scaffold928	588806	G	T	Och02	*OreG0026809*
scaffold928	589139	C	T	Och01,Och02	*OreG0026809*
scaffold928	589574	T	A	NA	*OreG0026809*

scaffold928	589606	C	T	Och01	*OreG0026809*
scaffold928	590521	T	A	NA	*OreG0026809*
scaffold928	590677	T	G	Och02	*OreG0026809*
scaffold928	590725	A	G	NA	*OreG0026809*
scaffold928	590825	C	A	Ore08	*OreG0026809*
scaffold928	597089	A	G	Och01	*OreG0026810*
scaffold928	599244	A	G	Och03,Och04	*OreG0026810*
scaffold928	714231	G	C	Ore01,Ore02,Ore03,Ore04,Ore05,Ore06,Ore07,Ore08,Ore09,Ore10,Ore11,Ore12,Ore13,Ore14	*OreG0026818*
scaffold928	717431	G	C	Och02	*OreG0026818*
scaffold928	717907	A	T	Och01,Och02,Och03,Och04,Och05,Och06,Och07,Och08,Och09,Och10,Och11,Och12,Och13,Och14,Ore02,Ore03,Ore04,Ore05,Ore06,Ore07,Ore08,Ore09,Ore10,Ore11,Ore12,Ore13,Ore14	*OreG0026818*
scaffold928	719085	G	T	NA	*OreG0026818*
scaffold928	720600	T	C	NA	*OreG0026818*
scaffold476	196120	A	G	Ore06,Ore09,Ore14	*OreG0022756*
scaffold476	261879	G	T	NA	*OreG0022760*
scaffold476	265381	C	T	Och01,Och02,Och03,Och04,Och05,Och06,Och07,Och08,Och09,Och10,Och11,Och12,Och13,Och14,Ore01,Ore02,Ore03,Ore04,Ore05,Ore06,Ore07,Ore08,Ore09,Ore10,Ore11,Ore12,Ore13,Ore14	*OreG0022761*
scaffold476	349658	C	T	Och02,Och03,Och04,Och05,Och06,Och12,Och13	*OreG0022767*
scaffold476	386724	C	T	Och02,Och06	*OreG0022769*
scaffold476	390537	C	T	Och01,Och02,Och03,Och04,Och05,Och06,Och07,Och08,Och09,Och10,Och11,Och12,Och13,Och14,Ore01,Ore02,Ore03,Ore04,Ore05,Ore06,Ore07,Ore08,Ore09,Ore10,Ore11,Ore12,Ore13,Ore14	*OreG0022771*
scaffold476	441695	T	C	NA	*OreG0022775*
scaffold476	450908	G	T	NA	*OreG0022776*
scaffold476	458589	G	A	Ore01,Ore02,Ore03,Ore04,Ore05,Ore06,Ore07,Ore08,Ore09,Ore10,Ore11,Ore12,Ore13,Ore14	*OreG0022777*
scaffold476	459858	A	C	Ore01,Ore02,Ore03,Ore04,Ore05,Ore06,Ore07,Ore08,Ore09,Ore10,Ore11,Ore12,Ore13,Ore14	*OreG0022777*
scaffold476	466073	A	T	Ore01,Ore02,Ore03,Ore04,Ore05,Ore06,Ore07,Ore08,Ore09,Ore10,Ore11,Ore12,Ore13,Ore14	*OreG0022778*
scaffold476	503473	A	C	Och05	*OreG0022780*
scaffold476	510193	G	T	NA	*OreG0022780*
scaffold476	510481	C	T	Och01,Och02,Och03,Och04,Och05,Och06,Och07,Och08,Och09,Och10,Och11,Och12,Och13,Och14	*OreG0022780*
scaffold476	511711	A	T	NA	*OreG0022780*
scaffold476	591810	G	A	Ore01,Ore02,Ore03,Ore04,Ore05,Ore06,Ore07,Ore08,Ore09,Ore10,Ore11,Ore12,Ore13,Ore14	*OreG0022784*
scaffold476	592090	A	G	Ore01,Ore02,Ore03,Ore04,Ore05,Ore06,Ore07,Ore08,Ore09,Ore10,Ore11,Ore12,Ore13,Ore14	*OreG0022784*
scaffold476	736435	A	T	Och01,Och02,Och03,Och04,Och05,Och06,Och07,Och08,Och09,Och10,Och11,Och12,Och13,Och14	*OreG0022786*
scaffold476	743734	T	G	Ore01,Ore02,Ore03,Ore04,Ore05,Ore06,Ore07,Ore08,Ore09,Ore10,Ore11,Ore12,Ore13,Ore14	*OreG0022786*
scaffold476	1309908	C	T	NA	*OreG0022792*
scaffold476	1310042	G	A	Och01,Och02,Och03,Och04,Och05,Och06,Och08,Och09,Och10,Och11,Och12,Och13	*OreG0022792*
scaffold476	1310060	C	T	Och01,Och02,Och03,Och04,Och05,Och06,Och08,Och09,Och10,Och11,Och12,Och13,Ore01,Ore02,Ore03,Ore04,Ore05,Ore06,Ore07,Ore08,Ore09,Ore10,Ore11,Ore12,Ore13,Ore14	*OreG0022792*
scaffold476	1310118	C	T	Och01,Och02,Och03,Och04,Och05,Och06,Och07,Och08,Och09,Och10,Och11,Och12,Och13,Och14,Ore01,Ore02,Ore03,Ore04,Ore05,Ore06,Ore07,Ore08,Ore09,Ore10,Ore11,Ore12,Ore13,Ore14	*OreG0022792*
scaffold476	1322551	C	T	Ore01,Ore02,Ore03,Ore04,Ore05,Ore06,Ore07,Ore08,Ore09,Ore10,Ore11,Ore12,Ore13,Ore14	*OreG0022794*
scaffold476	1323651	C	G	NA	*OreG0022794*
scaffold476	1323890	A	T	Ore08,Ore09,Ore10	*OreG0022794*
scaffold476	1324770	C	T	NA	*OreG0022794*
scaffold476	1325125	C	A	Och01,Och02,Och03,Och04,Och05,Och06,Och07,Och08,Och09,Och10,Och11,Och12,Och13,Och14	*OreG0022794*
scaffold476	1377003	T	G	NA	*OreG0022797*
scaffold476	1385959	G	A	Och01,Och02,Och03,Och04,Och05,Och06,Och08,Och09,Och10,Och11,Och12,Och13	*OreG0022798*
scaffold476	1386280	A	G	Ore01,Ore02,Ore03,Ore04,Ore05,Ore06,Ore07,Ore08,Ore09,Ore10,Ore11,Ore12,Ore13,Ore14	*OreG0022798*
scaffold476	1386327	G	C	Och13	*OreG0022798*
scaffold476	1422405	A	T	Och13	*OreG0022802*
scaffold476	1433819	G	A	Och01,Och02,Och03,Och04,Och05,Och06,Och07,Och08,Och09,Och10,Och11,Och12,Och13	*OreG0022803*
scaffold476	1452044	G	A	NA	*OreG0022805*
scaffold476	1462080	T	A	NA	*OreG0022807*
scaffold476	1462132	G	T	NA	*OreG0022807*
scaffold471	12113	G	A	Ore01,Ore02,Ore03,Ore04,Ore05,Ore06,Ore07,Ore08,Ore09,Ore10,Ore11,Ore12,Ore13,Ore14	*OreG0022519*

scaffold471	13093	T	G	NA	*OreG0022519*
scaffold471	13450	A	T	NA	*OreG0022519*
scaffold471	13682	C	A	Och06	*OreG0022519*
scaffold471	24708	G	A	Och11	*OreG0022520*
scaffold471	33886	A	C	Ore01,Ore02,Ore03,Ore04,Ore05,Ore06,Ore07,Ore08,Ore09,Ore10,Ore11,Ore12,Ore13,Ore14	*OreG0022521*
scaffold471	57744	G	A	Och02,Och03,Och04,Och05,Och06,Och07,Och08,Och09,Och10,Och11,Och12,Och13	*OreG0022523*
scaffold471	57804	G	T	Ore01,Ore02,Ore03,Ore04,Ore05,Ore06,Ore07,Ore08,Ore09,Ore10,Ore11,Ore12,Ore13,Ore14	*OreG0022523*
scaffold471	58229	T	C	Och02,Och03,Och04,Och05,Och06,Och07,Och08,Och09,Och10,Och11,Och12,Och13	*OreG0022523*
scaffold471	58434	G	T	Och01,Och02,Och03,Och04,Och05,Och06,Och07,Och08,Och09,Och10,Och11,Och12,Och13,Och14,Ore01,Ore02,Ore03,Ore04,Ore05,Ore06,Ore07,Ore08,Ore09,Ore10,Ore11,Ore12,Ore13,Ore14	*OreG0022523*
scaffold471	69772	A	C	Och01,Och02,Och03,Och04,Och05,Och06,Och07,Och08,Och09,Och10,Och11,Och12,Och13,Och14,Ore01,Ore02,Ore03,Ore04,Ore05,Ore06,Ore07,Ore08,Ore09,Ore10,Ore11,Ore12,Ore13,Ore14	*OreG0022525*
scaffold471	72163	G	A	NA	*OreG0022526*
scaffold471	95410	A	T	Ore01,Ore02,Ore03,Ore04,Ore05,Ore06,Ore07,Ore08,Ore09,Ore10,Ore11,Ore12,Ore13,Ore14	*OreG0022530*
scaffold471	96710	C	A	NA	*OreG0022530*
scaffold471	138642	G	C	Och01,Och06	*OreG0022535*
scaffold471	169996	G	A	NA	*OreG0022538*
scaffold471	170097	G	A	NA	*OreG0022538*
scaffold471	170376	G	A	NA	*OreG0022538*
scaffold471	187930	G	C	NA	*OreG0022541*
scaffold471	189205	A	T	Och13	*OreG0022541*
scaffold471	189913	T	A	Ore03,Ore04,Ore05,Ore07,Ore08,Ore09,Ore11,Ore12	*OreG0022541*
scaffold471	190289	C	A	NA	*OreG0022541*
scaffold471	190308	C	T	Och06	*OreG0022541*
scaffold471	190893	A	G	Ore03,Ore04,Ore05,Ore07,Ore08,Ore09,Ore11,Ore12,Ore14	*OreG0022541*
scaffold471	190905	G	T	NA	*OreG0022541*
scaffold471	191857	T	C	Och01,Och02,Och03,Och04,Och05,Och06,Och07,Och08,Och09,Och10,Och11,Och12,Och13,Och14	*OreG0022541*
scaffold471	273280	T	C	NA	*OreG0022546*
scaffold471	288922	G	T	Ore01,Ore02,Ore03,Ore04,Ore05,Ore06,Ore07,Ore08,Ore09,Ore10,Ore11,Ore12,Ore13,Ore14	*OreG0022547*
scaffold471	322891	T	A	NA	*OreG0022553*
scaffold471	330172	T	C	NA	*OreG0022554*
scaffold471	330199	C	T	NA	*OreG0022554*
scaffold471	330202	C	T	NA	*OreG0022554*
scaffold471	330205	G	A	NA	*OreG0022554*
scaffold471	331969	C	A	NA	*OreG0022554*
scaffold471	332020	C	T	NA	*OreG0022554*
scaffold471	332032	A	G	NA	*OreG0022554*
scaffold471	349527	T	A	Och01,Och02,Och03,Och04,Och05,Och06,Och07,Och08,Och09,Och10,Och11,Och12,Och13,Och14	*OreG0022556*
scaffold471	359248	T	A	Och01,Och02,Och03,Och04,Och05,Och06,Och07,Och08,Och09,Och10,Och11,Och12,Och13,Och14	*OreG0022558*
scaffold471	369346	T	A	Och01,Och02,Och03,Och04,Och05,Och06,Och07,Och08,Och09,Och10,Och11,Och12,Och13,Och14,Ore01,Ore02,Ore03,Ore04,Ore05,Ore06,Ore07,Ore08,Ore09,Ore10,Ore11,Ore12,Ore13,Ore14	*OreG0022558*
scaffold471	395787	G	A	Ore01,Ore02,Ore03,Ore04,Ore05,Ore06,Ore07,Ore08,Ore09,Ore10,Ore11,Ore12,Ore13,Ore14	*OreG0022560*
scaffold471	396012	C	T	NA	*OreG0022560*
scaffold471	403176	G	A	NA	*OreG0022562*
scaffold471	403454	G	A	Och09,Och10	*OreG0022562*
scaffold471	443500	G	C	Ore01,Ore02,Ore03,Ore04,Ore05,Ore06,Ore07,Ore08,Ore09,Ore10,Ore11,Ore12,Ore13,Ore14	*OreG0022565*
scaffold471	778416	G	A	Och13	*OreG0022585*
scaffold471	837308	C	T	NA	*OreG0022589*
scaffold471	858805	T	A	Och01	*OreG0022592*
scaffold471	869431	G	A	NA	*OreG0022592*
scaffold471	880325	G	T	Ore04,Ore05	*OreG0022597*
scaffold471	914202	G	A	NA	*OreG0022599*
scaffold471	917584	G	A	NA	*OreG0022600*
scaffold471	917632	G	A	NA	*OreG0022600*
scaffold471	917645	T	A	NA	*OreG0022600*
scaffold471	917978	C	A	Och11,Ore03	*OreG0022600*
scaffold471	918994	A	T	Ore03	*OreG0022600*
scaffold471	919086	G	T	Ore02,Ore04,Ore05	*OreG0022600*
scaffold471	919167	G	A	NA	*OreG0022600*
scaffold471	919197	C	G	NA	*OreG0022600*
scaffold471	934116	G	A	Och13	*OreG0022602*

scaffold471	934329	G	A	Och13	*OreG0022602*
scaffold471	961651	G	A	Och01,Och02,Och03,Och04,Och05,Och06,Och07,Och08,Och09,Och10,Och11,Och12,Och13	*OreG0022604*
scaffold471	985273	T	C	Ore01,Ore02,Ore03,Ore04,Ore05,Ore06,Ore07,Ore08,Ore09,Ore10,Ore11,Ore12,Ore13,Ore14	*OreG0022609*
scaffold471	1007895	G	A	NA	*OreG0022612*
scaffold471	1029008	T	C	Och12	*OreG0022616*
scaffold471	1051561	T	A	Och08	*OreG0022618*
scaffold471	1051806	T	G	Och01,Och02,Och03,Och04,Och05,Och06,Och07,Och08,Och09,Och10,Och11,Och12,Och13,Och14,Ore01,Ore02,Ore03,Ore04,Ore05,Ore06,Ore07,Ore08,Ore09,Ore10,Ore11,Ore12,Ore13,Ore14	*OreG0022618*
scaffold471	1051917	C	T	NA	*OreG0022618*
scaffold471	1052204	T	G	NA	*OreG0022618*
scaffold471	1052233	G	T	NA	*OreG0022618*
scaffold471	1069863	C	T	NA	*OreG0022619*
scaffold471	1070173	T	C	Ore02,Ore03,Ore09,Ore10,Ore14	*OreG0022619*
scaffold471	1093908	G	A	Och02,Och03,Och04,Och05,Och12,Och13	*OreG0022621*
scaffold471	1093970	T	G	Ore02,Ore03,Ore09,Ore10,Ore14	*OreG0022621*
scaffold471	1093998	T	G	Ore02,Ore03,Ore09,Ore10,Ore14	*OreG0022621*
scaffold471	1094065	C	G	Och08	*OreG0022621*
scaffold471	1094109	T	A	Ore01,Ore04,Ore05	*OreG0022621*
scaffold471	1094235	G	T	Och08	*OreG0022621*
scaffold471	1102121	T	C	NA	*OreG0022623*
scaffold471	1105285	A	T	Ore04,Ore05	*OreG0022624*
scaffold471	1131345	G	A	Och14	*OreG0022627*
scaffold471	1131395	G	A	NA	*OreG0022627*
scaffold471	1131425	A	T	Ore04,Ore05	*OreG0022627*
scaffold471	1137139	C	A	Och08	*OreG0022628*
scaffold471	1142269	T	G	Och01,Och02,Och03,Och04,Och05,Och06,Och08,Och11,Och12,Och13,Och14	*OreG0022629*
scaffold471	1143497	T	A	Ore02,Ore03,Ore04,Ore05,Ore07,Ore09,Ore10,Ore11,Ore12,Ore13,Ore14	*OreG0022629*
scaffold471	1148634	G	A	Ore02,Ore03,Ore04,Ore05,Ore07,Ore09,Ore10,Ore11,Ore12,Ore13,Ore14	*OreG0022630*
scaffold471	1149413	A	T	Och01,Och06,Och08,Och11,Och12,Och14	*OreG0022630*
scaffold471	1161157	G	A	Och01,Och02,Och03,Och04,Och05,Och06,Och08,Och11,Och12,Och13,Och14	*OreG0022633*
scaffold471	1161199	A	T	Och08	*OreG0022633*
scaffold471	1161213	T	A	Och01,Och02,Och03,Och05,Och06,Och08,Och11,Och12,Och13,Och14	*OreG0022633*
scaffold471	1161520	A	C	Ore02,Ore04,Ore05,Ore07,Ore09,Ore10,Ore11,Ore12,Ore13,Ore14	*OreG0022633*
scaffold471	1161580	C	A	NA	*OreG0022633*
scaffold471	1196845	T	G	Och13,Ore01,Ore02,Ore03,Ore04,Ore05,Ore06,Ore07,Ore08,Ore09,Ore10,Ore11,Ore12,Ore13,Ore14	*OreG0022634*
scaffold471	1196878	G	C	Och01,Och03,Och04,Och05,Och06,Och07,Och08,Och09,Och10,Och11,Och13,Och14,Ore01,Ore02,Ore03,Ore04,Ore05,Ore06,Ore07,Ore08,Ore09,Ore10,Ore11,Ore12,Ore13,Ore14	*OreG0022634*
scaffold471	1197109	T	G	NA	*OreG0022634*
scaffold471	1203133	C	T	NA	*OreG0022634*
scaffold471	1206967	G	A	NA	*OreG0022635*
scaffold471	1252372	C	T	NA	*OreG0022636*
scaffold471	1252444	G	A	NA	*OreG0022636*
scaffold471	1310562	T	C	Och07	*OreG0022641*
scaffold471	1333661	C	A	Och06,Och08,Och09,Och10	*OreG0022645*
scaffold471	1433729	G	A	NA	*OreG0022649*
scaffold471	1560409	A	G	Och01,Och02,Och03,Och04,Och05,Och06,Och07,Och08,Och09,Och10,Och11,Och12,Och13,Och14,Ore01,Ore02,Ore03,Ore04,Ore05,Ore06,Ore07,Ore08,Ore09,Ore10,Ore11,Ore12,Ore13,Ore14	*OreG0022661*
scaffold471	1560439	C	T	Och01,Och02,Och03,Och04,Och05,Och06,Och07,Och08,Och09,Och10,Och11,Och12,Och13,Och14,Ore01,Ore02,Ore03,Ore04,Ore05,Ore06,Ore07,Ore08,Ore09,Ore10,Ore11,Ore12,Ore13,Ore14	*OreG0022661*
scaffold471	1560472	T	A	Och01,Och02,Och03,Och04,Och05,Och06,Och07,Och08,Och09,Och10,Och11,Och12,Och13,Och14	*OreG0022661*
scaffold471	1560730	T	A	NA	*OreG0022661*
scaffold471	1560797	C	T	Ore01,Ore02,Ore03,Ore04,Ore05,Ore06,Ore07,Ore08,Ore09,Ore10,Ore11,Ore12,Ore13,Ore14	*OreG0022661*
scaffold471	1561871	C	G	Och01,Och02,Och03,Och04,Och05,Och06,Och07,Och08,Och09,Och10,Och11,Och12,Och13,Och14,Ore01,Ore02,Ore03,Ore04,Ore05,Ore06,Ore07,Ore08,Ore09,Ore10,Ore11,Ore12,Ore13,Ore14	*OreG0022661*
scaffold471	1562051	C	A	Och01	*OreG0022661*
scaffold471	1562252	T	A	Och01,Och02,Och03,Och04,Och05,Och06,Och07,Och08,Och09,Och10,Och11,Och12,Och13,Och14	*OreG0022661*
scaffold471	1598056	A	T	Och01,Och02,Och03,Och04,Och05,Och06,Och07,Och08,Och09,Och10,Och11,Och12,Och13,Och14	*OreG0022664*
scaffold471	1602355	C	T	NA	*OreG0022665*
scaffold471	1612519	G	A	Ore01,Ore08,Ore09	*OreG0022666*
scaffold471	1622020	G	A	Och01,Och02,Och03,Och04,Och05,Och06,Och07,Och08,Och09,Och10,Och11,Och12,Och13,Och14	*OreG0022668*
scaffold471	1622125	G	A	NA	*OreG0022668*

scaffold471	1622192	C	T	Och01,Och02,Och03,Och04,Och05,Och06,Och07,Och08,Och09,Och10,Och11,Och12,Och13,Och14	*OreG0022668*
scaffold471	1622355	C	T	Och06,Och07,Ore01,Ore02,Ore03,Ore04,Ore05,Ore06,Ore07,Ore08,Ore09,Ore10,Ore11,Ore12,Ore13,Ore14	*OreG0022668*
scaffold471	1631047	C	T	Ore01,Ore02,Ore03,Ore04,Ore05,Ore06,Ore07,Ore08,Ore09,Ore10,Ore11,Ore12,Ore13,Ore14	*OreG0022668*
scaffold471	1631056	C	T	Ore01,Ore02,Ore03,Ore04,Ore05,Ore06,Ore07,Ore08,Ore09,Ore10,Ore11,Ore12,Ore13,Ore14	*OreG0022668*
scaffold471	1631076	G	A	Ore01,Ore02,Ore03,Ore04,Ore05,Ore06,Ore07,Ore08,Ore09,Ore10,Ore11,Ore12,Ore13,Ore14	*OreG0022668*
scaffold471	1631092	C	T	NA	*OreG0022668*
scaffold471	1631119	C	T	NA	*OreG0022668*
scaffold471	1639301	C	T	NA	*OreG0022670*
scaffold471	1650744	C	A	Ore01,Ore02,Ore03,Ore04,Ore05,Ore06,Ore07,Ore08,Ore09,Ore10,Ore11,Ore12,Ore13,Ore14	*OreG0022671*
scaffold471	1658784	C	G	Ore01,Ore02,Ore03,Ore04,Ore05,Ore06,Ore07,Ore08,Ore09,Ore10,Ore11,Ore12,Ore13,Ore14	*OreG0022672*
scaffold471	1658835	C	A	NA	*OreG0022672*
scaffold471	1691671	C	T	NA	*OreG0022674*
scaffold471	1691749	G	T	Ore01,Ore02,Ore03,Ore04,Ore05,Ore06,Ore07,Ore08,Ore09,Ore10,Ore11,Ore12,Ore13,Ore14	*OreG0022674*
scaffold471	1694090	T	C	Och08	*OreG0022674*
scaffold471	1754605	A	C	Ore01,Ore02,Ore03,Ore04,Ore05,Ore06,Ore07,Ore08,Ore09,Ore10,Ore11,Ore12,Ore13,Ore14	*OreG0022677*
scaffold471	1836155	G	T	Och01,Och08,Och14	*OreG0022682*
scaffold471	1839117	C	T	Och02,Och04,Och06,Och09,Och10,Och11	*OreG0022683*
scaffold471	1845025	T	C	Och02,Och04,Och06,Och09,Och10,Och11	*OreG0022684*
scaffold471	1845122	A	T	NA	*OreG0022684*
scaffold471	1845352	C	T	Och01,Och08	*OreG0022684*
scaffold471	1845455	A	C	Och02,Och04,Och06,Och09,Och10,Och11	*OreG0022684*
scaffold471	1904099	G	T	NA	*OreG0022687*
scaffold471	1914555	T	A	NA	*OreG0022688*
scaffold471	1915100	A	C	Och08	*OreG0022688*
scaffold471	1944382	T	C	NA	*OreG0022691*
scaffold471	1961343	C	T	Ore01,Ore02,Ore03,Ore04,Ore05,Ore06,Ore07,Ore08,Ore09,Ore10,Ore11,Ore12,Ore13,Ore14	*OreG0022692*
scaffold471	2006137	C	A	Och11	*OreG0022696*
scaffold471	2006204	A	C	Och11	*OreG0022696*
scaffold471	2020223	C	T	Och01,Och02,Och03,Och04,Och05,Och06,Och07,Och08,Och09,Och10,Och11,Och12,Och13,Och14	*OreG0022697*
scaffold471	2026979	C	T	Och01,Och02,Och03,Och04,Och05,Och06,Och07,Och08,Och09,Och10,Och11,Och12,Och13,Och14	*OreG0022697*
scaffold471	2056761	G	T	Ore01,Ore02,Ore03,Ore04,Ore05,Ore06,Ore07,Ore08,Ore09,Ore10,Ore11,Ore12,Ore13,Ore14	*OreG0022700*
scaffold471	2062470	G	T	NA	*OreG0022701*
scaffold471	2069964	A	T	Ore01,Ore02,Ore03,Ore04,Ore05,Ore06,Ore07,Ore08,Ore09,Ore10,Ore11,Ore12,Ore13,Ore14	*OreG0022702*
scaffold471	2101955	A	T	Ore02,Ore03,Ore04,Ore05,Ore07,Ore09,Ore10,Ore11,Ore12,Ore13,Ore14	*OreG0022706*
scaffold471	2102171	C	T	Ore02,Ore03,Ore04,Ore05,Ore07,Ore09,Ore10,Ore11,Ore12,Ore13,Ore14	*OreG0022706*
scaffold471	2102177	C	T	NA	*OreG0022706*
scaffold471	2102634	G	C	NA	*OreG0022706*
scaffold471	2131713	A	C	Ore02,Ore03,Ore09,Ore10,Ore14	*OreG0022710*
scaffold471	2139499	G	A	NA	*OreG0022711*
scaffold471	2139532	G	A	NA	*OreG0022711*
scaffold471	2139538	A	G	NA	*OreG0022711*
scaffold471	2141218	C	T	Och01,Och02,Och03,Och04,Och05,Och06,Och07,Och08,Och09,Och10,Och11,Och12,Och13,Och14	*OreG0022711*
scaffold471	2163595	C	A	Och01,Och02,Och03,Och04,Och05,Och06,Och07,Och08,Och09,Och10,Och11,Och12,Och13,Och14,Ore01,Ore02,Ore03,Ore04,Ore05,Ore06,Ore07,Ore08,Ore09,Ore10,Ore11,Ore12,Ore13,Ore14	*OreG0022713*
scaffold471	2163925	T	C	Och01,Och02,Och03,Och04,Och05,Och06,Och07,Och08,Och09,Och10,Och11,Och12,Och13,Och14,Ore01,Ore02,Ore03,Ore04,Ore05,Ore06,Ore07,Ore08,Ore09,Ore10,Ore11,Ore12,Ore13,Ore14	*OreG0022713*
scaffold471	2167178	T	A	NA	*OreG0022713*
scaffold471	2180307	C	T	NA	*OreG0022714*
scaffold471	2206535	A	G	NA	*OreG0022718*
scaffold471	2207747	C	T	NA	*OreG0022718*
scaffold471	2207801	G	T	NA	*OreG0022718*
scaffold471	2208793	G	A	NA	*OreG0022718*
scaffold471	2208937	C	T	NA	*OreG0022718*
scaffold471	2208942	T	G	NA	*OreG0022718*
scaffold471	2209086	G	C	NA	*OreG0022718*
scaffold471	2209108	G	T	NA	*OreG0022718*
scaffold471	2226607	G	T	Och01,Och02,Och03,Och04,Och05,Och06,Och07,Och08,Och09,Och10,Och11,Och12,Och13,Och14	*OreG0022720*

scaffold471	2226760	G	T	Och01,Och02,Och03,Och04,Och05,Och06,Och07,Och08,Och09,Och10,Och11,Och12,Och13,Och14,Ore01,Ore02,Ore03,Ore04,Ore05,Ore06,Ore07,Ore08,Ore09,Ore10,Ore11,Ore12,Ore13,Ore14	*OreG0022720*
scaffold471	2226988	G	T	NA	*OreG0022720*
scaffold471	2226995	A	G	NA	*OreG0022720*
scaffold471	2229049	C	T	NA	*OreG0022720*
scaffold471	2269088	T	C	Och01,Och02,Och03,Och04,Och05,Och06,Och07,Och08,Och09,Och10,Och11,Och12,Och13,Och14,Ore01,Ore02,Ore03,Ore04,Ore05,Ore06,Ore07,Ore08,Ore09,Ore10,Ore11,Ore12,Ore13,Ore14	*OreG0022723*
scaffold471	2364291	A	C	Och01,Och02,Och03,Och04,Och05,Och06,Och07,Och08,Och09,Och10,Och11,Och12,Och13,Och14,Ore01,Ore02,Ore03,Ore04,Ore05,Ore06,Ore07,Ore08,Ore09,Ore10,Ore11,Ore12,Ore13,Ore14	*OreG0022727*
scaffold471	2364421	G	A	Ore09	*OreG0022727*
scaffold471	2364544	C	A	Ore09	*OreG0022727*
scaffold471	2379156	A	T	Och01,Och02,Och03,Och04,Och05,Och06,Och07,Och08,Och09,Och10,Och11,Och12,Och13,Och14,Ore01,Ore02,Ore03,Ore04,Ore05,Ore06,Ore07,Ore08,Ore09,Ore10,Ore11,Ore12,Ore13,Ore14	*OreG0022728*
scaffold471	2379270	C	T	Ore09	*OreG0022728*
scaffold471	2379563	C	A	NA	*OreG0022728*
scaffold471	2379767	G	A	Ore01,Ore02,Ore03,Ore04,Ore05,Ore06,Ore07,Ore08,Ore09,Ore10,Ore11,Ore12,Ore13,Ore14	*OreG0022728*
scaffold471	2450532	G	A	Och08,Och14	*OreG0022732*
scaffold471	2450535	T	A	Ore01,Ore02,Ore03,Ore04,Ore05,Ore06,Ore07,Ore08,Ore09,Ore10,Ore11,Ore12,Ore13,Ore14	*OreG0022732*
scaffold471	2450626	T	G	NA	*OreG0022732*
scaffold471	2460313	G	A	Ore01,Ore02,Ore03,Ore04,Ore05,Ore06,Ore07,Ore08,Ore09,Ore10,Ore11,Ore12,Ore13,Ore14	*OreG0022733*
scaffold471	2547277	A	G	NA	*OreG0022737*
scaffold471	2608367	C	G	Och02,Och04,Och06	*OreG0022739*
scaffold471	2608543	G	T	Och01,Och02,Och03,Och04,Och05,Och06,Och07,Och08,Och09,Och10,Och11,Och12,Och13,Och14,Ore01,Ore02,Ore03,Ore04,Ore05,Ore06,Ore07,Ore08,Ore09,Ore10,Ore11,Ore12,Ore13,Ore14	*OreG0022739*
scaffold1405	1641	C	A	Och06,Och09,Och10	*OreG0002153*
scaffold451	60137	G	A	NA	*OreG0020568*
scaffold451	116963	C	T	Och01,Och02,Och03,Och04,Och05,Och06,Och07,Och08,Och09,Och10,Och11,Och12,Och13,Och14,Ore01,Ore02,Ore03,Ore04,Ore05,Ore06,Ore07,Ore08,Ore09,Ore10,Ore11,Ore12,Ore13,Ore14	*OreG0020572*
scaffold451	118996	G	T	NA	*OreG0020572*
scaffold451	119257	C	A	Ore01,Ore02,Ore03,Ore04,Ore05,Ore06,Ore07,Ore08,Ore09,Ore10,Ore11,Ore12,Ore13,Ore14	*OreG0020572*
scaffold451	122588	C	G	NA	*OreG0020572*
scaffold451	147478	C	A	Och11	*OreG0020572*
scaffold451	192636	A	T	Och01,Och11,Och14	*OreG0020575*
scaffold451	222579	G	A	Ore01,Ore02,Ore03,Ore04,Ore05,Ore06,Ore07,Ore08,Ore09,Ore10,Ore11,Ore12,Ore13,Ore14	*OreG0020578*
scaffold451	279201	T	G	Ore01,Ore03,Ore04,Ore05,Ore07,Ore08,Ore10,Ore11,Ore12,Ore13,Ore14	*OreG0020586*
scaffold451	301241	G	C	Och07,Och08,Och09,Och10,Och11,Och14	*OreG0020589*
scaffold451	307892	A	C	NA	*OreG0020590*
scaffold451	308226	C	G	Ore01,Ore02,Ore03,Ore04,Ore05,Ore06,Ore07,Ore08,Ore09,Ore10,Ore11,Ore12,Ore13,Ore14	*OreG0020590*
scaffold451	312124	G	C	Ore01,Ore02,Ore03,Ore04,Ore05,Ore06,Ore07,Ore08,Ore09,Ore10,Ore11,Ore12,Ore13,Ore14	*OreG0020591*
scaffold451	317941	C	T	Ore01,Ore02,Ore03,Ore04,Ore05,Ore06,Ore07,Ore08,Ore09,Ore10,Ore11,Ore12,Ore13,Ore14	*OreG0020592*
scaffold451	318445	G	C	Och06	*OreG0020592*
scaffold451	318941	T	C	NA	*OreG0020592*
scaffold451	330651	C	T	Och01,Och02,Och03,Och04,Och05,Och06,Och07,Och08,Och09,Och10,Och11,Och12,Och13,Och14,Ore01,Ore02,Ore03,Ore04,Ore05,Ore06,Ore07,Ore08,Ore09,Ore10,Ore11,Ore12,Ore13,Ore14	*OreG0020593*
scaffold451	330655	G	A	Och01,Och02,Och03,Och04,Och05,Och06,Och07,Och08,Och09,Och10,Och11,Och12,Och13,Och14,Ore01,Ore02,Ore03,Ore04,Ore05,Ore06,Ore07,Ore08,Ore09,Ore10,Ore11,Ore12,Ore13,Ore14	*OreG0020593*
scaffold451	330677	C	T	Och01,Och02,Och03,Och04,Och05,Och06,Och07,Och08,Och09,Och10,Och11,Och12,Och13,Och14,Ore01,Ore02,Ore03,Ore04,Ore05,Ore06,Ore07,Ore08,Ore09,Ore10,Ore11,Ore12,Ore13,Ore14	*OreG0020593*
scaffold451	330696	G	A	Och01,Och02,Och03,Och04,Och05,Och06,Och07,Och08,Och09,Och10,Och11,Och12,Och13,Och14,Ore01,Ore02,Ore03,Ore04,Ore05,Ore06,Ore07,Ore08,Ore09,Ore10,Ore11,Ore12,Ore13,Ore14	*OreG0020593*
scaffold451	330748	C	T	NA	*OreG0020593*
scaffold451	330771	T	A	Och01,Och02,Och03,Och04,Och05,Och06,Och07,Och08,Och09,Och10,Och11,Och12,Och13,Och14	*OreG0020593*
scaffold451	337639	C	T	Och06	*OreG0020594*
scaffold451	350403	G	T	Ore01,Ore02,Ore03,Ore04,Ore05,Ore06,Ore07,Ore08,Ore09,Ore10,Ore11,Ore12,Ore13,Ore14	*OreG0020595*
scaffold451	378161	A	T	Ore01,Ore02,Ore03,Ore04,Ore05,Ore06,Ore07,Ore08,Ore09,Ore10,Ore11,Ore12,Ore13,Ore14	*OreG0020596*

scaffold451	378501	A	T	Och01,Och02,Och03,Och04,Och05,Och06,Och07,Och09,Och10,Och11,Och12,Och13,Och14	*OreG0020596*
scaffold451	421890	C	T	Och03,Och04,Och06	*OreG0020601*
scaffold451	422052	G	A	Och03,Och04,Och06	*OreG0020601*
scaffold451	443311	T	C	Och03,Och04,Och06	*OreG0020602*
scaffold451	443318	A	T	Och03,Och04,Och06,Och07,Och09,Och11,Och12,Och14,Ore01,Ore02,Ore03,Ore04,Ore05,Ore06,Ore07,Ore08,Ore09,Ore10,Ore11,Ore12,Ore13,Ore14	*OreG0020602*
scaffold451	455115	C	G	Och01,Och02,Och03,Och04,Och05,Och06,Och07,Och08,Och09,Och10,Och11,Och12,Och13,Och14,Ore01,Ore02,Ore03,Ore04,Ore05,Ore06,Ore07,Ore08,Ore09,Ore10,Ore11,Ore12,Ore13,Ore14	*OreG0020603*
scaffold451	463495	C	T	Och03,Och04,Och06	*OreG0020604*
scaffold451	474046	G	A	Och01,Och02,Och03,Och04,Och05,Och06,Och07,Och08,Och09,Och10,Och11,Och12,Och13,Och14,Ore01,Ore02,Ore03,Ore04,Ore05,Ore06,Ore07,Ore08,Ore09,Ore10,Ore11,Ore12,Ore13,Ore14	*OreG0020606*
scaffold451	474338	G	T	Och01,Och02,Och03,Och04,Och05,Och06,Och07,Och08,Och12,Och13,Och14	*OreG0020606*
scaffold451	480539	C	T	Och01,Och02,Och03,Och04,Och05,Och06,Och07,Och08,Och09,Och10,Och11,Och12,Och13,Och14	*OreG0020607*
scaffold451	497955	C	T	Och02,Och03,Och04,Och05,Och06,Och12,Och13	*OreG0020610*
scaffold451	499955	T	C	NA	*OreG0020610*
scaffold451	508112	T	A	Och01,Och02,Och03,Och04,Och05,Och06,Och07,Och08,Och09,Och10,Och11,Och12,Och13,Och14,Ore01,Ore02,Ore03,Ore04,Ore05,Ore06,Ore07,Ore08,Ore09,Ore10,Ore11,Ore12,Ore13,Ore14	*OreG0020610*
scaffold451	508553	T	C	Och01,Och02,Och03,Och04,Och05,Och06,Och07,Och08,Och09,Och10,Och11,Och12,Och13,Och14	*OreG0020610*
scaffold451	524550	T	A	Och01,Och02,Och03,Och04,Och05,Och06,Och07,Och08,Och09,Och10,Och11,Och12,Och13,Och14,Ore01,Ore02,Ore03,Ore04,Ore05,Ore06,Ore07,Ore08,Ore09,Ore10,Ore11,Ore12,Ore13,Ore14	*OreG0020611*
scaffold451	533767	C	A	Ore01,Ore02,Ore03,Ore04,Ore05,Ore06,Ore07,Ore08,Ore09,Ore10,Ore11,Ore12,Ore13,Ore14	*OreG0020612*
scaffold451	553240	A	T	Ore01,Ore02,Ore03,Ore04,Ore05,Ore06,Ore07,Ore08,Ore09,Ore10,Ore11,Ore12,Ore13,Ore14	*OreG0020615*
scaffold451	553249	T	G	Ore01,Ore02,Ore07,Ore14	*OreG0020615*
scaffold451	554795	C	A	NA	*OreG0020615*
scaffold451	590363	A	G	NA	*OreG0020617*
scaffold451	596290	C	A	Och01,Och02,Och03,Och04,Och05,Och06,Och07,Och08,Och09,Och10,Och11,Och12,Och13,Och14	*OreG0020617*
scaffold451	606241	T	A	Och02,Och06,Och08	*OreG0020618*
scaffold451	606246	A	G	NA	*OreG0020618*
scaffold451	637978	A	C	Ore01,Ore02,Ore03,Ore04,Ore05,Ore06,Ore07,Ore08,Ore09,Ore10,Ore11,Ore12,Ore13,Ore14	*OreG0020619*
scaffold451	638388	T	C	Ore01,Ore02,Ore03,Ore04,Ore05,Ore06,Ore07,Ore08,Ore09,Ore10,Ore11,Ore12,Ore13,Ore14	*OreG0020619*
scaffold451	639727	C	A	Och01,Och02,Och03,Och04,Och05,Och06,Och07,Och08,Och09,Och10,Och11,Och12,Och13,Och14,Ore01,Ore02,Ore03,Ore04,Ore05,Ore06,Ore07,Ore08,Ore09,Ore10,Ore11,Ore12,Ore13,Ore14	*OreG0020619*
scaffold451	663096	C	G	Och01,Och02,Och03,Och04,Och05,Och06,Och07,Och08,Och09,Och10,Och11,Och12,Och13,Och14,Ore01,Ore02,Ore03,Ore04,Ore05,Ore06,Ore07,Ore08,Ore09,Ore10,Ore11,Ore12,Ore13,Ore14	*OreG0020622*
scaffold451	663365	T	A	Och01,Och02,Och03,Och04,Och05,Och06,Och07,Och08,Och09,Och10,Och11,Och12,Och13,Och14	*OreG0020622*
scaffold451	690090	A	C	Och01,Och02,Och03,Och04,Och05,Och06,Och07,Och08,Och09,Och10,Och11,Och12,Och13,Och14	*OreG0020625*
scaffold451	702637	G	T	NA	*OreG0020626*
scaffold451	713087	C	G	NA	*OreG0020627*
scaffold451	713259	G	A	Ore01,Ore02,Ore03,Ore04,Ore05,Ore06,Ore07,Ore08,Ore09,Ore10,Ore11,Ore12,Ore13,Ore14	*OreG0020627*
scaffold451	713280	C	T	Och01,Och02,Och03,Och04,Och05,Och06,Och07,Och08,Och09,Och10,Och11,Och12,Och13,Och14	*OreG0020627*
scaffold451	713394	C	T	Ore01,Ore02,Ore03,Ore04,Ore05,Ore06,Ore07,Ore08,Ore09,Ore10,Ore11,Ore12,Ore13,Ore14	*OreG0020627*
scaffold451	713399	A	C	Och01,Och02,Och03,Och04,Och05,Och06,Och07,Och08,Och09,Och10,Och11,Och12,Och13,Och14	*OreG0020627*
scaffold451	713718	G	A	Ore01,Ore02,Ore03,Ore04,Ore05,Ore06,Ore07,Ore08,Ore09,Ore10,Ore11,Ore12,Ore13,Ore14	*OreG0020627*
scaffold451	713835	G	A	Och01,Och02,Och03,Och04,Och05,Och06,Och07,Och08,Och09,Och10,Och11,Och12,Och13,Och14	*OreG0020628*
scaffold451	713865	A	G	Och01,Och02,Och03,Och04,Och05,Och06,Och07,Och08,Och09,Och10,Och11,Och12,Och13,Och14	*OreG0020628*
scaffold451	740340	A	C	Och01,Och02,Och03,Och04,Och05,Och06,Och07,Och08,Och09,Och10,Och11,Och12,Och13,Och14,Ore01,Ore02,Ore03,Ore04,Ore05,Ore06,Ore07,Ore08,Ore09,Ore10,Ore11,Ore12,Ore13,Ore14	*OreG0020630*
scaffold451	751855	G	T	NA	*OreG0020631*
scaffold451	755530	A	G	Ore01,Ore02,Ore03,Ore04,Ore05,Ore06,Ore07,Ore08,Ore09,Ore10,Ore11,Ore12,Ore13,Ore14	*OreG0020632*
scaffold451	760733	A	T	NA	*OreG0020633*

scaffold451	760751	C	T	Och01,Och02,Och03,Och04,Och05,Och06,Och07,Och08,Och09,Och10,Och11,Och12,Och13,Och14	*OreG0020633*
scaffold451	760779	A	C	NA	*OreG0020633*
scaffold451	778652	C	T	Och02,Och03,Och04,Och05,Och06,Och08,Och11,Och12,Och13	*OreG0020635*
scaffold451	785519	C	G	NA	*OreG0020635*
scaffold451	805734	G	A	NA	*OreG0020636*
scaffold451	809583	T	C	NA	*OreG0020636*
scaffold451	932397	C	T	Och02,Och06	*OreG0020639*
scaffold451	933414	T	C	Och14	*OreG0020639*
scaffold451	955797	C	T	NA	*OreG0020641*
scaffold451	965688	G	A	Ore01,Ore02,Ore03,Ore04,Ore05,Ore06,Ore07,Ore08,Ore09,Ore10,Ore11,Ore12,Ore13,Ore14	*OreG0020643*
scaffold451	965792	G	C	Och01,Och02,Och03,Och04,Och05,Och06,Och07,Och08,Och09,Och10,Och11,Och12,Och13,Och14	*OreG0020643*
scaffold451	965823	C	A	Ore01,Ore02,Ore03,Ore04,Ore05,Ore06,Ore07,Ore08,Ore09,Ore10,Ore11,Ore12,Ore13,Ore14	*OreG0020643*
scaffold451	966234	T	A	NA	*OreG0020643*
scaffold451	966306	C	T	Ore01,Ore02,Ore03,Ore04,Ore05,Ore06,Ore07,Ore08,Ore09,Ore10,Ore11,Ore12,Ore13,Ore14	*OreG0020643*
scaffold451	966478	G	A	Ore01,Ore02,Ore03,Ore04,Ore05,Ore06,Ore07,Ore08,Ore09,Ore10,Ore11,Ore12,Ore13,Ore14	*OreG0020643*
scaffold451	966527	A	C	Ore01,Ore02,Ore03,Ore04,Ore05,Ore06,Ore07,Ore08,Ore09,Ore10,Ore11,Ore12,Ore13,Ore14	*OreG0020643*
scaffold451	966556	T	G	Ore01,Ore02,Ore03,Ore04,Ore05,Ore06,Ore07,Ore08,Ore09,Ore10,Ore11,Ore12,Ore13,Ore14	*OreG0020643*
scaffold451	975303	G	C	Ore01,Ore02,Ore03,Ore04,Ore05,Ore06,Ore07,Ore08,Ore09,Ore10,Ore11,Ore12,Ore13,Ore14	*OreG0020645*
scaffold451	975380	T	C	Och01	*OreG0020645*
scaffold451	975672	A	T	Och01,Och02,Och03,Och04,Och05,Och06,Och07,Och08,Och09,Och10,Och11,Och12,Och13,Och14	*OreG0020645*
scaffold451	975933	A	C	Ore01,Ore02,Ore03,Ore04,Ore05,Ore06,Ore07,Ore08,Ore09,Ore10,Ore11,Ore12,Ore13,Ore14	*OreG0020645*
scaffold451	977085	G	A	Och02,Och06,Och12	*OreG0020646*
scaffold451	977804	C	T	NA	*OreG0020646*
scaffold451	988176	T	G	NA	*OreG0020648*
scaffold451	1004418	C	T	Och02,Och03,Och04,Och05,Och06,Och07,Och12,Och13,Och14	*OreG0020650*
scaffold451	1004679	G	T	NA	*OreG0020650*
scaffold451	1004862	C	A	Och02,Och03,Och04,Och05,Och06,Och07,Och12,Och13,Och14	*OreG0020650*
scaffold451	1005253	T	A	NA	*OreG0020650*
scaffold451	1005513	A	G	NA	*OreG0020650*
scaffold451	1012707	C	A	Och01,Och02,Och03,Och04,Och05,Och06,Och07,Och08,Och12,Och13,Och14	*OreG0020651*
scaffold451	1035399	G	T	Och11	*OreG0020653*
scaffold451	1058846	C	A	Och01,Och02,Och03,Och04,Och05,Och06,Och07,Och08,Och09,Och10,Och11,Och12,Och13,Och14,Ore01,Ore02,Ore03,Ore04,Ore05,Ore06,Ore07,Ore08,Ore09,Ore10,Ore11,Ore12,Ore13,Ore14	*OreG0020655*
scaffold451	1087099	A	G	Och11	*OreG0020656*
scaffold451	1095471	A	C	NA	*OreG0020657*
scaffold451	1095571	T	C	NA	*OreG0020657*
scaffold451	1095588	C	G	NA	*OreG0020657*
scaffold451	1095729	G	A	NA	*OreG0020657*
scaffold451	1095914	C	G	NA	*OreG0020657*
scaffold451	1130420	T	G	Ore01,Ore02,Ore03,Ore04,Ore05,Ore06,Ore07,Ore08,Ore09,Ore10,Ore11,Ore12,Ore13,Ore14	*OreG0020662*
scaffold451	1141721	C	A	NA	*OreG0020664*
scaffold451	1151406	C	G	NA	*OreG0020665*
scaffold451	1169926	G	A	NA	*OreG0020666*
scaffold451	1179171	T	C	Ore01,Ore02,Ore03,Ore04,Ore05,Ore06,Ore07,Ore08,Ore09,Ore10,Ore11,Ore12,Ore13,Ore14	*OreG0020666*
scaffold451	1195713	C	T	NA	*OreG0020669*
scaffold451	1195751	A	T	NA	*OreG0020669*
scaffold451	1195761	G	A	NA	*OreG0020669*
scaffold451	1217673	C	A	Ore01,Ore02,Ore03,Ore04,Ore05,Ore06,Ore07,Ore08,Ore09,Ore10,Ore11,Ore12,Ore13,Ore14	*OreG0020671*
scaffold451	1217936	C	A	NA	*OreG0020671*
scaffold451	1218205	G	A	NA	*OreG0020671*
scaffold451	1237807	G	A	Ore01,Ore02,Ore03,Ore04,Ore05,Ore06,Ore07,Ore08,Ore09,Ore10,Ore11,Ore12,Ore13,Ore14	*OreG0020673*
scaffold451	1237830	A	G	NA	*OreG0020673*
scaffold451	1237953	G	A	NA	*OreG0020673*
scaffold451	1238136	C	T	Och01,Och02,Och03,Och04,Och05,Och06,Och07,Och08,Och09,Och10,Och11,Och12,Och13,Och14,Ore01,Ore02,Ore03,Ore04,Ore05,Ore06,Ore07,Ore08,Ore09,Ore10,Ore11,Ore12,Ore13,Ore14	*OreG0020673*
scaffold451	1238580	T	G	Och01,Och02,Och03,Och04,Och05,Och06,Och07,Och08,Och09,Och10,Och11,Och12,Och13,Och14,Ore01,Ore02,Ore03,Ore04,Ore05,Ore06,Ore07,Ore08,Ore09,Ore10,Ore11,Ore12,Ore13,Ore14	*OreG0020674*

scaffold451	1238725	C	T	Och01,Och02,Och03,Och04,Och05,Och06,Och07,Och08,Och09,Och10,Och11,Och12,Och13,Och14,Ore01,Ore02,Ore03,Ore04,Ore05,Ore06,Ore07,Ore08,Ore09,Ore10,Ore11,Ore12,Ore13,Ore14	*OreG0020674*
scaffold451	1238785	A	G	NA	*OreG0020674*
scaffold451	1238872	G	T	NA	*OreG0020674*
scaffold451	1238945	A	T	Och01,Och02,Och03,Och04,Och05,Och06,Och07,Och08,Och09,Och10,Och11,Och12,Och13,Och14,Ore01,Ore02,Ore03,Ore04,Ore05,Ore06,Ore07,Ore08,Ore09,Ore10,Ore11,Ore12,Ore13,Ore14	*OreG0020674*
scaffold451	1239117	C	T	Ore01,Ore02,Ore03,Ore04,Ore05,Ore06,Ore07,Ore08,Ore09,Ore10,Ore11,Ore12,Ore13,Ore14	*OreG0020674*
scaffold451	1239127	T	C	Och01,Och02,Och03,Och04,Och05,Och06,Och07,Och08,Och09,Och10,Och11,Och12,Och14	*OreG0020674*
scaffold451	1239142	T	A	Ore01,Ore02,Ore03,Ore04,Ore05,Ore06,Ore07,Ore08,Ore09,Ore10,Ore11,Ore12,Ore13,Ore14	*OreG0020674*
scaffold451	1239145	C	T	Ore01,Ore02,Ore03,Ore04,Ore05,Ore06,Ore07,Ore08,Ore09,Ore10,Ore11,Ore12,Ore13,Ore14	*OreG0020674*
scaffold451	1239552	A	T	Ore01,Ore02,Ore03,Ore04,Ore05,Ore06,Ore07,Ore08,Ore09,Ore10,Ore11,Ore12,Ore13,Ore14	*OreG0020674*
scaffold451	1239555	A	C	NA	*OreG0020674*
scaffold451	1239606	T	C	Ore01,Ore02,Ore03,Ore04,Ore05,Ore06,Ore07,Ore08,Ore09,Ore10,Ore11,Ore12,Ore13,Ore14	*OreG0020674*
scaffold451	1239651	G	A	Ore01,Ore02,Ore03,Ore04,Ore05,Ore06,Ore07,Ore08,Ore09,Ore10,Ore11,Ore12,Ore13,Ore14	*OreG0020674*
scaffold451	1239669	A	G	Ore01,Ore02,Ore03,Ore04,Ore05,Ore06,Ore07,Ore08,Ore09,Ore10,Ore11,Ore12,Ore13,Ore14	*OreG0020674*
scaffold451	1242034	G	A	Ore01,Ore02,Ore03,Ore04,Ore05,Ore06,Ore07,Ore08,Ore09,Ore10,Ore11,Ore12,Ore13,Ore14	*OreG0020675*
scaffold451	1242343	G	A	Ore01,Ore02,Ore03,Ore04,Ore05,Ore06,Ore07,Ore08,Ore09,Ore10,Ore11,Ore12,Ore13,Ore14	*OreG0020675*
scaffold451	1243862	G	T	Ore01,Ore02,Ore03,Ore04,Ore05,Ore06,Ore07,Ore08,Ore09,Ore10,Ore11,Ore12,Ore13,Ore14	*OreG0020675*
scaffold451	1245754	A	G	NA	*OreG0020676*
scaffold451	1246286	A	C	NA	*OreG0020676*
scaffold451	1246844	C	A	NA	*OreG0020677*
scaffold451	1246865	A	G	NA	*OreG0020677*
scaffold451	1249252	A	C	Och03	*OreG0020678*
scaffold451	1249282	C	T	NA	*OreG0020678*
scaffold451	1249315	C	T	Och02,Och06	*OreG0020678*
scaffold451	1255097	A	G	Ore01,Ore02,Ore03,Ore04,Ore05,Ore06,Ore07,Ore08,Ore09,Ore10,Ore11,Ore12,Ore13,Ore14	*OreG0020681*
scaffold451	1255133	T	A	Ore01,Ore02,Ore03,Ore04,Ore05,Ore06,Ore07,Ore08,Ore09,Ore10,Ore11,Ore12,Ore13,Ore14	*OreG0020681*
scaffold451	1255294	T	G	Ore01,Ore02,Ore03,Ore04,Ore05,Ore06,Ore07,Ore08,Ore09,Ore10,Ore11,Ore12,Ore13,Ore14	*OreG0020681*
scaffold451	1255717	G	A	Och01,Och02,Och03,Och04,Och05,Och06,Och07,Och08,Och11,Och12,Och13,Och14	*OreG0020681*
scaffold451	1256089	A	G	NA	*OreG0020681*
scaffold451	1256101	C	T	Och01,Och02,Och03,Och04,Och05,Och06,Och07,Och08,Och11,Och12,Och13,Och14	*OreG0020681*
scaffold451	1260272	A	T	NA	*OreG0020682*
scaffold451	1262874	T	A	Och01,Och02,Och03,Och04,Och05,Och06,Och07,Och08,Och11,Och12,Och13,Och14	*OreG0020683*
scaffold451	1262928	G	T	Och01,Och02,Och03,Och04,Och05,Och06,Och07,Och08,Och11,Och12,Och13,Och14	*OreG0020683*
scaffold451	1262992	T	A	Och01,Och02,Och03,Och04,Och05,Och06,Och07,Och08,Och11,Och12,Och13,Och14	*OreG0020683*
scaffold451	1264053	G	A	NA	*OreG0020683*
scaffold451	1264132	C	A	NA	*OreG0020683*
scaffold451	1268536	C	G	Ore01,Ore02,Ore03,Ore04,Ore05,Ore06,Ore07,Ore08,Ore09,Ore10,Ore11,Ore12,Ore13,Ore14	*OreG0020684*
scaffold451	1268987	A	T	NA	*OreG0020684*
scaffold451	1269006	G	C	NA	*OreG0020684*
scaffold451	1269068	T	C	Och02,Och03,Och04,Och05,Och06,Och12,Och13	*OreG0020684*
scaffold451	1282967	T	A	NA	*OreG0020686*
scaffold451	1284801	G	T	Och11	*OreG0020687*
scaffold451	1285108	A	G	Och02,Och03,Och04,Och05,Och06,Och07,Och12,Och13	*OreG0020687*
scaffold451	1290963	A	G	Och02,Och03,Och04,Och05,Och06,Och07,Och12,Och13	*OreG0020688*
scaffold451	1307595	G	A	NA	*OreG0020690*
scaffold451	1307988	G	A	Och01,Och02,Och03,Och04,Och05,Och06,Och07,Och08,Och09,Och10,Och11,Och12,Och13,Och14,Ore01,Ore02,Ore03,Ore04,Ore05,Ore06,Ore07,Ore08,Ore09,Ore10,Ore11,Ore12,Ore13,Ore14	*OreG0020690*
scaffold451	1326331	C	T	NA	*OreG0020691*
scaffold451	1334498	T	C	Ore01,Ore02,Ore03,Ore04,Ore05,Ore06,Ore07,Ore08,Ore09,Ore10,Ore11,Ore12,Ore13,Ore14	*OreG0020691*
scaffold451	1334666	C	T	NA	*OreG0020691*

scaffold451	1338282	C	A	Och01,Och02,Och03,Och04,Och05,Och06,Och07,Och08,Och09,Och10,Och11,Och12,Och13,Och14,Ore01,Ore02,Ore03,Ore04,Ore05,Ore06,Ore07,Ore08,Ore09,Ore10,Ore11,Ore12,Ore13,Ore14	*OreG0020692*
scaffold451	1338953	G	A	Och01,Och02,Och03,Och04,Och05,Och06,Och07,Och08,Och09,Och10,Och11,Och12,Och13,Och14,Ore01,Ore02,Ore03,Ore04,Ore05,Ore06,Ore07,Ore08,Ore09,Ore10,Ore11,Ore12,Ore13,Ore14	*OreG0020692*
scaffold451	1350094	G	A	Ore01,Ore02,Ore03,Ore04,Ore05,Ore06,Ore07,Ore08,Ore09,Ore10,Ore11,Ore12,Ore13,Ore14	*OreG0020693*
scaffold451	1350132	A	C	Ore01,Ore02,Ore03,Ore04,Ore05,Ore06,Ore07,Ore08,Ore09,Ore10,Ore11,Ore12,Ore13,Ore14	*OreG0020693*
scaffold451	1355123	C	T	Och01,Och02,Och03,Och04,Och05,Och06,Och07,Och08,Och09,Och10,Och11,Och12,Och13,Och14,Ore01,Ore02,Ore03,Ore04,Ore05,Ore06,Ore07,Ore08,Ore09,Ore10,Ore11,Ore12,Ore13,Ore14	*OreG0020695*
scaffold451	1355714	C	A	Ore06,Ore09	*OreG0020695*
scaffold451	1355991	A	G	Ore01,Ore02,Ore03,Ore04,Ore05,Ore06,Ore07,Ore08,Ore09,Ore10,Ore11,Ore12,Ore13,Ore14	*OreG0020695*
scaffold451	1381108	A	T	NA	*OreG0020698*
scaffold451	1381335	C	T	NA	*OreG0020698*
scaffold451	1399566	T	G	Och01,Och02,Och03,Och04,Och05,Och06,Och07,Och08,Och09,Och10,Och11,Och12,Och13,Och14	*OreG0020699*
scaffold451	1401511	T	G	NA	*OreG0020699*
scaffold451	1404506	G	A	NA	*OreG0020699*
scaffold451	1421443	G	A	NA	*OreG0020701*
scaffold451	1440257	C	T	NA	*OreG0020703*
scaffold451	1442394	A	T	NA	*OreG0020703*
scaffold451	1513170	G	A	Ore01,Ore02,Ore03,Ore04,Ore05,Ore06,Ore07,Ore08,Ore09,Ore10,Ore11,Ore12,Ore13,Ore14	*OreG0020706*
scaffold451	1638599	G	A	NA	*OreG0020718*
scaffold451	1644575	G	T	Ore01,Ore02,Ore07,Ore14	*OreG0020719*
scaffold451	1645911	T	A	Och02,Och03,Och04,Och05,Och06,Och07,Och08,Och09,Och10,Och11,Och12,Och13	*OreG0020719*
scaffold451	1649535	T	A	Och02,Och03,Och04,Och05,Och06,Och07,Och08,Och09,Och10,Och11,Och12,Och13	*OreG0020720*
scaffold451	1649633	T	A	Och01,Ore01,Ore02,Ore03,Ore04,Ore05,Ore06,Ore07,Ore08,Ore09,Ore10,Ore11,Ore12,Ore13,Ore14	*OreG0020720*
scaffold451	1649876	T	A	Ore01,Ore02,Ore03,Ore04,Ore05,Ore06,Ore07,Ore08,Ore09,Ore10,Ore11,Ore12,Ore13,Ore14	*OreG0020720*
scaffold451	1670995	G	T	NA	*OreG0020722*
scaffold451	1689237	A	T	NA	*OreG0020723*
scaffold451	1711373	A	T	Och01,Och02,Och03,Och04,Och05,Och06,Och07,Och08,Och09,Och10,Och11,Och12,Och13,Och14,Ore01,Ore02,Ore03,Ore04,Ore05,Ore06,Ore07,Ore08,Ore09,Ore10,Ore11,Ore12,Ore13,Ore14	*OreG0020725*
scaffold451	1743805	C	T	NA	*OreG0020727*
scaffold451	1743901	A	G	NA	*OreG0020727*
scaffold451	1753419	T	C	Ore03,Ore07,Ore08,Ore10,Ore11,Ore12,Ore13,Ore14	*OreG0020729*
scaffold451	1783423	A	T	Ore01,Ore02,Ore03,Ore04,Ore05,Ore06,Ore07,Ore08,Ore09,Ore10,Ore11,Ore12,Ore13,Ore14	*OreG0020731*
scaffold451	1799947	T	A	NA	*OreG0020733*
scaffold451	1818813	C	T	Och01,Och02,Och03,Och04,Och05,Och06,Och07,Och12,Och13,Och14	*OreG0020736*
scaffold451	1824433	G	A	Och02,Och03,Och04,Och05,Och06,Och12,Och13	*OreG0020736*
scaffold451	1843271	A	G	Och02,Och06	*OreG0020738*
scaffold451	1843310	T	A	NA	*OreG0020738*
scaffold451	1866034	A	G	Och09,Och10	*OreG0020741*
scaffold451	1866176	C	T	Och01	*OreG0020741*
scaffold451	1903319	C	A	NA	*OreG0020742*
scaffold451	1926712	A	T	Och01	*OreG0020746*
scaffold451	1926757	G	A	Och07,Och08	*OreG0020746*
scaffold451	1926781	T	A	Och01	*OreG0020746*
scaffold451	1986474	C	T	Ore01,Ore02,Ore03,Ore04,Ore05,Ore06,Ore07,Ore08,Ore09,Ore10,Ore11,Ore12,Ore13,Ore14	*OreG0020753*
scaffold451	2003546	A	T	Och01,Och02,Och03,Och04,Och05,Och06,Och07,Och08,Och09,Och10,Och11,Och12,Och13,Och14,Ore01,Ore02,Ore03,Ore04,Ore05,Ore06,Ore07,Ore08,Ore09,Ore10,Ore11,Ore12,Ore13,Ore14	*OreG0020756*
scaffold451	2030510	C	A	NA	*OreG0020759*
scaffold451	2032099	G	A	NA	*OreG0020759*
scaffold451	2032117	G	A	NA	*OreG0020759*
scaffold451	2032209	G	T	NA	*OreG0020759*
scaffold451	2039226	A	G	Och07	*OreG0020761*
scaffold451	2053186	T	C	Och02,Och03,Och04,Och05,Och06,Och12,Och13	*OreG0020763*
scaffold451	2104751	G	A	Ore06,Ore09	*OreG0020763*
scaffold451	2120988	G	A	Och02,Och03,Och04,Och05,Och06,Och07,Och08,Och12,Och13,Ore07,Ore14	*OreG0020764*
scaffold451	2121305	C	T	NA	*OreG0020764*
scaffold451	2122720	G	C	Och10	*OreG0020764*
scaffold451	2122974	G	T	Och09,Och10	*OreG0020764*
scaffold451	2123063	C	A	NA	*OreG0020764*

scaffold451	2123176	C	A	Ore07,Ore14	*OreG0020764*
scaffold451	2123607	A	G	NA	*OreG0020764*
scaffold451	2131360	T	C	NA	*OreG0020766*
scaffold451	2131578	T	A	NA	*OreG0020766*
scaffold451	2141894	G	C	Ore01,Ore02,Ore03,Ore06,Ore07,Ore08,Ore09,Ore10,Ore11,Ore12,Ore13,Ore14	*OreG0020767*
scaffold451	2152386	C	T	NA	*OreG0020769*
scaffold451	2160128	C	T	Och02,Och06	*OreG0020771*
scaffold451	2162792	C	A	NA	*OreG0020772*
scaffold451	2163436	C	T	NA	*OreG0020772*
scaffold451	2167560	G	A	Och01,Och02,Och03,Och04,Och05,Och06,Och07,Och08,Och09,Och10,Och11,Och12,Och13,Och14	*OreG0020773*
scaffold451	2167606	C	A	NA	*OreG0020773*
scaffold451	2335947	C	G	NA	*OreG0020778*
scaffold451	2342052	G	A	NA	*OreG0020780*
scaffold451	2400110	A	G	Och01,Och02,Och03,Och04,Och05,Och06,Och07,Och08,Och09,Och10,Och11,Och12,Och13,Och14,Ore01,Ore02,Ore03,Ore04,Ore05,Ore06,Ore07,Ore08,Ore09,Ore10,Ore11,Ore12,Ore13,Ore14	*OreG0020783*
scaffold451	2404401	A	G	NA	*OreG0020783*
scaffold451	2454437	A	T	Och01,Och02,Och03,Och04,Och05,Och06,Och07,Och08,Och09,Och10,Och11,Och12,Och13,Och14,Ore01,Ore02,Ore03,Ore04,Ore05,Ore06,Ore07,Ore08,Ore09,Ore10,Ore11,Ore12,Ore13,Ore14	*OreG0020791*
scaffold451	2457362	C	T	NA	*OreG0020791*
scaffold451	2504041	C	T	NA	*OreG0020797*
scaffold451	2589083	C	G	Ore01,Ore02,Ore03,Ore04,Ore05,Ore06,Ore07,Ore08,Ore09,Ore10,Ore11,Ore12,Ore13,Ore14	*OreG0020805*
scaffold451	2589091	A	C	Och14	*OreG0020805*
scaffold451	2590864	C	T	NA	*OreG0020805*
scaffold451	2591496	T	A	Ore01,Ore02,Ore03,Ore04,Ore05,Ore06,Ore07,Ore08,Ore09,Ore10,Ore11,Ore12,Ore13,Ore14	*OreG0020805*
scaffold451	2591585	C	A	Ore01,Ore02,Ore03,Ore04,Ore05,Ore06,Ore07,Ore08,Ore09,Ore10,Ore11,Ore12,Ore13,Ore14	*OreG0020805*
scaffold451	2591593	A	G	Ore01,Ore02,Ore03,Ore04,Ore05,Ore06,Ore07,Ore08,Ore09,Ore10,Ore11,Ore12,Ore13,Ore14	*OreG0020805*
scaffold451	2591614	A	C	Och01,Och02,Och03,Och04,Och05,Och06,Och07,Och09,Och10,Och11,Och12,Och13,Och14	*OreG0020805*
scaffold451	2620129	T	A	Och01,Och02,Och03,Och04,Och05,Och06,Och07,Och08,Och09,Och10,Och11,Och12,Och13,Och14,Ore01,Ore02,Ore03,Ore04,Ore05,Ore06,Ore07,Ore08,Ore09,Ore10,Ore11,Ore12,Ore13,Ore14	*OreG0020808*
scaffold451	2620275	G	A	Och02,Och06	*OreG0020808*
scaffold451	2640517	G	T	NA	*OreG0020809*
scaffold451	2643068	T	C	Och01,Och02,Och03,Och04,Och05,Och06,Och07,Och08,Och09,Och10,Och11,Och12,Och13,Och14	*OreG0020809*
scaffold451	2694514	A	T	Och01,Och02,Och03,Och04,Och05,Och06,Och07,Och08,Och09,Och10,Och11,Och12,Och13,Och14	*OreG0020814*
scaffold451	2695051	G	T	Och01,Och02,Och03,Och04,Och05,Och06,Och07,Och08,Och09,Och10,Och11,Och12,Och13,Och14,Ore01,Ore02,Ore03,Ore04,Ore05,Ore06,Ore07,Ore08,Ore09,Ore10,Ore11,Ore12,Ore13,Ore14	*OreG0020814*
scaffold451	2701900	G	A	Ore01,Ore02,Ore03,Ore06,Ore07,Ore08,Ore09,Ore10,Ore11,Ore12,Ore13,Ore14	*OreG0020812*
scaffold451	2707781	C	T	Och01,Och02,Och03,Och04,Och05,Och06,Och07,Och08,Och09,Och10,Och11,Och12,Och13,Och14	*OreG0020815*
scaffold451	2717246	A	T	Ore01,Ore02,Ore03,Ore04,Ore05,Ore06,Ore07,Ore08,Ore09,Ore10,Ore11,Ore12,Ore13,Ore14	*OreG0020817*
scaffold451	2718139	A	G	Och01	*OreG0020817*
scaffold451	2739231	G	T	NA	*OreG0020820*
scaffold451	2739771	T	A	NA	*OreG0020820*
scaffold451	2749916	A	G	NA	*OreG0020822*
scaffold451	2792978	C	A	NA	*OreG0020827*
scaffold451	2795143	A	T	NA	*OreG0020828*
scaffold451	2795283	T	A	Ore01,Ore02,Ore03,Ore06,Ore07,Ore08,Ore09,Ore10,Ore11,Ore12,Ore13,Ore14	*OreG0020828*
scaffold451	2795903	C	G	Ore01,Ore02,Ore03,Ore04,Ore05,Ore06,Ore07,Ore08,Ore09,Ore10,Ore11,Ore12,Ore13,Ore14	*OreG0020828*
scaffold451	2807472	C	A	NA	*OreG0020830*
scaffold451	2807485	C	A	NA	*OreG0020830*
scaffold451	2807503	G	A	NA	*OreG0020830*
scaffold451	2807737	G	T	NA	*OreG0020830*
scaffold451	2807805	C	T	NA	*OreG0020830*
scaffold451	2807814	T	C	NA	*OreG0020830*
scaffold451	2874392	T	A	Ore01,Ore02,Ore03,Ore04,Ore05,Ore06,Ore07,Ore08,Ore09,Ore10,Ore11,Ore12,Ore13,Ore14	*OreG0020833*
scaffold451	2874695	G	T	Ore01,Ore02,Ore03,Ore04,Ore05,Ore06,Ore07,Ore08,Ore09,Ore10,Ore11,Ore12,Ore13,Ore14	*OreG0020833*
scaffold451	2883285	C	A	NA	*OreG0020835*
scaffold451	2883513	T	G	NA	*OreG0020835*
scaffold451	2883659	A	C	Och01,Och02,Och03,Och04,Och05,Och06,Och07,Och08,Och09,Och10,Och11,Och12,Och13,Och14	*OreG0020835*

scaffold451	2883702	C	T	NA	*OreG0020835*
scaffold451	2900653	C	A	NA	*OreG0020838*
scaffold451	2911359	T	A	Och01,Och02,Och03,Och04,Och05,Och06,Och07,Och08,Och09,Och10,Och11,Och12,Och13,Och14	*OreG0020842*
scaffold451	2911514	C	T	Och01,Och02,Och03,Och04,Och05,Och06,Och07,Och08,Och09,Och10,Och11,Och12,Och13,Och14,Ore01,Ore02,Ore03,Ore04,Ore05,Ore06,Ore07,Ore08,Ore09,Ore10,Ore11,Ore12,Ore13,Ore14	*OreG0020842*
scaffold451	2940593	A	G	Och01,Och02,Och03,Och04,Och05,Och06,Och07,Och08,Och09,Och10,Och11,Och12,Och13,Och14	*OreG0020845*
scaffold451	2989541	C	G	NA	*OreG0020850*
scaffold451	3035557	A	C	Och01,Och02,Och03,Och04,Och05,Och06,Och07,Och08,Och09,Och10,Och11,Och12,Och13,Och14	*OreG0020857*
scaffold451	3038355	T	C	Och01	*OreG0020858*
scaffold451	3038370	A	G	Och01,Och02,Och03,Och04,Och05,Och06,Och07,Och08,Och09,Och10,Och11,Och12,Och13,Och14,Ore01,Ore02,Ore03,Ore04,Ore05,Ore06,Ore07,Ore08,Ore09,Ore10,Ore11,Ore12,Ore13,Ore14	*OreG0020858*
scaffold451	3042745	G	T	Ore01,Ore02,Ore03,Ore04,Ore05,Ore06,Ore07,Ore08,Ore09,Ore10,Ore11,Ore12,Ore13,Ore14	*OreG0020859*
scaffold451	3097192	T	G	NA	*OreG0020865*
scaffold451	3119920	A	T	Och01,Och02,Och03,Och04,Och05,Och06,Och07,Och08,Och09,Och10,Och11,Och12,Och13,Och14	*OreG0020867*
scaffold451	3130864	A	G	NA	*OreG0020868*
scaffold451	3137650	G	A	Ore01,Ore02,Ore03,Ore04,Ore05,Ore06,Ore07,Ore08,Ore09,Ore10,Ore11,Ore12,Ore13,Ore14	*OreG0020870*
scaffold451	3150696	T	C	Och01,Och02,Och03,Och04,Och05,Och06,Och07,Och08,Och09,Och10,Och11,Och12,Och13,Och14,Ore01,Ore02,Ore03,Ore04,Ore05,Ore06,Ore07,Ore08,Ore09,Ore10,Ore11,Ore12,Ore13,Ore14	*OreG0020875*
scaffold451	3152204	T	C	Och01,Och02,Och03,Och04,Och05,Och06,Och07,Och08,Och09,Och10,Och11,Och12,Och13,Och14	*OreG0020875*
scaffold451	3161259	A	T	Och01,Och02,Och03,Och04,Och05,Och06,Och07,Och08,Och09,Och10,Och11,Och12,Och13,Och14,Ore01,Ore02,Ore03,Ore04,Ore05,Ore06,Ore07,Ore08,Ore09,Ore10,Ore11,Ore12,Ore13,Ore14	*OreG0020876*
scaffold451	3185678	G	T	Och02,Och06,Och09,Och10,Och11	*OreG0020879*
scaffold451	3207154	G	C	Ore07,Ore14	*OreG0020882*
scaffold451	3208355	T	G	Och08,Och14	*OreG0020883*
scaffold451	3223578	T	G	NA	*OreG0020885*
scaffold451	3224061	T	A	Och02,Och03,Och04,Och05,Och06,Och07,Och11,Och12,Och13,Och14	*OreG0020885*
scaffold451	3224231	A	G	NA	*OreG0020885*
scaffold451	3250576	C	A	Och01,Och02,Och06,Och07,Och08,Och09,Och10,Och11,Och14	*OreG0020887*
scaffold451	3250906	G	A	NA	*OreG0020887*
scaffold451	3258707	T	C	Och02,Och03,Och04,Och05,Och06,Och07,Och09,Och10,Och11,Och12,Och13,Och14	*OreG0020888*
scaffold451	3290837	G	A	Ore02,Ore04,Ore05,Ore08,Ore09,Ore10,Ore11,Ore12,Ore13	*OreG0020893*
scaffold451	3330740	G	C	NA	*OreG0020898*
scaffold451	3331513	T	C	NA	*OreG0020898*
scaffold451	3331528	T	A	NA	*OreG0020898*
scaffold451	3332618	T	G	Ore01,Ore02,Ore03,Ore04,Ore05,Ore06,Ore07,Ore08,Ore09,Ore10,Ore11,Ore12,Ore13,Ore14	*OreG0020899*
scaffold451	3333054	G	A	NA	*OreG0020899*
scaffold451	3367095	G	A	Och01,Och02,Och03,Och04,Och05,Och06,Och07,Och08,Och09,Och10,Och11,Och12,Och13,Och14	*OreG0020902*
scaffold451	3377413	G	A	Och02,Och06	*OreG0020903*
scaffold451	3377445	A	G	NA	*OreG0020903*
scaffold451	3377495	C	A	Ore01,Ore02,Ore03,Ore04,Ore05,Ore06,Ore07,Ore08,Ore09,Ore10,Ore11,Ore12,Ore13,Ore14	*OreG0020903*
scaffold451	3377934	C	A	NA	*OreG0020903*
scaffold451	3377985	A	G	Ore01,Ore02,Ore03,Ore04,Ore05,Ore06,Ore07,Ore08,Ore09,Ore10,Ore11,Ore12,Ore13,Ore14	*OreG0020903*
scaffold451	3378626	C	G	Och02,Och06,Och07,Och09,Och10,Och11,Och14	*OreG0020903*
scaffold451	3378704	A	T	NA	*OreG0020903*
scaffold451	3411553	C	T	Ore01,Ore02,Ore03,Ore04,Ore05,Ore06,Ore07,Ore08,Ore09,Ore10,Ore11,Ore12,Ore13,Ore14	*OreG0020905*
scaffold451	3418117	A	G	NA	*OreG0020906*
scaffold451	3418650	C	T	Ore01,Ore02,Ore03,Ore04,Ore05,Ore06,Ore07,Ore08,Ore09,Ore10,Ore11,Ore12,Ore13,Ore14	*OreG0020906*
scaffold451	3491609	C	A	NA	*OreG0020912*
scaffold451	3491630	C	G	NA	*OreG0020912*
scaffold451	3491929	G	C	NA	*OreG0020912*
scaffold451	3496626	G	A	Och01,Och02,Och03,Och04,Och05,Och06,Och07,Och08,Och09,Och10,Och11,Och12,Och13,Och14,Ore01,Ore02,Ore03,Ore04,Ore05,Ore06,Ore07,Ore08,Ore09,Ore10,Ore11,Ore12,Ore13,Ore14	*OreG0020913*
scaffold451	3510046	T	A	NA	*OreG0020914*
scaffold451	3510055	G	A	NA	*OreG0020914*
scaffold451	3510066	C	G	NA	*OreG0020914*
scaffold451	3540902	T	A	NA	*OreG0020917*

scaffold451	3541217	C	T	Och14	*OreG0020917*
scaffold451	3558812	G	C	Ore01,Ore02,Ore03,Ore04,Ore05,Ore06,Ore07,Ore08,Ore09,Ore10,Ore11,Ore12,Ore13,Ore14	*OreG0020918*
scaffold48	19503	A	G	Och02,Och05	*OreG0022810*
scaffold48	31685	G	A	Och01,Och02,Och03,Och04,Och05,Och06,Och07,Och08,Och09,Och10,Och11,Och12,Och13,Och14,Ore01,Ore02,Ore03,Ore04,Ore05,Ore06,Ore07,Ore08,Ore09,Ore10,Ore11,Ore12,Ore13,Ore14	*OreG0022811*
scaffold48	31992	G	A	Och01,Och02,Och03,Och04,Och05,Och06,Och07,Och08,Och10,Och11,Och12,Och13,Och14	*OreG0022811*
scaffold48	56725	T	C	Och01,Och02,Och03,Och04,Och05,Och06,Och07,Och08,Och09,Och10,Och11,Och12,Och13,Och14,Ore01,Ore02,Ore03,Ore04,Ore05,Ore06,Ore07,Ore08,Ore09,Ore10,Ore11,Ore12,Ore13,Ore14	*OreG0022816*
scaffold48	62757	T	C	NA	*OreG0022817*
scaffold48	63160	A	C	Och01,Och02,Och03,Och04,Och05,Och06,Och07,Och08,Och09,Och10,Och11,Och12,Och13,Och14,Ore01,Ore02,Ore03,Ore04,Ore05,Ore06,Ore07,Ore08,Ore09,Ore10,Ore11,Ore12,Ore13,Ore14	*OreG0022817*
scaffold48	63185	C	A	Och01,Och07,Och08	*OreG0022817*
scaffold48	63441	G	A	NA	*OreG0022817*
scaffold48	63917	T	A	Och03,Och05,Och06,Ore01,Ore02,Ore03,Ore04,Ore05,Ore06,Ore07,Ore08,Ore09,Ore10,Ore11,Ore12,Ore13,Ore14	*OreG0022817*
scaffold48	82484	T	A	Och01,Och02,Och03,Och04,Och05,Och06,Och07,Och08,Och09,Och10,Och11,Och12,Och13,Och14	*OreG0022822*
scaffold48	98324	A	T	Ore01,Ore02,Ore03,Ore04,Ore05,Ore06,Ore07,Ore08,Ore09,Ore10,Ore11,Ore12,Ore13,Ore14	*OreG0022823*
scaffold48	113273	C	A	NA	*OreG0022824*
scaffold48	146407	A	G	NA	*OreG0022825*
scaffold48	151533	C	T	Och02,Och03	*OreG0022826*
scaffold48	153479	A	T	Ore02,Ore06,Ore11,Ore12	*OreG0022827*
scaffold48	176511	A	G	NA	*OreG0022829*
scaffold48	183627	A	T	Och01,Och02,Och03,Och04,Och05,Och06,Och07,Och08,Och09,Och10,Och11,Och12,Och13,Och14,Ore01,Ore02,Ore03,Ore04,Ore05,Ore06,Ore07,Ore08,Ore09,Ore10,Ore11,Ore12,Ore13,Ore14	*OreG0022831*
scaffold48	194800	G	T	Ore01,Ore02,Ore03,Ore04,Ore05,Ore06,Ore07,Ore08,Ore09,Ore10,Ore11,Ore12,Ore13,Ore14	*OreG0022832*
scaffold48	194827	A	G	NA	*OreG0022832*
scaffold48	259549	G	T	NA	*OreG0022837*
scaffold48	376017	G	A	Ore01,Ore02,Ore03,Ore04,Ore05,Ore06,Ore07,Ore08,Ore09,Ore10,Ore11,Ore12,Ore13,Ore14	*OreG0022841*
scaffold48	380178	T	A	Ore02,Ore04,Ore05,Ore06,Ore11,Ore12	*OreG0022843*
scaffold48	403464	A	T	Och02,Och03,Och04,Och05,Och06,Och07,Och08,Och09,Och10,Och11,Och12,Och13,Och14	*OreG0022844*
scaffold48	480930	C	T	Ore02,Ore04,Ore05,Ore06,Ore11,Ore12	*OreG0022845*
scaffold48	480939	C	T	Ore02,Ore04,Ore05,Ore06,Ore11,Ore12	*OreG0022845*
scaffold48	480941	T	G	Och01,Och02,Och03,Och04,Och05,Och06,Och07,Och08,Och09,Och10,Och11,Och12,Och13,Och14,Ore01,Ore02,Ore03,Ore04,Ore05,Ore06,Ore07,Ore08,Ore09,Ore10,Ore11,Ore12,Ore13,Ore14	*OreG0022845*
scaffold48	484860	T	C	Och01,Och05,Och12	*OreG0022845*
scaffold48	484870	C	T	Ore03	*OreG0022845*
scaffold48	491055	T	G	NA	*OreG0022848*
scaffold48	513902	T	C	Och02,Och03,Och07,Och08	*OreG0022851*
scaffold48	514442	C	A	NA	*OreG0022851*
scaffold48	514520	T	C	Ore02,Ore04,Ore05,Ore06,Ore11,Ore12	*OreG0022851*
scaffold48	546322	G	T	Ore02,Ore06,Ore11,Ore12	*OreG0022854*
scaffold48	608398	A	T	Och07,Och08,Och09,Och10	*OreG0022859*
scaffold48	640792	T	C	Och01,Och02,Och03,Och04,Och05,Och06,Och09,Och10,Och11,Och12,Och13,Ore01,Ore02,Ore03,Ore04,Ore05,Ore06,Ore07,Ore08,Ore09,Ore10,Ore11,Ore12,Ore13,Ore14	*OreG0022863*
scaffold48	649494	C	T	NA	*OreG0022864*
scaffold48	649758	A	G	NA	*OreG0022864*
scaffold48	650615	G	T	NA	*OreG0022864*
scaffold48	650999	C	G	NA	*OreG0022864*
scaffold48	674788	T	C	NA	*OreG0022865*
scaffold48	674848	T	C	NA	*OreG0022865*
scaffold48	674864	A	T	NA	*OreG0022865*
scaffold48	674946	T	C	NA	*OreG0022865*
scaffold48	674979	C	T	NA	*OreG0022865*
scaffold48	674985	G	A	Ore02,Ore06,Ore11,Ore12	*OreG0022865*
scaffold48	675037	C	A	NA	*OreG0022865*
scaffold48	681886	A	G	NA	*OreG0022867*
scaffold48	700847	C	T	NA	*OreG0022870*
scaffold48	725541	G	C	NA	*OreG0022873*
scaffold48	725586	T	A	NA	*OreG0022873*
scaffold48	725688	G	A	NA	*OreG0022873*
scaffold48	726480	T	A	Ore02,Ore04,Ore05,Ore06,Ore11,Ore12	*OreG0022873*
scaffold48	726964	A	C	Ore02,Ore04,Ore05,Ore06,Ore11,Ore12	*OreG0022873*

scaffold48	735124	A	G	NA	*OreG0022874*
scaffold48	750368	G	T	NA	*OreG0022876*
scaffold48	750373	G	C	Och08,Ore01,Ore02,Ore03,Ore04,Ore05,Ore06,Ore07,Ore08,Ore09,Ore10,Ore11,Ore12,Ore13,Ore14	*OreG0022876*
scaffold48	751017	G	C	Ore02,Ore04,Ore05,Ore06,Ore11,Ore12	*OreG0022876*
scaffold48	751052	T	C	Ore02,Ore04,Ore05,Ore06,Ore11,Ore12	*OreG0022876*
scaffold48	751561	G	C	NA	*OreG0022877*
scaffold48	751577	A	C	NA	*OreG0022877*
scaffold48	751817	C	T	NA	*OreG0022878*
scaffold48	752012	A	G	NA	*OreG0022878*
scaffold48	752249	G	T	Och01,Och02,Och03,Och04,Och05,Och06,Och07,Och08,Och09,Och10,Och11,Och12,Och13,Och14,Ore01,Ore02,Ore03,Ore04,Ore05,Ore06,Ore07,Ore08,Ore09,Ore10,Ore11,Ore12,Ore13,Ore14	*OreG0022878*
scaffold48	752269	G	T	Och01,Och02,Och03,Och04,Och05,Och06,Och07,Och08,Och11,Och12,Och13,Ore01,Ore02,Ore03,Ore04,Ore05,Ore06,Ore07,Ore08,Ore09,Ore10,Ore11,Ore12,Ore13,Ore14	*OreG0022878*
scaffold48	752542	G	C	NA	*OreG0022878*
scaffold48	752689	G	T	Och01,Och02,Och03,Och04,Och05,Och06,Och07,Och08,Och11,Och12,Och13,Ore01,Ore02,Ore03,Ore04,Ore05,Ore06,Ore07,Ore08,Ore09,Ore10,Ore11,Ore12,Ore13,Ore14	*OreG0022878*
scaffold48	752980	T	A	NA	*OreG0022878*
scaffold48	753066	G	A	Ore03	*OreG0022878*
scaffold48	753103	T	C	Ore02,Ore04,Ore05,Ore06,Ore11,Ore12	*OreG0022878*
scaffold48	753207	T	G	NA	*OreG0022878*
scaffold48	753214	T	A	Och01,Och02,Och03,Och04,Och05,Och06,Och07,Och08,Och11,Och12,Och13,Ore01,Ore02,Ore03,Ore04,Ore05,Ore06,Ore07,Ore08,Ore09,Ore10,Ore11,Ore12,Ore13,Ore14	*OreG0022878*
scaffold48	753219	C	G	Och01,Och02,Och03,Och04,Och05,Och06,Och07,Och08,Och11,Och12,Och13,Ore01,Ore02,Ore03,Ore04,Ore05,Ore06,Ore07,Ore08,Ore09,Ore10,Ore11,Ore12,Ore13,Ore14	*OreG0022878*
scaffold48	753247	A	C	Och01,Och02,Och03,Och04,Och05,Och06,Och07,Och08,Och11,Och12,Och13,Ore01,Ore02,Ore03,Ore04,Ore05,Ore06,Ore07,Ore08,Ore09,Ore10,Ore11,Ore12,Ore13,Ore14	*OreG0022878*
scaffold48	753398	C	A	NA	*OreG0022878*
scaffold48	753426	T	C	NA	*OreG0022878*
scaffold48	753651	G	A	NA	*OreG0022878*
scaffold48	763015	G	A	Och01,Och02,Och03,Och04,Och05,Och06,Och07,Och08,Och09,Och10,Och11,Och12,Och13,Och14,Ore01,Ore02,Ore03,Ore04,Ore05,Ore06,Ore07,Ore08,Ore09,Ore10,Ore11,Ore12,Ore13,Ore14	*OreG0022881*
scaffold48	763141	A	G	Och01,Och02,Och03,Och04,Och05,Och06,Och07,Och08,Och09,Och10,Och11,Och12,Och13,Och14,Ore01,Ore02,Ore03,Ore04,Ore05,Ore06,Ore07,Ore08,Ore09,Ore10,Ore11,Ore12,Ore13,Ore14	*OreG0022881*
scaffold48	763293	G	A	Och01,Och04,Och05,Och06,Och07,Och08,Och09,Och10,Och11,Och12,Och13,Och14	*OreG0022881*
scaffold48	763684	A	G	Och01,Och02,Och03,Och04,Och05,Och06,Och07,Och08,Och09,Och10,Och11,Och12,Och13,Och14,Ore01,Ore02,Ore03,Ore04,Ore05,Ore06,Ore07,Ore08,Ore09,Ore10,Ore11,Ore12,Ore13,Ore14	*OreG0022881*
scaffold48	764237	A	T	NA	*OreG0022881*
scaffold48	764240	A	G	NA	*OreG0022881*
scaffold48	764339	G	T	Ore01,Ore02,Ore03,Ore04,Ore05,Ore06,Ore07,Ore08,Ore09,Ore10,Ore11,Ore12,Ore13,Ore14	*OreG0022881*
scaffold48	767375	T	A	Och02,Och03,Och04,Och05,Och06,Och12,Och13,Och14	*OreG0022882*
scaffold48	767480	T	C	Och01,Och07,Och09,Och10,Ore01,Ore02,Ore03,Ore04,Ore05,Ore06,Ore07,Ore08,Ore09,Ore10,Ore11,Ore12,Ore13,Ore14	*OreG0022882*
scaffold48	767541	G	A	Och02,Och03,Och04,Och05,Och06,Och12,Och13	*OreG0022882*
scaffold48	767796	T	C	Ore02,Ore06,Ore11,Ore12	*OreG0022882*
scaffold48	768249	G	A	Och02,Och03,Och04,Och05,Och06,Och12,Och13,Och14,Ore01,Ore02,Ore03,Ore04,Ore05,Ore06,Ore07,Ore08,Ore09,Ore10,Ore11,Ore12,Ore13,Ore14	*OreG0022883*
scaffold48	768280	T	G	Och01,Och07,Och09,Och10	*OreG0022883*
scaffold48	768281	G	T	Och01,Och07,Och09,Och10	*OreG0022883*
scaffold48	770059	T	C	NA	*OreG0022884*
scaffold48	770108	T	G	Ore01,Ore02,Ore03,Ore04,Ore05,Ore06,Ore07,Ore08,Ore09,Ore10,Ore11,Ore12,Ore13,Ore14	*OreG0022884*
scaffold48	786296	G	C	NA	*OreG0022886*
scaffold48	869258	T	G	Och02,Och03,Och04,Och05,Och06,Och12,Och13	*OreG0022889*
scaffold48	870430	T	C	Ore01,Ore02,Ore03,Ore04,Ore05,Ore06,Ore07,Ore08,Ore09,Ore10,Ore11,Ore12,Ore13,Ore14	*OreG0022889*
scaffold48	900705	A	T	NA	*OreG0022890*
scaffold48	901833	T	C	NA	*OreG0022890*
scaffold48	914744	G	A	Och02,Och03,Och04,Och05,Och06,Och12,Och13	*OreG0022892*
scaffold48	919173	C	G	Ore01,Ore02,Ore03,Ore04,Ore05,Ore06,Ore07,Ore08,Ore09,Ore10,Ore11,Ore12,Ore13,Ore14	*OreG0022892*
scaffold48	919198	A	C	Ore01,Ore02,Ore03,Ore04,Ore05,Ore06,Ore07,Ore08,Ore09,Ore10,Ore11,Ore12,Ore13,Ore14	*OreG0022892*
scaffold48	951414	G	A	NA	*OreG0022893*

scaffold48	959966	C	T	NA	*OreG0022894*
scaffold48	959972	T	G	NA	*OreG0022894*
scaffold48	960014	C	G	NA	*OreG0022894*
scaffold48	981094	G	A	NA	*OreG0022894*
scaffold48	981153	T	C	NA	*OreG0022894*
scaffold48	982164	C	T	NA	*OreG0022894*
scaffold48	989537	T	C	NA	*OreG0022895*
scaffold48	1001415	G	A	Ore01,Ore02,Ore03,Ore04,Ore05,Ore06,Ore07,Ore08,Ore09,Ore10,Ore11,Ore12,Ore13,Ore14	*OreG0022897*
scaffold48	1002460	A	G	Och01,Och02,Och03,Och04,Och05,Och06,Och07,Och08,Och09,Och10,Och11,Och12,Och13,Och14,Ore01,Ore02,Ore03,Ore04,Ore05,Ore06,Ore07,Ore08,Ore09,Ore10,Ore11,Ore12,Ore13,Ore14	*OreG0022897*
scaffold48	1006075	C	T	Och05,Och12	*OreG0022898*
scaffold48	1016076	C	A	Och01	*OreG0022899*
scaffold48	1048582	T	A	Och01,Och02,Och03,Och04,Och05,Och06,Och07,Och08,Och09,Och10,Och11,Och12,Och13,Och14,Ore01,Ore02,Ore03,Ore04,Ore05,Ore06,Ore07,Ore08,Ore09,Ore10,Ore11,Ore12,Ore13,Ore14	*OreG0022904*
scaffold48	1048996	T	C	NA	*OreG0022904*
scaffold48	1049055	A	C	NA	*OreG0022904*
scaffold48	1049086	C	A	NA	*OreG0022904*
scaffold48	1049377	T	A	NA	*OreG0022904*
scaffold48	1101797	C	T	NA	*OreG0022907*
scaffold48	1171362	C	T	Ore01,Ore02,Ore03,Ore04,Ore05,Ore06,Ore07,Ore08,Ore09,Ore10,Ore11,Ore12,Ore13,Ore14	*OreG0022910*
scaffold48	1171584	C	G	Ore01,Ore02,Ore03,Ore04,Ore05,Ore06,Ore07,Ore08,Ore09,Ore10,Ore11,Ore12,Ore13,Ore14	*OreG0022910*
scaffold48	1172065	G	C	Ore01,Ore02,Ore03,Ore04,Ore05,Ore06,Ore07,Ore08,Ore09,Ore10,Ore11,Ore12,Ore13,Ore14	*OreG0022910*
scaffold48	1172088	T	A	Och01,Och02,Och03,Och04,Och05,Och06,Och07,Och08,Och09,Och10,Och11,Och12,Och13,Och14,Ore01,Ore02,Ore03,Ore04,Ore05,Ore06,Ore07,Ore08,Ore09,Ore10,Ore11,Ore12,Ore13,Ore14	*OreG0022910*
scaffold48	1172104	C	T	Ore01,Ore02,Ore03,Ore04,Ore05,Ore06,Ore07,Ore08,Ore09,Ore10,Ore11,Ore12,Ore13,Ore14	*OreG0022910*
scaffold48	1172112	T	C	NA	*OreG0022910*
scaffold48	1172242	C	G	NA	*OreG0022910*
scaffold48	1172440	A	G	NA	*OreG0022910*
scaffold48	1172958	G	A	Och02,Och05,Och07,Och09,Och10,Och11,Och12,Och13	*OreG0022910*
scaffold48	1173313	T	C	NA	*OreG0022910*
scaffold48	1173612	G	A	Och02,Och05,Och07,Och09,Och10,Och11,Och12,Och13	*OreG0022910*
scaffold48	1174181	G	T	NA	*OreG0022910*
scaffold48	1174200	G	A	NA	*OreG0022910*
scaffold48	1174338	C	A	NA	*OreG0022910*
scaffold48	1263632	A	T	Ore01,Ore02,Ore03,Ore04,Ore05,Ore06,Ore07,Ore08,Ore09,Ore10,Ore11,Ore12,Ore13,Ore14	*OreG0022912*
scaffold48	1269527	A	T	NA	*OreG0022912*
scaffold48	1275570	A	T	NA	*OreG0022912*
scaffold48	1306231	C	T	Och01,Och02,Och03,Och04,Och05,Och06,Och07,Och08,Och09,Och10,Och11,Och12,Och13,Och14	*OreG0022913*
scaffold48	1306233	G	A	Och01,Och02,Och03,Och04,Och05,Och06,Och07,Och08,Och09,Och10,Och11,Och12,Och13,Och14	*OreG0022913*
scaffold48	1324640	G	A	NA	*OreG0022914*
scaffold48	1325185	C	G	NA	*OreG0022914*
scaffold48	1325371	G	A	NA	*OreG0022914*
scaffold48	1379246	C	A	Ore01,Ore02,Ore03,Ore04,Ore05,Ore06,Ore07,Ore08,Ore09,Ore10,Ore11,Ore12,Ore13,Ore14	*OreG0022915*
scaffold48	1380081	A	G	NA	*OreG0022916*
scaffold48	1380316	A	C	Och01,Och02,Och03,Och04,Och05,Och06,Och07,Och08,Och09,Och10,Och11,Och12,Och13,Och14,Ore01,Ore02,Ore03,Ore04,Ore05,Ore06,Ore07,Ore08,Ore09,Ore10,Ore11,Ore12,Ore13,Ore14	*OreG0022916*
scaffold48	1381866	G	A	Ore01,Ore02,Ore03,Ore04,Ore05,Ore06,Ore07,Ore08,Ore09,Ore10,Ore11,Ore12,Ore13,Ore14	*OreG0022916*
scaffold48	1694825	G	T	Ore01,Ore02,Ore03,Ore04,Ore05,Ore06,Ore07,Ore08,Ore09,Ore10,Ore11,Ore12,Ore13,Ore14	*OreG0022920*
scaffold48	1696815	A	C	NA	*OreG0022920*
scaffold48	1783610	C	A	NA	*OreG0022922*
scaffold48	1792801	G	A	Och01,Och02,Och03,Och04,Och05,Och06,Och07,Och08,Och09,Och10,Och11,Och12,Och13,Och14	*OreG0022922*
scaffold48	1857921	A	G	NA	*OreG0022924*
scaffold48	1997597	G	T	Och12,Och13	*OreG0022927*
scaffold48	2102031	T	C	Och12	*OreG0022930*
scaffold48	2108146	A	C	Och01,Och02,Och03,Och04,Och05,Och06,Och07,Och08,Och09,Och10,Och11,Och12,Och13,Och14	*OreG0022932*
scaffold48	2109061	C	A	Och01,Och02,Och03,Och04,Och05,Och06,Och07,Och08,Och09,Och10,Och11,Och12,Och13,Och14	*OreG0022933*
scaffold48	2109064	G	A	NA	*OreG0022933*

scaffold48	2109083	C	G	Och02,Och14	*OreG0022933*
scaffold48	2109370	C	T	Ore01,Ore02,Ore03,Ore04,Ore05,Ore06,Ore07,Ore08,Ore09,Ore10,Ore11,Ore12,Ore13,Ore14	*OreG0022933*
scaffold48	2109407	T	G	Och01,Och02,Och03,Och04,Och05,Och06,Och07,Och08,Och09,Och10,Och11,Och12,Och13,Och14	*OreG0022933*
scaffold48	2109894	T	C	Ore01,Ore02,Ore03,Ore04,Ore05,Ore06,Ore07,Ore08,Ore09,Ore10,Ore11,Ore12,Ore13,Ore14	*OreG0022933*
scaffold48	2109939	T	C	Och08,Och12	*OreG0022933*
scaffold48	2109944	C	A	Ore01,Ore02,Ore03,Ore04,Ore05,Ore06,Ore07,Ore08,Ore09,Ore10,Ore11,Ore12,Ore13,Ore14	*OreG0022933*
scaffold48	2109983	T	C	Och01,Och02,Och03,Och04,Och05,Och06,Och07,Och08,Och09,Och10,Och11,Och12,Och13,Och14	*OreG0022933*
scaffold48	2192745	C	T	NA	*OreG0022941*
scaffold48	2280551	C	T	NA	*OreG0022948*
scaffold48	2284715	G	C	NA	*OreG0022949*
scaffold48	2290839	A	T	NA	*OreG0022949*
scaffold48	2299014	C	G	Och07	*OreG0022950*
scaffold48	2299218	C	G	Och07,Och08	*OreG0022950*
scaffold48	2329050	G	T	NA	*OreG0022951*
scaffold48	2329117	C	A	Och01,Och03,Och04,Och05,Och07,Och08,Och11,Och12,Och13	*OreG0022951*
scaffold48	2340775	G	T	Och01,Och02,Och03,Och04,Och05,Och06,Och07,Och08,Och09,Och10,Och11,Och12,Och13,Och14,Ore01,Ore02,Ore03,Ore04,Ore05,Ore06,Ore07,Ore08,Ore09,Ore10,Ore11,Ore12,Ore13,Ore14	*OreG0022952*
scaffold48	2365547	G	T	Och08,Och14	*OreG0022954*
scaffold48	2381398	G	T	NA	*OreG0022956*
scaffold48	2423465	A	G	NA	*OreG0022961*
scaffold48	2423688	A	C	NA	*OreG0022961*
scaffold48	2423735	A	G	NA	*OreG0022961*
scaffold48	2466715	G	C	Och01,Och09,Och10,Och11	*OreG0022965*
scaffold48	2466725	A	G	Ore01,Ore02,Ore03,Ore04,Ore05,Ore06,Ore07,Ore08,Ore09,Ore10,Ore11,Ore12,Ore13,Ore14	*OreG0022965*
scaffold48	2514008	T	C	Och01,Och02,Och03,Och04,Och05,Och06,Och07,Och08,Och09,Och10,Och11,Och12,Och13,Och14,Ore01,Ore02,Ore03,Ore04,Ore05,Ore06,Ore07,Ore08,Ore09,Ore10,Ore11,Ore12,Ore13,Ore14	*OreG0022969*
scaffold48	2514149	G	C	NA	*OreG0022969*
scaffold48	2514177	A	G	Och01,Och02,Och03,Och04,Och05,Och06,Och07,Och09,Och10,Och11,Och12,Och13,Och14	*OreG0022969*
scaffold48	2566795	A	G	NA	*OreG0022971*
scaffold48	2585474	A	C	NA	*OreG0022972*
scaffold48	2586495	G	A	NA	*OreG0022972*
scaffold48	2588256	C	T	NA	*OreG0022972*
scaffold48	2593616	G	A	Och01,Och02,Och03,Och04,Och05,Och06,Och07,Och08,Och09,Och10,Och11,Och12,Och13,Och14	*OreG0022973*
scaffold48	2620382	G	A	Ore01,Ore02,Ore03,Ore04,Ore05,Ore06,Ore07,Ore08,Ore09,Ore10,Ore11,Ore12,Ore13,Ore14	*OreG0022974*
scaffold48	2621520	T	A	NA	*OreG0022974*
scaffold48	2621700	A	G	NA	*OreG0022974*
scaffold48	2623125	C	G	NA	*OreG0022974*
scaffold48	2623412	G	T	NA	*OreG0022974*
scaffold48	2625109	C	T	Och01,Och02,Och03,Och04,Och05,Och06,Och07,Och08,Och09,Och10,Och11,Och12,Och13,Och14,Ore01,Ore02,Ore03,Ore04,Ore05,Ore06,Ore07,Ore08,Ore09,Ore10,Ore11,Ore12,Ore13,Ore14	*OreG0022974*
scaffold48	2653800	C	T	NA	*OreG0022977*
scaffold48	2663803	A	T	Och01,Och02,Och03,Och04,Och05,Och06,Och07,Och08,Och09,Och10,Och11,Och12,Och13,Och14,Ore01,Ore02,Ore03,Ore04,Ore05,Ore06,Ore07,Ore08,Ore09,Ore10,Ore11,Ore12,Ore13,Ore14	*OreG0022978*
scaffold48	2665331	C	A	Och01,Och02,Och03,Och04,Och05,Och06,Och07,Och08,Och09,Och10,Och11,Och12,Och13,Och14,Ore01,Ore02,Ore03,Ore04,Ore05,Ore06,Ore07,Ore08,Ore09,Ore10,Ore11,Ore12,Ore13,Ore14	*OreG0022978*
scaffold48	2667762	T	A	Och01,Och02,Och03,Och04,Och05,Och06,Och07,Och08,Och09,Och10,Och11,Och12,Och13,Och14,Ore01,Ore02,Ore03,Ore04,Ore05,Ore06,Ore07,Ore08,Ore09,Ore10,Ore11,Ore12,Ore13,Ore14	*OreG0022979*
scaffold331	62817	G	A	NA	*OreG0011353*
scaffold331	63330	A	G	NA	*OreG0011353*
scaffold331	63639	A	T	NA	*OreG0011353*
scaffold331	63961	C	T	NA	*OreG0011353*
scaffold331	63994	T	C	NA	*OreG0011353*
scaffold331	71057	A	T	Och01,Och02,Och03,Och04,Och05,Och06,Och07,Och08,Och09,Och10,Och12,Och13,Och14	*OreG0011355*
scaffold331	71230	C	T	Och01,Och02,Och03,Och04,Och05,Och06,Och07,Och08,Och09,Och10,Och11,Och12,Och13,Och14,Ore01,Ore02,Ore03,Ore04,Ore05,Ore06,Ore07,Ore08,Ore09,Ore10,Ore11,Ore12,Ore13,Ore14	*OreG0011355*
scaffold331	112641	G	T	Och02,Och03,Och04,Och05,Och06,Och11,Och12,Och13,Och14	*OreG0011360*
scaffold331	112661	T	A	Och07	*OreG0011360*

scaffold331	113674	G	C	Ore01,Ore02,Ore03,Ore04,Ore05,Ore06,Ore07,Ore08,Ore09,Ore10,Ore11,Ore12,Ore13,Ore14	*OreG0011360*
scaffold331	113924	T	A	NA	*OreG0011360*
scaffold331	113960	C	A	NA	*OreG0011361*
scaffold331	114391	G	A	Ore01,Ore02,Ore03,Ore04,Ore05,Ore06,Ore07,Ore08,Ore09,Ore10,Ore11,Ore12,Ore13,Ore14	*OreG0011361*
scaffold331	177249	G	A	Ore04,Ore05	*OreG0011367*
scaffold331	179224	C	T	NA	*OreG0011367*
scaffold331	279131	G	A	Och01,Och02,Och03,Och04,Och05,Och06,Och07,Och08,Och09,Och10,Och11,Och12,Och13,Och14	*OreG0011373*
scaffold331	289268	T	A	Och01,Och02,Och03,Och04,Och05,Och06,Och07,Och08,Och09,Och10,Och11,Och12,Och13,Och14,Ore01,Ore02,Ore03,Ore04,Ore05,Ore06,Ore07,Ore08,Ore09,Ore10,Ore11,Ore12,Ore13,Ore14	*OreG0011374*
scaffold331	289357	G	A	Och11	*OreG0011374*
scaffold331	293116	T	A	Och01,Och02,Och03,Och04,Och05,Och06,Och07,Och08,Och09,Och10,Och11,Och12,Och13,Och14,Ore01,Ore02,Ore03,Ore04,Ore05,Ore06,Ore07,Ore08,Ore09,Ore10,Ore11,Ore12,Ore13,Ore14	*OreG0011375*
scaffold331	293847	G	A	Och06	*OreG0011375*
scaffold331	294348	T	G	Och08,Och11	*OreG0011375*
scaffold331	298600	A	G	Ore03	*OreG0011376*
scaffold331	298648	G	T	Och04,Och06,Och09,Och10,Och11,Och13,Och14	*OreG0011376*
scaffold331	298764	C	T	Och01,Och02,Och03,Och04,Och05,Och06,Och07,Och08,Och09,Och10,Och11,Och12,Och13,Och14	*OreG0011376*
scaffold331	306442	G	A	Och01,Och04,Och06,Och09,Och10,Och11,Och14	*OreG0011378*
scaffold331	329569	A	T	Och01,Och02,Och03,Och04,Och05,Och06,Och07,Och08,Och09,Och10,Och11,Och12,Och13,Och14,Ore01,Ore02,Ore03,Ore04,Ore05,Ore06,Ore07,Ore08,Ore09,Ore10,Ore11,Ore12,Ore13,Ore14	*OreG0011379*
scaffold331	330624	C	A	Och13	*OreG0011379*
scaffold331	351756	C	G	NA	*OreG0011382*
scaffold331	351782	T	C	NA	*OreG0011382*
scaffold331	351789	T	C	NA	*OreG0011382*
scaffold331	351867	A	G	NA	*OreG0011382*
scaffold331	353841	C	T	NA	*OreG0011382*
scaffold331	361981	C	A	Ore01,Ore02,Ore03,Ore04,Ore05,Ore06,Ore07,Ore08,Ore09,Ore10,Ore11,Ore12,Ore13,Ore14	*OreG0011383*
scaffold331	361996	C	T	Och01,Och02,Och03,Och04,Och05,Och06,Och12,Och13	*OreG0011383*
scaffold331	393279	T	C	Och02,Och03,Och06,Och07,Och08,Och09,Och10,Och11,Och12,Och14	*OreG0011386*
scaffold331	399112	C	T	Ore04,Ore05	*OreG0011387*
scaffold331	399636	T	C	NA	*OreG0011387*
scaffold331	399680	T	C	NA	*OreG0011387*
scaffold331	407768	T	G	Och01,Och02,Och03,Och04,Och05,Och06,Och07,Och08,Och09,Och10,Och11,Och12,Och13,Och14,Ore01,Ore02,Ore03,Ore04,Ore05,Ore06,Ore07,Ore08,Ore09,Ore10,Ore11,Ore12,Ore13,Ore14	*OreG0011388*
scaffold331	409430	T	A	Och01,Och05,Och09,Och10,Och11,Och13,Och14	*OreG0011388*
scaffold331	409542	T	A	Och06	*OreG0011388*
scaffold331	409548	C	T	Och06	*OreG0011388*
scaffold331	412171	C	T	Och06	*OreG0011389*
scaffold331	412438	T	C	Ore04,Ore05	*OreG0011389*
scaffold331	413899	G	C	NA	*OreG0011389*
scaffold331	422374	T	C	Ore01,Ore02,Ore03,Ore04,Ore05,Ore06,Ore07,Ore08,Ore09,Ore10,Ore11,Ore12,Ore13,Ore14	*OreG0011391*
scaffold331	456230	T	G	Ore01,Ore02,Ore03,Ore04,Ore05,Ore06,Ore07,Ore08,Ore09,Ore10,Ore11,Ore12,Ore13,Ore14	*OreG0011396*
scaffold331	476112	C	A	NA	*OreG0011399*
scaffold331	476241	G	A	Ore01,Ore02,Ore03,Ore04,Ore05,Ore06,Ore07,Ore08,Ore09,Ore10,Ore11,Ore12,Ore13,Ore14	*OreG0011399*
scaffold331	476309	G	A	NA	*OreG0011399*
scaffold331	476337	C	A	Och01,Och02,Och03,Och04,Och05,Och06,Och07,Och08,Och09,Och10,Och11,Och12,Och13,Och14,Ore01,Ore02,Ore03,Ore04,Ore05,Ore06,Ore07,Ore08,Ore09,Ore10,Ore11,Ore12,Ore13,Ore14	*OreG0011399*
scaffold331	531702	T	A	Och01,Och02,Och03,Och04,Och05,Och06,Och07,Och08,Och09,Och10,Och11,Och12,Och13,Och14,Ore01,Ore02,Ore03,Ore04,Ore05,Ore06,Ore07,Ore08,Ore09,Ore10,Ore11,Ore12,Ore13,Ore14	*OreG0011403*
scaffold331	654878	A	T	NA	*OreG0011410*
scaffold331	654900	G	T	NA	*OreG0011410*
scaffold331	656641	T	A	Och02,Och03,Och04,Och06,Ore01,Ore02,Ore04,Ore05,Ore07,Ore09,Ore14	*OreG0011410*
scaffold331	669665	A	G	Och10,Och11	*OreG0011411*
scaffold331	689221	C	A	NA	*OreG0011417*
scaffold331	689355	G	A	Ore01,Ore02,Ore03,Ore04,Ore05,Ore06,Ore07,Ore08,Ore09,Ore10,Ore11,Ore12,Ore13,Ore14	*OreG0011417*
scaffold331	723880	C	T	Och06	*OreG0011423*
scaffold331	747830	T	A	NA	*OreG0011427*
scaffold331	748123	A	C	NA	*OreG0011427*
scaffold331	748319	G	A	NA	*OreG0011427*
scaffold331	748346	C	G	NA	*OreG0011427*

scaffold331	777941	A	G	NA	*OreG0011429*
scaffold331	797131	A	G	NA	*OreG0011430*
scaffold331	803579	C	T	NA	*OreG0011432*
scaffold331	815550	C	T	NA	*OreG0011434*
scaffold331	815555	T	A	NA	*OreG0011434*
scaffold331	837467	C	T	NA	*OreG0011438*
scaffold331	855191	G	T	Och11	*OreG0011440*
scaffold331	855299	G	A	Och01,Och02,Och03,Och04,Och05,Och06,Och07,Och08,Och09,Och10,Och11,Och12,Och13,Och14,Ore01,Ore02,Ore04,Ore05,Ore07,Ore09,Ore14	*OreG0011440*
scaffold331	855342	T	A	Och01,Och02,Och03,Och04,Och05,Och06,Och07,Och08,Och09,Och10,Och11,Och12,Och13,Och14,Ore01,Ore02,Ore03,Ore04,Ore05,Ore06,Ore07,Ore08,Ore09,Ore10,Ore11,Ore12,Ore13,Ore14	*OreG0011440*
scaffold331	855347	C	A	NA	*OreG0011440*
scaffold331	855479	C	A	Och01,Och02,Och03,Och04,Och05,Och06,Och07,Och08,Och09,Och10,Och11,Och12,Och13,Och14,Ore01,Ore02,Ore04,Ore05,Ore07,Ore09,Ore14	*OreG0011440*
scaffold331	1169794	A	T	Och12	*OreG0011446*
scaffold331	1243538	A	T	Och01,Och02,Och03,Och04,Och05,Och06,Och07,Och08,Och12,Och13,Ore01,Ore02,Ore03,Ore04,Ore05,Ore06,Ore07,Ore08,Ore09,Ore10,Ore11,Ore12,Ore13,Ore14	*OreG0011450*
scaffold331	1244851	C	T	Ore01,Ore02,Ore03,Ore04,Ore05,Ore06,Ore07,Ore08,Ore09,Ore10,Ore11,Ore12,Ore13,Ore14	*OreG0011450*
scaffold331	1259830	A	T	Och01,Och02,Och03,Och04,Och05,Och06,Och07,Och08,Och09,Och10,Och11,Och12,Och13,Och14,Ore01,Ore02,Ore03,Ore04,Ore05,Ore06,Ore07,Ore08,Ore09,Ore10,Ore11,Ore12,Ore13,Ore14	*OreG0011452*
scaffold331	1260843	T	G	Och01,Och02,Och03,Och04,Och05,Och06,Och07,Och09,Och10,Och11,Och12,Och13,Och14	*OreG0011452*
scaffold331	1300662	A	T	Ore01,Ore02,Ore03,Ore04,Ore05,Ore06,Ore07,Ore08,Ore09,Ore10,Ore11,Ore12,Ore13,Ore14	*OreG0011456*
scaffold331	1300791	T	C	Och02,Och06	*OreG0011456*
scaffold331	1303367	A	T	NA	*OreG0011457*
scaffold331	1311609	T	C	Och05	*OreG0011458*
scaffold331	1345947	G	A	NA	*OreG0011460*
scaffold331	1426584	G	C	Och01,Och02,Och03,Och04,Och05,Och06,Och07,Och08,Och09,Och10,Och11,Och12,Och13,Och14	*OreG0011469*
scaffold331	1485660	T	C	Ore01,Ore02,Ore03,Ore04,Ore05,Ore06,Ore07,Ore08,Ore09,Ore10,Ore11,Ore12,Ore13,Ore14	*OreG0011472*
scaffold331	1533679	G	T	NA	*OreG0011475*
scaffold331	1545489	C	T	Ore03,Ore06,Ore09,Ore11,Ore13,Ore14	*OreG0011476*
scaffold403	72842	G	A	Och14	*OreG0019655*
scaffold403	73010	G	A	NA	*OreG0019655*
scaffold403	73298	G	A	NA	*OreG0019655*
scaffold403	73337	T	C	NA	*OreG0019655*
scaffold403	74933	G	A	NA	*OreG0019655*
scaffold403	75008	C	T	NA	*OreG0019655*
scaffold403	91057	T	C	NA	*OreG0019656*
scaffold403	91361	C	T	Och02,Och04,Och06,Ore02,Ore04,Ore05,Ore07,Ore09,Ore12,Ore14	*OreG0019656*
scaffold403	129572	G	A	Och02,Och04,Och06	*OreG0019660*
scaffold403	158480	G	A	Och02,Och03,Och04,Och05,Och06,Och12,Och13	*OreG0019664*
scaffold403	162885	C	T	NA	*OreG0019664*
scaffold403	231152	C	T	Och01,Och02,Och03,Och04,Och05,Och06,Och07,Och08,Och09,Och10,Och11,Och12,Och13,Och14,Ore01,Ore02,Ore03,Ore04,Ore05,Ore06,Ore07,Ore08,Ore09,Ore10,Ore11,Ore12,Ore13,Ore14	*OreG0019668*
scaffold403	231225	C	T	Och02,Och03,Och04,Och05,Och06,Och12,Och13	*OreG0019668*
scaffold403	231853	C	T	Och01,Och02,Och03,Och04,Och05,Och06,Och07,Och08,Och09,Och10,Och11,Och12,Och13,Och14,Ore01,Ore02,Ore03,Ore04,Ore05,Ore06,Ore07,Ore08,Ore09,Ore10,Ore11,Ore12,Ore13,Ore14	*OreG0019668*
scaffold403	235845	T	C	NA	*OreG0019668*
scaffold403	236045	A	T	Ore01,Ore02,Ore03,Ore04,Ore05,Ore06,Ore07,Ore08,Ore09,Ore10,Ore11,Ore12,Ore13,Ore14	*OreG0019668*
scaffold403	241857	T	A	Och01,Och02,Och03,Och04,Och05,Och06,Och07,Och08,Och09,Och10,Och11,Och12,Och13,Och14,Ore01,Ore02,Ore03,Ore04,Ore05,Ore06,Ore07,Ore08,Ore09,Ore10,Ore11,Ore12,Ore13,Ore14	*OreG0019668*
scaffold403	242008	A	G	Ore01,Ore02,Ore03,Ore04,Ore05,Ore06,Ore07,Ore08,Ore09,Ore10,Ore11,Ore12,Ore13,Ore14	*OreG0019668*
scaffold403	247131	C	A	Och01,Och07,Och08,Och11	*OreG0019668*
scaffold403	247205	G	A	Och01,Och07,Och08,Och11	*OreG0019668*
scaffold403	266891	C	T	NA	*OreG0019670*
scaffold403	266961	G	T	Och02,Och04,Och06	*OreG0019670*
scaffold403	270735	G	A	NA	*OreG0019671*
scaffold403	274641	A	C	Ore01,Ore02,Ore03,Ore04,Ore05,Ore06,Ore07,Ore08,Ore09,Ore10,Ore11,Ore12,Ore13,Ore14	*OreG0019672*
scaffold403	277531	G	A	NA	*OreG0019673*
scaffold403	423142	G	T	NA	*OreG0019679*
scaffold403	433767	T	G	Och03,Och06,Och07,Och08,Och09,Och10	*OreG0019680*
scaffold403	433838	A	G	NA	*OreG0019680*
scaffold403	433860	C	G	NA	*OreG0019680*

scaffold403	434106	A	T	NA	*OreG0019680*
scaffold403	595040	G	T	Och08	*OreG0019686*
scaffold403	595058	T	G	Och08	*OreG0019686*
scaffold403	595144	T	G	Och08	*OreG0019686*
scaffold403	673443	G	T	NA	*OreG0019696*
scaffold403	673569	G	T	Och06	*OreG0019696*
scaffold403	684540	C	G	NA	*OreG0019697*
scaffold403	731240	G	A	Och01,Och02,Och03,Och04,Och05,Och06,Och07,Och08,Och09,Och10,Och11,Och12,Och13,Och14,Ore01,Ore02,Ore03,Ore04,Ore05,Ore06,Ore07,Ore08,Ore09,Ore10,Ore11,Ore12,Ore13,Ore14	*OreG0019699*
scaffold403	740998	C	T	Och01,Och02,Och03,Och04,Och05,Och06,Och07,Och08,Och09,Och10,Och11,Och12,Och13,Och14,Ore01,Ore02,Ore03,Ore04,Ore05,Ore06,Ore07,Ore08,Ore09,Ore10,Ore11,Ore12,Ore13,Ore14	*OreG0019700*
scaffold403	769976	A	T	Och01,Och11	*OreG0019702*
scaffold403	784103	G	T	Och01,Och02,Och03,Och04,Och05,Och06,Och07,Och08,Och09,Och10,Och11,Och12,Och13,Och14,Ore01,Ore02,Ore03,Ore04,Ore05,Ore06,Ore07,Ore08,Ore09,Ore10,Ore11,Ore12,Ore13,Ore14	*OreG0019703*
scaffold403	825969	T	C	NA	*OreG0019705*
scaffold403	853122	G	T	Ore01,Ore02,Ore03,Ore04,Ore05,Ore06,Ore07,Ore08,Ore09,Ore10,Ore11,Ore12,Ore13,Ore14	*OreG0019708*
scaffold403	853618	G	C	Och14	*OreG0019708*
scaffold403	855040	C	T	NA	*OreG0019708*
scaffold403	859602	G	A	Och07	*OreG0019708*
scaffold403	872566	G	C	NA	*OreG0019709*
scaffold403	875655	C	T	Och01	*OreG0019709*
scaffold403	885792	A	T	NA	*OreG0019710*
scaffold403	894298	G	T	Ore01,Ore02,Ore03,Ore04,Ore05,Ore06,Ore07,Ore08,Ore09,Ore10,Ore11,Ore12,Ore13,Ore14	*OreG0019711*
scaffold403	895081	T	G	Ore01,Ore02,Ore03,Ore04,Ore05,Ore06,Ore07,Ore08,Ore09,Ore10,Ore11,Ore12,Ore13,Ore14	*OreG0019711*
scaffold403	896230	C	G	NA	*OreG0019711*
scaffold403	975061	C	G	NA	*OreG0019716*
scaffold403	1015290	A	C	Och01,Och02,Och03,Och04,Och05,Och06,Och07,Och08,Och09,Och10,Och11,Och12,Och13,Och14,Ore01,Ore02,Ore03,Ore04,Ore05,Ore06,Ore07,Ore08,Ore09,Ore10,Ore11,Ore12,Ore13,Ore14	*OreG0019720*
scaffold403	1046718	T	A	Och01	*OreG0019725*
scaffold403	1046753	A	T	NA	*OreG0019725*
scaffold403	1059199	G	A	Ore01,Ore02,Ore03,Ore04,Ore05,Ore06,Ore07,Ore08,Ore09,Ore10,Ore11,Ore12,Ore13,Ore14	*OreG0019726*
scaffold403	1062230	T	G	Och01,Och02,Och03,Och04,Och05,Och06,Och07,Och08,Och09,Och10,Och11,Och12,Och13,Och14,Ore01,Ore02,Ore03,Ore04,Ore05,Ore06,Ore07,Ore08,Ore09,Ore10,Ore11,Ore12,Ore13,Ore14	*OreG0019727*
scaffold403	1081114	G	A	NA	*OreG0019728*
scaffold403	1081206	G	A	NA	*OreG0019728*
scaffold403	1082961	C	T	NA	*OreG0019728*
scaffold403	1083704	C	T	Ore01,Ore02,Ore03,Ore04,Ore05,Ore06,Ore07,Ore08,Ore09,Ore10,Ore11,Ore12,Ore13,Ore14	*OreG0019728*
scaffold403	1083706	C	T	Och08	*OreG0019728*
scaffold403	1110572	T	G	Och01,Och02,Och03,Och04,Och05,Och06,Och07,Och08,Och09,Och10,Och11,Och12,Och13,Och14	*OreG0019731*
scaffold403	1110642	A	C	Ore01,Ore02,Ore03,Ore04,Ore05,Ore06,Ore07,Ore08,Ore09,Ore10,Ore11,Ore12,Ore13,Ore14	*OreG0019731*
scaffold403	1110741	A	G	Ore01,Ore03,Ore06,Ore08,Ore10,Ore11,Ore13	*OreG0019731*
scaffold403	1110869	T	A	Och01,Och02,Och03,Och04,Och05,Och06,Och07,Och08,Och09,Och10,Och11,Och12,Och13,Och14	*OreG0019731*
scaffold403	1110877	T	C	NA	*OreG0019731*
scaffold403	1111333	G	A	NA	*OreG0019732*
scaffold403	1112154	G	A	Och01,Och02,Och03,Och04,Och05,Och06,Och07,Och08,Och09,Och10,Och11,Och12,Och13,Och14	*OreG0019732*
scaffold403	1112182	A	T	Och01,Och02,Och03,Och04,Och05,Och06,Och07,Och08,Och09,Och10,Och11,Och12,Och13,Och14	*OreG0019732*
scaffold403	1112214	C	T	Ore01,Ore02,Ore03,Ore04,Ore05,Ore06,Ore07,Ore08,Ore09,Ore10,Ore11,Ore12,Ore13,Ore14	*OreG0019732*
scaffold403	1179301	C	T	NA	*OreG0019738*
scaffold403	1180524	C	A	NA	*OreG0019738*
scaffold403	1234667	C	G	Och09,Och10	*OreG0019743*
scaffold403	1235316	G	A	Och01,Och02,Och03,Och04,Och05,Och06,Och07,Och08,Och09,Och10,Och11,Och12,Och13,Och14,Ore01,Ore02,Ore03,Ore04,Ore05,Ore06,Ore07,Ore08,Ore09,Ore10,Ore11,Ore12,Ore13,Ore14	*OreG0019743*
scaffold403	1235680	A	G	Ore01,Ore02,Ore03,Ore04,Ore05,Ore06,Ore07,Ore08,Ore09,Ore10,Ore11,Ore12,Ore13,Ore14	*OreG0019743*
scaffold403	1237582	G	A	Och06	*OreG0019741*
scaffold403	1237672	G	A	Ore01,Ore02,Ore03,Ore04,Ore05,Ore06,Ore07,Ore08,Ore09,Ore10,Ore11,Ore12,Ore13,Ore14	*OreG0019741*
scaffold403	1245381	T	A	NA	*OreG0019741*

scaffold403	1245815	G	T	Ore01,Ore03,Ore06,Ore08,Ore10,Ore11,Ore13	*OreG0019741*
scaffold403	1245817	C	G	NA	*OreG0019741*
scaffold403	1246180	G	T	Och01,Och02,Och03,Och04,Och05,Och06,Och07,Och08,Och11,Och12,Och13,Och14	*OreG0019741*
scaffold403	1254373	T	C	Och01,Och05,Och07,Och09,Och10,Och11,Och12,Och13	*OreG0019744*
scaffold403	1254507	G	C	Och06	*OreG0019744*
scaffold403	1295327	C	T	Och01,Och02,Och03,Och04,Och05,Och06,Och07,Och08,Och09,Och10,Och11,Och12,Och13,Och14	*OreG0019747*
scaffold403	1314150	G	A	NA	*OreG0019752*
scaffold403	1314159	C	T	NA	*OreG0019752*
scaffold403	1337066	C	T	Och01,Och02,Och03,Och04,Och05,Och06,Och07,Och08,Och09,Och10,Och11,Och13,Och14	*OreG0019759*
scaffold403	1359531	C	G	Och01,Och02,Och03,Och04,Och05,Och06,Och07,Och08,Och09,Och10,Och11,Och12,Och13,Och14	*OreG0019761*
scaffold403	1359856	G	A	Och01,Och02,Och03,Och04,Och05,Och06,Och07,Och08,Och09,Och10,Och11,Och12,Och13,Och14	*OreG0019761*
scaffold403	1359884	C	T	Och01,Och02,Och03,Och04,Och05,Och06,Och07,Och08,Och09,Och10,Och11,Och12,Och13,Och14,Ore01,Ore02,Ore03,Ore04,Ore05,Ore06,Ore07,Ore08,Ore09,Ore10,Ore11,Ore12,Ore13,Ore14	*OreG0019761*
scaffold403	1360709	T	C	Och12,Och13	*OreG0019761*
scaffold403	1360730	G	A	Och12,Och13	*OreG0019761*
scaffold403	1400046	C	T	NA	*OreG0019766*
scaffold403	1405153	G	C	Och01,Och02,Och03,Och04,Och05,Och06,Och07,Och08,Och09,Och10,Och11,Och12,Och13,Och14,Ore01,Ore02,Ore03,Ore04,Ore05,Ore06,Ore07,Ore08,Ore09,Ore10,Ore11,Ore12,Ore13,Ore14	*OreG0019767*
scaffold403	1548308	C	T	Ore01,Ore02,Ore03,Ore04,Ore05,Ore06,Ore07,Ore08,Ore09,Ore10,Ore11,Ore12,Ore13,Ore14	*OreG0019774*
scaffold403	1628039	T	G	Och01,Och02,Och03,Och04,Och05,Och06,Och07,Och08,Och09,Och10,Och11,Och12,Och13,Och14	*OreG0019775*
scaffold403	1645197	T	C	Ore01,Ore02,Ore03,Ore04,Ore05,Ore06,Ore07,Ore08,Ore09,Ore10,Ore11,Ore12,Ore13,Ore14	*OreG0019777*
scaffold403	1665882	G	C	NA	*OreG0019779*
scaffold403	1666485	G	A	NA	*OreG0019779*
scaffold403	1666587	A	T	Och02,Och03,Och04,Och05,Och06,Och12,Och13,Och14	*OreG0019779*
scaffold403	1686543	G	A	NA	*OreG0019781*
scaffold403	1687568	C	T	Ore04,Ore05,Ore13	*OreG0019781*
scaffold403	1805388	G	T	Ore01,Ore02,Ore03,Ore04,Ore05,Ore06,Ore07,Ore08,Ore09,Ore10,Ore11,Ore12,Ore13,Ore14	*OreG0019783*
scaffold403	1833257	C	A	NA	*OreG0019784*
scaffold403	1843950	A	G	Och14	*OreG0019786*
scaffold403	1852407	G	T	Ore03	*OreG0019787*
scaffold403	1853357	T	G	Och04,Och06	*OreG0019787*
scaffold403	1854745	G	A	Och08	*OreG0019787*
scaffold403	1854857	T	A	NA	*OreG0019787*
scaffold403	1932840	G	A	Ore02,Ore04,Ore05,Ore13	*OreG0019791*
scaffold403	1958429	T	A	Ore01,Ore02,Ore03,Ore04,Ore05,Ore06,Ore07,Ore08,Ore09,Ore10,Ore11,Ore12,Ore13,Ore14	*OreG0019795*
scaffold403	1961915	C	A	Och01,Och02,Och03,Och04,Och05,Och06,Och07,Och08,Och09,Och10,Och11,Och12,Och13,Och14,Ore01,Ore02,Ore03,Ore04,Ore05,Ore06,Ore07,Ore08,Ore09,Ore10,Ore11,Ore12,Ore13,Ore14	*OreG0019795*
scaffold403	1971601	G	A	NA	*OreG0019795*
scaffold403	2046653	C	G	Ore01,Ore02,Ore03,Ore04,Ore05,Ore06,Ore07,Ore08,Ore09,Ore10,Ore11,Ore12,Ore13,Ore14	*OreG0019798*
scaffold403	2047316	A	T	Ore01,Ore02,Ore03,Ore04,Ore05,Ore06,Ore07,Ore08,Ore09,Ore10,Ore11,Ore12,Ore13,Ore14	*OreG0019798*
scaffold403	2047352	A	T	Ore01,Ore02,Ore03,Ore04,Ore05,Ore06,Ore07,Ore08,Ore09,Ore10,Ore11,Ore12,Ore13,Ore14	*OreG0019798*
scaffold403	2047380	T	A	NA	*OreG0019798*
scaffold403	2047514	C	G	Och11	*OreG0019798*
scaffold403	2047923	A	G	Och04,Och06	*OreG0019798*
scaffold403	2048244	T	G	Ore01,Ore02,Ore03,Ore04,Ore05,Ore06,Ore07,Ore08,Ore09,Ore10,Ore11,Ore12,Ore13,Ore14	*OreG0019798*
scaffold403	2048646	T	C	Och02,Och03,Och04,Och05,Och06,Och12,Och13	*OreG0019798*
scaffold403	2179653	A	G	NA	*OreG0019805*
scaffold403	2193332	C	A	NA	*OreG0019806*
scaffold403	2197875	A	G	NA	*OreG0019807*
scaffold403	2198095	C	A	Ore02,Ore04,Ore05,Ore13	*OreG0019807*
scaffold403	2198204	T	G	Ore01,Ore03	*OreG0019807*
scaffold403	2231868	G	A	Och01,Och02,Och03,Och04,Och05,Och06,Och07,Och08,Och09,Och10,Och11,Och12,Och13,Och14	*OreG0019810*
scaffold403	2232194	C	T	Ore01,Ore02,Ore03,Ore04,Ore05,Ore06,Ore07,Ore08,Ore09,Ore10,Ore11,Ore12,Ore13,Ore14	*OreG0019810*
scaffold403	2232285	T	G	Och01,Och02,Och03,Och04,Och05,Och06,Och07,Och08,Och09,Och10,Och11,Och12,Och13,Och14	*OreG0019810*

scaffold403	2233018	C	G	Och01,Och02,Och03,Och04,Och05,Och06,Och07,Och08,Och09,Och10,Och11,Och12,Och13,Och14,Ore01,Ore02,Ore03,Ore04,Ore05,Ore06,Ore07,Ore08,Ore09,Ore10,Ore11,Ore12,Ore13,Ore14	*OreG0019810*
scaffold403	2233469	C	T	Och01,Och02,Och03,Och04,Och05,Och06,Och07,Och08,Och09,Och10,Och11,Och12,Och13,Och14	*OreG0019810*
scaffold403	2233493	C	T	Och11	*OreG0019810*
scaffold403	2233775	G	A	Och01,Och02,Och03,Och04,Och05,Och06,Och07,Och08,Och09,Och10,Och11,Och12,Och13,Och14	*OreG0019810*
scaffold403	2233877	T	C	Och01,Och02,Och03,Och04,Och05,Och06,Och07,Och08,Och09,Och10,Och11,Och12,Och13,Och14	*OreG0019810*
scaffold403	2234048	T	A	Ore01,Ore02,Ore03,Ore04,Ore05,Ore06,Ore07,Ore08,Ore09,Ore10,Ore11,Ore12,Ore13,Ore14	*OreG0019810*
scaffold403	2234161	C	T	Ore01,Ore02,Ore03,Ore04,Ore05,Ore06,Ore07,Ore08,Ore09,Ore10,Ore11,Ore12,Ore13,Ore14	*OreG0019810*
scaffold403	2234171	G	T	Ore01,Ore02,Ore03,Ore04,Ore05,Ore06,Ore07,Ore08,Ore09,Ore10,Ore11,Ore12,Ore13,Ore14	*OreG0019810*
scaffold403	2246675	G	A	NA	*OreG0019812*
scaffold403	2414086	G	T	NA	*OreG0019822*
scaffold403	2415172	G	A	NA	*OreG0019822*
scaffold403	2418572	C	T	NA	*OreG0019822*
scaffold403	2418656	G	A	NA	*OreG0019822*
scaffold403	2418665	A	T	NA	*OreG0019822*
scaffold403	2420556	A	G	Ore01,Ore02,Ore03,Ore04,Ore05,Ore06,Ore07,Ore08,Ore09,Ore10,Ore11,Ore12,Ore13,Ore14	*OreG0019822*
scaffold403	2429587	G	A	NA	*OreG0019822*
scaffold403	2429639	G	A	NA	*OreG0019822*
scaffold403	2468079	A	T	Och14	*OreG0019825*
scaffold403	2484406	C	T	NA	*OreG0019827*
scaffold403	2484413	C	T	Och11	*OreG0019827*
scaffold403	2484529	G	A	Och01,Och02,Och03,Och04,Och05,Och06,Och07,Och08,Och09,Och10,Och11,Och12,Och13,Och14	*OreG0019827*
scaffold403	2493471	G	A	Och01,Och02,Och03,Och04,Och05,Och06,Och07,Och08,Och09,Och10,Och11,Och12,Och13,Och14	*OreG0019828*
scaffold403	2495062	C	T	Och07	*OreG0019828*
scaffold403	2499760	T	C	NA	*OreG0019829*
scaffold403	2501627	T	G	Och01,Och02,Och03,Och04,Och05,Och06,Och07,Och08,Och09,Och10,Och11,Och12,Och13,Och14,Ore01,Ore02,Ore03,Ore04,Ore05,Ore06,Ore07,Ore08,Ore09,Ore10,Ore11,Ore12,Ore13,Ore14	*OreG0019829*
scaffold403	2502204	A	G	Och01,Och02,Och03,Och04,Och05,Och06,Och08,Och09,Och10,Och11,Och12,Och13,Och14,Ore01,Ore02,Ore03,Ore04,Ore05,Ore06,Ore07,Ore08,Ore09,Ore10,Ore11,Ore12,Ore13,Ore14	*OreG0019829*
scaffold403	2502436	C	T	Och01,Och02,Och03,Och04,Och05,Och06,Och08,Och09,Och10,Och11,Och12,Och13,Och14	*OreG0019829*
scaffold403	2554977	G	C	Och09,Ore01,Ore02,Ore03,Ore04,Ore05,Ore06,Ore07,Ore08,Ore09,Ore10,Ore11,Ore12,Ore13,Ore14	*OreG0019832*
scaffold403	2555166	G	T	Och04,Och06	*OreG0019832*
scaffold403	2582835	T	G	NA	*OreG0019833*
scaffold403	2584422	A	C	Och13,Ore01,Ore02,Ore03,Ore04,Ore05,Ore06,Ore07,Ore08,Ore09,Ore10,Ore11,Ore12,Ore13,Ore14	*OreG0019833*
scaffold403	2595273	T	C	NA	*OreG0019834*
scaffold403	2595278	G	T	NA	*OreG0019834*
scaffold403	2595495	G	C	Ore01,Ore02,Ore03,Ore04,Ore05,Ore06,Ore07,Ore08,Ore09,Ore10,Ore11,Ore12,Ore13,Ore14	*OreG0019834*
scaffold403	2599638	G	A	NA	*OreG0019834*
scaffold403	2599662	G	A	NA	*OreG0019834*
scaffold403	2599816	C	A	NA	*OreG0019834*
scaffold403	2617224	C	T	Och01,Och04,Och06	*OreG0019834*
scaffold403	2617238	C	T	NA	*OreG0019834*
scaffold403	2655640	G	A	Ore01,Ore02,Ore03,Ore04,Ore05,Ore06,Ore07,Ore08,Ore09,Ore10,Ore11,Ore12,Ore13,Ore14	*OreG0019837*
scaffold403	2656021	T	A	Och01,Och02,Och03,Och04,Och05,Och06,Och07,Och08,Och09,Och10,Och11,Och12,Och13,Och14,Ore01,Ore02,Ore03,Ore04,Ore05,Ore06,Ore07,Ore08,Ore09,Ore10,Ore11,Ore12,Ore13,Ore14	*OreG0019837*
scaffold403	2656171	T	A	NA	*OreG0019837*
scaffold403	2656407	T	C	Och01,Och02,Och03,Och04,Och05,Och06,Och07,Och08,Och09,Och10,Och11,Och12,Och13,Och14	*OreG0019837*
scaffold403	2684682	T	G	NA	*OreG0019838*
scaffold403	2759308	G	T	Ore01,Ore03,Ore04,Ore05,Ore07,Ore08,Ore10,Ore11,Ore12,Ore14	*OreG0019840*
scaffold403	2760183	A	G	NA	*OreG0019840*
scaffold403	2760886	A	T	Och01,Och02,Och03,Och04,Och05,Och06,Och07,Och08,Och09,Och10,Och11,Och12,Och13,Och14,Ore01,Ore02,Ore03,Ore04,Ore05,Ore06,Ore07,Ore08,Ore09,Ore10,Ore11,Ore12,Ore13,Ore14	*OreG0019840*
scaffold403	2784949	A	C	Och14	*OreG0019843*
scaffold403	2789112	C	T	Och02,Och03,Och04,Och05,Och06,Och11,Och12,Och13,Och14	*OreG0019843*
scaffold403	2789232	T	C	NA	*OreG0019843*

scaffold403	2793360	C	G	Och14	*OreG0019844*
scaffold403	2877171	G	C	NA	*OreG0019852*
scaffold403	2878302	G	A	NA	*OreG0019852*
scaffold403	2934492	A	C	Och01,Och02,Och03,Och04,Och05,Och06,Och07,Och08,Och09,Och10,Och11,Och12,Och13,Och14	*OreG0019855*
scaffold403	2935593	A	C	Ore01,Ore02,Ore03,Ore04,Ore05,Ore06,Ore07,Ore08,Ore09,Ore10,Ore11,Ore12,Ore13,Ore14	*OreG0019855*
scaffold403	2979102	G	T	NA	*OreG0019862*
scaffold403	2979433	C	T	Ore01,Ore02,Ore03,Ore04,Ore05,Ore06,Ore07,Ore08,Ore09,Ore10,Ore11,Ore12,Ore13,Ore14	*OreG0019862*
scaffold970	1783	T	A	Och09,Och10	*OreG0027034*
scaffold970	72908	G	A	Och01,Och02,Och03,Och04,Och05,Och06,Och07,Och08,Och09,Och10,Och11,Och12,Och13,Och14,Ore01,Ore02,Ore03,Ore04,Ore05,Ore06,Ore07,Ore08,Ore09,Ore10,Ore11,Ore12,Ore13,Ore14	*OreG0027044*
scaffold970	149599	T	A	NA	*OreG0027045*
scaffold970	151276	G	A	NA	*OreG0027045*
scaffold970	202041	G	C	NA	*OreG0027046*
scaffold970	214353	G	T	Och11	*OreG0027047*
scaffold970	218306	C	T	Och01,Och05,Och09,Och10,Och11,Och12	*OreG0027047*
scaffold970	218687	C	T	Och01,Och05,Och09,Och10,Och11,Och12	*OreG0027047*
scaffold970	233566	A	T	Och04,Och06,Och07,Och08,Och12,Och13	*OreG0027049*
scaffold970	260445	C	A	Och01	*OreG0027053*
scaffold970	284347	G	A	NA	*OreG0027057*
scaffold970	284357	A	T	NA	*OreG0027057*
scaffold970	351754	T	A	Ore01,Ore02,Ore03,Ore04,Ore05,Ore06,Ore07,Ore08,Ore09,Ore10,Ore11,Ore12,Ore13,Ore14	*OreG0027064*
scaffold970	351842	G	C	Ore01,Ore02,Ore03,Ore06,Ore07,Ore08,Ore09,Ore10,Ore11,Ore12,Ore13,Ore14	*OreG0027064*
scaffold970	352011	C	A	NA	*OreG0027064*
scaffold970	352354	C	G	NA	*OreG0027064*
scaffold970	352401	C	T	Och07,Och08,Och11	*OreG0027064*
scaffold970	358060	C	T	Och01,Och02,Och03,Och04,Och05,Och06,Och07,Och08,Och09,Och10,Och11,Och12,Och13,Och14	*OreG0027066*
scaffold970	358090	C	T	Och01,Och02,Och03,Och04,Och05,Och06,Och07,Och08,Och09,Och10,Och11,Och12,Och13,Och14	*OreG0027066*
scaffold915	13472	C	T	Ore02,Ore03,Ore06,Ore07,Ore08,Ore09,Ore10,Ore13,Ore14	*OreG0026524*
scaffold915	14205	C	G	Och01,Och02,Och03,Och04,Och05,Och06,Och07,Och08,Och09,Och10,Och11,Och12,Och13,Och14,Ore01,Ore02,Ore03,Ore04,Ore05,Ore06,Ore07,Ore08,Ore09,Ore10,Ore11,Ore12,Ore13,Ore14	*OreG0026524*
scaffold915	14312	A	T	Och01,Och02,Och03,Och04,Och05,Och06,Och07,Och08,Och09,Och10,Och11,Och12,Och13,Och14,Ore01,Ore02,Ore03,Ore04,Ore05,Ore06,Ore07,Ore08,Ore09,Ore10,Ore11,Ore12,Ore13,Ore14	*OreG0026524*
scaffold915	21903	A	G	NA	*OreG0026525*
scaffold915	22608	G	A	NA	*OreG0026525*
scaffold915	24932	C	T	Ore01,Ore02,Ore03,Ore04,Ore05,Ore06,Ore07,Ore08,Ore09,Ore10,Ore11,Ore12,Ore13,Ore14	*OreG0026525*
scaffold915	125792	G	T	NA	*OreG0026536*
scaffold915	126258	C	G	NA	*OreG0026536*
scaffold915	126293	T	A	NA	*OreG0026536*
scaffold915	126898	G	T	NA	*OreG0026536*
scaffold915	127154	A	T	NA	*OreG0026536*
scaffold915	137914	A	G	NA	*OreG0026537*
scaffold915	137923	G	A	NA	*OreG0026537*
scaffold915	138002	G	A	NA	*OreG0026537*
scaffold915	139063	T	C	NA	*OreG0026537*
scaffold915	159251	C	T	NA	*OreG0026538*
scaffold915	177384	C	T	Och01,Och04,Och06,Och12,Och13,Ore01,Ore02,Ore03,Ore04,Ore05,Ore06,Ore07,Ore08,Ore09,Ore10,Ore11,Ore12,Ore13,Ore14	*OreG0026542*
scaffold915	177396	T	C	NA	*OreG0026542*
scaffold915	177602	T	C	Och01,Och04,Och12,Och13	*OreG0026542*
scaffold915	179694	T	C	NA	*OreG0026542*
scaffold915	179901	G	T	NA	*OreG0026542*
scaffold915	180015	T	C	NA	*OreG0026542*
scaffold915	196013	C	T	Och01,Och14	*OreG0026545*
scaffold915	196043	C	T	Och01,Och02,Och03,Och04,Och05,Och06,Och07,Och08,Och09,Och10,Och11,Och12,Och13,Och14	*OreG0026545*
scaffold915	196419	A	G	Och07,Och08,Och09,Och11	*OreG0026545*
scaffold915	204892	G	A	Ore06,Ore07,Ore08,Ore13,Ore14	*OreG0026547*
scaffold915	221043	C	A	Och01,Och02,Och03,Och04,Och05,Och06,Och07,Och08,Och09,Och10,Och11,Och12,Och13,Och14	*OreG0026547*
scaffold915	231792	T	A	Och01,Och02,Och03,Och04,Och05,Och06,Och07,Och08,Och09,Och10,Och11,Och12,Och13,Och14,Ore04,Ore05,Ore06,Ore07,Ore08,Ore12,Ore13,Ore14	*OreG0026549*
scaffold915	312464	T	A	NA	*OreG0026552*
scaffold915	312494	C	G	NA	*OreG0026552*
scaffold915	312536	G	A	NA	*OreG0026552*
scaffold915	315175	A	C	NA	*OreG0026552*

scaffold915	417440	C	T	NA	*OreG0026559*
scaffold915	428672	G	A	NA	*OreG0026560*
scaffold915	441091	G	A	Och01,Och02,Och03,Och04,Och05,Och06,Och07,Och08,Och09,Och10,Och11,Och12,Och13,Och14	*OreG0026562*
scaffold915	450285	T	A	Och01,Och02,Och03,Och04,Och05,Och06,Och07,Och08,Och09,Och10,Och11,Och12,Och13,Och14,Ore04,Ore05,Ore06,Ore07,Ore08,Ore12,Ore13,Ore14	*OreG0026564*
scaffold915	452694	T	C	Och12,Och13	*OreG0026564*
scaffold915	456156	T	A	Och14,Ore01,Ore02,Ore07,Ore11,Ore12,Ore13	*OreG0026564*
scaffold915	456510	C	G	NA	*OreG0026564*
scaffold915	563595	G	A	Ore01,Ore03,Ore04,Ore05,Ore06,Ore07,Ore10,Ore13	*OreG0026571*
scaffold915	563599	A	T	Ore01,Ore03,Ore04,Ore05,Ore06,Ore07,Ore10,Ore13	*OreG0026571*
scaffold915	563688	A	G	NA	*OreG0026571*
scaffold915	571561	T	C	Och01,Och02,Och03,Och04,Och05,Och06,Och07,Och08,Och09,Och10,Och11,Och12,Och13,Och14,Ore01,Ore02,Ore03,Ore04,Ore05,Ore06,Ore07,Ore08,Ore09,Ore10,Ore11,Ore12,Ore13,Ore14	*OreG0026573*
scaffold915	573862	A	C	Och01,Och02,Och03,Och04,Och05,Och06,Och07,Och08,Och09,Och10,Och11,Och12,Och13,Och14,Ore01,Ore02,Ore03,Ore04,Ore05,Ore06,Ore07,Ore08,Ore09,Ore10,Ore11,Ore12,Ore13,Ore14	*OreG0026573*
scaffold915	573937	C	A	NA	*OreG0026573*
scaffold915	592551	C	T	Och05	*OreG0026575*
scaffold915	607197	A	T	NA	*OreG0026576*
scaffold915	607788	T	G	Och01,Och02,Och03,Och04,Och05,Och06,Och07,Och08,Och09,Och10,Och11,Och12,Och13,Och14,Ore01,Ore02,Ore03,Ore04,Ore05,Ore06,Ore07,Ore08,Ore09,Ore10,Ore11,Ore12,Ore13,Ore14	*OreG0026576*
scaffold915	608085	C	A	Ore03,Ore04,Ore05,Ore10	*OreG0026576*
scaffold915	616428	C	T	Och01,Och02,Och03,Och04,Och05,Och06,Och07,Och08,Och09,Och10,Och11,Och12,Och13,Och14,Ore01,Ore02,Ore03,Ore04,Ore05,Ore06,Ore07,Ore08,Ore09,Ore10,Ore11,Ore12,Ore13,Ore14	*OreG0026578*
scaffold915	654477	T	G	Och13	*OreG0026586*
scaffold915	670154	G	A	Och08,Och09,Och10	*OreG0026591*
scaffold915	670188	A	G	NA	*OreG0026591*
scaffold915	676130	T	C	Och03,Och05,Och13	*OreG0026591*
scaffold915	694129	C	T	Och08,Och09,Och10	*OreG0026593*
scaffold915	694211	C	T	NA	*OreG0026593*
scaffold915	701189	G	A	NA	*OreG0026593*
scaffold915	703943	C	A	Ore01,Ore02,Ore03,Ore04,Ore05,Ore06,Ore07,Ore08,Ore09,Ore10,Ore11,Ore12,Ore13,Ore14	*OreG0026594*
scaffold915	715388	G	T	Och02,Och04,Och05,Och07,Och08,Och09,Och10,Och11,Och12,Och13,Och14	*OreG0026596*
scaffold915	720314	C	T	NA	*OreG0026596*
scaffold915	748673	T	C	NA	*OreG0026599*
scaffold915	757827	G	A	Och01	*OreG0026601*
scaffold915	758684	C	A	NA	*OreG0026601*
scaffold915	758969	T	A	Och06	*OreG0026601*
scaffold915	778254	A	C	NA	*OreG0026603*
scaffold915	791304	T	G	Och03,Och08,Och09,Och10,Och11,Och13,Och14,Ore01,Ore02,Ore03,Ore04,Ore05,Ore06,Ore07,Ore08,Ore09,Ore10,Ore11,Ore12,Ore13,Ore14	*OreG0026604*
scaffold915	795310	G	A	Ore01,Ore02,Ore03,Ore04,Ore05,Ore06,Ore07,Ore08,Ore09,Ore10,Ore11,Ore12,Ore13,Ore14	*OreG0026604*
scaffold915	797540	C	T	NA	*OreG0026604*
scaffold915	806877	C	T	NA	*OreG0026605*
scaffold915	818969	T	A	NA	*OreG0026607*
scaffold915	855200	G	A	NA	*OreG0026612*
scaffold915	855276	A	T	NA	*OreG0026612*
scaffold915	864394	G	A	NA	*OreG0026612*
scaffold915	875352	C	G	Och03,Och13	*OreG0026614*
scaffold915	926219	T	A	NA	*OreG0026621*
scaffold915	983081	A	G	NA	*OreG0026626*
scaffold915	1050869	C	T	Och14	*OreG0026633*
scaffold97	78905	G	C	Ore01,Ore04,Ore05,Ore08,Ore09,Ore11,Ore12	*OreG0026952*
scaffold97	79264	A	C	Ore01,Ore02,Ore03,Ore04,Ore05,Ore06,Ore07,Ore08,Ore09,Ore10,Ore11,Ore12,Ore13,Ore14	*OreG0026952*
scaffold97	83888	G	A	Och01,Och02,Och03,Och04,Och05,Och06,Och07,Och08,Och09,Och10,Och11,Och12,Och13,Och14	*OreG0026952*
scaffold97	130337	G	A	Ore01,Ore02,Ore03,Ore04,Ore05,Ore06,Ore07,Ore08,Ore09,Ore10,Ore11,Ore12,Ore13,Ore14	*OreG0026955*
scaffold97	130347	C	A	Ore01,Ore02,Ore03,Ore04,Ore05,Ore06,Ore07,Ore08,Ore09,Ore10,Ore11,Ore12,Ore13,Ore14	*OreG0026955*
scaffold97	187255	T	G	NA	*OreG0026958*
scaffold97	210472	C	T	Och01,Och02,Och03,Och04,Och05,Och06,Och07,Och08,Och09,Och10,Och11,Och12,Och13,Och14	*OreG0026960*
scaffold97	212250	A	T	Och01,Och02,Och03,Och04,Och05,Och06,Och07,Och08,Och09,Och10,Och11,Och12,Och13,Och14	*OreG0026960*
scaffold97	231114	G	C	Och07,Och08	*OreG0026961*
scaffold97	414159	C	A	Ore01,Ore02,Ore03,Ore04,Ore05,Ore06,Ore07,Ore08,Ore09,Ore10,Ore11,Ore12,Ore13,Ore14	*OreG0026977*

scaffold97	414192	G	A	Ore01,Ore02,Ore03,Ore04,Ore05,Ore06,Ore07,Ore08,Ore09,Ore10,Ore11,Ore12,Ore13,Ore14	*OreG0026977*
scaffold97	490367	C	T	NA	*OreG0026981*
scaffold97	640027	G	A	Och01,Och02,Och03,Och04,Och05,Och06,Och07,Och12,Och13	*OreG0026984*
scaffold97	642600	A	C	Och01,Och02,Och03,Och04,Och05,Och06,Och07,Och08,Och09,Och10,Och11,Och12,Och13,Och14,Ore01,Ore02,Ore03,Ore04,Ore05,Ore06,Ore07,Ore08,Ore09,Ore10,Ore11,Ore12,Ore13,Ore14	*OreG0026984*
scaffold97	642789	T	A	NA	*OreG0026984*
scaffold97	668199	A	T	Och01,Och03,Och04,Och07	*OreG0026985*
scaffold97	871288	G	A	Och01	*OreG0026993*
scaffold97	871311	G	A	NA	*OreG0026993*
scaffold97	1304934	A	G	Och01,Och02,Och03,Och04,Och05,Och06,Och07,Och08,Och09,Och10,Och11,Och12,Och13,Och14,Ore01,Ore02,Ore03,Ore04,Ore05,Ore06,Ore07,Ore08,Ore09,Ore10,Ore11,Ore12,Ore13,Ore14	*OreG0027006*
scaffold97	1305785	T	C	NA	*OreG0027006*
scaffold97	1306086	G	A	Ore01,Ore02,Ore03,Ore04,Ore05,Ore06,Ore07,Ore08,Ore09,Ore10,Ore11,Ore12,Ore13,Ore14	*OreG0027006*
scaffold97	1728432	G	A	Och01,Ore01,Ore02,Ore03,Ore04,Ore05,Ore06,Ore07,Ore08,Ore09,Ore10,Ore11,Ore12,Ore13,Ore14	*OreG0027015*
scaffold97	1829197	G	A	Och02,Och04,Och05,Och06,Och07,Och08,Och09,Och10,Och11,Och12,Och13,Och14	*OreG0027016*
scaffold97	1915909	A	G	NA	*OreG0027019*
scaffold97	1916598	C	T	Och02,Och03,Och04,Och05,Och06,Och07,Och08,Och09,Och10,Och11,Och12,Och13,Och14	*OreG0027020*
scaffold97	1969393	G	A	Och01,Och02,Och03,Och04,Och05,Och06,Och07,Och08,Och09,Och10,Och11,Och12,Och13,Och14,Ore01,Ore02,Ore03,Ore04,Ore05,Ore06,Ore07,Ore08,Ore09,Ore10,Ore11,Ore12,Ore13,Ore14	*OreG0027022*
scaffold97	2026423	T	C	NA	*OreG0027023*
scaffold97	2100751	C	A	Och02,Och03,Och04,Och05,Och06,Och07,Och08,Och09,Och10,Och11,Och12,Och13,Och14	*OreG0027025*
scaffold97	2100853	C	G	Och01,Och02,Och03,Och04,Och05,Och06,Och07,Och08,Och09,Och10,Och11,Och12,Och13,Och14,Ore01,Ore02,Ore03,Ore04,Ore05,Ore06,Ore07,Ore08,Ore09,Ore10,Ore11,Ore12,Ore13,Ore14	*OreG0027025*
scaffold97	2100864	A	T	NA	*OreG0027025*
scaffold97	2100883	C	T	Och01,Och02,Och03,Och04,Och05,Och06,Och07,Och08,Och09,Och10,Och11,Och12,Och13,Och14,Ore01,Ore02,Ore03,Ore04,Ore05,Ore06,Ore07,Ore08,Ore09,Ore10,Ore11,Ore12,Ore13,Ore14	*OreG0027025*
scaffold97	2101234	C	T	NA	*OreG0027025*
scaffold97	2101366	T	A	Ore01,Ore02,Ore03,Ore04,Ore05,Ore06,Ore07,Ore08,Ore09,Ore10,Ore11,Ore12,Ore13,Ore14	*OreG0027025*
scaffold97	2101411	C	A	NA	*OreG0027025*
scaffold97	2101429	A	G	NA	*OreG0027025*
scaffold97	2101842	C	T	NA	*OreG0027025*
scaffold936	96878	A	T	Och02,Och04,Och12,Och13,Ore02,Ore03,Ore04,Ore05,Ore08,Ore09,Ore10,Ore12,Ore14	*OreG0026823*
scaffold936	350807	G	A	NA	*OreG0026838*
scaffold936	350987	C	T	Och13	*OreG0026838*
scaffold936	351315	G	A	Och08	*OreG0026838*
scaffold936	351446	C	T	NA	*OreG0026838*
scaffold936	351736	C	G	NA	*OreG0026838*
scaffold936	351766	C	A	NA	*OreG0026838*
scaffold936	351981	T	A	NA	*OreG0026838*
scaffold936	367062	T	C	NA	*OreG0026839*
scaffold936	367092	G	A	NA	*OreG0026839*
scaffold936	367404	T	G	Och02,Och04,Och05,Och09,Och10,Och12,Och13	*OreG0026839*
scaffold936	371167	G	A	NA	*OreG0026839*
scaffold936	371208	G	A	NA	*OreG0026839*
scaffold936	375059	T	C	NA	*OreG0026839*
scaffold936	375888	A	T	NA	*OreG0026839*
scaffold936	378086	C	T	Och08	*OreG0026840*
scaffold936	380754	T	G	NA	*OreG0026841*
scaffold936	380848	C	T	Och02,Och04,Och05,Och08,Och09,Och10,Och11,Och12,Och13	*OreG0026841*
scaffold936	381584	C	T	Och08	*OreG0026841*
scaffold936	382689	T	C	Och02,Och04,Och05,Och09,Och10,Och12,Och13	*OreG0026841*
scaffold936	387819	T	C	Och08	*OreG0026843*
scaffold936	391706	A	T	Ore10	*OreG0026843*
scaffold936	392872	A	G	Och01,Och02,Och03,Och04,Och05,Och06,Och07,Och08,Och09,Och10,Och11,Och12,Och13,Och14,Ore01,Ore02,Ore03,Ore04,Ore05,Ore06,Ore07,Ore08,Ore09,Ore10,Ore11,Ore12,Ore13,Ore14	*OreG0026843*
scaffold936	398790	C	T	Och01,Och02,Och03,Och04,Och05,Och06,Och07,Och08,Och09,Och10,Och11,Och12,Och13,Och14,Ore01,Ore02,Ore03,Ore04,Ore05,Ore06,Ore07,Ore08,Ore09,Ore10,Ore11,Ore12,Ore13,Ore14	*OreG0026844*
scaffold936	399200	G	A	Och09,Och10,Och13	*OreG0026844*
scaffold936	399731	A	T	Och09,Och10,Och13	*OreG0026844*
scaffold936	399949	A	C	NA	*OreG0026844*

scaffold936	412332	G	A	NA	*OreG0026845*
scaffold936	416692	G	A	Ore08,Ore09,Ore12,Ore14	*OreG0026846*
scaffold936	416838	A	T	Ore08,Ore09,Ore12,Ore14	*OreG0026846*
scaffold936	423281	G	A	NA	*OreG0026847*
scaffold936	433245	G	A	NA	*OreG0026849*
scaffold936	451789	A	T	NA	*OreG0026852*
scaffold936	452214	T	G	Och05,Och08,Och13	*OreG0026852*
scaffold936	453085	T	G	NA	*OreG0026852*
scaffold936	453112	C	T	NA	*OreG0026852*
scaffold936	455523	A	G	Och09,Och10,Och11	*OreG0026852*
scaffold936	503041	C	T	Och01,Och02,Och03,Och04,Och05,Och06,Och07,Och08,Och09,Och10,Och11,Och12,Och13,Och14	*OreG0026856*
scaffold936	552426	C	T	NA	*OreG0026859*
scaffold936	577627	G	A	NA	*OreG0026864*
scaffold936	579090	C	T	NA	*OreG0026864*
scaffold936	579112	A	G	Och01,Och03,Och08,Och09,Och10,Och11,Och13,Och14,Ore01,Ore02,Ore03,Ore04,Ore05,Ore06,Ore07,Ore08,Ore09,Ore10,Ore11,Ore12,Ore13,Ore14	*OreG0026864*
scaffold936	580210	C	T	NA	*OreG0026864*
scaffold936	580237	G	T	Och01,Och03,Och08,Och09,Och10,Och11,Och13,Och14,Ore01,Ore02,Ore03,Ore04,Ore05,Ore06,Ore07,Ore08,Ore09,Ore10,Ore11,Ore12,Ore13,Ore14	*OreG0026864*
scaffold936	583681	G	T	Och01,Och02,Och03,Och04,Och05,Och06,Och07,Och08,Och09,Och10,Och11,Och12,Och13,Och14,Ore01,Ore02,Ore03,Ore04,Ore05,Ore06,Ore07,Ore08,Ore09,Ore10,Ore11,Ore12,Ore13,Ore14	*OreG0026864*
scaffold936	602555	T	C	Och08,Och14	*OreG0026866*
scaffold936	603362	T	C	Och05,Och09,Och10,Och11	*OreG0026866*
scaffold936	603478	A	C	NA	*OreG0026866*
scaffold936	605338	G	A	Och08,Och14,Ore02,Ore06,Ore07,Ore10,Ore11,Ore13	*OreG0026867*
scaffold936	605863	T	G	Och09,Och10,Och11	*OreG0026867*
scaffold936	606720	C	T	Och08,Och14,Ore02,Ore06,Ore07,Ore10,Ore11,Ore13	*OreG0026867*
scaffold936	609165	G	A	Och08,Och14	*OreG0026867*
scaffold936	752595	C	G	NA	*OreG0026869*
scaffold936	763402	A	T	NA	*OreG0026870*
scaffold936	776785	G	T	NA	*OreG0026871*
scaffold936	790861	T	A	Och06,Ore01	*OreG0026871*
scaffold936	796956	T	G	Och08,Och09,Och10,Ore02,Ore10	*OreG0026872*
scaffold936	797475	C	T	Och02,Och04,Och05,Och12,Och13,Och14	*OreG0026872*
scaffold936	895039	T	C	NA	*OreG0026883*
scaffold936	945250	C	A	NA	*OreG0026886*
scaffold936	945516	A	T	NA	*OreG0026886*
scaffold936	945638	A	T	NA	*OreG0026886*
scaffold936	945727	A	G	NA	*OreG0026886*
scaffold936	945754	G	T	NA	*OreG0026886*
scaffold936	1079625	G	T	NA	*OreG0026889*
scaffold936	1080427	T	C	NA	*OreG0026889*
scaffold936	1080436	A	C	NA	*OreG0026889*
scaffold936	1080460	T	C	NA	*OreG0026889*
scaffold936	1081764	T	C	NA	*OreG0026889*
scaffold936	1085493	C	T	Och02,Och03,Och04,Och05,Och06,Och08,Och09,Och10,Och12,Och13	*OreG0026890*
scaffold936	1085984	T	C	Och01,Och02,Och03,Och04,Och05,Och06,Och07,Och08,Och09,Och10,Och11,Och12,Och13,Och14,Ore01,Ore02,Ore03,Ore04,Ore05,Ore06,Ore07,Ore08,Ore09,Ore10,Ore11,Ore12,Ore13,Ore14	*OreG0026890*
scaffold936	1106845	G	A	Och05	*OreG0026893*
scaffold936	1106937	G	A	NA	*OreG0026893*
scaffold936	1117237	G	T	Ore10	*OreG0026894*
scaffold936	1121213	T	G	Och05	*OreG0026895*
scaffold936	1121390	T	A	NA	*OreG0026895*
scaffold936	1138709	G	A	Och05	*OreG0026898*
scaffold936	1140053	T	C	NA	*OreG0026898*
scaffold936	1141802	G	A	NA	*OreG0026898*
scaffold936	1144701	T	G	NA	*OreG0026898*
scaffold936	1144744	C	T	NA	*OreG0026898*
scaffold936	1145735	C	T	NA	*OreG0026898*
scaffold936	1239516	G	C	Och01,Och02,Och06,Och09,Och10,Och14,Ore01,Ore02,Ore03,Ore04,Ore05,Ore06,Ore07,Ore08,Ore09,Ore10,Ore11,Ore12,Ore13,Ore14	*OreG0026903*
scaffold936	1239652	C	A	Och01,Och02,Och06,Och09,Och10,Och14,Ore01,Ore02,Ore03,Ore04,Ore05,Ore06,Ore07,Ore08,Ore09,Ore10,Ore11,Ore12,Ore13,Ore14	*OreG0026903*
scaffold936	1239759	C	T	NA	*OreG0026903*
scaffold936	1240345	A	G	Och01,Och02,Och06,Och09,Och10,Och14,Ore01,Ore02,Ore03,Ore04,Ore05,Ore06,Ore07,Ore08,Ore09,Ore10,Ore11,Ore12,Ore13,Ore14	*OreG0026904*
scaffold936	1241859	G	A	NA	*OreG0026905*
scaffold936	1241992	G	A	NA	*OreG0026905*
scaffold936	1242132	A	G	NA	*OreG0026905*
scaffold936	1242141	G	A	NA	*OreG0026905*
scaffold936	1242289	T	A	NA	*OreG0026905*

scaffold936	1261833	C	T	NA	*OreG0026907*
scaffold936	1262247	C	T	NA	*OreG0026907*
scaffold936	1305874	G	A	NA	*OreG0026912*
scaffold936	1307967	T	A	NA	*OreG0026912*
scaffold936	1308073	C	T	NA	*OreG0026912*
scaffold936	1308081	A	C	NA	*OreG0026912*
scaffold936	1316017	C	T	Och08	*OreG0026913*
scaffold936	1316218	C	T	NA	*OreG0026913*
scaffold936	1435366	A	G	Och01,Och02,Och03,Och04,Och05,Och06,Och07,Och08,Och09,Och10,Och11,Och12,Och13,Och14,Ore01,Ore02,Ore03,Ore04,Ore05,Ore06,Ore07,Ore08,Ore09,Ore10,Ore11,Ore12,Ore13,Ore14	*OreG0026924*
scaffold1302	181464	G	A	Och06	*OreG0001993*
scaffold1739	63290	G	A	Ore04,Ore05	*OreG0003534*
scaffold1739	63625	A	G	NA	*OreG0003534*
scaffold1739	83730	G	T	NA	*OreG0003537*
scaffold1739	84889	T	C	NA	*OreG0003537*
scaffold1739	101540	A	T	Och01,Och02,Och03,Och04,Och05,Och06,Och07,Och08,Och09,Och10,Och11,Och12,Och13,Och14,Ore01,Ore02,Ore03,Ore04,Ore05,Ore06,Ore07,Ore08,Ore09,Ore10,Ore11,Ore12,Ore13,Ore14	*OreG0003538*
scaffold1739	103261	C	A	Ore01,Ore02,Ore03,Ore04,Ore05,Ore06,Ore07,Ore08,Ore09,Ore10,Ore11,Ore12,Ore13,Ore14	*OreG0003538*
scaffold1739	104609	G	T	Och02,Och03,Och04,Och05,Och06,Och12,Och13	*OreG0003538*
scaffold1739	104617	C	T	NA	*OreG0003538*
scaffold1739	106797	T	A	Och12,Och13	*OreG0003539*
scaffold1739	106806	C	T	Och02,Och03,Och04,Och05,Och06,Och08,Och09,Och10,Och11,Och12,Och13,Och14	*OreG0003539*
scaffold1739	107144	T	G	NA	*OreG0003539*
scaffold1739	107187	T	C	Och01,Och02,Och03,Och04,Och05,Och06,Och07,Och08,Och09,Och10,Och11,Och12,Och13,Och14,Ore01,Ore02,Ore03,Ore04,Ore05,Ore06,Ore07,Ore08,Ore09,Ore10,Ore11,Ore12,Ore13,Ore14	*OreG0003539*
scaffold1739	109263	G	A	Ore01,Ore02,Ore03,Ore04,Ore05,Ore06,Ore07,Ore08,Ore09,Ore10,Ore11,Ore12,Ore13,Ore14	*OreG0003539*
scaffold1739	111698	C	T	Och02,Och03,Och04,Och05,Och06,Och12,Och13	*OreG0003539*
scaffold1739	111709	A	G	Ore01,Ore02,Ore03,Ore04,Ore05,Ore06,Ore07,Ore08,Ore09,Ore10,Ore11,Ore12,Ore13,Ore14	*OreG0003539*
scaffold1739	139663	T	A	NA	*OreG0003541*
scaffold1739	139687	G	T	NA	*OreG0003541*
scaffold718	1111	G	T	Och02,Och03,Och04,Och05,Och06,Och12,Och13,Och14	*OreG0024643*
scaffold718	1227	T	C	Och01,Och02,Och03,Och04,Och05,Och06,Och07,Och08,Och09,Och10,Och11,Och12,Och13,Och14,Ore01,Ore02,Ore03,Ore04,Ore05,Ore06,Ore07,Ore08,Ore09,Ore10,Ore11,Ore12,Ore13,Ore14	*OreG0024643*
scaffold718	144628	G	A	Ore01,Ore02,Ore03,Ore04,Ore05,Ore06,Ore07,Ore08,Ore09,Ore10,Ore11,Ore12,Ore13,Ore14	*OreG0024648*
scaffold718	144847	C	T	Ore01,Ore04,Ore05,Ore11	*OreG0024648*
scaffold718	144937	C	T	NA	*OreG0024648*
scaffold718	145918	A	G	Och01,Och02,Och03,Och04,Och05,Och06,Och07,Och08,Och09,Och10,Och11,Och12,Och13,Och14	*OreG0024648*
scaffold718	154444	G	A	NA	*OreG0024650*
scaffold718	154502	A	G	NA	*OreG0024650*
scaffold718	154854	G	A	NA	*OreG0024650*
scaffold718	155875	C	T	NA	*OreG0024650*
scaffold718	156110	C	T	NA	*OreG0024650*
scaffold718	156134	A	T	NA	*OreG0024650*
scaffold718	161056	G	A	NA	*OreG0024651*
scaffold718	164092	G	A	NA	*OreG0024651*
scaffold718	265868	G	A	Och12,Och13	*OreG0024654*
scaffold718	268349	C	T	Och01,Och02,Och03,Och04,Och05,Och06,Och07,Och08,Och09,Och10,Och11,Och12,Och13,Och14	*OreG0024654*
scaffold718	269299	G	A	Ore01,Ore02,Ore03,Ore04,Ore05,Ore06,Ore07,Ore08,Ore09,Ore10,Ore11,Ore12,Ore13,Ore14	*OreG0024654*
scaffold718	308643	T	A	Och01,Och02,Och03,Och04,Och05,Och06,Och07,Och08,Och09,Och10,Och11,Och12,Och13,Och14,Ore01,Ore02,Ore03,Ore04,Ore05,Ore06,Ore07,Ore08,Ore09,Ore10,Ore11,Ore12,Ore13,Ore14	*OreG0024657*
scaffold718	387120	A	T	Ore01,Ore02,Ore03,Ore04,Ore05,Ore06,Ore07,Ore08,Ore09,Ore10,Ore11,Ore12,Ore13,Ore14	*OreG0024662*
scaffold718	520695	C	T	NA	*OreG0024670*
scaffold718	535106	T	C	NA	*OreG0024671*
scaffold718	569379	G	A	NA	*OreG0024673*
scaffold718	569724	C	G	NA	*OreG0024673*
scaffold718	569910	A	G	Ore01,Ore02,Ore03,Ore04,Ore05,Ore06,Ore07,Ore08,Ore09,Ore10,Ore11,Ore12,Ore13,Ore14	*OreG0024673*
scaffold718	570016	T	A	NA	*OreG0024673*
scaffold718	570301	C	T	NA	*OreG0024673*
scaffold718	603962	T	C	NA	*OreG0024674*
scaffold718	607136	T	A	NA	*OreG0024674*

scaffold718	607178	T	G	NA	*OreG0024674*
scaffold718	607924	T	C	Och01,Och02,Och03,Och04,Och05,Och06,Och07,Och08,Och09,Och10,Och11,Och12,Och13,Och14	*OreG0024674*
scaffold718	618866	A	G	Och08	*OreG0024675*
scaffold718	712637	T	G	Och08	*OreG0024685*
scaffold718	714246	A	C	Och01,Och02,Och03,Och04,Och05,Och06,Och07,Och08,Och09,Och10,Och11,Och12,Och13,Och14,Ore01,Ore02,Ore03,Ore04,Ore05,Ore06,Ore07,Ore08,Ore09,Ore10,Ore11,Ore12,Ore13,Ore14	*OreG0024685*
scaffold718	715448	C	A	NA	*OreG0024685*
scaffold718	764124	G	T	NA	*OreG0024689*
scaffold718	765470	C	T	NA	*OreG0024689*
scaffold718	803406	G	T	Och02,Och03,Och04,Och05,Och06,Och07,Och08,Och09,Och10,Och11,Och12,Och13,Och14	*OreG0024693*
scaffold718	925643	T	A	Ore01,Ore02,Ore03,Ore04,Ore05,Ore06,Ore07,Ore08,Ore09,Ore10,Ore11,Ore12,Ore13,Ore14	*OreG0024704*
scaffold718	927396	A	T	NA	*OreG0024704*
scaffold718	936676	T	C	Och01,Och02,Och03,Och04,Och05,Och06,Och07,Och08,Och09,Och10,Och11,Och12,Och13,Och14,Ore01,Ore02,Ore03,Ore04,Ore05,Ore06,Ore07,Ore08,Ore09,Ore10,Ore11,Ore12,Ore13,Ore14	*OreG0024705*
scaffold718	1109877	G	A	NA	*OreG0024707*
scaffold718	1109914	C	A	NA	*OreG0024707*
scaffold718	1110637	G	A	NA	*OreG0024707*
scaffold718	1110880	G	A	NA	*OreG0024707*
scaffold718	1123001	T	A	NA	*OreG0024709*
scaffold718	1150590	G	C	NA	*OreG0024711*
scaffold718	1151029	C	T	NA	*OreG0024711*
scaffold718	1151034	G	A	NA	*OreG0024711*
scaffold718	1190567	G	T	Ore01,Ore02,Ore03,Ore04,Ore05,Ore06,Ore07,Ore08,Ore09,Ore10,Ore11,Ore12,Ore13,Ore14	*OreG0024715*
scaffold718	1191513	C	G	Och01,Och02,Och03,Och04,Och05,Och06,Och07,Och08,Och09,Och10,Och11,Och12,Och13,Och14	*OreG0024715*
scaffold718	1196609	T	A	Ore06,Ore07,Ore10,Ore11,Ore14	*OreG0024716*
scaffold718	1199637	G	A	NA	*OreG0024717*
scaffold718	1199764	C	A	Ore06,Ore07,Ore10,Ore11,Ore14	*OreG0024717*
scaffold718	1199769	C	A	NA	*OreG0024717*
scaffold718	1201484	G	T	NA	*OreG0024717*
scaffold718	1262346	T	A	Och07,Och08	*OreG0024723*
scaffold718	1263320	T	G	NA	*OreG0024723*
scaffold718	1266010	A	T	NA	*OreG0024724*
scaffold718	1266440	T	C	Ore01,Ore02,Ore03,Ore06,Ore07,Ore08,Ore09,Ore10,Ore11,Ore12,Ore13,Ore14	*OreG0024724*
scaffold718	1268602	T	A	Ore01,Ore02,Ore03,Ore06,Ore07,Ore08,Ore09,Ore10,Ore11,Ore12,Ore13,Ore14	*OreG0024724*
scaffold718	1309562	A	T	NA	*OreG0024730*
scaffold718	1329589	G	A	Ore01,Ore02,Ore03,Ore04,Ore05,Ore06,Ore07,Ore08,Ore09,Ore10,Ore11,Ore12,Ore13,Ore14	*OreG0024732*
scaffold718	1377980	G	A	NA	*OreG0024737*
scaffold718	1378226	G	A	NA	*OreG0024737*
scaffold718	1378254	C	T	NA	*OreG0024737*
scaffold718	1424352	T	C	Ore06,Ore07,Ore10,Ore11,Ore14	*OreG0024739*
scaffold718	1451893	A	G	NA	*OreG0024743*
scaffold718	1454508	A	G	Och01,Och02,Och03,Och04,Och05,Och06,Och07,Och08,Och09,Och10,Och11,Och12,Och13,Och14	*OreG0024745*
scaffold718	1454595	G	A	NA	*OreG0024745*
scaffold718	1454622	A	C	Ore01,Ore02,Ore03,Ore04,Ore05,Ore06,Ore07,Ore08,Ore09,Ore10,Ore11,Ore12,Ore13,Ore14	*OreG0024745*
scaffold718	1454635	A	G	Och01,Och02,Och03,Och04,Och05,Och06,Och07,Och08,Och09,Och10,Och11,Och12,Och13,Och14	*OreG0024745*
scaffold718	1454703	A	T	Och01,Och07,Och08,Och09,Och10,Och11,Och14	*OreG0024745*
scaffold718	1461338	G	C	Och02,Och05,Och12,Och13	*OreG0024746*
scaffold718	1464109	G	C	NA	*OreG0024747*
scaffold718	1464129	G	A	NA	*OreG0024747*
scaffold718	1556459	G	A	Och01,Och02,Och03,Och04,Och05,Och06,Och07,Och08,Och09,Och10,Och11,Och12,Och13,Och14,Ore01,Ore02,Ore03,Ore04,Ore05,Ore06,Ore07,Ore08,Ore09,Ore10,Ore11,Ore12,Ore13,Ore14	*OreG0024760*
scaffold718	1556462	G	A	Och03,Och06,Och12,Och13	*OreG0024760*
scaffold718	1558938	C	G	NA	*OreG0024760*
scaffold718	1622696	A	T	Och01	*OreG0024767*
scaffold718	1622741	T	A	NA	*OreG0024767*
scaffold718	1622752	C	G	NA	*OreG0024767*
scaffold718	1622758	G	T	NA	*OreG0024767*
scaffold718	1622778	C	T	Och01,Och02,Och03,Och04,Och05,Och06,Och07,Och08,Och09,Och10,Och11,Och12,Och13,Och14	*OreG0024767*
scaffold718	1622805	C	T	NA	*OreG0024767*
scaffold718	1622879	G	A	Och03,Och06,Och12,Och13	*OreG0024767*
scaffold718	1626459	A	C	NA	*OreG0024769*
scaffold718	1626855	C	T	Ore09	*OreG0024769*

scaffold718	1627005	G	T	NA	*OreG0024769*
scaffold718	1629025	T	C	NA	*OreG0024769*
scaffold718	1629048	C	T	NA	*OreG0024769*
scaffold718	1629067	G	T	NA	*OreG0024769*
scaffold718	1673982	C	A	Ore01,Ore02,Ore03,Ore04,Ore05,Ore06,Ore07,Ore08,Ore09,Ore10,Ore11,Ore12,Ore13,Ore14	*OreG0024773*
scaffold718	1697345	C	A	Ore01,Ore02,Ore03,Ore04,Ore05,Ore06,Ore07,Ore08,Ore09,Ore10,Ore11,Ore12,Ore13,Ore14	*OreG0024774*
scaffold718	1705991	C	T	NA	*OreG0024776*
scaffold718	1706247	C	T	NA	*OreG0024776*
scaffold718	1707281	A	C	NA	*OreG0024776*
scaffold718	1708745	C	T	NA	*OreG0024776*
scaffold718	1708834	C	A	NA	*OreG0024776*
scaffold718	1708983	C	A	NA	*OreG0024776*
scaffold718	1709253	C	T	NA	*OreG0024776*
scaffold718	1709909	G	A	NA	*OreG0024776*
scaffold718	1709912	G	C	Ore01,Ore04,Ore07,Ore09,Ore11,Ore12,Ore14	*OreG0024776*
scaffold718	1709927	G	A	NA	*OreG0024776*
scaffold718	1770311	G	A	NA	*OreG0024781*
scaffold718	1770521	G	T	Ore01,Ore02,Ore03,Ore04,Ore05,Ore06,Ore07,Ore08,Ore09,Ore10,Ore11,Ore12,Ore13,Ore14	*OreG0024781*
scaffold718	1832213	T	G	Och09,Och10,Och11	*OreG0024786*
scaffold718	1838147	G	A	NA	*OreG0024788*
scaffold718	1842212	C	A	NA	*OreG0024788*
scaffold718	1855758	T	G	Och01,Och02,Och03,Och04,Och05,Och06,Och07,Och08,Och09,Och10,Och11,Och12,Och13,Och14,Ore01,Ore02,Ore03,Ore04,Ore05,Ore06,Ore07,Ore08,Ore09,Ore10,Ore11,Ore12,Ore13,Ore14	*OreG0024790*
scaffold718	1857207	A	T	NA	*OreG0024790*
scaffold718	1871590	T	C	NA	*OreG0024791*
scaffold718	1876250	C	A	Ore04,Ore05	*OreG0024791*
scaffold718	1883162	G	A	NA	*OreG0024791*
scaffold718	1883295	C	T	NA	*OreG0024791*
scaffold718	1886149	C	A	NA	*OreG0024791*
scaffold718	1917545	C	T	NA	*OreG0024792*
scaffold718	1917700	A	G	NA	*OreG0024792*
scaffold718	1926864	T	C	NA	*OreG0024793*
scaffold718	1926930	C	T	NA	*OreG0024793*
scaffold718	1926942	A	G	NA	*OreG0024793*
scaffold718	1927043	G	C	NA	*OreG0024793*
scaffold718	1928581	A	T	Och06	*OreG0024793*
scaffold718	1932238	C	T	NA	*OreG0024793*
scaffold718	2055243	C	G	NA	*OreG0024807*
scaffold718	2055268	T	A	NA	*OreG0024807*
scaffold718	2055460	C	T	NA	*OreG0024807*
scaffold718	2055485	A	T	NA	*OreG0024807*
scaffold718	2055572	A	G	NA	*OreG0024807*
scaffold718	2100457	A	T	NA	*OreG0024811*
scaffold718	2112162	C	G	NA	*OreG0024813*
scaffold718	2112348	C	T	Och01,Och07,Och11,Och14	*OreG0024813*
scaffold718	2112399	A	G	Och02,Och03,Och04,Och05,Och06,Och12,Och13	*OreG0024813*
scaffold718	2149123	C	T	NA	*OreG0024818*
scaffold994	103319	C	T	Och01,Och02,Och03,Och04,Och05,Och06,Och07,Och08,Och09,Och10,Och11,Och12,Och13,Och14,Ore01,Ore02,Ore03,Ore04,Ore05,Ore06,Ore07,Ore08,Ore09,Ore10,Ore11,Ore12,Ore13,Ore14	*OreG0027254*
scaffold994	106594	C	T	Och01,Och02,Och03,Och04,Och05,Och06,Och07,Och08,Och09,Och10,Och11,Och12,Och13,Och14	*OreG0027254*
scaffold994	109906	G	A	Ore01,Ore02,Ore03,Ore04,Ore05,Ore06,Ore07,Ore08,Ore09,Ore10,Ore11,Ore12,Ore13,Ore14	*OreG0027255*
scaffold994	110659	C	T	Och04	*OreG0027255*
scaffold994	111868	C	T	NA	*OreG0027255*
scaffold994	154583	G	A	NA	*OreG0027256*
scaffold85	47067	C	G	Och01,Och02,Och03,Och04,Och05,Och06,Och07,Och08,Och09,Och10,Och11,Och12,Och13,Och14,Ore01,Ore02,Ore03,Ore04,Ore05,Ore06,Ore07,Ore08,Ore09,Ore10,Ore11,Ore12,Ore13,Ore14	*OreG0025413*
scaffold85	97201	C	T	Ore01,Ore02,Ore03,Ore04,Ore05,Ore06,Ore07,Ore08,Ore09,Ore10,Ore11,Ore12,Ore13,Ore14	*OreG0025418*
scaffold85	146257	G	T	NA	*OreG0025421*
scaffold85	177708	G	T	Och01,Och02,Och03,Och04,Och05,Och06,Och07,Och08,Och09,Och10,Och11,Och12,Och13,Och14,Ore01,Ore02,Ore03,Ore04,Ore05,Ore06,Ore07,Ore08,Ore09,Ore10,Ore11,Ore12,Ore13,Ore14	*OreG0025423*
scaffold85	177951	A	C	Och01,Och02,Och03,Och04,Och05,Och06,Och07,Och08,Och09,Och10,Och11,Och12,Och13,Och14,Ore01,Ore02,Ore03,Ore04,Ore05,Ore06,Ore07,Ore08,Ore09,Ore10,Ore11,Ore12,Ore13,Ore14	*OreG0025423*
scaffold85	180853	G	T	Och11	*OreG0025423*
scaffold85	183381	T	A	Och14	*OreG0025423*

scaffold85	184424	T	G	Och01,Och02,Och03,Och04,Och05,Och06,Och07,Och08,Och09,Och10,Och11,Och12,Och13,Och14,Ore01,Ore02,Ore03,Ore04,Ore05,Ore06,Ore07,Ore08,Ore09,Ore10,Ore11,Ore12,Ore13,Ore14	*OreG0025423*
scaffold85	308871	C	G	Och01,Och02,Och03,Och04,Och05,Och06,Och07,Och08,Och09,Och10,Och11,Och12,Och13,Och14,Ore01,Ore02,Ore03,Ore04,Ore05,Ore06,Ore07,Ore08,Ore09,Ore10,Ore11,Ore12,Ore13,Ore14	*OreG0025429*
scaffold85	309568	T	A	NA	*OreG0025429*
scaffold85	342586	C	T	NA	*OreG0025430*
scaffold85	559194	T	C	Och01,Och02,Och03,Och04,Och05,Och06,Och07,Och08,Och09,Och10,Och11,Och12,Och13,Och14,Ore01,Ore02,Ore03,Ore04,Ore05,Ore06,Ore07,Ore08,Ore09,Ore10,Ore11,Ore12,Ore13,Ore14	*OreG0025434*
scaffold85	686499	C	A	Och11	*OreG0025437*
scaffold85	743222	C	A	NA	*OreG0025442*
scaffold85	802575	C	G	Och01,Och02,Och03,Och04,Och05,Och06,Och07,Och08,Och09,Och10,Och11,Och12,Och13,Och14,Ore01,Ore02,Ore03,Ore04,Ore05,Ore06,Ore07,Ore08,Ore09,Ore10,Ore11,Ore12,Ore13,Ore14	*OreG0025445*
scaffold85	817936	A	T	Och02,Och03,Och04,Och05,Och06,Och12,Och13	*OreG0025445*
scaffold85	928867	G	A	Ore02,Ore03,Ore06,Ore07,Ore08,Ore09,Ore10,Ore11,Ore12,Ore13,Ore14	*OreG0025448*
scaffold85	928922	C	T	Och11	*OreG0025448*
scaffold85	973874	C	G	Och01,Och02,Och03,Och04,Och05,Och06,Och07,Och08,Och09,Och10,Och11,Och12,Och13,Och14,Ore01,Ore02,Ore03,Ore04,Ore05,Ore06,Ore07,Ore08,Ore09,Ore10,Ore11,Ore12,Ore13,Ore14	*OreG0025450*
scaffold85	975957	G	C	NA	*OreG0025451*
scaffold85	1002774	C	T	NA	*OreG0025453*
scaffold85	1002801	A	T	Och01,Och02,Och03,Och04,Och05,Och06,Och07,Och08,Och09,Och10,Och11,Och12,Och13,Och14,Ore01,Ore02,Ore03,Ore04,Ore05,Ore06,Ore07,Ore08,Ore09,Ore10,Ore11,Ore12,Ore13,Ore14	*OreG0025453*
scaffold85	1018223	G	A	NA	*OreG0025454*
scaffold85	1087928	G	T	Och11	*OreG0025456*
scaffold85	1088036	T	A	NA	*OreG0025456*
scaffold85	1177847	C	A	Och01,Och02,Och03,Och04,Och05,Och06,Och07,Och08,Och09,Och10,Och11,Och12,Och13,Och14,Ore01,Ore02,Ore03,Ore04,Ore05,Ore06,Ore07,Ore08,Ore09,Ore10,Ore11,Ore12,Ore13,Ore14	*OreG0025459*
scaffold85	1218702	G	A	Ore01,Ore02,Ore03,Ore04,Ore05,Ore06,Ore07,Ore08,Ore09,Ore10,Ore11,Ore12,Ore13,Ore14	*OreG0025460*
scaffold85	1275867	G	T	NA	*OreG0025461*
scaffold85	1282922	T	C	Och06,Och09,Och10	*OreG0025461*
scaffold85	1288975	C	T	NA	*OreG0025461*
scaffold85	1289192	C	G	Ore01,Ore02,Ore03,Ore04,Ore05,Ore06,Ore07,Ore08,Ore09,Ore10,Ore11,Ore12,Ore13,Ore14	*OreG0025461*
scaffold85	1289782	T	C	NA	*OreG0025461*
scaffold85	1289866	C	T	NA	*OreG0025461*
scaffold85	1318989	G	A	Och09	*OreG0025462*
scaffold85	1337319	C	T	NA	*OreG0025464*
scaffold85	1338423	G	A	NA	*OreG0025464*
scaffold85	1344484	C	T	Och11	*OreG0025464*
scaffold85	1363577	C	A	Ore01,Ore02,Ore03,Ore04,Ore05,Ore06,Ore07,Ore08,Ore09,Ore10,Ore11,Ore12,Ore13,Ore14	*OreG0025465*
scaffold85	1363669	G	A	NA	*OreG0025465*
scaffold85	1363801	T	A	NA	*OreG0025465*
scaffold85	1371500	G	A	NA	*OreG0025466*
scaffold85	1371602	A	C	NA	*OreG0025466*
scaffold85	1412522	T	A	Ore01,Ore02,Ore03,Ore04,Ore05,Ore06,Ore07,Ore08,Ore09,Ore10,Ore11,Ore12,Ore13,Ore14	*OreG0025469*
scaffold85	1412874	G	C	Och04,Och05,Och07,Och09,Och10,Och11,Och12,Och14	*OreG0025469*
scaffold85	1413090	A	T	NA	*OreG0025469*
scaffold85	1434440	C	G	Och02,Och03,Och04,Och05,Och06,Och07,Och08,Och09,Och10,Och11,Och12,Och13,Och14	*OreG0025471*
scaffold85	1434736	A	G	Och01,Och02,Och03,Och04,Och05,Och06,Och07,Och08,Och09,Och10,Och11,Och12,Och13,Och14,Ore01,Ore02,Ore03,Ore04,Ore05,Ore06,Ore07,Ore08,Ore09,Ore10,Ore11,Ore12,Ore13,Ore14	*OreG0025471*
scaffold85	1512175	G	A	NA	*OreG0025474*
scaffold85	1522427	C	T	NA	*OreG0025474*
scaffold85	1560097	T	C	Och01,Och02,Och03,Och04,Och05,Och06,Och07,Och08,Och09,Och10,Och11,Och12,Och13,Och14,Ore01,Ore02,Ore03,Ore04,Ore05,Ore06,Ore07,Ore08,Ore09,Ore10,Ore11,Ore12,Ore13,Ore14	*OreG0025476*
scaffold85	1564618	T	A	NA	*OreG0025477*
scaffold85	1564699	T	C	NA	*OreG0025477*
scaffold85	1565939	T	C	Och01,Och02,Och03,Och04,Och05,Och06,Och07,Och08,Och09,Och10,Och11,Och12,Och13,Och14,Ore01,Ore02,Ore03,Ore04,Ore05,Ore06,Ore07,Ore08,Ore09,Ore10,Ore11,Ore12,Ore13,Ore14	*OreG0025477*
scaffold85	1568704	A	T	NA	*OreG0025478*
scaffold85	1572869	A	G	Och05,Och12	*OreG0025478*
scaffold85	1586350	A	T	Ore01,Ore02,Ore03,Ore04,Ore05,Ore06,Ore07,Ore08,Ore09,Ore10,Ore11,Ore12,Ore13,Ore14	*OreG0025480*

scaffold85	1610366	G	T	Ore03	*OreG0025483*
scaffold85	1610436	G	A	Ore02,Ore06,Ore11,Ore12	*OreG0025483*
scaffold85	1611167	G	A	Ore03	*OreG0025483*
scaffold85	1611170	G	C	NA	*OreG0025483*
scaffold85	1618656	A	G	Och02,Och03,Och04,Och05,Och06,Och08,Och09,Och10,Och11,Och12,Och13,Och14	*OreG0025484*
scaffold85	1619866	G	T	NA	*OreG0025484*
scaffold85	1625429	A	C	Ore02,Ore06,Ore11,Ore12	*OreG0025485*
scaffold85	1625562	G	T	Ore01,Ore02,Ore03,Ore04,Ore05,Ore06,Ore07,Ore08,Ore09,Ore10,Ore11,Ore12,Ore13,Ore14	*OreG0025485*
scaffold85	1629252	A	G	Och01,Och02,Och03,Och04,Och05,Och06,Och07,Och08,Och09,Och10,Och11,Och12,Och13,Och14	*OreG0025486*
scaffold85	1644381	C	T	NA	*OreG0025488*
scaffold85	1644462	G	A	NA	*OreG0025488*
scaffold85	1644492	C	T	NA	*OreG0025488*
scaffold85	1660932	C	T	Ore03	*OreG0025491*
scaffold85	1736333	T	A	Ore02,Ore06,Ore11,Ore12	*OreG0025495*
scaffold85	1736928	C	T	NA	*OreG0025495*
scaffold85	1737021	T	C	Och12,Och13,Ore02,Ore06,Ore11,Ore12	*OreG0025495*
scaffold85	1800578	T	A	NA	*OreG0025499*
scaffold85	1801499	G	T	Ore03	*OreG0025499*
scaffold85	1847989	T	A	NA	*OreG0025507*
scaffold85	1848493	T	C	Och06,Och12,Och13	*OreG0025507*
scaffold85	1848795	G	T	NA	*OreG0025507*
scaffold85	1849594	G	A	NA	*OreG0025507*
scaffold85	1872054	T	C	Och01,Och02,Och03,Och04,Och05,Och06,Och11,Och12,Och13,Och14	*OreG0025508*
scaffold85	1876446	T	C	NA	*OreG0025509*
scaffold85	1877042	A	T	NA	*OreG0025509*
scaffold85	1877145	T	A	Och11	*OreG0025509*
scaffold85	1891024	A	G	Och11	*OreG0025511*
scaffold85	1900702	T	A	Ore02,Ore06,Ore11,Ore12	*OreG0025512*
scaffold85	1901789	T	A	Och01,Och02,Och03,Och04,Och05,Och06,Och07,Och08,Och09,Och10,Och11,Och12,Och13,Och14,Ore01,Ore02,Ore03,Ore04,Ore05,Ore06,Ore07,Ore08,Ore09,Ore10,Ore11,Ore12,Ore13,Ore14	*OreG0025512*
scaffold85	1934716	A	T	Och01,Och02,Och03,Och04,Och05,Och06,Och07,Och08,Och09,Och10,Och11,Och12,Och13,Och14,Ore01,Ore02,Ore03,Ore04,Ore05,Ore06,Ore07,Ore08,Ore09,Ore10,Ore11,Ore12,Ore13,Ore14	*OreG0025515*
scaffold85	1975108	C	T	Och01,Och02,Och03,Och04,Och05,Och06,Och07,Och08,Och09,Och10,Och11,Och12,Och13,Och14	*OreG0025519*
scaffold85	1975159	G	A	NA	*OreG0025519*
scaffold85	1975211	G	T	Och01	*OreG0025519*
scaffold85	2038626	T	C	NA	*OreG0025523*
scaffold85	2318484	G	C	Och12	*OreG0025532*
scaffold85	2321476	C	T	Och01,Och02,Och03,Och04,Och05,Och06,Och07,Och08,Och09,Och10,Och11,Och12,Och13,Och14,Ore01,Ore02,Ore03,Ore04,Ore05,Ore06,Ore07,Ore08,Ore09,Ore10,Ore11,Ore12,Ore13,Ore14	*OreG0025532*
scaffold85	2325563	C	T	NA	*OreG0025533*
scaffold85	2363323	A	T	Och02,Och03,Och04,Och05,Och06,Och12,Och13	*OreG0025538*
scaffold85	2456513	G	A	NA	*OreG0025548*
scaffold85	2464290	T	A	Och01,Och02,Och03,Och04,Och05,Och06,Och08,Och12,Och13	*OreG0025548*
scaffold85	2489175	T	G	NA	*OreG0025549*
scaffold85	2489492	A	T	NA	*OreG0025549*
scaffold85	2489984	T	C	NA	*OreG0025549*
scaffold85	2489998	G	C	Ore01,Ore02,Ore03,Ore04,Ore05,Ore06,Ore07,Ore08,Ore09,Ore10,Ore11,Ore12,Ore13,Ore14	*OreG0025549*
scaffold85	2520408	A	C	Och01,Och02,Och03,Och04,Och05,Och06,Och07,Och08,Och09,Och10,Och11,Och12,Och13,Och14	*OreG0025550*
scaffold85	2527532	A	G	NA	*OreG0025552*
scaffold85	2538047	C	T	Och06,Och12,Och13	*OreG0025553*
scaffold85	2558934	G	A	Och07	*OreG0025555*
scaffold85	2561188	T	C	Ore01,Ore02,Ore03,Ore04,Ore05,Ore06,Ore07,Ore08,Ore09,Ore10,Ore11,Ore12,Ore13,Ore14	*OreG0025555*
scaffold85	2565361	G	A	NA	*OreG0025556*
scaffold85	2575249	C	A	NA	*OreG0025557*
scaffold85	2575598	G	A	Och01,Och02,Och03,Och04,Och05,Och06,Och07,Och08,Och09,Och10,Och11,Och12,Och13,Och14,Ore01,Ore02,Ore03,Ore04,Ore05,Ore06,Ore07,Ore08,Ore09,Ore10,Ore11,Ore12,Ore13,Ore14	*OreG0025557*
scaffold85	2589212	G	T	Och01,Och02,Och03,Och04,Och05,Och06,Och07,Och08,Och09,Och10,Och11,Och12,Och13,Och14	*OreG0025558*
scaffold85	2612476	T	C	Ore01,Ore02,Ore03,Ore04,Ore05,Ore06,Ore07,Ore08,Ore09,Ore10,Ore11,Ore12,Ore13,Ore14	*OreG0025560*
scaffold85	2623716	C	A	Och01,Och02,Och03,Och04,Och05,Och06,Och07,Och08,Och09,Och10,Och11,Och12,Och13,Och14	*OreG0025562*
scaffold85	2627228	C	A	Ore01,Ore02,Ore03,Ore04,Ore05,Ore06,Ore07,Ore08,Ore09,Ore10,Ore11,Ore12,Ore13,Ore14	*OreG0025562*

scaffold85	2633489	G	C	Ore01,Ore02,Ore03,Ore04,Ore05,Ore06,Ore07,Ore08,Ore09,Ore10,Ore11,Ore12,Ore13,Ore14	*OreG0025563*
scaffold85	2634593	C	G	Och01,Och02,Och03,Och04,Och05,Och06,Och07,Och08,Och09,Och10,Och11,Och12,Och13,Och14	*OreG0025563*
scaffold85	2634598	C	T	NA	*OreG0025563*
scaffold85	2634668	C	T	NA	*OreG0025563*
scaffold85	2634833	C	A	Och01,Och02,Och03,Och04,Och05,Och06,Och07,Och08,Och09,Och10,Och11,Och12,Och13,Och14,Ore01,Ore02,Ore03,Ore04,Ore05,Ore06,Ore07,Ore08,Ore09,Ore10,Ore11,Ore12,Ore13,Ore14	*OreG0025563*
scaffold85	2634928	C	T	Ore01,Ore02,Ore03,Ore04,Ore05,Ore06,Ore07,Ore08,Ore09,Ore10,Ore11,Ore12,Ore13,Ore14	*OreG0025563*
scaffold85	2635159	G	T	NA	*OreG0025563*
scaffold85	2641014	A	G	Och01	*OreG0025564*
scaffold85	2645320	A	T	Och01	*OreG0025565*
scaffold85	2645387	A	T	Och01,Och02,Och03,Och04	*OreG0025565*
scaffold85	2645401	G	T	Ore03	*OreG0025565*
scaffold85	2645408	C	A	Och01,Och02,Och03,Och04	*OreG0025565*
scaffold85	2693584	C	T	Ore01,Ore02,Ore03,Ore04,Ore05,Ore06,Ore07,Ore08,Ore09,Ore10,Ore11,Ore12,Ore13,Ore14	*OreG0025570*
scaffold85	2767549	G	A	Och14	*OreG0025576*
scaffold85	2769975	T	G	Och01,Och02,Och03,Och04,Och05,Och06,Och07,Och09,Och10,Och11,Och12,Och13	*OreG0025576*
scaffold85	2802921	T	C	Och01	*OreG0025581*
scaffold85	2803636	T	C	Ore01,Ore02,Ore03,Ore04,Ore05,Ore06,Ore07,Ore08,Ore09,Ore10,Ore11,Ore12,Ore13,Ore14	*OreG0025581*
scaffold85	2857982	G	A	NA	*OreG0025582*
scaffold85	2858118	A	G	NA	*OreG0025582*
scaffold85	2880869	G	T	Och01,Och02,Och03,Och04,Och05,Och06,Och07,Och08,Och09,Och10,Och11,Och12,Och13,Och14,Ore01,Ore02,Ore03,Ore04,Ore05,Ore06,Ore07,Ore08,Ore09,Ore10,Ore11,Ore12,Ore13,Ore14	*OreG0025583*
scaffold85	2884509	T	C	Och11	*OreG0025584*
scaffold85	2884620	A	T	NA	*OreG0025584*
scaffold85	2884701	A	G	Ore01,Ore02,Ore03,Ore04,Ore05,Ore06,Ore07,Ore08,Ore09,Ore10,Ore11,Ore12,Ore13,Ore14	*OreG0025584*
scaffold85	2887982	C	A	Ore02,Ore06,Ore11,Ore12	*OreG0025585*
scaffold85	2999747	T	C	Ore01,Ore02,Ore03,Ore04,Ore05,Ore06,Ore07,Ore08,Ore09,Ore10,Ore11,Ore12,Ore13,Ore14	*OreG0025594*
scaffold85	3003573	A	C	NA	*OreG0025595*
scaffold85	3042250	G	A	Och12	*OreG0025599*
scaffold85	3042888	C	T	NA	*OreG0025599*
scaffold85	3043167	G	T	NA	*OreG0025599*
scaffold85	3044665	G	A	NA	*OreG0025599*
scaffold85	3112230	A	T	Och01,Och02,Och03,Och04,Och05,Och06,Och07,Och08,Och09,Och10,Och11,Och12,Och13,Och14,Ore01,Ore02,Ore03,Ore04,Ore05,Ore06,Ore07,Ore08,Ore09,Ore10,Ore11,Ore12,Ore13,Ore14	*OreG0025604*
scaffold85	3161483	C	T	Och05,Och12	*OreG0025606*
scaffold85	3278795	C	A	NA	*OreG0025614*
scaffold85	3297221	T	G	Och01,Och02,Och03,Och04,Och05,Och06,Och07,Och08,Och09,Och10,Och11,Och12,Och13,Och14	*OreG0025616*
scaffold85	3312741	T	C	Ore01,Ore02,Ore03,Ore04,Ore05,Ore06,Ore07,Ore08,Ore09,Ore10,Ore11,Ore12,Ore13,Ore14	*OreG0025618*
scaffold85	3312992	G	T	NA	*OreG0025618*
scaffold85	3348050	A	C	NA	*OreG0025623*
scaffold85	3407760	A	C	Och01,Och02,Och03,Och04,Och05,Och06,Och07,Och08,Och09,Och10,Och11,Och12,Och13,Och14,Ore01,Ore02,Ore03,Ore04,Ore05,Ore06,Ore07,Ore08,Ore09,Ore10,Ore11,Ore12,Ore13,Ore14	*OreG0025629*
scaffold85	3427055	C	T	NA	*OreG0025631*
scaffold85	3427058	C	T	NA	*OreG0025631*
scaffold85	3427079	G	A	NA	*OreG0025631*
scaffold85	3436271	C	T	NA	*OreG0025631*
scaffold85	3446264	G	T	NA	*OreG0025633*
scaffold85	3448247	A	T	Och11	*OreG0025633*
scaffold85	3450217	C	G	Och07,Och08,Och11	*OreG0025634*
scaffold85	3455946	A	T	Och02,Och03,Och04,Och05,Och06,Och07,Och08,Och11,Och12,Och13,Och14	*OreG0025635*
scaffold85	3456075	C	G	Och02,Och03,Och04,Och05,Och06,Och12,Och13	*OreG0025635*
scaffold85	3461895	T	A	NA	*OreG0025637*
scaffold85	3461916	G	T	NA	*OreG0025637*
scaffold85	3462005	A	T	NA	*OreG0025637*
scaffold85	3462210	A	T	NA	*OreG0025637*
scaffold85	3462387	C	T	Ore01,Ore02,Ore03,Ore04,Ore05,Ore06,Ore07,Ore08,Ore09,Ore10,Ore11,Ore12,Ore13,Ore14	*OreG0025637*
scaffold85	3462393	C	T	NA	*OreG0025637*
scaffold85	3463227	T	C	NA	*OreG0025637*
scaffold85	3463239	A	T	NA	*OreG0025637*
scaffold85	3463616	G	T	NA	*OreG0025637*

scaffold85	3502347	G	A	NA	*OreG0025641*
scaffold85	3503104	T	C	Ore01,Ore02,Ore03,Ore04,Ore05,Ore06,Ore07,Ore08,Ore09,Ore10,Ore11,Ore12,Ore13,Ore14	*OreG0025641*
scaffold85	3517667	A	T	Och01,Och02,Och03,Och04,Och05,Och06,Och07,Och08,Och09,Och10,Och11,Och12,Och13,Och14,Ore01,Ore02,Ore03,Ore04,Ore05,Ore06,Ore07,Ore08,Ore09,Ore10,Ore11,Ore12,Ore13,Ore14	*OreG0025642*
scaffold85	3571072	G	A	NA	*OreG0025644*
scaffold85	3610520	G	A	Ore01,Ore02,Ore03,Ore04,Ore05,Ore06,Ore07,Ore08,Ore09,Ore10,Ore11,Ore12,Ore13,Ore14	*OreG0025648*
scaffold85	3611202	G	A	NA	*OreG0025648*
scaffold85	3656297	G	A	Och11	*OreG0025654*
scaffold85	3657612	T	C	NA	*OreG0025654*
scaffold85	3667232	G	C	Och02,Och03,Och04,Och05,Och06,Och07,Och08,Och09,Och10,Och11,Och12,Och13,Och14	*OreG0025655*
scaffold85	3705547	A	G	Och02,Och03,Och04,Och05,Och06,Och12,Och13	*OreG0025657*
scaffold85	3705593	C	T	Och02,Och03,Och04,Och05,Och06,Och12,Och13	*OreG0025657*
scaffold85	3705605	C	T	NA	*OreG0025657*
scaffold85	3705623	C	G	Och02,Och03,Och04,Och05,Och06,Och12,Och13	*OreG0025657*
scaffold85	3705820	A	T	Och07,Och08,Och11	*OreG0025657*
scaffold85	3705835	G	A	Och07,Och08,Och11	*OreG0025657*
scaffold85	3706079	C	T	NA	*OreG0025658*
scaffold85	3707496	G	A	Och07,Och08,Och11	*OreG0025659*
scaffold85	3728023	A	T	Och11	*OreG0025662*
scaffold85	3737274	A	T	NA	*OreG0025663*
scaffold85	3737424	C	T	NA	*OreG0025663*
scaffold85	3737707	A	T	NA	*OreG0025663*
scaffold85	3752542	C	G	NA	*OreG0025664*
scaffold85	3757580	A	G	NA	*OreG0025665*
scaffold85	3782497	C	T	NA	*OreG0025668*
scaffold85	3782616	A	G	Och01,Och06,Och07,Och08,Och09,Och10,Och11,Och13	*OreG0025668*
scaffold85	3782914	G	A	Och14	*OreG0025668*
scaffold85	3783216	T	A	Och14	*OreG0025668*
scaffold85	3783442	C	G	NA	*OreG0025668*
scaffold85	3818413	G	A	Och01,Och11	*OreG0025671*
scaffold85	3818611	C	T	Och01	*OreG0025671*
scaffold85	3818913	A	G	Och14	*OreG0025671*
scaffold85	3819099	A	G	NA	*OreG0025671*
scaffold85	3875920	C	T	Och01,Och02,Och03,Och04,Och05,Och06,Och07,Och08,Och09,Och10,Och11,Och12,Och13	*OreG0025675*
scaffold1894	2373	T	C	Och12,Och13	*OreG0004819*
scaffold1894	3366	C	T	NA	*OreG0004819*
scaffold1410	32642	C	T	Ore01,Ore02,Ore03,Ore06,Ore08,Ore09,Ore10,Ore11,Ore12,Ore14	*OreG0002357*
scaffold1410	38790	C	T	Ore01,Ore02,Ore03,Ore04,Ore05,Ore06,Ore07,Ore08,Ore09,Ore10,Ore11,Ore12,Ore13,Ore14	*OreG0002358*
scaffold1410	39353	G	A	NA	*OreG0002358*
scaffold1410	39397	T	A	Ore01,Ore02,Ore03,Ore04,Ore05,Ore06,Ore07,Ore08,Ore09,Ore10,Ore11,Ore12,Ore13,Ore14	*OreG0002358*
scaffold470	28932	C	T	NA	*OreG0022360*
scaffold470	28977	A	C	Och01,Och02,Och03,Och04,Och05,Och06,Och07,Och08,Och12,Och13	*OreG0022360*
scaffold470	29177	A	T	Ore01,Ore02,Ore06,Ore08,Ore09,Ore10,Ore14	*OreG0022360*
scaffold470	29191	C	A	Och01,Och02,Och03,Och04,Och05,Och06,Och07,Och08,Och09,Och10,Och11,Och12,Och13	*OreG0022360*
scaffold470	29258	G	A	Ore01,Ore02,Ore06,Ore08,Ore09,Ore10,Ore14	*OreG0022360*
scaffold470	63767	G	C	Ore01,Ore02,Ore06,Ore08,Ore09,Ore10,Ore14	*OreG0022361*
scaffold470	82710	A	C	NA	*OreG0022363*
scaffold470	83505	C	G	NA	*OreG0022363*
scaffold470	92111	C	A	Och01,Och02,Och03,Och04,Och05,Och06,Och07,Och08,Och09,Och10,Och11,Och12,Och13,Och14,Ore01,Ore02,Ore03,Ore04,Ore05,Ore06,Ore07,Ore08,Ore09,Ore10,Ore11,Ore12,Ore13,Ore14	*OreG0022364*
scaffold470	92130	C	T	Och01,Och02,Och03,Och04,Och05,Och06,Och07,Och08,Och09,Och10,Och11,Och12,Och13,Och14,Ore01,Ore02,Ore03,Ore04,Ore05,Ore06,Ore07,Ore08,Ore09,Ore10,Ore11,Ore12,Ore13,Ore14	*OreG0022364*
scaffold470	95715	G	A	NA	*OreG0022365*
scaffold470	96052	G	C	Ore04,Ore05	*OreG0022365*
scaffold470	96326	C	T	Och08	*OreG0022365*
scaffold470	96377	G	A	Och01,Och02,Och03,Och04,Och05,Och06,Och08,Och12,Och13	*OreG0022365*
scaffold470	96436	T	G	NA	*OreG0022365*
scaffold470	97054	T	G	Ore02,Ore03,Ore09,Ore10,Ore14	*OreG0022365*
scaffold470	97866	T	C	NA	*OreG0022365*
scaffold470	99906	C	T	Ore01,Ore02,Ore03,Ore04,Ore05,Ore06,Ore07,Ore08,Ore09,Ore10,Ore11,Ore12,Ore13,Ore14	*OreG0022366*
scaffold470	148481	A	C	Och01,Och02,Och03,Och04,Och05,Och06,Och08,Och12,Och13	*OreG0022370*
scaffold470	148946	C	T	Och11	*OreG0022371*

scaffold470	168538	G	T	Och01,Och02,Och03,Och04,Och05,Och06,Och07,Och08,Och09,Och10,Och11,Och12,Och13,Och14,Ore01,Ore02,Ore03,Ore04,Ore05,Ore06,Ore07,Ore08,Ore09,Ore10,Ore11,Ore12,Ore13,Ore14	*OreG0022374*
scaffold470	187397	A	T	Ore01,Ore02,Ore03,Ore04,Ore05,Ore06,Ore07,Ore08,Ore09,Ore10,Ore11,Ore12,Ore13,Ore14	*OreG0022377*
scaffold470	187429	C	A	Ore02,Ore09,Ore10,Ore14	*OreG0022377*
scaffold470	187640	G	A	Och01,Och02,Och03,Och04,Och05,Och06,Och07,Och08,Och09,Och10,Och11,Och12,Och13,Och14,Ore01,Ore02,Ore03,Ore04,Ore05,Ore06,Ore07,Ore08,Ore09,Ore10,Ore11,Ore12,Ore13,Ore14	*OreG0022377*
scaffold470	187667	C	A	Ore01,Ore02,Ore03,Ore04,Ore05,Ore06,Ore07,Ore08,Ore09,Ore10,Ore11,Ore12,Ore13,Ore14	*OreG0022377*
scaffold470	188123	A	G	NA	*OreG0022378*
scaffold470	188234	A	G	Och01,Och02,Och03,Och04,Och05,Och06,Och07,Och08,Och12,Och13	*OreG0022378*
scaffold470	193421	C	A	Ore01,Ore02,Ore03,Ore04,Ore05,Ore06,Ore07,Ore08,Ore09,Ore10,Ore11,Ore12,Ore13,Ore14	*OreG0022379*
scaffold470	206109	A	G	NA	*OreG0022382*
scaffold470	206378	A	C	NA	*OreG0022382*
scaffold470	213689	C	A	NA	*OreG0022383*
scaffold470	213707	G	A	NA	*OreG0022383*
scaffold470	214202	C	A	NA	*OreG0022383*
scaffold470	226296	G	A	NA	*OreG0022385*
scaffold470	239212	T	A	Och01,Och02,Och03,Och04,Och05,Och06,Och07,Och08,Och09,Och10,Och11,Och12,Och13,Och14,Ore01,Ore02,Ore03,Ore04,Ore05,Ore06,Ore07,Ore08,Ore09,Ore10,Ore11,Ore12,Ore13,Ore14	*OreG0022386*
scaffold470	286681	G	C	Ore01,Ore02,Ore03,Ore04,Ore05,Ore06,Ore07,Ore08,Ore09,Ore10,Ore11,Ore12,Ore13,Ore14	*OreG0022390*
scaffold470	286891	A	G	NA	*OreG0022390*
scaffold470	287012	C	A	NA	*OreG0022390*
scaffold470	287542	G	C	NA	*OreG0022390*
scaffold470	308929	A	G	Och01,Och09,Och10,Ore01,Ore02,Ore03,Ore04,Ore05,Ore06,Ore07,Ore08,Ore09,Ore10,Ore11,Ore12,Ore13,Ore14	*OreG0022392*
scaffold470	308949	A	G	Och01,Och09,Och10	*OreG0022392*
scaffold470	309013	C	A	Och03,Och05,Och07,Och08,Och12,Och13	*OreG0022392*
scaffold470	309663	C	T	Och01,Och02,Och03,Och04,Och05,Och06,Och07,Och08,Och09,Och10,Och11,Och12,Och13,Och14	*OreG0022392*
scaffold470	322425	T	C	Och03,Och05,Och07,Och08,Och12,Och13	*OreG0022395*
scaffold470	322968	C	T	Ore01,Ore02,Ore03,Ore04,Ore05,Ore06,Ore07,Ore08,Ore09,Ore10,Ore11,Ore12,Ore13,Ore14	*OreG0022395*
scaffold470	323016	C	T	Ore01,Ore02,Ore03,Ore04,Ore05,Ore06,Ore07,Ore08,Ore09,Ore10,Ore11,Ore12,Ore13,Ore14	*OreG0022395*
scaffold470	323108	C	T	NA	*OreG0022395*
scaffold470	323180	G	T	Ore01,Ore02,Ore03,Ore04,Ore05,Ore06,Ore07,Ore08,Ore09,Ore10,Ore11,Ore12,Ore13,Ore14	*OreG0022395*
scaffold470	323190	C	T	NA	*OreG0022395*
scaffold470	323277	C	T	Ore01,Ore02,Ore03,Ore04,Ore05,Ore06,Ore07,Ore08,Ore09,Ore10,Ore11,Ore12,Ore13,Ore14	*OreG0022395*
scaffold470	324215	T	C	Ore02,Ore09,Ore10,Ore14	*OreG0022395*
scaffold470	324240	A	G	Och01	*OreG0022395*
scaffold470	324266	A	T	Ore02,Ore03,Ore09,Ore10,Ore14	*OreG0022395*
scaffold470	324458	T	C	NA	*OreG0022395*
scaffold470	324518	G	A	Ore02,Ore09,Ore10,Ore14	*OreG0022395*
scaffold470	324540	A	C	Ore02,Ore03,Ore09,Ore10,Ore14	*OreG0022395*
scaffold470	326866	G	C	Och02,Och03,Och04,Och05,Och06,Och08,Och09,Och10,Och12,Och13	*OreG0022396*
scaffold470	348208	T	C	Ore04,Ore05	*OreG0022398*
scaffold470	384233	G	C	NA	*OreG0022399*
scaffold470	390009	A	T	Och01,Och02,Och03,Och04,Och05,Och06,Och07,Och08,Och09,Och10,Och11,Och12,Och13,Och14	*OreG0022400*
scaffold470	390038	C	A	Ore01,Ore02,Ore03,Ore04,Ore05,Ore06,Ore07,Ore08,Ore09,Ore10,Ore11,Ore12,Ore13,Ore14	*OreG0022400*
scaffold470	390046	C	T	NA	*OreG0022400*
scaffold470	390117	A	C	NA	*OreG0022400*
scaffold470	390197	G	A	Och01,Och02,Och03,Och04,Och05,Och06,Och07,Och08,Och09,Och10,Och11,Och12,Och13,Och14	*OreG0022401*
scaffold470	390255	A	G	Ore01,Ore02,Ore03,Ore04,Ore05,Ore06,Ore07,Ore08,Ore09,Ore10,Ore11,Ore12,Ore13,Ore14	*OreG0022401*
scaffold470	390713	C	T	Och01,Och02,Och03,Och04,Och05,Och06,Och07,Och08,Och09,Och10,Och11,Och12,Och13,Och14	*OreG0022402*
scaffold470	390714	A	T	NA	*OreG0022402*
scaffold470	390731	C	T	Och08	*OreG0022402*
scaffold470	390799	C	A	Och01,Och02,Och03,Och04,Och05,Och06,Och07,Och08,Och09,Och10,Och11,Och12,Och13,Och14,Ore01,Ore02,Ore03,Ore04,Ore05,Ore06,Ore07,Ore08,Ore09,Ore10,Ore11,Ore12,Ore13,Ore14	*OreG0022402*
scaffold470	394443	G	A	NA	*OreG0022403*
scaffold470	511486	A	G	Ore01,Ore02,Ore03,Ore06,Ore07,Ore08,Ore09,Ore10,Ore11,Ore12,Ore13,Ore14	*OreG0022416*
scaffold470	511556	C	A	NA	*OreG0022416*

scaffold470	511921	G	T	NA	*OreG0022416*
scaffold470	512370	A	T	NA	*OreG0022416*
scaffold470	570284	A	T	Och01,Och06	*OreG0022422*
scaffold470	579744	G	T	Och01,Och02,Och03,Och04,Och05,Och06,Och07,Och08,Och09,Och10,Och11,Och12,Och13,Och14	*OreG0022423*
scaffold470	591165	A	C	Ore01,Ore02,Ore03,Ore04,Ore05,Ore06,Ore07,Ore08,Ore09,Ore10,Ore11,Ore12,Ore13,Ore14	*OreG0022424*
scaffold470	646309	A	G	NA	*OreG0022429*
scaffold470	683154	C	A	Ore01,Ore02,Ore03,Ore04,Ore05,Ore06,Ore07,Ore08,Ore09,Ore10,Ore11,Ore12,Ore13,Ore14	*OreG0022433*
scaffold470	709106	T	G	Och01,Och02,Och03,Och04,Och05,Och06,Och07,Och08,Och09,Och10,Och11,Och12,Och13,Och14,Ore01,Ore02,Ore03,Ore04,Ore05,Ore06,Ore07,Ore08,Ore09,Ore10,Ore11,Ore12,Ore13,Ore14	*OreG0022435*
scaffold470	709314	C	T	Ore04,Ore05	*OreG0022435*
scaffold470	742506	G	T	NA	*OreG0022438*
scaffold470	768144	T	A	Ore01,Ore02,Ore03,Ore04,Ore05,Ore06,Ore07,Ore08,Ore09,Ore10,Ore11,Ore12,Ore13,Ore14	*OreG0022440*
scaffold470	768216	C	T	Och01,Och02,Och03,Och04,Och05,Och06,Och07,Och08,Och09,Och10,Och11,Och12,Och13,Och14	*OreG0022440*
scaffold470	769077	C	T	NA	*OreG0022440*
scaffold470	769534	G	T	NA	*OreG0022440*
scaffold470	789546	T	C	NA	*OreG0022443*
scaffold470	789554	C	T	Ore01,Ore02,Ore03,Ore04,Ore05,Ore06,Ore07,Ore08,Ore09,Ore10,Ore11,Ore12,Ore13,Ore14	*OreG0022443*
scaffold470	819396	C	T	NA	*OreG0022444*
scaffold470	820794	C	A	Ore01,Ore02,Ore03,Ore04,Ore05,Ore06,Ore07,Ore08,Ore09,Ore10,Ore11,Ore12,Ore13,Ore14	*OreG0022444*
scaffold470	851090	C	A	Ore01,Ore02,Ore03,Ore04,Ore05,Ore06,Ore07,Ore08,Ore09,Ore10,Ore11,Ore12,Ore13,Ore14	*OreG0022447*
scaffold470	882099	T	A	NA	*OreG0022453*
scaffold470	883877	C	T	NA	*OreG0022453*
scaffold470	883931	G	A	NA	*OreG0022453*
scaffold470	883937	A	T	NA	*OreG0022453*
scaffold470	903730	C	G	NA	*OreG0022455*
scaffold470	904112	A	C	NA	*OreG0022455*
scaffold470	970746	G	A	Och06	*OreG0022460*
scaffold470	970867	T	A	Och06	*OreG0022460*
scaffold470	970934	C	T	Och01,Och02,Och03,Och04,Och05,Och06,Och07,Och08,Och09,Och10,Och11,Och12,Och13,Och14,Ore01,Ore02,Ore03,Ore04,Ore05,Ore06,Ore07,Ore08,Ore09,Ore10,Ore11,Ore12,Ore13,Ore14	*OreG0022460*
scaffold470	971137	T	C	Och06	*OreG0022460*
scaffold470	971162	A	T	Och06,Ore01,Ore02,Ore03,Ore04,Ore05,Ore06,Ore07,Ore08,Ore09,Ore10,Ore11,Ore12,Ore13,Ore14	*OreG0022460*
scaffold470	971457	C	T	Och01,Och02,Och03,Och04,Och05,Och06,Och07,Och08,Och09,Och10,Och11,Och12,Och13,Och14,Ore01,Ore02,Ore03,Ore04,Ore05,Ore06,Ore07,Ore08,Ore09,Ore10,Ore11,Ore12,Ore13,Ore14	*OreG0022460*
scaffold470	999162	C	T	NA	*OreG0022462*
scaffold470	999899	G	A	Och12	*OreG0022462*
scaffold470	1025959	C	T	Ore09	*OreG0022463*
scaffold470	1027292	T	A	Och06	*OreG0022463*
scaffold470	1030290	G	A	Och01,Och02,Och03,Och04,Och05,Och06,Och07,Och08,Och09,Och10,Och11,Och12,Och13,Och14,Ore01,Ore02,Ore03,Ore04,Ore05,Ore06,Ore07,Ore08,Ore09,Ore10,Ore11,Ore12,Ore13,Ore14	*OreG0022464*
scaffold470	1030443	T	A	Och01,Och02,Och03,Och04,Och05,Och06,Och07,Och08,Och09,Och10,Och11,Och12,Och13,Och14,Ore01,Ore02,Ore03,Ore04,Ore05,Ore06,Ore07,Ore08,Ore09,Ore10,Ore11,Ore12,Ore13,Ore14	*OreG0022464*
scaffold470	1031797	A	G	NA	*OreG0022464*
scaffold470	1031827	T	A	NA	*OreG0022464*
scaffold470	1035915	T	C	Och03,Och05,Och08,Och09,Och10,Och11,Och12,Och13,Och14	*OreG0022465*
scaffold470	1035987	T	C	NA	*OreG0022465*
scaffold470	1036139	C	T	NA	*OreG0022465*
scaffold470	1036342	G	A	NA	*OreG0022465*
scaffold470	1036588	G	A	NA	*OreG0022465*
scaffold470	1037057	G	A	NA	*OreG0022465*
scaffold470	1037060	A	G	NA	*OreG0022465*
scaffold470	1037152	T	A	NA	*OreG0022465*
scaffold470	1037172	C	A	NA	*OreG0022465*
scaffold470	1037191	T	A	NA	*OreG0022465*
scaffold470	1047399	C	T	NA	*OreG0022466*
scaffold470	1047624	C	T	NA	*OreG0022466*
scaffold470	1048008	T	A	Ore01,Ore02,Ore03,Ore04,Ore05,Ore06,Ore07,Ore08,Ore09,Ore10,Ore11,Ore12,Ore13,Ore14	*OreG0022466*
scaffold470	1050060	C	G	Ore06,Ore08	*OreG0022467*
scaffold470	1050079	C	T	Och01,Och02,Och03,Och04,Och05,Och06,Och07,Och08,Och09,Och10,Och11,Och12,Och13,Och14	*OreG0022467*

scaffold470	1050857	C	T	Och08	*OreG0022467*
scaffold470	1050906	G	T	Och06	*OreG0022467*
scaffold470	1056013	T	G	NA	*OreG0022468*
scaffold470	1056176	C	A	NA	*OreG0022468*
scaffold470	1056472	C	T	NA	*OreG0022469*
scaffold470	1089854	T	C	Och01,Och02,Och03,Och04,Och05,Och06,Och07,Och08,Och09,Och10,Och11,Och12,Och13,Och14,Ore01,Ore02,Ore03,Ore04,Ore05,Ore06,Ore07,Ore08,Ore09,Ore10,Ore11,Ore12,Ore13,Ore14	*OreG0022473*
scaffold470	1095307	C	A	Och01,Och02,Och03,Och04,Och05,Och06,Och07,Och08,Och09,Och10,Och11,Och12,Och13,Och14	*OreG0022474*
scaffold470	1095490	G	A	NA	*OreG0022474*
scaffold470	1095603	G	T	NA	*OreG0022474*
scaffold470	1100509	A	T	NA	*OreG0022475*
scaffold470	1100923	G	A	NA	*OreG0022475*
scaffold470	1104060	T	A	Ore01,Ore02,Ore03,Ore04,Ore05,Ore06,Ore07,Ore08,Ore09,Ore10,Ore11,Ore12,Ore13,Ore14	*OreG0022476*
scaffold470	1111780	C	G	NA	*OreG0022478*
scaffold470	1188138	T	G	NA	*OreG0022483*
scaffold470	1188687	A	G	Och01,Och02,Och04,Och06,Och07,Och08,Och14	*OreG0022483*
scaffold470	1192179	C	T	NA	*OreG0022483*
scaffold470	1195892	A	G	NA	*OreG0022483*
scaffold470	1196153	A	G	Och01,Och02,Och03,Och04,Och05,Och06,Och07,Och08,Och09,Och10,Och11,Och12,Och13,Och14,Ore01,Ore02,Ore03,Ore04,Ore05,Ore06,Ore07,Ore08,Ore09,Ore10,Ore11,Ore12,Ore13,Ore14	*OreG0022483*
scaffold470	1205513	C	A	Och14	*OreG0022485*
scaffold470	1212537	C	T	NA	*OreG0022486*
scaffold470	1236578	T	C	Och02,Och04,Och06,Och08,Och14	*OreG0022488*
scaffold470	1263082	G	T	Och01,Och02,Och03,Och04,Och05,Och06,Och07,Och08,Och09,Och10,Och11,Och12,Och13,Och14	*OreG0022491*
scaffold470	1263113	C	T	Ore01,Ore02,Ore03,Ore04,Ore05,Ore06,Ore07,Ore08,Ore09,Ore10,Ore11,Ore12,Ore13,Ore14	*OreG0022491*
scaffold470	1278755	C	T	Ore01,Ore08,Ore09,Ore11,Ore12,Ore13	*OreG0022492*
scaffold470	1278777	C	G	Och01,Och02,Och03,Och04,Och05,Och06,Och07,Och08,Och09,Och10,Och11,Och12,Och13,Och14,Ore01,Ore02,Ore03,Ore04,Ore05,Ore06,Ore07,Ore08,Ore09,Ore10,Ore11,Ore12,Ore13,Ore14	*OreG0022492*
scaffold470	1278843	T	C	Ore01,Ore02,Ore03,Ore04,Ore05,Ore06,Ore07,Ore08,Ore09,Ore10,Ore11,Ore12,Ore13,Ore14	*OreG0022492*
scaffold470	1279070	A	T	Och01,Och02,Och03,Och04,Och05,Och06,Och07,Och08,Och09,Och10,Och11,Och12,Och13,Och14,Ore01,Ore02,Ore03,Ore04,Ore05,Ore06,Ore07,Ore08,Ore09,Ore10,Ore11,Ore12,Ore13,Ore14	*OreG0022492*
scaffold470	1279194	C	T	Ore01,Ore02,Ore03,Ore04,Ore05,Ore06,Ore07,Ore08,Ore09,Ore10,Ore11,Ore12,Ore13,Ore14	*OreG0022492*
scaffold470	1279230	C	T	Ore01,Ore02,Ore03,Ore04,Ore05,Ore06,Ore07,Ore08,Ore09,Ore10,Ore11,Ore12,Ore13,Ore14	*OreG0022492*
scaffold470	1279546	A	T	NA	*OreG0022492*
scaffold470	1279554	C	A	Och01,Och02,Och03,Och04,Och05,Och06,Och07,Och08,Och09,Och10,Och11,Och12,Och13,Och14,Ore01,Ore02,Ore03,Ore04,Ore05,Ore06,Ore07,Ore08,Ore09,Ore10,Ore11,Ore12,Ore13,Ore14	*OreG0022492*
scaffold470	1279558	A	G	Och01,Och02,Och03,Och04,Och05,Och06,Och07,Och08,Och09,Och10,Och11,Och12,Och13,Och14	*OreG0022492*
scaffold470	1279579	A	G	NA	*OreG0022492*
scaffold470	1287664	C	T	Ore01,Ore02,Ore03,Ore04,Ore05,Ore06,Ore07,Ore08,Ore09,Ore10,Ore11,Ore12,Ore13,Ore14	*OreG0022493*
scaffold470	1287766	C	T	Och01,Och02,Och03,Och04,Och05,Och06,Och07,Och08,Och09,Och10,Och11,Och12,Och13,Och14	*OreG0022493*
scaffold470	1288451	G	T	Och01,Och08	*OreG0022493*
scaffold470	1303618	T	A	Och06	*OreG0022494*
scaffold470	1331472	C	T	NA	*OreG0022497*
scaffold470	1331947	T	A	NA	*OreG0022497*
scaffold470	1332279	A	G	Ore01,Ore02,Ore03,Ore04,Ore05,Ore06,Ore07,Ore08,Ore09,Ore10,Ore11,Ore12,Ore13,Ore14	*OreG0022497*
scaffold470	1333371	C	G	NA	*OreG0022497*
scaffold470	1333423	T	C	NA	*OreG0022497*
scaffold470	1336268	G	A	NA	*OreG0022498*
scaffold470	1336413	T	G	NA	*OreG0022498*
scaffold470	1336901	G	C	NA	*OreG0022498*
scaffold470	1354154	G	A	Och01,Och09,Och10,Och11	*OreG0022503*
scaffold470	1354224	T	C	Och01,Och02,Och03,Och04,Och05,Och06,Och07,Och08,Och09,Och10,Och11,Och12,Och13,Och14,Ore01,Ore02,Ore03,Ore04,Ore05,Ore06,Ore07,Ore08,Ore09,Ore10,Ore11,Ore12,Ore13,Ore14	*OreG0022503*
scaffold470	1354413	C	T	Ore01,Ore02,Ore03,Ore04,Ore05,Ore06,Ore07,Ore08,Ore09,Ore10,Ore11,Ore12,Ore13,Ore14	*OreG0022503*
scaffold470	1356454	C	G	NA	*OreG0022504*
scaffold470	1363775	G	A	NA	*OreG0022506*

scaffold470	1364839	G	A	Ore01,Ore02,Ore03,Ore04,Ore05,Ore06,Ore07,Ore08,Ore09,Ore10,Ore11,Ore12,Ore13,Ore14	*OreG0022507*
scaffold470	1369027	C	T	Ore01,Ore02,Ore03,Ore04,Ore05,Ore06,Ore07,Ore08,Ore09,Ore10,Ore11,Ore12,Ore13,Ore14	*OreG0022507*
scaffold470	1371395	G	A	Och01,Och02,Och03,Och04,Och05,Och06,Och07,Och08,Och09,Och10,Och11,Och12,Och13,Och14	*OreG0022507*
scaffold470	1388230	G	A	Och08	*OreG0022510*
scaffold470	1402586	C	A	Och01,Och02,Och03,Och04,Och05,Och06,Och07,Och08,Och09,Och10,Och11,Och12,Och13,Och14	*OreG0022511*
scaffold470	1403085	C	A	Och01,Och02,Och03,Och04,Och05,Och06,Och07,Och08,Och09,Och10,Och11,Och12,Och13,Och14,Ore01,Ore02,Ore03,Ore04,Ore05,Ore06,Ore07,Ore08,Ore09,Ore10,Ore11,Ore12,Ore13,Ore14	*OreG0022511*
scaffold470	1510524	A	G	Och03,Och05,Och11,Och13,Och14	*OreG0022517*
scaffold470	1510540	C	T	Ore01,Ore02,Ore03,Ore04,Ore05,Ore06,Ore07,Ore08,Ore09,Ore10,Ore11,Ore12,Ore13,Ore14	*OreG0022517*
scaffold470	1511389	A	G	Ore08	*OreG0022517*
scaffold470	1511397	C	A	Ore06,Ore09,Ore10,Ore14	*OreG0022517*
scaffold470	1511463	G	A	Och05,Och11,Och14	*OreG0022517*
scaffold1041	12700	A	G	NA	*OreG0000202*
scaffold1041	12703	G	A	NA	*OreG0000202*
scaffold1041	12749	G	T	NA	*OreG0000202*
scaffold1041	298412	C	A	Och01,Och02,Och03,Och04,Och05,Och06,Och07,Och08,Och09,Och10,Och11,Och12,Och13,Och14,Ore01,Ore02,Ore03,Ore04,Ore05,Ore06,Ore07,Ore08,Ore09,Ore10,Ore11,Ore12,Ore13,Ore14	*OreG0000215*
scaffold1041	300926	C	T	NA	*OreG0000215*
scaffold1978	20326	G	A	NA	*OreG0005000*
scaffold1978	20713	C	T	NA	*OreG0005000*
scaffold1978	28913	A	G	NA	*OreG0005002*
scaffold1978	30050	A	T	NA	*OreG0005002*
scaffold1856	3979	A	C	Och08	*OreG0004273*
scaffold2042	1773	G	A	Och08	*OreG0005147*
scaffold1292	45249	G	A	NA	*OreG0001967*
scaffold1292	168526	C	T	NA	*OreG0001974*
scaffold1292	171868	G	A	NA	*OreG0001974*
scaffold1292	171999	G	A	NA	*OreG0001974*
scaffold1292	172103	A	G	Ore03,Ore08,Ore09,Ore10,Ore14	*OreG0001974*
scaffold1292	172452	A	G	NA	*OreG0001974*
scaffold1292	253770	G	T	Ore01,Ore02,Ore03,Ore04,Ore05,Ore06,Ore07,Ore08,Ore09,Ore10,Ore11,Ore12,Ore13,Ore14	*OreG0001975*
scaffold1292	270882	C	T	Och01,Och02,Och03,Och04,Och05,Och06,Och07,Och08,Och09,Och10,Och11,Och12,Och13,Och14	*OreG0001976*
scaffold1292	287408	C	T	Ore01,Ore02,Ore03,Ore04,Ore05,Ore06,Ore07,Ore08,Ore09,Ore10,Ore11,Ore12,Ore13,Ore14	*OreG0001977*
scaffold33	10556	A	T	Och01	*OreG0011039*
scaffold33	33391	A	G	Och02,Och03,Och04,Och05,Och06,Och07,Och08,Och09,Och10,Och11,Och12,Och13,Och14	*OreG0011041*
scaffold33	34024	T	C	Ore01,Ore02,Ore03,Ore04,Ore05,Ore06,Ore07,Ore08,Ore09,Ore10,Ore11,Ore12,Ore13,Ore14	*OreG0011041*
scaffold33	268623	T	A	Och01,Och02,Och03,Och04,Och05,Och06,Och07,Och08,Och09,Och10,Och11,Och12,Och13,Och14	*OreG0011049*
scaffold33	318151	C	A	Och02,Och03,Och04,Och05,Och06,Och12,Och13	*OreG0011052*
scaffold33	323630	C	T	Och01	*OreG0011052*
scaffold33	324036	A	G	Och02,Och06	*OreG0011052*
scaffold33	325246	C	T	Ore01,Ore02,Ore03,Ore04,Ore05,Ore06,Ore07,Ore08,Ore09,Ore10,Ore11,Ore12,Ore13,Ore14	*OreG0011052*
scaffold33	328055	C	A	Och01,Och02,Och03,Och04,Och05,Och06,Och07,Och08,Och09,Och10,Och11,Och12,Och13,Och14,Ore01,Ore02,Ore03,Ore04,Ore05,Ore06,Ore07,Ore08,Ore09,Ore10,Ore11,Ore12,Ore13,Ore14	*OreG0011053*
scaffold33	330109	G	T	NA	*OreG0011053*
scaffold33	387349	G	A	NA	*OreG0011055*
scaffold33	387537	G	A	NA	*OreG0011055*
scaffold33	387694	A	C	Och01,Och02,Och03,Och04,Och05,Och06,Och07,Och08,Och09,Och10,Och11,Och12,Och13,Och14,Ore01,Ore02,Ore03,Ore04,Ore05,Ore06,Ore07,Ore08,Ore09,Ore10,Ore11,Ore12,Ore13,Ore14	*OreG0011055*
scaffold33	388183	A	C	Och01,Och02,Och06,Och07	*OreG0011055*
scaffold33	407632	G	A	NA	*OreG0011056*
scaffold33	451689	C	G	Ore01,Ore02,Ore03,Ore04,Ore05,Ore06,Ore07,Ore08,Ore09,Ore10,Ore11,Ore12,Ore13,Ore14	*OreG0011058*
scaffold33	451800	C	A	Ore01,Ore02,Ore03,Ore04,Ore05,Ore06,Ore07,Ore08,Ore09,Ore10,Ore11,Ore12,Ore13,Ore14	*OreG0011058*
scaffold33	452619	T	C	NA	*OreG0011058*
scaffold33	452784	T	C	Ore01,Ore02,Ore03,Ore04,Ore05,Ore06,Ore07,Ore08,Ore09,Ore10,Ore11,Ore12,Ore13,Ore14	*OreG0011058*
scaffold33	452823	C	T	Och01,Och02,Och03,Och04,Och05,Och06,Och07,Och08,Och09,Och10,Och11,Och12,Och13,Och14	*OreG0011058*

scaffold33	511446	G	A	NA	*OreG0011061*
scaffold33	514126	G	A	NA	*OreG0011061*
scaffold33	518964	G	A	Och01	*OreG0011061*
scaffold33	533595	T	G	NA	*OreG0011062*
scaffold33	533857	T	G	NA	*OreG0011062*
scaffold33	534275	G	A	NA	*OreG0011062*
scaffold33	534313	C	T	Ore07,Ore08	*OreG0011062*
scaffold33	534485	G	A	NA	*OreG0011062*
scaffold33	534508	G	T	NA	*OreG0011062*
scaffold33	534513	C	A	NA	*OreG0011062*
scaffold33	534684	C	T	NA	*OreG0011062*
scaffold33	568250	G	A	NA	*OreG0011064*
scaffold33	568329	C	T	NA	*OreG0011064*
scaffold33	568335	T	A	NA	*OreG0011064*
scaffold33	568341	C	T	NA	*OreG0011064*
scaffold33	568725	G	A	NA	*OreG0011064*
scaffold33	568727	T	C	NA	*OreG0011064*
scaffold33	568788	G	T	NA	*OreG0011064*
scaffold33	570253	G	A	NA	*OreG0011064*
scaffold33	570257	C	T	NA	*OreG0011064*
scaffold33	570443	C	T	NA	*OreG0011064*
scaffold33	570494	T	A	NA	*OreG0011064*
scaffold33	570518	A	G	NA	*OreG0011064*
scaffold33	577371	C	A	NA	*OreG0011065*
scaffold33	577429	A	G	Och02,Och06,Och07	*OreG0011065*
scaffold33	579041	A	G	NA	*OreG0011065*
scaffold33	605466	T	A	Och02	*OreG0011065*
scaffold33	699127	G	T	Ore01,Ore02,Ore03,Ore04,Ore05,Ore06,Ore07,Ore08,Ore09,Ore10,Ore11,Ore12,Ore13,Ore14	*OreG0011069*
scaffold33	701567	C	T	Ore01,Ore02,Ore03,Ore04,Ore05,Ore06,Ore07,Ore08,Ore09,Ore10,Ore11,Ore12,Ore13,Ore14	*OreG0011069*
scaffold33	713106	A	T	Ore01,Ore02,Ore03,Ore04,Ore05,Ore06,Ore07,Ore08,Ore09,Ore10,Ore11,Ore12,Ore13,Ore14	*OreG0011071*
scaffold33	725590	T	A	NA	*OreG0011073*
scaffold33	799423	C	T	Ore14	*OreG0011077*
scaffold33	844622	G	A	NA	*OreG0011078*
scaffold33	844763	T	A	NA	*OreG0011078*
scaffold33	886464	C	T	Och01,Och02,Och03,Och04,Och05,Och06,Och07,Och08,Och09,Och10,Och11,Och12,Och13,Och14	*OreG0011081*
scaffold33	897230	A	C	Ore01,Ore02,Ore03,Ore04,Ore05,Ore06,Ore07,Ore08,Ore09,Ore10,Ore11,Ore12,Ore13,Ore14	*OreG0011082*
scaffold33	900594	G	C	NA	*OreG0011083*
scaffold33	932790	G	A	NA	*OreG0011085*
scaffold33	974372	T	C	NA	*OreG0011087*
scaffold33	987610	A	T	Ore01,Ore02,Ore03,Ore04,Ore05,Ore06,Ore07,Ore08,Ore09,Ore10,Ore11,Ore12,Ore13,Ore14	*OreG0011088*
scaffold33	988109	T	G	NA	*OreG0011088*
scaffold33	988119	C	T	Ore01,Ore02,Ore03,Ore04,Ore05,Ore06,Ore07,Ore08,Ore09,Ore10,Ore11,Ore12,Ore13,Ore14	*OreG0011088*
scaffold33	988211	G	A	NA	*OreG0011089*
scaffold33	988286	A	C	NA	*OreG0011089*
scaffold33	988328	T	A	Och01,Och02,Och03,Och04,Och05,Och06,Och07,Och08,Och09,Och10,Och11,Och12,Och13,Och14	*OreG0011089*
scaffold33	1004098	C	T	Och01,Och02,Och03,Och04,Och05,Och06,Och07,Och08,Och09,Och10,Och11,Och12,Och13,Och14	*OreG0011093*
scaffold33	1004212	T	A	Ore01,Ore02,Ore03,Ore04,Ore05,Ore06,Ore07,Ore08,Ore09,Ore10,Ore11,Ore12,Ore13,Ore14	*OreG0011093*
scaffold33	1004249	G	A	Och01,Och02,Och03,Och04,Och05,Och06,Och07,Och08,Och09,Och10,Och11,Och12,Och13,Och14	*OreG0011093*
scaffold33	1004404	G	A	NA	*OreG0011093*
scaffold33	1004438	G	A	Och02,Och06	*OreG0011093*
scaffold33	1004440	C	T	Och02,Och06	*OreG0011093*
scaffold33	1102261	C	T	Ore01,Ore02,Ore03,Ore04,Ore05,Ore06,Ore07,Ore08,Ore09,Ore10,Ore11,Ore12,Ore13,Ore14	*OreG0011099*
scaffold33	1103788	G	A	Och01,Och02,Och03,Och04,Och05,Och06,Och07,Och08,Och09,Och10,Och11,Och12,Och13,Och14,Ore01,Ore02,Ore03,Ore04,Ore05,Ore06,Ore07,Ore08,Ore09,Ore10,Ore11,Ore12,Ore13,Ore14	*OreG0011099*
scaffold33	1103807	T	C	Ore01,Ore02,Ore03,Ore04,Ore05,Ore06,Ore07,Ore08,Ore09,Ore10,Ore11,Ore12,Ore13,Ore14	*OreG0011099*
scaffold33	1137430	G	A	Ore01,Ore02,Ore03,Ore04,Ore05,Ore06,Ore07,Ore08,Ore09,Ore10,Ore11,Ore12,Ore13,Ore14	*OreG0011100*
scaffold33	1159922	C	T	Och01,Och02,Och03,Och04,Och05,Och06,Och07,Och08,Och09,Och10,Och11,Och12,Och13,Och14	*OreG0011102*
scaffold33	1166283	A	G	NA	*OreG0011103*
scaffold33	1189032	G	A	NA	*OreG0011107*

scaffold33	1293883	A	G	NA	*OreG0011111*
scaffold33	1298296	G	A	Och01,Och02,Och03,Och04,Och05,Och06,Och12,Och13,Och14	*OreG0011112*
scaffold33	1318463	G	T	Och01,Och02,Och03,Och04,Och05,Och06,Och12,Och13,Och14	*OreG0011113*
scaffold33	1321421	C	T	NA	*OreG0011114*
scaffold33	1321820	G	T	NA	*OreG0011114*
scaffold33	1323971	G	A	Ore01,Ore02,Ore03,Ore04,Ore05,Ore06,Ore07,Ore08,Ore09,Ore10,Ore11,Ore12,Ore13,Ore14	*OreG0011114*
scaffold33	1363219	G	A	NA	*OreG0011115*
scaffold33	1376099	G	A	NA	*OreG0011115*
scaffold33	1473796	C	T	NA	*OreG0011119*
scaffold33	1496441	G	T	Och01,Och02,Och03,Och04,Och05,Och06,Och07,Och08,Och09,Och10,Och11,Och12,Och13,Och14,Ore01,Ore02,Ore03,Ore04,Ore05,Ore06,Ore07,Ore08,Ore09,Ore10,Ore11,Ore12,Ore13,Ore14	*OreG0011120*
scaffold33	1605647	C	T	NA	*OreG0011126*
scaffold33	1605851	C	T	Ore04,Ore05,Ore09	*OreG0011126*
scaffold33	1659947	T	A	Ore07,Ore08	*OreG0011131*
scaffold33	1660784	A	T	NA	*OreG0011131*
scaffold33	1664814	C	T	Och02,Och06,Och08	*OreG0011131*
scaffold33	1664974	C	T	Och02,Och06	*OreG0011131*
scaffold33	1695021	G	A	Och01,Och02,Och03,Och04,Och05,Och06,Och07,Och08,Och09,Och10,Och11,Och12,Och13,Och14,Ore01,Ore02,Ore03,Ore04,Ore05,Ore06,Ore07,Ore08,Ore09,Ore10,Ore11,Ore12,Ore13,Ore14	*OreG0011137*
scaffold33	1695343	G	C	Och01,Och02,Och03,Och04,Och05,Och06,Och07,Och08,Och09,Och10,Och11,Och12,Och13,Och14	*OreG0011137*
scaffold33	1720590	C	T	NA	*OreG0011144*
scaffold33	1782038	C	T	Och02,Och06	*OreG0011145*
scaffold33	1845753	A	T	Och01,Och02,Och03,Och04,Och05,Och06,Och07,Och08,Och09,Och10,Och11,Och12,Och13,Och14,Ore01,Ore02,Ore03,Ore04,Ore05,Ore06,Ore07,Ore08,Ore09,Ore10,Ore11,Ore12,Ore13,Ore14	*OreG0011151*
scaffold33	1845833	T	G	NA	*OreG0011151*
scaffold33	1846151	T	G	Och11	*OreG0011151*
scaffold33	1846845	G	A	Ore07,Ore08	*OreG0011151*
scaffold33	1847187	T	A	NA	*OreG0011151*
scaffold33	1852343	G	T	Och01,Och11	*OreG0011152*
scaffold33	1910476	G	A	Ore01,Ore02,Ore03,Ore04,Ore05,Ore06,Ore07,Ore08,Ore09,Ore10,Ore11,Ore12,Ore13,Ore14	*OreG0011154*
scaffold33	1930721	A	T	Och01,Och02,Och03,Och04,Och05,Och06,Och07,Och08,Och09,Och10,Och11,Och12,Och13,Och14	*OreG0011155*
scaffold33	1981969	C	T	NA	*OreG0011157*
scaffold33	2010635	A	G	NA	*OreG0011158*
scaffold33	2029472	A	G	NA	*OreG0011159*
scaffold33	2244465	T	C	NA	*OreG0011163*
scaffold33	2244742	T	A	NA	*OreG0011163*
scaffold33	2245011	C	T	NA	*OreG0011163*
scaffold33	2260927	C	T	NA	*OreG0011172*
scaffold33	2261106	C	T	NA	*OreG0011172*
scaffold33	2261217	C	T	NA	*OreG0011172*
scaffold33	2264269	G	A	NA	*OreG0011172*
scaffold33	2294223	G	A	Och02,Och03,Och04,Och05,Och06,Och08,Och12,Och13	*OreG0011173*
scaffold33	2303162	T	A	Ore07,Ore08,Ore14	*OreG0011174*
scaffold33	2338472	C	T	Ore01,Ore02,Ore03,Ore04,Ore05,Ore06,Ore07,Ore08,Ore09,Ore10,Ore11,Ore12,Ore13,Ore14	*OreG0011178*
scaffold33	2357657	T	C	Och01,Och02,Och03,Och04,Och05,Och06,Och07,Och08,Och09,Och10,Och11,Och12,Och13,Och14,Ore01,Ore02,Ore03,Ore04,Ore05,Ore06,Ore07,Ore08,Ore09,Ore10,Ore11,Ore12,Ore13,Ore14	*OreG0011179*
scaffold33	2357687	G	A	Ore01,Ore02,Ore03,Ore04,Ore05,Ore06,Ore07,Ore08,Ore09,Ore10,Ore11,Ore12,Ore13,Ore14	*OreG0011179*
scaffold33	2458819	T	A	NA	*OreG0011183*
scaffold33	2463657	G	A	NA	*OreG0011183*
scaffold33	2486374	T	C	Och01,Och02,Och06	*OreG0011185*
scaffold33	2486388	T	A	Ore01,Ore02,Ore08	*OreG0011185*
scaffold33	2486545	G	A	Och01,Och02,Och03,Och04,Och05,Och06,Och07,Och08,Och09,Och10,Och11,Och12,Och13,Och14,Ore01,Ore02,Ore03,Ore04,Ore05,Ore06,Ore07,Ore08,Ore09,Ore10,Ore11,Ore12,Ore13,Ore14	*OreG0011185*
scaffold33	2487286	G	T	Ore01,Ore02,Ore03,Ore04,Ore05,Ore06,Ore07,Ore08,Ore09,Ore10,Ore11,Ore12,Ore13,Ore14	*OreG0011185*
scaffold33	2487355	G	C	Ore01,Ore02,Ore03,Ore04,Ore05,Ore06,Ore07,Ore08,Ore09,Ore10,Ore11,Ore12,Ore13,Ore14	*OreG0011185*
scaffold33	2500720	A	G	Och01,Och02,Och03,Och04,Och05,Och06,Och07,Och08,Och09,Och10,Och11,Och12,Och13,Och14,Ore01,Ore02,Ore03,Ore04,Ore05,Ore06,Ore07,Ore08,Ore09,Ore10,Ore11,Ore12,Ore13,Ore14	*OreG0011188*
scaffold33	2500820	G	T	NA	*OreG0011188*
scaffold33	2500824	A	T	Ore01,Ore02,Ore03,Ore04,Ore05,Ore06,Ore07,Ore08,Ore09,Ore10,Ore11,Ore12,Ore13,Ore14	*OreG0011188*
scaffold33	2506885	T	C	NA	*OreG0011190*

scaffold33	2508518	C	T	Och01,Och02,Och03,Och04,Och05,Och06,Och07,Och08,Och09,Och10,Och11,Och12,Och13,Och14	*OreG0011190*
scaffold33	2508656	C	T	Och01,Och02,Och03,Och04,Och05,Och06,Och12,Och13,Och14	*OreG0011190*
scaffold33	2508711	C	A	Ore01,Ore02,Ore03,Ore04,Ore05,Ore08,Ore09,Ore10,Ore14	*OreG0011190*
scaffold33	2508719	G	A	Ore01,Ore02,Ore03,Ore04,Ore05,Ore06,Ore07,Ore08,Ore09,Ore10,Ore11,Ore12,Ore13,Ore14	*OreG0011190*
scaffold33	2537965	C	A	Ore01,Ore02,Ore03,Ore04,Ore05,Ore06,Ore07,Ore08,Ore09,Ore10,Ore11,Ore12,Ore13,Ore14	*OreG0011191*
scaffold33	2538169	C	A	Och07,Och09,Och10,Och11	*OreG0011191*
scaffold33	2548995	C	T	Och07,Och09,Och10	*OreG0011193*
scaffold33	2549016	G	A	Och01,Och02,Och06	*OreG0011193*
scaffold33	2561329	T	C	Och01,Och02,Och03,Och04,Och05,Och06,Och07,Och08,Och09,Och10,Och11,Och12,Och13,Och14,Ore01,Ore02,Ore03,Ore04,Ore05,Ore06,Ore07,Ore08,Ore09,Ore10,Ore11,Ore12,Ore13,Ore14	*OreG0011195*
scaffold33	2587281	C	T	NA	*OreG0011195*
scaffold33	2601352	T	A	Och01,Och02,Och03,Och04,Och05,Och06,Och07,Och08,Och09,Och10,Och11,Och12,Och13,Och14	*OreG0011197*
scaffold33	2602297	G	T	Och01,Och02,Och03,Och04,Och05,Och06,Och07,Och08,Och09,Och10,Och11,Och12,Och13,Och14	*OreG0011197*
scaffold33	2608775	C	T	NA	*OreG0011197*
scaffold33	2610486	A	T	Ore01,Ore02,Ore03,Ore04,Ore05,Ore06,Ore07,Ore08,Ore09,Ore10,Ore11,Ore12,Ore13,Ore14	*OreG0011198*
scaffold33	2613519	A	T	Ore01,Ore02,Ore03,Ore04,Ore05,Ore06,Ore07,Ore08,Ore09,Ore10,Ore11,Ore12,Ore13,Ore14	*OreG0011198*
scaffold33	2613916	G	A	Och01,Och02,Och06,Och07,Och09,Och10,Och11	*OreG0011198*
scaffold33	2784828	C	G	Och01,Och02,Och05,Och06,Och12,Och13	*OreG0011200*
scaffold33	2899096	A	T	Och02,Och06	*OreG0011204*
scaffold33	2900866	G	C	Ore03,Ore08,Ore10,Ore14	*OreG0011204*
scaffold33	2903891	G	T	Och01,Och02,Och03,Och04,Och05,Och06,Och07,Och08,Och09,Och10,Och11,Och12,Och13,Och14,Ore01,Ore02,Ore03,Ore04,Ore05,Ore06,Ore07,Ore08,Ore09,Ore10,Ore11,Ore12,Ore13,Ore14	*OreG0011205*
scaffold33	2903975	T	C	Ore01,Ore02,Ore03,Ore04,Ore05,Ore06,Ore07,Ore08,Ore09,Ore10,Ore11,Ore12,Ore13,Ore14	*OreG0011205*
scaffold33	2914132	C	A	Och01,Och02,Och03,Och04,Och05,Och06,Och07,Och08,Och09,Och10,Och11,Och12,Och13,Och14	*OreG0011206*
scaffold33	2914138	C	T	Och02,Och03,Och04,Och05,Och06,Och08,Och09,Och10,Och11,Och12,Och13	*OreG0011206*
scaffold33	2914251	C	T	Och01,Och02,Och03,Och04,Och05,Och06,Och07,Och08,Och09,Och10,Och11,Och12,Och13,Och14,Ore01,Ore02,Ore03,Ore04,Ore05,Ore06,Ore07,Ore08,Ore09,Ore10,Ore11,Ore12,Ore13,Ore14	*OreG0011206*
scaffold33	2934265	T	A	Och01,Och02,Och03,Och04,Och05,Och06,Och07,Och08,Och09,Och10,Och11,Och12,Och13,Och14	*OreG0011207*
scaffold33	2935057	A	T	Och02,Och03,Och04,Och05,Och06,Och09,Och10,Och11,Och12,Och13	*OreG0011207*
scaffold33	2937412	A	G	Ore01,Ore02,Ore03,Ore04,Ore05,Ore06,Ore07,Ore08,Ore09,Ore10,Ore11,Ore12,Ore13,Ore14	*OreG0011208*
scaffold33	2964499	G	A	Och01,Och02,Och03,Och04,Och05,Och06,Och07,Och08,Och09,Och10,Och11,Och12,Och13,Och14	*OreG0011210*
scaffold33	3003113	C	T	NA	*OreG0011213*
scaffold33	3188414	C	G	Ore01,Ore02,Ore03,Ore04,Ore05,Ore06,Ore07,Ore08,Ore09,Ore10,Ore11,Ore12,Ore13,Ore14	*OreG0011222*
scaffold33	3316655	A	G	Och01,Och02,Och03,Och04,Och05,Och06,Och07,Och08,Och09,Och10,Och11,Och12,Och13,Och14	*OreG0011234*
scaffold33	3373857	T	C	NA	*OreG0011236*
scaffold33	3373911	G	A	NA	*OreG0011236*
scaffold33	3374254	G	A	NA	*OreG0011236*
scaffold33	3421133	G	A	Och01,Och02,Och03,Och04,Och05,Och06,Och07,Och08,Och09,Och10,Och11,Och12,Och13,Och14	*OreG0011238*
scaffold33	3421146	T	G	NA	*OreG0011238*
scaffold33	3422001	A	T	Och01,Och02,Och03,Och04,Och05,Och06,Och07,Och08,Och09,Och10,Och11,Och12,Och13,Och14,Ore01,Ore02,Ore03,Ore04,Ore05,Ore06,Ore07,Ore08,Ore09,Ore10,Ore11,Ore12,Ore13,Ore14	*OreG0011238*
scaffold33	3428057	T	A	NA	*OreG0011239*
scaffold33	3638357	G	A	NA	*OreG0011244*
scaffold33	3654788	G	C	Och02,Och06	*OreG0011245*
scaffold33	3655059	G	C	NA	*OreG0011245*
scaffold33	3656421	C	T	Ore01,Ore02,Ore03,Ore04,Ore05,Ore06,Ore07,Ore08,Ore09,Ore10,Ore11,Ore12,Ore13,Ore14	*OreG0011245*
scaffold33	3656468	G	T	Och02,Och06	*OreG0011245*
scaffold33	3757066	T	A	Och01,Och02,Och03,Och04,Och05,Och06,Och07,Och08,Och09,Och10,Och11,Och12,Och13,Och14,Ore01,Ore02,Ore03,Ore04,Ore05,Ore06,Ore07,Ore08,Ore09,Ore10,Ore11,Ore12,Ore13,Ore14	*OreG0011255*
scaffold33	3757609	C	G	Och01,Och02,Och03,Och04,Och05,Och06,Och07,Och08,Och09,Och10,Och11,Och12,Och13,Och14,Ore01,Ore02,Ore03,Ore04,Ore05,Ore06,Ore07,Ore08,Ore09,Ore10,Ore11,Ore12,Ore13,Ore14	*OreG0011255*
scaffold33	3759163	G	A	Och01,Och02,Och03,Och04,Och05,Och06,Och07,Och08,Och09,Och10,Och11,Och12,Och13,Och14,Ore01,Ore02,Ore03,Ore04,Ore05,Ore06,Ore07,Ore08,Ore09,Ore10,Ore11,Ore12,Ore13,Ore14	*OreG0011255*

scaffold33	3828795	C	T	Och01,Och02,Och03,Och04,Och05,Och06,Och07,Och08,Och09,Och10,Och11,Och12,Och13,Och14,Ore01,Ore02,Ore03,Ore04,Ore05,Ore06,Ore07,Ore08,Ore09,Ore10,Ore11,Ore12,Ore13,Ore14	*OreG0011256*
scaffold33	4070653	T	A	Ore09	*OreG0011261*
scaffold33	4071887	C	T	NA	*OreG0011261*
scaffold33	4073797	G	A	Och01,Och02,Och03,Och04,Och05,Och06,Och08,Och12,Och13,Och14	*OreG0011261*
scaffold33	4080177	C	T	NA	*OreG0011261*
scaffold33	4080187	A	C	Och01,Och02,Och03,Och04,Och05,Och06,Och07,Och08,Och09,Och10,Och11,Och12,Och13,Och14,Ore01,Ore02,Ore03,Ore04,Ore05,Ore06,Ore07,Ore08,Ore09,Ore10,Ore11,Ore12,Ore13,Ore14	*OreG0011261*
scaffold33	4103683	C	G	NA	*OreG0011262*
scaffold33	4108203	A	G	NA	*OreG0011262*
scaffold33	4134453	G	A	NA	*OreG0011263*
scaffold33	4140144	C	T	Och01,Och02,Och03,Och04,Och05,Och06,Och07,Och08,Och09,Och10,Och11,Och12,Och13,Och14	*OreG0011264*
scaffold33	4214884	A	G	Och01,Och02,Och03,Och04,Och05,Och06,Och07,Och08,Och09,Och10,Och11,Och12,Och13,Och14,Ore01,Ore02,Ore03,Ore04,Ore05,Ore06,Ore07,Ore08,Ore09,Ore10,Ore11,Ore12,Ore13,Ore14	*OreG0011265*
scaffold33	4215633	A	G	Och01,Och02,Och03,Och04,Och05,Och06,Och07,Och08,Och09,Och10,Och11,Och12,Och13,Och14	*OreG0011265*
scaffold1849	48175	C	T	NA	*OreG0003972*
scaffold1849	49025	G	A	NA	*OreG0003972*
scaffold1849	180054	C	T	NA	*OreG0003978*
scaffold1849	180109	G	A	NA	*OreG0003978*
scaffold1000	52203	G	T	NA	*OreG0000002*
scaffold1000	52315	C	T	Och01,Och02,Och03,Och04,Och05,Och06,Och07,Och08,Och09,Och10,Och11,Och12,Och13,Och14,Ore01,Ore02,Ore03,Ore04,Ore05,Ore06,Ore07,Ore08,Ore09,Ore10,Ore11,Ore12,Ore13,Ore14	*OreG0000002*
scaffold1000	64927	T	A	Och01,Och02,Och03,Och04,Och05,Och06,Och09,Och10,Och11,Och12,Och13,Och14	*OreG0000004*
scaffold1000	64931	C	T	Och01,Och02,Och03,Och04,Och05,Och06,Och09,Och10,Och11,Och12,Och13,Och14	*OreG0000004*
scaffold1000	65728	T	C	NA	*OreG0000004*
scaffold1000	65740	A	G	Ore01,Ore02,Ore03,Ore04,Ore05,Ore06,Ore07,Ore08,Ore09,Ore10,Ore11,Ore12,Ore13,Ore14	*OreG0000004*
scaffold1000	104282	C	A	Och01,Och02,Och03,Och04,Och05,Och06,Och09,Och10,Och11,Och12,Och13,Och14	*OreG0000006*
scaffold1000	106007	A	G	Och02,Och03,Och04,Och05,Och06,Och09,Och10,Och11,Och12,Och13,Och14	*OreG0000006*
scaffold1000	144343	G	T	NA	*OreG0000009*
scaffold1000	146202	C	T	NA	*OreG0000009*
scaffold1000	148941	C	T	Ore01,Ore02,Ore03,Ore04,Ore05,Ore06,Ore07,Ore08,Ore09,Ore10,Ore11,Ore12,Ore13,Ore14	*OreG0000010*
scaffold1000	149275	A	G	Ore08	*OreG0000010*
scaffold1000	194594	A	G	NA	*OreG0000015*
scaffold1000	194940	T	C	NA	*OreG0000015*
scaffold1000	196113	C	A	NA	*OreG0000015*
scaffold1000	211566	C	T	NA	*OreG0000016*
scaffold1000	333995	T	C	Och10,Och14	*OreG0000022*
scaffold1000	358094	G	A	NA	*OreG0000023*
scaffold1000	358128	A	T	NA	*OreG0000023*
scaffold1000	358444	C	T	Och01,Och02,Och06,Och07,Och08,Och09,Och10,Och11,Och14,Ore01	*OreG0000023*
scaffold1000	358450	G	A	Och01,Och02,Och06,Och07,Och08,Och09,Och10,Och11,Och14,Ore01	*OreG0000023*
scaffold1000	358477	C	T	Och01,Och02,Och06,Och07,Och08,Och09,Och10,Och11,Och14,Ore01	*OreG0000023*
scaffold1000	358917	T	G	Och01,Och02,Och06,Och07,Och08,Och09,Och10,Och11,Och14,Ore01	*OreG0000023*
scaffold1000	359032	C	T	Ore11	*OreG0000023*
scaffold1000	434240	G	C	Ore01,Ore02,Ore08	*OreG0000027*
scaffold1000	439572	G	T	NA	*OreG0000028*
scaffold1000	448076	A	G	NA	*OreG0000030*
scaffold1000	512981	T	C	Ore01,Ore02,Ore03,Ore04,Ore05,Ore06,Ore07,Ore08,Ore09,Ore10,Ore11,Ore12,Ore13,Ore14	*OreG0000036*
scaffold1000	513650	G	A	Och01,Och02,Och03,Och04,Och05,Och06,Och07,Och08,Och09,Och10,Och11,Och12,Och13,Och14,Ore01,Ore02,Ore03,Ore04,Ore05,Ore06,Ore07,Ore08,Ore09,Ore10,Ore11,Ore12,Ore13,Ore14	*OreG0000036*
scaffold1000	514181	C	T	Och01,Och02,Och05,Och07,Och08,Och09,Och10,Och11,Och12,Och13,Och14	*OreG0000036*
scaffold1000	514390	G	T	Ore01,Ore02,Ore03,Ore04,Ore05,Ore06,Ore07,Ore08,Ore09,Ore10,Ore11,Ore12,Ore13,Ore14	*OreG0000036*
scaffold1000	614117	G	T	Ore03,Ore09,Ore13	*OreG0000042*
scaffold1000	658223	G	C	NA	*OreG0000044*
scaffold1000	658253	G	C	Ore01,Ore02,Ore03,Ore04,Ore05,Ore06,Ore07,Ore08,Ore09,Ore10,Ore11,Ore12,Ore13,Ore14	*OreG0000044*
scaffold1000	658269	G	A	NA	*OreG0000044*
scaffold1000	658340	A	T	NA	*OreG0000044*
scaffold1021	75582	G	C	Ore01,Ore02,Ore03,Ore04,Ore05,Ore06,Ore07,Ore08,Ore09,Ore10,Ore11,Ore12,Ore13,Ore14	*OreG0000066*

scaffold1021	75693	T	A	Och01,Och02,Och03,Och04,Och05,Och06,Och07,Och08,Och09,Och10,Och11,Och12,Och13,Och14	*OreG0000066*
scaffold1021	244480	C	G	Och01,Och02,Och03,Och04,Och05,Och06,Och07,Och08,Och09,Och10,Och11,Och12,Och13,Och14,Ore01,Ore02,Ore03,Ore04,Ore05,Ore06,Ore07,Ore08,Ore09,Ore10,Ore11,Ore12,Ore13,Ore14	*OreG0000070*
scaffold1021	245519	G	T	Och01,Och02,Och03,Och04,Och05,Och06,Och07,Och08,Och09,Och10,Och11,Och12,Och13,Och14,Ore01,Ore02,Ore03,Ore04,Ore05,Ore06,Ore07,Ore08,Ore09,Ore10,Ore11,Ore12,Ore13,Ore14	*OreG0000070*
scaffold1021	245819	T	C	NA	*OreG0000070*
scaffold1021	245830	G	A	NA	*OreG0000070*
scaffold1021	246113	T	C	NA	*OreG0000070*
scaffold1021	246167	G	A	NA	*OreG0000070*
scaffold1021	310248	A	T	NA	*OreG0000073*
scaffold1021	310818	T	C	NA	*OreG0000073*
scaffold1021	311082	C	A	NA	*OreG0000073*
scaffold1021	311085	G	C	NA	*OreG0000073*
scaffold1021	311105	G	A	NA	*OreG0000073*
scaffold1021	392160	A	G	NA	*OreG0000075*
scaffold1021	392670	A	G	NA	*OreG0000075*
scaffold1021	504236	C	G	NA	*OreG0000076*
scaffold1021	935845	A	G	Och01	*OreG0000080*
scaffold1021	935944	G	A	Och08	*OreG0000080*
scaffold1021	936092	C	A	Ore01,Ore02,Ore03,Ore04,Ore05,Ore06,Ore07,Ore08,Ore09,Ore10,Ore11,Ore12,Ore13,Ore14	*OreG0000080*
scaffold1021	936164	G	C	Och02,Och03,Och04,Och05,Och06,Och07,Och08,Och09,Och10,Och11,Och12,Och13,Och14	*OreG0000080*
scaffold1021	936173	A	G	NA	*OreG0000080*
scaffold1021	936202	C	T	Ore01,Ore02,Ore03,Ore04,Ore05,Ore06,Ore07,Ore08,Ore09,Ore10,Ore11,Ore12,Ore13,Ore14	*OreG0000080*
scaffold1021	936404	C	G	Ore01,Ore02,Ore03,Ore04,Ore05,Ore06,Ore07,Ore08,Ore09,Ore10,Ore11,Ore12,Ore13,Ore14	*OreG0000080*
scaffold1021	936439	C	A	Och02,Och03,Och04,Och05,Och06,Och07,Och08,Och09,Och10,Och11,Och12,Och13,Och14,Ore01,Ore02,Ore03,Ore04,Ore05,Ore06,Ore07,Ore08,Ore09,Ore10,Ore11,Ore12,Ore13,Ore14	*OreG0000080*
scaffold1021	959231	A	G	Och02,Och03,Och04,Och05,Och06,Och07,Och08,Och09,Och10,Och11,Och12,Och13,Och14	*OreG0000081*
scaffold1021	959737	T	A	Och02,Och03,Och04,Och05,Och06,Och07,Och08,Och09,Och10,Och11,Och12,Och13,Och14,Ore01,Ore02,Ore03,Ore04,Ore05,Ore06,Ore07,Ore08,Ore09,Ore10,Ore11,Ore12,Ore13,Ore14	*OreG0000081*
scaffold1021	1029293	A	G	NA	*OreG0000082*
scaffold1021	1029315	G	A	Och06	*OreG0000082*
scaffold1021	1070050	G	A	Ore01,Ore02,Ore03,Ore04,Ore05,Ore06,Ore07,Ore08,Ore09,Ore10,Ore11,Ore12,Ore13,Ore14	*OreG0000083*
scaffold1021	1070067	C	T	NA	*OreG0000083*
scaffold1021	1070283	G	A	NA	*OreG0000083*
scaffold1021	1070289	C	G	Ore01,Ore02,Ore03,Ore04,Ore05,Ore06,Ore07,Ore08,Ore09,Ore10,Ore11,Ore12,Ore13,Ore14	*OreG0000083*
scaffold1021	1070460	G	C	Och09	*OreG0000083*
scaffold1021	1070464	C	T	NA	*OreG0000083*
scaffold1021	1070517	T	A	Ore08,Ore09	*OreG0000083*
scaffold1021	1098815	T	A	NA	*OreG0000085*
scaffold1021	1098870	C	T	NA	*OreG0000085*
scaffold1021	1100442	C	T	NA	*OreG0000085*
scaffold1021	1106006	C	A	NA	*OreG0000086*
scaffold1021	1106081	C	T	NA	*OreG0000086*
scaffold1021	1106712	T	G	NA	*OreG0000086*
scaffold1021	1131153	G	A	Och02,Och03,Och04,Och05,Och06,Och08,Och09,Och10,Och11,Och12,Och13,Och14	*OreG0000087*
scaffold1021	1131864	C	T	Och02,Och03,Och04,Och05,Och06,Och08,Och09,Och10,Och11,Och12,Och13,Och14	*OreG0000087*
scaffold1021	1285382	A	G	Och01,Och02,Och03,Och04,Och05,Och06,Och07,Och08,Och09,Och10,Och11,Och12,Och13,Och14,Ore01,Ore02,Ore03,Ore04,Ore05,Ore06,Ore07,Ore08,Ore09,Ore10,Ore11,Ore12,Ore13,Ore14	*OreG0000091*
scaffold1021	1490532	A	G	Och08	*OreG0000096*
scaffold1021	1536071	C	A	Och06	*OreG0000101*
scaffold1021	1728799	G	C	Ore01,Ore02,Ore03,Ore04,Ore05,Ore06,Ore07,Ore08,Ore09,Ore10,Ore11,Ore12,Ore13,Ore14	*OreG0000108*
scaffold1021	1974641	C	A	NA	*OreG0000115*
scaffold1021	2024995	T	C	Och01,Och02,Och03,Och04,Och05,Och06,Och07,Och08,Och09,Och10,Och11,Och12,Och13,Och14	*OreG0000117*
scaffold1021	2025243	G	A	NA	*OreG0000117*
scaffold1021	2132202	C	A	Och08	*OreG0000126*
scaffold1021	2132394	C	A	NA	*OreG0000126*
scaffold1021	2132505	G	A	NA	*OreG0000126*

scaffold1021	2149570	G	T	Ore01,Ore02,Ore03,Ore04,Ore05,Ore06,Ore07,Ore08,Ore09,Ore10,Ore11,Ore12,Ore13,Ore14	*OreG0000127*
scaffold1021	2287058	C	T	Och01,Och02,Och03,Och04,Och05,Och06,Och07,Och08,Och09,Och10,Och11,Och12,Och13,Och14,Ore01,Ore02,Ore03,Ore04,Ore05,Ore06,Ore07,Ore08,Ore09,Ore10,Ore11,Ore12,Ore13,Ore14	*OreG0000133*
scaffold1021	2287175	A	T	Och01,Och02,Och03,Och04,Och05,Och06,Och07,Och08,Och09,Och10,Och11,Och12,Och13,Och14	*OreG0000133*
scaffold1021	2308889	C	T	NA	*OreG0000135*
scaffold2156	2188	A	T	NA	*OreG0006055*
scaffold586	63361	C	T	Och01,Och02,Och03,Och04,Och05,Och06,Och07,Och08,Och09,Och10,Och11,Och12,Och13,Och14	*OreG0023806*
scaffold586	122703	A	G	NA	*OreG0023809*
scaffold586	130245	C	T	Och01,Och02,Och03,Och04,Och05,Och06,Och07,Och08,Och09,Och10,Och11,Och12,Och13,Och14	*OreG0023810*
scaffold586	130278	C	G	Och06	*OreG0023810*
scaffold586	132049	A	G	Ore01,Ore02,Ore03,Ore04,Ore05,Ore06,Ore07,Ore08,Ore09,Ore10,Ore11,Ore12,Ore13,Ore14	*OreG0023810*
scaffold586	147758	G	A	Och08	*OreG0023811*
scaffold586	152298	T	A	NA	*OreG0023812*
scaffold586	152676	C	A	NA	*OreG0023812*
scaffold586	152908	A	C	Och01,Och02,Och03,Och04,Och05,Och06,Och07,Och08,Och09,Och10,Och11,Och12,Och13,Och14	*OreG0023812*
scaffold586	152969	A	G	Ore01,Ore02,Ore03,Ore04,Ore05,Ore06,Ore07,Ore08,Ore09,Ore10,Ore11,Ore12,Ore13,Ore14	*OreG0023812*
scaffold586	153875	G	A	NA	*OreG0023812*
scaffold586	159535	C	T	Ore01,Ore02,Ore03,Ore04,Ore05,Ore06,Ore07,Ore08,Ore09,Ore10,Ore11,Ore12,Ore13,Ore14	*OreG0023814*
scaffold586	200397	G	A	Ore04,Ore05	*OreG0023816*
scaffold586	205623	G	C	Och06	*OreG0023816*
scaffold586	206986	T	G	NA	*OreG0023816*
scaffold586	218618	C	T	Och01,Och02,Och03,Och04,Och05,Och06,Och07,Och08,Och09,Och10,Och11,Och12,Och13,Och14	*OreG0023817*
scaffold586	225982	C	T	NA	*OreG0023819*
scaffold586	227918	A	G	NA	*OreG0023819*
scaffold586	234935	A	G	Ore01,Ore02,Ore03,Ore04,Ore05,Ore06,Ore07,Ore08,Ore09,Ore10,Ore11,Ore12,Ore13,Ore14	*OreG0023820*
scaffold586	265349	A	G	Ore01,Ore02,Ore03,Ore04,Ore05,Ore06,Ore07,Ore08,Ore09,Ore10,Ore11,Ore12,Ore13,Ore14	*OreG0023823*
scaffold586	289082	C	T	Ore01,Ore02,Ore03,Ore04,Ore05,Ore06,Ore07,Ore08,Ore09,Ore10,Ore11,Ore12,Ore13,Ore14	*OreG0023826*
scaffold586	332180	G	T	Ore01,Ore02,Ore03,Ore04,Ore05,Ore06,Ore07,Ore08,Ore09,Ore10,Ore11,Ore12,Ore13,Ore14	*OreG0023833*
scaffold586	333416	G	A	NA	*OreG0023833*
scaffold586	334212	G	T	NA	*OreG0023833*
scaffold586	352952	C	T	NA	*OreG0023835*
scaffold586	375067	A	G	Ore03,Ore04,Ore05,Ore06,Ore07,Ore10,Ore13,Ore14	*OreG0023837*
scaffold586	377988	C	T	Ore01,Ore02,Ore03,Ore04,Ore05,Ore06,Ore07,Ore08,Ore09,Ore10,Ore11,Ore12,Ore13,Ore14	*OreG0023838*
scaffold586	408029	C	T	NA	*OreG0023839*
scaffold586	421093	T	A	NA	*OreG0023843*
scaffold586	430453	T	G	Ore01,Ore02,Ore03,Ore04,Ore05,Ore06,Ore07,Ore08,Ore09,Ore10,Ore11,Ore12,Ore13,Ore14	*OreG0023845*
scaffold586	430704	A	C	Och01,Och02,Och03,Och04,Och05,Och06,Och07,Och08,Och09,Och10,Och11,Och12,Och13,Och14,Ore01,Ore02,Ore03,Ore04,Ore05,Ore06,Ore07,Ore08,Ore09,Ore10,Ore11,Ore12,Ore13,Ore14	*OreG0023845*
scaffold586	437918	C	T	Och01,Och02,Och04,Och06,Och07,Och08,Och09,Och10,Och11	*OreG0023846*
scaffold586	447749	T	C	NA	*OreG0023848*
scaffold586	447861	A	G	Och01,Och02,Och03,Och04,Och05,Och06,Och07,Och08,Och09,Och10,Och11,Och12,Och13,Och14,Ore01,Ore02,Ore03,Ore04,Ore05,Ore06,Ore07,Ore08,Ore09,Ore10,Ore11,Ore12,Ore13,Ore14	*OreG0023848*
scaffold586	456939	T	C	NA	*OreG0023850*
scaffold586	504129	T	A	NA	*OreG0023854*
scaffold586	507176	G	T	Och01,Och02,Och03,Och04,Och05,Och06,Och07,Och08,Och09,Och10,Och11,Och12,Och13,Och14,Ore01,Ore02,Ore03,Ore04,Ore05,Ore06,Ore07,Ore08,Ore09,Ore10,Ore11,Ore12,Ore13,Ore14	*OreG0023855*
scaffold586	511942	C	T	Och01,Och02,Och03,Och04,Och05,Och06,Och07,Och08,Och09,Och10,Och11,Och12,Och13,Och14	*OreG0023856*
scaffold586	535332	C	T	Ore01,Ore02,Ore03,Ore04,Ore05,Ore06,Ore07,Ore08,Ore09,Ore10,Ore11,Ore12,Ore13,Ore14	*OreG0023858*
scaffold586	537737	T	C	NA	*OreG0023859*
scaffold586	562422	A	C	NA	*OreG0023862*
scaffold586	586683	A	C	NA	*OreG0023866*
scaffold586	586845	G	T	NA	*OreG0023866*
scaffold586	586992	G	T	NA	*OreG0023866*
scaffold586	587164	T	A	NA	*OreG0023866*
scaffold586	609967	G	A	NA	*OreG0023869*

scaffold586	611621	T	A	Och01,Och02,Och03,Och04,Och05,Och06,Och07,Och08,Och09,Och10,Och11,Och12,Och13,Och14,Ore01,Ore02,Ore03,Ore04,Ore05,Ore06,Ore07,Ore08,Ore09,Ore10,Ore11,Ore12,Ore13,Ore14	*OreG0023869*
scaffold586	612649	G	A	Och08,Och11	*OreG0023869*
scaffold586	615520	G	T	NA	*OreG0023869*
scaffold586	651719	C	T	NA	*OreG0023874*
scaffold586	729401	T	A	Och08,Och09,Och10,Och11	*OreG0023878*
scaffold586	729877	G	T	NA	*OreG0023878*
scaffold586	729892	C	G	Och01,Och02,Och03,Och04,Och05,Och06,Och07,Och08,Och09,Och10,Och11,Och12,Och13,Och14,Ore01,Ore02,Ore03,Ore04,Ore05,Ore06,Ore07,Ore08,Ore09,Ore10,Ore11,Ore12,Ore13,Ore14	*OreG0023878*
scaffold586	758170	T	G	Och08,Och09,Och10,Och11,Och14	*OreG0023882*
scaffold586	760152	T	C	Och06,Ore01,Ore08,Ore09,Ore11,Ore12	*OreG0023883*
scaffold586	760326	C	T	NA	*OreG0023883*
scaffold586	771896	G	T	Och01	*OreG0023885*
scaffold586	778742	T	G	Och01	*OreG0023886*
scaffold586	802412	T	A	Och01,Och03,Och05,Och08,Och09,Och10,Och11,Och12,Och13,Och14	*OreG0023888*
scaffold586	819556	A	G	NA	*OreG0023892*
scaffold586	819561	A	G	Och08,Och11	*OreG0023892*
scaffold586	820011	C	T	NA	*OreG0023892*
scaffold586	820061	G	T	NA	*OreG0023892*
scaffold586	820068	C	A	NA	*OreG0023892*
scaffold586	827314	A	C	NA	*OreG0023894*
scaffold586	836562	G	A	NA	*OreG0023896*
scaffold586	891644	G	A	NA	*OreG0023901*
scaffold586	891681	G	A	Och08	*OreG0023901*
scaffold586	891776	A	T	Och06,Och08,Och14	*OreG0023901*
scaffold586	891785	T	G	Och01,Och02,Och03,Och04,Och05,Och06,Och07,Och08,Och09,Och10,Och11,Och12,Och13,Och14,Ore01,Ore02,Ore03,Ore04,Ore05,Ore06,Ore07,Ore08,Ore09,Ore10,Ore11,Ore12,Ore13,Ore14	*OreG0023901*
scaffold586	916041	A	T	Och01,Och02,Och03,Och04,Och05,Och06,Och07,Och08,Och09,Och10,Och11,Och12,Och13,Och14,Ore01,Ore02,Ore03,Ore04,Ore05,Ore06,Ore07,Ore08,Ore09,Ore10,Ore11,Ore12,Ore13,Ore14	*OreG0023902*
scaffold586	945353	T	A	Och06	*OreG0023907*
scaffold586	955068	C	T	NA	*OreG0023908*
scaffold586	963408	C	T	NA	*OreG0023910*
scaffold586	967047	C	T	Ore01,Ore02,Ore03,Ore04,Ore05,Ore06,Ore07,Ore08,Ore09,Ore10,Ore11,Ore12,Ore13,Ore14	*OreG0023911*
scaffold586	1046109	C	T	NA	*OreG0023916*
scaffold586	1046145	C	T	Och08	*OreG0023916*
scaffold586	1049497	T	G	Ore01,Ore02,Ore03,Ore04,Ore05,Ore06,Ore07,Ore08,Ore09,Ore10,Ore11,Ore12,Ore13,Ore14	*OreG0023917*
scaffold586	1049604	A	G	Ore01,Ore02,Ore03,Ore04,Ore05,Ore06,Ore07,Ore08,Ore09,Ore10,Ore11,Ore12,Ore13,Ore14	*OreG0023917*
scaffold586	1049723	C	A	Och01,Och02,Och04,Och06,Och08,Och09,Och10	*OreG0023917*
scaffold586	1050616	G	T	Ore01,Ore02,Ore03,Ore04,Ore05,Ore06,Ore07,Ore08,Ore09,Ore10,Ore11,Ore12,Ore13,Ore14	*OreG0023919*
scaffold586	1051029	C	T	Ore01,Ore02,Ore03,Ore04,Ore05,Ore06,Ore07,Ore08,Ore09,Ore10,Ore11,Ore12,Ore13,Ore14	*OreG0023919*
scaffold586	1051133	A	G	Ore01,Ore02,Ore03,Ore04,Ore05,Ore06,Ore07,Ore08,Ore09,Ore10,Ore11,Ore12,Ore13,Ore14	*OreG0023919*
scaffold586	1051227	A	C	NA	*OreG0023919*
scaffold586	1055001	G	T	Och01,Och02,Och03,Och04,Och05,Och06,Och07,Och08,Och09,Och10,Och11,Och12,Och13,Och14,Ore01,Ore02,Ore03,Ore04,Ore05,Ore06,Ore07,Ore08,Ore09,Ore10,Ore11,Ore12,Ore13,Ore14	*OreG0023920*
scaffold586	1108666	A	G	Och02,Och03,Och04,Och05,Och06,Och12,Och13,Och14	*OreG0023923*
scaffold586	1158477	C	T	Och02,Och03,Och04,Och05,Och06,Och07,Och08,Och09,Och10,Och11,Och12,Och13,Och14	*OreG0023930*
scaffold586	1159453	G	A	NA	*OreG0023931*
scaffold586	1163056	C	T	Och01,Och02,Och03,Och04,Och05,Och06,Och07,Och08,Och09,Och10,Och11,Och12,Och13,Och14,Ore01,Ore02,Ore03,Ore04,Ore05,Ore06,Ore07,Ore08,Ore09,Ore10,Ore11,Ore12,Ore13,Ore14	*OreG0023932*
scaffold586	1188226	A	T	Ore06	*OreG0023934*
scaffold586	1188668	C	T	NA	*OreG0023934*
scaffold586	1191628	C	A	Och01,Och02,Och03,Och04,Och05,Och06,Och08,Och09,Och10,Och11,Och12,Och13,Och14	*OreG0023935*
scaffold586	1198573	G	A	Och08	*OreG0023936*
scaffold586	1199003	T	C	NA	*OreG0023936*
scaffold586	1220641	G	A	Och01,Och02,Och03,Och04,Och05,Och06,Och07,Och08,Och12,Och13,Och14	*OreG0023938*
scaffold586	1253227	A	G	Och01,Och02,Och03,Och04,Och05,Och06,Och07,Och08,Och09,Och10,Och11,Och12,Och13,Och14	*OreG0023939*
scaffold586	1256722	G	A	Och01,Och02,Och03,Och04,Och05,Och06,Och07,Och08,Och09,Och10,Och11,Och12,Och13,Och14,Ore01,Ore02,Ore03,Ore04,Ore05,Ore06,Ore07,Ore08,Ore09,Ore10,Ore11,Ore12,Ore13,Ore14	*OreG0023939*

scaffold586	1261201	C	A	Ore01,Ore02,Ore03,Ore04,Ore05,Ore06,Ore07,Ore08,Ore09,Ore10,Ore11,Ore12,Ore13,Ore14	*OreG0023940*
scaffold586	1284608	C	T	Ore01,Ore02,Ore03,Ore04,Ore05,Ore06,Ore07,Ore08,Ore09,Ore10,Ore11,Ore12,Ore13,Ore14	*OreG0023941*
scaffold586	1297165	G	C	NA	*OreG0023942*
scaffold586	1305170	C	A	Och06	*OreG0023943*
scaffold586	1315716	G	T	Och01,Och02,Och03,Och04,Och05,Och06,Och07,Och08,Och09,Och10,Och11,Och12,Och13,Och14	*OreG0023944*
scaffold586	1321601	G	A	NA	*OreG0023946*
scaffold586	1322564	A	G	Ore01,Ore04,Ore05,Ore08,Ore09,Ore11,Ore12	*OreG0023946*
scaffold586	1472579	A	T	Ore01,Ore02,Ore03,Ore04,Ore05,Ore06,Ore07,Ore08,Ore09,Ore10,Ore11,Ore12,Ore13,Ore14	*OreG0023947*
scaffold586	1475724	G	A	Och01,Och02,Och04,Och06,Och07,Och08,Och09,Och10,Och11,Och14	*OreG0023948*
scaffold586	1476322	G	A	Och08,Och14	*OreG0023948*
scaffold586	1476512	T	G	NA	*OreG0023948*
scaffold586	1476799	T	C	NA	*OreG0023948*
scaffold586	1477113	G	A	NA	*OreG0023948*
scaffold586	1477523	T	G	NA	*OreG0023948*
scaffold586	1483302	T	C	Och01,Och02,Och03,Och04,Och05,Och06,Och07,Och08,Och09,Och10,Och11,Och12,Och13,Och14,Ore01,Ore02,Ore03,Ore04,Ore05,Ore06,Ore07,Ore08,Ore09,Ore10,Ore11,Ore12,Ore13,Ore14	*OreG0023949*
scaffold586	1483440	T	C	Och01,Och02,Och03,Och04,Och05,Och06,Och07,Och08,Och09,Och10,Och11,Och12,Och13,Och14,Ore01,Ore02,Ore03,Ore04,Ore05,Ore06,Ore07,Ore08,Ore09,Ore10,Ore11,Ore12,Ore13,Ore14	*OreG0023949*
scaffold586	1484997	A	G	Och06	*OreG0023949*
scaffold586	1485078	A	G	NA	*OreG0023949*
scaffold586	1486478	C	G	Och03,Och05,Och07,Och08,Och09,Och10,Och11,Och12,Och13,Och14	*OreG0023950*
scaffold586	1500032	A	G	Ore01,Ore02,Ore03,Ore04,Ore05,Ore06,Ore07,Ore08,Ore09,Ore10,Ore11,Ore12,Ore13,Ore14	*OreG0023953*
scaffold586	1501258	T	C	NA	*OreG0023953*
scaffold586	1538517	C	T	Och01,Och02,Och03,Och04,Och05,Och06,Och07,Och08,Och09,Och10,Och11,Och12,Och13,Och14	*OreG0023956*
scaffold586	1538754	C	A	Och01,Och02,Och03,Och04,Och05,Och06,Och07,Och08,Och09,Och10,Och11,Och12,Och13,Och14	*OreG0023956*
scaffold586	1538786	G	C	Ore01,Ore02,Ore03,Ore04,Ore05,Ore06,Ore07,Ore08,Ore09,Ore10,Ore11,Ore12,Ore13,Ore14	*OreG0023956*
scaffold586	1555564	A	T	Och01	*OreG0023959*
scaffold586	1577140	G	A	Ore01,Ore02,Ore03,Ore06,Ore07,Ore08,Ore09,Ore10,Ore11,Ore12,Ore13,Ore14	*OreG0023966*
scaffold586	1577155	A	C	Ore07,Ore13	*OreG0023966*
scaffold586	1577158	T	C	NA	*OreG0023966*
scaffold586	1611273	T	C	NA	*OreG0023970*
scaffold586	1611334	G	A	NA	*OreG0023970*
scaffold586	1611364	G	A	Och01,Och02,Och03,Och04,Och05,Och06,Och07,Och08,Och09,Och10,Och11,Och12,Och13,Och14	*OreG0023970*
scaffold586	1611372	C	T	Ore01,Ore02,Ore03,Ore04,Ore05,Ore06,Ore07,Ore08,Ore09,Ore10,Ore11,Ore12,Ore13,Ore14	*OreG0023970*
scaffold586	1691060	C	A	Och08	*OreG0023973*
scaffold586	1691092	A	G	Och01,Och02,Och03,Och04,Och05,Och06,Och07,Och08,Och09,Och10,Och11,Och12,Och13,Och14,Ore01,Ore02,Ore03,Ore04,Ore05,Ore06,Ore07,Ore08,Ore09,Ore10,Ore11,Ore12,Ore13,Ore14	*OreG0023973*
scaffold586	1691453	C	A	Och01,Och02,Och03,Och04,Och05,Och06,Och07,Och08,Och09,Och10,Och11,Och12,Och13,Och14,Ore01,Ore02,Ore03,Ore04,Ore05,Ore06,Ore07,Ore08,Ore09,Ore10,Ore11,Ore12,Ore13,Ore14	*OreG0023973*
scaffold586	1691624	G	A	Och01,Och02,Och03,Och04,Och05,Och06,Och07,Och08,Och09,Och10,Och11,Och12,Och13,Och14,Ore01,Ore02,Ore03,Ore04,Ore05,Ore06,Ore07,Ore08,Ore09,Ore10,Ore11,Ore12,Ore13,Ore14	*OreG0023973*
scaffold586	1691893	G	A	Och01,Och08	*OreG0023973*
scaffold586	1757082	A	G	Och01,Och02,Och03,Och04,Och05,Och06,Och07,Och08,Och09,Och10,Och11,Och12,Och13,Och14,Ore01,Ore02,Ore03,Ore04,Ore05,Ore06,Ore07,Ore08,Ore09,Ore10,Ore11,Ore12,Ore13,Ore14	*OreG0023980*
scaffold586	1758883	C	T	NA	*OreG0023980*
scaffold586	1758896	G	T	NA	*OreG0023980*
scaffold586	1769712	G	A	Och08	*OreG0023981*
scaffold586	1770201	A	T	Och01,Och02,Och03,Och04,Och05,Och06,Och07,Och08,Och09,Och10,Och11,Och12,Och13,Och14	*OreG0023981*
scaffold586	1794325	T	C	NA	*OreG0023983*
scaffold586	1794666	A	C	Och08	*OreG0023983*
scaffold586	1795015	G	A	NA	*OreG0023983*
scaffold586	1919316	C	T	Ore01,Ore02,Ore03,Ore04,Ore05,Ore06,Ore07,Ore08,Ore09,Ore10,Ore11,Ore12,Ore13,Ore14	*OreG0023993*
scaffold586	1919382	A	T	NA	*OreG0023993*
scaffold586	1938826	T	C	Och01,Och02,Och03,Och04,Och05,Och06,Och07,Och08,Och09,Och10,Och11,Och12,Och13,Och14	*OreG0023996*
scaffold586	1948135	C	G	NA	*OreG0023997*
scaffold586	1956635	A	T	Ore01,Ore02,Ore03,Ore04,Ore05,Ore06,Ore07,Ore08,Ore09,Ore10,Ore11,Ore12,Ore13,Ore14	*OreG0023999*

scaffold586	1957242	G	T	Och02,Och04,Och06	*OreG0023999*
scaffold586	1965123	T	C	NA	*OreG0024000*
scaffold586	1965540	G	A	Ore01,Ore02,Ore03,Ore04,Ore05,Ore06,Ore07,Ore08,Ore09,Ore10,Ore11,Ore12,Ore13,Ore14	*OreG0024000*
scaffold586	1966285	A	C	Och02,Och04,Och06	*OreG0024000*
scaffold586	1988058	A	G	Ore01,Ore02,Ore03,Ore04,Ore05,Ore06,Ore07,Ore08,Ore09,Ore10,Ore11,Ore12,Ore13,Ore14	*OreG0024003*
scaffold586	1989794	C	T	NA	*OreG0024003*
scaffold586	1990512	C	A	NA	*OreG0024003*
scaffold586	1990634	C	A	NA	*OreG0024003*
scaffold586	2070068	A	G	NA	*OreG0024007*
scaffold586	2070137	G	A	NA	*OreG0024007*
scaffold586	2184346	T	C	NA	*OreG0024011*
scaffold586	2184555	T	G	NA	*OreG0024011*
scaffold586	2184583	G	A	NA	*OreG0024011*
scaffold586	2215469	A	C	Ore01,Ore02,Ore03,Ore04,Ore05,Ore06,Ore07,Ore08,Ore09,Ore10,Ore11,Ore12,Ore13,Ore14	*OreG0024012*
scaffold586	2215502	C	T	Och01,Och02,Och03,Och04,Och05,Och06,Och07,Och08,Och09,Och10,Och11,Och12,Och13,Och14	*OreG0024012*
scaffold586	2247883	C	T	NA	*OreG0024017*
scaffold586	2248951	A	T	Ore01,Ore02,Ore03,Ore04,Ore05,Ore06,Ore07,Ore08,Ore09,Ore10,Ore11,Ore12,Ore13,Ore14	*OreG0024017*
scaffold586	2251018	C	T	NA	*OreG0024017*
scaffold586	2271865	C	A	NA	*OreG0024019*
scaffold586	2283948	G	C	Och01,Och02,Och03,Och04,Och05,Och06,Och07,Och08,Och09,Och10,Och11,Och12,Och13,Och14,Ore01,Ore02,Ore03,Ore04,Ore05,Ore06,Ore07,Ore08,Ore09,Ore10,Ore11,Ore12,Ore13,Ore14	*OreG0024021*
scaffold586	2284002	G	A	Ore01,Ore02,Ore03,Ore04,Ore05,Ore06,Ore07,Ore08,Ore09,Ore10,Ore11,Ore12,Ore13,Ore14	*OreG0024021*
scaffold586	2344962	C	A	NA	*OreG0024026*
scaffold586	2384083	G	A	Och01,Och09,Och10	*OreG0024028*
scaffold586	2384216	G	T	Och01,Och02,Och03,Och04,Och05,Och06,Och07,Och08,Och09,Och10,Och11,Och12,Och13,Och14,Ore01,Ore02,Ore03,Ore04,Ore05,Ore06,Ore07,Ore08,Ore09,Ore10,Ore11,Ore12,Ore13,Ore14	*OreG0024028*
scaffold586	2449477	T	C	Och01,Och09,Och10	*OreG0024035*
scaffold586	2449498	G	A	Och01,Och09,Och10	*OreG0024035*
scaffold586	2450480	C	T	NA	*OreG0024035*
scaffold586	2451805	C	T	NA	*OreG0024035*
scaffold586	2452003	T	C	NA	*OreG0024035*
scaffold586	2472339	C	T	NA	*OreG0024037*
scaffold586	2537116	T	A	Ore02,Ore03,Ore06,Ore07,Ore09,Ore10,Ore11,Ore12,Ore13,Ore14	*OreG0024043*
scaffold586	2537367	T	A	NA	*OreG0024043*
scaffold1028	66405	G	T	Och01,Och02,Och03,Och04,Och05,Och06,Och07,Och08,Och09,Och10,Och11,Och12,Och13,Och14,Ore01,Ore02,Ore03,Ore04,Ore05,Ore06,Ore07,Ore08,Ore09,Ore10,Ore11,Ore12,Ore13,Ore14	*OreG0000138*
scaffold1028	100587	C	A	Ore01,Ore02,Ore03,Ore04,Ore05,Ore06,Ore07,Ore08,Ore09,Ore10,Ore11,Ore12,Ore13,Ore14	*OreG0000139*
scaffold1028	191007	G	A	Ore11	*OreG0000139*
scaffold1028	214742	C	G	Och01,Och02,Och03,Och04,Och05,Och06,Och07,Och08,Och09,Och10,Och11,Och12,Och13,Och14,Ore01,Ore02,Ore03,Ore04,Ore05,Ore06,Ore07,Ore08,Ore09,Ore10,Ore11,Ore12,Ore13,Ore14	*OreG0000140*
scaffold1028	357737	A	G	Och07	*OreG0000143*
scaffold1028	387957	T	G	Ore01,Ore02,Ore03,Ore04,Ore05,Ore06,Ore07,Ore08,Ore09,Ore10,Ore11,Ore12,Ore13,Ore14	*OreG0000144*
scaffold1028	875654	C	T	NA	*OreG0000147*
scaffold1028	892653	C	G	NA	*OreG0000148*
scaffold1028	892753	G	A	NA	*OreG0000148*
scaffold1028	940840	T	C	NA	*OreG0000151*
scaffold1028	942271	T	C	NA	*OreG0000151*
scaffold1028	943376	C	A	NA	*OreG0000151*
scaffold1028	943447	T	G	NA	*OreG0000151*
scaffold1028	945543	C	T	NA	*OreG0000151*
scaffold1028	982844	G	C	Och07,Och08,Och09,Och10,Och11,Och13	*OreG0000157*
scaffold1028	1003704	C	A	Och01,Och02,Och03,Och04,Och05,Och06,Och07,Och08,Och09,Och10,Och11,Och12,Och13,Och14,Ore01,Ore02,Ore03,Ore04,Ore05,Ore06,Ore07,Ore08,Ore09,Ore10,Ore11,Ore12,Ore13,Ore14	*OreG0000159*
scaffold1028	1020166	T	A	Ore01,Ore02,Ore03,Ore04,Ore05,Ore06,Ore07,Ore08,Ore09,Ore10,Ore11,Ore12,Ore13,Ore14	*OreG0000162*
scaffold1028	1024816	C	A	Och07,Och08,Och13	*OreG0000163*
scaffold1028	1024855	C	G	Ore08,Ore09,Ore10	*OreG0000163*
scaffold1028	1034245	G	A	NA	*OreG0000164*
scaffold1028	1034447	C	G	Och01,Och02,Och03,Och04,Och05,Och06,Och07,Och08,Och09,Och10,Och11,Och12,Och13,Och14,Ore01,Ore02,Ore03,Ore04,Ore05,Ore06,Ore07,Ore08,Ore09,Ore10,Ore11,Ore12,Ore13,Ore14	*OreG0000164*
scaffold1028	1077329	G	A	Och07,Och08,Och09,Och10,Och11,Och13	*OreG0000166*

scaffold1028	1077372	G	A	Och01,Och02,Och03,Och04,Och05,Och06,Och07,Och08,Och09,Och10,Och11,Och12,Och13,Och14,Ore01,Ore02,Ore03,Ore04,Ore05,Ore06,Ore07,Ore08,Ore09,Ore10,Ore11,Ore12,Ore13,Ore14	*OreG0000166*
scaffold1028	1077489	C	T	Och01,Och02,Och03,Och04,Och05,Och06,Och07,Och08,Och09,Och10,Och11,Och12,Och13,Och14	*OreG0000166*
scaffold1028	1142349	A	T	Och01,Och02,Och03,Och04,Och05,Och06,Och07,Och08,Och09,Och10,Och11,Och12,Och13,Och14,Ore01,Ore02,Ore03,Ore04,Ore05,Ore06,Ore07,Ore08,Ore09,Ore10,Ore11,Ore12,Ore13,Ore14	*OreG0000171*
scaffold1028	1142370	G	A	Ore01,Ore02,Ore03,Ore04,Ore05,Ore06,Ore07,Ore08,Ore09,Ore10,Ore11,Ore12,Ore13,Ore14	*OreG0000171*
scaffold1028	1142415	G	A	Ore01,Ore02,Ore03,Ore04,Ore05,Ore06,Ore07,Ore08,Ore09,Ore10,Ore11,Ore12,Ore13,Ore14	*OreG0000171*
scaffold1028	1142884	G	A	Och01,Och02,Och03,Och04,Och05,Och06,Och07,Och08,Och09,Och10,Och11,Och12,Och13,Och14,Ore01,Ore02,Ore03,Ore04,Ore05,Ore06,Ore07,Ore08,Ore09,Ore10,Ore11,Ore12,Ore13,Ore14	*OreG0000171*
scaffold1028	1142890	G	A	Ore01,Ore02,Ore03,Ore06,Ore07,Ore08,Ore09,Ore10,Ore11,Ore12,Ore13,Ore14	*OreG0000171*
scaffold1028	1148059	C	T	Och04	*OreG0000172*
scaffold1028	1148725	G	T	Ore01,Ore02,Ore03,Ore04,Ore05,Ore06,Ore07,Ore08,Ore09,Ore10,Ore11,Ore12,Ore13,Ore14	*OreG0000172*
scaffold1028	1154069	C	A	Ore01,Ore04,Ore05	*OreG0000174*
scaffold1028	1157281	T	A	Ore01,Ore02,Ore03,Ore04,Ore05,Ore06,Ore07,Ore08,Ore09,Ore10,Ore11,Ore12,Ore13,Ore14	*OreG0000174*
scaffold1028	1159209	C	A	NA	*OreG0000174*
scaffold1028	1160802	C	G	NA	*OreG0000174*
scaffold1028	1226065	C	T	Och01,Och02,Och03,Och04,Och05,Och06,Och07,Och08,Och09,Och10,Och11,Och12,Och13,Och14	*OreG0000185*
scaffold1028	1226302	G	A	Ore01,Ore02,Ore03,Ore04,Ore05,Ore06,Ore07,Ore08,Ore09,Ore10,Ore11,Ore12,Ore13,Ore14	*OreG0000185*
scaffold1028	1229092	G	T	Ore01,Ore02,Ore03,Ore04,Ore05,Ore06,Ore07,Ore08,Ore09,Ore10,Ore11,Ore12,Ore13,Ore14	*OreG0000185*
scaffold1028	1229222	A	T	Och01,Och02,Och03,Och04,Och05,Och06,Och07,Och08,Och09,Och10,Och11,Och12,Och13,Och14,Ore01,Ore02,Ore03,Ore04,Ore05,Ore06,Ore07,Ore08,Ore09,Ore10,Ore11,Ore12,Ore13,Ore14	*OreG0000185*
scaffold1028	1229582	A	G	NA	*OreG0000185*
scaffold1028	1229697	A	T	Och01,Och02,Och03,Och04,Och05,Och06,Och07,Och08,Och09,Och10,Och11,Och12,Och13,Och14,Ore01,Ore02,Ore03,Ore04,Ore05,Ore06,Ore07,Ore08,Ore09,Ore10,Ore11,Ore12,Ore13,Ore14	*OreG0000185*
scaffold1028	1251705	C	T	Ore08,Ore09,Ore10	*OreG0000186*
scaffold1028	1252793	G	A	Ore01	*OreG0000186*
scaffold1028	1258003	T	G	Och09,Och10,Och14	*OreG0000187*
scaffold1028	1258830	A	T	Och01,Och02,Och03,Och04,Och05,Och06,Och07,Och08,Och09,Och10,Och11,Och12,Och13,Och14,Ore01,Ore02,Ore03,Ore04,Ore05,Ore06,Ore07,Ore08,Ore09,Ore10,Ore11,Ore12,Ore13,Ore14	*OreG0000187*
scaffold1028	1258941	G	A	NA	*OreG0000187*
scaffold1028	1261866	A	G	Ore01,Ore02,Ore03,Ore04,Ore05,Ore06,Ore07,Ore08,Ore09,Ore10,Ore11,Ore12,Ore13,Ore14	*OreG0000188*
scaffold1028	1274422	C	G	Och01,Och02,Och03,Och04,Och05,Och06,Och07,Och08,Och09,Och10,Och11,Och12,Och13,Och14,Ore01,Ore02,Ore03,Ore04,Ore05,Ore06,Ore07,Ore08,Ore09,Ore10,Ore11,Ore12,Ore13,Ore14	*OreG0000190*
scaffold1028	1275029	C	T	Och04	*OreG0000190*
scaffold1028	1283873	C	A	NA	*OreG0000192*
scaffold1028	1297558	G	C	Ore01,Ore02,Ore03,Ore04,Ore05,Ore06,Ore07,Ore08,Ore09,Ore10,Ore11,Ore12,Ore13,Ore14	*OreG0000194*
scaffold1028	1297627	T	A	Och13	*OreG0000194*
scaffold1028	1297956	T	G	Och01,Och02,Och03,Och04,Och05,Och06,Och07,Och08,Och09,Och10,Och11,Och12,Och13,Och14,Ore01,Ore02,Ore03,Ore04,Ore05,Ore06,Ore07,Ore08,Ore09,Ore10,Ore11,Ore12,Ore13,Ore14	*OreG0000194*
scaffold1028	1298716	G	A	NA	*OreG0000194*
scaffold1028	1301472	C	T	NA	*OreG0000194*
scaffold1028	1301502	A	T	Och01,Och02,Och03,Och04,Och05,Och06,Och07,Och08,Och09,Och10,Och11,Och12,Och13,Och14,Ore01,Ore02,Ore03,Ore04,Ore05,Ore06,Ore07,Ore08,Ore09,Ore10,Ore11,Ore12,Ore13,Ore14	*OreG0000194*
scaffold1028	1302932	T	C	Och01,Och02,Och03,Och04,Och05,Och06,Och07,Och09,Och10,Och12,Och13,Och14	*OreG0000194*
scaffold1028	1303107	C	T	Och08	*OreG0000194*
scaffold1028	1306153	C	T	Och09,Och10	*OreG0000194*
scaffold1028	1309022	A	T	Och01,Och02,Och03,Och04,Och05,Och06,Och07,Och09,Och10,Och12,Och13,Och14	*OreG0000195*
scaffold1028	1309103	A	G	Ore01,Ore02,Ore03,Ore04,Ore05,Ore06,Ore07,Ore08,Ore09,Ore10,Ore11,Ore12,Ore13,Ore14	*OreG0000195*
scaffold1028	1309256	G	T	Ore01,Ore02,Ore03,Ore04,Ore05,Ore06,Ore07,Ore08,Ore09,Ore10,Ore11,Ore12,Ore13,Ore14	*OreG0000195*
scaffold1028	1309793	A	G	Och13	*OreG0000195*
scaffold1028	1321232	G	A	Och08	*OreG0000197*
scaffold1028	1347785	C	T	Och03,Och04,Och05,Och09,Och10,Och12,Och13	*OreG0000200*

scaffold1433	8243	G	T	Och01,Och02,Och03,Och04,Och05,Och06,Och07,Och08,Och09,Och10,Och11,Och12,Och13,Och14	*OreG0002362*
scaffold1433	9482	C	T	NA	*OreG0002362*
scaffold1433	11616	T	G	Och01,Och02,Och03,Och04,Och05,Och06,Och07,Och08,Och09,Och10,Och11,Och12,Och13,Och14,Ore01,Ore02,Ore03,Ore04,Ore05,Ore06,Ore07,Ore08,Ore09,Ore10,Ore11,Ore12,Ore13,Ore14	*OreG0002363*
scaffold1433	13109	C	A	NA	*OreG0002363*
scaffold1433	37322	T	A	Och03	*OreG0002364*
scaffold1433	51776	T	C	Och01,Och02,Och03,Och04,Och05,Och06,Och07,Och08,Och09,Och10,Och11,Och12,Och13,Och14,Ore01,Ore02,Ore03,Ore04,Ore05,Ore06,Ore07,Ore08,Ore09,Ore10,Ore11,Ore12,Ore13,Ore14	*OreG0002366*
scaffold1433	55628	T	A	NA	*OreG0002366*
scaffold1433	136240	A	G	Och12,Och13	*OreG0002372*
scaffold1433	184529	G	T	NA	*OreG0002376*
scaffold1433	209448	C	T	Ore01,Ore02,Ore03,Ore04,Ore05,Ore06,Ore07,Ore08,Ore09,Ore10,Ore11,Ore12,Ore13,Ore14	*OreG0002377*
scaffold1433	210869	A	G	Ore01,Ore02,Ore03,Ore04,Ore05,Ore06,Ore07,Ore08,Ore09,Ore10,Ore11,Ore12,Ore13,Ore14	*OreG0002379*
scaffold1433	210917	A	G	Och01,Och02,Och03,Och04,Och05,Och06,Och07,Och08,Och09,Och10,Och11,Och12,Och13,Och14	*OreG0002379*
scaffold1433	288538	C	T	NA	*OreG0002389*
scaffold1433	289789	A	G	NA	*OreG0002389*
scaffold1433	332124	G	T	Och01,Och02,Och03,Och04,Och05,Och06,Och07,Och08,Och09,Och10,Och11,Och12,Och13,Och14	*OreG0002391*
scaffold2205	20128	C	T	NA	*OreG0006246*
scaffold2205	20190	G	A	NA	*OreG0006246*
scaffold2205	20347	C	T	Och08,Och09,Och10,Och11,Och14	*OreG0006246*
scaffold2205	20352	G	A	NA	*OreG0006246*
scaffold2205	20371	T	C	Ore10	*OreG0006246*
scaffold2205	20410	G	A	NA	*OreG0006246*
scaffold309	1208668	G	A	NA	*OreG0008873*
scaffold309	1208952	C	G	Och01,Och02,Och03,Och04,Och05,Och06,Och07,Och08,Och09,Och10,Och11,Och12,Och13,Och14	*OreG0008873*
scaffold1174	88735	G	A	Och01,Och02,Och03,Och04,Och05,Och06,Och07,Och08,Och09,Och10,Och11,Och12,Och13,Och14,Ore01,Ore02,Ore03,Ore04,Ore05,Ore06,Ore07,Ore08,Ore09,Ore10,Ore11,Ore12,Ore13,Ore14	*OreG0001016*
scaffold1174	161528	A	T	Och06	*OreG0001022*
scaffold1174	218109	C	A	NA	*OreG0001024*
scaffold1174	231309	T	C	NA	*OreG0001025*
scaffold1174	260934	A	T	Ore01,Ore02,Ore03,Ore04,Ore05,Ore06,Ore07,Ore08,Ore09,Ore10,Ore11,Ore12,Ore13,Ore14	*OreG0001026*
scaffold1174	360601	T	A	Och06,Och11	*OreG0001031*
scaffold1174	373952	T	C	Och01,Och02,Och03,Och04,Och05,Och06,Och07,Och08,Och09,Och10,Och11,Och12,Och13,Och14	*OreG0001032*
scaffold1174	374497	T	C	Och01,Och02,Och03,Och04,Och05,Och06,Och07,Och08,Och09,Och10,Och11,Och12,Och13,Och14	*OreG0001032*
scaffold1174	385379	A	T	Och01,Och02,Och03,Och04,Och05,Och06,Och07,Och08,Och09,Och10,Och11,Och12,Och13,Och14,Ore01,Ore02,Ore03,Ore04,Ore05,Ore06,Ore07,Ore08,Ore09,Ore10,Ore11,Ore12,Ore13,Ore14	*OreG0001035*
scaffold1174	422010	T	C	Och01,Och02,Och03,Och04,Och05,Och06,Och07,Och08,Och09,Och10,Och11,Och12,Och13,Och14	*OreG0001038*
scaffold1174	477992	T	A	NA	*OreG0001042*
scaffold1174	608908	T	A	Och11	*OreG0001048*
scaffold1174	609186	G	T	Och06	*OreG0001048*
scaffold1174	609404	C	A	NA	*OreG0001048*
scaffold1174	609408	C	T	NA	*OreG0001048*
scaffold1174	702089	G	T	Och11	*OreG0001058*
scaffold1174	707793	C	T	Och01,Och02,Och03,Och04,Och05,Och06,Och07,Och08,Och09,Och10,Och11,Och12,Och13,Och14,Ore01,Ore02,Ore03,Ore04,Ore05,Ore06,Ore07,Ore08,Ore09,Ore10,Ore11,Ore12,Ore13,Ore14	*OreG0001059*
scaffold1174	713479	C	T	NA	*OreG0001059*
scaffold1174	738168	G	A	NA	*OreG0001064*
scaffold1174	790932	C	T	NA	*OreG0001069*
scaffold1174	790933	A	T	NA	*OreG0001069*
scaffold1174	795188	C	T	NA	*OreG0001069*
scaffold1174	795363	C	T	NA	*OreG0001069*
scaffold1174	807340	C	G	Och06	*OreG0001070*
scaffold1174	807436	A	T	Och01,Och02,Och03,Och04,Och05,Och06,Och07,Och08,Och09,Och10,Och11,Och12,Och13,Och14,Ore01,Ore02,Ore03,Ore04,Ore05,Ore06,Ore07,Ore08,Ore09,Ore10,Ore11,Ore12,Ore13,Ore14	*OreG0001070*
scaffold1174	808819	T	C	Ore01,Ore02,Ore03,Ore04,Ore05,Ore06,Ore07,Ore08,Ore09,Ore10,Ore11,Ore12,Ore13,Ore14	*OreG0001071*
scaffold1764	12123	T	C	NA	*OreG0003905*
scaffold1764	12482	G	A	NA	*OreG0003905*
scaffold1764	12494	C	T	NA	*OreG0003905*

scaffold1186	99375	G	C	Ore01,Ore02,Ore03,Ore04,Ore05,Ore06,Ore07,Ore08,Ore09,Ore10,Ore11,Ore12,Ore13,Ore14	*OreG0001324*
scaffold1186	99825	C	A	NA	*OreG0001324*
scaffold1186	109780	T	A	Och01,Och02,Och03,Och04,Och05,Och06,Och07,Och08,Och09,Och10,Och11,Och12,Och13,Och14	*OreG0001326*
scaffold1186	110964	T	C	Och01,Och02,Och03,Och04,Och05,Och06,Och07,Och08,Och09,Och10,Och11,Och12,Och13,Och14	*OreG0001326*
scaffold1186	119960	T	A	NA	*OreG0001327*
scaffold1186	119987	G	T	NA	*OreG0001327*
scaffold1186	126606	T	G	NA	*OreG0001328*
scaffold1186	127670	G	A	NA	*OreG0001328*
scaffold1186	322944	T	A	NA	*OreG0001344*
scaffold1186	324222	T	C	NA	*OreG0001344*
scaffold1186	324301	A	T	NA	*OreG0001344*
scaffold1186	324326	G	A	NA	*OreG0001344*
scaffold1186	324551	G	C	NA	*OreG0001344*
scaffold1186	324613	C	A	NA	*OreG0001344*
scaffold1186	326081	C	G	NA	*OreG0001344*
scaffold1186	326671	A	G	NA	*OreG0001344*
scaffold1186	407220	A	G	NA	*OreG0001347*
scaffold1903	30112	C	T	Och02,Och05,Och06,Och08,Och13,Och14	*OreG0004825*
scaffold1903	30118	C	T	Och01,Och02,Och03,Och04,Och05,Och06,Och07,Och08,Och09,Och10,Och11,Och12,Och13,Och14,Ore01,Ore02,Ore03,Ore04,Ore05,Ore06,Ore07,Ore08,Ore09,Ore10,Ore11,Ore12,Ore13,Ore14	*OreG0004825*
scaffold1903	30425	C	A	Och01,Och02,Och03,Och04,Och05,Och06,Och07,Och08,Och09,Och10,Och11,Och12,Och13,Och14,Ore01,Ore02,Ore03,Ore04,Ore05,Ore06,Ore07,Ore08,Ore09,Ore10,Ore11,Ore12,Ore13,Ore14	*OreG0004825*
scaffold1903	30520	A	G	NA	*OreG0004825*
scaffold1903	30640	T	C	NA	*OreG0004825*
scaffold1903	30691	C	T	Och01,Och02,Och03,Och04,Och05,Och06,Och07,Och09,Och10,Och11,Och12,Och13,Och14	*OreG0004825*
scaffold1903	30704	G	C	Och01,Och02,Och03,Och04,Och05,Och06,Och07,Och08,Och09,Och10,Och11,Och12,Och13,Och14,Ore01,Ore02,Ore03,Ore04,Ore05,Ore06,Ore07,Ore08,Ore09,Ore10,Ore11,Ore12,Ore13,Ore14	*OreG0004825*
scaffold1903	30880	C	T	NA	*OreG0004825*
scaffold1903	50725	C	T	Och02,Och03,Och04,Och05,Och06,Och07,Och08,Och09,Och10,Och11,Och12,Och13,Och14	*OreG0004827*
scaffold1903	50731	G	A	NA	*OreG0004827*
scaffold1903	56957	T	C	NA	*OreG0004828*
scaffold1903	57014	T	A	NA	*OreG0004828*
scaffold1903	57101	C	T	NA	*OreG0004828*
scaffold1903	57220	A	T	NA	*OreG0004828*
scaffold1903	57273	G	T	Ore01,Ore03,Ore04,Ore05,Ore10,Ore11,Ore12,Ore13,Ore14	*OreG0004828*
scaffold1903	57335	A	T	NA	*OreG0004828*
scaffold1903	132272	G	A	NA	*OreG0004836*
scaffold1903	135136	G	A	Och01,Och02,Och03,Och04,Och05,Och06,Och07,Och08,Och09,Och10,Och11,Och12,Och13,Och14,Ore01,Ore02,Ore03,Ore04,Ore05,Ore06,Ore07,Ore08,Ore09,Ore10,Ore11,Ore12,Ore13,Ore14	*OreG0004836*
scaffold1903	159247	C	T	NA	*OreG0004839*
scaffold1903	161967	C	T	Och05	*OreG0004839*
scaffold1903	166139	C	T	Ore01,Ore02,Ore03,Ore04,Ore05,Ore06,Ore07,Ore08,Ore09,Ore10,Ore11,Ore12,Ore13,Ore14	*OreG0004840*
scaffold1903	166223	G	T	Och01,Och02,Och03,Och04,Och05,Och06,Och07,Och08,Och09,Och10,Och11,Och12,Och13,Och14	*OreG0004840*
scaffold1903	173260	G	C	NA	*OreG0004841*
scaffold1903	179608	C	T	Ore01,Ore02,Ore03,Ore04,Ore05,Ore06,Ore07,Ore08,Ore09,Ore10,Ore11,Ore12,Ore13,Ore14	*OreG0004842*
scaffold1903	180350	C	T	NA	*OreG0004842*
scaffold1903	180562	G	A	Ore01,Ore02,Ore03,Ore04,Ore05,Ore06,Ore07,Ore08,Ore09,Ore10,Ore11,Ore12,Ore13,Ore14	*OreG0004842*
scaffold1903	190776	G	C	NA	*OreG0004844*
scaffold1903	218181	T	A	NA	*OreG0004850*
scaffold1903	218800	G	A	Ore01,Ore02,Ore03,Ore04,Ore05,Ore06,Ore07,Ore08,Ore09,Ore10,Ore11,Ore12,Ore13,Ore14	*OreG0004850*
scaffold1903	218847	T	A	Och01,Och02,Och03,Och04,Och05,Och06,Och07,Och08,Och09,Och10,Och11,Och12,Och13,Och14,Ore01,Ore02,Ore03,Ore04,Ore05,Ore06,Ore07,Ore08,Ore09,Ore10,Ore11,Ore12,Ore13,Ore14	*OreG0004850*
scaffold1903	218907	C	A	NA	*OreG0004850*
scaffold1903	233188	A	T	NA	*OreG0004853*
scaffold1903	245657	G	A	Och01,Och02,Och03,Och04,Och05,Och06,Och07,Och08,Och09,Och10,Och12,Och13,Och14	*OreG0004855*
scaffold1903	302167	G	A	NA	*OreG0004864*
scaffold1903	302695	G	A	Ore01,Ore03,Ore04,Ore05,Ore10,Ore11,Ore12,Ore13,Ore14	*OreG0004864*
scaffold1903	305951	T	A	NA	*OreG0004864*
scaffold1903	308517	A	T	NA	*OreG0004865*

scaffold1903	310590	A	C	NA	*OreG0004865*
scaffold1903	361669	A	T	Och01,Och02,Och03,Och04,Och05,Och06,Och07,Och08,Och09,Och10,Och11,Och12,Och13,Och14	*OreG0004871*
scaffold1903	378288	C	T	Och01,Och02,Och03,Och04,Och05,Och06,Och07,Och08,Och09,Och10,Och11,Och12,Och13,Och14	*OreG0004876*
scaffold1903	378561	G	A	Ore01,Ore03,Ore04,Ore05,Ore10,Ore11,Ore12,Ore13,Ore14	*OreG0004876*
scaffold1903	378758	T	C	Ore01,Ore02,Ore03,Ore04,Ore05,Ore06,Ore07,Ore08,Ore09,Ore10,Ore11,Ore12,Ore13,Ore14	*OreG0004876*
scaffold1903	379255	T	A	Ore01,Ore02,Ore03,Ore04,Ore05,Ore06,Ore07,Ore08,Ore09,Ore10,Ore11,Ore12,Ore13,Ore14	*OreG0004876*
scaffold1903	379763	G	T	Och01,Och02,Och03,Och04,Och05,Och06,Och07,Och08,Och09,Och10,Och11,Och12,Och13,Och14,Ore01,Ore02,Ore03,Ore04,Ore05,Ore06,Ore07,Ore08,Ore09,Ore10,Ore11,Ore12,Ore13,Ore14	*OreG0004876*
scaffold1903	391320	T	A	Ore01,Ore02,Ore03,Ore04,Ore05,Ore06,Ore07,Ore08,Ore09,Ore10,Ore11,Ore12,Ore13,Ore14	*OreG0004879*
scaffold1903	393357	C	G	Och01,Och02,Och03,Och04,Och05,Och06,Och07,Och08,Och09,Och10,Och11,Och12,Och13,Och14,Ore01,Ore02,Ore03,Ore04,Ore05,Ore06,Ore07,Ore08,Ore09,Ore10,Ore11,Ore12,Ore13,Ore14	*OreG0004879*
scaffold1903	400677	A	C	Och06	*OreG0004881*
scaffold1903	400966	C	T	Och03,Och04,Och06,Och12	*OreG0004881*
scaffold1903	455640	G	T	NA	*OreG0004886*
scaffold1903	476339	C	G	Och01,Och05	*OreG0004889*
scaffold1903	476358	C	T	NA	*OreG0004889*
scaffold1903	476628	C	A	Och01,Och05	*OreG0004889*
scaffold1903	476684	C	T	NA	*OreG0004889*
scaffold1903	476744	C	T	Och08,Och11,Och14	*OreG0004889*
scaffold1903	476832	T	C	Och01,Och02,Och03,Och04,Och05,Och06,Och07,Och08,Och09,Och10,Och11,Och12,Och13,Och14	*OreG0004889*
scaffold1903	476841	A	G	Ore01,Ore02,Ore03,Ore04,Ore05,Ore06,Ore07,Ore08,Ore09,Ore10,Ore11,Ore12,Ore13,Ore14	*OreG0004889*
scaffold1903	477159	A	G	NA	*OreG0004889*
scaffold1903	498892	T	A	Och01,Och02,Och03,Och04,Och05,Och06,Och07,Och08,Och09,Och10,Och11,Och12,Och13,Och14,Ore01,Ore02,Ore03,Ore04,Ore05,Ore06,Ore08,Ore09,Ore10,Ore11,Ore12,Ore13,Ore14	*OreG0004893*
scaffold1903	499261	T	A	Och01,Och02,Och03,Och04,Och05,Och06,Och07,Och08,Och09,Och10,Och11,Och12,Och13,Och14,Ore01,Ore02,Ore03,Ore04,Ore05,Ore06,Ore07,Ore08,Ore09,Ore10,Ore11,Ore12,Ore13,Ore14	*OreG0004893*
scaffold1903	504859	C	T	Och02,Och06	*OreG0004894*
scaffold1903	514039	T	C	NA	*OreG0004896*
scaffold705	90863	A	G	NA	*OreG0024367*
scaffold705	170730	G	C	Ore03	*OreG0024371*
scaffold705	174535	T	A	Och11	*OreG0024372*
scaffold705	275541	T	C	Ore03	*OreG0024374*
scaffold705	275617	A	T	Ore03	*OreG0024374*
scaffold705	275645	G	T	Ore03	*OreG0024374*
scaffold705	305830	C	A	Ore03	*OreG0024375*
scaffold705	305843	C	G	Och04,Och06,Och13	*OreG0024375*
scaffold705	305878	C	T	NA	*OreG0024375*
scaffold705	305905	G	T	Och01,Och02,Och03,Och04,Och05,Och06,Och07,Och08,Och09,Och10,Och11,Och12,Och13,Och14	*OreG0024375*
scaffold705	305980	A	T	Ore03	*OreG0024375*
scaffold705	306349	C	T	Och06,Och13	*OreG0024375*
scaffold705	306700	G	A	Och01,Och07,Och09,Och10,Och11,Och14	*OreG0024375*
scaffold705	306847	A	T	NA	*OreG0024375*
scaffold705	362513	G	C	NA	*OreG0024379*
scaffold705	363305	C	G	NA	*OreG0024379*
scaffold705	363605	G	C	Ore01,Ore02,Ore03,Ore04,Ore05,Ore06,Ore07,Ore08,Ore09,Ore10,Ore11,Ore12,Ore13,Ore14	*OreG0024379*
scaffold705	363612	A	G	NA	*OreG0024379*
scaffold705	370592	A	T	Och01,Och02,Och03,Och04,Och05,Och06,Och07,Och08,Och09,Och10,Och11,Och12,Och13,Och14,Ore01,Ore02,Ore03,Ore04,Ore05,Ore06,Ore07,Ore08,Ore09,Ore10,Ore11,Ore12,Ore13,Ore14	*OreG0024380*
scaffold705	421904	G	A	Och07,Och09,Och10,Och11	*OreG0024385*
scaffold705	422940	A	T	NA	*OreG0024385*
scaffold705	520819	G	C	NA	*OreG0024390*
scaffold705	576851	C	T	Ore01,Ore02,Ore03,Ore04,Ore05,Ore06,Ore07,Ore08,Ore09,Ore10,Ore11,Ore12,Ore13,Ore14	*OreG0024393*
scaffold705	579547	G	A	Ore01,Ore02,Ore04,Ore05,Ore06,Ore07,Ore08,Ore11,Ore12	*OreG0024394*
scaffold705	612938	T	C	Och01,Och02,Och03,Och04,Och05,Och06,Och07,Och08,Och09,Och10,Och11,Och12,Och13,Och14,Ore01,Ore02,Ore03,Ore04,Ore05,Ore06,Ore07,Ore08,Ore09,Ore10,Ore11,Ore12,Ore13,Ore14	*OreG0024396*
scaffold705	637112	G	A	NA	*OreG0024399*
scaffold705	637200	G	A	NA	*OreG0024399*
scaffold705	637211	G	A	NA	*OreG0024399*
scaffold705	637278	C	T	NA	*OreG0024399*

scaffold705	637329	C	T	NA	*OreG0024399*
scaffold705	637433	A	G	NA	*OreG0024399*
scaffold705	637499	G	A	NA	*OreG0024399*
scaffold705	637649	G	T	NA	*OreG0024399*
scaffold705	637941	C	T	Och13	*OreG0024399*
scaffold705	637995	C	T	NA	*OreG0024399*
scaffold705	638004	C	T	NA	*OreG0024399*
scaffold705	642777	C	A	Ore01,Ore02,Ore03,Ore04,Ore05,Ore06,Ore07,Ore08,Ore09,Ore10,Ore11,Ore12,Ore13,Ore14	*OreG0024400*
scaffold705	644383	T	A	Ore01,Ore02,Ore03,Ore04,Ore05,Ore06,Ore07,Ore08,Ore09,Ore10,Ore11,Ore12,Ore13,Ore14	*OreG0024400*
scaffold705	697404	A	T	Och07,Och08,Och14	*OreG0024403*
scaffold705	697836	C	T	NA	*OreG0024403*
scaffold705	740635	A	G	Och01,Och04,Och06,Och09,Och10,Och13	*OreG0024405*
scaffold705	807408	A	G	NA	*OreG0024406*
scaffold705	807410	T	A	NA	*OreG0024406*
scaffold705	807423	G	T	NA	*OreG0024406*
scaffold705	807465	G	T	NA	*OreG0024406*
scaffold705	807672	A	G	NA	*OreG0024406*
scaffold705	833644	T	G	Och07,Och08,Och14	*OreG0024408*
scaffold705	833678	C	G	NA	*OreG0024408*
scaffold705	833700	G	A	NA	*OreG0024408*
scaffold705	833854	C	A	NA	*OreG0024408*
scaffold705	833918	G	T	NA	*OreG0024408*
scaffold705	834059	T	A	NA	*OreG0024408*
scaffold705	834207	G	C	Och07,Och08,Och14,Ore01,Ore02,Ore03,Ore04,Ore05,Ore06,Ore07,Ore08,Ore09,Ore10,Ore11,Ore12,Ore13,Ore14	*OreG0024408*
scaffold705	834215	C	T	NA	*OreG0024408*
scaffold705	834231	G	C	NA	*OreG0024408*
scaffold705	834320	A	T	Ore02,Ore04,Ore05	*OreG0024408*
scaffold705	834392	A	C	NA	*OreG0024408*
scaffold705	893958	G	C	NA	*OreG0024413*
scaffold705	895525	A	G	Och01,Och02,Och03,Och04,Och05,Och06,Och07,Och08,Och09,Och10,Och11,Och12,Och13,Och14	*OreG0024413*
scaffold705	951986	T	C	Ore01,Ore02,Ore03,Ore04,Ore05,Ore06,Ore07,Ore08,Ore09,Ore10,Ore11,Ore12,Ore13,Ore14	*OreG0024424*
scaffold705	960419	T	A	Ore01,Ore02,Ore03,Ore04,Ore05,Ore06,Ore07,Ore08,Ore09,Ore10,Ore11,Ore12,Ore13,Ore14	*OreG0024425*
scaffold705	964387	C	A	Och01,Och02,Och03,Och04,Och05,Och06,Och07,Och08,Och09,Och10,Och11,Och12,Och13,Och14,Ore01,Ore02,Ore03,Ore04,Ore05,Ore06,Ore07,Ore08,Ore09,Ore10,Ore11,Ore12,Ore13,Ore14	*OreG0024426*
scaffold705	964465	A	C	Och01,Och02,Och03,Och04,Och05,Och06,Och07,Och08,Och12,Och13,Och14	*OreG0024426*
scaffold705	964487	C	T	Och01,Och02,Och03,Och04,Och05,Och06,Och07,Och08,Och12,Och13,Och14	*OreG0024426*
scaffold705	964528	A	G	Ore01,Ore02,Ore03,Ore04,Ore05,Ore06,Ore07,Ore08,Ore09,Ore10,Ore11,Ore12,Ore13,Ore14	*OreG0024426*
scaffold705	976311	T	C	Ore01,Ore02,Ore03,Ore04,Ore05,Ore06,Ore07,Ore08,Ore09,Ore10,Ore11,Ore12,Ore13,Ore14	*OreG0024428*
scaffold705	1006196	C	T	Ore01,Ore02,Ore03,Ore04,Ore05,Ore06,Ore07,Ore08,Ore09,Ore10,Ore11,Ore12,Ore13,Ore14	*OreG0024432*
scaffold705	1007264	C	T	Och01,Och02,Och03,Och04,Och05,Och06,Och07,Och08,Och09,Och10,Och11,Och12,Och13,Och14	*OreG0024432*
scaffold705	1016896	C	T	Och01,Och02,Och03,Och04,Och05,Och06,Och07,Och08,Och09,Och10,Och11,Och12,Och13,Och14,Ore01,Ore02,Ore03,Ore04,Ore05,Ore06,Ore07,Ore08,Ore09,Ore10,Ore11,Ore12,Ore13,Ore14	*OreG0024436*
scaffold705	1017296	G	T	Ore01,Ore02,Ore03,Ore04,Ore05,Ore06,Ore07,Ore08,Ore09,Ore10,Ore11,Ore12,Ore13,Ore14	*OreG0024436*
scaffold705	1108233	C	A	NA	*OreG0024438*
scaffold705	1108364	A	T	NA	*OreG0024438*
scaffold705	1108699	G	A	NA	*OreG0024438*
scaffold705	1109288	C	T	NA	*OreG0024438*
scaffold705	1109502	G	A	NA	*OreG0024438*
scaffold705	1109554	G	A	NA	*OreG0024438*
scaffold705	1127502	G	T	Och01,Och08,Och11,Och14	*OreG0024439*
scaffold705	1134364	C	A	Och01,Och02,Och03,Och04,Och05,Och06,Och07,Och08,Och09,Och10,Och11,Och12,Och13,Och14,Ore01,Ore02,Ore03,Ore04,Ore05,Ore06,Ore07,Ore08,Ore09,Ore10,Ore11,Ore12,Ore13,Ore14	*OreG0024439*
scaffold705	1148482	T	C	NA	*OreG0024442*
scaffold705	1160599	C	T	Och06,Ore02,Ore04,Ore05,Ore13	*OreG0024442*
scaffold705	1178843	G	A	NA	*OreG0024445*
scaffold705	1179002	G	T	Och01	*OreG0024445*
scaffold705	1179167	G	T	NA	*OreG0024445*
scaffold705	1179169	A	G	NA	*OreG0024445*
scaffold705	1179466	G	A	NA	*OreG0024445*
scaffold705	1179545	C	A	NA	*OreG0024445*

scaffold705	1179663	G	T	Och02,Och03,Och06,Och09,Och10,Och11,Och12,Och14,Ore01,Ore02,Ore03,Ore04,Ore05,Ore06,Ore07,Ore08,Ore09,Ore10,Ore11,Ore12,Ore13,Ore14	*OreG0024445*
scaffold705	1184899	T	A	Och01,Och02,Och03,Och04,Och05,Och06,Och07,Och08,Och09,Och10,Och11,Och12,Och13,Och14	*OreG0024447*
scaffold705	1219976	A	C	Och01,Och02,Och03,Och04,Och05,Och06,Och07,Och08,Och09,Och10,Och11,Och12,Och13,Och14	*OreG0024449*
scaffold705	1315344	A	T	NA	*OreG0024459*
scaffold705	1315421	G	A	NA	*OreG0024459*
scaffold705	1316943	C	G	Och02,Och03,Och04,Och05,Och06,Och08,Och09,Och10,Och11,Och12,Och13,Och14	*OreG0024459*
scaffold705	1365637	A	T	NA	*OreG0024463*
scaffold705	1386640	G	A	Och01,Och04,Och06,Och09,Och10,Och11,Och13,Och14	*OreG0024465*
scaffold705	1388496	A	G	NA	*OreG0024465*
scaffold705	1388733	A	C	Ore01,Ore02,Ore03,Ore04,Ore05,Ore06,Ore07,Ore08,Ore09,Ore10,Ore11,Ore12,Ore13,Ore14	*OreG0024465*
scaffold705	1397945	C	T	Och06	*OreG0024466*
scaffold705	1401729	C	T	Och06,Och09,Och10,Och11,Och14	*OreG0024467*
scaffold705	1401870	C	A	Ore02,Ore04,Ore05,Ore06,Ore07,Ore08,Ore09,Ore10,Ore11,Ore12,Ore14	*OreG0024467*
scaffold705	1402034	C	A	NA	*OreG0024467*
scaffold705	1402106	A	T	NA	*OreG0024467*
scaffold705	1402314	C	A	NA	*OreG0024467*
scaffold705	1402556	G	A	NA	*OreG0024467*
scaffold705	1402608	G	C	NA	*OreG0024467*
scaffold705	1402631	G	C	NA	*OreG0024467*
scaffold705	1407348	A	G	NA	*OreG0024469*
scaffold705	1438101	C	A	Och11	*OreG0024471*
scaffold705	1443580	T	A	Och01,Och02,Och03,Och04,Och05,Och06,Och07,Och08,Och09,Och10,Och11,Och12,Och13,Och14,Ore01,Ore02,Ore03,Ore04,Ore05,Ore06,Ore07,Ore08,Ore09,Ore10,Ore11,Ore12,Ore13,Ore14	*OreG0024472*
scaffold705	1453049	G	A	NA	*OreG0024474*
scaffold705	1453271	G	C	NA	*OreG0024474*
scaffold705	1465390	C	T	Och02,Och03,Och06,Och11,Och12,Och14	*OreG0024475*
scaffold705	1465616	T	C	NA	*OreG0024475*
scaffold705	1480751	A	C	NA	*OreG0024477*
scaffold705	1517872	A	G	Ore01,Ore02,Ore03,Ore04,Ore05,Ore06,Ore07,Ore08,Ore09,Ore10,Ore11,Ore12,Ore13,Ore14	*OreG0024481*
scaffold705	1517882	A	C	Ore01,Ore02,Ore03,Ore04,Ore05,Ore06,Ore07,Ore08,Ore09,Ore10,Ore11,Ore12,Ore13,Ore14	*OreG0024481*
scaffold705	1528328	G	A	Och02,Och03,Och04,Och05,Och06,Och12,Och13	*OreG0024482*
scaffold705	1529320	C	T	Och02,Och03,Och04,Och05,Och06,Och12,Och13	*OreG0024482*
scaffold705	1531012	T	C	Ore01,Ore02,Ore03,Ore04,Ore05,Ore06,Ore07,Ore08,Ore09,Ore10,Ore11,Ore12,Ore13,Ore14	*OreG0024482*
scaffold705	1532033	C	A	NA	*OreG0024482*
scaffold705	1532049	A	T	Ore01,Ore02,Ore03,Ore04,Ore05,Ore06,Ore07,Ore08,Ore09,Ore10,Ore11,Ore12,Ore13,Ore14	*OreG0024482*
scaffold705	1549464	A	C	Och01,Och09,Och10,Och11	*OreG0024483*
scaffold705	1566400	G	C	NA	*OreG0024485*
scaffold705	1622959	G	A	NA	*OreG0024493*
scaffold705	1628733	C	T	NA	*OreG0024494*
scaffold705	1634992	C	T	Och11	*OreG0024495*
scaffold705	1635433	A	T	NA	*OreG0024495*
scaffold705	1635478	C	T	Ore01,Ore02,Ore03,Ore04,Ore05,Ore06,Ore07,Ore08,Ore09,Ore10,Ore11,Ore12,Ore13,Ore14	*OreG0024495*
scaffold705	1635561	A	C	NA	*OreG0024495*
scaffold705	1635672	G	A	Och04,Och06,Och07,Och11,Och13	*OreG0024495*
scaffold705	1640104	A	G	NA	*OreG0024496*
scaffold705	1641762	T	C	NA	*OreG0024496*
scaffold705	1641784	C	A	NA	*OreG0024496*
scaffold705	1644495	C	T	Ore01,Ore02,Ore03,Ore04,Ore05,Ore06,Ore07,Ore08,Ore09,Ore10,Ore11,Ore12,Ore13,Ore14	*OreG0024497*
scaffold705	1645451	A	C	Och10,Och11	*OreG0024497*
scaffold705	1645607	G	A	Och11	*OreG0024497*
scaffold705	1645736	G	T	NA	*OreG0024498*
scaffold705	1645738	G	C	NA	*OreG0024498*
scaffold705	1645770	G	A	Och04,Och06,Och07,Och11,Och13,Och14	*OreG0024498*
scaffold705	1646178	C	T	Ore01,Ore02,Ore03,Ore04,Ore05,Ore06,Ore07,Ore08,Ore09,Ore10,Ore11,Ore12,Ore13,Ore14	*OreG0024498*
scaffold705	1646352	G	A	NA	*OreG0024498*
scaffold705	1656217	T	C	Ore01,Ore02,Ore03,Ore04,Ore05,Ore06,Ore07,Ore08,Ore09,Ore10,Ore11,Ore12,Ore13,Ore14	*OreG0024500*
scaffold705	1661837	A	T	NA	*OreG0024500*
scaffold705	1663023	G	C	Ore01,Ore02,Ore03,Ore04,Ore05,Ore06,Ore07,Ore08,Ore09,Ore10,Ore11,Ore12,Ore13,Ore14	*OreG0024501*
scaffold705	1663215	T	A	Och04,Och06,Och07,Och11,Och13	*OreG0024501*
scaffold705	1664270	G	C	Och04,Och11,Och13	*OreG0024502*

scaffold705	1664614	C	T	Och01,Och02,Och03,Och04,Och05,Och06,Och07,Och08,Och09,Och10,Och11,Och12,Och13,Och14,Ore01,Ore02,Ore03,Ore04,Ore05,Ore06,Ore07,Ore08,Ore09,Ore10,Ore11,Ore12,Ore13,Ore14	*OreG0024502*
scaffold705	1664644	C	T	Ore01,Ore02,Ore03,Ore04,Ore05,Ore06,Ore07,Ore08,Ore09,Ore10,Ore11,Ore12,Ore13,Ore14	*OreG0024502*
scaffold705	1664681	C	G	NA	*OreG0024502*
scaffold705	1664703	T	A	Och04,Och06,Och07,Och11,Och13	*OreG0024502*
scaffold705	1669902	G	A	NA	*OreG0024503*
scaffold705	1688903	G	T	Och04,Och06,Och13	*OreG0024506*
scaffold705	1689014	C	T	Ore01,Ore02,Ore03,Ore04,Ore05,Ore06,Ore07,Ore08,Ore09,Ore10,Ore11,Ore12,Ore13,Ore14	*OreG0024506*
scaffold705	1689016	G	A	Ore01,Ore02,Ore03,Ore04,Ore05,Ore06,Ore07,Ore08,Ore09,Ore10,Ore11,Ore12,Ore13,Ore14	*OreG0024506*
scaffold705	1689025	C	T	Ore01,Ore02,Ore03,Ore04,Ore05,Ore06,Ore07,Ore08,Ore09,Ore10,Ore11,Ore12,Ore13,Ore14	*OreG0024506*
scaffold705	1689134	G	A	Ore01,Ore02,Ore03,Ore04,Ore05,Ore06,Ore07,Ore08,Ore09,Ore10,Ore11,Ore12,Ore13,Ore14	*OreG0024506*
scaffold705	1689906	G	A	Ore01,Ore02,Ore03,Ore04,Ore05,Ore06,Ore07,Ore08,Ore09,Ore10,Ore11,Ore12,Ore13,Ore14	*OreG0024506*
scaffold705	1689999	G	A	Ore01,Ore02,Ore03,Ore04,Ore05,Ore06,Ore07,Ore08,Ore09,Ore10,Ore11,Ore12,Ore13,Ore14	*OreG0024506*
scaffold705	1690164	G	A	NA	*OreG0024506*
scaffold705	1690756	C	T	NA	*OreG0024506*
scaffold705	1690791	C	T	Ore01,Ore02,Ore04,Ore05,Ore06,Ore07,Ore08,Ore09,Ore10,Ore11,Ore12,Ore14	*OreG0024506*
scaffold705	1694841	C	T	Och01,Och02,Och03,Och04,Och05,Och06,Och07,Och08,Och09,Och10,Och11,Och12,Och13,Och14,Ore01,Ore02,Ore03,Ore04,Ore05,Ore06,Ore07,Ore08,Ore09,Ore10,Ore11,Ore12,Ore13,Ore14	*OreG0024508*
scaffold705	1694854	C	T	Och04,Och06,Och07,Och11,Och13	*OreG0024508*
scaffold705	1694863	G	A	NA	*OreG0024508*
scaffold705	1694908	C	T	NA	*OreG0024508*
scaffold705	1695355	A	G	NA	*OreG0024508*
scaffold705	1698840	C	A	Och04,Och06,Och07,Och11,Och13	*OreG0024509*
scaffold705	1699039	G	A	Ore01,Ore02,Ore04,Ore05,Ore06,Ore07,Ore08,Ore09,Ore10,Ore11,Ore12,Ore14	*OreG0024509*
scaffold705	1699046	C	T	Och04,Och06,Och07,Och11,Och13	*OreG0024509*
scaffold705	1699522	C	A	NA	*OreG0024509*
scaffold705	1713576	G	C	NA	*OreG0024512*
scaffold705	1714244	C	T	NA	*OreG0024512*
scaffold705	1714280	G	A	Och06,Och13	*OreG0024512*
scaffold705	1722490	C	T	Ore01,Ore02,Ore03,Ore04,Ore05,Ore06,Ore07,Ore08,Ore09,Ore10,Ore11,Ore12,Ore13,Ore14	*OreG0024514*
scaffold705	1722627	A	G	Ore01,Ore02,Ore03,Ore04,Ore05,Ore06,Ore07,Ore08,Ore09,Ore10,Ore11,Ore12,Ore13,Ore14	*OreG0024514*
scaffold705	1722865	A	G	NA	*OreG0024514*
scaffold705	1723418	G	A	NA	*OreG0024514*
scaffold705	1723517	T	A	NA	*OreG0024514*
scaffold705	1723533	G	T	NA	*OreG0024514*
scaffold705	1723568	C	A	Och04,Och06,Och13	*OreG0024514*
scaffold705	1723671	T	C	NA	*OreG0024514*
scaffold705	1723731	T	C	Och04,Och07,Ore01,Ore02,Ore03,Ore04,Ore05,Ore06,Ore07,Ore08,Ore09,Ore10,Ore11,Ore12,Ore13,Ore14	*OreG0024514*
scaffold705	1729813	C	A	Ore01,Ore02,Ore03,Ore04,Ore05,Ore06,Ore07,Ore08,Ore09,Ore10,Ore11,Ore12,Ore13,Ore14	*OreG0024515*
scaffold705	1729978	T	C	Och11	*OreG0024515*
scaffold705	1730002	A	T	Och07	*OreG0024515*
scaffold705	1730857	A	C	Och01,Och02,Och03,Och04,Och05,Och06,Och07,Och08,Och09,Och10,Och11,Och12,Och13,Och14,Ore01,Ore02,Ore03,Ore04,Ore05,Ore06,Ore07,Ore08,Ore09,Ore10,Ore11,Ore12,Ore13,Ore14	*OreG0024516*
scaffold705	1730893	G	T	NA	*OreG0024516*
scaffold705	1780612	C	A	NA	*OreG0024520*
scaffold705	1780647	T	G	NA	*OreG0024520*
scaffold705	1780779	C	T	NA	*OreG0024520*
scaffold705	1781719	T	G	NA	*OreG0024520*
scaffold705	1782536	G	A	NA	*OreG0024520*
scaffold705	1790802	T	A	Ore02,Ore04,Ore05	*OreG0024521*
scaffold705	1815371	C	T	Ore01,Ore02,Ore03,Ore04,Ore05,Ore06,Ore07,Ore08,Ore09,Ore10,Ore11,Ore12,Ore13,Ore14	*OreG0024523*
scaffold705	1821102	T	A	NA	*OreG0024524*
scaffold705	1821276	C	T	NA	*OreG0024524*
scaffold705	1821315	A	G	Ore01,Ore02,Ore03,Ore04,Ore05,Ore06,Ore07,Ore08,Ore09,Ore10,Ore11,Ore12,Ore13,Ore14	*OreG0024524*
scaffold705	1821342	A	G	NA	*OreG0024524*
scaffold705	1821384	G	A	Och01	*OreG0024524*
scaffold705	1821486	T	C	NA	*OreG0024524*
scaffold705	1821516	T	C	NA	*OreG0024524*
scaffold705	1821813	A	G	Och04,Och06,Och13	*OreG0024524*

scaffold705	1822230	T	C	NA	*OreG0024524*
scaffold705	1822359	T	C	NA	*OreG0024524*
scaffold705	1822601	G	A	NA	*OreG0024524*
scaffold705	1822751	T	C	NA	*OreG0024524*
scaffold705	1822877	A	G	Ore02,Ore04,Ore05,Ore13	*OreG0024524*
scaffold705	1827612	T	A	Och01	*OreG0024525*
scaffold705	1828385	G	A	Och01,Och02,Och03,Och04,Och05,Och06,Och07,Och08,Och09,Och10,Och11,Och12,Och13,Och14,Ore01,Ore02,Ore03,Ore04,Ore05,Ore06,Ore07,Ore08,Ore09,Ore10,Ore11,Ore12,Ore13,Ore14	*OreG0024525*
scaffold705	1828479	G	T	Ore01,Ore02,Ore03,Ore04,Ore05,Ore06,Ore07,Ore08,Ore09,Ore10,Ore11,Ore12,Ore13,Ore14	*OreG0024525*
scaffold705	1829919	A	T	Och01,Och02,Och03,Och04,Och05,Och06,Och07,Och08,Och09,Och10,Och11,Och12,Och13,Och14,Ore01,Ore02,Ore03,Ore04,Ore05,Ore06,Ore07,Ore08,Ore09,Ore10,Ore11,Ore12,Ore13,Ore14	*OreG0024525*
scaffold705	1832812	T	C	NA	*OreG0024525*
scaffold705	1887849	T	C	Ore01,Ore02,Ore03,Ore04,Ore05,Ore06,Ore07,Ore08,Ore09,Ore10,Ore11,Ore12,Ore13,Ore14	*OreG0024532*
scaffold705	1901738	G	A	NA	*OreG0024533*
scaffold705	1902701	G	T	Ore01,Ore02,Ore03,Ore04,Ore05,Ore06,Ore07,Ore08,Ore09,Ore10,Ore11,Ore12,Ore13,Ore14	*OreG0024533*
scaffold705	1911264	G	A	Och01,Och02,Och03,Och04,Och05,Och06,Och07,Och08,Och09,Och10,Och11,Och12,Och13	*OreG0024535*
scaffold705	1911333	T	C	Och04,Och06,Och13	*OreG0024535*
scaffold705	1911363	T	C	NA	*OreG0024535*
scaffold705	1911643	G	A	Ore01,Ore02,Ore03,Ore04,Ore05,Ore06,Ore07,Ore08,Ore09,Ore10,Ore11,Ore12,Ore13,Ore14	*OreG0024535*
scaffold705	1913131	T	C	NA	*OreG0024536*
scaffold705	1913192	A	T	NA	*OreG0024536*
scaffold705	1914852	G	C	Och01,Och02,Och03,Och04,Och05,Och06,Och07,Och08,Och09,Och10,Och11,Och12,Och13	*OreG0024537*
scaffold705	1914941	G	A	Och01,Och02,Och03,Och04,Och05,Och06,Och07,Och08,Och09,Och10,Och11,Och12,Och13,Och14,Ore01,Ore02,Ore03,Ore04,Ore05,Ore06,Ore07,Ore08,Ore09,Ore10,Ore11,Ore12,Ore13,Ore14	*OreG0024537*
scaffold705	1914954	T	G	Och01,Och02,Och03,Och04,Och05,Och06,Och07,Och08,Och09,Och10,Och11,Och12,Och13,Och14,Ore01,Ore02,Ore03,Ore04,Ore05,Ore06,Ore07,Ore08,Ore09,Ore10,Ore11,Ore12,Ore13,Ore14	*OreG0024537*
scaffold705	1915044	T	C	Och01,Och02,Och03,Och04,Och05,Och06,Och07,Och08,Och09,Och10,Och11,Och12,Och13	*OreG0024537*
scaffold705	1936566	A	G	Och04,Och06,Och13	*OreG0024540*
scaffold705	1954812	C	T	Ore01,Ore02,Ore03,Ore04,Ore05,Ore06,Ore07,Ore08,Ore09,Ore10,Ore11,Ore12,Ore13,Ore14	*OreG0024543*
scaffold705	1954824	T	C	NA	*OreG0024543*
scaffold705	1955160	G	A	Ore01,Ore02,Ore03,Ore04,Ore05,Ore06,Ore07,Ore08,Ore09,Ore10,Ore11,Ore12,Ore13,Ore14	*OreG0024543*
scaffold705	1957308	C	A	Och01,Och02,Och03,Och04,Och05,Och06,Och07,Och08,Och09,Och10,Och11,Och12,Och13,Och14	*OreG0024544*
scaffold705	1958254	G	A	Ore01,Ore02,Ore03,Ore04,Ore05,Ore06,Ore07,Ore08,Ore09,Ore10,Ore11,Ore12,Ore13,Ore14	*OreG0024544*
scaffold705	1958996	A	T	Och04,Och06,Och11,Och13	*OreG0024544*
scaffold705	1959960	G	A	NA	*OreG0024544*
scaffold705	1961720	T	C	Ore01,Ore02,Ore03,Ore04,Ore05,Ore06,Ore07,Ore08,Ore09,Ore10,Ore11,Ore12,Ore13,Ore14	*OreG0024545*
scaffold705	1961954	G	A	Ore01,Ore02,Ore03,Ore04,Ore05,Ore06,Ore07,Ore08,Ore09,Ore10,Ore11,Ore12,Ore13,Ore14	*OreG0024545*
scaffold705	1961975	C	G	Och01,Och02,Och03,Och04,Och05,Och06,Och07,Och08,Och09,Och10,Och11,Och12,Och13,Och14,Ore01,Ore02,Ore03,Ore04,Ore05,Ore06,Ore07,Ore08,Ore09,Ore10,Ore11,Ore12,Ore13,Ore14	*OreG0024545*
scaffold705	1962650	G	C	Och01,Och02,Och03,Och04,Och05,Och06,Och07,Och08,Och09,Och10,Och11,Och12,Och13,Och14	*OreG0024545*
scaffold705	1964550	C	T	Och01,Och02,Och03,Och04,Och05,Och06,Och07,Och08,Och09,Och10,Och11,Och12,Och13,Och14,Ore01,Ore02,Ore03,Ore04,Ore05,Ore06,Ore07,Ore08,Ore09,Ore10,Ore11,Ore12,Ore13,Ore14	*OreG0024545*
scaffold705	1964588	G	A	Och01,Och02,Och03,Och04,Och05,Och06,Och07,Och08,Och09,Och10,Och11,Och12,Och13,Och14	*OreG0024545*
scaffold705	1968558	G	A	Och01,Och02,Och03,Och04,Och05,Och06,Och07,Och08,Och09,Och10,Och11,Och12,Och13,Och14	*OreG0024545*
scaffold705	1969780	C	T	Ore01,Ore02,Ore03,Ore04,Ore05,Ore06,Ore07,Ore08,Ore09,Ore10,Ore11,Ore12,Ore13,Ore14	*OreG0024545*
scaffold705	1971070	C	G	NA	*OreG0024545*
scaffold705	1971181	A	G	Och01,Och02,Och03,Och04,Och05,Och06,Och07,Och08,Och09,Och10,Och11,Och12,Och13,Och14	*OreG0024545*
scaffold705	1976307	A	C	Ore01,Ore02,Ore03,Ore04,Ore05,Ore06,Ore07,Ore08,Ore09,Ore10,Ore11,Ore12,Ore13,Ore14	*OreG0024546*
scaffold705	1976490	T	C	Och07,Och08	*OreG0024546*
scaffold705	1976559	A	T	NA	*OreG0024546*
scaffold705	1976635	T	C	NA	*OreG0024546*

scaffold705	1976643	C	T	Och07	*OreG0024546*
scaffold705	1977057	G	C	Och01,Och11	*OreG0024546*
scaffold705	1977062	G	C	NA	*OreG0024546*
scaffold705	1977161	T	C	Och01,Och11	*OreG0024546*
scaffold705	1977186	C	A	Och04,Och06,Och13	*OreG0024546*
scaffold705	1977213	C	A	NA	*OreG0024546*
scaffold705	1977293	G	C	Och11	*OreG0024546*
scaffold705	1977466	C	A	NA	*OreG0024546*
scaffold705	1977678	T	G	NA	*OreG0024546*
scaffold705	1978351	G	T	NA	*OreG0024546*
scaffold705	1978365	C	G	Och04,Och06,Och13	*OreG0024546*
scaffold705	1979376	A	T	Och07,Och08	*OreG0024546*
scaffold705	1981295	G	A	Och07,Och08	*OreG0024546*
scaffold705	1982139	T	C	Och01,Och11	*OreG0024546*
scaffold705	1983064	T	G	NA	*OreG0024546*
scaffold705	1993966	C	T	Och01,Och02,Och03,Och04,Och05,Och06,Och07,Och08,Och09,Och10,Och11,Och12,Och13,Och14	*OreG0024547*
scaffold705	2012642	G	A	NA	*OreG0024550*
scaffold705	2032782	C	T	Och01,Och02,Och03,Och04,Och05,Och06,Och07,Och08,Och09,Och10,Och11,Och12,Och13,Och14,Ore01,Ore02,Ore03,Ore04,Ore05,Ore06,Ore07,Ore08,Ore09,Ore10,Ore11,Ore12,Ore13,Ore14	*OreG0024551*
scaffold705	2073487	T	A	NA	*OreG0024554*
scaffold705	2114897	A	C	Och07	*OreG0024559*
scaffold705	2120128	A	T	Ore01,Ore02,Ore03,Ore04,Ore05,Ore06,Ore07,Ore08,Ore09,Ore10,Ore11,Ore12,Ore13,Ore14	*OreG0024560*
scaffold705	2127444	G	A	Och01,Och02,Och03,Och04,Och05,Och06,Och07,Och08,Och09,Och10,Och11,Och12,Och13,Och14	*OreG0024561*
scaffold705	2128175	T	C	NA	*OreG0024561*
scaffold705	2131415	C	T	Och01,Och02,Och03,Och04,Och05,Och06,Och07,Och08,Och09,Och10,Och11,Och12,Och13,Och14	*OreG0024562*
scaffold705	2131664	C	T	Ore01,Ore02,Ore03,Ore04,Ore05,Ore06,Ore07,Ore08,Ore09,Ore10,Ore11,Ore12,Ore13,Ore14	*OreG0024562*
scaffold705	2131714	G	A	Och01,Och02,Och03,Och04,Och05,Och06,Och07,Och08,Och09,Och10,Och11,Och12,Och13,Och14	*OreG0024562*
scaffold705	2131732	G	A	Ore01,Ore02,Ore03,Ore04,Ore05,Ore06,Ore07,Ore08,Ore09,Ore10,Ore11,Ore12,Ore13,Ore14	*OreG0024562*
scaffold705	2132493	C	T	NA	*OreG0024562*
scaffold705	2133327	G	A	NA	*OreG0024562*
scaffold705	2133360	G	A	NA	*OreG0024562*
scaffold705	2133369	C	T	Och01,Och02,Och03,Och04,Och05,Och06,Och07,Och08,Och09,Och10,Och11,Och12,Och13,Och14,Ore01,Ore02,Ore03,Ore04,Ore05,Ore06,Ore07,Ore08,Ore09,Ore10,Ore11,Ore12,Ore13,Ore14	*OreG0024562*
scaffold705	2164866	C	T	Ore01	*OreG0024566*
scaffold705	2189837	C	G	Och01,Och02,Och03,Och04,Och05,Och06,Och07,Och08,Och09,Och10,Och11,Och12,Och13,Och14	*OreG0024569*
scaffold705	2190012	A	G	Och01,Och02,Och03,Och04,Och05,Och06,Och07,Och08,Och09,Och10,Och11,Och12,Och13,Och14	*OreG0024569*
scaffold705	2211088	C	A	Och01,Och04,Och06,Och13	*OreG0024572*
scaffold705	2211379	A	C	Och01,Och04,Och06,Och13	*OreG0024572*
scaffold705	2219537	G	A	Ore01,Ore02,Ore03,Ore04,Ore05,Ore06,Ore07,Ore08,Ore09,Ore10,Ore11,Ore12,Ore13,Ore14	*OreG0024573*
scaffold705	2226098	T	A	Och01,Och02,Och03,Och04,Och05,Och06,Och07,Och08,Och09,Och10,Och11,Och12,Och13,Och14	*OreG0024574*
scaffold705	2246895	A	T	Ore02,Ore04,Ore05	*OreG0024576*
scaffold705	2256153	T	C	Ore02,Ore04,Ore05	*OreG0024576*
scaffold705	2257532	A	C	NA	*OreG0024576*
scaffold705	2257681	T	A	Ore01,Ore03	*OreG0024576*
scaffold705	2264767	C	T	Ore01,Ore02,Ore03,Ore04,Ore05,Ore06,Ore07,Ore08,Ore09,Ore10,Ore11,Ore12,Ore13,Ore14	*OreG0024577*
scaffold705	2264777	A	T	Och01,Och02,Och03,Och04,Och05,Och06,Och07,Och08,Och09,Och10,Och11,Och12,Och13,Och14,Ore01,Ore02,Ore03,Ore04,Ore05,Ore06,Ore07,Ore08,Ore09,Ore10,Ore11,Ore12,Ore13,Ore14	*OreG0024577*
scaffold705	2284037	G	A	Ore01	*OreG0024580*
scaffold705	2284379	T	A	Och06	*OreG0024580*
scaffold705	2292318	G	A	Ore01,Ore02,Ore03,Ore04,Ore05,Ore06,Ore07,Ore08,Ore09,Ore10,Ore11,Ore12,Ore13,Ore14	*OreG0024580*
scaffold705	2293700	G	T	NA	*OreG0024580*
scaffold705	2322243	A	C	NA	*OreG0024583*
scaffold705	2322291	A	G	Och01,Och02,Och03,Och04,Och05,Och06,Och07,Och08,Och09,Och10,Och11,Och12,Och13,Och14,Ore01,Ore02,Ore03,Ore04,Ore05,Ore06,Ore07,Ore08,Ore09,Ore10,Ore11,Ore12,Ore13,Ore14	*OreG0024583*
scaffold705	2338255	G	A	Och11	*OreG0024586*
scaffold705	2339439	G	A	Ore01,Ore02,Ore03,Ore04,Ore05,Ore06,Ore07,Ore08,Ore09,Ore10,Ore11,Ore12,Ore13,Ore14	*OreG0024586*
scaffold705	2339714	C	G	Ore03	*OreG0024586*

scaffold705	2340638	C	T	Och11	*OreG0024586*
scaffold705	2341951	T	G	NA	*OreG0024586*
scaffold705	2356343	G	A	Och02,Och03,Och04,Och05,Och06,Och07,Och12,Och13	*OreG0024589*
scaffold705	2393078	T	A	Och01,Och02,Och03,Och04,Och05,Och06,Och07,Och08,Och09,Och10,Och11,Och12,Och13,Och14	*OreG0024595*
scaffold705	2395610	G	A	NA	*OreG0024595*
scaffold705	2400020	G	T	NA	*OreG0024596*
scaffold705	2403846	T	C	NA	*OreG0024597*
scaffold705	2404162	A	G	Och05	*OreG0024597*
scaffold705	2404921	G	A	NA	*OreG0024597*
scaffold705	2404992	C	A	Och11	*OreG0024597*
scaffold705	2420943	C	T	Och06,Och07,Och08,Och11	*OreG0024598*
scaffold705	2421259	A	G	Och06,Och07,Och08,Och11	*OreG0024598*
scaffold705	2421280	G	A	Och03,Och06,Och08,Och11	*OreG0024598*
scaffold705	2486746	G	A	NA	*OreG0024606*
scaffold705	2486837	G	T	NA	*OreG0024606*
scaffold705	2486863	G	C	NA	*OreG0024606*
scaffold705	2486881	C	T	NA	*OreG0024606*
scaffold705	2487466	T	C	NA	*OreG0024606*
scaffold705	2487473	G	C	NA	*OreG0024606*
scaffold705	2517092	G	T	Och01,Och02,Och03,Och04,Och05,Och06,Och07,Och08,Och09,Och10,Och11,Och12,Och13,Och14,Ore01,Ore02,Ore03,Ore04,Ore05,Ore06,Ore07,Ore08,Ore09,Ore10,Ore11,Ore12,Ore13,Ore14	*OreG0024609*
scaffold705	2524815	A	G	Och02,Och03,Och04,Och05,Och06,Och07,Och08,Och09,Och10,Och11,Och12,Och13,Och14	*OreG0024610*
scaffold705	2538328	T	G	Och01,Och06	*OreG0024615*
scaffold705	2538348	C	A	Ore01,Ore02,Ore03,Ore04,Ore05,Ore06,Ore07,Ore08,Ore09,Ore10,Ore11,Ore12,Ore13,Ore14	*OreG0024615*
scaffold705	2538392	A	G	NA	*OreG0024615*
scaffold705	2538564	T	A	Ore01,Ore02,Ore03,Ore04,Ore05,Ore06,Ore07,Ore08,Ore09,Ore10,Ore11,Ore12,Ore13,Ore14	*OreG0024615*
scaffold705	2538576	G	A	Ore01,Ore02,Ore03,Ore04,Ore05,Ore06,Ore07,Ore08,Ore09,Ore10,Ore11,Ore12,Ore13,Ore14	*OreG0024615*
scaffold705	2539675	T	A	Ore01,Ore02,Ore03,Ore04,Ore05,Ore06,Ore07,Ore08,Ore09,Ore10,Ore11,Ore12,Ore13,Ore14	*OreG0024615*
scaffold705	2546389	A	G	Och01,Och02,Och03,Och04,Och05,Och06,Och07,Och08,Och09,Och10,Och11,Och12,Och13,Och14	*OreG0024616*
scaffold705	2546398	A	C	Och01,Och02,Och03,Och04,Och05,Och06,Och07,Och08,Och09,Och10,Och11,Och12,Och13,Och14	*OreG0024616*
scaffold705	2633633	T	G	Och04,Och06,Och13	*OreG0024617*
scaffold705	2654644	C	T	NA	*OreG0024620*
scaffold705	2673621	C	T	Och01,Och02,Och03,Och04,Och05,Och06,Och07,Och08,Och09,Och10,Och11,Och12,Och13,Och14	*OreG0024622*
scaffold705	2706943	A	T	Ore04,Ore05,Ore13	*OreG0024625*
scaffold705	2706992	G	A	Ore06,Ore09	*OreG0024625*
scaffold705	2716740	C	T	Och01,Och02,Och03,Och04,Och05,Och06,Och07,Och08,Och09,Och10,Och11,Och12,Och13,Och14,Ore01,Ore02,Ore03,Ore04,Ore05,Ore06,Ore07,Ore08,Ore09,Ore10,Ore11,Ore12,Ore13,Ore14	*OreG0024626*
scaffold705	2720248	C	A	Ore06,Ore09	*OreG0024627*
scaffold705	2765772	A	G	NA	*OreG0024630*
scaffold705	2766663	T	C	Och01,Och02,Och03,Och04,Och05,Och06,Och07,Och08,Och09,Och10,Och11,Och12,Och13,Och14,Ore01,Ore02,Ore03,Ore04,Ore05,Ore06,Ore07,Ore08,Ore09,Ore10,Ore11,Ore12,Ore13,Ore14	*OreG0024630*
scaffold705	2767384	C	T	NA	*OreG0024630*
scaffold705	2768163	T	C	NA	*OreG0024630*
scaffold705	2776325	A	T	Och01,Och02,Och03,Och04,Och05,Och06,Och07,Och08,Och09,Och10,Och11,Och12,Och13,Och14,Ore01,Ore02,Ore03,Ore04,Ore05,Ore06,Ore07,Ore08,Ore09,Ore10,Ore11,Ore12,Ore13,Ore14	*OreG0024631*
scaffold705	2776499	T	C	NA	*OreG0024631*
scaffold705	2777067	C	T	Och11	*OreG0024631*
scaffold705	2792941	A	G	Och11	*OreG0024633*
scaffold705	2827115	T	A	NA	*OreG0024636*
scaffold2523	2923	G	C	Och07	*OreG0007783*
scaffold2523	3034	T	A	Och12,Och13,Och14,Ore02,Ore03,Ore06,Ore07,Ore09,Ore10,Ore11,Ore12,Ore13,Ore14	*OreG0007783*
scaffold2523	3118	C	A	NA	*OreG0007783*
scaffold273	144124	C	T	Och03	*OreG0007934*
scaffold273	145165	C	A	NA	*OreG0007934*
scaffold273	154485	G	T	NA	*OreG0007934*
scaffold273	166014	C	T	Ore01,Ore02,Ore04,Ore05,Ore06,Ore07,Ore08,Ore09,Ore10,Ore11,Ore12,Ore13,Ore14	*OreG0007937*
scaffold273	166045	G	T	NA	*OreG0007937*
scaffold273	194243	C	A	Och12,Och13	*OreG0007939*
scaffold273	255549	T	C	Ore01,Ore02,Ore04,Ore05,Ore06,Ore07,Ore08,Ore09,Ore10,Ore11,Ore12,Ore13,Ore14	*OreG0007942*

scaffold273	256158	G	A	NA	*OreG0007942*
scaffold273	256571	T	C	Ore01,Ore02,Ore04,Ore05,Ore06,Ore07,Ore08,Ore09,Ore10,Ore11,Ore12,Ore13,Ore14	*OreG0007942*
scaffold273	267290	A	C	NA	*OreG0007944*
scaffold273	269307	T	C	NA	*OreG0007944*
scaffold273	269481	G	A	Ore01,Ore02,Ore04,Ore05,Ore06,Ore07,Ore08,Ore09,Ore10,Ore11,Ore12,Ore13,Ore14	*OreG0007944*
scaffold273	269484	T	A	NA	*OreG0007944*
scaffold273	271417	G	T	NA	*OreG0007944*
scaffold273	328670	C	T	NA	*OreG0007946*
scaffold273	546881	C	T	Och12,Och13,Ore01,Ore02,Ore03,Ore06,Ore07,Ore08,Ore09,Ore10,Ore11,Ore12,Ore13,Ore14	*OreG0007955*
scaffold273	599840	A	G	NA	*OreG0007962*
scaffold273	600465	C	T	Och02,Och05,Och09,Och10,Och11,Och14	*OreG0007962*
scaffold273	601199	T	C	NA	*OreG0007962*
scaffold273	618762	C	A	Och01,Och02,Och03,Och04,Och05,Och06,Och07,Och08,Och09,Och10,Och11,Och12,Och13,Och14,Ore01,Ore02,Ore03,Ore04,Ore05,Ore06,Ore07,Ore08,Ore09,Ore10,Ore11,Ore12,Ore13,Ore14	*OreG0007965*
scaffold273	618779	T	G	Och01,Och02,Och03,Och04,Och05,Och06,Och07,Och08,Och09,Och10,Och11,Och12,Och13,Och14,Ore01,Ore02,Ore03,Ore04,Ore05,Ore06,Ore07,Ore08,Ore09,Ore10,Ore11,Ore12,Ore13,Ore14	*OreG0007965*
scaffold273	618783	A	G	Och01	*OreG0007965*
scaffold273	636679	G	T	Och14,Ore14	*OreG0007967*
scaffold273	637489	C	T	Och02,Och05	*OreG0007967*
scaffold273	637589	A	G	Och02,Och05	*OreG0007967*
scaffold273	638445	G	A	NA	*OreG0007967*
scaffold273	638523	A	T	Och12,Och13,Ore01,Ore11	*OreG0007967*
scaffold273	866885	A	C	NA	*OreG0007982*
scaffold273	967778	A	G	NA	*OreG0007992*
scaffold273	1035710	T	A	Och01,Och02,Och03,Och04,Och05,Och06,Och07,Och08,Och09,Och10,Och11,Och12,Och13,Och14,Ore01,Ore02,Ore03,Ore04,Ore05,Ore06,Ore07,Ore08,Ore09,Ore10,Ore11,Ore12,Ore13,Ore14	*OreG0007997*
scaffold273	1051518	A	G	Och14	*OreG0007998*
scaffold273	1065272	T	G	NA	*OreG0008000*
scaffold273	1065278	C	A	NA	*OreG0008000*
scaffold273	1065766	G	A	NA	*OreG0008000*
scaffold273	1065949	A	G	NA	*OreG0008000*
scaffold273	1066024	G	A	NA	*OreG0008000*
scaffold273	1066229	G	A	NA	*OreG0008000*
scaffold273	1066241	G	T	NA	*OreG0008000*
scaffold273	1066315	A	T	NA	*OreG0008000*
scaffold273	1121986	C	T	NA	*OreG0008005*
scaffold273	1135621	T	G	NA	*OreG0008009*
scaffold273	1135638	A	G	Och01,Och02,Och03,Och04,Och05,Och06,Och07,Och08,Och09,Och10,Och11,Och12,Och13,Och14	*OreG0008009*
scaffold273	1233200	C	T	NA	*OreG0008026*
scaffold273	1359834	T	C	Ore01,Ore02,Ore03,Ore04,Ore05,Ore06,Ore07,Ore08,Ore09,Ore10,Ore11,Ore12,Ore13,Ore14	*OreG0008036*
scaffold273	1392904	A	T	Ore01,Ore02,Ore08,Ore11,Ore12	*OreG0008039*
scaffold273	1433007	T	G	NA	*OreG0008045*
scaffold273	1462610	A	T	Ore03	*OreG0008050*
scaffold273	1469837	A	C	NA	*OreG0008050*
scaffold273	1470258	A	C	Ore01,Ore02,Ore03,Ore04,Ore05,Ore06,Ore07,Ore08,Ore09,Ore10,Ore11,Ore12,Ore13,Ore14	*OreG0008050*
scaffold273	1544938	C	T	Och01,Och02,Och03,Och04,Och05,Och06,Och07,Och08,Och09,Och10,Och11,Och12,Och13,Och14,Ore01,Ore02,Ore03,Ore04,Ore05,Ore06,Ore07,Ore08,Ore09,Ore10,Ore11,Ore12,Ore13,Ore14	*OreG0008057*
scaffold273	1550293	G	T	NA	*OreG0008059*
scaffold273	1582219	T	A	NA	*OreG0008061*
scaffold273	1585873	C	A	Och01,Och02,Och03,Och04,Och05,Och06,Och07,Och08,Och09,Och10,Och11,Och12,Och13,Och14,Ore01,Ore02,Ore03,Ore04,Ore05,Ore06,Ore07,Ore08,Ore09,Ore10,Ore11,Ore12,Ore13,Ore14	*OreG0008061*
scaffold273	1604245	G	T	NA	*OreG0008063*
scaffold273	1604260	G	T	NA	*OreG0008063*
scaffold273	1634391	C	G	Ore01,Ore02,Ore04,Ore05,Ore08,Ore09,Ore11,Ore12,Ore14	*OreG0008067*
scaffold273	1676536	G	T	NA	*OreG0008069*
scaffold273	1684800	C	A	NA	*OreG0008070*
scaffold273	1847983	G	T	Och01,Och02,Och03,Och04,Och05,Och06,Och07,Och08,Och09,Och10,Och11,Och12,Och13,Och14	*OreG0008080*
scaffold273	1868745	T	C	Ore01,Ore02,Ore03,Ore04,Ore05,Ore06,Ore07,Ore08,Ore09,Ore10,Ore11,Ore12,Ore13,Ore14	*OreG0008084*
scaffold273	1938361	G	A	NA	*OreG0008088*
scaffold273	1941252	A	T	Ore01,Ore02,Ore03,Ore04,Ore05,Ore06,Ore07,Ore08,Ore09,Ore10,Ore11,Ore12,Ore13,Ore14	*OreG0008088*
scaffold273	1942731	T	G	NA	*OreG0008089*

scaffold273	1942782	G	A	NA	*OreG0008089*
scaffold273	1943115	G	A	Och01,Och02,Och03,Och04,Och05,Och06,Och07,Och08,Och09,Och10,Och11,Och12,Och13,Och14	*OreG0008089*
scaffold273	1943289	G	A	NA	*OreG0008089*
scaffold273	1943961	G	A	NA	*OreG0008089*
scaffold273	1944105	C	T	Och01,Och02,Och03,Och04,Och05,Och06,Och07,Och08,Och09,Och10,Och11,Och12,Och13,Och14	*OreG0008089*
scaffold273	2017849	A	G	NA	*OreG0008096*
scaffold273	2056901	C	G	Ore01,Ore02,Ore03,Ore04,Ore05,Ore06,Ore07,Ore08,Ore09,Ore10,Ore11,Ore12,Ore13,Ore14	*OreG0008099*
scaffold273	2072472	G	C	NA	*OreG0008100*
scaffold273	2080103	T	C	Ore01,Ore02,Ore03,Ore04,Ore05,Ore06,Ore07,Ore08,Ore09,Ore10,Ore11,Ore12,Ore13,Ore14	*OreG0008102*
scaffold273	2094640	G	A	Och01,Och02,Och03,Och04,Och05,Och06,Och07,Och08,Och09,Och10,Och11,Och12,Och13,Och14,Ore01,Ore02,Ore03,Ore04,Ore05,Ore06,Ore07,Ore08,Ore09,Ore10,Ore11,Ore12,Ore13,Ore14	*OreG0008105*
scaffold273	2109042	C	A	NA	*OreG0008107*
scaffold273	2148606	T	C	NA	*OreG0008110*
scaffold273	2148647	G	A	NA	*OreG0008110*
scaffold273	2238155	T	A	Och01,Och02,Och03,Och04,Och05,Och06,Och07,Och08,Och09,Och10,Och11,Och12,Och13	*OreG0008116*
scaffold273	2260873	C	A	Och01,Och02,Och03,Och04,Och05,Och06,Och07,Och08,Och09,Och10,Och11,Och12,Och13,Och14,Ore01,Ore02,Ore03,Ore04,Ore05,Ore06,Ore07,Ore08,Ore09,Ore10,Ore11,Ore12,Ore13,Ore14	*OreG0008119*
scaffold273	2261866	C	T	NA	*OreG0008119*
scaffold273	2262888	G	T	Och02,Och03,Och04,Och06,Och12,Och13	*OreG0008119*
scaffold273	2489664	A	C	Ore01,Ore02,Ore03,Ore04,Ore05,Ore06,Ore07,Ore08,Ore09,Ore10,Ore11,Ore12,Ore13,Ore14	*OreG0008137*
scaffold273	2512192	A	T	Och01,Och02,Och03,Och04,Och05,Och06,Och07,Och08,Och09,Och10,Och11,Och12,Och13,Och14,Ore01,Ore02,Ore03,Ore04,Ore05,Ore06,Ore07,Ore08,Ore09,Ore10,Ore11,Ore12,Ore13,Ore14	*OreG0008141*
scaffold273	2512282	G	A	NA	*OreG0008141*
scaffold273	2513267	T	C	NA	*OreG0008141*
scaffold273	2514613	G	A	NA	*OreG0008141*
scaffold273	2515101	C	A	NA	*OreG0008141*
scaffold273	2515111	T	G	NA	*OreG0008141*
scaffold273	2515161	A	G	NA	*OreG0008141*
scaffold273	2644164	G	A	NA	*OreG0008151*
scaffold273	2707807	T	G	Och01,Och02,Och03,Och04,Och05,Och06,Och07,Och08,Och09,Och10,Och11,Och12,Och13,Och14	*OreG0008153*
scaffold273	2708331	T	A	Och01,Och02,Och03,Och04,Och05,Och06,Och07,Och08,Och09,Och10,Och11,Och12,Och13,Och14	*OreG0008153*
scaffold273	2728654	C	T	Och12,Och13	*OreG0008153*
scaffold273	2836790	A	C	NA	*OreG0008155*
scaffold273	2837542	G	A	NA	*OreG0008155*
scaffold273	2837581	C	A	Och01,Och02,Och03,Och04,Och05,Och06,Och07,Och08,Och09,Och10,Och11,Och12,Och13,Och14	*OreG0008155*
scaffold273	3032067	A	G	Ore01,Ore02,Ore03,Ore04,Ore05,Ore06,Ore07,Ore08,Ore09,Ore10,Ore11,Ore12,Ore13,Ore14	*OreG0008160*
scaffold273	3033711	C	A	Och01,Och02,Och03,Och04,Och05,Och06,Och07,Och08,Och09,Och10,Och11,Och12,Och13,Och14,Ore01,Ore02,Ore03,Ore04,Ore05,Ore06,Ore07,Ore08,Ore09,Ore10,Ore11,Ore12,Ore13,Ore14	*OreG0008160*
scaffold273	3047165	C	T	Och01,Och02,Och03,Och04,Och05,Och06,Och07,Och08,Och09,Och10,Och11,Och12,Och13,Och14	*OreG0008161*
scaffold273	3047948	C	A	Ore01,Ore02,Ore03,Ore04,Ore05,Ore06,Ore07,Ore08,Ore09,Ore10,Ore11,Ore12,Ore13,Ore14	*OreG0008161*
scaffold273	3294044	G	A	NA	*OreG0008163*
scaffold273	3295094	G	T	Och01,Och02,Och03,Och04,Och05,Och06,Och07,Och08,Och09,Och10,Och11,Och12,Och13,Och14,Ore01,Ore02,Ore03,Ore04,Ore05,Ore06,Ore07,Ore08,Ore09,Ore10,Ore11,Ore12,Ore13,Ore14	*OreG0008163*
scaffold273	3387122	A	G	NA	*OreG0008165*
scaffold273	3447500	C	T	NA	*OreG0008168*
scaffold273	3510279	T	A	Ore01,Ore02,Ore03,Ore04,Ore05,Ore06,Ore07,Ore08,Ore09,Ore10,Ore11,Ore12,Ore13,Ore14	*OreG0008169*
scaffold273	3512574	C	T	NA	*OreG0008169*
scaffold273	3527860	A	T	Och09,Och10,Och11,Och12,Och13	*OreG0008169*
scaffold273	3627997	C	A	Och03	*OreG0008170*
scaffold273	3629134	A	G	Och12,Och13	*OreG0008170*
scaffold273	3629500	C	T	Och09,Och10,Och11,Och13,Och14	*OreG0008170*
scaffold273	3630875	G	A	NA	*OreG0008170*
scaffold273	3631033	T	A	Ore01,Ore02,Ore03,Ore04,Ore05,Ore06,Ore07,Ore08,Ore09,Ore10,Ore11,Ore12,Ore13,Ore14	*OreG0008170*
scaffold273	3840323	G	A	Och01,Och02,Och03,Och04,Och05,Och06,Och07,Och08,Och12,Och13,Och14,Ore01,Ore02,Ore03,Ore04,Ore05,Ore06,Ore07,Ore08,Ore09,Ore10,Ore11,Ore12,Ore13,Ore14	*OreG0008171*
scaffold273	3840959	T	A	NA	*OreG0008171*

scaffold273	4279234	A	C	Och12	*OreG0008180*
scaffold273	4279502	C	T	Och01	*OreG0008180*
scaffold273	4287537	C	T	NA	*OreG0008180*
scaffold273	4287610	C	T	NA	*OreG0008180*
scaffold273	4404262	C	A	NA	*OreG0008182*
scaffold273	4432169	T	A	Och01	*OreG0008183*
scaffold273	4511051	C	T	Och01,Och02,Och03,Och04,Och05,Och06,Och07,Och08,Och09,Och10,Och11,Och12,Och13,Och14	*OreG0008187*
scaffold273	4511147	C	T	Och01,Och02,Och03,Och04,Och05,Och06,Och07,Och08,Och09,Och10,Och11,Och12,Och13,Och14	*OreG0008187*
scaffold273	4828461	G	A	Och09,Och10,Och11,Och12,Och13	*OreG0008196*
scaffold273	4830523	G	C	Ore01,Ore02,Ore03,Ore04,Ore05,Ore06,Ore07,Ore08,Ore09,Ore10,Ore11,Ore12,Ore13,Ore14	*OreG0008196*
scaffold273	4830528	G	A	Ore01,Ore02,Ore03,Ore04,Ore05,Ore06,Ore07,Ore08,Ore09,Ore10,Ore11,Ore12,Ore13,Ore14	*OreG0008196*
scaffold273	4892384	G	A	Och01,Och02,Och03,Och04,Och05,Och06,Och07,Och08,Och09,Och10,Och11,Och12,Och13,Och14	*OreG0008197*
scaffold273	4893031	G	T	NA	*OreG0008197*
scaffold273	4894735	G	A	NA	*OreG0008197*
scaffold273	4894939	C	G	Och01,Och02,Och03,Och04,Och05,Och06,Och07,Och08,Och09,Och10,Och11,Och12,Och13,Och14	*OreG0008197*
scaffold273	4931775	A	T	NA	*OreG0008198*
scaffold273	4932197	C	T	Och01,Och02,Och03,Och04,Och05,Och06,Och07,Och08,Och09,Och10,Och11,Och12,Och13,Och14	*OreG0008198*
scaffold273	4932249	A	T	Ore01,Ore02,Ore03,Ore04,Ore05,Ore06,Ore07,Ore08,Ore09,Ore10,Ore11,Ore12,Ore13,Ore14	*OreG0008198*
scaffold273	4943730	C	T	Ore01,Ore02,Ore03,Ore04,Ore05,Ore06,Ore07,Ore08,Ore09,Ore10,Ore11,Ore12,Ore13,Ore14	*OreG0008199*
scaffold273	4943840	G	A	Och01,Och02,Och03,Och04,Och05,Och06,Och07,Och08,Och09,Och10,Och11,Och12,Och13,Och14	*OreG0008199*
scaffold273	4944349	C	A	Ore01,Ore02,Ore03,Ore04,Ore05,Ore06,Ore07,Ore08,Ore09,Ore10,Ore11,Ore12,Ore13,Ore14	*OreG0008200*
scaffold273	4944357	G	A	Och01,Och02,Och03,Och04,Och05,Och06,Och07,Och08,Och09,Och10,Och11,Och12,Och13,Och14	*OreG0008200*
scaffold273	4944449	T	A	Ore01,Ore02,Ore03,Ore04,Ore05,Ore06,Ore07,Ore08,Ore09,Ore10,Ore11,Ore12,Ore13,Ore14	*OreG0008200*
scaffold273	5097959	G	A	NA	*OreG0008204*
scaffold273	5097986	A	G	NA	*OreG0008204*
scaffold273	5154316	C	T	Och01,Och02,Och03,Och04,Och05,Och06,Och07,Och08,Och09,Och10,Och11,Och12,Och13,Och14	*OreG0008206*
scaffold273	5154551	G	T	NA	*OreG0008206*
scaffold273	5393071	G	T	Och12,Och13	*OreG0008212*
scaffold273	5394962	C	T	Och03,Och07,Och14	*OreG0008212*
scaffold273	5464704	C	G	Ore01,Ore02,Ore03,Ore04,Ore05,Ore06,Ore07,Ore08,Ore09,Ore10,Ore11,Ore12,Ore13,Ore14	*OreG0008213*
scaffold273	5561030	C	A	Och12,Och13	*OreG0008216*
scaffold273	5567976	G	A	Och01,Och02,Och03,Och04,Och05,Och06,Och07,Och08,Och09,Och10,Och11,Och12,Och13,Och14	*OreG0008217*
scaffold273	5568030	C	G	Och01,Och02,Och03,Och04,Och05,Och06,Och07,Och08,Och09,Och10,Och11,Och12,Och13,Och14,Ore01,Ore02,Ore03,Ore04,Ore05,Ore06,Ore07,Ore08,Ore09,Ore10,Ore11,Ore12,Ore13,Ore14	*OreG0008217*
scaffold273	5582724	T	A	NA	*OreG0008218*
scaffold273	5583054	G	C	NA	*OreG0008218*
scaffold273	5583090	T	G	NA	*OreG0008218*
scaffold273	5583129	T	G	Och01,Och04,Och05,Och06,Och07,Och08,Och09,Och10,Och11,Och12,Och13	*OreG0008218*
scaffold273	5583544	T	C	NA	*OreG0008218*
scaffold273	5584333	A	G	Och01,Och02,Och03,Och04,Och05,Och06,Och07,Och08,Och09,Och10,Och11,Och12,Och13,Och14	*OreG0008218*
scaffold273	5584361	T	C	Ore01,Ore02,Ore03,Ore04,Ore05,Ore06,Ore07,Ore08,Ore09,Ore10,Ore11,Ore12,Ore13,Ore14	*OreG0008218*
scaffold273	5584610	A	G	NA	*OreG0008218*
scaffold273	5584834	T	A	NA	*OreG0008218*
scaffold273	5585042	T	G	Och01,Och12,Och13	*OreG0008218*
scaffold273	5641763	G	A	NA	*OreG0008220*
scaffold273	5641783	C	G	NA	*OreG0008220*
scaffold273	5641972	A	C	NA	*OreG0008220*
scaffold273	5756858	A	C	Och12,Och13	*OreG0008223*
scaffold273	5757637	C	T	Och02,Och08,Och12,Och13,Och14,Ore01,Ore02,Ore03,Ore04,Ore05,Ore06,Ore07,Ore08,Ore09,Ore10,Ore11,Ore12,Ore13,Ore14	*OreG0008223*
scaffold273	5759355	T	A	NA	*OreG0008224*
scaffold273	5760686	G	A	Och01,Och07	*OreG0008224*
scaffold273	5805544	C	A	NA	*OreG0008228*
scaffold273	5805780	G	A	NA	*OreG0008228*
scaffold273	5806626	G	T	NA	*OreG0008228*
scaffold273	5809637	T	G	NA	*OreG0008228*

scaffold273	5812405	A	T	Ore01,Ore02,Ore03,Ore04,Ore05,Ore06,Ore07,Ore08,Ore09,Ore10,Ore11,Ore12,Ore13,Ore14	*OreG0008229*
scaffold273	5813013	T	C	Ore01,Ore02,Ore03,Ore04,Ore05,Ore06,Ore07,Ore08,Ore09,Ore10,Ore11,Ore12,Ore13,Ore14	*OreG0008229*
scaffold273	5855773	G	A	Och12,Och13	*OreG0008230*
scaffold273	5855797	C	A	NA	*OreG0008230*
scaffold273	5856108	A	C	NA	*OreG0008230*
scaffold273	5856317	A	G	Och01,Och02,Och03,Och04,Och05,Och06,Och07,Och08,Och09,Och11,Och12,Och13,Och14,Ore01,Ore02,Ore03,Ore04,Ore05,Ore06,Ore07,Ore08,Ore09,Ore10,Ore11,Ore12,Ore13,Ore14	*OreG0008230*
scaffold273	5857039	G	T	Och01,Och02,Och03,Och06,Och07,Och08,Och09,Och10,Och11,Och14	*OreG0008230*
scaffold273	5857045	A	G	NA	*OreG0008230*
scaffold273	5857459	T	C	Ore01,Ore02,Ore03,Ore04,Ore05,Ore06,Ore07,Ore08,Ore09,Ore10,Ore11,Ore12,Ore13,Ore14	*OreG0008230*
scaffold273	5857840	G	A	Och01,Och02,Och03,Och04,Och05,Och06,Och07,Och08,Och09,Och10,Och11,Och12,Och13,Och14,Ore01,Ore02,Ore03,Ore04,Ore05,Ore06,Ore07,Ore08,Ore09,Ore10,Ore11,Ore12,Ore13,Ore14	*OreG0008230*
scaffold273	5857846	G	T	Ore01,Ore02,Ore03,Ore04,Ore05,Ore06,Ore07,Ore08,Ore09,Ore10,Ore11,Ore12,Ore13,Ore14	*OreG0008230*
scaffold273	5858068	G	A	Och12,Och13	*OreG0008230*
scaffold273	5858580	G	A	Och01,Och02,Och03,Och04,Och05,Och06,Och07,Och08,Och09,Och10,Och11,Och12,Och13,Och14	*OreG0008230*
scaffold273	5923008	G	A	Ore01,Ore02,Ore03,Ore04,Ore05,Ore06,Ore07,Ore08,Ore09,Ore10,Ore11,Ore12,Ore13,Ore14	*OreG0008232*
scaffold273	6174850	G	A	NA	*OreG0008238*
scaffold273	6178613	G	A	Ore06,Ore07,Ore08,Ore13,Ore14	*OreG0008238*
scaffold273	6268649	C	T	Och01	*OreG0008241*
scaffold273	6299005	C	G	Och03,Och14	*OreG0008241*
scaffold273	6299665	T	G	NA	*OreG0008241*
scaffold273	6299683	G	A	Ore01,Ore02,Ore03,Ore04,Ore05,Ore06,Ore07,Ore08,Ore09,Ore10,Ore11,Ore12,Ore13,Ore14	*OreG0008241*
scaffold273	6489809	C	T	Ore01,Ore02,Ore03,Ore04,Ore05,Ore06,Ore07,Ore08,Ore09,Ore10,Ore11,Ore12,Ore13,Ore14	*OreG0008248*
scaffold273	6490329	C	T	NA	*OreG0008248*
scaffold273	6490437	C	T	Ore01,Ore02,Ore03,Ore04,Ore05,Ore06,Ore07,Ore08,Ore09,Ore10,Ore11,Ore12,Ore13,Ore14	*OreG0008248*
scaffold273	6566178	A	G	Ore06,Ore07,Ore08,Ore13,Ore14	*OreG0008250*
scaffold273	6803440	G	A	Och12,Och13	*OreG0008259*
scaffold273	6809996	C	T	Och01,Och02,Och03,Och04,Och05,Och06,Och07,Och08,Och09,Och10,Och11,Och12,Och13,Och14,Ore01,Ore02,Ore03,Ore04,Ore05,Ore06,Ore07,Ore08,Ore09,Ore10,Ore11,Ore12,Ore13,Ore14	*OreG0008259*
scaffold273	6811243	A	C	Ore01,Ore02,Ore03,Ore04,Ore05,Ore06,Ore07,Ore08,Ore09,Ore10,Ore11,Ore12,Ore13,Ore14	*OreG0008259*
scaffold273	6811278	A	T	Och12,Och13	*OreG0008259*
scaffold273	6854449	G	T	NA	*OreG0008262*
scaffold273	6888603	G	A	Och01,Och02,Och03,Och04,Och05,Och06,Och07,Och08,Och09,Och10,Och11,Och12,Och13,Och14	*OreG0008264*
scaffold273	6889460	G	A	NA	*OreG0008264*
scaffold273	6891743	T	A	Och01,Och02,Och03,Och04,Och05,Och06,Och07,Och08,Och09,Och10,Och11,Och12,Och13,Och14	*OreG0008265*
scaffold273	7052753	A	T	NA	*OreG0008270*
scaffold273	7052822	A	T	Och03,Och04,Och05,Och12,Och13	*OreG0008270*
scaffold273	7063169	C	A	Och01,Och02,Och03,Och04,Och05,Och06,Och07,Och08,Och09,Och10,Och11,Och12,Och13,Och14,Ore01,Ore02,Ore03,Ore04,Ore05,Ore06,Ore07,Ore08,Ore09,Ore10,Ore11,Ore12,Ore13,Ore14	*OreG0008271*
scaffold273	7143105	G	A	Och03	*OreG0008277*
scaffold273	7143748	T	C	Och01,Och08,Ore01,Ore02,Ore03,Ore04,Ore05,Ore06,Ore07,Ore08,Ore09,Ore10,Ore11,Ore12,Ore13,Ore14	*OreG0008277*
scaffold273	7144138	C	A	Och03,Och04,Och05,Och12,Och13	*OreG0008277*
scaffold273	7144204	A	T	Och03,Och04,Och05,Och12,Och13	*OreG0008277*
scaffold273	7237423	T	G	Ore01,Ore02,Ore03,Ore04,Ore05,Ore06,Ore07,Ore08,Ore09,Ore10,Ore11,Ore12,Ore13,Ore14	*OreG0008280*
scaffold273	7239039	C	G	Och12,Och13	*OreG0008280*
scaffold273	7239295	G	A	Och03	*OreG0008280*
scaffold1384	570	G	A	NA	*OreG0002069*
scaffold1384	580	C	T	NA	*OreG0002069*
scaffold1384	768	C	A	NA	*OreG0002069*
scaffold1384	1098	C	T	NA	*OreG0002069*
scaffold1384	1140	G	A	NA	*OreG0002069*
scaffold1384	161975	A	T	NA	*OreG0002074*
scaffold1384	171326	T	A	Ore01,Ore02,Ore06,Ore07,Ore08,Ore09,Ore11,Ore12,Ore13,Ore14	*OreG0002076*
scaffold1384	172900	T	C	NA	*OreG0002076*
scaffold1384	205912	T	C	Och01	*OreG0002081*
scaffold1384	222280	G	C	NA	*OreG0002083*
scaffold1384	222879	A	T	NA	*OreG0002083*

scaffold1384	222904	T	G	NA	*OreG0002083*
scaffold1384	224457	A	G	Och07,Och11	*OreG0002083*
scaffold1384	249360	G	T	Och01,Och02,Och03,Och04,Och05,Och06,Och07,Och08,Och09,Och10,Och11,Och12,Och13,Och14,Ore01,Ore02,Ore03,Ore04,Ore05,Ore06,Ore07,Ore08,Ore09,Ore10,Ore11,Ore12,Ore13,Ore14	*OreG0002086*
scaffold1384	249496	G	A	Ore01,Ore02,Ore03,Ore04,Ore05,Ore06,Ore07,Ore08,Ore09,Ore10,Ore11,Ore12,Ore13,Ore14	*OreG0002086*
scaffold1384	250788	A	C	Ore02,Ore06,Ore08,Ore09,Ore12,Ore14	*OreG0002086*
scaffold1384	272617	G	A	NA	*OreG0002089*
scaffold1384	275224	A	T	Och02,Och03,Och04,Och05,Och06,Och07,Och08,Och09,Och10,Och11,Och12,Och13	*OreG0002089*
scaffold1384	325769	T	C	Och01,Och02,Och03,Och04,Och05,Och06,Och07,Och08,Och09,Och10,Och11,Och12,Och13,Och14	*OreG0002094*
scaffold1384	334209	C	T	NA	*OreG0002094*
scaffold1384	334215	G	A	Ore01,Ore02,Ore03,Ore04,Ore05,Ore06,Ore07,Ore08,Ore09,Ore10,Ore11,Ore12,Ore13,Ore14	*OreG0002094*
scaffold1384	339797	T	C	Ore01,Ore02,Ore03,Ore04,Ore05,Ore06,Ore07,Ore08,Ore09,Ore10,Ore11,Ore12,Ore13,Ore14	*OreG0002095*
scaffold1384	340046	A	T	NA	*OreG0002095*
scaffold1384	385613	T	C	NA	*OreG0002100*
scaffold1384	506816	G	T	Och01,Och02,Och03,Och04,Och05,Och06,Och07,Och08,Och09,Och10,Och11,Och12,Och13,Och14,Ore01,Ore02,Ore03,Ore04,Ore05,Ore06,Ore07,Ore08,Ore09,Ore10,Ore11,Ore12,Ore13,Ore14	*OreG0002110*
scaffold1384	509410	G	C	NA	*OreG0002110*
scaffold1384	611050	G	C	NA	*OreG0002121*
scaffold1384	638784	A	G	Och02,Och03,Och04,Och06,Ore01,Ore02,Ore03,Ore04,Ore05,Ore06,Ore07,Ore08,Ore09,Ore10,Ore11,Ore12,Ore13,Ore14	*OreG0002125*
scaffold1384	639113	T	C	NA	*OreG0002126*
scaffold1384	699501	C	G	NA	*OreG0002130*
scaffold1384	699572	G	T	Och01,Och02,Och03,Och04,Och05,Och06,Och07,Och08,Och09,Och10,Och11,Och12,Och13,Och14	*OreG0002130*
scaffold1801	5883	A	T	Och09,Och10	*OreG0003908*
scaffold1801	16868	T	C	Och01,Och02,Och03,Och04,Och05,Och06,Och07,Och08,Och09,Och10,Och11,Och12,Och13,Och14,Ore01,Ore02,Ore03,Ore04,Ore05,Ore06,Ore07,Ore08,Ore09,Ore10,Ore11,Ore12,Ore13,Ore14	*OreG0003909*
scaffold1801	16883	T	G	Och13	*OreG0003909*
scaffold1801	17015	C	G	NA	*OreG0003909*
scaffold1801	17026	T	C	Och13	*OreG0003909*
scaffold1801	23040	A	T	NA	*OreG0003910*
scaffold1801	23176	C	A	NA	*OreG0003910*
scaffold1801	23179	C	T	NA	*OreG0003910*
scaffold1801	23314	C	A	Ore09,Ore13,Ore14	*OreG0003910*
scaffold1801	23514	G	A	Och02,Och03,Och06,Och07,Och08,Och09,Och10,Och11,Och14	*OreG0003910*
scaffold1801	23529	C	T	Och02,Och03,Och06,Och07,Och08,Och09,Och10,Och11,Och14	*OreG0003910*
scaffold1801	110698	C	T	NA	*OreG0003917*
scaffold1801	112893	G	A	Ore09,Ore13,Ore14	*OreG0003917*
scaffold1801	183671	A	T	Och13	*OreG0003923*
scaffold1801	196199	T	C	Och01,Och02,Och03,Och04,Och05,Och06,Och07,Och08,Och09,Och10,Och11,Och12,Och13,Och14,Ore01,Ore02,Ore03,Ore04,Ore05,Ore06,Ore07,Ore08,Ore09,Ore10,Ore11,Ore12,Ore13,Ore14	*OreG0003925*
scaffold1801	198716	T	G	NA	*OreG0003925*
scaffold1801	201666	C	T	Och07,Och08,Och11	*OreG0003925*
scaffold1801	210683	T	G	Ore09,Ore13,Ore14	*OreG0003925*
scaffold1801	232653	T	C	NA	*OreG0003926*
scaffold1801	234107	T	C	Och01,Och02,Och03,Och04,Och05,Och06,Och07,Och08,Och09,Och10,Och11,Och12,Och13,Och14,Ore01,Ore02,Ore03,Ore04,Ore05,Ore06,Ore07,Ore08,Ore09,Ore10,Ore11,Ore12,Ore13,Ore14	*OreG0003926*
scaffold1801	234319	C	G	NA	*OreG0003926*
scaffold1801	234365	A	G	Och13	*OreG0003926*
scaffold1801	234436	A	T	NA	*OreG0003926*
scaffold1801	234442	G	T	Ore01,Ore02,Ore03,Ore04,Ore05,Ore06,Ore07,Ore08,Ore09,Ore10,Ore11,Ore12,Ore13,Ore14	*OreG0003926*
scaffold1801	234699	G	A	NA	*OreG0003926*
scaffold1801	234904	A	G	Och06	*OreG0003926*
scaffold1801	234966	G	A	Och06	*OreG0003926*
scaffold1801	235000	A	G	Och01,Och02,Och03,Och04,Och05,Och06,Och07,Och08,Och09,Och10,Och11,Och12,Och13,Och14,Ore01,Ore02,Ore03,Ore04,Ore05,Ore06,Ore07,Ore08,Ore09,Ore10,Ore11,Ore12,Ore13,Ore14	*OreG0003926*
scaffold1801	235422	G	A	Ore09,Ore13,Ore14	*OreG0003926*
scaffold1801	241163	T	C	Ore09,Ore13,Ore14	*OreG0003927*
scaffold1801	241224	C	G	NA	*OreG0003927*
scaffold1801	304809	T	A	NA	*OreG0003931*
scaffold1801	310005	T	G	Och01,Och02,Och03,Och04,Och05,Och06,Och07,Och08,Och09,Och10,Och11,Och12,Och13,Och14	*OreG0003932*

scaffold1801	331678	G	A	Ore01,Ore02,Ore03,Ore04,Ore05,Ore06,Ore07,Ore08,Ore09,Ore10,Ore11,Ore12,Ore13,Ore14	*OreG0003934*
scaffold1801	345121	G	C	Ore01,Ore02,Ore03,Ore04,Ore05,Ore06,Ore07,Ore08,Ore09,Ore10,Ore11,Ore12,Ore13,Ore14	*OreG0003935*
scaffold1801	345224	G	A	Och01,Och02,Och03,Och04,Och05,Och06,Och07,Och08,Och09,Och10,Och11,Och12,Och13,Och14,Ore01,Ore02,Ore03,Ore04,Ore05,Ore06,Ore07,Ore08,Ore09,Ore10,Ore11,Ore12,Ore13,Ore14	*OreG0003935*
scaffold1801	372231	G	A	Och01,Och02,Och03,Och04,Och05,Och06,Och07,Och08,Och09,Och10,Och11,Och12,Och13,Och14	*OreG0003937*
scaffold1801	372250	C	T	Ore01,Ore02,Ore04,Ore05,Ore07,Ore09,Ore13,Ore14	*OreG0003937*
scaffold1801	562692	C	T	NA	*OreG0003950*
scaffold1801	570833	G	A	Och01,Och02,Och03,Och04,Och05,Och06,Och07,Och08,Och09,Och10,Och11,Och12,Och13,Och14,Ore01,Ore02,Ore03,Ore04,Ore05,Ore06,Ore07,Ore08,Ore09,Ore10,Ore11,Ore12,Ore13,Ore14	*OreG0003952*
scaffold1801	579708	A	T	NA	*OreG0003953*
scaffold1801	582731	A	G	NA	*OreG0003953*
scaffold1801	589928	A	G	Och02,Och03,Och04,Och05,Och06,Och08,Och09,Och10,Och12,Och13,Och14	*OreG0003954*
scaffold1801	590099	C	T	NA	*OreG0003954*
scaffold1801	590863	G	A	NA	*OreG0003955*
scaffold1801	601202	A	T	Och06	*OreG0003956*
scaffold1801	601218	A	T	NA	*OreG0003956*
scaffold1801	601287	C	T	Ore01,Ore02,Ore04,Ore05,Ore07,Ore09,Ore13,Ore14	*OreG0003956*
scaffold1801	687762	G	A	Och11	*OreG0003961*
scaffold1801	689207	A	G	NA	*OreG0003961*
scaffold650	477892	T	A	Och02,Och04,Och06	*OreG0024277*
scaffold650	628952	C	T	NA	*OreG0024284*
scaffold650	943291	G	T	Och01,Och02,Och03,Och04,Och05,Och06,Och07,Och08,Och09,Och10,Och11,Och12,Och13,Och14	*OreG0024291*
scaffold650	943435	A	T	Och01,Och02,Och03,Och04,Och05,Och06,Och07,Och08,Och09,Och10,Och11,Och12,Och13,Och14	*OreG0024291*
scaffold650	986545	A	C	Ore01,Ore02,Ore03,Ore04,Ore05,Ore06,Ore07,Ore08,Ore09,Ore10,Ore11,Ore12,Ore13,Ore14	*OreG0024293*
scaffold650	986619	A	G	NA	*OreG0024293*
scaffold650	1009682	G	A	Och14	*OreG0024295*
scaffold650	1010069	T	G	NA	*OreG0024295*
scaffold650	1010303	T	A	Och01,Och02,Och03,Och04,Och05,Och06,Och07,Och08,Och09,Och10,Och11,Och12,Och13,Och14	*OreG0024295*
scaffold650	1013015	T	A	Ore01,Ore02,Ore03,Ore04,Ore05,Ore06,Ore07,Ore08,Ore09,Ore10,Ore11,Ore12,Ore13,Ore14	*OreG0024297*
scaffold650	1014455	G	A	Och01,Och02,Och03,Och04,Och05,Och06,Och07,Och08,Och09,Och10,Och11,Och12,Och13,Och14,Ore01,Ore02,Ore03,Ore04,Ore05,Ore06,Ore07,Ore08,Ore09,Ore10,Ore11,Ore12,Ore13,Ore14	*OreG0024297*
scaffold650	1016882	T	C	Och01,Och02,Och03,Och04,Och05,Och06,Och07,Och08,Och09,Och10,Och11,Och12,Och13,Och14,Ore01,Ore02,Ore03,Ore04,Ore05,Ore06,Ore07,Ore08,Ore09,Ore10,Ore11,Ore12,Ore13,Ore14	*OreG0024297*
scaffold650	1017110	T	G	Ore01,Ore02,Ore03,Ore04,Ore05,Ore06,Ore07,Ore08,Ore09,Ore10,Ore11,Ore12,Ore13,Ore14	*OreG0024297*
scaffold650	1018164	T	A	Och01,Och02,Och03,Och04,Och05,Och06,Och07,Och08,Och09,Och10,Och11,Och12,Och13,Och14,Ore01,Ore02,Ore03,Ore04,Ore05,Ore06,Ore07,Ore08,Ore09,Ore10,Ore11,Ore12,Ore13,Ore14	*OreG0024297*
scaffold650	1052240	C	T	Och01,Och02,Och03,Och04,Och05,Och06,Och07,Och08,Och09,Och10,Och11,Och12,Och13,Och14	*OreG0024298*
scaffold650	1052326	G	A	Och01,Och02,Och03,Och04,Och05,Och06,Och07,Och08,Och09,Och10,Och11,Och12,Och13,Och14	*OreG0024298*
scaffold650	1052417	C	T	Och01,Och02,Och03,Och04,Och05,Och06,Och07,Och08,Och09,Och10,Och11,Och12,Och13,Och14	*OreG0024298*
scaffold650	1052428	C	T	NA	*OreG0024298*
scaffold650	1071406	C	T	Och01,Och02,Och03,Och04,Och05,Och06,Och07,Och08,Och09,Och10,Och11,Och12,Och13,Och14	*OreG0024299*
scaffold650	1159218	C	G	Och01,Och02,Och03,Och04,Och05,Och06,Och07,Och08,Och09,Och10,Och11,Och12,Och13,Och14,Ore01,Ore02,Ore03,Ore04,Ore05,Ore06,Ore07,Ore08,Ore09,Ore10,Ore11,Ore12,Ore13,Ore14	*OreG0024300*
scaffold650	1174015	T	A	Ore01,Ore02,Ore03,Ore04,Ore05,Ore06,Ore07,Ore08,Ore09,Ore10,Ore11,Ore12,Ore13,Ore14	*OreG0024301*
scaffold650	1283384	G	T	Ore01,Ore02,Ore03,Ore04,Ore05,Ore06,Ore07,Ore08,Ore09,Ore10,Ore11,Ore12,Ore13,Ore14	*OreG0024303*
scaffold650	1293070	A	G	Ore01,Ore02,Ore03,Ore04,Ore05,Ore06,Ore07,Ore08,Ore09,Ore10,Ore11,Ore12,Ore13,Ore14	*OreG0024304*
scaffold650	1293073	A	C	Ore03,Ore08,Ore09,Ore10,Ore14	*OreG0024304*
scaffold650	1602647	C	T	NA	*OreG0024310*
scaffold650	1610793	T	C	NA	*OreG0024311*
scaffold650	1611054	T	A	NA	*OreG0024311*
scaffold650	1628769	A	C	Ore01,Ore02,Ore03,Ore04,Ore05,Ore06,Ore07,Ore08,Ore09,Ore10,Ore11,Ore12,Ore13,Ore14	*OreG0024313*
scaffold650	1628916	G	T	Ore01,Ore02,Ore03,Ore04,Ore05,Ore06,Ore07,Ore08,Ore09,Ore10,Ore11,Ore12,Ore13,Ore14	*OreG0024313*

scaffold650	1651863	G	A	Och13	*OreG0024314*
scaffold650	1652194	C	T	Och01,Och02,Och03,Och04,Och05,Och06,Och07,Och08,Och09,Och10,Och11,Och12,Och13,Och14	*OreG0024314*
scaffold650	1652220	T	C	NA	*OreG0024314*
scaffold650	1652502	G	A	Ore01,Ore02,Ore03,Ore06,Ore07,Ore08,Ore09,Ore10,Ore11,Ore12,Ore13,Ore14	*OreG0024315*
scaffold650	1659496	A	G	NA	*OreG0024318*
scaffold650	1811643	G	A	NA	*OreG0024323*
scaffold650	1820920	G	A	Och01,Och02,Och03,Och04,Och05,Och06,Och07,Och08,Och09,Och10,Och11,Och12,Och13,Och14,Ore01,Ore02,Ore03,Ore04,Ore05,Ore06,Ore07,Ore08,Ore09,Ore10,Ore11,Ore12,Ore13,Ore14	*OreG0024323*
scaffold650	1839520	C	A	NA	*OreG0024323*
scaffold650	1845712	C	A	NA	*OreG0024323*
scaffold650	1950920	T	A	NA	*OreG0024325*
scaffold650	1952032	A	T	NA	*OreG0024325*
scaffold650	1964899	A	T	NA	*OreG0024327*
scaffold650	1965815	C	A	NA	*OreG0024327*
scaffold650	1979806	G	C	Och01,Och02,Och03,Och04,Och05,Och06,Och07,Och08,Och09,Och10,Och11,Och12,Och13,Och14,Ore01,Ore02,Ore03,Ore04,Ore05,Ore06,Ore07,Ore08,Ore09,Ore10,Ore11,Ore12,Ore13,Ore14	*OreG0024328*
scaffold650	1979922	G	T	NA	*OreG0024328*
scaffold650	1979999	T	C	NA	*OreG0024328*
scaffold650	1980077	A	G	Ore01,Ore02,Ore03,Ore04,Ore05,Ore06,Ore07,Ore08,Ore09,Ore10,Ore11,Ore12,Ore13,Ore14	*OreG0024328*
scaffold650	1980272	A	C	Och01,Och02,Och03,Och04,Och05,Och06,Och07,Och08,Och09,Och10,Och11,Och12,Och13,Och14,Ore01,Ore02,Ore03,Ore04,Ore05,Ore06,Ore07,Ore08,Ore09,Ore10,Ore11,Ore12,Ore13,Ore14	*OreG0024328*
scaffold650	2004174	T	A	Och07	*OreG0024329*
scaffold650	2009275	G	T	Och07	*OreG0024330*
scaffold650	2095119	C	A	Och07	*OreG0024334*
scaffold650	2095156	G	T	NA	*OreG0024334*
scaffold650	2095296	G	A	NA	*OreG0024334*
scaffold650	2095348	T	C	Och02,Och04,Och06,Och08	*OreG0024334*
scaffold650	2095644	G	A	NA	*OreG0024334*
scaffold650	2095893	C	T	Och02,Och04,Och06,Och08	*OreG0024334*
scaffold650	2095998	G	C	NA	*OreG0024334*
scaffold650	2098611	G	A	Och02,Och04,Och06,Och08	*OreG0024335*
scaffold650	2098638	G	T	Ore01,Ore02,Ore03,Ore04,Ore05,Ore06,Ore07,Ore08,Ore09,Ore10,Ore11,Ore12,Ore13,Ore14	*OreG0024335*
scaffold650	2120440	C	A	Och11	*OreG0024336*
scaffold650	2120533	C	A	Och01,Och02,Och03,Och04,Och05,Och06,Och08,Och09,Och10,Och11,Och12,Och13,Och14,Ore01,Ore02,Ore03,Ore04,Ore05,Ore06,Ore07,Ore08,Ore09,Ore10,Ore11,Ore12,Ore13,Ore14	*OreG0024336*
scaffold650	2120568	G	A	NA	*OreG0024336*
scaffold650	2120572	G	A	Ore01,Ore02,Ore03,Ore04,Ore05,Ore06,Ore07,Ore08,Ore09,Ore10,Ore11,Ore12,Ore13,Ore14	*OreG0024336*
scaffold650	2120709	G	A	Ore01,Ore02,Ore03,Ore04,Ore05,Ore06,Ore07,Ore08,Ore09,Ore10,Ore11,Ore12,Ore13,Ore14	*OreG0024337*
scaffold650	2121274	G	T	Ore01,Ore02,Ore03,Ore04,Ore05,Ore06,Ore07,Ore08,Ore09,Ore10,Ore11,Ore12,Ore13,Ore14	*OreG0024337*
scaffold650	2121521	G	A	Och01,Och02,Och04,Och05,Och06,Och07,Och08,Och09,Och10,Och11,Och12,Och13,Och14	*OreG0024337*
scaffold650	2121528	G	A	Och01,Och02,Och03,Och04,Och05,Och06,Och07,Och08,Och09,Och10,Och11,Och12,Och13,Och14,Ore01,Ore02,Ore03,Ore04,Ore05,Ore06,Ore07,Ore08,Ore09,Ore10,Ore11,Ore12,Ore13,Ore14	*OreG0024337*
scaffold650	2121567	T	G	Ore01,Ore02,Ore03,Ore04,Ore05,Ore06,Ore07,Ore08,Ore09,Ore10,Ore11,Ore12,Ore13,Ore14	*OreG0024337*
scaffold650	2123401	G	A	Och07	*OreG0024338*
scaffold650	2123410	C	T	Och07	*OreG0024338*
scaffold650	2123458	C	T	Och02,Och04,Och06,Och08	*OreG0024338*
scaffold650	2123482	T	G	Ore01,Ore02,Ore03,Ore04,Ore05,Ore06,Ore07,Ore08,Ore09,Ore10,Ore11,Ore12,Ore13,Ore14	*OreG0024338*
scaffold650	2123508	G	A	Och07	*OreG0024338*
scaffold650	2183574	G	C	NA	*OreG0024341*
scaffold650	2212229	C	A	NA	*OreG0024341*
scaffold650	2217634	A	T	Och07	*OreG0024341*
scaffold650	2238143	C	T	NA	*OreG0024342*
scaffold650	2238195	T	C	Ore03,Ore08,Ore09,Ore10,Ore14	*OreG0024342*
scaffold650	2294133	G	A	Och02,Och04,Och06,Och08	*OreG0024346*
scaffold1936	26727	G	A	NA	*OreG0004908*
scaffold1936	32352	G	A	Och01,Och02,Och03,Och04,Och05,Och06,Och07,Och08,Och09,Och10,Och11,Och12,Och13,Och14,Ore01,Ore02,Ore03,Ore04,Ore05,Ore06,Ore07,Ore08,Ore09,Ore10,Ore11,Ore12,Ore13,Ore14	*OreG0004909*
scaffold1936	35580	A	G	Ore01,Ore02,Ore03,Ore04,Ore05,Ore06,Ore07,Ore08,Ore09,Ore10,Ore11,Ore12,Ore13,Ore14	*OreG0004909*

scaffold1936	39111	G	A	Ore01,Ore02,Ore03,Ore04,Ore05,Ore06,Ore07,Ore08,Ore09,Ore10,Ore11,Ore12,Ore13,Ore14	*OreG0004909*
scaffold1936	70460	A	G	Ore01,Ore02,Ore03,Ore04,Ore05,Ore06,Ore07,Ore08,Ore09,Ore10,Ore11,Ore12,Ore13,Ore14	*OreG0004910*
scaffold1936	96317	C	T	NA	*OreG0004911*
scaffold1936	97580	C	T	Och01,Och02,Och03,Och04,Och05,Och06,Och07,Och08,Och09,Och10,Och11,Och12,Och13,Och14,Ore01,Ore02,Ore03,Ore04,Ore05,Ore06,Ore07,Ore08,Ore09,Ore10,Ore11,Ore12,Ore13,Ore14	*OreG0004911*
scaffold1936	98706	T	C	NA	*OreG0004911*
scaffold1936	108867	G	A	NA	*OreG0004912*
scaffold1936	324964	G	A	Ore01,Ore02,Ore03,Ore04,Ore05,Ore06,Ore07,Ore08,Ore09,Ore10,Ore11,Ore12,Ore13,Ore14	*OreG0004915*
scaffold1936	325009	G	A	NA	*OreG0004915*
scaffold1936	325109	C	A	Och02,Och03,Och04,Och05,Och06,Och09,Och10,Och11,Och12,Och13,Och14	*OreG0004915*
scaffold1936	438877	C	A	NA	*OreG0004916*
scaffold1936	669608	A	G	NA	*OreG0004919*
scaffold1936	704402	A	C	Och12,Och13	*OreG0004921*
scaffold1936	705691	G	A	Och01,Och12,Och13,Ore01,Ore02,Ore03,Ore04,Ore05,Ore06,Ore07,Ore08,Ore09,Ore10,Ore11,Ore12,Ore13,Ore14	*OreG0004921*
scaffold1936	724734	C	T	Och02	*OreG0004922*
scaffold1936	727274	A	G	NA	*OreG0004922*
scaffold1936	824757	G	A	NA	*OreG0004927*
scaffold1936	825506	G	A	Och12,Och13	*OreG0004927*
scaffold1936	826236	A	G	Och02,Och03,Och04,Och05,Och06,Och09,Och10,Och11,Och12,Och13	*OreG0004927*
scaffold1936	826356	T	C	NA	*OreG0004927*
scaffold1625	75572	T	G	Ore01,Ore02,Ore03,Ore04,Ore05,Ore06,Ore07,Ore08,Ore09,Ore10,Ore11,Ore12,Ore13,Ore14	*OreG0003286*
scaffold1625	82587	G	A	Och01,Och02,Och03,Och04,Och05,Och06,Och07,Och08,Och09,Och10,Och11,Och12,Och13,Och14,Ore01,Ore02,Ore03,Ore04,Ore05,Ore06,Ore07,Ore08,Ore09,Ore10,Ore11,Ore12,Ore13,Ore14	*OreG0003287*
scaffold1625	91908	G	A	NA	*OreG0003288*
scaffold1625	195169	C	T	Och01,Och02,Och03,Och04,Och05,Och06,Och07,Och08,Och09,Och10,Och11,Och12,Och13,Och14	*OreG0003292*
scaffold1625	212282	C	A	Och01,Och14	*OreG0003295*
scaffold1625	229995	A	C	Och13	*OreG0003297*
scaffold1625	250355	C	G	Ore08,Ore09,Ore10	*OreG0003298*
scaffold1625	289412	T	C	NA	*OreG0003300*
scaffold1625	328411	G	T	NA	*OreG0003302*
scaffold1625	351707	G	A	Och01	*OreG0003307*
scaffold1625	352638	T	C	Ore01,Ore02,Ore03,Ore04,Ore05,Ore06,Ore07,Ore08,Ore09,Ore10,Ore11,Ore12,Ore13,Ore14	*OreG0003307*
scaffold1625	390730	T	A	Och01,Och02,Och03,Och04,Och05,Och06,Och07,Och08,Och09,Och10,Och11,Och12,Och13,Och14,Ore01,Ore02,Ore03,Ore04,Ore05,Ore06,Ore07,Ore08,Ore09,Ore10,Ore11,Ore12,Ore13,Ore14	*OreG0003310*
scaffold1625	413459	G	A	Och04	*OreG0003311*
scaffold1625	413748	G	A	Och13	*OreG0003311*
scaffold1625	418218	C	T	NA	*OreG0003312*
scaffold1625	474448	C	T	Ore01,Ore02,Ore03,Ore04,Ore05,Ore06,Ore07,Ore08,Ore09,Ore10,Ore11,Ore12,Ore13,Ore14	*OreG0003316*
scaffold1625	482522	C	A	Och03,Och06,Och07,Och08,Och09,Och12,Och13,Och14	*OreG0003317*
scaffold1625	488189	A	G	NA	*OreG0003319*
scaffold1625	488199	C	T	NA	*OreG0003319*
scaffold1625	488238	A	T	Ore01,Ore02,Ore03,Ore04,Ore05,Ore06,Ore07,Ore08,Ore09,Ore10,Ore11,Ore12,Ore13,Ore14	*OreG0003319*
scaffold1625	488271	A	T	Och01,Och02,Och03,Och04,Och05,Och06,Och07,Och08,Och09,Och10,Och11,Och12,Och13	*OreG0003319*
scaffold1625	492142	C	T	NA	*OreG0003320*
scaffold1625	517563	G	T	Och01,Och02,Och03,Och04,Och05,Och06,Och07,Och08,Och09,Och10,Och11,Och12,Och13,Och14	*OreG0003324*
scaffold1625	518646	A	T	Och01,Och02,Och04,Och06,Och08,Och14	*OreG0003324*
scaffold1625	519368	G	A	Ore01,Ore02,Ore03,Ore06,Ore07,Ore08,Ore09,Ore10,Ore11,Ore12,Ore13,Ore14	*OreG0003324*
scaffold1625	540314	C	T	Och01,Och02,Och04,Och06,Och08	*OreG0003326*
scaffold1625	628857	T	A	NA	*OreG0003336*
scaffold1625	637130	T	C	Och01,Och02,Och03,Och04,Och05,Och06,Och07,Och08,Och09,Och10,Och11,Och12,Och13,Och14	*OreG0003337*
scaffold1625	638993	C	A	NA	*OreG0003337*
scaffold1625	641280	G	T	Ore04,Ore05	*OreG0003337*
scaffold1625	641437	C	A	NA	*OreG0003337*
scaffold1625	662615	G	A	Och09,Och10	*OreG0003339*
scaffold1625	663599	C	T	Och02,Och03,Och04,Och05,Och06,Och07,Och08,Och12,Och13,Och14	*OreG0003339*
scaffold1625	671583	T	G	Och01,Och02,Och03,Och04,Och05,Och06,Och07,Och08,Och09,Och10,Och11,Och12,Och13,Och14,Ore01,Ore02,Ore03,Ore04,Ore05,Ore06,Ore07,Ore08,Ore09,Ore10,Ore11,Ore12,Ore13,Ore14	*OreG0003340*
scaffold1625	676822	G	A	Och01,Och09,Och10,Och11	*OreG0003341*
scaffold2696	180290	C	A	Ore02,Ore03,Ore04,Ore05,Ore06,Ore07,Ore08,Ore09,Ore10,Ore11,Ore12,Ore14	*OreG0007905*

scaffold2696	180365	A	G	Ore02,Ore03,Ore04,Ore05,Ore06,Ore07,Ore08,Ore09,Ore10,Ore11,Ore12,Ore14	*OreG0007905*
scaffold2696	180387	C	T	Ore02,Ore03,Ore04,Ore05,Ore06,Ore07,Ore08,Ore09,Ore10,Ore11,Ore12,Ore14	*OreG0007905*
scaffold2696	180429	C	T	Ore02,Ore03,Ore04,Ore05,Ore06,Ore07,Ore08,Ore09,Ore10,Ore11,Ore12,Ore14	*OreG0007905*
scaffold1520	117461	G	T	NA	*OreG0003254*
scaffold1520	166376	A	G	Och08	*OreG0003258*
scaffold1520	166448	A	T	NA	*OreG0003258*
scaffold1520	170717	A	T	NA	*OreG0003259*
scaffold1520	175348	C	T	Och02,Och04,Och06	*OreG0003260*
scaffold1520	176310	C	G	Och01,Och02,Och03,Och04,Och05,Och06,Och07,Och08,Och09,Och10,Och11,Och12,Och13,Och14,Ore01,Ore02,Ore03,Ore04,Ore05,Ore06,Ore07,Ore08,Ore09,Ore10,Ore11,Ore12,Ore13,Ore14	*OreG0003260*
scaffold1520	179441	G	T	Och01,Och02,Och03,Och04,Och05,Och06,Och07,Och08,Och09,Och10,Och11,Och12,Och13,Och14,Ore01,Ore02,Ore03,Ore04,Ore05,Ore06,Ore07,Ore08,Ore09,Ore10,Ore11,Ore12,Ore13,Ore14	*OreG0003260*
scaffold1520	180122	G	C	NA	*OreG0003261*
scaffold1520	180373	C	G	NA	*OreG0003261*
scaffold1520	181218	C	G	NA	*OreG0003262*
scaffold1520	181251	A	G	NA	*OreG0003262*
scaffold1520	199234	T	G	Och07,Och08	*OreG0003264*
scaffold2790	2124	A	C	NA	*OreG0008297*
scaffold2790	4527	T	C	Och08	*OreG0008297*
scaffold2709	22258	C	T	Och01,Och02,Och03,Och04,Och05,Och06,Och07,Och08,Och09,Och10,Och11,Och12,Och13,Och14,Ore01,Ore02,Ore03,Ore04,Ore05,Ore06,Ore07,Ore08,Ore09,Ore10,Ore11,Ore12,Ore13,Ore14	*OreG0007920*
scaffold2709	37725	C	A	Ore01,Ore02,Ore03,Ore04,Ore05,Ore06,Ore07,Ore08,Ore09,Ore10,Ore11,Ore12,Ore13,Ore14	*OreG0007924*
scaffold2709	48626	A	G	Och01,Och02,Och03,Och04,Och05,Och06,Och07,Och08,Och09,Och10,Och11,Och12,Och13,Och14,Ore01,Ore02,Ore03,Ore04,Ore05,Ore06,Ore07,Ore08,Ore09,Ore10,Ore11,Ore12,Ore13,Ore14	*OreG0007925*
scaffold2709	76905	G	A	NA	*OreG0007927*
scaffold2709	77149	G	A	Ore01,Ore02,Ore03,Ore04,Ore05,Ore06,Ore07,Ore08,Ore09,Ore10,Ore11,Ore12,Ore13,Ore14	*OreG0007927*
scaffold2709	77530	G	T	Och01,Och02,Och03,Och04,Och05,Och06,Och07,Och08,Och09,Och10,Och11,Och12,Och13,Och14	*OreG0007927*
scaffold2709	80442	G	T	Och01,Och02,Och03,Och04,Och05,Och06,Och07,Och08,Och09,Och10,Och11,Och12,Och13,Och14	*OreG0007927*
scaffold1244	26271	G	A	NA	*OreG0001803*
scaffold1244	26485	A	T	Och09,Och10	*OreG0001803*
scaffold1244	86041	G	A	NA	*OreG0001807*
scaffold1244	167104	G	T	NA	*OreG0001812*
scaffold1244	228036	T	A	Ore01,Ore02,Ore03,Ore04,Ore05,Ore06,Ore07,Ore08,Ore09,Ore10,Ore11,Ore12,Ore13,Ore14	*OreG0001816*
scaffold1244	246714	G	A	NA	*OreG0001818*
scaffold1244	247546	G	A	Och01,Och02,Och03,Och04,Och05,Och06,Och07,Och08,Och09,Och10,Och11,Och12,Och13,Och14,Ore01,Ore02,Ore03,Ore04,Ore05,Ore06,Ore07,Ore08,Ore09,Ore10,Ore11,Ore12,Ore13,Ore14	*OreG0001818*
scaffold1244	251428	C	T	Ore01,Ore02,Ore03,Ore04,Ore05,Ore06,Ore07,Ore08,Ore09,Ore10,Ore11,Ore12,Ore13,Ore14	*OreG0001819*
scaffold1244	251430	T	G	Och01,Och02,Och03,Och04,Och05,Och06,Och07,Och08,Och09,Och10,Och11,Och12,Och13,Och14	*OreG0001819*
scaffold1244	253301	A	T	Ore01,Ore02,Ore03,Ore04,Ore05,Ore06,Ore07,Ore08,Ore09,Ore10,Ore11,Ore12,Ore13,Ore14	*OreG0001819*
scaffold1244	253424	G	C	Ore01,Ore02,Ore03,Ore04,Ore05,Ore06,Ore07,Ore08,Ore09,Ore10,Ore11,Ore12,Ore13,Ore14	*OreG0001819*
scaffold1244	297851	G	A	NA	*OreG0001823*
scaffold1244	301477	T	A	NA	*OreG0001823*
scaffold1244	305949	T	A	Ore01,Ore02,Ore03,Ore04,Ore05,Ore06,Ore07,Ore08,Ore09,Ore10,Ore11,Ore12,Ore13,Ore14	*OreG0001824*
scaffold1244	367861	G	A	Ore07,Ore08,Ore11,Ore12,Ore13,Ore14	*OreG0001826*
scaffold1244	371589	C	G	Och01,Och02,Och03,Och04,Och05,Och06,Och07,Och08,Och09,Och10,Och11,Och12,Och13,Och14	*OreG0001827*
scaffold1244	373515	G	T	NA	*OreG0001827*
scaffold1244	375508	T	C	Och06	*OreG0001827*
scaffold1244	402155	A	T	NA	*OreG0001829*
scaffold1244	414348	G	A	Och01,Och02,Och03,Och04,Och05,Och06,Och07,Och08,Och09,Och10,Och11,Och12,Och13,Och14,Ore01,Ore02,Ore03,Ore04,Ore05,Ore06,Ore07,Ore08,Ore09,Ore10,Ore11,Ore12,Ore13,Ore14	*OreG0001829*
scaffold1244	415448	A	T	Och01,Och02,Och03,Och04,Och05,Och06,Och07,Och08,Och09,Och10,Och11,Och12,Och13,Och14,Ore01,Ore02,Ore03,Ore04,Ore05,Ore06,Ore07,Ore08,Ore09,Ore10,Ore11,Ore12,Ore13,Ore14	*OreG0001829*
scaffold1244	415457	A	T	Ore07,Ore08,Ore14	*OreG0001829*
scaffold1244	417115	T	A	NA	*OreG0001830*
scaffold1244	417723	T	C	NA	*OreG0001830*
scaffold1244	417751	G	A	NA	*OreG0001830*
scaffold1244	417755	C	T	NA	*OreG0001830*

scaffold1244	421979	A	G	Ore01,Ore02,Ore03,Ore04,Ore05,Ore06,Ore07,Ore08,Ore09,Ore10,Ore11,Ore12,Ore13,Ore14	*OreG0001830*
scaffold1244	451007	A	T	Ore07,Ore08,Ore14	*OreG0001833*
scaffold1244	454427	C	T	Och01	*OreG0001834*
scaffold1244	542133	C	A	NA	*OreG0001842*
scaffold1244	568621	T	A	NA	*OreG0001845*
scaffold1244	568963	T	C	NA	*OreG0001845*
scaffold1244	570140	T	G	NA	*OreG0001845*
scaffold1244	585050	A	T	Ore01,Ore02,Ore03,Ore04,Ore05,Ore06,Ore07,Ore08,Ore09,Ore10,Ore11,Ore12,Ore13,Ore14	*OreG0001848*
scaffold1244	606058	G	A	NA	*OreG0001853*
scaffold1244	606252	T	G	NA	*OreG0001853*
scaffold1244	657555	T	A	Och01,Och02,Och03,Och04,Och05,Och06,Och07,Och08,Och12,Och13,Och14	*OreG0001861*
scaffold1244	657662	C	A	Och01,Och02,Och03,Och04,Och05,Och06,Och07,Och08,Och12,Och13,Och14	*OreG0001861*
scaffold1244	657717	T	C	Och01,Och02,Och03,Och04,Och05,Och06,Och07,Och08,Och12,Och13,Och14	*OreG0001861*
scaffold1244	657863	T	C	NA	*OreG0001861*
scaffold1244	677500	C	T	NA	*OreG0001864*
scaffold1244	678717	T	C	NA	*OreG0001864*
scaffold1244	702436	G	T	Ore06	*OreG0001866*
scaffold1244	715975	T	A	Och01,Och02,Och03,Och04,Och05,Och06,Och07,Och08,Och09,Och10,Och11,Och12,Och13,Och14,Ore01,Ore02,Ore03,Ore04,Ore05,Ore06,Ore07,Ore08,Ore09,Ore10,Ore11,Ore12,Ore13,Ore14	*OreG0001868*
scaffold1244	716439	A	T	Och01,Och02,Och03,Och04,Och05,Och06,Och07,Och08,Och09,Och10,Och11,Och12,Och13,Och14,Ore01,Ore02,Ore03,Ore04,Ore05,Ore06,Ore07,Ore08,Ore09,Ore10,Ore11,Ore12,Ore13,Ore14	*OreG0001868*
scaffold1244	717943	T	A	Och01,Och02,Och03,Och04,Och05,Och06,Och07,Och08,Och09,Och10,Och11,Och12,Och13,Och14,Ore01,Ore02,Ore03,Ore04,Ore05,Ore06,Ore07,Ore08,Ore09,Ore10,Ore11,Ore12,Ore13,Ore14	*OreG0001868*
scaffold1244	757218	A	G	NA	*OreG0001870*
scaffold1244	757410	C	G	NA	*OreG0001870*
scaffold1244	757785	C	T	NA	*OreG0001870*
scaffold1244	772518	G	A	Och03,Och05,Och12,Och13	*OreG0001874*
scaffold1244	797374	C	A	Ore01,Ore02,Ore03,Ore04,Ore05,Ore06,Ore07,Ore08,Ore09,Ore10,Ore11,Ore12,Ore13,Ore14	*OreG0001878*
scaffold1244	800431	G	A	Ore02	*OreG0001880*
scaffold1244	814260	T	C	Ore01,Ore03,Ore04,Ore05,Ore09,Ore10,Ore11	*OreG0001882*
scaffold1244	814325	T	A	Och01,Och02,Och03,Och04,Och05,Och06,Och07,Och08,Och09,Och10,Och11,Och12,Och13,Och14,Ore01,Ore02,Ore03,Ore04,Ore05,Ore06,Ore07,Ore08,Ore09,Ore10,Ore11,Ore12,Ore13,Ore14	*OreG0001882*
scaffold1244	814648	T	C	NA	*OreG0001882*
scaffold1244	836587	A	T	NA	*OreG0001885*
scaffold1244	844043	T	C	NA	*OreG0001885*
scaffold1244	862181	A	G	Och08,Och14	*OreG0001888*
scaffold1244	862508	G	T	NA	*OreG0001888*
scaffold1244	892492	A	G	Och01,Och02,Och03,Och04,Och05,Och06,Och07,Och08,Och09,Och10,Och11,Och12,Och13,Och14,Ore01,Ore02,Ore03,Ore04,Ore05,Ore06,Ore07,Ore08,Ore09,Ore10,Ore11,Ore12,Ore13,Ore14	*OreG0001891*
scaffold1244	1076245	C	T	NA	*OreG0001904*
scaffold1244	1087959	T	C	NA	*OreG0001905*
scaffold1244	1094643	G	A	Och05,Och08,Och12,Och13	*OreG0001906*
scaffold1244	1094865	T	G	NA	*OreG0001906*
scaffold1244	1114602	C	A	NA	*OreG0001908*
scaffold1244	1118382	G	T	Och01,Och02,Och03,Och04,Och05,Och06,Och07,Och08,Och09,Och10,Och11,Och12,Och13,Och14	*OreG0001909*
scaffold1244	1118465	T	C	NA	*OreG0001909*
scaffold1244	1122481	T	C	NA	*OreG0001910*
scaffold1244	1141214	A	C	Och13	*OreG0001911*
scaffold1244	1158217	C	A	Ore01,Ore02,Ore03,Ore04,Ore05,Ore06,Ore07,Ore08,Ore09,Ore10,Ore11,Ore12,Ore13,Ore14	*OreG0001912*
scaffold1244	1166830	A	G	Och02,Och03,Och04,Och05,Och06,Och07,Och08,Och09,Och10,Och11,Och12,Och13,Och14	*OreG0001913*
scaffold1244	1167248	G	T	Och01,Och04,Och13,Och14	*OreG0001913*
scaffold1244	1183200	A	G	Och01,Och02,Och03,Och04,Och05,Och06,Och07,Och08,Och09,Och10,Och11,Och12,Och13,Och14,Ore01,Ore02,Ore03,Ore04,Ore05,Ore06,Ore07,Ore08,Ore09,Ore10,Ore11,Ore12,Ore13,Ore14	*OreG0001915*
scaffold1244	1211612	T	A	Och01,Och02,Och03,Och04,Och06,Och07,Och08,Och12,Och13	*OreG0001917*
scaffold1244	1216315	G	A	Ore01,Ore03,Ore04,Ore05,Ore09,Ore10,Ore13,Ore14	*OreG0001918*
scaffold1244	1217000	C	G	Och02,Och08	*OreG0001918*
scaffold1244	1229204	C	T	Ore01,Ore09	*OreG0001919*
scaffold1244	1229443	T	C	NA	*OreG0001919*
scaffold1244	1241985	T	C	Och03	*OreG0001920*
scaffold1244	1309664	G	A	Och01,Och02,Och03,Och04,Och05,Och06,Och07,Och08,Och09,Och10,Och11,Och12,Och13,Och14	*OreG0001927*
scaffold1244	1310019	A	T	NA	*OreG0001927*

scaffold1244	1310870	C	G	Och01,Och02,Och03,Och04,Och05,Och06,Och07,Och08,Och09,Och10,Och11,Och12,Och13,Och14	*OreG0001927*
scaffold1244	1328128	C	A	Och01	*OreG0001930*
scaffold1244	1412991	A	G	Och01,Och02,Och03,Och04,Och05,Och06,Och07,Och08,Och09,Och10,Och11,Och12,Och13,Och14,Ore01,Ore09	*OreG0001938*
scaffold1244	1413208	G	A	Och01,Och02,Och03,Och04,Och05,Och06,Och07,Och08,Och09,Och10,Och11,Och12,Och13,Och14,Ore01,Ore02,Ore03,Ore04,Ore05,Ore06,Ore07,Ore08,Ore09,Ore10,Ore11,Ore12,Ore13,Ore14	*OreG0001938*
scaffold1244	1413355	C	T	Ore01,Ore09	*OreG0001938*
scaffold1244	1413504	A	T	Och01,Och02,Och03,Och04,Och05,Och06,Och07,Och08,Och09,Och10,Och11,Och12,Och13,Och14,Ore01,Ore02,Ore03,Ore04,Ore05,Ore06,Ore07,Ore08,Ore09,Ore10,Ore11,Ore12,Ore13,Ore14	*OreG0001938*
scaffold1244	1424286	C	T	Och01,Och03,Och04,Och05,Och07,Och08,Och12,Och13,Och14	*OreG0001939*
scaffold1244	1450531	C	T	Ore01,Ore09	*OreG0001942*
scaffold1244	1450620	G	A	Ore01,Ore02,Ore03,Ore04,Ore05,Ore06,Ore07,Ore08,Ore09,Ore10,Ore11,Ore12,Ore13,Ore14	*OreG0001942*
scaffold1244	1450909	T	G	Ore01,Ore09	*OreG0001942*
scaffold1244	1451371	T	A	NA	*OreG0001942*
scaffold1244	1454562	G	A	NA	*OreG0001943*
scaffold1244	1455869	C	G	Ore01,Ore02,Ore03,Ore04,Ore05,Ore06,Ore07,Ore08,Ore09,Ore10,Ore11,Ore12,Ore13,Ore14	*OreG0001943*
scaffold1244	1456276	G	T	Och01,Och02,Och03,Och04,Och05,Och06,Och07,Och08,Och09,Och10,Och11,Och12,Och13,Och14,Ore01,Ore02,Ore03,Ore04,Ore05,Ore06,Ore07,Ore08,Ore09,Ore10,Ore11,Ore12,Ore13,Ore14	*OreG0001943*
scaffold1244	1495387	A	G	Och01,Och02,Och03,Och04,Och05,Och06,Och07,Och08,Och09,Och10,Och11,Och12,Och13,Och14,Ore01,Ore02,Ore03,Ore04,Ore05,Ore06,Ore07,Ore08,Ore09,Ore10,Ore11,Ore12,Ore13,Ore14	*OreG0001948*
scaffold1244	1499379	C	T	NA	*OreG0001948*
scaffold1244	1499385	A	G	Och04,Och13,Och14	*OreG0001948*
scaffold2769	21255	A	G	NA	*OreG0008293*
scaffold2769	21282	T	C	Ore01	*OreG0008293*
scaffold2769	21290	G	A	Ore01	*OreG0008293*
scaffold74	46992	A	T	Ore07,Ore08,Ore14	*OreG0024824*
scaffold74	48351	G	A	Och14	*OreG0024825*
scaffold74	49345	A	C	Och01,Och07,Och08,Och11,Och14,Ore01,Ore02,Ore03,Ore04,Ore05,Ore06,Ore07,Ore08,Ore09,Ore10,Ore11,Ore12,Ore13,Ore14	*OreG0024826*
scaffold74	59821	C	T	Och02,Och06	*OreG0024828*
scaffold74	137770	T	C	NA	*OreG0024829*
scaffold74	211043	T	C	Ore07,Ore08,Ore14	*OreG0024836*
scaffold74	221849	A	G	NA	*OreG0024840*
scaffold74	267796	T	A	Och01,Och02,Och03,Och04,Och05,Och06,Och07,Och08,Och09,Och10,Och11,Och12,Och13,Och14,Ore01,Ore02,Ore03,Ore04,Ore05,Ore06,Ore07,Ore08,Ore09,Ore10,Ore11,Ore12,Ore13,Ore14	*OreG0024844*
scaffold74	298090	T	C	Och01,Och02,Och03,Och04,Och05,Och06,Och07,Och08,Och09,Och10,Och11,Och12,Och13,Och14	*OreG0024845*
scaffold74	323226	C	T	Ore01,Ore02,Ore04,Ore05,Ore06,Ore07,Ore08,Ore09,Ore10,Ore11,Ore12,Ore13,Ore14	*OreG0024848*
scaffold74	323414	A	T	NA	*OreG0024848*
scaffold74	323436	C	A	NA	*OreG0024848*
scaffold74	323453	G	A	NA	*OreG0024848*
scaffold74	323693	G	A	Och01,Och03,Och05,Och07,Och08,Och09,Och10,Och12,Och13,Och14	*OreG0024848*
scaffold74	323700	A	C	Och01,Och02,Och03,Och04,Och05,Och06,Och07,Och08,Och09,Och10,Och11,Och12,Och13,Och14,Ore01,Ore02,Ore03,Ore04,Ore05,Ore06,Ore07,Ore08,Ore09,Ore10,Ore11,Ore12,Ore13,Ore14	*OreG0024848*
scaffold74	323912	G	T	NA	*OreG0024848*
scaffold74	324102	C	T	NA	*OreG0024848*
scaffold74	326113	C	A	NA	*OreG0024849*
scaffold74	326554	T	C	Och01,Och02,Och03,Och04,Och05,Och06,Och07,Och08,Och09,Och10,Och11,Och12,Och13,Och14,Ore01,Ore02,Ore03,Ore04,Ore05,Ore06,Ore07,Ore08,Ore09,Ore10,Ore11,Ore12,Ore13,Ore14	*OreG0024849*
scaffold74	326563	G	A	Och12,Och13	*OreG0024849*
scaffold74	333414	T	A	Och12,Och13	*OreG0024850*
scaffold74	334478	G	C	NA	*OreG0024850*
scaffold74	365076	T	G	NA	*OreG0024855*
scaffold74	365126	T	A	NA	*OreG0024855*
scaffold74	365129	C	T	NA	*OreG0024855*
scaffold74	365840	G	A	NA	*OreG0024855*
scaffold74	366272	T	C	Och09,Och10,Och11,Och14	*OreG0024855*
scaffold74	366495	T	A	NA	*OreG0024855*
scaffold74	366524	C	T	NA	*OreG0024855*
scaffold74	366743	G	A	NA	*OreG0024855*
scaffold74	369164	C	T	NA	*OreG0024856*
scaffold74	369340	C	T	NA	*OreG0024856*
scaffold74	369533	C	A	Ore06,Ore07,Ore08,Ore10,Ore12,Ore13,Ore14	*OreG0024856*
scaffold74	369540	C	A	Och03	*OreG0024856*

scaffold74	369545	T	C	NA	*OreG0024856*
scaffold74	369583	T	G	Och01,Och02,Och03,Och06,Och07,Och09,Och10,Och11,Och14,Ore01,Ore02,Ore03,Ore04,Ore05,Ore06,Ore07,Ore08,Ore09,Ore10,Ore11,Ore12,Ore13,Ore14	*OreG0024856*
scaffold74	369610	T	C	Och01,Och02,Och03,Och06,Och07,Och09,Och10,Och11,Och14	*OreG0024856*
scaffold74	370778	A	C	NA	*OreG0024856*
scaffold74	370804	G	A	NA	*OreG0024856*
scaffold74	370924	G	C	NA	*OreG0024856*
scaffold74	371077	T	C	Och14	*OreG0024856*
scaffold74	371243	T	C	NA	*OreG0024856*
scaffold74	412917	C	T	NA	*OreG0024857*
scaffold74	413222	T	A	Och01,Och02,Och03,Och04,Och06,Och07,Och08,Och09,Och10,Och11,Och14,Ore01,Ore02,Ore03,Ore04,Ore05,Ore06,Ore07,Ore08,Ore09,Ore10,Ore11,Ore12,Ore13,Ore14	*OreG0024857*
scaffold74	414247	C	G	Och01,Och02,Och03,Och04,Och05,Och06,Och07,Och08,Och09,Och10,Och11,Och12,Och13,Och14,Ore01,Ore02,Ore03,Ore04,Ore05,Ore06,Ore07,Ore08,Ore09,Ore10,Ore11,Ore12,Ore13,Ore14	*OreG0024857*
scaffold74	417348	A	G	Och01,Och02,Och03,Och04,Och05,Och06,Och07,Och08,Och09,Och10,Och11,Och12,Och13,Och14,Ore01,Ore02,Ore03,Ore04,Ore05,Ore06,Ore07,Ore08,Ore09,Ore10,Ore11,Ore12,Ore13,Ore14	*OreG0024857*
scaffold74	417767	A	T	Och01,Och02,Och03,Och04,Och05,Och06,Och07,Och08,Och09,Och10,Och11,Och12,Och13,Och14,Ore01,Ore02,Ore03,Ore04,Ore05,Ore06,Ore07,Ore08,Ore09,Ore10,Ore11,Ore12,Ore13,Ore14	*OreG0024857*
scaffold74	425898	G	A	Och12,Och13	*OreG0024862*
scaffold74	426383	G	A	NA	*OreG0024862*
scaffold74	426387	G	A	NA	*OreG0024862*
scaffold74	426521	C	G	Och12,Och13	*OreG0024862*
scaffold74	455529	A	G	NA	*OreG0024865*
scaffold74	501095	T	C	Och01,Och02,Och03,Och04,Och05,Och06,Och07,Och08,Och09,Och10,Och11,Och12,Och13,Och14,Ore01,Ore02,Ore03,Ore04,Ore05,Ore06,Ore07,Ore08,Ore09,Ore10,Ore11,Ore12,Ore13,Ore14	*OreG0024870*
scaffold74	562108	C	A	Och01,Och02,Och03,Och04,Och05,Och06,Och07,Och08,Och09,Och10,Och11,Och12,Och13,Och14	*OreG0024877*
scaffold74	562611	A	C	Och01,Och02,Och03,Och04,Och05,Och06,Och07,Och08,Och09,Och10,Och11,Och12,Och13,Och14	*OreG0024877*
scaffold74	568393	C	A	NA	*OreG0024877*
scaffold74	577908	G	T	Och12,Och13	*OreG0024877*
scaffold74	584066	G	A	NA	*OreG0024878*
scaffold74	598169	C	T	Ore01,Ore02,Ore03,Ore04,Ore05,Ore06,Ore07,Ore08,Ore09,Ore10,Ore11,Ore12,Ore13,Ore14	*OreG0024879*
scaffold74	598780	G	A	NA	*OreG0024879*
scaffold74	617602	T	A	Och01,Och02,Och03,Och04,Och05,Och06,Och07,Och08,Och09,Och10,Och11,Och12,Och13,Och14	*OreG0024882*
scaffold74	617705	C	T	NA	*OreG0024882*
scaffold74	636834	C	T	Ore01,Ore02,Ore03,Ore04,Ore05,Ore06,Ore07,Ore08,Ore09,Ore10,Ore11,Ore12,Ore13,Ore14	*OreG0024885*
scaffold74	636850	G	T	Och12,Och13	*OreG0024885*
scaffold74	636882	G	T	Ore01,Ore02,Ore03,Ore04,Ore05,Ore06,Ore07,Ore08,Ore09,Ore10,Ore11,Ore12,Ore13,Ore14	*OreG0024885*
scaffold74	636949	C	G	NA	*OreG0024885*
scaffold74	636963	A	G	NA	*OreG0024885*
scaffold74	637153	C	T	Ore01,Ore02,Ore03,Ore04,Ore05,Ore06,Ore07,Ore08,Ore09,Ore10,Ore11,Ore12,Ore13,Ore14	*OreG0024885*
scaffold74	637317	G	C	NA	*OreG0024885*
scaffold74	637396	T	C	Och12,Och13	*OreG0024885*
scaffold74	637507	G	C	NA	*OreG0024886*
scaffold74	637526	G	C	Och12,Och13	*OreG0024886*
scaffold74	643601	T	A	NA	*OreG0024887*
scaffold74	692807	C	T	NA	*OreG0024893*
scaffold74	692957	G	C	NA	*OreG0024893*
scaffold74	713223	G	T	Och01,Och02,Och03,Och04,Och05,Och06,Och07,Och08,Och09,Och10,Och11,Och12,Och13,Och14	*OreG0024895*
scaffold74	769971	C	T	Och01,Och02,Och03,Och04,Och05,Och06,Och07,Och08,Och09,Och10,Och11,Och12,Och13,Och14	*OreG0024899*
scaffold74	772262	G	A	Ore01,Ore02,Ore03,Ore04,Ore05,Ore06,Ore07,Ore08,Ore09,Ore10,Ore11,Ore12,Ore13,Ore14	*OreG0024899*
scaffold74	774872	G	A	Ore01,Ore02,Ore03,Ore04,Ore05,Ore06,Ore07,Ore08,Ore09,Ore10,Ore11,Ore12,Ore13,Ore14	*OreG0024899*
scaffold74	781306	T	C	Ore01,Ore02,Ore03,Ore04,Ore05,Ore06,Ore07,Ore08,Ore09,Ore10,Ore11,Ore12,Ore13,Ore14	*OreG0024900*
scaffold74	787160	G	T	Ore01,Ore02,Ore03,Ore04,Ore05,Ore06,Ore07,Ore08,Ore09,Ore10,Ore11,Ore12,Ore13,Ore14	*OreG0024902*
scaffold74	828217	C	G	Och01,Och02,Och03,Och04,Och05,Och06,Och07,Och08,Och09,Och10,Och11,Och12,Och13,Och14,Ore01,Ore02,Ore03,Ore04,Ore05,Ore06,Ore07,Ore08,Ore09,Ore10,Ore11,Ore12,Ore13,Ore14	*OreG0024906*
scaffold74	836487	A	T	Och11	*OreG0024907*
scaffold74	840103	G	T	NA	*OreG0024908*

scaffold74	841520	C	T	Och01,Och02,Och03,Och04,Och05,Och06,Och07,Och08,Och09,Och10,Och11,Och12,Och13,Och14,Ore01,Ore02,Ore03,Ore04,Ore05,Ore06,Ore07,Ore08,Ore09,Ore10,Ore11,Ore12,Ore13,Ore14	*OreG0024908*
scaffold74	852325	G	A	Ore01,Ore02,Ore03,Ore04,Ore05,Ore06,Ore07,Ore08,Ore09,Ore10,Ore11,Ore12,Ore13,Ore14	*OreG0024910*
scaffold74	852330	G	A	Och01	*OreG0024910*
scaffold74	870913	T	G	Ore01,Ore04,Ore05,Ore11	*OreG0024913*
scaffold74	875278	T	A	NA	*OreG0024913*
scaffold74	888186	A	G	Och01,Och02,Och03,Och04,Och05,Och06,Och07,Och08,Och09,Och10,Och11,Och12,Och13,Och14,Ore01,Ore02,Ore03,Ore04,Ore05,Ore06,Ore07,Ore08,Ore09,Ore10,Ore11,Ore12,Ore13,Ore14	*OreG0024916*
scaffold74	888207	G	T	NA	*OreG0024916*
scaffold74	892676	G	A	Ore01,Ore02,Ore03,Ore04,Ore05,Ore06,Ore07,Ore08,Ore09,Ore10,Ore11,Ore12,Ore13,Ore14	*OreG0024918*
scaffold74	892706	C	T	NA	*OreG0024918*
scaffold74	914956	A	G	NA	*OreG0024921*
scaffold74	915229	C	T	NA	*OreG0024921*
scaffold74	922882	A	G	NA	*OreG0024923*
scaffold74	923129	C	T	Och01,Och04,Och12,Och13	*OreG0024923*
scaffold74	923131	G	A	Och03,Och09,Och10	*OreG0024923*
scaffold74	923468	C	A	Och01,Och04,Och12,Och13	*OreG0024924*
scaffold74	923531	G	A	NA	*OreG0024924*
scaffold74	923605	C	G	NA	*OreG0024924*
scaffold74	923609	A	T	NA	*OreG0024924*
scaffold74	923648	G	A	Och01,Och04,Och12,Och13	*OreG0024924*
scaffold74	927497	C	T	Ore03,Ore06,Ore07,Ore08,Ore10,Ore12,Ore13,Ore14	*OreG0024925*
scaffold74	927550	A	G	Ore03,Ore06,Ore07,Ore08,Ore10,Ore12,Ore13,Ore14	*OreG0024925*
scaffold74	927608	T	A	Ore03,Ore06,Ore07,Ore08,Ore10,Ore12,Ore13,Ore14	*OreG0024925*
scaffold74	927892	A	T	Ore03,Ore06,Ore07,Ore08,Ore10,Ore12,Ore13,Ore14	*OreG0024926*
scaffold74	927910	G	A	Och01,Och02,Och03,Och04,Och05,Och06,Och07,Och08,Och09,Och10,Och11,Och12,Och13,Och14	*OreG0024926*
scaffold74	927961	G	A	Och01,Och02,Och03,Och04,Och05,Och06,Och07,Och08,Och09,Och10,Och11,Och12,Och13,Och14,Ore03,Ore06,Ore07,Ore08,Ore10,Ore12,Ore13,Ore14	*OreG0024926*
scaffold74	951130	C	G	NA	*OreG0024929*
scaffold74	951160	T	G	NA	*OreG0024929*
scaffold74	951688	A	G	Och08	*OreG0024929*
scaffold74	952012	A	G	Ore01,Ore02,Ore03,Ore04,Ore05,Ore06,Ore07,Ore08,Ore09,Ore10,Ore11,Ore12,Ore13,Ore14	*OreG0024929*
scaffold74	1008535	C	T	NA	*OreG0024934*
scaffold74	1019330	G	T	Ore01,Ore02,Ore03,Ore04,Ore05,Ore06,Ore07,Ore08,Ore09,Ore10,Ore11,Ore12,Ore13,Ore14	*OreG0024935*
scaffold74	1019346	G	C	Ore01,Ore02,Ore04,Ore05,Ore06,Ore07,Ore08,Ore09,Ore10,Ore11,Ore12,Ore13,Ore14	*OreG0024935*
scaffold74	1019514	C	T	Och01,Och02,Och03,Och04,Och05,Och06,Och07,Och08,Och09,Och10,Och11,Och12,Och13,Och14,Ore01,Ore02,Ore03,Ore04,Ore05,Ore06,Ore07,Ore08,Ore09,Ore10,Ore11,Ore12,Ore13,Ore14	*OreG0024935*
scaffold74	1019642	C	T	NA	*OreG0024935*
scaffold74	1020023	C	A	NA	*OreG0024935*
scaffold74	1020593	T	A	NA	*OreG0024935*
scaffold74	1021023	C	A	NA	*OreG0024935*
scaffold74	1051887	C	T	NA	*OreG0024937*
scaffold74	1113499	C	T	Ore01,Ore02,Ore03,Ore04,Ore05,Ore06,Ore07,Ore08,Ore09,Ore10,Ore11,Ore12,Ore13,Ore14	*OreG0024941*
scaffold74	1113565	A	G	Ore01,Ore02,Ore03,Ore04,Ore05,Ore06,Ore07,Ore08,Ore09,Ore10,Ore11,Ore12,Ore13,Ore14	*OreG0024941*
scaffold74	1113870	G	A	Och01,Och02,Och03,Och04,Och05,Och06,Och07,Och08,Och09,Och10,Och11,Och12,Och13,Och14	*OreG0024941*
scaffold74	1113997	G	T	Och01,Och02,Och03,Och04,Och05,Och06,Och07,Och08,Och09,Och10,Och11,Och12,Och13,Och14	*OreG0024941*
scaffold74	1114927	C	T	Och01,Och02,Och03,Och04,Och05,Och06,Och07,Och08,Och09,Och10,Och11,Och12,Och13,Och14	*OreG0024941*
scaffold74	1115958	T	A	Och03	*OreG0024942*
scaffold74	1116877	A	G	Och01,Och02,Och03,Och04,Och05,Och06,Och07,Och08,Och09,Och10,Och11,Och12,Och13,Och14,Ore01,Ore02,Ore03,Ore04,Ore05,Ore06,Ore07,Ore08,Ore09,Ore10,Ore11,Ore12,Ore13,Ore14	*OreG0024942*
scaffold74	1116925	G	T	Och01,Och02,Och03,Och04,Och05,Och06,Och07,Och08,Och09,Och10,Och11,Och12,Och13,Och14,Ore01,Ore02,Ore03,Ore04,Ore05,Ore06,Ore07,Ore08,Ore09,Ore10,Ore11,Ore12,Ore13,Ore14	*OreG0024942*
scaffold74	1116985	A	G	Och01,Och02,Och03,Och04,Och05,Och06,Och07,Och08,Och09,Och10,Och11,Och12,Och13,Och14,Ore01,Ore02,Ore03,Ore04,Ore05,Ore06,Ore07,Ore08,Ore09,Ore10,Ore11,Ore12,Ore13,Ore14	*OreG0024942*
scaffold74	1118143	T	C	NA	*OreG0024943*
scaffold74	1118410	A	C	NA	*OreG0024943*
scaffold74	1119134	C	A	Och01,Och02,Och03,Och04,Och05,Och06,Och07,Och08,Och09,Och10,Och11,Och12,Och13,Och14	*OreG0024943*

scaffold74	1119812	G	A	Ore01,Ore02,Ore03,Ore04,Ore05,Ore06,Ore07,Ore08,Ore09,Ore10,Ore11,Ore12,Ore13,Ore14	*OreG0024943*
scaffold74	1126359	G	A	Och01,Och02,Och03,Och04,Och05,Och06,Och07,Och08,Och09,Och10,Och11,Och12,Och13,Och14	*OreG0024943*
scaffold74	1126428	C	A	Ore01,Ore04,Ore05,Ore11	*OreG0024943*
scaffold74	1154937	A	T	NA	*OreG0024944*
scaffold74	1156469	G	C	NA	*OreG0024944*
scaffold74	1177337	G	A	NA	*OreG0024947*
scaffold74	1183776	G	T	Och08	*OreG0024948*
scaffold74	1233037	A	T	NA	*OreG0024954*
scaffold74	1233152	G	T	NA	*OreG0024954*
scaffold74	1233159	C	G	NA	*OreG0024954*
scaffold74	1233766	C	A	NA	*OreG0024954*
scaffold74	1241874	C	T	NA	*OreG0024955*
scaffold74	1256944	T	C	Och02,Och03,Och04,Och05,Och06,Och08,Och09,Och10,Och11,Och12,Och13,Och14	*OreG0024958*
scaffold74	1260885	G	T	Och01,Och02,Och03,Och04,Och05,Och06,Och07,Och08,Och09,Och10,Och11,Och12,Och13,Och14	*OreG0024959*
scaffold74	1268105	A	G	Ore03,Ore06,Ore07,Ore08,Ore10,Ore12,Ore13,Ore14	*OreG0024962*
scaffold74	1268336	T	A	Ore01,Ore02,Ore03,Ore04,Ore05,Ore06,Ore07,Ore08,Ore09,Ore10,Ore11,Ore12,Ore13,Ore14	*OreG0024962*
scaffold74	1320251	C	A	NA	*OreG0024966*
scaffold74	1379027	T	A	Och01,Och02,Och03,Och04,Och05,Och06,Och07,Och08,Och09,Och10,Och11,Och12,Och13,Och14,Ore01,Ore02,Ore03,Ore04,Ore05,Ore06,Ore07,Ore08,Ore09,Ore10,Ore11,Ore12,Ore13,Ore14	*OreG0024971*
scaffold74	1408438	G	A	NA	*OreG0024974*
scaffold74	1426746	T	C	Och12,Och13	*OreG0024975*
scaffold74	1427006	G	T	NA	*OreG0024975*
scaffold74	1427439	T	C	Och01,Och02,Och03,Och04,Och05,Och06,Och07,Och08,Och09,Och10,Och11,Och12,Och13,Och14,Ore01,Ore02,Ore03,Ore04,Ore05,Ore06,Ore07,Ore08,Ore09,Ore10,Ore11,Ore12,Ore13,Ore14	*OreG0024975*
scaffold74	1431579	T	C	Ore03,Ore06,Ore07,Ore08,Ore10,Ore12,Ore13,Ore14	*OreG0024976*
scaffold74	1431772	A	C	Och03,Och04,Och05,Och11,Och12,Och13	*OreG0024976*
scaffold74	1440751	C	T	Och01,Och02,Och03,Och04,Och05,Och06,Och07,Och08,Och09,Och10,Och11,Och12,Och13	*OreG0024978*
scaffold74	1440774	C	T	Och14	*OreG0024978*
scaffold74	1465247	T	G	Och12,Och13	*OreG0024981*
scaffold74	1539452	T	C	NA	*OreG0024985*
scaffold74	1554005	G	T	Och02,Och03,Och04,Och05,Och06,Och07,Och08,Och09,Och10,Och11,Och12,Och13,Och14	*OreG0024988*
scaffold74	1557238	T	A	Ore01,Ore04,Ore05,Ore11	*OreG0024988*
scaffold74	1564758	A	T	NA	*OreG0024989*
scaffold74	1663603	C	T	Och01	*OreG0024996*
scaffold74	1663654	C	T	Och02,Och03,Och04,Och05,Och06,Och12,Och13	*OreG0024996*
scaffold2890	10479	C	T	Och01,Och02,Och03,Och04,Och05,Och06,Och07,Och08,Och09,Och10,Och11,Och12,Och13,Och14,Ore01,Ore02,Ore03,Ore04,Ore05,Ore06,Ore07,Ore08,Ore09,Ore10,Ore11,Ore12,Ore13,Ore14	*OreG0008310*
scaffold2890	10527	C	G	Och01,Och02,Och03,Och04,Och05,Och06,Och07,Och08,Och09,Och10,Och11,Och12,Och13,Och14,Ore01,Ore02,Ore03,Ore04,Ore05,Ore06,Ore07,Ore08,Ore09,Ore10,Ore11,Ore12,Ore13,Ore14	*OreG0008310*
scaffold2890	13971	C	T	Ore01,Ore02,Ore03,Ore04,Ore05,Ore06,Ore07,Ore08,Ore09,Ore10,Ore11,Ore12,Ore13,Ore14	*OreG0008310*
scaffold2890	16677	G	A	Ore01,Ore02,Ore03,Ore04,Ore05,Ore06,Ore07,Ore08,Ore09,Ore10,Ore11,Ore12,Ore13,Ore14	*OreG0008311*
scaffold2213	108253	T	G	Och01,Och02,Och03,Och04,Och05,Och06,Och07,Och08,Och09,Och10,Och11,Och12,Och13,Och14	*OreG0006256*
scaffold2213	108667	C	A	Ore01,Ore02,Ore03,Ore04,Ore05,Ore06,Ore07,Ore08,Ore09,Ore10,Ore11,Ore12,Ore13,Ore14	*OreG0006256*
scaffold2213	108954	C	T	NA	*OreG0006256*
scaffold2213	109072	A	T	Ore08,Ore09	*OreG0006256*
scaffold2213	109084	C	T	NA	*OreG0006256*
scaffold2213	109738	G	C	NA	*OreG0006256*
scaffold2213	112323	G	A	NA	*OreG0006256*
scaffold2213	114239	C	T	NA	*OreG0006256*
scaffold2213	123410	G	A	NA	*OreG0006256*
scaffold2213	123721	A	G	NA	*OreG0006256*
scaffold2213	123928	G	A	NA	*OreG0006256*
scaffold2213	136500	G	A	Ore01,Ore02,Ore03,Ore04,Ore05,Ore06,Ore07,Ore08,Ore09,Ore10,Ore11,Ore12,Ore13,Ore14	*OreG0006257*
scaffold2213	236035	G	C	NA	*OreG0006258*
scaffold2213	236117	A	T	Ore01,Ore02,Ore03,Ore04,Ore05,Ore06,Ore07,Ore08,Ore09,Ore10,Ore11,Ore12,Ore13,Ore14	*OreG0006258*
scaffold2213	282527	C	T	Och01,Och02,Och03,Och04,Och05,Och06,Och07,Och08,Och09,Och10,Och11,Och12,Och13,Och14	*OreG0006259*

scaffold2213	501917	A	G	Ore01,Ore02,Ore03,Ore04,Ore05,Ore06,Ore07,Ore08,Ore09,Ore10,Ore11,Ore12,Ore13,Ore14	*OreG0006260*
scaffold2213	502096	A	T	Och03,Och05,Och12,Och13	*OreG0006260*
scaffold2213	502126	A	T	NA	*OreG0006260*
scaffold2213	502458	G	T	Och01,Och02,Och03,Och04,Och05,Och06,Och07,Och08,Och09,Och10,Och11,Och12,Och13,Och14	*OreG0006260*
scaffold2213	502483	G	A	Och08	*OreG0006260*
scaffold2213	646757	G	A	Och01,Och02,Och03,Och04,Och05,Och06,Och07,Och08,Och09,Och10,Och11,Och12,Och13,Och14	*OreG0006266*
scaffold2213	660649	G	A	Och01,Och02,Och03,Och04,Och05,Och06,Och07,Och08,Och09,Och10,Och11,Och12,Och13,Och14,Ore01,Ore02,Ore03,Ore04,Ore05,Ore06,Ore07,Ore08,Ore09,Ore10,Ore11,Ore12,Ore13,Ore14	*OreG0006267*
scaffold2213	674215	C	A	Ore01,Ore02,Ore03,Ore04,Ore05,Ore06,Ore07,Ore08,Ore09,Ore10,Ore11,Ore12,Ore13,Ore14	*OreG0006268*
scaffold2213	704760	G	T	NA	*OreG0006271*
scaffold2213	707238	G	A	Ore01,Ore02,Ore03,Ore04,Ore05,Ore06,Ore07,Ore08,Ore09,Ore10,Ore11,Ore12,Ore13,Ore14	*OreG0006272*
scaffold2213	719489	G	T	NA	*OreG0006274*
scaffold2213	731245	G	A	Ore01,Ore02,Ore03,Ore04,Ore05,Ore06,Ore07,Ore08,Ore09,Ore10,Ore11,Ore12,Ore13,Ore14	*OreG0006275*
scaffold2213	733035	C	G	NA	*OreG0006275*
scaffold2213	821469	G	A	NA	*OreG0006282*
scaffold2213	840223	C	A	Och01,Och02,Och03,Och04,Och05,Och06,Och07,Och08,Och09,Och10,Och11,Och12,Och13,Och14,Ore01,Ore02,Ore03,Ore04,Ore05,Ore06,Ore07,Ore08,Ore09,Ore10,Ore11,Ore12,Ore13,Ore14	*OreG0006284*
scaffold2213	849529	G	A	Och12	*OreG0006285*
scaffold2193	76496	A	G	NA	*OreG0006208*
scaffold2193	89973	C	T	NA	*OreG0006209*
scaffold2193	96517	T	G	NA	*OreG0006210*
scaffold2193	98277	A	T	Ore01,Ore02,Ore03,Ore04,Ore05,Ore06,Ore07,Ore08,Ore09,Ore10,Ore11,Ore12,Ore13,Ore14	*OreG0006211*
scaffold2193	98569	G	A	Ore01,Ore02,Ore03,Ore04,Ore05,Ore06,Ore07,Ore08,Ore09,Ore10,Ore11,Ore12,Ore13,Ore14	*OreG0006211*
scaffold2193	98589	T	C	Ore07,Ore08,Ore14	*OreG0006211*
scaffold2193	207915	C	T	NA	*OreG0006218*
scaffold2193	207942	T	C	NA	*OreG0006218*
scaffold2193	207954	A	G	NA	*OreG0006218*
scaffold2193	208684	A	G	NA	*OreG0006218*
scaffold2193	239876	C	T	NA	*OreG0006224*
scaffold2173	9078	C	A	Ore02,Ore03,Ore06,Ore07,Ore09,Ore10,Ore11,Ore12,Ore13,Ore14	*OreG0006056*
scaffold2173	9984	A	T	Och01,Och09,Och10,Och11,Och12,Och13	*OreG0006056*
scaffold2173	23347	C	T	Ore01,Ore02,Ore03,Ore04,Ore05,Ore06,Ore07,Ore08,Ore09,Ore10,Ore11,Ore12,Ore13,Ore14	*OreG0006059*
scaffold2173	23633	G	T	Och11	*OreG0006059*
scaffold2173	23691	C	G	Och11	*OreG0006059*
scaffold2173	29180	G	A	Ore02,Ore03,Ore06,Ore07,Ore09,Ore10,Ore11,Ore12,Ore13,Ore14	*OreG0006060*
scaffold2173	49995	C	A	Och01,Och09,Och10,Och12,Och13,Och14	*OreG0006063*
scaffold2173	68170	T	A	Och01,Och02,Och03,Och04,Och05,Och06,Och07,Och08,Och09,Och10,Och11,Och12,Och13,Och14,Ore01,Ore02,Ore03,Ore04,Ore05,Ore06,Ore07,Ore08,Ore09,Ore10,Ore11,Ore12,Ore13,Ore14	*OreG0006067*
scaffold2173	70628	G	T	Och01,Och02,Och03,Och04,Och05,Och06,Och07,Och08,Och09,Och10,Och11,Och12,Och13,Och14,Ore01,Ore02,Ore03,Ore04,Ore05,Ore06,Ore07,Ore08,Ore09,Ore10,Ore11,Ore12,Ore13,Ore14	*OreG0006067*
scaffold2173	104910	G	A	Ore01,Ore02,Ore03,Ore04,Ore05,Ore06,Ore07,Ore08,Ore09,Ore10,Ore11,Ore12,Ore13,Ore14	*OreG0006069*
scaffold2173	112487	A	G	Och01,Och02,Och03,Och04,Och05,Och06,Och07,Och08,Och09,Och10,Och11,Och12,Och13,Och14,Ore01,Ore02,Ore03,Ore04,Ore05,Ore06,Ore07,Ore08,Ore09,Ore10,Ore11,Ore12,Ore13,Ore14	*OreG0006070*
scaffold2173	112635	T	A	Och01,Och02,Och03,Och04,Och05,Och06,Och07,Och08,Och09,Och10,Och11,Och12,Och13,Och14,Ore01,Ore02,Ore03,Ore04,Ore05,Ore06,Ore07,Ore08,Ore09,Ore10,Ore11,Ore12,Ore13,Ore14	*OreG0006070*
scaffold2173	113020	A	G	NA	*OreG0006070*
scaffold2173	142891	C	G	Och01,Och02,Och03,Och04,Och05,Och06,Och07,Och08,Och09,Och10,Och11,Och12,Och13,Och14,Ore01,Ore02,Ore03,Ore04,Ore05,Ore06,Ore07,Ore08,Ore09,Ore10,Ore11,Ore12,Ore13,Ore14	*OreG0006074*
scaffold2173	150764	A	G	Och08	*OreG0006075*
scaffold2173	161427	G	A	NA	*OreG0006077*
scaffold2173	161514	T	A	NA	*OreG0006077*
scaffold2173	161662	G	A	NA	*OreG0006077*
scaffold2173	161792	C	T	NA	*OreG0006077*
scaffold2173	161820	C	T	NA	*OreG0006077*
scaffold2173	161828	C	T	Ore01,Ore02,Ore03,Ore04,Ore05,Ore06,Ore07,Ore08,Ore09,Ore10,Ore11,Ore12,Ore13,Ore14	*OreG0006077*
scaffold2173	164592	C	T	NA	*OreG0006077*
scaffold2173	164728	A	C	NA	*OreG0006077*

scaffold2173	164925	G	C	NA	*OreG0006077*
scaffold2173	165263	G	T	NA	*OreG0006077*
scaffold2173	165451	A	G	NA	*OreG0006077*
scaffold2173	166055	C	A	NA	*OreG0006077*
scaffold2173	166789	G	T	NA	*OreG0006077*
scaffold2173	167294	C	T	NA	*OreG0006077*
scaffold2173	167306	C	T	NA	*OreG0006077*
scaffold2173	167312	G	C	NA	*OreG0006077*
scaffold2173	184825	C	T	NA	*OreG0006079*
scaffold2173	184914	A	G	NA	*OreG0006079*
scaffold2173	187358	A	G	NA	*OreG0006079*
scaffold2173	188010	T	G	Och03,Och06,Ore01,Ore02,Ore03,Ore04,Ore05,Ore06,Ore07,Ore08,Ore09,Ore10,Ore11,Ore12,Ore13,Ore14	*OreG0006079*
scaffold2173	188016	C	A	Och03,Och06,Ore01,Ore02,Ore03,Ore04,Ore05,Ore06,Ore07,Ore08,Ore09,Ore10,Ore11,Ore12,Ore13,Ore14	*OreG0006079*
scaffold2173	189501	A	G	Ore02,Ore03,Ore06,Ore07,Ore09,Ore10,Ore11,Ore12,Ore13,Ore14	*OreG0006079*
scaffold2173	192014	T	C	NA	*OreG0006079*
scaffold2173	193124	T	G	Och01,Och09,Och10,Och12,Och13,Och14	*OreG0006079*
scaffold2173	195307	T	A	NA	*OreG0006079*
scaffold2173	196211	A	G	Och01,Och09,Och10,Och12,Och13,Och14	*OreG0006079*
scaffold2173	196236	T	A	Och01,Och09,Och10,Och12,Och13	*OreG0006079*
scaffold2173	205895	T	G	Och09,Och10,Och12,Och13	*OreG0006081*
scaffold2173	206061	A	G	NA	*OreG0006081*
scaffold2173	209030	C	A	NA	*OreG0006082*
scaffold2173	224230	T	A	NA	*OreG0006085*
scaffold2173	224297	C	T	Och01,Och02,Och03,Och04,Och05,Och06,Och07,Och08,Och09,Och10,Och11,Och12,Och13,Ore01,Ore02,Ore03,Ore04,Ore05,Ore06,Ore07,Ore08,Ore09,Ore10,Ore11,Ore12,Ore13,Ore14	*OreG0006085*
scaffold2173	261423	A	C	NA	*OreG0006089*
scaffold2173	261908	T	A	Och14	*OreG0006089*
scaffold2173	262238	G	A	Och09,Och10,Ore02,Ore03,Ore06,Ore07,Ore09,Ore10,Ore11,Ore12,Ore13,Ore14	*OreG0006089*
scaffold2173	263596	G	A	Och01,Och02,Och03,Och04,Och05,Och06,Och08,Och09,Och10,Och11,Och12,Och13,Ore01,Ore02,Ore03,Ore04,Ore05,Ore06,Ore07,Ore08,Ore09,Ore10,Ore11,Ore12,Ore13,Ore14	*OreG0006090*
scaffold2173	263908	T	C	Och01,Och05,Och08,Och12,Och13	*OreG0006090*
scaffold2173	265157	T	A	NA	*OreG0006090*
scaffold2173	265950	T	C	NA	*OreG0006090*
scaffold2173	266297	A	T	Och01,Och02,Och03,Och04,Och05,Och06,Och07,Och08,Och09,Och10,Och11,Och12,Och13,Och14,Ore01,Ore02,Ore03,Ore04,Ore05,Ore06,Ore07,Ore08,Ore09,Ore10,Ore11,Ore12,Ore13,Ore14	*OreG0006090*
scaffold2173	272102	A	T	NA	*OreG0006091*
scaffold2173	272114	G	T	NA	*OreG0006091*
scaffold2173	272731	C	T	NA	*OreG0006091*
scaffold2173	273219	G	A	NA	*OreG0006091*
scaffold2173	273574	C	A	NA	*OreG0006091*
scaffold2173	273589	G	T	NA	*OreG0006091*
scaffold2173	274086	C	T	NA	*OreG0006091*
scaffold2173	275718	T	G	NA	*OreG0006091*
scaffold2173	275789	C	A	NA	*OreG0006091*
scaffold2173	275982	A	G	NA	*OreG0006091*
scaffold2173	275994	A	G	NA	*OreG0006091*
scaffold2173	276091	C	G	NA	*OreG0006091*
scaffold2173	276450	A	G	NA	*OreG0006091*
scaffold2173	276470	G	T	NA	*OreG0006091*
scaffold2173	276537	A	G	NA	*OreG0006091*
scaffold2173	276660	G	T	NA	*OreG0006091*
scaffold2173	276707	C	G	NA	*OreG0006091*
scaffold2173	276724	A	T	NA	*OreG0006091*
scaffold2173	276922	T	C	NA	*OreG0006091*
scaffold2173	277191	G	A	NA	*OreG0006091*
scaffold2173	277233	A	G	NA	*OreG0006091*
scaffold2173	282806	G	A	Och09,Och10,Och11	*OreG0006092*
scaffold2173	283411	C	T	NA	*OreG0006092*
scaffold2173	283501	G	A	NA	*OreG0006092*
scaffold2173	283505	C	T	NA	*OreG0006092*
scaffold2173	283555	C	A	NA	*OreG0006092*
scaffold2173	283999	A	T	NA	*OreG0006092*
scaffold2173	284018	T	C	NA	*OreG0006092*
scaffold2173	284035	A	G	NA	*OreG0006092*
scaffold2173	284050	A	G	NA	*OreG0006092*
scaffold2173	284406	A	G	Och09,Och10	*OreG0006092*
scaffold2173	284416	C	A	Ore02,Ore03,Ore06,Ore07,Ore09,Ore10,Ore11,Ore12,Ore13,Ore14	*OreG0006092*
scaffold2173	284496	A	C	Och01,Och07,Ore02,Ore03,Ore06,Ore07,Ore09,Ore10,Ore11,Ore12,Ore13,Ore14	*OreG0006092*
scaffold2173	284690	C	T	NA	*OreG0006092*

scaffold2173	284731	T	C	Och02,Och04,Och06,Och12,Och13	*OreG0006092*
scaffold2173	285524	A	T	Ore02,Ore03,Ore06,Ore07,Ore09,Ore10,Ore11,Ore12,Ore13,Ore14	*OreG0006092*
scaffold2173	331632	G	A	NA	*OreG0006095*
scaffold2173	331969	G	T	Och06	*OreG0006095*
scaffold2173	335527	C	G	Ore02,Ore03,Ore06,Ore07,Ore09,Ore10,Ore11,Ore12,Ore13,Ore14	*OreG0006095*
scaffold2173	336209	A	G	NA	*OreG0006095*
scaffold2173	336956	C	G	NA	*OreG0006096*
scaffold2173	378710	G	T	NA	*OreG0006100*
scaffold2173	381505	G	A	NA	*OreG0006101*
scaffold2173	390242	A	G	NA	*OreG0006102*
scaffold2173	413636	C	T	NA	*OreG0006104*
scaffold2173	413648	G	A	NA	*OreG0006104*
scaffold2173	413687	G	A	NA	*OreG0006104*
scaffold2173	414209	G	A	Ore01,Ore02,Ore03,Ore04,Ore05,Ore06,Ore07,Ore08,Ore09,Ore10,Ore11,Ore12,Ore13,Ore14	*OreG0006104*
scaffold2173	431120	A	C	Och01,Och03,Och07,Och09,Och10,Och14	*OreG0006106*
scaffold2173	431288	C	A	Och01,Och03,Och07,Och09,Och10,Och14	*OreG0006106*
scaffold2173	431291	T	A	Ore01,Ore02,Ore03,Ore04,Ore05,Ore06,Ore07,Ore08,Ore09,Ore10,Ore11,Ore12,Ore13,Ore14	*OreG0006106*
scaffold2173	431347	A	T	Ore01,Ore02,Ore03,Ore04,Ore05,Ore06,Ore07,Ore08,Ore09,Ore10,Ore11,Ore12,Ore13,Ore14	*OreG0006106*
scaffold2173	431351	G	A	Och01,Och03,Och07,Och09,Och10,Och14	*OreG0006106*
scaffold2173	431358	G	A	Och01,Och03,Och07,Och09,Och10,Och14	*OreG0006106*
scaffold2173	431499	C	T	Och01,Och03,Och07,Och09,Och10,Och14	*OreG0006107*
scaffold2173	431504	G	A	Ore01,Ore02,Ore03,Ore04,Ore05,Ore06,Ore07,Ore08,Ore09,Ore10,Ore11,Ore12,Ore13,Ore14	*OreG0006107*
scaffold2173	431561	A	T	NA	*OreG0006107*
scaffold2173	431694	G	A	Och01,Och03,Och07,Och09,Och10,Och14,Ore02,Ore06,Ore07,Ore11,Ore12,Ore13,Ore14	*OreG0006107*
scaffold2173	431696	C	T	Ore02,Ore06,Ore07,Ore11,Ore12,Ore13,Ore14	*OreG0006107*
scaffold2173	431711	A	G	Och02,Och04,Och12,Och13	*OreG0006107*
scaffold2173	431775	T	C	NA	*OreG0006107*
scaffold2173	431822	T	C	NA	*OreG0006107*
scaffold2173	436600	C	T	Ore02,Ore06,Ore07,Ore11,Ore12,Ore13,Ore14	*OreG0006108*
scaffold2173	442788	A	G	NA	*OreG0006109*
scaffold2173	443212	T	G	Och01,Och02,Och04,Och06,Och07,Och12,Och13	*OreG0006109*
scaffold2173	443280	C	T	Och01,Och02,Och04,Och06,Och07,Och12,Och13	*OreG0006109*
scaffold2173	444041	C	G	Och01	*OreG0006109*
scaffold2173	445122	T	G	Och01,Och02,Och03,Och04,Och05,Och06,Och07,Och08,Och09,Och10,Och11,Och12,Och13,Och14,Ore01,Ore02,Ore03,Ore04,Ore05,Ore06,Ore07,Ore08,Ore09,Ore10,Ore11,Ore12,Ore13,Ore14	*OreG0006109*
scaffold2173	454998	C	A	Och01	*OreG0006112*
scaffold2173	455853	C	T	Och01	*OreG0006112*
scaffold2173	468061	A	C	Och01,Och02,Och03,Och04,Och05,Och06,Och07,Och08,Och09,Och10,Och11,Och12,Och13,Och14	*OreG0006113*
scaffold2173	494159	C	T	NA	*OreG0006116*
scaffold2173	559956	C	A	Och01,Och02,Och03,Och04,Och05,Och06,Och07,Och08,Och09,Och10,Och11,Och12,Och13,Och14	*OreG0006124*
scaffold2173	559958	G	T	Och01,Och02,Och03,Och04,Och05,Och06,Och07,Och08,Och09,Och10,Och11,Och12,Och13,Och14	*OreG0006124*
scaffold2173	560686	G	A	Och02,Och04,Och06,Och12,Och13	*OreG0006124*
scaffold2173	562755	C	A	Och01,Och02,Och03,Och04,Och05,Och06,Och07,Och08,Och09,Och10,Och11,Och12,Och13,Och14,Ore01,Ore02,Ore03,Ore04,Ore05,Ore06,Ore07,Ore08,Ore09,Ore10,Ore11,Ore12,Ore13,Ore14	*OreG0006124*
scaffold2173	562821	T	C	Ore01,Ore02,Ore03,Ore04,Ore05,Ore06,Ore07,Ore08,Ore09,Ore10,Ore11,Ore12,Ore13,Ore14	*OreG0006124*
scaffold2173	580959	G	A	Och01,Och02,Och03,Och04,Och05,Och06,Och07,Och08,Och09,Och10,Och11,Och12,Och13,Och14	*OreG0006126*
scaffold2173	582859	T	A	NA	*OreG0006126*
scaffold2173	593099	A	T	Och01,Och02,Och03,Och04,Och05,Och06,Och07,Och08,Och09,Och10,Och11,Och12,Och13,Och14	*OreG0006127*
scaffold2173	594535	C	T	Ore01,Ore02,Ore03,Ore04,Ore05,Ore06,Ore07,Ore08,Ore09,Ore10,Ore11,Ore12,Ore13,Ore14	*OreG0006127*
scaffold2173	594676	C	T	Och01,Och02,Och03,Och04,Och05,Och06,Och07,Och08,Och09,Och10,Och11,Och12,Och13,Och14	*OreG0006127*
scaffold2173	596306	G	T	Ore01,Ore02,Ore03,Ore04,Ore05,Ore06,Ore07,Ore08,Ore09,Ore10,Ore11,Ore12,Ore13,Ore14	*OreG0006127*
scaffold2173	620508	A	T	NA	*OreG0006129*
scaffold2173	620513	G	A	NA	*OreG0006129*
scaffold2173	628307	A	G	Ore02,Ore03,Ore06,Ore07,Ore09,Ore10,Ore11,Ore12,Ore13,Ore14	*OreG0006130*
scaffold2760	23356	T	A	NA	*OreG0008284*
scaffold2760	24050	G	T	NA	*OreG0008284*
scaffold2760	24193	G	A	NA	*OreG0008284*
scaffold2760	24255	T	A	NA	*OreG0008284*
scaffold2760	24276	A	G	NA	*OreG0008284*

scaffold2058	7197	C	T	Och01	*OreG0005153*
scaffold2058	7227	A	G	Och01	*OreG0005153*
scaffold2058	7603	C	G	NA	*OreG0005153*
scaffold2058	95948	C	T	Och01,Och02,Och03,Och04,Och05,Och06,Och07,Och08,Och09,Och10,Och11,Och12,Och13,Och14	*OreG0005156*
scaffold2058	106783	T	A	NA	*OreG0005157*
scaffold2058	123758	A	T	NA	*OreG0005159*
scaffold2058	130640	G	A	Och05,Och07,Och13	*OreG0005161*
scaffold2058	130860	A	G	NA	*OreG0005161*
scaffold2058	130896	A	T	NA	*OreG0005161*
scaffold2058	131826	A	C	NA	*OreG0005161*
scaffold2058	132869	T	C	NA	*OreG0005161*
scaffold2058	156789	G	A	NA	*OreG0005163*
scaffold2058	157475	T	G	Ore01,Ore02,Ore03,Ore04,Ore05,Ore06,Ore07,Ore08,Ore09,Ore10,Ore11,Ore12,Ore13,Ore14	*OreG0005163*
scaffold2058	162958	T	A	Ore06,Ore08	*OreG0005164*
scaffold2058	162993	C	T	Och01,Och02,Och03,Och04,Och05,Och06,Och07,Och08,Och09,Och10,Och11,Och12,Och13,Och14,Ore01,Ore02,Ore03,Ore04,Ore05,Ore06,Ore07,Ore08,Ore09,Ore10,Ore11,Ore12,Ore13,Ore14	*OreG0005164*
scaffold2058	164144	C	T	Ore01,Ore02,Ore06,Ore07,Ore08,Ore10,Ore11,Ore12,Ore13,Ore14	*OreG0005164*
scaffold2058	174805	C	A	Ore06,Ore08	*OreG0005166*
scaffold2058	184272	C	T	Och01,Och02,Och03,Och04,Och05,Och06,Och07,Och08,Och09,Och10,Och11,Och12,Och13,Och14	*OreG0005168*
scaffold2058	184970	G	A	Och02,Och08	*OreG0005168*
scaffold2058	185673	C	T	Ore06,Ore08	*OreG0005168*
scaffold2058	191164	G	A	NA	*OreG0005169*
scaffold2058	191251	G	T	Och01,Och02,Och03,Och04,Och05,Och06,Och07,Och08,Och09,Och10,Och11,Och12,Och13,Och14	*OreG0005169*
scaffold2058	191330	A	G	Och01,Och02,Och03,Och04,Och05,Och06,Och07,Och08,Och09,Och10,Och11,Och12,Och13,Och14	*OreG0005169*
scaffold2058	191581	G	C	Och05,Och07,Och09,Och10,Och13	*OreG0005170*
scaffold2058	191625	G	A	Ore01,Ore02,Ore03,Ore04,Ore05,Ore06,Ore07,Ore08,Ore09,Ore10,Ore11,Ore12,Ore13,Ore14	*OreG0005170*
scaffold2058	192121	C	G	Och05,Och07,Och09,Och10,Och13	*OreG0005170*
scaffold2058	192272	G	A	Ore01,Ore02,Ore03,Ore04,Ore05,Ore06,Ore07,Ore08,Ore09,Ore10,Ore11,Ore12,Ore13,Ore14	*OreG0005170*
scaffold2058	192817	C	G	Ore01	*OreG0005170*
scaffold2058	215222	G	C	NA	*OreG0005174*
scaffold2058	215747	A	T	Ore01,Ore02,Ore03,Ore04,Ore05,Ore06,Ore07,Ore08,Ore09,Ore10,Ore11,Ore12,Ore13,Ore14	*OreG0005174*
scaffold2058	217761	G	C	Och05,Och08,Och09,Och10,Och11,Och13,Och14	*OreG0005174*
scaffold2058	219629	G	T	NA	*OreG0005174*
scaffold2058	219970	G	C	NA	*OreG0005174*
scaffold2058	221070	G	A	NA	*OreG0005175*
scaffold2058	221521	G	A	Och09,Och10	*OreG0005175*
scaffold2058	233391	T	G	Och02,Och06	*OreG0005177*
scaffold2058	233856	C	A	Och02,Och06	*OreG0005177*
scaffold2058	234349	A	C	Och02,Och06	*OreG0005177*
scaffold2058	234700	A	C	Ore01,Ore02,Ore03,Ore04,Ore05,Ore06,Ore07,Ore08,Ore09,Ore10,Ore11,Ore12,Ore13,Ore14	*OreG0005177*
scaffold2058	281172	G	C	Och05,Och13	*OreG0005183*
scaffold2058	281589	T	A	Och02,Och06	*OreG0005183*
scaffold2058	282299	G	T	Ore01,Ore02,Ore03,Ore04,Ore05,Ore06,Ore07,Ore08,Ore09,Ore10,Ore11,Ore12,Ore13,Ore14	*OreG0005183*
scaffold2058	282482	C	T	Och05,Och13	*OreG0005183*
scaffold2058	282543	C	A	Ore01	*OreG0005183*
scaffold2058	283108	A	T	NA	*OreG0005183*
scaffold2058	294600	G	A	Ore01,Ore02,Ore03,Ore04,Ore05,Ore06,Ore07,Ore08,Ore09,Ore10,Ore11,Ore12,Ore13,Ore14	*OreG0005185*
scaffold2058	297838	C	A	Och07	*OreG0005185*
scaffold2058	310510	C	G	Och01,Och02,Och03,Och04,Och05,Och06,Och07,Och08,Och09,Och10,Och11,Och12,Och13,Och14,Ore01,Ore02,Ore03,Ore04,Ore05,Ore06,Ore07,Ore08,Ore09,Ore10,Ore11,Ore12,Ore13,Ore14	*OreG0005188*
scaffold2058	323574	A	G	Ore01,Ore02,Ore03,Ore04,Ore05,Ore06,Ore07,Ore08,Ore09,Ore10,Ore11,Ore12,Ore13,Ore14	*OreG0005190*
scaffold2058	336254	C	T	Ore01,Ore02,Ore03,Ore04,Ore05,Ore06,Ore07,Ore08,Ore09,Ore10,Ore11,Ore12,Ore13,Ore14	*OreG0005192*
scaffold2058	336309	T	A	Och02,Och03,Och04,Och05,Och06,Och08,Och12,Och13,Och14	*OreG0005192*
scaffold2058	336452	T	A	Och01,Och02,Och03,Och04,Och05,Och06,Och07,Och08,Och09,Och10,Och11,Och12,Och13,Och14,Ore01,Ore02,Ore03,Ore04,Ore05,Ore06,Ore07,Ore08,Ore09,Ore10,Ore11,Ore12,Ore13,Ore14	*OreG0005192*
scaffold2058	336512	G	C	Ore01,Ore02,Ore03,Ore04,Ore05,Ore06,Ore07,Ore08,Ore09,Ore10,Ore11,Ore12,Ore13,Ore14	*OreG0005192*
scaffold2058	350227	G	A	NA	*OreG0005194*

scaffold2058	362154	C	T	Ore01,Ore02,Ore03,Ore04,Ore05,Ore06,Ore07,Ore08,Ore09,Ore10,Ore11,Ore12,Ore13,Ore14	*OreG0005195*
scaffold2058	363613	C	A	NA	*OreG0005195*
scaffold2058	368823	G	C	NA	*OreG0005196*
scaffold2058	368886	G	A	NA	*OreG0005196*
scaffold2058	369004	G	C	Ore01,Ore02,Ore06,Ore07,Ore08,Ore10,Ore11,Ore12,Ore13,Ore14	*OreG0005196*
scaffold2058	378316	C	A	Och08,Och14	*OreG0005197*
scaffold2058	378426	G	T	Och09,Och10,Ore01,Ore02,Ore03,Ore04,Ore05,Ore06,Ore07,Ore08,Ore09,Ore10,Ore11,Ore12,Ore13,Ore14	*OreG0005197*
scaffold2058	380100	G	T	Och02,Och09,Och10	*OreG0005197*
scaffold2058	431973	G	C	Och01,Och02,Och03,Och04,Och05,Och06,Och07,Och08,Och09,Och10,Och11,Och12,Och13,Och14,Ore01,Ore02,Ore03,Ore04,Ore05,Ore06,Ore07,Ore08,Ore09,Ore10,Ore11,Ore12,Ore13,Ore14	*OreG0005202*
scaffold2058	435035	A	C	Ore01,Ore02,Ore03,Ore04,Ore05,Ore06,Ore07,Ore08,Ore09,Ore10,Ore11,Ore12,Ore13,Ore14	*OreG0005203*
scaffold2058	435146	G	A	NA	*OreG0005203*
scaffold2058	435207	A	G	Ore01,Ore02,Ore03,Ore04,Ore05,Ore06,Ore07,Ore08,Ore09,Ore10,Ore11,Ore12,Ore13,Ore14	*OreG0005203*
scaffold2058	436200	A	C	Ore01,Ore02,Ore03,Ore04,Ore05,Ore06,Ore07,Ore08,Ore09,Ore10,Ore11,Ore12,Ore13,Ore14	*OreG0005203*
scaffold2058	453845	G	A	Och08,Och09,Och10,Och11	*OreG0005206*
scaffold2058	456432	C	T	Och01,Och02,Och03,Och04,Och05,Och06,Och07,Och08,Och09,Och10,Och11,Och12,Och13,Och14	*OreG0005207*
scaffold2058	456738	G	T	Och02,Och03,Och04,Och05,Och12,Och13	*OreG0005207*
scaffold2058	458298	T	C	Ore01,Ore02,Ore03,Ore04,Ore05,Ore06,Ore07,Ore08,Ore09,Ore10,Ore11,Ore12,Ore13,Ore14	*OreG0005208*
scaffold2058	472215	C	T	Och02,Och03,Och04,Och05,Och12,Och13	*OreG0005210*
scaffold2058	474641	G	T	NA	*OreG0005210*
scaffold2058	477397	G	A	Och01,Och02,Och03,Och04,Och05,Och06,Och07,Och09,Och10,Och12,Och13,Och14	*OreG0005211*
scaffold2058	493381	T	A	Och01,Och02,Och03,Och04,Och05,Och06,Och07,Och08,Och09,Och10,Och11,Och12,Och13,Och14,Ore01,Ore02,Ore03,Ore04,Ore05,Ore06,Ore07,Ore08,Ore09,Ore10,Ore11,Ore12,Ore13,Ore14	*OreG0005214*
scaffold2058	493740	G	C	NA	*OreG0005214*
scaffold2058	494125	T	G	NA	*OreG0005214*
scaffold2058	511643	G	C	Och01,Och02,Och03,Och04,Och05,Och06,Och07,Och08,Och09,Och10,Och11,Och12,Och13,Och14,Ore01,Ore02,Ore03,Ore04,Ore05,Ore06,Ore07,Ore08,Ore09,Ore10,Ore11,Ore12,Ore13,Ore14	*OreG0005215*
scaffold2058	511778	T	C	Ore01	*OreG0005215*
scaffold2058	536278	A	C	Ore01	*OreG0005219*
scaffold2058	536333	C	T	NA	*OreG0005219*
scaffold2058	536650	G	A	NA	*OreG0005219*
scaffold2058	536711	C	T	Och05,Och13	*OreG0005219*
scaffold2058	536725	C	T	NA	*OreG0005219*
scaffold2058	547460	C	T	Och01,Och02,Och03,Och04,Och05,Och06,Och07,Och08,Och09,Och10,Och11,Och12,Och13,Och14,Ore01,Ore02,Ore03,Ore04,Ore05,Ore06,Ore07,Ore08,Ore09,Ore10,Ore11,Ore12,Ore13,Ore14	*OreG0005221*
scaffold2058	547729	G	A	NA	*OreG0005221*
scaffold2058	551287	G	A	NA	*OreG0005221*
scaffold2058	572674	G	A	Och02	*OreG0005224*
scaffold2058	580167	C	T	Ore01,Ore02,Ore03,Ore04,Ore05,Ore06,Ore07,Ore08,Ore09,Ore10,Ore11,Ore12,Ore13,Ore14	*OreG0005225*
scaffold2058	580197	A	T	Och01,Och02,Och03,Och04,Och05,Och06,Och07,Och08,Och09,Och10,Och11,Och12,Och13,Och14	*OreG0005225*
scaffold2058	602973	T	G	Ore01,Ore02,Ore03,Ore04,Ore05,Ore06,Ore07,Ore08,Ore09,Ore10,Ore11,Ore12,Ore13,Ore14	*OreG0005228*
scaffold2058	635020	C	A	NA	*OreG0005230*
scaffold2058	636795	G	A	NA	*OreG0005231*
scaffold2058	636804	T	G	NA	*OreG0005231*
scaffold2058	637055	A	T	NA	*OreG0005231*
scaffold2058	649200	A	T	Ore01	*OreG0005233*
scaffold2058	649488	G	A	NA	*OreG0005233*
scaffold2058	666216	G	T	NA	*OreG0005234*
scaffold2058	669537	C	T	Och01,Och02,Och03,Och04,Och05,Och06,Och07,Och08,Och09,Och10,Och11,Och12,Och13,Och14	*OreG0005234*
scaffold1943	69954	T	C	NA	*OreG0004953*
scaffold1943	136283	C	T	NA	*OreG0004961*
scaffold1943	136469	A	C	Och02,Och03	*OreG0004961*
scaffold1943	138669	C	A	NA	*OreG0004962*
scaffold1943	156174	C	A	Ore02,Ore03,Ore04,Ore05,Ore06,Ore08,Ore09,Ore10,Ore11,Ore12,Ore14	*OreG0004964*
scaffold1943	170659	C	T	NA	*OreG0004967*
scaffold1943	173386	C	T	NA	*OreG0004968*
scaffold1943	173473	A	G	NA	*OreG0004968*
scaffold1943	174208	C	T	NA	*OreG0004968*
scaffold1943	182398	C	T	Ore02,Ore03,Ore06,Ore08,Ore09,Ore10,Ore11,Ore12,Ore14	*OreG0004969*

scaffold1943	187840	T	A	Och02,Och03,Och11,Och14	*OreG0004970*
scaffold1943	188371	A	T	Och01,Och08,Ore01,Ore02,Ore03,Ore04,Ore05,Ore06,Ore07,Ore08,Ore09,Ore10,Ore11,Ore12,Ore13,Ore14	*OreG0004970*
scaffold1943	188844	G	T	NA	*OreG0004970*
scaffold1943	220458	A	G	NA	*OreG0004972*
scaffold1943	252127	C	G	NA	*OreG0004974*
scaffold1943	255392	C	T	Och09,Och10,Och11	*OreG0004974*
scaffold1943	294634	G	T	NA	*OreG0004978*
scaffold1943	314033	C	A	Och01,Och02,Och03,Och04,Och05,Och06,Och08,Och09,Och10,Och11,Och12,Och13,Och14	*OreG0004981*
scaffold1943	314060	T	G	Och01,Och02,Och03,Och04,Och05,Och06,Och08,Och09,Och10,Och11,Och12,Och13,Och14	*OreG0004981*
scaffold1943	314262	A	T	NA	*OreG0004981*
scaffold1943	315137	A	G	Och01,Och02,Och03,Och04,Och05,Och06,Och08,Och09,Och10,Och11,Och12,Och13,Och14,Ore01,Ore02,Ore03,Ore04,Ore05,Ore06,Ore07,Ore08,Ore09,Ore10,Ore11,Ore12,Ore13,Ore14	*OreG0004981*
scaffold1943	343439	T	C	NA	*OreG0004985*
scaffold1943	343664	T	C	Och01,Och02,Och03,Och04,Och05,Och06,Och07,Och08,Och09,Och10,Och11,Och12,Och13,Och14,Ore01,Ore02,Ore03,Ore04,Ore05,Ore06,Ore07,Ore08,Ore09,Ore10,Ore11,Ore12,Ore13,Ore14	*OreG0004985*
scaffold1943	343699	A	T	NA	*OreG0004985*
scaffold1943	343987	T	A	Och01,Och02,Och03,Och04,Och05,Och06,Och07,Och08,Och09,Och10,Och11,Och12,Och13,Och14	*OreG0004985*
scaffold1943	344023	C	A	NA	*OreG0004985*
scaffold1943	344080	C	T	NA	*OreG0004985*
scaffold1943	344234	C	T	Och01,Och02,Och03,Och04,Och05,Och06,Och07,Och08,Och09,Och10,Och11,Och12,Och13,Och14	*OreG0004985*
scaffold1943	344494	C	T	Ore02,Ore03,Ore06,Ore08,Ore09,Ore10,Ore11,Ore12,Ore14	*OreG0004985*
scaffold1943	345509	G	T	NA	*OreG0004986*
scaffold1943	345963	A	G	Och01,Och02,Och03,Och04,Och05,Och06,Och07,Och08,Och09,Och10,Och11,Och12,Och13,Och14,Ore01,Ore02,Ore03,Ore04,Ore05,Ore06,Ore07,Ore08,Ore09,Ore10,Ore11,Ore12,Ore13,Ore14	*OreG0004986*
scaffold1943	347039	A	C	Och01,Och02,Och03,Och04,Och05,Och06,Och07,Och08,Och09,Och10,Och11,Och12,Och13,Och14	*OreG0004986*
scaffold1943	429572	A	T	NA	*OreG0004988*
scaffold1943	439979	C	A	Ore01,Ore02,Ore03,Ore04,Ore05,Ore06,Ore07,Ore08,Ore09,Ore10,Ore11,Ore12,Ore13,Ore14	*OreG0004989*
scaffold987	61666	A	G	NA	*OreG0027072*
scaffold987	61734	G	A	NA	*OreG0027072*
scaffold987	70918	G	T	Ore02,Ore06,Ore08,Ore09,Ore12,Ore14	*OreG0027074*
scaffold987	71984	G	A	NA	*OreG0027074*
scaffold987	79569	G	C	Och01,Och02,Och03,Och04,Och05,Och06,Och07,Och08,Och09,Och10,Och11,Och12,Och13,Och14,Ore01,Ore02,Ore03,Ore04,Ore05,Ore06,Ore07,Ore08,Ore09,Ore10,Ore11,Ore12,Ore13,Ore14	*OreG0027075*
scaffold987	155956	A	T	Och01,Och02,Och03,Och04,Och05,Och06,Och07,Och08,Och09,Och10,Och11,Och12,Och13,Och14,Ore01,Ore02,Ore03,Ore04,Ore05,Ore06,Ore07,Ore08,Ore09,Ore10,Ore11,Ore12,Ore13,Ore14	*OreG0027083*
scaffold987	177090	A	T	Och01,Och02,Och03,Och04,Och05,Och06,Och07,Och08,Och09,Och10,Och11,Och12,Och13,Och14,Ore01,Ore02,Ore03,Ore04,Ore05,Ore06,Ore07,Ore08,Ore09,Ore10,Ore11,Ore12,Ore13,Ore14	*OreG0027086*
scaffold987	177264	A	T	Ore01,Ore02,Ore03,Ore04,Ore05,Ore06,Ore07,Ore08,Ore09,Ore10,Ore11,Ore12,Ore13,Ore14	*OreG0027086*
scaffold987	232215	G	A	Och01,Och02,Och03,Och04,Och05,Och06,Och07,Och08,Och09,Och10,Och11,Och12,Och13,Och14,Ore01,Ore02,Ore03,Ore04,Ore05,Ore06,Ore07,Ore08,Ore09,Ore10,Ore11,Ore12,Ore13,Ore14	*OreG0027092*
scaffold987	232366	A	G	Ore02,Ore04,Ore05,Ore06,Ore08,Ore09,Ore12,Ore14	*OreG0027092*
scaffold987	233082	T	C	NA	*OreG0027092*
scaffold987	249782	C	G	Ore01,Ore02,Ore04,Ore05,Ore06,Ore07,Ore08,Ore09,Ore11,Ore12,Ore13,Ore14	*OreG0027094*
scaffold987	260827	G	T	Och01,Och02,Och03,Och04,Och05,Och06,Och07,Och08,Och09,Och10,Och11,Och12,Och13,Och14,Ore01,Ore02,Ore03,Ore04,Ore05,Ore06,Ore07,Ore08,Ore09,Ore10,Ore11,Ore12,Ore13,Ore14	*OreG0027095*
scaffold987	262470	A	C	Och08	*OreG0027095*
scaffold987	262498	T	G	Och01	*OreG0027095*
scaffold987	262591	G	C	Och01,Och02,Och03,Och04,Och05,Och06,Och07,Och08,Och09,Och10,Och11,Och12,Och13,Och14,Ore01,Ore02,Ore03,Ore04,Ore05,Ore06,Ore07,Ore08,Ore09,Ore10,Ore11,Ore12,Ore13,Ore14	*OreG0027095*
scaffold987	262813	C	A	NA	*OreG0027095*
scaffold987	291507	A	T	Och01,Och02,Och03,Och04,Och05,Och06,Och07,Och08,Och09,Och10,Och11,Och12,Och13,Och14	*OreG0027098*
scaffold987	361540	G	C	Ore01,Ore02,Ore03,Ore04,Ore05,Ore06,Ore07,Ore08,Ore09,Ore10,Ore11,Ore12,Ore13,Ore14	*OreG0027106*
scaffold987	361557	T	C	Ore01,Ore02,Ore03,Ore04,Ore05,Ore06,Ore07,Ore08,Ore09,Ore10,Ore11,Ore12,Ore13,Ore14	*OreG0027106*
scaffold987	361564	A	C	NA	*OreG0027106*
scaffold987	369681	G	A	NA	*OreG0027107*

scaffold987	388753	T	A	Ore01,Ore02,Ore03,Ore04,Ore05,Ore06,Ore07,Ore08,Ore09,Ore10,Ore11,Ore12,Ore13,Ore14	*OreG0027108*
scaffold987	390352	G	A	Och05,Och07,Och08,Och09,Och10,Och12	*OreG0027108*
scaffold987	393515	A	C	Och01,Och02,Och03,Och04,Och05,Och06,Och07,Och08,Och09,Och10,Och11,Och12,Och13,Och14,Ore06,Ore09,Ore11,Ore13,Ore14	*OreG0027109*
scaffold987	394515	T	C	Och09,Och10	*OreG0027111*
scaffold987	420916	G	C	Ore01,Ore02,Ore03,Ore04,Ore05,Ore06,Ore07,Ore08,Ore09,Ore10,Ore11,Ore12,Ore13,Ore14	*OreG0027113*
scaffold987	453852	C	G	NA	*OreG0027119*
scaffold987	453864	C	A	NA	*OreG0027119*
scaffold987	454376	A	T	NA	*OreG0027119*
scaffold987	454750	A	C	NA	*OreG0027119*
scaffold987	456376	A	C	Och02,Och03,Och04,Och05,Och06,Och07,Och09,Och10,Och12,Och13,Och14,Ore01,Ore02,Ore03,Ore04,Ore05,Ore06,Ore07,Ore08,Ore09,Ore10,Ore11,Ore12,Ore13,Ore14	*OreG0027119*
scaffold987	456379	C	T	NA	*OreG0027119*
scaffold987	457686	C	G	NA	*OreG0027119*
scaffold987	498766	A	T	Ore01,Ore02,Ore03,Ore04,Ore05,Ore06,Ore07,Ore08,Ore09,Ore10,Ore11,Ore12,Ore13,Ore14	*OreG0027123*
scaffold987	508974	A	G	Och01,Och05,Och09,Och10,Och12	*OreG0027124*
scaffold987	522735	C	A	NA	*OreG0027126*
scaffold987	523865	T	C	NA	*OreG0027126*
scaffold987	523964	A	T	NA	*OreG0027126*
scaffold987	528418	T	A	NA	*OreG0027127*
scaffold987	676616	T	C	NA	*OreG0027131*
scaffold987	680579	C	A	Och01,Och02,Och03,Och04,Och05,Och06,Och07,Och08,Och09,Och10,Och11,Och12,Och13,Och14	*OreG0027131*
scaffold987	730183	C	A	Och02,Och03,Och04,Och05,Och06,Och07,Och12,Och13	*OreG0027133*
scaffold987	908454	A	G	Och01,Och02,Och03,Och04,Och05,Och06,Och07,Och08,Och09,Och10,Och11,Och12,Och13	*OreG0027143*
scaffold987	1044511	G	A	Och04,Och06,Och13	*OreG0027147*
scaffold987	1044950	A	C	Och14	*OreG0027147*
scaffold987	1045084	A	C	Och01,Och02,Och03,Och04,Och05,Och06,Och07,Och08,Och09,Och10,Och11,Och12,Och13,Och14,Ore01,Ore02,Ore03,Ore04,Ore05,Ore06,Ore07,Ore08,Ore09,Ore10,Ore11,Ore12,Ore13,Ore14	*OreG0027147*
scaffold987	1045475	G	A	NA	*OreG0027147*
scaffold987	1099792	G	A	NA	*OreG0027150*
scaffold987	1227081	T	G	Och09,Och10,Och11	*OreG0027156*
scaffold987	1228093	T	C	NA	*OreG0027156*
scaffold987	1457201	A	T	Och11	*OreG0027166*
scaffold987	1469987	C	T	NA	*OreG0027168*
scaffold987	1470038	A	G	NA	*OreG0027168*
scaffold987	1470272	A	T	Ore03	*OreG0027168*
scaffold987	1470403	A	G	Ore01,Ore02,Ore03,Ore04,Ore05,Ore06,Ore07,Ore08,Ore09,Ore10,Ore11,Ore12,Ore13,Ore14	*OreG0027168*
scaffold987	1470634	C	A	Ore02,Ore04,Ore05	*OreG0027168*
scaffold987	1470641	G	A	Ore01,Ore02,Ore03,Ore04,Ore05,Ore06,Ore07,Ore08,Ore09,Ore10,Ore11,Ore12,Ore13,Ore14	*OreG0027168*
scaffold987	1470778	A	G	NA	*OreG0027168*
scaffold987	1470826	T	C	Ore01,Ore02,Ore03,Ore04,Ore05,Ore06,Ore07,Ore08,Ore09,Ore10,Ore11,Ore12,Ore13,Ore14	*OreG0027168*
scaffold987	1497482	C	T	NA	*OreG0027171*
scaffold987	1497562	T	C	Och01,Och02,Och03,Och04,Och05,Och06,Och07,Och08,Och09,Och10,Och11,Och12,Och13,Och14	*OreG0027171*
scaffold987	1502423	T	A	Och01,Och02,Och03,Och04,Och05,Och06,Och07,Och08,Och09,Och10,Och11,Och12,Och13,Och14	*OreG0027171*
scaffold987	1504397	G	A	NA	*OreG0027171*
scaffold987	1504433	C	T	NA	*OreG0027171*
scaffold987	1504438	G	A	NA	*OreG0027171*
scaffold987	1504447	C	T	NA	*OreG0027171*
scaffold987	1504628	C	T	NA	*OreG0027171*
scaffold987	1504804	G	A	Ore01,Ore02,Ore03,Ore04,Ore05,Ore06,Ore07,Ore08,Ore09,Ore10,Ore11,Ore12,Ore13,Ore14	*OreG0027171*
scaffold987	1657722	C	A	NA	*OreG0027175*
scaffold987	1658864	C	T	NA	*OreG0027175*
scaffold987	1682227	C	G	NA	*OreG0027179*
scaffold987	1749770	T	A	NA	*OreG0027182*
scaffold987	2016733	C	A	NA	*OreG0027193*
scaffold987	2019430	T	C	Och01	*OreG0027193*
scaffold987	2030578	C	T	NA	*OreG0027195*
scaffold987	2142603	G	A	Ore01,Ore02,Ore03,Ore04,Ore05,Ore06,Ore07,Ore08,Ore09,Ore10,Ore11,Ore12,Ore13,Ore14	*OreG0027202*
scaffold987	2142811	T	G	NA	*OreG0027202*
scaffold987	2142820	G	T	Och03	*OreG0027202*

scaffold987	2241973	C	G	Ore01,Ore02,Ore03,Ore04,Ore05,Ore06,Ore07,Ore08,Ore09,Ore10,Ore11,Ore12,Ore13,Ore14	*OreG0027207*
scaffold987	2242025	A	G	Och01,Och02,Och03,Och04,Och05,Och06,Och07,Och08,Och09,Och10,Och11,Och12,Och13,Och14,Ore01,Ore02,Ore03,Ore04,Ore05,Ore06,Ore07,Ore08,Ore09,Ore10,Ore11,Ore12,Ore13,Ore14	*OreG0027207*
scaffold987	2245741	G	A	NA	*OreG0027207*
scaffold987	2246351	T	A	Och01,Och02,Och03,Och04,Och05,Och06,Och07,Och08,Och09,Och10,Och11,Och12,Och13,Och14	*OreG0027207*
scaffold987	2247627	A	G	Och07,Och09,Och10,Och11,Och14	*OreG0027207*
scaffold987	2337146	C	T	Ore01,Ore04,Ore05,Ore11	*OreG0027212*
scaffold987	2374840	T	A	NA	*OreG0027213*
scaffold987	2399922	G	A	Och01,Och02,Och03,Och04,Och05,Och06,Och07,Och08,Och09,Och10,Och11,Och12,Och13,Och14,Ore01,Ore02,Ore03,Ore04,Ore05,Ore06,Ore07,Ore08,Ore09,Ore10,Ore11,Ore12,Ore13,Ore14	*OreG0027215*
scaffold987	2423645	G	T	Och04,Och12,Och13	*OreG0027218*
scaffold987	2423945	T	A	NA	*OreG0027218*
scaffold987	2424333	A	G	NA	*OreG0027218*
scaffold987	2424572	G	A	NA	*OreG0027218*
scaffold987	2437906	C	T	Ore06,Ore07,Ore08,Ore13,Ore14	*OreG0027219*
scaffold987	2438411	G	T	Ore06,Ore07,Ore08,Ore13,Ore14	*OreG0027219*
scaffold987	2581894	G	A	NA	*OreG0027225*
scaffold987	2582383	G	A	NA	*OreG0027225*
scaffold987	2582531	C	T	NA	*OreG0027225*
scaffold987	2596171	T	C	NA	*OreG0027227*
scaffold987	2685265	G	A	Och14	*OreG0027234*
scaffold987	2704621	T	C	NA	*OreG0027239*
scaffold987	2712203	G	T	Och01,Och02,Och03,Och04,Och05,Och06,Och07,Och08,Och09,Och10,Och11,Och12,Och13,Och14,Ore01,Ore02,Ore03,Ore04,Ore05,Ore06,Ore07,Ore08,Ore09,Ore10,Ore11,Ore12,Ore13,Ore14	*OreG0027240*
scaffold987	2713065	C	A	Och01,Och02,Och03,Och04,Och05,Och06,Och07,Och08,Och09,Och10,Och11,Och12,Och13,Och14	*OreG0027240*
scaffold987	2716707	T	C	Och01,Och02,Och03,Och04,Och05,Och06,Och08,Och09,Och10,Och11,Och12,Och13,Och14	*OreG0027240*
scaffold987	2736973	A	G	NA	*OreG0027242*
scaffold987	2741898	C	T	Ore01,Ore02,Ore03,Ore04,Ore05,Ore06,Ore07,Ore08,Ore09,Ore10,Ore11,Ore12,Ore13,Ore14	*OreG0027242*
scaffold987	2742750	T	C	Och01,Och02,Och03,Och04,Och05,Och06,Och07,Och08,Och09,Och10,Och11,Och12,Och13,Och14	*OreG0027242*
scaffold987	2742831	T	G	NA	*OreG0027242*
scaffold987	2743488	T	C	Ore01,Ore04,Ore05,Ore11	*OreG0027242*
scaffold987	2743583	C	A	Ore06,Ore07,Ore08,Ore13,Ore14	*OreG0027242*
scaffold987	2743597	T	A	Och01,Och02,Och03,Och04,Och05,Och06,Och07,Och08,Och09,Och10,Och11,Och12,Och13,Och14,Ore01,Ore02,Ore03,Ore04,Ore05,Ore06,Ore07,Ore08,Ore09,Ore10,Ore11,Ore12,Ore13,Ore14	*OreG0027242*
scaffold987	2762033	C	A	Och02,Och03,Och04,Och05,Och06,Och07,Och09,Och10,Och11,Och12,Och13,Och14	*OreG0027243*
scaffold987	2762837	A	C	NA	*OreG0027243*
scaffold987	2764716	C	A	NA	*OreG0027244*
scaffold987	2778213	T	C	Och03,Och05,Och07,Och08,Och12,Och13	*OreG0027245*
scaffold987	2780422	G	C	Och01,Och02,Och03,Och04,Och05,Och06,Och07,Och08,Och09,Och10,Och11,Och12,Och13,Och14,Ore01,Ore02,Ore03,Ore04,Ore05,Ore06,Ore07,Ore08,Ore09,Ore10,Ore11,Ore12,Ore13,Ore14	*OreG0027245*
scaffold987	2798643	C	A	Och01,Och04,Och06,Och07,Och08,Och09,Och10,Och11,Och12,Och13,Och14	*OreG0027247*
scaffold987	2798889	G	T	NA	*OreG0027247*
scaffold987	2799342	G	A	NA	*OreG0027247*
scaffold987	2799552	C	T	NA	*OreG0027247*
scaffold987	2800236	C	G	Och01	*OreG0027247*
scaffold2239	32564	C	T	NA	*OreG0006292*
scaffold2239	32661	A	T	NA	*OreG0006292*
scaffold2239	32714	G	A	NA	*OreG0006292*
scaffold2239	32720	C	T	NA	*OreG0006292*
scaffold2239	33720	T	A	Och05	*OreG0006292*
scaffold2239	33733	A	G	NA	*OreG0006292*
scaffold2239	33763	T	C	NA	*OreG0006292*
scaffold2239	61692	C	T	NA	*OreG0006297*
scaffold2239	61702	G	A	Och12	*OreG0006297*
scaffold2239	61939	T	A	NA	*OreG0006297*
scaffold2239	65894	T	A	NA	*OreG0006298*
scaffold2239	65928	A	G	NA	*OreG0006298*
scaffold2239	69102	G	A	Ore01,Ore02,Ore03,Ore04,Ore05,Ore06,Ore07,Ore08,Ore09,Ore10,Ore11,Ore12,Ore13,Ore14	*OreG0006299*
scaffold2239	69968	C	A	Ore01,Ore02,Ore03,Ore04,Ore05,Ore06,Ore07,Ore08,Ore09,Ore10,Ore11,Ore12,Ore13,Ore14	*OreG0006299*
scaffold2239	71467	G	C	Och01,Och02,Och03,Och04,Och05,Och06,Och07,Och08,Och09,Och10,Och11,Och12,Och13,Och14	*OreG0006300*

scaffold2239	72740	C	T	Och02,Och03,Och04,Och05,Och06,Och12,Och13	*OreG0006300*
scaffold2239	73411	A	G	Och11,Ore01,Ore02,Ore03,Ore04,Ore05,Ore06,Ore07,Ore08,Ore09,Ore10,Ore11,Ore12,Ore13,Ore14	*OreG0006300*
scaffold2239	73625	C	T	Och01,Och02,Och03,Och04,Och05,Och06,Och07,Och08,Och09,Och10,Och11,Och12,Och13,Och14,Ore01,Ore02,Ore03,Ore04,Ore05,Ore06,Ore07,Ore08,Ore09,Ore10,Ore11,Ore12,Ore13,Ore14	*OreG0006300*
scaffold2239	74830	A	G	Och02,Och03,Och04,Och05,Och06,Och12,Och13	*OreG0006300*
scaffold2239	84850	T	C	Ore01,Ore02,Ore03,Ore04,Ore05,Ore06,Ore07,Ore08,Ore09,Ore10,Ore11,Ore12,Ore13,Ore14	*OreG0006301*
scaffold2239	91893	A	G	Ore01,Ore02,Ore03,Ore04,Ore05,Ore06,Ore07,Ore08,Ore09,Ore10,Ore11,Ore12,Ore13,Ore14	*OreG0006302*
scaffold2239	143460	G	A	Ore01,Ore02,Ore03,Ore04,Ore05,Ore06,Ore07,Ore08,Ore09,Ore10,Ore11,Ore12,Ore13,Ore14	*OreG0006308*
scaffold2239	143842	T	C	NA	*OreG0006308*
scaffold2239	147851	T	G	Ore01,Ore02,Ore03,Ore04,Ore05,Ore06,Ore07,Ore08,Ore09,Ore10,Ore11,Ore12,Ore13,Ore14	*OreG0006308*
scaffold2239	182866	T	C	Och01,Och02,Och03,Och04,Och05,Och06,Och07,Och08,Och09,Och10,Och11,Och12,Och13,Och14	*OreG0006309*
scaffold2239	184401	C	T	NA	*OreG0006309*
scaffold2239	203589	C	A	Ore01,Ore02,Ore03,Ore04,Ore05,Ore06,Ore07,Ore08,Ore09,Ore10,Ore11,Ore12,Ore13,Ore14	*OreG0006310*
scaffold2239	203901	C	T	NA	*OreG0006310*
scaffold2239	204766	C	T	Och01,Och02,Och03,Och04,Och05,Och06,Och07,Och08,Och09,Och10,Och11,Och12,Och13,Och14	*OreG0006310*
scaffold2239	240778	T	A	Och01,Och02,Och03,Och04,Och05,Och06,Och07,Och08,Och09,Och10,Och11,Och12,Och13,Och14,Ore01,Ore02,Ore03,Ore04,Ore05,Ore06,Ore07,Ore08,Ore09,Ore10,Ore11,Ore12,Ore13,Ore14	*OreG0006313*
scaffold2239	240993	G	A	NA	*OreG0006313*
scaffold2239	241159	T	G	Ore01,Ore02,Ore03,Ore04,Ore05,Ore06,Ore07,Ore08,Ore09,Ore10,Ore11,Ore12,Ore13,Ore14	*OreG0006313*
scaffold2239	253202	T	A	Ore01,Ore02,Ore03,Ore04,Ore05,Ore06,Ore07,Ore08,Ore09,Ore10,Ore11,Ore12,Ore13,Ore14	*OreG0006314*
scaffold2239	269985	T	A	Och08,Och11	*OreG0006318*
scaffold2239	271343	A	T	Och11	*OreG0006318*
scaffold2239	288767	T	C	Och04,Och06,Och12	*OreG0006319*
scaffold2239	301459	C	G	Ore01,Ore02,Ore03,Ore04,Ore05,Ore06,Ore07,Ore08,Ore09,Ore10,Ore11,Ore12,Ore13,Ore14	*OreG0006321*
scaffold2239	302788	C	G	NA	*OreG0006321*
scaffold2239	310816	G	A	NA	*OreG0006322*
scaffold2239	311704	G	A	Och01,Och02,Och03,Och04,Och05,Och06,Och08,Och12,Och13	*OreG0006322*
scaffold2239	332490	C	G	NA	*OreG0006323*
scaffold2239	335281	C	T	Och01,Och02,Och03,Och04,Och05,Och06,Och07,Och08,Och09,Och10,Och11,Och12,Och13,Och14	*OreG0006323*
scaffold2239	336205	C	T	Ore01,Ore06,Ore08,Ore10,Ore14	*OreG0006323*
scaffold2239	338710	G	A	NA	*OreG0006324*
scaffold2239	348177	C	G	Och02	*OreG0006325*
scaffold2239	349133	G	A	Och01,Och02,Och03,Och04,Och05,Och06,Och07,Och08,Och09,Och10,Och11,Och12,Och13,Och14	*OreG0006325*
scaffold2239	349268	C	A	NA	*OreG0006325*
scaffold2239	363070	G	A	Och02	*OreG0006327*
scaffold2239	368950	C	T	NA	*OreG0006328*
scaffold2239	384016	C	A	Och05,Och09,Och10	*OreG0006332*
scaffold2239	385077	C	A	Och05	*OreG0006332*
scaffold2239	472988	C	A	NA	*OreG0006346*
scaffold2239	473353	C	T	NA	*OreG0006346*
scaffold2239	475399	T	C	NA	*OreG0006347*
scaffold2239	475752	G	A	NA	*OreG0006347*
scaffold2239	476322	G	A	NA	*OreG0006347*
scaffold2239	477719	T	C	NA	*OreG0006348*
scaffold2239	477722	A	G	NA	*OreG0006348*
scaffold2239	477784	T	C	NA	*OreG0006348*
scaffold2239	478120	G	A	NA	*OreG0006348*
scaffold2239	478678	T	A	NA	*OreG0006348*
scaffold2239	479839	C	T	NA	*OreG0006349*
scaffold2239	479886	T	C	NA	*OreG0006349*
scaffold2239	489640	G	A	Och01,Och02,Och06,Och09,Och10,Och14	*OreG0006350*
scaffold2239	499497	G	A	Och05	*OreG0006351*
scaffold2239	502420	G	A	Ore01,Ore02,Ore03,Ore04,Ore05,Ore06,Ore07,Ore08,Ore09,Ore10,Ore11,Ore12,Ore13,Ore14	*OreG0006351*
scaffold2239	502685	C	T	Ore01,Ore02,Ore03,Ore04,Ore05,Ore06,Ore07,Ore08,Ore09,Ore10,Ore11,Ore12,Ore13,Ore14	*OreG0006351*
scaffold2239	506000	C	T	Och01,Och02,Och06,Och09,Och10,Och14	*OreG0006351*
scaffold2239	511093	G	A	Och01,Och02,Och06,Och09,Och10,Och14	*OreG0006352*
scaffold2239	516957	G	T	Och01,Och02,Och06,Och09,Och10,Och14	*OreG0006353*
scaffold2239	517266	T	C	Och01,Och02,Och06,Och09,Och10,Och14	*OreG0006353*

scaffold2239	544668	T	C	NA	*OreG0006357*
scaffold2239	544748	T	C	Och01,Och02,Och06,Och09,Och10,Och14	*OreG0006357*
scaffold2239	557437	G	A	NA	*OreG0006359*
scaffold2239	569383	G	A	NA	*OreG0006362*
scaffold2239	599245	T	G	Ore01,Ore02,Ore03,Ore04,Ore05,Ore06,Ore07,Ore08,Ore09,Ore10,Ore11,Ore12,Ore13,Ore14	*OreG0006369*
scaffold2239	599551	C	T	NA	*OreG0006369*
scaffold2239	599578	T	C	NA	*OreG0006369*
scaffold1758	20808	G	A	NA	*OreG0003617*
scaffold1758	20880	G	A	Ore01,Ore02,Ore03,Ore04,Ore05,Ore06,Ore07,Ore08,Ore09,Ore10,Ore11,Ore12,Ore13,Ore14	*OreG0003617*
scaffold1758	26310	G	A	Och08,Och11	*OreG0003617*
scaffold1758	29735	T	C	NA	*OreG0003618*
scaffold1758	29771	C	T	Och01,Och02,Och03,Och04,Och05,Och06,Och07,Och08,Och09,Och10,Och11,Och12,Och13,Och14,Ore01,Ore02,Ore03,Ore04,Ore05,Ore06,Ore07,Ore08,Ore09,Ore10,Ore11,Ore12,Ore13,Ore14	*OreG0003618*
scaffold1758	56643	C	T	Och01,Och02,Och03,Och04,Och05,Och06,Och07,Och08,Och09,Och10,Och11,Och12,Och13,Och14	*OreG0003623*
scaffold1758	56746	A	C	Ore01,Ore02,Ore03,Ore04,Ore05,Ore06,Ore07,Ore08,Ore09,Ore10,Ore11,Ore12,Ore13,Ore14	*OreG0003623*
scaffold1758	73196	G	A	NA	*OreG0003627*
scaffold1758	103384	G	A	Och06,Och12	*OreG0003632*
scaffold1758	121242	G	A	Och08	*OreG0003634*
scaffold1758	129273	G	T	NA	*OreG0003635*
scaffold1758	133933	C	T	Och01,Och06,Och07,Och08,Och11,Och12,Och14	*OreG0003637*
scaffold1758	133970	A	T	NA	*OreG0003637*
scaffold1758	134089	G	A	NA	*OreG0003637*
scaffold1758	134463	A	C	NA	*OreG0003637*
scaffold1758	136882	C	T	NA	*OreG0003638*
scaffold1758	137036	C	T	NA	*OreG0003638*
scaffold1758	137066	C	T	NA	*OreG0003638*
scaffold1758	137093	C	T	NA	*OreG0003638*
scaffold1758	138210	C	T	NA	*OreG0003638*
scaffold1758	138225	C	A	NA	*OreG0003638*
scaffold1758	138960	C	T	NA	*OreG0003638*
scaffold1758	138974	G	A	NA	*OreG0003638*
scaffold1758	153819	G	A	Ore02,Ore09,Ore10,Ore14	*OreG0003641*
scaffold1758	153994	C	T	NA	*OreG0003641*
scaffold1758	154021	C	T	Ore01,Ore02,Ore04,Ore05,Ore06,Ore07,Ore08,Ore09,Ore10,Ore11,Ore12,Ore13,Ore14	*OreG0003641*
scaffold1758	154961	C	G	Och08,Ore01,Ore02,Ore03,Ore04,Ore05,Ore06,Ore07,Ore08,Ore09,Ore10,Ore11,Ore12,Ore13,Ore14	*OreG0003641*
scaffold1758	155003	C	T	NA	*OreG0003641*
scaffold1758	155029	C	G	NA	*OreG0003641*
scaffold1758	155093	C	T	NA	*OreG0003641*
scaffold1758	155786	T	A	Och06,Och07,Och09,Och10,Och12,Ore01,Ore02,Ore04,Ore05,Ore06,Ore07,Ore08,Ore09,Ore10,Ore11,Ore12,Ore13,Ore14	*OreG0003641*
scaffold1758	155830	G	C	Och08,Och11	*OreG0003641*
scaffold1758	192280	C	G	NA	*OreG0003646*
scaffold1758	194976	A	G	Ore01,Ore02,Ore04,Ore05,Ore06,Ore07,Ore08,Ore09,Ore10,Ore11,Ore12,Ore13,Ore14	*OreG0003647*
scaffold1758	197626	G	C	NA	*OreG0003647*
scaffold1758	197840	T	C	Och08	*OreG0003647*
scaffold1758	200224	A	T	NA	*OreG0003648*
scaffold1758	229775	T	A	Ore01,Ore02,Ore03,Ore04,Ore05,Ore06,Ore07,Ore08,Ore09,Ore10,Ore11,Ore12,Ore13,Ore14	*OreG0003652*
scaffold1758	230061	G	C	Och01,Och06,Och07,Och08,Och09,Och10,Och12	*OreG0003652*
scaffold1758	230789	G	T	Och12	*OreG0003652*
scaffold1758	230810	T	C	Och01,Och09,Och12	*OreG0003652*
scaffold1758	230816	C	T	Och01,Och09,Och12	*OreG0003652*
scaffold1758	230833	G	A	Och01,Och09	*OreG0003652*
scaffold1758	230848	G	A	Och01,Och09	*OreG0003652*
scaffold1758	230873	C	T	Och01,Och09	*OreG0003652*
scaffold1758	230950	G	T	NA	*OreG0003652*
scaffold1758	230963	T	G	Ore01,Ore02,Ore03,Ore04,Ore05,Ore06,Ore07,Ore08,Ore09,Ore10,Ore11,Ore12,Ore13,Ore14	*OreG0003652*
scaffold1758	231092	T	A	NA	*OreG0003652*
scaffold1758	231103	C	T	Och01,Och07,Och08,Och09	*OreG0003652*
scaffold1758	235328	A	G	Och01,Och06,Och07,Och08,Och09,Och10,Och12	*OreG0003653*
scaffold1758	258808	C	A	NA	*OreG0003653*
scaffold1758	294917	G	C	Och08,Och09,Och10	*OreG0003658*
scaffold1758	298029	C	G	NA	*OreG0003659*
scaffold1758	299515	T	A	Ore01,Ore02,Ore03,Ore04,Ore05,Ore06,Ore07,Ore08,Ore09,Ore10,Ore11,Ore12,Ore13,Ore14	*OreG0003659*
scaffold1758	300456	C	T	Och12	*OreG0003659*

scaffold1758	301491	T	A	NA	*OreG0003659*
scaffold1758	305532	A	T	Ore08,Ore09,Ore14	*OreG0003660*
scaffold1758	309474	A	G	NA	*OreG0003661*
scaffold1758	312953	A	T	Och01,Och02,Och03,Och04,Och05,Och06,Och07,Och08,Och09,Och10,Och11,Och12,Och13,Och14,Ore01,Ore02,Ore03,Ore04,Ore05,Ore06,Ore07,Ore08,Ore09,Ore10,Ore11,Ore12,Ore13,Ore14	*OreG0003661*
scaffold1758	337844	C	T	Och07	*OreG0003666*
scaffold1758	339119	A	T	Och01,Och02,Och03,Och04,Och05,Och06,Och07,Och08,Och09,Och10,Och11,Och12,Och13,Och14	*OreG0003666*
scaffold1758	349807	C	A	Och01,Och02,Och03,Och04,Och05,Och06,Och07,Och08,Och09,Och10,Och11,Och12,Och13,Och14	*OreG0003668*
scaffold1758	351795	C	A	NA	*OreG0003668*
scaffold1758	353803	T	C	Och07	*OreG0003668*
scaffold1758	356480	T	A	Ore01,Ore03,Ore04,Ore05,Ore08,Ore09,Ore11,Ore14	*OreG0003669*
scaffold1758	382750	G	C	NA	*OreG0003671*
scaffold1758	384431	A	T	NA	*OreG0003672*
scaffold1758	393155	A	G	NA	*OreG0003673*
scaffold1758	393388	C	A	Ore01,Ore02,Ore03,Ore04,Ore05,Ore06,Ore07,Ore08,Ore09,Ore10,Ore11,Ore12,Ore13,Ore14	*OreG0003673*
scaffold1758	393820	T	C	Ore01,Ore02,Ore03,Ore04,Ore05,Ore06,Ore07,Ore08,Ore09,Ore10,Ore11,Ore12,Ore13,Ore14	*OreG0003673*
scaffold1758	406096	T	C	Och12	*OreG0003674*
scaffold1758	424448	G	C	NA	*OreG0003675*
scaffold1758	428129	C	T	NA	*OreG0003675*
scaffold1758	429593	G	T	NA	*OreG0003675*
scaffold1758	461161	C	T	Och12	*OreG0003677*
scaffold1758	461996	G	A	NA	*OreG0003677*
scaffold1758	462764	C	A	Och08,Och11	*OreG0003677*
scaffold1758	463578	A	C	Och01,Och08,Och09,Och10,Och11	*OreG0003677*
scaffold1758	467977	C	A	NA	*OreG0003677*
scaffold1758	467990	A	T	NA	*OreG0003677*
scaffold1758	469683	C	A	Och01,Och02,Och03,Och04,Och05,Och06,Och08,Och09,Och10,Och11,Och12,Och13,Och14	*OreG0003678*
scaffold1758	469899	G	A	Ore01,Ore02,Ore03,Ore04,Ore05,Ore06,Ore07,Ore08,Ore09,Ore10,Ore11,Ore12,Ore13,Ore14	*OreG0003678*
scaffold1758	476906	T	A	Ore01,Ore02,Ore03,Ore04,Ore05,Ore06,Ore07,Ore08,Ore09,Ore10,Ore11,Ore12,Ore13,Ore14	*OreG0003679*
scaffold1758	480255	T	C	NA	*OreG0003680*
scaffold1758	480697	T	C	NA	*OreG0003680*
scaffold1758	480849	A	G	NA	*OreG0003680*
scaffold1758	481299	T	G	NA	*OreG0003680*
scaffold1758	481491	T	C	Ore08,Ore09,Ore14	*OreG0003680*
scaffold1758	489980	A	T	NA	*OreG0003682*
scaffold1758	490173	C	T	NA	*OreG0003682*
scaffold1758	491121	C	A	Och01,Och02,Och03,Och04,Och05,Och07,Och08,Och09,Och10,Och11,Och12,Och13,Och14	*OreG0003682*
scaffold1758	491443	C	T	Ore01,Ore02,Ore03,Ore04,Ore05,Ore06,Ore07,Ore08,Ore09,Ore10,Ore11,Ore12,Ore13,Ore14	*OreG0003682*
scaffold1758	495068	A	G	Och01,Och02,Och03,Och04,Och05,Och07,Och08,Och09,Och10,Och11,Och12,Och13,Och14	*OreG0003683*
scaffold1758	495109	C	T	Och01,Och02,Och03,Och04,Och05,Och07,Och08,Och09,Och10,Och11,Och12,Och13,Och14	*OreG0003683*
scaffold1758	495152	C	T	NA	*OreG0003683*
scaffold1758	495444	G	C	NA	*OreG0003683*
scaffold1758	505819	T	A	NA	*OreG0003686*
scaffold1758	512095	T	G	Och08	*OreG0003688*
scaffold1758	512128	C	A	Och01,Och02,Och03,Och04,Och05,Och06,Och08,Och09,Och10,Och11,Och12,Och13,Och14,Ore01,Ore02,Ore03,Ore04,Ore05,Ore06,Ore07,Ore08,Ore09,Ore10,Ore11,Ore12,Ore13,Ore14	*OreG0003688*
scaffold1758	512158	C	T	Och12	*OreG0003688*
scaffold1758	512569	T	G	NA	*OreG0003688*
scaffold1758	512913	T	A	Ore01,Ore02,Ore03,Ore04,Ore05,Ore06,Ore07,Ore08,Ore09,Ore10,Ore11,Ore12,Ore13,Ore14	*OreG0003688*
scaffold1758	513155	A	T	NA	*OreG0003688*
scaffold1758	515050	G	C	Och07,Ore01,Ore02,Ore03,Ore04,Ore05,Ore06,Ore07,Ore08,Ore09,Ore10,Ore11,Ore12,Ore13,Ore14	*OreG0003689*
scaffold1758	528294	A	G	NA	*OreG0003690*
scaffold1758	528571	G	C	NA	*OreG0003690*
scaffold1758	528617	G	A	NA	*OreG0003690*
scaffold1758	528622	T	C	NA	*OreG0003690*
scaffold1758	528857	T	C	Ore01,Ore02,Ore03,Ore04,Ore05,Ore06,Ore07,Ore08,Ore09,Ore10,Ore11,Ore12,Ore13,Ore14	*OreG0003690*
scaffold1758	529294	G	T	NA	*OreG0003690*
scaffold1758	529310	C	A	NA	*OreG0003690*
scaffold1758	529314	G	T	NA	*OreG0003690*

scaffold1758	529319	C	G	NA	*OreG0003690*
scaffold1758	529356	C	G	NA	*OreG0003690*
scaffold1758	556785	A	T	NA	*OreG0003694*
scaffold1758	557359	G	A	Ore01,Ore02,Ore03,Ore04,Ore05,Ore06,Ore07,Ore08,Ore09,Ore10,Ore11,Ore12,Ore13,Ore14	*OreG0003694*
scaffold1758	569898	C	T	NA	*OreG0003697*
scaffold1758	600361	C	G	NA	*OreG0003702*
scaffold1758	600647	G	A	NA	*OreG0003702*
scaffold1758	600673	A	G	NA	*OreG0003702*
scaffold1758	600778	A	G	NA	*OreG0003702*
scaffold1758	622898	G	T	Och09,Och10	*OreG0003705*
scaffold1758	623488	G	A	Ore08,Ore09,Ore14	*OreG0003705*
scaffold1758	623810	C	T	Ore07,Ore13	*OreG0003705*
scaffold1758	628362	G	A	NA	*OreG0003706*
scaffold1758	639344	A	C	NA	*OreG0003708*
scaffold1758	649739	G	A	NA	*OreG0003711*
scaffold1758	678105	C	T	Och12	*OreG0003716*
scaffold1758	682670	C	T	Och12	*OreG0003717*
scaffold1758	683481	G	T	NA	*OreG0003717*
scaffold1758	695978	G	A	Och01,Och09,Och10,Och12	*OreG0003721*
scaffold1758	696827	C	T	Ore01,Ore02,Ore03,Ore04,Ore05,Ore06,Ore07,Ore08,Ore09,Ore10,Ore11,Ore12,Ore13,Ore14	*OreG0003721*
scaffold1758	697785	G	T	NA	*OreG0003721*
scaffold1758	702563	G	T	NA	*OreG0003722*
scaffold1758	703376	T	C	NA	*OreG0003722*
scaffold1758	703405	G	C	Ore01,Ore02,Ore03,Ore04,Ore05,Ore06,Ore07,Ore08,Ore09,Ore10,Ore11,Ore12,Ore13,Ore14	*OreG0003722*
scaffold1758	704066	C	A	NA	*OreG0003722*
scaffold1758	705159	A	T	Ore01,Ore02,Ore03,Ore04,Ore05,Ore06,Ore07,Ore08,Ore09,Ore10,Ore11,Ore12,Ore13,Ore14	*OreG0003722*
scaffold1758	705540	G	A	Och08,Och11,Och14	*OreG0003722*
scaffold1758	705588	C	A	Och08,Och11,Och14	*OreG0003722*
scaffold1758	705966	C	T	NA	*OreG0003722*
scaffold1758	706413	T	A	Ore01,Ore04,Ore05,Ore08,Ore09,Ore11,Ore14	*OreG0003722*
scaffold1758	706425	A	C	Och12	*OreG0003722*
scaffold1758	709073	C	A	NA	*OreG0003723*
scaffold1758	727728	G	A	NA	*OreG0003725*
scaffold1758	775879	T	A	NA	*OreG0003731*
scaffold1758	776513	A	G	NA	*OreG0003731*
scaffold1758	776725	C	T	NA	*OreG0003731*
scaffold1758	776899	A	G	NA	*OreG0003731*
scaffold1758	832531	C	G	NA	*OreG0003739*
scaffold1758	832901	C	A	NA	*OreG0003739*
scaffold1758	832930	C	A	NA	*OreG0003739*
scaffold1758	834406	C	T	NA	*OreG0003739*
scaffold1758	834695	T	C	Och01,Och02,Och03,Och04,Och05,Och06,Och07,Och08,Och09,Och10,Och11,Och12,Och13,Och14,Ore02,Ore06,Ore08,Ore09,Ore10,Ore12,Ore14	*OreG0003739*
scaffold1758	835111	T	G	NA	*OreG0003739*
scaffold1758	846462	T	C	NA	*OreG0003740*
scaffold1758	855155	T	C	NA	*OreG0003741*
scaffold1758	855391	A	T	Och01,Och02,Och03,Och04,Och05,Och06,Och07,Och08,Och09,Och10,Och11,Och12,Och13,Och14,Ore01,Ore02,Ore03,Ore04,Ore05,Ore06,Ore07,Ore08,Ore09,Ore10,Ore11,Ore12,Ore13,Ore14	*OreG0003741*
scaffold1758	894775	G	C	Och01,Och02,Och03,Och04,Och05,Och06,Och07,Och08,Och09,Och10,Och11,Och12,Och13,Och14	*OreG0003743*
scaffold1758	913456	C	T	NA	*OreG0003746*
scaffold1758	927611	A	G	Ore01,Ore02,Ore03,Ore04,Ore05,Ore06,Ore07,Ore08,Ore09,Ore10,Ore11,Ore12,Ore13,Ore14	*OreG0003748*
scaffold1758	927905	G	A	NA	*OreG0003748*
scaffold1758	928130	G	A	NA	*OreG0003748*
scaffold1758	930403	T	G	Ore01,Ore02,Ore03,Ore04,Ore05,Ore06,Ore07,Ore08,Ore09,Ore10,Ore11,Ore12,Ore13,Ore14	*OreG0003748*
scaffold1758	934542	C	T	Och02,Och03,Och04,Och05,Och06,Och08,Och11,Och12,Och13	*OreG0003748*
scaffold1758	939423	A	C	NA	*OreG0003749*
scaffold1758	964398	G	T	Och01,Och02,Och03,Och04,Och05,Och06,Och07,Och08,Och09,Och10,Och11,Och12,Och13,Och14,Ore01,Ore02,Ore03,Ore04,Ore05,Ore06,Ore07,Ore08,Ore09,Ore10,Ore11,Ore12,Ore13,Ore14	*OreG0003752*
scaffold1758	968947	G	T	NA	*OreG0003753*
scaffold1758	971673	A	G	Och12,Och14	*OreG0003755*
scaffold1758	971853	C	G	Och12	*OreG0003755*
scaffold1758	971989	A	G	NA	*OreG0003755*
scaffold1758	974400	T	C	NA	*OreG0003756*
scaffold1758	976929	G	A	Och01,Och07,Och08,Och09,Och10,Och11	*OreG0003756*
scaffold1758	978458	A	C	NA	*OreG0003756*
scaffold1758	979936	T	A	Och08	*OreG0003756*

scaffold1758	981871	C	G	Och08	*OreG0003756*
scaffold1758	982423	G	A	Och12	*OreG0003756*
scaffold1758	1002064	C	T	Och08,Och09,Och10	*OreG0003761*
scaffold1758	1004612	C	T	Och01,Och02,Och03,Och04,Och05,Och06,Och07,Och08,Och09,Och10,Och11,Och12,Och13,Och14	*OreG0003763*
scaffold1758	1007139	C	G	Och02,Och03,Och04,Och05,Och12,Och13	*OreG0003762*
scaffold1758	1009069	G	A	NA	*OreG0003762*
scaffold1758	1044483	G	C	Och02,Och03,Och04,Och05,Och06,Och08,Och11,Och12,Och13	*OreG0003768*
scaffold1758	1060691	C	T	Och01,Och02,Och03,Och04,Och05,Och06,Och07,Och08,Och09,Och10,Och11,Och12,Och13,Och14,Ore01,Ore02,Ore03,Ore04,Ore05,Ore06,Ore07,Ore08,Ore09,Ore10,Ore11,Ore12,Ore13,Ore14	*OreG0003770*
scaffold1758	1084683	T	C	NA	*OreG0003771*
scaffold1758	1092780	T	A	NA	*OreG0003772*
scaffold1758	1137042	A	G	Ore01,Ore02,Ore03,Ore04,Ore05,Ore06,Ore07,Ore08,Ore09,Ore10,Ore11,Ore12,Ore13,Ore14	*OreG0003773*
scaffold1758	1159936	T	C	NA	*OreG0003776*
scaffold1758	1176263	T	G	Och01,Och02,Och03,Och04,Och05,Och06,Och07,Och08,Och09,Och10,Och11,Och12,Och13,Och14	*OreG0003777*
scaffold1758	1179829	G	A	Och02,Och03,Och04,Och11	*OreG0003778*
scaffold1758	1190059	T	C	Och02,Och03,Och04,Och09,Och10,Och11	*OreG0003780*
scaffold1758	1190307	C	G	Och01,Och08,Och14	*OreG0003780*
scaffold1758	1190973	G	A	NA	*OreG0003780*
scaffold1758	1191233	C	G	NA	*OreG0003780*
scaffold1758	1194736	G	T	Ore01,Ore02,Ore03,Ore04,Ore05,Ore06,Ore07,Ore08,Ore09,Ore10,Ore11,Ore12,Ore13,Ore14	*OreG0003781*
scaffold1758	1204529	C	G	Och01,Och02,Och03,Och04,Och05,Och06,Och07,Och08,Och09,Och10,Och11,Och12,Och13,Och14	*OreG0003783*
scaffold1758	1204725	T	C	NA	*OreG0003783*
scaffold1758	1245652	T	G	NA	*OreG0003792*
scaffold1758	1275606	G	A	NA	*OreG0003797*
scaffold1758	1276030	G	C	Och13	*OreG0003797*
scaffold1758	1276183	T	G	NA	*OreG0003797*
scaffold1758	1294229	G	A	NA	*OreG0003802*
scaffold1758	1295042	A	G	NA	*OreG0003802*
scaffold1758	1327669	T	G	Ore01,Ore02,Ore03,Ore04,Ore05,Ore06,Ore07,Ore08,Ore09,Ore10,Ore11,Ore12,Ore13,Ore14	*OreG0003806*
scaffold1758	1347684	T	A	Och08	*OreG0003808*
scaffold1758	1352588	C	T	Och08	*OreG0003810*
scaffold1758	1352757	G	T	Och08	*OreG0003810*
scaffold1758	1358143	C	T	NA	*OreG0003811*
scaffold1758	1362257	G	C	Och01,Och02,Och03,Och04,Och05,Och06,Och07,Och08,Och09,Och10,Och11,Och12,Och13,Och14,Ore01,Ore02,Ore03,Ore04,Ore05,Ore06,Ore07,Ore08,Ore09,Ore10,Ore11,Ore12,Ore13,Ore14	*OreG0003812*
scaffold1758	1362485	G	A	Och01,Och02,Och03,Och04,Och05,Och06,Och07,Och08,Och09,Och10,Och11,Och12,Och13,Och14,Ore01,Ore02,Ore03,Ore04,Ore05,Ore06,Ore07,Ore08,Ore09,Ore10,Ore11,Ore12,Ore13,Ore14	*OreG0003812*
scaffold1758	1367222	A	G	Och01,Och02,Och03,Och04,Och05,Och06,Och07,Och08,Och09,Och10,Och11,Och12,Och13,Och14,Ore01,Ore02,Ore03,Ore04,Ore05,Ore06,Ore07,Ore08,Ore09,Ore10,Ore11,Ore12,Ore13,Ore14	*OreG0003813*
scaffold1758	1367462	T	C	NA	*OreG0003813*
scaffold1758	1397684	G	T	Och07,Och08	*OreG0003819*
scaffold1758	1398170	C	T	Ore01,Ore02,Ore03,Ore04,Ore05,Ore06,Ore07,Ore08,Ore09,Ore10,Ore11,Ore12,Ore13,Ore14	*OreG0003819*
scaffold1758	1418730	G	T	NA	*OreG0003822*
scaffold1758	1418934	G	A	NA	*OreG0003822*
scaffold1758	1442097	G	C	Ore01,Ore02,Ore03,Ore04,Ore05,Ore06,Ore07,Ore08,Ore09,Ore10,Ore11,Ore12,Ore13,Ore14	*OreG0003825*
scaffold1758	1443902	C	T	Ore01,Ore02,Ore03,Ore04,Ore05,Ore06,Ore07,Ore08,Ore09,Ore10,Ore11,Ore12,Ore13,Ore14	*OreG0003825*
scaffold1758	1446715	G	A	Och01	*OreG0003825*
scaffold1758	1449659	C	A	NA	*OreG0003825*
scaffold1758	1451851	G	A	Och05	*OreG0003825*
scaffold1758	1453223	T	C	Och01,Och02,Och03,Och04,Och05,Och06,Och07,Och08,Och09,Och10,Och11,Och12,Och13,Och14,Ore01,Ore02,Ore03,Ore04,Ore05,Ore06,Ore07,Ore08,Ore09,Ore10,Ore11,Ore12,Ore13,Ore14	*OreG0003825*
scaffold1758	1453961	T	A	Och01	*OreG0003825*
scaffold1758	1463576	C	A	Ore01,Ore09	*OreG0003826*
scaffold1758	1572875	G	A	Och01,Och02,Och03,Och04,Och05,Och06,Och07,Och08,Och09,Och10,Och11,Och12,Och13,Och14,Ore01,Ore02,Ore03,Ore04,Ore05,Ore06,Ore07,Ore08,Ore09,Ore10,Ore11,Ore12,Ore13,Ore14	*OreG0003834*
scaffold1758	1573034	C	A	Ore01,Ore02,Ore03,Ore04,Ore05,Ore06,Ore07,Ore08,Ore09,Ore10,Ore11,Ore12,Ore13,Ore14	*OreG0003834*
scaffold1758	1575880	G	A	Och01,Och07,Och08,Och14	*OreG0003835*
scaffold1758	1578191	C	T	Och01,Och07,Och08,Och14	*OreG0003835*
scaffold1758	1583104	C	A	NA	*OreG0003835*

scaffold1758	1588903	G	A	Och13	*OreG0003836*
scaffold1758	1610485	C	T	Ore01,Ore03,Ore04,Ore05,Ore09,Ore10,Ore13,Ore14	*OreG0003839*
scaffold1758	1615444	T	C	Och01,Och02,Och03,Och04,Och05,Och06,Och07,Och08,Och09,Och10,Och11,Och12,Och13,Och14	*OreG0003840*
scaffold1758	1648257	T	A	Ore04,Ore05	*OreG0003842*
scaffold1758	1655434	A	C	NA	*OreG0003843*
scaffold1758	1659437	C	T	Ore01,Ore02,Ore03,Ore04,Ore05,Ore06,Ore07,Ore08,Ore09,Ore10,Ore11,Ore12,Ore13,Ore14	*OreG0003844*
scaffold1758	1659752	T	A	Och05,Och08	*OreG0003844*
scaffold1758	1659884	C	T	NA	*OreG0003844*
scaffold1758	1660133	T	A	Och01,Och02,Och03,Och04,Och05,Och06,Och07,Och08,Och09,Och10,Och11,Och12,Och13,Och14,Ore01,Ore02,Ore03,Ore04,Ore05,Ore06,Ore07,Ore08,Ore09,Ore10,Ore11,Ore12,Ore13,Ore14	*OreG0003844*
scaffold1758	1660646	A	G	NA	*OreG0003844*
scaffold1758	1743597	C	T	Och05	*OreG0003854*
scaffold1758	1757683	A	G	Ore01,Ore02,Ore03,Ore04,Ore05,Ore06,Ore07,Ore08,Ore09,Ore10,Ore11,Ore12,Ore13,Ore14	*OreG0003857*
scaffold1758	1758100	C	G	Och01,Och02,Och03,Och04,Och05,Och06,Och07,Och08,Och09,Och10,Och11,Och12,Och13,Och14	*OreG0003857*
scaffold1758	1770455	T	C	NA	*OreG0003859*
scaffold1758	1773269	G	C	Ore01,Ore02,Ore03,Ore04,Ore05,Ore06,Ore07,Ore08,Ore09,Ore10,Ore11,Ore12,Ore13,Ore14	*OreG0003859*
scaffold1758	1775804	A	T	Ore01,Ore03,Ore04,Ore05,Ore09,Ore10,Ore13,Ore14	*OreG0003860*
scaffold1758	1782045	T	A	Och01,Och02,Och03,Och04,Och05,Och06,Och07,Och08,Och09,Och10,Och11,Och12,Och13,Och14,Ore01,Ore02,Ore03,Ore04,Ore05,Ore06,Ore07,Ore08,Ore09,Ore10,Ore11,Ore12,Ore13,Ore14	*OreG0003861*
scaffold1758	1782487	C	G	NA	*OreG0003861*
scaffold1758	1793542	A	G	Och08	*OreG0003864*
scaffold1758	1794503	A	G	Ore01,Ore09	*OreG0003864*
scaffold1758	1794803	G	A	Och08	*OreG0003864*
scaffold1758	1795671	G	A	Ore01,Ore02,Ore03,Ore04,Ore05,Ore06,Ore07,Ore08,Ore09,Ore10,Ore11,Ore12,Ore13,Ore14	*OreG0003864*
scaffold1758	1818489	T	C	NA	*OreG0003867*
scaffold1758	1819539	G	T	Ore01,Ore02,Ore03,Ore04,Ore05,Ore06,Ore07,Ore08,Ore09,Ore10,Ore11,Ore12,Ore13,Ore14	*OreG0003867*
scaffold1758	1824879	G	A	NA	*OreG0003868*
scaffold1758	1824892	G	T	NA	*OreG0003868*
scaffold1758	1825479	G	T	Ore01,Ore02,Ore03,Ore04,Ore05,Ore06,Ore07,Ore08,Ore09,Ore10,Ore11,Ore12,Ore13,Ore14	*OreG0003868*
scaffold1758	1827179	T	C	NA	*OreG0003869*
scaffold1758	1827510	C	A	Och01,Och02,Och03,Och04,Och05,Och06,Och07,Och08,Och09,Och10,Och11,Och12,Och13,Och14,Ore01,Ore02,Ore03,Ore04,Ore05,Ore06,Ore07,Ore08,Ore09,Ore10,Ore11,Ore12,Ore13,Ore14	*OreG0003869*
scaffold1758	1860335	C	T	Ore01,Ore09	*OreG0003871*
scaffold1758	1865600	C	T	Och01,Och02,Och03,Och04,Och05,Och06,Och07,Och08,Och09,Och10,Och11,Och12,Och13,Och14	*OreG0003872*
scaffold1758	1865642	C	T	NA	*OreG0003872*
scaffold1758	1867093	G	A	NA	*OreG0003872*
scaffold1758	1896686	G	T	NA	*OreG0003874*
scaffold1758	1905225	T	A	Och01,Och02,Och03,Och04,Och05,Och06,Och07,Och08,Och09,Och10,Och11,Och12,Och13,Och14,Ore01,Ore02,Ore03,Ore04,Ore05,Ore06,Ore07,Ore08,Ore09,Ore10,Ore11,Ore12,Ore13,Ore14	*OreG0003875*
scaffold1758	1939491	C	T	Ore01,Ore02,Ore03,Ore04,Ore05,Ore06,Ore07,Ore08,Ore09,Ore10,Ore11,Ore12,Ore13,Ore14	*OreG0003879*
scaffold1758	1939798	G	A	NA	*OreG0003879*
scaffold1758	1939867	C	A	Och02,Och04,Och06,Och07,Och08,Och13	*OreG0003879*
scaffold1758	1939997	T	C	Och01,Och02,Och03,Och04,Och05,Och06,Och07,Och08,Och09,Och10,Och11,Och12,Och13,Och14,Ore01,Ore02,Ore03,Ore04,Ore05,Ore06,Ore07,Ore08,Ore09,Ore10,Ore11,Ore12,Ore13,Ore14	*OreG0003879*
scaffold1758	1953420	C	T	NA	*OreG0003881*
scaffold1758	1961398	C	A	Ore01,Ore02,Ore03,Ore04,Ore05,Ore06,Ore07,Ore08,Ore09,Ore10,Ore11,Ore12,Ore13,Ore14	*OreG0003883*
scaffold1758	1972206	A	G	Och13	*OreG0003884*
scaffold1758	1978431	T	G	NA	*OreG0003885*
scaffold1758	1988111	T	C	Och08	*OreG0003889*
scaffold1758	2006939	A	T	Ore01,Ore09	*OreG0003891*
scaffold1758	2007531	G	T	Och04,Och13	*OreG0003891*
scaffold1758	2007587	T	C	NA	*OreG0003891*
scaffold1758	2007839	G	A	NA	*OreG0003891*
scaffold1758	2008223	C	T	NA	*OreG0003891*
scaffold1758	2013755	C	A	NA	*OreG0003892*
scaffold1758	2022068	T	C	Ore01,Ore02,Ore03,Ore04,Ore05,Ore06,Ore07,Ore08,Ore09,Ore10,Ore11,Ore12,Ore13,Ore14	*OreG0003894*
scaffold1758	2022125	A	G	Och01,Och02,Och03,Och04,Och05,Och06,Och07,Och08,Och09,Och10,Och11,Och12,Och13,Och14	*OreG0003894*

scaffold1758	2061001	G	T	NA	*OreG0003899*
scaffold1758	2118799	G	A	NA	*OreG0003902*
scaffold1758	2122816	G	A	NA	*OreG0003902*
scaffold1758	2122870	A	T	NA	*OreG0003902*
scaffold3374	1757	G	A	Ore01,Ore02,Ore03,Ore04,Ore05,Ore06,Ore07,Ore08,Ore09,Ore10,Ore11,Ore13,Ore14	*OreG0018566*
scaffold3374	2084	C	T	NA	*OreG0018566*
scaffold3374	3512	T	A	NA	*OreG0018566*
scaffold3454	8036	T	A	Ore03,Ore06,Ore09	*OreG0018614*
scaffold2142	82920	A	T	Och01,Och02,Och03,Och04,Och05,Och06,Och07,Och08,Och09,Och10,Och11,Och12,Och13,Och14,Ore01,Ore02,Ore03,Ore04,Ore05,Ore06,Ore07,Ore08,Ore09,Ore10,Ore11,Ore12,Ore13,Ore14	*OreG0006012*
scaffold2142	154115	G	T	Och14,Ore01,Ore02,Ore03,Ore04,Ore05,Ore06,Ore07,Ore08,Ore09,Ore10,Ore11,Ore12,Ore13,Ore14	*OreG0006020*
scaffold2142	159104	C	A	NA	*OreG0006021*
scaffold2142	159157	C	A	Och01,Och04,Och05,Och06,Och08,Och09,Och10,Och12,Och13	*OreG0006021*
scaffold2142	159410	C	A	NA	*OreG0006021*
scaffold2142	159808	C	T	Ore01,Ore02,Ore04,Ore05,Ore06,Ore10,Ore11,Ore12	*OreG0006021*
scaffold2142	164881	C	A	NA	*OreG0006022*
scaffold2142	169452	G	A	Och01,Och02,Och03,Och04,Och05,Och06,Och07,Och08,Och09,Och10,Och11,Och12,Och13,Och14	*OreG0006023*
scaffold2142	265029	T	G	Och01,Och02,Och03,Och04,Och05,Och06,Och07,Och08,Och09,Och10,Och11,Och12,Och13,Och14	*OreG0006033*
scaffold2142	340965	C	A	Ore01,Ore02,Ore03,Ore04,Ore05,Ore06,Ore07,Ore08,Ore09,Ore10,Ore11,Ore12,Ore13,Ore14	*OreG0006042*
scaffold2142	341164	T	A	Och01,Och02,Och03,Och04,Och05,Och06,Och07,Och08,Och09,Och10,Och11,Och12,Och13,Och14,Ore01,Ore02,Ore03,Ore04,Ore05,Ore06,Ore07,Ore08,Ore09,Ore10,Ore11,Ore12,Ore13,Ore14	*OreG0006042*
scaffold2142	349702	A	T	NA	*OreG0006044*
scaffold2142	358777	G	A	Ore06,Ore11	*OreG0006045*
scaffold2142	364775	A	C	Och01,Och02,Och03,Och04,Och05,Och06,Och07,Och08,Och09,Och10,Och11,Och12,Och13,Och14	*OreG0006045*
scaffold2142	365396	A	G	Och01,Och02,Och03,Och04,Och05,Och06,Och07,Och08,Och09,Och10,Och11,Och12,Och13,Och14	*OreG0006045*
scaffold2142	369021	T	G	Ore01,Ore02,Ore03,Ore04,Ore05,Ore06,Ore07,Ore08,Ore09,Ore10,Ore11,Ore12,Ore13,Ore14	*OreG0006046*
scaffold2142	369130	G	A	Och09,Och10	*OreG0006046*
scaffold2142	404225	A	G	NA	*OreG0006048*
scaffold2142	423980	G	A	Ore01,Ore02,Ore03,Ore04,Ore05,Ore06,Ore07,Ore08,Ore09,Ore10,Ore11,Ore12,Ore13,Ore14	*OreG0006049*
scaffold2142	425145	C	G	NA	*OreG0006049*
scaffold917	20382	A	G	Ore01,Ore02,Ore03,Ore04,Ore05,Ore06,Ore07,Ore08,Ore09,Ore10,Ore11,Ore12,Ore13,Ore14	*OreG0026645*
scaffold917	52536	C	A	NA	*OreG0026647*
scaffold917	55157	G	T	Ore01,Ore02,Ore03,Ore04,Ore05,Ore06,Ore07,Ore08,Ore09,Ore10,Ore11,Ore12,Ore13,Ore14	*OreG0026647*
scaffold917	98509	T	C	Ore01,Ore02,Ore03,Ore04,Ore05,Ore06,Ore07,Ore08,Ore09,Ore10,Ore11,Ore12,Ore13,Ore14	*OreG0026648*
scaffold917	213389	A	G	Ore01,Ore02,Ore03,Ore04,Ore05,Ore06,Ore07,Ore08,Ore09,Ore10,Ore11,Ore12,Ore13,Ore14	*OreG0026649*
scaffold917	241143	C	A	Och01	*OreG0026650*
scaffold917	464859	C	G	Och01,Och02,Och03,Och04,Och05,Och06,Och07,Och08,Och09,Och10,Och11,Och12,Och13,Och14	*OreG0026651*
scaffold917	673681	G	C	Ore01,Ore02,Ore03,Ore04,Ore05,Ore06,Ore07,Ore08,Ore09,Ore10,Ore11,Ore12,Ore13,Ore14	*OreG0026655*
scaffold917	676557	C	A	Och01,Och02,Och03,Och04,Och05,Och06,Och07,Och08,Och09,Och10,Och11,Och12,Och13,Och14,Ore01,Ore02,Ore03,Ore04,Ore05,Ore06,Ore07,Ore08,Ore09,Ore10,Ore11,Ore12,Ore13,Ore14	*OreG0026655*
scaffold917	676673	T	A	Och01,Och11	*OreG0026655*
scaffold917	714748	T	A	NA	*OreG0026656*
scaffold917	764073	T	C	Ore01,Ore02,Ore03,Ore04,Ore05,Ore06,Ore07,Ore08,Ore09,Ore10,Ore11,Ore12,Ore13,Ore14	*OreG0026660*
scaffold917	804617	G	A	Ore01,Ore02,Ore03,Ore04,Ore05,Ore06,Ore07,Ore08,Ore09,Ore10,Ore11,Ore12,Ore13,Ore14	*OreG0026663*
scaffold917	856251	T	G	NA	*OreG0026666*
scaffold917	856300	C	A	NA	*OreG0026666*
scaffold917	856527	G	T	NA	*OreG0026666*
scaffold917	956925	C	T	NA	*OreG0026670*
scaffold917	957054	C	T	Och01,Och02,Och03,Och04,Och05,Och06,Och07,Och08,Och09,Och10,Och11,Och12,Och13,Och14	*OreG0026670*
scaffold917	1096140	C	T	Och01,Och02,Och03,Och04,Och05,Och06,Och07,Och08,Och09,Och10,Och11,Och12,Och13,Och14	*OreG0026677*
scaffold917	1096468	G	A	Och01,Och07,Och08,Och11,Och14	*OreG0026677*
scaffold917	1097343	G	T	Och02,Och03,Och04,Och05,Och06,Och12,Och13	*OreG0026678*
scaffold917	1656693	T	C	Och01,Och02,Och03,Och04,Och05,Och06,Och07,Och08,Och09,Och10,Och11,Och12,Och13,Och14	*OreG0026692*

scaffold917	1659803	G	A	Ore01,Ore02,Ore03,Ore04,Ore05,Ore06,Ore07,Ore08,Ore09,Ore10,Ore11,Ore12,Ore13,Ore14	*OreG0026693*
scaffold917	1762737	G	A	NA	*OreG0026698*
scaffold917	1847772	G	A	Och04,Och06,Och13	*OreG0026699*
scaffold917	2088623	C	T	NA	*OreG0026709*
scaffold917	2095181	T	C	NA	*OreG0026709*
scaffold917	2215643	C	T	NA	*OreG0026713*
scaffold917	2341546	G	A	NA	*OreG0026717*
scaffold917	2511100	G	A	Ore01,Ore02,Ore03,Ore04,Ore05,Ore06,Ore07,Ore08,Ore09,Ore10,Ore11,Ore12,Ore13,Ore14	*OreG0026718*
scaffold917	2596933	T	A	Och01,Och02,Och03,Och04,Och05,Och06,Och07,Och08,Och09,Och10,Och11,Och12,Och13,Och14,Ore01,Ore02,Ore03,Ore04,Ore05,Ore06,Ore07,Ore08,Ore09,Ore10,Ore11,Ore12,Ore13,Ore14	*OreG0026723*
scaffold917	2694293	A	G	Och04,Och06,Och13	*OreG0026729*
scaffold917	2709856	C	T	Och01,Och02,Och03,Och04,Och05,Och06,Och07,Och08,Och09,Och10,Och11,Och12,Och13,Och14,Ore01,Ore02,Ore03,Ore04,Ore05,Ore06,Ore07,Ore08,Ore09,Ore10,Ore11,Ore12,Ore13,Ore14	*OreG0026730*
scaffold917	2750975	C	T	Ore01,Ore02,Ore03,Ore04,Ore05,Ore06,Ore07,Ore08,Ore09,Ore10,Ore11,Ore12,Ore13,Ore14	*OreG0026732*
scaffold917	2824417	G	A	Och04,Och06,Och13,Och14	*OreG0026734*
scaffold917	2842863	C	T	Ore04,Ore05,Ore13	*OreG0026735*
scaffold917	2843030	T	A	Ore01,Ore03,Ore06,Ore09	*OreG0026735*
scaffold917	2847427	C	A	Och01,Och02,Och03,Och04,Och05,Och06,Och07,Och08,Och09,Och10,Och11,Och12,Och13,Och14,Ore01,Ore02,Ore03,Ore04,Ore05,Ore06,Ore07,Ore08,Ore09,Ore10,Ore11,Ore12,Ore13,Ore14	*OreG0026735*
scaffold917	2904383	A	G	Och01	*OreG0026737*
scaffold917	2904936	G	C	NA	*OreG0026737*
scaffold917	2925267	A	G	NA	*OreG0026739*
scaffold917	2925342	C	T	Och04,Och06,Och07,Och08,Och11,Och13,Ore01,Ore02,Ore03,Ore04,Ore05,Ore06,Ore07,Ore08,Ore09,Ore10,Ore11,Ore12,Ore13,Ore14	*OreG0026739*
scaffold917	2925878	G	T	NA	*OreG0026739*
scaffold917	2990097	A	T	NA	*OreG0026741*
scaffold917	2990310	A	T	Och11	*OreG0026741*
scaffold917	2995057	C	G	NA	*OreG0026742*
scaffold917	3048034	A	G	NA	*OreG0026744*
scaffold917	3048479	A	G	Och01,Och02,Och03,Och04,Och05,Och06,Och07,Och08,Och09,Och10,Och11,Och12,Och13,Och14	*OreG0026744*
scaffold917	3123568	G	T	Och01,Och02,Och03,Och04,Och05,Och06,Och07,Och08,Och09,Och10,Och11,Och12,Och13,Och14	*OreG0026751*
scaffold917	3235154	C	T	NA	*OreG0026754*
scaffold917	3235571	C	T	Ore04,Ore05,Ore13	*OreG0026754*
scaffold917	3391668	G	T	Och01,Och02,Och03,Och04,Och05,Och06,Och07,Och08,Och09,Och10,Och11,Och12,Och13,Och14,Ore01,Ore02,Ore03,Ore04,Ore05,Ore06,Ore07,Ore08,Ore09,Ore10,Ore11,Ore12,Ore13,Ore14	*OreG0026763*
scaffold917	3446793	G	T	Och04,Och06,Och13	*OreG0026764*
scaffold917	3448857	C	T	NA	*OreG0026764*
scaffold917	3453469	G	A	NA	*OreG0026765*
scaffold917	3454718	G	A	NA	*OreG0026765*
scaffold917	3571377	G	A	NA	*OreG0026772*
scaffold917	3571385	C	T	NA	*OreG0026772*
scaffold2001	678197	C	A	Och01,Och02,Och03,Och04,Och05,Och06,Och07,Och08,Och09,Och10,Och11,Och12,Och13,Och14	*OreG0005081*
scaffold2001	685658	T	G	NA	*OreG0005083*
scaffold2001	686193	A	T	NA	*OreG0005083*
scaffold2001	701344	A	C	Och01,Och02,Och03,Och04,Och05,Och06,Och07,Och08,Och09,Och10,Och11,Och12,Och13,Och14	*OreG0005085*
scaffold2001	704315	C	T	Och01,Och02,Och03,Och04,Och05,Och06,Och07,Och08,Och09,Och10,Och11,Och12,Och13,Och14	*OreG0005085*
scaffold2001	704570	A	G	Ore03,Ore07,Ore08,Ore10,Ore14	*OreG0005085*
scaffold2001	704957	T	C	NA	*OreG0005085*
scaffold2001	705038	A	T	NA	*OreG0005085*
scaffold2001	726300	G	A	Ore01,Ore02,Ore03,Ore04,Ore05,Ore06,Ore07,Ore08,Ore09,Ore10,Ore11,Ore12,Ore13,Ore14	*OreG0005088*
scaffold2001	726306	G	A	Ore01,Ore02,Ore03,Ore04,Ore05,Ore06,Ore07,Ore08,Ore09,Ore10,Ore11,Ore12,Ore13,Ore14	*OreG0005088*
scaffold2001	727092	G	A	Ore01,Ore02,Ore03,Ore04,Ore05,Ore07,Ore08,Ore09,Ore10,Ore14	*OreG0005088*
scaffold2001	727192	C	T	Ore01,Ore02,Ore03,Ore04,Ore05,Ore06,Ore07,Ore08,Ore09,Ore10,Ore11,Ore12,Ore13,Ore14	*OreG0005088*
scaffold2001	727209	G	A	Och01,Och02,Och03,Och04,Och05,Och06,Och07,Och08,Och09,Och10,Och11,Och12,Och13,Och14,Ore01,Ore02,Ore03,Ore04,Ore05,Ore06,Ore07,Ore08,Ore09,Ore10,Ore11,Ore12,Ore13,Ore14	*OreG0005088*
scaffold2001	728107	C	A	NA	*OreG0005088*
scaffold2001	728654	G	A	Ore04,Ore05,Ore09	*OreG0005088*
scaffold2001	734010	A	G	Och01,Och02,Och03,Och04,Och05,Och06,Och07,Och08,Och09,Och10,Och11,Och12,Och13,Och14	*OreG0005088*

scaffold2001	734307	G	T	NA	*OreG0005088*
scaffold2001	734332	T	G	Ore01,Ore02,Ore03,Ore04,Ore05,Ore06,Ore07,Ore08,Ore09,Ore10,Ore11,Ore12,Ore13,Ore14	*OreG0005088*
scaffold2001	734857	C	A	Och01,Och02,Och03,Och04,Och05,Och06,Och07,Och08,Och09,Och10,Och11,Och12,Och13,Och14	*OreG0005088*
scaffold2001	871988	T	G	Och01,Och02,Och03,Och04,Och05,Och06,Och07,Och08,Och09,Och10,Och11,Och12,Och13,Och14	*OreG0005097*
scaffold2001	895856	A	T	Ore01,Ore02,Ore03,Ore04,Ore05,Ore06,Ore07,Ore08,Ore09,Ore10,Ore11,Ore12,Ore13,Ore14	*OreG0005100*
scaffold2001	901468	C	T	Och01,Och14	*OreG0005101*
scaffold2001	904169	C	A	Och02,Och06	*OreG0005101*
scaffold2001	904645	A	G	Och01,Och02,Och03,Och04,Och05,Och06,Och07,Och08,Och09,Och10,Och11,Och12,Och13,Och14,Ore01,Ore02,Ore03,Ore04,Ore05,Ore06,Ore07,Ore08,Ore09,Ore10,Ore11,Ore12,Ore13,Ore14	*OreG0005101*
scaffold2001	932309	G	T	Ore01,Ore02,Ore03,Ore04,Ore05,Ore06,Ore07,Ore08,Ore09,Ore10,Ore11,Ore12,Ore13,Ore14	*OreG0005103*
scaffold2001	936610	T	G	Ore01,Ore02,Ore03,Ore04,Ore05,Ore06,Ore07,Ore08,Ore09,Ore10,Ore11,Ore12,Ore13,Ore14	*OreG0005103*
scaffold2001	938392	C	T	Och08	*OreG0005104*
scaffold2001	939462	G	C	Och01,Och02,Och03,Och04,Och05,Och06,Och07,Och08,Och09,Och10,Och11,Och12,Och13,Och14,Ore01,Ore02,Ore03,Ore04,Ore05,Ore06,Ore07,Ore08,Ore09,Ore10,Ore11,Ore12,Ore13,Ore14	*OreG0005104*
scaffold2001	963063	T	A	Ore01,Ore02,Ore03,Ore04,Ore05,Ore06,Ore07,Ore08,Ore09,Ore10,Ore11,Ore12,Ore13,Ore14	*OreG0005106*
scaffold2001	1057034	A	G	NA	*OreG0005113*
scaffold2001	1067065	C	T	Och02,Och03,Och04,Och05,Och06,Och12,Och13	*OreG0005114*
scaffold2001	1100060	A	G	Och01,Och02,Och06	*OreG0005121*
scaffold2001	1100069	G	A	Och01,Och02,Och03,Och04,Och05,Och06,Och07,Och08,Och09,Och10,Och11,Och12,Och13,Och14	*OreG0005121*
scaffold2001	1109258	T	G	Och08,Och09,Och10,Ore01,Ore02,Ore03,Ore04,Ore05,Ore06,Ore07,Ore08,Ore09,Ore10,Ore11,Ore12,Ore13,Ore14	*OreG0005122*
scaffold2001	1109313	C	G	NA	*OreG0005122*
scaffold2001	1109562	A	T	Ore01,Ore02,Ore03,Ore04,Ore05,Ore06,Ore07,Ore08,Ore09,Ore10,Ore11,Ore12,Ore13,Ore14	*OreG0005122*
scaffold2001	1109635	C	A	Ore01,Ore02,Ore03,Ore04,Ore05,Ore06,Ore07,Ore08,Ore09,Ore10,Ore11,Ore12,Ore13,Ore14	*OreG0005122*
scaffold2001	1109662	A	T	NA	*OreG0005122*
scaffold2001	1109791	C	A	Ore01,Ore02,Ore03,Ore04,Ore05,Ore06,Ore07,Ore08,Ore09,Ore10,Ore11,Ore12,Ore13,Ore14	*OreG0005122*
scaffold2001	1109939	A	T	Ore01,Ore02,Ore03,Ore04,Ore05,Ore06,Ore07,Ore08,Ore09,Ore10,Ore11,Ore12,Ore13,Ore14	*OreG0005122*
scaffold2001	1113231	A	T	Ore01,Ore02,Ore03,Ore04,Ore05,Ore06,Ore07,Ore08,Ore09,Ore10,Ore11,Ore12,Ore13,Ore14	*OreG0005123*
scaffold2001	1129063	G	A	Och08	*OreG0005125*
scaffold2001	1167832	T	G	Ore01,Ore02,Ore03,Ore04,Ore05,Ore06,Ore07,Ore08,Ore09,Ore10,Ore11,Ore12,Ore13,Ore14	*OreG0005127*
scaffold2001	1167922	C	A	Och08	*OreG0005127*
scaffold2001	1182416	C	T	Ore03,Ore07,Ore08,Ore10,Ore11,Ore12,Ore13,Ore14	*OreG0005128*
scaffold2001	1183365	G	A	NA	*OreG0005128*
scaffold2001	1386257	A	G	Ore01,Ore02,Ore03,Ore04,Ore05,Ore06,Ore07,Ore08,Ore09,Ore10,Ore11,Ore12,Ore13,Ore14	*OreG0005141*
scaffold2001	1408472	G	T	Och01,Och13	*OreG0005142*
scaffold2001	1408496	C	T	Och02,Och05,Och06,Och07,Och08,Och09,Och10,Och11,Och13	*OreG0005142*
scaffold2001	1408809	A	G	Och01,Och02,Och03,Och04,Och05,Och06,Och07,Och08,Och09,Och10,Och11,Och12,Och13,Och14,Ore01,Ore02,Ore03,Ore04,Ore05,Ore06,Ore07,Ore08,Ore09,Ore10,Ore11,Ore12,Ore13,Ore14	*OreG0005142*
scaffold2001	1493797	G	A	NA	*OreG0005145*
scaffold3228	74204	G	A	NA	*OreG0010420*
scaffold3874	5231	T	A	NA	*OreG0019570*
scaffold1062	135480	G	A	NA	*OreG0000252*
scaffold1062	138296	T	C	Ore01,Ore02,Ore03,Ore04,Ore05,Ore06,Ore07,Ore08,Ore09,Ore10,Ore11,Ore12,Ore13,Ore14	*OreG0000253*
scaffold1062	138304	G	A	Ore01,Ore02,Ore03,Ore04,Ore05,Ore06,Ore07,Ore08,Ore09,Ore10,Ore11,Ore12,Ore13,Ore14	*OreG0000253*
scaffold1062	138314	C	A	Och03,Och04,Och06	*OreG0000253*
scaffold1062	180405	T	C	NA	*OreG0000258*
scaffold1062	180749	T	A	NA	*OreG0000258*
scaffold1062	180998	G	A	Och01,Och02,Och03,Och04,Och05,Och06,Och07,Och08,Och09,Och10,Och11,Och12,Och13,Och14,Ore01,Ore02,Ore03,Ore04,Ore05,Ore06,Ore07,Ore08,Ore09,Ore10,Ore11,Ore12,Ore13,Ore14	*OreG0000258*
scaffold1062	239450	T	C	Och02,Och03,Och04,Och05,Och06,Och07,Och08,Och11,Och12,Och13,Och14	*OreG0000263*
scaffold1062	294341	G	T	Och01,Och02,Och03,Och04,Och05,Och06,Och07,Och08,Och09,Och10,Och11,Och12,Och13,Och14,Ore01,Ore02,Ore03,Ore04,Ore05,Ore06,Ore07,Ore08,Ore09,Ore10,Ore11,Ore12,Ore13,Ore14	*OreG0000272*
scaffold1062	344773	G	C	Ore01,Ore02,Ore03,Ore04,Ore05,Ore06,Ore07,Ore08,Ore09,Ore10,Ore11,Ore12,Ore13,Ore14	*OreG0000275*

scaffold1062	344873	G	C	Och03,Och09,Och10,Och11	*OreG0000275*
scaffold1062	350168	C	A	Och01,Och02,Och03,Och04,Och05,Och06,Och07,Och08,Och09,Och10,Och11,Och12,Och13,Och14	*OreG0000276*
scaffold1062	362762	G	T	Ore01,Ore02,Ore03,Ore04,Ore05,Ore06,Ore07,Ore08,Ore09,Ore10,Ore11,Ore12,Ore13,Ore14	*OreG0000279*
scaffold1062	374220	G	T	NA	*OreG0000279*
scaffold1062	374639	C	G	Och01,Och02,Och03,Och04,Och05,Och07,Och08,Och09,Och10,Och11,Och12,Och13,Och14	*OreG0000279*
scaffold1062	395015	A	T	Och03,Och07	*OreG0000281*
scaffold1062	406660	G	T	Ore01,Ore02,Ore03,Ore04,Ore05,Ore06,Ore07,Ore08,Ore09,Ore10,Ore11,Ore12,Ore13,Ore14	*OreG0000283*
scaffold1062	406973	C	T	Och01,Och02,Och03,Och04,Och05,Och06,Och07,Och08,Och09,Och10,Och11,Och12,Och13,Och14,Ore01,Ore02,Ore03,Ore04,Ore05,Ore06,Ore07,Ore08,Ore09,Ore10,Ore11,Ore12,Ore13,Ore14	*OreG0000283*
scaffold1062	408789	A	G	Ore01,Ore02,Ore03,Ore04,Ore05,Ore06,Ore07,Ore08,Ore09,Ore10,Ore11,Ore12,Ore13,Ore14	*OreG0000283*
scaffold1062	409039	T	C	Ore01,Ore02,Ore03,Ore04,Ore05,Ore06,Ore07,Ore08,Ore09,Ore10,Ore11,Ore12,Ore13,Ore14	*OreG0000283*
scaffold1062	410195	T	C	NA	*OreG0000283*
scaffold1062	412971	A	T	NA	*OreG0000284*
scaffold1062	414579	A	G	Ore01,Ore02,Ore03,Ore04,Ore05,Ore06,Ore07,Ore08,Ore09,Ore10,Ore11,Ore12,Ore13,Ore14	*OreG0000284*
scaffold1062	414770	G	C	Ore01,Ore02,Ore03,Ore04,Ore05,Ore06,Ore07,Ore08,Ore09,Ore10,Ore11,Ore12,Ore13,Ore14	*OreG0000284*
scaffold1062	414938	T	A	Ore01,Ore02,Ore03,Ore04,Ore05,Ore06,Ore07,Ore08,Ore09,Ore10,Ore11,Ore12,Ore13,Ore14	*OreG0000284*
scaffold1062	428450	T	G	Ore01,Ore02,Ore03,Ore04,Ore05,Ore06,Ore07,Ore08,Ore09,Ore10,Ore11,Ore12,Ore13,Ore14	*OreG0000286*
scaffold1062	470892	C	A	Och01,Och02,Och03,Och04,Och05,Och06,Och07,Och08,Och09,Och10,Och11,Och12,Och13,Och14,Ore01,Ore02,Ore03,Ore04,Ore05,Ore06,Ore07,Ore08,Ore09,Ore10,Ore11,Ore12,Ore13,Ore14	*OreG0000290*
scaffold1062	488754	C	A	NA	*OreG0000292*
scaffold1062	488813	G	A	NA	*OreG0000292*
scaffold1062	492236	T	A	NA	*OreG0000292*
scaffold1062	510107	G	A	Ore13	*OreG0000294*
scaffold1062	650462	C	T	Ore01,Ore02,Ore03,Ore04,Ore05,Ore06,Ore07,Ore08,Ore09,Ore10,Ore11,Ore12,Ore13,Ore14	*OreG0000310*
scaffold1062	673842	G	A	Och01,Och02,Och03,Och04,Och05,Och06,Och07,Och08,Och09,Och10,Och11,Och12,Och13,Och14,Ore01,Ore02,Ore03,Ore04,Ore05,Ore06,Ore07,Ore08,Ore09,Ore10,Ore11,Ore12,Ore13,Ore14	*OreG0000313*
scaffold1062	673921	G	A	Ore01,Ore02,Ore03,Ore04,Ore05,Ore06,Ore07,Ore08,Ore09,Ore10,Ore11,Ore12,Ore13,Ore14	*OreG0000313*
scaffold1062	674046	C	T	Ore01,Ore02,Ore03,Ore04,Ore05,Ore06,Ore07,Ore08,Ore09,Ore10,Ore11,Ore12,Ore13,Ore14	*OreG0000313*
scaffold1062	674500	G	A	NA	*OreG0000313*
scaffold1062	675357	C	A	NA	*OreG0000313*
scaffold1062	675641	T	C	Ore01,Ore02,Ore03,Ore04,Ore05,Ore06,Ore07,Ore08,Ore09,Ore10,Ore11,Ore12,Ore13,Ore14	*OreG0000313*
scaffold1062	682906	C	T	Och01,Och02,Och03,Och04,Och05,Och06,Och07,Och08,Och09,Och10,Och11,Och12,Och13,Och14	*OreG0000314*
scaffold1062	682941	A	T	Ore01,Ore02,Ore03,Ore04,Ore05,Ore06,Ore07,Ore08,Ore09,Ore10,Ore11,Ore12,Ore13,Ore14	*OreG0000314*
scaffold1062	685633	C	A	Och01,Och02,Och03,Och04,Och05,Och06,Och07,Och08,Och09,Och10,Och11,Och12,Och13,Och14	*OreG0000315*
scaffold1062	702150	A	G	NA	*OreG0000317*
scaffold1062	702331	G	A	NA	*OreG0000317*
scaffold1062	702424	C	T	Ore01,Ore02,Ore03,Ore04,Ore05,Ore06,Ore07,Ore08,Ore09,Ore10,Ore11,Ore12,Ore13,Ore14	*OreG0000317*
scaffold1062	702489	A	T	NA	*OreG0000317*
scaffold1062	703113	G	A	Och01,Och02,Och03,Och04,Och05,Och06,Och07,Och08,Och09,Och10,Och11,Och12,Och13,Och14	*OreG0000317*
scaffold1062	712348	G	A	NA	*OreG0000319*
scaffold1062	718599	T	G	Och01,Och02,Och03,Och04,Och05,Och06,Och07,Och08,Och09,Och10,Och11,Och12,Och13,Och14,Ore01,Ore02,Ore03,Ore04,Ore05,Ore06,Ore07,Ore08,Ore09,Ore10,Ore11,Ore12,Ore13,Ore14	*OreG0000320*
scaffold1062	720895	T	C	NA	*OreG0000320*
scaffold1062	732494	A	C	NA	*OreG0000321*
scaffold1062	794654	C	G	Och01,Och02,Och03,Och04,Och05,Och06,Och07,Och08,Och09,Och10,Och11,Och12,Och13,Och14,Ore01,Ore02,Ore03,Ore04,Ore05,Ore06,Ore07,Ore08,Ore09,Ore10,Ore11,Ore12,Ore13,Ore14	*OreG0000326*
scaffold1062	811711	T	C	Och01,Och02,Och03,Och04,Och05,Och06,Och07,Och08,Och09,Och10,Och11,Och12,Och13,Och14,Ore01,Ore02,Ore03,Ore04,Ore05,Ore06,Ore07,Ore08,Ore09,Ore10,Ore11,Ore12,Ore13,Ore14	*OreG0000329*
scaffold1062	812285	C	A	NA	*OreG0000329*
scaffold1062	812554	C	A	Ore01,Ore02,Ore03,Ore04,Ore05,Ore06,Ore07,Ore08,Ore09,Ore10,Ore11,Ore12,Ore13,Ore14	*OreG0000329*

scaffold1062	813635	T	C	Och01,Och02,Och03,Och04,Och05,Och06,Och07,Och08,Och09,Och10,Och11,Och12,Och13,Och14,Ore01,Ore02,Ore03,Ore04,Ore05,Ore06,Ore07,Ore08,Ore09,Ore10,Ore11,Ore12,Ore13,Ore14	*OreG0000329*
scaffold1062	818075	A	C	Och01,Och02,Och03,Och04,Och05,Och06,Och07,Och08,Och09,Och10,Och11,Och12,Och13,Och14,Ore01,Ore02,Ore03,Ore04,Ore05,Ore06,Ore07,Ore08,Ore09,Ore10,Ore11,Ore12,Ore13,Ore14	*OreG0000330*
scaffold1062	859674	C	T	NA	*OreG0000332*
scaffold1062	866718	A	T	NA	*OreG0000334*
scaffold1062	867832	C	G	NA	*OreG0000334*
scaffold1062	888835	G	A	Och01,Och02,Och03,Och04,Och05,Och06,Och07,Och08,Och09,Och10,Och11,Och12,Och13,Och14,Ore01,Ore02,Ore03,Ore04,Ore05,Ore06,Ore07,Ore08,Ore09,Ore10,Ore11,Ore12,Ore13,Ore14	*OreG0000339*
scaffold1062	929247	G	A	Och01,Och02,Och03,Och04,Och05,Och06,Och07,Och08,Och09,Och10,Och11,Och12,Och13,Och14	*OreG0000345*
scaffold1062	951177	G	C	NA	*OreG0000347*
scaffold1062	952093	C	G	NA	*OreG0000347*
scaffold1062	952509	A	T	NA	*OreG0000347*
scaffold1062	968207	G	A	Och01,Och02,Och03,Och04,Och05,Och06,Och07,Och08,Och09,Och10,Och11,Och12,Och13,Och14,Ore01,Ore02,Ore03,Ore04,Ore05,Ore06,Ore07,Ore08,Ore09,Ore10,Ore11,Ore12,Ore13,Ore14	*OreG0000349*
scaffold1062	968246	G	C	Ore01,Ore03	*OreG0000349*
scaffold1062	968297	G	A	Och01,Och02,Och03,Och04,Och05,Och06,Och07,Och08,Och09,Och10,Och11,Och12,Och13,Och14,Ore01,Ore02,Ore03,Ore04,Ore05,Ore06,Ore07,Ore08,Ore09,Ore10,Ore11,Ore12,Ore13,Ore14	*OreG0000349*
scaffold1062	993197	C	A	Ore01,Ore02,Ore03,Ore04,Ore05,Ore06,Ore07,Ore08,Ore09,Ore10,Ore11,Ore12,Ore13,Ore14	*OreG0000350*
scaffold1062	1002811	T	G	NA	*OreG0000353*
scaffold1062	1002875	T	C	Ore01,Ore03	*OreG0000353*
scaffold1062	1003271	A	G	Ore01,Ore02,Ore03,Ore04,Ore05,Ore06,Ore07,Ore08,Ore09,Ore10,Ore11,Ore12,Ore13,Ore14	*OreG0000353*
scaffold1062	1006249	A	C	Ore01,Ore02,Ore03,Ore04,Ore05,Ore06,Ore07,Ore08,Ore09,Ore10,Ore11,Ore12,Ore13,Ore14	*OreG0000354*
scaffold1062	1070808	G	A	Och04,Och05,Och12,Och13,Och14	*OreG0000358*
scaffold1062	1071676	T	A	Ore01,Ore02,Ore03,Ore04,Ore05,Ore06,Ore07,Ore08,Ore09,Ore10,Ore11,Ore12,Ore13,Ore14	*OreG0000358*
scaffold1062	1071678	C	A	NA	*OreG0000358*
scaffold1062	1072014	C	G	Och09,Och10	*OreG0000358*
scaffold1062	1087804	C	G	Och01,Och02,Och03,Och04,Och05,Och06,Och07,Och08,Och09,Och10,Och11,Och12,Och13,Och14,Ore01,Ore02,Ore03,Ore04,Ore05,Ore06,Ore07,Ore08,Ore09,Ore10,Ore11,Ore12,Ore13,Ore14	*OreG0000360*
scaffold1062	1104205	T	G	NA	*OreG0000362*
scaffold1062	1125299	C	T	NA	*OreG0000364*
scaffold1062	1152381	C	A	NA	*OreG0000365*
scaffold1062	1169712	G	T	Ore01,Ore02,Ore03,Ore04,Ore05,Ore06,Ore07,Ore08,Ore09,Ore10,Ore11,Ore12,Ore13,Ore14	*OreG0000367*
scaffold1062	1169831	C	G	NA	*OreG0000367*
scaffold1062	1176314	G	A	Och01,Och02,Och03,Och04,Och05,Och06,Och07,Och08,Och12,Och13,Och14	*OreG0000368*
scaffold1062	1176786	G	T	NA	*OreG0000368*
scaffold1062	1237805	G	T	Ore01,Ore02,Ore03,Ore04,Ore05,Ore06,Ore07,Ore08,Ore09,Ore10,Ore11,Ore12,Ore13,Ore14	*OreG0000376*
scaffold1062	1278144	C	A	Ore01,Ore02,Ore03,Ore04,Ore05,Ore06,Ore07,Ore08,Ore09,Ore10,Ore11,Ore12,Ore13,Ore14	*OreG0000380*
scaffold1062	1294647	C	G	NA	*OreG0000382*
scaffold1062	1341273	A	T	Och04,Och06	*OreG0000386*
scaffold1062	1341708	C	T	Och01,Och07,Och08,Och09,Och10,Och11,Och14	*OreG0000386*
scaffold1062	1342005	A	C	NA	*OreG0000386*
scaffold1062	1342050	A	G	NA	*OreG0000386*
scaffold1062	1344643	C	T	Och01,Och02,Och03,Och04,Och05,Och06,Och07,Och08,Och09,Och10,Och11,Och12,Och13,Och14	*OreG0000387*
scaffold1062	1356980	T	C	Ore04,Ore05	*OreG0000389*
scaffold1062	1416716	T	C	Och01,Och02,Och03,Och04,Och05,Och06,Och07,Och08,Och09,Och10,Och11,Och12,Och13,Och14,Ore01,Ore02,Ore03,Ore04,Ore05,Ore06,Ore07,Ore08,Ore09,Ore10,Ore11,Ore12,Ore13,Ore14	*OreG0000390*
scaffold1062	1428358	G	A	Och04,Och06	*OreG0000392*
scaffold1062	1483186	C	T	NA	*OreG0000396*
scaffold1062	1484315	C	T	NA	*OreG0000396*
scaffold1062	1484364	C	T	NA	*OreG0000396*
scaffold1062	1485821	A	G	NA	*OreG0000396*
scaffold1062	1486041	C	T	NA	*OreG0000396*
scaffold1062	1486196	T	G	NA	*OreG0000396*
scaffold1062	1486283	T	G	NA	*OreG0000396*
scaffold1062	1631076	C	T	NA	*OreG0000405*
scaffold1062	1632523	G	A	NA	*OreG0000405*
scaffold1062	1633251	C	T	NA	*OreG0000405*
scaffold1062	1651947	C	T	NA	*OreG0000407*

scaffold1062	1666318	C	T	Ore01,Ore02,Ore03,Ore04,Ore05,Ore06,Ore07,Ore08,Ore09,Ore10,Ore11,Ore12,Ore13,Ore14	*OreG0000408*
scaffold1062	1666340	C	T	NA	*OreG0000408*
scaffold1062	1669358	T	C	NA	*OreG0000408*
scaffold1062	1669434	G	A	Ore01,Ore02,Ore03,Ore04,Ore05,Ore06,Ore07,Ore08,Ore09,Ore10,Ore11,Ore12,Ore13,Ore14	*OreG0000408*
scaffold1062	1671671	A	G	Ore04,Ore05	*OreG0000408*
scaffold1062	1707649	A	T	NA	*OreG0000409*
scaffold1062	1915926	C	T	Och08	*OreG0000411*
scaffold1062	1915968	A	C	Ore04,Ore05	*OreG0000411*
scaffold1062	1917726	C	T	Och04,Och06,Ore01,Ore02,Ore03,Ore04,Ore05,Ore06,Ore07,Ore08,Ore09,Ore10,Ore11,Ore12,Ore13,Ore14	*OreG0000412*
scaffold1062	1923765	G	C	NA	*OreG0000413*
scaffold1062	1956070	G	A	Och01,Och07	*OreG0000415*
scaffold1062	1956591	C	G	Ore01,Ore02,Ore03,Ore04,Ore05,Ore06,Ore07,Ore08,Ore09,Ore10,Ore11,Ore12,Ore13,Ore14	*OreG0000415*
scaffold1062	1959713	A	G	Och01,Och02,Och03,Och04,Och05,Och06,Och07,Och08,Och09,Och10,Och11,Och12,Och13,Och14	*OreG0000416*
scaffold1062	1959773	C	T	Och07	*OreG0000416*
scaffold1062	1966084	G	A	Och04,Och06,Och14	*OreG0000418*
scaffold1062	1984959	A	T	Och01,Och02,Och03,Och04,Och05,Och06,Och07,Och08,Och09,Och10,Och11,Och12,Och13,Och14	*OreG0000420*
scaffold1062	1988419	T	A	Ore03	*OreG0000420*
scaffold1062	1989329	C	T	NA	*OreG0000420*
scaffold1062	1989362	G	A	Och04,Och06	*OreG0000420*
scaffold1062	2005037	A	T	Ore01,Ore02,Ore03,Ore04,Ore05,Ore06,Ore07,Ore08,Ore09,Ore10,Ore11,Ore12,Ore13,Ore14	*OreG0000421*
scaffold1062	2063243	G	T	Och04,Och06	*OreG0000426*
scaffold1062	2102100	G	T	Och08,Och11	*OreG0000430*
scaffold1062	2106106	C	T	Ore02,Ore04,Ore05	*OreG0000431*
scaffold1062	2116406	A	G	Ore04,Ore05,Ore13	*OreG0000433*
scaffold1062	2140037	C	T	Ore04,Ore05	*OreG0000437*
scaffold1062	2148486	A	T	NA	*OreG0000438*
scaffold1062	2159019	T	C	NA	*OreG0000439*
scaffold1062	2176137	T	C	Ore04,Ore05	*OreG0000440*
scaffold1062	2176181	A	C	Och14	*OreG0000440*
scaffold1062	2176442	T	A	Och14	*OreG0000440*
scaffold1062	2219845	C	G	NA	*OreG0000447*
scaffold1062	2233920	A	G	Ore03	*OreG0000448*
scaffold1062	2235674	G	A	NA	*OreG0000448*
scaffold1062	2237343	G	A	NA	*OreG0000450*
scaffold1062	2238606	G	T	NA	*OreG0000450*
scaffold1062	2249049	T	C	Och04,Och06,Och09,Och10,Och11	*OreG0000451*
scaffold1062	2266367	G	A	Och04,Och06	*OreG0000453*
scaffold1062	2317752	T	C	NA	*OreG0000456*
scaffold1062	2318508	A	G	Ore03	*OreG0000456*
scaffold1062	2443375	T	G	Och07,Och09,Och10,Och11,Ore01,Ore02,Ore03,Ore04,Ore05,Ore06,Ore07,Ore08,Ore09,Ore10,Ore11,Ore12,Ore13,Ore14	*OreG0000459*
scaffold1062	2452391	G	A	NA	*OreG0000460*
scaffold1062	2469717	A	G	Och02,Och03,Och04,Och05,Och06,Och07,Och09,Och10,Och11,Och12,Och13	*OreG0000464*
scaffold1062	2486112	G	T	Och01,Och02,Och03,Och04,Och05,Och06,Och07,Och08,Och09,Och10,Och11,Och12,Och13,Och14,Ore01,Ore02,Ore03,Ore04,Ore05,Ore06,Ore07,Ore08,Ore09,Ore10,Ore11,Ore12,Ore13,Ore14	*OreG0000465*
scaffold1062	2486216	C	T	Och11	*OreG0000465*
scaffold1062	2532342	C	G	NA	*OreG0000474*
scaffold1062	2551178	T	C	Och02,Och03,Och04,Och05,Och06,Och07,Och08,Och09,Och10,Och11,Och12,Och13,Och14	*OreG0000476*
scaffold1062	2551244	G	T	Och01,Och02,Och03,Och04,Och05,Och06,Och07,Och08,Och09,Och10,Och11,Och12,Och13,Och14,Ore01,Ore02,Ore03,Ore04,Ore05,Ore06,Ore07,Ore08,Ore09,Ore10,Ore11,Ore12,Ore13,Ore14	*OreG0000476*
scaffold1062	2551365	C	T	Ore02,Ore03,Ore04,Ore05,Ore06,Ore07,Ore08,Ore09,Ore10,Ore11,Ore12,Ore13,Ore14	*OreG0000476*
scaffold1062	2551370	T	A	Och01,Och02,Och03,Och04,Och05,Och06,Och07,Och08,Och09,Och10,Och11,Och12,Och13,Och14,Ore01,Ore02,Ore03,Ore04,Ore05,Ore06,Ore07,Ore08,Ore09,Ore10,Ore11,Ore12,Ore13,Ore14	*OreG0000476*
scaffold1062	2551376	A	G	NA	*OreG0000476*
scaffold1062	2551463	A	T	NA	*OreG0000476*
scaffold1062	2552170	A	G	Och02,Och03,Och04,Och05,Och06,Och07,Och08,Och09,Och10,Och11,Och12,Och13,Och14	*OreG0000476*
scaffold1062	2552179	T	C	Ore01,Ore02,Ore03,Ore04,Ore05,Ore06,Ore07,Ore08,Ore09,Ore10,Ore11,Ore12,Ore13,Ore14	*OreG0000476*
scaffold1062	2552396	G	A	Ore01,Ore02,Ore03,Ore04,Ore05,Ore06,Ore07,Ore08,Ore09,Ore10,Ore11,Ore12,Ore13,Ore14	*OreG0000476*
scaffold1062	2552986	A	G	Ore01,Ore02,Ore03,Ore04,Ore05,Ore06,Ore07,Ore08,Ore09,Ore10,Ore11,Ore12,Ore13,Ore14	*OreG0000476*

scaffold1062	2564184	T	A	Och01,Och02,Och03,Och04,Och05,Och06,Och07,Och08,Och09,Och10,Och11,Och12,Och13,Och14,Ore01,Ore02,Ore03,Ore04,Ore05,Ore06,Ore07,Ore08,Ore09,Ore10,Ore11,Ore12,Ore13,Ore14	*OreG0000478*
scaffold1062	2564193	C	G	Ore01,Ore02,Ore03,Ore04,Ore05,Ore06,Ore07,Ore08,Ore09,Ore10,Ore11,Ore12,Ore13,Ore14	*OreG0000478*
scaffold1062	2566842	C	A	NA	*OreG0000478*
scaffold1062	2584181	T	C	NA	*OreG0000481*
scaffold1062	2673248	G	A	NA	*OreG0000492*
scaffold1062	2685533	C	A	Ore06,Ore09	*OreG0000493*
scaffold1062	2687713	G	A	Och02,Och03,Och04,Och05,Och06,Och09,Och10,Och11,Och12,Och13	*OreG0000493*
scaffold1062	2687872	T	G	Och02,Och03,Och04,Och05,Och06,Och09,Och10,Och11,Och12,Och13	*OreG0000493*
scaffold1062	2688007	T	C	Och02,Och03,Och04,Och05,Och06,Och09,Och10,Och11,Och12,Och13	*OreG0000493*
scaffold1062	2688183	G	A	Och02,Och03,Och04,Och05,Och06,Och09,Och10,Och11,Och12,Och13	*OreG0000493*
scaffold1062	2688406	A	G	NA	*OreG0000493*
scaffold1062	2688721	G	A	NA	*OreG0000493*
scaffold1062	2689275	C	T	NA	*OreG0000493*
scaffold1062	2689362	T	A	NA	*OreG0000493*
scaffold1062	2689428	A	T	NA	*OreG0000493*
scaffold1062	2747872	T	A	NA	*OreG0000494*
scaffold1062	2753073	A	G	Och01,Och02,Och03,Och04,Och05,Och06,Och07,Och08,Och09,Och10,Och11,Och12,Och13,Och14	*OreG0000495*
scaffold1062	2753212	C	T	Och01,Och02,Och03,Och04,Och05,Och06,Och07,Och08,Och09,Och10,Och11,Och12,Och13,Och14	*OreG0000495*
scaffold1062	2763128	T	C	NA	*OreG0000497*
scaffold1062	2763200	C	T	NA	*OreG0000497*
scaffold1062	2773262	C	A	Ore03,Ore06,Ore09	*OreG0000501*
scaffold1062	2773285	A	G	NA	*OreG0000501*
scaffold1062	2773292	G	C	Ore03,Ore06,Ore09	*OreG0000501*
scaffold1062	2773296	C	T	Och01,Och02,Och03,Och04,Och05,Och06,Och07,Och08,Och09,Och10,Och11,Och12,Och13,Och14,Ore01,Ore02,Ore03,Ore04,Ore05,Ore06,Ore07,Ore08,Ore09,Ore10,Ore11,Ore12,Ore13,Ore14	*OreG0000501*
scaffold1062	2773339	A	G	Ore01,Ore02,Ore03,Ore04,Ore05,Ore06,Ore07,Ore08,Ore09,Ore10,Ore11,Ore12,Ore13,Ore14	*OreG0000501*
scaffold1062	2773508	T	G	Ore04,Ore05	*OreG0000501*
scaffold1062	2787097	C	T	Ore01,Ore02,Ore03,Ore04,Ore05,Ore06,Ore07,Ore08,Ore09,Ore10,Ore11,Ore12,Ore13,Ore14	*OreG0000502*
scaffold1062	2787103	C	T	NA	*OreG0000502*
scaffold1062	2789537	T	A	Ore01,Ore02,Ore03,Ore04,Ore05,Ore06,Ore07,Ore08,Ore09,Ore10,Ore11,Ore12,Ore13,Ore14	*OreG0000503*
scaffold1062	2799139	C	A	NA	*OreG0000504*
scaffold1062	2799198	G	A	NA	*OreG0000504*
scaffold1062	2799234	G	A	NA	*OreG0000504*
scaffold1062	2799305	G	A	NA	*OreG0000504*
scaffold1062	2802924	G	T	NA	*OreG0000505*
scaffold1062	2812005	C	T	Och11,Ore01,Ore02,Ore03,Ore04,Ore05,Ore06,Ore07,Ore08,Ore09,Ore10,Ore11,Ore12,Ore13,Ore14	*OreG0000506*
scaffold1062	2828515	A	C	Och05	*OreG0000509*
scaffold1062	2829106	C	T	NA	*OreG0000509*
scaffold1062	2833213	T	C	Och01,Och02,Och03,Och04,Och05,Och06,Och07,Och08,Och09,Och10,Och11,Och12,Och13,Och14,Ore01,Ore02,Ore03,Ore04,Ore05,Ore06,Ore07,Ore08,Ore09,Ore10,Ore11,Ore12,Ore13,Ore14	*OreG0000510*
scaffold1062	2833225	C	A	Och11	*OreG0000510*
scaffold1062	2836455	G	C	Och01,Och02,Och03,Och04,Och05,Och06,Och07,Och08,Och09,Och10,Och11,Och12,Och13,Och14,Ore01,Ore02,Ore03,Ore04,Ore05,Ore06,Ore07,Ore08,Ore09,Ore10,Ore11,Ore12,Ore13,Ore14	*OreG0000511*
scaffold1062	2837191	A	G	Och02,Och03,Och04,Och05,Och06,Och07,Och08,Och09,Och10,Och11,Och12,Och13,Och14,Ore01,Ore02,Ore03,Ore04,Ore05,Ore06,Ore07,Ore08,Ore09,Ore10,Ore11,Ore12,Ore13,Ore14	*OreG0000511*
scaffold1062	2849493	C	T	Och01,Och02,Och03,Och04,Och05,Och06,Och07,Och08,Och09,Och10,Och11,Och12,Och13,Och14,Ore01,Ore02,Ore03,Ore04,Ore05,Ore06,Ore07,Ore08,Ore09,Ore10,Ore11,Ore12,Ore13,Ore14	*OreG0000514*
scaffold1062	2855449	A	G	NA	*OreG0000515*
scaffold1062	2866226	G	A	NA	*OreG0000518*
scaffold1062	2866618	G	A	NA	*OreG0000518*
scaffold1062	2923362	T	A	NA	*OreG0000526*
scaffold1062	2923505	G	A	NA	*OreG0000526*
scaffold1062	2937011	C	T	Och01,Och02,Och03,Och04,Och05,Och06,Och07,Och08,Och09,Och10,Och11,Och12,Och13,Och14,Ore01,Ore02,Ore03,Ore04,Ore05,Ore06,Ore07,Ore08,Ore09,Ore10,Ore11,Ore12,Ore13,Ore14	*OreG0000529*
scaffold1062	2938564	A	G	NA	*OreG0000529*
scaffold1062	2959848	G	C	Ore01,Ore02,Ore03,Ore04,Ore05,Ore06,Ore07,Ore08,Ore09,Ore10,Ore11,Ore12,Ore13,Ore14	*OreG0000530*
scaffold1062	2965454	C	G	NA	*OreG0000531*
scaffold1062	3001292	A	C	NA	*OreG0000535*

scaffold1062	3001437	C	T	Ore01,Ore02,Ore03,Ore04,Ore05,Ore06,Ore07,Ore08,Ore09,Ore10,Ore11,Ore12,Ore13,Ore14	*OreG0000535*
scaffold1062	3010196	C	T	Och01,Och02,Och03,Och04,Och05,Och06,Och07,Och08,Och09,Och10,Och11,Och12,Och13,Och14,Ore01,Ore02,Ore03,Ore04,Ore05,Ore06,Ore07,Ore08,Ore09,Ore10,Ore11,Ore12,Ore13,Ore14	*OreG0000537*
scaffold1062	3012804	G	C	NA	*OreG0000537*
scaffold1062	3025743	C	G	Ore01,Ore02,Ore03,Ore04,Ore05,Ore06,Ore07,Ore08,Ore09,Ore10,Ore11,Ore12,Ore13,Ore14	*OreG0000539*
scaffold1062	3050326	T	A	Ore01,Ore03,Ore06,Ore09	*OreG0000543*
scaffold1062	3053467	A	G	Ore01,Ore02,Ore03,Ore04,Ore05,Ore06,Ore07,Ore08,Ore09,Ore10,Ore11,Ore12,Ore13,Ore14	*OreG0000543*
scaffold1062	3123823	C	A	NA	*OreG0000549*
scaffold1062	3126957	G	A	Och01,Och02,Och03,Och04,Och05,Och06,Och07,Och08,Och09,Och10,Och11,Och12,Och13,Och14	*OreG0000549*
scaffold1062	3168999	A	T	Och06,Och11	*OreG0000553*
scaffold1062	3172319	C	T	Och01,Och02,Och03,Och04,Och05,Och06,Och07,Och08,Och09,Och10,Och11,Och12,Och13,Och14,Ore01,Ore02,Ore03,Ore04,Ore05,Ore06,Ore07,Ore08,Ore09,Ore10,Ore11,Ore12,Ore13,Ore14	*OreG0000553*
scaffold1062	3176711	G	A	Och04,Och06,Och11,Och13	*OreG0000554*
scaffold1062	3241977	G	A	Ore01,Ore02,Ore03,Ore04,Ore05,Ore06,Ore07,Ore08,Ore09,Ore10,Ore11,Ore12,Ore13,Ore14	*OreG0000559*
scaffold1062	3277498	C	A	NA	*OreG0000563*
scaffold1062	3297330	C	T	Ore01,Ore02,Ore04,Ore05,Ore06,Ore07,Ore08,Ore10,Ore11,Ore12,Ore14	*OreG0000564*
scaffold1062	3307260	A	G	Och06	*OreG0000565*
scaffold1062	3344106	C	T	Och01,Och02,Och03,Och04,Och05,Och06,Och08,Och12,Och13,Och14	*OreG0000570*
scaffold1062	3345976	C	T	NA	*OreG0000570*
scaffold1062	3349309	G	A	NA	*OreG0000571*
scaffold1062	3397070	C	A	NA	*OreG0000575*
scaffold1062	3398754	A	G	Ore01,Ore06,Ore08	*OreG0000575*
scaffold1062	3407070	C	A	Ore01,Ore02,Ore03,Ore04,Ore05,Ore06,Ore07,Ore08,Ore09,Ore10,Ore11,Ore12,Ore13,Ore14	*OreG0000576*
scaffold1062	3419085	C	T	Och01,Och02,Och03,Och04,Och05,Och06,Och07,Och08,Och09,Och10,Och11,Och12,Och13,Och14	*OreG0000577*
scaffold1062	3419087	T	A	Och01,Och02,Och03,Och04,Och05,Och06,Och07,Och08,Och09,Och10,Och11,Och12,Och13,Och14	*OreG0000577*
scaffold1062	3426187	G	T	Ore04,Ore05,Ore13	*OreG0000577*
scaffold1062	3453461	T	C	NA	*OreG0000579*
scaffold1062	3516178	C	A	Ore04,Ore05	*OreG0000585*
scaffold1062	3516425	C	A	Och06	*OreG0000585*
scaffold1062	3516795	C	T	NA	*OreG0000585*
scaffold1062	3517179	C	T	NA	*OreG0000585*
scaffold1062	3552903	A	T	NA	*OreG0000586*
scaffold1062	3559103	G	T	Ore09	*OreG0000587*
scaffold1062	3559258	C	A	NA	*OreG0000587*
scaffold1062	3562581	G	A	NA	*OreG0000588*
scaffold1062	3566731	T	A	NA	*OreG0000589*
scaffold1062	3592445	G	A	Och01,Och02,Och03,Och04,Och05,Och06,Och07,Och08,Och09,Och10,Och11,Och12,Och13,Och14,Ore01,Ore02,Ore03,Ore04,Ore05,Ore06,Ore07,Ore08,Ore09,Ore10,Ore11,Ore12,Ore13,Ore14	*OreG0000592*
scaffold1062	3592530	C	T	Och11	*OreG0000592*
scaffold1062	3596966	C	T	NA	*OreG0000593*
scaffold1062	3597697	G	A	Ore04,Ore05	*OreG0000593*
scaffold1062	3605410	A	G	NA	*OreG0000594*
scaffold1062	3605422	G	C	Och02,Och03,Och06,Och12	*OreG0000594*
scaffold1062	3605716	T	C	Och02,Och03,Och06,Och12	*OreG0000594*
scaffold1062	3642019	G	A	Ore03,Ore06,Ore09	*OreG0000597*
scaffold1062	3731609	T	A	Och01,Och02,Och03,Och04,Och05,Och06,Och07,Och08,Och09,Och10,Och11,Och12,Och13,Och14,Ore01,Ore02,Ore03,Ore04,Ore05,Ore06,Ore07,Ore08,Ore09,Ore10,Ore11,Ore12,Ore13,Ore14	*OreG0000601*
scaffold1062	3731649	C	T	Och01,Och11	*OreG0000601*
scaffold1062	3731685	C	T	NA	*OreG0000601*
scaffold1062	3742614	G	A	Och01,Och02,Och03,Och04,Och05,Och06,Och08,Och09,Och10,Och11,Och12,Och13	*OreG0000602*
scaffold1062	3742694	C	A	Ore01,Ore02,Ore03,Ore04,Ore05,Ore06,Ore07,Ore08,Ore09,Ore10,Ore11,Ore12,Ore13,Ore14	*OreG0000602*
scaffold1062	3743045	C	A	Och01,Och02,Och03,Och04,Och05,Och06,Och07,Och08,Och09,Och10,Och11,Och12,Och13	*OreG0000602*
scaffold1062	3780115	G	A	Och02,Och03,Och04,Och05,Och06,Och11,Och12,Och13	*OreG0000604*
scaffold1062	3780220	A	C	Och02,Och03,Och04,Och05,Och06,Och11,Och12,Och13	*OreG0000604*
scaffold1062	3828309	C	T	Och01,Och02,Och03,Och04,Och05,Och06,Och07,Och08,Och09,Och10,Och11,Och12,Och13,Och14	*OreG0000606*
scaffold1062	3941855	G	T	NA	*OreG0000612*
scaffold1062	3959361	G	T	Och01,Och02,Och03,Och04,Och05,Och06,Och07,Och08,Och09,Och10,Och11,Och12,Och13,Och14	*OreG0000615*

scaffold1062	3959380	C	T	Ore01,Ore02,Ore03,Ore04,Ore05,Ore06,Ore07,Ore08,Ore09,Ore10,Ore11,Ore12,Ore13,Ore14	*OreG0000615*
scaffold1062	3959395	C	T	Och01,Och02,Och03,Och04,Och05,Och06,Och07,Och08,Och09,Och10,Och11,Och12,Och13,Och14	*OreG0000615*
scaffold1062	3959467	G	A	Och01,Och02,Och03,Och04,Och05,Och06,Och07,Och08,Och09,Och10,Och11,Och12,Och13,Och14	*OreG0000615*
scaffold1062	3959647	A	T	Och07	*OreG0000615*
scaffold1062	3959651	C	G	Och01,Och02,Och03,Och04,Och05,Och06,Och07,Och08,Och09,Och10,Och11,Och12,Och13,Och14	*OreG0000615*
scaffold1062	3960262	C	T	Och01,Och04,Och06,Och07,Och08,Och13	*OreG0000615*
scaffold1062	3960274	C	T	Och11	*OreG0000615*
scaffold1062	3960283	G	C	Och04	*OreG0000615*
scaffold1062	3961159	C	T	Och04	*OreG0000615*
scaffold1997	130616	C	T	Och01,Och02,Och03,Och04,Och05,Och06,Och07,Och08,Och09,Och10,Och11,Och12,Och13,Och14	*OreG0005005*
scaffold1997	311450	A	T	Och01,Och02,Och03,Och04,Och05,Och06,Och07,Och08,Och09,Och10,Och11,Och12,Och13,Och14	*OreG0005007*
scaffold1997	499218	A	G	Och01,Och02,Och03,Och04,Och05,Och06,Och07,Och08,Och09,Och10,Och11,Och12,Och13,Och14	*OreG0005009*
scaffold1997	667929	C	T	Ore01,Ore02,Ore03,Ore04,Ore05,Ore06,Ore07,Ore08,Ore09,Ore10,Ore11,Ore12,Ore13,Ore14	*OreG0005012*
scaffold1997	669015	T	C	Och02	*OreG0005012*
scaffold1997	686114	A	G	Ore01,Ore02,Ore03,Ore04,Ore05,Ore06,Ore07,Ore08,Ore09,Ore10,Ore11,Ore12,Ore13,Ore14	*OreG0005013*
scaffold1997	686151	C	A	Och01,Och02,Och03,Och04,Och05,Och06,Och07,Och08,Och09,Och10,Och11,Och12,Och13,Och14	*OreG0005013*
scaffold1997	740580	T	G	NA	*OreG0005015*
scaffold1997	740583	C	A	Och01,Och07,Och08,Och12,Och13	*OreG0005015*
scaffold1997	740718	G	T	Och01,Och02,Och03,Och04,Och05,Och06,Och07,Och08,Och09,Och10,Och11,Och12,Och13,Och14	*OreG0005015*
scaffold1997	872579	G	A	NA	*OreG0005017*
scaffold1997	874471	A	T	Och01,Och02,Och03,Och04,Och05,Och06,Och07,Och08,Och09,Och10,Och11,Och12,Och13,Och14	*OreG0005018*
scaffold1997	875420	C	A	Och01,Och02,Och03,Och04,Och05,Och06,Och07,Och08,Och09,Och10,Och11,Och12,Och13,Och14,Ore01,Ore02,Ore03,Ore04,Ore05,Ore06,Ore07,Ore08,Ore09,Ore10,Ore11,Ore12,Ore13,Ore14	*OreG0005018*
scaffold1997	915880	G	A	NA	*OreG0005020*
scaffold1997	1060686	G	A	Ore01,Ore02,Ore03,Ore04,Ore05,Ore06,Ore07,Ore08,Ore09,Ore10,Ore11,Ore12,Ore13,Ore14	*OreG0005022*
scaffold1997	1070155	G	A	Och01,Och02,Och03,Och04,Och05,Och06,Och07,Och08,Och09,Och10,Och11,Och12,Och13	*OreG0005022*
scaffold1997	1263939	C	G	NA	*OreG0005025*
scaffold1997	1265342	T	C	Ore01,Ore02,Ore03,Ore04,Ore05,Ore06,Ore07,Ore08,Ore09,Ore10,Ore11,Ore12,Ore13,Ore14	*OreG0005025*
scaffold1997	1342581	T	G	NA	*OreG0005026*
scaffold1997	1343369	C	T	Och01,Och02,Och03,Och04,Och05,Och06,Och07,Och08,Och09,Och10,Och11,Och12,Och13,Och14,Ore01,Ore02,Ore03,Ore04,Ore05,Ore06,Ore07,Ore08,Ore09,Ore10,Ore11,Ore12,Ore13,Ore14	*OreG0005026*
scaffold1997	1347399	A	C	NA	*OreG0005026*
scaffold1997	1370876	C	T	Och01,Och05,Och12,Och13	*OreG0005027*
scaffold1997	1382933	A	T	NA	*OreG0005028*
scaffold1997	1392781	G	A	Och01,Och02,Och03,Och04,Och05,Och06,Och07,Och08,Och09,Och10,Och11,Och12,Och13,Och14,Ore01,Ore02,Ore03,Ore04,Ore05,Ore06,Ore07,Ore08,Ore09,Ore10,Ore11,Ore12,Ore13,Ore14	*OreG0005029*
scaffold1997	1553947	A	G	Och01,Och02,Och03,Och04,Och05,Och06,Och07,Och08,Och09,Och10,Och11,Och12,Och13,Och14,Ore01,Ore02,Ore03,Ore04,Ore05,Ore06,Ore07,Ore08,Ore09,Ore10,Ore11,Ore12,Ore13,Ore14	*OreG0005032*
scaffold1997	1574737	T	A	Och01,Och02,Och03,Och04,Och05,Och06,Och07,Och08,Och09,Och10,Och11,Och12,Och13,Och14,Ore01,Ore02,Ore03,Ore04,Ore05,Ore06,Ore07,Ore08,Ore09,Ore10,Ore11,Ore12,Ore13,Ore14	*OreG0005034*
scaffold1997	1574947	T	C	Och09,Och10,Och11,Och12,Och13,Och14	*OreG0005034*
scaffold1997	1828218	G	A	Och12,Och13	*OreG0005040*
scaffold1997	1867109	T	A	NA	*OreG0005041*
scaffold1997	1942225	C	T	Och01,Och02,Och03,Och04,Och05,Och06,Och07,Och08,Och09,Och10,Och11,Och12,Och13,Och14,Ore01,Ore02,Ore03,Ore04,Ore05,Ore06,Ore07,Ore08,Ore09,Ore10,Ore11,Ore12,Ore13,Ore14	*OreG0005046*
scaffold1997	2015196	G	T	Och01,Och02,Och03,Och04,Och05,Och06,Och07,Och08,Och09,Och10,Och11,Och12,Och13,Och14,Ore01,Ore02,Ore03,Ore04,Ore05,Ore06,Ore07,Ore08,Ore09,Ore10,Ore11,Ore12,Ore13,Ore14	*OreG0005049*
scaffold1997	2051123	A	C	Och01,Och02,Och03,Och04,Och05,Och06,Och07,Och08,Och09,Och10,Och11,Och12,Och13,Och14	*OreG0005051*
scaffold1997	2162092	T	G	NA	*OreG0005053*
scaffold1997	2162252	T	G	NA	*OreG0005053*
scaffold1997	2162304	T	A	NA	*OreG0005053*
scaffold1997	2162323	C	T	NA	*OreG0005053*
scaffold1997	2162911	C	T	NA	*OreG0005053*

scaffold1997	2171446	C	A	NA	*OreG0005054*
scaffold1457	359845	A	G	Och01	*OreG0002409*
scaffold1457	384865	A	G	Ore01,Ore02,Ore03,Ore04,Ore05,Ore06,Ore07,Ore08,Ore09,Ore10,Ore11,Ore12,Ore13,Ore14	*OreG0002410*
scaffold1457	429445	T	C	Och01,Och02,Och03,Och04,Och05,Och06,Och07,Och08,Och09,Och10,Och11,Och12,Och13,Och14	*OreG0002413*
scaffold1457	583161	G	A	Ore01,Ore02,Ore03,Ore04,Ore05,Ore06,Ore07,Ore08,Ore09,Ore10,Ore11,Ore12,Ore13,Ore14	*OreG0002416*
scaffold1457	717036	T	G	Ore01,Ore02,Ore03,Ore04,Ore05,Ore06,Ore07,Ore08,Ore09,Ore10,Ore11,Ore12,Ore13,Ore14	*OreG0002420*
scaffold1457	717070	C	T	Och04,Och06,Och08,Och13	*OreG0002420*
scaffold1457	717162	G	A	Ore01,Ore02,Ore03,Ore04,Ore05,Ore06,Ore07,Ore08,Ore09,Ore10,Ore11,Ore12,Ore13,Ore14	*OreG0002420*
scaffold1457	729394	T	C	Ore01,Ore02,Ore03,Ore04,Ore05,Ore06,Ore07,Ore08,Ore09,Ore10,Ore11,Ore12,Ore13,Ore14	*OreG0002420*
scaffold1457	869964	C	T	NA	*OreG0002427*
scaffold1457	981415	T	A	NA	*OreG0002428*
scaffold1457	1167581	C	A	NA	*OreG0002429*
scaffold1457	1248794	C	T	Ore01,Ore02,Ore03,Ore04,Ore05,Ore06,Ore07,Ore08,Ore09,Ore10,Ore11,Ore12,Ore13,Ore14	*OreG0002433*
scaffold1457	1249214	G	A	Och01,Och02,Och03,Och04,Och05,Och06,Och07,Och08,Och09,Och10,Och11,Och12,Och13,Och14,Ore01,Ore02,Ore03,Ore04,Ore05,Ore06,Ore07,Ore08,Ore09,Ore10,Ore11,Ore12,Ore13,Ore14	*OreG0002433*
scaffold1457	1249379	C	T	Och04,Och06,Och13	*OreG0002433*
scaffold1457	1250124	C	T	NA	*OreG0002433*
scaffold1457	1257632	G	T	Och01,Och02,Och03,Och04,Och05,Och06,Och07,Och08,Och09,Och10,Och11,Och12,Och13,Och14,Ore01,Ore02,Ore03,Ore04,Ore05,Ore06,Ore07,Ore08,Ore09,Ore10,Ore11,Ore12,Ore13,Ore14	*OreG0002433*
scaffold1457	1555861	T	A	Och01,Och02,Och03,Och04,Och05,Och06,Och07,Och08,Och09,Och10,Och11,Och12,Och13,Och14,Ore01,Ore02,Ore03,Ore04,Ore05,Ore06,Ore07,Ore08,Ore09,Ore10,Ore11,Ore12,Ore13,Ore14	*OreG0002437*
scaffold1457	1611414	C	T	NA	*OreG0002442*
scaffold1457	1611666	C	G	Och01,Och02,Och03,Och04,Och05,Och06,Och07,Och08,Och09,Och10,Och11,Och12,Och13,Och14	*OreG0002442*
scaffold1457	1611680	T	G	NA	*OreG0002442*
scaffold1457	1703276	A	T	Och01,Och02,Och03,Och04,Och05,Och06,Och07,Och08,Och09,Och10,Och11,Och12,Och13,Och14	*OreG0002452*
scaffold1457	1703470	G	A	Och14	*OreG0002452*
scaffold1457	1703513	G	A	NA	*OreG0002452*
scaffold1457	1703636	T	C	NA	*OreG0002452*
scaffold1457	1707003	G	A	Och01,Och02,Och03,Och04,Och05,Och06,Och07,Och08,Och09,Och10,Och11,Och12,Och13	*OreG0002453*
scaffold1457	1707016	C	G	Och01,Och02,Och03,Och04,Och05,Och06,Och07,Och08,Och09,Och10,Och11,Och12,Och13	*OreG0002453*
scaffold1457	1707048	C	G	NA	*OreG0002453*
scaffold1457	1708035	G	A	NA	*OreG0002454*
scaffold1457	1708362	G	A	NA	*OreG0002455*
scaffold1457	1708384	C	T	NA	*OreG0002455*
scaffold1457	1713009	G	C	Och01,Och02,Och03,Och04,Och05,Och06,Och07,Och08,Och09,Och10,Och11,Och12,Och13,Och14,Ore01,Ore02,Ore03,Ore04,Ore05,Ore06,Ore07,Ore08,Ore09,Ore10,Ore11,Ore12,Ore13,Ore14	*OreG0002456*
scaffold1457	1715294	T	C	Och01,Och02,Och03,Och04,Och05,Och06,Och08,Och09,Och10,Och11,Och12,Och13,Och14	*OreG0002456*
scaffold1457	1743386	A	T	NA	*OreG0002458*
scaffold1457	1798199	C	T	NA	*OreG0002463*
scaffold1457	1826974	A	T	Ore02,Ore07,Ore09,Ore12,Ore14	*OreG0002465*
scaffold1457	1907375	T	A	Och01,Och02,Och03,Och04,Och05,Och06,Och07,Och08,Och09,Och10,Och11,Och12,Och13,Och14,Ore01,Ore02,Ore03,Ore04,Ore05,Ore06,Ore07,Ore08,Ore09,Ore10,Ore11,Ore12,Ore13,Ore14	*OreG0002469*
scaffold1457	1911826	A	G	NA	*OreG0002470*
scaffold1457	1911940	C	T	NA	*OreG0002470*
scaffold1457	1912512	C	T	NA	*OreG0002470*
scaffold1457	1956761	T	A	Och02,Och03,Och04,Och05,Och06,Och09,Och10,Och11,Och12,Och13	*OreG0002478*
scaffold1457	1968484	C	T	NA	*OreG0002480*
scaffold1457	1969257	T	C	NA	*OreG0002480*
scaffold1457	2036711	T	A	Och02,Och03,Och04,Och05,Och06,Och07,Och08,Och09,Och10,Och11,Och12,Och13,Och14	*OreG0002486*
scaffold1457	2046680	C	T	NA	*OreG0002488*
scaffold1457	2051518	A	G	Och02,Och04,Och06	*OreG0002489*
scaffold1457	2051531	A	G	NA	*OreG0002489*
scaffold1457	2051657	T	C	Och02,Och04,Och06	*OreG0002489*
scaffold1457	2051782	A	C	Och02,Och04,Och06	*OreG0002489*
scaffold1457	2051965	G	T	Och02,Och03,Och04,Och05,Och06,Och07,Och08,Och09,Och10,Och11,Och12,Och13,Och14	*OreG0002489*
scaffold1457	2052049	G	A	Ore12	*OreG0002489*

scaffold1457	2065016	G	T	Och02,Och03,Och04,Och05,Och06,Och07,Och08,Och09,Och10,Och11,Och12,Och13,Och14	*OreG0002491*
scaffold1457	2089314	T	G	Och06,Ore01,Ore02,Ore03,Ore04,Ore05,Ore06,Ore07,Ore08,Ore09,Ore10,Ore11,Ore12,Ore13,Ore14	*OreG0002494*
scaffold1457	2090088	G	C	Och06,Ore01,Ore02,Ore03,Ore04,Ore05,Ore06,Ore07,Ore08,Ore09,Ore10,Ore11,Ore12,Ore13,Ore14	*OreG0002494*
scaffold1457	2090171	T	G	NA	*OreG0002494*
scaffold1457	2091039	C	A	Ore01,Ore02,Ore03,Ore04,Ore05,Ore06,Ore07,Ore08,Ore09,Ore10,Ore11,Ore12,Ore13,Ore14	*OreG0002494*
scaffold1457	2091776	C	T	Ore01,Ore02,Ore03,Ore04,Ore05,Ore06,Ore07,Ore08,Ore09,Ore10,Ore11,Ore12,Ore13,Ore14	*OreG0002494*
scaffold1457	2091814	A	T	NA	*OreG0002494*
scaffold1457	2092072	C	T	Ore01,Ore02,Ore03,Ore04,Ore05,Ore06,Ore07,Ore08,Ore09,Ore10,Ore11,Ore12,Ore13,Ore14	*OreG0002494*
scaffold1457	2092636	C	G	NA	*OreG0002494*
scaffold1457	2093164	G	A	NA	*OreG0002494*
scaffold1457	2093205	G	A	NA	*OreG0002494*
scaffold1457	2094736	C	G	Och06,Ore04,Ore05,Ore08	*OreG0002495*
scaffold1457	2095078	G	T	Och06,Ore04,Ore05,Ore08	*OreG0002495*
scaffold1457	2095187	T	A	Och06	*OreG0002495*
scaffold1457	2095684	T	C	Och06	*OreG0002495*
scaffold1457	2095846	T	A	NA	*OreG0002495*
scaffold1457	2130722	A	C	NA	*OreG0002498*
scaffold1457	2133869	T	A	NA	*OreG0002499*
scaffold1457	2134514	C	T	Och05,Och12,Och13,Och14	*OreG0002499*
scaffold1457	2137159	T	C	Och01	*OreG0002500*
scaffold1457	2137822	A	G	NA	*OreG0002500*
scaffold1457	2144243	T	G	Och01,Och02,Och03,Och04,Och05,Och06,Och07,Och08,Och09,Och10,Och11,Och12,Och13,Och14,Ore01,Ore02,Ore03,Ore04,Ore05,Ore06,Ore07,Ore08,Ore09,Ore10,Ore11,Ore12,Ore13,Ore14	*OreG0002502*
scaffold1457	2164126	T	G	Och01,Och02,Och03,Och04,Och05,Och06,Och07,Och08,Och09,Och10,Och11,Och12,Och13,Och14	*OreG0002505*
scaffold1457	2237698	T	A	Och06,Och14	*OreG0002509*
scaffold1457	2253621	G	T	NA	*OreG0002510*
scaffold1457	2255092	A	T	NA	*OreG0002510*
scaffold1457	2280245	G	C	NA	*OreG0002514*
scaffold1457	2422571	A	G	NA	*OreG0002518*
scaffold1457	2422580	C	G	NA	*OreG0002518*
scaffold1457	2422589	C	A	NA	*OreG0002518*
scaffold3213	11709	G	C	NA	*OreG0010391*
scaffold3213	12020	C	T	NA	*OreG0010391*
scaffold3213	12266	C	G	NA	*OreG0010391*
scaffold3213	21232	A	G	NA	*OreG0010393*
scaffold3213	28192	T	G	NA	*OreG0010394*
scaffold3213	42233	T	A	Och01,Och02,Och03,Och04,Och05,Och06,Och07,Och08,Och09,Och10,Och11,Och12,Och13,Och14,Ore01,Ore02,Ore03,Ore04,Ore05,Ore06,Ore07,Ore08,Ore09,Ore10,Ore11,Ore12,Ore13,Ore14	*OreG0010397*
scaffold3213	42356	A	G	Och01,Och02,Och03,Och04,Och05,Och06,Och07,Och08,Och09,Och10,Och11,Och12,Och13,Och14,Ore01,Ore02,Ore03,Ore04,Ore05,Ore06,Ore07,Ore08,Ore09,Ore10,Ore11,Ore12,Ore13,Ore14	*OreG0010397*
scaffold3213	61089	A	G	Ore01,Ore02,Ore03,Ore04,Ore05,Ore06,Ore07,Ore08,Ore09,Ore10,Ore11,Ore12,Ore13,Ore14	*OreG0010399*
scaffold3213	83956	T	G	Och09,Och10,Ore04,Ore05,Ore08	*OreG0010402*
scaffold3213	84037	G	A	Och09,Och10	*OreG0010402*
scaffold3213	157994	A	T	Och01,Och03,Och05,Och07,Och08,Och09,Och10,Och11,Och12,Och13,Och14,Ore01,Ore02,Ore03,Ore04,Ore05,Ore06,Ore07,Ore08,Ore09,Ore10,Ore11,Ore12,Ore13,Ore14	*OreG0010404*
scaffold3213	158010	G	A	NA	*OreG0010404*
scaffold3213	158051	C	A	NA	*OreG0010404*
scaffold2814	61400	G	A	NA	*OreG0008303*
scaffold2814	61450	G	A	NA	*OreG0008303*
scaffold2814	62749	G	C	Ore02,Ore03,Ore06,Ore07,Ore09,Ore10,Ore11,Ore12,Ore13,Ore14	*OreG0008303*
scaffold2814	63943	C	T	NA	*OreG0008304*
scaffold4594	3373	G	C	NA	*OreG0021798*
scaffold346	3762	C	G	NA	*OreG0018615*
scaffold346	21571	T	C	Och01,Och07	*OreG0018616*
scaffold346	261688	T	A	Ore01,Ore02,Ore03,Ore04,Ore05,Ore06,Ore07,Ore08,Ore09,Ore10,Ore11,Ore12,Ore13,Ore14	*OreG0018627*
scaffold346	298344	A	C	Och11	*OreG0018631*
scaffold346	298376	G	A	Och11	*OreG0018631*
scaffold346	317383	C	T	Och14	*OreG0018633*
scaffold346	456866	T	G	Ore01,Ore02,Ore03,Ore04,Ore05,Ore06,Ore07,Ore08,Ore09,Ore10,Ore11,Ore12,Ore13,Ore14	*OreG0018638*
scaffold346	457433	G	A	NA	*OreG0018638*
scaffold346	457760	T	C	Och09,Och10	*OreG0018638*

scaffold346	511137	A	T	Ore01,Ore02,Ore03,Ore04,Ore05,Ore06,Ore07,Ore08,Ore09,Ore10,Ore11,Ore12,Ore13,Ore14	*OreG0018639*
scaffold346	511209	C	T	NA	*OreG0018639*
scaffold346	516439	C	T	Och02,Och03,Och06,Och07,Och08,Och09,Och10,Och14	*OreG0018640*
scaffold346	517073	G	T	Ore01,Ore02,Ore03,Ore04,Ore05,Ore06,Ore07,Ore08,Ore09,Ore10,Ore11,Ore12,Ore13,Ore14	*OreG0018640*
scaffold346	580076	G	A	NA	*OreG0018644*
scaffold346	588257	T	A	Och14	*OreG0018645*
scaffold346	625723	A	G	NA	*OreG0018648*
scaffold346	627376	G	T	NA	*OreG0018649*
scaffold346	628103	A	G	NA	*OreG0018649*
scaffold346	807554	C	T	Och01,Och02,Och03,Och04,Och05,Och06,Och07,Och08,Och09,Och10,Och11,Och12,Och13,Och14,Ore01,Ore02,Ore03,Ore04,Ore05,Ore06,Ore07,Ore08,Ore09,Ore10,Ore11,Ore12,Ore13,Ore14	*OreG0018654*
scaffold346	807717	C	T	Ore01,Ore02,Ore03,Ore04,Ore05,Ore06,Ore07,Ore08,Ore09,Ore10,Ore11,Ore12,Ore13,Ore14	*OreG0018654*
scaffold346	807965	A	G	Och01,Och02,Och03,Och04,Och05,Och06,Och07,Och08,Och09,Och10,Och11,Och12,Och13,Och14,Ore01,Ore02,Ore03,Ore04,Ore05,Ore06,Ore07,Ore08,Ore09,Ore10,Ore11,Ore12,Ore13,Ore14	*OreG0018654*
scaffold346	913086	A	G	Ore01,Ore02,Ore03,Ore04,Ore05,Ore06,Ore07,Ore08,Ore09,Ore10,Ore11,Ore12,Ore13,Ore14	*OreG0018656*
scaffold346	1263734	G	A	NA	*OreG0018661*
scaffold346	1338964	G	C	NA	*OreG0018663*
scaffold346	1341577	T	C	Och01,Och02,Och03,Och04,Och05,Och06,Och07,Och08,Och09,Och10,Och11,Och12,Och13,Och14	*OreG0018663*
scaffold346	1535155	A	C	Ore01,Ore02,Ore03,Ore04,Ore05,Ore06,Ore07,Ore08,Ore09,Ore10,Ore11,Ore12,Ore13,Ore14	*OreG0018672*
scaffold346	1565613	T	A	Ore01,Ore02,Ore03,Ore04,Ore05,Ore06,Ore07,Ore08,Ore09,Ore10,Ore11,Ore12,Ore13,Ore14	*OreG0018677*
scaffold346	1644309	G	T	NA	*OreG0018681*
scaffold346	1669335	T	A	NA	*OreG0018683*
scaffold346	1669340	C	T	NA	*OreG0018683*
scaffold346	1669386	A	C	NA	*OreG0018683*
scaffold346	1669476	C	A	NA	*OreG0018683*
scaffold346	1682508	C	T	NA	*OreG0018685*
scaffold346	1684340	T	A	NA	*OreG0018686*
scaffold346	1684425	A	T	NA	*OreG0018686*
scaffold346	1689199	T	C	Och06	*OreG0018687*
scaffold346	1982582	C	G	Och01,Och02,Och03,Och04,Och05,Och06,Och07,Och08,Och09,Och10,Och11,Och12,Och13,Och14,Ore01,Ore02,Ore03,Ore04,Ore05,Ore06,Ore07,Ore08,Ore09,Ore10,Ore11,Ore12,Ore13,Ore14	*OreG0018694*
scaffold346	2060984	T	A	NA	*OreG0018703*
scaffold346	2077773	C	G	Och01,Och02,Och03,Och04,Och05,Och06,Och07,Och08,Och09,Och10,Och11,Och12,Och13,Och14	*OreG0018705*
scaffold346	2086604	C	A	NA	*OreG0018706*
scaffold346	2087387	G	A	Och02,Och03,Och04,Och05,Och06,Och07,Och08,Och09,Och10,Och11,Och12,Och13,Och14	*OreG0018706*
scaffold346	2093039	C	A	NA	*OreG0018707*
scaffold346	2093487	C	A	NA	*OreG0018707*
scaffold346	2116925	T	C	Och01,Och02,Och03,Och04,Och05,Och06,Och07,Och08,Och09,Och10,Och11,Och12,Och13,Och14,Ore01,Ore02,Ore03,Ore04,Ore05,Ore06,Ore07,Ore08,Ore09,Ore10,Ore11,Ore12,Ore13,Ore14	*OreG0018711*
scaffold346	2116936	G	A	Och01,Och02,Och03,Och04,Och05,Och06,Och07,Och08,Och09,Och10,Och11,Och12,Och13,Och14	*OreG0018711*
scaffold346	2116946	T	G	Och01,Och02,Och03,Och04,Och05,Och06,Och07,Och08,Och09,Och10,Och11,Och12,Och13,Och14	*OreG0018711*
scaffold346	2116948	G	A	Och01,Och02,Och03,Och04,Och05,Och06,Och07,Och08,Och09,Och10,Och11,Och12,Och13,Och14	*OreG0018711*
scaffold346	2134258	G	A	NA	*OreG0018713*
scaffold346	2134272	C	T	Ore04,Ore05	*OreG0018713*
scaffold346	2159337	G	A	Och01,Och02,Och03,Och04,Och05,Och06,Och07,Och08,Och09,Och10,Och11,Och12,Och13,Och14	*OreG0018716*
scaffold346	2159990	T	C	Ore01,Ore02,Ore03,Ore04,Ore05,Ore06,Ore07,Ore08,Ore09,Ore10,Ore11,Ore12,Ore13,Ore14	*OreG0018716*
scaffold346	2161736	A	G	Och01,Och02,Och03,Och04,Och05,Och06,Och07,Och08,Och09,Och10,Och11,Och12,Och13,Och14	*OreG0018716*
scaffold346	2181327	C	T	Och12	*OreG0018717*
scaffold346	2272045	T	C	Ore01,Ore02,Ore03,Ore04,Ore05,Ore06,Ore07,Ore08,Ore09,Ore10,Ore11,Ore12,Ore13,Ore14	*OreG0018729*
scaffold346	2272223	A	G	NA	*OreG0018729*
scaffold346	2334100	T	A	NA	*OreG0018733*
scaffold346	2339671	G	C	Och01,Och02,Och03,Och04,Och05,Och06,Och07,Och08,Och09,Och10,Och11,Och12,Och13,Och14,Ore01,Ore02,Ore03,Ore04,Ore05,Ore06,Ore07,Ore08,Ore09,Ore10,Ore11,Ore12,Ore13,Ore14	*OreG0018734*

scaffold346	2351204	A	G	Och01,Och02,Och03,Och04,Och05,Och06,Och07,Och08,Och09,Och10,Och11,Och12,Och13,Och14,Ore01,Ore02,Ore03,Ore04,Ore05,Ore06,Ore07,Ore08,Ore09,Ore10,Ore11,Ore12,Ore13,Ore14	*OreG0018735*
scaffold346	2417477	G	T	Ore01,Ore02,Ore03,Ore04,Ore05,Ore06,Ore07,Ore08,Ore09,Ore10,Ore11,Ore12,Ore13,Ore14	*OreG0018741*
scaffold346	2421771	G	T	NA	*OreG0018742*
scaffold346	2459276	G	A	NA	*OreG0018748*
scaffold346	2523470	C	T	NA	*OreG0018755*
scaffold346	2607002	T	A	NA	*OreG0018760*
scaffold346	2649990	G	T	Och06	*OreG0018765*
scaffold346	2651560	A	T	Och01,Och02,Och03,Och04,Och05,Och06,Och07,Och08,Och09,Och10,Och11,Och12,Och13,Och14,Ore01,Ore02,Ore03,Ore04,Ore05,Ore06,Ore07,Ore08,Ore09,Ore10,Ore11,Ore12,Ore13,Ore14	*OreG0018765*
scaffold346	2670388	C	A	NA	*OreG0018766*
scaffold4509	3769	A	T	Ore01,Ore02,Ore07,Ore08,Ore09,Ore12,Ore13	*OreG0020560*
scaffold4509	26580	G	C	NA	*OreG0020561*
scaffold4173	15397	G	A	NA	*OreG0020089*
scaffold4173	18827	G	A	NA	*OreG0020089*
scaffold4173	51029	C	T	Och07	*OreG0020092*
scaffold4681	32502	T	G	NA	*OreG0022355*
scaffold4681	33935	G	A	NA	*OreG0022356*
scaffold4681	36852	T	G	NA	*OreG0022357*
scaffold3757	19991	T	C	NA	*OreG0019083*
scaffold3757	20151	T	A	NA	*OreG0019083*
scaffold4913	2215	A	G	Och01,Och02,Och03,Och04,Och05,Och06,Och07,Och08,Och09,Och10,Och11,Och12,Och13,Och14,Ore01,Ore02,Ore03,Ore04,Ore05,Ore06,Ore07,Ore08,Ore09,Ore10,Ore11,Ore12,Ore13,Ore14	*OreG0023026*
scaffold4913	2260	A	C	Och01,Och02,Och03,Och05,Och07,Och08,Och11,Och12,Och13,Och14,Ore01,Ore02,Ore03,Ore04,Ore05,Ore06,Ore07,Ore08,Ore09,Ore10,Ore11,Ore12,Ore13,Ore14	*OreG0023026*
scaffold1220	111834	T	C	NA	*OreG0001365*
scaffold1220	152938	T	C	Och11	*OreG0001368*
scaffold1220	250443	C	T	Ore01,Ore02,Ore03,Ore04,Ore05,Ore06,Ore07,Ore08,Ore09,Ore10,Ore11,Ore12,Ore13,Ore14	*OreG0001375*
scaffold1220	250476	C	T	NA	*OreG0001375*
scaffold1220	250487	T	C	Och02,Och03,Och04,Och05,Och06,Och12,Och13	*OreG0001375*
scaffold1220	250757	C	T	NA	*OreG0001375*
scaffold1220	252910	A	G	NA	*OreG0001375*
scaffold1220	252990	C	T	Och02,Och03,Och04,Och05,Och06,Och12,Och13	*OreG0001375*
scaffold1220	253045	T	A	Och02,Och03,Och04,Och05,Och06,Och12,Och13	*OreG0001375*
scaffold1220	256302	T	C	NA	*OreG0001375*
scaffold1220	256344	G	A	NA	*OreG0001375*
scaffold1220	256348	C	T	NA	*OreG0001375*
scaffold1220	264704	G	T	NA	*OreG0001376*
scaffold1220	279559	T	A	NA	*OreG0001378*
scaffold1220	289068	C	T	NA	*OreG0001381*
scaffold1220	311952	A	T	NA	*OreG0001383*
scaffold1220	313787	A	G	NA	*OreG0001383*
scaffold1220	313793	G	A	NA	*OreG0001383*
scaffold1220	315734	A	T	NA	*OreG0001383*
scaffold1220	397409	G	T	NA	*OreG0001394*
scaffold1220	397472	A	T	Ore01,Ore02,Ore03,Ore06,Ore07,Ore08,Ore09,Ore10,Ore11,Ore12,Ore13,Ore14	*OreG0001394*
scaffold1220	397534	C	G	NA	*OreG0001394*
scaffold1220	397556	G	A	NA	*OreG0001394*
scaffold1220	439110	A	G	NA	*OreG0001398*
scaffold1220	439799	C	T	Och02,Och03,Och04,Och05,Och06,Och08,Och09,Och10,Och11,Och12,Och13,Och14,Ore01,Ore02,Ore03,Ore04,Ore05,Ore06,Ore07,Ore08,Ore09,Ore10,Ore11,Ore12,Ore13,Ore14	*OreG0001398*
scaffold1220	441779	A	T	Och12,Och13	*OreG0001398*
scaffold1220	442278	C	G	Och12,Och13	*OreG0001398*
scaffold1220	445183	C	G	NA	*OreG0001398*
scaffold1220	469864	A	G	Och14	*OreG0001401*
scaffold1220	471104	G	A	NA	*OreG0001401*
scaffold1220	475740	A	T	NA	*OreG0001401*
scaffold1220	480038	C	T	NA	*OreG0001402*
scaffold1220	480067	C	A	NA	*OreG0001402*
scaffold1220	480189	T	A	NA	*OreG0001402*
scaffold1220	492238	A	G	NA	*OreG0001404*
scaffold1220	492724	A	G	NA	*OreG0001404*
scaffold1220	492784	G	C	NA	*OreG0001404*
scaffold1220	492858	T	A	NA	*OreG0001404*
scaffold1220	493608	G	A	NA	*OreG0001404*
scaffold1220	493684	C	A	NA	*OreG0001404*
scaffold1220	493703	C	A	NA	*OreG0001404*
scaffold1220	493759	C	A	NA	*OreG0001404*

scaffold1220	493833	C	T	NA	*OreG0001404*
scaffold1220	495231	T	G	Ore02	*OreG0001405*
scaffold1220	495310	A	T	NA	*OreG0001405*
scaffold1220	496165	C	T	NA	*OreG0001405*
scaffold1220	496186	C	A	NA	*OreG0001405*
scaffold1220	496975	G	A	Ore02	*OreG0001405*
scaffold1220	503067	G	A	Och01,Och02,Och03,Och04,Och05,Och06,Och07,Och08,Och09,Och10,Och11,Och12,Och13,Och14	*OreG0001406*
scaffold1220	503298	A	T	NA	*OreG0001406*
scaffold1220	510661	T	C	Och01	*OreG0001407*
scaffold1220	515560	C	T	Och01,Och02,Och03,Och04,Och05,Och06,Och07,Och08,Och09,Och10,Och11,Och12,Och13,Och14,Ore01,Ore02,Ore03,Ore04,Ore05,Ore06,Ore07,Ore08,Ore09,Ore10,Ore11,Ore12,Ore13,Ore14	*OreG0001409*
scaffold1220	515640	C	T	Och03	*OreG0001409*
scaffold1220	515802	T	C	NA	*OreG0001409*
scaffold1220	515914	T	C	Och03	*OreG0001409*
scaffold1220	515962	C	T	Ore01,Ore02,Ore03,Ore04,Ore05,Ore06,Ore07,Ore08,Ore09,Ore10,Ore11,Ore12,Ore13,Ore14	*OreG0001409*
scaffold1220	515974	T	A	Och12,Och13	*OreG0001409*
scaffold1220	516129	T	A	Och03	*OreG0001409*
scaffold1220	516201	C	T	Ore01,Ore02,Ore03,Ore04,Ore05,Ore06,Ore07,Ore08,Ore09,Ore10,Ore11,Ore12,Ore13,Ore14	*OreG0001409*
scaffold1220	516678	G	T	Ore01,Ore02,Ore03,Ore04,Ore05,Ore06,Ore07,Ore08,Ore09,Ore10,Ore11,Ore12,Ore13,Ore14	*OreG0001409*
scaffold1220	535209	G	A	NA	*OreG0001412*
scaffold1220	572523	A	C	Ore01,Ore02,Ore03,Ore04,Ore05,Ore06,Ore07,Ore08,Ore09,Ore10,Ore11,Ore12,Ore13,Ore14	*OreG0001414*
scaffold1220	573024	C	T	Och01,Och02,Och03,Och04,Och05,Och06,Och07,Och08,Och09,Och10,Och11,Och12,Och13,Och14	*OreG0001414*
scaffold1220	574187	A	T	NA	*OreG0001414*
scaffold1220	574228	C	G	Och08,Och12,Och13,Och14	*OreG0001414*
scaffold1220	580098	C	T	NA	*OreG0001415*
scaffold1220	580776	C	A	Ore01,Ore02,Ore03,Ore04,Ore05,Ore06,Ore07,Ore08,Ore09,Ore10,Ore11,Ore12,Ore13,Ore14	*OreG0001415*
scaffold1220	582685	C	T	Och01,Och02,Och03,Och04,Och05,Och06,Och07,Och08,Och09,Och10,Och11,Och12,Och13,Och14,Ore01,Ore02,Ore03,Ore04,Ore05,Ore06,Ore07,Ore08,Ore09,Ore10,Ore11,Ore12,Ore13,Ore14	*OreG0001415*
scaffold1220	605800	A	G	NA	*OreG0001420*
scaffold1220	605830	A	C	NA	*OreG0001420*
scaffold1220	606019	T	A	NA	*OreG0001420*
scaffold1220	660511	G	T	Ore04,Ore05,Ore06,Ore07,Ore08,Ore12,Ore13,Ore14	*OreG0001425*
scaffold1220	660845	G	A	Och03,Och05,Och07,Och09,Och10,Och11,Och12,Och13	*OreG0001425*
scaffold1220	703130	T	C	NA	*OreG0001429*
scaffold1220	789267	G	A	Och03	*OreG0001431*
scaffold1220	789490	C	T	NA	*OreG0001431*
scaffold1220	789777	C	T	Och03,Och05,Och07,Och09,Och10,Och11,Och12,Och13	*OreG0001431*
scaffold1220	816272	C	T	Och12,Och13	*OreG0001432*
scaffold1220	860918	G	C	NA	*OreG0001433*
scaffold1220	905243	G	T	NA	*OreG0001437*
scaffold1220	921645	T	A	Ore01,Ore02,Ore03,Ore04,Ore05,Ore06,Ore07,Ore08,Ore09,Ore10,Ore11,Ore12,Ore13,Ore14	*OreG0001437*
scaffold1220	924716	T	A	NA	*OreG0001438*
scaffold1220	996039	T	A	Och02,Och03,Och04,Och05,Och06,Och08,Och12,Och13	*OreG0001444*
scaffold1220	997572	A	G	Och01,Och02,Och03,Och04,Och05,Och06,Och07,Och08,Och09,Och10,Och11,Och12,Och13,Och14,Ore01,Ore02,Ore03,Ore04,Ore05,Ore06,Ore07,Ore08,Ore09,Ore10,Ore11,Ore12,Ore13,Ore14	*OreG0001444*
scaffold1220	998757	T	C	Och02,Och03,Och04,Och05,Och06,Och07,Och08,Och09,Och10,Och11,Och12,Och13,Och14	*OreG0001444*
scaffold1220	1002331	G	T	Ore06,Ore07,Ore08,Ore13,Ore14	*OreG0001444*
scaffold1220	1028379	A	T	NA	*OreG0001447*
scaffold1220	1032290	C	A	Och01,Och02,Och04,Och08,Och12,Och13	*OreG0001447*
scaffold1220	1032308	C	G	Och01,Och04,Och08,Och12,Och13	*OreG0001447*
scaffold1220	1044323	G	A	NA	*OreG0001448*
scaffold1220	1044394	A	G	NA	*OreG0001448*
scaffold1220	1044559	G	T	Och01,Och02,Och03,Och04,Och05,Och06,Och07,Och08,Och09,Och10,Och11,Och12,Och13,Och14,Ore01,Ore02,Ore03,Ore04,Ore05,Ore06,Ore07,Ore08,Ore09,Ore10,Ore11,Ore12,Ore13,Ore14	*OreG0001448*
scaffold1220	1054332	T	A	NA	*OreG0001449*
scaffold1220	1055185	G	A	NA	*OreG0001449*
scaffold1220	1055273	G	C	NA	*OreG0001449*
scaffold1220	1059717	A	G	NA	*OreG0001450*
scaffold1220	1059927	T	A	NA	*OreG0001450*
scaffold1220	1060381	A	T	NA	*OreG0001450*
scaffold1220	1073868	C	T	Ore01,Ore02,Ore03,Ore04,Ore05,Ore06,Ore07,Ore08,Ore09,Ore10,Ore11,Ore12,Ore13,Ore14	*OreG0001451*

scaffold1220	1073953	T	G	Och03	*OreG0001451*
scaffold1220	1074294	T	C	NA	*OreG0001452*
scaffold1220	1078333	C	T	Ore01,Ore02,Ore03,Ore04,Ore05,Ore06,Ore07,Ore08,Ore09,Ore10,Ore11,Ore12,Ore13,Ore14	*OreG0001453*
scaffold1220	1078434	G	A	NA	*OreG0001453*
scaffold1220	1078743	A	G	NA	*OreG0001453*
scaffold1220	1078839	C	T	Ore01,Ore02,Ore03,Ore04,Ore05,Ore06,Ore07,Ore08,Ore09,Ore10,Ore11,Ore12,Ore13,Ore14	*OreG0001453*
scaffold1220	1181593	G	A	Och01,Och02,Och03,Och04,Och05,Och06,Och07,Och08,Och09,Och10,Och11,Och12,Och13,Och14,Ore01,Ore02,Ore03,Ore04,Ore05,Ore06,Ore07,Ore08,Ore09,Ore10,Ore11,Ore12,Ore13,Ore14	*OreG0001466*
scaffold1220	1182684	A	G	Och01,Och02,Och03,Och04,Och05,Och06,Och07,Och09,Och10,Och11,Och12,Och13,Och14	*OreG0001466*
scaffold1220	1201857	G	T	NA	*OreG0001467*
scaffold1220	1225322	G	A	Och01,Och02,Och03,Och04,Och05,Och06,Och07,Och08,Och09,Och10,Och11,Och12,Och13,Och14,Ore01,Ore02,Ore03,Ore04,Ore05,Ore06,Ore07,Ore08,Ore09,Ore10,Ore11,Ore12,Ore13,Ore14	*OreG0001468*
scaffold1220	1243073	T	C	NA	*OreG0001470*
scaffold1220	1258167	G	C	Och01,Och02,Och03,Och04,Och05,Och06,Och07,Och08,Och09,Och10,Och11,Och12,Och13,Och14,Ore01,Ore02,Ore03,Ore04,Ore05,Ore06,Ore07,Ore08,Ore09,Ore10,Ore11,Ore12,Ore13,Ore14	*OreG0001473*
scaffold1220	1314049	C	T	NA	*OreG0001478*
scaffold1220	1343879	C	A	Och06,Och11	*OreG0001480*
scaffold1220	1366172	G	A	Och01,Och02,Och03,Och04,Och05,Och06,Och07,Och08,Och09,Och10,Och11,Och12,Och13,Och14,Ore01,Ore02,Ore03,Ore04,Ore05,Ore06,Ore07,Ore08,Ore09,Ore10,Ore11,Ore12,Ore13,Ore14	*OreG0001482*
scaffold1220	1369528	A	T	NA	*OreG0001482*
scaffold1220	1497028	A	G	Ore01,Ore02,Ore03,Ore04,Ore05,Ore06,Ore07,Ore08,Ore09,Ore10,Ore11,Ore12,Ore13,Ore14	*OreG0001492*
scaffold1220	1500861	G	C	NA	*OreG0001493*
scaffold1220	1505681	G	T	NA	*OreG0001495*
scaffold1220	1506593	A	G	NA	*OreG0001496*
scaffold1220	1511859	A	G	NA	*OreG0001497*
scaffold1220	1512014	T	C	NA	*OreG0001497*
scaffold1220	1512471	G	A	NA	*OreG0001497*
scaffold1220	1516100	T	C	NA	*OreG0001498*
scaffold1220	1516187	G	C	Och01,Och02,Och03,Och04,Och05,Och06,Och08,Och12,Och13,Och14	*OreG0001498*
scaffold1220	1516253	C	G	NA	*OreG0001498*
scaffold1220	1517081	A	G	NA	*OreG0001498*
scaffold1220	1517252	G	T	NA	*OreG0001498*
scaffold1220	1520022	A	G	NA	*OreG0001499*
scaffold1220	1520563	C	A	NA	*OreG0001499*
scaffold1220	1520878	C	T	NA	*OreG0001499*
scaffold1220	1520907	G	A	NA	*OreG0001499*
scaffold1220	1521712	C	T	NA	*OreG0001499*
scaffold1220	1521766	T	C	NA	*OreG0001499*
scaffold1220	1533616	A	G	Ore01,Ore02,Ore04,Ore05,Ore06,Ore07,Ore13	*OreG0001500*
scaffold1220	1538016	G	A	Och06,Och07	*OreG0001501*
scaffold1220	1561874	C	G	NA	*OreG0001503*
scaffold1220	1583434	A	G	NA	*OreG0001506*
scaffold1220	1583437	T	C	Ore01,Ore03,Ore04,Ore05,Ore06,Ore08,Ore09,Ore11,Ore13	*OreG0001506*
scaffold1220	1583755	T	A	Ore01,Ore02,Ore03,Ore04,Ore05,Ore06,Ore07,Ore08,Ore09,Ore10,Ore11,Ore12,Ore13,Ore14	*OreG0001506*
scaffold1220	1584381	C	A	Och01,Och02,Och03,Och04,Och05,Och06,Och07,Och08,Och09,Och10,Och11,Och12,Och13,Och14	*OreG0001506*
scaffold1220	1600650	C	T	NA	*OreG0001509*
scaffold1220	1600664	T	C	NA	*OreG0001509*
scaffold1220	1602575	C	A	Och02,Och03,Och04,Och05,Och06,Och12,Och13	*OreG0001509*
scaffold1220	1603067	A	G	Och12	*OreG0001509*
scaffold1220	1603084	G	A	Och01,Och02,Och03,Och04,Och05,Och06,Och07,Och08,Och09,Och10,Och11,Och12,Och13,Och14	*OreG0001509*
scaffold1220	1603140	G	C	Och01,Och02,Och03,Och04,Och05,Och06,Och07,Och08,Och09,Och10,Och11,Och12,Och13,Och14	*OreG0001509*
scaffold1220	1651098	A	T	Och01,Och02,Och03,Och04,Och05,Och06,Och07,Och08,Och09,Och10,Och11,Och12,Och13,Och14,Ore01,Ore02,Ore03,Ore04,Ore05,Ore06,Ore07,Ore08,Ore09,Ore10,Ore11,Ore12,Ore13,Ore14	*OreG0001512*
scaffold1220	1689264	C	A	NA	*OreG0001514*
scaffold1220	1699709	T	C	NA	*OreG0001515*
scaffold1220	1700343	C	T	Ore09	*OreG0001515*
scaffold1220	1701176	C	A	Ore09	*OreG0001515*
scaffold1220	1708980	G	T	Ore01,Ore02,Ore03,Ore04,Ore05,Ore06,Ore07,Ore08,Ore09,Ore10,Ore11,Ore12,Ore13,Ore14	*OreG0001516*
scaffold1220	1751054	T	A	NA	*OreG0001519*
scaffold1220	1761985	G	T	NA	*OreG0001519*

scaffold1220	1797761	T	A	Ore01,Ore02,Ore03,Ore04,Ore05,Ore06,Ore07,Ore08,Ore09,Ore10,Ore11,Ore12,Ore13,Ore14	*OreG0001525*
scaffold1220	1802448	C	T	Och14	*OreG0001526*
scaffold1220	1808633	G	C	Ore01,Ore02,Ore03,Ore04,Ore05,Ore06,Ore07,Ore08,Ore09,Ore10,Ore11,Ore12,Ore13,Ore14	*OreG0001526*
scaffold1220	1814504	A	G	Ore01,Ore02,Ore03,Ore04,Ore05,Ore06,Ore07,Ore08,Ore09,Ore10,Ore11,Ore12,Ore13,Ore14	*OreG0001526*
scaffold1220	1847231	A	T	NA	*OreG0001529*
scaffold1220	1972206	C	G	Och12	*OreG0001541*
scaffold1220	1989609	C	T	NA	*OreG0001542*
scaffold1220	1993304	C	A	Ore01,Ore07	*OreG0001542*
scaffold1220	2003720	C	T	Ore01,Ore03,Ore07,Ore08,Ore10,Ore11,Ore12,Ore14	*OreG0001543*
scaffold1220	2005329	T	A	NA	*OreG0001543*
scaffold1220	2030639	G	C	NA	*OreG0001545*
scaffold1220	2046675	A	T	Ore01,Ore02,Ore03,Ore04,Ore05,Ore06,Ore07,Ore08,Ore09,Ore10,Ore11,Ore12,Ore13,Ore14	*OreG0001545*
scaffold1220	2046994	C	T	Och03,Och04	*OreG0001545*
scaffold1220	2069956	T	C	NA	*OreG0001547*
scaffold1220	2070863	C	G	Och01,Och03,Och04,Och05,Och07,Och09,Och10,Och11,Och12,Och13,Och14	*OreG0001548*
scaffold1220	2072323	C	A	Och01,Och03,Och04,Och05,Och07,Och09,Och10,Och11,Och12,Och13,Och14	*OreG0001548*
scaffold1220	2107214	T	A	Ore01,Ore02,Ore03,Ore04,Ore05,Ore06,Ore07,Ore08,Ore09,Ore10,Ore11,Ore12,Ore13,Ore14	*OreG0001551*
scaffold1220	2107220	C	T	Ore01,Ore02,Ore03,Ore04,Ore05,Ore06,Ore07,Ore08,Ore09,Ore10,Ore11,Ore12,Ore13,Ore14	*OreG0001551*
scaffold1220	2107260	C	T	Ore01,Ore02,Ore03,Ore04,Ore05,Ore06,Ore07,Ore08,Ore09,Ore10,Ore11,Ore12,Ore13,Ore14	*OreG0001551*
scaffold1220	2164188	T	G	Ore01,Ore02,Ore03,Ore04,Ore05,Ore06,Ore07,Ore08,Ore09,Ore10,Ore11,Ore12,Ore13,Ore14	*OreG0001554*
scaffold1220	2179172	C	T	Och01	*OreG0001555*
scaffold1220	2186571	C	T	Ore01,Ore02,Ore03,Ore04,Ore05,Ore06,Ore07,Ore08,Ore09,Ore10,Ore11,Ore12,Ore13,Ore14	*OreG0001556*
scaffold1220	2186933	A	C	Och01,Och02,Och05,Och07,Och08,Och09,Och10,Och11,Och12,Och13,Och14	*OreG0001556*
scaffold1220	2194015	G	A	NA	*OreG0001557*
scaffold1220	2211645	C	T	Och01,Och02,Och03,Och04,Och05,Och06,Och07,Och08,Och09,Och10,Och11,Och12,Och13,Och14,Ore01,Ore02,Ore03,Ore04,Ore05,Ore06,Ore07,Ore08,Ore09,Ore10,Ore11,Ore12,Ore13,Ore14	*OreG0001557*
scaffold1220	2241039	G	A	NA	*OreG0001557*
scaffold1220	2278998	C	A	NA	*OreG0001558*
scaffold1220	2292711	A	C	Och01,Och03,Och04,Och05,Och12,Och13	*OreG0001559*
scaffold1220	2375106	G	C	Ore03,Ore08,Ore10,Ore11,Ore12,Ore14	*OreG0001563*
scaffold1220	2451706	C	T	Och03,Och04	*OreG0001567*
scaffold1220	2456150	T	C	NA	*OreG0001568*
scaffold1220	2456153	C	T	Och01,Och02,Och03,Och04,Och05,Och06,Och07,Och08,Och09,Och10,Och11,Och12,Och13,Och14,Ore01,Ore02,Ore03,Ore04,Ore05,Ore06,Ore07,Ore08,Ore09,Ore10,Ore11,Ore12,Ore13,Ore14	*OreG0001568*
scaffold1220	2456986	G	A	Och01,Och07,Och08,Och12,Och14	*OreG0001568*
scaffold1220	2457016	C	T	Ore01,Ore02,Ore03,Ore04,Ore05,Ore06,Ore07,Ore08,Ore09,Ore10,Ore11,Ore12,Ore13,Ore14	*OreG0001568*
scaffold1220	2477915	T	C	Och01,Och02,Och03,Och04,Och05,Och06,Och07,Och08,Och09,Och10,Och11,Och12,Och13,Och14,Ore01,Ore02,Ore03,Ore04,Ore05,Ore06,Ore07,Ore08,Ore09,Ore10,Ore11,Ore12,Ore13,Ore14	*OreG0001570*
scaffold1220	2480879	G	A	NA	*OreG0001570*
scaffold1220	2504974	A	T	Och09,Och10	*OreG0001573*
scaffold1220	2506764	T	A	NA	*OreG0001573*
scaffold1220	2506771	T	C	NA	*OreG0001573*
scaffold1220	2552923	C	T	Ore01,Ore02,Ore03,Ore04,Ore05,Ore06,Ore07,Ore08,Ore09,Ore10,Ore11,Ore12,Ore13,Ore14	*OreG0001574*
scaffold1220	2570522	T	A	Och06	*OreG0001575*
scaffold1220	2593064	T	A	NA	*OreG0001576*
scaffold1220	2616356	A	T	Och01,Och02,Och03,Och04,Och05,Och06,Och07,Och08,Och09,Och10,Och11,Och12,Och13,Och14,Ore01,Ore02,Ore03,Ore04,Ore05,Ore06,Ore07,Ore08,Ore09,Ore10,Ore11,Ore12,Ore13,Ore14	*OreG0001579*
scaffold1220	2647096	G	A	NA	*OreG0001581*
scaffold1220	2652146	A	G	NA	*OreG0001581*
scaffold1220	2660947	T	C	Och01,Och02,Och03,Och04,Och05,Och06,Och07,Och08,Och09,Och10,Och11,Och12,Och13,Och14	*OreG0001583*
scaffold1220	2669591	G	A	Och08	*OreG0001584*
scaffold1220	2669634	A	T	Och02,Och03,Och04,Och05,Och06,Och07,Och08,Och12,Och13,Ore01,Ore02,Ore03,Ore04,Ore05,Ore06,Ore07,Ore08,Ore09,Ore10,Ore11,Ore12,Ore13,Ore14	*OreG0001584*
scaffold1220	2685355	A	T	NA	*OreG0001585*
scaffold1220	2685935	G	C	Och01,Och02,Och03,Och04,Och05,Och06,Och07,Och08,Och09,Och10,Och11,Och12,Och13,Och14,Ore01,Ore02,Ore03,Ore04,Ore05,Ore06,Ore07,Ore08,Ore09,Ore10,Ore11,Ore12,Ore13,Ore14	*OreG0001585*
scaffold1220	2702010	C	T	Ore01,Ore02,Ore03,Ore04,Ore05,Ore06,Ore07,Ore08,Ore09,Ore10,Ore11,Ore12,Ore13,Ore14	*OreG0001586*

scaffold1220	2726093	C	T	NA	*OreG0001590*
scaffold1220	2761368	T	A	NA	*OreG0001592*
scaffold1220	2761562	A	G	NA	*OreG0001592*
scaffold1220	2780439	T	A	Ore01,Ore02,Ore03,Ore04,Ore05,Ore06,Ore07,Ore08,Ore09,Ore10,Ore11,Ore12,Ore13,Ore14	*OreG0001593*
scaffold1220	2795872	C	T	Och01	*OreG0001594*
scaffold1220	2805992	T	A	Och01	*OreG0001594*
scaffold1220	2811911	G	C	Ore10,Ore14	*OreG0001598*
scaffold1220	2812569	C	G	NA	*OreG0001598*
scaffold1220	2836917	A	G	NA	*OreG0001600*
scaffold1220	2836947	C	T	Och03,Och04,Och05,Och12,Och13	*OreG0001600*
scaffold1220	2837037	A	G	Och01,Och02,Och03,Och04,Och05,Och06,Och07,Och08,Och09,Och10,Och11,Och12,Och13,Och14	*OreG0001600*
scaffold1220	2838349	C	G	Ore10,Ore14	*OreG0001600*
scaffold1220	2838713	T	G	NA	*OreG0001600*
scaffold1220	2838841	A	T	Och03,Och04,Och05,Och12,Och13	*OreG0001600*
scaffold1220	2838994	G	A	Och01,Och07,Och14	*OreG0001600*
scaffold1220	2839016	G	A	NA	*OreG0001600*
scaffold1220	2839037	T	C	NA	*OreG0001600*
scaffold1220	2846157	T	A	Ore03	*OreG0001601*
scaffold1220	2898434	G	A	NA	*OreG0001608*
scaffold1220	2898835	G	T	Och08	*OreG0001608*
scaffold1220	2909989	C	T	NA	*OreG0001609*
scaffold1220	3001947	C	T	NA	*OreG0001614*
scaffold1220	3009785	G	A	Och06,Och14	*OreG0001614*
scaffold1220	3027628	T	A	Och01,Och02,Och03,Och04,Och05,Och06,Och07,Och08,Och09,Och10,Och11,Och12,Och13,Och14,Ore01,Ore02,Ore03,Ore04,Ore05,Ore06,Ore07,Ore08,Ore09,Ore10,Ore11,Ore12,Ore13,Ore14	*OreG0001617*
scaffold1220	3031764	A	G	Och02,Och03,Och04,Och05,Och06,Och07,Och12,Och13	*OreG0001617*
scaffold1220	3056891	T	C	Och04,Och11	*OreG0001620*
scaffold1220	3061263	A	G	Ore03,Ore04,Ore05,Ore09,Ore13	*OreG0001621*
scaffold1220	3115720	G	T	NA	*OreG0001624*
scaffold1220	3129140	C	T	NA	*OreG0001625*
scaffold2483	532142	G	C	NA	*OreG0007473*
scaffold2483	532161	C	A	NA	*OreG0007473*
scaffold1468	28178	G	A	NA	*OreG0002617*
scaffold1468	28204	T	A	NA	*OreG0002617*
scaffold1468	32694	G	A	NA	*OreG0002617*
scaffold1468	43011	G	A	Och02,Och03,Och04,Och05,Och06,Och07,Och09,Och10,Och12,Och13	*OreG0002619*
scaffold1468	43240	A	G	Ore01,Ore02,Ore03,Ore04,Ore05,Ore06,Ore07,Ore08,Ore09,Ore10,Ore11,Ore12,Ore13,Ore14	*OreG0002619*
scaffold1468	44008	T	A	NA	*OreG0002619*
scaffold1468	44592	G	A	NA	*OreG0002619*
scaffold1468	44613	T	C	NA	*OreG0002619*
scaffold1468	53636	T	A	NA	*OreG0002620*
scaffold1468	59897	T	A	NA	*OreG0002620*
scaffold1468	78052	C	T	NA	*OreG0002623*
scaffold1468	99649	C	A	Och08	*OreG0002625*
scaffold1468	102170	G	C	Ore04,Ore05,Ore06,Ore11,Ore13	*OreG0002625*
scaffold1468	121691	C	T	NA	*OreG0002627*
scaffold1468	123801	G	T	NA	*OreG0002627*
scaffold1468	123964	C	T	NA	*OreG0002627*
scaffold1468	146699	C	T	NA	*OreG0002629*
scaffold1468	147351	A	T	Och01,Och02,Och03,Och04,Och05,Och06,Och07,Och08,Och09,Och10,Och11,Och12,Och13,Och14,Ore01,Ore02,Ore03,Ore04,Ore05,Ore06,Ore07,Ore08,Ore09,Ore10,Ore11,Ore12,Ore13,Ore14	*OreG0002629*
scaffold1468	150740	C	T	Och07	*OreG0002630*
scaffold1468	151585	A	G	NA	*OreG0002630*
scaffold1468	184783	C	T	NA	*OreG0002634*
scaffold1468	217104	T	A	Ore01,Ore02,Ore03,Ore04,Ore05,Ore06,Ore07,Ore08,Ore09,Ore10,Ore11,Ore12,Ore13,Ore14	*OreG0002638*
scaffold1468	252821	G	A	Ore10,Ore14	*OreG0002642*
scaffold1468	252827	C	T	Och01,Och02,Och03,Och04,Och05,Och06,Och07,Och08,Och09,Och10,Och11,Och12,Och13,Och14	*OreG0002642*
scaffold1468	252962	G	A	NA	*OreG0002642*
scaffold1468	253731	A	C	Ore01,Ore02,Ore03,Ore04,Ore05,Ore06,Ore07,Ore08,Ore09,Ore10,Ore11,Ore12,Ore13,Ore14	*OreG0002642*
scaffold1468	267805	A	G	Och01,Och02,Och03,Och04,Och05,Och06,Och07,Och08,Och09,Och10,Och11,Och12,Och13,Och14,Ore01,Ore02,Ore03,Ore04,Ore05,Ore06,Ore07,Ore08,Ore09,Ore10,Ore11,Ore12,Ore13,Ore14	*OreG0002645*
scaffold1468	314802	G	A	Och11,Och14	*OreG0002650*
scaffold1468	340804	A	G	Och02,Och03,Och04,Och05,Och07,Och08,Och09,Och10,Och11,Och12,Och13,Och14	*OreG0002654*
scaffold1468	341205	G	T	Ore01,Ore02,Ore03,Ore04,Ore05,Ore06,Ore07,Ore08,Ore09,Ore10,Ore11,Ore12,Ore13,Ore14	*OreG0002654*

scaffold1468	341235	A	G	Och01,Och02,Och03,Och04,Och05,Och06,Och07,Och08,Och09,Och10,Och11,Och12,Och13,Och14,Ore01,Ore02,Ore03,Ore04,Ore05,Ore06,Ore07,Ore08,Ore09,Ore10,Ore11,Ore12,Ore13,Ore14	*OreG0002654*
scaffold1468	346798	G	T	NA	*OreG0002655*
scaffold1468	353924	G	A	NA	*OreG0002657*
scaffold1468	355144	G	C	NA	*OreG0002657*
scaffold1468	355919	G	A	Ore03,Ore04,Ore05,Ore06,Ore09,Ore11,Ore13	*OreG0002657*
scaffold1468	356284	T	A	Och01,Och02,Och03,Och04,Och05,Och06,Och07,Och08,Och09,Och10,Och11,Och12,Och13,Och14,Ore01,Ore02,Ore03,Ore04,Ore05,Ore06,Ore07,Ore08,Ore09,Ore10,Ore11,Ore12,Ore13,Ore14	*OreG0002657*
scaffold1468	360845	T	C	Och12	*OreG0002658*
scaffold1468	362023	C	A	NA	*OreG0002658*
scaffold1468	362143	C	T	NA	*OreG0002658*
scaffold1468	363897	C	T	Och01,Och02,Och03,Och04,Och05,Och06,Och07,Och08,Och09,Och10,Och11,Och12,Och13,Och14,Ore01,Ore02,Ore03,Ore04,Ore05,Ore06,Ore07,Ore08,Ore09,Ore10,Ore11,Ore12,Ore13,Ore14	*OreG0002658*
scaffold1468	374296	T	A	Och01,Ore01,Ore02,Ore03,Ore04,Ore05,Ore06,Ore07,Ore08,Ore09,Ore10,Ore11,Ore12,Ore13,Ore14	*OreG0002659*
scaffold1468	374510	G	A	NA	*OreG0002659*
scaffold1468	374634	G	C	Och02,Och03,Och04,Och05,Och07,Och09,Och10,Och11,Och12,Och13,Och14	*OreG0002659*
scaffold1468	386922	A	G	Ore01,Ore02,Ore03,Ore04,Ore05,Ore06,Ore07,Ore08,Ore09,Ore10,Ore11,Ore12,Ore13,Ore14	*OreG0002661*
scaffold1468	389053	C	A	NA	*OreG0002661*
scaffold1468	390482	A	G	Ore11	*OreG0002661*
scaffold1468	390719	A	G	NA	*OreG0002661*
scaffold1468	398348	G	A	Och02,Och03,Och04,Och05,Och12,Och13,Ore01,Ore02,Ore03,Ore04,Ore05,Ore06,Ore07,Ore08,Ore09,Ore10,Ore11,Ore12,Ore13,Ore14	*OreG0002662*
scaffold1468	406110	A	T	Ore01,Ore02,Ore03,Ore04,Ore05,Ore06,Ore07,Ore08,Ore09,Ore10,Ore11,Ore12,Ore13,Ore14	*OreG0002663*
scaffold1468	407741	T	C	NA	*OreG0002663*
scaffold1468	407886	T	G	NA	*OreG0002663*
scaffold1468	481164	T	C	NA	*OreG0002670*
scaffold1468	481248	C	G	Och06,Och12	*OreG0002670*
scaffold1468	505940	G	A	NA	*OreG0002672*
scaffold1468	506803	C	T	NA	*OreG0002672*
scaffold1468	506814	G	T	NA	*OreG0002672*
scaffold1468	506825	T	A	NA	*OreG0002672*
scaffold1468	506891	A	G	NA	*OreG0002672*
scaffold1468	506912	A	G	NA	*OreG0002672*
scaffold1468	506939	C	A	NA	*OreG0002672*
scaffold1468	519107	G	T	Och01,Och02,Och03,Och04,Och05,Och06,Och07,Och08,Och09,Och10,Och11,Och12,Och13,Och14,Ore01,Ore02,Ore03,Ore04,Ore05,Ore06,Ore07,Ore08,Ore09,Ore10,Ore11,Ore12,Ore13,Ore14	*OreG0002674*
scaffold1468	563012	C	T	Ore01,Ore02,Ore03,Ore04,Ore05,Ore06,Ore07,Ore08,Ore09,Ore10,Ore11,Ore12,Ore13,Ore14	*OreG0002680*
scaffold1468	656893	A	G	Ore01,Ore02,Ore03,Ore04,Ore05,Ore06,Ore07,Ore08,Ore09,Ore10,Ore11,Ore12,Ore13,Ore14	*OreG0002690*
scaffold1468	658088	G	T	NA	*OreG0002690*
scaffold1468	686676	T	A	Ore01,Ore02,Ore03,Ore04,Ore05,Ore06,Ore07,Ore08,Ore09,Ore10,Ore11,Ore12,Ore13,Ore14	*OreG0002693*
scaffold1468	687576	G	A	Och08	*OreG0002693*
scaffold1468	688412	G	A	Och08	*OreG0002693*
scaffold1468	689561	G	A	Och01,Och02,Och03,Och04,Och05,Och06,Och07,Och08,Och09,Och10,Och11,Och12,Och13,Och14	*OreG0002693*
scaffold1468	691015	C	A	NA	*OreG0002693*
scaffold1468	718868	A	T	NA	*OreG0002697*
scaffold1468	719249	T	C	Och01,Och02,Och03,Och04,Och05,Och06,Och07,Och08,Och09,Och10,Och11,Och12,Och13,Och14	*OreG0002697*
scaffold1468	719441	C	T	Ore01,Ore02,Ore03,Ore04,Ore05,Ore06,Ore07,Ore08,Ore09,Ore10,Ore11,Ore12,Ore13,Ore14	*OreG0002697*
scaffold1468	721029	A	C	Och01,Och02,Och03,Och04,Och05,Och06,Och07,Och08,Och09,Och10,Och11,Och12,Och13,Och14	*OreG0002698*
scaffold1468	724654	G	C	Och01,Och02,Och03,Och04,Och05,Och06,Och07,Och08,Och09,Och10,Och11,Och12,Och13,Och14,Ore01,Ore02,Ore03,Ore04,Ore05,Ore06,Ore07,Ore08,Ore09,Ore10,Ore11,Ore12,Ore13,Ore14	*OreG0002699*
scaffold1468	725064	T	C	Och01,Och02,Och03,Och04,Och05,Och06,Och07,Och08,Och09,Och10,Och11,Och12,Och13,Och14	*OreG0002699*
scaffold1468	725157	A	T	Och01,Och02,Och03,Och04,Och05,Och06,Och07,Och08,Och09,Och10,Och11,Och12,Och13,Och14	*OreG0002699*
scaffold1468	725277	G	C	Ore01,Ore02,Ore03,Ore04,Ore05,Ore06,Ore07,Ore08,Ore09,Ore10,Ore11,Ore12,Ore13,Ore14	*OreG0002699*
scaffold1468	725723	T	C	Och01,Och02,Och03,Och04,Och05,Och06,Och07,Och08,Och09,Och10,Och11,Och12,Och13,Och14	*OreG0002699*
scaffold1468	725938	A	C	NA	*OreG0002699*
scaffold1468	726864	T	G	NA	*OreG0002699*

scaffold1468	734096	A	G	Ore01,Ore02,Ore03,Ore04,Ore05,Ore06,Ore07,Ore08,Ore09,Ore10,Ore11,Ore12,Ore13,Ore14	*OreG0002701*
scaffold1468	747367	T	G	Ore01,Ore02,Ore03,Ore04,Ore05,Ore06,Ore07,Ore08,Ore09,Ore10,Ore11,Ore12,Ore13,Ore14	*OreG0002703*
scaffold1468	747847	C	T	Och01,Och02,Och03,Och04,Och05,Och06,Och07,Och08,Och09,Och10,Och11,Och12,Och13,Och14	*OreG0002703*
scaffold1468	759415	C	T	NA	*OreG0002705*
scaffold1468	760336	C	G	NA	*OreG0002705*
scaffold1468	760390	C	T	NA	*OreG0002705*
scaffold1468	760824	C	G	NA	*OreG0002705*
scaffold1468	760856	G	A	NA	*OreG0002705*
scaffold1468	769514	T	A	NA	*OreG0002706*
scaffold1468	769565	A	G	NA	*OreG0002706*
scaffold1468	783819	T	G	Och01,Och07,Och14	*OreG0002709*
scaffold1468	783897	T	A	Och01,Och07,Och09,Och10,Och14	*OreG0002709*
scaffold1468	783989	C	A	Och01,Och07,Och09,Och10,Och14	*OreG0002709*
scaffold1468	797635	A	T	Och01,Och02,Och03,Och04,Och05,Och06,Och07,Och08,Och09,Och10,Och11,Och12,Och13,Och14	*OreG0002711*
scaffold1468	804122	T	A	NA	*OreG0002713*
scaffold1468	804571	G	C	NA	*OreG0002713*
scaffold1468	846884	T	C	NA	*OreG0002715*
scaffold1468	847258	G	C	Ore07,Ore08	*OreG0002715*
scaffold1468	861190	A	T	Ore01,Ore02,Ore03,Ore04,Ore05,Ore06,Ore07,Ore08,Ore09,Ore10,Ore11,Ore12,Ore13,Ore14	*OreG0002717*
scaffold1468	861385	C	T	Och02,Och03,Och04,Och05,Och06,Och09,Och10,Och12,Och13	*OreG0002717*
scaffold1468	861478	T	C	Och01,Och02,Och03,Och04,Och05,Och06,Och07,Och08,Och09,Och10,Och11,Och12,Och13,Och14,Ore01,Ore02,Ore03,Ore04,Ore05,Ore06,Ore07,Ore08,Ore09,Ore10,Ore11,Ore12,Ore13,Ore14	*OreG0002717*
scaffold1468	861771	G	T	Ore01,Ore02,Ore03,Ore04,Ore05,Ore06,Ore07,Ore08,Ore09,Ore10,Ore11,Ore12,Ore13,Ore14	*OreG0002717*
scaffold1468	890186	T	A	NA	*OreG0002719*
scaffold1468	890994	T	G	Ore01,Ore02,Ore03,Ore04,Ore05,Ore06,Ore07,Ore08,Ore09,Ore10,Ore11,Ore12,Ore13,Ore14	*OreG0002719*
scaffold1468	891116	G	C	Ore01,Ore02,Ore03,Ore04,Ore05,Ore06,Ore07,Ore08,Ore09,Ore10,Ore11,Ore12,Ore13,Ore14	*OreG0002719*
scaffold1468	892183	G	C	Ore01,Ore02,Ore03,Ore04,Ore05,Ore06,Ore07,Ore08,Ore09,Ore10,Ore11,Ore12,Ore13,Ore14	*OreG0002719*
scaffold1468	894319	C	G	Ore07,Ore08	*OreG0002719*
scaffold1468	902065	A	G	Och01,Och02,Och03,Och04,Och05,Och06,Och07,Och08,Och09,Och10,Och11,Och12,Och13,Och14,Ore01,Ore02,Ore03,Ore04,Ore05,Ore06,Ore07,Ore08,Ore09,Ore10,Ore11,Ore12,Ore13,Ore14	*OreG0002720*
scaffold1468	907373	A	T	Och01,Och02,Och03,Och04,Och05,Och06,Och07,Och08,Och09,Och10,Och11,Och12,Och13,Och14	*OreG0002720*
scaffold1468	919409	A	T	Och07,Och12	*OreG0002722*
scaffold1468	928609	T	A	Och06,Och12	*OreG0002724*
scaffold1468	931297	G	A	Och06,Och12	*OreG0002724*
scaffold1468	933373	T	C	Och01,Och02,Och03,Och04,Och05,Och06,Och07,Och08,Och09,Och10,Och11,Och12,Och13,Och14,Ore01,Ore02,Ore03,Ore04,Ore05,Ore06,Ore07,Ore08,Ore09,Ore10,Ore11,Ore12,Ore13,Ore14	*OreG0002724*
scaffold1468	942465	C	A	NA	*OreG0002725*
scaffold1468	942468	A	G	Och02,Och03,Och04,Och05,Och09,Och10,Och12,Och13,Och14	*OreG0002725*
scaffold1468	966407	G	A	NA	*OreG0002729*
scaffold1468	968122	C	T	NA	*OreG0002731*
scaffold1468	969202	C	A	Och01,Och02,Och03,Och04,Och05,Och06,Och07,Och08,Och09,Och10,Och11,Och12,Och13,Och14,Ore01,Ore02,Ore03,Ore04,Ore05,Ore06,Ore07,Ore08,Ore09,Ore10,Ore11,Ore12,Ore13,Ore14	*OreG0002731*
scaffold1468	969796	C	A	NA	*OreG0002731*
scaffold1468	971535	A	G	NA	*OreG0002730*
scaffold1468	971830	T	C	NA	*OreG0002730*
scaffold1468	991278	G	A	NA	*OreG0002734*
scaffold1468	991285	T	G	NA	*OreG0002734*
scaffold1468	1011264	C	G	Och07	*OreG0002737*
scaffold1468	1011367	A	C	Och07	*OreG0002737*
scaffold1468	1017314	G	A	NA	*OreG0002737*
scaffold1468	1017959	C	A	NA	*OreG0002737*
scaffold1468	1049243	G	A	NA	*OreG0002740*
scaffold1468	1054679	A	T	Ore01,Ore02,Ore03,Ore04,Ore05,Ore06,Ore07,Ore08,Ore09,Ore10,Ore11,Ore12,Ore13,Ore14	*OreG0002741*
scaffold1468	1060930	G	A	Och01,Och02,Och03,Och04,Och05,Och06,Och07,Och08,Och09,Och10,Och11,Och12,Och13,Och14,Ore01,Ore02,Ore03,Ore04,Ore05,Ore06,Ore07,Ore08,Ore09,Ore10,Ore11,Ore12,Ore13,Ore14	*OreG0002742*
scaffold1468	1087789	C	T	NA	*OreG0002746*
scaffold1468	1099138	C	T	Och12	*OreG0002746*
scaffold1468	1104214	T	C	Ore07,Ore08	*OreG0002748*
scaffold1468	1128488	G	T	Ore03,Ore09,Ore11	*OreG0002751*

scaffold1468	1128583	G	T	Ore07,Ore08	*OreG0002751*
scaffold1468	1132259	C	T	NA	*OreG0002752*
scaffold1468	1139994	C	G	Och01,Och02,Och03,Och04,Och05,Och06,Och07,Och08,Och09,Och10,Och11,Och12,Och13,Och14,Ore01,Ore02,Ore03,Ore04,Ore05,Ore06,Ore07,Ore08,Ore09,Ore10,Ore11,Ore12,Ore13,Ore14	*OreG0002754*
scaffold1468	1160920	A	G	NA	*OreG0002757*
scaffold1468	1160936	C	T	NA	*OreG0002757*
scaffold1468	1161963	T	A	Och06,Och08,Och12	*OreG0002757*
scaffold1468	1162009	C	T	NA	*OreG0002757*
scaffold1468	1162284	C	T	NA	*OreG0002757*
scaffold1468	1162512	C	A	NA	*OreG0002757*
scaffold1468	1162638	T	A	NA	*OreG0002757*
scaffold1468	1162857	T	C	NA	*OreG0002757*
scaffold1468	1167372	T	C	Ore01,Ore02,Ore03,Ore04,Ore05,Ore06,Ore07,Ore08,Ore09,Ore10,Ore11,Ore12,Ore13,Ore14	*OreG0002759*
scaffold1468	1167376	T	A	NA	*OreG0002759*
scaffold1468	1167612	T	C	Och01	*OreG0002759*
scaffold1468	1258357	C	T	NA	*OreG0002771*
scaffold1468	1258696	C	T	Ore01,Ore02,Ore03,Ore04,Ore05,Ore06,Ore07,Ore08,Ore09,Ore10,Ore11,Ore12,Ore13,Ore14	*OreG0002771*
scaffold1468	1259213	C	G	NA	*OreG0002771*
scaffold1468	1259378	A	T	NA	*OreG0002771*
scaffold1468	1278331	G	A	NA	*OreG0002776*
scaffold1468	1289232	C	T	NA	*OreG0002779*
scaffold1468	1290609	C	T	Och08	*OreG0002779*
scaffold1468	1290698	A	T	Och01,Och02,Och03,Och04,Och05,Och06,Och07,Och08,Och09,Och10,Och11,Och12,Och13,Och14,Ore01,Ore02,Ore03,Ore04,Ore05,Ore06,Ore07,Ore08,Ore09,Ore10,Ore11,Ore12,Ore13,Ore14	*OreG0002779*
scaffold1468	1290738	T	A	Och01,Och06,Och07,Och09,Och10,Och12,Och14	*OreG0002779*
scaffold1468	1291152	C	T	Och07,Och12	*OreG0002779*
scaffold1468	1291604	G	A	NA	*OreG0002779*
scaffold1468	1295508	A	T	NA	*OreG0002780*
scaffold1468	1296291	C	T	NA	*OreG0002780*
scaffold1468	1296302	C	T	NA	*OreG0002780*
scaffold1468	1361815	T	C	NA	*OreG0002783*
scaffold1468	1362480	C	G	Och06,Och12	*OreG0002783*
scaffold1468	1362870	C	T	Och06,Och12	*OreG0002783*
scaffold1468	1412327	A	T	Och01,Och02,Och03,Och04,Och05,Och06,Och07,Och08,Och09,Och10,Och11,Och12,Och13,Och14,Ore01,Ore02,Ore03,Ore04,Ore05,Ore06,Ore07,Ore08,Ore09,Ore10,Ore11,Ore12,Ore13,Ore14	*OreG0002793*
scaffold1468	1413548	C	G	Och01,Och02,Och03,Och04,Och05,Och06,Och07,Och08,Och09,Och10,Och11,Och12,Och13,Och14	*OreG0002793*
scaffold1468	1415008	G	A	Och01,Och02,Och03,Och04,Och05,Och06,Och07,Och08,Och09,Och10,Och11,Och12,Och13,Och14,Ore01,Ore02,Ore03,Ore04,Ore05,Ore06,Ore07,Ore08,Ore09,Ore10,Ore11,Ore12,Ore13,Ore14	*OreG0002794*
scaffold1468	1443427	A	G	Ore01,Ore02,Ore03,Ore04,Ore05,Ore06,Ore07,Ore08,Ore09,Ore10,Ore11,Ore12,Ore13,Ore14	*OreG0002797*
scaffold1468	1443607	A	G	Och01,Och02,Och03,Och04,Och05,Och06,Och07,Och08,Och09,Och10,Och11,Och12,Och13,Och14,Ore01,Ore02,Ore03,Ore04,Ore05,Ore06,Ore07,Ore08,Ore09,Ore10,Ore11,Ore12,Ore13,Ore14	*OreG0002797*
scaffold1468	1443856	G	C	NA	*OreG0002797*
scaffold1468	1443979	G	T	Och01,Och02,Och03,Och04,Och05,Och06,Och07,Och08,Och09,Och10,Och11,Och12,Och13,Och14,Ore01,Ore02,Ore03,Ore04,Ore05,Ore06,Ore07,Ore08,Ore09,Ore10,Ore11,Ore12,Ore13,Ore14	*OreG0002797*
scaffold1468	1447478	A	G	Och01,Och02,Och03,Och04,Och05,Och06,Och07,Och08,Och09,Och10,Och11,Och12,Och13,Och14	*OreG0002798*
scaffold1468	1448095	G	T	Ore11	*OreG0002798*
scaffold1468	1448120	C	T	NA	*OreG0002798*
scaffold1468	1448349	G	A	Ore01,Ore02,Ore03,Ore04,Ore05,Ore06,Ore07,Ore08,Ore09,Ore10,Ore11,Ore12,Ore13,Ore14	*OreG0002798*
scaffold1468	1448390	C	T	Och01,Och02,Och03,Och04,Och05,Och06,Och07,Och08,Och09,Och10,Och11,Och12,Och13,Och14	*OreG0002798*
scaffold1468	1451500	T	C	Ore01,Ore02,Ore03,Ore04,Ore05,Ore06,Ore07,Ore08,Ore09,Ore10,Ore11,Ore12,Ore13,Ore14	*OreG0002799*
scaffold1468	1451764	A	G	NA	*OreG0002799*
scaffold1468	1452059	T	A	NA	*OreG0002799*
scaffold1468	1452262	G	T	NA	*OreG0002799*
scaffold1468	1452281	A	G	NA	*OreG0002799*
scaffold1468	1452284	C	A	NA	*OreG0002799*
scaffold1468	1453040	A	T	Ore01,Ore02,Ore03,Ore04,Ore05,Ore06,Ore07,Ore08,Ore09,Ore10,Ore11,Ore12,Ore13,Ore14	*OreG0002799*
scaffold1468	1471829	A	T	NA	*OreG0002802*
scaffold1468	1472386	C	T	NA	*OreG0002802*

scaffold1468	1509728	G	C	Och01,Och02,Och03,Och04,Och05,Och06,Och07,Och08,Och09,Och10,Och11,Och12,Och13,Och14,Ore01,Ore02,Ore03,Ore04,Ore05,Ore06,Ore07,Ore08,Ore09,Ore10,Ore11,Ore12,Ore13,Ore14	*OreG0002804*
scaffold1468	1516204	T	C	NA	*OreG0002805*
scaffold1468	1516285	C	T	NA	*OreG0002805*
scaffold1468	1516750	C	A	Ore01,Ore02,Ore03,Ore04,Ore05,Ore06,Ore07,Ore08,Ore09,Ore10,Ore11,Ore12,Ore13,Ore14	*OreG0002805*
scaffold1468	1517749	G	T	Och01,Och02,Och03,Och04,Och05,Och06,Och07,Och08,Och09,Och10,Och11,Och12,Och13,Och14,Ore01,Ore02,Ore03,Ore04,Ore05,Ore06,Ore07,Ore08,Ore09,Ore10,Ore11,Ore12,Ore13,Ore14	*OreG0002805*
scaffold1468	1525454	C	A	NA	*OreG0002806*
scaffold1468	1525908	T	A	Och01,Och02,Och03,Och04,Och05,Och06,Och07,Och08,Och09,Och10,Och11,Och12,Och13,Och14,Ore01,Ore02,Ore03,Ore04,Ore05,Ore06,Ore07,Ore08,Ore09,Ore10,Ore11,Ore12,Ore13,Ore14	*OreG0002806*
scaffold1468	1526462	A	C	Och01,Och02,Och03,Och04,Och05,Och06,Och07,Och08,Och09,Och10,Och11,Och12,Och13,Och14,Ore01,Ore02,Ore03,Ore04,Ore05,Ore06,Ore07,Ore08,Ore09,Ore10,Ore11,Ore12,Ore13,Ore14	*OreG0002806*
scaffold1468	1526695	C	A	Ore07,Ore08	*OreG0002806*
scaffold1468	1528194	G	C	Och08	*OreG0002807*
scaffold1468	1528271	A	C	Och08	*OreG0002807*
scaffold1468	1528749	T	C	Och08	*OreG0002807*
scaffold1468	1529571	C	T	NA	*OreG0002807*
scaffold1468	1529646	C	T	Ore11	*OreG0002807*
scaffold1468	1534546	T	C	NA	*OreG0002808*
scaffold1468	1538029	T	C	Och12	*OreG0002809*
scaffold1468	1551326	C	T	Och01,Och02,Och03,Och04,Och05,Och06,Och07,Och08,Och09,Och10,Och11,Och12,Och13,Och14,Ore01,Ore02,Ore03,Ore04,Ore05,Ore06,Ore07,Ore08,Ore09,Ore10,Ore11,Ore12,Ore13,Ore14	*OreG0002812*
scaffold1468	1551761	C	A	Ore11	*OreG0002812*
scaffold1468	1564551	C	T	Och01,Och02,Och03,Och04,Och05,Och06,Och07,Och08,Och09,Och10,Och11,Och12,Och13,Och14,Ore01,Ore02,Ore03,Ore04,Ore05,Ore06,Ore07,Ore08,Ore09,Ore10,Ore11,Ore12,Ore13,Ore14	*OreG0002813*
scaffold1468	1607966	C	G	NA	*OreG0002818*
scaffold1468	1608088	T	C	Och12	*OreG0002818*
scaffold1468	1613563	C	T	Ore07,Ore08	*OreG0002819*
scaffold1468	1638520	T	A	NA	*OreG0002821*
scaffold1468	1649792	A	G	NA	*OreG0002822*
scaffold1468	1649820	G	T	Och01,Och02,Och03,Och04,Och05,Och06,Och07,Och08,Och09,Och10,Och11,Och12,Och13,Och14	*OreG0002822*
scaffold1468	1670627	A	G	Och06,Och12	*OreG0002825*
scaffold1468	1670660	T	C	NA	*OreG0002825*
scaffold1468	1670716	G	C	NA	*OreG0002825*
scaffold1468	1670942	A	G	NA	*OreG0002825*
scaffold1468	1671072	A	T	Och01,Och02,Och03,Och04,Och05,Och06,Och07,Och08,Och09,Och10,Och11,Och12,Och13,Och14	*OreG0002825*
scaffold1468	1671149	T	A	Och01,Och02,Och03,Och04,Och05,Och06,Och07,Och08,Och09,Och10,Och11,Och12,Och13,Och14	*OreG0002825*
scaffold1468	1671260	C	A	NA	*OreG0002825*
scaffold1468	1684490	C	A	NA	*OreG0002828*
scaffold1468	1696357	G	A	NA	*OreG0002830*
scaffold1468	1696630	C	T	Ore01,Ore02,Ore03,Ore04,Ore05,Ore06,Ore07,Ore08,Ore09,Ore10,Ore11,Ore12,Ore13,Ore14	*OreG0002830*
scaffold1468	1696668	A	T	Och07	*OreG0002830*
scaffold1468	1720631	G	A	NA	*OreG0002833*
scaffold1468	1721873	A	G	NA	*OreG0002833*
scaffold1468	1721955	C	A	NA	*OreG0002833*
scaffold1468	1722008	T	C	NA	*OreG0002833*
scaffold1468	1722207	T	A	Ore01,Ore02,Ore03,Ore04,Ore05,Ore06,Ore07,Ore08,Ore09,Ore10,Ore11,Ore12,Ore13,Ore14	*OreG0002833*
scaffold1468	1752567	G	A	NA	*OreG0002835*
scaffold1468	1769796	T	C	Och01,Och02,Och03,Och04,Och05,Och06,Och07,Och08,Och09,Och10,Och11,Och12,Och13,Och14,Ore01,Ore02,Ore03,Ore04,Ore05,Ore06,Ore07,Ore08,Ore09,Ore10,Ore11,Ore12,Ore13,Ore14	*OreG0002836*
scaffold1468	1770694	T	G	NA	*OreG0002836*
scaffold1468	1780630	T	G	Ore01,Ore02,Ore03,Ore04,Ore05,Ore06,Ore07,Ore08,Ore09,Ore10,Ore11,Ore12,Ore13,Ore14	*OreG0002838*
scaffold1468	1809727	C	T	Ore01,Ore02,Ore03,Ore04,Ore05,Ore06,Ore07,Ore08,Ore09,Ore10,Ore11,Ore12,Ore13,Ore14	*OreG0002841*
scaffold1468	1813575	T	A	NA	*OreG0002841*
scaffold1468	1872965	C	G	Och01,Och02,Och03,Och04,Och05,Och06,Och07,Och08,Och09,Och10,Och11,Och12,Och13,Och14,Ore01,Ore02,Ore03,Ore04,Ore05,Ore06,Ore07,Ore08,Ore09,Ore10,Ore11,Ore12,Ore13,Ore14	*OreG0002845*
scaffold1468	1877123	G	C	Och01,Och02,Och03,Och04,Och05,Och06,Och07,Och08,Och09,Och10,Och11,Och12,Och13,Och14,Ore01,Ore02,Ore03,Ore04,Ore05,Ore06,Ore07,Ore08,Ore09,Ore10,Ore11,Ore12,Ore13,Ore14	*OreG0002846*

scaffold1468	1892040	A	C	Och01,Och02,Och03,Och04,Och05,Och06,Och07,Och08,Och09,Och10,Och11,Och12,Och13,Och14,Ore01,Ore02,Ore03,Ore04,Ore05,Ore06,Ore07,Ore08,Ore09,Ore10,Ore11,Ore12,Ore13,Ore14	*OreG0002848*
scaffold1468	1904031	A	G	NA	*OreG0002850*
scaffold1468	1904211	A	G	NA	*OreG0002850*
scaffold1468	1907754	T	C	NA	*OreG0002851*
scaffold1468	1956972	T	C	NA	*OreG0002856*
scaffold1468	1976143	T	C	Ore01,Ore02,Ore03,Ore04,Ore05,Ore06,Ore07,Ore08,Ore09,Ore10,Ore11,Ore12,Ore13,Ore14	*OreG0002857*
scaffold1468	2043925	C	G	Ore01,Ore02,Ore03,Ore04,Ore05,Ore06,Ore07,Ore08,Ore09,Ore10,Ore11,Ore12,Ore13,Ore14	*OreG0002864*
scaffold1468	2044113	G	A	NA	*OreG0002864*
scaffold1468	2044494	G	A	Ore01,Ore02,Ore03,Ore04,Ore05,Ore06,Ore07,Ore08,Ore09,Ore10,Ore11,Ore12,Ore13,Ore14	*OreG0002864*
scaffold1468	2044588	C	T	Och01,Och02,Och03,Och04,Och05,Och06,Och07,Och08,Och09,Och10,Och11,Och12,Och13,Och14	*OreG0002864*
scaffold1468	2049318	G	A	Ore01,Ore02,Ore03,Ore04,Ore05,Ore06,Ore07,Ore08,Ore09,Ore10,Ore11,Ore12,Ore13,Ore14	*OreG0002865*
scaffold1468	2049564	T	A	Och01,Och02,Och03,Och04,Och05,Och06,Och07,Och08,Och09,Och10,Och11,Och12,Och13,Och14,Ore01,Ore02,Ore03,Ore04,Ore05,Ore06,Ore07,Ore08,Ore09,Ore10,Ore11,Ore12,Ore13,Ore14	*OreG0002866*
scaffold1468	2049569	A	C	Och01,Och02,Och03,Och04,Och05,Och06,Och07,Och08,Och09,Och10,Och11,Och12,Och13,Och14,Ore01,Ore02,Ore03,Ore04,Ore05,Ore06,Ore07,Ore08,Ore09,Ore10,Ore11,Ore12,Ore13,Ore14	*OreG0002866*
scaffold1468	2055120	C	T	Och01,Och02,Och03,Och04,Och05,Och06,Och07,Och08,Och09,Och10,Och11,Och12,Och13,Och14,Ore01,Ore02,Ore03,Ore04,Ore05,Ore06,Ore07,Ore08,Ore09,Ore10,Ore11,Ore12,Ore13,Ore14	*OreG0002867*
scaffold1468	2055179	A	G	Och01,Och02,Och03,Och04,Och05,Och06,Och07,Och08,Och09,Och10,Och11,Och12,Och13,Och14,Ore01,Ore02,Ore03,Ore04,Ore05,Ore06,Ore07,Ore08,Ore09,Ore10,Ore11,Ore12,Ore13,Ore14	*OreG0002867*
scaffold1468	2055219	C	T	Och02,Och03,Och04,Och05,Och06,Och07,Och08,Och11,Och12,Och13	*OreG0002867*
scaffold1468	2055301	T	A	Ore01,Ore02,Ore03,Ore04,Ore05,Ore06,Ore07,Ore08,Ore09,Ore10,Ore11,Ore12,Ore13,Ore14	*OreG0002867*
scaffold1468	2056100	C	A	Och01	*OreG0002867*
scaffold1468	2057248	G	T	Ore01,Ore02,Ore03,Ore04,Ore05,Ore06,Ore07,Ore08,Ore09,Ore10,Ore11,Ore12,Ore13,Ore14	*OreG0002867*
scaffold1468	2057409	C	T	Ore01,Ore02,Ore03,Ore04,Ore05,Ore06,Ore07,Ore08,Ore09,Ore10,Ore11,Ore12,Ore13,Ore14	*OreG0002867*
scaffold1468	2073416	C	T	NA	*OreG0002870*
scaffold1468	2073903	C	T	Ore01,Ore07,Ore08,Ore10,Ore13	*OreG0002870*
scaffold1468	2078052	G	T	Och01	*OreG0002870*
scaffold1468	2079617	G	A	Och02,Och03,Och04,Och05,Och06,Och07,Och08,Och11,Och12,Och13,Och14	*OreG0002870*
scaffold1468	2081143	T	C	Ore01,Ore07,Ore08,Ore10,Ore13	*OreG0002871*
scaffold1468	2096632	T	A	Och01,Och02,Och03,Och04,Och05,Och06,Och07,Och08,Och09,Och10,Och11,Och12,Och13,Och14	*OreG0002874*
scaffold1468	2123509	T	C	NA	*OreG0002875*
scaffold1468	2126258	T	A	Och01,Och02,Och03,Och04,Och05,Och06,Och07,Och08,Och09,Och10,Och11,Och12,Och13,Och14	*OreG0002876*
scaffold1468	2184539	T	G	Ore01,Ore02,Ore03,Ore04,Ore05,Ore06,Ore07,Ore08,Ore09,Ore10,Ore11,Ore12,Ore13,Ore14	*OreG0002881*
scaffold1468	2270843	C	T	Och02,Och04,Och05,Och07,Och12,Och13,Och14	*OreG0002892*
scaffold1468	2300946	A	G	Ore07,Ore08,Ore13	*OreG0002895*
scaffold1468	2338536	T	C	NA	*OreG0002898*
scaffold1468	2338551	G	A	Ore01,Ore02,Ore03,Ore04,Ore05,Ore06,Ore07,Ore08,Ore09,Ore10,Ore11,Ore12,Ore13,Ore14	*OreG0002898*
scaffold1468	2338567	C	G	Och01,Och02,Och03,Och04,Och05,Och06,Och07,Och08,Och09,Och10,Och11,Och12,Och13,Och14,Ore01,Ore02,Ore03,Ore04,Ore05,Ore06,Ore07,Ore08,Ore09,Ore10,Ore11,Ore12,Ore13,Ore14	*OreG0002898*
scaffold1468	2338603	C	T	Och01,Och02,Och03,Och04,Och05,Och06,Och09,Och10,Och12,Och13,Och14	*OreG0002898*
scaffold1468	2338621	C	A	Och01	*OreG0002898*
scaffold1468	2338756	G	T	Ore01,Ore02,Ore03,Ore04,Ore05,Ore06,Ore07,Ore08,Ore09,Ore10,Ore11,Ore12,Ore13,Ore14	*OreG0002898*
scaffold1468	2361503	G	A	Ore01,Ore02,Ore03,Ore04,Ore05,Ore06,Ore07,Ore08,Ore09,Ore10,Ore11,Ore12,Ore13,Ore14	*OreG0002899*
scaffold1468	2381475	G	T	Och06,Och12	*OreG0002901*
scaffold1468	2389745	C	T	Ore11	*OreG0002903*
scaffold1468	2389854	C	T	Ore01,Ore02,Ore03,Ore04,Ore05,Ore06,Ore07,Ore08,Ore09,Ore10,Ore11,Ore12,Ore13,Ore14	*OreG0002903*
scaffold1468	2389868	G	A	Och06,Och12	*OreG0002903*
scaffold1468	2389899	T	C	Och06,Och12	*OreG0002903*
scaffold1468	2389926	G	A	Och06,Och12	*OreG0002903*
scaffold1468	2389965	A	T	Ore01,Ore02,Ore03,Ore04,Ore05,Ore06,Ore07,Ore08,Ore09,Ore10,Ore11,Ore12,Ore13,Ore14	*OreG0002903*
scaffold1468	2389973	G	A	Och06,Och12,Ore01,Ore02,Ore03,Ore04,Ore05,Ore06,Ore07,Ore08,Ore09,Ore10,Ore11,Ore12,Ore13,Ore14	*OreG0002903*

scaffold1468	2389977	T	C	Ore01,Ore02,Ore03,Ore04,Ore05,Ore06,Ore07,Ore08,Ore09,Ore10,Ore11,Ore12,Ore13,Ore14	*OreG0002903*
scaffold1468	2389989	G	A	Och01,Och14	*OreG0002903*
scaffold1468	2390456	A	G	Och06,Och12	*OreG0002904*
scaffold1468	2390488	A	T	NA	*OreG0002904*
scaffold1468	2397385	G	A	NA	*OreG0002905*
scaffold1468	2397846	A	T	Och06,Och12	*OreG0002905*
scaffold1468	2397993	G	C	NA	*OreG0002905*
scaffold1468	2398063	C	A	Och02,Och03,Och04,Och05,Och06,Och08,Och11,Och12,Och13,Och14	*OreG0002905*
scaffold1468	2398066	G	A	NA	*OreG0002905*
scaffold1468	2405168	C	T	NA	*OreG0002902*
scaffold1468	2405182	G	A	Ore07,Ore08,Ore13	*OreG0002902*
scaffold1468	2405311	G	A	Ore11	*OreG0002902*
scaffold1468	2405335	G	A	Ore11	*OreG0002902*
scaffold1468	2427784	A	C	NA	*OreG0002906*
scaffold1468	2432698	A	G	NA	*OreG0002907*
scaffold1468	2433013	G	A	NA	*OreG0002907*
scaffold1468	2464722	T	A	Ore01,Ore02,Ore03,Ore04,Ore05,Ore06,Ore07,Ore08,Ore09,Ore10,Ore11,Ore12,Ore13,Ore14	*OreG0002908*
scaffold1468	2464845	G	T	Och01,Och02,Och03,Och04,Och05,Och06,Och07,Och08,Och09,Och10,Och11,Och12,Och13,Och14,Ore01,Ore02,Ore03,Ore04,Ore05,Ore06,Ore07,Ore08,Ore09,Ore10,Ore11,Ore12,Ore13,Ore14	*OreG0002908*
scaffold1468	2465575	C	T	Ore01,Ore02,Ore03,Ore04,Ore05,Ore06,Ore07,Ore08,Ore09,Ore10,Ore11,Ore12,Ore13,Ore14	*OreG0002908*
scaffold1468	2465631	G	A	Ore01,Ore02,Ore03,Ore04,Ore05,Ore06,Ore07,Ore08,Ore09,Ore10,Ore11,Ore12,Ore13,Ore14	*OreG0002908*
scaffold1468	2475359	T	C	NA	*OreG0002910*
scaffold1468	2475504	A	G	NA	*OreG0002910*
scaffold1468	2475514	C	T	Och01,Och02,Och03,Och04,Och05,Och06,Och07,Och08,Och09,Och10,Och11,Och12,Och13,Och14	*OreG0002910*
scaffold1468	2475721	C	A	Ore07,Ore08,Ore13	*OreG0002910*
scaffold1468	2475743	G	T	NA	*OreG0002910*
scaffold1468	2476106	C	T	NA	*OreG0002910*
scaffold1468	2503126	C	T	Och02,Och03,Och04,Och05,Och06,Och07,Och09,Och10,Och11,Och12,Och13,Och14	*OreG0002913*
scaffold1468	2506417	G	C	Ore01,Ore02,Ore03,Ore04,Ore05,Ore06,Ore07,Ore08,Ore09,Ore10,Ore11,Ore12,Ore13,Ore14	*OreG0002913*
scaffold1468	2529660	G	C	NA	*OreG0002916*
scaffold1468	2540536	G	A	NA	*OreG0002918*
scaffold1468	2554936	T	A	Ore01,Ore02,Ore03,Ore04,Ore05,Ore06,Ore07,Ore08,Ore09,Ore10,Ore11,Ore12,Ore13,Ore14	*OreG0002920*
scaffold1468	2554952	G	A	NA	*OreG0002920*
scaffold1468	2555056	T	A	Ore01,Ore02,Ore03,Ore04,Ore05,Ore06,Ore07,Ore08,Ore09,Ore10,Ore11,Ore12,Ore13,Ore14	*OreG0002920*
scaffold1468	2555234	T	A	Och01,Och02,Och03,Och04,Och05,Och06,Och07,Och08,Och09,Och10,Och11,Och12,Och13,Och14	*OreG0002920*
scaffold1468	2555720	G	C	NA	*OreG0002921*
scaffold1468	2556530	C	A	Och02,Och03,Och04,Och05,Och06,Och07,Och08,Och09,Och10,Och11,Och12,Och13,Och14	*OreG0002921*
scaffold1468	2561554	A	C	Och01,Och02,Och03,Och04,Och05,Och06,Och07,Och08,Och09,Och10,Och11,Och12,Och13,Och14,Ore01,Ore02,Ore03,Ore04,Ore05,Ore06,Ore07,Ore08,Ore09,Ore10,Ore11,Ore12,Ore13,Ore14	*OreG0002922*
scaffold1468	2565275	T	C	Ore01,Ore02,Ore03,Ore04,Ore05,Ore06,Ore07,Ore08,Ore09,Ore10,Ore11,Ore12,Ore13,Ore14	*OreG0002922*
scaffold1468	2575315	C	A	Och02,Och03,Och04,Och05,Och06,Och07,Och08,Och11,Och12,Och13,Och14	*OreG0002924*
scaffold1468	2575542	G	A	NA	*OreG0002924*
scaffold1468	2577010	G	C	Och01,Och02,Och03,Och04,Och05,Och06,Och07,Och08,Och09,Och10,Och11,Och12,Och13,Och14	*OreG0002924*
scaffold1468	2577586	C	T	Ore01,Ore02,Ore03,Ore04,Ore05,Ore06,Ore07,Ore08,Ore09,Ore10,Ore11,Ore12,Ore13,Ore14	*OreG0002924*
scaffold1468	2584055	C	T	Ore11	*OreG0002925*
scaffold1468	2594337	T	A	Och02,Och03,Och04,Och05,Och06,Och07,Och08,Och09,Och10,Och12,Och13	*OreG0002927*
scaffold1468	2594965	T	A	Ore01,Ore02,Ore03,Ore04,Ore05,Ore06,Ore07,Ore08,Ore09,Ore10,Ore11,Ore12,Ore13,Ore14	*OreG0002927*
scaffold1468	2600774	G	C	Och01,Och02,Och03,Och04,Och05,Och06,Och07,Och08,Och09,Och10,Och11,Och12,Och13,Och14,Ore01,Ore02,Ore03,Ore04,Ore05,Ore06,Ore07,Ore08,Ore09,Ore10,Ore11,Ore12,Ore13,Ore14	*OreG0002928*
scaffold1468	2606285	A	T	NA	*OreG0002929*
scaffold1468	2606322	T	C	Ore01,Ore02,Ore03,Ore04,Ore05,Ore06,Ore07,Ore08,Ore09,Ore10,Ore11,Ore12,Ore13,Ore14	*OreG0002929*
scaffold1468	2606345	G	A	NA	*OreG0002929*
scaffold1468	2630972	T	A	Och06,Och12	*OreG0002930*
scaffold1468	2631056	G	C	Och01,Och02,Och03,Och04,Och05,Och06,Och07,Och12,Och13,Och14	*OreG0002930*
scaffold1468	2631233	T	C	NA	*OreG0002930*
scaffold1468	2631299	T	C	Ore07,Ore08,Ore13	*OreG0002930*

scaffold1468	2631374	A	T	NA	*OreG0002930*
scaffold1468	2654840	A	T	NA	*OreG0002933*
scaffold1468	2657343	G	A	Och06,Och12	*OreG0002934*
scaffold1468	2659234	C	G	NA	*OreG0002934*
scaffold1468	2673574	A	G	Ore01,Ore02,Ore03,Ore04,Ore05,Ore06,Ore07,Ore08,Ore09,Ore10,Ore11,Ore12,Ore13,Ore14	*OreG0002935*
scaffold1468	2676659	C	G	Och01,Och02,Och03,Och04,Och05,Och06,Och07,Och08,Och09,Och10,Och11,Och12,Och13,Och14,Ore01,Ore02,Ore03,Ore04,Ore05,Ore06,Ore07,Ore08,Ore09,Ore10,Ore11,Ore12,Ore13,Ore14	*OreG0002935*
scaffold1468	2683031	G	A	Ore01,Ore02,Ore03,Ore04,Ore05,Ore06,Ore07,Ore08,Ore09,Ore10,Ore11,Ore12,Ore13,Ore14	*OreG0002936*
scaffold1468	2698692	G	T	NA	*OreG0002939*
scaffold1468	2723947	G	A	Ore01,Ore02,Ore03,Ore04,Ore05,Ore06,Ore07,Ore08,Ore09,Ore10,Ore11,Ore12,Ore13,Ore14	*OreG0002943*
scaffold1468	2735736	C	T	Och01,Och02,Och03,Och04,Och05,Och06,Och07,Och08,Och09,Och10,Och11,Och12,Och13,Och14,Ore01,Ore02,Ore03,Ore04,Ore05,Ore06,Ore07,Ore08,Ore09,Ore10,Ore11,Ore12,Ore13,Ore14	*OreG0002944*
scaffold1468	2750198	T	A	Och01,Och02,Och03,Och04,Och05,Och06,Och07,Och08,Och09,Och10,Och11,Och12,Och13,Och14,Ore01,Ore02,Ore03,Ore04,Ore05,Ore06,Ore07,Ore08,Ore09,Ore10,Ore11,Ore12,Ore13,Ore14	*OreG0002946*
scaffold1468	2766831	A	G	NA	*OreG0002949*
scaffold1468	2776964	T	A	Och09,Och10	*OreG0002950*
scaffold1468	2784531	A	G	Ore01,Ore02,Ore03,Ore04,Ore05,Ore06,Ore07,Ore08,Ore09,Ore10,Ore11,Ore12,Ore13,Ore14	*OreG0002950*
scaffold1468	2828661	T	A	NA	*OreG0002954*
scaffold1468	2828714	C	T	NA	*OreG0002954*
scaffold1468	2828801	G	A	NA	*OreG0002954*
scaffold1468	2828869	G	C	NA	*OreG0002954*
scaffold1468	2828905	G	A	NA	*OreG0002954*
scaffold1468	2828939	T	C	NA	*OreG0002954*
scaffold1468	2829221	G	T	NA	*OreG0002954*
scaffold1468	2829861	A	C	NA	*OreG0002954*
scaffold1468	2830566	A	G	NA	*OreG0002954*
scaffold1468	2833238	A	G	NA	*OreG0002954*
scaffold1468	2845028	C	A	NA	*OreG0002956*
scaffold1468	2847819	G	C	Ore01,Ore02,Ore03,Ore04,Ore05,Ore06,Ore07,Ore08,Ore09,Ore10,Ore11,Ore12,Ore13,Ore14	*OreG0002956*
scaffold1468	2853874	G	C	Ore01,Ore02,Ore03,Ore04,Ore05,Ore06,Ore07,Ore08,Ore09,Ore10,Ore11,Ore12,Ore13,Ore14	*OreG0002958*
scaffold1468	2853916	G	T	Ore01,Ore02,Ore03,Ore04,Ore05,Ore06,Ore07,Ore08,Ore09,Ore10,Ore11,Ore12,Ore13,Ore14	*OreG0002958*
scaffold1468	2853937	C	A	Och01,Och02,Och03,Och04,Och05,Och06,Och07,Och08,Och09,Och10,Och11,Och12,Och13,Och14,Ore01,Ore02,Ore03,Ore04,Ore05,Ore06,Ore07,Ore08,Ore09,Ore10,Ore11,Ore12,Ore13,Ore14	*OreG0002958*
scaffold1468	2853970	T	G	Ore01,Ore02,Ore03,Ore04,Ore05,Ore06,Ore07,Ore08,Ore09,Ore10,Ore11,Ore12,Ore13,Ore14	*OreG0002958*
scaffold1468	2858449	G	T	Och07	*OreG0002959*
scaffold1468	2858487	A	T	Och01,Och02,Och03,Och04,Och05,Och06,Och07,Och08,Och09,Och10,Och11,Och12,Och13,Och14,Ore01,Ore02,Ore03,Ore04,Ore05,Ore06,Ore07,Ore08,Ore09,Ore10,Ore11,Ore12,Ore13,Ore14	*OreG0002959*
scaffold1468	2858538	G	T	NA	*OreG0002959*
scaffold1468	2867115	C	T	Ore01	*OreG0002961*
scaffold1468	2867294	C	T	Och06,Och12	*OreG0002961*
scaffold1468	2867304	C	G	NA	*OreG0002961*
scaffold1468	2867845	T	C	Och01,Och02,Och03,Och04,Och05,Och06,Och07,Och08,Och09,Och10,Och11,Och12,Och13,Och14	*OreG0002961*
scaffold1468	2867899	G	A	Ore02,Ore04,Ore05,Ore06,Ore07,Ore08,Ore11,Ore12,Ore13	*OreG0002961*
scaffold1468	2867914	C	A	Ore01	*OreG0002961*
scaffold1468	2868030	C	T	Ore11	*OreG0002961*
scaffold1468	2868097	C	T	NA	*OreG0002961*
scaffold1468	2876792	C	T	Och01,Och02,Och03,Och04,Och05,Och06,Och07,Och08,Och09,Och10,Och11,Och12,Och13,Och14	*OreG0002964*
scaffold1468	2876866	C	T	NA	*OreG0002964*
scaffold1468	2880002	A	T	Och01,Och02,Och03,Och04,Och05,Och06,Och09,Och10,Och11,Och12,Och13,Och14	*OreG0002965*
scaffold1468	2895169	C	T	Ore01,Ore02,Ore03,Ore04,Ore05,Ore06,Ore07,Ore08,Ore09,Ore10,Ore11,Ore12,Ore13,Ore14	*OreG0002968*
scaffold1468	2901660	C	A	Ore01,Ore02,Ore03,Ore04,Ore05,Ore06,Ore07,Ore08,Ore09,Ore10,Ore11,Ore12,Ore13,Ore14	*OreG0002969*
scaffold1468	2925308	C	G	Och01,Och02,Och03,Och04,Och05,Och06,Och07,Och08,Och09,Och10,Och11,Och12,Och13,Och14,Ore01,Ore02,Ore03,Ore04,Ore05,Ore06,Ore07,Ore08,Ore09,Ore10,Ore11,Ore12,Ore13,Ore14	*OreG0002973*
scaffold1468	2927953	C	A	Ore01,Ore02,Ore03,Ore04,Ore05,Ore06,Ore07,Ore08,Ore09,Ore10,Ore11,Ore12,Ore13,Ore14	*OreG0002974*
scaffold1468	2942001	T	C	NA	*OreG0002979*
scaffold1468	2942818	A	G	NA	*OreG0002979*

scaffold1468	2942905	G	A	NA	*OreG0002979*
scaffold1468	2942935	G	A	NA	*OreG0002979*
scaffold1468	2942985	G	A	NA	*OreG0002979*
scaffold1468	2942994	G	A	NA	*OreG0002979*
scaffold1468	2943006	A	C	NA	*OreG0002979*
scaffold1468	2943021	A	G	NA	*OreG0002979*
scaffold1468	2949766	C	A	Och06,Och12	*OreG0002980*
scaffold1468	2950348	C	A	Och01	*OreG0002980*
scaffold1468	2951054	G	A	Ore11	*OreG0002980*
scaffold1468	2957468	A	G	Och01,Och02,Och03,Och04,Och05,Och06,Och07,Och08,Och09,Och10,Och11,Och12,Och13,Och14,Ore01,Ore02,Ore03,Ore04,Ore05,Ore06,Ore07,Ore08,Ore09,Ore10,Ore11,Ore12,Ore13,Ore14	*OreG0002981*
scaffold1468	2971924	A	G	Ore07,Ore08,Ore13	*OreG0002984*
scaffold1468	2972378	T	C	Ore07,Ore08,Ore13	*OreG0002984*
scaffold1468	2972712	G	T	NA	*OreG0002984*
scaffold1468	2991774	T	A	Och06,Och12	*OreG0002987*
scaffold1468	3005521	T	G	NA	*OreG0002989*
scaffold1468	3008340	C	T	Ore01,Ore02,Ore03,Ore04,Ore05,Ore06,Ore07,Ore08,Ore09,Ore10,Ore11,Ore12,Ore13,Ore14	*OreG0002989*
scaffold1468	3012688	T	A	Ore01,Ore03,Ore09,Ore11,Ore14	*OreG0002990*
scaffold1468	3043600	G	A	Och01,Och02,Och03,Och04,Och05,Och06,Och07,Och08,Och09,Och10,Och11,Och12,Och13,Och14,Ore01,Ore02,Ore03,Ore04,Ore05,Ore06,Ore07,Ore08,Ore09,Ore10,Ore11,Ore12,Ore13,Ore14	*OreG0002993*
scaffold1468	3060367	C	T	Och01,Och02,Och03,Och04,Och05,Och06,Och07,Och08,Och09,Och10,Och11,Och12,Och13,Och14	*OreG0002995*
scaffold1468	3062200	C	T	Och06,Och12	*OreG0002995*
scaffold1468	3110297	G	A	Ore01,Ore02,Ore03,Ore04,Ore05,Ore06,Ore07,Ore08,Ore09,Ore10,Ore11,Ore12,Ore13,Ore14	*OreG0003000*
scaffold1468	3110374	C	A	Och01	*OreG0003000*
scaffold1468	3119500	G	A	NA	*OreG0003001*
scaffold1468	3120027	C	A	Ore07,Ore08,Ore13	*OreG0003001*
scaffold1468	3124399	T	A	Och01,Och02,Och03,Och04,Och05,Och06,Och07,Och08,Och09,Och10,Och11,Och12,Och13,Och14,Ore01,Ore02,Ore03,Ore04,Ore05,Ore06,Ore07,Ore08,Ore09,Ore10,Ore11,Ore12,Ore13,Ore14	*OreG0003002*
scaffold1468	3130645	C	T	NA	*OreG0003003*
scaffold1468	3135894	C	G	NA	*OreG0003004*
scaffold1468	3195029	A	G	NA	*OreG0003010*
scaffold1468	3230742	A	G	NA	*OreG0003016*
scaffold1468	3230986	C	A	Ore07,Ore08,Ore13	*OreG0003016*
scaffold1468	3231028	C	T	Och01,Och02,Och03,Och04,Och05,Och06,Och07,Och08,Och09,Och10,Och11,Och12,Och13,Och14	*OreG0003016*
scaffold1468	3231409	A	G	NA	*OreG0003017*
scaffold1468	3231419	A	T	NA	*OreG0003017*
scaffold1468	3231481	A	C	Och02,Och03,Och04,Och05,Och06,Och09,Och10,Och12,Och13	*OreG0003017*
scaffold1468	3231938	T	A	Och02,Och03,Och04,Och05,Och06,Och09,Och10,Och12,Och13,Ore01,Ore02,Ore03,Ore04,Ore05,Ore06,Ore07,Ore08,Ore09,Ore10,Ore11,Ore12,Ore13,Ore14	*OreG0003017*
scaffold1468	3237437	T	A	Ore03,Ore09,Ore11,Ore14	*OreG0003018*
scaffold1468	3310734	T	C	NA	*OreG0003028*
scaffold1468	3310928	G	A	NA	*OreG0003028*
scaffold1468	3311432	C	T	Och01,Och02,Och03,Och04,Och05,Och06,Och07,Och08,Och09,Och10,Och11,Och12,Och13,Och14	*OreG0003028*
scaffold1468	3311909	C	T	Och01,Och02,Och03,Och04,Och05,Och06,Och07,Och08,Och09,Och10,Och11,Och12,Och13,Och14,Ore01,Ore02,Ore03,Ore04,Ore05,Ore06,Ore07,Ore08,Ore09,Ore10,Ore11,Ore12,Ore13,Ore14	*OreG0003028*
scaffold1468	3312540	T	A	NA	*OreG0003028*
scaffold1468	3373679	C	T	Och14	*OreG0003041*
scaffold1468	3376642	C	T	NA	*OreG0003041*
scaffold1468	3414041	G	T	NA	*OreG0003047*
scaffold1468	3430145	G	A	Och01,Och02,Och03,Och04,Och05,Och06,Och07,Och08,Och09,Och10,Och11,Och12,Och13,Och14	*OreG0003049*
scaffold1468	3431457	T	C	Ore01,Ore02,Ore03,Ore04,Ore05,Ore06,Ore07,Ore08,Ore09,Ore10,Ore11,Ore12,Ore13,Ore14	*OreG0003049*
scaffold1468	3431589	G	A	Ore01,Ore02,Ore03,Ore04,Ore05,Ore06,Ore07,Ore08,Ore09,Ore10,Ore11,Ore12,Ore13,Ore14	*OreG0003049*
scaffold1468	3431604	A	G	Och01,Och02,Och03,Och04,Och05,Och06,Och07,Och08,Och09,Och10,Och11,Och12,Och13,Och14,Ore01,Ore02,Ore03,Ore04,Ore05,Ore06,Ore07,Ore08,Ore09,Ore10,Ore11,Ore12,Ore13,Ore14	*OreG0003049*
scaffold1468	3432840	G	T	Och01,Och02,Och03,Och04,Och05,Och06,Och07,Och08,Och09,Och10,Och11,Och12,Och13,Och14	*OreG0003049*
scaffold1468	3432956	A	C	NA	*OreG0003049*
scaffold1468	3447928	C	T	Och01,Och02,Och03,Och04,Och05,Och06,Och07,Och08,Och09,Och10,Och11,Och12,Och13,Och14	*OreG0003052*
scaffold1468	3507260	T	C	Ore07,Ore08,Ore13	*OreG0003057*

scaffold1468	3507503	G	A	Och01,Och02,Och03,Och04,Och05,Och06,Och07,Och08,Och09,Och10,Och11,Och12,Och13,Och14,Ore01,Ore02,Ore03,Ore04,Ore05,Ore06,Ore07,Ore08,Ore09,Ore10,Ore11,Ore12,Ore13,Ore14	*OreG0003057*
scaffold1468	3549147	G	A	NA	*OreG0003061*
scaffold1468	3552020	A	T	NA	*OreG0003061*
scaffold1468	3579810	G	T	Ore01,Ore02,Ore03,Ore04,Ore05,Ore06,Ore07,Ore08,Ore09,Ore10,Ore11,Ore12,Ore13,Ore14	*OreG0003064*
scaffold1468	3581415	A	G	Ore01,Ore02,Ore03,Ore04,Ore05,Ore06,Ore07,Ore08,Ore09,Ore10,Ore11,Ore12,Ore13,Ore14	*OreG0003064*
scaffold1468	3617425	A	G	Och07	*OreG0003069*
scaffold1468	3625839	C	T	NA	*OreG0003071*
scaffold1468	3626931	G	T	NA	*OreG0003072*
scaffold1468	3630450	C	T	Ore01,Ore02,Ore03,Ore04,Ore05,Ore06,Ore07,Ore08,Ore09,Ore10,Ore11,Ore12,Ore13,Ore14	*OreG0003072*
scaffold1468	3639201	C	T	Och02,Och03,Och04,Och05,Och06,Och07,Och12,Och13	*OreG0003073*
scaffold1468	3689179	G	T	Och01,Och02,Och03,Och04,Och05,Och06,Och07,Och08,Och09,Och10,Och11,Och12,Och13,Och14,Ore01,Ore02,Ore03,Ore04,Ore05,Ore06,Ore07,Ore08,Ore09,Ore10,Ore11,Ore12,Ore13,Ore14	*OreG0003078*
scaffold1468	3690570	A	T	Och01,Och02,Och03,Och04,Och05,Och06,Och07,Och08,Och09,Och10,Och11,Och12,Och13,Och14,Ore01,Ore02,Ore03,Ore04,Ore05,Ore06,Ore07,Ore08,Ore09,Ore10,Ore11,Ore12,Ore13,Ore14	*OreG0003078*
scaffold1468	3702108	G	C	NA	*OreG0003081*
scaffold1468	3704267	T	C	NA	*OreG0003082*
scaffold1468	3705737	C	T	NA	*OreG0003082*
scaffold1468	3706967	A	G	Och01,Och08,Och09,Och10,Och11	*OreG0003082*
scaffold1468	3720717	G	A	NA	*OreG0003085*
scaffold1468	3753337	C	G	Och07	*OreG0003090*
scaffold1468	3753425	C	G	NA	*OreG0003090*
scaffold1468	3753437	G	A	NA	*OreG0003090*
scaffold1468	3753440	C	G	NA	*OreG0003090*
scaffold1468	3756620	C	T	NA	*OreG0003091*
scaffold1468	3758434	A	T	Ore01,Ore02,Ore03,Ore04,Ore05,Ore06,Ore07,Ore08,Ore09,Ore10,Ore11,Ore12,Ore13,Ore14	*OreG0003092*
scaffold1468	3758500	A	G	Ore07,Ore08,Ore13	*OreG0003092*
scaffold1468	3774956	C	T	Ore07,Ore08,Ore13	*OreG0003094*
scaffold1468	3778248	C	T	Ore01,Ore02,Ore03,Ore04,Ore05,Ore06,Ore07,Ore08,Ore09,Ore10,Ore11,Ore12,Ore13,Ore14	*OreG0003095*
scaffold1468	3778277	C	T	Och01,Och06,Och07,Och08,Och11,Och14	*OreG0003095*
scaffold1468	3778381	G	T	Och06	*OreG0003095*
scaffold1468	3799577	G	C	Ore07,Ore08,Ore13	*OreG0003097*
scaffold1468	3808973	A	T	Ore01,Ore02,Ore03,Ore04,Ore05,Ore06,Ore07,Ore08,Ore09,Ore10,Ore11,Ore12,Ore13,Ore14	*OreG0003098*
scaffold1468	3836136	G	T	Och07	*OreG0003101*
scaffold1468	3836149	A	G	NA	*OreG0003101*
scaffold1468	3836254	T	G	NA	*OreG0003101*
scaffold1468	3836278	A	T	NA	*OreG0003101*
scaffold1468	3836629	C	T	Och01,Och02,Och03,Och04,Och05,Och06,Och07,Och08,Och09,Och10,Och12,Och13	*OreG0003101*
scaffold1468	3836653	T	C	NA	*OreG0003101*
scaffold1468	3836742	T	G	Och01,Och02,Och03,Och04,Och05,Och06,Och07,Och08,Och09,Och10,Och12,Och13	*OreG0003101*
scaffold1468	3838153	G	T	Och01,Och02,Och03,Och04,Och05,Och06,Och07,Och08,Och09,Och10,Och11,Och12,Och13,Och14,Ore01,Ore02,Ore03,Ore04,Ore05,Ore06,Ore07,Ore08,Ore09,Ore10,Ore11,Ore12,Ore13,Ore14	*OreG0003102*
scaffold1468	3838276	G	A	Ore01,Ore02,Ore03,Ore04,Ore05,Ore06,Ore07,Ore08,Ore09,Ore10,Ore11,Ore12,Ore13,Ore14	*OreG0003102*
scaffold1468	3838313	T	A	Ore01,Ore02,Ore03,Ore04,Ore05,Ore06,Ore07,Ore08,Ore09,Ore10,Ore11,Ore12,Ore13,Ore14	*OreG0003102*
scaffold1468	3840286	A	T	Ore14	*OreG0003103*
scaffold1468	3840851	C	T	NA	*OreG0003103*
scaffold1468	3846613	G	C	Och01,Och02,Och03,Och04,Och05,Och06,Och07,Och09,Och10,Och11,Och12,Och13,Och14	*OreG0003104*
scaffold1468	3857724	C	T	Och01,Och02,Och03,Och04,Och05,Och06,Och07,Och08,Och09,Och10,Och11,Och12,Och13,Och14,Ore01,Ore02,Ore03,Ore04,Ore05,Ore06,Ore07,Ore08,Ore09,Ore10,Ore11,Ore12,Ore13,Ore14	*OreG0003106*
scaffold1468	3862654	G	C	NA	*OreG0003106*
scaffold1468	3863526	T	A	NA	*OreG0003106*
scaffold1468	3874666	A	T	Ore01,Ore02,Ore03,Ore04,Ore05,Ore06,Ore07,Ore08,Ore09,Ore10,Ore11,Ore12,Ore13,Ore14	*OreG0003110*
scaffold1468	3883452	G	A	NA	*OreG0003112*
scaffold1468	3884074	C	T	Ore01,Ore07,Ore08,Ore12,Ore13	*OreG0003112*
scaffold1468	3884138	G	C	NA	*OreG0003112*
scaffold1468	3884659	C	T	NA	*OreG0003112*
scaffold1468	3915543	T	A	Och02,Och06	*OreG0003115*
scaffold1468	3915964	A	T	NA	*OreG0003115*

scaffold1468	3916083	G	A	NA	*OreG0003115*
scaffold1468	3916782	G	A	NA	*OreG0003115*
scaffold1468	3936676	G	A	Och01,Och02,Och03,Och04,Och05,Och06,Och07,Och08,Och09,Och10,Och11,Och12,Och13,Och14,Ore01,Ore02,Ore03,Ore04,Ore05,Ore06,Ore07,Ore08,Ore09,Ore10,Ore11,Ore12,Ore13,Ore14	*OreG0003117*
scaffold1468	3936793	C	T	NA	*OreG0003117*
scaffold1468	3938205	T	A	Och02,Och06	*OreG0003118*
scaffold1468	3939505	G	C	Ore01,Ore02,Ore03,Ore04,Ore05,Ore06,Ore07,Ore08,Ore09,Ore10,Ore11,Ore12,Ore13,Ore14	*OreG0003118*
scaffold1468	3961676	A	C	NA	*OreG0003120*
scaffold1468	3963096	C	T	Och02,Och03,Och04,Och05,Och06,Och12,Och13	*OreG0003120*
scaffold1468	3964086	G	A	Ore02,Ore03,Ore04,Ore05,Ore06,Ore07,Ore08,Ore09,Ore10,Ore13,Ore14	*OreG0003120*
scaffold1468	3974444	T	C	NA	*OreG0003122*
scaffold1468	3975213	A	G	Ore14	*OreG0003122*
scaffold1468	3975672	C	A	Ore01,Ore02,Ore03,Ore04,Ore05,Ore06,Ore07,Ore08,Ore09,Ore10,Ore11,Ore12,Ore13,Ore14	*OreG0003122*
scaffold1468	3975706	A	C	NA	*OreG0003122*
scaffold1468	3975710	G	A	Ore01,Ore02,Ore03,Ore04,Ore05,Ore06,Ore07,Ore08,Ore09,Ore10,Ore11,Ore12,Ore13,Ore14	*OreG0003122*
scaffold1468	4009025	T	A	NA	*OreG0003129*
scaffold1468	4010238	A	C	Och01,Och02,Och03,Och04,Och05,Och06,Och07,Och08,Och09,Och10,Och11,Och12,Och13,Och14,Ore01,Ore02,Ore03,Ore04,Ore05,Ore06,Ore07,Ore08,Ore09,Ore10,Ore11,Ore12,Ore13,Ore14	*OreG0003129*
scaffold1468	4011987	G	A	NA	*OreG0003129*
scaffold1468	4018059	A	C	Ore01,Ore02,Ore03,Ore04,Ore05,Ore06,Ore07,Ore08,Ore09,Ore10,Ore11,Ore12,Ore13,Ore14	*OreG0003130*
scaffold1468	4022084	G	A	NA	*OreG0003130*
scaffold1468	4031011	A	T	NA	*OreG0003132*
scaffold1468	4031050	G	T	Och01,Och02,Och03,Och04,Och05,Och06,Och07,Och08,Och09,Och10,Och11,Och12,Och13,Och14	*OreG0003132*
scaffold1468	4032262	G	T	Ore14	*OreG0003132*
scaffold1468	4033935	T	C	NA	*OreG0003132*
scaffold1468	4040007	T	C	NA	*OreG0003134*
scaffold1468	4040430	G	A	Och14	*OreG0003134*
scaffold1468	4040457	C	T	NA	*OreG0003134*
scaffold1468	4040622	C	G	Och02,Och06	*OreG0003134*
scaffold1468	4040624	C	T	Ore01,Ore02,Ore03,Ore04,Ore05,Ore06,Ore07,Ore08,Ore09,Ore10,Ore11,Ore12,Ore13,Ore14	*OreG0003134*
scaffold1468	4041271	A	G	NA	*OreG0003134*
scaffold1468	4041336	A	G	Ore01,Ore02,Ore03,Ore04,Ore05,Ore06,Ore07,Ore08,Ore09,Ore10,Ore11,Ore12,Ore13,Ore14	*OreG0003134*
scaffold1468	4041343	T	C	NA	*OreG0003134*
scaffold1468	4041363	T	C	Och02,Och03,Och04,Och05,Och06,Och07,Och08,Och09,Och10,Och11,Och12,Och13,Och14	*OreG0003134*
scaffold1468	4041375	T	C	NA	*OreG0003134*
scaffold1468	4041529	C	T	Ore01,Ore02,Ore03,Ore04,Ore05,Ore06,Ore07,Ore08,Ore09,Ore10,Ore11,Ore12,Ore13,Ore14	*OreG0003134*
scaffold1468	4041549	G	A	Och02,Och03,Och04,Och05,Och06,Och07,Och08,Och09,Och10,Och11,Och12,Och13,Och14	*OreG0003134*
scaffold1468	4041556	C	T	Och02,Och03,Och04,Och05,Och06,Och07,Och08,Och09,Och10,Och11,Och12,Och13,Och14	*OreG0003134*
scaffold1468	4041650	A	T	NA	*OreG0003134*
scaffold1468	4069230	G	A	NA	*OreG0003140*
scaffold1468	4070817	C	T	Ore01,Ore02,Ore03,Ore04,Ore05,Ore06,Ore07,Ore08,Ore09,Ore10,Ore11,Ore12,Ore13,Ore14	*OreG0003140*
scaffold1468	4080248	C	G	Och01,Och02,Och06,Och07,Och09,Och10,Och14	*OreG0003142*
scaffold1468	4080345	C	A	Och01,Och02,Och03,Och04,Och05,Och06,Och07,Och08,Och09,Och10,Och11,Och12,Och13,Och14	*OreG0003142*
scaffold1468	4092045	G	A	NA	*OreG0003146*
scaffold1468	4103925	C	G	Och02,Och06,Och07,Och08,Och09,Och10	*OreG0003149*
scaffold1468	4104485	G	T	Och01,Och02,Och03,Och04,Och05,Och06,Och07,Och08,Och09,Och10,Och11,Och12,Och13,Och14,Ore01,Ore02,Ore03,Ore04,Ore05,Ore06,Ore07,Ore08,Ore09,Ore10,Ore11,Ore12,Ore13,Ore14	*OreG0003149*
scaffold1468	4105860	G	A	Och01,Och02,Och03,Och04,Och05,Och06,Och07,Och08,Och09,Och10,Och11,Och12,Och13,Och14,Ore01,Ore02,Ore03,Ore04,Ore05,Ore06,Ore07,Ore08,Ore09,Ore10,Ore11,Ore12,Ore13,Ore14	*OreG0003150*
scaffold1468	4111980	A	C	Ore01,Ore02,Ore03,Ore04,Ore05,Ore06,Ore07,Ore08,Ore09,Ore10,Ore11,Ore12,Ore13,Ore14	*OreG0003151*
scaffold1468	4112006	C	A	Och07,Och09,Och10	*OreG0003151*
scaffold1468	4125360	C	A	NA	*OreG0003153*
scaffold1468	4136262	G	C	NA	*OreG0003155*
scaffold1468	4155376	T	A	Och07	*OreG0003157*
scaffold1468	4155604	A	G	Och01,Och08	*OreG0003157*
scaffold1468	4155655	A	G	Och01,Och08	*OreG0003157*
scaffold1468	4155883	C	G	Ore14	*OreG0003157*

scaffold1468	4159019	G	A	Och02,Och06,Och07,Ore01,Ore02,Ore03,Ore04,Ore05,Ore06,Ore07,Ore08,Ore09,Ore10,Ore11,Ore12,Ore13,Ore14	*OreG0003158*
scaffold1468	4177106	C	T	NA	*OreG0003163*
scaffold1468	4177134	G	T	Och01,Och02,Och03,Och04,Och05,Och06,Och07,Och08,Och09,Och10,Och11,Och12,Och13,Och14,Ore01,Ore02,Ore03,Ore04,Ore05,Ore06,Ore07,Ore08,Ore09,Ore10,Ore11,Ore12,Ore13,Ore14	*OreG0003163*
scaffold1468	4177980	C	T	Och11,Ore01,Ore02,Ore03,Ore04,Ore05,Ore06,Ore07,Ore08,Ore09,Ore10,Ore11,Ore12,Ore13,Ore14	*OreG0003163*
scaffold1468	4178082	A	C	Och01	*OreG0003163*
scaffold1468	4178084	G	A	Och01	*OreG0003163*
scaffold1468	4178115	G	A	Och11,Ore01,Ore02,Ore03,Ore04,Ore05,Ore06,Ore07,Ore08,Ore09,Ore10,Ore11,Ore12,Ore13,Ore14	*OreG0003163*
scaffold1468	4178658	A	C	Och01	*OreG0003163*
scaffold1468	4184044	C	G	Ore07,Ore08,Ore13	*OreG0003164*
scaffold1468	4184251	G	T	Och01	*OreG0003164*
scaffold1468	4184904	A	G	Och01,Och08,Och14	*OreG0003164*
scaffold1468	4187657	C	T	Och02,Och06,Och11,Ore01,Ore02,Ore03,Ore04,Ore05,Ore06,Ore07,Ore08,Ore09,Ore10,Ore11,Ore12,Ore13,Ore14	*OreG0003165*
scaffold1468	4187976	T	A	NA	*OreG0003165*
scaffold1468	4187994	C	T	Ore07,Ore08,Ore13	*OreG0003165*
scaffold1468	4188011	C	T	Ore06,Ore14	*OreG0003165*
scaffold1468	4216490	A	C	NA	*OreG0003171*
scaffold1468	4245190	G	A	Och01,Och02,Och06	*OreG0003174*
scaffold1468	4245383	G	T	Och09,Och10,Och11	*OreG0003174*
scaffold1468	4247393	T	C	Och01,Och02,Och03,Och04,Och05,Och06,Och07,Och08,Och09,Och10,Och11,Och12,Och13,Och14,Ore01,Ore02,Ore03,Ore04,Ore05,Ore06,Ore07,Ore08,Ore09,Ore10,Ore11,Ore12,Ore13,Ore14	*OreG0003174*
scaffold1468	4250643	T	A	Och01,Och02,Och03,Och04,Och05,Och06,Och08,Och12,Och13,Och14	*OreG0003175*
scaffold1468	4253142	G	A	NA	*OreG0003175*
scaffold1468	4298545	A	T	Och01,Och07	*OreG0003181*
scaffold1468	4298809	C	A	Och02,Och03,Och04,Och05,Och06,Och08,Och12,Och13,Och14	*OreG0003181*
scaffold1468	4305792	A	T	Och02,Och12,Och13,Och14	*OreG0003182*
scaffold1468	4305829	T	G	NA	*OreG0003182*
scaffold1468	4305865	A	G	Ore07,Ore08,Ore13	*OreG0003182*
scaffold1468	4305919	C	G	Och02	*OreG0003182*
scaffold1468	4348662	C	G	Och02,Och03,Och04,Och05,Och06,Och07,Och08,Och09,Och10,Och11,Och12,Och13,Och14	*OreG0003184*
scaffold1468	4371883	G	T	NA	*OreG0003189*
scaffold1468	4372094	T	C	Ore14	*OreG0003189*
scaffold1468	4374602	C	T	Och02,Och03,Och04,Och05,Och12,Och13	*OreG0003190*
scaffold1468	4377394	T	A	Och07,Ore14	*OreG0003190*
scaffold1468	4377742	T	A	NA	*OreG0003190*
scaffold1468	4379945	G	T	Ore01,Ore07,Ore08,Ore12,Ore13	*OreG0003190*
scaffold1468	4379979	T	A	Ore01,Ore07,Ore08,Ore12,Ore13	*OreG0003190*
scaffold1468	4380233	A	G	Ore14	*OreG0003190*
scaffold1468	4381654	G	C	NA	*OreG0003190*
scaffold1468	4381699	A	G	Och07	*OreG0003190*
scaffold1468	4392474	A	G	Och07,Och08,Och11,Och14	*OreG0003191*
scaffold1468	4392782	G	A	NA	*OreG0003191*
scaffold1468	4393859	G	T	Och01,Och02,Och03,Och04,Och05,Och06,Och07,Och08,Och09,Och10,Och11,Och12,Och13,Och14,Ore01,Ore02,Ore03,Ore04,Ore05,Ore06,Ore07,Ore08,Ore09,Ore10,Ore11,Ore12,Ore13,Ore14	*OreG0003191*
scaffold1468	4413782	A	T	Ore01,Ore02,Ore03,Ore04,Ore05,Ore06,Ore07,Ore08,Ore09,Ore10,Ore11,Ore12,Ore13,Ore14	*OreG0003194*
scaffold1468	4415601	A	T	NA	*OreG0003194*
scaffold1468	4415603	A	C	Och02,Och04,Och06,Och07	*OreG0003194*
scaffold1468	4415630	T	C	Ore01	*OreG0003194*
scaffold1468	4415969	C	T	NA	*OreG0003194*
scaffold1468	4424619	C	T	NA	*OreG0003196*
scaffold1468	4425719	G	A	NA	*OreG0003196*
scaffold1468	4427559	A	G	NA	*OreG0003196*
scaffold1468	4427585	T	C	NA	*OreG0003196*
scaffold1468	4436338	G	A	NA	*OreG0003197*
scaffold1468	4437215	T	C	Ore14	*OreG0003197*
scaffold1468	4438267	T	A	NA	*OreG0003197*
scaffold1468	4439990	T	G	NA	*OreG0003197*
scaffold1468	4442145	T	C	NA	*OreG0003197*
scaffold1468	4457708	A	T	NA	*OreG0003200*
scaffold1468	4479425	T	A	NA	*OreG0003204*
scaffold1468	4484926	G	T	Och02	*OreG0003205*
scaffold1468	4484946	G	A	Och07,Och09,Och10	*OreG0003205*
scaffold1468	4485336	C	T	NA	*OreG0003205*
scaffold1468	4485445	C	T	Och07,Och09,Och10,Ore01,Ore02,Ore03,Ore04,Ore05,Ore06,Ore07,Ore08,Ore09,Ore10,Ore11,Ore12,Ore13,Ore14	*OreG0003205*
scaffold1468	4485651	G	T	Och02	*OreG0003205*

scaffold1468	4514611	A	G	Och01,Och02,Och03,Och04,Och05,Och06,Och07,Och08,Och09,Och10,Och11,Och12,Och13,Och14,Ore01,Ore02,Ore03,Ore04,Ore05,Ore06,Ore07,Ore08,Ore09,Ore10,Ore11,Ore12,Ore13,Ore14	*OreG0003206*
scaffold1468	4514863	C	T	NA	*OreG0003206*
scaffold1468	4516063	C	G	Ore02,Ore03,Ore04,Ore05,Ore06,Ore07,Ore08,Ore09,Ore10,Ore13,Ore14	*OreG0003206*
scaffold1468	4527038	C	T	NA	*OreG0003208*
scaffold1468	4527523	G	A	Och02	*OreG0003208*
scaffold1468	4527712	C	A	Ore14	*OreG0003208*
scaffold1468	4528148	A	T	Och02,Och03,Och04,Och05,Och06,Och07,Och09,Och10,Och11,Och12,Och13,Ore01,Ore02,Ore03,Ore04,Ore05,Ore06,Ore07,Ore08,Ore09,Ore10,Ore11,Ore12,Ore13,Ore14	*OreG0003208*
scaffold1468	4528224	G	A	Och02,Och03,Och04,Och05,Och06,Och07,Och09,Och10,Och11,Och12,Och13,Ore01,Ore02,Ore03,Ore04,Ore05,Ore06,Ore07,Ore08,Ore09,Ore10,Ore11,Ore12,Ore13,Ore14	*OreG0003208*
scaffold1468	4528342	C	T	Och02,Och03,Och04,Och05,Och06,Och07,Och09,Och10,Och11,Och12,Och13,Ore01,Ore02,Ore03,Ore04,Ore05,Ore06,Ore07,Ore08,Ore09,Ore10,Ore11,Ore12,Ore13,Ore14	*OreG0003208*
scaffold1468	4528459	C	T	Och02,Och03,Och04,Och05,Och06,Och07,Och09,Och10,Och11,Och12,Och13	*OreG0003208*
scaffold1468	4528846	C	T	Ore02,Ore03,Ore04,Ore05,Ore06,Ore07,Ore08,Ore09,Ore10,Ore13,Ore14	*OreG0003208*
scaffold1468	4528876	G	T	NA	*OreG0003208*
scaffold1468	4529164	T	G	Och02,Och03,Och04,Och05,Och06,Och07,Och09,Och10,Och11,Och12,Och13,Ore01,Ore02,Ore03,Ore04,Ore05,Ore06,Ore07,Ore08,Ore09,Ore10,Ore11,Ore12,Ore13,Ore14	*OreG0003208*
scaffold1468	4529290	G	A	NA	*OreG0003208*
scaffold1468	4529442	C	T	NA	*OreG0003208*
scaffold1468	4529539	T	C	NA	*OreG0003208*
scaffold1468	4529770	G	C	NA	*OreG0003208*
scaffold1468	4529802	G	A	Och11	*OreG0003208*
scaffold1468	4529905	G	A	NA	*OreG0003208*
scaffold1468	4529962	T	C	NA	*OreG0003208*
scaffold1468	4536383	C	A	NA	*OreG0003207*
scaffold1468	4538304	G	C	NA	*OreG0003207*
scaffold1468	4542627	C	T	Ore02,Ore04,Ore05,Ore10,Ore11,Ore12,Ore14	*OreG0003209*
scaffold1468	4547446	A	G	Och02,Och03,Och04,Och05,Och06,Och08,Och12,Och13,Och14	*OreG0003210*
scaffold1468	4547501	C	A	Och02,Och06,Och14	*OreG0003210*
scaffold1468	4580783	C	A	NA	*OreG0003216*
scaffold1468	4580823	G	A	NA	*OreG0003216*
scaffold1468	4581622	G	A	NA	*OreG0003216*
scaffold1468	4583046	G	A	NA	*OreG0003216*
scaffold1468	4596533	G	A	NA	*OreG0003217*
scaffold1468	4596637	C	T	NA	*OreG0003217*
scaffold1468	4612849	G	A	NA	*OreG0003219*
scaffold1468	4622505	A	G	NA	*OreG0003220*
scaffold1468	4637930	T	G	NA	*OreG0003222*
scaffold1468	4638562	T	A	NA	*OreG0003222*
scaffold1468	4639851	G	C	Och01,Och02,Och03,Och04,Och05,Och06,Och07,Och08,Och09,Och10,Och11,Och12,Och13,Och14,Ore01,Ore02,Ore03,Ore04,Ore05,Ore06,Ore07,Ore08,Ore09,Ore10,Ore11,Ore12,Ore13,Ore14	*OreG0003222*
scaffold1468	4640324	G	A	Och02,Och05,Och14	*OreG0003222*
scaffold1468	4640363	T	C	Och01,Och02,Och03,Och04,Och05,Och06,Och07,Och08,Och09,Och10,Och11,Och12,Och13,Och14,Ore01,Ore02,Ore03,Ore04,Ore05,Ore06,Ore07,Ore08,Ore09,Ore10,Ore11,Ore12,Ore13,Ore14	*OreG0003222*
scaffold1468	4640760	A	T	Och01,Och02,Och06,Och08,Och09,Och10,Och11,Och14	*OreG0003222*
scaffold1468	4656862	G	T	Ore10,Ore14	*OreG0003224*
scaffold1468	4659708	C	G	NA	*OreG0003225*
scaffold1468	4661202	C	G	NA	*OreG0003225*
scaffold1468	4664873	G	A	NA	*OreG0003226*
scaffold1468	4664904	G	T	Ore06,Ore11,Ore14	*OreG0003226*
scaffold1468	4680065	C	A	Och01,Och02,Och03,Och04,Och05,Och06,Och07,Och08,Och09,Och10,Och11,Och12,Och13,Och14,Ore01,Ore02,Ore03,Ore04,Ore05,Ore06,Ore07,Ore08,Ore09,Ore10,Ore11,Ore12,Ore13,Ore14	*OreG0003229*
scaffold1468	4694620	T	A	NA	*OreG0003231*
scaffold1468	4695422	C	A	NA	*OreG0003231*
scaffold1468	4695596	C	G	Ore06,Ore14	*OreG0003231*
scaffold1468	4695749	T	C	Ore01,Ore02,Ore03,Ore04,Ore05,Ore06,Ore07,Ore08,Ore09,Ore10,Ore11,Ore12,Ore13,Ore14	*OreG0003231*
scaffold1468	4698973	C	T	Ore02,Ore04,Ore05,Ore06,Ore07,Ore08,Ore10,Ore13,Ore14	*OreG0003231*
scaffold1468	4715198	A	C	Och02	*OreG0003234*
scaffold1468	4730349	C	T	NA	*OreG0003237*
scaffold1468	4740496	T	C	Ore06,Ore14	*OreG0003238*
scaffold1468	4772054	C	T	NA	*OreG0003242*
scaffold1468	4773887	G	T	Ore01,Ore02,Ore03,Ore04,Ore05,Ore06,Ore07,Ore08,Ore09,Ore10,Ore11,Ore12,Ore13,Ore14	*OreG0003242*
scaffold1468	4774079	G	T	NA	*OreG0003242*
scaffold1468	4774518	G	T	NA	*OreG0003242*

scaffold1468	4775847	T	C	NA	*OreG0003242*
scaffold1468	4776144	G	A	Och01,Och02,Och03,Och04,Och05,Och06,Och07,Och08,Och09,Och10,Och11,Och12,Och13,Och14,Ore01,Ore02,Ore03,Ore04,Ore05,Ore06,Ore07,Ore08,Ore09,Ore10,Ore11,Ore12,Ore13,Ore14	*OreG0003242*
scaffold1468	4808517	A	G	Och01,Och02,Och03,Och04,Och05,Och06,Och07,Och08,Och09,Och10,Och11,Och12,Och13,Och14,Ore01,Ore02,Ore03,Ore04,Ore05,Ore06,Ore07,Ore08,Ore09,Ore10,Ore11,Ore12,Ore13,Ore14	*OreG0003246*
scaffold1468	4814096	G	A	Och01,Och02,Och03,Och04,Och05,Och06,Och07,Och08,Och09,Och10,Och11,Och12,Och13,Och14	*OreG0003247*
scaffold1468	4819575	G	A	Ore01,Ore02,Ore03,Ore04,Ore05,Ore06,Ore07,Ore08,Ore09,Ore10,Ore11,Ore12,Ore13,Ore14	*OreG0003247*
scaffold5614	2960	C	G	NA	*OreG0023618*
scaffold1231	15164	T	C	Och02,Och03,Och04,Och06	*OreG0001631*
scaffold1231	15904	A	G	NA	*OreG0001631*
scaffold1231	16056	C	A	NA	*OreG0001631*
scaffold1231	37566	T	C	Och01,Och02,Och03,Och04,Och05,Och06,Och07,Och08,Och09,Och10,Och11,Och12,Och13,Och14,Ore01,Ore02,Ore03,Ore04,Ore05,Ore06,Ore07,Ore08,Ore09,Ore10,Ore11,Ore12,Ore13,Ore14	*OreG0001634*
scaffold1231	91848	T	C	NA	*OreG0001640*
scaffold1231	91875	G	T	NA	*OreG0001640*
scaffold1231	94705	C	T	NA	*OreG0001641*
scaffold1231	129683	G	T	Och01,Och02,Och03,Och04,Och05,Och06,Och07,Och08,Och09,Och10,Och11,Och12,Och13,Och14	*OreG0001644*
scaffold1231	129713	G	T	Och01,Och02,Och03,Och04,Och05,Och06,Och07,Och08,Och09,Och10,Och11,Och12,Och13,Och14	*OreG0001644*
scaffold1231	129841	A	T	Och08	*OreG0001644*
scaffold1231	134047	A	C	NA	*OreG0001645*
scaffold1231	134119	T	A	Och01,Och02,Och03,Och04,Och05,Och06,Och07,Och08,Och09,Och10,Och11,Och12,Och13,Och14,Ore01,Ore02,Ore03,Ore04,Ore05,Ore06,Ore07,Ore08,Ore09,Ore10,Ore11,Ore12,Ore13,Ore14	*OreG0001645*
scaffold1231	134401	A	T	Och01,Och02,Och03,Och04,Och05,Och06,Och07,Och08,Och09,Och10,Och11,Och12,Och13,Och14	*OreG0001645*
scaffold1231	134427	G	T	Ore01,Ore02,Ore03,Ore04,Ore05,Ore06,Ore07,Ore08,Ore09,Ore10,Ore11,Ore12,Ore13,Ore14	*OreG0001645*
scaffold1231	138060	A	G	NA	*OreG0001646*
scaffold1231	138070	C	T	NA	*OreG0001646*
scaffold1231	169147	G	A	NA	*OreG0001649*
scaffold1231	169554	C	T	Och02,Och03,Och04,Och05,Och08,Och12,Och13	*OreG0001649*
scaffold1231	169748	A	C	Och02,Och03,Och04,Och05,Och08,Och12,Och13	*OreG0001649*
scaffold1231	169844	T	C	Ore04,Ore05	*OreG0001649*
scaffold1231	169874	A	G	Och01,Och09,Och10,Och11,Och14	*OreG0001649*
scaffold1231	170049	C	T	Och09,Och10	*OreG0001649*
scaffold1231	170110	G	C	Och01,Och09,Och10,Och11,Och14	*OreG0001649*
scaffold1231	170241	C	T	Ore01,Ore02,Ore03,Ore04,Ore05,Ore06,Ore07,Ore08,Ore09,Ore10,Ore11,Ore12,Ore13,Ore14	*OreG0001649*
scaffold1231	183699	A	G	NA	*OreG0001652*
scaffold1231	190995	G	T	Och01,Och02,Och03,Och04,Och05,Och06,Och07,Och08,Och11,Och12,Och13,Och14	*OreG0001653*
scaffold1231	191608	A	T	NA	*OreG0001653*
scaffold1231	191979	G	A	Och02,Och03,Och04,Och05,Och12,Och13	*OreG0001653*
scaffold1231	197262	A	T	NA	*OreG0001654*
scaffold1231	221780	G	T	NA	*OreG0001658*
scaffold1231	221816	G	A	NA	*OreG0001658*
scaffold1231	228305	C	T	NA	*OreG0001659*
scaffold1231	238725	G	A	NA	*OreG0001661*
scaffold1231	238806	G	A	Och01,Och02,Och03,Och04,Och05,Och06,Och07,Och08,Och09,Och10,Och11,Och12,Och13,Och14,Ore01,Ore02,Ore03,Ore04,Ore05,Ore06,Ore07,Ore08,Ore09,Ore10,Ore11,Ore12,Ore13,Ore14	*OreG0001661*
scaffold1231	249987	C	A	Och07,Och08,Och12,Och14	*OreG0001662*
scaffold1231	255145	T	C	Ore01,Ore02,Ore06,Ore07,Ore08,Ore09,Ore10,Ore11,Ore12,Ore13,Ore14	*OreG0001663*
scaffold1231	256366	C	T	Och01,Och02,Och03,Och04,Och05,Och06,Och07,Och08,Och09,Och10,Och11,Och12,Och13,Och14,Ore01,Ore02,Ore03,Ore04,Ore05,Ore06,Ore07,Ore08,Ore09,Ore10,Ore11,Ore12,Ore13,Ore14	*OreG0001663*
scaffold1231	258115	C	T	Ore01,Ore02,Ore03,Ore04,Ore05,Ore06,Ore07,Ore08,Ore09,Ore10,Ore11,Ore12,Ore13,Ore14	*OreG0001663*
scaffold1231	258443	A	T	NA	*OreG0001663*
scaffold1231	263721	A	T	Och14	*OreG0001664*
scaffold1231	264778	G	C	NA	*OreG0001664*
scaffold1231	282261	G	T	NA	*OreG0001668*
scaffold1231	282414	G	T	NA	*OreG0001668*
scaffold1231	282878	A	G	NA	*OreG0001668*
scaffold1231	290353	C	T	NA	*OreG0001670*
scaffold1231	297583	G	C	Och01,Och02,Och03,Och04,Och05,Och06,Och07,Och08,Och09,Och10,Och11,Och12,Och13,Och14,Ore01,Ore02,Ore03,Ore04,Ore05,Ore06,Ore07,Ore08,Ore09,Ore10,Ore11,Ore12,Ore13,Ore14	*OreG0001671*

scaffold1231	297749	T	A	Och01,Och02,Och03,Och04,Och05,Och06,Och07,Och08,Och09,Och10,Och11,Och12,Och13,Och14	*OreG0001671*
scaffold1231	305621	G	T	Och01,Och02,Och03,Och04,Och05,Och06,Och07,Och08,Och09,Och10,Och11,Och12,Och13,Och14,Ore01,Ore02,Ore03,Ore04,Ore05,Ore06,Ore07,Ore08,Ore09,Ore10,Ore11,Ore12,Ore13,Ore14	*OreG0001672*
scaffold1231	305682	G	C	Och01,Och02,Och03,Och04,Och05,Och06,Och07,Och08,Och09,Och10,Och11,Och12,Och13,Och14,Ore01,Ore02,Ore03,Ore04,Ore05,Ore06,Ore07,Ore08,Ore09,Ore10,Ore11,Ore12,Ore13,Ore14	*OreG0001672*
scaffold1231	332028	G	C	NA	*OreG0001674*
scaffold1231	335812	C	T	NA	*OreG0001675*
scaffold1231	339916	T	A	NA	*OreG0001676*
scaffold1231	339940	A	G	Och06,Och07,Och12	*OreG0001676*
scaffold1231	340194	A	C	Och01	*OreG0001676*
scaffold1231	343246	C	T	Ore01,Ore02,Ore03,Ore04,Ore05,Ore06,Ore07,Ore08,Ore09,Ore10,Ore11,Ore12,Ore13,Ore14	*OreG0001676*
scaffold1231	418397	C	T	Och01,Och02,Och03,Och04,Och05,Och06,Och07,Och08,Och09,Och10,Och11,Och12,Och13,Och14,Ore01,Ore02,Ore03,Ore04,Ore05,Ore06,Ore07,Ore08,Ore09,Ore10,Ore11,Ore12,Ore13,Ore14	*OreG0001683*
scaffold1231	419200	A	T	Och01,Och02,Och03,Och04,Och05,Och06,Och07,Och08,Och09,Och10,Och11,Och12,Och13,Och14,Ore01,Ore02,Ore03,Ore04,Ore05,Ore06,Ore07,Ore08,Ore09,Ore10,Ore11,Ore12,Ore13,Ore14	*OreG0001683*
scaffold1231	434986	G	A	Ore01,Ore02,Ore03,Ore04,Ore05,Ore06,Ore07,Ore08,Ore09,Ore10,Ore11,Ore12,Ore13,Ore14	*OreG0001684*
scaffold1231	436256	C	T	Och09,Och10	*OreG0001684*
scaffold1231	505484	G	A	NA	*OreG0001689*
scaffold1231	505492	C	A	NA	*OreG0001689*
scaffold1231	679819	T	A	NA	*OreG0001702*
scaffold1231	696222	T	C	Ore03,Ore08,Ore09,Ore10,Ore14	*OreG0001703*
scaffold1231	696435	C	A	Och04,Och09,Och10	*OreG0001703*
scaffold1231	696768	T	A	Och01,Och02,Och03,Och04,Och05,Och06,Och07,Och08,Och09,Och10,Och11,Och12,Och13,Och14,Ore01,Ore02,Ore03,Ore04,Ore05,Ore06,Ore07,Ore08,Ore09,Ore10,Ore11,Ore12,Ore13,Ore14	*OreG0001703*
scaffold1231	696867	T	C	Ore01,Ore02,Ore03,Ore04,Ore05,Ore06,Ore07,Ore08,Ore09,Ore10,Ore11,Ore12,Ore13,Ore14	*OreG0001703*
scaffold1231	697089	G	A	Och02,Och03,Och04,Och05,Och06,Och07,Och08,Och09,Och10,Och11,Och12,Och13,Och14	*OreG0001703*
scaffold1231	747891	T	C	NA	*OreG0001705*
scaffold1231	750289	T	G	Ore01,Ore02,Ore03,Ore04,Ore05,Ore06,Ore07,Ore08,Ore09,Ore10,Ore11,Ore12,Ore13,Ore14	*OreG0001705*
scaffold1231	792320	G	T	Och13	*OreG0001711*
scaffold1231	793590	C	T	NA	*OreG0001711*
scaffold1231	828677	C	G	NA	*OreG0001715*
scaffold1231	828680	G	T	NA	*OreG0001715*
scaffold1231	828698	T	C	Och02,Och03,Och04,Och05,Och06,Och07,Och08,Och12,Och13,Och14	*OreG0001715*
scaffold1231	832310	T	C	NA	*OreG0001716*
scaffold1231	840821	C	G	Och08,Och14	*OreG0001717*
scaffold1231	846608	G	A	Och09,Och10	*OreG0001718*
scaffold1231	846806	G	C	Ore01,Ore02,Ore03,Ore04,Ore05,Ore06,Ore07,Ore08,Ore09,Ore10,Ore11,Ore12,Ore13,Ore14	*OreG0001718*
scaffold1231	857219	T	G	NA	*OreG0001720*
scaffold1231	857243	T	C	NA	*OreG0001720*
scaffold1231	857287	G	T	NA	*OreG0001720*
scaffold1231	917385	G	T	NA	*OreG0001722*
scaffold1231	917913	T	C	Och02,Och03,Och04,Och05,Och06,Och12,Och13,Och14	*OreG0001722*
scaffold1231	917934	A	G	Och01,Och09,Och10,Och11	*OreG0001722*
scaffold1231	918695	A	T	NA	*OreG0001722*
scaffold1231	967906	T	C	NA	*OreG0001727*
scaffold1231	968767	C	A	NA	*OreG0001727*
scaffold1231	968922	A	G	NA	*OreG0001727*
scaffold1231	972325	T	A	Ore01,Ore02,Ore03,Ore04,Ore05,Ore06,Ore07,Ore08,Ore09,Ore10,Ore11,Ore12,Ore13,Ore14	*OreG0001727*
scaffold1231	973632	C	A	Ore03,Ore08,Ore09,Ore10,Ore14	*OreG0001727*
scaffold1231	974160	C	T	Och01	*OreG0001727*
scaffold1231	983277	A	G	Och01,Och02,Och04,Och06	*OreG0001729*
scaffold1231	983439	T	C	NA	*OreG0001729*
scaffold1231	996561	T	C	Och04	*OreG0001731*
scaffold1231	1022701	C	T	NA	*OreG0001734*
scaffold1231	1077830	T	A	Och08	*OreG0001739*
scaffold1231	1135774	A	T	Och01,Och02,Och03,Och04,Och05,Och06,Och07,Och08,Och09,Och10,Och11,Och12,Och13,Och14,Ore01,Ore02,Ore03,Ore04,Ore05,Ore06,Ore07,Ore08,Ore09,Ore10,Ore11,Ore12,Ore13,Ore14	*OreG0001745*
scaffold1231	1136272	G	C	NA	*OreG0001745*
scaffold1231	1138748	T	A	Och01,Och02,Och03,Och04,Och05,Och06,Och07,Och08,Och09,Och10,Och11,Och12,Och13,Och14,Ore01,Ore02,Ore03,Ore04,Ore05,Ore06,Ore07,Ore08,Ore09,Ore10,Ore11,Ore12,Ore13,Ore14	*OreG0001745*

scaffold1231	1139594	T	A	Och09,Och10	*OreG0001745*
scaffold1231	1160288	C	A	Och01,Och02,Och03,Och04,Och05,Och06,Och07,Och08,Och09,Och10,Och11,Och12,Och13,Och14,Ore01,Ore02,Ore03,Ore04,Ore05,Ore06,Ore07,Ore08,Ore09,Ore10,Ore11,Ore12,Ore13,Ore14	*OreG0001747*
scaffold1231	1163598	T	A	Ore01,Ore02,Ore03,Ore04,Ore05,Ore06,Ore07,Ore08,Ore09,Ore10,Ore11,Ore12,Ore13,Ore14	*OreG0001749*
scaffold1231	1164190	G	T	Ore01,Ore02,Ore03,Ore06,Ore07,Ore08,Ore09,Ore10,Ore11,Ore12,Ore13,Ore14	*OreG0001749*
scaffold1231	1164210	A	G	Ore01,Ore02,Ore03,Ore04,Ore05,Ore06,Ore07,Ore08,Ore09,Ore10,Ore11,Ore12,Ore13,Ore14	*OreG0001749*
scaffold1231	1164375	G	A	Och01,Och02,Och03,Och04,Och05,Och06,Och08,Och09,Och10,Och11,Och12,Och13,Och14	*OreG0001749*
scaffold1231	1165103	A	C	NA	*OreG0001749*
scaffold1231	1165291	A	G	NA	*OreG0001749*
scaffold1231	1165383	T	C	Och01,Och02,Och03,Och04,Och05,Och06,Och07,Och08,Och09,Och10,Och11,Och12,Och13,Och14,Ore01,Ore02,Ore03,Ore04,Ore05,Ore06,Ore07,Ore08,Ore09,Ore10,Ore11,Ore12,Ore13,Ore14	*OreG0001749*
scaffold1231	1165780	T	A	Och01,Och02,Och04,Och05,Och06,Och07,Och08,Och09,Och10,Och11,Och12,Och13,Och14	*OreG0001749*
scaffold1231	1171648	C	G	Ore01,Ore02,Ore03,Ore04,Ore05,Ore06,Ore07,Ore08,Ore09,Ore10,Ore11,Ore12,Ore13,Ore14	*OreG0001750*
scaffold1231	1172751	G	A	NA	*OreG0001750*
scaffold1231	1264624	C	T	Och01,Och02,Och03,Och04,Och05,Och06,Och07,Och08,Och09,Och10,Och11,Och12,Och13,Och14,Ore01,Ore02,Ore03,Ore04,Ore05,Ore06,Ore07,Ore08,Ore09,Ore10,Ore11,Ore12,Ore13,Ore14	*OreG0001756*
scaffold1231	1268792	A	T	NA	*OreG0001757*
scaffold1231	1270977	G	A	Ore01,Ore02,Ore03,Ore04,Ore05,Ore06,Ore07,Ore08,Ore09,Ore10,Ore11,Ore12,Ore13,Ore14	*OreG0001758*
scaffold1231	1291366	G	T	Och01,Och13	*OreG0001759*
scaffold1231	1326641	A	T	Och01,Och02,Och03,Och04,Och05,Och06,Och07,Och08,Och09,Och10,Och11,Och12,Och13,Och14	*OreG0001764*
scaffold1231	1327775	A	T	Ore01,Ore02,Ore03,Ore04,Ore05,Ore06,Ore07,Ore08,Ore09,Ore10,Ore11,Ore12,Ore13,Ore14	*OreG0001764*
scaffold1231	1328471	C	A	Och04	*OreG0001764*
scaffold1231	1329077	T	A	Ore03,Ore08,Ore09,Ore10,Ore14	*OreG0001764*
scaffold1231	1331851	C	T	Ore01,Ore02,Ore03,Ore04,Ore05,Ore06,Ore07,Ore08,Ore09,Ore10,Ore11,Ore12,Ore13,Ore14	*OreG0001766*
scaffold1231	1332070	G	A	Ore03,Ore08,Ore09,Ore10,Ore14	*OreG0001766*
scaffold1231	1333556	G	A	Ore01,Ore02,Ore03,Ore04,Ore05,Ore06,Ore07,Ore08,Ore09,Ore10,Ore11,Ore12,Ore13,Ore14	*OreG0001768*
scaffold1231	1334361	A	T	Och01,Och02,Och03,Och04,Och05,Och06,Och07,Och08,Och09,Och10,Och11,Och12,Och13,Och14,Ore01,Ore02,Ore03,Ore04,Ore05,Ore06,Ore07,Ore08,Ore09,Ore10,Ore11,Ore12,Ore13,Ore14	*OreG0001769*
scaffold1231	1334371	T	C	Och01,Och02,Och03,Och04,Och05,Och06,Och07,Och08,Och09,Och10,Och11,Och12,Och13,Och14,Ore01,Ore02,Ore03,Ore04,Ore05,Ore06,Ore07,Ore08,Ore09,Ore10,Ore11,Ore12,Ore13,Ore14	*OreG0001769*
scaffold1231	1368904	G	A	NA	*OreG0001770*
scaffold1231	1490599	A	T	Och01,Och02,Och03,Och04,Och05,Och06,Och07,Och08,Och09,Och10,Och11,Och12,Och13,Och14	*OreG0001772*
scaffold1231	1490645	C	T	Och01,Och02,Och03,Och04,Och05,Och06,Och07,Och08,Och09,Och10,Och11,Och12,Och13,Och14	*OreG0001772*
scaffold1231	1649821	C	A	Ore01,Ore02,Ore03,Ore04,Ore05,Ore06,Ore07,Ore08,Ore09,Ore10,Ore11,Ore12,Ore13,Ore14	*OreG0001782*
scaffold1231	1664661	C	T	Ore01,Ore02,Ore03,Ore04,Ore05,Ore06,Ore07,Ore08,Ore09,Ore10,Ore11,Ore12,Ore13,Ore14	*OreG0001784*
scaffold1231	1694325	G	T	NA	*OreG0001786*
scaffold1231	1694773	A	G	NA	*OreG0001786*
scaffold1231	1708323	A	T	NA	*OreG0001787*
scaffold1231	1715870	C	T	Och02,Och03,Och04,Och05,Och06,Och08,Och09,Och10,Och11,Och12,Och13	*OreG0001790*
scaffold1231	1722355	G	T	Ore03,Ore08,Ore09,Ore10,Ore14	*OreG0001791*
scaffold1231	1722369	A	G	Ore01,Ore02,Ore03,Ore04,Ore05,Ore06,Ore07,Ore08,Ore09,Ore10,Ore11,Ore12,Ore13,Ore14	*OreG0001791*
scaffold1231	1728111	A	G	Och01,Och02,Och03,Och04,Och05,Och06,Och07,Och08,Och09,Och10,Och11,Och12,Och13,Och14,Ore01,Ore02,Ore03,Ore04,Ore05,Ore06,Ore07,Ore08,Ore09,Ore10,Ore11,Ore12,Ore13,Ore14	*OreG0001793*
scaffold1231	1743035	G	A	NA	*OreG0001794*
scaffold1231	1743263	C	T	Och01,Och02,Och03,Och04,Och05,Och06,Och07,Och08,Och09,Och10,Och11,Och12,Och13,Och14	*OreG0001794*
scaffold1231	1744127	A	T	Och01,Och02,Och03,Och04,Och05,Och06,Och07,Och08,Och09,Och10,Och11,Och12,Och13,Och14	*OreG0001794*
scaffold1231	1744451	G	A	Och01,Och02,Och03,Och04,Och05,Och06,Och07,Och08,Och09,Och10,Och11,Och12,Och13,Och14	*OreG0001794*
scaffold1231	1744501	T	C	Ore01,Ore02,Ore03,Ore04,Ore05,Ore06,Ore07,Ore08,Ore09,Ore10,Ore11,Ore12,Ore13,Ore14	*OreG0001794*
scaffold1231	1774460	T	A	NA	*OreG0001796*
scaffold4109	37377	C	G	NA	*OreG0019869*
scaffold4109	37380	C	A	Och02,Och03,Och04,Och05,Och06,Och12,Och13	*OreG0019869*

scaffold4109	59423	C	A	Och01,Och02,Och03,Och04,Och05,Och06,Och07,Och08,Och09,Och10,Och11,Och12,Och13,Och14,Ore01,Ore02,Ore03,Ore04,Ore05,Ore06,Ore07,Ore08,Ore09,Ore10,Ore11,Ore12,Ore13,Ore14	*OreG0019872*
scaffold4109	59440	A	G	Och01,Och07,Och09,Och10,Och11	*OreG0019872*
scaffold4109	59463	T	C	NA	*OreG0019872*
scaffold4109	59475	G	C	Ore01,Ore02,Ore03,Ore04,Ore05,Ore06,Ore07,Ore08,Ore09,Ore10,Ore11,Ore12,Ore13,Ore14	*OreG0019872*
scaffold4109	59499	T	C	Ore04,Ore05	*OreG0019872*
scaffold4109	119283	C	T	NA	*OreG0019879*
scaffold4109	128842	G	A	Och01,Och02,Och03,Och04,Och05,Och06,Och07,Och08,Och09,Och10,Och11,Och12,Och13,Och14	*OreG0019882*
scaffold4109	128866	T	C	NA	*OreG0019882*
scaffold4109	158269	T	C	NA	*OreG0019884*
scaffold4109	163050	A	G	Ore04,Ore05	*OreG0019885*
scaffold4109	185425	G	T	Ore01,Ore02,Ore03,Ore04,Ore05,Ore06,Ore07,Ore08,Ore09,Ore10,Ore11,Ore12,Ore13,Ore14	*OreG0019888*
scaffold4109	186713	G	A	Och01,Och02,Och03,Och04,Och05,Och06,Och07,Och08,Och09,Och10,Och11,Och12,Och13,Och14	*OreG0019889*
scaffold4109	187426	A	C	Och01,Och02,Och03,Och04,Och05,Och06,Och07,Och08,Och09,Och10,Och11,Och12,Och13,Och14	*OreG0019889*
scaffold4109	187444	G	T	Och01,Och02,Och03,Och04,Och05,Och06,Och07,Och08,Och09,Och10,Och11,Och12,Och13,Och14	*OreG0019889*
scaffold4109	187471	C	T	NA	*OreG0019889*
scaffold4109	193852	G	A	NA	*OreG0019892*
scaffold4109	205457	C	A	Och11	*OreG0019894*
scaffold4109	205808	C	T	Ore03,Ore09,Ore10,Ore12,Ore14	*OreG0019894*
scaffold4109	212394	G	T	NA	*OreG0019895*
scaffold4109	212620	C	G	Ore01,Ore02,Ore03,Ore04,Ore05,Ore06,Ore07,Ore08,Ore09,Ore10,Ore11,Ore12,Ore13,Ore14	*OreG0019895*
scaffold4109	213526	A	G	NA	*OreG0019895*
scaffold4109	213638	C	G	Och01,Och02,Och03,Och04,Och05,Och06,Och07,Och08,Och09,Och10,Och11,Och12,Och13,Och14,Ore01,Ore02,Ore03,Ore04,Ore05,Ore06,Ore07,Ore08,Ore09,Ore10,Ore11,Ore12,Ore13,Ore14	*OreG0019895*
scaffold4109	249956	T	A	Och01,Och02,Och03,Och04,Och05,Och06,Och07,Och08,Och09,Och10,Och11,Och12,Och13,Och14,Ore01,Ore02,Ore03,Ore04,Ore05,Ore06,Ore07,Ore08,Ore09,Ore10,Ore11,Ore12,Ore13,Ore14	*OreG0019899*
scaffold4109	250414	T	C	Ore03,Ore04,Ore05,Ore09,Ore10,Ore12,Ore14	*OreG0019899*
scaffold4109	251883	T	C	Ore01,Ore02,Ore03,Ore04,Ore05,Ore06,Ore07,Ore08,Ore09,Ore10,Ore11,Ore12,Ore13,Ore14	*OreG0019899*
scaffold4109	268996	T	C	Och01,Och02,Och03,Och04,Och05,Och06,Och07,Och08,Och09,Och10,Och11,Och12,Och13,Och14,Ore01,Ore02,Ore03,Ore04,Ore05,Ore06,Ore07,Ore08,Ore09,Ore10,Ore11,Ore12,Ore13,Ore14	*OreG0019900*
scaffold4109	270183	C	A	Ore01,Ore02,Ore03,Ore04,Ore05,Ore06,Ore07,Ore08,Ore09,Ore10,Ore11,Ore12,Ore13,Ore14	*OreG0019900*
scaffold4109	289037	C	G	NA	*OreG0019905*
scaffold4109	312845	A	C	NA	*OreG0019907*
scaffold4109	312851	G	A	Ore01,Ore02,Ore03,Ore06,Ore07,Ore08,Ore09,Ore10,Ore11,Ore12,Ore13,Ore14	*OreG0019907*
scaffold4109	312869	C	A	Ore01,Ore02,Ore03,Ore06,Ore07,Ore08,Ore09,Ore10,Ore11,Ore12,Ore13,Ore14	*OreG0019907*
scaffold4109	318064	T	A	Och02,Och03,Och04,Och05,Och06,Och12,Och13,Ore04,Ore05	*OreG0019908*
scaffold4109	331402	A	T	Och14	*OreG0019909*
scaffold4109	384304	C	A	Och02,Och03,Och04,Och05,Och06,Och07,Och08,Och09,Och10,Och12,Och13,Och14,Ore03,Ore09,Ore10,Ore12,Ore14	*OreG0019918*
scaffold4109	404270	G	C	NA	*OreG0019919*
scaffold4109	412163	T	A	NA	*OreG0019919*
scaffold4109	412170	C	T	NA	*OreG0019919*
scaffold4109	412194	G	A	NA	*OreG0019919*
scaffold4109	412514	G	A	Och01,Och02,Och03,Och04,Och05,Och06,Och07,Och08,Och09,Och10,Och11,Och12,Och13,Och14	*OreG0019919*
scaffold4109	412626	T	C	NA	*OreG0019919*
scaffold4109	412892	T	A	Ore04,Ore05	*OreG0019919*
scaffold4109	413027	A	T	Och01	*OreG0019919*
scaffold4109	413031	T	C	NA	*OreG0019919*
scaffold4109	477548	G	A	Och09,Och10,Och11	*OreG0019925*
scaffold4109	477584	G	A	Och01,Och02,Och03,Och04,Och05,Och06,Och07,Och08,Och09,Och10,Och11,Och12,Och13,Och14,Ore01,Ore02,Ore03,Ore04,Ore05,Ore06,Ore07,Ore08,Ore09,Ore10,Ore11,Ore12,Ore13,Ore14	*OreG0019925*
scaffold4109	477844	A	G	Och14,Ore03,Ore04,Ore05,Ore09,Ore10,Ore12,Ore14	*OreG0019925*
scaffold4109	478044	A	T	Och01,Och02,Och03,Och04,Och05,Och06,Och07,Och08,Och09,Och10,Och11,Och12,Och13,Och14,Ore01,Ore02,Ore03,Ore04,Ore05,Ore06,Ore07,Ore08,Ore09,Ore10,Ore11,Ore12,Ore13,Ore14	*OreG0019925*
scaffold4109	478056	G	C	Och14	*OreG0019925*
scaffold4109	478068	G	A	Och01,Och02,Och03,Och04,Och05,Och06,Och07,Och08,Och09,Och10,Och11,Och12,Och13,Och14,Ore01,Ore02,Ore03,Ore04,Ore05,Ore06,Ore07,Ore08,Ore09,Ore10,Ore11,Ore12,Ore13,Ore14	*OreG0019925*
scaffold4109	478144	T	A	Ore03,Ore04,Ore05,Ore09,Ore10,Ore12,Ore14	*OreG0019925*

scaffold4109	478414	C	A	Och14	*OreG0019925*
scaffold4109	478444	A	C	Och01,Och02,Och03,Och04,Och06,Och07,Och08,Och09,Och10,Och11,Och12,Och13,Och14,Ore01,Ore02,Ore03,Ore04,Ore05,Ore06,Ore07,Ore08,Ore09,Ore10,Ore11,Ore12,Ore13,Ore14	*OreG0019925*
scaffold4109	488837	A	G	NA	*OreG0019926*
scaffold4109	508307	G	A	NA	*OreG0019927*
scaffold4109	508595	T	C	NA	*OreG0019927*
scaffold4109	524445	G	C	NA	*OreG0019929*
scaffold4109	531463	C	T	NA	*OreG0019929*
scaffold4109	531514	C	T	Och13	*OreG0019929*
scaffold5880	4671	A	G	NA	*OreG0024045*
scaffold5280	60324	C	A	Och08	*OreG0023578*
scaffold5280	62099	G	A	Och01,Och02,Och03,Och04,Och05,Och06,Och07,Och08,Och09,Och10,Och11,Och12,Och13,Och14,Ore01,Ore02,Ore03,Ore04,Ore05,Ore06,Ore07,Ore08,Ore09,Ore10,Ore11,Ore12,Ore13,Ore14	*OreG0023578*
scaffold5280	64720	T	A	Och08	*OreG0023578*
scaffold5695	24488	C	T	Och01	*OreG0023670*
scaffold3691	85084	C	T	Ore03,Ore09,Ore14	*OreG0019074*
scaffold3691	85113	C	A	NA	*OreG0019074*
scaffold3691	85129	T	C	Och05,Och07,Och12,Och13,Och14	*OreG0019074*
scaffold3662	55182	G	A	NA	*OreG0019034*
scaffold3662	63739	G	C	Och01,Och02,Och03,Och04,Och05,Och06,Och07,Och08,Och09,Och10,Och11,Och12,Och13,Och14,Ore01,Ore02,Ore03,Ore04,Ore05,Ore06,Ore07,Ore08,Ore09,Ore10,Ore11,Ore12,Ore13,Ore14	*OreG0019035*
scaffold3662	78667	G	A	NA	*OreG0019035*
scaffold3662	232831	A	G	Ore01,Ore02,Ore03,Ore04,Ore05,Ore06,Ore07,Ore08,Ore09,Ore10,Ore11,Ore12,Ore13,Ore14	*OreG0019042*
scaffold3662	289129	G	T	NA	*OreG0019045*
scaffold3662	346144	A	T	Och01,Och02,Och03,Och04,Och05,Och06,Och07,Och08,Och09,Och10,Och11,Och12,Och13,Och14,Ore01,Ore02,Ore03,Ore04,Ore05,Ore06,Ore07,Ore08,Ore09,Ore10,Ore11,Ore12,Ore13,Ore14	*OreG0019047*
scaffold3662	375363	C	T	Ore01,Ore02,Ore03,Ore04,Ore05,Ore06,Ore07,Ore08,Ore09,Ore10,Ore11,Ore12,Ore13,Ore14	*OreG0019049*
scaffold3662	386031	A	T	Ore01,Ore02,Ore03,Ore04,Ore05,Ore06,Ore07,Ore08,Ore09,Ore10,Ore11,Ore12,Ore13,Ore14	*OreG0019050*
scaffold3662	397767	C	T	NA	*OreG0019052*
scaffold3662	397783	C	A	Och02,Och04,Och06,Och07,Och08,Och09,Och10,Och11,Och14	*OreG0019052*
scaffold3662	397839	C	A	Och02,Och04,Och06,Och07,Och08,Och09,Och10,Och11,Och14	*OreG0019052*
scaffold3662	397883	T	A	NA	*OreG0019052*
scaffold3662	398045	G	C	Och02,Och04,Och06,Och07,Och08,Och09,Och10,Och11,Och14	*OreG0019052*
scaffold3662	398154	C	A	Ore01,Ore02,Ore03,Ore04,Ore05,Ore06,Ore07,Ore08,Ore09,Ore10,Ore11,Ore12,Ore13,Ore14	*OreG0019052*
scaffold3662	398255	G	A	Och02,Och04,Och06,Och07,Och08,Och09,Och10,Och11,Och14	*OreG0019052*
scaffold3662	398744	A	G	NA	*OreG0019052*
scaffold3662	398933	T	C	Och07	*OreG0019052*
scaffold3662	399000	A	T	Och02,Och04,Och06,Och07,Och08,Och09,Och10,Och11,Och14	*OreG0019052*
scaffold3662	400334	C	T	Och01,Och02,Och03,Och04,Och05,Och06,Och07,Och08,Och09,Och10,Och11,Och12,Och13,Och14,Ore01,Ore02,Ore03,Ore04,Ore05,Ore06,Ore07,Ore08,Ore09,Ore10,Ore11,Ore12,Ore13,Ore14	*OreG0019053*
scaffold3662	408908	C	G	Ore04,Ore05	*OreG0019054*
scaffold3662	417625	T	A	Ore03,Ore08,Ore09,Ore10,Ore14	*OreG0019055*
scaffold3662	418538	G	T	Ore04,Ore05	*OreG0019055*
scaffold3662	418541	G	C	Ore03,Ore08,Ore09,Ore10,Ore14	*OreG0019055*
scaffold2546	521370	T	A	Och01,Och02,Och03,Och04,Och05,Och06,Och07,Och09,Och10,Och11,Och12,Och13,Och14	*OreG0007800*
scaffold2546	535431	A	C	Och01,Och02,Och03,Och04,Och05,Och06,Och07,Och08,Och09,Och10,Och11,Och12,Och13,Och14,Ore01,Ore02,Ore03,Ore04,Ore05,Ore06,Ore07,Ore08,Ore09,Ore10,Ore11,Ore12,Ore13,Ore14	*OreG0007802*
scaffold2546	566760	C	G	NA	*OreG0007804*
scaffold2546	566883	C	A	NA	*OreG0007804*
scaffold2546	567347	G	A	NA	*OreG0007804*
scaffold1157	66582	G	A	Och03	*OreG0000802*
scaffold1157	69673	G	C	Och09,Och10	*OreG0000802*
scaffold1157	79937	C	A	NA	*OreG0000803*
scaffold1157	83104	C	T	Och02,Och03,Och04,Och06,Och13	*OreG0000804*
scaffold1157	83556	A	T	Och03	*OreG0000804*
scaffold1157	84055	C	A	NA	*OreG0000804*
scaffold1157	85729	T	A	Ore09,Ore10	*OreG0000804*
scaffold1157	99522	C	A	NA	*OreG0000806*
scaffold1157	100802	C	A	NA	*OreG0000807*
scaffold1157	102416	T	C	NA	*OreG0000807*
scaffold1157	103647	C	T	Och03	*OreG0000808*
scaffold1157	103700	T	A	Och14	*OreG0000808*
scaffold1157	103815	C	A	NA	*OreG0000808*
scaffold1157	103847	G	A	Och03	*OreG0000808*

scaffold1157	125336	A	T	NA	*OreG0000811*
scaffold1157	152218	T	C	Och03	*OreG0000813*
scaffold1157	152978	A	G	Och01,Och02,Och03,Och04,Och05,Och06,Och07,Och08,Och09,Och10,Och11,Och12,Och13,Och14,Ore01,Ore02,Ore03,Ore04,Ore05,Ore06,Ore07,Ore08,Ore09,Ore10,Ore11,Ore12,Ore13,Ore14	*OreG0000814*
scaffold1157	157630	T	A	Ore04,Ore05	*OreG0000816*
scaffold1157	167079	T	C	Ore04,Ore05	*OreG0000818*
scaffold1157	211315	C	G	NA	*OreG0000825*
scaffold1157	214620	A	T	NA	*OreG0000826*
scaffold1157	214998	G	C	Och03,Och05	*OreG0000826*
scaffold1157	217132	T	G	NA	*OreG0000826*
scaffold1157	217360	C	A	Och01,Och02,Och03,Och04,Och05,Och06,Och07,Och08,Och09,Och10,Och11,Och12,Och13,Och14	*OreG0000826*
scaffold1157	221557	G	A	NA	*OreG0000827*
scaffold1157	228337	C	T	Ore02,Ore04,Ore05,Ore08,Ore09,Ore10,Ore11,Ore13	*OreG0000828*
scaffold1157	228504	C	T	Ore02,Ore04,Ore05,Ore08,Ore09,Ore10,Ore11,Ore13	*OreG0000828*
scaffold1157	230544	C	G	NA	*OreG0000828*
scaffold1157	235373	T	A	Och03,Och05,Och07,Och08	*OreG0000829*
scaffold1157	235816	G	A	NA	*OreG0000829*
scaffold1157	250523	C	T	NA	*OreG0000832*
scaffold1157	254717	G	A	Och01,Och02,Och03,Och04,Och05,Och06,Och09,Och10,Och11,Och12,Och13	*OreG0000832*
scaffold1157	255119	G	A	Ore01,Ore02,Ore03,Ore04,Ore05,Ore06,Ore07,Ore08,Ore09,Ore10,Ore11,Ore12,Ore13,Ore14	*OreG0000832*
scaffold1157	275943	A	G	NA	*OreG0000836*
scaffold1157	280826	G	T	NA	*OreG0000837*
scaffold1157	280891	G	A	NA	*OreG0000837*
scaffold1157	281129	A	T	Och03	*OreG0000837*
scaffold1157	281384	T	G	NA	*OreG0000837*
scaffold1157	281620	A	C	Och01,Och02,Och03,Och04,Och05,Och06,Och07,Och08,Och09,Och10,Och11,Och12,Och13,Och14	*OreG0000837*
scaffold1157	296677	G	T	Och03	*OreG0000839*
scaffold1157	297069	G	C	Och03	*OreG0000839*
scaffold1157	297257	T	G	Och01,Och02,Och03,Och04,Och05,Och06,Och07,Och08,Och09,Och10,Och11,Och12,Och13,Och14	*OreG0000839*
scaffold1157	297808	C	A	NA	*OreG0000839*
scaffold1157	298393	C	T	Ore04,Ore05,Ore09,Ore10	*OreG0000839*
scaffold1157	348021	C	A	NA	*OreG0000845*
scaffold1157	348316	C	T	NA	*OreG0000845*
scaffold1157	348357	C	T	NA	*OreG0000845*
scaffold1157	348363	C	T	NA	*OreG0000845*
scaffold1157	348403	C	T	Och14	*OreG0000845*
scaffold1157	348414	G	T	Och14	*OreG0000845*
scaffold1157	348695	C	T	NA	*OreG0000845*
scaffold1157	348724	C	T	NA	*OreG0000845*
scaffold1157	350042	T	A	NA	*OreG0000845*
scaffold1157	353851	G	A	NA	*OreG0000846*
scaffold1157	354148	C	T	NA	*OreG0000846*
scaffold1157	356034	A	T	NA	*OreG0000846*
scaffold1157	378781	G	A	NA	*OreG0000849*
scaffold1157	399212	G	A	NA	*OreG0000851*
scaffold1157	419354	C	T	NA	*OreG0000855*
scaffold1157	419375	G	A	NA	*OreG0000855*
scaffold1157	419570	C	A	NA	*OreG0000855*
scaffold1157	419627	C	T	NA	*OreG0000855*
scaffold1157	426073	T	C	NA	*OreG0000856*
scaffold1157	465525	C	T	NA	*OreG0000861*
scaffold1157	488605	C	A	Och07,Och08,Och11	*OreG0000863*
scaffold1157	499314	T	A	Och01,Och02,Och03,Och04,Och05,Och06,Och07,Och08,Och09,Och10,Och11,Och12,Och14	*OreG0000864*
scaffold1157	510927	G	A	NA	*OreG0000866*
scaffold1157	510955	T	C	NA	*OreG0000866*
scaffold1157	548947	T	G	Och01,Och02,Och03,Och04,Och05,Och06,Och07,Och08,Och09,Och10,Och11,Och12,Och13,Och14,Ore01,Ore02,Ore03,Ore04,Ore05,Ore06,Ore07,Ore08,Ore09,Ore10,Ore11,Ore12,Ore13,Ore14	*OreG0000869*
scaffold1157	584141	A	C	NA	*OreG0000869*
scaffold1157	604172	G	A	Ore01,Ore04,Ore05	*OreG0000872*
scaffold1157	604752	G	T	NA	*OreG0000872*
scaffold1157	605714	T	C	Ore01,Ore02,Ore03,Ore04,Ore05,Ore06,Ore07,Ore08,Ore09,Ore10,Ore11,Ore12,Ore13,Ore14	*OreG0000872*
scaffold1157	605786	C	T	Ore01,Ore02,Ore03,Ore04,Ore05,Ore06,Ore07,Ore08,Ore09,Ore10,Ore11,Ore12,Ore13,Ore14	*OreG0000872*
scaffold1157	611805	T	A	NA	*OreG0000873*
scaffold1157	611886	C	T	NA	*OreG0000873*
scaffold1157	633264	G	C	NA	*OreG0000877*

scaffold1157	633575	T	G	NA	*OreG0000877*
scaffold1157	656668	A	G	NA	*OreG0000880*
scaffold1157	659832	G	A	NA	*OreG0000881*
scaffold1157	659877	T	C	Och03,Och09,Och10	*OreG0000881*
scaffold1157	661554	G	C	NA	*OreG0000882*
scaffold1157	665668	C	A	NA	*OreG0000883*
scaffold1157	678323	G	A	NA	*OreG0000885*
scaffold1157	678941	C	G	NA	*OreG0000885*
scaffold1157	786484	C	T	Och01,Och02,Och03,Och04,Och05,Och06,Och07,Och08,Och09,Och10,Och11,Och12,Och13,Och14	*OreG0000888*
scaffold1157	790288	C	A	Och01,Och02,Och03,Och04,Och05,Och06,Och07,Och08,Och09,Och10,Och11,Och12,Och13,Och14,Ore01,Ore02,Ore03,Ore04,Ore05,Ore06,Ore07,Ore08,Ore09,Ore10,Ore11,Ore12,Ore13,Ore14	*OreG0000889*
scaffold1157	790530	A	G	Ore01,Ore02,Ore03,Ore04,Ore05,Ore06,Ore07,Ore08,Ore09,Ore10,Ore11,Ore12,Ore13,Ore14	*OreG0000889*
scaffold1157	790842	A	G	NA	*OreG0000889*
scaffold1157	791118	C	T	Och01,Och02,Och03,Och04,Och05,Och06,Och07,Och08,Och09,Och10,Och11,Och12,Och13,Och14,Ore01,Ore02,Ore03,Ore04,Ore05,Ore06,Ore07,Ore08,Ore09,Ore10,Ore11,Ore12,Ore13,Ore14	*OreG0000889*
scaffold1157	796673	T	C	Ore01,Ore02,Ore03,Ore04,Ore05,Ore06,Ore07,Ore08,Ore09,Ore10,Ore11,Ore12,Ore13,Ore14	*OreG0000890*
scaffold1157	797840	C	T	NA	*OreG0000890*
scaffold1157	798580	T	C	NA	*OreG0000890*
scaffold1157	805298	T	C	NA	*OreG0000891*
scaffold1157	805361	T	A	NA	*OreG0000891*
scaffold1157	805433	C	T	NA	*OreG0000891*
scaffold1157	833492	C	T	Ore01,Ore02,Ore03,Ore04,Ore05,Ore06,Ore07,Ore08,Ore09,Ore10,Ore11,Ore12,Ore13,Ore14	*OreG0000895*
scaffold1157	833591	G	A	Och01,Och02,Och03,Och04,Och05,Och06,Och07,Och08,Och12,Och13,Och14	*OreG0000895*
scaffold1157	839042	T	A	Ore03,Ore09,Ore10,Ore12,Ore14	*OreG0000896*
scaffold1157	923153	T	A	Och03	*OreG0000902*
scaffold1157	928995	T	A	Och01,Och07	*OreG0000902*
scaffold1157	928999	T	G	Och01,Och07	*OreG0000902*
scaffold1157	929806	G	C	Och09,Och10	*OreG0000902*
scaffold1157	929827	G	A	Och03,Och05,Och14	*OreG0000902*
scaffold1157	941435	A	G	NA	*OreG0000905*
scaffold1157	941916	C	T	NA	*OreG0000905*
scaffold1157	941928	C	T	NA	*OreG0000905*
scaffold1157	941930	A	G	NA	*OreG0000905*
scaffold1157	941948	A	G	NA	*OreG0000905*
scaffold1157	944722	G	A	NA	*OreG0000906*
scaffold1157	948594	G	A	NA	*OreG0000906*
scaffold1157	968605	T	A	NA	*OreG0000910*
scaffold1157	985003	C	T	NA	*OreG0000911*
scaffold1157	985107	T	C	NA	*OreG0000911*
scaffold1157	1014862	C	G	Och01,Och02,Och03,Och04,Och05,Och06,Och07,Och08,Och09,Och10,Och11,Och12,Och13,Och14,Ore01,Ore02,Ore03,Ore04,Ore05,Ore06,Ore07,Ore08,Ore09,Ore10,Ore11,Ore12,Ore13,Ore14	*OreG0000914*
scaffold1157	1052801	A	G	Ore01,Ore02,Ore03,Ore04,Ore05,Ore06,Ore07,Ore08,Ore09,Ore10,Ore11,Ore12,Ore13,Ore14	*OreG0000917*
scaffold1157	1052993	G	C	Och01,Och02,Och03,Och04,Och05,Och06,Och07,Och08,Och09,Och10,Och11,Och12,Och13,Och14	*OreG0000917*
scaffold1157	1064237	A	T	NA	*OreG0000918*
scaffold1157	1065179	G	A	NA	*OreG0000918*
scaffold1157	1065468	C	T	Och01,Och02,Och03,Och04,Och05,Och06,Och07,Och08,Och09,Och10,Och11,Och12,Och13,Och14,Ore01,Ore02,Ore03,Ore04,Ore05,Ore06,Ore07,Ore08,Ore09,Ore10,Ore11,Ore12,Ore13,Ore14	*OreG0000918*
scaffold1157	1098975	T	C	Och01,Och02,Och03,Och04,Och05,Och06,Och07,Och08,Och09,Och10,Och11,Och12,Och13,Och14	*OreG0000921*
scaffold1157	1122495	T	G	NA	*OreG0000923*
scaffold1157	1138703	C	A	NA	*OreG0000925*
scaffold1157	1171259	C	T	Ore01,Ore02,Ore03,Ore04,Ore05,Ore06,Ore07,Ore08,Ore09,Ore10,Ore11,Ore12,Ore13,Ore14	*OreG0000928*
scaffold1157	1171931	A	T	Ore03,Ore09,Ore10,Ore12,Ore14	*OreG0000928*
scaffold1157	1173338	T	C	Och03,Och05	*OreG0000928*
scaffold1157	1173440	C	T	NA	*OreG0000928*
scaffold1157	1173454	G	A	Och08	*OreG0000928*
scaffold1157	1173470	C	T	Ore01,Ore02,Ore03,Ore04,Ore05,Ore06,Ore07,Ore08,Ore09,Ore10,Ore11,Ore12,Ore13,Ore14	*OreG0000928*
scaffold1157	1173524	C	T	Ore01,Ore02,Ore03,Ore04,Ore05,Ore06,Ore07,Ore08,Ore09,Ore10,Ore11,Ore12,Ore13,Ore14	*OreG0000928*
scaffold1157	1173705	C	G	Ore03,Ore09,Ore10,Ore12,Ore14	*OreG0000928*
scaffold1157	1173954	G	A	NA	*OreG0000928*
scaffold1157	1173993	G	T	Ore04,Ore05	*OreG0000928*
scaffold1157	1183333	C	A	NA	*OreG0000930*

scaffold1157	1183434	C	T	Och01,Och02,Och03,Och04,Och05,Och06,Och07,Och08,Och09,Och10,Och11,Och12,Och13,Och14,Ore01,Ore02,Ore03,Ore04,Ore05,Ore06,Ore07,Ore08,Ore09,Ore10,Ore11,Ore12,Ore13,Ore14	*OreG0000930*
scaffold1157	1183511	G	T	Och03	*OreG0000930*
scaffold1157	1224716	G	A	Ore04,Ore05	*OreG0000933*
scaffold1157	1225270	T	A	Och01,Och02,Och03,Och04,Och06,Och07,Och13,Och14	*OreG0000934*
scaffold1157	1252638	G	A	Och03	*OreG0000938*
scaffold1157	1253410	T	C	NA	*OreG0000938*
scaffold1157	1255033	C	G	Och01,Och02,Och03,Och04,Och05,Och06,Och07,Och08,Och09,Och10,Och11,Och12,Och13,Och14,Ore01,Ore02,Ore03,Ore04,Ore05,Ore06,Ore07,Ore08,Ore09,Ore10,Ore11,Ore12,Ore13,Ore14	*OreG0000940*
scaffold1157	1277778	G	A	Och12	*OreG0000942*
scaffold1157	1277791	C	T	Och01,Och02,Och03,Och04,Och05,Och06,Och07,Och08,Och09,Och10,Och11,Och12,Och13,Och14,Ore01,Ore02,Ore03,Ore04,Ore05,Ore06,Ore07,Ore08,Ore09,Ore10,Ore11,Ore12,Ore13,Ore14	*OreG0000942*
scaffold1157	1278432	C	T	Och01,Och02,Och03,Och04,Och05,Och06,Och07,Och08,Och09,Och10,Och11,Och12,Och13,Och14,Ore01,Ore02,Ore03,Ore04,Ore05,Ore06,Ore07,Ore08,Ore09,Ore10,Ore11,Ore12,Ore13,Ore14	*OreG0000942*
scaffold1157	1281842	G	A	NA	*OreG0000943*
scaffold1157	1282199	G	A	NA	*OreG0000943*
scaffold1157	1291626	C	T	NA	*OreG0000944*
scaffold1157	1307562	A	G	Ore01,Ore02,Ore03,Ore04,Ore05,Ore06,Ore07,Ore08,Ore09,Ore10,Ore11,Ore12,Ore13,Ore14	*OreG0000946*
scaffold1157	1307617	C	T	Ore01,Ore02,Ore03,Ore04,Ore05,Ore06,Ore07,Ore08,Ore09,Ore10,Ore11,Ore12,Ore13,Ore14	*OreG0000946*
scaffold1157	1307716	G	A	Och01,Och02,Och03,Och04,Och05,Och06,Och07,Och08,Och09,Och10,Och11,Och12,Och13,Och14	*OreG0000946*
scaffold1157	1326582	C	G	Och01,Och02,Och03,Och04,Och05,Och06,Och07,Och08,Och09,Och10,Och11,Och12,Och13,Och14,Ore01,Ore02,Ore03,Ore04,Ore05,Ore06,Ore07,Ore08,Ore09,Ore10,Ore11,Ore12,Ore13,Ore14	*OreG0000947*
scaffold1157	1326658	C	G	Ore01,Ore02,Ore03,Ore04,Ore05,Ore06,Ore07,Ore08,Ore09,Ore10,Ore11,Ore12,Ore13,Ore14	*OreG0000947*
scaffold1157	1326692	A	T	Och02,Och03,Och04,Och06,Och07,Och08,Och13,Och14	*OreG0000947*
scaffold1157	1327255	G	T	NA	*OreG0000947*
scaffold1157	1353869	G	T	Ore04,Ore05	*OreG0000949*
scaffold1157	1372489	T	A	Ore01,Ore02,Ore03,Ore04,Ore05,Ore06,Ore07,Ore08,Ore09,Ore10,Ore11,Ore12,Ore13,Ore14	*OreG0000952*
scaffold1157	1376060	T	A	Och01,Och02,Och03,Och04,Och06,Och08,Och13	*OreG0000952*
scaffold1157	1376983	A	C	Ore01,Ore02,Ore03,Ore04,Ore05,Ore06,Ore07,Ore08,Ore09,Ore10,Ore11,Ore12,Ore13,Ore14	*OreG0000952*
scaffold1157	1391526	A	T	Ore01,Ore02,Ore03,Ore06,Ore07,Ore08,Ore09,Ore10,Ore11,Ore12,Ore13,Ore14	*OreG0000954*
scaffold1157	1407738	C	A	Ore04,Ore05	*OreG0000955*
scaffold1157	1408220	A	C	NA	*OreG0000955*
scaffold1157	1412939	T	A	Ore01,Ore02,Ore03,Ore04,Ore05,Ore06,Ore07,Ore08,Ore09,Ore10,Ore11,Ore12,Ore13,Ore14	*OreG0000956*
scaffold1157	1556738	C	T	Och09,Och10	*OreG0000967*
scaffold1157	1563612	C	T	Ore01,Ore02,Ore03,Ore04,Ore05,Ore06,Ore07,Ore08,Ore09,Ore10,Ore11,Ore12,Ore13,Ore14	*OreG0000968*
scaffold1157	1563868	G	A	Ore01,Ore02,Ore03,Ore04,Ore05,Ore06,Ore07,Ore08,Ore09,Ore10,Ore11,Ore12,Ore13,Ore14	*OreG0000968*
scaffold1157	1651397	T	C	Och07	*OreG0000972*
scaffold1157	1655391	C	T	Och01,Och02,Och03,Och04,Och05,Och06,Och07,Och08,Och09,Och10,Och11,Och12,Och13,Och14	*OreG0000972*
scaffold1157	1801094	G	T	Och06,Och08,Och13	*OreG0000981*
scaffold1157	1802033	C	T	NA	*OreG0000981*
scaffold1157	1822790	G	C	Och01,Och02,Och03,Och04,Och05,Och06,Och07,Och08,Och09,Och10,Och11,Och12,Och13,Och14,Ore01,Ore02,Ore03,Ore04,Ore05,Ore06,Ore07,Ore08,Ore09,Ore10,Ore11,Ore12,Ore13,Ore14	*OreG0000983*
scaffold1157	1900357	C	T	Ore01,Ore02,Ore03,Ore04,Ore05,Ore06,Ore07,Ore08,Ore09,Ore10,Ore11,Ore12,Ore13,Ore14	*OreG0000988*
scaffold1157	1903765	C	T	Ore01,Ore02,Ore03,Ore04,Ore05,Ore06,Ore07,Ore08,Ore09,Ore10,Ore11,Ore12,Ore13,Ore14	*OreG0000988*
scaffold1157	1908787	C	T	NA	*OreG0000989*
scaffold1157	2080610	T	A	Och01,Och02,Och03,Och04,Och05,Och06,Och07,Och08,Och11,Och12,Och13,Och14	*OreG0000991*
scaffold1157	2080658	C	A	NA	*OreG0000991*
scaffold1157	2135100	A	G	Och02,Och03,Och06,Och07,Och08,Och09,Och10,Och14	*OreG0000996*
scaffold1157	2136407	C	T	NA	*OreG0000996*
scaffold1157	2136544	C	T	Och01,Och09,Och10,Och11	*OreG0000996*
scaffold1157	2136767	G	A	Ore02,Ore03,Ore06,Ore07,Ore08,Ore09,Ore10,Ore11,Ore12,Ore13,Ore14	*OreG0000997*
scaffold1157	2160664	C	T	NA	*OreG0000999*
scaffold1157	2160670	G	A	NA	*OreG0000999*
scaffold1157	2160772	G	T	NA	*OreG0000999*
scaffold1157	2161051	T	A	NA	*OreG0000999*
scaffold1157	2161135	T	C	NA	*OreG0000999*

scaffold1157	2237058	G	A	NA	*OreG0001001*
scaffold1157	2243273	G	A	Och02,Och04,Och06,Och08	*OreG0001001*
scaffold1157	2253823	T	G	NA	*OreG0001001*
scaffold1157	2337626	T	C	NA	*OreG0001003*
scaffold1157	2345102	C	T	Ore01,Ore02,Ore03,Ore04,Ore05,Ore06,Ore07,Ore08,Ore09,Ore10,Ore11,Ore12,Ore13,Ore14	*OreG0001003*
scaffold1157	2365078	C	G	Och01,Och02,Och03,Och04,Och05,Och06,Och07,Och08,Och09,Och10,Och11,Och12,Och13,Och14,Ore01,Ore02,Ore03,Ore04,Ore05,Ore06,Ore07,Ore08,Ore09,Ore10,Ore11,Ore12,Ore13,Ore14	*OreG0001004*
scaffold1157	2492081	T	C	NA	*OreG0001006*
scaffold1157	2492295	G	T	Ore04,Ore05	*OreG0001006*
scaffold1157	2492316	C	T	Ore04,Ore05	*OreG0001006*
scaffold1157	2529378	A	G	Ore01,Ore02,Ore03,Ore04,Ore05,Ore06,Ore07,Ore08,Ore09,Ore10,Ore11,Ore12,Ore13,Ore14	*OreG0001007*
scaffold6632	644	G	T	NA	*OreG0024348*
scaffold6632	2958	G	A	NA	*OreG0024349*
scaffold6632	3087	A	T	Ore02,Ore03,Ore06,Ore07,Ore09,Ore10,Ore11,Ore12,Ore13,Ore14	*OreG0024349*
scaffold6632	3101	A	G	Och01,Och02,Och03,Och04,Och05,Och06,Och07,Och08,Och09,Och11,Och12,Och13,Och14,Ore01,Ore02,Ore03,Ore04,Ore05,Ore06,Ore07,Ore08,Ore09,Ore10,Ore11,Ore12,Ore13,Ore14	*OreG0024349*
scaffold5055	6179	G	A	NA	*OreG0023531*
scaffold5055	46833	C	T	NA	*OreG0023534*
scaffold5055	99948	G	T	NA	*OreG0023535*
scaffold5055	103674	G	A	NA	*OreG0023535*
scaffold5055	122268	C	T	NA	*OreG0023536*
scaffold5055	122596	A	G	Och02,Och04,Och08,Och09,Och10,Och11,Och12,Och14,Ore01,Ore02,Ore03,Ore04,Ore05,Ore06,Ore07,Ore08,Ore09,Ore10,Ore11,Ore12,Ore13,Ore14	*OreG0023536*
scaffold5055	124860	A	T	Och07,Och08	*OreG0023536*
scaffold5055	124864	C	T	Och12,Och13	*OreG0023536*
scaffold5055	130863	T	C	NA	*OreG0023537*
scaffold5055	130873	G	T	NA	*OreG0023537*
scaffold5055	146494	A	G	Ore01,Ore02,Ore03,Ore04,Ore05,Ore06,Ore07,Ore08,Ore09,Ore10,Ore11,Ore12,Ore13,Ore14	*OreG0023539*
scaffold5055	344350	C	T	Och01,Och02,Och03,Och04,Och05,Och06,Och07,Och08,Och09,Och10,Och11,Och12,Och13,Och14,Ore01,Ore02,Ore03,Ore04,Ore05,Ore06,Ore07,Ore08,Ore09,Ore10,Ore11,Ore12,Ore13,Ore14	*OreG0023550*
scaffold5055	344948	G	A	Och01,Och02,Och03,Och04,Och05,Och06,Och07,Och08,Och09,Och10,Och11,Och12,Och13,Och14,Ore01,Ore02,Ore03,Ore04,Ore05,Ore06,Ore07,Ore08,Ore09,Ore10,Ore11,Ore12,Ore13,Ore14	*OreG0023550*
scaffold5055	355480	C	T	NA	*OreG0023551*
scaffold1891	15440	G	A	NA	*OreG0004624*
scaffold1891	67868	T	C	Ore02,Ore03,Ore04,Ore05,Ore06,Ore07,Ore08,Ore09,Ore10,Ore11,Ore12,Ore13,Ore14	*OreG0004625*
scaffold1891	187365	T	G	NA	*OreG0004630*
scaffold1891	193871	T	A	Och01,Och02,Och03,Och04,Och05,Och06,Och07,Och08,Och09,Och10,Och11,Och12,Och13,Och14,Ore01,Ore02,Ore03,Ore04,Ore05,Ore06,Ore07,Ore08,Ore09,Ore10,Ore11,Ore12,Ore13,Ore14	*OreG0004631*
scaffold1891	223587	A	G	Och01,Och02,Och03,Och04,Och05,Och06,Och07,Och08,Och09,Och10,Och11,Och12,Och13,Och14,Ore01,Ore02,Ore03,Ore04,Ore05,Ore06,Ore07,Ore08,Ore09,Ore10,Ore11,Ore12,Ore13,Ore14	*OreG0004633*
scaffold1891	250777	A	T	Och04,Och06	*OreG0004634*
scaffold1891	258253	C	A	Och01,Och02,Och03,Och04,Och05,Och06,Och07,Och08,Och09,Och10,Och11,Och12,Och13,Och14	*OreG0004636*
scaffold1891	263180	C	T	Och01,Och02,Och03,Och04,Och05,Och06,Och07,Och08,Och11,Och12,Och13,Och14	*OreG0004636*
scaffold1891	265665	C	T	NA	*OreG0004637*
scaffold1891	290783	G	T	Och04,Och06	*OreG0004638*
scaffold1891	292817	C	T	Och01,Och02,Och03,Och04,Och05,Och06,Och07,Och08,Och09,Och10,Och11,Och12,Och13,Och14,Ore01,Ore02,Ore03,Ore04,Ore05,Ore06,Ore07,Ore08,Ore09,Ore10,Ore11,Ore12,Ore13,Ore14	*OreG0004638*
scaffold1891	420958	A	T	NA	*OreG0004647*
scaffold1891	421070	G	A	NA	*OreG0004647*
scaffold1891	437137	A	G	NA	*OreG0004648*
scaffold1891	437436	G	C	Och01,Och02,Och03,Och04,Och05,Och06,Och07,Och08,Och09,Och10,Och11,Och12,Och13,Och14,Ore01,Ore02,Ore03,Ore04,Ore05,Ore06,Ore07,Ore08,Ore09,Ore10,Ore11,Ore12,Ore13,Ore14	*OreG0004648*
scaffold1891	437443	T	C	Och01,Och02,Och03,Och04,Och05,Och06,Och07,Och08,Och09,Och10,Och11,Och12,Och13,Och14	*OreG0004648*
scaffold1891	481504	G	A	Ore03	*OreG0004652*
scaffold1891	484710	G	A	NA	*OreG0004652*
scaffold1891	573171	G	A	Ore01,Ore02,Ore03,Ore04,Ore05,Ore06,Ore07,Ore08,Ore09,Ore10,Ore11,Ore12,Ore13,Ore14	*OreG0004655*
scaffold1891	575064	G	A	Ore01,Ore02,Ore03,Ore04,Ore05,Ore06,Ore07,Ore08,Ore09,Ore10,Ore11,Ore12,Ore13,Ore14	*OreG0004655*
scaffold1891	599455	G	A	Ore02,Ore03,Ore04,Ore05,Ore06,Ore07,Ore08,Ore09,Ore10,Ore11,Ore12,Ore13,Ore14	*OreG0004658*

scaffold1891	608439	C	A	Och02,Och03,Och04,Och05,Och06,Och12,Och13,Ore02,Ore03,Ore04,Ore05,Ore06,Ore07,Ore08,Ore09,Ore10,Ore11,Ore12,Ore13,Ore14	*OreG0004658*
scaffold1891	825970	G	A	Och11	*OreG0004670*
scaffold1891	825982	C	T	Och02,Och03,Och04,Och05,Och06,Och12,Och13	*OreG0004670*
scaffold1891	825984	G	A	Och01,Och02,Och03,Och04,Och05,Och06,Och07,Och12,Och13,Och14	*OreG0004670*
scaffold1891	879226	G	T	NA	*OreG0004672*
scaffold1891	879311	G	A	NA	*OreG0004672*
scaffold1891	966794	T	C	NA	*OreG0004674*
scaffold1891	979622	A	T	NA	*OreG0004674*
scaffold1891	981153	A	G	NA	*OreG0004674*
scaffold1891	1008519	C	G	NA	*OreG0004675*
scaffold1891	1026324	T	A	Och11	*OreG0004678*
scaffold1891	1026346	A	T	NA	*OreG0004678*
scaffold1891	1026472	T	C	Ore01,Ore02,Ore03,Ore04,Ore05,Ore06,Ore07,Ore08,Ore09,Ore10,Ore11,Ore12,Ore13,Ore14	*OreG0004678*
scaffold1891	1030452	C	T	Och01,Och02,Och03,Och04,Och05,Och06,Och07,Och08,Och09,Och10,Och11,Och12,Och13,Och14,Ore01,Ore02,Ore03,Ore04,Ore05,Ore06,Ore07,Ore08,Ore09,Ore10,Ore11,Ore12,Ore13,Ore14	*OreG0004679*
scaffold1891	1055249	C	A	Och01,Och02,Och03,Och04,Och05,Och06,Och07,Och08,Och09,Och10,Och11,Och12,Och13,Och14,Ore01,Ore02,Ore03,Ore04,Ore05,Ore06,Ore07,Ore08,Ore09,Ore10,Ore11,Ore12,Ore13,Ore14	*OreG0004681*
scaffold1891	1055523	C	A	Och01,Och04,Och05,Och08,Och09,Och10,Och11,Och12	*OreG0004681*
scaffold1891	1055773	A	C	Och01,Och02,Och03,Och04,Och05,Och06,Och07,Och08,Och09,Och10,Och11,Och12,Och13,Och14,Ore01,Ore02,Ore03,Ore04,Ore05,Ore06,Ore07,Ore08,Ore09,Ore10,Ore11,Ore12,Ore13,Ore14	*OreG0004681*
scaffold1891	1121337	C	T	NA	*OreG0004682*
scaffold1891	1133574	G	T	Och01,Och02,Och03,Och04,Och05,Och06,Och07,Och08,Och09,Och10,Och11,Och12,Och13	*OreG0004683*
scaffold1891	1156571	G	T	Och01,Och02,Och03,Och04,Och05,Och06,Och07,Och08,Och09,Och10,Och11,Och12,Och13,Och14	*OreG0004684*
scaffold1891	1183842	C	T	NA	*OreG0004686*
scaffold1891	1184506	C	T	NA	*OreG0004686*
scaffold1891	1231930	C	G	Ore01,Ore02,Ore03,Ore04,Ore05,Ore06,Ore07,Ore08,Ore09,Ore10,Ore11,Ore12,Ore13,Ore14	*OreG0004687*
scaffold1891	1233696	G	T	NA	*OreG0004687*
scaffold1891	1239317	G	A	NA	*OreG0004688*
scaffold1891	1271341	T	G	Och01,Och02,Och03,Och04,Och05,Och06,Och07,Och08,Och09,Och10,Och11,Och12,Och13,Och14,Ore01,Ore02,Ore03,Ore04,Ore05,Ore06,Ore07,Ore08,Ore09,Ore10,Ore11,Ore12,Ore13,Ore14	*OreG0004690*
scaffold1891	1272185	C	A	Och01,Och02,Och03,Och04,Och05,Och06,Och07,Och08,Och09,Och10,Och11,Och12,Och13,Och14	*OreG0004691*
scaffold1891	1272385	G	A	Ore01,Ore02,Ore03,Ore04,Ore05,Ore06,Ore07,Ore08,Ore09,Ore10,Ore11,Ore12,Ore13,Ore14	*OreG0004691*
scaffold1891	1272439	A	G	NA	*OreG0004691*
scaffold1891	1281721	C	G	NA	*OreG0004692*
scaffold1891	1289873	A	G	NA	*OreG0004693*
scaffold1891	1291400	G	C	NA	*OreG0004693*
scaffold1891	1291469	C	G	NA	*OreG0004693*
scaffold1891	1291813	C	A	Och02,Och03,Och07	*OreG0004693*
scaffold1891	1302788	G	A	Och01,Och02,Och03,Och04,Och05,Och06,Och07,Och08,Och09,Och10,Och11,Och12,Och13,Och14,Ore01,Ore02,Ore03,Ore04,Ore05,Ore06,Ore07,Ore08,Ore09,Ore10,Ore11,Ore12,Ore13,Ore14	*OreG0004694*
scaffold1891	1302833	G	A	NA	*OreG0004694*
scaffold1891	1317565	G	A	NA	*OreG0004695*
scaffold1891	1349668	A	G	Och01,Och02,Och03,Och04,Och05,Och06,Och07,Och08,Och09,Och10,Och11,Och12,Och13,Och14	*OreG0004699*
scaffold1891	1349692	G	T	Och01,Och02,Och03,Och04,Och05,Och06,Och07,Och08,Och09,Och10,Och11,Och12,Och13,Och14,Ore01,Ore02,Ore03,Ore04,Ore05,Ore06,Ore07,Ore08,Ore09,Ore10,Ore11,Ore12,Ore13,Ore14	*OreG0004699*
scaffold1891	1349724	G	C	NA	*OreG0004699*
scaffold1891	1407063	C	A	Ore01,Ore02,Ore03,Ore04,Ore05,Ore06,Ore07,Ore08,Ore09,Ore10,Ore11,Ore12,Ore14	*OreG0004703*
scaffold1891	1455339	C	T	Och01,Och02,Och03,Och04,Och05,Och06,Och07,Och08,Och09,Och10,Och11,Och12,Och13,Och14	*OreG0004705*
scaffold1891	1455621	T	C	Och01,Och02,Och03,Och04,Och05,Och06,Och07,Och08,Och09,Och10,Och11,Och12,Och13,Och14	*OreG0004705*
scaffold1891	1457658	A	T	Ore01,Ore03,Ore06,Ore09	*OreG0004705*
scaffold1891	1457679	A	G	Ore01,Ore02,Ore03,Ore04,Ore05,Ore06,Ore07,Ore08,Ore09,Ore10,Ore11,Ore12,Ore13,Ore14	*OreG0004705*
scaffold1891	1466445	C	A	Ore01,Ore03,Ore06,Ore09	*OreG0004707*
scaffold1891	1467928	A	G	NA	*OreG0004707*
scaffold1891	1476302	C	T	Ore04,Ore05,Ore13	*OreG0004709*
scaffold1891	1476549	G	T	NA	*OreG0004709*
scaffold1891	1480505	T	C	Ore01,Ore02,Ore03,Ore04,Ore05,Ore06,Ore07,Ore08,Ore09,Ore10,Ore11,Ore12,Ore13,Ore14	*OreG0004709*

scaffold1891	1493768	T	A	Ore01,Ore02,Ore03,Ore04,Ore05,Ore06,Ore07,Ore08,Ore09,Ore10,Ore11,Ore12,Ore13,Ore14	*OreG0004711*
scaffold1891	1517649	T	A	Och01,Och02,Och03,Och04,Och05,Och06,Och07,Och08,Och09,Och10,Och11,Och12,Och13,Och14	*OreG0004712*
scaffold1891	1518375	T	A	NA	*OreG0004712*
scaffold1891	1524744	G	A	Och02,Och04,Och05	*OreG0004713*
scaffold1891	1524895	G	C	NA	*OreG0004713*
scaffold1891	1524978	C	T	NA	*OreG0004713*
scaffold1891	1525029	T	G	NA	*OreG0004713*
scaffold1891	1525187	C	T	NA	*OreG0004713*
scaffold1891	1525620	G	A	NA	*OreG0004713*
scaffold1891	1578782	T	C	NA	*OreG0004718*
scaffold1891	1578797	T	G	Ore01,Ore02,Ore03,Ore04,Ore05,Ore06,Ore07,Ore08,Ore09,Ore10,Ore11,Ore12,Ore13,Ore14	*OreG0004718*
scaffold1891	1602679	C	T	NA	*OreG0004720*
scaffold1891	1602704	G	A	Ore01,Ore02,Ore03,Ore04,Ore05,Ore06,Ore07,Ore08,Ore09,Ore10,Ore11,Ore12,Ore13,Ore14	*OreG0004720*
scaffold1891	1634856	G	A	Och11	*OreG0004721*
scaffold1891	1649819	A	G	Och01,Och02,Och03,Och04,Och05,Och06,Och07,Och08,Och09,Och10,Och11,Och12,Och13,Och14,Ore01,Ore02,Ore03,Ore04,Ore05,Ore06,Ore07,Ore08,Ore09,Ore10,Ore11,Ore12,Ore13,Ore14	*OreG0004723*
scaffold1891	1656645	T	A	Och01,Och02,Och03,Och04,Och05,Och06,Och07,Och08,Och09,Och10,Och11,Och12,Och13,Och14,Ore01,Ore02,Ore03,Ore04,Ore05,Ore06,Ore07,Ore08,Ore09,Ore10,Ore11,Ore12,Ore13,Ore14	*OreG0004724*
scaffold1891	1684354	C	T	Ore01,Ore02,Ore03,Ore04,Ore05,Ore06,Ore07,Ore08,Ore09,Ore10,Ore11,Ore12,Ore13,Ore14	*OreG0004726*
scaffold1891	1684406	T	C	Och02,Och03,Och04,Och05,Och06,Och12,Och13	*OreG0004726*
scaffold1891	1684466	A	G	NA	*OreG0004726*
scaffold1891	1730592	A	G	NA	*OreG0004729*
scaffold1891	1730644	A	T	NA	*OreG0004729*
scaffold1891	1730745	G	A	NA	*OreG0004729*
scaffold1891	1730781	G	C	NA	*OreG0004729*
scaffold1891	1730862	C	T	NA	*OreG0004729*
scaffold1891	1730941	A	G	NA	*OreG0004729*
scaffold1891	1731146	C	A	NA	*OreG0004729*
scaffold1891	1758248	T	A	NA	*OreG0004732*
scaffold1891	1763737	G	C	Och11	*OreG0004732*
scaffold1891	1777418	T	A	NA	*OreG0004732*
scaffold1891	1810610	A	T	NA	*OreG0004734*
scaffold1891	1839325	G	T	Och01,Och02,Och03,Och04,Och05,Och06,Och07,Och08,Och09,Och10,Och11,Och12,Och13,Och14,Ore01,Ore02,Ore03,Ore04,Ore05,Ore06,Ore07,Ore08,Ore09,Ore10,Ore11,Ore12,Ore13,Ore14	*OreG0004737*
scaffold1891	1866621	G	A	Och09,Och10,Och11	*OreG0004742*
scaffold1891	1866722	G	T	NA	*OreG0004742*
scaffold1891	1869444	T	A	Och01,Och04,Och09,Och10,Och11	*OreG0004743*
scaffold1891	1869753	G	A	Ore01,Ore02,Ore03,Ore04,Ore05,Ore06,Ore07,Ore08,Ore09,Ore10,Ore11,Ore12,Ore13,Ore14	*OreG0004743*
scaffold1891	1949531	A	T	NA	*OreG0004750*
scaffold1891	1972809	T	C	NA	*OreG0004754*
scaffold1891	2001848	T	C	NA	*OreG0004757*
scaffold1891	2007136	G	A	NA	*OreG0004758*
scaffold1891	2007198	C	A	Och01,Och03,Och07,Och09,Och10,Och11,Och12	*OreG0004758*
scaffold1891	2007387	C	T	NA	*OreG0004758*
scaffold1891	2011460	T	C	NA	*OreG0004758*
scaffold1891	2012969	A	C	Ore01,Ore02,Ore03,Ore04,Ore05,Ore06,Ore07,Ore08,Ore09,Ore10,Ore11,Ore12,Ore13,Ore14	*OreG0004758*
scaffold1891	2075312	T	A	Ore01,Ore02,Ore03,Ore04,Ore05,Ore06,Ore07,Ore08,Ore09,Ore10,Ore11,Ore12,Ore13,Ore14	*OreG0004761*
scaffold1891	2153140	A	G	Och01,Och02,Och03,Och04,Och05,Och06,Och07,Och08,Och09,Och10,Och11,Och12,Och13,Och14,Ore01,Ore02,Ore03,Ore04,Ore05,Ore06,Ore07,Ore08,Ore09,Ore10,Ore11,Ore12,Ore13,Ore14	*OreG0004763*
scaffold1891	2153232	G	A	Ore01,Ore02,Ore03,Ore04,Ore05,Ore06,Ore07,Ore08,Ore09,Ore10,Ore11,Ore12,Ore13,Ore14	*OreG0004763*
scaffold1891	2184415	A	G	NA	*OreG0004765*
scaffold1891	2184425	A	G	Ore01,Ore02,Ore03,Ore04,Ore05,Ore06,Ore07,Ore08,Ore09,Ore10,Ore11,Ore12,Ore13,Ore14	*OreG0004765*
scaffold1891	2184456	C	A	Ore01,Ore02,Ore03,Ore04,Ore05,Ore06,Ore07,Ore08,Ore09,Ore10,Ore11,Ore12,Ore13,Ore14	*OreG0004765*
scaffold1891	2371925	G	A	Ore01,Ore02,Ore03,Ore04,Ore05,Ore06,Ore07,Ore08,Ore09,Ore10,Ore11,Ore12,Ore13,Ore14	*OreG0004774*
scaffold1891	2372191	G	A	Och01,Och02,Och03,Och04,Och05,Och06,Och08,Och09,Och10,Och11,Och12,Och13	*OreG0004774*
scaffold1891	2372502	G	C	NA	*OreG0004774*
scaffold1891	2393354	C	A	NA	*OreG0004776*
scaffold1891	2393377	G	C	NA	*OreG0004776*

scaffold1891	2401676	G	T	Ore04,Ore05	*OreG0004777*
scaffold1891	2532333	T	G	Ore01,Ore02,Ore03,Ore04,Ore05,Ore06,Ore07,Ore08,Ore09,Ore10,Ore11,Ore12,Ore13,Ore14	*OreG0004782*
scaffold1891	2688618	C	T	Och01,Och02,Och03,Och04,Och05,Och06,Och07,Och08,Och09,Och10,Och11,Och12,Och13,Och14	*OreG0004787*
scaffold1891	2688736	G	T	Ore01,Ore02,Ore03,Ore04,Ore05,Ore06,Ore07,Ore08,Ore09,Ore10,Ore11,Ore12,Ore13,Ore14	*OreG0004787*
scaffold1891	2703860	A	T	Ore01,Ore02,Ore03,Ore04,Ore05,Ore06,Ore07,Ore08,Ore09,Ore10,Ore11,Ore12,Ore13,Ore14	*OreG0004789*
scaffold1891	2758883	C	T	Och04	*OreG0004791*
scaffold1891	2777355	C	T	Ore04,Ore05	*OreG0004791*
scaffold1891	2780344	C	A	Och01,Och02,Och03,Och04,Och05,Och06,Och07,Och08,Och09,Och10,Och11,Och12,Och13,Och14,Ore01,Ore02,Ore03,Ore04,Ore05,Ore06,Ore07,Ore08,Ore09,Ore10,Ore11,Ore12,Ore13,Ore14	*OreG0004791*
scaffold1891	2780584	G	C	Och01,Och07	*OreG0004791*
scaffold1891	2786310	G	A	Och11	*OreG0004791*
scaffold1891	2786368	A	G	NA	*OreG0004791*
scaffold1891	2787683	A	T	Och02,Och03,Och04,Och05,Och06,Och11,Och12,Och13	*OreG0004791*
scaffold1891	2788228	A	T	Ore04,Ore05	*OreG0004791*
scaffold1891	2790031	G	A	NA	*OreG0004791*
scaffold1891	2794785	A	G	NA	*OreG0004791*
scaffold1891	2795245	T	C	Och01,Och07	*OreG0004791*
scaffold1891	2888365	C	T	Och01,Och07	*OreG0004794*
scaffold1891	2903243	C	T	NA	*OreG0004795*
scaffold1891	2903540	G	A	Och01,Och02,Och03,Och04,Och05,Och06,Och07,Och08,Och09,Och10,Och11,Och12,Och13,Och14,Ore01,Ore02,Ore03,Ore04,Ore05,Ore06,Ore07,Ore08,Ore09,Ore10,Ore11,Ore12,Ore13,Ore14	*OreG0004795*
scaffold1891	2928309	G	A	Ore01,Ore02,Ore03,Ore04,Ore05,Ore06,Ore07,Ore08,Ore09,Ore10,Ore11,Ore12,Ore13,Ore14	*OreG0004796*
scaffold1891	2928541	C	T	Ore01,Ore02,Ore03,Ore04,Ore05,Ore06,Ore07,Ore08,Ore09,Ore10,Ore11,Ore12,Ore13,Ore14	*OreG0004796*
scaffold1891	3027017	A	C	Och01,Och04,Och06,Och07,Och09,Och10,Och11,Och13	*OreG0004798*
scaffold1891	3148024	G	T	Och01,Och02,Och03,Och04,Och05,Och06,Och07,Och08,Och09,Och10,Och11,Och12,Och13,Och14,Ore01,Ore02,Ore03,Ore04,Ore05,Ore06,Ore07,Ore08,Ore09,Ore10,Ore11,Ore12,Ore13,Ore14	*OreG0004801*
scaffold1891	3283771	G	C	Och01,Och02,Och03,Och04,Och05,Och06,Och07,Och08,Och09,Och10,Och11,Och12,Och13,Och14,Ore01,Ore02,Ore03,Ore04,Ore05,Ore06,Ore07,Ore08,Ore09,Ore10,Ore11,Ore12,Ore13,Ore14	*OreG0004809*
scaffold1891	3307049	A	G	Ore06,Ore07,Ore08,Ore13,Ore14	*OreG0004810*
scaffold1891	3370863	A	T	NA	*OreG0004812*
scaffold1891	3385043	T	G	Ore01,Ore02,Ore03,Ore04,Ore05,Ore06,Ore07,Ore08,Ore09,Ore10,Ore11,Ore12,Ore13,Ore14	*OreG0004813*
scaffold1891	3387010	A	G	Ore01,Ore11	*OreG0004813*
scaffold1891	3387278	G	T	NA	*OreG0004813*
scaffold1891	3387425	T	A	NA	*OreG0004813*
scaffold1891	3387442	G	C	Ore01,Ore02,Ore03,Ore04,Ore05,Ore06,Ore07,Ore08,Ore09,Ore10,Ore11,Ore12,Ore13,Ore14	*OreG0004813*
scaffold1891	3399140	T	A	Och01,Och02,Och03,Och04,Och05,Och06,Och07,Och08,Och09,Och10,Och11,Och12,Och13,Och14,Ore01,Ore02,Ore03,Ore04,Ore05,Ore06,Ore07,Ore08,Ore09,Ore10,Ore11,Ore12,Ore13,Ore14	*OreG0004814*
scaffold1891	3402758	A	C	Och01,Och02,Och03,Och04,Och05,Och06,Och07,Och08,Och09,Och10,Och11,Och12,Och13,Och14,Ore01,Ore02,Ore03,Ore04,Ore05,Ore06,Ore07,Ore08,Ore09,Ore10,Ore11,Ore12,Ore13,Ore14	*OreG0004814*
scaffold1891	3429181	A	G	Och01,Och02,Och03,Och04,Och05,Och06,Och07,Och08,Och09,Och10,Och11,Och12,Och13,Och14,Ore01,Ore02,Ore03,Ore04,Ore05,Ore06,Ore07,Ore08,Ore09,Ore10,Ore11,Ore12,Ore13,Ore14	*OreG0004816*
scaffold1891	3430288	C	T	Och02,Och03,Och06	*OreG0004816*
scaffold1891	3430368	A	G	NA	*OreG0004816*
scaffold6319	34857	A	C	NA	*OreG0024262*
scaffold6319	35277	C	A	NA	*OreG0024262*
scaffold3426	85720	T	A	Och01,Och02,Och03,Och04,Och05,Och06,Och07,Och08,Och09,Och10,Och11,Och12,Och13,Och14,Ore01,Ore02,Ore03,Ore04,Ore05,Ore06,Ore07,Ore08,Ore09,Ore10,Ore11,Ore12,Ore13,Ore14	*OreG0018572*
scaffold3426	91551	T	C	NA	*OreG0018573*
scaffold3426	92162	G	A	Ore03,Ore04,Ore05,Ore09,Ore13	*OreG0018573*
scaffold3426	302135	T	A	NA	*OreG0018590*
scaffold3426	324526	C	T	NA	*OreG0018592*
scaffold3426	324674	T	C	NA	*OreG0018592*
scaffold3426	325072	G	A	NA	*OreG0018592*
scaffold3426	325187	G	A	NA	*OreG0018592*
scaffold3426	325208	G	T	NA	*OreG0018592*
scaffold3426	326386	G	T	NA	*OreG0018592*
scaffold3426	326391	G	A	NA	*OreG0018592*
scaffold3426	326502	A	T	NA	*OreG0018592*
scaffold3426	326550	G	C	NA	*OreG0018592*

scaffold3426	369264	T	C	NA	*OreG0018593*
scaffold3426	409959	A	G	Och01,Och09,Och10	*OreG0018594*
scaffold3426	411747	G	A	NA	*OreG0018595*
scaffold3426	423893	G	A	Och01,Och02,Och03,Och04,Och05,Och06,Och07,Och08,Och09,Och10,Och11,Och12,Och13,Och14,Ore01,Ore02,Ore03,Ore04,Ore05,Ore06,Ore07,Ore08,Ore09,Ore10,Ore11,Ore12,Ore13,Ore14	*OreG0018596*
scaffold3426	424244	A	T	Och04,Ore01,Ore02,Ore03,Ore04,Ore05,Ore06,Ore07,Ore08,Ore09,Ore10,Ore11,Ore12,Ore13,Ore14	*OreG0018596*
scaffold3426	424322	G	A	Och01,Och02,Och03,Och04,Och05,Och06,Och07,Och08,Och09,Och10,Och11,Och12,Och13,Och14,Ore01,Ore02,Ore03,Ore04,Ore05,Ore06,Ore07,Ore08,Ore09,Ore10,Ore11,Ore12,Ore13,Ore14	*OreG0018596*
scaffold3426	424328	C	T	NA	*OreG0018596*
scaffold3426	424337	G	A	Och01,Och02,Och03,Och04,Och05,Och06,Och07,Och08,Och09,Och10,Och11,Och12,Och13,Och14,Ore01,Ore02,Ore03,Ore04,Ore05,Ore06,Ore07,Ore08,Ore09,Ore10,Ore11,Ore12,Ore13,Ore14	*OreG0018596*
scaffold3426	446448	T	A	Och01,Och02,Och03,Och04,Och05,Och06,Och07,Och08,Och09,Och10,Och11,Och12,Och13,Och14,Ore01,Ore02,Ore03,Ore04,Ore05,Ore06,Ore07,Ore08,Ore09,Ore10,Ore11,Ore12,Ore13,Ore14	*OreG0018597*
scaffold4159	3737	A	C	NA	*OreG0019932*
scaffold4159	4044	C	G	NA	*OreG0019932*
scaffold4159	9234	G	A	Och03,Och05	*OreG0019933*
scaffold4159	9300	T	C	NA	*OreG0019933*
scaffold4159	12306	C	T	Och07,Och08,Och09,Och10,Och12,Och13,Ore01,Ore02,Ore03,Ore04,Ore05,Ore06,Ore07,Ore08,Ore09,Ore10,Ore11,Ore12,Ore13,Ore14	*OreG0019934*
scaffold4159	12370	T	C	NA	*OreG0019934*
scaffold4159	12495	T	A	NA	*OreG0019934*
scaffold4159	41480	C	T	Och02,Och03,Och04,Och05,Och06	*OreG0019936*
scaffold4159	41545	G	T	Och01,Och03,Och05,Och07,Och08,Och09,Och10,Och12,Och13,Och14,Ore01,Ore02,Ore03,Ore04,Ore05,Ore06,Ore07,Ore08,Ore09,Ore10,Ore11,Ore12,Ore13,Ore14	*OreG0019936*
scaffold4159	41847	A	G	Och03,Och05	*OreG0019936*
scaffold4159	41849	T	G	Och01,Och02,Och03,Och04,Och05,Och06,Och07,Och08,Och09,Och10,Och11,Och12,Och13,Och14,Ore01,Ore02,Ore03,Ore04,Ore05,Ore06,Ore07,Ore08,Ore09,Ore10,Ore11,Ore12,Ore13,Ore14	*OreG0019936*
scaffold4159	46010	T	G	Och01,Och07,Och08,Och09,Och10,Och12,Och13,Och14,Ore01,Ore02,Ore03,Ore04,Ore05,Ore06,Ore07,Ore08,Ore09,Ore10,Ore11,Ore12,Ore13,Ore14	*OreG0019937*
scaffold4159	46396	C	T	Och03,Och05	*OreG0019937*
scaffold4159	46429	T	A	NA	*OreG0019937*
scaffold4159	46567	T	C	Och01,Och07,Och08,Och09,Och10,Och12,Och13,Och14,Ore01,Ore02,Ore03,Ore04,Ore05,Ore06,Ore07,Ore08,Ore09,Ore10,Ore11,Ore12,Ore13,Ore14	*OreG0019938*
scaffold4159	47649	C	T	Och02,Och03,Och04,Och05,Och06	*OreG0019938*
scaffold4159	53955	G	A	Och02,Och04,Och06	*OreG0019939*
scaffold4159	54269	T	C	Och01,Och02,Och03,Och04,Och05,Och06,Och07,Och08,Och09,Och10,Och11,Och12,Och13,Och14,Ore01,Ore02,Ore03,Ore04,Ore05,Ore06,Ore07,Ore08,Ore09,Ore10,Ore11,Ore12,Ore13,Ore14	*OreG0019940*
scaffold4159	54355	T	G	NA	*OreG0019940*
scaffold4159	64581	G	A	Ore06,Ore07,Ore08,Ore14	*OreG0019942*
scaffold4159	75206	C	A	NA	*OreG0019943*
scaffold4159	88533	T	G	NA	*OreG0019944*
scaffold4159	88599	C	A	NA	*OreG0019944*
scaffold4159	88653	G	A	NA	*OreG0019944*
scaffold4159	88677	G	T	NA	*OreG0019944*
scaffold4159	98574	G	A	NA	*OreG0019944*
scaffold4159	101023	C	T	Och02,Och03,Och04,Och05,Och06	*OreG0019945*
scaffold4159	101112	G	A	NA	*OreG0019945*
scaffold4159	109993	T	C	NA	*OreG0019947*
scaffold4159	110183	G	A	NA	*OreG0019947*
scaffold4159	118242	A	T	NA	*OreG0019948*
scaffold4159	168640	T	G	NA	*OreG0019952*
scaffold4159	169229	T	C	Och01,Och02,Och03,Och04,Och05,Och06,Och09,Och10,Och11,Och12,Och13,Och14,Ore06,Ore07,Ore08,Ore12,Ore13,Ore14	*OreG0019952*
scaffold4159	176882	C	T	Och01	*OreG0019953*
scaffold4159	176927	G	A	Ore06,Ore07,Ore08,Ore12,Ore13,Ore14	*OreG0019953*
scaffold4159	177154	T	G	Ore01,Ore04,Ore05	*OreG0019953*
scaffold4159	177531	A	G	Och03,Och05,Och11	*OreG0019953*
scaffold4159	177806	C	T	Och03,Och05	*OreG0019953*
scaffold4159	183592	G	A	Och01,Och02,Och03,Och04,Och05,Och06,Och11,Och12,Och13,Och14	*OreG0019953*
scaffold4159	184099	G	A	Och01,Och03,Och05,Och09,Och10,Och11	*OreG0019953*
scaffold4159	185434	G	A	NA	*OreG0019953*
scaffold4159	185768	T	A	NA	*OreG0019953*
scaffold4159	187213	C	T	Och03,Och05	*OreG0019954*
scaffold4159	191250	G	A	Och01,Och02,Och03,Och04,Och05,Och06,Och09,Och11,Och12,Och13,Och14,Ore01,Ore02,Ore03,Ore04,Ore05,Ore06,Ore07,Ore08,Ore09,Ore10,Ore11,Ore12,Ore13,Ore14	*OreG0019955*
scaffold4159	191281	C	T	NA	*OreG0019955*
scaffold4159	191461	G	A	NA	*OreG0019955*

scaffold4159	192022	T	G	NA	*OreG0019955*
scaffold4159	192989	G	T	NA	*OreG0019955*
scaffold4159	207997	G	A	NA	*OreG0019955*
scaffold4159	208036	G	A	NA	*OreG0019955*
scaffold4159	209661	C	A	NA	*OreG0019955*
scaffold4159	209743	A	C	NA	*OreG0019955*
scaffold4159	217258	T	C	Och01,Och02,Och03,Och04,Och05,Och06,Och07,Och08,Och09,Och10,Och11,Och12,Och13,Och14,Ore01,Ore02,Ore03,Ore04,Ore05,Ore06,Ore07,Ore08,Ore09,Ore10,Ore11,Ore12,Ore13,Ore14	*OreG0019956*
scaffold4159	217711	G	A	Och01,Och02,Och03,Och04,Och05,Och06,Och07,Och08,Och09,Och10,Och11,Och12,Och13,Och14,Ore01,Ore02,Ore03,Ore04,Ore05,Ore06,Ore07,Ore08,Ore09,Ore10,Ore11,Ore12,Ore13,Ore14	*OreG0019956*
scaffold4159	220011	A	G	Och03,Och05	*OreG0019957*
scaffold4159	220350	A	T	Och12,Och13	*OreG0019957*
scaffold4159	220369	G	A	Och03,Och05	*OreG0019957*
scaffold4159	224132	G	A	Och03,Och05	*OreG0019957*
scaffold4159	225936	C	G	Och01,Och02,Och03,Och04,Och05,Och06	*OreG0019957*
scaffold4159	226161	T	C	Ore06,Ore07,Ore08,Ore14	*OreG0019957*
scaffold4159	226287	T	C	Och12,Och13,Ore01,Ore02,Ore03,Ore04,Ore05,Ore06,Ore07,Ore08,Ore09,Ore10,Ore11,Ore12,Ore13,Ore14	*OreG0019957*
scaffold4159	226469	G	T	Och03,Och05	*OreG0019957*
scaffold4159	233109	C	T	NA	*OreG0019958*
scaffold4159	237722	G	A	Och03,Och05	*OreG0019958*
scaffold4159	237917	T	C	Och01	*OreG0019958*
scaffold4159	238063	C	G	Och03,Och05	*OreG0019958*
scaffold4159	242681	C	G	Och01,Och02,Och03,Och04,Och05,Och06	*OreG0019959*
scaffold4159	242729	C	T	Och12,Och13,Ore01,Ore02,Ore03,Ore04,Ore05,Ore06,Ore07,Ore08,Ore09,Ore10,Ore11,Ore12,Ore13,Ore14	*OreG0019959*
scaffold4159	242747	C	T	Ore04,Ore05,Ore06,Ore07,Ore08,Ore12,Ore13,Ore14	*OreG0019959*
scaffold4159	242830	T	C	Ore04,Ore05,Ore06,Ore07,Ore08,Ore12,Ore13,Ore14	*OreG0019959*
scaffold4159	242875	C	A	Och12,Och13,Ore01,Ore02,Ore03,Ore04,Ore05,Ore06,Ore07,Ore08,Ore09,Ore10,Ore11,Ore12,Ore13,Ore14	*OreG0019959*
scaffold4159	242881	T	G	Och12,Och13,Ore01,Ore02,Ore03,Ore04,Ore05,Ore06,Ore07,Ore08,Ore09,Ore10,Ore11,Ore12,Ore13,Ore14	*OreG0019959*
scaffold4159	242891	A	C	Och12,Och13,Ore01,Ore02,Ore03,Ore04,Ore05,Ore06,Ore07,Ore08,Ore09,Ore10,Ore11,Ore12,Ore13,Ore14	*OreG0019959*
scaffold4159	242906	G	A	Och01,Och02,Och03,Och04,Och05,Och06	*OreG0019959*
scaffold4159	243111	A	G	Och12,Och13	*OreG0019959*
scaffold4159	243203	C	T	Och12,Och13,Ore01,Ore02,Ore03,Ore04,Ore05,Ore06,Ore07,Ore08,Ore09,Ore10,Ore11,Ore12,Ore13,Ore14	*OreG0019959*
scaffold4159	243433	G	A	Och12,Och13,Ore01,Ore02,Ore03,Ore04,Ore05,Ore06,Ore07,Ore08,Ore09,Ore10,Ore11,Ore12,Ore13,Ore14	*OreG0019959*
scaffold4159	243772	C	T	Och01,Och02,Och03,Och04,Och05,Och06	*OreG0019959*
scaffold4159	243793	C	T	Och01,Och02,Och03,Och04,Och05,Och06,Och07,Och08,Och09,Och10,Och11,Och12,Och13,Och14,Ore01,Ore02,Ore03,Ore04,Ore05,Ore06,Ore07,Ore08,Ore09,Ore10,Ore11,Ore12,Ore13,Ore14	*OreG0019959*
scaffold4159	243799	C	T	Och12,Och13,Ore01,Ore02,Ore03,Ore04,Ore05,Ore06,Ore07,Ore08,Ore09,Ore10,Ore11,Ore12,Ore13,Ore14	*OreG0019959*
scaffold4159	243823	G	A	Och01,Och02,Och03,Och04,Och05,Och06,Och07,Och08,Och09,Och10,Och11,Och12,Och13,Och14,Ore01,Ore02,Ore03,Ore04,Ore05,Ore06,Ore07,Ore08,Ore09,Ore10,Ore11,Ore12,Ore13,Ore14	*OreG0019959*
scaffold4159	243921	C	A	Och01,Och02,Och03,Och04,Och05,Och06	*OreG0019959*
scaffold4159	244559	G	A	Och01,Och02,Och03,Och04,Och05,Och06	*OreG0019959*
scaffold4159	244927	G	A	Och01,Och02,Och03,Och04,Och05,Och06	*OreG0019959*
scaffold4159	245017	C	A	Och01,Och02,Och03,Och04,Och05,Och06	*OreG0019959*
scaffold4159	245065	G	A	Och12,Och13,Ore01,Ore02,Ore03,Ore04,Ore05,Ore06,Ore07,Ore08,Ore09,Ore10,Ore11,Ore12,Ore13,Ore14	*OreG0019959*
scaffold4159	245078	T	C	Och12,Och13	*OreG0019959*
scaffold4159	245135	G	A	Och12,Och13,Ore01,Ore02,Ore03,Ore04,Ore05,Ore06,Ore07,Ore08,Ore09,Ore10,Ore11,Ore12,Ore13,Ore14	*OreG0019959*
scaffold4159	245188	G	A	Och01,Och02,Och03,Och04,Och05,Och06	*OreG0019959*
scaffold4159	245213	C	T	NA	*OreG0019959*
scaffold4159	245234	C	T	Ore04,Ore05,Ore06,Ore07,Ore08,Ore12,Ore13,Ore14	*OreG0019959*
scaffold4159	245503	C	T	Och12,Och13,Ore01,Ore02,Ore03,Ore04,Ore05,Ore06,Ore07,Ore08,Ore09,Ore10,Ore11,Ore12,Ore13,Ore14	*OreG0019959*
scaffold4159	245626	T	C	Och12,Och13	*OreG0019959*
scaffold4159	245632	G	A	Och12,Och13,Ore01,Ore02,Ore03,Ore04,Ore05,Ore06,Ore07,Ore08,Ore09,Ore10,Ore11,Ore12,Ore13,Ore14	*OreG0019959*
scaffold4159	245752	T	C	Och12,Och13,Ore01,Ore02,Ore03,Ore04,Ore05,Ore06,Ore07,Ore08,Ore09,Ore10,Ore11,Ore12,Ore13,Ore14	*OreG0019959*
scaffold4159	245803	G	A	Och01	*OreG0019959*
scaffold4159	254152	C	G	Och07,Och08,Och12,Och13,Ore01,Ore02,Ore03,Ore04,Ore05,Ore06,Ore07,Ore08,Ore09,Ore10,Ore11,Ore12,Ore13,Ore14	*OreG0019961*
scaffold4159	254202	T	C	Och03,Och05	*OreG0019961*
scaffold4159	254230	G	C	Och01	*OreG0019961*

scaffold4159	254314	A	T	NA	*OreG0019961*
scaffold4159	256425	T	C	Och03,Och05	*OreG0019961*
scaffold4159	256975	G	A	Och03,Och05	*OreG0019961*
scaffold4159	256999	G	A	Och03,Och05	*OreG0019961*
scaffold4159	257003	C	T	Och01	*OreG0019961*
scaffold4159	257005	G	A	Och03,Och05	*OreG0019961*
scaffold4159	257247	G	A	Och01	*OreG0019961*
scaffold4159	258678	T	C	Och03,Och05	*OreG0019961*
scaffold4159	258688	G	A	Och01	*OreG0019961*
scaffold4159	259213	T	C	Och01	*OreG0019961*
scaffold4159	259409	C	T	NA	*OreG0019961*
scaffold4159	267493	T	G	NA	*OreG0019962*
scaffold4159	268019	C	T	Och01,Och02,Och03,Och04,Och05,Och06,Och07,Och08,Och09,Och10,Och11,Och12,Och13,Och14,Ore01,Ore02,Ore03,Ore04,Ore05,Ore06,Ore07,Ore08,Ore09,Ore10,Ore11,Ore12,Ore13,Ore14	*OreG0019962*
scaffold4159	283124	C	T	Och07,Och09,Och10,Och11,Och12,Och13,Ore01,Ore02,Ore03,Ore04,Ore05,Ore06,Ore07,Ore08,Ore09,Ore10,Ore11,Ore12,Ore13,Ore14	*OreG0019964*
scaffold4159	283165	G	A	Och01,Och02,Och03,Och04,Och05,Och06,Och07,Och08,Och09,Och10,Och11,Och12,Och13,Och14,Ore01,Ore02,Ore03,Ore04,Ore05,Ore06,Ore07,Ore08,Ore09,Ore10,Ore11,Ore12,Ore13,Ore14	*OreG0019964*
scaffold4159	283190	C	T	Och01,Och03,Och05	*OreG0019964*
scaffold4159	283256	G	A	Och12,Och13	*OreG0019964*
scaffold4159	283261	T	C	Och01,Och03,Och05	*OreG0019964*
scaffold4159	283292	C	T	Och12,Och13	*OreG0019964*
scaffold4159	283303	G	A	Ore01,Ore02,Ore03,Ore04,Ore05,Ore06,Ore07,Ore08,Ore09,Ore10,Ore11,Ore12,Ore13,Ore14	*OreG0019964*
scaffold4159	283325	G	A	Och07	*OreG0019964*
scaffold4159	287991	T	A	Och02,Och03,Och04,Och05,Och06,Och12,Och13	*OreG0019965*
scaffold4159	288327	C	T	Och01,Och02,Och03,Och04,Och05,Och06,Och09,Och10,Och12,Och13	*OreG0019965*
scaffold4159	288966	G	T	Och12,Och13	*OreG0019965*
scaffold4159	290403	T	C	NA	*OreG0019965*
scaffold4159	298257	T	A	Och01,Och02,Och03,Och04,Och05,Och06,Och07,Och09,Och10,Och11,Och12,Och13,Och14,Ore01,Ore02,Ore03,Ore04,Ore05,Ore06,Ore07,Ore08,Ore09,Ore10,Ore11,Ore12,Ore13,Ore14	*OreG0019966*
scaffold4159	301787	C	A	Och09,Och10,Och12,Och13	*OreG0019967*
scaffold4159	319196	T	A	Och02,Och03,Och04,Och05,Och06	*OreG0019970*
scaffold4159	320099	T	A	Och02,Och03,Och04,Och05,Och06,Ore01,Ore02,Ore03,Ore04,Ore05,Ore06,Ore07,Ore08,Ore09,Ore10,Ore11,Ore12,Ore13,Ore14	*OreG0019970*
scaffold4159	320124	G	C	Ore06,Ore07,Ore08,Ore14	*OreG0019970*
scaffold4159	326244	A	T	NA	*OreG0019971*
scaffold4159	335086	G	A	Ore02,Ore03,Ore06,Ore07,Ore08,Ore09,Ore10,Ore11,Ore14	*OreG0019973*
scaffold4159	338574	A	G	NA	*OreG0019973*
scaffold4159	339547	T	A	Och01,Och02,Och03,Och04,Och05,Och06,Och07,Och08,Och12,Och13	*OreG0019973*
scaffold4159	341721	G	T	Ore02,Ore03,Ore06,Ore07,Ore08,Ore09,Ore10,Ore11,Ore14	*OreG0019973*
scaffold4159	396017	G	A	NA	*OreG0019978*
scaffold4159	396166	G	T	Ore01,Ore02,Ore03,Ore04,Ore05,Ore06,Ore07,Ore08,Ore09,Ore10,Ore11,Ore12,Ore13,Ore14	*OreG0019978*
scaffold4159	405092	G	A	NA	*OreG0019980*
scaffold4159	410797	G	A	Ore01,Ore02,Ore03,Ore04,Ore05,Ore06,Ore07,Ore08,Ore09,Ore10,Ore11,Ore12,Ore13,Ore14	*OreG0019980*
scaffold4159	413884	A	G	NA	*OreG0019981*
scaffold4159	415974	C	T	Och08,Och11	*OreG0019982*
scaffold4159	451992	G	A	NA	*OreG0019986*
scaffold4159	455087	A	T	Ore06,Ore07,Ore08,Ore12,Ore13,Ore14	*OreG0019987*
scaffold4159	463916	C	T	NA	*OreG0019988*
scaffold4159	488603	A	C	NA	*OreG0019991*
scaffold4159	491538	A	G	Och01,Och02,Och03,Och04,Och05,Och06,Och07,Och08,Och09,Och10,Och11,Och14,Ore01,Ore02,Ore03,Ore04,Ore05,Ore06,Ore07,Ore08,Ore09,Ore10,Ore11,Ore12,Ore13,Ore14	*OreG0019992*
scaffold4159	491653	T	C	Och01,Och02,Och03,Och04,Och05,Och06,Och07,Och08,Och09,Och10,Och11,Och12,Och13,Och14,Ore01,Ore02,Ore03,Ore04,Ore05,Ore06,Ore07,Ore08,Ore09,Ore10,Ore11,Ore12,Ore13,Ore14	*OreG0019992*
scaffold4159	547332	T	A	NA	*OreG0019997*
scaffold4159	554063	C	A	NA	*OreG0019998*
scaffold4159	554072	G	A	NA	*OreG0019998*
scaffold4159	554074	G	A	NA	*OreG0019998*
scaffold4159	554092	C	T	NA	*OreG0019998*
scaffold4159	560719	C	T	NA	*OreG0019999*
scaffold4159	561380	G	T	Och01,Och02,Och03,Och04,Och05,Och06,Och07,Och08,Och09,Och10,Och11,Och12,Och13,Och14,Ore01,Ore02,Ore03,Ore04,Ore05,Ore06,Ore07,Ore08,Ore09,Ore10,Ore11,Ore12,Ore13,Ore14	*OreG0019999*
scaffold4159	565910	G	T	Och01,Och02,Och03,Och04,Och05,Och06,Och07,Och08,Och09,Och10,Och11,Och12,Och13,Och14,Ore01,Ore02,Ore03,Ore04,Ore05,Ore06,Ore07,Ore08,Ore09,Ore10,Ore11,Ore12,Ore13,Ore14	*OreG0020000*
scaffold4159	566695	A	C	NA	*OreG0020000*

scaffold4159	590966	A	T	Och01,Och12,Och13	*OreG0020002*
scaffold4159	638826	A	G	NA	*OreG0020005*
scaffold4159	645522	C	T	NA	*OreG0020006*
scaffold4159	654062	G	A	Och01,Och02,Och03,Och04,Och05,Och06,Och07,Och08,Och09,Och10,Och11,Och12,Och13,Och14	*OreG0020007*
scaffold4159	708870	A	G	Och01,Och02,Och03,Och04,Och05,Och06,Och07,Och08,Och09,Och10,Och11,Och12,Och13,Och14	*OreG0020010*
scaffold4159	718028	C	A	Och01	*OreG0020011*
scaffold4159	718271	T	C	Och01,Och02,Och03,Och04,Och05,Och06,Och07,Och08,Och09,Och10,Och11,Och12,Och13,Och14,Ore01,Ore02,Ore03,Ore04,Ore05,Ore06,Ore07,Ore08,Ore09,Ore10,Ore11,Ore12,Ore13,Ore14	*OreG0020011*
scaffold4159	718734	G	A	Och01,Och02,Och03,Och04,Och05,Och06,Och07,Och08,Och09,Och10,Och11,Och12,Och13,Och14,Ore01,Ore02,Ore03,Ore04,Ore05,Ore06,Ore07,Ore08,Ore09,Ore10,Ore11,Ore12,Ore13,Ore14	*OreG0020011*
scaffold4159	719928	A	C	Ore01,Ore02,Ore03,Ore04,Ore05,Ore06,Ore07,Ore08,Ore09,Ore10,Ore11,Ore12,Ore13,Ore14	*OreG0020011*
scaffold4159	734964	G	T	NA	*OreG0020015*
scaffold4159	735156	A	G	Och01,Och02,Och03,Och04,Och05,Och06,Och07,Och08,Och09,Och10,Och11,Och12,Och13,Och14	*OreG0020015*
scaffold4159	742422	C	T	Och01,Och02,Och03,Och04,Och05,Och06,Och07,Och08,Och09,Och10,Och11,Och12,Och13,Och14,Ore01,Ore02,Ore03,Ore04,Ore05,Ore06,Ore07,Ore08,Ore09,Ore10,Ore11,Ore12,Ore13,Ore14	*OreG0020016*
scaffold4159	753120	T	C	NA	*OreG0020018*
scaffold4159	753415	G	A	NA	*OreG0020018*
scaffold4159	766393	G	C	NA	*OreG0020021*
scaffold4159	767766	C	G	NA	*OreG0020021*
scaffold4159	788982	G	T	NA	*OreG0020024*
scaffold4159	809854	T	C	NA	*OreG0020026*
scaffold4159	812396	A	T	Och07,Och08,Och11	*OreG0020026*
scaffold4159	859593	G	A	Och01	*OreG0020028*
scaffold4159	874271	T	C	Och01,Och02,Och03,Och04,Och05,Och06,Och07,Och08,Och09,Och10,Och11,Och12,Och13,Och14	*OreG0020030*
scaffold4159	874299	T	A	Och01,Och02,Och03,Och04,Och05,Och06,Och07,Och08,Och09,Och10,Och11,Och12,Och13,Och14	*OreG0020030*
scaffold4159	874347	C	T	Och01,Och02,Och03,Och04,Och05,Och06,Och07,Och08,Och09,Och10,Och11,Och12,Och13,Och14,Ore01,Ore02,Ore03,Ore04,Ore05,Ore06,Ore07,Ore08,Ore09,Ore10,Ore11,Ore12,Ore13,Ore14	*OreG0020030*
scaffold4159	874449	G	A	NA	*OreG0020030*
scaffold4159	891995	A	C	Ore06,Ore07,Ore08	*OreG0020033*
scaffold4159	913397	A	T	Och01,Och02,Och03,Och04,Och05,Och06,Och07,Och08,Och09,Och10,Och11,Och12,Och13,Och14,Ore01,Ore02,Ore03,Ore04,Ore05,Ore06,Ore07,Ore08,Ore09,Ore10,Ore11,Ore12,Ore13,Ore14	*OreG0020034*
scaffold4159	915517	A	C	NA	*OreG0020034*
scaffold4159	919310	A	G	Och02,Och03,Och05	*OreG0020035*
scaffold4159	919402	T	G	Ore01,Ore02,Ore03,Ore04,Ore05,Ore06,Ore07,Ore08,Ore09,Ore10,Ore11,Ore12,Ore13,Ore14	*OreG0020035*
scaffold4159	919481	C	T	Och01,Och02,Och03,Och04,Och05,Och06,Och07,Och08,Och09,Och10,Och11,Och12,Och13,Och14,Ore01,Ore02,Ore03,Ore04,Ore05,Ore06,Ore07,Ore08,Ore09,Ore10,Ore11,Ore12,Ore13,Ore14	*OreG0020035*
scaffold4159	933732	G	C	NA	*OreG0020036*
scaffold4159	937234	T	A	Ore02,Ore06,Ore07,Ore08,Ore11	*OreG0020037*
scaffold4159	940217	A	T	Och12,Och13	*OreG0020038*
scaffold4159	941118	G	T	NA	*OreG0020038*
scaffold4159	953954	T	C	NA	*OreG0020040*
scaffold4159	964342	C	T	Och01,Och02,Och03,Och04,Och05,Och06,Och07,Och08,Och09,Och10,Och11,Och12,Och13,Och14,Ore01,Ore02,Ore03,Ore04,Ore05,Ore06,Ore07,Ore08,Ore09,Ore10,Ore11,Ore12,Ore13,Ore14	*OreG0020042*
scaffold4159	964813	C	G	NA	*OreG0020042*
scaffold4159	965642	A	C	Och12,Och13	*OreG0020042*
scaffold4159	977398	A	C	NA	*OreG0020044*
scaffold4159	981836	C	T	Och01,Och02,Och03,Och04,Och05,Och06,Och07,Och08,Och09,Och10,Och11,Och12,Och13,Och14,Ore01,Ore02,Ore03,Ore04,Ore05,Ore06,Ore07,Ore08,Ore09,Ore10,Ore11,Ore12,Ore13,Ore14	*OreG0020045*
scaffold4159	994672	A	T	NA	*OreG0020047*
scaffold4159	1003949	A	C	NA	*OreG0020048*
scaffold4159	1004773	A	T	Ore02,Ore06,Ore07,Ore08,Ore11	*OreG0020048*
scaffold4159	1041209	A	T	NA	*OreG0020053*
scaffold4159	1054550	A	G	NA	*OreG0020055*
scaffold4159	1055263	C	T	Ore01,Ore02,Ore03,Ore04,Ore05,Ore06,Ore07,Ore08,Ore09,Ore10,Ore11,Ore12,Ore13,Ore14	*OreG0020055*
scaffold4159	1055495	T	A	Och01,Och02,Och03,Och04,Och05,Och06,Och07,Och08,Och09,Och10,Och11,Och12,Och13,Och14,Ore01,Ore02,Ore03,Ore04,Ore05,Ore06,Ore07,Ore08,Ore09,Ore10,Ore11,Ore12,Ore13,Ore14	*OreG0020055*
scaffold4159	1073619	A	C	Och01,Och02,Och03,Och04,Och05,Och06,Och07,Och08,Och09,Och10,Och11,Och12,Och13,Och14,Ore01,Ore02,Ore03,Ore04,Ore05,Ore06,Ore07,Ore08,Ore09,Ore10,Ore11,Ore12,Ore13,Ore14	*OreG0020057*

scaffold4159	1091532	C	G	Ore01,Ore02,Ore03,Ore04,Ore05,Ore06,Ore07,Ore08,Ore09,Ore10,Ore11,Ore12,Ore13,Ore14	*OreG0020060*
scaffold4159	1099341	G	A	Ore01,Ore02,Ore03,Ore04,Ore05,Ore06,Ore07,Ore08,Ore09,Ore10,Ore11,Ore12,Ore13,Ore14	*OreG0020061*
scaffold4159	1107183	A	T	Och01	*OreG0020062*
scaffold4159	1132819	G	T	Ore01,Ore02,Ore03,Ore04,Ore05,Ore06,Ore07,Ore08,Ore09,Ore10,Ore11,Ore12,Ore13,Ore14	*OreG0020066*
scaffold4159	1133653	A	G	Ore01,Ore02,Ore03,Ore04,Ore05,Ore06,Ore07,Ore08,Ore09,Ore10,Ore11,Ore12,Ore13,Ore14	*OreG0020067*
scaffold4159	1169873	G	A	Och01,Och02,Och03,Och04,Och05,Och06,Och07,Och08,Och09,Och10,Och11,Och12,Och13,Och14	*OreG0020072*
scaffold4159	1170128	T	C	Och01,Och02,Och03,Och04,Och05,Och06,Och07,Och09,Och10,Och11,Och12,Och13,Och14	*OreG0020072*
scaffold4159	1170251	T	C	Och01,Och02,Och03,Och04,Och05,Och06,Och07,Och08,Och09,Och10,Och11,Och12,Och13,Och14	*OreG0020072*
scaffold4159	1170350	T	A	Och01,Och03,Och05,Och12,Och13	*OreG0020072*
scaffold4159	1170494	T	C	Ore01,Ore02,Ore03,Ore04,Ore05,Ore06,Ore07,Ore08,Ore09,Ore10,Ore11,Ore12,Ore13,Ore14	*OreG0020072*
scaffold4159	1190896	C	A	Och12,Och13,Och14	*OreG0020077*
scaffold4159	1191942	A	T	NA	*OreG0020077*
scaffold4159	1213673	C	A	Ore01,Ore02,Ore03,Ore04,Ore05,Ore06,Ore07,Ore08,Ore09,Ore10,Ore11,Ore12,Ore13,Ore14	*OreG0020079*
scaffold4159	1238517	G	C	Ore01,Ore02,Ore03,Ore04,Ore05,Ore06,Ore07,Ore08,Ore09,Ore10,Ore11,Ore12,Ore13,Ore14	*OreG0020082*
scaffold8486	5571	C	A	NA	*OreG0025410*
scaffold8486	5672	G	T	NA	*OreG0025410*
scaffold8486	5688	A	T	NA	*OreG0025410*
scaffold8486	5753	G	A	NA	*OreG0025410*
scaffold8486	6032	C	T	NA	*OreG0025410*
scaffold998	157683	A	G	Och01,Och07,Och09,Och10,Och11,Ore08,Ore09,Ore11,Ore12	*OreG0027264*
scaffold998	158641	A	C	Och06,Ore01,Ore02,Ore03,Ore04,Ore05,Ore06,Ore07,Ore08,Ore09,Ore10,Ore11,Ore12,Ore13,Ore14	*OreG0027264*
scaffold998	179138	G	A	Och01,Och11	*OreG0027268*
scaffold998	193968	G	A	NA	*OreG0027270*
scaffold998	215612	G	A	NA	*OreG0027273*
scaffold998	215622	T	A	NA	*OreG0027273*
scaffold998	215879	T	G	Och01	*OreG0027273*
scaffold998	234004	A	G	NA	*OreG0027276*
scaffold998	234064	C	A	NA	*OreG0027276*
scaffold998	243351	C	A	Och02,Och03,Och04,Och05,Och06,Och07,Och08,Och09,Och10,Och11,Och12,Och13,Och14,Ore01,Ore02,Ore03,Ore04,Ore05,Ore06,Ore07,Ore08,Ore09,Ore10,Ore11,Ore12,Ore13,Ore14	*OreG0027278*
scaffold998	243486	A	T	Och01,Och02,Och03,Och04,Och05,Och06,Och07,Och08,Och09,Och10,Och11,Och12,Och13,Och14,Ore01,Ore02,Ore03,Ore04,Ore05,Ore06,Ore07,Ore08,Ore09,Ore10,Ore11,Ore12,Ore13,Ore14	*OreG0027278*
scaffold998	248024	A	G	Ore02	*OreG0027279*
scaffold998	279234	C	A	NA	*OreG0027281*
scaffold998	330070	A	T	Och01,Och02,Och03,Och04,Och05,Och06,Och07,Och08,Och09,Och10,Och11,Och12,Och13,Och14,Ore01,Ore02,Ore03,Ore04,Ore05,Ore06,Ore07,Ore08,Ore09,Ore10,Ore11,Ore12,Ore13,Ore14	*OreG0027286*
scaffold998	333133	G	A	NA	*OreG0027287*
scaffold998	341156	A	T	Och07,Och08	*OreG0027288*
scaffold998	341802	T	G	Ore01,Ore02,Ore03,Ore06,Ore07,Ore08,Ore09,Ore10,Ore11,Ore12,Ore13,Ore14	*OreG0027288*
scaffold998	345919	G	T	NA	*OreG0027288*
scaffold998	349936	G	T	Och03,Och05,Och07,Och08,Och09,Och10,Och12,Och13,Och14	*OreG0027289*
scaffold998	350439	T	C	Ore01,Ore02,Ore03,Ore04,Ore05,Ore06,Ore07,Ore08,Ore09,Ore10,Ore11,Ore12,Ore13,Ore14	*OreG0027289*
scaffold998	350901	G	C	Och01,Och02,Och03,Och04,Och05,Och06,Och07,Och08,Och09,Och10,Och11,Och12,Och13,Och14,Ore01,Ore02,Ore03,Ore04,Ore05,Ore06,Ore07,Ore08,Ore09,Ore10,Ore11,Ore12,Ore13,Ore14	*OreG0027289*
scaffold998	352287	C	G	Ore01,Ore02,Ore03,Ore04,Ore05,Ore06,Ore07,Ore08,Ore09,Ore10,Ore11,Ore12,Ore13,Ore14	*OreG0027289*
scaffold998	462805	G	T	NA	*OreG0027295*
scaffold998	463171	A	G	Och08	*OreG0027295*
scaffold998	522313	G	A	Ore01,Ore02,Ore03,Ore06,Ore07,Ore08,Ore09,Ore10,Ore11,Ore12,Ore13,Ore14	*OreG0027299*
scaffold998	522316	A	T	Och01,Och02,Och03,Och04,Och05,Och06,Och07,Och09,Och10,Och11,Och12,Och13,Och14,Ore01,Ore02,Ore03,Ore04,Ore05,Ore06,Ore07,Ore08,Ore09,Ore10,Ore11,Ore12,Ore13,Ore14	*OreG0027299*
scaffold998	522968	C	A	Ore01,Ore02,Ore03,Ore04,Ore05,Ore06,Ore07,Ore08,Ore09,Ore10,Ore11,Ore12,Ore13,Ore14	*OreG0027299*
scaffold998	523124	C	T	Och08	*OreG0027299*
scaffold998	526025	A	G	Ore01,Ore02,Ore03,Ore04,Ore05,Ore06,Ore07,Ore08,Ore09,Ore10,Ore11,Ore12,Ore13,Ore14	*OreG0027301*
scaffold998	526070	C	T	Och01,Och06	*OreG0027301*
scaffold998	526115	C	T	Ore01,Ore02,Ore03,Ore04,Ore05,Ore06,Ore07,Ore08,Ore09,Ore10,Ore11,Ore12,Ore13,Ore14	*OreG0027301*

scaffold998	526153	C	T	Ore01,Ore02,Ore03,Ore04,Ore05,Ore06,Ore07,Ore08,Ore09,Ore10,Ore11,Ore12,Ore13,Ore14	*OreG0027301*
scaffold998	527873	G	T	NA	*OreG0027302*
scaffold998	535222	A	G	Ore01,Ore02,Ore03,Ore04,Ore05,Ore06,Ore07,Ore08,Ore09,Ore10,Ore11,Ore12,Ore13,Ore14	*OreG0027304*
scaffold998	537447	G	A	Och06	*OreG0027305*
scaffold998	539534	C	T	Och06	*OreG0027306*
scaffold998	549326	C	G	Och01,Och02,Och03,Och04,Och05,Och06,Och07,Och09,Och10,Och11,Och12,Och13,Och14	*OreG0027308*
scaffold998	549592	C	T	NA	*OreG0027308*
scaffold998	549826	T	A	NA	*OreG0027308*
scaffold998	549936	C	T	NA	*OreG0027308*
scaffold998	550155	G	A	Ore04,Ore05	*OreG0027308*
scaffold998	550186	C	T	NA	*OreG0027308*
scaffold998	626033	A	T	Ore01,Ore02,Ore03,Ore04,Ore05,Ore06,Ore07,Ore08,Ore09,Ore10,Ore11,Ore12,Ore13,Ore14	*OreG0027313*
scaffold998	626146	C	G	NA	*OreG0027313*
scaffold998	626555	G	A	NA	*OreG0027313*
scaffold998	663123	C	A	Och01,Och02,Och03,Och04,Och05,Och06,Och07,Och08,Och09,Och10,Och11,Och12,Och13,Och14,Ore01,Ore02,Ore03,Ore04,Ore05,Ore06,Ore07,Ore08,Ore09,Ore10,Ore11,Ore12,Ore13,Ore14	*OreG0027317*
scaffold998	684762	G	A	NA	*OreG0027319*
scaffold998	684782	A	G	NA	*OreG0027319*
scaffold998	684884	G	T	NA	*OreG0027319*
scaffold998	704122	G	A	Och09	*OreG0027321*
scaffold998	704124	A	C	NA	*OreG0027321*
scaffold998	704157	A	G	NA	*OreG0027321*
scaffold998	704214	C	T	Ore01,Ore02,Ore03,Ore04,Ore05,Ore06,Ore07,Ore08,Ore09,Ore10,Ore11,Ore12,Ore13,Ore14	*OreG0027321*
scaffold998	704298	C	T	Och03,Och05,Och07,Och08,Och09,Och12,Och13	*OreG0027321*
scaffold998	719579	C	G	Och01,Och02,Och06,Och07,Och08,Och09,Och12,Och13,Och14,Ore04,Ore05	*OreG0027322*
scaffold998	720592	A	T	Och01,Och02,Och03,Och04,Och05,Och06,Och07,Och08,Och09,Och10,Och11,Och12,Och13,Och14,Ore01,Ore02,Ore03,Ore04,Ore05,Ore06,Ore07,Ore08,Ore09,Ore10,Ore11,Ore12,Ore13,Ore14	*OreG0027322*
scaffold998	736585	T	A	Ore01,Ore02,Ore03,Ore04,Ore05,Ore06,Ore07,Ore08,Ore09,Ore10,Ore11,Ore12,Ore13,Ore14	*OreG0027324*
scaffold998	748712	C	A	NA	*OreG0027326*
scaffold998	749755	G	A	Ore01,Ore02,Ore03,Ore04,Ore05,Ore06,Ore07,Ore08,Ore09,Ore10,Ore11,Ore12,Ore13,Ore14	*OreG0027326*
scaffold998	749792	A	T	Ore01,Ore02,Ore03,Ore04,Ore05,Ore06,Ore07,Ore08,Ore09,Ore10,Ore11,Ore12,Ore13,Ore14	*OreG0027326*
scaffold998	750279	A	G	NA	*OreG0027326*
scaffold998	813093	A	G	Ore01,Ore02,Ore03,Ore04,Ore05,Ore06,Ore07,Ore08,Ore09,Ore10,Ore11,Ore12,Ore13,Ore14	*OreG0027332*
scaffold998	826127	T	C	Och01,Och02,Och03,Och04,Och05,Och06,Och07,Och08,Och09,Och10,Och11,Och12,Och13,Och14,Ore01,Ore02,Ore03,Ore04,Ore05,Ore06,Ore07,Ore08,Ore09,Ore10,Ore11,Ore12,Ore13,Ore14	*OreG0027333*
scaffold998	827353	T	G	Och01,Och02,Och03,Och04,Och05,Och06,Och07,Och08,Och09,Och10,Och11,Och12,Och13,Och14,Ore01,Ore02,Ore03,Ore04,Ore05,Ore06,Ore07,Ore08,Ore09,Ore10,Ore11,Ore12,Ore13,Ore14	*OreG0027333*
scaffold998	856138	C	A	Och01,Och02,Och03,Och04,Och05,Och06,Och07,Och08,Och09,Och10,Och11,Och12,Och13,Och14,Ore01,Ore02,Ore03,Ore04,Ore05,Ore06,Ore07,Ore08,Ore09,Ore10,Ore11,Ore12,Ore13,Ore14	*OreG0027337*
scaffold998	858006	A	G	Ore01,Ore02,Ore03,Ore04,Ore05,Ore06,Ore07,Ore08,Ore09,Ore10,Ore11,Ore12,Ore13,Ore14	*OreG0027337*
scaffold998	861618	G	T	Och01,Och02,Och03,Och04,Och05,Och06,Och07,Och08,Och09,Och10,Och11,Och12,Och13,Och14,Ore01,Ore02,Ore03,Ore04,Ore05,Ore06,Ore07,Ore08,Ore09,Ore10,Ore11,Ore12,Ore13,Ore14	*OreG0027337*
scaffold998	895141	C	A	Och01,Och02,Och03,Och04,Och05,Och06,Och07,Och08,Och09,Och10,Och11,Och12,Och13,Och14,Ore01,Ore02,Ore03,Ore04,Ore05,Ore06,Ore07,Ore08,Ore09,Ore10,Ore11,Ore12,Ore13,Ore14	*OreG0027342*
scaffold998	909621	C	A	Och08	*OreG0027344*
scaffold998	910519	T	A	Och01,Och02,Och03,Och04,Och05,Och06,Och12,Och13	*OreG0027344*
scaffold998	912639	G	T	NA	*OreG0027345*
scaffold998	915029	A	T	NA	*OreG0027345*
scaffold998	916574	A	G	Och01,Och02,Och03,Och04,Och05,Och06,Och07,Och08,Och09,Och10,Och11,Och12,Och13,Och14	*OreG0027345*
scaffold998	916844	C	T	NA	*OreG0027345*
scaffold998	952923	C	G	Och06,Och07,Och08,Och14	*OreG0027353*
scaffold998	964963	A	T	NA	*OreG0027355*
scaffold998	971556	G	C	Ore02,Ore10	*OreG0027356*
scaffold998	980801	T	A	Och01,Och06,Och07,Och08,Och14	*OreG0027357*
scaffold998	992272	A	G	Ore02,Ore10	*OreG0027359*
scaffold998	992514	G	A	NA	*OreG0027359*

scaffold998	1000735	A	T	Och01,Och02,Och04,Och05,Och06,Och07,Och08,Och09,Och10,Och11,Och12,Och13,Och14	*OreG0027361*
scaffold998	1019681	C	T	NA	*OreG0027362*
scaffold998	1021950	G	A	NA	*OreG0027362*
scaffold998	1038148	G	A	Ore01,Ore02,Ore03,Ore04,Ore05,Ore06,Ore07,Ore08,Ore09,Ore10,Ore11,Ore12,Ore13,Ore14	*OreG0027364*
scaffold998	1045419	A	G	Ore01,Ore02,Ore03,Ore04,Ore05,Ore06,Ore07,Ore08,Ore09,Ore10,Ore11,Ore12,Ore13,Ore14	*OreG0027365*
scaffold998	1045734	A	T	Ore01,Ore02,Ore03,Ore04,Ore05,Ore06,Ore07,Ore08,Ore09,Ore10,Ore11,Ore12,Ore13,Ore14	*OreG0027365*
scaffold998	1069393	T	G	Ore01,Ore02,Ore03,Ore04,Ore05,Ore06,Ore07,Ore08,Ore09,Ore10,Ore11,Ore12,Ore13,Ore14	*OreG0027369*
scaffold998	1070851	G	A	Och02,Och06	*OreG0027369*
scaffold998	1071856	C	T	Och01,Och02,Och03,Och04,Och05,Och06,Och07,Och08,Och09,Och10,Och11,Och12,Och13,Och14,Ore01,Ore02,Ore03,Ore04,Ore05,Ore06,Ore07,Ore08,Ore09,Ore10,Ore11,Ore12,Ore13,Ore14	*OreG0027369*
scaffold998	1117839	T	A	NA	*OreG0027376*
scaffold998	1122153	T	C	Och01,Och02,Och03,Och04,Och05,Och06,Och07,Och08,Och09,Och10,Och11,Och12,Och13,Och14	*OreG0027378*
scaffold998	1125479	A	G	Och01,Och02,Och03,Och04,Och05,Och06,Och07,Och08,Och09,Och10,Och11,Och12,Och13,Och14,Ore01,Ore02,Ore03,Ore04,Ore05,Ore06,Ore07,Ore08,Ore09,Ore10,Ore11,Ore12,Ore13,Ore14	*OreG0027379*
scaffold998	1129065	T	G	Och01,Och02,Och03,Och04,Och05,Och06,Och07,Och08,Och09,Och10,Och11,Och12,Och13,Och14	*OreG0027380*
scaffold998	1159259	A	C	NA	*OreG0027384*
scaffold998	1160063	C	T	NA	*OreG0027384*
scaffold998	1186983	G	A	NA	*OreG0027390*
scaffold998	1243858	T	A	Och08	*OreG0027398*
scaffold998	1243886	C	T	Och01,Och07,Och08,Och09,Och10,Och11	*OreG0027398*
scaffold998	1249724	T	A	NA	*OreG0027400*
scaffold998	1249729	T	C	NA	*OreG0027400*
scaffold998	1261777	T	A	Ore01,Ore02,Ore03,Ore04,Ore05,Ore06,Ore07,Ore08,Ore09,Ore10,Ore11,Ore12,Ore13,Ore14	*OreG0027402*
scaffold998	1284770	G	T	Och08	*OreG0027406*
scaffold998	1287578	A	G	Och01,Och02,Och03,Och04,Och05,Och06,Och07,Och08,Och09,Och10,Och11,Och12,Och13,Och14,Ore01,Ore02,Ore03,Ore04,Ore05,Ore06,Ore07,Ore08,Ore09,Ore10,Ore11,Ore12,Ore13,Ore14	*OreG0027407*
scaffold998	1312669	G	A	Och08	*OreG0027413*
scaffold998	1363145	C	T	NA	*OreG0027420*
scaffold998	1375839	G	C	NA	*OreG0027421*
scaffold998	1386200	G	A	NA	*OreG0027422*
scaffold998	1386573	G	A	Och01,Och02,Och03,Och04,Och05,Och06,Och07,Och08,Och09,Och10,Och11,Och12,Och13,Och14,Ore01,Ore02,Ore03,Ore04,Ore05,Ore06,Ore07,Ore08,Ore09,Ore10,Ore11,Ore12,Ore13,Ore14	*OreG0027422*
scaffold998	1387363	C	G	Och01,Och02,Och03,Och04,Och05,Och06,Och07,Och08,Och09,Och10,Och11,Och12,Och13,Och14,Ore01,Ore02,Ore03,Ore04,Ore05,Ore06,Ore07,Ore08,Ore09,Ore10,Ore11,Ore12,Ore13,Ore14	*OreG0027422*
scaffold998	1418362	G	C	Och01,Och02,Och03,Och04,Och05,Och06,Och07,Och08,Och09,Och10,Och11,Och12,Och13,Och14,Ore01,Ore02,Ore03,Ore04,Ore05,Ore06,Ore07,Ore08,Ore09,Ore10,Ore11,Ore12,Ore13,Ore14	*OreG0027426*
scaffold998	1429906	T	A	Och01,Och06,Och08	*OreG0027427*
scaffold998	1443677	T	C	NA	*OreG0027429*
scaffold998	1443706	C	T	Ore02,Ore10	*OreG0027429*
scaffold998	1444090	C	T	NA	*OreG0027429*
scaffold998	1523619	A	T	Och08	*OreG0027437*
scaffold998	1523708	G	T	NA	*OreG0027437*
scaffold998	1525993	A	T	Och01,Och02,Och03,Och04,Och05,Och06,Och07,Och08,Och09,Och10,Och11,Och12,Och13,Och14,Ore01,Ore02,Ore03,Ore04,Ore05,Ore06,Ore07,Ore08,Ore09,Ore10,Ore11,Ore12,Ore13,Ore14	*OreG0027437*
scaffold998	1548646	G	T	Ore01,Ore02,Ore03,Ore04,Ore05,Ore06,Ore07,Ore08,Ore09,Ore10,Ore11,Ore12,Ore13,Ore14	*OreG0027441*
scaffold998	1562742	G	C	Och01,Och02,Och03,Och04,Och05,Och06,Och07,Och08,Och09,Och10,Och11,Och12,Och13,Och14	*OreG0027443*
scaffold998	1566986	G	A	Ore01,Ore02,Ore03,Ore04,Ore05,Ore06,Ore07,Ore08,Ore09,Ore10,Ore11,Ore12,Ore13,Ore14	*OreG0027443*
scaffold998	1569889	G	T	NA	*OreG0027444*
scaffold998	1570096	C	T	Och01,Och02,Och03,Och04,Och05,Och06,Och07,Och08,Och12,Och13,Och14	*OreG0027444*
scaffold998	1570120	A	G	NA	*OreG0027444*
scaffold998	1570176	C	A	NA	*OreG0027444*
scaffold998	1570344	C	A	NA	*OreG0027444*
scaffold998	1570465	G	A	NA	*OreG0027444*
scaffold998	1570558	G	T	NA	*OreG0027444*
scaffold998	1570648	A	T	NA	*OreG0027444*
scaffold998	1570990	A	C	NA	*OreG0027444*
scaffold998	1572581	C	T	NA	*OreG0027445*

scaffold998	1582329	G	T	NA	*OreG0027446*
scaffold998	1625840	C	T	Ore01,Ore02,Ore03,Ore04,Ore05,Ore06,Ore07,Ore08,Ore09,Ore10,Ore11,Ore12,Ore13,Ore14	*OreG0027452*
scaffold998	1625877	G	A	NA	*OreG0027452*
scaffold998	1626432	G	A	Ore01,Ore02,Ore03,Ore04,Ore05,Ore06,Ore07,Ore08,Ore09,Ore10,Ore11,Ore12,Ore13,Ore14	*OreG0027452*
scaffold998	1628710	C	T	NA	*OreG0027452*
scaffold998	1629383	T	C	NA	*OreG0027452*
scaffold998	1630113	G	T	Och01	*OreG0027452*
scaffold998	1679691	T	A	NA	*OreG0027457*
scaffold998	1679699	G	A	Och14	*OreG0027457*
scaffold998	1680342	A	C	NA	*OreG0027457*
scaffold998	1681030	T	G	Och01,Och02,Och03,Och04,Och05,Och06,Och07,Och08,Och09,Och10,Och11,Och12,Och13,Och14,Ore01,Ore02,Ore03,Ore04,Ore05,Ore06,Ore07,Ore08,Ore09,Ore10,Ore11,Ore12,Ore13,Ore14	*OreG0027457*
scaffold998	1690079	G	T	NA	*OreG0027458*
scaffold998	1690082	G	A	Och01,Och02,Och03,Och04,Och05,Och06,Och08,Och12,Och13	*OreG0027458*
scaffold998	1690584	C	G	Ore01,Ore02,Ore03,Ore04,Ore05,Ore06,Ore07,Ore08,Ore09,Ore10,Ore11,Ore12,Ore13,Ore14	*OreG0027459*
scaffold998	1693275	C	A	Och08,Ore01,Ore02,Ore03,Ore04,Ore05,Ore06,Ore07,Ore08,Ore09,Ore10,Ore11,Ore12,Ore13,Ore14	*OreG0027460*
scaffold998	1695868	C	T	NA	*OreG0027460*
scaffold998	1701713	C	G	Ore01,Ore02,Ore03,Ore04,Ore05,Ore06,Ore07,Ore08,Ore09,Ore10,Ore11,Ore12,Ore13,Ore14	*OreG0027461*
scaffold998	1784848	C	T	Ore01,Ore02,Ore03,Ore04,Ore05,Ore06,Ore07,Ore08,Ore09,Ore10,Ore11,Ore12,Ore13,Ore14	*OreG0027474*
scaffold998	1784856	C	T	NA	*OreG0027474*
scaffold998	1859198	T	A	NA	*OreG0027482*
scaffold998	1877284	A	T	Och01,Och02,Och03,Och04,Och05,Och06,Och07,Och08,Och09,Och10,Och11,Och12,Och13,Och14,Ore01,Ore02,Ore03,Ore04,Ore05,Ore06,Ore07,Ore08,Ore09,Ore10,Ore11,Ore12,Ore13,Ore14	*OreG0027485*
scaffold998	1942819	T	G	NA	*OreG0027489*
scaffold998	1947470	C	T	Ore02,Ore10	*OreG0027490*
scaffold998	1947508	C	A	Och08	*OreG0027490*
scaffold998	1950604	G	A	Och01,Och02,Och03,Och04,Och05,Och06,Och07,Och08,Och09,Och10,Och11,Och12,Och13,Och14	*OreG0027491*
scaffold998	1950624	A	G	NA	*OreG0027491*
scaffold998	1970394	G	T	NA	*OreG0027494*
scaffold998	1983652	C	A	Ore04,Ore05	*OreG0027499*
scaffold998	1987985	G	A	NA	*OreG0027500*
scaffold998	2017388	T	G	Och01,Och02,Och03,Och04,Och05,Och06,Och07,Och08,Och09,Och10,Och11,Och12,Och13,Och14	*OreG0027503*
scaffold998	2017427	G	C	Och01,Och02,Och03,Och04,Och05,Och06,Och07,Och08,Och09,Och10,Och11,Och12,Och13,Och14	*OreG0027503*
scaffold998	2017429	A	G	Och01,Och02,Och03,Och04,Och05,Och06,Och07,Och08,Och09,Och10,Och11,Och12,Och13	*OreG0027503*
scaffold998	2022739	C	A	NA	*OreG0027504*
scaffold998	2024607	G	A	NA	*OreG0027504*
scaffold998	2025820	T	A	Ore01,Ore02,Ore03,Ore04,Ore05,Ore06,Ore07,Ore08,Ore09,Ore10,Ore11,Ore12,Ore13,Ore14	*OreG0027504*
scaffold998	2042891	C	A	NA	*OreG0027506*
scaffold998	2044973	G	A	Ore04,Ore05	*OreG0027506*
scaffold998	2067109	C	T	NA	*OreG0027510*
scaffold998	2067126	A	C	NA	*OreG0027510*
scaffold998	2067175	T	A	Ore01,Ore02,Ore03,Ore04,Ore05,Ore06,Ore07,Ore08,Ore09,Ore10,Ore11,Ore12,Ore13,Ore14	*OreG0027510*
scaffold998	2067223	T	G	Och06,Och08	*OreG0027510*
scaffold998	2067270	A	G	NA	*OreG0027510*
scaffold998	2067274	C	T	Ore01,Ore02,Ore03,Ore04,Ore05,Ore06,Ore07,Ore08,Ore09,Ore10,Ore11,Ore12,Ore13,Ore14	*OreG0027510*
scaffold998	2067629	G	A	NA	*OreG0027510*
scaffold998	2067778	C	T	NA	*OreG0027510*
scaffold998	2067994	A	T	Och01,Och02,Och03,Och04,Och05,Och06,Och07,Och08,Och09,Och10,Och11,Och12,Och13,Och14,Ore01,Ore02,Ore03,Ore04,Ore05,Ore06,Ore07,Ore08,Ore09,Ore10,Ore11,Ore12,Ore13,Ore14	*OreG0027510*
scaffold998	2079757	T	A	Och01,Och02,Och03,Och04,Och05,Och06,Och07,Och08,Och09,Och10,Och11,Och12,Och13,Och14	*OreG0027513*
scaffold998	2079841	A	G	Och01	*OreG0027513*
scaffold998	2079933	T	A	Ore01,Ore02,Ore03,Ore04,Ore05,Ore06,Ore07,Ore08,Ore09,Ore10,Ore11,Ore12,Ore13,Ore14	*OreG0027513*
scaffold998	2080324	C	T	Och08	*OreG0027513*
scaffold998	2080372	C	T	Ore01,Ore02,Ore03,Ore04,Ore05,Ore06,Ore07,Ore08,Ore09,Ore10,Ore11,Ore12,Ore13,Ore14	*OreG0027513*
scaffold998	2107112	A	G	NA	*OreG0027517*
scaffold998	2108458	G	A	NA	*OreG0027517*

scaffold998	2118207	C	G	Och07,Och08,Och11,Och14	*OreG0027519*
scaffold998	2118987	A	T	NA	*OreG0027519*
scaffold998	2119703	A	G	NA	*OreG0027519*
scaffold998	2121896	A	G	Ore04,Ore05	*OreG0027520*
scaffold998	2121913	G	A	Och01	*OreG0027520*
scaffold998	2121920	C	T	NA	*OreG0027520*
scaffold998	2121931	G	T	NA	*OreG0027520*
scaffold998	2121935	C	A	Och02,Och03,Och04,Och05,Och06,Och08,Och12,Och13	*OreG0027520*
scaffold998	2121941	C	A	Ore04,Ore05	*OreG0027520*
scaffold998	2121998	C	T	NA	*OreG0027520*
scaffold998	2122045	G	T	NA	*OreG0027520*
scaffold998	2122363	T	C	NA	*OreG0027521*
scaffold998	2122477	C	T	NA	*OreG0027521*
scaffold998	2122489	C	T	Ore01,Ore02,Ore03,Ore04,Ore05,Ore06,Ore07,Ore08,Ore09,Ore10,Ore11,Ore12,Ore13,Ore14	*OreG0027521*
scaffold998	2122516	C	A	Och12	*OreG0027521*
scaffold998	2133854	G	T	Ore01,Ore02,Ore03,Ore04,Ore05,Ore06,Ore07,Ore08,Ore09,Ore10,Ore11,Ore12,Ore13,Ore14	*OreG0027523*
scaffold998	2152360	T	C	NA	*OreG0027525*
scaffold998	2158054	C	T	NA	*OreG0027528*
scaffold998	2158593	C	T	NA	*OreG0027528*
scaffold998	2158609	C	A	NA	*OreG0027528*
scaffold998	2170457	G	A	NA	*OreG0027529*
scaffold998	2180679	C	T	Och01	*OreG0027530*
scaffold998	2215981	A	T	Ore01,Ore02,Ore03,Ore04,Ore05,Ore06,Ore07,Ore08,Ore09,Ore10,Ore11,Ore12,Ore13,Ore14	*OreG0027534*
scaffold998	2216671	G	A	Och01,Och02,Och03,Och04,Och05,Och06,Och07,Och08,Och09,Och10,Och11,Och12,Och13,Och14,Ore01,Ore02,Ore03,Ore04,Ore05,Ore06,Ore07,Ore08,Ore09,Ore10,Ore11,Ore12,Ore13,Ore14	*OreG0027534*
scaffold998	2242410	A	G	NA	*OreG0027536*
scaffold998	2243022	A	T	NA	*OreG0027536*
scaffold998	2244866	T	C	NA	*OreG0027536*
scaffold998	2259800	T	A	NA	*OreG0027539*
scaffold998	2300399	G	T	Och02,Och03,Och04,Och05,Och06,Och07,Och08,Och09,Och10,Och11,Och12,Och13,Och14	*OreG0027543*
scaffold998	2340987	G	A	Och02,Och07	*OreG0027548*
scaffold998	2342626	G	A	Och01,Och02,Och03,Och04,Och05,Och06,Och07,Och08,Och09,Och10,Och11,Och12,Och13,Och14	*OreG0027548*
scaffold998	2375552	T	A	NA	*OreG0027552*
scaffold998	2375651	A	T	NA	*OreG0027552*
scaffold998	2375656	G	C	NA	*OreG0027552*
scaffold998	2375675	A	G	NA	*OreG0027552*
scaffold998	2375725	C	G	NA	*OreG0027552*
scaffold998	2375759	A	C	NA	*OreG0027552*
scaffold998	2375765	A	T	NA	*OreG0027552*
scaffold998	2375884	G	A	NA	*OreG0027552*
scaffold998	2375926	G	T	NA	*OreG0027552*
scaffold998	2376151	A	G	NA	*OreG0027552*
scaffold998	2376433	A	G	NA	*OreG0027552*
scaffold998	2376536	T	C	NA	*OreG0027552*
scaffold998	2376548	G	A	NA	*OreG0027552*
scaffold998	2376851	C	G	NA	*OreG0027552*
scaffold998	2377036	C	A	NA	*OreG0027552*
scaffold998	2377076	A	C	NA	*OreG0027552*
scaffold998	2377091	A	T	NA	*OreG0027552*
scaffold998	2377102	G	A	NA	*OreG0027552*
scaffold998	2377334	G	C	NA	*OreG0027552*
scaffold998	2377478	G	A	NA	*OreG0027552*
scaffold998	2377601	T	G	NA	*OreG0027552*
scaffold998	2377643	C	A	NA	*OreG0027552*
scaffold998	2383903	A	T	NA	*OreG0027553*
scaffold998	2384245	G	T	NA	*OreG0027553*
scaffold998	2384311	G	T	NA	*OreG0027553*
scaffold998	2384449	G	A	Och08,Och14	*OreG0027553*
scaffold998	2384560	A	T	Och02	*OreG0027553*
scaffold998	2384569	G	T	NA	*OreG0027553*
scaffold998	2384749	A	G	NA	*OreG0027553*
scaffold998	2385174	C	G	Och02,Och08,Och14,Ore01,Ore02,Ore03,Ore04,Ore05,Ore06,Ore07,Ore08,Ore09,Ore10,Ore11,Ore12,Ore13,Ore14	*OreG0027553*
scaffold998	2385356	A	T	NA	*OreG0027553*
scaffold998	2385787	T	C	NA	*OreG0027553*
scaffold998	2385833	G	A	Ore02,Ore10	*OreG0027553*
scaffold998	2389240	A	G	NA	*OreG0027554*
scaffold998	2389245	A	G	NA	*OreG0027554*

scaffold998	2389840	A	G	NA	*OreG0027554*
scaffold998	2389896	G	A	NA	*OreG0027554*
scaffold998	2389906	T	A	Och03,Och08,Och12	*OreG0027554*
scaffold998	2390016	G	A	NA	*OreG0027554*
scaffold998	2390098	A	T	NA	*OreG0027554*
scaffold998	2390102	A	C	NA	*OreG0027554*
scaffold998	2390197	C	A	NA	*OreG0027554*
scaffold998	2390212	T	C	NA	*OreG0027554*
scaffold998	2390442	G	C	Och03,Och08,Och09,Och10,Och11,Ore01,Ore02,Ore03,Ore04,Ore05,Ore06,Ore07,Ore08,Ore09,Ore10,Ore11,Ore12,Ore13,Ore14	*OreG0027554*
scaffold998	2390514	C	T	NA	*OreG0027554*
scaffold998	2391148	C	T	NA	*OreG0027554*
scaffold998	2391440	G	T	NA	*OreG0027554*
scaffold998	2441038	C	A	NA	*OreG0027559*
scaffold998	2441053	G	C	Ore02,Ore10,Ore11,Ore12,Ore13	*OreG0027559*
scaffold998	2441243	T	C	Och01,Och08	*OreG0027559*
scaffold998	2444604	G	A	Och01,Och02,Och03,Och04,Och05,Och06,Och07,Och08,Och09,Och10,Och11,Och12,Och13,Och14,Ore01,Ore02,Ore03,Ore04,Ore05,Ore06,Ore07,Ore08,Ore09,Ore10,Ore11,Ore12,Ore13,Ore14	*OreG0027560*
scaffold998	2445538	C	G	Och01,Och02,Och03,Och04,Och05,Och06,Och07,Och08,Och09,Och10,Och11,Och12,Och13,Och14,Ore01,Ore02,Ore03,Ore04,Ore05,Ore06,Ore07,Ore08,Ore09,Ore10,Ore11,Ore12,Ore13,Ore14	*OreG0027560*
scaffold998	2452810	G	A	NA	*OreG0027562*
scaffold998	2462960	T	G	Och03,Och06	*OreG0027563*
scaffold998	2463142	C	T	Och08,Ore04,Ore05	*OreG0027563*
scaffold998	2463160	T	G	Och01,Och07	*OreG0027563*
scaffold998	2463168	G	T	Och02,Och03,Och04,Och05,Och06,Och11,Och12,Och13,Och14,Ore02,Ore10,Ore11,Ore12,Ore13	*OreG0027563*
scaffold998	2464554	C	A	Ore02,Ore10,Ore11,Ore12,Ore13	*OreG0027563*
scaffold998	2481389	G	T	NA	*OreG0027565*
scaffold998	2502196	T	A	NA	*OreG0027568*
scaffold998	2503330	T	A	NA	*OreG0027568*
scaffold998	2533776	T	A	Ore02,Ore10	*OreG0027574*
scaffold998	2534943	C	A	NA	*OreG0027574*
scaffold998	2536038	G	A	Och01,Och02,Och03,Och04,Och05,Och06,Och07,Och08,Och09,Och10,Och11,Och12,Och13,Och14,Ore01,Ore02,Ore03,Ore04,Ore05,Ore06,Ore07,Ore08,Ore09,Ore10,Ore11,Ore12,Ore13,Ore14	*OreG0027574*
scaffold998	2536079	A	C	NA	*OreG0027574*
scaffold998	2546449	A	C	NA	*OreG0027576*
scaffold998	2560942	G	A	Och01,Och02,Och03,Och04,Och05,Och06,Och07,Och08,Och09,Och10,Och11,Och12,Och13,Och14	*OreG0027578*
scaffold998	2562782	T	C	NA	*OreG0027578*
scaffold998	2563029	C	A	NA	*OreG0027578*
scaffold998	2569603	G	A	NA	*OreG0027579*
scaffold998	2607522	T	C	Ore01,Ore02,Ore03,Ore04,Ore05,Ore06,Ore07,Ore08,Ore09,Ore10,Ore11,Ore12,Ore13,Ore14	*OreG0027582*
scaffold998	2616524	C	G	Och01,Och02,Och03,Och04,Och05,Och06,Och07,Och08,Och11,Och12,Och13,Och14	*OreG0027584*
scaffold998	2616680	G	A	NA	*OreG0027584*
scaffold998	2617006	C	T	Ore02,Ore10	*OreG0027584*
scaffold998	2617061	A	T	NA	*OreG0027584*
scaffold998	2617706	T	A	Och01,Och02,Och03,Och04,Och05,Och06,Och07,Och08,Och09,Och10,Och11,Och12,Och13,Och14,Ore01,Ore02,Ore03,Ore04,Ore05,Ore06,Ore07,Ore08,Ore09,Ore10,Ore11,Ore12,Ore13,Ore14	*OreG0027584*
scaffold998	2654922	T	A	NA	*OreG0027590*
scaffold998	2656524	G	A	NA	*OreG0027590*
scaffold998	2656694	A	C	Och01,Och02,Och03,Och04,Och05,Och06,Och07,Och08,Och09,Och10,Och11,Och12,Och13,Och14,Ore01,Ore02,Ore03,Ore04,Ore05,Ore06,Ore07,Ore08,Ore09,Ore10,Ore11,Ore12,Ore13,Ore14	*OreG0027590*
scaffold998	2658905	A	T	Ore01,Ore02,Ore03,Ore04,Ore05,Ore06,Ore07,Ore08,Ore09,Ore10,Ore14	*OreG0027590*
scaffold998	2659068	C	G	Och01,Och02,Och03,Och04,Och05,Och06,Och07,Och08,Och09,Och10,Och11,Och12,Och13,Och14,Ore01,Ore02,Ore03,Ore04,Ore05,Ore06,Ore07,Ore08,Ore09,Ore10,Ore11,Ore12,Ore13,Ore14	*OreG0027590*
scaffold998	2682315	G	A	Ore01,Ore02,Ore03,Ore04,Ore05,Ore06,Ore07,Ore08,Ore09,Ore10,Ore11,Ore12,Ore13,Ore14	*OreG0027593*
scaffold998	2722109	C	G	Och03,Och06	*OreG0027598*
scaffold998	2722202	G	A	NA	*OreG0027598*
scaffold998	2722206	C	T	Och08	*OreG0027598*
scaffold998	2722214	C	G	Och01,Och02,Och03,Och04,Och05,Och06,Och07,Och08,Och12,Och13,Och14	*OreG0027598*
scaffold998	2722227	G	A	NA	*OreG0027598*
scaffold998	2747183	G	C	Och14,Ore02,Ore03,Ore07,Ore10,Ore11,Ore13	*OreG0027600*
scaffold998	2833489	C	A	Och01,Och02,Och03,Och04,Och05,Och06,Och07,Och08,Och09,Och10,Och11,Och12,Och13,Och14,Ore01,Ore02,Ore03,Ore04,Ore05,Ore06,Ore07,Ore08,Ore09,Ore10,Ore11,Ore12,Ore13,Ore14	*OreG0027607*

scaffold998	2850592	G	A	Och01,Och02,Och03,Och04,Och05,Och06,Och07,Och08,Och09,Och10,Och11,Och12,Och13,Och14,Ore01,Ore02,Ore03,Ore04,Ore05,Ore06,Ore07,Ore08,Ore09,Ore10,Ore11,Ore12,Ore13,Ore14	*OreG0027609*
scaffold998	2850883	A	G	Ore04,Ore05	*OreG0027609*
scaffold998	2851210	T	C	Och03,Och06,Och07	*OreG0027609*
scaffold998	2855006	T	C	NA	*OreG0027610*
scaffold998	2855388	C	A	Och01,Och02,Och03,Och04,Och05,Och06,Och07,Och08,Och09,Och10,Och11,Och12,Och13,Och14,Ore01,Ore02,Ore03,Ore04,Ore05,Ore06,Ore07,Ore08,Ore09,Ore10,Ore11,Ore12,Ore13,Ore14	*OreG0027610*
scaffold998	2855452	G	C	Ore01,Ore02,Ore03,Ore04,Ore05,Ore06,Ore07,Ore08,Ore09,Ore10,Ore11,Ore12,Ore13,Ore14	*OreG0027610*
scaffold998	2855648	G	T	Och01,Och02,Och03,Och04,Och05,Och06,Och07,Och08,Och09,Och10,Och11,Och12,Och13,Och14,Ore01,Ore02,Ore03,Ore04,Ore05,Ore06,Ore07,Ore08,Ore09,Ore10,Ore11,Ore12,Ore13,Ore14	*OreG0027610*
scaffold998	2863379	G	T	NA	*OreG0027611*
scaffold998	3194675	C	T	Och13	*OreG0027628*
scaffold998	3330350	G	A	Och01,Och02,Och03,Och04,Och05,Och06,Och07,Och08,Och09,Och10,Och11,Och12,Och13,Och14,Ore01,Ore02,Ore03,Ore04,Ore05,Ore06,Ore07,Ore08,Ore09,Ore10,Ore11,Ore12,Ore13,Ore14	*OreG0027639*
scaffold998	3330408	C	A	Ore01,Ore02,Ore03,Ore04,Ore05,Ore06,Ore07,Ore08,Ore09,Ore10,Ore11,Ore12,Ore13,Ore14	*OreG0027639*
scaffold998	3330597	C	A	Ore01,Ore02,Ore03,Ore04,Ore05,Ore06,Ore07,Ore08,Ore09,Ore10,Ore11,Ore12,Ore13,Ore14	*OreG0027639*
scaffold998	3363354	G	A	Och07,Och08,Och09,Och10,Och11,Och12,Och14	*OreG0027643*
scaffold998	3370053	A	C	Och01,Och02,Och03,Och04,Och05,Och06,Och07,Och08,Och09,Och10,Och11,Och12,Och13,Och14	*OreG0027644*
scaffold998	3413769	A	G	Ore01,Ore02,Ore03,Ore04,Ore05,Ore06,Ore07,Ore08,Ore09,Ore10,Ore11,Ore12,Ore13,Ore14	*OreG0027646*
scaffold998	3425377	C	T	NA	*OreG0027647*
scaffold998	3470568	C	A	Och01,Och02,Och03,Och04,Och06,Och07,Och08,Och09,Och10,Och11,Och13,Och14	*OreG0027652*
scaffold998	3470598	A	C	Ore04,Ore05	*OreG0027652*
scaffold998	3470679	C	T	Och01,Och02,Och03,Och04,Och06,Och07,Och08,Och09,Och10,Och11,Och13,Och14	*OreG0027652*
scaffold998	3470689	G	T	Ore03,Ore09,Ore10,Ore12,Ore14	*OreG0027652*
scaffold998	3470691	T	G	Ore03,Ore09,Ore10,Ore12,Ore14	*OreG0027652*
scaffold998	3481371	C	A	Och09,Och10,Och11	*OreG0027654*
scaffold998	3481437	T	A	NA	*OreG0027654*
scaffold998	3539110	G	T	Och01,Och02,Och03,Och04,Och05,Och06,Och07,Och08,Och09,Och10,Och11,Och12,Och13,Och14,Ore01,Ore02,Ore03,Ore04,Ore05,Ore06,Ore07,Ore08,Ore09,Ore10,Ore11,Ore12,Ore13,Ore14	*OreG0027659*
scaffold998	3570735	T	G	Ore01,Ore02,Ore03,Ore04,Ore05,Ore06,Ore07,Ore08,Ore09,Ore10,Ore11,Ore12,Ore13,Ore14	*OreG0027662*
scaffold998	3570808	G	T	NA	*OreG0027662*
scaffold998	3579926	A	C	Ore02,Ore08,Ore09,Ore10,Ore11,Ore13	*OreG0027663*
scaffold998	3645411	C	T	Och01,Och02,Och03,Och04,Och05,Och06,Och07,Och08,Och09,Och10,Och11,Och12,Och13,Och14	*OreG0027671*
scaffold998	3645589	T	C	Och07	*OreG0027671*
scaffold998	3645672	A	C	NA	*OreG0027671*
scaffold998	3651898	C	G	Och02,Och03,Och04,Och05,Och06,Och07,Och08,Och09,Och10,Och11,Och12,Och13,Och14	*OreG0027672*
scaffold998	3652294	T	A	Ore01,Ore02,Ore03,Ore04,Ore05,Ore06,Ore07,Ore08,Ore09,Ore10,Ore11,Ore12,Ore13,Ore14	*OreG0027672*
scaffold998	3740733	C	G	Ore01,Ore02,Ore03,Ore04,Ore05,Ore06,Ore07,Ore08,Ore09,Ore10,Ore11,Ore12,Ore13,Ore14	*OreG0027674*
scaffold998	3745512	G	A	NA	*OreG0027674*
scaffold998	3747841	A	T	Ore01,Ore02,Ore03,Ore04,Ore05,Ore06,Ore07,Ore08,Ore09,Ore10,Ore11,Ore12,Ore13,Ore14	*OreG0027674*
scaffold998	3802679	G	A	Och01,Och02,Och03,Och04,Och05,Och06,Och07,Och08,Och09,Och10,Och11,Och12,Och13,Och14	*OreG0027679*
scaffold998	3812857	C	G	Ore01,Ore02,Ore03,Ore04,Ore05,Ore06,Ore07,Ore08,Ore09,Ore10,Ore11,Ore12,Ore13,Ore14	*OreG0027679*
scaffold998	3893188	C	A	NA	*OreG0027687*
scaffold998	3912212	C	T	NA	*OreG0027688*
scaffold998	3917581	T	A	NA	*OreG0027689*
scaffold998	3922389	C	T	NA	*OreG0027690*
scaffold998	3927362	T	G	Ore01,Ore02,Ore03,Ore04,Ore05,Ore06,Ore07,Ore08,Ore09,Ore10,Ore11,Ore12,Ore13,Ore14	*OreG0027690*
scaffold998	3953359	G	A	Och01	*OreG0027693*
scaffold998	4003750	G	A	Och03,Och07,Och11	*OreG0027698*
scaffold998	4016831	C	A	Och01	*OreG0027699*
scaffold998	4018073	C	G	Ore01,Ore02,Ore03,Ore04,Ore05,Ore06,Ore07,Ore08,Ore09,Ore10,Ore11,Ore12,Ore13,Ore14	*OreG0027699*
scaffold998	4066083	A	G	NA	*OreG0027708*
scaffold998	4070476	A	C	NA	*OreG0027708*
scaffold998	4070690	A	T	Och03	*OreG0027708*

scaffold998	4072059	T	G	NA	*OreG0027708*
scaffold998	4078294	A	T	Och03	*OreG0027710*
scaffold998	4110521	G	T	NA	*OreG0027713*
scaffold998	4110569	C	G	Ore01,Ore02,Ore03,Ore04,Ore05,Ore06,Ore07,Ore08,Ore09,Ore10,Ore11,Ore12,Ore13,Ore14	*OreG0027713*
scaffold998	4110603	G	C	NA	*OreG0027713*
scaffold998	4110796	C	T	NA	*OreG0027713*
scaffold998	4110799	C	T	Och03	*OreG0027713*
scaffold998	4110919	A	T	Och12	*OreG0027713*
scaffold998	4111039	G	A	Ore01,Ore02,Ore03,Ore04,Ore05,Ore06,Ore07,Ore08,Ore09,Ore10,Ore11,Ore12,Ore13,Ore14	*OreG0027713*
scaffold998	4151634	C	T	Och02,Och03,Och04,Och05,Och06,Och07,Och08,Och12,Och13,Och14	*OreG0027715*
scaffold998	4151895	C	T	Och02,Och03,Och04,Och05,Och06,Och07,Och08,Och13,Och14	*OreG0027715*
scaffold998	4151912	G	A	Och03,Och06,Och14	*OreG0027715*
scaffold998	4152993	G	T	Och02,Och03,Och04,Och05,Och06,Och07,Och08,Och12,Och13,Och14	*OreG0027715*
scaffold998	4153029	G	A	Och02,Och03,Och04,Och05,Och06,Och07,Och08,Och12,Och13,Och14	*OreG0027715*
scaffold998	4153089	C	T	Och01	*OreG0027715*
scaffold998	4156047	A	G	Och02,Och03,Och04,Och05,Och06,Och07,Och08,Och12,Och13,Och14	*OreG0027716*
scaffold998	4156095	G	A	Och02,Och03,Och04,Och05,Och06,Och07,Och08,Och12,Och13,Och14	*OreG0027716*
scaffold998	4194006	G	A	Och02,Och03,Och04,Och06,Och09,Och10,Och13	*OreG0027720*
scaffold998	4194089	T	A	Och02,Och03,Och04,Och06,Och09,Och10,Och13,Ore03,Ore09,Ore10,Ore12,Ore14	*OreG0027720*
scaffold998	4221683	T	A	Och03	*OreG0027721*
scaffold998	4221745	G	A	Ore09,Ore10,Ore13	*OreG0027721*
scaffold998	4247600	G	A	Ore01,Ore02,Ore03,Ore04,Ore05,Ore06,Ore07,Ore08,Ore09,Ore10,Ore11,Ore12,Ore13,Ore14	*OreG0027724*
scaffold998	4247700	C	T	NA	*OreG0027724*
scaffold998	4273383	A	T	Och03	*OreG0027728*
scaffold998	4275944	C	A	NA	*OreG0027729*
scaffold998	4278679	G	A	NA	*OreG0027729*
scaffold998	4279812	C	A	Ore01,Ore02,Ore03,Ore04,Ore05,Ore06,Ore07,Ore08,Ore09,Ore10,Ore11,Ore12,Ore13,Ore14	*OreG0027729*
scaffold998	4288478	G	A	Ore01,Ore02,Ore03,Ore06,Ore07,Ore08,Ore09,Ore10,Ore11,Ore12,Ore13,Ore14	*OreG0027730*
scaffold998	4288540	A	G	Ore01,Ore02,Ore03,Ore04,Ore05,Ore06,Ore07,Ore08,Ore09,Ore10,Ore11,Ore12,Ore13,Ore14	*OreG0027730*
scaffold998	4288597	A	T	NA	*OreG0027730*
scaffold998	4288915	G	A	Ore02,Ore04,Ore05,Ore08,Ore09,Ore10,Ore11,Ore13	*OreG0027730*
scaffold998	4289068	A	G	Och03	*OreG0027730*
scaffold998	4289074	C	G	Och01,Och02,Och03,Och04,Och05,Och06,Och07,Och08,Och09,Och10,Och11,Och12,Och13	*OreG0027730*
scaffold998	4289272	G	A	NA	*OreG0027730*
scaffold998	4289356	T	C	Och03	*OreG0027730*
scaffold998	4289713	G	C	Och01,Och02,Och03,Och04,Och05,Och06,Och07,Och08,Och09,Och10,Och11,Och12,Och13,Och14,Ore01,Ore02,Ore03,Ore04,Ore05,Ore06,Ore07,Ore08,Ore09,Ore10,Ore11,Ore12,Ore13,Ore14	*OreG0027730*
scaffold998	4290428	C	T	Och01,Och02,Och03,Och04,Och05,Och06,Och07,Och08,Och09,Och10,Och11,Och12,Och13,Och14	*OreG0027730*
scaffold998	4294552	C	A	Och03,Och05,Och08	*OreG0027731*
scaffold998	4294654	C	T	Ore01,Ore02,Ore03,Ore04,Ore05,Ore06,Ore07,Ore08,Ore09,Ore10,Ore11,Ore12,Ore13,Ore14	*OreG0027731*
scaffold998	4294725	C	T	Och01,Och02,Och03,Och04,Och05,Och06,Och07,Och08,Och09,Och10,Och11,Och12,Och13,Och14,Ore01,Ore02,Ore03,Ore04,Ore05,Ore06,Ore07,Ore08,Ore09,Ore10,Ore11,Ore12,Ore13,Ore14	*OreG0027731*
scaffold998	4295056	C	A	NA	*OreG0027731*
scaffold998	4296797	A	G	Ore01,Ore02,Ore03,Ore04,Ore05,Ore06,Ore07,Ore08,Ore09,Ore10,Ore11,Ore12,Ore13,Ore14	*OreG0027731*
scaffold998	4300731	A	G	Ore01,Ore02,Ore03,Ore04,Ore05,Ore06,Ore07,Ore08,Ore09,Ore10,Ore11,Ore12,Ore13,Ore14	*OreG0027732*
scaffold998	4300890	G	A	Och01,Och02,Och03,Och04,Och05,Och06,Och07,Och08,Och09,Och10,Och11,Och12,Och13,Och14,Ore01,Ore02,Ore03,Ore04,Ore05,Ore06,Ore07,Ore08,Ore09,Ore10,Ore11,Ore12,Ore13,Ore14	*OreG0027732*
scaffold998	4301313	A	G	Ore01,Ore02,Ore03,Ore04,Ore05,Ore06,Ore07,Ore08,Ore09,Ore10,Ore11,Ore12,Ore13,Ore14	*OreG0027732*
scaffold998	4355628	A	G	Och02,Och03,Och04,Och05,Och06,Och07,Och08,Och11,Och12,Och13	*OreG0027742*
scaffold998	4355659	A	G	NA	*OreG0027742*
scaffold998	4355713	A	G	Och01,Och02,Och03,Och04,Och05,Och06,Och07,Och08,Och09,Och10,Och11,Och12,Och13,Och14,Ore01,Ore02,Ore03,Ore04,Ore05,Ore06,Ore07,Ore08,Ore09,Ore10,Ore11,Ore12,Ore13,Ore14	*OreG0027742*
scaffold998	4377863	C	A	Och01,Och02,Och03,Och04,Och05,Och06,Och07,Och08,Och09,Och10,Och11,Och12,Och13,Och14,Ore01,Ore02,Ore03,Ore04,Ore05,Ore06,Ore07,Ore08,Ore09,Ore10,Ore11,Ore12,Ore13,Ore14	*OreG0027745*
scaffold998	4394107	C	T	NA	*OreG0027748*
scaffold998	4397269	A	G	NA	*OreG0027748*
scaffold998	4411812	C	T	Och03,Och05,Och08,Och09,Och10,Och11,Ore01,Ore02,Ore03,Ore04,Ore05,Ore06,Ore07,Ore08,Ore09,Ore10,Ore11,Ore12,Ore13,Ore14	*OreG0027750*
scaffold998	4411817	A	G	Och14	*OreG0027750*

scaffold998	4430806	T	A	Och14	*OreG0027753*
scaffold998	4430955	G	A	Och02,Och03,Och04,Och05,Och06,Och12,Och13	*OreG0027753*
scaffold998	4430977	C	T	NA	*OreG0027753*
scaffold998	4431034	G	A	NA	*OreG0027753*
scaffold998	4431091	T	C	NA	*OreG0027753*
scaffold998	4431130	C	A	NA	*OreG0027753*
scaffold998	4431187	C	T	NA	*OreG0027753*
scaffold998	4431210	G	A	Och02,Och03,Och04,Och05,Och06,Och12,Och13	*OreG0027753*
scaffold998	4431241	A	C	NA	*OreG0027753*
scaffold998	4432355	C	A	NA	*OreG0027753*
scaffold998	4453354	C	A	NA	*OreG0027756*
scaffold5701	46606	G	T	NA	*OreG0023674*
scaffold5701	47070	G	A	Och02,Och03,Och04,Och06,Och09,Och10,Och12,Och13,Ore01,Ore02,Ore03,Ore04,Ore05,Ore06,Ore07,Ore08,Ore09,Ore10,Ore11,Ore12,Ore13,Ore14	*OreG0023674*
scaffold5701	49375	G	C	NA	*OreG0023674*
scaffold5701	49510	G	A	Ore02,Ore03,Ore04,Ore05,Ore08,Ore09,Ore10,Ore12,Ore14	*OreG0023674*
scaffold5701	49518	C	T	NA	*OreG0023674*
scaffold5701	49621	T	G	NA	*OreG0023674*
scaffold5701	53703	C	T	Ore02,Ore03,Ore04,Ore05,Ore08,Ore09,Ore10,Ore12,Ore14	*OreG0023674*
scaffold5701	53714	G	T	NA	*OreG0023674*
scaffold5701	54831	A	T	NA	*OreG0023674*
scaffold3610	38078	C	A	NA	*OreG0018942*
scaffold3610	38483	A	G	Och01,Och02,Och03,Och04,Och05,Och06,Och07,Och08,Och09,Och10,Och11,Och12,Och13,Och14,Ore01,Ore02,Ore03,Ore04,Ore05,Ore06,Ore07,Ore08,Ore09,Ore10,Ore11,Ore12,Ore13,Ore14	*OreG0018942*
scaffold3610	38495	A	T	Och01,Och03,Och11	*OreG0018942*
scaffold3610	91659	C	T	NA	*OreG0018948*
scaffold3610	91751	G	A	NA	*OreG0018948*
scaffold3610	106309	T	A	Ore01,Ore02,Ore03,Ore04,Ore05,Ore06,Ore07,Ore08,Ore09,Ore10,Ore11,Ore12,Ore13,Ore14	*OreG0018952*
scaffold3610	106507	C	T	Ore01,Ore02,Ore03,Ore04,Ore05,Ore06,Ore07,Ore08,Ore09,Ore10,Ore11,Ore12,Ore13,Ore14	*OreG0018952*
scaffold3610	113124	A	T	Ore01,Ore04,Ore05,Ore11	*OreG0018953*
scaffold3610	125812	A	G	NA	*OreG0018955*
scaffold3610	131300	G	A	NA	*OreG0018956*
scaffold3610	155710	A	T	Och01,Och07,Och08,Och09,Och10,Och11,Och12,Och13	*OreG0018958*
scaffold3610	156491	G	A	Ore01,Ore02,Ore03,Ore04,Ore05,Ore06,Ore07,Ore08,Ore09,Ore10,Ore11,Ore12,Ore13,Ore14	*OreG0018958*
scaffold3610	156493	A	C	Och12,Och13	*OreG0018958*
scaffold3610	164549	A	T	NA	*OreG0018959*
scaffold3610	178710	T	A	Ore03,Ore06,Ore07,Ore08,Ore10,Ore12,Ore13,Ore14	*OreG0018961*
scaffold3610	229138	G	A	NA	*OreG0018965*
scaffold3610	235671	C	T	Och02,Och03,Och04,Och05,Och06,Och08,Och12,Och13	*OreG0018967*
scaffold3610	236491	T	G	NA	*OreG0018967*
scaffold3610	236871	G	C	Och01	*OreG0018967*
scaffold3610	263696	G	T	NA	*OreG0018972*
scaffold3610	266033	T	A	Och01,Och02,Och03,Och04,Och05,Och06,Och07,Och08,Och09,Och10,Och11,Och12,Och13,Och14,Ore01,Ore02,Ore03,Ore04,Ore05,Ore06,Ore07,Ore08,Ore09,Ore10,Ore11,Ore12,Ore13,Ore14	*OreG0018972*
scaffold3610	268300	T	A	Och01,Och02,Och03,Och04,Och05,Och06,Och07,Och08,Och09,Och10,Och11,Och12,Och13,Och14,Ore01,Ore02,Ore03,Ore04,Ore05,Ore06,Ore07,Ore08,Ore09,Ore10,Ore11,Ore12,Ore13,Ore14	*OreG0018972*
scaffold3610	268520	C	A	Och01,Och02,Och03,Och04,Och05,Och06,Och07,Och08,Och09,Och10,Och11,Och12,Och13,Och14,Ore01,Ore02,Ore03,Ore04,Ore05,Ore06,Ore07,Ore08,Ore09,Ore10,Ore11,Ore12,Ore13,Ore14	*OreG0018972*
scaffold3610	268525	T	A	NA	*OreG0018972*
scaffold3610	273417	G	A	Ore01,Ore02,Ore03,Ore04,Ore05,Ore06,Ore07,Ore08,Ore09,Ore10,Ore11,Ore12,Ore13,Ore14	*OreG0018973*
scaffold3610	293351	C	A	NA	*OreG0018975*
scaffold3610	293353	A	G	NA	*OreG0018975*
scaffold3610	308070	T	A	Och01,Och02,Och03,Och04,Och05,Och06,Och07,Och08,Och09,Och10,Och11,Och12,Och13,Och14,Ore01,Ore02,Ore03,Ore04,Ore05,Ore06,Ore07,Ore08,Ore09,Ore10,Ore11,Ore12,Ore13,Ore14	*OreG0018976*
scaffold3610	387939	T	A	NA	*OreG0018978*
scaffold3610	387978	C	T	NA	*OreG0018978*
scaffold3610	389578	A	T	NA	*OreG0018978*
scaffold3610	415461	T	A	NA	*OreG0018981*
scaffold3610	444914	C	A	NA	*OreG0018983*
scaffold3610	459977	G	A	Ore01,Ore04,Ore05,Ore11	*OreG0018984*
scaffold3610	460802	G	C	Ore01,Ore02,Ore03,Ore04,Ore05,Ore06,Ore07,Ore08,Ore09,Ore10,Ore11,Ore12,Ore13,Ore14	*OreG0018984*
scaffold3610	474607	G	A	Ore01,Ore02,Ore03,Ore04,Ore05,Ore06,Ore07,Ore08,Ore09,Ore10,Ore11,Ore12,Ore13,Ore14	*OreG0018987*
scaffold3610	474875	A	G	Och01,Och02,Och03,Och04,Och05,Och06,Och07,Och08,Och09,Och10,Och11,Och12,Och13,Och14	*OreG0018987*

scaffold3610	505770	C	A	NA	*OreG0018990*
scaffold3610	506439	C	T	Ore01,Ore02,Ore03,Ore04,Ore05,Ore06,Ore07,Ore08,Ore09,Ore10,Ore11,Ore12,Ore13,Ore14	*OreG0018990*
scaffold3610	507000	G	T	Och07	*OreG0018990*
scaffold3610	513583	T	A	Ore06,Ore07,Ore08,Ore10,Ore12,Ore13,Ore14	*OreG0018992*
scaffold3610	529002	G	A	NA	*OreG0018993*
scaffold3610	529126	T	C	Och01,Och02,Och03,Och04,Och05,Och06,Och07,Och08,Och09,Och10,Och11,Och12,Och13,Och14,Ore01,Ore02,Ore03,Ore04,Ore05,Ore06,Ore07,Ore08,Ore09,Ore10,Ore11,Ore12,Ore13,Ore14	*OreG0018993*
scaffold3610	529191	G	C	Och01,Och02,Och03,Och04,Och05,Och06,Och07,Och08,Och09,Och10,Och11,Och12,Och13,Och14,Ore01,Ore02,Ore03,Ore04,Ore05,Ore06,Ore07,Ore08,Ore09,Ore10,Ore11,Ore12,Ore13,Ore14	*OreG0018993*
scaffold3610	529340	C	A	Och01,Och02,Och03,Och04,Och05,Och06,Och07,Och08,Och09,Och10,Och11,Och12,Och13,Och14,Ore01,Ore02,Ore03,Ore04,Ore05,Ore06,Ore07,Ore08,Ore09,Ore10,Ore11,Ore12,Ore13,Ore14	*OreG0018993*
scaffold3610	529353	T	C	NA	*OreG0018993*
scaffold3610	529464	T	C	NA	*OreG0018993*
scaffold3610	529992	C	A	NA	*OreG0018993*
scaffold3610	530024	T	A	NA	*OreG0018993*
scaffold3610	531969	T	A	Och12,Och13	*OreG0018993*
scaffold3610	684013	T	A	Och01,Och02,Och03,Och04,Och05,Och07,Och08,Och09,Och10,Och11,Och12,Och13,Och14,Ore01,Ore02,Ore03,Ore04,Ore05,Ore06,Ore07,Ore08,Ore09,Ore10,Ore11,Ore12,Ore13,Ore14	*OreG0018994*
scaffold3610	686829	C	T	Och12,Och13	*OreG0018994*
scaffold3610	733944	G	C	Och01,Och02,Och03,Och04,Och05,Och06,Och07,Och08,Och09,Och10,Och11,Och12,Och13,Och14	*OreG0019000*
scaffold3610	736572	T	A	Och01,Och02,Och03,Och04,Och05,Och06,Och07,Och08,Och09,Och10,Och11,Och12,Och13,Och14,Ore01,Ore02,Ore03,Ore04,Ore05,Ore06,Ore07,Ore08,Ore09,Ore10,Ore11,Ore12,Ore13,Ore14	*OreG0019001*
scaffold3610	737046	A	G	Ore01,Ore02,Ore03,Ore04,Ore05,Ore06,Ore07,Ore08,Ore09,Ore10,Ore11,Ore12,Ore13,Ore14	*OreG0019001*
scaffold3610	738522	G	T	Ore01,Ore02,Ore03,Ore04,Ore05,Ore06,Ore07,Ore08,Ore09,Ore10,Ore11,Ore12,Ore13,Ore14	*OreG0019001*
scaffold3610	766217	C	T	Ore01,Ore02,Ore03,Ore04,Ore05,Ore06,Ore07,Ore08,Ore09,Ore10,Ore11,Ore12,Ore13,Ore14	*OreG0019007*
scaffold3610	772524	A	G	Och03	*OreG0019008*
scaffold3610	852281	G	C	NA	*OreG0019014*
scaffold3610	889167	C	T	Ore01,Ore04,Ore05,Ore11	*OreG0019017*
scaffold3610	905862	G	A	Och01,Och02,Och03,Och04,Och05,Och06,Och07,Och08,Och09,Och10,Och11,Och12,Och13,Och14,Ore01,Ore02,Ore03,Ore04,Ore05,Ore06,Ore07,Ore08,Ore09,Ore10,Ore11,Ore12,Ore13,Ore14	*OreG0019018*
scaffold3610	910910	C	T	Ore01,Ore02,Ore03,Ore04,Ore05,Ore06,Ore07,Ore08,Ore09,Ore10,Ore11,Ore12,Ore13,Ore14	*OreG0019019*
scaffold3610	955780	A	T	NA	*OreG0019024*
scaffold3610	968055	C	G	Ore03,Ore06,Ore07,Ore08,Ore10,Ore12,Ore13,Ore14	*OreG0019025*
scaffold3610	968093	A	G	Ore01,Ore02,Ore03,Ore04,Ore05,Ore06,Ore07,Ore08,Ore09,Ore10,Ore11,Ore12,Ore13,Ore14	*OreG0019025*
scaffold3610	975587	G	T	Och01,Och02,Och03,Och04,Och05,Och06,Och07,Och08,Och11,Och12,Och13,Och14	*OreG0019028*
scaffold3610	975591	G	A	Och01,Och07	*OreG0019028*
scaffold3610	975606	C	G	NA	*OreG0019028*
scaffold3610	975909	A	C	Och01,Och02,Och03,Och04,Och05,Och06,Och07,Och08,Och09,Och10,Och11,Och12,Och13,Och14,Ore06,Ore07,Ore08,Ore10,Ore12,Ore13,Ore14	*OreG0019028*
scaffold3610	976125	A	G	Och01,Och02,Och03,Och04,Och05,Och06,Och07,Och08,Och11,Och12,Och13,Och14	*OreG0019028*
scaffold7557	4859	G	T	Och12,Och13	*OreG0025280*
scaffold7557	56528	C	T	NA	*OreG0025283*
scaffold7557	56600	A	C	Och01,Och02,Och03,Och04,Och05,Och06,Och07,Och08,Och09,Och10,Och11,Och12,Och13,Och14,Ore01,Ore02,Ore03,Ore04,Ore05,Ore06,Ore07,Ore08,Ore09,Ore10,Ore11,Ore12,Ore13,Ore14	*OreG0025283*
scaffold7557	63723	G	A	NA	*OreG0025284*
scaffold4392	79810	G	A	Ore02,Ore03,Ore04,Ore05,Ore08,Ore09,Ore10,Ore12,Ore14	*OreG0020349*
scaffold4392	85871	C	T	Ore02,Ore03,Ore04,Ore05,Ore08,Ore09,Ore10,Ore12,Ore14	*OreG0020350*
scaffold4392	102279	G	T	Och01,Och02,Och03,Och04,Och06,Och09,Och10,Och12,Och13,Ore01,Ore02,Ore03,Ore04,Ore05,Ore06,Ore07,Ore08,Ore09,Ore10,Ore11,Ore12,Ore13,Ore14	*OreG0020351*
scaffold4392	109293	T	G	NA	*OreG0020351*
scaffold10278	10796	C	T	NA	*OreG0000136*
scaffold10278	10808	C	G	NA	*OreG0000136*
scaffold10278	10988	C	T	Och03,Och04,Och05,Och06,Och12,Och13	*OreG0000136*
scaffold10278	11032	C	T	NA	*OreG0000136*
scaffold10278	15808	G	T	Och04,Och06	*OreG0000136*
scaffold11409	1156	G	T	Ore05,Ore09,Ore13	*OreG0000799*
scaffold11409	1190	C	A	NA	*OreG0000799*
scaffold11409	1306	A	T	NA	*OreG0000799*
scaffold2136	50667	C	T	NA	*OreG0005248*

scaffold2136	52700	T	G	NA	*OreG0005248*
scaffold2136	53257	G	A	NA	*OreG0005248*
scaffold2136	78284	T	G	Ore01,Ore02,Ore03,Ore04,Ore05,Ore06,Ore07,Ore08,Ore09,Ore10,Ore11,Ore12,Ore13,Ore14	*OreG0005251*
scaffold2136	78504	C	A	Ore01,Ore02,Ore03,Ore04,Ore05,Ore06,Ore07,Ore08,Ore09,Ore10,Ore11,Ore12,Ore13,Ore14	*OreG0005251*
scaffold2136	78548	T	A	NA	*OreG0005251*
scaffold2136	78572	C	A	NA	*OreG0005251*
scaffold2136	78752	A	G	NA	*OreG0005251*
scaffold2136	78892	G	A	Och07	*OreG0005251*
scaffold2136	93898	A	G	NA	*OreG0005254*
scaffold2136	109536	C	G	NA	*OreG0005255*
scaffold2136	117868	A	G	Och01,Och02,Och03,Och04,Och05,Och06,Och07,Och08,Och09,Och10,Och11,Och12,Och13,Och14,Ore01,Ore02,Ore03,Ore04,Ore05,Ore06,Ore07,Ore08,Ore09,Ore10,Ore11,Ore12,Ore13,Ore14	*OreG0005256*
scaffold2136	118239	A	T	Och01,Och02,Och03,Och04,Och05,Och06,Och07,Och08,Och09,Och10,Och11,Och12,Och13,Och14,Ore01,Ore02,Ore03,Ore04,Ore05,Ore06,Ore07,Ore08,Ore09,Ore10,Ore11,Ore12,Ore13,Ore14	*OreG0005256*
scaffold2136	118782	C	G	Och01,Och02,Och03,Och04,Och05,Och06,Och07,Och08,Och09,Och10,Och11,Och12,Och13,Och14,Ore01,Ore02,Ore03,Ore04,Ore05,Ore06,Ore07,Ore08,Ore09,Ore10,Ore11,Ore12,Ore13,Ore14	*OreG0005256*
scaffold2136	125426	G	A	Ore01,Ore02,Ore03,Ore04,Ore05,Ore06,Ore07,Ore08,Ore09,Ore10,Ore11,Ore12,Ore13,Ore14	*OreG0005257*
scaffold2136	127369	G	T	Och04,Och05,Och07,Och08,Och09,Och10,Och12,Och13,Och14	*OreG0005257*
scaffold2136	134569	T	A	Och04,Och05,Och07,Och08,Och09,Och10,Och12,Och13,Och14	*OreG0005258*
scaffold2136	139166	G	A	NA	*OreG0005259*
scaffold2136	139343	G	A	NA	*OreG0005259*
scaffold2136	139606	C	T	NA	*OreG0005259*
scaffold2136	140191	A	C	Och01,Och02,Och03,Och04,Och05,Och06,Och07,Och08,Och09,Och10,Och11,Och12,Och13,Och14,Ore01,Ore02,Ore03,Ore04,Ore05,Ore06,Ore07,Ore08,Ore09,Ore10,Ore11,Ore12,Ore13,Ore14	*OreG0005259*
scaffold2136	153346	A	G	Och11	*OreG0005261*
scaffold2136	153500	C	T	Och11	*OreG0005261*
scaffold2136	159147	G	A	NA	*OreG0005262*
scaffold2136	159461	G	A	NA	*OreG0005262*
scaffold2136	159546	G	A	NA	*OreG0005262*
scaffold2136	159623	T	A	NA	*OreG0005262*
scaffold2136	160298	G	A	NA	*OreG0005262*
scaffold2136	160323	G	A	NA	*OreG0005262*
scaffold2136	160407	A	G	NA	*OreG0005262*
scaffold2136	160464	G	A	NA	*OreG0005262*
scaffold2136	160525	G	T	NA	*OreG0005262*
scaffold2136	160584	T	A	NA	*OreG0005262*
scaffold2136	162691	T	C	NA	*OreG0005262*
scaffold2136	220044	T	A	NA	*OreG0005270*
scaffold2136	229307	C	T	Och13,Ore01,Ore02,Ore03,Ore04,Ore05,Ore06,Ore07,Ore08,Ore09,Ore10,Ore11,Ore12,Ore13,Ore14	*OreG0005271*
scaffold2136	269260	G	C	Och01,Och02,Och03,Och04,Och05,Och06,Och07,Och08,Och09,Och10,Och11,Och12,Och13,Och14,Ore01,Ore02,Ore03,Ore04,Ore05,Ore06,Ore07,Ore08,Ore09,Ore10,Ore11,Ore12,Ore13,Ore14	*OreG0005275*
scaffold2136	270331	A	T	NA	*OreG0005275*
scaffold2136	270389	T	G	Ore01,Ore02,Ore03,Ore04,Ore05,Ore06,Ore07,Ore08,Ore09,Ore10,Ore11,Ore12,Ore13,Ore14	*OreG0005275*
scaffold2136	280591	C	T	NA	*OreG0005278*
scaffold2136	283878	C	T	Och01,Och08	*OreG0005278*
scaffold2136	284605	T	C	NA	*OreG0005278*
scaffold2136	284787	C	T	NA	*OreG0005278*
scaffold2136	296083	A	G	Och01,Och08	*OreG0005280*
scaffold2136	296993	A	T	NA	*OreG0005280*
scaffold2136	297412	G	T	NA	*OreG0005280*
scaffold2136	297427	T	C	Och03,Och12	*OreG0005280*
scaffold2136	297749	C	A	Ore01,Ore02,Ore03,Ore04,Ore05,Ore06,Ore07,Ore08,Ore09,Ore10,Ore11,Ore12,Ore13,Ore14	*OreG0005280*
scaffold2136	309091	A	G	Och02,Och06,Och09,Och10,Och11,Och14	*OreG0005281*
scaffold2136	310066	T	C	NA	*OreG0005281*
scaffold2136	310103	A	C	NA	*OreG0005281*
scaffold2136	310120	A	G	Och01,Och02,Och04,Och05,Och06,Och07,Och08,Och09,Och10,Och11,Och12,Och13,Och14,Ore01,Ore02,Ore03,Ore04,Ore05,Ore06,Ore07,Ore08,Ore09,Ore10,Ore11,Ore12,Ore13,Ore14	*OreG0005281*
scaffold2136	310258	G	T	NA	*OreG0005281*
scaffold2136	310610	T	C	NA	*OreG0005282*
scaffold2136	329804	G	C	Ore01,Ore02,Ore03,Ore04,Ore05,Ore06,Ore07,Ore08,Ore09,Ore10,Ore11,Ore12,Ore13,Ore14	*OreG0005285*
scaffold2136	331320	C	T	NA	*OreG0005285*
scaffold2136	380191	T	A	NA	*OreG0005289*

scaffold2136	437121	A	T	Ore01,Ore02,Ore03,Ore04,Ore05,Ore06,Ore07,Ore08,Ore09,Ore10,Ore11,Ore12,Ore13,Ore14	*OreG0005296*
scaffold2136	454083	A	T	NA	*OreG0005298*
scaffold2136	458690	A	G	Och01,Och02,Och03,Och04,Och05,Och06,Och07,Och08,Och09,Och10,Och11,Och12,Och13,Och14	*OreG0005298*
scaffold2136	474058	G	A	NA	*OreG0005301*
scaffold2136	488720	C	T	NA	*OreG0005303*
scaffold2136	498444	A	T	Och01,Och02,Och03,Och04,Och05,Och06,Och07,Och08,Och09,Och10,Och11,Och12,Och13,Och14,Ore01,Ore02,Ore03,Ore04,Ore05,Ore06,Ore07,Ore08,Ore09,Ore10,Ore11,Ore12,Ore13,Ore14	*OreG0005304*
scaffold2136	541967	T	C	NA	*OreG0005309*
scaffold2136	552485	G	A	NA	*OreG0005310*
scaffold2136	552498	T	C	NA	*OreG0005310*
scaffold2136	555289	G	A	Och02	*OreG0005310*
scaffold2136	555295	A	G	Ore01,Ore02,Ore03,Ore04,Ore05,Ore06,Ore07,Ore08,Ore09,Ore10,Ore11,Ore12,Ore13,Ore14	*OreG0005310*
scaffold2136	555391	C	T	NA	*OreG0005310*
scaffold2136	556744	T	C	Och02,Och06,Och09,Och10	*OreG0005311*
scaffold2136	556747	T	G	NA	*OreG0005311*
scaffold2136	557112	G	T	NA	*OreG0005311*
scaffold2136	572764	G	A	Och02,Och06	*OreG0005312*
scaffold2136	572777	T	C	NA	*OreG0005312*
scaffold2136	574577	T	C	NA	*OreG0005312*
scaffold2136	574715	G	A	NA	*OreG0005312*
scaffold2136	575450	C	T	Ore01,Ore02,Ore03,Ore04,Ore05,Ore06,Ore07,Ore08,Ore09,Ore10,Ore11,Ore12,Ore13,Ore14	*OreG0005312*
scaffold2136	575908	A	C	NA	*OreG0005312*
scaffold2136	584085	T	G	NA	*OreG0005313*
scaffold2136	629976	T	A	Ore01,Ore02,Ore03,Ore04,Ore05,Ore06,Ore07,Ore08,Ore09,Ore10,Ore11,Ore12,Ore13,Ore14	*OreG0005316*
scaffold2136	629983	C	A	Och01,Och02,Och03,Och04,Och05,Och06,Och07,Och08,Och09,Och10,Och11,Och12,Och13,Och14	*OreG0005316*
scaffold2136	633681	T	C	Ore01,Ore02,Ore03,Ore04,Ore05,Ore06,Ore07,Ore08,Ore09,Ore10,Ore11,Ore12,Ore13,Ore14	*OreG0005317*
scaffold2136	635713	A	T	Ore01,Ore02,Ore03,Ore04,Ore05,Ore06,Ore07,Ore08,Ore09,Ore10,Ore11,Ore12,Ore13,Ore14	*OreG0005317*
scaffold2136	636147	G	T	Och01,Och02,Och03,Och04,Och05,Och06,Och07,Och08,Och09,Och10,Och11,Och12,Och13,Och14	*OreG0005317*
scaffold2136	637293	C	T	Ore01,Ore02,Ore03,Ore04,Ore05,Ore06,Ore07,Ore08,Ore09,Ore10,Ore11,Ore12,Ore13,Ore14	*OreG0005317*
scaffold2136	637971	C	T	Ore01,Ore02,Ore03,Ore04,Ore05,Ore06,Ore07,Ore08,Ore09,Ore10,Ore11,Ore12,Ore13,Ore14	*OreG0005317*
scaffold2136	638024	C	T	NA	*OreG0005317*
scaffold2136	641003	G	T	Ore01,Ore02,Ore03,Ore04,Ore05,Ore06,Ore07,Ore08,Ore09,Ore10,Ore11,Ore12,Ore13,Ore14	*OreG0005317*
scaffold2136	641565	T	A	NA	*OreG0005317*
scaffold2136	641681	C	T	NA	*OreG0005317*
scaffold2136	641768	T	C	Och01,Och02,Och03,Och04,Och05,Och06,Och12,Och13	*OreG0005317*
scaffold2136	641770	A	C	Och01,Och02,Och03,Och04,Och05,Och06,Och12,Och13	*OreG0005317*
scaffold2136	650315	G	A	Och01,Och02,Och03,Och04,Och05,Och06,Och07,Och08,Och09,Och10,Och11,Och12,Och13,Och14	*OreG0005319*
scaffold2136	650780	C	A	Ore01,Ore02,Ore03,Ore04,Ore05,Ore06,Ore07,Ore08,Ore09,Ore10,Ore11,Ore12,Ore13,Ore14	*OreG0005319*
scaffold2136	712564	C	T	NA	*OreG0005326*
scaffold2136	712874	C	T	NA	*OreG0005326*
scaffold2136	723067	G	A	NA	*OreG0005328*
scaffold2136	737096	C	T	Och01,Och02,Och03,Och04,Och05,Och06,Och07,Och08,Och09,Och10,Och11,Och12,Och13,Och14,Ore01,Ore02,Ore03,Ore04,Ore05,Ore06,Ore07,Ore08,Ore09,Ore10,Ore11,Ore12,Ore13,Ore14	*OreG0005330*
scaffold2136	739586	G	A	NA	*OreG0005330*
scaffold2136	793969	C	G	Ore01,Ore02,Ore03,Ore04,Ore05,Ore06,Ore07,Ore08,Ore09,Ore10,Ore11,Ore12,Ore13,Ore14	*OreG0005337*
scaffold2136	808549	G	T	NA	*OreG0005338*
scaffold2136	808656	G	T	NA	*OreG0005338*
scaffold2136	808955	A	T	NA	*OreG0005338*
scaffold2136	809101	G	A	NA	*OreG0005338*
scaffold2136	810112	G	A	NA	*OreG0005338*
scaffold2136	815851	A	G	NA	*OreG0005339*
scaffold2136	826213	A	T	NA	*OreG0005340*
scaffold2136	826716	G	A	Ore01,Ore02,Ore03,Ore04,Ore05,Ore06,Ore07,Ore08,Ore09,Ore10,Ore11,Ore12,Ore13,Ore14	*OreG0005340*
scaffold2136	827179	A	G	Och01,Och02,Och03,Och04,Och05,Och06,Och07,Och08,Och09,Och10,Och11,Och12,Och13,Och14	*OreG0005340*
scaffold2136	827245	G	A	NA	*OreG0005340*

scaffold2136	828276	C	A	Ore01,Ore02,Ore03,Ore04,Ore05,Ore06,Ore07,Ore08,Ore09,Ore10,Ore11,Ore12,Ore13,Ore14	*OreG0005340*
scaffold2136	833321	C	T	NA	*OreG0005341*
scaffold2136	833808	T	G	Och01,Och02,Och03,Och04,Och05,Och06,Och07,Och08,Och09,Och10,Och11,Och12,Och13,Och14,Ore01,Ore02,Ore03,Ore04,Ore05,Ore06,Ore07,Ore08,Ore09,Ore10,Ore11,Ore12,Ore13,Ore14	*OreG0005341*
scaffold2136	834441	A	C	NA	*OreG0005341*
scaffold2136	843646	T	C	Och01,Och02,Och03,Och04,Och05,Och06,Och07,Och08,Och09,Och10,Och11,Och12,Och13,Och14,Ore01,Ore02,Ore03,Ore04,Ore05,Ore06,Ore07,Ore08,Ore09,Ore10,Ore11,Ore12,Ore13,Ore14	*OreG0005342*
scaffold2136	846415	A	T	NA	*OreG0005342*
scaffold2136	854269	T	G	NA	*OreG0005344*
scaffold2136	880574	G	A	NA	*OreG0005345*
scaffold2136	880592	C	T	NA	*OreG0005345*
scaffold2136	886770	T	A	NA	*OreG0005347*
scaffold2136	887110	A	G	Och02,Och06,Och08,Ore01,Ore02,Ore03,Ore04,Ore05,Ore06,Ore07,Ore08,Ore09,Ore10,Ore11,Ore12,Ore13,Ore14	*OreG0005347*
scaffold2136	888384	A	C	Och01,Och02,Och03,Och04,Och05,Och06,Och07,Och08,Och09,Och10,Och11,Och12,Och13,Och14,Ore01,Ore02,Ore03,Ore04,Ore05,Ore06,Ore07,Ore08,Ore09,Ore10,Ore11,Ore12,Ore13,Ore14	*OreG0005347*
scaffold2136	888606	T	C	Och07	*OreG0005347*
scaffold2136	888720	G	T	Och01,Och02,Och03,Och04,Och05,Och06,Och07,Och08,Och09,Och10,Och11,Och12,Och13,Och14	*OreG0005347*
scaffold2136	891688	G	C	Och01,Och02,Och03,Och04,Och05,Och06,Och07,Och08,Och09,Och10,Och11,Och12,Och13,Och14,Ore01,Ore02,Ore03,Ore04,Ore05,Ore06,Ore07,Ore08,Ore09,Ore10,Ore11,Ore12,Ore13,Ore14	*OreG0005347*
scaffold2136	891736	A	G	NA	*OreG0005347*
scaffold2136	891739	G	T	NA	*OreG0005347*
scaffold2136	905906	T	G	Ore01,Ore02,Ore03,Ore04,Ore05,Ore06,Ore07,Ore08,Ore09,Ore10,Ore11,Ore12,Ore13,Ore14	*OreG0005348*
scaffold2136	906884	C	T	NA	*OreG0005348*
scaffold2136	907156	A	G	Ore01,Ore02,Ore03,Ore04,Ore05,Ore06,Ore07,Ore08,Ore09,Ore10,Ore11,Ore12,Ore13,Ore14	*OreG0005348*
scaffold2136	911318	T	A	Och01,Och02,Och03,Och04,Och05,Och06,Och07,Och08,Och09,Och10,Och11,Och12,Och13,Och14,Ore01,Ore02,Ore03,Ore04,Ore05,Ore06,Ore07,Ore08,Ore09,Ore10,Ore11,Ore12,Ore13,Ore14	*OreG0005349*
scaffold2136	949082	C	T	Ore01,Ore02,Ore03,Ore04,Ore05,Ore06,Ore07,Ore08,Ore09,Ore10,Ore11,Ore12,Ore13,Ore14	*OreG0005351*
scaffold2136	953557	C	T	NA	*OreG0005352*
scaffold2136	956181	C	G	Och01,Och02,Och03,Och04,Och05,Och06,Och07,Och08,Och09,Och10,Och11,Och12,Och13,Och14	*OreG0005353*
scaffold2136	956183	C	G	Och01,Och02,Och03,Och04,Och05,Och06,Och07,Och09,Och10,Och11,Och12,Och13,Och14	*OreG0005353*
scaffold2136	956280	A	G	NA	*OreG0005353*
scaffold2136	956462	T	A	Ore01,Ore02,Ore03,Ore04,Ore05,Ore06,Ore07,Ore08,Ore09,Ore10,Ore11,Ore12,Ore13,Ore14	*OreG0005353*
scaffold2136	956669	C	A	NA	*OreG0005353*
scaffold2136	957173	A	G	Och01,Och02,Och03,Och04,Och05,Och06,Och07,Och08,Och09,Och10,Och11,Och12,Och13,Och14	*OreG0005353*
scaffold2136	957284	A	T	Ore01,Ore02,Ore03,Ore04,Ore05,Ore06,Ore07,Ore08,Ore09,Ore10,Ore11,Ore12,Ore13,Ore14	*OreG0005353*
scaffold2136	960149	T	A	NA	*OreG0005354*
scaffold2136	964881	A	T	NA	*OreG0005355*
scaffold2136	976919	C	T	NA	*OreG0005356*
scaffold2136	980158	C	T	Och01,Och03,Och07,Och09,Och10,Och11,Och13,Och14	*OreG0005356*
scaffold2136	991181	A	T	Ore01,Ore02,Ore03,Ore04,Ore05,Ore06,Ore07,Ore08,Ore09,Ore10,Ore11,Ore12,Ore13,Ore14	*OreG0005357*
scaffold2136	1024324	T	C	NA	*OreG0005364*
scaffold2136	1024950	A	C	Och01,Och02,Och03,Och04,Och05,Och06,Och07,Och08,Och09,Och10,Och11,Och12,Och13,Och14,Ore01,Ore02,Ore03,Ore04,Ore05,Ore06,Ore07,Ore08,Ore09,Ore10,Ore11,Ore12,Ore13,Ore14	*OreG0005364*
scaffold2136	1025459	T	A	Och01,Och02,Och03,Och04,Och05,Och06,Och07,Och08,Och09,Och10,Och11,Och12,Och13,Och14	*OreG0005364*
scaffold2136	1035073	A	C	Och01,Och02,Och03,Och04,Och05,Och06,Och07,Och08,Och09,Och10,Och11,Och12,Och13,Och14,Ore01,Ore02,Ore03,Ore04,Ore05,Ore06,Ore07,Ore08,Ore09,Ore10,Ore11,Ore12,Ore13,Ore14	*OreG0005366*
scaffold2136	1059005	G	T	NA	*OreG0005369*
scaffold2136	1064655	T	C	Och01,Och02,Och03,Och04,Och05,Och06,Och07,Och08,Och09,Och10,Och11,Och12,Och13,Och14	*OreG0005370*
scaffold2136	1064677	C	T	Och01,Och02,Och03,Och04,Och05,Och06,Och07,Och08,Och09,Och10,Och11,Och12,Och13,Och14	*OreG0005370*
scaffold2136	1064818	C	A	Och01,Och02,Och03,Och04,Och05,Och06,Och07,Och08,Och09,Och10,Och11,Och12,Och13,Och14	*OreG0005370*
scaffold2136	1067167	C	G	NA	*OreG0005371*
scaffold2136	1067374	C	T	Ore01,Ore02,Ore03,Ore04,Ore05,Ore06,Ore07,Ore08,Ore09,Ore10,Ore11,Ore12,Ore13,Ore14	*OreG0005371*

scaffold2136	1067428	G	A	NA	*OreG0005371*
scaffold2136	1067454	G	C	Ore01,Ore02,Ore03,Ore04,Ore05,Ore06,Ore07,Ore08,Ore09,Ore10,Ore11,Ore12,Ore13,Ore14	*OreG0005371*
scaffold2136	1067548	A	G	NA	*OreG0005371*
scaffold2136	1073257	C	T	NA	*OreG0005372*
scaffold2136	1074510	G	T	NA	*OreG0005373*
scaffold2136	1074663	G	A	Och02,Och06	*OreG0005373*
scaffold2136	1074762	G	A	Och01,Och02,Och03,Och04,Och05,Och06,Och07,Och08,Och09,Och10,Och11,Och12,Och13,Och14	*OreG0005373*
scaffold2136	1074831	A	T	Och01,Och02,Och03,Och04,Och05,Och06,Och07,Och08,Och09,Och10,Och11,Och12,Och13,Och14	*OreG0005373*
scaffold2136	1074873	C	T	Och01,Och02,Och03,Och04,Och05,Och06,Och07,Och08,Och09,Och10,Och11,Och12,Och13,Och14	*OreG0005373*
scaffold2136	1074900	T	G	Och01,Och02,Och03,Och04,Och05,Och06,Och07,Och12,Och13	*OreG0005373*
scaffold2136	1077284	C	A	NA	*OreG0005374*
scaffold2136	1077289	G	A	Och08,Och09,Och10	*OreG0005374*
scaffold2136	1077316	C	T	Och01,Och02,Och03,Och04,Och05,Och06,Och07,Och08,Och09,Och10,Och11,Och12,Och13,Och14	*OreG0005374*
scaffold2136	1077380	T	A	Och01,Och02,Och03,Och04,Och05,Och06,Och07,Och08,Och09,Och10,Och11,Och12,Och13,Och14	*OreG0005374*
scaffold2136	1077385	G	A	Och01,Och02,Och03,Och04,Och05,Och06,Och07,Och08,Och09,Och10,Och11,Och12,Och13,Och14	*OreG0005374*
scaffold2136	1077398	C	T	Och01,Och02,Och03,Och04,Och05,Och06,Och07,Och08,Och09,Och10,Och11,Och12,Och13,Och14	*OreG0005374*
scaffold2136	1078513	G	A	Och01,Och02,Och03,Och04,Och05,Och06,Och07,Och08,Och09,Och10,Och11,Och12,Och13,Och14,Ore01,Ore02,Ore03,Ore04,Ore05,Ore06,Ore07,Ore08,Ore09,Ore10,Ore11,Ore12,Ore13,Ore14	*OreG0005375*
scaffold2136	1078538	G	T	NA	*OreG0005375*
scaffold2136	1078556	C	T	Och01,Och02,Och03,Och04,Och05,Och06,Och07,Och08,Och09,Och10,Och11,Och12,Och13,Och14	*OreG0005375*
scaffold2136	1078637	C	T	Ore01,Ore02,Ore03,Ore04,Ore05,Ore06,Ore07,Ore08,Ore09,Ore10,Ore11,Ore12,Ore13,Ore14	*OreG0005375*
scaffold2136	1078679	C	T	NA	*OreG0005375*
scaffold2136	1082983	C	A	Och02,Och06	*OreG0005376*
scaffold2136	1083105	C	T	NA	*OreG0005376*
scaffold2136	1088164	G	A	NA	*OreG0005377*
scaffold2136	1112935	G	T	Och01,Och03,Och04,Och05,Och06,Och07,Och08,Och09,Och10,Och11,Och12,Och13,Och14	*OreG0005380*
scaffold2136	1121223	G	A	NA	*OreG0005381*
scaffold2136	1146101	A	G	NA	*OreG0005383*
scaffold2136	1146130	A	G	NA	*OreG0005383*
scaffold2136	1146460	C	T	NA	*OreG0005383*
scaffold2136	1146526	G	A	Ore01,Ore02,Ore03,Ore04,Ore05,Ore06,Ore07,Ore08,Ore09,Ore10,Ore11,Ore12,Ore13,Ore14	*OreG0005383*
scaffold2136	1152352	A	C	NA	*OreG0005385*
scaffold2136	1153809	G	A	NA	*OreG0005385*
scaffold2136	1154944	A	G	NA	*OreG0005385*
scaffold2136	1158237	T	G	Och01,Och02,Och03,Och04,Och05,Och06,Och07,Och08,Och09,Och10,Och11,Och12,Och13,Och14,Ore01,Ore02,Ore03,Ore04,Ore05,Ore06,Ore07,Ore08,Ore09,Ore10,Ore11,Ore12,Ore13,Ore14	*OreG0005387*
scaffold2136	1186266	T	A	NA	*OreG0005390*
scaffold2136	1186965	A	G	Och07	*OreG0005390*
scaffold2136	1189266	C	T	NA	*OreG0005390*
scaffold2136	1194035	A	T	Ore01,Ore02,Ore03,Ore04,Ore05,Ore06,Ore07,Ore08,Ore09,Ore10,Ore11,Ore12,Ore13,Ore14	*OreG0005391*
scaffold2136	1197851	G	A	Och07	*OreG0005391*
scaffold2136	1234372	C	T	NA	*OreG0005398*
scaffold2136	1237332	G	C	Och07,Och08,Ore01,Ore03,Ore08,Ore12	*OreG0005399*
scaffold2136	1243263	A	T	Och03,Och07,Och08,Och09,Och10,Och11,Och13,Och14,Ore01,Ore02,Ore03,Ore04,Ore05,Ore06,Ore07,Ore08,Ore09,Ore10,Ore11,Ore12,Ore13,Ore14	*OreG0005400*
scaffold2136	1243272	C	T	NA	*OreG0005400*
scaffold2136	1243343	C	T	NA	*OreG0005400*
scaffold2136	1243376	C	T	NA	*OreG0005400*
scaffold2136	1243458	C	A	NA	*OreG0005400*
scaffold2136	1243466	G	A	NA	*OreG0005400*
scaffold2136	1243493	G	A	Ore01,Ore02,Ore03,Ore04,Ore05,Ore06,Ore07,Ore08,Ore09,Ore10,Ore11,Ore12,Ore13,Ore14	*OreG0005400*
scaffold2136	1244307	G	C	NA	*OreG0005400*
scaffold2136	1244383	G	A	NA	*OreG0005400*
scaffold2136	1244392	C	T	NA	*OreG0005400*
scaffold2136	1244420	C	T	Och03,Och07,Och08,Och09,Och10,Och11,Och13,Och14	*OreG0005400*
scaffold2136	1248266	G	A	NA	*OreG0005401*
scaffold2136	1248330	C	T	Ore01,Ore02,Ore03,Ore04,Ore05,Ore06,Ore07,Ore08,Ore09,Ore10,Ore11,Ore12,Ore13,Ore14	*OreG0005401*
scaffold2136	1248404	G	A	Ore01,Ore03,Ore08,Ore12	*OreG0005401*

scaffold2136	1248796	G	A	NA	*OreG0005401*
scaffold2136	1249036	G	C	NA	*OreG0005401*
scaffold2136	1251210	C	A	NA	*OreG0005402*
scaffold2136	1252182	A	G	NA	*OreG0005402*
scaffold2136	1253343	G	T	Och02,Och03,Och04,Och05,Och06,Och07,Och08,Och09,Och10,Och11,Och12,Och13	*OreG0005403*
scaffold2136	1253625	G	A	NA	*OreG0005403*
scaffold2136	1254156	C	T	NA	*OreG0005403*
scaffold2136	1254395	A	T	Och02,Och06,Och08	*OreG0005403*
scaffold2136	1257860	G	A	Och02,Och04,Och05,Och07,Och12	*OreG0005404*
scaffold2136	1258451	C	T	NA	*OreG0005404*
scaffold2136	1258764	G	C	NA	*OreG0005404*
scaffold2136	1260158	C	T	NA	*OreG0005405*
scaffold2136	1260340	G	T	NA	*OreG0005405*
scaffold2136	2852184	G	T	NA	*OreG0005626*
scaffold2136	2852188	A	G	NA	*OreG0005626*
scaffold2136	2856443	G	A	NA	*OreG0005628*
scaffold2136	2856470	G	A	NA	*OreG0005628*
scaffold2136	2856505	T	A	NA	*OreG0005628*
scaffold2136	2858555	T	C	NA	*OreG0005629*
scaffold2136	2859507	C	T	Och01,Och02,Och03,Och04,Och05,Och06,Och07,Och08,Och09,Och10,Och11,Och12,Och13,Och14,Ore01,Ore02,Ore03,Ore04,Ore05,Ore06,Ore07,Ore08,Ore09,Ore10,Ore11,Ore12,Ore13,Ore14	*OreG0005629*
scaffold2136	2872504	C	T	NA	*OreG0005627*
scaffold2136	2872728	C	A	NA	*OreG0005627*
scaffold2136	2892935	C	T	NA	*OreG0005632*
scaffold2136	2898218	G	A	Ore01,Ore03,Ore12	*OreG0005633*
scaffold2136	2898300	A	G	Ore01,Ore02,Ore03,Ore04,Ore05,Ore06,Ore07,Ore08,Ore09,Ore10,Ore11,Ore12,Ore13,Ore14	*OreG0005633*
scaffold2136	2899060	G	C	Och03,Och08,Och13,Och14	*OreG0005633*
scaffold2136	2900164	C	T	Och08	*OreG0005633*
scaffold2136	2907406	C	T	Och01,Och02,Och03,Och04,Och05,Och06,Och07,Och08,Och09,Och10,Och11,Och12,Och13,Och14	*OreG0005635*
scaffold2136	2907591	C	T	Och01,Och02,Och03,Och04,Och05,Och06,Och07,Och08,Och09,Och10,Och11,Och12,Och13,Och14	*OreG0005635*
scaffold2136	2908643	T	G	Ore01,Ore03,Ore12	*OreG0005635*
scaffold2136	2933430	C	T	NA	*OreG0005639*
scaffold2136	2944602	G	A	Och01	*OreG0005642*
scaffold2136	2944725	C	T	Och01,Och02,Och03,Och04,Och05,Och06,Och07,Och08,Och09,Och10,Och11,Och12,Och13,Och14	*OreG0005642*
scaffold2136	2954606	G	T	NA	*OreG0005643*
scaffold2136	2960244	C	A	Och01,Och02,Och03,Och04,Och05,Och06,Och07,Och08,Och09,Och10,Och11,Och12,Och13,Och14	*OreG0005644*
scaffold2136	2960381	A	G	Ore01,Ore02,Ore03,Ore04,Ore05,Ore06,Ore07,Ore08,Ore09,Ore10,Ore11,Ore12,Ore13,Ore14	*OreG0005644*
scaffold2136	2960620	T	C	NA	*OreG0005644*
scaffold2136	2979572	C	G	NA	*OreG0005647*
scaffold2136	2992866	G	T	NA	*OreG0005649*
scaffold2136	2993776	A	C	NA	*OreG0005649*
scaffold2136	3024907	G	A	Och03,Och13	*OreG0005653*
scaffold2136	3030511	T	A	Och01,Och02,Och03,Och04,Och05,Och06,Och07,Och08,Och09,Och10,Och11,Och12,Och13,Och14,Ore01,Ore02,Ore03,Ore04,Ore05,Ore06,Ore07,Ore08,Ore09,Ore10,Ore11,Ore12,Ore13,Ore14	*OreG0005654*
scaffold2136	3032911	G	A	Och08,Och14	*OreG0005655*
scaffold2136	3038789	G	T	Och07,Och14	*OreG0005656*
scaffold2136	3038909	A	G	NA	*OreG0005656*
scaffold2136	3038975	T	C	Ore01,Ore02,Ore03,Ore04,Ore05,Ore06,Ore07,Ore08,Ore09,Ore10,Ore11,Ore12,Ore13,Ore14	*OreG0005656*
scaffold2136	3039343	A	G	Ore08,Ore11	*OreG0005656*
scaffold2136	3051779	T	G	NA	*OreG0005658*
scaffold2136	3057685	A	T	Och01,Och02,Och03,Och04,Och05,Och06,Och07,Och08,Och09,Och10,Och11,Och12,Och13,Och14	*OreG0005659*
scaffold2136	3057842	C	G	Ore01,Ore02,Ore03,Ore04,Ore05,Ore06,Ore07,Ore08,Ore09,Ore10,Ore11,Ore12,Ore13,Ore14	*OreG0005659*
scaffold2136	3061762	G	T	NA	*OreG0005660*
scaffold2136	3062017	T	A	Ore01,Ore02,Ore03,Ore04,Ore05,Ore06,Ore07,Ore08,Ore09,Ore10,Ore11,Ore12,Ore13,Ore14	*OreG0005660*
scaffold2136	3065586	G	C	Och02	*OreG0005661*
scaffold2136	3088527	C	T	Och01,Och02,Och03,Och04,Och05,Och06,Och07,Och08,Och09,Och10,Och11,Och12,Och13,Och14,Ore01,Ore02,Ore03,Ore04,Ore05,Ore06,Ore07,Ore08,Ore09,Ore10,Ore11,Ore12,Ore13,Ore14	*OreG0005663*
scaffold2136	3089446	G	T	Och01,Och02,Och03,Och04,Och05,Och06,Och07,Och08,Och09,Och10,Och11,Och12,Och13,Och14,Ore01,Ore02,Ore03,Ore04,Ore05,Ore06,Ore07,Ore08,Ore09,Ore10,Ore11,Ore12,Ore13,Ore14	*OreG0005663*
scaffold2136	3089465	T	C	NA	*OreG0005663*

scaffold2136	3107660	T	C	Och02	*OreG0005665*
scaffold2136	3143421	C	T	Och01,Och02,Och06,Och07,Och08,Och11,Och14	*OreG0005668*
scaffold2136	3143769	G	T	Ore01,Ore02,Ore03,Ore04,Ore05,Ore06,Ore07,Ore08,Ore09,Ore10,Ore11,Ore12,Ore13,Ore14	*OreG0005668*
scaffold2136	3145458	G	T	NA	*OreG0005669*
scaffold2136	3145799	C	G	NA	*OreG0005669*
scaffold2136	3166582	C	G	Och02,Och06	*OreG0005673*
scaffold2136	3170098	A	T	Ore02,Ore04,Ore05,Ore06,Ore07,Ore08,Ore09,Ore10,Ore11,Ore12,Ore13,Ore14	*OreG0005674*
scaffold2136	3195655	C	G	Och01,Och02,Och03,Och04,Och05,Och06,Och07,Och08,Och09,Och10,Och11,Och12,Och13,Och14,Ore01,Ore02,Ore03,Ore04,Ore05,Ore06,Ore07,Ore08,Ore09,Ore10,Ore11,Ore12,Ore13,Ore14	*OreG0005677*
scaffold2136	3195693	C	T	NA	*OreG0005677*
scaffold2136	3195905	A	T	NA	*OreG0005677*
scaffold2136	3196436	C	T	Ore01,Ore02,Ore03,Ore04,Ore05,Ore06,Ore07,Ore08,Ore09,Ore10,Ore11,Ore12,Ore13,Ore14	*OreG0005677*
scaffold2136	3228777	T	C	Och01,Och03,Och07,Och09,Och10,Och14	*OreG0005679*
scaffold2136	3254078	C	A	NA	*OreG0005682*
scaffold2136	3254780	C	A	NA	*OreG0005682*
scaffold2136	3255605	A	G	Och01,Och02,Och03,Och04,Och05,Och06,Och07,Och08,Och09,Och10,Och11,Och12,Och13,Och14	*OreG0005682*
scaffold2136	3257724	T	G	NA	*OreG0005682*
scaffold2136	3265103	A	T	NA	*OreG0005683*
scaffold2136	3267679	G	A	NA	*OreG0005683*
scaffold2136	3272338	T	G	NA	*OreG0005683*
scaffold2136	3272724	T	A	NA	*OreG0005684*
scaffold2136	3272888	A	G	NA	*OreG0005684*
scaffold2136	3273541	T	C	NA	*OreG0005684*
scaffold2136	3273566	C	T	NA	*OreG0005684*
scaffold2136	3294895	C	T	NA	*OreG0005687*
scaffold2136	3295504	A	T	NA	*OreG0005687*
scaffold2136	3295646	T	A	NA	*OreG0005687*
scaffold2136	3319198	C	T	Ore07	*OreG0005692*
scaffold2136	3324176	A	G	Och01,Och02,Och03,Och04,Och05,Och06,Och07,Och08,Och09,Och10,Och11,Och12,Och13,Och14	*OreG0005693*
scaffold2136	3324445	A	G	Ore01,Ore02,Ore03,Ore04,Ore05,Ore06,Ore07,Ore08,Ore09,Ore10,Ore11,Ore12,Ore13,Ore14	*OreG0005693*
scaffold2136	3324611	T	C	Ore01,Ore02,Ore03,Ore04,Ore05,Ore06,Ore07,Ore08,Ore09,Ore10,Ore11,Ore12,Ore13,Ore14	*OreG0005693*
scaffold2136	3324778	G	A	Ore01,Ore02,Ore03,Ore04,Ore05,Ore06,Ore07,Ore08,Ore09,Ore10,Ore11,Ore12,Ore13,Ore14	*OreG0005693*
scaffold2136	3324797	C	G	NA	*OreG0005693*
scaffold2136	3336162	C	T	Ore01,Ore02,Ore03,Ore04,Ore05,Ore06,Ore07,Ore08,Ore09,Ore10,Ore11,Ore12,Ore13,Ore14	*OreG0005695*
scaffold2136	3345837	A	C	Ore01,Ore02,Ore03,Ore04,Ore05,Ore06,Ore07,Ore08,Ore09,Ore10,Ore11,Ore12,Ore13,Ore14	*OreG0005698*
scaffold2136	3346021	C	T	NA	*OreG0005698*
scaffold2136	3350235	C	T	NA	*OreG0005698*
scaffold2136	3351506	C	G	NA	*OreG0005698*
scaffold2136	3395685	T	C	Ore01,Ore02,Ore03,Ore04,Ore05,Ore06,Ore07,Ore08,Ore09,Ore10,Ore11,Ore12,Ore13,Ore14	*OreG0005706*
scaffold2136	3395701	G	C	Och01,Och07,Och09,Och10,Och11,Och14	*OreG0005706*
scaffold2136	3396779	G	T	Och01,Och02,Och03,Och04,Och05,Och06,Och07,Och08,Och09,Och10,Och11,Och12,Och13,Och14	*OreG0005706*
scaffold2136	3404114	G	T	Och01,Och03,Och07,Och08,Och09,Och10,Och11,Och14	*OreG0005707*
scaffold2136	3404971	A	C	NA	*OreG0005707*
scaffold2136	3405595	C	T	Och01,Och02,Och03,Och04,Och05,Och06,Och07,Och08,Och09,Och10,Och11,Och12,Och13,Och14	*OreG0005707*
scaffold2136	3407817	T	G	NA	*OreG0005708*
scaffold2136	3407822	A	G	NA	*OreG0005708*
scaffold2136	3412976	T	C	NA	*OreG0005709*
scaffold2136	3415185	G	A	Ore02,Ore04,Ore05,Ore06,Ore07,Ore09,Ore10,Ore12,Ore13,Ore14	*OreG0005709*
scaffold2136	3420075	T	A	Och01	*OreG0005710*
scaffold2136	3433896	G	A	Och01,Och02,Och03,Och04,Och05,Och06,Och07,Och08,Och09,Och10,Och11,Och12,Och13,Och14	*OreG0005713*
scaffold2136	3433971	A	G	Ore01,Ore02,Ore03,Ore04,Ore05,Ore06,Ore07,Ore08,Ore09,Ore10,Ore11,Ore12,Ore13,Ore14	*OreG0005713*
scaffold2136	3446184	A	G	NA	*OreG0005715*
scaffold2136	3490428	C	T	NA	*OreG0005719*
scaffold2136	3491118	T	G	Och01,Och02,Och03,Och04,Och05,Och06,Och07,Och08,Och09,Och10,Och11,Och13,Och14	*OreG0005719*
scaffold2136	3492630	C	A	Och01,Och02,Och03,Och04,Och05,Och06,Och07,Och08,Och09,Och10,Och11,Och12,Och13,Och14	*OreG0005719*
scaffold2136	3513707	A	G	Och02,Och04,Och05,Och08,Och12,Och13	*OreG0005722*
scaffold2136	3519883	C	T	NA	*OreG0005723*
scaffold2136	3528709	C	A	NA	*OreG0005724*

scaffold2136	3543414	A	G	NA	*OreG0005726*
scaffold2136	3543471	A	G	Och02,Och04,Och05,Och07,Och08,Och09,Och10,Och11,Och12,Och13,Och14	*OreG0005726*
scaffold2136	3556084	T	C	Och02,Och09,Och10,Och11	*OreG0005728*
scaffold2136	3559477	A	G	NA	*OreG0005728*
scaffold2136	3577423	G	A	NA	*OreG0005732*
scaffold2136	3603479	A	T	Och09,Och10	*OreG0005736*
scaffold2136	3604231	G	A	Och09,Och10	*OreG0005736*
scaffold2136	3604361	T	G	Och01	*OreG0005736*
scaffold2136	3614275	A	G	Och01,Och02,Och03,Och04,Och05,Och06,Och08,Och11,Och12,Och13,Och14	*OreG0005735*
scaffold2136	3614433	C	T	Och01	*OreG0005735*
scaffold2136	3619477	C	G	Ore01,Ore02,Ore03,Ore04,Ore05,Ore06,Ore07,Ore08,Ore09,Ore10,Ore11,Ore12,Ore13,Ore14	*OreG0005737*
scaffold2136	3622189	C	A	Ore01,Ore02,Ore03,Ore04,Ore05,Ore06,Ore07,Ore08,Ore09,Ore10,Ore11,Ore12,Ore13,Ore14	*OreG0005737*
scaffold2136	3623340	G	C	NA	*OreG0005737*
scaffold2136	3625037	G	T	Ore01,Ore02,Ore03,Ore04,Ore05,Ore06,Ore07,Ore08,Ore09,Ore10,Ore11,Ore12,Ore13,Ore14	*OreG0005738*
scaffold2136	3625298	T	A	NA	*OreG0005738*
scaffold2136	3626513	G	A	NA	*OreG0005738*
scaffold2136	3650525	A	T	NA	*OreG0005743*
scaffold2136	3650949	G	A	NA	*OreG0005743*
scaffold2136	3651058	G	A	NA	*OreG0005743*
scaffold2136	3653507	G	A	NA	*OreG0005743*
scaffold2136	3654268	G	A	NA	*OreG0005743*
scaffold2136	3654280	G	A	NA	*OreG0005743*
scaffold2136	3654423	A	T	Och01,Och08,Och12	*OreG0005743*
scaffold2136	3654617	G	T	Ore02,Ore04,Ore05,Ore06,Ore07,Ore08,Ore09	*OreG0005743*
scaffold2136	3654695	A	G	Ore02,Ore04,Ore05,Ore06,Ore07,Ore08,Ore09	*OreG0005743*
scaffold2136	3668816	G	T	NA	*OreG0005745*
scaffold2136	3669583	A	G	Och01,Och02,Och03,Och04,Och05,Och06,Och07,Och08,Och09,Och10,Och11,Och12,Och13,Och14	*OreG0005745*
scaffold2136	3676898	G	T	NA	*OreG0005747*
scaffold2136	3679700	A	C	NA	*OreG0005748*
scaffold2136	3679719	G	A	NA	*OreG0005748*
scaffold2136	3679816	G	A	Och01,Och02,Och06,Och14	*OreG0005748*
scaffold2136	3679825	G	A	Och01,Och02,Och06,Och14	*OreG0005748*
scaffold2136	3680146	C	T	Och01,Och02,Och06,Och09,Och10,Och14	*OreG0005749*
scaffold2136	3680241	C	A	NA	*OreG0005749*
scaffold2136	3680253	A	G	Och01,Och02,Och06,Och09,Och10,Och14	*OreG0005749*
scaffold2136	3681061	G	A	Ore01,Ore02,Ore03,Ore04,Ore05,Ore06,Ore07,Ore08,Ore09,Ore10,Ore11,Ore12,Ore13,Ore14	*OreG0005750*
scaffold2136	3681232	C	G	NA	*OreG0005751*
scaffold2136	3681235	T	C	Ore01,Ore02,Ore03,Ore04,Ore05,Ore06,Ore07,Ore08,Ore09,Ore10,Ore11,Ore12,Ore13,Ore14	*OreG0005751*
scaffold2136	3681259	C	T	Ore01,Ore02,Ore03,Ore04,Ore05,Ore06,Ore07,Ore08,Ore09,Ore10,Ore11,Ore12,Ore13,Ore14	*OreG0005751*
scaffold2136	3681303	A	T	Ore12,Ore14	*OreG0005751*
scaffold2136	3684723	G	T	Och01,Och02,Och03,Och04,Och05,Och06,Och07,Och08,Och09,Och10,Och11,Och12,Och13,Och14,Ore01,Ore02,Ore03,Ore04,Ore05,Ore06,Ore07,Ore08,Ore09,Ore10,Ore11,Ore12,Ore13,Ore14	*OreG0005752*
scaffold2136	3686121	A	T	Och01,Och02,Och03,Och04,Och05,Och06,Och07,Och08,Och09,Och10,Och11,Och12,Och13,Och14,Ore01,Ore02,Ore03,Ore04,Ore05,Ore06,Ore07,Ore08,Ore09,Ore10,Ore11,Ore12,Ore13,Ore14	*OreG0005752*
scaffold2136	3686433	C	T	NA	*OreG0005752*
scaffold2136	3691714	G	A	Och11	*OreG0005754*
scaffold2136	3691782	C	T	NA	*OreG0005754*
scaffold2136	3694881	T	G	NA	*OreG0005755*
scaffold2136	3697005	A	C	Ore01,Ore02,Ore03,Ore04,Ore05,Ore06,Ore07,Ore08,Ore09,Ore10,Ore11,Ore12,Ore13,Ore14	*OreG0005756*
scaffold2136	3697194	A	T	Ore01,Ore02,Ore03,Ore04,Ore05,Ore06,Ore07,Ore08,Ore09,Ore10,Ore11,Ore12,Ore13,Ore14	*OreG0005756*
scaffold2136	3697310	C	G	Ore01,Ore02,Ore03,Ore04,Ore05,Ore06,Ore07,Ore08,Ore09,Ore10,Ore11,Ore12,Ore13,Ore14	*OreG0005756*
scaffold2136	3703450	G	T	Ore01,Ore02,Ore03,Ore04,Ore05,Ore06,Ore07,Ore08,Ore09,Ore10,Ore11,Ore12,Ore13,Ore14	*OreG0005759*
scaffold2136	3704788	C	G	NA	*OreG0005760*
scaffold2136	3704842	T	C	NA	*OreG0005760*
scaffold2136	3704844	A	G	NA	*OreG0005760*
scaffold2136	3705192	A	T	NA	*OreG0005760*
scaffold2136	3705196	C	T	NA	*OreG0005760*
scaffold2136	3706197	A	G	NA	*OreG0005760*
scaffold2136	3706963	G	A	NA	*OreG0005760*
scaffold2136	3710395	C	T	Ore01,Ore02,Ore03,Ore04,Ore05,Ore06,Ore07,Ore08,Ore09,Ore10,Ore11,Ore12,Ore13,Ore14	*OreG0005760*
scaffold2136	3714960	G	A	Och02	*OreG0005761*

scaffold2136	3769650	G	A	NA	*OreG0005764*
scaffold2136	3770073	C	A	Ore08,Ore11	*OreG0005764*
scaffold2136	3770202	C	A	NA	*OreG0005764*
scaffold2136	3802815	G	A	Och01,Och02,Och03,Och04,Och05,Och06,Och07,Och08,Och09,Och10,Och11,Och12,Och13,Och14,Ore01,Ore02,Ore03,Ore04,Ore05,Ore06,Ore07,Ore08,Ore09,Ore10,Ore11,Ore12,Ore13,Ore14	*OreG0005767*
scaffold2136	3805713	G	A	NA	*OreG0005768*
scaffold2136	3805893	C	G	Och01,Och02,Och03,Och04,Och05,Och06,Och07,Och08,Och09,Och10,Och11,Och12,Och13,Och14	*OreG0005768*
scaffold2136	3807424	G	T	NA	*OreG0005768*
scaffold2136	3809925	T	C	Ore01,Ore02,Ore03,Ore04,Ore05,Ore06,Ore07,Ore08,Ore09,Ore10,Ore11,Ore12,Ore13,Ore14	*OreG0005769*
scaffold2136	3815201	G	C	NA	*OreG0005770*
scaffold2136	3815274	T	G	NA	*OreG0005770*
scaffold2136	3855753	C	A	Och01,Och02,Och03,Och04,Och05,Och06,Och09,Och10,Och12,Och13	*OreG0005779*
scaffold2136	3861993	T	C	Och01,Och02,Och03,Och04,Och05,Och06,Och07,Och08,Och09,Och10,Och11,Och12,Och13,Och14,Ore01,Ore02,Ore03,Ore04,Ore05,Ore06,Ore07,Ore08,Ore09,Ore10,Ore11,Ore12,Ore13,Ore14	*OreG0005781*
scaffold2136	3866547	C	T	Och01,Och02,Och03,Och04,Och05,Och06,Och07,Och08,Och09,Och10,Och11,Och12,Och13,Och14	*OreG0005783*
scaffold2136	3868604	T	A	Och01	*OreG0005783*
scaffold2136	3881780	A	C	Och01	*OreG0005784*
scaffold2136	3887521	A	G	Ore01,Ore02,Ore03,Ore04,Ore05,Ore06,Ore07,Ore08,Ore09,Ore10,Ore11,Ore12,Ore13,Ore14	*OreG0005784*
scaffold2136	3891807	A	G	Och01,Och02,Och03,Och04,Och05,Och06,Och07,Och08,Och09,Och10,Och11,Och12,Och13,Och14	*OreG0005785*
scaffold2136	3932857	G	T	Och07	*OreG0005788*
scaffold2136	3944905	T	C	NA	*OreG0005789*
scaffold2136	3946133	A	T	NA	*OreG0005789*
scaffold2136	3949831	C	T	NA	*OreG0005790*
scaffold2136	3950566	C	G	Och01,Och02,Och03,Och04,Och05,Och06,Och07,Och08,Och09,Och10,Och11,Och12,Och13,Och14,Ore01,Ore02,Ore03,Ore04,Ore05,Ore06,Ore07,Ore08,Ore09,Ore10,Ore11,Ore12,Ore13,Ore14	*OreG0005790*
scaffold2136	3951170	G	C	NA	*OreG0005790*
scaffold2136	3952016	A	T	Och01,Och02,Och03,Och04,Och05,Och06,Och07,Och08,Och09,Och10,Och11,Och12,Och13,Och14	*OreG0005791*
scaffold2136	3952064	A	G	Och01,Och02,Och03,Och04,Och05,Och06,Och07,Och08,Och09,Och10,Och11,Och12,Och13,Och14	*OreG0005791*
scaffold2136	3952070	G	A	Och01,Och02,Och03,Och04,Och05,Och06,Och07,Och08,Och09,Och10,Och11,Och12,Och13,Och14	*OreG0005791*
scaffold2136	3952076	A	G	NA	*OreG0005791*
scaffold2136	3952449	C	T	Och01,Och02,Och03,Och04,Och05,Och06,Och07,Och08,Och09,Och10,Och11,Och12,Och13,Och14	*OreG0005791*
scaffold2136	3952733	T	A	Och01,Och02,Och03,Och04,Och05,Och06,Och07,Och08,Och09,Och10,Och11,Och12,Och13,Och14	*OreG0005791*
scaffold2136	3952748	C	A	Ore01,Ore02,Ore03,Ore04,Ore05,Ore06,Ore07,Ore08,Ore09,Ore10,Ore11,Ore12,Ore13,Ore14	*OreG0005791*
scaffold2136	3953592	G	A	Och01,Och02,Och03,Och04,Och05,Och06,Och07,Och08,Och09,Och10,Och11,Och12,Och13,Och14	*OreG0005791*
scaffold2136	3954997	C	G	NA	*OreG0005792*
scaffold2136	3955408	C	T	NA	*OreG0005792*
scaffold2136	3955439	C	G	NA	*OreG0005792*
scaffold2136	3955501	C	T	NA	*OreG0005792*
scaffold2136	3955674	G	A	Ore01,Ore02,Ore03,Ore04,Ore05,Ore06,Ore07,Ore08,Ore09,Ore10,Ore11,Ore12,Ore13,Ore14	*OreG0005792*
scaffold2136	3955729	T	A	NA	*OreG0005792*
scaffold2136	3955833	A	C	NA	*OreG0005792*
scaffold2136	3956499	G	A	Och01,Och02,Och03,Och04,Och05,Och06,Och07,Och08,Och09,Och10,Och11,Och12,Och13,Och14	*OreG0005792*
scaffold2136	3978022	C	A	Och01,Och02,Och03,Och04,Och05,Och06,Och07,Och08,Och09,Och10,Och11,Och12,Och13,Och14	*OreG0005795*
scaffold2136	4019766	C	T	Och11	*OreG0005800*
scaffold2136	4025957	G	A	NA	*OreG0005801*
scaffold2136	4034508	C	T	NA	*OreG0005802*
scaffold2136	4041872	C	T	Och01,Och02,Och03,Och04,Och05,Och06,Och07,Och08,Och09,Och10,Och11,Och12,Och13,Och14,Ore01,Ore02,Ore03,Ore04,Ore05,Ore06,Ore07,Ore08,Ore09,Ore10,Ore11,Ore12,Ore13,Ore14	*OreG0005803*
scaffold2136	4041899	G	A	Och03,Och07,Och08,Och09,Och10,Och11,Och14,Ore01,Ore02,Ore03,Ore04,Ore05,Ore06,Ore07,Ore08,Ore09,Ore10,Ore11,Ore12,Ore13,Ore14	*OreG0005803*
scaffold2136	4045980	G	A	Och03,Och07,Och08,Och09,Och10,Och11,Och14,Ore01,Ore02,Ore03,Ore04,Ore05,Ore06,Ore07,Ore08,Ore09,Ore10,Ore11,Ore12,Ore13,Ore14	*OreG0005804*
scaffold2136	4046338	T	C	Ore01,Ore02,Ore03,Ore04,Ore05,Ore06,Ore07,Ore08,Ore09,Ore10,Ore11,Ore12,Ore13,Ore14	*OreG0005804*
scaffold2136	4046524	T	A	Ore02,Ore04,Ore05,Ore06,Ore07,Ore08,Ore09,Ore10,Ore11,Ore12,Ore13,Ore14	*OreG0005804*
scaffold2136	4046533	G	A	NA	*OreG0005804*
scaffold2136	4046565	G	A	NA	*OreG0005804*

scaffold2136	4046614	C	T	NA	*OreG0005804*
scaffold2136	4060819	C	G	Och11	*OreG0005806*
scaffold2136	4063107	G	A	NA	*OreG0005806*
scaffold2136	4088929	G	T	Och01,Och02,Och03,Och04,Och05,Och06,Och07,Och08,Och09,Och10,Och11,Och12,Och13,Och14	*OreG0005810*
scaffold2136	4097589	C	T	Och02,Och06,Och14	*OreG0005811*
scaffold2136	4097807	G	A	Och02,Och14	*OreG0005811*
scaffold2136	4097829	A	G	NA	*OreG0005811*
scaffold2136	4098084	C	T	NA	*OreG0005811*
scaffold2136	4098143	C	T	Och01,Och02,Och03,Och04,Och05,Och06,Och07,Och08,Och09,Och10,Och11,Och12,Och13,Och14,Ore01,Ore02,Ore03,Ore04,Ore05,Ore06,Ore07,Ore08,Ore09,Ore10,Ore11,Ore12,Ore13,Ore14	*OreG0005811*
scaffold2136	4098176	C	T	Ore12,Ore14	*OreG0005811*
scaffold2136	4098273	G	A	NA	*OreG0005811*
scaffold2136	4098276	G	A	Och01,Och07,Och08,Och09,Och10,Och11	*OreG0005811*
scaffold2136	4098327	G	A	NA	*OreG0005811*
scaffold2136	4098416	G	C	NA	*OreG0005811*
scaffold2136	4098522	C	T	Och02,Och06,Och14	*OreG0005811*
scaffold2136	4098546	G	A	Och02,Och06,Och14	*OreG0005811*
scaffold2136	4098588	C	T	Och02,Och06,Och14	*OreG0005811*
scaffold2136	4098666	G	A	NA	*OreG0005811*
scaffold2136	4101203	G	A	Ore01,Ore02,Ore03,Ore04,Ore05,Ore06,Ore07,Ore08,Ore09,Ore10,Ore11,Ore12,Ore13,Ore14	*OreG0005812*
scaffold2136	4101356	G	C	Och01,Och02,Och04,Och05,Och07,Och09,Och10,Och11,Och12,Och13,Och14,Ore01,Ore02,Ore03,Ore04,Ore05,Ore06,Ore07,Ore08,Ore09,Ore10,Ore11,Ore12,Ore13,Ore14	*OreG0005812*
scaffold2136	4111911	C	T	NA	*OreG0005813*
scaffold2136	4179771	A	T	Och01,Och02,Och03,Och04,Och05,Och06,Och07,Och08,Och09,Och10,Och11,Och12,Och13,Och14,Ore01,Ore02,Ore03,Ore04,Ore05,Ore06,Ore07,Ore08,Ore09,Ore10,Ore11,Ore12,Ore13,Ore14	*OreG0005821*
scaffold2136	4179797	T	G	NA	*OreG0005821*
scaffold2136	4193545	T	C	NA	*OreG0005823*
scaffold2136	4215488	C	T	NA	*OreG0005825*
scaffold2136	4215758	A	C	Ore01,Ore02,Ore03,Ore04,Ore05,Ore06,Ore07,Ore08,Ore09,Ore10,Ore11,Ore12,Ore13,Ore14	*OreG0005825*
scaffold2136	4252082	G	A	Och02	*OreG0005832*
scaffold2136	4322183	G	A	NA	*OreG0005840*
scaffold2136	4337410	A	C	Och01,Och02,Och03,Och04,Och05,Och06,Och07,Och08,Och09,Och10,Och11,Och12,Och13,Och14,Ore01,Ore02,Ore03,Ore04,Ore05,Ore06,Ore07,Ore08,Ore09,Ore10,Ore11,Ore12,Ore13,Ore14	*OreG0005842*
scaffold2136	4343574	G	C	NA	*OreG0005843*
scaffold2136	4388488	G	A	Och02	*OreG0005849*
scaffold2136	4395884	G	T	Ore01,Ore02,Ore03,Ore04,Ore05,Ore06,Ore07,Ore08,Ore09,Ore10,Ore11,Ore12,Ore13,Ore14	*OreG0005850*
scaffold2136	4399519	G	A	Och02,Och03,Och04,Och05,Och06,Och07,Och09,Och10,Och11,Och12,Och13,Och14	*OreG0005850*
scaffold2136	4400972	G	C	Och01	*OreG0005850*
scaffold2136	4409529	C	G	Ore01,Ore02,Ore03,Ore04,Ore05,Ore06,Ore07,Ore08,Ore09,Ore10,Ore11,Ore12,Ore13,Ore14	*OreG0005853*
scaffold2136	4412006	G	T	Och08	*OreG0005853*
scaffold2136	4419452	C	A	Ore02,Ore04,Ore05,Ore06,Ore07,Ore09,Ore10,Ore12,Ore13,Ore14	*OreG0005853*
scaffold2136	4419520	G	A	Och02	*OreG0005853*
scaffold2136	4421214	G	A	NA	*OreG0005853*
scaffold2136	4437744	A	G	Ore12,Ore14	*OreG0005855*
scaffold2136	4441003	C	T	NA	*OreG0005856*
scaffold2136	4450135	C	G	NA	*OreG0005857*
scaffold2136	4466202	T	C	NA	*OreG0005859*
scaffold2136	4466447	A	G	Och02,Och03,Och04,Och05,Och06,Och09,Och10,Och12,Och13	*OreG0005859*
scaffold2136	4466890	G	A	Och01,Och02,Och03,Och04,Och05,Och06,Och07,Och08,Och09,Och10,Och11,Och12,Och13,Och14,Ore01,Ore02,Ore03,Ore04,Ore05,Ore06,Ore07,Ore08,Ore09,Ore10,Ore11,Ore12,Ore13,Ore14	*OreG0005859*
scaffold2136	4466916	G	T	NA	*OreG0005859*
scaffold2136	4466919	A	T	Ore01,Ore02,Ore03,Ore04,Ore05,Ore06,Ore07,Ore08,Ore09,Ore10,Ore11,Ore12,Ore13,Ore14	*OreG0005859*
scaffold2136	4484460	C	A	NA	*OreG0005861*
scaffold2136	4484636	A	T	NA	*OreG0005861*
scaffold2136	4491649	G	T	Och01,Och02,Och03,Och04,Och05,Och06,Och07,Och08,Och09,Och10,Och11,Och12,Och13,Och14,Ore01,Ore02,Ore03,Ore04,Ore05,Ore06,Ore07,Ore08,Ore09,Ore10,Ore11,Ore12,Ore13,Ore14	*OreG0005862*
scaffold2136	4508172	G	C	Ore01,Ore02,Ore03,Ore04,Ore05,Ore06,Ore07,Ore09,Ore10,Ore12,Ore13,Ore14	*OreG0005863*
scaffold2136	4511743	G	A	Och01,Och02,Och03,Och04,Och05,Och06,Och07,Och08,Och09,Och10,Och11,Och12,Och13,Och14,Ore01,Ore02,Ore03,Ore04,Ore05,Ore06,Ore07,Ore08,Ore09,Ore10,Ore11,Ore12,Ore13,Ore14	*OreG0005864*
scaffold2136	4512326	C	A	Ore01,Ore02,Ore03,Ore04,Ore05,Ore06,Ore07,Ore08,Ore09,Ore10,Ore11,Ore12,Ore13,Ore14	*OreG0005864*

scaffold2136	4553778	A	G	Ore01,Ore02,Ore03,Ore04,Ore05,Ore06,Ore07,Ore08,Ore09,Ore10,Ore11,Ore12,Ore13,Ore14	*OreG0005868*
scaffold2136	4580347	T	C	Och01	*OreG0005868*
scaffold2136	4581812	C	T	Och01,Och03,Och08,Och09,Och10,Och14	*OreG0005868*
scaffold2136	4581965	A	C	Ore01,Ore02,Ore03,Ore04,Ore05,Ore06,Ore07,Ore08,Ore09,Ore10,Ore11,Ore12,Ore13,Ore14	*OreG0005868*
scaffold2136	4593394	G	A	NA	*OreG0005869*
scaffold2136	4599147	C	A	NA	*OreG0005870*
scaffold2136	4610359	G	C	Och02	*OreG0005871*
scaffold2136	4610363	G	A	NA	*OreG0005871*
scaffold2136	4612207	C	T	Och02	*OreG0005872*
scaffold2136	4614687	C	T	Ore01,Ore02,Ore04,Ore05,Ore06,Ore07,Ore08,Ore09,Ore10,Ore11,Ore12,Ore13,Ore14	*OreG0005873*
scaffold2136	4615386	G	T	NA	*OreG0005873*
scaffold2136	4615419	A	G	NA	*OreG0005873*
scaffold2136	4618129	C	T	NA	*OreG0005874*
scaffold2136	4618893	G	A	Och01,Och02,Och03,Och04,Och05,Och06,Och07,Och08,Och09,Och10,Och11,Och12,Och13,Och14,Ore01,Ore02,Ore03,Ore04,Ore05,Ore06,Ore07,Ore08,Ore09,Ore10,Ore11,Ore12,Ore13,Ore14	*OreG0005874*
scaffold2136	4619224	A	T	NA	*OreG0005874*
scaffold2136	4626532	C	A	NA	*OreG0005876*
scaffold2136	4627202	C	A	Ore01,Ore02,Ore03,Ore04,Ore05,Ore06,Ore07,Ore08,Ore09,Ore10,Ore11,Ore12,Ore13,Ore14	*OreG0005877*
scaffold2136	4631213	G	C	Och02	*OreG0005879*
scaffold2136	4631660	C	G	Ore01,Ore02,Ore03,Ore04,Ore05,Ore06,Ore07,Ore08,Ore09,Ore10,Ore11,Ore12,Ore13,Ore14	*OreG0005879*
scaffold2136	4650441	A	T	NA	*OreG0005882*
scaffold2136	4650447	T	C	NA	*OreG0005882*
scaffold2136	4656043	G	A	NA	*OreG0005883*
scaffold2136	4659427	C	T	Och01,Och02,Och03,Och04,Och05,Och06,Och07,Och08,Och09,Och10,Och11,Och12,Och13	*OreG0005884*
scaffold2136	4662131	G	A	NA	*OreG0005885*
scaffold2136	4665606	A	C	NA	*OreG0005886*
scaffold2136	4666205	G	C	Och01	*OreG0005886*
scaffold2136	4668108	T	A	NA	*OreG0005887*
scaffold2136	4824341	C	A	Och01,Och02,Och03,Och04,Och05,Och06,Och07,Och08,Och09,Och10,Och11,Och12,Och13,Och14,Ore01,Ore02,Ore03,Ore04,Ore05,Ore06,Ore07,Ore08,Ore09,Ore10,Ore11,Ore12,Ore13,Ore14	*OreG0005896*
scaffold2136	4824560	G	T	Ore08,Ore11	*OreG0005896*
scaffold2136	4829512	A	T	Och01,Och02,Och03,Och04,Och05,Och06,Och07,Och08,Och09,Och10,Och11,Och12,Och13,Och14,Ore01,Ore02,Ore03,Ore04,Ore05,Ore06,Ore07,Ore08,Ore09,Ore10,Ore11,Ore12,Ore13,Ore14	*OreG0005897*
scaffold2136	4829912	G	T	Ore01,Ore02,Ore03,Ore04,Ore05,Ore06,Ore07,Ore08,Ore09,Ore10,Ore11,Ore12,Ore13,Ore14	*OreG0005897*
scaffold2136	4830895	G	T	Ore01,Ore02,Ore03,Ore04,Ore05,Ore06,Ore07,Ore08,Ore09,Ore10,Ore11,Ore12,Ore13,Ore14	*OreG0005897*
scaffold2136	4830948	A	G	Och01,Och02,Och03,Och04,Och05,Och06,Och07,Och08,Och09,Och10,Och11,Och12,Och13,Och14	*OreG0005897*
scaffold2136	4831125	C	G	Och01,Och02,Och03,Och04,Och05,Och06,Och07,Och08,Och09,Och10,Och11,Och12,Och13,Och14	*OreG0005897*
scaffold2136	4845397	G	T	Ore12,Ore14	*OreG0005900*
scaffold2136	4888945	C	A	NA	*OreG0005911*
scaffold2136	4895636	T	C	Ore01,Ore02,Ore03,Ore04,Ore05,Ore06,Ore07,Ore08,Ore09,Ore10,Ore11,Ore12,Ore13,Ore14	*OreG0005913*
scaffold2136	4897110	C	G	Och01,Och02,Och03,Och04,Och05,Och06,Och07,Och08,Och09,Och10,Och11,Och12,Och13,Och14,Ore01,Ore02,Ore03,Ore04,Ore05,Ore06,Ore07,Ore08,Ore09,Ore10,Ore11,Ore12,Ore13,Ore14	*OreG0005913*
scaffold2136	4915974	G	T	Ore01,Ore02,Ore03,Ore04,Ore05,Ore06,Ore07,Ore08,Ore09,Ore10,Ore11,Ore12,Ore13,Ore14	*OreG0005915*
scaffold2136	4915984	A	T	Och01,Och02,Och03,Och04,Och05,Och06,Och07,Och08,Och09,Och10,Och11,Och12,Och13,Och14,Ore01,Ore02,Ore03,Ore04,Ore05,Ore06,Ore07,Ore08,Ore09,Ore10,Ore11,Ore12,Ore13,Ore14	*OreG0005915*
scaffold2136	4916213	G	A	Och01,Och02,Och03,Och04,Och05,Och06,Och07,Och08,Och09,Och10,Och11,Och12,Och13,Och14,Ore01,Ore02,Ore03,Ore04,Ore05,Ore06,Ore07,Ore08,Ore09,Ore10,Ore11,Ore12,Ore13,Ore14	*OreG0005915*
scaffold2136	4916257	T	A	Ore01,Ore02,Ore03,Ore04,Ore05,Ore06,Ore07,Ore08,Ore09,Ore10,Ore11,Ore12,Ore13,Ore14	*OreG0005915*
scaffold2136	4916615	A	G	NA	*OreG0005915*
scaffold2136	4917487	A	G	NA	*OreG0005915*
scaffold2136	4917559	C	T	NA	*OreG0005915*
scaffold2136	4917700	G	A	NA	*OreG0005915*
scaffold2136	4918351	G	T	NA	*OreG0005915*
scaffold2136	4918420	G	A	NA	*OreG0005915*
scaffold2136	4918687	G	T	Ore12,Ore14	*OreG0005915*
scaffold2136	4921284	C	T	NA	*OreG0005915*

scaffold2136	4959262	C	A	NA	*OreG0005919*
scaffold2136	4962391	C	A	Och03,Och07,Och08,Och09,Och10,Och11	*OreG0005920*
scaffold2136	4984406	G	T	Och02,Och07	*OreG0005922*
scaffold2136	4987943	A	G	NA	*OreG0005923*
scaffold2136	5224117	G	C	NA	*OreG0005940*
scaffold2136	5418678	T	A	NA	*OreG0005948*
scaffold2136	5421450	G	T	NA	*OreG0005948*
scaffold2136	5542778	A	C	NA	*OreG0005953*
scaffold2136	5542855	G	A	NA	*OreG0005953*
scaffold2136	5542898	G	A	Och03,Och04,Och05,Och08,Och09,Och10,Och11,Och12,Och13,Och14	*OreG0005953*
scaffold2136	5543038	A	G	NA	*OreG0005953*
scaffold2136	5543137	G	A	NA	*OreG0005953*
scaffold2136	5543200	C	A	Och04,Och14	*OreG0005953*
scaffold2136	5543350	G	A	NA	*OreG0005953*
scaffold2136	5580551	C	G	NA	*OreG0005957*
scaffold2136	5580615	G	A	Och01,Och02,Och03,Och04,Och05,Och06,Och07,Och08,Och09,Och10,Och11,Och12,Och13,Och14	*OreG0005957*
scaffold2136	5580993	C	A	NA	*OreG0005957*
scaffold2136	5581030	C	T	NA	*OreG0005957*
scaffold2136	5683881	A	T	Ore01,Ore02,Ore03,Ore04,Ore05,Ore06,Ore07,Ore08,Ore09,Ore10,Ore11,Ore12,Ore13,Ore14	*OreG0005960*
scaffold2136	5693957	A	T	NA	*OreG0005961*
scaffold2136	5696426	A	G	Och01,Och02,Och03,Och04,Och05,Och06,Och07,Och08,Och09,Och10,Och11,Och12,Och13,Och14,Ore01,Ore02,Ore03,Ore04,Ore05,Ore06,Ore07,Ore08,Ore09,Ore10,Ore11,Ore12,Ore13,Ore14	*OreG0005962*
scaffold2136	5697954	T	C	Och01,Och02,Och03,Och04,Och05,Och06,Och07,Och08,Och09,Och10,Och11,Och12,Och13,Och14,Ore01,Ore02,Ore03,Ore04,Ore05,Ore06,Ore07,Ore08,Ore09,Ore10,Ore11,Ore12,Ore13,Ore14	*OreG0005962*
scaffold2136	5729100	T	C	Och03,Och04	*OreG0005964*
scaffold2136	5729113	G	C	NA	*OreG0005964*
scaffold2136	5729179	A	G	Och01,Och02,Och03,Och04,Och05,Och06,Och07,Och08,Och09,Och10,Och11,Och12,Och13,Och14,Ore01,Ore02,Ore03,Ore04,Ore05,Ore06,Ore07,Ore08,Ore09,Ore10,Ore11,Ore12,Ore13,Ore14	*OreG0005964*
scaffold2136	5733554	C	T	Ore07,Ore08,Ore11,Ore12,Ore13	*OreG0005965*
scaffold2136	5733599	C	A	Och01,Och02,Och03,Och04,Och05,Och06,Och07,Och08,Och09,Och10,Och11,Och12,Och13,Och14,Ore04,Ore05,Ore09	*OreG0005965*
scaffold2136	5733650	C	T	Ore04,Ore05,Ore09	*OreG0005965*
scaffold2136	5733682	A	G	Ore04,Ore05,Ore09	*OreG0005965*
scaffold2136	5733994	A	G	NA	*OreG0005965*
scaffold2136	5734679	G	A	Ore07,Ore08,Ore11,Ore12,Ore13	*OreG0005965*
scaffold2136	5735886	G	A	Och01,Och02,Och03,Och04,Och05,Och06,Och07,Och08,Och09,Och10,Och11,Och12,Och13,Och14,Ore01,Ore02,Ore03,Ore04,Ore05,Ore06,Ore07,Ore08,Ore09,Ore10,Ore11,Ore12,Ore13,Ore14	*OreG0005965*
scaffold2136	5737070	C	T	Och01,Och03,Och04	*OreG0005968*
scaffold2136	5737098	A	G	Och01,Och03,Och04	*OreG0005968*
scaffold2136	5737200	A	T	Ore07,Ore08,Ore11,Ore12,Ore13	*OreG0005968*
scaffold2136	5737357	G	T	Ore08	*OreG0005968*
scaffold2136	5740364	C	T	Och01,Och03,Och04	*OreG0005969*
scaffold2136	5740567	G	A	Ore07,Ore08,Ore11,Ore12,Ore13	*OreG0005969*
scaffold2136	5740957	G	A	Och02,Och06,Och09,Och10,Och11,Och14	*OreG0005969*
scaffold2136	5759038	C	A	Och02,Och06,Och09,Och10,Och11,Och14	*OreG0005970*
scaffold2136	5759260	G	T	Ore07,Ore08,Ore11,Ore12,Ore13	*OreG0005970*
scaffold2136	5759993	G	T	NA	*OreG0005971*
scaffold2136	5760000	T	A	NA	*OreG0005971*
scaffold2136	5760048	A	T	NA	*OreG0005971*
scaffold2136	5760075	T	C	NA	*OreG0005971*
scaffold2136	5760315	A	T	NA	*OreG0005971*
scaffold2136	5769320	C	T	Och02,Och06,Och09,Och10,Och11,Och14	*OreG0005972*
scaffold2136	5769691	G	T	Och02,Och06,Och09,Och10,Och11,Och14	*OreG0005972*
scaffold2136	5769707	G	A	Och01,Och03,Och04	*OreG0005972*
scaffold2136	5769761	G	A	Och02,Och06,Och09,Och10,Och11,Och14	*OreG0005972*
scaffold2136	5769808	G	A	Och02,Och06,Och09,Och10,Och11,Och14	*OreG0005972*
scaffold2136	5801034	A	G	Ore04,Ore05,Ore09	*OreG0005973*
scaffold2136	5801184	C	T	Och02	*OreG0005973*
scaffold2136	5801520	G	T	Och02,Och06,Och09,Och10,Och11,Och14	*OreG0005973*
scaffold2136	5801529	T	C	NA	*OreG0005973*
scaffold2136	5844714	T	A	NA	*OreG0005976*
scaffold2136	5893927	G	A	NA	*OreG0005978*
scaffold2136	5916367	A	C	NA	*OreG0005980*
scaffold2136	5919032	C	A	Och02,Och03,Och05,Och06,Och09,Och10,Och11,Och12,Och13	*OreG0005981*
scaffold2136	5929958	G	A	Och01	*OreG0005982*
scaffold2136	5939049	T	A	NA	*OreG0005981*
scaffold2136	5939117	G	A	Ore08	*OreG0005981*
scaffold2136	5978471	C	T	Och02,Och03,Och04,Och05,Och06,Och12,Och13,Och14	*OreG0005986*

scaffold2136	5985072	G	A	NA	*OreG0005988*
scaffold2136	5985085	G	T	Ore03,Ore07,Ore08,Ore10,Ore11,Ore12,Ore13,Ore14	*OreG0005988*
scaffold2136	5985093	C	T	Och01	*OreG0005988*
scaffold2136	5985100	T	G	Och01	*OreG0005988*
scaffold2136	5987025	G	T	Och14,Ore08	*OreG0005988*
scaffold2136	5987148	G	T	NA	*OreG0005988*
scaffold2136	5987231	T	A	NA	*OreG0005988*
scaffold2136	5987282	G	A	Och01,Och02,Och06,Ore04,Ore05,Ore06,Ore09	*OreG0005988*
scaffold2136	5987289	C	T	Och09,Och10,Och11,Och14	*OreG0005988*
scaffold2136	5987575	G	A	NA	*OreG0005988*
scaffold2136	5987710	C	A	Och02,Och06	*OreG0005988*
scaffold2136	6002117	T	C	Och01,Och02,Och03,Och04,Och05,Och06,Och07,Och08,Och09,Och10,Och11,Och12,Och13,Och14,Ore01,Ore02,Ore03,Ore04,Ore05,Ore06,Ore07,Ore08,Ore09,Ore10,Ore11,Ore12,Ore13,Ore14	*OreG0005989*
scaffold2136	6002139	C	T	Och09,Och10,Och11	*OreG0005989*
scaffold2136	6002153	T	G	Och01,Och02,Och03,Och04,Och05,Och06,Och07,Och08,Och09,Och10,Och11,Och12,Och13,Och14	*OreG0005989*
scaffold2136	6002511	C	T	Och01	*OreG0005989*
scaffold2136	6002547	G	A	Ore08	*OreG0005989*
scaffold2136	6002646	G	A	Och09,Och10,Och11	*OreG0005989*
scaffold2136	6007371	A	T	Och09,Och10,Och11	*OreG0005989*
scaffold2136	6007451	A	T	Och01	*OreG0005989*
scaffold2136	6022492	T	A	Ore08	*OreG0005991*
scaffold2136	6027244	C	T	NA	*OreG0005992*
scaffold2136	6034745	T	C	Och01	*OreG0005993*
scaffold2136	6035124	G	T	Ore08	*OreG0005993*
scaffold2136	6035446	A	G	Och02,Och06	*OreG0005993*
scaffold2136	6046497	C	A	Och02,Och06	*OreG0005994*
scaffold2136	6046732	A	T	NA	*OreG0005994*
scaffold2136	6046871	G	T	Ore04,Ore05,Ore09	*OreG0005994*
scaffold2136	6052211	T	C	Och02,Och06	*OreG0005995*
scaffold2136	6052310	T	G	Och01,Och02,Och03,Och04,Och05,Och06,Och07,Och08,Och09,Och10,Och11,Och12,Och13,Och14	*OreG0005995*
scaffold2136	6089081	G	A	NA	*OreG0005999*
scaffold2136	6099972	A	T	Och01,Och02,Och03,Och04,Och05,Och06,Och07,Och08,Och09,Och10,Och11,Och12,Och13,Och14,Ore07,Ore08,Ore11,Ore12,Ore13	*OreG0006001*
scaffold2136	6099991	G	A	NA	*OreG0006001*
scaffold2136	6100135	T	G	Ore04,Ore05,Ore09	*OreG0006001*
scaffold2136	6100668	T	A	Ore04,Ore05,Ore09	*OreG0006001*
scaffold2136	6101517	G	A	Ore04,Ore05,Ore09	*OreG0006001*
scaffold2136	6101895	G	A	NA	*OreG0006001*
scaffold2136	6101974	G	A	NA	*OreG0006001*
scaffold2136	6104336	C	T	Och01,Och02,Och05,Och06,Och12,Och13	*OreG0006002*
scaffold2136	6104451	T	A	Ore04,Ore05,Ore09	*OreG0006002*
scaffold2136	6112290	T	G	Ore04,Ore05,Ore09	*OreG0006003*
scaffold2136	6116780	T	A	NA	*OreG0006004*
scaffold2136	6131825	C	T	NA	*OreG0006006*
scaffold12583	2533	A	C	Ore01,Ore02,Ore03,Ore04,Ore05,Ore06,Ore07,Ore08,Ore09,Ore10,Ore11,Ore12,Ore13,Ore14	*OreG0001952*
scaffold2276	56467	A	T	Ore02,Ore03,Ore07,Ore09,Ore10,Ore13,Ore14	*OreG0006375*
scaffold2276	108378	G	C	NA	*OreG0006378*
scaffold2276	116954	A	G	Och05	*OreG0006379*
scaffold2276	125329	T	G	Och07	*OreG0006380*
scaffold2276	129359	A	T	Och09,Och10	*OreG0006381*
scaffold2276	134390	T	C	Och01,Och02,Och03,Och04,Och05,Och06,Och07,Och08,Och09,Och10,Och11,Och12,Och13,Och14	*OreG0006381*
scaffold2276	143925	C	T	Och01,Och02,Och03,Och04,Och05,Och06,Och07,Och08,Och09,Och10,Och11,Och12,Och13,Och14,Ore01,Ore02,Ore03,Ore04,Ore05,Ore06,Ore07,Ore08,Ore09,Ore10,Ore11,Ore12,Ore13,Ore14	*OreG0006383*
scaffold2276	147784	C	T	NA	*OreG0006383*
scaffold2276	148307	C	T	NA	*OreG0006383*
scaffold2276	150805	C	A	Ore01,Ore02,Ore03,Ore04,Ore05,Ore06,Ore07,Ore08,Ore09,Ore10,Ore11,Ore12,Ore13,Ore14	*OreG0006384*
scaffold2276	151211	C	T	Och01,Och02,Och06,Och11,Och13	*OreG0006384*
scaffold2276	155135	G	A	NA	*OreG0006385*
scaffold2276	155176	G	A	NA	*OreG0006385*
scaffold2276	155392	A	T	NA	*OreG0006385*
scaffold2276	155510	A	G	NA	*OreG0006385*
scaffold2276	155513	C	T	NA	*OreG0006385*
scaffold2276	173254	G	A	Och02,Och03,Och04,Och05,Och06,Och12,Och13	*OreG0006387*
scaffold2276	175517	G	A	Och05,Och14	*OreG0006388*
scaffold2276	175525	A	G	NA	*OreG0006388*
scaffold2276	175550	C	T	Ore07,Ore10,Ore13,Ore14	*OreG0006388*
scaffold2276	176073	C	A	Och05	*OreG0006389*

scaffold2276	176167	C	T	Och08,Och14,Ore07,Ore10,Ore13,Ore14	*OreG0006389*
scaffold2276	176272	G	T	Och14	*OreG0006389*
scaffold2276	176299	C	A	Och02,Och06,Och13	*OreG0006389*
scaffold2276	182964	A	G	Ore01,Ore02,Ore03,Ore04,Ore05,Ore06,Ore07,Ore08,Ore09,Ore10,Ore11,Ore12,Ore13,Ore14	*OreG0006390*
scaffold2276	185345	C	T	Ore01,Ore02,Ore03,Ore04,Ore05,Ore06,Ore07,Ore08,Ore09,Ore10,Ore11,Ore12,Ore13,Ore14	*OreG0006391*
scaffold2276	189145	C	A	Ore01,Ore02,Ore03,Ore04,Ore05,Ore06,Ore07,Ore08,Ore09,Ore10,Ore11,Ore12,Ore13,Ore14	*OreG0006392*
scaffold2276	189434	G	A	Och01,Och02,Och03,Och04,Och05,Och06,Och07,Och08,Och09,Och10,Och11,Och12,Och13,Och14	*OreG0006392*
scaffold2276	189482	G	C	NA	*OreG0006392*
scaffold2276	189843	C	T	NA	*OreG0006392*
scaffold2276	190250	A	T	NA	*OreG0006392*
scaffold2276	190268	G	C	Ore01,Ore02,Ore03,Ore04,Ore05,Ore06,Ore07,Ore08,Ore09,Ore10,Ore11,Ore12,Ore13,Ore14	*OreG0006392*
scaffold2276	190382	G	A	NA	*OreG0006392*
scaffold2276	193999	T	C	Och01,Och02,Och03,Och04,Och05,Och06,Och07,Och08,Och09,Och10,Och11,Och12,Och13,Och14,Ore01,Ore02,Ore03,Ore04,Ore05,Ore06,Ore07,Ore08,Ore09,Ore10,Ore11,Ore12,Ore13,Ore14	*OreG0006393*
scaffold2276	207665	G	T	Och01,Och02,Och03,Och04,Och05,Och06,Och09,Och10,Och11,Och12,Och13	*OreG0006395*
scaffold2276	209284	G	T	Och01,Och02,Och03,Och04,Och05,Och06,Och07,Och08,Och09,Och10,Och11,Och12,Och13,Och14	*OreG0006395*
scaffold2276	209737	A	G	NA	*OreG0006395*
scaffold2276	211399	C	T	NA	*OreG0006395*
scaffold2276	221509	G	A	NA	*OreG0006397*
scaffold2276	235460	T	G	NA	*OreG0006400*
scaffold2276	256961	G	A	Ore01,Ore07,Ore10,Ore13,Ore14	*OreG0006403*
scaffold2276	258184	T	A	Och01,Och02,Och03,Och04,Och05,Och06,Och07,Och08,Och09,Och10,Och11,Och12,Och13,Och14,Ore01,Ore02,Ore03,Ore04,Ore05,Ore06,Ore07,Ore08,Ore09,Ore10,Ore11,Ore12,Ore13,Ore14	*OreG0006403*
scaffold2276	268775	A	T	NA	*OreG0006404*
scaffold2276	280791	T	A	NA	*OreG0006406*
scaffold2276	280794	G	T	Och01,Ore01,Ore02,Ore03,Ore04,Ore05,Ore06,Ore07,Ore08,Ore09,Ore10,Ore11,Ore12,Ore13,Ore14	*OreG0006406*
scaffold2276	281998	G	A	NA	*OreG0006406*
scaffold2276	282034	T	A	NA	*OreG0006406*
scaffold2276	284170	G	A	NA	*OreG0006406*
scaffold2276	304437	C	T	NA	*OreG0006408*
scaffold2276	308009	C	G	Och09,Och10,Och11	*OreG0006409*
scaffold2276	308341	T	G	Ore01,Ore02,Ore03,Ore04,Ore05,Ore06,Ore07,Ore08,Ore09,Ore10,Ore11,Ore12,Ore13,Ore14	*OreG0006409*
scaffold2276	346621	G	C	NA	*OreG0006415*
scaffold2276	346842	G	C	Och01,Och02,Och03,Och04,Och05,Och06,Och07,Och08,Och09,Och10,Och11,Och12,Och13,Och14	*OreG0006415*
scaffold2276	355406	G	C	Och05,Och07	*OreG0006417*
scaffold2276	355427	C	T	NA	*OreG0006417*
scaffold2276	357162	T	A	NA	*OreG0006417*
scaffold2276	358176	A	G	NA	*OreG0006418*
scaffold2276	358201	G	A	NA	*OreG0006418*
scaffold2276	358231	A	G	Ore01,Ore02,Ore03,Ore04,Ore05,Ore06,Ore07,Ore08,Ore09,Ore10,Ore11,Ore12,Ore13,Ore14	*OreG0006418*
scaffold2276	358275	A	G	NA	*OreG0006418*
scaffold2276	368655	A	C	Och01,Och02,Och03,Och04,Och05,Och06,Och07,Och08,Och09,Och10,Och11,Och12,Och13,Och14	*OreG0006420*
scaffold2276	368753	G	A	NA	*OreG0006420*
scaffold2276	368982	A	G	NA	*OreG0006420*
scaffold2276	386762	A	T	Ore01,Ore02,Ore03,Ore04,Ore05,Ore06,Ore07,Ore08,Ore09,Ore10,Ore11,Ore12,Ore13,Ore14	*OreG0006424*
scaffold2276	387303	T	C	Och05,Ore01,Ore02,Ore03,Ore04,Ore05,Ore06,Ore07,Ore08,Ore09,Ore10,Ore11,Ore12,Ore13,Ore14	*OreG0006424*
scaffold2276	402515	A	C	Ore01,Ore02,Ore03,Ore04,Ore05,Ore06,Ore07,Ore08,Ore09,Ore10,Ore11,Ore12,Ore13,Ore14	*OreG0006426*
scaffold2276	408810	A	G	Ore01,Ore02,Ore03,Ore04,Ore05,Ore06,Ore07,Ore08,Ore09,Ore10,Ore11,Ore12,Ore13,Ore14	*OreG0006427*
scaffold2276	409157	T	A	Och01,Och02,Och03,Och04,Och05,Och06,Och07,Och08,Och09,Och10,Och11,Och12,Och13,Och14,Ore01,Ore02,Ore03,Ore04,Ore05,Ore06,Ore07,Ore08,Ore09,Ore10,Ore11,Ore12,Ore13,Ore14	*OreG0006427*
scaffold2276	413441	G	A	Ore01,Ore02,Ore03,Ore04,Ore05,Ore06,Ore07,Ore08,Ore09,Ore10,Ore11,Ore12,Ore13,Ore14	*OreG0006427*
scaffold2276	414962	G	T	NA	*OreG0006427*
scaffold2276	415262	C	T	NA	*OreG0006427*
scaffold2276	416862	A	T	NA	*OreG0006427*
scaffold2276	417012	T	C	Och01,Och02,Och03,Och04,Och05,Och06,Och07,Och08,Och09,Och10,Och11,Och12,Och13,Och14	*OreG0006427*

scaffold2276	431723	C	G	NA	*OreG0006428*
scaffold2276	457862	C	T	NA	*OreG0006431*
scaffold2276	467072	A	T	Och01,Och02,Och03,Och04,Och05,Och06,Och07,Och08,Och09,Och10,Och11,Och12,Och13,Och14,Ore01,Ore02,Ore03,Ore04,Ore05,Ore06,Ore07,Ore08,Ore09,Ore10,Ore11,Ore12,Ore13,Ore14	*OreG0006432*
scaffold2276	467429	A	T	Och02,Och06,Och13	*OreG0006432*
scaffold2276	474481	C	T	Och02,Och06,Och13	*OreG0006433*
scaffold2276	498442	G	A	NA	*OreG0006435*
scaffold2276	498526	C	T	Och05	*OreG0006435*
scaffold2276	515295	C	T	Ore01,Ore02,Ore03,Ore04,Ore05,Ore06,Ore07,Ore08,Ore09,Ore10,Ore11,Ore12,Ore13,Ore14	*OreG0006436*
scaffold2276	515536	C	G	Och05	*OreG0006436*
scaffold2276	524858	A	C	Ore01,Ore02,Ore03,Ore04,Ore05,Ore06,Ore07,Ore08,Ore09,Ore10,Ore11,Ore12,Ore13,Ore14	*OreG0006440*
scaffold2276	524863	T	A	Och05,Ore01,Ore02,Ore03,Ore04,Ore05,Ore06,Ore07,Ore08,Ore09,Ore10,Ore11,Ore12,Ore13,Ore14	*OreG0006440*
scaffold2276	524927	T	C	Och02,Och06,Och11,Och13	*OreG0006440*
scaffold2276	524954	T	C	Och05,Ore01,Ore02,Ore03,Ore04,Ore05,Ore06,Ore07,Ore08,Ore09,Ore10,Ore11,Ore12,Ore13,Ore14	*OreG0006440*
scaffold2276	525193	C	T	Och05,Ore01,Ore02,Ore03,Ore04,Ore05,Ore06,Ore07,Ore08,Ore09,Ore10,Ore11,Ore12,Ore13,Ore14	*OreG0006440*
scaffold2276	525208	G	T	Ore01,Ore02,Ore03,Ore04,Ore05,Ore06,Ore07,Ore08,Ore09,Ore10,Ore11,Ore12,Ore13,Ore14	*OreG0006440*
scaffold2276	538211	G	A	Och02,Och03,Och04,Och05,Och06,Och08,Och11,Och12,Och13,Och14	*OreG0006443*
scaffold2276	538279	C	A	Ore04,Ore05,Ore06,Ore08,Ore11,Ore12	*OreG0006443*
scaffold2276	541746	G	T	Och02,Och03,Och04,Och05,Och06,Och08,Och11,Och12,Och13,Och14	*OreG0006442*
scaffold2276	541825	G	A	Ore01,Ore02,Ore03,Ore04,Ore05,Ore06,Ore07,Ore08,Ore09,Ore10,Ore11,Ore12,Ore13,Ore14	*OreG0006442*
scaffold2276	554695	G	A	NA	*OreG0006446*
scaffold2276	563313	A	G	Ore01,Ore02,Ore03,Ore04,Ore05,Ore06,Ore07,Ore08,Ore09,Ore10,Ore11,Ore12,Ore13,Ore14	*OreG0006445*
scaffold2276	566093	G	A	NA	*OreG0006447*
scaffold2276	566129	A	G	NA	*OreG0006447*
scaffold2276	566603	G	A	Och01,Och02,Och03,Och04,Och05,Och06,Och07,Och08,Och09,Och10,Och11,Och12,Och13,Och14,Ore01,Ore02,Ore03,Ore04,Ore05,Ore06,Ore07,Ore08,Ore09,Ore10,Ore11,Ore12,Ore13,Ore14	*OreG0006447*
scaffold2276	581211	T	A	NA	*OreG0006450*
scaffold2276	583850	C	T	NA	*OreG0006450*
scaffold2276	585713	G	C	Ore01,Ore02,Ore03,Ore04,Ore05,Ore06,Ore07,Ore08,Ore09,Ore10,Ore11,Ore12,Ore13,Ore14	*OreG0006451*
scaffold2276	588229	T	C	Och01,Och02,Och03,Och04,Och05,Och06,Och07,Och08,Och09,Och10,Och11,Och12,Och13,Och14	*OreG0006451*
scaffold2276	588522	G	A	NA	*OreG0006451*
scaffold2276	593198	G	A	Ore01,Ore02,Ore03,Ore04,Ore05,Ore06,Ore07,Ore08,Ore09,Ore10,Ore11,Ore12,Ore13,Ore14	*OreG0006451*
scaffold2276	605206	C	T	NA	*OreG0006453*
scaffold2276	606600	T	A	Och01,Och02,Och03,Och04,Och05,Och06,Och07,Och08,Och09,Och10,Och11,Och12,Och13,Och14	*OreG0006453*
scaffold2276	636402	G	C	Ore01,Ore02,Ore03,Ore04,Ore05,Ore06,Ore07,Ore08,Ore09,Ore10,Ore11,Ore12,Ore13,Ore14	*OreG0006458*
scaffold2276	667972	C	A	NA	*OreG0006464*
scaffold2276	667974	T	C	NA	*OreG0006464*
scaffold2276	668081	G	T	NA	*OreG0006464*
scaffold2276	679096	C	T	NA	*OreG0006465*
scaffold2276	681250	C	T	NA	*OreG0006465*
scaffold2276	683018	C	T	Ore01,Ore02,Ore03,Ore04,Ore05,Ore06,Ore07,Ore08,Ore09,Ore10,Ore11,Ore12,Ore13,Ore14	*OreG0006465*
scaffold2276	683168	T	C	NA	*OreG0006465*
scaffold2276	683197	G	A	NA	*OreG0006465*
scaffold2276	727575	A	G	Ore04,Ore05,Ore06,Ore08,Ore11,Ore12	*OreG0006468*
scaffold2276	736410	C	T	Ore04,Ore05,Ore06,Ore08,Ore11,Ore12	*OreG0006471*
scaffold2276	741042	T	A	NA	*OreG0006472*
scaffold2276	744043	G	A	NA	*OreG0006473*
scaffold2276	773397	A	C	Och01,Och02,Och06,Och13	*OreG0006478*
scaffold2276	773910	C	A	Ore07,Ore10,Ore13,Ore14	*OreG0006478*
scaffold2276	774031	A	T	Och01,Och02,Och06,Och13	*OreG0006478*
scaffold2276	774155	A	G	NA	*OreG0006478*
scaffold2276	774205	A	T	Ore01,Ore02,Ore03,Ore04,Ore05,Ore06,Ore07,Ore08,Ore09,Ore10,Ore11,Ore12,Ore13,Ore14	*OreG0006478*
scaffold2276	774905	A	C	Och01,Och02,Och03,Och04,Och05,Och06,Och07,Och08,Och09,Och10,Och11,Och12,Och13,Och14,Ore01,Ore02,Ore03,Ore04,Ore05,Ore06,Ore07,Ore08,Ore09,Ore10,Ore11,Ore12,Ore13,Ore14	*OreG0006479*
scaffold2276	775097	T	G	Och01,Och02,Och03,Och04,Och05,Och06,Och07,Och08,Och09,Och10,Och11,Och12,Och13,Och14,Ore01,Ore02,Ore03,Ore04,Ore05,Ore06,Ore07,Ore08,Ore09,Ore10,Ore11,Ore12,Ore13,Ore14	*OreG0006479*

scaffold2276	777492	T	A	Ore01,Ore02,Ore03,Ore04,Ore05,Ore06,Ore07,Ore08,Ore09,Ore10,Ore11,Ore12,Ore13,Ore14	*OreG0006480*
scaffold2276	801694	T	G	NA	*OreG0006483*
scaffold2276	805006	G	A	NA	*OreG0006484*
scaffold2276	812064	T	C	Och01,Och02,Och03,Och04,Och05,Och06,Och07,Och08,Och09,Och10,Och11,Och12,Och13,Och14,Ore01,Ore02,Ore03,Ore04,Ore05,Ore06,Ore07,Ore08,Ore09,Ore10,Ore11,Ore12,Ore13,Ore14	*OreG0006486*
scaffold2276	812130	C	T	Och07	*OreG0006486*
scaffold2276	819028	A	G	NA	*OreG0006488*
scaffold2276	819270	C	T	NA	*OreG0006488*
scaffold2276	819424	G	A	NA	*OreG0006488*
scaffold2276	825770	C	A	Och09,Och10	*OreG0006489*
scaffold2276	827078	G	A	Och05	*OreG0006490*
scaffold2276	827296	T	C	Och05	*OreG0006490*
scaffold2276	847643	C	T	NA	*OreG0006492*
scaffold2276	859839	G	T	NA	*OreG0006494*
scaffold2276	861839	A	G	NA	*OreG0006495*
scaffold2276	896717	C	T	Och01,Och02,Och03,Och04,Och05,Och06,Och07,Och08,Och09,Och10,Och11,Och12,Och13,Och14	*OreG0006500*
scaffold2276	913991	T	A	NA	*OreG0006502*
scaffold2276	924760	T	C	Ore07,Ore10,Ore13,Ore14	*OreG0006505*
scaffold2276	926560	G	A	NA	*OreG0006505*
scaffold2276	930191	A	G	NA	*OreG0006506*
scaffold2276	930443	A	G	Och01,Och02,Och03,Och04,Och05,Och06,Och07,Och12,Och13,Och14,Ore01,Ore02,Ore03,Ore04,Ore05,Ore06,Ore07,Ore08,Ore09,Ore10,Ore11,Ore12,Ore13,Ore14	*OreG0006506*
scaffold2276	931188	G	T	NA	*OreG0006506*
scaffold2276	933246	G	T	Och01,Och02,Och03,Och04,Och05,Och06,Och07,Och08,Och09,Och10,Och11,Och12,Och13,Och14,Ore01,Ore02,Ore03,Ore04,Ore05,Ore06,Ore07,Ore08,Ore09,Ore10,Ore11,Ore12,Ore13,Ore14	*OreG0006507*
scaffold2276	936773	T	G	Och01,Och02,Och03,Och04,Och05,Och06,Och07,Och08,Och09,Och10,Och11,Och12,Och13,Och14,Ore01,Ore02,Ore03,Ore04,Ore05,Ore06,Ore07,Ore08,Ore09,Ore10,Ore11,Ore12,Ore13,Ore14	*OreG0006508*
scaffold2276	964532	T	C	NA	*OreG0006513*
scaffold2276	964657	A	T	NA	*OreG0006513*
scaffold2276	978467	T	C	Ore01,Ore02,Ore03,Ore04,Ore05,Ore06,Ore07,Ore08,Ore09,Ore10,Ore11,Ore12,Ore13,Ore14	*OreG0006514*
scaffold2276	994219	A	T	Och08,Och09,Och10	*OreG0006516*
scaffold2276	994920	A	T	Och08,Och09,Och10	*OreG0006516*
scaffold2276	994924	T	A	Och01,Och02,Och03,Och04,Och05,Och06,Och07,Och08,Och09,Och10,Och11,Och12,Och13,Och14,Ore01,Ore02,Ore03,Ore04,Ore05,Ore06,Ore07,Ore08,Ore09,Ore10,Ore11,Ore12,Ore13,Ore14	*OreG0006516*
scaffold2276	998324	C	T	Och01,Och02,Och03,Och04,Och05,Och06,Och07,Och08,Och09,Och10,Och11,Och12,Och13,Och14,Ore01,Ore02,Ore03,Ore04,Ore05,Ore06,Ore07,Ore08,Ore09,Ore10,Ore11,Ore12,Ore13,Ore14	*OreG0006517*
scaffold2276	998672	C	T	Ore02,Ore03,Ore07,Ore09,Ore10,Ore13,Ore14	*OreG0006517*
scaffold2276	999674	C	T	NA	*OreG0006517*
scaffold2276	1001687	C	T	Ore02,Ore03,Ore05,Ore06,Ore07,Ore09,Ore10,Ore13,Ore14	*OreG0006518*
scaffold2276	1002747	T	C	Och05	*OreG0006518*
scaffold2276	1004458	A	T	NA	*OreG0006518*
scaffold2276	1006516	A	C	Ore01,Ore02,Ore03,Ore04,Ore05,Ore06,Ore07,Ore08,Ore09,Ore10,Ore11,Ore12,Ore13,Ore14	*OreG0006518*
scaffold2276	1025036	A	T	NA	*OreG0006520*
scaffold2276	1026611	G	A	Och02,Och06,Och13	*OreG0006521*
scaffold2276	1027067	A	C	Ore01,Ore02,Ore03,Ore04,Ore05,Ore06,Ore07,Ore08,Ore09,Ore10,Ore11,Ore12,Ore13,Ore14	*OreG0006521*
scaffold2276	1102623	C	T	Och01,Och02,Och06,Och07,Och09,Och10,Och11,Och13	*OreG0006529*
scaffold2276	1109058	C	A	NA	*OreG0006530*
scaffold2276	1117768	C	A	Och01,Och02,Och03,Och04,Och05,Och06,Och07,Och08,Och09,Och10,Och11,Och12,Och13,Och14	*OreG0006531*
scaffold2276	1117854	T	C	NA	*OreG0006531*
scaffold2276	1117887	T	C	Och01,Och02,Och03,Och04,Och05,Och06,Och07,Och08,Och09,Och10,Och11,Och12,Och13,Och14	*OreG0006531*
scaffold2276	1124356	T	C	Och01,Och02,Och03,Och04,Och05,Och06,Och07,Och08,Och09,Och10,Och11,Och12,Och13,Och14,Ore01,Ore02,Ore03,Ore04,Ore05,Ore06,Ore07,Ore08,Ore09,Ore10,Ore11,Ore12,Ore13,Ore14	*OreG0006533*
scaffold2276	1131566	T	G	Ore01,Ore02,Ore03,Ore04,Ore05,Ore06,Ore07,Ore08,Ore09,Ore10,Ore11,Ore12,Ore13,Ore14	*OreG0006534*
scaffold2276	1131683	A	T	Ore01,Ore02,Ore03,Ore04,Ore05,Ore06,Ore07,Ore08,Ore09,Ore10,Ore11,Ore12,Ore13,Ore14	*OreG0006534*
scaffold2276	1131818	T	A	Ore01,Ore02,Ore03,Ore04,Ore05,Ore06,Ore07,Ore08,Ore09,Ore10,Ore11,Ore12,Ore13,Ore14	*OreG0006534*
scaffold2276	1132084	G	A	Och01,Och02,Och03,Och04,Och05,Och06,Och07,Och08,Och09,Och10,Och11,Och12,Och13,Och14	*OreG0006534*
scaffold2276	1132719	C	G	NA	*OreG0006534*

scaffold2276	1132796	T	C	Och01,Och02,Och03,Och04,Och05,Och06,Och07,Och08,Och09,Och10,Och11,Och12,Och13,Och14	*OreG0006534*
scaffold2276	1133640	C	G	NA	*OreG0006534*
scaffold2276	1134977	C	T	NA	*OreG0006535*
scaffold2276	1135028	T	C	NA	*OreG0006535*
scaffold2276	1135162	G	A	Ore01,Ore02,Ore03,Ore04,Ore05,Ore06,Ore07,Ore08,Ore09,Ore10,Ore11,Ore12,Ore13,Ore14	*OreG0006535*
scaffold2276	1135498	A	T	Och01,Och02,Och03,Och04,Och05,Och06,Och07,Och08,Och09,Och10,Och11,Och12,Och13,Och14	*OreG0006535*
scaffold2276	1135530	G	C	NA	*OreG0006535*
scaffold2276	1135663	T	G	Och05	*OreG0006535*
scaffold2276	1135750	A	C	Och01,Och02,Och03,Och04,Och05,Och06,Och07,Och08,Och09,Och10,Och11,Och12,Och13,Och14	*OreG0006535*
scaffold2276	1136111	A	G	Ore01,Ore02,Ore03,Ore04,Ore05,Ore06,Ore07,Ore08,Ore09,Ore10,Ore11,Ore12,Ore13,Ore14	*OreG0006535*
scaffold2276	1136167	C	G	Ore01,Ore02,Ore03,Ore04,Ore05,Ore06,Ore07,Ore08,Ore09,Ore10,Ore11,Ore12,Ore13,Ore14	*OreG0006535*
scaffold2276	1136198	T	C	NA	*OreG0006535*
scaffold2276	1136215	G	A	NA	*OreG0006535*
scaffold2276	1136771	T	C	Ore01,Ore02,Ore03,Ore04,Ore05,Ore06,Ore07,Ore08,Ore09,Ore10,Ore11,Ore12,Ore13,Ore14	*OreG0006535*
scaffold2276	1136891	T	C	NA	*OreG0006535*
scaffold2276	1136984	C	G	Och01,Och02,Och03,Och04,Och05,Och06,Och07,Och09,Och10,Och11,Och12,Och13,Och14	*OreG0006535*
scaffold2276	1157083	G	A	Och01,Och02,Och03,Och04,Och05,Och06,Och07,Och08,Och09,Och10,Och11,Och12,Och13,Och14,Ore01,Ore02,Ore03,Ore04,Ore05,Ore06,Ore07,Ore08,Ore09,Ore10,Ore11,Ore12,Ore13,Ore14	*OreG0006539*
scaffold2276	1162278	T	G	NA	*OreG0006540*
scaffold2276	1192614	C	T	NA	*OreG0006546*
scaffold2276	1211689	C	A	Och07,Och08,Och09,Och10,Och11,Och14,Ore01,Ore02,Ore03,Ore04,Ore05,Ore06,Ore07,Ore08,Ore09,Ore10,Ore11,Ore12,Ore13,Ore14	*OreG0006551*
scaffold2276	1212125	T	C	NA	*OreG0006551*
scaffold2276	1214047	G	A	Och07	*OreG0006551*
scaffold2276	1229552	G	A	Och11	*OreG0006553*
scaffold2276	1254916	G	T	NA	*OreG0006556*
scaffold2276	1274116	G	A	Och05	*OreG0006559*
scaffold2276	1300560	C	T	Och02,Och06,Och07,Och09,Och10,Och11,Och13	*OreG0006562*
scaffold2276	1300767	C	G	Ore10,Ore13,Ore14	*OreG0006562*
scaffold2276	1414281	C	A	Ore01,Ore02,Ore03,Ore04,Ore05,Ore06,Ore07,Ore08,Ore09,Ore10,Ore11,Ore12,Ore13,Ore14	*OreG0006572*
scaffold2276	1430153	C	G	Och01,Och02,Och03,Och04,Och05,Och06,Och07,Och08,Och09,Och10,Och11,Och12,Och13,Och14	*OreG0006575*
scaffold2276	1430674	A	G	Och01,Och02,Och03,Och04,Och05,Och06,Och07,Och08,Och09,Och10,Och11,Och12,Och13,Och14	*OreG0006575*
scaffold2276	1439054	C	A	Ore01,Ore02,Ore03,Ore04,Ore05,Ore06,Ore07,Ore08,Ore09,Ore10,Ore11,Ore12,Ore13,Ore14	*OreG0006577*
scaffold2276	1448552	T	A	Och01,Och11	*OreG0006578*
scaffold2276	1550884	G	T	NA	*OreG0006590*
scaffold2276	1551675	C	A	NA	*OreG0006590*
scaffold2276	1552204	G	T	Ore01,Ore02,Ore03,Ore04,Ore05,Ore06,Ore07,Ore08,Ore09,Ore10,Ore11,Ore12,Ore13,Ore14	*OreG0006590*
scaffold2276	1552245	G	A	NA	*OreG0006590*
scaffold2276	1552400	G	A	Och01,Och02,Och03,Och04,Och05,Och06,Och07,Och08,Och09,Och10,Och11,Och12,Och13,Och14,Ore01,Ore02,Ore03,Ore04,Ore05,Ore06,Ore07,Ore08,Ore09,Ore10,Ore11,Ore12,Ore13,Ore14	*OreG0006590*
scaffold2276	1556169	A	T	NA	*OreG0006591*
scaffold2276	1558080	C	T	Och01,Och02,Och03,Och04,Och05,Och06,Och07,Och08,Och09,Och10,Och11,Och12,Och13,Och14,Ore01,Ore02,Ore03,Ore04,Ore05,Ore06,Ore07,Ore08,Ore09,Ore10,Ore11,Ore12,Ore13,Ore14	*OreG0006591*
scaffold2276	1577981	G	A	NA	*OreG0006594*
scaffold2276	1587561	C	A	NA	*OreG0006595*
scaffold2276	1588113	T	G	NA	*OreG0006595*
scaffold2276	1588137	T	A	Och01,Och02,Och03,Och04,Och05,Och06,Och07,Och08,Och09,Och10,Och11,Och12,Och13,Och14	*OreG0006595*
scaffold2276	1590142	G	A	NA	*OreG0006596*
scaffold2276	1591970	G	A	NA	*OreG0006596*
scaffold2276	1592554	A	G	NA	*OreG0006596*
scaffold2276	1593864	T	A	Och01,Och02,Och03,Och04,Och05,Och06,Och07,Och08,Och09,Och10,Och11,Och12,Och13,Och14,Ore01,Ore02,Ore03,Ore04,Ore05,Ore06,Ore07,Ore08,Ore09,Ore10,Ore11,Ore12,Ore13,Ore14	*OreG0006596*
scaffold2276	1596709	C	T	Och01,Och02,Och06,Och13	*OreG0006596*
scaffold2276	1596849	T	C	Ore02,Ore03,Ore07,Ore09,Ore10,Ore13,Ore14	*OreG0006596*
scaffold2276	1620750	C	T	Ore01,Ore02,Ore03,Ore04,Ore05,Ore06,Ore07,Ore08,Ore09,Ore10,Ore11,Ore12,Ore13,Ore14	*OreG0006600*
scaffold2276	1621201	G	A	Ore01,Ore02,Ore03,Ore04,Ore05,Ore06,Ore07,Ore08,Ore09,Ore10,Ore11,Ore12,Ore13,Ore14	*OreG0006601*

scaffold2276	1621255	C	G	Och02,Och06,Och13	*OreG0006601*
scaffold2276	1621427	A	T	Ore01,Ore02,Ore03,Ore04,Ore05,Ore06,Ore07,Ore08,Ore09,Ore10,Ore11,Ore12,Ore13,Ore14	*OreG0006601*
scaffold2276	1621559	C	G	Och05	*OreG0006601*
scaffold2276	1628829	C	G	Ore01,Ore02,Ore03,Ore04,Ore05,Ore06,Ore07,Ore08,Ore09,Ore10,Ore11,Ore12,Ore13,Ore14	*OreG0006602*
scaffold2276	1636938	G	T	Och01,Och02,Och03,Och04,Och05,Och06,Och07,Och08,Och09,Och10,Och11,Och12,Och13,Och14,Ore01,Ore02,Ore03,Ore04,Ore05,Ore06,Ore07,Ore08,Ore09,Ore10,Ore11,Ore12,Ore13,Ore14	*OreG0006603*
scaffold2276	1646158	C	T	NA	*OreG0006604*
scaffold2276	1669620	A	G	Och01	*OreG0006606*
scaffold2276	1681727	A	G	Och01,Och02,Och03,Och04,Och05,Och06,Och07,Och08,Och09,Och10,Och11,Och12,Och13,Och14,Ore01,Ore02,Ore03,Ore04,Ore05,Ore06,Ore07,Ore08,Ore09,Ore10,Ore11,Ore12,Ore13,Ore14	*OreG0006608*
scaffold2276	1722207	C	T	NA	*OreG0006610*
scaffold2276	1732513	T	A	Ore04,Ore05,Ore08,Ore11,Ore12	*OreG0006612*
scaffold2276	1743134	A	G	Och02,Och06,Och13	*OreG0006614*
scaffold2276	1745923	C	T	Ore10,Ore13,Ore14	*OreG0006615*
scaffold2276	1757160	G	A	NA	*OreG0006618*
scaffold2276	1771837	A	T	Och01,Och11,Och14	*OreG0006619*
scaffold2276	1772752	A	T	Och02,Och03,Och04,Och05,Och06,Och12,Och13,Ore01,Ore02,Ore03,Ore04,Ore05,Ore06,Ore07,Ore08,Ore09,Ore10,Ore11,Ore12,Ore13,Ore14	*OreG0006619*
scaffold2276	1772826	T	C	NA	*OreG0006619*
scaffold2276	1773216	G	T	Och01,Och11,Och14	*OreG0006619*
scaffold2276	1773991	C	A	Och02,Och06,Och13	*OreG0006619*
scaffold2276	1777691	G	A	Och02,Och06,Och13	*OreG0006620*
scaffold2276	1779675	G	A	Och01,Och02,Och03,Och04,Och05,Och06,Och07,Och08,Och09,Och10,Och11,Och12,Och13,Och14	*OreG0006620*
scaffold2276	1818349	A	C	Och01,Och02,Och03,Och04,Och05,Och06,Och07,Och08,Och09,Och10,Och11,Och12,Och13,Och14,Ore01,Ore02,Ore03,Ore04,Ore05,Ore06,Ore07,Ore08,Ore09,Ore10,Ore11,Ore12,Ore13,Ore14	*OreG0006624*
scaffold2276	1818673	G	T	Och01,Och02,Och03,Och04,Och05,Och06,Och07,Och08,Och09,Och10,Och11,Och12,Och13,Och14,Ore01,Ore02,Ore03,Ore04,Ore05,Ore06,Ore07,Ore08,Ore09,Ore10,Ore11,Ore12,Ore13,Ore14	*OreG0006624*
scaffold2276	1818868	C	T	Och01,Och02,Och03,Och04,Och05,Och06,Och07,Och08,Och09,Och10,Och11,Och12,Och13,Och14,Ore01,Ore02,Ore03,Ore04,Ore05,Ore06,Ore07,Ore08,Ore09,Ore10,Ore11,Ore12,Ore13,Ore14	*OreG0006624*
scaffold2276	1818884	G	T	Och01,Och02,Och03,Och04,Och05,Och06,Och07,Och08,Och09,Och10,Och11,Och12,Och13,Och14	*OreG0006624*
scaffold2276	1839406	A	T	Ore01,Ore02,Ore03,Ore04,Ore05,Ore06,Ore07,Ore08,Ore09,Ore10,Ore11,Ore12,Ore13,Ore14	*OreG0006627*
scaffold2276	1845493	A	G	Och02,Och03,Och04,Och05,Och06,Och08,Och09,Och10,Och11,Och12,Och13	*OreG0006628*
scaffold2276	1869667	G	T	NA	*OreG0006632*
scaffold2276	1870802	T	A	Och02,Och06,Och13	*OreG0006632*
scaffold2276	1870845	G	T	Ore01,Ore02,Ore03,Ore04,Ore05,Ore06,Ore07,Ore08,Ore09,Ore10,Ore11,Ore12,Ore13,Ore14	*OreG0006632*
scaffold2276	1921623	C	T	Ore01,Ore02,Ore03,Ore04,Ore05,Ore06,Ore07,Ore08,Ore09,Ore10,Ore11,Ore12,Ore13,Ore14	*OreG0006639*
scaffold2276	1940649	C	T	NA	*OreG0006641*
scaffold2276	1989363	G	T	NA	*OreG0006643*
scaffold2276	1997184	G	A	NA	*OreG0006644*
scaffold2276	1999010	T	C	Och01	*OreG0006644*
scaffold2276	2016357	C	T	Och02,Och06,Och13	*OreG0006646*
scaffold2276	2016507	C	G	Och01,Och02,Och03,Och04,Och05,Och06,Och08,Och11,Och12,Och13,Och14	*OreG0006646*
scaffold2276	2016739	C	G	Ore01,Ore02,Ore03,Ore04,Ore05,Ore06,Ore07,Ore08,Ore09,Ore10,Ore11,Ore12,Ore13,Ore14	*OreG0006646*
scaffold2276	2017074	T	C	Och01,Och02,Och03,Och04,Och05,Och06,Och07,Och08,Och09,Och10,Och11,Och12,Och13,Och14	*OreG0006646*
scaffold2276	2017368	C	G	Och01,Och02,Och03,Och04,Och05,Och06,Och08,Och11,Och12,Och13	*OreG0006646*
scaffold2276	2022094	C	A	NA	*OreG0006647*
scaffold2276	2026749	T	G	Ore01,Ore02,Ore03,Ore04,Ore05,Ore06,Ore07,Ore08,Ore09,Ore10,Ore11,Ore12,Ore13,Ore14	*OreG0006648*
scaffold2276	2060988	G	T	NA	*OreG0006651*
scaffold2276	2104205	T	G	Och01,Och02,Och03,Och04,Och05,Och06,Och07,Och08,Och09,Och10,Och11,Och12,Och13,Och14	*OreG0006658*
scaffold2276	2108752	C	A	Ore08,Ore12	*OreG0006659*
scaffold2276	2109458	G	A	Ore01,Ore02,Ore03,Ore06,Ore07,Ore08,Ore09,Ore10,Ore11,Ore12,Ore13,Ore14	*OreG0006659*
scaffold2276	2119223	G	T	Och01,Och02,Och03,Och04,Och05,Och06,Och07,Och08,Och09,Och10,Och11,Och12,Och13,Och14,Ore01,Ore02,Ore03,Ore04,Ore05,Ore06,Ore07,Ore08,Ore09,Ore10,Ore11,Ore12,Ore13,Ore14	*OreG0006661*
scaffold2276	2119966	C	T	Ore10,Ore13,Ore14	*OreG0006661*
scaffold2276	2120944	A	C	NA	*OreG0006661*
scaffold2276	2160016	C	T	Och01,Och02,Och03,Och04,Och05,Och06,Och07,Och08,Och09,Och10,Och11,Och12,Och13,Och14,Ore01,Ore02,Ore03,Ore04,Ore05,Ore06,Ore07,Ore08,Ore09,Ore10,Ore11,Ore12,Ore13,Ore14	*OreG0006663*

scaffold2276	2195252	G	T	NA	*OreG0006667*
scaffold2276	2202279	G	A	Och01,Och02,Och03,Och04,Och05,Och06,Och07,Och08,Och09,Och10,Och11,Och12,Och13,Och14	*OreG0006668*
scaffold2276	2205843	G	T	NA	*OreG0006669*
scaffold2276	2216075	T	A	Och05	*OreG0006671*
scaffold2276	2230819	G	C	Och01,Och02,Och03,Och04,Och05,Och06,Och07,Och08,Och09,Och10,Och11,Och12,Och13,Och14	*OreG0006672*
scaffold2276	2277496	T	G	Och01,Och02,Och03,Och04,Och05,Och06,Och07,Och08,Och09,Och10,Och11,Och12,Och13,Och14	*OreG0006678*
scaffold2276	2278200	A	T	Och01,Och02,Och03,Och04,Och05,Och06,Och07,Och08,Och09,Och10,Och11,Och12,Och13,Och14	*OreG0006678*
scaffold2276	2309548	T	C	NA	*OreG0006680*
scaffold2276	2314490	C	A	Ore06,Ore10,Ore13,Ore14	*OreG0006681*
scaffold2276	2314824	T	A	Ore08,Ore12	*OreG0006681*
scaffold2276	2316339	T	A	Och05	*OreG0006681*
scaffold2276	2335589	C	A	NA	*OreG0006684*
scaffold2276	2348307	T	A	NA	*OreG0006685*
scaffold2276	2348552	A	G	NA	*OreG0006685*
scaffold2276	2353833	C	A	Och07,Och08,Och14	*OreG0006686*
scaffold2276	2382370	C	T	Och01	*OreG0006688*
scaffold2276	2383132	A	G	Ore06,Ore10,Ore13,Ore14	*OreG0006688*
scaffold2276	2383155	T	C	Och01,Och02,Och03,Och04,Och05,Och06,Och07,Och08,Och09,Och10,Och11,Och12,Och13,Och14	*OreG0006688*
scaffold2276	2383257	T	C	Och01,Och02,Och03,Och04,Och05,Och06,Och07,Och08,Och09,Och10,Och11,Och12,Och13,Och14,Ore01,Ore02,Ore03,Ore04,Ore05,Ore06,Ore07,Ore08,Ore09,Ore10,Ore11,Ore12,Ore13,Ore14	*OreG0006688*
scaffold2276	2383275	G	A	Och01,Och02,Och03,Och04,Och05,Och06,Och07,Och08,Och09,Och10,Och11,Och12,Och13,Och14,Ore01,Ore02,Ore03,Ore04,Ore05,Ore06,Ore07,Ore08,Ore09,Ore10,Ore11,Ore12,Ore13,Ore14	*OreG0006688*
scaffold2276	2383324	C	T	NA	*OreG0006688*
scaffold2276	2383366	G	T	Och01,Och02,Och03,Och04,Och05,Och06,Och07,Och08,Och09,Och10,Och11,Och12,Och13,Och14,Ore01,Ore02,Ore03,Ore04,Ore05,Ore06,Ore07,Ore08,Ore09,Ore10,Ore11,Ore12,Ore13,Ore14	*OreG0006688*
scaffold2276	2383605	A	G	Ore01,Ore02,Ore03,Ore04,Ore05,Ore06,Ore07,Ore08,Ore09,Ore10,Ore11,Ore12,Ore13,Ore14	*OreG0006688*
scaffold2276	2384143	G	T	Ore01,Ore02,Ore03,Ore04,Ore05,Ore06,Ore07,Ore08,Ore09,Ore10,Ore11,Ore12,Ore13,Ore14	*OreG0006688*
scaffold2276	2384175	A	G	Ore01,Ore02,Ore03,Ore04,Ore05,Ore06,Ore07,Ore08,Ore09,Ore10,Ore11,Ore12,Ore13,Ore14	*OreG0006688*
scaffold2276	2385488	C	T	Och01,Och02,Och03,Och04,Och05,Och06,Och07,Och08,Och09,Och10,Och11,Och12,Och13,Och14,Ore01,Ore02,Ore03,Ore04,Ore05,Ore06,Ore07,Ore08,Ore09,Ore10,Ore11,Ore12,Ore13,Ore14	*OreG0006688*
scaffold2276	2385511	G	A	Och01,Och02,Och03,Och04,Och05,Och06,Och07,Och08,Och09,Och10,Och11,Och12,Och13,Och14,Ore01,Ore02,Ore03,Ore04,Ore05,Ore06,Ore07,Ore08,Ore09,Ore10,Ore11,Ore12,Ore13,Ore14	*OreG0006688*
scaffold2276	2385659	T	G	Och07,Och14	*OreG0006688*
scaffold2276	2385954	A	T	Och07,Och14	*OreG0006688*
scaffold2276	2385974	G	A	Och01,Och02,Och03,Och04,Och05,Och06,Och07,Och08,Och09,Och10,Och11,Och12,Och13,Och14,Ore01,Ore02,Ore03,Ore04,Ore05,Ore06,Ore07,Ore08,Ore09,Ore10,Ore11,Ore12,Ore13,Ore14	*OreG0006688*
scaffold2276	2394305	T	A	Och01,Och02,Och03,Och04,Och05,Och06,Och07,Och08,Och09,Och10,Och11,Och12,Och13,Och14,Ore01,Ore02,Ore03,Ore04,Ore05,Ore06,Ore07,Ore08,Ore09,Ore10,Ore11,Ore12,Ore13,Ore14	*OreG0006690*
scaffold2276	2453199	C	T	NA	*OreG0006693*
scaffold2276	2466843	A	G	Och05	*OreG0006694*
scaffold2276	2467025	A	T	NA	*OreG0006694*
scaffold2276	2482673	A	T	Ore01,Ore02,Ore03,Ore06,Ore07,Ore08,Ore09,Ore10,Ore11,Ore12,Ore13,Ore14	*OreG0006696*
scaffold2276	2484731	C	T	Ore01,Ore02,Ore03,Ore04,Ore05,Ore06,Ore07,Ore08,Ore09,Ore10,Ore11,Ore12,Ore13,Ore14	*OreG0006696*
scaffold2276	2494736	G	A	Och01,Och02,Och03,Och04,Och05,Och06,Och07,Och08,Och09,Och10,Och11,Och12,Och13,Och14	*OreG0006698*
scaffold2276	2496161	G	C	NA	*OreG0006698*
scaffold2276	2500079	A	T	NA	*OreG0006699*
scaffold2276	2500725	G	A	Ore08,Ore12	*OreG0006699*
scaffold2276	2500729	C	G	Och01,Och02,Och03,Och04,Och05,Och06,Och07,Och08,Och09,Och10,Och11,Och12,Och13,Och14,Ore01,Ore02,Ore03,Ore04,Ore05,Ore06,Ore07,Ore08,Ore09,Ore10,Ore11,Ore12,Ore13,Ore14	*OreG0006699*
scaffold2276	2500777	C	A	NA	*OreG0006699*
scaffold2276	2501761	C	T	NA	*OreG0006699*
scaffold2276	2502062	A	T	Och01,Och05	*OreG0006699*
scaffold2276	2502575	C	A	Och01,Och02,Och03,Och04,Och05,Och06,Och07,Och08,Och09,Och10,Och11,Och12,Och13,Och14	*OreG0006699*
scaffold2276	2502940	G	A	Och01,Och05	*OreG0006699*
scaffold2276	2502970	G	A	Och02,Och06,Och13	*OreG0006699*
scaffold2276	2503001	G	A	Och01	*OreG0006699*
scaffold2276	2503527	G	A	NA	*OreG0006699*

scaffold2276	2503591	C	A	NA	*OreG0006699*
scaffold2276	2505037	C	A	Och02,Och06,Och13	*OreG0006699*
scaffold2276	2514168	T	C	NA	*OreG0006700*
scaffold2276	2514420	A	G	Och01,Och02,Och03,Och04,Och05,Och06,Och07,Och09,Och10,Och11,Och12,Och13	*OreG0006700*
scaffold2276	2545638	C	T	Och02,Och06,Och13	*OreG0006703*
scaffold2276	2545960	G	A	NA	*OreG0006703*
scaffold2276	2549876	A	G	Ore01,Ore02,Ore03,Ore04,Ore05,Ore06,Ore07,Ore08,Ore09,Ore10,Ore11,Ore12,Ore13,Ore14	*OreG0006703*
scaffold2276	2549975	T	C	Och01,Och02,Och03,Och04,Och05,Och06,Och07,Och08,Och09,Och10,Och12,Och13,Och14	*OreG0006703*
scaffold2276	2570826	C	T	Ore01,Ore02,Ore03,Ore04,Ore05,Ore06,Ore07,Ore08,Ore09,Ore10,Ore11,Ore12,Ore13,Ore14	*OreG0006705*
scaffold2276	2571458	G	A	Och01,Och02,Och03,Och04,Och05,Och06,Och07,Och08,Och09,Och10,Och11,Och12,Och13,Och14	*OreG0006705*
scaffold2276	2578380	G	A	NA	*OreG0006706*
scaffold2276	2636602	C	A	Ore01,Ore02,Ore03,Ore04,Ore05,Ore06,Ore07,Ore08,Ore09,Ore10,Ore11,Ore12,Ore13,Ore14	*OreG0006710*
scaffold2276	2658348	G	A	Ore01,Ore02,Ore03,Ore04,Ore05,Ore06,Ore07,Ore08,Ore09,Ore10,Ore11,Ore12,Ore13,Ore14	*OreG0006712*
scaffold2276	2658665	G	C	Och01,Och02,Och03,Och04,Och05,Och06,Och07,Och08,Och09,Och10,Och11,Och12,Och13,Och14	*OreG0006712*
scaffold2276	2711397	C	A	Ore01,Ore02,Ore03,Ore04,Ore05,Ore06,Ore07,Ore08,Ore09,Ore10,Ore11,Ore12,Ore13,Ore14	*OreG0006714*
scaffold2276	2715926	C	T	NA	*OreG0006714*
scaffold2276	2718166	A	C	Och01,Och02,Och03,Och04,Och05,Och06,Och07,Och08,Och09,Och10,Och11,Och12,Och13,Och14,Ore01,Ore02,Ore03,Ore04,Ore05,Ore06,Ore07,Ore08,Ore09,Ore10,Ore11,Ore12,Ore13,Ore14	*OreG0006714*
scaffold2276	2752756	G	T	Och01,Och02,Och03,Och04,Och05,Och06,Och07,Och08,Och09,Och10,Och11,Och12,Och13,Och14,Ore01,Ore02,Ore03,Ore04,Ore05,Ore06,Ore07,Ore08,Ore09,Ore10,Ore11,Ore12,Ore13,Ore14	*OreG0006719*
scaffold2276	2768922	C	A	Ore08,Ore12	*OreG0006720*
scaffold2276	2776382	A	G	Och01,Och11,Och14	*OreG0006721*
scaffold2276	2784466	C	A	NA	*OreG0006723*
scaffold2276	2787543	T	A	Och01,Och02,Och03,Och04,Och05,Och06,Och07,Och08,Och09,Och10,Och11,Och12,Och13,Och14,Ore01,Ore02,Ore03,Ore04,Ore05,Ore06,Ore07,Ore08,Ore09,Ore10,Ore11,Ore12,Ore13,Ore14	*OreG0006723*
scaffold2276	2790257	A	T	Ore01,Ore02,Ore03,Ore04,Ore05,Ore06,Ore07,Ore08,Ore09,Ore10,Ore11,Ore12,Ore13,Ore14	*OreG0006723*
scaffold2276	2814020	C	G	Ore06,Ore10,Ore13,Ore14	*OreG0006727*
scaffold2276	2840148	A	G	NA	*OreG0006729*
scaffold2276	2844781	G	A	Och01,Och02,Och03,Och04,Och05,Och06,Och12,Och13,Och14	*OreG0006730*
scaffold2276	2844794	T	A	Ore04,Ore05,Ore08,Ore12	*OreG0006730*
scaffold2276	2844958	G	T	NA	*OreG0006730*
scaffold2276	2845235	C	T	Ore01,Ore02,Ore03,Ore04,Ore05,Ore06,Ore07,Ore08,Ore09,Ore10,Ore11,Ore12,Ore13,Ore14	*OreG0006730*
scaffold2276	2845253	C	T	NA	*OreG0006730*
scaffold2276	2846931	G	A	Ore08,Ore12	*OreG0006730*
scaffold2276	2848300	C	T	NA	*OreG0006730*
scaffold2276	2848529	A	T	Och01,Och02,Och03,Och04,Och05,Och06,Och07,Och08,Och09,Och10,Och11,Och12,Och13,Och14	*OreG0006730*
scaffold2276	2850890	C	G	NA	*OreG0006731*
scaffold2276	2851965	G	C	Och02,Och03,Och04,Och05,Och06,Och12,Och13	*OreG0006731*
scaffold2276	2852325	C	A	NA	*OreG0006731*
scaffold2276	2857255	C	G	Och01,Och02,Och03,Och04,Och05,Och06,Och07,Och08,Och09,Och10,Och11,Och12,Och13,Och14,Ore01,Ore02,Ore03,Ore04,Ore05,Ore06,Ore07,Ore08,Ore09,Ore10,Ore11,Ore12,Ore13,Ore14	*OreG0006732*
scaffold2276	2868861	C	T	NA	*OreG0006733*
scaffold2276	2886879	A	G	NA	*OreG0006734*
scaffold2276	2886924	A	T	Och01,Och02,Och03,Och04,Och05,Och06,Och07,Och08,Och09,Och10,Och11,Och12,Och13,Och14,Ore01,Ore02,Ore03,Ore04,Ore05,Ore06,Ore07,Ore08,Ore09,Ore10,Ore11,Ore12,Ore13,Ore14	*OreG0006734*
scaffold2276	2915507	G	T	Och01,Och02,Och03,Och04,Och05,Och06,Och07,Och08,Och09,Och10,Och11,Och12,Och13,Och14,Ore01,Ore02,Ore03,Ore04,Ore05,Ore06,Ore07,Ore08,Ore09,Ore10,Ore11,Ore12,Ore13,Ore14	*OreG0006738*
scaffold2276	2916486	C	A	NA	*OreG0006738*
scaffold2276	2931434	T	C	NA	*OreG0006739*
scaffold2276	2939644	G	A	Och02,Och06,Och13	*OreG0006740*
scaffold2276	2939751	A	C	NA	*OreG0006740*
scaffold2276	2991191	C	T	Och01,Och02,Och03,Och04,Och05,Och06,Och07,Och08,Och09,Och10,Och11,Och12,Och13,Och14,Ore01,Ore02,Ore03,Ore04,Ore05,Ore06,Ore07,Ore08,Ore09,Ore10,Ore11,Ore12,Ore13,Ore14	*OreG0006745*
scaffold2276	2993507	C	T	NA	*OreG0006745*
scaffold2276	3023337	C	G	Och01,Och05,Och09,Och10	*OreG0006749*
scaffold2276	3030101	C	T	NA	*OreG0006750*

scaffold2276	3031157	G	T	Och11	*OreG0006750*
scaffold2276	3031171	C	T	NA	*OreG0006750*
scaffold2276	3038978	T	G	Och01,Och02,Och03,Och04,Och05,Och06,Och07,Och08,Och09,Och10,Och11,Och12,Och13,Och14,Ore01,Ore02,Ore03,Ore04,Ore05,Ore06,Ore07,Ore08,Ore09,Ore10,Ore11,Ore12,Ore13,Ore14	*OreG0006752*
scaffold2276	3060112	A	G	Och01,Och02,Och03,Och04,Och05,Och06,Och07,Och09,Och10,Och11,Och12,Och13,Och14	*OreG0006755*
scaffold2276	3061554	C	G	NA	*OreG0006756*
scaffold2276	3061792	C	G	Ore01,Ore02,Ore03,Ore04,Ore05,Ore06,Ore07,Ore08,Ore09,Ore10,Ore11,Ore12,Ore13,Ore14	*OreG0006756*
scaffold2276	3061819	C	G	NA	*OreG0006756*
scaffold2276	3061831	T	A	NA	*OreG0006756*
scaffold2276	3077531	G	C	NA	*OreG0006759*
scaffold2276	3098246	A	T	NA	*OreG0006761*
scaffold2276	3107285	T	C	Och01,Och02,Och03,Och04,Och05,Och06,Och07,Och08,Och09,Och10,Och11,Och12,Och13,Och14,Ore01,Ore02,Ore03,Ore04,Ore05,Ore06,Ore07,Ore08,Ore09,Ore10,Ore11,Ore12,Ore13,Ore14	*OreG0006763*
scaffold2276	3109953	C	T	Och01,Och02,Och03,Och04,Och05,Och06,Och07,Och08,Och09,Och10,Och11,Och12,Och13,Och14,Ore01,Ore02,Ore03,Ore04,Ore05,Ore06,Ore07,Ore08,Ore09,Ore10,Ore11,Ore12,Ore13,Ore14	*OreG0006763*
scaffold2276	3125528	G	C	Och01,Och02,Och03,Och04,Och05,Och06,Och07,Och08,Och09,Och10,Och11,Och12,Och13,Och14,Ore01,Ore02,Ore03,Ore04,Ore05,Ore06,Ore07,Ore08,Ore09,Ore10,Ore11,Ore12,Ore13,Ore14	*OreG0006765*
scaffold2276	3166938	C	A	NA	*OreG0006768*
scaffold2276	3169784	A	G	Och02,Och06,Och13	*OreG0006768*
scaffold2276	3254523	C	G	Ore08,Ore12	*OreG0006782*
scaffold2276	3257347	A	T	NA	*OreG0006782*
scaffold2276	3261171	T	C	Och01,Och02,Och03,Och04,Och05,Och06,Och07,Och08,Och09,Och10,Och11,Och12,Och13,Och14,Ore01,Ore02,Ore03,Ore04,Ore05,Ore06,Ore07,Ore08,Ore09,Ore10,Ore11,Ore12,Ore13,Ore14	*OreG0006783*
scaffold2276	3266629	T	C	NA	*OreG0006784*
scaffold2276	3267597	C	A	Ore06,Ore10,Ore13,Ore14	*OreG0006784*
scaffold2276	3285408	C	T	Och09,Och10	*OreG0006787*
scaffold2276	3315912	T	C	Och01,Och02,Och03,Och04,Och05,Och06,Och07,Och08,Och09,Och10,Och11,Och12,Och13,Och14	*OreG0006791*
scaffold2276	3335807	T	C	Ore04,Ore05,Ore08,Ore12	*OreG0006794*
scaffold2276	3365107	A	G	Och01,Och02,Och03,Och04,Och05,Och06,Och07,Och08,Och09,Och10,Och11,Och12,Och13,Och14	*OreG0006800*
scaffold2276	3366243	C	T	Ore01,Ore02,Ore03,Ore04,Ore05,Ore06,Ore07,Ore08,Ore09,Ore10,Ore11,Ore12,Ore13,Ore14	*OreG0006800*
scaffold2276	3366738	C	T	Och02,Och03,Och04,Och05,Och06,Och12,Och13	*OreG0006800*
scaffold2276	3366945	G	T	NA	*OreG0006800*
scaffold2276	3389434	C	T	NA	*OreG0006801*
scaffold2276	3389524	T	C	Ore01,Ore02,Ore03,Ore04,Ore05,Ore06,Ore07,Ore08,Ore09,Ore10,Ore11,Ore12,Ore13,Ore14	*OreG0006801*
scaffold2276	3393502	C	G	NA	*OreG0006802*
scaffold2276	3394159	C	T	NA	*OreG0006802*
scaffold2276	3399339	T	G	NA	*OreG0006803*
scaffold2276	3482649	C	G	NA	*OreG0006811*
scaffold2276	3504572	G	A	Ore01,Ore02,Ore03,Ore04,Ore05,Ore06,Ore07,Ore08,Ore09,Ore10,Ore11,Ore12,Ore13,Ore14	*OreG0006812*
scaffold2276	3522959	A	G	Och02,Och03,Och04,Och05,Och06,Och12,Och13,Och14	*OreG0006814*
scaffold2276	3523028	T	C	NA	*OreG0006814*
scaffold2276	3548666	A	G	Och02,Och06,Och13	*OreG0006817*
scaffold2276	3559262	C	T	NA	*OreG0006818*
scaffold2276	3581850	G	T	NA	*OreG0006820*
scaffold2276	3617531	G	T	Och14	*OreG0006823*
scaffold2276	3617546	T	C	Ore01,Ore02,Ore03,Ore04,Ore05,Ore06,Ore07,Ore08,Ore09,Ore10,Ore11,Ore12,Ore13,Ore14	*OreG0006823*
scaffold2276	3649447	G	A	Ore01,Ore02,Ore03,Ore04,Ore05,Ore06,Ore07,Ore08,Ore09,Ore10,Ore11,Ore12,Ore13,Ore14	*OreG0006828*
scaffold2276	3657809	G	A	Och01,Och02,Och03,Och04,Och05,Och06,Och07,Och08,Och09,Och10,Och11,Och12,Och13,Och14,Ore01,Ore02,Ore03,Ore04,Ore05,Ore06,Ore07,Ore08,Ore09,Ore10,Ore11,Ore12,Ore13,Ore14	*OreG0006828*
scaffold2276	3659552	A	T	Och01,Och02,Och03,Och04,Och05,Och06,Och08,Och11,Och12,Och13	*OreG0006829*
scaffold2276	3660509	C	G	Och05	*OreG0006829*
scaffold2276	3669006	A	C	NA	*OreG0006830*
scaffold2276	3677926	C	T	Och01,Och02,Och03,Och04,Och05,Och06,Och07,Och08,Och09,Och10,Och11,Och12,Och13,Och14	*OreG0006832*
scaffold2276	3678161	G	T	Ore01,Ore02,Ore03,Ore04,Ore05,Ore06,Ore07,Ore08,Ore09,Ore10,Ore11,Ore12,Ore13,Ore14	*OreG0006832*
scaffold2276	3678231	G	T	Ore01,Ore02,Ore03,Ore04,Ore05,Ore06,Ore07,Ore08,Ore09,Ore10,Ore11,Ore12,Ore13,Ore14	*OreG0006832*
scaffold2276	3679744	G	C	Och01,Och02,Och03,Och04,Och05,Och06,Och07,Och08,Och09,Och10,Och11,Och12,Och13,Och14	*OreG0006833*

scaffold2276	3679773	A	T	Ore08,Ore12	*OreG0006833*
scaffold2276	3679973	A	C	Och01,Och02,Och03,Och04,Och05,Och06,Och07,Och08,Och09,Och10,Och11,Och12,Och13,Och14,Ore01,Ore02,Ore03,Ore04,Ore05,Ore06,Ore07,Ore08,Ore09,Ore10,Ore11,Ore12,Ore13,Ore14	*OreG0006834*
scaffold2276	3691574	A	G	Ore01,Ore02,Ore03,Ore04,Ore05,Ore06,Ore07,Ore08,Ore09,Ore10,Ore11,Ore12,Ore13,Ore14	*OreG0006836*
scaffold2276	3695395	T	C	Och07	*OreG0006837*
scaffold2276	3778683	G	T	Och01,Och02,Och03,Och04,Och05,Och06,Och07,Och08,Och09,Och10,Och11,Och12,Och13,Och14,Ore01,Ore02,Ore03,Ore04,Ore05,Ore06,Ore07,Ore08,Ore09,Ore10,Ore11,Ore12,Ore13,Ore14	*OreG0006844*
scaffold2276	3778695	A	G	Och01,Och02,Och03,Och04,Och05,Och06,Och07,Och08,Och09,Och10,Och11,Och12,Och13,Och14	*OreG0006844*
scaffold2276	3855601	A	T	Ore04,Ore05,Ore08	*OreG0006852*
scaffold2276	3885289	C	T	Och01,Och02,Och03,Och04,Och05,Och06,Och07,Och08,Och09,Och10,Och11,Och12,Och13,Och14	*OreG0006854*
scaffold2276	3894317	T	G	Och01,Och02,Och03,Och04,Och05,Och06,Och07,Och08,Och09,Och10,Och11,Och12,Och13,Och14,Ore01,Ore02,Ore03,Ore04,Ore05,Ore06,Ore07,Ore08,Ore09,Ore10,Ore11,Ore12,Ore13,Ore14	*OreG0006857*
scaffold2276	4018596	C	T	Ore01,Ore02,Ore03,Ore04,Ore05,Ore06,Ore07,Ore08,Ore09,Ore10,Ore11,Ore12,Ore13,Ore14	*OreG0006867*
scaffold2276	4041332	G	A	NA	*OreG0006870*
scaffold2276	4041642	T	G	Och01,Och02,Och03,Och04,Och05,Och06,Och07,Och08,Och09,Och10,Och11,Och12,Och13,Och14	*OreG0006870*
scaffold2276	4076245	T	G	NA	*OreG0006874*
scaffold2276	4146470	T	C	Ore01,Ore02,Ore03,Ore04,Ore05,Ore06,Ore07,Ore08,Ore09,Ore10,Ore11,Ore12,Ore13,Ore14	*OreG0006884*
scaffold2276	4165186	T	C	NA	*OreG0006887*
scaffold2276	4165581	G	A	NA	*OreG0006887*
scaffold2276	4523654	T	C	NA	*OreG0006927*
scaffold175	14845	T	A	Och01,Och02,Och03,Och04,Och05,Och06,Och07,Och08,Och09,Och10,Och11,Och12,Och13,Och14	*OreG0003543*
scaffold175	29789	C	T	NA	*OreG0003545*
scaffold175	51808	C	T	Ore01,Ore02,Ore03,Ore04,Ore05,Ore06,Ore07,Ore08,Ore09,Ore10,Ore11,Ore12,Ore13,Ore14	*OreG0003546*
scaffold175	62956	G	A	Ore01,Ore02,Ore03,Ore04,Ore05,Ore06,Ore07,Ore08,Ore09,Ore10,Ore11,Ore12,Ore13,Ore14	*OreG0003548*
scaffold175	63002	T	C	NA	*OreG0003548*
scaffold175	63044	C	T	Ore01,Ore02,Ore03,Ore04,Ore05,Ore06,Ore07,Ore08,Ore09,Ore10,Ore11,Ore12,Ore13,Ore14	*OreG0003548*
scaffold175	63283	G	A	Ore01,Ore02,Ore03,Ore04,Ore05,Ore06,Ore07,Ore08,Ore09,Ore10,Ore11,Ore12,Ore13,Ore14	*OreG0003548*
scaffold175	63494	T	G	NA	*OreG0003548*
scaffold175	63523	G	T	Och01	*OreG0003548*
scaffold175	64165	A	T	Och13	*OreG0003549*
scaffold175	64183	A	G	Och01,Och04	*OreG0003549*
scaffold175	64241	G	C	NA	*OreG0003549*
scaffold175	70490	G	A	Och01	*OreG0003547*
scaffold175	71150	G	T	Ore02,Ore03,Ore06,Ore07,Ore08,Ore09,Ore10,Ore11,Ore12,Ore13,Ore14	*OreG0003547*
scaffold175	71262	T	C	NA	*OreG0003547*
scaffold175	71268	G	T	Ore01,Ore02,Ore03,Ore04,Ore05,Ore06,Ore07,Ore08,Ore09,Ore10,Ore11,Ore12,Ore13,Ore14	*OreG0003547*
scaffold175	95147	A	C	Och01	*OreG0003550*
scaffold175	95156	T	C	NA	*OreG0003550*
scaffold175	95320	G	C	Och01,Och02,Och03,Och04,Och05,Och06,Och07,Och08,Och09,Och10,Och11,Och12,Och13,Och14	*OreG0003550*
scaffold175	263462	T	A	Och01,Och02,Och03,Och04,Och05,Och06,Och07,Och08,Och09,Och10,Och11,Och12,Och13,Och14,Ore01,Ore02,Ore03,Ore04,Ore05,Ore06,Ore07,Ore08,Ore09,Ore10,Ore11,Ore12,Ore13,Ore14	*OreG0003555*
scaffold175	264702	A	T	Och09,Och10	*OreG0003557*
scaffold175	264744	C	A	Ore01,Ore02,Ore03,Ore04,Ore05,Ore06,Ore07,Ore08,Ore09,Ore10,Ore11,Ore12,Ore13,Ore14	*OreG0003557*
scaffold175	274745	C	T	Och01,Och02,Och03,Och04,Och05,Och06,Och07,Och08,Och09,Och10,Och11,Och12,Och13,Och14,Ore01,Ore02,Ore03,Ore04,Ore05,Ore06,Ore07,Ore08,Ore09,Ore10,Ore11,Ore12,Ore13,Ore14	*OreG0003559*
scaffold175	298161	G	T	Och01,Och02,Och03,Och04,Och05,Och06,Och07,Och08,Och09,Och10,Och11,Och12,Och13,Och14	*OreG0003562*
scaffold175	331158	A	C	Ore01,Ore02,Ore03,Ore04,Ore05,Ore06,Ore07,Ore08,Ore09,Ore10,Ore11,Ore12,Ore13,Ore14	*OreG0003566*
scaffold175	331725	A	T	Och01,Och09,Och10,Och11	*OreG0003566*
scaffold175	335911	G	T	Och01,Och02,Och03,Och04,Och05,Och06,Och07,Och08,Och09,Och10,Och11,Och12,Och13,Och14	*OreG0003567*
scaffold175	336093	C	A	Och01,Och02,Och03,Och04,Och05,Och06,Och07,Och08,Och09,Och10,Och11,Och12,Och13,Och14,Ore01,Ore02,Ore03,Ore04,Ore05,Ore06,Ore07,Ore08,Ore09,Ore10,Ore11,Ore12,Ore13,Ore14	*OreG0003567*
scaffold175	338874	T	C	Och01,Och03,Och04,Och05,Och09,Och10,Och12,Och13	*OreG0003568*

scaffold175	339022	C	T	Och01,Och02,Och03,Och04,Och05,Och06,Och07,Och08,Och09,Och10,Och11,Och12,Och13,Och14	*OreG0003568*
scaffold175	339048	G	A	Ore01,Ore02,Ore03,Ore04,Ore05,Ore06,Ore07,Ore08,Ore09,Ore10,Ore11,Ore12,Ore13,Ore14	*OreG0003568*
scaffold175	339052	A	T	Och01,Och02,Och03,Och04,Och05,Och06,Och07,Och08,Och09,Och10,Och11,Och12,Och13,Och14	*OreG0003568*
scaffold175	349214	C	A	Och01,Och02,Och03,Och04,Och05,Och06,Och07,Och08,Och09,Och10,Och11,Och12,Och13,Och14,Ore01,Ore02,Ore03,Ore04,Ore05,Ore06,Ore07,Ore08,Ore09,Ore10,Ore11,Ore12,Ore13,Ore14	*OreG0003570*
scaffold175	370913	C	G	NA	*OreG0003571*
scaffold175	392611	G	C	Och07,Och09,Och10	*OreG0003577*
scaffold175	401469	G	A	Och01,Och02,Och03,Och04,Och05,Och06,Och07,Och08,Och09,Och10,Och11,Och12,Och13,Och14,Ore01,Ore02,Ore03,Ore04,Ore05,Ore06,Ore07,Ore08,Ore09,Ore10,Ore11,Ore12,Ore13,Ore14	*OreG0003578*
scaffold175	401640	G	A	NA	*OreG0003578*
scaffold175	404580	A	G	NA	*OreG0003578*
scaffold175	405286	A	T	Och14	*OreG0003578*
scaffold175	420631	A	C	NA	*OreG0003579*
scaffold175	446333	T	A	Och01,Och02,Och03,Och04,Och05,Och06,Och07,Och08,Och09,Och10,Och11,Och12,Och13,Och14,Ore01,Ore02,Ore03,Ore04,Ore05,Ore06,Ore07,Ore08,Ore09,Ore10,Ore11,Ore12,Ore13,Ore14	*OreG0003581*
scaffold175	468193	C	A	NA	*OreG0003583*
scaffold175	468214	A	G	NA	*OreG0003583*
scaffold175	488448	G	T	Ore01,Ore02,Ore03,Ore04,Ore05,Ore06,Ore07,Ore08,Ore09,Ore10,Ore11,Ore12,Ore13,Ore14	*OreG0003585*
scaffold175	616007	C	G	NA	*OreG0003590*
scaffold175	681806	G	A	Och01,Och02,Och03,Och04,Och05,Och06,Och07,Och08,Och09,Och10,Och11,Och12,Och13,Och14,Ore01,Ore02,Ore03,Ore04,Ore05,Ore06,Ore07,Ore08,Ore09,Ore10,Ore11,Ore12,Ore13,Ore14	*OreG0003596*
scaffold175	681917	C	T	Och02,Och03,Och04,Och05,Och06,Och07,Och08,Och09,Och10,Och11,Och12,Och13,Och14	*OreG0003596*
scaffold175	682155	C	T	Och01	*OreG0003596*
scaffold175	682215	C	T	Och01,Och02,Och03,Och04,Och05,Och06,Och07,Och08,Och09,Och10,Och11,Och12,Och13,Och14	*OreG0003596*
scaffold175	682431	A	G	Och01,Och02,Och03,Och04,Och05,Och06,Och07,Och08,Och09,Och10,Och11,Och12,Och13,Och14	*OreG0003596*
scaffold175	691307	C	A	NA	*OreG0003597*
scaffold175	691366	A	G	NA	*OreG0003597*
scaffold175	691432	C	T	NA	*OreG0003597*
scaffold175	691480	A	G	NA	*OreG0003597*
scaffold175	691502	C	T	NA	*OreG0003597*
scaffold175	691759	A	C	NA	*OreG0003597*
scaffold175	725424	C	G	NA	*OreG0003600*
scaffold175	725480	G	A	Och04	*OreG0003600*
scaffold175	740063	T	G	NA	*OreG0003601*
scaffold175	740994	C	T	Ore01,Ore02,Ore03,Ore04,Ore05,Ore06,Ore07,Ore08,Ore09,Ore10,Ore11,Ore12,Ore13,Ore14	*OreG0003601*
scaffold175	932297	G	A	NA	*OreG0003606*
scaffold175	979699	C	A	Och01,Ore01,Ore02,Ore03,Ore04,Ore05,Ore06,Ore07,Ore08,Ore09,Ore10,Ore11,Ore12,Ore13,Ore14	*OreG0003611*
scaffold175	979792	G	T	Och01	*OreG0003611*
scaffold175	979945	G	C	Och14	*OreG0003611*
scaffold175	979972	A	G	Och07,Och08,Och13	*OreG0003611*
scaffold175	980829	A	G	NA	*OreG0003611*
scaffold175	980861	A	C	Och06,Och07,Och08,Och13	*OreG0003611*
scaffold175	981146	A	G	NA	*OreG0003611*
scaffold175	981155	A	C	NA	*OreG0003611*
scaffold175	1019113	G	A	NA	*OreG0003614*
scaffold175	1020737	C	T	NA	*OreG0003614*
scaffold4400	82489	G	T	Och01,Och02,Och03,Och04,Och05,Och06,Och07,Och08,Och09,Och10,Och11,Och12,Och13,Och14,Ore01,Ore02,Ore03,Ore04,Ore05,Ore06,Ore07,Ore08,Ore09,Ore10,Ore11,Ore12,Ore13,Ore14	*OreG0020358*
scaffold4400	82900	C	A	NA	*OreG0020358*
scaffold4400	85778	A	G	Och02,Och03,Och04,Och05,Och06,Och12,Och13	*OreG0020359*
scaffold4400	86456	C	T	Och09,Och10	*OreG0020359*
scaffold4400	86751	C	A	NA	*OreG0020359*
scaffold4400	86774	C	A	NA	*OreG0020359*
scaffold4400	86787	C	T	NA	*OreG0020359*
scaffold4400	86899	C	G	Ore01,Ore02,Ore03,Ore04,Ore05,Ore06,Ore07,Ore08,Ore09,Ore10,Ore11,Ore12,Ore13,Ore14	*OreG0020359*
scaffold4400	87837	A	T	Ore01,Ore02,Ore03,Ore04,Ore05,Ore06,Ore07,Ore08,Ore09,Ore10,Ore11,Ore12,Ore13,Ore14	*OreG0020360*
scaffold4400	100273	G	A	NA	*OreG0020362*
scaffold4400	100280	G	A	NA	*OreG0020362*
scaffold4400	101158	G	A	Och01	*OreG0020362*

scaffold4400	107655	G	C	NA	*OreG0020363*
scaffold4400	112235	C	T	Och01,Och02,Och03,Och04,Och05,Och06,Och07,Och08,Och09,Och10,Och11,Och12,Och13,Och14,Ore01,Ore02,Ore03,Ore04,Ore05,Ore06,Ore07,Ore08,Ore09,Ore10,Ore11,Ore12,Ore13,Ore14	*OreG0020364*
scaffold4400	115887	A	G	NA	*OreG0020364*
scaffold4400	118314	C	T	Och01,Och02,Och03,Och04,Och05,Och06,Och07,Och08,Och09,Och10,Och11,Och12,Och13,Och14,Ore01,Ore02,Ore03,Ore04,Ore05,Ore06,Ore07,Ore08,Ore09,Ore10,Ore11,Ore12,Ore13,Ore14	*OreG0020364*
scaffold4400	125406	G	C	Och08	*OreG0020364*
scaffold4400	125471	G	T	NA	*OreG0020364*
scaffold4400	130255	A	G	Ore01	*OreG0020365*
scaffold4400	130586	C	T	Ore01,Ore02,Ore03,Ore04,Ore05,Ore06,Ore07,Ore08,Ore09,Ore10,Ore11,Ore12,Ore13,Ore14	*OreG0020365*
scaffold4400	161762	C	A	Och01,Och02,Och03,Och04,Och05,Och06,Och07,Och08,Och09,Och10,Och11,Och12,Och13,Och14	*OreG0020369*
scaffold4400	161903	C	A	Och09,Och10	*OreG0020369*
scaffold4400	169695	C	G	Och01,Och04,Och09,Och10	*OreG0020370*
scaffold4400	171855	G	A	Och01,Och02,Och03,Och04,Och05,Och06,Och07,Och08,Och09,Och10,Och11,Och12,Och13,Och14	*OreG0020370*
scaffold4400	175173	G	C	NA	*OreG0020370*
scaffold4400	175176	A	T	NA	*OreG0020370*
scaffold4400	176881	A	G	Och01,Och02,Och03,Och04,Och05,Och06,Och07,Och08,Och09,Och10,Och11,Och12,Och13,Och14	*OreG0020370*
scaffold4400	184646	A	T	Och08	*OreG0020370*
scaffold4400	188048	C	T	NA	*OreG0020371*
scaffold4400	190597	T	C	Och08	*OreG0020372*
scaffold4400	191418	G	T	Ore01,Ore02,Ore03,Ore04,Ore05,Ore06,Ore07,Ore08,Ore09,Ore10,Ore11,Ore12,Ore13,Ore14	*OreG0020372*
scaffold4400	236136	G	C	NA	*OreG0020376*
scaffold4400	285683	C	T	NA	*OreG0020377*
scaffold4400	286548	C	T	NA	*OreG0020377*
scaffold4400	286566	G	A	Och01,Och02,Och03,Och04,Och05,Och06,Och07,Och08,Och09,Och10,Och11,Och12,Och13,Och14,Ore01,Ore02,Ore03,Ore04,Ore05,Ore06,Ore07,Ore08,Ore09,Ore10,Ore11,Ore12,Ore13,Ore14	*OreG0020377*
scaffold4400	286580	G	A	Ore01,Ore02,Ore03,Ore04,Ore05,Ore06,Ore07,Ore08,Ore09,Ore10,Ore11,Ore12,Ore13,Ore14	*OreG0020377*
scaffold4400	367553	C	T	NA	*OreG0020378*
scaffold4400	367922	G	T	Och01,Och02,Och03,Och04,Och05,Och06,Och07,Och08,Och09,Och10,Och11,Och12,Och13,Och14,Ore01,Ore02,Ore03,Ore04,Ore05,Ore06,Ore07,Ore08,Ore09,Ore10,Ore11,Ore12,Ore13,Ore14	*OreG0020378*
scaffold4400	551284	C	G	Ore01,Ore02,Ore03,Ore04,Ore05,Ore06,Ore07,Ore08,Ore09,Ore10,Ore11,Ore12,Ore13,Ore14	*OreG0020381*
scaffold4400	552371	A	G	Och02	*OreG0020381*
scaffold4400	576388	C	G	Ore01,Ore02,Ore03,Ore04,Ore05,Ore06,Ore07,Ore08,Ore09,Ore10,Ore11,Ore12,Ore13,Ore14	*OreG0020382*
scaffold4400	576630	A	T	Och01,Och02,Och03,Och04,Och05,Och06,Och07,Och08,Och09,Och10,Och11,Och12,Och13,Och14,Ore01,Ore02,Ore03,Ore04,Ore05,Ore06,Ore07,Ore08,Ore09,Ore10,Ore11,Ore12,Ore13,Ore14	*OreG0020382*
scaffold4400	577071	A	C	Ore01,Ore02,Ore03,Ore04,Ore05,Ore06,Ore07,Ore08,Ore09,Ore10,Ore11,Ore12,Ore13,Ore14	*OreG0020382*
scaffold4400	593569	G	A	Och01,Och02,Och03,Och04,Och05,Och06,Och07,Och08,Och09,Och10,Och11,Och12,Och13,Och14,Ore01,Ore02,Ore03,Ore04,Ore05,Ore06,Ore07,Ore08,Ore09,Ore10,Ore11,Ore12,Ore13,Ore14	*OreG0020383*
scaffold4400	593597	A	C	Och01,Och02,Och03,Och04,Och05,Och06,Och07,Och08,Och09,Och10,Och11,Och12,Och13,Och14	*OreG0020383*
scaffold4400	594444	T	A	Och01,Och02,Och03,Och04,Och05,Och06,Och07,Och08,Och09,Och10,Och11,Och12,Och13,Och14,Ore01,Ore02,Ore03,Ore04,Ore05,Ore06,Ore07,Ore08,Ore09,Ore10,Ore11,Ore12,Ore13,Ore14	*OreG0020383*
scaffold4400	594466	G	A	Ore04,Ore05,Ore06,Ore07,Ore10,Ore14	*OreG0020383*
scaffold4400	594521	T	C	Och01,Och02,Och03,Och04,Och05,Och06,Och07,Och08,Och09,Och10,Och11,Och12,Och13,Och14	*OreG0020383*
scaffold4400	594536	C	T	Och01,Och02,Och03,Och04,Och05,Och06,Och07,Och08,Och09,Och10,Och11,Och12,Och13,Och14,Ore01,Ore02,Ore03,Ore04,Ore05,Ore06,Ore07,Ore08,Ore09,Ore10,Ore11,Ore12,Ore13,Ore14	*OreG0020383*
scaffold4400	594544	C	T	Och01,Och02,Och03,Och04,Och05,Och06,Och07,Och08,Och09,Och10,Och11,Och12,Och13,Och14,Ore01,Ore02,Ore03,Ore04,Ore05,Ore06,Ore07,Ore08,Ore09,Ore10,Ore11,Ore12,Ore13,Ore14	*OreG0020383*
scaffold4400	594948	C	T	Ore01,Ore02,Ore03,Ore04,Ore05,Ore06,Ore07,Ore08,Ore09,Ore10,Ore11,Ore12,Ore13,Ore14	*OreG0020383*
scaffold4400	595032	A	T	NA	*OreG0020383*
scaffold4400	638141	C	T	NA	*OreG0020384*
scaffold4400	638231	C	T	NA	*OreG0020384*
scaffold4400	649162	C	A	NA	*OreG0020384*
scaffold4400	661556	C	T	NA	*OreG0020384*
scaffold4400	668941	C	T	NA	*OreG0020384*
scaffold4400	710373	G	C	NA	*OreG0020386*

scaffold4400	719885	C	A	Och01,Och02,Och03,Och04,Och05,Och06,Och07,Och08,Och09,Och10,Och11,Och12,Och13,Och14	*OreG0020386*
scaffold4400	759266	A	G	Och01,Och02,Och08	*OreG0020388*
scaffold4400	777103	G	A	Och02,Och03,Och04,Och05,Och06,Och07,Och12,Och13	*OreG0020389*
scaffold4400	797516	G	T	NA	*OreG0020390*
scaffold4400	802683	G	C	NA	*OreG0020391*
scaffold4400	821181	G	T	Ore01,Ore02,Ore03,Ore04,Ore05,Ore06,Ore07,Ore08,Ore09,Ore10,Ore11,Ore12,Ore13,Ore14	*OreG0020393*
scaffold4400	838931	G	T	Och01	*OreG0020395*
scaffold4400	873176	C	T	Ore01,Ore02,Ore03,Ore04,Ore05,Ore06,Ore07,Ore08,Ore09,Ore10,Ore11,Ore12,Ore13,Ore14	*OreG0020397*
scaffold4400	931036	T	A	Och01,Och02,Och03,Och04,Och05,Och06,Och07,Och08,Och09,Och10,Och11,Och12,Och13,Och14,Ore01,Ore02,Ore03,Ore04,Ore05,Ore06,Ore07,Ore08,Ore09,Ore10,Ore11,Ore12,Ore13,Ore14	*OreG0020399*
scaffold4400	944254	C	T	Och01,Och02,Och03,Och04,Och05,Och06,Och07,Och08,Och09,Och10,Och11,Och12,Och13,Och14,Ore01,Ore02,Ore03,Ore04,Ore05,Ore06,Ore07,Ore08,Ore09,Ore10,Ore11,Ore12,Ore13,Ore14	*OreG0020399*
scaffold4400	1032086	C	T	NA	*OreG0020400*
scaffold4400	1033534	C	G	Ore01,Ore02,Ore03,Ore04,Ore05,Ore06,Ore07,Ore08,Ore09,Ore10,Ore11,Ore12,Ore13,Ore14	*OreG0020400*
scaffold4400	1040464	A	T	NA	*OreG0020401*
scaffold4400	1045081	G	A	Ore01,Ore02,Ore03,Ore06,Ore07,Ore08,Ore09,Ore10,Ore11,Ore12,Ore13,Ore14	*OreG0020401*
scaffold4400	1051529	C	T	Och02,Och09,Och10,Och11	*OreG0020401*
scaffold4400	1054780	C	T	NA	*OreG0020401*
scaffold4400	1059251	A	T	Ore01,Ore02,Ore03,Ore04,Ore05,Ore06,Ore07,Ore08,Ore09,Ore10,Ore11,Ore12,Ore13,Ore14	*OreG0020403*
scaffold4400	1059284	A	T	Och01,Och02,Och03,Och04,Och05,Och06,Och07,Och08,Och09,Och10,Och11,Och12,Och13,Och14	*OreG0020403*
scaffold4400	1068956	G	T	Och14	*OreG0020405*
scaffold4400	1070393	A	C	Ore01,Ore02,Ore03,Ore04,Ore05,Ore06,Ore07,Ore08,Ore09,Ore10,Ore11,Ore12,Ore13,Ore14	*OreG0020405*
scaffold4400	1096024	C	T	NA	*OreG0020406*
scaffold4400	1142069	T	A	Ore01,Ore02,Ore03,Ore04,Ore05,Ore06,Ore07,Ore08,Ore09,Ore10,Ore11,Ore12,Ore13,Ore14	*OreG0020411*
scaffold4400	1165400	A	G	NA	*OreG0020412*
scaffold4400	1169086	A	T	Och12,Och13	*OreG0020413*
scaffold4400	1204010	T	A	NA	*OreG0020419*
scaffold4400	1222483	G	A	NA	*OreG0020422*
scaffold4400	1223270	T	A	Och12,Och13	*OreG0020422*
scaffold4400	1223390	G	T	Och03	*OreG0020422*
scaffold4400	1224149	A	G	Ore01,Ore04,Ore05	*OreG0020422*
scaffold4400	1224233	T	A	Och03	*OreG0020422*
scaffold4400	1231526	C	T	Ore01,Ore02,Ore03,Ore04,Ore05,Ore06,Ore07,Ore08,Ore09,Ore10,Ore11,Ore12,Ore13,Ore14	*OreG0020424*
scaffold4400	1237117	C	G	Och02,Och03,Och04,Och05,Och06,Och12,Och13	*OreG0020426*
scaffold4400	1238108	A	G	Och11	*OreG0020426*
scaffold4400	1238200	T	G	Ore06,Ore07,Ore10,Ore12,Ore13	*OreG0020426*
scaffold4400	1238843	A	G	Och01,Och02,Och03,Och04,Och05,Och06,Och07,Och08,Och09,Och10,Och11,Och12,Och13,Och14	*OreG0020426*
scaffold4400	1240714	G	A	Och01,Och02,Och03,Och04,Och05,Och06,Och07,Och08,Och09,Och10,Och11,Och12,Och13,Och14	*OreG0020426*
scaffold4400	1240810	C	A	Och03	*OreG0020426*
scaffold4400	1264316	C	A	Och01,Och02,Och03,Och04,Och05,Och06,Och07,Och08,Och09,Och10,Och11,Och12,Och13,Och14	*OreG0020428*
scaffold4400	1265379	T	C	NA	*OreG0020428*
scaffold4400	1283166	A	G	NA	*OreG0020430*
scaffold4400	1283910	G	A	Och07,Och09,Och10,Och11,Och12,Och13	*OreG0020430*
scaffold4400	1303895	G	T	Ore01,Ore02,Ore03,Ore04,Ore05,Ore06,Ore07,Ore08,Ore09,Ore10,Ore11,Ore12,Ore13,Ore14	*OreG0020432*
scaffold4400	1335020	A	G	NA	*OreG0020435*
scaffold4400	1396065	G	T	NA	*OreG0020439*
scaffold4400	1396076	C	T	Och01,Och02,Och03,Och04,Och05,Och06,Och07,Och08,Och09,Och10,Och11,Och12,Och13,Och14,Ore01,Ore02,Ore03,Ore04,Ore05,Ore06,Ore07,Ore08,Ore09,Ore10,Ore11,Ore12,Ore13,Ore14	*OreG0020439*
scaffold4400	1410650	A	C	Och01,Och02,Och03,Och04,Och05,Och06,Och07,Och08,Och09,Och10,Och11,Och12,Och13,Och14	*OreG0020440*
scaffold4400	1523861	T	C	Och01,Och02,Och03,Och04,Och05,Och06,Och07,Och08,Och09,Och10,Och11,Och12,Och13,Och14,Ore01,Ore02,Ore03,Ore04,Ore05,Ore06,Ore07,Ore08,Ore09,Ore10,Ore11,Ore12,Ore13,Ore14	*OreG0020443*
scaffold4400	1523906	G	T	Ore01,Ore02,Ore03,Ore04,Ore05,Ore06,Ore07,Ore08,Ore09,Ore10,Ore11,Ore12,Ore13,Ore14	*OreG0020443*
scaffold4400	1574096	T	A	Och01,Och02,Och03,Och04,Och05,Och06,Och07,Och08,Och11,Och12,Och13,Och14	*OreG0020446*
scaffold4400	1588109	C	T	Och03,Och05,Och12,Och13	*OreG0020447*
scaffold4400	1588161	C	T	Och03,Och05,Och12,Och13	*OreG0020447*

scaffold4400	1588301	C	T	Ore01,Ore02,Ore03,Ore04,Ore05,Ore06,Ore07,Ore08,Ore09,Ore10,Ore11,Ore12,Ore13,Ore14	*OreG0020447*
scaffold4400	1588380	T	C	Och03,Och05,Och12,Och13	*OreG0020447*
scaffold4400	1588486	T	A	Ore01,Ore02,Ore03,Ore04,Ore05,Ore06,Ore07,Ore08,Ore09,Ore10,Ore11,Ore12,Ore13,Ore14	*OreG0020447*
scaffold4400	1588920	A	T	Och02,Och03,Och04,Och05,Och06,Och07,Och09,Och10,Och11,Och12,Och13	*OreG0020447*
scaffold4400	1604301	A	T	NA	*OreG0020449*
scaffold4400	1614554	G	A	Och01,Och02,Och03,Och04,Och05,Och06,Och07,Och08,Och09,Och10,Och11,Och12,Och13,Och14	*OreG0020449*
scaffold4400	1642361	G	A	NA	*OreG0020451*
scaffold4400	1642998	G	A	Ore01,Ore04,Ore05,Ore08,Ore09,Ore11,Ore12	*OreG0020451*
scaffold4400	1757848	G	A	NA	*OreG0020455*
scaffold4400	1758399	T	C	Och08,Och09,Och10,Och11	*OreG0020455*
scaffold4400	1803928	G	A	Och02,Och03,Och04,Och06,Och12,Och13	*OreG0020458*
scaffold4400	1804217	G	A	Och01,Och02,Och03,Och04,Och05,Och06,Och07,Och08,Och12,Och13,Och14	*OreG0020458*
scaffold4400	1804221	C	T	Och08	*OreG0020458*
scaffold4400	1884514	C	G	NA	*OreG0020463*
scaffold4400	1901308	G	A	NA	*OreG0020464*
scaffold4400	1905069	G	A	Och01,Och02,Och03,Och04,Och05,Och06,Och07,Och08,Och09,Och10,Och11,Och12,Och13,Och14,Ore01,Ore02,Ore03,Ore04,Ore05,Ore06,Ore07,Ore08,Ore09,Ore10,Ore11,Ore12,Ore13,Ore14	*OreG0020465*
scaffold4400	2064655	G	C	Ore01,Ore02,Ore03,Ore04,Ore05,Ore06,Ore07,Ore08,Ore09,Ore10,Ore11,Ore12,Ore13,Ore14	*OreG0020473*
scaffold4400	2068151	C	T	Ore01,Ore02,Ore03,Ore04,Ore05,Ore06,Ore07,Ore08,Ore09,Ore10,Ore11,Ore12,Ore13,Ore14	*OreG0020473*
scaffold4400	2159978	T	C	NA	*OreG0020478*
scaffold4400	2216712	T	A	NA	*OreG0020481*
scaffold4400	2223039	C	T	Ore08,Ore09,Ore11,Ore12	*OreG0020481*
scaffold4400	2227568	C	T	NA	*OreG0020482*
scaffold4400	2227607	T	C	Och01,Och02,Och03,Och04,Och05,Och06,Och07,Och08,Och09,Och10,Och11,Och12,Och13,Och14	*OreG0020482*
scaffold4400	2228451	C	A	NA	*OreG0020482*
scaffold4400	2258084	C	A	Och08	*OreG0020483*
scaffold4400	2258156	A	G	NA	*OreG0020483*
scaffold10469	229667	C	T	NA	*OreG0000225*
scaffold10469	251274	G	T	Och01,Och02,Och03,Och04,Och05,Och06,Och07,Och08,Och09,Och10,Och11,Och12,Och13,Och14,Ore01,Ore02,Ore03,Ore04,Ore05,Ore06,Ore07,Ore08,Ore09,Ore10,Ore11,Ore12,Ore13,Ore14	*OreG0000226*
scaffold10469	328333	C	T	Och06,Och07,Och13	*OreG0000231*
scaffold10469	328351	G	A	Och06,Och07,Och13	*OreG0000231*
scaffold10469	328525	G	A	Och11	*OreG0000231*
scaffold10469	328536	G	A	Och11	*OreG0000231*
scaffold10469	339291	T	C	NA	*OreG0000234*
scaffold10469	339295	C	T	NA	*OreG0000234*
scaffold10469	339307	C	T	NA	*OreG0000234*
scaffold15727	23710	G	A	Och07,Och08,Och09,Och10,Och11	*OreG0003275*
scaffold15727	23741	A	G	Och07,Och08,Och09,Och10,Och11	*OreG0003275*
scaffold15727	24175	G	A	Och07,Och08,Och09,Och10,Och11	*OreG0003275*
scaffold15727	24190	T	A	Och01,Och02,Och06,Ore01,Ore02,Ore03,Ore04,Ore05,Ore06,Ore07,Ore08,Ore09,Ore10,Ore11,Ore12,Ore13,Ore14	*OreG0003275*
scaffold15727	24194	G	A	Och07,Och08,Och09,Och10,Och11	*OreG0003275*
scaffold16583	11972	G	T	Och01	*OreG0003459*
scaffold16552	29481	A	T	NA	*OreG0003456*
scaffold16552	29559	C	T	NA	*OreG0003456*
scaffold16552	45761	G	A	NA	*OreG0003457*
scaffold8846	168855	G	A	Ore01,Ore02,Ore03,Ore04,Ore05,Ore06,Ore07,Ore08,Ore09,Ore10,Ore11,Ore12,Ore13,Ore14	*OreG0025819*
scaffold8846	176220	G	C	NA	*OreG0025819*
scaffold16506	21004	G	T	Ore04,Ore05	*OreG0003437*
scaffold16506	21059	A	G	Ore03,Ore06,Ore09	*OreG0003437*
scaffold16506	24545	T	C	NA	*OreG0003437*
scaffold16506	32023	A	G	Och01,Och04,Och06,Och09,Och10,Och11,Och13	*OreG0003438*
scaffold16506	32140	G	A	Och01,Och02,Och03,Och04,Och05,Och06,Och07,Och08,Och09,Och10,Och11,Och12,Och13,Och14,Ore01,Ore02,Ore03,Ore04,Ore05,Ore06,Ore07,Ore08,Ore09,Ore10,Ore11,Ore12,Ore13,Ore14	*OreG0003438*
scaffold16506	32210	G	A	Och01,Och02,Och03,Och04,Och05,Och06,Och07,Och08,Och09,Och10,Och11,Och12,Och13,Och14,Ore01,Ore02,Ore03,Ore04,Ore05,Ore06,Ore07,Ore08,Ore09,Ore10,Ore11,Ore12,Ore13,Ore14	*OreG0003438*
scaffold16506	32335	A	G	Ore01,Ore02,Ore03,Ore04,Ore05,Ore06,Ore07,Ore08,Ore09,Ore10,Ore11,Ore12,Ore13,Ore14	*OreG0003438*
scaffold16506	32444	C	A	NA	*OreG0003438*
scaffold16506	32533	A	G	NA	*OreG0003438*
scaffold16506	36614	A	G	NA	*OreG0003439*
scaffold16506	58453	C	A	Och04,Och06,Och07,Och13,Och14	*OreG0003441*
scaffold16506	125161	T	A	Och01,Och08,Och11,Och14	*OreG0003449*

scaffold16506	125818	A	G	Ore01,Ore02,Ore03,Ore04,Ore05,Ore06,Ore07,Ore08,Ore09,Ore10,Ore11,Ore12,Ore13,Ore14	*OreG0003449*
scaffold16506	126904	T	C	NA	*OreG0003450*
scaffold16506	136091	C	T	Och01,Och02,Och03,Och04,Och05,Och06,Och07,Och08,Och09,Och10,Och11,Och12,Och13,Och14,Ore01,Ore02,Ore03,Ore04,Ore05,Ore06,Ore07,Ore08,Ore09,Ore10,Ore11,Ore12,Ore13,Ore14	*OreG0003452*
scaffold16506	136204	A	G	Och01,Och02,Och03,Och04,Och05,Och06,Och07,Och08,Och09,Och10,Och11,Och12,Och13,Och14,Ore01,Ore02,Ore03,Ore04,Ore05,Ore06,Ore07,Ore08,Ore09,Ore10,Ore11,Ore12,Ore13,Ore14	*OreG0003452*
scaffold16506	136345	A	T	Och01,Och02,Och03,Och04,Och05,Och06,Och07,Och08,Och09,Och10,Och11,Och12,Och13,Och14,Ore01,Ore02,Ore03,Ore04,Ore05,Ore06,Ore07,Ore08,Ore09,Ore10,Ore11,Ore12,Ore13,Ore14	*OreG0003452*
scaffold16506	136348	T	A	Och07,Och08,Och09,Och10,Och11	*OreG0003452*
scaffold16506	136415	C	G	Och01,Och02,Och03,Och04,Och05,Och06,Och07,Och08,Och09,Och10,Och11,Och12,Och13,Och14,Ore01,Ore02,Ore03,Ore04,Ore05,Ore06,Ore07,Ore08,Ore09,Ore10,Ore11,Ore12,Ore13,Ore14	*OreG0003452*
scaffold16506	165775	A	G	NA	*OreG0003455*
scaffold16506	168913	A	G	NA	*OreG0003455*
scaffold16506	168924	G	A	NA	*OreG0003455*
scaffold14584	81650	G	A	NA	*OreG0002525*
scaffold14584	88779	A	C	Och01,Och14	*OreG0002526*
scaffold14584	88974	C	G	Och01,Och14	*OreG0002526*
scaffold14584	89982	C	T	Och02,Och03,Och04,Och05,Och06,Och07,Och09,Och10,Och11,Och12,Och13,Ore01,Ore02,Ore03,Ore04,Ore05,Ore06,Ore07,Ore08,Ore09,Ore10,Ore11,Ore12,Ore13,Ore14	*OreG0002526*
scaffold14584	95460	A	G	NA	*OreG0002527*
scaffold14584	113503	C	T	NA	*OreG0002528*
scaffold14584	127140	C	G	Och01,Och14	*OreG0002529*
scaffold14584	127159	T	C	Och01,Och03,Och04,Och05,Och07,Och08,Och09,Och10,Och11,Och12,Och13,Och14,Ore01,Ore02,Ore03,Ore04,Ore05,Ore06,Ore07,Ore08,Ore09,Ore10,Ore11,Ore12,Ore13,Ore14	*OreG0002529*
scaffold14584	129285	C	A	NA	*OreG0002530*
scaffold14584	144869	A	T	NA	*OreG0002532*
scaffold14584	144908	G	A	NA	*OreG0002532*
scaffold14584	144987	C	G	NA	*OreG0002532*
scaffold14584	145019	A	G	NA	*OreG0002532*
scaffold14584	166357	C	A	NA	*OreG0002536*
scaffold14584	166504	T	A	Ore04,Ore05	*OreG0002536*
scaffold14584	198711	C	A	NA	*OreG0002539*
scaffold14584	198856	T	C	Och01,Och02,Och03,Och04,Och05,Och06,Och07,Och08,Och09,Och10,Och11,Och12,Och13,Och14,Ore01,Ore02,Ore03,Ore04,Ore05,Ore06,Ore07,Ore08,Ore09,Ore10,Ore11,Ore12,Ore13,Ore14	*OreG0002539*
scaffold14584	199128	C	T	Och01,Och02,Och03,Och04,Och05,Och06,Och07,Och08,Och09,Och10,Och11,Och12,Och13,Och14,Ore01,Ore02,Ore03,Ore04,Ore05,Ore06,Ore07,Ore08,Ore09,Ore10,Ore11,Ore12,Ore13,Ore14	*OreG0002539*
scaffold14584	199831	C	G	Ore01,Ore02,Ore03,Ore04,Ore05,Ore06,Ore07,Ore08,Ore09,Ore10,Ore11,Ore12,Ore13,Ore14	*OreG0002539*
scaffold14584	200110	C	T	Ore01,Ore02,Ore03,Ore04,Ore05,Ore06,Ore07,Ore08,Ore09,Ore10,Ore11,Ore12,Ore13,Ore14	*OreG0002539*
scaffold14584	207015	T	C	NA	*OreG0002541*
scaffold14584	209437	A	T	NA	*OreG0002541*
scaffold14584	220269	G	A	Ore01,Ore02,Ore03,Ore04,Ore05,Ore06,Ore07,Ore08,Ore09,Ore10,Ore11,Ore12,Ore13,Ore14	*OreG0002543*
scaffold14584	220623	C	T	NA	*OreG0002543*
scaffold14584	220820	A	G	NA	*OreG0002543*
scaffold14584	221079	C	A	Ore04,Ore05	*OreG0002543*
scaffold14584	221325	C	T	Ore01,Ore02,Ore03,Ore04,Ore05,Ore06,Ore07,Ore08,Ore09,Ore10,Ore11,Ore12,Ore13,Ore14	*OreG0002543*
scaffold14584	237693	C	T	Och01,Och02,Och03,Och04,Och05,Och06,Och07,Och08,Och09,Och10,Och11,Och12,Och13,Och14,Ore01,Ore02,Ore03,Ore04,Ore05,Ore06,Ore07,Ore08,Ore09,Ore10,Ore11,Ore12,Ore13,Ore14	*OreG0002545*
scaffold14584	248167	A	T	NA	*OreG0002546*
scaffold14584	268137	C	A	Och02,Och04,Och13,Ore01,Ore02,Ore03,Ore04,Ore05,Ore06,Ore07,Ore08,Ore09,Ore10,Ore11,Ore12,Ore13,Ore14	*OreG0002549*
scaffold14584	268342	C	G	NA	*OreG0002549*
scaffold14584	270375	G	T	NA	*OreG0002551*
scaffold14584	270382	T	G	Och07,Och09,Och10,Och11	*OreG0002551*
scaffold14584	270493	G	T	Ore09,Ore13	*OreG0002551*
scaffold14584	270504	A	T	Och09,Och10,Och11	*OreG0002551*
scaffold14584	270605	G	A	Och05	*OreG0002551*
scaffold14584	293742	A	G	NA	*OreG0002554*
scaffold14584	293814	G	A	NA	*OreG0002554*
scaffold14584	294031	T	C	Och03,Och05	*OreG0002554*

scaffold14584	295896	G	A	Och01,Och02,Och03,Och04,Och05,Och06,Och07,Och08,Och09,Och10,Och11,Och12,Och13,Och14,Ore01,Ore02,Ore03,Ore04,Ore05,Ore06,Ore07,Ore08,Ore09,Ore10,Ore11,Ore12,Ore13,Ore14	*OreG0002554*
scaffold7534	27671	G	T	NA	*OreG0025026*
scaffold7534	29748	C	G	Ore01,Ore02,Ore03,Ore04,Ore05,Ore06,Ore07,Ore08,Ore09,Ore10,Ore11,Ore12,Ore13,Ore14	*OreG0025026*
scaffold7534	72919	G	T	NA	*OreG0025029*
scaffold7534	139233	T	G	Och01,Och02,Och03,Och04,Och05,Och06,Och07,Och08,Och09,Och10,Och11,Och12,Och13,Och14,Ore01,Ore02,Ore03,Ore04,Ore05,Ore06,Ore07,Ore08,Ore09,Ore10,Ore11,Ore12,Ore13,Ore14	*OreG0025035*
scaffold7534	202891	G	A	Och08,Och09,Och10,Ore01,Ore02,Ore03,Ore04,Ore05,Ore06,Ore07,Ore08,Ore09,Ore10,Ore11,Ore12,Ore13,Ore14	*OreG0025040*
scaffold7534	202962	G	A	NA	*OreG0025040*
scaffold7534	203642	C	T	Ore02,Ore06,Ore07,Ore11,Ore13	*OreG0025040*
scaffold7534	204768	C	T	NA	*OreG0025040*
scaffold7534	205022	C	G	Och08,Och09,Och10,Och11,Ore02,Ore06,Ore07,Ore11,Ore13	*OreG0025040*
scaffold7534	205323	A	T	NA	*OreG0025040*
scaffold7534	206364	T	A	Och02,Och12	*OreG0025040*
scaffold7534	216619	T	C	NA	*OreG0025041*
scaffold7534	219196	C	T	NA	*OreG0025041*
scaffold7534	219264	T	A	NA	*OreG0025041*
scaffold7534	268601	A	G	Och02,Och04,Och05,Och12,Och13,Och14	*OreG0025044*
scaffold7534	269176	G	A	NA	*OreG0025044*
scaffold7534	270184	C	T	Ore01,Ore02,Ore03,Ore04,Ore05,Ore06,Ore07,Ore08,Ore09,Ore10,Ore11,Ore12,Ore13,Ore14	*OreG0025044*
scaffold7534	270457	T	G	Ore01,Ore02,Ore03,Ore04,Ore05,Ore06,Ore07,Ore08,Ore09,Ore10,Ore11,Ore12,Ore13,Ore14	*OreG0025044*
scaffold7534	270756	G	A	Och02,Och04,Och05,Och12,Och13,Och14	*OreG0025044*
scaffold7534	345338	A	G	Ore01,Ore02,Ore03,Ore04,Ore05,Ore06,Ore07,Ore08,Ore09,Ore10,Ore11,Ore12,Ore13,Ore14	*OreG0025048*
scaffold7534	382930	C	T	Och02,Och12,Och13,Och14	*OreG0025054*
scaffold7534	382969	A	G	NA	*OreG0025054*
scaffold7534	383395	C	T	Och01,Och02,Och03,Och04,Och05,Och06,Och07,Och08,Och09,Och10,Och11,Och12,Och13,Och14	*OreG0025054*
scaffold7534	383421	C	T	Och01,Och02,Och03,Och04,Och05,Och06,Och07,Och08,Och09,Och10,Och11,Och12,Och13,Och14	*OreG0025054*
scaffold7534	388733	T	G	Och05,Och07,Och13	*OreG0025055*
scaffold7534	396173	G	T	NA	*OreG0025056*
scaffold7534	399440	C	T	NA	*OreG0025058*
scaffold7534	413346	A	T	NA	*OreG0025059*
scaffold7534	414578	C	A	NA	*OreG0025059*
scaffold7534	415103	G	T	NA	*OreG0025059*
scaffold7534	428477	C	A	Ore01,Ore02,Ore03,Ore04,Ore05,Ore06,Ore07,Ore08,Ore09,Ore10,Ore11,Ore12,Ore13,Ore14	*OreG0025060*
scaffold7534	467299	A	T	Ore01,Ore02,Ore03,Ore04,Ore05,Ore06,Ore07,Ore08,Ore09,Ore10,Ore11,Ore12,Ore13,Ore14	*OreG0025066*
scaffold7534	477038	C	A	NA	*OreG0025067*
scaffold7534	484095	T	A	Och01,Och02,Och03,Och04,Och05,Och06,Och07,Och08,Och09,Och10,Och11,Och12,Och13,Och14,Ore01,Ore02,Ore03,Ore04,Ore05,Ore06,Ore07,Ore08,Ore09,Ore10,Ore11,Ore12,Ore13,Ore14	*OreG0025068*
scaffold7534	484140	G	A	Ore01,Ore02,Ore03,Ore04,Ore05,Ore06,Ore07,Ore08,Ore09,Ore10,Ore11,Ore12,Ore13,Ore14	*OreG0025068*
scaffold7534	484203	A	G	Ore01,Ore02,Ore03,Ore04,Ore05,Ore06,Ore07,Ore08,Ore09,Ore10,Ore11,Ore12,Ore13,Ore14	*OreG0025068*
scaffold7534	484311	G	T	Ore01,Ore02,Ore03,Ore04,Ore05,Ore06,Ore07,Ore08,Ore09,Ore10,Ore11,Ore12,Ore13,Ore14	*OreG0025068*
scaffold7534	484371	G	A	Och01,Och02,Och03,Och04,Och05,Och06,Och07,Och08,Och09,Och10,Och11,Och12,Och13,Och14,Ore01,Ore02,Ore03,Ore04,Ore05,Ore06,Ore07,Ore08,Ore09,Ore10,Ore11,Ore12,Ore13,Ore14	*OreG0025068*
scaffold7534	485070	G	T	Och01,Och02,Och03,Och04,Och05,Och06,Och07,Och08,Och09,Och10,Och11,Och12,Och13,Och14	*OreG0025069*
scaffold7534	485189	T	C	Och01,Och02,Och03,Och04,Och05,Och06,Och07,Och08,Och09,Och10,Och11,Och12,Och13,Och14	*OreG0025069*
scaffold7534	485323	T	A	NA	*OreG0025069*
scaffold7534	491761	C	T	Ore03,Ore04,Ore05	*OreG0025070*
scaffold7534	492199	G	C	Och01,Och02,Och03,Och04,Och05,Och06,Och07,Och08,Och09,Och10,Och11,Och12,Och13,Och14,Ore01,Ore02,Ore03,Ore04,Ore05,Ore06,Ore07,Ore08,Ore09,Ore10,Ore11,Ore12,Ore13,Ore14	*OreG0025070*
scaffold7534	492539	G	T	Ore01,Ore02,Ore03,Ore04,Ore05,Ore06,Ore07,Ore08,Ore09,Ore10,Ore11,Ore12,Ore13,Ore14	*OreG0025070*
scaffold7534	493416	A	T	Ore01,Ore02,Ore03,Ore04,Ore05,Ore06,Ore07,Ore08,Ore09,Ore10,Ore11,Ore12,Ore13,Ore14	*OreG0025070*
scaffold7534	493916	T	C	Och01,Och02,Och03,Och04,Och05,Och06,Och07,Och08,Och09,Och10,Och11,Och12,Och13,Och14	*OreG0025070*
scaffold7534	495084	T	C	Och01,Och02,Och03,Och04,Och05,Och06,Och07,Och08,Och09,Och10,Och11,Och12,Och13,Och14	*OreG0025070*

scaffold7534	516908	G	A	Och09,Och10	*OreG0025071*
scaffold7534	517154	G	T	Ore01,Ore02,Ore03,Ore04,Ore05,Ore06,Ore07,Ore08,Ore09,Ore10,Ore11,Ore12,Ore13,Ore14	*OreG0025071*
scaffold7534	517230	T	C	NA	*OreG0025071*
scaffold7534	557472	A	G	Och09,Och10	*OreG0025074*
scaffold7534	557996	G	A	Och01,Och02,Och03,Och04,Och05,Och06,Och07,Och08,Och09,Och10,Och11,Och12,Och13,Och14	*OreG0025074*
scaffold7534	612031	C	T	Ore02,Ore04,Ore05,Ore06,Ore07,Ore10,Ore11,Ore13	*OreG0025077*
scaffold7534	613803	G	A	NA	*OreG0025077*
scaffold7534	613805	G	T	Och01,Och02,Och03,Och04,Och05,Och06,Och07,Och08,Och09,Och10,Och11,Och12,Och13,Och14,Ore01,Ore02,Ore03,Ore04,Ore05,Ore06,Ore07,Ore08,Ore09,Ore10,Ore11,Ore12,Ore13,Ore14	*OreG0025077*
scaffold7534	635020	A	C	Och01,Och02,Och03,Och04,Och05,Och06,Och08,Och09,Och10,Och11,Och12,Och13,Och14	*OreG0025080*
scaffold7534	637952	G	T	NA	*OreG0025081*
scaffold7534	638003	T	C	NA	*OreG0025081*
scaffold7534	640851	G	C	Ore01,Ore02,Ore03,Ore04,Ore05,Ore06,Ore07,Ore08,Ore09,Ore10,Ore11,Ore12,Ore13,Ore14	*OreG0025082*
scaffold7534	659007	A	T	Och08	*OreG0025084*
scaffold7534	664164	T	C	NA	*OreG0025084*
scaffold7534	666468	C	A	Och01,Och02,Och03,Och04,Och05,Och06,Och07,Och08,Och12,Och13,Och14	*OreG0025084*
scaffold7534	666583	A	C	Och09,Och10	*OreG0025084*
scaffold7534	702265	C	A	Och01,Och06,Och07,Och08,Och12,Och14	*OreG0025087*
scaffold7534	719597	G	T	Och01,Och02,Och03,Och04,Och05,Och06,Och07,Och08,Och09,Och10,Och11,Och12,Och13,Och14,Ore01,Ore02,Ore03,Ore04,Ore05,Ore06,Ore07,Ore08,Ore09,Ore10,Ore11,Ore12,Ore13,Ore14	*OreG0025088*
scaffold7534	720400	A	G	Och01,Och02,Och03,Och04,Och05,Och06,Och07,Och08,Och09,Och10,Och11,Och12,Och13,Och14,Ore01,Ore02,Ore03,Ore04,Ore05,Ore06,Ore07,Ore08,Ore09,Ore10,Ore11,Ore12,Ore13,Ore14	*OreG0025088*
scaffold7534	745805	G	A	NA	*OreG0025090*
scaffold7534	778137	G	A	Och01,Och03,Och05,Och13	*OreG0025092*
scaffold7534	778935	A	G	Och01,Och02,Och03,Och04,Och05,Och06,Och07,Och08,Och09,Och10,Och11,Och12,Och13,Och14,Ore01,Ore02,Ore03,Ore04,Ore05,Ore06,Ore07,Ore08,Ore09,Ore10,Ore11,Ore12,Ore13,Ore14	*OreG0025092*
scaffold7534	779082	A	G	Och01,Och02,Och03,Och04,Och05,Och06,Och07,Och09,Och10,Och11,Och12,Och13,Ore03	*OreG0025092*
scaffold7534	784295	G	A	Och01,Och13	*OreG0025093*
scaffold7534	785631	G	A	NA	*OreG0025093*
scaffold7534	797662	G	T	NA	*OreG0025095*
scaffold7534	843475	C	T	NA	*OreG0025099*
scaffold7534	855981	C	T	Och08,Och13	*OreG0025100*
scaffold7534	856000	C	T	Ore01	*OreG0025100*
scaffold7534	856045	G	A	NA	*OreG0025100*
scaffold7534	859513	C	G	NA	*OreG0025100*
scaffold7534	859778	G	A	Och08,Och13	*OreG0025100*
scaffold7534	864645	A	T	NA	*OreG0025101*
scaffold7534	864799	C	T	NA	*OreG0025101*
scaffold7534	865971	C	T	Och13	*OreG0025101*
scaffold7534	874760	T	A	Och09,Och10,Och11	*OreG0025101*
scaffold7534	874829	C	T	NA	*OreG0025101*
scaffold7534	875135	G	T	NA	*OreG0025101*
scaffold7534	898059	T	C	Ore02,Ore06,Ore07,Ore11,Ore13	*OreG0025104*
scaffold7534	927466	G	A	NA	*OreG0025105*
scaffold7534	938834	T	C	NA	*OreG0025105*
scaffold7534	938849	C	G	NA	*OreG0025105*
scaffold7534	939133	T	C	NA	*OreG0025105*
scaffold7534	953348	T	C	NA	*OreG0025108*
scaffold7534	955885	C	A	NA	*OreG0025109*
scaffold7534	957795	C	A	NA	*OreG0025109*
scaffold7534	961697	G	T	Och02,Och03,Och04,Och05,Och06,Och08,Och09,Och10,Och11,Och12,Och13	*OreG0025110*
scaffold7534	961721	T	A	NA	*OreG0025110*
scaffold7534	961733	A	T	NA	*OreG0025110*
scaffold7534	962030	T	A	Och06,Och08	*OreG0025110*
scaffold7534	964778	G	A	NA	*OreG0025111*
scaffold7534	968147	C	T	Och02,Och03,Och04,Och06,Och08,Och13	*OreG0025112*
scaffold7534	1034951	C	A	NA	*OreG0025118*
scaffold7534	1046882	G	T	Och12	*OreG0025119*
scaffold7534	1058551	G	A	NA	*OreG0025119*
scaffold7534	1058560	C	T	NA	*OreG0025119*
scaffold7534	1058967	T	A	NA	*OreG0025119*
scaffold7534	1058976	C	T	NA	*OreG0025119*
scaffold7534	1059391	T	C	Och13	*OreG0025119*
scaffold7534	1080748	G	T	Ore02,Ore08,Ore09,Ore12,Ore14	*OreG0025122*
scaffold7534	1096425	G	A	NA	*OreG0025123*
scaffold7534	1104083	G	A	Och09,Och10	*OreG0025124*

scaffold7534	1104137	T	C	Och01,Och02,Och03,Och04,Och05,Och06,Och07,Och08,Och09,Och10,Och11,Och12,Och13,Och14,Ore01,Ore02,Ore03,Ore04,Ore05,Ore06,Ore07,Ore08,Ore09,Ore10,Ore11,Ore12,Ore13,Ore14	*OreG0025124*
scaffold7534	1111530	T	A	Och01,Och02,Och03,Och04,Och05,Och06,Och07,Och08,Och09,Och10,Och11,Och12,Och13,Och14,Ore01,Ore02,Ore03,Ore04,Ore05,Ore06,Ore07,Ore08,Ore09,Ore10,Ore11,Ore12,Ore13,Ore14	*OreG0025125*
scaffold7534	1182325	T	C	NA	*OreG0025129*
scaffold7534	1182820	T	C	NA	*OreG0025129*
scaffold7534	1182883	C	G	Och01,Och02,Och03,Och04,Och05,Och06,Och07,Och08,Och09,Och10,Och11,Och12,Och13,Och14,Ore01,Ore02,Ore03,Ore04,Ore05,Ore06,Ore07,Ore08,Ore09,Ore10,Ore11,Ore12,Ore13,Ore14	*OreG0025129*
scaffold7534	1192443	G	C	NA	*OreG0025130*
scaffold7534	1192996	T	A	Och01,Och02,Och03,Och04,Och05,Och06,Och07,Och08,Och09,Och10,Och11,Och12,Och13,Och14,Ore01,Ore02,Ore03,Ore04,Ore05,Ore06,Ore07,Ore08,Ore09,Ore10,Ore11,Ore12,Ore13,Ore14	*OreG0025130*
scaffold7534	1242235	G	T	Ore01,Ore02,Ore06,Ore07,Ore08,Ore09,Ore11,Ore12,Ore13,Ore14	*OreG0025134*
scaffold7534	1301859	C	A	Ore01,Ore02,Ore06,Ore07,Ore08,Ore09,Ore11,Ore12,Ore13,Ore14	*OreG0025137*
scaffold7534	1324337	A	G	Och01,Och02,Och03,Och04,Och05,Och06,Och07,Och08,Och09,Och10,Och11,Och12,Och13,Och14,Ore01,Ore02,Ore03,Ore04,Ore05,Ore06,Ore07,Ore08,Ore09,Ore10,Ore11,Ore12,Ore13,Ore14	*OreG0025139*
scaffold7534	1396774	C	T	Och01,Och02,Och03,Och04,Och05,Och06,Och07,Och08,Och09,Och10,Och11,Och12,Och13,Och14,Ore01,Ore02,Ore03,Ore04,Ore05,Ore06,Ore07,Ore08,Ore09,Ore10,Ore11,Ore12,Ore13,Ore14	*OreG0025145*
scaffold7534	1410065	C	T	NA	*OreG0025146*
scaffold7534	1410378	T	C	NA	*OreG0025146*
scaffold7534	1412064	C	G	NA	*OreG0025147*
scaffold7534	1419967	A	T	Ore01,Ore02,Ore06,Ore07,Ore08,Ore09,Ore11,Ore12,Ore13,Ore14	*OreG0025148*
scaffold7534	1428628	T	C	NA	*OreG0025149*
scaffold7534	1428740	G	C	NA	*OreG0025149*
scaffold7534	1428892	A	T	Ore01,Ore02,Ore06,Ore07,Ore08,Ore09,Ore11,Ore12,Ore13,Ore14	*OreG0025149*
scaffold7534	1429178	G	A	Och01,Och08,Och09,Och10	*OreG0025149*
scaffold7534	1463686	G	A	Och01,Och02,Och03,Och04,Och05,Och06,Och07,Och08,Och09,Och10,Och11,Och12,Och13,Och14	*OreG0025152*
scaffold7534	1495101	A	T	Och08	*OreG0025154*
scaffold7534	1515887	G	A	Ore01,Ore02,Ore06,Ore07,Ore08,Ore09,Ore11,Ore12,Ore13,Ore14	*OreG0025156*
scaffold7534	1549208	C	T	NA	*OreG0025161*
scaffold7534	1551632	G	A	NA	*OreG0025162*
scaffold7534	1551994	A	T	Och01,Och02,Och03,Och04,Och05,Och06,Och07,Och08,Och09,Och10,Och11,Och12,Och13,Och14,Ore01,Ore02,Ore03,Ore04,Ore05,Ore06,Ore07,Ore08,Ore09,Ore10,Ore11,Ore12,Ore13,Ore14	*OreG0025162*
scaffold7534	1573377	C	T	Och02,Och04,Och13	*OreG0025163*
scaffold7534	1573659	C	T	NA	*OreG0025163*
scaffold7534	1574205	C	T	Och01,Och02,Och03,Och04,Och05,Och06,Och07,Och08,Och09,Och10,Och11,Och12,Och13,Och14	*OreG0025163*
scaffold7534	1582983	T	A	NA	*OreG0025164*
scaffold7534	1583091	T	C	NA	*OreG0025164*
scaffold7534	1584653	T	A	NA	*OreG0025164*
scaffold7534	1585723	A	C	NA	*OreG0025164*
scaffold7534	1586684	T	A	Och01,Och02,Och03,Och04,Och05,Och06,Och07,Och08,Och09,Och10,Och11,Och12,Och13,Och14	*OreG0025164*
scaffold7534	1587856	T	G	Och06,Och09,Och10,Och11	*OreG0025164*
scaffold7534	1588606	A	C	NA	*OreG0025164*
scaffold7534	1600781	T	A	NA	*OreG0025165*
scaffold7534	1601386	G	A	NA	*OreG0025165*
scaffold7534	1605039	C	A	Och01,Och02,Och03,Och04,Och05,Och06,Och07,Och08,Och09,Och10,Och11,Och12,Och13,Och14	*OreG0025166*
scaffold7534	1605046	C	A	Och01,Och02,Och03,Och04,Och05,Och06,Och07,Och08,Och09,Och10,Och11,Och12,Och13,Och14	*OreG0025166*
scaffold7534	1605184	G	T	Och01,Och02,Och03,Och04,Och05,Och06,Och07,Och08,Och09,Och10,Och11,Och12,Och13,Och14	*OreG0025166*
scaffold7534	1606018	T	C	NA	*OreG0025166*
scaffold7534	1606044	C	A	Och01,Och02,Och03,Och04,Och05,Och06,Och07,Och08,Och09,Och10,Och11,Och12,Och13,Och14,Ore01,Ore02,Ore06,Ore07,Ore08,Ore09,Ore11,Ore12,Ore13,Ore14	*OreG0025166*
scaffold7534	1617686	A	C	Och06,Och08,Och09,Och10,Ore01,Ore02,Ore03,Ore04,Ore05,Ore06,Ore07,Ore08,Ore09,Ore10,Ore11,Ore12,Ore13,Ore14	*OreG0025167*
scaffold7534	1617883	A	T	Och01	*OreG0025167*
scaffold7534	1619851	G	A	Och01,Och02,Och03,Och04,Och05,Och06,Och07,Och08,Och09,Och10,Och11,Och12,Och13,Och14,Ore01,Ore02,Ore03,Ore04,Ore05,Ore06,Ore07,Ore08,Ore09,Ore10,Ore11,Ore12,Ore13,Ore14	*OreG0025167*
scaffold7534	1661841	T	C	NA	*OreG0025169*
scaffold7534	1675376	C	G	Och01,Och02,Och03,Och04,Och05,Och06,Och07,Och08,Och09,Och10,Och11,Och12,Och13,Och14	*OreG0025169*
scaffold7534	1690853	C	T	NA	*OreG0025170*
scaffold7534	1698350	A	T	Ore01,Ore02,Ore03,Ore04,Ore05,Ore06,Ore07,Ore08,Ore09,Ore10,Ore11,Ore12,Ore13,Ore14	*OreG0025171*

scaffold7534	1698473	C	A	NA	*OreG0025171*
scaffold7534	1776193	T	C	Och08,Och09,Och10	*OreG0025176*
scaffold7534	1776454	T	A	NA	*OreG0025176*
scaffold7534	1776732	T	G	NA	*OreG0025176*
scaffold7534	1776908	G	T	NA	*OreG0025176*
scaffold7534	1777084	G	C	NA	*OreG0025176*
scaffold7534	1777137	G	T	Och08,Och09,Och10	*OreG0025176*
scaffold7534	1777184	G	T	Och08,Och09,Och10	*OreG0025176*
scaffold7534	1777836	T	C	Ore02,Ore07,Ore11,Ore13	*OreG0025176*
scaffold7534	1816151	T	C	NA	*OreG0025177*
scaffold7534	1845724	C	A	NA	*OreG0025180*
scaffold7534	1845726	G	A	NA	*OreG0025180*
scaffold7534	1845729	G	A	NA	*OreG0025180*
scaffold7534	1857179	C	T	NA	*OreG0025181*
scaffold7534	1861517	A	T	Och08	*OreG0025182*
scaffold7534	1863419	G	T	Och01,Och02,Och03,Och04,Och05,Och06,Och07,Och08,Och09,Och10,Och11,Och12,Och13,Och14,Ore01,Ore02,Ore03,Ore04,Ore05,Ore06,Ore07,Ore08,Ore09,Ore10,Ore11,Ore12,Ore13,Ore14	*OreG0025182*
scaffold7534	1866230	G	T	NA	*OreG0025182*
scaffold7534	1868215	T	C	NA	*OreG0025182*
scaffold7534	1879006	A	T	Ore01,Ore02,Ore06,Ore07,Ore08,Ore09,Ore11,Ore12,Ore13,Ore14	*OreG0025184*
scaffold7534	1956993	G	A	Och01,Och02,Och03,Och04,Och05,Och06,Och07,Och08,Och09,Och10,Och11,Och12,Och13,Och14,Ore01,Ore02,Ore03,Ore04,Ore05,Ore06,Ore07,Ore08,Ore09,Ore10,Ore11,Ore12,Ore13,Ore14	*OreG0025192*
scaffold7534	1960235	T	G	Och02,Och03,Och04,Och05,Och06,Och08,Och12,Och13	*OreG0025193*
scaffold7534	1960256	C	A	Och01,Och02,Och03,Och04,Och05,Och06,Och07,Och08,Och09,Och10,Och11,Och12,Och13,Och14,Ore01,Ore02,Ore03,Ore04,Ore05,Ore06,Ore07,Ore08,Ore09,Ore10,Ore11,Ore12,Ore13,Ore14	*OreG0025193*
scaffold7534	1960277	T	C	Och09,Och10,Och11	*OreG0025193*
scaffold7534	1960384	T	A	Ore01,Ore02,Ore06,Ore07,Ore08,Ore09,Ore11,Ore12,Ore13,Ore14	*OreG0025193*
scaffold7534	1965193	T	A	NA	*OreG0025194*
scaffold7534	1965588	C	G	Och08	*OreG0025194*
scaffold7534	1980411	T	G	NA	*OreG0025195*
scaffold7534	1980420	C	T	NA	*OreG0025195*
scaffold7534	1980550	T	A	NA	*OreG0025195*
scaffold7534	1980657	C	T	NA	*OreG0025195*
scaffold7534	1980991	C	G	NA	*OreG0025195*
scaffold7534	1981018	T	A	NA	*OreG0025195*
scaffold7534	1991475	T	A	Ore01,Ore02,Ore06,Ore07,Ore08,Ore09,Ore11,Ore12,Ore13,Ore14	*OreG0025196*
scaffold7534	1995115	G	T	Ore01,Ore02,Ore06,Ore07,Ore08,Ore09,Ore11,Ore12,Ore13,Ore14	*OreG0025197*
scaffold7534	1995119	G	T	Ore01,Ore02,Ore06,Ore07,Ore08,Ore09,Ore11,Ore12,Ore13,Ore14	*OreG0025197*
scaffold7534	1996113	G	A	Och09,Och10	*OreG0025198*
scaffold7534	1996489	T	C	NA	*OreG0025198*
scaffold7534	1996697	G	A	Ore01,Ore02,Ore06,Ore07,Ore08,Ore09,Ore11,Ore12,Ore13,Ore14	*OreG0025198*
scaffold7534	1999508	G	A	NA	*OreG0025199*
scaffold7534	1999532	C	A	Och01,Och02,Och03,Och04,Och05,Och06,Och07,Och08,Och09,Och10,Och11,Och12,Och13,Och14,Ore01,Ore02,Ore03,Ore04,Ore05,Ore06,Ore07,Ore08,Ore09,Ore10,Ore11,Ore12,Ore13,Ore14	*OreG0025199*
scaffold7534	2023318	A	T	Och01,Och02,Och03,Och04,Och05,Och06,Och07,Och08,Och09,Och10,Och11,Och12,Och13,Och14,Ore01,Ore02,Ore03,Ore04,Ore05,Ore06,Ore07,Ore08,Ore09,Ore10,Ore11,Ore12,Ore13,Ore14	*OreG0025200*
scaffold7534	2060804	T	A	Och01,Och02,Och03,Och04,Och05,Och06,Och07,Och08,Och09,Och10,Och11,Och12,Och13,Och14	*OreG0025202*
scaffold7534	2060892	C	A	Och01,Och02,Och03,Och04,Och05,Och06,Och07,Och08,Och09,Och10,Och11,Och12,Och13,Och14,Ore01,Ore02,Ore03,Ore04,Ore05,Ore06,Ore07,Ore08,Ore09,Ore10,Ore11,Ore12,Ore13,Ore14	*OreG0025202*
scaffold7534	2061503	G	T	Ore01,Ore02,Ore06,Ore07,Ore08,Ore09,Ore11,Ore12,Ore13,Ore14	*OreG0025202*
scaffold7534	2087290	G	T	Och01,Och02,Och03,Och04,Och05,Och06,Och07,Och08,Och09,Och10,Och11,Och12,Och13,Och14	*OreG0025206*
scaffold7534	2190291	G	C	Och01	*OreG0025214*
scaffold7534	2191421	T	C	Och03,Och05,Och13	*OreG0025214*
scaffold7534	2211970	A	G	Och03,Och05,Och13	*OreG0025217*
scaffold7534	2212017	A	T	Och01,Och02,Och03,Och04,Och05,Och06,Och07,Och08,Och09,Och10,Och11,Och12,Och13,Och14,Ore01,Ore02,Ore03,Ore04,Ore05,Ore06,Ore07,Ore08,Ore09,Ore10,Ore11,Ore12,Ore13,Ore14	*OreG0025217*
scaffold7534	2212032	A	T	Ore01,Ore02,Ore06,Ore07,Ore08,Ore09,Ore11,Ore12,Ore13,Ore14	*OreG0025217*
scaffold7534	2223226	T	A	Och04,Och08,Och09,Och10,Och11	*OreG0025219*
scaffold7534	2255205	A	T	Ore01,Ore02,Ore06,Ore07,Ore08,Ore09,Ore11,Ore12,Ore13,Ore14	*OreG0025223*
scaffold7534	2257144	T	G	NA	*OreG0025224*
scaffold7534	2257196	G	C	NA	*OreG0025224*
scaffold7534	2257249	G	T	Ore01,Ore02,Ore06,Ore07,Ore08,Ore09,Ore11,Ore12,Ore13,Ore14	*OreG0025224*
scaffold7534	2257682	C	A	NA	*OreG0025224*
scaffold7534	2257727	T	G	NA	*OreG0025224*
scaffold7534	2258011	C	T	NA	*OreG0025224*
scaffold7534	2270438	T	C	NA	*OreG0025225*

scaffold7534	2271009	C	G	Och08	*OreG0025225*
scaffold7534	2276183	A	T	NA	*OreG0025226*
scaffold7534	2286090	G	A	Och01,Och02,Och03,Och04,Och05,Och06,Och07,Och08,Och09,Och10,Och11,Och12,Och13,Och14,Ore01,Ore02,Ore03,Ore04,Ore05,Ore06,Ore07,Ore08,Ore09,Ore10,Ore11,Ore12,Ore13,Ore14	*OreG0025227*
scaffold7534	2314917	T	G	NA	*OreG0025231*
scaffold7534	2325557	G	A	NA	*OreG0025234*
scaffold7534	2325774	C	T	NA	*OreG0025234*
scaffold7534	2328189	G	A	Och01	*OreG0025235*
scaffold7534	2341270	G	A	Ore01,Ore02,Ore03,Ore04,Ore05,Ore06,Ore07,Ore08,Ore09,Ore10,Ore11,Ore12,Ore13,Ore14	*OreG0025236*
scaffold7534	2390025	T	G	NA	*OreG0025240*
scaffold7534	2390028	C	T	Och01	*OreG0025240*
scaffold7534	2402524	C	T	NA	*OreG0025243*
scaffold7534	2413324	T	C	NA	*OreG0025241*
scaffold7534	2413337	A	T	Ore01,Ore02,Ore06,Ore07,Ore08,Ore09,Ore11,Ore12,Ore13,Ore14	*OreG0025241*
scaffold7534	2419292	G	C	NA	*OreG0025244*
scaffold7534	2426834	A	G	Ore01,Ore02,Ore06,Ore07,Ore08,Ore09,Ore11,Ore12,Ore13,Ore14	*OreG0025245*
scaffold7534	2427112	G	T	NA	*OreG0025245*
scaffold7534	2427716	G	A	NA	*OreG0025245*
scaffold7534	2427949	T	G	NA	*OreG0025245*
scaffold7534	2439336	G	A	Och08,Och11,Och14	*OreG0025246*
scaffold7534	2439378	T	C	Ore01,Ore02,Ore06,Ore07,Ore08,Ore09,Ore11,Ore12,Ore13,Ore14	*OreG0025246*
scaffold7534	2440005	T	C	Ore01,Ore02,Ore06,Ore07,Ore08,Ore09,Ore11,Ore12,Ore13,Ore14	*OreG0025246*
scaffold7534	2441519	A	T	Och01,Och02,Och04,Och05,Och07,Och12,Och13	*OreG0025246*
scaffold7534	2441592	A	T	Och01,Och02,Och03,Och04,Och05,Och06,Och07,Och08,Och09,Och10,Och11,Och12,Och13,Och14	*OreG0025246*
scaffold7534	2446104	C	A	Och01,Och02,Och04,Och05,Och07,Och08,Och12,Och13,Och14	*OreG0025247*
scaffold7534	2447758	G	A	NA	*OreG0025247*
scaffold7534	2453227	G	C	Och01	*OreG0025248*
scaffold7534	2476074	G	A	Och01,Och12	*OreG0025250*
scaffold7534	2571120	T	C	Ore01,Ore02,Ore06,Ore07,Ore08,Ore09,Ore11,Ore12,Ore13,Ore14	*OreG0025258*
scaffold7534	2603201	A	T	NA	*OreG0025264*
scaffold7534	2610656	A	C	Ore01,Ore02,Ore06,Ore07,Ore08,Ore09,Ore11,Ore12,Ore13,Ore14	*OreG0025266*
scaffold7534	2636757	G	T	Ore02,Ore09,Ore14	*OreG0025269*
scaffold7534	2641893	G	A	NA	*OreG0025269*
scaffold7534	2653323	G	A	NA	*OreG0025271*
scaffold7534	2654026	T	A	Ore01,Ore02,Ore03,Ore04,Ore05,Ore06,Ore07,Ore08,Ore09,Ore10,Ore11,Ore12,Ore13,Ore14	*OreG0025271*
scaffold7534	2656330	A	G	NA	*OreG0025271*
scaffold7534	2679124	T	C	NA	*OreG0025275*
scaffold7534	2679345	G	A	NA	*OreG0025275*
scaffold7534	2679478	T	C	NA	*OreG0025275*
scaffold7534	2688149	G	T	NA	*OreG0025275*
scaffold7534	2694271	C	A	NA	*OreG0025276*
scaffold7534	2695189	C	T	NA	*OreG0025276*
scaffold7534	2695261	T	G	NA	*OreG0025276*
scaffold7534	2695822	T	A	Och01,Och07,Och09,Och10,Ore01,Ore02,Ore06,Ore07,Ore08,Ore09,Ore11,Ore12,Ore13,Ore14	*OreG0025276*
scaffold7534	2696534	A	T	NA	*OreG0025276*
scaffold7534	2701774	T	C	NA	*OreG0025278*
scaffold7534	2701838	C	G	NA	*OreG0025278*
scaffold7534	2701870	T	C	NA	*OreG0025278*
scaffold4993	7922	A	G	NA	*OreG0023296*
scaffold4993	16155	T	A	Och01,Och02,Och03,Och04,Och05,Och06,Och07,Och08,Och09,Och10,Och11,Och12,Och13,Och14,Ore01,Ore02,Ore03,Ore04,Ore05,Ore06,Ore07,Ore08,Ore09,Ore10,Ore11,Ore12,Ore13,Ore14	*OreG0023297*
scaffold4993	51126	A	T	Och02,Och03,Och06	*OreG0023300*
scaffold4993	51413	A	T	NA	*OreG0023300*
scaffold4993	51566	T	C	Och01,Och02,Och03,Och04,Och05,Och06,Och07,Och08,Och09,Och10,Och11,Och12,Och13,Och14,Ore01,Ore02,Ore03,Ore04,Ore05,Ore06,Ore07,Ore08,Ore09,Ore10,Ore11,Ore12,Ore13,Ore14	*OreG0023300*
scaffold4993	51569	C	T	NA	*OreG0023300*
scaffold4993	69634	A	G	NA	*OreG0023301*
scaffold4993	69651	A	T	Och08,Och09,Och10,Och12,Och13	*OreG0023301*
scaffold4993	69732	A	T	NA	*OreG0023301*
scaffold4993	69813	G	T	Och01,Och02,Och03,Och06,Ore01,Ore02,Ore03,Ore04,Ore05,Ore06,Ore07,Ore08,Ore09,Ore10,Ore11,Ore12,Ore13,Ore14	*OreG0023301*
scaffold4993	69921	G	A	NA	*OreG0023301*
scaffold4993	70252	T	A	Ore01,Ore02,Ore03,Ore04,Ore05,Ore06,Ore07,Ore08,Ore09,Ore10,Ore11,Ore12,Ore13,Ore14	*OreG0023301*
scaffold4993	70443	C	A	Och08,Och12,Och13	*OreG0023301*
scaffold4993	75942	C	T	Och01	*OreG0023302*
scaffold4993	75948	C	G	NA	*OreG0023302*

scaffold4993	75963	T	C	NA	*OreG0023302*
scaffold4993	76026	C	T	NA	*OreG0023302*
scaffold4993	77061	C	A	Och01	*OreG0023302*
scaffold4993	77740	C	A	Och01,Och02,Och03,Och04,Och05,Och06,Och07,Och08,Och09,Och10,Och11,Och12,Och13,Och14,Ore01,Ore02,Ore03,Ore04,Ore05,Ore06,Ore07,Ore08,Ore09,Ore10,Ore11,Ore12,Ore13,Ore14	*OreG0023302*
scaffold4993	77906	C	A	NA	*OreG0023302*
scaffold4993	78127	G	A	Och01	*OreG0023302*
scaffold4993	124355	C	T	Och02,Och03,Och06	*OreG0023306*
scaffold4993	139341	G	A	Och01,Och02,Och03,Och04,Och05,Och06,Och07,Och08,Och09,Och10,Och11,Och12,Och13,Och14,Ore01,Ore02,Ore03,Ore04,Ore05,Ore06,Ore07,Ore08,Ore09,Ore10,Ore11,Ore12,Ore13,Ore14	*OreG0023308*
scaffold4993	163714	C	A	Ore01,Ore02,Ore03,Ore04,Ore05,Ore06,Ore07,Ore08,Ore09,Ore10,Ore11,Ore12,Ore13,Ore14	*OreG0023312*
scaffold4993	164116	C	A	Och01,Och02,Och03,Och04,Och05,Och06,Och07,Och08,Och09,Och10,Och11,Och12,Och13,Och14	*OreG0023312*
scaffold4993	164366	G	T	Och12,Och13	*OreG0023312*
scaffold4993	164440	T	G	Ore01,Ore02,Ore03,Ore04,Ore05,Ore06,Ore07,Ore08,Ore09,Ore10,Ore11,Ore12,Ore13,Ore14	*OreG0023312*
scaffold4993	169225	G	A	Ore06,Ore07,Ore08,Ore12,Ore13,Ore14	*OreG0023313*
scaffold4993	190326	G	C	NA	*OreG0023316*
scaffold4993	238812	G	A	Och04,Och12,Och13	*OreG0023320*
scaffold4993	268915	T	G	Ore01,Ore02,Ore03,Ore04,Ore05,Ore06,Ore07,Ore08,Ore09,Ore10,Ore11,Ore12,Ore13,Ore14	*OreG0023323*
scaffold4993	269477	G	T	NA	*OreG0023323*
scaffold4993	400389	T	C	NA	*OreG0023336*
scaffold4993	416289	C	T	NA	*OreG0023337*
scaffold4993	416482	T	A	Och01,Och03,Och05,Och08,Och09,Och10,Och12,Och13,Och14	*OreG0023337*
scaffold4993	417302	G	C	Och03,Och05,Och08,Och09,Och10,Och12,Och13,Och14	*OreG0023337*
scaffold4993	422966	T	G	Och01,Och12,Och13,Ore01,Ore02,Ore03,Ore04,Ore05,Ore06,Ore07,Ore08,Ore09,Ore10,Ore11,Ore12,Ore13,Ore14	*OreG0023338*
scaffold4993	435600	T	A	NA	*OreG0023339*
scaffold4993	435611	T	C	NA	*OreG0023339*
scaffold4993	456232	T	A	Och02,Och03,Och04,Och05,Och06,Och07,Och08,Och09,Och10,Och11,Och12,Och13,Och14	*OreG0023342*
scaffold4993	458362	T	A	Och01	*OreG0023342*
scaffold4993	500247	T	A	Och01,Och02,Och03,Och04,Och05,Och06,Och07,Och08,Och09,Och10,Och11,Och12,Och13,Och14,Ore01,Ore02,Ore03,Ore04,Ore05,Ore06,Ore07,Ore08,Ore09,Ore10,Ore11,Ore12,Ore13,Ore14	*OreG0023345*
scaffold4993	501300	G	A	NA	*OreG0023345*
scaffold4993	513143	G	A	Ore01,Ore02,Ore03,Ore04,Ore05,Ore06,Ore07,Ore08,Ore09,Ore10,Ore11,Ore12,Ore13,Ore14	*OreG0023346*
scaffold4993	543022	G	T	Ore01,Ore02,Ore03,Ore04,Ore05,Ore06,Ore07,Ore08,Ore09,Ore10,Ore11,Ore12,Ore13,Ore14	*OreG0023349*
scaffold4993	557076	C	A	Och03,Och05,Och12,Och13	*OreG0023350*
scaffold4993	651912	A	G	Och12,Och13	*OreG0023358*
scaffold4993	668689	C	T	Ore01,Ore02,Ore03,Ore04,Ore05,Ore06,Ore07,Ore08,Ore09,Ore10,Ore11,Ore12,Ore13,Ore14	*OreG0023360*
scaffold4993	694403	G	A	Och01,Och02,Och03,Och04,Och05,Och06,Och07,Och08,Och09,Och10,Och11,Och12,Och13,Och14	*OreG0023361*
scaffold4993	712138	C	G	Och03	*OreG0023363*
scaffold4993	713454	A	C	NA	*OreG0023363*
scaffold4993	720859	T	A	Ore01,Ore02,Ore03,Ore04,Ore05,Ore06,Ore07,Ore08,Ore09,Ore10,Ore11,Ore12,Ore13,Ore14	*OreG0023364*
scaffold4993	742700	C	G	NA	*OreG0023367*
scaffold4993	743145	T	A	Och01,Och02,Och03,Och04,Och05,Och06,Och07,Och08,Och09,Och10,Och11,Och12,Och13,Och14,Ore01,Ore02,Ore03,Ore04,Ore05,Ore06,Ore07,Ore08,Ore09,Ore10,Ore11,Ore12,Ore13,Ore14	*OreG0023367*
scaffold4993	743305	G	A	NA	*OreG0023367*
scaffold4993	877526	C	T	NA	*OreG0023379*
scaffold4993	903314	A	G	NA	*OreG0023382*
scaffold4993	928884	G	A	Och08,Ore01,Ore02,Ore03,Ore04,Ore05,Ore06,Ore07,Ore08,Ore09,Ore10,Ore11,Ore12,Ore13,Ore14	*OreG0023387*
scaffold4993	975088	G	T	Och01,Och02,Och03,Och04,Och05,Och06,Och07,Och08,Och09,Och10,Och11,Och12,Och13,Och14,Ore01,Ore02,Ore03,Ore04,Ore05,Ore06,Ore07,Ore08,Ore09,Ore10,Ore11,Ore12,Ore13,Ore14	*OreG0023391*
scaffold4993	976706	T	C	Ore01,Ore02,Ore03,Ore04,Ore05,Ore06,Ore07,Ore08,Ore09,Ore10,Ore11,Ore12,Ore13,Ore14	*OreG0023391*
scaffold4993	979863	C	A	Och01	*OreG0023392*
scaffold4993	995040	C	T	Och03	*OreG0023393*
scaffold4993	1030617	C	T	Och01,Och02,Och03,Och04,Och05,Och06,Och07,Och08,Och09,Och10,Och11,Och12,Och13,Och14	*OreG0023395*
scaffold4993	1072086	C	T	NA	*OreG0023397*
scaffold4993	1072357	A	G	Och12,Och13	*OreG0023397*
scaffold4993	1116155	C	T	NA	*OreG0023402*

scaffold4993	1126953	T	C	Ore01,Ore02,Ore03,Ore04,Ore05,Ore06,Ore07,Ore08,Ore09,Ore10,Ore11,Ore12,Ore13,Ore14	*OreG0023404*
scaffold4993	1134915	G	A	Och01,Och02,Och03,Och04,Och05,Och06,Och07,Och08,Och09,Och10,Och11,Och12,Och13,Och14	*OreG0023405*
scaffold4993	1142881	G	A	NA	*OreG0023406*
scaffold4993	1147267	T	C	Ore01,Ore02,Ore03,Ore04,Ore05,Ore06,Ore07,Ore08,Ore09,Ore10,Ore11,Ore12,Ore13,Ore14	*OreG0023406*
scaffold4993	1199253	T	C	NA	*OreG0023410*
scaffold4993	1219199	T	G	Och02,Och05,Och09,Och11,Och13	*OreG0023411*
scaffold4993	1219279	C	T	NA	*OreG0023411*
scaffold4993	1247818	A	C	NA	*OreG0023412*
scaffold4993	1247971	T	A	NA	*OreG0023412*
scaffold4993	1335947	A	T	Ore04,Ore05,Ore06,Ore07,Ore08,Ore12,Ore13,Ore14	*OreG0023414*
scaffold4993	1336205	C	G	Och12,Och13	*OreG0023414*
scaffold4993	1336373	C	A	Ore04,Ore05,Ore06,Ore07,Ore08,Ore12,Ore13,Ore14	*OreG0023414*
scaffold4993	1336400	C	T	Och12,Och13	*OreG0023414*
scaffold4993	1337297	T	C	NA	*OreG0023414*
scaffold4993	1337429	C	T	NA	*OreG0023414*
scaffold4993	1361986	C	T	NA	*OreG0023415*
scaffold4993	1361991	C	G	NA	*OreG0023415*
scaffold4993	1364313	T	A	NA	*OreG0023415*
scaffold4993	1421833	G	A	Ore01,Ore04,Ore05	*OreG0023420*
scaffold4993	1422126	C	G	Och01,Och02,Och03,Och04,Och05,Och06,Och07,Och08,Och09,Och10,Och11,Och12,Och13,Och14	*OreG0023420*
scaffold4993	1427923	C	T	Och01,Och02,Och03,Och04,Och05,Och06,Och07,Och08,Och09,Och10,Och11,Och12,Och13,Och14	*OreG0023421*
scaffold4993	1428696	G	A	NA	*OreG0023421*
scaffold4993	1478268	G	T	NA	*OreG0023424*
scaffold4993	1479133	C	T	NA	*OreG0023424*
scaffold4993	1493196	C	T	Ore01,Ore02,Ore03,Ore04,Ore05,Ore06,Ore07,Ore08,Ore09,Ore10,Ore11,Ore12,Ore13,Ore14	*OreG0023425*
scaffold4993	1496848	T	A	Ore01,Ore04,Ore05	*OreG0023426*
scaffold4993	1512723	T	A	NA	*OreG0023427*
scaffold4993	1521946	G	A	NA	*OreG0023429*
scaffold4993	1532393	G	A	Och02,Och03,Och06	*OreG0023429*
scaffold4993	1537842	T	A	Och02,Och03,Och06,Ore01,Ore02,Ore03,Ore04,Ore05,Ore06,Ore07,Ore08,Ore09,Ore10,Ore11,Ore12,Ore13,Ore14	*OreG0023429*
scaffold4993	1578592	C	G	Och01,Och02,Och03,Och04,Och05,Och06,Och07,Och08,Och09,Och10,Och11,Och12,Och13,Och14,Ore01,Ore02,Ore03,Ore04,Ore05,Ore06,Ore07,Ore08,Ore09,Ore10,Ore11,Ore12,Ore13,Ore14	*OreG0023432*
scaffold4993	1594629	A	G	Och03	*OreG0023433*
scaffold4993	1600179	C	T	NA	*OreG0023434*
scaffold4993	1657304	G	C	Och01,Och04,Och07,Och08,Och09,Och10,Och11,Och12,Och13,Och14	*OreG0023437*
scaffold4993	1664791	C	T	Och01,Och02,Och03,Och04,Och05,Och06,Och07,Och08,Och09,Och10,Och11,Och12,Och13,Och14,Ore01,Ore02,Ore03,Ore04,Ore05,Ore06,Ore07,Ore08,Ore09,Ore10,Ore11,Ore12,Ore13,Ore14	*OreG0023438*
scaffold4993	1678024	C	T	NA	*OreG0023441*
scaffold4993	1678444	C	A	NA	*OreG0023441*
scaffold4993	1693842	C	T	NA	*OreG0023442*
scaffold4993	1699148	T	A	Och01,Och02,Och03,Och04,Och05,Och06,Och07,Och08,Och09,Och10,Och11,Och12,Och13,Och14	*OreG0023443*
scaffold4993	1721949	C	T	NA	*OreG0023445*
scaffold4993	1722789	A	T	NA	*OreG0023445*
scaffold4993	1723133	G	A	Ore01,Ore02,Ore03,Ore04,Ore05,Ore06,Ore07,Ore08,Ore09,Ore10,Ore11,Ore12,Ore13,Ore14	*OreG0023445*
scaffold4993	1725871	G	A	Ore01,Ore02,Ore03,Ore04,Ore05,Ore06,Ore07,Ore08,Ore09,Ore10,Ore11,Ore12,Ore13,Ore14	*OreG0023446*
scaffold4993	1745308	C	A	NA	*OreG0023447*
scaffold4993	1757643	C	T	NA	*OreG0023449*
scaffold4993	1757711	G	A	NA	*OreG0023449*
scaffold4993	1757783	T	C	NA	*OreG0023449*
scaffold4993	1757932	T	C	NA	*OreG0023449*
scaffold4993	1758035	C	T	NA	*OreG0023449*
scaffold4993	1758202	A	G	NA	*OreG0023449*
scaffold4993	1758241	T	A	NA	*OreG0023449*
scaffold4993	1758373	A	G	NA	*OreG0023449*
scaffold4993	1758472	A	G	NA	*OreG0023449*
scaffold4993	1758569	G	A	NA	*OreG0023449*
scaffold4993	1758589	C	T	Och01,Och14	*OreG0023449*
scaffold4993	1758596	T	C	NA	*OreG0023449*
scaffold4993	1758665	C	A	NA	*OreG0023449*
scaffold4993	1758694	G	A	NA	*OreG0023449*
scaffold4993	1758696	C	T	NA	*OreG0023449*
scaffold4993	1758734	T	G	NA	*OreG0023449*
scaffold4993	1758755	C	A	NA	*OreG0023449*

scaffold4993	1758770	T	C	NA	*OreG0023449*
scaffold4993	1758788	T	C	NA	*OreG0023449*
scaffold4993	1783721	G	A	NA	*OreG0023451*
scaffold4993	1879084	G	A	NA	*OreG0023459*
scaffold4993	1879169	C	T	NA	*OreG0023459*
scaffold4993	1879421	C	A	NA	*OreG0023459*
scaffold4993	1879640	A	T	NA	*OreG0023459*
scaffold4993	1879661	T	C	NA	*OreG0023459*
scaffold4993	1879670	C	T	NA	*OreG0023459*
scaffold4993	1899434	A	C	NA	*OreG0023461*
scaffold4993	1899572	C	T	Ore01,Ore02,Ore03,Ore04,Ore05,Ore06,Ore07,Ore08,Ore09,Ore10,Ore11,Ore12,Ore13,Ore14	*OreG0023461*
scaffold4993	1899862	G	A	Ore03,Ore09,Ore10,Ore11	*OreG0023461*
scaffold4993	1901913	A	T	Och01,Och02,Och03,Och04,Och05,Och06,Och07,Och08,Och09,Och10,Och11,Och12,Och13,Och14,Ore01,Ore02,Ore03,Ore04,Ore05,Ore06,Ore07,Ore08,Ore09,Ore10,Ore11,Ore12,Ore13,Ore14	*OreG0023461*
scaffold4993	1906194	G	A	NA	*OreG0023462*
scaffold4993	1911998	G	A	Och01,Och02,Och03,Och04,Och05,Och06,Och07,Och08,Och09,Och10,Och11,Och12,Och13,Och14	*OreG0023464*
scaffold4993	1912721	A	G	NA	*OreG0023464*
scaffold4993	1912887	T	G	Och01,Och02,Och03,Och04,Och05,Och06,Och07,Och08,Och09,Och10,Och11,Och12,Och13,Och14	*OreG0023464*
scaffold4993	1921205	A	C	Ore01,Ore02,Ore03,Ore04,Ore05,Ore06,Ore07,Ore08,Ore09,Ore10,Ore11,Ore12,Ore13,Ore14	*OreG0023465*
scaffold4993	1921379	G	A	Och01,Och02,Och03,Och04,Och05,Och06,Och07,Och08,Och09,Och10,Och11,Och12,Och13,Och14	*OreG0023465*
scaffold4993	1921585	G	T	Ore04,Ore05	*OreG0023465*
scaffold4993	1921637	G	A	Och02,Och04	*OreG0023465*
scaffold4993	1922624	A	T	NA	*OreG0023465*
scaffold4993	2002651	C	A	Och01,Och02,Och03,Och04,Och05,Och06,Och07,Och08,Och09,Och10,Och11,Och12,Och13,Och14	*OreG0023472*
scaffold4993	2002831	A	T	Ore01,Ore02,Ore03,Ore04,Ore05,Ore06,Ore07,Ore08,Ore09,Ore10,Ore11,Ore12,Ore13,Ore14	*OreG0023472*
scaffold4993	2002909	A	T	NA	*OreG0023472*
scaffold4993	2003296	G	A	NA	*OreG0023472*
scaffold4993	2003431	G	A	Ore01,Ore02,Ore03,Ore04,Ore05,Ore06,Ore07,Ore08,Ore09,Ore10,Ore11,Ore12,Ore13,Ore14	*OreG0023472*
scaffold4993	2025033	G	C	Och01,Och02,Och03,Och04,Och05,Och06,Och07,Och08,Och09,Och10,Och11,Och12,Och13,Och14,Ore01,Ore02,Ore03,Ore04,Ore05,Ore06,Ore07,Ore08,Ore09,Ore10,Ore11,Ore12,Ore13,Ore14	*OreG0023473*
scaffold4993	2064732	G	C	NA	*OreG0023476*
scaffold4993	2079605	A	T	Och01,Och02,Och03,Och04,Och05,Och06,Och07,Och08,Och09,Och10,Och11,Och12,Och13,Och14,Ore01,Ore02,Ore03,Ore04,Ore05,Ore06,Ore07,Ore08,Ore09,Ore10,Ore11,Ore12,Ore13,Ore14	*OreG0023477*
scaffold4993	2083865	T	G	Ore01,Ore02,Ore03,Ore04,Ore05,Ore06,Ore07,Ore08,Ore09,Ore10,Ore11,Ore12,Ore13,Ore14	*OreG0023477*
scaffold4993	2098341	G	T	NA	*OreG0023479*
scaffold4993	2098707	C	T	NA	*OreG0023479*
scaffold4993	2098764	G	T	NA	*OreG0023479*
scaffold4993	2099078	G	T	NA	*OreG0023479*
scaffold4993	2099417	G	C	NA	*OreG0023479*
scaffold4993	2099604	A	C	NA	*OreG0023479*
scaffold4993	2099683	A	T	Ore01,Ore02,Ore03,Ore04,Ore05,Ore06,Ore07,Ore08,Ore09,Ore10,Ore11,Ore12,Ore13,Ore14	*OreG0023479*
scaffold4993	2099684	G	T	NA	*OreG0023479*
scaffold4993	2099831	G	A	NA	*OreG0023479*
scaffold4993	2101617	A	G	NA	*OreG0023480*
scaffold4993	2102723	G	A	NA	*OreG0023480*
scaffold4993	2104375	G	T	Ore07	*OreG0023481*
scaffold4993	2104582	G	A	Och10	*OreG0023482*
scaffold4993	2107969	T	C	NA	*OreG0023483*
scaffold4993	2108041	G	A	NA	*OreG0023483*
scaffold4993	2153384	C	G	NA	*OreG0023489*
scaffold4993	2156114	C	G	Ore01,Ore02,Ore03,Ore04,Ore05,Ore06,Ore07,Ore08,Ore09,Ore10,Ore11,Ore12,Ore13,Ore14	*OreG0023489*
scaffold4993	2180132	A	C	Och01,Och02,Och03,Och04,Och05,Och06,Och07,Och08,Och09,Och10,Och11,Och12,Och13,Och14,Ore01,Ore02,Ore03,Ore04,Ore05,Ore06,Ore07,Ore08,Ore09,Ore10,Ore11,Ore12,Ore13,Ore14	*OreG0023492*
scaffold4993	2180179	A	T	Ore01,Ore02,Ore03,Ore04,Ore05,Ore06,Ore07,Ore08,Ore09,Ore10,Ore11,Ore12,Ore13,Ore14	*OreG0023492*
scaffold4993	2180354	C	A	Och01,Och02,Och03,Och04,Och05,Och06,Och07,Och08,Och09,Och10,Och11,Och12,Och13,Och14,Ore01,Ore02,Ore03,Ore04,Ore05,Ore06,Ore07,Ore08,Ore09,Ore10,Ore11,Ore12,Ore13,Ore14	*OreG0023492*
scaffold4993	2180384	A	G	NA	*OreG0023492*
scaffold4993	2189339	G	A	Ore01,Ore02,Ore12	*OreG0023494*

scaffold4993	2189414	G	A	Och01,Och02,Och03,Och04,Och05,Och06,Och07,Och08,Och09,Och10,Och11,Och12,Och13,Och14,Ore01,Ore02,Ore03,Ore04,Ore05,Ore06,Ore07,Ore08,Ore09,Ore10,Ore11,Ore12,Ore13,Ore14	*OreG0023494*
scaffold4993	2189732	A	T	Ore01,Ore02,Ore03,Ore04,Ore05,Ore06,Ore07,Ore08,Ore09,Ore10,Ore11,Ore12,Ore13,Ore14	*OreG0023494*
scaffold4993	2189741	C	T	Och01,Och04,Och07,Och08,Och11,Och12,Och13	*OreG0023494*
scaffold4993	2239874	A	C	Och01,Och02,Och03,Och04,Och05,Och06,Och07,Och08,Och09,Och10,Och11,Och12,Och13,Och14,Ore01,Ore02,Ore03,Ore04,Ore05,Ore06,Ore07,Ore08,Ore09,Ore10,Ore11,Ore12,Ore13,Ore14	*OreG0023500*
scaffold4993	2239953	C	G	Och01,Och02,Och03,Och04,Och05,Och06,Och07,Och08,Och09,Och10,Och11,Och12,Och13,Och14,Ore01,Ore02,Ore03,Ore04,Ore05,Ore06,Ore07,Ore08,Ore09,Ore10,Ore11,Ore12,Ore13,Ore14	*OreG0023500*
scaffold4993	2247247	C	A	Och01,Och02,Och03,Och04,Och05,Och06,Och07,Och08,Och11,Och12,Och13,Och14	*OreG0023501*
scaffold4993	2247349	T	C	Och01,Och02,Och03,Och04,Och05,Och06,Och07,Och08,Och09,Och10,Och11,Och12,Och13,Och14,Ore01,Ore02,Ore03,Ore04,Ore05,Ore06,Ore07,Ore08,Ore09,Ore10,Ore11,Ore12,Ore13,Ore14	*OreG0023501*
scaffold4993	2251835	A	T	Ore01,Ore02,Ore03,Ore06,Ore07,Ore08,Ore09,Ore10,Ore11,Ore12,Ore13,Ore14	*OreG0023502*
scaffold4993	2254347	C	T	NA	*OreG0023502*
scaffold4993	2254881	G	A	NA	*OreG0023502*
scaffold4993	2254902	A	G	NA	*OreG0023502*
scaffold4993	2257012	C	A	NA	*OreG0023502*
scaffold4993	2259268	C	A	NA	*OreG0023502*
scaffold4993	2259271	A	T	NA	*OreG0023502*
scaffold4993	2262450	G	A	Och03	*OreG0023502*
scaffold4993	2263914	G	A	NA	*OreG0023502*
scaffold4993	2264790	C	A	NA	*OreG0023502*
scaffold4993	2334410	G	A	Och03	*OreG0023504*
scaffold4993	2350221	G	A	Och14	*OreG0023505*
scaffold4993	2436625	G	A	NA	*OreG0023512*
scaffold4993	2436627	G	A	Och01,Och04,Och09,Och10,Och11,Och12,Och13	*OreG0023512*
scaffold4993	2436718	T	C	NA	*OreG0023512*
scaffold4993	2439261	C	T	Ore04,Ore05,Ore14	*OreG0023513*
scaffold4993	2439431	G	A	Ore02	*OreG0023513*
scaffold4993	2441240	T	A	Och01,Och02,Och03,Och04,Och05,Och06,Och07,Och08,Och09,Och10,Och11,Och12,Och13,Och14,Ore01,Ore02,Ore03,Ore04,Ore05,Ore06,Ore07,Ore08,Ore09,Ore10,Ore11,Ore12,Ore13,Ore14	*OreG0023514*
scaffold4993	2441276	G	T	Och01,Och02,Och03,Och04,Och05,Och06,Och07,Och08,Och09,Och10,Och11,Och12,Och13,Och14,Ore01,Ore02,Ore03,Ore04,Ore05,Ore06,Ore07,Ore08,Ore09,Ore10,Ore11,Ore12,Ore13,Ore14	*OreG0023514*
scaffold4993	2442184	A	T	Och01,Och02,Och03,Och04,Och05,Och06,Och07,Och08,Och09,Och10,Och11,Och12,Och13,Och14,Ore01,Ore02,Ore03,Ore04,Ore05,Ore06,Ore07,Ore08,Ore09,Ore10,Ore11,Ore12,Ore13,Ore14	*OreG0023514*
scaffold4993	2469508	C	G	NA	*OreG0023516*
scaffold4993	2469575	A	T	NA	*OreG0023516*
scaffold4993	2469680	C	G	Och01,Och02,Och03,Och04,Och05,Och06,Och07,Och08,Och09,Och10,Och12,Och13,Och14,Ore01,Ore02,Ore03,Ore04,Ore05,Ore06,Ore07,Ore08,Ore09,Ore10,Ore11,Ore12,Ore13,Ore14	*OreG0023516*
scaffold4993	2470543	G	T	NA	*OreG0023516*
scaffold4993	2506361	C	T	Ore04,Ore05,Ore06,Ore07,Ore08,Ore12,Ore13,Ore14	*OreG0023518*
scaffold4993	2506511	G	T	NA	*OreG0023518*
scaffold4993	2506533	A	T	Ore06,Ore07,Ore08,Ore12,Ore13,Ore14	*OreG0023518*
scaffold4993	2506685	C	T	NA	*OreG0023518*
scaffold4993	2507436	T	A	NA	*OreG0023518*
scaffold4993	2507516	A	T	Och01,Och02,Och03,Och04,Och05,Och06,Och07,Och08,Och09,Och10,Och11,Och12,Och13,Och14,Ore01,Ore02,Ore03,Ore06,Ore07,Ore08,Ore09,Ore10,Ore11,Ore12,Ore13,Ore14	*OreG0023518*
scaffold4993	2509003	C	T	NA	*OreG0023519*
scaffold4993	2509005	G	A	Ore06,Ore07,Ore08,Ore12,Ore13,Ore14	*OreG0023519*
scaffold4993	2509008	A	G	NA	*OreG0023519*
scaffold4993	2511457	A	G	Och01,Och02,Och03,Och04,Och05,Och06,Och07,Och08,Och09,Och10,Och11,Och12,Och13,Och14,Ore01,Ore02,Ore03,Ore06,Ore07,Ore08,Ore09,Ore10,Ore11,Ore12,Ore13,Ore14	*OreG0023520*
scaffold4993	2511938	A	G	NA	*OreG0023521*
scaffold4993	2512000	C	A	NA	*OreG0023521*
scaffold4993	2512027	G	A	NA	*OreG0023521*
scaffold4993	2512088	C	T	NA	*OreG0023521*
scaffold4993	2512310	G	C	NA	*OreG0023521*
scaffold4993	2512409	C	T	NA	*OreG0023521*
scaffold4993	2512469	C	A	Och12,Och13	*OreG0023521*
scaffold4993	2512945	C	A	NA	*OreG0023521*
scaffold4993	2515731	C	T	NA	*OreG0023522*
scaffold4993	2516111	G	A	NA	*OreG0023522*
scaffold4993	2516442	A	C	NA	*OreG0023522*
scaffold4993	2520928	T	A	NA	*OreG0023523*

scaffold4993	2552788	C	T	Och01,Och04,Och08,Och09,Och10,Och11,Och12,Och13	*OreG0023525*
scaffold4993	2552794	A	G	Och03,Och05	*OreG0023525*
scaffold4993	2552800	G	T	Och12,Och13	*OreG0023525*
scaffold4993	2553542	C	T	Och03,Och05	*OreG0023525*
scaffold4993	2554018	G	A	Och03,Och05	*OreG0023525*
scaffold8682	133636	T	C	NA	*OreG0025784*
scaffold8682	134272	G	T	NA	*OreG0025784*
scaffold8682	134317	G	C	NA	*OreG0025784*
scaffold8682	134592	T	C	NA	*OreG0025784*
scaffold13403	35919	A	G	Och01,Och02,Och03,Och04,Och05,Och06,Och07,Och08,Och09,Och10,Och11,Och12,Och13,Och14,Ore01,Ore02,Ore03,Ore04,Ore05,Ore06,Ore07,Ore08,Ore09,Ore10,Ore11,Ore12,Ore13,Ore14	*OreG0002037*
scaffold13403	55309	G	C	Och08,Och11	*OreG0002038*
scaffold13403	68630	G	T	NA	*OreG0002040*
scaffold13403	70427	T	C	NA	*OreG0002041*
scaffold13403	70460	G	A	NA	*OreG0002041*
scaffold13403	83906	C	G	NA	*OreG0002042*
scaffold13403	85232	G	A	Och01,Och02,Och03,Och04,Och05,Och06,Och07,Och08,Och09,Och10,Och11,Och12,Och13,Och14,Ore01,Ore02,Ore03,Ore04,Ore05,Ore06,Ore07,Ore08,Ore09,Ore10,Ore11,Ore12,Ore13,Ore14	*OreG0002042*
scaffold13403	110568	C	A	Och01,Och02,Och03,Och04,Och05,Och06,Och07,Och08,Och09,Och10,Och11,Och12,Och13,Och14,Ore01,Ore02,Ore03,Ore04,Ore05,Ore06,Ore07,Ore08,Ore09,Ore10,Ore11,Ore12,Ore13,Ore14	*OreG0002044*
scaffold13403	116350	T	A	NA	*OreG0002044*
scaffold13403	119969	C	T	NA	*OreG0002044*
scaffold13403	119976	G	A	NA	*OreG0002044*
scaffold13403	120054	T	A	NA	*OreG0002044*
scaffold13403	121556	C	T	NA	*OreG0002044*
scaffold13403	169409	C	T	Och02,Och03,Och08,Och11,Och14,Ore01,Ore02,Ore03,Ore04,Ore05,Ore06,Ore07,Ore08,Ore09,Ore10,Ore11,Ore12,Ore13,Ore14	*OreG0002051*
scaffold13403	169424	C	T	Och02,Och03,Och08,Och11,Och14,Ore01,Ore02,Ore03,Ore04,Ore05,Ore06,Ore07,Ore08,Ore09,Ore10,Ore11,Ore12,Ore13,Ore14	*OreG0002051*
scaffold13403	171092	G	C	NA	*OreG0002051*
scaffold13403	229397	T	A	NA	*OreG0002056*
scaffold13403	246653	G	A	NA	*OreG0002059*
scaffold13403	246672	C	T	Ore01,Ore02,Ore03,Ore04,Ore05,Ore06,Ore07,Ore08,Ore09,Ore10,Ore11,Ore12,Ore13,Ore14	*OreG0002059*
scaffold13403	247530	A	C	Ore01,Ore02,Ore03,Ore04,Ore05,Ore06,Ore07,Ore08,Ore09,Ore10,Ore11,Ore12,Ore13,Ore14	*OreG0002059*
scaffold31006	2080	C	T	NA	*OreG0009366*
scaffold31006	2313	A	C	NA	*OreG0009366*
scaffold31006	2441	T	A	Och13	*OreG0009366*
scaffold31006	2452	A	T	NA	*OreG0009366*
scaffold31006	3447	G	A	NA	*OreG0009366*
scaffold31242	4591	G	C	NA	*OreG0009409*
scaffold31447	1526	T	A	Och14	*OreG0009427*
scaffold31447	1773	C	T	NA	*OreG0009427*
scaffold31457	1163	T	C	NA	*OreG0009428*
scaffold31457	1169	T	A	Och01,Och02,Och03,Och04,Och05,Och06,Och07,Och08,Och09,Och10,Och11,Och12,Och13,Och14,Ore01,Ore02,Ore03,Ore04,Ore05,Ore06,Ore07,Ore08,Ore09,Ore10,Ore11,Ore12,Ore13,Ore14	*OreG0009428*
scaffold31457	1257	A	T	Och08,Och14	*OreG0009428*
scaffold31457	2022	G	A	Ore02	*OreG0009428*
scaffold31457	2351	A	C	Ore01,Ore02,Ore06,Ore07,Ore08,Ore09,Ore11,Ore12,Ore13,Ore14	*OreG0009428*
scaffold31457	2402	T	A	NA	*OreG0009428*
scaffold31704	555	A	G	NA	*OreG0009709*
scaffold31704	836	G	C	NA	*OreG0009709*
scaffold31704	1114	G	C	Och13	*OreG0009709*
scaffold31704	1403	C	G	Och13	*OreG0009709*
scaffold31711	3841	C	A	Ore01,Ore02,Ore03,Ore04,Ore05,Ore06,Ore07,Ore08,Ore09,Ore10,Ore11,Ore12,Ore13,Ore14	*OreG0009712*
scaffold31711	3965	G	T	Ore01,Ore02,Ore03,Ore04,Ore05,Ore06,Ore07,Ore08,Ore09,Ore10,Ore11,Ore12,Ore13,Ore14	*OreG0009712*
scaffold31711	3995	C	T	Ore01,Ore02,Ore03,Ore04,Ore05,Ore06,Ore07,Ore08,Ore09,Ore10,Ore11,Ore12,Ore13,Ore14	*OreG0009712*
scaffold31782	6556	A	G	Och01,Och02,Och03,Och04,Och05,Och06,Och07,Och08,Och09,Och10,Och11,Och12,Och13,Och14	*OreG0009867*
scaffold31782	6684	A	T	Ore01,Ore02,Ore03,Ore04,Ore05,Ore06,Ore07,Ore08,Ore09,Ore10,Ore11,Ore12,Ore13,Ore14	*OreG0009867*
scaffold31782	14017	G	T	Ore01,Ore02,Ore03,Ore04,Ore05,Ore06,Ore07,Ore08,Ore09,Ore10,Ore11,Ore12,Ore13,Ore14	*OreG0009867*
scaffold31868	2111	T	C	Och01,Och02,Och03,Och04,Och05,Och06,Och07,Och08,Och09,Och10,Och11,Och12,Och13,Och14,Ore01,Ore02,Ore03,Ore04,Ore05,Ore06,Ore07,Ore08,Ore09,Ore10,Ore11,Ore12,Ore13,Ore14	*OreG0009906*

scaffold31868	4377	G	T	Och01,Och02,Och03,Och04,Och05,Och06,Och07,Och08,Och11,Och12,Och13,Och14	*OreG0009906*
scaffold31868	4485	A	T	Och01,Och02,Och03,Och04,Och05,Och06,Och07,Och08,Och09,Och11,Och12,Och13,Och14,Ore01,Ore02,Ore03,Ore04,Ore05,Ore06,Ore07,Ore08,Ore09,Ore10,Ore11,Ore12,Ore13,Ore14	*OreG0009906*
scaffold31868	5357	T	C	Ore01,Ore02,Ore03,Ore04,Ore05,Ore06,Ore07,Ore08,Ore09,Ore10,Ore11,Ore12,Ore13,Ore14	*OreG0009906*
scaffold31826	7408	G	T	Och01,Och02,Och03,Och04,Och05,Och06,Och07,Och08,Och09,Och10,Och11,Och12,Och13,Och14,Ore01,Ore02,Ore03,Ore04,Ore05,Ore06,Ore07,Ore08,Ore09,Ore10,Ore11,Ore12,Ore13,Ore14	*OreG0009871*
scaffold31826	7412	T	C	Ore01,Ore02,Ore03,Ore04,Ore05,Ore06,Ore07,Ore08,Ore09,Ore10,Ore11,Ore12,Ore13,Ore14	*OreG0009871*
scaffold3788	65773	A	C	Och01,Och05,Och08,Och12,Och13,Och14	*OreG0019095*
scaffold3788	66294	A	C	NA	*OreG0019095*
scaffold3788	71602	C	G	Och02,Och13,Ore01,Ore02,Ore03,Ore04,Ore05,Ore06,Ore07,Ore08,Ore09,Ore10,Ore11,Ore12,Ore13,Ore14	*OreG0019095*
scaffold3788	101561	A	G	Och08	*OreG0019099*
scaffold3788	131332	G	A	NA	*OreG0019103*
scaffold3788	132833	C	A	Och08	*OreG0019103*
scaffold3788	136230	G	A	Och01,Och02,Och03,Och04,Och05,Och06,Och07,Och08,Och09,Och10,Och11,Och12,Och13,Och14,Ore01,Ore02,Ore03,Ore04,Ore05,Ore06,Ore07,Ore08,Ore09,Ore10,Ore11,Ore12,Ore13,Ore14	*OreG0019104*
scaffold3788	147764	G	A	Och01,Och02,Och03,Och04,Och05,Och06,Och07,Och08,Och09,Och10,Och11,Och12,Och13,Och14,Ore01,Ore02,Ore03,Ore04,Ore05,Ore06,Ore07,Ore08,Ore09,Ore10,Ore11,Ore12,Ore13,Ore14	*OreG0019106*
scaffold3788	164465	C	T	NA	*OreG0019108*
scaffold3788	171365	G	A	Ore04,Ore05	*OreG0019109*
scaffold3788	171395	C	A	Och01,Och02,Och03,Och04,Och05,Och06,Och07,Och08,Och09,Och10,Och11,Och12,Och13,Och14	*OreG0019109*
scaffold3788	212466	G	C	NA	*OreG0019112*
scaffold3788	223812	A	G	Och03,Och05,Och07,Och08	*OreG0019113*
scaffold3788	263436	T	C	NA	*OreG0019117*
scaffold3788	263676	G	A	Och05	*OreG0019117*
scaffold3788	264725	C	T	Och01,Och02,Och03,Och04,Och05,Och06,Och07,Och08,Och12,Och13,Och14,Ore01,Ore02,Ore03,Ore04,Ore05,Ore06,Ore07,Ore08,Ore09,Ore10,Ore11,Ore12,Ore13,Ore14	*OreG0019117*
scaffold3788	265659	C	T	Och09,Och10,Och11	*OreG0019117*
scaffold3788	265678	T	A	Och01,Och04,Och13,Och14	*OreG0019117*
scaffold3788	267033	G	A	NA	*OreG0019117*
scaffold3788	280210	C	T	Ore04,Ore05	*OreG0019118*
scaffold31247	2534	G	A	NA	*OreG0009411*
scaffold31247	15930	C	G	NA	*OreG0009412*
scaffold31247	17759	C	T	Och01,Och02,Och03,Och04,Och05,Och06,Och07,Och08,Och09,Och10,Och11,Och12,Och13,Och14,Ore01,Ore02,Ore03,Ore04,Ore05,Ore06,Ore07,Ore08,Ore09,Ore10,Ore11,Ore12,Ore13,Ore14	*OreG0009412*
scaffold31247	18491	A	G	Och11	*OreG0009412*
scaffold31247	18515	A	G	Ore03,Ore06,Ore09,Ore11,Ore13,Ore14	*OreG0009412*
scaffold31247	18719	C	T	NA	*OreG0009412*
scaffold31247	22234	G	C	Och11	*OreG0009413*
scaffold31247	22298	T	A	Ore01,Ore02,Ore03,Ore04,Ore05,Ore06,Ore07,Ore08,Ore09,Ore10,Ore11,Ore12,Ore13,Ore14	*OreG0009413*
scaffold31247	23736	T	C	Ore04,Ore05	*OreG0009413*
scaffold31247	23958	G	A	Och01,Och02,Och03,Och04,Och05,Och06,Och07,Och08,Och09,Och10,Och11,Och12,Och13,Och14,Ore01,Ore02,Ore03,Ore04,Ore05,Ore06,Ore07,Ore08,Ore09,Ore10,Ore11,Ore12,Ore13,Ore14	*OreG0009413*
scaffold31247	24062	G	A	NA	*OreG0009413*
scaffold31247	34663	C	G	Ore01,Ore02,Ore03,Ore04,Ore05,Ore06,Ore07,Ore08,Ore09,Ore10,Ore11,Ore12,Ore13,Ore14	*OreG0009414*
scaffold31247	36384	C	T	NA	*OreG0009414*
scaffold31247	36390	G	A	Och01,Och02,Och03,Och04,Och05,Och06,Och07,Och08,Och09,Och10,Och11,Och12,Och13,Och14,Ore01,Ore02,Ore03,Ore04,Ore05,Ore06,Ore07,Ore08,Ore09,Ore10,Ore11,Ore12,Ore13,Ore14	*OreG0009414*
scaffold32542	4259	T	G	NA	*OreG0010610*
scaffold32542	4718	G	T	Och01,Och06,Och08,Och09,Och10,Och11	*OreG0010610*
scaffold32542	4949	G	T	NA	*OreG0010610*
scaffold32542	5334	C	T	NA	*OreG0010610*
scaffold32542	6269	G	C	NA	*OreG0010610*
scaffold32739	3785	G	A	NA	*OreG0011015*
scaffold32784	2665	C	A	NA	*OreG0011021*
scaffold31810	16816	C	T	NA	*OreG0009868*
scaffold32841	17784	C	A	NA	*OreG0011025*
scaffold32841	17833	A	T	NA	*OreG0011025*
scaffold32988	2898	T	C	NA	*OreG0011033*
scaffold32988	2910	G	T	NA	*OreG0011033*
scaffold32988	95421	G	T	NA	*OreG0011034*

scaffold32988	95434	C	T	NA	*OreG0011034*
scaffold32988	95683	G	T	NA	*OreG0011034*
scaffold32988	126957	T	G	NA	*OreG0011037*
scaffold32988	127059	T	C	NA	*OreG0011037*
scaffold32988	132710	G	A	NA	*OreG0011037*
scaffold32988	133797	T	C	NA	*OreG0011037*
scaffold31684	60655	C	A	Och03,Och09,Och10,Och11,Och12	*OreG0009646*
scaffold31684	64253	C	A	Och01,Och02,Och03,Och04,Och05,Och06,Och07,Och08,Och09,Och10,Och11,Och12,Och13,Och14,Ore01,Ore02,Ore03,Ore04,Ore05,Ore06,Ore07,Ore08,Ore09,Ore10,Ore11,Ore12,Ore13,Ore14	*OreG0009646*
scaffold31684	84420	T	G	Ore01,Ore02,Ore03,Ore04,Ore05,Ore06,Ore07,Ore08,Ore09,Ore10,Ore11,Ore12,Ore13,Ore14	*OreG0009648*
scaffold31684	86447	A	T	Och03,Och12	*OreG0009649*
scaffold31684	99186	G	T	NA	*OreG0009651*
scaffold31684	102023	A	G	NA	*OreG0009651*
scaffold31684	102153	C	T	Ore01,Ore02,Ore03,Ore04,Ore05,Ore06,Ore07,Ore08,Ore09,Ore10,Ore11,Ore12,Ore13,Ore14	*OreG0009651*
scaffold31684	102189	A	G	NA	*OreG0009651*
scaffold31684	102253	C	A	NA	*OreG0009651*
scaffold31684	102261	C	A	NA	*OreG0009651*
scaffold31684	102323	G	A	NA	*OreG0009651*
scaffold31684	102483	A	C	Ore01,Ore02,Ore03,Ore04,Ore05,Ore06,Ore07,Ore08,Ore09,Ore10,Ore11,Ore12,Ore14	*OreG0009651*
scaffold31684	102564	C	A	Och01	*OreG0009651*
scaffold31684	102609	C	T	Och09,Och10,Och11	*OreG0009651*
scaffold31684	102611	T	A	Ore01,Ore02,Ore03,Ore04,Ore05,Ore06,Ore07,Ore08,Ore09,Ore10,Ore11,Ore12,Ore13,Ore14	*OreG0009651*
scaffold31684	102635	A	G	NA	*OreG0009651*
scaffold31684	102647	A	G	Och01	*OreG0009651*
scaffold31684	120356	C	A	NA	*OreG0009665*
scaffold31684	147175	G	A	NA	*OreG0009667*
scaffold31684	148579	C	T	NA	*OreG0009667*
scaffold31684	165047	T	C	Och11	*OreG0009669*
scaffold31684	183216	G	A	Och04	*OreG0009670*
scaffold31684	195487	A	G	NA	*OreG0009672*
scaffold31684	210667	G	A	Och11	*OreG0009673*
scaffold31684	211972	A	G	NA	*OreG0009673*
scaffold31684	213868	T	A	Och01,Och02,Och03,Och04,Och05,Och06,Och07,Och08,Och09,Och10,Och11,Och12,Och13,Och14,Ore01,Ore02,Ore03,Ore04,Ore05,Ore06,Ore07,Ore08,Ore09,Ore10,Ore11,Ore12,Ore13,Ore14	*OreG0009674*
scaffold31684	214202	C	A	NA	*OreG0009674*
scaffold31684	266309	C	T	NA	*OreG0009679*
scaffold31684	278499	C	T	NA	*OreG0009681*
scaffold31684	324655	G	T	NA	*OreG0009686*
scaffold31684	324946	A	C	NA	*OreG0009686*
scaffold31684	328536	A	T	NA	*OreG0009686*
scaffold31684	328808	C	T	NA	*OreG0009686*
scaffold31684	333907	G	T	NA	*OreG0009686*
scaffold31684	334182	C	T	NA	*OreG0009686*
scaffold31684	334221	C	T	NA	*OreG0009686*
scaffold31684	378145	A	T	NA	*OreG0009690*
scaffold31684	378175	C	T	NA	*OreG0009690*
scaffold31684	378203	T	C	NA	*OreG0009690*
scaffold31684	378698	A	C	NA	*OreG0009690*
scaffold31684	395485	G	A	Och01,Och02,Och03,Och04,Och05,Och06,Och07,Och08,Och09,Och10,Och11,Och12,Och13,Och14,Ore01,Ore02,Ore03,Ore04,Ore05,Ore06,Ore07,Ore08,Ore09,Ore10,Ore11,Ore12,Ore13,Ore14	*OreG0009693*
scaffold31684	415316	T	A	Ore01,Ore02,Ore03,Ore04,Ore05,Ore06,Ore07,Ore08,Ore09,Ore10,Ore11,Ore12,Ore13,Ore14	*OreG0009697*
scaffold31684	437342	C	T	NA	*OreG0009700*
scaffold31684	437590	G	A	NA	*OreG0009700*
scaffold31684	445247	A	T	Ore01,Ore02,Ore03,Ore04,Ore05,Ore06,Ore07,Ore08,Ore09,Ore10,Ore11,Ore12,Ore13,Ore14	*OreG0009701*
scaffold31684	445269	C	T	NA	*OreG0009701*
scaffold31684	445277	G	T	NA	*OreG0009701*
scaffold31684	446731	A	C	Ore01,Ore02,Ore03,Ore04,Ore05,Ore06,Ore07,Ore08,Ore09,Ore10,Ore11,Ore12,Ore13,Ore14	*OreG0009702*
scaffold33104	54293	T	A	NA	*OreG0011811*
scaffold33104	57561	A	G	Ore01,Ore02,Ore03,Ore04,Ore05,Ore06,Ore07,Ore08,Ore09,Ore10,Ore11,Ore12,Ore13,Ore14	*OreG0011811*
scaffold33104	296520	C	T	Och07,Och08,Och09,Och10,Och11,Och14	*OreG0011813*
scaffold33104	296642	A	T	NA	*OreG0011813*
scaffold33104	297397	G	A	Och01,Och02,Och03,Och04,Och05,Och06,Och07,Och08,Och09,Och10,Och11,Och12,Och13,Och14	*OreG0011813*

scaffold33104	299922	T	A	Ore01,Ore02,Ore03,Ore04,Ore05,Ore06,Ore07,Ore08,Ore09,Ore10,Ore11,Ore12,Ore13,Ore14	*OreG0011813*
scaffold33104	528031	A	T	NA	*OreG0011816*
scaffold33104	528042	C	T	Och01,Och02,Och03,Och04,Och05,Och06,Och07,Och08,Och09,Och10,Och11,Och12,Och13,Och14	*OreG0011816*
scaffold31847	2769	G	A	NA	*OreG0009874*
scaffold31847	2805	G	T	NA	*OreG0009874*
scaffold33131	25622	G	C	NA	*OreG0013791*
scaffold33102	49497	C	G	Och01,Och02,Och03,Och04,Och05,Och06,Och07,Och08,Och09,Och10,Och11,Och12,Och13,Och14,Ore01,Ore02,Ore03,Ore04,Ore05,Ore06,Ore07,Ore08,Ore09,Ore10,Ore11,Ore12,Ore13,Ore14	*OreG0011646*
scaffold33102	50422	G	C	NA	*OreG0011646*
scaffold33102	56118	C	A	NA	*OreG0011647*
scaffold33102	94806	T	A	NA	*OreG0011653*
scaffold33102	96397	G	C	NA	*OreG0011653*
scaffold33102	96681	C	T	Ore01,Ore03,Ore09,Ore10,Ore11	*OreG0011653*
scaffold33102	105195	T	A	Och01,Och02,Och03,Och04,Och05,Och06,Och07,Och08,Och09,Och10,Och11,Och12,Och13,Och14,Ore01,Ore02,Ore03,Ore04,Ore05,Ore06,Ore07,Ore08,Ore09,Ore10,Ore11,Ore12,Ore13,Ore14	*OreG0011654*
scaffold33102	105216	T	G	Och01,Och02,Och03,Och04,Och05,Och06,Och07,Och08,Och09,Och10,Och11,Och12,Och13,Och14,Ore01,Ore02,Ore03,Ore04,Ore05,Ore06,Ore07,Ore08,Ore09,Ore10,Ore11,Ore12,Ore13,Ore14	*OreG0011654*
scaffold33102	106147	G	A	Och02,Och03,Och04,Och05,Och06,Och07,Och08,Och09,Och10,Och11,Och12,Och13,Och14,Ore01,Ore02,Ore03,Ore04,Ore05,Ore06,Ore07,Ore08,Ore09,Ore10,Ore11,Ore12,Ore13,Ore14	*OreG0011654*
scaffold33102	106168	G	A	NA	*OreG0011654*
scaffold33102	138269	A	G	Och02,Och03,Och04,Och05,Och06,Och09,Och10,Och11,Ore04,Ore05,Ore14	*OreG0011657*
scaffold33102	138705	C	A	Och09,Och10,Och11	*OreG0011657*
scaffold33102	139203	C	A	NA	*OreG0011657*
scaffold33102	139529	C	T	Och02,Och03,Och04,Och05,Och06,Och09,Och10,Och11,Ore04,Ore05,Ore14	*OreG0011657*
scaffold33102	159672	C	T	NA	*OreG0011661*
scaffold33102	161238	A	T	Och12,Och13	*OreG0011661*
scaffold33102	161261	T	G	Och12,Och13,Ore04,Ore05,Ore14	*OreG0011661*
scaffold33102	164312	C	A	Och01,Och02,Och03,Och04,Och05,Och06,Och07,Och08,Och09,Och10,Och11,Och12,Och13,Och14,Ore01,Ore02,Ore03,Ore04,Ore05,Ore06,Ore07,Ore08,Ore09,Ore10,Ore11,Ore12,Ore13,Ore14	*OreG0011661*
scaffold33102	164849	T	C	Och02,Och05	*OreG0011661*
scaffold33102	165087	A	G	Och02,Och05	*OreG0011661*
scaffold33102	165436	C	G	Ore01,Ore11	*OreG0011661*
scaffold33102	165478	A	T	Och12,Och13,Ore04,Ore05,Ore14	*OreG0011661*
scaffold33102	165486	G	A	Och01,Och02,Och03,Och04,Och05,Och06,Och07,Och08,Och09,Och10,Och11,Och12,Och13,Och14,Ore01,Ore02,Ore03,Ore04,Ore05,Ore06,Ore07,Ore08,Ore09,Ore10,Ore11,Ore12,Ore13,Ore14	*OreG0011661*
scaffold33102	165505	G	A	Och12,Och13,Ore04,Ore05,Ore14	*OreG0011661*
scaffold33102	167688	G	C	Och01,Och02,Och03,Och04,Och05,Och06,Och07,Och08,Och09,Och10,Och11,Och14,Ore01,Ore11	*OreG0011662*
scaffold33102	168117	A	G	NA	*OreG0011662*
scaffold33102	168345	C	T	Och01,Och02,Och03,Och04,Och05,Och06,Och07,Och08,Och09,Och10,Och11,Och12,Och13,Och14,Ore01,Ore02,Ore03,Ore04,Ore05,Ore06,Ore07,Ore08,Ore09,Ore10,Ore11,Ore12,Ore13,Ore14	*OreG0011662*
scaffold33102	170256	G	A	NA	*OreG0011662*
scaffold33102	188339	G	C	NA	*OreG0011665*
scaffold33102	194060	G	C	Och12,Och13,Ore01,Ore11	*OreG0011666*
scaffold33102	215837	T	A	Och12,Och13	*OreG0011670*
scaffold33102	218903	C	A	NA	*OreG0011670*
scaffold33102	219176	C	G	NA	*OreG0011670*
scaffold33125	17744	C	T	NA	*OreG0013600*
scaffold33125	18236	C	T	NA	*OreG0013600*
scaffold31969	101357	C	T	NA	*OreG0010260*
scaffold31969	108502	C	A	NA	*OreG0010262*
scaffold31969	135888	G	C	Och01,Och02,Och03,Och04,Och05,Och06,Och07,Och08,Och09,Och10,Och11,Och12,Och13,Och14	*OreG0010266*
scaffold31969	157211	C	T	Och01,Och09,Och10,Och11	*OreG0010269*
scaffold31969	174143	T	C	NA	*OreG0010272*
scaffold31969	185998	A	T	Och01,Och02,Och03,Och04,Och05,Och06,Och07,Och08,Och09,Och10,Och11,Och12,Och13,Och14,Ore01,Ore02,Ore03,Ore04,Ore05,Ore06,Ore07,Ore08,Ore09,Ore10,Ore11,Ore12,Ore13,Ore14	*OreG0010274*
scaffold31969	187691	T	C	Och01,Och02,Och03,Och04,Och05,Och06,Och07,Och08,Och09,Och10,Och11,Och12,Och13,Och14,Ore01,Ore02,Ore03,Ore04,Ore05,Ore06,Ore07,Ore08,Ore09,Ore10,Ore11,Ore12,Ore13,Ore14	*OreG0010274*
scaffold31969	193778	G	A	Och01,Och02,Och03,Och04,Och05,Och06,Och07,Och08,Och09,Och10,Och11,Och12,Och13,Och14	*OreG0010275*
scaffold31969	195451	A	G	Och01	*OreG0010275*
scaffold31969	195617	C	T	Ore01,Ore02,Ore03,Ore04,Ore05,Ore06,Ore07,Ore08,Ore09,Ore10,Ore11,Ore12,Ore13,Ore14	*OreG0010275*

scaffold31969	196902	G	T	Ore01,Ore02,Ore03,Ore04,Ore05,Ore06,Ore07,Ore08,Ore09,Ore10,Ore11,Ore12,Ore13,Ore14	*OreG0010276*
scaffold31969	215810	A	T	Och02,Och03,Och04,Och05,Och06,Och12,Och13	*OreG0010278*
scaffold31969	219077	G	T	Och01,Och02,Och03,Och04,Och05,Och06,Och07,Och09,Och10,Och11,Och12,Och13,Och14	*OreG0010278*
scaffold31969	251829	T	C	Ore01,Ore02,Ore03,Ore04,Ore05,Ore06,Ore07,Ore08,Ore09,Ore10,Ore11,Ore12,Ore13,Ore14	*OreG0010282*
scaffold31969	254681	A	T	NA	*OreG0010282*
scaffold31969	254819	A	C	Ore01,Ore02,Ore06,Ore08,Ore09,Ore10	*OreG0010282*
scaffold31969	260487	T	A	NA	*OreG0010283*
scaffold31969	263399	G	T	NA	*OreG0010283*
scaffold31969	268699	C	T	Ore01,Ore02,Ore03,Ore04,Ore05,Ore06,Ore07,Ore08,Ore09,Ore10,Ore11,Ore12,Ore13,Ore14	*OreG0010284*
scaffold31969	268916	T	C	Och03,Och12	*OreG0010284*
scaffold31969	269149	T	C	Och01,Och02,Och03,Och04,Och05,Och06,Och07,Och08,Och09,Och10,Och11,Och12,Och13,Och14	*OreG0010284*
scaffold31969	270824	C	T	Ore01,Ore02,Ore03,Ore04,Ore05,Ore06,Ore07,Ore08,Ore09,Ore10,Ore11,Ore12,Ore13,Ore14	*OreG0010284*
scaffold31969	275152	T	C	Ore01,Ore02,Ore03,Ore04,Ore05,Ore06,Ore07,Ore08,Ore09,Ore10,Ore11,Ore12,Ore13,Ore14	*OreG0010284*
scaffold31969	276297	A	G	Och03,Och08,Och09,Och10,Och11,Och12,Och14	*OreG0010284*
scaffold31969	278294	G	A	NA	*OreG0010285*
scaffold31969	283302	C	T	NA	*OreG0010286*
scaffold31969	283313	T	A	NA	*OreG0010286*
scaffold31969	307235	G	A	Och01,Och02,Och03,Och04,Och05,Och06,Och07,Och08,Och09,Och10,Och11,Och12,Och13,Och14,Ore01,Ore02,Ore03,Ore04,Ore05,Ore06,Ore07,Ore08,Ore09,Ore10,Ore11,Ore12,Ore13,Ore14	*OreG0010290*
scaffold31969	307242	T	G	NA	*OreG0010290*
scaffold31969	307722	G	T	Ore01,Ore02,Ore03,Ore04,Ore05,Ore06,Ore07,Ore08,Ore09,Ore10,Ore11,Ore12,Ore13,Ore14	*OreG0010290*
scaffold31969	307923	A	G	Och01,Och02,Och03,Och04,Och05,Och06,Och07,Och08,Och09,Och10,Och11,Och12,Och13,Och14,Ore01,Ore02,Ore03,Ore04,Ore05,Ore06,Ore07,Ore08,Ore09,Ore10,Ore11,Ore12,Ore13,Ore14	*OreG0010290*
scaffold31969	307931	G	T	NA	*OreG0010290*
scaffold31969	314954	A	T	Och02,Och03,Och04,Och05,Och06,Och07,Och08,Och09,Och10,Och11,Och12,Och13,Och14	*OreG0010291*
scaffold31969	314958	A	T	Ore01,Ore02,Ore03,Ore04,Ore05,Ore06,Ore07,Ore08,Ore09,Ore10,Ore11,Ore12,Ore13,Ore14	*OreG0010291*
scaffold31969	327433	A	T	Och01,Och09,Och10,Och11	*OreG0010293*
scaffold31969	327722	G	A	Ore01,Ore02,Ore03,Ore04,Ore05,Ore06,Ore07,Ore08,Ore09,Ore10,Ore11,Ore12,Ore13,Ore14	*OreG0010293*
scaffold31969	327900	G	A	NA	*OreG0010293*
scaffold31969	330242	T	G	Och02,Och04,Och05,Och13,Och14	*OreG0010293*
scaffold31969	330669	A	T	NA	*OreG0010293*
scaffold31969	340470	G	A	NA	*OreG0010294*
scaffold31969	377652	A	C	Och01,Och07	*OreG0010299*
scaffold31969	377771	G	C	Och01,Och02,Och03,Och04,Och05,Och06,Och07,Och08,Och09,Och10,Och11,Och12,Och13,Och14	*OreG0010299*
scaffold31969	377787	C	T	NA	*OreG0010299*
scaffold31969	397560	T	C	Ore01,Ore02,Ore03,Ore04,Ore05,Ore06,Ore07,Ore08,Ore09,Ore10,Ore11,Ore12,Ore13,Ore14	*OreG0010302*
scaffold31969	398263	C	A	Och08	*OreG0010302*
scaffold31969	411100	T	G	Och01	*OreG0010303*
scaffold31969	411630	C	T	Och01,Och02,Och03,Och04,Och05,Och06,Och07,Och08,Och09,Och10,Och11,Och12,Och13,Och14,Ore01,Ore02,Ore03,Ore04,Ore05,Ore06,Ore07,Ore08,Ore09,Ore10,Ore11,Ore12,Ore13,Ore14	*OreG0010303*
scaffold31969	412089	T	A	Ore01,Ore02,Ore06,Ore07,Ore08,Ore09,Ore10,Ore11,Ore12,Ore13	*OreG0010303*
scaffold31969	412193	T	C	Och02,Och03,Och04,Och05,Och06,Och07,Och12,Och13,Ore01,Ore02,Ore03,Ore04,Ore05,Ore06,Ore07,Ore08,Ore09,Ore10,Ore11,Ore12,Ore13,Ore14	*OreG0010303*
scaffold31969	413353	G	A	Ore01,Ore02,Ore06,Ore08,Ore09,Ore10	*OreG0010303*
scaffold31969	414328	T	G	NA	*OreG0010303*
scaffold31969	415862	T	C	NA	*OreG0010303*
scaffold31969	416238	A	G	Och06	*OreG0010303*
scaffold31969	422864	G	A	NA	*OreG0010304*
scaffold31969	431453	T	A	Och01,Och02,Och03,Och04,Och05,Och06,Och07,Och08,Och09,Och10,Och11,Och12,Och13,Och14	*OreG0010305*
scaffold31969	432879	G	A	NA	*OreG0010305*
scaffold31969	433070	C	T	NA	*OreG0010305*
scaffold31969	478300	C	A	NA	*OreG0010308*
scaffold31969	484914	G	A	Ore01,Ore02,Ore03,Ore04,Ore05,Ore06,Ore07,Ore08,Ore09,Ore10,Ore11,Ore12,Ore13,Ore14	*OreG0010309*
scaffold31969	487462	A	G	NA	*OreG0010310*
scaffold31969	493112	A	G	NA	*OreG0010311*
scaffold31969	502128	C	A	Ore01,Ore02,Ore06,Ore08,Ore09,Ore10	*OreG0010314*

scaffold31969	526246	T	G	Och01,Och02,Och03,Och04,Och05,Och06,Och07,Och08,Och09,Och10,Och11,Och12,Och13,Och14,Ore01,Ore02,Ore03,Ore04,Ore05,Ore06,Ore07,Ore08,Ore09,Ore10,Ore11,Ore12,Ore13,Ore14	*OreG0010316*
scaffold31969	526269	C	A	NA	*OreG0010316*
scaffold31969	540825	C	T	Och01,Och02,Och03,Och04,Och05,Och06,Och07,Och12,Och13	*OreG0010317*
scaffold31969	541523	A	T	NA	*OreG0010317*
scaffold31969	545078	T	C	NA	*OreG0010317*
scaffold31969	560635	A	T	NA	*OreG0010320*
scaffold31969	567870	T	A	Ore01,Ore02,Ore03,Ore04,Ore05,Ore06,Ore07,Ore08,Ore09,Ore10,Ore11,Ore12,Ore13,Ore14	*OreG0010321*
scaffold31969	568455	A	T	Och01,Och02,Och03,Och04,Och05,Och06,Och07,Och08,Och09,Och10,Och11,Och12,Och13,Och14,Ore01,Ore02,Ore03,Ore04,Ore05,Ore06,Ore07,Ore08,Ore09,Ore10,Ore11,Ore12,Ore13,Ore14	*OreG0010321*
scaffold31969	622611	C	T	NA	*OreG0010333*
scaffold31969	622626	A	T	Och07,Och08,Och14	*OreG0010333*
scaffold31969	627264	C	A	Och03,Och12	*OreG0010334*
scaffold31969	657920	C	A	NA	*OreG0010339*
scaffold31969	678663	G	T	Ore01,Ore02,Ore03,Ore04,Ore05,Ore06,Ore07,Ore08,Ore09,Ore10,Ore11,Ore12,Ore13,Ore14	*OreG0010344*
scaffold31969	679981	T	C	Och02,Och03,Och04,Och05,Och06,Och07,Och08,Och12,Och13	*OreG0010344*
scaffold31969	702186	T	A	Ore01,Ore02,Ore03,Ore04,Ore05,Ore06,Ore07,Ore08,Ore09,Ore10,Ore11,Ore12,Ore13,Ore14	*OreG0010346*
scaffold31969	724752	C	A	Ore01,Ore02,Ore03,Ore04,Ore05,Ore06,Ore08,Ore09,Ore10,Ore14	*OreG0010349*
scaffold31969	728037	C	T	Ore01,Ore02,Ore03,Ore04,Ore05,Ore06,Ore07,Ore08,Ore09,Ore10,Ore11,Ore12,Ore13,Ore14	*OreG0010350*
scaffold31969	728352	C	A	Och01,Och02,Och03,Och04,Och05,Och06,Och07,Och08,Och09,Och10,Och11,Och12,Och13,Och14,Ore01,Ore02,Ore03,Ore04,Ore05,Ore06,Ore07,Ore08,Ore09,Ore10,Ore11,Ore12,Ore13,Ore14	*OreG0010350*
scaffold31969	730283	T	C	NA	*OreG0010351*
scaffold31969	730532	A	T	NA	*OreG0010351*
scaffold31969	730577	C	A	NA	*OreG0010351*
scaffold31969	749113	G	T	NA	*OreG0010352*
scaffold31969	750922	G	A	NA	*OreG0010353*
scaffold31969	751360	A	G	NA	*OreG0010353*
scaffold31969	756716	C	T	Ore01,Ore02,Ore03,Ore04,Ore05,Ore06,Ore07,Ore08,Ore09,Ore10,Ore11,Ore12,Ore13,Ore14	*OreG0010353*
scaffold31969	759411	C	T	NA	*OreG0010353*
scaffold31969	768990	T	C	NA	*OreG0010355*
scaffold31969	770168	C	G	NA	*OreG0010355*
scaffold31969	777086	T	C	Och01,Och02,Och03,Och04,Och05,Och06,Och07,Och08,Och09,Och10,Och11,Och12,Och13,Och14,Ore01,Ore02,Ore03,Ore04,Ore05,Ore06,Ore07,Ore08,Ore09,Ore10,Ore11,Ore12,Ore13,Ore14	*OreG0010356*
scaffold31969	777142	T	G	Ore01,Ore02,Ore03,Ore04,Ore05,Ore06,Ore07,Ore08,Ore09,Ore10,Ore11,Ore12,Ore13,Ore14	*OreG0010356*
scaffold31969	777869	G	C	NA	*OreG0010356*
scaffold31969	779093	C	A	NA	*OreG0010356*
scaffold31969	780756	A	C	NA	*OreG0010356*
scaffold31969	860395	T	A	Och08	*OreG0010365*
scaffold31969	869670	G	C	NA	*OreG0010367*
scaffold31969	869690	A	G	NA	*OreG0010367*
scaffold31969	869894	A	G	Och01,Och07,Och08	*OreG0010367*
scaffold31969	900294	C	T	Ore01,Ore02,Ore03,Ore04,Ore05,Ore06,Ore07,Ore08,Ore09,Ore10,Ore11,Ore12,Ore13,Ore14	*OreG0010370*
scaffold31969	931378	G	A	NA	*OreG0010373*
scaffold31969	936540	A	T	NA	*OreG0010374*
scaffold31969	948402	T	C	Och13	*OreG0010376*
scaffold31969	949150	A	T	Och01,Och02,Och03,Och04,Och05,Och06,Och08,Och09,Och10,Och11,Och12,Och13,Och14	*OreG0010376*
scaffold31969	951172	A	C	Och13	*OreG0010376*
scaffold33138	8573	T	A	Och02,Och03,Och04,Och05,Och06,Och07,Och08,Och09,Och10,Och11,Och12,Och13,Och14,Ore01,Ore02,Ore03,Ore04,Ore05,Ore06,Ore07,Ore08,Ore09,Ore10,Ore11,Ore12,Ore13,Ore14	*OreG0013880*
scaffold33138	9623	G	T	Och02,Och03,Och04,Och05,Och06,Och07,Och08,Och09,Och10,Och11,Och12,Och13,Och14	*OreG0013880*
scaffold33111	9640	G	A	Ore01,Ore02,Ore03,Ore04,Ore05,Ore06,Ore07,Ore08,Ore09,Ore10,Ore11,Ore12,Ore13,Ore14	*OreG0012624*
scaffold33111	9730	G	A	Och01,Och02,Och03,Och04,Och05,Och06,Och07,Och08,Och09,Och10,Och11,Och12,Och13,Och14,Ore01,Ore02,Ore03,Ore04,Ore05,Ore06,Ore07,Ore08,Ore09,Ore10,Ore11,Ore12,Ore13,Ore14	*OreG0012624*
scaffold33111	9805	A	G	Ore02,Ore03,Ore09,Ore10,Ore14	*OreG0012624*
scaffold33111	9848	T	G	Ore01,Ore02,Ore03,Ore04,Ore05,Ore06,Ore07,Ore08,Ore09,Ore10,Ore11,Ore12,Ore13,Ore14	*OreG0012624*
scaffold33111	10788	T	A	NA	*OreG0012626*
scaffold33111	11330	A	C	NA	*OreG0012626*
scaffold33111	11338	T	C	Ore02,Ore03,Ore09,Ore10,Ore14	*OreG0012626*

scaffold33111	11389	C	T	Och02,Och03,Och04,Och05,Och06,Och12,Och13	*OreG0012626*
scaffold33111	11407	G	A	Och09,Och10,Och11,Ore01,Ore02,Ore03,Ore04,Ore05,Ore06,Ore07,Ore08,Ore09,Ore10,Ore11,Ore12,Ore13,Ore14	*OreG0012626*
scaffold33111	11409	G	A	Och09,Och10,Och11,Ore01,Ore02,Ore03,Ore04,Ore05,Ore06,Ore07,Ore08,Ore09,Ore10,Ore11,Ore12,Ore13,Ore14	*OreG0012626*
scaffold33111	11535	G	A	Och09,Och10,Och11,Ore01,Ore02,Ore03,Ore04,Ore05,Ore06,Ore07,Ore08,Ore09,Ore10,Ore11,Ore12,Ore13,Ore14	*OreG0012626*
scaffold33111	11811	T	C	Och09,Och10,Och11,Ore01,Ore02,Ore03,Ore04,Ore05,Ore06,Ore07,Ore08,Ore09,Ore10,Ore11,Ore12,Ore13,Ore14	*OreG0012626*
scaffold33111	12871	A	G	NA	*OreG0012625*
scaffold33111	40669	A	T	Och09,Och10,Och11	*OreG0012628*
scaffold33111	74334	G	A	NA	*OreG0012630*
scaffold33111	75687	C	T	Och09,Och10,Och11,Ore01,Ore02,Ore03,Ore04,Ore05,Ore06,Ore07,Ore08,Ore09,Ore10,Ore11,Ore12,Ore13,Ore14	*OreG0012630*
scaffold33111	85907	G	A	NA	*OreG0012631*
scaffold33111	116546	C	A	Ore01,Ore02,Ore03,Ore04,Ore05,Ore06,Ore07,Ore08,Ore09,Ore10,Ore11,Ore12,Ore13,Ore14	*OreG0012634*
scaffold33111	119237	C	T	Och13	*OreG0012634*
scaffold33111	127587	G	A	Ore04,Ore05	*OreG0012636*
scaffold33111	130018	A	C	NA	*OreG0012636*
scaffold33111	151258	T	A	Ore01,Ore02,Ore03,Ore04,Ore05,Ore06,Ore07,Ore08,Ore09,Ore10,Ore11,Ore12,Ore13,Ore14	*OreG0012639*
scaffold33111	153714	T	A	NA	*OreG0012639*
scaffold33111	153759	G	C	NA	*OreG0012639*
scaffold33111	154905	C	T	NA	*OreG0012639*
scaffold33111	155715	A	G	Ore02,Ore03,Ore09,Ore10,Ore14	*OreG0012639*
scaffold33111	160428	T	C	Och01,Och02,Och03,Och04,Och05,Och06,Och07,Och08,Och09,Och10,Och11,Och12,Och13,Och14,Ore01,Ore02,Ore03,Ore04,Ore05,Ore06,Ore07,Ore08,Ore09,Ore10,Ore11,Ore12,Ore13,Ore14	*OreG0012640*
scaffold33111	168537	C	T	Ore01,Ore02,Ore03,Ore04,Ore05,Ore06,Ore07,Ore08,Ore09,Ore10,Ore11,Ore12,Ore13,Ore14	*OreG0012641*
scaffold33111	171276	G	C	NA	*OreG0012642*
scaffold33111	186640	C	A	Och01,Och02,Och03,Och04,Och05,Och06,Och07,Och08,Och09,Och10,Och11,Och12,Och13,Och14,Ore01,Ore02,Ore03,Ore04,Ore05,Ore06,Ore07,Ore08,Ore09,Ore10,Ore11,Ore12,Ore13,Ore14	*OreG0012644*
scaffold33111	222126	C	A	Ore01,Ore02,Ore03,Ore04,Ore05,Ore06,Ore07,Ore08,Ore09,Ore10,Ore11,Ore12,Ore13,Ore14	*OreG0012647*
scaffold33111	250439	T	C	Och04	*OreG0012651*
scaffold33111	276592	T	A	NA	*OreG0012655*
scaffold33111	276958	C	A	Ore01,Ore02,Ore03,Ore04,Ore05,Ore06,Ore07,Ore08,Ore09,Ore10,Ore11,Ore12,Ore13,Ore14	*OreG0012655*
scaffold33111	298634	G	A	Och02,Och03,Och04,Och05,Och06,Och09,Och10,Och11,Och12,Och13,Och14	*OreG0012658*
scaffold33111	298637	C	T	Och09,Och10,Och11	*OreG0012658*
scaffold33111	298642	G	A	Och01,Och02,Och03,Och04,Och05,Och06,Och07,Och08,Och09,Och10,Och11,Och12,Och13,Och14,Ore01,Ore02,Ore03,Ore04,Ore05,Ore06,Ore07,Ore08,Ore09,Ore10,Ore11,Ore12,Ore13,Ore14	*OreG0012658*
scaffold33111	313648	A	C	Och03,Och09,Och10,Och11,Och13,Och14	*OreG0012660*
scaffold33111	365522	C	T	NA	*OreG0012665*
scaffold33111	418614	G	C	Och01,Och02,Och03,Och04,Och05,Och06,Och07,Och08,Och09,Och10,Och11,Och12,Och13,Och14	*OreG0012670*
scaffold33111	429237	T	G	NA	*OreG0012671*
scaffold33111	433309	G	A	NA	*OreG0012671*
scaffold33111	459951	A	C	NA	*OreG0012675*
scaffold33111	467707	C	T	Och01,Och02,Och03,Och04,Och05,Och06,Och07,Och08,Och09,Och10,Och11,Och12,Och13,Och14,Ore01,Ore02,Ore03,Ore04,Ore05,Ore06,Ore07,Ore08,Ore09,Ore10,Ore11,Ore12,Ore13,Ore14	*OreG0012677*
scaffold33111	510803	G	C	NA	*OreG0012679*
scaffold33111	528340	T	C	Och01	*OreG0012681*
scaffold33111	528672	G	A	NA	*OreG0012681*
scaffold33111	528840	C	T	Och07,Och13	*OreG0012681*
scaffold33111	529065	C	T	Och13	*OreG0012681*
scaffold33111	540094	G	T	Och01,Och02,Och03,Och04,Och05,Och06,Och07,Och08,Och09,Och10,Och11,Och12,Och13,Och14,Ore01,Ore02,Ore03,Ore04,Ore05,Ore06,Ore07,Ore08,Ore09,Ore10,Ore11,Ore12,Ore13,Ore14	*OreG0012683*
scaffold33111	557892	G	A	Och01,Och02,Och03,Och04,Och05,Och06,Och07,Och08,Och09,Och10,Och11,Och12,Och13,Och14	*OreG0012685*
scaffold33111	601469	C	A	Ore01,Ore02,Ore03,Ore04,Ore05,Ore06,Ore07,Ore08,Ore09,Ore10,Ore11,Ore12,Ore13,Ore14	*OreG0012690*
scaffold33111	601951	C	T	Och01,Och02,Och03,Och04,Och05,Och06,Och07,Och08,Och09,Och10,Och11,Och12,Och13,Och14	*OreG0012690*
scaffold33111	620764	G	A	NA	*OreG0012692*
scaffold33111	627909	G	A	Ore01,Ore02,Ore03,Ore04,Ore05,Ore06,Ore07,Ore08,Ore09,Ore10,Ore11,Ore12,Ore13,Ore14	*OreG0012694*
scaffold33111	628024	A	C	Och01,Och02,Och03,Och04,Och05,Och06,Och07,Och08,Och09,Och10,Och11,Och12,Och13,Och14	*OreG0012694*
scaffold33111	628606	A	G	NA	*OreG0012694*

scaffold33122	111280	G	T	NA	*OreG0013562*
scaffold31865	13057	C	T	Och01,Och02,Och03,Och04,Och05,Och06,Och07,Och08,Och09,Och10,Och11,Och12,Och13,Och14	*OreG0009877*
scaffold31865	13735	T	A	Ore01,Ore02,Ore03,Ore04,Ore05,Ore06,Ore07,Ore08,Ore09,Ore10,Ore11,Ore12,Ore13,Ore14	*OreG0009877*
scaffold31865	14010	C	T	Ore01,Ore02,Ore03,Ore04,Ore05,Ore06,Ore07,Ore08,Ore09,Ore10,Ore11,Ore12,Ore13,Ore14	*OreG0009877*
scaffold31865	14446	C	T	NA	*OreG0009877*
scaffold31865	14805	G	A	NA	*OreG0009877*
scaffold31865	14929	C	T	Ore01,Ore02,Ore03,Ore04,Ore05,Ore06,Ore07,Ore08,Ore09,Ore10,Ore11,Ore12,Ore13,Ore14	*OreG0009877*
scaffold31865	14941	T	C	NA	*OreG0009877*
scaffold31865	312859	A	G	NA	*OreG0009884*
scaffold31865	337837	G	T	Ore01,Ore02,Ore03,Ore04,Ore05,Ore06,Ore07,Ore08,Ore09,Ore10,Ore11,Ore12,Ore13,Ore14	*OreG0009888*
scaffold31865	606616	T	A	NA	*OreG0009896*
scaffold31865	642002	T	A	Och06,Och07,Och08,Och14	*OreG0009898*
scaffold31865	643822	G	A	Och01,Och02,Och03,Och04,Och05,Och06,Och07,Och08,Och09,Och10,Och11,Och12,Och13,Och14,Ore01,Ore02,Ore03,Ore04,Ore05,Ore06,Ore07,Ore08,Ore09,Ore10,Ore11,Ore12,Ore13,Ore14	*OreG0009898*
scaffold31865	662776	C	T	NA	*OreG0009898*
scaffold31865	708557	C	T	NA	*OreG0009899*
scaffold31865	710882	G	T	Och01,Och06,Och07,Och08,Och09,Och10,Och11,Och14	*OreG0009899*
scaffold31865	711707	A	G	Och01,Och02,Och03,Och04,Och05,Och06,Och07,Och08,Och09,Och10,Och11,Och12,Och13,Och14,Ore01,Ore02,Ore03,Ore04,Ore05,Ore06,Ore07,Ore08,Ore09,Ore10,Ore11,Ore12,Ore13,Ore14	*OreG0009899*
scaffold31865	723585	T	C	NA	*OreG0009900*
scaffold31865	743544	T	A	Och01,Och02,Och03,Och04,Och05,Och06,Och07,Och08,Och09,Och10,Och11,Och12,Och13,Och14	*OreG0009901*
scaffold31865	745446	T	G	Och02,Och03,Och11,Och12,Och13,Och14	*OreG0009901*
scaffold31865	746061	G	A	NA	*OreG0009901*
scaffold31583	11775	T	G	NA	*OreG0009435*
scaffold31583	23799	G	T	NA	*OreG0009436*
scaffold31583	37194	G	A	NA	*OreG0009437*
scaffold31583	37285	C	G	NA	*OreG0009437*
scaffold31583	37304	G	A	NA	*OreG0009437*
scaffold31583	37621	G	A	NA	*OreG0009437*
scaffold31583	37630	A	T	NA	*OreG0009437*
scaffold31583	85384	A	G	NA	*OreG0009443*
scaffold31583	113054	G	C	NA	*OreG0009447*
scaffold31583	113583	G	T	NA	*OreG0009447*
scaffold31583	125920	A	T	NA	*OreG0009449*
scaffold31583	127855	G	C	NA	*OreG0009449*
scaffold31583	164155	A	T	NA	*OreG0009454*
scaffold31583	194222	C	G	Och01,Och02,Och03,Och04,Och05,Och06,Och07,Och08,Och09,Och10,Och11,Och12,Och13,Och14,Ore01,Ore02,Ore03,Ore04,Ore05,Ore06,Ore07,Ore08,Ore09,Ore10,Ore11,Ore12,Ore13,Ore14	*OreG0009457*
scaffold31583	194992	A	T	Ore01,Ore02,Ore03,Ore04,Ore05,Ore06,Ore07,Ore08,Ore09,Ore10,Ore11,Ore12,Ore13,Ore14	*OreG0009457*
scaffold31583	195052	A	G	Ore01,Ore02,Ore03,Ore04,Ore05,Ore06,Ore07,Ore08,Ore09,Ore10,Ore11,Ore12,Ore13,Ore14	*OreG0009457*
scaffold31583	195337	C	T	NA	*OreG0009457*
scaffold31583	195630	G	T	Och09,Och10	*OreG0009457*
scaffold31583	196358	C	G	Och01,Och02,Och03,Och04,Och05,Och06,Och07,Och08,Och09,Och10,Och11,Och12,Och13,Och14,Ore01,Ore02,Ore03,Ore04,Ore05,Ore06,Ore07,Ore08,Ore09,Ore10,Ore11,Ore12,Ore13,Ore14	*OreG0009457*
scaffold31583	197502	G	C	Och04,Och05,Och12,Och13	*OreG0009458*
scaffold31583	210970	G	A	Ore06,Ore07,Ore09,Ore10,Ore11,Ore13,Ore14	*OreG0009460*
scaffold31583	211141	T	A	Ore01,Ore02,Ore03,Ore04,Ore05,Ore06,Ore07,Ore08,Ore09,Ore10,Ore11,Ore12,Ore13,Ore14	*OreG0009460*
scaffold31583	214214	A	G	Och04,Och05,Och07,Och08,Och12,Och13	*OreG0009460*
scaffold31583	242951	G	C	NA	*OreG0009463*
scaffold31583	258310	C	T	NA	*OreG0009464*
scaffold31583	271784	C	A	Och01,Och02,Och03,Och04,Och05,Och06,Och07,Och08,Och09,Och10,Och11,Och12,Och13	*OreG0009465*
scaffold31583	271911	T	A	Och02,Och06,Och07,Och08,Och14	*OreG0009465*
scaffold31583	272168	G	C	NA	*OreG0009465*
scaffold31583	273652	A	G	NA	*OreG0009465*
scaffold31583	281984	C	T	NA	*OreG0009467*
scaffold31583	298912	G	C	Och01,Och02,Och03,Och04,Och05,Och06,Och07,Och08,Och09,Och10,Och11,Och12,Och13,Och14,Ore01,Ore02,Ore03,Ore04,Ore05,Ore06,Ore07,Ore08,Ore09,Ore10,Ore11,Ore12,Ore13,Ore14	*OreG0009472*
scaffold31583	303192	G	A	NA	*OreG0009473*
scaffold31583	310181	C	A	Och01,Och02,Och06,Och07,Och08,Och09,Och10,Och11,Och14	*OreG0009474*

scaffold31583	332562	T	A	Ore01,Ore02,Ore03,Ore04,Ore05,Ore06,Ore07,Ore08,Ore09,Ore10,Ore11,Ore12,Ore13,Ore14	*OreG0009476*
scaffold31583	333769	C	T	NA	*OreG0009476*
scaffold31583	337482	C	T	Och01,Och02,Och03,Och04,Och05,Och06,Och11,Och12,Och13,Och14	*OreG0009477*
scaffold31583	342084	T	C	NA	*OreG0009478*
scaffold31583	346325	C	T	NA	*OreG0009478*
scaffold31583	346602	G	C	NA	*OreG0009478*
scaffold31583	346604	T	A	NA	*OreG0009478*
scaffold31583	380982	C	A	Ore01,Ore02,Ore03,Ore04,Ore05,Ore06,Ore07,Ore08,Ore09,Ore10,Ore11,Ore12,Ore13,Ore14	*OreG0009482*
scaffold31583	385381	C	A	Ore01,Ore02,Ore03,Ore04,Ore05,Ore06,Ore07,Ore08,Ore09,Ore10,Ore11,Ore12,Ore13,Ore14	*OreG0009484*
scaffold31583	390349	G	T	Ore01,Ore02,Ore03,Ore04,Ore05,Ore06,Ore07,Ore08,Ore09,Ore10,Ore11,Ore12,Ore13,Ore14	*OreG0009483*
scaffold31583	411347	G	A	NA	*OreG0009487*
scaffold31583	411542	C	T	Ore01,Ore02,Ore03,Ore04,Ore05,Ore06,Ore07,Ore08,Ore09,Ore10,Ore11,Ore12,Ore13,Ore14	*OreG0009487*
scaffold31583	477552	A	G	NA	*OreG0009492*
scaffold31583	490445	G	T	Ore06,Ore07,Ore09,Ore10,Ore11,Ore13,Ore14	*OreG0009495*
scaffold31583	492069	C	T	Och01,Och02,Och03,Och04,Och05,Och06,Och07,Och08,Och09,Och10,Och11,Och12,Och13,Och14,Ore01,Ore02,Ore03,Ore04,Ore05,Ore06,Ore07,Ore08,Ore09,Ore10,Ore11,Ore12,Ore13,Ore14	*OreG0009495*
scaffold31583	494522	G	A	NA	*OreG0009496*
scaffold31583	503247	G	A	Och01,Och02,Och03,Och04,Och05,Och06,Och08,Och12,Och13,Och14,Ore01,Ore02,Ore03,Ore04,Ore05,Ore06,Ore07,Ore08,Ore09,Ore10,Ore11,Ore12,Ore13,Ore14	*OreG0009498*
scaffold33124	130037	C	G	Och01,Och02,Och03,Och04,Och05,Och06,Och07,Och08,Och12,Och13,Och14	*OreG0013576*
scaffold33124	201105	G	A	Ore01,Ore02,Ore03,Ore04,Ore05,Ore06,Ore07,Ore08,Ore09,Ore10,Ore11,Ore12,Ore13,Ore14	*OreG0013582*
scaffold33124	217488	A	C	NA	*OreG0013584*
scaffold33124	217512	A	G	NA	*OreG0013584*
scaffold33124	218338	T	C	NA	*OreG0013584*
scaffold33124	218650	C	T	Och12,Och13	*OreG0013584*
scaffold33124	221700	T	C	Ore01,Ore02,Ore03,Ore04,Ore05,Ore06,Ore07,Ore08,Ore09,Ore10,Ore11,Ore12,Ore13,Ore14	*OreG0013585*
scaffold33124	262126	T	C	NA	*OreG0013589*
scaffold33124	264042	C	A	NA	*OreG0013589*
scaffold33124	272209	G	A	NA	*OreG0013590*
scaffold33124	272386	T	C	Ore01,Ore02,Ore03,Ore04,Ore05,Ore06,Ore07,Ore08,Ore09,Ore10,Ore11,Ore12,Ore13,Ore14	*OreG0013590*
scaffold33124	414628	C	T	NA	*OreG0013598*
scaffold33124	417070	C	T	Ore01,Ore04,Ore05,Ore11	*OreG0013598*
scaffold33124	417072	G	A	NA	*OreG0013598*
scaffold33124	420182	G	C	Och03	*OreG0013599*
scaffold31965	78339	T	A	NA	*OreG0010228*
scaffold31965	125163	G	A	Och01,Och02,Och03,Och04,Och05,Och06,Och07,Och08,Och09,Och10,Och11,Och12,Och13,Och14	*OreG0010229*
scaffold31965	240070	C	A	Och02,Och03,Och04,Och05,Och06,Och08,Och09,Och10,Och11,Och12,Och13	*OreG0010233*
scaffold31965	467733	C	T	NA	*OreG0010238*
scaffold31965	467880	C	A	NA	*OreG0010238*
scaffold31965	468104	G	A	Och01,Och02,Och03,Och04,Och05,Och06,Och07,Och08,Och09,Och10,Och11,Och12,Och13,Och14,Ore01,Ore02,Ore03,Ore04,Ore05,Ore06,Ore07,Ore08,Ore09,Ore10,Ore11,Ore12,Ore13,Ore14	*OreG0010238*
scaffold31965	500278	G	A	Och03,Och05,Och09,Och10,Och12,Och13	*OreG0010241*
scaffold31965	506333	C	T	Ore08,Ore09	*OreG0010241*
scaffold31965	506434	A	G	Och03,Och05,Och07,Och09,Och10,Och12,Och13	*OreG0010241*
scaffold31965	552500	A	T	Och01,Och02,Och03,Och04,Och05,Och06,Och07,Och08,Och09,Och10,Och11,Och12,Och13,Och14,Ore01,Ore02,Ore03,Ore04,Ore05,Ore06,Ore07,Ore08,Ore09,Ore10,Ore11,Ore12,Ore13,Ore14	*OreG0010244*
scaffold31965	553434	G	C	Och01,Och02,Och03,Och04,Och05,Och06,Och07,Och08,Och09,Och10,Och11,Och12,Och13,Och14	*OreG0010244*
scaffold31965	615034	T	C	Och02,Och03,Och04,Och05,Och06,Och08,Och09,Och10,Och12,Och13,Och14	*OreG0010245*
scaffold31965	674439	C	T	Ore01,Ore02,Ore03,Ore04,Ore05,Ore06,Ore07,Ore08,Ore09,Ore10,Ore11,Ore12,Ore13,Ore14	*OreG0010249*
scaffold33112	135614	T	C	Och01,Och02,Och03,Och04,Och05,Och06,Och07,Och08,Och09,Och10,Och11,Och12,Och13,Och14,Ore01,Ore02,Ore03,Ore04,Ore05,Ore06,Ore07,Ore08,Ore09,Ore10,Ore11,Ore12,Ore13,Ore14	*OreG0012700*
scaffold33112	273612	G	A	NA	*OreG0012704*
scaffold33112	278969	G	A	NA	*OreG0012704*
scaffold33112	385166	T	C	Och01,Och02,Och03,Och04,Och05,Och06,Och07,Och08,Och09,Och10,Och11,Och12,Och13,Och14,Ore01,Ore02,Ore03,Ore04,Ore05,Ore06,Ore07,Ore08,Ore09,Ore10,Ore11,Ore12,Ore13,Ore14	*OreG0012706*
scaffold33112	416685	T	A	NA	*OreG0012710*
scaffold33112	416886	T	G	NA	*OreG0012710*
scaffold33112	417471	T	C	NA	*OreG0012710*
scaffold33112	417478	T	A	NA	*OreG0012710*

scaffold33112	417558	G	T	NA	*OreG0012710*
scaffold31095	98100	G	A	NA	*OreG0009385*
scaffold31095	116767	A	G	Och01,Och12,Och13	*OreG0009386*
scaffold31095	116787	T	G	NA	*OreG0009386*
scaffold31095	116788	C	T	Och03,Och04,Och07,Och14	*OreG0009386*
scaffold31095	117751	A	G	Ore01,Ore02,Ore03,Ore04,Ore05,Ore06,Ore07,Ore08,Ore09,Ore10,Ore11,Ore12,Ore13,Ore14	*OreG0009386*
scaffold31095	117870	G	T	Och01,Och12,Och13	*OreG0009386*
scaffold31095	119946	A	G	Och03,Och04,Och07,Och14	*OreG0009386*
scaffold31095	120035	A	G	Och01,Och12,Och13,Ore01,Ore02,Ore03,Ore04,Ore05,Ore06,Ore07,Ore08,Ore09,Ore10,Ore11,Ore12,Ore13,Ore14	*OreG0009386*
scaffold31095	126162	C	T	Ore01,Ore02,Ore03,Ore04,Ore05,Ore06,Ore07,Ore08,Ore09,Ore10,Ore11,Ore12,Ore13,Ore14	*OreG0009387*
scaffold31095	138105	C	T	Ore01,Ore02,Ore03,Ore04,Ore05,Ore06,Ore07,Ore08,Ore09,Ore10,Ore11,Ore12,Ore13,Ore14	*OreG0009388*
scaffold31095	138289	C	T	NA	*OreG0009388*
scaffold31095	138304	G	A	NA	*OreG0009388*
scaffold31095	138352	C	T	Ore01,Ore02,Ore03,Ore04,Ore05,Ore06,Ore07,Ore08,Ore09,Ore10,Ore11,Ore12,Ore13,Ore14	*OreG0009388*
scaffold31095	212906	A	C	NA	*OreG0009392*
scaffold31095	258213	G	A	Och01,Och02,Och03,Och04,Och05,Och06,Och07,Och08,Och09,Och10,Och11,Och12,Och13,Och14,Ore01,Ore02,Ore03,Ore04,Ore05,Ore06,Ore07,Ore08,Ore09,Ore10,Ore11,Ore12,Ore13,Ore14	*OreG0009394*
scaffold31095	293220	C	T	Och05,Och12,Och13	*OreG0009397*
scaffold31095	338922	G	A	NA	*OreG0009399*
scaffold31095	340570	C	T	NA	*OreG0009399*
scaffold31095	348249	T	C	Och01,Och02,Och03,Och04,Och05,Och06,Och07,Och08,Och09,Och10,Och11,Och12,Och13,Och14,Ore01,Ore02,Ore03,Ore04,Ore05,Ore06,Ore07,Ore08,Ore09,Ore10,Ore11,Ore12,Ore13,Ore14	*OreG0009399*
scaffold31095	349176	C	A	NA	*OreG0009399*
scaffold31095	349286	T	C	NA	*OreG0009399*
scaffold31095	349349	T	C	NA	*OreG0009399*
scaffold33166	786	A	G	Ore01,Ore02,Ore03,Ore04,Ore05,Ore06,Ore07,Ore08,Ore09,Ore10,Ore11,Ore12,Ore13,Ore14	*OreG0014347*
scaffold33172	578	C	T	Och01,Och02,Och03,Och04,Och05,Och06,Och07,Och08,Och09,Och10,Och11,Och12,Och13,Och14	*OreG0014360*
scaffold33130	86416	G	A	Ore01,Ore02,Ore03,Ore04,Ore05,Ore06,Ore07,Ore08,Ore09,Ore10,Ore11,Ore12,Ore13,Ore14	*OreG0013782*
scaffold33130	94829	G	A	NA	*OreG0013784*
scaffold33130	94979	G	A	Och11	*OreG0013784*
scaffold33130	95151	G	A	Och01,Och02,Och03,Och04,Och05,Och06,Och07,Och09,Och10,Och11,Och12,Och13,Och14	*OreG0013784*
scaffold33130	95338	C	T	NA	*OreG0013784*
scaffold33130	95420	C	T	NA	*OreG0013784*
scaffold33130	95437	C	T	Och01,Och02,Och03,Och04,Och05,Och06,Och07,Och09,Och10,Och11,Och12,Och13,Och14	*OreG0013784*
scaffold33130	95648	C	T	NA	*OreG0013784*
scaffold33130	95795	A	T	Och11	*OreG0013784*
scaffold33130	95813	A	C	Och01,Och02,Och03,Och04,Och05,Och06,Och07,Och09,Och10,Och11,Och12,Och13,Och14	*OreG0013784*
scaffold33130	96791	C	A	NA	*OreG0013784*
scaffold33130	96827	C	T	Och01,Och02,Och03,Och04,Och05,Och06,Och07,Och09,Och10,Och11,Och12,Och13,Och14	*OreG0013784*
scaffold33130	97105	G	A	Och01,Och02,Och03,Och04,Och05,Och06,Och07,Och09,Och10,Och11,Och12,Och13,Och14	*OreG0013784*
scaffold33130	104619	T	C	NA	*OreG0013785*
scaffold33130	106945	A	G	NA	*OreG0013785*
scaffold33130	111590	G	A	Ore01,Ore02,Ore03,Ore04,Ore05,Ore06,Ore07,Ore08,Ore09,Ore10,Ore11,Ore12,Ore13,Ore14	*OreG0013786*
scaffold33130	111631	A	T	NA	*OreG0013786*
scaffold33130	111699	T	C	Och01,Och02,Och03,Och04,Och05,Och06,Och07,Och08,Och09,Och10,Och11,Och12,Och13,Och14,Ore01,Ore02,Ore03,Ore04,Ore05,Ore06,Ore07,Ore08,Ore09,Ore10,Ore11,Ore12,Ore13,Ore14	*OreG0013786*
scaffold33130	111809	A	C	Och01,Och02,Och03,Och04,Och05,Och06,Och07,Och08,Och09,Och10,Och11,Och12,Och13,Och14,Ore01,Ore02,Ore03,Ore04,Ore05,Ore06,Ore07,Ore08,Ore09,Ore10,Ore11,Ore12,Ore13,Ore14	*OreG0013786*
scaffold33130	111863	T	C	Och01,Och02,Och03,Och04,Och05,Och06,Och07,Och08,Och09,Och10,Och11,Och12,Och13,Och14,Ore01,Ore02,Ore03,Ore04,Ore05,Ore06,Ore07,Ore08,Ore09,Ore10,Ore11,Ore12,Ore13,Ore14	*OreG0013786*
scaffold33130	111875	A	G	Och01,Och02,Och03,Och04,Och05,Och06,Och07,Och08,Och09,Och10,Och11,Och12,Och13,Och14,Ore01,Ore02,Ore03,Ore04,Ore05,Ore06,Ore07,Ore08,Ore09,Ore10,Ore11,Ore12,Ore13,Ore14	*OreG0013786*
scaffold33130	112064	A	T	Och01,Och02,Och03,Och04,Och05,Och06,Och07,Och08,Och09,Och10,Och11,Och12,Och13,Och14	*OreG0013786*
scaffold33143	16630	A	C	Och01,Och02,Och03,Och04,Och05,Och06,Och07,Och08,Och09,Och10,Och11,Och12,Och13,Och14	*OreG0013994*

scaffold33143	120290	A	T	Och01,Och02,Och03,Och04,Och05,Och06,Och07,Och08,Och09,Och10,Och11,Och12,Och13,Och14	*OreG0014000*
scaffold33143	122638	G	A	Och14	*OreG0014000*
scaffold33143	152049	C	T	Och12,Och13	*OreG0014001*
scaffold33143	152210	C	T	NA	*OreG0014001*
scaffold33143	152672	C	G	NA	*OreG0014001*
scaffold33143	250669	T	A	NA	*OreG0014002*
scaffold33186	11978	A	C	Och01,Ore01,Ore02,Ore06,Ore07,Ore08,Ore09,Ore11,Ore12,Ore13,Ore14	*OreG0014707*
scaffold33186	17839	T	C	Och02,Och04,Och05,Och08,Och12,Och13,Och14	*OreG0014708*
scaffold33100	57397	A	T	NA	*OreG0011479*
scaffold33100	57726	T	C	Ore07,Ore10,Ore13,Ore14	*OreG0011479*
scaffold33100	84597	G	A	NA	*OreG0011481*
scaffold33100	84683	C	T	Och01,Och05,Och09,Och10,Och11,Och14	*OreG0011481*
scaffold33100	85435	G	A	Och05	*OreG0011481*
scaffold33100	85666	T	A	NA	*OreG0011481*
scaffold33100	88467	C	T	Och01,Och05,Och09,Och10,Och11,Och14	*OreG0011481*
scaffold33100	93275	T	C	Och02,Och06,Och13	*OreG0011482*
scaffold33100	94369	A	G	Ore01,Ore07,Ore10,Ore13,Ore14	*OreG0011482*
scaffold33100	95884	A	G	Och01	*OreG0011483*
scaffold33100	96507	G	T	Och09,Och10,Och14	*OreG0011483*
scaffold33100	96665	G	T	NA	*OreG0011483*
scaffold33100	135064	G	A	NA	*OreG0011489*
scaffold33100	135222	A	G	Och02,Och06,Och13,Ore07,Ore10,Ore13,Ore14	*OreG0011489*
scaffold33100	135451	G	A	Och02,Och06,Och13	*OreG0011489*
scaffold33100	136071	C	T	NA	*OreG0011489*
scaffold33100	136288	G	A	NA	*OreG0011489*
scaffold33100	136842	A	G	Och05	*OreG0011489*
scaffold33100	146352	T	A	Och02,Och03,Och04,Och05,Och06,Och12,Och13	*OreG0011491*
scaffold33100	159492	A	G	NA	*OreG0011493*
scaffold33100	161339	C	A	NA	*OreG0011493*
scaffold33100	161800	G	A	NA	*OreG0011493*
scaffold33100	166569	C	G	NA	*OreG0011493*
scaffold33100	166732	A	G	NA	*OreG0011493*
scaffold33100	186618	T	G	NA	*OreG0011497*
scaffold33100	187593	A	C	NA	*OreG0011497*
scaffold33100	190434	A	T	NA	*OreG0011498*
scaffold33100	190464	A	G	Och01,Och09,Och10,Och14	*OreG0011498*
scaffold33100	190610	T	A	NA	*OreG0011498*
scaffold33100	190744	T	G	NA	*OreG0011498*
scaffold33100	191008	C	A	NA	*OreG0011498*
scaffold33100	191097	A	G	NA	*OreG0011498*
scaffold33100	191185	T	C	NA	*OreG0011498*
scaffold33100	191200	A	G	NA	*OreG0011498*
scaffold33100	195031	G	T	NA	*OreG0011499*
scaffold33100	195080	G	T	NA	*OreG0011499*
scaffold33100	195113	G	T	NA	*OreG0011499*
scaffold33100	195145	A	T	NA	*OreG0011499*
scaffold33100	195154	A	G	NA	*OreG0011499*
scaffold33100	196022	G	C	NA	*OreG0011499*
scaffold33100	196028	A	G	NA	*OreG0011499*
scaffold33100	196055	C	G	NA	*OreG0011499*
scaffold33100	196070	G	T	NA	*OreG0011499*
scaffold33100	196080	A	T	NA	*OreG0011499*
scaffold33100	196089	T	C	NA	*OreG0011499*
scaffold33100	196100	G	A	NA	*OreG0011499*
scaffold33100	196109	A	G	NA	*OreG0011499*
scaffold33100	196623	C	T	NA	*OreG0011499*
scaffold33100	196710	C	G	NA	*OreG0011499*
scaffold33100	198497	T	C	NA	*OreG0011499*
scaffold33100	198946	A	T	NA	*OreG0011499*
scaffold33100	203492	G	A	Och01,Och02,Och03,Och04,Och05,Och06,Och07,Och08,Och09,Och10,Och11,Och12,Och13,Och14,Ore01,Ore02,Ore03,Ore04,Ore05,Ore06,Ore07,Ore08,Ore09,Ore10,Ore11,Ore12,Ore13,Ore14	*OreG0011499*
scaffold33100	244717	G	T	NA	*OreG0011506*
scaffold33100	245002	A	G	NA	*OreG0011506*
scaffold33100	250276	C	T	Och01,Och02,Och03,Och04,Och05,Och06,Och07,Och08,Och09,Och10,Och11,Och12,Och13,Och14,Ore01,Ore02,Ore03,Ore04,Ore05,Ore06,Ore07,Ore08,Ore09,Ore10,Ore11,Ore12,Ore13,Ore14	*OreG0011507*
scaffold33100	253069	T	A	NA	*OreG0011508*
scaffold33100	253173	T	G	NA	*OreG0011508*
scaffold33100	267628	T	A	Och02,Och03,Och04,Och05,Och06,Och07,Och08,Och09,Och10,Och11,Och12,Och13,Och14	*OreG0011510*
scaffold33100	270132	C	A	Ore01,Ore02,Ore03,Ore04,Ore05,Ore06,Ore07,Ore08,Ore09,Ore10,Ore11,Ore12,Ore13,Ore14	*OreG0011511*

scaffold33100	288362	A	T	Ore01,Ore02,Ore03,Ore04,Ore05,Ore06,Ore07,Ore08,Ore09,Ore10,Ore11,Ore12,Ore13,Ore14	*OreG0011514*
scaffold33100	290881	A	G	Ore01,Ore02,Ore03,Ore04,Ore05,Ore06,Ore07,Ore08,Ore09,Ore10,Ore11,Ore12,Ore13,Ore14	*OreG0011515*
scaffold33100	291215	A	C	Och02,Och03,Och04,Och05,Och06,Och08,Och09,Och10,Och11,Och12,Och13,Ore01,Ore02,Ore03,Ore04,Ore05,Ore06,Ore07,Ore08,Ore09,Ore10,Ore11,Ore12,Ore13,Ore14	*OreG0011515*
scaffold33100	291781	G	T	NA	*OreG0011515*
scaffold33100	291906	G	C	NA	*OreG0011515*
scaffold33100	291972	C	G	Och02,Och06,Och13	*OreG0011515*
scaffold33100	292018	A	G	NA	*OreG0011515*
scaffold33100	292063	C	T	Ore07,Ore10,Ore13,Ore14	*OreG0011515*
scaffold33100	293570	C	G	Och05	*OreG0011516*
scaffold33100	298761	T	C	Ore01,Ore07,Ore10,Ore13,Ore14	*OreG0011516*
scaffold33100	300081	C	A	NA	*OreG0011516*
scaffold33100	300098	G	T	Och07	*OreG0011516*
scaffold33100	313507	T	A	Ore01,Ore02,Ore03,Ore04,Ore05,Ore06,Ore07,Ore08,Ore09,Ore10,Ore11,Ore12,Ore13,Ore14	*OreG0011520*
scaffold33100	322704	G	C	Ore05	*OreG0011521*
scaffold33100	323914	C	T	Och01,Och02,Och03,Och04,Och05,Och06,Och07,Och08,Och09,Och10,Och11,Och12,Och13,Och14,Ore01,Ore02,Ore03,Ore04,Ore05,Ore06,Ore07,Ore08,Ore09,Ore10,Ore11,Ore12,Ore13,Ore14	*OreG0011521*
scaffold33100	338637	T	C	NA	*OreG0011524*
scaffold33100	342263	T	G	Och01,Och02,Och03,Och04,Och05,Och06,Och07,Och08,Och09,Och10,Och11,Och12,Och13,Och14,Ore01,Ore02,Ore03,Ore04,Ore05,Ore06,Ore07,Ore08,Ore09,Ore10,Ore11,Ore12,Ore13,Ore14	*OreG0011525*
scaffold33100	352180	C	G	Och01	*OreG0011526*
scaffold33100	354268	A	G	NA	*OreG0011527*
scaffold33100	354277	G	A	Och01	*OreG0011527*
scaffold33100	378182	G	A	NA	*OreG0011536*
scaffold33100	378224	G	T	Och01,Och02,Och03,Och04,Och05,Och06,Och07,Och08,Och09,Och10,Och11,Och12,Och13,Ore01,Ore02,Ore03,Ore04,Ore05,Ore06,Ore07,Ore08,Ore09,Ore10,Ore11,Ore12,Ore13,Ore14	*OreG0011536*
scaffold33100	378246	T	A	Och01,Och02,Och03,Och04,Och05,Och06,Och07,Och08,Och09,Och10,Och11,Och12,Och13,Ore01,Ore02,Ore03,Ore04,Ore05,Ore06,Ore07,Ore08,Ore09,Ore10,Ore11,Ore12,Ore13,Ore14	*OreG0011536*
scaffold33100	378270	C	T	NA	*OreG0011536*
scaffold33100	388868	G	A	Ore01,Ore07,Ore10,Ore13,Ore14	*OreG0011540*
scaffold33100	409351	A	C	NA	*OreG0011544*
scaffold33100	409698	C	A	Och02,Och03,Och04,Och05,Och06,Och07,Och08,Och09,Och10,Och11,Och12,Och13,Ore01,Ore02,Ore03,Ore04,Ore05,Ore06,Ore07,Ore08,Ore09,Ore10,Ore11,Ore12,Ore13,Ore14	*OreG0011544*
scaffold33100	410681	A	C	NA	*OreG0011544*
scaffold33100	416099	C	T	NA	*OreG0011545*
scaffold33100	416123	C	T	NA	*OreG0011545*
scaffold33100	416155	G	A	Ore01,Ore02,Ore03,Ore04,Ore05,Ore06,Ore07,Ore08,Ore09,Ore10,Ore11,Ore12,Ore13,Ore14	*OreG0011545*
scaffold33100	418137	A	G	Och02,Och03,Och04,Och05,Och06,Och12,Och13	*OreG0011545*
scaffold33100	433682	G	A	Och01	*OreG0011548*
scaffold33100	436422	G	T	Och02,Och06,Och13	*OreG0011549*
scaffold33100	437155	G	A	Ore01,Ore07,Ore13,Ore14	*OreG0011549*
scaffold33100	438646	A	G	Och05,Och08	*OreG0011549*
scaffold33100	440616	G	A	Ore01,Ore02,Ore03,Ore04,Ore05,Ore06,Ore07,Ore08,Ore09,Ore10,Ore11,Ore12,Ore13,Ore14	*OreG0011550*
scaffold33100	442626	G	T	Och01,Och02,Och03,Och04,Och05,Och06,Och07,Och08,Och09,Och10,Och11,Och12,Och13,Och14,Ore01,Ore02,Ore03,Ore04,Ore05,Ore06,Ore07,Ore08,Ore09,Ore10,Ore11,Ore12,Ore13,Ore14	*OreG0011551*
scaffold33100	531630	T	G	Och01,Och02,Och03,Och04,Och05,Och06,Och07,Och08,Och09,Och10,Och11,Och12,Och13,Och14,Ore01,Ore02,Ore03,Ore04,Ore05,Ore06,Ore07,Ore08,Ore09,Ore10,Ore11,Ore12,Ore13,Ore14	*OreG0011562*
scaffold33100	548144	A	G	NA	*OreG0011565*
scaffold33100	560541	C	G	NA	*OreG0011567*
scaffold33100	607236	C	T	NA	*OreG0011572*
scaffold33100	608028	T	G	Och01,Och02,Och03,Och04,Och05,Och06,Och07,Och08,Och09,Och10,Och11,Och12,Och13,Och14,Ore01,Ore02,Ore03,Ore04,Ore05,Ore06,Ore07,Ore08,Ore09,Ore10,Ore11,Ore12,Ore13,Ore14	*OreG0011572*
scaffold33100	609197	C	T	Och01,Och02,Och03,Och04,Och05,Och06,Och07,Och08,Och09,Och10,Och11,Och12,Och13,Och14,Ore01,Ore02,Ore03,Ore04,Ore05,Ore06,Ore07,Ore08,Ore09,Ore10,Ore11,Ore12,Ore13,Ore14	*OreG0011572*
scaffold33100	629165	A	T	Och01,Och02,Och03,Och04,Och05,Och06,Och07,Och08,Och09,Och10,Och11,Och12,Och13,Och14,Ore01,Ore02,Ore03,Ore04,Ore05,Ore06,Ore07,Ore08,Ore09,Ore10,Ore11,Ore12,Ore13,Ore14	*OreG0011575*
scaffold33100	665171	A	T	Och01,Och02,Och03,Och04,Och05,Och06,Och07,Och08,Och09,Och10,Och11,Och12,Och13,Och14,Ore01,Ore02,Ore03,Ore04,Ore05,Ore06,Ore07,Ore08,Ore09,Ore10,Ore11,Ore12,Ore13,Ore14	*OreG0011582*
scaffold33100	670593	G	C	NA	*OreG0011583*

scaffold33100	672655	C	A	NA	*OreG0011584*
scaffold33100	672911	G	T	Ore01,Ore02,Ore03,Ore04,Ore05,Ore06,Ore07,Ore08,Ore09,Ore10,Ore11,Ore12,Ore13,Ore14	*OreG0011584*
scaffold33100	680723	C	A	Och01,Och02,Och03,Och04,Och05,Och06,Och07,Och08,Och09,Och10,Och11,Och12,Och13,Och14,Ore01,Ore02,Ore03,Ore04,Ore05,Ore06,Ore07,Ore08,Ore09,Ore10,Ore11,Ore12,Ore13,Ore14	*OreG0011586*
scaffold33100	680883	G	A	Och01,Och02,Och03,Och04,Och05,Och06,Och07,Och08,Och09,Och10,Och11,Och12,Och13,Och14,Ore01,Ore02,Ore03,Ore04,Ore05,Ore06,Ore07,Ore08,Ore09,Ore10,Ore11,Ore12,Ore13,Ore14	*OreG0011586*
scaffold33100	686965	A	C	NA	*OreG0011588*
scaffold33100	687064	G	T	Och01	*OreG0011588*
scaffold33100	687175	T	C	Och02,Och03,Och04,Och05,Och06,Och07,Och11,Och12,Och13	*OreG0011588*
scaffold33100	687272	C	A	NA	*OreG0011588*
scaffold33100	693675	G	A	NA	*OreG0011589*
scaffold33100	693686	C	T	NA	*OreG0011589*
scaffold33100	693716	A	C	Ore01,Ore02,Ore03,Ore04,Ore05,Ore06,Ore07,Ore08,Ore09,Ore10,Ore11,Ore12,Ore13,Ore14	*OreG0011589*
scaffold33100	694495	G	A	Och01	*OreG0011589*
scaffold33100	697329	C	T	Och02,Och03,Och04,Och05,Och06,Och07,Och11,Och12,Och13	*OreG0011590*
scaffold33100	700721	C	T	NA	*OreG0011591*
scaffold33100	719670	C	A	Och01,Och02,Och03,Och04,Och05,Och06,Och07,Och08,Och09,Och10,Och11,Och12,Och13,Och14	*OreG0011592*
scaffold33100	767440	G	A	Ore01,Ore02,Ore03,Ore04,Ore05,Ore06,Ore07,Ore08,Ore09,Ore10,Ore11,Ore12,Ore13,Ore14	*OreG0011594*
scaffold33100	769371	A	C	Och01,Och02,Och03,Och04,Och05,Och06,Och07,Och08,Och09,Och10,Och11,Och12,Och13,Och14	*OreG0011594*
scaffold33100	779829	G	A	NA	*OreG0011595*
scaffold33100	779902	C	T	NA	*OreG0011595*
scaffold33100	780065	C	T	NA	*OreG0011595*
scaffold33100	782326	C	T	NA	*OreG0011595*
scaffold33100	782840	A	C	NA	*OreG0011595*
scaffold33100	782891	C	T	NA	*OreG0011595*
scaffold33100	816678	G	T	Och01,Och02,Och03,Och04,Och05,Och06,Och07,Och11,Och12,Och13,Och14	*OreG0011601*
scaffold33100	830220	G	A	NA	*OreG0011602*
scaffold33100	830247	A	T	NA	*OreG0011602*
scaffold33100	830694	T	A	NA	*OreG0011602*
scaffold33116	14298	A	G	Och01,Och02,Och03,Och04,Och05,Och06,Och07,Och08,Och09,Och10,Och11,Och12,Och13,Och14	*OreG0012942*
scaffold33116	16961	G	A	Ore01,Ore02,Ore03,Ore04,Ore05,Ore06,Ore07,Ore08,Ore09,Ore10,Ore11,Ore12,Ore13,Ore14	*OreG0012942*
scaffold33116	37604	T	C	Och01,Och02,Och03,Och04,Och05,Och06,Och07,Och08,Och09,Och10,Och11,Och12,Och13,Och14	*OreG0012946*
scaffold33116	37651	C	A	Och01,Och02,Och03,Och04,Och05,Och06,Och07,Och08,Och09,Och10,Och11,Och12,Och13,Och14	*OreG0012946*
scaffold33116	40577	T	C	Ore12,Ore14	*OreG0012947*
scaffold33116	69100	G	A	Ore01,Ore02,Ore03,Ore04,Ore05,Ore06,Ore07,Ore08,Ore09,Ore10,Ore11,Ore12,Ore13,Ore14	*OreG0012952*
scaffold33116	70259	C	T	NA	*OreG0012952*
scaffold33116	70285	A	T	Ore01,Ore02,Ore03,Ore04,Ore05,Ore06,Ore07,Ore08,Ore09,Ore10,Ore11,Ore12,Ore13,Ore14	*OreG0012952*
scaffold33116	90937	C	A	Ore01,Ore12,Ore14	*OreG0012955*
scaffold33116	121792	T	A	NA	*OreG0012961*
scaffold33116	130426	C	T	NA	*OreG0012962*
scaffold33116	136784	G	A	Och01,Och02,Och03,Och04,Och05,Och06,Och07,Och08,Och09,Och10,Och11,Och12,Och13,Och14,Ore01,Ore02,Ore03,Ore04,Ore05,Ore06,Ore07,Ore08,Ore09,Ore10,Ore11,Ore12,Ore13,Ore14	*OreG0012963*
scaffold33116	144108	C	G	NA	*OreG0012964*
scaffold33116	151569	G	A	Ore01,Ore02,Ore03,Ore04,Ore05,Ore06,Ore07,Ore08,Ore09,Ore10,Ore11,Ore12,Ore13,Ore14	*OreG0012965*
scaffold33116	157098	T	G	Och01,Och02,Och04,Och05,Och12,Och13	*OreG0012967*
scaffold33116	157152	C	G	NA	*OreG0012967*
scaffold33116	159055	T	C	NA	*OreG0012967*
scaffold33116	159317	C	T	Och02	*OreG0012967*
scaffold33116	164944	G	A	NA	*OreG0012968*
scaffold33116	164998	G	A	NA	*OreG0012968*
scaffold33116	169937	T	A	Och01,Och02,Och03,Och04,Och05,Och06,Och07,Och08,Och09,Och10,Och11,Och12,Och13,Och14	*OreG0012969*
scaffold33116	169951	G	T	Ore01,Ore02,Ore03,Ore04,Ore05,Ore06,Ore07,Ore08,Ore09,Ore10,Ore11,Ore12,Ore13,Ore14	*OreG0012969*
scaffold33116	170004	C	T	Och01,Och02,Och03,Och06,Och07,Och08,Och09,Och10,Och11	*OreG0012969*
scaffold33116	170172	G	A	Ore01,Ore02,Ore03,Ore04,Ore05,Ore06,Ore07,Ore08,Ore09,Ore10,Ore11,Ore12,Ore13,Ore14	*OreG0012969*
scaffold33116	170176	A	G	Ore01,Ore02,Ore03,Ore04,Ore05,Ore06,Ore07,Ore08,Ore09,Ore10,Ore11,Ore12,Ore13,Ore14	*OreG0012969*
scaffold33116	170501	T	A	Och01,Och02,Och06,Och07,Och09,Och10,Och11	*OreG0012969*

scaffold33116	170548	C	A	NA	*OreG0012969*
scaffold33116	170594	T	A	Och01,Och02,Och03,Och04,Och05,Och06,Och07,Och08,Och09,Och10,Och11,Och12,Och13,Och14,Ore01,Ore02,Ore03,Ore04,Ore05,Ore06,Ore07,Ore08,Ore09,Ore10,Ore11,Ore12,Ore13,Ore14	*OreG0012969*
scaffold33116	192785	A	T	Ore01,Ore02,Ore03,Ore04,Ore05,Ore06,Ore07,Ore08,Ore09,Ore10,Ore11,Ore12,Ore13,Ore14	*OreG0012972*
scaffold33116	215920	G	T	Och02,Och03,Och04,Och05,Och06,Och08,Och12,Och13	*OreG0012974*
scaffold33116	216575	G	A	NA	*OreG0012974*
scaffold33116	224652	G	A	NA	*OreG0012975*
scaffold33116	224806	C	T	NA	*OreG0012975*
scaffold33116	225466	A	T	Ore01,Ore02,Ore03,Ore04,Ore05,Ore06,Ore07,Ore08,Ore09,Ore10,Ore11,Ore12,Ore13,Ore14	*OreG0012975*
scaffold33116	228760	T	A	Och01,Och02,Och03,Och04,Och05,Och06,Och07,Och08,Och09,Och10,Och11,Och12,Och13,Och14,Ore01,Ore02,Ore03,Ore04,Ore05,Ore06,Ore07,Ore08,Ore09,Ore10,Ore11,Ore12,Ore13,Ore14	*OreG0012975*
scaffold33116	265306	A	G	Ore01,Ore02,Ore03,Ore04,Ore05,Ore06,Ore07,Ore08,Ore09,Ore10,Ore11,Ore12,Ore13,Ore14	*OreG0012979*
scaffold33116	265693	A	G	Och01,Och03,Och07,Och08,Och14	*OreG0012979*
scaffold33116	331961	A	T	Ore01,Ore02,Ore03,Ore04,Ore05,Ore06,Ore07,Ore08,Ore09,Ore10,Ore11,Ore12,Ore13,Ore14	*OreG0012990*
scaffold32492	10315	T	A	Och08,Och09,Och10	*OreG0010537*
scaffold32492	311630	G	A	NA	*OreG0010552*
scaffold32492	355209	C	T	NA	*OreG0010558*
scaffold32492	410445	G	A	NA	*OreG0010564*
scaffold32492	410457	A	G	Och06	*OreG0010564*
scaffold32492	430024	C	A	NA	*OreG0010566*
scaffold32492	440333	A	G	Och06	*OreG0010566*
scaffold32492	458363	A	T	NA	*OreG0010568*
scaffold32492	458501	A	T	NA	*OreG0010568*
scaffold32492	458513	G	A	NA	*OreG0010568*
scaffold32492	480012	G	A	Och01,Och02,Och03,Och04,Och05,Och06,Och07,Och08,Och09,Och10,Och11,Och12,Och13	*OreG0010569*
scaffold32492	553936	T	C	NA	*OreG0010574*
scaffold32492	558671	G	T	NA	*OreG0010574*
scaffold32492	559643	T	C	NA	*OreG0010574*
scaffold32492	644889	G	A	NA	*OreG0010576*
scaffold32492	644973	G	A	NA	*OreG0010576*
scaffold32492	645074	G	T	NA	*OreG0010576*
scaffold32492	724550	A	G	Och01,Och14	*OreG0010582*
scaffold32492	724698	C	T	Och01,Och09,Och10,Och11,Och14	*OreG0010582*
scaffold32492	725843	A	G	NA	*OreG0010582*
scaffold32492	773590	C	G	Och13,Ore01,Ore02,Ore03,Ore04,Ore05,Ore06,Ore07,Ore08,Ore09,Ore10,Ore11,Ore12,Ore13,Ore14	*OreG0010590*
scaffold32492	775079	C	T	NA	*OreG0010590*
scaffold32492	775798	G	A	Och13,Ore01,Ore02,Ore03,Ore04,Ore05,Ore06,Ore07,Ore08,Ore09,Ore10,Ore11,Ore12,Ore13,Ore14	*OreG0010590*
scaffold32492	807985	C	A	NA	*OreG0010595*
scaffold32492	808568	C	T	Och13	*OreG0010595*
scaffold32492	808976	C	T	Och11	*OreG0010595*
scaffold32492	849810	T	A	Och01,Och02,Och03,Och06,Och07,Och08,Och09,Och10,Och11,Och12,Och14	*OreG0010600*
scaffold32492	850238	G	A	Och13,Ore01,Ore02,Ore03,Ore04,Ore05,Ore06,Ore07,Ore08,Ore09,Ore10,Ore11,Ore12,Ore13,Ore14	*OreG0010600*
scaffold32492	856342	T	A	Ore01,Ore02,Ore03,Ore04,Ore05,Ore06,Ore07,Ore08,Ore09,Ore10,Ore11,Ore12,Ore13,Ore14	*OreG0010602*
scaffold32492	856382	A	G	NA	*OreG0010602*
scaffold32492	864730	C	T	Ore01,Ore02,Ore03,Ore04,Ore05,Ore06,Ore07,Ore08,Ore09,Ore10,Ore11,Ore12,Ore13,Ore14	*OreG0010603*
scaffold32492	865021	C	T	Och01,Och02,Och03,Och06,Och07,Och08,Och09,Och10,Och11,Och12,Och14	*OreG0010603*
scaffold32492	865105	C	A	Och01,Och02,Och03,Och06,Och07,Och08,Och09,Och10,Och11,Och12,Och14	*OreG0010603*
scaffold32492	865180	C	T	Ore01,Ore02,Ore03,Ore04,Ore05,Ore06,Ore07,Ore08,Ore09,Ore10,Ore11,Ore12,Ore13,Ore14	*OreG0010603*
scaffold91	964112	G	A	NA	*OreG0026051*
scaffold91	965051	G	A	NA	*OreG0026051*
scaffold91	965134	A	T	Och12,Och13	*OreG0026051*
scaffold91	965186	C	T	Och01,Och03,Och04,Och05,Och07,Och08,Och09,Och10,Och11,Och12,Och13,Och14	*OreG0026051*
scaffold91	965294	G	A	Och01,Och05,Och07,Och09,Och10,Och11,Och12,Och13,Och14	*OreG0026051*
scaffold91	3347117	G	T	NA	*OreG0026254*
scaffold91	3347750	G	T	NA	*OreG0026254*
scaffold91	3362579	C	G	Och01,Och02,Och03,Och04,Och05,Och06,Och07,Och08,Och09,Och10,Och11,Och12,Och13,Och14,Ore01,Ore02,Ore03,Ore04,Ore05,Ore06,Ore07,Ore08,Ore09,Ore10,Ore11,Ore12,Ore13,Ore14	*OreG0026255*
scaffold91	3375799	C	G	Och03,Och04,Och05,Och12,Och13	*OreG0026256*
scaffold91	3377133	C	T	Ore01,Ore02,Ore03,Ore04,Ore05,Ore06,Ore07,Ore08,Ore09,Ore10,Ore11,Ore12,Ore13,Ore14	*OreG0026256*

scaffold91	3381509	C	T	NA	*OreG0026257*
scaffold91	3385478	A	T	Och12,Och13	*OreG0026258*
scaffold91	3386495	T	G	NA	*OreG0026258*
scaffold91	3386529	C	G	Och12,Och13	*OreG0026258*
scaffold91	3427775	G	A	Och09,Och10,Och11	*OreG0026263*
scaffold91	3432203	G	A	Och12,Och13	*OreG0026265*
scaffold91	3444476	C	T	NA	*OreG0026266*
scaffold91	3446003	G	A	NA	*OreG0026266*
scaffold91	3447194	G	A	NA	*OreG0026266*
scaffold91	3488718	C	A	NA	*OreG0026270*
scaffold91	3488942	A	T	Och03,Och04,Och12,Och14	*OreG0026270*
scaffold91	3489595	A	G	NA	*OreG0026270*
scaffold91	3506879	C	A	NA	*OreG0026272*
scaffold91	3507751	A	G	NA	*OreG0026272*
scaffold91	3507771	C	T	NA	*OreG0026272*
scaffold91	3507822	G	A	NA	*OreG0026272*
scaffold91	3517040	C	T	NA	*OreG0026273*
scaffold91	3517045	G	A	NA	*OreG0026273*
scaffold91	3517770	G	A	NA	*OreG0026273*
scaffold91	3517812	A	C	NA	*OreG0026273*
scaffold91	3538148	T	A	NA	*OreG0026274*
scaffold91	3538186	A	T	NA	*OreG0026274*
scaffold91	3538219	A	T	NA	*OreG0026274*
scaffold91	3542083	G	C	NA	*OreG0026275*
scaffold91	3547874	C	A	NA	*OreG0026275*
scaffold91	3548425	G	A	Ore01,Ore02,Ore03,Ore04,Ore05,Ore06,Ore07,Ore08,Ore09,Ore10,Ore11,Ore12,Ore13,Ore14	*OreG0026275*
scaffold91	3548452	G	A	NA	*OreG0026275*
scaffold91	3548459	C	T	NA	*OreG0026275*
scaffold91	3548591	C	A	NA	*OreG0026275*
scaffold91	3548660	C	A	NA	*OreG0026275*
scaffold91	3553227	T	C	NA	*OreG0026276*
scaffold91	3553465	G	A	NA	*OreG0026276*
scaffold91	3553523	C	G	NA	*OreG0026276*
scaffold91	3555450	G	A	NA	*OreG0026276*
scaffold91	3558705	G	T	Och12	*OreG0026277*
scaffold91	3682840	G	A	NA	*OreG0026286*
scaffold91	3683137	C	T	Och12,Och13	*OreG0026286*
scaffold91	3684948	C	T	NA	*OreG0026287*
scaffold91	3684990	T	C	NA	*OreG0026287*
scaffold91	3693951	G	A	NA	*OreG0026289*
scaffold91	3693961	G	A	NA	*OreG0026289*
scaffold91	3693984	G	A	NA	*OreG0026289*
scaffold91	3742427	G	A	Ore01,Ore02,Ore03,Ore04,Ore05,Ore06,Ore07,Ore08,Ore09,Ore10,Ore11,Ore12,Ore13,Ore14	*OreG0026295*
scaffold91	3742556	C	T	Ore01,Ore02,Ore03,Ore04,Ore05,Ore06,Ore07,Ore08,Ore09,Ore10,Ore11,Ore12,Ore13,Ore14	*OreG0026295*
scaffold91	3742577	T	A	NA	*OreG0026295*
scaffold91	3744722	G	T	NA	*OreG0026295*
scaffold91	3744726	A	G	Och01,Och02	*OreG0026295*
scaffold91	3744934	C	A	NA	*OreG0026295*
scaffold91	3744980	T	C	Och01,Och02,Och03,Och04,Och05,Och06,Och07,Och08,Och09,Och10,Och11,Och12,Och13,Och14,Ore01,Ore02,Ore03,Ore04,Ore05,Ore06,Ore07,Ore08,Ore09,Ore10,Ore11,Ore12,Ore13,Ore14	*OreG0026295*
scaffold91	3753272	C	T	Och01,Och02,Och03,Och04,Och05,Och06,Och07,Och08,Och09,Och10,Och11,Och12,Och13,Och14	*OreG0026296*
scaffold91	3765233	G	C	Ore09	*OreG0026297*
scaffold91	3765300	C	T	Ore04,Ore05,Ore06,Ore07,Ore10,Ore11,Ore14	*OreG0026297*
scaffold91	3765308	G	C	Ore01,Ore02,Ore03,Ore04,Ore05,Ore06,Ore07,Ore08,Ore09,Ore10,Ore11,Ore12,Ore13,Ore14	*OreG0026297*
scaffold91	3765371	T	C	Ore09	*OreG0026297*
scaffold91	3776478	A	T	Ore01,Ore02,Ore03,Ore04,Ore05,Ore06,Ore07,Ore08,Ore09,Ore10,Ore11,Ore12,Ore13,Ore14	*OreG0026299*
scaffold91	3779139	T	A	Och01,Och02,Och03,Och04,Och05,Och06,Och07,Och08,Och09,Och10,Och11,Och12,Och13,Och14,Ore01,Ore02,Ore03,Ore04,Ore05,Ore06,Ore07,Ore08,Ore09,Ore10,Ore11,Ore12,Ore13,Ore14	*OreG0026299*
scaffold91	3791170	A	T	Ore09	*OreG0026300*
scaffold91	3815555	C	T	NA	*OreG0026304*
scaffold91	3815594	C	T	Ore01,Ore02,Ore03,Ore04,Ore05,Ore06,Ore07,Ore08,Ore09,Ore10,Ore11,Ore12,Ore13,Ore14	*OreG0026304*
scaffold91	3815746	T	A	Och03,Och04,Och05,Och12,Och13	*OreG0026304*
scaffold91	3815794	C	T	Ore01,Ore02,Ore03,Ore04,Ore05,Ore06,Ore07,Ore08,Ore09,Ore10,Ore11,Ore12,Ore13,Ore14	*OreG0026304*
scaffold91	3816997	T	G	Ore01,Ore02,Ore03,Ore04,Ore05,Ore06,Ore07,Ore08,Ore09,Ore10,Ore11,Ore12,Ore13,Ore14	*OreG0026304*

scaffold91	3817527	T	A	NA	*OreG0026304*
scaffold91	3817531	G	A	Och01,Och02,Och03,Och04,Och05,Och06,Och07,Och08,Och09,Och10,Och11,Och12,Och13,Och14,Ore01,Ore02,Ore03,Ore04,Ore05,Ore06,Ore07,Ore08,Ore09,Ore10,Ore11,Ore12,Ore13,Ore14	*OreG0026304*
scaffold91	3817597	G	A	Och01,Och02,Och03,Och04,Och05,Och06,Och07,Och08,Och09,Och10,Och11,Och12,Och13,Och14,Ore01,Ore02,Ore03,Ore04,Ore05,Ore06,Ore07,Ore08,Ore09,Ore10,Ore11,Ore12,Ore13,Ore14	*OreG0026304*
scaffold91	3820711	T	C	Ore02,Ore03,Ore06,Ore07,Ore08,Ore09,Ore10,Ore11,Ore12,Ore13,Ore14	*OreG0026305*
scaffold91	3821410	A	G	NA	*OreG0026305*
scaffold91	3829986	T	C	NA	*OreG0026307*
scaffold91	3834895	G	T	NA	*OreG0026308*
scaffold91	3839741	G	T	NA	*OreG0026310*
scaffold91	3839754	G	T	NA	*OreG0026310*
scaffold91	3841989	T	G	NA	*OreG0026311*
scaffold91	3842022	G	A	NA	*OreG0026311*
scaffold91	3897062	A	T	Och02,Och08	*OreG0026319*
scaffold91	3899329	T	G	NA	*OreG0026320*
scaffold91	3899343	T	C	NA	*OreG0026320*
scaffold91	3908331	A	G	NA	*OreG0026322*
scaffold91	3910449	T	A	Och01,Och02,Och03,Och04,Och05,Och06,Och07,Och08,Och09,Och10,Och11,Och12,Och13,Och14	*OreG0026323*
scaffold91	3910723	G	T	Och08	*OreG0026323*
scaffold91	3943080	C	T	Och12,Och13	*OreG0026326*
scaffold91	3967951	T	C	NA	*OreG0026329*
scaffold91	3970996	A	T	NA	*OreG0026329*
scaffold91	3985114	T	A	NA	*OreG0026330*
scaffold91	3985234	C	T	NA	*OreG0026330*
scaffold91	3987456	C	T	Och01,Och02,Och03,Och04,Och05,Och06,Och07,Och08,Och09,Och10,Och11,Och12,Och13,Och14,Ore01,Ore02,Ore03,Ore04,Ore05,Ore06,Ore07,Ore08,Ore09,Ore10,Ore11,Ore12,Ore13,Ore14	*OreG0026330*
scaffold91	4002441	T	A	Och01,Och02,Och03,Och04,Och05,Och06,Och07,Och08,Och09,Och10,Och11,Och12,Och13,Och14,Ore01,Ore02,Ore03,Ore04,Ore05,Ore06,Ore07,Ore08,Ore09,Ore10,Ore11,Ore12,Ore13,Ore14	*OreG0026331*
scaffold91	4163225	C	T	NA	*OreG0026344*
scaffold91	4182665	T	C	NA	*OreG0026346*
scaffold91	4204412	T	G	Och01,Och02,Och03,Och04,Och05,Och06,Och07,Och08,Och09,Och10,Och11,Och12,Och13,Och14,Ore01,Ore02,Ore03,Ore04,Ore05,Ore06,Ore07,Ore08,Ore09,Ore10,Ore11,Ore12,Ore13,Ore14	*OreG0026348*
scaffold91	4242595	A	G	NA	*OreG0026352*
scaffold91	4258248	T	C	Ore09	*OreG0026353*
scaffold91	4646378	T	A	Och13	*OreG0026356*
scaffold91	4824311	C	G	Och01,Och02,Och03,Och04,Och05,Och06,Och07,Och08,Och09,Och10,Och11,Och12,Och13,Och14,Ore01,Ore02,Ore03,Ore04,Ore05,Ore06,Ore07,Ore08,Ore09,Ore10,Ore11,Ore12,Ore13,Ore14	*OreG0026357*
scaffold91	4825457	T	A	Ore01,Ore02,Ore03,Ore04,Ore05,Ore06,Ore07,Ore08,Ore09,Ore10,Ore11,Ore12,Ore13,Ore14	*OreG0026357*
scaffold91	4825462	G	A	NA	*OreG0026357*
scaffold91	5172513	C	T	Och07,Och09,Och10,Och11	*OreG0026364*
scaffold91	5267903	T	C	NA	*OreG0026368*
scaffold91	5271350	T	A	NA	*OreG0026368*
scaffold91	5316839	T	A	Och07,Och08,Och09,Och10	*OreG0026370*
scaffold91	5317079	T	C	Och01,Och03,Och05,Och07,Och08,Och09,Och10,Och11,Och12,Och13,Och14	*OreG0026370*
scaffold91	5325008	G	A	Ore06	*OreG0026371*
scaffold91	5327245	C	A	NA	*OreG0026371*
scaffold91	5327265	C	T	Och01,Och02,Och03,Och04,Och05,Och06,Och07,Och08,Och09,Och10,Och11,Och12,Och13,Och14,Ore01,Ore02,Ore03,Ore04,Ore05,Ore06,Ore07,Ore08,Ore09,Ore10,Ore11,Ore12,Ore13,Ore14	*OreG0026371*
scaffold91	5327449	G	A	Och07,Och08,Och09,Och10	*OreG0026371*
scaffold91	5439048	C	G	NA	*OreG0026380*
scaffold91	5452841	G	C	NA	*OreG0026382*
scaffold91	5487067	T	C	Och01	*OreG0026386*
scaffold91	5487466	A	G	NA	*OreG0026386*
scaffold91	5487706	C	G	NA	*OreG0026386*
scaffold91	5488428	A	G	NA	*OreG0026386*
scaffold91	5488439	G	C	NA	*OreG0026386*
scaffold91	5620890	G	T	NA	*OreG0026390*
scaffold91	5620927	C	T	Och06	*OreG0026390*
scaffold91	5621670	G	A	NA	*OreG0026390*
scaffold91	5621743	T	C	Ore01,Ore02,Ore03,Ore04,Ore05,Ore06,Ore07,Ore08,Ore09,Ore10,Ore11,Ore12,Ore13,Ore14	*OreG0026390*
scaffold91	5629637	C	T	Och06	*OreG0026390*
scaffold91	5632311	C	A	NA	*OreG0026390*
scaffold91	5634474	G	T	Ore01,Ore02,Ore03,Ore04,Ore05,Ore06,Ore07,Ore08,Ore09,Ore10,Ore11,Ore12,Ore13,Ore14	*OreG0026390*
scaffold91	5787174	C	A	Och06	*OreG0026398*

scaffold91	5799776	A	T	Ore01,Ore02,Ore03,Ore04,Ore05,Ore06,Ore07,Ore08,Ore09,Ore10,Ore11,Ore12,Ore13,Ore14	*OreG0026400*
scaffold91	5800169	G	A	Och14	*OreG0026400*
scaffold91	5850117	A	G	Och06,Och08	*OreG0026404*
scaffold91	5865572	C	G	Och01,Och02,Och03,Och04,Och05,Och06,Och07,Och08,Och09,Och10,Och11,Och12,Och13,Och14,Ore01,Ore02,Ore03,Ore04,Ore05,Ore06,Ore07,Ore08,Ore09,Ore10,Ore11,Ore12,Ore13,Ore14	*OreG0026407*
scaffold91	5915530	A	T	Och01,Och02,Och03,Och04,Och05,Och06,Och07,Och08,Och09,Och10,Och11,Och12,Och13,Och14	*OreG0026409*
scaffold91	5919065	A	T	Och01,Och02,Och03,Och04,Och05,Och06,Och07,Och08,Och09,Och10,Och11,Och12,Och13,Och14,Ore01,Ore02,Ore03,Ore04,Ore05,Ore06,Ore07,Ore08,Ore09,Ore10,Ore11,Ore12,Ore13,Ore14	*OreG0026409*
scaffold91	5928653	C	A	Och14	*OreG0026411*
scaffold91	5929822	A	C	Och14	*OreG0026411*
scaffold91	5935391	C	G	NA	*OreG0026412*
scaffold91	5941359	G	A	NA	*OreG0026412*
scaffold91	6004753	C	G	NA	*OreG0026416*
scaffold91	6004815	C	T	NA	*OreG0026416*
scaffold91	6033306	G	A	NA	*OreG0026418*
scaffold91	6049555	C	T	Ore06,Ore07,Ore08,Ore14	*OreG0026419*
scaffold91	6050011	C	G	Ore06,Ore07,Ore08,Ore14	*OreG0026419*
scaffold91	6050220	C	T	Och03,Och05	*OreG0026420*
scaffold91	6050597	G	A	NA	*OreG0026420*
scaffold91	6067031	A	T	NA	*OreG0026422*
scaffold91	6067410	A	G	NA	*OreG0026422*
scaffold91	6090648	C	A	NA	*OreG0026424*
scaffold91	6102402	T	A	Ore01,Ore02,Ore03,Ore04,Ore05,Ore06,Ore07,Ore08,Ore09,Ore10,Ore11,Ore12,Ore13,Ore14	*OreG0026425*
scaffold91	6102756	C	A	Ore01,Ore02,Ore03,Ore04,Ore05,Ore06,Ore07,Ore08,Ore09,Ore10,Ore11,Ore12,Ore13,Ore14	*OreG0026425*
scaffold91	6142946	G	A	Ore01,Ore02,Ore03,Ore04,Ore05,Ore06,Ore07,Ore08,Ore09,Ore10,Ore11,Ore12,Ore13,Ore14	*OreG0026430*
scaffold91	6155212	C	A	Och02,Och03,Och04,Och05,Och06,Och07,Och08,Och09,Och10,Och11,Och12,Och13,Och14,Ore01,Ore02,Ore03,Ore04,Ore05,Ore06,Ore07,Ore08,Ore09,Ore10,Ore11,Ore12,Ore13,Ore14	*OreG0026431*
scaffold91	6187063	G	T	Och01,Och02,Och03,Och04,Och05,Och06,Och07,Och08,Och09,Och10,Och11,Och12,Och13,Och14	*OreG0026433*
scaffold91	6195270	G	T	NA	*OreG0026434*
scaffold91	6196271	T	C	NA	*OreG0026434*
scaffold91	6206496	T	C	NA	*OreG0026435*
scaffold91	6207214	C	A	Och12,Och13	*OreG0026436*
scaffold91	6208355	A	T	Och01,Och02,Och03,Och04,Och05,Och06,Och07,Och09,Och10,Och11,Ore06,Ore07,Ore08,Ore14	*OreG0026436*
scaffold91	6217069	C	A	NA	*OreG0026437*
scaffold91	6314410	C	A	NA	*OreG0026446*
scaffold91	6332792	T	G	Ore01,Ore02,Ore03,Ore04,Ore05,Ore06,Ore07,Ore08,Ore09,Ore10,Ore11,Ore12,Ore13,Ore14	*OreG0026447*
scaffold91	6339721	A	G	Och01,Och03,Och05,Och07,Och08,Och11,Och12,Och13	*OreG0026448*
scaffold91	6341644	G	C	Ore01,Ore02,Ore03,Ore04,Ore05,Ore06,Ore07,Ore08,Ore09,Ore10,Ore11,Ore12,Ore13,Ore14	*OreG0026448*
scaffold91	6348596	T	C	Och01,Och02,Och03,Och04,Och05,Och06,Och07,Och08,Och09,Och10,Och11,Och12,Och13,Och14,Ore01,Ore02,Ore03,Ore04,Ore05,Ore06,Ore07,Ore08,Ore09,Ore10,Ore11,Ore12,Ore13,Ore14	*OreG0026449*
scaffold91	6382849	C	T	NA	*OreG0026453*
scaffold91	6382899	G	C	NA	*OreG0026453*
scaffold91	6383486	A	T	NA	*OreG0026453*
scaffold91	6463662	A	T	NA	*OreG0026462*
scaffold91	6473981	G	A	Och03,Och05	*OreG0026465*
scaffold91	6476089	G	A	NA	*OreG0026465*
scaffold91	6538081	A	T	Ore04,Ore05,Ore06,Ore07,Ore08,Ore12,Ore13,Ore14	*OreG0026470*
scaffold91	6551475	C	A	NA	*OreG0026471*
scaffold91	6551692	C	T	NA	*OreG0026471*
scaffold91	6551778	G	A	NA	*OreG0026471*
scaffold91	6551838	G	A	NA	*OreG0026471*
scaffold91	6552349	G	A	NA	*OreG0026471*
scaffold91	6556992	G	T	NA	*OreG0026472*
scaffold91	6582492	G	C	Och12,Och13	*OreG0026473*
scaffold91	6636690	A	G	NA	*OreG0026480*
scaffold91	6636757	C	A	Och08	*OreG0026480*
scaffold91	6636825	G	T	Och08	*OreG0026480*
scaffold91	6755857	T	A	NA	*OreG0026489*
scaffold91	6755872	T	C	NA	*OreG0026489*
scaffold91	6756060	G	T	Ore01,Ore02,Ore03,Ore04,Ore05,Ore06,Ore07,Ore08,Ore09,Ore10,Ore11,Ore12,Ore13,Ore14	*OreG0026489*

scaffold91	6771027	A	G	Och01,Och02,Och03,Och04,Och05,Och06,Och07,Och08,Och09,Och10,Och11,Och12,Och13,Och14	*OreG0026491*
scaffold91	6782828	C	T	NA	*OreG0026495*
scaffold91	6788458	G	A	NA	*OreG0026496*
scaffold91	6788478	G	A	NA	*OreG0026496*
scaffold91	6788569	G	A	Och02,Och03,Och04,Och05,Och06,Och07,Och08,Och11,Och14	*OreG0026496*
scaffold91	6788625	T	G	NA	*OreG0026496*
scaffold91	6788638	C	A	NA	*OreG0026496*
scaffold91	6843881	C	T	Och07,Ore01,Ore02,Ore03,Ore04,Ore05,Ore06,Ore07,Ore08,Ore09,Ore10,Ore11,Ore12,Ore13,Ore14	*OreG0026499*
scaffold91	6844103	G	A	Och01	*OreG0026499*
scaffold91	6844131	C	T	Och02,Och03,Och04,Och05,Och06,Och08,Och11,Och14,Ore01,Ore02,Ore03,Ore04,Ore05,Ore06,Ore07,Ore08,Ore09,Ore10,Ore11,Ore12,Ore13,Ore14	*OreG0026499*
scaffold91	7030290	T	C	Och01	*OreG0026506*
scaffold91	7030784	A	G	Och01,Och02,Och03,Och04,Och05,Och06,Och07,Och08,Och09,Och10,Och11,Och12,Och13,Och14,Ore01,Ore02,Ore03,Ore04,Ore05,Ore06,Ore07,Ore08,Ore09,Ore10,Ore11,Ore12,Ore13,Ore14	*OreG0026506*
scaffold91	7031990	G	A	Och01,Och02,Och03,Och04,Och05,Och06,Och07,Och08,Och09,Och10,Och11,Och12,Och13,Och14,Ore01,Ore02,Ore03,Ore04,Ore05,Ore06,Ore07,Ore08,Ore09,Ore10,Ore11,Ore12,Ore13,Ore14	*OreG0026506*
scaffold91	7033280	T	C	Och01,Och02,Och03,Och04,Och05,Och06,Och14	*OreG0026506*
scaffold91	7033332	A	T	Ore06,Ore07,Ore08,Ore12,Ore13,Ore14	*OreG0026506*
scaffold91	7033393	G	C	Och07	*OreG0026506*
scaffold91	7033890	C	T	NA	*OreG0026507*
scaffold91	7035028	C	A	NA	*OreG0026507*
scaffold91	7035234	C	T	Och12,Och13	*OreG0026507*
scaffold91	7135153	A	T	NA	*OreG0026511*
scaffold91	7190506	A	T	Och14	*OreG0026517*
scaffold91	7190520	T	C	Ore01,Ore02,Ore03,Ore04,Ore05,Ore06,Ore07,Ore08,Ore09,Ore10,Ore11,Ore12,Ore13,Ore14	*OreG0026517*
scaffold91	7190934	T	A	Och01,Och02,Och03,Och04,Och05,Och06,Och07,Och08,Och09,Och10,Och11,Och12,Och13,Och14,Ore01,Ore02,Ore03,Ore04,Ore05,Ore06,Ore07,Ore08,Ore09,Ore10,Ore11,Ore12,Ore13,Ore14	*OreG0026517*
scaffold91	7191034	T	C	Och01,Och02,Och03,Och04,Och05,Och06,Och07,Och08,Och09,Och10,Och11,Och12,Och13,Och14,Ore01,Ore02,Ore03,Ore04,Ore05,Ore06,Ore07,Ore08,Ore09,Ore10,Ore11,Ore12,Ore13,Ore14	*OreG0026517*
scaffold91	7207614	C	A	Ore06,Ore07,Ore08,Ore12,Ore13,Ore14	*OreG0026519*
scaffold91	7238464	C	T	Och05,Och14	*OreG0026521*
scaffold33107	72310	A	G	NA	*OreG0012388*
scaffold33133	47920	T	A	Och01,Och02,Och03,Och04,Och05,Och06,Och07,Och08,Och09,Och10,Och11,Och12,Och13	*OreG0013801*
scaffold33133	48660	G	A	NA	*OreG0013801*
scaffold33133	50406	C	A	Ore03,Ore08,Ore10,Ore14	*OreG0013801*
scaffold33133	50634	A	C	NA	*OreG0013801*
scaffold33133	50799	G	A	Och01,Och02,Och03,Och04,Och05,Och06,Och07,Och08,Och09,Och10,Och11,Och12,Och13,Och14,Ore01,Ore02,Ore03,Ore04,Ore05,Ore06,Ore07,Ore08,Ore09,Ore10,Ore11,Ore12,Ore13,Ore14	*OreG0013801*
scaffold33133	50922	G	A	Ore01,Ore02,Ore03,Ore04,Ore05,Ore06,Ore07,Ore08,Ore09,Ore10,Ore11,Ore12,Ore13,Ore14	*OreG0013801*
scaffold33133	74868	A	T	NA	*OreG0013802*
scaffold33133	109348	A	T	Ore01,Ore02,Ore03,Ore04,Ore05,Ore06,Ore07,Ore08,Ore09,Ore10,Ore11,Ore12,Ore13,Ore14	*OreG0013803*
scaffold33133	118394	A	G	Och01,Och02,Och03,Och04,Och05,Och06,Och09,Och10,Och11,Och12,Och13,Och14	*OreG0013804*
scaffold33133	118413	A	G	Och02,Och03,Och04,Och05,Och06,Och12,Och13	*OreG0013804*
scaffold33133	120650	C	A	NA	*OreG0013805*
scaffold33133	120687	C	T	NA	*OreG0013805*
scaffold33133	120700	C	G	NA	*OreG0013805*
scaffold33133	152512	A	G	Och01,Och02,Och03,Och04,Och05,Och06,Och07,Och08,Och09,Och10,Och11,Och12,Och13,Och14,Ore01,Ore02,Ore03,Ore04,Ore05,Ore06,Ore07,Ore08,Ore09,Ore10,Ore11,Ore12,Ore13,Ore14	*OreG0013807*
scaffold33133	170164	G	T	NA	*OreG0013808*
scaffold33133	233511	A	T	Och01,Och02,Och03,Och04,Och05,Och06,Och07,Och08,Och09,Och10,Och11,Och12,Och13,Och14,Ore01,Ore02,Ore03,Ore04,Ore05,Ore06,Ore07,Ore08,Ore09,Ore10,Ore11,Ore12,Ore13,Ore14	*OreG0013811*
scaffold33133	235569	C	T	NA	*OreG0013811*
scaffold33133	241661	G	T	Och01,Och03,Och07,Och14	*OreG0013812*
scaffold33133	313704	G	T	Ore01,Ore02,Ore03,Ore04,Ore05,Ore06,Ore07,Ore08,Ore09,Ore10,Ore11,Ore12,Ore13,Ore14	*OreG0013813*
scaffold33133	349457	T	A	Och02,Och06	*OreG0013815*
scaffold33133	352963	T	C	NA	*OreG0013816*
scaffold33133	499559	G	C	Och01,Och02,Och03,Och04,Och05,Och06,Och07,Och08,Och09,Och10,Och11,Och12,Och13,Och14,Ore01,Ore02,Ore03,Ore04,Ore05,Ore06,Ore07,Ore08,Ore09,Ore10,Ore11,Ore12,Ore13,Ore14	*OreG0013822*

scaffold33133	625774	A	G	Ore01,Ore02,Ore03,Ore04,Ore05,Ore06,Ore07,Ore08,Ore09,Ore10,Ore11,Ore12,Ore13,Ore14	*OreG0013825*
scaffold28919	6236	G	C	NA	*OreG0008313*
scaffold28919	9133	T	G	NA	*OreG0008314*
scaffold28919	22455	A	G	NA	*OreG0008315*
scaffold28919	37403	C	T	Och01,Och02,Och03,Och04,Och05,Och06,Och07,Och08,Och09,Och10,Och11,Och12,Och13,Och14,Ore01,Ore02,Ore03,Ore04,Ore05,Ore06,Ore07,Ore08,Ore09,Ore10,Ore11,Ore12,Ore13,Ore14	*OreG0008316*
scaffold28919	41327	G	T	NA	*OreG0008316*
scaffold28919	41793	C	T	Ore02,Ore10,Ore11,Ore12,Ore13	*OreG0008316*
scaffold28919	43267	A	G	Och01,Och02,Och03,Och04,Och05,Och06,Och07,Och08,Och09,Och10,Och11,Och12,Och13,Och14	*OreG0008316*
scaffold28919	44124	C	G	Och01,Och02,Och03,Och04,Och05,Och06,Och07,Och08,Och10,Och11,Och12,Och13,Och14	*OreG0008316*
scaffold28919	45164	T	G	Och01,Och02,Och03,Och04,Och05,Och06,Och07,Och08,Och09,Och10,Och11,Och12,Och13,Och14	*OreG0008316*
scaffold28919	45816	T	A	Och01,Och02,Och03,Och04,Och05,Och06,Och07,Och08,Och09,Och10,Och11,Och12,Och13,Och14	*OreG0008316*
scaffold28919	47611	G	T	Ore02,Ore10,Ore11,Ore12,Ore13	*OreG0008316*
scaffold28919	55148	T	A	Och01,Och02,Och03,Och04,Och05,Och06,Och07,Och08,Och09,Och10,Och11,Och12,Och13,Och14,Ore01,Ore02,Ore03,Ore04,Ore05,Ore06,Ore07,Ore08,Ore09,Ore10,Ore11,Ore12,Ore13,Ore14	*OreG0008319*
scaffold28919	55500	T	A	NA	*OreG0008319*
scaffold28919	55502	G	T	NA	*OreG0008319*
scaffold28919	55540	C	A	Och01,Och02,Och03,Och04,Och05,Och06,Och08,Och12,Och13	*OreG0008319*
scaffold28919	55690	G	T	Ore01,Ore02,Ore03,Ore04,Ore05,Ore06,Ore07,Ore08,Ore09,Ore10,Ore11,Ore12,Ore13,Ore14	*OreG0008319*
scaffold28919	56141	T	C	Ore01,Ore02,Ore03,Ore04,Ore05,Ore06,Ore07,Ore08,Ore09,Ore10,Ore11,Ore12,Ore13,Ore14	*OreG0008319*
scaffold28919	56457	G	T	Och01,Och02,Och03,Och04,Och05,Och06,Och07,Och08,Och09,Och10,Och11,Och12,Och13,Och14	*OreG0008319*
scaffold28919	56925	C	T	Och01,Och02,Och03,Och04,Och05,Och06,Och07,Och08,Och09,Och10,Och11,Och12,Och13,Och14	*OreG0008319*
scaffold28919	56946	T	C	Ore01,Ore02,Ore03,Ore04,Ore05,Ore06,Ore07,Ore08,Ore09,Ore10,Ore11,Ore12,Ore13,Ore14	*OreG0008319*
scaffold28919	56948	G	C	Ore02,Ore10,Ore11,Ore12,Ore13	*OreG0008319*
scaffold28919	56957	T	C	Och01,Och02,Och03,Och04,Och05,Och06,Och07,Och08,Och09,Och10,Och11,Och12,Och13,Och14	*OreG0008319*
scaffold28919	56985	A	G	Och01,Och02,Och03,Och04,Och05,Och06,Och07,Och08,Och09,Och10,Och11,Och12,Och13,Och14,Ore01,Ore02,Ore03,Ore04,Ore05,Ore06,Ore07,Ore08,Ore09,Ore10,Ore11,Ore12,Ore13,Ore14	*OreG0008319*
scaffold28919	75838	G	A	Och01	*OreG0008322*
scaffold28919	87866	A	G	NA	*OreG0008323*
scaffold28919	90176	G	T	NA	*OreG0008323*
scaffold28919	90887	G	C	Och01	*OreG0008323*
scaffold28919	90911	A	T	NA	*OreG0008323*
scaffold28919	91702	C	T	NA	*OreG0008323*
scaffold28919	91708	G	A	NA	*OreG0008323*
scaffold28919	91899	G	C	NA	*OreG0008323*
scaffold28919	92330	A	G	NA	*OreG0008323*
scaffold28919	92500	G	A	NA	*OreG0008323*
scaffold28919	100220	T	G	NA	*OreG0008324*
scaffold28919	126510	T	C	Ore01,Ore02,Ore03,Ore04,Ore05,Ore06,Ore07,Ore08,Ore09,Ore10,Ore11,Ore12,Ore13,Ore14	*OreG0008328*
scaffold28919	126684	C	G	NA	*OreG0008328*
scaffold28919	126837	C	A	Ore01,Ore02,Ore03,Ore04,Ore05,Ore06,Ore07,Ore08,Ore09,Ore10,Ore11,Ore12,Ore13,Ore14	*OreG0008328*
scaffold28919	128144	C	T	Ore01,Ore02,Ore03,Ore04,Ore05,Ore06,Ore07,Ore08,Ore09,Ore10,Ore11,Ore12,Ore13,Ore14	*OreG0008329*
scaffold28919	128146	G	T	Och01,Och03,Och04,Och05,Och12,Och13	*OreG0008329*
scaffold28919	128150	C	T	Och14	*OreG0008329*
scaffold28919	161307	C	T	NA	*OreG0008333*
scaffold28919	213155	A	T	Ore01,Ore02,Ore03,Ore04,Ore05,Ore06,Ore07,Ore08,Ore09,Ore10,Ore11,Ore12,Ore13,Ore14	*OreG0008339*
scaffold28919	213165	C	G	Och01,Och02,Och03,Och04,Och05,Och06,Och07,Och08,Och09,Och10,Och11,Och12,Och13,Och14	*OreG0008339*
scaffold28919	264738	T	A	NA	*OreG0008345*
scaffold28919	285663	C	T	NA	*OreG0008349*
scaffold28919	285965	G	T	NA	*OreG0008349*
scaffold28919	289220	C	T	Ore01,Ore02,Ore03,Ore04,Ore05,Ore06,Ore07,Ore08,Ore09,Ore10,Ore11,Ore12,Ore13,Ore14	*OreG0008350*
scaffold28919	326001	C	T	NA	*OreG0008354*
scaffold28919	332932	C	T	Och01,Och04,Och05,Och12,Och13	*OreG0008356*
scaffold28919	354727	G	T	Och09,Och10,Och11	*OreG0008361*

scaffold28919	383159	T	A	Och01,Och02,Och03,Och04,Och05,Och06,Och07,Och08,Och09,Och10,Och11,Och12,Och13,Och14	*OreG0008365*
scaffold28919	387399	C	T	Ore01,Ore02,Ore03,Ore04,Ore05,Ore06,Ore07,Ore08,Ore09,Ore10,Ore11,Ore12,Ore13,Ore14	*OreG0008366*
scaffold28919	393327	T	A	Och01,Och02,Och03,Och04,Och05,Och06,Och07,Och08,Och09,Och10,Och11,Och12,Och13,Och14,Ore01,Ore02,Ore03,Ore04,Ore05,Ore06,Ore07,Ore08,Ore09,Ore10,Ore11,Ore12,Ore13,Ore14	*OreG0008367*
scaffold28919	399420	C	T	Och01,Och02,Och03,Och04,Och05,Och06,Och07,Och08,Och09,Och10,Och11,Och12,Och13,Och14,Ore01,Ore02,Ore03,Ore04,Ore05,Ore06,Ore07,Ore08,Ore09,Ore10,Ore11,Ore12,Ore13,Ore14	*OreG0008367*
scaffold28919	399497	A	T	Ore01,Ore02,Ore03,Ore04,Ore05,Ore06,Ore07,Ore08,Ore09,Ore10,Ore11,Ore12,Ore13,Ore14	*OreG0008367*
scaffold28919	431648	T	C	Och01,Och02,Och03,Och04,Och05,Och06,Och07,Och08,Och09,Och10,Och11,Och12,Och13,Och14	*OreG0008369*
scaffold28919	431676	C	A	Och01,Och02,Och03,Och04,Och05,Och06,Och07,Och08,Och09,Och10,Och11,Och12,Och13,Och14,Ore01,Ore02,Ore03,Ore04,Ore05,Ore06,Ore07,Ore08,Ore09,Ore10,Ore11,Ore12,Ore13,Ore14	*OreG0008369*
scaffold28919	455329	G	A	NA	*OreG0008370*
scaffold28919	458896	G	A	Och04,Och05,Och07,Och12,Och13,Och14	*OreG0008372*
scaffold28919	463942	G	A	NA	*OreG0008373*
scaffold28919	479403	C	G	NA	*OreG0008374*
scaffold28919	535548	G	C	Och01,Och02,Och03,Och04,Och05,Och06,Och07,Och08,Och09,Och10,Och11,Och12,Och13,Och14,Ore01,Ore02,Ore03,Ore04,Ore05,Ore06,Ore07,Ore08,Ore09,Ore10,Ore11,Ore12,Ore13,Ore14	*OreG0008380*
scaffold28919	537725	C	T	Ore01,Ore02,Ore03,Ore04,Ore05,Ore06,Ore07,Ore08,Ore09,Ore10,Ore11,Ore12,Ore13,Ore14	*OreG0008381*
scaffold28919	538816	T	C	Ore01,Ore02,Ore03,Ore04,Ore05,Ore06,Ore07,Ore08,Ore09,Ore10,Ore11,Ore12,Ore13,Ore14	*OreG0008381*
scaffold28919	539419	G	A	Och01,Och02,Och03,Och04,Och05,Och06,Och07,Och08,Och09,Och10,Och11,Och12,Och13,Och14	*OreG0008381*
scaffold28919	539679	G	A	Ore01,Ore02,Ore03,Ore04,Ore05,Ore06,Ore07,Ore08,Ore09,Ore10,Ore11,Ore12,Ore13,Ore14	*OreG0008381*
scaffold28919	542111	T	G	Och08	*OreG0008381*
scaffold28919	542125	C	A	Ore01,Ore03,Ore04,Ore05,Ore08,Ore09,Ore11,Ore12,Ore14	*OreG0008381*
scaffold28919	542182	T	G	Och08	*OreG0008381*
scaffold28919	542442	C	G	Och01,Och02,Och03,Och04,Och05,Och06,Och07,Och09,Och10,Och11,Och12,Och13	*OreG0008381*
scaffold28919	542449	C	T	Och01,Och02,Och03,Och04,Och05,Och06,Och07,Och09,Och10,Och11,Och12,Och13	*OreG0008381*
scaffold28919	542485	C	T	Och08	*OreG0008381*
scaffold28919	553100	T	C	Och01	*OreG0008382*
scaffold28919	579908	A	T	Ore01,Ore02,Ore03,Ore04,Ore05,Ore06,Ore07,Ore08,Ore09,Ore10,Ore11,Ore12,Ore13,Ore14	*OreG0008385*
scaffold28919	580850	C	A	NA	*OreG0008385*
scaffold28919	581480	C	T	NA	*OreG0008385*
scaffold28919	581533	A	G	NA	*OreG0008385*
scaffold28919	583360	C	A	NA	*OreG0008385*
scaffold28919	587745	C	T	NA	*OreG0008384*
scaffold28919	587800	A	T	Och01	*OreG0008384*
scaffold28919	592507	G	C	Ore01,Ore03,Ore04,Ore05,Ore08,Ore09,Ore11,Ore12,Ore14	*OreG0008388*
scaffold28919	607952	G	A	Ore11,Ore12,Ore14	*OreG0008390*
scaffold28919	608570	C	T	Och07,Och08,Och09,Och10,Och11,Och14	*OreG0008390*
scaffold28919	614042	C	A	Och01	*OreG0008391*
scaffold28919	624227	C	A	NA	*OreG0008393*
scaffold28919	624427	T	C	Och04,Och05,Och08,Och12,Och13	*OreG0008393*
scaffold28919	625227	G	A	Och01,Och02,Och03,Och04,Och05,Och06,Och07,Och08,Och09,Och10,Och11,Och12,Och13,Och14,Ore01,Ore02,Ore03,Ore04,Ore05,Ore06,Ore07,Ore08,Ore09,Ore10,Ore11,Ore12,Ore13,Ore14	*OreG0008393*
scaffold28919	629404	G	T	Ore11,Ore12,Ore14	*OreG0008394*
scaffold28919	645134	A	G	NA	*OreG0008395*
scaffold28919	646071	G	A	Och01,Och04,Och05,Och08,Och12,Och13	*OreG0008395*
scaffold28919	646113	C	T	Ore01,Ore02,Ore03,Ore04,Ore05,Ore06,Ore07,Ore08,Ore09,Ore10,Ore11,Ore12,Ore13,Ore14	*OreG0008395*
scaffold28919	665717	G	A	NA	*OreG0008398*
scaffold28919	690342	T	C	NA	*OreG0008402*
scaffold28919	696440	C	T	Och01,Och02,Och03,Och04,Och05,Och06,Och07,Och08,Och09,Och10,Och11,Och12,Och13,Och14,Ore01,Ore02,Ore03,Ore04,Ore05,Ore06,Ore07,Ore08,Ore09,Ore10,Ore11,Ore12,Ore13,Ore14	*OreG0008404*
scaffold28919	696713	C	A	Och01,Och02,Och03,Och04,Och05,Och06,Och07,Och08,Och09,Och10,Och11,Och12,Och13,Och14,Ore01,Ore02,Ore03,Ore04,Ore05,Ore06,Ore07,Ore08,Ore09,Ore10,Ore11,Ore12,Ore13,Ore14	*OreG0008404*
scaffold28919	700484	G	T	Ore01,Ore02,Ore03,Ore04,Ore05,Ore06,Ore07,Ore08,Ore09,Ore10,Ore11,Ore12,Ore13,Ore14	*OreG0008405*
scaffold28919	700632	A	G	NA	*OreG0008405*
scaffold28919	718474	T	C	Och08	*OreG0008408*
scaffold28919	718516	G	A	Ore11,Ore12,Ore14	*OreG0008408*

scaffold28919	718686	A	C	Och01,Och02,Och03,Och04,Och05,Och06,Och07,Och08,Och09,Och10,Och11,Och12,Och13,Och14	*OreG0008408*
scaffold28919	728114	T	G	Och01,Och02,Och03,Och04,Och05,Och06,Och07,Och08,Och09,Och10,Och11,Och12,Och13,Och14	*OreG0008409*
scaffold28919	729803	A	T	NA	*OreG0008410*
scaffold28919	729988	G	C	Ore11,Ore12,Ore14	*OreG0008410*
scaffold28919	730102	T	A	NA	*OreG0008410*
scaffold28919	730123	T	C	NA	*OreG0008410*
scaffold28919	730660	G	C	Och01,Och02,Och03,Och04,Och05,Och06,Och07,Och08,Och09,Och10,Och11,Och12,Och13,Och14	*OreG0008410*
scaffold28919	756708	G	A	NA	*OreG0008414*
scaffold28919	758433	T	A	Och02,Och03,Och04,Och05,Och06,Och07,Och08,Och09,Och10,Och11,Och12,Och13,Och14	*OreG0008414*
scaffold28919	758449	G	T	NA	*OreG0008414*
scaffold28919	791346	G	A	Och04,Och05,Och12,Och13,Och14	*OreG0008419*
scaffold28919	794863	T	G	Och02,Och03,Och04,Och05,Och06,Och07,Och08,Och09,Och10,Och11,Och12,Och13,Och14	*OreG0008421*
scaffold28919	820827	A	G	NA	*OreG0008426*
scaffold28919	820912	A	C	Ore01,Ore02,Ore03,Ore04,Ore05,Ore06,Ore07,Ore08,Ore09,Ore10,Ore11,Ore12,Ore13,Ore14	*OreG0008426*
scaffold28919	821056	C	A	NA	*OreG0008426*
scaffold28919	836314	A	T	Och01,Och02,Och03,Och04,Och05,Och06,Och07,Och08,Och09,Och10,Och11,Och12,Och13,Och14,Ore01,Ore02,Ore03,Ore04,Ore05,Ore06,Ore07,Ore08,Ore09,Ore10,Ore11,Ore12,Ore13,Ore14	*OreG0008429*
scaffold28919	847165	C	A	NA	*OreG0008431*
scaffold28919	847586	G	T	Ore01,Ore03,Ore08,Ore09,Ore11,Ore12,Ore14	*OreG0008431*
scaffold28919	854491	G	T	Och01,Och02,Och03,Och04,Och05,Och06,Och07,Och08,Och09,Och10,Och11,Och12,Och13,Och14	*OreG0008433*
scaffold28919	854562	T	G	Ore01,Ore02,Ore03,Ore04,Ore05,Ore06,Ore07,Ore08,Ore09,Ore10,Ore11,Ore12,Ore13,Ore14	*OreG0008433*
scaffold28919	930221	C	A	Och02,Och03,Och04,Och05,Och06,Och12,Och13	*OreG0008444*
scaffold28919	960493	T	G	NA	*OreG0008449*
scaffold28919	960610	T	C	Och01,Och02,Och03,Och04,Och05,Och06,Och07,Och08,Och09,Och10,Och11,Och12,Och13,Och14,Ore01,Ore02,Ore03,Ore04,Ore05,Ore06,Ore07,Ore08,Ore09,Ore10,Ore11,Ore12,Ore13,Ore14	*OreG0008449*
scaffold28919	969423	C	T	NA	*OreG0008451*
scaffold28919	975920	A	G	Och01,Och02,Och03,Och04,Och05,Och06,Och07,Och08,Och09,Och10,Och11,Och12,Och13,Och14,Ore01,Ore02,Ore03,Ore04,Ore05,Ore06,Ore07,Ore08,Ore09,Ore10,Ore11,Ore12,Ore13,Ore14	*OreG0008453*
scaffold28919	996794	A	T	NA	*OreG0008455*
scaffold28919	1000663	T	A	Och01,Och02,Och03,Och04,Och05,Och06,Och07,Och08,Och09,Och10,Och11,Och12,Och13,Och14,Ore01,Ore02,Ore03,Ore04,Ore05,Ore06,Ore07,Ore08,Ore09,Ore10,Ore11,Ore12,Ore13,Ore14	*OreG0008455*
scaffold28919	1011051	T	A	Och01,Och02,Och03,Och04,Och05,Och06,Och07,Och08,Och09,Och10,Och11,Och12,Och13,Och14	*OreG0008457*
scaffold28919	1011665	A	T	Och01,Och02,Och03,Och04,Och05,Och06,Och07,Och08,Och09,Och10,Och11,Och12,Och13,Och14,Ore01,Ore02,Ore03,Ore04,Ore05,Ore06,Ore07,Ore08,Ore09,Ore10,Ore11,Ore12,Ore13,Ore14	*OreG0008457*
scaffold28919	1014417	G	A	Ore01,Ore02,Ore03,Ore04,Ore05,Ore06,Ore07,Ore08,Ore09,Ore10,Ore11,Ore12,Ore13,Ore14	*OreG0008457*
scaffold28919	1160033	T	A	NA	*OreG0008473*
scaffold33097	17779	A	G	Ore04,Ore05	*OreG0011272*
scaffold33097	17873	A	T	Och01	*OreG0011272*
scaffold33097	21940	G	A	NA	*OreG0011273*
scaffold33097	23181	T	C	NA	*OreG0011273*
scaffold33097	25071	G	T	Och09,Och10	*OreG0011273*
scaffold33097	111298	G	T	NA	*OreG0011275*
scaffold33097	111307	G	T	NA	*OreG0011275*
scaffold33097	111487	A	G	NA	*OreG0011275*
scaffold33097	111752	A	T	NA	*OreG0011275*
scaffold33097	113885	G	C	NA	*OreG0011275*
scaffold33097	113887	C	T	NA	*OreG0011275*
scaffold33097	174458	G	A	Ore01,Ore02,Ore03,Ore04,Ore05,Ore06,Ore07,Ore08,Ore09,Ore10,Ore11,Ore12,Ore13,Ore14	*OreG0011279*
scaffold33097	174543	C	T	Ore01,Ore02,Ore03,Ore04,Ore05,Ore06,Ore07,Ore08,Ore09,Ore10,Ore11,Ore12,Ore13,Ore14	*OreG0011279*
scaffold33097	174723	C	T	Och03,Och13	*OreG0011279*
scaffold33097	174732	C	T	Ore01,Ore02,Ore03,Ore04,Ore05,Ore06,Ore07,Ore08,Ore09,Ore10,Ore11,Ore12,Ore13,Ore14	*OreG0011279*
scaffold33097	174758	G	A	Och01,Och02,Och03,Och04,Och05,Och06,Och07,Och08,Och09,Och10,Och11,Och12,Och13,Och14,Ore01,Ore02,Ore03,Ore04,Ore05,Ore06,Ore07,Ore08,Ore09,Ore10,Ore11,Ore12,Ore13,Ore14	*OreG0011279*
scaffold33097	174762	C	T	Ore01,Ore02,Ore03,Ore04,Ore05,Ore06,Ore07,Ore08,Ore09,Ore10,Ore11,Ore12,Ore13,Ore14	*OreG0011279*
scaffold33097	175117	A	G	Och09,Och10	*OreG0011279*

scaffold33097	175186	C	T	Och02,Och03,Och04,Och05,Och06,Och07,Och12,Och13,Ore01,Ore02,Ore03,Ore04,Ore05,Ore06,Ore07,Ore08,Ore09,Ore10,Ore11,Ore12,Ore13,Ore14	*OreG0011279*
scaffold33097	212925	G	C	Och02,Och03,Och04,Och06,Och07,Och08,Och09,Och10,Och11,Och13,Och14	*OreG0011284*
scaffold33097	434480	T	C	NA	*OreG0011291*
scaffold33097	438296	T	C	Ore02,Ore06,Ore07,Ore11,Ore13	*OreG0011291*
scaffold33097	484024	T	C	Och01,Och09,Och10	*OreG0011295*
scaffold33097	484918	A	T	Och01,Och02,Och03,Och04,Och05,Och06,Och07,Och08,Och09,Och10,Och11,Och12,Och13,Och14,Ore01,Ore02,Ore03,Ore04,Ore05,Ore06,Ore07,Ore08,Ore09,Ore10,Ore11,Ore12,Ore13,Ore14	*OreG0011295*
scaffold33097	492424	G	A	Och01,Och02,Och03,Och04,Och05,Och06,Och07,Och08,Och09,Och10,Och11,Och12,Och13,Och14,Ore01,Ore02,Ore03,Ore04,Ore05,Ore06,Ore07,Ore08,Ore09,Ore10,Ore11,Ore12,Ore13,Ore14	*OreG0011297*
scaffold33097	492467	C	A	NA	*OreG0011297*
scaffold33097	492469	G	A	Och05,Och13	*OreG0011297*
scaffold33097	492490	T	C	NA	*OreG0011297*
scaffold33097	492859	A	G	NA	*OreG0011297*
scaffold33097	554188	T	C	Och01,Och02,Och03,Och04,Och05,Och06,Och07,Och08,Och09,Och10,Och11,Och12,Och13,Och14,Ore01,Ore02,Ore03,Ore04,Ore05,Ore06,Ore07,Ore08,Ore09,Ore10,Ore11,Ore12,Ore13,Ore14	*OreG0011299*
scaffold33097	554362	G	A	Och01,Och02,Och03,Och04,Och05,Och06,Och07,Och08,Och09,Och10,Och11,Och12,Och13,Och14,Ore01,Ore02,Ore03,Ore04,Ore05,Ore06,Ore07,Ore08,Ore09,Ore10,Ore11,Ore12,Ore13,Ore14	*OreG0011299*
scaffold33097	568669	G	A	NA	*OreG0011300*
scaffold33097	587307	G	C	NA	*OreG0011303*
scaffold33097	587354	C	G	Ore01,Ore02,Ore03,Ore04,Ore05,Ore06,Ore07,Ore08,Ore09,Ore10,Ore11,Ore12,Ore13,Ore14	*OreG0011303*
scaffold33097	587357	C	A	Och07	*OreG0011303*
scaffold33097	620659	A	C	Och01,Och02,Och03,Och04,Och05,Och06,Och07,Och08,Och09,Och10,Och11,Och12,Och13,Och14,Ore01,Ore02,Ore03,Ore04,Ore05,Ore06,Ore07,Ore08,Ore09,Ore10,Ore11,Ore12,Ore13,Ore14	*OreG0011309*
scaffold33097	629664	C	A	Och08	*OreG0011311*
scaffold33097	632619	G	A	NA	*OreG0011311*
scaffold33097	658982	A	G	NA	*OreG0011313*
scaffold33097	735567	C	A	Och01,Och02,Och03,Och04,Och05,Och06,Och07,Och08,Och09,Och10,Och11,Och12,Och13,Och14	*OreG0011321*
scaffold33097	750765	C	T	Och01,Och14	*OreG0011322*
scaffold33097	750792	G	A	Och12	*OreG0011322*
scaffold33097	751126	C	T	NA	*OreG0011322*
scaffold33097	751189	T	C	NA	*OreG0011322*
scaffold33097	763351	T	A	Och01,Och02,Och03,Och04,Och05,Och06,Och07,Och08,Och09,Och10,Och11,Och12,Och13,Och14,Ore01,Ore02,Ore03,Ore04,Ore05,Ore06,Ore07,Ore08,Ore09,Ore10,Ore11,Ore12,Ore13,Ore14	*OreG0011323*
scaffold33097	763968	G	A	NA	*OreG0011323*
scaffold33097	764457	C	T	NA	*OreG0011323*
scaffold33097	765479	C	T	NA	*OreG0011324*
scaffold33097	765538	T	C	Och02,Och03,Och04,Och05,Och06,Och07,Och08,Och09,Och10,Och11,Och12,Och13,Och14	*OreG0011324*
scaffold33097	766083	G	T	NA	*OreG0011324*
scaffold33097	766145	T	C	NA	*OreG0011324*
scaffold33097	766514	C	A	NA	*OreG0011324*
scaffold33097	772650	C	G	Och02,Och03,Och04,Och05,Och06,Och07,Och08,Och09,Och10,Och11,Och12,Och13,Och14	*OreG0011325*
scaffold33097	777599	C	T	Och01,Och02,Och03,Och04,Och05,Och06,Och07,Och08,Och09,Och10,Och11,Och12,Och13,Och14,Ore01,Ore02,Ore03,Ore04,Ore05,Ore06,Ore07,Ore08,Ore09,Ore10,Ore11,Ore12,Ore13,Ore14	*OreG0011326*
scaffold33097	781861	T	C	Och13	*OreG0011326*
scaffold33097	862172	C	A	Ore01,Ore02,Ore03,Ore04,Ore05,Ore06,Ore07,Ore08,Ore09,Ore10,Ore11,Ore12,Ore13,Ore14	*OreG0011330*
scaffold33097	890231	G	A	NA	*OreG0011336*
scaffold33097	903833	G	A	NA	*OreG0011340*
scaffold33097	911359	G	A	NA	*OreG0011340*
scaffold33097	915785	T	A	Och01,Och08	*OreG0011341*
scaffold33097	917813	G	A	Och01,Och02,Och03,Och04,Och05,Och06,Och07,Och08,Och09,Och10,Och11,Och12,Och13,Och14	*OreG0011341*
scaffold33097	932793	G	A	NA	*OreG0011342*
scaffold33097	933161	T	C	NA	*OreG0011342*
scaffold33117	1027	C	T	NA	*OreG0012994*
scaffold33117	8776	C	T	Ore02,Ore03,Ore04,Ore05,Ore08,Ore09,Ore10,Ore13,Ore14	*OreG0012994*
scaffold33117	9456	G	T	Och01,Och02,Och03,Och04,Och05,Och06,Och07,Och08,Och09,Och10,Och11,Och12,Och13,Och14	*OreG0012994*
scaffold33117	10202	C	T	Ore02,Ore03,Ore04,Ore05,Ore08,Ore09,Ore10,Ore13,Ore14	*OreG0012994*
scaffold33117	14028	T	A	Och01,Och02,Och03,Och04,Och05,Och06,Och07,Och08,Och11,Och12,Och13,Och14	*OreG0012996*
scaffold33117	15207	T	A	Och01,Och02,Och03,Och04,Och05,Och06,Och07,Och08,Och11,Och12,Och13,Och14	*OreG0012996*

scaffold33117	34910	C	G	Och01,Och02,Och03,Och04,Och05,Och06,Och07,Och08,Och09,Och10,Och11,Och12,Och13,Och14,Ore01,Ore02,Ore03,Ore04,Ore05,Ore06,Ore07,Ore08,Ore09,Ore10,Ore11,Ore12,Ore13,Ore14	*OreG0013000*
scaffold33117	47374	A	T	NA	*OreG0013001*
scaffold33117	75094	T	C	Ore01,Ore02,Ore03,Ore04,Ore05,Ore06,Ore07,Ore08,Ore09,Ore10,Ore11,Ore12,Ore13,Ore14	*OreG0013004*
scaffold33117	91904	C	T	Ore01,Ore02,Ore03,Ore04,Ore05,Ore06,Ore07,Ore08,Ore09,Ore10,Ore11,Ore12,Ore13,Ore14	*OreG0013005*
scaffold33117	95773	C	T	NA	*OreG0013005*
scaffold33117	95854	C	T	NA	*OreG0013005*
scaffold33117	95859	G	A	NA	*OreG0013005*
scaffold33117	111590	C	T	Och01,Och02,Och03,Och04,Och05,Och06,Och07,Och08,Och09,Och10,Och11,Och12,Och13,Och14	*OreG0013007*
scaffold33117	115133	C	T	NA	*OreG0013007*
scaffold33117	115458	C	G	Och01,Och02,Och03,Och04,Och05,Och06,Och07,Och08,Och09,Och10,Och11,Och12,Och13,Och14	*OreG0013007*
scaffold33117	139950	A	G	Och01,Och09,Och10,Och11	*OreG0013009*
scaffold33117	142416	G	C	NA	*OreG0013010*
scaffold33117	145801	G	C	NA	*OreG0013011*
scaffold33117	145899	C	T	NA	*OreG0013011*
scaffold33117	170834	A	G	Och06	*OreG0013014*
scaffold33117	173151	A	G	Och01,Och02,Och03,Och04,Och05,Och06,Och07,Och08,Och09,Och10,Och11,Och12,Och13,Och14,Ore01,Ore02,Ore03,Ore04,Ore05,Ore06,Ore07,Ore08,Ore09,Ore10,Ore11,Ore12,Ore13,Ore14	*OreG0013014*
scaffold33117	173194	C	T	Och01,Och02,Och03,Och04,Och05,Och06,Och07,Och08,Och09,Och10,Och11,Och12,Och13,Och14,Ore01,Ore02,Ore03,Ore04,Ore05,Ore06,Ore07,Ore08,Ore09,Ore10,Ore11,Ore12,Ore13,Ore14	*OreG0013014*
scaffold33117	180017	C	T	Och08,Och09,Och10,Och11,Ore01,Ore02,Ore03,Ore04,Ore05,Ore06,Ore07,Ore08,Ore09,Ore10,Ore11,Ore12,Ore13,Ore14	*OreG0013015*
scaffold33117	213371	T	A	NA	*OreG0013019*
scaffold33117	213511	G	A	Ore01,Ore02,Ore03,Ore04,Ore05,Ore06,Ore07,Ore08,Ore09,Ore10,Ore11,Ore12,Ore13,Ore14	*OreG0013019*
scaffold33117	271152	A	G	NA	*OreG0013026*
scaffold33117	271194	T	G	Och01,Och02,Och03,Och04,Och05,Och06,Och07,Och08,Och09,Och10,Och11,Och12,Och13,Och14,Ore01,Ore02,Ore03,Ore04,Ore05,Ore06,Ore07,Ore08,Ore09,Ore10,Ore11,Ore12,Ore13,Ore14	*OreG0013026*
scaffold33117	281073	T	C	Ore01,Ore02,Ore03,Ore04,Ore05,Ore06,Ore07,Ore08,Ore09,Ore10,Ore11,Ore12,Ore13,Ore14	*OreG0013028*
scaffold33117	281804	A	G	Och01,Och02,Och03,Och04,Och05,Och06,Och07,Och08,Och09,Och10,Och11,Och12,Och13,Och14	*OreG0013028*
scaffold33117	290436	A	G	NA	*OreG0013029*
scaffold33117	291122	T	C	NA	*OreG0013029*
scaffold33117	292642	G	A	Ore01,Ore02,Ore03,Ore04,Ore05,Ore06,Ore07,Ore08,Ore09,Ore10,Ore11,Ore12,Ore13,Ore14	*OreG0013029*
scaffold33117	296823	A	T	Och11	*OreG0013030*
scaffold33117	354494	T	A	Och01,Och08,Och14	*OreG0013038*
scaffold33117	363934	G	T	NA	*OreG0013035*
scaffold33117	364026	G	A	NA	*OreG0013035*
scaffold33117	384443	C	T	Ore01,Ore02,Ore03,Ore04,Ore05,Ore06,Ore07,Ore08,Ore09,Ore10,Ore11,Ore12,Ore13,Ore14	*OreG0013041*
scaffold33117	386488	G	A	Ore02,Ore04,Ore05	*OreG0013042*
scaffold33117	390862	T	A	Och01,Och02,Och03,Och04,Och05,Och06,Och07,Och08,Och09,Och10,Och11,Och12,Och13,Och14,Ore01,Ore02,Ore03,Ore04,Ore05,Ore06,Ore07,Ore08,Ore09,Ore10,Ore11,Ore12,Ore13,Ore14	*OreG0013043*
scaffold33117	390934	G	A	NA	*OreG0013043*
scaffold33117	428478	A	G	Och01,Och02,Och03,Och04,Och05,Och06,Och07,Och08,Och09,Och10,Och11,Och12,Och13,Och14	*OreG0013047*
scaffold33117	483085	A	G	NA	*OreG0013050*
scaffold33117	493703	C	T	Ore01,Ore02,Ore03,Ore04,Ore05,Ore06,Ore07,Ore08,Ore09,Ore10,Ore11,Ore12,Ore13,Ore14	*OreG0013053*
scaffold33117	494483	C	A	Ore01,Ore02,Ore04,Ore05,Ore06,Ore07,Ore11,Ore12	*OreG0013053*
scaffold33117	505684	T	A	NA	*OreG0013054*
scaffold33117	524805	A	T	Och01,Och02,Och03,Och04,Och05,Och06,Och07,Och08,Och09,Och10,Och11,Och12,Och13,Och14,Ore01,Ore02,Ore03,Ore04,Ore05,Ore06,Ore07,Ore08,Ore09,Ore10,Ore11,Ore12,Ore13,Ore14	*OreG0013058*
scaffold33117	528684	C	T	Och07,Och14	*OreG0013058*
scaffold33117	537575	G	A	NA	*OreG0013059*
scaffold33117	539374	A	C	NA	*OreG0013059*
scaffold33117	543525	A	T	Ore01,Ore02,Ore03,Ore04,Ore05,Ore06,Ore07,Ore08,Ore09,Ore10,Ore11,Ore12,Ore13,Ore14	*OreG0013061*
scaffold33128	54516	G	A	NA	*OreG0013608*
scaffold33128	54621	T	C	NA	*OreG0013608*
scaffold33128	54654	T	C	NA	*OreG0013608*
scaffold33128	54667	G	A	NA	*OreG0013608*
scaffold33128	66193	A	T	NA	*OreG0013610*

scaffold33128	108411	C	A	NA	*OreG0013617*
scaffold33128	147707	T	G	Och09,Och10	*OreG0013622*
scaffold33128	147799	T	A	Ore01,Ore03,Ore04,Ore05	*OreG0013622*
scaffold33128	149256	G	T	Ore01,Ore03,Ore04,Ore05	*OreG0013622*
scaffold33128	149459	G	A	Och01	*OreG0013622*
scaffold33128	149685	T	C	Ore01,Ore03,Ore04,Ore05	*OreG0013622*
scaffold33128	149711	A	G	Och01,Och02,Och03,Och04,Och05,Och06,Och07,Och08,Och09,Och10,Och11,Och12,Och13,Och14	*OreG0013622*
scaffold33128	149781	G	A	Ore06,Ore08	*OreG0013622*
scaffold33128	149802	C	T	Och01,Och05,Och07,Och08,Och13,Och14	*OreG0013622*
scaffold33128	150103	C	T	Och05,Och13	*OreG0013622*
scaffold33128	150679	C	G	Och01,Och05,Och07,Och08,Och13,Och14	*OreG0013621*
scaffold33128	152402	A	G	Och01,Och02,Och03,Och04,Och05,Och06,Och07,Och08,Och09,Och10,Och11,Och12,Och13,Och14,Ore01,Ore02,Ore03,Ore04,Ore05,Ore06,Ore07,Ore08,Ore09,Ore10,Ore11,Ore12,Ore13,Ore14	*OreG0013621*
scaffold33128	164167	C	T	Och02,Och06,Och07,Och09,Och10,Ore01,Ore02,Ore03,Ore04,Ore05,Ore06,Ore07,Ore08,Ore09,Ore10,Ore11,Ore12,Ore13,Ore14	*OreG0013624*
scaffold33128	178308	G	T	Och09,Och10	*OreG0013626*
scaffold33128	182127	C	A	Och01,Och02,Och03,Och04,Och05,Och06,Och07,Och08,Och09,Och10,Och11,Och12,Och13,Och14,Ore01,Ore02,Ore03,Ore04,Ore05,Ore06,Ore07,Ore08,Ore09,Ore10,Ore11,Ore12,Ore13,Ore14	*OreG0013627*
scaffold33128	213241	A	T	Ore01	*OreG0013630*
scaffold33128	213474	T	A	Ore01,Ore02,Ore03,Ore04,Ore05,Ore06,Ore07,Ore08,Ore09,Ore10,Ore11,Ore12,Ore13,Ore14	*OreG0013630*
scaffold33128	213520	G	A	Och01,Och02,Och03,Och04,Och05,Och06,Och07,Och08,Och09,Och10,Och11,Och12,Och13,Och14,Ore01,Ore02,Ore03,Ore04,Ore05,Ore06,Ore07,Ore08,Ore09,Ore10,Ore11,Ore12,Ore13,Ore14	*OreG0013630*
scaffold33128	219082	T	C	Och01,Och02,Och03,Och04,Och05,Och06,Och07,Och08,Och09,Och10,Och11,Och12,Och13,Och14	*OreG0013631*
scaffold33128	219610	T	C	Och05,Och13	*OreG0013631*
scaffold33128	230855	A	G	NA	*OreG0013633*
scaffold33128	233804	T	G	Och01,Och02,Och03,Och04,Och05,Och06,Och07,Och08,Och09,Och10,Och11,Och12,Och13,Och14	*OreG0013634*
scaffold33128	233807	A	G	NA	*OreG0013634*
scaffold33128	233831	A	G	Ore02,Ore06,Ore07,Ore08,Ore09,Ore11,Ore12,Ore13	*OreG0013634*
scaffold33128	233883	A	G	Ore02,Ore06,Ore07,Ore08,Ore09,Ore11,Ore12,Ore13	*OreG0013634*
scaffold33128	233982	C	G	Ore01,Ore02,Ore03,Ore04,Ore05,Ore06,Ore07,Ore08,Ore09,Ore10,Ore11,Ore12,Ore13,Ore14	*OreG0013634*
scaffold33128	244307	A	G	Ore01,Ore02,Ore03,Ore04,Ore05,Ore06,Ore07,Ore08,Ore09,Ore10,Ore11,Ore12,Ore13,Ore14	*OreG0013637*
scaffold33128	244356	T	G	NA	*OreG0013637*
scaffold33128	251692	T	C	Och01,Och02,Och03,Och04,Och05,Och06,Och07,Och08,Och09,Och10,Och11,Och12,Och13,Och14,Ore01,Ore02,Ore03,Ore04,Ore05,Ore06,Ore07,Ore08,Ore09,Ore10,Ore11,Ore12,Ore13,Ore14	*OreG0013638*
scaffold33128	252389	C	T	Och02	*OreG0013638*
scaffold33128	252392	A	T	NA	*OreG0013638*
scaffold33128	253556	G	T	Och01,Och02,Och03,Och04,Och05,Och06,Och07,Och08,Och09,Och10,Och11,Och12,Och13,Och14,Ore01,Ore02,Ore03,Ore04,Ore05,Ore06,Ore07,Ore08,Ore09,Ore10,Ore11,Ore12,Ore13,Ore14	*OreG0013638*
scaffold33128	273696	G	A	Och01,Och05,Och09,Och10,Och13	*OreG0013640*
scaffold33128	305317	A	G	Och02,Och06	*OreG0013644*
scaffold33128	353698	C	G	Och03,Och04,Och05,Och08,Och12,Och13	*OreG0013651*
scaffold33128	358625	A	G	NA	*OreG0013652*
scaffold33128	361979	C	G	Och03,Och04,Och05,Och12,Och13	*OreG0013653*
scaffold33128	363877	G	T	NA	*OreG0013653*
scaffold33128	364703	A	T	Och08	*OreG0013653*
scaffold33128	366364	C	T	Ore01,Ore02,Ore03,Ore04,Ore05,Ore06,Ore07,Ore08,Ore09,Ore10,Ore11,Ore12,Ore13,Ore14	*OreG0013653*
scaffold33128	367096	T	C	Ore01,Ore02,Ore03,Ore04,Ore05,Ore06,Ore07,Ore08,Ore09,Ore10,Ore11,Ore12,Ore13,Ore14	*OreG0013653*
scaffold33128	377151	C	T	NA	*OreG0013654*
scaffold33128	377679	G	T	NA	*OreG0013654*
scaffold33128	378013	T	C	Och03,Och04,Och05,Och12,Och13	*OreG0013654*
scaffold33128	378748	T	G	NA	*OreG0013654*
scaffold33128	383652	C	A	Och01,Och02,Och03,Och04,Och05,Och06,Och07,Och08,Och09,Och10,Och11,Och12,Och13,Och14,Ore01,Ore02,Ore03,Ore04,Ore05,Ore06,Ore07,Ore08,Ore09,Ore10,Ore11,Ore12,Ore13,Ore14	*OreG0013655*
scaffold33128	397314	T	C	NA	*OreG0013657*
scaffold33128	397435	C	T	NA	*OreG0013657*
scaffold33128	399675	G	T	Ore01,Ore02,Ore03,Ore04,Ore05,Ore06,Ore07,Ore08,Ore09,Ore10,Ore11,Ore12,Ore13,Ore14	*OreG0013658*
scaffold33128	400258	T	G	NA	*OreG0013658*
scaffold33128	402738	G	C	NA	*OreG0013659*
scaffold33128	427016	C	T	NA	*OreG0013661*
scaffold33128	427050	A	G	NA	*OreG0013661*

scaffold33128	427463	T	A	NA	*OreG0013661*
scaffold33128	437721	G	A	NA	*OreG0013663*
scaffold33128	467545	T	C	Ore02,Ore10,Ore11,Ore12,Ore13	*OreG0013666*
scaffold33128	467926	G	C	NA	*OreG0013666*
scaffold33128	505075	T	A	Ore04,Ore05	*OreG0013673*
scaffold33128	505106	C	T	Och01,Och02,Och03,Och04,Och05,Och06,Och09,Och10,Och11,Och12,Och13,Ore01,Ore02,Ore03,Ore04,Ore05,Ore06,Ore07,Ore08,Ore09,Ore10,Ore11,Ore12,Ore13,Ore14	*OreG0013673*
scaffold33128	510640	T	G	NA	*OreG0013674*
scaffold33128	599844	T	G	Och01,Och02,Och03,Och04,Och05,Och06,Och07,Och08,Och09,Och10,Och11,Och12,Och13,Och14,Ore01,Ore02,Ore03,Ore04,Ore05,Ore06,Ore07,Ore08,Ore09,Ore10,Ore11,Ore12,Ore13,Ore14	*OreG0013679*
scaffold33128	600367	G	C	Och01,Och02,Och03,Och04,Och05,Och06,Och07,Och08,Och09,Och10,Och11,Och12,Och13,Och14,Ore01,Ore02,Ore03,Ore04,Ore05,Ore06,Ore07,Ore08,Ore09,Ore10,Ore11,Ore12,Ore13,Ore14	*OreG0013679*
scaffold33128	633653	T	C	Och08	*OreG0013681*
scaffold33128	639478	C	T	Ore01,Ore02,Ore03,Ore04,Ore05,Ore06,Ore07,Ore08,Ore09,Ore10,Ore11,Ore12,Ore13,Ore14	*OreG0013682*
scaffold33128	640240	C	T	Och01,Och02,Och03,Och04,Och05,Och06,Och07,Och08,Och09,Och10,Och11,Och12,Och13,Och14	*OreG0013682*
scaffold33128	683859	A	T	NA	*OreG0013687*
scaffold33128	685290	G	T	NA	*OreG0013687*
scaffold33128	697519	G	T	Ore04,Ore05	*OreG0013690*
scaffold33128	697926	C	T	Ore01,Ore02,Ore03,Ore04,Ore05,Ore06,Ore07,Ore08,Ore09,Ore10,Ore11,Ore12,Ore13,Ore14	*OreG0013690*
scaffold33128	698452	G	C	NA	*OreG0013690*
scaffold33128	757635	A	G	Och02,Och03,Och04,Och05,Och06,Och08,Och12,Och13,Och14	*OreG0013697*
scaffold33128	758262	A	T	Ore01,Ore02,Ore03,Ore04,Ore05,Ore06,Ore07,Ore08,Ore09,Ore10,Ore11,Ore12,Ore13,Ore14	*OreG0013697*
scaffold33128	768461	A	T	Och03,Och06	*OreG0013698*
scaffold33128	779023	C	A	NA	*OreG0013699*
scaffold33128	779153	C	T	NA	*OreG0013699*
scaffold33128	779687	G	A	NA	*OreG0013699*
scaffold33128	779757	A	G	NA	*OreG0013699*
scaffold33128	784182	C	A	NA	*OreG0013701*
scaffold33128	787536	C	T	Och08	*OreG0013701*
scaffold33128	788262	G	A	NA	*OreG0013701*
scaffold33128	791770	G	T	Ore01,Ore02,Ore03,Ore04,Ore05,Ore06,Ore07,Ore08,Ore09,Ore10,Ore11,Ore12,Ore13,Ore14	*OreG0013702*
scaffold33128	791928	A	T	NA	*OreG0013702*
scaffold33128	791938	C	G	Och01,Och02,Och03,Och04,Och05,Och06,Och07,Och08,Och09,Och10,Och11,Och12,Och13,Och14	*OreG0013702*
scaffold33128	792299	C	A	Och08	*OreG0013702*
scaffold33128	804565	T	C	Och01,Och03,Och06,Och08	*OreG0013704*
scaffold33128	806366	A	G	NA	*OreG0013704*
scaffold33128	813728	A	T	NA	*OreG0013705*
scaffold33128	820039	G	A	NA	*OreG0013705*
scaffold33128	833906	G	T	Och01,Och02,Och03,Och04,Och05,Och06,Och07,Och08,Och09,Och10,Och11,Och12,Och13,Och14,Ore01,Ore02,Ore03,Ore04,Ore05,Ore06,Ore07,Ore08,Ore09,Ore10,Ore11,Ore12,Ore13,Ore14	*OreG0013706*
scaffold33128	846259	G	A	Ore01,Ore02,Ore03,Ore04,Ore05,Ore06,Ore07,Ore08,Ore09,Ore10,Ore11,Ore12,Ore13,Ore14	*OreG0013707*
scaffold33128	859897	C	T	Och01,Och02,Och03,Och04,Och05,Och06,Och07,Och08,Och09,Och10,Och11,Och12,Och13,Och14	*OreG0013709*
scaffold33128	859900	G	A	Ore01,Ore02,Ore03,Ore04,Ore05,Ore06,Ore07,Ore08,Ore09,Ore10,Ore11,Ore12,Ore13,Ore14	*OreG0013709*
scaffold33128	859920	C	A	Och01,Och02,Och03,Och04,Och05,Och06,Och07,Och08,Och09,Och10,Och11,Och12,Och13,Och14,Ore01,Ore02,Ore03,Ore04,Ore05,Ore06,Ore07,Ore08,Ore09,Ore10,Ore11,Ore12,Ore13,Ore14	*OreG0013709*
scaffold33128	860434	G	A	NA	*OreG0013709*
scaffold33128	869811	T	C	NA	*OreG0013710*
scaffold33128	878723	C	A	Och03,Och06	*OreG0013710*
scaffold33128	881777	A	G	Och01,Och02,Och03,Och04,Och05,Och06,Och08,Och09,Och10,Och11,Och12,Och13	*OreG0013710*
scaffold33128	885758	C	T	Och01,Och02,Och03,Och04,Och05,Och06,Och07,Och08,Och09,Och10,Och11,Och12,Och13,Och14	*OreG0013710*
scaffold33128	893872	T	G	NA	*OreG0013711*
scaffold33128	915537	T	G	Ore02,Ore10,Ore11,Ore12,Ore13	*OreG0013713*
scaffold33128	916652	C	T	NA	*OreG0013713*
scaffold33128	918100	A	T	NA	*OreG0013713*
scaffold33128	918174	G	A	NA	*OreG0013713*
scaffold33128	1047579	A	T	NA	*OreG0013716*
scaffold33128	1136900	A	T	Och01,Och02,Och03,Och04,Och05,Och06,Och07,Och08,Och09,Och10,Och11,Och12,Och13,Och14	*OreG0013723*

scaffold33128	1174356	T	C	Och01,Och02,Och03,Och04,Och05,Och06,Och07,Och08,Och09,Och10,Och11,Och12,Och13,Och14	*OreG0013727*
scaffold33128	1194041	C	A	Och01,Och02,Och03,Och04,Och05,Och06,Och07,Och08,Och09,Och10,Och11,Och12,Och13,Och14,Ore01,Ore02,Ore03,Ore04,Ore05,Ore06,Ore07,Ore08,Ore09,Ore10,Ore11,Ore12,Ore13,Ore14	*OreG0013729*
scaffold33128	1197839	C	T	Och02,Och03,Och04,Och05,Och06,Och07,Och08,Och11,Och12,Och13,Och14	*OreG0013730*
scaffold33128	1201295	T	A	Ore01,Ore02,Ore03,Ore04,Ore05,Ore06,Ore07,Ore08,Ore09,Ore10,Ore11,Ore12,Ore13,Ore14	*OreG0013730*
scaffold33128	1228800	T	C	Ore01,Ore02,Ore03,Ore04,Ore05,Ore06,Ore07,Ore08,Ore09,Ore10,Ore11,Ore12,Ore13,Ore14	*OreG0013733*
scaffold33128	1258402	C	T	NA	*OreG0013735*
scaffold33128	1261928	C	A	Och08	*OreG0013735*
scaffold33128	1261967	T	C	Ore01,Ore02,Ore03,Ore04,Ore05,Ore06,Ore07,Ore08,Ore09,Ore10,Ore11,Ore12,Ore13,Ore14	*OreG0013735*
scaffold33128	1262309	A	G	Och02,Och03,Och04,Och05,Och06,Och08,Och12,Och13	*OreG0013735*
scaffold33128	1313027	A	T	Ore01,Ore02,Ore03,Ore04,Ore05,Ore06,Ore07,Ore08,Ore09,Ore10,Ore11,Ore12,Ore13,Ore14	*OreG0013741*
scaffold33128	1325457	A	G	NA	*OreG0013742*
scaffold33128	1335708	A	G	Och08	*OreG0013743*
scaffold33128	1339237	A	G	Och03,Och06	*OreG0013743*
scaffold33128	1350842	C	A	Och01,Och02,Och03,Och04,Och05,Och06,Och07,Och08,Och09,Och10,Och11,Och12,Och13,Och14,Ore01,Ore02,Ore03,Ore04,Ore05,Ore06,Ore07,Ore08,Ore09,Ore10,Ore11,Ore12,Ore13,Ore14	*OreG0013744*
scaffold33128	1498235	C	T	Och02,Och03,Och04,Och05,Och06,Och08,Och11,Och12,Och13,Och14,Ore01,Ore02,Ore03,Ore04,Ore05,Ore06,Ore07,Ore08,Ore09,Ore10,Ore11,Ore12,Ore13,Ore14	*OreG0013756*
scaffold33128	1498264	G	C	NA	*OreG0013756*
scaffold33128	1498306	T	A	NA	*OreG0013756*
scaffold33128	1499729	G	A	NA	*OreG0013756*
scaffold33128	1500536	C	A	NA	*OreG0013756*
scaffold33128	1522096	T	A	Och01,Och02,Och03,Och04,Och05,Och06,Och07,Och08,Och09,Och10,Och11,Och12,Och13,Och14,Ore01,Ore02,Ore03,Ore04,Ore05,Ore06,Ore07,Ore08,Ore09,Ore10,Ore11,Ore12,Ore13,Ore14	*OreG0013759*
scaffold33128	1522322	A	C	Ore01,Ore02,Ore03,Ore04,Ore05,Ore06,Ore07,Ore08,Ore09,Ore10,Ore11,Ore12,Ore13,Ore14	*OreG0013759*
scaffold33128	1529618	G	A	Ore01,Ore02,Ore03,Ore04,Ore05,Ore06,Ore07,Ore08,Ore09,Ore10,Ore11,Ore12,Ore13,Ore14	*OreG0013761*
scaffold33128	1547941	T	A	Och08	*OreG0013762*
scaffold33128	1557180	G	T	Och01,Och02,Och03,Och04,Och05,Och06,Och07,Och08,Och11,Och12,Och13,Ore01,Ore02,Ore03,Ore04,Ore05,Ore06,Ore07,Ore08,Ore09,Ore10,Ore11,Ore12,Ore13,Ore14	*OreG0013763*
scaffold33128	1583509	T	C	NA	*OreG0013764*
scaffold33128	1599025	C	T	Och02,Och03,Och04,Och05,Och06,Och07,Och09,Och10,Och11,Och12,Och13,Och14	*OreG0013770*
scaffold33128	1602346	C	T	Ore01,Ore02,Ore03,Ore04,Ore05,Ore06,Ore07,Ore08,Ore09,Ore10,Ore11,Ore12,Ore13,Ore14	*OreG0013771*
scaffold33128	1602973	C	A	Ore01,Ore02,Ore03,Ore04,Ore05,Ore06,Ore07,Ore08,Ore09,Ore10,Ore11,Ore12,Ore13,Ore14	*OreG0013771*
scaffold33128	1604407	C	T	NA	*OreG0013771*
scaffold33128	1604442	G	A	NA	*OreG0013771*
scaffold33128	1605553	A	T	Och07	*OreG0013771*
scaffold33128	1639821	T	C	NA	*OreG0013773*
scaffold33128	1673383	A	G	Ore04,Ore05	*OreG0013778*
scaffold33128	1684683	A	G	Ore01,Ore02,Ore03,Ore04,Ore05,Ore06,Ore07,Ore08,Ore09,Ore10,Ore11,Ore12,Ore13,Ore14	*OreG0013778*
scaffold33154	100020	C	A	Och08,Och11	*OreG0014174*
scaffold33154	490013	G	T	Och01,Och02,Och03,Och04,Och05,Och06,Och07,Och08,Och09,Och10,Och11,Och12,Och13,Och14,Ore01,Ore02,Ore03,Ore04,Ore05,Ore06,Ore07,Ore08,Ore09,Ore10,Ore11,Ore12,Ore13,Ore14	*OreG0014208*
scaffold33154	490247	T	C	Ore01,Ore02,Ore03,Ore04,Ore05,Ore06,Ore07,Ore08,Ore09,Ore10,Ore11,Ore12,Ore13,Ore14	*OreG0014208*
scaffold33154	491210	T	C	NA	*OreG0014208*
scaffold33154	491258	C	T	Och03,Och04	*OreG0014208*
scaffold33154	491683	C	T	Ore01,Ore02,Ore03,Ore04,Ore05,Ore06,Ore07,Ore08,Ore09,Ore10,Ore11,Ore12,Ore13,Ore14	*OreG0014208*
scaffold33154	491782	A	C	NA	*OreG0014208*
scaffold33154	492297	A	C	NA	*OreG0014208*
scaffold33154	492418	C	T	NA	*OreG0014208*
scaffold33154	492504	A	T	Ore02,Ore04,Ore05,Ore08,Ore11,Ore12,Ore14	*OreG0014208*
scaffold33154	521213	T	A	NA	*OreG0014211*
scaffold33154	521219	C	T	NA	*OreG0014211*
scaffold33154	521272	T	C	Ore01,Ore02,Ore03,Ore04,Ore05,Ore06,Ore07,Ore08,Ore09,Ore10,Ore11,Ore12,Ore13,Ore14	*OreG0014211*
scaffold33154	521312	C	A	NA	*OreG0014211*
scaffold33154	955454	C	A	Och01,Och02,Och04,Och06,Och07,Och08,Och09,Och10,Och11,Och12,Och13,Och14	*OreG0014245*
scaffold33154	957764	G	A	NA	*OreG0014245*

scaffold33179	33341	G	C	Och12	*OreG0014647*
scaffold2685	12549	C	G	Ore01,Ore02,Ore03,Ore04,Ore05,Ore06,Ore07,Ore08,Ore09,Ore10,Ore11,Ore12,Ore13,Ore14	*OreG0007838*
scaffold2685	13997	A	C	NA	*OreG0007838*
scaffold2685	25515	T	G	NA	*OreG0007839*
scaffold2685	25597	G	T	NA	*OreG0007839*
scaffold2685	26562	C	A	Ore01,Ore02,Ore03,Ore04,Ore05,Ore06,Ore07,Ore08,Ore09,Ore10,Ore11,Ore12,Ore13,Ore14	*OreG0007839*
scaffold2685	203048	G	C	Ore01,Ore02,Ore03,Ore04,Ore05,Ore06,Ore07,Ore08,Ore09,Ore10,Ore11,Ore12,Ore13,Ore14	*OreG0007856*
scaffold2685	210437	T	A	Ore02	*OreG0007858*
scaffold2685	317948	A	T	NA	*OreG0007867*
scaffold2685	318731	T	A	NA	*OreG0007867*
scaffold2685	319307	T	C	NA	*OreG0007867*
scaffold2685	401123	T	G	NA	*OreG0007872*
scaffold2685	401654	A	G	NA	*OreG0007872*
scaffold2685	417159	T	C	Ore02	*OreG0007873*
scaffold2685	417666	C	T	Ore01,Ore02,Ore03,Ore04,Ore05,Ore06,Ore07,Ore08,Ore09,Ore10,Ore11,Ore12,Ore13,Ore14	*OreG0007873*
scaffold2685	421193	A	T	Och01,Och02,Och03,Och04,Och05,Och06,Och07,Och08,Och09,Och10,Och11,Och12,Och13,Och14	*OreG0007873*
scaffold2685	565977	G	A	NA	*OreG0007885*
scaffold2685	565980	C	T	NA	*OreG0007885*
scaffold2685	566086	T	A	NA	*OreG0007885*
scaffold2685	566127	T	C	NA	*OreG0007885*
scaffold2685	594926	G	A	Och11	*OreG0007887*
scaffold2685	595041	G	A	Och01,Och05,Och06,Och07,Och08,Och09,Och10,Och11,Och12,Och14,Ore01,Ore02,Ore03,Ore04,Ore05,Ore06,Ore07,Ore08,Ore09,Ore10,Ore11,Ore12,Ore13,Ore14	*OreG0007887*
scaffold2685	595245	C	A	NA	*OreG0007887*
scaffold2685	595260	C	A	NA	*OreG0007887*
scaffold2685	595467	G	A	Ore02,Ore03,Ore06,Ore08,Ore09,Ore10,Ore11,Ore12,Ore14	*OreG0007887*
scaffold2685	597289	A	G	NA	*OreG0007887*
scaffold2685	597518	C	T	NA	*OreG0007887*
scaffold31091	119469	G	A	NA	*OreG0009374*
scaffold33159	76128	G	T	Och06	*OreG0014272*
scaffold33159	395496	G	T	Och01,Och02,Och03,Och04,Och05,Och06,Och07,Och08,Och09,Och10,Och11,Och12,Och13,Och14	*OreG0014320*
scaffold33208	31100	C	A	Och12,Och13	*OreG0016226*
scaffold33208	31200	G	A	Och12,Och13	*OreG0016226*
scaffold33208	78658	G	A	Och14	*OreG0016233*
scaffold33110	32827	C	A	Ore01,Ore02,Ore03,Ore04,Ore05,Ore06,Ore07,Ore08,Ore09,Ore10,Ore11,Ore12,Ore13,Ore14	*OreG0012397*
scaffold33110	32837	A	G	NA	*OreG0012397*
scaffold33110	56903	G	A	NA	*OreG0012400*
scaffold33110	56909	G	T	Och02,Och03,Och04,Och06,Och09,Och10,Och13	*OreG0012400*
scaffold33110	56938	C	A	Och01,Och02,Och03,Och04,Och05,Och06,Och07,Och08,Och09,Och10,Och11,Och12,Och13,Och14,Ore01,Ore02,Ore03,Ore04,Ore05,Ore06,Ore07,Ore08,Ore09,Ore10,Ore11,Ore12,Ore13,Ore14	*OreG0012400*
scaffold33110	63307	G	A	Ore01,Ore02,Ore03,Ore04,Ore05,Ore06,Ore07,Ore08,Ore09,Ore10,Ore11,Ore12,Ore13,Ore14	*OreG0012401*
scaffold33110	67825	A	G	Ore01,Ore02,Ore03,Ore04,Ore05,Ore06,Ore07,Ore08,Ore09,Ore10,Ore11,Ore12,Ore13,Ore14	*OreG0012402*
scaffold33110	68098	A	C	Och02,Och03,Och04,Och06,Och07,Och08,Och09,Och10,Och11,Och13	*OreG0012402*
scaffold33110	68376	T	C	Och02,Och03,Och04,Och06,Och07,Och08,Och09,Och10,Och11,Och13	*OreG0012402*
scaffold33110	69203	A	T	Och01	*OreG0012402*
scaffold33110	116707	A	C	NA	*OreG0012410*
scaffold33110	116781	C	A	NA	*OreG0012410*
scaffold33110	122666	G	T	Och01,Och02,Och03,Och04,Och05,Och06,Och07,Och08,Och09,Och10,Och11,Och12,Och13,Och14	*OreG0012411*
scaffold33110	128713	G	C	Ore01,Ore02,Ore03,Ore04,Ore05,Ore06,Ore07,Ore08,Ore09,Ore10,Ore11,Ore12,Ore13,Ore14	*OreG0012412*
scaffold33110	131125	T	A	Och07,Och08	*OreG0012412*
scaffold33110	150269	G	A	Ore02,Ore04,Ore05,Ore06,Ore07,Ore08,Ore11,Ore12,Ore13,Ore14	*OreG0012415*
scaffold33110	150280	C	T	Och01,Och02,Och03,Och04,Och05,Och06,Och07,Och08,Och09,Och10,Och11,Och12,Och13,Och14,Ore01,Ore02,Ore03,Ore04,Ore05,Ore06,Ore07,Ore08,Ore09,Ore10,Ore11,Ore12,Ore13,Ore14	*OreG0012415*
scaffold33110	175899	C	T	Ore01,Ore02,Ore03,Ore04,Ore05,Ore06,Ore07,Ore08,Ore09,Ore10,Ore11,Ore12,Ore13,Ore14	*OreG0012418*
scaffold33110	185997	G	A	Och02,Och03,Och04,Och05,Och06,Och12	*OreG0012419*
scaffold33110	186028	C	G	Och02,Och03,Och04,Och05,Och06,Och12	*OreG0012419*
scaffold33110	186116	C	A	Och01,Och07,Och08,Och09,Och10,Och11,Och14	*OreG0012419*
scaffold33110	186168	G	A	Och02,Och03,Och04,Och05,Och06,Och12,Och13	*OreG0012419*
scaffold33110	186186	G	C	NA	*OreG0012419*
scaffold33110	186219	T	C	Och02,Och03,Och04,Och05,Och06,Och12,Och13	*OreG0012419*
scaffold33110	186271	C	T	NA	*OreG0012419*

scaffold33110	186334	C	T	Ore01,Ore02,Ore03,Ore04,Ore05,Ore06,Ore07,Ore08,Ore09,Ore10,Ore11,Ore12,Ore13,Ore14	*OreG0012419*
scaffold33110	186336	G	T	Ore01,Ore02,Ore03,Ore04,Ore05,Ore06,Ore07,Ore08,Ore09,Ore10,Ore11,Ore12,Ore13,Ore14	*OreG0012419*
scaffold33110	186445	C	T	NA	*OreG0012419*
scaffold33110	186617	T	C	Och01	*OreG0012419*
scaffold33110	186630	C	T	Ore01,Ore02,Ore04,Ore05,Ore06,Ore07,Ore08,Ore11,Ore12,Ore13,Ore14	*OreG0012419*
scaffold33110	186839	C	T	Och07,Och09,Och10,Och11,Och14	*OreG0012419*
scaffold33110	186850	G	T	Och02,Och03,Och04,Och05,Och06,Och12,Och13	*OreG0012419*
scaffold33110	186860	G	T	Ore01,Ore02,Ore03,Ore04,Ore05,Ore06,Ore07,Ore08,Ore09,Ore10,Ore11,Ore12,Ore13,Ore14	*OreG0012419*
scaffold33110	186882	G	A	Ore01,Ore02,Ore03,Ore04,Ore05,Ore06,Ore07,Ore08,Ore09,Ore10,Ore11,Ore12,Ore13,Ore14	*OreG0012419*
scaffold33110	188050	T	A	Ore01,Ore02,Ore03,Ore04,Ore05,Ore06,Ore07,Ore08,Ore09,Ore10,Ore11,Ore12,Ore13,Ore14	*OreG0012420*
scaffold33110	188218	G	A	Ore01,Ore02,Ore04,Ore05,Ore06,Ore07,Ore08,Ore11,Ore12,Ore13,Ore14	*OreG0012420*
scaffold33110	200628	C	G	Ore01,Ore02,Ore03,Ore04,Ore05,Ore06,Ore07,Ore08,Ore09,Ore10,Ore11,Ore12,Ore13,Ore14	*OreG0012422*
scaffold33110	248781	T	A	Och02,Och03,Och04,Och06,Och13,Och14	*OreG0012430*
scaffold33110	264830	G	A	Och14	*OreG0012431*
scaffold33110	265341	G	A	Och02,Och03,Och04,Och05,Och06,Och09,Och10,Och12,Och13	*OreG0012431*
scaffold33110	266033	G	A	NA	*OreG0012431*
scaffold33110	271728	T	C	NA	*OreG0012433*
scaffold33110	332920	C	T	NA	*OreG0012439*
scaffold33110	333050	C	T	Ore01,Ore02,Ore03,Ore04,Ore05,Ore06,Ore07,Ore08,Ore09,Ore10,Ore11,Ore12,Ore13,Ore14	*OreG0012439*
scaffold33110	353051	C	T	NA	*OreG0012447*
scaffold33110	353419	C	A	NA	*OreG0012447*
scaffold33110	353535	C	G	NA	*OreG0012447*
scaffold33110	353781	G	A	NA	*OreG0012447*
scaffold33110	353963	C	A	NA	*OreG0012447*
scaffold33110	355961	A	G	NA	*OreG0012448*
scaffold33110	356136	G	A	NA	*OreG0012448*
scaffold33110	375377	A	G	NA	*OreG0012450*
scaffold33110	381463	T	C	NA	*OreG0012452*
scaffold33110	381505	C	T	Ore01,Ore02,Ore03,Ore04,Ore05,Ore06,Ore07,Ore08,Ore09,Ore10,Ore11,Ore12,Ore13,Ore14	*OreG0012452*
scaffold33110	389123	A	G	Och01,Och02,Och03,Och04,Och05,Och06,Och07,Och08,Och09,Och10,Och11,Och12,Och13,Och14	*OreG0012454*
scaffold33110	389147	A	G	Och01,Och02,Och03,Och04,Och05,Och06,Och07,Och08,Och09,Och10,Och11,Och12,Och13	*OreG0012454*
scaffold33110	394520	A	C	NA	*OreG0012455*
scaffold33110	417763	T	C	Ore01,Ore02,Ore03,Ore04,Ore05,Ore06,Ore07,Ore08,Ore09,Ore10,Ore11,Ore12,Ore13,Ore14	*OreG0012459*
scaffold33110	417788	A	G	Ore01,Ore02,Ore03,Ore04,Ore05,Ore06,Ore07,Ore08,Ore09,Ore10,Ore11,Ore12,Ore13,Ore14	*OreG0012459*
scaffold33110	417793	T	A	Och01,Och02,Och03,Och04,Och05,Och06,Och07,Och08,Och09,Och10,Och11,Och12,Och13,Och14	*OreG0012459*
scaffold33110	417965	G	A	Och07,Och11,Ore01,Ore02,Ore04,Ore05,Ore06,Ore07,Ore08,Ore11,Ore12,Ore13,Ore14	*OreG0012459*
scaffold33110	418082	T	A	Ore01,Ore02,Ore03,Ore04,Ore05,Ore06,Ore07,Ore08,Ore09,Ore10,Ore11,Ore12,Ore13,Ore14	*OreG0012459*
scaffold33110	423309	T	C	Och01,Och02,Och03,Och04,Och05,Och06,Och07,Och08,Och09,Och10,Och11,Och12,Och13,Och14,Ore01,Ore02,Ore03,Ore04,Ore05,Ore06,Ore07,Ore08,Ore09,Ore10,Ore11,Ore12,Ore13,Ore14	*OreG0012462*
scaffold33110	423627	T	C	Och01,Och02,Och03,Och04,Och05,Och06,Och07,Och08,Och09,Och10,Och11,Och12,Och13,Och14	*OreG0012462*
scaffold33110	424455	T	C	NA	*OreG0012462*
scaffold33110	443138	G	C	NA	*OreG0012465*
scaffold33110	443787	G	A	Och01,Och02,Och03,Och04,Och05,Och06,Och07,Och08,Och09,Och10,Och11,Och12,Och13,Och14	*OreG0012465*
scaffold33110	451919	G	T	Ore01,Ore02,Ore03,Ore04,Ore05,Ore06,Ore07,Ore08,Ore09,Ore10,Ore11,Ore12,Ore13,Ore14	*OreG0012467*
scaffold33110	452343	T	A	Och07	*OreG0012467*
scaffold33110	452356	C	A	Och01,Och02,Och03,Och04,Och05,Och06,Och07,Och08,Och09,Och10,Och11,Och12,Och13,Och14,Ore01,Ore02,Ore03,Ore04,Ore05,Ore06,Ore07,Ore08,Ore09,Ore10,Ore11,Ore12,Ore13,Ore14	*OreG0012467*
scaffold33110	452365	T	G	Och09,Och10	*OreG0012467*
scaffold33110	452389	C	A	Ore01,Ore02,Ore03,Ore04,Ore05,Ore06,Ore07,Ore08,Ore09,Ore10,Ore11,Ore12,Ore13,Ore14	*OreG0012467*
scaffold33110	469723	G	C	Och07	*OreG0012470*
scaffold33110	474151	C	T	Och07	*OreG0012471*
scaffold33110	475880	T	A	Ore02,Ore04,Ore05,Ore06,Ore07,Ore08,Ore11,Ore12,Ore13,Ore14	*OreG0012471*
scaffold33110	480753	C	A	Och02,Och03,Och04,Och05,Och06,Och09,Och10,Och12,Och13	*OreG0012472*
scaffold33110	492189	C	G	NA	*OreG0012474*

scaffold33110	516414	A	T	Och01,Och02,Och03,Och04,Och05,Och06,Och07,Och08,Och09,Och10,Och11,Och12,Och13,Och14,Ore01,Ore02,Ore03,Ore04,Ore05,Ore06,Ore07,Ore08,Ore09,Ore10,Ore11,Ore12,Ore13,Ore14	*OreG0012478*
scaffold33110	516795	A	G	Och02,Och03,Och04,Och05,Och06,Och12,Och13	*OreG0012478*
scaffold33110	709144	C	T	Och02,Och03,Och04,Och06,Och12,Och13	*OreG0012487*
scaffold33110	721037	C	T	NA	*OreG0012488*
scaffold33110	721372	G	A	NA	*OreG0012488*
scaffold33110	726940	T	A	Och02,Och03,Och04,Och06,Och13,Och14	*OreG0012489*
scaffold33110	727098	C	G	Och02,Och03,Och04,Och06,Och13	*OreG0012489*
scaffold33110	728527	T	A	NA	*OreG0012489*
scaffold33110	738121	G	A	NA	*OreG0012490*
scaffold33110	738795	T	A	Och01,Och02,Och03,Och04,Och05,Och06,Och07,Och08,Och09,Och10,Och11,Och12,Och13,Och14,Ore01,Ore02,Ore03,Ore04,Ore05,Ore06,Ore07,Ore08,Ore09,Ore10,Ore11,Ore12,Ore13,Ore14	*OreG0012490*
scaffold33110	739846	T	C	NA	*OreG0012490*
scaffold33110	740280	G	A	Och02,Och03,Och04,Och06,Och07,Och13	*OreG0012490*
scaffold33110	773396	C	A	Och01,Och02,Och03,Och04,Och05,Och06,Och07,Och08,Och09,Och10,Och11,Och12,Och13,Och14	*OreG0012495*
scaffold33110	776414	C	G	NA	*OreG0012495*
scaffold33110	780463	T	A	Ore01,Ore03,Ore04,Ore05,Ore08,Ore12	*OreG0012496*
scaffold33110	784947	A	T	NA	*OreG0012497*
scaffold33110	793276	C	G	Ore04,Ore05,Ore06,Ore07,Ore08,Ore14	*OreG0012498*
scaffold33110	811832	T	C	NA	*OreG0012501*
scaffold33110	823846	C	A	Och01,Och02,Och03,Och04,Och05,Och06,Och07,Och08,Och09,Och10,Och11,Och12,Och13,Och14,Ore01,Ore02,Ore03,Ore04,Ore05,Ore06,Ore07,Ore08,Ore09,Ore10,Ore11,Ore12,Ore13,Ore14	*OreG0012503*
scaffold33110	823880	A	C	Och01,Och02,Och03,Och04,Och05,Och06,Och07,Och08,Och09,Och10,Och11,Och12,Och13,Och14,Ore01,Ore02,Ore03,Ore04,Ore05,Ore06,Ore07,Ore08,Ore09,Ore10,Ore11,Ore12,Ore13,Ore14	*OreG0012503*
scaffold33110	832776	G	A	NA	*OreG0012504*
scaffold33110	832981	G	T	NA	*OreG0012504*
scaffold33110	833001	T	A	NA	*OreG0012504*
scaffold33110	833049	T	G	NA	*OreG0012504*
scaffold33110	833094	C	T	NA	*OreG0012504*
scaffold33110	838420	A	T	Och01,Och02,Och03,Och04,Och05,Och06,Och07,Och08,Och09,Och10,Och11,Och12,Och13,Och14,Ore01,Ore02,Ore03,Ore04,Ore05,Ore06,Ore07,Ore08,Ore09,Ore10,Ore11,Ore12,Ore13,Ore14	*OreG0012505*
scaffold33110	841423	C	T	Och01,Och02,Och03,Och04,Och05,Och06,Och07,Och08,Och09,Och10,Och11,Och12,Och13,Och14,Ore01,Ore02,Ore03,Ore04,Ore05,Ore06,Ore07,Ore08,Ore09,Ore10,Ore11,Ore12,Ore13,Ore14	*OreG0012505*
scaffold33110	843082	C	T	NA	*OreG0012506*
scaffold33110	843722	T	A	Och09,Och10,Och12	*OreG0012506*
scaffold33110	843752	A	C	NA	*OreG0012506*
scaffold33110	843794	A	G	Och09,Och10,Och12	*OreG0012506*
scaffold33110	844685	T	C	NA	*OreG0012506*
scaffold33110	845303	T	C	Och06,Och07,Och14	*OreG0012506*
scaffold33110	861618	T	A	Och01,Och02,Och03,Och04,Och05,Och06,Och07,Och08,Och09,Och10,Och11,Och12,Och13,Och14,Ore01,Ore02,Ore03,Ore04,Ore05,Ore06,Ore07,Ore08,Ore09,Ore10,Ore11,Ore12,Ore13,Ore14	*OreG0012510*
scaffold33110	862375	G	T	NA	*OreG0012510*
scaffold33110	869164	G	C	NA	*OreG0012511*
scaffold33110	873127	A	T	Ore03,Ore04,Ore05,Ore06,Ore07,Ore08,Ore14	*OreG0012511*
scaffold33110	878300	T	A	Och01,Och02,Och03,Och04,Och05,Och06,Och07,Och08,Och09,Och10,Och11,Och12,Och13,Och14	*OreG0012513*
scaffold33110	914368	T	C	Och08,Och09,Och10,Och11	*OreG0012516*
scaffold33110	914380	C	T	Ore01,Ore02,Ore03,Ore04,Ore05,Ore06,Ore07,Ore08,Ore09,Ore10,Ore11,Ore12,Ore13,Ore14	*OreG0012516*
scaffold33110	914780	C	A	NA	*OreG0012517*
scaffold33110	941031	A	G	Och01,Och07,Och08	*OreG0012522*
scaffold33110	941044	C	G	Ore02,Ore04,Ore05,Ore06,Ore07,Ore08,Ore11,Ore12,Ore13,Ore14	*OreG0012522*
scaffold33110	962458	T	A	NA	*OreG0012526*
scaffold33110	962787	G	T	NA	*OreG0012526*
scaffold33110	963796	C	T	NA	*OreG0012526*
scaffold33110	963998	C	T	NA	*OreG0012526*
scaffold33110	964019	A	G	NA	*OreG0012526*
scaffold33110	964241	C	T	NA	*OreG0012526*
scaffold33110	964988	A	G	NA	*OreG0012526*
scaffold33110	964990	A	C	NA	*OreG0012526*
scaffold33110	965706	T	A	Och02,Och03,Och04,Och06,Och07,Och09,Och10,Och12,Och13	*OreG0012526*
scaffold33110	979590	T	C	Ore04,Ore05,Ore06,Ore07,Ore08,Ore14	*OreG0012528*
scaffold33110	979685	C	T	NA	*OreG0012528*
scaffold33110	1014571	G	T	NA	*OreG0012533*
scaffold33110	1015269	G	A	NA	*OreG0012533*
scaffold33110	1015708	T	C	Ore01,Ore02,Ore03,Ore04,Ore05,Ore06,Ore07,Ore08,Ore09,Ore10,Ore11,Ore12,Ore13,Ore14	*OreG0012533*

scaffold33110	1028609	C	A	Ore03,Ore04,Ore05,Ore06,Ore07,Ore08,Ore14	*OreG0012533*
scaffold33110	1031616	A	T	NA	*OreG0012535*
scaffold33110	1033555	C	A	NA	*OreG0012536*
scaffold33110	1034141	C	T	Och01,Och02,Och03,Och04,Och05,Och06,Och07,Och08,Och09,Och10,Och11,Och12,Och13,Och14,Ore01,Ore02,Ore03,Ore04,Ore05,Ore06,Ore07,Ore08,Ore09,Ore10,Ore11,Ore12,Ore13,Ore14	*OreG0012536*
scaffold33110	1034901	T	A	Och01,Och02,Och03,Och04,Och05,Och06,Och07,Och08,Och09,Och10,Och11,Och12,Och13,Och14,Ore01,Ore02,Ore03,Ore04,Ore05,Ore06,Ore07,Ore08,Ore09,Ore10,Ore11,Ore12,Ore13,Ore14	*OreG0012536*
scaffold33110	1051008	A	T	Ore01,Ore03,Ore04,Ore05,Ore06,Ore07,Ore08,Ore14	*OreG0012540*
scaffold33110	1051146	C	T	NA	*OreG0012540*
scaffold33110	1051486	A	C	Ore01,Ore02,Ore03,Ore04,Ore05,Ore06,Ore07,Ore08,Ore09,Ore10,Ore11,Ore12,Ore13,Ore14	*OreG0012540*
scaffold33110	1051514	A	G	Ore01,Ore02,Ore03,Ore04,Ore05,Ore06,Ore07,Ore08,Ore09,Ore10,Ore11,Ore12,Ore13,Ore14	*OreG0012540*
scaffold33110	1054706	G	A	Och05,Och06,Och14	*OreG0012541*
scaffold33110	1054772	A	T	NA	*OreG0012541*
scaffold33110	1055524	C	T	NA	*OreG0012541*
scaffold33110	1067671	T	C	NA	*OreG0012542*
scaffold33110	1067808	T	A	NA	*OreG0012542*
scaffold33110	1068050	G	A	NA	*OreG0012542*
scaffold33110	1068173	A	G	NA	*OreG0012542*
scaffold33110	1068210	G	T	NA	*OreG0012542*
scaffold33110	1068834	A	G	NA	*OreG0012542*
scaffold33110	1069857	C	A	NA	*OreG0012542*
scaffold33110	1069869	C	T	NA	*OreG0012542*
scaffold33110	1069985	C	T	NA	*OreG0012542*
scaffold33110	1070274	G	A	NA	*OreG0012542*
scaffold33110	1070373	T	C	NA	*OreG0012542*
scaffold33110	1070439	G	C	NA	*OreG0012542*
scaffold33110	1070730	T	G	NA	*OreG0012542*
scaffold33110	1070818	G	T	NA	*OreG0012542*
scaffold33110	1074939	A	T	NA	*OreG0012543*
scaffold33110	1075099	A	C	Ore01,Ore03,Ore04,Ore05,Ore06,Ore07,Ore08,Ore14	*OreG0012543*
scaffold33110	1078403	A	G	Och05,Och06,Och14	*OreG0012543*
scaffold33110	1092178	T	G	NA	*OreG0012545*
scaffold33110	1094799	G	T	NA	*OreG0012546*
scaffold33110	1096726	G	T	NA	*OreG0012546*
scaffold33110	1108539	G	A	NA	*OreG0012548*
scaffold33110	1117564	T	C	Ore01,Ore02,Ore03,Ore04,Ore05,Ore06,Ore07,Ore08,Ore09,Ore10,Ore11,Ore12,Ore13,Ore14	*OreG0012550*
scaffold33110	1119791	G	A	NA	*OreG0012550*
scaffold33110	1122339	T	A	NA	*OreG0012551*
scaffold33110	1135066	T	A	Ore01,Ore02,Ore03,Ore04,Ore05,Ore06,Ore07,Ore08,Ore09,Ore10,Ore11,Ore12,Ore13,Ore14	*OreG0012555*
scaffold33110	1136650	G	A	Och01,Och02,Och03,Och04,Och05,Och06,Och07,Och08,Och09,Och10,Och11,Och12,Och13,Och14,Ore01,Ore02,Ore03,Ore04,Ore05,Ore06,Ore07,Ore08,Ore09,Ore10,Ore11,Ore12,Ore13,Ore14	*OreG0012555*
scaffold33110	1151028	G	C	Och01,Och02,Och03,Och04,Och05,Och06,Och07,Och08,Och09,Och10,Och11,Och12,Och13,Och14	*OreG0012557*
scaffold33110	1164296	A	C	Och01,Och02,Och03,Och04,Och05,Och06,Och07,Och08,Och09,Och10,Och11,Och12,Och13,Och14,Ore01,Ore02,Ore03,Ore04,Ore05,Ore06,Ore07,Ore08,Ore09,Ore10,Ore11,Ore12,Ore13,Ore14	*OreG0012559*
scaffold33110	1171595	G	T	NA	*OreG0012560*
scaffold33110	1193275	T	A	NA	*OreG0012565*
scaffold33110	1194746	G	T	NA	*OreG0012565*
scaffold33110	1209869	T	A	Ore01,Ore02,Ore03,Ore04,Ore05,Ore06,Ore07,Ore08,Ore09,Ore10,Ore11,Ore12,Ore13,Ore14	*OreG0012568*
scaffold33110	1228417	A	C	Och01,Och02,Och03,Och04,Och05,Och06,Och07,Och08,Och09,Och10,Och11,Och12,Och13,Och14	*OreG0012569*
scaffold33110	1228840	A	T	Och01,Och02,Och03,Och04,Och05,Och06,Och07,Och08,Och09,Och10,Och11,Och12,Och13,Och14	*OreG0012569*
scaffold33110	1229039	A	G	Och14	*OreG0012569*
scaffold33110	1248149	T	C	Och01,Och02,Och03,Och04,Och05,Och06,Och07,Och08,Och09,Och10,Och11,Och12,Och13,Och14,Ore01,Ore02,Ore03,Ore04,Ore05,Ore06,Ore07,Ore08,Ore09,Ore10,Ore11,Ore12,Ore13,Ore14	*OreG0012572*
scaffold33110	1293960	C	T	NA	*OreG0012577*
scaffold33110	1294723	G	C	Ore01,Ore04,Ore05	*OreG0012577*
scaffold33110	1301403	T	C	Och02,Och03,Och04,Och05,Och06,Och07,Och08,Och09,Och10,Och11,Och12,Och13,Och14,Ore01,Ore02,Ore03,Ore04,Ore05,Ore06,Ore07,Ore08,Ore09,Ore10,Ore11,Ore12,Ore13,Ore14	*OreG0012579*
scaffold33110	1324863	G	A	Ore01,Ore02,Ore03,Ore04,Ore05,Ore06,Ore07,Ore08,Ore09,Ore10,Ore11,Ore12,Ore13,Ore14	*OreG0012583*
scaffold33110	1361102	T	G	Ore01,Ore02,Ore03,Ore04,Ore05,Ore06,Ore07,Ore08,Ore09,Ore10,Ore11,Ore12,Ore13,Ore14	*OreG0012589*

scaffold33110	1361481	C	T	Och01	*OreG0012589*
scaffold33110	1362652	C	T	Och01,Och02,Och03,Och04,Och05,Och06,Och07,Och08,Och09,Och10,Och11,Och12,Och13,Och14,Ore01,Ore02,Ore03,Ore04,Ore05,Ore06,Ore07,Ore08,Ore09,Ore10,Ore11,Ore12,Ore13,Ore14	*OreG0012590*
scaffold33110	1362692	C	A	NA	*OreG0012590*
scaffold33110	1362743	T	A	Ore01,Ore02,Ore03,Ore04,Ore05,Ore06,Ore07,Ore08,Ore09,Ore10,Ore11,Ore12,Ore13,Ore14	*OreG0012590*
scaffold33110	1362755	T	G	Ore01,Ore02,Ore03,Ore04,Ore05,Ore06,Ore07,Ore08,Ore09,Ore10,Ore11,Ore12,Ore13,Ore14	*OreG0012590*
scaffold33110	1362769	A	T	Ore01,Ore02,Ore03,Ore04,Ore05,Ore06,Ore07,Ore08,Ore09,Ore10,Ore11,Ore12,Ore13,Ore14	*OreG0012590*
scaffold33110	1365600	G	C	NA	*OreG0012591*
scaffold33110	1369431	A	T	Och02,Och03,Och04,Och06,Och13	*OreG0012592*
scaffold33110	1369459	T	A	Ore01,Ore02,Ore03,Ore04,Ore05,Ore06,Ore07,Ore08,Ore09,Ore10,Ore11,Ore12,Ore13,Ore14	*OreG0012592*
scaffold33110	1372458	G	C	NA	*OreG0012593*
scaffold33110	1387511	A	C	Och01,Och02,Och03,Och04,Och05,Och06,Och07,Och08,Och09,Och10,Och11,Och12,Och13,Och14	*OreG0012595*
scaffold33110	1394704	C	T	Och01,Och02,Och03,Och04,Och05,Och06,Och07,Och08,Och09,Och10,Och11,Och12,Och13,Och14	*OreG0012596*
scaffold33110	1397618	G	A	NA	*OreG0012597*
scaffold33110	1399450	T	C	Ore03,Ore04,Ore05,Ore06,Ore07,Ore08,Ore14	*OreG0012598*
scaffold33110	1403737	T	G	Och14	*OreG0012599*
scaffold33110	1404398	T	C	NA	*OreG0012599*
scaffold33110	1407127	T	A	Och02,Och03,Och04,Och05,Och06,Och07,Och08,Och09,Och10,Och11,Och12,Och13,Och14	*OreG0012599*
scaffold33110	1435613	T	A	Och02,Och03,Och04,Och06,Och13	*OreG0012601*
scaffold33110	1435841	T	C	NA	*OreG0012601*
scaffold33110	1435936	G	T	Och01,Och02,Och03,Och04,Och05,Och06,Och07,Och08,Och09,Och10,Och11,Och12,Och13,Och14,Ore01,Ore02,Ore03,Ore04,Ore05,Ore06,Ore07,Ore08,Ore09,Ore10,Ore11,Ore12,Ore13,Ore14	*OreG0012601*
scaffold33110	1436098	C	T	NA	*OreG0012601*
scaffold33110	1458827	C	T	Ore04,Ore05	*OreG0012605*
scaffold33110	1459040	C	T	Och01,Och12,Och14	*OreG0012605*
scaffold33110	1463790	A	G	NA	*OreG0012607*
scaffold33110	1464123	C	A	NA	*OreG0012607*
scaffold33110	1473143	C	G	NA	*OreG0012609*
scaffold33110	1496837	T	A	NA	*OreG0012611*
scaffold33110	1497468	A	G	NA	*OreG0012611*
scaffold33110	1497477	G	A	NA	*OreG0012611*
scaffold33110	1497484	C	T	NA	*OreG0012611*
scaffold33110	1497486	G	A	NA	*OreG0012611*
scaffold33110	1497549	A	T	NA	*OreG0012611*
scaffold33110	1498086	G	C	NA	*OreG0012611*
scaffold33110	1498146	T	A	NA	*OreG0012611*
scaffold33110	1498352	C	T	NA	*OreG0012611*
scaffold33110	1498374	C	G	NA	*OreG0012611*
scaffold33110	1503508	T	C	NA	*OreG0012612*
scaffold33110	1505408	G	T	Ore01,Ore04,Ore05	*OreG0012613*
scaffold33110	1505686	A	G	Och07,Och08,Och12,Och14	*OreG0012613*
scaffold33110	1505759	G	C	Ore01,Ore02,Ore03,Ore04,Ore05,Ore06,Ore07,Ore08,Ore09,Ore10,Ore11,Ore12,Ore13,Ore14	*OreG0012613*
scaffold33110	1506572	A	T	Och07,Och08,Och12,Och14	*OreG0012613*
scaffold33110	1525135	C	A	Och01,Och02,Och03,Och04,Och05,Och06,Och07,Och08,Och09,Och10,Och11,Och12,Och13,Och14	*OreG0012617*
scaffold33110	1531148	C	G	Och01,Och02,Och03,Och04,Och05,Och06,Och07,Och08,Och09,Och10,Och11,Och12,Och13,Och14,Ore01,Ore02,Ore03,Ore04,Ore05,Ore06,Ore07,Ore08,Ore09,Ore10,Ore11,Ore12,Ore13,Ore14	*OreG0012618*
scaffold33110	1531648	C	G	Ore01,Ore02,Ore03,Ore04,Ore05,Ore06,Ore07,Ore08,Ore09,Ore10,Ore11,Ore12,Ore13,Ore14	*OreG0012618*
scaffold33110	1531845	C	A	Och01,Och02,Och03,Och04,Och05,Och06,Och07,Och08,Och09,Och10,Och11,Och12,Och13,Och14	*OreG0012618*
scaffold33110	1531954	C	T	Och01,Och02,Och03,Och04,Och05,Och06,Och07,Och08,Och09,Och10,Och11,Och12,Och13,Och14,Ore01,Ore02,Ore03,Ore04,Ore05,Ore06,Ore07,Ore08,Ore09,Ore10,Ore11,Ore12,Ore13,Ore14	*OreG0012618*
scaffold33110	1532014	C	T	Och01,Och02,Och03,Och04,Och05,Och06,Och07,Och08,Och09,Och10,Och11,Och12,Och13,Och14,Ore01,Ore02,Ore03,Ore04,Ore05,Ore06,Ore07,Ore08,Ore09,Ore10,Ore11,Ore12,Ore13,Ore14	*OreG0012618*
scaffold33110	1538880	A	G	NA	*OreG0012616*
scaffold33110	1539060	T	A	NA	*OreG0012616*
scaffold33110	1539225	A	G	NA	*OreG0012616*
scaffold33110	1539312	A	T	NA	*OreG0012616*
scaffold33110	1545767	C	A	Ore13	*OreG0012619*
scaffold33110	1545783	A	G	Och01,Och07,Och08,Och09,Och10,Och11,Och14	*OreG0012619*
scaffold33110	1545786	T	A	Ore13	*OreG0012619*

scaffold33110	1545897	G	C	Och01,Och02,Och03,Och04,Och05,Och06,Och07,Och08,Och09,Och10,Och11,Och12,Och13,Och14,Ore01,Ore02,Ore03,Ore04,Ore05,Ore06,Ore07,Ore08,Ore09,Ore10,Ore11,Ore12,Ore13,Ore14	*OreG0012619*
scaffold33110	1546503	C	T	Och01,Och02,Och03,Och04,Och05,Och06,Och07,Och08,Och09,Och10,Och11,Och12,Och13,Och14,Ore01,Ore02,Ore03,Ore04,Ore05,Ore06,Ore07,Ore08,Ore09,Ore10,Ore11,Ore12,Ore13,Ore14	*OreG0012620*
scaffold33110	1546750	G	C	Och02,Och03,Och04,Och06,Och13	*OreG0012620*
scaffold33110	1546815	C	T	Och01,Och02,Och03,Och04,Och05,Och06,Och07,Och08,Och09,Och10,Och11,Och12,Och13,Och14	*OreG0012620*
scaffold33110	1546863	G	T	Och01,Och07,Och08,Och09,Och10,Och11,Och14	*OreG0012620*
scaffold33110	1552415	T	C	NA	*OreG0012621*
scaffold33110	1552676	T	C	NA	*OreG0012621*
scaffold33110	1557390	C	G	Ore03,Ore04,Ore05,Ore06,Ore07,Ore08,Ore14	*OreG0012622*
scaffold33110	1557498	A	C	Ore13	*OreG0012622*
scaffold33120	28846	G	A	Och02,Och03,Och04,Och05,Och06,Och12,Och13	*OreG0013354*
scaffold33120	32696	C	A	NA	*OreG0013355*
scaffold33120	35736	T	C	Och01,Och02,Och03,Och04,Och05,Och06,Och07,Och08,Och09,Och10,Och11,Och12,Och13,Och14,Ore01,Ore02,Ore03,Ore04,Ore05,Ore06,Ore07,Ore08,Ore09,Ore10,Ore11,Ore12,Ore13,Ore14	*OreG0013356*
scaffold33120	78267	G	T	Ore01,Ore06,Ore07,Ore08,Ore10,Ore11,Ore14	*OreG0013359*
scaffold33120	78398	G	T	Och01	*OreG0013359*
scaffold33120	78462	G	T	NA	*OreG0013359*
scaffold33120	95910	T	C	Ore01,Ore02,Ore03,Ore04,Ore05,Ore06,Ore07,Ore08,Ore09,Ore10,Ore11,Ore12,Ore13,Ore14	*OreG0013362*
scaffold33120	95952	A	T	Och01,Och02,Och03,Och04,Och05,Och06,Och07,Och09,Och10,Och11,Och12,Och13,Och14,Ore01,Ore02,Ore03,Ore04,Ore05,Ore06,Ore07,Ore08,Ore09,Ore10,Ore11,Ore12,Ore13,Ore14	*OreG0013362*
scaffold33120	130334	A	C	NA	*OreG0013366*
scaffold33120	142771	G	T	NA	*OreG0013368*
scaffold33120	142814	T	C	Och01,Och02,Och03,Och04,Och05,Och06,Och07,Och08,Och09,Och10,Och11,Och12,Och13,Och14,Ore01,Ore02,Ore03,Ore04,Ore05,Ore06,Ore07,Ore08,Ore09,Ore10,Ore11,Ore12,Ore13,Ore14	*OreG0013368*
scaffold33120	147043	C	T	Ore01,Ore02,Ore03,Ore04,Ore05,Ore06,Ore07,Ore08,Ore09,Ore10,Ore11,Ore12,Ore13,Ore14	*OreG0013369*
scaffold33120	147564	G	A	Och01,Och02,Och03,Och04,Och05,Och06,Och07,Och08,Och09,Och10,Och11,Och12,Och13,Och14,Ore01,Ore02,Ore03,Ore04,Ore05,Ore06,Ore07,Ore08,Ore09,Ore10,Ore11,Ore12,Ore13,Ore14	*OreG0013369*
scaffold33120	147918	A	G	Och01,Och02,Och03,Och04,Och05,Och06,Och07,Och08,Och09,Och10,Och11,Och12,Och13,Och14	*OreG0013369*
scaffold33120	157908	A	C	Och05,Och08	*OreG0013371*
scaffold33120	158329	A	G	Och02	*OreG0013371*
scaffold33120	162108	T	A	Och01,Och02,Och03,Och04,Och05,Och06,Och07,Och08,Och09,Och10,Och11,Och12,Och13,Och14	*OreG0013372*
scaffold33120	173886	G	C	Ore01,Ore06,Ore07,Ore08,Ore10,Ore11,Ore14	*OreG0013374*
scaffold33120	175600	G	C	Ore01,Ore02,Ore03,Ore04,Ore05,Ore06,Ore07,Ore08,Ore09,Ore10,Ore11,Ore12,Ore13,Ore14	*OreG0013375*
scaffold33120	175929	A	T	NA	*OreG0013375*
scaffold33120	178609	C	A	NA	*OreG0013376*
scaffold33120	179237	C	T	NA	*OreG0013376*
scaffold33120	205634	A	G	Och01,Och02,Och03,Och04,Och05,Och06,Och07,Och08,Och09,Och10,Och11,Och12,Och13,Och14,Ore01,Ore02,Ore03,Ore04,Ore05,Ore06,Ore07,Ore08,Ore09,Ore10,Ore11,Ore12,Ore13,Ore14	*OreG0013379*
scaffold33120	206078	G	A	Och02,Och06	*OreG0013379*
scaffold33120	218494	C	T	NA	*OreG0013381*
scaffold33120	233442	C	A	Och01,Och11	*OreG0013383*
scaffold33120	246509	G	T	Och01	*OreG0013385*
scaffold33120	257622	A	C	Och01,Och02,Och03,Och04,Och05,Och06,Och07,Och08,Och09,Och10,Och11,Och12,Och13,Och14,Ore01,Ore02,Ore03,Ore04,Ore05,Ore06,Ore07,Ore08,Ore09,Ore10,Ore11,Ore12,Ore13,Ore14	*OreG0013387*
scaffold33120	265622	T	C	NA	*OreG0013390*
scaffold33120	266048	G	C	Ore01,Ore02,Ore03,Ore04,Ore05,Ore06,Ore07,Ore08,Ore09,Ore10,Ore11,Ore12,Ore13,Ore14	*OreG0013390*
scaffold33120	286242	G	A	NA	*OreG0013394*
scaffold33120	291361	C	A	Och01,Och02,Och03,Och04,Och05,Och06,Och09,Och10,Och11,Och12,Och13,Och14	*OreG0013395*
scaffold33120	313132	C	G	Ore01,Ore06,Ore07,Ore08,Ore10,Ore11,Ore14	*OreG0013396*
scaffold33120	316345	G	A	Ore01,Ore02,Ore03,Ore04,Ore05,Ore06,Ore07,Ore08,Ore09,Ore10,Ore11,Ore12,Ore13,Ore14	*OreG0013397*
scaffold33120	342302	C	G	Och05	*OreG0013400*
scaffold33120	350338	T	C	NA	*OreG0013401*
scaffold33120	371749	G	A	NA	*OreG0013402*
scaffold33120	373426	G	T	NA	*OreG0013402*
scaffold33120	386869	C	T	Ore01,Ore02,Ore03,Ore04,Ore05,Ore06,Ore07,Ore08,Ore09,Ore10,Ore11,Ore12,Ore13,Ore14	*OreG0013404*
scaffold33120	410855	A	G	Och05	*OreG0013406*

scaffold33120	414451	G	C	Ore01,Ore02,Ore03,Ore04,Ore05,Ore06,Ore07,Ore08,Ore09,Ore10,Ore11,Ore12,Ore13,Ore14	*OreG0013407*
scaffold33120	415077	T	A	NA	*OreG0013407*
scaffold33120	431591	A	T	Och05,Och11	*OreG0013409*
scaffold33120	431599	G	C	Ore01,Ore02,Ore03,Ore04,Ore05,Ore06,Ore07,Ore08,Ore09,Ore10,Ore11,Ore12,Ore13,Ore14	*OreG0013409*
scaffold33120	432854	G	A	Ore01,Ore02,Ore03,Ore04,Ore05,Ore06,Ore07,Ore08,Ore09,Ore10,Ore11,Ore12,Ore13,Ore14	*OreG0013409*
scaffold33120	434258	A	G	Ore01	*OreG0013409*
scaffold33120	434815	A	G	NA	*OreG0013410*
scaffold33120	434896	C	T	Och02	*OreG0013410*
scaffold33120	437548	A	C	Ore01,Ore02,Ore03,Ore04,Ore05,Ore06,Ore07,Ore08,Ore09,Ore10,Ore11,Ore12,Ore13,Ore14	*OreG0013412*
scaffold33120	438582	G	C	Och02	*OreG0013412*
scaffold33120	441728	C	T	Och05	*OreG0013414*
scaffold33120	442032	C	T	Ore06,Ore07,Ore08	*OreG0013414*
scaffold33120	457504	G	A	Och01,Och02,Och03,Och04,Och05,Och06,Och07,Och08,Och09,Och10,Och11,Och12,Och13,Och14,Ore01,Ore02,Ore03,Ore04,Ore05,Ore06,Ore07,Ore08,Ore09,Ore10,Ore11,Ore12,Ore13,Ore14	*OreG0013415*
scaffold33120	530493	C	A	NA	*OreG0013423*
scaffold33120	541518	C	A	NA	*OreG0013425*
scaffold33120	744072	G	A	Och02,Och03,Och04,Och05,Och06,Och07,Och09,Och10,Och11,Och12,Och13	*OreG0013447*
scaffold33120	756813	C	T	Ore01,Ore02,Ore03,Ore04,Ore05,Ore06,Ore07,Ore08,Ore09,Ore10,Ore11,Ore12,Ore13,Ore14	*OreG0013448*
scaffold33120	757031	C	A	Ore01,Ore02,Ore03,Ore04,Ore05,Ore06,Ore07,Ore08,Ore09,Ore10,Ore11,Ore12,Ore13,Ore14	*OreG0013448*
scaffold33120	757035	T	C	Och01,Och02,Och03,Och04,Och05,Och06,Och07,Och08,Och09,Och10,Och11,Och12,Och13,Och14,Ore01,Ore02,Ore03,Ore04,Ore05,Ore06,Ore07,Ore08,Ore09,Ore10,Ore11,Ore12,Ore13,Ore14	*OreG0013448*
scaffold33120	779086	C	T	NA	*OreG0013452*
scaffold33120	779253	A	G	NA	*OreG0013452*
scaffold33120	793785	C	T	Och05	*OreG0013453*
scaffold33120	827275	G	T	Och01	*OreG0013455*
scaffold33120	832539	T	C	Ore06,Ore07,Ore08	*OreG0013456*
scaffold33120	854509	A	C	Och01,Och02,Och03,Och04,Och05,Och06,Och07,Och08,Och09,Och10,Och11,Och12,Och13,Och14,Ore01,Ore02,Ore03,Ore04,Ore05,Ore06,Ore07,Ore08,Ore09,Ore10,Ore11,Ore12,Ore13,Ore14	*OreG0013459*
scaffold33120	864809	G	C	Och01,Och02,Och03,Och04,Och05,Och06,Och07,Och08,Och09,Och10,Och11,Och12,Och13,Och14,Ore01,Ore02,Ore03,Ore04,Ore05,Ore06,Ore07,Ore08,Ore09,Ore10,Ore11,Ore12,Ore13,Ore14	*OreG0013462*
scaffold33120	885088	G	A	Ore01,Ore02,Ore03,Ore04,Ore05,Ore06,Ore07,Ore08,Ore09,Ore10,Ore11,Ore12,Ore13,Ore14	*OreG0013464*
scaffold33120	900427	A	G	Och05	*OreG0013467*
scaffold33120	900710	C	T	Och05	*OreG0013467*
scaffold33120	903656	G	C	Och05	*OreG0013467*
scaffold33120	907084	A	G	NA	*OreG0013468*
scaffold33120	947427	T	G	Ore02,Ore06,Ore07,Ore08,Ore09	*OreG0013473*
scaffold33120	952201	T	C	Och05	*OreG0013474*
scaffold33120	972556	C	A	Och01,Och02,Och03,Och04,Och05,Och06,Och07,Och08,Och09,Och10,Och11,Och12,Och13,Och14	*OreG0013479*
scaffold33120	972710	G	T	Och02,Och03,Och04,Och05,Och06,Och08,Och09,Och10,Och11,Och12,Och13	*OreG0013479*
scaffold33120	972951	G	T	NA	*OreG0013479*
scaffold33120	973026	G	T	Och08,Och09,Och10,Och11	*OreG0013479*
scaffold33120	1003616	A	G	Ore01,Ore02,Ore03,Ore04,Ore05,Ore06,Ore07,Ore08,Ore09,Ore10,Ore11,Ore12,Ore13,Ore14	*OreG0013483*
scaffold33120	1050321	T	C	Och01,Och02,Och03,Och04,Och05,Och06,Och07,Och08,Och09,Och10,Och11,Och12,Och13,Och14	*OreG0013487*
scaffold33120	1053046	G	A	Ore06,Ore07,Ore08	*OreG0013488*
scaffold33120	1081145	C	T	Och01,Och02,Och03,Och04,Och05,Och06,Och07,Och08,Och09,Och10,Och11,Och12,Och13,Och14	*OreG0013492*
scaffold33120	1096463	G	A	Och01,Och02,Och03,Och04,Och05,Och06,Och07,Och08,Och09,Och10,Och11,Och12,Och13,Och14	*OreG0013493*
scaffold33120	1096499	G	A	Och01,Och02,Och03,Och04,Och05,Och06,Och07,Och08,Och09,Och10,Och11,Och12,Och13,Och14	*OreG0013493*
scaffold33120	1122700	A	G	Ore01,Ore02,Ore03,Ore04,Ore05,Ore06,Ore07,Ore08,Ore09,Ore10,Ore11,Ore12,Ore13,Ore14	*OreG0013496*
scaffold33120	1122900	G	A	Och01,Och02,Och03,Och04,Och05,Och06,Och07,Och08,Och09,Och10,Och11,Och12,Och13,Och14	*OreG0013496*
scaffold33120	1123286	G	T	Ore01,Ore02,Ore03,Ore04,Ore05,Ore06,Ore07,Ore08,Ore09,Ore10,Ore11,Ore12,Ore13,Ore14	*OreG0013496*
scaffold33120	1130545	T	A	Och01,Och02,Och03,Och04,Och05,Och06,Och07,Och08,Och09,Och10,Och11,Och12,Och13,Och14	*OreG0013497*
scaffold33120	1181060	G	A	NA	*OreG0013504*
scaffold33120	1183121	C	A	Och02	*OreG0013504*
scaffold33120	1183316	C	T	Och05	*OreG0013504*
scaffold33120	1184611	C	T	NA	*OreG0013504*

scaffold33120	1188567	G	A	Ore02,Ore04,Ore05,Ore06,Ore07,Ore08,Ore09	*OreG0013504*
scaffold33120	1205617	T	C	Ore01,Ore02,Ore03,Ore04,Ore05,Ore06,Ore07,Ore08,Ore09,Ore10,Ore11,Ore12,Ore13,Ore14	*OreG0013506*
scaffold33120	1205723	T	A	Och01,Och02,Och03,Och04,Och05,Och06,Och07,Och08,Och09,Och10,Och11,Och12,Och13,Och14,Ore01,Ore02,Ore03,Ore04,Ore05,Ore06,Ore07,Ore08,Ore09,Ore10,Ore11,Ore12,Ore13,Ore14	*OreG0013506*
scaffold33120	1210705	A	C	Ore01,Ore02,Ore03,Ore04,Ore05,Ore06,Ore07,Ore08,Ore09,Ore10,Ore11,Ore12,Ore13,Ore14	*OreG0013507*
scaffold33120	1210726	C	G	Ore01,Ore02,Ore03,Ore04,Ore05,Ore06,Ore07,Ore08,Ore09,Ore10,Ore11,Ore12,Ore13,Ore14	*OreG0013507*
scaffold33120	1210729	C	A	Ore01,Ore02,Ore03,Ore04,Ore05,Ore06,Ore07,Ore08,Ore09,Ore10,Ore11,Ore12,Ore13,Ore14	*OreG0013507*
scaffold33120	1214426	G	A	NA	*OreG0013508*
scaffold33120	1216110	C	T	NA	*OreG0013508*
scaffold33120	1235591	C	G	Ore01,Ore02,Ore03,Ore04,Ore05,Ore06,Ore07,Ore08,Ore09,Ore10,Ore11,Ore12,Ore13,Ore14	*OreG0013512*
scaffold33120	1235624	G	A	Och02	*OreG0013512*
scaffold33120	1235625	C	A	Och02	*OreG0013512*
scaffold33120	1236312	C	G	Och02	*OreG0013513*
scaffold33120	1237415	G	A	Ore01,Ore02,Ore03,Ore04,Ore05,Ore06,Ore07,Ore08,Ore09,Ore10,Ore11,Ore12,Ore13,Ore14	*OreG0013513*
scaffold33120	1280345	C	T	Och01,Och02,Och03,Och04,Och05,Och06,Och07,Och08,Och09,Och10,Och11,Och12,Och13,Och14,Ore01,Ore02,Ore03,Ore04,Ore05,Ore06,Ore07,Ore08,Ore09,Ore10,Ore11,Ore12,Ore13,Ore14	*OreG0013522*
scaffold33120	1281618	A	G	NA	*OreG0013522*
scaffold33120	1323506	C	A	Och02	*OreG0013529*
scaffold33120	1327926	T	C	Och01,Och02,Och03,Och04,Och05,Och06,Och07,Och08,Och09,Och10,Och11,Och12,Och13,Och14,Ore01,Ore02,Ore03,Ore04,Ore05,Ore06,Ore07,Ore08,Ore09,Ore10,Ore11,Ore12,Ore13,Ore14	*OreG0013530*
scaffold33120	1351637	T	A	Och01,Och02,Och03,Och04,Och05,Och06,Och07,Och08,Och09,Och10,Och11,Och12,Och13,Och14,Ore01,Ore02,Ore03,Ore04,Ore05,Ore06,Ore07,Ore08,Ore09,Ore10,Ore11,Ore12,Ore13,Ore14	*OreG0013533*
scaffold33120	1351833	T	A	NA	*OreG0013533*
scaffold33120	1382047	T	A	NA	*OreG0013537*
scaffold33120	1382182	C	G	NA	*OreG0013537*
scaffold33120	1386511	T	C	Och08	*OreG0013538*
scaffold33120	1386873	G	A	Och01,Och02,Och03,Och04,Och05,Och06,Och07,Och08,Och09,Och10,Och11,Och12,Och13,Och14,Ore01,Ore02,Ore03,Ore04,Ore05,Ore06,Ore07,Ore08,Ore09,Ore10,Ore11,Ore12,Ore13,Ore14	*OreG0013538*
scaffold33120	1415729	G	T	NA	*OreG0013543*
scaffold33120	1415777	C	A	NA	*OreG0013543*
scaffold33120	1416551	G	T	NA	*OreG0013543*
scaffold33120	1416594	A	G	NA	*OreG0013543*
scaffold33120	1430799	A	T	NA	*OreG0013546*
scaffold33120	1431836	T	G	NA	*OreG0013546*
scaffold33120	1460461	T	A	Och01,Och02,Och03,Och04,Och05,Och06,Och07,Och08,Och09,Och10,Och11,Och12,Och13,Och14	*OreG0013548*
scaffold33190	78689	G	T	NA	*OreG0014722*
scaffold33190	101727	G	A	NA	*OreG0014724*
scaffold33190	135116	T	C	Ore01,Ore02,Ore03,Ore04,Ore05,Ore06,Ore07,Ore08,Ore09,Ore10,Ore11,Ore12,Ore13,Ore14	*OreG0014727*
scaffold33190	135956	T	A	NA	*OreG0014727*
scaffold33190	136008	G	A	NA	*OreG0014727*
scaffold33190	136019	T	C	NA	*OreG0014727*
scaffold33190	138716	G	A	NA	*OreG0014727*
scaffold33190	338292	T	C	NA	*OreG0014736*
scaffold33106	34567	G	T	Ore01,Ore02,Ore03,Ore04,Ore05,Ore06,Ore07,Ore08,Ore09,Ore10,Ore11,Ore12,Ore13,Ore14	*OreG0012164*
scaffold33106	130181	T	C	Ore01,Ore02,Ore03,Ore04,Ore05,Ore06,Ore07,Ore08,Ore09,Ore10,Ore11,Ore12,Ore13,Ore14	*OreG0012170*
scaffold33106	130370	C	T	Ore01,Ore02,Ore03,Ore04,Ore05,Ore06,Ore07,Ore08,Ore09,Ore10,Ore11,Ore12,Ore13,Ore14	*OreG0012170*
scaffold33106	130444	G	A	Och04,Och06	*OreG0012170*
scaffold33106	130471	C	A	Och01,Och11	*OreG0012170*
scaffold33106	130655	G	T	Ore03	*OreG0012170*
scaffold33106	130786	C	T	Och01,Och11	*OreG0012170*
scaffold33106	131343	C	T	Och04,Och06	*OreG0012170*
scaffold33106	131475	G	A	Ore01,Ore02,Ore03,Ore04,Ore05,Ore06,Ore07,Ore08,Ore09,Ore10,Ore11,Ore12,Ore13,Ore14	*OreG0012170*
scaffold33106	131540	G	A	Och11	*OreG0012170*
scaffold33106	131556	T	A	Och01,Och11	*OreG0012170*
scaffold33106	132773	G	A	Ore01,Ore02,Ore03,Ore04,Ore05,Ore06,Ore07,Ore08,Ore09,Ore10,Ore11,Ore12,Ore13,Ore14	*OreG0012170*
scaffold33106	172150	C	A	Och01	*OreG0012175*
scaffold33106	187507	C	G	Ore04,Ore05	*OreG0012177*

scaffold33106	187609	C	G	Och01,Och02,Och03,Och04,Och05,Och06,Och07,Och08,Och09,Och10,Och11,Och12,Och13,Och14	*OreG0012177*
scaffold33106	187849	A	T	Ore01,Ore02,Ore03,Ore04,Ore05,Ore06,Ore07,Ore08,Ore09,Ore10,Ore11,Ore12,Ore13,Ore14	*OreG0012177*
scaffold33106	188028	A	C	Ore01,Ore02,Ore03,Ore04,Ore05,Ore06,Ore07,Ore08,Ore09,Ore10,Ore11,Ore12,Ore13,Ore14	*OreG0012177*
scaffold33106	188167	G	T	Och02,Och03,Och04,Och05,Och06,Och07,Och08,Och09,Och10,Och11,Och12,Och13,Och14	*OreG0012177*
scaffold33106	191354	T	C	Och01,Och02,Och03,Och04,Och05,Och06,Och07,Och08,Och09,Och10,Och11,Och12,Och13,Och14,Ore01,Ore02,Ore03,Ore04,Ore05,Ore06,Ore07,Ore08,Ore09,Ore10,Ore11,Ore12,Ore13,Ore14	*OreG0012177*
scaffold33106	191462	A	G	Och02,Och03,Och04,Och05,Och06,Och07,Och08,Och09,Och10,Och11,Och12,Och13,Och14	*OreG0012177*
scaffold33106	192731	A	G	Och01,Och02,Och03,Och04,Och05,Och06,Och07,Och08,Och09,Och10,Och11,Och12,Och13,Och14	*OreG0012177*
scaffold33106	201506	T	C	Ore03	*OreG0012179*
scaffold33106	211502	C	A	Och01	*OreG0012180*
scaffold33106	211510	A	G	Ore01,Ore02,Ore03,Ore04,Ore05,Ore06,Ore07,Ore08,Ore09,Ore10,Ore11,Ore12,Ore13,Ore14	*OreG0012180*
scaffold33106	211616	C	G	Och01,Och02,Och03,Och04,Och05,Och06,Och07,Och12,Och13,Och14	*OreG0012180*
scaffold33106	211898	G	A	Ore01,Ore02,Ore03,Ore04,Ore05,Ore06,Ore07,Ore08,Ore09,Ore10,Ore11,Ore12,Ore13,Ore14	*OreG0012180*
scaffold33106	212072	G	A	Och01,Och02,Och03,Och04,Och05,Och06,Och07,Och08,Och09,Och10,Och11,Och12,Och13,Och14,Ore01,Ore02,Ore03,Ore04,Ore05,Ore06,Ore07,Ore08,Ore09,Ore10,Ore11,Ore12,Ore13,Ore14	*OreG0012180*
scaffold33106	253569	G	A	NA	*OreG0012184*
scaffold33106	269136	T	A	Och09,Och10,Och11	*OreG0012185*
scaffold33106	269936	C	A	NA	*OreG0012185*
scaffold33106	317334	G	A	NA	*OreG0012188*
scaffold33106	329640	C	T	Och04,Och06	*OreG0012190*
scaffold33106	332654	C	G	NA	*OreG0012190*
scaffold33106	335334	A	C	NA	*OreG0012190*
scaffold33106	374525	A	T	Och14	*OreG0012195*
scaffold33106	378340	T	A	Och01,Och04,Och06	*OreG0012196*
scaffold33106	482312	G	C	Och04,Och06	*OreG0012201*
scaffold33106	482592	C	G	Och07,Och08,Och09,Och10,Och11,Ore01,Ore02,Ore03,Ore04,Ore05,Ore06,Ore07,Ore08,Ore09,Ore10,Ore11,Ore12,Ore13,Ore14	*OreG0012201*
scaffold33106	483477	C	A	Ore01,Ore02,Ore03,Ore04,Ore05,Ore06,Ore07,Ore08,Ore09,Ore10,Ore11,Ore12,Ore13,Ore14	*OreG0012201*
scaffold33106	535607	T	C	Och04,Och06,Och07	*OreG0012212*
scaffold33106	536028	A	C	NA	*OreG0012212*
scaffold33106	545771	T	A	Och01,Och02,Och03,Och04,Och05,Och06,Och07,Och08,Och09,Och10,Och11,Och12,Och13,Och14	*OreG0012213*
scaffold33106	578611	A	T	NA	*OreG0012217*
scaffold33106	578635	C	A	NA	*OreG0012217*
scaffold33106	578702	A	G	Och02,Och03,Och04,Och05,Och06,Och12,Och13	*OreG0012217*
scaffold33106	633896	G	T	Och14	*OreG0012223*
scaffold33106	634001	A	G	Och14	*OreG0012223*
scaffold33106	639467	C	T	NA	*OreG0012224*
scaffold33106	639524	C	G	Och02,Och03,Och04,Och05,Och06,Och07,Och08,Och09,Och10,Och11,Och12,Och13,Och14	*OreG0012224*
scaffold33106	639904	G	A	Ore02	*OreG0012224*
scaffold33106	640703	T	G	Ore01,Ore02,Ore03,Ore04,Ore05,Ore06,Ore07,Ore08,Ore09,Ore10,Ore11,Ore12,Ore13,Ore14	*OreG0012224*
scaffold33106	687058	A	G	NA	*OreG0012226*
scaffold33106	693718	C	T	Ore01,Ore02,Ore03,Ore04,Ore05,Ore06,Ore07,Ore08,Ore09,Ore10,Ore11,Ore12,Ore13,Ore14	*OreG0012227*
scaffold33106	693730	C	T	Och08,Och11	*OreG0012227*
scaffold33106	705436	G	A	NA	*OreG0012228*
scaffold33106	752543	A	T	NA	*OreG0012234*
scaffold33106	753595	T	G	NA	*OreG0012234*
scaffold33106	761649	G	C	NA	*OreG0012235*
scaffold33106	763848	C	A	Och01,Och02,Och03,Och04,Och05,Och06,Och07,Och08,Och09,Och10,Och11,Och12,Och13,Och14	*OreG0012235*
scaffold33106	788027	G	A	Och02,Och03,Och04,Och05,Och06,Och12,Och13	*OreG0012239*
scaffold33106	806857	C	T	Och01,Och02,Och03,Och04,Och05,Och06,Och07,Och08,Och09,Och10,Och11,Och12,Och13,Och14	*OreG0012241*
scaffold33106	815346	C	A	Och01,Och11	*OreG0012241*
scaffold33106	820036	C	G	Och01,Och02,Och03,Och04,Och05,Och06,Och07,Och08,Och09,Och10,Och11,Och12,Och13,Och14	*OreG0012242*
scaffold33106	820901	T	A	Ore01,Ore02,Ore03,Ore04,Ore05,Ore06,Ore07,Ore08,Ore09,Ore10,Ore11,Ore12,Ore13,Ore14	*OreG0012242*
scaffold33106	827178	A	T	Och01,Och02,Och03,Och04,Och05,Och06,Och07,Och08,Och09,Och10,Och11,Och12,Och13,Och14,Ore01,Ore02,Ore03,Ore04,Ore05,Ore06,Ore07,Ore08,Ore09,Ore10,Ore11,Ore12,Ore13,Ore14	*OreG0012243*

scaffold33106	836751	G	C	Ore01,Ore03,Ore06,Ore09	*OreG0012244*
scaffold33106	850408	T	C	Ore01,Ore02,Ore03,Ore04,Ore05,Ore06,Ore07,Ore08,Ore09,Ore10,Ore11,Ore12,Ore13,Ore14	*OreG0012245*
scaffold33106	850930	C	G	NA	*OreG0012245*
scaffold33106	852809	T	G	NA	*OreG0012246*
scaffold33106	852905	G	A	Ore01,Ore02,Ore03,Ore04,Ore05,Ore06,Ore07,Ore08,Ore09,Ore10,Ore11,Ore12,Ore13,Ore14	*OreG0012246*
scaffold33106	853133	G	A	Ore01,Ore02,Ore03,Ore04,Ore05,Ore06,Ore07,Ore08,Ore09,Ore10,Ore11,Ore12,Ore13,Ore14	*OreG0012246*
scaffold33106	866987	C	G	NA	*OreG0012247*
scaffold33106	867023	C	T	NA	*OreG0012247*
scaffold33106	895604	T	A	NA	*OreG0012248*
scaffold33106	896849	G	A	Och01,Och02,Och03,Och04,Och05,Och06,Och07,Och08,Och09,Och10,Och11,Och12,Och13,Och14	*OreG0012248*
scaffold33106	897353	G	A	NA	*OreG0012248*
scaffold33106	898761	A	T	Ore01,Ore02,Ore03,Ore04,Ore05,Ore06,Ore07,Ore08,Ore09,Ore10,Ore11,Ore12,Ore13,Ore14	*OreG0012249*
scaffold33106	907376	C	T	NA	*OreG0012250*
scaffold33106	907468	G	A	NA	*OreG0012250*
scaffold33106	907522	A	G	NA	*OreG0012250*
scaffold33106	926245	T	A	Och04,Och06,Och11,Och12,Ore01,Ore02,Ore03,Ore04,Ore05,Ore06,Ore07,Ore08,Ore09,Ore10,Ore11,Ore12,Ore13,Ore14	*OreG0012251*
scaffold33106	926778	T	G	NA	*OreG0012251*
scaffold33106	927289	T	G	NA	*OreG0012251*
scaffold33106	927537	A	G	NA	*OreG0012251*
scaffold33106	927587	T	A	Ore02,Ore03,Ore04,Ore05,Ore06,Ore07,Ore08,Ore09,Ore10,Ore11,Ore12,Ore13,Ore14	*OreG0012251*
scaffold33106	927675	G	T	NA	*OreG0012251*
scaffold33106	927857	C	A	NA	*OreG0012251*
scaffold33106	955873	G	A	Och01,Och02,Och03,Och04,Och05,Och06,Och07,Och08,Och09,Och10,Och11,Och12,Och13,Och14,Ore01,Ore03	*OreG0012255*
scaffold33106	959363	C	T	Ore01,Ore02,Ore03,Ore04,Ore05,Ore06,Ore07,Ore08,Ore09,Ore10,Ore11,Ore12,Ore13,Ore14	*OreG0012256*
scaffold33106	986343	G	A	Ore01,Ore02,Ore03,Ore04,Ore05,Ore06,Ore07,Ore08,Ore09,Ore10,Ore11,Ore12,Ore13,Ore14	*OreG0012257*
scaffold33106	1017624	A	C	Och01	*OreG0012259*
scaffold33106	1021333	A	C	NA	*OreG0012260*
scaffold33106	1021354	T	C	NA	*OreG0012260*
scaffold33106	1026066	A	G	NA	*OreG0012261*
scaffold33106	1026144	A	G	Och01,Och02,Och03,Och04,Och05,Och06,Och07,Och08,Och09,Och10,Och11,Och12,Och13,Och14,Ore01,Ore02,Ore03,Ore04,Ore05,Ore06,Ore07,Ore08,Ore09,Ore10,Ore11,Ore12,Ore13,Ore14	*OreG0012261*
scaffold33106	1117199	A	T	NA	*OreG0012265*
scaffold33106	1126163	G	A	Ore01,Ore02,Ore03,Ore04,Ore05,Ore06,Ore07,Ore08,Ore09,Ore10,Ore11,Ore12,Ore13,Ore14	*OreG0012266*
scaffold33106	1126567	T	C	Och01,Och09,Och10,Och11	*OreG0012266*
scaffold33106	1126594	T	C	Och01,Och11	*OreG0012266*
scaffold33106	1132593	G	A	NA	*OreG0012267*
scaffold33106	1201972	T	G	Ore01,Ore02,Ore03,Ore04,Ore05,Ore06,Ore07,Ore08,Ore09,Ore10,Ore11,Ore12,Ore13,Ore14	*OreG0012272*
scaffold33106	1202004	T	C	NA	*OreG0012272*
scaffold33106	1202066	C	T	Och01,Och09,Och10,Och11	*OreG0012272*
scaffold33106	1204130	C	T	NA	*OreG0012273*
scaffold33106	1205932	A	T	Ore01,Ore02,Ore03,Ore04,Ore05,Ore06,Ore07,Ore08,Ore09,Ore10,Ore11,Ore12,Ore13,Ore14	*OreG0012274*
scaffold33106	1206044	C	T	NA	*OreG0012274*
scaffold33106	1206068	G	A	NA	*OreG0012274*
scaffold33106	1206388	G	A	Och01,Och02,Och03,Och04,Och05,Och06,Och07,Och08,Och09,Och10,Och11,Och12,Och13,Och14,Ore01,Ore02,Ore03,Ore04,Ore05,Ore06,Ore07,Ore08,Ore09,Ore10,Ore11,Ore12,Ore13,Ore14	*OreG0012274*
scaffold33106	1207050	C	A	NA	*OreG0012274*
scaffold33106	1230045	G	C	Och07,Och08,Och11	*OreG0012277*
scaffold33106	1230320	A	T	NA	*OreG0012277*
scaffold33106	1248291	G	A	Och02,Och03,Och04,Och05,Och06,Och07,Och08,Och11,Och12,Och13,Och14	*OreG0012279*
scaffold33106	1276557	C	G	Och02,Och03,Och04,Och05,Och06,Och07,Och08,Och09,Och10,Och11,Och12,Och13,Och14	*OreG0012280*
scaffold33106	1276683	A	G	NA	*OreG0012280*
scaffold33106	1300958	T	A	NA	*OreG0012281*
scaffold33106	1301003	G	A	NA	*OreG0012281*
scaffold33106	1308806	G	T	NA	*OreG0012282*
scaffold33106	1309405	G	A	Och04,Och06,Och12	*OreG0012282*
scaffold33106	1331746	C	T	Och11	*OreG0012285*
scaffold33106	1337398	C	A	NA	*OreG0012286*
scaffold33106	1337543	C	T	Och11	*OreG0012286*
scaffold33106	1339570	A	T	NA	*OreG0012286*

scaffold33106	1340551	C	A	NA	*OreG0012286*
scaffold33106	1350732	C	T	Ore02,Ore04,Ore05,Ore10,Ore12,Ore13,Ore14	*OreG0012287*
scaffold33106	1354025	G	A	Och11	*OreG0012288*
scaffold33106	1354412	A	G	Och04,Och06,Och12	*OreG0012288*
scaffold33106	1354466	C	T	Och08	*OreG0012288*
scaffold33106	1354841	T	C	NA	*OreG0012288*
scaffold33106	1361234	G	A	NA	*OreG0012289*
scaffold33106	1386251	C	A	NA	*OreG0012292*
scaffold33106	1386327	G	T	NA	*OreG0012292*
scaffold33106	1387849	A	G	NA	*OreG0012292*
scaffold33106	1388059	A	G	NA	*OreG0012292*
scaffold33106	1396447	T	C	NA	*OreG0012294*
scaffold33106	1409533	C	T	NA	*OreG0012295*
scaffold33106	1416393	G	A	Ore01,Ore02,Ore03,Ore04,Ore05,Ore06,Ore07,Ore08,Ore09,Ore10,Ore11,Ore12,Ore13,Ore14	*OreG0012296*
scaffold33106	1424175	C	T	NA	*OreG0012297*
scaffold33106	1452769	G	T	Och11	*OreG0012299*
scaffold33106	1456925	A	T	Ore06,Ore09	*OreG0012300*
scaffold33106	1456937	C	T	Ore01,Ore02,Ore03,Ore04,Ore05,Ore06,Ore07,Ore08,Ore09,Ore10,Ore11,Ore12,Ore13,Ore14	*OreG0012300*
scaffold33106	1609151	G	A	NA	*OreG0012308*
scaffold33106	1611480	T	C	Och11	*OreG0012309*
scaffold33106	1624987	G	A	NA	*OreG0012311*
scaffold33106	1626011	G	A	Ore01,Ore02,Ore03,Ore04,Ore05,Ore06,Ore07,Ore08,Ore09,Ore10,Ore11,Ore12,Ore13,Ore14	*OreG0012312*
scaffold33106	1626221	G	A	Och02,Och03,Och04,Och05,Och06,Och07,Och08,Och09,Och10,Och11,Och12,Och13,Ore01,Ore02,Ore03,Ore04,Ore05,Ore06,Ore07,Ore08,Ore09,Ore10,Ore11,Ore12,Ore13,Ore14	*OreG0012312*
scaffold33106	1626310	G	T	Ore01,Ore02,Ore03,Ore04,Ore05,Ore06,Ore07,Ore08,Ore09,Ore10,Ore11,Ore12,Ore13,Ore14	*OreG0012312*
scaffold33106	1626356	G	A	Och04,Och06,Och12	*OreG0012312*
scaffold33106	1626386	T	A	NA	*OreG0012312*
scaffold33106	1626400	A	T	Och02,Och03,Och04,Och05,Och06,Och07,Och08,Och09,Och10,Och11,Och12,Och13,Ore01,Ore02,Ore03,Ore04,Ore05,Ore06,Ore07,Ore08,Ore09,Ore10,Ore11,Ore12,Ore13,Ore14	*OreG0012312*
scaffold33106	1626573	T	A	NA	*OreG0012312*
scaffold33106	1626591	A	G	NA	*OreG0012312*
scaffold33106	1627600	G	T	Ore01,Ore02,Ore03,Ore04,Ore05,Ore06,Ore07,Ore08,Ore09,Ore10,Ore11,Ore12,Ore13,Ore14	*OreG0012312*
scaffold33106	1649995	G	T	NA	*OreG0012314*
scaffold33106	1650470	A	T	Och01,Och02,Och04,Och05,Och08,Och09,Och10,Och11,Och12,Och13,Och14	*OreG0012314*
scaffold33106	1650607	A	G	NA	*OreG0012314*
scaffold33106	1650918	G	T	NA	*OreG0012314*
scaffold33106	1650937	A	G	NA	*OreG0012314*
scaffold33106	1688623	A	T	Ore01,Ore02,Ore03,Ore04,Ore05,Ore06,Ore07,Ore08,Ore09,Ore10,Ore11,Ore12,Ore13,Ore14	*OreG0012319*
scaffold33106	1704236	G	C	NA	*OreG0012322*
scaffold33106	1705120	A	G	NA	*OreG0012322*
scaffold33106	1714650	T	G	Och01,Och02,Och03,Och04,Och05,Och06,Och07,Och08,Och09,Och10,Och11,Och12,Och13,Och14,Ore01,Ore02,Ore03,Ore04,Ore05,Ore06,Ore07,Ore08,Ore09,Ore10,Ore11,Ore12,Ore13,Ore14	*OreG0012324*
scaffold33106	1715829	T	C	Och09,Och10,Och11	*OreG0012324*
scaffold33106	1715851	C	A	NA	*OreG0012324*
scaffold33106	1729521	G	C	NA	*OreG0012326*
scaffold33106	1732926	C	G	NA	*OreG0012327*
scaffold33106	1734655	C	A	NA	*OreG0012327*
scaffold33106	1734664	G	C	Och01,Och02,Och03,Och04,Och05,Och06,Och07,Och08,Och09,Och10,Och11,Och12,Och13,Och14	*OreG0012327*
scaffold33106	1735899	T	A	Och01,Och02,Och03,Och04,Och05,Och06,Och07,Och08,Och09,Och10,Och11,Och12,Och13,Och14	*OreG0012327*
scaffold33106	1736225	T	C	NA	*OreG0012327*
scaffold33106	1736540	C	T	NA	*OreG0012327*
scaffold33106	1736633	G	C	Ore01	*OreG0012327*
scaffold33106	1737020	T	A	NA	*OreG0012327*
scaffold33106	1737176	T	C	NA	*OreG0012327*
scaffold33106	1737330	A	G	NA	*OreG0012327*
scaffold33106	1737627	C	T	NA	*OreG0012327*
scaffold33106	1737635	A	G	NA	*OreG0012327*
scaffold33106	1737666	C	T	NA	*OreG0012327*
scaffold33106	1739414	T	A	NA	*OreG0012327*
scaffold33106	1739529	C	A	NA	*OreG0012327*
scaffold33106	1743717	C	T	Och01,Och02,Och03,Och04,Och05,Och06,Och07,Och08,Och09,Och10,Och11,Och12,Och13,Och14	*OreG0012328*
scaffold33106	1756749	C	G	NA	*OreG0012331*
scaffold33106	1757339	A	G	NA	*OreG0012331*

scaffold33106	1762487	T	C	Och01,Och02,Och03,Och04,Och05,Och06,Och07,Och08,Och09,Och10,Och11,Och12,Och13,Och14,Ore01,Ore02,Ore03,Ore04,Ore05,Ore06,Ore07,Ore08,Ore09,Ore10,Ore11,Ore12,Ore13,Ore14	*OreG0012332*
scaffold33106	1762540	G	C	Ore02,Ore04,Ore05,Ore10,Ore12,Ore13,Ore14	*OreG0012332*
scaffold33106	1774472	A	T	NA	*OreG0012333*
scaffold33106	1814511	C	T	Ore01,Ore02,Ore03,Ore04,Ore05,Ore06,Ore07,Ore08,Ore09,Ore10,Ore11,Ore12,Ore13,Ore14	*OreG0012337*
scaffold33106	1828768	G	T	NA	*OreG0012338*
scaffold33106	1828912	T	A	NA	*OreG0012339*
scaffold33106	1828999	T	C	NA	*OreG0012339*
scaffold33106	1829026	G	C	NA	*OreG0012339*
scaffold33106	1829045	C	G	NA	*OreG0012339*
scaffold33106	1829335	G	A	Och08	*OreG0012339*
scaffold33106	1874517	G	A	NA	*OreG0012343*
scaffold33106	1875176	C	T	NA	*OreG0012343*
scaffold33106	1875191	G	C	NA	*OreG0012343*
scaffold33106	1875365	C	G	Ore08	*OreG0012343*
scaffold33106	1875425	G	A	Ore02,Ore04,Ore05,Ore10,Ore11,Ore12,Ore13,Ore14	*OreG0012343*
scaffold33106	1875767	G	A	NA	*OreG0012343*
scaffold33106	1876435	G	C	Och09,Och10,Och11,Och14	*OreG0012343*
scaffold33106	1880192	C	G	Och01,Och02,Och03,Och04,Och05,Och06,Och07,Och08,Och09,Och10,Och11,Och12,Och13,Och14,Ore01,Ore02,Ore03,Ore04,Ore05,Ore06,Ore07,Ore08,Ore09,Ore10,Ore11,Ore12,Ore13,Ore14	*OreG0012344*
scaffold33106	1880283	C	T	NA	*OreG0012344*
scaffold33106	1880455	C	G	Och09,Och10,Och11	*OreG0012344*
scaffold33106	1881239	G	T	NA	*OreG0012344*
scaffold33106	1881342	T	C	Ore02,Ore04,Ore05,Ore10,Ore12,Ore13,Ore14	*OreG0012344*
scaffold33106	1881421	G	A	NA	*OreG0012344*
scaffold33106	1881566	G	A	NA	*OreG0012344*
scaffold33106	1881582	A	C	NA	*OreG0012344*
scaffold33106	1884600	A	C	NA	*OreG0012345*
scaffold33106	1973521	C	G	NA	*OreG0012354*
scaffold33106	2002993	A	G	NA	*OreG0012359*
scaffold33106	2017750	A	G	NA	*OreG0012362*
scaffold33106	2017944	C	T	NA	*OreG0012362*
scaffold33106	2019119	A	C	Och01,Och02,Och03,Och04,Och05,Och06,Och07,Och08,Och09,Och10,Och11,Och12,Och13,Och14,Ore01,Ore02,Ore03,Ore04,Ore05,Ore06,Ore07,Ore08,Ore09,Ore10,Ore11,Ore12,Ore13,Ore14	*OreG0012362*
scaffold33106	2019120	G	A	Ore01,Ore02,Ore03,Ore04,Ore05,Ore06,Ore07,Ore08,Ore09,Ore10,Ore11,Ore12,Ore13,Ore14	*OreG0012362*
scaffold33106	2052222	G	A	NA	*OreG0012365*
scaffold33106	2056288	G	A	Ore01,Ore02,Ore03,Ore04,Ore05,Ore06,Ore07,Ore08,Ore09,Ore10,Ore11,Ore12,Ore13,Ore14	*OreG0012366*
scaffold33106	2076729	G	A	Och01,Och02,Och03,Och04,Och05,Och06,Och07,Och08,Och09,Och10,Och11,Och12,Och13,Och14,Ore01,Ore02,Ore03,Ore04,Ore05,Ore06,Ore07,Ore08,Ore09,Ore10,Ore11,Ore12,Ore13,Ore14	*OreG0012367*
scaffold33106	2077057	G	C	NA	*OreG0012367*
scaffold33106	2081060	G	A	NA	*OreG0012368*
scaffold33106	2081276	C	T	Och11	*OreG0012368*
scaffold33106	2085441	T	A	Ore01,Ore02,Ore03,Ore04,Ore05,Ore06,Ore07,Ore08,Ore09,Ore10,Ore11,Ore12,Ore13,Ore14	*OreG0012368*
scaffold33106	2085630	T	A	NA	*OreG0012368*
scaffold33106	2122105	G	T	Och11,Ore01,Ore02,Ore03,Ore04,Ore05,Ore06,Ore07,Ore08,Ore09,Ore10,Ore11,Ore12,Ore13,Ore14	*OreG0012373*
scaffold33106	2122620	G	A	NA	*OreG0012373*
scaffold33106	2122780	T	C	Och07	*OreG0012373*
scaffold33106	2149033	G	C	Ore01,Ore02,Ore03,Ore04,Ore05,Ore06,Ore07,Ore08,Ore09,Ore10,Ore11,Ore12,Ore13,Ore14	*OreG0012375*
scaffold33101	5938	A	T	NA	*OreG0011604*
scaffold33101	10800	T	A	NA	*OreG0011605*
scaffold33101	10962	T	C	NA	*OreG0011605*
scaffold33101	10989	T	C	NA	*OreG0011605*
scaffold33101	17574	G	A	Ore01,Ore02,Ore03,Ore04,Ore05,Ore06,Ore07,Ore08,Ore09,Ore10,Ore11,Ore12,Ore13,Ore14	*OreG0011606*
scaffold33101	26560	C	A	NA	*OreG0011607*
scaffold33101	27036	A	T	Ore01,Ore02,Ore03,Ore04,Ore05,Ore06,Ore07,Ore08,Ore09,Ore10,Ore11,Ore12,Ore13,Ore14	*OreG0011607*
scaffold33101	40851	G	C	NA	*OreG0011610*
scaffold33101	47666	C	A	Ore01,Ore02,Ore03,Ore04,Ore05,Ore06,Ore07,Ore08,Ore09,Ore10,Ore11,Ore12,Ore13,Ore14	*OreG0011611*
scaffold33101	48274	T	A	Och01,Och02,Och03,Och04,Och05,Och06,Och07,Och08,Och09,Och10,Och11,Och12,Och13,Och14	*OreG0011611*
scaffold33101	51201	C	A	Och01,Och02,Och03,Och04,Och05,Och06,Och07,Och08,Och09,Och10,Och11,Och12,Och13,Och14	*OreG0011612*
scaffold33101	88100	G	A	Ore02,Ore04,Ore05,Ore06,Ore07,Ore08,Ore09,Ore10,Ore11,Ore12,Ore13,Ore14	*OreG0011616*

scaffold33101	113933	T	A	NA	*OreG0011620*
scaffold33101	153543	G	A	NA	*OreG0011623*
scaffold33101	155075	T	A	NA	*OreG0011623*
scaffold33101	193874	T	G	NA	*OreG0011627*
scaffold33101	226242	C	T	Och01,Och02,Och03,Och04,Och05,Och06,Och07,Och08,Och09,Och10,Och11,Och12,Och13,Och14	*OreG0011630*
scaffold33101	226638	A	T	Och01,Och02,Och03,Och04,Och05,Och06,Och07,Och08,Och09,Och10,Och11,Och12,Och13,Och14,Ore01,Ore02,Ore03,Ore04,Ore05,Ore06,Ore07,Ore08,Ore09,Ore10,Ore11,Ore12,Ore13,Ore14	*OreG0011630*
scaffold33101	256007	C	G	NA	*OreG0011633*
scaffold33101	262435	G	T	NA	*OreG0011634*
scaffold33101	282382	G	T	Och09,Och10	*OreG0011637*
scaffold33101	298593	T	C	Ore01,Ore02,Ore03,Ore04,Ore05,Ore06,Ore07,Ore08,Ore09,Ore10,Ore11,Ore12,Ore13,Ore14	*OreG0011639*
scaffold33201	35722	C	T	NA	*OreG0015824*
scaffold33201	35753	C	T	NA	*OreG0015824*
scaffold33201	35948	C	T	NA	*OreG0015824*
scaffold33201	36148	G	C	NA	*OreG0015824*
scaffold33201	36567	G	A	NA	*OreG0015824*
scaffold33201	37203	C	T	NA	*OreG0015824*
scaffold33201	37215	A	G	NA	*OreG0015824*
scaffold33201	37396	G	C	NA	*OreG0015824*
scaffold33201	37584	T	C	NA	*OreG0015824*
scaffold33201	37608	A	G	NA	*OreG0015824*
scaffold33201	37629	T	C	NA	*OreG0015824*
scaffold33201	38460	C	T	NA	*OreG0015824*
scaffold33201	38603	G	A	NA	*OreG0015824*
scaffold33201	39628	C	T	NA	*OreG0015824*
scaffold33201	40140	G	T	NA	*OreG0015824*
scaffold33201	53970	G	A	Ore01,Ore02,Ore03,Ore04,Ore05,Ore06,Ore07,Ore08,Ore09,Ore10,Ore11,Ore12,Ore13,Ore14	*OreG0015825*
scaffold33201	217986	G	A	NA	*OreG0015840*
scaffold33201	232690	G	T	Och06	*OreG0015841*
scaffold33201	232711	G	A	NA	*OreG0015841*
scaffold33105	56591	T	A	Och12,Och13	*OreG0011820*
scaffold33105	56860	G	A	Och02,Och03,Och06,Och09,Och10,Och11,Och14	*OreG0011820*
scaffold33105	56863	G	A	Och03	*OreG0011820*
scaffold33105	56872	G	A	Och08,Och12,Och13	*OreG0011820*
scaffold33105	64639	G	A	Och08,Och12,Och13	*OreG0011821*
scaffold33105	64697	G	A	Och02,Och03,Och06,Och09,Och10,Och11,Och14	*OreG0011821*
scaffold33105	70989	G	A	Ore01,Ore02,Ore03,Ore04,Ore05,Ore06,Ore07,Ore08,Ore09,Ore10,Ore11,Ore12,Ore13,Ore14	*OreG0011823*
scaffold33105	75477	C	A	Och08,Och12,Och13	*OreG0011823*
scaffold33105	75581	G	T	Och07,Och08,Och09,Och10,Och11,Och12,Och13	*OreG0011823*
scaffold33105	76653	G	A	NA	*OreG0011823*
scaffold33105	182783	C	G	NA	*OreG0011835*
scaffold33105	182789	A	G	Ore01,Ore02,Ore03,Ore04,Ore05,Ore06,Ore07,Ore08,Ore09,Ore10,Ore11,Ore12,Ore13,Ore14	*OreG0011835*
scaffold33105	190949	T	A	Ore01,Ore02,Ore03,Ore04,Ore05,Ore06,Ore07,Ore08,Ore09,Ore10,Ore11,Ore12,Ore13,Ore14	*OreG0011836*
scaffold33105	192447	T	C	Ore01,Ore02,Ore03,Ore04,Ore05,Ore06,Ore07,Ore08,Ore09,Ore10,Ore11,Ore12,Ore13,Ore14	*OreG0011836*
scaffold33105	241577	A	G	Och01,Och02,Och03,Och06,Och14	*OreG0011837*
scaffold33105	262070	C	A	Och01,Och02,Och03,Och04,Och05,Och06,Och07,Och08,Och09,Och10,Och11,Och12,Och13,Och14,Ore01,Ore02,Ore03,Ore04,Ore05,Ore06,Ore07,Ore08,Ore09,Ore10,Ore11,Ore12,Ore13,Ore14	*OreG0011841*
scaffold33105	262085	A	T	Och01,Och02,Och03,Och04,Och05,Och06,Och07,Och08,Och09,Och10,Och11,Och12,Och13,Och14,Ore01,Ore02,Ore03,Ore04,Ore05,Ore06,Ore07,Ore08,Ore09,Ore10,Ore11,Ore12,Ore13,Ore14	*OreG0011841*
scaffold33105	262272	A	C	Och01,Och02,Och03,Och04,Och05,Och06,Och07,Och08,Och09,Och10,Och11,Och12,Och13,Och14,Ore01,Ore02,Ore03,Ore04,Ore05,Ore06,Ore07,Ore08,Ore09,Ore10,Ore11,Ore12,Ore13,Ore14	*OreG0011841*
scaffold33105	270322	G	A	NA	*OreG0011842*
scaffold33105	298170	G	A	Ore01,Ore02,Ore04,Ore05,Ore06,Ore07,Ore08,Ore09,Ore10,Ore11,Ore12,Ore13,Ore14	*OreG0011846*
scaffold33105	298173	G	A	Ore01,Ore02,Ore03,Ore04,Ore05,Ore06,Ore07,Ore08,Ore09,Ore10,Ore11,Ore12,Ore13,Ore14	*OreG0011846*
scaffold33105	348963	G	T	NA	*OreG0011850*
scaffold33105	418041	C	G	NA	*OreG0011853*
scaffold33105	504405	C	A	Och01,Och02,Och03,Och04,Och05,Och06,Och07,Och08,Och09,Och10,Och11,Och12,Och13,Och14,Ore01,Ore02,Ore03,Ore04,Ore05,Ore06,Ore07,Ore08,Ore09,Ore10,Ore11,Ore12,Ore13,Ore14	*OreG0011860*
scaffold33105	570743	G	A	NA	*OreG0011863*
scaffold33105	614866	C	T	NA	*OreG0011866*
scaffold33105	616758	A	T	NA	*OreG0011866*

scaffold33105	630066	T	C	NA	*OreG0011866*
scaffold33105	651982	G	T	Och01,Och02,Och03,Och04,Och05,Och06,Och07,Och08,Och09,Och10,Och11,Och12,Och13,Och14,Ore01,Ore02,Ore03,Ore04,Ore05,Ore06,Ore07,Ore08,Ore09,Ore10,Ore11,Ore12,Ore13,Ore14	*OreG0011868*
scaffold33105	655374	A	T	Ore01,Ore04,Ore05,Ore11	*OreG0011868*
scaffold33105	696583	C	A	NA	*OreG0011870*
scaffold33105	697576	C	T	NA	*OreG0011870*
scaffold33105	770842	G	C	Och01,Och02,Och03,Och04,Och05,Och06,Och07,Och08,Och09,Och10,Och11,Och12,Och13,Och14	*OreG0011875*
scaffold33105	770848	C	T	Ore01,Ore02,Ore03,Ore04,Ore05,Ore06,Ore07,Ore08,Ore09,Ore10,Ore11,Ore12,Ore13,Ore14	*OreG0011875*
scaffold33105	771667	G	T	Ore01,Ore02,Ore03,Ore04,Ore05,Ore06,Ore07,Ore08,Ore09,Ore10,Ore11,Ore12,Ore13,Ore14	*OreG0011875*
scaffold33105	814748	G	T	NA	*OreG0011878*
scaffold33105	821482	A	G	Ore01,Ore04,Ore05,Ore11	*OreG0011880*
scaffold33105	821644	C	T	NA	*OreG0011880*
scaffold33105	835403	T	C	Och01,Och02,Och03,Och04,Och05,Och06,Och07,Och08,Och09,Och10,Och11,Och12,Och13,Och14,Ore01,Ore02,Ore03,Ore04,Ore05,Ore06,Ore07,Ore08,Ore09,Ore10,Ore11,Ore12,Ore13,Ore14	*OreG0011881*
scaffold33105	835427	A	C	Och03,Och04,Och05,Och07,Och08,Och09,Och10,Och11,Och12,Och13,Och14	*OreG0011881*
scaffold33105	835429	G	A	Och01,Och02,Och03,Och04,Och05,Och06,Och07,Och08,Och09,Och10,Och11,Och12,Och13,Och14,Ore01,Ore02,Ore03,Ore04,Ore05,Ore06,Ore07,Ore08,Ore09,Ore10,Ore11,Ore12,Ore13,Ore14	*OreG0011881*
scaffold33105	881235	A	T	Ore01,Ore02,Ore03,Ore04,Ore05,Ore06,Ore07,Ore08,Ore09,Ore10,Ore11,Ore12,Ore13,Ore14	*OreG0011886*
scaffold33105	881421	G	A	Och01,Och02,Och03,Och04,Och05,Och06,Och07,Och08,Och09,Och10,Och11,Och12,Och13,Och14,Ore01,Ore02,Ore03,Ore04,Ore05,Ore06,Ore07,Ore08,Ore09,Ore10,Ore11,Ore12,Ore13,Ore14	*OreG0011886*
scaffold33105	886678	G	A	Och01,Och02,Och03,Och04,Och05,Och06,Och07,Och08,Och09,Och10,Och11,Och12,Och13,Och14,Ore01,Ore02,Ore03,Ore04,Ore05,Ore06,Ore07,Ore08,Ore09,Ore10,Ore11,Ore12,Ore13,Ore14	*OreG0011887*
scaffold33105	952823	G	C	Ore01,Ore04,Ore05,Ore11	*OreG0011895*
scaffold33105	968422	C	T	Ore01,Ore02,Ore03,Ore04,Ore05,Ore06,Ore07,Ore08,Ore09,Ore10,Ore11,Ore12,Ore13,Ore14	*OreG0011896*
scaffold33105	968527	C	T	Och01,Och02,Och03,Och04,Och05,Och06,Och07,Och08,Och09,Och10,Och11,Och12,Och13,Och14,Ore06,Ore07,Ore08,Ore12,Ore13,Ore14	*OreG0011896*
scaffold33105	968773	C	T	Och01,Och02,Och03,Och04,Och05,Och06,Och07,Och08,Och09,Och10,Och11,Och12,Och13,Och14	*OreG0011896*
scaffold33105	968926	C	A	NA	*OreG0011896*
scaffold33105	968934	T	C	NA	*OreG0011896*
scaffold33105	968946	G	A	Ore01,Ore02,Ore03,Ore04,Ore05,Ore06,Ore07,Ore08,Ore09,Ore10,Ore11,Ore12,Ore13,Ore14	*OreG0011896*
scaffold33105	982459	C	T	Ore01,Ore02,Ore04,Ore05,Ore06,Ore07,Ore08,Ore09,Ore10,Ore11,Ore12,Ore13,Ore14	*OreG0011899*
scaffold33105	982610	T	A	Och08	*OreG0011899*
scaffold33105	983411	G	T	Och01,Och02,Och03,Och04,Och05,Och06,Och09,Och10,Och11,Och12,Och13,Och14	*OreG0011899*
scaffold33105	987222	A	G	NA	*OreG0011900*
scaffold33105	987542	A	T	NA	*OreG0011900*
scaffold33105	992021	C	G	NA	*OreG0011901*
scaffold33105	993319	G	T	Och03	*OreG0011901*
scaffold33105	999944	T	G	Och01,Och02,Och03,Och04,Och05,Och06,Och07,Och08,Och09,Och10,Och11,Och12,Och13,Och14,Ore01,Ore02,Ore03,Ore04,Ore05,Ore06,Ore07,Ore08,Ore09,Ore10,Ore11,Ore12,Ore13,Ore14	*OreG0011903*
scaffold33105	1005298	T	C	Och12,Och13	*OreG0011904*
scaffold33105	1030213	G	A	Och01,Och02,Och03,Och04,Och05,Och06,Och07,Och08,Och09,Och10,Och11,Och12,Och13,Och14,Ore01,Ore02,Ore03,Ore04,Ore05,Ore06,Ore07,Ore08,Ore09,Ore10,Ore11,Ore12,Ore13,Ore14	*OreG0011905*
scaffold33105	1114259	A	T	NA	*OreG0011908*
scaffold33105	1115006	G	A	NA	*OreG0011908*
scaffold33105	1118733	G	A	Ore06,Ore07,Ore08,Ore12,Ore13,Ore14	*OreG0011908*
scaffold33105	1118970	G	T	Och01,Och02,Och03,Och04,Och05,Och06,Och07,Och08,Och09,Och10,Och11,Och12,Och13,Och14	*OreG0011908*
scaffold33105	1119533	A	G	Ore01,Ore02,Ore03,Ore04,Ore05,Ore06,Ore07,Ore08,Ore09,Ore10,Ore11,Ore12,Ore13,Ore14	*OreG0011908*
scaffold33105	1119831	C	T	Och03,Ore01,Ore02,Ore03,Ore04,Ore05,Ore06,Ore07,Ore08,Ore09,Ore10,Ore11,Ore12,Ore13,Ore14	*OreG0011908*
scaffold33105	1119939	G	A	Ore06,Ore07,Ore08,Ore12,Ore13,Ore14	*OreG0011908*
scaffold33105	1128681	T	G	Och01,Och02,Och03,Och04,Och05,Och06,Och07,Och08,Och09,Och10,Och11,Och12,Och13,Och14	*OreG0011909*
scaffold33105	1143753	A	T	NA	*OreG0011911*
scaffold33105	1143849	G	A	Och03,Och08,Ore01,Ore02,Ore03,Ore04,Ore05,Ore06,Ore07,Ore08,Ore09,Ore10,Ore11,Ore12,Ore13,Ore14	*OreG0011911*
scaffold33105	1144723	T	G	Och01,Och02,Och03,Och04,Och05,Och06,Och07,Och08,Och09,Och10,Och11,Och12,Och13,Och14,Ore01,Ore02,Ore03,Ore04,Ore05,Ore06,Ore07,Ore08,Ore09,Ore10,Ore11,Ore12,Ore13,Ore14	*OreG0011911*

scaffold33105	1144933	T	G	Och01,Och02,Och03,Och04,Och05,Och06,Och07,Och08,Och09,Och10,Och11,Och12,Och13,Och14,Ore01,Ore02,Ore03,Ore04,Ore05,Ore06,Ore07,Ore08,Ore09,Ore10,Ore11,Ore12,Ore13,Ore14	*OreG0011911*
scaffold33105	1161217	T	C	NA	*OreG0011913*
scaffold33105	1164714	C	G	Ore01,Ore02,Ore03,Ore04,Ore05,Ore06,Ore07,Ore08,Ore09,Ore10,Ore11,Ore12,Ore13,Ore14	*OreG0011913*
scaffold33105	1181806	C	T	Ore01,Ore02,Ore03,Ore04,Ore05,Ore06,Ore07,Ore08,Ore09,Ore10,Ore11,Ore12,Ore13,Ore14	*OreG0011916*
scaffold33105	1203657	A	G	Och02,Och03,Och04,Och05,Och06,Och07,Och08,Och12,Och13,Och14	*OreG0011918*
scaffold33105	1306284	T	G	Och01	*OreG0011928*
scaffold33105	1307137	A	T	NA	*OreG0011928*
scaffold33105	1321611	G	A	NA	*OreG0011930*
scaffold33105	1321641	C	T	Ore01,Ore02,Ore03,Ore04,Ore05,Ore06,Ore07,Ore08,Ore09,Ore10,Ore11,Ore12,Ore13,Ore14	*OreG0011930*
scaffold33105	1324098	C	T	Och01	*OreG0011930*
scaffold33105	1324227	C	T	Ore01,Ore02,Ore03,Ore04,Ore05,Ore06,Ore07,Ore08,Ore09,Ore10,Ore11,Ore12,Ore13,Ore14	*OreG0011930*
scaffold33105	1325009	T	G	Ore01,Ore02,Ore03,Ore04,Ore05,Ore06,Ore07,Ore08,Ore09,Ore10,Ore11,Ore12,Ore13,Ore14	*OreG0011930*
scaffold33105	1326058	G	A	Och01,Och02,Och03,Och04,Och05,Och06,Och07,Och08,Och09,Och10,Och11,Och12,Och13,Och14	*OreG0011930*
scaffold33105	1327159	G	A	Ore06,Ore07,Ore08,Ore12,Ore13,Ore14	*OreG0011930*
scaffold33105	1327680	T	A	Ore01,Ore02,Ore03,Ore04,Ore05,Ore06,Ore07,Ore08,Ore09,Ore10,Ore11,Ore12,Ore13,Ore14	*OreG0011930*
scaffold33105	1327706	A	G	Och01,Och02,Och03,Och04,Och05,Och06,Och07,Och08,Och09,Och10,Och11,Och12,Och13,Och14	*OreG0011930*
scaffold33105	1328051	A	T	NA	*OreG0011930*
scaffold33105	1351054	G	T	NA	*OreG0011933*
scaffold33105	1387856	C	A	Och01,Och02,Och03,Och04,Och05,Och06,Och07,Och08,Och09,Och10,Och11,Och12,Och13,Och14	*OreG0011937*
scaffold33105	1388996	A	G	Och01,Och02,Och03,Och04,Och05,Och06,Och07,Och08,Och09,Och10,Och11,Och12,Och13,Och14	*OreG0011937*
scaffold33105	1389105	C	G	Och01,Och02,Och03,Och04,Och05,Och06,Och07,Och08,Och09,Och10,Och11,Och12,Och13,Och14,Ore01,Ore02,Ore03,Ore04,Ore05,Ore06,Ore07,Ore08,Ore09,Ore10,Ore11,Ore12,Ore13,Ore14	*OreG0011937*
scaffold33105	1406778	G	T	Och01,Och02,Och03,Och04,Och05,Och06,Och07,Och08,Och09,Och10,Och11,Och12,Och13,Och14,Ore01,Ore02,Ore03,Ore04,Ore05,Ore06,Ore07,Ore08,Ore09,Ore10,Ore11,Ore12,Ore13,Ore14	*OreG0011939*
scaffold33105	1431089	T	A	NA	*OreG0011942*
scaffold33105	1436462	G	A	NA	*OreG0011942*
scaffold33105	1570615	C	T	NA	*OreG0011954*
scaffold33105	1570617	G	A	NA	*OreG0011954*
scaffold33105	1570704	G	T	NA	*OreG0011954*
scaffold33105	1579490	A	T	Och01,Och02,Och03,Och04,Och05,Och06,Och07,Och08,Och09,Och10,Och11,Och12,Och13,Och14	*OreG0011955*
scaffold33105	1579517	C	A	NA	*OreG0011955*
scaffold33105	1602695	T	G	NA	*OreG0011958*
scaffold33105	1611351	A	G	Ore01,Ore04,Ore05,Ore11	*OreG0011958*
scaffold33105	1639142	G	A	NA	*OreG0011960*
scaffold33105	1640622	C	T	Ore01,Ore02,Ore03,Ore04,Ore05,Ore06,Ore07,Ore08,Ore09,Ore10,Ore11,Ore12,Ore13,Ore14	*OreG0011960*
scaffold33105	1649050	T	C	NA	*OreG0011961*
scaffold33105	1649210	G	C	NA	*OreG0011961*
scaffold33105	1651102	T	G	Ore01,Ore02,Ore03,Ore04,Ore05,Ore06,Ore07,Ore08,Ore09,Ore10,Ore11,Ore12,Ore13,Ore14	*OreG0011962*
scaffold33105	1652363	A	T	NA	*OreG0011962*
scaffold33105	1673143	C	A	Och03,Och04,Och05,Och12,Och13	*OreG0011963*
scaffold33105	1688364	C	T	Ore06,Ore07,Ore08,Ore10,Ore12,Ore13,Ore14	*OreG0011964*
scaffold33105	1729683	A	C	NA	*OreG0011968*
scaffold33105	1729706	C	T	NA	*OreG0011968*
scaffold33105	1730009	C	T	NA	*OreG0011969*
scaffold33105	1730021	C	T	NA	*OreG0011969*
scaffold33105	1730429	T	C	NA	*OreG0011969*
scaffold33105	1730480	C	G	NA	*OreG0011969*
scaffold33105	1730684	T	C	NA	*OreG0011969*
scaffold33105	1730772	G	T	Och02,Och03,Och04,Och06,Och13	*OreG0011969*
scaffold33105	1730774	A	C	NA	*OreG0011969*
scaffold33105	1730818	T	A	Ore13	*OreG0011969*
scaffold33105	1730835	G	T	NA	*OreG0011969*
scaffold33105	1730859	T	A	NA	*OreG0011969*
scaffold33105	1730868	C	T	NA	*OreG0011969*
scaffold33105	1732060	G	C	NA	*OreG0011969*
scaffold33105	1732068	T	G	NA	*OreG0011969*
scaffold33105	1732191	C	T	Ore13	*OreG0011969*
scaffold33105	1732253	A	T	NA	*OreG0011969*

scaffold33105	1732277	A	T	NA	*OreG0011969*
scaffold33105	1738910	C	G	NA	*OreG0011970*
scaffold33105	1738929	T	A	NA	*OreG0011970*
scaffold33105	1739175	C	A	NA	*OreG0011970*
scaffold33105	1739229	A	T	NA	*OreG0011970*
scaffold33105	1747439	G	T	NA	*OreG0011971*
scaffold33105	1747883	G	T	Och07	*OreG0011971*
scaffold33105	1830212	A	G	NA	*OreG0011972*
scaffold33105	1854706	G	T	NA	*OreG0011974*
scaffold33105	1858743	G	A	NA	*OreG0011975*
scaffold33105	1907756	G	C	NA	*OreG0011978*
scaffold33105	1945353	T	C	Och01,Och02,Och03,Och04,Och05,Och06,Och07,Och08,Och09,Och10,Och11,Och12,Och13,Och14,Ore01,Ore02,Ore03,Ore04,Ore05,Ore06,Ore07,Ore08,Ore09,Ore10,Ore11,Ore12,Ore13,Ore14	*OreG0011985*
scaffold33105	1957597	C	G	NA	*OreG0011986*
scaffold33105	1962077	C	T	NA	*OreG0011987*
scaffold33105	1971119	A	G	Ore04,Ore05,Ore12,Ore13	*OreG0011988*
scaffold33105	1971151	A	C	NA	*OreG0011988*
scaffold33105	1972644	C	G	Ore01,Ore02,Ore03,Ore04,Ore05,Ore06,Ore07,Ore08,Ore09,Ore10,Ore11,Ore12,Ore13,Ore14	*OreG0011988*
scaffold33105	1975884	A	G	Ore01,Ore02,Ore03,Ore04,Ore05,Ore06,Ore07,Ore08,Ore09,Ore10,Ore11,Ore12,Ore13,Ore14	*OreG0011989*
scaffold33105	2061738	C	A	Och01	*OreG0011997*
scaffold33105	2100147	C	A	NA	*OreG0012002*
scaffold33105	2116535	G	A	NA	*OreG0012004*
scaffold33105	2117369	G	T	NA	*OreG0012004*
scaffold33105	2124977	G	A	NA	*OreG0012006*
scaffold33105	2131050	A	T	Ore01,Ore02,Ore03,Ore04,Ore05,Ore06,Ore07,Ore08,Ore09,Ore10,Ore11,Ore12,Ore13,Ore14	*OreG0012007*
scaffold33105	2131134	A	T	NA	*OreG0012007*
scaffold33105	2147804	C	A	Och01	*OreG0012010*
scaffold33105	2162400	G	A	Och01,Och02,Och03,Och04,Och05,Och06,Och07,Och08,Och09,Och10,Och11,Och12,Och13,Och14,Ore01,Ore02,Ore03,Ore04,Ore05,Ore06,Ore07,Ore08,Ore09,Ore10,Ore11,Ore12,Ore13,Ore14	*OreG0012012*
scaffold33105	2162907	A	G	Ore01,Ore02,Ore03,Ore04,Ore05,Ore06,Ore07,Ore08,Ore09,Ore10,Ore11,Ore12,Ore13,Ore14	*OreG0012012*
scaffold33105	2172897	A	G	Ore01,Ore02,Ore03,Ore04,Ore05,Ore06,Ore07,Ore08,Ore09,Ore10,Ore11,Ore12,Ore13,Ore14	*OreG0012016*
scaffold33105	2192835	G	C	NA	*OreG0012018*
scaffold33105	2193358	T	A	Och01,Och02,Och03,Och04,Och05,Och06,Och07,Och08,Och09,Och10,Och11,Och12,Och13,Och14,Ore01,Ore02,Ore03,Ore04,Ore05,Ore06,Ore07,Ore08,Ore09,Ore10,Ore11,Ore12,Ore13,Ore14	*OreG0012018*
scaffold33105	2242383	A	G	Och01,Och02,Och03,Och04,Och05,Och06,Och07,Och08,Och09,Och10,Och11,Och12,Och13,Och14	*OreG0012026*
scaffold33105	2253562	C	T	NA	*OreG0012028*
scaffold33105	2253601	C	T	NA	*OreG0012028*
scaffold33105	2253604	C	G	NA	*OreG0012028*
scaffold33105	2253663	G	T	NA	*OreG0012028*
scaffold33105	2254170	G	A	NA	*OreG0012028*
scaffold33105	2254329	G	T	NA	*OreG0012028*
scaffold33105	2261933	C	A	Ore01,Ore02,Ore03,Ore04,Ore05,Ore06,Ore07,Ore08,Ore09,Ore10,Ore11,Ore12,Ore13,Ore14	*OreG0012029*
scaffold33105	2299971	T	C	Och01,Och02,Och03,Och04,Och05,Och06,Och07,Och08,Och09,Och10,Och11,Och12,Och13,Och14,Ore01,Ore02,Ore03,Ore04,Ore05,Ore06,Ore07,Ore08,Ore09,Ore10,Ore11,Ore12,Ore13,Ore14	*OreG0012035*
scaffold33105	2304581	C	A	NA	*OreG0012035*
scaffold33105	2323927	G	A	NA	*OreG0012038*
scaffold33105	2336811	A	T	NA	*OreG0012043*
scaffold33105	2336856	A	G	NA	*OreG0012043*
scaffold33105	2337334	C	T	Ore01,Ore06,Ore07,Ore08	*OreG0012043*
scaffold33105	2345991	A	C	Ore02,Ore03,Ore04,Ore05,Ore06,Ore07,Ore08,Ore09,Ore10,Ore11,Ore12,Ore13,Ore14	*OreG0012047*
scaffold33105	2346784	A	T	Och01,Och02,Och03,Och04,Och05,Och06,Och07,Och08,Och09,Och10,Och11,Och12,Och13,Och14,Ore01,Ore02,Ore03,Ore04,Ore05,Ore06,Ore07,Ore08,Ore09,Ore10,Ore11,Ore12,Ore13,Ore14	*OreG0012048*
scaffold33105	2346860	C	G	NA	*OreG0012048*
scaffold33105	2347155	A	C	Och01,Och02,Och03,Och04,Och05,Och06,Och07,Och08,Och09,Och10,Och11,Och12,Och13,Och14,Ore01,Ore02,Ore03,Ore04,Ore05,Ore06,Ore07,Ore08,Ore09,Ore10,Ore11,Ore12,Ore13,Ore14	*OreG0012048*
scaffold33105	2347164	C	T	Och01,Och02,Och03,Och04,Och05,Och06,Och07,Och08,Och09,Och10,Och11,Och12,Och13,Och14	*OreG0012048*
scaffold33105	2347182	G	A	NA	*OreG0012048*
scaffold33105	2347455	C	A	NA	*OreG0012048*
scaffold33105	2347502	G	A	NA	*OreG0012048*
scaffold33105	2367667	G	T	Och03	*OreG0012051*

scaffold33105	2368964	T	C	Ore01,Ore02,Ore03,Ore04,Ore05,Ore06,Ore07,Ore08,Ore09,Ore10,Ore11,Ore12,Ore13,Ore14	*OreG0012051*
scaffold33105	2369320	A	T	NA	*OreG0012051*
scaffold33105	2370357	G	A	Ore01,Ore02,Ore03,Ore04,Ore05,Ore06,Ore07,Ore08,Ore09,Ore10,Ore11,Ore12,Ore13,Ore14	*OreG0012051*
scaffold33105	2371786	C	T	NA	*OreG0012051*
scaffold33105	2371814	G	A	NA	*OreG0012051*
scaffold33105	2426198	G	A	Och07,Ore01,Ore02,Ore03,Ore04,Ore05,Ore06,Ore07,Ore08,Ore09,Ore10,Ore11,Ore12,Ore13,Ore14	*OreG0012055*
scaffold33105	2426430	C	G	Ore01,Ore02,Ore03,Ore04,Ore05,Ore06,Ore07,Ore08,Ore09,Ore10,Ore11,Ore12,Ore13,Ore14	*OreG0012055*
scaffold33105	2426694	T	C	Och01,Och02,Och03,Och04,Och05,Och06,Och07,Och08,Och09,Och10,Och11,Och12,Och13,Och14,Ore01,Ore02,Ore03,Ore04,Ore05,Ore06,Ore07,Ore08,Ore09,Ore10,Ore11,Ore12,Ore13,Ore14	*OreG0012055*
scaffold33105	2427269	C	A	Och01,Och02,Och03,Och04,Och05,Och06,Och07,Och08,Och09,Och10,Och11,Och12,Och13,Och14	*OreG0012055*
scaffold33105	2439569	T	A	Och01,Och02,Och03,Och04,Och05,Och06,Och07,Och08,Och09,Och10,Och11,Och12,Och13,Och14,Ore01,Ore02,Ore03,Ore04,Ore05,Ore06,Ore07,Ore08,Ore09,Ore10,Ore11,Ore12,Ore13,Ore14	*OreG0012057*
scaffold33105	2439587	G	C	Och01,Och02,Och03,Och04,Och05,Och06,Och07,Och08,Och09,Och10,Och11,Och12,Och13,Och14,Ore01,Ore02,Ore03,Ore04,Ore05,Ore06,Ore07,Ore08,Ore09,Ore10,Ore11,Ore12,Ore13,Ore14	*OreG0012057*
scaffold33105	2440553	C	A	Och01,Och02,Och03,Och04,Och05,Och06,Och07,Och08,Och09,Och10,Och11,Och12,Och13,Och14,Ore01,Ore02,Ore03,Ore04,Ore05,Ore06,Ore07,Ore08,Ore09,Ore10,Ore11,Ore12,Ore13,Ore14	*OreG0012057*
scaffold33105	2441117	G	T	Och01,Och02,Och03,Och04,Och05,Och06,Och07,Och08,Och09,Och10,Och11,Och12,Och13,Och14,Ore01,Ore02,Ore03,Ore04,Ore05,Ore06,Ore07,Ore08,Ore09,Ore10,Ore11,Ore12,Ore13,Ore14	*OreG0012057*
scaffold33105	2447996	G	A	Ore01,Ore02,Ore03,Ore04,Ore05,Ore06,Ore07,Ore08,Ore09,Ore10,Ore11,Ore12,Ore13,Ore14	*OreG0012058*
scaffold33105	2449608	C	T	Och03	*OreG0012058*
scaffold33105	2449651	T	C	Ore01,Ore02,Ore03,Ore04,Ore05,Ore06,Ore07,Ore08,Ore09,Ore10,Ore11,Ore12,Ore13,Ore14	*OreG0012058*
scaffold33105	2450804	G	T	Och03	*OreG0012058*
scaffold33105	2451834	C	T	Och01,Och02,Och03,Och04,Och05,Och06,Och07,Och08,Och09,Och10,Och11,Och12,Och13,Och14,Ore01,Ore02,Ore03,Ore04,Ore05,Ore06,Ore07,Ore08,Ore09,Ore10,Ore11,Ore12,Ore13,Ore14	*OreG0012058*
scaffold33105	2461193	T	A	Och01,Och02,Och03,Och04,Och05,Och06,Och07,Och08,Och09,Och10,Och11,Och12,Och13,Och14,Ore01,Ore02,Ore03,Ore04,Ore05,Ore06,Ore07,Ore08,Ore09,Ore10,Ore11,Ore12,Ore13,Ore14	*OreG0012060*
scaffold33105	2497142	G	A	Ore09,Ore10,Ore13	*OreG0012065*
scaffold33105	2513773	A	G	NA	*OreG0012068*
scaffold33105	2522153	A	T	Och09,Och10,Och12,Och14	*OreG0012070*
scaffold33105	2522294	A	G	Och01,Och03,Och07,Och08	*OreG0012070*
scaffold33105	2522369	T	C	Och09,Och10,Och12,Och14	*OreG0012070*
scaffold33105	2536157	G	T	Och01,Och02,Och03,Och04,Och05,Och06,Och07,Och08,Och09,Och10,Och11,Och12,Och13,Och14,Ore01,Ore02,Ore03,Ore04,Ore05,Ore06,Ore07,Ore08,Ore09,Ore10,Ore11,Ore12,Ore13,Ore14	*OreG0012072*
scaffold33105	2536161	T	G	Och01,Och02,Och03,Och04,Och05,Och06,Och07,Och08,Och09,Och10,Och11,Och12,Och13,Och14	*OreG0012072*
scaffold33105	2536508	C	T	Och07,Och08,Och12,Och14	*OreG0012072*
scaffold33105	2538936	T	C	Och07,Och08,Och12,Och14	*OreG0012073*
scaffold33105	2538956	G	A	Och01,Och02,Och03,Och04,Och05,Och06,Och07,Och08,Och09,Och10,Och11,Och12,Och13,Och14,Ore01,Ore02,Ore03,Ore04,Ore05,Ore06,Ore07,Ore08,Ore09,Ore10,Ore11,Ore12,Ore13,Ore14	*OreG0012073*
scaffold33105	2547461	A	G	Och08	*OreG0012074*
scaffold33105	2548596	C	A	Och01,Och02,Och03,Och04,Och05,Och06,Och07,Och08,Och09,Och10,Och11,Och12,Och13,Och14	*OreG0012074*
scaffold33105	2549498	G	T	Och03	*OreG0012074*
scaffold33105	2552834	A	G	Och01,Och02,Och03,Och04,Och05,Och06,Och07,Och08,Och09,Och10,Och11,Och12,Och13,Och14	*OreG0012075*
scaffold33105	2553770	C	T	NA	*OreG0012075*
scaffold33105	2553836	A	T	NA	*OreG0012075*
scaffold33105	2553978	C	A	Och03	*OreG0012075*
scaffold33105	2558688	G	C	Ore01,Ore02,Ore03,Ore04,Ore05,Ore06,Ore07,Ore08,Ore09,Ore10,Ore11,Ore12,Ore13,Ore14	*OreG0012076*
scaffold33105	2558768	A	T	Ore01,Ore02,Ore03,Ore04,Ore05,Ore06,Ore07,Ore08,Ore09,Ore10,Ore11,Ore12,Ore13,Ore14	*OreG0012076*
scaffold33105	2560411	C	T	Ore01,Ore02,Ore03,Ore04,Ore05,Ore06,Ore07,Ore08,Ore09,Ore10,Ore11,Ore12,Ore13,Ore14	*OreG0012077*
scaffold33105	2565448	T	A	Och01,Och02,Och03,Och04,Och05,Och06,Och07,Och08,Och09,Och10,Och11,Och12,Och13,Och14,Ore01,Ore02,Ore03,Ore04,Ore05,Ore06,Ore07,Ore08,Ore09,Ore10,Ore11,Ore12,Ore13,Ore14	*OreG0012078*
scaffold33105	2569026	T	A	Ore09,Ore10,Ore13	*OreG0012078*
scaffold33105	2579942	G	A	NA	*OreG0012079*

scaffold33105	2613151	G	A	Ore01,Ore02,Ore03,Ore04,Ore05,Ore06,Ore07,Ore08,Ore09,Ore10,Ore11,Ore12,Ore13,Ore14	*OreG0012083*
scaffold33105	2615477	C	T	Och03	*OreG0012083*
scaffold33105	2624555	G	C	Och01,Och02,Och03,Och04,Och05,Och06,Och07,Och08,Och09,Och10,Och11,Och12,Och13,Och14	*OreG0012084*
scaffold33105	2625084	T	A	Och01,Och02,Och03,Och04,Och05,Och06,Och07,Och08,Och09,Och10,Och11,Och12,Och13,Och14,Ore01,Ore02,Ore03,Ore04,Ore05,Ore06,Ore07,Ore08,Ore09,Ore10,Ore11,Ore12,Ore13,Ore14	*OreG0012084*
scaffold33105	2625092	T	C	Och01,Och02,Och03,Och04,Och05,Och06,Och07,Och08,Och09,Och10,Och11,Och12,Och13,Och14,Ore01,Ore02,Ore03,Ore04,Ore05,Ore06,Ore07,Ore08,Ore09,Ore10,Ore11,Ore12,Ore13,Ore14	*OreG0012084*
scaffold33105	2634668	T	C	NA	*OreG0012086*
scaffold33105	2634773	C	A	NA	*OreG0012086*
scaffold33105	2634958	A	T	Och03	*OreG0012086*
scaffold33105	2635072	T	A	Och01,Och02,Och03,Och04,Och05,Och06,Och07,Och08,Och09,Och10,Och11,Och12,Och13,Och14	*OreG0012086*
scaffold33105	2635283	C	T	NA	*OreG0012086*
scaffold33105	2635368	C	T	Och01	*OreG0012086*
scaffold33105	2636153	C	A	Och01,Och02,Och03,Och04,Och05,Och06,Och07,Och08,Och09,Och10,Och11,Och12,Och13,Och14	*OreG0012086*
scaffold33105	2636248	A	T	Och01,Och02,Och03,Och04,Och05,Och06,Och07,Och08,Och09,Och10,Och11,Och12,Och13,Och14,Ore01,Ore02,Ore03,Ore04,Ore05,Ore06,Ore07,Ore08,Ore09,Ore10,Ore11,Ore12,Ore13,Ore14	*OreG0012086*
scaffold33105	2655752	G	A	NA	*OreG0012088*
scaffold33105	2680229	T	A	Och01,Och02,Och03,Och04,Och05,Och06,Och07,Och08,Och09,Och10,Och11,Och12,Och13,Och14,Ore01,Ore02,Ore03,Ore04,Ore05,Ore06,Ore07,Ore08,Ore09,Ore10,Ore11,Ore12,Ore13,Ore14	*OreG0012091*
scaffold33105	2680805	C	A	NA	*OreG0012091*
scaffold33105	2689299	A	G	Ore06,Ore07,Ore08	*OreG0012092*
scaffold33105	2692590	G	A	Och01	*OreG0012093*
scaffold33105	2693241	C	G	Och01	*OreG0012093*
scaffold33105	2693283	C	T	Och01	*OreG0012093*
scaffold33105	2706401	G	T	Ore01,Ore02,Ore03,Ore04,Ore05,Ore06,Ore07,Ore08,Ore09,Ore10,Ore11,Ore12,Ore13,Ore14	*OreG0012094*
scaffold33105	2706443	T	C	NA	*OreG0012094*
scaffold33105	2709252	C	T	Ore01,Ore02,Ore03,Ore04,Ore05,Ore06,Ore07,Ore08,Ore09,Ore10,Ore11,Ore12,Ore13,Ore14	*OreG0012095*
scaffold33105	2714851	A	G	Och01,Och02,Och03,Och04,Och05,Och06,Och07,Och08,Och09,Och10,Och11,Och12,Och13,Och14,Ore01,Ore02,Ore03,Ore04,Ore05,Ore06,Ore07,Ore08,Ore09,Ore10,Ore11,Ore12,Ore13,Ore14	*OreG0012096*
scaffold33105	2714854	T	C	Och01,Och02,Och03,Och04,Och05,Och06,Och07,Och08,Och09,Och10,Och11,Och12,Och13,Och14,Ore01,Ore02,Ore03,Ore04,Ore05,Ore06,Ore07,Ore08,Ore09,Ore10,Ore11,Ore12,Ore13,Ore14	*OreG0012096*
scaffold33105	2723803	C	G	Och01,Och02,Och03,Och04,Och05,Och06,Och07,Och08,Och09,Och10,Och11,Och12,Och13,Och14	*OreG0012098*
scaffold33105	2723872	A	G	NA	*OreG0012098*
scaffold33105	2744763	A	T	NA	*OreG0012101*
scaffold33105	2753434	T	G	NA	*OreG0012102*
scaffold33105	2806535	A	T	NA	*OreG0012110*
scaffold33105	2806539	A	G	NA	*OreG0012110*
scaffold33105	2806556	T	A	NA	*OreG0012110*
scaffold33105	2806611	T	A	NA	*OreG0012110*
scaffold33105	2806968	G	A	NA	*OreG0012110*
scaffold33105	2826051	G	T	NA	*OreG0012113*
scaffold33105	2830616	G	C	Och01,Och02,Och03,Och04,Och05,Och06,Och07,Och08,Och09,Och10,Och11,Och12,Och13,Och14	*OreG0012115*
scaffold33105	2830698	T	C	Och01,Och02,Och03,Och04,Och05,Och06,Och07,Och08,Och09,Och10,Och11,Och12,Och13,Och14	*OreG0012115*
scaffold33105	2830817	C	G	Och03	*OreG0012115*
scaffold33105	2852174	G	A	NA	*OreG0012117*
scaffold33105	2873689	T	A	Ore01,Ore02,Ore03,Ore04,Ore05,Ore06,Ore07,Ore08,Ore09,Ore10,Ore11,Ore12,Ore13,Ore14	*OreG0012119*
scaffold33105	2873771	A	C	Och01,Och02,Och03,Och04,Och05,Och06,Och07,Och08,Och09,Och10,Och11,Och12,Och13,Och14	*OreG0012119*
scaffold33105	2873931	C	G	Ore01	*OreG0012119*
scaffold33105	2877460	C	T	Och01,Och02,Och03,Och04,Och05,Och06,Och07,Och08,Och09,Och10,Och11,Och12,Och13,Och14,Ore01,Ore02,Ore03,Ore04,Ore05,Ore06,Ore07,Ore08,Ore09,Ore10,Ore11,Ore12,Ore13,Ore14	*OreG0012120*
scaffold33105	2880056	C	G	Ore01,Ore02,Ore03,Ore04,Ore05,Ore06,Ore07,Ore08,Ore09,Ore10,Ore11,Ore12,Ore13,Ore14	*OreG0012120*
scaffold33105	2882535	A	G	Ore09,Ore10,Ore13	*OreG0012120*
scaffold33105	2884568	T	A	NA	*OreG0012121*
scaffold33105	2885402	A	G	NA	*OreG0012121*
scaffold33105	2905948	C	G	NA	*OreG0012125*
scaffold33105	2908834	G	C	NA	*OreG0012126*

scaffold33105	2957567	C	G	NA	*OreG0012134*
scaffold33105	2958056	G	C	Och01,Och02,Och03,Och04,Och05,Och06,Och07,Och08,Och09,Och10,Och11,Och12,Och13,Och14,Ore01,Ore02,Ore03,Ore04,Ore05,Ore06,Ore07,Ore08,Ore09,Ore10,Ore11,Ore12,Ore13,Ore14	*OreG0012134*
scaffold33105	2988234	A	T	NA	*OreG0012136*
scaffold33105	2989156	T	A	Och01,Och02,Och03,Och04,Och05,Och06,Och07,Och08,Och09,Och10,Och11,Och12,Och13,Och14,Ore01,Ore02,Ore03,Ore04,Ore05,Ore06,Ore07,Ore08,Ore09,Ore10,Ore11,Ore12,Ore13,Ore14	*OreG0012136*
scaffold33105	2989173	C	A	NA	*OreG0012136*
scaffold33105	2989245	T	C	Och07,Och08,Och09,Och10,Och12,Och14,Ore01,Ore02,Ore03,Ore04,Ore05,Ore06,Ore07,Ore08,Ore09,Ore10,Ore11,Ore12,Ore13,Ore14	*OreG0012136*
scaffold33105	3003510	C	G	Och07,Och08,Och11	*OreG0012135*
scaffold33105	3003637	C	T	NA	*OreG0012135*
scaffold33105	3003855	A	C	NA	*OreG0012135*
scaffold33105	3029083	T	G	Och01,Och02,Och03,Och04,Och05,Och06,Och07,Och08,Och09,Och10,Och11,Och12,Och13,Och14,Ore01,Ore02,Ore03,Ore04,Ore05,Ore06,Ore07,Ore08,Ore09,Ore10,Ore11,Ore12,Ore13,Ore14	*OreG0012139*
scaffold33105	3064611	G	C	NA	*OreG0012144*
scaffold33105	3084544	A	C	Ore01	*OreG0012147*
scaffold33105	3158775	G	T	NA	*OreG0012152*
scaffold33105	3158789	A	T	NA	*OreG0012152*
scaffold33105	3158834	G	T	NA	*OreG0012152*
scaffold33105	3158859	T	C	NA	*OreG0012152*
scaffold33105	3158967	C	T	NA	*OreG0012152*
scaffold33105	3159010	C	T	Och05,Och13	*OreG0012152*
scaffold33105	3159046	T	G	NA	*OreG0012152*
scaffold33105	3159061	G	A	NA	*OreG0012152*
scaffold33139	10153	G	A	Och01,Och02,Och03,Och04,Och05,Och06,Och07,Och08,Och09,Och10,Och11,Och12,Och13,Och14,Ore01,Ore02,Ore03,Ore04,Ore05,Ore06,Ore07,Ore08,Ore09,Ore10,Ore11,Ore12,Ore13,Ore14	*OreG0013881*
scaffold33139	11902	T	C	Och02,Och03,Och04,Och05,Och06,Och07,Och08,Och09,Och10,Och11,Och12,Och13,Och14	*OreG0013881*
scaffold33139	75194	C	T	Och02,Och07	*OreG0013883*
scaffold33139	75235	C	A	Ore01,Ore02,Ore03,Ore04,Ore05,Ore06,Ore07,Ore08,Ore09,Ore10,Ore11,Ore12,Ore13,Ore14	*OreG0013883*
scaffold33139	75282	C	A	Och02,Och07,Och14	*OreG0013883*
scaffold33139	121382	G	A	NA	*OreG0013886*
scaffold33139	121647	T	C	NA	*OreG0013886*
scaffold33139	210396	T	A	NA	*OreG0013891*
scaffold33139	210813	A	G	NA	*OreG0013891*
scaffold33139	210873	A	G	Ore01,Ore02,Ore03,Ore04,Ore05,Ore06,Ore07,Ore08,Ore09,Ore10,Ore11,Ore12,Ore13,Ore14	*OreG0013891*
scaffold33139	228101	T	G	Ore01,Ore02,Ore03,Ore04,Ore05,Ore06,Ore07,Ore08,Ore09,Ore10,Ore11,Ore12,Ore13,Ore14	*OreG0013894*
scaffold33139	228128	G	T	NA	*OreG0013894*
scaffold33139	228722	T	C	Och11	*OreG0013894*
scaffold33139	230714	T	A	Ore01,Ore02,Ore03,Ore04,Ore05,Ore06,Ore07,Ore08,Ore09,Ore10,Ore11,Ore12,Ore13,Ore14	*OreG0013894*
scaffold33139	233017	C	A	Ore01,Ore02,Ore03,Ore04,Ore05,Ore06,Ore07,Ore08,Ore09,Ore10,Ore11,Ore12,Ore13,Ore14	*OreG0013894*
scaffold33139	233173	T	C	Ore01,Ore02,Ore03,Ore04,Ore05,Ore06,Ore07,Ore08,Ore09,Ore10,Ore11,Ore12,Ore13,Ore14	*OreG0013894*
scaffold33139	241182	C	A	Och11	*OreG0013896*
scaffold33139	279737	C	A	NA	*OreG0013898*
scaffold33139	280720	T	C	NA	*OreG0013898*
scaffold33139	280751	A	G	NA	*OreG0013898*
scaffold33139	281038	G	C	Och01,Och02,Och03,Och04,Och05,Och06,Och07,Och08,Och09,Och10,Och11,Och12,Och13,Och14,Ore01,Ore02,Ore03,Ore04,Ore05,Ore06,Ore07,Ore08,Ore09,Ore10,Ore11,Ore12,Ore13,Ore14	*OreG0013898*
scaffold33139	281047	G	C	NA	*OreG0013898*
scaffold33139	285265	C	A	NA	*OreG0013899*
scaffold33139	307782	C	T	NA	*OreG0013903*
scaffold33139	319746	G	C	Och01,Och02,Och03,Och04,Och05,Och06,Och07,Och08,Och09,Och10,Och11,Och12,Och13,Och14,Ore01,Ore02,Ore03,Ore04,Ore05,Ore06,Ore07,Ore08,Ore09,Ore10,Ore11,Ore12,Ore13,Ore14	*OreG0013904*
scaffold33139	323549	G	C	NA	*OreG0013905*
scaffold33139	334519	G	T	NA	*OreG0013906*
scaffold33139	334756	A	T	NA	*OreG0013906*
scaffold33139	453157	C	G	NA	*OreG0013922*
scaffold33139	466184	C	T	Och01,Och02,Och03,Och04,Och05,Och06,Och07,Och08,Och09,Och10,Och11,Och12,Och13,Och14,Ore01,Ore02,Ore03,Ore04,Ore05,Ore06,Ore07,Ore08,Ore09,Ore10,Ore11,Ore12,Ore13,Ore14	*OreG0013924*
scaffold33139	480403	T	A	NA	*OreG0013927*
scaffold33139	481267	C	A	NA	*OreG0013928*
scaffold33139	484287	T	A	NA	*OreG0013928*

scaffold33139	495836	G	A	Och01,Och02,Och03,Och04,Och05,Och06,Och07,Och08,Och09,Och10,Och11,Och12,Och13,Och14,Ore01,Ore02,Ore03,Ore04,Ore05,Ore06,Ore07,Ore08,Ore09,Ore10,Ore11,Ore12,Ore13,Ore14	*OreG0013929*
scaffold33139	506916	G	T	Ore02,Ore04,Ore05,Ore08,Ore11,Ore12	*OreG0013931*
scaffold33139	507624	T	C	NA	*OreG0013931*
scaffold33139	507678	A	G	Ore03	*OreG0013931*
scaffold33139	509292	T	G	NA	*OreG0013931*
scaffold33139	513154	C	T	NA	*OreG0013931*
scaffold33139	514703	G	A	NA	*OreG0013931*
scaffold33139	514732	G	T	NA	*OreG0013931*
scaffold33139	538160	T	C	Ore01,Ore02,Ore03,Ore04,Ore05,Ore06,Ore07,Ore08,Ore09,Ore10,Ore11,Ore12,Ore13,Ore14	*OreG0013935*
scaffold33139	554452	G	A	Och01,Och02,Och03,Och04,Och05,Och06,Och07,Och08,Och09,Och10,Och11,Och12,Och13,Och14,Ore01,Ore02,Ore03,Ore04,Ore05,Ore06,Ore07,Ore08,Ore09,Ore10,Ore11,Ore12,Ore13,Ore14	*OreG0013936*
scaffold33139	561828	T	A	Och01,Och02,Och03,Och04,Och05,Och06,Och07,Och08,Och09,Och10,Och11,Och12,Och13,Och14	*OreG0013937*
scaffold33139	582760	G	C	NA	*OreG0013940*
scaffold33139	593982	C	T	NA	*OreG0013941*
scaffold33139	627761	T	C	NA	*OreG0013946*
scaffold33139	634684	G	C	NA	*OreG0013947*
scaffold33139	634850	G	A	NA	*OreG0013947*
scaffold33139	635704	T	C	Ore01,Ore02,Ore03,Ore04,Ore05,Ore06,Ore07,Ore08,Ore09,Ore10,Ore11,Ore12,Ore13,Ore14	*OreG0013947*
scaffold33139	638047	A	T	Och01,Och02,Och03,Och04,Och05,Och06,Och07,Och08,Och09,Och10,Och11,Och12,Och13,Och14,Ore01,Ore02,Ore03,Ore04,Ore05,Ore06,Ore07,Ore08,Ore09,Ore10,Ore11,Ore12,Ore13,Ore14	*OreG0013948*
scaffold33139	656992	C	T	NA	*OreG0013952*
scaffold33139	658744	G	A	Ore01,Ore02,Ore03,Ore04,Ore05,Ore06,Ore07,Ore08,Ore09,Ore10,Ore11,Ore12,Ore13,Ore14	*OreG0013952*
scaffold33139	665450	G	C	NA	*OreG0013953*
scaffold33139	665776	G	A	NA	*OreG0013953*
scaffold33139	666121	C	G	Och12	*OreG0013953*
scaffold33139	671112	C	T	NA	*OreG0013954*
scaffold33139	696880	G	A	Och01,Och07,Och09,Och10,Och11,Och14	*OreG0013955*
scaffold33139	702468	G	A	Ore01,Ore02,Ore03,Ore04,Ore05,Ore06,Ore07,Ore08,Ore09,Ore10,Ore11,Ore12,Ore13,Ore14	*OreG0013956*
scaffold33139	707670	C	T	Ore01,Ore02,Ore03,Ore04,Ore05,Ore06,Ore07,Ore08,Ore09,Ore10,Ore11,Ore12,Ore13,Ore14	*OreG0013956*
scaffold33139	708654	C	T	NA	*OreG0013956*
scaffold33139	709973	T	C	Ore01,Ore02,Ore03,Ore04,Ore05,Ore06,Ore07,Ore08,Ore09,Ore10,Ore11,Ore12,Ore13,Ore14	*OreG0013957*
scaffold33139	752495	T	G	NA	*OreG0013961*
scaffold33139	752522	G	T	Ore02,Ore08,Ore11,Ore12	*OreG0013961*
scaffold33139	752574	G	C	Ore01,Ore02,Ore03,Ore04,Ore05,Ore06,Ore07,Ore08,Ore09,Ore10,Ore11,Ore12,Ore13,Ore14	*OreG0013961*
scaffold33139	752829	G	T	NA	*OreG0013961*
scaffold33139	755801	G	T	NA	*OreG0013962*
scaffold33139	756507	C	T	Och01,Och02,Och03,Och04,Och05,Och06,Och07,Och08,Och09,Och10,Och11,Och12,Och13,Och14,Ore01,Ore02,Ore03,Ore04,Ore05,Ore06,Ore07,Ore08,Ore09,Ore10,Ore11,Ore12,Ore13,Ore14	*OreG0013962*
scaffold33139	786700	A	G	Och08	*OreG0013967*
scaffold33139	786756	T	A	Och02,Och03,Och04,Och06,Och12,Och13	*OreG0013967*
scaffold33139	786819	T	C	NA	*OreG0013967*
scaffold33139	786903	G	T	NA	*OreG0013967*
scaffold33139	795870	G	T	NA	*OreG0013968*
scaffold33139	798811	G	A	Ore02,Ore08,Ore11,Ore12	*OreG0013969*
scaffold33139	798830	A	C	Ore01,Ore02,Ore03,Ore04,Ore05,Ore06,Ore07,Ore08,Ore09,Ore10,Ore11,Ore12,Ore13,Ore14	*OreG0013969*
scaffold33139	822169	G	T	NA	*OreG0013971*
scaffold33139	822186	G	A	Och01,Och02,Och03,Och04,Och05,Och06,Och07,Och08,Och09,Och10,Och11,Och12,Och13,Och14,Ore01,Ore02,Ore03,Ore04,Ore05,Ore06,Ore07,Ore08,Ore09,Ore10,Ore11,Ore12,Ore13,Ore14	*OreG0013971*
scaffold33139	822196	C	A	NA	*OreG0013971*
scaffold33139	847524	G	T	Och01,Och02,Och03,Och04,Och05,Och06,Och07,Och08,Och09,Och10,Och11,Och12,Och13,Och14	*OreG0013973*
scaffold33139	852016	T	G	Och01,Och02,Och03,Och04,Och05,Och06,Och07,Och08,Och09,Och10,Och11,Och12,Och13,Och14,Ore01,Ore02,Ore03,Ore04,Ore05,Ore06,Ore07,Ore08,Ore09,Ore10,Ore11,Ore12,Ore13,Ore14	*OreG0013974*
scaffold33139	852079	G	A	Och14	*OreG0013974*
scaffold33139	864712	G	A	NA	*OreG0013977*
scaffold33139	865505	C	T	NA	*OreG0013977*
scaffold33139	867621	G	A	Och02,Och12	*OreG0013977*
scaffold33139	867942	T	C	Ore02,Ore04,Ore05,Ore06,Ore07,Ore08,Ore09,Ore10,Ore11,Ore12,Ore13,Ore14	*OreG0013977*
scaffold33139	869065	G	T	Och01,Och07	*OreG0013977*

scaffold33139	869843	A	T	NA	*OreG0013977*
scaffold33139	884472	G	A	NA	*OreG0013978*
scaffold33139	892985	T	G	NA	*OreG0013981*
scaffold33139	893498	A	T	NA	*OreG0013981*
scaffold33139	902696	C	A	Ore01,Ore02,Ore03,Ore04,Ore05,Ore06,Ore07,Ore08,Ore09,Ore10,Ore11,Ore12,Ore13,Ore14	*OreG0013984*
scaffold33139	904631	C	T	NA	*OreG0013984*
scaffold33139	909598	C	T	Ore01,Ore02,Ore03,Ore04,Ore05,Ore06,Ore07,Ore08,Ore09,Ore10,Ore11,Ore12,Ore13,Ore14	*OreG0013985*
scaffold33139	923044	G	A	Och01,Och02,Och03,Och04,Och05,Och06,Och07,Och08,Och09,Och10,Och11,Och12,Och13,Och14,Ore01,Ore02,Ore03,Ore04,Ore05,Ore06,Ore07,Ore08,Ore09,Ore10,Ore11,Ore12,Ore13,Ore14	*OreG0013986*
scaffold33139	928187	T	C	NA	*OreG0013987*
scaffold33139	941064	G	A	Ore01,Ore02,Ore03,Ore04,Ore05,Ore06,Ore07,Ore08,Ore09,Ore10,Ore11,Ore12,Ore13,Ore14	*OreG0013988*
scaffold33139	941138	G	T	Ore01,Ore02,Ore03,Ore04,Ore05,Ore06,Ore07,Ore08,Ore09,Ore10,Ore11,Ore12,Ore13,Ore14	*OreG0013988*
scaffold33139	941213	C	T	Ore01,Ore02,Ore03,Ore04,Ore05,Ore06,Ore07,Ore08,Ore09,Ore10,Ore11,Ore12,Ore13,Ore14	*OreG0013988*
scaffold31714	19505	G	A	Och01,Och04,Och05,Och09,Och10,Och12,Och13,Och14	*OreG0009716*
scaffold31714	21646	T	A	Och14	*OreG0009716*
scaffold31714	30622	T	C	NA	*OreG0009719*
scaffold31714	32173	C	A	NA	*OreG0009720*
scaffold31714	41624	A	T	NA	*OreG0009721*
scaffold31714	78637	C	A	NA	*OreG0009724*
scaffold31714	86790	G	A	Och01,Och02,Och03,Och04,Och05,Och06,Och07,Och08,Och09,Och10,Och11,Och12,Och13,Och14,Ore01,Ore02,Ore03,Ore04,Ore05,Ore06,Ore07,Ore08,Ore09,Ore10,Ore11,Ore12,Ore13,Ore14	*OreG0009727*
scaffold31714	89207	G	A	Ore01,Ore02,Ore03,Ore04,Ore05,Ore06,Ore07,Ore08,Ore09,Ore10,Ore11,Ore12,Ore13,Ore14	*OreG0009727*
scaffold31714	89766	T	A	Och01,Och02,Och03,Och04,Och05,Och06,Och07,Och08,Och09,Och10,Och11,Och12,Och13,Och14	*OreG0009727*
scaffold31714	91166	A	G	Och01,Och02,Och03,Och04,Och05,Och06,Och07,Och08,Och09,Och10,Och11,Och12,Och13,Och14,Ore01,Ore02,Ore03,Ore04,Ore05,Ore06,Ore07,Ore08,Ore09,Ore10,Ore11,Ore12,Ore13,Ore14	*OreG0009727*
scaffold31714	148147	T	A	Ore01,Ore02,Ore03,Ore04,Ore05,Ore06,Ore07,Ore08,Ore09,Ore10,Ore11,Ore12,Ore13,Ore14	*OreG0009732*
scaffold31714	201521	T	C	Och01,Och02,Och03,Och04,Och05,Och06,Och07,Och08,Och09,Och10,Och11,Och12,Och13,Och14,Ore01,Ore02,Ore03,Ore04,Ore05,Ore06,Ore07,Ore08,Ore09,Ore10,Ore11,Ore12,Ore13,Ore14	*OreG0009736*
scaffold31714	213333	G	C	NA	*OreG0009737*
scaffold31714	220199	T	C	NA	*OreG0009738*
scaffold31714	235539	G	T	Och04,Och05,Och08,Och12,Och13	*OreG0009740*
scaffold31714	239487	C	T	Och01,Och02,Och03,Och04,Och05,Och06,Och07,Och08,Och09,Och10,Och11,Och12,Och13,Och14,Ore01,Ore02,Ore03,Ore04,Ore05,Ore06,Ore07,Ore08,Ore09,Ore10,Ore11,Ore12,Ore13,Ore14	*OreG0009740*
scaffold31714	245738	G	A	NA	*OreG0009741*
scaffold31714	284011	C	T	NA	*OreG0009744*
scaffold31714	284269	G	A	NA	*OreG0009744*
scaffold31714	284481	C	T	Och02,Och03,Och04,Och05,Och06,Och08,Och12,Och13	*OreG0009744*
scaffold31714	284767	T	C	NA	*OreG0009744*
scaffold31714	284899	C	T	Och01,Och02,Och03,Och04,Och05,Och06,Och07,Och08,Och09,Och10,Och11,Och12,Och13,Och14	*OreG0009744*
scaffold31714	284982	T	C	Ore01,Ore02,Ore03,Ore06,Ore07,Ore08,Ore09,Ore10,Ore11,Ore12,Ore13,Ore14	*OreG0009744*
scaffold31714	290918	C	T	NA	*OreG0009745*
scaffold31714	291277	T	C	NA	*OreG0009745*
scaffold31714	314052	T	A	Och01,Och02,Och03,Och04,Och05,Och06,Och07,Och08,Och09,Och10,Och11,Och12,Och13,Och14,Ore01,Ore02,Ore03,Ore04,Ore05,Ore06,Ore07,Ore08,Ore09,Ore10,Ore11,Ore12,Ore13,Ore14	*OreG0009746*
scaffold31714	326429	C	A	NA	*OreG0009747*
scaffold31714	367619	G	A	Och02,Och06,Och14	*OreG0009750*
scaffold31714	394972	A	C	Och01,Och02,Och03,Och04,Och05,Och06,Och07,Och08,Och09,Och10,Och11,Och12,Och13,Och14,Ore01,Ore02,Ore03,Ore04,Ore05,Ore06,Ore07,Ore08,Ore09,Ore10,Ore11,Ore12,Ore13,Ore14	*OreG0009754*
scaffold31714	425490	C	T	NA	*OreG0009759*
scaffold31714	456942	A	G	NA	*OreG0009761*
scaffold31714	525799	C	A	Ore01,Ore02,Ore03,Ore04,Ore05,Ore06,Ore07,Ore08,Ore09,Ore10,Ore11,Ore12,Ore13,Ore14	*OreG0009768*
scaffold31714	584998	T	C	Och03,Och07,Och08,Och09,Och10,Och11,Och12,Och14,Ore01,Ore02,Ore03,Ore04,Ore05,Ore06,Ore07,Ore08,Ore09,Ore10,Ore11,Ore12,Ore13,Ore14	*OreG0009775*
scaffold31714	616243	G	A	Ore02	*OreG0009778*
scaffold31714	616247	C	T	NA	*OreG0009778*
scaffold31714	668611	T	C	Ore01,Ore02,Ore03,Ore04,Ore05,Ore06,Ore07,Ore08,Ore09,Ore10,Ore11,Ore12,Ore13,Ore14	*OreG0009781*
scaffold31714	713083	C	A	Och01,Och02,Och06,Ore01,Ore02,Ore03,Ore04,Ore05,Ore06,Ore07,Ore08,Ore09,Ore10,Ore11,Ore12,Ore13,Ore14	*OreG0009786*

scaffold31714	714694	G	A	Och01,Och02,Och06,Ore01,Ore02,Ore03,Ore04,Ore05,Ore06,Ore07,Ore08,Ore09,Ore10,Ore11,Ore12,Ore13,Ore14	*OreG0009786*
scaffold31714	717532	T	A	Och07,Och08,Och09,Och10,Och11,Och12,Och14	*OreG0009786*
scaffold31714	718067	C	G	Och09,Och10,Och12,Och14	*OreG0009786*
scaffold31714	739544	G	A	Ore01,Ore02,Ore03,Ore06,Ore07,Ore08,Ore09,Ore10,Ore11,Ore12,Ore13,Ore14	*OreG0009788*
scaffold31714	743403	G	A	NA	*OreG0009789*
scaffold31714	744620	A	T	Och12	*OreG0009789*
scaffold31714	748104	C	A	Ore01,Ore02,Ore03,Ore04,Ore05,Ore06,Ore07,Ore08,Ore09,Ore10,Ore11,Ore12,Ore13,Ore14	*OreG0009790*
scaffold31714	748523	A	G	NA	*OreG0009790*
scaffold31714	764308	T	G	NA	*OreG0009792*
scaffold31714	765368	G	A	Och01,Och02,Och03,Och04,Och05,Och06,Och07,Och08,Och09,Och10,Och11,Och12,Och13,Och14	*OreG0009792*
scaffold31714	765542	G	C	Och01,Och02,Och03,Och04,Och05,Och06,Och07,Och08,Och09,Och10,Och11,Och12,Och13,Och14,Ore01,Ore02,Ore03,Ore04,Ore05,Ore06,Ore07,Ore08,Ore09,Ore10,Ore11,Ore12,Ore13,Ore14	*OreG0009792*
scaffold31714	766089	C	G	Ore01,Ore02,Ore03,Ore06,Ore07,Ore08,Ore09,Ore10,Ore11,Ore12,Ore13,Ore14	*OreG0009792*
scaffold31714	766316	T	C	Och01,Och02,Och03,Och04,Och05,Och06,Och07,Och08,Och09,Och10,Och11,Och12,Och13,Och14,Ore01,Ore02,Ore03,Ore04,Ore05,Ore06,Ore07,Ore08,Ore09,Ore10,Ore11,Ore12,Ore13,Ore14	*OreG0009792*
scaffold31714	813458	C	T	Ore01,Ore02,Ore03,Ore04,Ore05,Ore06,Ore07,Ore08,Ore09,Ore10,Ore11,Ore12,Ore13,Ore14	*OreG0009799*
scaffold31714	831262	G	T	Och01,Och02,Och03,Och04,Och05,Och06,Och07,Och08,Och09,Och10,Och11,Och12,Och13,Och14,Ore01,Ore02,Ore03,Ore04,Ore05,Ore06,Ore07,Ore08,Ore09,Ore10,Ore11,Ore12,Ore13,Ore14	*OreG0009801*
scaffold31714	831475	C	A	Och01,Och02,Och03,Och04,Och05,Och06,Och07,Och08,Och09,Och10,Och11,Och12,Och13,Och14	*OreG0009801*
scaffold31714	845286	T	C	Och01,Och02,Och03,Och04,Och05,Och06,Och07,Och08,Och09,Och10,Och11,Och12,Och13,Och14	*OreG0009803*
scaffold31714	845477	A	C	NA	*OreG0009803*
scaffold31714	845837	G	C	NA	*OreG0009803*
scaffold31714	855666	A	C	Ore01,Ore02,Ore03,Ore04,Ore05,Ore06,Ore07,Ore08,Ore09,Ore10,Ore11,Ore12,Ore13,Ore14	*OreG0009804*
scaffold31714	855870	C	T	Och01,Och02,Och03,Och04,Och05,Och06,Och07,Och08,Och09,Och10,Och11,Och12,Och13,Och14	*OreG0009804*
scaffold31714	878621	G	C	Och01,Och02,Och03,Och04,Och05,Och06,Och07,Och08,Och09,Och10,Och11,Och12,Och13,Och14	*OreG0009808*
scaffold31714	878755	T	C	NA	*OreG0009808*
scaffold31714	878892	C	T	NA	*OreG0009808*
scaffold31714	879108	G	A	NA	*OreG0009808*
scaffold31714	879160	C	T	Och01,Och02,Och03,Och04,Och05,Och06,Och07,Och08,Och09,Och10,Och11,Och12,Och13,Och14	*OreG0009808*
scaffold31714	879204	G	A	Ore03,Ore04,Ore05	*OreG0009808*
scaffold31714	879292	C	G	NA	*OreG0009808*
scaffold31714	879302	C	A	Ore01,Ore02,Ore03,Ore04,Ore05,Ore06,Ore07,Ore08,Ore09,Ore10,Ore11,Ore12,Ore13,Ore14	*OreG0009808*
scaffold31714	880901	A	C	Och08	*OreG0009808*
scaffold31714	880982	A	C	Och12	*OreG0009808*
scaffold31714	880986	T	C	Ore03,Ore04,Ore05	*OreG0009808*
scaffold31714	940702	G	T	NA	*OreG0009812*
scaffold31714	942523	C	T	Och02,Och06,Och09,Och10,Och14	*OreG0009812*
scaffold31714	943255	G	A	Och01,Och12	*OreG0009812*
scaffold31714	1011135	C	A	Och01,Och02,Och03,Och04,Och05,Och06,Och07,Och08,Och09,Och10,Och11,Och12,Och13,Och14	*OreG0009818*
scaffold31714	1011392	G	A	Och01,Och02,Och03,Och04,Och05,Och06,Och07,Och08,Och09,Och10,Och11,Och12,Och13,Och14,Ore01,Ore02,Ore03,Ore04,Ore05,Ore06,Ore07,Ore08,Ore09,Ore10,Ore11,Ore12,Ore13,Ore14	*OreG0009818*
scaffold31714	1049975	G	C	NA	*OreG0009825*
scaffold31714	1050291	A	G	NA	*OreG0009825*
scaffold31714	1050548	T	C	NA	*OreG0009825*
scaffold31714	1050587	T	G	NA	*OreG0009825*
scaffold31714	1079384	T	G	Och03,Och07,Och08,Och09,Och10,Och11,Och12	*OreG0009829*
scaffold31714	1089004	T	C	Och02,Och03,Och04,Och05,Och06,Och07,Och08,Och09,Och10,Och11,Och12,Och13,Och14	*OreG0009831*
scaffold31714	1097049	C	A	Och01,Och02,Och03,Och04,Och05,Och06,Och07,Och08,Och09,Och10,Och11,Och12,Och13,Och14	*OreG0009832*
scaffold31714	1106751	A	C	Ore01,Ore02,Ore03,Ore04,Ore05,Ore06,Ore07,Ore08,Ore09,Ore10,Ore11,Ore12,Ore13,Ore14	*OreG0009834*
scaffold31714	1107105	T	A	NA	*OreG0009834*
scaffold31714	1108269	C	G	Och01,Och02,Och03,Och04,Och05,Och06,Och07,Och08,Och09,Och10,Och11,Och12,Och13,Och14,Ore01,Ore02,Ore03,Ore04,Ore05,Ore06,Ore07,Ore08,Ore09,Ore10,Ore11,Ore12,Ore13,Ore14	*OreG0009834*
scaffold31714	1115819	A	C	NA	*OreG0009837*
scaffold31714	1121712	T	C	NA	*OreG0009838*
scaffold31714	1122715	T	C	NA	*OreG0009838*

scaffold31714	1130275	A	G	Och01,Och02,Och03,Och04,Och05,Och06,Och07,Och08,Och09,Och10,Och11,Och12,Och13,Och14,Ore01,Ore02,Ore03,Ore04,Ore05,Ore06,Ore07,Ore08,Ore09,Ore10,Ore11,Ore12,Ore13,Ore14	*OreG0009839*
scaffold31714	1198520	C	A	Och01,Och02,Och03,Och04,Och05,Och06,Och07,Och08,Och09,Och10,Och11,Och12,Och13,Och14,Ore01,Ore02,Ore03,Ore04,Ore05,Ore06,Ore07,Ore08,Ore09,Ore10,Ore11,Ore12,Ore13,Ore14	*OreG0009846*
scaffold31714	1203464	T	C	Ore01,Ore02,Ore03,Ore04,Ore05,Ore06,Ore07,Ore08,Ore09,Ore10,Ore11,Ore12,Ore13,Ore14	*OreG0009847*
scaffold31714	1204268	C	T	Ore01,Ore02,Ore03,Ore04,Ore05,Ore06,Ore07,Ore08,Ore09,Ore10,Ore11,Ore12,Ore13,Ore14	*OreG0009847*
scaffold31714	1211599	G	T	NA	*OreG0009848*
scaffold31714	1225866	G	A	Och07,Och08,Och12,Och14	*OreG0009851*
scaffold31714	1244257	T	C	Och01,Och02,Och03,Och04,Och05,Och06,Och07,Och08,Och09,Och10,Och11,Och12,Och13,Och14,Ore01,Ore02,Ore03,Ore04,Ore05,Ore06,Ore07,Ore08,Ore09,Ore10,Ore11,Ore12,Ore13,Ore14	*OreG0009853*
scaffold31714	1268236	G	C	Ore02	*OreG0009856*
scaffold31714	1268491	C	T	NA	*OreG0009856*
scaffold31714	1273418	C	A	NA	*OreG0009856*
scaffold31714	1284981	G	A	Ore01,Ore02,Ore03,Ore04,Ore05,Ore06,Ore07,Ore08,Ore09,Ore10,Ore11,Ore12,Ore13,Ore14	*OreG0009857*
scaffold31714	1286613	T	A	Och06,Och14,Ore01,Ore02,Ore03,Ore04,Ore05,Ore06,Ore07,Ore08,Ore09,Ore10,Ore11,Ore12,Ore13,Ore14	*OreG0009857*
scaffold31714	1294792	T	G	Och01,Och02,Och03,Och04,Och05,Och06,Och07,Och08,Och09,Och10,Och11,Och12,Och13,Och14	*OreG0009857*
scaffold31714	1295203	A	G	Och01,Och02,Och03,Och04,Och05,Och06,Och07,Och08,Och09,Och10,Och11,Och12,Och13,Och14,Ore01,Ore02,Ore03,Ore04,Ore05,Ore06,Ore07,Ore08,Ore09,Ore10,Ore11,Ore12,Ore13,Ore14	*OreG0009857*
scaffold31714	1343703	A	G	NA	*OreG0009861*
scaffold33137	178044	G	A	Ore01,Ore02,Ore03,Ore04,Ore05,Ore06,Ore07,Ore08,Ore09,Ore10,Ore11,Ore12,Ore13,Ore14	*OreG0013839*
scaffold33137	197862	C	T	NA	*OreG0013842*
scaffold33137	198419	T	G	Och01,Och02,Och03,Och04,Och05,Och06,Och07,Och08,Och09,Och10,Och11,Och12,Och13,Och14,Ore01,Ore02,Ore03,Ore04,Ore05,Ore06,Ore07,Ore08,Ore09,Ore10,Ore11,Ore12,Ore13,Ore14	*OreG0013842*
scaffold33137	201314	G	T	Och01,Och02,Och03,Och04,Och05,Och06,Och07,Och08,Och09,Och10,Och11,Och12,Och13,Och14,Ore01,Ore02,Ore03,Ore04,Ore05,Ore06,Ore07,Ore08,Ore09,Ore10,Ore11,Ore12,Ore13,Ore14	*OreG0013842*
scaffold33137	201435	G	T	Och01,Och02,Och03,Och04,Och05,Och06,Och07,Och08,Och09,Och10,Och11,Och12,Och13,Och14,Ore01,Ore02,Ore03,Ore04,Ore05,Ore06,Ore07,Ore08,Ore09,Ore10,Ore11,Ore12,Ore13,Ore14	*OreG0013842*
scaffold33137	230098	C	A	NA	*OreG0013844*
scaffold33137	240375	C	A	Och01,Och02,Och03,Och04,Och05,Och06,Och07,Och08,Och09,Och10,Och11,Och12,Och13,Och14,Ore01,Ore02,Ore03,Ore04,Ore05,Ore06,Ore07,Ore08,Ore09,Ore10,Ore11,Ore12,Ore13,Ore14	*OreG0013843*
scaffold33137	256446	G	A	Ore01,Ore02,Ore03,Ore04,Ore05,Ore06,Ore07,Ore08,Ore09,Ore10,Ore11,Ore12,Ore13,Ore14	*OreG0013845*
scaffold33137	257415	C	T	NA	*OreG0013845*
scaffold33137	257439	A	C	NA	*OreG0013845*
scaffold33137	257454	C	T	Och03,Och04	*OreG0013845*
scaffold33137	257472	T	A	Ore01,Ore02,Ore03,Ore04,Ore05,Ore06,Ore07,Ore08,Ore09,Ore10,Ore11,Ore12,Ore13,Ore14	*OreG0013845*
scaffold33137	258673	T	G	Ore01,Ore02,Ore03,Ore04,Ore05,Ore06,Ore07,Ore08,Ore09,Ore10,Ore11,Ore12,Ore13,Ore14	*OreG0013845*
scaffold33137	264685	G	C	NA	*OreG0013846*
scaffold33137	264769	T	C	NA	*OreG0013846*
scaffold33137	264791	T	G	NA	*OreG0013846*
scaffold33137	265411	A	G	NA	*OreG0013846*
scaffold33137	265870	G	T	NA	*OreG0013846*
scaffold33137	266035	G	A	NA	*OreG0013846*
scaffold33137	266119	T	C	NA	*OreG0013846*
scaffold33137	268700	C	T	NA	*OreG0013847*
scaffold33137	272256	G	C	Ore01,Ore02,Ore03,Ore04,Ore05,Ore06,Ore07,Ore08,Ore09,Ore10,Ore11,Ore12,Ore13,Ore14	*OreG0013848*
scaffold33137	273462	A	T	Och01,Och02,Och03,Och04,Och05,Och06,Och07,Och08,Och09,Och10,Och11,Och12,Och13,Och14,Ore01,Ore02,Ore03,Ore04,Ore05,Ore06,Ore07,Ore08,Ore09,Ore10,Ore11,Ore12,Ore13,Ore14	*OreG0013848*
scaffold33137	274307	G	T	Ore08	*OreG0013848*
scaffold33137	293795	C	G	NA	*OreG0013851*
scaffold33137	295520	C	G	Och01,Och02,Och03,Och04,Och05,Och06,Och07,Och08,Och09,Och10,Och11,Och12,Och13,Och14,Ore01,Ore02,Ore03,Ore04,Ore05,Ore06,Ore07,Ore08,Ore09,Ore10,Ore11,Ore12,Ore13,Ore14	*OreG0013852*
scaffold33137	296712	A	G	Och03,Och04,Och08,Och14,Ore01,Ore02,Ore03,Ore04,Ore05,Ore06,Ore07,Ore08,Ore09,Ore10,Ore11,Ore12,Ore13,Ore14	*OreG0013852*
scaffold33137	374551	T	C	Ore01,Ore02,Ore03,Ore04,Ore05,Ore06,Ore07,Ore08,Ore09,Ore10,Ore11,Ore12,Ore13,Ore14	*OreG0013860*

scaffold33137	374584	C	A	Ore01,Ore02,Ore03,Ore04,Ore05,Ore06,Ore07,Ore08,Ore09,Ore10,Ore11,Ore12,Ore13,Ore14	*OreG0013860*
scaffold33137	374686	A	G	Och01,Och02,Och03,Och04,Och05,Och06,Och07,Och08,Och09,Och10,Och11,Och12,Och13,Och14,Ore01,Ore02,Ore03,Ore04,Ore05,Ore06,Ore07,Ore08,Ore09,Ore10,Ore11,Ore12,Ore13,Ore14	*OreG0013860*
scaffold33137	374690	T	C	Och01,Och02,Och03,Och04,Och05,Och06,Och07,Och08,Och09,Och10,Och11,Och12,Och13,Och14,Ore01,Ore02,Ore03,Ore04,Ore05,Ore06,Ore07,Ore08,Ore09,Ore10,Ore11,Ore12,Ore13,Ore14	*OreG0013860*
scaffold33137	374843	T	C	Och01,Och02,Och03,Och04,Och05,Och06,Och07,Och08,Och09,Och10,Och11,Och12,Och13,Och14,Ore01,Ore02,Ore03,Ore04,Ore05,Ore06,Ore07,Ore08,Ore09,Ore10,Ore11,Ore12,Ore13,Ore14	*OreG0013860*
scaffold33137	374858	A	G	Och01,Och02,Och03,Och04,Och05,Och06,Och07,Och08,Och09,Och10,Och11,Och12,Och13,Och14,Ore01,Ore02,Ore03,Ore04,Ore05,Ore06,Ore07,Ore08,Ore09,Ore10,Ore11,Ore12,Ore13,Ore14	*OreG0013860*
scaffold33137	505632	A	T	Ore01,Ore02,Ore08	*OreG0013867*
scaffold33137	578910	A	G	NA	*OreG0013870*
scaffold33137	625282	G	A	Ore04,Ore05,Ore06,Ore09	*OreG0013874*
scaffold33137	626898	G	T	Och08	*OreG0013874*
scaffold33137	627037	C	A	Ore04,Ore05,Ore06,Ore09	*OreG0013874*
scaffold33137	627208	C	T	Och01,Och02,Och03,Och04,Och05,Och06,Och07,Och08,Och09,Och10,Och11,Och12,Och13,Och14,Ore01,Ore02,Ore03,Ore04,Ore05,Ore06,Ore07,Ore08,Ore09,Ore10,Ore11,Ore12,Ore13,Ore14	*OreG0013874*
scaffold33137	631339	G	T	NA	*OreG0013875*
scaffold33137	637154	C	G	Ore04,Ore05,Ore06,Ore09	*OreG0013875*
scaffold33137	639487	A	T	Och02,Och03,Och04,Och05,Och06,Och08,Och09,Och10,Och11,Och12,Och13,Och14	*OreG0013875*
scaffold33202	18818	A	T	Och01,Och02,Och03,Och04,Och05,Och06,Och07,Och08,Och09,Och10,Och11,Och12,Och13,Och14,Ore01,Ore02,Ore03,Ore04,Ore05,Ore06,Ore07,Ore08,Ore09,Ore10,Ore11,Ore12,Ore13,Ore14	*OreG0015845*
scaffold33202	23496	C	G	NA	*OreG0015846*
scaffold33202	25649	A	G	NA	*OreG0015846*
scaffold33202	25684	A	T	NA	*OreG0015846*
scaffold33206	42082	G	A	NA	*OreG0015890*
scaffold33206	92854	G	T	Och01,Och02,Och03,Och04,Och05,Och06,Och07,Och08,Och09,Och10,Och11,Och12,Och13,Och14,Ore01,Ore02,Ore03,Ore04,Ore05,Ore06,Ore07,Ore08,Ore09,Ore10,Ore11,Ore12,Ore13,Ore14	*OreG0015893*
scaffold33206	95433	A	T	NA	*OreG0015893*
scaffold33206	97222	A	T	Ore01,Ore02,Ore03,Ore04,Ore05,Ore06,Ore07,Ore08,Ore09,Ore10,Ore11,Ore12,Ore13,Ore14	*OreG0015893*
scaffold33206	97385	G	C	NA	*OreG0015893*
scaffold33206	111054	C	A	Och05,Och07,Och09,Och11,Och12,Och13	*OreG0015895*
scaffold33206	122861	C	G	NA	*OreG0015899*
scaffold33206	122900	C	T	Ore01,Ore02,Ore03,Ore04,Ore05,Ore06,Ore07,Ore08,Ore09,Ore10,Ore11,Ore12,Ore13,Ore14	*OreG0015899*
scaffold33206	122954	C	G	Ore01,Ore02,Ore03,Ore04,Ore05,Ore06,Ore07,Ore08,Ore09,Ore10,Ore11,Ore12,Ore13,Ore14	*OreG0015899*
scaffold33206	123594	T	C	Och01,Och02,Och03,Och04,Och05,Och06,Och07,Och08,Och09,Och10,Och11,Och12,Och13,Och14	*OreG0015899*
scaffold33206	137314	G	A	Och01,Och02,Och03,Och04,Och05,Och06,Och07,Och08,Och09,Och10,Och11,Och12,Och13,Och14,Ore01,Ore02,Ore03,Ore04,Ore05,Ore06,Ore07,Ore08,Ore09,Ore10,Ore11,Ore12,Ore13,Ore14	*OreG0015901*
scaffold33206	287699	C	A	Ore01,Ore02,Ore03,Ore04,Ore05,Ore06,Ore07,Ore08,Ore09,Ore10,Ore11,Ore12,Ore13,Ore14	*OreG0015907*
scaffold33206	401527	C	A	NA	*OreG0015912*
scaffold33194	40046	A	G	NA	*OreG0015054*
scaffold33194	46839	A	C	Och11,Och14,Ore09,Ore14	*OreG0015054*
scaffold33194	123165	A	C	NA	*OreG0015063*
scaffold33194	163721	C	G	Och01,Och02,Och03,Och04,Och05,Och06,Och07,Och08,Och09,Och10,Och11,Och12,Och13,Och14,Ore01,Ore02,Ore03,Ore04,Ore05,Ore06,Ore07,Ore08,Ore09,Ore10,Ore11,Ore12,Ore13,Ore14	*OreG0015066*
scaffold33194	164217	G	A	Och01,Och02,Och03,Och04,Och05,Och06,Och07,Och09,Och10,Och11,Och12,Och13,Och14	*OreG0015066*
scaffold33194	226785	T	A	Och01,Och02,Och03,Och04,Och05,Och06,Och07,Och08,Och09,Och10,Och11,Och12,Och13,Och14,Ore01,Ore02,Ore03,Ore04,Ore05,Ore06,Ore07,Ore08,Ore09,Ore10,Ore11,Ore12,Ore13,Ore14	*OreG0015070*
scaffold33194	226958	C	T	Och01,Och02,Och03,Och04,Och05,Och06,Och07,Och08,Och09,Och10,Och11,Och12,Och13,Och14,Ore01,Ore02,Ore03,Ore04,Ore05,Ore06,Ore07,Ore08,Ore09,Ore10,Ore11,Ore12,Ore13,Ore14	*OreG0015070*
scaffold33194	233317	T	A	Och14	*OreG0015071*
scaffold33194	233868	T	C	Ore01,Ore02,Ore03,Ore04,Ore05,Ore06,Ore07,Ore08,Ore09,Ore10,Ore11,Ore12,Ore13,Ore14	*OreG0015071*
scaffold33194	357807	A	C	NA	*OreG0015081*
scaffold33194	363348	C	G	NA	*OreG0015081*
scaffold33194	369452	G	A	NA	*OreG0015083*
scaffold33194	369633	C	T	NA	*OreG0015083*
scaffold33194	370196	G	T	NA	*OreG0015083*

scaffold33194	370350	C	G	Och05,Och12,Och13,Och14	*OreG0015083*
scaffold33194	377792	G	T	Och14	*OreG0015084*
scaffold33194	377816	G	C	NA	*OreG0015084*
scaffold33194	387834	A	T	Och06,Och07	*OreG0015085*
scaffold33194	481252	C	T	NA	*OreG0015093*
scaffold33119	49794	C	T	Och05,Och08	*OreG0013081*
scaffold33119	53218	C	T	Och01,Och02,Och03,Och04,Och05,Och06,Och07,Och08,Och09,Och10,Och11,Och12,Och13,Och14,Ore01,Ore02,Ore03,Ore04,Ore05,Ore06,Ore07,Ore08,Ore09,Ore10,Ore11,Ore12,Ore13,Ore14	*OreG0013082*
scaffold33119	55527	T	G	NA	*OreG0013083*
scaffold33119	65605	T	A	Ore04,Ore05	*OreG0013085*
scaffold33119	65677	T	A	Och01,Och02,Och03,Och04,Och05,Och06,Och07,Och08,Och09,Och10,Och11,Och12,Och13	*OreG0013085*
scaffold33119	65949	C	A	NA	*OreG0013085*
scaffold33119	66224	G	A	Ore02,Ore07,Ore09,Ore10,Ore11,Ore12,Ore13,Ore14	*OreG0013085*
scaffold33119	66586	C	A	Och01,Och02,Och03,Och04,Och05,Och06,Och07,Och08,Och09,Och10,Och11,Och12,Och13,Och14	*OreG0013085*
scaffold33119	89476	C	T	Och02,Och03,Och04,Och05,Och12,Och13	*OreG0013090*
scaffold33119	89547	C	G	Ore02,Ore04,Ore05,Ore07,Ore09,Ore10,Ore11,Ore12,Ore13,Ore14	*OreG0013090*
scaffold33119	94106	T	A	Ore01,Ore02,Ore04,Ore05,Ore06,Ore07,Ore08,Ore09,Ore10,Ore11,Ore12,Ore13,Ore14	*OreG0013091*
scaffold33119	94810	G	C	NA	*OreG0013091*
scaffold33119	94958	T	C	Och01,Och02,Och03,Och04,Och05,Och06,Och07,Och08,Och09,Och10,Och11,Och12,Och13,Och14,Ore01,Ore02,Ore04,Ore05,Ore06,Ore07,Ore08,Ore09,Ore10,Ore11,Ore12,Ore13,Ore14	*OreG0013091*
scaffold33119	107005	A	T	Ore01,Ore02,Ore04,Ore05,Ore06,Ore07,Ore08,Ore09,Ore10,Ore11,Ore12,Ore13,Ore14	*OreG0013093*
scaffold33119	107746	G	C	NA	*OreG0013093*
scaffold33119	108016	A	G	Ore02,Ore07,Ore09,Ore10,Ore11,Ore12,Ore13,Ore14	*OreG0013093*
scaffold33119	112316	T	A	NA	*OreG0013094*
scaffold33119	118702	T	G	Och12,Och14	*OreG0013095*
scaffold33119	118985	G	A	NA	*OreG0013095*
scaffold33119	140318	G	C	Och01,Och02,Och03,Och04,Och05,Och06,Och07,Och08,Och09,Och10,Och11,Och12,Och13,Och14	*OreG0013100*
scaffold33119	171074	A	G	NA	*OreG0013104*
scaffold33119	187968	C	T	Och02,Och03,Och04,Och05,Och07,Och08,Och12,Och13,Och14,Ore01,Ore02,Ore03,Ore04,Ore05,Ore06,Ore07,Ore08,Ore09,Ore10,Ore11,Ore12,Ore13,Ore14	*OreG0013106*
scaffold33119	231459	G	A	NA	*OreG0013111*
scaffold33119	233704	A	T	NA	*OreG0013112*
scaffold33119	246006	C	G	NA	*OreG0013113*
scaffold33119	253206	A	T	Och07,Och08,Och11,Och12	*OreG0013114*
scaffold33119	253532	G	A	NA	*OreG0013114*
scaffold33119	255477	C	T	NA	*OreG0013115*
scaffold33119	256091	G	A	Och07,Och08,Och12	*OreG0013115*
scaffold33119	256092	G	C	Ore04,Ore05	*OreG0013115*
scaffold33119	256225	A	T	Och07,Och08,Och12	*OreG0013115*
scaffold33119	267821	G	A	NA	*OreG0013118*
scaffold33119	268133	G	A	NA	*OreG0013118*
scaffold33119	272774	C	A	Och01,Och02,Och03,Och04,Och05,Och06,Och07,Och08,Och09,Och10,Och11,Och12,Och13,Och14,Ore01,Ore02,Ore03,Ore04,Ore05,Ore06,Ore07,Ore08,Ore09,Ore10,Ore11,Ore12,Ore13,Ore14	*OreG0013120*
scaffold33119	273249	G	A	NA	*OreG0013120*
scaffold33119	274493	C	A	NA	*OreG0013121*
scaffold33119	278917	G	T	Och12	*OreG0013121*
scaffold33119	294045	A	G	NA	*OreG0013124*
scaffold33119	320978	C	A	Och01,Och08	*OreG0013128*
scaffold33119	322813	G	C	Ore01,Ore02,Ore03,Ore04,Ore05,Ore06,Ore07,Ore08,Ore09,Ore10,Ore11,Ore12,Ore13,Ore14	*OreG0013128*
scaffold33119	369400	G	A	Och01,Och02,Och03,Och04,Och05,Och06,Och07,Och08,Och09,Och10,Och11,Och12,Och13,Och14	*OreG0013131*
scaffold33119	407827	C	A	NA	*OreG0013133*
scaffold33119	425175	G	A	Ore01,Ore02,Ore03,Ore04,Ore05,Ore06,Ore07,Ore08,Ore09,Ore10,Ore11,Ore12,Ore13,Ore14	*OreG0013135*
scaffold33119	425389	G	C	Och02,Och03,Och04,Och05,Och06,Och08,Och09,Och10,Och12,Och13	*OreG0013135*
scaffold33119	425398	C	A	Ore01,Ore02,Ore03,Ore04,Ore05,Ore06,Ore07,Ore08,Ore09,Ore10,Ore11,Ore12,Ore13,Ore14	*OreG0013135*
scaffold33119	451283	T	C	NA	*OreG0013137*
scaffold33119	499975	C	T	Och05	*OreG0013146*
scaffold33119	505135	A	G	NA	*OreG0013147*
scaffold33119	511965	G	C	Och01,Och02,Och03,Och04,Och05,Och06,Och07,Och08,Och09,Och10,Och11,Och12,Och13,Och14,Ore01,Ore02,Ore03,Ore04,Ore05,Ore06,Ore07,Ore08,Ore09,Ore10,Ore11,Ore12,Ore13,Ore14	*OreG0013148*
scaffold33119	528387	C	T	NA	*OreG0013150*
scaffold33119	528727	C	T	NA	*OreG0013150*

scaffold33119	536419	C	A	Och01,Och02,Och03,Och04,Och05,Och06,Och07,Och08,Och09,Och10,Och11,Och12,Och13,Och14	*OreG0013151*
scaffold33119	569329	G	A	NA	*OreG0013153*
scaffold33119	581678	G	T	NA	*OreG0013154*
scaffold33119	581693	C	T	NA	*OreG0013154*
scaffold33119	581707	C	T	NA	*OreG0013154*
scaffold33119	584989	A	G	NA	*OreG0013154*
scaffold33119	617203	G	A	NA	*OreG0013158*
scaffold33119	621848	T	A	NA	*OreG0013159*
scaffold33119	630676	G	C	NA	*OreG0013160*
scaffold33119	717541	C	T	Och02,Och03,Och04,Och05,Och06,Och08,Och12,Och13	*OreG0013167*
scaffold33119	742344	T	A	NA	*OreG0013168*
scaffold33119	745033	T	C	NA	*OreG0013169*
scaffold33119	747728	G	A	Ore01,Ore02,Ore04,Ore05,Ore08,Ore11,Ore12,Ore14	*OreG0013169*
scaffold33119	747751	T	A	Ore01,Ore02,Ore03,Ore04,Ore05,Ore06,Ore07,Ore08,Ore09,Ore10,Ore11,Ore12,Ore13,Ore14	*OreG0013169*
scaffold33119	755181	G	A	NA	*OreG0013170*
scaffold33119	755554	A	T	Och01,Och02,Och03,Och04,Och05,Och06,Och07,Och08,Och09,Och10,Och11,Och12,Och13,Och14,Ore01,Ore02,Ore03,Ore04,Ore05,Ore06,Ore07,Ore08,Ore09,Ore10,Ore11,Ore12,Ore13,Ore14	*OreG0013170*
scaffold33119	762060	C	T	NA	*OreG0013171*
scaffold33119	762171	C	T	NA	*OreG0013171*
scaffold33119	762455	A	G	NA	*OreG0013171*
scaffold33119	762512	C	T	NA	*OreG0013171*
scaffold33119	762564	A	T	NA	*OreG0013171*
scaffold33119	762597	C	T	Och02,Och03,Och04,Och05,Och06,Och08,Och12,Och13	*OreG0013171*
scaffold33119	822836	T	C	Och02	*OreG0013176*
scaffold33119	822929	C	T	Ore01,Ore02,Ore03,Ore04,Ore05,Ore06,Ore07,Ore08,Ore09,Ore10,Ore11,Ore12,Ore13,Ore14	*OreG0013176*
scaffold33119	822953	T	C	Och02	*OreG0013176*
scaffold33119	823013	G	A	Ore01,Ore02,Ore03,Ore04,Ore05,Ore06,Ore07,Ore08,Ore09,Ore10,Ore11,Ore12,Ore13,Ore14	*OreG0013176*
scaffold33119	833431	T	A	NA	*OreG0013178*
scaffold33119	833762	C	A	NA	*OreG0013178*
scaffold33119	833837	G	T	NA	*OreG0013178*
scaffold33119	844585	C	A	NA	*OreG0013179*
scaffold33119	844978	A	G	NA	*OreG0013179*
scaffold33119	884712	T	C	Och01,Och02,Och05,Och07,Och08,Och09,Och10,Och11,Och14	*OreG0013182*
scaffold33119	888822	A	C	Ore01,Ore02,Ore03,Ore04,Ore05,Ore06,Ore07,Ore08,Ore09,Ore10,Ore11,Ore12,Ore13,Ore14	*OreG0013182*
scaffold33119	908671	C	T	NA	*OreG0013184*
scaffold33119	915968	G	C	Och03,Och04,Och12,Och13	*OreG0013184*
scaffold33119	936998	A	T	Och01,Och02,Och03,Och04,Och05,Och06,Och07,Och08,Och09,Och10,Och11,Och12,Och13,Och14	*OreG0013188*
scaffold33119	968030	A	C	Och07,Och09,Och10,Och11,Och14	*OreG0013190*
scaffold33119	979575	C	A	Och01,Och02,Och03,Och04,Och05,Och06,Och07,Och08,Och09,Och10,Och11,Och12,Och13,Och14	*OreG0013191*
scaffold33119	979617	T	A	Ore01,Ore02,Ore03,Ore04,Ore05,Ore06,Ore07,Ore08,Ore09,Ore10,Ore11,Ore12,Ore13,Ore14	*OreG0013191*
scaffold33119	979780	C	A	Ore01,Ore02,Ore03,Ore04,Ore05,Ore06,Ore07,Ore08,Ore09,Ore10,Ore11,Ore12,Ore13,Ore14	*OreG0013191*
scaffold33119	979787	T	C	NA	*OreG0013191*
scaffold33119	983592	A	T	NA	*OreG0013192*
scaffold33119	991042	C	T	Och01,Och02,Och03,Och04,Och05,Och06,Och07,Och08,Och09,Och10,Och11,Och12,Och13,Och14,Ore01,Ore02,Ore03,Ore04,Ore05,Ore06,Ore07,Ore08,Ore09,Ore10,Ore11,Ore12,Ore13,Ore14	*OreG0013193*
scaffold33119	996742	A	G	NA	*OreG0013194*
scaffold33119	1008938	A	T	Och06,Och07,Och08,Och14	*OreG0013196*
scaffold33119	1017166	G	A	NA	*OreG0013197*
scaffold33119	1018909	C	T	Och08	*OreG0013198*
scaffold33119	1018998	G	A	Och08	*OreG0013198*
scaffold33119	1019001	T	A	Ore01,Ore02,Ore04,Ore05,Ore08,Ore10,Ore11,Ore12,Ore14	*OreG0013198*
scaffold33119	1022401	G	A	Och01,Och02,Och03,Och04,Och05,Och06,Och07,Och08,Och09,Och10,Och11,Och12,Och13,Och14	*OreG0013198*
scaffold33119	1023709	G	A	Ore01,Ore02,Ore03,Ore04,Ore05,Ore06,Ore07,Ore08,Ore09,Ore10,Ore11,Ore12,Ore13,Ore14	*OreG0013198*
scaffold33119	1023970	G	A	NA	*OreG0013198*
scaffold33119	1024307	A	G	Och01,Och02,Och03,Och04,Och05,Och07,Och09,Och10,Och11,Och12,Och13,Och14	*OreG0013198*
scaffold33119	1024315	A	G	NA	*OreG0013198*
scaffold33119	1027201	T	G	Och02,Och03,Och04,Och05,Och06,Och07,Och08,Och12,Och13,Och14	*OreG0013198*
scaffold33119	1135382	C	T	Ore01,Ore02,Ore03,Ore04,Ore05,Ore06,Ore07,Ore08,Ore09,Ore10,Ore11,Ore12,Ore13,Ore14	*OreG0013205*
scaffold33119	1135412	G	A	NA	*OreG0013205*
scaffold33119	1136305	A	C	NA	*OreG0013205*

scaffold33119	1136952	G	A	Och07	*OreG0013205*
scaffold33119	1180486	A	G	NA	*OreG0013209*
scaffold33119	1182450	G	C	Och07,Och09,Och10,Och14	*OreG0013210*
scaffold33119	1203040	C	T	NA	*OreG0013215*
scaffold33119	1203270	C	T	NA	*OreG0013215*
scaffold33119	1265047	C	T	Ore01,Ore02,Ore03,Ore04,Ore05,Ore06,Ore07,Ore08,Ore09,Ore10,Ore11,Ore12,Ore13,Ore14	*OreG0013218*
scaffold33119	1266302	A	T	Och01,Och02,Och03,Och04,Och05,Och06,Och07,Och08,Och09,Och10,Och11,Och12,Och13,Och14,Ore01,Ore02,Ore03,Ore04,Ore05,Ore06,Ore07,Ore08,Ore09,Ore10,Ore11,Ore12,Ore13,Ore14	*OreG0013218*
scaffold33119	1299073	G	A	NA	*OreG0013222*
scaffold33119	1304467	G	A	NA	*OreG0013222*
scaffold33119	1350275	G	A	NA	*OreG0013225*
scaffold33119	1351315	A	T	NA	*OreG0013225*
scaffold33119	1351420	C	T	Och07	*OreG0013225*
scaffold33119	1351546	G	A	NA	*OreG0013225*
scaffold33119	1351563	T	C	NA	*OreG0013225*
scaffold33119	1358677	T	A	Ore03	*OreG0013226*
scaffold33119	1552902	A	T	Och01,Och02,Och03,Och04,Och05,Och06,Och07,Och08,Och09,Och10,Och11,Och12,Och13,Och14,Ore01,Ore02,Ore03,Ore04,Ore05,Ore06,Ore07,Ore08,Ore09,Ore10,Ore11,Ore12,Ore13,Ore14	*OreG0013232*
scaffold33119	1567128	T	G	NA	*OreG0013233*
scaffold33119	1595718	G	A	Och01,Och02,Och03,Och04,Och05,Och06,Och07,Och08,Och09,Och10,Och11,Och12,Och13,Och14,Ore01,Ore02,Ore03,Ore04,Ore05,Ore06,Ore07,Ore08,Ore09,Ore10,Ore11,Ore12,Ore13,Ore14	*OreG0013235*
scaffold33119	1601820	G	C	Ore01,Ore02,Ore03,Ore04,Ore05,Ore06,Ore07,Ore08,Ore09,Ore10,Ore11,Ore12,Ore13,Ore14	*OreG0013236*
scaffold33119	1601837	A	T	Och01,Och02,Och03,Och04,Och05,Och06,Och07,Och08,Och09,Och10,Och11,Och12,Och13,Och14	*OreG0013236*
scaffold33119	1601852	A	G	Ore01,Ore02,Ore03,Ore04,Ore05,Ore06,Ore07,Ore08,Ore09,Ore10,Ore11,Ore12,Ore13,Ore14	*OreG0013236*
scaffold33119	1602622	A	T	Ore01,Ore02,Ore03,Ore04,Ore05,Ore06,Ore07,Ore08,Ore09,Ore10,Ore11,Ore12,Ore13,Ore14	*OreG0013236*
scaffold33119	1603547	G	A	Ore01,Ore02,Ore03,Ore04,Ore05,Ore06,Ore07,Ore08,Ore09,Ore10,Ore11,Ore12,Ore13,Ore14	*OreG0013236*
scaffold33119	1603783	C	T	Ore01,Ore02,Ore03,Ore04,Ore05,Ore06,Ore07,Ore08,Ore09,Ore10,Ore11,Ore12,Ore13,Ore14	*OreG0013236*
scaffold33119	1604105	T	C	NA	*OreG0013236*
scaffold33119	1604241	G	A	NA	*OreG0013236*
scaffold33119	1604259	C	T	Och01,Och02,Och11	*OreG0013236*
scaffold33119	1609881	C	T	NA	*OreG0013236*
scaffold33119	1635068	C	G	NA	*OreG0013239*
scaffold33119	1635330	A	C	Och01,Och02,Och03,Och04,Och05,Och06,Och07,Och08,Och09,Och10,Och11,Och12,Och13,Och14	*OreG0013239*
scaffold33119	1648339	T	A	Och01,Och02,Och03,Och04,Och05,Och06,Och07,Och08,Och09,Och10,Och11,Och12,Och13,Och14	*OreG0013241*
scaffold33119	1666371	G	A	Och01,Och02,Och03,Och04,Och05,Och06,Och07,Och08,Och09,Och10,Och11,Och12,Och13,Och14	*OreG0013244*
scaffold33119	1791654	T	C	Och14	*OreG0013253*
scaffold33119	1838182	C	A	NA	*OreG0013257*
scaffold33119	1857694	G	A	NA	*OreG0013259*
scaffold33119	1862050	C	A	Och14	*OreG0013260*
scaffold33119	1862082	G	A	NA	*OreG0013260*
scaffold33119	1868939	T	C	Ore01,Ore02,Ore03,Ore04,Ore05,Ore06,Ore07,Ore08,Ore09,Ore10,Ore11,Ore12,Ore13,Ore14	*OreG0013261*
scaffold33119	1869079	T	A	Och01,Och02,Och03,Och04,Och05,Och06,Och07,Och08,Och09,Och10,Och11,Och12,Och13,Och14,Ore01,Ore02,Ore03,Ore04,Ore05,Ore06,Ore07,Ore08,Ore09,Ore10,Ore11,Ore12,Ore13,Ore14	*OreG0013261*
scaffold33119	1871412	A	G	NA	*OreG0013262*
scaffold33119	1872556	A	G	Och01,Och02,Och03,Och04,Och05,Och06,Och07,Och08,Och09,Och10,Och11,Och12,Och13,Och14,Ore01,Ore02,Ore03,Ore04,Ore05,Ore06,Ore07,Ore08,Ore09,Ore10,Ore11,Ore12,Ore13,Ore14	*OreG0013262*
scaffold33119	1907545	A	C	Och11	*OreG0013265*
scaffold33119	1907677	A	G	Ore01,Ore02,Ore03,Ore04,Ore05,Ore06,Ore07,Ore08,Ore09,Ore10,Ore11,Ore12,Ore13,Ore14	*OreG0013265*
scaffold33119	1907707	G	A	Och01,Och02,Och03,Och04,Och05,Och06,Och07,Och08,Och09,Och10,Och11,Och12,Och13,Och14,Ore01,Ore02,Ore03,Ore04,Ore05,Ore06,Ore07,Ore08,Ore09,Ore10,Ore11,Ore12,Ore13,Ore14	*OreG0013265*
scaffold33119	1907738	C	A	Ore01,Ore02,Ore03,Ore04,Ore05,Ore06,Ore07,Ore08,Ore09,Ore10,Ore11,Ore12,Ore13,Ore14	*OreG0013265*
scaffold33119	1908727	G	T	NA	*OreG0013265*
scaffold33119	1908753	C	T	Och01,Och02,Och03,Och04,Och05,Och06,Och07,Och08,Och09,Och10,Och11,Och12,Och13,Och14,Ore01,Ore02,Ore03,Ore04,Ore05,Ore06,Ore07,Ore08,Ore09,Ore10,Ore11,Ore12,Ore13,Ore14	*OreG0013265*
scaffold33119	1921693	C	A	NA	*OreG0013266*
scaffold33119	1928775	C	A	NA	*OreG0013267*

scaffold33119	1929063	G	A	Och01,Och02,Och03,Och04,Och05,Och06,Och07,Och08,Och09,Och10,Och11,Och12,Och13,Och14,Ore01,Ore02,Ore03,Ore04,Ore05,Ore06,Ore07,Ore08,Ore09,Ore10,Ore11,Ore12,Ore13,Ore14	*OreG0013267*
scaffold33119	1930119	G	A	NA	*OreG0013267*
scaffold33119	2011293	A	T	NA	*OreG0013271*
scaffold33119	2028553	G	C	Och01,Och02,Och03,Och04,Och05,Och06,Och07,Och08,Och09,Och10,Och11,Och12,Och13,Och14	*OreG0013273*
scaffold33119	2029737	G	A	NA	*OreG0013273*
scaffold33119	2029924	G	A	NA	*OreG0013273*
scaffold33119	2037663	G	A	Ore02,Ore04,Ore05,Ore08,Ore11,Ore12	*OreG0013274*
scaffold33119	2113806	C	A	Och06,Och07,Och09,Och10	*OreG0013282*
scaffold33119	2120088	T	A	Och01,Ore01,Ore02,Ore03,Ore04,Ore05,Ore06,Ore07,Ore08,Ore09,Ore10,Ore11,Ore12,Ore13,Ore14	*OreG0013283*
scaffold33119	2121759	C	A	Och08	*OreG0013283*
scaffold33119	2191326	C	G	Ore02,Ore04,Ore05,Ore06,Ore07,Ore08,Ore09,Ore10,Ore11,Ore12,Ore13,Ore14	*OreG0013292*
scaffold33119	2191612	G	C	NA	*OreG0013292*
scaffold33119	2192771	A	G	Och01,Och02,Och03,Och04,Och05,Och06,Och07,Och08,Och09,Och10,Och11,Och12,Och13,Och14,Ore01,Ore02,Ore03,Ore04,Ore05,Ore06,Ore07,Ore08,Ore09,Ore10,Ore11,Ore12,Ore13,Ore14	*OreG0013292*
scaffold33119	2193640	C	A	NA	*OreG0013292*
scaffold33119	2239851	T	C	NA	*OreG0013296*
scaffold33119	2242627	C	T	Och01,Och02,Och03,Och04,Och05,Och06,Och07,Och08,Och09,Och10,Och11,Och12,Och13,Och14,Ore01,Ore02,Ore03,Ore04,Ore05,Ore06,Ore07,Ore08,Ore09,Ore10,Ore11,Ore12,Ore13,Ore14	*OreG0013296*
scaffold33119	2246607	T	G	Och01,Och02,Och03,Och04,Och05,Och06,Och07,Och08,Och09,Och10,Och11,Och12,Och13,Och14	*OreG0013296*
scaffold33119	2305229	T	A	Och01,Och02,Och03,Och04,Och05,Och06,Och07,Och08,Och09,Och10,Och11,Och12,Och13,Och14,Ore01,Ore02,Ore03,Ore04,Ore05,Ore06,Ore07,Ore08,Ore09,Ore10,Ore11,Ore12,Ore13,Ore14	*OreG0013299*
scaffold33119	2308246	C	T	NA	*OreG0013300*
scaffold33119	2308729	G	A	Och09,Och12,Ore02,Ore04,Ore05,Ore08,Ore11,Ore12,Ore14	*OreG0013300*
scaffold33119	2315551	T	A	NA	*OreG0013301*
scaffold33119	2315732	A	G	NA	*OreG0013301*
scaffold33119	2315766	A	C	NA	*OreG0013301*
scaffold33119	2332749	G	A	NA	*OreG0013302*
scaffold33119	2333229	C	T	Och03,Och04,Och08,Och13	*OreG0013303*
scaffold33119	2333255	C	T	NA	*OreG0013303*
scaffold33119	2333585	G	A	NA	*OreG0013304*
scaffold33119	2342641	C	T	Och11,Ore01,Ore02,Ore04,Ore05,Ore08,Ore11,Ore12	*OreG0013305*
scaffold33119	2386458	C	G	NA	*OreG0013307*
scaffold33119	2386509	G	A	NA	*OreG0013307*
scaffold33119	2403903	G	T	Och01,Och02,Och03,Och04,Och05,Och06,Och07,Och08,Och09,Och10,Och11,Och12,Och13,Och14,Ore01,Ore02,Ore03,Ore04,Ore05,Ore06,Ore07,Ore08,Ore09,Ore10,Ore11,Ore12,Ore13,Ore14	*OreG0013309*
scaffold33119	2425957	T	C	Ore01,Ore02,Ore03,Ore04,Ore05,Ore06,Ore07,Ore08,Ore09,Ore10,Ore11,Ore12,Ore13,Ore14	*OreG0013315*
scaffold33119	2425975	G	A	NA	*OreG0013315*
scaffold33119	2426462	G	A	Och01,Och02,Och03,Och04,Och05,Och06,Och07,Och08,Och09,Och10,Och11,Och12,Och13,Och14,Ore01,Ore02,Ore03,Ore04,Ore05,Ore06,Ore07,Ore08,Ore09,Ore10,Ore11,Ore12,Ore13,Ore14	*OreG0013315*
scaffold33119	2455735	G	T	NA	*OreG0013317*
scaffold33119	2455768	G	A	NA	*OreG0013317*
scaffold33119	2484535	C	A	NA	*OreG0013322*
scaffold33119	2484724	C	T	Och01,Och07,Och08,Och09,Och10,Och11,Och14	*OreG0013322*
scaffold33119	2488668	T	C	NA	*OreG0013323*
scaffold33119	2490898	T	A	Och01,Och02,Och03,Och04,Och05,Och06,Och07,Och08,Och09,Och10,Och11,Och12,Och13,Och14	*OreG0013324*
scaffold33119	2495994	A	G	NA	*OreG0013325*
scaffold33119	2519640	C	T	Och01,Och02,Och03,Och04,Och05,Och06,Och07,Och08,Och09,Och10,Och11,Och12,Och13,Och14,Ore01,Ore02,Ore03,Ore04,Ore05,Ore06,Ore07,Ore08,Ore09,Ore10,Ore11,Ore12,Ore13,Ore14	*OreG0013327*
scaffold33119	2519831	A	C	Och01,Och02,Och03,Och04,Och05,Och06,Och07,Och08,Och09,Och10,Och11,Och12,Och13,Och14,Ore01,Ore02,Ore03,Ore04,Ore05,Ore06,Ore07,Ore08,Ore09,Ore10,Ore11,Ore12,Ore13,Ore14	*OreG0013327*
scaffold33119	2520874	T	C	Och01,Och08	*OreG0013327*
scaffold33119	2535417	C	T	Ore09	*OreG0013330*
scaffold33119	2554207	G	A	Ore01,Ore02,Ore03,Ore04,Ore05,Ore06,Ore07,Ore08,Ore09,Ore10,Ore11,Ore12,Ore13,Ore14	*OreG0013333*
scaffold33119	2561245	C	T	NA	*OreG0013335*
scaffold33119	2571959	C	G	Ore01,Ore02,Ore03,Ore04,Ore05,Ore06,Ore07,Ore08,Ore09,Ore10,Ore11,Ore12,Ore13,Ore14	*OreG0013336*
scaffold33119	2576044	G	A	NA	*OreG0013336*
scaffold33119	2695101	G	T	NA	*OreG0013347*
scaffold33119	2698288	C	A	Och08	*OreG0013348*

scaffold33119	2698440	A	G	Ore01,Ore02,Ore03,Ore04,Ore05,Ore06,Ore07,Ore08,Ore09,Ore10,Ore11,Ore12,Ore13,Ore14	*OreG0013348*
scaffold33119	2698557	T	C	Och13	*OreG0013348*
scaffold33119	2766718	G	C	Och01,Och02,Och03,Och04,Och05,Och06,Och07,Och08,Och09,Och10,Och11,Och12,Och13,Och14,Ore01,Ore02,Ore03,Ore04,Ore05,Ore06,Ore07,Ore08,Ore09,Ore10,Ore11,Ore12,Ore13,Ore14	*OreG0013352*
scaffold33217	36157	T	A	Och01,Och03,Och05,Och12,Och14,Ore01,Ore02,Ore03,Ore04,Ore05,Ore06,Ore07,Ore08,Ore09,Ore10,Ore11,Ore12,Ore13,Ore14	*OreG0016593*
scaffold33217	41815	C	T	Och05	*OreG0016593*
scaffold33217	41854	C	T	NA	*OreG0016593*
scaffold33217	52690	G	A	Ore01,Ore02,Ore03,Ore04,Ore05,Ore06,Ore07,Ore08,Ore09,Ore10,Ore11,Ore12,Ore13,Ore14	*OreG0016595*
scaffold33217	68257	T	A	Och01,Och02,Och03,Och04,Och05,Och06,Och07,Och08,Och09,Och10,Och11,Och12,Och13,Och14,Ore01,Ore02,Ore03,Ore04,Ore05,Ore06,Ore07,Ore08,Ore09,Ore10,Ore11,Ore12,Ore13,Ore14	*OreG0016598*
scaffold33217	80858	G	A	Och07,Och08,Och09,Och10,Och11	*OreG0016600*
scaffold33217	125930	A	T	NA	*OreG0016603*
scaffold33217	132656	G	A	Och13	*OreG0016604*
scaffold33217	143913	C	T	Och02,Och03,Och04,Och05,Och06,Och07,Och08,Och09,Och10,Och11,Och12,Och13,Och14,Ore01,Ore02,Ore03,Ore04,Ore05,Ore06,Ore07,Ore08,Ore09,Ore10,Ore11,Ore12,Ore13,Ore14	*OreG0016605*
scaffold33217	155130	G	C	Och03,Och05,Och06,Och12	*OreG0016607*
scaffold33217	155236	G	A	Och01	*OreG0016607*
scaffold33217	155269	C	T	Och13	*OreG0016607*
scaffold33217	155505	A	T	NA	*OreG0016607*
scaffold33217	173778	A	G	Och01,Och02,Och03,Och04,Och05,Och06,Och07,Och08,Och09,Och10,Och11,Och12,Och13,Och14,Ore01,Ore02,Ore03,Ore04,Ore05,Ore06,Ore07,Ore08,Ore09,Ore10,Ore11,Ore12,Ore13,Ore14	*OreG0016609*
scaffold33217	174001	T	A	NA	*OreG0016609*
scaffold33217	236946	T	A	Och05,Och07,Ore09,Ore13	*OreG0016613*
scaffold33217	237345	A	C	Ore09,Ore13	*OreG0016613*
scaffold33217	251590	G	A	NA	*OreG0016612*
scaffold33217	264869	C	T	Och06	*OreG0016616*
scaffold33217	349203	C	A	Och02,Och04,Och05,Och12,Och13	*OreG0016623*
scaffold33217	349246	C	A	Och01,Och02,Och03,Och04,Och05,Och06,Och07,Och08,Och09,Och10,Och11,Och12,Och13,Och14,Ore01,Ore02,Ore03,Ore04,Ore05,Ore06,Ore07,Ore08,Ore09,Ore10,Ore11,Ore12,Ore13,Ore14	*OreG0016623*
scaffold33217	372027	G	A	Ore09,Ore13	*OreG0016627*
scaffold33217	372117	G	A	Ore09,Ore13	*OreG0016627*
scaffold33217	373290	G	T	NA	*OreG0016627*
scaffold33217	373971	T	C	Ore09,Ore13	*OreG0016627*
scaffold33217	380446	G	T	Och01,Och02,Och03,Och04,Och05,Och06,Och07,Och08,Och09,Och10,Och11,Och12,Och13,Och14,Ore01,Ore02,Ore03,Ore04,Ore05,Ore06,Ore07,Ore08,Ore09,Ore10,Ore11,Ore12,Ore13,Ore14	*OreG0016629*
scaffold33217	380463	G	A	Ore04,Ore05	*OreG0016629*
scaffold33217	381978	G	A	Och01,Och02,Och03,Och04,Och05,Och06,Och07,Och08,Och09,Och10,Och11,Och12,Och13,Och14,Ore01,Ore02,Ore03,Ore04,Ore05,Ore06,Ore07,Ore08,Ore09,Ore10,Ore11,Ore12,Ore13,Ore14	*OreG0016629*
scaffold33217	406304	C	T	NA	*OreG0016634*
scaffold33217	406314	C	T	NA	*OreG0016634*
scaffold33217	406317	C	T	NA	*OreG0016634*
scaffold33217	409342	G	A	NA	*OreG0016634*
scaffold33103	7807	A	C	Ore01,Ore02,Ore03,Ore04,Ore05,Ore06,Ore07,Ore08,Ore09,Ore10,Ore11,Ore12,Ore13,Ore14	*OreG0011675*
scaffold33103	21924	C	G	NA	*OreG0011677*
scaffold33103	80072	G	A	Ore03,Ore04,Ore05	*OreG0011681*
scaffold33103	81142	C	T	Och01	*OreG0011681*
scaffold33103	81175	T	C	Ore04,Ore05	*OreG0011681*
scaffold33103	81577	G	T	NA	*OreG0011681*
scaffold33103	81635	A	G	NA	*OreG0011681*
scaffold33103	109197	G	T	Och01,Och02,Och03,Och04,Och05,Och06,Och07,Och08,Och09,Och10,Och11,Och12,Och13,Och14	*OreG0011682*
scaffold33103	115052	C	T	Och01,Och02,Och03,Och04,Och05,Och06,Och07,Och08,Och09,Och10,Och11,Och12,Och13	*OreG0011683*
scaffold33103	157258	A	C	Och01,Och02,Och03,Och04,Och05,Och06,Och07,Och08,Och09,Och10,Och11,Och12,Och13,Och14	*OreG0011688*
scaffold33103	157308	G	T	Ore01,Ore02,Ore03,Ore04,Ore05,Ore06,Ore07,Ore08,Ore09,Ore10,Ore11,Ore12,Ore13,Ore14	*OreG0011688*
scaffold33103	157687	C	T	Och14	*OreG0011688*
scaffold33103	158151	A	G	Och01,Och02,Och03,Och04,Och05,Och06,Och07,Och08,Och09,Och10,Och11,Och12,Och13,Och14	*OreG0011688*
scaffold33103	158346	A	G	Och12	*OreG0011688*
scaffold33103	167021	A	T	NA	*OreG0011691*
scaffold33103	171332	G	A	Ore04,Ore05	*OreG0011692*
scaffold33103	171467	G	T	Ore01,Ore04,Ore05	*OreG0011692*

scaffold33103	171766	T	G	NA	*OreG0011692*
scaffold33103	174242	C	T	Och01,Och02,Och03,Och04,Och05,Och06,Och07,Och08,Och09,Och10,Och11,Och12,Och13,Och14	*OreG0011692*
scaffold33103	217318	C	A	Ore01,Ore02,Ore03,Ore04,Ore05,Ore06,Ore07,Ore08,Ore09,Ore10,Ore11,Ore12,Ore13,Ore14	*OreG0011695*
scaffold33103	247469	G	C	NA	*OreG0011700*
scaffold33103	248330	C	A	Ore01,Ore02,Ore03,Ore04,Ore05,Ore06,Ore07,Ore08,Ore09,Ore10,Ore11,Ore12,Ore13,Ore14	*OreG0011701*
scaffold33103	248373	G	A	NA	*OreG0011701*
scaffold33103	249034	C	T	NA	*OreG0011701*
scaffold33103	249060	G	C	NA	*OreG0011701*
scaffold33103	249123	C	A	Och01,Och09,Och10,Och11,Och14	*OreG0011701*
scaffold33103	268310	A	G	Och01,Och02,Och03,Och04,Och05,Och06,Och07,Och08,Och09,Och10,Och11,Och12,Och13,Och14,Ore01,Ore02,Ore03,Ore04,Ore05,Ore06,Ore07,Ore08,Ore09,Ore10,Ore11,Ore12,Ore13,Ore14	*OreG0011705*
scaffold33103	268826	G	T	Och14	*OreG0011705*
scaffold33103	269318	A	G	Och01	*OreG0011705*
scaffold33103	269701	G	A	Ore01,Ore02,Ore03,Ore04,Ore05,Ore06,Ore07,Ore08,Ore09,Ore10,Ore11,Ore12,Ore13,Ore14	*OreG0011705*
scaffold33103	269795	G	A	NA	*OreG0011705*
scaffold33103	313896	G	T	Och01,Och02,Och03,Och04,Och05,Och06,Och07,Och08,Och09,Och10,Och11,Och12,Och13,Och14	*OreG0011711*
scaffold33103	338145	G	A	Och14	*OreG0011715*
scaffold33103	343082	C	A	Och01,Och02,Och03,Och04,Och05,Och06,Och07,Och08,Och09,Och10,Och11,Och12,Och13,Och14,Ore01,Ore02,Ore03,Ore04,Ore05,Ore06,Ore07,Ore08,Ore09,Ore10,Ore11,Ore12,Ore13,Ore14	*OreG0011716*
scaffold33103	344059	C	G	Och01,Och02,Och03,Och04,Och05,Och06,Och07,Och08,Och09,Och10,Och11,Och12,Och13,Och14,Ore01,Ore02,Ore03,Ore04,Ore05,Ore06,Ore07,Ore08,Ore09,Ore10,Ore11,Ore12,Ore13,Ore14	*OreG0011716*
scaffold33103	366216	C	T	Ore01,Ore03,Ore04,Ore05,Ore09,Ore10,Ore11	*OreG0011719*
scaffold33103	401656	A	T	Och12,Och13	*OreG0011722*
scaffold33103	417776	T	A	NA	*OreG0011724*
scaffold33103	436658	C	A	NA	*OreG0011726*
scaffold33103	436684	C	T	Och01,Och12,Och13	*OreG0011726*
scaffold33103	436742	T	A	NA	*OreG0011726*
scaffold33103	436758	G	A	NA	*OreG0011726*
scaffold33103	438049	G	A	Och02,Och03,Och06	*OreG0011726*
scaffold33103	438193	C	T	Ore01,Ore04,Ore05	*OreG0011726*
scaffold33103	471568	T	C	NA	*OreG0011730*
scaffold33103	472492	T	C	NA	*OreG0011730*
scaffold33103	472928	G	T	NA	*OreG0011730*
scaffold33103	473233	A	T	Och01,Och02,Och03,Och04,Och05,Och06,Och07,Och08,Och09,Och10,Och11,Och12,Och13,Och14,Ore01,Ore02,Ore03,Ore04,Ore05,Ore06,Ore07,Ore08,Ore09,Ore10,Ore11,Ore12,Ore13,Ore14	*OreG0011730*
scaffold33103	492103	T	C	NA	*OreG0011731*
scaffold33103	514308	T	A	Och01,Och02,Och03,Och04,Och05,Och06,Och07,Och08,Och09,Och10,Och11,Och12,Och13,Och14,Ore01,Ore02,Ore03,Ore04,Ore05,Ore06,Ore07,Ore08,Ore09,Ore10,Ore11,Ore12,Ore13,Ore14	*OreG0011734*
scaffold33103	514362	G	A	Och02,Och03,Och04,Och06,Och07,Och12,Och13,Och14	*OreG0011734*
scaffold33103	514620	A	G	NA	*OreG0011734*
scaffold33103	540867	G	A	Och01,Och02,Och03,Och04,Och05,Och06,Och07,Och08,Och09,Och10,Och11,Och12,Och13,Och14,Ore01,Ore02,Ore03,Ore04,Ore05,Ore06,Ore07,Ore08,Ore09,Ore10,Ore11,Ore12,Ore13,Ore14	*OreG0011736*
scaffold33103	546661	T	A	NA	*OreG0011739*
scaffold33103	564221	T	G	Ore01,Ore02,Ore03,Ore04,Ore05,Ore06,Ore07,Ore08,Ore09,Ore10,Ore11,Ore12,Ore13,Ore14	*OreG0011744*
scaffold33103	604040	T	A	NA	*OreG0011746*
scaffold33103	604120	T	C	Ore01,Ore02,Ore03,Ore04,Ore05,Ore06,Ore07,Ore08,Ore09,Ore10,Ore11,Ore12,Ore13,Ore14	*OreG0011746*
scaffold33103	617196	T	A	NA	*OreG0011748*
scaffold33103	627740	C	T	NA	*OreG0011749*
scaffold33103	628424	T	A	Och03,Och04,Och07,Och11,Och12,Och13	*OreG0011749*
scaffold33103	636894	T	A	NA	*OreG0011751*
scaffold33103	639083	G	A	Och01,Och02,Och03,Och04,Och05,Och06,Och07,Och08,Och09,Och10,Och11,Och12,Och13,Och14	*OreG0011751*
scaffold33103	639091	G	T	NA	*OreG0011751*
scaffold33103	641183	T	C	NA	*OreG0011751*
scaffold33103	745164	A	T	Ore01,Ore02,Ore03,Ore04,Ore05,Ore06,Ore07,Ore08,Ore09,Ore10,Ore11,Ore12,Ore13,Ore14	*OreG0011757*
scaffold33103	891697	T	G	Och01,Och02,Och03,Och04,Och05,Och06,Och07,Och08,Och09,Och10,Och11,Och12,Och13,Och14,Ore01,Ore02,Ore03,Ore04,Ore05,Ore06,Ore07,Ore08,Ore09,Ore10,Ore11,Ore12,Ore13,Ore14	*OreG0011760*
scaffold33103	894234	A	C	NA	*OreG0011761*
scaffold33103	894354	T	C	NA	*OreG0011761*

scaffold33103	894383	A	G	Och01,Och02,Och03,Och04,Och05,Och06,Och07,Och08,Och09,Och10,Och11,Och12,Och13,Och14	*OreG0011761*
scaffold33103	894456	C	T	Och01,Och02,Och05,Och07,Och08,Och09,Och10,Och11,Och14	*OreG0011761*
scaffold33103	894468	G	C	Ore01,Ore02,Ore03,Ore04,Ore05,Ore06,Ore07,Ore08,Ore09,Ore10,Ore11,Ore12,Ore13,Ore14	*OreG0011761*
scaffold33103	962504	A	G	NA	*OreG0011764*
scaffold33103	962537	G	A	NA	*OreG0011764*
scaffold33103	962543	T	C	NA	*OreG0011764*
scaffold33103	962791	C	G	Och02,Och03,Och05,Ore01,Ore02,Ore03,Ore04,Ore05,Ore06,Ore07,Ore08,Ore09,Ore10,Ore11,Ore12,Ore13,Ore14	*OreG0011764*
scaffold33103	963074	G	C	NA	*OreG0011764*
scaffold33103	963803	C	T	Ore01,Ore02,Ore03,Ore04,Ore05,Ore06,Ore07,Ore08,Ore09,Ore10,Ore11,Ore12,Ore13,Ore14	*OreG0011764*
scaffold33103	964325	A	G	NA	*OreG0011764*
scaffold33103	1001565	C	A	NA	*OreG0011765*
scaffold33103	1037725	A	T	Ore01,Ore02,Ore03,Ore04,Ore05,Ore06,Ore07,Ore08,Ore09,Ore10,Ore11,Ore12,Ore13,Ore14	*OreG0011766*
scaffold33103	1037845	T	C	Ore01,Ore02,Ore03,Ore04,Ore05,Ore06,Ore07,Ore08,Ore09,Ore10,Ore11,Ore12,Ore13,Ore14	*OreG0011766*
scaffold33103	1044214	G	T	NA	*OreG0011767*
scaffold33103	1044392	T	C	NA	*OreG0011767*
scaffold33103	1052264	A	T	NA	*OreG0011768*
scaffold33103	1066152	G	A	Och08	*OreG0011769*
scaffold33103	1066231	C	T	Och08	*OreG0011769*
scaffold33103	1066533	C	T	NA	*OreG0011769*
scaffold33103	1071408	C	A	Och08	*OreG0011769*
scaffold33103	1103348	A	G	Och01,Och02,Och03,Och04,Och05,Och06,Och07,Och08,Och09,Och10,Och11,Och12,Och13,Och14	*OreG0011771*
scaffold33103	1126347	T	C	NA	*OreG0011773*
scaffold33103	1169176	G	C	Och03,Och04,Och05,Och08,Och12,Och13	*OreG0011775*
scaffold33103	1171374	A	T	Och01,Och07,Och11	*OreG0011776*
scaffold33103	1217557	C	G	Och01,Och07	*OreG0011778*
scaffold33103	1228036	G	T	NA	*OreG0011779*
scaffold33103	1233261	T	A	NA	*OreG0011780*
scaffold33103	1233315	C	T	NA	*OreG0011780*
scaffold33103	1233518	A	G	NA	*OreG0011780*
scaffold33103	1234388	C	T	NA	*OreG0011780*
scaffold33103	1234599	T	C	NA	*OreG0011780*
scaffold33103	1234626	G	A	Och01,Och07	*OreG0011780*
scaffold33103	1234945	G	A	Och02,Och03,Och04,Och05,Och06,Och08,Och09,Och10,Och11,Och12,Och13,Och14	*OreG0011780*
scaffold33103	1243986	T	G	Och02,Och03,Och04,Och05,Och06,Och08,Och09,Och10,Och11,Och12,Och13,Och14	*OreG0011781*
scaffold33103	1384265	A	T	Och01,Och02,Och05,Och08,Och09,Och10,Och11,Ore01,Ore02,Ore03,Ore04,Ore05,Ore06,Ore07,Ore08,Ore09,Ore10,Ore11,Ore12,Ore13,Ore14	*OreG0011787*
scaffold33103	1384278	C	T	NA	*OreG0011787*
scaffold33103	1384542	T	A	NA	*OreG0011787*
scaffold33103	1384634	G	A	NA	*OreG0011787*
scaffold33103	1384716	T	C	Ore01,Ore02,Ore03,Ore04,Ore05,Ore06,Ore07,Ore08,Ore09,Ore10,Ore11,Ore12,Ore13,Ore14	*OreG0011787*
scaffold33103	1384781	T	A	NA	*OreG0011787*
scaffold33103	1384941	C	G	NA	*OreG0011787*
scaffold33103	1385006	G	A	NA	*OreG0011787*
scaffold33103	1402856	C	T	NA	*OreG0011788*
scaffold33103	1402936	C	T	NA	*OreG0011788*
scaffold33103	1402982	T	C	NA	*OreG0011788*
scaffold33103	1408361	C	T	NA	*OreG0011788*
scaffold33103	1409087	T	G	NA	*OreG0011788*
scaffold33103	1409625	C	T	NA	*OreG0011788*
scaffold33103	1417271	C	T	NA	*OreG0011789*
scaffold33103	1418955	T	G	NA	*OreG0011789*
scaffold33103	1431740	G	C	NA	*OreG0011790*
scaffold33103	1431834	C	T	NA	*OreG0011790*
scaffold33103	1432095	A	G	NA	*OreG0011790*
scaffold33103	1492970	C	A	Och03,Och04,Och12,Och13	*OreG0011794*
scaffold33103	1633684	C	T	NA	*OreG0011798*
scaffold33103	1692281	A	C	Och01,Och02,Och03,Och04,Och05,Och06,Och07,Och08,Och09,Och10,Och11,Och12,Och13,Och14,Ore01,Ore02,Ore03,Ore04,Ore05,Ore06,Ore07,Ore08,Ore09,Ore10,Ore11,Ore12,Ore13,Ore14	*OreG0011799*
scaffold33103	1693648	C	G	Och01,Och02,Och03,Och04,Och05,Och06,Och07,Och08,Och09,Och10,Och11,Och12,Och13,Och14,Ore01,Ore02,Ore03,Ore04,Ore05,Ore06,Ore07,Ore08,Ore09,Ore10,Ore11,Ore12,Ore13,Ore14	*OreG0011799*
scaffold33103	1695703	C	T	Och09,Och10	*OreG0011800*
scaffold33103	1947674	C	T	NA	*OreG0011804*
scaffold33103	1947947	G	A	NA	*OreG0011804*

scaffold33103	1948014	G	T	NA	*OreG0011804*
scaffold33103	1948088	A	T	NA	*OreG0011804*
scaffold33228	91837	G	T	NA	*OreG0017088*
scaffold33228	99750	G	T	Ore03,Ore04,Ore05,Ore09,Ore10,Ore13,Ore14	*OreG0017090*
scaffold33228	99936	C	T	NA	*OreG0017090*
scaffold33228	99984	C	T	NA	*OreG0017090*
scaffold33228	100003	G	T	Och05	*OreG0017090*
scaffold33228	100934	T	A	Ore03,Ore04,Ore05,Ore09,Ore10,Ore13,Ore14	*OreG0017090*
scaffold33228	101029	C	T	NA	*OreG0017090*
scaffold33228	102279	G	A	NA	*OreG0017091*
scaffold33175	60423	A	T	NA	*OreG0014387*
scaffold33175	60588	C	G	NA	*OreG0014387*
scaffold33175	61089	C	T	Ore02,Ore06,Ore08,Ore09,Ore12,Ore14	*OreG0014387*
scaffold33175	208938	T	C	Ore01,Ore02,Ore03,Ore04,Ore05,Ore06,Ore07,Ore08,Ore09,Ore10,Ore11,Ore12,Ore13,Ore14	*OreG0014411*
scaffold33175	639481	A	T	NA	*OreG0014462*
scaffold33175	639510	T	C	Och01,Och02,Och03,Och04,Och05,Och06,Och07,Och08,Och09,Och10,Och11,Och12,Och13,Och14	*OreG0014462*
scaffold33175	639530	T	A	Ore01,Ore02,Ore03,Ore04,Ore05,Ore06,Ore07,Ore08,Ore09,Ore10,Ore11,Ore12,Ore13,Ore14	*OreG0014462*
scaffold33175	641013	C	A	Och01,Och02,Och03,Och04,Och05,Och06,Och07,Och08,Och09,Och10,Och11,Och12,Och13,Och14,Ore01,Ore02,Ore03,Ore04,Ore05,Ore06,Ore07,Ore08,Ore09,Ore10,Ore11,Ore12,Ore13,Ore14	*OreG0014462*
scaffold33175	664103	C	A	NA	*OreG0014466*
scaffold33175	665215	G	C	Och01,Och02,Och03,Och04,Och05,Och06,Och07,Och08,Och09,Och10,Och11,Och12,Och13,Och14,Ore01,Ore02,Ore03,Ore04,Ore05,Ore06,Ore07,Ore08,Ore09,Ore10,Ore11,Ore12,Ore13,Ore14	*OreG0014466*
scaffold33175	705397	T	C	Och01,Och02,Och03,Och04,Och05,Och06,Och07,Och08,Och09,Och10,Och11,Och12,Och13,Och14	*OreG0014470*
scaffold33175	941779	G	C	Ore01,Ore02,Ore03,Ore04,Ore05,Ore06,Ore07,Ore08,Ore09,Ore10,Ore11,Ore12,Ore13,Ore14	*OreG0014497*
scaffold33175	1146347	C	T	NA	*OreG0014528*
scaffold33175	1149386	T	A	NA	*OreG0014528*
scaffold33175	1151309	G	C	Och01,Och02,Och03,Och04,Och05,Och06,Och07,Och08,Och09,Och10,Och11,Och12,Och13,Och14,Ore01,Ore02,Ore03,Ore04,Ore05,Ore06,Ore07,Ore08,Ore09,Ore10,Ore11,Ore12,Ore13,Ore14	*OreG0014528*
scaffold33175	1166678	G	A	Och01,Och02,Och03,Och04,Och05,Och06,Och09,Och10,Och11,Och12,Och13	*OreG0014531*
scaffold33175	1166887	G	A	NA	*OreG0014531*
scaffold33175	1167223	G	A	NA	*OreG0014531*
scaffold33175	1168256	T	C	Och01,Och02,Och03,Och04,Och05,Och06,Och07,Och08,Och09,Och10,Och11,Och12,Och13,Och14,Ore01,Ore02,Ore03,Ore04,Ore05,Ore06,Ore07,Ore08,Ore09,Ore10,Ore11,Ore12,Ore13,Ore14	*OreG0014531*
scaffold33175	1272018	C	A	Och01,Och02,Och03,Och04,Och05,Och06,Och07,Och08,Och09,Och10,Och11,Och12,Och13,Och14	*OreG0014545*
scaffold33175	1309400	G	A	NA	*OreG0014550*
scaffold33175	1376362	T	C	Och01,Och02,Och03,Och04,Och05,Och06,Och07,Och08,Och09,Och10,Och11,Och12,Och13,Och14	*OreG0014559*
scaffold33175	1376504	C	T	NA	*OreG0014559*
scaffold33175	1376552	C	T	Och05	*OreG0014559*
scaffold33175	1376702	C	T	Ore01,Ore02,Ore03,Ore04,Ore05,Ore06,Ore07,Ore08,Ore09,Ore10,Ore11,Ore12,Ore13,Ore14	*OreG0014559*
scaffold33175	1376954	T	G	Och01,Och02,Och03,Och04,Och05,Och06,Och07,Och08,Och09,Och10,Och11,Och12,Och13,Och14	*OreG0014559*
scaffold33175	1376980	C	T	Ore01,Ore02,Ore03,Ore04,Ore05,Ore06,Ore07,Ore08,Ore09,Ore10,Ore11,Ore12,Ore13,Ore14	*OreG0014559*
scaffold33175	1377076	C	T	Ore01,Ore02,Ore03,Ore04,Ore05,Ore06,Ore07,Ore08,Ore09,Ore10,Ore11,Ore12,Ore13,Ore14	*OreG0014559*
scaffold33175	1377520	C	G	Ore01,Ore02,Ore03,Ore04,Ore05,Ore06,Ore07,Ore08,Ore09,Ore10,Ore11,Ore12,Ore13,Ore14	*OreG0014559*
scaffold33175	1377754	C	T	Ore01,Ore02,Ore03,Ore04,Ore05,Ore06,Ore07,Ore08,Ore09,Ore10,Ore11,Ore12,Ore13,Ore14	*OreG0014559*
scaffold33175	1377802	C	T	Ore01,Ore02,Ore03,Ore04,Ore05,Ore06,Ore07,Ore08,Ore09,Ore10,Ore11,Ore12,Ore13,Ore14	*OreG0014559*
scaffold33175	1378441	A	G	Ore01,Ore02,Ore03,Ore04,Ore05,Ore06,Ore07,Ore08,Ore09,Ore10,Ore11,Ore12,Ore13,Ore14	*OreG0014560*
scaffold33175	1378530	A	G	Och01,Och02,Och03,Och04,Och05,Och06,Och07,Och08,Och09,Och10,Och11,Och12,Och13,Och14	*OreG0014560*
scaffold33175	1378536	T	C	Ore01,Ore06,Ore10,Ore14	*OreG0014560*
scaffold33175	1378548	G	A	Och05	*OreG0014560*
scaffold33175	1414292	G	C	Och01,Och02,Och03,Och04,Och05,Och06,Och07,Och08,Och09,Och10,Och11,Och12,Och13,Och14,Ore01,Ore02,Ore03,Ore04,Ore05,Ore06,Ore07,Ore08,Ore09,Ore10,Ore11,Ore12,Ore13,Ore14	*OreG0014567*
scaffold33175	1461840	G	A	NA	*OreG0014573*
scaffold33175	1585706	A	C	Och01,Och02,Och03,Och04,Och05,Och06,Och07,Och08,Och09,Och10,Och11,Och12,Och13,Och14,Ore01,Ore02,Ore03,Ore04,Ore05,Ore06,Ore07,Ore08,Ore09,Ore10,Ore11,Ore12,Ore13,Ore14	*OreG0014590*

scaffold33175	1626484	A	G	Ore01,Ore02,Ore03,Ore04,Ore05,Ore06,Ore07,Ore08,Ore09,Ore10,Ore11,Ore12,Ore13,Ore14	*OreG0014596*
scaffold33175	1630102	T	A	Och01,Och02,Och09,Och10,Och14	*OreG0014597*
scaffold33175	1630576	C	G	Och01,Och02,Och03,Och04,Och05,Och06,Och07,Och08,Och09,Och10,Och11,Och12,Och13,Och14	*OreG0014597*
scaffold33175	1631180	T	G	Och05	*OreG0014597*
scaffold33175	1727681	T	A	NA	*OreG0014614*
scaffold33175	1727759	C	A	NA	*OreG0014614*
scaffold33175	1844482	G	A	NA	*OreG0014621*
scaffold33223	16872	G	A	Och07	*OreG0016811*
scaffold33223	17015	C	T	Och07	*OreG0016811*
scaffold33223	18291	C	T	NA	*OreG0016811*
scaffold452	52246	T	A	Och06	*OreG0020925*
scaffold452	55346	G	A	NA	*OreG0020926*
scaffold452	55375	G	A	NA	*OreG0020926*
scaffold452	55417	C	T	NA	*OreG0020926*
scaffold452	55621	G	A	Ore01,Ore02,Ore04,Ore05,Ore07,Ore09,Ore14	*OreG0020926*
scaffold452	55790	G	T	NA	*OreG0020926*
scaffold452	55883	C	A	NA	*OreG0020926*
scaffold452	56728	G	T	NA	*OreG0020926*
scaffold452	56809	A	G	NA	*OreG0020926*
scaffold452	57147	A	T	NA	*OreG0020926*
scaffold452	57260	C	A	Och01,Och05,Och12,Och13	*OreG0020926*
scaffold452	57319	A	T	NA	*OreG0020926*
scaffold452	72258	C	T	NA	*OreG0020929*
scaffold452	72736	T	C	NA	*OreG0020929*
scaffold452	80208	A	G	NA	*OreG0020929*
scaffold452	88114	A	T	NA	*OreG0020929*
scaffold452	88401	G	A	Och11,Och14,Ore01,Ore02,Ore03,Ore04,Ore05,Ore06,Ore07,Ore08,Ore09,Ore10,Ore11,Ore12,Ore13,Ore14	*OreG0020929*
scaffold452	229212	C	T	NA	*OreG0020938*
scaffold452	233885	G	A	Och02,Och03,Och04,Och06,Och07,Och08,Och09,Och10,Och11,Och14	*OreG0020938*
scaffold452	251385	A	T	NA	*OreG0020939*
scaffold452	251915	G	T	Och06	*OreG0020939*
scaffold452	351064	A	T	Och01	*OreG0020943*
scaffold452	354042	C	A	Ore09,Ore14	*OreG0020944*
scaffold452	354179	T	A	Och02,Och03,Och04,Och06,Och08,Och11,Och14	*OreG0020944*
scaffold452	380478	A	G	Och01,Och02,Och03,Och04,Och05,Och06,Och07,Och08,Och09,Och10,Och11,Och12,Och13,Och14,Ore01,Ore02,Ore03,Ore04,Ore05,Ore06,Ore07,Ore08,Ore09,Ore10,Ore11,Ore12,Ore13,Ore14	*OreG0020945*
scaffold452	380572	G	A	NA	*OreG0020945*
scaffold452	512858	A	T	NA	*OreG0020949*
scaffold452	513212	A	T	Ore09,Ore14	*OreG0020949*
scaffold452	515218	A	G	NA	*OreG0020949*
scaffold452	516035	C	A	NA	*OreG0020949*
scaffold452	516156	C	G	Och06	*OreG0020949*
scaffold452	516437	T	A	Och02,Och03,Och06,Och11	*OreG0020949*
scaffold452	516997	A	T	Och02,Och03,Och06,Och11	*OreG0020949*
scaffold452	517147	G	A	Ore01,Ore02,Ore03,Ore04,Ore05,Ore06,Ore07,Ore08,Ore09,Ore10,Ore11,Ore12,Ore13,Ore14	*OreG0020949*
scaffold452	517149	A	G	Och01,Och02,Och03,Och04,Och05,Och06,Och07,Och08,Och09,Och10,Och11,Och12,Och13,Och14,Ore01,Ore02,Ore03,Ore04,Ore05,Ore06,Ore07,Ore08,Ore09,Ore10,Ore11,Ore12,Ore13,Ore14	*OreG0020949*
scaffold452	518674	G	A	Ore09,Ore14	*OreG0020950*
scaffold452	521407	T	G	Och01,Och02,Och03,Och04,Och05,Och06,Och07,Och08,Och09,Och10,Och11,Och12,Och13,Och14,Ore01,Ore02,Ore03,Ore04,Ore05,Ore06,Ore07,Ore08,Ore09,Ore10,Ore11,Ore12,Ore13,Ore14	*OreG0020950*
scaffold452	524977	C	T	Ore01,Ore02,Ore03,Ore04,Ore05,Ore06,Ore07,Ore08,Ore09,Ore10,Ore11,Ore12,Ore13,Ore14	*OreG0020952*
scaffold452	524987	C	A	Ore01,Ore02,Ore03,Ore04,Ore05,Ore06,Ore07,Ore08,Ore09,Ore10,Ore11,Ore12,Ore13,Ore14	*OreG0020952*
scaffold452	531235	T	G	Ore01,Ore02,Ore03,Ore04,Ore05,Ore06,Ore07,Ore08,Ore09,Ore10,Ore11,Ore12,Ore13,Ore14	*OreG0020953*
scaffold452	532218	A	C	Och14	*OreG0020953*
scaffold452	532588	T	A	Och01,Och02,Och03,Och04,Och05,Och06,Och07,Och08,Och09,Och10,Och11,Och12,Och13,Och14	*OreG0020953*
scaffold452	532603	A	T	Ore01,Ore02,Ore03,Ore04,Ore05,Ore06,Ore07,Ore08,Ore09,Ore10,Ore11,Ore12,Ore13,Ore14	*OreG0020953*
scaffold452	559628	G	A	NA	*OreG0020954*
scaffold452	577018	A	G	Ore09,Ore14	*OreG0020955*
scaffold452	596045	G	A	Och05,Och11,Och12,Och14,Ore01,Ore02,Ore03,Ore04,Ore05,Ore06,Ore07,Ore08,Ore09,Ore10,Ore11,Ore12,Ore13,Ore14	*OreG0020960*
scaffold452	600980	T	C	Och02,Och03,Och04,Och05,Och06,Och08,Och09,Och10,Och11,Och12,Och13,Och14	*OreG0020961*
scaffold452	612815	T	A	NA	*OreG0020962*

scaffold452	613364	C	T	Och11	*OreG0020963*
scaffold452	829705	C	T	NA	*OreG0020973*
scaffold452	829810	G	T	NA	*OreG0020973*
scaffold452	840793	C	T	NA	*OreG0020975*
scaffold452	840798	G	T	Och11	*OreG0020975*
scaffold452	840997	G	T	Och11	*OreG0020975*
scaffold452	1053147	T	C	NA	*OreG0020981*
scaffold452	1152043	G	A	Ore01,Ore02,Ore03,Ore04,Ore05,Ore06,Ore07,Ore08,Ore09,Ore10,Ore11,Ore12,Ore13,Ore14	*OreG0020985*
scaffold452	1162065	G	A	NA	*OreG0020986*
scaffold452	1162941	G	A	NA	*OreG0020986*
scaffold452	1180668	C	A	NA	*OreG0020991*
scaffold452	1184967	G	A	NA	*OreG0020992*
scaffold452	1185285	G	A	Ore03,Ore06,Ore09,Ore11,Ore13,Ore14	*OreG0020992*
scaffold452	1185339	C	T	NA	*OreG0020992*
scaffold452	1185375	C	T	NA	*OreG0020992*
scaffold452	1185379	T	A	NA	*OreG0020992*
scaffold452	1185861	G	A	NA	*OreG0020992*
scaffold452	1185874	C	A	NA	*OreG0020992*
scaffold452	1186508	T	A	Ore03,Ore06,Ore09,Ore11,Ore13,Ore14	*OreG0020992*
scaffold452	1202807	G	A	NA	*OreG0020993*
scaffold452	1202870	G	C	NA	*OreG0020993*
scaffold452	1202985	G	T	Och06	*OreG0020993*
scaffold452	1203570	G	T	NA	*OreG0020993*
scaffold452	1203665	T	G	NA	*OreG0020993*
scaffold452	1203716	G	T	NA	*OreG0020993*
scaffold452	1203728	C	T	NA	*OreG0020993*
scaffold452	1203878	T	C	Ore01,Ore02,Ore03,Ore04,Ore05,Ore06,Ore07,Ore08,Ore09,Ore10,Ore11,Ore12,Ore13,Ore14	*OreG0020993*
scaffold452	1204003	A	G	NA	*OreG0020993*
scaffold452	1287029	A	C	NA	*OreG0021000*
scaffold452	1324055	A	G	NA	*OreG0021004*
scaffold452	1324379	C	T	Och01,Och02,Och03,Och04,Och05,Och06,Och08,Och09,Och10,Och11,Och12,Och13,Och14	*OreG0021004*
scaffold452	1327883	C	T	Och07	*OreG0021005*
scaffold452	1327901	A	G	NA	*OreG0021005*
scaffold452	1327936	G	C	Och01,Och02,Och03,Och04,Och05,Och06,Och07,Och08,Och09,Och10,Och11,Och12,Och13,Och14,Ore01,Ore02,Ore03,Ore04,Ore05,Ore06,Ore07,Ore08,Ore09,Ore10,Ore11,Ore12,Ore13,Ore14	*OreG0021005*
scaffold452	1327974	A	C	Och01	*OreG0021005*
scaffold452	1442193	G	A	Och01,Och02,Och03,Och04,Och05,Och06,Och07,Och08,Och09,Och10,Och11,Och12,Och13,Och14,Ore01,Ore02,Ore03,Ore04,Ore05,Ore06,Ore07,Ore08,Ore09,Ore10,Ore11,Ore12,Ore13,Ore14	*OreG0021013*
scaffold452	1454815	G	T	NA	*OreG0021014*
scaffold452	1456289	C	T	Och01,Och02,Och03,Och04,Och05,Och06,Och07,Och08,Och09,Och10,Och11,Och12,Och13	*OreG0021014*
scaffold452	1456796	T	A	NA	*OreG0021014*
scaffold452	1461978	C	T	NA	*OreG0021015*
scaffold452	1464791	T	C	NA	*OreG0021016*
scaffold452	1475970	A	C	Och01,Och02,Och03,Och04,Och05,Och06,Och07,Och08,Och09,Och10,Och11,Och12,Och13,Och14,Ore01,Ore02,Ore03,Ore04,Ore05,Ore06,Ore07,Ore08,Ore09,Ore10,Ore11,Ore12,Ore13,Ore14	*OreG0021017*
scaffold452	1484141	C	T	NA	*OreG0021018*
scaffold452	1487118	G	T	NA	*OreG0021019*
scaffold452	1487273	A	T	NA	*OreG0021019*
scaffold452	1505114	C	A	Och01,Och02,Och03,Och04,Och05,Och06,Och07,Och08,Och09,Och10,Och11,Och12,Och13,Och14,Ore01,Ore02,Ore03,Ore04,Ore05,Ore06,Ore07,Ore08,Ore09,Ore10,Ore11,Ore12,Ore13,Ore14	*OreG0021022*
scaffold452	1506484	G	A	NA	*OreG0021022*
scaffold452	1508599	G	A	NA	*OreG0021022*
scaffold452	1508602	T	A	Och01,Och02,Och03,Och04,Och05,Och06,Och07,Och08,Och09,Och10,Och11,Och12,Och13,Och14,Ore01,Ore02,Ore03,Ore04,Ore05,Ore06,Ore07,Ore08,Ore09,Ore10,Ore11,Ore12,Ore13,Ore14	*OreG0021022*
scaffold452	1552373	T	C	Ore01,Ore02,Ore03,Ore04,Ore05,Ore06,Ore07,Ore08,Ore09,Ore10,Ore11,Ore12,Ore13,Ore14	*OreG0021027*
scaffold452	1572372	G	T	NA	*OreG0021028*
scaffold452	1594218	A	G	Ore01,Ore02,Ore03,Ore04,Ore05,Ore06,Ore07,Ore08,Ore09,Ore10,Ore11,Ore12,Ore13,Ore14	*OreG0021031*
scaffold452	1623988	C	A	Och01,Och07,Och14,Ore01,Ore02,Ore03,Ore04,Ore05,Ore06,Ore07,Ore08,Ore09,Ore10,Ore11,Ore12,Ore13,Ore14	*OreG0021033*
scaffold452	1624378	A	G	NA	*OreG0021033*
scaffold452	1642260	C	A	Och02,Och03,Och04,Och05,Och06,Och08,Och09,Och10,Och11,Och12,Och13,Och14,Ore01,Ore02,Ore03,Ore04,Ore05,Ore06,Ore07,Ore08,Ore09,Ore10,Ore11,Ore12,Ore13,Ore14	*OreG0021036*
scaffold452	1718772	C	T	NA	*OreG0021046*

scaffold452	1720071	C	T	NA	*OreG0021046*
scaffold452	1736360	A	T	NA	*OreG0021051*
scaffold452	1801097	G	C	Och06	*OreG0021055*
scaffold452	1801140	T	G	NA	*OreG0021055*
scaffold452	2080244	G	T	NA	*OreG0021061*
scaffold452	2117102	C	T	Ore03,Ore07,Ore08,Ore10,Ore14	*OreG0021063*
scaffold452	2117353	G	T	Och01,Och02,Och03,Och04,Och05,Och06,Och07,Och08,Och09,Och10,Och11,Och12,Och13,Och14	*OreG0021063*
scaffold452	2126025	C	T	Och01,Och05,Och07,Och08,Och09,Och10,Och12,Och13	*OreG0021065*
scaffold452	2126046	A	T	Ore01,Ore02,Ore03,Ore04,Ore05,Ore06,Ore07,Ore08,Ore09,Ore10,Ore11,Ore12,Ore13,Ore14	*OreG0021065*
scaffold452	2126112	G	T	Och01	*OreG0021065*
scaffold452	2126115	G	T	NA	*OreG0021065*
scaffold452	2144701	C	T	NA	*OreG0021067*
scaffold452	2171184	C	T	NA	*OreG0021068*
scaffold452	2172534	G	A	NA	*OreG0021068*
scaffold452	2172602	G	A	NA	*OreG0021068*
scaffold452	2172653	G	A	NA	*OreG0021068*
scaffold452	2175401	A	G	NA	*OreG0021068*
scaffold452	2178048	A	T	NA	*OreG0021069*
scaffold452	2182916	C	A	Och02,Och06,Och07,Och08,Och09,Och10	*OreG0021069*
scaffold452	2193221	G	A	Och06,Och07,Och08,Och09,Och10,Och11	*OreG0021071*
scaffold452	2202339	G	T	NA	*OreG0021072*
scaffold452	2216694	G	C	NA	*OreG0021074*
scaffold452	2222178	G	A	NA	*OreG0021075*
scaffold452	2240546	G	A	NA	*OreG0021077*
scaffold452	2241182	C	T	Och01,Och02,Och03,Och04,Och05,Och06,Och07,Och08,Och09,Och10,Och11,Och12,Och13,Och14	*OreG0021077*
scaffold452	2248514	G	A	Och01,Och02,Och03,Och04,Och05,Och06,Och08,Och09,Och10,Och11,Och12,Och13,Och14	*OreG0021078*
scaffold452	2251537	A	G	Och05,Och09,Och10,Och12,Och13,Och14	*OreG0021078*
scaffold452	2272337	A	G	Ore01,Ore02,Ore03,Ore04,Ore05,Ore06,Ore07,Ore08,Ore09,Ore10,Ore11,Ore12,Ore13,Ore14	*OreG0021079*
scaffold452	2366434	G	A	Och01,Och02,Och03,Och04,Och05,Och06,Och07,Och08,Och09,Och10,Och11,Och12,Och13,Och14,Ore01,Ore02,Ore03,Ore04,Ore05,Ore06,Ore07,Ore08,Ore09,Ore10,Ore11,Ore12,Ore13,Ore14	*OreG0021086*
scaffold452	2443559	A	T	NA	*OreG0021092*
scaffold452	2477575	T	C	NA	*OreG0021094*
scaffold452	2489065	A	T	Och01,Och02,Och03,Och04,Och05,Och06,Och07,Och08,Och09,Och10,Och11,Och12,Och13,Och14,Ore01,Ore02,Ore03,Ore04,Ore05,Ore06,Ore07,Ore08,Ore09,Ore10,Ore11,Ore12,Ore13,Ore14	*OreG0021096*
scaffold452	2490126	G	T	NA	*OreG0021096*
scaffold452	2538198	T	C	Ore01,Ore02,Ore03,Ore04,Ore05,Ore06,Ore07,Ore08,Ore09,Ore10,Ore11,Ore12,Ore13,Ore14	*OreG0021098*
scaffold452	2540596	A	T	Ore01,Ore02,Ore03,Ore04,Ore05,Ore06,Ore07,Ore08,Ore09,Ore10,Ore11,Ore12,Ore13,Ore14	*OreG0021098*
scaffold452	2544259	T	C	Och06	*OreG0021099*
scaffold452	2553905	G	A	Och01,Och02,Och03,Och04,Och05,Och06,Och07,Och08,Och09,Och10,Och11,Och12,Och13,Och14	*OreG0021100*
scaffold452	2559956	T	C	Och01,Och02,Och03,Och04,Och05,Och06,Och07,Och08,Och09,Och10,Och11,Och12,Och13,Och14	*OreG0021101*
scaffold452	2561403	A	G	Och01,Och02,Och03,Och04,Och05,Och06,Och07,Och08,Och09,Och10,Och11,Och12,Och13,Och14,Ore01,Ore02,Ore03,Ore04,Ore05,Ore06,Ore07,Ore08,Ore09,Ore10,Ore11,Ore12,Ore13,Ore14	*OreG0021101*
scaffold452	2688938	A	C	Ore07,Ore08,Ore14	*OreG0021108*
scaffold452	2691122	C	T	Ore01,Ore02,Ore03,Ore04,Ore05,Ore06,Ore07,Ore08,Ore09,Ore10,Ore11,Ore12,Ore13,Ore14	*OreG0021108*
scaffold452	2697501	G	A	NA	*OreG0021109*
scaffold452	2701502	T	C	Ore01,Ore02,Ore06,Ore07,Ore08,Ore11,Ore12,Ore13,Ore14	*OreG0021109*
scaffold452	2702577	G	A	Ore07,Ore14	*OreG0021109*
scaffold452	2702609	G	A	Ore07,Ore14	*OreG0021109*
scaffold452	2702741	T	C	NA	*OreG0021109*
scaffold452	2705163	G	C	NA	*OreG0021110*
scaffold452	2705223	G	A	Ore01,Ore02,Ore03,Ore04,Ore05,Ore06,Ore07,Ore08,Ore09,Ore10,Ore11,Ore12,Ore13,Ore14	*OreG0021110*
scaffold452	2787101	C	A	NA	*OreG0021115*
scaffold452	2860444	A	T	NA	*OreG0021124*
scaffold452	2863679	C	T	NA	*OreG0021125*
scaffold452	2864757	C	A	Och01,Och02,Och03,Och04,Och05,Och06,Och08,Och12,Och13,Och14	*OreG0021125*
scaffold452	2865043	T	A	NA	*OreG0021125*
scaffold452	2866048	T	C	NA	*OreG0021125*
scaffold452	2866148	C	T	NA	*OreG0021125*
scaffold452	2879253	C	T	NA	*OreG0021126*

scaffold452	2881376	A	T	Och01,Och02,Och03,Och04,Och05,Och06,Och07,Och08,Och09,Och10,Och11,Och12,Och13,Och14,Ore01,Ore02,Ore03,Ore04,Ore05,Ore06,Ore07,Ore08,Ore09,Ore10,Ore11,Ore12,Ore13,Ore14	*OreG0021126*
scaffold452	2883737	C	T	Och01	*OreG0021127*
scaffold452	2883744	C	T	NA	*OreG0021127*
scaffold452	2883768	C	T	NA	*OreG0021127*
scaffold452	2883990	C	T	NA	*OreG0021127*
scaffold452	2884011	C	T	Och01	*OreG0021127*
scaffold452	2884580	C	T	NA	*OreG0021127*
scaffold452	2884609	G	A	Ore01,Ore02,Ore03,Ore04,Ore05,Ore06,Ore07,Ore08,Ore09,Ore10,Ore11,Ore12,Ore13,Ore14	*OreG0021127*
scaffold452	2884643	G	A	Ore01,Ore02,Ore03,Ore04,Ore05,Ore06,Ore07,Ore08,Ore09,Ore10,Ore11,Ore12,Ore13,Ore14	*OreG0021127*
scaffold452	2886816	C	T	NA	*OreG0021127*
scaffold452	2910033	C	A	NA	*OreG0021128*
scaffold452	2910122	G	A	NA	*OreG0021128*
scaffold452	2910213	A	G	Och02,Och03,Och04,Och05,Och06,Och07,Och08,Och12,Och13	*OreG0021128*
scaffold452	2912112	G	A	Och01	*OreG0021128*
scaffold452	2912163	G	A	Och06	*OreG0021128*
scaffold452	2912196	G	A	Och02,Och03,Och04,Och05,Och06,Och07,Och08,Och12,Och13	*OreG0021128*
scaffold452	2912208	G	C	Och02,Och06	*OreG0021128*
scaffold452	2912362	C	A	NA	*OreG0021128*
scaffold452	2912399	G	T	Och02,Och03,Och04,Och05,Och06,Och07,Och08,Och12,Och13	*OreG0021128*
scaffold452	2919452	G	A	NA	*OreG0021129*
scaffold452	2922383	C	T	NA	*OreG0021129*
scaffold452	2937620	G	T	NA	*OreG0021130*
scaffold452	2937735	C	T	NA	*OreG0021130*
scaffold452	2945291	C	T	NA	*OreG0021131*
scaffold452	2960111	A	G	NA	*OreG0021134*
scaffold452	2961008	C	T	Och06	*OreG0021134*
scaffold452	2969910	A	C	Och01,Och02,Och03,Och04,Och05,Och06,Och07,Och08,Och09,Och10,Och11,Och12,Och13,Och14,Ore01,Ore02,Ore03,Ore04,Ore05,Ore06,Ore07,Ore08,Ore09,Ore10,Ore11,Ore12,Ore13,Ore14	*OreG0021135*
scaffold452	2987634	T	C	Och09,Och10	*OreG0021136*
scaffold452	2994320	C	A	Och01,Och02,Och03,Och04,Och05,Och06,Och07,Och08,Och09,Och10,Och11,Och12,Och13,Och14	*OreG0021138*
scaffold452	2999950	A	T	Ore03,Ore07,Ore10,Ore14	*OreG0021139*
scaffold452	3000967	T	A	Och06	*OreG0021139*
scaffold452	3003776	T	C	NA	*OreG0021140*
scaffold452	3008573	T	C	Och01	*OreG0021141*
scaffold452	3020162	A	G	Och01,Och02,Och03,Och04,Och05,Och06,Och07,Och08,Och09,Och10,Och11,Och12,Och13,Och14,Ore01,Ore02,Ore03,Ore04,Ore05,Ore06,Ore07,Ore08,Ore09,Ore10,Ore11,Ore12,Ore13,Ore14	*OreG0021143*
scaffold452	3028831	A	T	NA	*OreG0021144*
scaffold452	3028898	T	A	NA	*OreG0021144*
scaffold452	3063284	T	A	Och01,Och02,Och03,Och04,Och05,Och06,Och07,Och08,Och09,Och10,Och11,Och12,Och13,Och14,Ore01,Ore02,Ore03,Ore04,Ore05,Ore06,Ore07,Ore08,Ore09,Ore10,Ore11,Ore12,Ore13,Ore14	*OreG0021148*
scaffold452	3064871	G	A	Och01,Och02,Och03,Och04,Och05,Och06,Och07,Och08,Och09,Och10,Och11,Och12,Och13,Och14	*OreG0021148*
scaffold452	3064923	T	C	NA	*OreG0021148*
scaffold452	3069515	G	T	NA	*OreG0021147*
scaffold452	3070038	G	C	Och06	*OreG0021147*
scaffold452	3070442	T	G	NA	*OreG0021147*
scaffold452	3072746	C	G	Och02,Och03,Och04,Och05,Och06,Och08,Och09,Och10,Och11,Och12,Och13	*OreG0021147*
scaffold452	3129561	A	T	Ore01,Ore02,Ore03,Ore04,Ore05,Ore06,Ore07,Ore08,Ore09,Ore10,Ore11,Ore12,Ore13,Ore14	*OreG0021151*
scaffold452	3142372	G	T	Ore07,Ore14	*OreG0021155*
scaffold452	3146811	C	A	NA	*OreG0021156*
scaffold452	3149876	C	T	NA	*OreG0021157*
scaffold452	3152632	T	C	NA	*OreG0021157*
scaffold452	3153580	G	A	Och08,Och11,Och14	*OreG0021157*
scaffold452	3166906	T	C	NA	*OreG0021158*
scaffold452	3166944	A	T	Och14	*OreG0021158*
scaffold452	3171364	C	T	Ore01,Ore02,Ore03,Ore04,Ore05,Ore06,Ore07,Ore08,Ore09,Ore10,Ore11,Ore12,Ore13,Ore14	*OreG0021159*
scaffold452	3176924	C	A	NA	*OreG0021160*
scaffold452	3219986	A	G	Och01,Och02,Och03,Och04,Och05,Och06,Och07,Och08,Och09,Och10,Och11,Och12,Och13,Och14,Ore01,Ore02,Ore03,Ore04,Ore05,Ore06,Ore07,Ore08,Ore09,Ore10,Ore11,Ore12,Ore13,Ore14	*OreG0021161*
scaffold452	3317884	C	A	NA	*OreG0021163*
scaffold452	3320472	G	T	NA	*OreG0021163*
scaffold452	3357840	C	T	NA	*OreG0021167*
scaffold452	3361920	A	T	Och01,Och02,Och03,Och04,Och05,Och06,Och07,Och08,Och09,Och10,Och11,Och12,Och13,Och14	*OreG0021167*

scaffold452	3366345	A	C	NA	*OreG0021168*
scaffold452	3408826	C	T	NA	*OreG0021170*
scaffold452	3485275	G	T	Och02,Och03,Och04,Och06,Och08,Och09,Och10,Och12,Och13	*OreG0021177*
scaffold452	3502686	C	T	NA	*OreG0021179*
scaffold452	3506516	C	T	NA	*OreG0021179*
scaffold452	3507632	A	T	NA	*OreG0021179*
scaffold452	3513549	C	T	NA	*OreG0021180*
scaffold452	3513914	G	A	NA	*OreG0021180*
scaffold452	3611918	A	T	Ore01,Ore02,Ore03,Ore04,Ore05,Ore06,Ore07,Ore08,Ore09,Ore10,Ore11,Ore12,Ore13,Ore14	*OreG0021186*
scaffold452	3617093	A	T	NA	*OreG0021187*
scaffold452	3618872	T	G	NA	*OreG0021187*
scaffold452	3632908	C	T	Och01,Och02,Och03,Och04,Och05,Och06,Och07,Och08,Och09,Och10,Och11,Och12,Och13,Och14,Ore01,Ore02,Ore03,Ore04,Ore05,Ore06,Ore07,Ore08,Ore09,Ore10,Ore11,Ore12,Ore13,Ore14	*OreG0021189*
scaffold452	3633812	C	A	Ore01,Ore11	*OreG0021189*
scaffold452	3633831	G	C	NA	*OreG0021189*
scaffold452	3637678	T	G	Och01,Och02,Och03,Och04,Och05,Och06,Och07,Och08,Och09,Och10,Och11,Och12,Och13,Och14	*OreG0021190*
scaffold452	3637745	G	A	Ore01,Ore02,Ore03,Ore04,Ore05,Ore06,Ore07,Ore08,Ore09,Ore10,Ore11,Ore12,Ore13,Ore14	*OreG0021190*
scaffold452	3639072	A	T	Och11	*OreG0021190*
scaffold452	3639781	C	T	Och11	*OreG0021190*
scaffold452	3639857	A	T	NA	*OreG0021190*
scaffold452	3640651	C	T	NA	*OreG0021190*
scaffold452	3643077	T	A	NA	*OreG0021191*
scaffold452	3657549	T	C	Ore01,Ore02,Ore03,Ore04,Ore05,Ore06,Ore07,Ore08,Ore09,Ore10,Ore11,Ore12,Ore13,Ore14	*OreG0021192*
scaffold452	3657561	G	A	Och01,Och02,Och03,Och04,Och05,Och06,Och07,Och08,Och09,Och10,Och11,Och12,Och13,Och14	*OreG0021192*
scaffold452	3657603	G	T	Ore03,Ore09,Ore13	*OreG0021192*
scaffold452	3671948	A	T	Och01,Och02,Och03,Och04,Och05,Och06,Och07,Och08,Och09,Och10,Och11,Och12,Och13,Och14,Ore01,Ore02,Ore03,Ore04,Ore05,Ore06,Ore07,Ore08,Ore09,Ore10,Ore11,Ore12,Ore13,Ore14	*OreG0021193*
scaffold452	3680391	T	A	NA	*OreG0021194*
scaffold452	3730462	A	G	NA	*OreG0021198*
scaffold452	3739668	A	T	NA	*OreG0021199*
scaffold452	3818879	C	T	NA	*OreG0021203*
scaffold452	3818948	T	G	NA	*OreG0021203*
scaffold452	3818972	G	A	NA	*OreG0021203*
scaffold452	3819014	C	T	NA	*OreG0021203*
scaffold452	3819023	C	A	Ore01,Ore02,Ore03,Ore04,Ore05,Ore06,Ore07,Ore08,Ore09,Ore10,Ore11,Ore12,Ore13,Ore14	*OreG0021203*
scaffold452	3819173	T	A	Och02,Och11,Och12,Och13	*OreG0021203*
scaffold452	3819239	A	T	Ore01,Ore11	*OreG0021203*
scaffold452	3819316	C	A	NA	*OreG0021203*
scaffold452	3819495	C	T	Ore01,Ore02,Ore03,Ore04,Ore05,Ore06,Ore07,Ore08,Ore09,Ore10,Ore11,Ore12,Ore13,Ore14	*OreG0021204*
scaffold452	3819525	G	A	NA	*OreG0021204*
scaffold452	3819611	G	A	NA	*OreG0021204*
scaffold452	3820665	A	T	Ore01,Ore02,Ore03,Ore04,Ore05,Ore06,Ore07,Ore08,Ore09,Ore10,Ore11,Ore12,Ore13,Ore14	*OreG0021206*
scaffold452	3820831	C	T	NA	*OreG0021206*
scaffold452	3820857	A	G	NA	*OreG0021206*
scaffold452	3839326	A	G	Ore01,Ore02,Ore03,Ore04,Ore05,Ore06,Ore07,Ore08,Ore09,Ore10,Ore11,Ore12,Ore13,Ore14	*OreG0021207*
scaffold452	3839672	T	C	NA	*OreG0021207*
scaffold452	3861758	G	A	Och01,Och02,Och03,Och04,Och05,Och06,Och07,Och08,Och09,Och10,Och11,Och12,Och13,Och14,Ore01,Ore02,Ore03,Ore04,Ore05,Ore06,Ore07,Ore08,Ore09,Ore10,Ore11,Ore12,Ore13,Ore14	*OreG0021208*
scaffold452	3862509	G	A	Ore01,Ore11	*OreG0021208*
scaffold452	3888217	C	T	Och02,Och11,Och12,Och13	*OreG0021210*
scaffold452	3895090	C	T	Ore01,Ore02,Ore03,Ore04,Ore05,Ore06,Ore07,Ore08,Ore09,Ore10,Ore11,Ore12,Ore13,Ore14	*OreG0021211*
scaffold452	3895096	C	T	Och02,Och03,Och04,Och05,Och06,Och07,Och08,Och09,Och10,Och11,Och12,Och13,Och14	*OreG0021211*
scaffold452	3895098	G	A	NA	*OreG0021211*
scaffold452	3895159	C	T	NA	*OreG0021211*
scaffold452	3895263	C	A	NA	*OreG0021211*
scaffold452	3895284	C	A	Och04,Och05,Och06,Och07,Och08,Och09,Och10,Och12,Och14	*OreG0021211*
scaffold452	3895387	G	A	Ore01,Ore02,Ore03,Ore04,Ore05,Ore06,Ore07,Ore08,Ore09,Ore10,Ore11,Ore12,Ore13,Ore14	*OreG0021211*
scaffold452	3895882	C	A	Och02,Och03,Och04,Och05,Och06,Och07,Och08,Och09,Och10,Och11,Och12,Och13,Och14	*OreG0021211*
scaffold452	3895980	A	G	Och14	*OreG0021211*

scaffold452	3895982	A	T	Ore01,Ore02,Ore03,Ore04,Ore05,Ore06,Ore07,Ore08,Ore09,Ore10,Ore11,Ore12,Ore13,Ore14	*OreG0021211*
scaffold452	3896242	G	A	Och02,Och03,Och04,Och05,Och06,Och07,Och08,Och09,Och10,Och11,Och12,Och13,Och14	*OreG0021211*
scaffold452	3896390	G	T	Ore01,Ore02,Ore03,Ore04,Ore05,Ore06,Ore07,Ore08,Ore09,Ore10,Ore11,Ore12,Ore13,Ore14	*OreG0021211*
scaffold452	3914972	G	A	NA	*OreG0021213*
scaffold452	3919087	A	G	Och14	*OreG0021214*
scaffold452	3920307	G	T	Och14	*OreG0021214*
scaffold452	3924394	A	T	Och02,Och03,Och04,Och06,Och07,Och09,Och10,Och11,Och12,Och13,Ore01,Ore02,Ore03,Ore04,Ore05,Ore06,Ore07,Ore08,Ore09,Ore10,Ore11,Ore12,Ore13,Ore14	*OreG0021214*
scaffold452	3933016	C	T	NA	*OreG0021215*
scaffold452	3936511	G	C	Ore01,Ore11	*OreG0021216*
scaffold452	3936583	C	G	NA	*OreG0021216*
scaffold452	3936598	C	A	Och01,Och07,Och14	*OreG0021216*
scaffold452	3936715	G	A	Och01,Och07,Och14	*OreG0021216*
scaffold452	3936988	T	C	Och01,Och02,Och03,Och04,Och05,Och06,Och07,Och08,Och09,Och10,Och11,Och12,Och13,Och14,Ore01,Ore02,Ore03,Ore04,Ore05,Ore06,Ore07,Ore08,Ore09,Ore10,Ore11,Ore12,Ore13,Ore14	*OreG0021216*
scaffold452	3953564	C	T	NA	*OreG0021218*
scaffold452	3962229	G	A	Och06	*OreG0021219*
scaffold452	3977425	A	G	Och09,Och10,Och14	*OreG0021221*
scaffold452	3977463	T	A	NA	*OreG0021221*
scaffold452	4003699	C	A	NA	*OreG0021225*
scaffold452	4004193	T	A	Och01,Och02,Och03,Och04,Och05,Och06,Och07,Och08,Och09,Och10,Och11,Och12,Och13,Och14,Ore01,Ore02,Ore03,Ore04,Ore05,Ore06,Ore07,Ore08,Ore09,Ore10,Ore11,Ore12,Ore13,Ore14	*OreG0021225*
scaffold452	4004350	T	A	NA	*OreG0021225*
scaffold452	4004479	G	A	Och02,Och03,Och04,Och05,Och06,Och12,Och13	*OreG0021225*
scaffold452	4004756	G	A	Och02,Och03,Och04,Och05,Och06,Och12,Och13	*OreG0021225*
scaffold452	4033589	C	T	Ore01,Ore02,Ore03,Ore04,Ore05,Ore06,Ore07,Ore08,Ore09,Ore10,Ore11,Ore12,Ore13,Ore14	*OreG0021227*
scaffold452	4033595	T	A	NA	*OreG0021227*
scaffold452	4033612	C	T	NA	*OreG0021227*
scaffold452	4034612	A	G	Ore03,Ore09,Ore13	*OreG0021227*
scaffold452	4035125	G	A	NA	*OreG0021227*
scaffold452	4035140	A	G	Och01,Och02,Och03,Och04,Och05,Och06,Och07,Och08,Och09,Och10,Och11,Och12,Och13,Och14	*OreG0021227*
scaffold452	4043632	T	A	Och01,Och02,Och03,Och04,Och05,Och06,Och07,Och08,Och09,Och10,Och11,Och12,Och13,Och14,Ore01,Ore02,Ore03,Ore04,Ore05,Ore06,Ore07,Ore08,Ore09,Ore10,Ore11,Ore12,Ore13,Ore14	*OreG0021229*
scaffold452	4043950	G	T	Och01,Och02,Och03,Och04,Och05,Och06,Och07,Och09,Och10,Och11,Och12,Och13,Och14	*OreG0021229*
scaffold452	4043954	C	G	Ore01,Ore02,Ore03,Ore04,Ore05,Ore06,Ore07,Ore08,Ore09,Ore10,Ore11,Ore12,Ore13,Ore14	*OreG0021229*
scaffold452	4046775	T	G	NA	*OreG0021229*
scaffold452	4046934	A	G	NA	*OreG0021229*
scaffold452	4046958	G	A	NA	*OreG0021229*
scaffold452	4092200	C	T	Ore01,Ore02,Ore03,Ore04,Ore05,Ore06,Ore07,Ore08,Ore09,Ore10,Ore11,Ore12,Ore13,Ore14	*OreG0021231*
scaffold452	4092362	C	T	Och01,Och02,Och03,Och04,Och05,Och06,Och07,Och08,Och09,Och10,Och11,Och12,Och13,Och14	*OreG0021231*
scaffold452	4092442	C	T	Och01,Och02,Och03,Och04,Och05,Och06,Och07,Och08,Och09,Och10,Och11,Och12,Och13,Och14	*OreG0021231*
scaffold452	4130207	G	T	NA	*OreG0021235*
scaffold452	4144886	G	T	NA	*OreG0021236*
scaffold452	4145444	C	G	NA	*OreG0021236*
scaffold452	4153445	C	T	NA	*OreG0021237*
scaffold452	4153509	A	G	Och02,Och12,Och13	*OreG0021237*
scaffold452	4155072	C	A	NA	*OreG0021237*
scaffold452	4163478	C	A	NA	*OreG0021238*
scaffold452	4184138	C	G	NA	*OreG0021239*
scaffold452	4184333	A	G	NA	*OreG0021239*
scaffold452	4184461	A	C	Och01	*OreG0021239*
scaffold452	4184581	A	T	Och01,Och02,Och03,Och04,Och05,Och06,Och07,Och08,Och09,Och10,Och11,Och12,Och13,Och14	*OreG0021239*
scaffold452	4187643	C	T	Och02,Och09,Och10,Och11,Och12,Och13	*OreG0021240*
scaffold452	4213735	C	G	Ore01,Ore02,Ore03,Ore04,Ore05,Ore06,Ore07,Ore08,Ore09,Ore10,Ore11,Ore12,Ore13,Ore14	*OreG0021241*
scaffold452	4308171	G	A	NA	*OreG0021250*
scaffold452	4360299	G	A	Och01,Och02,Och03,Och04,Och05,Och06,Och07,Och08,Och09,Och10,Och11,Och12,Och13,Och14	*OreG0021254*
scaffold452	4398163	T	C	Och01,Och02,Och03,Och04,Och05,Och06,Och07,Och08,Och09,Och10,Och11,Och12,Och13,Och14,Ore01,Ore02,Ore03,Ore04,Ore05,Ore06,Ore07,Ore08,Ore09,Ore10,Ore11,Ore12,Ore13,Ore14	*OreG0021257*

scaffold452	4398503	G	T	Och01,Och02,Och03,Och04,Och05,Och06,Och07,Och08,Och09,Och10,Och11,Och12,Och13,Och14,Ore01,Ore02,Ore03,Ore04,Ore05,Ore06,Ore07,Ore08,Ore09,Ore10,Ore11,Ore12,Ore13,Ore14	*OreG0021257*
scaffold452	4410144	G	C	NA	*OreG0021258*
scaffold452	4412017	G	A	Ore01,Ore02,Ore03,Ore04,Ore05,Ore06,Ore07,Ore08,Ore09,Ore10,Ore11,Ore12,Ore13,Ore14	*OreG0021258*
scaffold452	4422088	T	A	NA	*OreG0021259*
scaffold452	4422128	T	G	Ore01,Ore02,Ore03,Ore04,Ore05,Ore06,Ore07,Ore08,Ore09,Ore10,Ore11,Ore12,Ore13,Ore14	*OreG0021259*
scaffold452	4435682	A	T	NA	*OreG0021261*
scaffold452	4477986	G	T	Och01	*OreG0021264*
scaffold452	4507417	G	T	NA	*OreG0021265*
scaffold452	4521871	T	C	NA	*OreG0021269*
scaffold452	4563214	G	T	Ore11	*OreG0021273*
scaffold452	4625375	C	T	Ore01,Ore02,Ore03,Ore04,Ore05,Ore06,Ore07,Ore08,Ore09,Ore10,Ore11,Ore12,Ore13,Ore14	*OreG0021277*
scaffold452	4692674	T	A	NA	*OreG0021284*
scaffold452	4700302	C	T	NA	*OreG0021285*
scaffold452	4752792	G	A	NA	*OreG0021287*
scaffold452	4785484	C	T	Ore02,Ore03,Ore04,Ore05,Ore06,Ore07,Ore08,Ore09,Ore10,Ore11,Ore12,Ore13,Ore14	*OreG0021293*
scaffold452	4815345	C	T	Ore01,Ore02,Ore03,Ore04,Ore05,Ore06,Ore07,Ore08,Ore09,Ore10,Ore11,Ore12,Ore13,Ore14	*OreG0021294*
scaffold452	4832678	C	A	NA	*OreG0021296*
scaffold452	4834180	A	G	NA	*OreG0021296*
scaffold452	4834593	A	G	Ore01,Ore02,Ore03,Ore04,Ore05,Ore06,Ore07,Ore08,Ore09,Ore10,Ore11,Ore12,Ore13,Ore14	*OreG0021296*
scaffold452	4835040	T	C	Ore01,Ore02,Ore03,Ore04,Ore05,Ore06,Ore07,Ore08,Ore09,Ore10,Ore11,Ore12,Ore13,Ore14	*OreG0021296*
scaffold452	4835185	T	C	NA	*OreG0021296*
scaffold452	4835709	A	T	NA	*OreG0021296*
scaffold452	4838703	A	T	Ore01,Ore02,Ore03,Ore04,Ore05,Ore06,Ore07,Ore08,Ore09,Ore10,Ore11,Ore12,Ore13,Ore14	*OreG0021297*
scaffold452	4839822	C	A	Och01,Och02,Och03,Och04,Och05,Och06,Och07,Och08,Och09,Och10,Och11,Och12,Och13,Och14	*OreG0021297*
scaffold452	4840135	C	A	Ore01,Ore02,Ore03,Ore04,Ore05,Ore06,Ore07,Ore08,Ore09,Ore10,Ore11,Ore12,Ore13,Ore14	*OreG0021297*
scaffold452	4840141	C	G	Ore01,Ore02,Ore03,Ore04,Ore05,Ore06,Ore07,Ore08,Ore09,Ore10,Ore11,Ore12,Ore13,Ore14	*OreG0021297*
scaffold452	4855529	C	A	Och01,Och02,Och03,Och04,Och05,Och06,Och07,Och08,Och09,Och10,Och11,Och12,Och13,Och14	*OreG0021298*
scaffold452	4855645	A	T	NA	*OreG0021298*
scaffold452	4855687	G	C	Och01,Och02,Och03,Och04,Och05,Och06,Och07,Och08,Och09,Och10,Och11,Och12,Och13,Och14	*OreG0021298*
scaffold452	4855938	G	A	Ore01,Ore02,Ore03,Ore04,Ore05,Ore06,Ore07,Ore08,Ore09,Ore10,Ore11,Ore12,Ore13,Ore14	*OreG0021298*
scaffold452	4864629	G	C	Ore01,Ore02,Ore03,Ore04,Ore05,Ore06,Ore07,Ore08,Ore09,Ore10,Ore11,Ore12,Ore13,Ore14	*OreG0021299*
scaffold452	4866235	G	A	Och02,Och03,Och04,Och05,Och06,Och07,Och08,Och11,Och12,Och13,Och14	*OreG0021299*
scaffold452	4867201	G	C	Ore02,Ore03,Ore04,Ore05,Ore06,Ore07,Ore08,Ore09,Ore10,Ore11,Ore12,Ore13,Ore14	*OreG0021299*
scaffold452	4874239	T	C	Och01,Och02,Och03,Och04,Och05,Och06,Och07,Och08,Och09,Och10,Och11,Och12,Och13,Och14	*OreG0021300*
scaffold452	4875118	A	G	NA	*OreG0021300*
scaffold452	4875127	C	T	Ore01,Ore02,Ore03,Ore04,Ore05,Ore06,Ore07,Ore08,Ore09,Ore10,Ore11,Ore12,Ore13,Ore14	*OreG0021300*
scaffold452	4880624	T	A	NA	*OreG0021301*
scaffold452	4880978	C	T	NA	*OreG0021301*
scaffold452	4881018	C	T	NA	*OreG0021301*
scaffold452	4945268	T	A	Och01	*OreG0021302*
scaffold452	4945452	A	T	NA	*OreG0021302*
scaffold452	4948223	T	C	NA	*OreG0021302*
scaffold452	4961752	C	T	Och02,Och03,Och04,Och05,Och06,Och07,Och08,Och11,Och12,Och13,Och14	*OreG0021303*
scaffold452	4962005	A	T	NA	*OreG0021303*
scaffold452	4962877	G	T	NA	*OreG0021303*
scaffold452	4965239	C	A	NA	*OreG0021303*
scaffold452	4965246	G	A	Och02,Och03,Och04,Och05,Och06,Och07,Och08,Och11,Och12,Och13,Och14	*OreG0021303*
scaffold452	5022948	A	G	NA	*OreG0021310*
scaffold452	5029711	G	A	Och01,Och02,Och03,Och04,Och05,Och06,Och07,Och08,Och09,Och10,Och11,Och12,Och13,Och14,Ore01,Ore02,Ore03,Ore04,Ore05,Ore06,Ore07,Ore08,Ore09,Ore10,Ore11,Ore12,Ore13,Ore14	*OreG0021311*
scaffold452	5029721	T	C	Ore03,Ore09,Ore13	*OreG0021311*
scaffold452	5037633	C	A	Och01,Och02,Och03,Och04,Och05,Och06,Och07,Och08,Och09,Och10,Och11,Och12,Och13,Och14	*OreG0021311*
scaffold452	5049208	T	C	Och01,Och02,Och03,Och04,Och05,Och06,Och07,Och08,Och09,Och10,Och11,Och12,Och13,Och14	*OreG0021312*

scaffold452	5060188	G	A	Och01,Och02,Och03,Och04,Och05,Och06,Och07,Och08,Och09,Och10,Och11,Och12,Och13,Och14	*OreG0021314*
scaffold452	5065862	G	T	Ore11	*OreG0021315*
scaffold452	5096300	G	T	Och01,Och02,Och03,Och04,Och05,Och06,Och07,Och08,Och09,Och10,Och11,Och12,Och13,Och14,Ore01,Ore02,Ore03,Ore04,Ore05,Ore06,Ore07,Ore08,Ore09,Ore10,Ore11,Ore12,Ore13,Ore14	*OreG0021316*
scaffold452	5097648	C	G	Ore01,Ore11	*OreG0021316*
scaffold452	5097699	A	C	Ore01,Ore11	*OreG0021316*
scaffold452	5133648	G	A	Och02,Och12,Och13	*OreG0021319*
scaffold452	5140596	A	T	Och02,Och12,Och13	*OreG0021320*
scaffold452	5164131	T	C	Och02,Och12,Och13	*OreG0021323*
scaffold452	5169565	A	C	Ore01,Ore02,Ore03,Ore04,Ore05,Ore06,Ore07,Ore08,Ore09,Ore10,Ore11,Ore12,Ore13,Ore14	*OreG0021323*
scaffold452	5171881	A	T	Och01,Och02,Och03,Och04,Och05,Och06,Och07,Och08,Och09,Och10,Och11,Och12,Och13,Och14,Ore01,Ore02,Ore03,Ore04,Ore05,Ore06,Ore07,Ore08,Ore09,Ore10,Ore11,Ore12,Ore13,Ore14	*OreG0021323*
scaffold452	5172490	T	C	Ore01,Ore02,Ore03,Ore04,Ore05,Ore06,Ore07,Ore08,Ore09,Ore10,Ore11,Ore12,Ore13,Ore14	*OreG0021323*
scaffold452	5172900	A	C	Och01,Och02,Och03,Och04,Och05,Och06,Och07,Och08,Och09,Och10,Och11,Och12,Och13,Och14,Ore01,Ore02,Ore03,Ore04,Ore05,Ore06,Ore07,Ore08,Ore09,Ore10,Ore11,Ore12,Ore13,Ore14	*OreG0021323*
scaffold452	5173854	C	T	Och01,Och02,Och03,Och04,Och05,Och06,Och07,Och08,Och09,Och10,Och11,Och12,Och13,Och14,Ore01,Ore02,Ore03,Ore04,Ore05,Ore06,Ore07,Ore08,Ore09,Ore10,Ore11,Ore12,Ore13,Ore14	*OreG0021323*
scaffold452	5174660	T	C	NA	*OreG0021323*
scaffold452	5178265	A	T	Och01,Och02,Och03,Och04,Och05,Och06,Och07,Och08,Och09,Och10,Och11,Och12,Och13,Och14	*OreG0021323*
scaffold452	5246834	A	T	Och02,Och05,Och12,Och13	*OreG0021328*
scaffold452	5316987	G	T	NA	*OreG0021335*
scaffold452	5328061	C	A	NA	*OreG0021337*
scaffold452	5351537	T	C	Och01,Och02,Och03,Och04,Och05,Och06,Och07,Och08,Och09,Och10,Och11,Och12,Och13,Och14,Ore01,Ore02,Ore03,Ore04,Ore05,Ore06,Ore07,Ore08,Ore09,Ore10,Ore11,Ore12,Ore13,Ore14	*OreG0021338*
scaffold452	5356068	G	A	NA	*OreG0021339*
scaffold452	5356360	G	A	NA	*OreG0021339*
scaffold452	5384616	G	C	NA	*OreG0021342*
scaffold452	5565432	C	T	Och01,Och02,Och03,Och04,Och05,Och06,Och07,Och08,Och09,Och10,Och11,Och12,Och13,Och14	*OreG0021347*
scaffold452	5590143	T	C	NA	*OreG0021345*
scaffold452	5600618	A	T	Ore01,Ore02,Ore03,Ore04,Ore05,Ore06,Ore07,Ore08,Ore09,Ore10,Ore11,Ore12,Ore13,Ore14	*OreG0021349*
scaffold452	5602225	G	T	Ore01,Ore02,Ore03,Ore04,Ore05,Ore06,Ore07,Ore08,Ore09,Ore10,Ore11,Ore12,Ore13,Ore14	*OreG0021349*
scaffold452	5603376	A	T	Och01,Och02,Och03,Och04,Och05,Och06,Och07,Och08,Och09,Och10,Och11,Och12,Och13,Och14	*OreG0021349*
scaffold452	5627115	A	T	NA	*OreG0021351*
scaffold452	5670560	G	A	NA	*OreG0021354*
scaffold452	5836866	G	A	Ore01,Ore02,Ore03,Ore04,Ore05,Ore06,Ore07,Ore08,Ore09,Ore10,Ore11,Ore12,Ore13,Ore14	*OreG0021361*
scaffold452	5836875	C	T	Ore01,Ore02,Ore03,Ore04,Ore05,Ore06,Ore07,Ore08,Ore09,Ore10,Ore11,Ore12,Ore13,Ore14	*OreG0021361*
scaffold452	5855885	A	G	Ore01,Ore02,Ore03,Ore04,Ore05,Ore06,Ore07,Ore08,Ore09,Ore10,Ore11,Ore12,Ore13,Ore14	*OreG0021363*
scaffold452	5856136	A	T	Och01,Och02,Och03,Och04,Och05,Och06,Och07,Och08,Och09,Och10,Och11,Och12,Och13,Och14,Ore01,Ore02,Ore03,Ore04,Ore05,Ore06,Ore07,Ore08,Ore09,Ore10,Ore11,Ore12,Ore13,Ore14	*OreG0021363*
scaffold452	5890933	C	T	NA	*OreG0021370*
scaffold452	5891943	A	G	NA	*OreG0021370*
scaffold452	5891968	G	T	Och01,Och02,Och03,Och04,Och05,Och06,Och07,Och08,Och09,Och10,Och11,Och12,Och13,Och14	*OreG0021370*
scaffold452	5931697	T	A	Ore01,Ore02,Ore03,Ore04,Ore05,Ore06,Ore07,Ore08,Ore09,Ore10,Ore11,Ore12,Ore13,Ore14	*OreG0021372*
scaffold452	5951150	A	G	Och01	*OreG0021374*
scaffold452	5992170	C	T	Och01,Och02,Och03,Och04,Och05,Och06,Och07,Och08,Och09,Och10,Och11,Och12,Och13,Och14	*OreG0021377*
scaffold452	6004055	C	T	Ore01,Ore02,Ore03,Ore04,Ore05,Ore06,Ore07,Ore08,Ore09,Ore10,Ore11,Ore12,Ore13,Ore14	*OreG0021378*
scaffold452	6004169	C	T	Och01,Och02,Och03,Och04,Och05,Och06,Och07,Och08,Och09,Och10,Och11,Och12,Och13,Och14	*OreG0021378*
scaffold452	6004190	C	T	NA	*OreG0021378*
scaffold452	6005645	G	A	NA	*OreG0021378*
scaffold452	6005656	G	A	Och01,Och02,Och03,Och04,Och05,Och06,Och07,Och08,Och09,Och10,Och11,Och12,Och13,Och14	*OreG0021378*
scaffold452	6005690	G	T	Ore01,Ore02,Ore03,Ore04,Ore05,Ore06,Ore07,Ore08,Ore09,Ore10,Ore11,Ore12,Ore13,Ore14	*OreG0021378*

scaffold452	6005696	C	T	Och07,Ore01,Ore02,Ore03,Ore04,Ore05,Ore06,Ore07,Ore08,Ore09,Ore10,Ore11,Ore12,Ore13,Ore14	*OreG0021378*
scaffold452	6005756	C	T	NA	*OreG0021378*
scaffold452	6005866	C	T	Ore01	*OreG0021378*
scaffold452	6101782	T	A	NA	*OreG0021385*
scaffold452	6103257	C	T	Och01,Och02,Och03,Och04,Och05,Och06,Och07,Och08,Och09,Och10,Och11,Och12,Och13,Och14,Ore01,Ore02,Ore03,Ore04,Ore05,Ore06,Ore07,Ore08,Ore09,Ore10,Ore11,Ore12,Ore13,Ore14	*OreG0021385*
scaffold452	6163573	G	T	NA	*OreG0021388*
scaffold452	6169650	A	T	NA	*OreG0021389*
scaffold452	6198845	C	G	Och01,Och02,Och03,Och04,Och05,Och06,Och07,Och08,Och09,Och10,Och11,Och12,Och13,Och14,Ore01,Ore02,Ore03,Ore04,Ore05,Ore06,Ore07,Ore08,Ore09,Ore10,Ore11,Ore12,Ore13,Ore14	*OreG0021392*
scaffold452	6214214	G	C	NA	*OreG0021393*
scaffold452	6214351	A	T	NA	*OreG0021393*
scaffold452	6236588	C	T	Och01,Och02,Och03,Och04,Och05,Och06,Och07,Och08,Och09,Och10,Och11,Och12,Och13,Och14,Ore01,Ore02,Ore03,Ore04,Ore05,Ore06,Ore07,Ore08,Ore09,Ore10,Ore11,Ore12,Ore13,Ore14	*OreG0021394*
scaffold452	6243057	A	C	Och01,Och02,Och03,Och04,Och05,Och06,Och07,Och08,Och09,Och10,Och11,Och12,Och13,Och14,Ore01,Ore02,Ore03,Ore04,Ore05,Ore06,Ore07,Ore08,Ore09,Ore10,Ore11,Ore12,Ore13,Ore14	*OreG0021395*
scaffold452	6252445	C	G	Och01,Och02,Och03,Och04,Och05,Och06,Och07,Och08,Och09,Och10,Och11,Och12,Och13,Och14,Ore01,Ore02,Ore03,Ore04,Ore05,Ore06,Ore07,Ore08,Ore09,Ore10,Ore11,Ore12,Ore13,Ore14	*OreG0021395*
scaffold452	6264591	A	T	NA	*OreG0021396*
scaffold452	6283093	T	A	Och01,Och02,Och03,Och04,Och05,Och06,Och07,Och08,Och09,Och10,Och11,Och12,Och13,Och14,Ore01,Ore02,Ore03,Ore04,Ore05,Ore06,Ore07,Ore08,Ore09,Ore10,Ore11,Ore12,Ore13,Ore14	*OreG0021397*
scaffold452	6286037	C	A	NA	*OreG0021397*
scaffold452	6286271	C	G	NA	*OreG0021397*
scaffold452	6304256	T	C	NA	*OreG0021400*
scaffold452	6306040	T	A	NA	*OreG0021400*
scaffold452	6387365	T	G	NA	*OreG0021407*
scaffold452	6388976	C	T	Och01,Och02,Och03,Och04,Och05,Och06,Och07,Och08,Och09,Och10,Och11,Och12,Och13,Och14	*OreG0021407*
scaffold452	6388992	G	A	Och01,Och02,Och03,Och04,Och05,Och06,Och07,Och08,Och09,Och10,Och11,Och12,Och13,Och14	*OreG0021407*
scaffold452	6389007	C	T	Och03,Och04	*OreG0021407*
scaffold452	6389038	A	T	Och01	*OreG0021407*
scaffold452	6403720	A	C	Ore01,Ore02,Ore03,Ore04,Ore05,Ore06,Ore07,Ore08,Ore09,Ore10,Ore11,Ore12,Ore13,Ore14	*OreG0021409*
scaffold452	6404729	C	G	Ore01,Ore02,Ore03,Ore04,Ore05,Ore06,Ore07,Ore08,Ore09,Ore10,Ore11,Ore12,Ore13,Ore14	*OreG0021409*
scaffold452	6404742	C	G	Och01,Och02,Och03,Och04,Och05,Och06,Och07,Och08,Och09,Och10,Och11,Och12,Och13,Och14,Ore01,Ore02,Ore03,Ore04,Ore05,Ore06,Ore07,Ore08,Ore09,Ore10,Ore11,Ore12,Ore13,Ore14	*OreG0021409*
scaffold452	6404864	A	G	Ore01,Ore02,Ore03,Ore04,Ore05,Ore06,Ore07,Ore08,Ore09,Ore10,Ore11,Ore12,Ore13,Ore14	*OreG0021409*
scaffold452	6437100	T	G	Och01,Och02,Och03,Och04,Och05,Och06,Och07,Och08,Och09,Och10,Och11,Och12,Och13,Och14,Ore01,Ore02,Ore03,Ore04,Ore05,Ore06,Ore07,Ore08,Ore09,Ore10,Ore11,Ore12,Ore13,Ore14	*OreG0021410*
scaffold452	6451799	T	G	NA	*OreG0021412*
scaffold452	6493935	A	T	NA	*OreG0021415*
scaffold452	6522482	G	C	NA	*OreG0021418*
scaffold452	6522740	T	A	NA	*OreG0021418*
scaffold452	6522785	G	A	NA	*OreG0021418*
scaffold452	6522836	G	T	NA	*OreG0021418*
scaffold452	6523507	G	A	NA	*OreG0021418*
scaffold452	6524119	G	A	NA	*OreG0021418*
scaffold452	6527636	T	G	Och01,Och02,Och03,Och04,Och05,Och06,Och07,Och08,Och09,Och10,Och11,Och12,Och13,Och14	*OreG0021418*
scaffold452	6528715	T	A	Och02,Och03,Och04,Och05,Och06,Och07,Och08,Och09,Och10,Och12,Och13,Och14	*OreG0021418*
scaffold452	6548167	C	T	Och01,Och02,Och03,Och04,Och05,Och06,Och07,Och08,Och09,Och10,Och11,Och12,Och13,Och14	*OreG0021419*
scaffold452	6548671	T	C	NA	*OreG0021419*
scaffold452	6548831	G	A	NA	*OreG0021419*
scaffold452	6548970	A	G	NA	*OreG0021419*
scaffold452	6549161	C	T	NA	*OreG0021419*
scaffold452	6549192	C	A	Ore01,Ore02,Ore03,Ore04,Ore05,Ore06,Ore07,Ore08,Ore09,Ore10,Ore11,Ore12,Ore13,Ore14	*OreG0021419*
scaffold452	6549521	A	T	NA	*OreG0021419*
scaffold452	6549778	G	A	NA	*OreG0021419*
scaffold452	6549810	A	G	NA	*OreG0021419*
scaffold452	6549823	G	A	NA	*OreG0021419*

scaffold452	6549891	T	G	NA	*OreG0021419*
scaffold452	6550069	A	G	NA	*OreG0021419*
scaffold452	6553414	C	T	NA	*OreG0021419*
scaffold452	6559829	A	G	Ore03	*OreG0021420*
scaffold452	6573333	T	C	Och02,Och03,Och04,Och05,Och06,Och07,Och08,Och09,Och10,Och11,Och12,Och13,Och14	*OreG0021423*
scaffold452	6574273	C	T	NA	*OreG0021423*
scaffold452	6575704	C	A	NA	*OreG0021424*
scaffold452	6624628	T	A	Och01,Och02,Och03,Och04,Och05,Och06,Och07,Och08,Och09,Och10,Och11,Och12,Och13,Och14,Ore01,Ore02,Ore03,Ore04,Ore05,Ore06,Ore07,Ore08,Ore09,Ore10,Ore11,Ore12,Ore13,Ore14	*OreG0021427*
scaffold452	6625003	C	T	NA	*OreG0021427*
scaffold452	6625063	G	A	NA	*OreG0021427*
scaffold452	6642956	A	T	NA	*OreG0021429*
scaffold452	6645853	A	T	Och01,Och02,Och03,Och04,Och05,Och06,Och07,Och08,Och09,Och10,Och11,Och12,Och13,Och14,Ore01,Ore02,Ore03,Ore04,Ore05,Ore06,Ore07,Ore08,Ore09,Ore10,Ore11,Ore12,Ore13,Ore14	*OreG0021429*
scaffold452	6657655	A	T	NA	*OreG0021430*
scaffold452	6670867	T	C	Och03,Och04	*OreG0021433*
scaffold452	6673046	C	A	NA	*OreG0021433*
scaffold452	6675220	A	G	Och01,Och02,Och03,Och04,Och05,Och06,Och07,Och08,Och09,Och10,Och11,Och12,Och13,Och14,Ore01,Ore02,Ore03,Ore04,Ore05,Ore06,Ore07,Ore08,Ore09,Ore10,Ore11,Ore12,Ore13,Ore14	*OreG0021434*
scaffold452	6700234	C	A	NA	*OreG0021436*
scaffold452	6729211	C	T	Och02,Och03,Och04,Och05,Och06,Och07,Och08,Och09,Och10,Och11,Och12,Och13,Och14	*OreG0021439*
scaffold452	6731633	G	C	Ore01,Ore02,Ore08	*OreG0021439*
scaffold452	6733840	C	T	NA	*OreG0021439*
scaffold452	6735161	C	T	NA	*OreG0021439*
scaffold452	6735916	C	A	NA	*OreG0021439*
scaffold452	6740566	G	A	NA	*OreG0021440*
scaffold452	6784695	T	C	Och01,Och02,Och06,Och09,Och10	*OreG0021445*
scaffold452	6816422	G	C	NA	*OreG0021447*
scaffold452	6824395	T	C	NA	*OreG0021447*
scaffold452	6824464	A	T	NA	*OreG0021447*
scaffold452	6833792	G	A	NA	*OreG0021448*
scaffold452	6850004	T	G	Ore01,Ore02,Ore03,Ore04,Ore05,Ore06,Ore07,Ore08,Ore09,Ore10,Ore11,Ore12,Ore13,Ore14	*OreG0021450*
scaffold452	6855626	C	G	Ore01,Ore02,Ore03,Ore04,Ore05,Ore06,Ore07,Ore08,Ore09,Ore10,Ore11,Ore12,Ore13,Ore14	*OreG0021452*
scaffold452	6858043	G	A	NA	*OreG0021452*
scaffold452	6863802	G	A	Ore01,Ore02,Ore03,Ore04,Ore05,Ore06,Ore07,Ore08,Ore09,Ore10,Ore11,Ore12,Ore13,Ore14	*OreG0021453*
scaffold452	6863840	A	G	Och03,Och04	*OreG0021453*
scaffold452	6864335	G	A	NA	*OreG0021453*
scaffold452	6864899	G	A	Och01	*OreG0021453*
scaffold452	6878811	G	T	Och01	*OreG0021454*
scaffold452	6879007	C	A	Och01,Och02,Och03,Och04,Och05,Och06,Och07,Och08,Och09,Och10,Och11,Och12,Och13,Och14	*OreG0021454*
scaffold452	6879153	T	A	Och01,Och02,Och03,Och04,Och05,Och06,Och07,Och08,Och09,Och10,Och11,Och12,Och13,Och14,Ore01,Ore02,Ore03,Ore04,Ore05,Ore06,Ore07,Ore08,Ore09,Ore10,Ore11,Ore12,Ore13,Ore14	*OreG0021454*
scaffold452	6879589	T	C	NA	*OreG0021454*
scaffold452	6884151	T	G	Och01,Och02,Och03,Och04,Och05,Och06,Och07,Och08,Och09,Och10,Och11,Och12,Och13,Och14	*OreG0021455*
scaffold452	6901421	C	T	Ore03,Ore07,Ore08,Ore10,Ore11,Ore12,Ore13,Ore14	*OreG0021456*
scaffold452	6915185	G	A	Och02,Och03,Och04,Och05,Och06,Och12,Och13	*OreG0021458*
scaffold452	6915245	T	C	Och02,Och03,Och04,Och05,Och06,Och07,Och08,Och09,Och10,Och11,Och12,Och13	*OreG0021458*
scaffold452	6915902	C	A	Och09,Och10,Och11	*OreG0021458*
scaffold452	6937448	C	T	Ore01,Ore02,Ore03,Ore04,Ore05,Ore06,Ore07,Ore08,Ore09,Ore10,Ore11,Ore12,Ore13,Ore14	*OreG0021460*
scaffold452	6937673	A	T	Ore01,Ore02,Ore03,Ore04,Ore05,Ore06,Ore07,Ore08,Ore09,Ore10,Ore11,Ore12,Ore13,Ore14	*OreG0021460*
scaffold452	6997110	A	T	Och01,Och02,Och03,Och04,Och05,Och06,Och07,Och08,Och09,Och10,Och11,Och12,Och13,Och14,Ore01,Ore02,Ore03,Ore04,Ore05,Ore06,Ore07,Ore08,Ore09,Ore10,Ore11,Ore12,Ore13,Ore14	*OreG0021464*
scaffold452	6997509	A	C	Ore01,Ore02,Ore03,Ore04,Ore05,Ore06,Ore07,Ore08,Ore09,Ore10,Ore11,Ore12,Ore13,Ore14	*OreG0021464*
scaffold452	7000469	C	A	Ore01,Ore02,Ore03,Ore04,Ore05,Ore06,Ore07,Ore08,Ore09,Ore10,Ore11,Ore12,Ore13,Ore14	*OreG0021466*
scaffold452	7016382	T	C	Ore01,Ore02,Ore03,Ore04,Ore05,Ore06,Ore07,Ore08,Ore09,Ore10,Ore11,Ore12,Ore13,Ore14	*OreG0021468*
scaffold452	7016405	G	T	NA	*OreG0021468*

scaffold452	7016811	C	T	Och01,Och02,Och03,Och04,Och05,Och06,Och07,Och08,Och09,Och10,Och11,Och12,Och13,Och14	*OreG0021468*
scaffold452	7020077	C	G	Ore01,Ore02,Ore03,Ore04,Ore05,Ore06,Ore07,Ore08,Ore09,Ore10,Ore11,Ore12,Ore13,Ore14	*OreG0021469*
scaffold452	7020436	G	T	Ore01,Ore02,Ore03,Ore04,Ore05,Ore06,Ore07,Ore08,Ore09,Ore10,Ore11,Ore12,Ore13,Ore14	*OreG0021470*
scaffold452	7065988	C	T	Och01,Och02,Och03,Och04,Och05,Och06,Och07,Och08,Och09,Och10,Och11,Och12,Och13,Och14	*OreG0021474*
scaffold452	7066540	C	G	NA	*OreG0021474*
scaffold452	7067137	C	G	Ore01,Ore02,Ore03,Ore04,Ore05,Ore06,Ore07,Ore08,Ore09,Ore10,Ore11,Ore12,Ore13,Ore14	*OreG0021474*
scaffold452	7071106	A	T	NA	*OreG0021475*
scaffold452	7097123	G	A	NA	*OreG0021477*
scaffold452	7105107	G	A	NA	*OreG0021478*
scaffold452	7105122	G	A	NA	*OreG0021478*
scaffold452	7106574	C	T	NA	*OreG0021478*
scaffold452	7135965	G	A	NA	*OreG0021481*
scaffold452	7244003	C	T	NA	*OreG0021485*
scaffold452	7244417	G	A	NA	*OreG0021485*
scaffold452	7244422	C	T	NA	*OreG0021485*
scaffold452	7244564	T	C	NA	*OreG0021485*
scaffold452	7245980	C	T	Och01,Och02,Och03,Och04,Och05,Och06,Och07,Och08,Och09,Och10,Och11,Och12,Och13,Och14	*OreG0021485*
scaffold452	7265859	C	T	Och01	*OreG0021487*
scaffold452	7266950	A	G	Ore01,Ore02,Ore03,Ore04,Ore05,Ore06,Ore07,Ore08,Ore09,Ore10,Ore11,Ore12,Ore13,Ore14	*OreG0021487*
scaffold452	7266978	A	T	Och01,Och02,Och03,Och04,Och05,Och06,Och07,Och08,Och09,Och10,Och11,Och12,Och13,Och14,Ore01,Ore02,Ore03,Ore04,Ore05,Ore06,Ore07,Ore08,Ore09,Ore10,Ore11,Ore12,Ore13,Ore14	*OreG0021487*
scaffold452	7267109	A	C	Och03,Och04,Och09,Och10,Och11	*OreG0021487*
scaffold452	7267130	G	A	Och01	*OreG0021487*
scaffold452	7278368	G	A	Och01,Och02,Och03,Och04,Och05,Och06,Och07,Och08,Och09,Och10,Och11,Och12,Och13,Och14,Ore01,Ore02,Ore03,Ore04,Ore05,Ore06,Ore07,Ore08,Ore09,Ore10,Ore11,Ore12,Ore13,Ore14	*OreG0021488*
scaffold452	7281328	C	T	Och01,Och02,Och03,Och04,Och05,Och06,Och07,Och08,Och09,Och10,Och11,Och12,Och13,Och14,Ore01,Ore02,Ore03,Ore04,Ore05,Ore06,Ore07,Ore08,Ore09,Ore10,Ore11,Ore12,Ore13,Ore14	*OreG0021488*
scaffold452	7281340	C	T	Och01,Och02,Och03,Och04,Och05,Och06,Och07,Och08,Och09,Och10,Och11,Och12,Och13,Och14,Ore01,Ore02,Ore03,Ore04,Ore05,Ore06,Ore07,Ore08,Ore09,Ore10,Ore11,Ore12,Ore13,Ore14	*OreG0021488*
scaffold452	7297010	T	C	Ore01,Ore02,Ore03,Ore04,Ore05,Ore06,Ore07,Ore08,Ore09,Ore10,Ore11,Ore12,Ore13,Ore14	*OreG0021489*
scaffold452	7303603	T	A	Och01,Och02,Och03,Och04,Och05,Och06,Och07,Och08,Och09,Och10,Och11,Och12,Och13,Och14,Ore01,Ore02,Ore03,Ore04,Ore05,Ore06,Ore07,Ore08,Ore09,Ore10,Ore11,Ore12,Ore13,Ore14	*OreG0021489*
scaffold452	7311581	C	T	Ore05	*OreG0021490*
scaffold452	7334538	G	T	Ore01,Ore02,Ore03,Ore04,Ore05,Ore06,Ore07,Ore08,Ore09,Ore10,Ore11,Ore12,Ore13,Ore14	*OreG0021492*
scaffold452	7336331	C	T	NA	*OreG0021492*
scaffold452	7344222	C	A	Och09,Och10	*OreG0021493*
scaffold452	7344247	C	T	Ore01,Ore02,Ore03,Ore04,Ore05,Ore06,Ore07,Ore08,Ore09,Ore10,Ore11,Ore12,Ore13,Ore14	*OreG0021493*
scaffold452	7351187	G	T	Ore01,Ore02,Ore03,Ore04,Ore05,Ore06,Ore07,Ore08,Ore09,Ore10,Ore11,Ore12,Ore13,Ore14	*OreG0021495*
scaffold452	7351931	T	C	NA	*OreG0021495*
scaffold452	7352820	G	T	Och01,Och07,Och08,Och09,Och10,Och11,Och14	*OreG0021495*
scaffold452	7468340	C	A	NA	*OreG0021503*
scaffold452	7468352	G	T	Och03,Och04	*OreG0021503*
scaffold452	7468449	A	G	Och14	*OreG0021503*
scaffold452	7468605	G	A	Och03,Och04,Och11	*OreG0021503*
scaffold452	7468736	A	T	Och03,Och04	*OreG0021503*
scaffold452	7468812	A	T	NA	*OreG0021503*
scaffold452	7468957	C	G	Och03,Och04	*OreG0021503*
scaffold452	7469111	G	A	NA	*OreG0021503*
scaffold452	7469330	G	A	NA	*OreG0021503*
scaffold452	7469514	T	C	NA	*OreG0021503*
scaffold452	7469946	A	T	NA	*OreG0021503*
scaffold452	7470095	G	A	NA	*OreG0021503*
scaffold452	7475109	C	A	NA	*OreG0021504*
scaffold452	7475177	C	T	NA	*OreG0021504*
scaffold452	7482098	G	A	NA	*OreG0021507*
scaffold452	7486780	A	G	NA	*OreG0021507*
scaffold452	7489383	G	A	Ore01,Ore02,Ore03,Ore04,Ore05,Ore06,Ore07,Ore08,Ore09,Ore10,Ore11,Ore12,Ore13,Ore14	*OreG0021507*
scaffold452	7500257	G	C	NA	*OreG0021508*

scaffold452	7500275	A	T	NA	*OreG0021508*
scaffold452	7544110	C	A	NA	*OreG0021510*
scaffold452	7615053	T	C	NA	*OreG0021517*
scaffold452	7626789	G	A	NA	*OreG0021518*
scaffold452	7627380	G	A	Ore06,Ore09,Ore14	*OreG0021518*
scaffold452	7632217	G	A	NA	*OreG0021518*
scaffold452	7649939	C	T	NA	*OreG0021519*
scaffold452	7662485	A	T	Och02,Och03,Och04,Och05,Och06,Och08,Och12,Och13	*OreG0021521*
scaffold452	7663070	A	G	NA	*OreG0021521*
scaffold452	7672624	C	T	Och09,Och10	*OreG0021523*
scaffold452	7673902	C	A	Och02,Och06	*OreG0021523*
scaffold452	7694829	T	A	Och01,Och10,Och14	*OreG0021525*
scaffold452	7695183	T	A	NA	*OreG0021525*
scaffold452	7696347	G	T	NA	*OreG0021525*
scaffold452	7696354	G	A	NA	*OreG0021525*
scaffold452	7731048	T	C	Ore01,Ore02,Ore03,Ore04,Ore05,Ore06,Ore07,Ore08,Ore09,Ore10,Ore11,Ore12,Ore13,Ore14	*OreG0021528*
scaffold452	7731387	A	T	NA	*OreG0021528*
scaffold452	7795531	A	G	NA	*OreG0021529*
scaffold452	7803544	T	C	NA	*OreG0021531*
scaffold452	7858333	C	A	NA	*OreG0021534*
scaffold452	7901542	C	T	Och01,Och02,Och03,Och04,Och05,Och06,Och07,Och08,Och09,Och10,Och11,Och12,Och13,Och14,Ore08	*OreG0021536*
scaffold452	7901570	C	T	Ore01,Ore02,Ore03,Ore04,Ore05,Ore06,Ore07,Ore08,Ore09,Ore10,Ore11,Ore12,Ore13,Ore14	*OreG0021536*
scaffold452	7915835	A	C	Och01,Och02,Och03,Och04,Och05,Och06,Och07,Och08,Och09,Och10,Och11,Och12,Och13,Och14	*OreG0021537*
scaffold452	7917200	T	C	Och01,Och02,Och03,Och04,Och05,Och06,Och07,Och08,Och09,Och10,Och11,Och12,Och13,Och14,Ore01,Ore02,Ore03,Ore04,Ore05,Ore06,Ore07,Ore08,Ore09,Ore10,Ore11,Ore12,Ore13,Ore14	*OreG0021537*
scaffold452	7962837	T	A	Ore01,Ore02,Ore03,Ore04,Ore05,Ore06,Ore07,Ore08,Ore09,Ore10,Ore11,Ore12,Ore13,Ore14	*OreG0021539*
scaffold452	7963099	C	T	Ore08	*OreG0021539*
scaffold452	7963459	G	A	NA	*OreG0021539*
scaffold452	7978207	G	A	Och01,Och02,Och03,Och04,Och05,Och06,Och07,Och08,Och09,Och10,Och11,Och12,Och13,Och14	*OreG0021541*
scaffold452	7992803	T	C	Och01,Och02,Och03,Och04,Och05,Och06,Och07,Och08,Och09,Och10,Och11,Och12,Och13,Och14	*OreG0021542*
scaffold452	8021620	C	G	Och01,Och02,Och03,Och04,Och05,Och06,Och07,Och08,Och09,Och10,Och11,Och12,Och13,Och14	*OreG0021545*
scaffold452	8025962	G	T	Och01,Och02,Och03,Och04,Och05,Och06,Och07,Och08,Och09,Och10,Och11,Och12,Och13,Och14,Ore01,Ore02,Ore03,Ore04,Ore05,Ore06,Ore07,Ore08,Ore09,Ore10,Ore11,Ore12,Ore13,Ore14	*OreG0021547*
scaffold452	8025980	A	G	Och01,Och02,Och03,Och04,Och05,Och06,Och07,Och08,Och09,Och10,Och11,Och12,Och13,Och14,Ore01,Ore02,Ore03,Ore04,Ore05,Ore06,Ore07,Ore08,Ore09,Ore10,Ore11,Ore12,Ore13,Ore14	*OreG0021547*
scaffold452	8026130	A	T	Ore01,Ore02,Ore03,Ore04,Ore05,Ore06,Ore07,Ore08,Ore09,Ore10,Ore11,Ore12,Ore13,Ore14	*OreG0021547*
scaffold452	8026278	T	A	Och01,Och02,Och03,Och04,Och05,Och06,Och07,Och08,Och09,Och10,Och11,Och12,Och13,Och14,Ore01,Ore02,Ore03,Ore04,Ore05,Ore06,Ore07,Ore08,Ore09,Ore10,Ore11,Ore12,Ore13,Ore14	*OreG0021547*
scaffold452	8026344	T	G	NA	*OreG0021547*
scaffold452	8126968	G	A	NA	*OreG0021553*
scaffold452	8139396	C	T	Ore01,Ore02,Ore08	*OreG0021554*
scaffold452	8139581	C	T	Ore01,Ore02,Ore08	*OreG0021554*
scaffold452	8139773	G	A	Ore01,Ore02,Ore08	*OreG0021554*
scaffold452	8139788	G	A	NA	*OreG0021554*
scaffold452	8139810	C	T	Ore01,Ore02,Ore03,Ore04,Ore05,Ore06,Ore07,Ore08,Ore09,Ore10,Ore11,Ore12,Ore13,Ore14	*OreG0021554*
scaffold452	8140267	C	A	NA	*OreG0021554*
scaffold452	8140599	C	T	NA	*OreG0021554*
scaffold452	8184031	G	T	NA	*OreG0021555*
scaffold452	8184089	A	G	Och03,Och04,Och13	*OreG0021555*
scaffold452	8184676	T	G	Och09,Och10,Och11	*OreG0021555*
scaffold452	8184999	G	T	NA	*OreG0021555*
scaffold452	8193800	T	A	Ore01,Ore02,Ore03,Ore04,Ore05,Ore06,Ore07,Ore08,Ore09,Ore10,Ore11,Ore12,Ore13,Ore14	*OreG0021556*
scaffold452	8197865	T	A	NA	*OreG0021556*
scaffold452	8218788	T	A	NA	*OreG0021559*
scaffold452	8226310	A	G	Och03,Och04,Och13	*OreG0021559*
scaffold452	8226313	A	G	Och01,Och09,Och10,Och11	*OreG0021559*
scaffold452	8232633	G	A	NA	*OreG0021560*
scaffold452	8360062	T	C	NA	*OreG0021564*
scaffold452	8360239	C	T	NA	*OreG0021564*
scaffold452	8360241	G	A	NA	*OreG0021564*

scaffold452	8366085	G	A	NA	*OreG0021564*
scaffold452	8367590	C	T	NA	*OreG0021564*
scaffold452	8370685	G	C	NA	*OreG0021565*
scaffold452	8370745	A	T	NA	*OreG0021565*
scaffold452	8370780	C	A	NA	*OreG0021565*
scaffold452	8370987	G	A	NA	*OreG0021565*
scaffold452	8371045	C	T	NA	*OreG0021565*
scaffold452	8371176	T	C	NA	*OreG0021565*
scaffold452	8371195	G	A	NA	*OreG0021565*
scaffold452	8371290	A	T	NA	*OreG0021565*
scaffold452	8371474	G	A	NA	*OreG0021565*
scaffold452	8394595	C	A	Och01,Och02,Och03,Och04,Och05,Och06,Och07,Och08,Och09,Och10,Och11,Och12,Och13,Och14,Ore01,Ore02,Ore03,Ore04,Ore05,Ore06,Ore07,Ore08,Ore09,Ore10,Ore11,Ore12,Ore13,Ore14	*OreG0021567*
scaffold452	8394940	C	A	Och01,Och02,Och03,Och04,Och05,Och06,Och07,Och08,Och09,Och10,Och11,Och12,Och13,Och14,Ore01,Ore02,Ore03,Ore04,Ore05,Ore06,Ore07,Ore08,Ore09,Ore10,Ore11,Ore12,Ore13,Ore14	*OreG0021567*
scaffold452	8396971	C	T	NA	*OreG0021567*
scaffold452	8513798	T	A	Ore01,Ore02,Ore08	*OreG0021576*
scaffold452	8513819	C	G	NA	*OreG0021576*
scaffold452	8515721	G	C	NA	*OreG0021577*
scaffold452	8516171	T	C	Och01,Och02,Och03,Och04,Och05,Och06,Och07,Och08,Och09,Och10,Och11,Och12,Och13,Och14,Ore01,Ore02,Ore03,Ore04,Ore05,Ore06,Ore07,Ore08,Ore09,Ore10,Ore11,Ore12,Ore13,Ore14	*OreG0021577*
scaffold452	8536457	C	A	Och01,Och02,Och03,Och04,Och05,Och06,Och07,Och08,Och09,Och10,Och11,Och12,Och13,Och14	*OreG0021579*
scaffold452	8544089	T	A	Och09,Och10	*OreG0021580*
scaffold452	8544094	C	A	Och01,Och02,Och03,Och04,Och05,Och06,Och07,Och08,Och09,Och10,Och11,Och12,Och13,Och14	*OreG0021580*
scaffold452	8544510	G	T	Och09,Och10	*OreG0021580*
scaffold452	8582516	C	T	Och01,Och02,Och03,Och04,Och05,Och06,Och07,Och08,Och09,Och10,Och11,Och12,Och13,Och14	*OreG0021584*
scaffold452	8582606	G	A	NA	*OreG0021584*
scaffold452	8604614	T	C	NA	*OreG0021587*
scaffold452	8639372	C	A	Ore08	*OreG0021590*
scaffold452	8648002	C	A	NA	*OreG0021591*
scaffold452	8663268	C	A	Ore01,Ore02,Ore03,Ore04,Ore05,Ore06,Ore07,Ore08,Ore09,Ore10,Ore11,Ore12,Ore13,Ore14	*OreG0021593*
scaffold452	8791948	T	G	NA	*OreG0021600*
scaffold452	8812100	T	C	NA	*OreG0021602*
scaffold452	8812130	G	T	NA	*OreG0021602*
scaffold452	8813960	G	A	NA	*OreG0021602*
scaffold452	8813987	C	T	NA	*OreG0021602*
scaffold452	8814114	G	A	NA	*OreG0021602*
scaffold452	8814417	G	A	NA	*OreG0021602*
scaffold452	8897734	C	T	Ore01,Ore02,Ore03,Ore04,Ore05,Ore06,Ore07,Ore08,Ore09,Ore10,Ore11,Ore12,Ore13,Ore14	*OreG0021608*
scaffold452	8928384	C	T	NA	*OreG0021610*
scaffold452	9003809	G	T	Och01,Och02,Och03,Och04,Och05,Och06,Och07,Och08,Och09,Och10,Och11,Och12,Och13,Och14,Ore01,Ore02,Ore03,Ore04,Ore05,Ore06,Ore07,Ore08,Ore09,Ore10,Ore11,Ore12,Ore13,Ore14	*OreG0021616*
scaffold452	9004400	A	G	NA	*OreG0021616*
scaffold452	9029962	G	T	Och01,Och02,Och03,Och04,Och05,Och06,Och08,Och09,Och10,Och11,Och12,Och13	*OreG0021619*
scaffold452	9030222	A	T	Och01,Och02,Och03,Och04,Och05,Och06,Och07,Och08,Och09,Och10,Och11,Och12,Och13,Och14,Ore01,Ore02,Ore03,Ore04,Ore05,Ore06,Ore07,Ore08,Ore09,Ore10,Ore11,Ore12,Ore13,Ore14	*OreG0021619*
scaffold452	9036944	A	G	NA	*OreG0021621*
scaffold452	9036983	G	A	NA	*OreG0021621*
scaffold452	9037029	G	A	NA	*OreG0021621*
scaffold452	9037059	T	C	NA	*OreG0021621*
scaffold452	9089223	C	G	Och08	*OreG0021625*
scaffold452	9089384	T	A	Och01,Och02,Och03,Och04,Och05,Och06,Och09,Och10,Och11,Och12,Och13	*OreG0021625*
scaffold452	9123623	T	A	NA	*OreG0021628*
scaffold452	9207355	G	A	NA	*OreG0021633*
scaffold452	9211705	C	T	Ore01,Ore02,Ore03,Ore04,Ore05,Ore06,Ore07,Ore08,Ore09,Ore10,Ore11,Ore12,Ore13,Ore14	*OreG0021635*
scaffold452	9215469	T	A	Ore01,Ore02,Ore03,Ore04,Ore05,Ore06,Ore07,Ore08,Ore09,Ore10,Ore11,Ore12,Ore13,Ore14	*OreG0021636*
scaffold452	9226486	C	A	Ore01,Ore02,Ore03,Ore04,Ore05,Ore06,Ore07,Ore08,Ore09,Ore10,Ore11,Ore12,Ore13,Ore14	*OreG0021639*
scaffold452	9265240	C	T	Ore01,Ore02,Ore03,Ore04,Ore05,Ore06,Ore07,Ore08,Ore09,Ore10,Ore11,Ore12,Ore13,Ore14	*OreG0021642*
scaffold452	9265252	G	A	Ore01,Ore02,Ore03,Ore04,Ore05,Ore06,Ore07,Ore08,Ore09,Ore10,Ore11,Ore12,Ore13,Ore14	*OreG0021642*

scaffold452	9265271	G	T	Ore01,Ore02,Ore03,Ore04,Ore05,Ore06,Ore07,Ore08,Ore09,Ore10,Ore11,Ore12,Ore13,Ore14	*OreG0021642*
scaffold452	9300924	C	A	Ore01,Ore02,Ore03,Ore04,Ore05,Ore06,Ore07,Ore08,Ore09,Ore10,Ore11,Ore12,Ore13,Ore14	*OreG0021646*
scaffold452	9300961	A	G	Ore08	*OreG0021646*
scaffold452	9301370	C	T	NA	*OreG0021646*
scaffold452	9306068	C	T	NA	*OreG0021647*
scaffold452	9330490	G	A	NA	*OreG0021647*
scaffold452	9330564	G	C	Och01,Och02,Och03,Och04,Och05,Och06,Och07,Och08,Och09,Och10,Och11,Och12,Och13,Och14	*OreG0021647*
scaffold452	9348223	A	G	Ore01,Ore02,Ore03,Ore04,Ore05,Ore06,Ore07,Ore08,Ore09,Ore10,Ore11,Ore12,Ore13,Ore14	*OreG0021648*
scaffold452	9368598	A	T	Ore01,Ore02,Ore03,Ore04,Ore05,Ore06,Ore07,Ore08,Ore09,Ore10,Ore11,Ore12,Ore13,Ore14	*OreG0021650*
scaffold452	9368634	C	A	Ore01,Ore02,Ore03,Ore04,Ore05,Ore06,Ore07,Ore08,Ore09,Ore10,Ore11,Ore12,Ore13,Ore14	*OreG0021650*
scaffold452	9368766	A	G	Och01,Och02,Och03,Och04,Och05,Och06,Och07,Och08,Och09,Och10,Och11,Och12,Och13,Och14	*OreG0021650*
scaffold452	9368790	C	T	Och09,Och10	*OreG0021650*
scaffold452	9368799	G	A	Och01,Och02,Och03,Och04,Och05,Och06,Och07,Och08,Och09,Och10,Och11,Och12,Och13,Och14,Ore01,Ore02,Ore03,Ore04,Ore05,Ore06,Ore07,Ore08,Ore09,Ore10,Ore11,Ore12,Ore13,Ore14	*OreG0021650*
scaffold452	9368810	G	A	NA	*OreG0021650*
scaffold452	9368847	G	A	Och01,Och02,Och03,Och04,Och05,Och06,Och07,Och08,Och09,Och10,Och11,Och12,Och13,Och14	*OreG0021650*
scaffold452	9368852	G	A	Och01,Och02,Och03,Och04,Och05,Och06,Och07,Och08,Och09,Och10,Och11,Och12,Och13,Och14	*OreG0021650*
scaffold452	9368888	G	A	Och07,Och12	*OreG0021650*
scaffold452	9368910	A	T	Ore01,Ore02,Ore03,Ore04,Ore05,Ore06,Ore07,Ore08,Ore09,Ore10,Ore11,Ore12,Ore13,Ore14	*OreG0021650*
scaffold452	9368927	T	C	Ore01,Ore02,Ore03,Ore04,Ore05,Ore06,Ore07,Ore08,Ore09,Ore10,Ore11,Ore12,Ore13,Ore14	*OreG0021650*
scaffold452	9368969	C	T	NA	*OreG0021650*
scaffold452	9387767	T	A	Och08	*OreG0021651*
scaffold452	9387791	C	T	Och09,Och10	*OreG0021651*
scaffold452	9402045	A	G	NA	*OreG0021652*
scaffold452	9435702	G	T	Och01,Och02,Och03,Och04,Och05,Och06,Och07,Och08,Och09,Och10,Och11,Och12,Och13,Och14,Ore01,Ore02,Ore03,Ore04,Ore05,Ore06,Ore07,Ore08,Ore09,Ore10,Ore11,Ore12,Ore13,Ore14	*OreG0021654*
scaffold452	9446318	A	T	Ore01,Ore02,Ore03,Ore04,Ore05,Ore06,Ore07,Ore08,Ore09,Ore10,Ore11,Ore12,Ore13,Ore14	*OreG0021655*
scaffold452	9457842	A	T	Och01,Och02,Och03,Och04,Och05,Och06,Och07,Och08,Och09,Och10,Och11,Och12,Och13,Och14	*OreG0021655*
scaffold452	9458544	G	A	Ore08	*OreG0021655*
scaffold452	9483340	C	T	NA	*OreG0021657*
scaffold452	9528933	T	G	Och09,Och10	*OreG0021663*
scaffold452	9535137	C	G	Ore01,Ore02,Ore03,Ore04,Ore05,Ore06,Ore07,Ore08,Ore09,Ore10,Ore11,Ore12,Ore13,Ore14	*OreG0021664*
scaffold452	9544622	A	T	NA	*OreG0021666*
scaffold452	9563639	T	A	Och01,Och02,Och03,Och04,Och05,Och06,Och07,Och08,Och09,Och10,Och11,Och12,Och13,Och14,Ore01,Ore02,Ore03,Ore04,Ore05,Ore06,Ore07,Ore08,Ore09,Ore10,Ore11,Ore12,Ore13,Ore14	*OreG0021668*
scaffold452	9573581	G	A	Ore01,Ore02,Ore03,Ore04,Ore05,Ore06,Ore07,Ore08,Ore09,Ore10,Ore11,Ore12,Ore13,Ore14	*OreG0021668*
scaffold452	9577945	G	T	NA	*OreG0021669*
scaffold452	9615125	A	C	Och02,Och03,Och04,Och05,Och06,Och12,Och13	*OreG0021670*
scaffold452	9624725	C	T	Och01,Och02,Och03,Och04,Och05,Och06,Och07,Och08,Och09,Och10,Och11,Och12,Och13,Och14	*OreG0021672*
scaffold452	9635636	G	A	Och01,Och02,Och03,Och04,Och05,Och06,Och07,Och08,Och09,Och10,Och11,Och12,Och13,Och14	*OreG0021674*
scaffold452	9655886	T	C	NA	*OreG0021675*
scaffold452	9677258	C	T	Och01,Och02,Och03,Och04,Och05,Och06,Och07,Och08,Och09,Och10,Och11,Och12,Och13,Och14	*OreG0021676*
scaffold452	9678299	G	A	NA	*OreG0021676*
scaffold452	9742241	A	G	Ore01,Ore02,Ore03,Ore04,Ore05,Ore06,Ore07,Ore08,Ore09,Ore10,Ore11,Ore12,Ore13,Ore14	*OreG0021679*
scaffold452	9751207	C	G	NA	*OreG0021680*
scaffold452	9771058	A	C	NA	*OreG0021681*
scaffold452	9801404	G	T	NA	*OreG0021682*
scaffold452	9801458	A	G	NA	*OreG0021682*
scaffold452	9820466	T	G	Och12	*OreG0021685*
scaffold452	9820512	C	G	Och12	*OreG0021685*
scaffold452	9824922	A	C	NA	*OreG0021686*
scaffold452	9826573	A	T	Och01,Och02,Och03,Och04,Och05,Och06,Och07,Och08,Och09,Och10,Och11,Och12,Och13,Och14,Ore01,Ore02,Ore03,Ore04,Ore05,Ore06,Ore07,Ore08,Ore09,Ore10,Ore11,Ore12,Ore13,Ore14	*OreG0021686*

scaffold452	9828399	A	C	Ore01,Ore02,Ore03,Ore04,Ore05,Ore06,Ore07,Ore08,Ore09,Ore10,Ore11,Ore12,Ore13,Ore14	*OreG0021687*
scaffold452	9828564	G	A	Och01,Och02,Och03,Och04,Och05,Och06,Och07,Och08,Och09,Och10,Och11,Och12,Och13,Och14	*OreG0021687*
scaffold452	9828705	A	C	Ore01,Ore02,Ore03,Ore04,Ore05,Ore06,Ore07,Ore08,Ore09,Ore10,Ore11,Ore12,Ore13,Ore14	*OreG0021687*
scaffold452	9828708	T	A	Och01,Och02,Och03,Och04,Och05,Och06,Och07,Och08,Och09,Och10,Och11,Och12,Och13,Och14	*OreG0021687*
scaffold452	9830683	T	C	Och02,Och03,Och04,Och05,Och06,Och08,Och09,Och10,Och11,Och12,Och13,Och14	*OreG0021688*
scaffold452	9831400	T	C	Och03,Och04,Och08,Och09,Och10,Och13	*OreG0021688*
scaffold452	9831904	G	T	NA	*OreG0021688*
scaffold452	9834158	A	T	NA	*OreG0021689*
scaffold452	1E+07	G	A	NA	*OreG0021704*
scaffold452	1E+07	G	T	Och09,Och10	*OreG0021704*
scaffold452	1E+07	C	A	Ore01,Ore02,Ore03,Ore04,Ore05,Ore06,Ore07,Ore08,Ore09,Ore10,Ore11,Ore12,Ore13,Ore14	*OreG0021708*
scaffold452	1E+07	T	G	NA	*OreG0021710*
scaffold452	1E+07	A	G	NA	*OreG0021710*
scaffold452	1E+07	G	A	Ore01,Ore02,Ore03,Ore04,Ore05,Ore06,Ore07,Ore08,Ore09,Ore10,Ore11,Ore12,Ore13,Ore14	*OreG0021710*
scaffold452	1E+07	C	A	NA	*OreG0021714*
scaffold452	1E+07	A	G	NA	*OreG0021715*
scaffold452	1E+07	T	A	NA	*OreG0021717*
scaffold452	1E+07	A	T	NA	*OreG0021719*
scaffold452	1E+07	G	C	Och01,Och02,Och06,Och07,Och08,Och09,Och10,Och12	*OreG0021719*
scaffold452	1E+07	A	C	Och01,Och02,Och03,Och04,Och05,Och06,Och07,Och08,Och09,Och10,Och11,Och12,Och13,Och14	*OreG0021719*
scaffold452	1E+07	G	A	NA	*OreG0021720*
scaffold452	1E+07	T	G	Och07,Och09,Och10	*OreG0021721*
scaffold452	1E+07	T	A	Och02,Och03,Och04,Och05,Och06,Och07,Och08,Och09,Och10,Och11,Och12,Och13,Och14	*OreG0021731*
scaffold452	1E+07	T	C	Ore01,Ore02,Ore03,Ore04,Ore05,Ore06,Ore07,Ore08,Ore09,Ore10,Ore11,Ore12,Ore13,Ore14	*OreG0021731*
scaffold452	1E+07	A	T	NA	*OreG0021731*
scaffold452	1E+07	C	A	Och02,Och03,Och04,Och05,Och06,Och07,Och08,Och09,Och10,Och11,Och12,Och13,Och14,Ore01,Ore02,Ore03,Ore04,Ore05,Ore06,Ore07,Ore08,Ore09,Ore10,Ore11,Ore12,Ore13,Ore14	*OreG0021731*
scaffold452	1E+07	G	T	Ore01,Ore02,Ore08	*OreG0021732*
scaffold452	1.1E+07	C	T	Och02,Och06,Och07,Och12	*OreG0021735*
scaffold452	1.1E+07	G	A	Och11,Och14	*OreG0021735*
scaffold452	1.1E+07	G	T	NA	*OreG0021736*
scaffold452	1.1E+07	C	A	NA	*OreG0021737*
scaffold452	1.1E+07	G	T	Ore04,Ore05	*OreG0021744*
scaffold452	1.1E+07	T	C	Och01,Och02,Och03,Och04,Och05,Och06,Och07,Och08,Och09,Och10,Och11,Och12,Och13,Och14,Ore01,Ore02,Ore03,Ore06,Ore07,Ore08,Ore09,Ore10,Ore11,Ore12,Ore13,Ore14	*OreG0021744*
scaffold452	1.1E+07	A	C	Och03,Och05,Och07,Och08,Och12,Och13,Och14,Ore01,Ore02,Ore03,Ore06,Ore07,Ore08,Ore09,Ore10,Ore11,Ore12,Ore13,Ore14	*OreG0021744*
scaffold452	1.1E+07	C	T	Ore04,Ore05	*OreG0021744*
scaffold452	1.1E+07	C	T	Och01,Och02,Och03,Och04,Och05,Och06,Och07,Och08,Och09,Och10,Och11,Och12,Och13,Och14,Ore01,Ore02,Ore03,Ore04,Ore05,Ore06,Ore07,Ore08,Ore09,Ore10,Ore11,Ore12,Ore13,Ore14	*OreG0021745*
scaffold452	1.1E+07	G	A	Och02,Och03,Och04,Och05,Och06,Och07,Och08,Och11,Och12,Och13,Och14,Ore04,Ore05	*OreG0021745*
scaffold452	1.1E+07	G	T	Och08	*OreG0021755*
scaffold452	1.1E+07	G	C	NA	*OreG0021756*
scaffold452	1.1E+07	C	A	NA	*OreG0021757*
scaffold452	1.1E+07	C	G	NA	*OreG0021757*
scaffold452	1.1E+07	C	T	NA	*OreG0021757*
scaffold452	1.1E+07	G	T	NA	*OreG0021757*
scaffold452	1.1E+07	C	T	Och08	*OreG0021757*
scaffold452	1.1E+07	G	T	Och01,Och06,Och09,Och10,Och11,Och14	*OreG0021757*
scaffold452	1.1E+07	A	G	NA	*OreG0021758*
scaffold452	1.1E+07	T	C	Och08	*OreG0021758*
scaffold452	1.1E+07	G	A	Och01,Och06,Och09,Och10,Och11,Och14	*OreG0021758*
scaffold452	1.1E+07	G	A	Ore04,Ore05	*OreG0021759*
scaffold452	1.1E+07	A	C	NA	*OreG0021759*
scaffold452	1.1E+07	G	A	Ore04,Ore05	*OreG0021759*
scaffold452	1.1E+07	G	A	Och08,Ore01,Ore02,Ore03,Ore04,Ore05,Ore06,Ore07,Ore08,Ore09,Ore10,Ore11,Ore12,Ore13,Ore14	*OreG0021759*
scaffold452	1.1E+07	T	A	NA	*OreG0021760*
scaffold452	1.1E+07	G	A	NA	*OreG0021761*
scaffold452	1.1E+07	A	T	NA	*OreG0021761*
scaffold452	1.1E+07	G	A	Och06,Och07,Och08,Och09,Och10,Och11,Och14	*OreG0021764*

scaffold452	1.1E+07	A	T	Och08	*OreG0021764*
scaffold452	1.1E+07	A	T	Ore01,Ore02,Ore03,Ore04,Ore05,Ore06,Ore07,Ore08,Ore09,Ore10,Ore11,Ore12,Ore13,Ore14	*OreG0021765*
scaffold452	1.1E+07	G	A	NA	*OreG0021766*
scaffold452	1.1E+07	T	A	NA	*OreG0021766*
scaffold452	1.1E+07	T	C	NA	*OreG0021766*
scaffold452	1.1E+07	C	T	Och06,Och07	*OreG0021766*
scaffold452	1.1E+07	A	T	Ore01,Ore02,Ore03,Ore04,Ore05,Ore06,Ore07,Ore08,Ore09,Ore10,Ore11,Ore12,Ore13,Ore14	*OreG0021766*
scaffold452	1.1E+07	T	A	NA	*OreG0021768*
scaffold452	1.1E+07	G	A	NA	*OreG0021772*
scaffold452	1.1E+07	C	G	Ore04,Ore05	*OreG0021772*
scaffold33118	210395	T	A	NA	*OreG0013062*
scaffold33118	210615	A	G	Ore01,Ore02,Ore03,Ore04,Ore05,Ore06,Ore07,Ore08,Ore09,Ore10,Ore11,Ore12,Ore13,Ore14	*OreG0013062*
scaffold33118	214853	A	T	Och01,Och02,Och03,Och04,Och05,Och06,Och07,Och08,Och09,Och10,Och11,Och12,Och13,Och14	*OreG0013062*
scaffold33118	603230	C	T	Och01,Och02,Och03,Och04,Och05,Och06,Och07,Och08,Och09,Och10,Och11,Och12,Och13,Och14,Ore01,Ore02,Ore03,Ore04,Ore05,Ore06,Ore07,Ore08,Ore09,Ore10,Ore11,Ore12,Ore13,Ore14	*OreG0013066*
scaffold33118	603263	A	G	Och01,Och02,Och03,Och04,Och05,Och06,Och07,Och08,Och09,Och10,Och11,Och12,Och13,Och14,Ore01,Ore02,Ore03,Ore04,Ore05,Ore06,Ore07,Ore08,Ore09,Ore10,Ore11,Ore12,Ore13,Ore14	*OreG0013066*
scaffold33118	608190	G	T	Och01,Och02,Och03,Och04,Och05,Och06,Och07,Och08,Och09,Och10,Och11,Och12,Och13,Och14	*OreG0013067*
scaffold33230	18116	C	T	NA	*OreG0017177*
scaffold33230	70798	C	G	Och01,Och02,Och03,Och04,Och05,Och06,Och07,Och08,Och09,Och10,Och11,Och12,Och13,Och14,Ore01,Ore02,Ore03,Ore04,Ore05,Ore06,Ore07,Ore08,Ore09,Ore10,Ore11,Ore12,Ore13,Ore14	*OreG0017179*
scaffold33230	70987	G	A	NA	*OreG0017179*
scaffold33230	70994	T	A	NA	*OreG0017179*
scaffold33230	71053	G	A	NA	*OreG0017179*
scaffold33230	73926	C	A	NA	*OreG0017179*
scaffold33210	35257	A	T	Ore03,Ore08,Ore09,Ore14	*OreG0016297*
scaffold33210	97325	A	T	Och01,Och02,Och03,Och04,Och05,Och06,Och07,Och08,Och09,Och10,Och11,Och12,Och13,Och14,Ore01,Ore02,Ore03,Ore04,Ore05,Ore06,Ore07,Ore08,Ore09,Ore10,Ore11,Ore12,Ore13,Ore14	*OreG0016300*
scaffold33210	99976	C	T	Och01,Och02,Och03,Och04,Och05,Och06,Och07,Och08,Och09,Och10,Och11,Och12,Och13,Och14,Ore01,Ore02,Ore03,Ore04,Ore05,Ore06,Ore07,Ore08,Ore09,Ore10,Ore11,Ore12,Ore13,Ore14	*OreG0016300*
scaffold33210	273699	A	T	Ore01,Ore02,Ore03,Ore04,Ore05,Ore06,Ore07,Ore08,Ore09,Ore10,Ore11,Ore12,Ore13,Ore14	*OreG0016309*
scaffold33210	313579	A	G	Och01,Och02,Och03,Och04,Och05,Och06,Och07,Och08,Och09,Och10,Och11,Och12,Och13,Och14	*OreG0016310*
scaffold33210	336624	G	T	Och01,Och02,Och03,Och04,Och05,Och06,Och07,Och08,Och09,Och10,Och11,Och12,Och13,Och14	*OreG0016311*
scaffold33210	338069	C	A	Och01,Och02,Och03,Och04,Och05,Och06,Och07,Och08,Och09,Och10,Och11,Och12,Och13,Och14	*OreG0016311*
scaffold33210	351474	T	A	NA	*OreG0016312*
scaffold33210	483959	G	T	Ore01,Ore02,Ore03,Ore04,Ore05,Ore06,Ore07,Ore08,Ore09,Ore10,Ore11,Ore12,Ore13,Ore14	*OreG0016322*
scaffold33210	483963	T	C	NA	*OreG0016322*
scaffold33210	484033	A	G	Ore01,Ore02,Ore03,Ore04,Ore05,Ore06,Ore07,Ore08,Ore09,Ore10,Ore11,Ore12,Ore13,Ore14	*OreG0016322*
scaffold33210	491417	G	A	Och01,Och02,Och03,Och04,Och05,Och06,Och07,Och08,Och09,Och10,Och11,Och12,Och13,Och14,Ore01,Ore02,Ore03,Ore04,Ore05,Ore06,Ore07,Ore08,Ore09,Ore10,Ore11,Ore12,Ore13,Ore14	*OreG0016323*
scaffold33210	491446	A	C	Ore01,Ore02,Ore03,Ore04,Ore05,Ore06,Ore07,Ore08,Ore09,Ore10,Ore11,Ore12,Ore13,Ore14	*OreG0016323*
scaffold33210	491514	C	T	Ore01,Ore02,Ore03,Ore04,Ore05,Ore06,Ore07,Ore08,Ore09,Ore10,Ore11,Ore12,Ore13,Ore14	*OreG0016323*
scaffold33210	491543	G	A	Och03,Och04,Och05,Och08,Och09,Och10,Och11,Och12,Och13,Och14	*OreG0016323*
scaffold33210	491637	G	A	Och01,Och02,Och03,Och04,Och05,Och06,Och07,Och08,Och09,Och10,Och11,Och12,Och13,Och14,Ore01,Ore02,Ore03,Ore04,Ore05,Ore06,Ore07,Ore08,Ore09,Ore10,Ore11,Ore12,Ore13,Ore14	*OreG0016323*
scaffold33210	491645	T	A	Ore01,Ore02,Ore03,Ore04,Ore05,Ore06,Ore07,Ore08,Ore09,Ore10,Ore11,Ore12,Ore13,Ore14	*OreG0016323*
scaffold33210	491693	G	A	Och01,Och02,Och03,Och04,Och05,Och06,Och07,Och08,Och09,Och10,Och11,Och12,Och13,Och14,Ore01,Ore02,Ore03,Ore04,Ore05,Ore06,Ore07,Ore08,Ore09,Ore10,Ore11,Ore12,Ore13,Ore14	*OreG0016323*
scaffold33210	491724	C	T	Och01,Och02,Och03,Och04,Och05,Och06,Och07,Och08,Och09,Och10,Och11,Och12,Och13,Och14	*OreG0016323*
scaffold33210	495226	G	T	Och01,Och02,Och03,Och04,Och05,Och06,Och07,Och08,Och09,Och10,Och11,Och12,Och13,Och14,Ore01,Ore02,Ore03,Ore04,Ore05,Ore06,Ore07,Ore08,Ore09,Ore10,Ore11,Ore12,Ore13,Ore14	*OreG0016324*
scaffold33210	495339	C	G	NA	*OreG0016324*

scaffold33210	501218	T	C	Och01,Och02,Och03,Och04,Och05,Och06,Och07,Och08,Och09,Och10,Och11,Och12,Och13,Och14,Ore01,Ore02,Ore03,Ore04,Ore05,Ore06,Ore07,Ore08,Ore09,Ore10,Ore11,Ore12,Ore13,Ore14	*OreG0016324*
scaffold33210	530230	C	T	Ore01,Ore02,Ore03,Ore04,Ore05,Ore06,Ore07,Ore08,Ore09,Ore10,Ore11,Ore12,Ore13,Ore14	*OreG0016325*
scaffold33210	640608	C	T	Ore03,Ore08,Ore09,Ore10,Ore14	*OreG0016329*
scaffold33210	688288	T	C	NA	*OreG0016332*
scaffold33210	688300	C	T	NA	*OreG0016332*
scaffold33210	695085	G	C	Och05	*OreG0016333*
scaffold33210	695086	C	T	Och01,Och02,Och03,Och04,Och05,Och06,Och07,Och08,Och09,Och10,Och11,Och12,Och13,Och14,Ore01,Ore02,Ore03,Ore04,Ore05,Ore06,Ore07,Ore08,Ore09,Ore10,Ore11,Ore12,Ore13,Ore14	*OreG0016333*
scaffold33210	695453	G	C	Ore01,Ore02,Ore03,Ore04,Ore05,Ore06,Ore07,Ore08,Ore09,Ore10,Ore11,Ore12,Ore13,Ore14	*OreG0016333*
scaffold33210	695467	G	A	Ore01,Ore02,Ore03,Ore04,Ore05,Ore06,Ore07,Ore08,Ore09,Ore10,Ore11,Ore12,Ore13,Ore14	*OreG0016333*
scaffold33210	696437	T	A	NA	*OreG0016334*
scaffold33210	707366	C	T	Och09,Och10	*OreG0016335*
scaffold33210	708862	G	A	NA	*OreG0016335*
scaffold33210	709002	C	T	NA	*OreG0016335*
scaffold33210	709030	C	T	NA	*OreG0016335*
scaffold33210	712590	C	T	Och09,Och10	*OreG0016336*
scaffold33210	712721	C	G	NA	*OreG0016336*
scaffold33210	712817	G	C	Och09,Och10	*OreG0016336*
scaffold33210	712967	G	A	Och09,Och10	*OreG0016336*
scaffold33210	713135	T	G	Och02,Och03,Och04,Och05,Och06,Och12,Och13	*OreG0016336*
scaffold33210	713181	C	T	NA	*OreG0016336*
scaffold33210	721762	G	A	Ore01,Ore02,Ore03,Ore04,Ore05,Ore06,Ore07,Ore08,Ore09,Ore10,Ore11,Ore12,Ore13,Ore14	*OreG0016337*
scaffold33210	721837	C	T	NA	*OreG0016337*
scaffold33210	722158	C	T	NA	*OreG0016337*
scaffold33210	728340	C	A	NA	*OreG0016338*
scaffold33225	71401	A	T	Ore01,Ore02,Ore03,Ore04,Ore05,Ore06,Ore07,Ore08,Ore09,Ore10,Ore11,Ore12,Ore13,Ore14	*OreG0017059*
scaffold33225	93763	T	A	NA	*OreG0017061*
scaffold33225	98940	A	G	Ore01,Ore02,Ore03,Ore04,Ore05,Ore06,Ore07,Ore08,Ore09,Ore10,Ore11,Ore12,Ore13,Ore14	*OreG0017062*
scaffold33225	98985	A	G	Ore01,Ore02,Ore03,Ore04,Ore05,Ore06,Ore07,Ore08,Ore09,Ore10,Ore11,Ore12,Ore13,Ore14	*OreG0017062*
scaffold33225	117963	G	T	NA	*OreG0017064*
scaffold33225	117988	C	T	NA	*OreG0017064*
scaffold33225	118221	G	T	NA	*OreG0017064*
scaffold33225	119123	C	T	NA	*OreG0017064*
scaffold33225	157377	C	T	NA	*OreG0017066*
scaffold33225	212348	C	T	NA	*OreG0017072*
scaffold33213	83582	G	A	Och06,Och08	*OreG0016430*
scaffold33213	166307	A	C	Ore02,Ore03,Ore04,Ore05,Ore08,Ore09,Ore10,Ore13,Ore14	*OreG0016434*
scaffold33213	166584	A	G	NA	*OreG0016434*
scaffold33213	208239	T	A	Och01,Och02,Och03,Och04,Och05,Och06,Och07,Och08,Och09,Och10,Och11,Och12,Och13,Och14,Ore01,Ore02,Ore03,Ore04,Ore05,Ore06,Ore07,Ore08,Ore09,Ore10,Ore11,Ore12,Ore13,Ore14	*OreG0016439*
scaffold33213	208712	A	T	Och06	*OreG0016439*
scaffold33213	275060	C	G	Ore04,Ore05	*OreG0016444*
scaffold33213	279449	C	T	NA	*OreG0016444*
scaffold33213	280286	A	G	Och06	*OreG0016444*
scaffold33213	292163	A	T	Och01,Och02,Och03,Och04,Och05,Och06,Och07,Och08,Och09,Och10,Och11,Och12,Och13,Och14,Ore01,Ore02,Ore03,Ore04,Ore05,Ore06,Ore07,Ore08,Ore09,Ore10,Ore11,Ore12,Ore13,Ore14	*OreG0016446*
scaffold33213	292248	C	G	Och01,Och02,Och03,Och04,Och05,Och06,Och07,Och08,Och09,Och10,Och11,Och12,Och13,Och14,Ore01,Ore02,Ore03,Ore04,Ore05,Ore06,Ore07,Ore08,Ore09,Ore10,Ore11,Ore12,Ore13,Ore14	*OreG0016446*
scaffold33213	294996	A	G	NA	*OreG0016446*
scaffold33213	295071	T	G	Ore04,Ore05	*OreG0016446*
scaffold33213	295703	T	A	Och01,Och02,Och03,Och04,Och05,Och06,Och07,Och08,Och09,Och10,Och11,Och12,Och13,Och14,Ore01,Ore02,Ore03,Ore04,Ore05,Ore06,Ore07,Ore08,Ore09,Ore10,Ore11,Ore12,Ore13,Ore14	*OreG0016446*
scaffold33213	301753	G	C	NA	*OreG0016448*
scaffold33213	301760	A	G	Ore03,Ore04,Ore05,Ore07,Ore08,Ore09,Ore11,Ore12,Ore14	*OreG0016448*
scaffold33213	310329	G	T	Och13	*OreG0016449*
scaffold33213	319617	G	T	NA	*OreG0016451*
scaffold33213	336720	T	G	NA	*OreG0016454*
scaffold33213	336723	T	A	Och01,Och02,Och03,Och04,Och05,Och06,Och07,Och08,Och11,Och12,Och13,Och14,Ore01,Ore02,Ore03,Ore04,Ore05,Ore06,Ore07,Ore08,Ore09,Ore10,Ore11,Ore12,Ore13,Ore14	*OreG0016454*
scaffold33213	449195	G	C	Och12,Och13	*OreG0016459*

scaffold33213	561182	C	T	Ore01,Ore02,Ore03,Ore04,Ore05,Ore06,Ore07,Ore08,Ore09,Ore10,Ore11,Ore12,Ore13,Ore14	*OreG0016462*
scaffold33213	562482	G	A	Ore01,Ore02,Ore03,Ore04,Ore05,Ore06,Ore07,Ore08,Ore09,Ore10,Ore11,Ore12,Ore13,Ore14	*OreG0016462*
scaffold33213	581544	A	T	Och01,Och02,Och03,Och04,Och05,Och06,Och07,Och08,Och09,Och10,Och11,Och12,Och13,Och14,Ore01,Ore02,Ore03,Ore04,Ore05,Ore06,Ore07,Ore08,Ore09,Ore10,Ore11,Ore12,Ore13,Ore14	*OreG0016465*
scaffold33226	60830	G	A	NA	*OreG0017083*
scaffold33241	19815	A	C	Och01,Och02,Och03,Och04,Och05,Och06,Och07,Och08,Och09,Och10,Och11,Och12,Och13,Och14,Ore01,Ore02,Ore03,Ore04,Ore05,Ore06,Ore07,Ore08,Ore09,Ore10,Ore11,Ore12,Ore13,Ore14	*OreG0017393*
scaffold33241	27227	C	T	NA	*OreG0017394*
scaffold33212	236298	C	G	NA	*OreG0016403*
scaffold33212	260168	C	T	Och01,Och02,Och03,Och04,Och05,Och06,Och07,Och08,Och09,Och10,Och11,Och12,Och13,Och14	*OreG0016404*
scaffold33212	260188	G	A	NA	*OreG0016404*
scaffold33212	281082	T	C	Ore04,Ore05	*OreG0016405*
scaffold33212	298308	G	T	Och01,Och02,Och03,Och04,Och05,Och06,Och07,Och08,Och09,Och10,Och11,Och12,Och13,Och14,Ore01,Ore02,Ore03,Ore04,Ore05,Ore06,Ore07,Ore08,Ore09,Ore10,Ore11,Ore12,Ore13,Ore14	*OreG0016406*
scaffold33212	300122	A	C	Och06,Och14	*OreG0016406*
scaffold33212	534165	G	C	NA	*OreG0016419*
scaffold33212	534324	G	A	Och04,Och05,Och11,Och12,Och13	*OreG0016419*
scaffold33212	534387	C	T	NA	*OreG0016419*
scaffold33212	542994	G	A	Ore04,Ore05	*OreG0016419*
scaffold33212	543039	G	A	Ore01,Ore02,Ore03,Ore04,Ore05,Ore06,Ore07,Ore08,Ore09,Ore10,Ore11,Ore12,Ore13,Ore14	*OreG0016419*
scaffold33212	544130	C	A	Och01	*OreG0016419*
scaffold33212	546577	G	C	Och02,Och07,Och14	*OreG0016421*
scaffold33212	547153	T	C	NA	*OreG0016421*
scaffold33212	563986	C	T	Och01	*OreG0016422*
scaffold33176	45012	G	A	NA	*OreG0014628*
scaffold33176	153451	C	A	Ore01,Ore02,Ore03,Ore04,Ore05,Ore07,Ore08,Ore09,Ore10,Ore14	*OreG0014637*
scaffold33176	153515	A	C	Ore01,Ore02,Ore03,Ore04,Ore05,Ore06,Ore07,Ore08,Ore09,Ore10,Ore11,Ore12,Ore13,Ore14	*OreG0014637*
scaffold33195	64421	C	T	Ore02,Ore03,Ore09,Ore10,Ore11,Ore12,Ore14	*OreG0015094*
scaffold33195	65214	G	T	NA	*OreG0015095*
scaffold33195	65940	T	A	NA	*OreG0015095*
scaffold33195	66251	C	G	Och01,Och02,Och03,Och04,Och05,Och07,Och12,Och13	*OreG0015095*
scaffold33195	66748	G	A	Och01,Och02,Och03,Och04,Och05,Och12,Och13	*OreG0015095*
scaffold33195	104555	C	A	NA	*OreG0015100*
scaffold33195	106449	C	T	NA	*OreG0015100*
scaffold33195	106566	T	A	NA	*OreG0015100*
scaffold33195	116499	T	C	NA	*OreG0015101*
scaffold33195	116759	A	G	NA	*OreG0015101*
scaffold33195	116786	G	A	NA	*OreG0015101*
scaffold33195	116790	C	T	NA	*OreG0015101*
scaffold33195	116802	T	A	NA	*OreG0015101*
scaffold33195	117416	G	A	NA	*OreG0015101*
scaffold33195	118298	T	C	NA	*OreG0015101*
scaffold33195	118560	T	C	NA	*OreG0015101*
scaffold33195	118731	C	A	NA	*OreG0015101*
scaffold33195	119893	G	C	Ore02,Ore03,Ore09,Ore10,Ore11,Ore12,Ore14	*OreG0015101*
scaffold33195	120136	C	A	NA	*OreG0015101*
scaffold33195	120217	G	T	Och02,Och03,Och04,Och05,Och06,Och07,Och09,Och10,Och11,Och12,Och13,Och14,Ore01,Ore02,Ore03,Ore04,Ore05,Ore06,Ore07,Ore08,Ore09,Ore10,Ore11,Ore12,Ore13,Ore14	*OreG0015101*
scaffold33195	241182	C	G	NA	*OreG0015111*
scaffold33195	241890	C	A	NA	*OreG0015111*
scaffold33195	242762	G	A	NA	*OreG0015111*
scaffold33195	355810	T	A	Och01,Och02,Och03,Och04,Och05,Och06,Och07,Och08,Och09,Och10,Och11,Och12,Och13,Och14	*OreG0015122*
scaffold33195	361994	G	T	Och01,Och02,Och03,Och04,Och05,Och06,Och07,Och08,Och09,Och10,Och11,Och12,Och13,Och14	*OreG0015123*
scaffold33195	380021	G	A	NA	*OreG0015126*
scaffold33195	383597	A	G	NA	*OreG0015127*
scaffold33195	403953	G	A	Och01,Och02,Och03,Och04,Och05,Och06,Och09,Och10,Och12,Och13	*OreG0015129*
scaffold33195	453169	G	A	Ore01,Ore02,Ore03,Ore04,Ore05,Ore06,Ore07,Ore08,Ore09,Ore10,Ore11,Ore12,Ore13,Ore14	*OreG0015134*
scaffold33195	454590	T	C	NA	*OreG0015135*
scaffold33195	454788	A	G	Och07	*OreG0015135*
scaffold33195	466215	G	T	NA	*OreG0015136*
scaffold33195	477583	C	G	Ore02,Ore03,Ore04,Ore05,Ore06,Ore07,Ore08,Ore09,Ore10,Ore11,Ore12,Ore13,Ore14	*OreG0015137*
scaffold33195	478278	T	C	NA	*OreG0015137*

scaffold33195	482838	A	T	Ore01,Ore02,Ore03,Ore04,Ore05,Ore06,Ore07,Ore08,Ore09,Ore10,Ore11,Ore12,Ore13,Ore14	*OreG0015137*
scaffold33195	499477	C	T	Och01,Och02,Och03,Och04,Och05,Och06,Och07,Och08,Och09,Och10,Och11,Och12,Och13,Och14	*OreG0015139*
scaffold33195	500026	A	G	NA	*OreG0015139*
scaffold33195	500269	T	A	Och01,Och02,Och03,Och04,Och05,Och06,Och07,Och08,Och09,Och10,Och11,Och12,Och13,Och14	*OreG0015139*
scaffold33195	500449	G	T	Ore01,Ore02,Ore03,Ore04,Ore05,Ore06,Ore07,Ore08,Ore09,Ore10,Ore11,Ore12,Ore13,Ore14	*OreG0015139*
scaffold33195	542200	G	A	NA	*OreG0015143*
scaffold33195	542302	G	A	NA	*OreG0015143*
scaffold33195	542315	C	T	NA	*OreG0015143*
scaffold33195	542734	A	C	NA	*OreG0015143*
scaffold33195	549425	G	A	Och11	*OreG0015144*
scaffold33195	550410	G	A	NA	*OreG0015144*
scaffold33195	558901	A	C	NA	*OreG0015145*
scaffold33195	561124	A	G	NA	*OreG0015145*
scaffold33195	583593	T	C	NA	*OreG0015147*
scaffold33195	584073	A	G	Och01,Och02,Och03,Och04,Och05,Och06,Och07,Och08,Och09,Och10,Och11,Och12,Och13,Och14,Ore01,Ore02,Ore03,Ore04,Ore05,Ore06,Ore07,Ore08,Ore09,Ore10,Ore11,Ore12,Ore13,Ore14	*OreG0015147*
scaffold33195	584100	C	G	Ore02,Ore03,Ore04,Ore05,Ore06,Ore07,Ore08,Ore09,Ore10,Ore11,Ore12,Ore13,Ore14	*OreG0015147*
scaffold33195	586401	T	G	Och01,Och06,Och07,Och08,Och11,Och12,Och14	*OreG0015147*
scaffold33195	606540	C	G	NA	*OreG0015150*
scaffold33195	617691	G	T	NA	*OreG0015151*
scaffold33236	22926	A	G	NA	*OreG0017271*
scaffold33236	23617	A	G	NA	*OreG0017271*
scaffold33236	24362	A	C	Ore01,Ore02,Ore03,Ore04,Ore05,Ore06,Ore07,Ore08,Ore09,Ore10,Ore11,Ore12,Ore13,Ore14	*OreG0017271*
scaffold33236	53241	G	C	NA	*OreG0017274*
scaffold33236	115469	C	G	Ore01,Ore02,Ore03,Ore04,Ore05,Ore06,Ore07,Ore08,Ore09,Ore10,Ore11,Ore12,Ore13,Ore14	*OreG0017277*
scaffold33236	115557	A	G	Och01,Och02,Och03,Och04,Och05,Och06,Och07,Och08,Och09,Och10,Och11,Och12,Och13,Och14	*OreG0017277*
scaffold33236	117018	G	A	Och01,Och02,Och03,Och04,Och05,Och06,Och07,Och08,Och09,Och10,Och11,Och12,Och13,Och14	*OreG0017279*
scaffold33236	135837	T	C	Och01,Och02,Och03,Och04,Och05,Och06,Och07,Och08,Och09,Och10,Och11,Och12,Och13,Och14,Ore01,Ore02,Ore03,Ore04,Ore05,Ore06,Ore07,Ore08,Ore09,Ore10,Ore11,Ore12,Ore13,Ore14	*OreG0017280*
scaffold33236	136056	A	T	NA	*OreG0017280*
scaffold33236	136134	A	T	Och01,Och02,Och03,Och04,Och05,Och06,Och07,Och08,Och09,Och10,Och11,Och12,Och13,Och14,Ore01,Ore02,Ore03,Ore04,Ore05,Ore06,Ore07,Ore08,Ore09,Ore10,Ore11,Ore12,Ore13,Ore14	*OreG0017280*
scaffold33182	25518	C	T	Ore09,Ore13,Ore14	*OreG0014653*
scaffold33182	101243	A	C	NA	*OreG0014657*
scaffold33182	313471	G	A	Och01,Och02,Och03,Och04,Och05,Och06,Och07,Och08,Och09,Och10,Och11,Och12,Och13,Och14,Ore01,Ore02,Ore03,Ore04,Ore05,Ore06,Ore07,Ore08,Ore09,Ore10,Ore11,Ore12,Ore13,Ore14	*OreG0014666*
scaffold33182	313567	G	A	NA	*OreG0014666*
scaffold33182	313659	T	C	NA	*OreG0014666*
scaffold33182	314846	G	A	Och02,Och03,Och04,Och05,Och06,Och09,Och10,Och11,Och12,Och13,Och14,Ore08,Ore10,Ore12	*OreG0014666*
scaffold33182	314879	G	A	NA	*OreG0014666*
scaffold33182	379178	G	A	Och06,Och13	*OreG0014669*
scaffold33182	379250	G	A	Och01,Och02,Och03,Och04,Och05,Och06,Och07,Och08,Och09,Och10,Och11,Och12,Och13,Och14,Ore01,Ore02,Ore03,Ore04,Ore05,Ore06,Ore07,Ore08,Ore09,Ore10,Ore11,Ore12,Ore13,Ore14	*OreG0014669*
scaffold33182	379528	G	T	Och01,Och02,Och03,Och04,Och05,Och06,Och07,Och08,Och09,Och10,Och11,Och12,Och13,Och14,Ore01,Ore02,Ore03,Ore04,Ore05,Ore06,Ore07,Ore08,Ore09,Ore10,Ore11,Ore12,Ore13,Ore14	*OreG0014669*
scaffold33182	379597	G	A	Och11	*OreG0014669*
scaffold33182	629898	G	A	NA	*OreG0014684*
scaffold33216	16116	G	T	Och01,Och02,Och03,Och04,Och05,Och06,Och07,Och08,Och09,Och10,Och11,Och12,Och13,Och14	*OreG0016472*
scaffold33216	16623	C	T	Ore01,Ore02,Ore03,Ore04,Ore05,Ore06,Ore07,Ore08,Ore09,Ore10,Ore11,Ore12,Ore13,Ore14	*OreG0016472*
scaffold33216	16628	T	C	Och01,Och02,Och03,Och04,Och05,Och06,Och07,Och08,Och09,Och10,Och11,Och12,Och13,Och14,Ore01,Ore02,Ore03,Ore04,Ore05,Ore06,Ore07,Ore08,Ore09,Ore10,Ore11,Ore12,Ore13,Ore14	*OreG0016472*
scaffold33216	16632	A	G	Och14	*OreG0016472*
scaffold33216	16789	G	A	NA	*OreG0016472*
scaffold33216	16794	A	G	Och06	*OreG0016472*
scaffold33216	16953	C	G	NA	*OreG0016472*
scaffold33216	17202	A	T	Ore01,Ore02,Ore03,Ore04,Ore05,Ore06,Ore07,Ore08,Ore09,Ore10,Ore11,Ore12,Ore13,Ore14	*OreG0016472*

scaffold33216	17541	G	A	Ore01,Ore02,Ore03,Ore06,Ore08,Ore09,Ore10,Ore14	*OreG0016472*
scaffold33216	56302	A	T	Och01,Och02,Och03,Och04,Och05,Och06,Och07,Och08,Och09,Och10,Och11,Och12,Och13,Och14,Ore01,Ore02,Ore03,Ore04,Ore05,Ore06,Ore07,Ore08,Ore09,Ore10,Ore11,Ore12,Ore13,Ore14	*OreG0016477*
scaffold33216	103191	C	G	Och02,Och03,Och04,Och05,Och06,Och12,Och13	*OreG0016479*
scaffold33216	133746	A	G	Och01	*OreG0016482*
scaffold33216	133873	C	T	NA	*OreG0016482*
scaffold33216	135558	T	G	Och01,Och02,Och03,Och04,Och05,Och06,Och07,Och08,Och09,Och10,Och11,Och12,Och13,Och14	*OreG0016484*
scaffold33216	156286	C	T	NA	*OreG0016486*
scaffold33216	156338	T	A	Och01,Och02,Och03,Och04,Och05,Och06,Och07,Och08,Och09,Och10,Och11,Och12,Och13,Och14,Ore01,Ore02,Ore03,Ore04,Ore05,Ore06,Ore07,Ore08,Ore09,Ore10,Ore11,Ore12,Ore13,Ore14	*OreG0016486*
scaffold33216	156859	G	A	NA	*OreG0016486*
scaffold33216	187542	C	T	NA	*OreG0016488*
scaffold33216	187546	G	A	Och07	*OreG0016488*
scaffold33216	187639	G	A	NA	*OreG0016488*
scaffold33216	193613	A	G	NA	*OreG0016489*
scaffold33216	194420	G	A	NA	*OreG0016489*
scaffold33216	194643	C	G	NA	*OreG0016489*
scaffold33216	196124	C	T	Och01,Och02,Och03,Och04,Och05,Och06,Och07,Och08,Och09,Och10,Och11,Och12,Och13,Och14	*OreG0016490*
scaffold33216	196253	T	C	NA	*OreG0016490*
scaffold33216	197629	G	A	Och01,Och06	*OreG0016491*
scaffold33216	197644	C	A	Och09,Och10,Och11	*OreG0016491*
scaffold33216	197701	C	G	Och09,Och10	*OreG0016491*
scaffold33216	222911	A	C	Ore01,Ore02,Ore06,Ore07,Ore08,Ore09,Ore10,Ore11,Ore12,Ore13	*OreG0016493*
scaffold33216	228191	G	T	NA	*OreG0016494*
scaffold33216	228221	G	A	Ore01,Ore02,Ore03,Ore04,Ore05,Ore06,Ore07,Ore08,Ore09,Ore10,Ore11,Ore12,Ore13,Ore14	*OreG0016494*
scaffold33216	320782	C	A	Och01,Och02,Och03,Och04,Och05,Och06,Och07,Och08,Och09,Och10,Och11,Och12,Och13,Och14	*OreG0016505*
scaffold33216	322964	G	A	Och06	*OreG0016506*
scaffold33216	323005	A	G	Ore01,Ore02,Ore03,Ore04,Ore05,Ore06,Ore07,Ore08,Ore09,Ore10,Ore11,Ore12,Ore13,Ore14	*OreG0016506*
scaffold33216	323778	T	C	Ore01,Ore02,Ore03,Ore04,Ore05,Ore06,Ore07,Ore08,Ore09,Ore10,Ore11,Ore12,Ore13,Ore14	*OreG0016507*
scaffold33216	323940	A	C	Ore01,Ore02,Ore03,Ore04,Ore05,Ore06,Ore07,Ore08,Ore09,Ore10,Ore11,Ore12,Ore13,Ore14	*OreG0016507*
scaffold33216	324105	C	T	NA	*OreG0016507*
scaffold33216	324109	A	G	NA	*OreG0016507*
scaffold33216	324303	C	A	Och01,Och02,Och03,Och04,Och05,Och06,Och07,Och08,Och09,Och10,Och11,Och12,Och13,Och14	*OreG0016508*
scaffold33216	324895	T	C	Ore01,Ore02,Ore03,Ore04,Ore05,Ore06,Ore07,Ore08,Ore09,Ore10,Ore11,Ore12,Ore13,Ore14	*OreG0016508*
scaffold33216	325209	A	C	NA	*OreG0016508*
scaffold33216	325241	T	C	Och01,Och02,Och03,Och04,Och05,Och06,Och07,Och08,Och09,Och10,Och11,Och12,Och13,Och14	*OreG0016508*
scaffold33216	331472	C	A	Och02,Och03,Och04,Och05,Och06,Och12,Och13,Och14	*OreG0016510*
scaffold33216	343705	G	A	NA	*OreG0016513*
scaffold33216	352000	A	T	Och06	*OreG0016514*
scaffold33216	378065	C	T	NA	*OreG0016517*
scaffold33216	378139	G	T	NA	*OreG0016517*
scaffold33216	382768	T	A	NA	*OreG0016518*
scaffold33216	382903	G	A	Och11	*OreG0016518*
scaffold33216	393695	G	T	Och14	*OreG0016519*
scaffold33216	405969	G	C	NA	*OreG0016520*
scaffold33216	405990	A	G	NA	*OreG0016520*
scaffold33216	407361	A	T	Och01,Och02,Och03,Och04,Och05,Och06,Och07,Och08,Och09,Och10,Och11,Och12,Och13,Och14	*OreG0016520*
scaffold33216	407633	G	A	NA	*OreG0016520*
scaffold33216	408270	A	T	Ore01,Ore02,Ore03,Ore04,Ore05,Ore06,Ore07,Ore08,Ore09,Ore10,Ore11,Ore12,Ore13,Ore14	*OreG0016520*
scaffold33216	408291	C	T	Och07,Och08,Och11	*OreG0016520*
scaffold33216	495150	T	C	Och01,Och02,Och03,Och04,Och05,Och06,Och07,Och08,Och09,Och10,Och11,Och12,Och13,Och14	*OreG0016529*
scaffold33216	495631	C	A	Ore01,Ore02,Ore06,Ore08,Ore09,Ore10	*OreG0016529*
scaffold33216	503890	G	C	Och07	*OreG0016531*
scaffold33216	504160	T	A	NA	*OreG0016531*
scaffold33216	506075	T	C	NA	*OreG0016532*
scaffold33216	507235	G	A	Och01,Och02,Och03,Och04,Och05,Och06,Och07,Och08,Och09,Och10,Och11,Och12,Och13,Och14,Ore01,Ore02,Ore03,Ore04,Ore05,Ore06,Ore07,Ore08,Ore09,Ore10,Ore11,Ore12,Ore13,Ore14	*OreG0016532*
scaffold33216	508095	T	C	NA	*OreG0016533*

scaffold33216	508609	C	A	Och01,Och02,Och03,Och04,Och05,Och06,Och07,Och08,Och09,Och10,Och11,Och12,Och13,Och14	*OreG0016533*
scaffold33216	510895	C	G	Ore01,Ore02,Ore03,Ore04,Ore05,Ore06,Ore07,Ore08,Ore09,Ore10,Ore11,Ore12,Ore13,Ore14	*OreG0016534*
scaffold33216	599565	A	G	Och01,Och02,Och03,Och04,Och05,Och06,Och07,Och08,Och09,Och10,Och11,Och12,Och13,Och14	*OreG0016542*
scaffold33216	602866	C	A	Och01,Och02,Och03,Och04,Och05,Och06,Och07,Och08,Och09,Och10,Och11,Och12,Och13,Och14	*OreG0016543*
scaffold33216	602911	G	A	Och02,Och03,Och04,Och05,Och06,Och08,Och09,Och10,Och11,Och12,Och13,Och14	*OreG0016543*
scaffold33216	617625	T	G	Ore01,Ore02,Ore03,Ore04,Ore05,Ore06,Ore07,Ore08,Ore09,Ore10,Ore11,Ore12,Ore13,Ore14	*OreG0016545*
scaffold33216	617814	A	G	NA	*OreG0016545*
scaffold33216	617901	A	G	Och01,Och02,Och03,Och04,Och05,Och06,Och07,Och08,Och09,Och10,Och11,Och12,Och13,Och14	*OreG0016545*
scaffold33216	649604	A	G	Ore01,Ore02,Ore03,Ore04,Ore05,Ore06,Ore07,Ore08,Ore09,Ore10,Ore11,Ore12,Ore13,Ore14	*OreG0016550*
scaffold33216	652554	G	A	NA	*OreG0016550*
scaffold33216	659322	A	T	NA	*OreG0016551*
scaffold33216	679852	G	A	NA	*OreG0016553*
scaffold33216	681783	G	A	Och01,Och02,Och03,Och04,Och05,Och06,Och07,Och08,Och09,Och10,Och11,Och12,Och13,Och14	*OreG0016553*
scaffold33216	694380	C	G	NA	*OreG0016554*
scaffold33216	704683	A	T	NA	*OreG0016556*
scaffold33216	707957	C	T	Ore01,Ore02,Ore04,Ore05,Ore06,Ore07,Ore08,Ore09,Ore10,Ore11,Ore12,Ore13	*OreG0016557*
scaffold33216	709332	G	A	NA	*OreG0016557*
scaffold33216	738398	A	C	NA	*OreG0016561*
scaffold33216	738455	T	C	Och06	*OreG0016561*
scaffold33216	800372	C	T	NA	*OreG0016567*
scaffold33216	800609	G	A	NA	*OreG0016567*
scaffold33216	800794	G	A	NA	*OreG0016567*
scaffold33216	800857	T	A	NA	*OreG0016567*
scaffold33216	814288	T	A	NA	*OreG0016569*
scaffold33216	816207	T	G	NA	*OreG0016569*
scaffold33216	832490	G	T	NA	*OreG0016572*
scaffold33216	835976	G	A	Och01,Och02,Och03,Och04,Och05,Och06,Och07,Och08,Och09,Och10,Och11,Och12,Och13,Och14,Ore01,Ore02,Ore03,Ore04,Ore05,Ore06,Ore07,Ore08,Ore09,Ore10,Ore11,Ore12,Ore13,Ore14	*OreG0016572*
scaffold33216	838389	A	C	Och01,Och02,Och03,Och04,Och05,Och06,Och07,Och08,Och09,Och10,Och11,Och12,Och13,Och14,Ore01,Ore02,Ore03,Ore04,Ore05,Ore06,Ore07,Ore08,Ore09,Ore10,Ore11,Ore12,Ore13,Ore14	*OreG0016572*
scaffold33216	851175	C	A	NA	*OreG0016575*
scaffold33216	851551	G	A	Och01,Och02,Och03,Och04,Och05,Och06,Och07,Och08,Och09,Och10,Och11,Och12,Och13,Och14,Ore01,Ore02,Ore03,Ore04,Ore05,Ore06,Ore07,Ore08,Ore09,Ore10,Ore11,Ore12,Ore13,Ore14	*OreG0016575*
scaffold33216	854821	T	C	NA	*OreG0016576*
scaffold33216	860809	G	A	NA	*OreG0016577*
scaffold33216	872649	G	T	Och01,Och06,Och12	*OreG0016579*
scaffold33216	872983	C	G	NA	*OreG0016579*
scaffold33216	873177	C	T	NA	*OreG0016579*
scaffold33216	888823	G	A	NA	*OreG0016581*
scaffold33216	896556	A	G	NA	*OreG0016582*
scaffold33216	897256	A	G	Och01,Och02,Och03,Och04,Och05,Och06,Och07,Och08,Och09,Och10,Och11,Och12,Och13,Och14,Ore01,Ore02,Ore03,Ore04,Ore05,Ore06,Ore07,Ore08,Ore09,Ore10,Ore11,Ore12,Ore13,Ore14	*OreG0016583*
scaffold33216	898330	A	G	Och01,Och02,Och03,Och04,Och05,Och06,Och07,Och08,Och09,Och10,Och11,Och12,Och13,Och14,Ore01,Ore02,Ore03,Ore04,Ore05,Ore06,Ore07,Ore08,Ore09,Ore10,Ore11,Ore12,Ore13,Ore14	*OreG0016583*
scaffold33216	898635	A	G	NA	*OreG0016583*
scaffold33216	904342	G	C	NA	*OreG0016585*
scaffold33216	913437	G	A	Och02,Och03,Och04,Och05,Och06,Och12,Och13	*OreG0016587*
scaffold33216	913459	C	T	Och08	*OreG0016587*
scaffold33216	918439	T	C	Och05,Och12	*OreG0016589*
scaffold33216	924326	C	G	Ore02,Ore03,Ore09,Ore10,Ore11,Ore12,Ore14	*OreG0016590*
scaffold33216	928023	G	T	Och14	*OreG0016591*
scaffold33113	18873	T	A	NA	*OreG0012712*
scaffold33113	19026	C	A	NA	*OreG0012712*
scaffold33113	48981	G	T	NA	*OreG0012715*
scaffold33113	48984	G	C	NA	*OreG0012715*
scaffold33113	83465	A	T	Och01,Och02,Och03,Och04,Och05,Och06,Och07,Och08,Och09,Och10,Och11,Och12,Och13,Och14,Ore01,Ore02,Ore03,Ore04,Ore05,Ore06,Ore07,Ore08,Ore09,Ore10,Ore11,Ore12,Ore13,Ore14	*OreG0012718*
scaffold33113	113351	G	A	NA	*OreG0012720*
scaffold33113	147095	G	C	NA	*OreG0012722*
scaffold33113	147371	G	A	NA	*OreG0012722*

scaffold33113	220193	G	T	Ore01,Ore02,Ore03,Ore04,Ore05,Ore06,Ore07,Ore08,Ore09,Ore10,Ore11,Ore12,Ore13,Ore14	*OreG0012729*
scaffold33113	247426	T	A	Och01,Och02,Och03,Och04,Och05,Och06,Och07,Och08,Och09,Och10,Och11,Och12,Och13,Och14,Ore01,Ore02,Ore03,Ore04,Ore05,Ore06,Ore07,Ore08,Ore09,Ore10,Ore11,Ore12,Ore13,Ore14	*OreG0012730*
scaffold33113	268933	G	A	Ore01,Ore02,Ore03,Ore04,Ore05,Ore06,Ore07,Ore08,Ore09,Ore10,Ore11,Ore12,Ore13,Ore14	*OreG0012733*
scaffold33113	268973	C	T	Ore01,Ore02,Ore03,Ore04,Ore05,Ore06,Ore07,Ore08,Ore09,Ore10,Ore11,Ore12,Ore13,Ore14	*OreG0012733*
scaffold33113	268981	A	G	Ore01,Ore02,Ore03,Ore04,Ore05,Ore06,Ore07,Ore08,Ore09,Ore10,Ore11,Ore12,Ore13,Ore14	*OreG0012733*
scaffold33113	268988	C	T	Ore01,Ore02,Ore03,Ore04,Ore05,Ore06,Ore07,Ore08,Ore09,Ore10,Ore11,Ore12,Ore13,Ore14	*OreG0012733*
scaffold33113	268993	G	A	Ore01,Ore02,Ore03,Ore04,Ore05,Ore06,Ore07,Ore08,Ore09,Ore10,Ore11,Ore12,Ore13,Ore14	*OreG0012733*
scaffold33113	269032	G	A	Ore01,Ore02,Ore03,Ore04,Ore05,Ore06,Ore07,Ore08,Ore09,Ore10,Ore11,Ore12,Ore13,Ore14	*OreG0012733*
scaffold33113	269339	C	T	Ore01,Ore02,Ore03,Ore04,Ore05,Ore06,Ore07,Ore08,Ore09,Ore10,Ore11,Ore12,Ore13,Ore14	*OreG0012733*
scaffold33113	269351	C	T	Ore01,Ore02,Ore03,Ore04,Ore05,Ore06,Ore07,Ore08,Ore09,Ore10,Ore11,Ore12,Ore13,Ore14	*OreG0012733*
scaffold33113	269384	C	T	Ore01,Ore02,Ore03,Ore04,Ore05,Ore06,Ore07,Ore08,Ore09,Ore10,Ore11,Ore12,Ore13,Ore14	*OreG0012733*
scaffold33113	269387	C	T	Ore01,Ore02,Ore03,Ore04,Ore05,Ore06,Ore07,Ore08,Ore09,Ore10,Ore11,Ore12,Ore13,Ore14	*OreG0012733*
scaffold33113	269749	C	T	Ore01,Ore02,Ore03,Ore04,Ore05,Ore06,Ore07,Ore08,Ore09,Ore10,Ore11,Ore12,Ore13,Ore14	*OreG0012734*
scaffold33113	372260	G	C	Och02,Och03,Och04,Och05,Och06,Och08,Och12,Och13,Och14	*OreG0012742*
scaffold33113	428329	A	G	Och02,Och04,Och06,Och09	*OreG0012744*
scaffold33113	456539	C	A	NA	*OreG0012745*
scaffold33113	457554	A	C	Ore08,Ore11,Ore12	*OreG0012745*
scaffold33113	477336	T	C	NA	*OreG0012747*
scaffold33113	478808	C	T	NA	*OreG0012747*
scaffold33113	570534	C	T	NA	*OreG0012753*
scaffold33113	571072	T	G	NA	*OreG0012753*
scaffold33113	574505	T	C	NA	*OreG0012753*
scaffold33113	603062	G	T	Och01,Och02,Och03,Och04,Och05,Och06,Och07,Och08,Och09,Och10,Och11,Och12,Och13,Och14	*OreG0012757*
scaffold33113	665488	T	C	NA	*OreG0012760*
scaffold33113	665647	C	A	Och06,Och07,Och09,Och10,Och11	*OreG0012760*
scaffold33113	665656	T	A	NA	*OreG0012760*
scaffold33113	667763	G	A	Och01,Och02,Och03,Och04,Och05,Och06,Och07,Och08,Och09,Och10,Och11,Och12,Och13,Och14,Ore01,Ore02,Ore03,Ore04,Ore05,Ore06,Ore07,Ore08,Ore09,Ore10,Ore11,Ore12,Ore13,Ore14	*OreG0012760*
scaffold33113	682605	T	C	Ore01,Ore02,Ore03,Ore04,Ore05,Ore06,Ore07,Ore08,Ore09,Ore10,Ore11,Ore12,Ore13,Ore14	*OreG0012761*
scaffold33113	708413	G	A	Och01,Och02,Och03,Och04,Och05,Och06,Och07,Och08,Och09,Och10,Och11,Och12,Och13,Och14	*OreG0012762*
scaffold33113	750045	C	G	NA	*OreG0012764*
scaffold33113	864000	T	G	Ore01,Ore02,Ore03,Ore04,Ore05,Ore06,Ore07,Ore08,Ore09,Ore10,Ore11,Ore12,Ore13,Ore14	*OreG0012771*
scaffold33113	897854	C	T	Och12	*OreG0012774*
scaffold33113	898452	C	A	NA	*OreG0012774*
scaffold33113	898643	G	A	Och01,Och02,Och03,Och04,Och05,Och06,Och07,Och08,Och12,Och13,Och14	*OreG0012774*
scaffold33113	910721	C	G	NA	*OreG0012775*
scaffold33113	910805	C	A	NA	*OreG0012775*
scaffold33113	911183	C	T	NA	*OreG0012775*
scaffold33113	912635	T	A	Och01,Och03,Och04,Och05,Och12,Och13	*OreG0012776*
scaffold33113	912689	C	G	Ore07,Ore10,Ore14	*OreG0012776*
scaffold33113	912699	C	T	Och01,Och02,Och03,Och04,Och05,Och06,Och12,Och13,Och14	*OreG0012776*
scaffold33113	912853	A	G	Ore01,Ore02,Ore03,Ore04,Ore05,Ore06,Ore07,Ore08,Ore09,Ore10,Ore11,Ore12,Ore13,Ore14	*OreG0012776*
scaffold33113	912934	C	A	NA	*OreG0012776*
scaffold33113	937611	T	A	Och12	*OreG0012777*
scaffold33113	937718	G	A	NA	*OreG0012777*
scaffold33113	942799	A	G	NA	*OreG0012779*
scaffold33113	942924	C	A	Och12	*OreG0012779*
scaffold33113	945186	T	A	NA	*OreG0012779*
scaffold33113	950639	C	G	NA	*OreG0012780*
scaffold33113	987382	G	A	NA	*OreG0012785*
scaffold33113	1026432	A	T	Och14	*OreG0012788*
scaffold33113	1027388	A	T	Ore01,Ore02,Ore03,Ore04,Ore05,Ore06,Ore07,Ore08,Ore09,Ore10,Ore11,Ore12,Ore13,Ore14	*OreG0012788*
scaffold33113	1076107	A	T	NA	*OreG0012794*

scaffold33113	1079553	T	C	Och01,Och02,Och03,Och04,Och05,Och06,Och07,Och08,Och09,Och10,Och11,Och12,Och13,Och14	*OreG0012795*
scaffold33113	1079879	G	T	NA	*OreG0012795*
scaffold33113	1080515	A	T	Och01,Och02,Och03,Och04,Och05,Och06,Och07,Och08,Och09,Och10,Och11,Och12,Och13,Och14,Ore01,Ore02,Ore03,Ore04,Ore05,Ore06,Ore07,Ore08,Ore09,Ore10,Ore11,Ore12,Ore13,Ore14	*OreG0012795*
scaffold33113	1081038	T	A	Och01,Och02,Och03,Och04,Och05,Och06,Och07,Och08,Och09,Och10,Och11,Och12,Och13,Och14,Ore01,Ore02,Ore03,Ore04,Ore05,Ore06,Ore07,Ore08,Ore09,Ore10,Ore11,Ore12,Ore13,Ore14	*OreG0012795*
scaffold33113	1081113	A	G	Ore01,Ore02,Ore03,Ore04,Ore05,Ore06,Ore07,Ore08,Ore09,Ore10,Ore11,Ore12,Ore13,Ore14	*OreG0012795*
scaffold33113	1081185	G	T	Och01,Och02,Och03,Och04,Och05,Och06,Och07,Och08,Och09,Och10,Och11,Och12,Och13,Och14,Ore01,Ore02,Ore03,Ore04,Ore05,Ore06,Ore07,Ore08,Ore09,Ore10,Ore11,Ore12,Ore13,Ore14	*OreG0012795*
scaffold33113	1081632	T	C	NA	*OreG0012795*
scaffold33113	1082012	G	C	Och01,Och02,Och03,Och04,Och05,Och06,Och07,Och08,Och09,Och10,Och11,Och12,Och13,Och14	*OreG0012795*
scaffold33113	1083345	C	A	Ore01,Ore02,Ore03,Ore04,Ore05,Ore06,Ore07,Ore08,Ore09,Ore10,Ore11,Ore12,Ore13,Ore14	*OreG0012796*
scaffold33113	1084328	T	A	NA	*OreG0012796*
scaffold33113	1148900	G	A	NA	*OreG0012801*
scaffold33113	1148951	G	A	NA	*OreG0012801*
scaffold33113	1188324	T	A	Ore01,Ore02,Ore03,Ore04,Ore05,Ore06,Ore07,Ore08,Ore09,Ore10,Ore11,Ore12,Ore13,Ore14	*OreG0012804*
scaffold33113	1188337	A	G	Och01,Och02,Och03,Och04,Och05,Och06,Och07,Och08,Och09,Och10,Och11,Och12,Och13,Och14	*OreG0012804*
scaffold33113	1188447	A	T	Ore01,Ore02,Ore03,Ore04,Ore05,Ore06,Ore07,Ore08,Ore09,Ore10,Ore11,Ore12,Ore13,Ore14	*OreG0012804*
scaffold33113	1199807	A	T	Och01,Och02,Och03,Och04,Och05,Och06,Och07,Och08,Och09,Och10,Och11,Och12,Och13,Och14,Ore01,Ore02,Ore03,Ore04,Ore05,Ore06,Ore07,Ore08,Ore09,Ore10,Ore11,Ore12,Ore13,Ore14	*OreG0012806*
scaffold33113	1200404	T	C	Ore01,Ore02,Ore03,Ore04,Ore05,Ore06,Ore07,Ore08,Ore09,Ore10,Ore11,Ore12,Ore13,Ore14	*OreG0012806*
scaffold33113	1247833	T	A	NA	*OreG0012809*
scaffold33113	1248352	T	A	Ore01,Ore02,Ore03,Ore04,Ore05,Ore06,Ore07,Ore08,Ore09,Ore10,Ore11,Ore12,Ore13,Ore14	*OreG0012809*
scaffold33113	1248442	G	T	Ore02	*OreG0012809*
scaffold33113	1248701	T	A	Ore01,Ore02,Ore03,Ore04,Ore05,Ore06,Ore07,Ore08,Ore09,Ore10,Ore11,Ore12,Ore13,Ore14	*OreG0012809*
scaffold33113	1267735	T	A	Ore01,Ore02,Ore03,Ore04,Ore05,Ore06,Ore07,Ore08,Ore09,Ore10,Ore11,Ore12,Ore13,Ore14	*OreG0012810*
scaffold33113	1267749	G	A	NA	*OreG0012810*
scaffold33113	1271633	C	A	Och02,Och03,Och04,Och05,Och06,Och07,Och08,Och09,Och10,Och11,Och12,Och13	*OreG0012811*
scaffold33113	1275861	C	T	Och01,Och02,Och03,Och04,Och05,Och06,Och07,Och08,Och09,Och10,Och11,Och12,Och13,Och14	*OreG0012812*
scaffold33113	1289462	A	G	Ore01,Ore02,Ore03,Ore04,Ore05,Ore06,Ore07,Ore08,Ore09,Ore10,Ore11,Ore12,Ore13,Ore14	*OreG0012814*
scaffold33113	1289737	A	T	NA	*OreG0012814*
scaffold33113	1292346	G	A	Och14	*OreG0012814*
scaffold33113	1297706	C	T	Ore01,Ore02,Ore03,Ore04,Ore05,Ore06,Ore07,Ore08,Ore09,Ore10,Ore11,Ore12,Ore13,Ore14	*OreG0012815*
scaffold33113	1301315	T	A	Ore01,Ore02,Ore03,Ore04,Ore05,Ore06,Ore07,Ore08,Ore09,Ore10,Ore11,Ore12,Ore13,Ore14	*OreG0012815*
scaffold33113	1301326	T	C	NA	*OreG0012815*
scaffold33113	1324794	A	G	NA	*OreG0012819*
scaffold33113	1324826	A	C	NA	*OreG0012819*
scaffold33113	1375411	C	T	NA	*OreG0012822*
scaffold33113	1375770	T	A	Och01,Och06,Och07,Och08,Och09,Och10,Och11,Och12,Och14	*OreG0012822*
scaffold33113	1376849	T	G	Och01,Och02,Och03,Och04,Och05,Och06,Och07,Och08,Och09,Och10,Och11,Och12,Och13,Och14,Ore01,Ore02,Ore03,Ore04,Ore05,Ore06,Ore07,Ore08,Ore09,Ore10,Ore11,Ore12,Ore13,Ore14	*OreG0012822*
scaffold33113	1376944	T	G	NA	*OreG0012822*
scaffold33113	1377512	G	A	Och01,Och02,Och03,Och04,Och05,Och06,Och07,Och08,Och09,Och10,Och11,Och12,Och13,Och14,Ore01,Ore02,Ore03,Ore04,Ore05,Ore06,Ore07,Ore08,Ore09,Ore10,Ore11,Ore12,Ore13,Ore14	*OreG0012822*
scaffold33113	1378017	A	C	Och01,Och02,Och03,Och04,Och05,Och06,Och07,Och08,Och09,Och10,Och11,Och12,Och13,Och14	*OreG0012822*
scaffold33113	1378457	C	G	Och12	*OreG0012822*
scaffold33113	1401089	C	G	Och01,Och02,Och03,Och04,Och05,Och06,Och07,Och08,Och09,Och10,Och11,Och12,Och13,Och14,Ore01,Ore02,Ore03,Ore04,Ore05,Ore06,Ore07,Ore08,Ore09,Ore10,Ore11,Ore12,Ore13,Ore14	*OreG0012824*
scaffold33113	1401373	A	T	Och01,Och02,Och03,Och04,Och05,Och06,Och07,Och08,Och09,Och10,Och11,Och12,Och13,Och14	*OreG0012824*
scaffold33113	1406085	C	G	NA	*OreG0012825*
scaffold33113	1406130	T	A	NA	*OreG0012825*

scaffold33113	1406363	G	T	Och12	*OreG0012825*
scaffold33113	1459849	C	A	Ore01,Ore02,Ore03,Ore04,Ore05,Ore06,Ore07,Ore08,Ore09,Ore10,Ore11,Ore12,Ore13,Ore14	*OreG0012829*
scaffold33113	1459938	G	A	NA	*OreG0012829*
scaffold33113	1460587	G	A	NA	*OreG0012829*
scaffold33113	1460686	T	A	Ore01,Ore02,Ore03,Ore04,Ore05,Ore06,Ore07,Ore08,Ore09,Ore10,Ore11,Ore12,Ore13,Ore14	*OreG0012829*
scaffold33113	1460859	T	G	NA	*OreG0012829*
scaffold33113	1498900	A	G	NA	*OreG0012831*
scaffold33113	1498991	G	A	NA	*OreG0012831*
scaffold33113	1528812	G	A	Och01,Och02,Och03,Och04,Och05,Och06,Och07,Och08,Och09,Och10,Och11,Och12,Och13,Och14,Ore01,Ore02,Ore03,Ore04,Ore05,Ore06,Ore07,Ore08,Ore09,Ore10,Ore11,Ore12,Ore13,Ore14	*OreG0012832*
scaffold33113	1547311	G	A	Och08,Och14	*OreG0012833*
scaffold33113	1547551	C	T	Och01,Och02,Och03,Och04,Och05,Och06,Och07,Och08,Och09,Och10,Och11,Och12,Och13,Och14,Ore01,Ore02,Ore03,Ore04,Ore05,Ore06,Ore07,Ore08,Ore09,Ore10,Ore11,Ore12,Ore13,Ore14	*OreG0012833*
scaffold33113	1567452	A	G	Ore01,Ore02,Ore03,Ore04,Ore05,Ore06,Ore07,Ore08,Ore09,Ore10,Ore11,Ore12,Ore13,Ore14	*OreG0012834*
scaffold33113	1598010	T	G	Ore01,Ore02,Ore03,Ore04,Ore05,Ore06,Ore07,Ore08,Ore09,Ore10,Ore11,Ore12,Ore13,Ore14	*OreG0012837*
scaffold33113	1607606	C	T	NA	*OreG0012838*
scaffold33113	1617946	C	T	NA	*OreG0012839*
scaffold33113	1636373	G	C	Och02,Och14	*OreG0012842*
scaffold33113	1636401	A	G	NA	*OreG0012842*
scaffold33113	1637263	A	C	NA	*OreG0012842*
scaffold33113	1645027	C	T	Och01,Och02,Och03,Och04,Och05,Och06,Och07,Och08,Och09,Och10,Och11,Och12,Och13,Och14,Ore01,Ore02,Ore03,Ore04,Ore05,Ore06,Ore07,Ore08,Ore09,Ore10,Ore11,Ore12,Ore13,Ore14	*OreG0012844*
scaffold33113	1645557	T	A	Och01,Och02,Och03,Och04,Och05,Och06,Och07,Och08,Och09,Och10,Och11,Och12,Och13,Och14,Ore01,Ore02,Ore03,Ore04,Ore05,Ore06,Ore07,Ore08,Ore09,Ore10,Ore11,Ore12,Ore13,Ore14	*OreG0012844*
scaffold33113	1697371	G	C	Ore01,Ore02,Ore03,Ore06,Ore07,Ore08,Ore09,Ore10,Ore11,Ore12,Ore13,Ore14	*OreG0012848*
scaffold33113	1778032	T	G	Och12,Och13	*OreG0012852*
scaffold33113	1787029	C	A	Och01,Och02,Och03,Och04,Och05,Och06,Och07,Och08,Och09,Och10,Och11,Och12,Och13,Och14,Ore01,Ore02,Ore03,Ore04,Ore05,Ore06,Ore07,Ore08,Ore09,Ore10,Ore11,Ore12,Ore13,Ore14	*OreG0012854*
scaffold33113	1788399	T	G	Och01,Och02,Och03,Och04,Och05,Och06,Och07,Och08,Och09,Och10,Och11,Och12,Och13,Och14,Ore01,Ore02,Ore03,Ore04,Ore05,Ore06,Ore07,Ore08,Ore09,Ore10,Ore11,Ore12,Ore13,Ore14	*OreG0012854*
scaffold33113	1788402	G	C	Och01,Och02,Och03,Och04,Och05,Och06,Och07,Och08,Och09,Och10,Och11,Och12,Och13,Och14,Ore01,Ore02,Ore03,Ore04,Ore05,Ore06,Ore07,Ore08,Ore09,Ore10,Ore11,Ore12,Ore13,Ore14	*OreG0012854*
scaffold33113	1794826	T	C	NA	*OreG0012854*
scaffold33113	1823365	A	T	NA	*OreG0012857*
scaffold33113	1872848	A	G	NA	*OreG0012865*
scaffold33113	1874518	G	A	NA	*OreG0012866*
scaffold33113	1879771	A	T	NA	*OreG0012868*
scaffold33113	1882331	G	A	NA	*OreG0012869*
scaffold33113	1889158	A	G	NA	*OreG0012871*
scaffold33113	1889632	G	T	NA	*OreG0012871*
scaffold33113	1889779	C	T	Ore01,Ore02,Ore03,Ore04,Ore05,Ore06,Ore07,Ore08,Ore09,Ore10,Ore11,Ore12,Ore13,Ore14	*OreG0012871*
scaffold33113	1891330	T	C	Och07,Och08	*OreG0012872*
scaffold33113	1891397	G	C	NA	*OreG0012872*
scaffold33113	1891399	A	T	NA	*OreG0012872*
scaffold33113	1893702	C	T	Ore02,Ore03,Ore06,Ore08,Ore09,Ore10,Ore12,Ore14	*OreG0012873*
scaffold33113	1894082	A	T	NA	*OreG0012873*
scaffold33113	1894262	A	G	Ore02,Ore03,Ore06,Ore08,Ore09,Ore10,Ore12,Ore14	*OreG0012873*
scaffold33113	1896479	C	T	Ore02,Ore03,Ore04,Ore05,Ore06,Ore08,Ore09,Ore10,Ore12,Ore14	*OreG0012874*
scaffold33113	1898269	T	C	NA	*OreG0012875*
scaffold33113	1898286	G	C	Ore01,Ore02,Ore03,Ore04,Ore05,Ore06,Ore07,Ore08,Ore09,Ore10,Ore11,Ore12,Ore13,Ore14	*OreG0012875*
scaffold33113	1898321	T	A	NA	*OreG0012875*
scaffold33113	1907434	T	A	Och12	*OreG0012877*
scaffold33113	1913757	G	A	NA	*OreG0012879*
scaffold33113	1917492	G	A	NA	*OreG0012880*
scaffold33113	1917510	C	T	NA	*OreG0012880*
scaffold33113	1917516	G	T	NA	*OreG0012880*
scaffold33113	1923908	T	A	NA	*OreG0012881*
scaffold33113	1923913	T	C	NA	*OreG0012881*
scaffold33113	1925576	C	T	NA	*OreG0012881*
scaffold33113	1925627	A	G	NA	*OreG0012881*
scaffold33113	1929245	C	A	Ore01,Ore02,Ore03,Ore04,Ore05,Ore06,Ore07,Ore08,Ore09,Ore10,Ore11,Ore12,Ore13,Ore14	*OreG0012882*

scaffold33113	1929316	T	C	Ore01,Ore02,Ore03,Ore04,Ore05,Ore06,Ore07,Ore08,Ore09,Ore10,Ore11,Ore12,Ore13,Ore14	*OreG0012882*
scaffold33113	1955993	T	A	Och01,Och02,Och03,Och04,Och05,Och06,Och07,Och08,Och09,Och10,Och11,Och12,Och13,Och14,Ore01,Ore02,Ore03,Ore04,Ore05,Ore06,Ore07,Ore08,Ore09,Ore10,Ore11,Ore12,Ore13,Ore14	*OreG0012887*
scaffold33113	1956076	G	C	Och02,Och03,Och04,Och05,Och06,Och07,Och08,Och09,Och10,Och11,Och12,Och13,Och14	*OreG0012887*
scaffold33113	1974671	C	T	Ore01,Ore02,Ore03,Ore04,Ore05,Ore06,Ore07,Ore08,Ore09,Ore10,Ore11,Ore12,Ore13,Ore14	*OreG0012888*
scaffold33113	1976715	G	A	Och01,Och02,Och03,Och04,Och05,Och06,Och07,Och08,Och09,Och10,Och11,Och12,Och13,Och14	*OreG0012888*
scaffold33113	1977272	G	T	Ore01,Ore02,Ore03,Ore04,Ore05,Ore06,Ore07,Ore08,Ore09,Ore10,Ore11,Ore12,Ore13,Ore14	*OreG0012888*
scaffold33113	1978368	A	T	NA	*OreG0012888*
scaffold33113	1992734	C	G	Ore01,Ore02,Ore03,Ore04,Ore05,Ore06,Ore07,Ore08,Ore09,Ore10,Ore11,Ore12,Ore13,Ore14	*OreG0012890*
scaffold33113	2028545	A	G	NA	*OreG0012892*
scaffold33113	2028581	A	T	Och01,Och02,Och03,Och04,Och05,Och06,Och07,Och09,Och10,Och11,Och12,Och13,Och14	*OreG0012892*
scaffold33113	2037669	G	T	Och01,Och02,Och03,Och04,Och05,Och06,Och07,Och08,Och09,Och10,Och11,Och12,Och13,Och14,Ore01,Ore02,Ore03,Ore04,Ore05,Ore06,Ore07,Ore08,Ore09,Ore10,Ore11,Ore12,Ore13,Ore14	*OreG0012893*
scaffold33113	2053506	C	T	Ore01,Ore02,Ore03,Ore06,Ore07,Ore08,Ore09,Ore10,Ore11,Ore12,Ore13,Ore14	*OreG0012894*
scaffold33113	2055949	C	G	NA	*OreG0012895*
scaffold33113	2100146	A	C	Och09,Och10	*OreG0012898*
scaffold33113	2112483	G	T	Och01,Och08	*OreG0012900*
scaffold33113	2127491	C	T	NA	*OreG0012901*
scaffold33113	2133485	A	T	Ore01,Ore02,Ore03,Ore06,Ore07,Ore08,Ore09,Ore10,Ore11,Ore12,Ore13,Ore14	*OreG0012902*
scaffold33113	2134518	C	T	Och11	*OreG0012902*
scaffold33113	2135694	A	G	Och07,Och08,Och09,Och10,Och11	*OreG0012902*
scaffold33113	2135744	C	A	NA	*OreG0012902*
scaffold33113	2137509	A	G	Och11	*OreG0012902*
scaffold33113	2138864	G	A	Och08	*OreG0012903*
scaffold33113	2138919	A	G	Och07,Och08,Och09,Och10,Och11	*OreG0012903*
scaffold33113	2144847	A	G	Och01	*OreG0012905*
scaffold33113	2148230	G	C	NA	*OreG0012905*
scaffold33113	2149388	A	T	Och01,Och02,Och03,Och04,Och05,Och06,Och07,Och08,Och09,Och10,Och11,Och12,Och13,Och14	*OreG0012905*
scaffold33113	2154902	T	A	NA	*OreG0012906*
scaffold33113	2154978	T	C	NA	*OreG0012906*
scaffold33113	2155097	G	A	NA	*OreG0012906*
scaffold33113	2155593	A	T	NA	*OreG0012906*
scaffold33113	2172028	G	A	Och01,Och02,Och03,Och04,Och05,Och06,Och07,Och08,Och09,Och10,Och11,Och12,Och13,Och14	*OreG0012908*
scaffold33113	2172031	G	A	Och08	*OreG0012908*
scaffold33113	2172060	T	C	Och01,Och02,Och03,Och04,Och05,Och06,Och07,Och08,Och09,Och10,Och11,Och12,Och13,Och14,Ore01,Ore02,Ore03,Ore04,Ore05,Ore06,Ore07,Ore08,Ore09,Ore10,Ore11,Ore12,Ore13,Ore14	*OreG0012908*
scaffold33113	2172091	T	A	Och01,Och02,Och03,Och04,Och05,Och06,Och07,Och08,Och09,Och10,Och11,Och12,Och13,Och14	*OreG0012908*
scaffold33113	2172136	C	T	NA	*OreG0012908*
scaffold33113	2172141	A	T	Ore01,Ore02,Ore03,Ore04,Ore05,Ore06,Ore07,Ore08,Ore09,Ore10,Ore11,Ore12,Ore13,Ore14	*OreG0012908*
scaffold33113	2257287	T	C	Ore02,Ore03,Ore04,Ore05,Ore06,Ore08,Ore09,Ore10,Ore12,Ore14	*OreG0012915*
scaffold33113	2285422	G	A	NA	*OreG0012916*
scaffold33113	2285570	G	A	NA	*OreG0012916*
scaffold33113	2323760	A	G	NA	*OreG0012917*
scaffold33113	2325089	G	A	Och01,Och02,Och03,Och04,Och05,Och06,Och07,Och08,Och09,Och10,Och11,Och12,Och13,Och14,Ore01,Ore02,Ore03,Ore04,Ore05,Ore06,Ore07,Ore08,Ore09,Ore10,Ore11,Ore12,Ore13,Ore14	*OreG0012918*
scaffold33113	2325371	G	T	NA	*OreG0012918*
scaffold33113	2325373	G	T	NA	*OreG0012918*
scaffold33113	2326121	G	C	Ore01,Ore02,Ore03,Ore04,Ore05,Ore06,Ore07,Ore08,Ore09,Ore10,Ore11,Ore12,Ore13,Ore14	*OreG0012918*
scaffold33113	2342018	G	T	Ore01,Ore02,Ore03,Ore04,Ore05,Ore06,Ore07,Ore08,Ore09,Ore10,Ore11,Ore12,Ore13,Ore14	*OreG0012920*
scaffold33113	2342851	C	G	NA	*OreG0012920*
scaffold33113	2344217	G	A	NA	*OreG0012920*
scaffold33113	2352291	C	T	Och01,Och02,Och03,Och04,Och05,Och06,Och07,Och08,Och09,Och10,Och11,Och12,Och13,Och14,Ore01,Ore02,Ore03,Ore04,Ore05,Ore06,Ore07,Ore08,Ore09,Ore10,Ore11,Ore12,Ore13,Ore14	*OreG0012921*
scaffold33113	2369921	G	A	Och08	*OreG0012923*
scaffold33113	2391580	C	A	NA	*OreG0012926*
scaffold33113	2392603	C	A	NA	*OreG0012926*
scaffold33113	2392734	A	T	NA	*OreG0012926*

scaffold33113	2399333	C	A	NA	*OreG0012927*
scaffold33113	2402567	G	T	Och05,Och08,Och13,Och14	*OreG0012927*
scaffold33113	2403971	A	G	NA	*OreG0012927*
scaffold33113	2404932	T	A	Och05,Och13	*OreG0012927*
scaffold33113	2415878	A	G	Ore01,Ore02,Ore03,Ore04,Ore05,Ore06,Ore07,Ore08,Ore09,Ore10,Ore11,Ore12,Ore13,Ore14	*OreG0012929*
scaffold33113	2416059	C	G	Och08	*OreG0012929*
scaffold33113	2416451	A	C	Ore02,Ore03,Ore06,Ore08,Ore09,Ore10,Ore12,Ore14	*OreG0012929*
scaffold33113	2418792	C	T	Ore02,Ore03,Ore04,Ore05,Ore06,Ore08,Ore09,Ore10,Ore12,Ore14	*OreG0012931*
scaffold33113	2418797	G	A	Ore01,Ore02,Ore03,Ore04,Ore05,Ore06,Ore07,Ore08,Ore09,Ore10,Ore11,Ore12,Ore13,Ore14	*OreG0012931*
scaffold33113	2419367	C	T	NA	*OreG0012931*
scaffold33113	2419427	C	T	Ore02,Ore03,Ore06,Ore08,Ore09,Ore10,Ore12,Ore14	*OreG0012931*
scaffold33113	2419784	T	A	NA	*OreG0012931*
scaffold33113	2420963	C	A	Och01,Och02,Och03,Och04,Och05,Och06,Och07,Och08,Och09,Och10,Och11,Och12,Och13,Och14,Ore01,Ore02,Ore03,Ore04,Ore05,Ore06,Ore07,Ore08,Ore09,Ore10,Ore11,Ore12,Ore13,Ore14	*OreG0012930*
scaffold33147	16690	T	G	Ore01,Ore02,Ore03,Ore04,Ore05,Ore06,Ore07,Ore08,Ore09,Ore10,Ore11,Ore12,Ore13,Ore14	*OreG0014008*
scaffold33147	57158	A	T	Och01,Och02,Och03,Och04,Och05,Och06,Och07,Och08,Och09,Och10,Och11,Och12,Och13,Och14,Ore01,Ore02,Ore03,Ore04,Ore05,Ore06,Ore07,Ore08,Ore09,Ore10,Ore11,Ore12,Ore13,Ore14	*OreG0014011*
scaffold33147	66140	T	C	NA	*OreG0014011*
scaffold33147	71674	A	T	Och01,Och02,Och03,Och04,Och05,Och06,Och07,Och08,Och09,Och10,Och11,Och12,Och13,Och14,Ore01,Ore02,Ore03,Ore04,Ore05,Ore06,Ore07,Ore08,Ore09,Ore10,Ore11,Ore12,Ore13,Ore14	*OreG0014011*
scaffold33147	80685	T	A	Och01,Och02,Och03,Och04,Och05,Och06,Och07,Och08,Och09,Och10,Och11,Och12,Och13,Och14	*OreG0014012*
scaffold33147	80824	C	T	NA	*OreG0014012*
scaffold33147	94525	G	A	NA	*OreG0014012*
scaffold33147	128034	G	T	NA	*OreG0014013*
scaffold33147	134490	G	C	Och01,Och02,Och03,Och04,Och05,Och06,Och12,Och13	*OreG0014014*
scaffold33147	145425	C	G	NA	*OreG0014015*
scaffold33147	148478	C	T	Och01,Och02,Och04,Och06,Och07,Och12	*OreG0014017*
scaffold33147	148502	C	T	NA	*OreG0014017*
scaffold33147	148617	T	G	Och01,Och02,Och03,Och04,Och05,Och06,Och07,Och08,Och09,Och10,Och11,Och12,Och13,Och14,Ore01,Ore02,Ore03,Ore04,Ore05,Ore06,Ore07,Ore08,Ore09,Ore10,Ore11,Ore12,Ore13,Ore14	*OreG0014017*
scaffold33147	161398	G	T	NA	*OreG0014018*
scaffold33147	162211	C	G	Och01,Och02,Och03,Och04,Och05,Och06,Och07,Och08,Och09,Och10,Och11,Och12,Och13,Ore01,Ore02,Ore03,Ore04,Ore05,Ore06,Ore07,Ore08,Ore09,Ore10,Ore11,Ore12,Ore13,Ore14	*OreG0014018*
scaffold33147	163919	C	T	NA	*OreG0014018*
scaffold33147	349485	A	G	NA	*OreG0014026*
scaffold33147	358368	C	T	Ore03,Ore08,Ore09,Ore10,Ore14	*OreG0014027*
scaffold33147	847725	T	A	Och01,Och02,Och03,Och04,Och05,Och06,Och07,Och08,Och09,Och10,Och11,Och12,Och13,Och14	*OreG0014058*
scaffold33147	847856	C	A	Och01,Och02,Och03,Och04,Och05,Och06,Och07,Och08,Och09,Och10,Och11,Och12,Och13,Och14	*OreG0014058*
scaffold33147	848808	A	G	Och08	*OreG0014058*
scaffold33147	1226332	A	G	NA	*OreG0014083*
scaffold33147	1228377	A	T	NA	*OreG0014083*
scaffold33147	1517978	C	A	NA	*OreG0014104*
scaffold33147	1659083	G	A	Ore04,Ore05	*OreG0014115*
scaffold33147	1659736	G	A	Ore03,Ore08,Ore09,Ore10,Ore14	*OreG0014115*
scaffold33147	1659968	C	T	Ore01,Ore02,Ore03,Ore04,Ore05,Ore06,Ore07,Ore08,Ore09,Ore10,Ore11,Ore12,Ore13,Ore14	*OreG0014115*
scaffold33147	1661637	C	T	Ore01,Ore02,Ore03,Ore04,Ore05,Ore06,Ore07,Ore08,Ore09,Ore10,Ore11,Ore12,Ore13,Ore14	*OreG0014115*
scaffold33147	1857710	G	T	Och01,Och02,Och03,Och04,Och05,Och06,Och07,Och08,Och09,Och10,Och11,Och12,Och13,Och14,Ore01,Ore02,Ore03,Ore04,Ore05,Ore06,Ore07,Ore08,Ore09,Ore10,Ore11,Ore12,Ore13,Ore14	*OreG0014133*
scaffold33147	1857766	G	T	Och02,Och04,Och06,Och07,Och09,Och10,Och11,Och14,Ore01,Ore02,Ore03,Ore04,Ore05,Ore06,Ore07,Ore08,Ore09,Ore10,Ore11,Ore12,Ore13,Ore14	*OreG0014133*
scaffold33147	1864124	G	A	NA	*OreG0014134*
scaffold33232	100197	G	C	Och01,Och08,Och14	*OreG0017184*
scaffold33232	100558	T	C	NA	*OreG0017184*
scaffold33232	116452	G	T	Ore01,Ore02,Ore03,Ore04,Ore05,Ore06,Ore07,Ore08,Ore09,Ore10,Ore11,Ore12,Ore13,Ore14	*OreG0017185*
scaffold33232	116767	C	T	Och01,Och02,Och03,Och04,Och05,Och06,Och07,Och08,Och09,Och10,Och11,Och12,Och13,Och14,Ore01,Ore02,Ore03,Ore04,Ore05,Ore06,Ore07,Ore08,Ore09,Ore10,Ore11,Ore12,Ore13,Ore14	*OreG0017185*
scaffold31892	3279	C	T	Och07	*OreG0009911*
scaffold31892	106298	G	T	NA	*OreG0009914*
scaffold31892	112339	T	A	NA	*OreG0009915*

scaffold31892	115536	C	A	Och12	*OreG0009915*
scaffold31892	270070	C	T	NA	*OreG0009926*
scaffold31892	270137	G	A	Och01,Och02,Och03,Och04,Och05,Och06,Och07,Och08,Och09,Och10,Och11,Och12,Och13,Och14,Ore01,Ore02,Ore03,Ore04,Ore05,Ore06,Ore07,Ore08,Ore09,Ore10,Ore11,Ore12,Ore13,Ore14	*OreG0009926*
scaffold31892	277429	A	G	NA	*OreG0009927*
scaffold31892	303833	C	T	Och01,Och02,Och03,Och04,Och05,Och06,Och07,Och08,Och09,Och10,Och11,Och12,Och13,Och14,Ore01,Ore02,Ore03,Ore04,Ore05,Ore06,Ore07,Ore08,Ore09,Ore10,Ore11,Ore12,Ore13,Ore14	*OreG0009929*
scaffold31892	335503	G	A	Ore01,Ore02,Ore03,Ore04,Ore05,Ore06,Ore07,Ore08,Ore09,Ore10,Ore11,Ore12,Ore13,Ore14	*OreG0009933*
scaffold31892	335614	T	G	Ore01,Ore02,Ore03,Ore04,Ore05,Ore06,Ore07,Ore08,Ore09,Ore10,Ore11,Ore12,Ore13,Ore14	*OreG0009933*
scaffold31892	335918	C	A	NA	*OreG0009933*
scaffold31892	395654	C	A	NA	*OreG0009936*
scaffold31892	395658	A	C	Och01,Och02,Och03,Och04,Och05,Och06,Och07,Och08,Och09,Och10,Och11,Och12,Och13,Och14	*OreG0009936*
scaffold31892	569112	A	G	Ore01,Ore02,Ore03,Ore04,Ore05,Ore06,Ore07,Ore08,Ore09,Ore10,Ore11,Ore12,Ore13,Ore14	*OreG0009945*
scaffold31892	569177	G	T	NA	*OreG0009945*
scaffold31892	569181	C	T	Ore01,Ore02,Ore03,Ore04,Ore05,Ore06,Ore07,Ore08,Ore09,Ore10,Ore11,Ore12,Ore13,Ore14	*OreG0009945*
scaffold31892	571574	A	G	NA	*OreG0009946*
scaffold31892	573073	C	T	NA	*OreG0009946*
scaffold31892	574096	C	T	Och01,Och02,Och03,Och04,Och05,Och06,Och07,Och08,Och09,Och10,Och11,Och12,Och13,Och14	*OreG0009946*
scaffold31892	588196	G	A	NA	*OreG0009947*
scaffold31892	594007	C	T	Ore01,Ore02,Ore03,Ore04,Ore05,Ore06,Ore07,Ore08,Ore09,Ore10,Ore11,Ore12,Ore13,Ore14	*OreG0009948*
scaffold31892	638604	C	T	Och01,Och02,Och03,Och04,Och05,Och06,Och07,Och08,Och09,Och10,Och11,Och13,Och14	*OreG0009950*
scaffold31892	703603	A	T	NA	*OreG0009955*
scaffold31892	706690	T	C	NA	*OreG0009956*
scaffold31892	710725	T	G	Ore01,Ore02,Ore03,Ore04,Ore05,Ore06,Ore07,Ore08,Ore09,Ore10,Ore11,Ore12,Ore13,Ore14	*OreG0009957*
scaffold31892	711255	G	A	NA	*OreG0009957*
scaffold31892	711331	G	C	Och01,Och02,Och03,Och04,Och05,Och06,Och07,Och08,Och12,Och13,Och14	*OreG0009957*
scaffold31892	725654	G	A	NA	*OreG0009960*
scaffold31892	725673	A	G	NA	*OreG0009960*
scaffold31892	813855	A	T	Ore01,Ore02,Ore03,Ore04,Ore05,Ore06,Ore07,Ore08,Ore09,Ore10,Ore11,Ore12,Ore13,Ore14	*OreG0009972*
scaffold31892	814080	C	T	Ore01,Ore02,Ore03,Ore04,Ore05,Ore06,Ore07,Ore08,Ore09,Ore10,Ore11,Ore12,Ore13,Ore14	*OreG0009972*
scaffold31892	871992	C	T	Och06,Och07,Och12,Och14	*OreG0009976*
scaffold31892	872501	C	A	Ore04,Ore05,Ore06,Ore07,Ore10,Ore14	*OreG0009976*
scaffold31892	874732	T	C	NA	*OreG0009976*
scaffold31892	892068	C	A	NA	*OreG0009978*
scaffold31892	926219	G	A	Och01,Och02,Och03,Och04,Och05,Och06,Och07,Och08,Och09,Och10,Och11,Och12,Och13,Och14,Ore01,Ore02,Ore03,Ore04,Ore05,Ore06,Ore07,Ore08,Ore09,Ore10,Ore11,Ore12,Ore13,Ore14	*OreG0009981*
scaffold31892	927544	G	A	Och09,Och10,Och11	*OreG0009982*
scaffold31892	927553	C	T	Och05,Och09,Och10,Och11,Och12,Och13,Och14	*OreG0009982*
scaffold31892	927562	C	T	NA	*OreG0009982*
scaffold31892	928006	C	A	Ore01,Ore02,Ore03,Ore04,Ore05,Ore06,Ore07,Ore08,Ore09,Ore10,Ore11,Ore12,Ore13,Ore14	*OreG0009982*
scaffold31892	928021	T	C	Och05,Och09,Och10,Och11,Och12,Och13,Och14	*OreG0009982*
scaffold31892	928361	G	T	Ore01,Ore02,Ore03,Ore04,Ore05,Ore06,Ore07,Ore08,Ore09,Ore10,Ore11,Ore12,Ore13,Ore14	*OreG0009982*
scaffold31892	954940	T	G	NA	*OreG0009984*
scaffold31892	955141	C	T	Och09,Och10,Och11	*OreG0009984*
scaffold31892	955234	A	T	Och06	*OreG0009984*
scaffold31892	956045	C	T	Och09,Och10,Och11	*OreG0009984*
scaffold31892	1001471	A	T	NA	*OreG0009987*
scaffold31892	1006233	G	T	Och05,Och12,Och13	*OreG0009988*
scaffold31892	1007616	C	A	Ore01,Ore02,Ore03,Ore04,Ore05,Ore06,Ore07,Ore08,Ore09,Ore10,Ore11,Ore12,Ore13,Ore14	*OreG0009988*
scaffold31892	1045146	C	T	Och09,Och10,Och11	*OreG0009990*
scaffold31892	1046195	G	A	Och09,Och10,Och11	*OreG0009990*
scaffold31892	1061237	C	T	NA	*OreG0009991*
scaffold31892	1061252	A	T	NA	*OreG0009991*
scaffold31892	1061288	A	T	NA	*OreG0009991*
scaffold31892	1084598	A	C	Ore01,Ore02,Ore03,Ore04,Ore05,Ore06,Ore07,Ore08,Ore09,Ore10,Ore11,Ore12,Ore13,Ore14	*OreG0009994*

scaffold31892	1092368	G	A	Och01,Och02,Och03,Och04,Och05,Och06,Och07,Och08,Och09,Och10,Och11,Och12,Och13,Och14,Ore01,Ore02,Ore03,Ore04,Ore05,Ore06,Ore07,Ore08,Ore09,Ore10,Ore11,Ore12,Ore13,Ore14	*OreG0009995*
scaffold31892	1102598	C	T	Och09,Och10,Och11	*OreG0009996*
scaffold31892	1102688	G	A	NA	*OreG0009996*
scaffold31892	1103373	C	A	NA	*OreG0009996*
scaffold31892	1157704	G	C	NA	*OreG0009999*
scaffold31892	1158120	G	T	Och05,Och12,Och13	*OreG0009999*
scaffold31892	1188338	C	T	NA	*OreG0010005*
scaffold31892	1189600	T	A	Och12	*OreG0010005*
scaffold31892	1189633	G	T	NA	*OreG0010005*
scaffold31892	1190204	G	T	NA	*OreG0010005*
scaffold31892	1270881	A	T	NA	*OreG0010013*
scaffold31892	1270913	C	A	NA	*OreG0010013*
scaffold31892	1271132	C	T	Och02	*OreG0010013*
scaffold31892	1271146	C	T	NA	*OreG0010013*
scaffold31892	1271198	T	A	Och14	*OreG0010013*
scaffold31892	1293846	G	A	NA	*OreG0010016*
scaffold31892	1293864	C	G	NA	*OreG0010016*
scaffold31892	1313394	A	G	NA	*OreG0010019*
scaffold31892	1320317	A	G	NA	*OreG0010019*
scaffold31892	1367170	C	T	Och01,Och02,Och03,Och04,Och05,Och06,Och07,Och08,Och09,Och10,Och11,Och12,Och13,Och14	*OreG0010022*
scaffold31892	1367912	G	A	NA	*OreG0010022*
scaffold31892	1372707	C	G	Ore01,Ore02,Ore03,Ore06,Ore07,Ore08,Ore09,Ore10,Ore11,Ore12,Ore13,Ore14	*OreG0010022*
scaffold31892	1383840	A	G	Och01,Och02,Och03,Och04,Och05,Och06,Och07,Och08,Och09,Och10,Och11,Och12,Och13,Och14,Ore01,Ore02,Ore03,Ore06,Ore07,Ore08,Ore09,Ore10,Ore11,Ore12,Ore13,Ore14	*OreG0010023*
scaffold31892	1384593	C	G	NA	*OreG0010023*
scaffold31892	1384962	C	T	Och12,Och13	*OreG0010023*
scaffold31892	1384998	T	C	Och01,Och02,Och03,Och04,Och05,Och06,Och07,Och08,Och09,Och10,Och11,Och12,Och13,Och14,Ore01,Ore02,Ore03,Ore04,Ore05,Ore06,Ore07,Ore08,Ore09,Ore10,Ore11,Ore12,Ore13,Ore14	*OreG0010023*
scaffold31892	1416034	A	T	NA	*OreG0010030*
scaffold31892	1423730	C	T	NA	*OreG0010031*
scaffold31892	1442107	A	T	Ore01,Ore02,Ore03,Ore04,Ore05,Ore06,Ore07,Ore08,Ore09,Ore10,Ore11,Ore12,Ore13,Ore14	*OreG0010035*
scaffold31892	1442152	T	C	Ore01,Ore02,Ore03,Ore04,Ore05,Ore06,Ore07,Ore08,Ore09,Ore10,Ore11,Ore12,Ore13,Ore14	*OreG0010035*
scaffold31892	1443874	A	C	Och01,Och02,Och03,Och04,Och05,Och06,Och07,Och08,Och09,Och10,Och11,Och12,Och13,Och14	*OreG0010035*
scaffold31892	1497488	C	A	Och01	*OreG0010037*
scaffold31892	1498522	G	T	Ore01,Ore02,Ore03,Ore04,Ore05,Ore06,Ore07,Ore08,Ore09,Ore10,Ore11,Ore12,Ore13,Ore14	*OreG0010037*
scaffold31892	1498616	G	A	NA	*OreG0010037*
scaffold31892	1521590	A	T	Ore01,Ore02,Ore03,Ore04,Ore05,Ore06,Ore07,Ore08,Ore09,Ore10,Ore11,Ore12,Ore13,Ore14	*OreG0010041*
scaffold31892	1563117	C	A	Ore01,Ore02,Ore03,Ore04,Ore05,Ore06,Ore07,Ore08,Ore09,Ore10,Ore11,Ore12,Ore13,Ore14	*OreG0010039*
scaffold31892	1580169	T	A	Och01,Och02,Och03,Och04,Och05,Och06,Och07,Och08,Och09,Och10,Och11,Och12,Och13,Och14	*OreG0010046*
scaffold31892	1603893	G	A	Och01,Och02,Och03,Och04,Och05,Och06,Och07,Och08,Och09,Och10,Och11,Och12,Och13,Och14,Ore01,Ore02,Ore03,Ore04,Ore05,Ore06,Ore07,Ore08,Ore09,Ore10,Ore11,Ore12,Ore13,Ore14	*OreG0010048*
scaffold31892	1608253	A	T	Och01,Och02,Och03,Och04,Och05,Och06,Och07,Och08,Och09,Och10,Och11,Och12,Och13,Och14,Ore01,Ore02,Ore03,Ore04,Ore05,Ore06,Ore07,Ore08,Ore09,Ore10,Ore11,Ore12,Ore13,Ore14	*OreG0010049*
scaffold31892	1654191	C	A	Ore01,Ore02,Ore03,Ore04,Ore05,Ore06,Ore07,Ore08,Ore09,Ore10,Ore11,Ore12,Ore13,Ore14	*OreG0010050*
scaffold31892	1662873	G	A	Och12,Och13	*OreG0010051*
scaffold31892	1665454	G	C	Och01,Och02,Och03,Och04,Och05,Och06,Och07,Och08,Och09,Och10,Och11,Och12,Och13,Och14,Ore01,Ore02,Ore03,Ore04,Ore05,Ore06,Ore07,Ore08,Ore09,Ore10,Ore11,Ore12,Ore13,Ore14	*OreG0010051*
scaffold31892	1675787	T	C	Ore01,Ore02,Ore03,Ore04,Ore05,Ore06,Ore07,Ore08,Ore09,Ore10,Ore11,Ore12,Ore13,Ore14	*OreG0010053*
scaffold31892	1683246	G	A	Ore01,Ore02,Ore03,Ore04,Ore05,Ore06,Ore07,Ore08,Ore09,Ore10,Ore11,Ore12,Ore13,Ore14	*OreG0010055*
scaffold31892	1683375	T	G	NA	*OreG0010055*
scaffold31892	1711430	T	G	NA	*OreG0010057*
scaffold31892	1711858	C	T	Och12,Och13	*OreG0010057*
scaffold31892	1716785	T	A	Och01,Och02,Och03,Och04,Och05,Och06,Och07,Och08,Och09,Och10,Och11,Och12,Och13,Och14	*OreG0010057*
scaffold31892	1742503	C	T	NA	*OreG0010061*
scaffold31892	1742704	G	A	NA	*OreG0010061*
scaffold31892	1743098	C	A	NA	*OreG0010061*

scaffold31892	1743124	G	A	NA	*OreG0010061*
scaffold31892	1743130	G	A	NA	*OreG0010061*
scaffold31892	1743138	G	A	NA	*OreG0010061*
scaffold31892	1743171	C	G	NA	*OreG0010061*
scaffold31892	1744063	G	C	NA	*OreG0010061*
scaffold31892	1769298	T	A	Och02,Och03,Och04,Och06	*OreG0010063*
scaffold31892	1769574	C	T	Och01,Och02,Och03,Och04,Och05,Och06,Och07,Och08,Och09,Och10,Och11,Och12,Och13,Och14	*OreG0010063*
scaffold31892	1769600	G	A	Och01,Och02,Och03,Och04,Och05,Och06,Och07,Och08,Och09,Och10,Och11,Och12,Och13,Och14	*OreG0010063*
scaffold31892	1769627	G	A	Och01,Och02,Och03,Och04,Och05,Och06,Och07,Och08,Och09,Och10,Och11,Och12,Och13,Och14	*OreG0010063*
scaffold31892	1769631	C	T	Och02	*OreG0010063*
scaffold31892	1769673	T	C	Och01,Och02,Och03,Och04,Och05,Och06,Och07,Och08,Och09,Och10,Och11,Och12,Och13,Och14	*OreG0010063*
scaffold31892	1778253	T	C	NA	*OreG0010065*
scaffold31892	1778346	G	A	NA	*OreG0010065*
scaffold31892	1778382	G	T	Ore06,Ore07,Ore10,Ore14	*OreG0010065*
scaffold31892	1778535	T	C	Ore06,Ore07,Ore08,Ore10,Ore11,Ore12,Ore14	*OreG0010065*
scaffold31892	1782027	G	A	NA	*OreG0010064*
scaffold31892	1799193	T	A	NA	*OreG0010066*
scaffold31892	1799725	T	G	Och01,Och02,Och03,Och04,Och05,Och06,Och07,Och08,Och09,Och10,Och11,Och12,Och13,Och14	*OreG0010066*
scaffold31892	1839391	A	G	Och01	*OreG0010068*
scaffold31892	1841773	G	A	NA	*OreG0010068*
scaffold31892	1877928	G	A	NA	*OreG0010072*
scaffold31892	1954667	C	T	NA	*OreG0010080*
scaffold31892	1954732	G	T	Och01,Och07,Och08,Och09,Och10,Och11,Och14	*OreG0010080*
scaffold31892	1954912	A	G	Ore01,Ore02,Ore03,Ore06,Ore07,Ore08,Ore09,Ore10,Ore11,Ore12,Ore13,Ore14	*OreG0010080*
scaffold31892	1976850	A	T	NA	*OreG0010082*
scaffold31892	1986446	T	C	Och01,Och02,Och03,Och04,Och05,Och06,Och07,Och08,Och09,Och10,Och11,Och12,Och13,Och14	*OreG0010084*
scaffold31892	1991672	T	C	NA	*OreG0010084*
scaffold31892	2020476	T	C	NA	*OreG0010088*
scaffold31892	2034154	A	T	Och01,Och02,Och03,Och04,Och06,Och07,Och08,Och09,Och10,Och11,Och14	*OreG0010090*
scaffold31892	2037022	C	T	NA	*OreG0010091*
scaffold31892	2037037	C	T	NA	*OreG0010091*
scaffold31892	2091384	T	A	NA	*OreG0010095*
scaffold31892	2139943	T	A	NA	*OreG0010096*
scaffold31892	2140009	A	G	Ore01,Ore02,Ore03,Ore04,Ore05,Ore06,Ore07,Ore08,Ore09,Ore10,Ore11,Ore12,Ore13,Ore14	*OreG0010096*
scaffold31892	2140336	C	A	Och01,Och02,Och03,Och04,Och05,Och06,Och07,Och08,Och09,Och10,Och11,Och12,Och13,Och14	*OreG0010096*
scaffold31892	2140830	C	G	Ore02,Ore08,Ore11,Ore12	*OreG0010096*
scaffold31892	2149808	G	A	Och01,Och02,Och03,Och04,Och05,Och06,Och07,Och08,Och09,Och10,Och11,Och12,Och13,Och14	*OreG0010097*
scaffold31892	2167821	G	T	NA	*OreG0010098*
scaffold31892	2168920	T	G	NA	*OreG0010098*
scaffold31892	2244407	G	A	NA	*OreG0010104*
scaffold31892	2245310	A	T	NA	*OreG0010104*
scaffold31892	2247095	C	T	Och01,Och02,Och03,Och04,Och05,Och06,Och07,Och08,Och09,Och10,Och11,Och12,Och13,Och14,Ore01,Ore02,Ore03,Ore04,Ore05,Ore06,Ore07,Ore08,Ore09,Ore10,Ore11,Ore12,Ore13,Ore14	*OreG0010104*
scaffold31892	2248202	A	T	Och01,Och02,Och03,Och04,Och05,Och06,Och07,Och08,Och09,Och10,Och11,Och12,Och13,Och14	*OreG0010104*
scaffold31892	2250022	A	T	Och08	*OreG0010104*
scaffold31892	2250025	A	T	NA	*OreG0010104*
scaffold31892	2306552	A	T	Och01,Och02,Och03,Och04,Och05,Och06,Och07,Och08,Och09,Och10,Och11,Och12,Och13,Och14,Ore01,Ore02,Ore03,Ore04,Ore05,Ore06,Ore07,Ore08,Ore09,Ore10,Ore11,Ore12,Ore13,Ore14	*OreG0010112*
scaffold31892	2343297	G	A	NA	*OreG0010115*
scaffold31892	2343315	T	C	Och12	*OreG0010115*
scaffold31892	2384186	T	G	Och01,Och02,Och03,Och04,Och05,Och06,Och07,Och08,Och09,Och10,Och11,Och12,Och13,Och14,Ore01,Ore02,Ore03,Ore04,Ore05,Ore06,Ore07,Ore08,Ore09,Ore10,Ore11,Ore12,Ore13,Ore14	*OreG0010119*
scaffold31892	2384234	T	C	Och02,Och03,Och04,Och05,Och06,Och07,Och08,Och09,Och10,Och11,Och14	*OreG0010119*
scaffold31892	2384716	G	A	Och12,Och13	*OreG0010119*
scaffold31892	2384745	G	C	NA	*OreG0010119*
scaffold31892	2390890	C	T	Och01,Och06,Och07,Och08,Och09,Och10,Och11,Och12,Och13,Och14	*OreG0010120*
scaffold31892	2392828	C	G	NA	*OreG0010121*
scaffold31892	2510534	C	T	NA	*OreG0010130*
scaffold31892	2511771	C	T	NA	*OreG0010130*
scaffold31892	2512360	G	T	Och01,Och02,Och03,Och04,Och05,Och06,Och07,Och08,Och09,Och10,Och11,Och12,Och13,Och14	*OreG0010130*

scaffold31892	2512477	G	A	Och01,Och02,Och03,Och04,Och05,Och06,Och07,Och08,Och09,Och10,Och11,Och12,Och13,Och14,Ore01,Ore02,Ore03,Ore04,Ore05,Ore06,Ore07,Ore08,Ore09,Ore10,Ore11,Ore12,Ore13,Ore14	*OreG0010130*
scaffold31892	2588687	A	G	NA	*OreG0010139*
scaffold31892	2628193	G	A	NA	*OreG0010142*
scaffold31892	2628556	A	G	Ore01,Ore02,Ore03,Ore04,Ore05,Ore06,Ore07,Ore08,Ore09,Ore10,Ore11,Ore12,Ore13,Ore14	*OreG0010142*
scaffold31892	2628705	A	G	Och01,Och02,Och03,Och04,Och05,Och06,Och07,Och09,Och10,Och11,Och12,Och13	*OreG0010142*
scaffold31892	2628778	G	C	NA	*OreG0010142*
scaffold31892	2629006	T	C	Och01,Och02,Och03,Och04,Och05,Och06,Och07,Och09,Och10,Och11,Och12,Och13	*OreG0010142*
scaffold31892	2629066	C	A	NA	*OreG0010142*
scaffold31892	2629357	G	C	NA	*OreG0010142*
scaffold31892	2629384	G	T	Och12,Och13	*OreG0010142*
scaffold31892	2632211	A	G	Och14	*OreG0010142*
scaffold31892	2651081	A	C	Och12,Och13	*OreG0010145*
scaffold31892	2653419	G	T	NA	*OreG0010145*
scaffold31892	2656513	C	A	Ore01,Ore03,Ore09,Ore13	*OreG0010146*
scaffold31892	2657310	A	C	Och02,Och05,Och07,Och12,Och13,Och14	*OreG0010146*
scaffold31892	2658769	C	G	Och01,Och02,Och03,Och04,Och05,Och06,Och07,Och08,Och09,Och10,Och11,Och12,Och13,Och14,Ore01,Ore02,Ore03,Ore04,Ore05,Ore06,Ore07,Ore08,Ore09,Ore10,Ore11,Ore12,Ore13,Ore14	*OreG0010146*
scaffold31892	2669846	T	A	Och01,Och02,Och03,Och04,Och05,Och06,Och07,Och08,Och09,Och10,Och11,Och12,Och13,Och14,Ore01,Ore02,Ore03,Ore04,Ore05,Ore06,Ore07,Ore08,Ore09,Ore10,Ore11,Ore12,Ore13,Ore14	*OreG0010147*
scaffold31892	2672129	C	T	Ore01,Ore02,Ore03,Ore04,Ore05,Ore06,Ore07,Ore08,Ore09,Ore10,Ore11,Ore12,Ore13,Ore14	*OreG0010148*
scaffold31892	2672191	C	T	Och02,Och05,Och09,Och10,Och11,Och12,Och13	*OreG0010148*
scaffold31892	2672219	G	T	NA	*OreG0010148*
scaffold31892	2672227	A	G	Ore01,Ore02,Ore03,Ore04,Ore05,Ore06,Ore07,Ore08,Ore09,Ore10,Ore11,Ore12,Ore13,Ore14	*OreG0010148*
scaffold31892	2672252	C	T	NA	*OreG0010148*
scaffold31892	2672734	C	T	Och01,Och02,Och03,Och04,Och05,Och06,Och07,Och08,Och09,Och10,Och11,Och12,Och13,Och14	*OreG0010148*
scaffold31892	2672911	C	T	Och01,Och02,Och03,Och04,Och05,Och06,Och07,Och08,Och09,Och10,Och11,Och12,Och13,Och14	*OreG0010148*
scaffold31892	2672946	G	A	Och02,Och05,Och09,Och10,Och11,Och12,Och13	*OreG0010148*
scaffold31892	2672994	G	T	NA	*OreG0010148*
scaffold31892	2673218	C	A	Och02,Och05,Och12,Och13	*OreG0010148*
scaffold31892	2673501	C	A	Och01,Och02,Och03,Och04,Och05,Och06,Och07,Och08,Och09,Och10,Och11,Och12,Och13,Och14	*OreG0010148*
scaffold31892	2673509	G	A	Och02,Och05,Och09,Och10,Och11,Och12,Och13,Ore01,Ore03,Ore09,Ore13	*OreG0010148*
scaffold31892	2673543	C	A	Och01,Och02,Och03,Och04,Och05,Och06,Och07,Och08,Och09,Och10,Och11,Och12,Och13,Och14	*OreG0010148*
scaffold31892	2677062	G	A	Och01,Och02,Och03,Och04,Och05,Och06,Och07,Och08,Och09,Och10,Och11,Och12,Och13,Och14	*OreG0010149*
scaffold31892	2677128	C	T	Ore01,Ore02,Ore03,Ore04,Ore05,Ore06,Ore07,Ore08,Ore09,Ore10,Ore11,Ore12,Ore13,Ore14	*OreG0010149*
scaffold31892	2677191	A	G	Och01,Och02,Och03,Och04,Och05,Och06,Och07,Och08,Och09,Och10,Och11,Och12,Och13,Och14	*OreG0010149*
scaffold31892	2677330	T	A	NA	*OreG0010150*
scaffold31892	2677407	C	T	Ore01,Ore02,Ore03,Ore04,Ore05,Ore06,Ore07,Ore08,Ore09,Ore10,Ore11,Ore12,Ore13,Ore14	*OreG0010150*
scaffold31892	2679645	G	A	Ore01,Ore02,Ore03,Ore04,Ore05,Ore06,Ore07,Ore08,Ore09,Ore10,Ore11,Ore12,Ore13,Ore14	*OreG0010150*
scaffold31892	2679683	A	G	NA	*OreG0010150*
scaffold31892	2679755	G	A	NA	*OreG0010150*
scaffold31892	2679757	A	T	Ore01,Ore02,Ore03,Ore04,Ore05,Ore06,Ore07,Ore08,Ore09,Ore10,Ore11,Ore12,Ore13,Ore14	*OreG0010150*
scaffold31892	2679825	G	A	Och13,Och14	*OreG0010150*
scaffold31892	2687701	A	T	NA	*OreG0010151*
scaffold31892	2688055	C	T	NA	*OreG0010151*
scaffold31892	2688130	C	T	Ore01,Ore02,Ore03,Ore04,Ore05,Ore06,Ore07,Ore08,Ore09,Ore10,Ore11,Ore12,Ore13,Ore14	*OreG0010151*
scaffold31892	2688234	T	A	NA	*OreG0010151*
scaffold31892	2688361	C	T	NA	*OreG0010151*
scaffold31892	2688381	G	A	Ore01,Ore02,Ore03,Ore04,Ore05,Ore06,Ore07,Ore08,Ore09,Ore10,Ore11,Ore12,Ore13,Ore14	*OreG0010151*
scaffold31892	2688705	A	T	NA	*OreG0010151*
scaffold31892	2688709	G	A	NA	*OreG0010151*
scaffold31892	2696858	A	C	Ore01,Ore02,Ore03,Ore04,Ore05,Ore06,Ore07,Ore08,Ore09,Ore10,Ore11,Ore12,Ore13,Ore14	*OreG0010152*
scaffold31892	2696876	C	A	NA	*OreG0010152*
scaffold31892	2729587	G	A	Och01	*OreG0010156*

scaffold31892	2730314	G	T	NA	*OreG0010156*
scaffold31892	2743212	T	C	Och03,Och04	*OreG0010157*
scaffold31892	2767934	C	G	NA	*OreG0010161*
scaffold31892	2768349	T	G	NA	*OreG0010161*
scaffold31892	2777952	T	C	NA	*OreG0010162*
scaffold31892	2787722	C	T	NA	*OreG0010163*
scaffold31892	2793402	A	T	Ore01,Ore02,Ore03,Ore04,Ore05,Ore06,Ore07,Ore08,Ore09,Ore10,Ore11,Ore12,Ore13,Ore14	*OreG0010163*
scaffold31892	2800633	C	T	Ore01,Ore02,Ore03,Ore04,Ore05,Ore06,Ore07,Ore08,Ore09,Ore10,Ore11,Ore12,Ore13,Ore14	*OreG0010163*
scaffold31892	2801896	C	T	NA	*OreG0010163*
scaffold31892	2812838	C	T	NA	*OreG0010164*
scaffold31892	2913100	G	A	Och08,Och12,Och13	*OreG0010170*
scaffold31892	2932117	C	T	Ore02,Ore08,Ore11,Ore12	*OreG0010171*
scaffold31892	2956478	A	T	Och01,Och02,Och03,Och04,Och05,Och06,Och07,Och08,Och09,Och10,Och11,Och12,Och13,Och14,Ore01,Ore02,Ore03,Ore04,Ore05,Ore06,Ore07,Ore08,Ore09,Ore10,Ore11,Ore12,Ore13,Ore14	*OreG0010173*
scaffold31892	2961509	G	T	Och01,Och02,Och03,Och04,Och05,Och06,Och07,Och08,Och09,Och10,Och11,Och12,Och13,Och14,Ore01,Ore02,Ore03,Ore04,Ore05,Ore06,Ore07,Ore08,Ore09,Ore10,Ore11,Ore12,Ore13,Ore14	*OreG0010174*
scaffold31892	2991691	C	G	Ore01,Ore02,Ore03,Ore04,Ore05,Ore06,Ore07,Ore08,Ore09,Ore10,Ore11,Ore12,Ore13,Ore14	*OreG0010176*
scaffold31892	3030282	G	T	Och12,Och13	*OreG0010180*
scaffold31892	3045738	T	A	NA	*OreG0010181*
scaffold31892	3137213	C	A	NA	*OreG0010185*
scaffold31892	3201131	G	C	Ore01,Ore02,Ore03,Ore04,Ore05,Ore06,Ore07,Ore08,Ore09,Ore10,Ore11,Ore12,Ore13,Ore14	*OreG0010187*
scaffold31892	3427821	G	A	Och12,Och13	*OreG0010196*
scaffold31892	3499882	G	T	NA	*OreG0010198*
scaffold31892	4051284	G	A	NA	*OreG0010210*
scaffold33218	32546	C	T	NA	*OreG0016638*
scaffold33218	75359	G	A	Och01,Och02,Och03,Och04,Och05,Och06,Och07,Och08,Och09,Och10,Och11,Och12,Och13,Och14,Ore01,Ore02,Ore03,Ore04,Ore05,Ore06,Ore07,Ore08,Ore09,Ore10,Ore11,Ore12,Ore13,Ore14	*OreG0016643*
scaffold33218	133191	C	A	Och13	*OreG0016650*
scaffold33218	135147	G	T	Och01,Och02,Och03,Och04,Och05,Och06,Och07,Och08,Och09,Och10,Och11,Och12,Och13,Och14,Ore01,Ore02,Ore03,Ore04,Ore05,Ore06,Ore07,Ore08,Ore09,Ore10,Ore11,Ore12,Ore13,Ore14	*OreG0016650*
scaffold33218	135160	G	A	Ore01,Ore02,Ore03,Ore04,Ore05,Ore06,Ore07,Ore08,Ore09,Ore10,Ore11,Ore12,Ore13,Ore14	*OreG0016650*
scaffold33218	135248	C	A	Och01,Och02,Och03,Och04,Och05,Och06,Och07,Och08,Och09,Och10,Och11,Och12,Och13,Och14,Ore01,Ore02,Ore03,Ore04,Ore05,Ore06,Ore07,Ore08,Ore09,Ore10,Ore11,Ore12,Ore13,Ore14	*OreG0016650*
scaffold33218	161860	G	T	Ore01,Ore03,Ore04,Ore05	*OreG0016652*
scaffold33218	167591	A	G	Och01,Och07,Och09,Och10,Och11	*OreG0016654*
scaffold33218	190437	G	T	Och13	*OreG0016657*
scaffold33218	190553	T	C	Och01,Och02,Och03,Och04,Och05,Och06,Och07,Och08,Och09,Och10,Och11,Och12,Och13,Och14,Ore01,Ore02,Ore03,Ore04,Ore05,Ore06,Ore07,Ore08,Ore09,Ore10,Ore11,Ore12,Ore13,Ore14	*OreG0016657*
scaffold33218	190835	C	T	Ore01,Ore02,Ore03,Ore04,Ore05,Ore06,Ore07,Ore08,Ore09,Ore10,Ore11,Ore12,Ore13,Ore14	*OreG0016657*
scaffold33218	193385	G	A	Och03,Och13	*OreG0016659*
scaffold33218	195367	C	T	NA	*OreG0016659*
scaffold33218	217540	C	T	Ore03,Ore04,Ore05	*OreG0016661*
scaffold33218	224236	C	T	NA	*OreG0016662*
scaffold33218	250191	A	T	NA	*OreG0016663*
scaffold33218	265199	G	A	Ore01,Ore02,Ore03,Ore04,Ore05,Ore06,Ore07,Ore08,Ore09,Ore10,Ore11,Ore12,Ore13,Ore14	*OreG0016665*
scaffold33218	266937	G	A	Och06	*OreG0016665*
scaffold33218	299106	A	G	NA	*OreG0016667*
scaffold33218	307079	G	C	NA	*OreG0016668*
scaffold33218	323419	C	A	Ore03,Ore04,Ore05	*OreG0016669*
scaffold33218	323649	A	G	NA	*OreG0016669*
scaffold33218	323656	G	A	NA	*OreG0016669*
scaffold33218	325123	T	G	Och09,Och10,Och11,Och13	*OreG0016669*
scaffold33218	326025	T	A	NA	*OreG0016669*
scaffold33218	327151	A	T	Och01,Och02,Och03,Och04,Och05,Och06,Och07,Och08,Och09,Och10,Och11,Och12,Och13,Och14	*OreG0016669*
scaffold33218	331122	G	A	NA	*OreG0016670*
scaffold33218	331625	A	G	NA	*OreG0016670*
scaffold33218	331727	T	A	Ore01,Ore02,Ore03,Ore04,Ore05,Ore06,Ore07,Ore08,Ore09,Ore10,Ore11,Ore12,Ore13,Ore14	*OreG0016670*
scaffold33218	382071	C	T	NA	*OreG0016676*
scaffold33218	406698	G	T	NA	*OreG0016678*

scaffold33218	413987	A	C	Och01,Och02,Och03,Och04,Och05,Och06,Och07,Och08,Och09,Och10,Och11,Och12,Och13,Och14,Ore01,Ore02,Ore03,Ore04,Ore05,Ore06,Ore07,Ore08,Ore09,Ore10,Ore11,Ore12,Ore13,Ore14	*OreG0016679*
scaffold33218	443195	T	A	Och13	*OreG0016683*
scaffold33218	460751	C	T	Och02,Och04,Och07,Och12,Och13	*OreG0016685*
scaffold33218	466981	T	C	NA	*OreG0016688*
scaffold33218	467066	C	T	Ore01,Ore02,Ore03,Ore04,Ore05,Ore06,Ore07,Ore08,Ore09,Ore10,Ore11,Ore12,Ore13,Ore14	*OreG0016688*
scaffold33218	474856	T	C	Ore01,Ore02,Ore03,Ore04,Ore05,Ore06,Ore07,Ore08,Ore09,Ore10,Ore11,Ore12,Ore13,Ore14	*OreG0016689*
scaffold33218	475416	C	G	NA	*OreG0016689*
scaffold33218	477832	A	G	Och01,Och02,Och03,Och04,Och05,Och06,Och07,Och08,Och09,Och10,Och11,Och12,Och13,Ore01,Ore02,Ore03,Ore04,Ore05,Ore06,Ore07,Ore08,Ore09,Ore10,Ore11,Ore12,Ore13,Ore14	*OreG0016690*
scaffold33218	478340	T	C	Ore01,Ore02,Ore03,Ore04,Ore05,Ore06,Ore07,Ore08,Ore09,Ore10,Ore11,Ore12,Ore13,Ore14	*OreG0016690*
scaffold33218	483474	A	T	Och01,Och02,Och03,Och04,Och05,Och06,Och07,Och08,Och09,Och10,Och11,Och12,Och13,Och14,Ore01,Ore02,Ore03,Ore04,Ore05,Ore06,Ore07,Ore08,Ore09,Ore10,Ore11,Ore12,Ore13,Ore14	*OreG0016690*
scaffold33218	485153	T	C	Ore01,Ore02,Ore03,Ore04,Ore05,Ore06,Ore07,Ore08,Ore09,Ore10,Ore11,Ore12,Ore13,Ore14	*OreG0016690*
scaffold33218	523228	T	C	Och01,Och06	*OreG0016697*
scaffold33218	542122	G	T	Ore01,Ore02,Ore03,Ore04,Ore05,Ore06,Ore07,Ore08,Ore09,Ore10,Ore11,Ore12,Ore13,Ore14	*OreG0016699*
scaffold33218	551452	G	A	Och13	*OreG0016700*
scaffold33218	557294	G	A	Ore01,Ore03,Ore04,Ore05	*OreG0016702*
scaffold33218	559329	G	A	Ore02	*OreG0016702*
scaffold33218	559587	T	G	Ore02	*OreG0016702*
scaffold33218	567582	G	T	Och01,Och02,Och03,Och04,Och05,Och06,Och07,Och08,Och09,Och10,Och11,Och12,Och13,Och14	*OreG0016704*
scaffold33218	567751	G	T	Och13	*OreG0016704*
scaffold33218	567826	A	C	Och01,Och02,Och03,Och04,Och05,Och06,Och07,Och08,Och09,Och10,Och11,Och12,Och13,Och14	*OreG0016704*
scaffold33218	569344	A	T	Ore02	*OreG0016704*
scaffold33218	569379	T	C	Och02,Och03,Och04,Och05,Och06,Och07,Och08,Och09,Och10,Och11,Och12,Och13,Och14	*OreG0016704*
scaffold33218	569754	T	G	NA	*OreG0016704*
scaffold33218	573969	C	G	NA	*OreG0016705*
scaffold33218	593355	A	G	NA	*OreG0016708*
scaffold33218	643158	G	A	Och07,Och13	*OreG0016713*
scaffold33218	646321	C	T	Och01,Och02,Och03,Och04,Och05,Och06,Och07,Och08,Och09,Och10,Och11,Och12,Och13,Och14,Ore01,Ore02,Ore03,Ore04,Ore05,Ore06,Ore07,Ore08,Ore09,Ore10,Ore11,Ore12,Ore13,Ore14	*OreG0016713*
scaffold33218	646488	A	G	Och01,Och02,Och03,Och04,Och05,Och06,Och07,Och08,Och09,Och10,Och11,Och12,Och13,Och14	*OreG0016713*
scaffold33218	660093	G	A	Och01	*OreG0016716*
scaffold33218	669774	C	T	NA	*OreG0016718*
scaffold33218	669807	A	G	Och01	*OreG0016718*
scaffold33218	671434	A	T	Ore01,Ore02,Ore03,Ore04,Ore05,Ore06,Ore07,Ore08,Ore09,Ore10,Ore11,Ore12,Ore13,Ore14	*OreG0016718*
scaffold33218	685970	C	G	Och01,Och02,Och03,Och04,Och05,Och06,Och07,Och08,Och09,Och10,Och11,Och12,Och13,Och14,Ore01,Ore02,Ore03,Ore04,Ore05,Ore06,Ore07,Ore08,Ore09,Ore10,Ore11,Ore12,Ore13,Ore14	*OreG0016720*
scaffold33218	709547	C	A	Ore01,Ore02,Ore03,Ore04,Ore05,Ore06,Ore07,Ore08,Ore09,Ore10,Ore11,Ore12,Ore13,Ore14	*OreG0016724*
scaffold33218	709591	G	A	Och01,Och02,Och03,Och04,Och05,Och06,Och07,Och08,Och09,Och10,Och11,Och12,Och13,Och14	*OreG0016724*
scaffold33218	709997	T	A	Och01,Och02,Och03,Och04,Och05,Och06,Och07,Och08,Och09,Och10,Och11,Och12,Och13,Och14	*OreG0016724*
scaffold33218	710048	C	A	Och01,Och02,Och03,Och04,Och05,Och06,Och07,Och08,Och09,Och10,Och11,Och12,Och13,Och14,Ore01,Ore02,Ore03,Ore04,Ore05,Ore06,Ore07,Ore08,Ore09,Ore10,Ore11,Ore12,Ore13,Ore14	*OreG0016724*
scaffold33218	763502	T	A	NA	*OreG0016731*
scaffold33218	763603	A	T	NA	*OreG0016731*
scaffold33218	766298	C	A	Och01,Och02,Och03,Och04,Och05,Och06,Och07,Och08,Och09,Och10,Och11,Och12,Och13,Och14,Ore01,Ore02,Ore03,Ore04,Ore05,Ore06,Ore07,Ore08,Ore09,Ore10,Ore11,Ore12,Ore13,Ore14	*OreG0016731*
scaffold33218	772696	T	A	NA	*OreG0016732*
scaffold33218	772908	G	A	NA	*OreG0016732*
scaffold33218	775971	A	C	Och01,Och02,Och03,Och04,Och05,Och06,Och07,Och08,Och09,Och10,Och11,Och12,Och13,Och14,Ore01,Ore02,Ore03,Ore04,Ore05,Ore06,Ore07,Ore08,Ore09,Ore10,Ore11,Ore12,Ore13,Ore14	*OreG0016733*
scaffold33218	779281	T	A	Ore02,Ore06,Ore07,Ore11,Ore13	*OreG0016733*
scaffold33218	782661	A	T	Och01,Och02,Och03,Och04,Och05,Och06,Och07,Och08,Och09,Och10,Och11,Och12,Och13,Och14,Ore01,Ore02,Ore03,Ore04,Ore05,Ore06,Ore07,Ore08,Ore09,Ore10,Ore11,Ore12,Ore13,Ore14	*OreG0016733*

scaffold33218	789019	G	C	NA	*OreG0016734*
scaffold33218	789107	G	A	Ore01,Ore02,Ore03,Ore04,Ore05,Ore06,Ore07,Ore08,Ore09,Ore10,Ore11,Ore12,Ore13,Ore14	*OreG0016734*
scaffold33218	790328	C	T	Ore03,Ore04,Ore05	*OreG0016734*
scaffold33218	791285	G	A	Ore03,Ore04,Ore05	*OreG0016734*
scaffold33218	793346	A	T	Ore01,Ore02,Ore03,Ore04,Ore05,Ore06,Ore07,Ore08,Ore09,Ore10,Ore11,Ore12,Ore13,Ore14	*OreG0016734*
scaffold33218	813609	A	T	Och01,Och02,Och03,Och04,Och05,Och06,Och07,Och08,Och09,Och10,Och11,Och12,Och13,Och14,Ore01,Ore02,Ore03,Ore04,Ore05,Ore06,Ore07,Ore08,Ore09,Ore10,Ore11,Ore12,Ore13,Ore14	*OreG0016737*
scaffold33218	813618	C	T	Ore01,Ore02,Ore03,Ore04,Ore05,Ore06,Ore07,Ore08,Ore09,Ore10,Ore11,Ore12,Ore13,Ore14	*OreG0016737*
scaffold33218	841457	C	G	Ore02,Ore06,Ore07,Ore11,Ore13	*OreG0016741*
scaffold33218	848127	C	T	Och13	*OreG0016743*
scaffold33249	53937	A	T	Och01,Och02,Och03,Och04,Och05,Och06,Och07,Och08,Och09,Och10,Och11,Och12,Och13,Och14,Ore01,Ore02,Ore03,Ore04,Ore05,Ore06,Ore07,Ore08,Ore09,Ore10,Ore11,Ore12,Ore13,Ore14	*OreG0017594*
scaffold33249	58967	A	G	NA	*OreG0017596*
scaffold33249	59364	T	C	Och01,Och02,Och03,Och04,Och05,Och06,Och07,Och08,Och09,Och10,Och11,Och12,Och13,Och14	*OreG0017596*
scaffold33249	125055	T	G	Ore04,Ore05	*OreG0017601*
scaffold33249	155380	C	T	NA	*OreG0017603*
scaffold33249	155821	G	A	Och03,Och05,Och08,Och14	*OreG0017603*
scaffold33270	9808	C	A	Och01,Och02,Och03,Och04,Och06,Och07,Och08,Och09,Och10,Och11,Och12	*OreG0018160*
scaffold33270	10033	C	G	Och01,Och02,Och03,Och04,Och06,Och07,Och08,Och09,Och10,Och11	*OreG0018160*
scaffold33270	10104	A	G	NA	*OreG0018160*
scaffold33259	97223	C	T	Ore14	*OreG0017953*
scaffold33259	98433	C	T	NA	*OreG0017953*
scaffold33246	40146	A	G	NA	*OreG0017440*
scaffold33246	80358	A	G	NA	*OreG0017442*
scaffold33246	80428	G	T	Och02,Och03,Och04,Och05,Och06,Och09,Och10,Och11,Och12,Och13,Och14,Ore08	*OreG0017442*
scaffold33246	80710	C	T	NA	*OreG0017442*
scaffold33246	80722	G	A	NA	*OreG0017442*
scaffold33246	85273	G	A	NA	*OreG0017443*
scaffold33246	86556	C	A	Och02,Och03,Och04,Och05,Och06,Och09,Och10,Och11,Och12,Och13,Och14,Ore08	*OreG0017443*
scaffold33246	86565	T	C	NA	*OreG0017443*
scaffold33246	90298	G	A	Ore08	*OreG0017444*
scaffold33246	97336	G	C	Ore08	*OreG0017445*
scaffold33258	365	A	G	NA	*OreG0017919*
scaffold33258	408	C	T	Ore01,Ore02,Ore03,Ore04,Ore05,Ore06,Ore07,Ore08,Ore09,Ore10,Ore11,Ore12,Ore13,Ore14	*OreG0017919*
scaffold33258	503	G	A	NA	*OreG0017919*
scaffold33258	647	G	C	Ore01,Ore02,Ore03,Ore04,Ore05,Ore06,Ore07,Ore08,Ore09,Ore10,Ore11,Ore12,Ore13,Ore14	*OreG0017919*
scaffold33258	661	C	T	Ore01,Ore02,Ore03,Ore04,Ore05,Ore06,Ore07,Ore08,Ore09,Ore10,Ore11,Ore12,Ore13,Ore14	*OreG0017919*
scaffold33258	679	A	T	NA	*OreG0017919*
scaffold33258	763	A	C	Ore01,Ore02,Ore03,Ore04,Ore05,Ore06,Ore07,Ore08,Ore09,Ore10,Ore11,Ore12,Ore13,Ore14	*OreG0017919*
scaffold33258	786	C	A	NA	*OreG0017919*
scaffold33258	889	T	C	Ore01,Ore02,Ore03,Ore04,Ore05,Ore06,Ore07,Ore08,Ore09,Ore10,Ore11,Ore12,Ore13,Ore14	*OreG0017919*
scaffold33258	921	G	A	Ore01,Ore04,Ore05	*OreG0017919*
scaffold33258	945	A	T	Och01,Och08	*OreG0017919*
scaffold33258	961	C	A	Och01,Och08	*OreG0017919*
scaffold33258	40804	G	T	Och09,Och10,Och11	*OreG0017924*
scaffold33258	48054	G	A	NA	*OreG0017925*
scaffold33258	55152	G	A	Ore01,Ore02,Ore03,Ore04,Ore05,Ore06,Ore07,Ore08,Ore09,Ore10,Ore11,Ore12,Ore13,Ore14	*OreG0017927*
scaffold33258	55272	T	G	Ore01,Ore03,Ore04,Ore05,Ore09,Ore10,Ore12,Ore13	*OreG0017927*
scaffold33258	55740	C	G	Och01,Och07,Och08,Och09,Och10,Och11,Ore01,Ore02,Ore03,Ore04,Ore05,Ore06,Ore07,Ore08,Ore09,Ore10,Ore11,Ore12,Ore13,Ore14	*OreG0017927*
scaffold33258	63496	T	C	NA	*OreG0017926*
scaffold33258	71509	G	C	Ore13	*OreG0017929*
scaffold33258	103761	T	C	Och01,Och07,Och08,Ore01,Ore02,Ore03,Ore04,Ore05,Ore06,Ore07,Ore08,Ore09,Ore10,Ore11,Ore12,Ore13,Ore14	*OreG0017932*
scaffold33258	105781	C	T	NA	*OreG0017933*
scaffold33258	106598	G	C	Ore04,Ore05	*OreG0017933*
scaffold33258	106671	C	T	Och12	*OreG0017933*
scaffold33258	107074	G	A	Ore01,Ore02,Ore03,Ore04,Ore05,Ore06,Ore07,Ore08,Ore09,Ore10,Ore11,Ore12,Ore13,Ore14	*OreG0017933*
scaffold33258	107173	T	G	Och02,Och03,Och04,Och05,Och06,Och12,Och13	*OreG0017933*

scaffold33258	108101	G	T	Och01,Och08,Ore01,Ore02,Ore03,Ore04,Ore05,Ore06,Ore07,Ore08,Ore09,Ore10,Ore11,Ore12,Ore13,Ore14	*OreG0017933*
scaffold33258	111342	G	T	Ore09,Ore10,Ore13	*OreG0017933*
scaffold33258	111453	G	T	Och02,Och03,Och04,Och05,Och06,Och12,Och13,Och14	*OreG0017933*
scaffold33258	120012	A	G	Ore04,Ore05,Ore06,Ore07,Ore08,Ore14	*OreG0017933*
scaffold33258	134444	T	A	Och03,Och04	*OreG0017936*
scaffold33258	134693	C	T	Ore01,Ore02,Ore03,Ore04,Ore05,Ore06,Ore07,Ore08,Ore09,Ore10,Ore11,Ore12,Ore13,Ore14	*OreG0017936*
scaffold33258	154640	C	A	NA	*OreG0017939*
scaffold33258	154655	T	G	Ore01,Ore02,Ore03,Ore04,Ore05,Ore06,Ore07,Ore08,Ore09,Ore10,Ore11,Ore12,Ore13,Ore14	*OreG0017939*
scaffold33258	154799	T	A	NA	*OreG0017939*
scaffold33258	155013	G	A	Och08,Och12	*OreG0017939*
scaffold33258	155028	T	A	Och01,Och02,Och03,Och04,Och05,Och06,Och07,Och08,Och09,Och10,Och11,Och12,Och13,Och14,Ore01,Ore02,Ore03,Ore04,Ore05,Ore06,Ore07,Ore08,Ore09,Ore10,Ore11,Ore12,Ore13,Ore14	*OreG0017939*
scaffold33258	155038	G	T	Och08,Och12	*OreG0017939*
scaffold33258	155494	C	G	Ore01,Ore02,Ore03,Ore04,Ore05,Ore06,Ore07,Ore08,Ore09,Ore10,Ore11,Ore12,Ore13,Ore14	*OreG0017939*
scaffold33258	155539	C	A	Ore01,Ore02,Ore03,Ore04,Ore05,Ore06,Ore07,Ore08,Ore09,Ore10,Ore11,Ore12,Ore13,Ore14	*OreG0017939*
scaffold33258	155574	A	C	Ore01,Ore02,Ore03,Ore04,Ore05,Ore06,Ore07,Ore08,Ore09,Ore10,Ore11,Ore12,Ore13,Ore14	*OreG0017939*
scaffold33258	155707	A	C	Och08,Och12	*OreG0017939*
scaffold33258	156183	C	T	NA	*OreG0017939*
scaffold33258	177246	C	G	NA	*OreG0017941*
scaffold33258	177653	C	T	NA	*OreG0017941*
scaffold33258	183338	T	G	Ore01,Ore02,Ore03,Ore04,Ore05,Ore06,Ore07,Ore08,Ore09,Ore10,Ore11,Ore12,Ore13,Ore14	*OreG0017943*
scaffold33258	183387	C	G	Ore01,Ore02,Ore03,Ore04,Ore05,Ore06,Ore07,Ore08,Ore09,Ore10,Ore11,Ore12,Ore13,Ore14	*OreG0017943*
scaffold33258	183436	C	A	NA	*OreG0017943*
scaffold33258	184338	T	C	NA	*OreG0017943*
scaffold33233	32278	C	T	Och01,Och02,Och03,Och04,Och05,Och06,Och07,Och08,Och09,Och10,Och11,Och12,Och13,Och14,Ore01,Ore02,Ore03,Ore04,Ore05,Ore06,Ore07,Ore08,Ore09,Ore10,Ore11,Ore12,Ore13,Ore14	*OreG0017189*
scaffold33233	162591	T	A	NA	*OreG0017193*
scaffold33233	163175	T	A	NA	*OreG0017193*
scaffold33233	163650	G	T	Ore01,Ore04,Ore05,Ore11	*OreG0017193*
scaffold33233	163713	G	T	Och01,Och02,Och03,Och04,Och05,Och06,Och07,Och08,Och09,Och10,Och11,Och12,Och13,Och14	*OreG0017193*
scaffold33233	169744	T	C	NA	*OreG0017194*
scaffold33233	205837	G	T	NA	*OreG0017196*
scaffold33233	212626	C	T	Ore06,Ore07,Ore08,Ore12,Ore13,Ore14	*OreG0017196*
scaffold33233	213070	T	A	Ore01,Ore02,Ore03,Ore04,Ore05,Ore06,Ore07,Ore08,Ore09,Ore10,Ore11,Ore12,Ore13,Ore14	*OreG0017196*
scaffold33233	218194	C	A	Och12,Och13	*OreG0017197*
scaffold33233	229375	C	T	Och01,Och02,Och03,Och04,Och05,Och06,Och08,Och09,Och10,Och11,Och12,Och13,Och14	*OreG0017197*
scaffold33233	238312	T	A	NA	*OreG0017197*
scaffold33233	239016	G	A	Och12,Och13	*OreG0017197*
scaffold33233	289680	G	T	Och01,Och02,Och03,Och04,Och05,Och06,Och07,Och08,Och09,Och10,Och11,Och12,Och13,Och14,Ore01,Ore02,Ore03,Ore04,Ore05,Ore06,Ore07,Ore08,Ore09,Ore10,Ore11,Ore12,Ore13,Ore14	*OreG0017199*
scaffold33233	289977	G	A	Och01,Och02,Och03,Och04,Och05,Och06,Och08,Och09,Och10,Och11,Och12,Och13,Och14	*OreG0017199*
scaffold33233	290000	A	G	Och01,Och03,Och04,Och05,Och07,Och08,Och09,Och10,Och11,Och12,Och13,Och14	*OreG0017199*
scaffold33233	290309	T	A	Och01,Och02,Och03,Och04,Och05,Och06,Och08,Och09,Och10,Och11,Och12,Och13,Och14	*OreG0017199*
scaffold33233	310057	A	C	Och01,Och02,Och03,Och04,Och05,Och06,Och07,Och08,Och09,Och10,Och11,Och12,Och13,Och14,Ore01,Ore02,Ore03,Ore04,Ore05,Ore06,Ore07,Ore08,Ore09,Ore10,Ore11,Ore12,Ore13,Ore14	*OreG0017200*
scaffold33233	319169	A	T	Och11	*OreG0017200*
scaffold33233	336900	G	C	NA	*OreG0017201*
scaffold33233	347938	T	C	NA	*OreG0017202*
scaffold33233	352924	A	T	NA	*OreG0017202*
scaffold33233	354201	G	C	Och11	*OreG0017202*
scaffold33233	372960	G	A	Och01,Och02,Och03,Och04,Och05,Och06,Och07,Och08,Och09,Och10,Och11,Och12,Och13,Och14	*OreG0017205*
scaffold33233	405685	A	C	Och11	*OreG0017206*
scaffold33233	489593	A	G	Och01,Och02,Och03,Och04,Och05,Och06,Och07,Och08,Och09,Och10,Och11,Och12,Och13,Och14,Ore01,Ore02,Ore03,Ore04,Ore05,Ore06,Ore07,Ore08,Ore09,Ore10,Ore11,Ore12,Ore13,Ore14	*OreG0017218*
scaffold33233	490031	A	T	NA	*OreG0017218*

scaffold33233	490107	G	A	NA	*OreG0017218*
scaffold33233	490145	G	A	NA	*OreG0017218*
scaffold33233	490466	G	C	NA	*OreG0017218*
scaffold33245	6639	G	A	NA	*OreG0017415*
scaffold33245	13825	T	C	NA	*OreG0017417*
scaffold33245	50391	C	T	Ore01,Ore02,Ore03,Ore04,Ore05,Ore06,Ore07,Ore08,Ore09,Ore10,Ore11,Ore12,Ore13,Ore14	*OreG0017420*
scaffold33245	70803	T	A	Och01,Och02,Och03,Och04,Och05,Och06,Och07,Och08,Och09,Och10,Och11,Och12,Och13,Och14,Ore01,Ore02,Ore03,Ore04,Ore05,Ore06,Ore07,Ore08,Ore09,Ore10,Ore11,Ore12,Ore13,Ore14	*OreG0017424*
scaffold33245	87310	A	G	Ore01,Ore02,Ore03,Ore04,Ore05,Ore06,Ore07,Ore08,Ore09,Ore10,Ore11,Ore12,Ore13,Ore14	*OreG0017425*
scaffold33245	249001	C	T	Ore01,Ore02,Ore03,Ore04,Ore05,Ore06,Ore07,Ore08,Ore09,Ore10,Ore11,Ore12,Ore13,Ore14	*OreG0017437*
scaffold33245	251193	T	C	Ore01,Ore02,Ore03,Ore04,Ore05,Ore06,Ore07,Ore08,Ore09,Ore10,Ore11,Ore12,Ore13,Ore14	*OreG0017437*
scaffold33245	255357	G	A	Ore01,Ore02,Ore03,Ore04,Ore05,Ore06,Ore07,Ore08,Ore09,Ore10,Ore11,Ore12,Ore13,Ore14	*OreG0017438*
scaffold3100	495972	A	T	Ore01,Ore02,Ore03,Ore04,Ore05,Ore06,Ore07,Ore08,Ore09,Ore10,Ore11,Ore12,Ore13,Ore14	*OreG0009148*
scaffold3100	509008	G	A	NA	*OreG0009150*
scaffold3100	546165	C	T	Ore01,Ore02,Ore03,Ore04,Ore05,Ore06,Ore07,Ore08,Ore09,Ore10,Ore11,Ore12,Ore13,Ore14	*OreG0009153*
scaffold3100	546295	C	T	Och01,Ore01,Ore02,Ore03,Ore04,Ore05,Ore06,Ore07,Ore08,Ore09,Ore10,Ore11,Ore12,Ore13,Ore14	*OreG0009153*
scaffold3100	548256	T	C	NA	*OreG0009154*
scaffold3100	569157	T	A	Och01,Och02,Och03,Och04,Och05,Och06,Och07,Och08,Och09,Och10,Och11,Och12,Och13	*OreG0009159*
scaffold3100	569812	G	T	Och01,Och02,Och03,Och04,Och05,Och06,Och07,Och08,Och09,Och10,Och11,Och12,Och13,Och14,Ore01,Ore02,Ore03,Ore04,Ore05,Ore06,Ore07,Ore08,Ore09,Ore10,Ore11,Ore12,Ore13,Ore14	*OreG0009159*
scaffold3100	571170	C	G	Och01,Och02,Och03,Och04,Och05,Och06,Och07,Och08,Och09,Och10,Och11,Och12,Och13	*OreG0009160*
scaffold3100	574376	G	A	NA	*OreG0009160*
scaffold3100	598049	G	A	Och01,Och02,Och03,Och04,Och05,Och06,Och09,Och10,Och12,Och13	*OreG0009162*
scaffold3100	598816	A	T	NA	*OreG0009162*
scaffold3100	652176	T	C	Ore01,Ore02,Ore03,Ore04,Ore05,Ore06,Ore07,Ore08,Ore09,Ore10,Ore11,Ore12,Ore13,Ore14	*OreG0009164*
scaffold3100	652557	T	G	NA	*OreG0009164*
scaffold3100	696234	T	A	NA	*OreG0009169*
scaffold3100	729260	C	G	Och07,Och08	*OreG0009174*
scaffold3100	737740	T	G	Ore01,Ore02,Ore03,Ore04,Ore05,Ore06,Ore07,Ore08,Ore09,Ore10,Ore11,Ore12,Ore13,Ore14	*OreG0009175*
scaffold3100	737743	C	A	Ore01,Ore02,Ore03,Ore04,Ore05,Ore06,Ore07,Ore08,Ore09,Ore10,Ore11,Ore12,Ore13,Ore14	*OreG0009175*
scaffold3100	771255	T	A	Och01,Och02,Och03,Och04,Och05,Och06,Och07,Och08,Och09,Och10,Och11,Och12,Och13,Och14,Ore01,Ore02,Ore03,Ore04,Ore05,Ore06,Ore07,Ore08,Ore09,Ore10,Ore11,Ore12,Ore13,Ore14	*OreG0009180*
scaffold3100	783646	T	C	NA	*OreG0009182*
scaffold3100	785499	A	T	NA	*OreG0009182*
scaffold3100	789315	C	A	NA	*OreG0009183*
scaffold3100	789745	A	C	Och14	*OreG0009183*
scaffold3100	790226	A	T	NA	*OreG0009183*
scaffold3100	791988	A	T	Och02,Och03,Och04,Och05,Och06,Och07,Och12,Och13,Och14	*OreG0009183*
scaffold3100	809414	G	C	Ore01,Ore02,Ore03,Ore04,Ore05,Ore06,Ore07,Ore08,Ore09,Ore10,Ore11,Ore12,Ore13,Ore14	*OreG0009184*
scaffold3100	813763	G	A	Och01,Och02,Och03,Och04,Och05,Och06,Och07,Och08,Och09,Och10,Och11,Och12,Och13,Och14,Ore01,Ore02,Ore03,Ore04,Ore05,Ore06,Ore07,Ore08,Ore09,Ore10,Ore11,Ore12,Ore13,Ore14	*OreG0009184*
scaffold3100	814510	C	T	Och01,Och02,Och03,Och04,Och05,Och06,Och07,Och08,Och09,Och10,Och11,Och12,Och13,Och14,Ore01,Ore02,Ore03,Ore04,Ore05,Ore06,Ore07,Ore08,Ore09,Ore10,Ore11,Ore12,Ore13,Ore14	*OreG0009184*
scaffold3100	828165	G	T	NA	*OreG0009186*
scaffold3100	828797	A	G	NA	*OreG0009186*
scaffold3100	926348	G	T	Och05	*OreG0009192*
scaffold3100	1135552	G	A	Och01,Och02,Och03,Och04,Och05,Och06,Och07,Och08,Och09,Och10,Och11,Och12,Och13,Och14,Ore01,Ore02,Ore03,Ore04,Ore05,Ore06,Ore07,Ore08,Ore09,Ore10,Ore11,Ore12,Ore13,Ore14	*OreG0009198*
scaffold3100	1135569	A	C	NA	*OreG0009198*
scaffold3100	1135874	T	G	Och01,Och02,Och03,Och04,Och05,Och06,Och07,Och08,Och09,Och10,Och11,Och12,Och13,Och14,Ore01,Ore02,Ore03,Ore04,Ore05,Ore06,Ore07,Ore08,Ore09,Ore10,Ore11,Ore12,Ore13,Ore14	*OreG0009198*
scaffold3100	1164982	C	T	Ore01,Ore02,Ore03,Ore04,Ore05,Ore06,Ore07,Ore08,Ore09,Ore10,Ore11,Ore12,Ore13,Ore14	*OreG0009199*
scaffold3100	1683555	C	A	NA	*OreG0009209*
scaffold3100	1691457	C	A	NA	*OreG0009209*

scaffold3100	1821099	C	G	NA	*OreG0009212*
scaffold3100	2395082	G	A	NA	*OreG0009221*
scaffold3100	2566487	T	C	Ore01,Ore02,Ore03,Ore04,Ore05,Ore06,Ore07,Ore08,Ore09,Ore10,Ore11,Ore12,Ore13,Ore14	*OreG0009222*
scaffold3100	2729318	G	A	Ore01,Ore02,Ore03,Ore04,Ore05,Ore06,Ore07,Ore08,Ore09,Ore10,Ore11,Ore12,Ore13,Ore14	*OreG0009224*
scaffold3100	2838067	C	G	Och06	*OreG0009229*
scaffold3100	2868484	T	A	NA	*OreG0009231*
scaffold3100	2893020	C	T	Ore01,Ore02,Ore03,Ore04,Ore05,Ore06,Ore07,Ore08,Ore09,Ore10,Ore11,Ore12,Ore13,Ore14	*OreG0009232*
scaffold3100	2926734	G	T	Och11	*OreG0009233*
scaffold3100	2962816	T	C	NA	*OreG0009235*
scaffold3100	2979043	C	A	Och08,Och11	*OreG0009237*
scaffold3100	2993915	A	G	Ore03,Ore06,Ore09,Ore11,Ore13,Ore14	*OreG0009238*
scaffold3100	3087966	G	A	Och08,Och11	*OreG0009240*
scaffold3100	3088107	G	C	Ore03,Ore06,Ore09,Ore11,Ore13,Ore14	*OreG0009240*
scaffold3100	3468222	C	T	Ore01,Ore02,Ore03,Ore04,Ore05,Ore06,Ore07,Ore08,Ore09,Ore10,Ore11,Ore12,Ore13,Ore14	*OreG0009245*
scaffold3100	3469012	A	C	Och01,Och02,Och03,Och04,Och05,Och06,Och07,Och08,Och09,Och10,Och11,Och12,Och13,Och14,Ore01,Ore02,Ore03,Ore04,Ore05,Ore06,Ore07,Ore08,Ore09,Ore10,Ore11,Ore12,Ore13,Ore14	*OreG0009245*
scaffold3100	3784878	T	A	Ore01,Ore02,Ore03,Ore04,Ore05,Ore06,Ore07,Ore08,Ore09,Ore10,Ore11,Ore12,Ore13,Ore14	*OreG0009255*
scaffold3100	3785103	C	T	Och01,Och02,Och03,Och04,Och05,Och06,Och07,Och08,Och09,Och10,Och11,Och12,Och13,Och14	*OreG0009255*
scaffold3100	3785184	C	T	Ore01,Ore02,Ore03,Ore04,Ore05,Ore06,Ore07,Ore08,Ore09,Ore10,Ore11,Ore12,Ore13,Ore14	*OreG0009255*
scaffold3100	3785256	T	C	Och01,Och02,Och03,Och04,Och05,Och06,Och07,Och08,Och09,Och10,Och11,Och12,Och13,Och14,Ore01,Ore02,Ore03,Ore04,Ore05,Ore06,Ore07,Ore08,Ore09,Ore10,Ore11,Ore12,Ore13,Ore14	*OreG0009255*
scaffold3100	3785316	C	T	Och01,Och02,Och03,Och04,Och05,Och06,Och07,Och08,Och09,Och10,Och11,Och12,Och13,Och14,Ore01,Ore02,Ore03,Ore04,Ore05,Ore06,Ore07,Ore08,Ore09,Ore10,Ore11,Ore12,Ore13,Ore14	*OreG0009255*
scaffold3100	3785628	C	A	Ore01,Ore02,Ore03,Ore04,Ore05,Ore06,Ore07,Ore08,Ore09,Ore10,Ore11,Ore12,Ore13,Ore14	*OreG0009255*
scaffold3100	3794168	G	A	Ore01,Ore02,Ore03,Ore04,Ore05,Ore06,Ore07,Ore08,Ore09,Ore10,Ore11,Ore12,Ore13,Ore14	*OreG0009255*
scaffold3100	3794204	C	T	Ore01,Ore02,Ore03,Ore04,Ore05,Ore06,Ore07,Ore08,Ore09,Ore10,Ore11,Ore12,Ore13,Ore14	*OreG0009255*
scaffold3100	3794218	A	G	Och01,Och02,Och03,Och04,Och05,Och06,Och07,Och08,Och09,Och10,Och11,Och12,Och13,Och14,Ore01,Ore02,Ore03,Ore04,Ore05,Ore06,Ore07,Ore08,Ore09,Ore10,Ore11,Ore12,Ore13,Ore14	*OreG0009255*
scaffold3100	3794222	A	G	Och01,Och02,Och03,Och04,Och05,Och06,Och07,Och08,Och09,Och10,Och11,Och12,Och13,Och14,Ore01,Ore02,Ore03,Ore04,Ore05,Ore06,Ore07,Ore08,Ore09,Ore10,Ore11,Ore12,Ore13,Ore14	*OreG0009255*
scaffold3100	3794241	C	A	Ore01,Ore02,Ore03,Ore04,Ore05,Ore06,Ore07,Ore08,Ore09,Ore10,Ore11,Ore12,Ore13,Ore14	*OreG0009255*
scaffold3100	4114449	T	C	NA	*OreG0009261*
scaffold3100	4115055	G	A	Och01	*OreG0009261*
scaffold3100	4163762	C	A	NA	*OreG0009262*
scaffold3100	4285793	G	C	NA	*OreG0009266*
scaffold3100	4285829	G	T	NA	*OreG0009266*
scaffold3100	4405461	A	G	NA	*OreG0009271*
scaffold3100	4406873	C	T	Ore01,Ore02,Ore03,Ore04,Ore05,Ore06,Ore07,Ore08,Ore09,Ore10,Ore11,Ore12,Ore13,Ore14	*OreG0009271*
scaffold3100	4407024	C	T	Och01,Och08	*OreG0009271*
scaffold3100	4407150	G	A	Ore01,Ore02,Ore03,Ore04,Ore05,Ore06,Ore07,Ore08,Ore09,Ore10,Ore11,Ore12,Ore13,Ore14	*OreG0009271*
scaffold3100	4407264	T	C	NA	*OreG0009271*
scaffold3100	4527447	C	A	Och01,Och02,Och03,Och04,Och05,Och06,Och07,Och08,Och09,Och10,Och11,Och12,Och13,Och14	*OreG0009273*
scaffold3100	4740425	C	T	Ore03,Ore04,Ore05,Ore09,Ore13	*OreG0009280*
scaffold3100	4786771	C	T	Och02,Och03,Och04,Och05,Och06,Och11,Och12,Och13	*OreG0009281*
scaffold3100	4818216	C	T	NA	*OreG0009282*
scaffold3100	4821983	G	T	NA	*OreG0009282*
scaffold3100	4823361	G	A	NA	*OreG0009283*
scaffold3100	4892287	G	A	NA	*OreG0009286*
scaffold3100	4922098	T	C	NA	*OreG0009286*
scaffold3100	4935962	T	A	NA	*OreG0009289*
scaffold3100	4936029	A	T	Ore01,Ore02,Ore03,Ore04,Ore05,Ore06,Ore07,Ore08,Ore09,Ore10,Ore11,Ore12,Ore13,Ore14	*OreG0009289*
scaffold3100	4936048	C	A	Ore01,Ore11	*OreG0009289*
scaffold3100	5017042	C	T	Ore01,Ore02,Ore03,Ore04,Ore05,Ore06,Ore07,Ore08,Ore09,Ore10,Ore11,Ore12,Ore13,Ore14	*OreG0009294*
scaffold3100	5022644	C	A	Ore01,Ore02,Ore03,Ore04,Ore05,Ore06,Ore07,Ore08,Ore09,Ore10,Ore11,Ore12,Ore13,Ore14	*OreG0009294*

scaffold3100	5182232	T	A	Och01,Och02,Och03,Och04,Och05,Och06,Och07,Och08,Och09,Och10,Och11,Och12,Och13,Och14,Ore01,Ore02,Ore03,Ore04,Ore05,Ore06,Ore07,Ore08,Ore09,Ore10,Ore11,Ore12,Ore13,Ore14	*OreG0009301*
scaffold3100	5233098	A	T	NA	*OreG0009303*
scaffold3100	5234153	C	T	Ore01,Ore02,Ore03,Ore04,Ore05,Ore06,Ore07,Ore08,Ore09,Ore10,Ore11,Ore12,Ore13,Ore14	*OreG0009303*
scaffold3100	5236478	G	A	NA	*OreG0009303*
scaffold3100	5269750	T	G	Ore11	*OreG0009304*
scaffold3100	5269810	T	A	Och01,Och02,Och03,Och04,Och05,Och06,Och07,Och08,Och09,Och10,Och11,Och12,Och13,Och14,Ore01,Ore02,Ore03,Ore04,Ore05,Ore06,Ore07,Ore08,Ore09,Ore10,Ore11,Ore12,Ore13,Ore14	*OreG0009304*
scaffold3100	5367250	G	C	NA	*OreG0009305*
scaffold3100	5405116	C	T	NA	*OreG0009309*
scaffold3100	5405148	T	C	NA	*OreG0009309*
scaffold3100	5405396	C	A	Och01,Och02,Och03,Och04,Och05,Och06,Och07,Och08,Och09,Och10,Och11,Och12,Och13,Och14,Ore01,Ore02,Ore03,Ore04,Ore05,Ore06,Ore07,Ore08,Ore09,Ore10,Ore11,Ore12,Ore13,Ore14	*OreG0009309*
scaffold3100	5416386	C	T	NA	*OreG0009310*
scaffold3100	5494108	A	C	Och01,Och02,Och03,Och04,Och05,Och06,Och07,Och08,Och09,Och10,Och11,Och12,Och13,Och14,Ore01,Ore02,Ore03,Ore04,Ore05,Ore06,Ore07,Ore08,Ore09,Ore10,Ore11,Ore12,Ore13,Ore14	*OreG0009312*
scaffold3100	5494146	A	G	Och01,Och02,Och03,Och04,Och05,Och06,Och07,Och08,Och09,Och10,Och11,Och12,Och13,Och14,Ore01,Ore02,Ore03,Ore04,Ore05,Ore06,Ore07,Ore08,Ore09,Ore10,Ore11,Ore12,Ore13,Ore14	*OreG0009312*
scaffold3100	5503553	A	G	NA	*OreG0009313*
scaffold3100	5504020	T	C	Och01,Och02,Och03,Och04,Och05,Och06,Och07,Och08,Och09,Och10,Och11,Och12,Och13,Och14,Ore01,Ore02,Ore03,Ore04,Ore05,Ore06,Ore07,Ore08,Ore09,Ore10,Ore11,Ore12,Ore13,Ore14	*OreG0009313*
scaffold3100	5506461	G	A	Ore03,Ore04,Ore05,Ore09,Ore13	*OreG0009314*
scaffold3100	5506471	T	C	NA	*OreG0009314*
scaffold3100	5542174	A	C	Ore11	*OreG0009316*
scaffold3100	5574825	A	T	Ore01,Ore02,Ore03,Ore04,Ore05,Ore06,Ore07,Ore08,Ore09,Ore10,Ore11,Ore12,Ore13,Ore14	*OreG0009318*
scaffold3100	5574853	G	T	Ore11	*OreG0009318*
scaffold3100	5574911	T	G	Och02,Och03,Och04,Och05,Och06,Och12,Och13,Och14	*OreG0009318*
scaffold3100	5575475	T	A	NA	*OreG0009318*
scaffold3100	5575601	T	A	Ore03,Ore04,Ore05,Ore09,Ore13	*OreG0009318*
scaffold3100	5583082	G	A	Ore11	*OreG0009319*
scaffold3100	5586499	G	A	NA	*OreG0009320*
scaffold3100	5609279	G	C	NA	*OreG0009322*
scaffold3100	5609448	C	G	Ore01,Ore02,Ore03,Ore04,Ore05,Ore06,Ore07,Ore08,Ore09,Ore10,Ore11,Ore12,Ore13,Ore14	*OreG0009322*
scaffold3100	5610652	C	A	NA	*OreG0009322*
scaffold3100	5704189	T	C	Och01,Och02,Och03,Och04,Och05,Och06,Och07,Och08,Och09,Och10,Och11,Och12,Och13,Och14	*OreG0009328*
scaffold3100	5704279	A	C	Och02,Och12,Och13,Och14	*OreG0009328*
scaffold3100	5791378	A	G	Och01,Och02,Och03,Och04,Och05,Och06,Och07,Och08,Och09,Och10,Och11,Och12,Och13,Och14	*OreG0009330*
scaffold3100	5825187	A	G	Och02,Och12,Och13	*OreG0009333*
scaffold3100	5826411	T	C	Ore01,Ore02,Ore03,Ore04,Ore05,Ore06,Ore07,Ore08,Ore09,Ore10,Ore11,Ore12,Ore13,Ore14	*OreG0009334*
scaffold3100	5826429	T	G	Ore01,Ore02,Ore03,Ore04,Ore05,Ore06,Ore07,Ore08,Ore09,Ore10,Ore11,Ore12,Ore13,Ore14	*OreG0009334*
scaffold3100	5827067	T	A	Ore01,Ore02,Ore03,Ore04,Ore05,Ore06,Ore07,Ore08,Ore09,Ore10,Ore11,Ore12,Ore13,Ore14	*OreG0009334*
scaffold3100	5907073	C	A	Ore01,Ore02,Ore03,Ore04,Ore05,Ore06,Ore07,Ore08,Ore09,Ore10,Ore11,Ore12,Ore13,Ore14	*OreG0009337*
scaffold3100	6002319	A	T	Ore01,Ore02,Ore03,Ore04,Ore05,Ore06,Ore07,Ore08,Ore09,Ore10,Ore11,Ore12,Ore13,Ore14	*OreG0009342*
scaffold3100	6002731	G	T	Ore01,Ore02,Ore03,Ore04,Ore05,Ore06,Ore07,Ore08,Ore09,Ore10,Ore11,Ore12,Ore13,Ore14	*OreG0009342*
scaffold3100	6002831	C	T	Och02,Och12,Och13	*OreG0009342*
scaffold3100	6007577	C	T	NA	*OreG0009343*
scaffold3100	6007604	C	T	NA	*OreG0009343*
scaffold3100	6008115	G	A	NA	*OreG0009343*
scaffold3100	6188773	G	T	NA	*OreG0009349*
scaffold3100	6189257	C	T	NA	*OreG0009349*
scaffold3100	6189448	G	A	NA	*OreG0009349*
scaffold3100	6189842	A	T	NA	*OreG0009349*
scaffold3100	6263283	C	T	NA	*OreG0009352*
scaffold3100	6263306	G	A	NA	*OreG0009352*
scaffold3100	6266389	T	A	Och01,Och02,Och03,Och04,Och05,Och06,Och07,Och08,Och09,Och10,Och11,Och12,Och13,Och14,Ore01,Ore02,Ore03,Ore04,Ore05,Ore06,Ore07,Ore08,Ore09,Ore10,Ore11,Ore12,Ore13,Ore14	*OreG0009353*

scaffold3100	6321296	G	A	Ore01,Ore02,Ore03,Ore04,Ore05,Ore06,Ore07,Ore08,Ore09,Ore10,Ore11,Ore12,Ore13,Ore14	*OreG0009355*
scaffold3100	6559585	G	T	Och01,Och02,Och03,Och04,Och05,Och06,Och07,Och08,Och09,Och10,Och11,Och12,Och13,Och14,Ore01,Ore02,Ore03,Ore04,Ore05,Ore06,Ore07,Ore08,Ore09,Ore10,Ore11,Ore12,Ore13,Ore14	*OreG0009363*
scaffold3100	6559626	G	A	Och01,Och02,Och03,Och04,Och05,Och06,Och07,Och08,Och09,Och10,Och11,Och12,Och13,Och14,Ore01,Ore02,Ore03,Ore04,Ore05,Ore06,Ore07,Ore08,Ore09,Ore10,Ore11,Ore12,Ore13,Ore14	*OreG0009363*
scaffold3100	6590412	C	T	NA	*OreG0009365*
scaffold33237	42904	T	C	NA	*OreG0017286*
scaffold33237	104498	A	T	NA	*OreG0017287*
scaffold33237	141456	C	G	NA	*OreG0017292*
scaffold33237	143774	G	A	NA	*OreG0017292*
scaffold33237	145281	C	G	NA	*OreG0017292*
scaffold33237	169259	C	A	NA	*OreG0017295*
scaffold33237	174768	C	T	NA	*OreG0017295*
scaffold33237	200257	T	C	NA	*OreG0017297*
scaffold33237	201660	T	A	NA	*OreG0017297*
scaffold33237	204992	G	A	NA	*OreG0017297*
scaffold33197	152469	A	G	NA	*OreG0015275*
scaffold33197	154724	C	A	NA	*OreG0015275*
scaffold33197	157498	G	T	Ore03,Ore04,Ore05,Ore09,Ore13	*OreG0015275*
scaffold33197	163667	G	A	Och01,Och02,Och03,Och04,Och05,Och06,Och07,Och08,Och09,Och10,Och11,Och12,Och13,Och14	*OreG0015275*
scaffold33197	164582	A	T	Och05,Och14	*OreG0015275*
scaffold33197	164640	G	A	Och01,Och02,Och03,Och04,Och05,Och06,Och07,Och08,Och09,Och10,Och11,Och12,Och13,Och14,Ore01,Ore02,Ore03,Ore04,Ore05,Ore06,Ore07,Ore08,Ore09,Ore10,Ore11,Ore12,Ore13,Ore14	*OreG0015275*
scaffold33197	176619	T	G	Och01,Och02,Och03,Och04,Och05,Och06,Och07,Och08,Och09,Och10,Och11,Och12,Och13,Och14,Ore01,Ore02,Ore03,Ore04,Ore05,Ore06,Ore07,Ore08,Ore09,Ore10,Ore11,Ore12,Ore13,Ore14	*OreG0015276*
scaffold33197	226932	C	A	NA	*OreG0015277*
scaffold33197	227937	G	A	NA	*OreG0015277*
scaffold33197	227971	C	T	Ore11	*OreG0015277*
scaffold33197	228033	T	A	Och05,Och14	*OreG0015277*
scaffold33197	253223	C	T	Ore01,Ore02,Ore03,Ore04,Ore05,Ore06,Ore07,Ore08,Ore09,Ore10,Ore11,Ore12,Ore13,Ore14	*OreG0015278*
scaffold33197	267252	G	C	NA	*OreG0015279*
scaffold33197	309037	A	T	NA	*OreG0015282*
scaffold33197	452002	A	C	Och02,Och12,Och13	*OreG0015289*
scaffold33197	452116	G	A	NA	*OreG0015289*
scaffold33197	560445	G	C	NA	*OreG0015291*
scaffold33197	581986	C	A	NA	*OreG0015295*
scaffold33197	583296	C	A	NA	*OreG0015295*
scaffold33197	620004	G	A	Ore01,Ore02,Ore03,Ore04,Ore05,Ore06,Ore07,Ore08,Ore09,Ore10,Ore11,Ore12,Ore13,Ore14	*OreG0015299*
scaffold33197	620072	G	T	Ore01,Ore02,Ore03,Ore04,Ore05,Ore06,Ore07,Ore08,Ore09,Ore10,Ore11,Ore12,Ore13,Ore14	*OreG0015299*
scaffold33197	629909	A	G	Och01,Och02,Och03,Och04,Och05,Och06,Och07,Och08,Och09,Och10,Och11,Och12,Och13,Och14	*OreG0015300*
scaffold33197	706051	C	T	Och01,Och02,Och03,Och04,Och05,Och06,Och07,Och08,Och09,Och10,Och11,Och12,Och13,Och14,Ore01,Ore02,Ore03,Ore04,Ore05,Ore06,Ore07,Ore08,Ore09,Ore10,Ore11,Ore12,Ore13,Ore14	*OreG0015302*
scaffold33197	806399	C	G	NA	*OreG0015307*
scaffold33197	812844	G	A	Ore01,Ore02,Ore03,Ore04,Ore05,Ore06,Ore07,Ore08,Ore09,Ore10,Ore11,Ore12,Ore13,Ore14	*OreG0015308*
scaffold33197	881704	C	T	NA	*OreG0015314*
scaffold33197	883048	A	T	Ore01,Ore02,Ore03,Ore04,Ore05,Ore06,Ore07,Ore08,Ore09,Ore10,Ore11,Ore12,Ore13,Ore14	*OreG0015314*
scaffold33197	888781	G	A	Ore01,Ore02,Ore03,Ore04,Ore05,Ore06,Ore07,Ore08,Ore09,Ore10,Ore11,Ore12,Ore13,Ore14	*OreG0015315*
scaffold33197	889167	A	G	Ore02,Ore03,Ore04,Ore05,Ore06,Ore07,Ore08,Ore09,Ore10,Ore11,Ore12,Ore13,Ore14	*OreG0015315*
scaffold33197	891764	C	T	Och01,Och02,Och03,Och04,Och05,Och06,Och07,Och08,Och09,Och10,Och11,Och12,Och13,Och14	*OreG0015315*
scaffold33197	895279	T	A	Ore01,Ore02,Ore03,Ore04,Ore05,Ore06,Ore07,Ore08,Ore09,Ore10,Ore11,Ore12,Ore13,Ore14	*OreG0015316*
scaffold33197	899777	G	A	Och01,Och02,Och03,Och04,Och05,Och06,Och07,Och08,Och09,Och10,Och11,Och12,Och13,Och14	*OreG0015317*
scaffold33197	899840	C	T	Och14	*OreG0015317*
scaffold33197	899858	C	T	Ore01,Ore02,Ore03,Ore04,Ore05,Ore06,Ore07,Ore08,Ore09,Ore10,Ore11,Ore12,Ore13,Ore14	*OreG0015317*
scaffold33197	969708	G	C	NA	*OreG0015319*
scaffold33197	1083052	G	A	Ore01,Ore02,Ore03,Ore04,Ore05,Ore06,Ore07,Ore08,Ore09,Ore10,Ore11,Ore12,Ore13,Ore14	*OreG0015322*

scaffold33197	1100199	C	T	NA	*OreG0015322*
scaffold33197	1100666	C	T	Ore01,Ore02,Ore03,Ore04,Ore05,Ore06,Ore07,Ore08,Ore09,Ore10,Ore11,Ore12,Ore13,Ore14	*OreG0015322*
scaffold33197	1100804	A	T	Ore11	*OreG0015322*
scaffold33197	1114318	G	A	NA	*OreG0015323*
scaffold33197	1129883	A	T	Ore01,Ore02,Ore03,Ore04,Ore05,Ore06,Ore07,Ore08,Ore09,Ore10,Ore11,Ore12,Ore13,Ore14	*OreG0015323*
scaffold33197	1331781	C	T	Och01	*OreG0015327*
scaffold33240	5702	A	T	Och01,Och06,Och11	*OreG0017354*
scaffold33240	6045	T	C	NA	*OreG0017355*
scaffold33240	6290	A	G	NA	*OreG0017355*
scaffold33240	6338	G	A	NA	*OreG0017355*
scaffold33240	10354	C	A	NA	*OreG0017356*
scaffold33240	10688	T	C	NA	*OreG0017356*
scaffold33240	10859	G	T	NA	*OreG0017356*
scaffold33240	11205	T	C	Och01,Och02,Och03,Och04,Och06,Och09,Och10,Och11,Och14,Ore01,Ore02,Ore03,Ore04,Ore05,Ore06,Ore07,Ore08,Ore09,Ore10,Ore11,Ore12,Ore13,Ore14	*OreG0017356*
scaffold33240	11238	A	G	NA	*OreG0017356*
scaffold33240	24385	G	A	NA	*OreG0017359*
scaffold33240	24391	A	G	NA	*OreG0017359*
scaffold33240	24899	A	G	NA	*OreG0017359*
scaffold33240	25095	C	T	NA	*OreG0017359*
scaffold33240	25160	G	T	NA	*OreG0017359*
scaffold33240	129951	T	A	NA	*OreG0017366*
scaffold33240	171591	C	A	NA	*OreG0017369*
scaffold33240	175478	G	A	NA	*OreG0017369*
scaffold33240	175714	G	A	NA	*OreG0017369*
scaffold33240	175759	T	C	NA	*OreG0017369*
scaffold33240	176083	G	A	NA	*OreG0017369*
scaffold33240	176167	T	C	NA	*OreG0017369*
scaffold33240	177617	G	T	NA	*OreG0017370*
scaffold33240	194316	A	G	NA	*OreG0017371*
scaffold33240	195645	G	A	NA	*OreG0017371*
scaffold33240	196919	A	G	Och11	*OreG0017371*
scaffold33240	197016	C	T	Och07,Och08,Och09,Och10	*OreG0017371*
scaffold33240	197253	A	G	NA	*OreG0017371*
scaffold33240	224779	A	T	NA	*OreG0017374*
scaffold33240	228602	C	T	NA	*OreG0017374*
scaffold33240	241491	G	T	Ore09,Ore13,Ore14	*OreG0017375*
scaffold33240	243659	G	A	Och01,Och07,Och08	*OreG0017375*
scaffold33240	243682	A	G	Ore03,Ore06,Ore09,Ore11,Ore13,Ore14	*OreG0017375*
scaffold33240	259587	T	C	Och11	*OreG0017376*
scaffold33240	264422	G	T	NA	*OreG0017378*
scaffold33240	264599	G	T	Och08	*OreG0017378*
scaffold33240	265436	C	G	Och06,Och11	*OreG0017378*
scaffold33240	265615	T	A	NA	*OreG0017378*
scaffold33240	265816	T	C	NA	*OreG0017378*
scaffold33240	265962	A	T	NA	*OreG0017378*
scaffold33240	266364	G	A	NA	*OreG0017378*
scaffold33240	266383	A	G	NA	*OreG0017378*
scaffold33240	266572	G	A	NA	*OreG0017378*
scaffold33240	270631	T	A	Och11	*OreG0017379*
scaffold33240	272977	G	A	NA	*OreG0017379*
scaffold33240	272989	C	T	NA	*OreG0017379*
scaffold33240	273148	G	A	NA	*OreG0017379*
scaffold33240	273153	C	G	NA	*OreG0017379*
scaffold33240	323079	G	T	Och06	*OreG0017381*
scaffold33240	365879	T	C	NA	*OreG0017385*
scaffold33240	434103	C	A	NA	*OreG0017391*
scaffold33209	58067	A	G	Och01,Och02,Och03,Och04,Och05,Och06,Och07,Och08,Och09,Och10,Och11,Och12,Och13,Och14,Ore01,Ore02,Ore03,Ore04,Ore05,Ore06,Ore07,Ore08,Ore09,Ore10,Ore11,Ore12,Ore13,Ore14	*OreG0016240*
scaffold33209	73330	A	G	NA	*OreG0016243*
scaffold33209	95147	C	T	Och02,Och04,Och06	*OreG0016247*
scaffold33209	95319	A	G	Ore01,Ore02,Ore03,Ore04,Ore05,Ore06,Ore07,Ore08,Ore09,Ore10,Ore11,Ore12,Ore13,Ore14	*OreG0016247*
scaffold33209	97268	C	T	Ore01,Ore02,Ore03,Ore04,Ore05,Ore06,Ore07,Ore08,Ore09,Ore10,Ore11,Ore12,Ore13,Ore14	*OreG0016249*
scaffold33209	97298	A	G	NA	*OreG0016249*
scaffold33209	98621	A	G	Och12,Ore01,Ore02,Ore03,Ore04,Ore05,Ore06,Ore07,Ore08,Ore09,Ore10,Ore11,Ore12,Ore13,Ore14	*OreG0016250*
scaffold33209	98671	C	A	Ore01,Ore02,Ore03,Ore04,Ore05,Ore06,Ore07,Ore08,Ore09,Ore10,Ore11,Ore12,Ore13,Ore14	*OreG0016250*

scaffold33209	98813	C	A	Och01,Och02,Och03,Och04,Och05,Och06,Och07,Och08,Och09,Och10,Och11,Och12,Och13,Och14,Ore01,Ore02,Ore03,Ore04,Ore05,Ore06,Ore07,Ore08,Ore09,Ore10,Ore11,Ore12,Ore13,Ore14	*OreG0016250*
scaffold33209	98984	G	C	Och01,Och02,Och03,Och04,Och05,Och06,Och07,Och08,Och09,Och10,Och11,Och12,Och13,Och14,Ore01,Ore02,Ore03,Ore04,Ore05,Ore06,Ore07,Ore08,Ore09,Ore10,Ore11,Ore12,Ore13,Ore14	*OreG0016250*
scaffold33209	176850	G	T	Och01,Och02,Och03,Och04,Och05,Och06,Och07,Och08,Och09,Och10,Och11,Och12,Och13,Och14,Ore01,Ore02,Ore03,Ore04,Ore05,Ore06,Ore07,Ore08,Ore09,Ore10,Ore11,Ore12,Ore13,Ore14	*OreG0016256*
scaffold33209	177135	C	T	Ore03,Ore08,Ore09,Ore10,Ore14	*OreG0016256*
scaffold33209	190209	T	A	NA	*OreG0016258*
scaffold33209	190560	G	C	Ore03,Ore08,Ore09,Ore10,Ore14	*OreG0016258*
scaffold33209	209669	T	C	NA	*OreG0016259*
scaffold33209	270449	C	G	Ore03,Ore08,Ore09,Ore10,Ore14	*OreG0016264*
scaffold33209	553610	C	A	Ore03,Ore08,Ore09,Ore10,Ore14	*OreG0016274*
scaffold33209	716626	C	G	NA	*OreG0016281*
scaffold33209	731663	G	A	Ore01,Ore02,Ore03,Ore04,Ore05,Ore06,Ore07,Ore08,Ore09,Ore10,Ore11,Ore12,Ore13,Ore14	*OreG0016284*
scaffold33209	731844	C	A	Och01,Och02,Och03,Och04,Och05,Och06,Och07,Och08,Och09,Och10,Och11,Och12,Och13,Och14,Ore01,Ore02,Ore03,Ore04,Ore05,Ore06,Ore07,Ore08,Ore09,Ore10,Ore11,Ore12,Ore13,Ore14	*OreG0016284*
scaffold33209	731864	C	G	Ore01,Ore02,Ore03,Ore04,Ore05,Ore06,Ore07,Ore08,Ore09,Ore10,Ore11,Ore12,Ore13,Ore14	*OreG0016284*
scaffold33209	755143	C	T	Ore02,Ore09,Ore10	*OreG0016287*
scaffold33209	755265	G	T	Och12,Och13	*OreG0016287*
scaffold33209	811576	C	T	Och01,Och02,Och03,Och04,Och05,Och06,Och07,Och08,Och09,Och10,Och11,Och12,Och13,Och14,Ore01,Ore02,Ore03,Ore04,Ore05,Ore06,Ore07,Ore08,Ore09,Ore10,Ore11,Ore12,Ore13,Ore14	*OreG0016295*
scaffold33204	232846	G	A	Ore01,Ore02,Ore03,Ore04,Ore05,Ore06,Ore07,Ore08,Ore09,Ore10,Ore11,Ore12,Ore13,Ore14	*OreG0015867*
scaffold33204	232920	A	G	Och01,Och02,Och03,Och04,Och05,Och06,Och07,Och08,Och09,Och10,Och11,Och12,Och13,Och14,Ore01,Ore02,Ore03,Ore04,Ore05,Ore06,Ore07,Ore08,Ore09,Ore10,Ore11,Ore12,Ore13,Ore14	*OreG0015867*
scaffold33204	553352	C	A	NA	*OreG0015878*
scaffold33204	581821	C	T	Och01,Och02,Och03,Och04,Och05,Och06,Och07,Och08,Och09,Och10,Och11,Och12,Och13,Och14	*OreG0015880*
scaffold33204	581848	T	A	Och01,Och02,Och03,Och04,Och05,Och06,Och07,Och08,Och09,Och10,Och11,Och12,Och13,Och14	*OreG0015880*
scaffold33204	582552	G	A	Och01,Och02,Och03,Och04,Och05,Och06,Och07,Och08,Och09,Och10,Och11,Och12,Och13,Och14	*OreG0015880*
scaffold33204	582571	G	A	Ore01,Ore02,Ore03,Ore04,Ore05,Ore06,Ore07,Ore08,Ore09,Ore10,Ore11,Ore12,Ore13,Ore14	*OreG0015880*
scaffold33204	582580	T	C	Och01,Och02,Och03,Och04,Och05,Och06,Och07,Och08,Och09,Och10,Och11,Och12,Och13,Och14,Ore01,Ore02,Ore03,Ore04,Ore05,Ore06,Ore07,Ore08,Ore09,Ore10,Ore11,Ore12,Ore13,Ore14	*OreG0015880*
scaffold33204	582622	C	T	Och01,Och02,Och03,Och04,Och05,Och06,Och07,Och08,Och09,Och10,Och11,Och12,Och13,Och14	*OreG0015880*
scaffold33204	582751	C	T	NA	*OreG0015880*
scaffold33204	582838	G	A	Och01,Och02,Och03,Och04,Och05,Och06,Och07,Och08,Och09,Och10,Och11,Och12,Och13,Och14	*OreG0015880*
scaffold33204	738360	T	C	Ore01,Ore02,Ore03,Ore04,Ore05,Ore06,Ore07,Ore08,Ore09,Ore10,Ore11,Ore12,Ore13,Ore14	*OreG0015883*
scaffold33204	739851	T	C	NA	*OreG0015883*
scaffold33204	740159	A	T	NA	*OreG0015883*
scaffold33204	748384	C	G	Och01,Och02,Och03,Och04,Och05,Och06,Och07,Och08,Och09,Och10,Och11,Och12,Och13,Och14	*OreG0015883*
scaffold33204	780525	C	A	NA	*OreG0015884*
scaffold33244	162632	T	C	Ore08	*OreG0017408*
scaffold33244	170345	A	T	NA	*OreG0017409*
scaffold33244	234101	T	A	NA	*OreG0017413*
scaffold33244	234116	C	A	NA	*OreG0017413*
scaffold33269	25497	A	G	Och01,Och02,Och03,Och04,Och05,Och06,Och07,Och08,Och09,Och10,Och11,Och12,Och13,Och14	*OreG0018147*
scaffold33269	25594	T	A	NA	*OreG0018147*
scaffold33269	25778	C	T	NA	*OreG0018147*
scaffold33269	55146	C	T	Och01,Och02,Och03,Och04,Och05,Och06,Och07,Och08,Och09,Och10,Och11,Och12,Och13,Och14,Ore01,Ore02,Ore03,Ore04,Ore05,Ore06,Ore07,Ore08,Ore09,Ore10,Ore11,Ore12,Ore13,Ore14	*OreG0018151*
scaffold33269	59068	C	T	NA	*OreG0018151*
scaffold33269	59107	T	C	NA	*OreG0018151*
scaffold33269	59394	T	C	NA	*OreG0018151*
scaffold33269	59488	T	C	NA	*OreG0018151*
scaffold33269	59884	T	A	NA	*OreG0018151*
scaffold33269	60024	T	C	Ore09	*OreG0018151*
scaffold33269	60196	C	T	NA	*OreG0018151*
scaffold33269	60265	G	A	NA	*OreG0018151*

scaffold33269	60510	G	A	NA	*OreG0018151*
scaffold33269	62087	T	A	NA	*OreG0018152*
scaffold33269	62203	C	A	NA	*OreG0018152*
scaffold33269	63480	G	A	NA	*OreG0018152*
scaffold33269	63511	G	A	NA	*OreG0018152*
scaffold33269	63688	G	A	NA	*OreG0018152*
scaffold33269	63730	G	A	NA	*OreG0018152*
scaffold33269	63733	A	T	NA	*OreG0018152*
scaffold33269	63750	T	A	NA	*OreG0018152*
scaffold33269	67426	C	A	Och01,Och02,Och03,Och04,Och05,Och06,Och07,Och08,Och09,Och10,Och11,Och12,Och13,Och14,Ore01,Ore02,Ore03,Ore04,Ore05,Ore06,Ore07,Ore08,Ore09,Ore10,Ore11,Ore12,Ore13,Ore14	*OreG0018152*
scaffold33269	82879	G	A	Och01,Och02,Och03,Och04,Och05,Och06,Och07,Och08,Och09,Och10,Och11,Och12,Och13,Och14	*OreG0018153*
scaffold33269	86229	A	C	NA	*OreG0018153*
scaffold33269	88270	G	T	NA	*OreG0018153*
scaffold33269	89941	A	G	Ore01,Ore02,Ore03,Ore04,Ore05,Ore06,Ore07,Ore08,Ore09,Ore10,Ore11,Ore12,Ore13,Ore14	*OreG0018153*
scaffold33269	95672	T	A	Och01,Och02,Och03,Och04,Och05,Och06,Och07,Och08,Och09,Och10,Och11,Och12,Och13,Och14,Ore01,Ore02,Ore03,Ore04,Ore05,Ore06,Ore07,Ore08,Ore09,Ore10,Ore11,Ore12,Ore13,Ore14	*OreG0018154*
scaffold33269	99270	A	T	Och03,Och12,Och13	*OreG0018154*
scaffold33269	118499	T	C	Och01	*OreG0018155*
scaffold33269	134656	C	T	Och07	*OreG0018158*
scaffold33266	970	T	C	NA	*OreG0018130*
scaffold33266	1086	C	A	NA	*OreG0018130*
scaffold33266	10543	C	A	NA	*OreG0018132*
scaffold33266	10688	A	T	NA	*OreG0018132*
scaffold33266	10759	G	A	NA	*OreG0018132*
scaffold33266	18599	T	A	NA	*OreG0018133*
scaffold33266	18686	T	C	NA	*OreG0018133*
scaffold33266	18688	T	C	NA	*OreG0018133*
scaffold33266	34634	C	A	Och02,Och03,Och04,Och05,Och06,Och08,Och09,Och10,Och12,Och13,Ore02,Ore06,Ore08,Ore09,Ore10,Ore12,Ore14	*OreG0018134*
scaffold33266	35145	C	T	NA	*OreG0018134*
scaffold33261	37254	G	T	NA	*OreG0017970*
scaffold33261	37922	A	G	Och06	*OreG0017970*
scaffold33261	40929	C	T	Och06	*OreG0017970*
scaffold33219	101756	C	T	NA	*OreG0016751*
scaffold33219	101998	C	A	Och02	*OreG0016751*
scaffold33219	142683	T	G	NA	*OreG0016752*
scaffold33219	196771	A	G	Ore01,Ore02,Ore03,Ore04,Ore05,Ore06,Ore07,Ore08,Ore09,Ore10,Ore11,Ore12,Ore13,Ore14	*OreG0016755*
scaffold33219	197161	A	G	NA	*OreG0016755*
scaffold33219	198507	G	T	Ore01,Ore02,Ore03,Ore04,Ore05,Ore06,Ore07,Ore08,Ore09,Ore10,Ore11,Ore12,Ore13,Ore14	*OreG0016755*
scaffold33219	201312	G	T	NA	*OreG0016755*
scaffold33219	201344	T	A	Ore01,Ore02,Ore03,Ore04,Ore05,Ore06,Ore07,Ore08,Ore09,Ore10,Ore11,Ore12,Ore13,Ore14	*OreG0016755*
scaffold33219	201351	G	C	Ore01,Ore02,Ore03,Ore04,Ore05,Ore06,Ore07,Ore08,Ore09,Ore10,Ore11,Ore12,Ore13,Ore14	*OreG0016755*
scaffold33219	201453	A	T	NA	*OreG0016755*
scaffold33219	234522	T	G	Och02,Och03,Och04,Och05,Och06,Och07,Och08,Och12,Och13,Och14	*OreG0016757*
scaffold33219	236133	C	T	NA	*OreG0016757*
scaffold33219	236189	T	G	Och02,Och03,Och04,Och05,Och06,Och07,Och08,Och09,Och10,Och11,Och12,Och13,Och14	*OreG0016757*
scaffold33219	236216	C	A	Ore01,Ore02,Ore03,Ore06,Ore07,Ore08,Ore09,Ore10,Ore11,Ore12,Ore13,Ore14	*OreG0016757*
scaffold33219	241832	G	A	NA	*OreG0016758*
scaffold33289	31606	T	A	Och11,Ore01,Ore02,Ore03,Ore04,Ore05,Ore06,Ore07,Ore08,Ore09,Ore10,Ore11,Ore12,Ore13,Ore14	*OreG0018458*
scaffold33207	28457	C	A	NA	*OreG0015917*
scaffold33207	78079	C	T	Och01,Och02,Och03,Och04,Och05,Och06,Och07,Och08,Och09,Och10,Och11,Och12,Och13,Och14,Ore01,Ore02,Ore03,Ore04,Ore05,Ore06,Ore07,Ore08,Ore09,Ore10,Ore11,Ore12,Ore13,Ore14	*OreG0015920*
scaffold33207	78472	T	C	Och01,Och02,Och03,Och04,Och05,Och07,Och08,Och09,Och10,Och11,Och12,Och13,Och14,Ore01,Ore02,Ore03,Ore04,Ore05,Ore06,Ore07,Ore08,Ore09,Ore10,Ore11,Ore12,Ore13,Ore14	*OreG0015920*
scaffold33207	78627	G	A	NA	*OreG0015920*
scaffold33207	78812	C	T	Och01,Och02,Och04,Och05,Och12,Och13	*OreG0015920*
scaffold33207	79311	C	T	Och08,Och09,Och10,Och11,Och14	*OreG0015921*
scaffold33207	79338	G	A	Och01,Och02,Och03,Och04,Och05,Och12,Och13	*OreG0015921*
scaffold33207	79353	A	G	NA	*OreG0015921*
scaffold33207	79548	C	G	Och01,Och02,Och04,Och05,Och07,Och08,Och09,Och10,Och11,Och12,Och13,Ore01,Ore02,Ore03,Ore04,Ore05,Ore06,Ore07,Ore08,Ore09,Ore10,Ore11,Ore12,Ore13,Ore14	*OreG0015921*

scaffold33207	79604	T	C	Och01,Och02,Och04,Och05,Och12,Och13	*OreG0015921*
scaffold33207	79658	C	T	Och01,Och02,Och04,Och05,Och12,Och13	*OreG0015921*
scaffold33207	79670	C	T	NA	*OreG0015921*
scaffold33207	79922	T	C	NA	*OreG0015921*
scaffold33207	104206	G	A	Och08,Och14	*OreG0015922*
scaffold33207	120985	C	G	Och01,Och02,Och03,Och04,Och05,Och06,Och08,Och09,Och10,Och11,Och12,Och13,Och14	*OreG0015924*
scaffold33207	122370	G	A	Ore01,Ore02,Ore03,Ore04,Ore05,Ore06,Ore07,Ore08,Ore09,Ore10,Ore11,Ore12,Ore13,Ore14	*OreG0015924*
scaffold33207	122373	A	C	Och02,Och03,Och04,Och05,Och06,Och12,Och13	*OreG0015924*
scaffold33207	122377	T	C	NA	*OreG0015924*
scaffold33207	129800	C	T	NA	*OreG0015925*
scaffold33207	154735	G	C	NA	*OreG0015928*
scaffold33207	160954	A	T	Ore01,Ore02,Ore04,Ore05,Ore06,Ore07,Ore08,Ore09,Ore10,Ore11,Ore12,Ore13	*OreG0015929*
scaffold33207	189884	C	G	NA	*OreG0015932*
scaffold33207	192035	G	T	NA	*OreG0015932*
scaffold33207	253136	A	T	NA	*OreG0015936*
scaffold33207	264739	T	A	NA	*OreG0015937*
scaffold33207	306492	C	T	Ore02,Ore03,Ore06,Ore07,Ore09,Ore10,Ore11,Ore12,Ore13,Ore14	*OreG0015940*
scaffold33207	347923	C	T	NA	*OreG0015947*
scaffold33207	347939	A	C	Och08,Och09,Och10	*OreG0015947*
scaffold33207	362891	C	T	Och01,Och02,Och03,Och04,Och05,Och06,Och07,Och08,Och09,Och10,Och11,Och12,Och13,Och14	*OreG0015949*
scaffold33207	376951	G	A	NA	*OreG0015952*
scaffold33207	386458	C	T	NA	*OreG0015953*
scaffold33207	399622	C	T	Ore01,Ore02,Ore03,Ore04,Ore05,Ore06,Ore07,Ore08,Ore09,Ore10,Ore11,Ore12,Ore13,Ore14	*OreG0015956*
scaffold33207	400397	C	A	Och01,Och02,Och03,Och04,Och05,Och06,Och07,Och08,Och09,Och10,Och11,Och12,Och13,Och14,Ore01,Ore02,Ore03,Ore04,Ore05,Ore06,Ore07,Ore08,Ore09,Ore10,Ore11,Ore12,Ore13,Ore14	*OreG0015956*
scaffold33207	405900	T	G	NA	*OreG0015957*
scaffold33207	406770	T	C	Och08,Och14	*OreG0015957*
scaffold33207	408210	C	A	Och01,Och02,Och03,Och04,Och05,Och06,Och07,Och08,Och09,Och10,Och11,Och12,Och13,Och14	*OreG0015957*
scaffold33207	408244	G	A	Ore01,Ore02,Ore03,Ore04,Ore05,Ore06,Ore07,Ore08,Ore09,Ore10,Ore11,Ore12,Ore13,Ore14	*OreG0015957*
scaffold33207	423368	A	G	Och01,Och02,Och03,Och04,Och05,Och06,Och07,Och08,Och09,Och10,Och11,Och12,Och13,Och14	*OreG0015959*
scaffold33207	423524	C	T	Ore01,Ore02,Ore03,Ore04,Ore05,Ore06,Ore07,Ore08,Ore09,Ore10,Ore11,Ore12,Ore13,Ore14	*OreG0015959*
scaffold33207	426732	C	A	Och01,Och09,Och10,Och11,Och12,Och13,Och14	*OreG0015960*
scaffold33207	426830	G	A	Och01,Och02,Och03,Och04,Och05,Och06,Och07,Och08,Och09,Och10,Och11,Och12,Och13,Och14,Ore01,Ore02,Ore03,Ore04,Ore05,Ore06,Ore07,Ore08,Ore09,Ore10,Ore11,Ore12,Ore13,Ore14	*OreG0015960*
scaffold33207	437234	G	T	Ore02,Ore03,Ore06,Ore07,Ore09,Ore10,Ore11,Ore12,Ore13,Ore14	*OreG0015961*
scaffold33207	504856	A	G	Och02,Och04,Och05,Och07,Och08,Och12,Och13	*OreG0015967*
scaffold33207	508944	A	G	Och01,Och09,Och10,Och11,Och14	*OreG0015967*
scaffold33207	541241	A	G	Ore02,Ore03,Ore04,Ore05,Ore06,Ore07,Ore09,Ore10,Ore11,Ore12,Ore13,Ore14	*OreG0015971*
scaffold33207	546266	G	T	Ore01,Ore02,Ore03,Ore04,Ore05,Ore06,Ore07,Ore08,Ore09,Ore10,Ore11,Ore12,Ore13,Ore14	*OreG0015972*
scaffold33207	546951	C	G	Ore01,Ore02,Ore03,Ore04,Ore05,Ore06,Ore07,Ore08,Ore09,Ore10,Ore11,Ore12,Ore13,Ore14	*OreG0015972*
scaffold33207	548345	C	T	Och01,Och02,Och03,Och04,Och05,Och06,Och07,Och08,Och09,Och10,Och11,Och12,Och13,Och14	*OreG0015972*
scaffold33207	548469	T	C	Och02,Och03,Och04,Och05,Och06,Och07,Och08,Och09,Och10,Och11,Och12,Och13,Och14	*OreG0015972*
scaffold33207	553283	G	C	Ore01,Ore02,Ore03,Ore04,Ore05,Ore06,Ore07,Ore08,Ore09,Ore10,Ore11,Ore12,Ore13,Ore14	*OreG0015973*
scaffold33207	567316	G	T	NA	*OreG0015974*
scaffold33207	575078	G	A	Och02,Och04,Och05,Och12,Och13,Och14	*OreG0015975*
scaffold33207	575450	C	A	Och06,Och07,Och09,Och10,Och14	*OreG0015975*
scaffold33207	575486	C	T	Och05,Och12,Och13	*OreG0015975*
scaffold33207	580443	C	G	Ore01,Ore02,Ore03,Ore04,Ore05,Ore06,Ore07,Ore08,Ore09,Ore10,Ore11,Ore12,Ore13,Ore14	*OreG0015977*
scaffold33207	580550	C	T	Och05,Och08,Och09,Och10,Och11,Och12,Och13	*OreG0015977*
scaffold33207	581051	C	T	NA	*OreG0015977*
scaffold33207	621917	T	C	NA	*OreG0015982*
scaffold33207	701210	C	A	NA	*OreG0015984*
scaffold33207	701276	T	A	Ore01,Ore02,Ore03,Ore04,Ore05,Ore06,Ore07,Ore08,Ore09,Ore10,Ore11,Ore12,Ore13,Ore14	*OreG0015984*
scaffold33207	706418	G	A	Ore01,Ore02,Ore03,Ore04,Ore05,Ore06,Ore07,Ore08,Ore09,Ore10,Ore11,Ore12,Ore13,Ore14	*OreG0015985*
scaffold33207	706423	T	A	Ore01,Ore02,Ore03,Ore04,Ore05,Ore06,Ore07,Ore08,Ore09,Ore10,Ore11,Ore12,Ore13,Ore14	*OreG0015985*
scaffold33207	720770	T	A	NA	*OreG0015987*

scaffold33207	738370	A	G	NA	*OreG0015989*
scaffold33207	738446	C	A	NA	*OreG0015989*
scaffold33207	738765	A	T	Och01,Och02,Och03,Och04,Och05,Och06,Och07,Och08,Och09,Och10,Och11,Och12,Och13,Och14,Ore01,Ore02,Ore03,Ore04,Ore05,Ore06,Ore07,Ore08,Ore09,Ore10,Ore11,Ore12,Ore13,Ore14	*OreG0015989*
scaffold33207	739017	C	T	Och01,Och02,Och03,Och04,Och05,Och06,Och07,Och08,Och09,Och10,Och11,Och12,Och13,Och14	*OreG0015989*
scaffold33207	739026	G	T	Ore04,Ore05	*OreG0015989*
scaffold33207	752972	A	G	Ore04,Ore05	*OreG0015992*
scaffold33207	807381	C	T	Och12,Och13	*OreG0016000*
scaffold33207	807826	T	A	Ore06,Ore07,Ore10,Ore12	*OreG0016000*
scaffold33207	826082	C	T	Och01,Och02,Och03,Och04,Och05,Och06,Och07,Och08,Och09,Och10,Och11,Och12,Och13,Och14,Ore01,Ore02,Ore03,Ore04,Ore05,Ore06,Ore07,Ore08,Ore09,Ore10,Ore11,Ore12,Ore13,Ore14	*OreG0016002*
scaffold33207	826750	G	T	NA	*OreG0016002*
scaffold33207	826832	C	T	NA	*OreG0016002*
scaffold33207	827038	G	A	Och01,Och02,Och03,Och04,Och05,Och06,Och07,Och08,Och09,Och10,Och11,Och12,Och13,Och14	*OreG0016002*
scaffold33207	827329	T	A	NA	*OreG0016002*
scaffold33207	827519	T	A	NA	*OreG0016002*
scaffold33207	830048	A	G	Och12,Och13	*OreG0016003*
scaffold33207	865608	T	A	NA	*OreG0016010*
scaffold33207	866391	G	T	NA	*OreG0016010*
scaffold33207	869972	T	C	Och01,Och02,Och03,Och04,Och05,Och06,Och07,Och08,Och09,Och10,Och11,Och12,Och13,Och14	*OreG0016010*
scaffold33207	870055	G	C	Och14	*OreG0016010*
scaffold33207	870119	G	C	Och01,Och02,Och03,Och04,Och05,Och06,Och07,Och08,Och09,Och10,Och11,Och12,Och13,Och14,Ore01,Ore02,Ore03,Ore04,Ore05,Ore06,Ore07,Ore08,Ore09,Ore10,Ore11,Ore12,Ore13,Ore14	*OreG0016010*
scaffold33207	870154	T	G	Och01,Och02,Och03,Och04,Och05,Och06,Och07,Och08,Och09,Och10,Och11,Och12,Och13,Och14	*OreG0016010*
scaffold33207	897410	A	T	Ore01,Ore02,Ore03,Ore04,Ore05,Ore06,Ore07,Ore08,Ore09,Ore10,Ore11,Ore12,Ore13,Ore14	*OreG0016012*
scaffold33207	911293	C	T	NA	*OreG0016015*
scaffold33207	911306	C	T	NA	*OreG0016015*
scaffold33207	920491	G	T	Ore04,Ore05	*OreG0016016*
scaffold33207	936680	C	T	NA	*OreG0016018*
scaffold33207	990499	C	A	Och01,Och02,Och03,Och04,Och05,Och06,Och07,Och08,Och09,Och10,Och11,Och12,Och13,Och14,Ore01,Ore02,Ore03,Ore04,Ore05,Ore06,Ore07,Ore08,Ore09,Ore10,Ore11,Ore12,Ore13,Ore14	*OreG0016027*
scaffold33207	1002429	C	A	NA	*OreG0016028*
scaffold33207	1020801	G	T	Och01,Och02,Och03,Och04,Och05,Och06,Och07,Och08,Och09,Och10,Och11,Och12,Och13,Och14,Ore01,Ore02,Ore03,Ore04,Ore05,Ore06,Ore07,Ore08,Ore09,Ore10,Ore11,Ore12,Ore13,Ore14	*OreG0016030*
scaffold33207	1051820	C	A	Och01,Och02,Och03,Och04,Och05,Och06,Och07,Och08,Och09,Och10,Och11,Och12,Och13,Och14	*OreG0016035*
scaffold33207	1069291	A	C	NA	*OreG0016037*
scaffold33207	1085790	A	G	Och01,Och02,Och03,Och04,Och05,Och06,Och07,Och08,Och09,Och10,Och11,Och12,Och13,Och14	*OreG0016039*
scaffold33207	1086103	A	G	Och01,Och03,Och05,Och08,Och09,Och10,Och12,Och13,Och14	*OreG0016039*
scaffold33207	1086828	G	A	Och01,Och02,Och03,Och04,Och05,Och06,Och07,Och08,Och09,Och10,Och11,Och12,Och13,Och14,Ore01,Ore02,Ore03,Ore04,Ore05,Ore06,Ore07,Ore08,Ore09,Ore10,Ore11,Ore12,Ore13,Ore14	*OreG0016039*
scaffold33207	1092268	A	G	NA	*OreG0016039*
scaffold33207	1100397	G	A	NA	*OreG0016040*
scaffold33207	1111110	C	A	Ore01,Ore02,Ore03,Ore04,Ore05,Ore06,Ore07,Ore08,Ore09,Ore10,Ore11,Ore12,Ore13,Ore14	*OreG0016043*
scaffold33207	1111119	C	G	Ore01,Ore02,Ore03,Ore04,Ore05,Ore06,Ore07,Ore08,Ore09,Ore10,Ore11,Ore12,Ore13,Ore14	*OreG0016043*
scaffold33207	1114074	G	C	NA	*OreG0016044*
scaffold33207	1114124	G	C	Och09,Och10	*OreG0016044*
scaffold33207	1115281	C	A	Och01,Och02,Och03,Och04,Och05,Och06,Och07,Och08,Och09,Och10,Och11,Och12,Och13,Och14,Ore01,Ore02,Ore03,Ore04,Ore05,Ore06,Ore07,Ore08,Ore09,Ore10,Ore11,Ore12,Ore13,Ore14	*OreG0016044*
scaffold33207	1115304	G	T	Ore03,Ore06,Ore07,Ore10,Ore12,Ore14	*OreG0016044*
scaffold33207	1115458	G	A	Ore03,Ore06,Ore07,Ore10,Ore12,Ore14	*OreG0016044*
scaffold33207	1122878	G	A	Och01,Och02,Och03,Och04,Och05,Och06,Och07,Och08,Och09,Och10,Och11,Och12,Och13,Och14,Ore01,Ore02,Ore03,Ore04,Ore05,Ore06,Ore07,Ore08,Ore09,Ore10,Ore11,Ore12,Ore13,Ore14	*OreG0016047*
scaffold33207	1122952	A	G	Ore03,Ore06,Ore07,Ore10,Ore12,Ore14	*OreG0016047*
scaffold33207	1123840	C	T	Och01,Och02,Och03,Och04,Och05,Och06,Och07,Och08,Och09,Och10,Och11,Och12,Och13,Och14,Ore01,Ore02,Ore03,Ore04,Ore05,Ore06,Ore07,Ore08,Ore09,Ore10,Ore11,Ore12,Ore13,Ore14	*OreG0016047*
scaffold33207	1136061	G	C	Ore01,Ore02,Ore03,Ore04,Ore05,Ore06,Ore07,Ore08,Ore09,Ore10,Ore11,Ore12,Ore13,Ore14	*OreG0016050*
scaffold33207	1139190	T	A	NA	*OreG0016050*

scaffold33207	1139679	C	G	Ore01,Ore02,Ore03,Ore04,Ore05,Ore06,Ore07,Ore08,Ore09,Ore10,Ore11,Ore12,Ore13,Ore14	*OreG0016050*
scaffold33207	1154034	A	G	NA	*OreG0016052*
scaffold33207	1169759	A	G	Och14	*OreG0016056*
scaffold33207	1173170	T	A	Och03	*OreG0016057*
scaffold33207	1173270	T	C	Och08,Och09,Och10	*OreG0016057*
scaffold33207	1173300	C	G	Ore03,Ore06,Ore07,Ore10,Ore12,Ore14	*OreG0016057*
scaffold33207	1173345	A	G	NA	*OreG0016057*
scaffold33207	1184041	C	T	Och01,Och02,Och03,Och04,Och05,Och06,Och07,Och08,Och09,Och10,Och11,Och12,Och13,Och14,Ore01,Ore02,Ore03,Ore04,Ore05,Ore06,Ore07,Ore08,Ore09,Ore10,Ore11,Ore12,Ore13,Ore14	*OreG0016060*
scaffold33207	1217602	C	T	Och07,Och08,Och09,Och10,Och12,Och13	*OreG0016064*
scaffold33207	1218536	C	G	Ore01,Ore03,Ore06,Ore07,Ore10,Ore12,Ore14	*OreG0016064*
scaffold33207	1218599	T	C	Och01,Och02,Och03,Och04,Och05,Och06,Och07,Och08,Och09,Och10,Och11,Och12,Och13,Och14,Ore01,Ore03,Ore06,Ore07,Ore10,Ore12,Ore14	*OreG0016064*
scaffold33207	1235930	G	C	NA	*OreG0016067*
scaffold33207	1244977	T	A	Och01,Och02,Och03,Och04,Och05,Och06,Och07,Och08,Och09,Och10,Och11,Och12,Och13,Och14	*OreG0016068*
scaffold33207	1245129	T	A	Ore01,Ore02,Ore03,Ore04,Ore05,Ore06,Ore07,Ore08,Ore09,Ore10,Ore11,Ore12,Ore13,Ore14	*OreG0016068*
scaffold33207	1259463	G	A	NA	*OreG0016070*
scaffold33207	1264944	G	A	Och01,Och02,Och03,Och04,Och05,Och06,Och07,Och08,Och09,Och10,Och11,Och12,Och13,Och14,Ore01,Ore02,Ore03,Ore04,Ore05,Ore06,Ore07,Ore08,Ore09,Ore10,Ore11,Ore12,Ore13,Ore14	*OreG0016071*
scaffold33207	1266543	C	A	Och03,Och09,Och10,Och11	*OreG0016071*
scaffold33207	1281316	C	A	Och09,Och10	*OreG0016076*
scaffold33207	1332923	T	C	Ore04,Ore05	*OreG0016082*
scaffold33207	1333382	T	G	NA	*OreG0016082*
scaffold33207	1345985	C	G	NA	*OreG0016083*
scaffold33207	1346074	G	A	Och01,Och02,Och03,Och04,Och05,Och06,Och07,Och08,Och09,Och10,Och11,Och12,Och13,Och14,Ore01,Ore02,Ore03,Ore04,Ore05,Ore06,Ore07,Ore08,Ore09,Ore10,Ore11,Ore12,Ore13,Ore14	*OreG0016083*
scaffold33207	1346335	C	T	NA	*OreG0016083*
scaffold33207	1415764	T	G	Ore04,Ore05	*OreG0016095*
scaffold33207	1417202	A	C	Och02,Och04,Och12,Och13	*OreG0016095*
scaffold33207	1417487	G	A	NA	*OreG0016095*
scaffold33207	1420966	T	A	Och12,Och13	*OreG0016095*
scaffold33207	1443543	G	A	Ore01,Ore02,Ore03,Ore04,Ore05,Ore06,Ore07,Ore08,Ore09,Ore10,Ore11,Ore12,Ore13,Ore14	*OreG0016100*
scaffold33207	1461055	G	A	NA	*OreG0016102*
scaffold33207	1485387	G	A	Och12,Och13	*OreG0016106*
scaffold33207	1523322	A	G	NA	*OreG0016113*
scaffold33207	1534129	C	T	NA	*OreG0016114*
scaffold33207	1534148	C	A	NA	*OreG0016114*
scaffold33207	1540166	G	C	Ore01,Ore02,Ore03,Ore04,Ore05,Ore06,Ore07,Ore08,Ore09,Ore10,Ore11,Ore12,Ore13,Ore14	*OreG0016115*
scaffold33207	1540330	T	C	Ore01,Ore02,Ore03,Ore04,Ore05,Ore06,Ore07,Ore08,Ore09,Ore10,Ore11,Ore12,Ore13,Ore14	*OreG0016115*
scaffold33207	1540379	G	T	NA	*OreG0016115*
scaffold33207	1540388	T	C	Och01,Och03,Och06,Och07,Och08,Och09,Och10,Och11	*OreG0016115*
scaffold33207	1540538	A	T	Och09	*OreG0016115*
scaffold33207	1540589	A	G	Och01,Och09	*OreG0016115*
scaffold33207	1540628	C	T	Ore04,Ore05	*OreG0016115*
scaffold33207	1541587	A	G	NA	*OreG0016115*
scaffold33207	1541710	G	A	Och01,Och02,Och03,Och04,Och05,Och06,Och07,Och08,Och09,Och10,Och11,Och12,Och13,Och14	*OreG0016115*
scaffold33207	1541795	A	T	Och06	*OreG0016115*
scaffold33207	1541916	A	C	NA	*OreG0016115*
scaffold33207	1557730	G	A	Ore03,Ore06,Ore07,Ore10,Ore12,Ore14	*OreG0016116*
scaffold33207	1563142	G	T	NA	*OreG0016116*
scaffold33207	1565286	T	A	Och01,Och02,Och03,Och04,Och05,Och06,Och07,Och08,Och09,Och10,Och11,Och12,Och13,Och14,Ore01,Ore02,Ore03,Ore04,Ore05,Ore06,Ore07,Ore08,Ore09,Ore10,Ore11,Ore12,Ore13,Ore14	*OreG0016116*
scaffold33207	1594812	T	C	NA	*OreG0016120*
scaffold33207	1594960	G	A	Och06,Och09,Och10	*OreG0016120*
scaffold33207	1595110	C	T	Och06,Och09,Och10	*OreG0016120*
scaffold33207	1595769	A	G	NA	*OreG0016120*
scaffold33207	1595790	A	C	Ore01,Ore02,Ore03,Ore04,Ore05,Ore06,Ore07,Ore08,Ore09,Ore10,Ore11,Ore12,Ore13,Ore14	*OreG0016120*
scaffold33207	1595995	T	C	Och05,Och07,Och08,Och12,Och13,Och14	*OreG0016120*
scaffold33207	1610945	G	C	Ore01,Ore02,Ore03,Ore04,Ore05,Ore06,Ore07,Ore08,Ore09,Ore10,Ore11,Ore12,Ore13,Ore14	*OreG0016123*
scaffold33207	1612329	G	T	Ore03,Ore06,Ore07,Ore10,Ore12,Ore14	*OreG0016124*
scaffold33207	1641252	A	G	NA	*OreG0016127*

scaffold33207	1662044	A	T	Och01,Och02,Och03,Och04,Och05,Och06,Och07,Och08,Och09,Och10,Och11,Och12,Och13,Och14,Ore01,Ore02,Ore03,Ore04,Ore05,Ore06,Ore07,Ore08,Ore09,Ore10,Ore11,Ore12,Ore13,Ore14	*OreG0016130*
scaffold33207	1662196	G	A	NA	*OreG0016130*
scaffold33207	1662671	T	A	NA	*OreG0016130*
scaffold33207	1675828	G	A	Och09,Och10	*OreG0016132*
scaffold33207	1676417	T	C	Och09,Och10	*OreG0016132*
scaffold33207	1712107	T	C	Och02,Och04,Och07,Och12,Och13,Och14	*OreG0016137*
scaffold33207	1712470	G	T	NA	*OreG0016138*
scaffold33207	1712643	C	A	Och02,Och12,Och14	*OreG0016138*
scaffold33207	1712931	C	T	Och01,Och02,Och03,Och04,Och05,Och06,Och07,Och08,Och09,Och10,Och11,Och12,Och13,Och14,Ore01,Ore02,Ore03,Ore04,Ore05,Ore06,Ore07,Ore08,Ore09,Ore10,Ore11,Ore12,Ore13,Ore14	*OreG0016138*
scaffold33207	1725210	G	T	Och01,Och02,Och03,Och04,Och05,Och06,Och07,Och08,Och09,Och10,Och11,Och12,Och13,Och14,Ore01,Ore02,Ore03,Ore04,Ore05,Ore06,Ore07,Ore08,Ore09,Ore10,Ore11,Ore12,Ore13,Ore14	*OreG0016140*
scaffold33207	1725996	C	T	Och01,Och02,Och03,Och04,Och05,Och06,Och07,Och08,Och09,Och10,Och11,Och12,Och13,Och14,Ore01,Ore02,Ore03,Ore04,Ore05,Ore06,Ore07,Ore08,Ore09,Ore10,Ore11,Ore12,Ore13,Ore14	*OreG0016140*
scaffold33207	1729315	A	G	Och01,Och02,Och03,Och04,Och05,Och06,Och07,Och08,Och09,Och10,Och11,Och12,Och13,Och14,Ore01,Ore02,Ore03,Ore04,Ore05,Ore06,Ore07,Ore08,Ore09,Ore10,Ore11,Ore12,Ore13,Ore14	*OreG0016141*
scaffold33207	1737043	G	A	Och01,Och02,Och03,Och04,Och05,Och06,Och07,Och08,Och09,Och10,Och11,Och12,Och13,Och14	*OreG0016142*
scaffold33207	1754033	G	C	Och01,Och02,Och03,Och04,Och05,Och06,Och07,Och08,Och09,Och10,Och11,Och12,Och13,Och14,Ore01,Ore02,Ore03,Ore04,Ore05,Ore06,Ore07,Ore08,Ore09,Ore10,Ore11,Ore12,Ore13,Ore14	*OreG0016144*
scaffold33207	1823819	C	T	NA	*OreG0016148*
scaffold33207	1863160	A	T	Och07	*OreG0016151*
scaffold33207	1864245	C	T	Ore01,Ore02,Ore03,Ore04,Ore05,Ore06,Ore07,Ore08,Ore09,Ore10,Ore11,Ore12,Ore13,Ore14	*OreG0016151*
scaffold33207	1864249	C	T	Ore01,Ore02,Ore03,Ore04,Ore05,Ore06,Ore07,Ore08,Ore09,Ore10,Ore11,Ore12,Ore13,Ore14	*OreG0016151*
scaffold33207	1864791	G	T	NA	*OreG0016151*
scaffold33207	1864827	G	C	NA	*OreG0016151*
scaffold33207	1974933	G	A	Och02,Och04,Och05,Och12,Och13	*OreG0016163*
scaffold33207	2017237	A	T	Och02,Och04,Och12	*OreG0016168*
scaffold33207	2017319	C	A	Och02,Och04,Och12,Och13	*OreG0016168*
scaffold33207	2017706	A	G	NA	*OreG0016168*
scaffold33207	2036244	T	A	NA	*OreG0016170*
scaffold33207	2050011	C	T	NA	*OreG0016172*
scaffold33207	2051292	G	T	NA	*OreG0016172*
scaffold33207	2053244	A	C	Och09,Och10	*OreG0016172*
scaffold33207	2054318	G	T	NA	*OreG0016172*
scaffold33207	2062084	T	C	Ore04,Ore05,Ore08	*OreG0016173*
scaffold33207	2067657	G	T	Ore03,Ore06,Ore07,Ore10,Ore12,Ore14	*OreG0016173*
scaffold33207	2070586	G	A	Och03,Och06,Och07,Och08,Och09,Och10,Och11	*OreG0016174*
scaffold33207	2102771	G	A	Och01,Och02,Och03,Och04,Och05,Och06,Och07,Och08,Och09,Och10,Och11,Och12,Och13,Och14,Ore01,Ore02,Ore03,Ore04,Ore05,Ore06,Ore07,Ore08,Ore09,Ore10,Ore11,Ore12,Ore13,Ore14	*OreG0016178*
scaffold33207	2103322	T	C	NA	*OreG0016178*
scaffold33207	2104212	C	T	Och12,Och13	*OreG0016179*
scaffold33207	2104898	G	A	NA	*OreG0016179*
scaffold33207	2104989	T	C	NA	*OreG0016179*
scaffold33207	2125946	T	C	Ore01,Ore02,Ore03,Ore04,Ore05,Ore06,Ore07,Ore08,Ore09,Ore10,Ore11,Ore12,Ore13,Ore14	*OreG0016181*
scaffold33207	2128524	T	A	NA	*OreG0016182*
scaffold33207	2128569	G	A	NA	*OreG0016182*
scaffold33207	2130087	G	A	NA	*OreG0016182*
scaffold33207	2135736	G	A	Och02,Och04,Och08,Och12,Och13	*OreG0016183*
scaffold33207	2135806	A	G	Och01,Och02,Och04,Och06,Och08,Och09,Och10,Och12,Och13,Och14	*OreG0016183*
scaffold33207	2135808	C	T	NA	*OreG0016183*
scaffold33207	2135824	T	C	NA	*OreG0016183*
scaffold33207	2135956	A	G	NA	*OreG0016183*
scaffold33207	2135959	T	A	Ore01,Ore02,Ore03,Ore04,Ore05,Ore06,Ore07,Ore08,Ore09,Ore10,Ore11,Ore12,Ore13,Ore14	*OreG0016183*
scaffold33207	2136815	C	T	Och02,Och04,Och08,Och12,Och13	*OreG0016184*
scaffold33207	2146009	C	T	NA	*OreG0016186*
scaffold33207	2147321	G	A	NA	*OreG0016186*
scaffold33207	2171506	T	A	NA	*OreG0016190*
scaffold33207	2172037	T	C	NA	*OreG0016190*
scaffold33207	2172390	G	T	NA	*OreG0016190*
scaffold33207	2347885	G	A	NA	*OreG0016205*
scaffold33207	2347912	T	A	NA	*OreG0016205*
scaffold33207	2348421	A	G	Och09,Och10,Och11	*OreG0016205*

scaffold33207	2348661	G	A	NA	*OreG0016205*
scaffold33207	2355876	C	T	NA	*OreG0016206*
scaffold33207	2401973	A	T	NA	*OreG0016210*
scaffold33207	2411801	A	T	NA	*OreG0016211*
scaffold33207	2436988	G	T	NA	*OreG0016213*
scaffold33207	2451575	G	C	NA	*OreG0016214*
scaffold33207	2451589	C	T	NA	*OreG0016214*
scaffold33207	2462608	G	T	Och12,Och13	*OreG0016215*
scaffold33207	2466680	A	G	Och03,Och05	*OreG0016216*
scaffold33207	2466965	G	T	Ore02	*OreG0016216*
scaffold33207	2470252	C	T	NA	*OreG0016217*
scaffold33207	2479744	G	A	NA	*OreG0016219*
scaffold33207	2480069	C	T	NA	*OreG0016219*
scaffold33207	2489091	G	A	Ore01,Ore02,Ore03,Ore04,Ore05,Ore06,Ore07,Ore08,Ore09,Ore10,Ore11,Ore12,Ore13,Ore14	*OreG0016221*
scaffold33207	2490646	T	A	NA	*OreG0016221*
scaffold33207	2494385	G	T	NA	*OreG0016222*
scaffold33207	2501966	C	G	Och01,Och02,Och03,Och04,Och05,Och06,Och07,Och08,Och09,Och10,Och11,Och12,Och13,Och14	*OreG0016223*
scaffold33207	2505103	C	T	Och01,Och02,Och03,Och04,Och05,Och06,Och07,Och08,Och09,Och10,Och11,Och12,Och13,Och14,Ore01,Ore02,Ore03,Ore04,Ore05,Ore06,Ore07,Ore08,Ore09,Ore10,Ore11,Ore12,Ore13,Ore14	*OreG0016224*
scaffold33207	2505321	C	T	Och01,Och02,Och03,Och04,Och05,Och06,Och07,Och08,Och09,Och10,Och11,Och12,Och13,Och14	*OreG0016224*
scaffold33207	2511217	A	T	NA	*OreG0016225*
scaffold33207	2511518	T	C	Ore01,Ore02,Ore03,Ore04,Ore05,Ore06,Ore07,Ore08,Ore09,Ore10,Ore11,Ore12,Ore13,Ore14	*OreG0016225*
scaffold33207	2512555	C	T	Och12,Och13	*OreG0016225*
scaffold2623	16432	T	C	Och02	*OreG0007828*
scaffold2623	17211	T	G	NA	*OreG0007828*
scaffold33275	19300	T	A	NA	*OreG0018258*
scaffold33275	19380	T	C	Och01,Och02,Och03,Och04,Och05,Och06,Och07,Och08,Och09,Och10,Och11,Och12,Och13,Och14	*OreG0018258*
scaffold33275	73727	C	G	Ore03,Ore04,Ore05,Ore09,Ore13	*OreG0018261*
scaffold33271	7683	C	T	Ore02,Ore03,Ore04,Ore05,Ore06,Ore07,Ore08,Ore09,Ore10,Ore11,Ore12,Ore14	*OreG0018163*
scaffold33271	7997	C	T	Ore02,Ore03,Ore04,Ore05,Ore06,Ore07,Ore08,Ore09,Ore10,Ore11,Ore12,Ore14	*OreG0018163*
scaffold33271	8009	C	T	Ore02,Ore03,Ore04,Ore05,Ore06,Ore07,Ore08,Ore09,Ore10,Ore11,Ore12,Ore14	*OreG0018163*
scaffold33267	36128	G	A	Och02,Och03,Och04,Och05,Och06,Och09,Och10,Och12,Och13,Ore03,Ore04,Ore05,Ore10	*OreG0018138*
scaffold33267	36143	C	T	Och02,Och03,Och04,Och05,Och06,Och09,Och10,Och12,Och13,Ore03,Ore04,Ore05,Ore10	*OreG0018138*
scaffold33267	36174	C	A	Och02,Och03,Och04,Och05,Och06,Och09,Och10,Och12,Och13,Ore03,Ore05,Ore10	*OreG0018138*
scaffold33267	36477	C	T	Och02,Och03,Och04,Och05,Och09,Och10,Och12,Och13,Ore05	*OreG0018138*
scaffold33267	36497	C	T	Och02,Och03,Och04,Och05,Och06,Och09,Och10,Och12,Och13,Ore05	*OreG0018138*
scaffold33267	38770	A	G	Och02,Och03,Och04,Och05,Och06,Och09,Och10,Och12,Och13,Ore03,Ore04,Ore05,Ore10	*OreG0018138*
scaffold33267	49003	A	C	Och02,Och03,Och04,Och05,Och06,Och09,Och10,Och12,Och13,Ore03,Ore04,Ore05,Ore10	*OreG0018139*
scaffold33267	49101	A	G	NA	*OreG0018139*
scaffold33267	49193	A	C	Och02,Och03,Och04,Och06,Och08,Och12,Och13,Och14	*OreG0018139*
scaffold33267	66261	G	C	NA	*OreG0018140*
scaffold33283	40163	G	C	NA	*OreG0018421*
scaffold33283	46541	C	A	NA	*OreG0018420*
scaffold33283	48477	G	A	Och01,Och02,Och03,Och04,Och05,Och06,Och07,Och08,Och09,Och10,Och11,Och12,Och13,Och14,Ore01,Ore02,Ore03,Ore04,Ore05,Ore06,Ore07,Ore08,Ore09,Ore10,Ore11,Ore12,Ore13,Ore14	*OreG0018420*
scaffold33283	112431	G	A	Ore02,Ore07,Ore08,Ore09,Ore12	*OreG0018426*
scaffold33283	115029	C	A	Ore04,Ore05	*OreG0018426*
scaffold33283	115806	C	G	Och09,Och10,Och11	*OreG0018426*
scaffold33283	116314	G	A	Och07,Och08,Ore04,Ore05	*OreG0018426*
scaffold33283	116883	T	C	Och02,Och13	*OreG0018426*
scaffold33198	22356	G	T	Ore01,Ore02,Ore03,Ore04,Ore05,Ore06,Ore07,Ore08,Ore09,Ore10,Ore11,Ore12,Ore13,Ore14	*OreG0015329*
scaffold33198	24061	G	C	Ore01,Ore02,Ore03,Ore04,Ore05,Ore06,Ore07,Ore08,Ore09,Ore10,Ore11,Ore12,Ore13,Ore14	*OreG0015329*
scaffold33198	24405	C	T	Och09,Och10	*OreG0015329*
scaffold33198	26114	G	C	Och02,Och03,Och04,Och05,Och06,Och07,Och12,Och13	*OreG0015329*
scaffold33198	105782	A	G	NA	*OreG0015332*
scaffold33198	116852	G	A	Och02,Och06,Och07,Och08,Och11	*OreG0015333*
scaffold33198	117260	A	G	Och09,Och10	*OreG0015333*
scaffold33198	117779	T	C	NA	*OreG0015333*
scaffold33198	117960	C	A	Och01,Och02,Och03,Och04,Och05,Och06,Och07,Och08,Och09,Och10,Och11,Och12,Och13,Och14,Ore01,Ore02,Ore03,Ore04,Ore05,Ore06,Ore07,Ore08,Ore09,Ore10,Ore11,Ore12,Ore13,Ore14	*OreG0015333*

scaffold33198	146030	G	T	Ore01,Ore02,Ore03,Ore04,Ore05,Ore06,Ore07,Ore08,Ore09,Ore10,Ore11,Ore12,Ore13,Ore14	*OreG0015336*
scaffold33198	149526	A	C	Och02,Och03,Och04,Och05,Och06,Och12,Och13	*OreG0015337*
scaffold33198	149577	G	T	Ore01,Ore02,Ore03,Ore04,Ore05,Ore06,Ore07,Ore08,Ore09,Ore10,Ore11,Ore12,Ore13,Ore14	*OreG0015337*
scaffold33198	149961	G	C	Ore01,Ore02,Ore03,Ore04,Ore05,Ore06,Ore07,Ore08,Ore09,Ore10,Ore11,Ore12,Ore13,Ore14	*OreG0015337*
scaffold33198	164491	T	G	NA	*OreG0015339*
scaffold33198	199524	C	T	Och01,Och02,Och03,Och04,Och05,Och06,Och07,Och09,Och10,Och11,Och12,Och13,Och14,Ore01,Ore02,Ore03,Ore04,Ore05,Ore06,Ore07,Ore08,Ore09,Ore10,Ore11,Ore12,Ore13,Ore14	*OreG0015341*
scaffold33198	199902	C	T	Och01,Och02,Och03,Och04,Och05,Och06,Och07,Och08,Och09,Och10,Och11,Och12,Och13,Och14,Ore01,Ore02,Ore03,Ore04,Ore05,Ore06,Ore07,Ore08,Ore09,Ore10,Ore11,Ore12,Ore13,Ore14	*OreG0015341*
scaffold33198	240193	G	C	Och01	*OreG0015343*
scaffold33198	272659	T	C	NA	*OreG0015346*
scaffold33198	299040	A	C	Ore01,Ore02,Ore03,Ore04,Ore05,Ore06,Ore07,Ore08,Ore09,Ore10,Ore11,Ore12,Ore13,Ore14	*OreG0015348*
scaffold33198	299135	A	C	Och01,Och02,Och03,Och04,Och05,Och06,Och07,Och08,Och09,Och10,Och11,Och12,Och13,Och14	*OreG0015348*
scaffold33198	299331	A	C	Och02,Och03,Och04,Och05,Och06,Och07,Och09,Och10,Och11,Och12,Och13	*OreG0015348*
scaffold33198	305656	A	C	Ore01,Ore02,Ore03,Ore04,Ore05,Ore06,Ore07,Ore08,Ore09,Ore10,Ore11,Ore12,Ore13,Ore14	*OreG0015348*
scaffold33198	339896	G	A	NA	*OreG0015350*
scaffold33198	355537	G	A	Ore01,Ore02,Ore03,Ore04,Ore05,Ore06,Ore07,Ore08,Ore09,Ore10,Ore11,Ore12,Ore13,Ore14	*OreG0015350*
scaffold33198	364579	C	T	NA	*OreG0015351*
scaffold33198	389116	T	C	Och01,Och02,Och03,Och04,Och05,Och06,Och07,Och08,Och09,Och10,Och11,Och12,Och13,Och14	*OreG0015353*
scaffold33198	396081	G	C	Ore08	*OreG0015353*
scaffold33198	412154	T	C	Och05,Och09,Och10	*OreG0015354*
scaffold33198	412214	T	C	Och05,Och09,Och10	*OreG0015354*
scaffold33198	442139	G	T	NA	*OreG0015357*
scaffold33198	447423	G	T	Och01,Och02,Och03,Och04,Och05,Och06,Och07,Och08,Och09,Och10,Och11,Och12,Och13,Och14,Ore01,Ore02,Ore03,Ore04,Ore05,Ore06,Ore07,Ore08,Ore09,Ore10,Ore11,Ore12,Ore13,Ore14	*OreG0015358*
scaffold33198	447989	G	T	NA	*OreG0015358*
scaffold33198	448007	C	T	NA	*OreG0015358*
scaffold33198	450640	G	T	Och02,Och03,Och04,Och05,Och06,Och07,Och09,Och10,Och12,Och13,Och14	*OreG0015358*
scaffold33198	488888	A	T	Ore01,Ore02,Ore03,Ore04,Ore05,Ore06,Ore07,Ore08,Ore09,Ore10,Ore11,Ore12,Ore13,Ore14	*OreG0015360*
scaffold33198	500865	T	A	Och01,Och02,Och03,Och04,Och05,Och06,Och07,Och08,Och09,Och10,Och11,Och12,Och13,Och14,Ore01,Ore02,Ore03,Ore04,Ore05,Ore06,Ore07,Ore08,Ore09,Ore10,Ore11,Ore12,Ore13,Ore14	*OreG0015361*
scaffold33198	538406	C	T	Och09,Och10	*OreG0015364*
scaffold33198	557478	T	A	NA	*OreG0015366*
scaffold33198	561120	A	T	Och09,Och10	*OreG0015366*
scaffold33198	620724	C	A	NA	*OreG0015373*
scaffold33198	623228	T	C	Ore02,Ore04,Ore05,Ore06,Ore08,Ore09,Ore11,Ore12,Ore13,Ore14	*OreG0015373*
scaffold33198	624560	G	T	Och01,Och02,Och07,Och09,Och10,Och11,Och12,Och13,Ore01,Ore02,Ore03,Ore04,Ore05,Ore06,Ore07,Ore08,Ore09,Ore10,Ore11,Ore12,Ore13,Ore14	*OreG0015373*
scaffold33198	634923	A	G	NA	*OreG0015374*
scaffold33198	643191	G	C	NA	*OreG0015376*
scaffold33198	653690	A	T	NA	*OreG0015376*
scaffold33198	711734	C	T	NA	*OreG0015382*
scaffold33198	713912	T	G	NA	*OreG0015382*
scaffold33198	759499	G	A	NA	*OreG0015387*
scaffold33198	856382	G	A	NA	*OreG0015388*
scaffold33198	859913	A	T	NA	*OreG0015388*
scaffold33198	861096	G	T	NA	*OreG0015388*
scaffold33198	863641	G	T	NA	*OreG0015388*
scaffold33198	863677	C	T	NA	*OreG0015388*
scaffold33198	869144	C	T	Och01	*OreG0015389*
scaffold33198	869150	T	C	Och01	*OreG0015389*
scaffold33198	875288	G	A	NA	*OreG0015390*
scaffold33198	875361	C	T	NA	*OreG0015390*
scaffold33198	875393	C	T	Och01	*OreG0015390*
scaffold33198	875414	T	A	Och01	*OreG0015390*
scaffold33198	875450	G	A	Och08,Ore01,Ore02,Ore03,Ore04,Ore05,Ore06,Ore07,Ore08,Ore09,Ore10,Ore11,Ore12,Ore13,Ore14	*OreG0015390*
scaffold33198	875465	T	C	Och01	*OreG0015390*
scaffold33198	875531	C	T	Och02,Och03,Och04,Och05,Och06,Och07,Och08,Och09,Och10,Och11,Och12,Och13,Ore01,Ore02,Ore03,Ore04,Ore05,Ore06,Ore07,Ore08,Ore09,Ore10,Ore11,Ore12,Ore13,Ore14	*OreG0015390*
scaffold33198	875555	C	A	Och01	*OreG0015390*

scaffold33198	890248	C	A	Och02,Och03,Och09,Och10,Och12,Och13	*OreG0015392*
scaffold33198	976987	C	T	NA	*OreG0015396*
scaffold33198	977013	C	A	NA	*OreG0015396*
scaffold33198	978984	A	G	NA	*OreG0015396*
scaffold33198	1016135	G	C	Och01,Och07	*OreG0015399*
scaffold33198	1016575	C	A	Och01,Och02,Och03,Och04,Och05,Och06,Och07,Och08,Och09,Och10,Och11,Och12,Och13,Och14,Ore01,Ore02,Ore03,Ore04,Ore05,Ore06,Ore07,Ore08,Ore09,Ore10,Ore11,Ore12,Ore13,Ore14	*OreG0015399*
scaffold33198	1035313	C	A	NA	*OreG0015403*
scaffold33198	1037068	A	T	NA	*OreG0015404*
scaffold33198	1037455	T	G	NA	*OreG0015404*
scaffold33198	1037491	T	A	NA	*OreG0015404*
scaffold33198	1037506	G	C	NA	*OreG0015404*
scaffold33198	1056866	C	T	Och01,Och02,Och03,Och04,Och05,Och06,Och07,Och08,Och09,Och10,Och11,Och12,Och13,Och14,Ore01,Ore02,Ore03,Ore04,Ore05,Ore06,Ore07,Ore08,Ore09,Ore10,Ore11,Ore12,Ore13,Ore14	*OreG0015409*
scaffold33198	1056918	C	G	Och08	*OreG0015409*
scaffold33198	1057064	T	A	NA	*OreG0015409*
scaffold33198	1057191	G	T	Och01,Och02,Och03,Och04,Och05,Och06,Och07,Och09,Och10,Och11,Och12,Och13,Och14	*OreG0015409*
scaffold33198	1083919	G	A	NA	*OreG0015412*
scaffold33198	1083949	G	A	NA	*OreG0015412*
scaffold33198	1084365	A	G	NA	*OreG0015412*
scaffold33198	1084389	A	G	NA	*OreG0015412*
scaffold33198	1084438	G	T	NA	*OreG0015412*
scaffold33198	1084455	T	A	NA	*OreG0015412*
scaffold33198	1121163	G	T	Och01,Och02,Och03,Och04,Och05,Och06,Och07,Och08,Och09,Och10,Och11,Och12,Och13,Och14	*OreG0015415*
scaffold33198	1121839	G	C	NA	*OreG0015415*
scaffold33198	1155536	A	T	Och02,Och06	*OreG0015422*
scaffold33198	1175735	G	T	NA	*OreG0015424*
scaffold33198	1199517	G	A	Ore01,Ore02,Ore03,Ore04,Ore05,Ore06,Ore07,Ore08,Ore09,Ore10,Ore11,Ore12,Ore13,Ore14	*OreG0015426*
scaffold33198	1282799	A	G	NA	*OreG0015430*
scaffold33198	1282986	G	T	Och01,Och02,Och03,Och04,Och05,Och06,Och07,Och08,Och09,Och10,Och11,Och12,Och13	*OreG0015430*
scaffold33198	1283002	A	T	Ore01,Ore02,Ore03,Ore04,Ore05,Ore07,Ore08,Ore09,Ore10,Ore14	*OreG0015430*
scaffold33198	1283105	C	G	Och02,Och06	*OreG0015430*
scaffold33198	1284632	A	T	NA	*OreG0015430*
scaffold33198	1289130	C	G	Och07,Och11,Ore01,Ore02,Ore03,Ore04,Ore05,Ore06,Ore07,Ore08,Ore09,Ore10,Ore11,Ore12,Ore13,Ore14	*OreG0015431*
scaffold33198	1291097	A	G	Och07,Och11	*OreG0015431*
scaffold33198	1291152	G	C	Och14,Ore01,Ore02,Ore03,Ore04,Ore05,Ore07,Ore08,Ore09,Ore10,Ore14	*OreG0015431*
scaffold33198	1312621	G	T	NA	*OreG0015433*
scaffold33198	1316554	G	A	Och02,Och06,Och14	*OreG0015434*
scaffold33198	1321660	G	A	Ore01,Ore02,Ore07,Ore08,Ore14	*OreG0015435*
scaffold33198	1326784	T	C	Ore01,Ore02,Ore03,Ore04,Ore05,Ore07,Ore08,Ore09,Ore10,Ore14	*OreG0015436*
scaffold33198	1327544	C	A	NA	*OreG0015436*
scaffold33198	1450718	C	G	NA	*OreG0015445*
scaffold33198	1470923	G	A	Och01,Och02,Och03,Och04,Och05,Och06,Och07,Och08,Och09,Och10,Och11,Och12,Och13,Och14	*OreG0015446*
scaffold33198	1472124	G	A	Och01,Och02,Och03,Och04,Och05,Och06,Och07,Och08,Och09,Och10,Och11,Och12,Och13,Och14,Ore01,Ore02,Ore03,Ore04,Ore05,Ore06,Ore07,Ore08,Ore09,Ore10,Ore11,Ore12,Ore13,Ore14	*OreG0015446*
scaffold33198	1495511	G	A	Och01,Och02,Och03,Och04,Och05,Och06,Och07,Och08,Och09,Och10,Och11,Och12,Och13,Och14,Ore01,Ore02,Ore03,Ore04,Ore05,Ore06,Ore07,Ore08,Ore09,Ore10,Ore11,Ore12,Ore13,Ore14	*OreG0015447*
scaffold33198	1515534	T	C	NA	*OreG0015449*
scaffold33234	119428	T	C	Och01,Och02,Och03,Och04,Och05,Och06,Och07,Och08,Och09,Och10,Och11,Och12,Och13,Och14	*OreG0017225*
scaffold33234	120010	C	T	NA	*OreG0017225*
scaffold33234	132545	T	A	Ore01,Ore02,Ore03,Ore04,Ore05,Ore06,Ore07,Ore08,Ore09,Ore10,Ore11,Ore12,Ore13,Ore14	*OreG0017227*
scaffold33234	133237	A	T	Och02,Och03,Och04,Och05,Och06,Och08,Och12,Och13,Och14	*OreG0017227*
scaffold33234	151596	T	C	Och02,Och03,Och04,Och05,Och06,Och07,Och08,Och09,Och10,Och11,Och12,Och13,Och14,Ore01,Ore02,Ore03,Ore04,Ore05,Ore06,Ore07,Ore08,Ore09,Ore10,Ore11,Ore12,Ore13,Ore14	*OreG0017229*
scaffold33234	168717	T	C	NA	*OreG0017230*
scaffold33234	196320	C	A	NA	*OreG0017234*
scaffold33234	215069	G	C	Ore01,Ore03,Ore07,Ore08,Ore09,Ore10,Ore11,Ore12,Ore14	*OreG0017237*
scaffold33234	216313	G	A	NA	*OreG0017237*
scaffold33234	226337	A	G	NA	*OreG0017239*
scaffold33234	226364	C	T	NA	*OreG0017239*
scaffold33234	226395	C	T	NA	*OreG0017239*

scaffold33234	237102	C	A	Och01,Och02,Och03,Och04,Och05,Och06,Och07,Och08,Och09,Och10,Och11,Och12,Och13,Och14	*OreG0017241*
scaffold33234	276785	C	T	NA	*OreG0017248*
scaffold33234	320407	C	A	Ore01,Ore02,Ore03,Ore04,Ore05,Ore06,Ore07,Ore08,Ore09,Ore10,Ore11,Ore12,Ore13,Ore14	*OreG0017256*
scaffold33234	391645	A	G	NA	*OreG0017262*
scaffold33234	392130	T	C	Och01,Och02,Och03,Och04,Och05,Och06,Och07,Och08,Och09,Och10,Och11,Och12,Och13,Och14,Ore01,Ore02,Ore03,Ore04,Ore05,Ore06,Ore07,Ore08,Ore09,Ore10,Ore11,Ore12,Ore13,Ore14	*OreG0017262*
scaffold33234	403802	G	T	Ore01,Ore02,Ore03,Ore04,Ore05,Ore06,Ore07,Ore08,Ore09,Ore10,Ore11,Ore12,Ore13,Ore14	*OreG0017263*
scaffold33234	405674	T	A	NA	*OreG0017263*
scaffold33234	406102	T	C	NA	*OreG0017263*
scaffold33234	425100	A	C	NA	*OreG0017265*
scaffold33234	425118	A	G	NA	*OreG0017265*
scaffold33234	425135	A	C	NA	*OreG0017265*
scaffold33234	425467	C	T	NA	*OreG0017265*
scaffold33234	432451	A	T	NA	*OreG0017265*
scaffold33234	432537	T	A	Ore06,Ore07	*OreG0017265*
scaffold33234	451137	T	C	NA	*OreG0017268*
scaffold33234	451145	G	A	NA	*OreG0017268*
scaffold33234	451181	C	T	NA	*OreG0017268*
scaffold33292	10927	C	T	NA	*OreG0018504*
scaffold33292	11134	G	A	Och01,Och02,Och03,Och04,Och05,Och06,Och07,Och08,Och09,Och10,Och11,Och12,Och13,Och14	*OreG0018504*
scaffold33292	11886	C	T	NA	*OreG0018504*
scaffold33292	11940	G	T	NA	*OreG0018504*
scaffold33292	11945	G	A	NA	*OreG0018504*
scaffold33292	16829	T	A	Och06	*OreG0018505*
scaffold33292	16895	T	C	Och04,Och05,Och11,Och12,Och13	*OreG0018505*
scaffold33292	16943	A	T	Och04,Och05,Och11,Och12,Och13	*OreG0018505*
scaffold33254	42435	G	T	Och01,Och02,Och03,Och04,Och05,Och06,Och07,Och08,Och09,Och10,Och11,Och12,Och13,Och14,Ore01,Ore02,Ore03,Ore04,Ore05,Ore06,Ore07,Ore08,Ore09,Ore10,Ore11,Ore12,Ore13,Ore14	*OreG0017886*
scaffold33254	84815	G	C	Och01,Och02,Och03,Och04,Och05,Och06,Och07,Och08,Och09,Och10,Och11,Och12,Och13,Och14	*OreG0017888*
scaffold33254	84871	T	C	Och01,Och02,Och03,Och04,Och05,Och06,Och07,Och08,Och09,Och10,Och11,Och12,Och13,Och14,Ore01,Ore02,Ore03,Ore04,Ore05,Ore06,Ore07,Ore08,Ore09,Ore10,Ore11,Ore12,Ore13,Ore14	*OreG0017888*
scaffold33254	85442	G	A	Ore11	*OreG0017888*
scaffold33254	85496	C	T	NA	*OreG0017888*
scaffold33254	85568	T	A	NA	*OreG0017888*
scaffold33254	85855	G	T	Och01,Och02,Och03,Och04,Och05,Och06,Och07,Och08,Och09,Och10,Och11,Och12,Och13,Och14	*OreG0017888*
scaffold33254	180112	T	A	Och01,Och02,Och03,Och04,Och05,Och06,Och07,Och08,Och09,Och10,Och11,Och12,Och13,Och14,Ore01,Ore02,Ore03,Ore04,Ore05,Ore06,Ore07,Ore08,Ore09,Ore10,Ore11,Ore12,Ore13,Ore14	*OreG0017892*
scaffold33254	217587	G	A	Ore11	*OreG0017893*
scaffold33254	283980	A	T	NA	*OreG0017895*
scaffold33265	38861	G	A	Och12,Och13	*OreG0018126*
scaffold33253	20037	G	A	NA	*OreG0017833*
scaffold33253	27451	A	T	Och01,Och02,Och03,Och04,Och05,Och06,Och07,Och08,Och09,Och10,Och11,Och12,Och13,Och14	*OreG0017834*
scaffold33253	43178	C	G	Ore01,Ore02,Ore03,Ore04,Ore05,Ore06,Ore07,Ore08,Ore09,Ore10,Ore11,Ore12,Ore13,Ore14	*OreG0017835*
scaffold33253	43421	T	C	Och01	*OreG0017835*
scaffold33253	43438	T	G	Och01,Och02,Och03,Och04,Och05,Och06,Och07,Och08,Och09,Och10,Och11,Och12,Och13,Och14,Ore01,Ore02,Ore03,Ore04,Ore05,Ore06,Ore07,Ore08,Ore09,Ore10,Ore11,Ore12,Ore13,Ore14	*OreG0017835*
scaffold33253	43532	C	T	NA	*OreG0017835*
scaffold33253	82993	G	C	Ore01,Ore02,Ore03,Ore04,Ore05,Ore06,Ore07,Ore08,Ore09,Ore10,Ore11,Ore12,Ore13,Ore14	*OreG0017837*
scaffold33253	115891	A	C	Och01,Och02,Och03,Och04,Och05,Och06,Och07,Och08,Och09,Och10,Och11,Och12,Och13,Och14,Ore01,Ore02,Ore03,Ore04,Ore05,Ore06,Ore07,Ore08,Ore09,Ore10,Ore11,Ore12,Ore13,Ore14	*OreG0017838*
scaffold33253	155337	T	C	NA	*OreG0017840*
scaffold33253	156068	T	C	Och01,Och02,Och03,Och04,Och05,Och06,Och07,Och08,Och09,Och10,Och11,Och12,Och13,Och14,Ore01,Ore02,Ore03,Ore04,Ore05,Ore06,Ore07,Ore08,Ore09,Ore10,Ore11,Ore12,Ore13,Ore14	*OreG0017840*
scaffold33253	156074	A	T	NA	*OreG0017840*
scaffold33253	156565	A	C	Ore01,Ore02,Ore03,Ore04,Ore05,Ore06,Ore07,Ore08,Ore09,Ore10,Ore11,Ore12,Ore13,Ore14	*OreG0017840*
scaffold33253	195079	T	C	Ore11	*OreG0017843*
scaffold33253	485778	T	G	Och01,Och07	*OreG0017848*
scaffold33253	485812	A	T	Ore11	*OreG0017848*

scaffold33253	485814	A	G	NA	*OreG0017848*
scaffold33253	515886	A	G	Och01,Och02,Och03,Och04,Och05,Och06,Och07,Och08,Och09,Och10,Och11,Och12,Och13,Och14	*OreG0017849*
scaffold33253	563722	C	T	NA	*OreG0017850*
scaffold33253	622592	C	G	Och01,Och02,Och03,Och04,Och05,Och06,Och07,Och08,Och09,Och10,Och11,Och12,Och13,Och14	*OreG0017853*
scaffold33253	648005	G	A	NA	*OreG0017853*
scaffold33253	648007	G	T	NA	*OreG0017853*
scaffold33253	648025	G	A	NA	*OreG0017853*
scaffold33253	712487	T	C	Ore03,Ore04,Ore05,Ore09,Ore13	*OreG0017854*
scaffold33253	749744	T	A	Och01,Och02,Och03,Och04,Och05,Och06,Och07,Och08,Och09,Och10,Och11,Och12,Och13,Och14,Ore01,Ore02,Ore03,Ore04,Ore05,Ore06,Ore07,Ore08,Ore09,Ore10,Ore11,Ore12,Ore13,Ore14	*OreG0017856*
scaffold33253	752017	G	A	NA	*OreG0017856*
scaffold33253	756691	A	G	NA	*OreG0017856*
scaffold33253	756766	G	A	NA	*OreG0017856*
scaffold33253	758438	C	T	NA	*OreG0017856*
scaffold33253	758619	G	C	NA	*OreG0017856*
scaffold33253	758634	C	T	NA	*OreG0017856*
scaffold33253	760668	T	C	Ore01,Ore02,Ore03,Ore04,Ore05,Ore06,Ore07,Ore08,Ore09,Ore10,Ore11,Ore12,Ore13,Ore14	*OreG0017856*
scaffold33253	761885	T	G	Ore01,Ore02,Ore03,Ore04,Ore05,Ore06,Ore07,Ore08,Ore09,Ore10,Ore11,Ore12,Ore13,Ore14	*OreG0017856*
scaffold33253	773324	C	T	NA	*OreG0017857*
scaffold33253	796021	C	T	NA	*OreG0017860*
scaffold33253	812834	A	C	Och01,Och02,Och03,Och04,Och05,Och06,Och07,Och08,Och09,Och10,Och11,Och12,Och13,Och14	*OreG0017862*
scaffold33253	813213	C	A	NA	*OreG0017862*
scaffold33253	830957	C	G	Ore04,Ore05	*OreG0017864*
scaffold33253	856295	T	C	Och01,Och02,Och03,Och04,Och05,Och06,Och07,Och08,Och09,Och10,Och11,Och12,Och13,Och14	*OreG0017865*
scaffold33253	857871	C	T	Ore01,Ore02,Ore03,Ore04,Ore05,Ore06,Ore07,Ore08,Ore09,Ore10,Ore11,Ore12,Ore13,Ore14	*OreG0017865*
scaffold33253	917522	C	T	Ore04,Ore05	*OreG0017870*
scaffold33253	917715	C	A	Och01,Och02,Och03,Och04,Och05,Och06,Och07,Och08,Och09,Och10,Och11,Och12,Och13,Och14,Ore01,Ore02,Ore03,Ore04,Ore05,Ore06,Ore07,Ore08,Ore09,Ore10,Ore11,Ore12,Ore13,Ore14	*OreG0017870*
scaffold33253	917722	C	A	NA	*OreG0017870*
scaffold33253	917934	A	G	NA	*OreG0017870*
scaffold33253	923800	A	G	Och01,Och02,Och03,Och04,Och05,Och06,Och07,Och08,Och09,Och10,Och11,Och12,Och14	*OreG0017871*
scaffold33253	926695	C	T	Och08	*OreG0017871*
scaffold33253	932993	G	T	NA	*OreG0017873*
scaffold33253	933011	G	A	Och08	*OreG0017873*
scaffold33253	982445	G	A	Ore02,Ore10,Ore11,Ore12,Ore13	*OreG0017879*
scaffold33253	990623	C	A	Ore04,Ore05	*OreG0017880*
scaffold33284	32714	G	A	NA	*OreG0018432*
scaffold33284	33771	G	A	NA	*OreG0018432*
scaffold33284	33864	C	A	NA	*OreG0018432*
scaffold33280	2513	A	C	Och09,Och10	*OreG0018415*
scaffold33260	47899	G	A	Och01,Och02,Och03,Och04,Och05,Och06,Och07,Och08,Och09,Och10,Och11,Och12,Och13,Och14,Ore01,Ore02,Ore03,Ore04,Ore05,Ore06,Ore07,Ore08,Ore09,Ore10,Ore11,Ore12,Ore13,Ore14	*OreG0017962*
scaffold33260	61830	A	T	Och01,Och02,Och03,Och04,Och05,Och06,Och07,Och08,Och09,Och10,Och11,Och12,Och13,Och14	*OreG0017964*
scaffold33279	10954	G	T	NA	*OreG0018372*
scaffold33279	11046	G	A	NA	*OreG0018372*
scaffold33279	11218	C	T	NA	*OreG0018372*
scaffold33279	11238	G	T	NA	*OreG0018372*
scaffold33279	39514	A	T	Ore01,Ore02,Ore03,Ore04,Ore05,Ore06,Ore07,Ore08,Ore09,Ore10,Ore11,Ore12,Ore13,Ore14	*OreG0018376*
scaffold33279	44896	G	A	NA	*OreG0018377*
scaffold33279	44973	C	G	NA	*OreG0018377*
scaffold33279	48861	T	G	Och01,Och02,Och03,Och04,Och05,Och06,Och07,Och08,Och09,Och10,Och11,Och12,Och13,Och14,Ore01,Ore02,Ore03,Ore04,Ore05,Ore06,Ore07,Ore08,Ore09,Ore10,Ore11,Ore12,Ore13,Ore14	*OreG0018378*
scaffold33279	104500	G	T	NA	*OreG0018382*
scaffold33279	146497	G	T	NA	*OreG0018385*
scaffold33279	146515	G	A	NA	*OreG0018385*
scaffold33279	151749	T	C	NA	*OreG0018385*
scaffold33279	151786	C	A	NA	*OreG0018385*
scaffold33279	175739	T	A	Ore02,Ore03,Ore09,Ore10,Ore11,Ore12,Ore14	*OreG0018387*
scaffold33279	179134	T	A	NA	*OreG0018387*
scaffold33279	193840	T	A	Ore02,Ore03,Ore04,Ore05,Ore06,Ore07,Ore08,Ore09,Ore10,Ore11,Ore12,Ore13,Ore14	*OreG0018389*

scaffold33279	195456	A	G	NA	*OreG0018389*
scaffold33279	204159	T	C	NA	*OreG0018390*
scaffold33279	204219	A	T	NA	*OreG0018390*
scaffold33279	204379	A	G	NA	*OreG0018390*
scaffold33279	204888	A	C	NA	*OreG0018390*
scaffold33279	205174	C	A	Ore02,Ore03,Ore04,Ore05,Ore06,Ore07,Ore08,Ore09,Ore10,Ore11,Ore12,Ore13,Ore14	*OreG0018390*
scaffold33279	205508	A	G	NA	*OreG0018390*
scaffold33279	218450	C	G	Och01,Och02,Och03,Och04,Och05,Och06,Och07,Och08,Och09,Och10,Och11,Och12,Och13,Och14	*OreG0018392*
scaffold33279	218485	T	C	Och01,Och11,Och12,Och13,Och14	*OreG0018392*
scaffold33279	218820	G	A	Och01,Och02,Och03,Och04,Och05,Och06,Och07,Och08,Och09,Och10,Och11,Och12,Och13,Och14	*OreG0018392*
scaffold33279	222314	C	T	NA	*OreG0018393*
scaffold33279	222341	G	T	NA	*OreG0018393*
scaffold33279	222352	C	T	NA	*OreG0018393*
scaffold33279	237799	A	T	NA	*OreG0018396*
scaffold33279	238145	G	T	NA	*OreG0018396*
scaffold33279	238184	C	T	NA	*OreG0018396*
scaffold33279	238421	T	C	NA	*OreG0018396*
scaffold33279	238487	T	A	NA	*OreG0018396*
scaffold33279	238880	T	A	Ore02,Ore03,Ore09,Ore10,Ore11,Ore12,Ore14	*OreG0018396*
scaffold33279	239255	A	G	NA	*OreG0018396*
scaffold33279	239315	C	T	NA	*OreG0018396*
scaffold33279	239327	C	A	NA	*OreG0018396*
scaffold33279	239686	G	A	NA	*OreG0018396*
scaffold33279	240527	A	G	NA	*OreG0018396*
scaffold33279	275762	T	A	NA	*OreG0018397*
scaffold33279	275819	G	C	NA	*OreG0018397*
scaffold33279	275875	C	T	Och01,Och02,Och03,Och04,Och05,Och06,Och07,Och08,Och09,Och10,Och11,Och12,Och13,Och14,Ore01,Ore02,Ore03,Ore04,Ore05,Ore06,Ore07,Ore08,Ore09,Ore10,Ore11,Ore12,Ore13,Ore14	*OreG0018397*
scaffold33279	275893	C	A	NA	*OreG0018397*
scaffold33279	288382	C	T	Ore02,Ore03,Ore04,Ore05,Ore06,Ore07,Ore08,Ore09,Ore10,Ore11,Ore12,Ore13,Ore14	*OreG0018399*
scaffold33279	291215	C	T	Och01,Och02,Och03,Och04,Och05,Och06,Och07,Och08,Och09,Och10,Och11,Och12,Och13,Och14,Ore02,Ore03,Ore04,Ore05,Ore06,Ore07,Ore08,Ore09,Ore10,Ore11,Ore12,Ore13,Ore14	*OreG0018400*
scaffold33279	292116	C	T	Och01,Och02,Och03,Och04,Och05,Och06,Och07,Och08,Och09,Och10,Och11,Och12,Och13	*OreG0018400*
scaffold33279	294933	G	T	Och06,Och12	*OreG0018400*
scaffold33279	295486	G	A	Ore02,Ore03,Ore04,Ore05,Ore06,Ore07,Ore08,Ore09,Ore10,Ore11,Ore12,Ore13,Ore14	*OreG0018400*
scaffold33279	301235	G	T	NA	*OreG0018401*
scaffold33279	303728	T	G	NA	*OreG0018401*
scaffold33279	309372	G	C	Ore02,Ore03,Ore04,Ore05,Ore06,Ore07,Ore08,Ore09,Ore10,Ore11,Ore12,Ore13,Ore14	*OreG0018401*
scaffold33279	315103	C	T	NA	*OreG0018402*
scaffold33279	315105	G	A	NA	*OreG0018402*
scaffold33279	324620	T	C	NA	*OreG0018403*
scaffold33279	324682	C	A	Och01,Och02,Och03,Och04,Och05,Och06,Och07,Och08,Och09,Och10,Och11,Och12,Och13,Och14,Ore01,Ore02,Ore03,Ore04,Ore05,Ore06,Ore07,Ore08,Ore09,Ore10,Ore11,Ore12,Ore13,Ore14	*OreG0018403*
scaffold33279	324884	A	T	Och01,Och02,Och03,Och04,Och05,Och06,Och07,Och08,Och09,Och10,Och11,Och12,Och13,Och14,Ore01,Ore02,Ore03,Ore04,Ore05,Ore06,Ore07,Ore08,Ore09,Ore10,Ore11,Ore12,Ore13,Ore14	*OreG0018403*
scaffold33279	325008	G	C	Ore01,Ore02,Ore03,Ore04,Ore05,Ore06,Ore07,Ore08,Ore09,Ore10,Ore11,Ore12,Ore13,Ore14	*OreG0018403*
scaffold33279	342378	A	G	Ore01,Ore02,Ore03,Ore04,Ore05,Ore06,Ore07,Ore08,Ore09,Ore10,Ore11,Ore12,Ore13,Ore14	*OreG0018405*
scaffold33279	343447	G	A	NA	*OreG0018405*
scaffold33279	365329	T	A	Ore01	*OreG0018410*
scaffold33279	365457	C	T	NA	*OreG0018410*
scaffold33279	365490	G	T	NA	*OreG0018410*
scaffold33279	365653	G	A	NA	*OreG0018410*
scaffold33279	366114	G	A	Ore01	*OreG0018410*
scaffold33279	369530	C	T	NA	*OreG0018411*
scaffold33247	8321	G	A	Och01,Och02,Och03,Och04,Och05,Och06,Och07,Och08,Och09,Och10,Och11,Och12,Och13,Och14,Ore01,Ore02,Ore03,Ore04,Ore05,Ore06,Ore07,Ore08,Ore09,Ore10,Ore11,Ore12,Ore13,Ore14	*OreG0017447*
scaffold33247	8390	G	T	Ore01,Ore02,Ore03,Ore04,Ore05,Ore06,Ore07,Ore08,Ore09,Ore10,Ore11,Ore12,Ore13,Ore14	*OreG0017447*
scaffold33247	12064	C	A	NA	*OreG0017448*
scaffold33247	46759	A	G	Och01,Och02,Och06,Och07,Och09,Och10,Och11,Och14,Ore01,Ore02,Ore03,Ore04,Ore05,Ore06,Ore07,Ore08,Ore09,Ore10,Ore11,Ore12,Ore13,Ore14	*OreG0017451*

scaffold33247	71461	A	C	Och04,Och05,Och12,Och13	*OreG0017454*
scaffold33247	71494	C	T	Och04,Och05,Och12,Och13	*OreG0017454*
scaffold33247	71579	G	A	NA	*OreG0017454*
scaffold33247	71636	T	A	Och04,Och05,Och12,Och13	*OreG0017454*
scaffold33247	71701	G	A	NA	*OreG0017454*
scaffold33247	71737	C	T	NA	*OreG0017454*
scaffold33247	104088	A	G	NA	*OreG0017457*
scaffold33247	107063	G	T	NA	*OreG0017458*
scaffold33247	115944	A	T	NA	*OreG0017459*
scaffold33247	116022	T	A	Och01,Och02,Och03,Och04,Och05,Och06,Och07,Och08,Och09,Och10,Och11,Och12,Och13,Och14,Ore01,Ore02,Ore03,Ore04,Ore05,Ore06,Ore07,Ore08,Ore09,Ore10,Ore11,Ore12,Ore13,Ore14	*OreG0017459*
scaffold33247	116087	T	C	Och01,Och02,Och03,Och04,Och05,Och06,Och07,Och08,Och09,Och10,Och11,Och12,Och13,Och14,Ore01,Ore02,Ore03,Ore04,Ore05,Ore06,Ore07,Ore08,Ore09,Ore10,Ore11,Ore12,Ore13,Ore14	*OreG0017459*
scaffold33247	116447	A	G	NA	*OreG0017459*
scaffold33247	116504	T	C	NA	*OreG0017459*
scaffold33247	116753	T	C	NA	*OreG0017459*
scaffold33247	131752	G	T	NA	*OreG0017460*
scaffold33247	181740	C	T	NA	*OreG0017464*
scaffold33247	205228	C	T	Ore01,Ore02,Ore03,Ore04,Ore05,Ore06,Ore07,Ore08,Ore09,Ore10,Ore11,Ore12,Ore13,Ore14	*OreG0017467*
scaffold33247	206126	T	G	NA	*OreG0017467*
scaffold33247	206165	G	C	Och02,Och03,Och04,Och05,Och06,Och08,Och09,Och10,Och12,Och13	*OreG0017467*
scaffold33247	226897	C	G	Ore01,Ore02,Ore03,Ore04,Ore05,Ore06,Ore07,Ore08,Ore09,Ore10,Ore11,Ore12,Ore13,Ore14	*OreG0017469*
scaffold33247	228877	G	A	Ore01,Ore02,Ore03,Ore04,Ore05,Ore06,Ore07,Ore08,Ore09,Ore10,Ore11,Ore12,Ore13,Ore14	*OreG0017469*
scaffold33247	262503	T	C	Ore01,Ore02,Ore03,Ore04,Ore05,Ore06,Ore07,Ore08,Ore09,Ore10,Ore11,Ore12,Ore13,Ore14	*OreG0017471*
scaffold33247	263716	C	T	NA	*OreG0017471*
scaffold33247	291342	C	T	Ore01,Ore03,Ore06,Ore07,Ore09,Ore10,Ore11,Ore13,Ore14	*OreG0017472*
scaffold33247	296208	A	C	Och01,Och02,Och03,Och04,Och05,Och06,Och07,Och08,Och09,Och10,Och11,Och12,Och13,Och14,Ore01,Ore02,Ore03,Ore04,Ore05,Ore06,Ore07,Ore08,Ore09,Ore10,Ore11,Ore12,Ore13,Ore14	*OreG0017474*
scaffold33247	324942	G	C	Och08,Och14	*OreG0017476*
scaffold33247	419526	G	A	NA	*OreG0017483*
scaffold33247	419657	C	T	NA	*OreG0017483*
scaffold33247	549124	C	A	NA	*OreG0017498*
scaffold33247	595511	C	A	NA	*OreG0017506*
scaffold33247	595775	A	G	NA	*OreG0017506*
scaffold33247	617844	A	G	NA	*OreG0017510*
scaffold33247	618964	T	C	NA	*OreG0017510*
scaffold33247	633412	A	G	Ore01,Ore02,Ore03,Ore04,Ore05,Ore06,Ore07,Ore08,Ore09,Ore10,Ore11,Ore12,Ore13,Ore14	*OreG0017512*
scaffold33247	644260	C	A	Ore06,Ore07,Ore09,Ore10,Ore11,Ore13,Ore14	*OreG0017514*
scaffold33247	657214	C	T	Ore06,Ore07,Ore09,Ore10,Ore11,Ore13,Ore14	*OreG0017515*
scaffold33247	659772	C	A	Ore06,Ore07,Ore09,Ore10,Ore11,Ore13,Ore14	*OreG0017515*
scaffold33247	680110	T	A	NA	*OreG0017521*
scaffold33247	704558	T	C	Ore01,Ore02,Ore03,Ore04,Ore05,Ore06,Ore07,Ore08,Ore09,Ore10,Ore11,Ore12,Ore13,Ore14	*OreG0017522*
scaffold33247	715731	A	T	NA	*OreG0017523*
scaffold33247	715934	C	T	NA	*OreG0017523*
scaffold33247	822054	C	G	NA	*OreG0017530*
scaffold33247	830419	G	T	NA	*OreG0017531*
scaffold33247	830546	A	T	NA	*OreG0017531*
scaffold33247	830557	A	G	NA	*OreG0017531*
scaffold33247	830731	A	G	NA	*OreG0017531*
scaffold33247	837393	G	A	NA	*OreG0017532*
scaffold33247	837411	G	T	NA	*OreG0017532*
scaffold33247	847937	G	A	NA	*OreG0017535*
scaffold33247	847946	G	A	Ore01,Ore02,Ore03,Ore04,Ore05,Ore06,Ore07,Ore08,Ore09,Ore10,Ore11,Ore12,Ore13,Ore14	*OreG0017535*
scaffold33264	1395	C	T	NA	*OreG0018009*
scaffold33264	17187	C	A	NA	*OreG0018011*
scaffold33264	49778	A	G	Och01	*OreG0018016*
scaffold33264	51268	A	T	Och01,Och02,Och06,Och08,Och09,Och10,Och11,Och12,Och14	*OreG0018016*
scaffold33264	51914	A	G	NA	*OreG0018017*
scaffold33264	88215	C	A	Och09,Och10	*OreG0018023*
scaffold33264	95782	G	T	Och02,Och06,Och09,Och10,Och12	*OreG0018024*
scaffold33264	138667	C	G	Ore06,Ore08	*OreG0018029*
scaffold33264	138717	C	A	NA	*OreG0018029*
scaffold33264	139246	A	G	NA	*OreG0018029*
scaffold33264	139255	C	T	Ore01,Ore02,Ore06,Ore07,Ore08,Ore10,Ore11,Ore12,Ore13,Ore14	*OreG0018029*
scaffold33264	139682	A	T	Och02,Och03,Och04,Och05,Och12,Och13	*OreG0018029*

scaffold33264	144804	T	A	Och01,Och02,Och03,Och04,Och05,Och06,Och07,Och08,Och09,Och10,Och11,Och12,Och13,Och14,Ore01,Ore02,Ore03,Ore04,Ore05,Ore06,Ore07,Ore08,Ore09,Ore10,Ore11,Ore12,Ore13,Ore14	*OreG0018030*
scaffold33264	162919	A	G	Och01,Och02,Och03,Och04,Och05,Och06,Och07,Och08,Och09,Och10,Och11,Och12,Och13,Och14,Ore01,Ore02,Ore03,Ore04,Ore05,Ore06,Ore07,Ore08,Ore09,Ore10,Ore11,Ore12,Ore13,Ore14	*OreG0018031*
scaffold33264	165301	C	A	Och01,Och02,Och03,Och04,Och05,Och06,Och07,Och08,Och09,Och10,Och11,Och12,Och13,Och14,Ore01,Ore02,Ore03,Ore04,Ore05,Ore06,Ore07,Ore08,Ore09,Ore10,Ore11,Ore12,Ore13,Ore14	*OreG0018032*
scaffold33264	183750	C	A	Och02,Och12	*OreG0018038*
scaffold33264	183900	C	T	NA	*OreG0018038*
scaffold33264	191724	A	C	NA	*OreG0018039*
scaffold33264	192386	G	A	NA	*OreG0018039*
scaffold33264	197489	T	A	NA	*OreG0018040*
scaffold33264	198139	G	A	NA	*OreG0018040*
scaffold33264	206518	A	G	NA	*OreG0018041*
scaffold33264	206768	T	G	Och01,Och02,Och03,Och04,Och05,Och06,Och07,Och08,Och09,Och10,Och11,Och12,Och13,Och14,Ore01,Ore02,Ore03,Ore04,Ore05,Ore06,Ore07,Ore08,Ore09,Ore10,Ore11,Ore12,Ore13,Ore14	*OreG0018041*
scaffold33264	207554	C	A	Ore01,Ore02,Ore03,Ore04,Ore05,Ore06,Ore07,Ore08,Ore09,Ore10,Ore11,Ore12,Ore13,Ore14	*OreG0018041*
scaffold33264	207983	A	G	Ore01,Ore02,Ore03,Ore04,Ore05,Ore06,Ore07,Ore08,Ore09,Ore10,Ore11,Ore12,Ore13,Ore14	*OreG0018041*
scaffold33264	208200	C	T	Ore01,Ore02,Ore03,Ore04,Ore05,Ore06,Ore07,Ore08,Ore09,Ore10,Ore11,Ore12,Ore13,Ore14	*OreG0018041*
scaffold33264	211580	C	G	Ore01	*OreG0018042*
scaffold33264	229170	C	A	NA	*OreG0018044*
scaffold33264	239192	A	T	Och01,Och02,Och03,Och04,Och05,Och06,Och07,Och08,Och09,Och10,Och11,Och12,Och13,Och14,Ore01,Ore02,Ore03,Ore04,Ore05,Ore06,Ore07,Ore08,Ore09,Ore10,Ore11,Ore12,Ore13,Ore14	*OreG0018046*
scaffold33264	253835	C	G	Ore01	*OreG0018048*
scaffold33264	264723	G	C	Och01,Och02,Och03,Och04,Och05,Och06,Och07,Och08,Och09,Och10,Och11,Och12,Och13,Och14,Ore01,Ore02,Ore03,Ore04,Ore05,Ore06,Ore07,Ore08,Ore09,Ore10,Ore11,Ore12,Ore13,Ore14	*OreG0018050*
scaffold33264	265994	A	T	NA	*OreG0018050*
scaffold33264	271449	C	T	Och02,Och03,Och04,Och05,Och06,Och07,Och09,Och10,Och12,Och13	*OreG0018052*
scaffold33264	271592	G	A	Ore01	*OreG0018052*
scaffold33264	271646	T	C	NA	*OreG0018052*
scaffold33264	287581	G	T	Ore01,Ore02,Ore03,Ore04,Ore05,Ore06,Ore07,Ore08,Ore09,Ore10,Ore11,Ore12,Ore13,Ore14	*OreG0018054*
scaffold33264	315313	T	C	NA	*OreG0018058*
scaffold33264	333101	C	A	Och01	*OreG0018060*
scaffold33264	334485	C	T	Och01,Och02,Och03,Och04,Och05,Och06,Och07,Och08,Och09,Och10,Och11,Och12,Och13,Och14,Ore01,Ore02,Ore03,Ore04,Ore05,Ore06,Ore07,Ore08,Ore09,Ore10,Ore11,Ore12,Ore13,Ore14	*OreG0018060*
scaffold33264	339381	T	C	Och05,Och13	*OreG0018061*
scaffold33264	341525	A	G	Ore01,Ore02,Ore03,Ore04,Ore05,Ore06,Ore07,Ore08,Ore09,Ore10,Ore11,Ore12,Ore13,Ore14	*OreG0018061*
scaffold33264	343967	C	A	NA	*OreG0018062*
scaffold33264	368195	G	T	NA	*OreG0018064*
scaffold33264	368256	G	C	Och01,Och02,Och03,Och04,Och05,Och06,Och07,Och08,Och09,Och10,Och11,Och12,Och13,Och14	*OreG0018064*
scaffold33264	384633	C	G	NA	*OreG0018066*
scaffold33264	477269	A	C	Ore06,Ore08	*OreG0018077*
scaffold33264	521944	A	C	Och01,Och02,Och03,Och04,Och05,Och06,Och07,Och08,Och09,Och10,Och11,Och12,Och13,Och14	*OreG0018080*
scaffold33264	522113	A	G	Ore01,Ore02,Ore03,Ore04,Ore05,Ore06,Ore07,Ore08,Ore09,Ore10,Ore11,Ore12,Ore13,Ore14	*OreG0018080*
scaffold33264	522643	A	G	Ore01,Ore02,Ore03,Ore04,Ore05,Ore06,Ore07,Ore08,Ore09,Ore10,Ore11,Ore12,Ore13,Ore14	*OreG0018080*
scaffold33264	610880	C	T	Ore01,Ore02,Ore03,Ore04,Ore05,Ore06,Ore07,Ore08,Ore09,Ore10,Ore11,Ore12,Ore13,Ore14	*OreG0018089*
scaffold33264	611314	C	T	Och02,Och12	*OreG0018089*
scaffold33264	617325	G	C	NA	*OreG0018090*
scaffold33264	622401	C	G	Och01,Och02,Och03,Och04,Och05,Och06,Och07,Och08,Och09,Och10,Och11,Och12,Och13,Och14	*OreG0018091*
scaffold33264	622873	G	C	NA	*OreG0018091*
scaffold33264	623915	T	G	NA	*OreG0018091*
scaffold33264	624128	G	T	NA	*OreG0018091*
scaffold33264	633775	T	G	Och02,Och03,Och04,Och05,Och06,Och09,Och10,Och11,Och12,Och13,Och14	*OreG0018094*
scaffold33264	634577	C	T	Ore01,Ore02,Ore03,Ore04,Ore05,Ore06,Ore07,Ore08,Ore09,Ore10,Ore11,Ore12,Ore13,Ore14	*OreG0018094*
scaffold33264	635169	G	T	NA	*OreG0018093*
scaffold33264	636438	C	A	NA	*OreG0018093*
scaffold33264	639848	G	A	Ore01,Ore02,Ore06,Ore07,Ore08,Ore10,Ore11,Ore12,Ore13,Ore14	*OreG0018095*

scaffold33264	640481	A	T	Ore01,Ore02,Ore03,Ore04,Ore05,Ore06,Ore07,Ore08,Ore09,Ore10,Ore11,Ore12,Ore13,Ore14	*OreG0018095*
scaffold33264	640560	A	T	Ore01,Ore02,Ore03,Ore04,Ore05,Ore06,Ore07,Ore08,Ore09,Ore10,Ore11,Ore12,Ore13,Ore14	*OreG0018095*
scaffold33264	640629	T	C	Och01,Och02,Och03,Och04,Och05,Och06,Och07,Och08,Och09,Och10,Och11,Och12,Och13,Och14,Ore01,Ore02,Ore03,Ore04,Ore05,Ore06,Ore07,Ore08,Ore09,Ore10,Ore11,Ore12,Ore13,Ore14	*OreG0018095*
scaffold33264	641667	C	T	Och01,Och02,Och03,Och04,Och05,Och06,Och07,Och08,Och09,Och10,Och11,Och12,Och13,Och14	*OreG0018095*
scaffold33264	641717	G	A	NA	*OreG0018095*
scaffold33264	642041	A	T	Och02,Och12	*OreG0018095*
scaffold33264	642070	T	A	Och01,Och02,Och03,Och04,Och05,Och06,Och07,Och08,Och09,Och10,Och11,Och12,Och13,Och14	*OreG0018095*
scaffold33264	660573	G	T	Ore01,Ore02,Ore03,Ore04,Ore05,Ore06,Ore07,Ore08,Ore09,Ore10,Ore11,Ore12,Ore13,Ore14	*OreG0018097*
scaffold33264	660894	C	T	Och01,Och02,Och03,Och04,Och05,Och06,Och07,Och08,Och09,Och10,Och11,Och12,Och13,Och14,Ore01,Ore02,Ore03,Ore04,Ore05,Ore06,Ore07,Ore08,Ore09,Ore10,Ore11,Ore12,Ore13,Ore14	*OreG0018097*
scaffold33264	663860	C	A	NA	*OreG0018097*
scaffold33264	696637	A	G	NA	*OreG0018101*
scaffold33264	705005	G	T	Och01,Och02,Och03,Och04,Och05,Och06,Och07,Och08,Och09,Och10,Och11,Och12,Och13,Och14,Ore01,Ore02,Ore03,Ore04,Ore05,Ore06,Ore07,Ore08,Ore09,Ore10,Ore11,Ore12,Ore13,Ore14	*OreG0018101*
scaffold33264	741481	A	G	Ore01,Ore02,Ore03,Ore04,Ore05,Ore06,Ore07,Ore08,Ore09,Ore10,Ore11,Ore12,Ore13,Ore14	*OreG0018107*
scaffold33264	743352	C	T	Och01,Och02,Och03,Och04,Och05,Och06,Och07,Och08,Och09,Och10,Och11,Och12,Och13,Och14	*OreG0018107*
scaffold33264	748698	T	G	Och02,Och06,Och12	*OreG0018108*
scaffold33264	758884	C	G	NA	*OreG0018110*
scaffold33264	758914	G	C	Ore01,Ore02,Ore03,Ore04,Ore05,Ore06,Ore07,Ore08,Ore09,Ore10,Ore11,Ore12,Ore13,Ore14	*OreG0018110*
scaffold33264	758973	C	T	Och01,Och05,Och07,Och13	*OreG0018110*
scaffold33264	790060	A	T	NA	*OreG0018113*
scaffold33264	790062	C	T	Ore01,Ore02,Ore03,Ore04,Ore05,Ore06,Ore07,Ore08,Ore09,Ore10,Ore11,Ore12,Ore13,Ore14	*OreG0018113*
scaffold33264	793491	G	A	NA	*OreG0018114*
scaffold33264	810320	G	A	NA	*OreG0018116*
scaffold33264	819754	T	C	Och01,Och02,Och03,Och04,Och05,Och06,Och07,Och08,Och09,Och10,Och11,Och12,Och13,Och14,Ore01,Ore02,Ore03,Ore04,Ore05,Ore06,Ore07,Ore08,Ore09,Ore10,Ore11,Ore12,Ore13,Ore14	*OreG0018117*
scaffold33264	825657	G	A	Och09,Och10	*OreG0018118*
scaffold33264	828009	C	T	NA	*OreG0018119*
scaffold33264	830928	G	A	Ore01,Ore02,Ore03,Ore04,Ore05,Ore06,Ore07,Ore08,Ore09,Ore10,Ore11,Ore12,Ore13,Ore14	*OreG0018120*
scaffold33264	834849	T	A	Och01,Och02,Och03,Och04,Och05,Och06,Och07,Och08,Och09,Och10,Och11,Och12,Och13,Och14,Ore01,Ore02,Ore03,Ore04,Ore05,Ore06,Ore07,Ore08,Ore09,Ore10,Ore11,Ore12,Ore13,Ore14	*OreG0018120*
scaffold33264	841309	C	T	NA	*OreG0018121*
scaffold33264	841464	G	A	Ore01,Ore03,Ore04,Ore05	*OreG0018121*
scaffold33264	841483	C	T	Ore01,Ore03,Ore04,Ore05,Ore09,Ore10,Ore14	*OreG0018121*
scaffold33264	841651	T	C	Och03,Och08,Och13,Och14	*OreG0018121*
scaffold33264	842046	C	G	NA	*OreG0018121*
scaffold33264	842369	G	T	Ore06	*OreG0018121*
scaffold33264	842417	T	A	NA	*OreG0018121*
scaffold33264	842566	G	A	NA	*OreG0018121*
scaffold33264	845365	A	T	Och02,Och03,Och04,Och05,Och06,Och12,Och13,Och14	*OreG0018122*
scaffold33239	78347	G	A	Och01,Och02,Och03,Och04,Och05,Och06,Och07,Och08,Och09,Och10,Och11,Och12,Och13,Och14,Ore01,Ore02,Ore03,Ore04,Ore05,Ore06,Ore07,Ore08,Ore09,Ore10,Ore11,Ore12,Ore13,Ore14	*OreG0017316*
scaffold33239	78849	C	A	Och01,Och02,Och03,Och04,Och05,Och06,Och07,Och08,Och09,Och10,Och11,Och12,Och13,Och14	*OreG0017316*
scaffold33239	107272	T	A	Ore01,Ore02,Ore03,Ore04,Ore05,Ore06,Ore07,Ore08,Ore09,Ore10,Ore11,Ore12,Ore13,Ore14	*OreG0017318*
scaffold33239	131691	G	A	Och01,Och02,Och03,Och04,Och05,Och06,Och07,Och08,Och09,Och10,Och11,Och12,Och13,Och14	*OreG0017320*
scaffold33239	190823	C	T	NA	*OreG0017323*
scaffold33239	247821	C	A	Och01,Och02,Och03,Och04,Och05,Och06,Och07,Och08,Och09,Och10,Och11,Och12,Och13,Och14,Ore01,Ore02,Ore03,Ore04,Ore05,Ore06,Ore07,Ore08,Ore09,Ore10,Ore11,Ore12,Ore13,Ore14	*OreG0017325*
scaffold33239	248110	G	A	Och04	*OreG0017325*
scaffold33239	248465	G	A	Och01,Och02,Och03,Och04,Och05,Och06,Och07,Och08,Och09,Och10,Och11,Och12,Och13,Och14,Ore01,Ore02,Ore03,Ore04,Ore05,Ore06,Ore07,Ore08,Ore09,Ore10,Ore11,Ore12,Ore13,Ore14	*OreG0017325*
scaffold33239	307757	G	A	Ore01,Ore02,Ore03,Ore04,Ore05,Ore06,Ore07,Ore08,Ore09,Ore10,Ore11,Ore12,Ore13,Ore14	*OreG0017328*

scaffold33239	307832	T	C	Och01,Och02,Och03,Och04,Och05,Och06,Och07,Och08,Och09,Och10,Och11,Och12,Och13,Och14,Ore01,Ore02,Ore03,Ore04,Ore05,Ore06,Ore07,Ore08,Ore09,Ore10,Ore11,Ore12,Ore13,Ore14	*OreG0017328*
scaffold33239	307985	C	A	Och01,Och02,Och03,Och04,Och05,Och06,Och07,Och08,Och09,Och10,Och11,Och12,Och13,Och14	*OreG0017328*
scaffold33239	312014	G	A	Ore01,Ore02,Ore03,Ore04,Ore05,Ore06,Ore07,Ore08,Ore09,Ore10,Ore11,Ore12,Ore13,Ore14	*OreG0017329*
scaffold33239	312092	G	A	Ore01,Ore02,Ore03,Ore04,Ore05,Ore06,Ore07,Ore08,Ore09,Ore10,Ore11,Ore12,Ore13,Ore14	*OreG0017329*
scaffold33239	350861	C	T	NA	*OreG0017334*
scaffold33239	352625	A	T	Ore03,Ore04,Ore05,Ore09,Ore13	*OreG0017335*
scaffold33239	352644	T	A	Ore11	*OreG0017335*
scaffold33239	352665	C	A	Ore01,Ore02,Ore03,Ore04,Ore05,Ore06,Ore07,Ore08,Ore09,Ore10,Ore11,Ore12,Ore13,Ore14	*OreG0017335*
scaffold33239	352874	G	T	Ore01,Ore02,Ore03,Ore04,Ore05,Ore06,Ore07,Ore08,Ore09,Ore10,Ore11,Ore12,Ore13,Ore14	*OreG0017335*
scaffold33239	422536	C	T	NA	*OreG0017341*
scaffold33239	422634	G	A	NA	*OreG0017341*
scaffold33239	452805	C	A	Och01,Och02,Och03,Och04,Och05,Och06,Och07,Och08,Och09,Och10,Och11,Och12,Och13,Och14	*OreG0017343*
scaffold33239	461685	G	T	NA	*OreG0017345*
scaffold33239	461832	G	A	NA	*OreG0017345*
scaffold33239	466120	T	A	Ore01,Ore02,Ore03,Ore04,Ore05,Ore06,Ore07,Ore08,Ore09,Ore10,Ore11,Ore12,Ore13,Ore14	*OreG0017345*
scaffold33239	484989	C	G	NA	*OreG0017347*
scaffold33239	585735	A	T	NA	*OreG0017349*
scaffold33239	592581	G	C	NA	*OreG0017350*
scaffold33239	592909	G	A	NA	*OreG0017350*
scaffold33239	668979	C	A	NA	*OreG0017351*
scaffold33229	15019	A	T	Och08	*OreG0017099*
scaffold33229	19851	G	C	Ore01,Ore02,Ore03,Ore04,Ore05,Ore06,Ore07,Ore08,Ore09,Ore10,Ore11,Ore12,Ore13,Ore14	*OreG0017099*
scaffold33229	19884	A	C	NA	*OreG0017099*
scaffold33229	19969	G	A	NA	*OreG0017099*
scaffold33229	69041	G	T	Och08	*OreG0017104*
scaffold33229	69604	G	A	Ore01,Ore02,Ore03,Ore04,Ore05,Ore06,Ore07,Ore08,Ore09,Ore10,Ore11,Ore12,Ore13,Ore14	*OreG0017104*
scaffold33229	69699	G	A	NA	*OreG0017104*
scaffold33229	69701	G	C	Ore01,Ore02,Ore03,Ore04,Ore05,Ore06,Ore07,Ore08,Ore09,Ore10,Ore11,Ore12,Ore13,Ore14	*OreG0017104*
scaffold33229	69769	T	A	Ore01,Ore02,Ore03,Ore04,Ore05,Ore06,Ore07,Ore08,Ore09,Ore10,Ore11,Ore12,Ore13,Ore14	*OreG0017104*
scaffold33229	69941	A	G	Ore04,Ore05	*OreG0017104*
scaffold33229	80052	G	A	NA	*OreG0017105*
scaffold33229	80453	C	G	Och01,Och02,Och13,Och14	*OreG0017105*
scaffold33229	80731	G	T	NA	*OreG0017105*
scaffold33229	141612	C	T	NA	*OreG0017115*
scaffold33229	141725	C	T	Och01,Och02,Och03,Och04,Och05,Och06,Och07,Och08,Och09,Och10,Och11,Och12,Och13,Och14,Ore01,Ore02,Ore03,Ore04,Ore05,Ore06,Ore07,Ore08,Ore09,Ore10,Ore11,Ore12,Ore13,Ore14	*OreG0017115*
scaffold33229	169392	C	G	Ore01,Ore02,Ore03,Ore04,Ore05,Ore06,Ore07,Ore08,Ore09,Ore10,Ore11,Ore12,Ore13,Ore14	*OreG0017118*
scaffold33229	169557	C	G	Ore01,Ore02,Ore03,Ore04,Ore05,Ore06,Ore07,Ore08,Ore09,Ore10,Ore11,Ore12,Ore13,Ore14	*OreG0017118*
scaffold33229	171772	G	A	NA	*OreG0017118*
scaffold33229	172273	T	C	Och01,Och02,Och03,Och04,Och05,Och06,Och07,Och08,Och09,Och10,Och11,Och12,Och13	*OreG0017118*
scaffold33229	178860	G	A	Och13	*OreG0017118*
scaffold33229	179353	T	A	Och03,Och07,Och08	*OreG0017118*
scaffold33229	179380	C	A	Och01,Och02,Och03,Och04,Och05,Och06,Och07,Och08,Och09,Och10,Och11,Och12,Och13,Och14,Ore01,Ore02,Ore03,Ore04,Ore05,Ore06,Ore07,Ore08,Ore09,Ore10,Ore11,Ore12,Ore13,Ore14	*OreG0017118*
scaffold33229	181986	C	T	Och07,Och08,Ore01,Ore02,Ore03,Ore04,Ore05,Ore06,Ore07,Ore08,Ore09,Ore10,Ore11,Ore12,Ore13,Ore14	*OreG0017118*
scaffold33229	188484	A	T	Och03,Och07,Och08	*OreG0017119*
scaffold33229	191232	G	A	NA	*OreG0017119*
scaffold33229	200968	A	T	Ore01,Ore02,Ore03,Ore04,Ore05,Ore06,Ore07,Ore08,Ore09,Ore10,Ore11,Ore12,Ore13,Ore14	*OreG0017119*
scaffold33229	250497	A	T	Och07,Och08	*OreG0017121*
scaffold33229	253625	A	C	Och13	*OreG0017123*
scaffold33229	255272	C	T	NA	*OreG0017123*
scaffold33229	256130	C	T	NA	*OreG0017123*
scaffold33229	328270	C	G	Och01,Och02,Och03,Och04,Och05,Och06,Och07,Och08,Och09,Och10,Och11,Och12,Och13,Och14,Ore01,Ore02,Ore03,Ore04,Ore05,Ore06,Ore07,Ore08,Ore09,Ore10,Ore11,Ore12,Ore13,Ore14	*OreG0017131*

scaffold33229	330082	G	C	NA	*OreG0017131*
scaffold33229	333783	C	T	Och01,Och02,Och03,Och04,Och05,Och06,Och07,Och08,Och09,Och10,Och11,Och12,Och13,Och14,Ore01,Ore02,Ore03,Ore04,Ore05,Ore06,Ore07,Ore08,Ore09,Ore10,Ore11,Ore12,Ore13,Ore14	*OreG0017131*
scaffold33229	341909	G	A	NA	*OreG0017132*
scaffold33229	347315	T	C	NA	*OreG0017132*
scaffold33229	423489	T	A	NA	*OreG0017140*
scaffold33229	423530	C	G	NA	*OreG0017140*
scaffold33229	423594	G	A	NA	*OreG0017140*
scaffold33229	423798	C	T	NA	*OreG0017140*
scaffold33229	423929	G	T	NA	*OreG0017140*
scaffold33229	424029	T	A	NA	*OreG0017140*
scaffold33229	424110	C	T	NA	*OreG0017140*
scaffold33229	424219	T	C	NA	*OreG0017140*
scaffold33229	424262	T	A	NA	*OreG0017140*
scaffold33229	424677	C	T	NA	*OreG0017140*
scaffold33229	424700	A	C	NA	*OreG0017140*
scaffold33229	444145	A	T	NA	*OreG0017146*
scaffold33229	444285	T	C	Och01,Och02,Och03,Och04,Och05,Och06,Och07,Och08,Och09,Och10,Och11,Och12,Och13,Och14,Ore01,Ore02,Ore03,Ore04,Ore05,Ore06,Ore07,Ore08,Ore09,Ore10,Ore11,Ore12,Ore13,Ore14	*OreG0017146*
scaffold33229	453187	C	A	NA	*OreG0017148*
scaffold33229	453254	T	A	Ore01,Ore03,Ore04,Ore05,Ore09,Ore10,Ore13,Ore14	*OreG0017148*
scaffold33229	453270	C	T	Och02,Och03,Och04,Och05,Och06,Och12,Och13	*OreG0017148*
scaffold33229	453436	C	G	NA	*OreG0017148*
scaffold33229	453520	A	G	Och02,Och03,Och04,Och05,Och06,Och07,Och08,Och09,Och10,Och11,Och12,Och13,Och14,Ore01,Ore02,Ore03,Ore04,Ore05,Ore06,Ore07,Ore08,Ore09,Ore10,Ore11,Ore12,Ore13,Ore14	*OreG0017148*
scaffold33229	453637	A	G	Och02,Och03,Och04,Och05,Och06,Och07,Och08,Och09,Och10,Och11,Och12,Och13,Och14,Ore01,Ore02,Ore03,Ore04,Ore05,Ore06,Ore07,Ore08,Ore09,Ore10,Ore11,Ore12,Ore13,Ore14	*OreG0017148*
scaffold33229	453703	C	T	NA	*OreG0017148*
scaffold33229	453775	G	T	Och08	*OreG0017148*
scaffold33229	455260	C	T	Och08,Och09,Och11	*OreG0017149*
scaffold33229	467627	C	T	NA	*OreG0017151*
scaffold33229	467742	T	G	NA	*OreG0017151*
scaffold33229	467830	G	T	NA	*OreG0017151*
scaffold33229	475521	G	T	NA	*OreG0017154*
scaffold33229	475832	A	T	NA	*OreG0017154*
scaffold33229	527533	G	C	NA	*OreG0017159*
scaffold33229	552197	G	A	Och06	*OreG0017165*
scaffold33229	552258	T	A	NA	*OreG0017165*
scaffold33229	554130	G	A	Och01,Och02,Och03,Och04,Och05,Och06,Och07,Och08,Och09,Och10,Och11,Och12,Och13,Och14,Ore01,Ore02,Ore03,Ore04,Ore05,Ore06,Ore07,Ore08,Ore09,Ore10,Ore11,Ore12,Ore13,Ore14	*OreG0017165*
scaffold33229	554236	C	A	Och01,Och02,Och03,Och04,Och05,Och06,Och07,Och08,Och09,Och10,Och11,Och12,Och13,Och14,Ore01,Ore02,Ore03,Ore04,Ore05,Ore06,Ore07,Ore08,Ore09,Ore10,Ore11,Ore12,Ore13,Ore14	*OreG0017165*
scaffold33229	554670	T	C	Och11	*OreG0017165*
scaffold33229	555953	C	T	Ore01,Ore02,Ore03,Ore04,Ore05,Ore06,Ore07,Ore08,Ore09,Ore10,Ore11,Ore12,Ore13,Ore14	*OreG0017165*
scaffold33229	556382	C	T	Och01,Ore01,Ore02,Ore03,Ore04,Ore05,Ore06,Ore07,Ore08,Ore09,Ore10,Ore11,Ore12,Ore13,Ore14	*OreG0017165*
scaffold33229	567103	T	G	Ore01,Ore02,Ore03,Ore04,Ore05,Ore06,Ore07,Ore08,Ore09,Ore10,Ore11,Ore12,Ore13,Ore14	*OreG0017167*
scaffold33229	568986	C	T	NA	*OreG0017167*
scaffold33229	569031	C	A	NA	*OreG0017167*
scaffold33229	569034	C	A	NA	*OreG0017167*
scaffold33229	569097	C	T	NA	*OreG0017167*
scaffold33229	569123	G	A	NA	*OreG0017167*
scaffold33229	569150	A	C	NA	*OreG0017167*
scaffold33229	573553	G	C	NA	*OreG0017169*
scaffold33229	573622	G	A	Och11	*OreG0017169*
scaffold33229	573934	G	A	Och02,Och03,Och04,Och05,Och06,Och07,Och08,Och12,Och13,Och14	*OreG0017169*
scaffold33229	624513	C	A	Ore02,Ore04,Ore05,Ore10,Ore12,Ore13,Ore14	*OreG0017173*
scaffold33229	625062	A	C	Och05	*OreG0017173*
scaffold33229	625075	G	A	Och05	*OreG0017173*
scaffold33211	185455	T	C	Och01,Och02,Och03,Och04,Och05,Och06,Och07,Och08,Och09,Och10,Och11,Och12,Och13,Och14,Ore01,Ore02,Ore03,Ore04,Ore05,Ore06,Ore07,Ore08,Ore09,Ore10,Ore11,Ore12,Ore13,Ore14	*OreG0016345*
scaffold33211	186588	G	A	Ore01,Ore02,Ore03,Ore04,Ore05,Ore06,Ore07,Ore08,Ore09,Ore10,Ore11,Ore12,Ore13,Ore14	*OreG0016345*
scaffold33211	202968	C	T	Och01,Och02,Och03,Och04,Och05,Och06,Och07,Och08,Och09,Och10,Och11,Och12,Och13,Och14,Ore01,Ore02,Ore03,Ore04,Ore05,Ore06,Ore07,Ore08,Ore09,Ore10,Ore11,Ore12,Ore13,Ore14	*OreG0016347*

scaffold33211	202977	C	T	Och01	*OreG0016347*
scaffold33211	203293	C	G	Och01,Och02,Och03,Och04,Och05,Och06,Och07,Och08,Och09,Och10,Och11,Och12,Och13,Och14,Ore01,Ore02,Ore03,Ore04,Ore05,Ore06,Ore07,Ore08,Ore09,Ore10,Ore11,Ore12,Ore13,Ore14	*OreG0016347*
scaffold33211	203411	C	G	Ore01,Ore02,Ore03,Ore04,Ore05,Ore06,Ore07,Ore08,Ore09,Ore10,Ore11,Ore12,Ore13,Ore14	*OreG0016347*
scaffold33211	203568	A	G	Och01,Och02,Och03,Och04,Och05,Och06,Och07,Och08,Och09,Och10,Och11,Och12,Och13,Och14,Ore01,Ore02,Ore03,Ore04,Ore05,Ore06,Ore07,Ore08,Ore09,Ore10,Ore11,Ore12,Ore13,Ore14	*OreG0016347*
scaffold33211	203658	A	C	Ore01,Ore02,Ore03,Ore04,Ore05,Ore06,Ore07,Ore08,Ore09,Ore10,Ore11,Ore12,Ore13,Ore14	*OreG0016347*
scaffold33211	203666	G	A	Och01,Och02,Och03,Och04,Och05,Och06,Och07,Och08,Och09,Och10,Och11,Och12,Och13,Och14,Ore01,Ore02,Ore03,Ore04,Ore05,Ore06,Ore07,Ore08,Ore09,Ore10,Ore11,Ore12,Ore13,Ore14	*OreG0016347*
scaffold33211	203672	G	T	Ore01,Ore02,Ore03,Ore04,Ore05,Ore06,Ore07,Ore08,Ore09,Ore10,Ore11,Ore12,Ore13,Ore14	*OreG0016347*
scaffold33211	203679	C	T	Och01,Och02,Och03,Och04,Och05,Och06,Och07,Och08,Och09,Och10,Och11,Och12,Och13,Och14,Ore01,Ore02,Ore03,Ore04,Ore05,Ore06,Ore07,Ore08,Ore09,Ore10,Ore11,Ore12,Ore13,Ore14	*OreG0016347*
scaffold33211	203919	G	A	NA	*OreG0016347*
scaffold33211	203926	G	T	Och01,Och08,Och09,Och10,Och11,Och14	*OreG0016347*
scaffold33211	204335	T	C	Ore01,Ore02,Ore03,Ore04,Ore05,Ore06,Ore07,Ore08,Ore09,Ore10,Ore11,Ore12,Ore13,Ore14	*OreG0016347*
scaffold33211	204339	C	T	Och01,Och02,Och03,Och04,Och05,Och06,Och07,Och08,Och09,Och10,Och11,Och12,Och13,Och14	*OreG0016347*
scaffold33211	204664	C	T	NA	*OreG0016347*
scaffold33211	326480	G	C	Ore01,Ore02,Ore03,Ore04,Ore05,Ore06,Ore07,Ore08,Ore09,Ore10,Ore11,Ore12,Ore13,Ore14	*OreG0016350*
scaffold33211	326634	C	T	Ore01,Ore02,Ore03,Ore04,Ore05,Ore06,Ore07,Ore08,Ore09,Ore10,Ore11,Ore12,Ore13,Ore14	*OreG0016350*
scaffold33211	326870	G	A	Ore01,Ore02,Ore03,Ore04,Ore05,Ore06,Ore07,Ore08,Ore09,Ore10,Ore11,Ore12,Ore13,Ore14	*OreG0016350*
scaffold33211	326948	C	T	Och01	*OreG0016350*
scaffold33211	354986	G	C	Ore01,Ore02,Ore03,Ore04,Ore05,Ore06,Ore07,Ore08,Ore09,Ore10,Ore11,Ore12,Ore13,Ore14	*OreG0016351*
scaffold33211	355109	A	T	Och01,Och02,Och03,Och04,Och05,Och06,Och07,Och08,Och09,Och10,Och11,Och12,Och13,Och14	*OreG0016351*
scaffold33211	355399	T	A	NA	*OreG0016351*
scaffold33211	390150	G	T	Och12,Och13	*OreG0016357*
scaffold33211	407940	A	T	Och01,Och02,Och03,Och04,Och05,Och06,Och07,Och08,Och09,Och10,Och11,Och12,Och13,Och14,Ore01,Ore02,Ore03,Ore04,Ore05,Ore06,Ore07,Ore08,Ore09,Ore10,Ore11,Ore12,Ore13,Ore14	*OreG0016358*
scaffold33211	408107	G	A	NA	*OreG0016358*
scaffold33211	417076	A	G	Och01,Och02,Och03,Och04,Och05,Och06,Och07,Och08,Och09,Och10,Och11,Och12,Och13,Och14,Ore01,Ore02,Ore03,Ore04,Ore05,Ore06,Ore07,Ore08,Ore09,Ore10,Ore11,Ore12,Ore13,Ore14	*OreG0016359*
scaffold33211	433661	T	C	NA	*OreG0016360*
scaffold33211	433667	C	A	Och01,Och02,Och03,Och04,Och06,Och07	*OreG0016360*
scaffold33211	457025	A	C	Ore01,Ore02,Ore03,Ore04,Ore05,Ore06,Ore07,Ore08,Ore09,Ore10,Ore11,Ore12,Ore13,Ore14	*OreG0016363*
scaffold33211	574519	G	A	Ore01,Ore02,Ore03,Ore04,Ore05,Ore06,Ore07,Ore08,Ore09,Ore10,Ore11,Ore12,Ore13,Ore14	*OreG0016369*
scaffold33211	659390	G	A	NA	*OreG0016376*
scaffold33211	659736	C	A	NA	*OreG0016376*
scaffold33211	659770	T	C	NA	*OreG0016376*
scaffold33211	681242	T	G	NA	*OreG0016377*
scaffold33211	688995	G	T	NA	*OreG0016379*
scaffold33211	689005	A	G	NA	*OreG0016379*
scaffold33211	691244	A	T	Och11	*OreG0016380*
scaffold33211	697454	C	A	Ore01,Ore02,Ore03,Ore04,Ore05,Ore06,Ore07,Ore08,Ore09,Ore10,Ore11,Ore12,Ore13,Ore14	*OreG0016381*
scaffold33211	697483	C	T	NA	*OreG0016381*
scaffold33211	771617	G	C	Ore06,Ore07,Ore10,Ore14	*OreG0016385*
scaffold33211	771629	G	A	Ore01,Ore02,Ore03,Ore04,Ore05,Ore06,Ore07,Ore08,Ore09,Ore10,Ore11,Ore12,Ore13,Ore14	*OreG0016385*
scaffold33211	771692	C	T	Ore01,Ore02,Ore03,Ore04,Ore05,Ore06,Ore07,Ore08,Ore09,Ore10,Ore11,Ore12,Ore13,Ore14	*OreG0016385*
scaffold33211	772226	G	A	NA	*OreG0016385*
scaffold33211	772589	A	G	Ore01,Ore02,Ore03,Ore04,Ore05,Ore06,Ore07,Ore08,Ore09,Ore10,Ore11,Ore12,Ore13,Ore14	*OreG0016385*
scaffold33211	1621449	G	A	Ore01,Ore02,Ore03,Ore04,Ore05,Ore06,Ore07,Ore08,Ore09,Ore10,Ore11,Ore12,Ore13,Ore14	*OreG0016391*
scaffold33211	1621500	C	T	Ore01,Ore02,Ore03,Ore04,Ore05,Ore06,Ore07,Ore08,Ore09,Ore10,Ore11,Ore12,Ore13,Ore14	*OreG0016391*
scaffold33211	1621758	C	G	NA	*OreG0016391*
scaffold33211	1623628	T	A	NA	*OreG0016391*

scaffold33211	1631123	G	A	Ore01,Ore02,Ore03,Ore04,Ore05,Ore06,Ore07,Ore08,Ore09,Ore10,Ore11,Ore12,Ore13,Ore14	*OreG0016391*
scaffold33211	1650786	C	T	Ore01,Ore02,Ore03,Ore04,Ore05,Ore06,Ore07,Ore08,Ore09,Ore10,Ore11,Ore12,Ore13,Ore14	*OreG0016392*
scaffold33211	1651049	C	T	Ore01,Ore02,Ore03,Ore04,Ore05,Ore06,Ore07,Ore08,Ore09,Ore10,Ore11,Ore12,Ore13,Ore14	*OreG0016392*
scaffold33211	1651149	C	A	Och01,Och02,Och03,Och04,Och05,Och06,Och07,Och08,Och09,Och10,Och11,Och12,Och13,Och14,Ore01,Ore02,Ore03,Ore04,Ore05,Ore06,Ore07,Ore08,Ore09,Ore10,Ore11,Ore12,Ore13,Ore14	*OreG0016392*
scaffold33211	1651168	G	A	Och01,Och02,Och03,Och04,Och05,Och06,Och07,Och08,Och09,Och10,Och11,Och12,Och13,Och14	*OreG0016392*
scaffold33200	4876	C	A	NA	*OreG0015666*
scaffold33200	12619	G	T	Ore09,Ore14	*OreG0015667*
scaffold33200	40412	A	T	NA	*OreG0015671*
scaffold33200	86170	T	C	NA	*OreG0015674*
scaffold33200	98724	A	T	Och03,Och05,Och06,Och07,Och08,Och12,Och13	*OreG0015676*
scaffold33200	102694	T	A	Och06	*OreG0015677*
scaffold33200	102805	C	A	NA	*OreG0015677*
scaffold33200	106453	G	A	Och01,Och02,Och03,Och04,Och05,Och06,Och07,Och08,Och09,Och10,Och11,Och12,Och13,Och14,Ore01,Ore02,Ore03,Ore04,Ore05,Ore06,Ore07,Ore08,Ore09,Ore10,Ore11,Ore12,Ore13,Ore14	*OreG0015678*
scaffold33200	109144	G	A	Och01,Och02,Och03,Och04,Och05,Och06,Och07,Och08,Och09,Och10,Och11,Och12,Och13,Och14,Ore01,Ore02,Ore03,Ore04,Ore05,Ore06,Ore07,Ore08,Ore09,Ore10,Ore11,Ore12,Ore13,Ore14	*OreG0015678*
scaffold33200	109701	G	A	Ore01,Ore02,Ore03,Ore04,Ore05,Ore06,Ore07,Ore08,Ore09,Ore10,Ore11,Ore12,Ore13,Ore14	*OreG0015678*
scaffold33200	114290	C	A	Ore04,Ore05	*OreG0015679*
scaffold33200	114442	G	A	NA	*OreG0015679*
scaffold33200	114494	A	T	Ore03,Ore06,Ore09,Ore10,Ore11,Ore13	*OreG0015679*
scaffold33200	177367	C	T	NA	*OreG0015681*
scaffold33200	177370	C	T	NA	*OreG0015681*
scaffold33200	177557	C	A	NA	*OreG0015681*
scaffold33200	177587	G	A	NA	*OreG0015681*
scaffold33200	177602	C	T	NA	*OreG0015681*
scaffold33200	185191	A	G	NA	*OreG0015682*
scaffold33200	227937	G	A	NA	*OreG0015685*
scaffold33200	266500	C	T	Och01,Och02,Och03,Och04,Och05,Och06,Och07,Och08,Och09,Och10,Och11,Och12,Och13,Och14,Ore01,Ore02,Ore03,Ore04,Ore05,Ore06,Ore07,Ore08,Ore09,Ore10,Ore11,Ore12,Ore13,Ore14	*OreG0015687*
scaffold33200	272135	G	A	Ore01,Ore02,Ore03,Ore04,Ore05,Ore06,Ore07,Ore08,Ore09,Ore10,Ore11,Ore12,Ore13,Ore14	*OreG0015688*
scaffold33200	278107	C	T	NA	*OreG0015689*
scaffold33200	313757	C	T	NA	*OreG0015691*
scaffold33200	314019	T	A	Ore03,Ore06,Ore09,Ore10,Ore11,Ore13	*OreG0015691*
scaffold33200	353849	T	A	NA	*OreG0015693*
scaffold33200	356170	T	A	Och01,Och02,Och03,Och04,Och05,Och06,Och07,Och08,Och09,Och10,Och11,Och12,Och13,Och14	*OreG0015693*
scaffold33200	356552	C	G	Ore01,Ore02,Ore04,Ore05,Ore07,Ore09,Ore12,Ore14	*OreG0015693*
scaffold33200	356645	C	A	Ore01,Ore02,Ore03,Ore04,Ore05,Ore06,Ore07,Ore08,Ore09,Ore10,Ore11,Ore12,Ore13,Ore14	*OreG0015693*
scaffold33200	434609	T	A	Ore01,Ore02,Ore03,Ore04,Ore05,Ore06,Ore07,Ore08,Ore09,Ore10,Ore11,Ore12,Ore13,Ore14	*OreG0015697*
scaffold33200	521862	G	A	Och06,Och09,Och10,Och11	*OreG0015703*
scaffold33200	522320	A	T	Ore01,Ore02,Ore03,Ore04,Ore05,Ore06,Ore07,Ore08,Ore09,Ore10,Ore11,Ore12,Ore13,Ore14	*OreG0015703*
scaffold33200	531814	A	C	NA	*OreG0015705*
scaffold33200	532131	G	A	NA	*OreG0015705*
scaffold33200	532161	C	T	Ore01,Ore02,Ore03,Ore04,Ore05,Ore06,Ore07,Ore08,Ore09,Ore10,Ore11,Ore12,Ore13,Ore14	*OreG0015705*
scaffold33200	532573	A	G	Och01,Och02,Och03,Och04,Och05,Och06,Och07,Och08,Och09,Och10,Och11,Och12,Och13,Och14,Ore01,Ore02,Ore03,Ore04,Ore05,Ore06,Ore07,Ore08,Ore09,Ore10,Ore11,Ore12,Ore13,Ore14	*OreG0015705*
scaffold33200	546211	T	C	NA	*OreG0015707*
scaffold33200	546373	A	T	Och06	*OreG0015707*
scaffold33200	546863	T	G	NA	*OreG0015707*
scaffold33200	547955	C	T	NA	*OreG0015707*
scaffold33200	547976	C	T	Och01,Och02,Och03,Och04,Och05,Och06,Och07,Och08,Och09,Och10,Och11,Och12,Och13,Och14,Ore01,Ore02,Ore03,Ore04,Ore05,Ore06,Ore07,Ore08,Ore09,Ore10,Ore11,Ore12,Ore13,Ore14	*OreG0015707*
scaffold33200	586925	A	G	NA	*OreG0015708*
scaffold33200	599520	T	C	NA	*OreG0015709*
scaffold33200	601390	G	A	NA	*OreG0015709*
scaffold33200	601453	A	C	NA	*OreG0015709*

scaffold33200	602430	G	A	Och01,Och02,Och03,Och04,Och05,Och06,Och07,Och08,Och09,Och10,Och11,Och12,Och13,Och14,Ore01,Ore02,Ore03,Ore04,Ore05,Ore06,Ore07,Ore08,Ore09,Ore10,Ore11,Ore12,Ore13,Ore14	*OreG0015709*
scaffold33200	624068	T	C	Ore01,Ore02,Ore03,Ore04,Ore05,Ore06,Ore07,Ore08,Ore09,Ore10,Ore11,Ore12,Ore13,Ore14	*OreG0015710*
scaffold33200	624978	A	G	Och02,Och04,Och07,Och09,Och10,Och11	*OreG0015711*
scaffold33200	624995	C	T	NA	*OreG0015711*
scaffold33200	625314	T	C	Och06	*OreG0015711*
scaffold33200	625601	C	T	Och06,Ore01,Ore02,Ore03,Ore04,Ore05,Ore06,Ore07,Ore08,Ore09,Ore10,Ore11,Ore12,Ore13,Ore14	*OreG0015711*
scaffold33200	625637	C	T	NA	*OreG0015711*
scaffold33200	626856	T	C	Ore01,Ore02,Ore03,Ore04,Ore05,Ore06,Ore07,Ore08,Ore09,Ore10,Ore11,Ore12,Ore13,Ore14	*OreG0015711*
scaffold33200	626931	C	T	Och01,Och06	*OreG0015711*
scaffold33200	744629	G	A	NA	*OreG0015715*
scaffold33200	751000	G	A	Ore01,Ore02,Ore03,Ore04,Ore05,Ore06,Ore07,Ore08,Ore09,Ore10,Ore11,Ore12,Ore13,Ore14	*OreG0015716*
scaffold33200	757872	G	A	NA	*OreG0015717*
scaffold33200	758268	G	A	Ore01,Ore02,Ore03,Ore04,Ore05,Ore06,Ore07,Ore08,Ore09,Ore10,Ore11,Ore12,Ore13,Ore14	*OreG0015717*
scaffold33200	761649	C	A	Ore01,Ore02,Ore03,Ore04,Ore05,Ore06,Ore07,Ore08,Ore09,Ore10,Ore11,Ore12,Ore13,Ore14	*OreG0015718*
scaffold33200	761843	G	A	NA	*OreG0015718*
scaffold33200	774548	C	A	Och01,Och02,Och03,Och04,Och05,Och06,Och07,Och08,Och09,Och10,Och11,Och12,Och13,Och14,Ore01,Ore02,Ore03,Ore04,Ore05,Ore06,Ore07,Ore08,Ore09,Ore10,Ore11,Ore12,Ore13,Ore14	*OreG0015719*
scaffold33200	775288	C	T	Och03,Och05,Och06,Och12,Och13	*OreG0015719*
scaffold33200	793700	A	G	NA	*OreG0015720*
scaffold33200	794738	G	A	NA	*OreG0015720*
scaffold33200	833007	C	T	NA	*OreG0015722*
scaffold33200	833668	G	A	Och02,Och04	*OreG0015722*
scaffold33200	833748	C	T	Och08	*OreG0015722*
scaffold33200	834039	A	G	Ore01,Ore02,Ore04,Ore05,Ore07,Ore09,Ore12,Ore14	*OreG0015722*
scaffold33200	848000	T	C	Och01,Och02,Och03,Och04,Och05,Och06,Och07,Och08,Och09,Och10,Och11,Och12,Och13,Och14,Ore01,Ore02,Ore03,Ore04,Ore05,Ore06,Ore07,Ore08,Ore09,Ore10,Ore11,Ore12,Ore13,Ore14	*OreG0015725*
scaffold33200	880507	G	A	NA	*OreG0015729*
scaffold33200	904270	C	A	NA	*OreG0015733*
scaffold33200	904465	G	A	NA	*OreG0015733*
scaffold33200	929566	T	G	Och01,Och02,Och03,Och04,Och05,Och06,Och07,Och08,Och09,Och10,Och11,Och12,Och13,Och14,Ore01,Ore02,Ore03,Ore04,Ore05,Ore06,Ore07,Ore08,Ore09,Ore10,Ore11,Ore12,Ore13,Ore14	*OreG0015735*
scaffold33200	939555	C	T	Och02,Och03,Och04,Och05,Och06,Och07,Och08,Och09,Och10,Och11,Och12,Och13,Och14	*OreG0015736*
scaffold33200	1012591	G	A	Och02,Och03,Och06,Och07,Och09,Och10,Och14	*OreG0015742*
scaffold33200	1062850	C	T	Ore01,Ore02,Ore03,Ore04,Ore05,Ore06,Ore07,Ore08,Ore09,Ore10,Ore11,Ore12,Ore13,Ore14	*OreG0015747*
scaffold33200	1065709	C	T	NA	*OreG0015748*
scaffold33200	1065717	T	C	NA	*OreG0015748*
scaffold33200	1065747	T	C	NA	*OreG0015748*
scaffold33200	1065808	A	C	NA	*OreG0015748*
scaffold33200	1065891	A	C	NA	*OreG0015748*
scaffold33200	1065940	T	C	NA	*OreG0015748*
scaffold33200	1065964	C	A	NA	*OreG0015748*
scaffold33200	1065970	G	A	NA	*OreG0015748*
scaffold33200	1065972	G	A	NA	*OreG0015748*
scaffold33200	1066057	C	A	NA	*OreG0015748*
scaffold33200	1066096	T	G	NA	*OreG0015748*
scaffold33200	1088837	T	C	Och07,Och09,Och10,Och11	*OreG0015752*
scaffold33200	1089240	T	A	Ore01,Ore02,Ore03,Ore04,Ore05,Ore06,Ore07,Ore08,Ore09,Ore10,Ore11,Ore12,Ore13,Ore14	*OreG0015752*
scaffold33200	1089358	A	C	Och02,Och03,Och04,Och05,Och06,Och07,Och08,Och09,Och10,Och11,Och12,Och13,Och14	*OreG0015752*
scaffold33200	1089363	A	G	Ore01,Ore02,Ore03,Ore04,Ore05,Ore06,Ore07,Ore08,Ore09,Ore10,Ore11,Ore12,Ore13,Ore14	*OreG0015752*
scaffold33200	1089367	C	T	NA	*OreG0015752*
scaffold33200	1089394	A	G	NA	*OreG0015752*
scaffold33200	1104364	C	A	Och01,Och02,Och03,Och04,Och05,Och06,Och07,Och08,Och09,Och10,Och11,Och12,Och13,Och14	*OreG0015755*
scaffold33200	1104908	G	A	NA	*OreG0015755*
scaffold33200	1107528	G	A	Och02,Och03,Och04,Och05,Och06,Och08,Och12,Och13	*OreG0015756*
scaffold33200	1107789	G	T	Och09,Och10,Och14,Ore01,Ore03,Ore04,Ore05,Ore08,Ore09,Ore11	*OreG0015756*
scaffold33200	1107818	C	T	Och02,Och03,Och04,Och05,Och06,Och08,Och12,Och13	*OreG0015756*
scaffold33200	1107890	G	C	NA	*OreG0015756*
scaffold33200	1107896	A	G	Och09,Och10,Och14,Ore01,Ore03,Ore04,Ore05,Ore08,Ore09,Ore11	*OreG0015756*

scaffold33200	1118786	C	A	NA	*OreG0015758*
scaffold33200	1144781	G	A	NA	*OreG0015760*
scaffold33200	1146888	T	G	NA	*OreG0015761*
scaffold33200	1147685	T	C	Och04,Och06,Och12,Och13	*OreG0015761*
scaffold33200	1162202	G	C	NA	*OreG0015763*
scaffold33200	1162423	C	A	Och01,Och02,Och03,Och06,Och07,Och08,Och09,Och10,Och11,Och14	*OreG0015763*
scaffold33200	1164012	A	C	Och01,Och02,Och03,Och04,Och05,Och06,Och07,Och08,Och09,Och10,Och11,Och12,Och13,Och14	*OreG0015764*
scaffold33200	1164029	A	T	Ore01,Ore02,Ore03,Ore04,Ore05,Ore06,Ore07,Ore08,Ore09,Ore10,Ore11,Ore12,Ore13,Ore14	*OreG0015764*
scaffold33200	1165478	C	G	Ore01,Ore02,Ore03,Ore04,Ore05,Ore06,Ore07,Ore08,Ore09,Ore10,Ore11,Ore12,Ore13,Ore14	*OreG0015764*
scaffold33200	1172619	G	A	Och12,Och13,Och14	*OreG0015766*
scaffold33200	1176057	A	C	Och01,Och02,Och03,Och04,Och05,Och06,Och07,Och08,Och09,Och10,Och11,Och12,Och13,Och14,Ore01,Ore02,Ore03,Ore04,Ore05,Ore06,Ore07,Ore08,Ore09,Ore10,Ore11,Ore12,Ore13,Ore14	*OreG0015766*
scaffold33200	1181880	C	T	Och01,Och02,Och03,Och04,Och05,Och06,Och07,Och08,Och09,Och10,Och11,Och12,Och13,Och14	*OreG0015767*
scaffold33200	1182053	C	G	Och01,Och02,Och03,Och04,Och05,Och06,Och07,Och08,Och09,Och10,Och11,Och12,Och13,Och14	*OreG0015767*
scaffold33200	1187904	T	G	NA	*OreG0015768*
scaffold33200	1208466	A	T	NA	*OreG0015769*
scaffold33200	1208600	C	G	NA	*OreG0015769*
scaffold33200	1250241	G	A	NA	*OreG0015773*
scaffold33200	1341202	G	T	Och01,Och02,Och03,Och04,Och05,Och06,Och07,Och08,Och09,Och10,Och11,Och12,Och13,Och14	*OreG0015781*
scaffold33200	1469023	T	G	Och01,Och02,Och03,Och04,Och05,Och06,Och07,Och08,Och09,Och10,Och11,Och12,Och13,Och14,Ore01,Ore02,Ore03,Ore04,Ore05,Ore06,Ore07,Ore08,Ore09,Ore10,Ore11,Ore12,Ore13,Ore14	*OreG0015786*
scaffold33200	1469271	T	A	NA	*OreG0015786*
scaffold33200	1469301	G	T	Och01,Och02,Och03,Och04,Och05,Och06,Och07,Och08,Och09,Och10,Och11,Och12,Och13,Och14,Ore01,Ore02,Ore03,Ore04,Ore05,Ore06,Ore07,Ore08,Ore09,Ore10,Ore11,Ore12,Ore13,Ore14	*OreG0015786*
scaffold33200	1469769	T	A	Ore01,Ore02,Ore03,Ore04,Ore05,Ore06,Ore07,Ore08,Ore09,Ore10,Ore11,Ore12,Ore13,Ore14	*OreG0015786*
scaffold33200	1470944	T	A	NA	*OreG0015786*
scaffold33200	1471766	T	C	NA	*OreG0015786*
scaffold33200	1471782	G	A	NA	*OreG0015786*
scaffold33200	1474169	G	T	Och11,Och12,Och13,Och14	*OreG0015786*
scaffold33200	1476594	T	C	NA	*OreG0015786*
scaffold33200	1481418	G	A	Ore01,Ore02,Ore03,Ore04,Ore05,Ore06,Ore07,Ore08,Ore09,Ore10,Ore11,Ore12,Ore13,Ore14	*OreG0015787*
scaffold33200	1481493	C	T	NA	*OreG0015787*
scaffold33200	1481510	C	G	NA	*OreG0015787*
scaffold33200	1512960	C	G	Och01	*OreG0015789*
scaffold33200	1542303	A	G	Och01	*OreG0015790*
scaffold33200	1542740	G	T	Och05,Och12,Och13	*OreG0015790*
scaffold33200	1542897	G	T	NA	*OreG0015790*
scaffold33200	1542960	C	A	NA	*OreG0015790*
scaffold33200	1543482	G	A	Och01,Och06,Och07,Och08	*OreG0015790*
scaffold33200	1554052	G	C	NA	*OreG0015792*
scaffold33200	1554190	T	C	NA	*OreG0015792*
scaffold33200	1573101	G	T	NA	*OreG0015794*
scaffold33200	1574211	A	C	Och05,Och12,Och13	*OreG0015794*
scaffold33200	1578886	C	A	NA	*OreG0015795*
scaffold33200	1579348	T	A	NA	*OreG0015795*
scaffold33200	1597434	A	C	NA	*OreG0015796*
scaffold33200	1599965	A	T	NA	*OreG0015796*
scaffold33200	1630869	G	C	NA	*OreG0015799*
scaffold33200	1714542	G	A	Och01,Och02,Och03,Och04,Och05,Och06,Och07,Och08,Och09,Och10,Och11,Och12,Och13,Och14	*OreG0015804*
scaffold33200	1733983	A	G	Och01,Och02,Och03,Och04,Och05,Och06,Och07,Och08,Och09,Och10,Och11,Och12,Och13,Och14	*OreG0015805*
scaffold33200	1734242	C	T	Och01,Och02,Och03,Och04,Och05,Och06,Och07,Och08,Och09,Och10,Och11,Och12,Och13,Och14	*OreG0015805*
scaffold33200	1734638	C	A	NA	*OreG0015805*
scaffold33200	1882507	G	A	Och01,Och02,Och03,Och04,Och05,Och06,Och07,Och08,Och09,Och10,Och11,Och12,Och13,Och14	*OreG0015807*
scaffold33200	1882597	G	T	Och01,Och02,Och03,Och04,Och05,Och06,Och07,Och08,Och09,Och10,Och11,Och12,Och13,Och14	*OreG0015807*
scaffold33200	1882616	C	T	Och01,Och02,Och03,Och04,Och05,Och06,Och07,Och08,Och09,Och10,Och11,Och12,Och13,Och14	*OreG0015807*
scaffold33200	1882955	G	C	NA	*OreG0015807*
scaffold33200	1885832	C	A	Ore01,Ore02,Ore03,Ore04,Ore05,Ore06,Ore07,Ore08,Ore09,Ore10,Ore11,Ore12,Ore13,Ore14	*OreG0015808*

scaffold33200	2162873	C	A	Ore01,Ore02,Ore03,Ore04,Ore05,Ore06,Ore07,Ore08,Ore09,Ore10,Ore11,Ore12,Ore13,Ore14	*OreG0015812*
scaffold33200	2715684	T	C	Ore01,Ore02,Ore03,Ore04,Ore05,Ore06,Ore07,Ore08,Ore09,Ore10,Ore11,Ore12,Ore13,Ore14	*OreG0015816*
scaffold33200	2717042	G	A	NA	*OreG0015816*
scaffold33200	2973539	C	T	Och01,Och02,Och03,Och04,Och05,Och06,Och07,Och08,Och09,Och10,Och11,Och12,Och13,Och14	*OreG0015817*
scaffold33200	2974559	G	C	Ore01,Ore02,Ore03,Ore04,Ore05,Ore06,Ore07,Ore08,Ore09,Ore10,Ore11,Ore12,Ore13,Ore14	*OreG0015817*
scaffold33200	2999486	A	G	Och01,Och02,Och03,Och04,Och05,Och06,Och07,Och08,Och09,Och10,Och11,Och12,Och13,Och14	*OreG0015817*
scaffold33200	3407276	G	A	Och08	*OreG0015819*
scaffold33200	3407354	A	G	Ore08	*OreG0015819*
scaffold33200	3407583	C	G	NA	*OreG0015819*
scaffold33200	3412313	C	T	NA	*OreG0015820*
scaffold33272	26866	T	C	Och01,Och02,Och03,Och04,Och05,Och06,Och07,Och08,Och09,Och10,Och11,Och12,Och13,Och14,Ore01,Ore02,Ore03,Ore04,Ore05,Ore06,Ore07,Ore08,Ore09,Ore10,Ore11,Ore12,Ore13,Ore14	*OreG0018171*
scaffold33272	82541	C	T	Ore01,Ore02,Ore03,Ore04,Ore05,Ore06,Ore07,Ore08,Ore09,Ore10,Ore11,Ore12,Ore13,Ore14	*OreG0018173*
scaffold33272	83304	G	C	NA	*OreG0018173*
scaffold33272	85220	T	A	Ore01,Ore02,Ore03,Ore04,Ore05,Ore06,Ore07,Ore08,Ore09,Ore10,Ore11,Ore12,Ore13,Ore14	*OreG0018174*
scaffold33272	129902	G	A	Och01,Och02,Och03,Och04,Och05,Och06,Och07,Och08,Och09,Och10,Och11,Och12,Och13,Och14	*OreG0018176*
scaffold33272	130349	G	A	Ore01,Ore02,Ore03,Ore04,Ore05,Ore06,Ore07,Ore08,Ore09,Ore10,Ore11,Ore12,Ore13,Ore14	*OreG0018176*
scaffold33272	144236	C	A	Ore01,Ore02,Ore03,Ore04,Ore05,Ore06,Ore07,Ore08,Ore09,Ore10,Ore11,Ore12,Ore13,Ore14	*OreG0018177*
scaffold33272	160203	A	T	NA	*OreG0018178*
scaffold33272	211892	A	G	Och01,Och04,Och06,Och13	*OreG0018184*
scaffold33272	211920	G	C	Ore01,Ore02,Ore03,Ore04,Ore05,Ore06,Ore07,Ore08,Ore09,Ore10,Ore11,Ore12,Ore13,Ore14	*OreG0018184*
scaffold33272	240446	G	T	Och01,Och11	*OreG0018188*
scaffold33272	250996	C	T	NA	*OreG0018189*
scaffold33272	254402	G	A	Och01,Och02,Och03,Och06,Och07,Och08,Och09,Och10,Och11,Och12	*OreG0018190*
scaffold33272	274543	C	G	Och01,Och02,Och03,Och04,Och05,Och06,Och07,Och08,Och09,Och10,Och11,Och12,Och13,Och14	*OreG0018192*
scaffold33272	274707	A	G	Och01,Och02,Och03,Och04,Och05,Och06,Och07,Och08,Och09,Och10,Och11,Och12,Och13,Och14,Ore01,Ore02,Ore03,Ore04,Ore05,Ore06,Ore07,Ore08,Ore09,Ore10,Ore11,Ore12,Ore13,Ore14	*OreG0018192*
scaffold33272	281101	C	A	NA	*OreG0018193*
scaffold33272	281320	A	C	Och06	*OreG0018193*
scaffold33272	281335	A	G	Och01,Och07,Och14	*OreG0018193*
scaffold33272	281353	G	T	Ore06	*OreG0018193*
scaffold33272	281636	T	C	NA	*OreG0018193*
scaffold33272	297442	G	T	NA	*OreG0018196*
scaffold33272	298279	G	A	Ore04,Ore05	*OreG0018196*
scaffold33272	300047	G	T	Och01,Och02,Och03,Och04,Och05,Och06,Och07,Och08,Och09,Och10,Och11,Och12,Och13,Och14,Ore01,Ore02,Ore03,Ore04,Ore05,Ore06,Ore07,Ore08,Ore09,Ore10,Ore11,Ore12,Ore13,Ore14	*OreG0018197*
scaffold33272	383120	C	G	Och02,Och03,Och04,Och05,Och06,Och09,Och10,Och11,Och12,Och13	*OreG0018203*
scaffold33272	383420	G	T	Och02,Och03,Och04,Och05,Och06,Och09,Och10,Och11,Och12,Och13	*OreG0018203*
scaffold33272	383524	T	G	NA	*OreG0018203*
scaffold33272	396564	C	T	Och01,Och02,Och03,Och04,Och05,Och06,Och07,Och08,Och09,Och10,Och11,Och12,Och13,Och14,Ore01,Ore02,Ore03,Ore04,Ore05,Ore06,Ore07,Ore08,Ore09,Ore10,Ore11,Ore12,Ore13,Ore14	*OreG0018206*
scaffold33272	396601	A	G	Och01,Och02,Och03,Och04,Och05,Och06,Och07,Och08,Och09,Och10,Och11,Och12,Och13,Och14	*OreG0018206*
scaffold33272	396657	T	A	NA	*OreG0018206*
scaffold33272	402246	C	T	NA	*OreG0018207*
scaffold33288	16962	A	T	NA	*OreG0018436*
scaffold33288	17335	T	C	Och01,Och02,Och03,Och04,Och05,Och06,Och07,Och09,Och10,Och11,Och12,Och13,Ore01,Ore02,Ore03,Ore04,Ore05,Ore06,Ore07,Ore08,Ore09,Ore10,Ore11,Ore12,Ore13,Ore14	*OreG0018436*
scaffold33288	17358	G	C	Och01,Och02,Och03,Och04,Och05,Och06,Och07,Och09,Och10,Och11,Och12,Och13	*OreG0018436*
scaffold33288	36741	T	C	Ore03,Ore06,Ore09,Ore11,Ore13,Ore14	*OreG0018440*
scaffold33290	51264	A	C	Och01,Och08	*OreG0018464*
scaffold33290	51267	G	A	NA	*OreG0018464*
scaffold33290	55265	G	A	NA	*OreG0018464*
scaffold33290	55299	C	A	NA	*OreG0018464*
scaffold33290	97090	T	A	Och01,Och02,Och03,Och04,Och05,Och06,Och07,Och08,Och09,Och10,Och11,Och12,Och13,Och14,Ore01,Ore02,Ore03,Ore04,Ore05,Ore06,Ore07,Ore08,Ore09,Ore10,Ore11,Ore12,Ore13,Ore14	*OreG0018473*
scaffold33290	97160	C	T	NA	*OreG0018473*

scaffold33290	97168	G	A	Och03,Och09,Och10,Och11	*OreG0018473*
scaffold33290	97214	C	T	Ore09,Ore10,Ore13	*OreG0018473*
scaffold33290	97253	A	G	NA	*OreG0018473*
scaffold33290	97360	A	G	NA	*OreG0018473*
scaffold33290	97513	A	C	NA	*OreG0018473*
scaffold33290	97622	G	C	NA	*OreG0018473*
scaffold33290	97647	G	T	Ore09,Ore10,Ore13	*OreG0018473*
scaffold33290	97682	C	A	Och01,Och02,Och03,Och04,Och05,Och06,Och07,Och08,Och09,Och10,Och11,Och12,Och13,Och14,Ore01,Ore02,Ore03,Ore04,Ore05,Ore06,Ore07,Ore08,Ore09,Ore10,Ore11,Ore12,Ore13,Ore14	*OreG0018473*
scaffold33290	97805	A	T	Och01,Och02,Och03,Och04,Och05,Och06,Och07,Och08,Och09,Och10,Och11,Och12,Och13,Och14	*OreG0018473*
scaffold33290	111594	T	C	Och01,Och02,Och03,Och04,Och05,Och06,Och07,Och08,Och09,Och10,Och11,Och12,Och13,Och14	*OreG0018474*
scaffold33290	112648	C	A	Och01,Och02,Och03,Och04,Och05,Och06,Och07,Och08,Och09,Och10,Och11,Och12,Och13,Och14,Ore01,Ore02,Ore03,Ore04,Ore05,Ore06,Ore07,Ore08,Ore09,Ore10,Ore11,Ore12,Ore13,Ore14	*OreG0018474*
scaffold33290	123139	C	T	Och01,Och02,Och03,Och04,Och05,Och06,Och07,Och08,Och09,Och10,Och11,Och12,Och13,Och14,Ore01,Ore02,Ore03,Ore04,Ore05,Ore06,Ore07,Ore08,Ore09,Ore10,Ore11,Ore12,Ore13,Ore14	*OreG0018477*
scaffold33290	128126	G	C	NA	*OreG0018478*
scaffold33290	128576	C	A	NA	*OreG0018478*
scaffold33290	129119	A	T	Och01,Och02,Och03,Och04,Och05,Och06,Och07,Och08,Och09,Och10,Och11,Och12,Och13,Och14,Ore01,Ore02,Ore03,Ore04,Ore05,Ore06,Ore07,Ore08,Ore09,Ore10,Ore11,Ore12,Ore13,Ore14	*OreG0018478*
scaffold33290	129150	G	T	NA	*OreG0018478*
scaffold33290	137509	C	A	NA	*OreG0018479*
scaffold33290	139931	A	G	NA	*OreG0018480*
scaffold33290	142804	C	T	Och01,Och03,Och05,Och07,Och08	*OreG0018480*
scaffold33290	148785	T	G	Och03	*OreG0018480*
scaffold33290	165401	G	T	Och12	*OreG0018484*
scaffold33290	178385	A	T	NA	*OreG0018487*
scaffold33290	178416	A	T	NA	*OreG0018487*
scaffold33290	198910	A	G	NA	*OreG0018491*
scaffold33290	199179	T	A	Ore01	*OreG0018491*
scaffold33290	201301	G	T	NA	*OreG0018491*
scaffold33290	230443	A	C	NA	*OreG0018494*
scaffold33290	250206	C	A	Och01,Och02,Och03,Och04,Och05,Och06,Och07,Och08,Och09,Och10,Och11,Och12,Och13,Och14,Ore01,Ore02,Ore03,Ore04,Ore05,Ore06,Ore07,Ore08,Ore09,Ore10,Ore11,Ore12,Ore13,Ore14	*OreG0018496*
scaffold33290	250918	T	C	Ore01,Ore02,Ore03,Ore04,Ore05,Ore06,Ore07,Ore08,Ore09,Ore10,Ore11,Ore12,Ore13,Ore14	*OreG0018496*
scaffold33290	257091	T	A	Och11,Och12	*OreG0018497*
scaffold33290	274767	C	A	Ore01,Ore02,Ore03,Ore04,Ore05,Ore06,Ore07,Ore08,Ore09,Ore10,Ore11,Ore12,Ore13,Ore14	*OreG0018500*
scaffold33290	275409	G	A	NA	*OreG0018500*
scaffold33290	277075	A	G	Ore01,Ore02,Ore03,Ore04,Ore05,Ore06,Ore07,Ore08,Ore09,Ore10,Ore11,Ore12,Ore13,Ore14	*OreG0018500*
scaffold33290	278189	T	A	NA	*OreG0018500*
scaffold33290	280554	C	G	NA	*OreG0018500*
scaffold33290	282364	C	T	Och02,Och03,Och04,Och06,Och13	*OreG0018500*
scaffold33238	1280	G	T	NA	*OreG0017302*
scaffold33238	12657	A	C	NA	*OreG0017303*
scaffold33238	38120	C	T	NA	*OreG0017304*
scaffold33238	52489	A	T	NA	*OreG0017306*
scaffold33238	200295	G	A	NA	*OreG0017311*
scaffold33274	23891	G	T	Och14	*OreG0018215*
scaffold33274	35689	G	T	NA	*OreG0018216*
scaffold33274	61510	C	A	NA	*OreG0018218*
scaffold33274	79007	G	A	Och11	*OreG0018219*
scaffold33274	85598	G	T	Och11	*OreG0018221*
scaffold33274	124819	G	T	Ore01,Ore02,Ore04,Ore05,Ore07,Ore09,Ore14	*OreG0018227*
scaffold33274	125006	G	T	Och06	*OreG0018227*
scaffold33274	125039	G	A	Och02,Och03,Och04,Och05,Och06,Och07,Och08,Och09,Och10,Och11,Och12,Och13,Och14	*OreG0018227*
scaffold33274	125421	T	G	NA	*OreG0018227*
scaffold33274	130603	A	G	Ore01,Ore02,Ore04,Ore05,Ore07,Ore09,Ore14	*OreG0018228*
scaffold33274	179693	G	A	NA	*OreG0018232*
scaffold33274	184048	C	T	Ore01,Ore02,Ore04,Ore05,Ore07,Ore09,Ore14	*OreG0018232*
scaffold33274	185210	T	A	Och14	*OreG0018232*
scaffold33274	208577	A	G	NA	*OreG0018233*
scaffold33274	210033	A	G	Och02,Och03,Och04,Och05,Och06,Och07,Och09,Och10,Och11,Och12,Och13,Och14,Ore04,Ore05	*OreG0018234*
scaffold33274	216719	C	T	NA	*OreG0018235*
scaffold33274	226992	G	A	NA	*OreG0018236*

scaffold33274	227050	G	A	NA	*OreG0018236*
scaffold33274	227080	T	G	Och08,Och11	*OreG0018236*
scaffold33274	253121	C	G	Och11	*OreG0018238*
scaffold33274	254181	C	T	Och01,Och02,Och03,Och04,Och05,Och06,Och07,Och08,Och09,Och10,Och11,Och12,Och13,Och14,Ore01,Ore02,Ore03,Ore04,Ore05,Ore06,Ore07,Ore08,Ore09,Ore10,Ore11,Ore12,Ore13,Ore14	*OreG0018238*
scaffold33274	260539	G	A	NA	*OreG0018239*
scaffold33274	261765	C	T	Och01,Och02,Och03,Och04,Och05,Och06,Och07,Och08,Och09,Och10,Och11,Och12,Och13,Och14,Ore01,Ore02,Ore03,Ore04,Ore05,Ore06,Ore07,Ore08,Ore09,Ore10,Ore11,Ore12,Ore13,Ore14	*OreG0018239*
scaffold33274	261885	A	G	Och01,Och02,Och03,Och04,Och05,Och06,Och07,Och08,Och11,Och12,Och13,Och14,Ore01,Ore02,Ore03,Ore04,Ore05,Ore06,Ore07,Ore08,Ore09,Ore10,Ore11,Ore12,Ore13,Ore14	*OreG0018239*
scaffold33274	285636	A	T	NA	*OreG0018243*
scaffold33274	287542	T	C	Och01,Och02,Och03,Och04,Och05,Och06,Och07,Och08,Och09,Och10,Och11,Och12,Och13,Och14,Ore01,Ore02,Ore03,Ore04,Ore05,Ore06,Ore07,Ore08,Ore09,Ore10,Ore11,Ore12,Ore13,Ore14	*OreG0018243*
scaffold33274	290253	A	G	NA	*OreG0018243*
scaffold33274	293132	A	G	Och14	*OreG0018243*
scaffold33274	297557	A	T	Ore09,Ore14	*OreG0018244*
scaffold33274	299001	G	A	NA	*OreG0018244*
scaffold33274	299563	G	T	Och06	*OreG0018244*
scaffold33274	299713	C	G	NA	*OreG0018244*
scaffold33274	368792	C	T	Ore01,Ore02,Ore03,Ore04,Ore05,Ore06,Ore07,Ore08,Ore09,Ore10,Ore11,Ore12,Ore13,Ore14	*OreG0018250*
scaffold33274	369016	C	T	Och11	*OreG0018250*
scaffold33274	369071	G	A	Och01,Och02,Och03,Och04,Och05,Och06,Och07,Och08,Och09,Och10,Och11,Och12,Och13,Och14,Ore01,Ore02,Ore03,Ore04,Ore05,Ore06,Ore07,Ore08,Ore09,Ore10,Ore11,Ore12,Ore13,Ore14	*OreG0018250*
scaffold33274	370862	C	T	NA	*OreG0018250*
scaffold33274	467627	G	C	Och02,Och03,Och04,Och06	*OreG0018253*
scaffold33274	469005	C	T	NA	*OreG0018253*
scaffold33274	469987	T	C	NA	*OreG0018253*
scaffold33274	470656	T	C	NA	*OreG0018253*
scaffold33274	501679	T	A	Och01,Och02,Och03,Och04,Och05,Och06,Och07,Och08,Och09,Och10,Och11,Och12,Och13,Och14,Ore01,Ore02,Ore03,Ore04,Ore05,Ore06,Ore07,Ore08,Ore09,Ore10,Ore11,Ore12,Ore13,Ore14	*OreG0018255*
scaffold33274	501930	C	T	NA	*OreG0018255*
scaffold33274	523394	G	T	Och01,Och02,Och03,Och04,Och05,Och06,Och07,Och08,Och09,Och10,Och12,Och13,Och14,Ore01,Ore02,Ore03,Ore04,Ore05,Ore06,Ore07,Ore08,Ore09,Ore10,Ore11,Ore12,Ore13,Ore14	*OreG0018257*
scaffold33193	162978	C	T	NA	*OreG0014756*
scaffold33193	162986	A	C	NA	*OreG0014756*
scaffold33193	162999	C	A	NA	*OreG0014756*
scaffold33193	163829	C	T	NA	*OreG0014756*
scaffold33193	163864	A	G	NA	*OreG0014756*
scaffold33193	165643	C	G	NA	*OreG0014756*
scaffold33193	214780	G	T	Och01,Och02,Och03,Och04,Och05,Och06,Och07,Och08,Och09,Och10,Och11,Och12,Och13,Och14,Ore01,Ore02,Ore03,Ore04,Ore05,Ore06,Ore07,Ore08,Ore09,Ore10,Ore11,Ore12,Ore13,Ore14	*OreG0014760*
scaffold33193	237534	C	G	Och03,Och05,Och08,Och12,Och13	*OreG0014762*
scaffold33193	290398	C	T	NA	*OreG0014766*
scaffold33193	368774	A	T	NA	*OreG0014776*
scaffold33193	384101	G	A	NA	*OreG0014777*
scaffold33193	423543	T	A	NA	*OreG0014780*
scaffold33193	430441	C	T	Och03,Och04,Och05,Och06,Och09,Och10,Och13	*OreG0014782*
scaffold33193	430679	C	A	NA	*OreG0014782*
scaffold33193	434861	C	A	NA	*OreG0014784*
scaffold33193	434896	T	A	Och01,Och02,Och03,Och04,Och05,Och06,Och07,Och08,Och09,Och10,Och11,Och12,Och13,Och14	*OreG0014784*
scaffold33193	466238	A	G	Och01,Och02,Och03,Och04,Och05,Och06,Och07,Och08,Och09,Och10,Och11,Och12,Och13,Och14	*OreG0014787*
scaffold33193	466636	C	T	Och09,Och10,Och11	*OreG0014787*
scaffold33193	467367	T	C	NA	*OreG0014787*
scaffold33193	469301	C	T	Och01,Och02,Och03,Och04,Och05,Och06,Och07,Och08,Och09,Och10,Och11,Och12,Och13,Och14	*OreG0014787*
scaffold33193	469330	T	C	NA	*OreG0014787*
scaffold33193	470352	A	G	Ore01,Ore02,Ore03,Ore04,Ore05,Ore06,Ore07,Ore08,Ore09,Ore10,Ore11,Ore12,Ore13,Ore14	*OreG0014787*
scaffold33193	479405	T	C	NA	*OreG0014789*
scaffold33193	479758	G	C	NA	*OreG0014789*
scaffold33193	566474	A	C	NA	*OreG0014799*
scaffold33193	566831	T	C	Och01,Och02,Och03,Och04,Och05,Och06,Och07,Och08,Och09,Och10,Och11,Och12,Och13,Och14	*OreG0014799*

scaffold33193	568912	C	T	Och01,Och02,Och03,Och04,Och05,Och06,Och07,Och08,Och09,Och10,Och11,Och12,Och13,Och14,Ore01,Ore02,Ore03,Ore04,Ore05,Ore06,Ore07,Ore08,Ore09,Ore10,Ore11,Ore12,Ore13,Ore14	*OreG0014800*
scaffold33193	603501	C	T	Och01	*OreG0014806*
scaffold33193	603614	T	C	NA	*OreG0014806*
scaffold33193	843381	A	T	Och01,Och02,Och03,Och04,Och05,Och06,Och07,Och08,Och09,Och10,Och11,Och12,Och13,Och14,Ore01,Ore02,Ore03,Ore04,Ore05,Ore06,Ore07,Ore08,Ore09,Ore10,Ore11,Ore12,Ore13,Ore14	*OreG0014832*
scaffold33193	917532	T	G	Och12	*OreG0014838*
scaffold33193	917984	G	A	NA	*OreG0014838*
scaffold33193	924269	G	A	Ore02,Ore04,Ore05,Ore06,Ore07,Ore08,Ore09,Ore10,Ore11,Ore12,Ore13,Ore14	*OreG0014839*
scaffold33193	961500	G	A	NA	*OreG0014846*
scaffold33193	961671	G	A	NA	*OreG0014846*
scaffold33193	998064	C	T	Och06,Och11,Ore01,Ore02,Ore03,Ore04,Ore05,Ore06,Ore07,Ore08,Ore09,Ore10,Ore11,Ore12,Ore13,Ore14	*OreG0014850*
scaffold33193	998367	G	T	Ore01,Ore02,Ore03,Ore04,Ore05,Ore06,Ore07,Ore08,Ore09,Ore10,Ore11,Ore12,Ore13,Ore14	*OreG0014850*
scaffold33193	998712	T	C	NA	*OreG0014850*
scaffold33193	998810	G	A	NA	*OreG0014850*
scaffold33193	998958	T	C	NA	*OreG0014850*
scaffold33193	999081	G	A	Och01,Och02,Och03,Och04,Och05,Och06,Och07,Och08,Och09,Och10,Och11,Och12,Och13,Och14,Ore01,Ore02,Ore03,Ore04,Ore05,Ore06,Ore07,Ore08,Ore09,Ore10,Ore11,Ore12,Ore13,Ore14	*OreG0014850*
scaffold33193	999686	T	G	NA	*OreG0014850*
scaffold33193	1000164	A	C	NA	*OreG0014850*
scaffold33193	1000172	G	A	NA	*OreG0014850*
scaffold33193	1000331	C	T	NA	*OreG0014850*
scaffold33193	1000340	C	T	NA	*OreG0014850*
scaffold33193	1000391	A	G	NA	*OreG0014850*
scaffold33193	1000397	G	T	NA	*OreG0014850*
scaffold33193	1000412	G	A	NA	*OreG0014850*
scaffold33193	1000469	T	A	NA	*OreG0014850*
scaffold33193	1000635	A	G	Ore01,Ore02,Ore03,Ore04,Ore05,Ore06,Ore07,Ore08,Ore09,Ore10,Ore11,Ore12,Ore13,Ore14	*OreG0014850*
scaffold33193	1036828	T	C	NA	*OreG0014852*
scaffold33193	1036915	A	C	NA	*OreG0014852*
scaffold33193	1037167	C	T	NA	*OreG0014852*
scaffold33193	1037190	G	A	NA	*OreG0014852*
scaffold33193	1037339	T	G	Och06,Och08,Och09,Och10,Och11,Och14,Ore01,Ore02,Ore03,Ore04,Ore05,Ore06,Ore07,Ore08,Ore09,Ore10,Ore11,Ore12,Ore13,Ore14	*OreG0014852*
scaffold33193	1037467	C	T	NA	*OreG0014852*
scaffold33193	1038980	G	C	NA	*OreG0014852*
scaffold33193	1040793	T	A	NA	*OreG0014853*
scaffold33193	1041261	A	T	NA	*OreG0014853*
scaffold33193	1041472	G	A	NA	*OreG0014853*
scaffold33193	1041481	C	T	NA	*OreG0014853*
scaffold33193	1104980	G	A	Ore02,Ore06,Ore07,Ore08,Ore09,Ore10,Ore11,Ore12,Ore13,Ore14	*OreG0014860*
scaffold33193	1105302	A	G	NA	*OreG0014860*
scaffold33193	1108923	A	G	Och12,Ore01,Ore02,Ore03,Ore04,Ore05,Ore06,Ore07,Ore08,Ore09,Ore10,Ore11,Ore12,Ore13,Ore14	*OreG0014860*
scaffold33193	1210274	A	G	NA	*OreG0014872*
scaffold33193	1210289	C	A	NA	*OreG0014872*
scaffold33193	1244145	G	T	Ore01,Ore02,Ore03,Ore04,Ore05,Ore06,Ore07,Ore08,Ore09,Ore10,Ore11,Ore12,Ore13,Ore14	*OreG0014875*
scaffold33193	1247843	A	T	NA	*OreG0014876*
scaffold33193	1248557	C	T	NA	*OreG0014876*
scaffold33193	1263397	A	C	NA	*OreG0014879*
scaffold33193	1264066	C	A	Ore01,Ore02,Ore03,Ore04,Ore05,Ore06,Ore07,Ore08,Ore09,Ore10,Ore11,Ore12,Ore13,Ore14	*OreG0014879*
scaffold33193	1396095	A	T	Ore02,Ore06,Ore07,Ore08,Ore09,Ore10,Ore11,Ore12,Ore13,Ore14	*OreG0014891*
scaffold33193	1410291	G	A	Ore02,Ore06,Ore07,Ore08,Ore09,Ore10,Ore11,Ore12,Ore13,Ore14	*OreG0014893*
scaffold33193	1486661	C	T	Och01	*OreG0014900*
scaffold33193	1498066	C	T	NA	*OreG0014904*
scaffold33193	1498132	C	T	NA	*OreG0014904*
scaffold33193	1498288	A	C	NA	*OreG0014904*
scaffold33193	1498315	T	A	NA	*OreG0014904*
scaffold33193	1498344	A	G	NA	*OreG0014904*
scaffold33193	1535718	C	A	NA	*OreG0014907*
scaffold33193	1535948	G	A	NA	*OreG0014907*
scaffold33193	1553068	T	A	Och01,Och02,Och03,Och04,Och05,Och06,Och07,Och08,Och09,Och10,Och11,Och12,Och13,Och14,Ore01,Ore02,Ore03,Ore04,Ore05,Ore06,Ore07,Ore08,Ore09,Ore10,Ore11,Ore12,Ore13,Ore14	*OreG0014909*
scaffold33193	1596562	A	G	NA	*OreG0014912*
scaffold33193	1599346	G	T	NA	*OreG0014912*
scaffold33193	1617185	T	C	NA	*OreG0014916*

scaffold33193	1617427	G	A	NA	*OreG0014916*
scaffold33193	1618267	C	A	Och14	*OreG0014916*
scaffold33193	1644780	T	C	NA	*OreG0014921*
scaffold33193	1646248	A	C	Och01,Och02,Och03,Och04,Och05,Och06,Och07,Och08,Och09,Och10,Och11,Och12,Och13,Och14,Ore01,Ore02,Ore03,Ore04,Ore05,Ore06,Ore07,Ore08,Ore09,Ore10,Ore11,Ore12,Ore13,Ore14	*OreG0014922*
scaffold33193	1646701	T	G	Och01,Och14	*OreG0014922*
scaffold33193	1647138	G	A	Och01,Och02,Och03,Och04,Och05,Och06,Och07,Och08,Och09,Och10,Och11,Och12,Och13,Och14,Ore01,Ore02,Ore03,Ore04,Ore05,Ore06,Ore07,Ore08,Ore09,Ore10,Ore11,Ore12,Ore13,Ore14	*OreG0014922*
scaffold33193	1648362	C	A	NA	*OreG0014923*
scaffold33193	1648547	C	G	Och01,Och02,Och03,Och04,Och05,Och06,Och07,Och08,Och09,Och10,Och11,Och12,Och13,Och14	*OreG0014923*
scaffold33193	1651502	T	G	Och01,Och02,Och03,Och04,Och05,Och06,Och07,Och08,Och09,Och10,Och11,Och12,Och13,Och14,Ore01,Ore02,Ore03,Ore04,Ore05,Ore06,Ore07,Ore08,Ore09,Ore10,Ore11,Ore12,Ore13,Ore14	*OreG0014923*
scaffold33193	1699002	T	C	NA	*OreG0014927*
scaffold33193	1699502	T	C	NA	*OreG0014927*
scaffold33193	1699543	C	T	NA	*OreG0014927*
scaffold33193	1706678	G	C	Och01,Och02,Och03,Och04,Och05,Och06,Och07,Och08,Och09,Och10,Och11,Och12,Och13,Och14	*OreG0014929*
scaffold33193	1706932	C	T	NA	*OreG0014929*
scaffold33193	1709677	A	G	NA	*OreG0014930*
scaffold33193	1710216	G	A	NA	*OreG0014930*
scaffold33193	1712348	T	G	NA	*OreG0014931*
scaffold33193	1776591	C	T	Och01,Och02,Och03,Och04,Och05,Och06,Och07,Och08,Och09,Och10,Och11,Och12,Och13,Och14	*OreG0014940*
scaffold33193	1818338	G	A	Och01,Och02,Och03,Och04,Och05,Och06,Och07,Och08,Och09,Och10,Och11,Och12,Och13,Och14,Ore01,Ore02,Ore03,Ore04,Ore05,Ore06,Ore07,Ore08,Ore09,Ore10,Ore11,Ore12,Ore13,Ore14	*OreG0014943*
scaffold33193	1818390	C	A	Och01,Och02,Och03,Och04,Och05,Och06,Och07,Och08,Och09,Och10,Och11,Och12,Och13,Och14,Ore01,Ore02,Ore03,Ore04,Ore05,Ore06,Ore07,Ore08,Ore09,Ore10,Ore11,Ore12,Ore13,Ore14	*OreG0014943*
scaffold33193	1819194	C	A	NA	*OreG0014943*
scaffold33193	1819228	T	C	Och06	*OreG0014943*
scaffold33193	1820404	G	A	Och12	*OreG0014943*
scaffold33193	1820466	T	C	Och01,Och02,Och03,Och04,Och05,Och06,Och07,Och08,Och09,Och10,Och11,Och12,Och13,Och14	*OreG0014943*
scaffold33193	1830154	A	C	NA	*OreG0014944*
scaffold33193	1830191	G	A	Och01,Och02,Och03,Och04,Och05,Och06,Och07,Och08,Och09,Och10,Och11,Och12,Och13,Och14	*OreG0014944*
scaffold33193	1831337	A	C	NA	*OreG0014944*
scaffold33193	1832382	A	T	NA	*OreG0014944*
scaffold33193	1834040	A	C	Och09,Och10	*OreG0014944*
scaffold33193	1865099	T	G	Och02,Och03,Och04,Och05,Och06,Och07,Och08,Och09,Och10,Och11,Och12,Och13,Och14	*OreG0014948*
scaffold33193	1873540	G	C	NA	*OreG0014949*
scaffold33193	1874717	C	T	Ore02,Ore04,Ore05,Ore06,Ore07,Ore08,Ore09,Ore10,Ore11,Ore12,Ore13,Ore14	*OreG0014949*
scaffold33193	1875982	A	T	Ore04,Ore05	*OreG0014949*
scaffold33193	1901525	G	T	Och12,Och14	*OreG0014952*
scaffold33193	1915515	G	T	Och12	*OreG0014956*
scaffold33193	1916218	C	T	NA	*OreG0014956*
scaffold33193	1965464	G	C	Ore01,Ore02,Ore03,Ore04,Ore05,Ore06,Ore07,Ore08,Ore09,Ore10,Ore11,Ore12,Ore13,Ore14	*OreG0014960*
scaffold33193	1966250	C	T	Och01	*OreG0014960*
scaffold33193	1967235	T	A	NA	*OreG0014960*
scaffold33193	1968443	C	A	Och01	*OreG0014960*
scaffold33193	1980920	C	T	NA	*OreG0014961*
scaffold33193	1981049	A	T	NA	*OreG0014961*
scaffold33193	1981093	A	G	NA	*OreG0014961*
scaffold33193	1981483	G	T	NA	*OreG0014961*
scaffold33193	2000786	G	A	NA	*OreG0014962*
scaffold33193	2053653	G	C	NA	*OreG0014967*
scaffold33193	2107690	G	T	Och12	*OreG0014971*
scaffold33193	2141476	C	T	Och14	*OreG0014976*
scaffold33193	2144944	T	A	Ore01,Ore02,Ore03,Ore04,Ore05,Ore06,Ore07,Ore08,Ore09,Ore10,Ore11,Ore12,Ore13,Ore14	*OreG0014977*
scaffold33193	2295755	G	A	NA	*OreG0014991*
scaffold33193	2344215	A	C	NA	*OreG0014997*
scaffold33193	2403867	G	A	NA	*OreG0015005*
scaffold33193	2435916	G	T	NA	*OreG0015010*
scaffold33193	2436390	C	T	NA	*OreG0015010*
scaffold33193	2436435	G	A	NA	*OreG0015010*
scaffold33193	2436464	T	A	Ore01,Ore02,Ore03,Ore04,Ore05,Ore06,Ore07,Ore08,Ore09,Ore10,Ore11,Ore12,Ore13,Ore14	*OreG0015010*

scaffold33193	2436515	C	T	Och01,Och02,Och03,Och04,Och05,Och06,Och07,Och08,Och09,Och10,Och11,Och12,Och13,Och14,Ore01,Ore02,Ore03,Ore04,Ore05,Ore06,Ore07,Ore08,Ore09,Ore10,Ore11,Ore12,Ore13,Ore14	*OreG0015010*
scaffold33193	2509569	G	A	NA	*OreG0015018*
scaffold33193	2530345	C	T	NA	*OreG0015020*
scaffold33193	2531013	C	T	NA	*OreG0015020*
scaffold33193	2531091	C	A	Ore03	*OreG0015020*
scaffold33193	2531387	A	C	Och01,Och02,Och03,Och04,Och05,Och06,Och07,Och08,Och09,Och10,Och11,Och12,Och13,Och14	*OreG0015020*
scaffold33193	2531917	G	C	NA	*OreG0015020*
scaffold33193	2534127	C	A	Ore01,Ore02,Ore03,Ore04,Ore05,Ore06,Ore07,Ore08,Ore09,Ore10,Ore11,Ore12,Ore13,Ore14	*OreG0015021*
scaffold33193	2535507	G	A	Och01,Och02,Och03,Och04,Och05,Och06,Och07,Och08,Och09,Och10,Och11,Och12,Och13,Och14,Ore01,Ore02,Ore03,Ore04,Ore05,Ore06,Ore07,Ore08,Ore09,Ore10,Ore11,Ore12,Ore13,Ore14	*OreG0015021*
scaffold33193	2536316	G	C	NA	*OreG0015021*
scaffold33193	2593043	T	G	Och01,Och02,Och03,Och04,Och05,Och06,Och07,Och08,Och09,Och10,Och11,Och12,Och13,Och14,Ore01,Ore02,Ore03,Ore04,Ore05,Ore06,Ore07,Ore08,Ore09,Ore10,Ore11,Ore12,Ore13,Ore14	*OreG0015026*
scaffold33193	2593231	G	A	NA	*OreG0015026*
scaffold33193	2603440	G	A	NA	*OreG0015030*
scaffold33193	2695589	G	A	NA	*OreG0015036*
scaffold33193	2713332	G	A	Och02,Och06,Och11	*OreG0015038*
scaffold33193	2713411	T	C	NA	*OreG0015038*
scaffold33193	2713812	T	A	Och08,Ore04,Ore11,Ore13	*OreG0015038*
scaffold33193	2758334	C	T	Och01,Och06	*OreG0015040*
scaffold33193	2758402	A	C	NA	*OreG0015040*
scaffold33193	2759974	A	C	NA	*OreG0015040*
scaffold33193	2760007	G	A	NA	*OreG0015040*
scaffold33193	2761931	C	T	Och01,Och02,Och04,Och06	*OreG0015040*
scaffold33193	2822516	G	A	NA	*OreG0015045*
scaffold33193	2850064	C	T	Och01,Och08,Och14	*OreG0015049*
scaffold33193	2856562	A	C	NA	*OreG0015051*
scaffold33263	224790	C	A	NA	*OreG0017981*
scaffold33263	224843	C	A	NA	*OreG0017981*
scaffold33263	238487	T	A	Ore01,Ore02,Ore03,Ore04,Ore05,Ore06,Ore07,Ore08,Ore09,Ore10,Ore11,Ore12,Ore13,Ore14	*OreG0017983*
scaffold33263	252805	T	C	NA	*OreG0017982*
scaffold33263	252909	G	C	Ore01,Ore02,Ore03,Ore04,Ore05,Ore06,Ore07,Ore08,Ore09,Ore10,Ore11,Ore12,Ore13,Ore14	*OreG0017982*
scaffold33263	262971	C	T	Ore01,Ore02,Ore03,Ore04,Ore05,Ore06,Ore07,Ore08,Ore09,Ore10,Ore11,Ore12,Ore13,Ore14	*OreG0017982*
scaffold33263	263037	A	G	Och01,Och02,Och03,Och04,Och05,Och06,Och07,Och08,Och09,Och10,Och11,Och12,Och13,Och14,Ore01,Ore02,Ore03,Ore04,Ore05,Ore06,Ore07,Ore08,Ore09,Ore10,Ore11,Ore12,Ore13,Ore14	*OreG0017982*
scaffold33263	383307	G	A	Och01,Och02,Och03,Och04,Och05,Och06,Och07,Och09,Och10,Och11,Och12,Och13	*OreG0017989*
scaffold33263	383469	A	G	Ore01,Ore02,Ore03,Ore04,Ore05,Ore06,Ore07,Ore08,Ore09,Ore10,Ore11,Ore12,Ore13,Ore14	*OreG0017989*
scaffold33263	383619	T	G	Ore01,Ore02,Ore03,Ore04,Ore05,Ore06,Ore07,Ore08,Ore09,Ore10,Ore11,Ore12,Ore13,Ore14	*OreG0017989*
scaffold33263	383686	A	T	Ore01,Ore02,Ore03,Ore04,Ore05,Ore06,Ore07,Ore08,Ore09,Ore10,Ore11,Ore12,Ore13,Ore14	*OreG0017989*
scaffold33263	384022	C	A	NA	*OreG0017989*
scaffold33263	448341	T	A	NA	*OreG0017995*
scaffold33263	477499	C	T	Och01,Och02,Och03,Och04,Och05,Och06,Och07,Och08,Och09,Och10,Och11,Och12,Och13,Och14,Ore01,Ore02,Ore03,Ore04,Ore05,Ore06,Ore07,Ore08,Ore09,Ore10,Ore11,Ore12,Ore13,Ore14	*OreG0017995*
scaffold33263	477783	C	A	NA	*OreG0017995*
scaffold33263	478271	G	A	Ore01,Ore02,Ore03,Ore04,Ore05,Ore06,Ore07,Ore08,Ore09,Ore10,Ore11,Ore12,Ore13,Ore14	*OreG0017995*
scaffold33263	503399	G	A	NA	*OreG0017996*
scaffold33263	510599	A	C	NA	*OreG0017997*
scaffold33263	511328	C	T	NA	*OreG0017997*
scaffold33263	511553	G	A	NA	*OreG0017997*
scaffold33263	511850	C	T	NA	*OreG0017997*
scaffold33263	512848	G	A	Ore01,Ore02,Ore03,Ore04,Ore05,Ore06,Ore07,Ore08,Ore09,Ore10,Ore11,Ore12,Ore13,Ore14	*OreG0017997*
scaffold33263	512930	C	A	NA	*OreG0017997*
scaffold33263	590633	G	A	NA	*OreG0018001*
scaffold33263	607635	G	A	NA	*OreG0018004*
scaffold33263	608410	C	T	Ore02,Ore04,Ore05,Ore06,Ore07,Ore09,Ore10,Ore13,Ore14	*OreG0018004*
scaffold33276	3279	A	T	Och01,Och02,Och03,Och04,Och05,Och06,Och07,Och08,Och12,Och13,Och14	*OreG0018263*
scaffold33276	4536	T	A	NA	*OreG0018263*
scaffold33276	51219	C	G	NA	*OreG0018264*

scaffold33276	67321	C	A	Och08	*OreG0018265*
scaffold33276	67415	A	G	Och01,Och02,Och03,Och04,Och05,Och06,Och07,Och08,Och12,Och13,Och14,Ore01,Ore02,Ore03,Ore04,Ore05,Ore06,Ore07,Ore08,Ore09,Ore10,Ore11,Ore12,Ore13,Or e14	*OreG0018265*
scaffold33276	68678	T	C	NA	*OreG0018265*
scaffold33276	69060	T	A	NA	*OreG0018265*
scaffold33276	86305	G	A	NA	*OreG0018266*
scaffold33276	103718	C	A	Ore01,Ore02,Ore03,Ore04,Ore05,Ore06,Ore07,Ore08,Ore09,Ore10,Ore11,Ore12,Ore 13,Ore14	*OreG0018267*
scaffold33276	113137	T	G	Och01,Och03,Och05,Och08,Och09,Och10,Och13	*OreG0018269*
scaffold33276	156044	G	A	Och02,Och03,Och04,Och05,Och06,Och12,Och13,Och14,Ore01,Ore02,Ore03,Ore04,Ore05,Ore06,Ore07,Ore08,Ore09,Ore10,Ore11,Ore12,Ore13,Ore14	*OreG0018271*
scaffold33276	173830	A	T	NA	*OreG0018272*
scaffold33276	205960	A	C	NA	*OreG0018276*
scaffold33276	207726	C	T	Och01	*OreG0018276*
scaffold33276	208902	C	A	NA	*OreG0018277*
scaffold33276	209033	A	G	NA	*OreG0018277*
scaffold33276	209247	T	C	NA	*OreG0018277*
scaffold33276	211188	T	A	NA	*OreG0018278*
scaffold33276	211924	T	C	NA	*OreG0018278*
scaffold33276	211936	T	C	NA	*OreG0018278*
scaffold33276	212362	A	T	Ore01,Ore02,Ore03,Ore04,Ore05,Ore06,Ore07,Ore08,Ore09,Ore10,Ore11,Ore12,Ore 13,Ore14	*OreG0018278*
scaffold33276	212444	T	A	NA	*OreG0018278*
scaffold33276	212446	A	G	NA	*OreG0018278*
scaffold33276	218556	T	G	NA	*OreG0018279*
scaffold33276	225887	T	G	Och01,Och05,Och07,Och08,Och09,Och10,Och13	*OreG0018280*
scaffold33276	226152	G	A	Ore02,Ore06,Ore07,Ore11,Ore13	*OreG0018280*
scaffold33276	262383	T	G	Och01,Och02,Och03,Och04,Och05,Och06,Och07,Och08,Och09,Och10,Och12,Och1 3	*OreG0018285*
scaffold33276	262398	A	G	Och01,Och02,Och03,Och04,Och05,Och06,Och07,Och08,Och09,Och10,Och11,Och1 2,Och13,Och14,Ore01,Ore02,Ore03,Ore04,Ore05,Ore06,Ore07,Ore08,Ore09,Ore10,Ore11,Ore12,Ore13,Ore14	*OreG0018285*
scaffold33276	378108	C	T	Och09,Och10,Och11	*OreG0018292*
scaffold33276	403638	T	C	Och13	*OreG0018295*
scaffold33276	429946	A	T	Och01,Och02,Och03,Och04,Och05,Och06,Och07,Och08,Och09,Och10,Och11,Och1 2,Och13,Och14,Ore01,Ore02,Ore03,Ore04,Ore05,Ore06,Ore07,Ore08,Ore09,Ore10,Ore11,Ore12,Ore13,Ore14	*OreG0018298*
scaffold33276	430459	T	C	Och09,Och10,Och13,Och14	*OreG0018298*
scaffold33276	447383	A	T	NA	*OreG0018300*
scaffold33276	447953	C	A	Och01,Och02,Och03,Och04,Och05,Och06,Och07,Och08,Och09,Och10,Och11,Och1 2,Och13,Och14,Ore01,Ore02,Ore03,Ore04,Ore05,Ore06,Ore07,Ore08,Ore09,Ore10,Ore11,Ore12,Ore13,Ore14	*OreG0018300*
scaffold33276	448136	A	T	Och08	*OreG0018300*
scaffold33276	458183	A	T	Och01,Och02,Och03,Och04,Och05,Och06,Och07,Och08,Och09,Och10,Och11,Och1 2,Och13,Och14,Ore01,Ore02,Ore03,Ore04,Ore05,Ore06,Ore07,Ore08,Ore09,Ore10,Ore11,Ore12,Ore13,Ore14	*OreG0018301*
scaffold33276	477254	G	A	NA	*OreG0018304*
scaffold33276	477344	G	A	Och01,Och02,Och03,Och04,Och05,Och06,Och07,Och08,Och09,Och10,Och11,Och1 2,Och13,Och14	*OreG0018304*
scaffold33276	483837	C	T	Och08	*OreG0018305*
scaffold33276	516107	C	A	NA	*OreG0018306*
scaffold33276	535336	G	T	Och01,Och02,Och03,Och04,Och05,Och06,Och07,Och08,Och09,Och10,Och11,Och1 2,Och13,Och14	*OreG0018307*
scaffold33276	553781	C	G	Ore01,Ore02,Ore03,Ore04,Ore05,Ore06,Ore07,Ore08,Ore09,Ore10,Ore11,Ore12,Ore 13,Ore14	*OreG0018310*
scaffold33276	625721	C	T	Och05	*OreG0018314*
scaffold33276	681564	G	C	Ore01,Ore02,Ore03,Ore04,Ore05,Ore06,Ore07,Ore08,Ore09,Ore10,Ore11,Ore12,Ore 13,Ore14	*OreG0018318*
scaffold33276	685540	T	C	NA	*OreG0018319*
scaffold33276	685606	A	G	NA	*OreG0018319*
scaffold33276	687198	G	A	NA	*OreG0018319*
scaffold33276	700943	C	G	Och01,Och02,Och03,Och04,Och05,Och06,Och07,Och08,Och09,Och10,Och11,Och1 2,Och13,Och14,Ore01,Ore02,Ore03,Ore04,Ore05,Ore06,Ore07,Ore08,Ore09,Ore10,Ore11,Ore12,Ore13,Ore14	*OreG0018321*
scaffold33276	720497	T	G	Ore01,Ore02,Ore03,Ore04,Ore05,Ore06,Ore07,Ore08,Ore09,Ore10,Ore11,Ore12,Ore 13,Ore14	*OreG0018322*
scaffold33276	729073	A	T	Och09,Och10,Och11	*OreG0018323*
scaffold33276	739937	G	A	Och08	*OreG0018325*
scaffold33276	740120	T	C	NA	*OreG0018325*
scaffold33276	740319	T	C	NA	*OreG0018325*
scaffold33276	740649	C	G	NA	*OreG0018325*
scaffold33276	748720	G	A	NA	*OreG0018327*
scaffold33276	752636	C	A	Ore01,Ore02,Ore03,Ore04,Ore05,Ore06,Ore07,Ore08,Ore09,Ore10,Ore11,Ore12,Ore 13,Ore14	*OreG0018328*

scaffold33276	849819	G	T	NA	*OreG0018332*
scaffold33276	850473	G	A	NA	*OreG0018332*
scaffold33276	852434	G	A	Och13	*OreG0018332*
scaffold33276	872619	C	T	NA	*OreG0018333*
scaffold33276	873244	C	G	Och01,Och02,Och03,Och04,Och05,Och06,Och08,Och09,Och10,Och11,Och12,Och13,Och14	*OreG0018333*
scaffold33276	897008	T	G	Ore06,Ore07,Ore10,Ore13	*OreG0018335*
scaffold33276	927042	G	C	Och13	*OreG0018340*
scaffold33276	930885	A	G	Och09,Och10,Och14	*OreG0018341*
scaffold33276	930894	C	T	NA	*OreG0018341*
scaffold33276	934603	G	A	NA	*OreG0018342*
scaffold33276	934862	T	C	NA	*OreG0018342*
scaffold33276	937378	T	C	Och09,Och10,Och14	*OreG0018342*
scaffold33276	941912	C	T	NA	*OreG0018343*
scaffold33276	976822	C	T	Ore01,Ore02,Ore03,Ore04,Ore05,Ore06,Ore07,Ore08,Ore09,Ore10,Ore11,Ore12,Ore13,Ore14	*OreG0018347*
scaffold33276	979029	G	T	Och01,Och02,Och03,Och04,Och05,Och06,Och07,Och08,Och09,Och10,Och11,Och12,Och13,Och14	*OreG0018347*
scaffold33276	979225	C	T	Ore01,Ore02,Ore03,Ore04,Ore05,Ore06,Ore07,Ore08,Ore09,Ore10,Ore11,Ore12,Ore13,Ore14	*OreG0018347*
scaffold33276	979657	C	T	Ore01,Ore02,Ore03,Ore04,Ore05,Ore06,Ore07,Ore08,Ore09,Ore10,Ore11,Ore12,Ore13,Ore14	*OreG0018347*
scaffold33276	1006021	C	A	NA	*OreG0018348*
scaffold33276	1006629	T	A	NA	*OreG0018348*
scaffold33276	1006851	G	C	NA	*OreG0018348*
scaffold33276	1006998	C	T	Och08	*OreG0018348*
scaffold33276	1058929	T	C	Och08	*OreG0018349*
scaffold33276	1058962	A	G	NA	*OreG0018349*
scaffold33276	1062679	A	T	Och01,Och02,Och03,Och04,Och05,Och06,Och07,Och08,Och09,Och10,Och11,Och12,Och13,Och14,Ore01,Ore02,Ore03,Ore04,Ore05,Ore06,Ore07,Ore08,Ore09,Ore10,Ore11,Ore12,Ore13,Ore14	*OreG0018350*
scaffold33276	1078899	G	T	Och01,Och02,Och03,Och04,Och05,Och06,Och07,Och08,Och09,Och10,Och11,Och12,Och13,Och14	*OreG0018352*
scaffold33276	1083182	C	T	Och01,Och02,Och03,Och04,Och05,Och06,Och07,Och08,Och09,Och10,Och11,Och12,Och13,Och14,Ore01,Ore02,Ore03,Ore04,Ore05,Ore06,Ore07,Ore08,Ore09,Ore10,Ore11,Ore12,Ore13,Ore14	*OreG0018352*
scaffold33276	1088580	G	A	Och02,Och04,Och12,Och13	*OreG0018354*
scaffold33276	1092196	T	C	Ore01,Ore02,Ore03,Ore04,Ore05,Ore06,Ore07,Ore08,Ore09,Ore10,Ore11,Ore12,Ore13,Ore14	*OreG0018355*
scaffold33276	1092316	C	T	NA	*OreG0018355*
scaffold33276	1106550	A	C	NA	*OreG0018358*
scaffold33276	1110498	G	C	Och01,Och02,Och03,Och04,Och05,Och06,Och07,Och08,Och09,Och10,Och11,Och12,Och13,Och14,Ore01,Ore02,Ore03,Ore04,Ore05,Ore06,Ore07,Ore08,Ore09,Ore10,Ore11,Ore12,Ore13,Ore14	*OreG0018359*
scaffold33276	1110918	C	A	Och01,Och02,Och03,Och04,Och05,Och06,Och07,Och08,Och09,Och10,Och11,Och12,Och13,Och14,Ore01,Ore02,Ore03,Ore04,Ore05,Ore06,Ore07,Ore08,Ore09,Ore10,Ore11,Ore12,Ore13,Ore14	*OreG0018359*
scaffold33276	1111140	A	C	Och01,Och02,Och03,Och04,Och05,Och06,Och07,Och08,Och09,Och10,Och11,Och12,Och13,Och14,Ore01,Ore02,Ore03,Ore04,Ore05,Ore06,Ore07,Ore08,Ore09,Ore10,Ore11,Ore12,Ore13,Ore14	*OreG0018359*
scaffold33276	1115834	G	C	NA	*OreG0018360*
scaffold33276	1115902	A	T	Ore01,Ore02,Ore03,Ore04,Ore05,Ore06,Ore07,Ore08,Ore09,Ore10,Ore11,Ore12,Ore13,Ore14	*OreG0018360*
scaffold33276	1117290	T	C	Ore01,Ore02,Ore03,Ore04,Ore05,Ore06,Ore07,Ore08,Ore09,Ore10,Ore11,Ore12,Ore13,Ore14	*OreG0018360*
scaffold33276	1117362	T	C	Ore01,Ore02,Ore03,Ore04,Ore05,Ore06,Ore07,Ore08,Ore09,Ore10,Ore11,Ore12,Ore13,Ore14	*OreG0018360*
scaffold33276	1117731	G	T	NA	*OreG0018360*
scaffold33276	1147396	C	G	Ore01,Ore02,Ore03,Ore04,Ore05,Ore06,Ore07,Ore08,Ore09,Ore10,Ore11,Ore12,Ore13,Ore14	*OreG0018365*
scaffold33276	1153252	C	A	Ore01,Ore02,Ore03,Ore04,Ore05,Ore06,Ore07,Ore08,Ore09,Ore10,Ore11,Ore12,Ore13,Ore14	*OreG0018365*
scaffold33276	1167768	A	T	Ore01,Ore02,Ore03,Ore04,Ore05,Ore06,Ore07,Ore08,Ore09,Ore10,Ore11,Ore12,Ore13,Ore14	*OreG0018368*
scaffold33276	1168114	A	G	Och01,Och02,Och03,Och04,Och05,Och06,Och07,Och08,Och09,Och10,Och11,Och12,Och13,Och14,Ore01,Ore02,Ore03,Ore04,Ore05,Ore06,Ore07,Ore08,Ore09,Ore10,Ore11,Ore12,Ore13,Ore14	*OreG0018368*
scaffold33276	1168538	C	A	Ore01,Ore02,Ore03,Ore04,Ore05,Ore06,Ore07,Ore08,Ore09,Ore10,Ore11,Ore12,Ore13,Ore14	*OreG0018368*
scaffold33276	1173460	G	C	Ore02,Ore03,Ore04,Ore05,Ore08,Ore09,Ore10,Ore12,Ore14	*OreG0018369*
scaffold33196	112167	T	A	NA	*OreG0015161*
scaffold33196	112362	A	T	Och13,Och14,Ore03	*OreG0015161*
scaffold33196	112375	C	T	Och02,Och03,Och06,Och12	*OreG0015161*
scaffold33196	112380	G	T	Och07,Och11	*OreG0015161*
scaffold33196	131639	A	G	NA	*OreG0015161*
scaffold33196	136690	T	A	Ore01,Ore02,Ore04,Ore05,Ore06,Ore07,Ore11,Ore12	*OreG0015162*

scaffold33196	137826	T	A	NA	*OreG0015162*
scaffold33196	137841	A	C	NA	*OreG0015162*
scaffold33196	138815	G	A	Ore03,Ore04,Ore05,Ore08,Ore10,Ore13,Ore14	*OreG0015162*
scaffold33196	139052	T	C	Ore01,Ore02,Ore03,Ore04,Ore05,Ore06,Ore07,Ore08,Ore09,Ore10,Ore11,Ore12,Ore13,Ore14	*OreG0015162*
scaffold33196	162031	A	G	Ore01,Ore02,Ore03,Ore04,Ore05,Ore06,Ore07,Ore08,Ore09,Ore10,Ore11,Ore12,Ore13,Ore14	*OreG0015165*
scaffold33196	162087	A	T	Och01,Och06,Och07,Och14	*OreG0015165*
scaffold33196	162108	G	A	Och05,Och11,Och13	*OreG0015165*
scaffold33196	162264	G	A	Och01,Och02,Och03,Och04,Och05,Och06,Och07,Och08,Och09,Och10,Och11,Och12,Och13,Och14	*OreG0015165*
scaffold33196	162303	C	T	Ore01,Ore02,Ore03,Ore04,Ore05,Ore06,Ore07,Ore08,Ore09,Ore10,Ore11,Ore12,Ore13,Ore14	*OreG0015165*
scaffold33196	162508	T	A	Ore01,Ore02,Ore03,Ore04,Ore05,Ore06,Ore07,Ore08,Ore09,Ore10,Ore11,Ore12,Ore13,Ore14	*OreG0015166*
scaffold33196	162516	C	A	Och05,Och11,Och13	*OreG0015166*
scaffold33196	162539	C	G	Och01,Och06,Och07,Och14	*OreG0015166*
scaffold33196	162559	G	A	Och01,Och06,Och07,Och14	*OreG0015166*
scaffold33196	217516	A	G	NA	*OreG0015174*
scaffold33196	300029	T	A	NA	*OreG0015186*
scaffold33196	305681	C	A	Ore01,Ore02,Ore03,Ore04,Ore05,Ore06,Ore07,Ore08,Ore09,Ore10,Ore11,Ore12,Ore13,Ore14	*OreG0015187*
scaffold33196	306271	G	A	Ore01,Ore02,Ore03,Ore04,Ore05,Ore06,Ore07,Ore08,Ore09,Ore10,Ore11,Ore12,Ore13,Ore14	*OreG0015187*
scaffold33196	350532	A	T	NA	*OreG0015191*
scaffold33196	376282	C	A	NA	*OreG0015193*
scaffold33196	397072	C	G	Och14	*OreG0015195*
scaffold33196	412107	C	G	NA	*OreG0015196*
scaffold33196	436585	T	C	NA	*OreG0015198*
scaffold33196	436738	T	A	NA	*OreG0015198*
scaffold33196	436754	C	A	NA	*OreG0015198*
scaffold33196	436774	C	T	NA	*OreG0015198*
scaffold33196	436816	T	A	NA	*OreG0015198*
scaffold33196	436818	T	C	NA	*OreG0015198*
scaffold33196	436824	A	T	NA	*OreG0015198*
scaffold33196	436834	C	T	NA	*OreG0015198*
scaffold33196	449173	C	T	Ore06	*OreG0015199*
scaffold33196	449539	C	T	Ore03	*OreG0015199*
scaffold33196	456324	C	T	NA	*OreG0015200*
scaffold33196	456887	T	C	Och14	*OreG0015200*
scaffold33196	456905	A	G	Och01,Och02,Och03,Och04,Och05,Och06,Och07,Och08,Och09,Och10,Och11,Och12,Och13,Och14	*OreG0015200*
scaffold33196	456915	A	G	NA	*OreG0015200*
scaffold33196	510258	G	T	Och04,Och06,Och13	*OreG0015204*
scaffold33196	510331	C	T	Och01,Och02,Och03,Och04,Och05,Och06,Och07,Och08,Och09,Och10,Och11,Och12,Och13,Och14,Ore01,Ore02,Ore03,Ore04,Ore05,Ore06,Ore07,Ore08,Ore09,Ore10,Ore11,Ore12,Ore13,Ore14	*OreG0015204*
scaffold33196	510519	G	A	NA	*OreG0015204*
scaffold33196	516056	G	A	NA	*OreG0015205*
scaffold33196	516134	C	A	NA	*OreG0015205*
scaffold33196	541891	G	C	Och01,Och02,Och03,Och04,Och05,Och06,Och07,Och08,Och09,Och10,Och11,Och12,Och13,Och14,Ore01,Ore02,Ore03,Ore04,Ore05,Ore06,Ore07,Ore08,Ore09,Ore10,Ore11,Ore12,Ore13,Ore14	*OreG0015206*
scaffold33196	562234	G	A	Och01,Och02,Och03,Och04,Och05,Och06,Och07,Och08,Och09,Och10,Och11,Och12,Och13,Och14	*OreG0015208*
scaffold33196	562299	A	G	Ore01,Ore02,Ore03,Ore04,Ore05,Ore06,Ore07,Ore08,Ore09,Ore10,Ore11,Ore12,Ore13,Ore14	*OreG0015208*
scaffold33196	589858	A	G	NA	*OreG0015211*
scaffold33196	681758	C	T	Ore04,Ore05	*OreG0015216*
scaffold33196	681764	C	A	Ore06	*OreG0015216*
scaffold33196	681784	G	T	Och11	*OreG0015216*
scaffold33196	693910	A	G	Och01,Och02,Och03,Och04,Och05,Och06,Och07,Och08,Och09,Och10,Och11,Och12,Och13,Och14	*OreG0015218*
scaffold33196	697521	A	G	NA	*OreG0015218*
scaffold33196	711312	C	T	NA	*OreG0015219*
scaffold33196	716869	G	A	Ore01,Ore02,Ore03,Ore04,Ore05,Ore06,Ore07,Ore08,Ore09,Ore10,Ore11,Ore12,Ore13,Ore14	*OreG0015219*
scaffold33196	731485	T	C	NA	*OreG0015220*
scaffold33196	750834	G	A	NA	*OreG0015221*
scaffold33196	755879	T	G	Och06	*OreG0015222*
scaffold33196	763916	C	T	NA	*OreG0015222*
scaffold33196	766300	G	T	Och01,Och02,Och03,Och04,Och05,Och06,Och07,Och08,Och09,Och10,Och11,Och12,Och13,Och14,Ore01,Ore02,Ore03,Ore04,Ore05,Ore06,Ore07,Ore08,Ore09,Ore10,Ore11,Ore12,Ore13,Ore14	*OreG0015222*
scaffold33196	766721	G	A	NA	*OreG0015222*

scaffold33196	768322	C	G	Och02,Och03,Och04,Och05,Och06,Och08,Och09,Och10,Och11,Och12,Och13,Och14	*OreG0015222*
scaffold33196	779534	C	T	NA	*OreG0015224*
scaffold33196	788348	C	T	NA	*OreG0015225*
scaffold33196	806028	G	C	NA	*OreG0015227*
scaffold33196	820412	T	A	Och01,Och13	*OreG0015230*
scaffold33196	821421	T	C	NA	*OreG0015230*
scaffold33196	821741	A	T	NA	*OreG0015230*
scaffold33196	821793	G	T	NA	*OreG0015230*
scaffold33196	827252	C	T	NA	*OreG0015231*
scaffold33196	832403	A	T	Ore01,Ore02,Ore03,Ore04,Ore05,Ore06,Ore07,Ore08,Ore09,Ore10,Ore11,Ore12,Ore13,Ore14	*OreG0015232*
scaffold33196	832713	G	T	Ore01,Ore02,Ore03,Ore04,Ore05,Ore06,Ore07,Ore08,Ore09,Ore10,Ore11,Ore12,Ore13,Ore14	*OreG0015232*
scaffold33196	846566	G	A	Och01,Och02,Och03,Och04,Och05,Och06,Och08,Och09,Och10,Och11,Och12,Och13,Ore01,Ore02,Ore03,Ore04,Ore05,Ore06,Ore07,Ore08,Ore09,Ore10,Ore11,Ore12,Ore13,Ore14	*OreG0015233*
scaffold33196	846579	G	A	NA	*OreG0015233*
scaffold33196	879926	T	A	Ore01,Ore02,Ore03,Ore04,Ore05,Ore06,Ore07,Ore08,Ore09,Ore10,Ore11,Ore12,Ore13,Ore14	*OreG0015238*
scaffold33196	888886	C	T	Och08,Och11	*OreG0015239*
scaffold33196	950127	C	A	Ore04,Ore05	*OreG0015243*
scaffold33196	1068354	G	A	Och01,Och04,Och06,Och07,Och13,Och14	*OreG0015248*
scaffold33196	1108577	G	T	Och01,Och02,Och03,Och04,Och05,Och06,Och07,Och08,Och09,Och10,Och11,Och12,Och13,Och14,Ore01,Ore02,Ore03,Ore04,Ore05,Ore06,Ore07,Ore08,Ore09,Ore10,Ore11,Ore12,Ore13,Ore14	*OreG0015251*
scaffold33196	1164856	C	T	NA	*OreG0015253*
scaffold33196	1164871	G	A	Och01,Och04,Och06,Och07,Och08,Och09,Och10,Och11,Och13,Och14	*OreG0015253*
scaffold33196	1209467	C	G	NA	*OreG0015255*
scaffold33196	1210693	C	T	Och01,Och02,Och03,Och04,Och05,Och06,Och07,Och08,Och09,Och10,Och11,Och12,Och13,Och14,Ore01,Ore02,Ore03,Ore04,Ore05,Ore06,Ore07,Ore08,Ore09,Ore10,Ore11,Ore12,Ore13,Ore14	*OreG0015255*
scaffold33196	1211345	A	T	Och01,Och02,Och03,Och04,Och05,Och06,Och07,Och08,Och09,Och10,Och11,Och12,Och13,Och14,Ore01,Ore02,Ore03,Ore04,Ore05,Ore06,Ore07,Ore08,Ore09,Ore10,Ore11,Ore12,Ore13,Ore14	*OreG0015255*
scaffold33196	1220846	T	C	NA	*OreG0015256*
scaffold33196	1239779	G	A	Ore01,Ore02,Ore03,Ore04,Ore05,Ore06,Ore07,Ore08,Ore09,Ore10,Ore11,Ore12,Ore13,Ore14	*OreG0015257*
scaffold33196	1307782	G	A	NA	*OreG0015262*
scaffold33196	1308764	C	G	NA	*OreG0015262*
scaffold33196	1308786	C	A	NA	*OreG0015262*
scaffold33196	1319657	A	T	Och01,Och02,Och03,Och04,Och05,Och06,Och07,Och08,Och09,Och10,Och11,Och12,Och13,Och14,Ore01,Ore02,Ore03,Ore04,Ore05,Ore06,Ore07,Ore08,Ore09,Ore10,Ore11,Ore12,Ore13,Ore14	*OreG0015264*
scaffold33196	1360736	A	T	Och01,Och02,Och03,Och04,Och05,Och06,Och07,Och08,Och09,Och10,Och11,Och12,Och13,Och14,Ore01,Ore02,Ore03,Ore04,Ore05,Ore06,Ore07,Ore08,Ore09,Ore10,Ore11,Ore12,Ore13,Ore14	*OreG0015265*
scaffold33196	1361646	T	C	Och01,Och02,Och03,Och04,Och05,Och06,Och07,Och08,Och09,Och10,Och11,Och12,Och13,Och14,Ore01,Ore02,Ore03,Ore04,Ore05,Ore06,Ore07,Ore08,Ore09,Ore10,Ore11,Ore12,Ore13,Ore14	*OreG0015265*
scaffold33196	1388683	G	A	NA	*OreG0015268*
scaffold33196	1389006	A	G	NA	*OreG0015268*
scaffold33196	1389112	C	T	NA	*OreG0015268*
scaffold33196	1389204	G	A	NA	*OreG0015268*
scaffold33196	1389220	T	A	NA	*OreG0015268*
scaffold33196	1389319	C	T	NA	*OreG0015268*
scaffold33196	1389331	C	T	NA	*OreG0015268*
scaffold33196	1390476	G	C	NA	*OreG0015268*
scaffold33196	1390725	C	A	NA	*OreG0015268*
scaffold33196	1432863	T	A	Och11	*OreG0015270*
scaffold33222	242955	G	A	NA	*OreG0016767*
scaffold33222	259439	G	C	Och01,Och02,Och03,Och04,Och05,Och06,Och07,Och08,Och09,Och10,Och11,Och12,Och13,Och14,Ore01,Ore02,Ore03,Ore04,Ore05,Ore06,Ore07,Ore08,Ore09,Ore10,Ore11,Ore12,Ore13,Ore14	*OreG0016769*
scaffold33222	259527	A	C	NA	*OreG0016769*
scaffold33222	284651	C	A	NA	*OreG0016770*
scaffold33222	297152	A	C	Och01,Och02,Och03,Och04,Och05,Och06,Och07,Och08,Och09,Och10,Och11,Och12,Och13,Och14,Ore01,Ore02,Ore03,Ore04,Ore05,Ore06,Ore07,Ore08,Ore09,Ore10,Ore11,Ore12,Ore13,Ore14	*OreG0016772*
scaffold33222	385946	A	G	Och01,Och02,Och03,Och04,Och05,Och06,Och07,Och08,Och09,Och10,Och11,Och12,Och13,Och14,Ore01,Ore02,Ore03,Ore04,Ore05,Ore06,Ore07,Ore08,Ore09,Ore10,Ore11,Ore12,Ore13,Ore14	*OreG0016780*
scaffold33222	418296	C	A	Och01,Och02,Och03,Och04,Och05,Och06,Och07,Och08,Och09,Och10,Och11,Och12,Och13,Och14,Ore01,Ore02,Ore03,Ore04,Ore05,Ore06,Ore07,Ore08,Ore09,Ore10,Ore11,Ore12,Ore13,Ore14	*OreG0016782*

scaffold33222	424241	T	A	NA	*OreG0016782*
scaffold33222	500612	G	A	Och01,Och02,Och03,Och04,Och05,Och06,Och07,Och08,Och09,Och10,Och11,Och12,Och13,Och14	*OreG0016785*
scaffold33222	500755	T	C	NA	*OreG0016785*
scaffold33222	545162	T	C	NA	*OreG0016790*
scaffold33222	545755	A	T	Ore02,Ore04,Ore05,Ore06,Ore07,Ore08,Ore09,Ore10,Ore11,Ore12,Ore13,Ore14	*OreG0016790*
scaffold33222	545758	A	G	NA	*OreG0016790*
scaffold33222	546042	G	A	Ore10,Ore14	*OreG0016790*
scaffold33222	546050	G	A	Och01,Och02,Och03,Och04,Och05,Och06,Och07,Och09,Och10,Och12,Och13,Och14	*OreG0016790*
scaffold33222	546081	T	C	Och03,Och04	*OreG0016790*
scaffold33222	546110	A	G	NA	*OreG0016790*
scaffold33222	546162	C	A	NA	*OreG0016790*
scaffold33222	546164	C	T	Och12	*OreG0016790*
scaffold33222	555320	G	T	Och01,Och02,Och03,Och04,Och05,Och06,Och07,Och08,Och09,Och10,Och11,Och12,Och13,Och14,Ore01,Ore02,Ore03,Ore04,Ore05,Ore06,Ore07,Ore08,Ore09,Ore10,Ore11,Ore12,Ore13,Ore14	*OreG0016792*
scaffold33222	570139	G	A	NA	*OreG0016793*
scaffold33222	585532	A	G	Och03,Och12	*OreG0016795*
scaffold33222	585738	G	A	Och01,Och02,Och03,Och04,Och05,Och06,Och07,Och08,Och09,Och10,Och11,Och12,Och13,Och14	*OreG0016795*
scaffold33222	585792	G	A	Och01,Och02,Och03,Och04,Och05,Och06,Och07,Och08,Och09,Och10,Och11,Och12,Och13,Och14	*OreG0016795*
scaffold33222	619502	G	T	NA	*OreG0016797*
scaffold33222	732931	A	T	NA	*OreG0016804*
scaffold33222	745663	A	G	Ore01,Ore02,Ore03,Ore04,Ore05,Ore06,Ore07,Ore08,Ore09,Ore10,Ore11,Ore12,Ore13,Ore14	*OreG0016804*
scaffold33222	762499	C	T	Och01,Och02,Och03,Och04,Och05,Och06,Och07,Och08,Och09,Och10,Och11,Och12,Och13,Och14,Ore01,Ore02,Ore03,Ore04,Ore05,Ore06,Ore07,Ore08,Ore09,Ore10,Ore11,Ore12,Ore13,Ore14	*OreG0016805*
scaffold33222	792494	A	C	Ore01,Ore02,Ore03,Ore04,Ore05,Ore06,Ore07,Ore08,Ore09,Ore10,Ore11,Ore12,Ore13,Ore14	*OreG0016806*
scaffold33222	792506	C	T	Ore01,Ore02,Ore03,Ore04,Ore05,Ore06,Ore07,Ore08,Ore09,Ore10,Ore11,Ore12,Ore13,Ore14	*OreG0016806*
scaffold33222	792650	A	G	Och01	*OreG0016806*
scaffold33222	811313	A	G	NA	*OreG0016807*
scaffold33222	812621	G	T	Ore01,Ore02,Ore03,Ore04,Ore05,Ore06,Ore07,Ore08,Ore09,Ore10,Ore11,Ore12,Ore13,Ore14	*OreG0016807*
scaffold33222	815408	A	T	Och09,Och10,Och11,Och14	*OreG0016807*
scaffold32660	22913	A	T	NA	*OreG0010617*
scaffold32660	23215	G	T	NA	*OreG0010617*
scaffold32660	23516	A	C	NA	*OreG0010617*
scaffold32660	91497	G	T	Ore01,Ore02,Ore03,Ore04,Ore05,Ore06,Ore07,Ore08,Ore09,Ore10,Ore11,Ore12,Ore13,Ore14	*OreG0010622*
scaffold32660	92818	G	C	NA	*OreG0010622*
scaffold32660	211838	C	T	Och01,Och02,Och03,Och04,Och05,Och06,Och07,Och08,Och09,Och10,Och11,Och12,Och13,Och14	*OreG0010628*
scaffold32660	482365	A	T	NA	*OreG0010641*
scaffold32660	482805	C	T	NA	*OreG0010641*
scaffold32660	482853	C	T	NA	*OreG0010641*
scaffold32660	489009	C	T	Ore01,Ore02,Ore03,Ore04,Ore05,Ore06,Ore07,Ore08,Ore09,Ore10,Ore11,Ore12,Ore13,Ore14	*OreG0010642*
scaffold32660	814147	C	T	NA	*OreG0010650*
scaffold32660	815605	G	A	NA	*OreG0010650*
scaffold32660	960114	T	G	Och01,Och02,Och03,Och04,Och05,Och06,Och07,Och08,Och09,Och10,Och11,Och12,Och13,Och14,Ore01,Ore02,Ore03,Ore04,Ore05,Ore06,Ore07,Ore08,Ore09,Ore10,Ore11,Ore12,Ore13,Ore14	*OreG0010657*
scaffold32660	960207	A	G	Ore01,Ore02,Ore03,Ore04,Ore05,Ore06,Ore07,Ore08,Ore09,Ore10,Ore11,Ore12,Ore13,Ore14	*OreG0010657*
scaffold32660	982772	T	G	Och01,Och02,Och03,Och04,Och05,Och06,Och07,Och08,Och09,Och10,Och11,Och12,Och13,Och14,Ore01,Ore02,Ore03,Ore04,Ore05,Ore06,Ore07,Ore08,Ore09,Ore10,Ore11,Ore12,Ore13,Ore14	*OreG0010658*
scaffold32660	983153	T	C	Och01,Och02,Och03,Och04,Och05,Och06,Och07,Och08,Och09,Och10,Och11,Och12,Och13,Och14,Ore01,Ore02,Ore03,Ore04,Ore05,Ore06,Ore07,Ore08,Ore09,Ore10,Ore11,Ore12,Ore13,Ore14	*OreG0010658*
scaffold32660	1047827	G	A	NA	*OreG0010660*
scaffold32660	1047875	G	A	Ore01,Ore02,Ore03,Ore04,Ore05,Ore06,Ore07,Ore08,Ore09,Ore10,Ore11,Ore12,Ore13,Ore14	*OreG0010660*
scaffold32660	1048079	G	A	NA	*OreG0010660*
scaffold32660	1048118	A	G	Ore01,Ore02,Ore03,Ore04,Ore05,Ore06,Ore07,Ore08,Ore09,Ore10,Ore11,Ore12,Ore13,Ore14	*OreG0010660*
scaffold32660	1146125	T	A	NA	*OreG0010662*
scaffold32660	1156032	A	T	Och07,Och08	*OreG0010662*
scaffold32660	1156064	C	T	NA	*OreG0010662*
scaffold32660	1156308	A	G	NA	*OreG0010662*

scaffold32660	1156342	C	A	NA	*OreG0010662*
scaffold32660	1258580	C	T	Och01,Och02,Och03,Och04,Och05,Och06,Och07,Och08,Och09,Och10,Och11,Och12,Och13,Och14	*OreG0010665*
scaffold32660	1258846	G	A	Och01,Och02,Och03,Och04,Och05,Och06,Och07,Och08,Och09,Och10,Och11,Och12,Och13,Och14	*OreG0010665*
scaffold32660	1258859	C	G	NA	*OreG0010665*
scaffold32660	1284237	G	C	Ore06	*OreG0010666*
scaffold32660	1396876	C	T	Ore01,Ore02,Ore03,Ore04,Ore05,Ore06,Ore07,Ore08,Ore09,Ore10,Ore11,Ore12,Ore13,Ore14	*OreG0010671*
scaffold32660	1700997	A	T	NA	*OreG0010677*
scaffold32660	1701826	C	T	Och01,Och02,Och03,Och04,Och05,Och06,Och07,Och08,Och09,Och10,Och11,Och12,Och13,Och14	*OreG0010677*
scaffold32660	1701833	A	T	Ore01,Ore02,Ore03,Ore04,Ore05,Ore06,Ore07,Ore08,Ore09,Ore10,Ore11,Ore12,Ore13,Ore14	*OreG0010677*
scaffold32660	1704766	C	A	NA	*OreG0010677*
scaffold32660	1709695	C	T	Och01,Och02,Och03,Och04,Och05,Och06,Och07,Och08,Och09,Och10,Och11,Och12,Och13,Och14	*OreG0010677*
scaffold32660	1792887	G	C	Och06	*OreG0010681*
scaffold32660	2020518	C	T	Och01,Och07,Och08	*OreG0010687*
scaffold32660	2020531	C	G	Och01	*OreG0010687*
scaffold32660	2020566	G	A	Ore01,Ore02,Ore03,Ore04,Ore05,Ore06,Ore07,Ore08,Ore09,Ore10,Ore11,Ore12,Ore13,Ore14	*OreG0010687*
scaffold32660	2101257	G	A	Ore01,Ore02,Ore03,Ore04,Ore05,Ore06,Ore07,Ore08,Ore09,Ore10,Ore11,Ore12,Ore13,Ore14	*OreG0010691*
scaffold32660	2101603	G	A	Ore01,Ore02,Ore03,Ore04,Ore05,Ore06,Ore07,Ore08,Ore09,Ore10,Ore11,Ore12,Ore13,Ore14	*OreG0010691*
scaffold32660	2259531	C	T	Och01,Och02,Och03,Och04,Och05,Och06,Och07,Och08,Och09,Och10,Och11,Och12,Och13,Och14,Ore01,Ore02,Ore03,Ore04,Ore05,Ore06,Ore07,Ore08,Ore09,Ore10,Ore11,Ore12,Ore13,Ore14	*OreG0010696*
scaffold32660	2259603	C	T	NA	*OreG0010696*
scaffold32660	2269490	G	A	Och06	*OreG0010696*
scaffold32660	2270525	C	T	Och03,Och05,Och09,Och10,Och12,Och13,Och14	*OreG0010696*
scaffold32660	2270528	C	T	NA	*OreG0010696*
scaffold32660	2270737	A	G	Och07,Och08	*OreG0010696*
scaffold32660	2322917	G	A	NA	*OreG0010699*
scaffold32660	2363065	A	T	NA	*OreG0010700*
scaffold32660	2363188	A	T	Och01,Och02,Och03,Och04,Och05,Och06,Och07,Och08,Och09,Och10,Och11,Och12,Och13,Och14,Ore01,Ore02,Ore03,Ore04,Ore05,Ore06,Ore07,Ore08,Ore09,Ore10,Ore11,Ore12,Ore13,Ore14	*OreG0010700*
scaffold32660	2366039	C	A	NA	*OreG0010700*
scaffold32660	2366729	C	T	Och06	*OreG0010700*
scaffold32660	2572367	C	T	NA	*OreG0010706*
scaffold32660	2581434	T	A	NA	*OreG0010707*
scaffold32660	2581653	G	A	NA	*OreG0010707*
scaffold32660	2581674	C	T	NA	*OreG0010707*
scaffold32660	2606764	C	T	NA	*OreG0010712*
scaffold32660	2606828	T	A	NA	*OreG0010712*
scaffold32660	2607259	A	G	NA	*OreG0010712*
scaffold32660	2607299	A	G	NA	*OreG0010712*
scaffold32660	2607494	T	C	NA	*OreG0010712*
scaffold32660	2607662	G	A	NA	*OreG0010712*
scaffold32660	2609676	T	C	NA	*OreG0010713*
scaffold32660	2610028	G	A	NA	*OreG0010714*
scaffold32660	2610164	G	T	NA	*OreG0010714*
scaffold32660	2610974	A	T	NA	*OreG0010715*
scaffold32660	2670563	A	T	NA	*OreG0010717*
scaffold32660	2670611	G	T	Och01,Och02,Och03,Och04,Och05,Och06,Och07,Och08,Och09,Och10,Och11,Och12,Och13,Och14	*OreG0010717*
scaffold32660	2670855	A	G	Och01,Och02,Och03,Och04,Och05,Och06,Och07,Och08,Och09,Och10,Och11,Och12,Och13,Och14	*OreG0010717*
scaffold32660	2675468	G	T	NA	*OreG0010718*
scaffold32660	2675907	G	C	NA	*OreG0010718*
scaffold32660	2675967	A	G	NA	*OreG0010718*
scaffold32660	2676030	C	A	Ore01,Ore03,Ore04,Ore05,Ore09,Ore10,Ore13,Ore14	*OreG0010718*
scaffold32660	2676054	T	C	NA	*OreG0010718*
scaffold32660	2676192	A	G	Och01,Och02,Och03,Och04,Och05,Och06,Och07,Och08,Och09,Och10,Och11,Och12,Och13,Och14,Ore01,Ore02,Ore03,Ore04,Ore05,Ore06,Ore07,Ore08,Ore09,Ore10,Ore11,Ore12,Ore13,Ore14	*OreG0010718*
scaffold32660	2680735	C	T	Och01,Och02,Och03,Och04,Och05,Och06,Och07,Och08,Och09,Och10,Och11,Och12,Och13,Och14	*OreG0010719*
scaffold32660	2680756	T	G	Och01,Och02,Och03,Och04,Och05,Och06,Och07,Och08,Och09,Och10,Och11,Och12,Och13,Och14	*OreG0010719*
scaffold32660	2681070	G	A	Och01,Och02,Och03,Och04,Och05,Och06,Och07,Och08,Och09,Och10,Och11,Och12,Och13,Och14	*OreG0010719*

scaffold32660	2681467	G	A	Och01,Och02,Och03,Och04,Och05,Och06,Och07,Och08,Och09,Och10,Och11,Och12,Och13,Och14	*OreG0010719*
scaffold32660	2698509	T	C	Och01,Och02,Och03,Och04,Och05,Och06,Och07,Och08,Och09,Och10,Och11,Och12,Och13,Och14	*OreG0010721*
scaffold32660	2698659	T	A	Och01,Och02,Och03,Och04,Och05,Och06,Och07,Och08,Och09,Och10,Och11,Och12,Och13,Och14,Ore01,Ore02,Ore03,Ore04,Ore05,Ore06,Ore07,Ore08,Ore09,Ore10,Ore11,Ore12,Ore13,Ore14	*OreG0010721*
scaffold32660	2698719	A	T	Ore01,Ore09	*OreG0010721*
scaffold32660	2698788	T	G	NA	*OreG0010721*
scaffold32660	2698964	G	T	Och13	*OreG0010721*
scaffold32660	2699113	T	G	Och14	*OreG0010721*
scaffold32660	2704568	G	A	Och02,Och03,Och04,Och05,Och06,Och07,Och08,Och09,Och10,Och11,Och12,Och13,Och14	*OreG0010722*
scaffold32660	2821114	T	A	NA	*OreG0010728*
scaffold32660	2822083	G	A	Ore01,Ore02,Ore03,Ore04,Ore05,Ore06,Ore07,Ore08,Ore09,Ore10,Ore11,Ore12,Ore13,Ore14	*OreG0010728*
scaffold32660	2822173	G	A	NA	*OreG0010728*
scaffold32660	2822178	G	A	Och01,Och02,Och03,Och04,Och05,Och06,Och07,Och08,Och09,Och10,Och11,Och12,Och13,Och14,Ore01,Ore02,Ore03,Ore04,Ore05,Ore06,Ore07,Ore08,Ore09,Ore10,Ore11,Ore12,Ore13,Ore14	*OreG0010728*
scaffold32660	2824938	G	A	Ore03,Ore09,Ore10,Ore12,Ore14	*OreG0010728*
scaffold32660	2826004	G	A	Ore03,Ore04,Ore05,Ore09,Ore10,Ore12,Ore14	*OreG0010728*
scaffold32660	2826459	A	G	Och01,Och03,Ore01,Ore02,Ore03,Ore04,Ore05,Ore06,Ore07,Ore08,Ore09,Ore10,Ore11,Ore12,Ore13,Ore14	*OreG0010728*
scaffold32660	2826896	G	A	NA	*OreG0010728*
scaffold32660	2879956	T	A	Ore04,Ore05	*OreG0010735*
scaffold32660	2880484	G	T	NA	*OreG0010735*
scaffold32660	2926235	A	G	Ore01,Ore02,Ore03,Ore04,Ore05,Ore06,Ore07,Ore08,Ore09,Ore10,Ore11,Ore12,Ore13,Ore14	*OreG0010737*
scaffold32660	2931632	G	T	NA	*OreG0010738*
scaffold32660	2941548	T	G	NA	*OreG0010739*
scaffold32660	2965817	C	A	NA	*OreG0010741*
scaffold32660	2969739	T	A	Och03	*OreG0010741*
scaffold32660	2994823	G	A	Ore01,Ore02,Ore03,Ore04,Ore05,Ore06,Ore07,Ore08,Ore09,Ore10,Ore11,Ore12,Ore13,Ore14	*OreG0010745*
scaffold32660	2997632	A	C	Och01,Och02,Och03,Och04,Och05,Och06,Och07,Och08,Och09,Och10,Och11,Och12,Och13,Och14,Ore01,Ore02,Ore03,Ore04,Ore05,Ore06,Ore07,Ore08,Ore09,Ore10,Ore11,Ore12,Ore13,Ore14	*OreG0010746*
scaffold32660	3005969	C	G	Och07,Och08,Och09,Och10,Och11,Och14	*OreG0010747*
scaffold32660	3019593	G	A	NA	*OreG0010749*
scaffold32660	3108185	A	C	Och01,Och02,Och03,Och04,Och05,Och06,Och07,Och08,Och09,Och10,Och11,Och12,Och13,Och14,Ore01,Ore02,Ore03,Ore04,Ore05,Ore06,Ore07,Ore08,Ore09,Ore10,Ore11,Ore12,Ore13,Ore14	*OreG0010755*
scaffold32660	3124452	C	T	NA	*OreG0010757*
scaffold32660	3146612	C	T	NA	*OreG0010758*
scaffold32660	3152605	C	G	NA	*OreG0010759*
scaffold32660	3152751	C	A	NA	*OreG0010759*
scaffold32660	3152762	C	A	Och03,Och13	*OreG0010759*
scaffold32660	3153202	G	A	NA	*OreG0010759*
scaffold32660	3153652	C	T	NA	*OreG0010759*
scaffold32660	3165834	G	C	Och07,Och09,Och10,Och11	*OreG0010760*
scaffold32660	3165940	C	T	Och01,Och02,Och03,Och04,Och05,Och06,Och07,Och08,Och09,Och10,Och11,Och12,Och13,Och14,Ore01,Ore02,Ore03,Ore04,Ore05,Ore06,Ore07,Ore08,Ore09,Ore10,Ore11,Ore12,Ore13,Ore14	*OreG0010760*
scaffold32660	3165981	G	C	Och01,Och02,Och03,Och04,Och05,Och06,Och07,Och08,Och09,Och10,Och11,Och12,Och13,Och14,Ore01,Ore02,Ore03,Ore04,Ore05,Ore06,Ore07,Ore08,Ore09,Ore10,Ore11,Ore12,Ore13,Ore14	*OreG0010760*
scaffold32660	3192952	C	A	NA	*OreG0010762*
scaffold32660	3193180	G	A	Och01,Och02,Och03,Och04,Och05,Och06,Och07,Och08,Och09,Och10,Och11,Och12,Och13,Och14,Ore01,Ore02,Ore03,Ore04,Ore05,Ore06,Ore07,Ore08,Ore09,Ore10,Ore11,Ore12,Ore13,Ore14	*OreG0010762*
scaffold32660	3209497	C	A	Ore01,Ore02,Ore03,Ore04,Ore05,Ore06,Ore07,Ore08,Ore09,Ore10,Ore11,Ore12,Ore13,Ore14	*OreG0010763*
scaffold32660	3209598	G	A	Ore01,Ore02,Ore03,Ore04,Ore05,Ore06,Ore07,Ore08,Ore09,Ore10,Ore11,Ore12,Ore13,Ore14	*OreG0010763*
scaffold32660	3214815	C	T	Och03,Och13	*OreG0010764*
scaffold32660	3214941	C	T	Ore01,Ore02,Ore03,Ore04,Ore05,Ore06,Ore07,Ore08,Ore09,Ore10,Ore11,Ore12,Ore13,Ore14	*OreG0010764*
scaffold32660	3215143	T	C	NA	*OreG0010764*
scaffold32660	3215228	T	A	NA	*OreG0010764*
scaffold32660	3215551	G	A	Och01,Och02,Och03,Och04,Och05,Och06,Och07,Och08,Och09,Och10,Och11,Och12,Och13,Och14	*OreG0010764*
scaffold32660	3219884	A	T	NA	*OreG0010765*
scaffold32660	3220270	G	T	Och03,Och05	*OreG0010765*

scaffold32660	3220320	T	A	Och01,Och02,Och03,Och04,Och05,Och06,Och07,Och08,Och09,Och10,Och11,Och12,Och13,Och14	*OreG0010765*
scaffold32660	3245421	T	G	Ore03,Ore04,Ore05,Ore09,Ore10,Ore12,Ore14	*OreG0010768*
scaffold32660	3297570	C	T	Och12	*OreG0010769*
scaffold32660	3297580	G	A	NA	*OreG0010769*
scaffold32660	3297586	G	A	Och12	*OreG0010769*
scaffold32660	3297952	C	T	Ore01,Ore02,Ore03,Ore05,Ore06,Ore07,Ore08,Ore09,Ore10,Ore11,Ore12,Ore13,Ore14	*OreG0010769*
scaffold32660	3302387	A	T	Och01,Och03,Och11	*OreG0010770*
scaffold32660	3302552	G	A	Och11	*OreG0010770*
scaffold32660	3313539	A	G	NA	*OreG0010771*
scaffold32660	3313782	C	T	Och01,Och03,Och07,Och14,Ore03,Ore04,Ore05,Ore09,Ore10,Ore12,Ore14	*OreG0010771*
scaffold32660	3313792	G	A	NA	*OreG0010771*
scaffold32660	3313852	C	T	Och12	*OreG0010771*
scaffold32660	3313876	C	T	NA	*OreG0010771*
scaffold32660	3313887	G	A	NA	*OreG0010771*
scaffold32660	3314232	T	A	NA	*OreG0010771*
scaffold32660	3314492	A	G	NA	*OreG0010771*
scaffold32660	3314691	G	A	Och03	*OreG0010771*
scaffold32660	3314706	T	A	Och12	*OreG0010771*
scaffold32660	3314742	G	A	Ore03,Ore04,Ore05,Ore09,Ore10,Ore12,Ore14	*OreG0010771*
scaffold32660	3314747	G	A	Och12	*OreG0010771*
scaffold32660	3314756	G	A	Och01,Och03,Och07,Och14	*OreG0010771*
scaffold32660	3314760	G	A	Ore03,Ore04,Ore05,Ore09,Ore10,Ore12,Ore14	*OreG0010771*
scaffold32660	3314775	G	A	Och03	*OreG0010771*
scaffold32660	3314804	G	A	Och03	*OreG0010771*
scaffold32660	3314837	G	A	Och03	*OreG0010771*
scaffold32660	3314847	T	C	Ore03,Ore04,Ore05,Ore09,Ore10,Ore12,Ore14	*OreG0010771*
scaffold32660	3315033	G	C	NA	*OreG0010772*
scaffold32660	3315138	C	T	Ore03,Ore04,Ore05,Ore09,Ore10,Ore12,Ore14	*OreG0010772*
scaffold32660	3315146	C	T	Och01,Och03,Och07,Och14	*OreG0010772*
scaffold32660	3328038	G	A	Ore03,Ore04,Ore05,Ore09,Ore10,Ore12,Ore14	*OreG0010773*
scaffold32660	3328058	G	T	NA	*OreG0010773*
scaffold32660	3328089	C	A	Och01,Och02,Och03,Och04,Och05,Och06,Och07,Och08,Och09,Och10,Och11,Och12,Och13,Och14,Ore01,Ore02,Ore03,Ore04,Ore05,Ore06,Ore07,Ore08,Ore09,Ore10,Ore11,Ore12,Ore13,Ore14	*OreG0010773*
scaffold32660	3332439	G	A	Ore03,Ore04,Ore05,Ore09,Ore10,Ore12,Ore14	*OreG0010774*
scaffold32660	3332468	T	C	NA	*OreG0010774*
scaffold32660	3332529	G	A	NA	*OreG0010774*
scaffold32660	3332576	C	A	NA	*OreG0010774*
scaffold32660	3342105	C	T	Ore01,Ore02,Ore03,Ore04,Ore05,Ore06,Ore07,Ore08,Ore09,Ore10,Ore11,Ore12,Ore13,Ore14	*OreG0010776*
scaffold32660	3364227	A	G	Ore01,Ore02,Ore03,Ore04,Ore05,Ore06,Ore07,Ore08,Ore09,Ore10,Ore11,Ore12,Ore13,Ore14	*OreG0010778*
scaffold32660	3364461	C	A	Och01,Och02,Och03,Och04,Och05,Och06,Och07,Och08,Och09,Och10,Och11,Och12,Och13,Och14	*OreG0010778*
scaffold32660	3364773	G	A	Ore01,Ore02,Ore03,Ore04,Ore05,Ore06,Ore07,Ore08,Ore09,Ore10,Ore11,Ore12,Ore13,Ore14	*OreG0010778*
scaffold32660	3364790	G	A	NA	*OreG0010778*
scaffold32660	3364806	G	A	NA	*OreG0010778*
scaffold32660	3364812	G	A	NA	*OreG0010778*
scaffold32660	3364853	G	A	Och11,Och12	*OreG0010778*
scaffold32660	3364932	G	A	NA	*OreG0010778*
scaffold32660	3367562	C	T	Ore03,Ore09,Ore10,Ore12,Ore14	*OreG0010779*
scaffold32660	3367612	G	T	NA	*OreG0010779*
scaffold32660	3368028	A	G	NA	*OreG0010779*
scaffold32660	3447399	A	G	Ore01,Ore02,Ore03,Ore04,Ore05,Ore06,Ore07,Ore08,Ore09,Ore10,Ore11,Ore12,Ore13,Ore14	*OreG0010786*
scaffold32660	3447531	G	T	Ore01,Ore02,Ore03,Ore04,Ore05,Ore06,Ore07,Ore08,Ore09,Ore10,Ore11,Ore12,Ore13,Ore14	*OreG0010786*
scaffold32660	3451620	A	G	Och02,Och03,Och04,Och05,Och06,Och07,Och09,Och10,Och12,Och13	*OreG0010788*
scaffold32660	3451639	T	A	Och02,Och03,Och04,Och05,Och06,Och07,Och12,Och13	*OreG0010788*
scaffold32660	3460874	A	T	Och02,Och03,Och04,Och05,Och06,Och07,Och12,Och13,Ore01,Ore02,Ore03,Ore04,Ore05,Ore06,Ore07,Ore08,Ore09,Ore10,Ore11,Ore12,Ore13,Ore14	*OreG0010790*
scaffold32660	3465602	A	T	NA	*OreG0010791*
scaffold32660	3469993	C	A	NA	*OreG0010791*
scaffold32660	3472544	G	A	Och03	*OreG0010792*
scaffold32660	3478990	C	A	Och01,Och02,Och03,Och04,Och05,Och06,Och07,Och08,Och09,Och10,Och11,Och12,Och13,Och14	*OreG0010792*
scaffold32660	3573341	T	C	Och11	*OreG0010801*
scaffold32660	3573432	C	A	NA	*OreG0010801*
scaffold32660	3573776	C	T	Ore01,Ore02,Ore03,Ore04,Ore05,Ore06,Ore07,Ore08,Ore09,Ore10,Ore11,Ore12,Ore13,Ore14	*OreG0010801*
scaffold32660	3573868	G	T	Och11	*OreG0010801*
scaffold32660	3573930	G	A	Och11	*OreG0010801*

scaffold32660	3573978	T	A	Och11	*OreG0010801*
scaffold32660	3574181	G	T	Ore03,Ore04,Ore05,Ore09,Ore10,Ore12,Ore14	*OreG0010801*
scaffold32660	3574185	G	T	Ore03,Ore04,Ore05,Ore09,Ore10,Ore12,Ore14	*OreG0010801*
scaffold32660	3574364	T	C	Ore03,Ore09,Ore10,Ore12,Ore14	*OreG0010801*
scaffold32660	3574383	G	C	Och02,Och03,Och04,Och06,Och07,Och08,Och13,Och14	*OreG0010801*
scaffold32660	3574481	G	A	NA	*OreG0010801*
scaffold32660	3574583	A	T	Och11	*OreG0010801*
scaffold32660	3574689	G	A	Och11	*OreG0010801*
scaffold32660	3574691	C	T	Ore01,Ore02,Ore03,Ore04,Ore05,Ore06,Ore07,Ore08,Ore09,Ore10,Ore11,Ore12,Ore13,Ore14	*OreG0010801*
scaffold32660	3580873	G	A	Ore01,Ore02,Ore03,Ore04,Ore05,Ore06,Ore07,Ore08,Ore09,Ore10,Ore11,Ore12,Ore13,Ore14	*OreG0010802*
scaffold32660	3583340	T	A	NA	*OreG0010802*
scaffold32660	3583865	T	A	NA	*OreG0010802*
scaffold32660	3656418	A	G	NA	*OreG0010805*
scaffold32660	3665582	C	T	Och01,Och02,Och03,Och04,Och05,Och06,Och07,Och08,Och09,Och10,Och11,Och12,Och13,Och14,Ore01,Ore02,Ore03,Ore04,Ore05,Ore06,Ore07,Ore08,Ore09,Ore10,Ore11,Ore12,Ore13,Ore14	*OreG0010805*
scaffold32660	3696958	T	C	Och03	*OreG0010806*
scaffold32660	3697003	C	T	Ore01,Ore02,Ore03,Ore04,Ore05,Ore06,Ore07,Ore08,Ore09,Ore10,Ore11,Ore12,Ore13,Ore14	*OreG0010806*
scaffold32660	3730899	G	A	NA	*OreG0010807*
scaffold32660	3730992	C	T	Ore01,Ore02,Ore03,Ore04,Ore05,Ore06,Ore07,Ore08,Ore09,Ore10,Ore11,Ore12,Ore13,Ore14	*OreG0010807*
scaffold32660	3731012	A	G	NA	*OreG0010807*
scaffold32660	3739290	G	A	Och14	*OreG0010807*
scaffold32660	3739344	C	T	Och14	*OreG0010807*
scaffold32660	3739362	G	A	Ore01,Ore02,Ore03,Ore04,Ore05,Ore06,Ore07,Ore08,Ore09,Ore10,Ore11,Ore12,Ore13,Ore14	*OreG0010807*
scaffold32660	3739410	G	A	Och14,Ore01,Ore02,Ore03,Ore04,Ore05,Ore06,Ore07,Ore08,Ore09,Ore10,Ore11,Ore12,Ore13,Ore14	*OreG0010807*
scaffold32660	3739431	G	A	Ore01,Ore02,Ore03,Ore04,Ore05,Ore06,Ore07,Ore08,Ore09,Ore10,Ore11,Ore12,Ore13,Ore14	*OreG0010807*
scaffold32660	3739434	C	T	Och01,Och03,Och05,Och09,Och10,Och11	*OreG0010807*
scaffold32660	3739552	G	A	Ore01,Ore02,Ore03,Ore04,Ore05,Ore06,Ore07,Ore08,Ore09,Ore10,Ore11,Ore12,Ore13,Ore14	*OreG0010808*
scaffold32660	3739740	C	A	Ore01,Ore02,Ore03,Ore04,Ore05,Ore06,Ore07,Ore08,Ore09,Ore10,Ore11,Ore12,Ore13,Ore14	*OreG0010808*
scaffold32660	3742698	C	T	Och14,Ore01,Ore02,Ore03,Ore04,Ore05,Ore06,Ore07,Ore08,Ore09,Ore10,Ore11,Ore12,Ore13,Ore14	*OreG0010809*
scaffold32660	3742709	G	A	NA	*OreG0010809*
scaffold32660	3742758	G	T	Och01,Och03,Och05,Och09,Och10,Och11	*OreG0010809*
scaffold32660	3742820	G	A	Och01,Och03,Och05,Och09,Och10,Och11	*OreG0010809*
scaffold32660	3749636	T	A	Och01,Och02,Och03,Och04,Och05,Och06,Och07,Och08,Och09,Och10,Och11,Och12,Och13,Och14	*OreG0010810*
scaffold32660	3764709	T	A	Och01,Och02,Och03,Och04,Och05,Och06,Och07,Och09,Och10,Och12,Och13,Ore01,Ore02,Ore03,Ore04,Ore05,Ore06,Ore07,Ore08,Ore09,Ore10,Ore11,Ore12,Ore13,Ore14	*OreG0010812*
scaffold32660	3776690	C	T	Och01,Och02,Och03,Och04,Och05,Och06,Och07,Och08,Och09,Och10,Och11,Och12,Och13,Och14,Ore01,Ore02,Ore03,Ore04,Ore05,Ore06,Ore07,Ore08,Ore09,Ore10,Ore11,Ore12,Ore13,Ore14	*OreG0010814*
scaffold32660	3776894	A	T	NA	*OreG0010814*
scaffold32660	3801900	C	T	Ore01,Ore02,Ore03,Ore04,Ore05,Ore06,Ore07,Ore08,Ore09,Ore10,Ore11,Ore12,Ore13,Ore14	*OreG0010815*
scaffold32660	3814612	A	T	Ore03,Ore09,Ore10,Ore12,Ore14	*OreG0010816*
scaffold32660	3830472	G	A	Ore01,Ore02,Ore03,Ore04,Ore05,Ore06,Ore07,Ore08,Ore09,Ore10,Ore11,Ore12,Ore13,Ore14	*OreG0010817*
scaffold32660	3853875	C	T	Och03,Och07	*OreG0010818*
scaffold32660	3854201	G	T	NA	*OreG0010818*
scaffold32660	3955149	G	A	NA	*OreG0010827*
scaffold32660	3978702	C	T	NA	*OreG0010829*
scaffold32660	3988756	G	A	Ore01,Ore02,Ore03,Ore04,Ore05,Ore06,Ore07,Ore08,Ore09,Ore10,Ore11,Ore12,Ore13,Ore14	*OreG0010831*
scaffold32660	3988994	T	G	NA	*OreG0010831*
scaffold32660	3992509	G	T	NA	*OreG0010832*
scaffold32660	3992594	T	C	NA	*OreG0010832*
scaffold32660	3993185	T	C	NA	*OreG0010832*
scaffold32660	3993554	G	T	Och01	*OreG0010832*
scaffold32660	4001475	C	A	Och07	*OreG0010833*
scaffold32660	4002298	A	G	Och07	*OreG0010833*
scaffold32660	4003190	A	G	Och01	*OreG0010834*
scaffold32660	4003194	C	T	Och01,Och02,Och03,Och04,Och05,Och06,Och07,Och08,Och09,Och10,Och11,Och12,Och13,Och14	*OreG0010834*
scaffold32660	4003202	C	G	Och12	*OreG0010834*
scaffold32660	4022430	T	C	Och08	*OreG0010836*

scaffold32660	4029283	A	G	NA	*OreG0010837*
scaffold32660	4032075	C	A	NA	*OreG0010837*
scaffold32660	4032380	T	C	Ore01,Ore02,Ore03,Ore04,Ore05,Ore06,Ore07,Ore08,Ore09,Ore10,Ore11,Ore12,Ore13,Ore14	*OreG0010837*
scaffold32660	4076416	C	T	NA	*OreG0010841*
scaffold32660	4081981	A	T	NA	*OreG0010842*
scaffold32660	4103112	T	C	NA	*OreG0010843*
scaffold32660	4103122	G	C	Ore01,Ore02,Ore03,Ore04,Ore05,Ore06,Ore07,Ore08,Ore09,Ore10,Ore11,Ore12,Ore13,Ore14	*OreG0010843*
scaffold32660	4104067	A	G	Ore01,Ore02,Ore03,Ore04,Ore05,Ore06,Ore07,Ore08,Ore09,Ore10,Ore11,Ore12,Ore13,Ore14	*OreG0010843*
scaffold32660	4104825	C	A	Och05	*OreG0010843*
scaffold32660	4104984	A	C	NA	*OreG0010843*
scaffold32660	4105174	A	G	Och05,Ore01,Ore02,Ore03,Ore04,Ore05,Ore06,Ore07,Ore08,Ore09,Ore10,Ore11,Ore12,Ore13,Ore14	*OreG0010843*
scaffold32660	4105330	G	T	Ore01,Ore02,Ore03,Ore04,Ore05,Ore06,Ore07,Ore08,Ore09,Ore10,Ore11,Ore12,Ore13,Ore14	*OreG0010843*
scaffold32660	4168096	G	A	Och01,Och02,Och03,Och04,Och05,Och06,Och07,Och08,Och09,Och10,Och11,Och12,Och13,Och14,Ore01,Ore02,Ore03,Ore04,Ore05,Ore06,Ore07,Ore08,Ore09,Ore10,Ore11,Ore12,Ore13,Ore14	*OreG0010849*
scaffold32660	4199743	C	A	Ore03,Ore04,Ore05,Ore08,Ore09,Ore10,Ore12,Ore13	*OreG0010851*
scaffold32660	4208696	A	G	Ore01,Ore02,Ore03,Ore04,Ore05,Ore06,Ore07,Ore08,Ore09,Ore10,Ore11,Ore12,Ore13,Ore14	*OreG0010852*
scaffold32660	4208813	C	A	NA	*OreG0010852*
scaffold32660	4208840	C	T	Och01,Och02,Och03,Och04,Och05,Och06,Och07,Och08,Och09,Och10,Och11,Och12,Och13,Och14,Ore01,Ore02,Ore03,Ore04,Ore05,Ore06,Ore07,Ore08,Ore09,Ore10,Ore11,Ore12,Ore13,Ore14	*OreG0010852*
scaffold32660	4208987	C	A	Ore01,Ore02,Ore03,Ore04,Ore05,Ore06,Ore07,Ore08,Ore09,Ore10,Ore11,Ore12,Ore13,Ore14	*OreG0010852*
scaffold32660	4234324	G	T	NA	*OreG0010855*
scaffold32660	4234420	T	A	Och01,Och02,Och03,Och04,Och05,Och06,Och07,Och08,Och09,Och10,Och11,Och12,Och13,Och14	*OreG0010855*
scaffold32660	4235510	G	A	Ore01,Ore02,Ore03,Ore04,Ore05,Ore06,Ore07,Ore08,Ore09,Ore10,Ore11,Ore12,Ore13,Ore14	*OreG0010855*
scaffold32660	4237608	A	G	Ore01,Ore02,Ore03,Ore04,Ore05,Ore06,Ore07,Ore08,Ore09,Ore10,Ore11,Ore12,Ore13,Ore14	*OreG0010855*
scaffold32660	4417053	G	A	NA	*OreG0010873*
scaffold32660	4421084	T	A	Och04	*OreG0010874*
scaffold32660	4445542	C	T	NA	*OreG0010876*
scaffold32660	4448668	C	A	Och02,Och03,Och04,Och05,Och06,Och07,Och08,Och09,Och10,Och11,Och12,Och13,Och14,Ore01,Ore02,Ore03,Ore04,Ore05,Ore06,Ore07,Ore08,Ore09,Ore10,Ore11,Ore12,Ore13,Ore14	*OreG0010876*
scaffold32660	4448680	G	T	NA	*OreG0010876*
scaffold32660	4448750	C	T	NA	*OreG0010876*
scaffold32660	4453819	C	T	NA	*OreG0010877*
scaffold32660	4457497	T	G	Och02,Och03,Och04,Och06,Och12,Och13,Och14	*OreG0010877*
scaffold32660	4459249	G	T	Ore01,Ore02,Ore03,Ore04,Ore05,Ore06,Ore07,Ore08,Ore09,Ore10,Ore11,Ore12,Ore13,Ore14	*OreG0010878*
scaffold32660	4471748	A	T	Och01,Och02,Och03,Och04,Och05,Och06,Och07,Och08,Och09,Och10,Och11,Och12,Och13,Och14,Ore01,Ore02,Ore03,Ore04,Ore05,Ore06,Ore07,Ore08,Ore09,Ore10,Ore11,Ore12,Ore13,Ore14	*OreG0010880*
scaffold32660	4475078	T	A	Och01,Och02,Och03,Och04,Och05,Och06,Och07,Och08,Och09,Och10,Och11,Och12,Och13,Och14,Ore01,Ore02,Ore03,Ore04,Ore05,Ore06,Ore07,Ore08,Ore09,Ore10,Ore11,Ore12,Ore13,Ore14	*OreG0010881*
scaffold32660	4486125	C	T	NA	*OreG0010882*
scaffold32660	4486173	G	A	NA	*OreG0010882*
scaffold32660	4487433	C	T	Och01,Och02,Och03,Och04,Och05,Och06,Och07,Och08,Och09,Och10,Och11,Och12,Och13,Och14	*OreG0010882*
scaffold32660	4490134	T	C	NA	*OreG0010882*
scaffold32660	4491172	C	T	Ore02,Ore03,Ore04,Ore05,Ore06,Ore07,Ore08,Ore09,Ore10,Ore11,Ore12,Ore13,Ore14	*OreG0010882*
scaffold32660	4496180	G	A	NA	*OreG0010883*
scaffold32660	4496973	A	T	Och01,Och02,Och03,Och04,Och05,Och06,Och07,Och12,Och13,Och14	*OreG0010883*
scaffold32660	4512038	A	G	Ore01,Ore02,Ore03,Ore04,Ore05,Ore06,Ore07,Ore08,Ore09,Ore10,Ore11,Ore12,Ore13,Ore14	*OreG0010885*
scaffold32660	4513837	A	C	NA	*OreG0010885*
scaffold32660	4513912	C	G	NA	*OreG0010885*
scaffold32660	4519945	C	T	NA	*OreG0010886*
scaffold32660	4520032	T	A	Och01,Och02,Och03,Och04,Och05,Och06,Och07,Och08,Och09,Och10,Och11,Och12,Och13,Och14,Ore01,Ore02,Ore03,Ore04,Ore05,Ore06,Ore07,Ore08,Ore09,Ore10,Ore11,Ore12,Ore13,Ore14	*OreG0010886*
scaffold32660	4691070	A	T	NA	*OreG0010898*
scaffold32660	4691388	C	A	NA	*OreG0010898*
scaffold32660	4701961	T	G	Ore01,Ore02,Ore03,Ore04,Ore05,Ore06,Ore07,Ore08,Ore09,Ore10,Ore11,Ore12,Ore13,Ore14	*OreG0010899*

scaffold32660	4703921	A	T	Ore02,Ore03,Ore04,Ore05,Ore06,Ore07,Ore08,Ore09,Ore10,Ore11,Ore12,Ore13,Ore14	*OreG0010902*
scaffold32660	4704011	T	C	Ore02,Ore03,Ore04,Ore05,Ore06,Ore07,Ore08,Ore09,Ore10,Ore11,Ore12,Ore13,Ore14	*OreG0010902*
scaffold32660	4704461	T	A	NA	*OreG0010902*
scaffold32660	4704541	T	C	NA	*OreG0010902*
scaffold32660	4705080	A	G	Och01,Och06,Och07,Och09,Och10,Och14	*OreG0010902*
scaffold32660	4732863	C	A	NA	*OreG0010904*
scaffold32660	4781624	G	A	NA	*OreG0010909*
scaffold32660	4781661	T	A	NA	*OreG0010909*
scaffold32660	4782395	G	A	NA	*OreG0010909*
scaffold32660	4782442	G	A	NA	*OreG0010909*
scaffold32660	4784360	C	T	NA	*OreG0010909*
scaffold32660	4807269	G	A	NA	*OreG0010913*
scaffold32660	4807420	T	C	NA	*OreG0010913*
scaffold32660	4807709	G	T	NA	*OreG0010913*
scaffold32660	4807814	C	A	NA	*OreG0010913*
scaffold32660	4807823	T	G	NA	*OreG0010913*
scaffold32660	4810629	G	T	Och01,Och02,Och03,Och04,Och05,Och06,Och07,Och08,Och09,Och10,Och11,Och12,Och13,Och14,Ore01,Ore02,Ore03,Ore04,Ore05,Ore06,Ore07,Ore08,Ore09,Ore10,Ore11,Ore12,Ore13,Ore14	*OreG0010914*
scaffold32660	4810658	A	T	Och01,Och02,Och03,Och04,Och05,Och06,Och07,Och08,Och09,Och10,Och11,Och12,Och13,Och14,Ore01,Ore02,Ore03,Ore04,Ore05,Ore06,Ore07,Ore08,Ore09,Ore10,Ore11,Ore12,Ore13,Ore14	*OreG0010914*
scaffold32660	4811425	T	A	Och01,Och02,Och03,Och04,Och05,Och06,Och07,Och08,Och09,Och10,Och11,Och12,Och13,Och14	*OreG0010914*
scaffold32660	4842871	C	T	NA	*OreG0010917*
scaffold32660	4855379	G	A	Och01,Och02,Och03,Och04,Och05,Och06,Och07,Och08,Och09,Och10,Och11,Och12,Och13,Och14	*OreG0010918*
scaffold32660	4855432	G	A	Och01,Och02,Och03,Och04,Och05,Och06,Och07,Och08,Och09,Och10,Och11,Och12,Och13,Och14,Ore01,Ore02,Ore03,Ore04,Ore05,Ore06,Ore07,Ore08,Ore09,Ore10,Ore11,Ore12,Ore13,Ore14	*OreG0010918*
scaffold32660	4855583	C	A	Och01,Och02,Och03,Och04,Och05,Och06,Och07,Och08,Och09,Och10,Och11,Och12,Och13,Och14,Ore01,Ore02,Ore03,Ore04,Ore05,Ore06,Ore07,Ore08,Ore09,Ore10,Ore11,Ore12,Ore13,Ore14	*OreG0010918*
scaffold32660	4855588	C	T	Ore01,Ore02,Ore03,Ore04,Ore05,Ore06,Ore07,Ore08,Ore09,Ore10,Ore11,Ore12,Ore13,Ore14	*OreG0010918*
scaffold32660	4858749	G	A	Ore01,Ore02,Ore03,Ore04,Ore05,Ore06,Ore07,Ore08,Ore09,Ore10,Ore11,Ore12,Ore13,Ore14	*OreG0010919*
scaffold32660	4858936	A	G	Ore01,Ore02,Ore03,Ore04,Ore05,Ore06,Ore07,Ore08,Ore09,Ore10,Ore11,Ore12,Ore13,Ore14	*OreG0010919*
scaffold32660	4858974	G	A	Och09,Och10,Och11	*OreG0010919*
scaffold32660	4859148	G	T	Och01,Och02,Och03,Och04,Och05,Och06,Och07,Och08,Och09,Och10,Och11,Och12,Och13,Och14	*OreG0010919*
scaffold32660	4859168	T	A	NA	*OreG0010919*
scaffold32660	4862350	G	A	Ore01,Ore02,Ore03,Ore04,Ore05,Ore06,Ore07,Ore08,Ore09,Ore10,Ore11,Ore12,Ore13,Ore14	*OreG0010920*
scaffold32660	4864185	G	A	Och01,Och02,Och03,Och04,Och05,Och06,Och07,Och08,Och09,Och10,Och11,Och12,Och13,Och14	*OreG0010920*
scaffold32660	4864187	G	A	Och01,Och02,Och03,Och04,Och05,Och06,Och07,Och08,Och09,Och10,Och11,Och12,Och13,Och14,Ore01,Ore02,Ore03,Ore04,Ore05,Ore06,Ore07,Ore08,Ore09,Ore10,Ore11,Ore12,Ore13,Ore14	*OreG0010920*
scaffold32660	4864197	C	T	NA	*OreG0010920*
scaffold32660	4889661	A	T	Och01,Och02,Och03,Och04,Och05,Och06,Och07,Och08,Och09,Och10,Och11,Och12,Och13,Och14,Ore01,Ore02,Ore03,Ore04,Ore05,Ore06,Ore07,Ore08,Ore09,Ore10,Ore11,Ore12,Ore13,Ore14	*OreG0010922*
scaffold32660	4890718	T	A	NA	*OreG0010922*
scaffold32660	4895266	G	A	Och01	*OreG0010922*
scaffold32660	4910439	C	T	NA	*OreG0010924*
scaffold32660	4930904	T	C	NA	*OreG0010925*
scaffold32660	5202357	G	A	Ore01,Ore02,Ore03,Ore04,Ore05,Ore06,Ore07,Ore08,Ore09,Ore10,Ore11,Ore12,Ore13,Ore14	*OreG0010928*
scaffold32660	5209705	G	T	Ore01	*OreG0010929*
scaffold32660	5209808	C	T	NA	*OreG0010929*
scaffold32660	5210092	A	T	NA	*OreG0010929*
scaffold32660	5210124	C	A	NA	*OreG0010929*
scaffold32660	5210138	A	T	NA	*OreG0010929*
scaffold32660	5210362	A	C	NA	*OreG0010929*
scaffold32660	5210617	G	A	NA	*OreG0010929*
scaffold32660	5210698	G	C	NA	*OreG0010929*
scaffold32660	5257960	C	G	NA	*OreG0010935*
scaffold32660	5258701	A	G	NA	*OreG0010935*
scaffold32660	5394119	G	A	NA	*OreG0010944*

scaffold32660	5394706	T	G	Och01,Och02,Och03,Och04,Och05,Och06,Och07,Och08,Och09,Och10,Och11,Och12,Och13,Och14,Ore01,Ore02,Ore03,Ore04,Ore05,Ore06,Ore07,Ore08,Ore09,Ore10,Ore11,Ore12,Ore13,Ore14	*OreG0010944*
scaffold32660	5405198	G	C	Och09,Och10	*OreG0010945*
scaffold32660	5405286	C	G	NA	*OreG0010945*
scaffold32660	5432670	A	G	NA	*OreG0010949*
scaffold32660	5437036	A	C	NA	*OreG0010950*
scaffold32660	5437349	C	T	NA	*OreG0010950*
scaffold32660	5446096	A	G	Och01,Och02,Och03,Och04,Och05,Och06,Och07,Och08,Och09,Och10,Och11,Och12,Och13,Och14,Ore01,Ore02,Ore03,Ore04,Ore05,Ore06,Ore07,Ore08,Ore09,Ore10,Ore11,Ore12,Ore13,Ore14	*OreG0010952*
scaffold32660	5446133	A	C	Ore01,Ore02,Ore03,Ore04,Ore05,Ore06,Ore07,Ore08,Ore09,Ore10,Ore11,Ore12,Ore13,Ore14	*OreG0010952*
scaffold32660	5446772	T	G	Ore01,Ore02,Ore03,Ore04,Ore05,Ore06,Ore07,Ore08,Ore09,Ore10,Ore11,Ore12,Ore13,Ore14	*OreG0010952*
scaffold32660	5466673	T	A	Och04	*OreG0010955*
scaffold32660	5469160	T	A	Och01,Och07,Och09,Och10,Och11,Och14	*OreG0010955*
scaffold32660	5546642	A	G	NA	*OreG0010959*
scaffold32660	5546857	A	G	Och09,Och10,Och11,Ore01,Ore02,Ore03,Ore04,Ore05,Ore06,Ore07,Ore08,Ore09,Ore10,Ore11,Ore12,Ore13,Ore14	*OreG0010959*
scaffold32660	5547088	G	C	Och09,Och10,Och11,Ore01,Ore02,Ore03,Ore04,Ore05,Ore06,Ore07,Ore08,Ore09,Ore10,Ore11,Ore12,Ore13,Ore14	*OreG0010959*
scaffold32660	5547595	G	T	Ore01,Ore02,Ore03,Ore04,Ore05,Ore06,Ore07,Ore08,Ore09,Ore10,Ore11,Ore12,Ore13,Ore14	*OreG0010959*
scaffold32660	5717823	A	G	Ore01,Ore02,Ore03,Ore04,Ore05,Ore06,Ore07,Ore08,Ore09,Ore10,Ore11,Ore12,Ore13,Ore14	*OreG0010968*
scaffold32660	5717887	C	A	Och02,Och03,Och05,Och08,Och12,Och13	*OreG0010968*
scaffold32660	5718069	G	A	Och02,Och03,Och04,Och08,Och12,Och13	*OreG0010968*
scaffold32660	5750640	C	T	Och01	*OreG0010970*
scaffold32660	5795644	G	A	Och01,Och02,Och03,Och04,Och05,Och06,Och07,Och08,Och09,Och10,Och11,Och12,Och13,Och14,Ore01,Ore02,Ore03,Ore04,Ore05,Ore06,Ore07,Ore08,Ore09,Ore10,Ore11,Ore12,Ore13,Ore14	*OreG0010972*
scaffold32660	5822169	T	G	NA	*OreG0010975*
scaffold32660	5822508	T	A	Och06,Och07,Och08,Och09,Och10,Och11,Och14,Ore01,Ore02,Ore03,Ore04,Ore05,Ore06,Ore07,Ore08,Ore09,Ore10,Ore11,Ore12,Ore13,Ore14	*OreG0010975*
scaffold32660	5828292	A	G	NA	*OreG0010976*
scaffold32660	5828304	T	A	NA	*OreG0010976*
scaffold32660	5828422	C	A	Ore01,Ore02,Ore03,Ore04,Ore05,Ore06,Ore07,Ore08,Ore09,Ore10,Ore11,Ore12,Ore13,Ore14	*OreG0010976*
scaffold32660	5828643	A	G	Ore01,Ore02,Ore03,Ore04,Ore05,Ore06,Ore07,Ore08,Ore09,Ore10,Ore11,Ore12,Ore13,Ore14	*OreG0010976*
scaffold32660	5841631	C	T	Och01,Och02,Och03,Och04,Och05,Och06,Och07,Och08,Och09,Och10,Och12,Och13	*OreG0010978*
scaffold32660	5841908	T	C	NA	*OreG0010978*
scaffold32660	5848662	C	T	Ore01,Ore02,Ore03,Ore04,Ore05,Ore06,Ore07,Ore08,Ore09,Ore10,Ore11,Ore12,Ore13,Ore14	*OreG0010978*
scaffold32660	5848789	G	A	NA	*OreG0010978*
scaffold32660	5855523	G	A	Ore01,Ore02,Ore03,Ore04,Ore05,Ore06,Ore07,Ore08,Ore09,Ore10,Ore11,Ore12,Ore13,Ore14	*OreG0010979*
scaffold32660	5855532	G	T	Ore03,Ore04,Ore05,Ore09,Ore13	*OreG0010979*
scaffold32660	5913010	G	A	NA	*OreG0010984*
scaffold32660	5913353	G	A	Ore01,Ore02,Ore03,Ore04,Ore05,Ore06,Ore07,Ore08,Ore09,Ore10,Ore11,Ore12,Ore13,Ore14	*OreG0010984*
scaffold32660	5913468	C	T	NA	*OreG0010984*
scaffold32660	5914029	A	T	NA	*OreG0010984*
scaffold32660	5924713	C	T	Och02,Och03,Och12,Och13	*OreG0010986*
scaffold32660	5926309	G	A	NA	*OreG0010986*
scaffold32660	6028079	G	A	Och01,Och02,Och03,Och04,Och05,Och06,Och07,Och08,Och09,Och10,Och11,Och12,Och13,Och14	*OreG0010991*
scaffold32660	6033471	G	C	Ore01,Ore02,Ore03,Ore04,Ore05,Ore06,Ore07,Ore08,Ore09,Ore10,Ore11,Ore12,Ore13,Ore14	*OreG0010992*
scaffold32660	6061089	A	G	NA	*OreG0010994*
scaffold32660	6065674	C	T	NA	*OreG0010995*
scaffold32660	6067097	A	T	NA	*OreG0010995*
scaffold32660	6067717	A	G	Ore01,Ore02,Ore03,Ore04,Ore05,Ore06,Ore07,Ore08,Ore09,Ore10,Ore11,Ore12,Ore13,Ore14	*OreG0010995*
scaffold32660	6067933	A	G	NA	*OreG0010995*
scaffold32660	6233260	G	C	Ore03,Ore04,Ore05,Ore09,Ore13	*OreG0011004*
scaffold32660	6264601	C	T	Och01,Och02,Och03,Och04,Och05,Och06,Och07,Och08,Och09,Och10,Och11,Och12,Och13,Och14	*OreG0011006*
scaffold33248	113360	G	A	NA	*OreG0017539*
scaffold33248	128188	G	C	Och01,Och02,Och03,Och04,Och05,Och06,Och07,Och08,Och09,Och10,Och11,Och12,Och13,Och14,Ore01,Ore02,Ore03,Ore04,Ore05,Ore06,Ore07,Ore08,Ore09,Ore10,Ore11,Ore12,Ore13,Ore14	*OreG0017540*
scaffold33248	202788	G	C	NA	*OreG0017543*
scaffold33248	203539	C	T	NA	*OreG0017543*

scaffold33248	204933	A	T	NA	*OreG0017543*
scaffold33248	209282	G	A	NA	*OreG0017543*
scaffold33248	211687	T	C	NA	*OreG0017543*
scaffold33248	278306	T	A	Ore04,Ore05	*OreG0017551*
scaffold33248	328135	C	T	NA	*OreG0017552*
scaffold33248	330118	G	T	Och08,Och14	*OreG0017552*
scaffold33248	442343	G	A	NA	*OreG0017558*
scaffold33248	459733	G	A	Och01,Och02,Och03,Och04,Och05,Och06,Och07,Och08,Och09,Och10,Och11,Och12,Och13,Och14	*OreG0017559*
scaffold33248	460695	G	A	Ore01,Ore02,Ore03,Ore04,Ore05,Ore06,Ore07,Ore08,Ore09,Ore10,Ore11,Ore12,Ore13,Ore14	*OreG0017559*
scaffold33248	460711	C	G	Och01,Och02,Och03,Och04,Och05,Och06,Och07,Och08,Och11,Och12,Och13	*OreG0017559*
scaffold33248	460741	A	T	Ore01,Ore02,Ore03,Ore04,Ore05,Ore06,Ore07,Ore08,Ore09,Ore10,Ore11,Ore12,Ore13,Ore14	*OreG0017559*
scaffold33248	568681	T	C	NA	*OreG0017562*
scaffold33248	571186	G	A	NA	*OreG0017562*
scaffold33248	663454	G	T	Ore04,Ore05	*OreG0017566*
scaffold33248	907931	G	A	Och01,Och02,Och04,Och05,Och06,Och07,Och08,Och09,Och10,Och12,Och13,Och14	*OreG0017575*
scaffold33248	927847	C	T	Ore01,Ore02,Ore03,Ore04,Ore05,Ore06,Ore07,Ore08,Ore09,Ore10,Ore11,Ore12,Ore13,Ore14	*OreG0017577*
scaffold33248	931347	A	G	NA	*OreG0017578*
scaffold33248	972233	G	A	Ore01,Ore02,Ore03,Ore04,Ore05,Ore06,Ore07,Ore08,Ore09,Ore10,Ore11,Ore12,Ore13,Ore14	*OreG0017579*
scaffold33248	986892	A	G	Och01,Och02,Och03,Och04,Och05,Och06,Och07,Och08,Och09,Och10,Och11,Och12,Och13,Och14	*OreG0017580*
scaffold33248	1033392	C	A	Och01,Och02,Och03,Och04,Och05,Och06,Och07,Och08,Och09,Och10,Och11,Och12,Och13,Och14,Ore01,Ore02,Ore03,Ore04,Ore05,Ore06,Ore07,Ore08,Ore09,Ore10,Ore11,Ore12,Ore13,Ore14	*OreG0017583*
scaffold33248	1033800	T	C	Ore01,Ore02,Ore03,Ore04,Ore05,Ore06,Ore07,Ore08,Ore09,Ore10,Ore11,Ore12,Ore13,Ore14	*OreG0017583*
scaffold33248	1033978	T	C	Och01	*OreG0017583*
scaffold33248	1088353	G	T	NA	*OreG0017586*
scaffold33248	1091869	A	G	NA	*OreG0017586*
scaffold33248	1091987	C	T	Och04,Och06,Och11,Och13	*OreG0017586*
scaffold33248	1092032	G	A	NA	*OreG0017586*
scaffold33248	1103169	G	C	NA	*OreG0017587*
scaffold33248	1103476	G	A	NA	*OreG0017587*
scaffold33248	1106104	C	A	Och04,Och06,Och07,Och09,Och10,Och11,Och13	*OreG0017587*
scaffold33248	1126663	G	C	NA	*OreG0017588*
scaffold33248	1128705	T	G	NA	*OreG0017588*
scaffold33250	55335	A	T	Ore03,Ore04,Ore05,Ore09,Ore13	*OreG0017607*
scaffold33250	148834	C	A	NA	*OreG0017609*
scaffold33250	148926	G	A	Och09,Och10	*OreG0017609*
scaffold33250	149125	G	T	Och01,Och02,Och03,Och04,Och05,Och06,Och07,Och08,Och09,Och10,Och11,Och12,Och13,Och14	*OreG0017609*
scaffold33250	298553	C	A	NA	*OreG0017614*
scaffold33250	465311	T	G	Ore01,Ore02,Ore03,Ore04,Ore05,Ore06,Ore07,Ore08,Ore09,Ore10,Ore11,Ore12,Ore13,Ore14	*OreG0017619*
scaffold33250	468088	A	G	Och09,Och10,Och14	*OreG0017619*
scaffold33250	477337	C	T	Och01,Och02,Och03,Och04,Och05,Och06,Och07,Och08,Och09,Och10,Och11,Och12,Och13,Och14	*OreG0017620*
scaffold33250	477780	C	T	NA	*OreG0017620*
scaffold33250	547334	G	A	NA	*OreG0017621*
scaffold33250	555198	C	T	Och01,Och02,Och03,Och04,Och05,Och06,Och07,Och08,Och09,Och10,Och11,Och12,Och13,Och14,Ore01,Ore02,Ore03,Ore04,Ore05,Ore06,Ore07,Ore08,Ore09,Ore10,Ore11,Ore12,Ore13,Ore14	*OreG0017622*
scaffold33250	555216	T	G	Och09,Och10,Och14	*OreG0017622*
scaffold33250	555266	A	T	Och01,Och02,Och03,Och04,Och05,Och06,Och07,Och08,Och09,Och10,Och11,Och12,Och13,Och14,Ore01,Ore02,Ore03,Ore04,Ore05,Ore06,Ore07,Ore08,Ore09,Ore10,Ore11,Ore12,Ore13,Ore14	*OreG0017622*
scaffold33250	555306	A	G	Ore01,Ore02,Ore03,Ore04,Ore05,Ore06,Ore07,Ore08,Ore09,Ore10,Ore11,Ore12,Ore13,Ore14	*OreG0017622*
scaffold33250	601909	G	A	NA	*OreG0017625*
scaffold33250	785811	A	G	Och01,Och02,Och03,Och04,Och05,Och06,Och07,Och08,Och09,Och10,Och11,Och12,Och13,Och14	*OreG0017630*
scaffold33250	785924	C	T	NA	*OreG0017630*
scaffold33250	787326	T	A	NA	*OreG0017631*
scaffold33250	801278	G	T	NA	*OreG0017632*
scaffold33250	803373	G	A	NA	*OreG0017632*
scaffold33250	803376	T	C	Och01,Och02,Och03,Och04,Och05,Och06,Och07,Och08,Och09,Och10,Och11,Och12,Och13,Och14	*OreG0017632*
scaffold33250	803472	T	C	Och01,Och06	*OreG0017632*
scaffold33250	861252	C	T	Ore03,Ore04,Ore05,Ore09,Ore13	*OreG0017633*
scaffold33250	875515	C	T	NA	*OreG0017634*

scaffold33250	949107	A	C	Och07	*OreG0017636*
scaffold33250	1329755	G	C	Och01,Och02,Och03,Och04,Och05,Och06,Och07,Och08,Och09,Och10,Och11,Och12,Och13,Och14,Ore01,Ore02,Ore03,Ore04,Ore05,Ore06,Ore07,Ore08,Ore09,Ore10,Ore11,Ore12,Ore13,Ore14	*OreG0017641*
scaffold33250	1381323	T	G	NA	*OreG0017644*
scaffold33250	1443040	A	G	NA	*OreG0017645*
scaffold33250	1830357	G	A	Ore01,Ore02,Ore03,Ore04,Ore05,Ore06,Ore07,Ore08,Ore09,Ore10,Ore11,Ore12,Ore13,Ore14	*OreG0017651*
scaffold33250	1840224	G	A	Ore01,Ore02,Ore03,Ore04,Ore05,Ore06,Ore07,Ore08,Ore09,Ore10,Ore11,Ore12,Ore13,Ore14	*OreG0017652*
scaffold33250	1841028	G	A	NA	*OreG0017652*
scaffold33250	1845746	T	G	Och01,Och02,Och03,Och04,Och05,Och06,Och07,Och08,Och09,Och10,Och11,Och12,Och13,Och14,Ore01,Ore02,Ore03,Ore04,Ore05,Ore06,Ore07,Ore08,Ore09,Ore10,Ore11,Ore12,Ore13,Ore14	*OreG0017653*
scaffold33250	1846677	T	A	Och06	*OreG0017653*
scaffold33250	1851568	G	A	Och13,Och14	*OreG0017654*
scaffold33250	1865048	G	T	Och13	*OreG0017656*
scaffold33250	1865100	T	G	NA	*OreG0017656*
scaffold33250	1865481	T	C	Och05,Och09,Och10,Och11,Och13,Och14	*OreG0017656*
scaffold33250	1865996	C	T	Ore01,Ore02,Ore03,Ore04,Ore05,Ore06,Ore07,Ore08,Ore09,Ore10,Ore11,Ore12,Ore13,Ore14	*OreG0017656*
scaffold33250	1866488	G	T	Ore01,Ore02,Ore03,Ore04,Ore05,Ore06,Ore07,Ore08,Ore09,Ore10,Ore11,Ore12,Ore13,Ore14	*OreG0017656*
scaffold33250	1880087	T	C	Ore01,Ore02,Ore03,Ore04,Ore05,Ore06,Ore07,Ore08,Ore09,Ore10,Ore11,Ore12,Ore13,Ore14	*OreG0017658*
scaffold33250	1909084	A	T	NA	*OreG0017662*
scaffold33250	1921970	C	T	NA	*OreG0017664*
scaffold33250	1968452	G	T	NA	*OreG0017668*
scaffold33250	2001103	C	T	Och01,Och02,Och03,Och04,Och05,Och06,Och07,Och08,Och09,Och10,Och11,Och12,Och13,Och14	*OreG0017673*
scaffold33250	2001573	A	C	Och01,Och02,Och03,Och04,Och05,Och06,Och07,Och08,Och09,Och10,Och11,Och12,Och13,Och14,Ore01,Ore02,Ore03,Ore04,Ore05,Ore06,Ore07,Ore08,Ore09,Ore10,Ore11,Ore12,Ore13,Ore14	*OreG0017673*
scaffold33250	2001606	C	A	Och01,Och02,Och03,Och04,Och05,Och06,Och07,Och08,Och09,Och10,Och11,Och12,Och13,Och14,Ore01,Ore02,Ore03,Ore04,Ore05,Ore06,Ore07,Ore08,Ore09,Ore10,Ore11,Ore12,Ore13,Ore14	*OreG0017673*
scaffold33250	2012517	A	G	Och01,Och02,Och03,Och04,Och05,Och06,Och07,Och08,Och09,Och10,Och11,Och12,Och13,Och14,Ore01,Ore02,Ore03,Ore04,Ore05,Ore06,Ore07,Ore08,Ore09,Ore10,Ore11,Ore12,Ore13,Ore14	*OreG0017675*
scaffold33250	2016956	A	G	Och01,Och02,Och03,Och04,Och05,Och06,Och07,Och08,Och09,Och10,Och11,Och12,Och13,Och14	*OreG0017677*
scaffold33250	2026688	A	G	Och01,Och02,Och03,Och04,Och05,Och06,Och07,Och08,Och09,Och10,Och11,Och12,Och13,Och14	*OreG0017678*
scaffold33250	2026795	C	A	Ore10	*OreG0017678*
scaffold33250	2026982	G	C	Ore01,Ore02,Ore03,Ore04,Ore05,Ore06,Ore07,Ore08,Ore09,Ore10,Ore11,Ore12,Ore13,Ore14	*OreG0017678*
scaffold33250	2027198	C	T	Ore01,Ore02,Ore03,Ore04,Ore05,Ore06,Ore07,Ore08,Ore09,Ore10,Ore11,Ore12,Ore13,Ore14	*OreG0017678*
scaffold33250	2027342	G	C	Ore01,Ore02,Ore03,Ore04,Ore05,Ore06,Ore07,Ore08,Ore09,Ore10,Ore11,Ore12,Ore13,Ore14	*OreG0017678*
scaffold33250	2027468	A	C	Och01,Och02,Och03,Och04,Och05,Och06,Och07,Och08,Och09,Och10,Och11,Och12,Och13,Och14	*OreG0017678*
scaffold33250	2027518	G	A	Och01,Och02,Och03,Och04,Och05,Och06,Och07,Och08,Och09,Och10,Och11,Och12,Och13,Och14	*OreG0017678*
scaffold33250	2087617	A	T	Ore01,Ore02,Ore03,Ore04,Ore05,Ore06,Ore07,Ore08,Ore09,Ore10,Ore11,Ore12,Ore13,Ore14	*OreG0017685*
scaffold33250	2097430	C	A	NA	*OreG0017686*
scaffold33250	2100397	A	C	Och07	*OreG0017686*
scaffold33250	2104648	C	T	Och09,Och10	*OreG0017687*
scaffold33250	2120064	G	T	Ore03,Ore04,Ore05,Ore10	*OreG0017689*
scaffold33250	2124835	A	G	NA	*OreG0017690*
scaffold33250	2128872	A	G	Och01,Och02,Och03,Och04,Och05,Och06,Och07,Och08,Och09,Och10,Och11,Och12,Och13,Och14,Ore01,Ore02,Ore03,Ore04,Ore05,Ore06,Ore07,Ore08,Ore09,Ore10,Ore11,Ore12,Ore13,Ore14	*OreG0017691*
scaffold33250	2129299	C	G	NA	*OreG0017691*
scaffold33250	2129591	T	A	Och01,Och02,Och03,Och04,Och05,Och06,Och07,Och08,Och09,Och10,Och11,Och12,Och13,Och14,Ore01,Ore02,Ore03,Ore04,Ore05,Ore06,Ore07,Ore08,Ore09,Ore10,Ore11,Ore12,Ore13,Ore14	*OreG0017691*
scaffold33250	2142063	A	T	Och07,Och08,Och09,Och10	*OreG0017693*
scaffold33250	2143877	G	A	Och01,Och02,Och03,Och04,Och05,Och06,Och07,Och08,Och09,Och10,Och11,Och12,Och13,Och14	*OreG0017693*
scaffold33250	2144252	G	A	NA	*OreG0017693*
scaffold33250	2144321	C	T	Ore04,Ore05	*OreG0017693*
scaffold33250	2144765	G	T	NA	*OreG0017693*
scaffold33250	2150839	C	T	Och08,Och09,Och10,Och11,Och14	*OreG0017694*
scaffold33250	2150944	T	C	NA	*OreG0017694*

scaffold33250	2153895	G	A	Ore01,Ore04,Ore05,Ore08,Ore09,Ore11,Ore12,Ore13,Ore14	*OreG0017694*
scaffold33250	2154353	G	A	Och08,Och09,Och10,Och11,Och14	*OreG0017694*
scaffold33250	2167178	A	G	Och02,Och03,Och04,Och05,Och06,Och07,Och08,Och12,Och13,Och14	*OreG0017697*
scaffold33250	2168530	A	T	Ore01,Ore02,Ore03,Ore04,Ore05,Ore06,Ore07,Ore08,Ore09,Ore10,Ore11,Ore12,Ore13,Ore14	*OreG0017697*
scaffold33250	2168637	T	A	NA	*OreG0017697*
scaffold33250	2169088	C	G	Ore04,Ore05,Ore08,Ore09,Ore12,Ore14	*OreG0017697*
scaffold33250	2198653	T	C	Och09,Och10,Och11	*OreG0017700*
scaffold33250	2248523	T	A	Ore01,Ore02,Ore03,Ore04,Ore05,Ore06,Ore07,Ore08,Ore09,Ore10,Ore11,Ore12,Ore13,Ore14	*OreG0017706*
scaffold33250	2249151	C	A	Och01	*OreG0017706*
scaffold33250	2262825	C	T	Och08	*OreG0017708*
scaffold33250	2263088	G	A	Ore01,Ore02,Ore03,Ore04,Ore05,Ore06,Ore07,Ore08,Ore09,Ore10,Ore11,Ore12,Ore13,Ore14	*OreG0017708*
scaffold33250	2272488	C	T	Och09,Och10,Och14	*OreG0017709*
scaffold33250	2273887	G	A	Och09,Och10,Och14	*OreG0017709*
scaffold33250	2279012	A	G	Och09,Och10,Och14	*OreG0017710*
scaffold33250	2283952	T	C	Och01,Och02,Och03,Och04,Och05,Och06,Och07,Och08,Och09,Och10,Och11,Och12,Och13,Och14	*OreG0017711*
scaffold33250	2312231	G	T	Och08	*OreG0017713*
scaffold33250	2325421	G	T	Och01,Och02,Och03,Och04,Och05,Och06,Och07,Och08,Och09,Och10,Och11,Och12,Och13,Och14,Ore01,Ore02,Ore03,Ore04,Ore05,Ore06,Ore07,Ore08,Ore09,Ore10,Ore11,Ore12,Ore13,Ore14	*OreG0017715*
scaffold33250	2326454	C	T	Och03,Och07,Och08,Och13	*OreG0017715*
scaffold33250	2327866	T	A	NA	*OreG0017715*
scaffold33250	2360300	T	G	NA	*OreG0017719*
scaffold33250	2365097	A	T	Ore01,Ore02,Ore03,Ore04,Ore05,Ore06,Ore07,Ore08,Ore09,Ore10,Ore11,Ore12,Ore13,Ore14	*OreG0017719*
scaffold33250	2365958	C	G	Och01,Och02,Och03,Och04,Och05,Och06,Och07,Och08,Och09,Och10,Och11,Och12,Och13,Och14	*OreG0017719*
scaffold33250	2366074	C	T	NA	*OreG0017719*
scaffold33250	2366230	G	A	Och01,Och02,Och03,Och04,Och05,Och06,Och07,Och08,Och09,Och10,Och11,Och12,Och13,Och14	*OreG0017719*
scaffold33250	2366717	T	A	Och01,Och02,Och03,Och04,Och05,Och06,Och07,Och08,Och09,Och10,Och11,Och12,Och13,Och14	*OreG0017719*
scaffold33250	2367908	C	T	NA	*OreG0017719*
scaffold33250	2381517	G	A	Och01,Och02,Och03,Och04,Och05,Och06,Och07,Och08,Och09,Och10,Och11,Och12,Och13,Och14	*OreG0017720*
scaffold33250	2382837	A	G	NA	*OreG0017720*
scaffold33250	2386756	C	T	NA	*OreG0017720*
scaffold33250	2394379	G	C	Och07	*OreG0017720*
scaffold33250	2449772	A	G	Ore03,Ore04,Ore05,Ore10	*OreG0017726*
scaffold33250	2454442	G	T	Och01,Och02,Och03,Och04,Och05,Och06,Och07,Och08,Och09,Och10,Och11,Och12,Och13,Och14	*OreG0017726*
scaffold33250	2479411	G	A	NA	*OreG0017729*
scaffold33250	2479442	G	A	NA	*OreG0017729*
scaffold33250	2536540	T	G	NA	*OreG0017737*
scaffold33250	2536872	C	T	Ore01,Ore02,Ore03,Ore04,Ore05,Ore06,Ore07,Ore08,Ore09,Ore10,Ore11,Ore12,Ore13,Ore14	*OreG0017737*
scaffold33250	2536894	C	T	Och01,Och05,Och13	*OreG0017737*
scaffold33250	2640023	G	C	NA	*OreG0017748*
scaffold33250	2640568	G	T	NA	*OreG0017748*
scaffold33250	2809870	A	G	NA	*OreG0017764*
scaffold33250	2824388	C	A	NA	*OreG0017765*
scaffold33250	2853716	C	T	Ore01,Ore02,Ore03,Ore04,Ore05,Ore06,Ore07,Ore08,Ore09,Ore10,Ore11,Ore12,Ore13,Ore14	*OreG0017768*
scaffold33250	2853799	G	A	NA	*OreG0017768*
scaffold33250	2877408	A	T	Och01,Och05,Och12,Och13	*OreG0017771*
scaffold33250	2877689	G	T	Och01,Och02,Och03,Och04,Och05,Och06,Och07,Och08,Och09,Och10,Och11,Och12,Och13,Och14,Ore01,Ore02,Ore03,Ore04,Ore05,Ore06,Ore07,Ore08,Ore09,Ore10,Ore11,Ore12,Ore13,Ore14	*OreG0017771*
scaffold33250	3068472	T	A	NA	*OreG0017780*
scaffold33250	3140743	C	T	NA	*OreG0017784*
scaffold33250	3193641	T	C	Ore01,Ore02,Ore03,Ore04,Ore05,Ore06,Ore07,Ore08,Ore09,Ore10,Ore11,Ore12,Ore13,Ore14	*OreG0017786*
scaffold33250	3195496	G	A	Ore01,Ore02,Ore03,Ore04,Ore05,Ore06,Ore07,Ore08,Ore09,Ore10,Ore11,Ore12,Ore13,Ore14	*OreG0017786*
scaffold33250	3214520	G	A	NA	*OreG0017787*
scaffold33250	3234867	A	T	NA	*OreG0017789*
scaffold33250	3234895	C	T	NA	*OreG0017789*
scaffold33250	3234897	G	A	NA	*OreG0017789*
scaffold33250	3235131	A	G	NA	*OreG0017789*
scaffold33250	3235147	C	T	NA	*OreG0017789*
scaffold33250	3336885	G	A	NA	*OreG0017795*
scaffold33250	3368419	T	C	NA	*OreG0017796*

scaffold33250	3368531	G	A	Och01,Och02,Och03,Och04,Och05,Och06,Och07,Och08,Och09,Och10,Och11,Och12,Och13,Och14,Ore01,Ore02,Ore03,Ore04,Ore05,Ore06,Ore07,Ore08,Ore09,Ore10,Ore11,Ore12,Ore13,Ore14	*OreG0017796*
scaffold33250	3369244	G	A	NA	*OreG0017796*
scaffold33250	3371152	C	T	Och01,Och02,Och03,Och04,Och06,Och08,Och09,Och10,Och11,Och12,Och13,Och14	*OreG0017796*
scaffold33250	3460747	T	A	NA	*OreG0017798*
scaffold33250	3460802	C	T	Och09,Och10,Och11,Och14	*OreG0017798*
scaffold33250	3624188	G	A	NA	*OreG0017805*
scaffold33250	3624190	C	T	NA	*OreG0017805*
scaffold33250	3624468	G	A	Och09,Och10,Och11,Och14,Ore01,Ore02,Ore03,Ore04,Ore05,Ore06,Ore07,Ore08,Ore09,Ore10,Ore11,Ore12,Ore13,Ore14	*OreG0017805*
scaffold33250	3838612	C	T	Och01,Och02,Och05,Och12,Och13	*OreG0017816*
scaffold33250	3909287	T	A	NA	*OreG0017820*
scaffold33250	3961158	G	A	Och01,Och02,Och03,Och04,Och05,Och06,Och07,Och08,Och09,Och10,Och11,Och12,Och13,Och14	*OreG0017823*
scaffold33224	79090	C	T	Och01,Och02,Och03,Och04,Och05,Och06,Och07,Och08,Och09,Och10,Och11,Och12,Och13,Och14,Ore01,Ore02,Ore03,Ore04,Ore05,Ore06,Ore07,Ore08,Ore09,Ore10,Ore11,Ore12,Ore13,Ore14	*OreG0016820*
scaffold33224	85587	C	A	NA	*OreG0016820*
scaffold33224	130278	G	A	Och09,Och10	*OreG0016826*
scaffold33224	136281	T	C	NA	*OreG0016827*
scaffold33224	228827	C	T	Och01,Och02,Och03,Och04,Och05,Och06,Och07,Och08,Och09,Och10,Och11,Och12,Och13,Och14,Ore01,Ore02,Ore03,Ore04,Ore05,Ore06,Ore07,Ore08,Ore09,Ore10,Ore11,Ore12,Ore13,Ore14	*OreG0016836*
scaffold33224	228871	T	G	Ore01,Ore02,Ore03,Ore04,Ore05,Ore06,Ore07,Ore08,Ore09,Ore10,Ore11,Ore12,Ore13,Ore14	*OreG0016836*
scaffold33224	230490	T	A	NA	*OreG0016836*
scaffold33224	230668	A	G	Och01,Och02,Och03,Och04,Och05,Och06,Och07,Och08,Och09,Och10,Och11,Och12,Och13,Och14,Ore01,Ore02,Ore03,Ore04,Ore05,Ore06,Ore07,Ore08,Ore09,Ore10,Ore11,Ore12,Ore13,Ore14	*OreG0016836*
scaffold33224	259556	C	A	NA	*OreG0016838*
scaffold33224	310136	A	T	Ore01,Ore02,Ore03,Ore04,Ore05,Ore06,Ore07,Ore08,Ore09,Ore10,Ore11,Ore12,Ore13,Ore14	*OreG0016843*
scaffold33224	315576	C	T	NA	*OreG0016844*
scaffold33224	315594	A	G	NA	*OreG0016844*
scaffold33224	315600	T	A	NA	*OreG0016844*
scaffold33224	317586	G	T	Och02,Och04,Och12,Och13	*OreG0016844*
scaffold33224	317600	G	T	NA	*OreG0016844*
scaffold33224	317754	A	T	Och02,Och04,Och12,Och13	*OreG0016844*
scaffold33224	321782	T	A	NA	*OreG0016845*
scaffold33224	322848	G	A	NA	*OreG0016845*
scaffold33224	322976	C	T	NA	*OreG0016845*
scaffold33224	323268	T	C	Och02,Och03,Och04,Och05,Och06,Och09,Och10,Och11,Och12,Och13,Och14	*OreG0016845*
scaffold33224	324006	C	G	NA	*OreG0016845*
scaffold33224	352265	C	A	Ore01,Ore02,Ore03,Ore04,Ore05,Ore06,Ore07,Ore08,Ore09,Ore10,Ore11,Ore12,Ore13,Ore14	*OreG0016847*
scaffold33224	354965	C	T	NA	*OreG0016848*
scaffold33224	394487	C	A	NA	*OreG0016854*
scaffold33224	394666	A	C	NA	*OreG0016854*
scaffold33224	394693	G	T	Och06	*OreG0016854*
scaffold33224	401226	C	A	NA	*OreG0016855*
scaffold33224	442651	T	G	NA	*OreG0016861*
scaffold33224	455029	T	A	NA	*OreG0016862*
scaffold33224	505458	A	G	NA	*OreG0016866*
scaffold33224	598500	T	C	NA	*OreG0016871*
scaffold33224	653606	T	A	NA	*OreG0016873*
scaffold33224	664641	G	A	Ore01,Ore02,Ore03,Ore04,Ore05,Ore06,Ore07,Ore08,Ore09,Ore10,Ore11,Ore12,Ore13,Ore14	*OreG0016874*
scaffold33224	670445	C	A	Och01,Och02,Och03,Och04,Och05,Och06,Och07,Och08,Och09,Och10,Och11,Och12,Och13,Och14,Ore01,Ore02,Ore03,Ore04,Ore05,Ore06,Ore07,Ore08,Ore09,Ore10,Ore11,Ore12,Ore13,Ore14	*OreG0016877*
scaffold33224	673208	C	A	NA	*OreG0016878*
scaffold33224	695723	A	G	Och01,Och08,Och09,Och10,Och11,Och14	*OreG0016882*
scaffold33224	702157	C	T	Ore01,Ore02,Ore03,Ore04,Ore05,Ore06,Ore07,Ore08,Ore09,Ore10,Ore11,Ore12,Ore13,Ore14	*OreG0016884*
scaffold33224	725042	G	A	Ore04,Ore05	*OreG0016886*
scaffold33224	726237	C	T	NA	*OreG0016886*
scaffold33224	732917	G	A	Och04,Och06	*OreG0016887*
scaffold33224	744211	A	G	Och02,Och03,Och04,Och05,Och06,Och12,Och13	*OreG0016888*
scaffold33224	744515	C	T	Och01	*OreG0016888*
scaffold33224	744548	C	T	Och11,Och14	*OreG0016888*
scaffold33224	744578	T	C	Och01	*OreG0016888*
scaffold33224	752141	A	C	Och11,Och14	*OreG0016889*

scaffold33224	752303	G	A	Ore01,Ore02,Ore03,Ore04,Ore05,Ore06,Ore07,Ore08,Ore09,Ore10,Ore11,Ore12,Ore13,Ore14	*OreG0016889*
scaffold33224	752364	C	T	Och11,Och14	*OreG0016889*
scaffold33224	808179	G	A	Ore01,Ore02,Ore03,Ore04,Ore05,Ore06,Ore07,Ore08,Ore09,Ore10,Ore11,Ore12,Ore13,Ore14	*OreG0016891*
scaffold33224	808182	G	A	Och01,Och02,Och03,Och04,Och05,Och06,Och07,Och08,Och09,Och10,Och11,Och12,Och13,Och14	*OreG0016891*
scaffold33224	818207	G	A	Ore03	*OreG0016893*
scaffold33224	868151	T	A	NA	*OreG0016897*
scaffold33224	895757	G	A	NA	*OreG0016899*
scaffold33224	895848	C	T	NA	*OreG0016899*
scaffold33224	895850	G	A	NA	*OreG0016899*
scaffold33224	896393	G	A	Ore04,Ore05	*OreG0016899*
scaffold33224	896631	G	A	NA	*OreG0016899*
scaffold33224	896684	G	A	NA	*OreG0016899*
scaffold33224	896703	G	A	NA	*OreG0016899*
scaffold33224	926249	A	G	Ore01,Ore02,Ore03,Ore04,Ore05,Ore06,Ore07,Ore08,Ore09,Ore10,Ore11,Ore12,Ore13,Ore14	*OreG0016901*
scaffold33224	940510	A	G	Och01,Och02,Och03,Och04,Och05,Och06,Och07,Och08,Och09,Och10,Och11,Och12,Och13,Och14,Ore01,Ore02,Ore03,Ore04,Ore05,Ore06,Ore07,Ore08,Ore09,Ore10,Ore11,Ore12,Ore13,Ore14	*OreG0016903*
scaffold33224	941226	T	C	Och01,Och02,Och03,Och04,Och05,Och06,Och07,Och08,Och09,Och10,Och11,Och12,Och13,Och14,Ore01,Ore02,Ore03,Ore04,Ore05,Ore06,Ore07,Ore08,Ore09,Ore10,Ore11,Ore12,Ore13,Ore14	*OreG0016903*
scaffold33224	965809	G	A	Ore02,Ore04,Ore05	*OreG0016903*
scaffold33224	989081	C	G	Och14	*OreG0016905*
scaffold33224	989795	T	C	Ore01,Ore02,Ore03,Ore04,Ore05,Ore06,Ore07,Ore08,Ore09,Ore10,Ore11,Ore12,Ore13,Ore14	*OreG0016905*
scaffold33224	990184	C	T	Och11	*OreG0016905*
scaffold33224	994552	T	C	Ore01,Ore02,Ore03,Ore04,Ore05,Ore06,Ore07,Ore08,Ore09,Ore10,Ore11,Ore12,Ore13,Ore14	*OreG0016905*
scaffold33224	999240	G	C	Ore01,Ore02,Ore03,Ore04,Ore05,Ore06,Ore07,Ore08,Ore09,Ore10,Ore11,Ore12,Ore13,Ore14	*OreG0016906*
scaffold33224	999437	C	T	NA	*OreG0016906*
scaffold33224	1161555	C	T	NA	*OreG0016912*
scaffold33224	1174490	A	C	NA	*OreG0016914*
scaffold33224	1180897	T	A	NA	*OreG0016914*
scaffold33224	1229715	C	T	NA	*OreG0016919*
scaffold33224	1237760	A	T	Ore01,Ore02,Ore03,Ore04,Ore05,Ore06,Ore07,Ore08,Ore09,Ore10,Ore11,Ore12,Ore13,Ore14	*OreG0016919*
scaffold33224	1237775	A	G	Och01,Och02,Och03,Och04,Och05,Och06,Och07,Och08,Och09,Och10,Och11,Och12,Och13,Och14,Ore01,Ore02,Ore03,Ore04,Ore05,Ore06,Ore07,Ore08,Ore09,Ore10,Ore11,Ore12,Ore13,Ore14	*OreG0016919*
scaffold33224	1250795	T	A	Och01,Och02,Och03,Och04,Och05,Och06,Och07,Och08,Och09,Och10,Och11,Och12,Och13,Och14	*OreG0016919*
scaffold33224	1307974	G	A	NA	*OreG0016937*
scaffold33224	1358565	G	A	NA	*OreG0016941*
scaffold33224	1388993	A	T	Och01,Och02,Och03,Och04,Och05,Och06,Och07,Och08,Och09,Och10,Och11,Och12,Och13,Och14,Ore01,Ore02,Ore03,Ore04,Ore05,Ore06,Ore07,Ore08,Ore09,Ore10,Ore11,Ore12,Ore13,Ore14	*OreG0016943*
scaffold33224	1396562	C	A	NA	*OreG0016944*
scaffold33224	1398094	C	A	NA	*OreG0016945*
scaffold33224	1400168	T	C	Och01,Och02,Och03,Och04,Och05,Och06,Och07,Och08,Och09,Och10,Och11,Och12,Och13,Och14,Ore01,Ore02,Ore03,Ore04,Ore05,Ore06,Ore07,Ore08,Ore09,Ore10,Ore11,Ore12,Ore13,Ore14	*OreG0016946*
scaffold33224	1400385	G	A	Ore03,Ore04,Ore05,Ore07,Ore08,Ore10,Ore11,Ore12,Ore13,Ore14	*OreG0016947*
scaffold33224	1400707	C	A	NA	*OreG0016947*
scaffold33224	1425726	T	C	NA	*OreG0016948*
scaffold33224	1428422	T	G	NA	*OreG0016949*
scaffold33224	1478596	A	G	Och01,Och02,Och03,Och04,Och05,Och06,Och07,Och08,Och09,Och10,Och11,Och12,Och13,Och14,Ore01,Ore02,Ore03,Ore04,Ore05,Ore06,Ore07,Ore08,Ore09,Ore10,Ore11,Ore12,Ore13,Ore14	*OreG0016951*
scaffold33224	1479037	C	A	Och01,Och02,Och03,Och04,Och05,Och06,Och07,Och08,Och09,Och10,Och11,Och12,Och13,Och14,Ore01,Ore02,Ore03,Ore04,Ore05,Ore06,Ore07,Ore08,Ore09,Ore10,Ore11,Ore12,Ore13,Ore14	*OreG0016951*
scaffold33224	1491854	G	A	Ore03	*OreG0016952*
scaffold33224	1566462	T	G	Ore01,Ore02,Ore03,Ore04,Ore05,Ore06,Ore07,Ore08,Ore09,Ore10,Ore11,Ore12,Ore13,Ore14	*OreG0016957*
scaffold33224	1566901	G	T	NA	*OreG0016957*
scaffold33224	1575698	C	T	NA	*OreG0016958*
scaffold33224	1578236	G	A	Ore01,Ore02,Ore03,Ore04,Ore05,Ore06,Ore07,Ore08,Ore09,Ore10,Ore11,Ore12,Ore13,Ore14	*OreG0016958*
scaffold33224	1655457	C	T	NA	*OreG0016964*
scaffold33224	1737487	C	T	NA	*OreG0016966*
scaffold33224	1769543	G	A	NA	*OreG0016968*

scaffold33224	1773330	T	A	Och01,Och02,Och03,Och04,Och05,Och06,Och07,Och08,Och09,Och10,Och11,Och12,Och13,Och14	*OreG0016968*
scaffold33224	1778050	A	T	Och08,Och11,Och14	*OreG0016969*
scaffold33224	1804056	G	A	Och01,Och02,Och03,Och04,Och05,Och06,Och07,Och08,Och09,Och10,Och11,Och12,Och13,Och14	*OreG0016970*
scaffold33224	1808010	G	A	NA	*OreG0016970*
scaffold33224	1839390	G	T	Och01,Och02,Och03,Och04,Och05,Och06,Och07,Och08,Och09,Och10,Och11,Och12,Och13,Och14	*OreG0016971*
scaffold33224	1839498	G	A	Ore01,Ore02,Ore03,Ore04,Ore05,Ore06,Ore07,Ore08,Ore09,Ore10,Ore11,Ore12,Ore13,Ore14	*OreG0016971*
scaffold33224	1963695	C	T	NA	*OreG0016976*
scaffold33224	1963755	G	A	Ore01,Ore02,Ore03,Ore04,Ore05,Ore06,Ore07,Ore08,Ore09,Ore10,Ore11,Ore12,Ore13,Ore14	*OreG0016976*
scaffold33224	2108285	A	T	Och11	*OreG0016981*
scaffold33224	2108918	T	A	Och01,Och02,Och03,Och04,Och05,Och06,Och07,Och08,Och09,Och10,Och11,Och12,Och13,Och14	*OreG0016981*
scaffold33224	2115643	T	A	Och11	*OreG0016981*
scaffold33224	2122690	A	C	Och11	*OreG0016981*
scaffold33224	2128655	G	A	NA	*OreG0016982*
scaffold33224	2129209	G	C	Och01,Och02,Och03,Och04,Och05,Och06,Och07,Och08,Och09,Och10,Och11,Och12,Och13,Och14	*OreG0016982*
scaffold33224	2129282	G	A	Och01,Och02,Och03,Och04,Och05,Och06,Och07,Och08,Och09,Och10,Och11,Och12,Och13,Och14	*OreG0016982*
scaffold33224	2136940	G	A	NA	*OreG0016983*
scaffold33224	2180639	G	A	NA	*OreG0016986*
scaffold33224	2193573	T	G	NA	*OreG0016988*
scaffold33224	2193627	G	C	Och11	*OreG0016988*
scaffold33224	2195121	C	A	Ore02,Ore04,Ore05,Ore12,Ore13	*OreG0016988*
scaffold33224	2196700	C	T	Och14	*OreG0016988*
scaffold33224	2201848	G	A	Ore02,Ore04,Ore05,Ore12,Ore13	*OreG0016989*
scaffold33224	2205795	T	C	Ore03	*OreG0016989*
scaffold33224	2205824	G	A	NA	*OreG0016989*
scaffold33224	2214537	C	T	Och01,Och02,Och03,Och04,Och05,Och06,Och07,Och08,Och09,Och10,Och11,Och13,Och14	*OreG0016990*
scaffold33224	2214893	G	C	Ore01,Ore02,Ore03,Ore04,Ore05,Ore06,Ore07,Ore08,Ore09,Ore10,Ore11,Ore12,Ore13,Ore14	*OreG0016990*
scaffold33224	2221093	G	T	Och04,Och06	*OreG0016990*
scaffold33224	2226301	C	T	Ore01,Ore02,Ore03,Ore04,Ore05,Ore06,Ore07,Ore08,Ore09,Ore10,Ore11,Ore12,Ore13,Ore14	*OreG0016990*
scaffold33224	2227325	C	T	Ore01,Ore02,Ore03,Ore04,Ore05,Ore06,Ore07,Ore08,Ore09,Ore10,Ore11,Ore12,Ore13,Ore14	*OreG0016991*
scaffold33224	2227422	T	G	Och01,Och02,Och03,Och04,Och05,Och06,Och07,Och08,Och09,Och10,Och11,Och12,Och13,Och14	*OreG0016991*
scaffold33224	2227511	A	T	Ore01,Ore02,Ore03,Ore04,Ore05,Ore06,Ore07,Ore08,Ore09,Ore10,Ore11,Ore12,Ore13,Ore14	*OreG0016991*
scaffold33224	2227521	G	C	Ore01,Ore02,Ore03,Ore04,Ore05,Ore06,Ore07,Ore08,Ore09,Ore10,Ore11,Ore12,Ore13,Ore14	*OreG0016991*
scaffold33224	2227971	C	G	Ore01,Ore02,Ore03,Ore04,Ore05,Ore06,Ore07,Ore08,Ore09,Ore10,Ore11,Ore12,Ore13,Ore14	*OreG0016991*
scaffold33224	2228094	T	A	Ore02,Ore04,Ore05,Ore12,Ore13	*OreG0016991*
scaffold33224	2228228	C	T	Ore01,Ore02,Ore03,Ore04,Ore05,Ore06,Ore07,Ore08,Ore09,Ore10,Ore11,Ore12,Ore13,Ore14	*OreG0016991*
scaffold33224	2228456	T	C	NA	*OreG0016991*
scaffold33224	2243800	C	A	Och01,Och02,Och03,Och04,Och05,Och06,Och07,Och08,Och09,Och10,Och11,Och12,Och13,Och14	*OreG0016992*
scaffold33224	2262463	A	G	NA	*OreG0016995*
scaffold33224	2263588	A	T	Ore01,Ore02,Ore03,Ore04,Ore05,Ore06,Ore07,Ore08,Ore09,Ore10,Ore11,Ore12,Ore13,Ore14	*OreG0016995*
scaffold33224	2273156	G	A	Ore03	*OreG0016997*
scaffold33224	2292442	C	T	Och02,Och03,Och04,Och05,Och06,Och12,Och13	*OreG0016997*
scaffold33224	2370694	G	A	NA	*OreG0017000*
scaffold33224	2398761	T	A	NA	*OreG0017002*
scaffold33224	2399056	C	A	NA	*OreG0017002*
scaffold33224	2403139	T	G	NA	*OreG0017003*
scaffold33224	2403243	T	A	NA	*OreG0017003*
scaffold33224	2403295	G	T	Ore01,Ore02,Ore03,Ore04,Ore05,Ore06,Ore07,Ore08,Ore09,Ore10,Ore11,Ore12,Ore13,Ore14	*OreG0017003*
scaffold33224	2403638	A	G	NA	*OreG0017003*
scaffold33224	2403641	G	A	NA	*OreG0017003*
scaffold33224	2406658	G	A	Och07	*OreG0017005*
scaffold33224	2415692	A	C	NA	*OreG0017006*
scaffold33224	2442928	T	G	Och01,Och02,Och03,Och04,Och05,Och06,Och07,Och08,Och09,Och10,Och11,Och12,Och13,Och14	*OreG0017008*
scaffold33224	2536412	T	A	Och04,Och06	*OreG0017012*

scaffold33224	2536513	A	T	Och01,Och02,Och03,Och04,Och05,Och06,Och07,Och08,Och09,Och10,Och11,Och12,Och13,Och14,Ore01,Ore02,Ore03,Ore04,Ore05,Ore06,Ore07,Ore08,Ore09,Ore10,Ore11,Ore12,Ore13,Ore14	*OreG0017012*
scaffold33224	2552402	G	T	Ore04,Ore05	*OreG0017013*
scaffold33224	2569147	C	A	Och01,Och11,Och14	*OreG0017015*
scaffold33224	2578107	C	A	NA	*OreG0017015*
scaffold33224	2646036	C	T	Ore01,Ore02,Ore03,Ore04,Ore05,Ore06,Ore07,Ore08,Ore09,Ore10,Ore11,Ore12,Ore13,Ore14	*OreG0017016*
scaffold33224	2674680	A	C	Ore04,Ore05	*OreG0017020*
scaffold33224	2675024	G	A	NA	*OreG0017020*
scaffold33224	2734645	A	T	NA	*OreG0017024*
scaffold33224	2734673	T	A	Och02,Och03,Och04,Och05,Och06,Och07,Och11,Och12,Och13	*OreG0017024*
scaffold33224	2764345	C	G	NA	*OreG0017026*
scaffold33224	2764584	G	A	NA	*OreG0017026*
scaffold33224	2764590	C	T	NA	*OreG0017026*
scaffold33224	2765084	T	A	NA	*OreG0017026*
scaffold33224	2765114	T	A	NA	*OreG0017026*
scaffold33224	2765324	T	A	NA	*OreG0017026*
scaffold33224	2765763	G	A	Ore01,Ore02,Ore03,Ore04,Ore05,Ore06,Ore07,Ore08,Ore09,Ore10,Ore11,Ore12,Ore13,Ore14	*OreG0017026*
scaffold33224	2765895	G	A	NA	*OreG0017026*
scaffold33224	2766379	G	A	NA	*OreG0017026*
scaffold33224	2766408	T	G	NA	*OreG0017026*
scaffold33224	2766550	C	G	NA	*OreG0017026*
scaffold33224	2766969	T	A	NA	*OreG0017026*
scaffold33224	2783123	C	A	Och01,Och02,Och03,Och04,Och05,Och06,Och07,Och08,Och09,Och10,Och11,Och12,Och13	*OreG0017027*
scaffold33224	2795314	C	T	NA	*OreG0017029*
scaffold33224	2796569	C	G	Ore01,Ore02,Ore03,Ore04,Ore05,Ore06,Ore07,Ore08,Ore09,Ore10,Ore11,Ore12,Ore13,Ore14	*OreG0017029*
scaffold33224	2904390	A	T	NA	*OreG0017035*
scaffold33224	2904934	G	A	NA	*OreG0017035*
scaffold33224	2922718	C	G	NA	*OreG0017036*
scaffold33224	2934617	G	A	Och01,Och02,Och03,Och04,Och05,Och06,Och07,Och08,Och09,Och10,Och11,Och12,Och13,Och14,Ore01,Ore02,Ore03,Ore04,Ore05,Ore06,Ore07,Ore08,Ore09,Ore10,Ore11,Ore12,Ore13,Ore14	*OreG0017037*
scaffold33224	2951487	C	T	Och14	*OreG0017038*
scaffold33224	2952577	G	T	NA	*OreG0017038*
scaffold33224	2952775	G	T	NA	*OreG0017038*
scaffold33224	3029552	A	G	Ore01,Ore02,Ore03,Ore04,Ore05,Ore06,Ore07,Ore08,Ore09,Ore10,Ore11,Ore12,Ore13,Ore14	*OreG0017041*
scaffold33224	3029716	T	C	Ore01,Ore02,Ore03,Ore04,Ore05,Ore06,Ore07,Ore08,Ore09,Ore10,Ore11,Ore12,Ore13,Ore14	*OreG0017041*
scaffold33224	3032591	G	T	Och08,Och11	*OreG0017041*
scaffold33224	3039754	G	A	NA	*OreG0017042*
scaffold33224	3040332	T	C	Och01,Och02,Och03,Och04,Och05,Och06,Och07,Och08,Och09,Och10,Och11,Och12,Och13,Och14	*OreG0017042*
scaffold33224	3040359	G	A	Och02,Och03,Och04,Och05,Och06,Och12,Och13	*OreG0017042*
scaffold33224	3040494	T	C	Ore02,Ore04,Ore05,Ore12,Ore13	*OreG0017042*
scaffold33224	3041072	T	C	Och07	*OreG0017043*
scaffold33224	3041126	A	T	NA	*OreG0017043*
scaffold33224	3046642	G	T	NA	*OreG0017044*
scaffold33224	3063897	C	A	Ore03	*OreG0017046*
scaffold33224	3144289	T	A	NA	*OreG0017049*
scaffold33224	3144329	T	A	NA	*OreG0017049*
scaffold33224	3173126	G	A	Och01,Och02,Och03,Och04,Och05,Och06,Och07,Och08,Och12,Och13	*OreG0017051*
scaffold33199	43855	G	A	NA	*OreG0015457*
scaffold33199	43867	C	T	Ore03,Ore04,Ore05,Ore09,Ore14	*OreG0015457*
scaffold33199	48712	C	T	NA	*OreG0015458*
scaffold33199	50383	T	C	Ore04,Ore05,Ore09,Ore14	*OreG0015458*
scaffold33199	50652	C	G	NA	*OreG0015458*
scaffold33199	57532	G	T	Och01,Och02,Och03,Och04,Och05,Och06,Och07,Och08,Och09,Och10,Och11,Och12,Och13,Och14,Ore01,Ore02,Ore03,Ore04,Ore05,Ore06,Ore07,Ore08,Ore09,Ore10,Ore11,Ore12,Ore13,Ore14	*OreG0015459*
scaffold33199	67146	C	A	Och14	*OreG0015461*
scaffold33199	67458	C	T	NA	*OreG0015461*
scaffold33199	68262	C	A	NA	*OreG0015461*
scaffold33199	68269	A	G	NA	*OreG0015461*
scaffold33199	68376	C	A	NA	*OreG0015461*
scaffold33199	74163	C	T	Och11	*OreG0015462*
scaffold33199	75673	G	T	Och01,Och02,Och03,Och04,Och05,Och06,Och07,Och08,Och09,Och10,Och11,Och12,Och13,Och14,Ore01,Ore02,Ore03,Ore04,Ore05,Ore06,Ore07,Ore08,Ore09,Ore10,Ore11,Ore12,Ore13,Ore14	*OreG0015462*
scaffold33199	88120	C	A	Ore01,Ore02,Ore03,Ore04,Ore05,Ore06,Ore07,Ore08,Ore09,Ore10,Ore11,Ore12,Ore13,Ore14	*OreG0015464*

scaffold33199	120179	G	A	Och01,Och02,Och03,Och04,Och05,Och06,Och07,Och08,Och09,Och10,Och11,Och12,Och13,Och14	*OreG0015467*
scaffold33199	125625	T	A	Ore09,Ore14	*OreG0015468*
scaffold33199	126036	C	G	Ore01,Ore02,Ore03,Ore04,Ore05,Ore06,Ore07,Ore08,Ore09,Ore10,Ore11,Ore12,Ore13,Ore14	*OreG0015468*
scaffold33199	133510	C	A	Ore03,Ore04,Ore05,Ore09,Ore14	*OreG0015469*
scaffold33199	149739	G	A	Och01,Och02,Och03,Och04,Och05,Och06,Och07,Och08,Och09,Och10,Och11,Och12,Och13,Och14,Ore01,Ore02,Ore03,Ore04,Ore05,Ore06,Ore07,Ore08,Ore09,Ore10,Ore11,Ore12,Ore13,Ore14	*OreG0015470*
scaffold33199	151854	G	C	Och01,Och02,Och03,Och04,Och05,Och06,Och07,Och08,Och09,Och10,Och11,Och12,Och13,Och14,Ore01,Ore02,Ore03,Ore04,Ore05,Ore06,Ore07,Ore08,Ore09,Ore10,Ore11,Ore12,Ore13,Ore14	*OreG0015470*
scaffold33199	156214	C	T	NA	*OreG0015471*
scaffold33199	158378	C	A	NA	*OreG0015472*
scaffold33199	159248	G	A	NA	*OreG0015472*
scaffold33199	160628	A	C	NA	*OreG0015472*
scaffold33199	160675	C	T	NA	*OreG0015472*
scaffold33199	160885	A	C	NA	*OreG0015472*
scaffold33199	165572	C	T	NA	*OreG0015474*
scaffold33199	165588	A	G	NA	*OreG0015474*
scaffold33199	165763	A	T	NA	*OreG0015474*
scaffold33199	165984	G	A	Ore01,Ore02,Ore03,Ore04,Ore05,Ore06,Ore07,Ore08,Ore09,Ore10,Ore11,Ore12,Ore13,Ore14	*OreG0015475*
scaffold33199	166110	C	A	NA	*OreG0015475*
scaffold33199	166116	C	T	NA	*OreG0015475*
scaffold33199	166172	G	A	NA	*OreG0015475*
scaffold33199	166208	G	A	NA	*OreG0015475*
scaffold33199	166527	C	T	Ore01,Ore02,Ore03,Ore04,Ore05,Ore06,Ore07,Ore08,Ore09,Ore10,Ore11,Ore12,Ore13,Ore14	*OreG0015475*
scaffold33199	170981	T	G	Och01,Och02,Och03,Och04,Och05,Och06,Och07,Och08,Och09,Och10,Och11,Och12,Och13,Och14,Ore01,Ore02,Ore03,Ore04,Ore05,Ore06,Ore07,Ore08,Ore09,Ore10,Ore11,Ore12,Ore13,Ore14	*OreG0015477*
scaffold33199	171047	C	T	Och03,Och06,Och07,Och08,Och09,Och10,Och12,Och13	*OreG0015477*
scaffold33199	171049	G	A	Ore01,Ore02,Ore03,Ore04,Ore05,Ore06,Ore07,Ore08,Ore09,Ore10,Ore11,Ore12,Ore13,Ore14	*OreG0015477*
scaffold33199	172237	G	T	Ore01,Ore02,Ore03,Ore04,Ore05,Ore06,Ore07,Ore08,Ore09,Ore10,Ore11,Ore12,Ore13,Ore14	*OreG0015477*
scaffold33199	337504	A	T	Och01,Och02,Och03,Och04,Och05,Och06,Och07,Och08,Och09,Och10,Och11,Och12,Och13,Och14,Ore01,Ore02,Ore03,Ore04,Ore05,Ore06,Ore07,Ore08,Ore09,Ore10,Ore11,Ore12,Ore13,Ore14	*OreG0015493*
scaffold33199	341497	C	T	NA	*OreG0015494*
scaffold33199	371298	T	C	NA	*OreG0015495*
scaffold33199	402760	A	C	NA	*OreG0015496*
scaffold33199	402775	T	C	Och01,Och02,Och03,Och04,Och05,Och06,Och07,Och08,Och09,Och10,Och11,Och12,Och13,Och14,Ore01,Ore02,Ore03,Ore04,Ore05,Ore06,Ore07,Ore08,Ore09,Ore10,Ore11,Ore12,Ore13,Ore14	*OreG0015496*
scaffold33199	402790	G	T	Ore02,Ore06,Ore07,Ore08,Ore09,Ore11,Ore12,Ore13,Ore14	*OreG0015496*
scaffold33199	465481	G	T	Och05,Och12,Och13	*OreG0015505*
scaffold33199	476773	G	A	NA	*OreG0015507*
scaffold33199	476872	G	A	NA	*OreG0015507*
scaffold33199	481148	C	T	Och07,Och08,Och09,Och10,Och11,Och12,Och13	*OreG0015508*
scaffold33199	506879	G	C	Ore01,Ore02,Ore03,Ore04,Ore05,Ore06,Ore07,Ore08,Ore09,Ore10,Ore11,Ore12,Ore13,Ore14	*OreG0015511*
scaffold33199	545962	T	A	NA	*OreG0015518*
scaffold33199	559451	T	G	Och12,Och13	*OreG0015520*
scaffold33199	559760	G	C	Ore01,Ore02,Ore03,Ore04,Ore05,Ore06,Ore07,Ore08,Ore09,Ore10,Ore11,Ore12,Ore13,Ore14	*OreG0015520*
scaffold33199	560502	A	T	NA	*OreG0015520*
scaffold33199	563722	G	T	NA	*OreG0015521*
scaffold33199	563755	A	G	NA	*OreG0015521*
scaffold33199	563989	A	G	NA	*OreG0015521*
scaffold33199	564428	A	G	NA	*OreG0015521*
scaffold33199	587817	G	A	Ore09,Ore14	*OreG0015523*
scaffold33199	592261	T	A	Och12,Och13	*OreG0015524*
scaffold33199	600086	G	A	NA	*OreG0015525*
scaffold33199	601989	A	G	Och02,Och04,Och06	*OreG0015525*
scaffold33199	602586	T	G	NA	*OreG0015525*
scaffold33199	618616	C	T	Och01,Och02,Och03,Och04,Och05,Och06,Och07,Och08,Och09,Och10,Och11,Och12,Och13,Och14,Ore01,Ore02,Ore03,Ore04,Ore05,Ore06,Ore07,Ore08,Ore09,Ore10,Ore11,Ore12,Ore13,Ore14	*OreG0015526*
scaffold33199	622615	A	T	Och01,Och02,Och03,Och04,Och05,Och06,Och07,Och08,Och09,Och10,Och11,Och12,Och13,Och14,Ore01,Ore02,Ore03,Ore04,Ore05,Ore06,Ore07,Ore08,Ore09,Ore10,Ore11,Ore12,Ore13,Ore14	*OreG0015526*
scaffold33199	623898	C	T	Och07	*OreG0015526*

scaffold33199	623977	A	T	Ore01,Ore02,Ore03,Ore04,Ore05,Ore06,Ore07,Ore08,Ore09,Ore10,Ore11,Ore12,Ore13,Ore14	*OreG0015526*
scaffold33199	668477	G	A	Och02,Och03,Och04,Och05,Och06,Och07,Och08,Och09,Och11,Och12,Och13,Och14	*OreG0015532*
scaffold33199	705255	G	A	Ore09,Ore14	*OreG0015538*
scaffold33199	705531	C	G	Ore01,Ore02,Ore03,Ore04,Ore05,Ore06,Ore07,Ore08,Ore09,Ore10,Ore11,Ore12,Ore13,Ore14	*OreG0015538*
scaffold33199	705533	C	T	NA	*OreG0015538*
scaffold33199	705909	T	A	Och05,Och12,Och13	*OreG0015538*
scaffold33199	716292	G	A	NA	*OreG0015540*
scaffold33199	716338	C	T	NA	*OreG0015540*
scaffold33199	716367	T	C	NA	*OreG0015540*
scaffold33199	716903	T	G	NA	*OreG0015540*
scaffold33199	717179	T	C	Och01,Och02,Och03,Och04,Och05,Och06,Och07,Och08,Och09,Och10,Och11,Och12,Och13,Och14	*OreG0015540*
scaffold33199	726534	A	T	Och01,Och02,Och03,Och04,Och05,Och06,Och07,Och08,Och09,Och10,Och11,Och12,Och13,Och14,Ore01,Ore02,Ore03,Ore04,Ore05,Ore06,Ore07,Ore08,Ore09,Ore10,Ore11,Ore12,Ore13,Ore14	*OreG0015542*
scaffold33199	743633	A	T	Och01,Och06	*OreG0015543*
scaffold33199	746126	A	T	Ore03,Ore04,Ore05,Ore09,Ore14	*OreG0015544*
scaffold33199	747766	C	T	Ore03,Ore04,Ore05,Ore09,Ore14	*OreG0015544*
scaffold33199	747811	T	C	Ore03,Ore04,Ore05,Ore09,Ore14	*OreG0015544*
scaffold33199	747859	G	A	Ore01,Ore02,Ore03,Ore04,Ore05,Ore06,Ore07,Ore08,Ore09,Ore10,Ore11,Ore12,Ore13,Ore14	*OreG0015544*
scaffold33199	747957	G	A	Ore03,Ore04,Ore05,Ore09,Ore14	*OreG0015544*
scaffold33199	747961	G	C	Och05,Och08,Och09,Och10,Och11,Och12,Och13	*OreG0015544*
scaffold33199	750458	T	C	Ore01,Ore02,Ore03,Ore04,Ore05,Ore06,Ore07,Ore08,Ore09,Ore10,Ore11,Ore12,Ore13,Ore14	*OreG0015545*
scaffold33199	751917	G	C	Och01,Och02,Och03,Och04,Och05,Och06,Och07,Och08,Och09,Och10,Och11,Och12,Och13,Och14	*OreG0015545*
scaffold33199	752153	C	T	NA	*OreG0015545*
scaffold33199	752327	A	G	NA	*OreG0015545*
scaffold33199	752519	C	T	Och01,Och02,Och03,Och04,Och05,Och06,Och07,Och08,Och09,Och10,Och11,Och12,Och13,Och14	*OreG0015545*
scaffold33199	758383	C	T	NA	*OreG0015546*
scaffold33199	758642	C	G	Ore03,Ore09,Ore14	*OreG0015546*
scaffold33199	758812	G	A	Och01,Och02,Och03,Och04,Och05,Och06,Och07,Och08,Och09,Och10,Och11,Och12,Och13,Och14,Ore01,Ore02,Ore03,Ore04,Ore05,Ore06,Ore07,Ore08,Ore09,Ore10,Ore11,Ore12,Ore13,Ore14	*OreG0015546*
scaffold33199	761806	T	G	Och01,Och02,Och03,Och04,Och05,Och06,Och07,Och08,Och09,Och10,Och11,Och12,Och13,Och14	*OreG0015547*
scaffold33199	761838	G	T	Och01,Och02,Och03,Och04,Och05,Och06,Och07,Och08,Och09,Och10,Och11,Och12,Och13,Och14	*OreG0015547*
scaffold33199	762256	G	A	Och01,Och02,Och03,Och04,Och05,Och06,Och07,Och08,Och09,Och10,Och11,Och12,Och13,Och14	*OreG0015547*
scaffold33199	765191	T	C	Och01,Och02,Och03,Och04,Och05,Och06,Och07,Och08,Och09,Och10,Och11,Och12,Och13,Och14,Ore01,Ore02,Ore03,Ore04,Ore05,Ore06,Ore07,Ore08,Ore09,Ore10,Ore11,Ore12,Ore13,Ore14	*OreG0015548*
scaffold33199	770026	G	T	NA	*OreG0015549*
scaffold33199	770247	G	T	NA	*OreG0015549*
scaffold33199	774801	G	C	Och01,Och02,Och03,Och04,Och05,Och06,Och07,Och08,Och09,Och10,Och11,Och12,Och13,Och14,Ore01,Ore02,Ore03,Ore04,Ore05,Ore06,Ore07,Ore08,Ore09,Ore10,Ore11,Ore12,Ore13,Ore14	*OreG0015550*
scaffold33199	774856	A	T	Och01,Och02,Och03,Och04,Och05,Och06,Och07,Och08,Och09,Och10,Och11,Och12,Och13,Och14,Ore01,Ore02,Ore03,Ore04,Ore05,Ore06,Ore07,Ore08,Ore09,Ore10,Ore11,Ore12,Ore13,Ore14	*OreG0015550*
scaffold33199	776330	C	G	Ore04,Ore05,Ore09,Ore14	*OreG0015550*
scaffold33199	776449	G	A	NA	*OreG0015550*
scaffold33199	799099	A	G	NA	*OreG0015553*
scaffold33199	810125	G	A	NA	*OreG0015557*
scaffold33199	845348	C	A	Och01	*OreG0015560*
scaffold33199	846448	T	G	NA	*OreG0015560*
scaffold33199	886504	G	T	NA	*OreG0015565*
scaffold33199	889198	A	T	Ore01,Ore10	*OreG0015566*
scaffold33199	890271	T	G	Och01,Och02,Och03,Och04,Och05,Och06,Och07,Och08,Och09,Och10,Och11,Och12,Och13,Och14,Ore01,Ore02,Ore03,Ore04,Ore05,Ore06,Ore07,Ore08,Ore09,Ore10,Ore11,Ore12,Ore13,Ore14	*OreG0015566*
scaffold33199	894472	G	T	Och01,Och02,Och03,Och04,Och05,Och06,Och07,Och08,Och09,Och10,Och11,Och12,Och13,Och14	*OreG0015567*
scaffold33199	900772	T	C	NA	*OreG0015568*
scaffold33199	901630	T	C	NA	*OreG0015568*
scaffold33199	902057	C	T	NA	*OreG0015568*
scaffold33199	943442	A	T	NA	*OreG0015574*
scaffold33199	943901	C	T	Ore03,Ore09,Ore14	*OreG0015574*
scaffold33199	949011	G	A	NA	*OreG0015575*

scaffold33199	949026	T	C	NA	*OreG0015575*
scaffold33199	949367	G	T	NA	*OreG0015575*
scaffold33199	949424	G	T	NA	*OreG0015575*
scaffold33199	949435	C	G	NA	*OreG0015575*
scaffold33199	949450	G	A	NA	*OreG0015575*
scaffold33199	949925	C	G	NA	*OreG0015575*
scaffold33199	949982	T	A	NA	*OreG0015575*
scaffold33199	959042	T	A	Och01,Och02,Och04,Och06,Och07,Och08,Och09,Och10,Och11,Och14,Ore01,Ore02,Ore03,Ore04,Ore05,Ore06,Ore07,Ore08,Ore09,Ore10,Ore11,Ore12,Ore13,Ore14	*OreG0015576*
scaffold33199	1025597	G	A	Och01,Och02,Och03,Och04,Och05,Och06,Och07,Och08,Och09,Och10,Och11,Och12,Och13,Och14	*OreG0015584*
scaffold33199	1025766	G	A	Och01,Och02,Och03,Och04,Och05,Och06,Och07,Och08,Och09,Och10,Och11,Och12,Och13,Och14	*OreG0015584*
scaffold33199	1037213	A	G	Ore01,Ore02,Ore03,Ore04,Ore05,Ore06,Ore07,Ore08,Ore09,Ore10,Ore11,Ore12,Ore13,Ore14	*OreG0015586*
scaffold33199	1037229	C	A	NA	*OreG0015586*
scaffold33199	1041313	G	C	Och11	*OreG0015586*
scaffold33199	1041914	T	G	Ore03,Ore09,Ore14	*OreG0015586*
scaffold33199	1043246	C	A	Och01,Och06,Och07,Och08,Och09,Och10,Och11,Och14	*OreG0015586*
scaffold33199	1062707	C	A	Ore01,Ore02,Ore03,Ore04,Ore05,Ore06,Ore07,Ore08,Ore09,Ore10,Ore11,Ore12,Ore13,Ore14	*OreG0015587*
scaffold33199	1075672	T	A	Och01,Och02,Och03,Och04,Och05,Och06,Och07,Och08,Och09,Och10,Och11,Och12,Och13,Och14	*OreG0015588*
scaffold33199	1086778	A	G	Och03,Och06,Och12,Och13	*OreG0015590*
scaffold33199	1092260	G	A	NA	*OreG0015590*
scaffold33199	1092550	G	C	Ore03,Ore09,Ore14	*OreG0015590*
scaffold33199	1092607	T	C	NA	*OreG0015590*
scaffold33199	1093139	A	T	Ore01,Ore02,Ore03,Ore06,Ore07,Ore08,Ore09,Ore10,Ore11,Ore12,Ore13,Ore14	*OreG0015590*
scaffold33199	1105392	G	C	Ore01,Ore02,Ore03,Ore04,Ore05,Ore06,Ore07,Ore08,Ore09,Ore10,Ore11,Ore12,Ore13,Ore14	*OreG0015592*
scaffold33199	1105399	C	A	Och06	*OreG0015592*
scaffold33199	1111808	G	T	Och06	*OreG0015593*
scaffold33199	1113214	A	G	Och01,Och02,Och03,Och04,Och05,Och06,Och07,Och08,Och09,Och10,Och11,Och12,Och13	*OreG0015593*
scaffold33199	1123270	A	G	NA	*OreG0015594*
scaffold33199	1150802	T	G	NA	*OreG0015596*
scaffold33199	1150957	T	C	Och01,Och02,Och03,Och04,Och05,Och06,Och07,Och08,Och09,Och10,Och11,Och12,Och13,Och14,Ore01,Ore02,Ore03,Ore04,Ore05,Ore06,Ore07,Ore08,Ore09,Ore10,Ore11,Ore12,Ore13,Ore14	*OreG0015596*
scaffold33199	1170443	G	C	Och07,Och09,Och10	*OreG0015600*
scaffold33199	1171222	T	G	Och06	*OreG0015600*
scaffold33199	1183515	T	A	NA	*OreG0015602*
scaffold33199	1183534	G	A	NA	*OreG0015602*
scaffold33199	1183855	G	A	Och12,Och13	*OreG0015602*
scaffold33199	1205883	A	T	Ore01,Ore02,Ore03,Ore04,Ore05,Ore06,Ore07,Ore08,Ore09,Ore10,Ore11,Ore12,Ore13,Ore14	*OreG0015605*
scaffold33199	1205952	T	C	Och01,Och02,Och03,Och04,Och05,Och06,Och07,Och08,Och09,Och10,Och11,Och12,Och13,Och14	*OreG0015605*
scaffold33199	1206011	C	G	NA	*OreG0015605*
scaffold33199	1206528	A	G	Och01,Och02,Och03,Och04,Och05,Och06,Och07,Och08,Och09,Och10,Och11,Och12,Och13,Och14,Ore01,Ore02,Ore03,Ore04,Ore05,Ore06,Ore07,Ore08,Ore09,Ore10,Ore11,Ore12,Ore13,Ore14	*OreG0015605*
scaffold33199	1207798	T	G	Och01,Och02,Och03,Och04,Och05,Och06,Och07,Och08,Och09,Och10,Och11,Och12,Och13,Och14,Ore01,Ore02,Ore03,Ore04,Ore05,Ore06,Ore07,Ore08,Ore09,Ore10,Ore11,Ore12,Ore13,Ore14	*OreG0015606*
scaffold33199	1208231	A	G	NA	*OreG0015606*
scaffold33199	1213080	C	A	Och01,Och02,Och03,Och04,Och05,Och06,Och07,Och08,Och09,Och10,Och11,Och12,Och13,Och14,Ore01,Ore02,Ore03,Ore04,Ore05,Ore06,Ore07,Ore08,Ore09,Ore10,Ore11,Ore12,Ore13,Ore14	*OreG0015607*
scaffold33199	1231165	G	A	Och12,Och13,Ore01,Ore02,Ore03,Ore04,Ore05,Ore06,Ore07,Ore08,Ore09,Ore10,Ore11,Ore12,Ore13,Ore14	*OreG0015609*
scaffold33199	1235048	T	C	Och01,Och02,Och03,Och04,Och05,Och06,Och07,Och08,Och09,Och10,Och11,Och12,Och13,Och14,Ore01,Ore02,Ore03,Ore04,Ore05,Ore06,Ore07,Ore08,Ore09,Ore10,Ore11,Ore12,Ore13,Ore14	*OreG0015610*
scaffold33199	1251506	T	C	NA	*OreG0015612*
scaffold33199	1251510	A	G	NA	*OreG0015612*
scaffold33199	1251666	G	C	NA	*OreG0015612*
scaffold33199	1251707	A	T	NA	*OreG0015612*
scaffold33199	1253596	C	T	NA	*OreG0015612*
scaffold33199	1254154	T	C	NA	*OreG0015612*
scaffold33199	1254180	C	G	NA	*OreG0015612*
scaffold33199	1254702	A	T	NA	*OreG0015612*
scaffold33199	1254714	G	A	NA	*OreG0015612*
scaffold33199	1254717	G	A	NA	*OreG0015612*
scaffold33199	1254726	G	A	NA	*OreG0015612*

scaffold33199	1254744	A	G	NA	*OreG0015612*
scaffold33199	1283214	C	T	Och12,Och13,Ore01,Ore02,Ore03,Ore04,Ore05,Ore06,Ore07,Ore08,Ore09,Ore10,Ore11,Ore12,Ore13,Ore14	*OreG0015616*
scaffold33199	1285457	A	G	NA	*OreG0015616*
scaffold33199	1292209	G	A	Och08,Och11,Och14	*OreG0015619*
scaffold33199	1296948	G	A	Och01,Och06	*OreG0015620*
scaffold33199	1330694	A	T	Och12,Och13,Ore01,Ore02,Ore03,Ore04,Ore05,Ore06,Ore07,Ore08,Ore09,Ore10,Ore11,Ore12,Ore13,Ore14	*OreG0015627*
scaffold33199	1330698	T	C	Ore03,Ore09,Ore14	*OreG0015627*
scaffold33199	1331208	T	A	Och12,Och13,Ore06,Ore07	*OreG0015627*
scaffold33199	1335863	T	A	Och12,Och13,Ore06,Ore07	*OreG0015629*
scaffold33199	1350526	T	G	Och12,Och13,Ore01,Ore02,Ore03,Ore04,Ore05,Ore06,Ore07,Ore08,Ore09,Ore10,Ore11,Ore12,Ore13,Ore14	*OreG0015631*
scaffold33199	1361971	G	T	Och08,Och09,Och10,Och11	*OreG0015632*
scaffold33199	1363369	C	A	Och01,Och02,Och03,Och04,Och05,Och06,Och07,Och08,Och09,Och10,Och11,Och12,Och13,Och14,Ore01,Ore02,Ore03,Ore04,Ore05,Ore06,Ore07,Ore08,Ore09,Ore10,Ore11,Ore12,Ore13,Ore14	*OreG0015632*
scaffold33199	1376666	G	A	Och12,Och13,Ore06,Ore07	*OreG0015634*
scaffold33199	1402669	C	T	Och12,Och13,Ore01,Ore02,Ore03,Ore04,Ore05,Ore06,Ore07,Ore08,Ore09,Ore10,Ore11,Ore12,Ore13,Ore14	*OreG0015637*
scaffold33199	1405596	T	A	Och12,Och13	*OreG0015638*
scaffold33199	1413763	T	C	Och01,Och02,Och04,Och06,Och07,Och08,Och14	*OreG0015639*
scaffold33199	1414008	T	C	Och01,Och02,Och03,Och04,Och05,Och06,Och07,Och08,Och09,Och10,Och11,Och12,Och13,Och14,Ore01,Ore02,Ore03,Ore04,Ore05,Ore06,Ore07,Ore08,Ore09,Ore10,Ore11,Ore12,Ore13,Ore14	*OreG0015639*
scaffold33199	1433104	T	G	Och12,Och13,Ore01,Ore02,Ore03,Ore04,Ore05,Ore06,Ore07,Ore08,Ore09,Ore10,Ore11,Ore12,Ore13,Ore14	*OreG0015642*
scaffold33199	1470859	G	A	NA	*OreG0015647*
scaffold33199	1471315	C	T	NA	*OreG0015647*
scaffold33199	1473749	C	T	NA	*OreG0015647*
scaffold33199	1473857	G	A	NA	*OreG0015647*
scaffold33199	1616035	T	C	NA	*OreG0015652*
scaffold33199	1623960	T	G	Ore06,Ore07	*OreG0015654*
scaffold33199	1657296	G	T	NA	*OreG0015658*
scaffold33199	1657941	A	G	NA	*OreG0015658*
scaffold33199	1658163	C	A	Och01,Och02,Och03,Och04,Och05,Och06,Och07,Och08,Och09,Och10,Och11,Och12,Och13,Och14,Ore01,Ore02,Ore03,Ore04,Ore05,Ore06,Ore07,Ore08,Ore09,Ore10,Ore11,Ore12,Ore13,Ore14	*OreG0015658*
scaffold33199	1681019	C	A	Och06,Och08,Och09,Och10,Och11	*OreG0015659*
scaffold33199	1725830	T	C	Och06,Och08,Och09,Och10,Och11	*OreG0015664*
scaffold33199	1729644	G	A	NA	*OreG0015664*
scaffold38	29893	T	A	Och01,Och02,Och03,Och04,Och05,Och06,Och07,Och08,Och09,Och10,Och11,Och12,Och13,Och14,Ore01,Ore02,Ore03,Ore04,Ore05,Ore06,Ore07,Ore08,Ore09,Ore10,Ore11,Ore12,Ore13,Ore14	*OreG0019125*
scaffold38	33909	T	A	Ore01,Ore02,Ore03,Ore04,Ore05,Ore06,Ore07,Ore08,Ore09,Ore10,Ore11,Ore12,Ore13,Ore14	*OreG0019125*
scaffold38	53084	T	A	NA	*OreG0019127*
scaffold38	53088	A	G	NA	*OreG0019127*
scaffold38	53098	G	A	NA	*OreG0019127*
scaffold38	60253	C	G	NA	*OreG0019127*
scaffold38	78202	G	A	Och01,Och02,Och03,Och04,Och05,Och06,Och07,Och08,Och09,Och10,Och11,Och12,Och13,Och14	*OreG0019130*
scaffold38	78331	A	T	NA	*OreG0019130*
scaffold38	121097	A	T	NA	*OreG0019132*
scaffold38	141231	A	G	NA	*OreG0019133*
scaffold38	162222	G	A	NA	*OreG0019135*
scaffold38	163207	C	T	NA	*OreG0019135*
scaffold38	169058	G	A	NA	*OreG0019137*
scaffold38	170037	A	C	NA	*OreG0019137*
scaffold38	170363	T	A	NA	*OreG0019137*
scaffold38	170653	C	T	NA	*OreG0019137*
scaffold38	188164	C	A	Och01,Och02,Och03,Och04,Och05,Och06,Och07,Och08,Och09,Och10,Och11,Och12,Och13,Och14,Ore01,Ore02,Ore03,Ore04,Ore05,Ore06,Ore07,Ore08,Ore09,Ore10,Ore11,Ore12,Ore13,Ore14	*OreG0019138*
scaffold38	189687	C	A	NA	*OreG0019138*
scaffold38	201773	G	A	NA	*OreG0019138*
scaffold38	203643	A	G	NA	*OreG0019138*
scaffold38	204500	G	A	NA	*OreG0019138*
scaffold38	205379	C	T	NA	*OreG0019138*
scaffold38	209309	C	T	NA	*OreG0019138*
scaffold38	215377	G	T	NA	*OreG0019138*
scaffold38	216414	A	C	NA	*OreG0019138*
scaffold38	216496	A	T	NA	*OreG0019138*
scaffold38	241710	C	G	NA	*OreG0019140*

scaffold38	245419	G	T	Och01,Och02,Och03,Och04,Och05,Och06,Och07,Och08,Och09,Och10,Och11,Och12,Och13,Och14,Ore01,Ore02,Ore03,Ore04,Ore05,Ore06,Ore07,Ore08,Ore09,Ore10,Ore11,Ore12,Ore13,Ore14	*OreG0019140*
scaffold38	245433	G	A	Och01,Och02,Och03,Och04,Och05,Och06,Och07,Och08,Och09,Och10,Och11,Och12,Och13,Och14,Ore01,Ore02,Ore03,Ore04,Ore05,Ore06,Ore07,Ore08,Ore09,Ore10,Ore11,Ore12,Ore13,Ore14	*OreG0019140*
scaffold38	271693	A	C	NA	*OreG0019142*
scaffold38	274599	C	T	NA	*OreG0019142*
scaffold38	274602	C	A	NA	*OreG0019142*
scaffold38	276652	G	A	NA	*OreG0019142*
scaffold38	276896	T	C	NA	*OreG0019142*
scaffold38	276973	G	T	NA	*OreG0019142*
scaffold38	292235	G	A	Och01,Och02,Och03,Och04,Och05,Och06,Och07,Och08,Och12,Och13,Och14	*OreG0019144*
scaffold38	295680	G	A	Ore11	*OreG0019144*
scaffold38	297174	T	C	Och01	*OreG0019144*
scaffold38	467184	A	G	Och01,Och09,Och10,Och11	*OreG0019153*
scaffold38	467885	T	C	Och01,Och02,Och03,Och04,Och05,Och06,Och07,Och08,Och09,Och10,Och11,Och12,Och13,Och14,Ore01,Ore02,Ore03,Ore04,Ore05,Ore06,Ore07,Ore08,Ore09,Ore10,Ore11,Ore12,Ore13,Ore14	*OreG0019153*
scaffold38	480964	A	T	NA	*OreG0019155*
scaffold38	481487	C	T	Ore11	*OreG0019155*
scaffold38	491932	C	A	Ore03,Ore04,Ore05,Ore09,Ore13	*OreG0019156*
scaffold38	525262	G	A	Och04	*OreG0019157*
scaffold38	637782	G	A	Ore01,Ore02,Ore03,Ore04,Ore05,Ore06,Ore07,Ore08,Ore09,Ore10,Ore11,Ore12,Ore13,Ore14	*OreG0019166*
scaffold38	644284	T	C	NA	*OreG0019167*
scaffold38	644330	A	C	NA	*OreG0019167*
scaffold38	651958	G	C	NA	*OreG0019169*
scaffold38	701052	G	T	Och02,Och03,Och12,Och13	*OreG0019171*
scaffold38	701184	G	T	NA	*OreG0019171*
scaffold38	701207	A	T	Ore11	*OreG0019171*
scaffold38	701319	A	G	NA	*OreG0019171*
scaffold38	701528	C	A	Ore03,Ore04,Ore05,Ore09,Ore13	*OreG0019171*
scaffold38	701775	T	G	Och01,Och02,Och03,Och05,Och07,Och08,Och09,Och10,Och11,Och12,Och13,Och14	*OreG0019171*
scaffold38	701935	G	T	NA	*OreG0019171*
scaffold38	743084	T	A	Ore01,Ore02,Ore03,Ore04,Ore05,Ore06,Ore07,Ore08,Ore09,Ore10,Ore11,Ore12,Ore13,Ore14	*OreG0019175*
scaffold38	743154	T	G	Och01,Och02,Och03,Och04,Och05,Och06,Och07,Och08,Och09,Och10,Och11,Och12,Och13,Och14,Ore01,Ore02,Ore03,Ore04,Ore05,Ore06,Ore07,Ore08,Ore09,Ore10,Ore11,Ore12,Ore13,Ore14	*OreG0019175*
scaffold38	747095	A	T	NA	*OreG0019177*
scaffold38	747152	A	C	Ore01,Ore02,Ore03,Ore04,Ore05,Ore06,Ore07,Ore08,Ore09,Ore10,Ore11,Ore12,Ore13,Ore14	*OreG0019177*
scaffold38	747510	T	C	Ore01,Ore02,Ore03,Ore04,Ore05,Ore06,Ore07,Ore08,Ore09,Ore10,Ore11,Ore12,Ore13,Ore14	*OreG0019177*
scaffold38	793858	C	T	Ore03,Ore04,Ore05,Ore09,Ore13	*OreG0019188*
scaffold38	793930	A	C	Ore03,Ore04,Ore05,Ore09,Ore13	*OreG0019188*
scaffold38	829773	A	T	NA	*OreG0019192*
scaffold38	833254	G	C	Ore01,Ore02,Ore03,Ore04,Ore05,Ore06,Ore07,Ore08,Ore09,Ore10,Ore11,Ore12,Ore13,Ore14	*OreG0019193*
scaffold38	866847	C	T	Och01,Och02,Och03,Och04,Och05,Och06,Och07,Och08,Och09,Och10,Och11,Och12,Och13,Och14,Ore01,Ore02,Ore03,Ore04,Ore05,Ore06,Ore07,Ore08,Ore09,Ore10,Ore11,Ore12,Ore13,Ore14	*OreG0019196*
scaffold38	867280	C	A	Och01,Och11	*OreG0019196*
scaffold38	867319	A	G	Ore01,Ore02,Ore03,Ore04,Ore05,Ore06,Ore07,Ore08,Ore09,Ore10,Ore11,Ore12,Ore13,Ore14	*OreG0019196*
scaffold38	867640	C	T	Ore01,Ore02,Ore03,Ore04,Ore05,Ore06,Ore07,Ore08,Ore09,Ore10,Ore11,Ore12,Ore13,Ore14	*OreG0019196*
scaffold38	868122	G	A	Ore01,Ore02,Ore03,Ore04,Ore05,Ore06,Ore07,Ore08,Ore09,Ore10,Ore11,Ore12,Ore13,Ore14	*OreG0019196*
scaffold38	868131	T	C	NA	*OreG0019196*
scaffold38	868371	T	C	NA	*OreG0019196*
scaffold38	869299	A	G	NA	*OreG0019197*
scaffold38	869468	C	G	Och01,Och02,Och03,Och04,Och05,Och06,Och07,Och08,Och09,Och10,Och11,Och12,Och13,Och14,Ore01,Ore02,Ore03,Ore04,Ore05,Ore06,Ore07,Ore08,Ore09,Ore10,Ore11,Ore12,Ore13,Ore14	*OreG0019197*
scaffold38	869536	C	A	NA	*OreG0019197*
scaffold38	869842	T	C	NA	*OreG0019197*
scaffold38	879207	A	C	NA	*OreG0019198*
scaffold38	879216	A	C	NA	*OreG0019198*
scaffold38	879368	C	A	Och01,Och02,Och03,Och04,Och05,Och06,Och07,Och08,Och09,Och10,Och11,Och12,Och13,Och14,Ore01,Ore02,Ore03,Ore04,Ore05,Ore06,Ore07,Ore08,Ore09,Ore10,Ore11,Ore12,Ore13,Ore14	*OreG0019198*

scaffold38	879624	C	G	Ore01,Ore02,Ore03,Ore04,Ore05,Ore06,Ore07,Ore08,Ore09,Ore10,Ore11,Ore12,Ore13,Ore14	*OreG0019198*
scaffold38	879676	G	C	Och01,Och02,Och03,Och04,Och05,Och06,Och07,Och08,Och09,Och10,Och11,Och12,Och13,Och14,Ore01,Ore02,Ore03,Ore04,Ore05,Ore06,Ore07,Ore08,Ore09,Ore10,Ore11,Ore12,Ore13,Ore14	*OreG0019198*
scaffold38	880236	A	C	NA	*OreG0019198*
scaffold38	909341	A	T	NA	*OreG0019200*
scaffold38	917003	T	C	Ore01,Ore02,Ore03,Ore04,Ore05,Ore06,Ore07,Ore08,Ore09,Ore10,Ore11,Ore12,Ore13,Ore14	*OreG0019202*
scaffold38	917052	T	G	Och01,Och11	*OreG0019202*
scaffold38	917124	G	A	Ore01,Ore02,Ore03,Ore04,Ore05,Ore06,Ore07,Ore08,Ore09,Ore10,Ore11,Ore12,Ore13,Ore14	*OreG0019202*
scaffold38	917313	A	G	NA	*OreG0019202*
scaffold38	917318	A	T	Ore01,Ore02,Ore03,Ore04,Ore05,Ore06,Ore07,Ore08,Ore09,Ore10,Ore11,Ore12,Ore13,Ore14	*OreG0019202*
scaffold38	917325	T	A	Och02,Och03,Och04,Och06,Och07,Och12,Och13	*OreG0019202*
scaffold38	980215	C	A	NA	*OreG0019205*
scaffold38	1031467	A	G	NA	*OreG0019208*
scaffold38	1031748	A	G	NA	*OreG0019208*
scaffold38	1033199	A	G	Ore11	*OreG0019208*
scaffold38	1034549	C	T	Och01,Och02,Och03,Och04,Och05,Och06,Och07,Och08,Och09,Och10,Och11,Och12,Och13,Och14	*OreG0019208*
scaffold38	1036589	T	C	NA	*OreG0019208*
scaffold38	1036826	G	A	NA	*OreG0019208*
scaffold38	1036997	A	T	Och01,Och02,Och03,Och04,Och05,Och06,Och07,Och08,Och09,Och10,Och11,Och14,Ore01,Ore02,Ore03,Ore04,Ore05,Ore06,Ore07,Ore08,Ore09,Ore10,Ore11,Ore12,Ore13,Ore14	*OreG0019208*
scaffold38	1037072	G	A	NA	*OreG0019208*
scaffold38	1037453	A	G	NA	*OreG0019208*
scaffold38	1044597	C	A	NA	*OreG0019209*
scaffold38	1048631	G	C	NA	*OreG0019210*
scaffold38	1060468	G	A	NA	*OreG0019211*
scaffold38	1071326	A	T	NA	*OreG0019212*
scaffold38	1074215	G	A	NA	*OreG0019212*
scaffold38	1213459	G	A	Och01,Och02,Och03,Och04,Och05,Och06,Och07,Och08,Och09,Och10,Och11,Och12,Och13,Och14,Ore01,Ore02,Ore03,Ore04,Ore05,Ore06,Ore07,Ore08,Ore09,Ore10,Ore11,Ore12,Ore13,Ore14	*OreG0019214*
scaffold38	1226332	G	A	Ore01,Ore02,Ore03,Ore04,Ore05,Ore06,Ore07,Ore08,Ore09,Ore10,Ore11,Ore12,Ore13,Ore14	*OreG0019215*
scaffold38	1226369	C	T	Och01,Och06,Och08,Och09,Och10,Och11	*OreG0019215*
scaffold38	1246046	G	C	NA	*OreG0019217*
scaffold38	1274473	T	C	Och01,Och06,Och08	*OreG0019219*
scaffold38	1355714	G	C	Och14	*OreG0019227*
scaffold38	1356500	T	C	NA	*OreG0019227*
scaffold38	1395112	C	T	NA	*OreG0019230*
scaffold38	1534226	G	A	Och01,Och02,Och03,Och04,Och05,Och06,Och07,Och08,Och09,Och10,Och11,Och12,Och13,Och14,Ore01,Ore02,Ore03,Ore04,Ore05,Ore06,Ore07,Ore08,Ore09,Ore10,Ore11,Ore12,Ore13,Ore14	*OreG0019244*
scaffold38	1573887	A	T	NA	*OreG0019247*
scaffold38	1573901	A	T	Och01	*OreG0019247*
scaffold38	1573947	G	T	Och01,Och02,Och03,Och04,Och05,Och06,Och07,Och08,Och09,Och10,Och11,Och12,Och13,Och14,Ore01,Ore02,Ore03,Ore04,Ore05,Ore06,Ore07,Ore08,Ore09,Ore10,Ore11,Ore12,Ore13,Ore14	*OreG0019247*
scaffold38	1634961	T	A	NA	*OreG0019249*
scaffold38	1663436	C	T	NA	*OreG0019253*
scaffold38	1663490	G	T	NA	*OreG0019253*
scaffold38	1664122	G	A	Ore01,Ore02,Ore03,Ore04,Ore05,Ore06,Ore07,Ore08,Ore09,Ore10,Ore11,Ore12,Ore13,Ore14	*OreG0019253*
scaffold38	1732092	C	T	Och01,Och07,Och08,Och09,Och10,Och11,Och13,Och14	*OreG0019258*
scaffold38	1763222	T	G	Och01,Och02,Och03,Och04,Och05,Och06,Och07,Och08,Och09,Och10,Och11,Och12,Och13,Och14	*OreG0019258*
scaffold38	1787610	C	T	Ore01,Ore02,Ore03,Ore04,Ore05,Ore06,Ore07,Ore08,Ore09,Ore10,Ore11,Ore12,Ore13,Ore14	*OreG0019260*
scaffold38	1787853	T	A	Och01,Och02,Och03,Och04,Och05,Och06,Och07,Och08,Och09,Och10,Och11,Och12,Och13,Och14	*OreG0019260*
scaffold38	1787900	A	G	NA	*OreG0019260*
scaffold38	1787943	G	T	Och07,Och09,Och10,Och11,Och13	*OreG0019260*
scaffold38	1788221	G	A	Ore01,Ore02,Ore03,Ore04,Ore05,Ore06,Ore07,Ore08,Ore09,Ore10,Ore11,Ore12,Ore13,Ore14	*OreG0019260*
scaffold38	1788227	T	C	Och07,Och09,Och10,Och11,Och13	*OreG0019260*
scaffold38	1810192	G	T	NA	*OreG0019262*
scaffold38	1819426	G	T	Och01,Och02,Och03,Och04,Och05,Och06,Och07,Och08,Och09,Och10,Och11,Och12,Och13,Och14,Ore01,Ore02,Ore03,Ore04,Ore05,Ore06,Ore07,Ore08,Ore09,Ore10,Ore11,Ore12,Ore13,Ore14	*OreG0019263*
scaffold38	1829165	G	T	NA	*OreG0019264*

scaffold38	1829348	T	A	Och01,Och02,Och03,Och04,Och05,Och06,Och07,Och08,Och09,Och10,Och11,Och12,Och13,Och14,Ore01,Ore02,Ore03,Ore04,Ore05,Ore06,Ore07,Ore08,Ore09,Ore10,Ore11,Ore12,Ore13,Ore14	*OreG0019264*
scaffold38	1877476	C	A	Och13	*OreG0019266*
scaffold38	2095491	T	C	NA	*OreG0019274*
scaffold38	2095578	T	C	NA	*OreG0019274*
scaffold38	2206476	T	C	Och07	*OreG0019276*
scaffold38	2206877	C	T	NA	*OreG0019276*
scaffold38	2343568	A	G	Ore01,Ore02,Ore03,Ore04,Ore05,Ore06,Ore07,Ore08,Ore09,Ore10,Ore11,Ore12,Ore13,Ore14	*OreG0019277*
scaffold38	2343792	A	C	Och01,Och02,Och03,Och04,Och05,Och06,Och07,Och08,Och09,Och10,Och11,Och12,Och13,Och14	*OreG0019277*
scaffold38	2343844	G	C	Och01,Och02,Och03,Och04,Och05,Och06,Och07,Och08,Och09,Och10,Och11,Och12,Och13,Och14	*OreG0019277*
scaffold38	2343888	G	A	Och01,Och02,Och03,Och04,Och05,Och06,Och07,Och08,Och09,Och10,Och11,Och12,Och13,Och14,Ore01,Ore02,Ore03,Ore04,Ore05,Ore06,Ore07,Ore08,Ore09,Ore10,Ore11,Ore12,Ore13,Ore14	*OreG0019277*
scaffold38	2343889	C	G	Och01,Och02,Och03,Och04,Och05,Och06,Och07,Och08,Och09,Och10,Och11,Och12,Och13,Och14,Ore01,Ore02,Ore03,Ore04,Ore05,Ore06,Ore07,Ore08,Ore09,Ore10,Ore11,Ore12,Ore13,Ore14	*OreG0019277*
scaffold38	2399909	C	T	Och01,Och02,Och03,Och04,Och05,Och06,Och07,Och08,Och09,Och10,Och11,Och12,Och13,Och14	*OreG0019279*
scaffold38	2399969	G	T	Och01,Och03,Och04,Och07,Och08,Och09,Och10,Och11,Och14	*OreG0019279*
scaffold38	2582890	A	G	Och01,Och02,Och03,Och04,Och05,Och06,Och07,Och08,Och09,Och10,Och11,Och13,Och14	*OreG0019283*
scaffold38	2858911	G	C	Och01,Och03,Och04,Och14	*OreG0019287*
scaffold38	3155563	G	T	Och02,Och06	*OreG0019292*
scaffold38	3155893	G	A	Och01,Och02,Och03,Och04,Och05,Och06,Och07,Och08,Och09,Och10,Och11,Och12,Och13,Och14,Ore01,Ore02,Ore03,Ore04,Ore05,Ore06,Ore07,Ore08,Ore09,Ore10,Ore11,Ore12,Ore13,Ore14	*OreG0019292*
scaffold38	3163846	G	A	NA	*OreG0019292*
scaffold38	3165447	A	G	NA	*OreG0019292*
scaffold38	3169446	A	G	NA	*OreG0019293*
scaffold38	3204910	T	A	Och03,Och04	*OreG0019295*
scaffold38	3204952	G	A	NA	*OreG0019295*
scaffold38	3245205	G	A	NA	*OreG0019297*
scaffold38	3255619	C	T	Och01,Och02,Och03,Och04,Och05,Och06,Och07,Och08,Och09,Och10,Och11,Och12,Och13,Och14	*OreG0019297*
scaffold38	3324094	T	C	Och01,Och02,Och03,Och04,Och05,Och06,Och07,Och08,Och09,Och10,Och11,Och12,Och13,Och14	*OreG0019298*
scaffold38	3341633	G	C	Ore01,Ore02,Ore04,Ore05,Ore14	*OreG0019302*
scaffold38	3346226	C	T	Och01,Och02,Och03,Och04,Och05,Och06,Och07,Och08,Och09,Och10,Och11,Och12,Och13,Och14,Ore01,Ore02,Ore03,Ore04,Ore05,Ore06,Ore07,Ore08,Ore09,Ore10,Ore11,Ore12,Ore13,Ore14	*OreG0019303*
scaffold38	3346247	A	G	NA	*OreG0019303*
scaffold38	3354925	C	G	Och01,Och02,Och03,Och04,Och05,Och06,Och07,Och08,Och09,Och10,Och11,Och12,Och13,Och14,Ore01,Ore02,Ore03,Ore04,Ore05,Ore06,Ore07,Ore08,Ore09,Ore10,Ore11,Ore12,Ore13,Ore14	*OreG0019303*
scaffold38	3355769	C	T	Och03	*OreG0019303*
scaffold38	3383508	T	G	NA	*OreG0019305*
scaffold38	3400151	C	A	Och01,Och02,Och03,Och04,Och05,Och06,Och07,Och08,Och09,Och10,Och11,Och12,Och13,Och14	*OreG0019306*
scaffold38	3422694	A	G	NA	*OreG0019309*
scaffold38	3429721	C	A	NA	*OreG0019310*
scaffold38	3437993	C	T	NA	*OreG0019312*
scaffold38	3438479	A	G	NA	*OreG0019312*
scaffold38	3438608	A	G	NA	*OreG0019312*
scaffold38	3438623	C	T	NA	*OreG0019312*
scaffold38	3438737	G	A	NA	*OreG0019312*
scaffold38	3438753	C	A	NA	*OreG0019312*
scaffold38	3438888	C	A	NA	*OreG0019312*
scaffold38	3438924	A	C	NA	*OreG0019312*
scaffold38	3438934	C	G	NA	*OreG0019312*
scaffold38	3466925	A	T	Ore01,Ore02,Ore03,Ore04,Ore05,Ore06,Ore07,Ore08,Ore09,Ore10,Ore11,Ore12,Ore13,Ore14	*OreG0019317*
scaffold38	3466943	G	A	Ore01,Ore02,Ore03,Ore04,Ore05,Ore06,Ore07,Ore08,Ore09,Ore10,Ore11,Ore12,Ore13,Ore14	*OreG0019317*
scaffold38	3466968	A	T	Och01,Och02,Och03,Och04,Och05,Och06,Och07,Och08,Och09,Och10,Och12,Och13,Och14	*OreG0019317*
scaffold38	3498907	C	G	NA	*OreG0019321*
scaffold38	3499067	A	T	NA	*OreG0019321*
scaffold38	3499108	C	A	Och14	*OreG0019321*
scaffold38	3500359	C	A	NA	*OreG0019321*
scaffold38	3500445	A	T	NA	*OreG0019321*
scaffold38	3500451	C	T	NA	*OreG0019321*

scaffold38	3500453	G	T	Och14	*OreG0019321*
scaffold38	3500469	C	T	Och14	*OreG0019321*
scaffold38	3509317	C	T	Och01,Och03,Och05,Och07,Och08,Och09,Och10,Och11,Och12,Och13,Ore01,Ore02,Ore03,Ore04,Ore05,Ore06,Ore07,Ore08,Ore09,Ore10,Ore11,Ore12,Ore13,Ore14	*OreG0019322*
scaffold38	3512377	T	A	Ore01,Ore02,Ore03,Ore04,Ore05,Ore06,Ore07,Ore08,Ore09,Ore10,Ore11,Ore12,Ore13,Ore14	*OreG0019322*
scaffold38	3514979	A	T	Ore01,Ore03,Ore04,Ore05,Ore08,Ore09,Ore11	*OreG0019323*
scaffold38	3520684	T	A	Och14	*OreG0019323*
scaffold38	3551784	C	A	Ore01,Ore02,Ore03,Ore04,Ore05,Ore06,Ore07,Ore08,Ore09,Ore10,Ore11,Ore12,Ore13,Ore14	*OreG0019326*
scaffold38	3557516	G	A	NA	*OreG0019327*
scaffold38	3571025	T	C	NA	*OreG0019328*
scaffold38	3571216	T	A	Och01,Och02,Och03,Och04,Och05,Och06,Och07,Och08,Och09,Och10,Och11,Och12,Och13,Och14,Ore01,Ore02,Ore03,Ore04,Ore05,Ore06,Ore07,Ore08,Ore09,Ore10,Ore11,Ore12,Ore13,Ore14	*OreG0019328*
scaffold38	3571219	T	A	NA	*OreG0019328*
scaffold38	3572754	C	T	NA	*OreG0019328*
scaffold38	3577242	C	T	Och01,Och02,Och03,Och04,Och05,Och06,Och07,Och08,Och09,Och10,Och11,Och12,Och13,Och14,Ore01,Ore02,Ore03,Ore04,Ore05,Ore06,Ore07,Ore08,Ore09,Ore10,Ore11,Ore12,Ore13,Ore14	*OreG0019329*
scaffold38	3577501	A	C	NA	*OreG0019329*
scaffold38	3577638	T	C	Ore01,Ore02,Ore03,Ore04,Ore05,Ore06,Ore07,Ore08,Ore09,Ore10,Ore11,Ore12,Ore13,Ore14	*OreG0019329*
scaffold38	3579557	C	A	Och01,Och02,Och03,Och04,Och05,Och06,Och07,Och09,Och10,Och11,Och12,Och13,Och14,Ore01,Ore02,Ore03,Ore04,Ore05,Ore06,Ore07,Ore08,Ore09,Ore10,Ore11,Ore12,Ore13,Ore14	*OreG0019329*
scaffold38	3599992	G	A	Ore01,Ore02,Ore04,Ore05,Ore14	*OreG0019334*
scaffold38	3607739	G	A	NA	*OreG0019337*
scaffold38	3628606	A	G	Och12,Och13	*OreG0019340*
scaffold38	3628706	C	A	Och02,Och03,Och04,Och05,Och06,Och12,Och13	*OreG0019340*
scaffold38	3701129	T	A	Och01,Och02,Och03,Och04,Och05,Och06,Och07,Och08,Och09,Och10,Och11,Och12,Och13,Och14	*OreG0019352*
scaffold38	3702018	G	A	NA	*OreG0019352*
scaffold38	3704276	G	A	Ore01,Ore02,Ore03,Ore04,Ore05,Ore06,Ore07,Ore08,Ore09,Ore10,Ore11,Ore12,Ore13,Ore14	*OreG0019353*
scaffold38	3704281	G	T	Och12,Och13	*OreG0019353*
scaffold38	3704488	A	G	NA	*OreG0019353*
scaffold38	3705880	T	C	NA	*OreG0019353*
scaffold38	3705915	G	T	Och01,Och02,Och03,Och04,Och05,Och06,Och07,Och08,Och09,Och10,Och11,Och12,Och13,Och14	*OreG0019353*
scaffold38	3720406	C	G	Och08	*OreG0019354*
scaffold38	3720751	T	C	Och01,Och02,Och03,Och04,Och05,Och06,Och07,Och08,Och09,Och10,Och11,Och12,Och13,Och14,Ore01,Ore02,Ore03,Ore04,Ore05,Ore06,Ore07,Ore08,Ore09,Ore10,Ore11,Ore12,Ore13,Ore14	*OreG0019354*
scaffold38	3725973	C	A	Ore01,Ore02,Ore03,Ore04,Ore05,Ore06,Ore07,Ore08,Ore09,Ore10,Ore11,Ore12,Ore13,Ore14	*OreG0019356*
scaffold38	3727079	T	A	NA	*OreG0019356*
scaffold38	3727297	A	G	Och01,Och03,Och05,Och08,Och12,Och13,Och14,Ore01,Ore02,Ore03,Ore04,Ore05,Ore06,Ore07,Ore08,Ore09,Ore10,Ore11,Ore12,Ore13,Ore14	*OreG0019356*
scaffold38	3727418	T	C	Och03,Och05,Och12,Och13,Och14,Ore01,Ore02,Ore03,Ore04,Ore05,Ore06,Ore07,Ore08,Ore09,Ore10,Ore11,Ore12,Ore13,Ore14	*OreG0019356*
scaffold38	3729384	G	T	NA	*OreG0019355*
scaffold38	3729397	T	C	NA	*OreG0019355*
scaffold38	3729817	A	G	NA	*OreG0019355*
scaffold38	3729988	C	G	NA	*OreG0019355*
scaffold38	3730094	A	G	Och01,Och02,Och04,Och05,Och09,Och10,Och12,Och13,Och14	*OreG0019355*
scaffold38	3741183	C	T	NA	*OreG0019357*
scaffold38	3742260	T	C	NA	*OreG0019357*
scaffold38	3742454	C	T	Och01,Och02,Och03,Och04,Och05,Och06,Och07,Och08,Och09,Och10,Och11,Och12,Och13,Och14,Ore01,Ore02,Ore03,Ore04,Ore05,Ore06,Ore07,Ore08,Ore09,Ore10,Ore11,Ore12,Ore13,Ore14	*OreG0019357*
scaffold38	3788402	T	C	NA	*OreG0019362*
scaffold38	3788636	A	T	Ore01,Ore04,Ore05	*OreG0019362*
scaffold38	3816100	C	T	NA	*OreG0019364*
scaffold38	3841121	A	G	NA	*OreG0019366*
scaffold38	3843767	G	A	Och01,Och02,Och03,Och04,Och05,Och06,Och07,Och08,Och09,Och10,Och11,Och12,Och13,Och14,Ore01,Ore02,Ore03,Ore04,Ore05,Ore06,Ore07,Ore08,Ore09,Ore10,Ore11,Ore12,Ore13,Ore14	*OreG0019366*
scaffold38	3901683	G	A	Ore01,Ore02,Ore04,Ore05,Ore14	*OreG0019372*
scaffold38	3942035	G	A	Och01,Och04,Och06,Och07,Och08,Och09,Och10,Och11,Och12,Och13	*OreG0019376*
scaffold38	3958648	C	A	Och01,Och02,Och03,Och04,Och05,Och06,Och07,Och08,Och09,Och10,Och11,Och12,Och13,Och14	*OreG0019377*
scaffold38	3982428	G	A	NA	*OreG0019380*
scaffold38	3998754	A	G	Och01,Och02,Och03,Och04,Och05,Och06,Och07,Och08,Och09,Och10,Och11,Och12,Och13,Och14	*OreG0019382*

scaffold38	3998756	A	G	Ore01,Ore02,Ore03,Ore04,Ore05,Ore06,Ore07,Ore08,Ore09,Ore10,Ore11,Ore12,Ore13,Ore14	*OreG0019382*
scaffold38	3998799	T	C	NA	*OreG0019382*
scaffold38	3998888	G	A	Ore01,Ore02,Ore03,Ore04,Ore05,Ore06,Ore07,Ore08,Ore09,Ore10,Ore11,Ore12,Ore13,Ore14	*OreG0019382*
scaffold38	3998954	C	A	Ore01,Ore02,Ore03,Ore04,Ore05,Ore06,Ore07,Ore08,Ore09,Ore10,Ore11,Ore12,Ore13,Ore14	*OreG0019382*
scaffold38	4009415	C	A	Ore01,Ore02,Ore04,Ore05,Ore14	*OreG0019383*
scaffold38	4023355	C	T	Och01,Och02,Och03,Och04,Och05,Och06,Och07,Och08,Och09,Och10,Och11,Och12,Och13,Och14,Ore01,Ore02,Ore03,Ore04,Ore05,Ore06,Ore07,Ore08,Ore09,Ore10,Ore11,Ore12,Ore13,Ore14	*OreG0019384*
scaffold38	4049397	G	C	NA	*OreG0019386*
scaffold38	4049523	G	T	NA	*OreG0019386*
scaffold38	4118857	A	C	Och01,Och02,Och03,Och04,Och05,Och06,Och07,Och08,Och09,Och10,Och11,Och12,Och13,Och14,Ore01,Ore02,Ore03,Ore04,Ore05,Ore06,Ore07,Ore08,Ore09,Ore10,Ore11,Ore12,Ore13,Ore14	*OreG0019389*
scaffold38	4119168	A	T	Och01,Och03,Och09,Och10	*OreG0019389*
scaffold38	4121465	C	A	Och12,Och13	*OreG0019390*
scaffold38	4239175	C	T	NA	*OreG0019400*
scaffold38	4245427	C	G	Och01,Och02,Och03,Och04,Och05,Och06,Och07,Och08,Och09,Och10,Och11,Och12,Och13,Och14	*OreG0019400*
scaffold38	4245600	C	A	Och01,Och02,Och03,Och04,Och05,Och06,Och07,Och08,Och09,Och10,Och11,Och12,Och13,Och14,Ore01,Ore02,Ore03,Ore04,Ore05,Ore06,Ore07,Ore08,Ore09,Ore10,Ore11,Ore12,Ore13,Ore14	*OreG0019400*
scaffold38	4281411	C	G	NA	*OreG0019403*
scaffold38	4291852	T	C	Ore01,Ore02,Ore04,Ore05,Ore06,Ore07,Ore11,Ore12	*OreG0019403*
scaffold38	4293520	C	A	NA	*OreG0019403*
scaffold38	4300402	C	T	Ore01,Ore02,Ore03,Ore04,Ore05,Ore06,Ore07,Ore08,Ore09,Ore10,Ore11,Ore12,Ore13,Ore14	*OreG0019404*
scaffold38	4318706	C	A	Och01,Och02,Och03,Och04,Och05,Och06,Och07,Och08,Och09,Och10,Och11,Och12,Och13,Och14	*OreG0019405*
scaffold38	4330166	G	A	NA	*OreG0019408*
scaffold38	4336430	G	T	NA	*OreG0019408*
scaffold38	4430507	C	T	NA	*OreG0019411*
scaffold38	4431947	G	A	Ore02,Ore04,Ore05	*OreG0019411*
scaffold38	4434241	G	C	Och05	*OreG0019412*
scaffold38	4434361	T	A	Ore03	*OreG0019412*
scaffold38	4434446	G	A	Och02,Och03,Och04,Och05,Och06,Och12,Och13	*OreG0019412*
scaffold38	4477413	G	T	Ore02,Ore04,Ore05	*OreG0019416*
scaffold38	4485474	T	C	NA	*OreG0019417*
scaffold38	4525972	T	A	NA	*OreG0019421*
scaffold38	4533054	G	A	Och06,Och07,Och08,Och14	*OreG0019422*
scaffold38	4535713	C	T	Och01,Och02,Och03,Och04,Och05,Och06,Och07,Och08,Och09,Och10,Och11,Och12,Och13,Och14,Ore01,Ore02,Ore03,Ore04,Ore05,Ore06,Ore07,Ore08,Ore09,Ore10,Ore11,Ore12,Ore13,Ore14	*OreG0019423*
scaffold38	4535779	C	T	Och01	*OreG0019423*
scaffold38	4541634	G	A	Ore01,Ore02,Ore03,Ore04,Ore05,Ore06,Ore07,Ore08,Ore09,Ore10,Ore11,Ore12,Ore13,Ore14	*OreG0019424*
