# Supplementary material for: Genomic effects of population collapse in a critically endangered ironwood tree Ostrya rehderiana
Source: Nat Commun. 2018 Dec 21;9:5449. doi: 10.1038/s41467-018-07913-4 (PMC6303402; doi:10.1038/s41467-018-07913-4)
Supplement: Supplementary file 5 — Supplementary Data 2 [file 41467_2018_7913_MOESM5_ESM.pdf]

Supplementary Data file 2. The loss of function variant sites (LOF)

Chr	Pos	Ancestral	Derived	Individuals carries homozygous LoF	Gene affectd
scaffold188	3685	T	A	Och01,Och02,Och03,Och04,Och05,Och06,Och07,Och08,Och09,Och10,Och11,Och12,Och13,Och14	*OreG0004622*
scaffold220	4883	C	T	NA	*OreG0006227*
scaffold220	23661	A	T	Och05,Och13	*OreG0006231*
scaffold220	61866	C	T	NA	*OreG0006239*
scaffold220	63383	C	A	NA	*OreG0006239*
scaffold55	17844	G	A	Ore06,Ore11	*OreG0023610*
scaffold344	15521	C	T	Ore03	*OreG0018609*
scaffold270	137001	C	T	Och01,Och02,Och03,Och04,Och06,Och07,Och08,Och09,Och10,Och13	*OreG0007913*
scaffold333	483537	C	T	Och01,Och02,Och03,Och04,Och05,Och06,Och07,Och08,Och09,Och10,Och11,Och12,Och13,Och14	*OreG0018513*
scaffold333	717501	C	T	NA	*OreG0018519*
scaffold333	809216	A	T	NA	*OreG0018527*
scaffold333	837527	C	T	NA	*OreG0018530*
scaffold333	837642	G	A	NA	*OreG0018530*
scaffold243	94252	T	A	NA	*OreG0007253*
scaffold243	94335	C	T	NA	*OreG0007253*
scaffold243	110678	C	T	Och11	*OreG0007255*
scaffold243	194382	G	A	NA	*OreG0007265*
scaffold243	195006	C	A	NA	*OreG0007265*
scaffold399	140878	C	T	NA	*OreG0019584*
scaffold35	205850	C	A	NA	*OreG0018794*
scaffold35	209751	C	T	NA	*OreG0018796*
scaffold35	292375	G	T	NA	*OreG0018806*
scaffold35	292378	C	T	NA	*OreG0018806*
scaffold35	292412	G	A	NA	*OreG0018806*
scaffold35	416068	C	A	NA	*OreG0018817*
scaffold35	571879	C	T	NA	*OreG0018833*
scaffold35	629638	T	A	Och14	*OreG0018838*
scaffold35	751996	A	T	NA	*OreG0018846*
scaffold35	754317	C	A	NA	*OreG0018846*
scaffold35	888422	G	A	NA	*OreG0018861*
scaffold35	888511	C	T	NA	*OreG0018861*
scaffold35	970838	G	A	NA	*OreG0018872*
scaffold35	1023054	C	T	Och01,Och02,Och03,Och04,Och05,Och06,Och07,Och08,Och09,Och10,Och11,Och12,Och13,Och14	*OreG0018877*
scaffold35	1051190	G	A	Ore06,Ore07	*OreG0018879*
scaffold35	1319392	C	T	NA	*OreG0018897*
scaffold35	1370208	A	T	NA	*OreG0018903*
scaffold436	21179	C	A	NA	*OreG0020347*
scaffold53	397250	C	T	Ore01,Ore02,Ore03,Ore04,Ore05,Ore06,Ore07,Ore08,Ore09,Ore10,Ore11,Ore12,Ore13,Ore14	*OreG0023590*
scaffold53	897939	G	C	Och01,Och02,Och03,Och04,Och05,Och06,Och07,Och08,Och09,Och10,Och11,Och12,Och13,Och14	*OreG0023598*
scaffold385	4286	C	A	NA	*OreG0019436*
scaffold385	30977	C	T	Och01,Och02,Och03,Och04,Och05,Och06,Och07,Och08,Och09,Och10,Och11,Och12,Och13	*OreG0019439*
scaffold385	120274	G	T	Och06	*OreG0019449*
scaffold385	258563	T	A	Ore01,Ore02,Ore03,Ore04,Ore05,Ore06,Ore07,Ore08,Ore09,Ore10,Ore11,Ore12,Ore13,Ore14	*OreG0019464*
scaffold385	261175	G	T	Och01,Och02,Och03,Och04,Och05,Och06,Och07,Och08,Och09,Och10,Och11,Och12,Och13,Och14,Ore01,Ore02,Ore03,Ore04,Ore05,Ore06,Ore07,Ore08,Ore09,Ore10,Ore11,Ore12,Ore13,Ore14	*OreG0019465*
scaffold385	261419	G	A	Och14	*OreG0019465*
scaffold385	416863	C	A	NA	*OreG0019480*
scaffold385	622620	C	T	NA	*OreG0019493*
scaffold385	736887	A	C	Ore01,Ore02,Ore03,Ore04,Ore05,Ore06,Ore07,Ore08,Ore09,Ore10,Ore11,Ore12,Ore13,Ore14	*OreG0019505*
scaffold248	38234	G	A	NA	*OreG0007348*
scaffold248	492599	G	A	Och01,Och02,Och03,Och04,Och05,Och06,Och07,Och08,Och09,Och10,Och11,Och12,Och13,Och14	*OreG0007379*
scaffold248	494021	C	T	Och01,Och02,Och03,Och04,Och05,Och06,Och07,Och08,Och09,Och10,Och11,Och12,Och13,Och14	*OreG0007380*
scaffold248	495450	T	A	Och12	*OreG0007381*
scaffold248	524087	C	T	Och01,Och02,Och03,Och04,Och05,Och06,Och07,Och08,Och09,Och10,Och11,Och12,Och13,Och14	*OreG0007384*
scaffold248	611867	T	G	NA	*OreG0007393*
scaffold248	620864	G	A	NA	*OreG0007393*
scaffold248	709347	G	A	NA	*OreG0007398*
scaffold248	718260	A	T	NA	*OreG0007400*
scaffold316	92240	C	T	NA	*OreG0009512*
scaffold316	105204	C	T	NA	*OreG0009514*
scaffold316	261723	G	A	Och05	*OreG0009530*

scaffold316	274474	A	T	Ore04,Ore05,Ore06,Ore11	*OreG0009531*
scaffold316	301583	A	T	Och05	*OreG0009535*
scaffold316	310725	G	A	NA	*OreG0009535*
scaffold316	315380	G	A	Och08,Och13	*OreG0009536*
scaffold316	382125	T	A	Ore01,Ore02,Ore03,Ore04,Ore05,Ore06,Ore07,Ore08,Ore09,Ore10,Ore11,Ore12,Ore13,Ore14	*OreG0009547*
scaffold316	424580	A	T	NA	*OreG0009551*
scaffold316	432547	C	T	Och04,Och05,Och13	*OreG0009552*
scaffold316	432730	C	T	NA	*OreG0009552*
scaffold316	708633	G	A	Ore04,Ore05	*OreG0009582*
scaffold316	977368	C	A	NA	*OreG0009596*
scaffold316	1095204	G	C	Ore01,Ore02,Ore03,Ore04,Ore05,Ore06,Ore07,Ore08,Ore09,Ore10,Ore11,Ore12,Ore13,Ore14	*OreG0009612*
scaffold316	1100928	G	T	NA	*OreG0009612*
scaffold316	1114551	A	T	NA	*OreG0009613*
scaffold316	1158815	G	T	Och01,Och02,Och03,Och04,Och05,Och06,Och07,Och08,Och09,Och10,Och11,Och12,Och13,Och14	*OreG0009616*
scaffold316	1163981	C	A	Och01,Och02,Och03,Och04,Och05,Och06,Och07,Och08,Och09,Och10,Och11,Och12,Och13,Och14	*OreG0009617*
scaffold316	1176965	T	G	NA	*OreG0009620*
scaffold316	1253043	G	A	Och03,Och06,Och07,Och08,Och11,Och12,Och13	*OreG0009628*
scaffold316	1283455	A	T	Och01,Och02,Och04,Och06,Och07,Och08,Och09,Och10,Och11	*OreG0009631*
scaffold17	11457	C	T	NA	*OreG0003464*
scaffold17	291376	G	A	Ore01,Ore02,Ore03,Ore04,Ore05,Ore06,Ore07,Ore08,Ore09,Ore10,Ore11,Ore12,Ore13,Ore14	*OreG0003484*
scaffold17	625425	C	T	NA	*OreG0003526*
scaffold109	320996	G	A	NA	*OreG0000642*
scaffold109	430682	G	A	NA	*OreG0000652*
scaffold109	494746	G	A	NA	*OreG0000658*
scaffold109	502256	A	T	NA	*OreG0000660*
scaffold109	628479	C	A	NA	*OreG0000670*
scaffold109	633191	A	T	NA	*OreG0000672*
scaffold109	919229	G	A	NA	*OreG0000697*
scaffold109	996988	G	A	NA	*OreG0000718*
scaffold109	1088191	G	A	Ore01,Ore09	*OreG0000726*
scaffold109	1355489	A	C	NA	*OreG0000760*
scaffold109	1478540	C	T	Ore01,Ore02,Ore03,Ore04,Ore05,Ore06,Ore07,Ore08,Ore09,Ore10,Ore11,Ore12,Ore13,Ore14	*OreG0000768*
scaffold109	1488404	T	A	Och01,Och02,Och03,Och04,Och05,Och06,Och07,Och08,Och09,Och10,Och11,Och12,Och13,Och14	*OreG0000770*
scaffold109	1701536	A	T	Och01,Och06,Och07,Och08,Och09,Och10,Och11,Och14	*OreG0000782*
scaffold109	1704324	C	T	Och01,Och02,Och03,Och04,Och05,Och06,Och07,Och08,Och09,Och10,Och11,Och12,Och13,Och14	*OreG0000782*
scaffold45	21428	G	A	Och01,Och02,Och03,Och04,Och05,Och06,Och07,Och08,Och09,Och10,Och11,Och12,Och13,Och14,Ore01,Ore02,Ore03,Ore04,Ore05,Ore06,Ore07,Ore08,Ore09,Ore10,Ore11,Ore12,Ore13,Ore14	*OreG0020494*
scaffold45	300379	G	A	NA	*OreG0020529*
scaffold45	300917	C	T	NA	*OreG0020529*
scaffold45	308582	C	A	NA	*OreG0020530*
scaffold45	308704	G	T	NA	*OreG0020530*
scaffold45	317038	G	A	NA	*OreG0020533*
scaffold45	319958	A	C	Och01,Och02,Och03,Och04,Och05,Och06,Och07,Och08,Och09,Och10,Och11,Och12,Och13,Och14,Ore01,Ore02,Ore03,Ore04,Ore05,Ore06,Ore07,Ore08,Ore09,Ore10,Ore11,Ore12,Ore13,Ore14	*OreG0020534*
scaffold45	342642	C	A	NA	*OreG0020536*
scaffold45	353374	G	A	Och01	*OreG0020539*
scaffold3	74911	C	A	Ore06,Ore08,Ore09	*OreG0008601*
scaffold3	143286	C	A	Och13	*OreG0008612*
scaffold3	145613	G	A	Och07,Och13	*OreG0008612*
scaffold3	235131	G	T	Och08	*OreG0008626*
scaffold3	254881	A	T	Och01,Och02,Och03,Och04,Och05,Och06,Och08,Och12,Och13,Och14	*OreG0008630*
scaffold3	282710	T	A	Ore01,Ore02,Ore03,Ore04,Ore05,Ore06,Ore07,Ore08,Ore09,Ore10,Ore11,Ore12,Ore13,Ore14	*OreG0008632*
scaffold3	475571	A	T	NA	*OreG0008656*
scaffold3	566118	G	A	Och01,Och02,Och03,Och04,Och05,Och06,Och07,Och08,Och09,Och10,Och11,Och12,Och13,Och14	*OreG0008669*
scaffold3	728397	C	A	NA	*OreG0008692*
scaffold3	819261	G	A	NA	*OreG0008701*
scaffold3	836926	C	A	Ore01,Ore02,Ore03,Ore04,Ore05,Ore06,Ore07,Ore08,Ore09,Ore10,Ore11,Ore12,Ore13,Ore14	*OreG0008701*
scaffold3	1138728	A	T	NA	*OreG0008730*
scaffold3	1144056	G	T	NA	*OreG0008731*
scaffold3	1185134	A	C	Och01	*OreG0008736*
scaffold3	1360631	G	T	Och09,Och10	*OreG0008750*

scaffold3	1394754	C	T	Ore01,Ore02,Ore03,Ore04,Ore05,Ore06,Ore07,Ore08,Ore09,Ore10,Ore11,Ore12,Ore13,Ore14	*OreG0008754*
scaffold3	1469116	G	A	Och01,Och02,Och03,Och04,Och05,Och06,Och07,Och08,Och09,Och10,Och11,Och12,Och13,Och14,Ore01,Ore02,Ore03,Ore04,Ore05,Ore06,Ore07,Ore08,Ore09,Ore10,Ore11,Ore12,Ore13,Ore14	*OreG0008762*
scaffold3	1469131	C	A	Och01,Och02,Och03,Och04,Och05,Och06,Och07,Och08,Och09,Och10,Och11,Och12,Och13,Och14	*OreG0008762*
scaffold323	50695	G	A	NA	*OreG0010425*
scaffold323	371910	C	T	Ore04,Ore05	*OreG0010446*
scaffold323	371976	C	T	Ore04,Ore05	*OreG0010446*
scaffold323	511301	C	T	Och08	*OreG0010453*
scaffold323	511514	C	T	Och06,Och08	*OreG0010453*
scaffold323	518933	C	A	NA	*OreG0010454*
scaffold323	617917	G	T	NA	*OreG0010464*
scaffold323	1098461	C	T	NA	*OreG0010498*
scaffold323	1108731	C	T	NA	*OreG0010499*
scaffold323	1191632	C	T	NA	*OreG0010505*
scaffold323	1293602	C	T	NA	*OreG0010520*
scaffold323	1303437	T	A	NA	*OreG0010521*
scaffold323	1309861	G	A	Och01,Och02,Och03,Och04,Och05,Och06,Och07,Och08,Och09,Och10,Och11,Och12,Och13,Och14,Ore01,Ore02,Ore03,Ore04,Ore05,Ore06,Ore07,Ore08,Ore09,Ore10,Ore11,Ore12,Ore13,Ore14	*OreG0010522*
scaffold323	1313359	G	A	NA	*OreG0010522*
scaffold323	1360027	G	A	Ore02,Ore06,Ore08,Ore09,Ore10,Ore12,Ore14	*OreG0010530*
scaffold218	513567	G	C	NA	*OreG0006144*
scaffold218	517598	G	A	NA	*OreG0006144*
scaffold218	755925	C	T	Och01,Och02,Och03,Och04,Och05,Och06,Och07,Och08,Och09,Och10,Och11,Och12,Och13,Och14	*OreG0006158*
scaffold218	802188	G	T	Ore01,Ore02,Ore03,Ore04,Ore05,Ore06,Ore07,Ore08,Ore09,Ore10,Ore11,Ore12,Ore13,Ore14	*OreG0006159*
scaffold218	870508	G	T	NA	*OreG0006163*
scaffold218	870835	G	T	NA	*OreG0006163*
scaffold218	903773	T	G	Ore01,Ore02,Ore03,Ore04,Ore05,Ore06,Ore07,Ore08,Ore09,Ore10,Ore11,Ore12,Ore13,Ore14	*OreG0006165*
scaffold218	1004923	G	A	Och01,Och02,Och03,Och04,Och05,Och06,Och07,Och08,Och09,Och10,Och11,Och12,Och13,Och14,Ore01,Ore02,Ore03,Ore04,Ore05,Ore06,Ore07,Ore08,Ore09,Ore10,Ore11,Ore12,Ore13,Ore14	*OreG0006173*
scaffold218	1028167	A	T	NA	*OreG0006177*
scaffold218	1079125	A	T	Och03	*OreG0006178*
scaffold664	4927	G	T	Och01,Och02,Och03,Och04,Och05,Och06,Och07,Och08,Och10,Och11,Och12,Och13,Och14	*OreG0024350*
scaffold25	126984	A	C	NA	*OreG0007541*
scaffold25	188376	G	T	Och05,Och13,Och14	*OreG0007547*
scaffold25	247554	C	T	Och01,Och04	*OreG0007550*
scaffold25	262107	G	A	Ore03,Ore04,Ore05,Ore09,Ore13	*OreG0007552*
scaffold25	278477	C	T	Och01,Och02,Och03,Och04,Och05,Och06,Och07,Och08,Och09,Och10,Och11,Och12,Och13,Och14	*OreG0007554*
scaffold25	915958	C	T	Och01,Och02,Och03,Och04,Och05,Och06,Och11,Och12,Och13	*OreG0007592*
scaffold25	915959	C	T	Och01,Och02,Och03,Och04,Och05,Och06,Och11,Och12,Och13	*OreG0007592*
scaffold25	962837	A	T	NA	*OreG0007596*
scaffold25	1043328	C	A	NA	*OreG0007607*
scaffold25	1043358	G	A	NA	*OreG0007607*
scaffold25	1059122	T	A	NA	*OreG0007609*
scaffold25	1139158	T	A	NA	*OreG0007613*
scaffold585	106975	A	T	NA	*OreG0023797*
scaffold585	168300	C	A	NA	*OreG0023801*
scaffold585	168303	C	A	NA	*OreG0023801*
scaffold232	6553	G	A	Ore01,Ore02,Ore03,Ore04,Ore05,Ore06,Ore07,Ore08,Ore09,Ore10,Ore11,Ore12,Ore13,Ore14	*OreG0006996*
scaffold232	48923	G	A	NA	*OreG0006998*
scaffold232	48968	G	A	NA	*OreG0006998*
scaffold232	49085	G	A	NA	*OreG0006998*
scaffold232	49100	G	A	NA	*OreG0006998*
scaffold232	49347	C	A	NA	*OreG0006998*
scaffold232	51904	G	A	NA	*OreG0006998*
scaffold232	51907	C	A	Och01,Och02,Och03,Och06	*OreG0006998*
scaffold232	58898	G	T	NA	*OreG0007000*
scaffold232	59533	T	A	NA	*OreG0007000*
scaffold232	77708	G	A	Och02,Och03,Och04,Och05,Och06,Och07,Och08,Och09,Och10,Och11,Och12,Och13,Och14,Ore01,Ore02,Ore03,Ore04,Ore05,Ore06,Ore07,Ore08,Ore09,Ore10,Ore11,Ore12,Ore13,Ore14	*OreG0007005*
scaffold232	77747	G	A	Och01,Och02,Och03,Och04,Och05,Och06,Och07,Och08,Och09,Och10,Och11,Och12,Och13,Och14,Ore01,Ore02,Ore03,Ore04,Ore05,Ore06,Ore07,Ore08,Ore09,Ore10,Ore11,Ore12,Ore13,Ore14	*OreG0007005*

scaffold232	80448	G	A	Och07,Och08,Och09,Och10,Och11,Och12,Och13,Och14,Ore01,Ore02,Ore03,Ore04,Ore05,Ore06,Ore07,Ore08,Ore09,Ore10,Ore11,Ore12,Ore13,Ore14	*OreG0007006*
scaffold232	129512	A	T	Och12	*OreG0007012*
scaffold232	206381	C	T	Och12,Och13	*OreG0007018*
scaffold232	224352	C	T	Ore01,Ore11	*OreG0007021*
scaffold232	477019	A	T	Och01,Och02,Och03,Och04,Och05,Och06,Och07,Och08,Och09,Och10,Och11,Och12,Och13,Och14	*OreG0007032*
scaffold232	477049	C	T	Och01,Och02,Och03,Och04,Och05,Och06,Och07,Och08,Och09,Och10,Och11,Och12,Och13,Och14	*OreG0007032*
scaffold232	477825	G	A	Och02,Och03,Och06	*OreG0007033*
scaffold232	933917	G	A	NA	*OreG0007057*
scaffold232	984676	G	A	Och08	*OreG0007065*
scaffold232	1027366	G	T	Och05,Och12,Och13	*OreG0007073*
scaffold232	1172288	C	A	NA	*OreG0007089*
scaffold232	1180583	T	A	NA	*OreG0007091*
scaffold232	1243845	G	A	NA	*OreG0007102*
scaffold232	1311772	T	A	NA	*OreG0007113*
scaffold490	104860	A	C	NA	*OreG0022995*
scaffold490	307972	A	T	Och02	*OreG0023013*
scaffold490	313387	T	A	NA	*OreG0023014*
scaffold490	417014	T	A	NA	*OreG0023023*
scaffold386	272212	G	T	Och01,Och02,Och03,Och04,Och05,Och06,Och07,Och08,Och09,Och10,Och11,Och12,Och13,Och14,Ore01,Ore02,Ore03,Ore04,Ore05,Ore06,Ore07,Ore08,Ore09,Ore10,Ore11,Ore12,Ore13,Ore14	*OreG0019538*
scaffold386	1005518	C	T	NA	*OreG0019544*
scaffold187	86120	G	T	NA	*OreG0004279*
scaffold187	118412	C	T	NA	*OreG0004280*
scaffold187	118538	A	T	NA	*OreG0004280*
scaffold187	121838	C	T	NA	*OreG0004280*
scaffold187	147928	G	A	NA	*OreG0004281*
scaffold187	224079	C	T	NA	*OreG0004287*
scaffold187	330456	G	A	NA	*OreG0004301*
scaffold187	398088	C	A	NA	*OreG0004308*
scaffold187	431839	T	A	Och01,Och02,Och03,Och04,Och05,Och06,Och07,Och08,Och09,Och10,Och11,Och12,Och13,Och14	*OreG0004312*
scaffold187	544984	T	A	NA	*OreG0004321*
scaffold187	545218	G	A	NA	*OreG0004321*
scaffold187	984382	C	T	Och01,Och02,Och03,Och04,Och05,Och06,Och07,Och08,Och09,Och10,Och11,Och12,Och13,Och14	*OreG0004353*
scaffold187	1330974	A	T	NA	*OreG0004378*
scaffold187	1331219	G	C	Och01	*OreG0004378*
scaffold187	1331944	T	A	NA	*OreG0004378*
scaffold187	1451366	T	A	NA	*OreG0004389*
scaffold187	1524039	C	T	Och02,Och03,Och04,Och05,Och06,Och07,Och08,Och09,Och10,Och11,Och12,Och13,Och14	*OreG0004394*
scaffold187	1914582	G	T	Ore01,Ore02,Ore03,Ore04,Ore05,Ore06,Ore07,Ore08,Ore09,Ore10,Ore11,Ore12,Ore13,Ore14	*OreG0004437*
scaffold187	1954945	T	A	NA	*OreG0004438*
scaffold187	2001551	C	A	NA	*OreG0004441*
scaffold187	2190660	G	T	NA	*OreG0004460*
scaffold187	2195377	C	T	Och07	*OreG0004461*
scaffold187	2409885	G	T	Ore01,Ore02,Ore03,Ore04,Ore05,Ore06,Ore07,Ore08,Ore09,Ore10,Ore11,Ore12,Ore13,Ore14	*OreG0004485*
scaffold187	2471096	A	T	Och03,Och13	*OreG0004490*
scaffold187	2547726	C	T	NA	*OreG0004497*
scaffold187	2856078	C	T	Och02,Och03,Och04,Och05,Och06,Och12,Och13	*OreG0004518*
scaffold187	2884439	C	G	NA	*OreG0004524*
scaffold187	3182361	A	T	NA	*OreG0004557*
scaffold187	3195801	G	A	Och01,Och02,Och03,Och04,Och05,Och06,Och07,Och08,Och09,Och10,Och11,Och12,Och13,Och14	*OreG0004558*
scaffold187	3213549	G	T	Ore01,Ore08	*OreG0004560*
scaffold187	3213567	C	T	Och14	*OreG0004560*
scaffold187	3389351	C	T	Och04,Och05,Och12,Och13	*OreG0004578*
scaffold187	3389366	A	T	Ore01,Ore02,Ore03,Ore04,Ore05,Ore06,Ore07,Ore08,Ore09,Ore10,Ore11,Ore12,Ore13,Ore14	*OreG0004578*
scaffold187	3414614	A	T	Ore01,Ore02,Ore03,Ore04,Ore05,Ore06,Ore07,Ore08,Ore09,Ore10,Ore11,Ore12,Ore13,Ore14	*OreG0004583*
scaffold187	3598177	T	A	NA	*OreG0004601*
scaffold187	3650283	G	A	Och09,Och10,Och11	*OreG0004606*
scaffold187	3650894	G	T	Och09	*OreG0004606*
scaffold187	3725930	G	T	Och01,Och02,Och03,Och04,Och05,Och06,Och07,Och08,Och09,Och10,Och11,Och12,Och13,Och14	*OreG0004616*
scaffold141	103129	G	A	Och01,Och02,Och03,Och04,Och05,Och06,Och07,Och08,Och09,Och10,Och11,Och12,Och13,Och14	*OreG0002169*
scaffold141	103777	G	A	Och01,Och02,Och03,Och04,Och05,Och06,Och09,Och10,Och11,Och12,Och13	*OreG0002169*

scaffold141	196367	G	A	Ore01,Ore02,Ore03,Ore04,Ore05,Ore06,Ore07,Ore08,Ore09,Ore10,Ore11,Ore12,Ore13,Ore14	*OreG0002178*
scaffold141	258101	T	A	NA	*OreG0002187*
scaffold141	301020	C	T	Och01,Och02,Och03,Och04,Och05,Och06,Och07,Och08,Och09,Och10,Och11,Och12,Och13,Och14,Ore01,Ore02,Ore03,Ore04,Ore05,Ore06,Ore07,Ore08,Ore09,Ore10,Ore11,Ore12,Ore13,Ore14	*OreG0002192*
scaffold141	449828	C	A	NA	*OreG0002208*
scaffold141	532707	G	A	NA	*OreG0002217*
scaffold141	669131	A	T	Och01,Och02,Och03,Och04,Och05,Och06,Och07,Och08,Och09,Och10,Och11,Och12,Och13,Och14,Ore01,Ore02,Ore03,Ore04,Ore05,Ore06,Ore07,Ore08,Ore09,Ore10,Ore11,Ore12,Ore13,Ore14	*OreG0002237*
scaffold141	700310	C	T	Och08	*OreG0002241*
scaffold141	1404483	C	T	Och01,Och02,Och03,Och04,Och05,Och06,Och07,Och08,Och09,Och10,Och11,Och12,Och13,Och14,Ore01,Ore02,Ore03,Ore04,Ore05,Ore06,Ore07,Ore08,Ore09,Ore10,Ore11,Ore12,Ore13,Ore14	*OreG0002315*
scaffold141	1405968	G	A	Ore01,Ore02,Ore03,Ore04,Ore05,Ore06,Ore07,Ore08,Ore09,Ore10,Ore11,Ore12,Ore13,Ore14	*OreG0002317*
scaffold141	1555085	A	T	Och01,Och02,Och03,Och04,Och05,Och06,Och07,Och08,Och09,Och10,Och11,Och12,Och13,Och14,Ore01,Ore02,Ore03,Ore04,Ore05,Ore06,Ore07,Ore08,Ore09,Ore10,Ore11,Ore12,Ore13,Ore14	*OreG0002323*
scaffold141	1658882	G	A	NA	*OreG0002330*
scaffold141	1673583	G	A	Och01,Och02,Och03,Och04,Och05,Och06,Och07,Och08,Och09,Och10,Och11,Och12,Och13,Och14	*OreG0002332*
scaffold141	1704678	C	T	Ore01,Ore02,Ore03,Ore04,Ore05,Ore06,Ore07,Ore08,Ore09,Ore10,Ore11,Ore12,Ore13,Ore14	*OreG0002334*
scaffold118	69338	A	T	Ore01,Ore02,Ore03,Ore04,Ore05,Ore06,Ore07,Ore08,Ore09,Ore10,Ore11,Ore12,Ore13,Ore14	*OreG0001082*
scaffold118	75873	T	A	NA	*OreG0001083*
scaffold118	141472	T	A	NA	*OreG0001096*
scaffold118	476939	G	A	NA	*OreG0001131*
scaffold118	784093	G	C	NA	*OreG0001163*
scaffold118	842128	G	T	Och08	*OreG0001171*
scaffold118	1214499	C	A	Och01,Och02,Och03,Och04,Och05,Och06,Och07,Och08,Och09,Och10,Och11,Och12,Och13,Och14,Ore01,Ore02,Ore03,Ore04,Ore05,Ore06,Ore07,Ore08,Ore09,Ore10,Ore11,Ore12,Ore13,Ore14	*OreG0001216*
scaffold118	1417621	A	T	Och02,Och09,Och10,Och11,Och14	*OreG0001240*
scaffold118	1426093	G	A	NA	*OreG0001243*
scaffold118	1586094	T	A	NA	*OreG0001258*
scaffold118	1744512	C	A	Och03	*OreG0001264*
scaffold118	1992572	G	A	NA	*OreG0001293*
scaffold118	1992856	C	T	NA	*OreG0001293*
scaffold118	1993360	C	T	NA	*OreG0001293*
scaffold118	2007627	C	T	NA	*OreG0001294*
scaffold118	2054426	C	T	NA	*OreG0001300*
scaffold118	2090558	C	G	NA	*OreG0001305*
scaffold118	2091225	C	T	NA	*OreG0001305*
scaffold76	5844	C	T	NA	*OreG0025293*
scaffold76	6879	G	A	NA	*OreG0025293*
scaffold76	38705	C	T	Och01,Och02,Och03,Och04,Och05,Och06,Och07,Och08,Och09,Och10,Och11,Och12,Och13,Och14	*OreG0025295*
scaffold76	43107	C	T	NA	*OreG0025296*
scaffold76	171231	G	A	NA	*OreG0025305*
scaffold76	171322	C	T	NA	*OreG0025305*
scaffold76	171367	A	T	NA	*OreG0025305*
scaffold76	186723	C	T	NA	*OreG0025307*
scaffold76	187132	G	A	Och13	*OreG0025307*
scaffold76	187287	G	C	Och02,Och06	*OreG0025307*
scaffold76	206097	G	T	NA	*OreG0025310*
scaffold76	679012	C	A	NA	*OreG0025352*
scaffold76	684494	T	A	Ore03,Ore07,Ore08,Ore10,Ore14	*OreG0025353*
scaffold76	1135106	C	T	NA	*OreG0025385*
scaffold164	484920	G	A	NA	*OreG0003380*
scaffold164	502474	C	A	Och01,Och02,Och03,Och04,Och05,Och06,Och07,Och08,Och09,Och10,Och11,Och12,Och13,Och14	*OreG0003382*
scaffold164	537984	T	A	Ore04,Ore05	*OreG0003386*
scaffold164	544060	C	T	NA	*OreG0003387*
scaffold164	745077	A	T	NA	*OreG0003399*
scaffold164	746250	C	T	Och04,Och06,Och13,Och14	*OreG0003399*
scaffold164	754782	A	C	NA	*OreG0003400*
scaffold164	754872	A	T	NA	*OreG0003400*
scaffold164	768775	A	T	Och01,Och02,Och03,Och04,Och05,Och06,Och07,Och08,Och09,Och10,Och11,Och12,Och13,Och14	*OreG0003401*
scaffold164	992626	G	A	NA	*OreG0003423*
scaffold565	113540	C	T	Och01,Och02,Och03,Och04,Och05,Och06,Och07,Och08,Och09,Och10,Och11,Och12,Och13,Och14	*OreG0023626*

scaffold565	418566	C	A	NA	*OreG0023642*
scaffold565	493126	A	T	NA	*OreG0023649*
scaffold565	511847	G	A	NA	*OreG0023653*
scaffold565	511883	T	A	NA	*OreG0023653*
scaffold565	611106	C	A	NA	*OreG0023663*
scaffold565	611133	T	A	NA	*OreG0023663*
scaffold565	611589	C	A	NA	*OreG0023663*
scaffold146	63064	A	T	Och01,Och02,Och03,Och04,Och05,Och06,Och07,Och08,Och09,Och10,Och11,Och12,Och13,Och14,Ore01,Ore02,Ore03,Ore04,Ore05,Ore06,Ore07,Ore08,Ore09,Ore10,Ore11,Ore12,Ore13,Ore14	*OreG0002562*
scaffold146	95053	G	T	Och01,Och08	*OreG0002565*
scaffold146	103757	G	T	Och12,Och13	*OreG0002566*
scaffold146	170170	A	T	NA	*OreG0002570*
scaffold146	630815	G	T	NA	*OreG0002598*
scaffold146	739942	T	A	Ore01,Ore02,Ore03,Ore04,Ore05,Ore06,Ore07,Ore08,Ore09,Ore10,Ore11,Ore12,Ore13,Ore14	*OreG0002608*
scaffold250	128080	G	T	NA	*OreG0007633*
scaffold250	128521	G	A	NA	*OreG0007633*
scaffold250	183632	C	T	Och01	*OreG0007635*
scaffold250	185905	C	T	Och01,Och02,Och03,Och04,Och05,Och06,Och07,Och09,Och11,Och12,Och13,Och14	*OreG0007635*
scaffold250	303996	C	T	Och13	*OreG0007647*
scaffold250	304291	C	T	Och02,Och03,Och04,Och05,Och07,Och08,Och11,Och13,Och14	*OreG0007647*
scaffold250	478200	C	A	NA	*OreG0007658*
scaffold250	537712	G	A	NA	*OreG0007663*
scaffold250	976429	C	T	NA	*OreG0007691*
scaffold250	1021208	C	T	NA	*OreG0007698*
scaffold250	1084702	C	A	Och01,Och02,Och03,Och04,Och05,Och06,Och07,Och08,Och09,Och10,Och11,Och12,Och13,Och14	*OreG0007706*
scaffold250	1091273	C	T	NA	*OreG0007706*
scaffold250	1091329	G	C	Och01,Och02,Och03,Och04,Och05,Och06,Och07,Och08,Och09,Och10,Och11,Och12,Och13,Och14	*OreG0007706*
scaffold250	1243215	G	A	Och01,Och02,Och03,Och04,Och05,Och06,Och07,Och08,Och09,Och10,Och11,Och12,Och13,Och14,Ore01,Ore02,Ore03,Ore04,Ore05,Ore06,Ore07,Ore08,Ore09,Ore10,Ore11,Ore12,Ore13,Ore14	*OreG0007724*
scaffold250	1323316	C	A	NA	*OreG0007733*
scaffold250	1333316	C	A	Ore02,Ore07,Ore09,Ore10,Ore11,Ore12,Ore13,Ore14	*OreG0007735*
scaffold250	1386519	C	T	Och01,Och02,Och03,Och04,Och05,Och06,Och07,Och08,Och09,Och10,Och11,Och12,Och13,Och14,Ore01,Ore02,Ore03,Ore04,Ore05,Ore06,Ore07,Ore08,Ore09,Ore10,Ore11,Ore12,Ore13,Ore14	*OreG0007742*
scaffold250	1435084	A	T	NA	*OreG0007746*
scaffold250	1440518	G	T	Ore02,Ore09,Ore10,Ore14	*OreG0007747*
scaffold250	1718833	G	T	Och08	*OreG0007769*
scaffold250	1753290	G	A	NA	*OreG0007775*
scaffold579	243644	G	A	NA	*OreG0023705*
scaffold579	243799	C	T	NA	*OreG0023705*
scaffold579	245772	G	T	Och08,Ore09	*OreG0023705*
scaffold579	472592	G	T	Ore03,Ore10,Ore13	*OreG0023730*
scaffold579	472595	G	T	Ore01,Ore02,Ore03,Ore04,Ore05,Ore06,Ore07,Ore08,Ore09,Ore10,Ore11,Ore12,Ore13,Ore14	*OreG0023730*
scaffold579	509392	C	A	NA	*OreG0023739*
scaffold579	550363	C	T	Och01,Och02,Och03,Och04,Och05,Och06,Och07,Och08,Och09,Och10,Och11,Och12,Och13,Och14	*OreG0023744*
scaffold579	642264	C	T	Och01,Och02,Och03,Och04,Och05,Och06,Och07,Och08,Och09,Och10,Och11,Och12,Och13,Och14,Ore01,Ore02,Ore03,Ore04,Ore05,Ore06,Ore07,Ore08,Ore09,Ore10,Ore11,Ore12,Ore13,Ore14	*OreG0023751*
scaffold579	833668	C	A	NA	*OreG0023773*
scaffold579	859215	C	T	NA	*OreG0023777*
scaffold579	859264	C	T	NA	*OreG0023777*
scaffold579	859526	G	A	NA	*OreG0023777*
scaffold579	901245	C	T	NA	*OreG0023781*
scaffold579	959417	G	T	NA	*OreG0023786*
scaffold579	995445	C	T	NA	*OreG0023789*
scaffold741	10261	T	A	Ore03,Ore04,Ore05,Ore08,Ore09,Ore14	*OreG0024997*
scaffold741	72530	A	T	NA	*OreG0025004*
scaffold741	76577	G	A	Ore05	*OreG0025005*
scaffold741	89251	C	T	Ore01,Ore02,Ore03,Ore04,Ore05,Ore06,Ore07,Ore08,Ore09,Ore10,Ore11,Ore12,Ore13,Ore14	*OreG0025007*
scaffold741	163795	C	A	NA	*OreG0025014*
scaffold61	132777	C	T	NA	*OreG0024163*
scaffold61	387880	C	A	NA	*OreG0024180*
scaffold61	392632	C	A	Och06	*OreG0024181*
scaffold61	392869	C	A	Och01,Och02,Och03,Och04,Och05,Och06,Och07,Och08,Och09,Och10,Och11,Och12,Och13,Och14,Ore01,Ore02,Ore03,Ore04,Ore05,Ore06,Ore07,Ore08,Ore09,Ore10,Ore11,Ore12,Ore13,Ore14	*OreG0024181*

scaffold61	433681	C	T	NA	*OreG0024185*
scaffold460	80904	C	T	Och09,Och10,Ore01,Ore03,Ore04,Ore05,Ore09,Ore10,Ore11	*OreG0021801*
scaffold460	177688	G	A	NA	*OreG0021802*
scaffold460	388283	G	A	Ore01,Ore02,Ore03,Ore04,Ore05,Ore06,Ore07,Ore08,Ore09,Ore10,Ore11,Ore12,Ore13,Ore14	*OreG0021812*
scaffold460	473309	C	A	Och01,Och02,Och03,Och04,Och05,Och06,Och07,Och08,Och09,Och10,Och11,Och12,Och13,Och14,Ore01,Ore02,Ore03,Ore04,Ore05,Ore06,Ore07,Ore08,Ore09,Ore10,Ore11,Ore12,Ore13,Ore14	*OreG0021819*
scaffold460	488186	G	A	Och13	*OreG0021821*
scaffold460	532873	C	A	NA	*OreG0021825*
scaffold460	532920	G	C	NA	*OreG0021825*
scaffold460	641106	A	T	NA	*OreG0021834*
scaffold460	680060	G	A	NA	*OreG0021838*
scaffold460	838998	C	T	Och02,Och13,Ore01,Ore02,Ore03,Ore04,Ore05,Ore06,Ore07,Ore08,Ore09,Ore10,Ore11,Ore12,Ore13,Ore14	*OreG0021845*
scaffold460	900024	T	A	Och05,Och13	*OreG0021852*
scaffold460	1140669	C	T	NA	*OreG0021861*
scaffold460	1367377	G	A	NA	*OreG0021875*
scaffold460	1383455	G	C	Ore02,Ore04,Ore05,Ore06,Ore07,Ore10,Ore11,Ore13	*OreG0021876*
scaffold4	490007	A	T	Och01,Och02,Och03,Och04,Och05,Och06,Och12,Och13	*OreG0019603*
scaffold4	843873	G	A	Och01,Och02,Och03,Och04,Och05,Och06,Och07,Och08,Och09,Och10,Och11,Och12,Och13,Och14,Ore01,Ore02,Ore03,Ore04,Ore05,Ore06,Ore07,Ore08,Ore09,Ore10,Ore11,Ore12,Ore13,Ore14	*OreG0019617*
scaffold4	893831	G	A	NA	*OreG0019620*
scaffold4	1298410	A	C	NA	*OreG0019632*
scaffold4	1354590	T	G	Och01,Och02,Och03,Och04,Och05,Och06,Och07,Och08,Och09,Och10,Och11,Och12,Och13,Och14,Ore01,Ore02,Ore03,Ore04,Ore05,Ore06,Ore07,Ore08,Ore09,Ore10,Ore11,Ore12,Ore13,Ore14	*OreG0019634*
scaffold4	1568284	C	T	Och02,Och04,Och06,Och07,Och09,Och10,Och11	*OreG0019642*
scaffold4	1573144	C	T	Ore01,Ore02,Ore03,Ore04,Ore05,Ore06,Ore07,Ore08,Ore09,Ore10,Ore11,Ore12,Ore13,Ore14	*OreG0019642*
scaffold4	1638755	T	A	NA	*OreG0019646*
scaffold4	1676354	C	T	Och02,Och03,Och04,Och05,Och06,Och12,Och13	*OreG0019648*
scaffold4	1676516	C	T	Ore01,Ore02,Ore03,Ore04,Ore05,Ore06,Ore07,Ore08,Ore09,Ore10,Ore11,Ore12,Ore13,Ore14	*OreG0019649*
scaffold424	68868	C	A	NA	*OreG0020108*
scaffold424	148028	G	A	Och01,Och02,Och04,Och07,Och08,Och09,Och10,Och11,Och12,Och13,Och14	*OreG0020119*
scaffold424	445750	T	A	NA	*OreG0020156*
scaffold424	445919	G	T	NA	*OreG0020156*
scaffold424	887386	G	A	Och01,Och02,Och03,Och04,Och05,Och06,Och07,Och08,Och09,Och10,Och11,Och12,Och13,Och14,Ore01,Ore02,Ore03,Ore04,Ore05,Ore06,Ore07,Ore08,Ore09,Ore10,Ore11,Ore12,Ore13,Ore14	*OreG0020178*
scaffold424	888327	C	A	NA	*OreG0020178*
scaffold424	965719	G	C	NA	*OreG0020186*
scaffold424	1053714	G	A	NA	*OreG0020193*
scaffold424	1102456	G	T	Och01,Och02,Och03,Och04,Och05,Och06,Och07,Och08,Och09,Och10,Och11,Och12,Och13,Och14,Ore01,Ore02,Ore03,Ore04,Ore05,Ore06,Ore07,Ore08,Ore09,Ore10,Ore11,Ore12,Ore13,Ore14	*OreG0020197*
scaffold424	1180381	G	T	NA	*OreG0020206*
scaffold424	1244697	G	A	NA	*OreG0020214*
scaffold424	1464368	T	G	Ore01,Ore02,Ore03,Ore04,Ore05,Ore06,Ore07,Ore08,Ore09,Ore10,Ore11,Ore12,Ore13,Ore14	*OreG0020236*
scaffold424	1482934	A	T	Ore01,Ore02,Ore03,Ore04,Ore05,Ore06,Ore07,Ore08,Ore09,Ore10,Ore11,Ore12,Ore13,Ore14	*OreG0020237*
scaffold424	1497011	G	T	NA	*OreG0020239*
scaffold424	1662078	A	T	Och01,Och02,Och03,Och04,Och05,Och06,Och07,Och08,Och12,Och13,Och14	*OreG0020257*
scaffold424	1785400	G	A	Och02,Och03,Och04,Och07,Och09,Och10,Och11,Och12,Och13	*OreG0020271*
scaffold424	2035783	G	T	Och01	*OreG0020291*
scaffold424	2176518	A	C	Och05,Och14	*OreG0020305*
scaffold424	2338316	G	A	NA	*OreG0020325*
scaffold424	2345337	G	A	NA	*OreG0020326*
scaffold424	2345579	C	A	Och14	*OreG0020326*
scaffold424	2345805	G	A	Och06	*OreG0020326*
scaffold424	2353395	C	T	NA	*OreG0020328*
scaffold424	2354094	C	T	Och14	*OreG0020328*
scaffold424	2356087	C	T	Och14	*OreG0020328*
scaffold618	67704	C	A	NA	*OreG0024204*
scaffold618	267529	G	A	NA	*OreG0024221*
scaffold951	87925	C	T	NA	*OreG0026941*
scaffold524	43884	G	A	NA	*OreG0023565*
scaffold524	43886	A	T	NA	*OreG0023565*
scaffold606	30086	C	A	NA	*OreG0024054*
scaffold606	33695	T	A	Och01,Och02,Och03,Och04,Och05,Och06,Och07,Och08,Och09,Och10,Och11,Och12,Och13,Och14,Ore01,Ore02,Ore03,Ore04,Ore05,Ore06,Ore07,Ore08,Ore09,Ore10,Ore11,Ore12,Ore13,Ore14	*OreG0024054*

scaffold606	120288	G	T	NA	*OreG0024065*
scaffold606	262976	C	T	NA	*OreG0024085*
scaffold606	433299	G	T	NA	*OreG0024102*
scaffold606	545233	C	A	NA	*OreG0024120*
scaffold185	60228	C	T	NA	*OreG0003983*
scaffold185	77565	C	T	Och01,Och02,Och03,Och04,Och05,Och06,Och07,Och08,Och09,Och10,Och11,Och12,Och13,Och14,Ore01,Ore02,Ore03,Ore04,Ore05,Ore06,Ore07,Ore08,Ore09,Ore10,Ore11,Ore12,Ore13,Ore14	*OreG0003986*
scaffold185	83651	G	A	Och08,Och11,Och14	*OreG0003988*
scaffold185	83657	C	A	Och12,Och13	*OreG0003988*
scaffold185	83774	G	A	Och12,Och13	*OreG0003988*
scaffold185	112200	C	A	NA	*OreG0003993*
scaffold185	126029	G	T	NA	*OreG0003995*
scaffold185	132072	C	A	Och01,Och02,Och03,Och04,Och05,Och06,Och07,Och08,Och09,Och10,Och11,Och12,Och13,Och14,Ore02,Ore03,Ore06,Ore07,Ore09,Ore10,Ore11,Ore12,Ore13,Ore14	*OreG0003995*
scaffold185	250203	C	T	Och03,Och06,Och08,Och11	*OreG0004013*
scaffold185	271333	G	T	Och03,Och06	*OreG0004018*
scaffold185	272504	T	A	NA	*OreG0004018*
scaffold185	273557	G	T	NA	*OreG0004018*
scaffold185	282532	G	A	NA	*OreG0004020*
scaffold185	402327	T	A	Och01,Och02,Och03,Och04,Och05,Och06,Och07,Och08,Och09,Och10,Och11,Och12,Och13,Och14,Ore01,Ore02,Ore03,Ore04,Ore05,Ore06,Ore07,Ore08,Ore09,Ore10,Ore11,Ore12,Ore13,Ore14	*OreG0004037*
scaffold185	610018	C	T	Och12,Och13	*OreG0004053*
scaffold185	871787	G	A	Ore06,Ore07,Ore08,Ore13,Ore14	*OreG0004062*
scaffold185	916836	C	A	Och02,Och03,Och06	*OreG0004066*
scaffold185	1080478	C	T	Och01	*OreG0004070*
scaffold185	1401827	C	T	Och01,Ore01,Ore02,Ore03,Ore04,Ore05,Ore06,Ore07,Ore08,Ore09,Ore10,Ore11,Ore12,Ore13,Ore14	*OreG0004079*
scaffold185	1410315	C	A	Ore06,Ore07,Ore08,Ore13,Ore14	*OreG0004080*
scaffold185	1410364	G	T	NA	*OreG0004080*
scaffold185	1508312	A	T	Och01,Och02,Och03,Och04,Och05,Och06,Och07,Och08,Och09,Och10,Och11,Och12,Och13,Och14	*OreG0004089*
scaffold185	1648616	C	T	Och07,Och08,Och12,Och13,Och14	*OreG0004092*
scaffold185	1857036	C	T	NA	*OreG0004099*
scaffold185	1911782	C	T	NA	*OreG0004101*
scaffold185	1969072	C	T	NA	*OreG0004105*
scaffold185	2079490	C	T	NA	*OreG0004108*
scaffold185	2079919	C	T	NA	*OreG0004108*
scaffold185	2821027	A	T	NA	*OreG0004149*
scaffold185	2823393	C	G	Och12,Och13	*OreG0004150*
scaffold185	2823650	C	T	Och12,Och13	*OreG0004150*
scaffold185	2836975	C	G	NA	*OreG0004153*
scaffold185	2881934	G	A	NA	*OreG0004162*
scaffold185	2891830	C	T	NA	*OreG0004163*
scaffold185	2904405	A	T	NA	*OreG0004166*
scaffold185	3034856	G	T	Och01,Och02,Och03,Och04,Och05,Och06,Och07,Och08,Och09,Och10,Och11,Och12,Och13,Och14	*OreG0004182*
scaffold185	3343966	G	T	NA	*OreG0004218*
scaffold185	3407390	G	A	Och03,Och06	*OreG0004225*
scaffold185	3812352	C	T	NA	*OreG0004259*
scaffold733	2091	G	A	Och01,Och02,Och03,Och04,Och05,Och06,Och07,Och08,Och09,Och10,Och11,Och12,Och13,Och14	*OreG0024820*
scaffold494	5046	G	A	NA	*OreG0023027*
scaffold494	258482	C	A	NA	*OreG0023057*
scaffold494	260510	G	A	Och02,Och03,Och04,Och05,Och06,Och12,Och13	*OreG0023057*
scaffold494	439692	G	A	Och01,Och02,Och03,Och04,Och05,Och06,Och07,Och08,Och09,Och10,Och11,Och12,Och13,Och14	*OreG0023076*
scaffold494	492184	A	T	NA	*OreG0023082*
scaffold494	495876	C	T	NA	*OreG0023082*
scaffold494	496582	C	A	Ore12,Ore14	*OreG0023082*
scaffold494	496993	C	T	NA	*OreG0023082*
scaffold494	614929	G	A	Och08	*OreG0023101*
scaffold494	631513	G	A	NA	*OreG0023104*
scaffold494	744677	G	T	NA	*OreG0023118*
scaffold494	787477	T	A	NA	*OreG0023123*
scaffold494	880713	C	T	NA	*OreG0023133*
scaffold494	1411817	G	C	Och05	*OreG0023202*
scaffold494	1431754	A	T	Ore01,Ore02,Ore03,Ore04,Ore05,Ore06,Ore07,Ore08,Ore09,Ore10,Ore11,Ore12,Ore13,Ore14	*OreG0023205*
scaffold494	1432239	G	A	NA	*OreG0023205*
scaffold494	1432257	C	A	Och01,Och02,Och03,Och04,Och05,Och06,Och07,Och08,Och09,Och10,Och12,Och13	*OreG0023205*
scaffold494	1452791	G	C	Och01,Och02,Och03,Och04,Och05,Och06,Och07,Och08,Och09,Och10,Och11,Och12,Och13,Och14	*OreG0023208*

scaffold494	1564038	G	A	NA	*OreG0023225*
scaffold494	1697804	C	T	NA	*OreG0023246*
scaffold494	1864227	G	T	Och06	*OreG0023269*
scaffold494	1903978	T	A	Och06,Och08,Och14	*OreG0023273*
scaffold494	1904032	G	A	Och04,Och05,Och12,Och13	*OreG0023273*
scaffold494	1904078	G	T	Och04,Och05,Och12,Och13	*OreG0023273*
scaffold494	1910421	G	T	Och04,Och05,Och12,Och13	*OreG0023274*
scaffold494	1911275	A	T	Och06,Och08,Och14	*OreG0023274*
scaffold494	1911415	C	A	Ore09,Ore13,Ore14	*OreG0023274*
scaffold494	2127443	C	T	NA	*OreG0023293*
scaffold465	197865	G	T	Och01	*OreG0021911*
scaffold465	374112	G	A	NA	*OreG0021928*
scaffold465	398392	C	T	Och03,Och05,Och08	*OreG0021929*
scaffold465	587599	C	T	NA	*OreG0021937*
scaffold465	791178	T	A	NA	*OreG0021954*
scaffold465	878933	G	T	NA	*OreG0021963*
scaffold465	919747	A	T	Och01,Och02,Och03,Och04,Och05,Och06,Och07,Och08,Och09,Och10,Och11,Och12,Och13,Och14,Ore01,Ore02,Ore03,Ore04,Ore05,Ore06,Ore07,Ore08,Ore09,Ore10,Ore11,Ore12,Ore13,Ore14	*OreG0021969*
scaffold465	1019189	C	A	Och03,Och04,Och05,Och06,Och07,Och08,Och09,Och10,Och11,Och12,Och14	*OreG0021981*
scaffold465	1029368	G	A	NA	*OreG0021984*
scaffold465	1313719	C	T	Och01,Och02,Och03,Och04,Och05,Och06,Och07,Och08,Och09,Och10,Och11,Och12,Och13,Och14,Ore01,Ore02,Ore03,Ore04,Ore05,Ore06,Ore07,Ore08,Ore09,Ore10,Ore11,Ore12,Ore13,Ore14	*OreG0022019*
scaffold465	1430909	G	A	NA	*OreG0022025*
scaffold465	1474773	T	A	Och01,Ore01,Ore02,Ore03,Ore04,Ore05,Ore06,Ore07,Ore08,Ore09,Ore10,Ore11,Ore12,Ore13,Ore14	*OreG0022029*
scaffold465	1481250	G	A	NA	*OreG0022033*
scaffold465	1627992	G	A	Och01,Och02,Och03,Och04,Och05,Och06,Och08,Och09,Och10,Och11,Och12,Och13,Och14	*OreG0022046*
scaffold465	1646909	T	A	Och01,Och02,Och03,Och04,Och05,Och06,Och07,Och08,Och09,Och10,Och11,Och12,Och13,Och14,Ore01,Ore02,Ore03,Ore04,Ore05,Ore06,Ore07,Ore08,Ore09,Ore10,Ore11,Ore12,Ore13,Ore14	*OreG0022048*
scaffold465	2017405	C	T	Och06,Och11,Och13	*OreG0022076*
scaffold465	2112847	C	A	Och01,Och07,Och08,Och11	*OreG0022081*
scaffold465	2114340	G	C	NA	*OreG0022081*
scaffold465	2168371	A	T	NA	*OreG0022086*
scaffold465	2168401	C	T	NA	*OreG0022086*
scaffold465	2203274	A	C	NA	*OreG0022089*
scaffold466	111973	C	T	Och01	*OreG0022105*
scaffold466	167966	C	A	NA	*OreG0022116*
scaffold466	178700	G	A	NA	*OreG0022118*
scaffold466	307399	G	A	NA	*OreG0022131*
scaffold466	547285	G	A	NA	*OreG0022164*
scaffold466	602883	A	T	NA	*OreG0022172*
scaffold466	700312	T	A	Ore01,Ore02,Ore03,Ore04,Ore05,Ore06,Ore07,Ore08,Ore09,Ore10,Ore11,Ore12,Ore13,Ore14	*OreG0022183*
scaffold466	713916	T	A	Ore01,Ore02,Ore03,Ore04,Ore05,Ore06,Ore07,Ore08,Ore09,Ore10,Ore11,Ore12,Ore13,Ore14	*OreG0022186*
scaffold466	744732	G	T	Och01,Och06	*OreG0022194*
scaffold466	749087	G	T	NA	*OreG0022195*
scaffold466	749206	G	A	NA	*OreG0022195*
scaffold466	749479	G	A	NA	*OreG0022195*
scaffold466	780050	G	A	Ore03,Ore04,Ore05,Ore09,Ore14	*OreG0022197*
scaffold466	819075	G	A	NA	*OreG0022204*
scaffold466	916829	C	T	Och01,Och02,Och03,Och04,Och05,Och06,Och07,Och08,Och09,Och10,Och11,Och12,Och13,Och14,Ore01,Ore02,Ore03,Ore04,Ore05,Ore06,Ore07,Ore08,Ore09,Ore10,Ore11,Ore12,Ore13,Ore14	*OreG0022215*
scaffold466	975895	T	A	Och02,Och03,Och04,Och05,Och06,Och09,Och10,Och11,Och12,Och13,Och14	*OreG0022218*
scaffold466	1002366	C	T	Och01,Och02,Och03,Och04,Och05,Och06,Och07,Och08,Och09,Och10,Och11,Och12,Och13,Och14,Ore01,Ore02,Ore03,Ore04,Ore05,Ore06,Ore07,Ore08,Ore09,Ore10,Ore11,Ore12,Ore13,Ore14	*OreG0022221*
scaffold466	1190786	G	T	NA	*OreG0022234*
scaffold466	1564385	G	A	NA	*OreG0022258*
scaffold466	1667450	G	A	Och01,Och02,Och03,Och04,Och05,Och06,Och07,Och08,Och09,Och10,Och11,Och12,Och13,Och14	*OreG0022266*
scaffold466	1705535	C	T	NA	*OreG0022273*
scaffold466	1773409	G	T	NA	*OreG0022283*
scaffold466	1834129	G	A	Ore01,Ore02,Ore03,Ore04,Ore05,Ore06,Ore07,Ore08,Ore09,Ore10,Ore11,Ore12,Ore13,Ore14	*OreG0022294*
scaffold466	1850660	C	T	Och02,Och03,Och04,Och06,Och13	*OreG0022296*
scaffold466	1924400	G	T	NA	*OreG0022307*
scaffold466	1927557	G	T	NA	*OreG0022307*
scaffold466	1931276	G	A	NA	*OreG0022308*
scaffold466	1936196	A	T	NA	*OreG0022308*

scaffold466	1936205	G	T	NA	*OreG0022308*
scaffold299	100389	A	T	Och01,Och02,Och03,Och04,Och05,Och06,Och07,Och08,Och09,Och10,Och11,Och12,Och13,Och14,Ore01,Ore02,Ore03,Ore04,Ore05,Ore06,Ore07,Ore08,Ore09,Ore10,Ore11,Ore12,Ore13,Ore14	*OreG0008580*
scaffold299	100495	G	T	Och01,Och03,Och04,Och07	*OreG0008580*
scaffold299	109835	C	T	Och01,Och02,Och03,Och04,Och05,Och06,Och07,Och08,Och09,Och10,Och11,Och12,Och13,Och14	*OreG0008581*
scaffold299	276682	G	A	Och01,Och02,Och03,Och04,Och05,Och06,Och07,Och08,Och09,Och10,Och11,Och12,Och13,Och14,Ore01,Ore02,Ore03,Ore04,Ore05,Ore06,Ore07,Ore08,Ore09,Ore10,Ore11,Ore12,Ore13,Ore14	*OreG0008583*
scaffold1318	16552	C	T	NA	*OreG0001995*
scaffold928	299229	G	A	Och01,Och02,Och03,Och04,Och05,Och06,Och07,Och08,Och09,Och10,Och11,Och12,Och13,Och14,Ore01,Ore02,Ore03,Ore04,Ore05,Ore06,Ore07,Ore08,Ore09,Ore10,Ore11,Ore12,Ore13,Ore14	*OreG0026793*
scaffold928	319487	A	T	NA	*OreG0026794*
scaffold928	454239	G	T	NA	*OreG0026799*
scaffold928	520118	A	T	NA	*OreG0026804*
scaffold928	523441	G	A	NA	*OreG0026805*
scaffold928	554900	C	T	Och02,Och03,Och04,Och05,Och06,Och09,Och10,Och11,Och12,Och13,Och14	*OreG0026806*
scaffold928	555020	C	T	Och02	*OreG0026806*
scaffold928	590663	T	A	Och02	*OreG0026809*
scaffold928	663573	G	A	Och01,Och03,Och04	*OreG0026814*
scaffold928	664032	C	T	NA	*OreG0026814*
scaffold476	387204	G	T	Och01,Och02,Och03,Och04,Och05,Och06,Och07,Och08,Och09,Och10,Och11,Och12,Och13,Och14	*OreG0022770*
scaffold476	406941	G	T	NA	*OreG0022771*
scaffold476	842438	G	A	Och01,Och02,Och03,Och04,Och05,Och06,Och07,Och08,Och09,Och10,Och11,Och12,Och13,Och14	*OreG0022787*
scaffold476	842566	G	T	Och01,Och02,Och03,Och04,Och05,Och06,Och07,Och08,Och09,Och10,Och11,Och12,Och13,Och14	*OreG0022787*
scaffold476	1279589	C	T	NA	*OreG0022790*
scaffold476	1363342	C	A	NA	*OreG0022795*
scaffold471	12559	A	T	NA	*OreG0022519*
scaffold471	83209	T	A	Och02,Och03,Och04,Och05,Och06,Och07,Och08,Och09,Och10,Och11,Och12,Och13,Och14	*OreG0022528*
scaffold471	91265	G	A	NA	*OreG0022529*
scaffold471	91266	G	A	NA	*OreG0022529*
scaffold471	170072	G	T	NA	*OreG0022538*
scaffold471	331356	C	T	NA	*OreG0022554*
scaffold471	343337	T	A	Och01,Och02,Och03,Och04,Och05,Och06,Och07,Och08,Och09,Och10,Och11,Och12,Och13,Och14,Ore01,Ore02,Ore03,Ore04,Ore05,Ore06,Ore07,Ore08,Ore09,Ore10,Ore11,Ore12,Ore13,Ore14	*OreG0022555*
scaffold471	484293	G	C	NA	*OreG0022569*
scaffold471	572726	A	T	NA	*OreG0022572*
scaffold471	715437	C	T	Och02,Och03,Och06,Och07,Och08,Och09,Och10,Och11,Och12,Och14	*OreG0022581*
scaffold471	838073	G	A	NA	*OreG0022589*
scaffold471	859909	G	T	NA	*OreG0022592*
scaffold471	917726	C	A	Ore03	*OreG0022600*
scaffold471	1054146	T	A	Och01,Och02,Och03,Och04,Och05,Och08,Och12,Och13,Ore01,Ore02,Ore03,Ore04,Ore05,Ore06,Ore07,Ore08,Ore09,Ore10,Ore11,Ore12,Ore13,Ore14	*OreG0022618*
scaffold471	1093020	G	A	Och02,Och03,Och04,Och05,Och12,Och13	*OreG0022621*
scaffold471	1094553	G	A	NA	*OreG0022621*
scaffold471	1094764	G	A	NA	*OreG0022621*
scaffold471	1114836	G	A	Och02,Och03,Och04,Och05,Och12,Och13	*OreG0022626*
scaffold471	1276073	G	A	Ore01,Ore02,Ore03,Ore04,Ore05,Ore06,Ore07,Ore08,Ore09,Ore10,Ore11,Ore12,Ore13,Ore14	*OreG0022639*
scaffold471	1276157	C	A	Ore01,Ore02,Ore03,Ore04,Ore05,Ore06,Ore07,Ore08,Ore09,Ore10,Ore11,Ore12,Ore13,Ore14	*OreG0022639*
scaffold471	1356955	G	A	Ore01,Ore02,Ore03,Ore04,Ore05,Ore06,Ore07,Ore08,Ore09,Ore10,Ore11,Ore12,Ore13,Ore14	*OreG0022647*
scaffold471	1631085	A	T	NA	*OreG0022668*
scaffold471	1903828	G	A	Och01,Och02,Och03,Och04,Och05,Och06,Och07,Och08,Och09,Och10,Och11,Och12,Och13,Och14,Ore01,Ore02,Ore03,Ore04,Ore05,Ore06,Ore07,Ore08,Ore09,Ore10,Ore11,Ore12,Ore13,Ore14	*OreG0022687*
scaffold471	1991801	G	A	NA	*OreG0022695*
scaffold471	2002250	G	T	Och01,Och02,Och03,Och04,Och05,Och06,Och07,Och08,Och09,Och10,Och11,Och12,Och13,Och14	*OreG0022696*
scaffold471	2180356	G	A	NA	*OreG0022714*
scaffold471	2206595	T	G	NA	*OreG0022718*
scaffold471	2207793	G	A	NA	*OreG0022718*
scaffold471	2207876	G	A	NA	*OreG0022718*
scaffold471	2208351	C	A	NA	*OreG0022718*
scaffold471	2235689	T	A	Och01,Och02,Och03,Och04,Och05,Och06,Och07,Och08,Och09,Och10,Och11,Och12,Och13,Och14	*OreG0022721*

scaffold471	2235923	C	T	Och01,Och02,Och03,Och04,Och05,Och06,Och07,Och08,Och09,Och10,Och11,Och12,Och13,Och14	*OreG0022721*
scaffold471	2260232	C	T	NA	*OreG0022722*
scaffold451	4002	G	A	Och01,Och02,Och03,Och04,Och05,Och06,Och07,Och08,Och09,Och10,Och11,Och12,Och13,Och14	*OreG0020563*
scaffold451	96500	C	T	NA	*OreG0020571*
scaffold451	193493	C	A	NA	*OreG0020575*
scaffold451	272039	T	G	Och06	*OreG0020584*
scaffold451	443299	T	G	NA	*OreG0020602*
scaffold451	713137	C	T	Och01,Och02,Och03,Och04,Och05,Och06,Och07,Och08,Och09,Och10,Och11,Och12,Och13,Och14	*OreG0020627*
scaffold451	713247	G	A	Och01,Och02,Och03,Och04,Och05,Och06,Och07,Och08,Och09,Och10,Och11,Och12,Och13,Och14	*OreG0020627*
scaffold451	713258	C	T	NA	*OreG0020627*
scaffold451	751774	G	T	Och01,Och02,Och03,Och04,Och05,Och06,Och07,Och08,Och09,Och10,Och11,Och12,Och13,Och14,Ore01,Ore02,Ore03,Ore04,Ore05,Ore06,Ore07,Ore08,Ore09,Ore10,Ore11,Ore12,Ore13,Ore14	*OreG0020631*
scaffold451	1004538	G	A	Och02,Och03,Och04,Och12,Och13,Och14	*OreG0020650*
scaffold451	1004825	C	T	NA	*OreG0020650*
scaffold451	1238097	G	T	NA	*OreG0020673*
scaffold451	1238801	C	A	Och01,Och02,Och03,Och04,Och05,Och06,Och07,Och08,Och09,Och10,Och11,Och12,Och13,Och14	*OreG0020674*
scaffold451	1249264	G	A	NA	*OreG0020678*
scaffold451	1255019	G	A	Och01,Och02,Och03,Och04,Och05,Och06,Och07,Och08,Och11,Och12,Och13,Och14	*OreG0020681*
scaffold451	1256342	C	A	Och01,Och02,Och03,Och04,Och05,Och06,Och07,Och08,Och11,Och12,Och13,Och14	*OreG0020681*
scaffold451	1265326	G	A	NA	*OreG0020683*
scaffold451	1290804	G	A	Och01,Och02,Och03,Och04,Och05,Och06,Och07,Och08,Och12,Och13,Och14	*OreG0020688*
scaffold451	1291446	G	A	NA	*OreG0020688*
scaffold451	1291476	T	A	NA	*OreG0020688*
scaffold451	1338451	A	T	Och01,Och02,Och03,Och04,Och05,Och06,Och07,Och08,Och09,Och10,Och11,Och12,Och13,Och14,Ore01,Ore02,Ore03,Ore04,Ore05,Ore06,Ore07,Ore08,Ore09,Ore10,Ore11,Ore12,Ore13,Ore14	*OreG0020692*
scaffold451	1351754	G	A	Och02,Och03,Och04,Och05,Och06,Och07,Och08,Och12,Och13	*OreG0020694*
scaffold451	1649889	G	A	Och01	*OreG0020720*
scaffold451	1787515	T	G	NA	*OreG0020731*
scaffold451	1788177	G	A	NA	*OreG0020731*
scaffold451	2053872	G	T	NA	*OreG0020763*
scaffold451	2108399	G	A	Och01,Och02,Och03,Och04,Och05,Och06,Och09,Och10,Och12,Och13	*OreG0020763*
scaffold451	2336589	C	T	Ore07,Ore14	*OreG0020778*
scaffold451	2504068	G	A	NA	*OreG0020797*
scaffold451	2739242	C	T	NA	*OreG0020820*
scaffold451	2783257	G	A	NA	*OreG0020824*
scaffold451	2864126	T	G	NA	*OreG0020832*
scaffold451	2883805	T	G	Ore01,Ore02,Ore03,Ore06,Ore07,Ore08,Ore09,Ore10,Ore11,Ore12,Ore13,Ore14	*OreG0020835*
scaffold451	3138952	A	T	Ore01,Ore02,Ore03,Ore04,Ore05,Ore06,Ore07,Ore08,Ore09,Ore10,Ore11,Ore12,Ore13,Ore14	*OreG0020871*
scaffold451	3185929	G	T	Och02,Och06	*OreG0020879*
scaffold451	3206995	G	A	Och01,Och02,Och03,Och04,Och05,Och06,Och07,Och08,Och09,Och10,Och11,Och12,Och13,Och14	*OreG0020882*
scaffold451	3207171	G	C	Och01	*OreG0020882*
scaffold451	3290655	G	A	NA	*OreG0020893*
scaffold451	3290859	G	A	Och01,Och02,Och03,Och04,Och05,Och06,Och07,Och08,Och09,Och10,Och11,Och12,Och13,Och14	*OreG0020893*
scaffold451	3294820	C	T	Och02,Och03,Och04,Och05,Och06,Och08,Och09,Och10,Och12,Och13,Och14	*OreG0020894*
scaffold451	3301021	T	G	Ore09	*OreG0020895*
scaffold451	3329472	G	T	Och01,Och02,Och03,Och04,Och05,Och06,Och07,Och08,Och09,Och10,Och11,Och12,Och13,Och14	*OreG0020897*
scaffold451	3378012	G	T	NA	*OreG0020903*
scaffold1183	10651	G	A	NA	*OreG0001323*
scaffold48	19204	G	A	NA	*OreG0022810*
scaffold48	194814	G	T	Och05,Och12	*OreG0022832*
scaffold48	221644	C	A	NA	*OreG0022835*
scaffold48	379790	C	T	Och02,Och03,Och07,Och09,Och10,Och11,Ore01,Ore02,Ore03,Ore04,Ore05,Ore06,Ore07,Ore08,Ore09,Ore10,Ore11,Ore12,Ore13,Ore14	*OreG0022842*
scaffold48	478984	C	T	Ore03	*OreG0022845*
scaffold48	479398	G	T	Ore02,Ore04,Ore05,Ore06,Ore11,Ore12	*OreG0022845*
scaffold48	700947	C	A	NA	*OreG0022870*
scaffold48	719123	G	T	NA	*OreG0022872*
scaffold48	725197	T	A	NA	*OreG0022873*
scaffold48	725941	C	G	NA	*OreG0022873*
scaffold48	726103	G	A	Och08	*OreG0022873*
scaffold48	747332	C	T	NA	*OreG0022875*
scaffold48	752543	G	A	NA	*OreG0022878*

scaffold48	753255	C	T	NA	*OreG0022878*
scaffold48	753597	C	T	NA	*OreG0022878*
scaffold48	764224	A	T	NA	*OreG0022881*
scaffold48	768245	A	T	Och14	*OreG0022883*
scaffold48	907681	G	A	NA	*OreG0022890*
scaffold48	919138	C	A	NA	*OreG0022892*
scaffold48	1038046	T	A	Och01,Och02,Och03,Och04,Och05,Och06,Och07,Och08,Och09,Och10,Och11,Och12,Och13,Och14,Ore01,Ore02,Ore03,Ore04,Ore05,Ore06,Ore07,Ore08,Ore09,Ore10,Ore11,Ore12,Ore13,Ore14	*OreG0022902*
scaffold48	1039766	G	T	NA	*OreG0022902*
scaffold48	1040456	C	T	NA	*OreG0022902*
scaffold48	1041576	C	T	NA	*OreG0022902*
scaffold48	2113717	G	A	Och02,Och07,Och14	*OreG0022933*
scaffold48	2229898	G	A	Och12	*OreG0022945*
scaffold48	2230097	C	T	NA	*OreG0022945*
scaffold48	2315425	C	T	NA	*OreG0022950*
scaffold48	2318230	T	A	NA	*OreG0022950*
scaffold48	2318530	G	T	NA	*OreG0022950*
scaffold48	2566978	C	T	Och02,Och03,Och04,Och05,Och06,Och08,Och09,Och10,Och11,Och12,Och13,Och14	*OreG0022971*
scaffold48	2654574	C	T	Ore01,Ore02,Ore03,Ore04,Ore05,Ore06,Ore07,Ore08,Ore09,Ore10,Ore11,Ore12,Ore13,Ore14	*OreG0022977*
scaffold48	2665075	C	T	Och01	*OreG0022978*
scaffold331	22404	G	A	NA	*OreG0011348*
scaffold331	83201	C	A	Och01,Och02,Och03,Och06,Och07,Och08,Och09,Och10,Och11,Och12,Och14	*OreG0011356*
scaffold331	176216	C	A	Och01,Och02,Och03,Och04,Och05,Och06,Och07,Och08,Och09,Och10,Och11,Och12,Och13,Och14,Ore01,Ore02,Ore03,Ore04,Ore05,Ore06,Ore07,Ore08,Ore09,Ore10,Ore11,Ore12,Ore13,Ore14	*OreG0011366*
scaffold331	425684	G	T	NA	*OreG0011392*
scaffold331	426889	C	A	Och01,Och04,Och06,Och07,Och08,Och09,Och11,Och13,Och14	*OreG0011392*
scaffold331	610839	G	A	NA	*OreG0011409*
scaffold331	655677	G	A	NA	*OreG0011410*
scaffold331	748108	A	T	NA	*OreG0011427*
scaffold331	748242	G	T	NA	*OreG0011427*
scaffold331	1385368	C	T	Ore06,Ore09,Ore10,Ore14	*OreG0011463*
scaffold331	1385595	C	T	NA	*OreG0011463*
scaffold331	1413999	T	A	Och01,Och02,Och03,Och04,Och05,Och06,Och07,Och08,Och09,Och10,Och11,Och12,Och13,Och14	*OreG0011467*
scaffold331	1462449	C	A	Och01,Och02,Och03,Och04,Och05,Och06,Och08,Och10,Och11,Och12,Och13,Och14	*OreG0011471*
scaffold403	159514	G	A	NA	*OreG0019664*
scaffold403	278095	G	A	NA	*OreG0019673*
scaffold403	278135	C	T	Och08	*OreG0019673*
scaffold403	347568	C	T	Och02,Och03,Och04,Och05,Och06,Och08,Och09,Och10,Och12,Och13	*OreG0019677*
scaffold403	434610	C	T	NA	*OreG0019680*
scaffold403	699816	T	A	Och01,Och02,Och03,Och04,Och05,Och06,Och07,Och08,Och09,Och10,Och11,Och12,Och13,Och14,Ore01,Ore02,Ore03,Ore04,Ore05,Ore06,Ore07,Ore08,Ore09,Ore10,Ore11,Ore12,Ore13,Ore14	*OreG0019698*
scaffold403	991283	C	A	Ore04,Ore05	*OreG0019718*
scaffold403	1015353	C	A	Och01,Och02,Och03,Och04,Och05,Och06,Och07,Och08,Och09,Och10,Och11,Och12,Och13,Och14,Ore01,Ore02,Ore03,Ore04,Ore05,Ore06,Ore07,Ore08,Ore09,Ore10,Ore11,Ore12,Ore13,Ore14	*OreG0019720*
scaffold403	1111411	G	A	Och01	*OreG0019732*
scaffold403	1665880	C	T	NA	*OreG0019779*
scaffold403	1837483	G	A	Ore03	*OreG0019785*
scaffold403	1837786	C	A	NA	*OreG0019785*
scaffold403	1948035	G	T	Ore02,Ore04,Ore05,Ore13	*OreG0019794*
scaffold403	2037295	C	T	Ore02,Ore04,Ore05,Ore13	*OreG0019797*
scaffold403	2281892	C	T	Ore01,Ore02,Ore03,Ore04,Ore05,Ore06,Ore07,Ore08,Ore09,Ore10,Ore11,Ore12,Ore13,Ore14	*OreG0019816*
scaffold403	2387343	C	A	NA	*OreG0019819*
scaffold403	2483951	G	A	Och01,Och02,Och03,Och04,Och05,Och06,Och07,Och08,Och09,Och10,Och11,Och12,Och13,Och14	*OreG0019827*
scaffold403	2492504	G	T	NA	*OreG0019828*
scaffold403	2858069	A	C	Ore01,Ore03,Ore06,Ore09,Ore12	*OreG0019850*
scaffold403	2858382	C	A	NA	*OreG0019850*
scaffold970	149647	T	A	NA	*OreG0027045*
scaffold970	237368	G	T	NA	*OreG0027050*
scaffold970	245204	G	A	Och07,Och11	*OreG0027052*
scaffold915	59144	G	A	NA	*OreG0026530*
scaffold915	138124	A	T	NA	*OreG0026537*
scaffold915	160623	C	T	Och01,Och02,Och03,Och04,Och05,Och06,Och07,Och08,Och09,Och10,Och11,Och12,Och13,Och14,Ore01,Ore02,Ore03,Ore04,Ore05,Ore06,Ore07,Ore08,Ore09,Ore10,Ore11,Ore12,Ore13,Ore14	*OreG0026539*

scaffold915	230298	A	T	Och01,Och02,Och03,Och04,Och05,Och06,Och07,Och08,Och09,Och10,Och11,Och12,Och13,Och14,Ore01,Ore02,Ore03,Ore04,Ore05,Ore06,Ore07,Ore08,Ore09,Ore10,Ore11,Ore12,Ore13,Ore14	*OreG0026549*
scaffold915	456268	G	A	NA	*OreG0026564*
scaffold915	456480	G	T	NA	*OreG0026564*
scaffold915	701381	G	T	NA	*OreG0026593*
scaffold915	929978	C	A	NA	*OreG0026622*
scaffold915	930088	A	T	Och05,Och08,Och09,Och10,Och11,Ore10	*OreG0026622*
scaffold915	930359	G	A	Och08,Och13,Ore10	*OreG0026622*
scaffold97	23051	A	T	NA	*OreG0026949*
scaffold97	752125	G	A	Och01,Och02,Och03,Och04,Och05,Och06,Och07,Och08,Och09,Och10,Och11,Och12,Och13,Och14	*OreG0026989*
scaffold97	752146	G	A	Och01,Och02,Och03,Och04,Och05,Och06,Och07,Och08,Och09,Och10,Och11,Och12,Och13,Och14	*OreG0026989*
scaffold97	841011	A	T	Ore01,Ore02,Ore03,Ore04,Ore05,Ore06,Ore07,Ore08,Ore09,Ore10,Ore11,Ore12,Ore13,Ore14	*OreG0026992*
scaffold97	885754	C	A	NA	*OreG0026995*
scaffold97	1159382	C	T	NA	*OreG0027003*
scaffold97	1305787	G	A	NA	*OreG0027006*
scaffold97	1728742	C	T	NA	*OreG0027015*
scaffold97	1829337	T	G	Och02,Och03,Och04,Och05,Och06,Och07,Och08,Och09,Och10,Och11,Och12,Och14	*OreG0027016*
scaffold97	2025800	C	A	Och02,Och06	*OreG0027023*
scaffold936	298873	T	A	NA	*OreG0026834*
scaffold936	339961	G	A	NA	*OreG0026835*
scaffold936	340027	T	A	Och13	*OreG0026835*
scaffold936	340069	G	A	Och07	*OreG0026835*
scaffold936	350637	G	A	NA	*OreG0026838*
scaffold936	351771	G	A	NA	*OreG0026838*
scaffold936	351903	G	A	NA	*OreG0026838*
scaffold936	352386	T	A	NA	*OreG0026838*
scaffold936	352392	G	A	NA	*OreG0026838*
scaffold936	360531	C	A	NA	*OreG0026834*
scaffold936	360963	T	A	NA	*OreG0026834*
scaffold936	375897	G	A	NA	*OreG0026839*
scaffold936	394221	G	A	Ore08,Ore09,Ore12,Ore14	*OreG0026843*
scaffold936	430608	G	A	Och03,Och05,Och08,Och13	*OreG0026848*
scaffold936	444831	C	T	NA	*OreG0026850*
scaffold936	778772	G	A	NA	*OreG0026871*
scaffold936	1080475	G	A	NA	*OreG0026889*
scaffold936	1085693	C	T	Och01,Och02,Och03,Och04,Och05,Och06,Och07,Och08,Och09,Och10,Och11,Och12,Och13,Och14,Ore01,Ore02,Ore03,Ore04,Ore05,Ore06,Ore07,Ore08,Ore09,Ore10,Ore11,Ore12,Ore13,Ore14	*OreG0026890*
scaffold936	1141827	C	A	NA	*OreG0026898*
scaffold936	1146068	G	A	NA	*OreG0026898*
scaffold936	1218464	T	G	Och02,Och04,Och12,Och13	*OreG0026900*
scaffold936	1239641	C	A	NA	*OreG0026903*
scaffold936	1262378	C	T	NA	*OreG0026907*
scaffold936	1262552	C	T	Ore02,Ore03,Ore06,Ore07,Ore08,Ore09,Ore10,Ore11,Ore12,Ore13,Ore14	*OreG0026907*
scaffold936	1263333	C	A	NA	*OreG0026907*
scaffold1739	83790	C	G	NA	*OreG0003537*
scaffold1739	83834	C	T	NA	*OreG0003537*
scaffold1739	84106	G	A	NA	*OreG0003537*
scaffold1739	107134	C	A	Och12,Och13	*OreG0003539*
scaffold718	154505	C	A	NA	*OreG0024650*
scaffold718	154876	C	T	NA	*OreG0024650*
scaffold718	307366	T	A	NA	*OreG0024657*
scaffold718	742999	G	A	Och01,Och02,Och03,Och04,Och05,Och06,Och07,Och08,Och09,Och10,Och11,Och12,Och13,Och14	*OreG0024686*
scaffold718	857200	T	A	Och01,Och02,Och03,Och04,Och05,Och06,Och07,Och08,Och09,Och10,Och11,Och12,Och13,Och14,Ore01,Ore02,Ore03,Ore04,Ore05,Ore06,Ore07,Ore08,Ore09,Ore10,Ore11,Ore12,Ore13,Ore14	*OreG0024698*
scaffold718	873963	G	A	NA	*OreG0024700*
scaffold718	1150615	C	T	NA	*OreG0024711*
scaffold718	1159298	A	T	NA	*OreG0024712*
scaffold718	1243836	G	A	Och02,Och04,Och12,Och13	*OreG0024720*
scaffold718	1247295	T	A	NA	*OreG0024721*
scaffold718	1341917	C	A	NA	*OreG0024734*
scaffold718	1488974	G	T	NA	*OreG0024751*
scaffold718	1622790	G	A	Och01,Och07,Och08,Och09,Och10,Och11	*OreG0024767*
scaffold718	1628863	G	A	NA	*OreG0024769*
scaffold718	1632704	G	C	Och12	*OreG0024769*
scaffold718	1641681	T	A	NA	*OreG0024770*
scaffold718	1705707	G	A	NA	*OreG0024776*

scaffold718	1708896	G	A	NA	*OreG0024776*
scaffold718	1710257	C	T	NA	*OreG0024776*
scaffold718	2055028	A	C	NA	*OreG0024807*
scaffold85	148434	G	A	NA	*OreG0025422*
scaffold85	401668	C	T	Ore02,Ore03,Ore06,Ore07,Ore08,Ore09,Ore10,Ore11,Ore12,Ore13,Ore14	*OreG0025431*
scaffold85	928891	G	C	NA	*OreG0025448*
scaffold85	928943	G	A	Och01,Och02,Och03,Och04,Och05,Och06,Och07,Och08,Och11,Och12,Och13	*OreG0025448*
scaffold85	975726	C	T	Och01,Och02,Och03,Och04,Och05,Och06,Och07,Och08,Och09,Och10,Och11,Och12,Och13,Och14	*OreG0025451*
scaffold85	1002779	C	T	NA	*OreG0025453*
scaffold85	1371599	A	T	NA	*OreG0025466*
scaffold85	1587067	G	A	NA	*OreG0025480*
scaffold85	1610293	C	A	Och01,Och02,Och03,Och04,Och05,Och06,Och07,Och08,Och09,Och10,Och11,Och12,Och13,Och14	*OreG0025483*
scaffold85	1644617	C	T	NA	*OreG0025488*
scaffold85	1644641	C	T	NA	*OreG0025488*
scaffold85	1707289	C	T	NA	*OreG0025491*
scaffold85	1712451	C	T	Och02,Och03,Och04,Och05,Och06,Och07,Och08,Och12,Och13,Och14	*OreG0025492*
scaffold85	1712660	G	A	NA	*OreG0025492*
scaffold85	1723974	C	A	Och07,Och08	*OreG0025494*
scaffold85	1737096	C	A	Och06,Och12,Ore02,Ore04,Ore06,Ore11,Ore12	*OreG0025495*
scaffold85	1809143	C	T	NA	*OreG0025500*
scaffold85	1809506	G	T	Och02,Och03,Och04,Och07,Och08,Och11,Och14	*OreG0025500*
scaffold85	1825190	C	A	NA	*OreG0025504*
scaffold85	1849606	G	A	NA	*OreG0025507*
scaffold85	1877059	G	A	NA	*OreG0025509*
scaffold85	1974979	G	T	Och01	*OreG0025519*
scaffold85	1991038	C	A	Och01	*OreG0025521*
scaffold85	2034855	T	A	NA	*OreG0025523*
scaffold85	2194889	C	A	NA	*OreG0025526*
scaffold85	2521707	A	T	Och01,Och02,Och03,Och04,Och05,Och06,Och07,Och08,Och09,Och10,Och11,Och12,Och13,Och14	*OreG0025551*
scaffold85	2719293	C	T	NA	*OreG0025575*
scaffold85	2719374	C	T	NA	*OreG0025575*
scaffold85	2780999	C	T	NA	*OreG0025577*
scaffold85	2781083	C	T	Och02,Och03,Och04,Och05,Och06,Och07,Och08,Och09,Och10,Och11,Och12,Och13,Och14	*OreG0025577*
scaffold85	2924804	C	T	NA	*OreG0025588*
scaffold85	3042470	C	G	Och01,Och02,Och03,Och04,Och05,Och06,Och07,Och08,Och09,Och10,Och11,Och12,Och13,Och14,Ore01,Ore02,Ore03,Ore04,Ore05,Ore06,Ore07,Ore08,Ore09,Ore10,Ore11,Ore12,Ore13,Ore14	*OreG0025599*
scaffold85	3075292	A	T	Ore02,Ore04,Ore05,Ore06,Ore11,Ore12	*OreG0025602*
scaffold85	3319761	G	A	Och11	*OreG0025619*
scaffold85	3320454	C	A	NA	*OreG0025619*
scaffold85	3422630	G	A	NA	*OreG0025630*
scaffold85	3426992	G	A	NA	*OreG0025631*
scaffold85	3619294	G	A	NA	*OreG0025650*
scaffold85	3705368	G	A	NA	*OreG0025657*
scaffold85	3707421	A	T	Och06,Och13	*OreG0025659*
scaffold85	3725424	C	A	NA	*OreG0025662*
scaffold85	3755798	G	A	NA	*OreG0025664*
scaffold85	3785422	G	T	NA	*OreG0025668*
scaffold85	3785482	G	T	NA	*OreG0025668*
scaffold85	3797853	C	T	Och01,Och02,Och03,Och04,Och05,Och06,Och07,Och08,Och09,Och10,Och11,Och12,Och13	*OreG0025670*
scaffold85	3814743	G	T	Och05,Och12	*OreG0025671*
scaffold85	3818386	C	A	Och01,Och11	*OreG0025671*
scaffold85	3861257	C	A	NA	*OreG0025674*
scaffold85	4060414	G	A	NA	*OreG0025695*
scaffold85	4060627	C	A	NA	*OreG0025695*
scaffold85	4061614	G	A	NA	*OreG0025695*
scaffold85	4061698	G	A	NA	*OreG0025695*
scaffold85	4111505	A	C	NA	*OreG0025699*
scaffold85	4120786	C	A	NA	*OreG0025700*
scaffold85	4187780	G	A	NA	*OreG0025708*
scaffold85	4187915	G	A	NA	*OreG0025708*
scaffold85	4359761	G	T	Och09,Och10	*OreG0025712*
scaffold85	4386749	C	A	Och02,Och03,Och04,Och05,Och12	*OreG0025713*
scaffold85	4631258	C	T	Och01,Och02,Och03,Och04,Och05,Och06,Och07,Och08,Och09,Och10,Och11,Och12,Och13,Och14,Ore01,Ore02,Ore03,Ore04,Ore05,Ore06,Ore07,Ore08,Ore09,Ore10,Ore11,Ore12,Ore13,Ore14	*OreG0025733*
scaffold85	4634848	G	A	Och08	*OreG0025735*
scaffold85	4706613	C	T	NA	*OreG0025742*
scaffold85	4850672	A	T	NA	*OreG0025753*

scaffold85	4894412	C	T	Och02,Och03,Och04,Och05,Och06,Och07,Och08,Och09,Och10,Och11,Och12,Och13,Och14	*OreG0025757*
scaffold85	4894574	C	T	NA	*OreG0025757*
scaffold85	4897900	T	G	Ore01,Ore02,Ore03,Ore04,Ore05,Ore06,Ore07,Ore08,Ore09,Ore10,Ore11,Ore12,Ore13,Ore14	*OreG0025758*
scaffold1410	39254	T	A	NA	*OreG0002358*
scaffold470	28980	G	A	NA	*OreG0022360*
scaffold470	185101	C	A	Och01,Och02,Och03,Och04,Och05,Och06,Och07,Och08,Och12,Och13	*OreG0022376*
scaffold470	187341	T	A	Och01,Och02,Och03,Och04,Och05,Och06,Och07,Och08,Och12,Och13	*OreG0022376*
scaffold470	271341	C	T	NA	*OreG0022389*
scaffold470	285820	G	T	NA	*OreG0022390*
scaffold470	324234	C	A	Och02,Och03,Och04,Och05,Och06,Och07,Och08,Och09,Och10,Och11,Och12,Och13,Och14	*OreG0022395*
scaffold470	324604	C	T	Och02,Och03,Och04,Och05,Och06,Och08,Och09,Och10,Och12,Och13	*OreG0022395*
scaffold470	389983	G	A	Och01,Och02,Och03,Och04,Och05,Och06,Och07,Och08,Och09,Och10,Och11,Och12,Och13,Och14	*OreG0022400*
scaffold470	390061	G	A	NA	*OreG0022400*
scaffold470	390127	G	A	Och01,Och02,Och03,Och04,Och05,Och06,Och07,Och08,Och09,Och10,Och11,Och12,Och13,Och14	*OreG0022400*
scaffold470	390189	G	A	Och03,Och05,Och08,Och12,Och13	*OreG0022401*
scaffold470	419042	G	A	NA	*OreG0022406*
scaffold470	419498	C	T	NA	*OreG0022406*
scaffold470	557994	C	A	Och01,Och02,Och03,Och04,Och05,Och06,Och07,Och08,Och09,Och10,Och11,Och12,Och13,Och14	*OreG0022421*
scaffold470	558045	G	A	Och01,Och02,Och03,Och04,Och05,Och06,Och09,Och10,Och11,Och12,Och13,Och14	*OreG0022421*
scaffold470	558129	G	A	Och02,Och03,Och04,Och05,Och06,Och09,Och10,Och12,Och13	*OreG0022421*
scaffold470	569826	C	A	Ore01,Ore02,Ore03,Ore04,Ore05,Ore06,Ore07,Ore08,Ore09,Ore10,Ore11,Ore12,Ore13,Ore14	*OreG0022422*
scaffold470	827681	A	T	NA	*OreG0022445*
scaffold470	883419	T	A	NA	*OreG0022453*
scaffold470	1036255	G	T	NA	*OreG0022465*
scaffold470	1048845	T	A	NA	*OreG0022466*
scaffold470	1050588	C	T	NA	*OreG0022467*
scaffold470	1100522	G	A	Och14	*OreG0022475*
scaffold470	1278987	C	A	NA	*OreG0022492*
scaffold470	1303617	A	T	Och06	*OreG0022494*
scaffold470	1336894	T	A	NA	*OreG0022498*
scaffold470	1445191	A	T	NA	*OreG0022513*
scaffold470	1585044	A	C	Och01,Och02,Och03,Och04,Och05,Och06,Och07,Och08,Och09,Och10,Och11,Och12,Och13,Och14,Ore01,Ore02,Ore03,Ore04,Ore05,Ore06,Ore07,Ore08,Ore09,Ore10,Ore11,Ore12,Ore13,Ore14	*OreG0022517*
scaffold1292	45331	C	A	NA	*OreG0001967*
scaffold1292	287580	C	T	Och07	*OreG0001977*
scaffold1292	355555	G	T	Ore01,Ore02,Ore03,Ore04,Ore05,Ore06,Ore07,Ore08,Ore09,Ore10,Ore11,Ore12,Ore13,Ore14	*OreG0001979*
scaffold1292	355605	G	A	Ore01,Ore02,Ore03,Ore04,Ore05,Ore06,Ore07,Ore08,Ore09,Ore10,Ore11,Ore12,Ore13,Ore14	*OreG0001979*
scaffold1292	370545	G	A	NA	*OreG0001982*
scaffold33	10555	G	T	Och01	*OreG0011039*
scaffold33	509745	G	A	Och09	*OreG0011060*
scaffold33	799369	C	T	NA	*OreG0011077*
scaffold33	934221	G	A	NA	*OreG0011085*
scaffold33	1520125	G	A	NA	*OreG0011122*
scaffold33	1605653	G	A	NA	*OreG0011126*
scaffold33	1659923	T	A	NA	*OreG0011131*
scaffold33	1676327	C	T	Och01,Och02,Och03,Och04,Och05,Och06,Och07,Och08,Och09,Och10,Och11,Och12,Och13,Och14,Ore01,Ore02,Ore03,Ore04,Ore05,Ore06,Ore07,Ore08,Ore09,Ore10,Ore11,Ore12,Ore13,Ore14	*OreG0011134*
scaffold33	1687157	G	A	NA	*OreG0011135*
scaffold33	1787968	G	T	Ore01,Ore02,Ore03,Ore04,Ore05,Ore06,Ore07,Ore08,Ore09,Ore10,Ore11,Ore12,Ore13,Ore14	*OreG0011147*
scaffold33	1788065	T	A	NA	*OreG0011147*
scaffold33	2022704	C	T	Och07	*OreG0011160*
scaffold33	2305952	G	T	Ore07,Ore08,Ore14	*OreG0011175*
scaffold33	2393368	C	T	NA	*OreG0011181*
scaffold33	2463666	G	A	NA	*OreG0011183*
scaffold33	2704070	A	T	Ore08	*OreG0011199*
scaffold33	2914009	G	A	Ore01,Ore02,Ore03,Ore04,Ore05,Ore06,Ore07,Ore08,Ore09,Ore10,Ore11,Ore12,Ore13,Ore14	*OreG0011206*
scaffold33	2914249	C	A	Och01,Och14	*OreG0011206*
scaffold33	3655096	G	T	NA	*OreG0011245*
scaffold33	3662847	C	T	NA	*OreG0011246*
scaffold33	3662935	G	A	NA	*OreG0011246*
scaffold33	3663183	C	T	Och06	*OreG0011247*

scaffold33	3663240	C	T	Och02,Och06,Och07	*OreG0011247*
scaffold33	3663519	C	T	Och03,Och04,Och14	*OreG0011247*
scaffold33	3663732	C	T	NA	*OreG0011247*
scaffold33	3663842	C	T	NA	*OreG0011248*
scaffold33	3663889	G	A	NA	*OreG0011248*
scaffold33	3664326	G	T	NA	*OreG0011248*
scaffold33	3707653	G	T	NA	*OreG0011251*
scaffold33	3721468	C	T	Och02,Och06	*OreG0011254*
scaffold33	3721516	C	T	Och02,Och06	*OreG0011254*
scaffold33	3889271	C	T	Ore01,Ore02,Ore03,Ore04,Ore05,Ore06,Ore07,Ore08,Ore09,Ore10,Ore11,Ore12,Ore13,Ore14	*OreG0011258*
scaffold1849	176644	G	T	NA	*OreG0003977*
scaffold1849	181940	G	A	NA	*OreG0003979*
scaffold1000	65868	G	T	NA	*OreG0000004*
scaffold1000	334036	A	T	NA	*OreG0000022*
scaffold1000	358155	G	A	NA	*OreG0000023*
scaffold1000	358157	C	T	NA	*OreG0000023*
scaffold1000	408447	C	T	NA	*OreG0000025*
scaffold1000	418043	G	T	NA	*OreG0000026*
scaffold1000	418140	T	G	Och02,Och03,Och04,Och05,Och06,Och12,Och13	*OreG0000026*
scaffold1000	443323	G	A	Ore01,Ore02,Ore03,Ore04,Ore05,Ore06,Ore07,Ore08,Ore09,Ore10,Ore11,Ore12,Ore13,Ore14	*OreG0000029*
scaffold1000	660197	C	T	NA	*OreG0000045*
scaffold1000	680287	C	T	Och02,Och03,Och04,Och06,Och07,Och08,Och09,Och10,Och11,Och12,Och13,Och14	*OreG0000047*
scaffold1000	681960	C	T	Och02,Och03,Och04,Och06,Och07,Och08,Och09,Och10,Och11,Och12,Och13,Och14	*OreG0000047*
scaffold1000	682126	G	A	NA	*OreG0000047*
scaffold1000	682285	G	A	Ore02,Ore03,Ore04,Ore05,Ore06,Ore07,Ore08,Ore09,Ore10,Ore11,Ore12,Ore13,Ore14	*OreG0000047*
scaffold1021	252259	C	T	Och06	*OreG0000071*
scaffold1021	326509	C	A	NA	*OreG0000074*
scaffold1021	1098748	G	T	NA	*OreG0000085*
scaffold1021	1232479	C	T	Och01,Och02,Och03,Och04,Och05,Och06,Och07,Och08,Och09,Och10,Och11,Och12,Och13,Och14	*OreG0000090*
scaffold1021	1425404	C	T	NA	*OreG0000094*
scaffold1021	1533915	T	A	Ore01,Ore08,Ore09,Ore11,Ore12	*OreG0000101*
scaffold1021	1723886	G	T	NA	*OreG0000107*
scaffold1021	2091135	C	T	Och01,Och02,Och03,Och04,Och05,Och06,Och07,Och08,Och09,Och10,Och11,Och12,Och13,Och14	*OreG0000122*
scaffold1021	2144599	C	T	Och08	*OreG0000128*
scaffold586	131431	C	T	Och01,Och02,Och03,Och04,Och05,Och06,Och07,Och08,Och09,Och10,Och11,Och12,Och13,Och14	*OreG0023810*
scaffold586	230637	G	T	NA	*OreG0023819*
scaffold586	318664	T	A	Och03,Och05,Och08,Och12,Och13,Och14	*OreG0023831*
scaffold586	327964	G	C	NA	*OreG0023832*
scaffold586	367229	C	T	Och01,Och02,Och04,Och06,Och07,Och09,Och10,Och11,Och14	*OreG0023836*
scaffold586	399141	G	T	Och01,Och02,Och03,Och04,Och05,Och06,Och07,Och08,Och09,Och10,Och11,Och12,Och13,Och14,Ore01,Ore02,Ore03,Ore04,Ore05,Ore06,Ore07,Ore08,Ore09,Ore10,Ore11,Ore12,Ore13,Ore14	*OreG0023840*
scaffold586	399232	C	A	Och01,Och02,Och03,Och04,Och05,Och06,Och07,Och08,Och09,Och10,Och11,Och12,Och13,Och14	*OreG0023840*
scaffold586	400075	C	T	Och01,Och02,Och03,Och04,Och05,Och06,Och07,Och08,Och09,Och10,Och11,Och12,Och13,Och14	*OreG0023841*
scaffold586	410400	G	A	Och01,Och02,Och03,Och04,Och05,Och06,Och07,Och08,Och09,Och10,Och11,Och12,Och13,Och14	*OreG0023839*
scaffold586	584270	T	A	NA	*OreG0023866*
scaffold586	759941	C	A	Och08,Och09,Och10,Och11,Och14	*OreG0023883*
scaffold586	771449	C	T	NA	*OreG0023884*
scaffold586	836602	C	A	NA	*OreG0023896*
scaffold586	967683	T	A	Ore01,Ore02,Ore03,Ore04,Ore05,Ore06,Ore07,Ore08,Ore09,Ore10,Ore11,Ore12,Ore13,Ore14	*OreG0023911*
scaffold586	1108498	G	A	NA	*OreG0023923*
scaffold586	1188557	C	A	Och01,Och02,Och03,Och04,Och05,Och06,Och08,Och09,Och10,Och11,Och12,Och13,Och14	*OreG0023934*
scaffold586	1191913	C	A	NA	*OreG0023935*
scaffold586	1318925	G	A	Och01,Och02,Och03,Och04,Och05,Och06,Och07,Och08,Och09,Och10,Och11,Och12,Och13,Och14	*OreG0023945*
scaffold586	1483380	C	T	Och06,Och08,Och14,Ore01,Ore02,Ore03,Ore04,Ore05,Ore06,Ore07,Ore08,Ore09,Ore10,Ore11,Ore12,Ore13,Ore14	*OreG0023949*
scaffold586	1483720	C	T	NA	*OreG0023949*
scaffold586	1924178	G	A	Ore01,Ore02,Ore03,Ore04,Ore05,Ore06,Ore07,Ore08,Ore09,Ore10,Ore11,Ore12,Ore13,Ore14	*OreG0023995*
scaffold586	1990947	C	T	NA	*OreG0024003*
scaffold586	2184069	G	T	NA	*OreG0024011*

scaffold586	2284118	G	T	Och07,Och08	*OreG0024021*
scaffold586	2284206	G	A	Och01,Och02,Och03,Och04,Och05,Och06,Och07,Och08,Och09,Och10,Och11,Och12,Och13,Och14	*OreG0024021*
scaffold586	2284353	G	A	Och01,Och02,Och03,Och04,Och05,Och06,Och07,Och08,Och09,Och10,Och11,Och12,Och13,Och14	*OreG0024021*
scaffold586	2434361	G	T	Och01,Och07,Och08,Och09,Och10,Och11	*OreG0024032*
scaffold586	2536703	G	A	NA	*OreG0024043*
scaffold1028	875519	C	T	Ore01,Ore02,Ore03,Ore04,Ore05,Ore06,Ore07,Ore08,Ore09,Ore10,Ore11,Ore12,Ore13,Ore14	*OreG0000147*
scaffold1028	944465	G	A	NA	*OreG0000151*
scaffold1028	946310	C	T	NA	*OreG0000151*
scaffold1028	1137699	C	T	Och04	*OreG0000171*
scaffold1028	1137786	C	T	Och04	*OreG0000171*
scaffold1028	1142345	C	T	Och07,Och08,Och09,Och10,Och11,Och13	*OreG0000171*
scaffold1028	1142414	C	T	NA	*OreG0000171*
scaffold1028	1275725	G	T	Ore01,Ore02,Ore03,Ore04,Ore05,Ore06,Ore07,Ore08,Ore09,Ore10,Ore11,Ore12,Ore13,Ore14	*OreG0000191*
scaffold1028	1314746	G	T	Och01,Och02,Och03,Och04,Och05,Och06,Och07,Och08,Och09,Och10,Och11,Och12,Och13,Och14,Ore01,Ore02,Ore03,Ore04,Ore05,Ore06,Ore07,Ore08,Ore09,Ore10,Ore11,Ore12,Ore13,Ore14	*OreG0000196*
scaffold1028	1320779	G	A	NA	*OreG0000197*
scaffold1433	92807	A	T	Och01,Och02,Och03,Och04,Och05,Och06,Och07,Och08,Och09,Och10,Och11,Och12,Och13,Och14,Ore01,Ore02,Ore03,Ore04,Ore05,Ore06,Ore07,Ore08,Ore09,Ore10,Ore11,Ore12,Ore13,Ore14	*OreG0002369*
scaffold1433	102941	G	A	Och02,Och03,Och04,Och05,Och06,Och07,Och09,Och10,Och11,Och12,Och13,Och14	*OreG0002370*
scaffold1433	103455	G	A	Och09,Och10,Och11	*OreG0002371*
scaffold1433	103486	C	T	NA	*OreG0002371*
scaffold1433	244397	C	A	Och01,Och02,Och03,Och04,Och05,Och06,Och07,Och08,Och09,Och10,Och11,Och12,Och13,Och14,Ore01,Ore02,Ore03,Ore04,Ore05,Ore06,Ore07,Ore08,Ore09,Ore10,Ore11,Ore12,Ore13,Ore14	*OreG0002383*
scaffold1433	244411	T	A	Ore01,Ore02,Ore03,Ore04,Ore05,Ore06,Ore07,Ore08,Ore09,Ore10,Ore11,Ore12,Ore13,Ore14	*OreG0002383*
scaffold309	249611	T	A	NA	*OreG0008831*
scaffold309	484421	G	A	Och01,Och02,Och03,Och04,Och05,Och06,Och07,Och08,Och09,Och10,Och11,Och12,Och13,Och14	*OreG0008836*
scaffold309	683104	G	T	Och01,Och02,Och03,Och04,Och05,Och06,Och07,Och08,Och09,Och10,Och11,Och12,Och13,Och14	*OreG0008850*
scaffold309	814269	C	T	Och11	*OreG0008854*
scaffold309	962980	G	A	Och01,Och02,Och03,Och04,Och05,Och06,Och07,Och08,Och09,Och10,Och11,Och12,Och13,Och14	*OreG0008859*
scaffold309	1003121	C	A	NA	*OreG0008861*
scaffold309	1060464	G	A	Och01,Och02,Och03,Och04,Och05,Och06,Och07,Och08,Och09,Och10,Och11,Och12,Och13,Och14	*OreG0008863*
scaffold309	1332691	G	A	Ore01,Ore02,Ore03,Ore04,Ore05,Ore06,Ore07,Ore08,Ore09,Ore10,Ore11,Ore12,Ore13,Ore14	*OreG0008884*
scaffold309	1333667	C	T	NA	*OreG0008884*
scaffold309	1460641	C	A	NA	*OreG0008887*
scaffold309	1515094	C	T	Och02,Och03,Och04,Och05,Och06,Och07,Och08,Och10,Och11,Och12,Och13,Och14	*OreG0008889*
scaffold309	1515121	C	T	Och01,Och02,Och03,Och04,Och05,Och06,Och07,Och08,Och10,Och11,Och12,Och13,Och14	*OreG0008889*
scaffold309	1556247	C	T	NA	*OreG0008892*
scaffold309	1617290	G	T	Och01,Och02,Och03,Och04,Och05,Och06,Och07,Och08,Och09,Och10,Och11,Och12,Och13,Och14	*OreG0008894*
scaffold309	1758366	G	A	Och01,Och02,Och03,Och04,Och05,Och06,Och07,Och08,Och09,Och10,Och11,Och12,Och13,Och14	*OreG0008903*
scaffold309	1995874	G	A	Och04	*OreG0008924*
scaffold309	1996171	C	A	NA	*OreG0008924*
scaffold309	2008247	G	A	NA	*OreG0008926*
scaffold309	2008289	C	A	NA	*OreG0008926*
scaffold309	2008793	C	A	NA	*OreG0008926*
scaffold309	2156057	C	T	NA	*OreG0008931*
scaffold309	2156794	G	A	Ore03,Ore08,Ore09,Ore10,Ore14	*OreG0008931*
scaffold309	2161396	G	T	NA	*OreG0008931*
scaffold309	2188425	C	T	NA	*OreG0008933*
scaffold309	2305266	G	T	NA	*OreG0008938*
scaffold309	2314579	G	A	NA	*OreG0008938*
scaffold309	2320481	G	A	NA	*OreG0008938*
scaffold309	2362153	G	T	Och01,Och02,Och03,Och04,Och05,Och06,Och07,Och08,Och09,Och10,Och11,Och12,Och13,Och14	*OreG0008940*
scaffold309	2616696	C	T	NA	*OreG0008967*
scaffold309	2645062	G	A	Och03,Och07,Och09,Och10,Och11	*OreG0008970*
scaffold309	2648256	G	A	NA	*OreG0008970*
scaffold309	2648390	C	T	Och07,Och09,Och10,Och11	*OreG0008970*

scaffold309	2709759	C	T	Och12,Ore01,Ore02,Ore03,Ore04,Ore05,Ore06,Ore07,Ore08,Ore09,Ore10,Ore11,Ore12,Ore13,Ore14	*OreG0008976*
scaffold309	2879895	C	A	Och02,Och03,Och04,Och05,Och06,Och08,Och09,Och10,Och11,Och12,Och13,Och14	*OreG0008995*
scaffold309	2913155	T	G	Och01,Och02,Och03,Och04,Och05,Och06,Och07,Och08,Och09,Och10,Och11,Och12,Och13,Och14,Ore01,Ore02,Ore03,Ore04,Ore05,Ore06,Ore07,Ore08,Ore09,Ore10,Ore11,Ore12,Ore13,Ore14	*OreG0008996*
scaffold309	2929722	G	A	NA	*OreG0008999*
scaffold309	3094034	T	A	Ore01,Ore02,Ore03,Ore04,Ore05,Ore06,Ore07,Ore08,Ore09,Ore10,Ore11,Ore12,Ore13,Ore14	*OreG0009011*
scaffold309	3212412	A	C	Och01,Och02,Och03,Och04,Och05,Och06,Och07,Och08,Och09,Och10,Och11,Och12,Och13,Och14,Ore01,Ore02,Ore03,Ore04,Ore05,Ore06,Ore07,Ore08,Ore09,Ore10,Ore11,Ore12,Ore13,Ore14	*OreG0009024*
scaffold309	3377610	A	T	Och14	*OreG0009040*
scaffold309	3582387	G	A	NA	*OreG0009055*
scaffold309	3607141	C	T	Och14	*OreG0009058*
scaffold309	3608151	G	A	NA	*OreG0009058*
scaffold309	3630811	C	T	NA	*OreG0009060*
scaffold309	4037259	G	T	NA	*OreG0009080*
scaffold1174	515445	G	T	NA	*OreG0001043*
scaffold1174	588601	G	A	Och01,Och02,Och03,Och04,Och05,Och07,Och08,Och09,Och10,Och11,Och12,Och13,Och14	*OreG0001046*
scaffold1174	713433	G	C	NA	*OreG0001059*
scaffold1174	786936	T	A	NA	*OreG0001068*
scaffold1174	795072	G	A	NA	*OreG0001069*
scaffold1186	99021	C	T	NA	*OreG0001324*
scaffold1186	139004	T	A	Och06	*OreG0001329*
scaffold1186	161270	C	T	NA	*OreG0001331*
scaffold1186	324267	G	A	NA	*OreG0001344*
scaffold2360	11462	G	A	NA	*OreG0007115*
scaffold1903	57147	T	A	Och06	*OreG0004828*
scaffold1903	90485	G	A	NA	*OreG0004831*
scaffold1903	102978	C	T	NA	*OreG0004833*
scaffold1903	255980	G	A	NA	*OreG0004857*
scaffold1903	330679	G	A	Och01,Och02,Och03,Och04,Och05,Och06,Och07,Och08,Och09,Och10,Och11,Och12,Och13,Och14,Ore01,Ore02,Ore03,Ore04,Ore05,Ore06,Ore07,Ore08,Ore09,Ore10,Ore11,Ore12,Ore13,Ore14	*OreG0004868*
scaffold1903	412952	T	G	Och01,Och02,Och03,Och04,Och05,Och06,Och07,Och08,Och09,Och10,Och11,Och12,Och13,Och14,Ore01,Ore02,Ore03,Ore04,Ore05,Ore06,Ore07,Ore08,Ore09,Ore10,Ore11,Ore12,Ore13,Ore14	*OreG0004882*
scaffold705	153183	C	T	Och01,Och02,Och03,Och04,Och05,Och06,Och07,Och08,Och09,Och10,Och11,Och12,Och13,Och14,Ore01,Ore02,Ore03,Ore04,Ore05,Ore06,Ore07,Ore08,Ore09,Ore10,Ore11,Ore12,Ore13,Ore14	*OreG0024369*
scaffold705	171252	C	T	Och01,Och02,Och03,Och04,Och05,Och06,Och07,Och09,Och10,Och11,Och12,Och13	*OreG0024372*
scaffold705	180826	C	T	Och04,Och06,Och11,Och13	*OreG0024372*
scaffold705	355688	G	A	Ore02,Ore04,Ore05	*OreG0024378*
scaffold705	362893	G	A	NA	*OreG0024379*
scaffold705	637087	A	C	NA	*OreG0024399*
scaffold705	807691	G	A	Och01,Och02,Och03,Och04,Och05,Och06,Och07,Och08,Och09,Och10,Och11,Och12,Och13,Och14,Ore01,Ore02,Ore03,Ore04,Ore05,Ore06,Ore07,Ore08,Ore09,Ore10,Ore11,Ore12,Ore13,Ore14	*OreG0024406*
scaffold705	807706	T	A	NA	*OreG0024406*
scaffold705	833539	C	T	NA	*OreG0024408*
scaffold705	833540	C	T	NA	*OreG0024408*
scaffold705	1108879	G	A	NA	*OreG0024438*
scaffold705	1480252	G	T	NA	*OreG0024477*
scaffold705	1535265	A	C	NA	*OreG0024482*
scaffold705	1635484	G	A	Och04,Och06,Och07,Och11,Och13	*OreG0024495*
scaffold705	1635595	G	A	Och04,Och06,Och07,Och11,Och13	*OreG0024495*
scaffold705	1640397	C	T	NA	*OreG0024496*
scaffold705	1641751	C	A	NA	*OreG0024496*
scaffold705	1664719	C	A	NA	*OreG0024502*
scaffold705	1667992	C	A	Och04,Och07,Och13	*OreG0024502*
scaffold705	1668016	C	A	NA	*OreG0024502*
scaffold705	1668129	C	T	NA	*OreG0024502*
scaffold705	1671181	C	A	Och04,Och06,Och07,Och11,Och13,Och14	*OreG0024503*
scaffold705	1685353	C	A	Och01,Och02,Och03,Och04,Och05,Och06,Och07,Och08,Och09,Och10,Och11,Och12,Och13,Och14,Ore01,Ore02,Ore03,Ore04,Ore05,Ore06,Ore07,Ore08,Ore09,Ore10,Ore11,Ore12,Ore13,Ore14	*OreG0024505*
scaffold705	1693959	C	T	NA	*OreG0024507*
scaffold705	1694005	G	A	NA	*OreG0024507*
scaffold705	1694592	A	C	Ore01,Ore02,Ore03,Ore04,Ore05,Ore06,Ore07,Ore08,Ore09,Ore10,Ore11,Ore12,Ore13,Ore14	*OreG0024507*
scaffold705	1723281	A	T	NA	*OreG0024514*

scaffold705	1723622	C	T	Och04,Och06,Och13	*OreG0024514*
scaffold705	1730895	C	T	Och11,Och14	*OreG0024516*
scaffold705	1770534	T	A	Och01,Och02,Och03,Och04,Och05,Och06,Och07,Och08,Och09,Och10,Och11,Och12,Och13,Och14,Ore01,Ore02,Ore03,Ore04,Ore05,Ore06,Ore07,Ore08,Ore09,Ore10,Ore11,Ore12,Ore13,Ore14	*OreG0024519*
scaffold705	1770569	A	T	Ore02,Ore04,Ore05,Ore13	*OreG0024519*
scaffold705	1780569	A	T	NA	*OreG0024520*
scaffold705	1780798	G	A	NA	*OreG0024520*
scaffold705	1911238	G	A	NA	*OreG0024535*
scaffold705	1911403	G	A	NA	*OreG0024535*
scaffold705	1914972	C	A	NA	*OreG0024537*
scaffold705	2110040	G	T	Och01,Och02,Och03,Och04,Och05,Och06,Och07,Och08,Och09,Och10,Och11,Och12,Och14	*OreG0024558*
scaffold705	2202786	C	A	NA	*OreG0024571*
scaffold705	2332690	C	T	Och01,Och02,Och03,Och04,Och05,Och06,Och07,Och08,Och09,Och10,Och11,Och12,Och13,Och14,Ore01,Ore02,Ore03,Ore04,Ore05,Ore06,Ore07,Ore08,Ore09,Ore10,Ore11,Ore12,Ore13,Ore14	*OreG0024585*
scaffold705	2404995	G	T	NA	*OreG0024597*
scaffold705	2746333	C	T	NA	*OreG0024628*
scaffold2523	3177	G	T	NA	*OreG0007783*
scaffold2523	3199	T	A	NA	*OreG0007783*
scaffold273	75717	T	A	Ore01,Ore02,Ore03,Ore04,Ore05,Ore06,Ore07,Ore08,Ore09,Ore10,Ore11,Ore12,Ore13,Ore14	*OreG0007930*
scaffold273	75766	C	T	Och01,Och12,Och13	*OreG0007930*
scaffold273	145248	C	T	NA	*OreG0007934*
scaffold273	169004	C	T	NA	*OreG0007938*
scaffold273	231561	T	A	NA	*OreG0007941*
scaffold273	258194	G	C	NA	*OreG0007943*
scaffold273	305029	C	T	NA	*OreG0007945*
scaffold273	432929	G	A	NA	*OreG0007949*
scaffold273	546222	T	A	Och02,Och05	*OreG0007955*
scaffold273	597593	C	G	Och01	*OreG0007962*
scaffold273	618535	A	C	Och01,Och02,Och03,Och04,Och05,Och06,Och07,Och08,Och09,Och10,Och11,Och12,Och13,Och14,Ore01,Ore02,Ore03,Ore04,Ore05,Ore06,Ore07,Ore08,Ore09,Ore10,Ore11,Ore12,Ore13,Ore14	*OreG0007965*
scaffold273	1066037	C	A	NA	*OreG0008000*
scaffold273	1122016	C	A	NA	*OreG0008005*
scaffold273	1229330	A	T	Ore02,Ore03,Ore06,Ore08,Ore09,Ore10,Ore11,Ore12,Ore14	*OreG0008025*
scaffold273	1350722	C	T	Och01,Och02,Och03,Och04,Och05,Och06,Och07,Och08,Och09,Och10,Och11,Och12,Och13,Och14	*OreG0008034*
scaffold273	1351081	C	A	NA	*OreG0008034*
scaffold273	1392911	C	A	NA	*OreG0008039*
scaffold273	1403767	C	A	Och01,Och02,Och03,Och04,Och05,Och06,Och07,Och08,Och09,Och10,Och11,Och12,Och13,Och14	*OreG0008042*
scaffold273	1666765	T	A	Och02,Och03,Och04,Och05,Och06,Och07,Och08,Och09,Och10,Och11,Och12,Och13,Och14,Ore01,Ore02,Ore03,Ore04,Ore05,Ore06,Ore07,Ore08,Ore09,Ore10,Ore11,Ore12,Ore13,Ore14	*OreG0008068*
scaffold273	1666822	T	A	Och02,Och03,Och04,Och05,Och06,Och07,Och08,Och09,Och10,Och11,Och12,Och13,Och14,Ore01,Ore02,Ore03,Ore04,Ore05,Ore06,Ore07,Ore08,Ore09,Ore10,Ore11,Ore12,Ore13,Ore14	*OreG0008068*
scaffold273	1756298	G	A	NA	*OreG0008074*
scaffold273	1756520	C	A	Och01	*OreG0008074*
scaffold273	1837852	G	T	Och06,Och14	*OreG0008079*
scaffold273	2073044	G	T	NA	*OreG0008100*
scaffold273	2073077	G	T	NA	*OreG0008100*
scaffold273	2174988	T	A	NA	*OreG0008112*
scaffold273	2251640	G	A	Ore01,Ore02,Ore03,Ore04,Ore05,Ore06,Ore07,Ore08,Ore09,Ore10,Ore11,Ore12,Ore13,Ore14	*OreG0008118*
scaffold273	2255335	C	A	NA	*OreG0008118*
scaffold273	2273966	C	A	Och01,Och02,Och03,Och04,Och05,Och06,Och07,Och08,Och09,Och10,Och11,Och12,Och13,Och14,Ore01,Ore02,Ore03,Ore04,Ore05,Ore06,Ore07,Ore08,Ore09,Ore10,Ore11,Ore12,Ore13,Ore14	*OreG0008120*
scaffold273	2355339	T	A	Ore01,Ore02,Ore03,Ore04,Ore05,Ore06,Ore07,Ore08,Ore09,Ore10,Ore11,Ore12,Ore13,Ore14	*OreG0008127*
scaffold273	2644380	C	T	Och01,Och02,Och03,Och04,Och05,Och06,Och07,Och08,Och09,Och10,Och11,Och12,Och13,Och14	*OreG0008151*
scaffold273	2815732	C	A	Ore03,Ore06,Ore07,Ore08,Ore13,Ore14	*OreG0008154*
scaffold273	2913257	C	T	Och01,Och02,Och03,Och04,Och05,Och06,Och07,Och08,Och09,Och10,Och11,Och12,Och13,Och14,Ore01,Ore02,Ore03,Ore04,Ore05,Ore06,Ore07,Ore08,Ore09,Ore10,Ore11,Ore12,Ore13,Ore14	*OreG0008157*
scaffold273	4404300	C	T	NA	*OreG0008182*
scaffold273	4577893	G	T	Och01,Och02,Och03,Och04,Och05,Och06,Och07,Och08,Och09,Och10,Och11,Och12,Och13,Och14	*OreG0008190*
scaffold273	4577999	G	A	Och01,Och02,Och03,Och04,Och05,Och06,Och07,Och08,Och09,Och10,Och11,Och12,Och13,Och14	*OreG0008190*

scaffold273	4578000	G	A	Och01,Och02,Och03,Och04,Och05,Och06,Och07,Och08,Och09,Och10,Och11,Och12,Och13,Och14	*OreG0008190*
scaffold273	4578004	C	T	NA	*OreG0008190*
scaffold273	4593259	G	A	Och01,Och02,Och03,Och04,Och05,Och06,Och07,Och08,Och09,Och10,Och11,Och12,Och13,Och14	*OreG0008192*
scaffold273	4830914	C	G	Och01,Och02,Och03,Och04,Och05,Och06,Och07,Och08,Och09,Och10,Och11,Och12,Och13,Och14	*OreG0008196*
scaffold273	5098332	C	A	NA	*OreG0008204*
scaffold273	5855593	C	T	NA	*OreG0008230*
scaffold273	6105989	C	T	Ore01,Ore11	*OreG0008237*
scaffold273	6249193	C	T	NA	*OreG0008240*
scaffold273	6330923	C	T	Och01	*OreG0008243*
scaffold273	6356514	C	A	Och01	*OreG0008244*
scaffold2461	17780	C	T	NA	*OreG0007277*
scaffold1384	293248	G	A	Och01,Och02,Och03,Och04,Och05,Och06,Och07,Och08,Och09,Och10,Och11,Och12,Och13,Och14,Ore01,Ore02,Ore03,Ore04,Ore05,Ore06,Ore07,Ore08,Ore09,Ore10,Ore11,Ore12,Ore13,Ore14	*OreG0002091*
scaffold1384	444782	G	A	NA	*OreG0002104*
scaffold1384	444798	C	T	NA	*OreG0002104*
scaffold1384	626271	C	T	Och01,Och02,Och03,Och04,Och05,Och06,Och07,Och08,Och09,Och10,Och11,Och12,Och13	*OreG0002123*
scaffold1384	654192	T	A	NA	*OreG0002128*
scaffold1801	111895	C	T	NA	*OreG0003917*
scaffold1801	115026	T	G	Och01,Och02,Och03,Och04,Och05,Och06,Och07,Och08,Och09,Och10,Och11,Och12,Och13,Och14	*OreG0003917*
scaffold1801	198930	C	T	NA	*OreG0003925*
scaffold1801	236855	A	T	Och01,Och02,Och03,Och04,Och05,Och06,Och07,Och08,Och09,Och10,Och11,Och12,Och13,Och14,Ore01,Ore02,Ore03,Ore04,Ore05,Ore06,Ore07,Ore08,Ore09,Ore10,Ore11,Ore12,Ore13,Ore14	*OreG0003927*
scaffold1801	372132	C	T	Och01,Och02,Och03,Och04,Och05,Och06,Och07,Och08,Och09,Och10,Och11,Och12,Och13,Och14	*OreG0003937*
scaffold1801	372319	T	A	Och01,Och02,Och03,Och04,Och05,Och06,Och07,Och08,Och09,Och10,Och11,Och12,Och13,Och14	*OreG0003937*
scaffold1801	435107	C	T	NA	*OreG0003941*
scaffold1801	590171	C	T	NA	*OreG0003954*
scaffold1801	590199	G	A	NA	*OreG0003954*
scaffold1801	590631	G	C	NA	*OreG0003954*
scaffold1801	632091	G	T	Och11	*OreG0003958*
scaffold650	829877	C	T	Och01,Och02,Och03,Och04,Och05,Och06,Och07,Och08,Och09,Och10,Och11,Och12,Och13,Och14	*OreG0024290*
scaffold650	830231	C	T	Och01,Och02,Och03,Och04,Och05,Och06,Och07,Och08,Och09,Och10,Och11,Och12,Och13,Och14	*OreG0024290*
scaffold650	830368	G	T	Och02,Och03,Och04,Och05,Och06,Och07,Och12,Och13,Och14	*OreG0024290*
scaffold650	830464	A	T	Och04	*OreG0024290*
scaffold650	953970	G	T	NA	*OreG0024291*
scaffold650	1052003	C	T	Och01,Och02,Och03,Och04,Och05,Och06,Och07,Och08,Och09,Och10,Och11,Och12,Och13,Och14	*OreG0024298*
scaffold650	1088091	G	A	Ore03,Ore08,Ore09,Ore10,Ore14	*OreG0024299*
scaffold650	1093943	C	T	NA	*OreG0024299*
scaffold650	1652950	C	A	NA	*OreG0024316*
scaffold650	1655157	G	T	NA	*OreG0024316*
scaffold650	1704391	T	A	Och01,Och02,Och03,Och04,Och05,Och06,Och07,Och08,Och09,Och10,Och11,Och12,Och13,Och14,Ore01,Ore02,Ore03,Ore04,Ore05,Ore06,Ore07,Ore08,Ore09,Ore10,Ore11,Ore12,Ore13,Ore14	*OreG0024320*
scaffold650	2025528	G	A	NA	*OreG0024331*
scaffold650	2030635	C	A	Och07	*OreG0024333*
scaffold650	2121461	C	T	NA	*OreG0024337*
scaffold650	2271258	C	A	Och07	*OreG0024344*
scaffold1936	55815	C	A	Och01	*OreG0004909*
scaffold1936	195697	G	A	Och01,Ore06,Ore07,Ore10,Ore14	*OreG0004913*
scaffold1936	701957	C	T	Och02	*OreG0004920*
scaffold1625	59739	T	A	Och01,Och02,Och03,Och04,Och05,Och06,Och07,Och08,Och09,Och10,Och11,Och12,Och13,Och14,Ore01,Ore02,Ore03,Ore04,Ore05,Ore06,Ore07,Ore08,Ore09,Ore10,Ore11,Ore12,Ore13,Ore14	*OreG0003283*
scaffold1625	334865	C	G	Och01,Och02,Och03,Och04,Och05,Och06,Och07,Och08,Och09,Och10,Och11,Och12,Och13,Och14,Ore01,Ore02,Ore03,Ore04,Ore05,Ore06,Ore07,Ore08,Ore09,Ore10,Ore11,Ore12,Ore13,Ore14	*OreG0003304*
scaffold1625	369142	A	T	Och09,Och10	*OreG0003308*
scaffold1625	418142	G	A	NA	*OreG0003312*
scaffold1625	418277	T	G	NA	*OreG0003312*
scaffold1625	474410	G	T	NA	*OreG0003316*
scaffold1625	474823	G	T	NA	*OreG0003316*
scaffold1625	488099	G	T	NA	*OreG0003319*
scaffold1625	517464	T	A	Och01,Och02,Och04,Och06	*OreG0003324*
scaffold2696	39169	G	A	Och01,Och03,Och06	*OreG0007895*

scaffold1520	123907	G	C	Och02,Och04,Och06,Och07,Och09,Och10	*OreG0003255*
scaffold1520	129455	G	T	NA	*OreG0003255*
scaffold1520	130697	G	A	NA	*OreG0003255*
scaffold1520	131167	C	A	NA	*OreG0003255*
scaffold1520	131288	G	T	NA	*OreG0003255*
scaffold1520	132355	C	A	Och07,Och08	*OreG0003255*
scaffold1520	132407	A	C	Och01,Och03,Och06	*OreG0003255*
scaffold1520	180147	C	A	NA	*OreG0003261*
scaffold2709	99959	T	G	NA	*OreG0007928*
scaffold1244	35923	C	T	NA	*OreG0001804*
scaffold1244	192851	C	T	NA	*OreG0001813*
scaffold1244	271431	C	T	NA	*OreG0001820*
scaffold1244	417112	C	T	NA	*OreG0001830*
scaffold1244	480600	G	A	Och01,Och02,Och03,Och04,Och05,Och06,Och07,Och08,Och09,Och10,Och11,Och12,Och13,Och14	*OreG0001837*
scaffold1244	568132	G	T	NA	*OreG0001845*
scaffold1244	570192	T	A	NA	*OreG0001845*
scaffold1244	585018	C	A	Och01,Och02,Och03,Och04,Och05,Och06,Och07,Och11,Och12,Och13	*OreG0001848*
scaffold1244	657727	G	A	Och01,Och02,Och03,Och04,Och05,Och06,Och07,Och08,Och12,Och13,Och14	*OreG0001861*
scaffold1244	712442	G	A	Och06,Och11,Och14	*OreG0001867*
scaffold1244	764477	C	T	NA	*OreG0001871*
scaffold1244	773256	A	T	NA	*OreG0001874*
scaffold1244	798573	T	A	Och03	*OreG0001879*
scaffold1244	1303011	G	T	NA	*OreG0001925*
scaffold1244	1450725	C	T	Och01	*OreG0001942*
scaffold1244	1458156	G	T	NA	*OreG0001943*
scaffold74	221792	G	A	NA	*OreG0024840*
scaffold74	306394	G	C	Och01,Och02,Och03,Och06	*OreG0024846*
scaffold74	370008	T	A	NA	*OreG0024856*
scaffold74	370963	C	T	NA	*OreG0024856*
scaffold74	506878	C	G	NA	*OreG0024871*
scaffold74	543588	C	T	NA	*OreG0024874*
scaffold74	666004	C	T	Och08	*OreG0024890*
scaffold74	666005	C	T	Och08	*OreG0024890*
scaffold74	851280	G	T	Och01,Och02,Och03,Och04,Och05,Och06,Och07,Och08,Och09,Och10,Och11,Och12,Och13,Och14	*OreG0024910*
scaffold74	892701	T	A	Och02	*OreG0024918*
scaffold74	915122	C	T	NA	*OreG0024921*
scaffold74	1020789	G	A	NA	*OreG0024935*
scaffold74	1181237	C	T	NA	*OreG0024948*
scaffold74	1223376	T	A	Och12,Och13	*OreG0024953*
scaffold74	1378689	G	A	Och12,Och13	*OreG0024971*
scaffold2213	627728	C	T	NA	*OreG0006265*
scaffold2193	207901	G	A	NA	*OreG0006218*
scaffold2464	14169	A	C	Och02	*OreG0007279*
scaffold2464	72748	G	A	Ore01,Ore02,Ore03,Ore04,Ore05,Ore06,Ore07,Ore08,Ore09,Ore10,Ore11,Ore12,Ore13,Ore14	*OreG0007285*
scaffold2464	92728	T	A	NA	*OreG0007288*
scaffold2464	169003	A	C	NA	*OreG0007298*
scaffold2464	212138	C	G	Och13	*OreG0007305*
scaffold2464	214861	G	A	Och09	*OreG0007306*
scaffold2464	214989	C	T	Och12	*OreG0007306*
scaffold2464	234180	G	C	NA	*OreG0007308*
scaffold2464	237544	C	T	NA	*OreG0007310*
scaffold2464	253352	C	T	Och02,Och03,Och04,Och05,Och06,Och07,Och08,Och09,Och10,Och11,Och12,Och13	*OreG0007313*
scaffold2464	307157	G	C	Och01,Och02,Och03,Och04,Och05,Och06,Och07,Och08,Och09,Och10,Och11,Och12,Och13,Och14,Ore01,Ore02,Ore03,Ore04,Ore05,Ore06,Ore07,Ore08,Ore09,Ore10,Ore11,Ore12,Ore13,Ore14	*OreG0007321*
scaffold2173	64669	T	A	NA	*OreG0006065*
scaffold2173	70763	C	A	Och09,Och10	*OreG0006067*
scaffold2173	161592	C	T	NA	*OreG0006077*
scaffold2173	161797	T	A	NA	*OreG0006077*
scaffold2173	161850	G	A	NA	*OreG0006077*
scaffold2173	161888	A	T	NA	*OreG0006077*
scaffold2173	209385	G	T	NA	*OreG0006082*
scaffold2173	272037	C	A	NA	*OreG0006091*
scaffold2173	273526	G	A	NA	*OreG0006091*
scaffold2173	273568	G	A	NA	*OreG0006091*
scaffold2173	282756	C	A	NA	*OreG0006092*
scaffold2173	282813	C	A	NA	*OreG0006092*
scaffold2173	283959	G	C	NA	*OreG0006092*
scaffold2173	284033	C	A	NA	*OreG0006092*
scaffold2173	431193	C	A	Och01,Och03,Och07,Och09,Och10,Och14	*OreG0006106*

scaffold2173	564085	C	A	NA	*OreG0006124*
scaffold2173	644953	A	T	NA	*OreG0006132*
scaffold2058	7180	G	A	NA	*OreG0005153*
scaffold2058	56748	C	T	Och01	*OreG0005155*
scaffold2058	130883	C	T	NA	*OreG0005161*
scaffold2058	167104	A	T	Och01,Och02,Och03,Och04,Och05,Och06,Och07,Och08,Och09,Och10,Och11,Och12,Och13,Och14	*OreG0005165*
scaffold2058	167108	T	A	Och01,Och02,Och03,Och04,Och05,Och06,Och07,Och08,Och09,Och10,Och11,Och12,Och13,Och14	*OreG0005165*
scaffold2058	168423	T	A	Och01,Och02,Och03,Och04,Och05,Och06,Och07,Och08,Och09,Och10,Och11,Och12,Och13,Och14	*OreG0005165*
scaffold2058	174635	C	A	NA	*OreG0005166*
scaffold2058	191623	C	T	Och01,Och02,Och03,Och04,Och05,Och06,Och07,Och08,Och09,Och10,Och11,Och12,Och13,Och14,Ore01,Ore02,Ore03,Ore04,Ore05,Ore06,Ore07,Ore08,Ore09,Ore10,Ore11,Ore12,Ore13,Ore14	*OreG0005170*
scaffold2058	221008	G	A	NA	*OreG0005175*
scaffold2058	271432	G	A	Och09,Och10	*OreG0005182*
scaffold2058	455240	C	T	NA	*OreG0005207*
scaffold2058	456395	C	T	Och01,Och07,Och08,Och09,Och10,Och11	*OreG0005207*
scaffold2058	536354	C	A	Och05,Och13	*OreG0005219*
scaffold2058	536843	C	A	NA	*OreG0005219*
scaffold2058	639355	C	A	Och01,Och02,Och03,Och04,Och05,Och06,Och07,Och08,Och09,Och10,Och11,Och12,Och13,Och14	*OreG0005232*
scaffold1943	85047	C	T	NA	*OreG0004954*
scaffold1943	136225	A	T	NA	*OreG0004961*
scaffold1943	136434	G	C	Och02,Och03	*OreG0004961*
scaffold1943	140860	A	T	Och06,Och13	*OreG0004962*
scaffold1943	170876	C	T	NA	*OreG0004967*
scaffold1943	170929	G	A	NA	*OreG0004967*
scaffold1943	187272	G	T	NA	*OreG0004970*
scaffold1943	304556	C	T	NA	*OreG0004979*
scaffold1943	343705	A	T	Ore02,Ore03,Ore06,Ore08,Ore09,Ore10,Ore11,Ore12,Ore14	*OreG0004985*
scaffold987	323286	C	T	Och14	*OreG0027103*
scaffold987	429729	G	A	NA	*OreG0027115*
scaffold987	453861	C	A	NA	*OreG0027119*
scaffold987	527284	C	T	NA	*OreG0027127*
scaffold987	700453	G	A	Och01,Och02,Och03,Och04,Och05,Och06,Och07,Och08,Och09,Och10,Och11,Och12,Och13,Och14	*OreG0027132*
scaffold987	895432	G	A	Ore01,Ore02,Ore03,Ore04,Ore05,Ore06,Ore07,Ore08,Ore09,Ore10,Ore11,Ore12,Ore13,Ore14	*OreG0027141*
scaffold987	895441	C	A	Ore02,Ore03,Ore04,Ore05,Ore06,Ore07,Ore08,Ore09,Ore10,Ore11,Ore12,Ore14	*OreG0027141*
scaffold987	1462264	G	T	NA	*OreG0027166*
scaffold987	1463400	C	T	NA	*OreG0027166*
scaffold987	1470129	G	T	NA	*OreG0027168*
scaffold987	1494351	C	T	Ore01,Ore02,Ore03,Ore04,Ore05,Ore06,Ore07,Ore08,Ore09,Ore10,Ore11,Ore12,Ore13,Ore14	*OreG0027170*
scaffold987	1504421	G	A	NA	*OreG0027171*
scaffold987	1672449	C	A	Ore01,Ore02,Ore03,Ore04,Ore05,Ore06,Ore07,Ore08,Ore09,Ore10,Ore11,Ore12,Ore13,Ore14	*OreG0027177*
scaffold987	1911637	A	C	Ore03,Ore06,Ore07,Ore08,Ore13,Ore14	*OreG0027189*
scaffold987	1915382	G	A	Och12,Och13	*OreG0027189*
scaffold987	2411013	C	T	Och03,Och09,Och10,Och11,Och14	*OreG0027216*
scaffold987	2703278	G	A	Ore01,Ore04,Ore05,Ore11	*OreG0027239*
scaffold987	2799287	G	T	NA	*OreG0027247*
scaffold987	2799521	C	T	NA	*OreG0027247*
scaffold2239	32265	C	T	NA	*OreG0006292*
scaffold2239	63497	C	T	NA	*OreG0006297*
scaffold2239	63535	T	A	NA	*OreG0006297*
scaffold2239	107406	C	A	NA	*OreG0006303*
scaffold2239	475245	A	C	NA	*OreG0006347*
scaffold2239	475403	C	T	NA	*OreG0006347*
scaffold2239	475750	G	A	NA	*OreG0006347*
scaffold2239	477778	A	T	NA	*OreG0006348*
scaffold2239	521412	G	T	NA	*OreG0006354*
scaffold2239	568774	C	G	Och01,Och02,Och06,Och09,Och10,Och14	*OreG0006362*
scaffold2239	569380	G	A	NA	*OreG0006362*
scaffold1758	138245	C	T	NA	*OreG0003638*
scaffold1758	197654	T	A	NA	*OreG0003647*
scaffold1758	299191	G	C	NA	*OreG0003659*
scaffold1758	299488	C	T	NA	*OreG0003659*
scaffold1758	490015	C	T	NA	*OreG0003682*
scaffold1758	490176	C	A	NA	*OreG0003682*
scaffold1758	490280	G	T	NA	*OreG0003682*

scaffold1758	512129	C	G	Och01,Och02,Och03,Och04,Och05,Och06,Och07,Och08,Och09,Och10,Och11,Och12,Och13,Och14,Ore01,Ore02,Ore03,Ore04,Ore05,Ore06,Ore07,Ore08,Ore09,Ore10,Ore11,Ore12,Ore13,Ore14	*OreG0003688*
scaffold1758	597540	C	T	NA	*OreG0003701*
scaffold1758	650759	C	T	NA	*OreG0003711*
scaffold1758	657603	C	T	NA	*OreG0003713*
scaffold1758	694471	C	T	Ore01,Ore04,Ore05,Ore08,Ore09,Ore11,Ore14	*OreG0003720*
scaffold1758	705533	G	A	Och12	*OreG0003722*
scaffold1758	924665	C	A	Och01,Och02,Och03,Och04,Och05,Och06,Och07,Och08,Och09,Och10,Och11,Och12,Och13,Och14,Ore01,Ore02,Ore03,Ore04,Ore05,Ore06,Ore07,Ore08,Ore09,Ore10,Ore11,Ore12,Ore13,Ore14	*OreG0003747*
scaffold1758	1057405	G	A	Och02,Och03,Och04,Och05,Och12,Och13,Och14,Ore01,Ore02,Ore03,Ore04,Ore05,Ore06,Ore07,Ore08,Ore09,Ore10,Ore11,Ore12,Ore13,Ore14	*OreG0003770*
scaffold1758	1236026	G	T	NA	*OreG0003791*
scaffold1758	1236076	C	T	NA	*OreG0003791*
scaffold1758	1236704	G	T	Ore01,Ore02,Ore03,Ore04,Ore05,Ore06,Ore07,Ore08,Ore09,Ore10,Ore11,Ore12,Ore13,Ore14	*OreG0003791*
scaffold1758	1276145	G	A	NA	*OreG0003797*
scaffold1758	1418958	C	T	NA	*OreG0003822*
scaffold1758	1439525	G	T	Och01	*OreG0003825*
scaffold1758	1477660	G	A	Ore04,Ore05	*OreG0003827*
scaffold1758	1600356	T	A	Ore01,Ore02,Ore03,Ore04,Ore05,Ore06,Ore07,Ore08,Ore09,Ore10,Ore11,Ore12,Ore13,Ore14	*OreG0003837*
scaffold1758	1665455	T	A	Och01,Och02,Och03,Och04,Och05,Och06,Och07,Och08,Och09,Och10,Och11,Och12,Och13,Och14,Ore01,Ore02,Ore03,Ore04,Ore05,Ore06,Ore07,Ore08,Ore09,Ore10,Ore11,Ore12,Ore13,Ore14	*OreG0003845*
scaffold1758	1758407	C	A	NA	*OreG0003857*
scaffold1758	1775183	G	T	NA	*OreG0003860*
scaffold1758	1824707	A	T	Ore01,Ore02,Ore03,Ore04,Ore05,Ore06,Ore07,Ore08,Ore09,Ore10,Ore11,Ore12,Ore13,Ore14	*OreG0003868*
scaffold1758	1987470	T	A	Och08	*OreG0003888*
scaffold1758	1987556	T	A	Och01,Och02,Och03,Och04,Och05,Och06,Och07,Och08,Och09,Och10,Och11,Och12,Och13,Och14,Ore01,Ore02,Ore03,Ore04,Ore05,Ore06,Ore07,Ore08,Ore09,Ore10,Ore11,Ore12,Ore13,Ore14	*OreG0003889*
scaffold1758	2022637	C	T	Och03	*OreG0003894*
scaffold1758	2044244	G	T	Och01,Och02,Och03,Och04,Och05,Och06,Och07,Och08,Och09,Och10,Och11,Och12,Och13,Och14	*OreG0003897*
scaffold2142	76164	A	T	NA	*OreG0006011*
scaffold917	464671	C	A	Och01,Och11	*OreG0026651*
scaffold917	465036	A	T	Ore01,Ore02,Ore03,Ore04,Ore05,Ore06,Ore07,Ore08,Ore09,Ore10,Ore11,Ore12,Ore13,Ore14	*OreG0026651*
scaffold917	804398	A	C	Ore01,Ore02,Ore03,Ore04,Ore05,Ore06,Ore07,Ore08,Ore09,Ore10,Ore11,Ore12,Ore13,Ore14	*OreG0026663*
scaffold917	1093100	C	T	Och01,Och02,Och03,Och04,Och05,Och06,Och07,Och08,Och09,Och10,Och11,Och12,Och13,Och14	*OreG0026677*
scaffold917	1642788	G	A	Och01,Och02,Och03,Och04,Och05,Och06,Och07,Och08,Och09,Och10,Och11,Och12,Och13,Och14	*OreG0026688*
scaffold917	1647963	G	T	Och01,Och08,Och11,Och14	*OreG0026688*
scaffold917	1656689	C	T	Och01,Och02,Och03,Och04,Och05,Och06,Och07,Och08,Och09,Och10,Och11,Och12,Och13,Och14	*OreG0026692*
scaffold917	1659050	T	A	Och01,Och02,Och04,Och07,Och09,Och11,Och12,Och13,Och14	*OreG0026692*
scaffold917	1773391	G	A	NA	*OreG0026698*
scaffold917	2615184	T	A	Och01,Och02,Och03,Och04,Och05,Och06,Och07,Och08,Och09,Och10,Och11,Och12,Och13,Och14	*OreG0026724*
scaffold917	2925266	G	T	NA	*OreG0026739*
scaffold917	3335892	C	T	Och01,Och02,Och03,Och04,Och05,Och06,Och07,Och08,Och09,Och10,Och11,Och12,Och13,Och14	*OreG0026759*
scaffold917	3336000	C	T	Och01,Och02,Och03,Och04,Och05,Och06,Och07,Och08,Och09,Och10,Och11,Och12,Och13,Och14	*OreG0026759*
scaffold917	3336084	C	T	Och01,Och02,Och03,Och04,Och05,Och06,Och07,Och08,Och09,Och10,Och11,Och12,Och13,Och14	*OreG0026759*
scaffold917	3374279	G	A	Och02,Och03,Och04,Och05,Och06,Och07,Och12,Och13	*OreG0026761*
scaffold917	3557097	C	T	Och08,Och09,Och10,Och11	*OreG0026771*
scaffold917	3583332	G	T	Och01,Och08,Och09,Och10,Och11,Och14,Ore01,Ore02,Ore03,Ore04,Ore05,Ore06,Ore07,Ore08,Ore09,Ore10,Ore11,Ore12,Ore13,Ore14	*OreG0026774*
scaffold2001	320959	C	T	Ore03,Ore08,Ore09,Ore10,Ore14	*OreG0005062*
scaffold2001	521978	C	T	NA	*OreG0005071*
scaffold2001	524294	C	T	NA	*OreG0005071*
scaffold2001	677515	C	T	NA	*OreG0005081*
scaffold2001	736949	G	T	Och08,Och11,Och14	*OreG0005088*
scaffold2001	857554	C	T	Ore01,Ore02,Ore03,Ore04,Ore05,Ore06,Ore07,Ore08,Ore09,Ore10,Ore11,Ore12,Ore13,Ore14	*OreG0005096*
scaffold2001	888071	G	A	Ore07,Ore08,Ore14	*OreG0005099*
scaffold2001	904566	G	T	Och01,Och02,Och03,Och04,Och05,Och06,Och07,Och08,Och09,Och10,Och11,Och12,Och13,Och14	*OreG0005101*

scaffold2001	1058802	A	T	Och01,Och02,Och03,Och04,Och05,Och06,Och07,Och08,Och09,Och10,Och11,Och12,Och13,Och14,Ore01,Ore02,Ore03,Ore04,Ore05,Ore06,Ore07,Ore08,Ore09,Ore10,Ore11,Ore12,Ore13,Ore14	*OreG0005114*
scaffold2001	1100037	G	A	Och08	*OreG0005121*
scaffold2001	1109215	T	A	Och01,Och02,Och06,Och07	*OreG0005122*
scaffold2001	1109711	A	T	Och08	*OreG0005122*
scaffold2001	1357061	C	A	Och02,Och04,Och06	*OreG0005138*
scaffold2001	1376330	C	A	Och13	*OreG0005140*
scaffold3228	64168	G	A	NA	*OreG0010418*
scaffold3228	72637	T	A	NA	*OreG0010419*
scaffold2398	94537	C	A	NA	*OreG0007125*
scaffold2398	241406	T	A	Och06	*OreG0007134*
scaffold2398	446248	C	T	NA	*OreG0007163*
scaffold2398	454941	C	A	Ore01,Ore02,Ore03,Ore04,Ore05,Ore06,Ore07,Ore08,Ore09,Ore10,Ore11,Ore12,Ore13,Ore14	*OreG0007164*
scaffold2398	752071	A	T	NA	*OreG0007203*
scaffold2398	752104	G	T	Och13	*OreG0007203*
scaffold2398	832386	A	T	Ore01,Ore02,Ore03,Ore04,Ore05,Ore06,Ore07,Ore08,Ore09,Ore10,Ore11,Ore12,Ore13,Ore14	*OreG0007213*
scaffold2398	928539	T	G	Och14	*OreG0007224*
scaffold2398	1022440	T	A	Och01,Och02,Och03,Och04,Och05,Och06,Och07,Och08,Och09,Och10,Och11,Och12,Och13,Och14,Ore01,Ore02,Ore03,Ore04,Ore05,Ore06,Ore07,Ore08,Ore09,Ore10,Ore11,Ore12,Ore13,Ore14	*OreG0007235*
scaffold1062	275972	C	T	Och14	*OreG0000268*
scaffold1062	284475	C	A	Ore01,Ore02,Ore03,Ore04,Ore05,Ore06,Ore07,Ore08,Ore09,Ore10,Ore11,Ore12,Ore13,Ore14	*OreG0000270*
scaffold1062	375747	T	A	NA	*OreG0000279*
scaffold1062	682901	C	A	Och01,Och02,Och03,Och04,Och05,Och06,Och07,Och08,Och09,Och10,Och11,Och12,Och13,Och14	*OreG0000314*
scaffold1062	1294820	A	T	Och04,Och06	*OreG0000381*
scaffold1062	1484295	G	A	NA	*OreG0000396*
scaffold1062	1486152	G	A	NA	*OreG0000396*
scaffold1062	1637958	T	A	Och02,Och03,Och04,Och05,Och06,Och11,Och12,Och13,Och14	*OreG0000406*
scaffold1062	1641093	G	A	Och02,Och03,Och04,Och05,Och06,Och07,Och11,Och12,Och13,Och14	*OreG0000406*
scaffold1062	1669418	G	T	Och02,Och03,Och04,Och05,Och06,Och07,Och11,Och12,Och13,Och14	*OreG0000408*
scaffold1062	1671438	G	T	NA	*OreG0000408*
scaffold1062	1916241	G	A	Och01,Och02,Och03,Och04,Och05,Och06,Och07,Och08,Och09,Och10,Och11,Och12,Och13,Och14,Ore01,Ore02,Ore03,Ore04,Ore05,Ore06,Ore07,Ore08,Ore09,Ore10,Ore11,Ore12,Ore13,Ore14	*OreG0000411*
scaffold1062	1917811	A	T	NA	*OreG0000412*
scaffold1062	1960791	G	T	NA	*OreG0000417*
scaffold1062	2005386	A	T	Ore03	*OreG0000421*
scaffold1062	2022806	T	A	Ore01,Ore02,Ore03,Ore04,Ore05,Ore06,Ore07,Ore08,Ore09,Ore10,Ore11,Ore12,Ore13,Ore14	*OreG0000423*
scaffold1062	2050724	C	T	Och01,Och02,Och03,Och04,Och05,Och06,Och07,Och08,Och09,Och10,Och11,Och12,Och13,Och14	*OreG0000425*
scaffold1062	2176170	T	A	NA	*OreG0000440*
scaffold1062	2179304	G	A	Och02,Och03,Och04,Och06,Och09,Och10,Och11	*OreG0000441*
scaffold1062	2249050	C	A	Och04,Och06,Och09,Och10,Och11	*OreG0000451*
scaffold1062	2446864	T	A	Och01,Och07,Och09,Och10,Och11,Ore01,Ore02,Ore03,Ore04,Ore05,Ore06,Ore07,Ore08,Ore09,Ore10,Ore11,Ore12,Ore13,Ore14	*OreG0000459*
scaffold1062	2469887	T	A	NA	*OreG0000464*
scaffold1062	2486407	C	T	Ore01,Ore02,Ore03,Ore04,Ore05,Ore06,Ore07,Ore08,Ore09,Ore10,Ore11,Ore12,Ore13,Ore14	*OreG0000465*
scaffold1062	2551083	C	A	NA	*OreG0000476*
scaffold1062	2552395	C	T	Och02,Och03,Och04,Och05,Och06,Och07,Och08,Och09,Och10,Och11,Och12,Och13,Och14	*OreG0000476*
scaffold1062	2648091	T	A	Och01,Och02,Och03,Och04,Och05,Och06,Och07,Och08,Och09,Och10,Och11,Och12,Och13,Och14,Ore01,Ore02,Ore03,Ore04,Ore05,Ore06,Ore07,Ore08,Ore09,Ore10,Ore11,Ore12,Ore13,Ore14	*OreG0000490*
scaffold1062	2673238	G	T	Och05,Och08,Och14	*OreG0000492*
scaffold1062	2673804	C	T	NA	*OreG0000492*
scaffold1062	2680192	G	A	Och01,Och02,Och03,Och04,Och05,Och06,Och07,Och08,Och09,Och10,Och11,Och12,Och13,Och14	*OreG0000492*
scaffold1062	2680598	G	T	Och02,Och03,Och04,Och05,Och06,Och09,Och10,Och11,Och12,Och13	*OreG0000492*
scaffold1062	2681096	C	A	Ore04,Ore05	*OreG0000492*
scaffold1062	2681175	A	T	NA	*OreG0000492*
scaffold1062	2763956	G	A	NA	*OreG0000497*
scaffold1062	2763957	G	A	NA	*OreG0000497*
scaffold1062	2774516	G	T	Och01,Och02,Och03,Och04,Och05,Och06,Och07,Och08,Och09,Och10,Och11,Och12,Och13,Och14	*OreG0000501*
scaffold1062	2881426	C	T	NA	*OreG0000520*
scaffold1062	2888503	C	A	Ore01,Ore02,Ore03,Ore04,Ore05,Ore06,Ore07,Ore08,Ore09,Ore10,Ore11,Ore12,Ore13,Ore14	*OreG0000521*

scaffold1062	3049145	C	T	Ore01,Ore02,Ore03,Ore04,Ore05,Ore06,Ore07,Ore08,Ore09,Ore10,Ore11,Ore12,Ore13,Ore14	*OreG0000542*
scaffold1062	3049150	G	A	Och01,Och02,Och03,Och04,Och05,Och06,Och07,Och08,Och09,Och10,Och11,Och12,Och13,Och14	*OreG0000542*
scaffold1062	3106792	C	T	NA	*OreG0000547*
scaffold1062	3192429	C	A	NA	*OreG0000557*
scaffold1062	3292788	T	A	Ore01,Ore03,Ore06,Ore09	*OreG0000564*
scaffold1062	3426186	G	A	Ore04,Ore05,Ore13	*OreG0000577*
scaffold1062	3592193	A	T	NA	*OreG0000592*
scaffold1062	3592796	G	T	NA	*OreG0000592*
scaffold1062	3674061	C	T	Och11	*OreG0000598*
scaffold1062	3891393	C	A	NA	*OreG0000610*
scaffold1997	740731	G	A	Och01,Och02,Och03,Och04,Och05,Och06,Och07,Och08,Och09,Och10,Och11,Och12,Och13,Och14	*OreG0005015*
scaffold1997	1342582	C	A	NA	*OreG0005026*
scaffold1997	2029564	C	A	Och01,Och02,Och03,Och04,Och05,Och06,Och07,Och08,Och09,Och10,Och11,Och12,Och13,Och14	*OreG0005050*
scaffold1997	2029838	G	T	Och01,Och02,Och03,Och04,Och05,Och06,Och07,Och08,Och09,Och10,Och11,Och12,Och13,Och14	*OreG0005050*
scaffold1997	2030396	G	A	Och01,Och02,Och03,Och04,Och05,Och06,Och07,Och08,Och09,Och10,Och11,Och12,Och13,Och14	*OreG0005050*
scaffold1457	207063	G	A	Och01,Och02,Och03,Och04,Och05,Och06,Och08,Och09,Och10,Och11,Och12,Och13	*OreG0002405*
scaffold1457	207092	C	T	Ore01,Ore02,Ore03,Ore04,Ore05,Ore06,Ore07,Ore08,Ore09,Ore10,Ore11,Ore12,Ore13,Ore14	*OreG0002405*
scaffold1457	773057	C	T	Ore01,Ore02,Ore03,Ore04,Ore05,Ore06,Ore07,Ore08,Ore09,Ore10,Ore11,Ore12,Ore13,Ore14	*OreG0002423*
scaffold1457	788905	G	A	NA	*OreG0002425*
scaffold1457	788958	G	T	NA	*OreG0002425*
scaffold1457	789470	C	T	Och01,Och02,Och03,Och04,Och05,Och06,Och07,Och08,Och09,Och10,Och11,Och12,Och13,Och14	*OreG0002425*
scaffold1457	1641340	A	C	Och01,Och02,Och03,Och04,Och05,Och06,Och07,Och08,Och09,Och10,Och11,Och12,Och13,Och14,Ore01,Ore02,Ore03,Ore04,Ore05,Ore06,Ore07,Ore08,Ore09,Ore10,Ore11,Ore12,Ore13,Ore14	*OreG0002446*
scaffold1457	1643465	G	A	NA	*OreG0002447*
scaffold1457	1782463	C	T	NA	*OreG0002461*
scaffold1457	1783214	T	A	Och01,Och02,Och03,Och04,Och05,Och06,Och07,Och08,Och09,Och10,Och11,Och12,Och13,Och14	*OreG0002461*
scaffold1457	1882787	C	T	Ore01,Ore02,Ore03,Ore04,Ore05,Ore06,Ore07,Ore08,Ore09,Ore10,Ore11,Ore12,Ore13,Ore14	*OreG0002467*
scaffold1457	1912013	C	A	NA	*OreG0002470*
scaffold1457	1913394	T	A	NA	*OreG0002470*
scaffold1457	1931227	C	A	NA	*OreG0002473*
scaffold1457	1969248	T	A	NA	*OreG0002480*
scaffold1457	2124880	G	A	Och03,Och06	*OreG0002497*
scaffold1457	2134362	C	T	Och01	*OreG0002499*
scaffold1457	2134613	C	A	Och05,Och12,Och14	*OreG0002499*
scaffold1457	2273278	T	A	NA	*OreG0002513*
scaffold3213	12268	A	T	NA	*OreG0010391*
scaffold3213	21235	C	A	NA	*OreG0010393*
scaffold346	317732	T	A	Och01,Och02,Och03,Och04,Och06,Och07,Och09,Och10,Och11	*OreG0018633*
scaffold346	580477	G	T	NA	*OreG0018644*
scaffold346	580501	G	T	NA	*OreG0018644*
scaffold346	1337879	C	A	Och01	*OreG0018663*
scaffold346	1408091	A	C	NA	*OreG0018665*
scaffold346	1470717	C	T	Och01,Och06,Och09,Och10,Ore01,Ore03,Ore06,Ore10,Ore11,Ore13	*OreG0018668*
scaffold346	1471001	C	A	Och01,Och09,Och10	*OreG0018668*
scaffold346	1669471	C	T	NA	*OreG0018683*
scaffold346	1992971	G	T	NA	*OreG0018695*
scaffold346	2093133	G	A	NA	*OreG0018707*
scaffold346	2439482	C	T	NA	*OreG0018745*
scaffold346	2442561	G	C	NA	*OreG0018747*
scaffold346	2486490	G	A	Och01,Och02,Och03,Och04,Och05,Och06,Och07,Och08,Och09,Och10,Och11,Och12,Och13,Och14	*OreG0018751*
scaffold346	2503922	C	T	NA	*OreG0018753*
scaffold346	2523265	C	A	NA	*OreG0018755*
scaffold4173	30881	C	T	Och01,Och09,Och10	*OreG0020090*
scaffold4173	34386	G	A	Och01	*OreG0020090*
scaffold4173	34758	G	A	NA	*OreG0020090*
scaffold4681	43326	A	T	NA	*OreG0022357*
scaffold4681	43381	G	A	NA	*OreG0022357*
scaffold3757	19897	G	A	NA	*OreG0019083*
scaffold1220	231539	G	A	Ore01,Ore02,Ore03,Ore04,Ore05,Ore06,Ore07,Ore08,Ore09,Ore10,Ore11,Ore12,Ore13,Ore14	*OreG0001374*
scaffold1220	296725	C	T	NA	*OreG0001382*

scaffold1220	325138	C	T	NA	*OreG0001385*
scaffold1220	357104	C	T	NA	*OreG0001391*
scaffold1220	435213	T	G	NA	*OreG0001397*
scaffold1220	439800	G	A	NA	*OreG0001398*
scaffold1220	444521	G	A	Och09,Och10,Och11	*OreG0001398*
scaffold1220	463694	G	C	Och03,Och05,Och08,Och12,Och13	*OreG0001400*
scaffold1220	480174	C	A	NA	*OreG0001402*
scaffold1220	492949	C	A	NA	*OreG0001404*
scaffold1220	493680	G	T	NA	*OreG0001404*
scaffold1220	513314	G	A	Och03,Och06	*OreG0001408*
scaffold1220	532751	G	T	NA	*OreG0001412*
scaffold1220	703200	G	A	NA	*OreG0001429*
scaffold1220	1005610	G	T	NA	*OreG0001445*
scaffold1220	1414132	G	A	Och01,Och02,Och03,Och04,Och05,Och06,Och07,Och08,Och09,Och10,Och11,Och12,Och13,Och14	*OreG0001485*
scaffold1220	1505105	T	A	Och02,Och05,Och12,Och13	*OreG0001494*
scaffold1220	1505399	G	T	Och12,Och13	*OreG0001494*
scaffold1220	1506703	C	T	NA	*OreG0001496*
scaffold1220	1516140	C	A	NA	*OreG0001498*
scaffold1220	1526370	C	T	Och01,Och02,Och03,Och04,Och05,Och06,Och07,Och08,Och09,Och10,Och11,Och12,Och13,Och14,Ore01,Ore02,Ore03,Ore04,Ore05,Ore06,Ore07,Ore08,Ore09,Ore10,Ore11,Ore12,Ore13,Ore14	*OreG0001500*
scaffold1220	1784171	C	T	NA	*OreG0001524*
scaffold1220	2455631	G	A	NA	*OreG0001568*
scaffold1220	2493907	C	A	NA	*OreG0001572*
scaffold1220	2810449	C	G	NA	*OreG0001597*
scaffold1220	2854165	C	T	Och07	*OreG0001604*
scaffold1220	2854220	T	A	NA	*OreG0001604*
scaffold1220	2868220	C	G	Ore01,Ore02,Ore03,Ore04,Ore05,Ore06,Ore07,Ore08,Ore09,Ore10,Ore11,Ore12,Ore13,Ore14	*OreG0001606*
scaffold1220	2905483	A	T	NA	*OreG0001608*
scaffold1220	2973023	G	A	Och01,Och02,Och03,Och04,Och05,Och06,Och07,Och08,Och09,Och10,Och11,Och12,Och13,Och14,Ore01,Ore02,Ore03,Ore04,Ore05,Ore06,Ore07,Ore08,Ore09,Ore10,Ore11,Ore12,Ore13,Ore14	*OreG0001612*
scaffold1220	2973118	G	T	Och02,Och03,Och05,Och11,Och12,Och13,Och14	*OreG0001612*
scaffold2483	89794	G	A	Ore02,Ore10,Ore11,Ore12,Ore13,Ore14	*OreG0007419*
scaffold2483	97088	C	T	NA	*OreG0007421*
scaffold2483	97831	T	A	Och08,Och14,Ore01,Ore02,Ore03,Ore04,Ore05,Ore06,Ore07,Ore08,Ore09,Ore10,Ore11,Ore12,Ore13,Ore14	*OreG0007421*
scaffold2483	100463	C	A	NA	*OreG0007422*
scaffold2483	104813	G	A	Ore04,Ore05	*OreG0007423*
scaffold2483	170590	C	A	NA	*OreG0007432*
scaffold2483	207950	A	C	Ore01,Ore02,Ore03,Ore04,Ore05,Ore06,Ore07,Ore08,Ore09,Ore10,Ore11,Ore12,Ore13,Ore14	*OreG0007437*
scaffold2483	442386	G	T	NA	*OreG0007461*
scaffold2483	442418	C	A	NA	*OreG0007461*
scaffold2483	480123	G	A	Och01,Och02,Och03,Och04,Och05,Och06,Och07,Och08,Och09,Och10,Och11,Och12,Och13,Och14,Ore01,Ore02,Ore03,Ore04,Ore05,Ore06,Ore07,Ore08,Ore09,Ore10,Ore11,Ore12,Ore13,Ore14	*OreG0007466*
scaffold2483	594635	G	A	Och01,Och02,Och03,Och04,Och05,Och06,Och07,Och08,Och09,Och10,Och11,Och12,Och13,Och14	*OreG0007481*
scaffold2483	822360	C	T	Och01,Och02,Och03,Och04,Och05,Och06,Och07,Och08,Och09,Och10,Och11,Och12,Och13,Och14,Ore01,Ore02,Ore03,Ore04,Ore05,Ore06,Ore07,Ore08,Ore09,Ore10,Ore11,Ore12,Ore13,Ore14	*OreG0007505*
scaffold2483	850349	G	T	NA	*OreG0007509*
scaffold2483	962872	C	T	Och01	*OreG0007516*
scaffold2483	1085628	A	C	Ore01,Ore02,Ore03,Ore04,Ore05,Ore06,Ore07,Ore08,Ore09,Ore10,Ore11,Ore12,Ore13,Ore14	*OreG0007527*
scaffold2483	1105244	C	T	Och01,Och02,Och03,Och04,Och05,Och06,Och07,Och08,Och09,Och10,Och11,Och12,Och13,Och14,Ore01,Ore02,Ore03,Ore04,Ore05,Ore06,Ore07,Ore08,Ore09,Ore10,Ore11,Ore12,Ore13,Ore14	*OreG0007531*
scaffold2483	1105494	G	A	NA	*OreG0007532*
scaffold1468	174740	C	G	Och01,Och02,Och03,Och04,Och05,Och06,Och07,Och08,Och09,Och10,Och11,Och12,Och13,Och14	*OreG0002632*
scaffold1468	174750	G	A	Och01,Och02,Och03,Och04,Och05,Och06,Och07,Och08,Och09,Och10,Och11,Och12,Och13,Och14	*OreG0002632*
scaffold1468	317559	C	G	NA	*OreG0002651*
scaffold1468	686675	C	T	Och01,Och02,Och03,Och04,Och05,Och06,Och07,Och08,Och09,Och10,Och11,Och12,Och13,Och14	*OreG0002693*
scaffold1468	726311	C	T	Och01,Och02,Och03,Och04,Och05,Och06,Och07,Och08,Och09,Och10,Och11,Och12,Och13,Och14,Ore01,Ore02,Ore03,Ore04,Ore05,Ore06,Ore07,Ore08,Ore09,Ore10,Ore11,Ore12,Ore13,Ore14	*OreG0002699*
scaffold1468	758888	G	A	NA	*OreG0002705*
scaffold1468	992517	A	T	Ore01,Ore02,Ore03,Ore04,Ore05,Ore06,Ore07,Ore08,Ore09,Ore10,Ore11,Ore12,Ore13,Ore14	*OreG0002734*

scaffold1468	1058905	T	A	Och01,Och02,Och03,Och04,Och05,Och06,Och07,Och08,Och09,Och10,Och11,Och12,Och13,Och14	*OreG0002742*
scaffold1468	1058913	A	C	Ore01,Ore02,Ore03,Ore04,Ore05,Ore06,Ore07,Ore08,Ore09,Ore10,Ore11,Ore12,Ore13,Ore14	*OreG0002742*
scaffold1468	1060109	A	T	Och01,Och02,Och03,Och04,Och05,Och06,Och07,Och08,Och09,Och10,Och11,Och12,Och13,Och14,Ore01,Ore02,Ore03,Ore04,Ore05,Ore06,Ore07,Ore08,Ore09,Ore10,Ore11,Ore12,Ore13,Ore14	*OreG0002742*
scaffold1468	1160793	G	T	Och01,Och02,Och03,Och04,Och05,Och06,Och07,Och08,Och09,Och10,Och11,Och12,Och13,Och14	*OreG0002757*
scaffold1468	1164600	C	A	NA	*OreG0002758*
scaffold1468	1178085	G	T	NA	*OreG0002761*
scaffold1468	1199060	G	A	NA	*OreG0002763*
scaffold1468	1215118	G	A	NA	*OreG0002766*
scaffold1468	1221267	T	A	NA	*OreG0002767*
scaffold1468	1258355	G	A	NA	*OreG0002771*
scaffold1468	1312774	G	A	NA	*OreG0002781*
scaffold1468	1385169	G	T	Och02,Och03,Och04,Och05,Och12,Och13	*OreG0002787*
scaffold1468	1386228	C	A	Och09,Och10,Och14	*OreG0002788*
scaffold1468	1407362	C	T	NA	*OreG0002791*
scaffold1468	1408017	T	A	NA	*OreG0002791*
scaffold1468	1408043	C	T	NA	*OreG0002791*
scaffold1468	1409969	A	T	Och01,Och02,Och03,Och04,Och05,Och06,Och07,Och08,Och09,Och10,Och11,Och12,Och13,Och14,Ore01,Ore02,Ore03,Ore04,Ore05,Ore06,Ore07,Ore08,Ore09,Ore10,Ore11,Ore12,Ore13,Ore14	*OreG0002792*
scaffold1468	1447392	C	A	Ore11	*OreG0002798*
scaffold1468	1451776	C	T	NA	*OreG0002799*
scaffold1468	1452112	C	T	NA	*OreG0002799*
scaffold1468	1452233	T	A	NA	*OreG0002799*
scaffold1468	1542215	G	T	Ore07,Ore08	*OreG0002809*
scaffold1468	1550722	C	T	Och01,Och02,Och03,Och04,Och05,Och06,Och07,Och08,Och09,Och10,Och11,Och12,Och13,Och14,Ore01,Ore02,Ore03,Ore04,Ore05,Ore06,Ore07,Ore08,Ore09,Ore10,Ore11,Ore12,Ore13,Ore14	*OreG0002812*
scaffold1468	1773647	C	T	Ore07,Ore08,Ore13	*OreG0002837*
scaffold1468	1903600	G	A	NA	*OreG0002850*
scaffold1468	1903990	C	A	NA	*OreG0002850*
scaffold1468	2055728	G	T	Och02,Och03,Och04,Och05,Och06,Och07,Och08,Och11,Och12,Och13	*OreG0002867*
scaffold1468	2165778	C	T	Och01,Och02,Och03,Och06,Och09,Och11,Och13,Och14	*OreG0002878*
scaffold1468	2166461	G	A	Och01,Och02,Och04,Och05,Och06,Och08,Och10,Och11,Och12,Och14	*OreG0002879*
scaffold1468	2184652	C	T	NA	*OreG0002881*
scaffold1468	2336010	G	T	Och01	*OreG0002898*
scaffold1468	2390458	G	T	Och06,Och12	*OreG0002904*
scaffold1468	2397357	G	T	Och13	*OreG0002905*
scaffold1468	2398227	C	T	NA	*OreG0002905*
scaffold1468	2464965	G	T	Ore11	*OreG0002908*
scaffold1468	2523887	T	A	Ore07,Ore08	*OreG0002916*
scaffold1468	2524667	T	A	Ore07,Ore08,Ore13	*OreG0002916*
scaffold1468	2524674	C	T	Ore07,Ore08,Ore13	*OreG0002916*
scaffold1468	2631089	C	A	Och01,Och02,Och03,Och04,Och05,Och06,Och07,Och08,Och09,Och10,Och11,Och12,Och13,Och14	*OreG0002930*
scaffold1468	2719666	C	T	NA	*OreG0002942*
scaffold1468	2736185	A	T	Och01,Och02,Och03,Och04,Och05,Och06,Och07,Och08,Och09,Och10,Och11,Och12,Och13,Och14,Ore01,Ore02,Ore03,Ore04,Ore05,Ore06,Ore07,Ore08,Ore09,Ore10,Ore11,Ore12,Ore13,Ore14	*OreG0002944*
scaffold1468	2766108	G	A	Och01,Och02,Och03,Och04,Och05,Och06,Och07,Och08,Och09,Och10,Och11,Och12,Och13,Och14,Ore01,Ore02,Ore03,Ore04,Ore05,Ore06,Ore07,Ore08,Ore09,Ore10,Ore11,Ore12,Ore13,Ore14	*OreG0002948*
scaffold1468	2835860	G	C	NA	*OreG0002955*
scaffold1468	2857844	C	A	NA	*OreG0002959*
scaffold1468	2867045	A	T	Ore02,Ore04,Ore05,Ore06,Ore07,Ore08,Ore11,Ore12,Ore13	*OreG0002961*
scaffold1468	2881802	T	A	Och01,Och02,Och03,Och04,Och05,Och06,Och07,Och08,Och09,Och10,Och11,Och12,Och13,Och14,Ore01,Ore02,Ore03,Ore04,Ore05,Ore06,Ore07,Ore08,Ore09,Ore10,Ore11,Ore12,Ore13,Ore14	*OreG0002965*
scaffold1468	2886211	G	T	Och06,Och12	*OreG0002966*
scaffold1468	2887261	C	T	NA	*OreG0002966*
scaffold1468	2941521	G	A	NA	*OreG0002979*
scaffold1468	2941975	G	A	NA	*OreG0002979*
scaffold1468	2942816	C	T	NA	*OreG0002979*
scaffold1468	2971651	G	T	Och06,Och12	*OreG0002984*
scaffold1468	3209539	C	A	Och01,Och02,Och03,Och04,Och05,Och06,Och07,Och08,Och09,Och10,Och11,Och12,Och13,Och14	*OreG0003012*
scaffold1468	3236548	G	T	NA	*OreG0003018*
scaffold1468	3304110	C	A	Och01,Och06,Och08,Och09,Och10,Och11	*OreG0003026*
scaffold1468	3356140	T	A	Och01,Och02,Och03,Och04,Och05,Och06,Och07,Och08,Och09,Och10,Och11,Och12,Och13,Och14,Ore01,Ore02,Ore03,Ore04,Ore05,Ore06,Ore07,Ore08,Ore09,Ore10,Ore11,Ore12,Ore13,Ore14	*OreG0003037*

scaffold1468	3429998	G	A	Och08,Och14	*OreG0003049*
scaffold1468	3430083	C	T	Och08,Och14	*OreG0003049*
scaffold1468	3477725	G	T	NA	*OreG0003056*
scaffold1468	3507030	C	A	Och01,Och02,Och03,Och04,Och05,Och06,Och07,Och08,Och09,Och10,Och11,Och12,Och13,Och14	*OreG0003057*
scaffold1468	3581275	T	A	Ore01,Ore02,Ore03,Ore04,Ore05,Ore06,Ore07,Ore08,Ore09,Ore10,Ore11,Ore12,Ore13,Ore14	*OreG0003064*
scaffold1468	3594997	C	T	Och01,Och02,Och03,Och04,Och05,Och06,Och07,Och08,Och09,Och10,Och11,Och12,Och13,Och14,Ore01,Ore02,Ore03,Ore04,Ore05,Ore06,Ore07,Ore08,Ore09,Ore10,Ore11,Ore12,Ore13,Ore14	*OreG0003066*
scaffold1468	3868214	G	T	Och01,Och02,Och03,Och04,Och05,Och06,Och07,Och08,Och09,Och10,Och11,Och12,Och13,Och14,Ore01,Ore02,Ore03,Ore04,Ore05,Ore06,Ore07,Ore08,Ore09,Ore10,Ore11,Ore12,Ore13,Ore14	*OreG0003107*
scaffold1468	3937674	G	T	Och07,Och08,Och09,Och10	*OreG0003118*
scaffold1468	3939666	T	A	NA	*OreG0003118*
scaffold1468	3988300	C	T	NA	*OreG0003124*
scaffold1468	4155485	T	A	Ore07,Ore08,Ore13	*OreG0003157*
scaffold1468	4161496	C	A	Och01,Och02,Och03,Och04,Och05,Och06,Och07,Och08,Och09,Och10,Och11,Och12,Och13,Och14	*OreG0003159*
scaffold1468	4275478	A	T	Och01,Och02,Och03,Och04,Och05,Och06,Och07,Och08,Och09,Och10,Och11,Och12,Och13,Och14,Ore01,Ore02,Ore03,Ore04,Ore05,Ore06,Ore07,Ore08,Ore09,Ore10,Ore11,Ore12,Ore13,Ore14	*OreG0003177*
scaffold1468	4298507	G	A	Och01	*OreG0003181*
scaffold1468	4299332	C	T	Ore01,Ore02,Ore03,Ore04,Ore05,Ore06,Ore07,Ore08,Ore09,Ore10,Ore11,Ore12,Ore13,Ore14	*OreG0003182*
scaffold1468	4303689	G	A	Ore02,Ore03,Ore04,Ore05,Ore06,Ore07,Ore08,Ore09,Ore10,Ore13,Ore14	*OreG0003182*
scaffold1468	4330751	T	G	NA	*OreG0003183*
scaffold1468	4415655	G	A	NA	*OreG0003194*
scaffold1468	4437060	A	T	Och01,Och02,Och03,Och04,Och05,Och07,Och08,Och09,Och10,Och11,Och12,Och13,Ore01,Ore02,Ore03,Ore04,Ore05,Ore06,Ore07,Ore08,Ore09,Ore10,Ore11,Ore12,Ore13,Ore14	*OreG0003197*
scaffold1468	4528509	C	T	NA	*OreG0003208*
scaffold1468	4528744	C	T	Och02,Och03,Och04,Och05,Och06,Och07,Och09,Och10,Och11,Och12,Och13	*OreG0003208*
scaffold1468	4528780	G	T	NA	*OreG0003208*
scaffold1468	4538285	A	T	NA	*OreG0003207*
scaffold1468	4572008	G	A	NA	*OreG0003215*
scaffold1468	4585952	T	A	Ore02,Ore04,Ore05,Ore06,Ore07,Ore08,Ore10,Ore11,Ore12,Ore13,Ore14	*OreG0003217*
scaffold1468	4610555	G	T	Ore14	*OreG0003219*
scaffold1468	4639879	T	A	Och01,Och02,Och03,Och04,Och05,Och06,Och07,Och08,Och09,Och10,Och11,Och12,Och13,Och14,Ore01,Ore02,Ore03,Ore04,Ore05,Ore06,Ore07,Ore08,Ore09,Ore10,Ore11,Ore12,Ore13,Ore14	*OreG0003222*
scaffold1468	4640261	A	T	NA	*OreG0003222*
scaffold1468	4815758	C	T	NA	*OreG0003247*
scaffold1468	4818996	G	A	NA	*OreG0003247*
scaffold1468	4819022	G	T	NA	*OreG0003247*
scaffold3087	104795	G	A	Ore01,Ore02,Ore03,Ore04,Ore05,Ore06,Ore07,Ore08,Ore09,Ore10,Ore11,Ore12,Ore13,Ore14	*OreG0008795*
scaffold3087	214954	C	A	Och11	*OreG0008809*
scaffold1231	59365	C	T	NA	*OreG0001637*
scaffold1231	91811	A	T	NA	*OreG0001640*
scaffold1231	138064	T	A	NA	*OreG0001646*
scaffold1231	169110	C	T	Och01,Och09,Och10,Och11,Och14	*OreG0001649*
scaffold1231	284965	T	A	Och01,Och02,Och03,Och04,Och05,Och06,Och07,Och08,Och09,Och10,Och11,Och12,Och13,Och14,Ore01,Ore02,Ore03,Ore04,Ore05,Ore06,Ore07,Ore08,Ore09,Ore10,Ore11,Ore12,Ore13,Ore14	*OreG0001669*
scaffold1231	768120	C	A	NA	*OreG0001707*
scaffold1231	807059	C	T	Ore01,Ore02,Ore03,Ore04,Ore05,Ore06,Ore07,Ore08,Ore09,Ore10,Ore11,Ore12,Ore13,Ore14	*OreG0001713*
scaffold1231	829718	T	G	NA	*OreG0001715*
scaffold1231	917387	A	T	Och09,Och10,Och11	*OreG0001722*
scaffold1231	917924	C	T	Och04	*OreG0001722*
scaffold1231	917935	T	A	Och02,Och03,Och04,Och05,Och06,Och12,Och13,Och14	*OreG0001722*
scaffold1231	996517	G	A	NA	*OreG0001731*
scaffold1231	1132749	G	A	Ore01,Ore02,Ore03,Ore04,Ore05,Ore06,Ore07,Ore08,Ore09,Ore10,Ore11,Ore12,Ore13,Ore14	*OreG0001744*
scaffold1231	1165742	C	A	NA	*OreG0001749*
scaffold1231	1236170	A	T	Och01,Och02,Och03,Och04,Och05,Och06,Och07,Och08,Och09,Och10,Och11,Och12,Och13,Och14	*OreG0001754*
scaffold1231	1368886	C	T	NA	*OreG0001770*
scaffold1231	1381684	G	A	Ore01,Ore02,Ore03,Ore04,Ore05,Ore06,Ore07,Ore08,Ore09,Ore10,Ore11,Ore12,Ore13,Ore14	*OreG0001771*
scaffold1231	1381771	G	A	Och04	*OreG0001771*
scaffold1231	1643271	C	T	NA	*OreG0001781*

scaffold1231	1728347	T	G	Och01,Och02,Och03,Och04,Och05,Och06,Och07,Och08,Och09,Och10,Och11,Och12,Och13,Och14,Ore01,Ore02,Ore03,Ore04,Ore05,Ore06,Ore07,Ore08,Ore09,Ore10,Ore11,Ore12,Ore13,Ore14	*OreG0001793*
scaffold1231	1744511	G	A	NA	*OreG0001794*
scaffold4109	58496	G	T	Ore01,Ore02,Ore03,Ore04,Ore05,Ore06,Ore07,Ore08,Ore09,Ore10,Ore11,Ore12,Ore13,Ore14	*OreG0019872*
scaffold4109	65107	G	A	Ore04,Ore05	*OreG0019873*
scaffold4109	67800	G	T	Och01,Och07,Och09,Och10,Och11	*OreG0019874*
scaffold4109	67809	A	T	Ore01,Ore02,Ore03,Ore06,Ore07,Ore08,Ore09,Ore10,Ore11,Ore12,Ore13,Ore14	*OreG0019874*
scaffold4109	69013	C	T	NA	*OreG0019874*
scaffold4109	69088	C	T	NA	*OreG0019874*
scaffold4109	187746	G	T	NA	*OreG0019890*
scaffold4109	284132	C	T	Ore01,Ore02,Ore03,Ore04,Ore05,Ore06,Ore07,Ore08,Ore09,Ore10,Ore11,Ore12,Ore13,Ore14	*OreG0019903*
scaffold4109	284140	C	A	NA	*OreG0019903*
scaffold4109	289605	C	A	NA	*OreG0019905*
scaffold4109	315531	C	T	NA	*OreG0019907*
scaffold4109	375013	G	T	Ore03,Ore09,Ore10,Ore12,Ore14	*OreG0019916*
scaffold4109	404283	G	A	NA	*OreG0019919*
scaffold4109	412110	T	A	NA	*OreG0019919*
scaffold4109	412893	G	A	Ore04,Ore05	*OreG0019919*
scaffold4109	517653	G	T	NA	*OreG0019928*
scaffold4109	530606	A	T	NA	*OreG0019929*
scaffold4109	530663	A	T	NA	*OreG0019929*
scaffold5880	9136	C	A	NA	*OreG0024046*
scaffold5280	11488	A	T	NA	*OreG0023573*
scaffold3662	78760	G	T	NA	*OreG0019035*
scaffold3662	116309	T	A	NA	*OreG0019037*
scaffold3662	277624	G	A	Och03,Och05,Och12,Och13	*OreG0019044*
scaffold3662	399104	C	T	NA	*OreG0019052*
scaffold2546	182837	G	T	Och07	*OreG0007786*
scaffold2546	710745	G	A	NA	*OreG0007811*
scaffold2546	710780	C	T	NA	*OreG0007811*
scaffold1157	137959	A	T	Och01,Och02,Och03,Och04,Och05,Och06,Och07,Och08,Och09,Och10,Och11,Och12,Och13,Och14,Ore01,Ore02,Ore03,Ore04,Ore05,Ore06,Ore07,Ore08,Ore09,Ore10,Ore11,Ore12,Ore13,Ore14	*OreG0000812*
scaffold1157	233705	C	A	Och01,Och02,Och03,Och04,Och05,Och06,Och07,Och08,Och09,Och10,Och11,Och12,Och13,Och14	*OreG0000828*
scaffold1157	235353	T	A	Ore09,Ore10,Ore12,Ore14	*OreG0000829*
scaffold1157	343373	A	T	NA	*OreG0000844*
scaffold1157	348767	G	A	Och14	*OreG0000845*
scaffold1157	349055	T	A	NA	*OreG0000845*
scaffold1157	356023	G	A	NA	*OreG0000846*
scaffold1157	361456	A	C	NA	*OreG0000847*
scaffold1157	418369	G	T	NA	*OreG0000855*
scaffold1157	453538	C	T	NA	*OreG0000860*
scaffold1157	503910	G	A	Och02,Och03,Och04,Och05,Och06,Och12,Och13,Ore01,Ore02,Ore03,Ore04,Ore05,Ore06,Ore07,Ore08,Ore09,Ore10,Ore11,Ore12,Ore13,Ore14	*OreG0000865*
scaffold1157	611801	C	T	NA	*OreG0000873*
scaffold1157	678409	C	T	NA	*OreG0000885*
scaffold1157	800391	G	T	Och08,Och12	*OreG0000890*
scaffold1157	805856	C	T	Och01,Och03,Och05,Och09,Och10	*OreG0000891*
scaffold1157	833493	G	A	NA	*OreG0000895*
scaffold1157	833494	G	A	Och01,Och02,Och03,Och04,Och05,Och06,Och07,Och08,Och12,Och13,Och14	*OreG0000895*
scaffold1157	1071187	C	T	Och01,Och02,Och03,Och04,Och05,Och06,Och07,Och08,Och09,Och10,Och11,Och12,Och13,Och14	*OreG0000919*
scaffold1157	1227343	G	A	NA	*OreG0000935*
scaffold1157	1238170	G	T	NA	*OreG0000937*
scaffold1157	1278952	T	G	NA	*OreG0000942*
scaffold1157	1281601	C	T	NA	*OreG0000943*
scaffold1157	1282461	T	A	NA	*OreG0000943*
scaffold1157	1282634	G	T	NA	*OreG0000943*
scaffold1157	1291394	A	T	NA	*OreG0000944*
scaffold1157	1372478	G	A	Ore01,Ore02,Ore03,Ore04,Ore05,Ore06,Ore07,Ore08,Ore09,Ore10,Ore11,Ore12,Ore13,Ore14	*OreG0000952*
scaffold1157	1461582	G	A	Och01,Och02,Och03,Och04,Och05,Och06,Och07,Och08,Och09,Och10,Och11,Och12,Och13,Och14	*OreG0000960*
scaffold1157	1563613	G	A	Och02,Och03,Och06	*OreG0000968*
scaffold1157	1563614	G	A	Och04,Och05,Och12	*OreG0000968*
scaffold1157	1761047	C	T	NA	*OreG0000979*
scaffold1157	2136160	G	T	NA	*OreG0000996*
scaffold1157	2160930	A	T	NA	*OreG0000999*
scaffold1157	2161009	C	A	NA	*OreG0000999*
scaffold1157	2237133	T	A	NA	*OreG0001001*

scaffold6632	2059	C	T	NA	*OreG0024349*
scaffold5055	16855	G	A	Och02,Och04	*OreG0023531*
scaffold5055	99825	G	A	Och01,Och02,Och03,Och04,Och05,Och06,Och07,Och08,Och09,Och10,Och11,Och12,Och13,Och14,Ore01,Ore02,Ore03,Ore04,Ore05,Ore06,Ore07,Ore08,Ore09,Ore10,Ore11,Ore12,Ore13,Ore14	*OreG0023535*
scaffold5055	154957	A	T	Och01,Och02,Och03,Och04,Och05,Och06,Och07,Och08,Och09,Och10,Och11,Och12,Och13,Och14,Ore01,Ore02,Ore03,Ore04,Ore05,Ore06,Ore07,Ore08,Ore09,Ore10,Ore11,Ore12,Ore13,Ore14	*OreG0023541*
scaffold5055	342193	T	G	Och01	*OreG0023550*
scaffold5055	355550	G	A	NA	*OreG0023551*
scaffold1891	166602	C	T	NA	*OreG0004628*
scaffold1891	167926	G	A	Och01,Och02,Och03,Och04,Och05,Och06,Och07,Och08,Och09,Och10,Och11,Och12,Och13,Och14	*OreG0004629*
scaffold1891	228368	C	A	Ore01,Ore02,Ore03,Ore04,Ore05,Ore06,Ore07,Ore08,Ore09,Ore10,Ore11,Ore12,Ore13,Ore14	*OreG0004633*
scaffold1891	264354	C	T	NA	*OreG0004636*
scaffold1891	409408	G	A	Och01,Och06,Och08,Och09,Och10,Och11,Och12,Och14	*OreG0004646*
scaffold1891	409419	C	T	NA	*OreG0004646*
scaffold1891	574665	C	A	Och01,Och02,Och03,Och04,Och05,Och06,Och07,Och08,Och09,Och10,Och11,Och12,Och13,Och14,Ore01,Ore02,Ore03,Ore04,Ore05,Ore06,Ore07,Ore08,Ore09,Ore10,Ore11,Ore12,Ore13,Ore14	*OreG0004655*
scaffold1891	825969	C	T	Och02,Och03,Och04,Och05,Och06,Och12,Och13	*OreG0004670*
scaffold1891	1030365	C	A	NA	*OreG0004679*
scaffold1891	1335135	T	A	Ore01,Ore02,Ore03,Ore04,Ore05,Ore06,Ore07,Ore08,Ore09,Ore10,Ore11,Ore12,Ore13,Ore14	*OreG0004696*
scaffold1891	1524817	G	A	NA	*OreG0004713*
scaffold1891	1731066	A	T	NA	*OreG0004729*
scaffold1891	1763401	G	T	NA	*OreG0004732*
scaffold1891	1763809	G	A	Och01,Och02,Och03,Och04,Och05,Och06,Och07,Och08,Och09,Och10,Och11,Och12,Och13,Och14,Ore01,Ore02,Ore03,Ore04,Ore05,Ore06,Ore07,Ore08,Ore09,Ore10,Ore11,Ore12,Ore13,Ore14	*OreG0004732*
scaffold1891	1855628	G	A	Och04	*OreG0004740*
scaffold1891	2139134	C	T	NA	*OreG0004762*
scaffold1891	2368717	C	T	Ore01,Ore02,Ore03,Ore04,Ore05,Ore06,Ore07,Ore08,Ore09,Ore10,Ore11,Ore12,Ore13,Ore14	*OreG0004772*
scaffold1891	2465927	G	A	Ore01,Ore02,Ore03,Ore04,Ore05,Ore06,Ore07,Ore08,Ore09,Ore10,Ore11,Ore12,Ore13,Ore14	*OreG0004778*
scaffold1891	2806165	G	A	Och01,Och02,Och03,Och04,Och05,Och06,Och07,Och08,Och09,Och10,Och11,Och12,Och13,Och14,Ore01,Ore02,Ore03,Ore04,Ore05,Ore06,Ore07,Ore08,Ore09,Ore10,Ore11,Ore12,Ore13,Ore14	*OreG0004792*
scaffold1891	2806368	G	T	NA	*OreG0004792*
scaffold6319	50730	G	A	NA	*OreG0024263*
scaffold3426	191216	G	T	Och09,Och10	*OreG0018578*
scaffold3426	326423	G	A	NA	*OreG0018592*
scaffold3426	369253	C	A	NA	*OreG0018593*
scaffold4159	46378	T	A	NA	*OreG0019937*
scaffold4159	58291	C	A	NA	*OreG0019941*
scaffold4159	75165	G	A	NA	*OreG0019943*
scaffold4159	109991	T	A	NA	*OreG0019947*
scaffold4159	191507	G	A	NA	*OreG0019955*
scaffold4159	215531	C	T	NA	*OreG0019956*
scaffold4159	220465	G	A	NA	*OreG0019957*
scaffold4159	226203	G	A	Och03,Och05	*OreG0019957*
scaffold4159	243112	C	A	Och12,Och13	*OreG0019959*
scaffold4159	243860	T	A	Och01,Och02,Och03,Och04,Och05,Och06	*OreG0019959*
scaffold4159	244898	G	A	Och12,Och13,Ore01,Ore02,Ore03,Ore04,Ore05,Ore06,Ore07,Ore08,Ore09,Ore10,Ore11,Ore12,Ore13,Ore14	*OreG0019959*
scaffold4159	254528	G	T	NA	*OreG0019961*
scaffold4159	256981	C	T	Och01	*OreG0019961*
scaffold4159	279362	A	T	Och07	*OreG0019963*
scaffold4159	283425	G	A	Och03,Och05	*OreG0019964*
scaffold4159	302506	C	T	NA	*OreG0019967*
scaffold4159	331849	G	A	Ore04,Ore05	*OreG0019972*
scaffold4159	364272	T	A	Och01,Och12,Och13	*OreG0019977*
scaffold4159	398379	A	T	Och01,Och02,Och03,Och04,Och05,Och06,Och07,Och08,Och09,Och10,Och11,Och12,Och13,Och14,Ore01,Ore02,Ore03,Ore04,Ore05,Ore06,Ore07,Ore08,Ore09,Ore10,Ore11,Ore12,Ore13,Ore14	*OreG0019978*
scaffold4159	491597	G	T	Ore06,Ore07,Ore08	*OreG0019992*
scaffold4159	499976	C	T	Och03,Och05	*OreG0019993*
scaffold4159	940419	C	A	Och01,Och02,Och03,Och04,Och05,Och06,Och07,Och08,Och09,Och10,Och11,Och12,Och13,Och14	*OreG0020038*
scaffold4159	1090480	G	A	Ore01,Ore02,Ore03,Ore04,Ore05,Ore06,Ore07,Ore08,Ore09,Ore10,Ore11,Ore12,Ore13,Ore14	*OreG0020060*
scaffold4159	1107487	T	A	NA	*OreG0020062*
scaffold4159	1129094	C	A	Och08,Och09,Och10,Och12,Och13	*OreG0020065*

scaffold4159	1139399	G	A	NA	*OreG0020068*
scaffold4159	1274269	G	T	Och03,Och05,Och07,Och08,Och09,Och10,Och11,Och12,Och13,Ore01,Ore02,Ore03,Ore04,Ore05,Ore06,Ore07,Ore08,Ore09,Ore10,Ore11,Ore12,Ore13,Ore14	*OreG0020084*
scaffold998	146450	T	A	NA	*OreG0027262*
scaffold998	215408	G	A	NA	*OreG0027273*
scaffold998	223104	A	T	NA	*OreG0027275*
scaffold998	339720	C	T	NA	*OreG0027288*
scaffold998	354105	G	A	NA	*OreG0027290*
scaffold998	354118	A	T	Och07,Och08	*OreG0027290*
scaffold998	385324	C	T	Och08	*OreG0027294*
scaffold998	385448	G	A	Och07,Och08	*OreG0027294*
scaffold998	385522	C	T	NA	*OreG0027294*
scaffold998	492341	G	T	Och01,Och02,Och03,Och04,Och05,Och06,Och07,Och08,Och09,Och10,Och11,Och12,Och13,Och14,Ore01,Ore02,Ore03,Ore04,Ore05,Ore06,Ore07,Ore08,Ore09,Ore10,Ore11,Ore12,Ore13,Ore14	*OreG0027297*
scaffold998	521794	C	T	Och01,Och02,Och03,Och04,Och05,Och06,Och07,Och09,Och10,Och11,Och12,Och13,Och14,Ore01,Ore02,Ore03,Ore04,Ore05,Ore06,Ore07,Ore08,Ore09,Ore10,Ore11,Ore12,Ore13,Ore14	*OreG0027299*
scaffold998	523021	C	T	Och01,Och02,Och03,Och04,Och05,Och06,Och07,Och08,Och09,Och10,Och11,Och12,Och13,Och14,Ore01,Ore02,Ore03,Ore04,Ore05,Ore06,Ore07,Ore08,Ore09,Ore10,Ore11,Ore12,Ore13,Ore14	*OreG0027299*
scaffold998	526154	G	A	Ore01,Ore02,Ore03,Ore04,Ore05,Ore06,Ore07,Ore08,Ore09,Ore10,Ore11,Ore12,Ore13,Ore14	*OreG0027301*
scaffold998	550161	G	T	NA	*OreG0027308*
scaffold998	736582	C	A	NA	*OreG0027324*
scaffold998	952506	C	A	NA	*OreG0027353*
scaffold998	970388	G	T	Och01,Och02,Och03,Och04,Och05,Och06,Och07,Och08,Och09,Och10,Och11,Och12,Och13,Och14,Ore01,Ore02,Ore03,Ore04,Ore05,Ore06,Ore07,Ore08,Ore09,Ore10,Ore11,Ore12,Ore13,Ore14	*OreG0027356*
scaffold998	1029920	G	A	Och01,Och08,Och14	*OreG0027363*
scaffold998	1170452	A	T	Och01,Och02,Och03,Och04,Och05,Och06,Och07,Och08,Och09,Och10,Och11,Och12,Och13,Och14,Ore01,Ore02,Ore03,Ore04,Ore05,Ore06,Ore07,Ore08,Ore09,Ore10,Ore11,Ore12,Ore13,Ore14	*OreG0027386*
scaffold998	1170551	C	T	Och01,Och02,Och03,Och04,Och05,Och06,Och07,Och08,Och09,Och10,Och11,Och12,Och13,Och14	*OreG0027386*
scaffold998	1393464	G	C	NA	*OreG0027423*
scaffold998	1504723	C	T	NA	*OreG0027435*
scaffold998	1504771	C	T	NA	*OreG0027435*
scaffold998	1548571	C	T	Ore01,Ore02,Ore03,Ore04,Ore05,Ore06,Ore07,Ore08,Ore09,Ore10,Ore11,Ore12,Ore13,Ore14	*OreG0027441*
scaffold998	1569767	G	A	NA	*OreG0027444*
scaffold998	1570331	G	A	NA	*OreG0027444*
scaffold998	1680049	C	T	NA	*OreG0027457*
scaffold998	1849029	C	T	Och01,Och14	*OreG0027481*
scaffold998	1947150	C	T	NA	*OreG0027490*
scaffold998	1957329	G	T	Ore02,Ore10	*OreG0027491*
scaffold998	2067208	G	A	Och08	*OreG0027510*
scaffold998	2122440	C	T	Och12	*OreG0027521*
scaffold998	2376145	A	T	NA	*OreG0027552*
scaffold998	2391268	G	A	NA	*OreG0027554*
scaffold998	2486379	G	A	NA	*OreG0027566*
scaffold998	2722185	G	A	NA	*OreG0027598*
scaffold998	2742820	G	A	NA	*OreG0027599*
scaffold998	2758929	T	A	NA	*OreG0027601*
scaffold998	2759475	C	A	Och01,Och02,Och03,Och04,Och05,Och06,Och07,Och08,Och09,Och10,Och11,Och12,Och13,Och14,Ore01,Ore02,Ore03,Ore04,Ore05,Ore06,Ore07,Ore08,Ore09,Ore10,Ore11,Ore12,Ore13,Ore14	*OreG0027602*
scaffold998	2773992	C	T	NA	*OreG0027605*
scaffold998	2855282	C	T	Och03,Och06,Och07	*OreG0027610*
scaffold998	2857436	C	A	NA	*OreG0027610*
scaffold998	2857657	C	T	NA	*OreG0027610*
scaffold998	2863255	C	T	NA	*OreG0027611*
scaffold998	2967561	C	T	NA	*OreG0027619*
scaffold998	3215242	C	T	Och05,Och13	*OreG0027631*
scaffold998	3425287	G	A	Och01,Och02,Och03,Och04,Och05,Och06,Och07,Och08,Och09,Och10,Och11,Och12,Och13,Och14,Ore01,Ore02,Ore03,Ore04,Ore05,Ore06,Ore07,Ore08,Ore09,Ore10,Ore11,Ore12,Ore13,Ore14	*OreG0027647*
scaffold998	3470502	C	T	Och03,Och07,Och08,Och09,Och10,Och11,Och14	*OreG0027652*
scaffold998	3475051	G	T	Och01,Och02,Och03,Och04,Och06,Och07,Och08,Och09,Och10,Och11,Och13,Och14	*OreG0027652*
scaffold998	3586243	G	T	Och01,Och02,Och03,Och04,Och05,Och06,Och07,Och08,Och09,Och10,Och11,Och12,Och13,Och14,Ore01,Ore02,Ore03,Ore04,Ore05,Ore06,Ore07,Ore08,Ore09,Ore10,Ore11,Ore12,Ore13,Ore14	*OreG0027665*
scaffold998	3651656	T	A	NA	*OreG0027672*
scaffold998	3847355	C	A	Och01	*OreG0027682*

scaffold998	3983910	G	T	NA	*OreG0027696*
scaffold998	3984018	C	T	Och01,Ore02,Ore08,Ore09,Ore10,Ore11,Ore13	*OreG0027696*
scaffold998	3984039	C	T	Ore02,Ore08,Ore09,Ore10,Ore11,Ore13	*OreG0027696*
scaffold998	3984476	G	A	NA	*OreG0027696*
scaffold998	4289666	C	A	NA	*OreG0027730*
scaffold998	4423331	C	T	NA	*OreG0027752*
scaffold998	4423837	C	A	Och02,Och03,Och04,Och05,Och06,Och12,Och13	*OreG0027752*
scaffold998	4430596	C	T	Och14	*OreG0027753*
scaffold998	4431071	C	A	Och14	*OreG0027753*
scaffold998	4431191	C	G	Och14	*OreG0027753*
scaffold998	4432208	C	T	NA	*OreG0027753*
scaffold998	4441882	C	T	Ore04,Ore05	*OreG0027754*
scaffold998	4441930	C	T	NA	*OreG0027754*
scaffold998	4443272	G	A	NA	*OreG0027754*
scaffold998	4443609	C	T	Och02,Och03,Och04,Och05,Och06,Och08,Och09,Och10,Och11,Och12,Och13	*OreG0027754*
scaffold998	4443639	C	T	NA	*OreG0027754*
scaffold998	4544833	C	T	Och02,Och03,Och04,Och05,Och06,Och07,Och09,Och10,Och11,Och12,Och13,Och14	*OreG0027773*
scaffold998	4592996	T	A	Ore01,Ore02,Ore03,Ore04,Ore05,Ore06,Ore07,Ore08,Ore09,Ore10,Ore11,Ore12,Ore13,Ore14	*OreG0027777*
scaffold998	4757959	A	T	NA	*OreG0027790*
scaffold998	4875446	T	A	Och01,Och02,Och03,Och04,Och05,Och06,Och07,Och08,Och09,Och10,Och11,Och12,Och13,Och14,Ore01,Ore02,Ore03,Ore04,Ore05,Ore06,Ore07,Ore08,Ore09,Ore10,Ore11,Ore12,Ore13,Ore14	*OreG0027803*
scaffold998	4876654	G	T	Och01,Och02,Och03,Och04,Och05,Och06,Och07,Och08,Och09,Och10,Och11,Och12,Och13,Och14,Ore01,Ore02,Ore03,Ore04,Ore05,Ore06,Ore07,Ore08,Ore09,Ore10,Ore11,Ore12,Ore13,Ore14	*OreG0027804*
scaffold3610	121848	C	T	Och01,Och02,Och03,Och04,Och05,Och06,Och07,Och08,Och09,Och10,Och11,Och12,Och13,Och14,Ore01,Ore02,Ore03,Ore04,Ore05,Ore06,Ore07,Ore08,Ore09,Ore10,Ore11,Ore12,Ore13,Ore14	*OreG0018954*
scaffold3610	121875	C	T	Och01,Och02,Och03,Och04,Och05,Och06,Och07,Och08,Och09,Och10,Och11,Och12,Och13,Och14,Ore01,Ore02,Ore03,Ore04,Ore05,Ore06,Ore07,Ore08,Ore09,Ore10,Ore11,Ore12,Ore13,Ore14	*OreG0018954*
scaffold3610	132075	A	T	Ore01,Ore02,Ore03,Ore04,Ore05,Ore06,Ore07,Ore08,Ore09,Ore10,Ore11,Ore12,Ore13,Ore14	*OreG0018956*
scaffold3610	444824	C	A	NA	*OreG0018983*
scaffold3610	772437	G	A	Och14	*OreG0019008*
scaffold3610	842790	G	A	NA	*OreG0019013*
scaffold3610	889168	G	A	Och11	*OreG0019017*
scaffold3610	889169	G	A	Och11	*OreG0019017*
scaffold7557	87511	G	A	Och03	*OreG0025288*
scaffold4392	79519	C	T	Ore02,Ore10	*OreG0020349*
scaffold11692	629	G	A	Ore03,Ore07,Ore08,Ore10	*OreG0001013*
scaffold11899	4198	C	A	Och01,Och02,Och03,Och04,Och05,Och06,Och07,Och08,Och09,Och10,Och11,Och12,Och13,Och14	*OreG0001348*
scaffold2136	78409	C	A	NA	*OreG0005251*
scaffold2136	81319	G	A	Och01,Och02,Och03,Och04,Och05,Och06,Och07,Och08,Och09,Och10,Och11,Och12,Och13,Och14	*OreG0005252*
scaffold2136	81500	C	T	NA	*OreG0005252*
scaffold2136	109830	C	A	NA	*OreG0005255*
scaffold2136	159537	G	A	Ore01,Ore02,Ore03,Ore04,Ore05,Ore06,Ore07,Ore08,Ore09,Ore10,Ore11,Ore12,Ore13,Ore14	*OreG0005262*
scaffold2136	229251	G	A	Och03,Och08,Och09,Och10,Och11,Och14	*OreG0005271*
scaffold2136	245044	T	A	Ore01,Ore02,Ore03,Ore04,Ore05,Ore06,Ore07,Ore08,Ore09,Ore10,Ore11,Ore12,Ore13,Ore14	*OreG0005272*
scaffold2136	245328	C	T	Ore01,Ore02,Ore03,Ore04,Ore05,Ore06,Ore07,Ore08,Ore09,Ore10,Ore11,Ore12,Ore13,Ore14	*OreG0005272*
scaffold2136	261744	C	G	Och01,Och02,Och03,Och04,Och05,Och06,Och07,Och08,Och09,Och10,Och11,Och12,Och13,Och14,Ore01,Ore02,Ore03,Ore04,Ore05,Ore06,Ore07,Ore08,Ore09,Ore10,Ore11,Ore12,Ore13,Ore14	*OreG0005275*
scaffold2136	309276	A	T	Och01,Och02,Och03,Och04,Och05,Och06,Och07,Och09,Och10,Och11,Och12,Och13,Och14	*OreG0005281*
scaffold2136	310134	A	T	Och01,Och02,Och03,Och04,Och05,Och06,Och07,Och09,Och10,Och11,Och12,Och13,Och14	*OreG0005281*
scaffold2136	808879	C	T	NA	*OreG0005338*
scaffold2136	826394	C	T	NA	*OreG0005340*
scaffold2136	833249	T	A	Och01,Och02,Och03,Och04,Och05,Och06,Och07,Och08,Och09,Och10,Och11,Och12,Och13,Och14,Ore01,Ore02,Ore03,Ore04,Ore05,Ore06,Ore07,Ore08,Ore09,Ore10,Ore11,Ore12,Ore13,Ore14	*OreG0005341*
scaffold2136	853741	G	T	Och11	*OreG0005343*
scaffold2136	956926	G	T	NA	*OreG0005353*
scaffold2136	961182	C	T	Ore01,Ore02,Ore03,Ore04,Ore05,Ore06,Ore07,Ore08,Ore09,Ore10,Ore11,Ore12,Ore13,Ore14	*OreG0005354*
scaffold2136	1067214	G	T	NA	*OreG0005371*

scaffold2136	1067411	G	A	Och01,Och02,Och03,Och04,Och05,Och06,Och07,Och08,Och09,Och10,Och11,Och12,Och13,Och14	*OreG0005371*
scaffold2136	1206741	A	T	NA	*OreG0005394*
scaffold2136	1243535	A	T	NA	*OreG0005400*
scaffold2136	1248285	G	A	NA	*OreG0005401*
scaffold2136	1248513	C	A	NA	*OreG0005401*
scaffold2136	1350240	C	G	NA	*OreG0005422*
scaffold2136	1352317	G	A	Och09,Och10,Och14	*OreG0005424*
scaffold2136	1388235	T	A	Och02,Och03,Och04,Och05,Och06,Och07,Och11,Och12,Och13	*OreG0005429*
scaffold2136	1455298	G	A	Och01,Och14	*OreG0005437*
scaffold2136	1458582	G	A	Och01,Och03,Och09,Och10,Och11,Och13,Och14	*OreG0005438*
scaffold2136	1458779	G	A	Och08	*OreG0005438*
scaffold2136	1462624	G	A	Och01,Och14	*OreG0005439*
scaffold2136	1473335	C	T	Ore01,Ore02,Ore03,Ore04,Ore05,Ore06,Ore07,Ore08,Ore09,Ore10,Ore11,Ore12,Ore13,Ore14	*OreG0005440*
scaffold2136	1473389	C	T	Och08	*OreG0005440*
scaffold2136	1473427	G	A	Och14	*OreG0005440*
scaffold2136	1542604	C	A	Ore08,Ore12	*OreG0005449*
scaffold2136	1570569	C	T	NA	*OreG0005452*
scaffold2136	1958356	C	T	NA	*OreG0005501*
scaffold2136	1973976	C	A	Ore01,Ore02,Ore03,Ore04,Ore05,Ore06,Ore07,Ore08,Ore09,Ore10,Ore11,Ore12,Ore13,Ore14	*OreG0005505*
scaffold2136	2052721	G	A	NA	*OreG0005514*
scaffold2136	2052938	C	T	NA	*OreG0005514*
scaffold2136	2052939	C	T	NA	*OreG0005514*
scaffold2136	2211498	C	T	Och02,Och03,Och04,Och05,Och06,Och08,Och12,Och13	*OreG0005534*
scaffold2136	2211639	G	T	NA	*OreG0005534*
scaffold2136	2218740	C	T	Och01,Och02,Och03,Och04,Och05,Och06,Och07,Och08,Och09,Och10,Och11,Och12,Och13,Och14	*OreG0005535*
scaffold2136	2219489	C	T	Ore01,Ore02,Ore03,Ore04,Ore05,Ore06,Ore07,Ore08,Ore09,Ore10,Ore11,Ore12,Ore13,Ore14	*OreG0005536*
scaffold2136	2219495	C	T	Och02,Och03,Och04,Och05,Och06,Och08,Och12,Och13,Och14,Ore01,Ore02,Ore03,Ore04,Ore05,Ore06,Ore07,Ore08,Ore09,Ore10,Ore11,Ore12,Ore13,Ore14	*OreG0005536*
scaffold2136	2312658	C	A	NA	*OreG0005548*
scaffold2136	2356150	C	A	Och01,Och08,Och11	*OreG0005558*
scaffold2136	2556471	G	A	Och01,Och02,Och03,Och04,Och05,Och06,Och07,Och08,Och09,Och10,Och11,Och12,Och13,Och14	*OreG0005584*
scaffold2136	2561534	G	T	Och01,Och02,Och03,Och04,Och05,Och06,Och07,Och08,Och09,Och10,Och11,Och12,Och13,Och14	*OreG0005586*
scaffold2136	2652061	G	T	Och01,Och02,Och03,Och04,Och05,Och06,Och07,Och08,Och09,Och10,Och11,Och12,Och13,Och14	*OreG0005600*
scaffold2136	2724386	G	T	NA	*OreG0005607*
scaffold2136	2726020	A	C	Och01,Och02,Och03,Och04,Och05,Och06,Och07,Och08,Och09,Och10,Och11,Och12,Och13,Och14	*OreG0005607*
scaffold2136	2829241	C	T	Och01,Och02,Och03,Och04,Och05,Och06,Och07,Och08,Och09,Och10,Och11,Och12,Och13,Och14	*OreG0005622*
scaffold2136	2856433	C	T	NA	*OreG0005628*
scaffold2136	3062808	G	T	NA	*OreG0005660*
scaffold2136	3089793	A	T	NA	*OreG0005663*
scaffold2136	3159876	G	A	Och01,Och02,Och03,Och04,Och05,Och06,Och07,Och08,Och09,Och10,Och11,Och12,Och13,Och14	*OreG0005672*
scaffold2136	3178988	C	T	NA	*OreG0005675*
scaffold2136	3372596	G	A	Och01,Och02,Och03,Och04,Och05,Och06,Och07,Och08,Och09,Och10,Och11,Och12,Och13,Och14,Ore01,Ore02,Ore03,Ore04,Ore05,Ore06,Ore07,Ore08,Ore09,Ore10,Ore11,Ore12,Ore13,Ore14	*OreG0005701*
scaffold2136	3412900	G	T	Och01	*OreG0005709*
scaffold2136	3561971	C	T	Ore01,Ore02,Ore03,Ore04,Ore05,Ore06,Ore07,Ore08,Ore09,Ore10,Ore11,Ore12,Ore13,Ore14	*OreG0005729*
scaffold2136	3570022	C	T	Och01,Och02,Och03,Och04,Och05,Och06,Och07,Och09,Och10,Och11,Och12,Och13	*OreG0005731*
scaffold2136	3638149	G	A	NA	*OreG0005740*
scaffold2136	3674840	C	T	NA	*OreG0005746*
scaffold2136	3953099	G	A	Och01,Och02,Och03,Och04,Och05,Och06,Och07,Och08,Och09,Och10,Och11,Och12,Och13,Och14	*OreG0005791*
scaffold2136	3954880	C	A	NA	*OreG0005792*
scaffold2136	3954882	C	T	NA	*OreG0005792*
scaffold2136	3955441	G	A	NA	*OreG0005792*
scaffold2136	3955736	A	T	NA	*OreG0005792*
scaffold2136	4041927	G	T	Och03,Och07,Och08,Och09,Och10,Och11,Och14	*OreG0005803*
scaffold2136	4182366	A	T	Och08,Och14	*OreG0005821*
scaffold2136	4336443	C	T	NA	*OreG0005842*
scaffold2136	4360344	G	T	Och02	*OreG0005844*
scaffold2136	4440630	C	T	NA	*OreG0005855*
scaffold2136	4466166	C	A	NA	*OreG0005859*
scaffold2136	4730208	C	G	NA	*OreG0005888*

scaffold2136	5733596	G	A	Ore04,Ore05,Ore09	*OreG0005965*
scaffold2136	5769304	C	T	Och01	*OreG0005972*
scaffold2136	5916236	G	T	NA	*OreG0005980*
scaffold2136	5929681	C	T	NA	*OreG0005982*
scaffold2136	5939058	G	A	Och02,Och03,Och04,Och05,Och06,Och12,Och13	*OreG0005981*
scaffold2136	6031818	T	A	Och01	*OreG0005993*
scaffold2136	6032154	C	T	Och01,Och08	*OreG0005993*
scaffold2136	6037360	G	A	Och02,Och06	*OreG0005994*
scaffold2136	6082688	C	G	Och02,Och06	*OreG0005998*
scaffold2136	6101377	T	A	NA	*OreG0006001*
scaffold9992	34184	T	A	NA	*OreG0027812*
scaffold9992	46420	G	T	NA	*OreG0027813*
scaffold9992	134698	G	C	NA	*OreG0027819*
scaffold9992	252015	G	A	NA	*OreG0027830*
scaffold2276	151567	C	A	Och01,Och02,Och06,Och11,Och13	*OreG0006384*
scaffold2276	155264	T	A	NA	*OreG0006385*
scaffold2276	176301	C	T	Och02,Och06,Och13	*OreG0006389*
scaffold2276	176316	G	T	Och08,Och14	*OreG0006389*
scaffold2276	189802	G	A	NA	*OreG0006392*
scaffold2276	189803	G	A	NA	*OreG0006392*
scaffold2276	190157	C	T	NA	*OreG0006392*
scaffold2276	190202	G	T	NA	*OreG0006392*
scaffold2276	223366	G	A	Ore04,Ore05,Ore06,Ore08,Ore11,Ore12	*OreG0006398*
scaffold2276	356917	G	T	NA	*OreG0006417*
scaffold2276	370749	C	A	Och01,Och02,Och03,Och04,Och05,Och06,Och07,Och08,Och09,Och10,Och11,Och12,Och13,Och14,Ore01,Ore02,Ore03,Ore04,Ore05,Ore06,Ore07,Ore08,Ore09,Ore10,Ore11,Ore12,Ore13,Ore14	*OreG0006421*
scaffold2276	386852	G	T	Ore01,Ore02,Ore03,Ore04,Ore05,Ore06,Ore07,Ore08,Ore09,Ore10,Ore11,Ore12,Ore13,Ore14	*OreG0006424*
scaffold2276	387731	T	A	Ore07,Ore10,Ore13,Ore14	*OreG0006424*
scaffold2276	389284	G	T	Och02,Och06,Och07,Och08,Och09,Och10,Och11,Och13,Och14	*OreG0006424*
scaffold2276	457619	G	A	NA	*OreG0006431*
scaffold2276	522320	C	T	NA	*OreG0006438*
scaffold2276	522930	G	A	NA	*OreG0006438*
scaffold2276	524861	C	A	Och02,Och06,Och11,Och13	*OreG0006440*
scaffold2276	525164	T	A	Och02,Och06,Och11,Och13	*OreG0006440*
scaffold2276	554040	C	T	Och05	*OreG0006446*
scaffold2276	566850	C	A	Och02,Och06,Och07,Och09,Och10,Och13	*OreG0006447*
scaffold2276	599544	T	A	NA	*OreG0006452*
scaffold2276	634187	C	A	NA	*OreG0006458*
scaffold2276	651683	T	A	Och01,Och02,Och03,Och04,Och05,Och06,Och07,Och08,Och09,Och10,Och11,Och12,Och13,Och14,Ore01,Ore02,Ore03,Ore04,Ore05,Ore06,Ore07,Ore08,Ore09,Ore10,Ore11,Ore12,Ore13,Ore14	*OreG0006461*
scaffold2276	652915	C	A	Och01,Och02,Och03,Och04,Och05,Och06,Och07,Och08,Och09,Och10,Och11,Och12,Och13	*OreG0006461*
scaffold2276	668023	G	A	NA	*OreG0006464*
scaffold2276	668085	C	T	NA	*OreG0006464*
scaffold2276	876954	C	A	NA	*OreG0006497*
scaffold2276	930385	A	T	NA	*OreG0006506*
scaffold2276	984881	T	A	NA	*OreG0006515*
scaffold2276	985108	C	T	NA	*OreG0006515*
scaffold2276	1125159	T	A	Och01,Och02,Och03,Och04,Och05,Och06,Och07,Och08,Och09,Och10,Och11,Och12,Och13,Och14,Ore01,Ore02,Ore03,Ore04,Ore05,Ore06,Ore07,Ore08,Ore09,Ore10,Ore11,Ore12,Ore13,Ore14	*OreG0006533*
scaffold2276	1318509	G	A	Och01,Och02,Och03,Och04,Och05,Och06,Och07,Och08,Och09,Och10,Och11,Och12,Och13,Och14,Ore01,Ore02,Ore03,Ore04,Ore05,Ore06,Ore07,Ore08,Ore09,Ore10,Ore11,Ore12,Ore13,Ore14	*OreG0006564*
scaffold2276	1318578	C	A	Och01,Och02,Och03,Och04,Och05,Och06,Och07,Och08,Och09,Och10,Och11,Och12,Och13,Och14	*OreG0006564*
scaffold2276	1318647	G	A	Och02,Och03,Och04,Och05,Och06,Och12,Och13	*OreG0006564*
scaffold2276	1393976	G	T	NA	*OreG0006570*
scaffold2276	1394163	T	G	Och01,Och02,Och03,Och04,Och05,Och06,Och07,Och08,Och09,Och10,Och11,Och12,Och13,Och14,Ore01,Ore02,Ore03,Ore04,Ore05,Ore06,Ore07,Ore08,Ore09,Ore10,Ore11,Ore12,Ore13,Ore14	*OreG0006570*
scaffold2276	1449040	G	T	Och01,Och02,Och03,Och04,Och05,Och06,Och07,Och08,Och09,Och10,Och11,Och12,Och13,Och14	*OreG0006578*
scaffold2276	1538231	C	T	Ore01,Ore02,Ore03,Ore04,Ore05,Ore06,Ore07,Ore08,Ore09,Ore10,Ore11,Ore12,Ore13,Ore14	*OreG0006587*
scaffold2276	1585621	G	A	Och02,Och06,Och13	*OreG0006595*
scaffold2276	1866295	C	T	NA	*OreG0006631*
scaffold2276	1975150	T	A	Och02,Och03,Och04,Och05,Och06,Och07,Och12,Och13	*OreG0006642*
scaffold2276	2060551	C	G	NA	*OreG0006651*
scaffold2276	2083525	G	A	NA	*OreG0006654*
scaffold2276	2083531	C	A	NA	*OreG0006654*
scaffold2276	2314874	G	A	NA	*OreG0006681*

scaffold2276	2481379	C	A	Och09,Och10	*OreG0006696*
scaffold2276	2505048	C	G	Och05	*OreG0006699*
scaffold2276	2520868	C	T	Och01,Och02,Och03,Och04,Och05,Och06,Och07,Och08,Och09,Och10,Och11,Och12,Och13,Och14	*OreG0006701*
scaffold2276	2532136	G	T	NA	*OreG0006702*
scaffold2276	2751863	C	T	Ore04,Ore05,Ore08,Ore12	*OreG0006719*
scaffold2276	2848975	G	T	NA	*OreG0006730*
scaffold2276	3032930	G	A	Ore01,Ore02,Ore03,Ore04,Ore05,Ore06,Ore07,Ore08,Ore09,Ore10,Ore11,Ore12,Ore13,Ore14	*OreG0006751*
scaffold2276	3039118	A	C	Och01,Och02,Och03,Och04,Och05,Och06,Och07,Och08,Och09,Och10,Och11,Och12,Och13,Och14,Ore01,Ore02,Ore03,Ore04,Ore05,Ore06,Ore07,Ore08,Ore09,Ore10,Ore11,Ore12,Ore13,Ore14	*OreG0006752*
scaffold2276	3062447	G	A	NA	*OreG0006757*
scaffold2276	3071808	C	T	NA	*OreG0006758*
scaffold2276	3088943	G	T	Och02,Och03,Och04,Och05,Och06,Och12,Och13	*OreG0006760*
scaffold2276	3102092	A	T	Och01,Och02,Och03,Och04,Och05,Och06,Och07,Och08,Och09,Och10,Och11,Och12,Och13,Och14,Ore01,Ore02,Ore03,Ore04,Ore05,Ore06,Ore07,Ore08,Ore09,Ore10,Ore11,Ore12,Ore13,Ore14	*OreG0006762*
scaffold2276	3167323	T	A	Och09,Och10	*OreG0006768*
scaffold2276	3173049	C	A	Och01,Och02,Och03,Och04,Och05,Och06,Och07,Och09,Och10,Och11,Och12,Och13	*OreG0006770*
scaffold2276	3175053	C	A	Och01,Och02,Och03,Och04,Och05,Och06,Och12,Och13	*OreG0006771*
scaffold2276	3182755	C	T	Och05	*OreG0006772*
scaffold2276	3379736	G	T	Och01,Och02,Och03,Och04,Och05,Och06,Och07,Och08,Och09,Och10,Och11,Och12,Och13,Och14	*OreG0006801*
scaffold2276	3428166	G	A	NA	*OreG0006806*
scaffold2276	3679711	A	T	Och01,Och02,Och03,Och04,Och05,Och06,Och07,Och08,Och09,Och10,Och11,Och12,Och13,Och14	*OreG0006833*
scaffold2276	3795683	C	A	Och05	*OreG0006845*
scaffold2276	3824395	G	A	Och01,Och02,Och03,Och04,Och05,Och06,Och07,Och08,Och09,Och10,Och11,Och12,Och13,Och14,Ore01,Ore02,Ore03,Ore04,Ore05,Ore06,Ore07,Ore08,Ore09,Ore10,Ore11,Ore12,Ore13,Ore14	*OreG0006848*
scaffold2276	4203829	G	A	Och09,Och10	*OreG0006891*
scaffold175	298252	G	A	NA	*OreG0003562*
scaffold175	681908	C	T	Och01,Och02,Och03,Och04,Och05,Och06,Och07,Och08,Och09,Och10,Och11,Och12,Och13,Och14	*OreG0003596*
scaffold175	682409	C	T	Och07,Och14	*OreG0003596*
scaffold175	691298	G	A	NA	*OreG0003597*
scaffold175	691403	G	A	NA	*OreG0003597*
scaffold175	691469	G	A	NA	*OreG0003597*
scaffold175	691533	C	T	NA	*OreG0003597*
scaffold175	691826	G	A	NA	*OreG0003597*
scaffold175	698684	C	T	Och01	*OreG0003598*
scaffold175	725412	T	A	Ore01,Ore02,Ore03,Ore04,Ore05,Ore06,Ore07,Ore08,Ore09,Ore10,Ore11,Ore12,Ore13,Ore14	*OreG0003600*
scaffold175	869414	G	A	NA	*OreG0003604*
scaffold175	931992	C	T	Och01,Och02,Och03,Och04,Och05,Och06,Och07,Och08,Och09,Och10,Och11,Och12,Och13,Och14	*OreG0003606*
scaffold175	1003642	C	T	Och06	*OreG0003613*
scaffold175	1020529	C	A	NA	*OreG0003614*
scaffold4400	86891	G	C	NA	*OreG0020359*
scaffold4400	184748	C	G	Och01,Och02,Och03,Och04,Och05,Och06,Och07,Och08,Och09,Och10,Och11,Och12,Och13,Och14	*OreG0020370*
scaffold4400	190598	G	A	Ore08,Ore09,Ore10	*OreG0020372*
scaffold4400	593573	G	A	Och01,Och02,Och03,Och04,Och05,Och06,Och07,Och08,Och09,Och10,Och11,Och12,Och13,Och14	*OreG0020383*
scaffold4400	797192	G	A	NA	*OreG0020390*
scaffold4400	866175	C	A	Och07	*OreG0020396*
scaffold4400	1031053	G	A	NA	*OreG0020400*
scaffold4400	1061443	G	A	Och01,Och02,Och03,Och04,Och05,Och06,Och07,Och08,Och09,Och10,Och11,Och12,Och13,Och14	*OreG0020403*
scaffold4400	1238757	G	A	Och01	*OreG0020426*
scaffold4400	1238937	C	A	NA	*OreG0020426*
scaffold4400	1314428	C	T	Och01,Och02,Och03,Och04,Och05,Och06,Och07,Och08,Och09,Och10,Och11,Och12,Och13,Och14	*OreG0020434*
scaffold4400	1323609	C	A	Och08,Och13	*OreG0020435*
scaffold4400	1324353	G	A	Och03	*OreG0020435*
scaffold4400	1328132	C	T	Ore06,Ore07,Ore10,Ore12,Ore13	*OreG0020435*
scaffold4400	1369021	C	T	NA	*OreG0020437*
scaffold4400	1587965	C	T	Och03,Och05,Och12,Och13	*OreG0020447*
scaffold4400	1643829	G	T	NA	*OreG0020451*
scaffold4400	1922891	G	A	Och01,Och02,Och03,Och04,Och05,Och06,Och08,Och09,Och11,Och12,Och13	*OreG0020466*
scaffold4400	2064279	C	A	Ore01,Ore02,Ore03,Ore04,Ore05,Ore06,Ore07,Ore08,Ore09,Ore10,Ore11,Ore12,Ore13,Ore14	*OreG0020473*

scaffold4400	2150992	A	T	Och01,Och02,Och03,Och04,Och05,Och06,Och07,Och08,Och09,Och10,Och11,Och12,Och13,Och14	*OreG0020477*
scaffold4400	2227801	G	T	NA	*OreG0020482*
scaffold10469	264137	C	T	Och01,Och02,Och03,Och04,Och05,Och06,Och07,Och08,Och09,Och10,Och11,Och12,Och13,Och14,Ore01,Ore02,Ore03,Ore04,Ore05,Ore06,Ore07,Ore08,Ore09,Ore10,Ore11,Ore12,Ore13,Ore14	*OreG0000228*
scaffold15727	7407	C	T	Och07,Och08,Och09,Och10,Och11	*OreG0003274*
scaffold15727	23623	G	T	Och07,Och08,Och09,Och10,Och11	*OreG0003275*
scaffold15727	24368	G	A	Och02,Och06	*OreG0003275*
scaffold9076	199816	G	A	Och01,Och03,Och05,Och07,Och08,Och09,Och10,Och11,Och12,Och13,Och14	*OreG0025920*
scaffold9076	215853	A	C	Och01	*OreG0025923*
scaffold9076	306233	T	A	Och01,Och02,Och03,Och04,Och05,Och06,Och07,Och08,Och09,Och10,Och11,Och12,Och13,Och14,Ore01,Ore02,Ore03,Ore04,Ore05,Ore06,Ore07,Ore08,Ore09,Ore10,Ore11,Ore12,Ore13,Ore14	*OreG0025929*
scaffold9076	351317	C	T	Och01,Och02,Och03,Och04,Och05,Och06,Och07,Och08,Och09,Och10,Och11,Och12,Och13,Och14,Ore01,Ore02,Ore03,Ore04,Ore05,Ore06,Ore07,Ore08,Ore09,Ore10,Ore11,Ore12,Ore13,Ore14	*OreG0025933*
scaffold9076	912237	C	A	Och06	*OreG0025988*
scaffold9076	937380	G	T	NA	*OreG0025992*
scaffold9076	959088	C	T	NA	*OreG0025994*
scaffold8846	178454	A	T	Och02,Och03,Och04,Och05,Och06,Och07,Och08,Och12,Och13	*OreG0025820*
scaffold8846	301900	C	T	NA	*OreG0025838*
scaffold8846	372126	C	A	NA	*OreG0025849*
scaffold8846	414056	G	A	NA	*OreG0025856*
scaffold8846	580801	G	A	Ore02,Ore03,Ore06,Ore08,Ore09,Ore10,Ore12,Ore14	*OreG0025873*
scaffold8846	618477	G	A	NA	*OreG0025878*
scaffold8846	640124	G	A	Och01,Och08,Och12	*OreG0025881*
scaffold8846	676088	C	A	Och05,Och08,Och13	*OreG0025883*
scaffold8846	697109	T	A	NA	*OreG0025885*
scaffold8846	744576	C	T	Ore02,Ore06,Ore08,Ore09,Ore12,Ore14	*OreG0025896*
scaffold8846	744642	C	T	Och02,Och03,Och04,Och05,Och06,Och07,Och08,Och09,Och10,Och12,Och13	*OreG0025896*
scaffold16506	26775	A	T	NA	*OreG0003437*
scaffold16506	36180	G	A	Och01,Och06,Och07	*OreG0003439*
scaffold16506	135646	G	T	Och01,Och02,Och03,Och04,Och05,Och06,Och07,Och08,Och09,Och10,Och11,Och12,Och13,Och14,Ore01,Ore02,Ore03,Ore04,Ore05,Ore06,Ore07,Ore08,Ore09,Ore10,Ore11,Ore12,Ore13,Ore14	*OreG0003452*
scaffold16506	159415	C	A	NA	*OreG0003454*
scaffold16506	168889	T	A	NA	*OreG0003455*
scaffold14584	145026	T	A	NA	*OreG0002532*
scaffold14584	237968	C	T	Ore01,Ore02,Ore03,Ore04,Ore05,Ore06,Ore07,Ore08,Ore09,Ore10,Ore11,Ore12,Ore13,Ore14	*OreG0002545*
scaffold14584	270422	C	T	Ore04,Ore05	*OreG0002551*
scaffold14584	359662	G	T	NA	*OreG0002559*
scaffold7534	128979	C	A	Ore01,Ore02,Ore03,Ore04,Ore05,Ore06,Ore07,Ore08,Ore09,Ore10,Ore11,Ore12,Ore13,Ore14	*OreG0025033*
scaffold7534	132743	C	T	NA	*OreG0025034*
scaffold7534	133347	G	T	NA	*OreG0025034*
scaffold7534	133374	C	T	Och01,Och02,Och03,Och04,Och05,Och06,Och07,Och08,Och09,Och10,Och11,Och12,Och13,Och14	*OreG0025034*
scaffold7534	133672	A	T	NA	*OreG0025034*
scaffold7534	139174	G	A	Och01,Och02,Och03,Och04,Och05,Och06,Och07,Och08,Och09,Och10,Och11,Och12,Och13,Och14,Ore01,Ore02,Ore03,Ore04,Ore05,Ore06,Ore07,Ore08,Ore09,Ore10,Ore11,Ore12,Ore13,Ore14	*OreG0025035*
scaffold7534	154867	A	T	Ore01,Ore02,Ore03,Ore04,Ore05,Ore06,Ore07,Ore08,Ore09,Ore10,Ore11,Ore12,Ore13,Ore14	*OreG0025036*
scaffold7534	202932	C	T	Och02,Och04,Och05,Och12,Och13,Och14	*OreG0025040*
scaffold7534	266855	C	A	Och01,Och08,Och09,Och10,Och11	*OreG0025044*
scaffold7534	266894	G	A	Och01,Och08,Och09,Och10,Och11	*OreG0025044*
scaffold7534	270627	G	A	Och02,Och04,Och05,Och12,Och13,Och14	*OreG0025044*
scaffold7534	399033	G	A	Och09,Och10,Och11	*OreG0025058*
scaffold7534	819036	G	T	Och08	*OreG0025097*
scaffold7534	843488	G	A	NA	*OreG0025099*
scaffold7534	859320	C	A	Och08,Och13	*OreG0025100*
scaffold7534	859323	G	A	Och08,Och13	*OreG0025100*
scaffold7534	859706	G	A	Och01,Och09,Och10,Och11	*OreG0025100*
scaffold7534	875189	C	T	NA	*OreG0025101*
scaffold7534	939168	A	T	NA	*OreG0025105*
scaffold7534	951250	G	A	NA	*OreG0025107*
scaffold7534	955869	A	C	NA	*OreG0025109*
scaffold7534	1179244	G	A	NA	*OreG0025129*
scaffold7534	1197033	C	T	NA	*OreG0025131*
scaffold7534	1238960	C	T	NA	*OreG0025134*
scaffold7534	1314579	G	T	Och01,Och02,Och03,Och04,Och05,Och06,Och07,Och08,Och09,Och10,Och11,Och12,Och13,Och14,Ore01,Ore02,Ore03,Ore04,Ore05,Ore06,Ore07,Ore08,Ore09,Ore10,Ore11,Ore12,Ore13,Ore14	*OreG0025138*

scaffold7534	1330326	C	A	NA	*OreG0025140*
scaffold7534	1391726	C	T	NA	*OreG0025144*
scaffold7534	1412356	A	T	NA	*OreG0025147*
scaffold7534	1816190	C	T	NA	*OreG0025177*
scaffold7534	2020184	T	A	NA	*OreG0025200*
scaffold7534	2257396	C	A	Och01,Och07	*OreG0025224*
scaffold7534	2301823	G	T	NA	*OreG0025229*
scaffold7534	2351303	C	A	Och01,Och13	*OreG0025237*
scaffold7534	2387234	A	T	NA	*OreG0025240*
scaffold7534	2455516	A	T	Och01,Och02,Och03,Och04,Och05,Och06,Och07,Och09,Och10,Och12,Och13	*OreG0025248*
scaffold7534	2543388	G	A	Och01,Och14	*OreG0025254*
scaffold7534	2652229	C	T	NA	*OreG0025271*
scaffold7534	2680005	A	T	NA	*OreG0025275*
scaffold4993	70271	G	A	NA	*OreG0023301*
scaffold4993	76693	C	T	NA	*OreG0023302*
scaffold4993	78095	G	A	NA	*OreG0023302*
scaffold4993	147246	C	A	NA	*OreG0023309*
scaffold4993	164469	C	T	Och01,Och02,Och04,Och05,Och06,Och08,Och12,Och13,Och14	*OreG0023312*
scaffold4993	184857	T	G	Och01,Och02,Och03,Och04,Och05,Och06,Och08,Och09,Och10,Och11,Och12,Och13,Och14	*OreG0023315*
scaffold4993	210211	G	A	Och03	*OreG0023318*
scaffold4993	210253	G	A	Och01,Och02,Och03,Och04,Och05,Och06,Och12,Och13	*OreG0023318*
scaffold4993	210442	G	A	Och01,Och02,Och03,Och04,Och05,Och06,Och07,Och12,Och13	*OreG0023318*
scaffold4993	269191	C	T	NA	*OreG0023323*
scaffold4993	668581	G	T	Och01,Och02,Och03,Och04,Och05,Och06,Och07,Och08,Och09,Och10,Och11,Och12,Och13,Och14	*OreG0023360*
scaffold4993	694134	C	T	NA	*OreG0023361*
scaffold4993	720857	A	T	Ore01,Ore02,Ore03,Ore04,Ore05,Ore06,Ore07,Ore08,Ore09,Ore10,Ore11,Ore12,Ore13,Ore14	*OreG0023364*
scaffold4993	928993	G	A	NA	*OreG0023387*
scaffold4993	975203	T	A	Och01,Och02,Och03,Och04,Och05,Och06,Och07,Och08,Och09,Och10,Och11,Och12,Och13,Och14	*OreG0023391*
scaffold4993	976729	A	T	Och01,Och02,Och03,Och04,Och05,Och06,Och07,Och08,Och09,Och10,Och11,Och12,Och13,Och14,Ore01,Ore02,Ore03,Ore04,Ore05,Ore06,Ore07,Ore08,Ore09,Ore10,Ore11,Ore12,Ore13,Ore14	*OreG0023391*
scaffold4993	1163133	T	A	Och09,Och10,Och11	*OreG0023407*
scaffold4993	1407063	C	T	Och01,Och02,Och03,Och04,Och05,Och06,Och07,Och08,Och09,Och10,Och11,Och12,Och13,Och14	*OreG0023419*
scaffold4993	1429173	A	T	Och08,Och09,Och10	*OreG0023421*
scaffold4993	1520770	C	A	Och01,Och08,Och09,Och10,Och11,Och12,Och13	*OreG0023429*
scaffold4993	1725291	A	T	NA	*OreG0023446*
scaffold4993	1758857	T	A	NA	*OreG0023449*
scaffold4993	1879111	C	T	NA	*OreG0023459*
scaffold4993	1879205	T	G	NA	*OreG0023459*
scaffold4993	1879355	G	A	NA	*OreG0023459*
scaffold4993	1907271	G	T	Och01,Och02,Och03,Och04,Och05,Och06,Och07,Och08,Och09,Och10,Och11,Och12,Och13,Och14	*OreG0023463*
scaffold4993	1912256	G	T	Och01,Och02,Och03,Och04,Och05,Och06,Och07,Och08,Och09,Och10,Och11,Och12,Och13,Och14	*OreG0023464*
scaffold4993	1995062	C	T	Och07,Och09,Och10,Och11,Och14	*OreG0023471*
scaffold4993	2061279	G	A	Och12,Och13	*OreG0023476*
scaffold4993	2258665	C	A	NA	*OreG0023502*
scaffold4993	2292861	C	T	Och02,Och03,Och04,Och05,Och06,Och07,Och08,Och09,Och10,Och11,Och12,Och13,Och14	*OreG0023503*
scaffold4993	2506666	T	A	Och12,Och13	*OreG0023518*
scaffold4993	2511284	G	A	Och01,Och02,Och03,Och04,Och05,Och06,Och07,Och08,Och09,Och10,Och11,Och12,Och13,Och14,Ore01,Ore02,Ore03,Ore06,Ore07,Ore08,Ore09,Ore10,Ore11,Ore12,Ore13,Ore14	*OreG0023520*
scaffold4993	2512485	C	A	NA	*OreG0023521*
scaffold4993	2512696	A	T	NA	*OreG0023521*
scaffold8682	23645	G	T	NA	*OreG0025772*
scaffold8682	26373	C	T	NA	*OreG0025773*
scaffold8682	26490	C	T	NA	*OreG0025773*
scaffold8682	40568	C	A	NA	*OreG0025774*
scaffold8682	228070	C	A	Och08	*OreG0025793*
scaffold8682	254338	G	A	Och01,Och02,Och03,Och04,Och05,Och06,Och07,Och08,Och09,Och10,Och11,Och12,Och13,Och14	*OreG0025797*
scaffold13403	127687	G	C	NA	*OreG0002045*
scaffold13403	128004	G	A	NA	*OreG0002045*
scaffold13403	167455	G	A	NA	*OreG0002051*
scaffold13403	168924	G	T	NA	*OreG0002051*
scaffold13403	169355	G	A	NA	*OreG0002051*
scaffold31256	956	A	T	Och01,Och02,Och03,Och04,Och05,Och06,Och07,Och08,Och09,Och10,Och11,Och12,Och13,Och14,Ore01,Ore02,Ore03,Ore04,Ore05,Ore06,Ore07,Ore08,Ore09,Ore10,Ore11,Ore12,Ore13,Ore14	*OreG0009415*

scaffold31457	1994	G	A	NA	*OreG0009428*
scaffold31457	2122	G	A	Ore02	*OreG0009428*
scaffold31911	3753	C	T	NA	*OreG0010219*
scaffold31911	11366	G	A	Och01,Och02,Och03,Och04,Och05,Och06,Och07,Och08,Och09,Och10,Och11,Och12,Och13,Och14	*OreG0010220*
scaffold31911	11464	C	T	Och01,Och02,Och03,Och04,Och05,Och06,Och07,Och08,Och09,Och10,Och11,Och12,Och13,Och14	*OreG0010220*
scaffold3788	21961	G	A	NA	*OreG0019089*
scaffold3788	148256	A	C	NA	*OreG0019106*
scaffold32542	5295	A	T	Och01,Och02,Och03,Och04,Och05,Och06,Och07,Och08,Och09,Och10,Och11,Och12,Och13,Och14,Ore01,Ore02,Ore03,Ore04,Ore05,Ore06,Ore07,Ore08,Ore09,Ore10,Ore11,Ore12,Ore13,Ore14	*OreG0010610*
scaffold32778	985	G	T	NA	*OreG0011020*
scaffold32778	1448	T	A	NA	*OreG0011020*
scaffold32778	1714	C	T	NA	*OreG0011020*
scaffold32778	2845	C	T	NA	*OreG0011020*
scaffold23099	49745	T	A	Och05,Och08	*OreG0006946*
scaffold23099	50245	G	T	Och08	*OreG0006946*
scaffold23099	65504	T	A	NA	*OreG0006948*
scaffold23099	69580	C	G	NA	*OreG0006948*
scaffold23099	92880	C	T	Och01,Och02,Och03,Och04,Och05,Och06,Och07,Och08,Och09,Och10,Och11,Och12,Och13,Och14,Ore01,Ore02,Ore03,Ore04,Ore05,Ore06,Ore07,Ore08,Ore09,Ore10,Ore11,Ore12,Ore13,Ore14	*OreG0006950*
scaffold23099	97199	C	T	Och08	*OreG0006951*
scaffold23099	99093	C	T	Och01	*OreG0006951*
scaffold23099	102806	A	T	Och05	*OreG0006953*
scaffold23099	183139	C	A	NA	*OreG0006963*
scaffold23099	207488	C	A	NA	*OreG0006967*
scaffold23099	286088	G	A	Och02,Och03,Och04,Och08,Och09,Och10,Och12,Och13	*OreG0006975*
scaffold23099	339088	G	A	Och09,Och11,Och14	*OreG0006980*
scaffold23099	345477	T	A	NA	*OreG0006983*
scaffold23099	348645	C	T	NA	*OreG0006984*
scaffold32988	95022	C	T	NA	*OreG0011034*
scaffold32988	95793	G	T	NA	*OreG0011034*
scaffold32988	95808	A	T	NA	*OreG0011034*
scaffold31684	92869	G	A	NA	*OreG0009650*
scaffold31684	102293	G	T	Och09	*OreG0009651*
scaffold31684	189398	C	T	Och04,Och08	*OreG0009671*
scaffold31684	278545	G	A	NA	*OreG0009681*
scaffold31684	346539	C	T	NA	*OreG0009687*
scaffold31684	348043	G	A	NA	*OreG0009688*
scaffold31684	355683	G	C	NA	*OreG0009688*
scaffold31684	356570	T	A	NA	*OreG0009688*
scaffold31684	362535	T	A	NA	*OreG0009688*
scaffold31684	367292	G	A	NA	*OreG0009688*
scaffold31684	369685	G	A	NA	*OreG0009688*
scaffold31684	370942	G	A	NA	*OreG0009688*
scaffold31684	415254	C	T	NA	*OreG0009697*
scaffold31684	415290	C	T	NA	*OreG0009697*
scaffold31684	429351	G	A	Och01,Och02,Och03,Och04,Och05,Och06,Och07,Och08,Och09,Och10,Och11,Och12,Och13,Och14,Ore01,Ore02,Ore03,Ore04,Ore05,Ore06,Ore07,Ore08,Ore09,Ore10,Ore11,Ore12,Ore13,Ore14	*OreG0009699*
scaffold31684	445275	C	T	NA	*OreG0009701*
scaffold31684	463182	T	A	NA	*OreG0009703*
scaffold33104	44250	C	T	Ore01,Ore02,Ore03,Ore04,Ore05,Ore06,Ore07,Ore08,Ore09,Ore10,Ore11,Ore12,Ore13,Ore14	*OreG0011811*
scaffold33102	107369	C	A	NA	*OreG0011655*
scaffold33102	138239	G	T	NA	*OreG0011657*
scaffold33102	161237	C	T	Och12,Och13	*OreG0011661*
scaffold33102	161276	C	T	Och09,Och10,Och11	*OreG0011661*
scaffold31969	258280	T	A	Och01,Och02,Och03,Och04,Och05,Och06,Och08,Och09,Och10,Och11,Och12,Och13,Och14	*OreG0010283*
scaffold31969	327547	C	T	Och01,Och09,Och10,Och11	*OreG0010293*
scaffold31969	358278	C	A	Och01,Och02,Och03,Och04,Och05,Och06,Och07,Och08,Och09,Och10,Och11,Och12,Och13,Och14,Ore01,Ore02,Ore03,Ore04,Ore05,Ore06,Ore07,Ore08,Ore09,Ore10,Ore11,Ore12,Ore13,Ore14	*OreG0010297*
scaffold31969	572942	C	T	NA	*OreG0010321*
scaffold31969	600019	C	A	Och03,Och12	*OreG0010327*
scaffold31969	604788	C	A	NA	*OreG0010328*
scaffold31969	730531	A	T	NA	*OreG0010351*
scaffold31969	748874	G	A	NA	*OreG0010352*
scaffold33111	11370	G	T	NA	*OreG0012626*
scaffold33111	120863	G	T	Och01,Och02,Och03,Och04,Och05,Och06,Och07,Och08,Och09,Och10,Och11,Och12,Och13,Och14,Ore01,Ore02,Ore03,Ore04,Ore05,Ore06,Ore07,Ore08,Ore09,Ore10,Ore11,Ore12,Ore13,Ore14	*OreG0012635*

scaffold33111	264852	A	T	Och01,Och04,Och06,Och07,Och14	*OreG0012653*
scaffold33111	298808	G	A	Och09,Och10,Och11	*OreG0012658*
scaffold33148	8688	G	T	NA	*OreG0014150*
scaffold31865	7384	C	A	Och01,Och04,Och05,Och07,Och08,Och09,Och10,Och11	*OreG0009875*
scaffold31865	15599	G	A	NA	*OreG0009877*
scaffold31865	101113	A	T	Ore01,Ore02,Ore03,Ore04,Ore05,Ore06,Ore07,Ore08,Ore09,Ore10,Ore11,Ore12,Ore13,Ore14	*OreG0009881*
scaffold31865	786861	C	T	NA	*OreG0009903*
scaffold31583	89633	C	A	NA	*OreG0009444*
scaffold31583	200890	A	C	Och01,Och02,Och03,Och04,Och05,Och06,Och07,Och08,Och09,Och10,Och11,Och12,Och13,Och14,Ore01,Ore02,Ore03,Ore04,Ore05,Ore06,Ore07,Ore08,Ore09,Ore10,Ore11,Ore12,Ore13,Ore14	*OreG0009458*
scaffold31583	209777	T	A	Och01,Och02,Och03,Och04,Och05,Och06,Och07,Och08,Och09,Och10,Och11,Och12,Och13,Och14	*OreG0009459*
scaffold31583	209840	T	A	Ore01,Ore02,Ore03,Ore04,Ore05,Ore06,Ore07,Ore08,Ore09,Ore10,Ore11,Ore12,Ore13,Ore14	*OreG0009459*
scaffold31583	211036	A	T	Och01	*OreG0009460*
scaffold31583	346793	T	A	NA	*OreG0009478*
scaffold31583	502675	C	T	Och01	*OreG0009498*
scaffold33124	133024	T	A	NA	*OreG0013577*
scaffold33124	230770	C	T	Och12,Och13,Och14	*OreG0013586*
scaffold33124	244963	A	T	NA	*OreG0013588*
scaffold33124	273834	C	T	Och12,Och13,Och14	*OreG0013591*
scaffold33124	315148	C	T	NA	*OreG0013594*
scaffold31965	278675	C	T	Och01,Och02,Och03,Och04,Och05,Och06,Och07,Och08,Och09,Och10,Och11,Och12,Och13,Och14	*OreG0010235*
scaffold31965	278709	G	A	NA	*OreG0010235*
scaffold31965	551560	G	A	NA	*OreG0010243*
scaffold33112	46507	G	T	Och02,Och05,Och12,Och13	*OreG0012699*
scaffold33112	416708	G	T	NA	*OreG0012710*
scaffold33112	416720	G	C	NA	*OreG0012710*
scaffold31095	50676	C	T	Och01,Och02,Och03,Och04,Och05,Och06,Och07,Och08,Och09,Och10,Och11,Och12,Och13,Och14,Ore01,Ore02,Ore03,Ore04,Ore05,Ore06,Ore07,Ore08,Ore09,Ore10,Ore11,Ore12,Ore13,Ore14	*OreG0009382*
scaffold31095	51299	C	A	NA	*OreG0009382*
scaffold31095	51482	C	A	NA	*OreG0009382*
scaffold31095	201035	G	A	NA	*OreG0009391*
scaffold31095	257632	G	C	Och01,Och05,Och07,Och08,Och09,Och10,Och11,Och12,Och13,Och14	*OreG0009393*
scaffold31095	258347	C	T	Och01,Och02,Och03,Och04,Och05,Och06,Och07,Och08,Och09,Och10,Och11,Och12,Och13,Och14,Ore01,Ore02,Ore03,Ore04,Ore05,Ore06,Ore07,Ore08,Ore09,Ore10,Ore11,Ore12,Ore13,Ore14	*OreG0009394*
scaffold33163	6610	C	A	Och01,Och02,Och03,Och04,Och05,Och06,Och07,Och08,Och09,Och10,Och11,Och12,Och13,Och14	*OreG0014337*
scaffold33163	13771	C	T	NA	*OreG0014338*
scaffold33163	16370	G	A	NA	*OreG0014339*
scaffold33151	4417	G	A	NA	*OreG0014156*
scaffold33130	92603	G	A	Och01,Och02,Och03,Och04,Och05,Och06,Och07,Och09,Och10,Och11,Och12,Och13,Och14	*OreG0013783*
scaffold33130	92636	G	A	NA	*OreG0013783*
scaffold33130	95655	G	A	NA	*OreG0013784*
scaffold33130	96542	G	A	NA	*OreG0013784*
scaffold33130	111778	G	C	NA	*OreG0013786*
scaffold33143	10817	C	T	Ore01,Ore02,Ore03,Ore04,Ore05,Ore06,Ore07,Ore08,Ore09,Ore10,Ore11,Ore12,Ore13,Ore14	*OreG0013993*
scaffold33143	87639	T	A	Ore01,Ore02,Ore03,Ore04,Ore05,Ore06,Ore07,Ore08,Ore09,Ore10,Ore11,Ore12,Ore13,Ore14	*OreG0013998*
scaffold33143	87879	G	A	NA	*OreG0013998*
scaffold33100	100011	C	A	Och05,Och09,Och10	*OreG0011484*
scaffold33100	136595	A	C	Och01,Och02,Och03,Och04,Och05,Och06,Och07,Och08,Och09,Och10,Och11,Och12,Och13,Och14,Ore01,Ore02,Ore03,Ore04,Ore05,Ore06,Ore07,Ore08,Ore09,Ore10,Ore11,Ore12,Ore13,Ore14	*OreG0011489*
scaffold33100	137029	T	A	NA	*OreG0011489*
scaffold33100	153706	G	A	NA	*OreG0011492*
scaffold33100	154113	C	T	NA	*OreG0011492*
scaffold33100	416040	C	T	NA	*OreG0011545*
scaffold33100	440926	G	A	NA	*OreG0011550*
scaffold33100	451109	C	A	Och01,Och02,Och03,Och04,Och05,Och06,Och07,Och08,Och09,Och10,Och11,Och12,Och13,Och14,Ore01,Ore02,Ore03,Ore04,Ore05,Ore06,Ore07,Ore08,Ore09,Ore10,Ore11,Ore12,Ore13,Ore14	*OreG0011552*
scaffold33100	470281	C	T	NA	*OreG0011554*
scaffold33100	485774	C	T	Och01,Och02,Och03,Och04,Och05,Och06,Och07,Och08,Och09,Och10,Och11,Och12,Och13,Och14,Ore01,Ore02,Ore03,Ore04,Ore05,Ore06,Ore07,Ore08,Ore09,Ore10,Ore11,Ore12,Ore13,Ore14	*OreG0011555*
scaffold33100	685393	C	T	NA	*OreG0011587*
scaffold33100	693388	G	A	Och01	*OreG0011589*

scaffold33100	696033	T	A	Och01	*OreG0011589*
scaffold33100	696040	A	T	NA	*OreG0011589*
scaffold33116	32930	C	A	Och01,Och02,Och03,Och04,Och05,Och06,Och07,Och08,Och09,Och10,Och11,Och12,Och13,Och14	*OreG0012945*
scaffold33116	113563	T	A	Och02,Och03,Och04,Och05,Och06,Och08,Och12,Och13	*OreG0012959*
scaffold33116	215919	G	A	Och02,Och03,Och04,Och05,Och06,Och08,Och12,Och13	*OreG0012974*
scaffold33116	265541	G	A	Och02	*OreG0012979*
scaffold33116	323567	T	G	Och01,Och03,Och07,Och08,Och14	*OreG0012989*
scaffold33116	327041	C	A	Och01,Och02,Och03,Och04,Och05,Och06,Och07,Och08,Och09,Och10,Och11,Och12,Och13,Och14	*OreG0012989*
scaffold33116	410040	G	T	NA	*OreG0012993*
scaffold32492	86731	C	A	NA	*OreG0010543*
scaffold32492	355180	C	T	NA	*OreG0010558*
scaffold32492	375492	A	T	NA	*OreG0010561*
scaffold32492	440370	G	A	Och01,Och02,Och03,Och04,Och05,Och06,Och07,Och08,Och09,Och10,Och11,Och12,Och13,Och14,Ore01,Ore02,Ore03,Ore04,Ore05,Ore06,Ore07,Ore08,Ore09,Ore10,Ore11,Ore12,Ore13,Ore14	*OreG0010566*
scaffold32492	685925	C	T	NA	*OreG0010579*
scaffold32492	685926	C	T	NA	*OreG0010579*
scaffold91	23265	C	T	NA	*OreG0025996*
scaffold91	23746	G	A	Ore06,Ore07,Ore10,Ore14	*OreG0025997*
scaffold91	49344	C	A	Ore04,Ore05	*OreG0026000*
scaffold91	189391	G	A	NA	*OreG0026005*
scaffold91	415443	G	A	Och08,Och11	*OreG0026012*
scaffold91	415520	C	T	NA	*OreG0026012*
scaffold91	633660	C	A	NA	*OreG0026019*
scaffold91	655204	C	T	Och01,Och02,Och03,Och04,Och05,Och06,Och07,Och08,Och09,Och10,Och11,Och12,Och13,Och14,Ore01,Ore02,Ore03,Ore04,Ore05,Ore06,Ore07,Ore08,Ore09,Ore10,Ore11,Ore12,Ore13,Ore14	*OreG0026020*
scaffold91	659126	G	A	Och01,Och02,Och03,Och04,Och05,Och06,Och07,Och08,Och09,Och10,Och11,Och12,Och13,Och14,Ore01,Ore02,Ore03,Ore04,Ore05,Ore06,Ore07,Ore08,Ore09,Ore10,Ore11,Ore12,Ore13,Ore14	*OreG0026022*
scaffold91	695204	C	T	NA	*OreG0026028*
scaffold91	695205	C	T	NA	*OreG0026028*
scaffold91	782578	G	A	Och01,Och02,Och03,Och04,Och05,Och06,Och07,Och08,Och09,Och10,Och11,Och12,Och13,Och14,Ore01,Ore02,Ore03,Ore04,Ore05,Ore06,Ore07,Ore08,Ore09,Ore10,Ore11,Ore12,Ore13,Ore14	*OreG0026036*
scaffold91	871864	G	T	NA	*OreG0026044*
scaffold91	894354	G	A	NA	*OreG0026046*
scaffold91	974181	G	A	Ore04,Ore05	*OreG0026052*
scaffold91	976009	C	A	Och05,Och09,Och10,Och11,Och12,Och13,Och14	*OreG0026053*
scaffold91	1045013	G	A	Och09,Och10,Och11	*OreG0026060*
scaffold91	1223442	A	T	NA	*OreG0026071*
scaffold91	1352385	C	T	NA	*OreG0026079*
scaffold91	1471440	G	A	Och01,Och02,Och03,Och04,Och05,Och06,Och07,Och08,Och09,Och10,Och11,Och12,Och13,Och14	*OreG0026089*
scaffold91	1476549	C	T	Och01,Och02,Och03,Och04,Och05,Och06,Och07,Och08,Och09,Och10,Och11,Och12,Och13,Och14	*OreG0026091*
scaffold91	1504891	A	T	Ore01,Ore02,Ore03,Ore04,Ore05,Ore06,Ore07,Ore08,Ore09,Ore10,Ore11,Ore12,Ore13,Ore14	*OreG0026095*
scaffold91	1619167	A	T	Och01,Och02,Och03,Och04,Och05,Och06,Och07,Och08,Och09,Och10,Och11,Och12,Och13,Och14	*OreG0026102*
scaffold91	1721089	T	A	Och01,Och02,Och03,Och04,Och05,Och06,Och07,Och08,Och09,Och10,Och11,Och12,Och13,Och14,Ore01,Ore02,Ore03,Ore04,Ore05,Ore06,Ore07,Ore08,Ore09,Ore10,Ore11,Ore12,Ore13,Ore14	*OreG0026112*
scaffold91	1863108	G	T	Och02,Och05,Och08,Och12,Och13,Och14	*OreG0026124*
scaffold91	1896400	G	A	NA	*OreG0026126*
scaffold91	1897143	C	A	NA	*OreG0026126*
scaffold91	2038064	C	T	NA	*OreG0026139*
scaffold91	2038088	C	T	Och01,Och02,Och03,Och04,Och05,Och06,Och07,Och08,Och09,Och10,Och11,Och12,Och13,Och14	*OreG0026139*
scaffold91	2092633	C	A	NA	*OreG0026145*
scaffold91	2150001	G	T	NA	*OreG0026154*
scaffold91	2394086	G	T	NA	*OreG0026172*
scaffold91	2400060	C	T	NA	*OreG0026173*
scaffold91	2493106	G	C	Ore01,Ore02,Ore03,Ore04,Ore05,Ore06,Ore07,Ore08,Ore09,Ore10,Ore11,Ore12,Ore13,Ore14	*OreG0026182*
scaffold91	2496419	C	A	NA	*OreG0026182*
scaffold91	2535245	C	T	Och14	*OreG0026184*
scaffold91	2535857	C	T	Och14	*OreG0026184*
scaffold91	2675186	G	A	NA	*OreG0026191*
scaffold91	2688743	C	T	NA	*OreG0026192*
scaffold91	3214672	G	T	NA	*OreG0026243*
scaffold91	3427716	G	T	NA	*OreG0026263*
scaffold91	3459463	C	T	Och12,Och13	*OreG0026267*

scaffold91	3459747	A	T	Och09,Och10,Och11	*OreG0026267*
scaffold91	3459919	C	T	Och01,Och02,Och03,Och04,Och05,Och06,Och07,Och08,Och09,Och10,Och11,Och12,Och13,Och14	*OreG0026267*
scaffold91	3459946	C	T	NA	*OreG0026267*
scaffold91	3459964	C	T	Och01,Och02,Och03,Och04,Och05,Och06,Och07,Och08,Och09,Och10,Och11,Och12,Och13,Och14	*OreG0026267*
scaffold91	3460530	G	A	NA	*OreG0026268*
scaffold91	3460889	C	T	Och01,Och02,Och03,Och04,Och05,Och06,Och07,Och08,Och09,Och10,Och11,Och12,Och13,Och14	*OreG0026268*
scaffold91	3500243	T	A	Ore06,Ore10,Ore11	*OreG0026271*
scaffold91	3517058	C	A	NA	*OreG0026273*
scaffold91	3553882	T	A	NA	*OreG0026276*
scaffold91	3555370	G	A	NA	*OreG0026276*
scaffold91	3641774	C	A	NA	*OreG0026284*
scaffold91	3647642	G	A	NA	*OreG0026285*
scaffold91	3689342	T	A	NA	*OreG0026288*
scaffold91	3744803	A	T	Och01,Och02	*OreG0026295*
scaffold91	3772997	C	T	Och12,Och13	*OreG0026298*
scaffold91	3815372	C	A	NA	*OreG0026304*
scaffold91	3816639	C	T	NA	*OreG0026304*
scaffold91	3816922	C	T	Och01,Och02,Och03,Och04,Och05,Och06,Och07,Och08,Och09,Och10,Och11,Och12,Och13,Och14	*OreG0026304*
scaffold91	3894810	C	T	Och06,Och12,Och13	*OreG0026318*
scaffold91	3897090	C	T	NA	*OreG0026319*
scaffold91	3908736	A	T	Och01,Och02,Och03,Och04,Och05,Och06,Och07,Och08,Och09,Och10,Och11,Och12,Och13,Och14,Ore01,Ore02,Ore03,Ore04,Ore05,Ore06,Ore07,Ore08,Ore09,Ore10,Ore11,Ore12,Ore13,Ore14	*OreG0026322*
scaffold91	5115392	G	A	Och01,Och02,Och03,Och04,Och05,Och06,Och07,Och08,Och09,Och10,Och11,Och12,Och13,Och14	*OreG0026362*
scaffold91	5171579	G	A	Och01,Och02,Och03,Och04,Och05,Och06,Och07,Och08,Och09,Och10,Och11,Och12,Och13,Och14	*OreG0026363*
scaffold91	5264368	C	A	NA	*OreG0026368*
scaffold91	5334791	G	T	Och07,Och08,Och09,Och10	*OreG0026373*
scaffold91	5423549	C	T	Och01,Och02,Och04,Och13,Och14	*OreG0026378*
scaffold91	5447462	G	A	Och01,Och02,Och03,Och04,Och05,Och07,Och08,Och09,Och10,Och11,Och12,Och13,Och14	*OreG0026381*
scaffold91	5634633	C	T	Ore01,Ore02,Ore03,Ore04,Ore05,Ore06,Ore07,Ore08,Ore09,Ore10,Ore11,Ore12,Ore13,Ore14	*OreG0026390*
scaffold91	5719089	T	A	NA	*OreG0026394*
scaffold91	5855018	C	T	NA	*OreG0026405*
scaffold91	6049717	G	A	Och01,Och03,Och05,Och08,Och09,Och10,Och12,Och13,Och14	*OreG0026419*
scaffold91	6281780	G	A	Och01,Och02,Och03,Och04,Och05,Och06,Och07,Och08,Och09,Och10,Och11,Och12,Och13,Och14,Ore01,Ore02,Ore03,Ore04,Ore05,Ore06,Ore07,Ore08,Ore09,Ore10,Ore11,Ore12,Ore13,Ore14	*OreG0026443*
scaffold91	6551858	C	T	NA	*OreG0026471*
scaffold91	6631920	A	T	Och01,Och02,Och03,Och04,Och05,Och06,Och07,Och08,Och09,Och10,Och11,Och12,Och13,Och14,Ore01,Ore02,Ore03,Ore04,Ore05,Ore06,Ore07,Ore08,Ore09,Ore10,Ore11,Ore12,Ore13,Ore14	*OreG0026478*
scaffold91	6787858	C	G	Och11	*OreG0026496*
scaffold91	6843883	T	A	Och01	*OreG0026499*
scaffold91	7139287	C	A	Ore01,Ore02,Ore03,Ore04,Ore05,Ore06,Ore07,Ore08,Ore09,Ore10,Ore11,Ore12,Ore13,Ore14	*OreG0026513*
scaffold33107	72027	A	T	NA	*OreG0012388*
scaffold33107	72124	A	C	Och08	*OreG0012388*
scaffold33107	72143	T	A	NA	*OreG0012388*
scaffold33107	72176	C	A	NA	*OreG0012388*
scaffold33162	14795	C	T	NA	*OreG0014330*
scaffold33162	15068	G	T	NA	*OreG0014330*
scaffold33162	36896	C	T	NA	*OreG0014333*
scaffold33162	41984	C	T	Och08	*OreG0014335*
scaffold33162	56036	C	G	NA	*OreG0014336*
scaffold33133	35597	C	A	NA	*OreG0013800*
scaffold33133	47806	A	C	Och01,Och02,Och03,Och04,Och05,Och06,Och07,Och08,Och09,Och10,Och11,Och12,Och13,Och14	*OreG0013801*
scaffold33133	75282	C	T	Och01,Och02,Och03,Och04,Och05,Och06,Och07,Och08,Och09,Och10,Och11,Och12,Och13,Och14	*OreG0013802*
scaffold33133	431116	C	T	NA	*OreG0013818*
scaffold28919	50726	G	A	Och01,Och02,Och03,Och04,Och05,Och06,Och07,Och08,Och09,Och10,Och11,Och12,Och13,Och14	*OreG0008318*
scaffold28919	51096	G	A	Och01,Och02,Och03,Och04,Och05,Och06,Och07,Och08,Och09,Och10,Och11,Och12,Och13,Och14	*OreG0008318*
scaffold28919	87974	A	C	NA	*OreG0008323*
scaffold28919	90631	G	A	NA	*OreG0008323*
scaffold28919	126799	G	A	Och01,Och02,Och03,Och04,Och05,Och06,Och07,Och08,Och09,Och10,Och11,Och12,Och13,Och14	*OreG0008328*

scaffold28919	128252	C	A	Ore04,Ore05	*OreG0008329*
scaffold28919	128296	G	T	NA	*OreG0008329*
scaffold28919	128528	T	A	NA	*OreG0008329*
scaffold28919	231799	A	C	Ore01,Ore02,Ore03,Ore04,Ore05,Ore06,Ore07,Ore08,Ore09,Ore10,Ore11,Ore12,Ore13,Ore14	*OreG0008343*
scaffold28919	231843	G	A	Ore01,Ore02,Ore03,Ore04,Ore05,Ore06,Ore07,Ore08,Ore09,Ore10,Ore11,Ore12,Ore13,Ore14	*OreG0008343*
scaffold28919	231855	T	A	Ore01,Ore02,Ore03,Ore04,Ore05,Ore06,Ore07,Ore08,Ore09,Ore10,Ore11,Ore12,Ore13,Ore14	*OreG0008343*
scaffold28919	383157	T	A	Och01,Och02,Och03,Och04,Och05,Och06,Och07,Och08,Och09,Och10,Och11,Och12,Och13,Och14	*OreG0008365*
scaffold28919	539721	A	T	NA	*OreG0008381*
scaffold28919	540541	G	T	Och08	*OreG0008381*
scaffold28919	585188	C	T	Och12	*OreG0008387*
scaffold28919	614374	C	T	Och03,Och04,Och05,Och12,Och13	*OreG0008391*
scaffold28919	718820	G	A	NA	*OreG0008408*
scaffold28919	718832	C	A	Och02,Och03,Och04,Och05,Och06,Och08,Och09,Och10,Och11,Och12,Och13,Och14	*OreG0008408*
scaffold28919	726334	C	T	Ore04,Ore05,Ore06,Ore07	*OreG0008409*
scaffold28919	778588	C	A	Och14	*OreG0008417*
scaffold28919	785341	G	A	Och02,Och03,Och04,Och05,Och06,Och07,Och08,Och09,Och10,Och11,Och12,Och13,Och14,Ore01,Ore02,Ore03,Ore04,Ore05,Ore06,Ore07,Ore08,Ore09,Ore10,Ore11,Ore12,Ore13,Ore14	*OreG0008418*
scaffold28919	854569	G	A	Och01	*OreG0008433*
scaffold28919	896004	G	A	NA	*OreG0008439*
scaffold28919	1021184	T	A	NA	*OreG0008459*
scaffold28919	1042330	A	T	Ore01,Ore02,Ore03,Ore04,Ore05,Ore06,Ore07,Ore08,Ore09,Ore10,Ore11,Ore12,Ore13,Ore14	*OreG0008462*
scaffold28919	1123085	G	A	Och01,Och03,Och05,Och08,Och11,Och12,Och13	*OreG0008469*
scaffold28919	1159575	C	A	NA	*OreG0008473*
scaffold28919	1191327	G	T	NA	*OreG0008474*
scaffold28919	1206443	C	T	NA	*OreG0008477*
scaffold28919	1644948	G	T	Och01,Och02,Och03,Och04,Och05,Och06,Och07,Och08,Och09,Och10,Och11,Och12,Och13,Och14,Ore01,Ore02,Ore03,Ore04,Ore05,Ore06,Ore07,Ore08,Ore09,Ore10,Ore11,Ore12,Ore13,Ore14	*OreG0008502*
scaffold33097	175363	G	A	Och02,Och03,Och04,Och05,Och06,Och07,Och12,Och13	*OreG0011280*
scaffold33097	213234	G	T	Ore04,Ore05	*OreG0011284*
scaffold33097	632607	G	A	NA	*OreG0011311*
scaffold33097	632822	C	T	NA	*OreG0011311*
scaffold33097	764432	T	A	NA	*OreG0011323*
scaffold33097	765444	A	T	NA	*OreG0011324*
scaffold33097	864167	G	A	Och02,Och03,Och04,Och05,Och06,Och07,Och08,Och09,Och10,Och11,Och12,Och14	*OreG0011332*
scaffold33097	868483	G	A	Och08	*OreG0011333*
scaffold33097	869198	C	A	NA	*OreG0011333*
scaffold33097	869291	G	A	Och02,Och03,Och04,Och05,Och06,Och07,Och08,Och09,Och10,Och11,Och12,Och14	*OreG0011333*
scaffold33097	870106	G	A	Och02,Och03,Och06,Och07,Och08,Och09,Och10,Och11,Och12,Och14	*OreG0011333*
scaffold33097	873886	G	A	Och02,Och03,Och04,Och05,Och06,Och07,Och08,Och09,Och10,Och11,Och12,Och14	*OreG0011333*
scaffold33097	874832	G	A	Och07	*OreG0011333*
scaffold33097	890511	G	A	NA	*OreG0011336*
scaffold33097	906131	A	C	NA	*OreG0011340*
scaffold33097	911501	G	A	NA	*OreG0011340*
scaffold33097	928137	C	T	Och01,Och02,Och03,Och04,Och05,Och06,Och07,Och08,Och09,Och10,Och11,Och12,Och13,Och14	*OreG0011342*
scaffold33117	126309	A	T	Ore01,Ore02,Ore03,Ore04,Ore05,Ore06,Ore07,Ore08,Ore09,Ore10,Ore11,Ore12,Ore13,Ore14	*OreG0013007*
scaffold33117	235748	C	T	Ore01,Ore02,Ore03,Ore04,Ore05,Ore06,Ore07,Ore08,Ore09,Ore10,Ore11,Ore12,Ore13,Ore14	*OreG0013022*
scaffold33117	303232	A	T	Och01,Och02,Och03,Och04,Och05,Och06,Och07,Och08,Och09,Och10,Och11,Och12,Och13,Och14	*OreG0013031*
scaffold33117	308842	G	T	NA	*OreG0013032*
scaffold33117	308851	C	T	NA	*OreG0013032*
scaffold33117	381883	G	T	NA	*OreG0013040*
scaffold33117	386814	G	T	NA	*OreG0013042*
scaffold33117	395047	A	T	NA	*OreG0013044*
scaffold33117	412224	C	T	Och09,Och10,Och11	*OreG0013046*
scaffold33117	535252	A	T	Och01,Och05	*OreG0013059*
scaffold33117	542001	G	A	NA	*OreG0013060*
scaffold33128	70205	G	T	NA	*OreG0013612*
scaffold33128	70215	G	A	Och02,Och03,Och04,Och05,Och06,Och07,Och08,Och09,Och10,Och11,Och12,Och13,Och14	*OreG0013612*
scaffold33128	149735	A	T	Ore06,Ore08	*OreG0013622*

scaffold33128	182150	G	A	Och01,Och02,Och03,Och04,Och05,Och06,Och07,Och08,Och09,Och10,Och11,Och12,Och13,Och14,Ore01,Ore02,Ore03,Ore04,Ore05,Ore06,Ore07,Ore08,Ore09,Ore10,Ore11,Ore12,Ore13,Ore14	*OreG0013627*
scaffold33128	377097	C	T	NA	*OreG0013654*
scaffold33128	390345	T	G	Och01,Och02,Och03,Och04,Och05,Och06,Och07,Och08,Och09,Och10,Och11,Och12,Och13,Och14,Ore01,Ore02,Ore03,Ore04,Ore05,Ore06,Ore07,Ore08,Ore09,Ore10,Ore11,Ore12,Ore13,Ore14	*OreG0013656*
scaffold33128	399866	G	T	NA	*OreG0013658*
scaffold33128	403677	G	A	NA	*OreG0013659*
scaffold33128	469386	G	A	Och03,Och04,Och05,Och12,Och13	*OreG0013667*
scaffold33128	1137297	G	A	Och01,Och02,Och03,Och04,Och05,Och06,Och07,Och08,Och09,Och10,Och11,Och12,Och13,Och14	*OreG0013723*
scaffold33128	1228840	T	A	Och01,Och02,Och03,Och04,Och05,Och06,Och07,Och08,Och09,Och10,Och11,Och12,Och13,Och14	*OreG0013733*
scaffold33128	1289899	A	T	Och08	*OreG0013738*
scaffold33128	1496321	T	A	NA	*OreG0013757*
scaffold33128	1604472	C	T	NA	*OreG0013771*
scaffold33128	1668382	T	A	NA	*OreG0013777*
scaffold33154	64843	C	T	NA	*OreG0014170*
scaffold33154	182820	G	A	Och01,Och02,Och03,Och04,Och05,Och06,Och07,Och08,Och09,Och10,Och11,Och12,Och13,Och14	*OreG0014177*
scaffold33154	221486	T	G	NA	*OreG0014179*
scaffold33154	223409	A	T	Och01,Och02,Och03,Och04,Och05,Och06,Och07,Och08,Och09,Och10,Och11,Och12,Och13,Och14,Ore01,Ore02,Ore03,Ore04,Ore05,Ore06,Ore07,Ore08,Ore09,Ore10,Ore11,Ore12,Ore13,Ore14	*OreG0014180*
scaffold33154	392894	A	T	Och02,Och03,Och04,Och05,Och06,Och12,Och13,Och14	*OreG0014196*
scaffold33154	418190	C	A	NA	*OreG0014199*
scaffold33154	521224	G	T	NA	*OreG0014211*
scaffold33154	581060	C	T	NA	*OreG0014215*
scaffold33154	585557	G	T	NA	*OreG0014216*
scaffold33154	699325	C	A	Och02,Och03,Och04,Och05,Och06,Och07,Och08,Och09,Och10,Och11,Och12,Och13,Och14	*OreG0014226*
scaffold33154	727269	C	T	NA	*OreG0014227*
scaffold33154	727329	C	T	NA	*OreG0014227*
scaffold33154	935546	G	A	NA	*OreG0014243*
scaffold33154	948941	C	T	NA	*OreG0014244*
scaffold33154	960170	C	T	Och01,Och02,Och03,Och04,Och05,Och06,Och11,Och12,Och13	*OreG0014245*
scaffold2685	231297	C	A	Och01,Och02,Och03,Och04,Och05,Och06,Och07,Och08,Och09,Och10,Och11,Och12,Och13,Och14	*OreG0007860*
scaffold2685	566049	C	T	NA	*OreG0007885*
scaffold2685	576073	T	G	Och01,Och02,Och03,Och04,Och05,Och06,Och07,Och08,Och09,Och10,Och11,Och12,Och13,Och14,Ore01,Ore02,Ore03,Ore04,Ore05,Ore06,Ore07,Ore08,Ore09,Ore10,Ore11,Ore12,Ore13,Ore14	*OreG0007885*
scaffold2685	596175	C	T	Och09,Och10	*OreG0007887*
scaffold2685	598789	C	T	NA	*OreG0007887*
scaffold33159	61814	G	A	NA	*OreG0014270*
scaffold33159	61862	T	A	NA	*OreG0014270*
scaffold33159	141639	G	T	NA	*OreG0014279*
scaffold33159	188441	A	C	Och01,Och02,Och03,Och04,Och05,Och06,Och07,Och08,Och09,Och10,Och11,Och12,Och13,Och14,Ore01,Ore02,Ore03,Ore04,Ore05,Ore06,Ore07,Ore08,Ore09,Ore10,Ore11,Ore12,Ore13,Ore14	*OreG0014285*
scaffold33159	227003	C	T	NA	*OreG0014292*
scaffold33208	29975	C	A	Och01,Och02,Och03,Och04,Och05,Och06,Och07,Och08,Och09,Och10,Och11,Och12,Och13,Och14	*OreG0016226*
scaffold33208	41263	C	T	Och01,Och03,Och07,Och08,Och09,Och10,Och11	*OreG0016227*
scaffold33208	48376	C	T	Och01,Och02,Och03,Och04,Och05,Och06,Och07,Och09,Och10,Och11,Och12,Och13	*OreG0016228*
scaffold33208	65112	C	T	Ore01,Ore02,Ore03,Ore04,Ore05,Ore06,Ore07,Ore08,Ore09,Ore10,Ore11,Ore12,Ore13,Ore14	*OreG0016231*
scaffold33208	78670	G	A	NA	*OreG0016233*
scaffold33110	32732	G	A	NA	*OreG0012397*
scaffold33110	66112	C	T	Och07,Och08	*OreG0012402*
scaffold33110	187697	C	A	Och01,Och02,Och03,Och04,Och05,Och06,Och12,Och13	*OreG0012419*
scaffold33110	216746	G	A	Och01,Och02,Och03,Och04,Och05,Och06,Och07,Och08,Och09,Och10,Och11,Och12,Och13,Och14,Ore01,Ore02,Ore03,Ore04,Ore05,Ore06,Ore07,Ore08,Ore09,Ore10,Ore11,Ore12,Ore13,Ore14	*OreG0012425*
scaffold33110	277075	C	T	NA	*OreG0012434*
scaffold33110	391045	A	T	Ore01,Ore02,Ore03,Ore04,Ore05,Ore06,Ore07,Ore08,Ore09,Ore10,Ore11,Ore12,Ore13,Ore14	*OreG0012454*
scaffold33110	423084	G	T	Och07,Och11,Och14	*OreG0012462*
scaffold33110	423488	C	T	Och01,Och02,Och03,Och04,Och05,Och06,Och07,Och08,Och09,Och10,Och11,Och12,Och13,Och14	*OreG0012462*
scaffold33110	709103	G	A	NA	*OreG0012487*
scaffold33110	784946	G	T	NA	*OreG0012497*

scaffold33110	847975	G	A	Ore01,Ore02,Ore03,Ore04,Ore05,Ore06,Ore07,Ore08,Ore09,Ore10,Ore11,Ore12,Ore13,Ore14	*OreG0012507*
scaffold33110	878070	G	A	NA	*OreG0012513*
scaffold33110	914787	A	T	Och07,Och08,Och09,Och10,Och11	*OreG0012517*
scaffold33110	1031576	A	T	NA	*OreG0012535*
scaffold33110	1041185	G	A	Och01,Och02,Och03,Och04,Och05,Och06,Och07,Och08,Och09,Och10,Och11,Och12,Och13,Och14	*OreG0012538*
scaffold33110	1086788	C	A	NA	*OreG0012544*
scaffold33110	1088656	T	A	Ore04,Ore05,Ore06,Ore07,Ore08,Ore14	*OreG0012544*
scaffold33110	1092179	G	A	NA	*OreG0012545*
scaffold33110	1108722	T	A	Och01,Och02,Och03,Och04,Och05,Och06,Och07,Och08,Och09,Och10,Och11,Och12,Och13,Och14,Ore01,Ore02,Ore03,Ore04,Ore05,Ore06,Ore07,Ore08,Ore09,Ore10,Ore11,Ore12,Ore13,Ore14	*OreG0012548*
scaffold33110	1110365	C	T	NA	*OreG0012548*
scaffold33110	1113230	A	T	NA	*OreG0012549*
scaffold33110	1166552	C	T	NA	*OreG0012559*
scaffold33110	1286943	A	T	Och02,Och03,Och04,Och05,Och06,Och12,Och13,Och14	*OreG0012576*
scaffold33110	1362636	T	A	Och01,Och02,Och03,Och04,Och06,Och13,Och14	*OreG0012590*
scaffold33110	1435205	G	A	NA	*OreG0012601*
scaffold33110	1474748	C	T	NA	*OreG0012610*
scaffold33110	1476124	C	A	Ore01,Ore02,Ore03,Ore04,Ore05,Ore06,Ore07,Ore08,Ore09,Ore10,Ore11,Ore12,Ore13,Ore14	*OreG0012610*
scaffold33110	1498213	G	A	NA	*OreG0012611*
scaffold33120	207059	C	A	Ore01,Ore02,Ore03,Ore04,Ore05,Ore06,Ore07,Ore08,Ore09,Ore10,Ore11,Ore12,Ore13,Ore14	*OreG0013379*
scaffold33120	371752	T	A	NA	*OreG0013402*
scaffold33120	434234	T	A	Ore06,Ore07,Ore08	*OreG0013409*
scaffold33120	541515	G	A	NA	*OreG0013425*
scaffold33120	542372	C	A	Och01,Och02,Och03,Och04,Och05,Och06,Och07,Och08,Och09,Och10,Och11,Och12,Och13,Och14,Ore01,Ore02,Ore03,Ore04,Ore05,Ore06,Ore07,Ore08,Ore09,Ore10,Ore11,Ore12,Ore13,Ore14	*OreG0013426*
scaffold33120	554972	T	A	NA	*OreG0013430*
scaffold33120	696777	T	A	NA	*OreG0013438*
scaffold33120	826304	C	A	Och01,Och05	*OreG0013455*
scaffold33120	950669	C	A	Och01,Och02,Och03,Och04,Och05,Och06,Och07,Och08,Och09,Och10,Och11,Och12,Och13,Och14,Ore01,Ore02,Ore03,Ore04,Ore05,Ore06,Ore07,Ore08,Ore09,Ore10,Ore11,Ore12,Ore13,Ore14	*OreG0013474*
scaffold33120	963918	C	T	Ore01,Ore02,Ore03,Ore04,Ore05,Ore06,Ore07,Ore08,Ore09,Ore10,Ore11,Ore12,Ore13,Ore14	*OreG0013478*
scaffold33120	1090451	C	T	Och01,Och02,Och03,Och04,Och05,Och06,Och08,Och09,Och10,Och11,Och12,Och13,Och14	*OreG0013492*
scaffold33120	1212946	C	A	Och01,Och02,Och03,Och04,Och05,Och06,Och07,Och08,Och09,Och10,Och11,Och12,Och13,Och14	*OreG0013508*
scaffold33120	1351490	T	A	Och01,Och02,Och03,Och04,Och05,Och06,Och07,Och08,Och09,Och10,Och11,Och12,Och13,Och14,Ore01,Ore02,Ore03,Ore04,Ore05,Ore06,Ore07,Ore08,Ore09,Ore10,Ore11,Ore12,Ore13,Ore14	*OreG0013533*
scaffold33120	1390742	C	T	Och01,Och02,Och03,Och04,Och05,Och06,Och07,Och08,Och09,Och10,Och11,Och12,Och13,Och14,Ore01,Ore02,Ore03,Ore04,Ore05,Ore06,Ore07,Ore08,Ore09,Ore10,Ore11,Ore12,Ore13,Ore14	*OreG0013538*
scaffold33120	1417788	C	T	NA	*OreG0013543*
scaffold33120	1417916	T	A	NA	*OreG0013543*
scaffold33120	1496701	A	T	NA	*OreG0013549*
scaffold33190	20855	G	T	NA	*OreG0014720*
scaffold33190	21335	G	A	NA	*OreG0014720*
scaffold33190	135182	C	A	NA	*OreG0014727*
scaffold33190	163864	C	T	NA	*OreG0014728*
scaffold33106	109192	C	T	NA	*OreG0012168*
scaffold33106	130482	C	A	Och01	*OreG0012170*
scaffold33106	131184	C	A	Och02,Och03,Och04,Och05,Och06,Och12,Och13,Och14	*OreG0012170*
scaffold33106	499599	T	G	NA	*OreG0012204*
scaffold33106	509807	A	C	Och01,Och04,Och06,Och14	*OreG0012208*
scaffold33106	606314	G	T	Ore02	*OreG0012219*
scaffold33106	660324	G	A	NA	*OreG0012225*
scaffold33106	781290	G	A	Ore01,Ore02,Ore03,Ore04,Ore05,Ore06,Ore07,Ore08,Ore09,Ore10,Ore11,Ore12,Ore13,Ore14	*OreG0012238*
scaffold33106	895637	G	T	NA	*OreG0012248*
scaffold33106	895687	T	G	Och01,Och02,Och03,Och04,Och05,Och06,Och07,Och08,Och09,Och10,Och11,Och12,Och13,Och14,Ore01,Ore02,Ore03,Ore04,Ore05,Ore06,Ore07,Ore08,Ore09,Ore10,Ore11,Ore12,Ore13,Ore14	*OreG0012248*
scaffold33106	907959	A	C	Och01,Och02,Och03,Och04,Och05,Och06,Och07,Och08,Och09,Och10,Och11,Och12,Och13,Och14,Ore01,Ore02,Ore03,Ore04,Ore05,Ore06,Ore07,Ore08,Ore09,Ore10,Ore11,Ore12,Ore13,Ore14	*OreG0012250*
scaffold33106	926288	C	A	NA	*OreG0012251*
scaffold33106	1157701	C	T	NA	*OreG0012269*
scaffold33106	1201949	A	T	Ore02	*OreG0012272*

scaffold33106	1326623	G	A	NA	*OreG0012284*
scaffold33106	1354305	C	T	NA	*OreG0012288*
scaffold33106	1354487	G	A	NA	*OreG0012288*
scaffold33106	1400630	C	T	NA	*OreG0012294*
scaffold33106	1483268	C	A	NA	*OreG0012302*
scaffold33106	1483303	G	A	NA	*OreG0012302*
scaffold33106	1562400	C	T	Och02,Och03,Och04,Och05,Och06,Och12,Och13	*OreG0012303*
scaffold33106	1621509	G	T	Ore07,Ore08,Ore11	*OreG0012310*
scaffold33106	1650940	C	A	NA	*OreG0012314*
scaffold33106	1704390	A	T	NA	*OreG0012322*
scaffold33106	1720898	G	A	Och01,Och02,Och03,Och04,Och05,Och06,Och07,Och08,Och09,Och10,Och11,Och12,Och13,Och14	*OreG0012325*
scaffold33106	1721759	C	A	Och04,Och06,Och12	*OreG0012325*
scaffold33106	1736652	C	A	NA	*OreG0012327*
scaffold33106	1828672	C	A	NA	*OreG0012338*
scaffold33106	1833643	C	A	NA	*OreG0012340*
scaffold33106	1833668	A	C	NA	*OreG0012340*
scaffold33101	166552	A	T	Ore01,Ore02,Ore03,Ore04,Ore05,Ore06,Ore07,Ore08,Ore09,Ore10,Ore11,Ore12,Ore13,Ore14	*OreG0011625*
scaffold33105	64856	G	A	Och08,Och12,Och13	*OreG0011821*
scaffold33105	276198	G	A	Ore01,Ore02,Ore03,Ore04,Ore05,Ore06,Ore07,Ore08,Ore09,Ore10,Ore11,Ore12,Ore13,Ore14	*OreG0011843*
scaffold33105	280983	T	A	NA	*OreG0011844*
scaffold33105	286429	C	T	Ore01,Ore02,Ore03,Ore04,Ore05,Ore06,Ore07,Ore08,Ore09,Ore10,Ore11,Ore12,Ore13,Ore14	*OreG0011845*
scaffold33105	687585	C	T	Och01	*OreG0011869*
scaffold33105	817660	G	T	NA	*OreG0011879*
scaffold33105	819223	C	G	NA	*OreG0011880*
scaffold33105	941478	C	T	NA	*OreG0011894*
scaffold33105	968887	C	A	Och01,Och02,Och03,Och04,Och05,Och06,Och07,Och08,Och09,Och10,Och11,Och12,Och13,Och14	*OreG0011896*
scaffold33105	1310640	T	A	Och01,Och02,Och03,Och04,Och05,Och06,Och07,Och08,Och09,Och10,Och11,Och12,Och13,Och14	*OreG0011929*
scaffold33105	1332159	G	T	Ore01,Ore02,Ore04,Ore05,Ore06,Ore07,Ore08,Ore09,Ore10,Ore11,Ore12,Ore13,Ore14	*OreG0011931*
scaffold33105	1730338	C	T	Ore13	*OreG0011969*
scaffold33105	1730725	C	T	Ore13	*OreG0011969*
scaffold33105	1746313	G	A	NA	*OreG0011971*
scaffold33105	1747871	C	T	NA	*OreG0011971*
scaffold33105	1830320	A	T	NA	*OreG0011972*
scaffold33105	1911349	C	A	Och01,Och02,Och04,Och05,Och06,Och07,Och09,Och10,Och11,Och12,Och13,Och14	*OreG0011979*
scaffold33105	2076601	A	T	NA	*OreG0012000*
scaffold33105	2135095	G	A	NA	*OreG0012008*
scaffold33105	2208023	G	A	Och01,Och02,Och03,Och04,Och05,Och06,Och07,Och08,Och09,Och10,Och11,Och12,Och13,Och14,Ore01,Ore02,Ore03,Ore04,Ore05,Ore06,Ore07,Ore08,Ore09,Ore10,Ore11,Ore12,Ore13,Ore14	*OreG0012021*
scaffold33105	2253582	G	T	NA	*OreG0012028*
scaffold33105	2314098	C	T	NA	*OreG0012037*
scaffold33105	2369319	C	T	NA	*OreG0012051*
scaffold33105	2447247	C	T	Och01,Och02,Och03,Och04,Och05,Och06,Och07,Och08,Och09,Och10,Och11,Och12,Och13,Och14	*OreG0012058*
scaffold33105	2447397	G	T	Och01,Och02,Och03,Och04,Och05,Och06,Och07,Och08,Och09,Och10,Och11,Och12,Och13,Och14,Ore01,Ore02,Ore03,Ore04,Ore05,Ore06,Ore07,Ore08,Ore09,Ore10,Ore11,Ore12,Ore13,Ore14	*OreG0012058*
scaffold33105	2451088	T	A	Och03	*OreG0012058*
scaffold33105	2451894	C	A	NA	*OreG0012058*
scaffold33105	2477628	C	T	Ore01,Ore02,Ore03,Ore04,Ore05,Ore06,Ore07,Ore08,Ore09,Ore10,Ore11,Ore12,Ore13,Ore14	*OreG0012061*
scaffold33105	2717938	C	T	Ore01,Ore02,Ore03,Ore04,Ore05,Ore06,Ore07,Ore08,Ore09,Ore10,Ore11,Ore12,Ore13,Ore14	*OreG0012097*
scaffold33105	2740373	G	A	NA	*OreG0012101*
scaffold33105	2741117	A	T	Och01,Och02,Och03,Och04,Och05,Och06,Och07,Och08,Och09,Och10,Och11,Och12,Och13,Och14,Ore01,Ore02,Ore03,Ore04,Ore05,Ore06,Ore07,Ore08,Ore09,Ore10,Ore11,Ore12,Ore13,Ore14	*OreG0012101*
scaffold33105	2806873	A	T	NA	*OreG0012110*
scaffold33105	2825878	A	T	NA	*OreG0012113*
scaffold33105	2831838	A	T	Och01,Och02,Och03,Och04,Och05,Och06,Och07,Och08,Och09,Och10,Och11,Och12,Och13,Och14	*OreG0012115*
scaffold33105	2957094	G	C	NA	*OreG0012134*
scaffold33105	3051103	C	A	NA	*OreG0012143*
scaffold33105	3227184	C	T	NA	*OreG0012156*
scaffold33139	75208	C	A	Ore01,Ore02,Ore03,Ore04,Ore05,Ore06,Ore07,Ore08,Ore09,Ore10,Ore11,Ore12,Ore13,Ore14	*OreG0013883*

scaffold33139	237422	C	G	Ore01,Ore02,Ore03,Ore04,Ore05,Ore06,Ore07,Ore08,Ore09,Ore10,Ore11,Ore12,Ore13,Ore14	*OreG0013895*
scaffold33139	470442	C	A	NA	*OreG0013925*
scaffold33139	495800	G	T	Ore03	*OreG0013929*
scaffold33139	634955	G	T	NA	*OreG0013947*
scaffold33139	786361	G	A	Ore01,Ore02,Ore03,Ore04,Ore05,Ore06,Ore07,Ore08,Ore09,Ore10,Ore11,Ore12,Ore13,Ore14	*OreG0013967*
scaffold33139	822220	T	G	Och05,Och12	*OreG0013971*
scaffold33139	822222	C	T	Ore01,Ore02,Ore03,Ore06,Ore07,Ore08,Ore09,Ore10,Ore11,Ore12,Ore13,Ore14	*OreG0013971*
scaffold31714	283888	G	T	NA	*OreG0009744*
scaffold31714	283908	G	T	NA	*OreG0009744*
scaffold31714	312522	G	A	NA	*OreG0009746*
scaffold31714	335054	C	T	NA	*OreG0009748*
scaffold31714	391895	G	T	Och01,Och02,Och03,Och04,Och05,Och06,Och07,Och08,Och09,Och10,Och11,Och12,Och13,Och14	*OreG0009754*
scaffold31714	531468	C	T	NA	*OreG0009769*
scaffold31714	584828	G	A	NA	*OreG0009775*
scaffold31714	668501	G	A	NA	*OreG0009781*
scaffold31714	726362	C	A	NA	*OreG0009787*
scaffold31714	741482	C	T	NA	*OreG0009788*
scaffold31714	764309	G	A	NA	*OreG0009792*
scaffold31714	803552	C	A	Och01,Och08	*OreG0009797*
scaffold31714	856072	G	A	Och01,Och02,Och03,Och04,Och05,Och06,Och07,Och08,Och09,Och10,Och11,Och12,Och13,Och14,Ore01,Ore02,Ore03,Ore04,Ore05,Ore06,Ore07,Ore08,Ore09,Ore10,Ore11,Ore12,Ore13,Ore14	*OreG0009804*
scaffold31714	879004	G	A	Och08	*OreG0009808*
scaffold31714	1108438	A	T	NA	*OreG0009835*
scaffold31714	1188520	G	A	Och01,Och02,Och03,Och04,Och05,Och06,Och07,Och08,Och09,Och10,Och11,Och12,Och13,Och14,Ore01,Ore02,Ore03,Ore04,Ore05,Ore06,Ore07,Ore08,Ore09,Ore10,Ore11,Ore12,Ore13,Ore14	*OreG0009845*
scaffold31714	1221882	A	T	NA	*OreG0009850*
scaffold31714	1243000	G	T	NA	*OreG0009852*
scaffold31714	1315740	C	A	Och12	*OreG0009858*
scaffold33137	237244	G	T	Och03,Och04	*OreG0013843*
scaffold33137	240652	G	A	NA	*OreG0013843*
scaffold33137	256488	G	A	NA	*OreG0013845*
scaffold33137	256589	C	T	NA	*OreG0013845*
scaffold33137	258414	G	T	NA	*OreG0013845*
scaffold33137	265137	G	T	NA	*OreG0013846*
scaffold33137	308172	G	A	Ore01,Ore02,Ore03,Ore04,Ore05,Ore06,Ore07,Ore08,Ore09,Ore10,Ore11,Ore12,Ore13,Ore14	*OreG0013855*
scaffold33137	314210	C	T	NA	*OreG0013855*
scaffold33137	505468	G	A	NA	*OreG0013867*
scaffold33206	419587	C	T	Och01,Och02,Och03,Och04,Och05,Och06,Och07,Och08,Och09,Och10,Och11,Och12,Och13,Och14	*OreG0015914*
scaffold33206	419593	C	T	Och01,Och02,Och03,Och04,Och05,Och06,Och07,Och08,Och09,Och10,Och11,Och12,Och13,Och14	*OreG0015914*
scaffold33194	182807	C	A	NA	*OreG0015068*
scaffold33194	355431	G	A	Och14	*OreG0015080*
scaffold33194	366235	T	A	NA	*OreG0015083*
scaffold33194	377876	T	A	Och01,Och02,Och03,Och04,Och05,Och06,Och07,Och08,Och09,Och10,Och11,Och12,Och13,Och14,Ore01,Ore02,Ore03,Ore04,Ore05,Ore06,Ore07,Ore08,Ore09,Ore10,Ore11,Ore12,Ore13,Ore14	*OreG0015084*
scaffold33194	387659	C	T	Och07	*OreG0015085*
scaffold33194	387904	G	T	Och06,Och07	*OreG0015085*
scaffold33194	397457	A	T	NA	*OreG0015086*
scaffold33194	481256	G	A	NA	*OreG0015093*
scaffold33119	14837	G	T	NA	*OreG0013078*
scaffold33119	75756	G	A	NA	*OreG0013086*
scaffold33119	134778	C	T	Och01,Och02,Och03,Och04,Och05,Och06,Och07,Och08,Och09,Och10,Och11,Och12,Och13,Och14,Ore01,Ore02,Ore03,Ore04,Ore05,Ore06,Ore07,Ore08,Ore09,Ore10,Ore11,Ore12,Ore13,Ore14	*OreG0013098*
scaffold33119	262927	C	T	NA	*OreG0013116*
scaffold33119	264983	T	A	NA	*OreG0013117*
scaffold33119	630755	G	A	NA	*OreG0013160*
scaffold33119	847742	T	A	Och01,Och02,Och03,Och04,Och06,Och12,Och13	*OreG0013180*
scaffold33119	1133776	G	C	NA	*OreG0013205*
scaffold33119	1180487	T	A	NA	*OreG0013209*
scaffold33119	1215235	C	A	Och01,Och02,Och03,Och04,Och05,Och06,Och07,Och08,Och09,Och10,Och11,Och12,Och13,Och14	*OreG0013216*
scaffold33119	1215241	G	A	NA	*OreG0013216*
scaffold33119	1314471	C	T	Och01,Och02,Och03,Och04,Och05,Och06,Och07,Och08,Och09,Och10,Och11,Och12,Och13,Och14	*OreG0013223*
scaffold33119	1604135	C	T	Och01,Och02,Och03,Och04,Och05,Och06,Och07,Och08,Och09,Och10,Och11,Och12,Och13,Och14	*OreG0013236*

scaffold33119	1607701	C	T	Och01,Och02,Och03,Och04,Och05,Och06,Och07,Och08,Och09,Och10,Och11,Och12,Och13,Och14	*OreG0013236*
scaffold33119	2228778	G	T	Och06,Och08,Och14	*OreG0013295*
scaffold33119	2333315	C	T	NA	*OreG0013303*
scaffold33119	2342703	C	T	NA	*OreG0013305*
scaffold33119	2343073	G	A	NA	*OreG0013305*
scaffold33119	2409706	G	A	Ore01,Ore02,Ore03,Ore04,Ore05,Ore06,Ore07,Ore08,Ore09,Ore10,Ore11,Ore12,Ore13,Ore14	*OreG0013311*
scaffold33119	2516986	C	T	Och09,Och10,Och11	*OreG0013326*
scaffold33119	2517109	C	T	Och01,Och08	*OreG0013326*
scaffold33217	46638	G	A	Och05,Ore03,Ore04,Ore05,Ore09,Ore10,Ore13,Ore14	*OreG0016594*
scaffold33217	49749	T	A	Och01,Och05,Och14	*OreG0016594*
scaffold33217	173475	G	A	Ore04,Ore05	*OreG0016609*
scaffold33103	48270	T	G	Och01,Och02,Och03,Och04,Och05,Och06,Och07,Och08,Och09,Och10,Och11,Och12,Och13,Och14	*OreG0011679*
scaffold33103	80015	G	A	NA	*OreG0011681*
scaffold33103	81193	A	T	NA	*OreG0011681*
scaffold33103	217675	T	A	NA	*OreG0011695*
scaffold33103	234491	T	A	Och01,Och05,Och14	*OreG0011696*
scaffold33103	234765	C	T	NA	*OreG0011696*
scaffold33103	236320	T	A	Ore02,Ore11,Ore13	*OreG0011697*
scaffold33103	237932	G	A	Ore02,Ore11,Ore13	*OreG0011698*
scaffold33103	238011	T	A	Och07	*OreG0011698*
scaffold33103	238927	A	T	Och01,Och14	*OreG0011699*
scaffold33103	331278	G	C	Ore01,Ore02,Ore03,Ore04,Ore05,Ore06,Ore07,Ore08,Ore09,Ore10,Ore11,Ore12,Ore13,Ore14	*OreG0011713*
scaffold33103	369571	G	T	Och07,Och09,Och10,Och11,Och12,Och13,Och14,Ore01,Ore02,Ore03,Ore04,Ore05,Ore06,Ore07,Ore08,Ore09,Ore10,Ore11,Ore12,Ore13,Ore14	*OreG0011720*
scaffold33103	514465	T	A	Och01,Och02,Och03,Och04,Och05,Och06,Och07,Och08,Och09,Och10,Och11,Och12,Och13,Och14,Ore01,Ore02,Ore03,Ore04,Ore05,Ore06,Ore07,Ore08,Ore09,Ore10,Ore11,Ore12,Ore13,Ore14	*OreG0011734*
scaffold33103	514567	G	A	Och02,Och03,Och04,Och06,Och07,Och12,Och13,Och14	*OreG0011734*
scaffold33103	604087	A	T	Och01,Och07,Och08,Och09,Och10,Och11	*OreG0011746*
scaffold33103	606145	G	A	Och01,Och02,Och03,Och04,Och05,Och06,Och07,Och08,Och09,Och10,Och11,Och12,Och13,Och14	*OreG0011747*
scaffold33103	1229134	C	A	Och02,Och03,Och04,Och05,Och06,Och08,Och09,Och10,Och11,Och12,Och13,Och14	*OreG0011779*
scaffold33103	1384673	C	T	Och01,Och02,Och05,Och08,Och09,Och10,Och11	*OreG0011787*
scaffold33103	2016089	A	T	Och01,Och06,Och09,Och10,Och13	*OreG0011809*
scaffold33228	100067	G	T	NA	*OreG0017090*
scaffold33175	168735	G	T	Och01,Och02,Och03,Och04,Och05,Och06,Och07,Och08,Och09,Och10,Och11,Och12,Och13,Och14	*OreG0014403*
scaffold33175	168859	T	G	Och07,Och08,Och11	*OreG0014403*
scaffold33175	169122	G	T	Och01,Och02,Och03,Och04,Och05,Och06,Och07,Och08,Och09,Och10,Och11,Och12,Och13,Och14	*OreG0014403*
scaffold33175	181004	C	A	NA	*OreG0014406*
scaffold33175	181118	G	A	NA	*OreG0014406*
scaffold33175	190437	T	A	NA	*OreG0014409*
scaffold33175	195824	C	T	Och08	*OreG0014410*
scaffold33175	227899	C	A	Ore01,Ore02,Ore03,Ore04,Ore05,Ore06,Ore07,Ore08,Ore09,Ore10,Ore11,Ore12,Ore13,Ore14	*OreG0014414*
scaffold33175	273886	A	T	NA	*OreG0014420*
scaffold33175	324463	G	A	NA	*OreG0014425*
scaffold33175	333293	C	T	NA	*OreG0014426*
scaffold33175	397188	C	A	NA	*OreG0014434*
scaffold33175	407636	C	T	Och01,Och02,Och03,Och04,Och05,Och06,Och07,Och08,Och09,Och11,Och12,Och13,Och14	*OreG0014436*
scaffold33175	640498	A	T	NA	*OreG0014462*
scaffold33175	741942	C	T	NA	*OreG0014475*
scaffold33175	783439	G	A	Och05,Och13	*OreG0014479*
scaffold33175	783796	G	A	Och05,Och13	*OreG0014479*
scaffold33175	792514	A	C	NA	*OreG0014481*
scaffold33175	853077	C	T	NA	*OreG0014486*
scaffold33175	890243	G	A	Och07	*OreG0014490*
scaffold33175	1005110	C	G	Och01,Och02,Och03,Och04,Och05,Och06,Och07,Och08,Och09,Och10,Och11,Och12,Och13,Och14,Ore01,Ore02,Ore03,Ore04,Ore05,Ore06,Ore07,Ore08,Ore09,Ore10,Ore11,Ore12,Ore13,Ore14	*OreG0014505*
scaffold33175	1038486	G	T	Och01,Och14	*OreG0014510*
scaffold33175	1144377	C	A	NA	*OreG0014527*
scaffold33175	1301915	G	A	NA	*OreG0014548*
scaffold33175	1377389	C	T	Och09,Och10	*OreG0014559*
scaffold33175	1384365	G	T	Och01,Och02,Och03,Och04,Och05,Och06,Och07,Och08,Och09,Och10,Och11,Och12,Och13,Och14	*OreG0014563*
scaffold33175	1461820	G	A	NA	*OreG0014573*
scaffold33175	1613673	T	A	NA	*OreG0014593*

scaffold33175	1857084	C	T	NA	*OreG0014624*
scaffold33220	60777	C	T	NA	*OreG0016759*
scaffold452	511566	G	A	Ore08,Ore10,Ore12	*OreG0020949*
scaffold452	618232	G	A	NA	*OreG0020964*
scaffold452	618628	G	A	Och02,Och03,Och04,Och05,Och06,Och08,Och12,Och13,Och14	*OreG0020964*
scaffold452	873556	G	A	Och06	*OreG0020977*
scaffold452	1172948	A	T	Ore01,Ore02,Ore03,Ore04,Ore05,Ore06,Ore07,Ore08,Ore09,Ore10,Ore11,Ore12,Ore13,Ore14	*OreG0020988*
scaffold452	1185846	C	T	Och08,Och14	*OreG0020992*
scaffold452	1251720	C	A	Och01,Och02,Och03,Och04,Och05,Och06,Och07,Och08,Och09,Och10,Och11,Och12,Och13,Och14,Ore01,Ore02,Ore03,Ore04,Ore05,Ore06,Ore07,Ore08,Ore09,Ore10,Ore11,Ore12,Ore13,Ore14	*OreG0020998*
scaffold452	1316364	C	T	NA	*OreG0021002*
scaffold452	1318919	A	T	NA	*OreG0021002*
scaffold452	1323939	C	A	Och01,Och02,Och03,Och04,Och05,Och06,Och07,Och08,Och09,Och10,Och11,Och12,Och13,Och14	*OreG0021003*
scaffold452	1511573	T	A	Och01,Och02,Och03,Och04,Och05,Och06,Och07,Och08,Och09,Och10,Och11,Och12,Och13,Och14	*OreG0021023*
scaffold452	1584344	A	T	NA	*OreG0021029*
scaffold452	1735213	G	A	Och09,Och10,Och14,Ore01,Ore02,Ore06,Ore08,Ore09,Ore10	*OreG0021050*
scaffold452	1800711	G	T	Och14	*OreG0021055*
scaffold452	2177713	C	A	NA	*OreG0021069*
scaffold452	2214606	G	T	Och11	*OreG0021074*
scaffold452	2374053	C	A	Och01,Och02,Och03,Och04,Och05,Och06,Och07,Och08,Och09,Och10,Och11,Och12,Och13,Och14,Ore01,Ore02,Ore03,Ore04,Ore05,Ore06,Ore07,Ore08,Ore09,Ore10,Ore11,Ore12,Ore13,Ore14	*OreG0021087*
scaffold452	2453793	C	T	Och01,Och07,Och14	*OreG0021093*
scaffold452	2455929	C	A	NA	*OreG0021093*
scaffold452	2554309	G	T	Och06	*OreG0021100*
scaffold452	2583055	T	A	NA	*OreG0021103*
scaffold452	2909818	G	T	NA	*OreG0021128*
scaffold452	2916244	A	C	NA	*OreG0021129*
scaffold452	3151487	C	T	Och03,Och04,Och06	*OreG0021157*
scaffold452	3169761	C	A	Och05,Och12,Och13	*OreG0021159*
scaffold452	3819323	G	T	Och02,Och11,Och12,Och13	*OreG0021203*
scaffold452	3861686	G	T	Och01,Och14	*OreG0021208*
scaffold452	3861693	G	A	Och14	*OreG0021208*
scaffold452	3895246	C	A	NA	*OreG0021211*
scaffold452	3895832	A	T	NA	*OreG0021211*
scaffold452	3896781	A	T	NA	*OreG0021211*
scaffold452	3897917	C	T	NA	*OreG0021211*
scaffold452	3973697	T	A	Och01,Och02,Och03,Och04,Och05,Och06,Och07,Och08,Och09,Och10,Och11,Och12,Och13,Och14,Ore01,Ore02,Ore03,Ore04,Ore05,Ore06,Ore07,Ore08,Ore09,Ore10,Ore11,Ore12,Ore13,Ore14	*OreG0021221*
scaffold452	3975441	C	T	Och09,Och10,Och14	*OreG0021221*
scaffold452	4046998	G	A	NA	*OreG0021229*
scaffold452	4140968	G	T	NA	*OreG0021236*
scaffold452	4148597	G	A	Och01,Och02,Och03,Och04,Och05,Och06,Och07,Och08,Och09,Och10,Och11,Och12,Och13,Och14,Ore01,Ore02,Ore03,Ore04,Ore05,Ore06,Ore07,Ore08,Ore09,Ore10,Ore11,Ore12,Ore13,Ore14	*OreG0021236*
scaffold452	4295009	A	T	Och01,Och02,Och03,Och04,Och05,Och06,Och07,Och08,Och09,Och10,Och11,Och12,Och13,Och14,Ore01,Ore02,Ore03,Ore04,Ore05,Ore06,Ore07,Ore08,Ore09,Ore10,Ore11,Ore12,Ore13,Ore14	*OreG0021248*
scaffold452	4412016	C	T	Ore01,Ore02,Ore03,Ore04,Ore05,Ore06,Ore07,Ore08,Ore09,Ore10,Ore11,Ore12,Ore13,Ore14	*OreG0021258*
scaffold452	4443017	G	A	Och01,Och02,Och03,Och04,Och05,Och06,Och07,Och08,Och09,Och10,Och11,Och12,Och13,Och14	*OreG0021262*
scaffold452	4443161	C	T	Och01,Och02,Och03,Och04,Och05,Och06,Och07,Och08,Och09,Och10,Och11,Och12,Och13,Och14	*OreG0021262*
scaffold452	4563189	C	T	Och01,Och02,Och03,Och04,Och05,Och06,Och07,Och09,Och10,Och12,Och13,Och14	*OreG0021273*
scaffold452	4688832	G	A	NA	*OreG0021283*
scaffold452	4688891	C	T	Och07	*OreG0021283*
scaffold452	4723548	G	T	Och01,Och02,Och03,Och04,Och05,Och06,Och07,Och08,Och09,Och10,Och11,Och12,Och13,Och14	*OreG0021286*
scaffold452	4773616	G	T	Och01,Och02,Och03,Och04,Och05,Och06,Och07,Och08,Och09,Och10,Och11,Och12,Och13,Och14	*OreG0021291*
scaffold452	4874549	C	A	NA	*OreG0021300*
scaffold452	4875016	A	T	NA	*OreG0021300*
scaffold452	4880790	C	A	NA	*OreG0021301*
scaffold452	4881012	G	A	NA	*OreG0021301*
scaffold452	5002907	G	A	Ore01,Ore02,Ore03,Ore04,Ore05,Ore06,Ore07,Ore08,Ore09,Ore10,Ore11,Ore12,Ore13,Ore14	*OreG0021308*
scaffold452	5267985	C	G	Ore03	*OreG0021331*
scaffold452	5328313	T	A	NA	*OreG0021337*

scaffold452	5351398	G	T	NA	*OreG0021338*
scaffold452	5386353	C	T	NA	*OreG0021342*
scaffold452	5562142	G	A	Och01,Och02,Och03,Och04,Och05,Och06,Och07,Och08,Och09,Och10,Och11,Och12,Och13,Och14,Ore01,Ore02,Ore03,Ore04,Ore05,Ore06,Ore07,Ore08,Ore09,Ore10,Ore11,Ore12,Ore13,Ore14	*OreG0021346*
scaffold452	5562186	C	T	Och01,Och02,Och03,Och04,Och05,Och06,Och07,Och08,Och09,Och10,Och11,Och12,Och13,Och14	*OreG0021346*
scaffold452	5595701	C	G	Och07,Och08,Och09,Och10,Och11,Och14	*OreG0021345*
scaffold452	5631118	C	A	NA	*OreG0021352*
scaffold452	6286007	T	A	NA	*OreG0021397*
scaffold452	6289123	C	T	NA	*OreG0021398*
scaffold452	6855938	T	A	Och01,Och02,Och03,Och04,Och05,Och06,Och07,Och08,Och09,Och10,Och11,Och12,Och13,Och14	*OreG0021452*
scaffold452	6857957	T	A	NA	*OreG0021452*
scaffold452	7100832	T	A	NA	*OreG0021478*
scaffold452	7267071	G	A	Och01,Och02,Och03,Och04,Och05,Och06,Och07,Och08,Och09,Och10,Och11,Och12,Och13,Och14	*OreG0021487*
scaffold452	7283160	C	T	Och03,Och04,Och09,Och10	*OreG0021488*
scaffold452	7344432	C	T	Och01,Och02,Och03,Och04,Och05,Och06,Och07,Och08,Och09,Och10,Och11,Och12,Och13,Och14	*OreG0021493*
scaffold452	7468193	C	T	NA	*OreG0021503*
scaffold452	7469300	C	T	NA	*OreG0021503*
scaffold452	7469436	C	G	NA	*OreG0021503*
scaffold452	7469692	T	A	NA	*OreG0021503*
scaffold452	7469912	A	T	Och02,Och03,Och04,Och05,Och06,Och09,Och10,Och12,Och13,Och14,Ore01,Ore02,Ore03,Ore04,Ore05,Ore06,Ore07,Ore08,Ore09,Ore10,Ore11,Ore12,Ore13,Ore14	*OreG0021503*
scaffold452	7469987	C	T	NA	*OreG0021503*
scaffold452	7472175	A	T	Och09,Och10	*OreG0021503*
scaffold452	7797885	C	T	Och11	*OreG0021530*
scaffold452	8140608	C	A	NA	*OreG0021554*
scaffold452	8218789	T	A	NA	*OreG0021559*
scaffold452	8256424	C	A	NA	*OreG0021563*
scaffold452	8367615	T	A	NA	*OreG0021564*
scaffold452	8640229	T	A	Ore01,Ore02,Ore03,Ore04,Ore05,Ore06,Ore07,Ore08,Ore09,Ore10,Ore11,Ore12,Ore13,Ore14	*OreG0021590*
scaffold452	9191254	C	T	Och12	*OreG0021632*
scaffold452	9265342	C	T	NA	*OreG0021642*
scaffold452	9368462	C	T	Och01,Och02,Och03,Och04,Och05,Och06,Och07,Och08,Och09,Och10,Och11,Och12,Och13,Och14	*OreG0021650*
scaffold452	9368654	C	T	NA	*OreG0021650*
scaffold452	9831616	G	T	NA	*OreG0021688*
scaffold452	1E+07	G	A	Och14	*OreG0021723*
scaffold452	1.1E+07	G	A	Ore01,Ore02,Ore03,Ore04,Ore05,Ore06,Ore07,Ore08,Ore09,Ore10,Ore11,Ore12,Ore13,Ore14	*OreG0021735*
scaffold452	1.1E+07	T	A	Och01,Och02,Och03,Och04,Och05,Och06,Och07,Och08,Och09,Och10,Och11,Och12,Och13,Och14,Ore01,Ore02,Ore03,Ore04,Ore05,Ore06,Ore07,Ore08,Ore09,Ore10,Ore11,Ore12,Ore13,Ore14	*OreG0021744*
scaffold452	1.1E+07	G	T	Och08	*OreG0021750*
scaffold452	1.1E+07	C	A	Och01,Och06,Och09,Och10,Och11,Och14	*OreG0021755*
scaffold452	1.1E+07	A	T	Och08	*OreG0021758*
scaffold452	1.1E+07	C	A	Och08	*OreG0021759*
scaffold452	1.1E+07	G	A	NA	*OreG0021759*
scaffold452	1.1E+07	C	T	NA	*OreG0021760*
scaffold452	1.1E+07	C	T	NA	*OreG0021766*
scaffold33118	210346	C	G	NA	*OreG0013062*
scaffold33118	989106	A	T	Och02	*OreG0013072*
scaffold33230	11203	C	T	NA	*OreG0017175*
scaffold33230	22401	G	A	Och02,Och03,Och04,Och05,Och06,Och07,Och08,Och12,Och13,Och14	*OreG0017178*
scaffold33230	70945	C	T	NA	*OreG0017179*
scaffold33210	68027	C	T	NA	*OreG0016298*
scaffold33210	421314	G	A	Och01,Och02,Och03,Och04,Och05,Och06,Och07,Och08,Och09,Och10,Och11,Och12,Och13,Och14	*OreG0016317*
scaffold33210	421635	G	A	Och01,Och02,Och03,Och04,Och05,Och06,Och07,Och08,Och09,Och10,Och11,Och12,Och13,Och14	*OreG0016317*
scaffold33210	484689	G	A	Ore03,Ore08,Ore09,Ore10,Ore14	*OreG0016322*
scaffold33210	491574	G	A	Och01,Och02,Och03,Och04,Och05,Och06,Och07,Och08,Och09,Och10,Och11,Och12,Och13,Och14	*OreG0016323*
scaffold33210	695077	A	T	Och05	*OreG0016333*
scaffold33210	709179	G	T	NA	*OreG0016335*
scaffold33210	712644	G	A	Och09,Och10	*OreG0016336*
scaffold33210	737916	T	A	Och02,Och03,Och04,Och05,Och06,Och07,Och09,Och10,Och13,Och14	*OreG0016339*
scaffold33225	99007	T	A	Och01,Och02,Och03,Och04,Och05,Och06,Och07,Och08,Och09,Och10,Och11,Och12,Och13,Och14	*OreG0017062*
scaffold33225	117645	C	T	NA	*OreG0017064*
scaffold33225	117681	C	T	NA	*OreG0017064*

scaffold33225	118197	G	T	NA	*OreG0017064*
scaffold33225	118467	A	T	NA	*OreG0017064*
scaffold33213	106479	G	T	NA	*OreG0016431*
scaffold33213	319302	G	T	Och01,Och02,Och03,Och04,Och05,Och06,Och07,Och08,Och09,Och10,Och11,Och12,Och13,Och14	*OreG0016451*
scaffold33213	433847	C	T	NA	*OreG0016458*
scaffold33213	434328	C	T	Och12,Och13	*OreG0016458*
scaffold33213	573959	C	T	Och01,Och02,Och03,Och04,Och05,Och06,Och07,Och08,Och09,Och10,Och11,Och12,Och13,Och14,Ore01,Ore02,Ore03,Ore04,Ore05,Ore06,Ore07,Ore08,Ore09,Ore10,Ore11,Ore12,Ore13,Ore14	*OreG0016464*
scaffold33213	574238	C	T	Och04	*OreG0016464*
scaffold33226	18278	T	A	NA	*OreG0017081*
scaffold33212	112199	G	A	NA	*OreG0016398*
scaffold33212	534367	C	A	Och11	*OreG0016419*
scaffold33176	155173	C	A	Och01,Och02,Och03,Och04,Och05,Och06,Och07,Och08,Och09,Och10,Och11,Och12,Och13,Och14	*OreG0014637*
scaffold33195	204035	C	T	NA	*OreG0015109*
scaffold33195	403960	G	A	Och01,Och02,Och03,Och04,Och05,Och06,Och09,Och10,Och12,Och13	*OreG0015129*
scaffold33195	410032	A	T	NA	*OreG0015129*
scaffold33195	452673	C	A	Och14	*OreG0015134*
scaffold33195	456199	G	A	Och01,Och02,Och03,Och04,Och05,Och06,Och07,Och08,Och09,Och10,Och11,Och12,Och13,Och14,Ore01,Ore02,Ore03,Ore04,Ore05,Ore06,Ore07,Ore08,Ore09,Ore10,Ore11,Ore12,Ore13,Ore14	*OreG0015135*
scaffold33195	542317	G	T	NA	*OreG0015143*
scaffold33236	40023	A	C	Och01,Och02,Och03,Och04,Och05,Och06,Och07,Och08,Och09,Och10,Och11,Och12,Och13,Och14,Ore01,Ore02,Ore03,Ore04,Ore05,Ore06,Ore07,Ore08,Ore09,Ore10,Ore11,Ore12,Ore13,Ore14	*OreG0017273*
scaffold33236	115735	G	A	NA	*OreG0017277*
scaffold33236	226897	T	G	NA	*OreG0017285*
scaffold33236	227156	C	T	NA	*OreG0017285*
scaffold33236	227536	C	T	NA	*OreG0017285*
scaffold33236	227782	C	T	NA	*OreG0017285*
scaffold33182	69085	C	T	NA	*OreG0014655*
scaffold33182	69127	A	T	NA	*OreG0014655*
scaffold33182	91052	G	T	NA	*OreG0014656*
scaffold33182	253955	A	T	NA	*OreG0014663*
scaffold33216	187779	A	T	NA	*OreG0016488*
scaffold33216	187967	C	T	Och01,Och06	*OreG0016488*
scaffold33216	188235	C	T	Och02,Och03,Och04,Och05,Och06,Och07,Och08,Och09,Och10,Och11,Och12,Och13,Och14	*OreG0016488*
scaffold33216	189160	C	T	Och02,Och03,Och04,Och05,Och06,Och07,Och08,Och09,Och10,Och11,Och12,Och13,Och14	*OreG0016488*
scaffold33216	189214	G	T	NA	*OreG0016488*
scaffold33216	192942	C	T	Och09,Och10,Och11	*OreG0016489*
scaffold33216	193840	G	T	Och09,Och10	*OreG0016489*
scaffold33216	194214	G	A	Och01,Och06	*OreG0016489*
scaffold33216	194386	G	T	Och09,Och10,Och11	*OreG0016489*
scaffold33216	195825	G	A	Och01,Och06	*OreG0016490*
scaffold33216	195861	G	A	Och09,Och10,Och11	*OreG0016490*
scaffold33216	196142	C	T	Och09,Och10,Och11	*OreG0016490*
scaffold33216	196221	G	A	Och02,Och04,Och05,Och06,Och13	*OreG0016490*
scaffold33216	197603	G	A	Och01,Och06	*OreG0016491*
scaffold33216	201695	G	A	NA	*OreG0016491*
scaffold33216	332009	A	T	NA	*OreG0016510*
scaffold33216	373762	A	T	NA	*OreG0016517*
scaffold33216	374355	C	A	Och01,Och02,Och03,Och04,Och05,Och06,Och07,Och08,Och09,Och10,Och11,Och12,Och13,Och14,Ore01,Ore02,Ore03,Ore04,Ore05,Ore06,Ore07,Ore08,Ore09,Ore10,Ore11,Ore12,Ore13,Ore14	*OreG0016517*
scaffold33216	507857	C	T	Ore01,Ore02,Ore03,Ore04,Ore05,Ore06,Ore07,Ore08,Ore09,Ore10,Ore11,Ore12,Ore13,Ore14	*OreG0016533*
scaffold33216	616988	G	T	Och01	*OreG0016545*
scaffold33216	748799	C	T	Och03,Och07,Och12,Och13	*OreG0016562*
scaffold33216	800497	C	T	NA	*OreG0016567*
scaffold33216	856283	G	A	NA	*OreG0016576*
scaffold33216	872726	A	T	NA	*OreG0016579*
scaffold33216	892346	G	A	NA	*OreG0016582*
scaffold33113	41850	T	A	NA	*OreG0012714*
scaffold33113	49306	C	A	NA	*OreG0012715*
scaffold33113	59971	C	T	NA	*OreG0012717*
scaffold33113	203636	C	A	Och01,Och02,Och03,Och04,Och05,Och06,Och07,Och08,Och09,Och10,Och11,Och12,Och13,Och14	*OreG0012726*
scaffold33113	222154	C	T	NA	*OreG0012729*
scaffold33113	278473	G	T	Och02,Och03,Och04,Och05,Och06,Och08,Och09,Och10,Och11,Och12,Och13	*OreG0012734*
scaffold33113	278561	C	A	Och02,Och03,Och04,Och05,Och06,Och08,Och09,Och10,Och11,Och12,Och13	*OreG0012734*
scaffold33113	304496	C	A	NA	*OreG0012737*

scaffold33113	945067	C	T	Och12	*OreG0012779*
scaffold33113	1000274	G	T	Och01,Och02,Och03,Och04,Och05,Och06,Och07,Och08,Och09,Och10,Och11,Och12,Och13,Och14	*OreG0012786*
scaffold33113	1050142	C	A	Och01,Och02,Och03,Och04,Och05,Och06,Och07,Och08,Och09,Och10,Och11,Och12,Och13,Och14,Ore01,Ore02,Ore03,Ore04,Ore05,Ore06,Ore07,Ore08,Ore09,Ore10,Ore11,Ore12,Ore13,Ore14	*OreG0012791*
scaffold33113	1176711	A	T	Och01,Och02,Och03,Och04,Och05,Och06,Och07,Och08,Och09,Och10,Och11,Och12,Och13,Och14	*OreG0012803*
scaffold33113	1276926	C	T	Och01,Och02,Och03,Och04,Och05,Och06,Och07,Och08,Och09,Och10,Och11,Och12,Och13,Och14,Ore01,Ore02,Ore03,Ore04,Ore05,Ore06,Ore07,Ore08,Ore09,Ore10,Ore11,Ore12,Ore13,Ore14	*OreG0012812*
scaffold33113	1292504	A	T	NA	*OreG0012814*
scaffold33113	1376692	C	T	Och01,Och02,Och03,Och04,Och05,Och06,Och07,Och08,Och09,Och10,Och11,Och12,Och13,Och14	*OreG0012822*
scaffold33113	1642966	G	T	NA	*OreG0012843*
scaffold33113	1786403	C	A	Och01,Och02,Och03,Och04,Och05,Och06,Och07,Och08,Och09,Och10,Och11,Och12,Och13,Och14	*OreG0012853*
scaffold33113	1825245	G	A	Ore01,Ore02,Ore03,Ore04,Ore05,Ore06,Ore07,Ore08,Ore09,Ore10,Ore11,Ore12,Ore13,Ore14	*OreG0012858*
scaffold33113	1889634	G	T	NA	*OreG0012871*
scaffold33113	1891333	G	A	NA	*OreG0012872*
scaffold33113	1913956	G	A	NA	*OreG0012879*
scaffold33113	1927855	G	A	NA	*OreG0012882*
scaffold33113	2155727	G	T	NA	*OreG0012906*
scaffold33113	2415250	C	A	Och01,Och02,Och03,Och04,Och05,Och06,Och07,Och08,Och09,Och10,Och11,Och12,Och13,Och14,Ore01,Ore02,Ore03,Ore04,Ore05,Ore06,Ore07,Ore08,Ore09,Ore10,Ore11,Ore12,Ore13,Ore14	*OreG0012929*
scaffold33113	2440966	T	A	Och01,Och02,Och03,Och04,Och05,Och06,Och07,Och08,Och09,Och10,Och11,Och12,Och13,Och14,Ore01,Ore02,Ore03,Ore04,Ore05,Ore06,Ore07,Ore08,Ore09,Ore10,Ore11,Ore12,Ore13,Ore14	*OreG0012933*
scaffold33147	27912	T	A	Och07,Och14	*OreG0014009*
scaffold33147	171299	A	T	NA	*OreG0014019*
scaffold33147	310464	A	T	Ore03,Ore08,Ore09,Ore10,Ore14	*OreG0014025*
scaffold33147	379863	G	A	Ore01,Ore02,Ore03,Ore04,Ore05,Ore06,Ore07,Ore08,Ore09,Ore10,Ore11,Ore12,Ore13,Ore14	*OreG0014031*
scaffold33147	423819	G	A	Och01,Och02,Och03,Och04,Och05,Och06,Och07,Och08,Och09,Och10,Och11,Och12,Och13	*OreG0014036*
scaffold33147	466704	A	T	NA	*OreG0014040*
scaffold33147	795591	G	C	Och01,Och02,Och03,Och04,Och05,Och06,Och07,Och08,Och09,Och10,Och11,Och12,Och13,Och14,Ore01,Ore02,Ore03,Ore04,Ore05,Ore06,Ore07,Ore08,Ore09,Ore10,Ore11,Ore12,Ore13,Ore14	*OreG0014054*
scaffold33147	981203	G	A	Och01,Och02,Och03,Och04,Och05,Och06,Och07,Och08,Och09,Och10,Och11,Och12,Och13,Och14	*OreG0014069*
scaffold33147	1039232	C	A	NA	*OreG0014073*
scaffold33147	1565486	C	T	Ore01,Ore02,Ore03,Ore04,Ore05,Ore06,Ore07,Ore08,Ore09,Ore10,Ore11,Ore12,Ore13,Ore14	*OreG0014108*
scaffold33147	1565725	C	T	Och01	*OreG0014108*
scaffold33147	1849300	C	T	Ore04,Ore05	*OreG0014131*
scaffold33147	1934334	T	A	Och02,Och04,Och06,Och07,Och09,Och10,Och11,Och14	*OreG0014139*
scaffold33147	2070942	G	A	Och08	*OreG0014146*
scaffold31892	336872	G	T	NA	*OreG0009933*
scaffold31892	422077	A	C	NA	*OreG0009937*
scaffold31892	525415	A	C	Och01,Och02,Och03,Och04,Och05,Och06,Och07,Och08,Och09,Och10,Och11,Och12,Och13,Och14	*OreG0009941*
scaffold31892	641025	C	T	Ore01,Ore02,Ore03,Ore04,Ore05,Ore06,Ore07,Ore08,Ore09,Ore10,Ore11,Ore12,Ore13,Ore14	*OreG0009951*
scaffold31892	708185	A	C	NA	*OreG0009957*
scaffold31892	813843	A	T	NA	*OreG0009972*
scaffold31892	921470	C	T	Och02,Och03,Och04,Och05,Och06,Och08,Och09,Och10,Och11,Och12,Och13,Och14	*OreG0009980*
scaffold31892	1001450	C	T	NA	*OreG0009987*
scaffold31892	1163048	C	A	NA	*OreG0010000*
scaffold31892	1163151	T	A	NA	*OreG0010000*
scaffold31892	1169995	G	T	Och01,Och02,Och03,Och04,Och05,Och06,Och07,Och08,Och09,Och10,Och11,Och12,Och13,Och14	*OreG0010001*
scaffold31892	1232038	T	A	NA	*OreG0010010*
scaffold31892	1261903	C	G	NA	*OreG0010012*
scaffold31892	1270984	C	T	NA	*OreG0010013*
scaffold31892	1562924	C	A	Och01,Och02,Och03,Och04,Och05,Och06,Och07,Och08,Och09,Och10,Och11,Och12,Och13,Och14,Ore01,Ore02,Ore03,Ore04,Ore05,Ore06,Ore07,Ore08,Ore09,Ore10,Ore11,Ore12,Ore13,Ore14	*OreG0010039*
scaffold31892	1606907	C	G	Och01,Och02,Och03,Och04,Och05,Och06,Och07,Och08,Och09,Och10,Och11,Och12,Och13,Och14	*OreG0010049*
scaffold31892	1607709	T	G	Och01,Och02,Och03,Och04,Och05,Och06,Och07,Och08,Och09,Och10,Och11,Och12,Och13,Och14	*OreG0010049*

scaffold31892	1654238	C	T	Och01,Och02,Och03,Och04,Och05,Och06,Och07,Och08,Och09,Och10,Och11,Och12,Och13,Och14	*OreG0010050*
scaffold31892	1654361	C	T	NA	*OreG0010050*
scaffold31892	1654367	C	T	NA	*OreG0010050*
scaffold31892	1655053	C	T	Ore01,Ore02,Ore03,Ore04,Ore05,Ore06,Ore07,Ore08,Ore09,Ore10,Ore11,Ore12,Ore13,Ore14	*OreG0010050*
scaffold31892	1703283	G	A	NA	*OreG0010056*
scaffold31892	1711425	C	A	Och12,Och13	*OreG0010057*
scaffold31892	1719767	G	A	NA	*OreG0010058*
scaffold31892	1719980	C	A	NA	*OreG0010058*
scaffold31892	1736149	G	A	NA	*OreG0010060*
scaffold31892	1736239	C	A	NA	*OreG0010060*
scaffold31892	1736933	C	T	NA	*OreG0010060*
scaffold31892	1736936	A	T	NA	*OreG0010060*
scaffold31892	1742515	T	A	NA	*OreG0010061*
scaffold31892	1743136	G	A	NA	*OreG0010061*
scaffold31892	1750460	T	A	NA	*OreG0010062*
scaffold31892	1754266	G	A	NA	*OreG0010062*
scaffold31892	1754484	G	A	NA	*OreG0010062*
scaffold31892	1754559	G	A	NA	*OreG0010062*
scaffold31892	1754598	G	A	NA	*OreG0010062*
scaffold31892	1756562	C	A	NA	*OreG0010062*
scaffold31892	1869237	C	T	Ore01,Ore02,Ore03,Ore04,Ore05,Ore06,Ore07,Ore08,Ore09,Ore10,Ore11,Ore12,Ore13,Ore14	*OreG0010071*
scaffold31892	1899332	G	A	NA	*OreG0010073*
scaffold31892	2034141	C	T	Och01,Och02,Och03,Och04,Och06,Och07,Och08,Och09,Och10,Och11,Och14	*OreG0010090*
scaffold31892	2035177	G	T	Och12,Och13	*OreG0010090*
scaffold31892	2285421	A	C	NA	*OreG0010108*
scaffold31892	2330610	C	T	NA	*OreG0010114*
scaffold31892	2568839	G	A	NA	*OreG0010137*
scaffold31892	2628945	T	A	Och01,Och02,Och03,Och04,Och05,Och06,Och07,Och08,Och09,Och10,Och11,Och12,Och13,Och14,Ore01,Ore02,Ore03,Ore04,Ore05,Ore06,Ore07,Ore08,Ore09,Ore10,Ore11,Ore12,Ore13,Ore14	*OreG0010142*
scaffold31892	2629054	C	T	Och01,Och02,Och03,Och04,Och05,Och06,Och07,Och09,Och10,Och11,Och12,Och13	*OreG0010142*
scaffold31892	2673471	G	A	NA	*OreG0010148*
scaffold31892	2677232	C	T	Och02,Och05,Och09,Och10,Och11,Och12,Och13	*OreG0010149*
scaffold31892	2677345	G	A	Och02,Och05,Och09,Och10,Och11,Och12,Och13	*OreG0010150*
scaffold31892	2679622	C	T	NA	*OreG0010150*
scaffold31892	2768158	T	G	NA	*OreG0010161*
scaffold31892	2932288	C	T	NA	*OreG0010171*
scaffold31892	2996660	G	A	Ore01,Ore02,Ore03,Ore04,Ore05,Ore06,Ore07,Ore08,Ore09,Ore10,Ore11,Ore12,Ore13,Ore14	*OreG0010177*
scaffold31892	3089112	C	A	Och12,Och13	*OreG0010183*
scaffold31892	4097674	C	T	Och01,Och02,Och03,Och04,Och05,Och06,Och07,Och08,Och09,Och10,Och11,Och12,Och13,Och14	*OreG0010212*
scaffold33218	135146	A	T	Och01,Och02,Och03,Och04,Och05,Och06,Och07,Och08,Och09,Och10,Och11,Och12,Och13,Och14,Ore01,Ore02,Ore03,Ore04,Ore05,Ore06,Ore07,Ore08,Ore09,Ore10,Ore11,Ore12,Ore13,Ore14	*OreG0016650*
scaffold33218	190869	T	G	NA	*OreG0016657*
scaffold33218	190881	C	A	Och09,Och10	*OreG0016657*
scaffold33218	299377	C	A	Och14	*OreG0016667*
scaffold33218	365965	G	A	NA	*OreG0016673*
scaffold33218	365977	G	A	NA	*OreG0016673*
scaffold33218	548695	C	T	Och01,Och02,Och03,Och04,Och05,Och06,Och07,Och08,Och09,Och10,Och11,Och12,Och13,Och14	*OreG0016700*
scaffold33218	790285	C	A	NA	*OreG0016734*
scaffold33218	834143	A	T	Och01,Och02,Och03,Och04,Och05,Och06,Och07,Och08,Och09,Och10,Och11,Och12,Och13,Och14	*OreG0016740*
scaffold33249	53068	C	T	NA	*OreG0017593*
scaffold33249	54002	C	T	Och01,Och02,Och03,Och04,Och05,Och06,Och07,Och08,Och09,Och10,Och11,Och12,Och13,Och14	*OreG0017594*
scaffold33249	59667	A	T	Och12,Och13	*OreG0017596*
scaffold33258	466	G	T	NA	*OreG0017919*
scaffold33258	47503	G	A	NA	*OreG0017925*
scaffold33258	103927	G	A	Ore04,Ore05,Ore06,Ore07,Ore08,Ore14	*OreG0017932*
scaffold33258	154610	A	T	Ore01,Ore02,Ore03,Ore04,Ore05,Ore06,Ore07,Ore08,Ore09,Ore10,Ore11,Ore12,Ore13,Ore14	*OreG0017939*
scaffold33258	154650	C	A	Och12	*OreG0017939*
scaffold33258	154720	A	T	NA	*OreG0017939*
scaffold33258	154742	A	T	Och02,Och03,Och04,Och05,Och06,Och07,Och08,Och12,Och13,Och14	*OreG0017939*
scaffold33258	156067	C	A	NA	*OreG0017939*
scaffold33258	156393	G	C	NA	*OreG0017939*
scaffold33258	175838	C	T	Ore04,Ore05	*OreG0017942*

scaffold33258	188880	C	T	Och01,Och02,Och03,Och04,Och05,Och06,Och07,Och08,Och09,Och10,Och11,Och12,Och13,Och14,Ore01,Ore02,Ore03,Ore04,Ore05,Ore06,Ore07,Ore08,Ore09,Ore10,Ore11,Ore12,Ore13,Ore14	*OreG0017944*
scaffold33233	161301	G	T	NA	*OreG0017193*
scaffold33233	290067	C	A	Och01,Och02,Och03,Och04,Och05,Och06,Och08,Och09,Och10,Och11,Och12,Och13,Och14	*OreG0017199*
scaffold33245	55758	C	T	Och07,Och09,Och10,Och14	*OreG0017421*
scaffold33245	55889	G	A	NA	*OreG0017421*
scaffold33245	56432	C	T	NA	*OreG0017422*
scaffold33245	56510	C	T	Och14	*OreG0017422*
scaffold33245	156980	G	A	Och01,Och02,Och04,Och06,Och08,Och09,Och10,Och11,Och14	*OreG0017432*
scaffold33245	157046	T	A	Och01,Och02,Och04,Och06,Och08,Och09,Och10,Och11,Och14	*OreG0017432*
scaffold33245	251139	C	T	Ore04,Ore05	*OreG0017437*
scaffold3100	70733	C	T	Och02,Och05,Och06,Och13	*OreG0009088*
scaffold3100	70741	G	A	Och02,Och05,Och06,Och13	*OreG0009088*
scaffold3100	146531	G	A	NA	*OreG0009099*
scaffold3100	147092	G	A	Och14	*OreG0009099*
scaffold3100	156791	C	A	NA	*OreG0009101*
scaffold3100	163482	T	A	Ore01,Ore02,Ore03,Ore04,Ore05,Ore06,Ore07,Ore08,Ore09,Ore10,Ore11,Ore12,Ore13,Ore14	*OreG0009102*
scaffold3100	169178	G	T	NA	*OreG0009103*
scaffold3100	347617	C	T	Och03,Och04,Och05,Och06,Och07,Och08,Och09,Och10,Och11,Och12,Och13,Och14	*OreG0009126*
scaffold3100	405344	G	T	Och03,Och04,Och06,Och12	*OreG0009133*
scaffold3100	421725	C	T	Och06	*OreG0009137*
scaffold3100	421939	T	A	Och07,Och08,Och11	*OreG0009137*
scaffold3100	422265	G	T	NA	*OreG0009137*
scaffold3100	445128	C	T	NA	*OreG0009139*
scaffold3100	457684	C	T	Och01,Och02,Och05,Och06,Och07,Och09,Och10,Och11,Och12,Och13	*OreG0009142*
scaffold3100	484662	G	T	NA	*OreG0009145*
scaffold3100	564483	T	A	Och02,Och03,Och04,Och05,Och06,Och07,Och08,Och09,Och10,Och11,Och12,Och13,Och14	*OreG0009158*
scaffold3100	737087	C	A	Och01,Och02,Och03,Och04,Och05,Och06,Och07,Och08,Och09,Och10,Och11,Och12,Och13,Och14	*OreG0009175*
scaffold3100	789750	G	T	Och06	*OreG0009183*
scaffold3100	1164945	C	A	Och01,Och02,Och03,Och04,Och05,Och06,Och07,Och08,Och11,Och12,Och13	*OreG0009199*
scaffold3100	1852324	C	T	Och01,Och02,Och03,Och04,Och05,Och06,Och07,Och08,Och09,Och10,Och11,Och12,Och13,Och14	*OreG0009214*
scaffold3100	2340171	C	T	NA	*OreG0009220*
scaffold3100	2565164	G	A	NA	*OreG0009222*
scaffold3100	2568101	T	A	Ore01,Ore02,Ore03,Ore04,Ore05,Ore06,Ore07,Ore08,Ore09,Ore10,Ore11,Ore12,Ore13,Ore14	*OreG0009222*
scaffold3100	3088434	G	A	Och02,Och03,Och06,Och14	*OreG0009240*
scaffold3100	3519155	C	T	NA	*OreG0009246*
scaffold3100	4526188	G	A	Ore11	*OreG0009273*
scaffold3100	4527449	G	A	NA	*OreG0009273*
scaffold3100	5083943	T	G	Ore03,Ore04,Ore05,Ore09,Ore13	*OreG0009296*
scaffold3100	5083988	T	A	Och03,Och04	*OreG0009296*
scaffold3100	5159141	T	A	Ore03,Ore04,Ore05,Ore09,Ore13	*OreG0009299*
scaffold3100	5575578	C	A	NA	*OreG0009318*
scaffold3100	5610259	G	A	NA	*OreG0009322*
scaffold3100	5825240	A	T	Och01,Och07,Och08,Och09,Och10,Och11,Och14	*OreG0009333*
scaffold3100	5852089	G	A	Ore01,Ore02,Ore03,Ore04,Ore05,Ore06,Ore07,Ore08,Ore09,Ore10,Ore11,Ore12,Ore13,Ore14	*OreG0009335*
scaffold3100	5908150	C	T	NA	*OreG0009337*
scaffold3100	5985093	G	T	Och02,Och12,Och13	*OreG0009341*
scaffold3100	6189451	C	T	NA	*OreG0009349*
scaffold3100	6321306	G	A	Och01,Och02,Och03,Och04,Och05,Och06,Och07,Och08,Och09,Och10,Och11,Och12,Och13,Och14	*OreG0009355*
scaffold33237	104464	G	T	NA	*OreG0017287*
scaffold33237	200175	C	T	NA	*OreG0017297*
scaffold33237	211831	C	A	Och01	*OreG0017298*
scaffold33197	452175	C	T	Och01,Och14	*OreG0015289*
scaffold33197	620025	G	C	Ore01,Ore02,Ore03,Ore04,Ore05,Ore06,Ore07,Ore08,Ore09,Ore10,Ore11,Ore12,Ore13,Ore14	*OreG0015299*
scaffold33197	854797	A	T	NA	*OreG0015313*
scaffold33197	897893	G	A	Och01,Och02,Och03,Och04,Och05,Och06,Och07,Och08,Och09,Och10,Och11,Och12,Och13,Och14	*OreG0015316*
scaffold33240	5880	G	A	Ore01,Ore02,Ore03,Ore04,Ore05,Ore06,Ore07,Ore08,Ore09,Ore11,Ore12,Ore13,Ore14	*OreG0017355*
scaffold33240	22004	G	T	NA	*OreG0017358*
scaffold33240	343478	C	T	Och04	*OreG0017383*
scaffold33240	434120	C	T	NA	*OreG0017391*
scaffold33209	82800	T	A	Och02,Och03,Och04,Och05,Och06,Och12,Och13,Och14	*OreG0016244*
scaffold33209	97262	T	A	Och02,Och04,Och06,Och07,Och08,Och11,Och14	*OreG0016249*

scaffold33209	98990	C	A	NA	*OreG0016250*
scaffold33209	167827	G	T	NA	*OreG0016255*
scaffold33209	180362	G	A	Och01,Och02,Och03,Och04,Och05,Och06,Och07,Och08,Och09,Och10,Och11,Och12,Och13,Och14	*OreG0016257*
scaffold33209	247739	C	G	NA	*OreG0016260*
scaffold33209	261944	A	C	NA	*OreG0016263*
scaffold33209	754082	T	A	Och04	*OreG0016287*
scaffold33204	266903	C	T	NA	*OreG0015869*
scaffold33204	783340	G	A	Ore02,Ore03,Ore04,Ore05,Ore06,Ore07,Ore08,Ore09,Ore10,Ore11,Ore12,Ore14	*OreG0015885*
scaffold33269	63697	G	A	NA	*OreG0018152*
scaffold33266	10900	C	T	NA	*OreG0018132*
scaffold33266	11326	G	A	NA	*OreG0018132*
scaffold33261	33983	G	T	NA	*OreG0017969*
scaffold33207	78509	C	A	Och01,Och02,Och03,Och04,Och05,Och06,Och12,Och13	*OreG0015920*
scaffold33207	78826	C	T	NA	*OreG0015920*
scaffold33207	79726	C	T	Ore02,Ore09,Ore10	*OreG0015921*
scaffold33207	264754	C	T	NA	*OreG0015937*
scaffold33207	266741	C	A	NA	*OreG0015938*
scaffold33207	266777	G	A	Och02,Och04,Och06,Och07,Och08,Och11,Och12,Och13,Och14,Ore01,Ore02,Ore03,Ore04,Ore05,Ore06,Ore07,Ore09,Ore10,Ore11,Ore12,Ore13,Ore14	*OreG0015938*
scaffold33207	267477	T	A	NA	*OreG0015938*
scaffold33207	268437	C	A	NA	*OreG0015938*
scaffold33207	269617	C	A	NA	*OreG0015938*
scaffold33207	269629	G	A	NA	*OreG0015938*
scaffold33207	270754	G	A	NA	*OreG0015938*
scaffold33207	272668	C	A	NA	*OreG0015938*
scaffold33207	272701	C	A	NA	*OreG0015938*
scaffold33207	368218	G	T	Och01,Och02,Och03,Och04,Och05,Och06,Och07,Och08,Och09,Och10,Och11,Och12,Och13,Och14,Ore01,Ore02,Ore03,Ore04,Ore05,Ore06,Ore07,Ore08,Ore09,Ore10,Ore11,Ore12,Ore13,Ore14	*OreG0015951*
scaffold33207	540026	C	T	NA	*OreG0015971*
scaffold33207	553317	G	A	Ore01,Ore02,Ore03,Ore04,Ore05,Ore06,Ore07,Ore08,Ore09,Ore10,Ore11,Ore12,Ore13,Ore14	*OreG0015973*
scaffold33207	605793	C	T	NA	*OreG0015980*
scaffold33207	622314	C	A	NA	*OreG0015982*
scaffold33207	738392	C	A	Och02,Och03,Och04,Och05,Och06,Och07,Och09,Och10,Och11,Och12,Och13,Och14	*OreG0015989*
scaffold33207	909098	A	T	Och09,Och10,Och12,Och13	*OreG0016014*
scaffold33207	1152371	C	T	NA	*OreG0016052*
scaffold33207	1160860	T	G	Ore01,Ore02,Ore03,Ore04,Ore05,Ore06,Ore07,Ore08,Ore09,Ore10,Ore11,Ore12,Ore13,Ore14	*OreG0016053*
scaffold33207	1169454	C	A	Och12,Och13	*OreG0016056*
scaffold33207	1169474	G	T	Och01,Och02,Och03,Och04,Och05,Och06,Och07,Och08,Och09,Och10,Och11,Och12,Och13,Och14,Ore01,Ore02,Ore03,Ore04,Ore05,Ore06,Ore07,Ore08,Ore09,Ore10,Ore11,Ore12,Ore13,Ore14	*OreG0016056*
scaffold33207	1280539	G	T	Och01,Och02,Och03,Och04,Och05,Och06,Och07,Och08,Och09,Och10,Och11,Och12,Och13,Och14	*OreG0016076*
scaffold33207	1285323	G	A	Ore04,Ore05,Ore08	*OreG0016078*
scaffold33207	1418436	C	T	NA	*OreG0016095*
scaffold33207	1435400	C	T	Ore01,Ore02,Ore03,Ore04,Ore05,Ore06,Ore07,Ore08,Ore09,Ore10,Ore11,Ore12,Ore13,Ore14	*OreG0016099*
scaffold33207	1492742	C	T	NA	*OreG0016108*
scaffold33207	1662590	G	A	Och06	*OreG0016130*
scaffold33207	1662591	G	A	Och06	*OreG0016130*
scaffold33207	1725430	G	A	Och09,Och10	*OreG0016140*
scaffold33207	1855517	C	A	Ore04,Ore05,Ore08	*OreG0016150*
scaffold33207	1863221	G	A	NA	*OreG0016151*
scaffold33207	1994209	T	G	Ore01,Ore02,Ore03,Ore04,Ore05,Ore06,Ore07,Ore08,Ore09,Ore10,Ore11,Ore12,Ore13,Ore14	*OreG0016165*
scaffold33207	2046973	G	A	Ore01,Ore02,Ore03,Ore04,Ore05,Ore06,Ore07,Ore08,Ore09,Ore10,Ore11,Ore12,Ore13,Ore14	*OreG0016172*
scaffold33207	2112310	G	T	Och01	*OreG0016180*
scaffold2623	72090	A	T	NA	*OreG0007832*
scaffold33275	19368	C	T	NA	*OreG0018258*
scaffold33267	36029	A	T	Och02,Och03,Och04,Och06,Och09,Och10,Och12,Och13,Ore03,Ore04,Ore05,Ore10	*OreG0018138*
scaffold33267	40197	A	C	Ore01,Ore08,Ore09,Ore11,Ore12	*OreG0018138*
scaffold33267	40207	G	A	Och02,Och03,Och04,Och05,Och06,Och09,Och10,Och12,Och13,Ore03,Ore04,Ore05,Ore10	*OreG0018138*
scaffold33283	85125	C	A	NA	*OreG0018423*
scaffold33283	85826	A	C	NA	*OreG0018423*
scaffold33198	333678	T	A	Och01,Och02,Och03,Och04,Och05,Och06,Och07,Och08,Och09,Och10,Och11,Och12,Och13,Och14,Ore01,Ore02,Ore03,Ore04,Ore05,Ore06,Ore07,Ore08,Ore09,Ore10,Ore11,Ore12,Ore13,Ore14	*OreG0015350*
scaffold33198	412264	A	T	Ore01,Ore02,Ore03,Ore04,Ore05,Ore06,Ore07,Ore08,Ore09,Ore10,Ore11,Ore12,Ore13,Ore14	*OreG0015354*

scaffold33198	595330	T	A	NA	*OreG0015369*
scaffold33198	704696	G	T	NA	*OreG0015381*
scaffold33198	727530	C	A	NA	*OreG0015383*
scaffold33198	752825	G	T	Och01,Och02,Och03,Och04,Och05,Och06,Och07,Och08,Och09,Och10,Och11,Och12,Och13,Och14,Ore01,Ore02,Ore03,Ore04,Ore05,Ore06,Ore07,Ore08,Ore09,Ore10,Ore11,Ore12,Ore13,Ore14	*OreG0015387*
scaffold33198	861061	G	A	NA	*OreG0015388*
scaffold33198	861789	A	T	NA	*OreG0015388*
scaffold33198	976672	T	A	NA	*OreG0015396*
scaffold33198	986800	C	A	NA	*OreG0015397*
scaffold33198	988774	G	T	Ore02,Ore04,Ore05,Ore08,Ore09,Ore11,Ore12,Ore14	*OreG0015397*
scaffold33198	1139434	C	T	NA	*OreG0015418*
scaffold33234	33174	G	A	NA	*OreG0017220*
scaffold33254	377074	C	T	NA	*OreG0017897*
scaffold33254	395599	G	A	NA	*OreG0017898*
scaffold33254	396016	C	T	Och01,Och02,Och03,Och04,Och05,Och06,Och07,Och08,Och09,Och10,Och11,Och12,Och13,Och14	*OreG0017898*
scaffold33265	15602	G	A	NA	*OreG0018123*
scaffold33265	38799	T	A	NA	*OreG0018126*
scaffold33253	758592	C	A	NA	*OreG0017856*
scaffold33253	1011061	G	A	Ore01,Ore02,Ore03,Ore04,Ore05,Ore06,Ore07,Ore08,Ore09,Ore10,Ore11,Ore12,Ore13,Ore14	*OreG0017882*
scaffold33284	33050	C	T	NA	*OreG0018432*
scaffold33280	3480	C	T	NA	*OreG0018415*
scaffold33279	102503	A	C	Och01,Och02,Och03,Och04,Och05,Och06,Och07,Och08,Och09,Och10,Och11,Och12,Och13,Och14,Ore01,Ore02,Ore03,Ore04,Ore05,Ore06,Ore07,Ore08,Ore09,Ore10,Ore11,Ore12,Ore13,Ore14	*OreG0018382*
scaffold33279	152131	C	A	NA	*OreG0018385*
scaffold33279	232240	C	T	NA	*OreG0018394*
scaffold33279	234009	C	T	Ore02,Ore03,Ore09,Ore10,Ore11,Ore12,Ore14	*OreG0018395*
scaffold33279	239629	C	A	NA	*OreG0018396*
scaffold33279	276930	C	T	Och01,Och12	*OreG0018398*
scaffold33279	289245	C	T	NA	*OreG0018399*
scaffold33279	356617	G	A	NA	*OreG0018408*
scaffold33279	356839	G	A	NA	*OreG0018408*
scaffold33279	365512	G	A	NA	*OreG0018410*
scaffold33247	269708	G	A	Och01,Och02,Och03,Och04,Och05,Och06,Och08,Och12,Och13,Och14	*OreG0017473*
scaffold33247	378170	G	A	Och01,Och02,Och03,Och04,Och05,Och06,Och07,Och08,Och09,Och10,Och11,Och12,Och13,Och14,Ore01,Ore02,Ore03,Ore04,Ore05,Ore06,Ore07,Ore08,Ore09,Ore10,Ore11,Ore12,Ore13,Ore14	*OreG0017480*
scaffold33247	378194	G	A	Och02,Och03,Och04,Och05,Och06,Och07,Och09,Och10,Och11,Och12,Och13,Och14	*OreG0017480*
scaffold33247	378201	G	T	Och01,Och02,Och03,Och04,Och05,Och06,Och07,Och08,Och09,Och10,Och11,Och12,Och13,Och14	*OreG0017480*
scaffold33247	659635	A	C	NA	*OreG0017515*
scaffold33247	662323	A	T	Och01,Och02,Och03,Och04,Och05,Och06,Och07,Och08,Och09,Och10,Och11,Och12,Och13,Och14,Ore01,Ore02,Ore03,Ore04,Ore05,Ore06,Ore07,Ore08,Ore09,Ore10,Ore11,Ore12,Ore13,Ore14	*OreG0017516*
scaffold33247	663081	C	T	Ore01	*OreG0017516*
scaffold33247	673021	C	T	NA	*OreG0017519*
scaffold33247	722595	A	T	Och01	*OreG0017523*
scaffold33247	767901	C	A	NA	*OreG0017528*
scaffold33264	1674	C	T	NA	*OreG0018009*
scaffold33264	139571	G	A	Och02,Och03,Och04,Och05,Och12,Och13	*OreG0018029*
scaffold33264	260378	A	C	Ore01,Ore02,Ore03,Ore04,Ore05,Ore06,Ore07,Ore08,Ore09,Ore10,Ore11,Ore12,Ore13,Ore14	*OreG0018049*
scaffold33264	271794	T	A	Och02,Och09,Och10,Och12	*OreG0018052*
scaffold33264	546566	C	A	Och05,Och13	*OreG0018085*
scaffold33264	703651	C	T	NA	*OreG0018101*
scaffold33264	758986	T	A	Och02,Och06,Och12	*OreG0018110*
scaffold33264	759357	C	T	Ore06,Ore08	*OreG0018110*
scaffold33239	328716	C	T	Och01,Och02,Och03,Och04,Och05,Och06,Och07,Och08,Och09,Och10,Och11,Och12,Och13,Och14	*OreG0017331*
scaffold33229	69965	C	T	NA	*OreG0017104*
scaffold33229	168990	A	C	Och01,Och02,Och03,Och04,Och05,Och06,Och07,Och08,Och09,Och10,Och11,Och12,Och13	*OreG0017118*
scaffold33229	250300	G	A	Och02,Och03,Och04,Och05,Och06,Och07,Och08,Och09,Och10,Och11,Och12,Och13	*OreG0017121*
scaffold33229	320546	G	T	Och02,Och05,Och07,Och08,Och09,Och10,Och11,Och12,Och13,Och14	*OreG0017129*
scaffold33229	321330	G	A	NA	*OreG0017130*
scaffold33229	418450	C	A	Och01,Och02,Och03,Och04,Och05,Och06,Och07,Och08,Och09,Och10,Och11,Och12,Och13,Och14,Ore01,Ore02,Ore03,Ore04,Ore05,Ore06,Ore07,Ore08,Ore09,Ore10,Ore11,Ore12,Ore13,Ore14	*OreG0017139*
scaffold33229	438087	G	A	NA	*OreG0017145*
scaffold33229	448210	C	T	NA	*OreG0017147*

scaffold33229	459311	C	T	NA	*OreG0017150*
scaffold33229	467585	C	T	NA	*OreG0017151*
scaffold33229	533012	A	T	Ore01,Ore02,Ore03,Ore04,Ore05,Ore06,Ore07,Ore08,Ore09,Ore10,Ore11,Ore12,Ore13,Ore14	*OreG0017160*
scaffold33229	546347	C	T	Och14	*OreG0017164*
scaffold33229	619051	T	A	NA	*OreG0017172*
scaffold33211	186487	G	A	NA	*OreG0016345*
scaffold33211	202459	C	T	Och01,Och02,Och03,Och04,Och05,Och06,Och07,Och08,Och09,Och10,Och11,Och12,Och13,Och14	*OreG0016347*
scaffold33211	204359	C	T	Och01,Och02,Och03,Och04,Och05,Och06,Och07,Och08,Och09,Och10,Och11,Och12,Och13,Och14,Ore01,Ore02,Ore03,Ore04,Ore05,Ore06,Ore07,Ore08,Ore09,Ore10,Ore11,Ore12,Ore13,Ore14	*OreG0016347*
scaffold33211	380295	A	T	Ore06,Ore07,Ore10,Ore14	*OreG0016356*
scaffold33211	677270	A	T	NA	*OreG0016377*
scaffold33211	685402	A	T	Och01,Och02,Och03,Och04,Och05,Och06,Och07,Och09,Och10,Och11,Och12,Och13,Och14	*OreG0016378*
scaffold33211	1123791	G	A	Och12,Och13	*OreG0016388*
scaffold33200	35880	T	G	Och01,Och02,Och03,Och04,Och05,Och06,Och08,Och09,Och10,Och11,Och12,Och13	*OreG0015671*
scaffold33200	85442	A	T	NA	*OreG0015674*
scaffold33200	174493	G	A	NA	*OreG0015680*
scaffold33200	174660	G	A	NA	*OreG0015680*
scaffold33200	177329	G	A	NA	*OreG0015681*
scaffold33200	181090	C	A	NA	*OreG0015682*
scaffold33200	211898	G	A	NA	*OreG0015684*
scaffold33200	226473	A	T	NA	*OreG0015685*
scaffold33200	257200	C	A	NA	*OreG0015686*
scaffold33200	394220	C	T	Ore01,Ore02,Ore03,Ore04,Ore05,Ore06,Ore07,Ore08,Ore09,Ore10,Ore11,Ore12,Ore13,Ore14	*OreG0015695*
scaffold33200	504082	G	A	NA	*OreG0015701*
scaffold33200	623594	T	G	NA	*OreG0015710*
scaffold33200	754509	C	T	Ore01,Ore02,Ore03,Ore04,Ore05,Ore06,Ore07,Ore08,Ore09,Ore10,Ore11,Ore12,Ore13,Ore14	*OreG0015716*
scaffold33200	896204	T	G	Och01,Och09,Och10,Och11,Ore01,Ore02,Ore03,Ore04,Ore05,Ore06,Ore07,Ore08,Ore09,Ore10,Ore11,Ore12,Ore13,Ore14	*OreG0015732*
scaffold33200	929488	C	A	NA	*OreG0015735*
scaffold33200	1021434	C	A	NA	*OreG0015743*
scaffold33200	1085135	T	A	NA	*OreG0015751*
scaffold33200	1087329	C	A	NA	*OreG0015751*
scaffold33200	1089328	T	A	Och04,Och06,Och12,Och13	*OreG0015752*
scaffold33200	1187905	C	A	NA	*OreG0015768*
scaffold33200	1303966	A	T	NA	*OreG0015778*
scaffold33200	1315525	T	A	Och01,Och02,Och03,Och04,Och05,Och06,Och07,Och08,Och09,Och10,Och11,Och12,Och13,Och14	*OreG0015779*
scaffold33200	1542061	T	A	NA	*OreG0015790*
scaffold33200	1542622	A	T	Och07	*OreG0015790*
scaffold33200	1752841	C	T	Och01,Och02,Och03,Och04,Och05,Och06,Och07,Och08,Och09,Och10,Och11,Och12,Och13,Och14	*OreG0015806*
scaffold33200	2125988	C	T	Och01,Och02,Och03,Och04,Och05,Och06,Och07,Och08,Och09,Och10,Och11,Och12,Och13,Och14	*OreG0015811*
scaffold33200	3410185	C	T	NA	*OreG0015820*
scaffold33200	3410299	A	T	NA	*OreG0015820*
scaffold33200	3410404	C	T	Och02	*OreG0015820*
scaffold33290	62335	G	A	Och03,Och13	*OreG0018467*
scaffold33290	97782	C	A	NA	*OreG0018473*
scaffold33290	189300	G	T	NA	*OreG0018490*
scaffold33290	189630	C	A	NA	*OreG0018490*
scaffold33290	257086	G	A	NA	*OreG0018497*
scaffold33238	4924	C	A	NA	*OreG0017302*
scaffold33238	57215	G	T	NA	*OreG0017306*
scaffold33274	79111	C	T	Ore09,Ore13,Ore14	*OreG0018219*
scaffold33274	83815	C	T	NA	*OreG0018220*
scaffold33274	93282	A	T	Ore04,Ore05	*OreG0018222*
scaffold33274	219909	G	A	Ore04,Ore05	*OreG0018235*
scaffold33274	226935	C	T	Och11	*OreG0018236*
scaffold33274	227309	A	T	NA	*OreG0018236*
scaffold33274	244282	G	A	Ore09,Ore14	*OreG0018237*
scaffold33274	272779	C	T	NA	*OreG0018240*
scaffold33274	519625	A	T	Ore04,Ore05	*OreG0018256*
scaffold33193	238058	C	T	NA	*OreG0014762*
scaffold33193	430483	G	T	NA	*OreG0014782*
scaffold33193	430553	C	G	Och01,Och02,Och03,Och04,Och05,Och06,Och07,Och08,Och09,Och10,Och11,Och12,Och13,Och14,Ore01,Ore02,Ore03,Ore04,Ore05,Ore06,Ore07,Ore08,Ore09,Ore10,Ore11,Ore12,Ore13,Ore14	*OreG0014782*
scaffold33193	469361	G	A	NA	*OreG0014787*

scaffold33193	556423	C	A	NA	*OreG0014798*
scaffold33193	818663	G	A	NA	*OreG0014828*
scaffold33193	818732	G	A	NA	*OreG0014828*
scaffold33193	829542	G	T	NA	*OreG0014830*
scaffold33193	844937	T	A	Och01,Och02,Och03,Och04,Och05,Och06,Och07,Och08,Och09,Och10,Och11,Och12,Och13,Och14,Ore01,Ore02,Ore03,Ore04,Ore05,Ore06,Ore07,Ore08,Ore09,Ore10,Ore11,Ore12,Ore13,Ore14	*OreG0014832*
scaffold33193	866338	G	T	Ore02,Ore06,Ore07,Ore08,Ore09,Ore10,Ore11,Ore12,Ore13,Ore14	*OreG0014833*
scaffold33193	910569	G	A	Ore01,Ore02,Ore03,Ore04,Ore05,Ore06,Ore07,Ore08,Ore09,Ore10,Ore11,Ore12,Ore13,Ore14	*OreG0014837*
scaffold33193	913315	C	T	Och14	*OreG0014837*
scaffold33193	973855	C	T	Och01,Och02,Och03,Och04,Och05,Och06,Och07,Och08,Och12,Och13,Och14,Ore01,Ore02,Ore03,Ore04,Ore05,Ore06,Ore07,Ore08,Ore09,Ore10,Ore11,Ore12,Ore13,Ore14	*OreG0014847*
scaffold33193	1000254	T	A	NA	*OreG0014850*
scaffold33193	1036303	G	A	NA	*OreG0014852*
scaffold33193	1041664	G	A	NA	*OreG0014853*
scaffold33193	1241047	C	A	NA	*OreG0014875*
scaffold33193	1534907	C	A	NA	*OreG0014906*
scaffold33193	1619511	G	T	Och14	*OreG0014916*
scaffold33193	1623171	A	T	NA	*OreG0014917*
scaffold33193	1801399	C	T	NA	*OreG0014942*
scaffold33193	1865015	G	A	NA	*OreG0014948*
scaffold33193	1969519	C	T	Ore04,Ore05	*OreG0014960*
scaffold33193	2595856	G	A	NA	*OreG0015028*
scaffold33193	2599570	C	T	Och01	*OreG0015029*
scaffold33193	2702052	G	T	NA	*OreG0015037*
scaffold33193	2763691	G	A	NA	*OreG0015041*
scaffold33263	224565	C	A	NA	*OreG0017981*
scaffold33263	429795	T	A	NA	*OreG0017993*
scaffold33276	67702	C	T	NA	*OreG0018265*
scaffold33276	139640	G	A	NA	*OreG0018270*
scaffold33276	150221	G	T	Och01,Och02,Och03,Och04,Och05,Och06,Och07,Och08,Och09,Och10,Och11,Och12,Och13,Och14,Ore01,Ore02,Ore03,Ore04,Ore05,Ore06,Ore07,Ore08,Ore09,Ore10,Ore11,Ore12,Ore13,Ore14	*OreG0018271*
scaffold33276	314446	C	A	Och01,Och02,Och03,Och04,Och05,Och06,Och07,Och08,Och09,Och10,Och11,Och12,Och13,Och14,Ore01,Ore02,Ore03,Ore04,Ore05,Ore06,Ore07,Ore08,Ore09,Ore10,Ore11,Ore12,Ore13,Ore14	*OreG0018288*
scaffold33276	590068	A	T	NA	*OreG0018312*
scaffold33276	931002	T	A	Och09,Och10,Och14	*OreG0018341*
scaffold33276	1006099	G	A	Och01,Och03,Och14	*OreG0018348*
scaffold33276	1092265	A	T	NA	*OreG0018355*
scaffold33196	83422	C	T	Ore01,Ore02,Ore03,Ore04,Ore05,Ore06,Ore07,Ore08,Ore09,Ore10,Ore11,Ore12,Ore13,Ore14	*OreG0015160*
scaffold33196	112237	G	A	Och11	*OreG0015161*
scaffold33196	137857	A	T	NA	*OreG0015162*
scaffold33196	161895	A	T	Och05,Och11,Och13	*OreG0015165*
scaffold33196	161974	C	A	Och01,Och02,Och03,Och04,Och05,Och06,Och07,Och08,Och09,Och10,Och11,Och12,Och13,Och14	*OreG0015165*
scaffold33196	162289	G	A	Och01,Och02,Och03,Och04,Och05,Och06,Och07,Och08,Och09,Och10,Och11,Och12,Och13,Och14	*OreG0015165*
scaffold33196	176177	C	A	Och06,Och14	*OreG0015171*
scaffold33196	176537	A	T	Och05,Och11,Och13	*OreG0015171*
scaffold33196	176559	G	C	Och06,Och07,Och14	*OreG0015171*
scaffold33196	244126	T	A	Och01,Och02,Och03,Och04,Och05,Och06,Och07,Och08,Och09,Och10,Och11,Och12,Och13,Och14,Ore01,Ore02,Ore03,Ore04,Ore05,Ore06,Ore07,Ore08,Ore09,Ore10,Ore11,Ore12,Ore13,Ore14	*OreG0015177*
scaffold33196	247494	C	T	Och05,Och07,Och08,Och13	*OreG0015178*
scaffold33196	261211	G	A	NA	*OreG0015182*
scaffold33196	624147	G	A	Och02,Och03,Och04,Och05,Och06,Och09,Och10,Och11,Och12,Och13	*OreG0015213*
scaffold33196	716868	C	T	Ore01,Ore02,Ore03,Ore04,Ore05,Ore06,Ore07,Ore08,Ore09,Ore10,Ore11,Ore12,Ore13,Ore14	*OreG0015219*
scaffold33196	858398	G	A	Och01,Och02,Och03,Och04,Och05,Och06,Och07,Och08,Och09,Och10,Och11,Och12,Och13,Och14,Ore01,Ore02,Ore03,Ore04,Ore05,Ore06,Ore07,Ore08,Ore09,Ore10,Ore11,Ore12,Ore13,Ore14	*OreG0015235*
scaffold33196	969847	G	A	NA	*OreG0015245*
scaffold33196	1085386	T	A	Och01,Och02,Och03,Och04,Och05,Och06,Och07,Och08,Och09,Och10,Och11,Och12,Och13,Och14	*OreG0015250*
scaffold33222	249453	C	T	NA	*OreG0016768*
scaffold33222	250512	G	T	NA	*OreG0016768*
scaffold33222	251283	G	T	NA	*OreG0016768*
scaffold33222	792384	T	A	Och01,Och02,Och03,Och04,Och05,Och06,Och07,Och09,Och10,Och11,Och12,Och13,Och14	*OreG0016806*
scaffold32660	190200	C	T	NA	*OreG0010626*
scaffold32660	321466	G	A	NA	*OreG0010635*

scaffold32660	400910	G	A	NA	*OreG0010637*
scaffold32660	511486	G	A	NA	*OreG0010645*
scaffold32660	818651	G	T	NA	*OreG0010650*
scaffold32660	1047772	G	T	Ore04,Ore05	*OreG0010660*
scaffold32660	1151036	C	T	NA	*OreG0010662*
scaffold32660	1156286	C	T	NA	*OreG0010662*
scaffold32660	1258352	T	A	Och01,Och02,Och03,Och04,Och05,Och06,Och07,Och08,Och09,Och10,Och11,Och12,Och13,Och14	*OreG0010665*
scaffold32660	1327005	G	A	Och01,Och06,Och07,Och08,Och14	*OreG0010667*
scaffold32660	1328158	A	T	Och01,Och02,Och03,Och04,Och05,Och06,Och07,Och08,Och09,Och10,Och11,Och12,Och13,Och14,Ore01,Ore02,Ore03,Ore04,Ore05,Ore06,Ore07,Ore08,Ore09,Ore10,Ore11,Ore12,Ore13,Ore14	*OreG0010667*
scaffold32660	1759624	C	T	NA	*OreG0010680*
scaffold32660	1759645	C	T	NA	*OreG0010680*
scaffold32660	2094586	C	A	Och01,Och03,Och05,Och07,Och08,Och09,Och10,Och11,Och12,Och13	*OreG0010692*
scaffold32660	2581633	T	A	NA	*OreG0010707*
scaffold32660	2596519	C	T	NA	*OreG0010711*
scaffold32660	2607450	G	A	NA	*OreG0010712*
scaffold32660	2607454	C	T	NA	*OreG0010712*
scaffold32660	2670755	A	T	NA	*OreG0010717*
scaffold32660	2670800	G	T	Och13	*OreG0010717*
scaffold32660	2807899	T	A	Och14	*OreG0010726*
scaffold32660	2822125	G	A	NA	*OreG0010728*
scaffold32660	2823588	G	T	Och02,Och03,Och04,Och05,Och06,Och07,Och08,Och09,Och10,Och11,Och12,Och13	*OreG0010728*
scaffold32660	2826862	G	T	NA	*OreG0010728*
scaffold32660	2844026	C	A	NA	*OreG0010730*
scaffold32660	2983911	G	A	NA	*OreG0010742*
scaffold32660	2984068	T	A	NA	*OreG0010742*
scaffold32660	3165756	C	T	Och01,Och02,Och03,Och04,Och05,Och06,Och07,Och08,Och09,Och10,Och11,Och12,Och13,Och14,Ore01,Ore02,Ore03,Ore04,Ore05,Ore06,Ore07,Ore08,Ore09,Ore10,Ore11,Ore12,Ore13,Ore14	*OreG0010760*
scaffold32660	3165986	T	A	Och07,Och09,Och10,Och11	*OreG0010760*
scaffold32660	3167437	G	T	Ore04,Ore05	*OreG0010760*
scaffold32660	3215079	C	A	NA	*OreG0010764*
scaffold32660	3215505	G	T	NA	*OreG0010764*
scaffold32660	3220271	A	T	Och03,Och05	*OreG0010765*
scaffold32660	3298050	G	T	Ore01,Ore02,Ore03,Ore04,Ore05,Ore06,Ore07,Ore08,Ore09,Ore10,Ore11,Ore12,Ore13,Ore14	*OreG0010769*
scaffold32660	3313777	T	A	NA	*OreG0010771*
scaffold32660	3314714	C	T	NA	*OreG0010771*
scaffold32660	3364712	G	T	Och12	*OreG0010778*
scaffold32660	3364781	C	T	NA	*OreG0010778*
scaffold32660	3370234	T	A	Ore04,Ore05	*OreG0010780*
scaffold32660	3370540	C	A	Ore04,Ore05	*OreG0010780*
scaffold32660	3499851	G	T	Ore01,Ore02,Ore03,Ore04,Ore05,Ore06,Ore07,Ore08,Ore09,Ore10,Ore11,Ore12,Ore13,Ore14	*OreG0010795*
scaffold32660	3573777	G	A	Och11	*OreG0010801*
scaffold32660	3574184	C	T	Och11,Ore03,Ore04,Ore05,Ore09,Ore10,Ore12,Ore14	*OreG0010801*
scaffold32660	3580815	A	T	NA	*OreG0010802*
scaffold32660	3603622	C	T	Och01,Och02,Och03,Och04,Och05,Och06,Och07,Och08,Och09,Och10,Och11,Och12,Och13,Och14,Ore01,Ore02,Ore03,Ore04,Ore05,Ore06,Ore07,Ore08,Ore09,Ore10,Ore11,Ore12,Ore13,Ore14	*OreG0010804*
scaffold32660	3665440	A	T	NA	*OreG0010805*
scaffold32660	3696854	G	A	Ore04,Ore05	*OreG0010806*
scaffold32660	3739453	C	A	NA	*OreG0010807*
scaffold32660	3739543	G	A	Och01,Och03,Och05,Och09,Och10,Och11,Ore04,Ore05	*OreG0010808*
scaffold32660	3739727	G	C	Och08,Och14	*OreG0010808*
scaffold32660	3814725	G	A	NA	*OreG0010816*
scaffold32660	4003128	T	A	NA	*OreG0010834*
scaffold32660	4003147	G	T	Och01,Och02,Och03,Och04,Och05,Och06,Och07,Och08,Och09,Och10,Och11,Och12,Och13,Och14	*OreG0010834*
scaffold32660	4017182	C	T	Och01,Och02,Och03,Och04,Och05,Och06,Och07,Och08,Och09,Och10,Och11,Och12,Och13,Och14,Ore01,Ore02,Ore03,Ore04,Ore05,Ore06,Ore07,Ore08,Ore09,Ore10,Ore11,Ore12,Ore13,Ore14	*OreG0010835*
scaffold32660	4047815	G	A	NA	*OreG0010838*
scaffold32660	4118003	G	T	NA	*OreG0010845*
scaffold32660	4140675	C	T	NA	*OreG0010847*
scaffold32660	4182628	C	T	Och01,Och02,Och03,Och04,Och05,Och06,Och07,Och08,Och09,Och10,Och11,Och12,Och13,Och14	*OreG0010849*
scaffold32660	4209201	G	A	Ore02,Ore03,Ore04,Ore05,Ore06,Ore07,Ore08,Ore09,Ore10,Ore11,Ore12,Ore13,Ore14	*OreG0010852*
scaffold32660	4428984	G	A	NA	*OreG0010874*
scaffold32660	4445452	G	A	NA	*OreG0010876*
scaffold32660	4445473	G	A	NA	*OreG0010876*

scaffold32660	4446882	G	A	NA	*OreG0010876*
scaffold32660	4453966	A	T	NA	*OreG0010877*
scaffold32660	4457411	C	T	NA	*OreG0010877*
scaffold32660	4477342	C	T	NA	*OreG0010881*
scaffold32660	4704855	C	T	Och01,Och02,Och03,Och04,Och05,Och06,Och07,Och08,Och09,Och10,Och11,Och12,Och13,Och14	*OreG0010902*
scaffold32660	4787034	G	A	Och02,Och03,Och04,Och05,Och06,Och07,Och08,Och09,Och10,Och11,Och12,Och13	*OreG0010911*
scaffold32660	4848066	C	T	Och09,Och10	*OreG0010917*
scaffold32660	4855552	G	T	NA	*OreG0010918*
scaffold32660	4859079	C	T	Ore03,Ore04,Ore05,Ore09,Ore13	*OreG0010919*
scaffold32660	4859156	G	A	Och09,Och10,Och11	*OreG0010919*
scaffold32660	4862109	G	A	Och01,Och02,Och03,Och04,Och05,Och06,Och07,Och08,Och09,Och10,Och11,Och12,Och13,Och14,Ore01,Ore02,Ore03,Ore04,Ore05,Ore06,Ore07,Ore08,Ore09,Ore10,Ore11,Ore12,Ore13,Ore14	*OreG0010920*
scaffold32660	4864256	C	T	NA	*OreG0010920*
scaffold32660	4893958	C	T	NA	*OreG0010922*
scaffold32660	4909445	C	A	Och01,Och02,Och03,Och04,Och05,Och06,Och07,Och08,Och09,Och10,Och11,Och12,Och13,Och14	*OreG0010924*
scaffold32660	5209797	C	T	NA	*OreG0010929*
scaffold32660	5210708	T	A	NA	*OreG0010929*
scaffold32660	5218969	C	A	NA	*OreG0010930*
scaffold32660	5270204	G	T	NA	*OreG0010937*
scaffold32660	5546855	G	A	NA	*OreG0010959*
scaffold32660	5607435	C	T	NA	*OreG0010962*
scaffold32660	5741054	T	A	NA	*OreG0010969*
scaffold32660	5912954	G	A	NA	*OreG0010984*
scaffold32660	6301343	G	A	NA	*OreG0011008*
scaffold33248	257955	G	A	Ore01,Ore02,Ore03,Ore04,Ore05,Ore06,Ore07,Ore08,Ore09,Ore10,Ore11,Ore12,Ore13,Ore14	*OreG0017549*
scaffold33248	266313	A	T	Och01,Och02,Och03,Och04,Och05,Och06,Och07,Och08,Och09,Och10,Och11,Och12,Och13,Och14,Ore01,Ore02,Ore03,Ore04,Ore05,Ore06,Ore07,Ore08,Ore09,Ore10,Ore11,Ore12,Ore13,Ore14	*OreG0017550*
scaffold33248	328160	T	A	NA	*OreG0017552*
scaffold33248	484144	C	T	NA	*OreG0017560*
scaffold33248	582238	C	G	Och11	*OreG0017563*
scaffold33248	777405	C	T	Och01,Och02,Och03,Och04,Och05,Och06,Och07,Och08,Och09,Och10,Och11,Och12,Och13,Och14	*OreG0017571*
scaffold33248	907421	C	T	Och02,Och03,Och04,Och05,Och06,Och07,Och08,Och09,Och10,Och12,Och13	*OreG0017575*
scaffold33248	907684	C	T	Och01	*OreG0017575*
scaffold33248	907714	C	T	Och01	*OreG0017575*
scaffold33248	1088580	C	T	Ore02,Ore03,Ore04,Ore05,Ore06,Ore07,Ore08,Ore09,Ore10,Ore11,Ore12,Ore14	*OreG0017586*
scaffold33248	1090961	C	T	Och01	*OreG0017586*
scaffold33250	1865997	G	A	NA	*OreG0017656*
scaffold33250	2222101	G	T	Och01,Och02,Och03,Och04,Och05,Och06,Och07,Och08,Och09,Och10,Och11,Och12,Och13,Och14	*OreG0017703*
scaffold33250	2263976	T	A	Och01,Och02,Och03,Och04,Och05,Och06,Och07,Och08,Och09,Och10,Och11,Och12,Och13,Och14,Ore01,Ore02,Ore03,Ore04,Ore05,Ore06,Ore07,Ore08,Ore09,Ore10,Ore11,Ore12,Ore13,Ore14	*OreG0017707*
scaffold33250	2411172	G	A	Och03,Och04,Och05,Och06,Och07,Och08,Och09,Och10,Och12,Och13,Och14	*OreG0017724*
scaffold33250	2771753	C	A	Och06,Och07,Och08,Och09,Och10,Och11,Och12,Och13	*OreG0017762*
scaffold33250	2860451	C	A	Och01,Och02,Och03,Och04,Och05,Och06,Och07,Och08,Och09,Och10,Och11,Och12,Och13,Och14	*OreG0017769*
scaffold33250	2934245	G	A	NA	*OreG0017774*
scaffold33250	3060496	G	A	Och01,Och02,Och03,Och04,Och05,Och06,Och07,Och08,Och09,Och10,Och11,Och12,Och13,Och14	*OreG0017779*
scaffold33250	3084125	C	T	Och07,Och08,Och12,Och13	*OreG0017781*
scaffold33250	3215178	C	T	NA	*OreG0017787*
scaffold33250	3235116	C	T	NA	*OreG0017789*
scaffold33250	3521610	C	A	Och01,Och02,Och03,Och04,Och05,Och06,Och07,Och08,Och09,Och10,Och11,Och12,Och13,Och14	*OreG0017800*
scaffold33224	107698	G	T	NA	*OreG0016823*
scaffold33224	109554	T	G	Ore02	*OreG0016823*
scaffold33224	109827	T	A	NA	*OreG0016823*
scaffold33224	111727	G	T	Och01,Och08,Och09,Och10	*OreG0016823*
scaffold33224	113166	C	A	NA	*OreG0016823*
scaffold33224	114673	C	T	NA	*OreG0016823*
scaffold33224	263546	C	T	Och12	*OreG0016839*
scaffold33224	316870	C	T	NA	*OreG0016844*
scaffold33224	320939	C	T	NA	*OreG0016844*
scaffold33224	341840	G	A	NA	*OreG0016847*
scaffold33224	344446	G	A	NA	*OreG0016847*
scaffold33224	345850	A	T	NA	*OreG0016847*
scaffold33224	442495	G	T	NA	*OreG0016861*
scaffold33224	442556	C	A	NA	*OreG0016861*

scaffold33224	664679	A	T	NA	*OreG0016874*
scaffold33224	993603	C	T	Och11	*OreG0016905*
scaffold33224	1173706	C	T	Ore01,Ore02,Ore03,Ore04,Ore05,Ore06,Ore07,Ore08,Ore09,Ore10,Ore11,Ore12,Ore13,Ore14	*OreG0016913*
scaffold33224	1288630	G	T	NA	*OreG0016928*
scaffold33224	1326878	T	A	Ore01,Ore02,Ore03,Ore04,Ore05,Ore06,Ore07,Ore08,Ore09,Ore10,Ore11,Ore12,Ore13,Ore14	*OreG0016939*
scaffold33224	1641227	A	T	NA	*OreG0016963*
scaffold33224	1892065	G	T	NA	*OreG0016973*
scaffold33224	2085824	T	A	Och01,Och02,Och03,Och04,Och05,Och06,Och07,Och08,Och09,Och10,Och11,Och12,Och13,Och14	*OreG0016980*
scaffold33224	2085866	C	A	Och01,Och02,Och03,Och04,Och05,Och06,Och08,Och12,Och13,Och14	*OreG0016980*
scaffold33224	2147205	A	T	Och01,Och04,Och06,Och07,Och09,Och10,Och11,Och14	*OreG0016985*
scaffold33224	2147230	C	T	Ore01,Ore02,Ore03,Ore04,Ore05,Ore06,Ore07,Ore08,Ore09,Ore10,Ore11,Ore12,Ore13,Ore14	*OreG0016985*
scaffold33224	2147303	G	A	Och07,Och09,Och10,Och11	*OreG0016985*
scaffold33224	2214404	C	T	Och11	*OreG0016990*
scaffold33224	2228547	G	A	Och04,Och06	*OreG0016991*
scaffold33224	2403099	C	G	NA	*OreG0017003*
scaffold33224	2404841	C	T	NA	*OreG0017004*
scaffold33224	2406756	T	A	NA	*OreG0017005*
scaffold33224	2570439	G	T	NA	*OreG0017015*
scaffold33224	2765877	C	A	NA	*OreG0017026*
scaffold33224	3039602	C	T	Och02,Och03,Och04,Och05,Och06,Och07,Och08,Och09,Och10,Och11,Och12,Och13,Och14	*OreG0017042*
scaffold33199	43892	C	A	Och01,Och02,Och03,Och04,Och05,Och06,Och07,Och08,Och09,Och10,Och11,Och12,Och13,Och14	*OreG0015457*
scaffold33199	165606	G	A	Ore04,Ore05,Ore09,Ore14	*OreG0015474*
scaffold33199	273781	C	A	Och05,Och09,Och10,Och11,Och12,Och13	*OreG0015486*
scaffold33199	287496	G	T	NA	*OreG0015487*
scaffold33199	287504	C	A	NA	*OreG0015487*
scaffold33199	373024	T	A	NA	*OreG0015495*
scaffold33199	405438	C	T	NA	*OreG0015496*
scaffold33199	432537	C	A	NA	*OreG0015500*
scaffold33199	481246	C	T	Och05,Och09,Och10,Och11,Och12,Och13,Och14	*OreG0015508*
scaffold33199	481292	G	A	Och05,Och07,Och09,Och10,Och11,Och12,Och13,Och14	*OreG0015508*
scaffold33199	521590	C	G	NA	*OreG0015513*
scaffold33199	530799	G	T	Och01	*OreG0015516*
scaffold33199	564495	A	T	NA	*OreG0015521*
scaffold33199	761732	G	A	NA	*OreG0015547*
scaffold33199	766792	C	T	Och01,Och03,Och06,Och07,Och08,Och09,Och10,Och11,Och12,Och13	*OreG0015548*
scaffold33199	798556	C	T	Och01,Och02,Och03,Och04,Och05,Och06,Och07,Och08,Och09,Och10,Och11,Och12,Och13,Och14	*OreG0015553*
scaffold33199	927416	A	T	Och06	*OreG0015572*
scaffold33199	1029904	C	T	Och01,Och02,Och03,Och04,Och05,Och06,Och07,Och08,Och09,Och10,Och11,Och12,Och13,Och14,Ore01,Ore02,Ore03,Ore04,Ore05,Ore06,Ore07,Ore08,Ore09,Ore10,Ore11,Ore12,Ore13,Ore14	*OreG0015585*
scaffold33199	1100173	G	A	Ore01,Ore02,Ore03,Ore04,Ore05,Ore06,Ore07,Ore08,Ore09,Ore10,Ore11,Ore12,Ore13,Ore14	*OreG0015591*
scaffold33199	1251190	A	C	NA	*OreG0015612*
scaffold33199	1281416	T	A	NA	*OreG0015616*
scaffold33199	1487917	G	A	Och01	*OreG0015648*
scaffold33199	1488370	G	A	NA	*OreG0015648*
scaffold33199	1623961	C	A	Ore06,Ore07	*OreG0015654*
scaffold38	76512	C	T	Och08	*OreG0019129*
scaffold38	165374	G	A	Och08,Och11	*OreG0019136*
scaffold38	170036	C	T	NA	*OreG0019137*
scaffold38	203649	C	T	NA	*OreG0019138*
scaffold38	204310	A	T	NA	*OreG0019138*
scaffold38	215456	C	A	NA	*OreG0019138*
scaffold38	446878	T	A	NA	*OreG0019152*
scaffold38	467008	C	A	NA	*OreG0019153*
scaffold38	481428	G	A	Ore11	*OreG0019155*
scaffold38	703796	G	A	Ore01,Ore02,Ore03,Ore04,Ore05,Ore06,Ore07,Ore08,Ore09,Ore10,Ore11,Ore12,Ore13,Ore14	*OreG0019172*
scaffold38	780983	C	G	Och01,Och02,Och03,Och04,Och05,Och06,Och07,Och08,Och09,Och10,Och11,Och12,Och13,Och14	*OreG0019186*
scaffold38	867415	G	A	NA	*OreG0019196*
scaffold38	867697	C	A	Och01,Och11	*OreG0019196*
scaffold38	868347	G	T	Och01,Och11	*OreG0019196*
scaffold38	916931	C	T	Och02,Och03,Och04,Och06,Och07,Och12,Och13,Och14	*OreG0019202*
scaffold38	1634948	G	A	NA	*OreG0019249*
scaffold38	1634957	G	A	NA	*OreG0019249*

scaffold38	3341632	C	T	Och01,Och02,Och03,Och04,Och05,Och06,Och07,Och08,Och09,Och10,Och11,Och12,Och13,Och14,Ore01,Ore02,Ore03,Ore04,Ore05,Ore06,Ore07,Ore08,Ore09,Ore10,Ore11,Ore12,Ore13,Ore14	*OreG0019302*
scaffold38	3438807	G	A	NA	*OreG0019312*
scaffold38	3438853	G	T	NA	*OreG0019312*
scaffold38	3439226	C	T	NA	*OreG0019312*
scaffold38	3467672	G	T	Och01,Och02,Och03,Och04,Och05,Och06,Och07,Och08,Och09,Och10,Och11,Och12,Och13,Och14	*OreG0019317*
scaffold38	3599843	G	A	NA	*OreG0019334*
scaffold38	3599849	G	A	NA	*OreG0019334*
scaffold38	3610935	C	T	NA	*OreG0019338*
scaffold38	3611553	C	T	NA	*OreG0019338*
scaffold38	3701331	G	A	Och12,Och13	*OreG0019352*
scaffold38	3726009	G	A	NA	*OreG0019356*
scaffold38	3729608	C	A	NA	*OreG0019355*
scaffold38	3742008	C	A	Och04,Och06,Och12,Och13,Och14	*OreG0019357*
scaffold38	3765536	C	T	NA	*OreG0019360*
scaffold38	3975545	C	A	NA	*OreG0019379*
scaffold38	3998993	C	T	Och01,Och02,Och03,Och04,Och05,Och06,Och07,Och08,Och09,Och10,Och11,Och12,Och13,Och14,Ore01,Ore02,Ore03,Ore04,Ore05,Ore06,Ore07,Ore08,Ore09,Ore10,Ore11,Ore12,Ore13,Ore14	*OreG0019382*
scaffold38	4118928	G	A	Och04,Och07,Och12,Och13,Och14	*OreG0019389*
scaffold38	4190610	G	A	NA	*OreG0019394*
scaffold38	4306331	C	T	Och01,Och02,Och03,Och04,Och05,Och06,Och07,Och08,Och09,Och10,Och11,Och12,Och13,Och14,Ore01,Ore02,Ore03,Ore04,Ore05,Ore06,Ore07,Ore08,Ore09,Ore10,Ore11,Ore12,Ore13,Ore14	*OreG0019404*
scaffold38	4442266	G	T	NA	*OreG0019413*
scaffold38	4442326	C	T	NA	*OreG0019413*
scaffold33174	176515	C	T	Och01,Och02,Och03,Och04,Och05,Och06,Och07,Och08,Och09,Och10,Och11,Och12,Och13	*OreG0014366*
scaffold33174	178566	C	T	Och02,Och03,Och04,Och05,Och06,Och07,Och08,Och09,Och10,Och11,Och12,Och13	*OreG0014366*
scaffold33174	178810	C	T	Och02,Och03,Och04,Och05,Och06,Och07,Och08,Och09,Och10,Och11,Och12,Och13	*OreG0014366*
scaffold33174	265640	C	T	Och02,Och03,Och04,Och05,Och06,Och07,Och08,Och09,Och10,Och11,Och12,Och13,Och14	*OreG0014369*
scaffold33174	265719	G	A	Och02,Och03,Och04,Och05,Och06,Och07,Och08,Och09,Och10,Och11,Och12,Och13,Och14	*OreG0014369*
