# Supplementary material for: Genomic effects of population collapse in a critically endangered ironwood tree Ostrya rehderiana
Source: Nat Commun. 2018 Dec 21;9:5449. doi: 10.1038/s41467-018-07913-4 (PMC6303402; doi:10.1038/s41467-018-07913-4)
Supplement: Supplementary file 6 — Reporting Summary [file 41467_2018_7913_MOESM6_ESM.pdf]

nature research

Corresponding author(s): Jianqquan Liu

Reporting Summary

Nature Research wishes to improve the reproducibility of the work that we publish. This form provides structure for consistency and transparency in reporting. For further information on Nature Research policies, see Authors & Referees and the Editorial Policy Checklist.

Statistical parameters

When statistical analyses are reported, confirm that the following items are present in the relevant location (e.g. figure legend, table legend, main text, or Methods section).

n/a	Confirmed	
☐	☒	The exact sample size (n) for each experimental group/condition, given as a discrete number and unit of measurement
☐	☒	An indication of whether measurements were taken from distinct samples or whether the same sample was measured repeatedly
☐	☒	The statistical test(s) used AND whether they are one- or two-sided<br>*Only common tests should be described solely by name; describe more complex techniques in the Methods section.*
☒	☐	A description of all covariates tested
☒	☐	A description of any assumptions or corrections, such as tests of normality and adjustment for multiple comparisons
☐	☒	A full description of the statistics including central tendency (e.g. means) or other basic estimates (e.g. regression coefficient) AND variation (e.g. standard deviation) or associated estimates of uncertainty (e.g. confidence intervals)
☐	☒	For null hypothesis testing, the test statistic (e.g. F, t, r) with confidence intervals, effect sizes, degrees of freedom and P value noted<br>*Give P values as exact values whenever suitable.*
☐	☒	For Bayesian analysis, information on the choice of priors and Markov chain Monte Carlo settings
☒	☐	For hierarchical and complex designs, identification of the appropriate level for tests and full reporting of outcomes
☒	☐	Estimates of effect sizes (e.g. Cohen's d, Pearson's r), indicating how they were calculated
☐	☒	Clearly defined error bars<br>*State explicitly what error bars represent (e.g. SD, SE, CI)*

Our web collection on statistics for biologists may be useful.

Software and code

Policy information about availability of computer code

Data collection	All softwares used here have been listed in the Methods section.
Data analysis	All softwares we used in this study have been listed in the Methods section.

For manuscripts utilizing custom algorithms or software that are central to the research but not yet described in published literature, software must be made available to editors/reviewers upon request. We strongly encourage code deposition in a community repository (e.g. GitHub). See the Nature Research guidelines for submitting code & software for further information.

Data

Policy information about availability of data

All manuscripts must include a data availability statement. This statement should provide the following information, where applicable:

- Accession codes, unique identifiers, or web links for publicly available datasets
- A list of figures that have associated raw data
- A description of any restrictions on data availability

The WGS projects have been deposited at NCBI GenBank under BioProject ID PRJNA428013 for O. rehderiana and BioProject ID PRJNA428014 for O. chinensis. The genomic sequencing data and transcriptomic raw data have been deposited in the NCBI Sequence Read Archive (SRA) under BioProject ID PRJNA428015 and PRJNA428018, respectively.

Field-specific reporting

Please select the best fit for your research. If you are not sure, read the appropriate sections before making your selection.

☐ Life sciences ☐ Behavioural & social sciences ☒ Ecological, evolutionary & environmental sciences

For a reference copy of the document with all sections, see nature.com/authors/policies/ReportingSummary-flat.pdf

Ecological, evolutionary & environmental sciences study design

All studies must disclose on these points even when the disclosure is negative.

Study description	We examined genomic changes within the extremely dangered Ostrya rehderiana and the widespread congener O. chinensis
Research sample	We collected all the wild Ostrya rehderiana individuals and nine additional young trees. The sample size of Ostrya chinensis were also selected as 14 to be the same as Ostrya rehderiana.
Sampling strategy	We sampled five very old individuals currently residing in a single wild population and nine young progenies, which were planted from successfully germinated seeds intentionally collected from the old stand (s). We also sampled 14 trees of the widespread O. chinensis from the wide populations.
Data collection	We collected leaves of the sampled trees in the summer without harm to trees. We observed the developed cymules of the capkins.
Timing and spatial scale	We observed the developed cymules of the capkins in May.
Data exclusions	No data was excluded.
Reproducibility	n/a
Randomization	n/a
Blinding	n/a

Did the study involve field work? ☒ Yes ☐ No

Field work, collection and transport

Field conditions	We collected the fresh leaves for extracting DNAs in summer without affects from temperature
Location	Tianmushan Mountain, ZheJiang Province
Access and import/export	All the Ostrya rehderiana samples were collected with the agreements and helps of the Zhejiang Tianmushan National Nature Reserve Management Bureau. All the Ostrya chinensis samples were collected from it's wild habitats.
Disturbance	No harm to the normal growth of trees when collecting leaves for extracting DNAs

Reporting for specific materials, systems and methods

Materials & experimental systems

n/a	Involved in the study	
☐	☒	Unique biological materials
☒	☐	Antibodies
☒	☐	Eukaryotic cell lines
☒	☐	Palaeontology
☒	☐	Animals and other organisms
☒	☐	Human research participants

Methods

n/a	Involved in the study	
☒	☐	ChIP-seq
☒	☐	Flow cytometry
☒	☐	MRI-based neuroimaging

Unique biological materials

Policy information about availability of materials

Obtaining unique materials	All the Ostrya rehderiana samples were collected with the agreements and helps of the Zhejiang Tianmushan National Nature Reserve Management Bureau. All the Ostrya chinensis samples were collected from it's wild habitats.
